# Supplementary material for: Systematic literature review reveals suboptimal use of chemical probes in cell-based biomedical research
Source: Nat Commun. 2023 Jun 3;14:3228. doi: 10.1038/s41467-023-38952-1 (PMC10239480; doi:10.1038/s41467-023-38952-1)
Supplement: Supplementary file 1 — Supplementary Information [file 41467_2023_38952_MOESM1_ESM.pdf]

Systematic literature review reveals suboptimal use of chemical probes in cell-based biomedical research

Jayden Sterling[1], Jennifer R Baker[2], Adam McCluskey[2], Lenka Munoz[1*]

[1] Faculty of Medicine and Health, Charles Perkins Centre, The University of Sydney, NSW 2006, Australia
[2] Discipline of Chemistry, School of Environmental and Life Sciences, The University of Newcastle, Callaghan, NSW 2308, Australia
*Corresponding author: lenka.munoz@sydney.edu.au

Content

Supplementary Note 1. Reasons for exclusions of articles and eligibility criteria. **Page 2**
Supplementary Note 2. Citations for UNC1999 publications. **Page 9**
Supplementary Note 3. Citations for UNC0638 publications. **Page 17**
Supplementary Note 4. Citations for GSK-J4 publications. **Page 27**
Supplementary Note 5. Citations for A-485 publications. **Page 32**
Supplementary Note 6. Citations for AMG900 publications. **Page 38**
Supplementary Note 7. Citations for AZD1152 publications. **Page 53**
Supplementary Note 8. Citations for AZD2014 publications. **Page 63**
Supplementary Note 9. Citations for THZ1 publications. **Page 75**

Supplementary Figure 1. Overview of EZH2 chemical probes. **Page 3**
Supplementary Figure 2. PRISMA flow diagram: UNC1999 **Page 4**
Supplementary Figure 3. Overview of G9a/GLP and KDM6 chemical probes. **Page 10**
Supplementary Figure 4. PRISMA flow diagram: UNC0638. **Page 11**
Supplementary Figure 5. PRISMA flow diagram: GSK-J4. **Page 18**
Supplementary Figure 6. Overview of CREBBP/p300 chemical probes. **Page 28**
Supplementary Figure 7. PRISMA flow diagram: A-485. **Page 29**
Supplementary Figure 8. Overview of Aurora and mTOR chemical probes. **Page 33**
Supplementary Figure 9. PRISMA flow diagram: AMG900. **Page 34**
Supplementary Figure 10. PRISMA flow diagram: AZD1152. **Page 39**
Supplementary Figure 11. PRISMA flow diagram: AZD2014. **Page 54**
Supplementary Figure 12. Overview of CDK7 and CDK12/13 chemical probes. **Page 64**
Supplementary Figure 13. PRISMA flow diagram: THZ1. **Page 65**

Supplementary Table 1. List of excluded articles using UNC1999. **Page 5**
Supplementary Table 2. Overview of eligible publications using UNC1999. **Page 7**
Supplementary Table 3. List of excluded articles using UNC0638. **Page 12**
Supplementary Table 4. Overview of eligible publications using UNC0638. **Page 14**
Supplementary Table 5. List of excluded articles using GSK-J4. **Page 19**
Supplementary Table 6. Overview of eligible publications using GSK-J4. **Page 21**
Supplementary Table 7. List of excluded articles using A-485. **Page 30**
Supplementary Table 8. Overview of eligible publications using A-485. **Page 31**
Supplementary Table 9. List of excluded articles using AMG900. **Page 35**
Supplementary Table 10. Overview of eligible publications using AMG900. **Page 37**
Supplementary Table 11. List of excluded articles using AZD1152. **Page 40**
Supplementary Table 12. Overview of eligible publications using AZD1152. **Page 46**
Supplementary Table 13. List of excluded articles using AZD2014. **Page 55**
Supplementary Table 14. Overview of eligible publications using AZD2014. **Page 59**
Supplementary Table 15. List of excluded articles using THZ1. **Page 66**
Supplementary Table 16. Overview of eligible publications using THZ1. **Page 68**

Supplementary Note 1. Reasons for exclusions of articles and eligibility criteria for inclusion of publications.

Reasons for exclusion
1) Articles not written in English.
2) Articles whose full-text were not available via University of Sydney library access.
3) Articles describing the discovery and characterisation of a given chemical probe, or medicinal chemistry/SAR articles using the probe as a lead compound.
4) Articles in which the chemical probe was used to generate chemical probe-resistant clones and/or studies lacking mechanistic experiments
5) Articles containing only *in vivo* (animal/human tissue) data and/or clinical trial articles.
6) Reviews, commentaries, editorials, letters to the editors, conference proceedings, pre-print and similar.

Eligibility criteria for inclusion
1) Publication presents at least one figure panel using the chemical probe in a cell-based assay

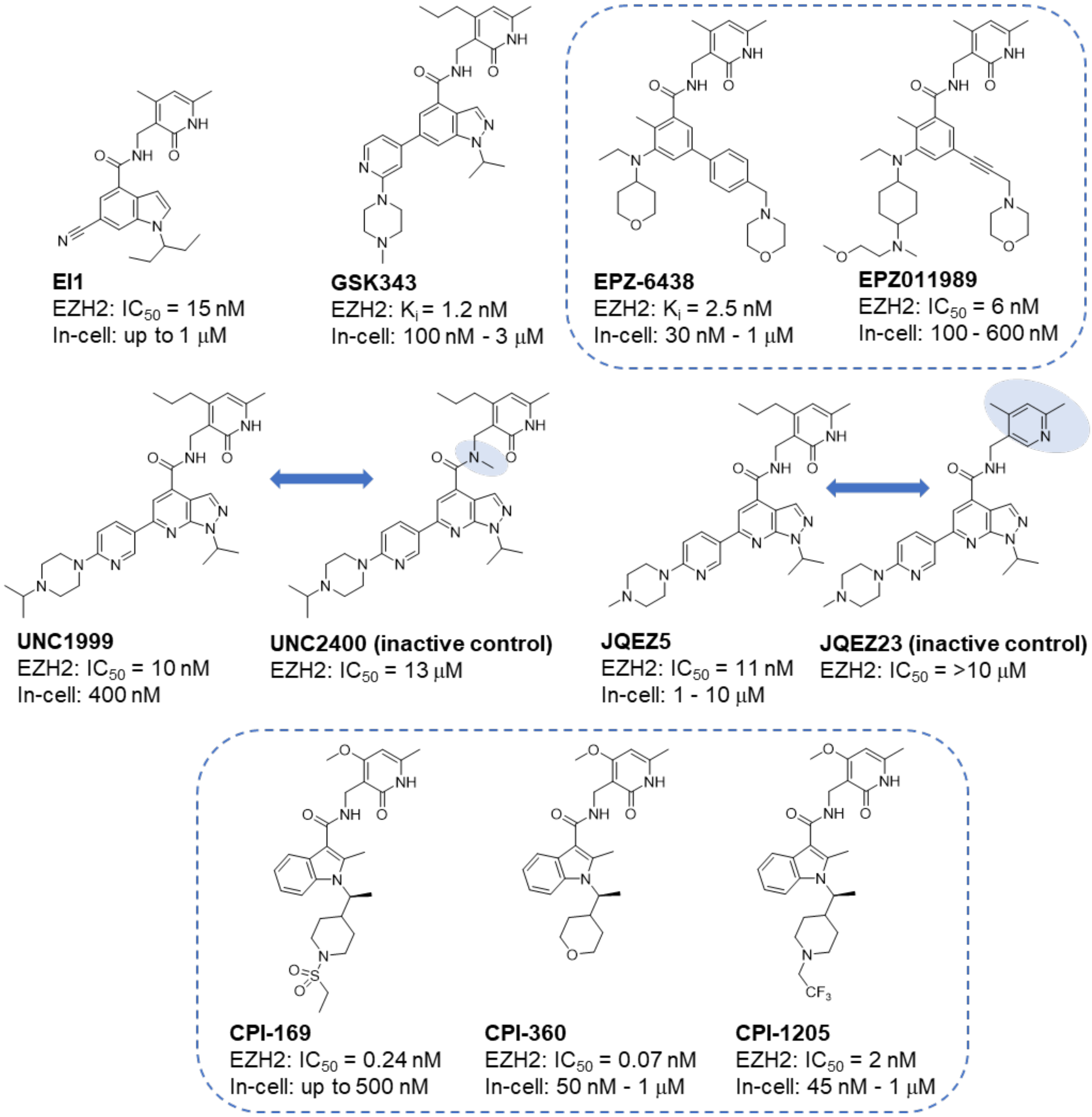

Supplementary Figure 1. Structures, biochemical potency (K_i/IC_{50}) and recommended in-cell concentration of EZH2 targeting chemical probes listed on the Chemical Probes Portal (www.chemicalprobes.org). Dashed lines group structurally related chemical probes that should not be used together as orthogonal tools (*e.g.,* EPZ-6438 can be used as an orthogonal chemical probe with UNC1999, but not with EPZ011989). Bidirectional arrows link target-inactive analogues and shaded fields highlight the structural changes leading to decreased EZH2 targeting.

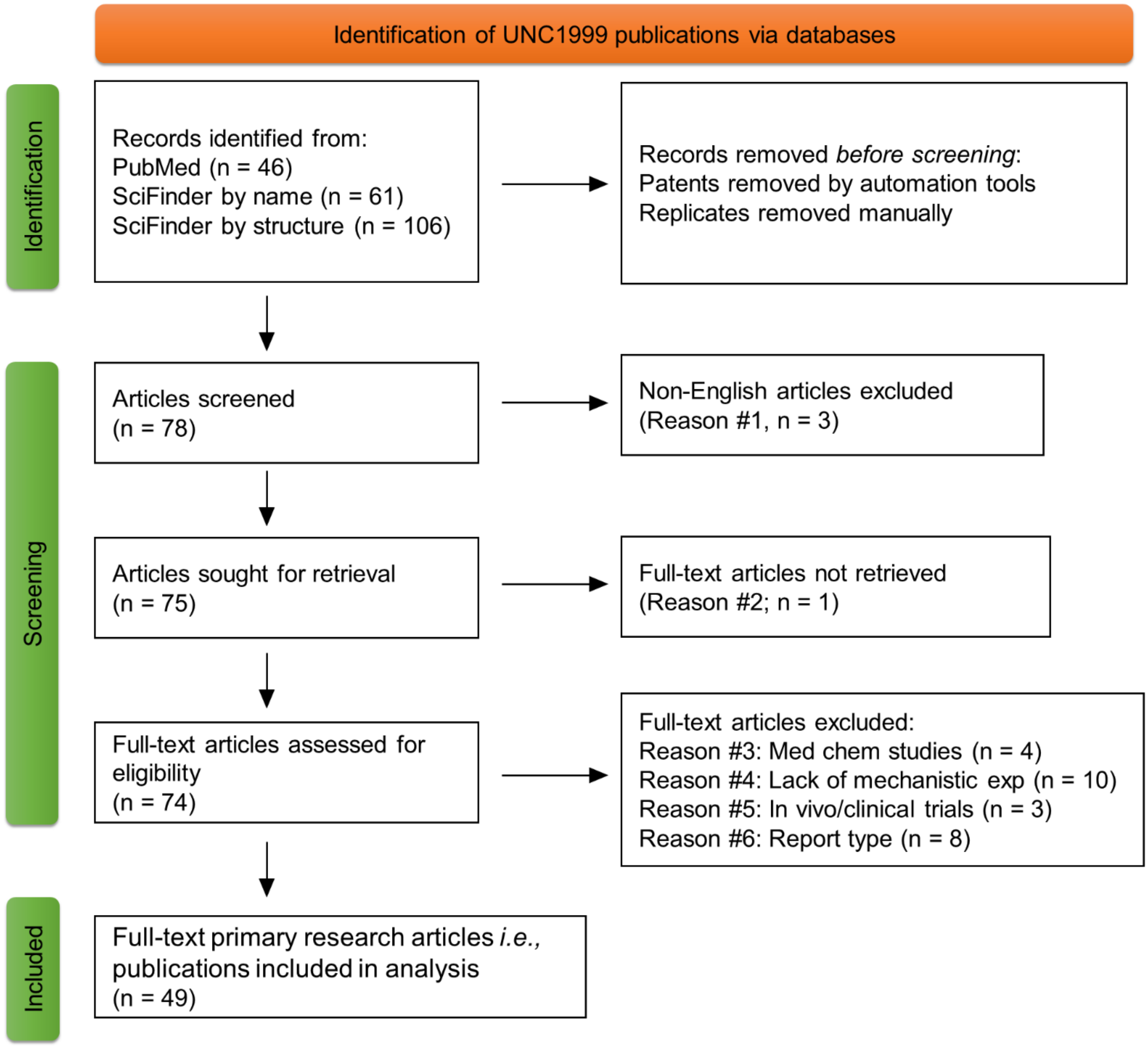

Supplementary Figure 2. PRISMA flow diagram summarising identification of publications using the EZH2 chemical probe UNC1999.

Supplementary Table 1. List of excluded articles using the EZH2 probe UNC1999.

	Title (PMID)	Reason for exclusion
1	[Novel epigenetic therapies for multiple myeloma] (PMID: 33967157)	1) Non-English publication
2	[Effects of histone methyltransferase inhibitors on the survival, apoptosis and cell cycle of Raji cells] (PMID: 31631594)	1) Non-English publication
3	Effects of EZH2 inhibitor combined with cisplatin on proliferation and apoptosis of non-small cell lung cancer A549 cells (No PMID; DOI: 10.3969/j.issn.1005-9202.2016.23.029)	1) Non-English publication
4	Development of a UPLC-MS/MS method for determination of a dual EZH1/2 inhibitor UNC1999 in rat plasma (PMID: 34841882)	2) No access
5	^{18}F-Labeled PET probe targeting enhancer of zeste homologue 2 (EZH2) for cancer imaging (PMID: 30891136)	3) Development of [^{18}F]-labelled EZH2 probes
6	An orally bioavailable chemical probe of the lysine methyltransferases EZH2 and EZH1 (PMID: 23614352)	3) Discovery of UNC1999 / UNC2400
7	Structure-activity relationship studies for enhancer of zeste homologue 2 (EZH2) and enhancer of zeste homologue 1 (EZH1) inhibitors (PMID: 27468126)	3) SAR of UNC1999 analogues
8	Identification of novel EZH2 inhibitors through pharmacophore-based virtual screening and biological assays (PMID: 27289323)	3) SAR study of new EZH1/2 inhibitors.
9	Identification of novel Ebola virus inhibitors using biologically contained virus (PMID: 35337896)	4) No mechanistic exp. - screen (>4000 compounds) for Ebola virus inhibitors, no follow up experiments with UNC1999.
10	Combined inhibition of JAK/STAT pathway and lysine-specific demethylase 1 as a therapeutic strategy in CSF3R/CEBPA mutant acute myeloid leukemia (PMID: 32471953)	4) No mechanistic exp. - cell viability screen only
11	The BRD9/7 inhibitor TP-472 blocks melanoma tumor growth by suppressing ECM-mediated oncogenic signaling and inducing apoptosis (PMID: 34771678)	4) No mechanistic exp. - cell viability screen only
12	Dissecting the role of novel EZH2 inhibitors in primary glioblastoma cell cultures: effects on proliferation, epithelial-mesenchymal transition, migration, and on the pro-inflammatory phenotype (PMID: 31791385)	4) No mechanistic experiments
13	Dual inhibition of enhancer of zeste homolog 1/2 overactivates WNT signaling to deplete cancer stem cells in multiple myeloma (PMID: 30343511)	4) No mechanistic experiments
14	BET-bromodomain and EZH2 inhibitor–treated chronic GVHD mice have blunted germinal centers with distinct transcriptomes (PMID: 35226736)	4) No mechanistic experiments
15	Discovery of a first-in-class EZH2 selective degrader (PMID: 31819273)	4) No mechanistic experiments
16	Cell panel profiling reveals conserved therapeutic clusters and differentiates the mechanism of action of different PI3K/mTOR, aurora kinase and EZH2 inhibitors (PMID: 27587489)	4) No mechanistic experiments - 122 epigenetic inhibitors tested; no follow up
17	Permissive epigenomes endow reprogramming competence to transcriptional regulators (PMID: 32807969)	4) No mechanistic experiments - 17 compounds identified as hits incl UNC1999; no follow up
18	Activity Comparison of Epigenetic Modulators against the Hemoprotozoan Parasites Babesia divergens and Plasmodium falciparum (PMID: 33599488)	4) No mechanistic exp. - Parasites growth
19	The role of the histone methyltransferase EZH2 in liver inflammation and fibrosis in STAM NASH mice (PMID: 32370249)	5) In vivo study
20	Enhancer of Zeste Homolog 2 inhibition stimulates bone formation and mitigates bone loss caused by ovariectomy in skeletally mature mice (PMID: 27758858)	5) In vivo study
21	Fate mapping of human glioblastoma reveals an invariant stem cell hierarchy (PMID: 28854171)	5) In vivo study
22	The miR-125a and miR-320c are potential tumor suppressor microRNAs epigenetically silenced by the polycomb repressive complex 2 in multiple myeloma (PMID: 28664185)	6) Commentary
23	Discovery of selective inhibitors for lysine methyltransferases EZH2/EZH1 and SETD8 (No PMID, DOI or ISSN available, AN: 2015:1328182)	6) Conference proceedings
24	Chemical probes for the lysine methyltransferases G9a and EZH2 (No PMID, DOI or ISSN available, AN: 2015:1328166)	6) Conference proceedings

25	Manipulation of chromatin to enhance CRISPR activity (No PMID, DOI or ISSN available, AN: 2017:1932488)	6) Conference proceedings
26	Evaluating small molecule histone inhibitors with high resolution mass spectrometry and 3D cell cultures (No PMID, DOI or ISSN available, AN: 2016:294990)	6) Conference proceedings
27	Histone modifications and active gene expression are associated with enhanced CRISPR activity in de-silenced chromatin (No PMID available, DOI: https://doi.org/10.1101/228601)	6) Pre-print
28	Six years (2012-2018) of researches on catalytic EZH2 inhibitors: the boom of the 2-pyridone compounds (PMID: 30338896)	6) Review
29	Targeting EZH2 and PRC2 dependence as novel anticancer therapy (PMID: 26027790)	6) Review

Supplementary Table 2. Overview of eligible publications using the EZH2 probe UNC1999 and compliance (**in blue**) with recommendations to use UNC1999 up to 400 nM (https://www.chemicalprobes.org/unc1999?q=UNC1999) or 3 µM (https://www.thesgc.org/chemical-probes/UNC1999), validate results with the inactive compound UNC2400 and orthogonal EZH2 inhibitors. Citations are sourced from SciFinder (January 2023).

	UNC1999	UNC2400	Orthogonal inhibitors	Title (PMID)	Cites
1	0.1 µM	No	GSK343, GSK126, EPZ005687, EPZ-6438 (100 nM)	ABCB1 and ABCG2 restrict the brain penetration of a panel of novel EZH2-Inhibitors (PMID: 25868794)	46
2	0.1-1 µM	No	None	EZH2 inhibition enhances the efficacy of an EGFR inhibitor in suppressing colon cancer cells (PMID: 25535899)	37
3	0.1-5 µM	No	GSK343 (5 µM)	An inhibitor screen identifies histone-modifying enzymes as mediators of polymer-mediated transgene expression from plasmid DNA (PMID: 29964136)	7
4	0.1-6 µM	No	None	Increased EZH2 expression in prostate cancer is associated with metastatic recurrence following external beam radiotherapy (PMID: 31104332)	20
5	0.25-1 µM	No	None	Palmitate-triggered COX2/PGE2-related hyperinflammation in dual-stressed pdl fibroblasts is mediated by repressive H3K27 trimethylation (PMID: 35326406)	1
6	0.5 µM	No	None	Uropathogenic E. coli (UPEC) infection induces proliferation through enhancer of zeste homologue 2 (EZH2) (PMID: 26964089)	7
7	0.5 - 3 µM	Yes	GSK126 (2 µM)	Selective inhibition of EZH2 and EZH1 enzymatic activity by a small molecule suppresses MLL-rearranged leukemia (PMID: 25395428)	161
8	1 - 2 µM	Yes	GSK343 (2 µM)	Genome-wide profiling of histone H3 lysine 27 and lysine 4 trimethylation in multiple myeloma reveals the importance of Polycomb gene targeting and highlights EZH2 as a potential therapeutic target (PMID: 26755663)	49
9	1 µM	No	GSK126, EPZ-5687 (1 µM)	Targeting EZH2-mediated methylation of histone 3 inhibits proliferation of pediatric acute monocytic leukemia cells in vitro (PMID: 33978549)	3
10	1 µM	No	GSK126 (1 µM)	EZH1/2 inhibition augments the anti-tumor effects of sorafenib in hepatocellular carcinoma. (PMID: 34725436)	9
11	1 µM	No	None	NOTCH and EZH2 collaborate to repress PTEN expression in breast cancer (PMID: 33750924)	7
12	1 µM	No	GSK126 (1 µM)	EZH1/2 inhibitors favor ILC3 development from human HSPC-CD34 + cells (PMID: 33467134)	6
13	1 µM	No	None	Contribution of DNA methylation and EZH2 in SRBC down-regulation in gastric cancer (PMID: 32676814)	2
14	1 µM	No	None	A distinct metabolic response characterizes sensitivity to EZH2 inhibition in multiple myeloma (PMID: 33579905)	8
15	1 µM	Yes	None	XIST loss impairs mammary stem cell differentiation and increases tumorigenicity through Mediator hyperactivation (PMID: 35597241)	2
16	1 µM	No	GSK343 (1 µM)	TRACE generates fluorescent human reporter cell lines to characterize epigenetic pathways (PMID: 34963054)	2
17	1 µM	No	None	A mass spectrometry-based assay using metabolic labeling to rapidly monitor chromatin accessibility of modified histone proteins (PMID: 31541121)	19
18	1 µM & 4 µM	No	None	EZH2 inhibition in multiple myeloma downregulates myeloma associated oncogenes and upregulates microRNAs with potential tumor suppressor functions (PMID: 28052011)	39
19	1 µM & 5 µM	No	GSK126, EPZ6438 (1 - 5 µM)	Mechanisms of resistance to EZH2 inhibitors in diffuse large B-cell lymphomas (PMID: 29572378)	67

20	1 µM & 5 µM	No	CPI-169 (40 µM), CPI-1205, EI1, EPZ-6438, SK126, GSK503, PF-06726304 (all 5 µM)	Multiple pharmacological inhibitors targeting the epigenetic suppressor enhancer of zeste homolog 2 (exh2) accelerate osteoblast differentiation (PMID: 33940225)	14
21	1.25-5 µM	No	None	Polycomb EZH1 regulates cell cycle/5-fluorouracil sensitivity of neuroblastoma cells in concert with MYCN (PMID: 36052716)	0
22	1-5 µM	No	None	Histone methyltransferase inhibition has a cytotoxic impact on transformed mast cells: implications for mastocytosis (PMID: 32366397)	1
23	1-50 µM	No	GSK126, GSK343, EPZ-6438, EPZ011989, EI1 (1 - 50 µM)	Chromatin remodeling controls Kaposi's sarcoma-associated herpesvirus reactivation from latency (PMID: 30212584)	26
24	0.5-5 µM	No	GSK343 (0.5 - 5 µM)	Evaluation of EED inhibitors as a class of PRC-2 targeted small molecules for HIV latency reversal (PMID: 32366397)	7
25	2-3 µM	No	None	Mammary molecular portraits reveal lineage-specific features and progenitor cell vulnerabilities (PMID: 29921600)	17
26	2 - 5 µM	Yes	GSK343, GSK126, EPZ-6438 (2 - 3 µM)	Small molecule epigenetic screen identifies novel EZH2 and HDAC inhibitors that target glioblastoma brain tumor-initiating cells (PMID: 27449082)	26
27	2 - 6 µM	No	GSK503 (4 - 10 µM)	Overexpression of EZH2 in conjunctival melanoma offers a new therapeutic target (PMID: 29732557)	12
28	2 - 10 µM	No	GSK343, EPZ005687 (2-10 µM)	EZH2-mediated epigenetic silencing of miR-29/miR-30 targets LOXL4 and contributes to tumorigenesis, metastasis, and immune microenvironment remodeling in breast cancer (PMID: 32754259)	27
29	2 - 10 µM	No	EPZ005687 (2 - 8 µM)	Targeting EZH1/2 induces cell cycle arrest and inhibits cell proliferation through reactivation of p57 CDKN1C and TP53INP1 in mantle cell lymphoma (PMID: 31565482)	6
30	2.5 µM & 5 µM	No	GSK343 (5-10 µM)	EZH2 inhibitors transcriptionally upregulate cytotoxic autophagy and cytoprotective unfolded protein response in human colorectal cancer cells (PMID: 27648357)	31
31	3 µM	No	None	Inhibition of polycomb repressor complex 2 ameliorates neointimal hyperplasia by suppressing trimethylation of H3K27 in vascular smooth muscle cells (PMID: 31162630)	9
32	3 µM	Yes	None	Targeting bivalency de-represses Indian Hedgehog and inhibits self-renewal of colorectal cancer-initiating cells (PMID: 30926792)	21
33	5 µM	No	None	EZH2 inhibits autophagic cell death of aortic vascular smooth muscle cells to affect aortic dissection (PMID: 29416002)	43
34	5 µM	No	GSK126 (1.25-5 µM)	Akt inhibition synergizes with polycomb repressive complex 2 inhibition in the treatment of multiple myeloma (PMID: 31571328)	11
35	5 µM	No	GSK126 (5 µM)	Dual inhibition of EZH2 and EZH1 sensitizes PRC2-dependent tumors to proteasome inhibition (PMID: 28490465)	45
36	5 µM	No	None	Multicellular tumor spheroids combined with mass spectrometric histone analysis to evaluate epigenetic drugs (PMID: 28194967)	24
37	5 µM	No	None	Dual inhibition of H3K9me2 and H3K27me3 promotes tumor cell senescence without triggering the secretion of SASP (PMID: 35409271)	1
38	5 µM	No	GSK126 (5 µM),	Sustained expression of FMR1 mRNA from reactivated fragile X syndrome alleles after treatment with small	25

			GSK343 (5 μM)	molecules that prevent trimethylation of H3K27 (PMID: 27378697)	
39	5 μM	No	None	Engineered multivalent sensors to detect coexisting histone modifications in living stem cells (PMID: 29174541)	26
40	5 μM	Yes	None	Combinatorial anticancer drug screen identifies off-target effects<br>of epigenetic chemical probes (PMID: 36084291)	0
41	5 μM	No	None	BRD4770 functions as a novel ferroptosis inhibitor to protect against aortic dissection (PMID: 35149187)	11
42	10 μM	No	None	Site-directed targeting of transcriptional activation-associated proteins to repressed chromatin restores CRISPR activity (PMID: 31967103)	3
43	10 μM	No	GSK126, GSK503, EPZ6438 (unclear)	EZH2-mediated H3K27me3 is a predictive biomarker and therapeutic target in uveal melanoma (PMID: 36276954)	0
44	5 - 10 μM	No	GSK343 (2.5 -10 μM)	Enhanced calcium signal induces NK cell degranulation but inhibits its cytotoxic activity (PMID: 34911773)	1
45	10 - 30 μM	No	GSK343 (10 - 30 μM)	Response and resistance to CDK12 inhibition in aggressive B-cell lymphomas (PMID: 34162179)	1
46	15 μM	No	None	MicroRNA-20a suppresses tumor proliferation and metastasis in hepatocellular carcinoma by directly targeting EZH1. (PMID: 34976797)	4
47	100 μM	No	None	EZH2 inhibition suppresses bladder cancer cell growth and metastasis via the JAK2/STAT3 signaling pathway (PMID: 31289569)	21
48	Not provided	No	None	EZH2 targeting to improve the sensitivity of acquired radio-resistance bladder cancer cells. (PMID: 34952334)	3
49	Not provided	No	None	SOX8 affects tumoral SPARC expression by regulating EZH2 to attenuate effectiveness of albumin-bound paclitaxel in PDAC (PMID: 35173526)	1
Compliance					
**Fully: 18 (37%)[a]**<br>**Partially: 14 (29%)[b]**		6 (12%)	23 (47%)		
Non-Compliance					
**17 (35%)** (total is 101% due to rounding up)		43 (88%)	26 (53%)		

a *Probe's concentration below the recommended in-cell maximum in all figures.*
b *Probe's concentration below the recommended in-cell maximum in some, but not all figures.*

Supplementary Note 2. Citations for UNC1999 publications included in the systematic review.
Citations for 49 publications using UNC1999: 909

Citations for 32 publications with a compliant UNC1999 concentration: 689
Citations for 17 publications with a non-compliant UNC1999 concentration: 220

Citations for 6 publications using inactive compound UNC2400: 259
Citations for 43 publications not using inactive compound UNC2400: 650

Citations for 23 publications using orthogonal inhibitors: 582
Citations for 26 publications not using orthogonal inhibitors: 327

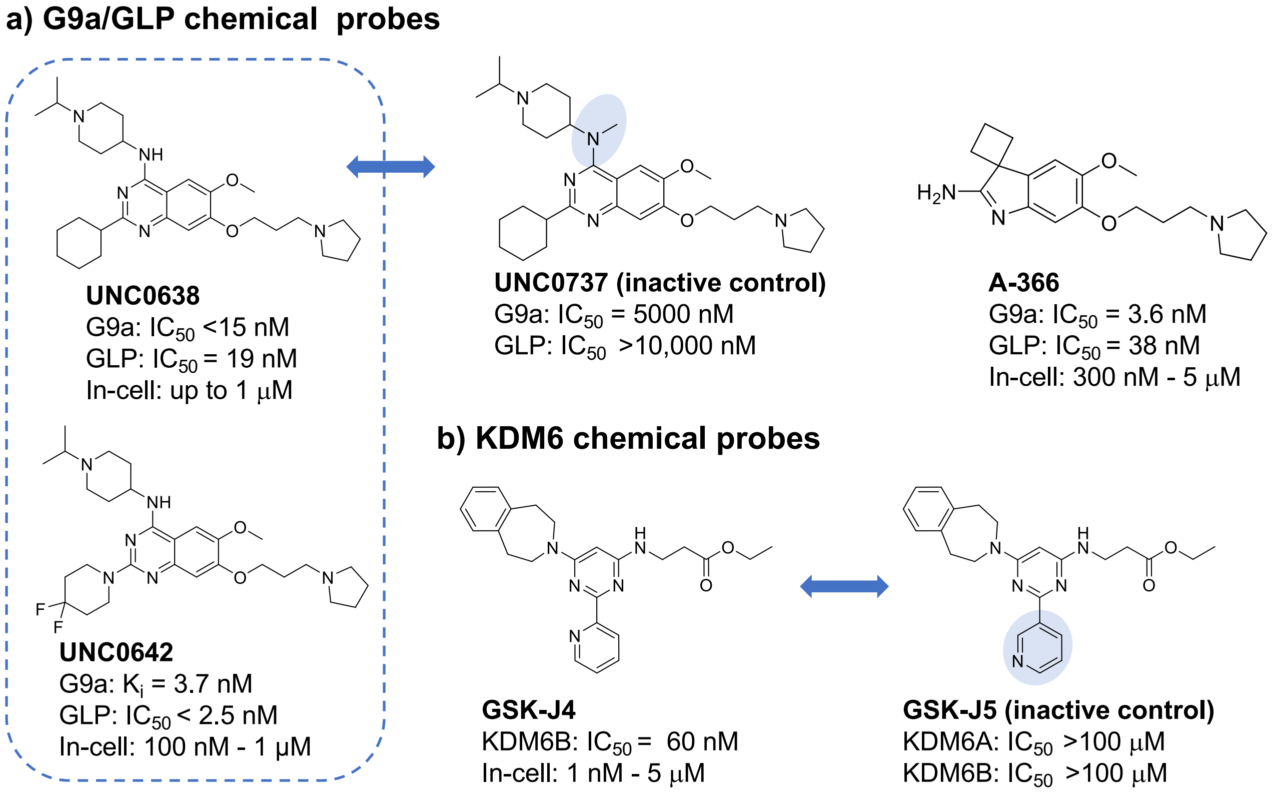

Supplementary Figure 3. Structures, biochemical potency (K_i/IC_{50}) and recommended in-cell concentrations of **a)** G9a/GLP and **b)** KDM6 chemical probes as listed on the Chemical Probes Portal (www.chemicalprobes.org). Dashed lines group structurally related chemical probes that should <u>not</u> be used together as orthogonal tools (*e.g.,* UNC0642 can be used as an orthogonal chemical probe with A-366, but not with UNC0638). Bidirectional arrows link target-inactive analogues and shaded fields highlight the structural changes leading to decreased G9a/GLP or KDM6 engagement.

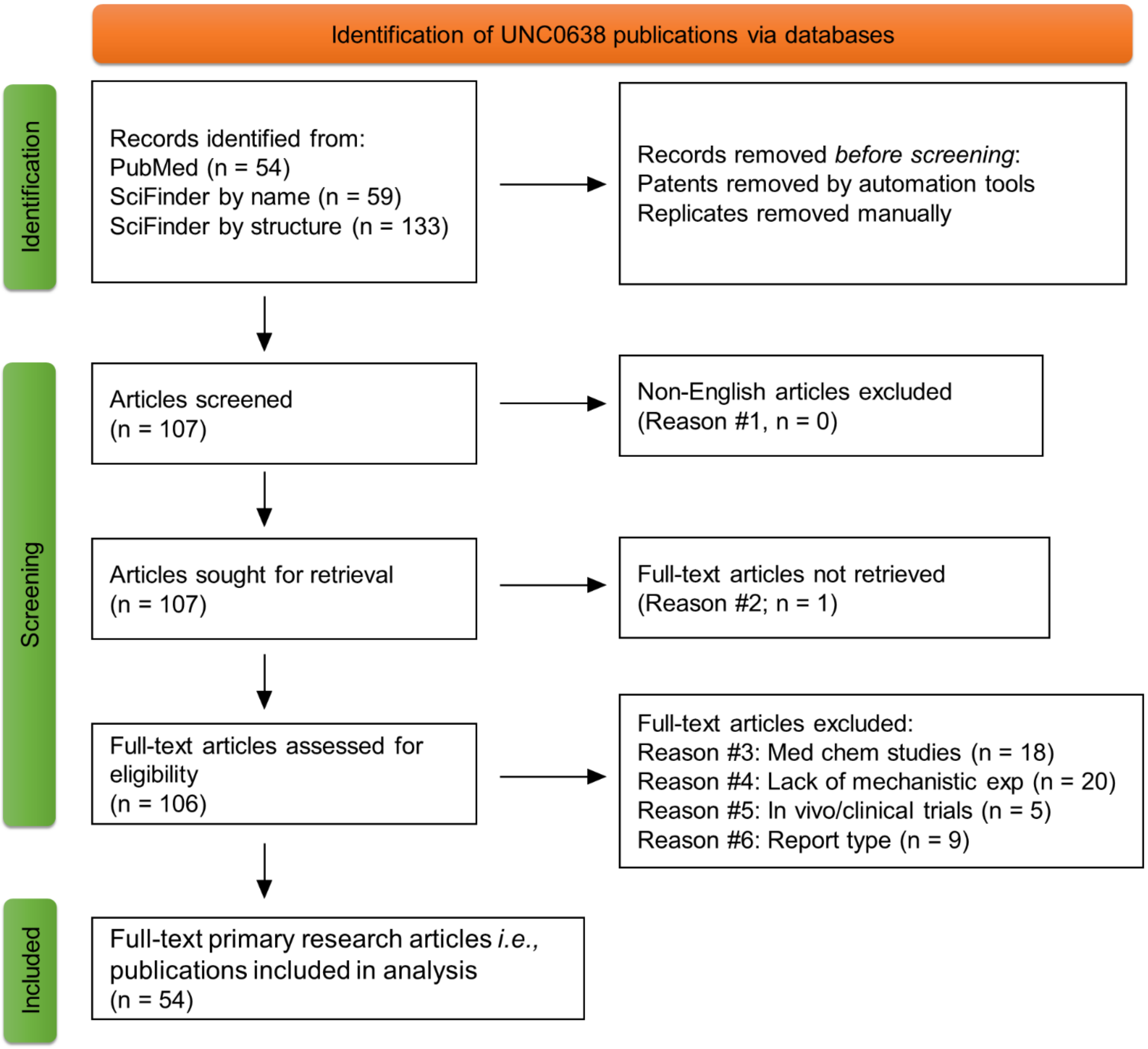

Supplementary Figure 4. PRISMA flow diagram summarising identification of publications using the G9a/GLP chemical probe UNC0638.

Supplementary Table 3. List of excluded articles using the G9a/GLP probe UNC0638.

	Title (PMID)	Reason for exclusion
1	The discovery of novel histone lysine methyltransferase G9a inhibitors (part 1): molecular design based on a series of substituted 2,4-diamino-7-aminoalkoxyquinazoline by molecular-docking-guided 3D quantitative structure-activity relationship studies (PMID: 24151879)	2) No access
2	A chemical probe selectively inhibits G9a and GLP methyltransferase activity in cells (PMID: 21743462)	3) Discovery of UNC0638
3	Optimization of cellular activity of G9a inhibitors 7-aminoalkoxy-quinazolines (PMID: 217807990)	3) Medicinal chemistry
4	Structure-activity relationship studies of G9a-like protein (GLP) inhibitors (PMID: 28662962)	3) Medicinal chemistry
5	Properly substituted analogues of BIX-01294 lose inhibition of G9a histone methyltransferase and gain selective anti-DNA methyltransferase 3A activity (PMID: 24810902)	3) Medicinal chemistry
6	7-Aminoalkoxy-quinazolines from epigenetic focused libraries are potent and selective inhibitors of DNA methyltransferase 1 (PMID: 35566242)	3) Medicinal chemistry
7	Discovery of a potent and selective fragment-like inhibitor of methyllysine reader protein Spindlin 1 (SPIN1) (PMID: 31260300)	3) Medicinal chemistry
8	Discovery of the first-in-class G9a/GLP covalent inhibitors (PMID: 3573668)	3) Medicinal chemistry
9	Discovery of a potent histone deacetylase (HDAC) 3/6 selective dual inhibitor (PMID: 31627059)	3) Medicinal chemistry
10	Identification of novel quinazoline derivatives as potent antiplasmodial agents (PMID: 30366254)	3) Medicinal chemistry
11	Discovery of Reversible DNA Methyltransferase and Lysine Methyltransferase G9a Inhibitors with Antitumoral in Vivo Efficacy (PMID: 29953809)	3) Medicinal chemistry
12	Discovery of a novel chemotype of histone lysine methyltransferase EHMT1/2 (GLP/G9a) inhibitors: Rational design, synthesis, biological Evaluation, and co-crystal Structure (PMID:30753076)	3) Medicinal chemistry
13	Discovery of potent and selective inhibitors for G9a-like protein (GLP) lysine methyltransferase (PMID: 28135087)	3) Medicinal chemistry
14	Mimicking H3 substrate arginine in the design of G9a lysine methyltransferase inhibitors for cancer therapy: A computational study for structure-based drug design (PMID: 33718701)	3) Medicinal chemistry
15	Novel SAR for quinazoline inhibitors of EHMT1 and EHMT2 (PMID: 31350126)	3) Medicinal chemistry
16	Discovery, design and synthesis of 6H-anthra[1,9-cd]isoxazol-6-one scaffold as G9a inhibitor through a combination of shape-based virtual screening and structure-based molecular modification (PMID: 27720557)	3) Medicinal chemistry
17	A chemical tool for in vitro and in vivo precipitation of lysine methyltransferase G9a (PMID: 24443078)	3) Medicinal chemistry
18	Discovery of an in vivo chemical probe of the lysine methyltransferases G9a and GLP (PMID: 24102134)	3) Medicinal chemistry
19	Selective, small-molecule co-factor binding site inhibition of a Su(var)3-9, enhancer of Zeste, Trithorax domain containing lysine methyltransferase (PMID: 31415173)	3) Medicinal chemistry
20	An inhibitor screen identifies histone-modifying enzymes as mediators of polymer-mediated<br>transgene expression from plasmid DNA (PMID: 29964136)	4) No mechanistic exp. - dose-response screen only
21	Identification of a novel autophagy-related prognostic signature and small molecule drugs for glioblastoma by bioinformatics (PMID: 35550147)	4) No mechanistic exp. - Bioinformatic study
22	Identification of novel quinoline inhibitor for EHMT2/G9a through virtual screening (PMID: 31756401)	4) No mechanistic exp. - Computational study
23	Strategy to target the substrate binding site of SET domain protein methyltransferases (PMID: 23410263)	4) No mechanistic exp. - Computational study
24	Detailed exploration around 4-aminoquinolines chemical space to navigate the lysine methyltransferase G9a and DNA methyltransferase biological spaces (PMID: 29890830)	4) No mechanistic exp. - Computational study
25	Efficient rescue of a newly classified Ebinur lake orthobunyavirus with GFP reporter and its application in rapid antiviral screening (PMID: 36150523)	4) No mechanistic exp. - Dose response anti-viral screen only

26	Bioluminescent cell-based NAD(P)/NAD(P)H assays for rapid dinucleotide measurement and inhibitor screening (PMID: 25506801)	4) No mechanistic exp. - Dose-response screen only
27	TR-FRET cellular assays for interrogating posttranslational modifications of histone H3 (PMID: 21972037)	4) No mechanistic exp. - Methodology study
28	Identification of six hub genes and analysis of their correlation with drug sensitivity in acute myeloid leukemia through bioinformatics (PMID: 35116245)	4) No mechanistic exp. - no cell-based experiments
29	High-throughput brain activity mapping and machine learning as a foundation for systems<br>Neuropharmacology (PMID: 30510233)	4) No mechanistic exp. - no cell-based experiments
30	Polymyxins and quinazolines are LSD1/KDM1A inhibitors with unusual structural features (PMID: 27626075)	4) No mechanistic exp. - no cell-based experiments
31	Phenotypic-screening generates active novel fetal globin-inducers that downregulate Bcl11a in a monkey model (PMID: 31751536)	4) No mechanistic exp. - no cell-based experiments
32	Hypoxia induces HIF1α-dependent epigenetic vulnerability in triple negative breast cancer to confer immune effector dysfunction and resistance to anti-PD-1 immunotherapy (PMID: 3580558)	4) Probed not used
33	Visualizing nuclear RNAi activity in single living human cells (PMID: 29073029)	4) Probed not used
34	Treatment of donor cells with recombinant KDM4D protein improves preimplantation development of cloned ovine embryos (PMID: 29766333)	4) Probed not used
35	HIV signaling through CD4 and CCR5 activates Rho family GTPases that are required for optimal infection of primary CD4+ T cells (PMID: 28114951)	4) No mechanistic exp. - dose-response viability
36	Genetically encoded molecular biosensors to image histone methylation in living animals (PMID: 25506787)	4) No mechanistic exp. - Probe used for validating real-time methylation
37	Histone methyltransferase G9a inhibitor-loaded redox-responsive nanoparticles for pancreatic ductal adenocarcinoma therapy (PMID: 32729861)	4) No mechanistic exp. - Nanoparticle delivery study
38	JAK1/2 and BCL2 inhibitors synergize to counteract bone marrow stromal cell-induced protection of AML (PMID: 28619982)	4) No mechanistic exp. – probe used only in screen
39	Discovery of novel drug sensitivities in T-PLL by high-throughput ex vivo drug testing and mutation profiling (PMID: 28804127)	4) No mechanistic exp. – probe used only in screen
40	An augmentation in histone demethylation at lysine nine residues elicits vision impairment following traumatic brain injury (PMID: 30790655)	5) In vivo study
41	Inhibition of histone methyltransferase G9a attenuates liver cancer initiation by sensitizing DNA-damaged hepatocytes to p53-induced apoptosis (PMID: 33468997)	5) In vivo study
42	Threshold inhibition of methyltransferase G9a/Glp exacerbates neuropathic hypersensitivity through mediating GRIN2B methylation (No PMID; DOI: 10.15354/si.19.ar910)	5) In vivo study
43	Effects of environmental conditions on nephron number: 13emethyla maternal disease and epigenetic regulation in renal development (PMID: 33923831)	5) Ex vivo study
44	Reduced and highly diverse peripheral HIV-1 reservoir in virally suppressed patients infected with non-B HIV-1 strains in Uganda (PMID: 35033105)	5) Study in human cohort
45	Chemical probes for the lysine methyltransferases G9a and EZH2 (No PMID, DOI or ISSN available, AN: 2015:1328166)	6) Conference proceeding
46	Stereoselective formation of gliotoxin isomers (No PMID, DOI or ISSN available, AN: 2014:379481)	6) Conference proceeding
47	Discovery of a first-in-class G9a/GLP covalent inhibitor (No PMID, DOI or ISSN available, AN: 2022:3010552)	6) Conference proceedings
48	Discovery of a potent and selective fragment-like inhibitor of SPIN1 (No PMID, DOI or ISSN available, AN: 2019:1501424)	6) Conference proceedings
49	A chemical probe selectively inhibits G9a and GLP methyltransferase activity in cells [Erratum to document cited in CA155:320969] (No PMID available, DOI: 10.1038/nchembio0911-648c)	6) Erratum
50	Small molecule inhibitors of G9a reactivate the maternal PWS genes in Prader-Willi-Syndrome patient derived neural stem cells and differentiated neurons (No PMID available, DOI: 10.1101/640938)	6) Preprint
51	Chromatin looping as a target for altering erythroid gene expression (PMID: 26918894)	6) Review
52	Chemical probes: sharpen your epigenetic tools (PMID: 21769094)	6) Review
53	Regulation, genomics, and clinical characteristics of cuproptosis regulators in pan-cancer (PMID: 36387247)	6) Review

Supplementary Table 4. Overview of eligible publications using the G9a/GLP probe UNC0638 and compliance (**in blue**) with recommendations to use UNC0638 up to 250 nM (ref[32]) and validate results with the inactive control compound UNC0737 and orthogonal G9a/GLP inhibitors. Citations are sourced from SciFinder (January 2023).

	UNC0638	UNC0737	Orthogonal inhibitors	Title (PMID)	Cites
1	1 nM - 1 µM Most assays: 100 nM	No	None	Effects of the histone methyltransferase inhibitor UNC0638 on histone H3K9 dimethylation of cultured ovine somatic cells and development of resulting early cloned embryos (PMID: 24467723)	11
2	10 nM & 5 µM	No	None	α-Synuclein enhances histone H3 lysine-9 dimethylation and H3K9me2-dependent transcriptional responses (PMID: 27808254)	35
3	50 nM - 250 nM	No	BIX01294 (14 nM - 10 µM)	EHMT1 and EHMT2 inhibition induces fetal hemoglobin expression (PMID: 26320100)	69
4	100 nM	No	BIX01294 (5 µM)	Multiple histone lysine methyltransferases are required for the establishment and maintenance of HIV-1 latency (PMID: 28246360)	77
5	100 nM - 250 nM	No	None	UNC0638 induces high levels of fetal hemoglobin expression in β-thalassemia/HbE erythroid progenitor cells (PMID: 32567028)	7
6	100 nM, 200 nM & 3 µM	No	None	Interaction of BARD1 and HP1 is required for BRCA1 retention at sites of DNA damage (PMID: 25634209)	70
7	0.1 - 0.5 µM	No	BIX01294 (0.1 - 1 µM)	Targeting p53 and histone methyltransferases restores exhausted CD8+ T cells in HCV infection (PMID: 32001678)	38
8	0.12 µM	No	None	Estimating intraclonal heterogeneity and subpopulation changes from bulk expression profiles in CMap (PMID: 35688486)	1
9	125 nM	No	None	HRI depletion cooperates with pharmacologic inducers to elevate fetal hemoglobin and reduce sickle cell formation (PMID: 32956454)	10
10	150 nM	No	BIX01294 (500 nM)	Epigenetic regulation by G9a/GLP complex ameliorates amyloid-beta 1-42 induced deficits in long-term plasticity and synaptic tagging/capture in hippocampal pyramidal neurons (PMID: 28665013)	25
11	150 nM	No	BIX01294 (500 nM)	Inhibition of G9a/GLP complex promotes long-term potentiation and synaptic tagging/capture in hippocampal CA1 pyramidal neurons (PMID: 27252354)	16
12	250 nM	No	None	Catalytic inhibition of H3K9me2 writers disturbs epigenetic marks during bovine nuclear reprogramming (PMID: 32661262)	3
13	250 nM	No	None	Dicer promotes genome stability via the bromodomain transcriptional co-activator BRD4 (PMID: 35194019)	2
14	250 nM - 750 nM	No	None	SETDB1 mediated histone H3 lysine 9 methylation suppresses MLL-fusion target expression and leukemic transformation (PMID: 33054052)	11
15	250 nM - 1 µM	No	UNC2249 (not provided)	A chromatin activity based chemoproteomic approach reveals a transcriptional repressome for gene-specific silencing (PMID: 25502336)	20
16	250 nM - 4 µM	No	UNC0642 (4 µM) BIX01294 (10 µM)	Targeting the histone methyltransferase G9a activates imprinted genes and improves survival of a mouse model of Prader-Willi syndrome (PMID: 28024084)	67
17	0.3 - 1 µM	No	None	Report and application of a tool compound data set (PMID: 29035535)	5
18	300 nM - 20 µM	No	None	Targeting histone methyltransferase G9a inhibits growth and Wnt signaling pathway by epigenetically regulating HP1α and APC2 gene expression in non-small cell lung cancer (PMID: 30348169)	42

19	0.375 - 3 µM	No	UNC0642 (2.5 - 10 µM), BIX-01294 (1 - 2 µM), A-366 (20 - 40 µM)	Targeting euchromatic histone lysine methyltransferases sensitizes colorectal cancer to histone deacetylase inhibitors (PMID: 35666536)	1
20	500 nM	No	None	Reactivation of endogenous retroviral elements via treatment with DNMT- and HDAC-inhibitors (PMID: 29633898)	32
21	500 nM - 1 µM	No	None	UNC0638, a G9a inhibitor, suppresses epithelial-mesenchymal transition-mediated cellular migration and invasion in triple negative breast cancer (PMID: 29207160)	21
22	500 nM - 1 µM	No	None	The methyltransferase G9a regulates HoxA9-dependent transcription in AML (PMID: 24532712)	101
23	500 nM - 2 µM	Yes	None	G9a/GLP-dependent histone H3K9me2 patterning during human hematopoietic stem cell lineage commitment (PMID: 23105005)	98
24	0.5 - 2 µM	No	BIX01294 (0.5 - 2 uM)	A disproportionate impact of G9a methyltransferase deficiency on the X chromosome (PMID: 34168040)	2
25	0.5 - 8 µM	No	BIX01294 (0.5 - 4 µM)	Degron protease blockade sensor to image epigenetic histone protein methylation in cells and living animals (PMID: 25489787)	7
26	1 µM	No	None	The combination of G9a histone methyltransferase inhibitors with erythropoietin protects heart against damage from acute myocardial infarction (PMID: 32774698)	8
27	1 µM	No	None	Transcriptional selectivity of epigenetic therapy in cancer (PMID: 27879268)	39
28	1 µM	No	A-366 (10 µM)	G9a inhibition potentiates the anti-tumour activity of DNA double-strand break inducing agents by impairing DNA repair independent of p53 status (PMID: 27431310)	28
29	1 µM	No	UNC0642 (1 µM)	Dual EZH2 and G9a inhibition suppresses multiple myeloma cell proliferation by regulating the interferon signal and IRF4-MYC axis (PMID: 33436557)	23
30	1 µM	No	UNC0642; A-366 (1 µM), UNC0648 (1 - 10 µM)	Dual G9A/EZH2 inhibition stimulates antitumor immune response in ovarian high-grade serous carcinoma (PMID: 35131874)	6
31	1 µM	No	None	Inhibition of G9a methyltransferase stimulates fetal hemoglobin production by facilitating LCR/γ-globin looping (PMID: 25979948)	54
32	1 - 10 µM	No	UNC0631, BIX01294, UNC0642 (1 - 10 µM)	Activity comparison of epigenetic modulators against the Hemoprotozoan parasites Babesia divergens and Plasmodium falciparum (PMID: 33599488)	6
33	1.25 - 5 µM	No	BIX01294 (0.5 - 5 µM)	G9a/GLP targeting in MM promotes autophagy-associated apoptosis and boosts proteasome inhibitor-mediated cell death (PMID: 33938943)	7
34	1.5 - 20 µM	No	None	Histone methyltransferase G9a promotes the development of renal cancer through epigenetic silencing of tumor suppressor gene SPINK5 (PMID: 34336110)	6
35	2 µM	No	BIX01294 (2 - 10 µM)	Inhibition of H3K9 methyltransferases G9a/GLP prevents ototoxicity and ongoing hair cell death (PMID: 23429292)	33
36	2 µM	No	BIX01294 (1 - 5 µM)	Epigenetic inhibitors target multiple stages of Plasmodium falciparum parasites (PMID: 32047203)	31

37	2 - 4 µM	No	BIX01294 (2 - 5 µM)	HeLa TI cell-based assay as a new approach to screen for chemicals able to reactivate the expression of epigenetically silenced genes (PMID: 34115770)	3
38	2 - 20 µM	No	None	Histone deacetylase inhibitors containing a benzamide functional group and a pyridyl cap are preferentially effective human immunodeficiency virus-1 latency-reversing agents in primary resting CD4 T cells (PMID: 28113052)	14
39	2.5 - 5 µM	No	None	Deregulation of Wnt/β-catenin signaling through genetic or epigenetic alterations in human neuroendocrine tumors (PMID: 23354304)	60
40	2.5 - 5 µM	No	None	G9a promotes invasion and metastasis of non-small cell lung cancer through enhancing focal adhesion kinase activation via NF-κB signaling pathway (PMID: 33298547)	11
41	3 µM	No	None	Class I histone deacetylase inhibitors inhibit the retention of BRCA1 and 53BP1 at the site of DNA damage (PMID: 26053117)	23
42	4 µM	No	None	Epigenetic therapy combination of UNC0638 and CI-994 suppresses breast cancer via epigenetic remodeling of BIRC5 and GADD45A (PMID: 34798471)	4
43	5 µM	No	BIX01294 (0.5 - 2.5 µM)	Inhibition of euchromatic histone methyltransferase 1 and 2 sensitizes chronic myeloid leukemia cells to interferon treatment (PMID: 25079219)	8
44	5 µM	No	None	Inhibition of EHMT2 induces a robust antiviral response against foot-and-mouth disease and vesicular stomatitis virus infections in bovine cells (PMID: 26418342)	7
45	5 µM	No	None	Histone methyltransferase inhibition has a cytotoxic impact on transformed mast cells: implications for mastocytosis (PMID: 32366397)	1
46	5 µM	No	None	M33 condenses chromatin through nuclear body formation and methylation of both histone H3 lysine 9 and lysine 27 (PMID: 34274396)	0
47	5 - 10 µM	No	None	Targeting EHMT2 reverses EGFR-TKI resistance in NSCLC by epigenetically regulating the PTEN/AKT signaling pathway (PMID: 29374157)	41
48	5 - 10 µM	No	UNC0642 (unclear) BIX01294 (3 µM)	Increased efficacy of histone methyltransferase G9a inhibitors against MYCN-amplified neuroblastoma (PMID: 32537432)	12
49	8 - 12 µM	No	None	Generation of induced pluripotent stem cells from a female patient with a Xq27.3-q28 deletion to establish disease models and identify therapies (PMID: 32608992)	3
50	10 µM	No	None	Deletion of histone methyltransferase G9a suppresses mutant Kras-driven pancreatic carcinogenesis (PMID: 33099471)	7
51	20 µM	No	None	The SUV39H1 inhibitor chaetocin induces differentiation and shows synergistic cytotoxicity with other epigenetic drugs in acute myeloid leukemia cells (PMID: 25978433)	57
52	5 - 640 µg	No	BIX01294 (0.625 - 80.0 µg)	Threshold effect of G9a/Glp on peripheral nerve injury-induced hypersensitivity (PMID: 28814147)	4
53	Not provided	No	UNC0642, UNC0631 (not provided)	An EHMT2/NFYA-ALDH2 signaling axis modulates the RAF pathway to regulate paclitaxel resistance in lung cancer (PMID: 35477569)	1
54	Not provided	No	UNC0224, UNC0321 (not provided)	Engineering lineage potency and plasticity of stem cells using epigenetic molecules (PMID: 30389989)	5
Compliance					
**Fully: 9 (17%)**[a]		1 (2%)	23 (43%)		

**Partially: 7 (13%)**[b]				
Non-Compliance				
**38 (70%)**	53 (98%)	31 (57%)		

a *Probe's concentration below the recommended in-cell maximum in all figures.*
b *Probe's concentration below the recommended in-cell maximum in some, but not all figures.*

Supplementary Note 3. Citations for UNC0638 publications included in the systematic review.
Citations for 54 publications using UNC0638: 1,273

Citations for 16 publications with a compliant UN0638 concentration: 462
Citations for 38 publications with a non-compliant UNC0638 concentration: 811

Citations for 1 publication using inactive compound UNC0737: 98
Citations for 53 publications not using inactive compound UNC0737: 1,175

Citations for 23 publications using orthogonal G9a/GLP inhibitors: 489
Citations for 31 publications not using orthogonal G9a/GLP inhibitors: 784

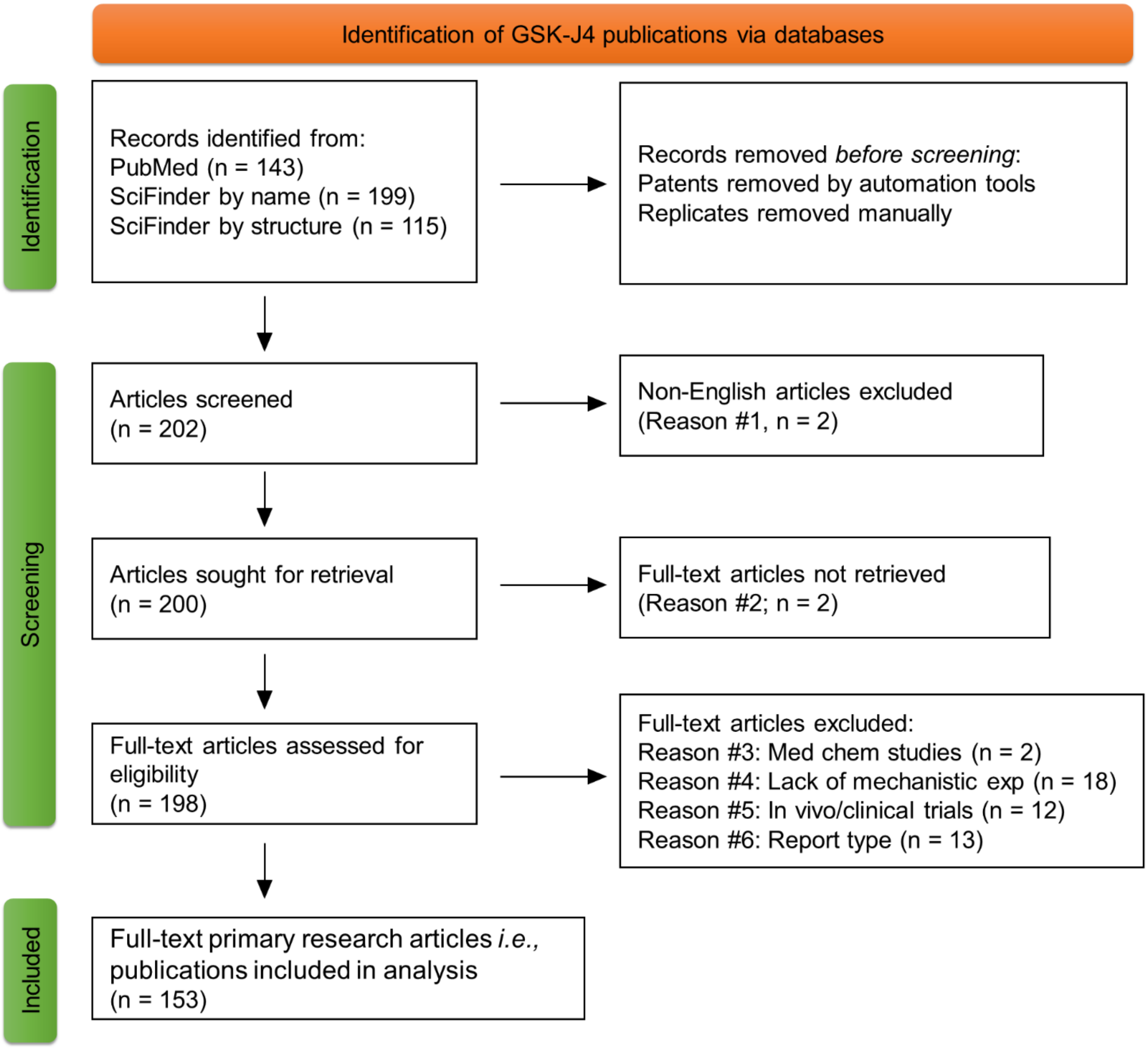

Supplementary Figure 5. PRISMA flow diagram summarising identification of publications using the KDM6 chemical probe GSK-J4.

Supplementary Table 5. List of excluded articles using the KDM6 probe GSK-J4.

	Title (PMID)	Reason for exclusion
1	Mechanism of alendronate sodium combined with LPS induced inflammatory responses in Macrophages (No PMID; DOI: 10.13241/j.cnki.pmb.2020.09.003)	1) Non-English publication
2	Bioinformatics analysis of the clinical expression of APOC1 in papillary thyroid carcinoma (No PMID or DOI; ISSN: 1007-1520)	1) Non-English publication
3	Forskolin potentiates the effects of GSKJ4 in human acute myeloid leukemia cells through protein kinase A pathway (No PMID or DOI; ISSN: 1105-4999)	2) No access
4	Pharmaceutical interference of the EWS-FLI1-driven transcriptome by cotargeting H3K27ac and RNA polymerase activity in ewing sarcoma. (PMID: 34315769)	2) No access
5	A selective jumonji H3K27 demethylase inhibitor modulates the proinflammatory macrophage response. (PMID: 22842901)	3) GSK-J4 discovery
6	Design and discovery of new pyrimidine coupled nitrogen aromatic rings as chelating groups of JMJD3 inhibitors. (PMID: 26776360)	3) Medicinal chemistry
7	The BRD9/7 inhibitor TP-472 blocks melanoma tumor growth by suppressing ECM-mediated oncogenic signaling and inducing apoptosis (PMID: 34771678)	4) No mechanistic exp.
8	Identification of disulfiram as a potential antifungal drug by screening small molecular libraries (PMID: 33358402)	4) No mechanistic exp.- Antifungal assays
9	The N6-methylandenosine-related gene BIRC5 as a prognostic biomarker correlated with cell migration and immune cell infiltrates in low grade glioma (PMID: 35309512)	4) No mechanistic exp. - Bioinformatics study
10	Analysis of characteristic genes and ceRNA regulation mechanism of endometriosis based on full transcriptional sequencing (PMID: 35938015)	4) No mechanistic exp. - Bioinformatics study
11	Effect of histone demethylase Jmjd3 on odontogenic differentiation of human dental pulp cells (PMID: 32208897)	4) No GSK-J4 data
12	Differential kinase activity of ACVR1 G328V and R206H mutations with implications to possible TβRI cross-talk in diffuse intrinsic pontine glioma. (PMID: 32273545)	4) No mechanistic exp.
13	HDAC inhibition enhances the in vivo efficacy of MEK inhibitor therapy in Uveal melanoma (PMID: 31227503)	4) No mechanistic exp. – viability screen
14	Functionally defined therapeutic targets in diffuse intrinsic pontine glioma (PMID: 25939062)	4) No mechanistic exp.
15	In silico identification of small molecules as new Cdc25 inhibitors through the correlation between chemosensitivity and protein expression pattern (PMID: 33918281)	4) No mechanistic exp.
16	Discovery of novel drug sensitivities in T-PLL by high-throughput ex vivo drug testing and mutation profiling (PMID: 28804127)	4) No GSK-J4 data
17	Mechanical stretch aggravates vascular smooth muscle cell apoptosis and vascular remodeling by downregulating EZH2 (PMID: 35985452)	4) GSK-J4 not used in-cells
18	The memory of environmental chemical exposure in C. elegans is dependent on the jumonji demethylases jmjd-2 and jmjd-3/utx-1 (PMID: 29791850)	4) No mechanistic exp.
19	Epigenetic reprogramming of H3K27me3 and DNA methylation during leaf-to-callus transition in peach (PMID: 35937864)	4) No mechanistic exp.
20	Histone lysine demethylase inhibition reprograms prostate cancer metabolism and mechanics (PMID: 35944897)	4) No mechanistic exp.
21	Systematic chemical and molecular profiling of MLL-rearranged infant acute lymphoblastic leukemia reveals efficacy of romidepsin (PMID: 27443263)	4) No mechanistic exp.
22	Regulatory network analysis reveals the oncogenesis roles of feed-forward loops and therapeutic target in T-cell acute lymphoblastic leukemia (PMID: 30646895)	4) No mechanistic exp. - Analytical research study
23	HIV signaling through CD4 and CCR5 activates Rho family GTPases that are required for optimal infection of primary CD4+ T cells (PMID: 28114951)	4) No mechanistic exp.
24	Activity comparison of epigenetic Modulators against the Hemoprotozoan Parasites Babesia divergens and Plasmodium falciparum (PMID: 33599488)	4) No mechanistic exp.
25	The histone demethylase KDM6B in the medial prefrontal cortex epigenetically regulates cocaine reward memory (PMID: 30165076)	5) In vivo study
26	Activated Braf induces esophageal dilation and gastric epithelial hyperplasia in mice (PMID: 28973166)	5) In vivo study

27	Histone modification of pain-related gene expression in spinal cord neurons under a persistent postsurgical pain-like state by electrocautery (PMID: 34544461)	5) In vivo study
28	Jmjd3 is involved in the susceptibility to depression induced by maternal separation via enhancing the neuroinflammation in the prefrontal cortex and hippocampus of male rats (PMID: 32084453)	5) In vivo study
29	Upregulation of KDM6B contributes to lipopolysaccharide-induced anxiety-like behavior via modulation of VGLL4 in mice (PMID: 33865886)	5) In vivo study
30	New BRAF knock-in mice provide a pathogenetic mechanism of developmental defects and a therapeutic approach in cardio-facio-cutaneous syndrome (PMID: 25035421)	5) In vivo study
31	KDM6B epigenetically regulated-interleukin-6 expression in the dorsal root ganglia and spinal dorsal horn contributes to the development and maintenance of neuropathic pain following peripheral nerve injury in male rats (PMID: 34464689)	5) In vivo study
32	IL-4 alleviates ischaemia-reperfusion injury by inducing kupffer cells M2 polarization via STAT6-JMJD3 pathway after rat liver transplantation. (PMID: 32258110)	5) In vivo study
33	Histone demethylase UTX counteracts glucocorticoid deregulation of osteogenesis by modulating histone-dependent and -independent pathways (PMID: 28130569)	5) In vivo study
34	Effects of GSK-J4 on JMJD3 Histone Demethylase in Mouse Prostate Cancer Xenografts (PMID: 35430567)	5) In vivo study
35	Downregulation of interleukin-1 beta via Jmjd3 inhibition improves post-myocardial infarction depression (PMID: 35800354)	5) In vivo study
36	Novel pharmacological inhibition of JMJD3 improves necrotizing enterocolitis by attenuating the inflammatory response and ameliorating intestinal injury (PMID: 35803318)	5) In vivo study
37	An epigenetic therapy for diffuse intrinsic pontine gliomas. (PMID: 25473916)	6) Comment
38	Inhibition of demethylases by GSK-J1/J4 (PMID: 25279926)	6) Comment
39	Inhibition of demethylases by GSK-J1/J4 (PMID: 25279927)	6) Comment reply
40	Functionally defined therapeutic targets in diffuse intrinsic pontine glioma [Erratum to document cited in CA163:294035] (PMID: 26151328)	6) Erratum
41	Role of JMJD3 demethylase and its inhibitor GSK-J4 in regulation of MGMT, TRA2A, RPS6KA2, and U2AF1 genes in prostate cancer cell lines: (PMID: 32525734)	6) Letter to the editor
42	Role of UTX histone demethylase in regulation of MGMT, TRA2A, U2AF1, and RPS6KA2 genes in prostate cancer cell lines (PMID: 33337267)	6) Letter to the editor
43	JMJD3 promotes esophageal squamous cell carcinoma pathogenesis through epigenetic regulation of MYC (PMID: 32843613)	6) Letter to the editor
44	GSK J4 is a Novel Neuroprotective Compound in Parkinson's Disease Model (No PMID, DOI or ISSN, AN: 2018:1273085)	6) Dissertation
45	The role and prospect of JMJD3 in stem cells and cancer. (PMID: 31545292)	6) Review
46	Neuroblastoma and histone demethylation. (PMID: 30646895)	6) Review
47	Histone methylation: Achilles heel and powerful mediator of periodontal homeostasis (PMID: 32762486)	6) Review
48	Structural genomics in drug discovery: an overview (no PMID; DOI: 10.4103/jpp.JPP_117_18)	6) Review
49	Therapeutic potential of inhibiting histone 3 lysine 27 demethylases: a review of the literature (PMID: 35915507)	6) Review

Supplementary Table 6. Overview of eligible publications using the KDM6 probe GSK-J4 and compliance (**in blue**) with recommendations to use GSK-J4 up to 5 µM (https://www.chemicalprobes.org/gsk-j4?q=GSK-J4) or up to 10 µM (https://www.thesgc.org/chemical-probes/GSKJ1) and validate results with the inactive control compound GSK-J5. Citations are sourced from SciFinder (January 2023).

	GSK-J4	GSK-J5	Title (PMID)	Cites
1	10 - 25 nM	No	The histone demethylase inhibitor GSK-J4 limits inflammation through the induction of a tolerogenic phenotype on DCs (PMID: 27528513)	48
2	25 nM	No	The demethylase inhibitor GSK-J4 limits inflammatory colitis by promoting de novo synthesis of retinoic acid in dendritic cells (PMID: 33446666)	5
3	50 nM	No	The H3K27me3-demethylase KDM6A is suppressed in breast cancer stem-like cells, and enables the resolution of bivalency during the mesenchymal-epithelial transition (PMID: 29029452)	39
4	80 nM	No	The histone H3 lysine-27 demethylase Jmjd3 plays a critical role in specific regulation of Th17 cell differentiation (PMID: 25840993)	68
5	100 nM	No	JMJD3 aids in reprogramming of bone marrow progenitor cells to hepatic phenotype through epigenetic activation of hepatic transcription factors (PMID: 28328977)	7
6	0.1 - 0.5 µM	No	Dual G9A/EZH2 inhibition stimulates antitumor immune response in ovarian high-grade serous carcinoma (PMID: 35131874)	6
7	100 - 750 nM	No	Histone H3 lysine 27 trimethylation acts as an epigenetic barrier in porcine nuclear reprogramming (PMID: 26515777)	53
8	0.1 - 1 µM	No	Lysine demethylase inhibition protects pancreatic β cells from apoptosis and improves β-cell function (PMID: 28684291)	15
9	0.1 - 2.5 µM	No	KDM6B promotes activation of the oncogenic CDK4/6-pRB-E2F pathway by maintaining enhancer activity in MYCN-amplified neuroblastoma (PMID: 34893606)	6
10	0.1 - 5 µM	No	Jumonji domain containing-3 (JMJD3) inhibition attenuates IL-1β-induced chondrocytes damage in vitro and protects osteoarthritis cartilage in vivo (PMID: 32394143)	6
11	0.1 - 10 µM	No	GSK-J4, a specific histone lysine demethylase 6A inhibitor, ameliorates lipotoxicity to cardiomyocytes via preserving H3K27 methylation and reducing ferroptosis (PMID: 35722096)	0
12	175 nM	Yes	Jumonji inhibitors overcome radioresistance in cancer through changes in h3k4 methylation at double-strand breaks (PMID: 30355483)	39
13	180 nM - 5 µM	No	Combined targeting of mutant p53 and Jumonji family histone demethylase augments therapeutic efficacy of radiation in H3K27M DIPG (PMID: 31940975)	15
14	200 nM	No	The repositioning of epigenetic probes/inhibitors identifies new anti-schistosomal lead compounds and chemotherapeutic targets (PMID: 31730617)	16
15	250 nM	Yes	Inhibition of H3K27me3 demethylases promotes plasmablast formation (PMID: 34880105)	0
16	250 - 500 nM	No	Therapeutic effect of a histone demethylase inhibitor in Parkinson's disease (PMID: 33116116)	15
17	0.25 - 1 µM	No	Adipocyte-mediated epigenomic instability in human T-ALL cells is cytotoxic and phenocopied by epigenetic-modifying drugs (PMID: 36060800)	0
18	0.25 - 4 µM	No	Impact of H3K27 demethylase inhibitor GSKJ4 on NSCLC cells alone and in combination with metformin (PMID: 27793936)	25
19	0.5 - 1 µM	No	Targeting of histone demethylases KDM5A and KDM6B inhibits the proliferation of temozolomide-resistant glioblastoma cells (PMID: 31238504)	16
20	0.5 - 1 µM	No	SAPCD2 promotes neuroblastoma progression by altering the subcellular distribution of E2F7 (PMID: 35197448)	1
21	0.5 - 1.8 µM	No	X-linked histone H3K27 demethylase Kdm6A regulates sexually dimorphic differentiation of hypothalamic neurons (PMID: 34633482)	4

22	0.5 - 1.9 µM	No	The inhibitors of KDM4 and KDM6 histone lysine demethylases enhance the anti-growth effects of erlotinib and HS-173 in head and neck cancer cells (PMID: 34363938)	4
23	0.5 - 2 µM	No	GSKJ4, a selective Jumonji H3K27 demethylase inhibitor, effectively targets ovarian cancer stem cells (PMID: 26637876**)**	45
24	0.5 - 2 µM	No	SMARCA4 deficient tumours are vulnerable to KDM6A/UTX and KDM6B/JMJD3 blockade (PMID: 34262032)	9
25	0.5 - 5 µM	Yes	Inhibition of demethylase KDM6B sensitizes diffuse large B-cell lymphoma to chemotherapeutic drugs (PMID: 27742770)	40
26	0.5 - 10 µM	No	KDM6B counteracts EZH2-mediated suppression of IDFBP5 to confer resistance to PI3K/AKT inhibitor treatment in breast cancer (PMID: 29925528)	29
27	500 nM	No	KDM6B overexpression activates innate immune signaling and impairs hematopoiesis in mice (PMID: 30275007)	20
28	500 nM	No	Epigenetic remodeling through downregulation of polycomb repressive complex 2 mediates chemotherapy resistance in testicular germ cell tumors (PMID:31181810)	15
29	600 nM - 1 µM	Yes	Systematic identification of biomarker-driven drug combinations to overcome resistance (PMID: 35332332)	1
30	1 µM	No	H19-dependent transcriptional regulation of β3 and β4 integrins upon estrogen and hypoxia favors metastatic potential in prostate cancer (PMID: 31426484)	14
31	1 µM	No	Morphological screening of mesenchymal mammary tumor organoids to identify drugs that reverse epithelial-mesenchymal transition (PMID: 34253738)	10
32	1 µM	No	Cooperation between KDM6B overexpression and TET2 deficiency in the pathogenesis of chronic myelomonocytic leukemia (PMID: 35697791)	0
33	1 µM	No	Acquired resistance during adoptive cell therapy by transcriptional silencing of immunogenic antigens (PMID: 31413920)	10
34	1 µM	No	KDM6B is an androgen regulated gene and plays oncogenic roles by demethylating H3K27me3 at cyclin D1 promoter in prostate cancer (PMID: 33414463)	16
35	1 µM	No	Elevating H3K27me3 level sensitizes colorectal cancer to oxaliplatin (PMID: 31065671)	30
36	1 µM	No	Cystathionine-γ-lyase ameliorates the histone demethylase JMJD3-mediated autoimmune response in rheumatoid arthritis (PMID: 29844591)	27
37	1 µM	No	Histone H3K27 demethylase negatively controls the memory formation of antigen-stimulated CD8 + T cells (PMID: 30626691)	10
38	1 µM	No	Targeted inhibition of histone H3K27 demethylation is effective in high-risk neuroblastoma (PMID: 29769286)	47
39	1 µM	Yes	Taxane-platin-resistant lung cancers co-develop hypersensitivity to JumonjiC demethylase inhibitors (PMID: 28538184)	56
40	1 µM	No	HDAC8 prevents anthrax lethal toxin-induced cell cycle arrest through silencing PTEN in human monocytic THP-1 cells (PMID: 28509866)	12
41	1 µM	No	Histone demethylase JMJD3 downregulation protects against aberrant force-induced osteoarthritis through epigenetic control of NR4A1 (PMID: 35831280)	0
42	1 - 2 µM	No	Targeting histone acetylation dynamics and oncogenic transcription by catalytic P300/CBP inhibition (PMID: 34019788)	22
43	1 - 2 µM	No	Pharmacological targeting of KDM6A and KDM6B, as a novel therapeutic strategy for treating craniosynostosis in Saethre-Chotzen syndrome (PMID: 33298158)	6
44	1 - 2.5 µM	No	Pharmaceutical interference of the EWS-FLI1-driven transcriptome by cotargeting H3K27ac and RNA polymerase activity in ewing sarcoma (PMID: 34315769)	4
45	1 - 3 µM	No	HMGN1 plays a significant role in CRLF2 driven Down Syndrome leukemia and provides a potential therapeutic target in this high-risk cohort (PMID: 34857887)	2
46	1 - 5 µM	No	KDM6B-mediated histone demethylation of LDHA promotes lung metastasis of osteosarcoma (PMID: 33664867)	9
47	1 - 5 µM	No	KDM6B promotes ESCC cell proliferation and metastasis by facilitating C/EBPβ transcription (PMID: 34001062)	9

48	1 - 6 µM	No	Radiosensitization by histone H3 demethylase inhibition in diffuse intrinsic pontine glioma (PMID: 31227500)	28
49	1 - 10 µM	No	Heterochromatin protein 1γ deficiency decreases histone H3K27 methylation in mouse neurosphere neuronal genes (PMID: 31961023)	1
50	1 - 10 µM	No	Inhibition of the H3K27 demethylase UTX enhances the epigenetic silencing of HIV proviruses and induces HIV-1 DNA hypermethylation but fails to permanently block HIV reactivation (PMID: 34673825)	8
51	1 - 10 µM	No	The KDM inhibitor GSKJ4 triggers CREB downregulation via a protein kinase A and proteasome-dependent mechanism in human acute myeloid leukemia cells (PMID: 32582541)	8
52	1 - 10 µM	No	Screening of a neuronal cell model of tau pathology for therapeutic compounds (PMID: 30640040)	5
53	1 & 10 µM	No	Low HOX gene expression in PML-RARα-positive leukemia results from suppressed histone demethylation (PMID: 29224413)	10
54	1 - 10 µM	No	An IFNγ/STAT1/JMJD3 axis induces ZEB1 expression and promotes aggressiveness in lung adenocarcinoma (PMID: 33771881)	2
55	1 - 20 µM	No	Histone demethylase Jmjd3 regulates the osteogenic differentiation and cytokine expressions of periodontal ligament cells (PMID: 35790358)	0
56	1.25 - 5 µM	No	Synergy of GSK-J4 with doxorubicin in KRAS-mutant anaplastic thyroid cancer (PMID: 32477122)	5
57	1.25 - 10 µM	No	Therapeutic potential of GSK-J4, a histone demethylase KDM6B/JMJD3 inhibitor, for acute myeloid leukemia (PMID: 29594337)	48
58	1.3 - 5 µM	No	A comprehensive study of epigenetic alterations in hepatocellular carcinoma identifies potential therapeutic targets (PMID: 30880225)	53
59	1.8 µM	Yes	Inhibition of KDM6 activity during murine ESC differentiation induces DNA damage (PMID: 26759175)	21
60	2 µM	Yes	Histone H3K27me3 demethylases regulate human Th17 cell development and effector functions by impacting on metabolism (PMID: 32123118)	39
61	2 µM	Yes	Contrasting roles of histone 3 lysine 27 demethylases in acute lymphoblastic leukaemia (PMID: 25132549)	266
62	2 - 4 µM	No	Expression pattern of histone lysine-specific demethylase 6B in gastric cancer (PMID: 33968207)	0
63	2 - 5 µM	No	AURKA suppresses leukemic THP-1 cell differentiation through inhibition of the KDM6B pathway (PMID: 29477140)	14
64	2 - 5 µM	No	The lysine demethylase KDM5B regulates islet function and glucose homeostasis (PMID: 31467927)	8
65	2.5 - 5 µM	No	Therapeutically targeting head and neck squamous cell carcinoma through synergistic inhibition of LSD1 and JMJD3 by TCP and GSK-J1 (PMID: 31848446) **Note: this publication used mostly the non-cell permeable GSK-J1**	18
66	2.5 - 5 µM	No	Two old drugs, NVP-AEW541 and GSK-J4, repurposed against the Toxoplasma gondii RH strain (PMID: 32393321)	6
67	2 - 8 µM	No	JMJD3 suppresses tumor progression in oral tongue squamous cell carcinoma patients receiving surgical resection (PMID: 35855897)	0
68	2 - 8 µM	No	Overexpression of UTX promotes tumor progression in Oral tongue squamous cell carcinoma patients receiving surgical resection: a case control study (PMID: 34465286)	2
69	2 & 8 µM	No	The epigenetic regulation of OLIG2 by histone demethylase KDM6B in glioma cells (PMID: 35132566)	0
70	2.5 - 10 µM	No	H3K27me3 loss plays a vital role in CEMIP mediated carcinogenesis and progression of breast cancer with poor prognosis (PMID: 31846842)	40
71	2.5 & 10 µM	No	Histone demethylase KDM6B regulates human podocyte differentiation in vitro (PMID: 31138771)	3
72	2 - 20 µM	No	Inhibitor of H3K27 demethylase JMJD3/UTX GSK-J4 is a potential therapeutic option for castration resistant prostate cancer (PMID: 28977932)	40
73	3 - 6 µM	No	Pharmacologic inhibition of histone demethylation as a therapy for pediatric brainstem glioma (PMID: 25401693)	294
74	3.53 & 3.93 & 22.87 µM	No	A new metabolic gene signature in prostate cancer regulated by JMJD3 and EZH2 (PMID: 29805743)	24

75	3.75 - 30 µM	Yes	The antischistosomal potential of GSK-J4, an H3K27 demethylase inhibitor: insights from molecular modeling, transcriptomics and in vitro assays (PMID: 32178714)	10
76	4 µM	No	Abnormally elevated EZH2-mediated H3K27me3 enhances osteogenesis in aortic valve interstitial cells by inhibiting SOCS3 expression (PMID: 36455343)	4
77	4 µM	No	Jmjd3 Mediates Neuropathic Pain by Inducing Macrophage Infiltration and Activation in Lumbar Spinal Stenosis Animal Model (PMID: 34948220)	2
78	4 µM	No	GSK-J4 induces cell cycle arrest and apoptosis via ER stress and the synergism between GSK-J4 and decitabine in acute myeloid leukemia KG-1a cells (PMID: 32514253)	9
79	4 µM	No	Histone demethylase UTX is a therapeutic target for diabetic kidney disease (PMID: 30516825)	27
80	4 µM	No	GSKJ4 protects mice against early sepsis via reducing proinflammatory factors and up-regulating MiR-146a (PMID: 30337925)	25
81	4 µM	No	Inhibition of the histone H3K27 demethylase UTX enhances tumor cell radiosensitivity (PMID: 29483212)	22
82	4 µM	No	Inhibiting KDM6A Demethylase Represses Long Non-Coding RNA Hotairm1 Transcription in MDSC During Sepsis (PMID: 35185915)	3
83	4 & 8 µM	No	The pharmacological role of histone demethylase JMJD3 inhibitor GSK-J4 on glioma cells (PMID: 28978140)	31
84	5 µM	No	Nanoparticle enhanced combination therapy for stem-like progenitors defined by single-cell<br>transcriptomics in chemotherapy-resistant osteosarcoma (PMID: 32973147)	17
85	5 µM	No	Mucosal-associated invariant T cell effector function is an intrinsic cell property that can be<br>augmented by the metabolic cofactor a-ketoglutarate (PMID: 33597151)	3
86	5 µM	No	Dual regulation of histone methylation by mTOR complexes controls glioblastoma tumor cell growth via EZH2 and SAM (PMID: 32366675)	12
87	5 µM	No	Nuclear pore protein NUP210 depletion suppresses metastasis through heterochromatin-mediated disruption of tumor cell mechanical response (PMID: 34903738)	6
88	5 µM	No	P53 affects epigenetic signature on SOCS1 promoter in response to TLR4 inhibition (PMID: 33476981)	5
89	5 µM	No	Structure of nascent chromatin is essential for hematopoietic lineage specification (PMID: 28402853)	23
90	5 µM	No	Fingolimod alleviates cognitive deficit in Type 2 diabetes by promoting microglial M2 polarization via the pSTAT3-jmjd3 axis (PMID: 36385233)	0
91	5 µM	No	Histone H3K27 methyltransferase EZH2 and demethylase JMJD3 regulate hepatic stellate cells activation and liver fibrosis (PMID: 33391480)	24
92	5 µM	No	Palmitate-TLR4 signaling regulates the histone demethylase, JMJD3, in macrophages and impairs diabetic wound healing (PMID: 32662520)	18
93	5 µM	Yes	Inhibition of histone H3K27 demethylases selectively modulates inflammatory phenotypes of natural killer cells (PMID: 29301935)	49
94	5 - 10 µM	No	Pharmacological inhibition of core regulatory circuitry liquid-liquid phase separation suppresses metastasis and chemoresistance in osteosarcoma (PMID: 34432948)	9
95	5 - 10 µM	No	PRC2 epigenetically silences Th1-type chemokines to suppress effector T-cell trafficking in colon cancer (PMID: 26567139)	157
96	5 - 10 µM	No	Development and validation of the TGx-HDACi transcriptomic biomarker to detect histone deacetylase inhibitors in human TK6 cells (PMID: 33770205)	5 (Pubmed)
97	5 & 10 µM	No	Oncogenic KRAS sensitizes lung adenocarcinoma to GSK-J4-induced metabolic and oxidative stress (PMID: 31506334)	16
98	5 - 20 µM	No	KDM6A and KDM6B: Altered expression in malignant pleural mesothelioma (PMID: 28197626)	8
99	5 & 30 µM	No	A KDM6 inhibitor potently induces ATF4 and its target gene expression through HRI activation and by UTX inhibition (PMID: 33633164)	0

100	6 µM	No	Histone demethylase JMJD3 protects against renal fibrosis by suppressing TGFβ and Notch signaling and preserving PTEN expression (PMID: 33456568)	19
101	6 - 20 µM	No	GSKJ4, an H3K27me3 demethylase inhibitor, effectively suppresses the breast cancer stem cells (PMID: 28823831)	56
102	6 - 60 µM	Yes	Disruption of the Plasmodium falciparum life cycle through transcriptional reprogramming by inhibitors of jumonji demethylases (PMID: 32272012)	12
103	7.5 µM	No	Inhibition of H3K27me3 demethylases attenuates asthma by reversing the shift in airway smooth muscle phenotype (PMID: 30084510)	11
104	7 - 10 µM	No	Inhibition of H3K27me3 histone demethylase activity prevents the proliferative regeneration of Zebrafish lateral line neuromasts (PMID: 28348517)	12
105	7.5 - 20 µM	No	Targeted inhibition of KDM6 histone demethylases eradicates tumor-initiating cells via enhancer reprogramming in colorectal cancer (PMID: 32929331)	10
106	10 µM	No	Increased H3K27 trimethylation contributes to cone survival in a mouse model of cone dystrophy (PMID: 35810394)	0
107	10 µM	Yes	Upregulation of H3K27 demethylase KDM6 during respiratory syncytial virus infection enhances proinflammatory responses and immunopathology (PMID: 31748348)	17
108	10 µM	No	Forskolin sensitizes human acute myeloid leukemia cells to H3K27me2/3 demethylases GSKJ4 inhibitor via protein kinase A (PMID: 30079022)	11
109	10 µM	No	Lysyl oxidase expression is regulated by the H3K27 demethylase Jmjd3 in tumor-associated M2-like macrophages (PMID: 32231406)	4
110	10 µM	Yes	KDM6B-dependent chromatin remodeling underpins effective virus-specific CD8+ T cell differentiation (PMID: 33730567)	8
111	10 µM	No	A transcriptomic dataset used to derive biomarkers of chemically induced histone deacetylase inhibition (HDACi) in human TK6 cells (PMID: 34036128)	0
112	10 µM	No	Inhibitors of Jumonji C domain-containing histone lysine demethylases overcome cisplatin and paclitaxel resistance in non-small cell lung cancer through APC/Cdh1-dependent degradation of CtIP and PAF15 (PMID: 35100078)	1
113	10 µM	No	Unlocking the recovery potential: JMJD3 inhibition-mediated SAPK/JNK signaling inactivation supports endogenous oligodendrocyte-lineage commitment post mammalian spinal cord injury (PMID: 33428096)	0
114	10 µM	No	Transcriptional repression of ER through hMAPK dependent histone deacetylation by class I HDACs (PMID: 25129342)	12
115	10 µM	No	JMJD3 downregulates IL4i1 aggravating lipopolysaccharide-induced acute lung injury via H3K27 and H3K4 demethylation (PMID: 36537648)	0
116	10 µM	No	Stretch-induced tenomodulin expression promotes tenocyte migration via F-actin and chromatin remodeling (PMID: 34066472)	4
117	10 µM	No	LncRNA H19 regulates macrophage polarization and promotes Freund's complete adjuvant-induced arthritis by upregulating KDM6A (PMID: 33540246)	10
118	10 µM	No	Effects of chronic stress on depressive-like behaviors and JMJD3 expression in the prefrontal cortex and hippocampus of C57BL/6 and ob/ob mice (PMID: 33340793)	4
119	10 µM	No	Histone demethylase Jmjd3 modulates osteoblast apoptosis induced by tumor necrosis factor-alpha through directly targeting RASSF5. (PMID: 31092054)	4
120	10 µM	No	Jmjd3 regulates inflammasome activation and aggravates DSS-induced colitis in mice (PMID: 31971317)	21
121	10 µM	Yes	Upregulation of H3K27 demethylase KDM6 during RSV infection enhances pro-inflammatory responses and immunopathology. (PMID: 31748348)	17
122	10 µM	No	Epigenetic profiling identifies LIF as a super-enhancer-controlled regulator of stem cell-like properties in osteosarcoma. (PMID: 31615908)	20
123	10 µM	No	Antiproliferative effect of the histone demethylase inhibitor GSK-J4 in chondrosarcomas. (PMID: 31241814)	5

124	10 µM	No	Histone demethylase JMJD3 regulates fibroblast-like synoviocyte-mediated proliferation and joint destruction in rheumatoid arthritis. (PMID: 29481307)	33
125	10 µM	No	GSK-J4-mediated transcriptomic alterations in differentiating embryoid bodies. (PMID: 29047260)	5
126	10 µM	Yes	H3K27me3 demethylases regulate in vitro chondrogenesis and chondrocyte activity in osteoarthritis. (PMID: 27388528)	23
127	10 µM	No	Transcriptomic profiling and H3K27me3 distribution reveal both demethylase-dependent and independent regulation of developmental gene transcription in cell differentiation. (PMID: 26263556)	13
128	10 µM	No	Histone demethylation maintains Prdm14 and Tsix expression and represses xIst in embryonic stem cells. (PMID: 25993097)	10
129	10 µM	No	Combined action of FOXO1 and superoxide dismutase 3 promotes MDA-MB-231 cell migration (PMID: 35271779)	1
130	10 µM	No	JMJD3 inhibition protects against isoproterenol-induced cardiac hypertrophy by suppressing β-MHC expression. (PMID: 29753027)	17
131	10 µM	No	The histone demethylase KDM6B fine-tunes the host response to Streptococcus pneumoniae. (PMID: 33349663)	7
132	10 µM	Yes	Critical role of histone demethylase Jumonji domain-containing protein 3 in the regulation of neointima formation following vascular injury. (PMID: 29982434)	24
133	10 µM	No	RNA sequencing reveals resistance of TLR4 ligand-activated microglial cells to inflammation mediated by the selective jumonji H3K27 demethylase inhibitor. (PMID: 28747667)	23
134	10 - 30 µM	No	Inhibition of Histone H3 Lysine-27 Demethylase Activity Relieves Rheumatoid Arthritis Symptoms via Repression of IL6 Transcription in Macrophages (PMID: 35371061)	1
135	20 µM	No	Inhibition of H3K4 demethylation induces autophagy in cancer cell lines (PMID: 28800922)	20
136	20 µM	No	The TrxG complex mediates cytokine induced de novo enhancer formation in islets (PMID: 26505193)	6
137	25 µM	No	EZH2, JMJD3, and UTX epigenetically regulate hepatic plasticity inducing retro-differentiation and proliferation of liver cells. (PMID: 31285428)	16
138	30 µM	No	Lysine demethylase 6B regulates prostate cancer cell proliferation by controlling c-MYC expression. (PMID: 34862309)	3
139	30 µM	No	JMJD3 is involved in neutrophil membrane proteinase 3 overexpression during the hyperinflammatory response in early sepsis. (PMID: 29621735)	10
140	35 µM	No	A unique bipartite Polycomb signature regulates stimulus-response transcription during development (PMID: 33603234)	9
141	30 - 50 µM	No	Targeting P16INK4A in uterine serous carcinoma through inhibition of histone demethylation. (PMID: 30896884)	3
142	30 - 60 µM	No	Epigenomic regulation of schwann cell reprogramming in peripheral nerve injury. (PMID: 27581455)	48
143	40 µM	No	hsa-miR-199b-3p prevents the epithelial-mesenchymal transition and dysfunction of the renal tubule by regulating E-cadherin through targeting KDM6A in diabetic nephropathy. (PMID: 34257820)	5
144	50 µM	No	Inhibition of H3K27me3-specific histone demethylases JMJD3 and UTX blocks reactivation of herpes simplex virus 1 in trigeminal ganglion neurons. (PMID: 25552720)	34
145	50 µM	No	Lysine demethylase KDM6B regulates HIF-1α-mediated systemic and cellular responses to intermittent hypoxia (PMID: 34297635)	4
146	10 mM	No	The histone demethylase inhibitor GSK-J4 is a therapeutic target for the kidney fibrosis of diabetic kidney disease via DKK1 modulation (PMID: 36012674)	0
147	concentration not provided	No	UTX-1 regulates Tat-induced HIV-1 transactivation via changing the methylated status of histone H3 (PMID: 27671333)	8
148	concentration not provided	No	The fungal metabolite chaetocin is a sensitizer for pro-apoptotic therapies in glioblastoma (PMID: 31772153)	11
149	concentration not provided	No	Hypoxia induces HIF1α-dependent epigenetic vulnerability in triple negative breast cancer to confer immune effector dysfunction and resistance to anti-PD-1 immunotherapy (PMID: 35840558)	3

150	concentration not provided	No	Engineering lineage potency and plasticity of stem cells using epigenetic molecules (PMID: 30389989)	5
151	concentration not provided	No	KDM2B promotes cell viability by enhancing DNA damage response in canine hemangiosarcoma (PMID: 34023294)	6
152	concentration not provided	No	Activation of NFKB-JMJD3 signaling promotes bladder fibrosis via boosting bladder smooth muscle cell proliferation and collagen accumulation. (PMID: 31102789)	8
153	concentration not provided	No	Comprehensive profiling of JMJD3 in gastric cancer and its influence on patient survival. (PMID: 30696880)	17
Compliance				
Fully below 5 µM: 72 (47%)[a]<br>Fully between 5 - 10 µM: 52 (34%)[b]<br>Partially: 10 (7%)[c]		16 (11%)		
Non-Compliance				
19 (12%)		137 (89%)		

***a** Probe's concentration below the recommended 5 µM (Chemical Probes Portal) in all figures.*
***b** Probe's concentration above the recommended 5 µM (Chemical Probes Portal) but below the recommended 10 µM (SGC).*
***c** Probe's concentration below the recommended 10 µM SGC in-cell maximum in some but not all figures.*

Supplementary Note 4. Citations for GSK-J4 publications included in the systematic review.
Citations for 153 publications using GSK-J4: 2,890

Citations for 134 publications with a compliant GSK-J4 concentration: 2,674
Citations for 19 publications with a non-compliant GSK-J4 concentration: 216

Citations for 16 publications using inactive compound GSK-J5: 622
Citations for 137 publications not using inactive compound GSK-J5: 2,268

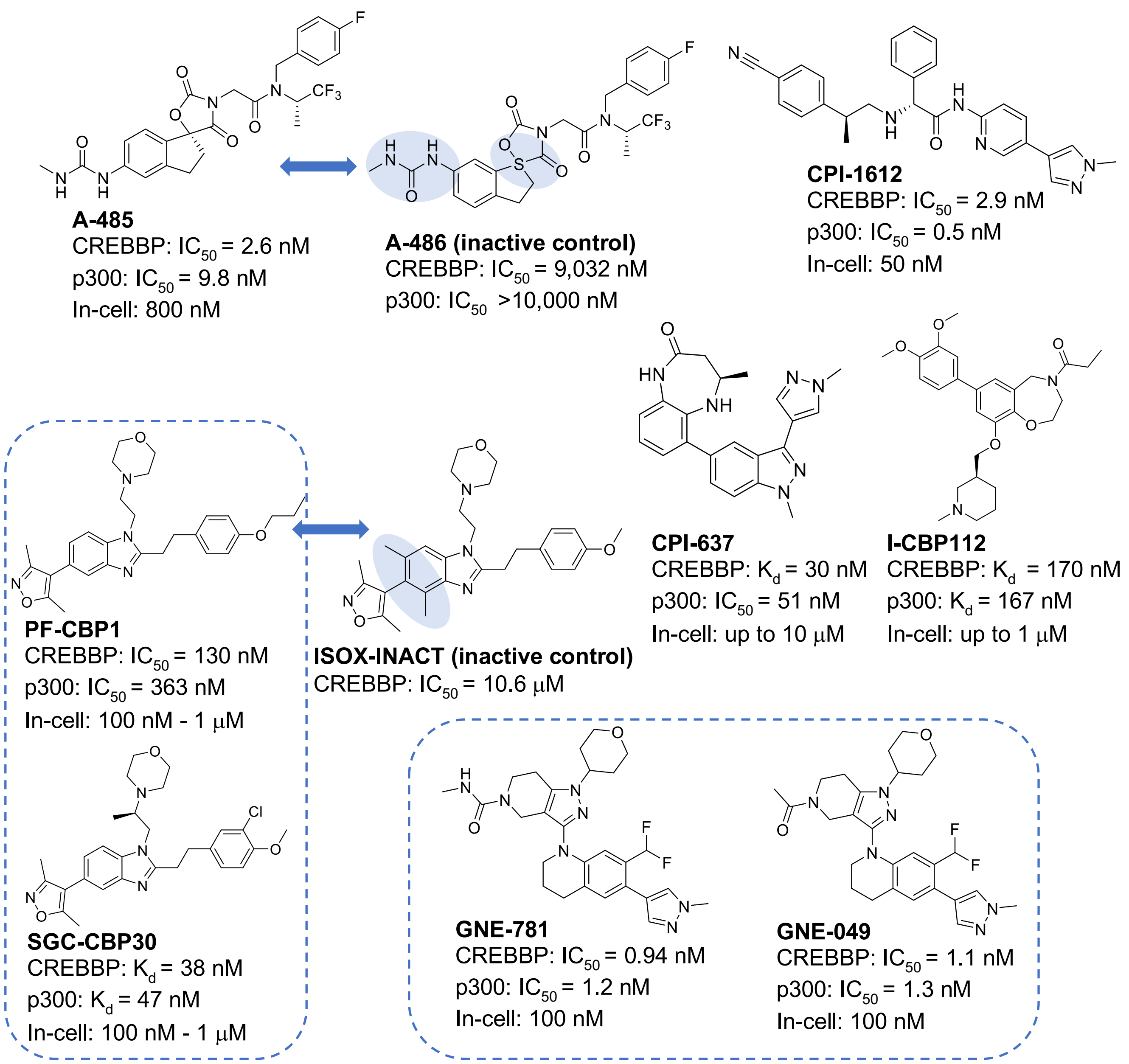

Supplementary Figure 6. Structures, biochemical potency (K_d/IC_{50}) and recommended in-cell concentrations of CREBBP/p300 chemical probes as listed on the Chemical Probes Portal (www.chemicalprobes.org). Dashed lines group structurally related chemical probes that should not be used together as orthogonal tools (*e.g.,* GNE-049 can be used as an orthogonal chemical probe with A-485, but not with GNE-781). Bidirectional arrows link target-inactive analogues and shaded fields highlight the structural changes leading to decreased CREBBP/p300 targeting.

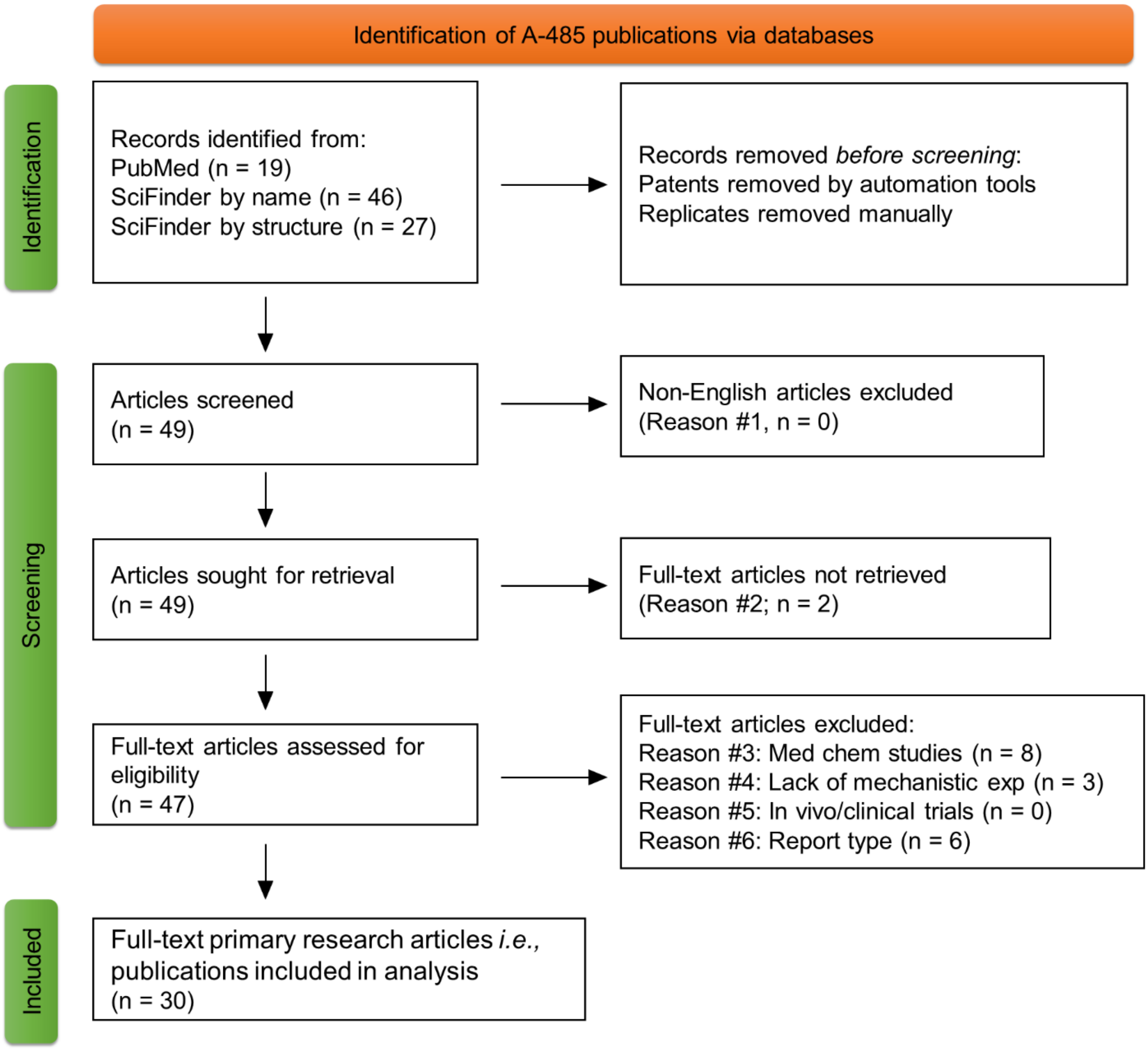

Supplementary Figure 7. PRISMA flow diagram summarising identification of publications using CREBBP/p300 chemical probe A-485.

Note: Pubmed search retrieved 90 publications but includes any publication that writes 'a 485' (e.g., a 485 nm wavelength, a 485 amino acid-long length). These 90 publications were manually reviewed for publications referring to probe A-485.

Supplementary Table 7. List of excluded articles using the CREBBP/p300 probe A-485.

	Title (PMID)	Reason for exclusion
1	Assays for validating histone acetyltransferase inhibitors (PMID: 32831305)	2) No access
2	Report of the first international symposium on NUT carcinoma (PMID: 35417004)	2) No access
3	Synthesis and biological evaluation of spirocyclic chromane derivatives as a potential treatment of prostate cancer (PMID: 34070610)	3) A-485 lead optimization
4	Discovery of a selective catalytic p300/CBP inhibitor that targets lineage-specific tumours (PMID: 28953875)	3) Discovery of A-485
5	Discovery of a potent and selective covalent p300/CBP inhibitor (PMID: 34055218)	3) Lead optimization
6	Discovery of spirohydantoins as selective, orally bioavailable inhibitors of p300/CBP histone acetyltransferases (PMID: 33631370)	3) Lead optimization
7	Identification of ligand linkage vectors for the development of p300/CBP degraders (PMID: 35814928)	3) Medicinal chemistry
8	Design, synthesis and biological evaluation of a novel spiro oxazolidinedione as potent p300/CBP HAT inhibitor for the treatment of ovarian cancer (PMID: 34801827)	3) Medicinal chemistry study
9	Discovery of spiro oxazolidinediones as selective, orally bioavailable inhibitors of p300/CBP histone acetyltransferases (PMID: 29348807)	3) Medicinal chemistry study
10	Combination targeting of the bromodomain and acetyltransferase active site of p300/CBP (PMID: 30924641)	3) Study primarily employed A-485rs, a 1:1 diastereomeric mixture of A-485
11	Dual-functional polyetheretherketone surface modification for regulating immunity and bone metabolism (no PMID; DOI: 10.1016/j.cej.2021.130806)	4) No mechanistic exp.: A-485 loaded onto an activated PEEK surface
12	Inactivation of the CIC-DUX4 oncogene through P300/CBP inhibition, a therapeutic approach for CIC-DUX4 sarcoma (PMID: 34642317)	4) No mechanistic exp.
13	Therapeutic targeting of ATR yields durable regressions in small cell lung cancers with high replication stress (PMID: 33848478)	4) No mechanistic exp.
14	Another one (of the "undruggable" targets) bites the dust: Discovery of a potent and selective inhibitor of the histone acetyl transferase p300/CBP (PMID: 29244478)	6) Commentary/Letter
15	Author Correction: Discovery of a selective catalytic p300/CBP inhibitor that targets lineage-specific tumours [Erratum to document cited in CA167:546652] (PMID: 29769713)	6) Erratum
16	Targeting cancer epigenetic pathways with small-molecule compounds: Therapeutic efficacy and combination therapies (PMID: 34102228)	6) Review
17	Histone acetyltransferases CBP/p300 in tumorigenesis and CBP/p300 inhibitors as promising novel anticancer agents (PMID: 35836809)	6) Review article
18	Anti-cancer agents repurposed for p300-HAT binding (No PMID, DOI or ISSN, AN: 2022:3061564)	6) Conference proceedings
19	Assay interference profiles of next-generation cell-active histone acetyltransferase inhibitors (No PMID, DPO or ISSN, AN: 2020:290416)	6) Conference proceedings

Supplementary Table 8. Overview of eligible publications using the CREBBP/p300 probe A-485 and compliance (**in blue**) with recommendations to use A-485 up to 800 nM (https://www.chemicalprobes.org/485?q=A-485; we consider publications using 1 µM A-485 as compliant), validate results with the inactive control compound A-486 and orthogonal inhibitors. Citations are sourced from SciFinder (January 2023).

	A-485	A-486	Orthogonal inhibitors	Title (PMID)	Cites
1	100 nM	No	GNE-781, dCBP-1 (both 100 nM)	Targeted degradation of the enhancer lysine acetyltransferases CBP and p300 (PMID: 33400925)	36
2	100 nM - 1 µM	No	GNE-049, SGC-CBP30 (0.1 - 10 µM)	Domain-independent inhibition of CBP/p300 attenuates α-synuclein aggregation (PMID: 34110772)	2
3	250 nM - 1 µM	Yes	I-CBP112, SGC-CBP30 (10 µM)	Therapeutic targeting of p300/CBP HAT domain for the treatment of NUT midline carcinoma (PMID: 32366905)	19
4	0.25 - 2 µM	No	None	H3K27 acetylation activated-CCS regulates autophagy and apoptosis of lung cancer by alleviating oxidative stress (PMID: 36402120)	0
5	250 nM - 3 µM	No	GNE-049 (0.25 - 1 µM)	Pharmacological inhibition of CBP/p300 blocks estrogen receptor alpha function through suppressing enhancer H3K27 acetylation in luminal breast cancer (PMID:34199844)	11
6	0.3 - 3 µM	No	C646 (3 - 10 µM)	Interplay between protein acetylation and ubiquitination controls MCL1 protein stability (PMID: 34758305)	4
7	0.3 & 3 µM	Yes	None	Targeting lineage-specific MITF pathway in human melanoma cell lines by A-485, the selective small-molecule inhibitor of p300/CBP (PMID: 30266801)	37
8	500 nM	No	None	Histone acetylation dynamics modulates chromatin conformation and allele-specific interactions at oncogenic loci (PMID: 33972799)	16
9	0.5 - 10 µM	No	None	Integrative genomic profiling uncovers therapeutic targets of acral melanoma in Asian populations (PMID: 35294533)	2
10	0.5 - 2 µM	No	None	p300/CBP inhibitor A-485 inhibits the differentiation of osteoclasts and protects against osteoporotic bone loss (PMID: 33626422)	3
11	0.8 µM	No	SGC-CBP30 (1 µM)<br>I-CBP 112 (3 µM)	The p300/CBP inhibitor A485 normalizes psoriatic fibroblast gene expression in vitro and reduces psoriasis-like skin inflammation in vivo (PMID: 36174717)	0
12	1 µM	No	A-241 (2 nM - 1 µM)	Targeting histone acetylation dynamics and oncogenic transcription by catalytic P300/CBP inhibition (PMID: 34019788)	22
13	1 µM	Yes	CBP30 (0.5 µM)<br>I-CBP112 (1 µM)	Bromodomain inhibition of the coactivators CBP/EP300 facilitate cellular reprogramming (PMID: 30962627)	44
14	1 µM	No	None	Targeted protein acetylation in cells using heterobifunctional molecules (PMID: 34592107)	12
15	1-10 µM	No	None	Merkel cell polyomavirus small T antigen is a viral transcription activator that is essential for viral genome maintenance (PMID: 36574443)	0
16	1-20 µM	No	None	Pharmacological targeting of CBP/p300 drives a redox/autophagy axis leading to senescence-induced growth arrest in non-small cell lung cancer cells (PMID: 36117234)	0
17	1.25 - 5 µM	No	None	The p300 inhibitor A-485 exerts antitumor activity in growth hormone pituitary adenoma (PMID: 35247260)	2
18	2.2 - 20 µM	No	SGC-CBP30, CPI-637 (2.2 - 20 µM)	p300/CBP inhibitor A-485 alleviates acute liver injury by regulating macrophage activation and polarization (PMID: 31754401)	27
19	3 µM	No	None	CBP/p300 HAT maintains the gene network critical for β cell identity and functional maturity (PMID: 33980820)	2
20	3 µM	No	CPI-637, SGC-CBP30 (0.01 - 10 µM)	P300/CBP inhibition sensitizes mantle cell lymphoma to PI3Kδ inhibitor idelalisib (PMID: 33850273)	4

21	3 µM	No	None	Selective inhibition of CBP/p300 HAT by A-485 results in suppression of lipogenesis and hepatic gluconeogenesis (PMID: 32917859)	10
22	3 µM	No	Cmpd-R (3 µM)<br>I-CBP112 (5 µM)	Time-resolved analysis reveals rapid dynamics and broad scope of the CBP/p300 acetylome (PMID: 29804834)	176
23	3 µM	No	None	TRPM3-induced gene transcription is under epigenetic control (PMID: 35890145)	0
24	5 µM	No	None	Histone modifications drive aberrant Notch3 expression/activity and growth in T-ALL (PMID: 31001470)	25
25	5 - 20 µM	No	None	A novel histone acetyltransferase inhibitor A485 improves sensitivity of non-small-cell lung carcinoma cells to TRAIL (PMID: 32173363)	12
26	10 µM	No	None	Promoter-specific changes in initiation, elongation, and homeostasis of histone H3 acetylation during CBP/p300 inhibition (PMID: 33704060)	9
27	10 µM	No	None	Large-scale RNAi screening uncovers therapeutic targets in the parasite Schistosoma mansoni (PMID: 32973031)	26
28	10 µM	No	CPI-637 (10 µM)	Analysis and therapeutic targeting of the EP300 and CREBBP acetyltransferases in anaplastic large cell lymphoma and Hodgkin lymphoma (PMID: 36456744)	0
29	Not provided	No	None	Acetylation of histone 3 promotes miR-29a expression and downregulates STAT3 in sepsis (PMID: 34615595)	0
30	Unclear	No	None	Identification of potent inhibitors of SARS-CoV-2 infection by combined pharmacological evaluation and cellular network prioritization (PMID: 35992305)	2
Compliance					
**8 (27%)** [a]<br>**8 (27%)** [b]		3 (10%)	12 (40%)		
Non-Compliance					
**14 (47%)**		27 (90%)	18 (60%)		

***a** Probe's concentration below the recommended in-cell maximum in all figures.*
***b** Probe's concentration below the recommended in-cell maximum in some, but not all figures.*

Supplementary Note 5. Citations for A-485 publications included in the systematic review.
Citations for 30 publications using A-485: 503

Citations for 16 publications with a compliant A-485 concentration: 208
Citations for 14 publications with a non-compliant A-485 concentration: 295

Citations for 3 publications using inactive compound A-486: 100
Citations for 27 publications not using inactive compound A-486: 403

Citations for 12 publications using orthogonal CREBBP/p300 inhibitors: 345
Citations for 18 publications not using orthogonal CREBBP/p300 inhibitors: 158

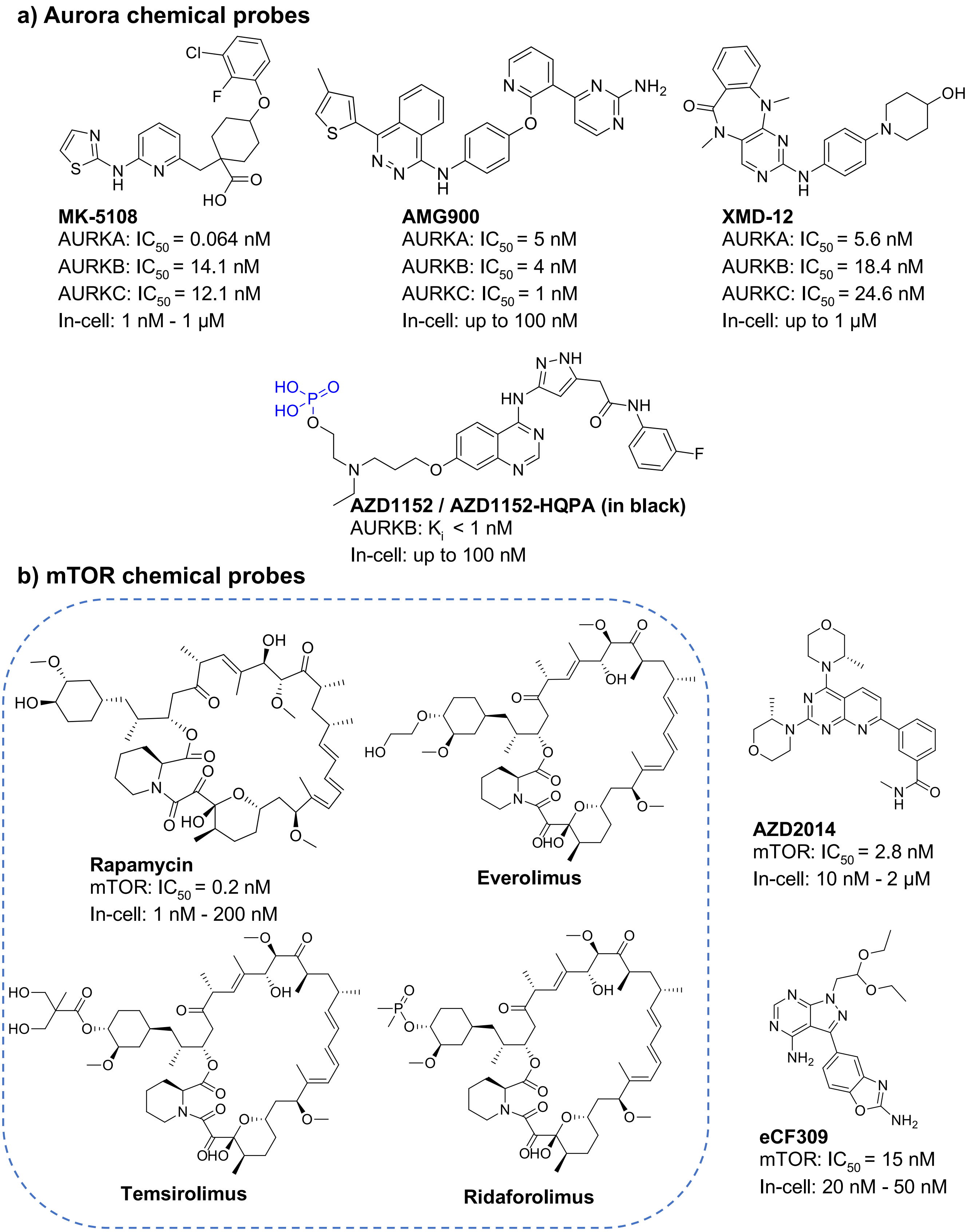

Supplementary Figure 8. Structures, biochemical potency (IC_{50}) and recommended in-cell concentrations of **a)** Aurora and **b)** mTOR chemical probes as listed on the Chemical Probes Portal (www.chemicalprobes.org). Note that everolimus, temsirolimus and ridaforolimus are not listed on the Chemical Probes Portal, but are included in the figure to demonstrate their structural similarity with rapamycin. Dashed lines group structurally related mTOR chemical probes that should not be used together as orthogonal tools.

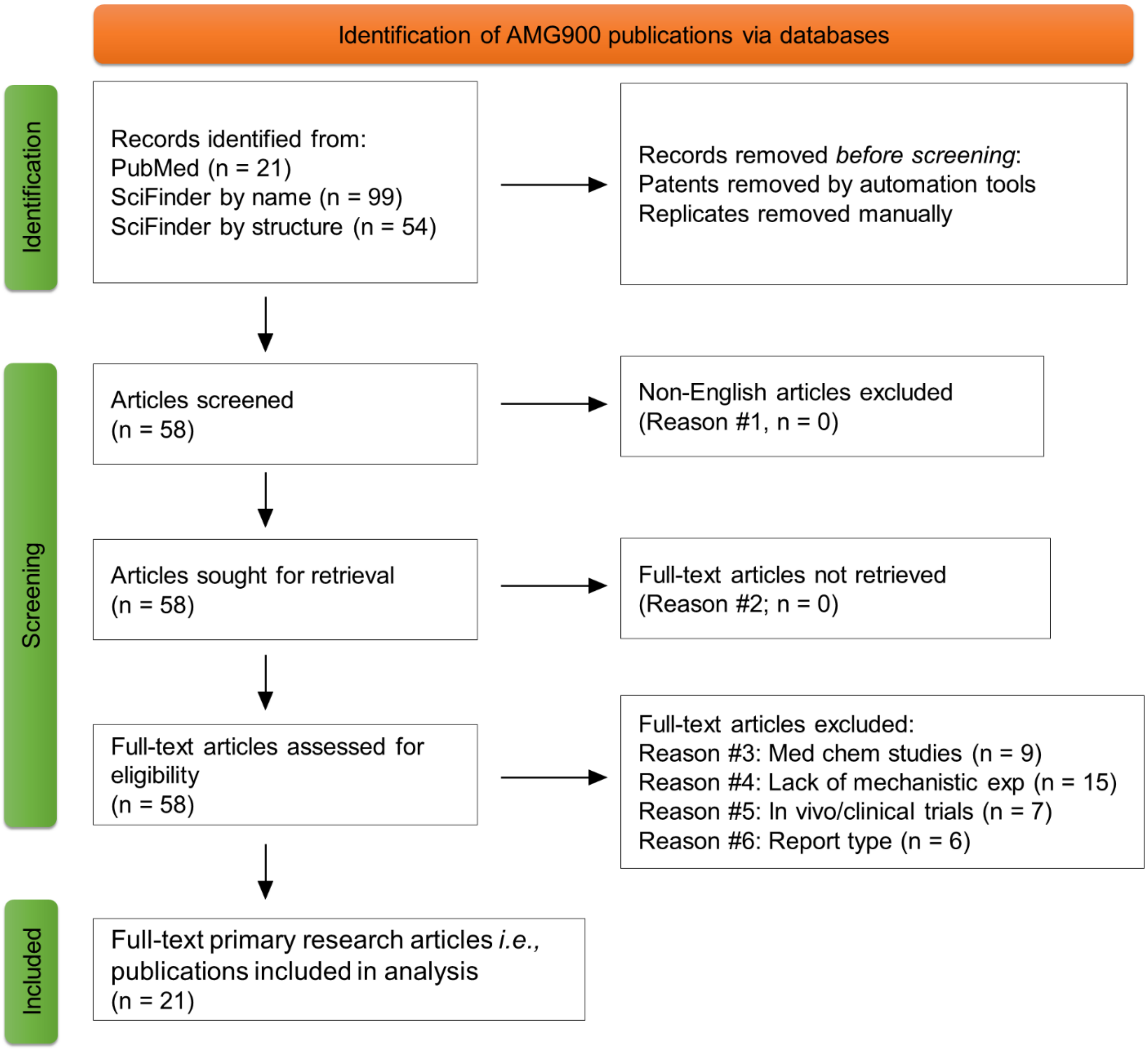

Supplementary Figure 9. PRISMA flow diagram summarising identification of publications using Aurora kinase chemical probe AMG900.

Supplementary Table 9. List of excluded articles using the Aurora kinase targeting probe AMG900.

	Title (PMID)	Reason for exclusion
1	In silico binding mode analysis (molecular docking studies) and absorption, distribution, metabolism and excretion prediction of some novel inhibitors of Aurora kinase A in clinical trials (No PMID; DOI: 10.14233/ajchem.2014.17175)	3) Medicinal chemistry /Computational study
2	Biologically relevant chemical space navigator: From patent and structure-activity relationship analysis to library acquisition and design (PMID: 23176522)	3) Medicinal chemistry /Computational study
3	Discovery of N-(4-(3-(2-aminopyrimidin-4-yl)pyridin-2-yloxy)phenyl)-4-(4-methylthiophen-2-yl)phthalazin-1-amine (AMG 900), a highly selective, orally bioavailable inhibitor of aurora kinases with activity against multidrug-resistant cancer cell lines (PMID: 25970324)	3) Discovery of AMG900
4	A combined approach based on 3D pharmacophore and docking for identification of new aurora A kinase inhibitors (No PMID; DOI: 10.1007/s00044-013-0747-5)	3) Medicinal chemistry / in silico study
5	Quantitative conformational profiling of kinase inhibitors reveals origins of selectivity for Aurora kinase activation states (PMID: 30518564)	3) Medicinal chemistry
6	Optimization and biological evaluation of nicotinamide derivatives as Aurora kinase inhibitors (PMID: 31307762)	3) Medicinal chemistry
7	New affinity probe targeting VEGF receptors for kinase inhibitor selectivity profiling by chemical proteomics (PMID: 24712744)	3) Medicinal chemistry
8	SAR and evaluation of novel 5H-benzo[c][1,8]naphthyridin-6-one analogs as Aurora kinase inhibitors (PMID: 23570792)	3) Medicinal chemistry
9	In silico identification of strong binders of the SARS-CoV-2 receptor-binding domain (PMID: 33130279)	3) Medicinal chemistry
10	Real-time luminescence enables continuous drug-response analysis in adherent and suspension cell lines (PMID: 35443861)	4) No mechanistic exp. - Assay protocol
11	Synthetic lethality of RB1 and aurora A is driven by stathmin-mediated disruption of microtubule dynamics (PMID: 33037191)	4) No mechanistic exp. - Dose response viability
12	Modeling and targeting of erythroleukemia by hematopoietic genome editing (PMID: 33512458; DOI: 10.1182/blood.2020009103)	4) Ex vivo study
13	Activity comparison of epigenetic modulators against the Hemoprotozoan parasites Babesia divergens and Plasmodium falciparum (PMID: 33599488)	4) No mechanistic exp. - Parasites growth
14	Cell panel profiling reveals conserved therapeutic clusters and differentiates the mechanism of action of different PI3K/mTOR, aurora kinase and EZH2 inhibitors (PMID: 27587489)	4) No mechanistic exp.
15	The new paradigm of network medicine to analyze breast cancer phenotypes (PMID: 32932728)	4) No mechanistic studies
16	Aurora kinase A inhibition induces synthetic lethality in SMAD4-deficient colorectal cancer cells via spindle assembly checkpoint activation (PMID: 35393542)	4) No mechanistic studies
17	High-throughput compound screen reveals mTOR inhibitors as potential therapeutics to reduce (auto)antibody production by human plasma cells (PMID: 31621069)	4) No mechanistic exp. with AMG900
18	Refinement of in vitro methods for identification of aldehyde oxidase substrates reveals metabolites of kinase inhibitors (PMID: 29615437)	4) No mechanistic exp. - AMG900 metabolism
19	Compound selectivity and target residence time of kinase inhibitors studied with surface plasmon resonance (PMID: 28043854)	4) No mechanistic exp. – AMG900 kinetics
20	RIPK1-dependent cell death: a novel target of the Aurora kinase inhibitor Tozasertib (VX-680) (PMID: 29434255)	4) No mechanistic exp.
21	Aneugen molecular mechanism assay: proof-of-concept with 27 reference chemicals (PMID: 31132080)	4) No mechanistic exp.
22	Interlaboratory evaluation of a multiplexed high information content in vitro genotoxicity assay (PMID: 28370322)	4) No mechanistic exp.
23	In vitro and in vivo pharmacokinetic characterizations of AMG 900, an orally bioavailable small molecule inhibitor of aurora kinases (PMID: 21294625)	4) No mechanistic exp.
24	Differential ABC transporter expression during hematopoiesis contributes to neutrophil-biased toxicity of Aurora kinase inhibitors (PMID: 36224199)	4) No mechanistic exp.
25	Definition of a novel cuproptosis-relevant lncRNA signature for uncovering distinct survival, genomic alterations, and treatment implications in lung adenocarcinoma (PMID: 36281357)	5) Clinical data

26	Experiments in the EpiDerm 3D skin in vitro model and minipigs in vivo indicate comparatively lower in vivo skin sensitivity of topically applied aneugenic compounds (PMID: 33481035)	5) Clinical study
27	A phase 1, first-in-human study of AMG 900, an orally administered pan-Aurora kinase inhibitor, in adult patients with advanced solid tumors (PMID: 29980894)	5) Clinical trial
28	A phase 1 study of AMG 900, an orally administered pan-aurora kinase inhibitor, in adult patients with acute myeloid leukemia (PMID: 28370201)	5) Clinical trial
29	AMG 900, a potent inhibitor of aurora kinases causes pharmacodynamic changes in p-Histone H3 immunoreactivity in human tumor xenografts and proliferating mouse tissues (PMID: 25367255)	5) In vivo study
30	LC-MS/MS bioanalytical method development for AMG 900: resolution of an isobaric interference in rodent in vivo studies (PMID: 23245248)	5) In vivo study
31	Gender effects on rat metabolism of AMG 900, an orally available small molecule Aurora kinase inhibitor (PMID: 22022868)	5) In vivo study
32	SnapShot: Kinase inhibitors II (PMID: 26000855)	6) Abstract only
33	Discovery of AMG 900, a highly selective, orally bioavailable inhibitor of Aurora kinases with efficacy in preclinical antitumor models and activity against multidrug-resistant cancer cell lines (No PMID, DOI or ISSN, AN: 2014:1304450)	6) Conference proceedings
34	Structural biology insight for the design of sub-type selective aurora kinase inhibitors (PMID: 25895501)	6) Review
35	Phthalazinone scaffold: Emerging tool in the development of target based novel anticancer agents (PMID: 32767957)	6) Review
36	Aurora kinase inhibitor patents and agents in clinical testing: an update (2011 - 2013) (PMID: 24965505)	6) Review
37	Aurora kinase inhibition: a new light in the sky? (PMID: 26111271)	6) Review

Supplementary Table 10. Overview of eligible publications using Aurora kinase targeting probe AMG900 and compliance (**in blue**) with recommendations to use AMG900 up to 100 nM (https://www.chemicalprobes.org/amg900?q=AMG-900) and validate results with at least one orthogonal probe. Citations are sourced from SciFinder (January 2023).

	AMG900	Orthogonal inhibitors	Title (PMID)	Cites
1	0 - 100 nM	None	Classification of in vitro genotoxicants using a novel multiplexed biomarker assay compared to the flow cytometric micronucleus test (PMID: 28940655)	13
2	0.01 - 100 nM	Barasertib (0.05-500 nM), Hesperadin (1 nM-10 µM)	A new imaging platform (iScreen) allows for the concurrent assessment of micronucleus induction and genotoxic mode of action in human A375 cells (PMID: 35703118)	0
3	50 pM - 1 µM	AZD1152-HQPA, MLN8054 (5 µM)	Dual targeting of aurora kinases with AMG 900 exhibits potent preclinical activity against acute myeloid leukemia with distinct post-mitotic outcomes (PMID: 30266802)	12
4	0.1 - 100 nM	None	An Aurora kinase inhibitor, AMG900, inhibits glioblastoma cell proliferation by disrupting mitotic progression (PMID: 30221846)	8
5	0.15625 - 2.5 nM	Hesperadin (3 - 50 nM) VX680 (6.25-100 nM)	Aneugen versus clastogen evaluation and oxidative stress-related mode-of-action assessment of genotoxic compounds using the toxtracker reporter assay (PMID: 32617558)	10
6	0.2 - 100 nM	Hesperadin (4 nM - 2 µM), VX-680 (0.01 - 5 µM)	TubulinTracker, a novel in vitro reporter assay to study intracellular microtubule dynamics, cell cycle progression, and aneugenicity (PMID: 35094094)	2
7	0.2 - 100 nM	None	Image analysis of mechanistic protein biomarkers for the characterization of genotoxicants:<br>Aneugens, clastogens, and reactive oxygen species inducers (No PMID; DOI: 10.1002/em.22374)	1
8	0.5 nM - 1 µM	AZD1152-HQPA, MK-5108 (0.5 nM - 1 µM)	Preclinical evaluation of the Aurora kinase inhibitors AMG 900, AZD1152-HQPA, and MK-5108 on SW-872 and 93T449 human liposarcoma cells (PMID: 29197031)	5
9	1 - 50 nM	None	Combining the pan-aurora kinase inhibitor AMG 900 with histone deacetylase inhibitors enhances antitumor activity in prostate cancer (PMID: 24989836)	22
10	1.5 - 50 nM	None	AMG 900, a small-molecule inhibitor of aurora kinases, potentiates the activity of microtubule-targeting agents in human metastatic breast cancer models (PMID: 23990115)	32
11	2.5 - 50 nM	None	The aurora kinase inhibitor AMG 900 increases apoptosis and induces chemosensitivity to anticancer drugs in the NCI-H295 adrenocortical carcinoma cell line (PMID: 28410270)	15
12	2.5 - 50 nM	None	Antitumour activity of AMG 900 alone or in combination with histone deacetylase inhibitor SaHa on medulloblastoma cell lines (PMID: 26000978)	5
13	2.5 - 160 nM	VX-680 (2.5 µM), MLN8237 (0.1 - 2.5 µM), AZD1152 (0.2 - 2.5 µM)	2-Phenoxy-3, 4′-bipyridine derivatives inhibit AURKB-dependent mitotic processes by disrupting its localization (PMID: 36413818)	2
14	10 nM	None	AMG 900, pan-Aurora kinase inhibitor, preferentially inhibits the proliferation of breast cancer cell lines with dysfunctional p53 (PMID: 24091768)	19
15	50 nM	ML8237 (50 nM)	Inhibition of Aurora kinase A activity enhances the antitumor response of beta-catenin blockade in human adrenocortical cancer cells (PMID: 33716050)	4
16	50 nM	None	Preclinical evaluation of AMG 900, a novel potent and highly selective pan-aurora kinase inhibitor with activity in taxane-resistant tumor cell lines (PMID: 20935223)	103
17	50 - 100 nM	None	Report and application of a tool compound data set (PMID: 29035535)	5
18	300 nM	AT9283 (50 nM)	AT9283 exhibits antiproliferative effect on tyrosine kinase inhibitor-sensitive and -resistant chronic myeloid leukemia cells by inhibition of Aurora A and Aurora B (PMID: 33000229)	2

19	3.435 - 13.74 µM	Artesunate (12.6 - 50 µM)	AMG900 as novel inhibitor of the translationally controlled tumor protein (PMID: 33259807)	3
20	66.4 nM & 11.2 µM	None	Pan Aurora Kinase Inhibitor: A Promising Targeted-Therapy in Dedifferentiated Liposarcomas With Differential Efficiency Depending on Sarcoma Molecular Profile (PMID: 32138169)	8
21	Unclear	VX680, PF03814735 (0 - 10 mM)	Biomarkers of DNA damage response improve in vitro micronucleus assays by revealing genotoxic mode of action and reducing the occurrence of irrelevant positive results (PMID: 34718711)	0
Compliance				
**14 (67%)** [a] **4 (19%)** [b]		10 (48%)		
Non-Compliance				
**3 (14%)**		11 (52%)		

a *Probe's concentration below the recommended in-cell maximum in all figures.* ***b*** *Probe's concentration below the recommended in-cell maximum in some, but not all figures.*

Supplementary Note 6. Citations for AMG900 publications included in the systematic review.
Citations for 21 publications using AMG900: 271

Citations for 18 publications with a compliant AMG900 concentration: 266
Citations for 3 publications with a non-compliant AMG900 concentration: 5

Citations for 10 publications using orthogonal Aurora kinase inhibitors: 40
Citations for 11 publications not using orthogonal Aurora kinase inhibitors: 231

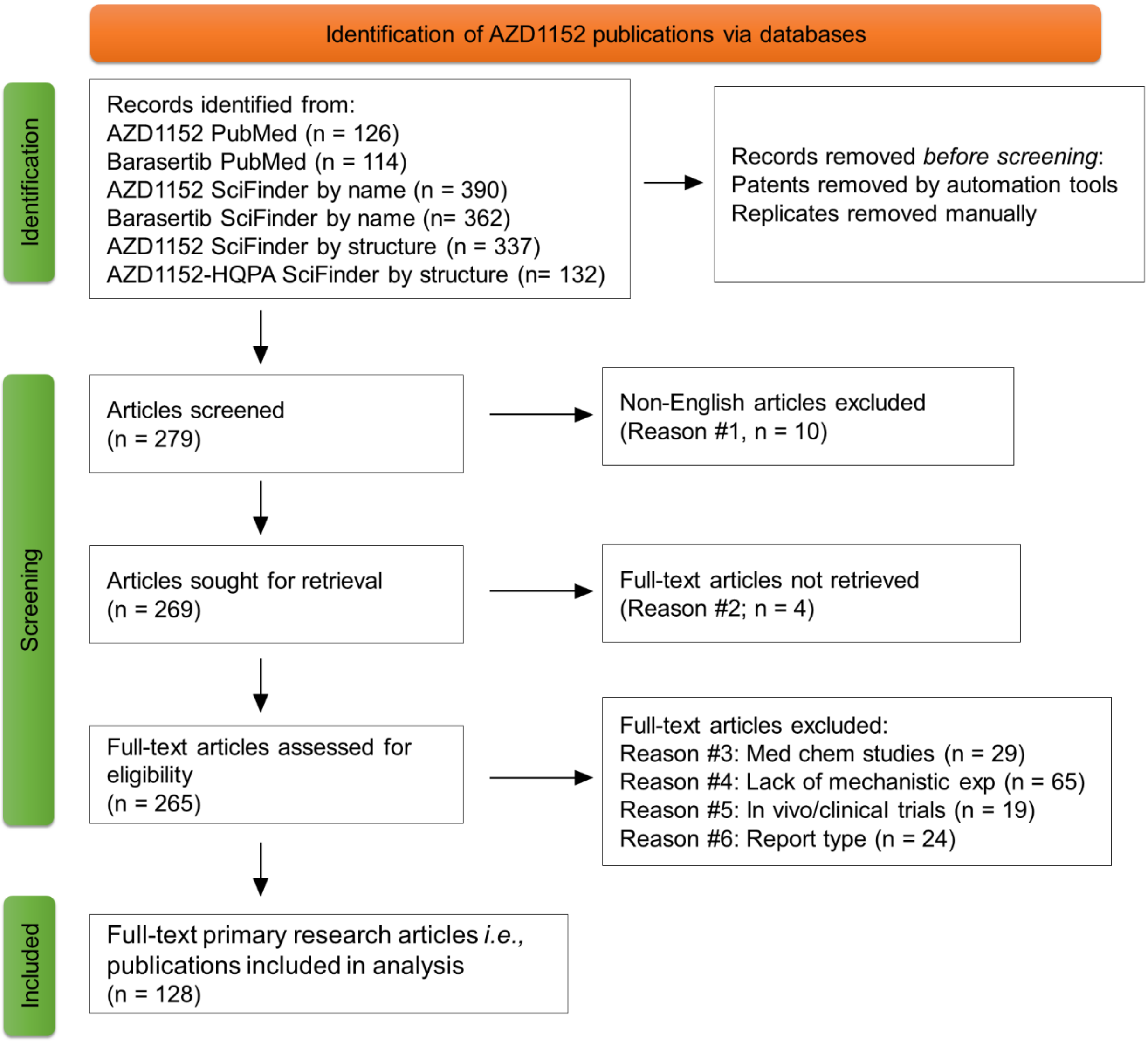

Supplementary Figure 10. PRISMA flow diagram summarising identification of publications using Aurora kinases targeting chemical probe AZD1152.

Supplementary Table 11. List of excluded articles using the Aurora kinase probe AZD1152.

	Title (PMID)	Reason for exclusion
1	Synthesis of Aurora-B kinase inhibitor AZD1152-HQPA No PMID or DOI; ISSN: 1001-9677)	1) Non-English
2	Inhibition effect of AZD1152-HQPA, a specific inhibitor of Aurora-B kinase, on human osteosarcoma cell line U2-OS cells (No PMID or DOI or ISSN, AN: 2011:420881)	1) Non-English
3	Discovery of a novel hydrogen sulfide donor and the study of its structure activity relationship (No PMID; DOI: 10.16519/j.cnki.1004-311x.2018.02.0032)	1) Non-English
4	The correlation between expression level of Aurora B and occurrence of cervical cancer (No PMID; DOI: 10.13417/j.gab.039.002367)	1) Non-English
5	Localization and function of Aurora-B in pig (Sus scrofa) oocytes (No PMID; DOI: 10.3969/j.issn.1674-7968.2014.06.003)	1) Non-English
6	Inhibitors of Aurora kinases (No PMID; DOI: 10.1016/j.pharma.2008.12.005)	1) Non-English
7	Possibility of aurora kinase inhibitors for AML (No PMID or DOI; ISSN: 2185-582X)	1) Non-English
8	Aurora kinase inhibitor (No PMID or DOI; ISSN: 0370-8241)	1) Non-English
9	Management and novel drugs for older patients with acute myeloid leukemia (No PMID or DOI; ISSN: 2185-582X)	1) Non-English
10	Effect of aurora kinase B inhibitor AZD1152 in the treatment of cisplatin-resistant ovarian carcinoma] (PMID: 23531251)	1) Non-English
11	Aurora kinase: A target for cancer therapy (No PMID or DOI; ISSN: 2230-9861)	2) No access
12	In vitro sensitivity profiling of neuroblastoma cells against a comprehensive small molecule kinase inhibitor library to identify agents for future therapeutic studies (PMID: 27875952)	2) No access
13	Exploring protein kinase inhibitors. Unveiling gemcitabine resistance in pancreatic cancer. Comments (No PMID; DOI: 10.1097/mpa.0b013e31823f3fcb)	2) No access
14	Targeting Aurora kinases in cancer treatment (No PMID; DOI: 10.2174/138945011798829410)	2) No access
15	Discovery, synthesis, and in vivo activity of a new class of pyrazoloquinazolines as selective inhibitors of aurora B kinase (PMID: 17373783)	3) Discovery of AZD1152
16	Structure-based drug design of novel aurora kinase A inhibitors: Structural basis for potency and specificity (PMID: 19140666)	3) Medicinal Chemistry
17	Structure-based discovery and bioactivity evaluation of novel Aurora-A kinase inhibitors as anticancer agents via docking-based comparative intermolecular contacts analysis (dbCICA) (PMID: 33353031)	3) Medicinal Chemistry
18	Epidermal growth factor receptor (EGFR) structure-based bioactive pharmacophore models for identifying next-generation inhibitors against clinically relevant EGFR mutations (PMID: 28303669)	3) Medicinal Chemistry
19	Development of o-chlorophenyl substituted pyrimidines as exceptionally potent aurora kinase inhibitors (PMID: 22803810)	3) Medicinal Chemistry
20	Quinazoline-benzimidazole hybrid as dual optical sensor for cyanide and Pb^{2+} ions and Aurora kinase inhibitor (No PMID; DOI: 10.1016/j.jphotochem.2015.05.025)	3) Medicinal Chemistry
21	Imidazo[4,5-b]pyridine derivatives as inhibitors of aurora kinases: Lead optimization studies toward the identification of an orally bioavailable preclinical development candidate (PMID: 20565112)	3) Medicinal Chemistry
22	Facile identification of dual FLT3-Aurora A inhibitors: A computer-guided drug design approach (PMID: 24665000)	3) Medicinal Chemistry
23	Identification of binding specificity-determining features in protein families (PMID: 22289061)	3) Medicinal Chemistry
24	Discovery of SP-96, the first non-ATP-competitive Aurora kinase B inhibitor, for reduced myelosuppression (PMID: 32717530)	3) Medicinal Chemistry
25	Identification of Aurora-A inhibitors by ligand and structure-based virtual screening (PMID: 27485892)	3) Medicinal Chemistry
26	Discovery of *N*-(4-(3-(2-Aminopyrimidin-4-yl)pyridine-2-yloxy)phenyl)-4-(4-methylthiophen-2-yl)phthalazine-1-amine (AMG900), a highly selective, orally bioavailable inhibitor of Aurora kinases with activity against multidrug-resistant cancer cell lines (PMID: 25970324)	3) Medicinal Chemistry – discovery of AMG900
27	Ligand efficiency based approach for efficient virtual screening of compound libraries (PMID: 24960626)	3) Medicinal Chemistry

28	Selectivity data: Assessment, predictions, concordance, and implications (PMID: 23937569)	3) Medicinal Chemistry
29	A specific pharmacophore model of Aurora B kinase inhibitors and virtual screening studies based on it (PMID: 19152640)	3) Medicinal Chemistry
30	Structural studies of B-type Aurora kinase inhibitors using computational methods (PMID: 20139908)	3) Medicinal Chemistry
31	Docking study and three-dimensional quantitative structure-activity relationship (3D-QSAR) analyses and novel molecular design of a series of 4-aminoquinazolines as inhibitors of Aurora B kinase (No PMID; DOI: 10.1002/cjoc.201180315)	3) Medicinal Chemistry
32	Prediction of biological activity of Aurora-A kinase inhibitors by multilinear regression analysis and support vector machine (PMID: 21421314)	3) Medicinal Chemistry
33	SAR and evaluation of novel 5*H*-benzo[c][1,8]naphthyridine-6-one analogs as Aurora kinase inhibitors (PMID: 23570792)	3) Medicinal Chemistry
34	Optimization and biological evaluation of nicotinamide derivatives as Aurora kinase inhibitors (PMID: 31307762)	3) Medicinal Chemistry
35	Discovery of 7-aryl-substituted (1,5-naphthyridin-4-yl)ureas as Aurora kinase inhibitors (PMID: 24273104)	3) Medicinal Chemistry
36	Discovery of selective aminothiazole Aurora kinase inhibitors (PMID: 18307303)	3) Medicinal Chemistry
37	Probing the structural requirements of A-type Aurora kinase inhibitors using 3D-QSAR and molecular docking analysis (PMID: 21670994)	3) Medicinal Chemistry
38	Analysis of kinase inhibitor selectivity using a thermodynamics-based partition index (PMID: 20459125)	3) Medicinal Chemistry
39	Two-stage model-based design of cancer phase I dose escalation trials: evaluation using the phase I program of barasertib (AZD1152) (PMID: 21626115)	3) Medicinal Chemistry
40	A novel cell-based, high-content assay for phosphorylation of Lats2 by Aurora A (PMID: 21788394)	3) Medicinal Chemistry
41	Selective aurora kinase inhibitors identified using a taxol-induced checkpoint sensitivity screen (PMID: 21992004)	3) Medicinal Chemistry
42	[14C]-AZD1152 drug substance manufacture: challenges of an IV-infusion dosed human mass balance study in patients (PMID: 27169761)	3) Medicinal Chemistry
43	Discovery of novel GMPS inhibitors of Candidatus Liberibacter Asiaticus by structure based design and enzyme kinetics (PMID: 34203217)	3) Medicinal Chemistry
44	RIPK1-dependent cell death: a novel target of the Aurora kinase inhibitor Tozasertib (VX-680) (PMID: 29434255)	4) AZD-1152 not used
45	Biochemical characterization of GSK1070916, a potent and selective inhibitor of Aurora B and Aurora C kinases with an extremely long residence time (PMID: 19284385)	4) AZD1152 not used in experiments
46	Preclinical Evaluation of AMG 900, a Novel Potent and Highly Selective Pan-Aurora Kinase Inhibitor with Activity in Taxane-Resistant Tumor Cell Lines (PMID: 20935223)	4) AZD1152 used to generate resistant cell clones
47	Identification of genes that confer tumor cell resistance to the aurora B kinase inhibitor, AZD1152 (PMID: 19188929)	4) AZD1152 used to generate resistant clones
48	Expression, purification, stability optimization and characterization of human Aurora B kinase domain from E. coli (PMID: 20699085)	4) No mechanistic exp - focus on an Aurora B plasmid construct
49	CEP-32496: A novel orally active $BRAF^{V600E}$ inhibitor with selective cellular and in vivo antitumor activity (PMID: 22319199)	4) No mechanistic exp with AZD1152
50	Structure of Aurora B-INCENP in complex with barasertib reveals a potential trans-inhibitory mechanism (PMID: 24598913)	4) No mechanistic exp - in silico study
51	Target inhibition networks: Predicting selective combinations of druggable targets to block cancer survival pathways (PMID: 24068907)	4) No mechanistic exp - model-based prediction approach to drug discovery
52	A novel in situ hydrophobic ion paring (HIP) formulation strategy for clinical product selection of a nanoparticle drug delivery system (PMID: 27001894)	4) No mechanistic exp - nanoparticle delivery of AZD1152-HQPA
53	Measuring the rate of in-vitro drug release from polymeric nanoparticles by ^{19}F solution state NMR spectroscopy (PMID: 34400184)	4) No mechanistic exp -- nanoparticle study
54	Evaluation of particle size techniques to support the development of manufacturing scale nanoparticles for application in pharmaceuticals (PMID: 32278922)	4) No mechanistic exp - nanoparticle study
55	A quantitative analysis of kinase inhibitor selectivity (PMID: 18183025)	4) No in-cell exp

56	Chemical signatures and new drug targets for gametocytocidal drug development (PMID: 24434750)	4) No mechanistic exp
57	CCT245718, a dual FLT3/Aurora A inhibitor overcomes D835Y-mediated resistance to FLT3 inhibitors in acute myeloid leukemia cells (PMID: 34446858)	4) No mechanistic exp
58	A theoretical entropy score as a single value to express inhibitor selectivity (PMID: 21486481)	4) No mechanistic exp
59	A new approach for prediction of tumor sensitivity to targeted drugs based on functional data (PMID: 23890326)	4) No mechanistic exp
60	QSAR modelling of SARS-CoV M^{pro} inhibitors identifies Sufugolix, Cenicriviroc, Proglumetacin, and other drugs as candidates for repurposing against SARS-CoV-2 (PMID: 33405340)	4) No mechanistic exp
61	Explainable artificial intelligence for precision medicine in acute myeloid leukemia (PMID: 36248800)	4) No mechanistic exp
62	Computationally predicting clinical drug combination efficacy with cancer cell line screens and independent drug action (PMID: 33203866)	4) No mechanistic exp
63	Butyrophilin-like 9 expression is associated with outcome in lung adenocarcinoma (PMID: 34635082)	4) No mechanistic exp
64	Machine learning models for the prediction of chemotherapy-induced peripheral neuropathy (PMID: 30617559)	4) No mechanistic exp
65	Combining machine learning systems and multiple docking simulation packages to improve docking prediction reliability for network pharmacology (PMID: 24391846)	4) No mechanistic exp
66	Using a gene network of pyroptosis to quantify the responses to immunotherapy and prognosis for neuroblastoma patients (PMID: 35401536)	4) No mechanistic exp
67	A molecular case report: Functional assay of tyrosine kinase inhibitors in cells from a patient's primary renal cell carcinoma (PMID: 23192268)	4) No mechanistic exp
68	Inhibition of Aurora-B kinase activity confers antitumor efficacy in preclinical mouse models of early and advanced gastrointestinal neoplasia (PMID: 22858681)	4) No mechanistic exp – dose response in viability assays only
69	Aurora B kinase as a therapeutic target in acute lymphoblastic leukemia (PMID: 32144432)	4) No mechanistic exp - Kinase inhibitor viability screen: 20-200 nM
70	Kinase pathway dependence in primary human leukemias determined by rapid inhibitor screening (PMID: 23087056)	4) No mechanistic exp - Viability screen
71	Aneugen molecular mechanism assay: Proof-of-concept with 27 reference chemicals (PMID: 31132080)	4) No mechanistic exp – toxicology study
72	IL15 agonists overcome the immunosuppressive effects of MEK inhibitors (PMID: 26980764)	4) No mechanistic exp with AZD1152
73	Profiling pathway-specific novel therapeutics in preclinical assessment for central nervous system atypical teratoid rhabdoid tumors (CNS ATRT): Favorable activity of targeting EGFR-ErbB2 signaling with lapatinib (PMID: 23375777)	4) No mechanistic exp - dose-response viability screen with AZD1152
74	JAK1/2 and BCL2 inhibitors synergize to counteract bone marrow stromal cell-induced protection of AML (PMID: 28619982)	4) No mechanistic exp with AZD1152-HQPA
75	Identification of selective cytotoxic and synthetic lethal drug responses in triple negative breast cancer cells (PMID: 27165605)	4) No mechanistic exp with AZD1152-HQPA
76	A high content clonogenic survival drug screen identifies MEK inhibitors as potent radiation sensitizers for KRAS mutant non-small-cell lung cancer (PMID: 24922006)	4) No mechanistic exp with AZD1152-HQPA
77	Ex vivo drug response profiling detects recurrent sensitivity patterns in drug-resistant acute lymphoblastic leukemia (PMID: 28122742)	4) No mechanistic exp with AZD1152-HQPA
78	System-level analysis of neuroblastoma tumor-initiating cells implicates AURKB as a novel drug target for neuroblastoma (PMID: 20651058)	4) No mechanistic exp with AZD1152
79	Comprehensive analysis of kinase inhibitor selectivity (PMID: 22037378)	4) No mechanistic exp
80	A cell cycle progression-derived gene signature to predict prognosis and therapeutic response in hepatocellular carcinoma (PMID: 34721731)	4) No mechanistic exp
81	RUNX1 mutations in blast-phase chronic myeloid leukemia associate with distinct phenotypes, transcriptional profiles, and drug responses (PMID: 32782381)	4) No mechanistic exp
82	Pan-transcriptome-based candidate therapeutic discovery for idiopathic pulmonary fibrosis (PMID: 33167785)	4) No mechanistic exp – analyses of gene expression signatures
83	BCL-XL blockage in TNBC models confers vulnerability to inhibition of specific cell cycle regulators (PMID: 34646365)	4) No mechanistic exp with AZD1152-HQPA

84	Sarcoma cell line screen of oncology drugs and investigational agents identifies patterns associated with gene and microRNA expression (PMID: 26351324)	4) No mechanistic exp with AZD1152-HQPA
85	Synthetic lethal interaction between the ESCRT paralog enzymes VPS4A and VPS4B in cancers harboring loss of chromosome 18q or 16q (PMID: 33326793)	4) No mechanistic exp with AZD1152-HQPA
86	Epigenome-wide DNA methylation analysis of small cell lung cancer cell lines suggests potential chemotherapy targets (PMID: 32586373)	4) No mechanistic exp - dose-response viability only
87	Aurora kinases in childhood acute leukemia: the promise of aurora B as therapeutic target (PMID: 22940834)	4) No mechanistic exp - dose-response viability only
88	Identifying cancer driver genes from functional genomics screens (PMID: 32083704)	4) No mechanistic exp - dose-response viability only
89	Aurora kinase B expression, its regulation and therapeutic targeting in human retinoblastoma (PMID: 33704359)	4) No mechanistic exp - dose-response viability only
90	Imaging colon cancer response following treatment with AZD1152: a preclinical analysis of [18F]fluoro-2-deoxyglucose and 3'-deoxy-3'-[18F]fluorothymidine imaging (PMID: 21245090)	4) No mechanistic exp - dose-response viability only
91	The target landscape of clinical kinase drugs (PMID: 29191878)	4) No mechanistic exp - kinobead profiling
92	Optimizing therapeutic effect of Aurora b inhibition in acute myeloid leukemia with AZD2811 nanoparticles (PMID: 28292940)	4) No mechanistic exp - nanoparticle delivery
93	Visualization of the distribution of nanoparticle-formulated AZD8211 in mouse tumour model using matrix-assisted laser desorption ionization mass spectrometry imaging (PMID: 32968211)	4) No mechanistic exp - nanoparticle delivery
94	Multi-modal molecular imaging maps the correlation between tumor microenvironments and nanomedicine distribution (PMID: 35265205)	4) No mechanistic exp - nanoparticle delivery
95	Real-time luminescence enables continuous drug-response analysis in adherent and suspension cell lines (PMID: 35443861)	4) No mechanistic exp with AZD1152-HQPA
96	Data-driven exploration of selectivity and off-target activities of designated chemical probes (PMID: 30249057)	4) No mechanistic exp - qualifying chemical probes
97	Activity comparison of epigenetic modulators against the Hemoprotozoan parasite Babesia divergens and Plasmodium falciparum (PMID: 33599488)	4) No mechanistic exp. - Parasites growth
98	In vitro and in vivo evaluation of kinase and protease inhibitors against Trypanosoma evansi (PMID: 35751782)	4) No mechanistic exp. - Parasites growth
99	Multiparametric cell-based assay for the evaluation of transcription inhibition by high-content imaging (PMID: 23307837)	4) No mechanistic exp with AZD1152
100	Predicting cell health phenotypes using image-based morphology profiling (PMID: 33534641)	4) No mechanistic exp – HTS image-based cell viability
101	Analytical method considerations regarding carryover for monophosphate prodrugs for in vivo samples by liquid chromatography-tandem mass spectroscopy (PMID: 31395357)	4) No mechanistic exp
102	High-throughput compound screening identifies navitoclax combined with irradiation as a candidate therapy for HPV-negative head and neck squamous cell carcinoma (PMID: 34285300)	4) No mechanistic exp with AZD1152-HQPA
103	A panel of isogenic human cancer cells suggests a therapeutic approach for cancers with inactivated p53 (PMID: 19225112)	4) No mechanistic exp with AZD1152
104	Combined inhibition of XIAP and BCL2 drives maximal therapeutic efficacy in genetically diverse aggressive acute myeloid leukemia (PMID: 35121960)	4) No mechanistic exp with AZD1152-HQPA
105	Integrative oncogene-dependency mapping identifies RIT1 vulnerabilities and synergies in lung cancer (PMID: 34373451)	4) No mechanistic exp with AZD1152-HQPA
106	Patient-derived models of acquired resistance can identify effective drug combinations for cancer (PMID: 25394791)	4) No mechanistic exp with AZD1152-HQPA
107	Viability fingerprint of glioblastoma cell lines: Roles of mitotic, proliferative, and epigenetic targets (PMID: 34645858)	4) No mechanistic exp with AZD1152-HQPA
108	Targeting vcp enhances anticancer activity of oncolytic virus m1 in hepatocellular carcinoma (PMID: 28835517)	4) No mechanistic exp with AZD1152-HQPA

109	Experiments in the EpiDerm 3D skin in vitro model and minipigs in vivo indicate comparatively lower in vivo skin sensitivity of topically applied aneugenic compounds (PMID: 33481035)	5) Clinical study
110	Clinical evaluation of AZD1152, an i.v. inhibitor of Aurora B kinase, in patients with solid malignant tumors (PMID: 20924078)	5) Clinical trial
111	A Phase I study to assess the safety, pharmacokinetics and efficacy of barasertib (AZD1152), an Aurora B kinase inhibitor, in Japanese patients with advanced acute myeloid leukemia (PMID: 21565405)	5) Clinical trial
112	Phase 1/2 study to assess the safety, efficacy, and pharmacokinetics of barasertib (AZD1152) in patients with advanced acute myeloid leukemia: (PMID: 21976672)	5) Clinical trial
113	Phase I study of barasertib (AZD1152), a selective inhibitor of Aurora B kinase, in patients with advanced solid tumors (PMID: 22661287)	5) Clinical trial
114	Phase I study of the Aurora B kinase inhibitor barasertib (AZD1152) to assess the pharmacokinetics, metabolism and excretion in patients with acute myeloid leukemia (PMID: 22864876)	5) Clinical trial
115	Stage I of a phase 2 study assessing the efficacy, safety, and tolerability of barasertib (AZD1152) versus low-dose cytosine arabinoside in elderly patients with acute myeloid leukemia (PMID: 23605952)	5) Clinical trial
116	Phase I study assessing the safety and tolerability of barasertib (AZD1152) with low-dose cytosine arabinoside in elderly patients with AML (PMID: 23763917)	5) Clinical trial
117	A phase II trial of AZD1152 in relapsed/refractory diffuse large B-cell lymphoma (PMID: 25721307)	5) Clinical trial
118	Biomarker-driven phase 2 umbrella trial study for patients with recurrent small cell lung cancer failing platinum-based chemotherapy (PMID: 32584426)	5) Clinical trial
119	A comparative assessment of preclinical chemotherapeutic response of tumors using quantitative non-Gaussian diffusion MRI (PMID: 27919785)	5) In vivo study
120	Simultaneous determination of AZD1152 (prodrug) and AZD1152-hydroxyquinazoline pyrazol anilide by reversed phase liquid chromatography (PMID: 19744901)	5) In vivo study
121	The MEK1/2 inhibitor, selumetinib (AZD6244; ARRY-142886), enhances anti-tumour efficacy when combined with conventional chemotherapeutic agents in human tumour xenograft models (PMID: 22343622)	5) In vivo study
122	Characterizing Tumor Response to Chemotherapy at Various Length Scales Using Temporal Diffusion Spectroscopy (PMID: 22911846)	5) In vivo study
123	Aurora kinase inhibitor nanoparticles target tumors with favorable therapeutic index in vivo (PMID: 26865565)	5) In vivo study
124	Salivary glands require Aurora Kinase B for regeneration after transient innate immune-mediated injury (PMID: 31383943)	5) In vivo study
125	Modeling Dose and Schedule Effects of AZD2811 Nanoparticles Targeting Aurora B Kinase for Treatment of Diffuse Large B-cell Lymphoma (PMID: 30872381)	5) In vivo study
126	Targeting aurora kinase B alleviates spinal microgliosis and neuropathic pain in a rat model of peripheral nerve injury (PMID: 31563141)	5) In vivo study
127	Aurora-B knockdown inhibits osteosarcoma metastasis by inducing autophagy via the mTOR/ULK1 pathway (PMID: 33292257)	5) In vivo study
128	Aurora kinase inhibitor AZD1152 negatively affects the growth and survival of HTLV-1-infected T lymphocytes in vitro [Retraction of document cited in CA154:351346] (No PMID; DOI: 10.1002/ijc.25178)	6) Retracted
129	Phosphoproteomic analysis of Aurora kinase inhibition in monopolar cytokinesis [Erratum to document cited in CA163327364] (PMID: 26561005)	6) Erratum
130	Correction: Bromodomain and extraterminal protein inhibition blocks growth of triple-negative breast cancers through the suppression of aurora kinases [Erratum to document cited in CA165:569636] (PMID: 32620694)	6) Erratum
131	Effects of AZD1152, a selective Aurora B kinase inhibitor, on Burkitt's and Hodgkin's lymphomas [Erratum to document cited in CA154:556930] (No PMID; DOI: 10.1016/j.bcp.2011.07.098)	6) Erratum
132	Therapeutic polymeric nanoparticles and the methods of making and using thereof: a patent evaluation of WO2015036792 (PMID: 27167102)	6) Patent evaluation
133	Aurora kinase inhibitor AZD1152 negatively affects the growth and survival of HTLV-1-infected T lymphocytes in vitro (PMID: 20091867)	6) Retracted
134	RETRACTED ARTICLE: Frequency of TP53 Mutations and its Impact on Drug Sensitivity in Acute Myeloid Leukemia? (PMID: 23543587)	6) Retracted

135	Design strategies, SAR, and mechanistic insight of Aurora kinase inhibitors in cancer (PMID: 33934503)	6) Review
136	Kinase Inhibitor 4 Minisymposium summary (PMID: 19589028)	6) Review
137	Aurora kinase inhibitors as anti-cancer therapy (PMID: 20016367)	6) Review
138	Novel agents and regimens for acute myeloid leukemia: 2009 ASH annual meeting highlights (PMID: 20416083)	6) Review
139	Aurora kinase inhibitor patents and agents in clinical testing: an update (2011 - 2013) (PMID: 24965505)	6) Review
140	Finding the optimal combination therapy for the treatment of newly diagnosed AML in older patients unfit for intensive therapy (PMID: 25577399)	6) Review
141	Structural Biology Insight for the Design of Sub-type Selective Aurora Kinase Inhibitors (PMID: 25895501)	6) Review
142	Aurora Kinase Inhibitors in Oncology Clinical Trials: Current State of the Progress (PMID: 26615129)	6) Review
143	Advances in targeted therapy for osteosarcoma based on molecular classification (PMID: 34022396)	6) Review
144	Are Accurins the cure for Aurora kinase inhibitors? (PMID: 26865564)	6) Review
145	Novel drugs for older patients with acute myeloid leukemia (PMID: 25142817)	6) Review
146	Targeting pan-essential genes in cancer: Challenges and opportunities (PMID: 33450197)	6) Review
147	Aurora kinase inhibitors: a new class of drugs targeting the regulatory mitotic system (PMID: 20045785)	6) Review
148	Emerging role of Aurora kinase inhibitors in chronic myeloid leukemia (No PMID; DOI: 10.3816/CLK.2007.n.025)	6) Review
149	Aurora kinase inhibitors: Potential molecular-targeted drugs for gynecological malignant tumours (review) (PMID: 31798873)	6) Review
150	SnapShot: Kinase inhibitors II (PMID: 26000855)	6) Snapshot
151	NPM-ALK expression levels identify two distinct subtypes of pediatric anaplastic large cell lymphoma (PMID: 27773932)	6) Letter to the editor

Supplementary Table 12. Overview of eligible publications using Aurora kinase probe AZD1152 and compliance (**in blue**) with recommendations to use AZD1152 up to 100 nM (https://www.chemicalprobes.org/azd1152?q=AZD1152) and validate results with at least one orthogonal inhibitor.

	AZD1152	Orthogonal inhibitors	Title (PMID)	Cites
1	75 pM - 300 pM	None	Aurora B expression modulates paclitaxel response in non-small cell lung cancer (PMID: 28095398)	29
2	0.1 nM	None	Dicer promotes genome stability via the bromodomain transcriptional co-activator BRD4 (PMID: 35194019)	2
3	0.5 - 25 nM	None	Overcoming cetuximab resistance in HNSCC: the role of AURKB and DUSP proteins (PMID: 25192874)	44
4	2 nM - 63 nM	GSK1070916 (20 nM)	High-throughput chemical screening identifies focal adhesion kinase and Aurora kinase B inhibition as a synergistic treatment combination in Ewing sarcoma (PMID: 30979745)	20
5	2 - 100 nM	None	AZD1152 negatively affects the growth of anaplastic thyroid carcinoma cells and enhances the effects of oncolytic virus dl922-947 (PMID: 21071467)	31
6	2.5 nM - 40 nM	None	Significance of AZD1152 as a potential treatment against Aurora B overexpression in acute promyelocytic leukemia (PMID: 27091351)	10
7	3 nM - 3 µM	None	Preclinical evaluation of M30 and M65 ELISAs as biomarkers of drug induced tumor cell death and antitumor activity (PMID: 18347133)	52
8	5 - 20 nM	None	RING1B recruits EWSR1-FLI1 and cooperates in the remodeling of chromatin necessary for Ewing sarcoma tumorigenesis (PMID: 33097530)	13
9	10 nM	None	Modeling the mitotic regulatory network identifies highly efficient anti-cancer drug combinations (PMID: 25418836)	2
10	10 - 50 nM	None	Vitamins C and K3: A powerful redox system for sensitizing leukemia lymphocytes to everolimus and barasertib (PMID: 29491065)	11
11	10 - 50 nM	None	Docosahexaenoic acid sensitizes leukemia lymphocytes to barasertib and everolimus by ROS-dependent mechanism without affecting the level of ROS and viability of normal lymphocytes (PMID: 27069145)	18
12	10 - 50 nM	None	2-Deoxy-D-glucose sensitizes cancer cells to barasertib and everolimus by ROS-independent mechanism(s) (PMID: 26637878)	9
13	20 - 100 nM	None	Aurora B kinase regulates the postmitotic endoreduplication checkpoint via phosphorylation of the retinoblastoma protein at serine 780. (PMID: 19225156)	73
14	50 nM	JNJ-7706621 (500 - 1500 nM)	Aurora kinase B is important for antiestrogen resistant cell growth and a potential biomarker for tamoxifen resistant breast cancer (PMID: 25885472)	20
15	50 nM - 1 µM	MLN8237 (10 nM-1 µM)	Selective inhibitors of aurora kinases inhibit proliferation, reduce cell viability and impair cell cycle progression in papillary thyroid carcinoma cells (PMID: 26753639)	6
16	50 nM & 1 µM	JNJ-7706621 (0.5 - 1.5 µM)	Aurora kinase A and B as new treatment targets in aromatase inhibitor-resistant breast cancer cells (PMID: 25667100)	29
17	60 nM	None	Enhanced radiosensitivity of androgen-resistant prostate cancer: AZD1152-mediated Aurora kinase B inhibition (PMID: 21222513)	25
18	60 nM	None	Close correlation between MEK/ERK and Aurora-B signaling pathways in sustaining tumorigenic potential and radioresistance of gynecological cancer cell lines (PMID: 24189697)	35

19	100 nM	None	Enhancing functional platelet release in vivo from in vitro-grown megakaryocytes using small molecule inhibitors (PMID: 29545255)	14
20	100 nM	None	A novel therapeutic combination sequentially targeting Aurora B and Bcl-xL in hepatocellular carcinoma (PMID: 25524010)	8
21	100 nM	MLN8237 (250 nM)	Bromodomain and extraterminal protein inhibition blocks growth of triple-negative breast cancers through the suppression of aurora kinases (PMID: 27650498)	41
22	100 nM	MLN8237 (50 nM)	Chromosome oscillation promotes Aurora A-dependent Hec1 phosphorylation and mitotic fidelity PMID: 33988677)	10
23	100 nM	MLN8237 (100 nM), Scoulerine (2-20 µM)	The phytochemical Scoulerine inhibits Aurora kinase activity to induce mitotic and cytokinetic defects (PMID: 34406008)	4
24	100 nM - 1 µM	MLN8237 (250 nM)	Effects of selective inhibitors of Aurora kinases on anaplastic thyroid carcinoma cell lines (PMID: 25074669)	24
25	0.1 - 10 µM	MLN8237 (0.5-10 µM), ZM447439 (1 - 20 µM)	A field guide to Aurora kinase inhibitors: an oocyte perspective (PMID: 36125382)	0
26	200 nM	MLN8054 (250 nM) VX-680 (1000 nM)	Inhibition of Aurora B kinase sensitizes a subset of human glioma cells to TRAIL concomitant with induction of TRAIL-R2 (PMID: 19079141)	26
27	200 nM	None	CNOT3 interacts with the Aurora B and MAPK/ERK kinases to promote survival of differentiating mesendodermal progenitor cells (PMID: 34613789)	0
28	200 nM	VX680 (0.001-10 µM), MLN8054 (0.001-5 µM)	Bcl-XL represents a druggable molecular vulnerability dusing aurora B inhibitor-mediated polyploidization PMID: 20616035)	34
29	0.2 - 5 µM	MLN8237 (100 nM - 5 µM), LY3285668 (1 nM - 5 µM)	Aurora A-selective inhibitor LY3295668 leads to dominant mitotic arrest, apoptosis in cancer cells, and shows potent preclinical antitumour efficacy (PMID: 31530649)	21
30	250 nM - 1 µM	MLN8237 (5 nM - 2 µM)	Preclinical testing of selective Aurora kinase inhibitors on a medullary thyroid carcinoma-derived cell line (PMID: 26215279)	5
31	400 nM	None	Histone H3 phosphorylation in GBM: a new rational to guide the use of kinase inhibitors in anti-GBM therapy (PMID: 25553095)	21
32	500 nM - 10 µM	None	AURKB promotes gastric cancer progression via activation of CCND1 expression (PMID: 31982864)	24
33	0.5 - 50 µM	None	Paradoxical implication of BAX/BAK in the persistence of tetraploid cells (PMID: 34725331)	1
34	1 µM	None	Aurora kinase B regulates axonal outgrowth and regeneration in the spinal motor neurons of developing zebrafish (PMID: 29468257)	12
35	1 µM	VX680 (400 nM)	Phosphoproteomic analysis of Aurora kinase inhibition in monopolar cytokinesis (PMID: 26270265)	9
36	1 µM	None	Aurora B induces epithelial-mesenchymal transition by stabilizing Snail1 to promote basal-like breast cancer metastasis (PMID: 31996785)	7
37	1 µM	MLN8237, SNS-314, AT9283, VX680, PHA-739358, PHA-680632, CYC116, CCT129202, ZM447439, JNJ-7706621, ENMD-2076, Aurora A Inhibitor 1 (1 µM)	Aurora kinases as druggable targets in pediatric leukemia: Heterogeneity in target modulation activities and cytotoxicity by diverse novel therapeutic agents (PMID: 25048812)	13

		hesperidin (0.1 - 1 µM)		
38	3 - 7 µM	None	Phosphorylation of histone H3 on Ser-10 by Aurora B is essential for chromosome condensation in porcine embryos during the first mitotic division (PMID: 28220245)	5
39	5 µM	ZM447439, MLN8054 (both 5 µM)	Aurora B and C kinases regulate chromosome desynapsis and segregation during mouse and human spermatogenesis (PMID: 33172986)	13
40	12.5 - 100 µg/ml (21.3 - 170.3 µM)	GSK-1070916, Hesperadin	Leishmania donovani Aurora kinase: A promising therapeutic target against visceral leishmaniasis (PMID: 27288586)	12
41	Unclear	MLN8237 (unclear), ZM447439 (0.25 - 4 µM)	Evolution of resistance to Aurora kinase B inhibitors in leukemia cells (PMID: 22359551)	11
	AZD1152-HQPA	Orthogonal inhibitors	Title (PMID)	
42	0.05 nM - 500 nM	AMG900 (0.01 - 100 nM), hesperadin (1 nM - 10 µM)	A new imaging platform (iScreen) allows for the concurrent assessment of micronucleus induction and genotoxic mode of action in human A375 cells (PMID: 35703118)	0
43	0.1 - 100 nM	MLN8237 (0.1 - 100 nM)	Suv420 enrichment at the centromere limits Aurora B localization and function (PMID: 34342353)	3
44	0.3 nM - 150 nM	None	Aurora B kinase phosphorylates and instigates degradation of p53 (PMID: 22611192)	117
45	0.5 nM - 200 nM	MLN8054, VX-680, MLN8237, MK-5108, MK-8745, ZM447439, hesperadin, GSK1070916	A cell biologist's field guide to Aurora kinase inhibitors (PMID: 26732741)	55
46	0.5 nM - 500 nM	None	Effects of AZD1152, a selective Aurora B kinase inhibitor, on Burkitt's and Hodgkin's lymphomas (PMID: 21371446)	17
47	0.5 nM - 500 nM	Alisertib (0.5 - 500 nM)	Selective inhibition of Aurora A and B kinases effectively induces cell cycle arrest in t(8;21) acute myeloid leukemia (PMID: 31207577)	6
48	500 pM - 1 µM	AMG900, MK-5108 (0.5 - 1000 nM)	Preclinical evaluation of the Aurora kinase inhibitors AMG 900, AZD1152-HQPA, and MK-5108 on SW-872 and 93T449 human liposarcoma cells (PMID: 29197031)	5
49	0.74 nM	Aurora A Inhibitor (6.8 nM), hesperidin (500 nM), JNJ-7706621 (18 nM), GSK1070916 (13 nM), ZM447439 (260 nM), PHA-680632 (270 nM)	Screening of compounds to identify novel epigenetic regulatory factors that affect innate immune memory in macrophages (PMID: 35115604)	3
50	1 nM	None	Indomethacin promotes apoptosis in gastric cancer cells through concomitant degradation of Survivin and Aurora B kinase proteins (PMID: 24874838)	13
51	1 nM	MLN8237 (5 nM) VX680 (2.5 nM)	The Aurora-kinase A Phe31-Ile polymorphism as possible predictor of response to treatment in head and neck squamous cell carcinoma (PMID: 29560108)	6
52	1 - 10 nM	None	AZD1152, a novel and selective aurora B kinase inhibitor, induces growth arrest, apoptosis, and sensitization for tubulin depolymerizing agent or topoisomerase II inhibitor in human acute leukemia cells in vitro and in vivo (PMID: 17495131)	217
53	1 - 100 nM	None	The selective Aurora B kinase inhibitor AZD1152 as a novel treatment for hepatocellular carcinoma (PMID: 19913935)	53

54	1 nM - 125 nM	None	Significance of Aurora B overexpression in hepatocellular carcinoma. Aurora B Overexpression in HCC. (PMID: 20799978)	93
55	1 nM - 500 nM	None	The selective Aurora B kinase inhibitor AZD1152 is a potential new treatment for multiple myeloma (PMID: 18076711)	50
56	2 nM - 30 µM	MLN8237 (0.1 - 0.5 µM), MK-5108 (5 nM - 33 µM), GSK1070916, VX680, PHA-739358 (5 nM-11.1 µM)	Loss of Aurora kinase signaling allows lung cancer cells to adopt endoreplication and form polyploid giant cancer cells that resist antimitotic drugs (PMID: 33172929)	13
57	2 nM - 100 nM	None	Barasertib (AZD1152), a small molecule Aurora B inhibitor, inhibits the growth of SCLC cell lines in vitro and in vivo (PMID: 27496133)	57
58	3 - 100 nM	None	p53 is critical for the Aurora B kinase inhibitor-mediated apoptosis in acute myelogenous leukemia cells (PMID: 20013323)	24
59	3 nM - 30 µM	None	Aurora B kinase inhibitor AZD1152: determinants of action and ability to enhance chemotherapeutics effectiveness in pancreatic and colon cancer (PMID: 21304529)	49
60	4 nM - 5 µM	VX680, CYC116, ENMD-2076, MLN8054, PHA-739358 (all 0-5 µM)	A robust high-content imaging approach for probing the mechanism of action and phenotypic outcomes of cell-cycle modulators (PMID: 21216932)	24
61	5 - 10 nM	None	Aurora kinase B inhibitor barasertib (AZD1152) inhibits glucose metabolism in gastric cancer cells (PMID: 30540594)	7
62	5 - 100 nM	None	Inhibiting the Aurora B kinase potently suppresses repopulation during fractionated irradiation of human lung cancer cell lines (PMID: 22381900)	20
63	5 - 500 nM	None	AZD1152-HQPA induces growth arrest and apoptosis in androgen-dependent prostate cancer cell line (LNCaP) via producing aneugenic micronuclei and polyploidy (PMID: 25277659)	12
64	5 - 500 nM	None	Reactive oxygen species generation and increase in mitochondrial copy number: new insight into the potential mechanism of cytotoxicity induced by aurora kinase inhibitor, AZD1152-HQPA (PMID: 28639950)	14
65	6.25 - 100 nM	MK5108, ZM447439 (both 250 nM - 10 µM), MLN8237 (50 - 1 µM)	P53 deficiency enhances mitotic arrest and slippage induced by pharmacological inhibition of Aurora kinases (PMID: 23955083)	50
66	8 - 1000 nM	AMG900, MLN8237, CYC-116, SNS-314, PHA-739358, ENMD-984693, VX680, JNJ-7706621 (10 pM - 100 µM)	Aurora B kinase is a potent and selective target in MYCN-driven neuroblastoma (PMID: 26497213)	35
67	10 nM	None	Epithelial cell transforming 2 and Aurora kinase B modulate formation of stress granule-containing transcripts from diverse cellular pathways in astrocytoma cells (PMID: 27106762)	7
68	10 nM	VX680 (1 µM)	A mixed modality approach towards Xi reactivation for Rett syndrome and other X-linked disorders (PMID: 29282321)	47

69	10 - 20 nM	None	Aurora kinase inhibitor AZD1152 has an additional effect of platinum on a sequential application at the human ovarian cancer cell line SKOV3 (PMID: 23389245)	8
70	10 - 50 nM	None	Divergent polypharmacology-driven cellular activity of structurally similar multi-kinase inhibitors through cumulative effects on individual targets (PMID: 31257184)	10
71	10 - 50 nM	None	Inhibition of the pentose-phosphate pathway selectively sensitizes leukemia lymphocytes to chemotherapeutics by ROS-independent mechanism (PMID: 27793928)	11
72	10 - 50 nM	None	Resveratrol modulates the redox-status and cytotoxicity of anticancer drugs by sensitizing leukemic lymphocytes and protecting normal lymphocytes (PMID: 31262901)	14
73	10 - 50 nM	None	Synergistic cytotoxicity of melatonin and new-generation anticancer drugs against leukemia lymphocytes but not normal lymphocytes (PMID: 28011485)	27
74	10 - 50 nM	Everolimus (5 µM)	Effect of alpha-tocopheryl succinate on the cytotoxicity of anticancer drugs towards leukemia lymphocytes (PMID: 34969764)	0
75	10 - 100 nM	None	The Aurora B kinase inhibitor AZD1152 sensitizes cancer cells to fractionated irradiation and induces mitotic catastrophe (PMID: 19755861)	43
76	10 - 100 nM	None	The FLT3 internal tandem duplication mutation is a secondary target of the aurora B kinase inhibitor AZD1152-HQPA in acute myelogenous leukemia cells (PMID: 20159992)	32
77	10 - 100 nM	MK-5108 (250 nM)	Co-inhibition of polo-like kinase 1 and Aurora kinases promotes mitotic catastrophe (PMID: 25871386)	15
78	10 - 100 nM	None	Inhibition of Aurora-B suppresses osteosarcoma cell migration and invasion (PMID: 24520245)	21
79	10 - 100 nM	None	The topoisomerase I poison CPT-11 enhances the effect of the aurora B kinase inhibitor AZD1152 both in vitro and in vivo (PMID: 19276280)	31
80	10 & 100 nM	ZM447439 (10 - 1000 nM)	Effects of the aurora kinase inhibitors AZD1152-HQPA and ZM447439 on growth arrest and polyploidy in acute myeloid leukemia cell lines and primary blasts (PMID: 18367484)	79
81	10 - 300 nM	VX680 (10 - 200 nM), MLN8237 (10 - 50 nM)	Genome-wide CRISPR screen uncovers a synergistic effect of combining Haspin and Aurora kinase B inhibition (PMID: 32300176)	9
82	10 - 1000 nM	None	Inhibitor of Aurora kinase B induces differentially cell death and polyploidy via DNA damage response pathways in neurological malignancy: Shedding new light on the challenge of resistance to AZD1152-HQPA (PMID: 25752998)	17
83	10 - 5 µM	None	A peptide-based positron emission tomography probe for in vivo detection of caspase activity in apoptotic cells (PMID: 24573549)	13
84	10 - 5 µM	Tozasertib, VX-680, PHA-739358, PHA-680632, Aurora A Kinase, MLN-8054 (0.01-5 µM)	Phenotypic fingerprinting of small molecule cell cycle kinase inhibitors for drug discovery (PMID: 20161832)	16
85	12.5 nM	MLN8237 (62.5 nM)<br>MK-5108 (125 nM)	Salt-inducible kinase 3 is a novel mitotic regulator and a target for enhancing antimitotic therapeutic-mediated cell death (PMID: 24743732)	14
86	12.5 - 100 nM	None	Analysis of Aurora B kinase in non-Hodgkin lymphoma (PMID: 19823168)	33
87	15 - 25 nM	None	Aurora B inhibitor barasertib and cytarabine exert a greater-than-additive cytotoxicity in acute myeloid leukemia cells (PMID: 23557198)	21

88	16 nM	None	Relapse-associated AURKB blunts the glucocorticoid sensitivity of B cell acute lymphoblastic leukemia (PMID: 30733284)	21
89	20 - 105 nM	None	Antineoplastic effects of an Aurora B kinase inhibitor in breast cancer (PMID: 20175926)	72
90	20 - 400 nM	ZM447439 (1 µM)	Enhancement of radiation response in p53-deficient cancer cells by the Aurora-B kinase inhibitor AZD1152 (PMID: 18084327)	76
91	20 nM - 1 µM	ZM447439 (2 µM)	Mitotic slippage and the subsequent cell fates after inhibition of Aurora B during tubulin-binding agent-induced mitotic arrest (PMID: 29196757)	25
92	25 - 800 nM	None	Sequential treatment with aurora B inhibitors enhances cisplatin-mediated apoptosis via c-Myc (PMID: 25411027)	5
93	25 nM - 10 µM	None	Aurora kinase B/C inhibition impairs malignant glioma growth in vivo (PMID: 22382783)	30
94	30 & 300 nM	None	Aurora kinase B inhibition reduces the proliferation of metastatic melanoma cells and enhances the response to chemotherapy (PMID: 25623468)	33
95	42 nM - 1 µM	None	Differential ABC transporter expression during hematopoiesis contributes to neutrophil-biased toxicity of Aurora kinase inhibitors (PMID: 36224199)	0
96	50 nM	GSK6000063A (150 nM)	Pharmacological inhibition of aurora-A but not aurora-B impairs interphase microtubule dynamics. (PMID: 19395863)	16
97	50 nM	None	Selective targeting of cancerous mitochondria and suppression of tumor growth using redox-active treatment adjuvant (PMID: 33204397)	8
98	50 nM	Alisertib (200 nM)	Combined TRIP13 and aurora kinase inhibition induces apoptosis in human papillomavirus-driven cancers (PMID: 35972731)	1
99	50 - 200 nM	S49076 (0.35 µM)	AURKB as a target in non-small cell lung cancer with acquired resistance to anti-EGFR therapy (PMID: 31000705)	68
100	50 - 500 nM	None	Aurora B is regulated by the mitogen-activated protein kinase/extracellular signal-regulated kinase (MAPK/ERK) signaling pathway and is a valuable potential target in melanoma cells (PMID: 22767597)	60
101	100 nM	None	In vitro evaluation of a combination treatment involving anticancer agents and an aurora kinase B inhibitor (PMID: 27895801)	4
102	100 nM	None	Mechanism of action and therapeutic efficacy of Aurora kinase B inhibition in MYC overexpressing medulloblastoma (PMID: 25739120)	19
103	100 nM	None	Human genome-wide RNAi screen reveals host factors required for enterovirus 71 replication (PMID: 27748395)	33
104	100 nM (most assays)<br>50 - 200 nM	None	Aurora B is dispensable for megakaryocyte polyploidization, but contributes to the endomitotic process (PMID: 20548097)	35
105	100 & 1000 nM	None	AZD1152 rapidly and negatively affects the growth and survival of human acute myeloid leukemia cells in vitro and in vivo (PMID: 19366807)	61
106	0.12 - 6 µM	None	Inhibition of Aurora kinase B activity disrupts development and differentiation of salivary glands (PMID: 33462217)	3
107	125 nM - 2 µM	MLN8237 (0.125 - 2 µM)	Inhibition of BRD4 triggers cellular senescence through suppressing Aurora kinases in oesophageal cancer cells (PMID: 32954665)	3
108	150 - 250 nM	None	Aurora B prevents aneuploidy via MAD2 during the first mitotic cleavage in oxidatively damaged embryos (PMID: 31264311)	3
109	200 nM	MK5108 (5 µM)	MPS1 inhibition primes immunogenicity of KRAS-LKB1 mutant lung cancer (PMID: 36150391)	2

110	250 - 1000 nM	None	The potential contribution of microRNAs in anti-cancer effects of Aurora kinase inhibitor (AZD1152-HQPA) (PMID: 30051358)	3
111	300 nM	None	AZD1152, a selective inhibitor of Aurora B kinase, inhibits human tumor xenograft growth by inducing apoptosis (PMID: 17575233)	300
112	300 nM	VX680, MLN8237 (300 nM)	Recurrent 8q24 rearrangement in blastic plasmacytoid dendritic cell neoplasm: association with immunoblastoid cytomorphology, MYC expression, and drug response (PMID: 29795241)	24
112	300 nM	None	BRAFV600E induces reversible mitotic arrest in human melanocytes via microrna-mediated suppression of AURKB (PMID: 34812139)	8
113	300 nM - 1 µM	None	P-glycoprotein and breast cancer resistance protein in acute myeloid leukaemia cells treated with the aurora-B kinase inhibitor barasertib-hQPA (PMID: 21679421)	14
114	500 nM	None	Janus face-like effects of Aurora B inhibition: antitumoral mode of action versus induction of aneuploid progeny (PMID: 27515963)	6
115	0.5 - 1 µM	22 Aurora kinase inhibitors (all 1 µM)	Chemical genetic screens identify kinase inhibitor combinations that target anti-apoptopic proteins for cancer therapy (PMID: 29608269)	10
116	1 µM	MLN8237, VX680, hesperidin, PHA-680632, PHA-739358, SNS-314, CYC116, TAK-901, AMG900 (all 1 µM)	High-throughput compound screen reveals mTOR inhibitors as potential therapeutics to reduce (auto)antibody production by human plasma cells (PMID: 31621069)	9
117	1 µM	None	Cytofluorometric assessment of cell cycle progression (PMID: 23296653)	5
118	1 µM	ZM-447439, hesperidin, PHA-739358, PHA-680632 CCT129202, CYC116, VX680, Aurora A Inhibitor I, MLN8237 (all 1 µM)	A chemical screening approach to identify novel key erythroid enuclaeation (PMID: 26569102)	8
119	1 - 5 µM	VX680 (0 - 10 µM)	Canine osteosarcoma cells exhibit resistance to aurora kinase inhibitors (PMID: 23410058)	3
120	1 - 5 µM	None	Inhibition of aurora kinase B attenuates fibroblast activation and pulmonary fibrosis (PMID: 32761869)	14
121	1.6 µM - 250 µM	None	Effect of the drug transporters ABCG2, Abcg2, ABCB1 and ABCC2 on the disposition, brain accumulation and myelotoxicity of the aurora kinase B inhibitor barasertib and its more active form barasertib-hydroxy-QPA (PMID: 23315030)	19
122	2 - 4 µM	None	Aurora B inhibitor barasertib prevents meiotic maturation and subsequent embryo development in pig oocytes (PMID: 26993175)	9
123	5 µM	AMG 900 (1 - 100 nM) MLN8054 (5 µM)	Dual targeting of Aurora kinases with AMG 900 exhibits potent preclinical activity against acute myeloid leukemia with distinct post-mitotic outcomes (PMID: 30266802)	12
124	5 µM	None	ALKBH5-mediated m6A-demethylation of USP1 regulated T-cell acute lymphoblastic leukemia cell glucocorticoid resistance by Aurora B (PMID: 34169564)	11
125	Not provided	ZM447439	Hypersensitivity to aurora kinase inhibitors in cells resistant against platinum- containing anticancer agents (PMID: 24521151)	9

126	Not provided	None	PLK1 and AURKB phosphorylate surviving differentially to affect proliferation in racially distinct triple-negative breast cancer (PMID: 36627281)	0
127	Unclear	None	Low-dose-rate irradiation suppresses the expression of cell-cycle-related genes, resulting in modification of sensitivity to anti-cancer drugs (PMID: 35159310)	1
128	Unclear	VX680, MLN8237 (Conc unclear)	Nf1-mutant tumors undergo transcriptome and kinome remodeling after inhibition of either mTOR or MEK (PMID: 32847978)	1
Compliance				
**55 (43%)**[a] **33 (26%)**[b]		50 (39%)		
Non-Compliance				
**40 (31%)**		78 (61%)		

a *Probe's concentration below the recommended in-cell maximum in all figures.*
b *Probe's concentration below the recommended in-cell maximum in some, but not all figures.*

Supplementary Note 7. Citations for AZD1152 publications included in the systematic review.
Citations for 128 publications using AZD1152/AZD1152-HQPA: 3,189

Citations for 88 publications with a compliant AZD1152/AZD1152-HQPA concentration: 2,507
Citations for 40 publications with a non-compliant AZD1152/AZD1152-HQPA concentration: 682

Citations for 50 publications using orthogonal Aurora kinase inhibitors: 935
Citations for 78 publications not using orthogonal Aurora kinase inhibitors: 2,254

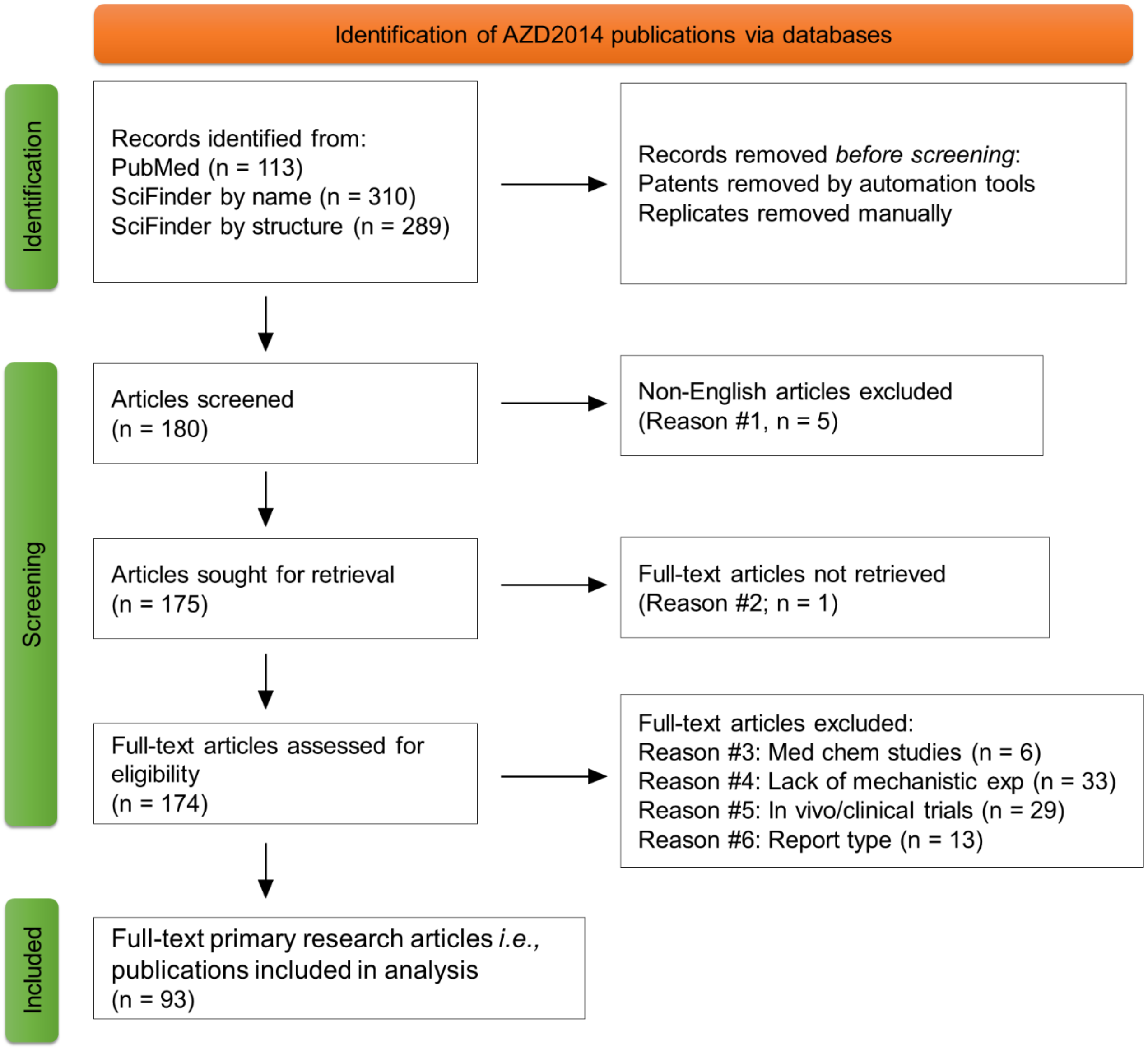

Supplementary Figure 11. PRISMA flow diagram summarising identification of publications using mTOR chemical probe AZD2014.

Supplementary Table 13. List of excluded articles using the mTOR probe AZD2014.

	Title (PMID)	Reason for exclusion
1	The dual mTORC1/2 inhibitor AZD2014 inhibits acute graft rejection in a rat liver transplantation model (PMID: 35527497)	1) Non-English publication
2	Antitumor effects of AZD2014, a dual mTORC1/2 inhibitor, against human hepatocellular carcinoma xenograft in nude mice (PMID: 34308856)	1) Non-English publication
3	Effect of mTOR inhibitor AZD2014 on proliferation of hepatoma HCC cells (No PMID; DOI: 10.3969/j.issn.1004-616x.2020.06.007)	1) Non-English publication
4	Dual mTORC1/2 inhibitor AZD2014 inhibits proliferation of HCCLM3 cells through induction of autophagy (No PMID or DOI; ISSN: 0577-7402)	1) Non-English publication
5	Optimization of synthesis process of 2-amino-6-chioronatenic acidnic acid (No PMID or DOI; ISSN: 1007-1865)	1) Non-English publication
6	Combination vistusertib and anastrozole shows benefit in endometrial cancer (PMID: 35593584)	2) No access to full article
7	Optimization of potent and selective dual mTORC1 and mTORC2 inhibitors: The discovery of AZD8055 and AZD2014 (PMID: 23375793)	3) Discovery of AZD2014
8	Design and development of tetrahydro-quinoline derivatives as dual mTOR-C1/C2 inhibitors for the treatment of lung cancer (PMID: 33280832)	3) Medicinal chemistry
9	Practical synthesis of Vistusertib (AZD2014), an ATP competitive mTOR inhibitor (no PMID; DOI: 10.1016/j.tetlet.2019.151333)	3) Medicinal chemistry
10	A conformational restriction strategy for the identification of a highly selective pyrimido-pyrrolooxazine mTOR inhibitor (PMID: 31465220)	3) Medicinal chemistry
11	4-(Difluoromethyl)-5-(4-((3*R*,5*S*)-3,5-dimethylmorpholino)-6-((*R*)-3-methylmorpholino)-1,3,5-triazin-2-yl)pyridin-2-amine (PQR626), a potent, orally available, and brain-penetrant mTOR inhibitor for the treatment of neurological disorders (PMID: 33166139)	3) Medicinal chemistry
12	Design, synthesis and antitumor evaluation of novel quinazoline analogs in hepatocellular carcinoma cell: Molecular docking study (PMID: 20599299)	3) Medicinal chemistry
13	3D Cell-based high-content screening (HCS) using a micropillar and microwell chip platform (PMID: 29889500)	4) Analytical chemistry/no mechanistic exp.
14	Discovery of novel autophagy inhibitors and their sensitization abilities for vincristine-resistant esophageal cancer cell line Eca109/VCR (PMID: 32207878)	4) No mechanistic exp.
15	Chemical proteomics reveals ferrochelatase as a common off-target of kinase inhibitors (PMID: 26863403)	4) No mechanistic exp.
16	Exploiting evolutionary steering to induce collateral drug sensitivity in cancer (PMID: 32317663)	4) AZD2014 not used
17	CC-223 inhibits human head and neck squamous cell carcinoma cell growth (PMID: 29402408)	4) AZD2014 not used
18	RAPTOR up-regulation contributes to resistance of renal cancer cells to PI3K-mTOR inhibition (PMID: 29389967)	4) Dose response only
19	Drug repurposing for paracoccidioidomycosis through a computational chemogenomics framework (PMID: 31244810)	4) No mechanistic exp.
20	Validation of a rapid liquid chromatography tandem mass spectrometric method for the quantitative analysis of vistusertib (PMID: 34735991)	4) No mechanistic exp. - Methodology study
21	Rituximab (anti-CD20)-modified AZD-2014-encapsulated nanoparticles killing of B lymphoma cells (PMID: 30198340)	4) No mechanistic exp. - Nano-carrier study
22	Synergistic drug combinations and machine learning for drug repurposing in chordoma (PMID: 32737414)	4) No *in vitro* experiments
23	Noninvasive MRI native T1 mapping detects response to MYCN-targeted therapies in the Th-MYCN model of neuroblastoma (PMID: 32595135)	4) No *in vitro* experiments
24	Identification of selective cytotoxic and synthetic lethal drug responses in triple negative breast cancer cells (PMID: 27165605)	4) No mechanistic exp. - cell viability screen
25	Palbociclib-based high-throughput combination drug screening identifies synergistic therapeutic options in HPV-negative head and neck squamous cell carcinoma (PMID: 35546399)	4) No mechanistic exp. using AZD2014
26	Ponatinib, lestaurtinib, and mTOR/PI3K inhibitors are promising repurposing candidates against Entamoeba histolytica (PMID: 34871094)	4) No mechanistic exp. using AZD2014
27	Sensitivity to PI3K and AKT inhibitors is mediated by divergent molecular mechanisms in subtypes of DLBCL (PMID: 28202458)	4) No mechanistic exp. using AZD2014

28	Drug repurposing screening identifies bortezomib and panobinostat as drugs targeting cancer associated fibroblasts (CAFs) by synergistic induction of apoptosis (PMID: 29349597)	4) No mechanistic exp. using AZD2014
29	High-throughput dynamic BH3 profiling may quickly and accurately predict effective therapies in solid tumors (PMID: 32546544)	4) No mechanistic exp. using AZD2014
30	Selective colony area method for heterogeneous patient-derived tumor cell lines in anti-cancer drug screening system (PMID: 30995234)	4) No mechanistic exp. using AZD2014
31	Drug efficacy comparison of 3D forming and preforming sphere models with a micropillar and microwell chip platform (PMID: 30753787)	4) No mechanistic exp. using AZD2014
32	Drug-dependent morphological transitions in spherical and worm-like polymeric micelles define stability and pharmacological performance of micellar drugs (PMID: 34841670)	4) No mechanistic exp. - micelle drug delivery system
33	Identification and analysis of DNA methylation-driven signatures for prognostic and immune<br>microenvironments evaluation in hepatocellular carcinoma (PMID: 36299585)	4) No mechanistic exp. using AZD2014
34	JAK1/2 and BCL2 inhibitors synergize to counteract bone marrow stromal cell-induced protection of AML (PMID: 28619982)	4) No mechanistic exp. using AZD2014
35	Enhanced MAPK signaling drives ETS1-mediated induction of miR-29b leading to downregulation of TET1 and changes in epigenetic modifications in a subset of lung SCC (PMID: 26776158)	4) No mechanistic exp. using AZD2014
36	Molecular taxonomy of systemic lupus erythematosus through data-driven patient stratification: molecular endotypes and cluster-tailored drugs (No PMID: DOI: 10.3389/fimmu.2022.860726)	4) No mechanistic exp. using AZD2014
37	Machine learning models for the prediction of chemotherapy-induced peripheral neuropathy (PMID: 30617559)	4) No mechanistic exp. using AZD2014
38	Extra-virgin olive oil contains a metabolo-epigenetic inhibitor of cancer stem cells (PMID: 29452350)	4) No mechanistic exp. using AZD2014
39	Reconciling selectivity trends from a comprehensive kinase inhibitor profiling campaign with known activity data (PMID: 30023860)	4) No mechanistic exp. using AZD2014
40	Ex vivo modelling of drug efficacy in a rare metastatic urachal carcinoma (PMID: 32576176)	4) No mechanistic exp. using AZD2014
41	Sustained mTORC1 activity during palbociclib-induced growth arrest triggers senescence in ER+ breast cancer cells (PMID: 33356791)	4) No mechanistic exp. using AZD2014
42	Context-dependent intravital imaging of therapeutic response using intramolecular FRET biosensors (PMID: 28435000)	4) No mechanistic exp. using AZD2014
43	Establishment and characterization of 18 human colorectal cancer cell lines (PMID: 32321971)	4) No mechanistic exp. using AZD2014
44	A cell-based high-throughput screen identifies inhibitors that overcome P-glycoprotein (Pgp)-mediated multidrug resistance (PMID: 32484843)	4) No mechanistic exp. using AZD2014
45	Pre-clinical activity of combined LSD1 and mTORC1 inhibition in MLL-translocated acute myeloid leukaemia (PMID: 31780813)	4) No mechanistic exp. using AZD2014
46	AKT/mTORC2 inhibition activates FOXO1 function in CLL cells reducing B-cell receptor-mediated survival (PMID: 30559170)	5) AZD2014 used *in vivo*
47	Safety and efficacy of the mTOR inhibitor, vistusertib, combined with anastrozole in patients with hormone receptor-positive recurrent or metastatic endometrial cancer (PMID: 35551299)	5) Clinical publication
48	Comprehensive genome profiling in patients with metastatic non-small cell lung cancer: the precision medicine phase 2 randomized SAFIR02-Lung trial (PMID: 35802649)	5) Clinical study
49	A phase I study investigating AZD8186, a potent and selective inhibitor of PI3Kβ/δ, in patients with advanced solid tumors (PMID: 35247924)	5) Clinical study
50	First-in-child phase I/II study of the dual mTORC1/2 inhibitor vistusertib (AZD2014) as monotherapy and in combination with topotecan-temozolomide in children with advanced malignancies: arms E and F of the AcSé-ESMART trial (PMID: 34543871)	5) Clinical study
51	A phase 1/2 study of the combination of acalabrutinib and vistusertib in patients with relapsed/refractory B-cell malignancies (PMID: 34269152)	5) Clinical study
52	An adaptive, biomarker-directed platform study of durvalumab in combination with targeted therapies in advanced urothelial cancer (PMID: 33941921)	5) Clinical study
53	Prospective experimental treatment of colorectal cancer patients based on organoid drug responses (PMID: 33887686)	5) Clinical study

54	Biomarker-driven phase 2 umbrella trial study for patients with recurrent small cell lung cancer failing platinum-based chemotherapy (PMID: 32584426)	5) Clinical study
55	A phase I study of vistusertib (dual mTORC1/2 inhibitor) in patients with previously treated glioblastoma multiforme: a CCTG study (PMID: 31707687)	5) Clinical study
56	Fulvestrant plus vistusertib vs fulvestrant plus everolimus vs fulvestrant alone for women with hormone receptor-positive metastatic breast cancer: The MANTA phase 2 randomized clinical trial (PMID: 31465093)	5) Clinical study
57	A phase II study to assess the safety and efficacy of the dual mTORC1/2 inhibitor vistusertib in relapsed, refractory DLBCL (PMID: 31385336)	5) Clinical study
58	Tumor genomic profiling guides patients with metastatic gastric cancer to targeted treatment: The VIKTORY umbrella trial (PMID: 31315834)	5) Clinical study
59	Phase I study of orally administered 14 Carbon-isotope labelled-vistusertib (AZD2014), a dual TORC1/2 kinase inhibitor, to assess the absorption, metabolism, excretion, and pharmacokinetics in patients with advanced solid malignancies (PMID: 30758651)	5) Clinical study
60	Vistusertib (dual m-TORC1/2 inhibitor) in combination with paclitaxel in patients with high-grade serous ovarian and squamous non-small-cell lung cancer (PMID: 30016392)	5) Clinical study
61	Rapamycin-insensitive companion of mTOR (RICTOR) amplification defines a subset of advanced gastric cancer and is sensitive to AZD2014-mediated mTORC1/2 inhibition (PMID: 28028034)	5) Clinical study
62	A randomised phase 2 study of AZD2014 versus everolimus in patients with VEGF-refractory metastatic clear cell renal cancer (PMID: 26364551)	5) Clinical study
63	A Phase 1 study of ADI-PEG20 (pegargiminase) combined with cisplatin and pemetrexed in ASS1- negative metastatic uveal melanoma (PMID: 35466524)	5) Clinical study
64	Ribosome biogenesis-based predictive biomarkers in endocrine therapy (Anastrozole) combined with mTOR inhibitor (Vistusertib) in endometrial cancer: translational study from the VICTORIA trial in collaboration with the GINECO group (PMID: 36370117)	5) Clinical study
65	Aberrant cholesterol metabolic signaling impairs antitumor immunosurveillance through natural killer T cell dysfunction in obese liver (PMID: 35595819)	5) In vivo study
66	Quantitation of vistusertib by UHPLC-MS/MS in rat plasma and its application to a pharmacokinetic study (PMID: 34470474)	5) In vivo study
67	The mTORC1 inhibitor rapamycin and the mTORC1/2 inhibitor AZD2014 impair the consolidation and persistence of contextual fear memory (PMID: 32601986)	*5) In vivo* study
68	Combined mTOR/MEK inhibition prevents proliferation and induces apoptosis in NF2-mutant tumors (PMID: 31298338)	5) *In vivo* study
69	Allosteric and ATP-competitive inhibitors of mTOR effectively suppress tumor progression-associated epithelial-mesenchymal transition in the kidneys of Tsc2+/− mice (PMID: 31207499)	5) *In vivo* study
70	Comparative toxicity evaluation of targeted anticancer therapeutics in embryonic zebrafish and sea urchin models (PMID: 30587022)	5) *In vivo* study
71	Combination of dual mTORC1/2 inhibition and immune-checkpoint blockade potentiates anti-tumour immunity (PMID: 30221055)	5) *In vivo* study
72	Selumetinib-based therapy in uveal melanoma patient-derived xenografts (PMID: 29774094)	5) *In vivo* study
73	In vivo E2F reporting reveals efficacious schedules of MEK1/2-CDK4/6 targeting and mTOR-S6 resistance mechanisms (PMID: 29496664)	5) *In vivo* study
74	First-in-human pharmacokinetic and pharmacodynamic study of the dual m-TORC 1/2 inhibitor, AZD2014 (PMID: 25805799)	5) *In vivo* study
75	Mastering the complex targeted therapy for non-small cell lung cancer (PMID: 32857949)	6) Commentary
76	Learning from BISCAY: The future of biomarker-based trial design in bladder cancer (PMID: 34256907)	6) Commentary
77	Correction to: Combination of mTORC1/2 inhibitor vistusertib plus fulvestrant in vitro and in vivo targets oestrogen receptor-positive endocrine-resistant breast cancer (PMID: 32005287)	6) Correction
78	Overcoming resistance to mTOR inhibition for enhanced strategies in clinical trials (PMID: 23631743)	6) Editorial piece
79	MEDI-573, alone or in combination with mammalian target of rapamycin inhibitors, targets the insulin-like growth factor pathway in sarcomas [Erratum to document cited in CA162:419021] (PMID: 26039569)	6) Erratum

80	One size doesn't fit all in Ph-like ALL (PMID: 28082291)	6) Letter
81	mTOR signaling and potential therapeutic targeting in meningioma (PMID: 35216092)	6) Review
82	Evolution of PIKK family kinase inhibitors: A new age cancer therapeutics (PMID: 32114443)	6) Review
83	Combined strategies with poly-(ADP-Ribose)-polymerase (PARP) inhibitors for the treatment of ovarian cancer: A literature review (PMID: 31374917)	6) Review
84	mTOR deregulation in oral cavity squamous cell carcinoma (PMID: 28730764)	6) Review
85	Ibrutinib inhibition of Bruton protein-tyrosine kinase (BTK) in the treatment of B cell neoplasms (PMID: 27641927)	6) Review
86	Catalytic mammalian target of rapamycin inhibitors as antineoplastic agents (PMID: 25747970)	6) Review
87	Time now to TORC the TORC? New developments in mTOR pathway inhibition in lymphoid malignancies (PMID: 24842496)	6) Review

Supplementary Table 14. Overview of eligible publications using the mTOR probe AZD2014 and compliance (**in blue**) with recommendations to use AZD2014 up to 2 µM (https://www.chemicalprobes.org/azd2014?q=AZD2014) and orthogonal mTOR inhibitors. Citations are sourced from SciFinder (January 2023).

	AZD2014	Orthogonal inhibitors	Title (PMID)	Cites
1	0.1 - 100 nM	AZD8055, INK128 (0.1 - 100 nM)	RICTOR amplification identifies a subgroup in small cell lung cancer and predicts response to drugs targeting mTOR (PMID: 27863413)	34
2	1 - 100 nM	None	Inhibition of Bcl-2 potentiates AZD-2014-induced anti-head and neck squamous cell carcinoma cell activity (PMID: 27343560)	10
3	1 - 100 nM	None	Dramatic suppression of colorectal cancer cell growth by the dual mTORC1 and mTORC2 inhibitor AZD-2014 (PMID: 24309100)	42
4	1 nM - 1 µM	INK-128, rapamycin, torin1 (1 nM - 1 µM), everolimus (1 µM)	HSP90 inhibitors induce GPNMB cell-surface expression by modulating lysosomal positioning and sensitize breast cancer cells to glembatumumab vedotin (PMID: 35110681)	1
5	2.5 - 500 nM	AZD8055 (250 nM), Torin2, rapamycin (both 500 nM)	Antiviral drug screen identifies DNA-damage response inhibitor as potent blocker of SARS-CoV-2 replication (PMID: 33784499)	43
6	3 - 300 nM	Rapamycin (20 nM - 1 µM)	A high-throughput kinome screen reveals serum/glucocorticoid-regulated kinase 1 as a therapeutic target for NF2-deficient meningiomas (PMID: 26219339)	37
7	3 nM - 1 µM	Everolimus (1 - 500 nM)	AZD2014, an inhibitor of mTORC1 and mTORC2, is highly effective in ER+ breast cancer when administered using intermittent or continuous schedules (PMID: 26358751)	90
8	4 - 250 nM	Everolimus (0 - 10 µM), AZD8055 (0 - 100 nM), BEZ235 (0 - 100 nM)	A genome-scale CRISPR screen identifies the ERBB and mTOR signaling networks as key determinants of response to PI3K inhibition in pancreatic cancer (PMID: 32371585)	8
9	10 nM	None	mTOR inhibition sensitizes human hepatocellular carcinoma cells to resminostat (PMID: 27311860)	9
10	10 nM	Everolimus (0.1 - 100 nM), AZD8055 (10 nM), KU-0063794 (100 nM)	Potentiation of growth inhibitory responses of the mTOR inhibitor everolimus by dual mTORC1/2 inhibitors in cultured breast cancer cell lines (PMID: 26148118)	28
11	10 nM	Rapamycin (10 ng/mL)	Rapamycin induces mitogen-activated protein (MAP) kinase phosphatase-1 (MKP-1) expression through activation of protein kinase B and mitogen-activated protein kinase kinase pathways (PMID: 24126911)	43
12	10 -100 nM	Rapamycin (100 nM)	AZD2014, a dual mTOR inhibitor, attenuates cardiac hypertrophy in vitro and in vivo (PMID: 34674743)	0
13	10 nM - 1 µM	Rapamycin (10 nM - 1 µM)	Potent antitumour of the mTORC1/2 dual inhibitor AZD2014 in docetaxel-sensitive and docetaxel-resistant castration-resistant prostate cancer cells (PMID: 33507584)	7
14	10 nM - 1 µM	Rapamycin (10 nM - 1 µM)	Dramatic antitumor effects of the dual mTORC1 and mTORC2 inhibitor AZD2014 in hepatocellular carcinoma (PMID: 25628925)	44
15	10 nM - 1 µM	Everolimus, NVP-BEZ235, rapamycin (0.1 nM - 1 µM), OSI-027 (10 nM - 1 µM)	Long-term acquired everolimus resistance in pancreatic neuroendocrine tumours can be overcome with novel PI3K-AKT-mTOR inhibitors (PMID: 26978006)	57
16	20 - 500 nM	None	18 F-meta-fluorobenzylguanidine (18 F-mFBG) to monitor changes in norepinephrine transporter expression in response to therapeutic intervention in neuroblastoma models (PMID: 33262374)	3
17	20 - 500 nM	None	F-metafluorobenzylguanidine (18F-mFBG) to monitor changes in norepinephrine transporter expression in response to therapeutic intervention in neuroblastoma models (PMID: 33262374)	3

18	25 nM	Rapamycin, everolimus, temsirolimus (1 - 5 µM)	SARS-CoV-2 infection rewires host cell metabolism and is potentially susceptible to mTORC1 inhibition (PMID: 33767183)	56
19	25 nM - 1 µM	None	An mTORC1/2 dual inhibitor, AZD2014, acts as a lysosomal function activator and enhances gemtuzumab ozogamicin-induced apoptosis in primary human leukemia cells (PMID: 31286402)	12
20	30 - 300 nM	None	Intermittent high-dose scheduling of AZD8835, a novel selective inhibitor of PI3Kα and PI3Kδ, demonstrates treatment strategies for PIK3CA-dependent breast cancers (PMID: 26839307)	34
21	50 - 100 nM	Rapamycin, everolimus (10 - 250 nM)	Pre-clinical evaluation of AZD-2014, a novel mTORC1/2 dual inhibitor, against renal cell carcinoma (PMID: 25444920)	90
22	50 - 500 nM	Rapamycin, AZD8055 (50 - 500 nM)	Synergistic anti-tumor effect of mTOR inhibitors with irinotecan on colon cancer cells (PMID: 31627299)	15
23	50 - 500 nM	None	Targeting mantle cell lymphoma metabolism and survival through simultaneous blockade of mTOR and nuclear transporter exportin-1 (PMID: 28388555)	6
24	50 nM - 1 µM	RAD001 (50 nM - 1 µM)	mTORC1/2 inhibitor served as a more ideal agent against the growth of mouse lymphocytic leukemia both in vitro and in vivo (PMID: 31519585)	3
25	50 nM - 10 µM	None	mTORC2 signaling drives the development and progression of pancreatic cancer (PMID: 27758884)	51
26	100 nM	None	Combination of mTORC1/2 inhibitor vistusertib plus fulvestrant in vitro and in vivo targets oestrogen receptor-positive endocrine-resistant breast cancer (PMID: 31801615)	5
27	100 nM	None	Targeting autophagy to modulate cell survival: a comparative analysis in cancer, normal and embryonic cells (PMID: 28664293)	10
28	100 nM	None	AZD2014 radiosensitizes oral squamous cell carcinoma by inhibiting AKT/mTOR axis and inducing G1/G2/M cell cycle arrest (PMID: 27031247)	15
29	100 nM	AZD8055 (100 nM)	Kinome RNAi screens reveal synergistic targeting of mTOR and FGFR1 pathways for treatment of lung cancer and HNSCC (PMID: 26359452)	46
30	100 nM	Everolimus (100 nM), rapamycin (25 nM), WYE-125132 (100 nM)	Screening reveals sterol derivatives with pro-differentiation, pro-survival, or potent cytotoxic effects on oligodendrocyte progenitor cells (PMID: 34232635)	3
31	100 nM	None	An mTOR and DNA-PK dual inhibitor CC-115 hinders non-small cell lung cancer cell growth (PMID: 35717530)	1
32	100 nM	None	mTOR inhibition amplifies the anti-lymphoma effect of PI3Kβ/δ blockage in diffuse large B-cell lymphoma (PMID: 36352190)	0
33	100 nM	Everolimus (1 µM)	Methuosis contributes to Jaspine-B-induced cell death (PMID: 35806262)	0
34	100 nM	Rapamycin (100 nM)	ADCK1 is a potential therapeutic target of osteosarcoma (PMID: 36371387)	0
35	100 nM	Everolimus, temsirolimus, rapamycin, OSI-027, INK-128, voxtalisib, palomid 529, VS-5584, gedatolisib, ICSN3250 (all 100 nM)	mTOR inhibition via displacement of phosphatidic acid induces enhanced cytotoxicity specifically in cancer cells (PMID: 30054335)	11
36	100 - 300 nM	None	Potential of the dual mTOR kinase inhibitor AZD2014 to overcome paclitaxel resistance in anaplastic thyroid carcinoma (PMID: 29790111)	13
37	100 - 800 nM	AZD8055 (50 - 400 nM)	Inducing and exploiting vulnerabilities for the treatment of liver cancer (PMID: 31578521)	148

38	100 - 1000 nM	None	Combined inhibition of mTOR and CDK4/6 is required for optimal blockade of E2F function and long term growth inhibition in estrogen receptor positive breast cancer (PMID: 29483206)	90
39	0.1 µM - 5 µM	None	Dual mTORC1/2 inhibitor AZD2014 diminishes myeloid-derived suppressor cells accumulation in ovarian cancer and delays tumor growth (PMID: 34560229)	2
40	0.1 - 10 µM	None	Landscapes of cellular phenotypic diversity in breast cancer xenografts and their impact on drug response (PMID: 33790302)	16
41	0.1 - 10 µM	Rapamycin (10 - 100 nM)	Investigation of the novel mTOR inhibitor AZD2014 in neuronal ischemia (PMID: 31100427)	5
42	0.125 - 1 µM	None	The novel combination of dual mTOR inhibitor AZD2014 and pan-PIM inhibitor AZD1208 inhibits growth in acute myeloid leukemia via HSF pathway suppression (PMID: 26473447)	28
43	200 nM	Rapamycin (200 nM)	Inhibition of mTORC1/C2 signaling improves anti-leukemia efficacy of JAK/STAT blockade in CRLF2 rearranged and/or JAK driven Philadelphia chromosome-like acute B-cell lymphoblastic leukemia (PMID: 29487712)	27
44	200 nM	OSI-027, AZD-8055 (200 nM)	MHY1485 ameliorates UV-induced skin cell damages via activating mTOR-Nrf2 signaling (PMID: 28061443)	8
45	200 nM	None	Synergistic induction of apoptosis by combination of BTK and dual mTORC1/2 inhibitors in diffuse large B cell lymphoma (PMID: 24970801)	39
46	200 nM	Torin1 (500 nM)	SHMT2 inhibition disrupts the TCF3 transcriptional survival program in Burkitt lymphoma (PMID: 34624079)	7
47	200 nM	BEZ235 (200 nM), rapamycin (100 nM), torin1 & MLN-0128 (200 nM)	PIK3CA mutations enable targeting of a breast tumor dependency through mTOR-mediated MCL-1 translation (PMID: 27974663)	36
48	200 - 400 nM	Rapamycin (200 nM)	Mechanisms underlying synergy between DNA topoisomerase I-targeted drugs and mTOR kinase inhibitors in NF1-associated malignant peripheral nerve sheath tumors (PMID: 31444410)	12
49	200 - 500 nM	Everolimus (100 - 400 nM), sapanisertib (100 - 400 nM)	Impact of baseline culture conditions of cancer organoids when determining therapeutic response and tumor heterogeneity (PMID: 35338174)	3
50	250 nM	None	Identification of differential PI3K pathway target dependencies in T-cell acute lymphoblastic leukemia through a large cancer cell panel screen (PMID: 26989080)	16
51	250 nM	Everolimus (100 nM)	Transforming growth factor-β (TGF-β) induces the expression of chondrogenesis-related genes through TGF-β receptor II (TGFRII)-AKT-mTOR signaling in primary cultured mouse precartilaginous stem cells (PMID: 24946212)	18
52	250 nM	None	Chemo-protective and regenerative effects of diarylheptanoids from the bark of black alder (Alnus glutinosa) in human normal keratinocytes (PMID: 26162555)	13
53	300 nM	Rapamycin (20 nM)	mTOR kinase inhibition disrupts neuregulin 1-ERBB3 autocrine signaling and sensitizes NF2-deficient meningioma cellular models to IGF1R inhibition (PMID: 33273014)	1
54	300 nM	None	Combination therapy with mTOR kinase inhibitor and dasatinib as a novel therapeutic strategy for vestibular schwannoma (PMID: 32144278)	8
55	300 nM	Rapamycin (20 nM)	EPH receptor signaling as a novel therapeutic target in NF2-deficient meningioma (PMID: 29982664)	14
56	300 nM	Everolimus (0 - 50 µM)	Diverse resistance mechanisms to the third-generation ALK inhibitor lorlatinib in alk-rearranged lung cancer (PMID: 31585938)	63
57	0.316 µM - 10 µM	Everolimus (50 nM - 5 µM)	Preclinical analysis of MTOR complex 1/2 inhibition in diffuse intrinsic pontine glioma (PMID: 29207163)	16
58	450 nM - 10 µM	BEZ235 (1.13 - 10 µM)	Implementation of a multiplex and quantitative proteomics platform for assessing protein lysates using DNA-barcoded antibodies (PMID: 29531020)	13
59	500 nM	Rapamycin (500 nM)	Therapeutic enhancement of verteporfin-mediated photodynamic therapy by mTOR inhibitors (PMID: 31769520)	3

60	500 nM	AZD8186 (250 nM)	Combined inhibition of PI3Kβ and mTOR inhibits growth of PTEN-null tumors (PMID: 30097489)	10
61	500 nM	Rapamycin (100 nM)	Intravital imaging to monitor therapeutic response in moving hypoxic regions resistant to PI3K pathway targeting in pancreatic cancer (PMID: 29898401)	50
62	500 nM	Rapamycin (30 nM)	MEDI-573, alone or in combination with mammalian target of rapamycin inhibitors, targets the insulin-like growth factor pathway in sarcomas (PMID: 25193511)	18
63	500 nM	Everolimus, LY294002 (500 nM)	Keratinocyte growth factor protects endometrial cells from oxygen glucose deprivation/re-oxygenation via activating Nrf2 signaling (PMID: 29709474)	22
64	500 nM - 1 µM	Rapamycin (20 nM), torin1 (250 nM), rapalink-1 (5 nM)	Reciprocal effects of mTOR inhibitors on pro-survival proteins dictate therapeutic responses in tuberous sclerosis complex (PMID: 36388985)	0
65	0.5 - 1 µM	None	Evaluation of the combination of the dual m-TORC1/2 inhibitor vistusertib (AZD2014) and paclitaxel in ovarian cancer models (PMID: 29371953)	15
66	0.5 - 1 µM	None	FBW7 suppression leads to SOX9 stabilization and increased malignancy in medulloblastoma (PMID: 27625374)	39
67	0.5 - 2 µM	Rapamycin (11.9 nM)	Influence of the novel ATP-competitive dual mTORC1/2 inhibitor AZD2014 on immune cell populations and heart allograft rejection (PMID: 28885497)	10
68	0.8 µM or 3.6 µM (unclear)	AZD8055, WYE-125132 (0.8 µM or 3.6 µM)	Pyrimidine inhibitors synergize with nucleoside analogues to block SARS-CoV-2 (PMID: 35130559)	45
69	1 µM	None	Inhibiting mTOR activity using AZD2014 increases autophagy in the mouse cerebral cortex (PMID: 33794244)	5
70	1 µM	BEZ235 (0.5 µM)	Synergistic effects of combination therapy with AKT and mTOR inhibitors on bladder cancer cells (PMID: 32325639)	6
71	1 µM	None	Enhanced autocrine FGF19/FGFR4 signaling drives the progression of lung squamous cell carcinoma, which responds to mTOR inhibitor AZD2104 (PMID: 32111983)	14
72	1 µM	Everolimus, rapamycin (1 - 5 µM)	Repurposing of mTOR complex inhibitors attenuates MCL-1 and sensitizes to PARP inhibition (PMID: 30201826)	11
73	1 µM	None	BEZ235 (PIK3/mTOR inhibitor) overcomes pazopanib resistance in patient-derived refractory soft tissue sarcoma cells (PMID: 27267837)	9
74	1 µM	Rapamycin (10 nM)	Phosphorylated ribosomal S6 (p-rpS6) as a post-treatment indicator of HER2 signalling targeted drug resistance (PMID: 26329528)	7
75	1 µM	Torin1 (250 nM), rapamycin (50 nM)	Macropinocytosis renders a subset of pancreatic tumor cells resistant to mTOR inhibition (PMID: 32101748)	16
76	1 µM	None	High-risk neuroblastoma with NF1 loss of function is targetable using SHP2 inhibition (PMID: 35905710)	1
77	1 µM	GDC0980 (10 µM)	Colon cancer organoids using monoclonal organoids established in four different lesions of one cancer patient reveal tumor heterogeneity and different real-time responsiveness to anti-cancer drugs (PMID: 35691158)	1
78	1 µM	Rapamycin (100 nM)	Dual blockade of the PI3K/Akt/mTOR pathway inhibits posttransplant Epstein-Barr virus B cell lymphomas and promotes allograft survival (PMID: 30549430)	12
79	1 - 4 µM	None	The mTORC1/mTORC2 inhibitor AZD2014 enhances the radiosensitivity of glioblastoma stem-like cells (PMID: 24311635)	76
80	1 - 5 µM	Rapamycin (2 - 8 µM)	Synergistic Effects between mTOR Complex 1/2 and Glycolysis Inhibitors in Non-Small-Cell Lung Carcinoma Cells (PMID: 26176608)	15
81	5 µM	None	Unspliced X-box-binding protein 1 (XBP1) protects endothelial cells from oxidative stress through interaction with histone deacetylase 3 (PMID: 25190803)	60

82	5 µM	None	Atractylenolide I inhibits colorectal cancer cell proliferation by affecting metabolism and stemness via AKT/mTOR signaling (PMID: 32135457)	20
83	7 - 11 µM	Rapamycin (10 µM) INK128 (7 - 16 µM)	Directly imaging the localisation and photosensitization properties of the pan-mTOR inhibitor, AZD2014, in living cancer cells (PMID: 33142217)	2
84	10 µM	Rapamycin, OSI-027, AZD8055 (0.1 - 10 µM)	Prolonged tau clearance and stress vulnerability rescue by pharmacological activation of autophagy in tauopathy neurons (PMID: 32591533)	53
85	10 µM	Everolimus, rapamycin (10 µM)	Somatic mutation profiling of vulvar cancer: Exploring therapeutic targets (PMID: 29980281)	29
86	20 µM	Everolimus, BEZ235, XL765, PF-05212384 (20 µM)	High throughput 3D cell migration assay using micropillar/microwell chips (PMID: 36014542)	0
87	Not clear	GDC-0349 (5 - 500 nM), rapamycin (100 nM)	GDC-0349 inhibits non-small cell lung cancer cell growth (PMID: 33154352)	12
88	Not provided	None	PI3k inhibitors (BKM120 and BYL719) as radiosensitizers for head and neck squamous cell carcinoma during radiotherapy (PMID: 33471836)	6
89	Not provided	None	Integrated pharmaco-proteogenomics defines two subgroups in isocitrate dehydrogenase wild-type glioblastoma with prognostic and therapeutic opportunities (PMID: 32620753)	23
90	Not provided	None	MiR-99a enhances the radiation sensitivity of non-small cell lung cancer by targeting mTOR (PMID: 29614485)	43
91	Not provided	None	Overproduction of IGF-2 drives a subset of colorectal cancer cells, which specifically respond to an anti-IGF therapeutic antibody and combination therapies (PMID: 27399333)	17
92	Unclear	None	Insights into significance of combined inhibition of MEK and m-TOR signalling output in KRAS mutant non-small-cell lung cancer (PMID: 27441499)	9
93	Unclear	AZD8055, everolimus	Dynamic BH3 profiling identifies active BH3 mimetic combinations in non-small cell lung cancer (PMID: 34315868)	4
Compliance				
**71 (76%)[a]**<br>**9 (10%)[b]**		56 (60%)		
Non-Compliance				
**13 (14%)**		37 (40%)		

a *Probe's concentration below the recommended in-cell maximum in all figures.*
b *Probe's concentration below the recommended in-cell maximum in some, but not all figures.*

Supplementary Note 8. Citations for AZD2014 publications included in the systematic review.
Citations for 93 publications using AZD2014: 2,076

Citations for 80 publications with a compliant AZD2014 concentration: 1,798
Citations for 13 publications with a non-compliant AZD2014 concentration: 278

Citations for 56 publications using orthogonal mTOR inhibitors: 1,313
Citations for 37 publications not using orthogonal mTOR inhibitors: 763

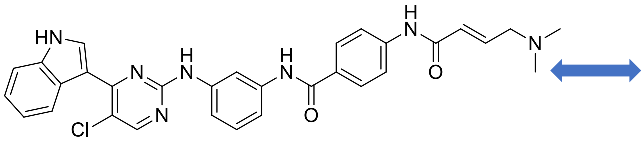

THZ1 (covalent inhibitor)
CDK7: IC_{50} = 3.2 nM
CDK12: IC_{50} = 371 nM
CDK13: IC_{50} = 225 nM
In-cell: 10 nM - 1 µM

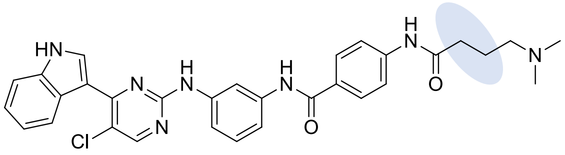

THZ1-R (inactive control: not covalent)
CDK7: IC_{50} = 146 nM

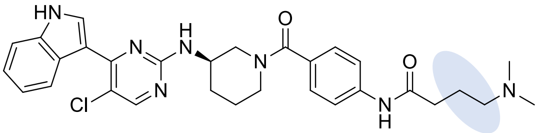

THZ531-R (inactive control: not covalent)

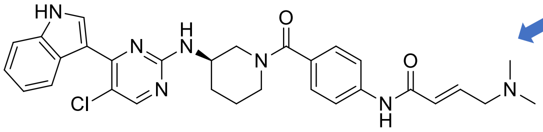

THZ531 (covalent inhibitor)
CDK12: IC_{50} = 158 nM
CDK13: IC_{50} = 69 nM
In-cell: 50 - 500 nM

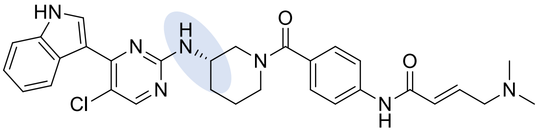

THZ532 (inactive control: enantiomer)

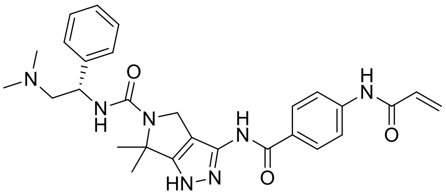

YKL-5-124 (covalent inhibitor)
CDK7: IC_{50} = 53.5 nM
In-cell: Not reported

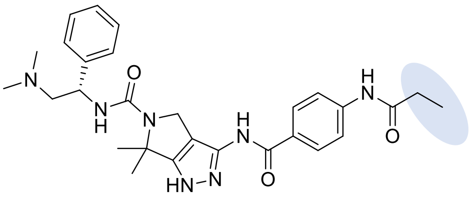

YKL-5-167 (inactive control: not covalent)
CDK7: IC_{50} > 500 nM

Supplementary Figure 12. Structures, biochemical potency (IC_{50}) and recommended in-cell concentrations of chemical probes targeting CDK7 and CDK12/13 as listed on the Chemical Probes Portal (www.chemicalprobes.org) and the Nathanael Gray Lab website https://graylab.stanford.edu/probe-resources/). Bidirectional arrows link target-inactive analogues and shaded fields highlight the structural changes leading to decreased CDK7/12/13 targeting.

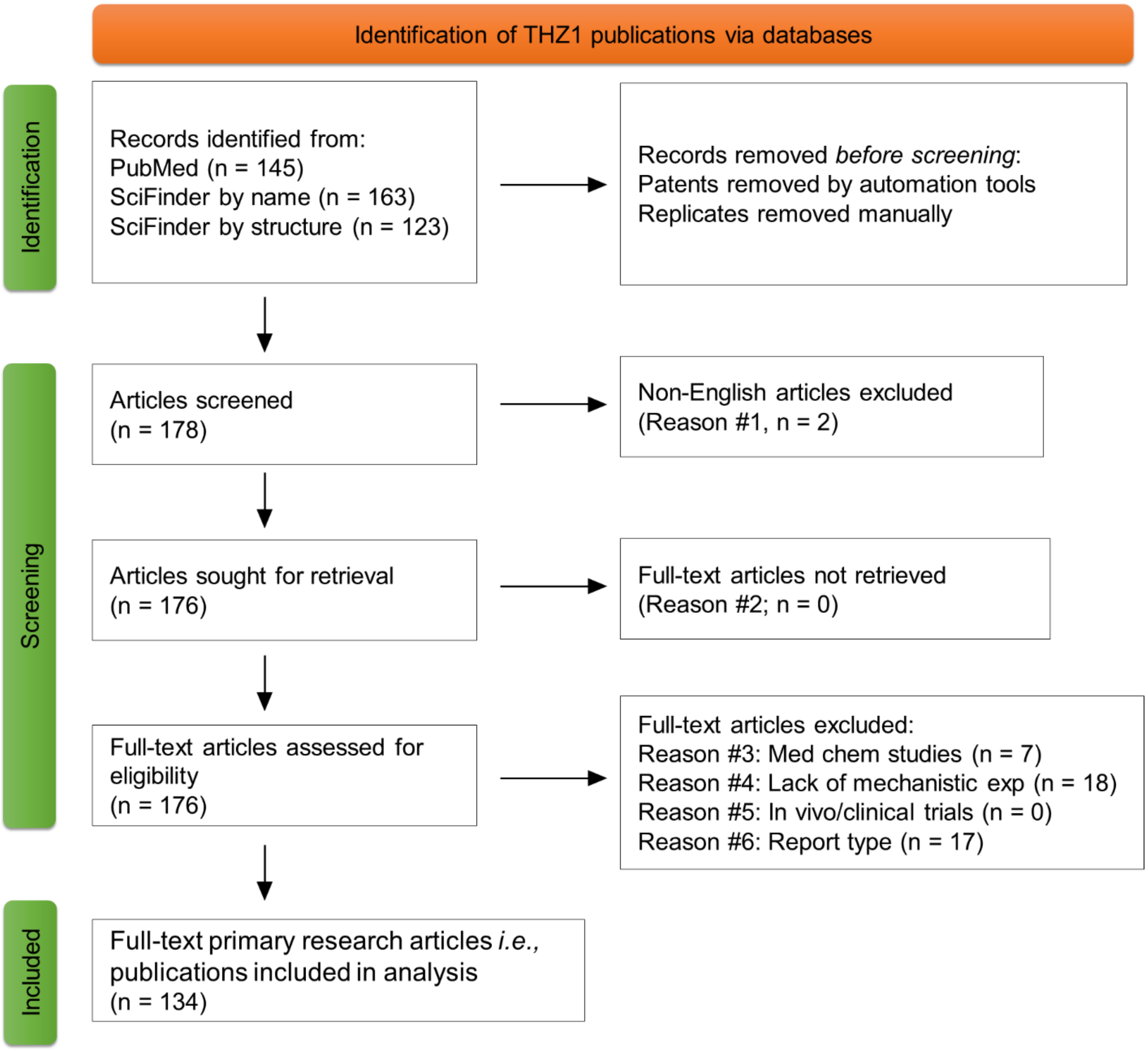

Supplementary Figure 13. PRISMA flow diagram summarising identification of publications using CDK7 chemical probe THZ1.

Note: Pubmed/SciFinder search keyword: THZ1 and "THZ-1", respectively (maximises number of papers retrieved). Pubmed search retrieved 145 publications including any publication that writes 'THz' (e.g., 0.3 THz - 1 THz range, 55 × 10^3 s^{-1} THz-1 mW^{-1}). These 145 publications were manually reviewed for publications referring to the probe THZ1. SciFinder keyword search retrieved 166 publications including any publication that writes 'THz' (e.g., 0.3 THz - 1 THz range, 55 × 10^3 s^{-1} THz-1 mW^{-1}). These 166 publications were manually reviewed for publications referring to the probe THZ1.

Supplementary Table 15. List of excluded articles using the CDK7 probe THZ1.

	Title (PMID)	Reason for exclusion
1	[Extracting THz absorption coefficient spectrum based on accurate determination of sample thickness] (PMID: 22715781)	1) Non-English
2	CDK7 inhibitor THZ1 increases the radiosensitivity of human glioma cell line U251 (No PMID or DOI; ISSN: 1671-7554)	1) Non-English
3	Targeting transcription regulation in cancer with a covalent CDK7 inhibitor (PMID: 25043025)	3) Discovery of THZ1& THZ1-R
4	Partitioning of cancer therapeutics in nuclear condensates (PMID: 32822580)	3) Fluorescently modified THZ1
5	Identification of CDK7 inhibitors from natural sources using pharmacoinformatics and molecular dynamics simulations (PMID: 34572383)	3) Medicinal chemistry
6	Synthesis and biological evaluation of new C-12(α/β)-(N-) sulfamoyl-phenylamino-14-deoxy-andrographolide derivatives as potent anti-cancer agents (PMID: 28527822)	3) Medicinal chemistry
7	Discovery of Novel N-(5-(Pyridin-3-yl)-1H-indazol-3-yl)benzamide derivatives as potent cyclin-dependent kinase 7 inhibitors for the treatment of autosomal dominant polycystic kidney disease (PMID: 36384292)	3) Medicinal chemistry
8	Leveraging gas-phase fragmentation pathways for improved identification and selective detection of targets modified by covalent probes (PMID: 28193034)	3) Medicinal chemistry
9	Discovery and characterization of SY-1365, a selective, covalent inhibitor of CDK7 (PMID: 31064851)	3) SAR analysis
10	Quantifying CDK inhibitor selectivity in live cells (PMID: 32488087)	4) No mechanistic exp.- cell viability & dose response only
11	Measurement and models accounting for cell death capture hidden variation in compound response (PMID: 32312951)	4) No mechanistic exp.- cell viability & dose response only
12	Multiplexed and reproducible high content screening of live and fixed cells using Dye Drop (PMID: 36376301)	4) No mechanistic exp. - dose response viability and cell cycle
13	The cryoelectron microscopy structure of the human CDK-activating kinase (PMID: 32855301)	4) No mechanistic exp.- crystallography study
14	Nanomaterial-facilitated cyclin-dependent kinase 7 inhibition suppresses gallbladder cancer progression via targeting transcriptional addiction (PMID: 34405985)	4) Nanoparticle delivery of THZ1
15	Targeting super-enhancers via nanoparticle-facilitated BRD4 and CDK7 inhibitors synergistically suppresses pancreatic ductal adenocarcinoma (PMID: 32274304)	4) Nanoparticle delivery of THZ1
16	Design and characterization of telecentric f- θ scanning lenses for broadband terahertz frequency systems (PMID: 33344001)	4) THZ1 not used
17	Vanadium dioxide-assisted broadband absorption and linear-to-circular polarization conversion based on a single metasurface design for the terahertz wave (PMID: 33114874)	4) THZ1 not used
18	Non-perturbing THZ generation at the Tsinghua University Accelerator Laboratory 31 MeV electron beamline (PMID: 30278713)	4) THZ1 not used
19	A single-crystal source of path-polarization entangled photons at non-degenerate wavelengths (PMID: 18575538)	4) THZ1 not used
20	Narrowband polarization-entangled photon pairs distributed over a WDM link for qubit networks (PMID: 19547007)	4) THZ1 not used
21	Computational drug repositioning identifies potentially active therapies for chordoma (PMID: 33017025)	4) THZ1 not used in mechanistic assays
22	Overcoming resistance to the THZ series of covalent transcriptional CDK inhibitors (PMID: 29276047)	4) THZ1 used to generate resistant cell clones
23	Patient-derived models recapitulate heterogeneity of molecular signatures and drug response in pediatric high-grade glioma (PMID: 34215733)	4) Used in dose-response screen, no further experiments
24	Therapeutic targeting of ATR yields durable regressions in small cell lung cancers with high replication stress (PMID: 33848478)	4) Used in dose-response screen, no further experiments

25	Modeling and targeting of erythroleukemia by hematopoietic genome editing (PMID: 33512458)	4) Used in dose-response screen, no further experiments
26	Refinement of in vitro methods for identification of aldehyde oxidase substrates reveals metabolites of kinase inhibitors (PMID: 29615437)	4) Used in dose-response screen, no further experiments
27	Functionally active cyclin-dependent kinase 9 is essential for porcine reproductive and respiratory syndrome virus subgenomic RNA synthesis (PMID: 33990004)	4) Used in screen, no further experiments
28	Androgen receptor interaction with mediator complex is enhanced in castration-resistant prostate cancer by CDK7 phosphorylation of MED1 (PMID: 31676563)	6) Commentary
29	Collapsing the tumor ecosystem: Preventing adaptive response to treatment by inhibiting transcription (PMID: 29311224)	6) Commentary
30	No driver behind the wheel? Targeting transcription in cancer (PMID: 26406367)	6) Commentary
31	Revisiting the function of CDK7 in transcription by virtue of a recently described TFIIH kinase inhibitor (PMID: 26295956)	6) Commentary
32	Treating transcriptional addiction in small cell lung cancer (PMID: 25490443)	6) Commentary
33	Inhibit globally, act locally: CDK7 inhibitors in cancer therapy (PMID: 25117707)	6) Commentary
34	Highly selective purine based covalent CDK12 inhibitors. (No PMID, DOI or ISSN, AN: 2018:1503628)	6) Conference abstract
35	Corrigendum: THZ1 targeting CDK7 suppresses STAT transcriptional activity and sensitizes T-cell lymphomas to BCL2 inhibitors (PMID: 28218232)	6) Corrigendum
36	A CRISPR screen identifies CDK7 as a therapeutic target in hepatocellular carcinoma (PMID: 29507396)	6) Letter to the editor
37	Comprehensive survey of CDK inhibitor selectivity in live cells with energy transfer probes (No PMID or DOI; ISSN: 2573-2293)	6) Preprint
38	Advances in targeted therapy for osteosarcoma based on molecular classification (PMID: 34022396)	6) Review
39	Cyclin-dependent kinase 7 inhibitor THZ1 in cancer therapy (PMID: 31891127)	6) Review
40	The essential and multifunctional TFIIH complex (PMID: 29664212)	6) Review
41	Targeting bromodomain and extraterminal proteins in breast cancer (PMID: 29154989)	6) Review
42	Nucleotide excision repair and transcriptional regulation: TFIIH and beyond (PMID: 27294439)	6) Review
43	Strategically targeting MYC in cancer (PMID: 27081479)	6) Review
44	CDK7 Inhibitors in cancer therapy: The sweet smell of success? (PMID: 32150405)	6) Review

Supplementary Table 16. Overview of eligible publications using the CDK7/12/13 probe THZ1 and compliance (**in blue**) with recommendations to use THZ1 up to 1 µM (https://www.chemicalprobes.org/thz1?q=THZ1), to validate data with an inactive control molecule THZ1-R and orthogonal inhibitors. Citations are sourced from SciFinder (January 2023), unless stated otherwise.

	THZ1	THZ1-R	Orthogonal inhibitors	Title (PMID)	Cites
1	0.1-500 nM	No	YKL-5-124, SY0351 (0.1 - 500 nM)	Transcriptional CDK inhibitors as potential treatment option for testicular germ cell tumors (PMID: 35406461)	0
2	0.1 nM - 1 µM	No	dinaciclib (CDK1/2/5/9), palbociclib (CDK4/6)	Characterizing CDK8/19 inhibitors through a NFκB-dependent cell-based assay (PMID: 31590445)	7
3	0.2 - 10 nM	No	THZ2 (40 - 240 nM) YKL-5-124 (50 nM) CT7001 (50 nM)	Transcriptional control of DNA repair networks by CDK7 regulates sensitivity to radiation in MYC-driven medulloblastoma (PMID: 33910002)	12
4	0.78 - 800 nM	No	None	Selective Inhibitors of Autophagy Reveal New Link between the Cell Cycle and Autophagy and Lead to Discovery of Novel Synergistic Drug Combinations (PMID: 36469692)	0
5	1 - 100 nM	No	None	SQLE inhibition suppresses the development of pancreatic ductal adenocarcinoma and enhances its sensitivity to chemotherapeutic agents in vitro (PMID: 35552960)	0
6	1 - 13 nM	No	None	Targeting CDK7 reverses tamoxifen resistance through regulating stemness in ER+ breast cancer (PMID: 35000145)	1
7	1 - 13 nM	No	None	Blockade of CDK7 reverses endocrine therapy resistance in breast cancer (PMID: 32340192)	8
8	1 - 200 nM	No	BS-181 (0.1 - 20 µM)	Therapeutic rationale to target highly expressed CDK7 conferring poor outcomes in triple-negative breast cancer (PMID: 28455421)	60
9	1 - 250 nM	Yes	YKL-1-116 (0.1 - 5 µM)	Targeting transcription in heart failure via CDK7/12/13 inhibition (PMID: 35896549)	0
10	1 nM - 1 µM	No	None	CDK7 inhibition synergizes with topoisomerase I inhibition in small cell lung cancer cells by inducing ubiquitin-mediated proteolysis of RNA polymerase II (PMID: 35830858)	1
11	1.56 - 50 nM	No	None	A combination strategy targeting enhancer plasticity exerts synergistic lethality against BETi-resistant leukemia cells (PMID: 32029739)	27
12	2 nM - 6.25 µM	Yes	THZ2 (333 nM)	CDK7-dependent transcriptional addiction in triple-negative breast cancer (PMID: 26406377)	30
13	3.125 nM - 200 nM	Yes	None	CDK7 inhibition is a novel therapeutic strategy against GBM both in vitro and in vivo (PMID: 30532595)	19
14	4 - 32 nM	No	None	Combination therapy with the CDK7 inhibitor and the tyrosine kinase inhibitor exerts synergistic anticancer effects against MYCN-amplified neuroblastoma (PMID: 32086952)	18
15	5 - 10 nM	No	None	Trib1 promotes acute myeloid leukemia progression by modulating the transcriptional programs of Hoxa9 (PMID: 32730594)	17
16	5 - 20 nM	No	Dinaciclib (CDK1/2/5/9; 5 - 20 nM); palbociclib (CDK4/6; 1 µM)	The individual effects of cyclin-dependent kinase inhibitors on head and neck cancer cells-A systematic analysis (PMID: 34063457)	1
17	5 nM - 1 µM	No	Palbociclib (CDK4/6);	Transcriptional targeting of oncogene addiction in medullary thyroid cancer (PMID: 30135308)	15

			dinaciclib (CDK1/2/5/9)		
18	10 - 20 nM	No	Abemaciclib (CDK4/6, 0.1 - 1 µM), dinaciclib (CDK1/2/5/9, 1 - 5 nM)	Preclinical head and neck squamous cell carcinoma models for combined targeted therapy approaches (PMID: 35626088)	0
19	10 - 50 nM	No	None	Myeloma-specific superenhancers affect genes of biological and clinical relevance in myeloma (PMID: 33579893)	5
20	10 - 50 nM	No	Flavopiridol (broad-spectrum CDK inhibitor)	Elevation of effective p53 expression sensitizes wild-type p53 breast cancer cells to CDK7 inhibitor THZ1 (PMID: 36058938)	0
21	10 - 100 nM	No	YPN-005 (10 - 100 nM)	Discovery of a novel CDK7 inhibitor YPN-005 in small cell lung cancer (PMID: 34224696)	3
22	10 - 100 nM	No	None	CDK7 is a reliable prognostic factor and novel therapeutic target in epithelial ovarian cancer (PMID: 31776040)	10
23	10 - 100 nM	No	YKL-1-116 (25 - 800 nM)	Activation of the p53 transcriptional program sensitizes cancer cells to Cdk7 inhibitors (PMID: 29020632)	51
24	10 - 160 nM	No	None	Pharmaceutical interference of the EWS-FLI1–driven transcriptome by cotargeting H3K27ac and RNA polymerase activity in Ewing Sarcoma (PMID: 34315769)	4
25	10 - 200 nM	No	None	Transcriptional dependencies in diffuse intrinsic pontine glioma (PMID: 28434841)	181
26	10 - 250 nM	No	None	CDK7 inhibition suppresses super-enhancer-linked oncogenic transcription in MYCN-driven cancer (PMID: 25416950)	394
27	10 - 500 nM	No	YKL-5-125 (1 - 500 nM)	BET and CDK inhibition reveal differences in the proliferation control of sympathetic ganglion neuroblasts and adrenal chromaffin cells (PMID: 35681734)	0
28	10 nM - 500 nM	No	YKL-1-116 (0.5 - 20 µM), THZ531 (50 nM - 5 µM)	Targeting MYC dependency in ovarian cancer through inhibition of CDK7 and CDK12/13 (PMID: 30422115)	90
29	10 nM - 1 µM	No	None	Efficacy of the CDK7 inhibitor on EMT-associated resistance to 3rd generation EGFR-TKIs in non-small cell lung cancer cell lines (PMID: 33287368)	9
30	10 - 1000 nM	No	None	Cdk7 is required for activity-dependent neuronal gene expression, long-lasting synaptic plasticity and long-term memory (PMID: 29163040)	10
31	10 nM - 1 µM	No	None	Tumors with TSC mutations are sensitive to CDK7 inhibition through NRF2 and glutathione depletion (PMID: 31506280)	12
32	10 nM - 1 µM	No	None	Carrier-free multifunctional nanomedicine for intraperitoneal disseminated ovarian cancer therapy (PMID: 35193583)	5
33	10 nM - 1 µM	No	None	Inhibition of cyclin-dependent kinase 7 suppresses human hepatocellular carcinoma by inducing apoptosis (PMID: 30145799)	16
34	10 nM - 5 µM	No	None	Genomic modelling of the ESR1 Y537S mutation for evaluating function and new therapeutic approaches for metastatic breast cancer (PMID: 27748765)	101
35	10 µM	No	LDC4297 (10 µM) milciclib (10 µM)	A high-throughput screen identifies that CDK7 activates glucose consumption in lung cancer cells (PMID: 31784510)	15

36	10 nM - 6.25 µM; 100 & 250 nM	No	None	CDK7 inhibition is effective in all the subtypes of breast cancer: Determinants of response and synergy with EGFR inhibition (PMID: 32155786)	16
37	12.5 - 100 nM	No	LDC4297 (2.5 - 5 µM)	Targeting mutated p53 dependency in triple-negative breast cancer cells through CDK7 inhibition (PMID: 34109118)	4
38	12.5 - 200 nM	No	None	CDK7 blockade suppresses super-enhancer-associated oncogenes in bladder cancer (PMID: 33905040)	2
39	12.5 - 25 nM	No	None	Combinational therapeutic targeting of BRD4 and CDK7 synergistically induces anticancer effects in head and neck squamous cell carcinoma (PMID: 31765738)	27
40	12.5 - 250 nM	No	THZ2	Systematic characterization of recurrent genomic alterations in cyclin-dependent kinases reveals potential therapeutic strategies for cancer treatment (PMID: 32668240)	13
41	12.5 - 250 nM	No	None	Super enhancer inhibitors suppress MYC driven transcriptional amplification and tumor progression in osteosarcoma (PMID: 29644114)	66
42	12.5 nM - 1 µM	Yes	CT7001 (125 nM - 5 µM)	CDK7 inhibition suppresses aberrant hedgehog pathway and overcomes resistance to smoothened antagonists (PMID: 31182587)	15
43	13 - 175 nM	No	None	CDK7 inhibition augments response to multidrug chemotherapy in pancreatic cancer (PMID: 35945614)	0
44	20 - 80 nM	No	TZH531 & THZ531R (50 - 500 nM)	EWS/FLI confers tumor cell synthetic lethality to CDK12 inhibition in Ewing sarcoma (PMID: 29358035)	80
45	20 - 200 nM	No	None	Targeting CDK7 suppresses super enhancer-linked inflammatory genes and alleviates CAR T cell-induced cytokine release syndrome (PMID: 33397398)	9
46	20 - 500 nM	No	None	Targeting CDK7 increases the stability of Snail to promote the dissemination of colorectal cancer (PMID: 30451989)	29
47	20 - 500 nM	No	None	High MITF expression is associated with super-enhancers and suppressed by CDK7 inhibition in melanoma (PMID: 29408204)	32
48	25 - 50 nM	No	None	Inhibition of retinoic acid receptor α phosphorylation represses the progression of triple-negative breast cancer via transactivating miR-3074-5p to target DHRS3 (PMID: 33902658)	2
49	25 - 100 nM	No	None	THZ1 targeting CDK7 suppresses c-KIT transcriptional activity in gastrointestinal stromal tumours (PMID: 36076237)	0
50	25 - 200 nM	No	None	Super-enhancer-associated MEIS1 promotes transcriptional dysregulation in Ewing sarcoma in co-operation with EWS-FLI1 (PMID: 30496486)	35
51	25 - 250 nM	No	None	SOX9 interacts with FOXC1 to activate MYC and regulate CDK7 inhibitor sensitivity in triple-negative breast cancer (PMID: 32398735)	16
52	25 - 300 nM	No	None	Targeting super-enhancer-associated oncogenes in oesophageal squamous cell carcinoma (PMID: 27196599)	134
53	25 - 400 nM	No	None	Characterization of super-enhancer-associated functional lncRNAs acting as ceRNAs in ESCC (PMID: 32460441)	12
54	25 - 400 nM	Yes	ICEC0942 (CDK7, 1 - 2.5 µM)	CDK7 inhibitor THZ1 inhibits MCL1 synthesis and drives cholangiocarcinoma apoptosis in	25

				combination with BCL2/BCL-XL inhibitor ABT-263 (PMID: 31399555)	
55	25 - 500 nM	No	None	Super-enhancer landscape reveals leukemia stem cell reliance on X-box binding protein 1 as a therapeutic vulnerability (PMID: 34550724)	7
56	25 - 600 nM	Yes	Flavopiridol (600 nM)	Targeting transcriptional addictions in small cell lung cancer with a covalent CDK7 inhibitor (PMID: 25490451)	295
57	25 - 800 nM	No	None	Covalent CDK7 inhibitor THZ1 inhibits myogenic differentiation (PMID: 30210638)	5
58	25 nM - 1 µM	No	YKL-5-124 (25 - 400 nM)	Synergistic anti-tumor effect of combining selective CDK7 and BRD4 inhibition in neuroblastoma (PMID: 35198433)	2
59	30 - 60 nM	No	None	Natural compound library screening identifies Sanguinarine chloride for the treatment of SCLC by upregulating CDKN1A (PMID: 35066462)	0
60	30 - 500 nM	No	None	Super-enhancer-driven metabolic reprogramming promotes cystogenesis in autosomal dominant polycystic kidney disease (PMID: 32694829)	11
61	31.25 nM - 1 µM	No	YKL-5-124 (62 nM - 19 µM) THZ531 (31 nM - 1 µM)	Development of a selective CDK7 covalent inhibitor reveals predominant cell-cycle phenotype (PMID: 30905681)	69
62	32 - 64 nM	No	None	JMJD6 is a tumorigenic factor and therapeutic target in neuroblastoma (PMID: 31346162)	48
63	35 nM	No	None	TBX2 is a neuroblastoma core regulatory circuitry component enhancing MYCN/FOXM1 reactivation of DREAM targets (PMID: 30451831)	48
64	40 nM - 5 µM	Yes	None	A chemoproteomic strategy for direct and proteome-wide covalent inhibitor target-site identification (PMID: 30518210)	48
65	50 nM	No	None	An integrative multi-omics approach uncovers the regulatory role of CDK7 and CDK4 in autophagy activation induced by silica nanoparticles (PMID: 32397800)	12
66	50 nM	No	Dinaciclib (25 nM)	Synthetic lethality of PARP inhibitors in combination with MYC blockade is independent of BRCA status in triple negative breast cancer (PMID: 29180466)	74
67	50 - 75 nM	No	THZ2, SY-1365	TGF-β/activin signaling promotes CDK7 inhibitor resistance in triple-negative breast cancer cells through upregulation of multidrug transporters (PMID: 34481843)	1
68	50 - 100 nM	No	Alvocidib (CDK9, 100 - 200 nM); dinaciclib (CDK9, 25 - 100 nM)	Vulnerability of drug-resistant EML4-ALK rearranged lung cancer to transcriptional inhibition (PMID: 32558295)	7
69	50 - 100 nM	No	None	CDK7 inhibitor suppresses tumor progression through blocking the cell cycle at the G2/M phase and inhibiting transcriptional activity in cervical cancer (PMID: 30962695)	16
70	50 - 100 nM	No	None	THZ1 reveals CDK7-dependent transcriptional addictions in pancreatic cancer (PMID: 30692639)	30
71	50 - 100 nM	No	None	THZ1 suppresses human non-small-cell lung cancer cells in vitro through interference with cancer metabolism (PMID: 30446732)	27
72	50 - 100 nM	No	Flavopiridol (pan-CDK, 50 nM)	Cyclin-dependent kinase 7 (CDK7)-mediated phosphorylation of the CDK9 activation loop promotes P-TEFb assembly with Tat and proviral HIV reactivation (PMID: 29743242)	29

73	50 - 100 nM	No	None	ER stress signaling promotes the survival of cancer "persister cells" tolerant to EGFR tyrosine kinase inhibitors (PMID: 29259014)	62
74	50 - 100 nM	No	None	Inhibition of MYC suppresses programmed cell death ligand-1 expression and enhances immunotherapy in triple-negative breast cancer (PMID: 36583862)	0 (Pubmed)
75	50 - 100 nM	No	None	Enhancer RNA commits osteogenesis via microRNA-3129 expression in human bone marrow-derived mesenchymal stem cells (PMID: 36114571)	0
76	50 - 200 nM	No	None	CDK7 inhibitor THZ1 enhances antiPD-1 therapy efficacy via the p38α/MYC/PD-L1 signaling in non-small cell lung cancer (PMID: 32690037)	29
77	50 - 200 nM	No	None	Inhibition of super enhancer downregulates the expression of KLF5 in basal-like breast cancers (PMID: 31360115)	18
78	50 - 200 nM	No	None	Antitumor effects of a covalent cyclin-dependent kinase 7 inhibitor in colorectal cancer (PMID: 30694816)	5
79	50 - 200 nM	No	THZ531 (250 - 750 nM)	CDK9 activity is critical for maintaining MDM4 overexpression in tumor cells (PMID: 32934219)	16
80	50 - 250 nM	No	None	Inhibition of the transcriptional kinase CDK7 overcomes therapeutic resistance in HER2-positive breast cancers (PMID: 31462705)	33
81	50 - 250 nM	No	None	BCL2 amplicon loss and transcriptional remodeling drives ABT-199 resistance in B cell lymphoma models (PMID: 31085176)	41
82	50 - 300 nM	No	None	Disruption of TFIIH activities generates a stress gene expression response and reveals possible new targets against cancer (PMID: 32543350)	2
83	50 - 400 nM	No	None	The covalent CDK7 inhibitor THZ1 potently induces apoptosis in multiple myeloma cells in vitro and in vivo (PMID: 31358538)	31
84	50 - 400 nM	No	Flavopiridol (pan-CDK, 400 nM); dinaciclib (CDK1/2/5/9, 25 nM)	The CDK7 inhibitor THZ1 alters RNA polymerase dynamics at the 5′ and 3′ ends of genes (PMID: 30805632)	19
85	50 - 500 nM	No	None	Suppression of angiogenesis by targeting cyclin-dependent kinase 7 in human umbilical vein endothelial cells and renal cell carcinoma: An in vitro and in vivo study (PMID: 31752390)	8
86	50 - 500 nM	No	None	Targeting super-enhancer-driven oncogenic transcription by CDK7 inhibition in anaplastic thyroid carcinoma (PMID: 30924726)	33
87	50 - 500 nM	No	None	Suppression of adaptive responses to targeted cancer therapy by transcriptional repression (PMID: 29054992)	65
88	50 - 500 nM	No	YKL-5-124, THZ531 (100 nM)	Blocking CDK7-mediated NOTCH1-cMYC signaling attenuates cancer stem cell activity in anaplastic thyroid cancer (PMID: 35822558)	0
89	50 - 800 nM	No	None	Anti-tumor drug THZ1 suppresses TGFβ2-mediated EMT in lens epithelial cells via Notch and TGFβ/Smad signaling pathway (PMID: 31333795)	10
90	50 nM - 1 µM	No	None	Epigenetic targeting of Mcl-1 is synthetically lethal with Bcl-xL/Bcl-2 inhibition in model systems of glioblastoma (PMID: 32752193)	10
91	50 nM - 1 µM	No	None	CDK7 inhibition as a promising therapeutic strategy for lung squamous cell carcinomas with a SOX2 amplification (PMID: 30838525)	11

92	50 nM - 1 µM	No	dinaciclib, flavopiridol	Preclinical efficacy and molecular mechanism of targeting CDK7-dependent transcriptional addiction in ovarian cancer (PMID: 28572168)	48
93	62.5 - 100 nM	No	None	CDK7/GRP78 signaling axis contributes to tumor growth and metastasis in osteosarcoma (PMID: 36042349)	0
94	100 nM	No	None	Identification of four enhancer-associated genes as risk signature for diffuse glioma patients (PMID: 34462884)	2
95	100 nM	No	CYC065 (CDK2/9, 3 µM)	Transcriptional CDK inhibitors, CYC065 and THZ1 promote Bim-dependent apoptosis in primary and recurrent GBM through cell cycle arrest and Mcl-1 downregulation (PMID: 34344865)	3
96	100 nM	No	CYC065 (CDK2/9, 3 µM)	Transcriptional CDK inhibitors CYC065 and THZ1 induce apoptosis in glioma stem cells derived from recurrent GBM (PMID: 34066147)	2
97	100 nM	No	None	Pharmacological inhibition of CDK7 by THZ1 impairs tumor growth in p53-mutated HNSCC (PMID: 33503275)	2
98	100 nM	No	None	Modulation of the Pol II CTD phosphorylation code by Rac1 and Cdc42 small GTPases in cultured human cancer cells and its implication for developing a synthetic-lethal cancer therapy (PMID: 32143485)	2
99	100 nM	No	None	Aberrant super-enhancer landscape in human hepatocellular carcinoma (PMID: 30723918)	53
100	100 nM	No	None	Essential role of endogenous prolactin and CDK7 in estrogen-induced upregulation of the prolactin receptor in breast cancer cells (PMID: 28423697)	12
101	100 nM	No	None	Super enhancer-regulated LINC00094 (SERLOC) upregulates the expression of MMP-1 and MMP-13 and promotes invasion of cutaneous squamous cell carcinoma (PMID: 36010973)	0
102	100 - 150 nM	No	None	Therapeutic targeting of CDK7 suppresses tumor progression in intrahepatic cholangiocarcinoma (PMID: 32174795)	10
103	100 - 200 nM	No	None	CDK7 regulates organ size and tumor growth by safeguarding the Hippo pathway effector Yki/Yap/Taz in the nucleus (PMID: 31857346)	32
104	100 - 200 nM	No	None	The covalent CDK7 inhibitor THZ1 enhances temsirolimus-induced cytotoxicity via autophagy suppression in human renal cell carcinoma (PMID: 31812697)	16
105	100 - 200 nM	Yes	dinaciclib (10 nM)	CDK7 inhibition suppresses castration-resistant prostate cancer through MED1 inactivation (PMID: 31466944)	42
106	100 - 200 nM	No	None	Cyclin-dependent kinase 7 is a therapeutic target in high-grade glioma (PMID: 28504693)	46
107	100 - 300 nM	No	None	Enhancer profiling identifies critical cancer genes and characterizes cell identity in adult T-cell leukemia (PMID: 28978570)	52
108	100 - 300 nM	No	None	Phosphoproteomics of primary cells reveals druggable kinase signatures in ovarian cancer (PMID: 28355574)	67
109	100 - 500 nM	No	None	Super-enhancers promote transcriptional dysregulation in nasopharyngeal carcinoma (PMID: 28951465)	69
110	100 - 500 nM	No	None	CDK7 inhibitor THZ1 induces the cell apoptosis of B-cell acute lymphocytic leukemia by perturbing cellular metabolism (PMID: 33889549)	5

111	0.1 - 1 µM	No	Compound 919278 (0.01 - 10 µM)	CDK12-mediated transcriptional regulation of noncanonical NF-κB components is essential for signaling (PMID: 30065029)	18
112	0.1 - 1.6 µM	No	None	CDK7 inhibition by THZ1 suppresses cancer stemness in both chemonaïve and chemoresistant urothelial carcinoma via the hedgehog signaling pathway (PMID: 33741425)	4
113	0.2 - 5 µM	No	None	Reimagining high-throughput profiling of reactive cysteines for cell-based screening of large electrophile libraries (PMID: 33398154)	50
114	200 nM	No	None	Converged DNA damage response renders human hepatocellular carcinoma sensitive to CDK7 inhibition (PMID: 35406486)	0
115	200 nM	No	None	Gene expression profile of THZ1-treated nasopharyngeal carcinoma cell lines indicates its involvement in the inhibition of the cell cycle (PMID: 35116274)	0
116	200 nM	No	None	Inhibition of CDK7 bypasses spindle assembly checkpoint via premature cyclin B degradation during oocyte meiosis (PMID: 27693251)	7
117	200 nM	No	None	Cyclin-dependent kinase 7 (CDK7) expression in human hepatocellular carcinoma: association with HCC progression, prognosis and cell proliferative capacity (No PMID; DOI: 10.21037/tcr.2018.04.15)	1
118	200 nM	No	None	Phosphatase PP2A enhances MCL-1 protein half-life in multiple myeloma cells (PMID: 33658484)	5
119	250 nM	No	HY-126251 (CDK9; 5 µM)	Targeting triple-negative breast cancer with combination therapy of EGFR CAR T cells and CDK7 inhibition (PMID: 33875483)	19
120	250 nM	No	THZ531 (1 µM), Palbociclib (1 µM), flavopiridol (10 - 200 nM)	Dynamic regulation of P-TEFb by 7SK snRNP is integral to the DNA damage response to regulate chemotherapy sensitivity (PMID: 36034227)	0
121	250 nM	No	None	Modulating Androgen Receptor-Driven Transcription in Prostate Cancer with Selective CDK9 Inhibitors (PMID: 33086052)	27
122	250 - 500 nM	No	None	THZ1, a covalent CDK7 inhibitor, enhances gemcitabine-induced cytotoxicity via suppression of Bcl-2 in urothelial carcinoma (PMID: 33520367)	2
123	250 - 500 nM	Yes	SNS-032	THZ1 targeting CDK7 suppresses STAT transcriptional activity and sensitizes T-cell lymphomas to BCL2 inhibitors (PMID: 28134252)	63
124	400 nM	No	ICEC0942 (1 µM)	ABC-transporter upregulation mediates resistance to the CDK7 inhibitors THZ1 and ICEC0942 (PMID: 31530935)	10
125	0.5 - 1 µM	No	SNS-032 (500 nM)	Pharmacological targeting of TFIIH suppresses KRAS mutant pancreatic ductal adenocarcinoma and synergizes with TRAIL (PMID: 35819261)	0 (Pubmed)
126	0.5 - 1 µM	No	THZ531 (0.01 pM - 10 mM)	Small-molecule targeting of brachyury transcription factor addiction in chordoma (PMID: 30664779)	70
127	0.5 - 1 µM	No	None	Global and transcription-coupled repair of 8-oxoG is initiated by nucleotide excision repair proteins (PMID: 35190564)	3
128	1 µM	No	THZ531 (0.1 - 1 µM)	CDK7 is a component of the integrated stress response regulating SNAT2 (SLC38A2)/System A adaptation in response to cellular amino acid deprivation (PMID: 30857869)	5

129	1 µM	No	None	Human TFIIH kinase CDK7 regulates transcription-associated chromatin modifications (PMID: 28768201)	83
130	1 µM	No	None	THZ1 reveals roles for Cdk7 in co-transcriptional capping and pausing (PMID: 26257281)	112
131	1 µM	No	None	CtIP-dependent nascent RNA expression flanking DNA breaks guides the choice of DNA repair pathway (PMID: 36085345)	0
132	1 µM & 10 µM	No	None	TFIIE orchestrates the recruitment of the TFIIH kinase module at promoter before release during transcription (PMID: 31064989)	21
133	concentration not provided	No	None	Transcription without XPB establishes a unified helicase-independent mechanism of promoter opening in eukaryotic gene expression (PMID: 28157507)	47
134	concentration not provided	No	None	Systematic analysis of drug vulnerabilities conferred by tumor suppressor loss (PMID: 31189115)	14
Compliance					
**124 (93%)**[a]<br>**7 (5%)**[b]		9 (7%)	42 (31%)		
Non-Compliance					
**3 (2%)**		125 (93%)	92 (69%)		

a *Probe's concentration below the recommended in-cell maximum in all figures.*
b *Probe's concentration below the recommended in-cell maximum in some, but not all figures.*

Supplementary Note 9. Citations for THZ1 publications included in the systematic review.
Citations for 134 publications using THZ1: 3,785

Citations for 131 publications with a compliant THZ1 concentration: 3,709
Citations for 3 publications with a non-compliant THZ1 concentration: 76

Citations for 9 publications using inactive compound THZ1-R: 537
Citations for 125 publications not using inactive compound THZ1-R: 3,248

Citations for 42 publications using orthogonal inhibitors: 1,213
Citations for 92 publications not using orthogonal inhibitors: 2,572
