# Supplementary material for: Systematic literature review reveals suboptimal use of chemical probes in cell-based biomedical research
Source: Nat Commun. 2023 Jun 3;14:3228. doi: 10.1038/s41467-023-38952-1 (PMC10239480; doi:10.1038/s41467-023-38952-1)
Supplement: Supplementary file 3 — Description of Additional Supplementary Files [file 41467_2023_38952_MOESM3_ESM.pdf]

File name: Supplementary Data 1

Description: Completed PRISMA checklists.

File name: Supplementary Data 2

Description: Results of literature database searches, containing all considered records.
