# Supplementary material for: Systematic literature review reveals suboptimal use of chemical probes in cell-based biomedical research
Source: Nat Commun. 2023 Jun 3;14:3228. doi: 10.1038/s41467-023-38952-1 (PMC10239480; doi:10.1038/s41467-023-38952-1)
Supplement: Supplementary file 6 — Reporting Summary [file 41467_2023_38952_MOESM6_ESM.pdf]

nature portfolio

Corresponding author(s): Lenka Munoz

Last updated by author(s): May 12, 2023

Reporting Summary

Nature Portfolio wishes to improve the reproducibility of the work that we publish. This form provides structure for consistency and transparency in reporting. For further information on Nature Portfolio policies, see our Editorial Policies and the Editorial Policy Checklist.

Statistics

For all statistical analyses, confirm that the following items are present in the figure legend, table legend, main text, or Methods section.

n/a	Confirmed	
☐	☒	The exact sample size (n) for each experimental group/condition, given as a discrete number and unit of measurement
☒	☐	A statement on whether measurements were taken from distinct samples or whether the same sample was measured repeatedly
☒	☐	The statistical test(s) used AND whether they are one- or two-sided<br>*Only common tests should be described solely by name; describe more complex techniques in the Methods section.*
☒	☐	A description of all covariates tested
☒	☐	A description of any assumptions or corrections, such as tests of normality and adjustment for multiple comparisons
☒	☐	A full description of the statistical parameters including central tendency (e.g. means) or other basic estimates (e.g. regression coefficient) AND variation (e.g. standard deviation) or associated estimates of uncertainty (e.g. confidence intervals)
☒	☐	For null hypothesis testing, the test statistic (e.g. F, t, r) with confidence intervals, effect sizes, degrees of freedom and P value noted<br>*Give P values as exact values whenever suitable.*
☒	☐	For Bayesian analysis, information on the choice of priors and Markov chain Monte Carlo settings
☒	☐	For hierarchical and complex designs, identification of the appropriate level for tests and full reporting of outcomes
☒	☐	Estimates of effect sizes (e.g. Cohen's d, Pearson's r), indicating how they were calculated

Our web collection on statistics for biologists contains articles on many of the points above.

Software and code

Policy information about availability of computer code

Data collection	PubMed, SciFinder, Chemical Probes Portal, Chemical Probes website of the Structural Genomics Consortium, Probe Miner
Data analysis	GraphPad Prism

For manuscripts utilizing custom algorithms or software that are central to the research but not yet described in published literature, software must be made available to editors and reviewers. We strongly encourage code deposition in a community repository (e.g. GitHub). See the Nature Portfolio guidelines for submitting code & software for further information.

Data

Policy information about availability of data

All manuscripts must include a data availability statement. This statement should provide the following information, where applicable:

- Accession codes, unique identifiers, or web links for publicly available datasets
- A description of any restrictions on data availability
- For clinical datasets or third party data, please ensure that the statement adheres to our policy

Databases used in this study: PubMed (https://pubmed.ncbi.nlm.nih.gov), SciFinder (https://scifinder.cas.org), Chemical Probes Portal (www.chemicalprobes.org), Chemical Probes website of the Structural Genomics Consortium (https://www.thesgc.org/chemical-probes), Probe Miner (https://probeminer.icr.ac.uk/)
Portals mentioned but not used: Donated Chemical Probes website (www.sgc-ffm.uni-frankfurt.de), Probes & Drugs database (www.probes-drugs.org

All data generated in this study are provided in this manuscript, Data Source and Supplementary Information.

Human research participants

Policy information about studies involving human research participants and Sex and Gender in Research.

Reporting on sex and gender	N/A
Population characteristics	N/A
Recruitment	N/A
Ethics oversight	N/A

Note that full information on the approval of the study protocol must also be provided in the manuscript.

Field-specific reporting

Please select the one below that is the best fit for your research. If you are not sure, read the appropriate sections before making your selection.

☒ Life sciences ☐ Behavioural & social sciences ☐ Ecological, evolutionary & environmental sciences

For a reference copy of the document with all sections, see nature.com/documents/nr-reporting-summary-flat.pdf

Life sciences study design

All studies must disclose on these points even when the disclosure is negative.

Sample size	Sample size reflects the number of publications eligible for the systematic review analysis. It was not predetermined, sample size reflect the number of records for each chemical probe and how many of these records met eligibility criteria.
Data exclusions	Exclusion criteria were predetermined at the beginning of the study. We excluded articles not written in English (Supplementary Note 1 - Reason #1) and articles whose full text were not available via University of Sydney library access (Reason #2). In the next step, we retrieved available full-text and English written articles. We excluded articles describing the discovery of a given chemical probe and/or medicinal chemistry articles using the probe as the lead compound (Reason #3); articles in which the chemical probe was used to generate chemical probe-resistant clones and/or articles lacking mechanistic experiments (Reason #4); articles containing only in vivo (animal/human tissue) data and/or clinical trials (Reason #5) and reviews, commentaries, editorials, letters to the editors, conference proceedings, pre-prints and similar (Reason #6).
Replication	Replication of this systematic review was not performed as the focus was on synthesizing existing research rather than generating new data.
Randomization	Randomization is not relevant/feasible in the systematic review analysis due to the nature of research performed - which was primarily observational and non-interventional.
Blinding	Blinding is not applicable to systematic reviews as they involve the synthesis and analysis of existing studies rather than the direct involvement of participants or researchers in data collection or interventions.

Reporting for specific materials, systems and methods

We require information from authors about some types of materials, experimental systems and methods used in many studies. Here, indicate whether each material, system or method listed is relevant to your study. If you are not sure if a list item applies to your research, read the appropriate section before selecting a response.

Materials & experimental systems

n/a	Involved in the study	
☒	☐	Antibodies
☒	☐	Eukaryotic cell lines
☒	☐	Palaeontology and archaeology
☒	☐	Animals and other organisms
☒	☐	Clinical data
☒	☐	Dual use research of concern

Methods

n/a	Involved in the study	
☒	☐	ChIP-seq
☒	☐	Flow cytometry
☒	☐	MRI-based neuroimaging
