# Supplementary material for: Systematic literature review reveals suboptimal use of chemical probes in cell-based biomedical research
Source: Nat Commun. 2023 Jun 3;14:3228. doi: 10.1038/s41467-023-38952-1 (PMC10239480; doi:10.1038/s41467-023-38952-1)
Supplement: Supplementary file 5 — Supplementary Data 2 [file 41467_2023_38952_MOESM5_ESM.pdf]

Results of PubMed search: keyword UNC1999

1: Konze KD, Ma A, Li F, Barsyte-Lovejoy D, Parton T, Macnevin CJ, Liu F, Gao C, Huang XP, Kuznetsova E, Rougie M, Jiang A, Pattenden SG, Norris JL, James LI, Roth BL, Brown PJ, Frye SV, Arrowsmith CH, Hahn KM, Wang GG, Vedadi M, Jin J. An orally bioavailable chemical probe of the Lysine Methyltransferases EZH2 and EZH1. ACS Chem Biol. 2013;8(6):1324-34. doi: 10.1021/cb400133j. Epub 2013 Apr 24. PMID: 23614352; PMCID: PMC3773059.

2: Xu B, On DM, Ma A, Parton T, Konze KD, Pattenden SG, Allison DF, Cai L, Rockowitz S, Liu S, Liu Y, Li F, Vedadi M, Frye SV, Garcia BA, Zheng D, Jin J, Wang GG. Selective inhibition of EZH2 and EZH1 enzymatic activity by a small molecule suppresses MLL-rearranged leukemia. Blood. 2015 Jan 8;125(2):346-57. doi: 10.1182/blood-2014-06-581082. Epub 2014 Nov 13. PMID: 25395428; PMCID: PMC4287641.

3: Katona BW, Liu Y, Ma A, Jin J, Hua X. EZH2 inhibition enhances the efficacy of an EGFR inhibitor in suppressing colon cancer cells. Cancer Biol Ther. 2014;15(12):1677-87. doi: 10.4161/15384047.2014.972776. PMID: 25535899; PMCID: PMC4622469.

4: Zhang P, de Gooijer MC, Buil LC, Beijnen JH, Li G, van Tellingen O. ABCB1 and ABCG2 restrict the brain penetration of a panel of novel EZH2-Inhibitors. Int J Cancer. 2015 Oct 15;137(8):2007-18. doi: 10.1002/ijc.29566. Epub 2015 Apr 24. PMID: 25868794.

5: Agarwal P, Alzrigat M, Párraga AA, Enroth S, Singh U, Ungerstedt J, Österborg A, Brown PJ, Ma A, Jin J, Nilsson K, Öberg F, Kalushkova A, Jernberg-Wiklund H. Genome-wide profiling of histone H3 lysine 27 and lysine 4 trimethylation in multiple myeloma reveals the importance of Polycomb gene targeting and highlights EZH2 as a potential therapeutic target. Oncotarget. 2016 Feb 9;7(6):6809-23. doi: 10.18632/oncotarget.6843. PMID: 26755663; PMCID: PMC4872750.

6: Ting K, Aitken KJ, Penna F, Samiei AN, Sidler M, Jiang JX, Ibrahim F, Tolg C, Delgado-Olguin P, Rosenblum N, Bägli DJ. Uropathogenic E. coli (UPEC) Infection Induces Proliferation through Enhancer of Zeste Homologue 2 (EZH2). PLoS One. 2016 Mar 10;11(3):e0149118. doi: 10.1371/journal.pone.0149118. PMID: 26964089; PMCID: PMC4786126.

7: Grinshtein N, Rioseco CC, Marcellus R, Uehling D, Aman A, Lun X, Muto O, Podmore L, Lever J, Shen Y, Blough MD, Cairncross GJ, Robbins SM, Jones SJ, Marra MA, Al-Awar R, Senger DL, Kaplan DR. Small molecule epigenetic screen identifies novel EZH2 and HDAC inhibitors that target glioblastoma brain tumor-initiating cells. Oncotarget. 2016 Sep 13;7(37):59360-59376. doi: 10.18632/oncotarget.10661. PMID: 27449082; PMCID: PMC5312317.

8: Yang X, Li F, Konze KD, Meslamani J, Ma A, Brown PJ, Zhou MM, Arrowsmith CH, Kaniskan HÜ, Vedadi M, Jin J. Structure-Activity Relationship Studies for Enhancer of Zeste Homologue 2 (EZH2) and Enhancer of Zeste Homologue 1 (EZH1) Inhibitors. J Med Chem. 2016 Aug 25;59(16):7617-33. doi: 10.1021/acs.jmedchem.6b00855. Epub 2016 Aug 11. PMID: 27468126; PMCID: PMC5003625.

9: Hsieh YY, Lo HL, Yang PM. EZH2 inhibitors transcriptionally upregulate cytotoxic autophagy and cytoprotective unfolded protein response in human colorectal cancer cells. Am J Cancer Res. 2016 Aug 1;6(8):1661-80. PMID: 27648357; PMCID: PMC5004071.

10: Alzrigat M, Párraga AA, Agarwal P, Zureigat H, Österborg A, Nahi H, Ma A, Jin J, Nilsson K, Öberg F, Kalushkova A, Jernberg-Wiklund H. EZH2 inhibition in multiple myeloma downregulates myeloma associated oncogenes and upregulates microRNAs with potential tumor suppressor functions. Oncotarget. 2017 Feb 7;8(6):10213-10224. doi: 10.18632/oncotarget.14378. PMID: 28052011; PMCID: PMC5354653.

11: Feist PE, Sidoli S, Liu X, Schroll MM, Rahmy S, Fujiwara R, Garcia BA, Hummon AB. Multicellular Tumor Spheroids Combined with Mass Spectrometric Histone Analysis To Evaluate Epigenetic Drugs. Anal Chem. 2017 Mar 7;89(5):2773-2781. doi: 10.1021/acs.analchem.6b03602. Epub 2017 Feb 21. PMID: 28194967; PMCID: PMC5371507.

12: Rizq O, Mimura N, Oshima M, Saraya A, Koide S, Kato Y, Aoyama K, Nakajima-Takagi Y, Wang C, Chiba T, Ma A, Jin J, Iseki T, Nakaseko C, Iwama A. Dual Inhibition of EZH2 and EZH1 Sensitizes PRC2-Dependent Tumors to Proteasome Inhibition. Clin Cancer Res. 2017 Aug 15;23(16):4817-4830. doi: 10.1158/1078-0432.CCR-16-2735. Epub 2017 May 10. PMID: 28490465; PMCID: PMC5562278.

13: Alzrigat M, Jernberg-Wiklund H. The miR-125a and miR-320c are potential tumor suppressor microRNAs epigenetically silenced by the polycomb repressive complex 2 in multiple myeloma. RNA Dis. 2017;4(2):e1529. doi: 10.14800/rd.1529. Epub 2017 Apr 3. PMID: 28664185; PMCID: PMC5485917.

14: Li R, Yi X, Wei X, Huo B, Guo X, Cheng C, Fang ZM, Wang J, Feng X, Zheng P, Su YS, Masau JF, Zhu XH, Jiang DS. EZH2 inhibits autophagic cell death of aortic vascular smooth muscle cells to affect aortic dissection. Cell Death Dis. 2018 Feb 7;9(2):180. doi: 10.1038/s41419-017-0213-2. PMID: 29416002; PMCID: PMC5833461.

15: Bisserier M, Wajapeyee N. Mechanisms of resistance to EZH2 inhibitors in diffuse large B-cell lymphomas. Blood. 2018 May 10;131(19):2125-2137. doi: 10.1182/blood-2017-08-804344. Epub 2018 Mar 23. PMID: 29572378; PMCID: PMC5946763.

16: Cao J, Pontes KC, Heijkants RC, Brouwer NJ, Groenewoud A, Jordanova ES, Marinkovic M, van Duinen S, Teunisse AF, Verdijk RM, Snaar-Jagalska E, Jochemsen AG, Jager MJ. Overexpression of EZH2 in conjunctival melanoma offers a new therapeutic target. J Pathol. 2018 Aug;245(4):433-444. doi: 10.1002/path.5094. Epub 2018 Jun 23. PMID: 29732557; PMCID: PMC6174981.

17: Fioravanti R, Stazi G, Zwergel C, Valente S, Mai A. Six Years (2012-2018) of Researches on Catalytic EZH2 Inhibitors: The Boom of the 2-Pyridone Compounds. Chem Rec. 2018 Dec;18(12):1818-1832. doi: 10.1002/tcr.201800091. Epub 2018 Oct 19. PMID: 30338896; PMCID: PMC7410397.

18: Nakagawa M, Fujita S, Katsumoto T, Yamagata K, Ogawara Y, Hattori A, Kagiyama Y, Honma D, Araki K, Inoue T, Kato A, Inaki K, Wada C, Ono Y, Yamamoto M, Miura O, Nakashima Y, Kitabayashi I. Dual inhibition of enhancer of zeste homolog 1/2 overactivates WNT signaling to deplete cancer stem cells in multiple myeloma. Cancer Sci. 2019 Jan;110(1):194-208. doi: 10.1111/cas.13840. Epub 2018 Nov 16. PMID: 30343511; PMCID: PMC6317945.

19: Yu L, Despotovic N, Kovacs MS, Pin CL, Luyt LG. ¹⁸F-Labeled PET Probe Targeting Enhancer of Zeste Homologue 2 (EZH2) for Cancer Imaging. ACS Med Chem Lett. 2019 Feb 27;10(3):334-340. doi: 10.1021/acsmedchemlett.8b00613. PMID:

30891136; PMCID: PMC6421531.

20: Lima-Fernandes E, Murison A, da Silva Medina T, Wang Y, Ma A, Leung C, Luciani GM, Haynes J, Pollett A, Zeller C, Duan S, Kreso A, Barsyte-Lovejoy D, Wouters BG, Jin J, Carvalho DD, Lupien M, Arrowsmith CH, O'Brien CA. Targeting bivalency de-represses Indian Hedgehog and inhibits self-renewal of colorectal cancer-initiating cells. Nat Commun. 2019 Mar 29;10(1):1436. doi: 10.1038/s41467-019-09309-4. PMID: 30926792; PMCID: PMC6441108.

21: Wu X, Scott H, Carlsson SV, Sjoberg DD, Cerundolo L, Lilja H, Prevo R, Rieunier G, Macaulay V, Higgins GS, Verrill CL, Lamb AD, Cunliffe VT, Bountra C, Hamdy FC, Bryant RJ. Increased EZH2 expression in prostate cancer is associated with metastatic recurrence following external beam radiotherapy. Prostate. 2019 Jul;79(10):1079-1089. doi: 10.1002/pros.23817. Epub 2019 May 18. PMID: 31104332; PMCID: PMC6563086.

22: Liang J, Li Q, Cai W, Zhang X, Yang B, Li X, Jiang S, Tian S, Zhang K, Song H, Ai D, Zhang X, Wang C, Zhu Y. Inhibition of polycomb repressor complex 2 ameliorates neointimal hyperplasia by suppressing trimethylation of H3K27 in vascular smooth muscle cells. Br J Pharmacol. 2019 Sep;176(17):3206-3219. doi: 10.1111/bph.14754. Epub 2019 Jul 19. PMID: 31162630; PMCID: PMC6692584.

23: Chen Z, Du Y, Liu X, Chen H, Weng X, Guo J, Wang M, Wang X, Wang L. EZH2 inhibition suppresses bladder cancer cell growth and metastasis via the JAK2/STAT3 signaling pathway. Oncol Lett. 2019 Jul;18(1):907-915. doi: 10.3892/ol.2019.10359. Epub 2019 May 14. PMID: 31289569; PMCID: PMC6539677.

24: Li W, Bi C, Han Y, Tian T, Wang X, Bao H, Xu X, Zhang X, Liu L, Zhang W, Gao H, Wang H, Zhang H, Meng B, Wang X, Fu K. Targeting EZH1/2 induces cell cycle arrest and inhibits cell proliferation through reactivation of p57^{CDKN1C} and TP53INP1 in mantle cell lymphoma. Cancer Biol Med. 2019 Aug;16(3):530-541. doi: 10.20892/j.issn.2095-3941.2018.0380. PMID: 31565482; PMCID: PMC6743615.

25: Rizk M, Rizq O, Oshima M, Nakajima-Takagi Y, Koide S, Saraya A, Isshiki Y, Chiba T, Yamazaki S, Ma A, Jin J, Iwama A, Mimura N. Akt inhibition synergizes with polycomb repressive complex 2 inhibition in the treatment of multiple myeloma. Cancer Sci. 2019 Dec;110(12):3695-3707. doi: 10.1111/cas.14207. Epub 2019 Oct 22. PMID: 31571328; PMCID: PMC6890440.

26: Xiao LR, Hou C, Zheng SJ, Guo B, Chen DN, Yan NH. [Effects of Histone Methyltransferase Inhibitors on the Survival, Apoptosis and Cell Cycle of Raji Cells]. Sichuan Da Xue Xue Bao Yi Xue Ban. 2019 May;50(3):305-310. Chinese. PMID: 31631594.

27: Stazi G, Taglieri L, Nicolai A, Romanelli A, Fioravanti R, Morrone S, Sabatino M, Ragno R, Taurone S, Nebbioso M, Carletti R, Artico M, Valente S, Scarpa S, Mai A. Dissecting the role of novel EZH2 inhibitors in primary glioblastoma cell cultures: effects on proliferation, epithelial-mesenchymal transition, migration, and on the pro-inflammatory phenotype. Clin Epigenetics. 2019 Dec 2;11(1):173. doi: 10.1186/s13148-019-0763-5. PMID: 31791385; PMCID: PMC6889222.

28: Daer R, Hamna F, Barrett CM, Haynes KA. Site-directed targeting of transcriptional activation-associated proteins to repressed chromatin restores CRISPR activity. APL Bioeng. 2020 Jan 14;4(1):016102. doi: 10.1063/1.5127302. PMID: 31967103; PMCID: PMC6960031.

29: Lee S, Woo DC, Kang J, Ra M, Kim KH, Lee SR, Choi DK, Lee H, Hong KB, Min SH, Lee Y, Yu JH. The Role of the Histone Methyltransferase EZH2 in Liver Inflammation and Fibrosis in STAM NASH Mice. Biology (Basel). 2020 May 2;9(5):93. doi: 10.3390/biology9050093. PMID: 32370249; PMCID: PMC7285133.

30: Rezaei S, Hosseinpourfeizi MA, Moaddab Y, Safaralizadeh R. Contribution of DNA methylation and EZH2 in SRBC down-regulation in gastric cancer. Mol Biol Rep. 2020 Aug;47(8):5721-5727. doi: 10.1007/s11033-020-05619-9. Epub 2020 Jul 16. PMID: 32676814.

31: Damele L, Amaro A, Serio A, Luchetti S, Pfeffer U, Mingari MC, Vitale C. EZH1/2 Inhibitors Favor ILC3 Development from Human HSPC-CD34⁺ Cells. Cancers (Basel). 2021 Jan 16;13(2):319. doi: 10.3390/cancers13020319. PMID: 33467134; PMCID: PMC7830003.

32: Nylund P, Atienza Párraga A, Haglöf J, De Bruyne E, Menu E, Garrido-Zabala B, Ma A, Jin J, Öberg F, Vanderkerken K, Kalushkova A, Jernberg-Wiklund H. A distinct metabolic response characterizes sensitivity to EZH2 inhibition in multiple myeloma. Cell Death Dis. 2021 Feb 12;12(2):167. doi: 10.1038/s41419-021-03447-8. PMID: 33579905; PMCID: PMC7881125.

33: Pappas K, Martin TC, Wolfe AL, Nguyen CB, Su T, Jin J, Hibshoosh H, Parsons R. NOTCH and EZH2 collaborate to repress PTEN expression in breast cancer. Commun Biol. 2021 Mar 9;4(1):312. doi: 10.1038/s42003-021-01825-8. PMID: 33750924; PMCID: PMC7943788.

34: Mimura N. [Novel epigenetic therapies for multiple myeloma]. Rinsho Ketsueki. 2021;62(4):314-320. Japanese. doi: 10.11406/rinketsu.62.314. PMID: 33967157.

35: Al-Ghabkari A, Narendran A. Targeting EZH2-mediated methylation of histone 3 inhibits proliferation of pediatric acute monocytic leukemia cells <i>in vitro</i>. Cancer Biol Ther. 2021 Apr 3;22(4):333-344. doi: 10.1080/15384047.2021.1902913. Epub 2021 May 12. PMID: 33978549; PMCID: PMC8183514.

36: Kusakabe Y, Chiba T, Oshima M, Koide S, Rizq O, Aoyama K, Ao J, Kaneko T, Kanzaki H, Kanayama K, Maeda T, Saito T, Nakagawa R, Kobayashi K, Kiyono S, Nakamura M, Ogasawara S, Suzuki E, Nakamoto S, Yasui S, Mikata R, Muroyama R, Kanda T, Maruyama H, Kato J, Mimura N, Ma A, Jin J, Zen Y, Otsuka M, Kaneda A, Iwama A, Kato N. EZH1/2 inhibition augments the anti-tumor effects of sorafenib in hepatocellular carcinoma. Sci Rep. 2021 Nov 1;11(1):21396. doi: 10.1038/s41598-021-00889-0. PMID: 34725436; PMCID: PMC8560765.

37: Zhou C, He A, Kang Q, Li C, Liu T, Cai W, Fang L, Ma S. Development of a UPLC-MS/MS method for determination of a dual EZH1/2 inhibitor UNC1999 in rat plasma. Bioanalysis. 2022 Jan;14(2):67-74. doi: 10.4155/bio-2021-0227. Epub 2021 Nov 29. PMID: 34841882.

38: Li Y, Yu M, Yin J, Yan H, Wang X. Enhanced Calcium Signal Induces NK Cell Degranulation but Inhibits Its Cytotoxic Activity. J Immunol. 2022 Jan 15;208(2):347-357. doi: 10.4049/jimmunol.2001141. Epub 2021 Dec 15. PMID: 34911773.

39: Zhang X, Ma X, Wang Q, Kong Z. EZH2 targeting to improve the sensitivity of acquired radio-resistance bladder cancer cells. Transl Oncol. 2022

Feb;16:101316. doi: 10.1016/j.tranon.2021.101316. Epub 2021 Dec 21. PMID: 34952334; PMCID: PMC8695351.

40: Zhang Q, Deng X, Tang X, You Y, Mei M, Liu D, Gui L, Cai Y, Xin X, He X, Huang J. MicroRNA-20a Suppresses Tumor Proliferation and Metastasis in Hepatocellular Carcinoma by Directly Targeting EZH1. Front Oncol. 2021 Dec 16;11:737986. doi: 10.3389/fonc.2021.737986. PMID: 34976797; PMCID: PMC8716374.

41: Yuan S, Xu J, Zhou B, Zhou Y, Lang M, Cao J, Liu Z, Yang S, Gao S, Hao J. SOX8 Affects Tumoral SPARC Expression by Regulating EZH2 to Attenuate Effectiveness of albumin-bound paclitaxel in PDAC. Int J Biol Sci. 2022 Jan 1;18(3):911-922. doi: 10.7150/ijbs.64752. PMID: 35173526; PMCID: PMC8771850.

42: Schuldt L, Reimann M, von Brandenstein K, Steinmetz J, Döding A, Schulze-Späte U, Jacobs C, Symmank J. Palmitate-Triggered COX2/PGE2-Related Hyperinflammation in Dual-Stressed PdL Fibroblasts Is Mediated by Repressive H3K27 Trimethylation. Cells. 2022 Mar 10;11(6):955. doi: 10.3390/cells11060955. PMID: 35326406; PMCID: PMC8946768.

43: Zhang N, Shang M, Li H, Wu L, Dong M, Huang B, Lu J, Zhang Y. Dual Inhibition of H3K9me2 and H3K27me3 Promotes Tumor Cell Senescence without Triggering the Secretion of SASP. Int J Mol Sci. 2022 Apr 1;23(7):3911. doi: 10.3390/ijms23073911. PMID: 35409271; PMCID: PMC8999616.

44: Shinno Y, Takenobu H, Sugino RP, Endo Y, Okada R, Haruta M, Satoh S, Mukae K, Shaliman D, Wada T, Akter J, Ando K, Nakazawa A, Yoshida H, Ohira M, Hishiki T, Kamijo T. Polycomb EZH1 regulates cell cycle/5-fluorouracil sensitivity of neuroblastoma cells in concert with MYCN. Cancer Sci. 2022 Dec;113(12):4193-4206. doi: 10.1111/cas.15555. Epub 2022 Oct 7. PMID: 36052716; PMCID: PMC9746046.

45: Barghout SH, Mann MK, Aman A, Yu Y, Alteen MG, Schimmer AD, Schapira M, Arrowsmith CH, Barsyte-Lovejoy D. Combinatorial Anticancer Drug Screen Identifies Off-Target Effects of Epigenetic Chemical Probes. ACS Chem Biol. 2022 Oct 21;17(10):2801-2816. doi: 10.1021/acschembio.2c00451. Epub 2022 Sep 9. PMID: 36084291.

46: Hou C, Xiao L, Ren X, Cheng L, Guo B, Zhang M, Yan N. <i>EZH2</i>-mediated H3K27me3 is a predictive biomarker and therapeutic target in uveal melanoma. Front Genet. 2022 Oct 6;13:1013475. doi: 10.3389/fgene.2022.1013475. PMID: 36276954; PMCID: PMC9582331.

47: Zhao Y, Cheng Y, Qu Y. The role of EZH2 as a potential therapeutic target in retinoblastoma. Exp Eye Res. 2023 Feb;227:109389. doi: 10.1016/j.exer.2023.109389. Epub 2023 Jan 18. PMID: 36669714.

CAS SciFinder^{n}

Task History

Initiating Search

January 11, 2023, 9:31PM

All: **UNC1999**

Search Tasks

Task	Search Type	View
Returned All Results	All	View Results
Exported: Returned Reference Results (61)	References	View Results

Copyright © 2023 American Chemical Society (ACS). All Rights Reserved.
Internal use only. Redistribution is subject to the terms of your SciFinder^{n} License Agreement and CAS information Use Policies.

CAS SciFinder^n

References (61)

View in SciFinder^n

1

Dual Inhibition of EZH2 and EZH1 Sensitizes PRC2-Dependent Tumors to Proteasome Inhibition

By: Rizq, Ola; Mimura, Naoya; Oshima, Motohiko; Saraya, Atsunori; Koide, Shuhei; Kato, Yuko; Aoyama, Kazumasa; Nakajima-Takagi, Yaeko; Wang, Changshan; Chiba, Tetsuhiro; et al
Clinical Cancer Research (2017), 23(16), 4817-4830 | Language: English, Database: CAplus and MEDLINE

Substances (8) Reactions (0) Cited By (45)

2

Small molecule epigenetic screen identifies novel EZH2 and HDAC inhibitors that target glioblastoma brain tumor-initiating cells.

By: Grinshtein, Natalie; Rioseco, Constanza C; Marcellus, Richard; Uehling, David; Aman, Ahmed; Lun, Xueqing; Muto, Osamu; Podmore, Lauren; Lever, Jake; Shen, Yaoqing; et al
Oncotarget (2016), 7(37), 59360-59376 | Language: English, Database: MEDLINE

Substance (1) Reactions (0) Cited By (26)

3

EZH2 inhibition in multiple myeloma downregulates myeloma associated oncogenes and upregulates microRNAs with potential tumor suppressor functions.

By: Alzrigat, Mohammad; Párraga, Alba Atienza; Agarwal, Prasoon; Zureigat, Hadil; Österborg, Anders; Nahi, Hareth; Ma, Anqi; Jin, Jian; Nilsson, Kenneth; Öberg, Fredrik; et al
Oncotarget (2017), 8(6), 10213-10224 | Language: English, Database: MEDLINE

Substance (1) Reactions (0) Cited By (39)

4

Development of a UPLC-MS/MS method for determination of a dual EZH1/2 inhibitor UNC1999 in rat plasma

By: Zhou, Chunling; He, Aiping; Kang, Qiang; Li, Chensen; Liu, Tianzhu; Cai, Wenting; Fang, Lina; Ma, Shuangcheng
Bioanalysis (2022), 14(2), 67-74 | Language: English, Database: CAplus and MEDLINE
Analytical Methods available

Substances (3) Reactions (0) Cited By (1)

5

Structure-Activity Relationship Studies for Enhancer of Zeste Homologue 2 (EZH2) and Enhancer of Zeste Homologue 1 (EZH1) Inhibitors

By: Yang, Xiaobao; Li, Fengling; Konze, Kyle D.; Meslamani, Jamel; Ma, Anqi; Brown, Peter J.; Zhou, Ming-Ming; Arrowsmith, Cheryl H.; Kaniskan, H. Umit; Vedadi, Masoud; et al
Journal of Medicinal Chemistry (2016), 59(16), 7617-7633 | Language: English, Database: CAplus and MEDLINE

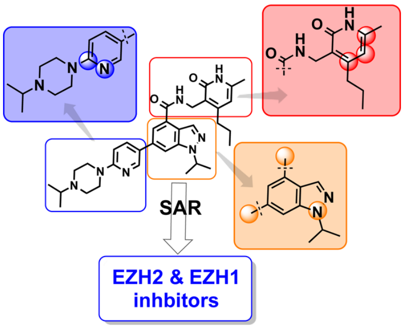

Substances (205) | Reactions (575) | Cited By (31)

6

Akt inhibition synergizes with polycomb repressive complex 2 inhibition in the treatment of multiple myeloma

By: Rizk, Mohamed ; Rizq, Ola; Oshima, Motohiko; Nakajima-Takagi, Yaeko; Koide, Shuhei; Saraya, Atsunori; Isshiki, Yusuke; Chiba, Tetsuhiro; Yamazaki, Satoshi; Ma, Anqi; et al
Cancer Science (2019), 110(12), 3695-3707 | Language: English, Database: CAplus and MEDLINE

Substances (7) | Reactions (0) | Cited By (11)

7

EZH2 inhibits autophagic cell death of aortic vascular smooth muscle cells to affect aortic dissection

By: Li, Rui; Yi, Xin; Wei, Xiang; Huo, Bo; Guo, Xian; Cheng, Cai; Fang, Ze-Min; Wang, Jing; Feng, Xin; Zheng, Ping; et al
Cell Death & Disease (2018), 9(2), 1-15 | Language: English, Database: CAplus and MEDLINE

Substances (15) | Reactions (0) | Cited By (43)

8

[Effects of Histone Methyltransferase Inhibitors on the Survival, Apoptosis and Cell Cycle of Raji Cells].

By: Xiao, Li-Rong; Hou, Chen; Zheng, Shi-Jie; Guo, Bo; Chen, Da-Nian; Yan, Nai-Hong
Sichuan da xue xue bao. Yi xue ban = Journal of Sichuan University. Medical science edition (2019), 50(3), 305-310 | Language: Chinese, Database: MEDLINE

Substances (0) | Reactions (0) | Cited By (0)

9

EZH1/2 inhibition augments the anti-tumor effects of sorafenib in hepatocellular carcinoma

By: Kusakabe, Yuko; Chiba, Tetsuhiro; Oshima, Motohiko; Koide, Shuhei; Rizq, Ola; Aoyama, Kazumasa; Ao, Junjie; Kaneko, Tatsuya; Kanzaki, Hiroaki; Kanayama, Kengo; et al
Scientific Reports (2021), 11(1), 21396 | Language: English, Database: CAplus and MEDLINE

Substances (6) | Reactions (0) | Cited By (9)

10

Contribution of DNA methylation and EZH2 in SRBC down-regulation in gastric cancer

By: Rezaei, Shiva; Hosseinpourfeizi, Mohammad Ali; Moaddab, Yaghoub; Safaralizadeh, Reza
Molecular Biology Reports (2020), 47(8), 5721-5727 | Language: English, Database: CAplus and MEDLINE

Substances (2) | Reactions (0) | Cited By (2)

11

Multicellular Tumor Spheroids Combined with Mass Spectrometric Histone Analysis to Evaluate Epigenetic Drugs

By: Feist, Peter E.; Sidoli, Simone; Liu, Xin; Schroll, Monica M.; Rahmy, Sharif; Fujiwara, Rina; Garcia, Benjamin A.; Hummon, Amanda B.
Analytical Chemistry (Washington, DC, United States) (2017), 89(5), 2773-2781 | Language: English, Database: CAplus and MEDLINE

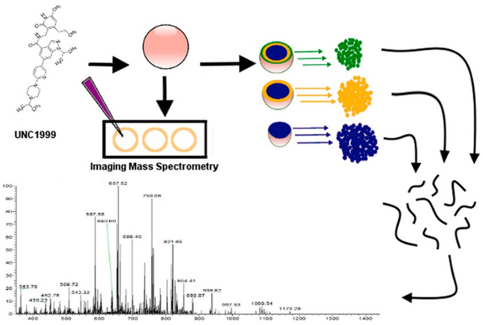

Substances (3) | Reactions (0) | Cited By (24)

12

Enhanced calcium signal induces NK cell degranulation but inhibits its cytotoxic activity

By: Li, Yang ; Yu, Minghang; Yin, Jie; Yan, Han; Wang, Xi
Journal of Immunology (2022), 208(2), 347-357, S1-S4 | Language: English, Database: CAplus and MEDLINE

Substances (4) | Reactions (0) | Cited By (1)

13

Overexpression of EZH2 in conjunctival melanoma offers a new therapeutic target

By: Cao, Jinfeng ; Pontes, Kelly C. S.; Heijkants, Renier C.; Brouwer, Niels J.; Groenewoud, Arwin; Jordanova, Ekaterina S.; Marinkovic, Marina; van Duinen, Sjoerd; Teunisse, Amina F. A. S.; Verdijk, Robert M.; et al
Journal of Pathology (2018), 245(4), 433-444 | Language: English, Database: CAplus and MEDLINE

Substances (3) | Reactions (0) | Cited By (12)

14

A distinct metabolic response characterizes sensitivity to EZH2 inhibition in multiple myeloma

By: Nylund, Patrick ; Atienza Parraga, Alba; Hagloef, Jakob; De Bruyne, Elke ; Menu, Eline ; Garrido-Zabala, Berta; Ma, Anqi; Jin, Jian ; Oeberg, Fredrik; Vanderkerken, Karin; et al
Cell Death & Disease (2021), 12(2), 167 | Language: English, Database: CAplus and MEDLINE

Substances (20) | Reactions (0) | Cited By (8)

15

Targeting bivalency de-represses Indian Hedgehog and inhibits self-renewal of colorectal cancer-initiating cells

By: Lima-Fernandes, Evelyne ; Murison, Alex; da Silva Medina, Tiago; Wang, Yadong; Ma, Anqi; Leung, Cherry; Luciani, Genna M. ; Haynes, Jennifer; Pollett, Aaron; Zeller, Constanze; et al
Nature Communications (2019), 10(1), 1-14 | Language: English, Database: CAplus and MEDLINE

Substances (2) | Reactions (0) | Cited By (21)

16

Application of compound UNC1999 in the preparation of insecticides

By: Kai, Zhenpeng; Chen, Shanshan
China, CN111066799 A 2020-04-28 | Language: Chinese, Database: CAplus

PatentPak available

Substance (1) | Reactions (0) | Cited By (0)

17

An Orally Bioavailable Chemical Probe of the Lysine Methyltransferases EZH2 and EZH1

By: Konze, Kyle D.; Ma, Anqi; Li, Fengling; Barsyte-Lovejoy, Dalia; Parton, Trevor; MacNevin, Christopher J.; Liu, Feng; Gao, Cen; Huang, Xi-Ping; Kuznetsova, Ekaterina; et al
ACS Chemical Biology (2013), 8(6), 1324-1334 | Language: English, Database: CAplus and MEDLINE

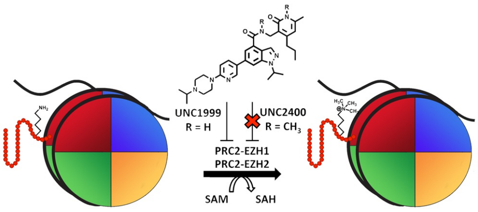

Substances (37) Reactions (61) Cited By (327)

18

Effects of EZH1/2 Inhibitor UNC1999 on Immunophenotypes of Peripheral Immune Cells

By: Liu Dan-ping; Chi Pei-dong; Mei Mei-hua; You Ying; Huang Jun-qi
Zhongshan Daxue Xuebao, Yixue Kexueban (2021), 42(4), 20-29 | Language: Chinese, Database: CAplus

Substances (0) Reactions (0) Cited By (0)

19

The effects of histone methyltransferase inhibitor UNC1999 on proliferation and migration of breast cancer cells

By: Zeng, Junjie; Zheng, Xiaodong
Linchuang Zhongliuxue Zazhi (2019), 24(1), 1-6 | Language: Chinese, Database: CAplus

Substances (0) Reactions (0) Cited By (0)

20

Selective inhibition of EZH2 and EZH1 enzymatic activity by a small molecule suppresses MLL-rearranged leukemia

By: Xu, Bowen; On, Doan M.; Ma, Anqi; Parton, Trevor; Konze, Kyle D.; Pattenden, Samantha G.; Allison, David F.; Cai, Ling; Rockowitz, Shira; Liu, Shichong; et al
Blood (2015), 125(2), 346-357 | Language: English, Database: CAplus and MEDLINE

Substances (7) Reactions (0) Cited By (161)

21

Application of substance UNC1999 for inhibiting enhancer of zeste homolog 1/2 (EZH1/2) in preparation of drug for improving vascular restenosis

By: Zhu, Yi; Ai, Ding; Wang, Chungui; Liang, Jing; Li, Qi
China, CN108524516 A 2018-09-14 | Language: Chinese, Database: CAplus

PatentPak available

Substance (1) Reactions (0) Cited By (0)

22

Genome-wide profiling of histone H3 lysine 27 and lysine 4 trimethylation in multiple myeloma reveals the importance of Polycomb gene targeting and highlights EZH2 as a potential therapeutic target.

By: Agarwal, Prasoon; Alzrigat, Mohammad; Párraga, Alba Atienza; Enroth, Stefan; Singh, Umashankar; Ungerstedt, Johanna; Österborg, Anders; Brown, Peter J; Ma, Anqi; Jin, Jian; et al
Oncotarget (2016), 7(6), 6809-23 | Language: English, Database: MEDLINE

Substance (1) Reactions (0) Cited By (49)

23

EZH2 inhibition enhances the efficacy of an EGFR inhibitor in suppressing colon cancer cells

By: Katona, Bryson W.; Liu, Yuanyuan; Ma, Anqi; Jin, Jian; Hua, Xianxin
Cancer Biology & Therapy (2014), 15(12), 1677-1687 | Language: English, Database: CAplus and MEDLINE

Substances (4) Reactions (0) Cited By (37)

24

EZH2 inhibition suppresses bladder cancer cell growth and metastasis via the JAK2/STAT3 signaling pathway

By: Chen, Zhiyuan; Du, Yang; Liu, Xiuheng; Chen, Hui; Weng, Xiaodong; Guo, Jia; Wang, Min; Wang, Xiao; Wang, Lei
Oncology Letters (2019), 18(1), 907-915 | Language: English, Database: CAplus and MEDLINE

Substances (0) Reactions (0) Cited By (21)

25

EZH2 inhibitors transcriptionally upregulate cytotoxic autophagy and cytoprotective unfolded protein response in human colorectal cancer cells

By: Hsieh, Yao-Yu; Lo, Hsiang-Ling; Yang, Pei-Ming
American Journal of Cancer Research (2016), 6(8), 1661-1680 | Language: English, Database: CAplus

Substances (0) Reactions (0) Cited By (31)

26

EZH2 inhibitors transcriptionally upregulate cytotoxic autophagy and cytoprotective unfolded protein response in human colorectal cancer cells.

By: Hsieh, Yao-Yu; Lo, Hsiang-Ling; Yang, Pei-Ming
American journal of cancer research (2016), 6(8), 1661-80 | Language: English, Database: MEDLINE

Substances (0) Reactions (0) Cited By (31)

27

Mechanisms of resistance to EZH2 inhibitors in diffuse large B-cell lymphomas

By: Bisserier, Malik; Wajapeyee, Narendra
Blood (2018), 131(19), 2125-2137 | Language: English, Database: CAplus and MEDLINE

Substances (15) Reactions (0) Cited By (67)

28

Targeting EZH1/2 induces cell cycle arrest and inhibits cell proliferation through reactivation of p57CDKN1C and TP53INP1 in mantle cell lymphoma

By: Li, Wei; Bi, Chengfeng; Han, Yating; Tian, Tian; Wang, Xianhuo; Bao, Huijing; Xu, Xiaoying; Zhang, Xuhan; Liu, Lu; Zhang, Weiwei; et al
Cancer Biology & Medicine (2019), 16(3), 530-547 | Language: English, Database: CAplus and MEDLINE

Substances (0) Reactions (0) Cited By (6)

29

Effects of EZH2 inhibitor combined with cisplatin on proliferation and apoptosis of non-small cell lung cancer A549 cells

By: Zhang, Yan-hua; Lu, Ying; Chen, Xia
Zhongguo Laonianxue Zazhi (2016), 36(23), 5839-5841 | Language: Chinese, Database: CAplus

Substances (4) Reactions (0) Cited By (0)

30

Polycomb inhibitors and therapeutic uses thereof

By: Elisabetta, Dejana; Malinverno, Matteo
World Intellectual Property Organization, WO2019115472 A1 2019-06-20 | Language: English, Database: CAplus

PatentPak available

Substances (7) Reactions (0) Cited By (0)

31

MicroRNA-20a Suppresses Tumor Proliferation and Metastasis in Hepatocellular Carcinoma by Directly Targeting EZH1.

By: Zhang, Qianqian; Deng, Xiaohong; Tang, Xiuxin; You, Ying; Mei, Meihua; Liu, Danping; Gui, Lian; Cai, Yan; Xin, Xiaoping; He, Xiaoshun; et al
Frontiers in oncology (2021), 11, 737986 | Language: English, Database: MEDLINE

Substances (0) Reactions (0) Cited By (4)

32

ABCB1 and ABCG2 restrict the brain penetration of a panel of novel EZH2-Inhibitors

By: Zhang, Ping; de Gooijer, Mark C.; Buil, Levi C. M.; Beijnen, Jos H.; Li, Gang; van Tellingen, Olaf
International Journal of Cancer (2015), 137(8), 2007-2018 | Language: English, Database: CAplus and MEDLINE

Substances (9) Reactions (0) Cited By (46)

33

Discovery of selective inhibitors for lysine methyltransferases EZH2/EZH1 and SETD8

By: Ma, Anqi; Xu, Bowen; On, Doan M.; Yu, Wenyu; Konze, Kyle D.; Butler, Kyle V.; Li, Fengling; Vedadi, Masoud; Brown, Peter J.; Wang, Gang G.; et al
Abstracts of Papers, 250th ACS National Meeting & Exposition, Boston, MA, United States, August 16-20, 2015 (2015), MEDI-311 | Language: English, Database: CAplus

Substances (0) Reactions (0) Cited By (0)

34

Dual Inhibition of H3K9me2 and H3K27me3 Promotes Tumor Cell Senescence without Triggering the Secretion of SASP

By: Zhang, Na ; Shang, Mengjie; Li, Hongxin; Wu, Lan; Dong, Meichen; Huang, Baiqu; Lu, Jun; Zhang, Yu
International Journal of Molecular Sciences (2022), 23(7), 3911 | Language: English, Database: CAplus and MEDLINE

Substances (7) Reactions (0) Cited By (1)

35

Six Years (2012-2018) of Researches on Catalytic EZH2 Inhibitors: The Boom of the 2-Pyridone Compounds

By: Fioravanti, Rossella; Stazi, Giulia; Zwergel, Clemens; Valente, Sergio; Mai, Antonello
Chemical Record (2018), 18(12), 1818-1832 | Language: English, Database: CAplus and MEDLINE

Substances (3) Reactions (0) Cited By (58)

36

Targeting EZH2-mediated methylation of histone 3 inhibits proliferation of pediatric acute monocytic leukemia cells in vitro

By: Al-Ghabkari, Abdulhameed ; Narendran, Aru
Cancer Biology & Therapy (2021), 22(4), 333-344 | Language: English, Database: CAplus and MEDLINE

Substances (11) | Reactions (0) | Cited By (3)

37

Site-directed targeting of transcriptional activation-associated proteins to repressed chromatin restores CRISPR activity

By: Daer, Rene ; Hamna, Fatima; Barrett, Cassandra M. ; Haynes, Karmella A.
APL Bioengineering (2020), 4(1), 016102 | Language: English, Database: CAplus and MEDLINE

Substance (1) | Reactions (0) | Cited By (3)

38

EZH2 targeting to improve the sensitivity of acquired radio-resistance bladder cancer cells

By: Zhang, Xiangyan; Ma, Xiangli; Wang, Quanxin; Kong, Zhaolu
Translational Oncology (2022), 16, 101316 | Language: English, Database: CAplus and MEDLINE

Substance (1) | Reactions (0) | Cited By (3)

39

EZH1/2 inhibitors favor ILC3 development from human HSPC-CD34$^+$ cells

By: Damele, Laura ; Amaro, Adriana; Serio, Alberto; Luchetti, Silvia; Pfeffer, Ulrich ; Mingari, Maria Cristina; Vitale, Chiara
Cancers (2021), 13(2), 319 | Language: English, Database: CAplus and MEDLINE

Substances (0) | Reactions (0) | Cited By (6)

40

[Novel epigenetic therapies for multiple myeloma].

By: Mimura, Naoya
[Rinsho ketsueki] The Japanese journal of clinical hematology (2021), 62(4), 314-320 | Language: Japanese, Database: MEDLINE

Substances (0) | Reactions (0) | Cited By (0)

41

Histone modifications and active gene expression are associated with enhanced CRISPR activity in de-silenced chromatin

By: Daer, Rene; Barrett, Cassandra M.; Haynes, Karmella A.
bioRxiv, Synthetic Biology (2018), 1-18 | Language: English, Database: CAplus

Substances (2) | Reactions (0) | Cited By (1)

42

Polycomb EZH1 regulates cell cycle/5-fluorouracil sensitivity of neuroblastoma cells in concert with MYCN

By: Shinno, Yoshitaka; Takenobu, Hisanori ; Sugino, Ryuichi P.; Endo, Yuki ; Okada, Ryu; Haruta, Masayuki; Satoh, Shunpei; Mukae, Kyosuke ; Shaliman, Dilibaerguli ; Wada, Tomoko; et al
Cancer Science (2022), 113(12), 4193-4206 | Language: English, Database: CAplus and MEDLINE

Substances (0) | Reactions (0) | Cited By (0)

43

Increased EZH2 expression in prostate cancer is associated with metastatic recurrence following external beam radiotherapy

By: Wu, Xiaoning; Scott, Helen; Carlsson, Sigrid V.; Sjoberg, Daniel D.; Cerundolo, Lucia; Lilja, Hans; Prevo, Remko; Rieunier, Guillaume; Macaulay, Valentine; Higgins, Geoffrey S.; et al
Prostate (Hoboken, NJ, United States) (2019), 79(10), 1079-1089 | Language: English, Database: CAplus and MEDLINE

Substance (1) | Reactions (0) | Cited By (20)

44

Inhibition of polycomb repressor complex 2 ameliorates neointimal hyperplasia by suppressing trimethylation of H3K27 in vascular smooth muscle cells

By: Liang, Jing; Li, Qi; Cai, Wenbin; Zhang, Xuejiao; Yang, Bing; Li, Xin; Jiang, Shuai; Tian, Shanshan; Zhang, Kai; Song, Hao; et al
British Journal of Pharmacology (2019), 176(17), 3206-3219 | Language: English, Database: CAplus and MEDLINE

Substances (6) | Reactions (0) | Cited By (9)

45

Chemical probes for the lysine methyltransferases G9a and EZH2

By: Konze, Kyle D.; Ma, Anqi; Pattenden, Samantha G.; Jin, Jian
Abstracts of Papers, 250th ACS National Meeting & Exposition, Boston, MA, United States, August 16-20, 2015 (2015), MEDI-295 | Language: English, Database: CAplus

Substances (0) | Reactions (0) | Cited By (0)

46

Methods and materials for promoting bone formation

By: Van Wijnen, Andre J.; Riester, Scott M.; Camilleri, Emily T.; Dudakovic, Amel; Thaler, Roman; Sperling, John W.
World Intellectual Property Organization, WO2016187394 A1 2016-11-24 | Language: English, Database: CAplus

PatentPak available

Substances (23) | Reactions (0) | Cited By (0)

47

Modulators of homeobox transcription factors, DUX4 (double homeobox 4) for regulation of muscle function

By: Rickard, Amanda; De La Garza, Anabel; Schmidt, Uli; Kiselyov, Alexander
World Intellectual Property Organization, WO2017201585 A1 2017-11-30 | Language: English, Database: CAplus

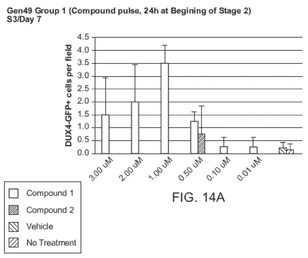

FIG. 14A

PatentPak available

Substances (241) | Reactions (0) | Cited By (3)

48

EZH2-mediated H3K27me3 is a predictive biomarker and therapeutic target in uveal melanoma

By: Hou, Chen; Xiao, Lirong; Ren, Xiang; Cheng, Lin; Guo, Bo; Zhang, Meixia; Yan, Naihong
Frontiers in Genetics (2022), 13, 1013475 | Language: English, Database: CAplus and MEDLINE

Substances (0) | Reactions (0) | Cited By (0)

49

The role of the histone methyltransferase EZH2 in liver inflammation and fibrosis in STAM NASH mice

By: Lee, Seul; Woo, Dong-Cheol; Kang, Jeeheon; Ra, Moonjin; Kim, Ki Hyun ; Lee, Seoung Rak; Choi, Dong Kyu; Lee, Heejin ; Hong, Ki Bum ; Min, Sang-Hyun; et al
Biology (Basel, Switzerland) (2020), 9(5), 93 | Language: English, Database: CAplus and MEDLINE

Substances (0) | Reactions (0) | Cited By (7)

50

SOX8 affects tumoral SPARC expression by regulating EZH2 to attenuate effectiveness of albumin-bound paclitaxel in PDAC

By: Yuan, Shuai; Xu, Jie; Zhou, Bodong; Zhou, Yizhang; Lang, Mingxiao; Cao, Junli; Liu, Zhe; Yang, Shengyu; Gao, Song; Hao, Jihui
International Journal of Biological Sciences (2022), 18(3), 911-922 | Language: English, Database: CAplus and MEDLINE

Substances (4) Reactions (0) Cited By (1)

51

Manipulation of chromatin to enhance CRISPR activity

By: Haynes, Karmella; Daer, Rene; Barrett, Cassandra
Abstracts, 69th Southeastern Regional Meeting of the American Chemical Society, Charlotte, NC, United States, November 7-11 (2017), SERMACS-428 | Language: English, Database: CAplus

Substances (0) Reactions (0) Cited By (0)

52

Uropathogenic E.coli (UPEC) infection induces proliferation through enhancer of zeste homologue 2 (EZH2)

By: Ting, Kenneth; Aitken, Karen J.; Penna, Frank; Samiei, Alaleh Najdi; Sidler, Martin; Jiang, Jia-Xin; Ibrahim, Fadi; Tolg, Cornelia; Delgado-Olguin, Paul; Rosenblum, Norman; et al
PLoS One (2016), 11(3), e0149118/1-e0149118/16 | Language: English, Database: CAplus and MEDLINE

Substance (1) Reactions (0) Cited By (7)

53

Application of EZH2 in preparing medicine for preventing or treating polycystic kidney disease

By: Li, Lin; Luo, Cheng; Wu, Ming; Qi, Na; Hu, Junchi; Lv, Jiayi; Li, Xin; Xue, Cheng; Song, Shuwei; Fu, Lili; et al
China, CN109966479 A 2019-07-05 | Language: Chinese, Database: CAplus

PatentPak available

Substances (16) Reactions (0) Cited By (0)

54

Dissecting the role of novel EZH2 inhibitors in primary glioblastoma cell cultures: effects on proliferation, epithelial-mesenchymal transition, migration, and on the pro-inflammatory phenotype

By: Stazi, Giulia; Taglieri, Ludovica; Nicolai, Alice; Romanelli, Annalisa; Fioravanti, Rossella; Morrone, Stefania; Sabatino, Manuela; Ragno, Rino; Taurone, Samanta; Nebbioso, Marcella; et al
Clinical Epigenetics (2019), 11(1), 173 | Language: English, Database: CAplus and MEDLINE

Substance (1) Reactions (0) Cited By (32)

55

NOTCH and EZH2 collaborate to repress PTEN expression in breast cancer

By: Pappas, Kyrie; Martin, Tiphaine C.; Wolfe, Andrew L.; Nguyen, Christie B.; Su, Tao; Jin, Jian; Hibshoosh, Hanina; Parsons, Ramon
Communications Biology (2021), 4(1), 312 | Language: English, Database: CAplus and MEDLINE

Substances (8) Reactions (0) Cited By (7)

56

^{18}F-Labeled PET Probe Targeting Enhancer of Zeste Homologue 2 (EZH2) for Cancer Imaging

By: Yu, Lihai; Despotovic, Nikola; Kovacs, Michael S.; Pin, Christopher L.; Luyt, Leonard G.
ACS Medicinal Chemistry Letters (2019), 10(3), 334-340 | Language: English, Database: CAplus and MEDLINE

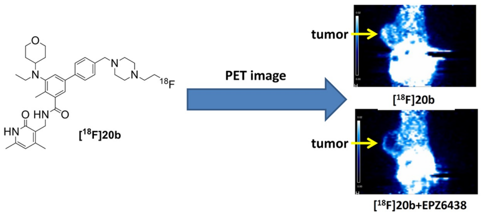

Substances (45) Reactions (111) Cited By (5)

57

The miR-125a and miR-320c are potential tumor suppressor microRNAs epigenetically silenced by the polycomb repressive complex 2 in multiple myeloma

By: Alzrigat, Mohammad; Jernberg-Wiklund, Helena
RNA & Disease (2017), 4(2), e1529/1-e1529/5 | Language: English, Database: CAplus and MEDLINE

Substances (0) Reactions (0) Cited By (18)

58

The miR-125a and miR-320c are potential tumor suppressor microRNAs epigenetically silenced by the polycomb repressive complex 2 in multiple myeloma

By: Alzrigat, Mohammad; Jernberg-Wiklund, Helena
RNA & Disease (2018), 5, e1529 | Language: English, Database: CAplus and MEDLINE

Substance (1) Reactions (0) Cited By (18)

59

Evaluating small molecule histone inhibitors with high resolution mass spectrometry and 3D cell cultures

By: Hummon, Amanda; Garcia, Benjamin; Sidoli, Simone; Schroll, Monica; Liu, Xin; Feist, Peter
Abstracts of Papers, 251st ACS National Meeting & Exposition, San Diego, CA, United States, March 13-17, 2016 (2016), ANYL-329 | Language: English, Database: CAplus

Substances (0) Reactions (0) Cited By (0)

60

Palmitate-Triggered COX2/PGE2-Related Hyperinflammation in Dual-Stressed PdL Fibroblasts Is Mediated by Repressive H3K27 Trimethylation

By: Schuldt, Lisa; Reimann, Michael; von Brandenstein, Katrin; Steinmetz, Julia; Doeding, Annika; Schulze-Spaete, Ulrike; Jacobs, Collin; Symmank, Judit
Cells (2022), 11(6), 955 | Language: English, Database: CAplus and MEDLINE

Substance (1) Reactions (0) Cited By (1)

61

Combinatorial Anticancer Drug Screen Identifies Off-Target Effects of Epigenetic Chemical Probes

By: Barghout, Samir H.; Mann, Mandeep K.; Aman, Ahmed; Yu, Yifan; Alteen, Matthew G.; Schimmer, Aaron D.; Schapira, Matthieu; Arrowsmith, Cheryl H.; Barsyte-Lovejoy, Dalia
ACS Chemical Biology (2022), 17(10), 2801-2816 | Language: English, Database: CAplus and MEDLINE

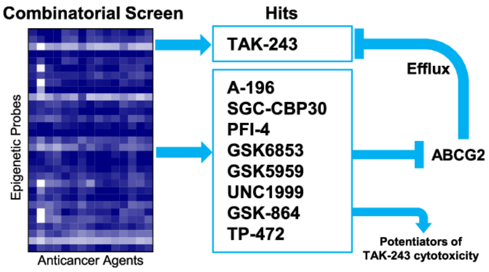

Substances (26) Reactions (0) Cited By (0)

Copyright © 2023 American Chemical Society (ACS). All Rights Reserved.
Copyright © 2023 U.S. National Library of Medicine.
Internal use only. Redistribution is subject to the terms of your SciFinderⁿ License Agreement and CAS information Use Policies.

SciFinder^n

Task History

Initiating Search

January 11, 2023, 9:34PM

Substances:

Filtered By:

Structure of UNC1999 (right)

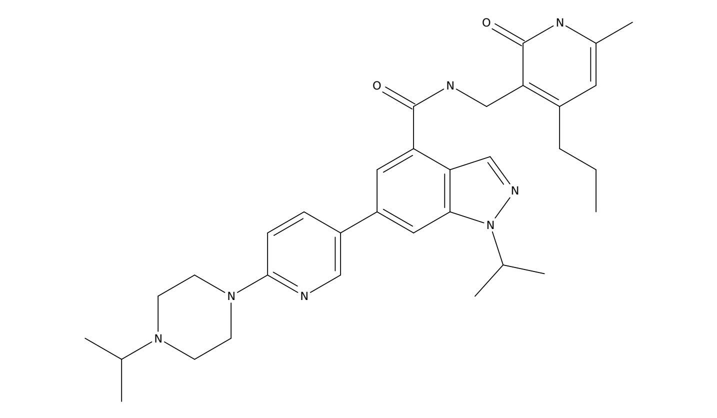

Structure Match: **As Drawn**

Search Tasks

Task	Search Type	View
Returned Substance Results + Filters (1)	Substances	View Results
Exported: Retrieved Related Reference Results (106)	References	View Results

Copyright © 2023 American Chemical Society (ACS). All Rights Reserved.
Internal use only. Redistribution is subject to the terms of your SciFinder^n License Agreement and CAS information Use Policies.

CAS SciFinderⁿ

References (106)

View in SciFinderⁿ

1

An Orally Bioavailable Chemical Probe of the Lysine Methyltransferases EZH2 and EZH1

By: Konze, Kyle D.; Ma, Anqi; Li, Fengling; Barsyte-Lovejoy, Dalia; Parton, Trevor; MacNevin, Christopher J.; Liu, Feng; Gao, Cen; Huang, Xi-Ping; Kuznetsova, Ekaterina; et al
ACS Chemical Biology (2013), 8(6), 1324-1334 | Language: English, Database: CAplus and MEDLINE

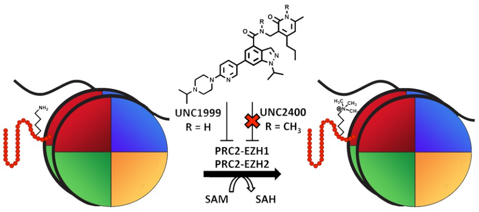

Substances (37) Reactions (61) Cited By (327)

2

Selective inhibition of EZH2 and EZH1 enzymatic activity by a small molecule suppresses MLL-rearranged leukemia

By: Xu, Bowen; On, Doan M.; Ma, Anqi; Parton, Trevor; Konze, Kyle D.; Pattenden, Samantha G.; Allison, David F.; Cai, Ling; Rockowitz, Shira; Liu, Shichong; et al
Blood (2015), 125(2), 346-357 | Language: English, Database: CAplus and MEDLINE

Substances (7) Reactions (0) Cited By (161)

3

Enhancer of Zeste Homolog 2 Inhibition Stimulates Bone Formation and Mitigates Bone Loss Caused by Ovariectomy in Skeletally Mature Mice

By: Dudakovic, Amel; Camilleri, Emily T.; Riester, Scott M.; Paradise, Christopher R.; Gluscevic, Martina; O'Toole, Thomas M.; Thaler, Roman; Evans, Jared M.; Yan, Huihuang; Subramaniam, Malayannan; et al
Journal of Biological Chemistry (2016), 291(47), 24594-24606 | Language: English, Database: CAplus and MEDLINE

Substances (4) Reactions (0) Cited By (58)

4

EZH2 inhibition enhances the efficacy of an EGFR inhibitor in suppressing colon cancer cells

By: Katona, Bryson W.; Liu, Yuanyuan; Ma, Anqi; Jin, Jian; Hua, Xianxin
Cancer Biology & Therapy (2014), 15(12), 1677-1687 | Language: English, Database: CAplus and MEDLINE

Substances (4) Reactions (0) Cited By (37)

5

The miR-125a and miR-320c are potential tumor suppressor microRNAs epigenetically silenced by the polycomb repressive complex 2 in multiple myeloma

By: Alzrigat, Mohammad; Jernberg-Wiklund, Helena
RNA & Disease (2018), 5, e1529 | Language: English, Database: CAplus and MEDLINE

Substance (1) Reactions (0) Cited By (18)

6

Application of compound UNC1999 in the preparation of insecticides

By: Kai, Zhenpeng; Chen, Shanshan
China, CN111066799 A 2020-04-28 | Language: Chinese, Database: CAplus

PatentPak available

Substance (1) Reactions (0) Cited By (0)

7

Application of substance UNC1999 for inhibiting enhancer of zeste homolog 1/2 (EZH1/2) in preparation of drug for improving vascular restenosis

By: Zhu, Yi; Ai, Ding; Wang, Chungui; Liang, Jing; Li, Qi
China, CN108524516 A 2018-09-14 | Language: Chinese, Database: CAplus

PatentPak available

Substance (1) Reactions (0) Cited By (0)

8

Histone modifications and active gene expression are associated with enhanced CRISPR activity in de-silenced chromatin

By: Daer, Rene; Barrett, Cassandra M.; Haynes, Karmella A.
bioRxiv, Synthetic Biology (2018), 1-18 | Language: English, Database: CAplus

Substances (2) Reactions (0) Cited By (1)

9

Development of a UPLC-MS/MS method for determination of a dual EZH1/2 inhibitor UNC1999 in rat plasma

By: Zhou, Chunling; He, Aiping; Kang, Qiang; Li, Chensen; Liu, Tianzhu; Cai, Wenting; Fang, Lina; Ma, Shuangcheng
Bioanalysis (2022), 14(2), 67-74 | Language: English, Database: CAplus and MEDLINE
Analytical Methods available

Substances (3) Reactions (0) Cited By (1)

10

Application of EZH2 inhibitor in preparation of drug for preventing or treating cardiovascular diseases caused by vascular smooth muscle cell proliferation

By: Jiang, Dingsheng; Wei, Xiang; Huo, Bo; Li, Rui; Zhu, Xuehai
China, CN108309978 A 2018-07-24 | Language: Chinese, Database: CAplus

PatentPak available

Substances (3) Reactions (0) Cited By (1)

11

Overexpression of EZH2 in conjunctival melanoma offers a new therapeutic target

By: Cao, Jinfeng ; Pontes, Kelly C. S.; Heijkants, Renier C.; Brouwer, Niels J.; Groenewoud, Arwin; Jordanova, Ekaterina S.; Marinkovic, Marina; van Duinen, Sjoerd; Teunisse, Amina F. A. S.; Verdijk, Robert M.; et al
Journal of Pathology (2018), 245(4), 433-444 | Language: English, Database: CAplus and MEDLINE

Substances (3) Reactions (0) Cited By (12)

12

Fate mapping of human glioblastoma reveals an invariant stem cell hierarchy

By: Lan, Xiaoyang; Jorg, David J.; Cavalli, Florence M. G.; Richards, Laura M.; Nguyen, Long V.; Vanner, Robert J.; Guilhamon, Paul; Lee, Lilian; Kushida, Michelle M.; Pellacani, Davide; et al
Nature (London, United Kingdom) (2017), 549(7671), 227-232 | Language: English, Database: CAplus and MEDLINE

Substances (3) Reactions (0) Cited By (223)

13

Multicellular Tumor Spheroids Combined with Mass Spectrometric Histone Analysis to Evaluate Epigenetic Drugs

By: Feist, Peter E.; Sidoli, Simone; Liu, Xin; Schroll, Monica M.; Rahmy, Sharif; Fujiwara, Rina; Garcia, Benjamin A.; Hummon, Amanda B.

Analytical Chemistry (Washington, DC, United States) (2017), 89(5), 2773-2781 | Language: English, Database: CAplus and MEDLINE

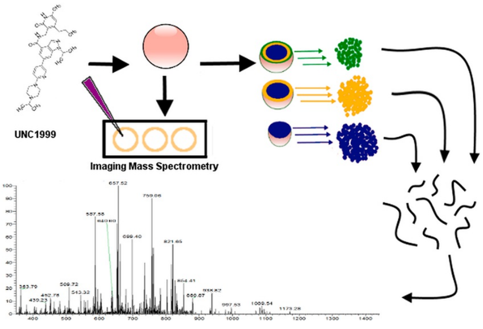

Substances (3) Reactions (0) Cited By (24)

14

Mechanisms of resistance to EZH2 inhibitors in diffuse large B-cell lymphomas

By: Bisserier, Malik; Wajapeyee, Narendra

Blood (2018), 131(19), 2125-2137 | Language: English, Database: CAplus and MEDLINE

Substances (15) Reactions (0) Cited By (67)

15

EZH2 inhibits autophagic cell death of aortic vascular smooth muscle cells to affect aortic dissection

By: Li, Rui; Yi, Xin; Wei, Xiang; Huo, Bo; Guo, Xian; Cheng, Cai; Fang, Ze-Min; Wang, Jing; Feng, Xin; Zheng, Ping; et al

Cell Death & Disease (2018), 9(2), 1-15 | Language: English, Database: CAplus and MEDLINE

Substances (15) Reactions (0) Cited By (43)

16

Application of an morphine inhibitor in relieving tolerance

By: Jiang, Chunyi; Liu, Wentao; Wan, Li

China, CN114642730 A 2022-06-21 | Language: Chinese, Database: CAplus

PatentPak available

Substances (4) Reactions (0) Cited By (0)

17

BET-bromodomain and EZH2 inhibitor-treated chronic GVHD mice have blunted germinal centers with distinct transcriptomes

By: Zaiken, Michael C.; Flynn, Ryan; Paz, Katelyn G.; Rhee, Stephanie Y.; Jin, Sujeong; Mohamed, Fathima A.; Saha, Asim; Thangavelu, Govindarajan; Park, Paul M. C.; Hemming, Matthew L.; et al
Blood (2022), 139(19), 2983-2997 | Language: English, Database: CAplus and MEDLINE

Substances (4) Reactions (0) Cited By (3)

18

Enhanced calcium signal induces NK cell degranulation but inhibits its cytotoxic activity

By: Li, Yang; Yu, Minghang; Yin, Jie; Yan, Han; Wang, Xi
Journal of Immunology (2022), 208(2), 347-357, S1-S4 | Language: English, Database: CAplus and MEDLINE

Substances (4) Reactions (0) Cited By (1)

19

SOX8 affects tumoral SPARC expression by regulating EZH2 to attenuate effectiveness of albumin-bound paclitaxel in PDAC

By: Yuan, Shuai; Xu, Jie; Zhou, Bodong; Zhou, Yizhang; Lang, Mingxiao; Cao, Junli; Liu, Zhe; Yang, Shengyu; Gao, Song; Hao, Jihui
International Journal of Biological Sciences (2022), 18(3), 911-922 | Language: English, Database: CAplus and MEDLINE

Substances (4) Reactions (0) Cited By (1)

20

Effects of EZH2 inhibitor combined with cisplatin on proliferation and apoptosis of non-small cell lung cancer A549 cells

By: Zhang, Yan-hua; Lu, Ying; Chen, Xia
Zhongguo Laonianxue Zazhi (2016), 36(23), 5839-5841 | Language: Chinese, Database: CAplus

Substances (4) Reactions (0) Cited By (0)

21

Targeting EZH2-mediated methylation of histone 3 inhibits proliferation of pediatric acute monocytic leukemia cells in vitro

By: Al-Ghabkari, Abdulhameed; Narendran, Aru
Cancer Biology & Therapy (2021), 22(4), 333-344 | Language: English, Database: CAplus and MEDLINE

Substances (11) Reactions (0) Cited By (3)

22

XIST loss impairs mammary stem cell differentiation and increases tumorigenicity through Mediator hyperactivation

By: Richart, Laia; Picod-Chedotel, Mary-Loup; Wassef, Michel; Macario, Manon; Aflaki, Setareh; Salvador, Marion A.; Hery, Tiphaine; Dauphin, Aurelien; Wicinski, Julien; Chevrier, Veronique; et al
Cell (Cambridge, MA, United States) (2022), 185(12), 2164-2183.e25 | Language: English, Database: CAplus and MEDLINE

Substances (5) Reactions (0) Cited By (2)

23

TRACE generates fluorescent human reporter cell lines to characterize epigenetic pathways

By: Tchasovnikarova, Iva A.; Marr, Sharon K. ; Damle, Manashree; Kingston, Robert E.
Molecular Cell (2022), 82(2), 479-491.e7 | Language: English, Database: CAplus and MEDLINE

Substances (5) Reactions (0) Cited By (2)

24

Compounds and methods for inhibiting cancers from neuronal cell lines via REST protein inhibition in ATRX mutant cells and use of EZH2 inhibitors

By: Bernstein, Emily; Qadeer, Zulekha; Hasson, Dan
World Intellectual Property Organization, WO2020247130 A1 2020-12-10 | Language: English, Database: CAplus

PatentPak available

Substances (5) Reactions (0) Cited By (0)

25

Preventing or treating viral infection by inhibition of the histone methyltransferase EZH1 or EZH2

By: Kristie, Thomas M.; Arbuckle, Jesse H.
World Intellectual Property Organization, WO2016178987 A2 2016-11-10 | Language: English, Database: CAplus

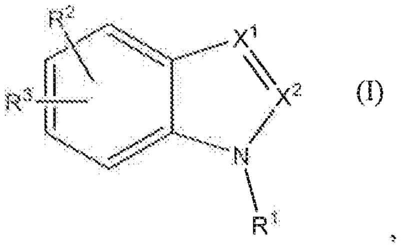

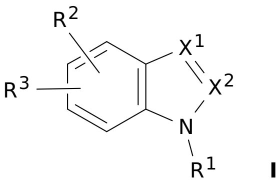

PatentPak available

Substances (5) | Reactions (0) | Cited By (0)

26

Method for the in vitro prognosis of individuals having multiple myeloma and method for the treatment thereof

By: Moreaux, Jerome; Herviou, Laurie; Kassambara, Alboukadel; Cavalli, Giacomo
European Patent Organization, EP3318642 A1 2018-05-09 | Language: English, Database: CAplus

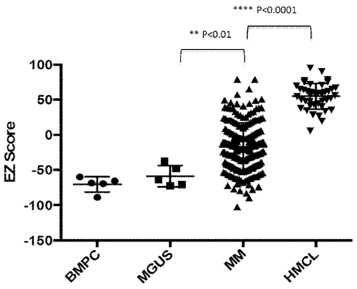

FIGURE 1

PatentPak available

Substances (13) | Reactions (0) | Cited By (1)

27

EZH1/2 inhibition augments the anti-tumor effects of sorafenib in hepatocellular carcinoma

By: Kusakabe, Yuko; Chiba, Tetsuhiro; Oshima, Motohiko; Koide, Shuhei; Rizq, Ola; Aoyama, Kazumasa; Ao, Junjie; Kaneko, Tatsuya; Kanzaki, Hiroaki; Kanayama, Kengo; et al
Scientific Reports (2021), 11(1), 21396 | Language: English, Database: CAplus and MEDLINE

Substances (6) | Reactions (0) | Cited By (9)

28

Inhibition of polycomb repressor complex 2 ameliorates neointimal hyperplasia by suppressing trimethylation of H3K27 in vascular smooth muscle cells

By: Liang, Jing; Li, Qi; Cai, Wenbin; Zhang, Xuejiao; Yang, Bing; Li, Xin; Jiang, Shuai; Tian, Shanshan; Zhang, Kai; Song, Hao; et al
British Journal of Pharmacology (2019), 176(17), 3206-3219 | Language: English, Database: CAplus and MEDLINE

Substances (6) | Reactions (0) | Cited By (9)

29

Discovery of a first-in-class EZH2 selective degrader

By: Ma, Anqi; Stratikopoulos, Elias; Park, Kwang-Su; Wei, Jieli; Martin, Tiphaine C.; Yang, Xiaobao; Schwarz, Megan; Leshchenko, Violetta; Rialdi, Alexander; Dale, Brandon; et al
Nature Chemical Biology (2020), 16(2), 214-222 | Language: English, Database: CAplus and MEDLINE

Substances (14) | Reactions (4) | Cited By (85)

30

Dual Inhibition of H3K9me2 and H3K27me3 Promotes Tumor Cell Senescence without Triggering the Secretion of SASP

By: Zhang, Na; Shang, Mengjie; Li, Hongxin; Wu, Lan; Dong, Meichen; Huang, Baiqu; Lu, Jun; Zhang, Yu
International Journal of Molecular Sciences (2022), 23(7), 3911 | Language: English, Database: CAplus and MEDLINE

Substances (7) | Reactions (0) | Cited By (1)

31

Multiple pharmacological inhibitors targeting the epigenetic suppressor enhancer of zeste homolog 2 (Ezh2) accelerate osteoblast differentiation

By: Galvan, M. Lizeth; Paradise, Christopher R.; Kubrova, Eva; Jerez, Sofia; Khani, Farzaneh; Thaler, Roman; Dudakovic, Amel; van Wijnen, Andre J.
Bone (New York, NY, United States) (2021), 150, 115993 | Language: English, Database: CAplus and MEDLINE

Substances (7) | Reactions (0) | Cited By (14)

32

Histone methyltransferase inhibition has a cytotoxic impact on transformed mast cells: implications for mastocytosis

By: Alanazi, Sultan; Melo, Fabio Rabelo; Pejler, Gunnar
Anticancer Research (2020), 40(5), 2525-2536 | Language: English, Database: CAplus and MEDLINE

Substances (7) | Reactions (0) | Cited By (1)

33

Polycomb inhibitors and therapeutic uses thereof

By: Elisabetta, Dejana; Malinverno, Matteo
World Intellectual Property Organization, WO2019115472 A1 2019-06-20 | Language: English, Database: CAplus

PatentPak available

Substances (7) Reactions (0) Cited By (0)

34

NOTCH and EZH2 collaborate to repress PTEN expression in breast cancer

By: Pappas, Kyrie; Martin, Tiphaine C. ; Wolfe, Andrew L.; Nguyen, Christie B.; Su, Tao; Jin, Jian ; Hibshoosh, Hanina; Parsons, Ramon
Communications Biology (2021), 4(1), 312 | Language: English, Database: CAplus and MEDLINE

Substances (8) Reactions (0) Cited By (7)

35

Evaluation of EED Inhibitors as a Class of PRC2-Targeted Small Molecules for HIV Latency Reversal

By: Turner, Anne-Marie W.; Dronamraju, Raghuvar; Potjewyd, Frances; James, Katherine S.; Winecoff, Daniel K.; Kirchherr, Jennifer L.; Archin, Nancie M.; Browne, Edward P.; Strahl, Brian D. ; Margolis, David M.; et al
ACS Infectious Diseases (2020), 6(7), 1719-1733 | Language: English, Database: CAplus and MEDLINE

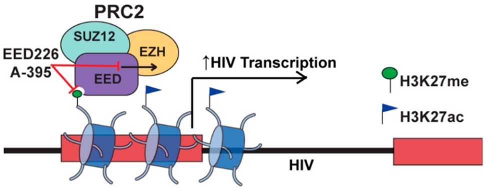

Substances (8) Reactions (0) Cited By (7)

36

Sustained expression of FMR1 mRNA from reactivated fragile X syndrome alleles after treatment with small molecules that prevent trimethylation of H3K27

By: Kumari, Daman; Usdin, Karen
Human Molecular Genetics (2016), 25(17), 3689-3698 | Language: English, Database: CAplus and MEDLINE

Substances (8) Reactions (0) Cited By (25)

37

A mass spectrometry-based assay using metabolic labeling to rapidly monitor chromatin accessibility of modified histone proteins

By: Sidoli, Simone; Lopes, Mariana; Lund, Peder J.; Goldman, Naomi; Fasolino, Maria; Coradin, Mariel; Kulej, Katarzyna; Bhanu, Natarajan V.; Vahedi, Golnaz; Garcia, Benjamin A.
Scientific Reports (2019), 9(1), 1-15 | Language: English, Database: CAplus and MEDLINE

Substances (9) Reactions (0) Cited By (19)

38

Targeting EZH2 and PRC2 dependence as novel anticancer therapy

By: Xu, Bowen; Konze, Kyle D.; Jin, Jian; Wang, Gang Greg
Experimental Hematology (New York, NY, United States) (2015), 43(8), 698-712 | Language: English, Database: CAplus and MEDLINE

Substances (9) Reactions (0) Cited By (80)

39

Methods of treating cancers overexpressing CARM1 with EZH2 inhibitors and platinum-based antineoplastic drugs

By: Zhang, Rugang; Karakashev, Sergey
World Intellectual Property Organization, WO2017192290 A1 2017-11-09 | Language: English, Database: CAplus

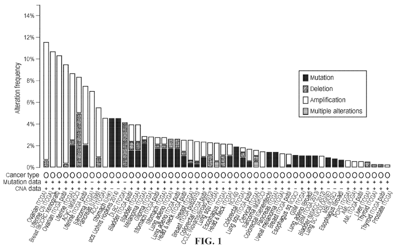

FIG. 1

PatentPak available

Substances (20) Reactions (0) Cited By (1)

40

Methods for predicting responsiveness of a cancer to an immunotherapeutic agent and methods of treating cancer

By: Shields, Bradley; Tackett, Alan; Byrum, Stephanie; Mahmoud, Fade; Shalin, Sara
World Intellectual Property Organization, WO2018145095 A1 2018-08-09 | Language: English, Database: CAplus

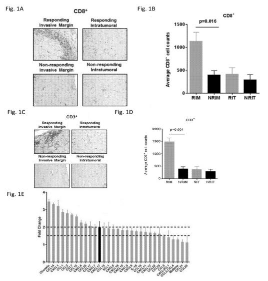

PatentPak available

Substances (10) Reactions (0) Cited By (1)

41

Methods and uses related to rhabdoid tumors

By: Huang, Annie; Torchia, Jonathon
United States, US20170298437 A1 2017-10-19 | Language: English, Database: CAplus

PatentPak available

Substances (10) Reactions (0) Cited By (0)

42

Methods for diagnosing and treating rhabdoid tumors

By: Huang, Annie; Torchia, Jonathon
Canada, CA2926943 A1 2017-10-13 | Language: English, Database: CAplus

PatentPak available

Substances (10) Reactions (0) Cited By (0)

43

Chromatin-activity-based chemoproteomic (ChaC) methods and systems for disease marker discovery and development

By: Chen, Xian
World Intellectual Property Organization, WO2015123326 A1 2015-08-20 | Language: English, Database: CAplus

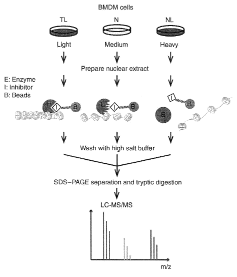

Fig.1

PatentPak available

Substances (10) | Reactions (0) | Cited By (0)

44

G9a inhibitors and EZH2 inhibitors as multifaceted COVID-19 therapeutics

By: Chen, Xian
World Intellectual Property Organization, WO2022104190 A1 2022-05-19 | Language: English, Database: CAplus

PatentPak available

Substances (11) | Reactions (0) | Cited By (0)

45

Combined inhibition of JAK/STAT pathway and lysine-specific demethylase 1 as a therapeutic strategy in CSF3R/CEBPA mutant acute myeloid leukemia

By: Braun, Theodore P. ; Coblentz, Cody; Smith, Brittany M.; Coleman, Daniel J.; Schonrock, Zachary ; Carratt, Sarah A.; Callahan, Rowan L.; Maniaci, Breanna ; Druker, Brian J.; Maxson, Julia E.
Proceedings of the National Academy of Sciences of the United States of America (2020), 117(24), 13670-13679 | Language: English, Database: CAplus and MEDLINE

Substances (11) | Reactions (0) | Cited By (13)

46

Chromatin remodeling controls Kaposi's sarcoma-associated herpesvirus reactivation from latency

By: Hopcraft, Sharon E. ; Pattenden, Samantha G. ; James, Lindsey I. ; Frye, Stephen ; Dittmer, Dirk P. ; Damania, Blossom
PLoS Pathogens (2018), 14(9), e1007267/1-e1007267/20 | Language: English, Database: CAplus and MEDLINE

Substances (11) | Reactions (0) | Cited By (26)

47

Preventing or treating viral infections by inhibition of the histone methyltransferase EZH1 or EZH2

By: Kristie, Thomas M.; Arbuckle, Jesse H.
United States, US20180071284 A1 2018-03-15 | Language: English, Database: CAplus

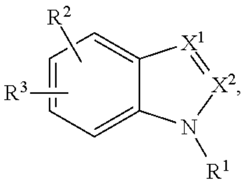

(I)

PatentPak available

Substances (11) | Reactions (0) | Cited By (0)

48

EZH2-mediated Epigenetic silencing of miR-29/miR-30 targets LOXL4 and contributes to tumorigenesis, metastasis, and immune microenvironment remodeling in breast cancer

By: Yin, Huilong; Wang, Yidi; Wu, Ye; Zhang, Xiang; Zhang, Xiaofang; Liu, Jun; Wang, Ting; Fan, Jing; Sun, Jianyong; Yang, Angang; et al
Theranostics (2020), 10(19), 8494-8512 | Language: English, Database: CAplus and MEDLINE

Substances (12) | Reactions (0) | Cited By (27)

49

GD2-upregulation by EZH2 inhibition in cancer therapy

By: Roessig, Claudia; Altvater, Bianca; Kailayangiri, Sareetha
World Intellectual Property Organization, WO2020165402 A1 2020-08-20 | Language: English, Database: CAplus

PatentPak available

Substances (12) | Reactions (0) | Cited By (1)

50

Combination therapies for cancer by modulation of histone methyl modifying enzymes

By: Arora, Shilpi; Balasubramanian, Srividya; Bradley, William D.; Trojer, Patrick
World Intellectual Property Organization, WO2017019721 A2 2017-02-02 | Language: English, Database: CAplus

FIG. 1

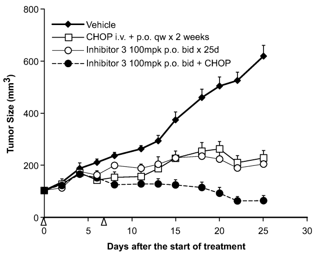

PatentPak available

Substances (34) | Reactions (0) | Cited By (1)

51

Structure-Activity Relationship Studies for Enhancer of Zeste Homologue 2 (EZH2) and Enhancer of Zeste Homologue 1 (EZH1) Inhibitors

By: Yang, Xiaobao; Li, Fengling; Konze, Kyle D.; Meslamani, Jamel; Ma, Anqi; Brown, Peter J.; Zhou, Ming-Ming; Arrowsmith, Cheryl H.; Kaniskan, H. Umit; Vedadi, Masoud; et al
Journal of Medicinal Chemistry (2016), 59(16), 7617-7633 | Language: English, Database: CAplus and MEDLINE

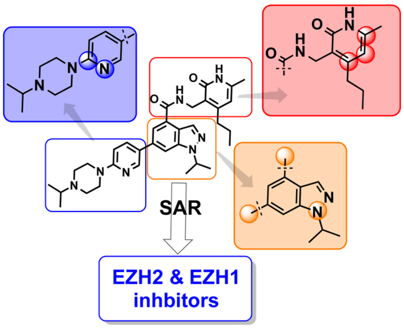

Substances (205) | Reactions (575) | Cited By (31)

52

The BRD9/7 Inhibitor TP-472 Blocks Melanoma Tumor Growth by Suppressing ECM-Mediated Oncogenic Signaling and Inducing Apoptosis

By: Mason, Lawrence David; Chava, Suresh; Reddi, Kiran Kumar; Gupta, Romi
Cancers (2021), 13(21), 5516 | Language: English, Database: CAplus and MEDLINE

Substances (13) | Reactions (0) | Cited By (5)

53

Method of prognosis of an individual having multiple myeloma to be sensitive to a treatment

By: Moreaux, Jerome; Alaterre, Elina; Cavalli, Giacomo
European Patent Organization, EP3995830 A1 2022-05-11 | Language: English, Database: CAplus

PatentPak available

Substances (15) Reactions (0) Cited By (0)

54

Application of EZH2 in preparing medicine for preventing or treating polycystic kidney disease

By: Li, Lin; Luo, Cheng; Wu, Ming; Qi, Na; Hu, Junchi; Lv, Jiayi; Li, Xin; Xue, Cheng; Song, Shuwei; Fu, Lili; et al
China, CN109966479 A 2019-07-05 | Language: Chinese, Database: CAplus

PatentPak available

Substances (16) Reactions (0) Cited By (0)

55

Response and resistance to CDK12 inhibition in aggressive B-cell lymphomas

By: Gao, Jing; Wang, Michelle Y.; Ren, Yuan; Lwin, Tint; Li, Tao; Yan, Joy C.; Sotomayor, Eduardo M.; Duckett, Derek R.; Shah, Bijal D.; Shain, Kenneth H.; et al
Haematologica (2022), 107(5), 1119-1130 | Language: English, Database: CAplus and MEDLINE

Substances (17) Reactions (0) Cited By (1)

56

Use of histone methyltransferase EZH2 inhibitors for the treatment of psoriasis

By: Kramer, Daniela; Mueller, Anne; Schulze Osthoff, Klaus
World Intellectual Property Organization, WO2021063737 A1 2021-04-08 | Language: English, Database: CAplus

PatentPak available

Substances (18) Reactions (0) Cited By (0)

57

Use of histone methyltransferase EZH2 inhibitors for the treatment of psoriasis

By: Kramer, Daniela; Mueller, Anne; Schulze Osthoff, Klaus
European Patent Organization, EP3797777 A1 2021-03-31 | Language: English, Database: CAplus

PatentPak available

Substances (18) Reactions (0) Cited By (0)

58

Inhibition of PRC2 subunits to treat eye disorders

By: Arsenijevic, Yvan; Mbefo, Kamdem Martial
World Intellectual Property Organization, WO2020011607 A1 2020-01-16 | Language: English, Database: CAplus

PatentPak available

Substances (18) Reactions (0) Cited By (1)

59

Permissive epigenomes endow reprogramming competence to transcriptional regulators

By: Kim, Kee-Pyo ; Choi, Jinmi; Yoon, Juyong; Bruder, Jan M.; Shin, Borami; Kim, Jonghun; Arauzo-Bravo, Marcos J. ; Han, Dong; Wu, Guangming; Han, Dong Wook; et al
Nature Chemical Biology (2021), 17(1), 47-56 | Language: English, Database: CAplus and MEDLINE

Substances (19) Reactions (0) Cited By (16)

60

Therapeutic treatment of select diffuse large B cell lymphoma (DLBCL) exhibiting distinct pathogenic mechanisms and outcomes

By: Chapuy, Bjoern; Shipp, Margaret; Stewart, Chip; Dunford, Andrew; Getz, Gad
United States, US20190292602 A1 2019-09-26 | Language: English, Database: CAplus

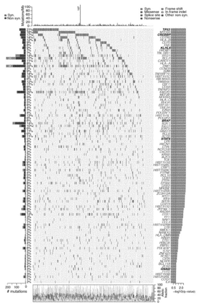

PatentPak available

Substances (19) Reactions (0) Cited By (0)

61

Engineered Multivalent Sensors to Detect Coexisting Histone Modifications in Living Stem Cells

By: Delachat, Aurore M.-F.; Guidotti, Nora; Bachmann, Andreas L.; Meireles-Filho, Antonio C. A.; Pick, Horst; Lechner, Carolin C.; Deluz, Cedric; Deplancke, Bart; Suter, David M.; Fierz, Beat
Cell Chemical Biology (2018), 25(1), 51-56.e6 | Language: English, Database: CAplus and MEDLINE

Substances (19) Reactions (0) Cited By (26)

62

A distinct metabolic response characterizes sensitivity to EZH2 inhibition in multiple myeloma

By: Nylund, Patrick ; Atienza Parraga, Alba; Hagloef, Jakob; De Bruyne, Elke ; Menu, Eline ; Garrido-Zabala, Berta; Ma, Anqi; Jin, Jian ; Oeberg, Fredrik; Vanderkerken, Karin; et al
Cell Death & Disease (2021), 12(2), 167 | Language: English, Database: CAplus and MEDLINE

Substances (20) Reactions (0) Cited By (8)

63

Use of combination of EZH2 inhibitors and immunodulators of regulatory T-cell function in treatment of cancer

By: Ghosh, Srimoyee; Lora, Jose M.
World Intellectual Property Organization, WO2017023671 A1 2017-02-09 | Language: English, Database: CAplus

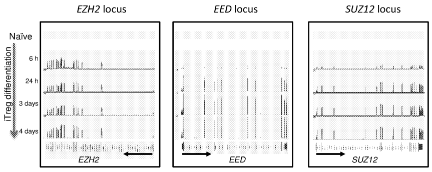

FIG. 1

PatentPak available

Substances (20) Reactions (0) Cited By (0)

64

Mammary molecular portraits reveal lineage-specific features and progenitor cell vulnerabilities

By: Casey, Alison E.; Sinha, Ankit; Singhania, Rajat; Livingstone, Julie ; Waterhouse, Paul ; Tharmapalan, Pirashaanthy; Cruickshank, Jennifer; Shehata, Mona; Drysdale, Erik; Fang, Hui; et al
Journal of Cell Biology (2018), 217(8), 2951-2974 | Language: English, Database: CAplus and MEDLINE

Substances (21) Reactions (0) Cited By (17)

65

BRD4770 functions as a novel ferroptosis inhibitor to protect against aortic dissection

By: Chen, Yue; Yi, Xin; Huo, Bo; He, Yi; Guo, Xian; Zhang, Zihao; Zhong, Xiaoxuan; Feng, Xin; Fang, Ze-Min; Zhu, Xue-Hai; et al
Pharmacological Research (2022), 177, 106122 | Language: English, Database: CAplus and MEDLINE

Substances (22) Reactions (0) Cited By (11)

66

Method for the in vitro prognosis of individuals having multiple myeloma and method for the treatment thereof

By: Moreaux, Jerome; Herviou, Laurie; Kassambara, Alboukadel; Cavalli, Giacomo
World Intellectual Property Organization, WO2018083086 A1 2018-05-11 | Language: English, Database: CAplus

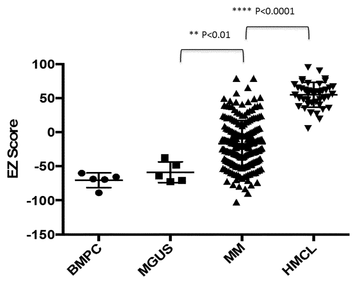

FIGURE 1

PatentPak available

Substances (22) | Reactions (0) | Cited By (1)

67

Combination therapies for cancer by modulation of histone methyl modifying enzymes

By: Arora, Shilpi; Balasubramanian, Srividya; Bradley, William D.; Trojer, Patrick
World Intellectual Property Organization, WO2017018975 A1 2017-02-02 | Language: English, Database: CAplus

PatentPak available

Substances (23) | Reactions (0) | Cited By (0)

68

Methods and materials for promoting bone formation

By: Van Wijnen, Andre J.; Riester, Scott M.; Camilleri, Emily T.; Dudakovic, Amel; Thaler, Roman; Sperling, John W.
World Intellectual Property Organization, WO2016187394 A1 2016-11-24 | Language: English, Database: CAplus

PatentPak available

Substances (23) | Reactions (0) | Cited By (0)

69

Methods of enhancing stem cell differentiation into beta cells

By: Pagliuca, Felicia; Harb, George; Gurtler, Mads; Gaglia, Jason; Poh, Yeh-Chuin; Ye, Lillian; Thiel, Austin; Yasin, Jihad; Thompson, Evrett

World Intellectual Property Organization, WO2019169351 A1 2019-09-06 | Language: English, Database: CAplus

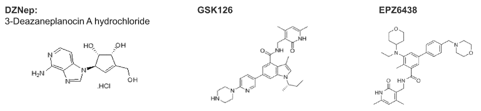

FIG. 1

PatentPak available

Substances (25) | Reactions (0) | Cited By (0)

70

Unimolecular micelles-nanoparticle platform for delivering drug to vascular adventitia via penbrush painting to prevent and treat intimal hyperplasia

By: Kent, Kenneth Craig; Guo, Lian-Wang; Shirasu, Takuro; Wang, Bowen; Gong, Shaoqin; Yodsanit, Nisakorn

World Intellectual Property Organization, WO2022246462 A1 2022-11-24 | Language: English, Database: CAplus

PatentPak available

Substances (50) | Reactions (0) | Cited By (0)

71

Panels and methods for treatment of diffuse large B-cell lymphoma

By: Chapuy, Bjoern; Stewart, Donald; Wood, Tim; Shipp, Margaret; Getz, Gad; Murakami, Mark; Lawton, Lee; Dunford, Andrew; Wienand, Kirsty

World Intellectual Property Organization, WO2022197930 A2 2022-09-22 | Language: English, Database: CAplus

PatentPak available

Substances (26) | Reactions (0) | Cited By (0)

72

Combinatorial Anticancer Drug Screen Identifies Off-Target Effects of Epigenetic Chemical Probes

By: Barghout, Samir H.; Mann, Mandeep K.; Aman, Ahmed; Yu, Yifan; Alteen, Matthew G.; Schimmer, Aaron D.; Schapira, Matthieu; Arrowsmith, Cheryl H.; Barsyte-Lovejoy, Dalia
ACS Chemical Biology (2022), 17(10), 2801-2816 | Language: English, Database: CAplus and MEDLINE

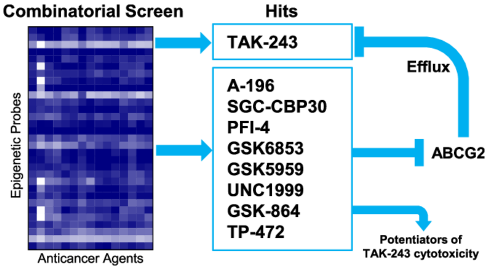

Substances (26) Reactions (0) Cited By (0)

73

Stromal-free methods for CD56+ Natural Killer cell differentiation from pluripotent stem cells for use in human transplant with decreased CD3+ T cells and increased expression of NK cell receptors for cancer immunotherapy

By: Jing, Ran; Daley, George Q.
World Intellectual Property Organization, WO2022187682 A2 2022-09-09 | Language: English, Database: CAplus

PatentPak available

Substances (76) Reactions (0) Cited By (0)

74

Compositions and methods of treating PIK3CA helical domain mutant cancers

By: Wang, Zhenghe; Hao, Yujun; Li, Yamu
United States, US20220193056 A1 2022-06-23 | Language: English, Database: CAplus

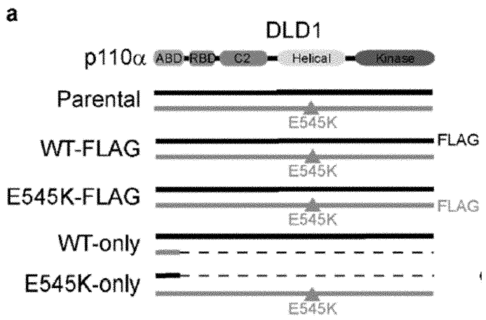

PatentPak available

Substances (82) Reactions (0) Cited By (0)

75

Identification of novel Ebola virus inhibitors using biologically contained virus

By: Vanmechelen, Bert ; Stroobants, Joren; Chiu, Winston; Schepers, Joost; Marchand, Arnaud; Chaltin, Patrick; Vermeire, Kurt; Maes, Piet
Antiviral Research (2022), 200, 105294 | Language: English, Database: CAplus and MEDLINE

Substances (29) Reactions (0) Cited By (1)

76

Small-molecule drug for treating diseases related to inner ear and intestinal epithelial tissue injuries

By: Deng, Hongkui; Qu, Molong; Xiong, Liang
World Intellectual Property Organization, WO2022042527 A1 2022-03-03 | Language: Chinese, Database: CAplus

PatentPak available

Substances (46) Reactions (0) Cited By (0)

77

Ex vivo gastrointestinal biopsy platform to evaluate multi-omic signatures for screening candidate therapeutics

By: Iliopoulos, Dimitrios; Karagiannidis, Iordanis
World Intellectual Property Organization, WO2022031954 A1 2022-02-10 | Language: English, Database: CAplus

PatentPak available

Substances (33) Reactions (0) Cited By (0)

78

Methods for treating cancer with combination therapies

By: Ahsan, Aarif; Bisht, Kamlesh; Bjorklund, Chad; Flynt, Jennifer Erin; Hsu, Chih-Chao; Jeyaraju, Danny Vijey; Ortiz-Estevez, Maria; Pierceall, William Edward; Thakurta, Anjan; Towfic, Fadi George
World Intellectual Property Organization, WO2021262962 A1 2021-12-30 | Language: English, Database: CAplus

PatentPak available

Substances (95) Reactions (0) Cited By (0)

79

Compositions and methods for treating or preventing cancer using deubiquitinase inhibitors in combination with other active agent

By: He, Chuan; Sun, Hui-Lung; Wei, Jiangbo; Zhu, Allen C.; Wang, Huanyu
World Intellectual Property Organization, WO2021178525 A1 2021-09-10 | Language: English, Database: CAplus

PatentPak available

Substances (71) Reactions (0) Cited By (0)

80

Stroma-free T cell differentiation from human pluripotent stem cells and uses thereof in cellular therapy

By: Daley, George Q.; Jing, Ran
World Intellectual Property Organization, WO2021150919 A1 2021-07-29 | Language: English, Database: CAplus

PatentPak available

Substances (46) Reactions (0) Cited By (0)

81

Single cell pathology analysis of tumor samples

By: Jackson, Hartland; Fischer, Jana; Bodenmiller, Bernd
World Intellectual Property Organization, WO2021144474 A1 2021-07-22 | Language: English, Database: CAplus

PatentPak available

Substances (97) Reactions (0) Cited By (0)

82

preparation of Ezh2 inhibitor, cdk4/6 inhibitor and mek inhibitors for treatment of cancer

By: Liao, Cheng; Zhang, Man; Lin, Kan; Zhu, Fuxiang
China, CN112870367 A 2021-06-01 | Language: Chinese, Database: CAplus

PatentPak available

Substances (52) Reactions (0) Cited By (0)

83

Methods of preparation of 2-(2,6-dioxopiperidin-3-yl)-4-((2-fluoro-4-((3-morpholinoazetidin-1-yl)methyl)benzyl)amino)isoindoline-1,3-dione and uses for treating a hematological cancers

By: Anton, Maria Soraya Carrancio; Fontanillo, Celia Fontanillo; Kasibhatla, Shailaja; Lopez-Girona, Antonia; Lu, Gang; Wang, Kai
United States, US20210116454 A1 2021-04-22 | Language: English, Database: CAplus

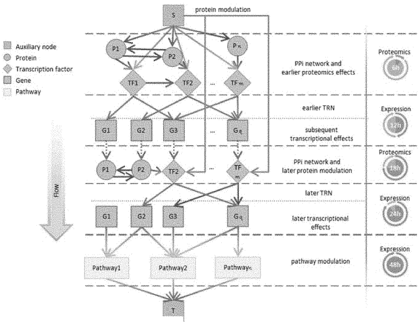

PatentPak available

Substances (74) Reactions (7) Cited By (0)

84

Substituted 4-aminoisoindoline-1,3-dione compounds and second active agents for combined use for treating hematological malignancies

By: Anton, Maria Soraya Carrancio; Buchholz, Tonia J.; Chang, Henry; Filvaroff, Ellen; Kasibhatla, Shailaja; Lopez-Girona, Antonia; Mohan, Adithi; Narla, Rama Krishna; Pourdehnad, Michael
United States, US20210113576 A1 2021-04-22 | Language: English, Database: CAplus

Sensitivity to Compound 1 single agent in DLBCL cell lines

sensitive to resistant

TMD-8	SU-DHL-2	SU-DHL-10	Pfeiffer	WSU-DLCL2	OCI-Ly10	Riva	U2932	SU-DHL-4	DB
58.67	108.4	123.7	288.2	362	403.5	496.3	577	585.3	670.8

PatentPak available

Substances (131) | Reactions (3) | Cited By (0)

85

Chemical small molecule composition and method for chemically inducing in vivo and in vitro direct reprogramming of fibroblasts into hepatocytes

By: Zhang, Peilin
World Intellectual Property Organization, WO2021047495 A1 2021-03-18 | Language: Chinese, Database: CAplus

PatentPak available

Substances (51) | Reactions (0) | Cited By (0)

86

Activity Comparison of Epigenetic Modulators against the Hemoprotozoan Parasites Babesia divergens and Plasmodium falciparum

By: Vanheer, Leen N.; Kafsack, Bjorn F. C.
ACS Infectious Diseases (2021), 7(8), 2277-2284 | Language: English, Database: CAplus and MEDLINE

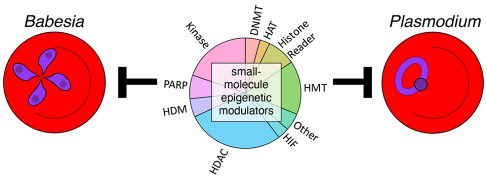

Substances (326) | Reactions (0) | Cited By (6)

87

Use of inhibitors of enhancer of zeste homolog 2 (EZH2) and methods of use thereof for treating cancer

By: Xu, Kexin; Chen, Chen-Hao; Liu, Xiaole Shirley; Brown, Myles
World Intellectual Property Organization, WO2020264580 A1 2020-12-30 | Language: English, Database: CAplus

PatentPak available

Substances (27) | Reactions (0) | Cited By (0)

88

Methods of sensitizing cancer cells to tumor necrosis factor-α (TNFα)-induced cell death with BET inhibitors

By: Bacac, Marina; Fauti, Tanja Andrea; Hogg, Simon John; Johnstone, Ricky Wayne; Ruefli-Brasse, Astrid Alexandra; Rohle, Daniel Alan; Wellinger, Lisa Christina
World Intellectual Property Organization, WO2020169698 A1 2020-08-27 | Language: English, Database: CAplus

PatentPak available

Substances (65) | Reactions (0) | Cited By (2)

89

Antiproliferative compounds and second active agents for combined use for treating multiple myeloma

By: Wong, Lilly L.
United States, US20200215060 A1 2020-07-09 | Language: English, Database: CAplus

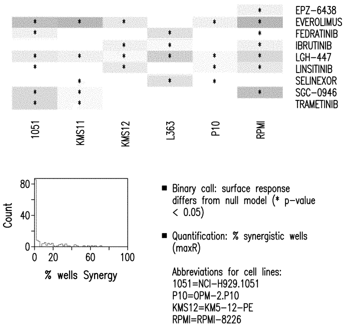

PatentPak available

Substances (76) | Reactions (42) | Cited By (0)

90

Methods using protein arginine methyltransferase (PRMT) inhibitors for the treatment and prevention of hematopoietic disorders and malignancies

By: Shlush, Liran; Abdel-Wahab, Omar
World Intellectual Property Organization, WO2020089892 A1 2020-05-07 | Language: English, Database: CAplus

PatentPak available

Substances (73) | Reactions (0) | Cited By (0)

91

Methods of combinatorial drug screening and use of therapeutic targets thereof in cancer treatment

By: Bernstein, Bradley E.; Doench, John G.; Najm, Fadi J.
World Intellectual Property Organization, WO2020033585 A1 2020-02-13 | Language: English, Database: CAplus

PatentPak available

Substances (109) | Reactions (0) | Cited By (3)

92

Compositions and methods for treating and preventing muscular disorders and dystrophies, steroid side effects, and inflammation

By: Heier, Christopher; Fiorillo, Alyson

World Intellectual Property Organization, WO2019232548 A2 2019-12-05 | Language: English, Database: CAplus

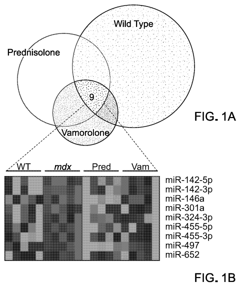

PatentPak available

Substances (95) Reactions (0) Cited By (1)

93

Methods of treating cancers overexpressing arginine methyltransferase CARM1 with enhancer of zeste homolog 2 (EZH2) inhibitors and a PARP inhibitor

By: Zhang, Rugang; Karakashev, Sergey

World Intellectual Property Organization, WO2019195443 A1 2019-10-10 | Language: English, Database: CAplus

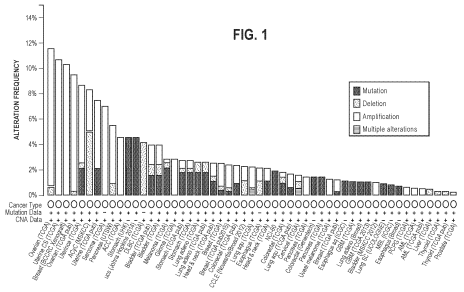

PatentPak available

Substances (47) Reactions (0) Cited By (1)

94

Genetic and pharmacological transcriptional upregulation of the repressed FXN gene as a therapeutic strategy for friedreich ataxia

By: Green, Michael R.; Fang, Minggang

World Intellectual Property Organization, WO2018160356 A1 2018-09-07 | Language: English, Database: CAplus

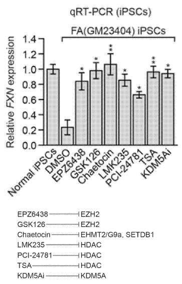

FIG. 1H

PatentPak available

Substances (65) | Reactions (0) | Cited By (1)

95

An inhibitor screen identifies histone-modifying enzymes as mediators of polymer-mediated transgene expression from plasmid DNA

By: Christensen, Matthew D.; Nitiyanandan, Rajeshwar; Meraji, Seyedehmelika; Daer, Rene; Godeshala, Sudhakar; Goklany, Sheba; Haynes, Karmella; Rege, Kaushal

Journal of Controlled Release (2018), 286, 210-223 | Language: English, Database: CAplus and MEDLINE

Substances (98) | Reactions (0) | Cited By (7)

96

Preparation of heterocyclic compounds as EZH2 ligands and their compositions and methods for treating EZH2-mediated cancer

By: Jin, Jian; Parsons, Ramon; Stratikopoulos, Ilias; Yang, Xiaobao

World Intellectual Property Organization, WO2018081530 A1 2018-05-03 | Language: English, Database: CAplus

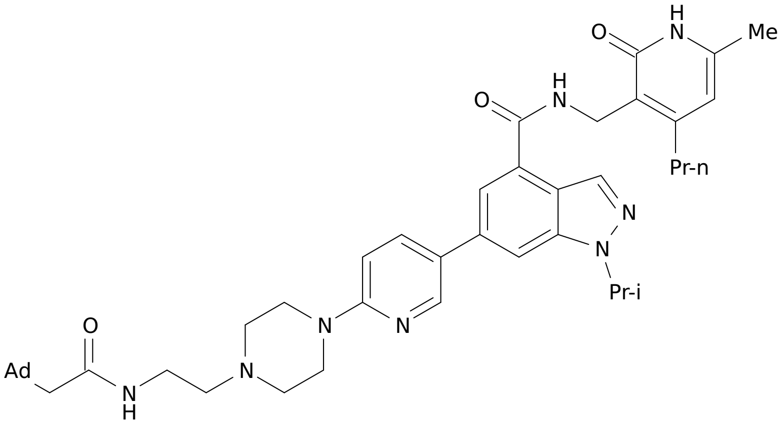

I

PatentPak available

Substances (355) | Reactions (581) | Cited By (1)

97

Treatment of cancers having alterations within the SWI/SNF chromatin remodeling complex

By: Fisher, David E.; Weng, Qing Yu; Kato, Shinichiro
World Intellectual Property Organization, WO2018049000 A1 2018-03-15 | Language: English, Database: CAplus

FIG. 2

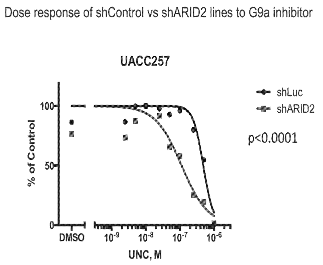

PatentPak available

Substances (46) | Reactions (0) | Cited By (1)

98

Immune cells derived from induced pluripotent stem cell

By: Vo, Linda Thuy; Daley, George Q.
World Intellectual Property Organization, WO2018048828 A1 2018-03-15 | Language: English, Database: CAplus

Fig. 17C

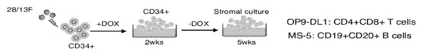

PatentPak available

Substances (31) | Reactions (0) | Cited By (0)

99

Methods of treating ARID1A-mutated cancers with histone deacetylase-6 (HDAC6) inhibitors and histone methyltransferase EZH2 inhibitors

By: Zhang, Rugang; Bitler, Benjamin
World Intellectual Property Organization, WO2018034801 A1 2018-02-22 | Language: English, Database: CAplus

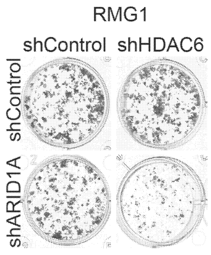

FIG. 4

PatentPak available

Substances (37) | Reactions (0) | Cited By (0)

100

Chromatin protective therapeutics and methods of targeting chromatin heterogeneity for treatment of cancer

By: Backman, Vadim; Szleifer, Igal; Almassalha, Luay; Chandler, John; Bauer, Greta; Gladstein, Scott
World Intellectual Property Organization, WO2018013942 A1 2018-01-18 | Language: English, Database: CAplus

PatentPak available

Substances (29) | Reactions (0) | Cited By (0)

101

Modulators of homeobox transcription factors, DUX4 (double homeobox 4) for regulation of muscle function

By: Rickard, Amanda; De La Garza, Anabel; Schmidt, Uli; Kiselyov, Alexander
World Intellectual Property Organization, WO2017201585 A1 2017-11-30 | Language: English, Database: CAplus

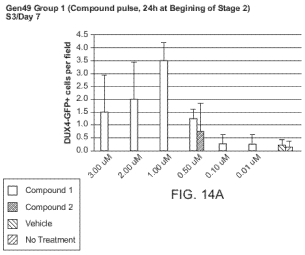

FIG. 14A

PatentPak available

Substances (241) | Reactions (0) | Cited By (3)

102

Preparation of histone methyltransferase EZH2 inhibitors useful for treatment of cancer, inflammation, and other diseases

By: Bradner, James E.; Paulk, Joshiawa; Qi, Jun

World Intellectual Property Organization, WO2017184999 A1 2017-10-26 | Language: English, Database: CAplus

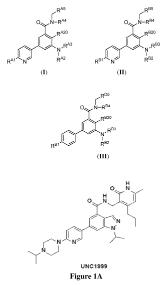

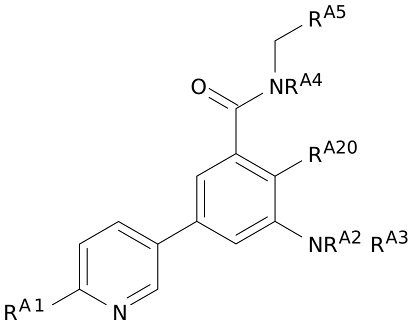

I

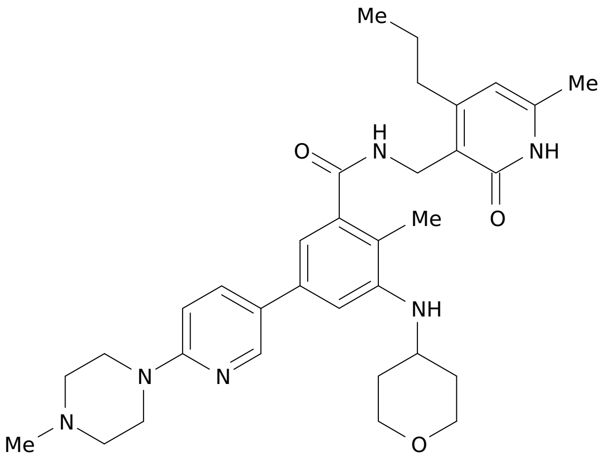

II

PatentPak available

Substances (47)   Reactions (65)   Cited By (5)

103

Compositions and methods for modulating FMR1 expression

By: Green, Michael R.; Fang, Minggang
World Intellectual Property Organization, WO2017049192 A1 2017-03-23 | Language: English, Database: CAplus

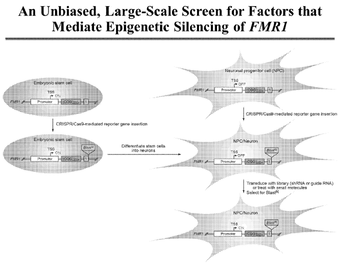

FIG. 5

PatentPak available

Substances (150) | Reactions (0) | Cited By (3)

104

Cell Panel Profiling Reveals Conserved Therapeutic Clusters and Differentiates the Mechanism of Action of Different PI3K/mTOR, Aurora Kinase and EZH2 Inhibitors

By: Uitdehaag, Joost C. M.; de Roos, Jeroen A. D. M.; Prinsen, Martine B. W.; Willemsen-Seegers, Nicole; de Vetter, Judith R. F.; Dylus, Jelle; van Doornmalen, Antoon M.; Kooijman, Jeffrey; Sawa, Masaaki; van Gerwen, Suzanne J. C.; et al
Molecular Cancer Therapeutics (2016), 15(12), 3097-3109 | Language: English, Database: CAplus and MEDLINE

Substances (130) | Reactions (0) | Cited By (13)

105

Identification of novel EZH2 inhibitors through pharmacophore-based virtual screening and biological assays

By: Wu, Yunlong ; Hu, Junchi; Ding, Hong; Chen, Limin; Zhang, Yuanyuan; Liu, Rongfeng; Xu, Pan; Du, Daohai; Lu, Wenchao; Liu, Jingqiu; et al
Bioorganic & Medicinal Chemistry Letters (2016), 26(15), 3813-3817 | Language: English, Database: CAplus and MEDLINE

Substances (88) | Reactions (0) | Cited By (16)

106

Antagonists of Setdb2 for use in the therapy of infectious diseases

By: Bergthaler, Andreas; Schliehe, Christopher
World Intellectual Property Organization, WO2016079321 A1 2016-05-26 | Language: English, Database: CAplus

PatentPak available

Substances (32) | Reactions (0) | Cited By (0)

Copyright © 2023 American Chemical Society (ACS). All Rights Reserved.
Copyright © 2023 U.S. National Library of Medicine.
Internal use only. Redistribution is subject to the terms of your SciFinder^n License Agreement and CAS information Use Policies.

Results of PubMed search: keyword UNC0648

1: Vedadi M, Barsyte-Lovejoy D, Liu F, Rival-Gervier S, Allali-Hassani A, Labrie V, Wigle TJ, Dimaggio PA, Wasney GA, Siarheyeva A, Dong A, Tempel W, Wang SC, Chen X, Chau I, Mangano TJ, Huang XP, Simpson CD, Pattenden SG, Norris JL, Kireev DB, Tripathy A, Edwards A, Roth BL, Janzen WP, Garcia BA, Petronis A, Ellis J, Brown PJ, Frye SV, Arrowsmith CH, Jin J. A chemical probe selectively inhibits G9a and GLP methyltransferase activity in cells. Nat Chem Biol. 2011 Jul 10;7(8):566-74. doi: 10.1038/nchembio.599. Erratum in: Nat Chem Biol. 2011 Sep;7(9):648. Erratum in: Nat Chem Biol. 2011;7(8):following 574. PMID: 21743462; PMCID: PMC3184254.

2: Hauser AT, Jung M. Chemical probes: sharpen your epigenetic tools. Nat Chem Biol. 2011 Jul 18;7(8):499-500. doi: 10.1038/nchembio.615. PMID: 21769094.

3: Machleidt T, Robers MB, Hermanson SB, Dudek JM, Bi K. TR-FRET cellular assays for interrogating posttranslational modifications of histone H3. J Biomol Screen. 2011 Dec;16(10):1236-46. doi: 10.1177/1087057111422943. Epub 2011 Oct 4. PMID: 21972037.

4: Chen X, Skutt-Kakaria K, Davison J, Ou YL, Choi E, Malik P, Loeb K, Wood B, Georges G, Torok-Storb B, Paddison PJ. G9a/GLP-dependent histone H3K9me2 patterning during human hematopoietic stem cell lineage commitment. Genes Dev. 2012 Nov 15;26(22):2499-511. doi: 10.1101/gad.200329.112. Epub 2012 Oct 26. PMID: 23105005; PMCID: PMC3505820.

5: Kim JT, Li J, Jang ER, Gulhati P, Rychahou PG, Napier DL, Wang C, Weiss HL, Lee EY, Anthony L, Townsend CM Jr, Liu C, Evers BM. Deregulation of Wnt/β-catenin signaling through genetic or epigenetic alterations in human neuroendocrine tumors. Carcinogenesis. 2013 May;34(5):953-61. doi: 10.1093/carcin/bgt018. Epub 2013 Jan 25. PMID: 23354304; PMCID: PMC3643417.

6: Yu H, Lin Q, Wang Y, He Y, Fu S, Jiang H, Yu Y, Sun S, Chen Y, Shou J, Li H. Inhibition of H3K9 methyltransferases G9a/GLP prevents ototoxicity and ongoing hair cell death. Cell Death Dis. 2013 Feb 21;4(2):e506. doi: 10.1038/cddis.2013.28. PMID: 23429292; PMCID: PMC3734817.

7: Liu F, Barsyte-Lovejoy D, Li F, Xiong Y, Korboukh V, Huang XP, Allali-Hassani A, Janzen WP, Roth BL, Frye SV, Arrowsmith CH, Brown PJ, Vedadi M, Jin J. Discovery of an in vivo chemical probe of the lysine methyltransferases G9a and GLP. J Med Chem. 2013 Nov 14;56(21):8931-42. doi: 10.1021/jm401480r. Epub 2013 Oct 31. PMID: 24102134; PMCID: PMC3880643.

8: Konze KD, Pattenden SG, Liu F, Barsyte-Lovejoy D, Li F, Simon JM, Davis IJ, Vedadi M, Jin J. A chemical tool for in vitro and in vivo precipitation of lysine methyltransferase G9a. ChemMedChem. 2014 Mar;9(3):549-53. doi: 10.1002/cmdc.201300450. Epub 2014 Jan 17. PMID: 24443078; PMCID: PMC4005005.

9: Fu L, Yan FX, An XR, Hou J. Effects of the histone methyltransferase inhibitor UNC0638 on histone H3K9 dimethylation of cultured ovine somatic cells and development of resulting early cloned embryos. Reprod Domest Anim. 2014 Apr;49(2):e21-5. doi: 10.1111/rda.12277. Epub 2014 Jan 28. PMID: 24467723.

10: Lehnertz B, Pabst C, Su L, Miller M, Liu F, Yi L, Zhang R, Krosl J, Yung E, Kirschner J, Rosten P, Underhill TM, Jin J, Hébert J, Sauvageau G, Humphries RK, Rossi FM. The methyltransferase G9a regulates HoxA9-dependent transcription in AML. Genes Dev. 2014 Feb 15;28(4):317-27. doi: 10.1101/gad.236794.113. PMID: 24532712; PMCID: PMC3937511.

11: Loh SW, Ng WL, Yeo KS, Lim YY, Ea CK. Inhibition of euchromatic histone methyltransferase 1 and 2 sensitizes chronic myeloid leukemia cells to interferon treatment. PLoS One. 2014 Jul 31;9(7):e103915. doi: 10.1371/journal.pone.0103915. PMID: 25079219; PMCID: PMC4117596.

12: Liu C, Yu Y, Liu F, Wei X, Wrobel JA, Gunawardena HP, Zhou L, Jin J, Chen X. A chromatin activity-based chemoproteomic approach reveals a transcriptional repressome for gene-specific silencing. Nat Commun. 2014 Dec 15;5:5733. doi: 10.1038/ncomms6733. PMID: 25502336; PMCID: PMC4360912.

13: Sekar TV, Foygel K, Gelovani JG, Paulmurugan R. Genetically encoded molecular biosensors to image histone methylation in living animals. Anal Chem. 2015 Jan 20;87(2):892-9. doi: 10.1021/ac502629r. Epub 2014 Dec 24. PMID: 25506787; PMCID: PMC4303335.

14: Wu W, Nishikawa H, Fukuda T, Vittal V, Asano M, Miyoshi Y, Klevit RE, Ohta T. Interaction of BARD1 and HP1 Is Required for BRCA1 Retention at Sites of DNA Damage. Cancer Res. 2015 Apr 1;75(7):1311-21. doi: 10.1158/0008-5472.CAN-14-2796. Epub 2015 Jan 29. PMID: 25634209; PMCID: PMC5003120.

15: Lai YS, Chen JY, Tsai HJ, Chen TY, Hung WC. The SUV39H1 inhibitor chaetocin induces differentiation and shows synergistic cytotoxicity with other epigenetic drugs in acute myeloid leukemia cells. Blood Cancer J. 2015 May 15;5(5):e313. doi: 10.1038/bcj.2015.37. PMID: 25978433; PMCID: PMC4476016.

16: Krivega I, Byrnes C, de Vasconcellos JF, Lee YT, Kaushal M, Dean A, Miller JL. Inhibition of G9a methyltransferase stimulates fetal hemoglobin production by facilitating LCR/γ-globin looping. Blood. 2015 Jul 30;126(5):665-72. doi: 10.1182/blood-2015-02-629972. Epub 2015 May 15. PMID: 25979948; PMCID: PMC4520881.

17: Fukuda T, Wu W, Okada M, Maeda I, Kojima Y, Hayami R, Miyoshi Y, Tsugawa K, Ohta T. Class I histone deacetylase inhibitors inhibit the retention of BRCA1 and 53BP1 at the site of DNA damage. Cancer Sci. 2015 Aug;106(8):1050-6. doi: 10.1111/cas.12717. Epub 2015 Jul 14. PMID: 26053117; PMCID: PMC4556395.

18: Renneville A, Van Galen P, Canver MC, McConkey M, Krill-Burger JM, Dorfman DM, Holson EB, Bernstein BE, Orkin SH, Bauer DE, Ebert BL. EHMT1 and EHMT2 inhibition induces fetal hemoglobin expression. Blood. 2015 Oct 15;126(16):1930-9. doi: 10.1182/blood-2015-06-649087. Epub 2015 Aug 28. PMID: 26320100; PMCID: PMC4608240.

19: Singh N, Ramĩrez-Carvajal L, de Los Santos T, Golding MC, Long CR. Inhibition of EHMT2 Induces a Robust Antiviral Response Against Foot-and-Mouth Disease and Vesicular Stomatitis Virus Infections in Bovine Cells. J Interferon Cytokine Res. 2016 Jan;36(1):37-47. doi: 10.1089/jir.2015.0006. Epub 2015 Sep 29. PMID: 26418342; PMCID: PMC4722570.

20: Krivega I, Dean A. Chromatin looping as a target for altering erythroid gene expression. Ann N Y Acad Sci. 2016 Mar;1368(1):31-9. doi: 10.1111/nyas.13012. Epub 2016 Feb 25. PMID: 26918894; PMCID: PMC4870130.

21: Sharma M, Razali NB, Sajikumar S. Inhibition of G9a/GLP Complex Promotes Long-Term Potentiation and Synaptic Tagging/Capture in Hippocampal CA1 Pyramidal Neurons. Cereb Cortex. 2017 Jun 1;27(6):3161-3171. doi: 10.1093/cercor/bhw170. PMID: 27252354.

22: Agarwal P, Jackson SP. G9a inhibition potentiates the anti-tumour activity of DNA double-strand break inducing agents by impairing DNA repair independent of p53 status. Cancer Lett. 2016 Oct 1;380(2):467-475. doi: 10.1016/j.canlet.2016.07.009. Epub 2016 Jul 16. PMID: 27431310; PMCID: PMC5011428.

23: Chen WL, Wang ZH, Feng TT, Li DD, Wang CH, Xu XL, Zhang XJ, You QD, Guo XK. Discovery, design and synthesis of 6H-anthra[1,9-cd]isoxazol-6-one scaffold as G9a inhibitor through a combination of shape-based virtual screening and structure-based molecular modification. Bioorg Med Chem. 2016 Nov 15;24(22):6102-6108. doi: 10.1016/j.bmc.2016.09.071. Epub 2016 Sep 30. PMID: 27720557.

24: Sugeno N, Jäckel S, Voigt A, Wassouf Z, Schulze-Hentrich J, Kahle PJ. α-Synuclein enhances histone H3 lysine-9 dimethylation and H3K9me2-dependent transcriptional responses. Sci Rep. 2016 Nov 3;6:36328. doi: 10.1038/srep36328. PMID: 27808254; PMCID: PMC5093762.

25: Sato T, Cesaroni M, Chung W, Panjarian S, Tran A, Madzo J, Okamoto Y, Zhang H, Chen X, Jelinek J, Issa JJ. Transcriptional Selectivity of Epigenetic Therapy in Cancer. Cancer Res. 2017 Jan 15;77(2):470-481. doi: 10.1158/0008-5472.CAN-16-0834. Epub 2016 Nov 22. PMID: 27879268; PMCID: PMC5243171.

26: Kim Y, Lee HM, Xiong Y, Sciaky N, Hulbert SW, Cao X, Everitt JI, Jin J, Roth BL, Jiang YH. Targeting the histone methyltransferase G9a activates imprinted genes and improves survival of a mouse model of Prader-Willi syndrome. Nat Med. 2017 Feb;23(2):213-222. doi: 10.1038/nm.4257. Epub 2016 Dec 26. PMID: 28024084; PMCID: PMC5589073.

27: Xiong Y, Li F, Babault N, Dong A, Zeng H, Wu H, Chen X, Arrowsmith CH, Brown PJ, Liu J, Vedadi M, Jin J. Discovery of Potent and Selective Inhibitors for G9a-Like Protein (GLP) Lysine Methyltransferase. J Med Chem. 2017 Mar 9;60(5):1876-1891. doi: 10.1021/acs.jmedchem.6b01645. Epub 2017 Feb 14. PMID: 28135087; PMCID: PMC5352984.

28: Nguyen K, Das B, Dobrowolski C, Karn J. Multiple Histone Lysine Methyltransferases Are Required for the Establishment and Maintenance of HIV-1 Latency. mBio. 2017 Feb 28;8(1):e00133-17. doi: 10.1128/mBio.00133-17. PMID: 28246360; PMCID: PMC5347344.

29: Sharma M, Dierkes T, Sajikumar S. Epigenetic regulation by G9a/GLP complex ameliorates amyloid-beta 1-42 induced deficits in long-term plasticity and synaptic tagging/capture in hippocampal pyramidal neurons. Aging Cell. 2017 Oct;16(5):1062-1072. doi: 10.1111/acel.12634. Epub 2017 Jun 30. PMID: 28665013; PMCID: PMC5595698.

30: Wang X, Shen X, Ma S, Liu Y, Xu S, Wu S, Wang F. Threshold Effect of G9a/Glp on Peripheral Nerve Injury Induced Hypersensitivity. Mol Pain. 2017 Jan-Dec;13:1744806917729305. doi: 10.1177/1744806917729305. PMID: 28814147; PMCID: PMC5588794.

31: Avivi S, Mor A, Dotan I, Tzadok S, Kanter I, Kinor N, Canaani D, Shav-Tal Y. Visualizing nuclear RNAi activity in single living human cells. Proc Natl Acad Sci U S A. 2017 Oct 17;114(42):E8837-E8846. doi: 10.1073/pnas.1707440114. Epub 2017 Oct 3. PMID: 29073029; PMCID: PMC5651755.

32: Liu XR, Zhou LH, Hu JX, Liu LM, Wan HP, Zhang XQ. UNC0638, a G9a inhibitor, suppresses epithelial-mesenchymal transition-mediated cellular migration and invasion in triple negative breast cancer. Mol Med Rep. 2018 Feb;17(2):2239-2244. doi: 10.3892/mmr.2017.8190. Epub 2017 Nov 29. PMID: 29207160; PMCID: PMC5783465.

33: Wang L, Dong X, Ren Y, Luo J, Liu P, Su D, Yang X. Targeting EHMT2 reverses EGFR-TKI resistance in NSCLC by epigenetically regulating the PTEN/AKT signaling pathway. Cell Death Dis. 2018 Jan 26;9(2):129. doi: 10.1038/s41419-017-0120-6. PMID: 29374157; PMCID: PMC5833639.

34: Zhang K, Wang J, Yang L, Yuan YC, Tong TR, Wu J, Yun X, Bonner M, Pangeni R, Liu Z, Yuchi T, Kim JY, Raz DJ. Targeting histone methyltransferase G9a inhibits growth and Wnt signaling pathway by epigenetically regulating HP1α and APC2 gene expression in non-small cell lung cancer. Mol Cancer. 2018 Oct 22;17(1):153. doi: 10.1186/s12943-018-0896-8. PMID: 30348169; PMCID: PMC6198520.

35: Gupta R, Saha P, Sen T, Sen N. An augmentation in histone dimethylation at lysine nine residues elicits vision impairment following traumatic brain injury. Free Radic Biol Med. 2019 Apr;134:630-643. doi: 10.1016/j.freeradbiomed.2019.02.015. Epub 2019 Feb 18. Erratum in: Free Radic Biol Med. 2019 May 20;136:184-185. PMID: 30790655; PMCID: PMC6588499.

36: Xiong Y, Greschik H, Johansson C, Seifert L, Bacher J, Park KS, Babault N, Martini M, Fagan V, Li F, Chau I, Christott T, Dilworth D, Barsyte-Lovejoy D, Vedadi M, Arrowsmith CH, Brennan P, Fedorov O, Jung M, Farnie G, Liu J, Oppermann U, Schüle R, Jin J. Discovery of a Potent and Selective Fragment-like Inhibitor of Methyllysine Reader Protein Spindlin 1 (SPIN1). J Med Chem. 2019 Oct 24;62(20):8996-9007. doi: 10.1021/acs.jmedchem.9b00522. Epub 2019 Jul 24. PMID: 31260300.

37: Leenders R, Zijlmans R, van Bree B, van de Sande M, Trivarelli F, Damen E, Wegert A, Müller D, Ehlert JE, Feger D, Heidemann-Dinger C, Kubbutat M, Schächtele C, Lenstra DC, Mecinović J, Müller G. Novel SAR for quinazoline inhibitors of EHMT1 and EHMT2. Bioorg Med Chem Lett. 2019 Sep 1;29(17):2516-2524. doi: 10.1016/j.bmcl.2019.06.012. Epub 2019 Jun 14. PMID: 31350126.

38: Bellamy J, Szemes M, Melegh Z, Dallosso A, Kollareddy M, Catchpoole D, Malik K. Increased Efficacy of Histone Methyltransferase G9a Inhibitors Against <i>MYCN</i>-Amplified Neuroblastoma. Front Oncol. 2020 May 27;10:818. doi: 10.3389/fonc.2020.00818. PMID: 32537432; PMCID: PMC7269128.

39: Nualkaew T, Khamphikham P, Pongpaksupasin P, Kaewsakulthong W, Songdej D, Paiboonsukwong K, Sripichai O, Engel JD, Hongeng S, Fucharoen S, Jearawiriyapaisarn N. UNC0638 induces high levels of fetal hemoglobin expression in β-thalassemia/HbE erythroid progenitor cells. Ann Hematol. 2020 Sep;99(9):2027-2036. doi: 10.1007/s00277-020-04136-w. Epub 2020 Jun 21. PMID: 32567028.

40: Watanabe N, Kitada K, Santostefano KE, Yokoyama A, Waldrop SM, Heldermon CD, Tachibana D, Koyama M, Meacham AM, Pacak CA, Terada N. Generation of Induced Pluripotent Stem Cells from a Female Patient with a Xq27.3-q28 Deletion to Establish Disease Models and Identify Therapies. Cell Reprogram. 2020 Aug;22(4):179-188. doi: 10.1089/cell.2020.0012. Epub 2020 Jun 30. PMID: 32608992; PMCID: PMC7415881.

41: Sampaio RV, Sangalli JR, De Bem THC, Ambrizi DR, Del Collado M, Bridi A, de Ávila ACFCM, Macabelli CH, de Jesus Oliveira L, da Silveira JC, Chiaratti MR, Perecin F, Bressan FF, Smith LC, Ross PJ, Meirelles FV. Catalytic inhibition of H3K9me2 writers disturbs epigenetic marks during bovine nuclear reprogramming. Sci Rep. 2020 Jul 13;10(1):11493. doi: 10.1038/s41598-020-67733-9. PMID: 32661262; PMCID: PMC7359371.

42: Wang JQ , Wang LY , Li SJ , Tong T , Wang L , Huang CS , Xu QC , Huang XT , Li JH , Wu J , Zhao W , Yin XY . Histone methyltransferase G9a inhibitor-loaded redox-responsive nanoparticles for pancreatic ductal adenocarcinoma therapy. Nanoscale. 2020 Aug 7;12(29):15767-15774. doi: 10.1039/d0nr03138k. Epub 2020 Jul 20. PMID: 32729861.

43: Sung PH, Luo CW, Chiang JY, Yip HK. The combination of G9a histone methyltransferase inhibitors with erythropoietin protects heart against damage from acute myocardial infarction. Am J Transl Res. 2020 Jul 15;12(7):3255-3271. PMID: 32774698; PMCID: PMC7407701.

44: Peslak SA, Khandros E, Huang P, Lan X, Geronimo CL, Grevet JD, Abdulmalik O, Zhang Z, Giardine BM, Keller CA, Shi J, Hardison RC, Blobel GA. HRI depletion cooperates with pharmacologic inducers to elevate fetal hemoglobin and reduce sickle cell formation. Blood Adv. 2020 Sep 22;4(18):4560-4572. doi: 10.1182/bloodadvances.2020002475. PMID: 32956454; PMCID: PMC7509868.

45: Ropa J, Saha N, Hu H, Peterson LF, Talpaz M, Muntean AG. SETDB1 mediated histone H3 lysine 9 methylation suppresses MLL-fusion target expression and leukemic transformation. Haematologica. 2020 Sep 1;105(9):2273-2285. doi: 10.3324/haematol.2019.223883. PMID: 33054052; PMCID: PMC7556517.

46: Kato H, Tateishi K, Fujiwara H, Ijichi H, Yamamoto K, Nakatsuka T, Kakiuchi M, Sano M, Kudo Y, Hayakawa Y, Nakagawa H, Tanaka Y, Otsuka M, Hirata Y, Tachibana M, Shinkai Y, Koike K. Deletion of Histone Methyltransferase G9a Suppresses Mutant Kras-driven Pancreatic Carcinogenesis. Cancer Genomics Proteomics. 2020 Nov-Dec;17(6):695-705. doi: 10.21873/cgp.20224. PMID: 33099471; PMCID: PMC7675651.

47: Nakatsuka T, Tateishi K, Kato H, Fujiwara H, Yamamoto K, Kudo Y, Nakagawa H, Tanaka Y, Ijichi H, Ikenoue T, Ishizawa T, Hasegawa K, Tachibana M, Shinkai Y, Koike K. Inhibition of histone methyltransferase G9a attenuates liver cancer initiation by sensitizing DNA-damaged hepatocytes to p53-induced apoptosis. Cell Death Dis. 2021 Jan 19;12(1):99. doi: 10.1038/s41419-020-03381-1. PMID: 33468997; PMCID: PMC7815717.

48: Chandar Charles MR, Li MC, Hsieh HP, Coumar MS. Mimicking H3 Substrate Arginine in the Design of G9a Lysine Methyltransferase Inhibitors for Cancer Therapy: A Computational Study for Structure-Based Drug Design. ACS Omega. 2021 Feb 22;6(9):6100-6111. doi: 10.1021/acsomega.0c04710. PMID: 33718701; PMCID: PMC7948220.

49: De Smedt E, Devin J, Muylaert C, Robert N, Requirand G, Vlummens P, Vincent L, Cartron G, Maes K, Moreaux J, De Bruyne E. G9a/GLP targeting in MM promotes autophagy-associated apoptosis and boosts proteasome inhibitor-mediated cell death. Blood Adv. 2021 May 11;5(9):2325-2338. doi: 10.1182/bloodadvances.2020003217. PMID: 33938943; PMCID: PMC8114552.

50: Maksimova V, Shalginskikh N, Vlasova O, Usalka O, Beizer A, Bugaeva P,

Fedorov D, Lizogub O, Lesovaya E, Katz R, Belitsky G, Kirsanov K, Yakubovskaya M. HeLa TI cell-based assay as a new approach to screen for chemicals able to reactivate the expression of epigenetically silenced genes. PLoS One. 2021 Jun 11;16(6):e0252504. doi: 10.1371/journal.pone.0252504. PMID: 34115770; PMCID: PMC8195432.

51: Li RG, Deng H, Liu XH, Chen ZY, Wan SS, Wang L. Histone Methyltransferase G9a Promotes the Development of Renal Cancer through Epigenetic Silencing of Tumor Suppressor Gene SPINK5. Oxid Med Cell Longev. 2021 Jul 13;2021:6650781. doi: 10.1155/2021/6650781. PMID: 34336110; PMCID: PMC8294961.

52: Lin HY, Wu HJ, Chen SY, Hou MF, Lin CS, Chu PY. Epigenetic therapy combination of UNC0638 and CI-994 suppresses breast cancer via epigenetic remodeling of BIRC5 and GADD45A. Biomed Pharmacother. 2022 Jan;145:112431. doi: 10.1016/j.biopha.2021.112431. Epub 2021 Nov 16. PMID: 34798471.

53: Ren N, Wang F, Zhao L, Wang S, Zhang G, Li J, Zhang B, Wang J, Bergeron E, Yuan Z, Xia H. Efficient rescue of a newly classified Ebinur lake orthobunyavirus with GFP reporter and its application in rapid antiviral screening. Antiviral Res. 2022 Nov;207:105421. doi: 10.1016/j.antiviral.2022.105421. Epub 2022 Sep 21. PMID: 36150523.

54: Zhou C, Li C, Zheng Y, Huang X. Regulation, genomics, and clinical characteristics of cuproptosis regulators in pan-cancer. Front Oncol. 2022 Oct 27;12:934076. doi: 10.3389/fonc.2022.934076. PMID: 36387247; PMCID: PMC9647015.

CAS SciFinder^{n}

Task History

Initiating Search

January 11, 2023, 9:37PM

All: **UNC0638**

Search Tasks

Task	Search Type	View
Returned All Results	All	View Results
Exported: Returned Reference Results (59)	References	View Results

Copyright © 2023 American Chemical Society (ACS). All Rights Reserved.
Internal use only. Redistribution is subject to the terms of your SciFinder^{n} License Agreement and CAS information Use Policies.

CAS SciFinderⁿ

References (59)

View in SciFinderⁿ

1

UNC0638, a G9a inhibitor, suppresses epithelial-mesenchymal transition-mediated cellular migration and invasion in triple negative breast cancer

By: Liu, Xiang-Rong; Zhou, Li-Hua; Hu, Jian-Xin; Liu, Li-Min; Wan, Hui-Ping; Zhang, Xi-Quan
Molecular Medicine Reports (2018), 17(2), 2239-2244 | Language: English, Database: CAplus and MEDLINE

Substance (1) | Reactions (0) | Cited By (21)

2

A chemical probe selectively inhibits G9a and GLP methyltransferase activity in cells

By: Vedadi, Masoud; Barsyte-Lovejoy, Dalia; Liu, Feng; Rival-Gervier, Sylvie; Allali-Hassani, Abdellah; Labrie, Viviane; Wigle, Tim J.; DiMaggio, Peter A.; Wasney, Gregory A.; Siarheyeva, Alena; et al
Nature Chemical Biology (2011), 7(8), 566-574 | Language: English, Database: CAplus and MEDLINE

Substances (17) | Reactions (24) | Cited By (388)

3

Effects of the Histone Methyltransferase Inhibitor UNC0638 on Histone H3K9 Dimethylation of Cultured Ovine Somatic Cells and Development of Resulting Early Cloned Embryos

By: Fu, L.; Yan, F.-X.; An, X.-R.; Hou, J.
Reproduction in Domestic Animals (2014), 49(2), e21-e25 | Language: English, Database: CAplus and MEDLINE

Substances (2) | Reactions (0) | Cited By (11)

4

UNC0638 induces high levels of fetal hemoglobin expression in β-thalassemia/HbE erythroid progenitor cells

By: Nualkaew, Tiwaporn; Khamphikham, Pinyaphat; Pongpaksupasin, Phitchapa; Kaewsakulthong, Woratree; Songdej, Duantida; Paiboonsukwong, Kittiphong; Sripichai, Orapan; Engel, James Douglas; Hongeng, Suradej; Fucharoen, Suthat; et al
Annals of Hematology (2020), 99(9), 2027-2036 | Language: English, Database: CAplus and MEDLINE

Substances (5) | Reactions (0) | Cited By (7)

5

Epigenetic therapy combination of UNC0638 and CI-994 suppresses breast cancer via epigenetic remodeling of BIRC5 and GADD45A

By: Lin, Hung-Yu; Wu, Hsing-Ju; Chen, Si-Yun; Hou, Ming-Feng; Lin, Chang-Shen; Chu, Pei-Yi
Biomedicine & Pharmacotherapy (2022), 145, 112431 | Language: English, Database: CAplus and MEDLINE

Substances (6) | Reactions (0) | Cited By (4)

6

Discovery of an in vivo chemical probe of the lysine methyltransferases G9a and GLP

By: Liu, Feng; Barsyte-Lovejoy, Dalia; Li, Fengling; Xiong, Yan; Korboukh, Victoria; Huang, Xi-Ping; Allali-Hassani, Abdellah; Janzen, William P.; Roth, Bryan L.; Frye, Stephen V.; et al
Journal of Medicinal Chemistry (2013), 56(21), 8931-8942 | Language: English, Database: CAplus and MEDLINE

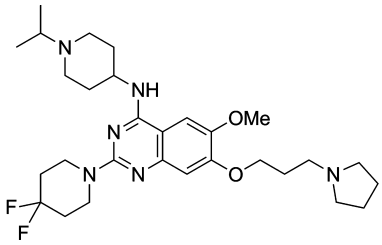

G9a and GLP: IC_{50} < 2.5 nM
> 2,000-fold selective over other methyltransferases
Improved in vivo PK properties

Substances (45) | Reactions (201) | Cited By (163)

7

EHMT1 and EHMT2 inhibition induces fetal hemoglobin expression

By: Renneville, Aline; Van Galen, Peter; Canver, Matthew C.; McConkey, Marie; Krill-Burger, John M.; Dorfman, David M.; Holson, Edward B.; Bernstein, Bradley E.; Orkin, Stuart H.; Bauer, Daniel E.; et al
Blood (2015), 126(16), 1930-1939 | Language: English, Database: CAplus and MEDLINE

Substances (11) | Reactions (0) | Cited By (69)

8

Inhibition of G9a methyltransferase stimulates fetal hemoglobin production by facilitating LCR/γ-globin looping

By: Krivega, Ivan; Byrnes, Colleen; de Vasconcellos, Jaira F.; Lee, Y. Terry; Kaushal, Megha; Dean, Ann; Miller, Jeffery L.
Blood (2015), 126(5), 665-672 | Language: English, Database: CAplus and MEDLINE

Substances (4) | Reactions (0) | Cited By (54)

9

Targeting the histone methyltransferase G9a activates imprinted genes and improves survival of a mouse model of Prader-Willi syndrome

By: Kim, Yuna; Lee, Hyeong-Min; Xiong, Yan; Sciaky, Noah; Hulbert, Samuel W.; Cao, Xinyu; Everitt, Jeffrey I.; Jin, Jian; Roth, Bryan L.; Jiang, Yong-hui
Nature Medicine (New York, NY, United States) (2017), 23(2), 213-222 | Language: English, Database: CAplus and MEDLINE

Substances (2) Reactions (0) Cited By (67)

10

G9a inhibition potentiates the anti-tumour activity of DNA double-strand break inducing agents by impairing DNA repair independent of p53 status

By: Agarwal, Pallavi; Jackson, Stephen P.
Cancer Letters (New York, NY, United States) (2016), 380(2), 467-475 | Language: English, Database: CAplus and MEDLINE

Substances (3) Reactions (0) Cited By (28)

11

Interaction of BARD1 and HP1 Is Required for BRCA1 Retention at Sites of DNA Damage

By: Wu, Wenwen; Nishikawa, Hiroyuki; Fukuda, Takayo; Vittal, Vinayak; Asano, Masahide; Miyoshi, Yasuo; Klevit, Rachel E.; Ohta, Tomohiko
Cancer Research (2015), 75(7), 1311-1321 | Language: English, Database: CAplus and MEDLINE

Substances (3) Reactions (0) Cited By (70)

12

G9a/GLP-dependent histone H3K9me2 patterning during human hematopoietic stem cell lineage commitment

By: Chen, Xiaoji; Skutt-Kakaria, Kyobi; Davison, Jerry; Ou, Yang-Li; Choi, Edward; Malik, Punam; Loeb, Keith; Wood, Brent; Georges, George; Torok-Storb, Beverly; et al
Genes & Development (2012), 26(22), 2499-2511 | Language: English, Database: CAplus and MEDLINE

Substances (3) Reactions (0) Cited By (98)

13

Histone methyltransferase G9a inhibitor-loaded redox-responsive nanoparticles for pancreatic ductal adenocarcinoma therapy

By: Wang, Jie-Qin; Wang, Li-Ying; Li, Shi-Jin; Tong, Tong; Wang, Li; Huang, Chen-Song; Xu, Qiong-Cong; Huang, Xi-Tai; Li, Jian-Hui; Wu, Jun; et al
Nanoscale (2020), 12(29), 15767-15774 | Language: English, Database: CAplus and MEDLINE

Substances (12) Reactions (0) Cited By (7)

14

Threshold effect of G9a/Glp on peripheral nerve injury-induced hypersensitivity

By: Wang, Xian; Shen, Xiaofeng; Ma, Shaolei; Liu, Yusheng; Xu, Shiqin; Wu, Shizheng; Wang, Fuzhou
Molecular Pain (2017), 13, 1744806917729305 | Language: English, Database: CAplus and MEDLINE

Substances (3) Reactions (0) Cited By (4)

15

A chemical probe selectively inhibits G9a and GLP methyltransferase activity in cells [Erratum to document cited in CA155:320969]

By: Vedadi, Masoud; Barsyte-Lovejoy, Dalia; Liu, Feng; Rival-Gervier, Sylvie; Allali-Hassani, Abdellah; Labrie, Viviane; Wigle, Tim J.; DiMaggio, Peter A.; Wasney, Gregory A.; Siarheyeva, Alena; et al
Nature Chemical Biology (2011), 7(9), 648 | Language: English, Database: CAplus

Substances (16) Reactions (7) Cited By (6)

16

Targeting histone methyltransferase G9a inhibits growth and Wnt signaling pathway by epigenetically regulating HP1α and APC2 gene expression in non-small cell lung cancer

By: Zhang, Keqiang ; Wang, Jinhui; Yang, Lu; Yuan, Yate-Ching; Tong, Tommy R.; Wu, Jun; Yun, Xinwei; Bonner, Melissa; Pangeni, Rajendra; Liu, Zheng; et al
Molecular Cancer (2018), 17, 153 | Language: English, Database: CAplus and MEDLINE

Substances (7) Reactions (0) Cited By (42)

17

Preparation method and application of nano medicine in treatment of pancreatic ductal carcinoma

By: Zhao, Wei; Wu, Jun; Yin, Xiaoyu
China, CN111643481 A 2020-09-11 | Language: Chinese, Database: CAplus

PatentPak available

Substances (5) Reactions (0) Cited By (0)

18

Catalytic inhibition of H3K9me2 writers disturbs epigenetic marks during bovine nuclear reprogramming

By: Sampaio, Rafael Vilar ; Sangalli, Juliano Rodrigues; De Bem, Tiago Henrique Camara ; Ambrizi, Dewison Ricardo; del Collado, Maite ; Bridi, Alessandra ; de Avila, Ana Clara Faquineli Cavalcante Mendes; Macabelli, Carolina Habermann ; de Jesus Oliveira, Lilian; da Silveira, Juliano Coelho ; et al
Scientific Reports (2020), 10(1), 11493 | Language: English, Database: CAplus and MEDLINE

Substances (8) Reactions (0) Cited By (3)

19

A Chemical Tool for In Vitro and In Vivo Precipitation of Lysine Methyltransferase G9a

By: Konze, Kyle D.; Pattenden, Samantha G.; Liu, Feng; Barsyte-Lovejoy, Dalia; Li, Fengling; Simon, Jeremy M.; Davis, Ian J.; Vedadi, Masoud; Jin, Jian
ChemMedChem (2014), 9(3), 549-553 | Language: English, Database: CAplus and MEDLINE

Substances (19) Reactions (47) Cited By (22)

20

Inhibition of H3K9 methyltransferases G9a/GLP prevents ototoxicity and ongoing hair cell death

By: Yu, H.; Lin, Q.; Wang, Y.; He, Y.; Fu, S.; Jiang, H.; Yu, Y.; Sun, S.; Chen, Y.; Shou, J.; et al
Cell Death & Disease (2013), 4(Feb.), e506 | Language: English, Database: CAplus and MEDLINE

Substances (4) Reactions (0) Cited By (33)

21

Class I histone deacetylase inhibitors inhibit the retention of BRCA1 and 53BP1 at the site of DNA damage

By: Fukuda, Takayo; Wu, Wenwen; Okada, Maiko; Maeda, Ichiro; Kojima, Yasuyuki; Hayami, Ryosuke; Miyoshi, Yasuo; Tsugawa, Ko-ichiro; Ohta, Tomohiko
Cancer Science (2015), 106(8), 1050-1056 | Language: English, Database: CAplus and MEDLINE

Substances (2) Reactions (0) Cited By (23)

22

A chromatin activity-based chemoproteomic approach reveals a transcriptional repressome for gene-specific silencing

By: Liu, Cui; Yu, Yanbao; Liu, Feng; Wei, Xin; Wrobel, John A.; Gunawardena, Harsha P.; Zhou, Li; Jin, Jian; Chen, Xian
Nature Communications (2014), 5, 5733 | Language: English, Database: CAplus and MEDLINE

Substances (3) Reactions (0) Cited By (20)

23

Discovery of Potent and Selective Inhibitors for G9a-Like Protein (GLP) Lysine Methyltransferase

By: Xiong, Yan; Li, Fengling; Babault, Nicolas; Dong, Aiping; Zeng, Hong; Wu, Hong; Chen, Xin; Arrowsmith, Cheryl H.; Brown, Peter J.; Liu, Jing; et al

Journal of Medicinal Chemistry (2017), 60(5), 1876-1891 | Language: English, Database: CAplus and MEDLINE

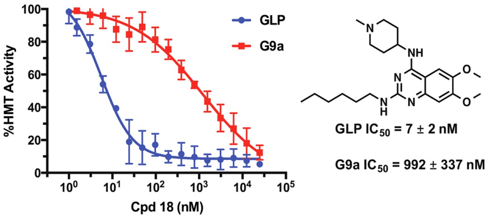

Substances (128) | Reactions (570) | Cited By (41)

24

HRI depletion cooperates with pharmacologic inducers to elevate fetalhemoglobin and reduce sickle cell formation

By: Peslak, Scott A.; Khandros, Eugene; Huang, Peng; Lan, Xianjiang; Geronimo, Carly L.; Grevet, Jeremy D.; Abdulmalik, Osheiza; Zhang, Zhe; Giardine, Belinda M.; Keller, Cheryl A.; et al

Blood Advances (2020), 4(18), 4560-4572 | Language: English, Database: CAplus and MEDLINE

Substances (2) | Reactions (0) | Cited By (10)

25

SETDB1 mediated histone H3 lysine 9 methylation suppresses MLL-fusion target expression and leukemic transformation

By: Ropa, James; Saha, Nirmalya; Hu, Hsiangyu; Peterson, Luke F.; Talpaz, Moshe; Muntean, Andrew G.

Haematologica (2020), 105(9), 2273-2285 | Language: English, Database: CAplus and MEDLINE

Substances (8) | Reactions (0) | Cited By (11)

26

Transcriptional Selectivity of Epigenetic Therapy in Cancer

By: Sato, Takahiro; Cesaroni, Matteo; Chung, Woonbok; Panjarian, Shoghag; Tran, Anthony; Madzo, Jozef; Okamoto, Yasuyuki; Zhang, Hanghang; Chen, Xiaowei; Jelinek, Jaroslav; et al

Cancer Research (2017), 77(2), 470-481 | Language: English, Database: CAplus and MEDLINE

Substances (12) | Reactions (0) | Cited By (39)

27

Discovery, design and synthesis of 6H-anthra[1,9-cd]isoxazol-6-one scaffold as G9a inhibitor through a combination of shape-based virtual screening and structure-based molecular modification

By: Chen, Wei-Lin; Wang, Zhi-Hui; Feng, Tao-Tao; Li, Dong-Dong; Wang, Chu-Hui; Xu, Xiao-Li; Zhang, Xiao-Jin; You, Qi-Dong; Guo, Xiao-Ke
Bioorganic & Medicinal Chemistry (2016), 24(22), 6102-6108 | Language: English, Database: CAplus and MEDLINE

Substances (71) Reactions (217) Cited By (22)

28

Inhibition of histone methyltransferase G9a attenuates liver cancer initiation by sensitizing DNA-damaged hepatocytes to p53-induced apoptosis

By: Nakatsuka, Takuma ; Tateishi, Keisuke; Kato, Hiroyuki; Fujiwara, Hiroaki; Yamamoto, Keisuke ; Kudo, Yotaro; Nakagawa, Hayato; Tanaka, Yasuo; Ijichi, Hideaki; Ikenoue, Tsuneo; et al
Cell Death & Disease (2021), 12(1), 99 | Language: English, Database: CAplus and MEDLINE

Substances (5) Reactions (0) Cited By (10)

29

The SUV39H1 inhibitor chaetocin induces differentiation and shows synergistic cytotoxicity with other epigenetic drugs in acute myeloid leukemia cells.

By: Lai, Y-S; Chen, J-Y; Tsai, H-J; Chen, T-Y; Hung, W-C
Blood cancer journal (2015), 5, e313 | Language: English, Database: MEDLINE

Substance (1) Reactions (0) Cited By (57)

30

Efficient rescue of a newly classified Ebinur lake orthobunyavirus with GFP reporter and its application in rapid antiviral screening

By: Ren, Nanjie; Wang, Fei; Zhao, Lu; Wang, Shunlong; Zhang, Guilin; Li, Jiaqi; Zhang, Bo; Wang, Jinglin; Bergeron, Eric; Yuan, Zhiming; et al
Antiviral Research (2022), 207, 105421 | Language: English, Database: CAplus and MEDLINE

Substances (12) Reactions (0) Cited By (0)

31

Deregulation of Wnt/β-catenin signaling through genetic or epigenetic alterations in human neuroendocrine tumors

By: Kim, Ji Tae; Li, Jing; Jang, Eun Ryoung; Gulhati, Pat; Rychahou, Piotr G.; Napier, Dana L.; Wang, Chi; Weiss, Heidi L.; Lee, Eun Y.; Anthony, Lowell; et al
Carcinogenesis (2013), 34(5), 953-961 | Language: English, Database: CAplus and MEDLINE

Substance (1) | Reactions (0) | Cited By (60)

32

The combination of G9a histone methyltransferase inhibitors with erythropoietin protects heart against damage from acute myocardial infarction.

By: Sung, Pei-Hsun; Luo, Chi-Wen; Chiang, John Y; Yip, Hon-Kan
American journal of translational research (2020), 12(7), 3255-3271 | Language: English, Database: MEDLINE

Substances (0) | Reactions (0) | Cited By (8)

33

Histone methyltransferase G9a promotes the development of renal cancer through epigenetic silencing of tumor suppressor gene SPINK5

By: Li, Ren-gui; Deng, Huan; Liu, Xiu-heng; Chen, Zhi-yuan; Wan, Shan-shan ; Wang, Lei
Oxidative Medicine and Cellular Longevity (2021), 6650781 | Language: English, Database: CAplus and MEDLINE

Substances (0) | Reactions (0) | Cited By (6)

34

Chemical probes for the lysine methyltransferases G9a and EZH2

By: Konze, Kyle D.; Ma, Anqi; Pattenden, Samantha G.; Jin, Jian
Abstracts of Papers, 250th ACS National Meeting & Exposition, Boston, MA, United States, August 16-20, 2015 (2015), MEDI-295 | Language: English, Database: CAplus

Substances (0) | Reactions (0) | Cited By (0)

35

Inhibition of EHMT2 Induces a Robust Antiviral Response Against Foot-and-Mouth Disease and Vesicular Stomatitis Virus Infections in Bovine Cells

By: Singh, Neetu; Ramirez-Carvajal, Lisbeth; de los Santos, Teresa; Golding, Michael C.; Long, Charles R.
Journal of Interferon & Cytokine Research (2016), 36(1), 37-47 | Language: English, Database: CAplus and MEDLINE

Substances (5) | Reactions (0) | Cited By (7)

36

multiple histone lysine methyltransferases are required for the establishment and maintenance of HIV-1 latency

By: Nguyen, Kien; Das, Biswajit; Dobrowolski, Curtis; Karn, Jonathan
mBio (2017), 8(1), e00133-17/1-e00133-17/15 | Language: English, Database: CAplus and MEDLINE

Substances (9) Reactions (0) Cited By (77)

37

Methods using G9a and/or G9a-like protein (GLP) methyltransferase inhibitors for treating a vascular leakage-associated disease or disorder

By: Wen, Xiao-Yan; Philip, Anju; Dossantos, Claudia; Marshall, John
World Intellectual Property Organization, WO2020154805 A1 2020-08-06 | Language: English, Database: CAplus

PatentPak available

Substances (6) Reactions (0) Cited By (0)

38

Discovery of a Potent and Selective Fragment-like Inhibitor of Methyllysine Reader Protein Spindlin 1 (SPIN1)

By: Xiong, Yan; Greschik, Holger; Johansson, Catrine; Seifert, Ludwig; Bacher, Johannes; Park, Kwang-su; Babault, Nicolas; Martini, Michael; Fagan, Vincent; Li, Fengling; et al
Journal of Medicinal Chemistry (2019), 62(20), 8996-9007 | Language: English, Database: CAplus and MEDLINE

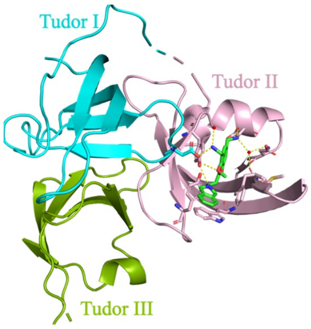

Substances (25) Reactions (122) Cited By (12)

39

The combination of G9a histone methyltransferase inhibitors with erythropoietin protects heart against damage from acute myocardial infarction

By: Sung, Pei-Hsun; Luo, Chi-Wen; Chiang, John Y.; Yip, Hon-Kan
American Journal of Translational Research (2020), 12(7), 3255-3271 | Language: English, Database: CAplus

Substances (0) Reactions (0) Cited By (7)

40

Deletion of histone methyltransferase G9a suppresses mutant Kras-driven pancreatic carcinogenesis

By: Kato, Hiroyuki; Tateishi, Keisuke; Fujiwara, Hiroaki; Ijichi, Hideaki; Yamamoto, Keisuke; Nakatsuka, Takuma; Kakiuchi, Miwako; Sano, Makoto; Kudo, Yotaro; Hayakawa, Yoku; et al
Cancer Genomics & Proteomics (2020), 17(6), 695-705 | Language: English, Database: CAplus and MEDLINE

Substances (2) Reactions (0) Cited By (7)

41

TR-FRET cellular assays for interrogating posttranslational modifications of histone H3

By: Machleidt, Thomas; Robers, Matthew B.; Hermanson, Spencer B.; Dudek, Jeanne M.; Bi, Kun
Journal of Biomolecular Screening (2011), 16(10), 1236-1246 | Language: English, Database: CAplus and MEDLINE

Substances (12) Reactions (0) Cited By (24)

42

α-Synuclein enhances histone H3 lysine-9 dimethylation and H3K9me2-dependent transcriptional responses

By: Sugeno, Naoto; Jaeckel, Sandra; Voigt, Aaron; Wassouf, Zinah; Schulze-Hentrich, Julia; Kahle, Philipp J.
Scientific Reports (2016), 6, 36328 | Language: English, Database: CAplus and MEDLINE

Substances (21) Reactions (0) Cited By (35)

43

Genetically encoded molecular biosensors to image histone methylation in living animals

By: Sekar, Thillai V.; Foygel, Kira; Gelovani, Juri G.; Paulmurugan, Ramasamy
Analytical Chemistry (Washington, DC, United States) (2015), 87(2), 892-899 | Language: English, Database: CAplus and MEDLINE

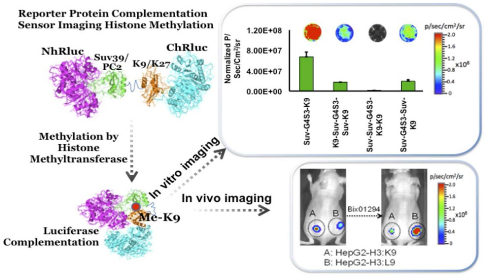

Substances (6) Reactions (0) Cited By (18)

44

Mimicking H3 Substrate Arginine in the Design of G9a Lysine Methyltransferase Inhibitors for Cancer Therapy: A Computational Study for Structure-Based Drug Design

By: Chandar Charles, M. Ramya; Li, Mu-Chun ; Hsieh, Hsing-Pang ; Coumar, Mohane Selvaraj
ACS Omega (2021), 6(9), 6100-6111 | Language: English, Database: CAplus and MEDLINE

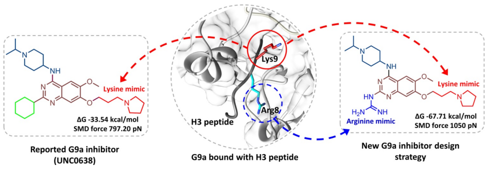

Substances (7) | Reactions (0) | Cited By (1)

45

Targeting EHMT2 reverses EGFR-TKI resistance in NSCLC by epigenetically regulating the PTEN/AKT signaling pathway

By: Wang, Lihui; Dong, Xiaoyu; Ren, Yong; Luo, Juanjuan; Liu, Pei; Su, Dongsheng; Yang, Xiaojun
Cell Death & Disease (2018), 9(2), 1-13 | Language: English, Database: CAplus and MEDLINE

Substances (17) | Reactions (0) | Cited By (41)

46

Generation of Induced Pluripotent Stem Cells from a Female Patient with a Xq27.3-q28 Deletion to Establish Disease Models and Identify Therapies

By: Watanabe, Noriko; Kitada, Kohei; Santostefano, Katherine E.; Yokoyama, Airi; Waldrop, Sara M.; Heldermon, Coy D.; Tachibana, Daisuke; Koyama, Masayasu; Meacham, Amy M.; Pacak, Christina A.; et al
Cellular Reprogramming (2020), 22(4), 179-188 | Language: English, Database: CAplus and MEDLINE

Substances (3) | Reactions (0) | Cited By (3)

47

Stereoselective formation of gliotoxin isomers

By: Blackhurst, Noah; Fultz, Micheal W.
Abstracts of Papers, 247th ACS National Meeting & Exposition, Dallas, TX, United States, March 16-20, 2014 (2014), CHED-1138 | Language: English, Database: CAplus

Substances (0) | Reactions (0) | Cited By (0)

48

Methods of treating cancer and preventing cancer drug resistance using histone methyltransferase G9a antagonists in combination with other chemotherapy agents

By: Classon, Marie; Guler, Gulfem Dilek; Pitti, Robert; Stephan, Jean-Philippe; Tindell, Charles Albert

World Intellectual Property Organization, WO2015200329 A1 2015-12-30 | Language: English, Database: CAplus

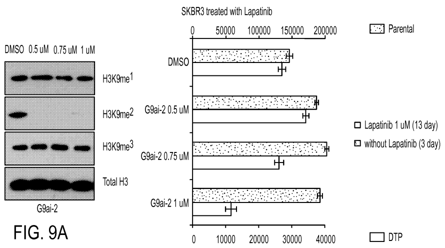

FIG. 9A FIG. 9B

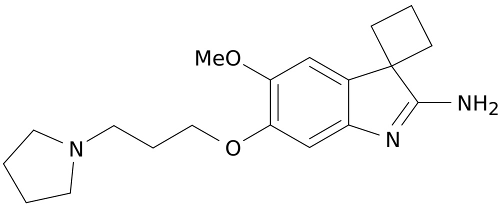

I

PatentPak available

Substances (28) Reactions (0) Cited By (0)

49

Discovery of a first-in-class G9a/GLP covalent inhibitor

By: Yan, Xiong; Park, Kwang-su; Velez, Julia; Yim, Hyerin; Liu, Jing; Jin, Jian

Abstracts of Papers, ACS Fall 2022, Chicago, IL, United States (2022), No pp. given | Language: English, Database: CAplus

Substances (0) Reactions (0) Cited By (0)

50

G9a/GLP targeting in MM promotes autophagy-associated apoptosis and boosts proteasome inhibitor-mediated cell death

By: De Smedt, Eva; Devin, Julie; Muylaert, Catharina; Robert, Nicolas; Requirand, Guilhem; Vlummens, Philip; Vincent, Laure; Cartron, Guillaume ; Maes, Ken ; Moreaux, Jerome ; et al

Blood Advances (2021), 5(9), 2325-2338 | Language: English, Database: CAplus and MEDLINE

Substances (0) Reactions (0) Cited By (7)

51

Preparation of quinazolin-4-amine derivatives as histone methyltransferase G9a inhibitors and methods for the treatment of Prader-willi syndrome

By: Jiang, Yong-Hui; Kim, Yuna; Lee, Hyeong-Min; Jin, Jian; Roth, Bryan L.

World Intellectual Property Organization, WO2017201199 A1 2017-11-23 | Language: English, Database: CAplus

UNC618

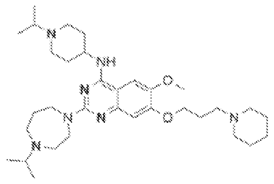

Figure 1C

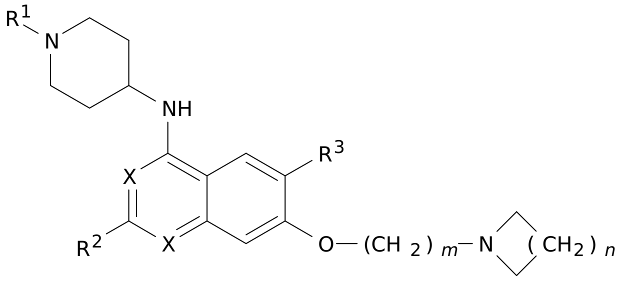

I

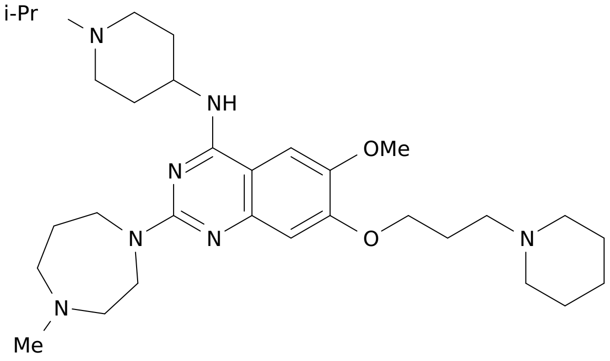

II

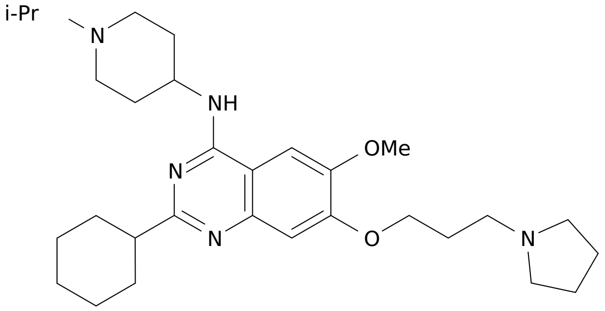

III

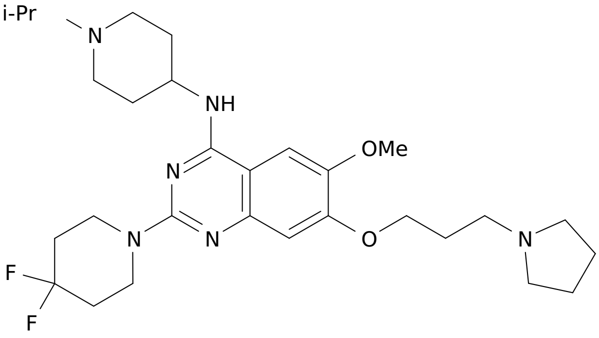

IV

PatentPak available

Substances (19) Reactions (4) Cited By (2)

52

Chemiluminescent detection of intracellular binding events by measuring β-galactosidase fusion protein abundance and stability

By: Raab, William; Wehrman, Thomas S.
United States, US20130203067 A1 2013-08-08 | Language: English, Database: CAplus

PatentPak available

Substances (36) Reactions (0) Cited By (1)

53

Increased Efficacy of Histone Methyltransferase G9a Inhibitors Against *MYCN*-Amplified Neuroblastoma.

By: Bellamy, Jacob; Szemes, Marianna; Melegh, Zsombor; Dallosso, Anthony; Kollareddy, Madhu; Catchpoole, Daniel; Malik, Karim
Frontiers in oncology (2020), 10, 818 | Language: English, Database: MEDLINE

Substances (0) Reactions (0) Cited By (12)

54

An augmentation in histone dimethylation at lysine nine residues elicits vision impairment following traumatic brain injury

By: Gupta, Rajaneesh; Saha, Pampa; Sen, Tanusree; Sen, Nilkantha
Free Radical Biology & Medicine (2019), 134, 630-643 | Language: English, Database: CAplus and MEDLINE

Substances (3) Reactions (0) Cited By (8)

55

Regulation, genomics, and clinical characteristics of cuproptosis regulators in pan-cancer.

By: Zhou, Cankun; Li, Chaomei; Zheng, Yuhua; Huang, Xiaobin
Frontiers in oncology (2022), 12, 934076 | Language: English, Database: MEDLINE

Substances (0) Reactions (0) Cited By (0)

56

HeLa TI cell-based assay as a new approach to screen for chemicals able to reactivate the expression of epigenetically silenced genes

By: Maksimova, Varvara ; Shalginskikh, Natalya; Vlasova, Olga; Usalka, Olga; Beizer, Anastasia; Bugaeva, Polina; Fedorov, Dmitry; Lizogub, Olga; Lesovaya, Ekaterina; Katz, Richard; et al
PLoS One (2021), 16(6), e0252504 | Language: English, Database: CAplus and MEDLINE

Substances (44) Reactions (0) Cited By (3)

57

Threshold inhibition of methyltransferase G9a/Glp exacerbates neuropathic hypersensi-tivity through mediating GRIN2B methylation

By: Wang, Xian; Wu, Haibo; Ma, Jianming; Xu, Shiqin; Li, Shengmei; Bao, Senzhu; Wang, Fuzhou
Science Insights (2019), 33-47 | Language: English, Database: CAplus

Substances (0) Reactions (0) Cited By (6)

58

Discovery of a potent and selective fragment-like inhibitor of SPIN1

By: Yan, Xiong; Greschik, Holger; Johansson, Catrine; Seifert, Ludwig; Bacher, Johannes; Park, Kwang-su; Babault, Nicolas; Martini, Michael; Fagan, Vincent; Li, Fengling; et al
Abstracts of Papers, ACS Fall 2019, San Diego, CA, United States (2019), MEDI-0243 | Language: English, Database: CAplus

Substances (0) Reactions (0) Cited By (0)

59

Dual G9A/EZH2 inhibition stimulates antitumor immune response in ovarian high-grade serous carcinoma

By: Spear, Sarah; Mirza, Hasan; Garner, Ian; McGarry, Lynn; Grundland-Freile, Fabio; Cheng, Zhao; Ennis, Darren P.; Iyer, Nayana; McNamara, Sophie; Natoli, Marina; et al
Molecular Cancer Therapeutics (2022), 21(4), 522-534 | Language: English, Database: CAplus and MEDLINE

Substances (0) Reactions (0) Cited By (6)

Copyright © 2023 American Chemical Society (ACS). All Rights Reserved.
Copyright © 2023 U.S. National Library of Medicine.
Internal use only. Redistribution is subject to the terms of your SciFinderⁿ License Agreement and CAS information Use Policies.

CAS SciFinder^{n}

Task History

Initiating Search

January 11, 2023, 9:40PM

All:

Structure of UNC0638
(right)

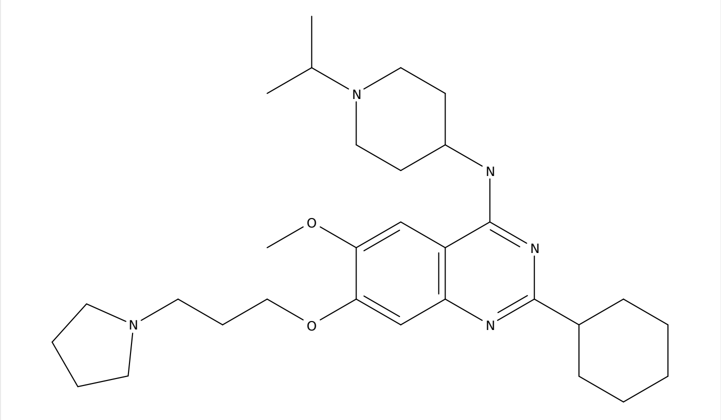

Search Tasks

Task	Search Type	View
Returned All Results	All	View Results
Exported: Returned Reference Results + Filters (132)<br>Filtered By:<br>Structure Match: As Drawn	References	View Results

Copyright © 2023 American Chemical Society (ACS). All Rights Reserved.
Internal use only. Redistribution is subject to the terms of your SciFinder^{n} License Agreement and CAS information Use Policies.

CAS SciFinder^{n}

References (132)

View in SciFinder^{n}

1

A chemical probe selectively inhibits G9a and GLP methyltransferase activity in cells

By: Vedadi, Masoud; Barsyte-Lovejoy, Dalia; Liu, Feng; Rival-Gervier, Sylvie; Allali-Hassani, Abdellah; Labrie, Viviane; Wigle, Tim J.; DiMaggio, Peter A.; Wasney, Gregory A.; Siarheyeva, Alena; et al
Nature Chemical Biology (2011), 7(8), 566-574 | Language: English, Database: CAplus and MEDLINE

Substances (17) Reactions (24) Cited By (388)

2

UNC0638, a G9a inhibitor, suppresses epithelial-mesenchymal transition-mediated cellular migration and invasion in triple negative breast cancer

By: Liu, Xiang-Rong; Zhou, Li-Hua; Hu, Jian-Xin; Liu, Li-Min; Wan, Hui-Ping; Zhang, Xi-Quan
Molecular Medicine Reports (2018), 17(2), 2239-2244 | Language: English, Database: CAplus and MEDLINE

Substance (1) Reactions (0) Cited By (21)

3

Inhibition of G9a methyltransferase stimulates fetal hemoglobin production by facilitating LCR/γ-globin looping

By: Krivega, Ivan; Byrnes, Colleen; de Vasconcellos, Jaira F.; Lee, Y. Terry; Kaushal, Megha; Dean, Ann; Miller, Jeffery L.
Blood (2015), 126(5), 665-672 | Language: English, Database: CAplus and MEDLINE

Substances (4) Reactions (0) Cited By (54)

4

Targeting histone methyltransferase G9a inhibits growth and Wnt signaling pathway by epigenetically regulating HP1α and APC2 gene expression in non-small cell lung cancer

By: Zhang, Keqiang ; Wang, Jinhui; Yang, Lu; Yuan, Yate-Ching; Tong, Tommy R.; Wu, Jun; Yun, Xinwei; Bonner, Melissa; Pangeni, Rajendra; Liu, Zheng; et al
Molecular Cancer (2018), 17, 153 | Language: English, Database: CAplus and MEDLINE

Substances (7) Reactions (0) Cited By (42)

5

G9a promotes invasion and metastasis of non-small cell lung cancer through enhancing focal adhesion kinase activation via NF-κB signaling pathway

By: Sun, Ting; Zhang, Keqiang; Pangeni, Rajendra P.; Wu, Jun; Li, Wendong; Du, Yong; Guo, Yuming; Chaurasiya, Shyambabu; Arvanitis, Leonidas; Raz, Dan J.
Molecular Cancer Research (2021), 19(3), 429-440 | Language: English, Database: CAplus and MEDLINE

Substances (2) Reactions (0) Cited By (11)

6

G9a inhibition potentiates the anti-tumour activity of DNA double-strand break inducing agents by impairing DNA repair independent of p53 status

By: Agarwal, Pallavi; Jackson, Stephen P.
Cancer Letters (New York, NY, United States) (2016), 380(2), 467-475 | Language: English, Database: CAplus and MEDLINE

Substances (3) Reactions (0) Cited By (28)

7

multiple histone lysine methyltransferases are required for the establishment and maintenance of HIV-1 latency

By: Nguyen, Kien; Das, Biswajit; Dobrowolski, Curtis; Karn, Jonathan
mBio (2017), 8(1), e00133-17/1-e00133-17/15 | Language: English, Database: CAplus and MEDLINE

Substances (9) Reactions (0) Cited By (77)

8

Discovery of an in vivo chemical probe of the lysine methyltransferases G9a and GLP

By: Liu, Feng; Barsyte-Lovejoy, Dalia; Li, Fengling; Xiong, Yan; Korboukh, Victoria; Huang, Xi-Ping; Allali-Hassani, Abdellah; Janzen, William P.; Roth, Bryan L.; Frye, Stephen V.; et al
Journal of Medicinal Chemistry (2013), 56(21), 8931-8942 | Language: English, Database: CAplus and MEDLINE

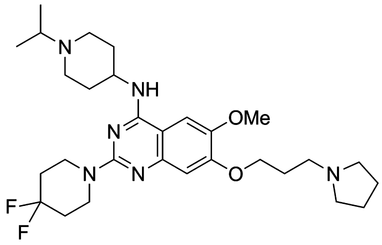

G9a and GLP: IC_{50} < 2.5 nM
> 2,000-fold selective over other methyltransferases
Improved in vivo PK properties

Substances (45) Reactions (201) Cited By (163)

9

Optimization of cellular activity of G9a inhibitors 7-aminoalkoxy-quinazolines

By: Liu, Feng; Barsyte-Lovejoy, Dalia; Allali-Hassani, Abdellah; He, Yunlong; Herold, J. Martin; Chen, Xin; Yates, Christopher M.; Frye, Stephen V.; Brown, Peter J.; Huang, Jing; et al

Journal of Medicinal Chemistry (2011), 54(17), 6139-6150 | Language: English, Database: CAplus and MEDLINE

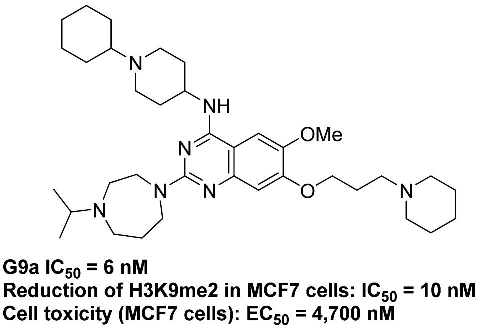

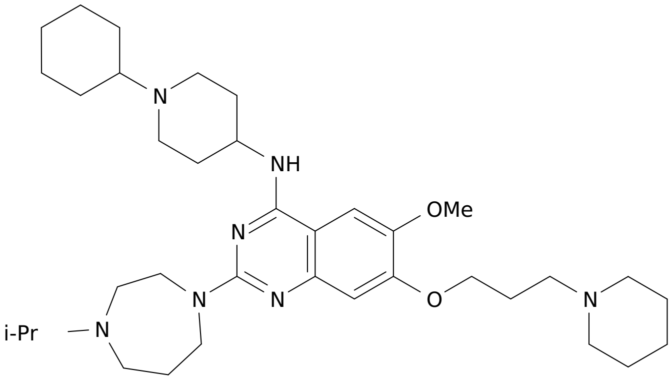

I

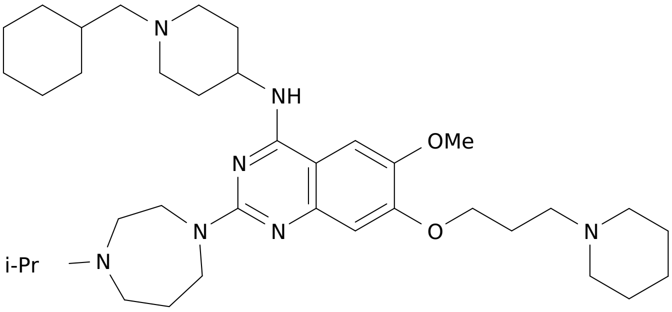

II

Substances (54) Reactions (105) Cited By (113)

10

Inhibition of histone methyltransferase G9a attenuates liver cancer initiation by sensitizing DNA-damaged hepatocytes to p53-induced apoptosis

By: Nakatsuka, Takuma ; Tateishi, Keisuke; Kato, Hiroyuki; Fujiwara, Hiroaki; Yamamoto, Keisuke ; Kudo, Yotaro; Nakagawa, Hayato; Tanaka, Yasuo; Ijichi, Hideaki; Ikenoue, Tsuneo; et al

Cell Death & Disease (2021), 12(1), 99 | Language: English, Database: CAplus and MEDLINE

Substances (5) Reactions (0) Cited By (10)

11

Estimating intraclonal heterogeneity and subpopulation changes from bulk expression profiles in CMap

By: Hsieh, Chiao-Yu; Tu, Ching-Chih; Hung, Jui-Hung
Life Science Alliance (2022), 5(10), e202101299 | Language: English, Database: CAplus and MEDLINE

Substances (3) Reactions (0) Cited By (1)

12

Threshold effect of G9a/Glp on peripheral nerve injury-induced hypersensitivity

By: Wang, Xian; Shen, Xiaofeng; Ma, Shaolei; Liu, Yusheng; Xu, Shiqin; Wu, Shizheng; Wang, Fuzhou
Molecular Pain (2017), 13, 1744806917729305 | Language: English, Database: CAplus and MEDLINE

Substances (3) Reactions (0) Cited By (4)

13

An augmentation in histone dimethylation at lysine nine residues elicits vision impairment following traumatic brain injury

By: Gupta, Rajaneesh; Saha, Pampa; Sen, Tanusree; Sen, Nilkantha
Free Radical Biology & Medicine (2019), 134, 630-643 | Language: English, Database: CAplus and MEDLINE

Substances (3) Reactions (0) Cited By (8)

14

Interaction of BARD1 and HP1 Is Required for BRCA1 Retention at Sites of DNA Damage

By: Wu, Wenwen; Nishikawa, Hiroyuki; Fukuda, Takayo; Vittal, Vinayak; Asano, Masahide; Miyoshi, Yasuo; Klevit, Rachel E.; Ohta, Tomohiko
Cancer Research (2015), 75(7), 1311-1321 | Language: English, Database: CAplus and MEDLINE

Substances (3) Reactions (0) Cited By (70)

15

Reduced and highly diverse peripheral HIV-1 reservoir in virally suppressed patients infected with non-B HIV-1 strains in Uganda

By: Joussef-Pina, Samira; Nankya, Immaculate; Nalukwago, Sophie; Baseke, Joy; Rwambuya, Sandra; Winner, Dane; Kyeyune, Fred; Chervenak, Keith; Thiel, Bonnie; Asaad, Robert; et al
Retrovirology (2022), 19(1), 1 | Language: English, Database: CAplus and MEDLINE

Substances (4) Reactions (0) Cited By (1)

16

Small molecule inhibitors of G9a reactivate the maternal PWS genes in Prader-Willi-Syndrome patient derived neural stem cells and differentiated neurons

By: Wu, Hao; Ng, Carrie; Villegas, Vivian; Chamberlain, Stormy; Cacace, Angela; Wallace, Owen
bioRxiv (2019), 1-19 | Language: English, Database: CAplus

Substances (4) | Reactions (0) | Cited By (1)

17

A disproportionate impact of G9a methyltransferase deficiency on the X chromosome

By: Szanto, Attila; Aguilar, Rodrigo ; Kesner, Barry; Blum, Roy; Wang, Danni; Cifuentes-Rojas, Catherine; del Rosario, Brian C.; Kis-Toth, Katalin; Lee, Jeannie T.
Genes & Development (2021), 35(13-14), 1035-1054 | Language: English, Database: CAplus and MEDLINE

Substances (5) | Reactions (0) | Cited By (2)

18

Dual EZH2 and G9a inhibition suppresses multiple myeloma cell proliferation by regulating the interferon signal and IRF4-MYC axis

By: Ishiguro, Kazuya; Kitajima, Hiroshi; Niinuma, Takeshi; Maruyama, Reo; Nishiyama, Naotaka; Ohtani, Hitoshi; Sudo, Gota; Toyota, Mutsumi; Sasaki, Hajime; Yamamoto, Eiichiro; et al
Cell Death Discovery (2021), 7(1), 7 | Language: English, Database: CAplus and MEDLINE

Substances (5) | Reactions (0) | Cited By (23)

19

Preparation method and application of nano medicine in treatment of pancreatic ductal carcinoma

By: Zhao, Wei; Wu, Jun; Yin, Xiaoyu
China, CN111643481 A 2020-09-11 | Language: Chinese, Database: CAplus

PatentPak available

Substances (5) | Reactions (0) | Cited By (0)

20

UNC0638 induces high levels of fetal hemoglobin expression in β-thalassemia/HbE erythroid progenitor cells

By: Nualkaew, Tiwaporn ; Khamphikham, Pinyaphat ; Pongpaksupasin, Phitchapa; Kaewsakulthong, Woratree; Songdej, Duantida; Paiboonsukwong, Kittiphong; Sripichai, Orapan; Engel, James Douglas; Hongeng, Suradej; Fucharoen, Suthat; et al
Annals of Hematology (2020), 99(9), 2027-2036 | Language: English, Database: CAplus and MEDLINE

Substances (5) | Reactions (0) | Cited By (7)

21

Compositions and methods for fetal hemoglobin production using a eIF2αK1 (eukaryotic translation initiation factor 2-alpha kinase 1) inhibitor for treating hemoglobinopathy or thalassemia

By: Blobel, Gerd; Grevet, Jeremy; Shi, Junwei; Vakoc, Christopher
World Intellectual Property Organization, WO2018140934 A1 2018-08-02 | Language: English, Database: CAplus

PatentPak available

Substances (5) | Reactions (0) | Cited By (0)

22

Report and Application of a Tool Compound Data Set

By: Butler, Kyle V.; MacDonald, Ian A.; Hathaway, Nathaniel A.; Jin, Jian
Journal of Chemical Information and Modeling (2017), 57(11), 2699-2706 | Language: English, Database: CAplus and MEDLINE

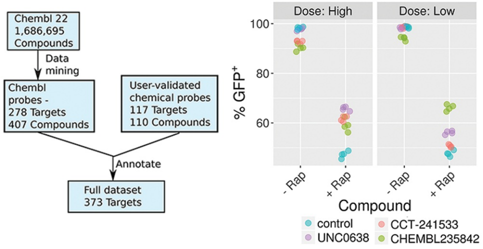

Substances (5) | Reactions (0) | Cited By (5)

23

Inhibition of euchromatic histone methyltransferase 1 and 2 sensitizes chronic myeloid leukemia cells to interferon treatment

By: Loh, Sheng Wei; Ng, Wei Lun; Yeo, Kok Siong; Lim, Yat-Yuen; Ea, Chee-Kwee
PLoS One (2014), 9(7), e103915/1-e103915/13, 13 pp. | Language: English, Database: CAplus and MEDLINE

Substances (5) | Reactions (0) | Cited By (8)

24

EHMT1 and EHMT2 inhibition induces fetal hemoglobin expression

By: Renneville, Aline; Van Galen, Peter; Canver, Matthew C.; McConkey, Marie; Krill-Burger, John M.; Dorfman, David M.; Holson, Edward B.; Bernstein, Bradley E.; Orkin, Stuart H.; Bauer, Daniel E.; et al
Blood (2015), 126(16), 1930-1939 | Language: English, Database: CAplus and MEDLINE

Substances (11) | Reactions (0) | Cited By (69)

25

Identification of novel quinoline inhibitor for EHMT2/G9a through virtual screening

By: Charles, M. Ramya Chandar; Mahesh, Arun; Lin, Shu-Yu; Hsieh, Hsing-Pang; Dhayalan, Arunkumar; Coumar, Mohane Selvaraj
Biochimie (2020), 168, 220-230 | Language: English, Database: CAplus and MEDLINE

Substances (11) Reactions (10) Cited By (8)

26

Reactivation of endogenous retroviral elements via treatment with DNMT- and HDAC-inhibitors

By: Daskalakis, Michael ; Brocks, David; Sheng, Yi-Hua; Islam, Saiful Md.; Ressnerova, Alzbeta; Assenov, Yassen ; Milde, Till; Oehme, Ina; Witt, Olaf; Goyal, Ashish ; et al
Cell Cycle (2018), 17(7), 811-822 | Language: English, Database: CAplus and MEDLINE

Substances (9) Reactions (0) Cited By (32)

27

Epigenetic therapy combination of UNC0638 and CI-994 suppresses breast cancer via epigenetic remodeling of BIRC5 and GADD45A

By: Lin, Hung-Yu; Wu, Hsing-Ju; Chen, Si-Yun; Hou, Ming-Feng; Lin, Chang-Shen; Chu, Pei-Yi
Biomedicine & Pharmacotherapy (2022), 145, 112431 | Language: English, Database: CAplus and MEDLINE

Substances (6) Reactions (0) Cited By (4)

28

Methods using G9a and/or G9a-like protein (GLP) methyltransferase inhibitors for treating a vascular leakage-associated disease or disorder

By: Wen, Xiao-Yan; Philip, Anju; Dossantos, Claudia; Marshall, John
World Intellectual Property Organization, WO2020154805 A1 2020-08-06 | Language: English, Database: CAplus

PatentPak available

Substances (6) Reactions (0) Cited By (0)

29

Genetically encoded molecular biosensors to image histone methylation in living animals

By: Sekar, Thillai V.; Foygel, Kira; Gelovani, Juri G.; Paulmurugan, Ramasamy
Analytical Chemistry (Washington, DC, United States) (2015), 87(2), 892-899 | Language: English, Database: CAplus and MEDLINE

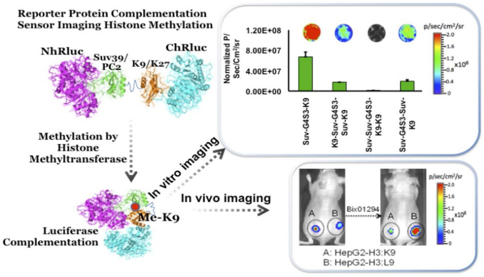

Substances (6) | Reactions (0) | Cited By (18)

30

Polymyxins and quinazolines are LSD1/KDM1A inhibitors with unusual structural features

By: Speranzini, Valentina; Rotili, Dante; Ciossani, Giuseppe; Pilotto, Simona; Marrocco, Biagina; Forgione, Mariantonietta; Lucidi, Alessia; Forneris, Federico; Mehdipour, Parinaz; Velankar, Sameer; et al
Science Advances (2016), 2(9), e1601017/1-e1601017/8 | Language: English, Database: CAplus and MEDLINE

Substances (10) | Reactions (0) | Cited By (43)

31

Mimicking H3 Substrate Arginine in the Design of G9a Lysine Methyltransferase Inhibitors for Cancer Therapy: A Computational Study for Structure-Based Drug Design

By: Chandar Charles, M. Ramya; Li, Mu-Chun; Hsieh, Hsing-Pang; Coumar, Mohane Selvaraj
ACS Omega (2021), 6(9), 6100-6111 | Language: English, Database: CAplus and MEDLINE

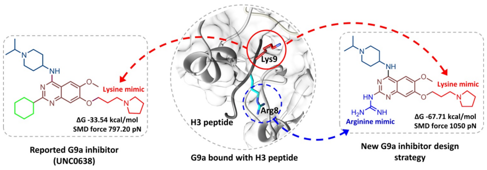

Substances (7) | Reactions (0) | Cited By (1)

32

Histone methyltransferase inhibition has a cytotoxic impact on transformed mast cells: implications for mastocytosis

By: Alanazi, Sultan; Melo, Fabio Rabelo; Pejler, Gunnar
Anticancer Research (2020), 40(5), 2525-2536 | Language: English, Database: CAplus and MEDLINE

Substances (7) | Reactions (0) | Cited By (1)

33

Methods and compositions relating to lung cell differentiation

By: Kim, Carla F.; Rowbotham, Samuel; Lee, Joo-Hyeon
World Intellectual Property Organization, WO2017218287 A1 2017-12-21 | Language: English, Database: CAplus

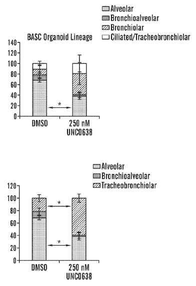

FIG. 5

PatentPak available

Substances (7) Reactions (0) Cited By (0)

34

Discovery of Reversible DNA Methyltransferase and Lysine Methyltransferase G9a Inhibitors with Antitumoral in Vivo Efficacy

By: Rabal, Obdulia ; San Jose-Eneriz, Edurne; Agirre, Xabier; Sanchez-Arias, Juan Antonio; Vilas-Zornoza, Amaia; Ugarte, Ana; de Miguel, Irene; Miranda, Estibaliz; Garate, Leire; Fraga, Mario; et al
Journal of Medicinal Chemistry (2018), 61(15), 6518-6545 | Language: English, Database: CAplus and MEDLINE

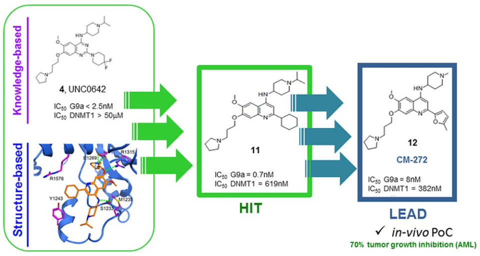

Substances (140) Reactions (448) Cited By (28)

35

Strategy to Target the Substrate Binding site of SET Domain Protein Methyltransferases

By: Nguyen, Kong T.; Li, Fengling; Poda, Gennadiy; Smil, David; Vedadi, Masoud; Schapira, Matthieu
Journal of Chemical Information and Modeling (2013), 53(3), 681-691 | Language: English, Database: CAplus and MEDLINE

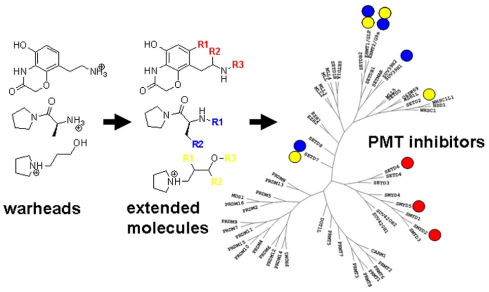

Substances (19) Reactions (0) Cited By (14)

36

SETDB1 mediated histone H3 lysine 9 methylation suppresses MLL-fusion target expression and leukemic transformation

By: Ropa, James; Saha, Nirmalya; Hu, Hsiangyu; Peterson, Luke F.; Talpaz, Moshe; Muntean, Andrew G.
Haematologica (2020), 105(9), 2273-2285 | Language: English, Database: CAplus and MEDLINE

Substances (8) Reactions (0) Cited By (11)

37

Catalytic inhibition of H3K9me2 writers disturbs epigenetic marks during bovine nuclear reprogramming

By: Sampaio, Rafael Vilar ; Sangalli, Juliano Rodrigues; De Bem, Tiago Henrique Camara ; Ambrizi, Dewison Ricardo; del Collado, Maite ; Bridi, Alessandra ; de Avila, Ana Clara Faquineli Cavalcante Mendes; Macabelli, Carolina Habermann ; de Jesus Oliveira, Lilian; da Silveira, Juliano Coelho ; et al
Scientific Reports (2020), 10(1), 11493 | Language: English, Database: CAplus and MEDLINE

Substances (8) Reactions (0) Cited By (3)

38

Treatment of donor cells with recombinant KDM4D protein improves preimplantation development of cloned ovine embryos

By: Zhang, Yumei; Wang, Qianqian; Liu, Kailing; Gao, Enen; Guan, Hong; Hou, Jian
Cytotechnology (2018), 70(5), 1469-1477 | Language: English, Database: CAplus and MEDLINE

Substances (8) Reactions (0) Cited By (9)

39

Structure-activity relationship studies of G9a-like protein (GLP) inhibitors

By: Xiong, Yan; Li, Fengling; Babault, Nicolas; Wu, Hong; Dong, Aiping; Zeng, Hong; Chen, Xin; Arrowsmith, Cheryl H.; Brown, Peter J.; Liu, Jing; et al
Bioorganic & Medicinal Chemistry (2017), 25(16), 4414-4423 | Language: English, Database: CAplus and MEDLINE

Substances (74) Reactions (60) Cited By (15)

40

A chemical probe selectively inhibits G9a and GLP methyltransferase activity in cells [Erratum to document cited in CA155:320969]

By: Vedadi, Masoud; Barsyte-Lovejoy, Dalia; Liu, Feng; Rival-Gervier, Sylvie; Allali-Hassani, Abdellah; Labrie, Viviane; Wigle, Tim J.; DiMaggio, Peter A.; Wasney, Gregory A.; Siarheyeva, Alena; et al
Nature Chemical Biology (2011), 7(9), 648 | Language: English, Database: CAplus

Substances (16) Reactions (7) Cited By (6)

41

Expansion of hematopoietic progenitor cells by histone methyltransferase g9a inhibition

By: Ugarte, Fernando
United States, US20160010059 A1 2016-01-14 | Language: English, Database: CAplus

PatentPak available Substances (9) Reactions (0) Cited By (0)

42

Methods for delaying differentiation of hematopoietic progenitor cells by histone methyltransferase G9a inhibition and therapeutic applications

By: Ugarte, Fernando
World Intellectual Property Organization, WO2014138488 A1 2014-09-12 | Language: English, Database: CAplus

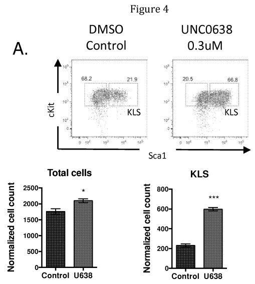

PatentPak available Substances (9) Reactions (0) Cited By (0)

43

M33 condenses chromatin through nuclear body formation and methylation of both histone H3 lysine 9 and lysine 27

By: Lin, Yu-Ru; Liu, You-Yu; Lan, Hsin-Chi; Shen, Chiung-Chyi; Yao, Ya-Li; Yang, Wen-Ming
Biochimica et Biophysica Acta, Molecular Cell Research (2021), 1868(11), 119100 | Language: English, Database: CAplus and MEDLINE

Substances (10) Reactions (0) Cited By (0)

44

Methods and uses related to rhabdoid tumors

By: Huang, Annie; Torchia, Jonathon
United States, US20170298437 A1 2017-10-19 | Language: English, Database: CAplus

PatentPak available

Substances (10) Reactions (0) Cited By (0)

45

Methods for diagnosing and treating rhabdoid tumors

By: Huang, Annie; Torchia, Jonathon
Canada, CA2926943 A1 2017-10-13 | Language: English, Database: CAplus

PatentPak available

Substances (10) Reactions (0) Cited By (0)

46

Chromatin-activity-based chemoproteomic (ChaC) methods and systems for disease marker discovery and development

By: Chen, Xian
World Intellectual Property Organization, WO2015123326 A1 2015-08-20 | Language: English, Database: CAplus

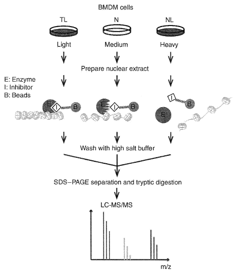

Fig.1

PatentPak available

Substances (10) Reactions (0) Cited By (0)

47

Properly substituted analogues of BIX-01294 lose inhibition of G9a histone methyltransferase and gain selective anti-DNA methyltransferase 3A activity

By: Rotili, Dante; Tarantino, Domenico; Marrocco, Biagina; Gros, Christina; Masson, Veronique; Poughon, Valerie; Ausseil, Frederic; Chang, Yanqi; Labella, Donatella; Cosconati, Sandro; et al
PLoS One (2014), 9(5), e96941/1-e96941/9, 9 pp. | Language: English, Database: CAplus and MEDLINE

Substances (39) Reactions (31) Cited By (24)

48

Efficient rescue of a newly classified Ebinur lake orthobunyavirus with GFP reporter and its application in rapid antiviral screening

By: Ren, Nanjie; Wang, Fei; Zhao, Lu; Wang, Shunlong; Zhang, Guilin; Li, Jiaqi; Zhang, Bo; Wang, Jinglin; Bergeron, Eric; Yuan, Zhiming; et al
Antiviral Research (2022), 207, 105421 | Language: English, Database: CAplus and MEDLINE

Substances (12) Reactions (0) Cited By (0)

49

Histone methyltransferase G9a inhibitor-loaded redox-responsive nanoparticles for pancreatic ductal adenocarcinoma therapy

By: Wang, Jie-Qin; Wang, Li-Ying; Li, Shi-Jin; Tong, Tong; Wang, Li; Huang, Chen-Song; Xu, Qiong-Cong; Huang, Xi-Tai; Li, Jian-Hui; Wu, Jun; et al
Nanoscale (2020), 12(29), 15767-15774 | Language: English, Database: CAplus and MEDLINE

Substances (12) Reactions (0) Cited By (7)

50

Transcriptional Selectivity of Epigenetic Therapy in Cancer

By: Sato, Takahiro; Cesaroni, Matteo; Chung, Woonbok; Panjarian, Shoghag; Tran, Anthony; Madzo, Jozef; Okamoto, Yasuyuki; Zhang, Hanghang; Chen, Xiaowei; Jelinek, Jaroslav; et al
Cancer Research (2017), 77(2), 470-481 | Language: English, Database: CAplus and MEDLINE

Substances (12) Reactions (0) Cited By (39)

51

Targeting euchromatic histone lysine methyltransferases sensitizes colorectal cancer to histone deacetylase inhibitors

By: Bamberg, Leonhard Valentin; Heigwer, Florian; Wandmacher, Anna Maxi; Singh, Ambika; Betge, Johannes; Rindtorff, Niklas; Werner, Johannes; Josten, Julia; Skabkina, Olga Valerievna; Hinsenkamp, Isabel; et al
International Journal of Cancer (2022), 151(9), 1586-1601 | Language: English, Database: CAplus and MEDLINE

Substances (13) Reactions (0) Cited By (1)

52

Hematopoietic stem cell compositions, methods of making and method of use in cellular therapy

By: Koechlein, Claire; Ostertag, Eric M.; Shedlock, Devon; Down, Julian
World Intellectual Property Organization, WO2020077360 A1 2020-04-16 | Language: English, Database: CAplus

PatentPak available

Substances (14) Reactions (0) Cited By (1)

53

Degron Protease Blockade Sensor to Image Epigenetic Histone Protein Methylation in Cells and Living Animals

By: Sekar, Thillai V.; Foygel, Kira; Devulapally, Rammohan; Paulmurugan, Ramasamy
ACS Chemical Biology (2015), 10(1), 165-174 | Language: English, Database: CAplus and MEDLINE

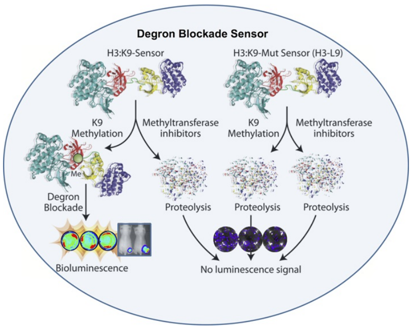

Substances (14) Reactions (0) Cited By (7)

54

Compositions and methods for increasing transposition frequency

By: Koechlein, Claire; Ostertag, Eric M.; Shedlock, Devon; Down, Julian
World Intellectual Property Organization, WO2020077357 A1 2020-04-16 | Language: English, Database: CAplus

PatentPak available

Substances (15) Reactions (0) Cited By (1)

55

Chemical induction of pluripotent stem cell formation

By: Deng, Hongkui; Zhao, Yang; Hou, Pingping; Li, Yanqin; Zhang, Xu; Liu, Chun; Guan, Jingyang; Li, Honggang
World Intellectual Property Organization, WO2015003643 A1 2015-01-15 | Language: English, Database: CAplus

PatentPak available

Substances (29) Reactions (0) Cited By (2)

56

7-Aminoalkoxy-Quinazolines from Epigenetic Focused Libraries Are Potent and Selective Inhibitors of DNA Methyltransferase 1

By: Medina-Franco, Jose L. ; Lopez-Lopez, Edgar; Martinez-Fernandez, Liliam P.
Molecules (2022), 27(9), 2892 | Language: English, Database: CAplus and MEDLINE

Substances (17) Reactions (0) Cited By (1)

57

Dicer promotes genome stability via the bromodomain transcriptional co-activator BRD4

By: Gutbrod, M. J. ; Roche, B.; Steinberg, J. I.; Lakhani, A. A.; Chang, K.; Schorn, A. J.; Martienssen, R. A.
Nature Communications (2022), 13(1), 1001 | Language: English, Database: CAplus and MEDLINE

Substances (17) Reactions (0) Cited By (2)

58

Targeting EHMT2 reverses EGFR-TKI resistance in NSCLC by epigenetically regulating the PTEN/AKT signaling pathway

By: Wang, Lihui; Dong, Xiaoyu; Ren, Yong; Luo, Juanjuan; Liu, Pei; Su, Dongsheng; Yang, Xiaojun
Cell Death & Disease (2018), 9(2), 1-13 | Language: English, Database: CAplus and MEDLINE

Substances (17) Reactions (0) Cited By (41)

59

Chromatin profiling assay to detect interacting proteins and characterize compounds interacting with said proteins

By: Drewes, Gerard; Bantscheff, Marcus; Dittmann, Antje; Rathert, Philipp
World Intellectual Property Organization, WO2013023770 A1 2013-02-21 | Language: English, Database: CAplus

PatentPak available

Substances (17) Reactions (0) Cited By (0)

60

Methods of combinatorial drug screening and use of therapeutic targets thereof in cancer treatment

By: Bernstein, Bradley E.; Doench, John G.; Najm, Fadi J.
World Intellectual Property Organization, WO2020033585 A1 2020-02-13 | Language: English, Database: CAplus

PatentPak available

Substances (109) | Reactions (0) | Cited By (3)

61

Discovery of a Novel Chemotype of Histone Lysine Methyltransferase EHMT1/2 (GLP/G9a) Inhibitors: Rational Design, Synthesis, Biological Evaluation, and Co-crystal Structure

By: Milite, Ciro; Feoli, Alessandra; Horton, John R.; Rescigno, Donatella; Cipriano, Alessandra; Pisapia, Vincenzo; Viviano, Monica; Pepe, Giacomo; Amendola, Giorgio; Novellino, Ettore; et al
Journal of Medicinal Chemistry (2019), 62(5), 2666-2689 | Language: English, Database: CAplus and MEDLINE

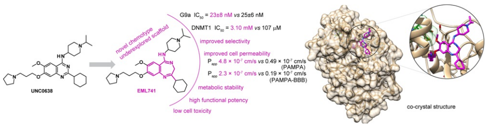

Substances (46) | Reactions (152) | Cited By (19)

62

Identification of novel quinazoline derivatives as potent antiplasmodial agents

By: Bouchut, Anne; Rotili, Dante; Pierrot, Christine; Valente, Sergio; Lafitte, Sophia; Schultz, Johan; Hoglund, Urban; Mazzone, Roberta; Lucidi, Alessia; Fabrizi, Giancarlo; et al
European Journal of Medicinal Chemistry (2019), 161, 277-291 | Language: English, Database: CAplus and MEDLINE

Substances (122) | Reactions (144) | Cited By (34)

63

Detailed Exploration around 4-Aminoquinolines Chemical Space to Navigate the Lysine Methyltransferase G9a and DNA Methyltransferase Biological Spaces

By: Rabal, Obdulia; Sanchez-Arias, Juan Antonio; San Jose-Eneriz, Edurne; Agirre, Xabier; de Miguel, Irene; Garate, Leire; Miranda, Estibaliz; Saez, Elena; Roa, Sergio; Martinez-Climent, Jose Angel; et al
Journal of Medicinal Chemistry (2018), 61(15), 6546-6573 | Language: English, Database: CAplus and MEDLINE

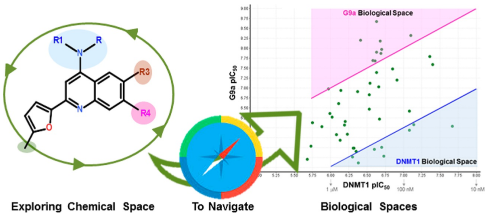

Substances (200) | Reactions (755) | Cited By (16)

64

preparation method of pluripotent stem cell with small molecular compounds

By: Deng, Hongkui; Zhao, Yang; Hou, Pingping; Li, Yanqin; Zhang, Xu; Liu, Chun; Guan, Jingyang; Li, Honggang; Zhao, Ting; Ye, Junqing; et al
China, CN104278008 A 2015-01-14 | Language: Chinese, Database: CAplus

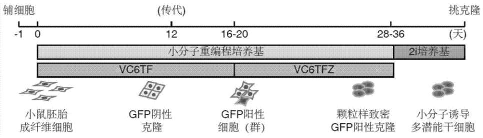

PatentPak available

Substances (138) | Reactions (0) | Cited By (4)

65

Homogenous thermal shift ligand binding assay

By: Treiber, Daniel K.; Menichelli, Elena
World Intellectual Property Organization, WO2016028804 A1 2016-02-25 | Language: English, Database: CAplus

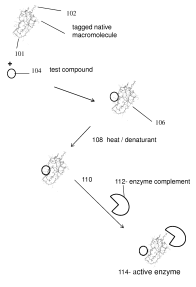

Figure 1

PatentPak available

Substances (18) | Reactions (0) | Cited By (0)

66

Targeting p53 and histone methyltransferases restores exhausted CD8+ T cells in HCV infection

By: Barili, Valeria; Fisicaro, Paola; Montanini, Barbara ; Acerbi, Greta; Filippi, Anita; Forleo, Giovanna; Romualdi, Chiara; Ferracin, Manuela ; Guerrieri, Francesca ; Pedrazzi, Giuseppe ; et al
Nature Communications (2020), 11(1), 604 | Language: English, Database: CAplus and MEDLINE

Substances (19) | Reactions (0) | Cited By (38)

67

Preparation of quinazolin-4-amine derivatives as histone methyltransferase G9a inhibitors and methods for the treatment of Prader-willi syndrome

By: Jiang, Yong-Hui; Kim, Yuna; Lee, Hyeong-Min; Jin, Jian; Roth, Bryan L.
World Intellectual Property Organization, WO2017201199 A1 2017-11-23 | Language: English, Database: CAplus

UNC618

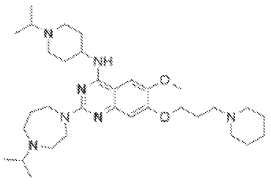

Figure 1C

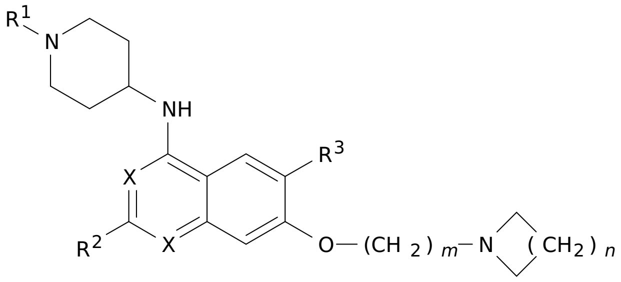

I

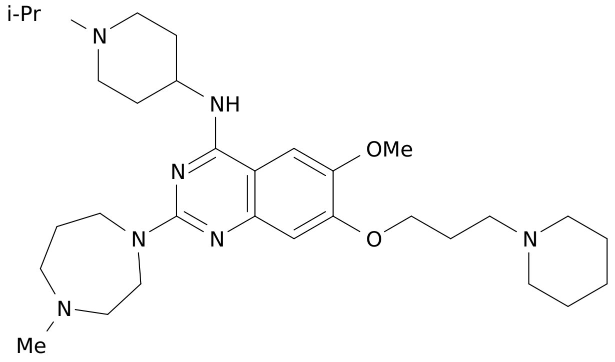

I I

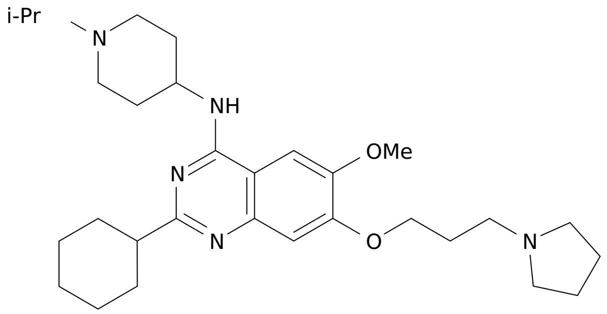

I I I

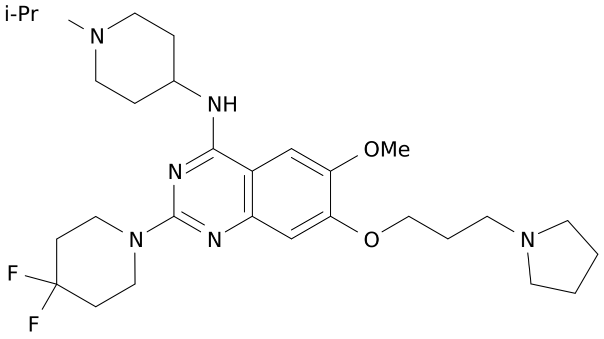

I V

PatentPak available

Substances (19) Reactions (4) Cited By (2)

68

Ex vivo expansion of human hematopoietic stem/progenitor cells

By: Chai, Li; Tatetsu, Hiro; Tenen, Daniel Geoffrey; Bradner, James Elliot; Federation, Alexander
World Intellectual Property Organization, WO2015148716 A1 2015-10-01 | Language: English, Database: CAplus

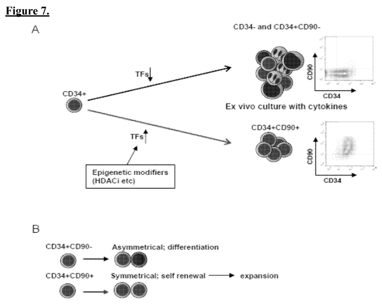

PatentPak available

Substances (20) | Reactions (0) | Cited By (3)

69

Homogeneous thermal shift ligand binding assay

By: Treiber, Daniel K.; Menichelli, Elena
United States, US20160054322 A1 2016-02-25 | Language: English, Database: CAplus

PatentPak available

Substances (22) | Reactions (0) | Cited By (0)

70

Hypoxia induces HIF1α-dependent epigenetic vulnerability in triple negative breast cancer to confer immune effector dysfunction and resistance to anti-PD-1 immunotherapy

By: Ma, Shijun ; Zhao, Yue; Lee, Wee Chyan; Ong, Li-Teng; Lee, Puay Leng; Jiang, Zemin; Oguz, Gokce; Niu, Zhitong; Liu, Min; Goh, Jian Yuan; et al
Nature Communications (2022), 13(1), 4118 | Language: English, Database: CAplus and MEDLINE

Substances (23) | Reactions (0) | Cited By (3)

71

Tooth movement promoter, and orthodontic treatment kit are minimally invasive and shortens treatment period

By: Saeki, Makio; Saito, Isao; Kakihara, Yoshito; Nakata, Juri; Akiba, Yosuke
Japan, JP2019131485 A 2019-08-08 | Language: Japanese, Database: CAplus

PatentPak available

Substances (23) | Reactions (0) | Cited By (0)

72

Homogeneous thermal shift ligand binding assay for measuring binding and cellular engagement of ligands with target proteins

By: Treiber, Daniel K.; Menichelli, Elena
World Intellectual Property Organization, WO2017030606 A1 2017-02-23 | Language: English, Database: CAplus

Figure 14

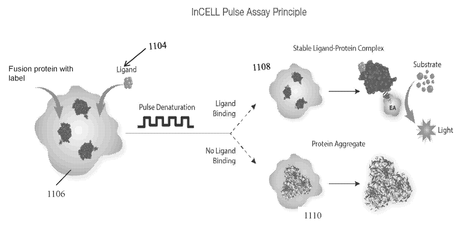

PatentPak available

Substances (24) | Reactions (0) | Cited By (0)

73

Effects of environmental conditions on nephron number: modeling maternal disease and epigenetic regulation in renal development

By: Fuhrmann, Lars ; Lindner, Saskia; Hauser, Alexander-Thomas; Hoese, Clemens; Kretz, Oliver; Cohen, Clemens D.; Lindenmeyer, Maja T.; Sippl, Wolfgang ; Jung, Manfred ; Huber, Tobias B.; et al
International Journal of Molecular Sciences (2021), 22(8), 4157 | Language: English, Database: CAplus and MEDLINE

Substances (25) | Reactions (0) | Cited By (2)

74

Methods of administering telomerase reverse transcriptase (TERT) activating therapies for treating premature aging diseases or neurodegenerative disorders

By: Depinho, Ronald A.; Wang, Y. Alan; Shim, Hong Seok; Horner, James W.
World Intellectual Property Organization, WO2020223475 A1 2020-11-05 | Language: English, Database: CAplus

PatentPak available

Substances (25) | Reactions (0) | Cited By (0)

75

Discovery of a Potent and Selective Fragment-like Inhibitor of Methyllysine Reader Protein Spindlin 1 (SPIN1)

By: Xiong, Yan; Greschik, Holger; Johansson, Catrine; Seifert, Ludwig; Bacher, Johannes; Park, Kwang-su; Babault, Nicolas; Martini, Michael; Fagan, Vincent; Li, Fengling; et al
Journal of Medicinal Chemistry (2019), 62(20), 8996-9007 | Language: English, Database: CAplus and MEDLINE

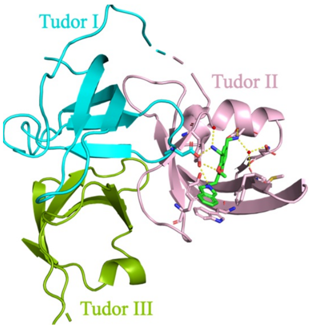

Substances (25) Reactions (122) Cited By (12)

76

Anti-HIV compositions and methods for reactivating latent immunodeficiency virus using a SMYD2 inhibitor

By: Ott, Melanie; Boehm, Daniela
United States, US20180002699 A1 2018-01-04 | Language: English, Database: CAplus

PatentPak available

Substances (25) Reactions (0) Cited By (0)

77

Stromal-free methods for CD56+ Natural Killer cell differentiation from pluripotent stem cells for use in human transplant with decreased CD3+ T cells and increased expression of NK cell receptors for cancer immunotherapy

By: Jing, Ran; Daley, George Q.
World Intellectual Property Organization, WO2022187682 A2 2022-09-09 | Language: English, Database: CAplus

PatentPak available

Substances (76) Reactions (0) Cited By (0)

78

Chemical micromolecule composition for chemically inducing fibroblasts to be reprogrammed into lung stem cells

By: Zhang, Peilin
World Intellectual Property Organization, WO2022179445 A1 2022-09-01 | Language: Chinese, Database: CAplus

PatentPak available

Substances (50) Reactions (0) Cited By (0)

79

Chemical micromolecule composition for chemically inducing fibroblasts to be reprogrammed into lung stem cells

By: Zhang, Peilin
China, CN114958726 A 2022-08-30 | Language: Chinese, Database: CAplus

PatentPak available

Substances (48) | Reactions (0) | Cited By (0)

80

Discovery of the first-in-class G9a/GLP covalent inhibitors

By: Park, Kwang-Su; Xiong, Yan; Yim, Hyerin; Velez, Julia; Babault, Nicolas; Kumar, Prashasti; Liu, Jing; Jin, Jian
Journal of Medicinal Chemistry (2022), 65(15), 10506-10522 | Language: English, Database: CAplus and MEDLINE

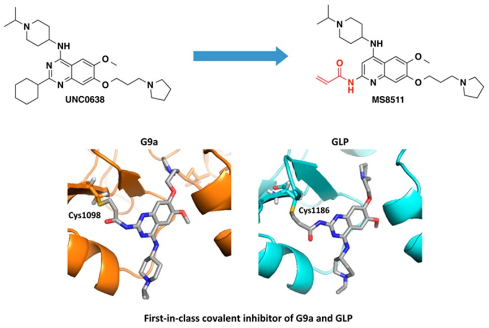

Substances (38) | Reactions (0) | Cited By (2)

81

Enhanced differentiation of beta cells

By: Carey, Bryce; Szymaniak, Aleksander; Thanos, Christopher
World Intellectual Property Organization, WO2022147056 A1 2022-07-07 | Language: English, Database: CAplus

PatentPak available

Substances (72) | Reactions (0) | Cited By (0)

82

An EHMT2/NFYA-ALDH2 signaling axis modulates the RAF pathway to regulate paclitaxel resistance in lung cancer

By: Wang, Wenjing; Wang, Jianmin; Liu, Shuai; Ren, Yong; Wang, Jingyu; Liu, Sen; Cui, Wei; Jia, Lina; Tang, Xing; Yang, Jingyu; et al
Molecular Cancer (2022), 21(1), 106 | Language: English, Database: CAplus and MEDLINE

Substances (29) | Reactions (0) | Cited By (1)

83

Identification of a novel autophagy-related prognostic signature and small molecule drugs for glioblastoma by bioinformatics

By: Wang, Dongjiao; Jiang, Yuxue; Wang, Tie; Wang, Zhe; Zou, Fei
BMC Medical Genomics (2022), 15(1), 111 | Language: English, Database: CAplus and MEDLINE

Substances (59) Reactions (0) Cited By (2)

84

Computational screening methods for identifying glutathione S-transferase P (GSTP1) inhibitors for treating pulmonary hypertension

By: Chan, Stephen Yu-Wah; Kim, Seungchan
World Intellectual Property Organization, WO2022103706 A1 2022-05-19 | Language: English, Database: CAplus

PatentPak available

Substances (264) Reactions (0) Cited By (0)

85

Preparation of substituted quinazolinyl compounds for the treatment of autoimmune diseases and cancer

By: Iliopoulos, Dimitrios; Ho, David G.; Karagiannidis, Iordanis; Nguyen, Phithi; Chalkia, Dimitra
World Intellectual Property Organization, WO2022031939 A1 2022-02-10 | Language: English, Database: CAplus

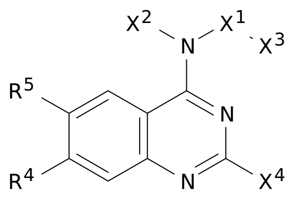

I

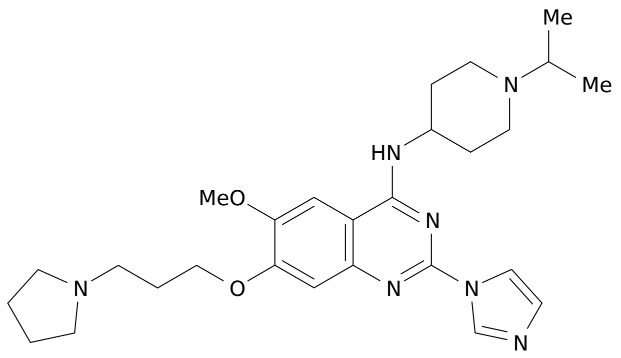

II

PatentPak available

Substances (331) Reactions (374) Cited By (0)

86

Stroma-free T cell differentiation from human pluripotent stem cells and uses thereof in cellular therapy

By: Daley, George Q.; Jing, Ran

World Intellectual Property Organization, WO2021150919 A1 2021-07-29 | Language: English, Database: CAplus

PatentPak available

Substances (46) Reactions (0) Cited By (0)

87

HeLa TI cell-based assay as a new approach to screen for chemicals able to reactivate the expression of epigenetically silenced genes

By: Maksimova, Varvara ; Shalginskikh, Natalya; Vlasova, Olga; Usalka, Olga; Beizer, Anastasia; Bugaeva, Polina; Fedorov, Dmitry; Lizogub, Olga; Lesovaya, Ekaterina; Katz, Richard; et al

PLoS One (2021), 16(6), e0252504 | Language: English, Database: CAplus and MEDLINE

Substances (44) Reactions (0) Cited By (3)

88

Identification of six hub genes and analysis of their correlation with drug sensitivity in acute myeloid leukemia through bioinformatics

By: Cai, Daxia; Liang, Jiajian; Cai, Xing-dong; Yang, Ying; Liu, Gexiu; Zhou, Fenling; He, Dongmei

Translational Cancer Research (2021), 10(1), 126-140 | Language: English, Database: CAplus and MEDLINE

Substances (87) Reactions (0) Cited By (0)

89

Chemical small molecule composition and method for chemically inducing in vivo and in vitro direct reprogramming of fibroblasts into hepatocytes

By: Zhang, Peilin

World Intellectual Property Organization, WO2021047495 A1 2021-03-18 | Language: Chinese, Database: CAplus

PatentPak available

Substances (51) Reactions (0) Cited By (0)

90

Activity Comparison of Epigenetic Modulators against the Hemoprotozoan Parasites Babesia divergens and Plasmodium falciparum

By: Vanheer, Leen N.; Kafsack, Bjorn F. C.
ACS Infectious Diseases (2021), 7(8), 2277-2284 | Language: English, Database: CAplus and MEDLINE

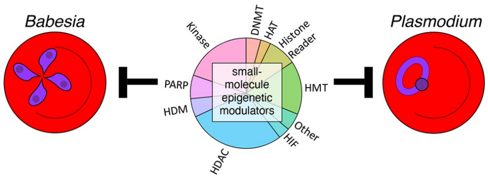

Substances (326) | Reactions (0) | Cited By (6)

91

Methods for treating small cell neuroendocrine (SCN) tumors and small-round-blue cell tumors (SRBCTs)

By: Graeber, Thomas G.; Balanis, Nikolas G.; Sheu, Katherine M.; Witte, Owen N.; Esedebe, Favour; Park, Jung Wook
World Intellectual Property Organization, WO2020243329 A1 2020-12-03 | Language: English, Database: CAplus

PatentPak available

Substances (259) | Reactions (0) | Cited By (0)

92

A method for treating SWI/SNF complex-deficient cancers comprising glutathione (GSH) metabolic pathway inhibitor

By: Ogiwara, Hideaki; Kohno, Takashi; Sasaki, Hiroki
World Intellectual Property Organization, WO2020138385 A1 2020-07-02 | Language: English, Database: CAplus

PatentPak available

Substances (334) | Reactions (0) | Cited By (0)

93

Epigenetic inhibitors target multiple stages of Plasmodium falciparum parasites

By: Coetzee, Nanika; von Gruning, Hilde; Opperman, Daniel; van der Watt, Mariette; Reader, Janette; Birkholtz, Lyn-Marie
Scientific Reports (2020), 10(1), 2355 | Language: English, Database: CAplus and MEDLINE

Substances (42) | Reactions (0) | Cited By (31)

94

Method and a system for analyzing neuropharmacology of a drug

By: Shi, Peng; Wang, Xin; Lin, Xudong
United States, US20200077947 A1 2020-03-12 | Language: English, Database: CAplus

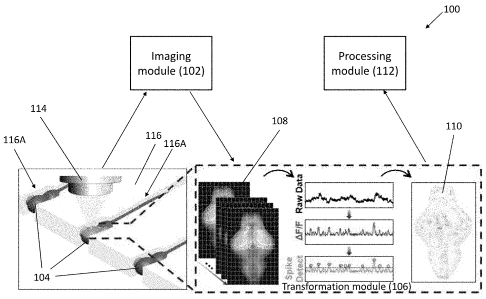

PatentPak available

Substances (297) | Reactions (0) | Cited By (0)

95

Biomarker detection for predicting response to cancer therapy

By: Lee, Jason; Al-Ejeh, Fares
World Intellectual Property Organization, WO2020047604 A1 2020-03-12 | Language: English, Database: CAplus

PatentPak available

Substances (43) | Reactions (0) | Cited By (1)

96

Phenotypic-screening generates active novel fetal globin-inducers that downregulate Bcl11a in a monkey model

By: Makino, Tomohiro ; Haruyama, Munetada; Katayama, Katsushi; Terashima, Hideki ; Tsunemi, Tomoyuki ; Miyazaki, Kazuo; Terakawa, Maki; Yamashiro, Kyoko; Yoshioka, Ryosuke; Maeda, Hiroaki
Biochemical Pharmacology (Amsterdam, Netherlands) (2020), 171, 113717 | Language: English, Database: CAplus and MEDLINE

Substances (30) | Reactions (24) | Cited By (3)

97

Methods and compounds for the treatment of genetic disease by modulating bean gene expression

By: Ansari, Aseem; Shah, Pratik
World Intellectual Property Organization, WO2019204781 A1 2019-10-24 | Language: English, Database: CAplus

PatentPak available

Substances (960) | Reactions (4) | Cited By (0)

98

Discovery of a potent histone deacetylase (HDAC) 3/6 selective dual inhibitor

By: Soumyanarayanan, Uttara; Ramanujulu, Pondy Murugappan; Mustafa, Nurulhuda; Haider, Shozeb; Fang Nee, Adina Huey; Tong, Jie Xin ; Tan, Kevin S. W.; Chng, Wee Joo; Dymock, Brian W.
European Journal of Medicinal Chemistry (2019), 184, 111755 | Language: English, Database: CAplus and MEDLINE

Substances (54) | Reactions (189) | Cited By (11)

99

Selective, Small-Molecule Co-Factor Binding Site Inhibition of a Su(var)3-9, Enhancer of Zeste, Trithorax Domain Containing Lysine Methyltransferase

By: Taylor, Alexandria P.; Swewczyk, Magdalena; Kennedy, Steven; Trush, Viacheslav V.; Wu, Hong; Zeng, Hong; Dong, Aiping; Ferreira de Freitas, Renato; Tatlock, John; Kumpf, Robert A.; et al
Journal of Medicinal Chemistry (2019), 62(17), 7669-7683 | Language: English, Database: CAplus and MEDLINE

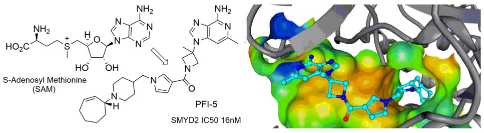

Substances (91) | Reactions (215) | Cited By (10)

100

Small molecule-induced method for directly reprogramming human fibroblasts into liver cells

By: Zhang, Peilin
China, CN109504650 A 2019-03-22 | Language: Chinese, Database: CAplus

PatentPak available

Substances (31) | Reactions (0) | Cited By (0)

101

Small molecule-induced method for directly reprogramming human fibroblasts into liver cells

By: Zhang, Peilin
World Intellectual Property Organization, WO2019052556 A1 2019-03-21 | Language: Chinese, Database: CAplus

PatentPak available

Substances (31) | Reactions (0) | Cited By (0)

102

Engineering Lineage Potency and Plasticity of Stem Cells using Epigenetic Molecules

By: Dhaliwal, Anandika; Pelka, Sandra; Gray, David S.; Moghe, Prabhas V.
Scientific Reports (2018), 8(1), 1-13 | Language: English, Database: CAplus and MEDLINE

Substances (71) | Reactions (0) | Cited By (5)

103

High-throughput brain activity mapping and machine learning as a foundation for systems neuropharmacology

By: Lin, Xudong; Duan, Xin; Jacobs, Claire; Ullmann, Jeremy; Chan, Chung-Yuen; Chen, Siya; Cheng, Shuk-Han ; Zhao, Wen-Ning; Poduri, Annapurna; Wang, Xin ; et al
Nature Communications (2018), 9(1), 1-12 | Language: English, Database: CAplus and MEDLINE

Substances (137) Reactions (0) Cited By (15)

104

An inhibitor screen identifies histone-modifying enzymes as mediators of polymer-mediated transgene expression from plasmid DNA

By: Christensen, Matthew D.; Nitiyanandan, Rajeshwar; Meraji, Seyedehmelika; Daer, Rene; Godeshala, Sudhakar; Goklany, Sheba; Haynes, Karmella; Rege, Kaushal
Journal of Controlled Release (2018), 286, 210-223 | Language: English, Database: CAplus and MEDLINE

Substances (98) Reactions (0) Cited By (7)

105

Treatment of cancers having alterations within the SWI/SNF chromatin remodeling complex

By: Fisher, David E.; Weng, Qing Yu; Kato, Shinichiro
World Intellectual Property Organization, WO2018049000 A1 2018-03-15 | Language: English, Database: CAplus

FIG. 2

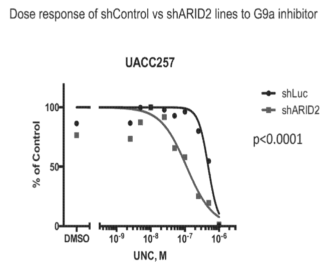

PatentPak available

Substances (46) Reactions (0) Cited By (1)

106

Immune cells derived from induced pluripotent stem cell

By: Vo, Linda Thuy; Daley, George Q.
World Intellectual Property Organization, WO2018048828 A1 2018-03-15 | Language: English, Database: CAplus

Fig. 17C

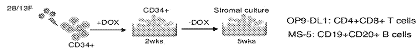

PatentPak available

Substances (31) | Reactions (0) | Cited By (0)

107

HIV signaling through CD4 and CCR5 activates Rho family GTPases that are required for optimal infection of primary CD4+ T cells

By: Lucera, Mark B.; Fleissner, Zach; Tabler, Caroline O.; Schlatzer, Daniela M.; Troyer, Zach; Tilton, John C.
Retrovirology (2017), 14, 4/1-4/13 | Language: English, Database: CAplus and MEDLINE

Substances (74) | Reactions (0) | Cited By (9)

108

Chromatin protective therapeutics and methods of targeting chromatin heterogeneity for treatment of cancer

By: Backman, Vadim; Szleifer, Igal; Almassalha, Luay; Chandler, John; Bauer, Greta; Gladstein, Scott
World Intellectual Property Organization, WO2018013942 A1 2018-01-18 | Language: English, Database: CAplus

PatentPak available

Substances (29) | Reactions (0) | Cited By (0)

109

Generation of human pluripotent stem cell derived functional beta cells showing a glucose-dependent mitochondrial respiration and two-phase insulin secretion response

By: Rieck, Sebastian; Rezania, Alireza
United States, US20170362572 A1 2017-12-21 | Language: English, Database: CAplus

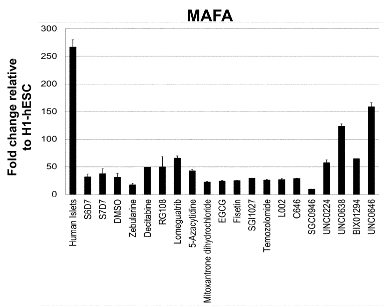

PatentPak available

Substances (30) Reactions (0) Cited By (0)

110

JAK1/2 and BCL2 inhibitors synergize to counteract bone marrow stromal cell-induced protection of AML

By: Karjalainen, Riikka; Pemovska, Tea; Popa, Mihaela; Liu, Minxia; Javarappa, Komal K.; Majumder, Muntasir M.; Yadav, Bhagwan; Tamborero, David; Tang, Jing; Bychkov, Dmitrii; et al
Blood (2017), 130(6), 789-802 | Language: English, Database: CAplus and MEDLINE

Substances (270) Reactions (0) Cited By (66)

111

Histone deacetylase inhibitors containing a benzamide functional group and a pyridyl cap are preferentially effective human immunodeficiency virus-1 latency-reversing agents in primary resting CD4^{+} T cells

By: Kobayashi, Yoshifumi; Gelinas, Celine; Dougherty, Joseph P.
Journal of General Virology (2017), 98(4), 799-809 | Language: English, Database: CAplus and MEDLINE

Substances (30) Reactions (0) Cited By (14)

112

Discovery of novel drug sensitivities in T-PLL by high-throughput ex vivo drug testing and mutation profiling

By: Andersson, E. I.; Putzer, S.; Yadav, B.; Dufva, O.; Khan, S.; He, L.; Sellner, L. ; Schrader, A.; Crispatzu, G.; Oles, M.; et al
Leukemia (2018), 32(3), 774-787 | Language: English, Database: CAplus and MEDLINE

Substances (65) Reactions (0) Cited By (60)

113

Reprogramming non-pluripotent cells into pluripotent stem cells using chemical inducers of pluripotency

By: Deng, Hongkui; Zhao, Yang; Zhao, Ting; Guan, Jingyang; Zhang, Xu; Fu, Yao; Ye, Junqing
World Intellectual Property Organization, WO2017091943 A1 2017-06-08 | Language: English, Database: CAplus

PatentPak available

Substances (27) Reactions (0) Cited By (2)

114

Reactivation of hiv-1 gene expression to treat persistent hiv infection

By: Van Lint, Carine; Rohr, Olivier; Bouchat, Sophie; Gatot, Jean-Stephane
United States, US20150023907 A1 2015-01-22 | Language: English, Database: CAplus

PatentPak available

Substances (51) Reactions (0) Cited By (0)

115

Modulation of reactivation of a latent virus

By: Roizman, Bernard; Zhou, Guoying; Du, Te
United States, US20160089376 A1 2016-03-31 | Language: English, Database: CAplus

PatentPak available

Substances (44) Reactions (0) Cited By (0)

116

Induction of cardiomyocyte formation from differentiated cells by small molecule inhibition of enzymes, growth factors, and signal transduction

By: Cao, Nan; Ding, Sheng
United States, US20160186141 A1 2016-06-30 | Language: English, Database: CAplus

PatentPak available

Substances (275) Reactions (0) Cited By (1)

117

Antagonists of Setdb2 for use in the therapy of infectious diseases

By: Bergthaler, Andreas; Schliehe, Christopher
World Intellectual Property Organization, WO2016079321 A1 2016-05-26 | Language: English, Database: CAplus

PatentPak available

Substances (32) Reactions (0) Cited By (0)

118

Thienopyrimidines and uses thereof for treating proliferative diseases such as cancers and an embryonic stem cell-based tumor cell model for drug screening and disease target identification

By: Tabar, Viviane; Funato, Kosuke

World Intellectual Property Organization, WO2016081732 A1 2016-05-26 | Language: English, Database: CAplus

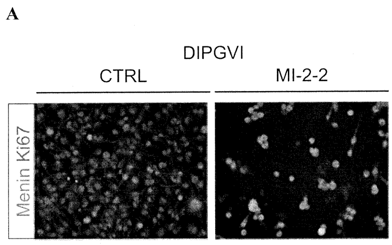

Figure 30

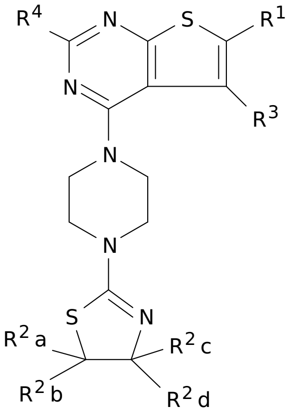

PatentPak available

Substances (104) Reactions (0) Cited By (0)

119

Increasing Transcription factor ATOH1 life to drive sensorineural hair cell differentiation

By: Edge, Albert; Cheng, Yen-Fu; Kempfle, Judith; Abdul-Aziz, Dunia

World Intellectual Property Organization, WO2016022776 A2 2016-02-11 | Language: English, Database: CAplus

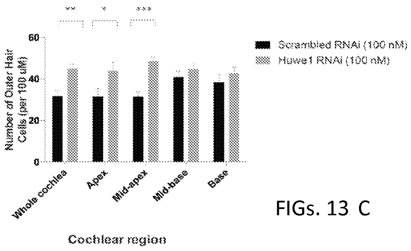

FIGs. 13 C

PatentPak available

Substances (52) Reactions (0) Cited By (0)

120

Methods of treating cancer and preventing cancer drug resistance using histone methyltransferase G9a antagonists in combination with other chemotherapy agents

By: Classon, Marie; Guler, Gulfem Dilek; Pitti, Robert; Stephan, Jean-Philippe; Tindell, Charles Albert

World Intellectual Property Organization, WO2015200329 A1 2015-12-30 | Language: English, Database: CAplus

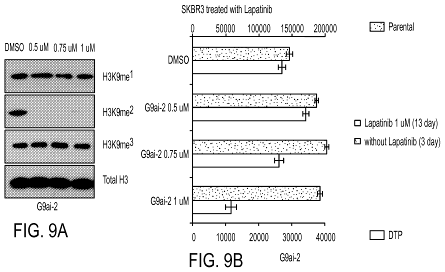

FIG. 9A

FIG. 9B

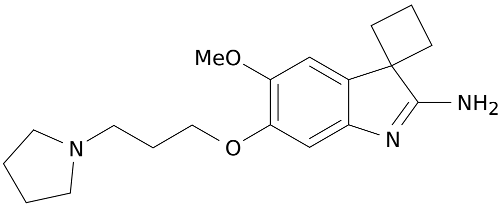

I

PatentPak available

Substances (28) Reactions (0) Cited By (0)

121

Preparation of aminoquinoline derivatives as dual inhibitors of histone methyltransferases and dna methyltransferases and pharmaceutical uses thereof

By: Aguirre Ena, Xabier; Oyarzabal Santamarina, Julen; Prosper Cardoso, Felipe; Rabal Gracia, Maria Obdulia; Rodriguez Madoz, Juan Roberto; San Jose Eneriz, Edurne
World Intellectual Property Organization, WO2015192981 A1 2015-12-23 | Language: English, Database: CAplus

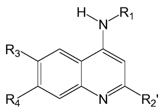
(I')

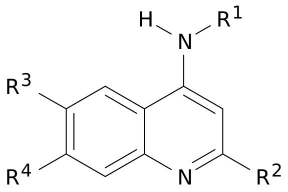
I

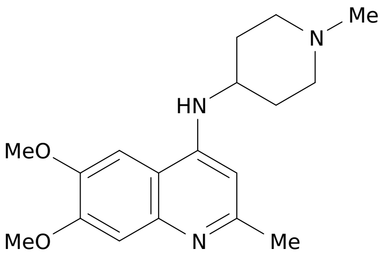
II

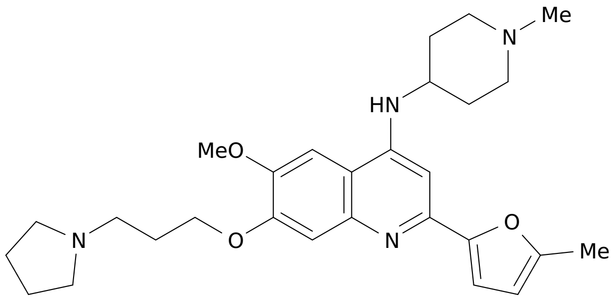
III

PatentPak available

Substances (240) Reactions (753) Cited By (4)

122

Methods and compositions comprising histone deacetylase (HDAC) inhibitors in combination with other therapeutics for treating subjects at risk for or with sensorineural hearing loss

By: Edge, Albert; Kempfle, Judith; Abdul-Aziz, Dunia
World Intellectual Property Organization, WO2015168149 A2 2015-11-05 | Language: English, Database: CAplus

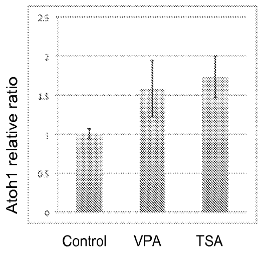

FIG. 15

PatentPak available

Substances (52) Reactions (0) Cited By (4)

123

Methods and compositions for expansion of stem cells and other cells and method to generate multipotent or immature cell from mature somatic cell

By: Ma, Yupo
United States, US20150250824 A1 2015-09-10 | Language: English, Database: CAplus

PatentPak available

Substances (30) Reactions (0) Cited By (0)

124

Pharmaceutical compositions for preparing cardiac progenitor cells for use in subjects suffering from a heart disease or condition

By: Cao, Nan; Ding, Sheng
World Intellectual Property Organization, WO2015038704 A1 2015-03-19 | Language: English, Database: CAplus

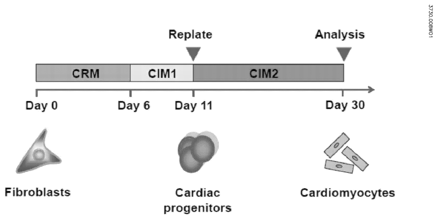

Fig 1

PatentPak available

Substances (284) Reactions (0) Cited By (1)

125

Epigenetic regulators of frataxin for use in treatment of Friedrich' s ataxia

By: Ozsolak, Fatih
World Intellectual Property Organization, WO2015023938 A1 2015-02-19 | Language: English, Database: CAplus

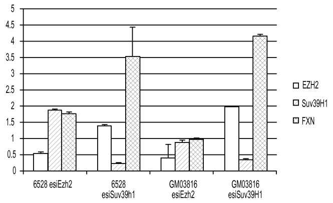

FIG. 3B

PatentPak available

Substances (268) | Reactions (0) | Cited By (1)

126

Bioluminescent Cell-Based NAD(P)/NAD(P)H Assays for Rapid Dinucleotide Measurement and Inhibitor Screening

By: Vidugiriene, Jolanta; Leippe, Donna; Sobol, Mary; Vidugiris, Gediminas; Zhou, Wenhui; Meisenheimer, Poncho; Gautam, Prson; Wennerberg, Krister; Cali, James J.
Assay and Drug Development Technologies (2014), 12(9-10), 514-526 | Language: English, Database: CAplus and MEDLINE

Substances (28) | Reactions (0) | Cited By (19)

127

Modulation of reactivation of a latent virus

By: Roizman, Bernard; Zhou, Guoying; Du, Te
World Intellectual Property Organization, WO2014185973 A2 2014-11-20 | Language: English, Database: CAplus

PatentPak available

Substances (44) | Reactions (0) | Cited By (1)

128

Transformation of drug cyclodextrin complex compositions into compositions of mixtures of lipid vesicle encapsulated drug and cyclodextrin drug complexes

By: Szoka, Francis C.; Noble, Charles O.; Hayes, Mark E.
World Intellectual Property Organization, WO2014121235 A2 2014-08-07 | Language: English, Database: CAplus

PatentPak available

Substances (244) | Reactions (0) | Cited By (0)

129

Remote loading of sparingly water-soluble drugs into liposomes

By: Hayes, Mark E.; Noble, Charles O.; Szoka, Francis C., Jr.
World Intellectual Property Organization, WO2014121211 A2 2014-08-07 | Language: English, Database: CAplus

PatentPak available

Substances (258) Reactions (0) Cited By (1)

130

The Discovery of Novel Histone Lysine Methyltransferase G9a Inhibitors (Part 1): Molecular Design Based on a Series of Substituted 2,4-Diamino-7- aminoalkoxyquinazoline by Molecular-Docking-Guided 3D Quantitative Structure-Activity Relationship Studies

By: Feng, Taotao; Wang, Hai; Zhang, Xiaojin; Sun, Haopeng; You, Qidong
Medicinal Chemistry (Sharjah, United Arab Emirates) (2014), 10(4), 426-440 | Language: English, Database: CAplus and MEDLINE

Substances (55) Reactions (0) Cited By (6)

131

Chemiluminescent detection of intracellular binding events by measuring β-galactosidase fusion protein abundance and stability

By: Raab, William; Wehrman, Thomas S.
United States, US20130203067 A1 2013-08-08 | Language: English, Database: CAplus

PatentPak available

Substances (36) Reactions (0) Cited By (1)

132

Reactivation of HIV-1 gene expression using DNA methylation inhibitors and histone methyltransferase inhibitors to treat persistent HIV infection

By: Van Lint, Carine; Rohr, Olivier; Bouchat, Sophie; Gatot, Jean-Stephane
World Intellectual Property Organization, WO2013050422 A1 2013-04-11 | Language: English, Database: CAplus

PatentPak available

Substances (51) Reactions (0) Cited By (1)

Copyright © 2023 American Chemical Society (ACS). All Rights Reserved.
Copyright © 2023 U.S. National Library of Medicine.
Internal use only. Redistribution is subject to the terms of your SciFinderⁿ License Agreement and CAS information Use Policies.

Results of PubMed search: keyword GSK-J4

1: Inoue S, Moriya M, Watanabe Y, Miyagawa-Tomita S, Niihori T, Oba D, Ono M, Kure S, Ogura T, Matsubara Y, Aoki Y. New BRAF knockin mice provide a pathogenetic mechanism of developmental defects and a therapeutic approach in cardio-facio-cutaneous syndrome. Hum Mol Genet. 2014 Dec 15;23(24):6553-66. doi: 10.1093/hmg/ddu376. Epub 2014 Jul 17. PMID: 25035421.

2: Ntziachristos P, Tsirigos A, Welstead GG, Trimarchi T, Bakogianni S, Xu L, Loizou E, Holmfeldt L, Strikoudis A, King B, Mullenders J, Becksfort J, Nedjic J, Paietta E, Tallman MS, Rowe JM, Tonon G, Satoh T, Kruidenier L, Prinjha R, Akira S, Van Vlierberghe P, Ferrando AA, Jaenisch R, Mullighan CG, Aifantis I. Contrasting roles of histone 3 lysine 27 demethylases in acute lymphoblastic leukaemia. Nature. 2014 Oct 23;514(7523):513-7. doi: 10.1038/nature13605. Epub 2014 Aug 17. PMID: 25132549; PMCID: PMC4209203.

3: Hashizume R, Andor N, Ihara Y, Lerner R, Gan H, Chen X, Fang D, Huang X, Tom MW, Ngo V, Solomon D, Mueller S, Paris PL, Zhang Z, Petritsch C, Gupta N, Waldman TA, James CD. Pharmacologic inhibition of histone demethylation as a therapy for pediatric brainstem glioma. Nat Med. 2014 Dec;20(12):1394-6. doi: 10.1038/nm.3716. Epub 2014 Nov 17. PMID: 25401693; PMCID: PMC4257862.

4: Messer HG, Jacobs D, Dhummakupt A, Bloom DC. Inhibition of H3K27me3-specific histone demethylases JMJD3 and UTX blocks reactivation of herpes simplex virus 1 in trigeminal ganglion neurons. J Virol. 2015 Mar;89(6):3417-20. doi: 10.1128/JVI.03052-14. Epub 2014 Dec 31. PMID: 25552720; PMCID: PMC4337550.

5: Liu Z, Cao W, Xu L, Chen X, Zhan Y, Yang Q, Liu S, Chen P, Jiang Y, Sun X, Tao Y, Hu Y, Li C, Wang Q, Wang Y, Chen CD, Shi Y, Zhang X. The histone H3 lysine-27 demethylase Jmjd3 plays a critical role in specific regulation of Th17 cell differentiation. J Mol Cell Biol. 2015 Dec;7(6):505-16. doi: 10.1093/jmcb/mjv022. Epub 2015 Apr 3. PMID: 25840993.

6: Grasso CS, Tang Y, Truffaux N, Berlow NE, Liu L, Debily MA, Quist MJ, Davis LE, Huang EC, Woo PJ, Ponnuswami A, Chen S, Johung TB, Sun W, Kogiso M, Du Y, Qi L, Huang Y, Hütt-Cabezas M, Warren KE, Le Dret L, Meltzer PS, Mao H, Quezado M, van Vuurden DG, Abraham J, Fouladi M, Svalina MN, Wang N, Hawkins C, Nazarian J, Alonso MM, Raabe EH, Hulleman E, Spellman PT, Li XN, Keller C, Pal R, Grill J, Monje M. Functionally defined therapeutic targets in diffuse intrinsic pontine glioma. Nat Med. 2015 Jun;21(6):555-9. doi: 10.1038/nm.3855. Epub 2015 May 4. Erratum in: Nat Med. 2015 Jul;21(7):827. PMID: 25939062; PMCID: PMC4862411.

7: Kamikawa YF, Donohoe ME. Histone demethylation maintains Prdm14 and Tsix expression and represses xIst in embryonic stem cells. PLoS One. 2015 May 20;10(5):e0125626. doi: 10.1371/journal.pone.0125626. PMID: 25993097; PMCID: PMC4439117.

8: Kang SC, Kim SK, Chai JC, Kim SH, Won KJ, Lee YS, Jung KH, Chai YG. Transcriptomic Profiling and H3K27me3 Distribution Reveal Both Demethylase-Dependent and Independent Regulation of Developmental Gene Transcription in Cell Differentiation. PLoS One. 2015 Aug 11;10(8):e0135276. doi: 10.1371/journal.pone.0135276. PMID: 26263556; PMCID: PMC4532468.

9: Tennant BR, Hurley P, Dhillon J, Gill A, Whiting C, Hoffman BG. The TrxG Complex Mediates Cytokine Induced De Novo Enhancer Formation in Islets. PLoS One. 2015 Oct 27;10(10):e0141470. doi: 10.1371/journal.pone.0141470. PMID: 26505193; PMCID: PMC4623983.

10: Xie B, Zhang H, Wei R, Li Q, Weng X, Kong Q, Liu Z. Histone H3 lysine 27 trimethylation acts as an epigenetic barrier in porcine nuclear reprogramming. Reproduction. 2016 Jan;151(1):9-16. doi: 10.1530/REP-15-0338. Epub 2015 Oct 29. PMID: 26515777.

11: Sakaki H, Okada M, Kuramoto K, Takeda H, Watarai H, Suzuki S, Seino S, Seino M, Ohta T, Nagase S, Kurachi H, Kitanaka C. GSKJ4, A Selective Jumonji H3K27 Demethylase Inhibitor, Effectively Targets Ovarian Cancer Stem Cells. Anticancer Res. 2015 Dec;35(12):6607-14. PMID: 26637876.

12: Hofstetter C, Kampka JM, Huppertz S, Weber H, Schlosser A, Müller AM, Becker M. Inhibition of KDM6 activity during murine ESC differentiation induces DNA damage. J Cell Sci. 2016 Feb 15;129(4):788-803. doi: 10.1242/jcs.175174. Epub 2016 Jan 12. PMID: 26759175.

13: Hu J, Wang X, Chen L, Huang M, Tang W, Zuo J, Liu YC, Shi Z, Liu R, Shen J, Xiong B. Design and discovery of new pyrimidine coupled nitrogen aromatic rings as chelating groups of JMJD3 inhibitors. Bioorg Med Chem Lett. 2016 Feb 1;26(3):721-725. doi: 10.1016/j.bmcl.2016.01.006. Epub 2016 Jan 7. PMID: 26776360.

14: Lulla RR, Saratsis AM, Hashizume R. Mutations in chromatin machinery and pediatric high-grade glioma. Sci Adv. 2016 Mar 18;2(3):e1501354. doi: 10.1126/sciadv.1501354. PMID: 27034984; PMCID: PMC4803494.

15: Yapp C, Carr AJ, Price A, Oppermann U, Snelling SJ. H3K27me3 demethylases regulate in vitro chondrogenesis and chondrocyte activity in osteoarthritis. Arthritis Res Ther. 2016 Jul 7;18(1):158. doi: 10.1186/s13075-016-1053-7. PMID: 27388528; PMCID: PMC4936015.

16: Doñas C, Carrasco M, Fritz M, Prado C, Tejón G, Osorio-Barrios F, Manríquez V, Reyes P, Pacheco R, Bono MR, Loyola A, Rosemblatt M. The histone demethylase inhibitor GSK-J4 limits inflammation through the induction of a tolerogenic phenotype on DCs. J Autoimmun. 2016 Dec;75:105-117. doi: 10.1016/j.jaut.2016.07.011. Epub 2016 Aug 12. PMID: 27528513.

17: Mathur R, Sehgal L, Havranek O, Köhrer S, Khashab T, Jain N, Burger JA, Neelapu SS, Davis RE, Samaniego F. Inhibition of demethylase KDM6B sensitizes diffuse large B-cell lymphoma to chemotherapeutic drugs. Haematologica. 2017 Feb;102(2):373-380. doi: 10.3324/haematol.2016.144964. Epub 2016 Oct 14. PMID: 27742770; PMCID: PMC5286945.

18: Watarai H, Okada M, Kuramoto K, Takeda H, Sakaki H, Suzuki S, Seino S, Oizumi H, Sadahiro M, Kitanaka C. Impact of H3K27 Demethylase Inhibitor GSKJ4 on NSCLC Cells Alone and in Combination with Metformin. Anticancer Res. 2016 Nov;36(11):6083-6092. doi: 10.21873/anticanres.11198. PMID: 27793936.

19: Wang FS, Lian WS, Lee MS, Weng WT, Huang YH, Chen YS, Sun YC, Wu SL, Chuang PC, Ko JY. Histone demethylase UTX counteracts glucocorticoid deregulation of osteogenesis by modulating histone-dependent and -independent pathways. J Mol Med (Berl). 2017 May;95(5):499-512. doi: 10.1007/s00109-017-1512-x. Epub 2017 Jan 27. PMID: 28130569.

20: Cregan S, Breslin M, Roche G, Wennstedt S, MacDonagh L, Albadri C, Gao Y, O'Byrne KJ, Cuffe S, Finn SP, Gray SG. Kdm6a and Kdm6b: Altered expression in malignant pleural mesothelioma. Int J Oncol. 2017 Mar;50(3):1044-1052. doi: 10.3892/ijo.2017.3870. Epub 2017 Feb 9. PMID: 28197626.

21: Kochat V, Equbal Z, Baligar P, Kumar V, Srivastava M, Mukhopadhyay A. JMJD3 aids in reprogramming of bone marrow progenitor cells to hepatic phenotype through epigenetic activation of hepatic transcription factors. PLoS One. 2017 Mar 22;12(3):e0173977. doi: 10.1371/journal.pone.0173977. PMID: 28328977; PMCID: PMC5362104.

22: Bao B, He Y, Tang D, Li W, Li H. Inhibition of H3K27me3 Histone Demethylase Activity Prevents the Proliferative Regeneration of Zebrafish Lateral Line Neuromasts. Front Mol Neurosci. 2017 Mar 13;10:51. doi: 10.3389/fnmol.2017.00051. PMID: 28348517; PMCID: PMC5346882.

23: Ha SD, Cho W, Kim SO. HDAC8 Prevents Anthrax Lethal Toxin-induced Cell Cycle Arrest through Silencing PTEN in Human Monocytic THP-1 Cells. Toxins (Basel). 2017 May 16;9(5):162. doi: 10.3390/toxins9050162. PMID: 28509866; PMCID: PMC5450710.

24: Dalvi MP, Wang L, Zhong R, Kollipara RK, Park H, Bayo J, Yenerall P, Zhou Y, Timmons BC, Rodriguez-Canales J, Behrens C, Mino B, Villalobos P, Parra ER, Suraokar M, Pataer A, Swisher SG, Kalhor N, Bhanu NV, Garcia BA, Heymach JV, Coombes K, Xie Y, Girard L, Gazdar AF, Kittler R, Wistuba II, Minna JD, Martinez ED. Taxane-Platin-Resistant Lung Cancers Co-develop Hypersensitivity to JumonjiC Demethylase Inhibitors. Cell Rep. 2017 May 23;19(8):1669-1684. doi: 10.1016/j.celrep.2017.04.077. PMID: 28538184; PMCID: PMC5531293.

25: Backe MB, Andersson JL, Bacos K, Christensen DP, Hansen JB, Dorosz JJ, Gajhede M, Dahlby T, Bysani M, Kristensen LH, Ling C, Olsen L, Mandrup-Poulsen T. Lysine demethylase inhibition protects pancreatic β cells from apoptosis and improves β-cell function. Mol Cell Endocrinol. 2018 Jan 15;460:47-56. doi: 10.1016/j.mce.2017.07.001. Epub 2017 Jul 4. PMID: 28684291.

26: Das A, Arifuzzaman S, Yoon T, Kim SH, Chai JC, Lee YS, Jung KH, Chai YG. RNA sequencing reveals resistance of TLR4 ligand-activated microglial cells to inflammation mediated by the selective jumonji H3K27 demethylase inhibitor. Sci Rep. 2017 Jul 26;7(1):6554. doi: 10.1038/s41598-017-06914-5. PMID: 28747667; PMCID: PMC5529413.

27: Yan N, Xu L, Wu X, Zhang L, Fei X, Cao Y, Zhang F. GSKJ4, an H3K27me3 demethylase inhibitor, effectively suppresses the breast cancer stem cells. Exp Cell Res. 2017 Oct 15;359(2):405-414. doi: 10.1016/j.yexcr.2017.08.024. Epub 2017 Aug 18. PMID: 28823831.

28: Inoue SI, Takahara S, Yoshikawa T, Niihori T, Yanai K, Matsubara Y, Aoki Y. Activated Braf induces esophageal dilation and gastric epithelial hyperplasia in mice. Hum Mol Genet. 2017 Dec 1;26(23):4715-4727. doi: 10.1093/hmg/ddx354. PMID: 28973166.

29: Morozov VM, Li Y, Clowers MM, Ishov AM. Inhibitor of H3K27 demethylase JMJD3/UTX GSK-J4 is a potential therapeutic option for castration resistant prostate cancer. Oncotarget. 2017 Jul 8;8(37):62131-62142. doi: 10.18632/oncotarget.19100. PMID: 28977932; PMCID: PMC5617492.

30: Sui A, Xu Y, Li Y, Hu Q, Wang Z, Zhang H, Yang J, Guo X, Zhao W. The pharmacological role of histone demethylase JMJD3 inhibitor GSK-J4 on glioma cells. Oncotarget. 2017 Aug 2;8(40):68591-68598. doi: 10.18632/oncotarget.19793. PMID: 28978140; PMCID: PMC5620280.

31: Taube JH, Sphyris N, Johnson KS, Reisenauer KN, Nesbit TA, Joseph R, Vijay GV, Sarkar TR, Bhangre NA, Song JJ, Chang JT, Lee MG, Soundararajan R, Mani SA. The H3K27me3-demethylase KDM6A is suppressed in breast cancer stem-like cells, and enables the resolution of bivalency during the mesenchymal-epithelial transition. Oncotarget. 2017 Jul 10;8(39):65548-65565. doi: 10.18632/oncotarget.19214. PMID: 29029452; PMCID: PMC5630352.

32: Mandal C, Kim SH, Kang SC, Chai JC, Lee YS, Jung KH, Chai YG. GSK-J4-Mediated Transcriptomic Alterations in Differentiating Embryoid Bodies. Mol Cells. 2017 Oct;40(10):737-751. doi: 10.14348/molcells.2017.0069. Epub 2017 Oct 17. PMID: 29047260; PMCID: PMC5682251.

33: Bergmann C, Brandt A, Merlevede B, Hallenberger L, Dees C, Wohlfahrt T, Pötter S, Zhang Y, Chen CW, Mallano T, Liang R, Kagwiria R, Kreuter A, Pantelaki I, Bozec A, Abraham D, Rieker R, Ramming A, Distler O, Schett G, Distler JHW. The histone demethylase Jumonji domain-containing protein 3 (JMJD3) regulates fibroblast activation in systemic sclerosis. Ann Rheum Dis. 2018 Jan;77(1):150-158. doi: 10.1136/annrheumdis-2017-211501. Epub 2017 Oct 25. PMID: 29070530.

34: Rejlova K, Musilova A, Kramarzova KS, Zaliova M, Fiser K, Alberich-Jorda M, Trka J, Starkova J. Low HOX gene expression in PML-RARα-positive leukemia results from suppressed histone demethylation. Epigenetics. 2018;13(1):73-84. doi: 10.1080/15592294.2017.1413517. Epub 2018 Feb 7. PMID: 29224413; PMCID: PMC5836981.

35: Cribbs A, Hookway ES, Wells G, Lindow M, Obad S, Oerum H, Prinjha RK, Athanasou N, Sowman A, Philpott M, Penn H, Soderstrom K, Feldmann M, Oppermann U. Inhibition of histone H3K27 demethylases selectively modulates inflammatory phenotypes of natural killer cells. J Biol Chem. 2018 Feb 16;293(7):2422-2437. doi: 10.1074/jbc.RA117.000698. Epub 2018 Jan 4. PMID: 29301935; PMCID: PMC5818173.

36: Park JW, Cho H, Oh H, Kim JY, Seo SB. AURKA Suppresses Leukemic THP-1 Cell Differentiation through Inhibition of the KDM6B Pathway. Mol Cells. 2018 May 31;41(5):444-453. doi: 10.14348/molcells.2018.2311. Epub 2018 Feb 23. PMID: 29477140; PMCID: PMC5974621.

37: Rath BH, Waung I, Camphausen K, Tofilon PJ. Inhibition of the Histone H3K27 Demethylase UTX Enhances Tumor Cell Radiosensitivity. Mol Cancer Ther. 2018 May;17(5):1070-1078. doi: 10.1158/1535-7163.MCT-17-1053. Epub 2018 Feb 26. PMID: 29483212; PMCID: PMC5932086.

38: Li Y, Zhang M, Sheng M, Zhang P, Chen Z, Xing W, Bai J, Cheng T, Yang FC, Zhou Y. Therapeutic potential of GSK-J4, a histone demethylase KDM6B/JMJD3 inhibitor, for acute myeloid leukemia. J Cancer Res Clin Oncol. 2018 Jun;144(6):1065-1077. doi: 10.1007/s00432-018-2631-7. Epub 2018 Mar 28. PMID: 29594337; PMCID: PMC5948279.

39: Chen Y, Liu Z, Pan T, Chen E, Mao E, Chen Y, Tan R, Wang X, Tian R, Liu J, Qu H. JMJD3 is involved in neutrophil membrane proteinase 3 overexpression during the hyperinflammatory response in early sepsis. Int Immunopharmacol. 2018 Jun;59:40-46. doi: 10.1016/j.intimp.2018.03.027. Epub 2018 Apr 3. PMID: 29621735.

40: Guo Z, Lu J, Li J, Wang P, Li Z, Zhong Y, Guo K, Wang J, Ye J, Liu P. JMJD3 inhibition protects against isoproterenol-induced cardiac hypertrophy by

suppressing β-MHC expression. Mol Cell Endocrinol. 2018 Dec 5;477:1-14. doi: 10.1016/j.mce.2018.05.009. Epub 2018 May 10. PMID: 29753027.

41: Lochmann TL, Powell KM, Ham J, Floros KV, Heisey DAR, Kurupi RIJ, Calbert ML, Ghotra MS, Greninger P, Dozmorov M, Gowda M, Souers AJ, Reynolds CP, Benes CH, Faber AC. Targeted inhibition of histone H3K27 demethylation is effective in high-risk neuroblastoma. Sci Transl Med. 2018 May 16;10(441):eaao4680. doi: 10.1126/scitranslmed.aao4680. PMID: 29769286; PMCID: PMC6200133.

42: Daures M, Idrissou M, Judes G, Rifaï K, Penault-Llorca F, Bignon YJ, Guy L, Bernard-Gallon D. A new metabolic gene signature in prostate cancer regulated by JMJD3 and EZH2. Oncotarget. 2018 May 4;9(34):23413-23425. doi: 10.18632/oncotarget.25182. PMID: 29805743; PMCID: PMC5955128.

43: Wu W, Qin M, Jia W, Huang Z, Li Z, Yang D, Huang M, Xiao C, Long F, Mao J, Moore PK, Liu X, Zhu YZ. Cystathionine-γ-lyase ameliorates the histone demethylase JMJD3-mediated autoimmune response in rheumatoid arthritis. Cell Mol Immunol. 2019 Aug;16(8):694-705. doi: 10.1038/s41423-018-0037-8. Epub 2018 May 29. PMID: 29844591; PMCID: PMC6804949.

44: Chen HJ, Huang RL, Liew PL, Su PH, Chen LY, Weng YC, Chang CC, Wang YC, Chan MW, Lai HC. GATA3 as a master regulator and therapeutic target in ovarian high-grade serous carcinoma stem cells. Int J Cancer. 2018 Dec 15;143(12):3106-3119. doi: 10.1002/ijc.31750. Epub 2018 Oct 3. PMID: 30006927.

45: Liu H, Peng L, So J, Tsang KH, Chong CH, Mak PHS, Chan KM, Chan SY. TSPYL2 Regulates the Expression of EZH2 Target Genes in Neurons. Mol Neurobiol. 2019 Apr;56(4):2640-2652. doi: 10.1007/s12035-018-1238-y. Epub 2018 Jul 26. PMID: 30051352; PMCID: PMC6459796.

46: Illiano M, Conte M, Sapio L, Nebbioso A, Spina A, Altucci L, Naviglio S. Forskolin Sensitizes Human Acute Myeloid Leukemia Cells to H3K27me2/3 Demethylases GSKJ4 Inhibitor via Protein Kinase A. Front Pharmacol. 2018 Jul 20;9:792. doi: 10.3389/fphar.2018.00792. PMID: 30079022; PMCID: PMC6063003.

47: Yu Q, Yu X, Zhao W, Zhu M, Wang Z, Zhang J, Huang M, Zeng X. Inhibition of H3K27me3 demethylases attenuates asthma by reversing the shift in airway smooth muscle phenotype. Clin Exp Allergy. 2018 Nov;48(11):1439-1452. doi: 10.1111/cea.13244. Epub 2018 Sep 4. PMID: 30084510.

48: Zhang YX, Akumuo RC, España RA, Yan CX, Gao WJ, Li YC. The histone demethylase KDM6B in the medial prefrontal cortex epigenetically regulates cocaine reward memory. Neuropharmacology. 2018 Oct;141:113-125. doi: 10.1016/j.neuropharm.2018.08.030. Epub 2018 Aug 27. PMID: 30165076; PMCID: PMC6170674.

49: Wei Y, Zheng H, Bao N, Jiang S, Bueso-Ramos CE, Khoury J, Class C, Lu Y, Lin K, Yang H, Ganan-Gomez I, Starczynowski DT, Do KA, Colla S, Garcia-Manero G. KDM6B overexpression activates innate immune signaling and impairs hematopoiesis in mice. Blood Adv. 2018 Oct 9;2(19):2491-2504. doi: 10.1182/bloodadvances.2018024166. PMID: 30275007; PMCID: PMC6177657.

50: Pan Y, Wang J, Xue Y, Zhao J, Li D, Zhang S, Li K, Hou Y, Fan H. GSKJ4 Protects Mice Against Early Sepsis via Reducing Proinflammatory Factors and Up-Regulating MiR-146a. Front Immunol. 2018 Oct 2;9:2272. doi: 10.3389/fimmu.2018.02272. PMID: 30337925; PMCID: PMC6179039.

51: Chen H, Huang Y, Zhu X, Liu C, Yuan Y, Su H, Zhang C, Liu C, Xiong M, Qu Y, Yun P, Zheng L, Huang K. Histone demethylase UTX is a therapeutic target for diabetic kidney disease. J Physiol. 2019 Mar;597(6):1643-1660. doi: 10.1113/JP277367. Epub 2018 Dec 25. PMID: 30516825; PMCID: PMC6418754.

52: Yamada T, Nabe S, Toriyama K, Suzuki J, Inoue K, Imai Y, Shiraishi A, Takenaka K, Yasukawa M, Yamashita M. Histone H3K27 Demethylase Negatively Controls the Memory Formation of Antigen-Stimulated CD8⁺ T Cells. J Immunol. 2019 Feb 15;202(4):1088-1098. doi: 10.4049/jimmunol.1801083. Epub 2019 Jan 9. PMID: 30626691.

53: Xia M, Zhang Q, Luo M, Li P, Wang Y, Lei Q, Guo AY. Regulatory network analysis reveals the oncogenesis roles of feed-forward loops and therapeutic target in T-cell acute lymphoblastic leukemia. BMC Med Genomics. 2019 Jan 15;12(1):8. doi: 10.1186/s12920-018-0469-0. PMID: 30646895; PMCID: PMC6332896.

54: Xu Z, Xia Y, Xiao Z, Jia Y, Li L, Jin Y, Zhao Q, Wan L, Yi T, Yu Y, Wen Q, Zhu Y, Qin B, Zhang F, Shen J. Comprehensive profiling of JMJD3 in gastric cancer and its influence on patient survival. Sci Rep. 2019 Jan 29;9(1):868. doi: 10.1038/s41598-018-37340-w. PMID: 30696880; PMCID: PMC6351656.

55: Bayo J, Fiore EJ, Dominguez LM, Real A, Malvicini M, Rizzo M, Atorrasagasti C, García MG, Argemi J, Martinez ED, Mazzolini GD. A comprehensive study of epigenetic alterations in hepatocellular carcinoma identifies potential therapeutic targets. J Hepatol. 2019 Jul;71(1):78-90. doi: 10.1016/j.jhep.2019.03.007. Epub 2019 Mar 15. PMID: 30880225.

56: Xiao Z, He Y, Liu C, Xiang L, Yi J, Wang M, Shen T, Shen L, Xue Y, Shi H, Liu P. Targeting P16INK4A in uterine serous carcinoma through inhibition of histone demethylation. Oncol Rep. 2019 May;41(5):2667-2678. doi: 10.3892/or.2019.7067. Epub 2019 Mar 14. PMID: 30896884; PMCID: PMC6448098.

57: Tottone L, Zhdanovskaya N, Carmona Pestaña Á, Zampieri M, Simeoni F, Lazzari S, Ruocco V, Pelullo M, Caiafa P, Felli MP, Checquolo S, Bellavia D, Talora C, Screpanti I, Palermo R. Histone Modifications Drive Aberrant Notch3 Expression/Activity and Growth in T-ALL. Front Oncol. 2019 Apr 3;9:198. doi: 10.3389/fonc.2019.00198. PMID: 31001470; PMCID: PMC6456714.

58: Wang Q, Chen X, Jiang Y, Liu S, Liu H, Sun X, Zhang H, Liu Z, Tao Y, Li C, Hu Y, Liu D, Ye D, Liu Y, Wang M, Zhang X. Elevating H3K27me3 level sensitizes colorectal cancer to oxaliplatin. J Mol Cell Biol. 2020 Feb 20;12(2):125-137. doi: 10.1093/jmcb/mjz032. PMID: 31065671; PMCID: PMC7109602.

59: Sun HY, Yang D, Mi J, Yu YQ, Qiu LH. Histone demethylase Jmjd3 modulates osteoblast apoptosis induced by tumor necrosis factor-alpha through directly targeting RASSF5. Connect Tissue Res. 2020 Nov;61(6):517-525. doi: 10.1080/03008207.2019.1620225. Epub 2019 May 27. PMID: 31092054.

60: Lai J, Ge M, Shen S, Yang L, Jin T, Cao D, Xu H, Zheng X, Qiu S, Wang K, Wei Q, Li H, Ai J. Activation of NFKB-JMJD3 signaling promotes bladder fibrosis via boosting bladder smooth muscle cell proliferation and collagen accumulation. Biochim Biophys Acta Mol Basis Dis. 2019 Sep 1;1865(9):2403-2410. doi: 10.1016/j.bbadis.2019.05.008. Epub 2019 May 16. PMID: 31102789.

61: Guo Y, Xiong Z, Guo X. Histone demethylase KDM6B regulates human podocyte differentiation <i>in vitro</i>. Biochem J. 2019 Jun 26;476(12):1741-1751. doi: 10.1042/BCJ20180968. PMID: 31138771.

62: Singh R, Fazal Z, Corbet AK, Bikorimana E, Rodriguez JC, Khan EM, Shahid K, Freemantle SJ, Spinella MJ. Epigenetic Remodeling through Downregulation of Polycomb Repressive Complex 2 Mediates Chemotherapy Resistance in Testicular Germ Cell Tumors. Cancers (Basel). 2019 Jun 8;11(6):796. doi: 10.3390/cancers11060796. PMID: 31181810; PMCID: PMC6627640.

63: Katagi H, Louis N, Unruh D, Sasaki T, He X, Zhang A, Ma Q, Piunti A, Shimazu Y, Lamano JB, Carcaboso AM, Tian X, Seluanov A, Gorbunova V, Laurie KL, Kondo A, Wadhwani NR, Lulla R, Goldman S, Venneti S, Becher OJ, Zou L, Shilatifard A, Hashizume R. Radiosensitization by Histone H3 Demethylase Inhibition in Diffuse Intrinsic Pontine Glioma. Clin Cancer Res. 2019 Sep 15;25(18):5572-5583. doi: 10.1158/1078-0432.CCR-18-3890. Epub 2019 Jun 21. PMID: 31227500; PMCID: PMC6744979.

64: Romani M, Daga A, Forlani A, Pistillo MP, Banelli B. Targeting of Histone Demethylases KDM5A and KDM6B Inhibits the Proliferation of Temozolomide-Resistant Glioblastoma Cells. Cancers (Basel). 2019 Jun 24;11(6):878. doi: 10.3390/cancers11060878. PMID: 31238504; PMCID: PMC6627323.

65: Lhuissier E, Aury-Landas J, Allas L, Boittin M, Boumediene K, Baugé C. Antiproliferative effect of the histone demethylase inhibitor GSK-J4 in chondrosarcomas. IUBMB Life. 2019 Nov;71(11):1711-1719. doi: 10.1002/iub.2110. Epub 2019 Jun 26. PMID: 31241814.

66: Pediconi N, Salerno D, Lupacchini L, Angrisani A, Peruzzi G, De Smaele E, Levrero M, Belloni L. EZH2, JMJD3, and UTX epigenetically regulate hepatic plasticity inducing retro-differentiation and proliferation of liver cells. Cell Death Dis. 2019 Jul 8;10(7):518. doi: 10.1038/s41419-019-1755-2. PMID: 31285428; PMCID: PMC6614397.

67: Backe MB, Jin C, Andreone L, Sankar A, Agger K, Helin K, Madsen AN, Poulsen SS, Bysani M, Bacos K, Ling C, Perone MJ, Holst B, Mandrup-Poulsen T. The Lysine Demethylase KDM5B Regulates Islet Function and Glucose Homeostasis. J Diabetes Res. 2019 Jul 28;2019:5451038. doi: 10.1155/2019/5451038. PMID: 31467927; PMCID: PMC6701283.

68: Hong BJ, Park WY, Kim HR, Moon JW, Lee HY, Park JH, Kim SK, Oh Y, Roe JS, Kim MY. Oncogenic KRAS Sensitizes Lung Adenocarcinoma to GSK-J4-Induced Metabolic and Oxidative Stress. Cancer Res. 2019 Nov 15;79(22):5849-5859. doi: 10.1158/0008-5472.CAN-18-3511. Epub 2019 Sep 10. PMID: 31506334.

69: Yin X, Yang S, Zhang M, Yue Y. The role and prospect of JMJD3 in stem cells and cancer. Biomed Pharmacother. 2019 Oct;118:109384. doi: 10.1016/j.biopha.2019.109384. Epub 2019 Sep 6. PMID: 31545292.

70: Lu B, He Y, He J, Wang L, Liu Z, Yang J, Gao Z, Lu G, Zou C, Zhao W. Epigenetic Profiling Identifies LIF as a Super-enhancer-Controlled Regulator of Stem Cell-like Properties in Osteosarcoma. Mol Cancer Res. 2020 Jan;18(1):57-67. doi: 10.1158/1541-7786.MCR-19-0470. Epub 2019 Oct 15. PMID: 31615908.

71: Whatley KCL, Padalino G, Whiteland H, Geyer KK, Hulme BJ, Chalmers IW, Forde-Thomas J, Ferla S, Brancale A, Hoffmann KF. The repositioning of epigenetic probes/inhibitors identifies new anti-schistosomal lead compounds and chemotherapeutic targets. PLoS Negl Trop Dis. 2019 Nov 15;13(11):e0007693. doi: 10.1371/journal.pntd.0007693. PMID: 31730617; PMCID: PMC6881072.

72: Hsieh IY, He J, Wang L, Lin B, Liang Z, Lu B, Chen W, Lu G, Li F, Lv W, Zhao W, Li J. H3K27me3 loss plays a vital role in CEMIP mediated carcinogenesis and progression of breast cancer with poor prognosis. Biomed Pharmacother. 2020 Mar;123:109728. doi: 10.1016/j.biopha.2019.109728. Epub 2019 Dec 14. PMID: 31846842.

73: Nikolaev A, Fiveash JB, Yang ES. Combined Targeting of Mutant p53 and Jumonji Family Histone Demethylase Augments Therapeutic Efficacy of Radiation in H3K27M DIPG. Int J Mol Sci. 2020 Jan 13;21(2):490. doi: 10.3390/ijms21020490. PMID: 31940975; PMCID: PMC7014308.

74: Naruse C, Abe K, Yoshihara T, Kato T, Nishiuchi T, Asano M. Heterochromatin protein 1γ deficiency decreases histone H3K27 methylation in mouse neurosphere neuronal genes. FASEB J. 2020 Mar;34(3):3956-3968. doi: 10.1096/fj.201900139R. Epub 2020 Jan 21. PMID: 31961023.

75: Wang R, Wang W, Xu J, Liu D, Wu H, Qin X, Jiang H, Pan F. Jmjd3 is involved in the susceptibility to depression induced by maternal separation via enhancing the neuroinflammation in the prefrontal cortex and hippocampus of male rats. Exp Neurol. 2020 Jun;328:113254. doi: 10.1016/j.expneurol.2020.113254. Epub 2020 Feb 19. PMID: 32084453.

76: Cribbs AP, Terlecki-Zaniewicz S, Philpott M, Baardman J, Ahern D, Lindow M, Obad S, Oerum H, Sampey B, Mander PK, Penn H, Wordsworth P, Bowness P, de Winther M, Prinjha RK, Feldmann M, Oppermann U. Histone H3K27me3 demethylases regulate human Th17 cell development and effector functions by impacting on metabolism. Proc Natl Acad Sci U S A. 2020 Mar 17;117(11):6056-6066. doi: 10.1073/pnas.1919893117. Epub 2020 Mar 2. PMID: 32123118; PMCID: PMC7084125.

77: Lobo-Silva J, Cabral FJ, Amaral MS, Miyasato PA, de Freitas RP, Pereira ASA, Khouri MI, Barbosa MMF, Ramos PIP, Leite LCC, Asojo OA, Nakano E, Verjovski-Almeida S, Farias LP. The antischistosomal potential of GSK-J4, an H3K27 demethylase inhibitor: insights from molecular modeling, transcriptomics and in vitro assays. Parasit Vectors. 2020 Mar 17;13(1):140. doi: 10.1186/s13071-020-4000-z. PMID: 32178714; PMCID: PMC7077139.

78: Deng M, Wang J, Wu H, Wang M, Cao D, Li J, Wu Y, Gong J. IL-4 Alleviates Ischaemia-Reperfusion Injury by Inducing Kupffer Cells M2 Polarization via STAT6-JMJD3 Pathway after Rat Liver Transplantation. Biomed Res Int. 2020 Mar 18;2020:2953068. doi: 10.1155/2020/2953068. PMID: 32258110; PMCID: PMC7104314.

79: Liu S, Wu M, Hua Q, Lu D, Tian Y, Yu H, Cheng L, Chen Y, Cao J, Hu X, Tan F. Two old drugs, NVP-AEW541 and GSK-J4, repurposed against the Toxoplasma gondii RH strain. Parasit Vectors. 2020 May 11;13(1):242. doi: 10.1186/s13071-020-04094-2. PMID: 32393321; PMCID: PMC7216583.

80: Jun Z, Xinmeng J, Yue L, Zhi W, Yan Z, Tieyi Y, Jiangan T. Jumonji domain containing-3 (JMJD3) inhibition attenuates IL-1β-induced chondrocytes damage in vitro and protects osteoarthritis cartilage in vivo. Inflamm Res. 2020 Jul;69(7):657-666. doi: 10.1007/s00011-020-01356-8. Epub 2020 May 11. PMID: 32394143.

81: Lin B, Lu B, Hsieh IY, Liang Z, Sun Z, Yi Y, Lv W, Zhao W, Li J. Synergy of GSK-J4 With Doxorubicin in KRAS-Mutant Anaplastic Thyroid Cancer. Front Pharmacol. 2020 May 13;11:632. doi: 10.3389/fphar.2020.00632. PMID: 32477122; PMCID: PMC7239034.

82: Chu X, Zhong L, Yu L, Xiong L, Li J, Dan W, Ye J, Liu C, Luo X, Liu B. GSK-J4 induces cell cycle arrest and apoptosis via ER stress and the synergism between GSK-J4 and decitabine in acute myeloid leukemia KG-1a cells. Cancer Cell Int. 2020 Jun 3;20:209. doi: 10.1186/s12935-020-01297-6. PMID: 32514253; PMCID: PMC7268296.

83: Sanchez A, El Ouardi D, Houfaf Khoufaf FZ, Idrissou M, Boisnier T, Penault-Llorca F, Bignon YJ, Guy L, Bernard-Gallon D. Role of JMJD3 Demethylase and Its Inhibitor GSK-J4 in Regulation of <i>MGMT</i>, <i>TRA2A</i>, <i>RPS6KA2</i>, and <i>U2AF1</i> Genes in Prostate Cancer Cell Lines. OMICS. 2020 Aug;24(8):505-507. doi: 10.1089/omi.2020.0054. Epub 2020 Jun 11. PMID: 32525734.

84: Illiano M, Conte M, Salzillo A, Ragone A, Spina A, Nebbioso A, Altucci L, Sapio L, Naviglio S. The KDM Inhibitor GSKJ4 Triggers CREB Downregulation via a Protein Kinase A and Proteasome-Dependent Mechanism in Human Acute Myeloid Leukemia Cells. Front Oncol. 2020 Jun 5;10:799. doi: 10.3389/fonc.2020.00799. PMID: 32582541; PMCID: PMC7289982.

85: Davis FM, denDekker A, Joshi AD, Wolf SJ, Audu C, Melvin WJ, Mangum K, Riordan MO, Kunkel SL, Gallagher KA. Palmitate-TLR4 signaling regulates the histone demethylase, JMJD3, in macrophages and impairs diabetic wound healing. Eur J Immunol. 2020 Dec;50(12):1929-1940. doi: 10.1002/eji.202048651. Epub 2020 Jul 20. PMID: 32662520; PMCID: PMC8127612.

86: Zhang J, Ying Y, Li M, Wang M, Huang X, Jia M, Zeng J, Ma C, Zhang Y, Li C, Wang X, Shu XS. Targeted inhibition of KDM6 histone demethylases eradicates tumor-initiating cells via enhancer reprogramming in colorectal cancer. Theranostics. 2020 Aug 8;10(22):10016-10030. doi: 10.7150/thno.47081. PMID: 32929331; PMCID: PMC7481431.

87: Mu MD, Qian ZM, Yang SX, Rong KL, Yung WH, Ke Y. Therapeutic effect of a histone demethylase inhibitor in Parkinson's disease. Cell Death Dis. 2020 Oct 28;11(10):927. doi: 10.1038/s41419-020-03105-5. PMID: 33116116; PMCID: PMC7595123.

88: Sapio L, Salzillo A, Ragone A, Illiano M, Spina A, Naviglio S. Targeting CREB in Cancer Therapy: A Key Candidate or One of Many? An Update. Cancers (Basel). 2020 Oct 28;12(11):3166. doi: 10.3390/cancers12113166. PMID: 33126560; PMCID: PMC7693618.

89: Pribadi C, Camp E, Cakouros D, Anderson P, Glackin C, Gronthos S. Pharmacological targeting of KDM6A and KDM6B, as a novel therapeutic strategy for treating craniosynostosis in Saethre-Chotzen syndrome. Stem Cell Res Ther. 2020 Dec 9;11(1):529. doi: 10.1186/s13287-020-02051-5. PMID: 33298158; PMCID: PMC7726873.

90: Wu H, Wang R, Qin X, Liu D, Wang W, Xu J, Jiang H, Pan F. Effects of chronic stress on depressive-like behaviors and JMJD3 expression in the prefrontal cortex and hippocampus of C57BL/6 and ob/ob mice. J Psychiatr Res. 2021 Jan;133:142-155. doi: 10.1016/j.jpsychires.2020.12.014. Epub 2020 Dec 13. PMID: 33340793.

91: Connor MG, Camarasa TMN, Patey E, Rasid O, Barrio L, Weight CM, Miller DP, Heyderman RS, Lamont RJ, Enninga J, Hamon MA. The histone demethylase KDM6B fine-tunes the host response to Streptococcus pneumoniae. Nat Microbiol. 2021 Feb;6(2):257-269. doi: 10.1038/s41564-020-00805-8. Epub 2020 Dec 21. PMID: 33349663.

92: Hao W, Qiao D, Han Y, Du N, Li X, Fan Y, Ge X, Zhang H. Identification of disulfiram as a potential antifungal drug by screening small molecular libraries. J Infect Chemother. 2021 May;27(5):696-701. doi: 10.1016/j.jiac.2020.12.012. Epub 2020 Dec 23. PMID: 33358402.

93: Jiang Y, Xiang C, Zhong F, Zhang Y, Wang L, Zhao Y, Wang J, Ding C, Jin L, He F, Wang H. Histone H3K27 methyltransferase EZH2 and demethylase JMJD3 regulate hepatic stellate cells activation and liver fibrosis. Theranostics. 2021 Jan 1;11(1):361-378. doi: 10.7150/thno.46360. PMID: 33391480; PMCID: PMC7681085.

94: Cao Z, Shi X, Tian F, Fang Y, Wu JB, Mrdenovic S, Nian X, Ji J, Xu H, Kong C, Xu Y, Chen X, Huang Y, Wei X, Yu Y, Yang B, Chung LWK, Wang F. KDM6B is an androgen regulated gene and plays oncogenic roles by demethylating H3K27me3 at cyclin D1 promoter in prostate cancer. Cell Death Dis. 2021 Jan 6;12(1):2. doi: 10.1038/s41419-020-03354-4. PMID: 33414463; PMCID: PMC7791132.

95: Doñas C, Neira J, Osorio-Barrios F, Carrasco M, Fernández D, Prado C, Loyola A, Pacheco R, Rosemblatt M. The demethylase inhibitor GSK-J4 limits inflammatory colitis by promoting de novo synthesis of retinoic acid in dendritic cells. Sci Rep. 2021 Jan 14;11(1):1342. doi: 10.1038/s41598-020-79122-3. PMID: 33446666; PMCID: PMC7809056.

96: Yu C, Xiong C, Tang J, Hou X, Liu N, Bayliss G, Zhuang S. Histone demethylase JMJD3 protects against renal fibrosis by suppressing TGFβ and Notch signaling and preserving PTEN expression. Theranostics. 2021 Jan 1;11(6):2706-2721. doi: 10.7150/thno.48679. PMID: 33456568; PMCID: PMC7806480.

97: Zhu X, Zhu Y, Ding C, Zhang W, Guan H, Li C, Lin X, Zhang Y, Huang C, Zhang L, Yu X, Zhang X, Zhu W. LncRNA H19 regulates macrophage polarization and promotes Freund's complete adjuvant-induced arthritis by upregulating KDM6A. Int Immunopharmacol. 2021 Apr;93:107402. doi: 10.1016/j.intimp.2021.107402. Epub 2021 Feb 1. PMID: 33540246.

98: Wu X, Li J, Chang K, Yang F, Jia Z, Sun C, Li Q, Xu Y. Histone H3 methyltransferase Ezh2 promotes white adipocytes but inhibits brown and beige adipocyte differentiation in mice. Biochim Biophys Acta Mol Cell Biol Lipids. 2021 Jun;1866(6):158901. doi: 10.1016/j.bbalip.2021.158901. Epub 2021 Feb 9. PMID: 33571671.

99: Kitajima S, Sun W, Lee KL, Ho JC, Oyadomari S, Okamoto T, Masai H, Poellinger L, Kato H. A KDM6 inhibitor potently induces ATF4 and its target gene expression through HRI activation and by UTX inhibition. Sci Rep. 2021 Feb 25;11(1):4538. doi: 10.1038/s41598-021-83857-y. PMID: 33633164; PMCID: PMC7907191.

100: Shentu Y, Tian Q, Yang J, Liu X, Han Y, Yang D, Zhang N, Fan X, Wang P, Ma J, Chen R, Li D, Liu S, Wang Y, Mao S, Gong Y, Du C, Fan J. Upregulation of KDM6B contributes to lipopolysaccharide-induced anxiety-like behavior via modulation of VGLL4 in mice. Behav Brain Res. 2021 Jun 25;408:113305. doi: 10.1016/j.bbr.2021.113305. Epub 2021 Apr 15. PMID: 33865886.

101: Qin M, Han F, Wu J, Gao FX, Li Y, Yan DX, He XM, Long Y, Tang XP, Ren DL, Gao Y, Dai TY. KDM6B promotes ESCC cell proliferation and metastasis by facilitating C/EBPβ transcription. BMC Cancer. 2021 May 17;21(1):559. doi: 10.1186/s12885-021-08282-w. Erratum in: BMC Cancer. 2021 Aug 5;21(1):893. PMID:

34001062; PMCID: PMC8130268.

102: Gulay KCM, Aoshima K, Shibata Y, Yasui H, Yan Q, Kobayashi A, Kimura T. KDM2B promotes cell viability by enhancing DNA damage response in canine hemangiosarcoma. J Genet Genomics. 2021 Jul 20;48(7):618-630. doi: 10.1016/j.jgg.2021.02.005. Epub 2021 Mar 10. PMID: 34023294.

103: Xu P, Deng B, Zhang B, Luo Q, Song G. Stretch-Induced Tenomodulin Expression Promotes Tenocyte Migration via F-Actin and Chromatin Remodeling. Int J Mol Sci. 2021 May 6;22(9):4928. doi: 10.3390/ijms22094928. PMID: 34066472; PMCID: PMC8124537.

104: Bai S, Xiong X, Tang B, Ji T, Li X, Qu X, Li W. hsa-miR-199b-3p Prevents the Epithelial-Mesenchymal Transition and Dysfunction of the Renal Tubule by Regulating E-cadherin through Targeting KDM6A in Diabetic Nephropathy. Oxid Med Cell Longev. 2021 Jun 27;2021:8814163. doi: 10.1155/2021/8814163. PMID: 34257820; PMCID: PMC8257373.

105: Romero OA, Vilarrubi A, Alburquerque-Bejar JJ, Gomez A, Andrades A, Trastulli D, Pros E, Setien F, Verdura S, Farré L, Martín-Tejera JF, Llabata P, Oaknin A, Saigi M, Piulats JM, Matias-Guiu X, Medina PP, Vidal A, Villanueva A, Sanchez-Cespedes M. SMARCA4 deficient tumours are vulnerable to KDM6A/UTX and KDM6B/JMJD3 blockade. Nat Commun. 2021 Jul 14;12(1):4319. doi: 10.1038/s41467-021-24618-3. PMID: 34262032; PMCID: PMC8280185.

106: Nanduri J, Wang N, Wang BL, Prabhakar NR. Lysine demethylase KDM6B regulates HIF-1α-mediated systemic and cellular responses to intermittent hypoxia. Physiol Genomics. 2021 Sep 1;53(9):385-394. doi: 10.1152/physiolgenomics.00045.2021. Epub 2021 Jul 23. PMID: 34297635; PMCID: PMC8887999.

107: Heisey DAR, Jacob S, Lochmann TL, Kurupi R, Ghotra MS, Calbert ML, Shende M, Maves YK, Koblinski JE, Dozmorov MG, Boikos SA, Benes CH, Faber AC. Pharmaceutical Interference of the EWS-FLI1-driven Transcriptome By Cotargeting H3K27ac and RNA Polymerase Activity in Ewing Sarcoma. Mol Cancer Ther. 2021 Oct;20(10):1868-1879. doi: 10.1158/1535-7163.MCT-20-0489. Epub 2021 Jul 26. PMID: 34315769.

108: Kleszcz R, Skalski M, Krajka-Kuźniak V, Paluszczak J. The inhibitors of KDM4 and KDM6 histone lysine demethylases enhance the anti-growth effects of erlotinib and HS-173 in head and neck cancer cells. Eur J Pharm Sci. 2021 Nov 1;166:105961. doi: 10.1016/j.ejps.2021.105961. Epub 2021 Aug 8. PMID: 34363938.

109: Lu B, Zou C, Yang M, He Y, He J, Zhang C, Chen S, Yu J, Liu KY, Cao Q, Zhao W. Pharmacological Inhibition of Core Regulatory Circuitry Liquid-liquid Phase Separation Suppresses Metastasis and Chemoresistance in Osteosarcoma. Adv Sci (Weinh). 2021 Oct;8(20):e2101895. doi: 10.1002/advs.202101895. Epub 2021 Aug 25. PMID: 34432948; PMCID: PMC8529446.

110: Li L, Bai L, Yang K, Zhang J, Gao Y, Jiang M, Yang Y, Zhang X, Wang L, Wang X, Qiao Y, Xu JT. KDM6B epigenetically regulated-interleukin-6 expression in the dorsal root ganglia and spinal dorsal horn contributes to the development and maintenance of neuropathic pain following peripheral nerve injury in male rats. Brain Behav Immun. 2021 Nov;98:265-282. doi: 10.1016/j.bbi.2021.08.231. Epub 2021 Aug 28. PMID: 34464689.

111: Chen YH, Chen CH, Chien CY, Su YY, Luo SD, Li SH. Overexpression of UTX

promotes tumor progression in Oral tongue squamous cell carcinoma patients receiving surgical resection: a case control study. BMC Cancer. 2021 Sep 1;21(1):979. doi: 10.1186/s12885-021-08726-3. PMID: 34465286; PMCID: PMC8408955.

112: Katsuda Y, Tanaka K, Mori T, Narita M, Takeshima H, Kondo T, Yamabe Y, Matsufuji M, Sato D, Hamada Y, Yamaguchi K, Ushijima T, Inada E, Kuzumaki N, Iseki M, Narita M. Histone modification of pain-related gene expression in spinal cord neurons under a persistent postsurgical pain-like state by electrocautery. Mol Brain. 2021 Sep 20;14(1):146. doi: 10.1186/s13041-021-00854-y. PMID: 34544461; PMCID: PMC8451106.

113: Cabrera Zapata LE, Cisternas CD, Sosa C, Garcia-Segura LM, Arevalo MA, Cambiasso MJ. X-linked histone H3K27 demethylase Kdm6a regulates sexually dimorphic differentiation of hypothalamic neurons. Cell Mol Life Sci. 2021 Nov;78(21-22):7043-7060. doi: 10.1007/s00018-021-03945-0. Epub 2021 Oct 11. PMID: 34633482; PMCID: PMC8558156.

114: Nguyen K, Dobrowolski C, Shukla M, Cho WK, Luttge B, Karn J. Inhibition of the H3K27 demethylase UTX enhances the epigenetic silencing of HIV proviruses and induces HIV-1 DNA hypermethylation but fails to permanently block HIV reactivation. PLoS Pathog. 2021 Oct 21;17(10):e1010014. doi: 10.1371/journal.ppat.1010014. PMID: 34673825; PMCID: PMC8562785.

115: Rana S, Maurya S, Mohapatra G, Singh S, Babar R, Chandrasekhar H, Chamoli G, Rathore D, Kshetrapal P, Srikanth CV. Activation of epigenetic regulator KDM6B by <i>Salmonella</i> Typhimurium enables chronic infections. Gut Microbes. 2021 Jan-Dec;13(1):1986665. doi: 10.1080/19490976.2021.1986665. PMID: 34696686; PMCID: PMC8555538.

116: Page EC, Heatley SL, Eadie LN, McClure BJ, de Bock CE, Omari S, Yeung DT, Hughes TP, Thomas PQ, White DL. HMGN1 plays a significant role in CRLF2 driven Down Syndrome leukemia and provides a potential therapeutic target in this high-risk cohort. Oncogene. 2022 Feb;41(6):797-808. doi: 10.1038/s41388-021-02126-4. Epub 2021 Dec 2. PMID: 34857887.

117: Yıldırım-Buharalıoğlu G. Lysine Demethylase 6B Regulates Prostate Cancer Cell Proliferation by Controlling c-MYC Expression. Mol Pharmacol. 2022 Feb;101(2):106-119. doi: 10.1124/molpharm.121.000372. Epub 2021 Dec 3. PMID: 34862309.

118: Kania AK, Guo M, Scharer CD, Boss JM. Inhibition of H3K27me3 Demethylases Promotes Plasmablast Formation. Immunohorizons. 2021 Dec 8;5(12):918-930. doi: 10.4049/immunohorizons.2000087. PMID: 34880105.

119: D'Oto A, Fang J, Jin H, Xu B, Singh S, Mullasseril A, Jones V, Abu-Zaid A, von Buttlar X, Cooke B, Hu D, Shohet J, Murphy AJ, Davidoff AM, Yang J. KDM6B promotes activation of the oncogenic CDK4/6-pRB-E2F pathway by maintaining enhancer activity in MYCN-amplified neuroblastoma. Nat Commun. 2021 Dec 10;12(1):7204. doi: 10.1038/s41467-021-27502-2. PMID: 34893606; PMCID: PMC8664842.

120: Lee J, Choi H, Park C, Jeon S, Yune T. Jmjd3 Mediates Neuropathic Pain by Inducing Macrophage Infiltration and Activation in Lumbar Spinal Stenosis Animal Model. Int J Mol Sci. 2021 Dec 14;22(24):13426. doi: 10.3390/ijms222413426. PMID: 34948220; PMCID: PMC8707917.

121: Sui A, Han B, Ren W, He W, Gao C, Han X, Liu S, Zhang Y, Qi X, Guo X. The

Epigenetic Regulation of OLIG2 by Histone Demethylase KDM6B in Glioma Cells. J Mol Neurosci. 2022 May;72(5):939-946. doi: 10.1007/s12031-022-01976-1. Epub 2022 Feb 7. PMID: 35132566.

122: Bah I, Youssef D, Yao ZQ, McCall CE, El Gazzar M. Inhibiting KDM6A Demethylase Represses Long Non-Coding RNA Hotairm1 Transcription in MDSC During Sepsis. Front Immunol. 2022 Jan 28;13:823660. doi: 10.3389/fimmu.2022.823660. PMID: 35185915; PMCID: PMC8851568.

123: Zhang ZM, Cao HB, Li ZH, Zhuo R, Tao YF, Li XL, Li G, Liao XM, Fang F, Xie Y, Wu D, Wang HR, Wang JW, Chen YL, Yu JJ, Jia SQ, Yang RD, Guo XY, Yang Y, Feng CX, Xu YY, Qian GH, Pan J. SAPCD2 promotes neuroblastoma progression by altering the subcellular distribution of E2F7. Cell Death Dis. 2022 Feb 23;13(2):174. doi: 10.1038/s41419-022-04624-z. PMID: 35197448; PMCID: PMC8866461.

124: Kamiya T, Yamaguchi Y, Oka M, Hara H. Combined action of FOXO1 and superoxide dismutase 3 promotes MDA-MB-231 cell migration. Free Radic Res. 2022 Jan;56(1):106-114. doi: 10.1080/10715762.2022.2049770. Epub 2022 Mar 10. PMID: 35271779.

125: Rees MG, Brenan L, do Carmo M, Duggan P, Bajrami B, Arciprete M, Boghossian A, Vaimberg E, Ferrara SJ, Lewis TA, Rosenberg D, Sangpo T, Roth JA, Kaushik VK, Piccioni F, Doench JG, Root DE, Johannessen CM. Systematic identification of biomarker-driven drug combinations to overcome resistance. Nat Chem Biol. 2022 Jun;18(6):615-624. doi: 10.1038/s41589-022-00996-7. Epub 2022 Mar 24. PMID: 35332332.

126: Zhao Z, Zhang Y, Gao D, Zhang Y, Han W, Xu X, Song Q, Zhao C, Yang J. Inhibition of Histone H3 Lysine-27 Demethylase Activity Relieves Rheumatoid Arthritis Symptoms <i>via</i> Repression of IL6 Transcription in Macrophages. Front Immunol. 2022 Mar 15;13:818070. doi: 10.3389/fimmu.2022.818070. PMID: 35371061; PMCID: PMC8965057.

127: Sanchez A, Penault-Llorca F, Bignon YJ, Guy L, Bernard-Gallon D. Effects of GSK-J4 on JMJD3 Histone Demethylase in Mouse Prostate Cancer Xenografts. Cancer Genomics Proteomics. 2022 May-Jun;19(3):339-349. doi: 10.21873/cgp.20324. PMID: 35430567; PMCID: PMC9016480.

128: Xu K, Liu X, Wen B, Liu Y, Zhang W, Hu X, Chen L, Hang W, Chen J. GSK-J4, a Specific Histone Lysine Demethylase 6A Inhibitor, Ameliorates Lipotoxicity to Cardiomyocytes <i>via</i> Preserving H3K27 Methylation and Reducing Ferroptosis. Front Cardiovasc Med. 2022 Jun 2;9:907747. doi: 10.3389/fcvm.2022.907747. PMID: 35722096; PMCID: PMC9200982.

129: Yu B, Wang R, Luo H, Yang D, Wang S, Yu Y, Okamura H, Qiu L. Histone Demethylase Jmjd3 Regulates the Osteogenic Differentiation and Cytokine Expressions of Periodontal Ligament Cells. Acta Med Okayama. 2022 Jun;76(3):281-290. doi: 10.18926/AMO/63722. PMID: 35790358.

130: Ma S, Xu L, Chen L, Sun X, Hu F, Gong Y, Yang R, Li J, Wang Q, Huang S, Zhou H, Wang J. Novel pharmacological inhibition of JMJD3 improves necrotizing enterocolitis by attenuating the inflammatory response and ameliorating intestinal injury. Biochem Pharmacol. 2022 Sep;203:115165. doi: 10.1016/j.bcp.2022.115165. Epub 2022 Jul 5. PMID: 35803318.

131: Miller AL, Fuller-Carter PI, Masarini K, Samardzija M, Carter KW, Rashwan R, Lim XR, Brunet AA, Chopra A, Ram R, Grimm C, Ueffing M, Carvalho LS,

Trifunović D. Increased H3K27 trimethylation contributes to cone survival in a mouse model of cone dystrophy. Cell Mol Life Sci. 2022 Jul 10;79(8):409. doi: 10.1007/s00018-022-04436-6. PMID: 35810394; PMCID: PMC9271452.

132: Jin Y, Liu Z, Li Z, Li H, Zhu C, Li R, Zhou T, Fang B. Histone demethylase JMJD3 downregulation protects against aberrant force-induced osteoarthritis through epigenetic control of NR4A1. Int J Oral Sci. 2022 Jul 14;14(1):34. doi: 10.1038/s41368-022-00190-4. PMID: 35831280; PMCID: PMC9279410.

133: Chen YH, Chen CH, Chien CY, Su YY, Luo SD, Li SH. JMJD3 suppresses tumor progression in oral tongue squamous cell carcinoma patients receiving surgical resection. PeerJ. 2022 Jul 13;10:e13759. doi: 10.7717/peerj.13759. PMID: 35855897; PMCID: PMC9288160.

134: Diaz-Bulnes P, Saiz ML, Corte-Iglesias V, Rodrigues-Diez RR, Bernardo Florez A, Ruiz Bernet C, Martin Martin C, Ruiz-Ortega M, Suarez-Alvarez B, López-Larrea C. Demethylation of H3K9 and H3K27 Contributes to the Tubular Renal Damage Triggered by Endoplasmic Reticulum Stress. Antioxidants (Basel). 2022 Jul 12;11(7):1355. doi: 10.3390/antiox11071355. PMID: 35883846; PMCID: PMC9312208.

135: Abu-Hanna J, Patel JA, Anastasakis E, Cohen R, Clapp LH, Loizidou M, Eddama MMR. Therapeutic potential of inhibiting histone 3 lysine 27 demethylases: a review of the literature. Clin Epigenetics. 2022 Aug 1;14(1):98. doi: 10.1186/s13148-022-01305-8. PMID: 35915507; PMCID: PMC9344682.

136: Zheng B, Liu J, Gao A, Chen X, Gao L, Liao L, Luo B, Ogutu CO, Han Y. Epigenetic reprogramming of H3K27me3 and DNA methylation during leaf-to-callus transition in peach. Hortic Res. 2022 Jun 3;9:uhac132. doi: 10.1093/hr/uhac132. PMID: 35937864; PMCID: PMC9350832.

137: Zhong HY, Yuan C, Liu XL, Wang QQ, Li X, Zhao YC, Li X, Liu DD, Zheng TF, Zhang M. Mechanical stretch aggravates vascular smooth muscle cell apoptosis and vascular remodeling by downregulating EZH2. Int J Biochem Cell Biol. 2022 Oct;151:106278. doi: 10.1016/j.biocel.2022.106278. Epub 2022 Aug 17. PMID: 35985452.

138: Hung PH, Hsu YC, Chen TH, Ho C, Lin CL. The Histone Demethylase Inhibitor GSK-J4 Is a Therapeutic Target for the Kidney Fibrosis of Diabetic Kidney Disease via DKK1 Modulation. Int J Mol Sci. 2022 Aug 20;23(16):9407. doi: 10.3390/ijms23169407. PMID: 36012674; PMCID: PMC9409090.

139: Lee M, Geitgey DK, Hamilton JAG, Boss JM, Scharer CD, Spangle JM, Haynes KA, Henry CJ. Adipocyte-mediated epigenomic instability in human T-ALL cells is cytotoxic and phenocopied by epigenetic-modifying drugs. Front Cell Dev Biol. 2022 Aug 19;10:909557. doi: 10.3389/fcell.2022.909557. PMID: 36060800; PMCID: PMC9438935.

140: Sood A, Fernandes V, Preeti K, Khot M, Khatri DK, Singh SB. Fingolimod Alleviates Cognitive Deficit in Type 2 Diabetes by Promoting Microglial M2 Polarization via the pSTAT3-jmjd3 Axis. Mol Neurobiol. 2023 Feb;60(2):901-922. doi: 10.1007/s12035-022-03120-x. Epub 2022 Nov 16. PMID: 36385233.

141: Xie K, Zeng J, Wen L, Peng X, Lin Z, Xian G, Guo Y, Yang X, Li P, Xu D, Zeng Q. Abnormally elevated EZH2-mediated H3K27me3 enhances osteogenesis in aortic valve interstitial cells by inhibiting SOCS3 expression. Atherosclerosis. 2023 Jan;364:1-9. doi: 10.1016/j.atherosclerosis.2022.11.017. Epub 2022 Nov 23. PMID: 36455343.

142: Deng H, Guo X, Feng N, Luo Y, Liu B, Liu S, Wu JI, Shi X. Targeting H3K27me3 demethylase to inhibit Shh signaling and cholesterol metabolism in medulloblastoma growth. Front Oncol. 2022 Dec 1;12:1057147. doi: 10.3389/fonc.2022.1057147. PMID: 36531063; PMCID: PMC9753684.

143: Gao Y, Wang N, Jia D. JMJD3 downregulates IL4i1 aggravating lipopolysaccharide-induced acute lung injury via H3K27 and H3K4 demethylation. Environ Toxicol. 2023 Mar;38(4):754-769. doi: 10.1002/tox.23725. Epub 2022 Dec 20. PMID: 36537648.

144: Dalpatraj N, Naik A, Thakur N. Combination Treatment of a Phytochemical and a Histone Demethylase Inhibitor-A Novel Approach towards Targeting TGFβ-Induced EMT, Invasion, and Migration in Prostate Cancer. Int J Mol Sci. 2023 Jan 17;24(3):1860. doi: 10.3390/ijms24031860. PMID: 36768182; PMCID: PMC9915876.

145: Lu X, Liu Y, Xu L, Liang H, Zhou X, Lei H, Sha L. Role of Jumonji domain-containing protein D3 and its inhibitor GSK-J4 in Hashimoto's thyroiditis. Open Med (Wars). 2023 Mar 1;18(1):20230659. doi: 10.1515/med-2023-0659. PMID: 36874364; PMCID: PMC9979002.

CAS SciFinder^n Task History

Initiating Search

January 11, 2023, 11:15PM

All: **GSK-J4**

Search Tasks

Task	Search Type	View
Returned All Results	All	View Results
Exported: Returned Reference Results (253)<br>Kept Selected Results	References	View Results

Copyright © 2023 American Chemical Society (ACS). All Rights Reserved.
Internal use only. Redistribution is subject to the terms of your SciFinder^n License Agreement and CAS information Use Policies.

CAS SciFinder^{n}

References (253)

View in SciFinder^{n}

1

Therapeutic potential of gsk-j4, a histone demethylase kdm6b/jmjd3 inhibitor, for acute myeloid leukemia

By: Li, Yunan; Zhang, Mingying; Sheng, Mengyao; Zhang, Peng; Chen, Zizhen; Xing, Wen; Bai, Jie; Cheng, Tao; Yang, Feng-Chun; Zhou, Yuan
Journal of Cancer Research and Clinical Oncology (2018), 144(6), 1065-1077 | Language: English, Database: CAplus and MEDLINE

Substances (3) Reactions (0) Cited By (48)

2

The histone demethylase inhibitor GSK-J4 limits inflammation through the induction of a tolerogenic phenotype on DCs

By: Donas, Cristian; Carrasco, Macarena; Fritz, Macarena; Prado, Carolina; Tejon, Gabriela; Osorio-Barrios, Francisco ; Manriquez, Valeria; Reyes, Paz; Pacheco, Rodrigo; Bono, Maria Rosa; et al
Journal of Autoimmunity (2016), 75, 105-117 | Language: English, Database: CAplus and MEDLINE

Substances (3) Reactions (0) Cited By (48)

3

Inhibitor of H3K27 demethylase JMJD3/UTX GSK-J4 is a potential therapeutic option for castration resistant prostate cancer.

By: Morozov, Viacheslav M; Li, Ying; Clowers, Matthew M; Ishov, Alexander M
Oncotarget (2017), 8(37), 62131-62142 | Language: English, Database: MEDLINE

Substances (0) Reactions (0) Cited By (40)

4

Inhibition of demethylases by GSK-J1/J4

By: Heinemann, Bo; Nielsen, Jesper Morten; Hudlebusch, Heidi Rye; Lees, Michael J.; Larsen, Dorthe Vang; Boesen, Thomas; Labelle, Marc; Gerlach, Lars-Ole; Birk, Peter; Helin, Kristian
Nature (London, United Kingdom) (2014), 514(7520), E1-E2 | Language: English, Database: CAplus and MEDLINE

Substances (4) Reactions (0) Cited By (135)

5

The pharmacological role of histone demethylase JMJD3 inhibitor GSK-J4 on glioma cells.

By: Sui, Aixia; Xu, Yongbing; Li, Yitong; Hu, Qilu; Wang, Zeyang; Zhang, Hongtao; Yang, Junjie; Guo, Xiaoqiang; Zhao, Wenqing
Oncotarget (2017), 8(40), 68591-68598 | Language: English, Database: MEDLINE

Substances (0) | Reactions (0) | Cited By (31)

6

GSK-J4 induces cell cycle arrest and apoptosis via ER stress and the synergism between GSK-J4 and decitabine in acute myeloid leukemia KG-1a cells

By: Chu, Xuan; Zhong, Liang; Yu, Lihua; Xiong, Ling; Li, Jian; Dan, Wenran; Ye, Jiao; Liu, Chen; Luo, Xu; Liu, Beizhong
Cancer Cell International (2020), 20(1), 209 | Language: English, Database: CAplus and MEDLINE

Substances (6) | Reactions (0) | Cited By (9)

7

The antischistosomal potential of GSK-J4, an H3K27 demethylase inhibitor: insights from molecular modeling, transcriptomics and in vitro assays

By: Lobo-Silva, Jessica; Cabral, Fernanda J.; Amaral, Murilo S.; Miyasato, Patricia A.; de Freitas, Rafaela Paula; Pereira, Adriana S. A.; Khouri, Mariana I.; Barbosa, Mayra M. F.; Ramos, Pablo I. P.; Leite, Luciana C. C.; et al
Parasites & Vectors (2020), 13(1), 140 | Language: English, Database: CAplus and MEDLINE

Substances (5) | Reactions (0) | Cited By (10)

8

Inhibition of demethylases by GSK-J1/J4

By: Kruidenier, Laurens; Chung, Chun-wa; Cheng, Zhongjun; Liddle, John; Che, KaHing; Joberty, Gerard; Bantscheff, Marcus; Bountra, Chas; Bridges, Angela; Diallo, Hawa; et al
Nature (London, United Kingdom) (2014), 514(7520), E2 | Language: English, Database: CAplus and MEDLINE

Substances (5) | Reactions (0) | Cited By (16)

9

Oncogenic KRAS sensitizes lung adenocarcinoma to GSK-J4-Induced metabolic and oxidative stress

By: Hong, Beom-Jin; Park, Woo-Yong; Kim, Hwa-Ryeon; Moon, Jin Woo; Lee, Ho Yeon; Park, Jun Hyung; Kim, Seon-Kyu; Oh, Youngbin; Roe, Jae-Seok; Kim, Mi-Young
Cancer Research (2019), 79(22), 5849-5859 | Language: English, Database: CAplus and MEDLINE

Substances (11) | Reactions (0) | Cited By (16)

10

The demethylase inhibitor GSK-J4 limits inflammatory colitis by promoting de novo synthesis of retinoic acid in dendritic cells

By: Donas, Cristian; Neira, Jocelyn; Osorio-Barrios, Francisco; Carrasco, Macarena; Fernandez, Dominique; Prado, Carolina; Loyola, Alejandra; Pacheco, Rodrigo; Rosemblatt, Mario
Scientific Reports (2021), 11(1), 1342 | Language: English, Database: CAplus and MEDLINE

Substances (7) Reactions (0) Cited By (5)

11

Antiproliferative effect of the histone demethylase inhibitor GSK-J4 in chondrosarcomas

By: Lhuissier, Eva; Aury-Landas, Juliette; Allas, Lyess; Boittin, Martine; Boumediene, Karim; Bauge, Catherine
IUBMB Life (2019), 71(11), 1711-1719 | Language: English, Database: CAplus and MEDLINE

Substances (2) Reactions (0) Cited By (5)

12

Role of JMJD3 Demethylase and Its Inhibitor GSK-J4 in Regulation of MGMT, TRA2A, RPS6KA2, and U2AF1 Genes in Prostate Cancer Cell Lines

By: Sanchez, Anna; El Ouardi, Driss; Houfaf Khoufaf, Fatma Zohra; Idrissou, Mouhamed; Boisnier, Tiphanie; Penault-Llorca, Frederique; Bignon, Yves-Jean; Guy, Laurent; Bernard-Gallon, Dominique
OMICS (2020), 24(8), 505-507 | Language: English, Database: CAplus and MEDLINE

Substances (3) Reactions (0) Cited By (4)

13

GSK-J4-mediated transcriptomic alterations in differentiating embryoid bodies

By: Mandal, Chanchal; Kim, Sun Hwa; Kang, Sung Chul; Chai, Jin Choul; Lee, Young Seek; Jung, Kyoung Hwa; Chai, Young Gyu
Molecules and Cells (2017), 40(10), 737-751 | Language: English, Database: CAplus and MEDLINE

Substance (1) Reactions (0) Cited By (5)

14

Inhibition of demethylase KDM6B sensitizes diffuse large B-cell lymphoma to chemotherapeutic drugs

By: Mathur, Rohit; Sehgal, Lalit; Havranek, Ondrej; Koehrer, Stefan; Khashab, Tamer; Jain, Neeraj; Burger, Jan A.; Neelapu, Sattva S.; Davis, R. Eric; Samaniego, Felipe
Haematologica (2017), 102(2), 373-380 | Language: English, Database: CAplus and MEDLINE

Substances (2) Reactions (0) Cited By (40)

15

Impact of H3K27 demethylase inhibitor GSKJ4 on NSCLC cells alone and in combination with metformin

By: Watarai, Hikaru; Okada, Masashi; Kuramoto, Kenta; Takeda, Hiroyuki; Sakaki, Hirotsugu; Suzuki, Shuhei; Seino, Shizuka; Oizumi, Hiroyuki; Sadahiro, Mitsuaki; Kitanaka, Chifumi

Anticancer Research (2016), 36(11), 6083-6092 | Language: English, Database: CAplus and MEDLINE

Substances (7) | Reactions (0) | Cited By (25)

16

Targeted inhibition of histone H3K27 demethylation is effective in high-risk neuroblastoma

By: Lochmann, Timothy L.; Powell, Krista M.; Ham, Jungoh; Floros, Konstantinos V.; Heisey, Daniel A. R.; Kurupi, Richard I. J.; Calbert, Marissa L.; Ghotra, Maninderjit S.; Greninger, Patricia; Dozmorov, Mikhail; et al

Science Translational Medicine (2018), 10(441), eaao4680/1-eaao4680/12 | Language: English, Database: CAplus and MEDLINE

Substances (5) | Reactions (0) | Cited By (47)

17

GSKJ4, a selective Jumonji H3K27 demethylase inhibitor, effectively targets ovarian cancer stem cells

By: Sakaki, Hirotsugu; Okada, Masashi; Kuramoto, Kenta; Takeda, Hiroyuki; Watarai, Hikaru; Suzuki, Shuhei; Seino, Shizuka; Seino, Manabu; Ohta, Tsuyoshi; Nagase, Satoru; et al

Anticancer Research (2015), 35(12), 6607-6614 | Language: English, Database: CAplus and MEDLINE

Substance (1) | Reactions (0) | Cited By (45)

18

GSKJ4, an H3K27me3 demethylase inhibitor, effectively suppresses the breast cancer stem cells

By: Yan, Ningning; Xu, Liang; Wu, Xiaobo; Zhang, Le; Fei, Xiaochun; Cao, Yali; Zhang, Fengchun

Experimental Cell Research (2017), 359(2), 405-414 | Language: English, Database: CAplus and MEDLINE

Substances (7) | Reactions (0) | Cited By (56)

19

The role and prospect of JMJD3 in stem cells and cancer

By: Yin, Xiaojiao; Yang, Siyu; Zhang, Mingyue; Yue, Ying

Biomedicine & Pharmacotherapy (2019), 118, 109384 | Language: English, Database: CAplus and MEDLINE

Substances (2) | Reactions (0) | Cited By (32)

20

Functionally defined therapeutic targets in diffuse intrinsic pontine glioma

By: Grasso, Catherine S.; Tang, Yujie; Truffaux, Nathalene; Berlow, Noah E.; Liu, Lining; Debily, Marie-Anne; Quist, Michael J.; Davis, Lara E.; Huang, Elaine C.; Woo, Pamelyn J.; et al
Nature Medicine (New York, NY, United States) (2015), 21(6), 555-559 | Language: English, Database: CAplus and MEDLINE

Substances (9) Reactions (0) Cited By (290)

21

Synergy of GSK-J4 with doxorubicin in KRAS-mutant anaplastic thyroid cancer

By: Lin, Bo; Lu, Bing; Hsieh, I-Yun; Liang, Zhen; Sun, Zicheng; Yi, Yang; Lv, Weiming; Zhao, Wei; Li, Jie
Frontiers in Pharmacology (2020), 11, 00632 | Language: English, Database: CAplus and MEDLINE

Substances (3) Reactions (0) Cited By (5)

22

Two old drugs, NVP-AEW541 and GSK-J4, repurposed against the Toxoplasma gondii RH strain

By: Liu, Shuxian; Wu, Mimi; Hua, Qianqian; Lu, Daiqiang; Tian, Yuan; Yu, Helin; Cheng, Linyan; Chen, Yinqi; Cao, Jiaxin; Hu, Xin; et al
Parasites & Vectors (2020), 13(1), 242 | Language: English, Database: CAplus and MEDLINE

Substances (18) Reactions (0) Cited By (6)

23

Taxane-Platin-Resistant Lung Cancers Co-develop Hypersensitivity to JumonjiC Demethylase Inhibitors

By: Dalvi, Maithili P.; Wang, Lei; Zhong, Rui; Kollipara, Rahul K.; Park, Hyunsil; Bayo, Juan; Yenerall, Paul; Zhou, Yunyun; Timmons, Brenda C.; Rodriguez-Canales, Jaime; et al
Cell Reports (2017), 19(8), 1669-1684 | Language: English, Database: CAplus and MEDLINE

Substances (8) Reactions (0) Cited By (56)

24

Pharmacologic inhibition of histone demethylation as a therapy for pediatric brainstem glioma

By: Hashizume, Rintaro; Andor, Noemi; Ihara, Yuichiro; Lerner, Robin; Gan, Haiyun; Chen, Xiaoyue; Fang, Dong; Huang, Xi; Tom, Maxwell W.; Ngo, Vy; et al
Nature Medicine (New York, NY, United States) (2014), 20(12), 1394-1396 | Language: English, Database: CAplus and MEDLINE

Substances (2) Reactions (0) Cited By (294)

25

Contrasting roles of histone 3 lysine 27 demethylases in acute lymphoblastic leukaemia

By: Ntziachristos, Panagiotis; Tsirigos, Aristotelis; Welstead, G. Grant; Trimarchi, Thomas; Bakogianni, Sofia; Xu, Luyao; Loizou, Evangelia; Holmfeldt, Linda; Strikoudis, Alexandros; King, Bryan; et al
Nature (London, United Kingdom) (2014), 514(7523), 513-517 | Language: English, Database: CAplus and MEDLINE

Substances (2) Reactions (0) Cited By (266)

26

H3K27me3 demethylases regulate in vitro chondrogenesis and chondrocyte activity in osteoarthritis

By: Yapp, Clarence; Carr, Andrew J.; Price, Andrew; Oppermann, Udo; Snelling, Sarah J. B.
Arthritis Research & Therapy (2016), 18, 158/1-158/10 | Language: English, Database: CAplus and MEDLINE

Substances (5) Reactions (0) Cited By (23)

27

Epigenetic drug histone demethylase inhibitor GSK-J4 for treating osteosarcoma

By: Zhao, Wei; Zou, Changye; Lu, Bing
China, CN108464984 A 2018-08-31 | Language: Chinese, Database: CAplus

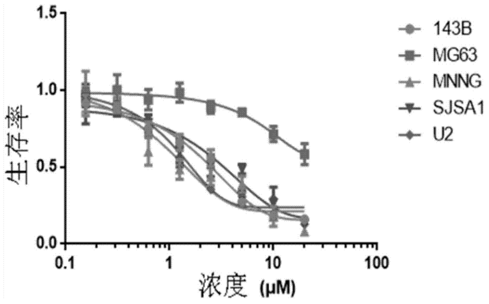

PatentPak available

Substances (5) Reactions (0) Cited By (0)

28

Effects of GSK-J4 on JMJD3 histone demethylase in mouse prostate cancer xenografts

By: Sanchez, Anna; Penault-Llorca, Frederique; Bignon, Yves-Jean; Guy, Laurent; Bernard-Gallon, Dominique
Cancer Genomics & Proteomics (2022), 19(3), 339-349 | Language: English, Database: CAplus and MEDLINE

Substances (7) Reactions (0) Cited By (0)

29

Radiosensitization by histone H3 Demethylase inhibition in diffuse intrinsic pontine glioma

By: Katagi, Hiroaki; Louis, Nundia; Unruh, Dusten; Sasaki, Takahiro; He, Xingyao; Zhang, Ali; Ma, Quanhong; Piunti, Andrea; Shimazu, Yosuke; Lamano, Jonathan B.; et al
Clinical Cancer Research (2019), 25(18), 5572-5583 | Language: English, Database: CAplus and MEDLINE

Substances (2) Reactions (0) Cited By (28)

30

A selective jumonji H3K27 demethylase inhibitor modulates the proinflammatory macrophage response

By: Kruidenier, Laurens; Chung, Chun-wa; Cheng, Zhongjun; Liddle, John; Che, Ka Hing; Joberty, Gerard; Bantscheff, Marcus; Bountra, Chas; Bridges, Angela; Diallo, Hawa; et al
Nature (London, United Kingdom) (2012), 488(7411), 404-408 | Language: English, Database: CAplus and MEDLINE

Substances (28) Reactions (53) Cited By (640)

31

The Histone Demethylase Inhibitor GSK-J4 Is a Therapeutic Target for the Kidney Fibrosis of Diabetic Kidney Disease via DKK1 Modulation

By: Hung, Peir-Haur; Hsu, Yung-Chien; Chen, Tsung-Hsien; Ho, Cheng; Lin, Chun-Liang
International Journal of Molecular Sciences (2022), 23(16), 9407 | Language: English, Database: CAplus and MEDLINE

Substances (5) Reactions (0) Cited By (0)

32

Upregulation of H3K27 demethylase KDM6 during respiratory syncytial virus infection enhances proinflammatory responses and immunopathology

By: Malinczak, Carrie-Anne; Rasky, Andrew J.; Fonseca, Wendy; Schaller, Matthew A.; Allen, Ronald M.; Ptaschinski, Catherine; Morris, Susan; Lukacs, Nicholas W.
Journal of Immunology (2020), 204(1), 159-168 | Language: English, Database: CAplus and MEDLINE

Substances (4) Reactions (0) Cited By (17)

33

The histone H3 lysine-27 demethylase Jmjd3 plays a critical role in specific regulation of Th17 cell differentiation

By: Liu, Zhi; Cao, Wei; Xu, Longxia; Chen, Xi; Zhan, Yu; Yang, Qian; Liu, Sanhong; Chen, Pengfei; Jiang, Yuhang; Sun, Xiaohua; et al
Journal of Molecular Cell Biology (Oxford, United Kingdom) (2015), 7(6), 505-516 | Language: English, Database: CAplus and MEDLINE

Substances (3) Reactions (0) Cited By (68)

34

RNA sequencing reveals resistance of TLR4 ligand-activated microglial cells to inflammation mediated by the selective jumonji H3K27 demethylase inhibitor

By: Das, Amitabh; Arifuzzaman, Sarder; Yoon, Taeho; Kim, Sun Hwa; Chai, Jin Choul; Lee, Young Seek; Jung, Kyoung Hwa; Chai, Young Gyu
Scientific Reports (2017), 7(1), 1-19 | Language: English, Database: CAplus and MEDLINE

Substances (9) Reactions (0) Cited By (23)

35

Histone demethylase UTX is a therapeutic target for diabetic kidney disease

By: Chen, Hong; Huang, Yixue; Zhu, Xiuqin; Liu, Chong; Yuan, Yangmian; Su, Hua; Zhang, Chun; Liu, Chengyu; Xiong, Mingrui; Qu, Yannan; et al
Journal of Physiology (Oxford, United Kingdom) (2019), 597(6), 1643-1660 | Language: English, Database: CAplus and MEDLINE

Substances (2) Reactions (0) Cited By (27)

36

Inhibition of H3K27me3 demethylases attenuates asthma by reversing the shift in airway smooth muscle phenotype

By: Yu, Qijun; Yu, Xiaowei; Zhao, Wenxue; Zhu, Manni; Wang, Zhengxia; Zhang, Jiaxiang; Huang, Mao ; Zeng, Xiaoning
Clinical & Experimental Allergy (2018), 48(11), 1439-1452 | Language: English, Database: CAplus and MEDLINE

Substances (5) Reactions (0) Cited By (11)

37

Lysine demethylase inhibition protects pancreatic β cells from apoptosis and improves β-cell function

By: Backe, Marie Balslev; Andersson, Jan Legaard; Bacos, Karl; Christensen, Dan Ploug; Hansen, Jakob Bondo; Dorosz, Jerzy Jozef; Gajhede, Michael; Dahlby, Tina; Bysani, Madhusudhan; Kristensen, Line Hyltoft; et al
Molecular and Cellular Endocrinology (2018), 460, 47-56 | Language: English, Database: CAplus and MEDLINE

Substances (7) Reactions (0) Cited By (15)

38

Cystathionine-γ-lyase ameliorates the histone demethylase JMJD3-mediated autoimmune response in rheumatoid arthritis

By: Wu, Weijun; Qin, Ming; Jia, Wanwan; Huang, Zheng; Li, Zhongzheng; Yang, Di; Huang, Mengwei; Xiao, Chenxi; Long, Fen; Mao, Jianchun; et al
Cellular & Molecular Immunology (2019), 16(8), 694-705 | Language: English, Database: CAplus and MEDLINE

Substances (4) Reactions (0) Cited By (27)

39

HDAC8 prevents anthrax lethal toxin-induced cell cycle arrest through silencing PTEN in human monocytic THP-1 cells

By: Ha, Soon-Duck; Cho, Woohyun; Kim, Sung Ouk
Toxins (2017), 9(5), 162/1-162/15 | Language: English, Database: CAplus and MEDLINE

Substances (3) Reactions (0) Cited By (12)

40

GSKJ4 protects mice against early sepsis via reducing proinflammatory factors and up-regulating MiR-146a

By: Pan, Yuchen; Wang, Jiali; Xue, Yaxian; Zhao, Jiaojiao; Li, Dan; Zhang, Shaolong; Li, Kuanyu; Hou, Yayi; Fan, Hongye
Frontiers in Immunology (2018), 9, 2272/1-2272/11 | Language: English, Database: CAplus and MEDLINE

Substances (2) Reactions (0) Cited By (25)

41

Histone H3K27me3 demethylases regulate human Th17 cell development and effector functions by impacting on metabolism

By: Cribbs, Adam P. ; Terlecki-Zaniewicz, Stefan; Philpott, Martin; Baardman, Jeroen; Ahern, David; Lindow, Morten; Obad, Susanna; Oerum, Henrik; Sampey, Brante; Mander, Palwinder K.; et al
Proceedings of the National Academy of Sciences of the United States of America (2020), 117(11), 6056-6066 | Language: English, Database: CAplus and MEDLINE

Substances (3) Reactions (0) Cited By (39)

42

Histone demethylase JMJD3 regulates fibroblast-like synoviocyte-mediated proliferation and joint destruction in rheumatoid arthritis

By: Jia, Wanwan; Wu, Weijun; Yang, Di; Xiao, Chenxi; Su, Zhenghua; Huang, Zheng; Li, Zhongzheng; Qin, Ming; Huang, Mengwei; Liu, Siyu; et al
FASEB Journal (2018), 32(7), 4031-4042 | Language: English, Database: CAplus and MEDLINE

Substances (7) Reactions (0) Cited By (33)

43

KDM2B promotes cell viability by enhancing DNA damage response in canine hemangiosarcoma

By: Gulay, Kevin Christian Montecillo ; Aoshima, Keisuke ; Shibata, Yuki ; Yasui, Hironobu ; Yan, Qin ; Kobayashi, Atsushi; Kimura, Takashi
Journal of Genetics and Genomics (2021), 48(7), 618-630 | Language: English, Database: CAplus and MEDLINE

Substances (2) Reactions (0) Cited By (6)

44

JMJD3 is involved in neutrophil membrane proteinase 3 overexpression during the hyperinflammatory response in early sepsis

By: Chen, Yang ; Liu, Zhaojun; Pan, Tingting; Chen, Erzhen; Mao, Enqiang; Chen, Ying; Tan, Ruoming; Wang, Xiaoli; Tian, Rui; Liu, Jialin ; et al
International Immunopharmacology (2018), 59, 40-46 | Language: English, Database: CAplus and MEDLINE

Substances (3) Reactions (0) Cited By (10)

45

PRC2 Epigenetically Silences Th1-Type Chemokines to Suppress Effector T-Cell Trafficking in Colon Cancer

By: Nagarsheth, Nisha; Peng, Dongjun; Kryczek, Ilona; Wu, Ke; Li, Wei; Zhao, Ende; Zhao, Lili; Wei, Shuang; Frankel, Timothy; Vatan, Linda; et al
Cancer Research (2016), 76(2), 275-282 | Language: English, Database: CAplus and MEDLINE

Substances (3) Reactions (0) Cited By (157)

46

Inhibition of H3K27me3-specific histone demethylases JMJD3 and UTX blocks reactivation of herpes simplex virus 1 in trigeminal ganglion neurons

By: Messer, Harald G. P.; Jacobs, Derek; Dhummakupt, Adit; Bloom, David C.
Journal of Virology (2015), 89(6), 3417-3420 | Language: English, Database: CAplus and MEDLINE

Substances (3) Reactions (0) Cited By (34)

47

Jumonji domain containing-3 (JMJD3) inhibition attenuates IL-1β-induced chondrocytes damage in vitro and protects osteoarthritis cartilage in vivo

By: Zhou, Jun; Jin, Xinmeng; Yue, Liu; Zhi, Wang; Yan, Zhang; Yang, Tieyi; Tang, Jiangan
Inflammation Research (2020), 69(7), 657-666 | Language: English, Database: CAplus and MEDLINE

Substances (5) Reactions (0) Cited By (6)

48

An epigenetic therapy for diffuse intrinsic pontine gliomas

By: Ramaswamy, Vijay; Remke, Marc; Taylor, Michael D.
Nature Medicine (New York, NY, United States) (2014), 20(12), 1378-1379 | Language: English, Database: CAplus and MEDLINE

Substances (2) Reactions (0) Cited By (24)

49

Inhibition of histone H3 lysine-27 demethylase activity relieves rheumatoid arthritis symptoms via repression of IL6 transcription in macrophages

By: Zhao, Zhan; Zhang, Yazhuo; Gao, Danling; Zhang, Yidan; Han, Wenwei; Xu, Ximing; Song, Qiaoling; Zhao, Chenyang; Yang, Jinbo
Frontiers in Immunology (2022), 13, 818070 | Language: English, Database: CAplus and MEDLINE

Substances (3) Reactions (0) Cited By (1)

50

Jmjd3 is involved in the susceptibility to depression induced by maternal separation via enhancing the neuroinflammation in the prefrontal cortex and hippocampus of male rats

By: Wang, Rui; Wang, Wei; Xu, Jingjing; Liu, Dexiang; Wu, Huiran; Qin, Xiaqing; Jiang, Hong; Pan, Fang
Experimental Neurology (2020), 328, 113254 | Language: English, Database: CAplus and MEDLINE

Substances (3) Reactions (0) Cited By (22)

51

Functionally defined therapeutic targets in diffuse intrinsic pontine glioma [Erratum to document cited in CA163:294035]

By: Grasso, Catherine S.; Tang, Yujie; Truffaux, Nathalene; Berlow, Noah E.; Liu, Lining; Debily, Marie-Anne; Quist, Michael J.; Davis, Lara E.; Huang, Elaine C.; Woo, Pamelyn J.; et al
Nature Medicine (New York, NY, United States) (2015), 21(7), 827 | Language: English, Database: CAplus and MEDLINE

Substances (9) Reactions (0) Cited By (73)

52

GSK-J4, a specific histone lysine demethylase 6A inhibitor, ameliorates lipotoxicity to cardiomyocytes via preserving H3K27 methylation and reducing ferroptosis

By: Xu, Kai; Liu, Xiang; Wen, Bin; Liu, Yazhou; Zhang, Wei; Hu, Xiaolin; Chen, Ling; Hang, Weijian; Chen, Juan
Frontiers in Cardiovascular Medicine (2022), 9, 907747 | Language: English, Database: CAplus and MEDLINE

Substances (0) Reactions (0) Cited By (0)

53

Targeted inhibition of KDM6 histone demethylases eradicates tumor-initiating cells via enhancer reprogramming in colorectal cancer

By: Zhang, Junbao; Ying, Ying; Li, Meiqi; Wang, Maolin; Huang, Xiaoyan; Jia, Min; Zeng, Junhui; Ma, Canjie; Zhang, Yixiang; Li, Chen; et al
Theranostics (2020), 10(22), 10016-10030 | Language: English, Database: CAplus and MEDLINE

Substances (4) Reactions (0) Cited By (10)

54

Comprehensive profiling of JMJD3 in gastric cancer and its influence on patient survival

By: Xu, Zhenyu; Xia, Yabin; Xiao, Zhangang; Jia, Yuliang; Li, Lina; Jin, Yan; Zhao, Qijie; Wan, Lin; Yi, Tao; Yu, Yangyang; et al
Scientific Reports (2019), 9(1), 1-10 | Language: English, Database: CAplus and MEDLINE

Substances (2) Reactions (0) Cited By (17)

55

The histone demethylase KDM6B fine-tunes the host response to Streptococcus pneumoniae

By: Connor, Michael G.; Camarasa, Tiphaine M. N.; Patey, Emma; Rasid, Orhan; Barrio, Laura; Weight, Caroline M.; Miller, Daniel P.; Heyderman, Robert S.; Lamont, Richard J.; Enninga, Jost; et al
Nature Microbiology (2021), 6(2), 257-269 | Language: English, Database: CAplus and MEDLINE

Substances (4) Reactions (0) Cited By (7)

56

Histone H3 lysine 27 trimethylation acts as an epigenetic barrier in porcine nuclear reprogramming

By: Xie, Bingteng; Zhang, Heng; Wei, Renyue; Li, Qiannan; Weng, Xiaogang; Kong, Qingran; Liu, Zhonghua
Reproduction (Bristol, United Kingdom) (2016), 151(1), 9-16 | Language: English, Database: CAplus and MEDLINE

Substances (4) Reactions (0) Cited By (53)

57

Transcriptomic profiling and H3K27me3 distribution reveal both demethylase- dependent and independent regulation of developmental gene transcription in cell differentiation

By: Chul, Kang Sung; Kye, Kim Se; Choul, Chai Jin; Hwa, Kim Sun; Won, Kyoung-Jae; Seek, Lee Young; Hwa, Jung Kyoung; Gyu, Chai Young
PLoS One (2015), 10(8), e0135276/1-e0135276/25 | Language: English, Database: CAplus and MEDLINE

Substances (4) Reactions (0) Cited By (13)

58

Forskolin sensitizes human acute myeloid leukemia cells to H3K27me2/3 demethylases GSKJ4 inhibitor via protein kinase A

By: Illiano, Michela; Conte, Mariarosaria; Sapio, Luigi; Nebbioso, Angela; Spina, Annamaria; Altucci, Lucia; Naviglio, Silvio
Frontiers in Pharmacology (2018), 9, 792/1-792/9 | Language: English, Database: CAplus and MEDLINE

Substances (11) Reactions (0) Cited By (11)

59

Inhibition of the H3K27 demethylase UTX enhances the epigenetic silencing of HIV proviruses and induces HIV-1 DNA hypermethylation but fails to permanently block HIV reactivation

By: Nguyen, Kien ; Dobrowolski, Curtis ; Shukla, Meenakshi; Cho, Won-Kyung; Luttge, Benjamin ; Karn, Jonathan
PLoS Pathogens (2021), 17(10), e1010014 | Language: English, Database: CAplus and MEDLINE

Substances (6) Reactions (0) Cited By (8)

60

KDM6B counteeacts EZH2-mediated suppression of IDFBP5 to confer resistance to PI3K/AKT inhibitor treatment in breast cancer

By: Wang, Wenyu; Lim, Keng Gat; Feng, Min; Bao, Yi; Lee, Puay Leng; Cai, Yu; Chen, Yufeng; Zhang, Hao; Marzese, Diego; Hoon, Dave S. B.; et al
Molecular Cancer Therapeutics (2018), 17(9), 1973-1983 | Language: English, Database: CAplus and MEDLINE

Substances (10) Reactions (0) Cited By (29)

61

KDM6B is an androgen regulated gene and plays oncogenic roles by demethylating H3K27me3 at cyclin D1 promoter in prostate cancer

By: Cao, Zhi ; Shi, Xiaolei; Tian, Feng; Fang, Yu; Wu, Jason Boyang ; Mrdenovic, Stefan; Nian, Xinwen; Ji, Jin; Xu, Huan; Kong, Chen; et al
Cell Death & Disease (2021), 12(1), 2 | Language: English, Database: CAplus and MEDLINE

Substances (5) Reactions (0) Cited By (16)

62

Nanoparticle enhanced combination therapy for stem-like progenitors defined by single-cell transcriptomics in chemotherapy-resistant osteosarcoma

By: Wang, Li; Huang, Xiaojia; You, Xinru; Yi, Tianqi; Lu, Bing; Liu, Jiali; Lu, Guohao; Ma, Minglin; Zou, Changye; Wu, Jun; et al
Signal Transduction and Targeted Therapy (2020), 5(1), 196 | Language: English, Database: CAplus and MEDLINE

Substances (3) | Reactions (0) | Cited By (17)

63

Kdm6a and Kdm6b: altered expression in malignant pleural mesothelioma

By: Cregan, Sian; Breslin, Maeve; Roche, Gerard; Wennstedt, Sigrid; MacDonagh, Lauren; Albadri, Cinaria; Gao, Yun; O'Byrne, Kenneth J.; Cuffe, Sinead; Finn, Stephen P.; et al
International Journal of Oncology (2017), 50(3), 1044-1052 | Language: English, Database: CAplus and MEDLINE

Substances (6) | Reactions (0) | Cited By (8)

64

Histone H3K27 demethylase negatively controls the memory formation of antigen-stimulated CD8 $^{+}$ T cells

By: Yamada, Takeshi; Nabe, Shogo; Toriyama, Koji; Suzuki, Junpei; Inoue, Kazuki; Imai, Yuuki; Shiraishi, Atsushi; Takenaka, Katsuto; Yasukawa, Masaki; Yamashita, Masakatsu
Journal of Immunology (2019), 202(4), 1088-1098, S1-S4 | Language: English, Database: CAplus and MEDLINE

Substances (4) | Reactions (0) | Cited By (10)

65

New BRAF knockin mice provide a pathogenetic mechanism of developmental defects and a therapeutic approach in cardio-facio-cutaneous syndrome

By: Inoue, Shin-ichi; Moriya, Mitsuji; Watanabe, Yusuke; Miyagawa-Tomita, Sachiko; Niihori, Tetsuya; Oba, Daiju; Ono, Masao; Kure, Shigeo; Ogura, Toshihiko; Matsubara, Yoichi; et al
Human Molecular Genetics (2014), 23(24), 6553-6566 | Language: English, Database: CAplus and MEDLINE

Substances (8) | Reactions (0) | Cited By (40)

66

The TrxG complex mediates cytokine induced de novo enhancer formation in islets

By: Tennant, Bryan R.; Hurley, Peter; Dhillon, Jasmine; Gill, Amol; Whiting, Cheryl; Hoffman, Brad G.
PLoS One (2015), 10(10), e0141470/1-e0141470/22 | Language: English, Database: CAplus and MEDLINE

Substances (12) | Reactions (0) | Cited By (6)

67

EZH2, JMJD3, and UTX epigenetically regulate hepatic plasticity inducing retro-differentiation and proliferation of liver cells

By: Pediconi, Natalia; Salerno, Debora; Lupacchini, Leonardo; Angrisani, Annapaola; Peruzzi, Giovanna ; De Smaele, Enrico ; Levrero, Massimo; Belloni, Laura
Cell Death & Disease (2019), 10(7), 1-13 | Language: English, Database: CAplus and MEDLINE

Substances (9) | Reactions (0) | Cited By (16)

68

The histone demethylase KDM6B in the medial prefrontal cortex epigenetically regulates cocaine reward memory

By: Zhang, Yu-Xiang; Akumuo, Rita C.; Espana, Rodrigo A.; Yan, Chun-Xia; Gao, Wen-Jun ; Li, Yan-Chun
Neuropharmacology (2018), 141, 113-125 | Language: English, Database: CAplus and MEDLINE

Substances (4) | Reactions (0) | Cited By (18)

69

HMGN1 plays a significant role in CRLF2 driven Down Syndrome leukemia and provides a potential therapeutic target in this high-risk cohort

By: Page, Elyse C. ; Heatley, Susan L. ; Eadie, Laura N.; McClure, Barbara J. ; de Bock, Charles E.; Omari, Sofia ; Yeung, David T.; Hughes, Timothy P.; Thomas, Paul Q.; White, Deborah L.
Oncogene (2022), 41(6), 797-808 | Language: English, Database: CAplus and MEDLINE

Substances (7) | Reactions (0) | Cited By (2)

70

Histone lysine demethylase inhibition reprograms prostate cancer metabolism and mechanics

By: Chianese, Ugo ; Papulino, Chiara; Passaro, Eugenia; Evers, Tom MJ.; Babaei, Mehrad; Toraldo, Antonella; De Marchi, Tommaso; Nimeus, Emma; Carafa, Vincenzo; Nicoletti, Maria Maddalena; et al
Molecular Metabolism (2022), 64, 101561 | Language: English, Database: CAplus and MEDLINE

Substances (4) | Reactions (0) | Cited By (0)

71

Lysyl oxidase expression is regulated by the H3K27 demethylase Jmjd3 in tumor-associated M2-like macrophages

By: Takemoto, Ryuhei; Kamiya, Tetsuro; Hara, Hirokazu; Adachi, Tetsuo
Journal of Clinical Biochemistry and Nutrition (2020), 66(2), 110-115 | Language: English, Database: CAplus and MEDLINE

Substances (8) | Reactions (0) | Cited By (4)

72

Application of micromolecular inhibitor for chemosensitivity of colorectal cancer

By: Jiang, Yuhang
China, CN109498629 A 2019-03-22 | Language: Chinese, Database: CAplus

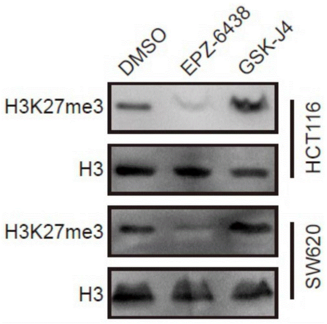

PatentPak available

Substances (3) Reactions (0) Cited By (0)

73

Pharmaceutical composition containing apatinib with synergistic anti-osteosarcoma effect and its application

By: Zhao, Wei; Zou, Changye
China, CN108186643 A 2018-06-22 | Language: Chinese, Database: CAplus

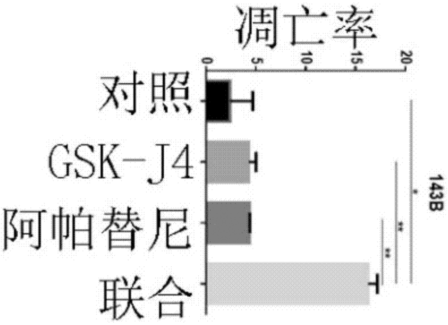

PatentPak available

Substances (3) Reactions (0) Cited By (0)

74

Lysine demethylase 6B regulates prostate cancer cell proliferation by controlling c-MYC expression

By: Yildirim-Buharalioglu, Gokce
Molecular Pharmacology (2022), 101(2), 106-119 | Language: English, Database: CAplus and MEDLINE

Substances (3) Reactions (0) Cited By (3)

75

JMJD3 aids in reprogramming of bone marrow progenitor cells to hepatic phenotype through epigenetic activation of hepatic transcription factors

By: Kochat, Veena; Equbal, Zaffar; Baligar, Prakash; Kumar, Vikash; Srivastava, Madhulika; Mukhopadhyay, Asok
PLoS One (2017), 12(3), e0173977/1-e0173977/23 | Language: English, Database: CAplus and MEDLINE

Substances (14) | Reactions (0) | Cited By (7)

76

Therapeutically targeting head and neck squamous cell carcinoma through synergistic inhibition of LSD1 and JMJD3 by TCP and GSK-J1

By: Zhang, Wei; Cheng, Jie; Diao, Pengfei; Wang, Dongmiao; Jiang, Hongbing; Wang, Yanling
British Journal of Cancer (2020), 122(4), 528-538 | Language: English, Database: CAplus and MEDLINE

Substances (4) | Reactions (0) | Cited By (18)

77

Critical role of histone demethylase Jumonji domain-containing protein 3 in the regulation of neointima formation following vascular injury

By: Luo, XiaoLing; Yang, Di; Wu, WeiJun; Long, Fen; Xiao, ChenXi; Qin, Ming; Law, Betty YuenKwan; Suguro, Rinkiko; Xu, Xin; Qu, LeFeng; et al
Cardiovascular Research (2018), 114(14), 1894-1906 | Language: English, Database: CAplus and MEDLINE

Substances (4) | Reactions (0) | Cited By (24)

78

Histone H3K27 methyltransferase EZH2 and demethylase JMJD3 regulate hepatic stellate cells activation and liver fibrosis

By: Jiang, Yan; Xiang, Chan; Zhong, Fan; Zhang, Yang; Wang, Liyan; Zhao, Yuanyuan; Wang, Jiucun; Ding, Chen; Jin, Li; He, Fuchu; et al
Theranostics (2021), 11(1), 361-378 | Language: English, Database: CAplus and MEDLINE

Substances (12) | Reactions (0) | Cited By (24)

79

Application of JMJD3 inhibitor in preparing medicine for preventing and treating pancreatitis

By: Wei, Xiawei; Zhang, Xiangxian; Wei, Yuquan
China, CN110339198 A 2019-10-18 | Language: Chinese, Database: CAplus

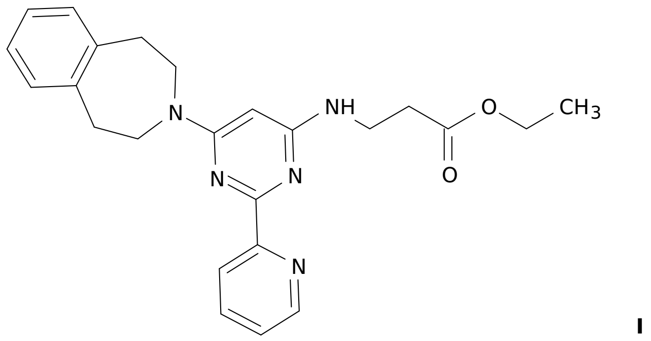

PatentPak available

Substances (4) Reactions (0) Cited By (0)

80

Preparation method of drug carrier and application thereof in treating osteosarcoma

By: Zhao, Wei; Wu, Jun; Wang, Li
China, CN110922587 A 2020-03-27 | Language: Chinese, Database: CAplus

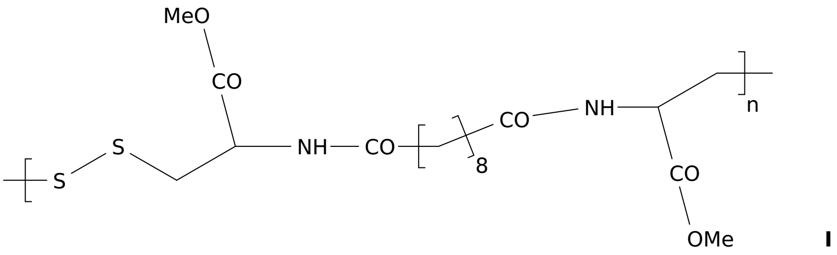

PatentPak available

Substances (9) Reaction (1) Cited By (0)

81

Targeting P16INK4A in uterine serous carcinoma through inhibition of histone demethylation

By: Xiao, Zhen; He, Yingying; Liu, Chongya; Xiang, Lin; Yi, Jingyan; Wang, Min; Shen, Tingting; Shen, Lanlin; Xue, Yijue; Shi, Hong; et al
Oncology Reports (2019), 41(5), 2667-2678 | Language: English, Database: CAplus and MEDLINE

Substances (4) Reactions (0) Cited By (3)

82

UTX-1 regulates Tat-induced HIV-1 transactivation via changing the methylated status of histone H3

By: Zhang, Hong-Sheng; Du, Guang-Yuan; Liu, Yang; Zhang, Zhong-Guo; Zhou, Zhen; Li, Hu; Dai, Ke-Qing; Yu, Xiao-Ying; Gou, Xiao-Meng
International Journal of Biochemistry & Cell Biology (2016), 80, 51-56 | Language: English, Database: CAplus and MEDLINE

Substances (4) Reactions (0) Cited By (8)

83

The repositioning of epigenetic probes/inhibitors identifies new anti-schistosomal lead compounds and chemotherapeutic targets

By: Whatley, Kezia C. L.; Padalino, Gilda; Whiteland, Helen; Geyer, Kathrin K.; Hulme, Benjamin J.; Chalmers, Iain W.; Forde-Thomas, Josephine; Ferla, Salvatore; Brancale, Andrea; Hoffmann, Karl F.
PLoS Neglected Tropical Diseases (2019), 13(11), e0007693/1-e0007693/24 | Language: English, Database: CAplus and MEDLINE

Substance (1) Reactions (0) Cited By (16)

84

KDM6B promotes activation of the oncogenic CDK4/6-pRB-E2F pathway by maintaining enhancer activity in MYCN-amplified neuroblastoma

By: D'Oto, Alexandra; Fang, Jie; Jin, Hongjian; Xu, Beisi ; Singh, Shivendra; Mullasseril, Anoushka; Jones, Victoria; Abu-Zaid, Ahmed; von Buttlar, Xinyu; Cooke, Bailey; et al
Nature Communications (2021), 12(1), 7204 | Language: English, Database: CAplus and MEDLINE

Substances (9) Reactions (0) Cited By (6)

85

Inhibition of the Histone H3K27 Demethylase UTX Enhances Tumor Cell Radiosensitivity

By: Rath, Barbara H.; Waung, Isabella; Camphausen, Kevin; Tofilon, Philip J.
Molecular Cancer Therapeutics (2018), 17(5), 1070-1078 | Language: English, Database: CAplus and MEDLINE

Substances (2) Reactions (0) Cited By (22)

86

The inhibitors of KDM4 and KDM6 histone lysine demethylases enhance the anti-growth effects of erlotinib and HS-173 in head and neck cancer cells

By: Kleszcz, Robert; Skalski, Marcin; Krajka-Kuzniak, Violetta; Paluszczak, Jaroslaw
European Journal of Pharmaceutical Sciences (2021), 166, 105961 | Language: English, Database: CAplus and MEDLINE

Substances (8) Reactions (0) Cited By (4)

87

JMJD3 inhibition protects against isoproterenol-induced cardiac hypertrophy by suppressing β-MHC expression

By: Guo, Zhen; Lu, Jing ; Li, Jingyan; Wang, Panxia; Li, Zhenzhen; Zhong, Yao; Guo, Kaiteng; Wang, Junjian; Ye, Jiantao; Liu, Peiqing
Molecular and Cellular Endocrinology (2018), 477, 1-14 | Language: English, Database: CAplus and MEDLINE

Substances (6) Reactions (0) Cited By (17)

88

Inhibition of H3K27me3 demethylases promotes plasmablast formation

By: Kania, Anna K. ; Guo, Muyao ; Scharer, Christopher D. ; Boss, Jeremy M.
ImmunoHorizons (2021), 5(12), 918-930 | Language: English, Database: CAplus and MEDLINE

Substance (1) Reactions (0) Cited By (0)

89

Compositions and methods for treating drug-tolerant glioblastoma

By: Liau, Brian B.; Patel, Anoop P.; Sievers, Cem; Bernstein, Bradley E.
World Intellectual Property Organization, WO2017099829 A1 2017-06-15 | Language: English, Database: CAplus

PatentPak available

Substances (7) Reactions (0) Cited By (2)

90

A KDM6 inhibitor potently induces ATF4 and its target gene expression through HRI activation and by UTX inhibition

By: Kitajima, Shojiro; Sun, Wendi; Lee, Kian Leong; Ho, Jolene Caifeng; Oyadomari, Seiichi; Okamoto, Takashi; Masai, Hisao; Poellinger, Lorenz; Kato, Hiroyuki
Scientific Reports (2021), 11(1), 4538 | Language: English, Database: CAplus and MEDLINE

Substances (5) Reactions (0) Cited By (0)

91

Identification of disulfiram as a potential antifungal drug by screening small molecular libraries

By: Hao, Weifeng; Qiao, Dan; Han, Ying; Du, Ning; Li, Xuefen; Fan, Yufeng; Ge, Xuejun; Zhang, Heyu
Journal of Infection and Chemotherapy (2021), 27(5), 696-701 | Language: English, Database: CAplus and MEDLINE
Analytical Methods available

Substances (8) Reactions (0) Cited By (10)

92

Pharmaceutical interference of the EWS-FLI1-driven transcriptome by cotargeting H3K27ac and RNA polymerase activity in ewing sarcoma

By: Heisey, Daniel A. R.; Jacob, Sheeba; Lochmann, Timothy L.; Kurupi, Richard; Ghotra, Maninderjit S.; Calbert, Marissa L.; Shende, Mayuri; Maves, Yuki Kato; Koblinski, Jennifer E.; Dozmorov, Mikhail G.; et al
Molecular Cancer Therapeutics (2021), 20(10), 1868-1879 | Language: English, Database: CAplus and MEDLINE

Substances (8) | Reactions (0) | Cited By (4)

93

KDM6B epigenetically regulated-interleukin-6 expression in the dorsal root ganglia and spinal dorsal horn contributes to the development and maintenance of neuropathic pain following peripheral nerve injury in male rats

By: Li, Liren; Bai, Liying; Yang, Kangli; Zhang, Jian; Gao, Yan; Jiang, Mingjun; Yang, Yin; Zhang, Xuan; Wang, Li; Wang, Xueli; et al
Brain, Behavior, and Immunity (2021), 98, 265-282 | Language: English, Database: CAplus and MEDLINE

Substances (4) | Reactions (0) | Cited By (3)

94

AURKA suppresses leukemic THP-1 cell differentiation through inhibition of the KDM6B pathway

By: Park, Jin Woo; Cho, Hana; Oh, Hyein; Kim, Ji-Young; Seo, Sang-Beom
Molecules and Cells (2018), 41(5), 444-453 | Language: English, Database: CAplus and MEDLINE

Substances (4) | Reactions (0) | Cited By (14)

95

SMARCA4 deficient tumours are vulnerable to KDM6A/UTX and KDM6B/JMJD3 blockade

By: Romero, Octavio A.; Vilarrubi, Andrea; Alburquerque-Bejar, Juan J.; Gomez, Antonio; Andrades, Alvaro; Trastulli, Deborah; Pros, Eva; Setien, Fernando; Verdura, Sara; Farre, Lourdes; et al
Nature Communications (2021), 12(1), 4319 | Language: English, Database: CAplus and MEDLINE

Substances (7) | Reactions (0) | Cited By (9)

96

KDM6B-dependent chromatin remodeling underpins effective virus-specific CD8+ T cell differentiation

By: Li, Jasmine; Hardy, Kristine; Olshansky, Moshe; Barugahare, Adele; Gearing, Linden J.; Prier, Julia E.; Sng, Xavier Y. X.; Nguyen, Michelle Ly Thai; Piovesan, Dana; Russ, Brendan E.; et al
Cell Reports (2021), 34(11), 108839 | Language: English, Database: CAplus and MEDLINE

Substances (9) | Reactions (0) | Cited By (8)

97

Histone modification of pain-related gene expression in spinal cord neurons under a persistent postsurgical pain-like state by electrocautery

By: Katsuda, Yosuke; Tanaka, Kenichi; Mori, Tomohisa; Narita, Michiko; Takeshima, Hideyuki; Kondo, Takashige; Yamabe, Yoshiyuki; Matsufuji, Misa; Sato, Daisuke; Hamada, Yusuke; et al
Molecular Brain (2021), 14(1), 146 | Language: English, Database: CAplus and MEDLINE

Substances (12) Reactions (0) Cited By (0)

98

Histone demethylase KDM6B regulates human podocyte differentiation in vitro

By: Guo, Yanyan; Xiong, Zuying; Guo, Xiaoqiang
Biochemical Journal (2019), 476(12), 1741-1751 | Language: English, Database: CAplus and MEDLINE

Substances (3) Reactions (0) Cited By (3)

99

Combined action of FOXO1 and superoxide dismutase 3 promotes MDA-MB-231 cell migration

By: Kamiya, Tetsuro ; Yamaguchi, Yuji; Oka, Manami; Hara, Hirokazu
Free Radical Research (2022), 56(1), 106-114 | Language: English, Database: CAplus and MEDLINE

Substances (3) Reactions (0) Cited By (1)

100

X-linked histone H3K27 demethylase Kdm6a regulates sexually dimorphic differentiation of hypothalamic neurons

By: Cabrera Zapata, Lucas E. ; Cisternas, Carla D. ; Sosa, Camila ; Garcia-Segura, Luis Miguel ; Arevalo, Maria Angeles ; Cambiasso, Maria Julia
Cellular and Molecular Life Sciences (2021), 78(21-22), 7043-7060 | Language: English, Database: CAplus and MEDLINE

Substances (4) Reactions (0) Cited By (4)

101

LncRNA H19 regulates macrophage polarization and promotes Freund's complete adjuvant-induced arthritis by upregulating KDM6A

By: Zhu, Xiaodong; Zhu, Ye; Ding, Chen; Zhang, Weiting; Guan, Huilin; Li, Chunmei; Lin, Xiao; Zhang, Yang; Huang, Chunyan; Zhang, Luyao; et al
International Immunopharmacology (2021), 93, 107402 | Language: English, Database: CAplus and MEDLINE

Substances (6) Reactions (0) Cited By (10)

102

H3K27me3 loss plays a vital role in CEMIP mediated carcinogenesis and progression of breast cancer with poor prognosis

By: Hsieh, I-yun; He, Jincan; Wang, Li ; Lin, Bo; Liang, Zhen; Lu, Bing; Chen, Weixin; Lu, Guohao; Li, Fuxi; Lv, Weiming; et al
Biomedicine & Pharmacotherapy (2020), 123, 109728 | Language: English, Database: CAplus and MEDLINE

Substance (1) | Reactions (0) | Cited By (40)

103

Jmjd3 Mediates Neuropathic Pain by Inducing Macrophage Infiltration and Activation in Lumbar Spinal Stenosis Animal Model

By: Lee, Jeeyoun; Choi, Haeyoung; Park, Chansol; Jeon, Sangryong; Yune, Taeyoung
International Journal of Molecular Sciences (2021), 22(24), 13426 | Language: English, Database: CAplus and MEDLINE

Substances (8) | Reactions (0) | Cited By (2)

104

Low HOX gene expression in PML-RARα-positive leukemia results from suppressed histone demethylation.

By: Rejlova, Katerina; Musilova, Alena; Kramarzova, Karolina Skvarova; Zaliova, Marketa; Fiser, Karel; Alberich-Jorda, Meritxell; Trka, Jan; Starkova, Julia
Epigenetics (2018), 13(1), 73-84 | Language: English, Database: MEDLINE

Substance (1) | Reactions (0) | Cited By (10)

105

Activation of NFKB-JMJD3 signaling promotes bladder fibrosis via boosting bladder smooth muscle cell proliferation and collagen accumulation

By: Lai, Junyu; Ge, Manqing; Shen, Sikui; Yang, Lu; Jin, Tao; Cao, Dehong; Xu, Hang; Zheng, Xiaonan; Qiu, Shi; Wang, Kunjie; et al
Biochimica et Biophysica Acta, Molecular Basis of Disease (2019), 1865(9), 2403-2410 | Language: English, Database: CAplus and MEDLINE

Substances (2) | Reactions (0) | Cited By (8)

106

Elevating H3K27me3 level sensitizes colorectal cancer to oxaliplatin

By: Wang, Qi; Chen, Xi; Jiang, Yuhang; Liu, Sanhong; Liu, Hanshao; Sun, Xiaohua; Zhang, Haohao; Liu, Zhi; Tao, Yu; Li, Cuifeng; et al
Journal of Molecular Cell Biology (Oxford, United Kingdom) (2020), 12(2), 125-137 | Language: English, Database: CAplus and MEDLINE

Substances (5) | Reactions (0) | Cited By (30)

107

Systematic identification of biomarker-driven drug combinations to overcome resistance

By: Rees, Matthew G. ; Brenan, Lisa; do Carmo, Mariana; Duggan, Patrick; Bajrami, Besnik; Arciprete, Michael; Boghossian, Andrew ; Vaimberg, Emma; Ferrara, Steven J.; Lewis, Timothy A.; et al
Nature Chemical Biology (2022), 18(6), 615-624 | Language: English, Database: CAplus and MEDLINE

Substances (18) | Reactions (0) | Cited By (1)

108

A transcriptomic dataset used to derive biomarkers of chemically induced histone deacetylase inhibition (HDACi) in human TK6 cells

By: Cho, Eunnara; Williams, Andrew; Yauk, Carole L.
Data in Brief (2021), 36, 107097 | Language: English, Database: CAplus and MEDLINE

Substances (11) | Reactions (0) | Cited By (0)

109

Histone Methylation: Achilles Heel and Powerful Mediator of Periodontal Homeostasis

By: Francis, M.; Gopinathan, G.; Foyle, D.; Fallah, P.; Gonzalez, M.; Luan, X.; Diekwisch, T. G. H.
Journal of Dental Research (2020), 99(12), 1332-1340 | Language: English, Database: CAplus and MEDLINE

Substances (11) | Reactions (0) | Cited By (9)

110

Pharmacological Inhibition of Core Regulatory Circuitry Liquid-liquid Phase Separation Suppresses Metastasis and Chemoresistance in Osteosarcoma

By: Lu, Bing; Zou, Changye; Yang, Meiling; He, Yangyang; He, Jincan; Zhang, Chuanxia; Chen, Siyun; Yu, Jiaming; Liu, Kilia Yun; Cao, Qi; et al
Advanced Science (Weinheim, Germany) (2021), 8(20), 2101895 | Language: English, Database: CAplus and MEDLINE

Substance (1) | Reactions (0) | Cited By (9)

111

Increased H3K27 trimethylation contributes to cone survival in a mouse model of cone dystrophy

By: Miller, Annie L.; Fuller-Carter, Paula I.; Masarini, Klaudija; Samardzija, Marijana; Carter, Kim W.; Rashwan, Rabab; Lim, Xin Ru; Brunet, Alicia A.; Chopra, Abha; Ram, Ramesh; et al
Cellular and Molecular Life Sciences (2022), 79(8), 409 | Language: English, Database: CAplus and MEDLINE

Substances (19) | Reactions (0) | Cited By (0)

112

Method and device for drug indication of acute myeloid leukemia

By: Guo, Anyuan; Lin, Shengyan; Chen, Zhichao; Miao, Yaru
China, CN107974506 A 2018-05-01 | Language: Chinese, Database: CAplus

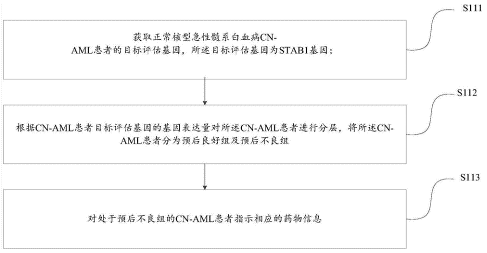

PatentPak available

Substances (10) | Reactions (0) | Cited By (0)

113

Inhibitors of Jumonji C domain-containing histone lysine demethylases overcome cisplatin and paclitaxel resistance in non-small cell lung cancer through APC/Cdh1-dependent degradation of CtIP and PAF15

By: Duan, Lei; Perez, Ricardo E.; Calhoun, Sarah; Maki, Carl G.
Cancer Biology & Therapy (2022), 23(1), 65-75 | Language: English, Database: CAplus and MEDLINE

Substances (10) | Reactions (0) | Cited By (1)

114

Mucosal-associated invariant T cell effector function is an intrinsic cell property that can be augmented by the metabolic cofactor a-ketoglutarate

By: Howson, Lauren J. ; Li, Jasmine; von Borstel, Anouk ; Barugahare, Adele ; Mak, Jeffrey Y. W. ; Fairlie, David P. ; McCluskey, James ; Turner, Stephen J. ; Davey, Martin S. ; Rossjohn, Jamie
Journal of Immunology (2021), 206(7), 1425-1440 | Language: English, Database: CAplus and MEDLINE

Substances (3) | Reactions (0) | Cited By (3)

115

Unlocking the Recovery Potential: JMJD3 Inhibition-Mediated SAPK/JNK Signaling Inactivation Supports Endogenous Oligodendrocyte-Lineage Commitment Post Mammalian Spinal Cord Injury

By: Zhang, Bo-Yin; Zhu, Qingsan; Ma, Yihang; Fan, Yang; Zhu, Yuhang; Chang, Pengyu
Neurochemical Research (2021), 46(4), 792-803 | Language: English, Database: CAplus and MEDLINE

Substances (6) | Reactions (0) | Cited By (0)

116

Dual regulation of histone methylation by mTOR complexes controls glioblastoma tumor cell growth via EZH2 and SAM

By: Harachi, Mio; Masui, Kenta; Honda, Hiroaki; Muragaki, Yoshihiro; Kawamata, Takakazu; Cavenee, Webster K.; Mischel, Paul S.; Shibata, Noriyuki
Molecular Cancer Research (2020), 18(8), 1142-1152 | Language: English, Database: CAplus and MEDLINE

Substances (4) | Reactions (0) | Cited By (12)

117

Disruption of the Plasmodium falciparum Life Cycle through Transcriptional Reprogramming by Inhibitors of Jumonji Demethylases

By: Matthews, Krista A.; Senagbe, Kossi M.; Notzel, Christopher; Gonzales, Christopher A.; Tong, Xinran; Rijo-Ferreira, Filipa; Bhanu, Natarajan V.; Miguel-Blanco, Celia; Lafuente-Monasterio, Maria Jose; Garcia, Benjamin A.; et al
ACS Infectious Diseases (2020), 6(5), 1058-1075 | Language: English, Database: CAplus and MEDLINE

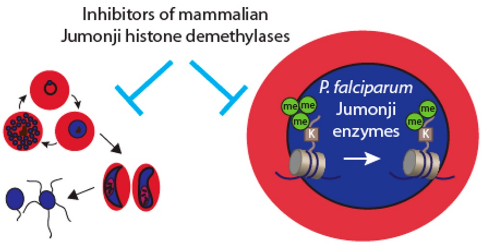

Substances (10) | Reactions (0) | Cited By (12)

118

H19-dependent transcriptional regulation of β3 and β4 integrins upon estrogen and hypoxia favors metastatic potential in prostate cancer

By: Bacci, Lorenza; Aiello, Aurora; Ripoli, Cristian; Loria, Rossella; Pugliese, Dario; Pierconti, Francesco; Rotili, Dante; Strigari, Lidia; Pinto, Francesco; Bassi, Pier Francesco; et al
International Journal of Molecular Sciences (2019), 20(16), 4012 | Language: English, Database: CAplus and MEDLINE

Substances (3) | Reactions (0) | Cited By (14)

119

Neuroblastoma and Histone Demethylation.

By: DuBois, Steven G; Park, Julie R
The New England journal of medicine (2018), 379(15), 1476-1477 | Language: English, Database: MEDLINE

Substances (0) | Reactions (0) | Cited By (2)

120

Preparation of model mouse for CFC (cardiofaciocutaneous) syndrome and establishment of method for treating CFC syndrome

By: Inoue, Shinichi; Aoki, Yoko; Matsubara, Yoichi
Japan, JP2015181456 A 2015-10-22 | Language: Japanese, Database: CAplus

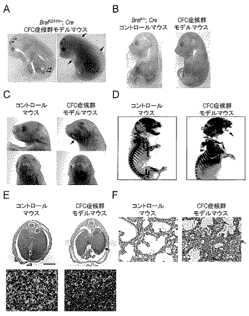

PatentPak available

Substances (9) | Reactions (0) | Cited By (0)

121

Inhibition of histone H3K27 demethylases selectively modulates inflammatory phenotypes of natural killer cells

By: Cribbs, Adam; Hookway, Edward S.; Wells, Graham; Lindow, Morten; Obad, Susanna; Oerum, Henrik; Prinjha, Rab K.; Athanasou, Nick; Sowman, Aneka; Philpott, Martin; et al
Journal of Biological Chemistry (2018), 293(7), 2422-2437 | Language: English, Database: CAplus and MEDLINE

Substances (3) | Reactions (0) | Cited By (49)

122

Application of JMJD3 inhibitor GSK-J4·HCl in preparing anti-colitis medicament

By: Zhu, Yizhun; Liu, Xinhua; Huang, Mengwei
China, CN110314164 A 2019-10-11 | Language: Chinese, Database: CAplus

PatentPak available

Substances (2) | Reactions (0) | Cited By (0)

123

GSK J4 is a Novel Neuroprotective Compound in Parkinson's Disease Model

By: Mu, Mingdao
2017, 79(5E), 181 pages | Language: English, Database: CAplus

Substances (0) | Reactions (0) | Cited By (0)

124

Forskolin potentiates the effects of GSKJ4 in human acute myeloid leukemia cells through protein kinase A pathway

By: Illiano, Michela; Conte, Mariarosaria; Sapio, Luigi; Nebbioso, Angela; Spina, Annamaria; Altucci, Lucia; Naviglio, Silvio
Pharmakeutike (2019), 31(2), 44-55 | Language: English, Database: CAplus

Substances (13) Reactions (0) Cited By (0)

125

Small molecule for specifically regulating iron metabolism in nerve cell and application thereof

By: Ke, Ya; Mo, Mingdao; Qian, Zhongming
China, CN110063959 A 2019-07-30 | Language: Chinese, Database: CAplus

PatentPak available

Substances (5) Reactions (0) Cited By (0)

126

The H3K27me3-demethylase KDM6A is suppressed in breast cancer stem-like cells, and enables the resolution of bivalency during the mesenchymal-epithelial transition.

By: Taube, Joseph H; Sphyris, Nathalie; Johnson, Kelsey S; Reisenauer, Keighley N; Nesbit, Taylor A; Joseph, Robiya; Vijay, Geraldine V; Sarkar, Tapasree R; Bhangre, Neeraja A; Song, Joon Jin; et al
Oncotarget (2017), 8(39), 65548-65565 | Language: English, Database: MEDLINE

Substances (0) Reactions (0) Cited By (39)

127

The BRD9/7 Inhibitor TP-472 Blocks Melanoma Tumor Growth by Suppressing ECM-Mediated Oncogenic Signaling and Inducing Apoptosis

By: Mason, Lawrence David; Chava, Suresh; Reddi, Kiran Kumar; Gupta, Romi
Cancers (2021), 13(21), 5516 | Language: English, Database: CAplus and MEDLINE

Substances (13) Reactions (0) Cited By (5)

128

Targeting histone acetylation dynamics and oncogenic transcription by catalytic P300/CBP inhibition

By: Hogg, Simon J.; Motorna, Olga; Cluse, Leonie A.; Johanson, Timothy M.; Coughlan, Hannah D.; Raviram, Ramya; Myers, Robert M.; Costacurta, Matteo; Todorovski, Izabela; Pijpers, Lizzy; et al
Molecular Cell (2021), 81(10), 2183-2200.e13 | Language: English, Database: CAplus and MEDLINE

Substances (13) Reactions (0) Cited By (22)

129

Upregulation of KDM6B contributes to lipopolysaccharide-induced anxiety-like behavior via modulation of VGLL4 in mice

By: Shentu, Yangping; Tian, Qiuyun; Yang, Jinge; Liu, Xiaoyuan; Han, Yujiao; Yang, Dichen; Zhang, Nan; Fan, Xiaofang; Wang, Ping; Ma, Jianshe; et al

Behavioural Brain Research (2021), 408, 113305 | Language: English, Database: CAplus and MEDLINE

Substances (3) Reactions (0) Cited By (3)

130

The Memory of Environmental Chemical Exposure in C. elegans Is Dependent on the Jumonji Demethylases jmjd-2 and jmjd-3/utx-1

By: Camacho, Jessica; Truong, Lisa; Kurt, Zeyneb; Chen, Yen-Wei; Morselli, Marco; Gutierrez, Gerardo; Pellegrini, Matteo; Yang, Xia; Allard, Patrick

Cell Reports (2018), 23(8), 2392-2404 | Language: English, Database: CAplus and MEDLINE

Substances (5) Reactions (0) Cited By (31)

131

Application of JMJD3 inhibitor GSK-J4·HCl in preparing medicine for treating rheumatoid arthritis

By: Zhu, Yizhun; Liu, Xinhua; Jia, Wanwan

China, CN108721300 A 2018-11-02 | Language: Chinese, Database: CAplus

PatentPak available

Substances (3) Reactions (0) Cited By (0)

132

Massively parallel combinatorial genetics for CRISPR

By: Lu, Timothy Kuan-Ta; Wong, Alan Siu Lun; Choi, Ching Gee

World Intellectual Property Organization, WO2016070037 A2 2016-05-06 | Language: English, Database: CAplus

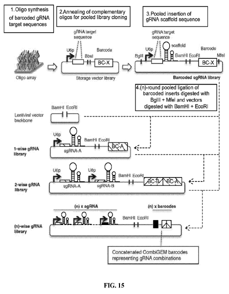

PatentPak available

Substances (4) Reactions (0) Cited By (5)

133

Methods of combinatorial drug screening and use of therapeutic targets thereof in cancer treatment

By: Bernstein, Bradley E.; Doench, John G.; Najm, Fadi J.
World Intellectual Property Organization, WO2020033585 A1 2020-02-13 | Language: English, Database: CAplus

PatentPak available

Substances (109) | Reactions (0) | Cited By (3)

134

Synergistic drug combinations predicted from genomic features and single-agent response profiles

By: Rees, Matthew; Johannessen, Cory
World Intellectual Property Organization, WO2019200224 A1 2019-10-17 | Language: English, Database: CAplus

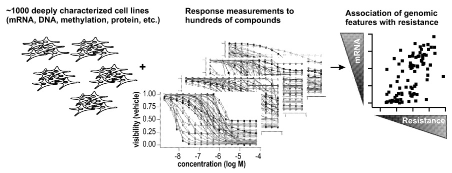

FIG. 1A

PatentPak available

Substances (134) | Reactions (0) | Cited By (3)

135

Treatment of cancers having alterations within the SWI/SNF chromatin remodeling complex

By: Fisher, David E.; Weng, Qing Yu; Kato, Shinichiro
World Intellectual Property Organization, WO2018049000 A1 2018-03-15 | Language: English, Database: CAplus

FIG. 2

Dose response of shControl vs shARID2 lines to G9a inhibitor

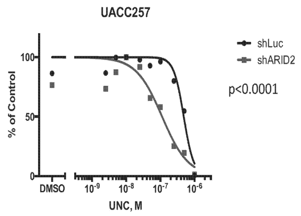

PatentPak available

Substances (46) | Reactions (0) | Cited By (1)

136

Use of Jumonji C demethylase inhibitors for the treatment and prevention of chemotherapy resistance and radioresistance in cancer

By: Dalvi, Maithili P.; Martinez, Elisabeth D.; Minna, John D.; Bayo-Fina, Juan; Das, Amit

World Intellectual Property Organization, WO2017190009 A1 2017-11-02 | Language: English, Database: CAplus

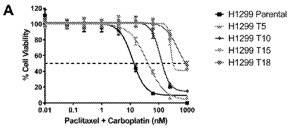

FIGS. 1A

PatentPak available

Substances (35) | Reactions (0) | Cited By (5)

137

Jumonji Inhibitors Overcome Radioresistance in Cancer through Changes in H3K4 Methylation at Double-Strand Breaks

By: Bayo, Juan; Tran, Tram Anh; Wang, Lei; Pena-Llopis, Samuel; Das, Amit K.; Martinez, Elisabeth D.

Cell Reports (2018), 25(4), 1040-1050.e5 | Language: English, Database: CAplus and MEDLINE

Substances (2) | Reactions (0) | Cited By (39)

138

Palmitate-TLR4 signaling regulates the histone demethylase, JMJD3, in macrophages and impairs diabetic wound healing

By: Davis, Frank M. ; denDekker, Aaron; Joshi, Amrita D.; Wolf, Sonya J.; Audu, Christopher; Melvin, William J.; Mangum, Kevin; Riordan, Mary O.; Kunkel, Steven L.; Gallagher, Katherine A.

European Journal of Immunology (2020), 50(12), 1929-1940 | Language: English, Database: CAplus and MEDLINE

Substances (4) | Reactions (0) | Cited By (18)

139

SAPCD2 promotes neuroblastoma progression by altering the subcellular distribution of E2F7

By: Zhang, Zi-Mu ; Cao, Hai-Bo; Li, Zhi-Heng; Zhuo, Ran; Tao, Yan-Fang; Li, Xiao-Lu; Li, Gen; Liao, Xin-Mei; Fang, Fang; Xie, Yi; et al

Cell Death & Disease (2022), 13(2), 174 | Language: English, Database: CAplus and MEDLINE

Substances (9) | Reactions (0) | Cited By (1)

140

Activated Braf induces esophageal dilation and gastric epithelial hyperplasia in mice

By: Inoue, Shin-Ichi; Takahara, Shingo; Yoshikawa, Takeo; Niihori, Tetsuya; Yanai, Kazuhiko; Matsubara, Yoichi; Aoki, Yoko
Human Molecular Genetics (2017), 26(23), 4715-4727 | Language: English, Database: CAplus and MEDLINE

Substances (12) Reactions (0) Cited By (11)

141

Drug-induced epigenetic remodeling to prevent fibrosis

By: Walker, Janice L.; Mazo, Alexander; Petruk, Svetlana; Menko, Sue A.
World Intellectual Property Organization, WO2018058034 A1 2018-03-29 | Language: English, Database: CAplus

PatentPak available

Substances (6) Reactions (0) Cited By (0)

142

Application of GSK J4 HCl in preparing drug for treating vascular intimal hyperplasia related vascular diseases

By: Zhu, Yizhun; Liu, Xinhua; Pan, Lilong; Luo, Xiaoling
China, CN107233346 A 2017-10-10 | Language: Chinese, Database: CAplus

PatentPak available

Substances (2) Reactions (0) Cited By (0)

143

H3K27 demethylase inhibitors in pediatric and juvenile osteoporosis

By: Wan, Mei; Cao, Xu
World Intellectual Property Organization, WO2019046378 A1 2019-03-07 | Language: English, Database: CAplus

PatentPak available

Substances (5) Reactions (0) Cited By (0)

144

A new metabolic gene signature in prostate cancer regulated by JMJD3 and EZH2.

By: Daures, Marine; Idrissou, Mouhamed; Judes, Gaëlle; Rifaï, Khaldoun; Penault-Llorca, Frédérique; Bignon, Yves-Jean; Guy, Laurent; Bernard-Gallon, Dominique
Oncotarget (2018), 9(34), 23413-23425 | Language: English, Database: MEDLINE

Substances (0) Reactions (0) Cited By (24)

145

Methods for treating T-cell acute lymphoblastic leukemia (T-ALL)

By: Aifantis, Iannis; Ntziachristos, Panagiotis
United States, US20170042904 A1 2017-02-16 | Language: English, Database: CAplus

PatentPak available

Substances (25) | Reactions (0) | Cited By (0)

146

Inhibiting KDM6A Demethylase Represses Long Non-Coding RNA Hotairm1 Transcription in MDSC During Sepsis

By: Bah, Isatou; Youssef, Dima; Yao, Zhi Q.; Mccall, Charles E.; El Gazzar, Mohamed
Frontiers in Immunology (2022), 13, 823660 | Language: English, Database: CAplus and MEDLINE

Substances (3) | Reactions (0) | Cited By (3)

147

Inhibition of H3K27me3 histone demethylase activity prevents the proliferative regeneration of zebrafish lateral line neuromasts

By: Bao, Beier; He, Yingzi; Tang, Dongmei; Li, Wenyan; Li, Huawei
Frontiers in Molecular Neuroscience (2017), 10, 51/1-51/12 | Language: English, Database: CAplus and MEDLINE

Substances (0) | Reactions (0) | Cited By (12)

148

Inhibition of KDM6 activity during murine ESC differentiation induces DNA damage

By: Hofstetter, Christine; Kampka, Justyna. M.; Huppertz, Sascha; Weber, Heike; Schlosser, Andreas; Mueller, Albrecht. M.; Becker, Matthias
Journal of Cell Science (2016), 129(4), 788-803 | Language: English, Database: CAplus and MEDLINE

Substances (4) | Reactions (0) | Cited By (21)

149

Micelle drug delivery system for targeting epigenetic modification site and preparation method thereof

By: Mo, Jingxin; Wei, Lili; Chen, Wei
China, CN108553418 A 2018-09-21 | Language: Chinese, Database: CAplus

PatentPak available

Substances (5) | Reactions (0) | Cited By (0)

150

KDM6B-mediated histone demethylation of LDHA promotes lung metastasis of osteosarcoma

By: Jiang, Yuhang; Li, Fengfeng; Gao, Bowen; Ma, Mengjun; Chen, Meng; Wu, Yanfeng; Zhang, Weidong; Sun, Yangbai; Liu, Sanhong; Shen, Huiyong
Theranostics (2021), 11(8), 3868-3881 | Language: English, Database: CAplus and MEDLINE

Substances (2) | Reactions (0) | Cited By (9)

151

KDM6B promotes ESCC cell proliferation and metastasis by facilitating C/EBPβ transcription

By: Qin, Mei; Han, Fei; Wu, Jian; Gao, Feng-xia; Li, Yuan; Yan, De-xin; He, Xue-mei; Long, Yang; Tang, Xiao-ping; Ren, De-lian; et al
BMC Cancer (2021), 21(1), 559 | Language: English, Database: CAplus and MEDLINE

Substance (1) | Reactions (0) | Cited By (9)

152

Application of H3K27me3 capable of regulating RUNX1 transcription in synthesis of steroid hormone in porcine ovarian granulosa cells

By: Zhong, Yuyi; Li, Jiaqi; Yuan, Xiaolong; Zhang, Zhe; Yun, Bing
China, CN111996194 A 2020-11-27 | Language: Chinese, Database: CAplus

PatentPak available

Substances (2) | Reactions (0) | Cited By (0)

153

Methods of diagnosing and treating cancer with microRNAs

By: Chung, Leland W.K.; Wang, Fubo; Sun, Yinghao
World Intellectual Property Organization, WO2017193008 A1 2017-11-09 | Language: English, Database: CAplus

FIG. 1B

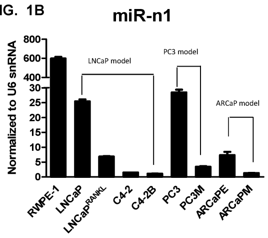

PatentPak available

Substances (18) | Reactions (0) | Cited By (0)

154

Novel anticancer compound and pharmaceutical composition comprising same as active ingredient

By: Kim, Yong Bae; Yun, Kyung Seop; Jang, Min Hee
World Intellectual Property Organization, WO2021020902 A1 2021-02-04 | Language: Korean, Database: CAplus

PatentPak available

Substances (19) | Reactions (0) | Cited By (0)

155

Application of H3K27me3 capable of regulating Ssc-miR-143-3p transcription in synthesis of steroid hormone in porcine ovarian granulosa cells

By: Zhong, Yuyi; Yuan, Xiaolong; Li, Jiaqi; Zhang, Zhe; Li, Nian
China, CN111979242 A 2020-11-24 | Language: Chinese, Database: CAplus

PatentPak available

Substances (3) | Reactions (0) | Cited By (0)

156

Acquired resistance during adoptive cell therapy by transcriptional silencing of immunogenic antigens

By: Wylie, Ben; Chee, Jonathan; Forbes, Catherine A.; Booth, Mitchell; Stone, Shane R.; Buzzai, Anthony; Abad, Ana; Foley, Bree; Cruickshank, Mark N.; Waithman, Jason
OncoImmunology (2019), 8(8), 1609874/1-1609874/11 | Language: English, Database: CAplus and MEDLINE

Substances (20) | Reactions (0) | Cited By (10)

157

Lysine demethylase KDM6B regulates HIF-1α-mediated systemic and cellular responses to intermittent hypoxia

By: Nanduri, Jayasri ; Wang, Ning; Wang, Benjamin L.; Prabhakar, Nanduri R.
Physiological Genomics (2021), 53(9), 385-394 | Language: English, Database: CAplus and MEDLINE

Substance (1) | Reactions (0) | Cited By (4)

158

Methods for treating glioblastoma with combination of KDM6B inhibitor and immunotherapy

By: Goswami, Sangeeta; Sharma, Padmanee; Allison, James
World Intellectual Property Organization, WO2021231405 A1 2021-11-18 | Language: English, Database: CAplus

PatentPak available

Substances (7) | Reactions (0) | Cited By (0)

159

The fungal metabolite chaetocin is a sensitizer for pro-apoptotic therapies in glioblastoma

By: Ozyerli-Goknar, Ezgi; Sur-Erdem, Ilknur; Seker, Fidan; Cingoz, Ahmet; Kayabolen, Alisan; Kahya-Yesil, Zeynep; Uyulur, Firat; Gezen, Melike; Tolay, Nazife; Erman, Batu; et al
Cell Death & Disease (2019), 10(12), 894 | Language: English, Database: CAplus and MEDLINE

Substances (21) Reactions (0) Cited By (11)

160

Morphological screening of mesenchymal mammary tumor organoids to identify drugs that reverse epithelial-mesenchymal transition

By: Zhao, Na; Powell, Reid T.; Yuan, Xueying; Bae, Goeun; Roarty, Kevin P.; Stossi, Fabio; Strempfl, Martina; Toneff, Michael J.; Johnson, Hannah L.; Mani, Sendurai A.; et al
Nature Communications (2021), 12(1), 4262 | Language: English, Database: CAplus and MEDLINE

Substances (22) Reactions (0) Cited By (10)

161

An IFNγ/STAT1/JMJD3 axis induces ZEB1 expression and promotes aggressiveness in lung adenocarcinoma

By: Yang, Jianjian; Wang, Xue; Huang, Bing; Liu, Rong; Xiong, Hui; Ye, Fan; Zeng, Chenxi; Fu, Xiangning; Li, Lequn
Molecular Cancer Research (2021), 19(7), 1234-1246 | Language: English, Database: CAplus and MEDLINE

Substances (7) Reactions (0) Cited By (2)

162

Inhibition of H3K4 demethylation induces autophagy in cancer cell lines

By: Wang, Zhen; Long, Qiao-Yun; Chen, Lin; Fan, Jia-Dong; Wang, Zhao-Ning; Li, Lian-Yun; Wu, Min; Chen, Xuefeng
Biochimica et Biophysica Acta, Molecular Cell Research (2017), 1864(12), 2428-2437 | Language: English, Database: CAplus and MEDLINE

Substances (12) Reactions (0) Cited By (20)

163

A unique bipartite Polycomb signature regulates stimulus-response transcription during development

By: Kitazawa, Taro; Machlab, Dania; Joshi, Onkar; Maiorano, Nicola; Kohler, Hubertus; Ducret, Sebastien; Kessler, Sandra; Gezelius, Henrik; Soneson, Charlotte; Papasaikas, Panagiotis; et al
Nature Genetics (2021), 53(3), 379-391 | Language: English, Database: CAplus and MEDLINE

Substances (6) Reactions (0) Cited By (8)

164

Systematic chemical and molecular profiling of MLL-rearranged infant acute lymphoblastic leukemia reveals efficacy of romidepsin

By: Cruickshank, M. N.; Ford, J.; Cheung, L. C.; Heng, J.; Singh, S.; Wells, J.; Failes, T. W.; Arndt, G. M.; Smithers, N.; Prinjha, R. K.; et al
Leukemia (2017), 31(1), 40-50 | Language: English, Database: CAplus and MEDLINE

Substances (32) Reactions (0) Cited By (18)

165

Analysis of characteristic genes and ceRNA regulation mechanism of endometriosis based on full transcriptional sequencing

By: Xie, Chengmao; Yin, Ziran; Liu, Yong
Frontiers in Genetics (2022), 13, 902329 | Language: English, Database: CAplus and MEDLINE

Substances (23) Reactions (0) Cited By (0)

166

Hypoxia induces HIF1α-dependent epigenetic vulnerability in triple negative breast cancer to confer immune effector dysfunction and resistance to anti-PD-1 immunotherapy

By: Ma, Shijun ; Zhao, Yue; Lee, Wee Chyan; Ong, Li-Teng; Lee, Puay Leng; Jiang, Zemin; Oguz, Gokce; Niu, Zhitong; Liu, Min; Goh, Jian Yuan; et al
Nature Communications (2022), 13(1), 4118 | Language: English, Database: CAplus and MEDLINE

Substances (23) Reactions (0) Cited By (3)

167

Combined Targeting of Mutant p53 and jumonji family histone demethylase augments therapeutic efficacy of radiation in H3K27M DIPG

By: Nikolaev, Anatoly; Fiveash, John B.; Yang, Eddy S.
International Journal of Molecular Sciences (2020), 21(2), 490 | Language: English, Database: CAplus and MEDLINE

Substances (2) Reactions (0) Cited By (15)

168

Cooperation between KDM6B overexpression and TET2 deficiency in the pathogenesis of chronic myelomonocytic leukemia

By: Wei, Yue; Kanagal-Shamanna, Rashmi ; Zheng, Hong; Bao, Naran; Lockyer, Pamela Pennington; Class, Caleb A. ; Darbaniyan, Faezeh; Lu, Yue; Lin, Kevin; Yang, Hui; et al
Leukemia (2022), 36(8), 2097-2107 | Language: English, Database: CAplus and MEDLINE

Substances (6) | Reactions (0) | Cited By (0)

169

The N6-methylandenosine-related gene BIRC5 as a prognostic biomarker correlated with cell migration and immune cell infiltrates in low grade glioma

By: Jiang, Xiulin; Shi, Yulin; Chen, Xi; Xu, Haitao; Huang, Xiaobin; Li, Lihua; Pu, Jun
Frontiers in Molecular Biosciences (2022), 9, 773662 | Language: English, Database: CAplus and MEDLINE

Substances (31) | Reactions (0) | Cited By (1)

170

Ex vivo gastrointestinal biopsy platform to evaluate multi-omic signatures for screening candidate therapeutics

By: Iliopoulos, Dimitrios; Karagiannidis, Iordanis
World Intellectual Property Organization, WO2022031954 A1 2022-02-10 | Language: English, Database: CAplus

PatentPak available

Substances (33) | Reactions (0) | Cited By (0)

171

Nuclear pore protein NUP210 depletion suppresses metastasis through heterochromatin-mediated disruption of tumor cell mechanical response

By: Amin, Ruhul ; Shukla, Anjali; Zhu, Jacqueline Jufen ; Kim, Sohyoung ; Wang, Ping; Tian, Simon Zhongyuan; Tran, Andy D. ; Paul, Debasish; Cappell, Steven D. ; Burkett, Sandra ; et al
Nature Communications (2021), 12(1), 7216 | Language: English, Database: CAplus and MEDLINE

Substances (3) | Reactions (0) | Cited By (6)

172

Compounds and compositions for treating, ameliorating, and/or preventing SARS-Cov-2 infection and/or complications thereof

By: Wilen, Craig
World Intellectual Property Organization, WO2021247367 A1 2021-12-09 | Language: English, Database: CAplus

PatentPak available

Substances (309) Reactions (0) Cited By (0)

173

Methods for induction of enteric neurons expressing NOS1 for use in cell therapy of enteric nervous system disorders and drug screening

By: Fattahi, Faranak
World Intellectual Property Organization, WO2021195439 A1 2021-09-30 | Language: English, Database: CAplus

PatentPak available

Substances (28) Reactions (0) Cited By (0)

174

Sequential targeting in crosslinking nano-theranostics for treating brain tumors

By: Li, Yuanpei; Wu, Hao; Lin, Tzu-Yin
World Intellectual Property Organization, WO2021126970 A1 2021-06-24 | Language: English, Database: CAplus

PatentPak available

Substances (58) Reactions (3) Cited By (0)

175

In silico identification of small molecules as new Cdc25 inhibitors through the correlation between chemosensitivity and protein expression pattern

By: Lauria, Antonino ; Martorana, Annamaria ; La Monica, Gabriele ; Mannino, Salvatore; Mannino, Giuseppe ; Peri, Daniele ; Gentile, Carla
International Journal of Molecular Sciences (2021), 22(7), 3714 | Language: English, Database: CAplus and MEDLINE

Substances (34) Reactions (0) Cited By (6)

176

KDM subfamily 6 protein inhibitor for use in the treatment of cancer

By: Sanchez Cespedes, Montse; Romero Ferraro, Octavio
World Intellectual Property Organization, WO2021105224 A1 2021-06-03 | Language: English, Database: CAplus

PatentPak available

Substances (7) Reactions (0) Cited By (0)

177

Activity Comparison of Epigenetic Modulators against the Hemoprotozoan Parasites Babesia divergens and Plasmodium falciparum

By: Vanheer, Leen N.; Kafsack, Bjorn F. C.
ACS Infectious Diseases (2021), 7(8), 2277-2284 | Language: English, Database: CAplus and MEDLINE

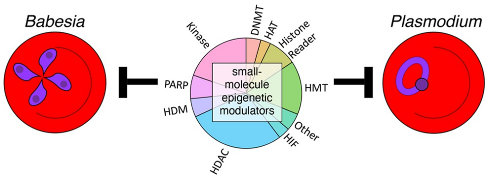

Substances (326) | Reactions (0) | Cited By (6)

178

P53 affects epigenetic signature on SOCS1 promoter in response to TLR4 inhibition

By: Sheikh, Touseef; Sen, Ellora
Cytokine+ (2021), 140, 155418 | Language: English, Database: CAplus and MEDLINE

Substances (3) | Reactions (0) | Cited By (5)

179

Screening methods for identifying therapeutic agents for treating chronic inflammatory injury, metaplasia, dysplasia and cancers of epithelial tissues

By: McKeon, Frank; Duleba, Marcin; Zhang, Yanting; Xie, Jingzhong; Xian, Wa; Vincent, Matthew
World Intellectual Property Organization, WO2020219963 A1 2020-10-29 | Language: English, Database: CAplus

PatentPak available

Substances (113) | Reactions (0) | Cited By (2)

180

Methods of sensitizing cancer cells to tumor necrosis factor-α (TNFα)-induced cell death with BET inhibitors

By: Bacac, Marina; Fauti, Tanja Andrea; Hogg, Simon John; Johnstone, Ricky Wayne; Ruefli-Brasse, Astrid Alexandra; Rohle, Daniel Alan; Wellinger, Lisa Christina
World Intellectual Property Organization, WO2020169698 A1 2020-08-27 | Language: English, Database: CAplus

PatentPak available

Substances (65) | Reactions (0) | Cited By (2)

181

Novel glia-like cells differentiated from somatic cells, preparation method therefor, cocktail composition, cell therapy for preventing or treating neurological disorders

By: Kim, Kyeong-Kyu; De, Debojyoti
World Intellectual Property Organization, WO2020141648 A1 2020-07-09 | Language: Korean, Database: CAplus

PatentPak available

Substances (61) Reactions (0) Cited By (0)

182

Methods using protein arginine methyltransferase (PRMT) inhibitors for the treatment and prevention of hematopoietic disorders and malignancies

By: Shlush, Liran; Abdel-Wahab, Omar
World Intellectual Property Organization, WO2020089892 A1 2020-05-07 | Language: English, Database: CAplus

PatentPak available

Substances (73) Reactions (0) Cited By (0)

183

Differential kinase activity of ACVR1 G328V and R206H mutations with implications to possible TβRI cross-talk in diffuse intrinsic pontine glioma

By: Cao, Hongnan ; Jin, Miao; Gao, Mu; Zhou, Hongyi; Tao, Yizhi Jane; Skolnick, Jeffrey
Scientific Reports (2020), 10(1), 6140 | Language: English, Database: CAplus and MEDLINE

Substances (2) Reactions (0) Cited By (4)

184

HDAC inhibition enhances the in vivo efficacy of MEK inhibitor therapy in Uveal melanoma

By: Faiao-Flores, Fernanda; Emmons, Michael F.; Durante, Michael A.; Kinose, Fumi; Saha, Biswarup; Fang, Bin; Koomen, John M.; Chellappan, Srikumar P.; Maria-Engler, Silvya Stuchi; Rix, Uwe; et al
Clinical Cancer Research (2019), 25(18), 5686-5701 | Language: English, Database: CAplus and MEDLINE

Substances (30) Reactions (0) Cited By (48)

185

Pluripotent stem cell microRNA and small mol. compound induction

By: Yu, Xiyong; Wang, Yigang; Liang, Jialiang
World Intellectual Property Organization, WO2020007036 A1 2020-01-09 | Language: Chinese, Database: CAplus

PatentPak available

Substances (95) Reactions (0) Cited By (0)

186

Compositions and methods for treating and preventing muscular disorders and dystrophies, steroid side effects, and inflammation

By: Heier, Christopher; Fiorillo, Alyson
World Intellectual Property Organization, WO2019232548 A2 2019-12-05 | Language: English, Database: CAplus

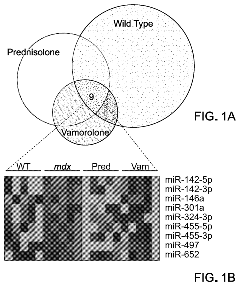

PatentPak available

Substances (95) Reactions (0) Cited By (1)

187

Composition and method to produce improved T cells with increased Ezh2 activity for treating cancer and chronic infection

By: Zhang, Yi; He, Shan
World Intellectual Property Organization, WO2019094404 A1 2019-05-16 | Language: English, Database: CAplus

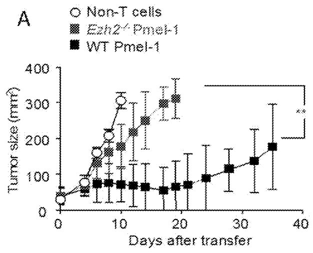

PatentPak available

Substances (92) Reactions (0) Cited By (0)

188

Methods for treating diseases and nerve injury by administering a composition comprising a myelination enhancing inhibitor

By: He, Xuelian; Lu, Qing; Zhang, Liguo
World Intellectual Property Organization, WO2019032652 A1 2019-02-14 | Language: English, Database: CAplus

FIG. 8

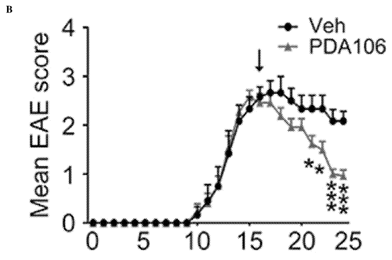

PatentPak available

Substances (33) Reactions (0) Cited By (0)

189

Engineering Lineage Potency and Plasticity of Stem Cells using Epigenetic Molecules

By: Dhaliwal, Anandika; Pelka, Sandra; Gray, David S.; Moghe, Prabhas V.
Scientific Reports (2018), 8(1), 1-13 | Language: English, Database: CAplus and MEDLINE

Substances (71) Reactions (0) Cited By (5)

190

Screening of a neuronal cell model of tau pathology for therapeutic compounds

By: Pickhardt, Marcus; Tassoni, Michele; Denner, Philip; Kurkowsky, Birgit; Kitanovic, Ana; Moehl, Christoph; Fava, Eugenio; Mandelkow, Eckhard
Neurobiology of Aging (2019), 76, 24-34 | Language: English, Database: CAplus and MEDLINE

Substances (36) Reactions (0) Cited By (5)

191

HIV signaling through CD4 and CCR5 activates Rho family GTPases that are required for optimal infection of primary CD4+ T cells

By: Lucera, Mark B.; Fleissner, Zach; Tabler, Caroline O.; Schlatzer, Daniela M.; Troyer, Zach; Tilton, John C.
Retrovirology (2017), 14, 4/1-4/13 | Language: English, Database: CAplus and MEDLINE

Substances (74) Reactions (0) Cited By (9)

192

Modulators of homeobox transcription factors, DUX4 (double homeobox 4) for regulation of muscle function

By: Rickard, Amanda; De La Garza, Anabel; Schmidt, Uli; Kiselyov, Alexander
World Intellectual Property Organization, WO2017201585 A1 2017-11-30 | Language: English, Database: CAplus

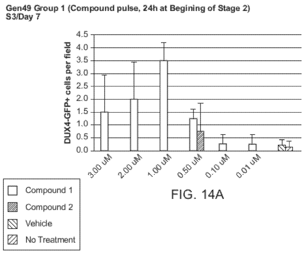

FIG. 14A

PatentPak available

Substances (241) | Reactions (0) | Cited By (3)

193

Retinoic acid receptor-α (RAR-R) agonists for the treatment of acute myelocytic leukemia (AML) and myelodysplastic syndrome (MDS)

By: McKeown, Michael Robert; Fiore, Christopher; Eaton, Matthew Lucas; Lee, Emily Payton; Fritz, Christian
World Intellectual Property Organization, WO2017177167 A1 2017-10-12 | Language: English, Database: CAplus

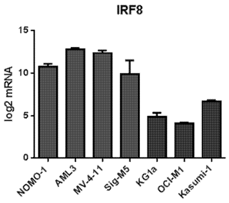

Figure 1

PatentPak available

Substances (31) | Reactions (0) | Cited By (0)

194

Discovery of novel drug sensitivities in T-PLL by high-throughput ex vivo drug testing and mutation profiling

By: Andersson, E. I.; Putzer, S.; Yadav, B.; Dufva, O.; Khan, S.; He, L.; Sellner, L. ; Schrader, A.; Crispatzu, G.; Oles, M.; et al
Leukemia (2018), 32(3), 774-787 | Language: English, Database: CAplus and MEDLINE

Substances (65) | Reactions (0) | Cited By (60)

195

Structure of Nascent Chromatin Is Essential for Hematopoietic Lineage Specification

By: Petruk, Svetlana; Mariani, Samanta A.; De Dominici, Marco; Porazzi, Patrizia; Minieri, Valentina; Cai, Jingli; Iacovitti, Lorraine; Flomenberg, Neal; Calabretta, Bruno; Mazo, Alexander
Cell Reports (2017), 19(2), 295-306 | Language: English, Database: CAplus and MEDLINE

Substances (11) Reactions (0) Cited By (23)

196

Compositions and methods for modulating FMR1 expression

By: Green, Michael R.; Fang, Minggang
World Intellectual Property Organization, WO2017049192 A1 2017-03-23 | Language: English, Database: CAplus

An Unbiased, Large-Scale Screen for Factors that Mediate Epigenetic Silencing of *FMR1*

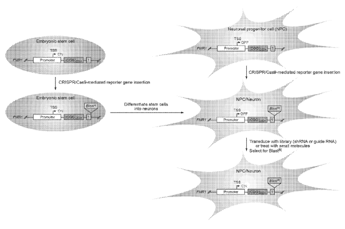

FIG. 5

PatentPak available

Substances (150) Reactions (0) Cited By (3)

197

Epigenetic silencing of N-myristoyltransferase 2 (NMT2) in cancer due to hypermethylation and treatment with inhibitors of NMT

By: Berthiaume, Luc G.; Beauchamp, Erwan
World Intellectual Property Organization, WO2017011907 A1 2017-01-26 | Language: English, Database: CAplus

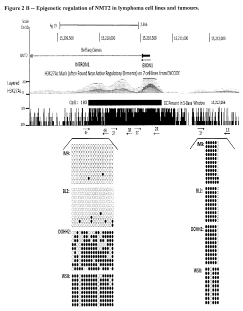

PatentPak available

Substances (76) Reactions (0) Cited By (3)

198

EZH2 inhibitors for treating lymphoma

By: Keilhack, Heike; Knutson, Sarah K.; Johnston, L. Danielle
World Intellectual Property Organization, WO2016201328 A1 2016-12-15 | Language: English, Database: CAplus

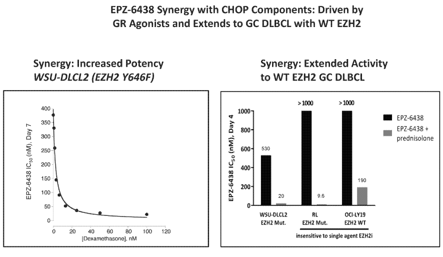

Figure 1A

Figure 1B

PatentPak available

Substances (43) | Reactions (0) | Cited By (3)

199

Epigenomic regulation of schwann cell reprogramming in peripheral nerve injury

By: Ma, Ki H.; Hung, Holly A.; Svaren, John
Journal of Neuroscience (2016), 36(35), 9135-9147 | Language: English, Database: CAplus and MEDLINE

Substances (5) | Reactions (0) | Cited By (48)

200

Methods of treating hepatitis B virus

By: Aguayo, Esmeralda; Appleby, Todd; Birkus, Gabriel; Cheng, Guofeng; Dornan, David; Kobayashi, Tetsuya; Mello, Christopher Charles; Schmitz, Uli; Willkom, Madeleine; Yu, Mei
World Intellectual Property Organization, WO2016168349 A1 2016-10-20 | Language: English, Database: CAplus

PatentPak available

Substances (51) | Reactions (0) | Cited By (1)

201

Methods for inhibiting alpha-v beta-3 expression on cancer stem cells and inhibiting progression to a cancer stem cell phenotype

By: Cheresh, David A.; Gozo, Maricel; Yebra, Mayra; Seguin, Laetitia
World Intellectual Property Organization, WO2016100858 A1 2016-06-23 | Language: English, Database: CAplus

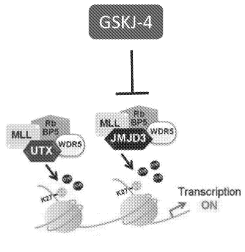

Fig. 11A

PatentPak available

Substances (33) | Reactions (0) | Cited By (2)

202

Method of predicting the tumor response to DNA methylation inhibitors and alternative therapeutic regimen for overcoming resistance

By: Agrawal, Khushboo; Dzubak, Petr; Frydrych, Ivo; Hajduch, Marian
World Intellectual Property Organization, WO2015144102 A1 2015-10-01 | Language: English, Database: CAplus

PatentPak available

Substances (20) | Reactions (0) | Cited By (1)

203

Method of predicting the tumor response to DNA methylation inhibitors and alternative therapeutic regimens for overcoming resistance

By: Agrawal, Khushboo; Dzubak, Petr; Hajduch, Marian; Frydrych, Ivo
European Patent Organization, EP2924125 A1 2015-09-30 | Language: English, Database: CAplus

PatentPak available

Substances (9) | Reactions (0) | Cited By (0)

204

Epigenetic regulators of frataxin for use in treatment of Friedrich' s ataxia

By: Ozsolak, Fatih
World Intellectual Property Organization, WO2015023938 A1 2015-02-19 | Language: English, Database: CAplus

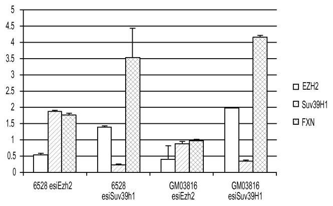

FIG. 3B

PatentPak available

Substances (268) | Reactions (0) | Cited By (1)

205

Pharmaceutical compositions and methods for identification, assessment, prevention, and treatment of cancer using histone H3K27me2 biomarkers and modulators

By: Lane, Andrew; Weinstock, David
World Intellectual Property Organization, WO2014190035 A2 2014-11-27 | Language: English, Database: CAplus

Figure 6

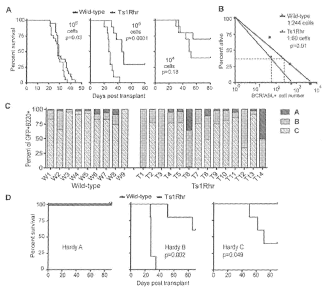

PatentPak available

Substances (5) | Reactions (0) | Cited By (0)

206

Transcriptional repression of ER through hMAPK dependent histone deacetylation by class I HDACs

By: Plotkin, Amy; Volmar, Claude-Henry; Wahlestedt, Claes; Ayad, Nagi; El-Ashry, Dorraya
Breast Cancer Research and Treatment (2014), 147(2), 249-263 | Language: English, Database: CAplus and MEDLINE

Substances (59) | Reactions (0) | Cited By (12)

207

Pyrmidinyl pyrimidines as affinity ligands for histone demethylases and their use identifying ligands and effectors of the demethylase

By: Atkinson, Stephen John; Barker, Michael David; Campbell, Matthew; Humphreys, Philip; Liddle, John; Sheppard, Robert John; Wilson, David; Joberty, Gerard

European Patent Organization, EP2592154 A1 2013-05-15 | Language: English, Database: CAplus

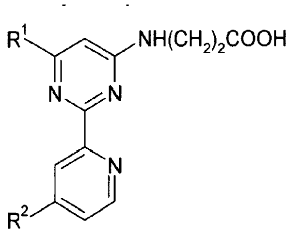

(I)

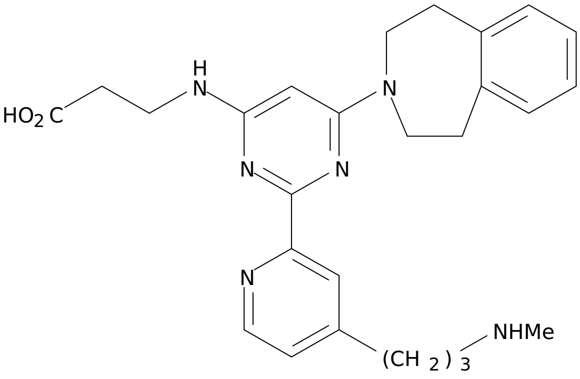

PatentPak available

Substances (56) | Reactions (137) | Cited By (1)

208

Preparation of N-(pyridinyl)pyrimidinyl-β-alanine derivatives for use as histone demethylase JMJD3 inhibitors

By: Atkinson, Stephen John; Barker, Michael David; Campbell, Matthew; Humphreys, Philip; Liddle, John; Sheppard, Robert John; Wilson, David

World Intellectual Property Organization, WO2012052390 A1 2012-04-26 | Language: English, Database: CAplus

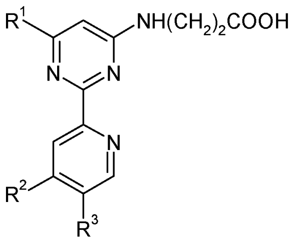

(I)

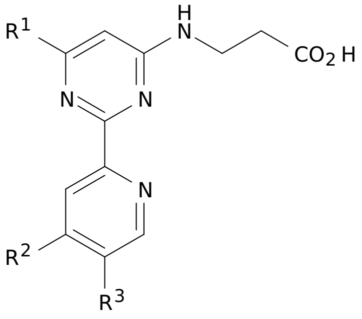

I

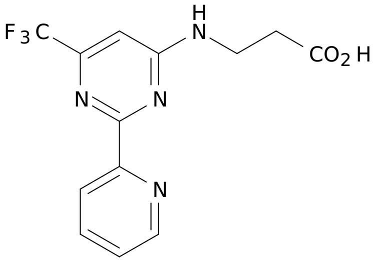

II

PatentPak available

Substances (387) | Reactions (688) | Cited By (3)

209

Histone demethylation maintains Prdm14 and Tsix expression and represses Xist in embryonic stem cells

By: Kamikawa, Yasunao F.; Donohoe, Mary E.

PLoS One (2015), 10(5), e0125626/1-e0125626/17 | Language: English, Database: CAplus and MEDLINE

Substances (9) | Reactions (0) | Cited By (10)

210

KDM6B overexpression activates innate immune signaling and impairs hematopoiesis in mice

By: Wei, Yue; Zheng, Hong; Bao, Naran; Jiang, Shan; Bueso-Ramos, Carlos E. ; Khoury, Joseph ; Class, Caleb ; Lu, Yue; Lin, Kevin; Yang, Hui; et al
Blood Advances (2018), 2(19), 2491-2504 | Language: English, Database: CAplus and MEDLINE

Substances (0) Reactions (0) Cited By (20)

211

Novel pharmacological inhibition of JMJD3 improves necrotizing enterocolitis by attenuating the inflammatory response and ameliorating intestinal injury

By: Ma, Shurong; Xu, Lingqi; Chen, Lulu; Sun, Xu; Hu, Fangjie; Gong, Yuan; Yang, Randong; Li, Jing; Wang, Qian; Huang, Shungen; et al
Biochemical Pharmacology (Amsterdam, Netherlands) (2022), 203, 115165 | Language: English, Database: CAplus and MEDLINE

Substance (1) Reactions (0) Cited By (0)

212

Therapeutic effect of a histone demethylase inhibitor in Parkinsons disease

By: Mu, Ming-Dao ; Qian, Zhong-Ming ; Yang, Sheng-Xi; Rong, Kang-Lin; Yung, Wing-Ho; Ke, Ya
Cell Death & Disease (2020), 11(10), 927 | Language: English, Database: CAplus and MEDLINE

Substances (0) Reactions (0) Cited By (15)

213

Pharmacological targeting of KDM6A and KDM6B, as a novel therapeutic strategy for treating craniosynostosis in Saethre-Chotzen syndrome

By: Pribadi, Clara; Camp, Esther; Cakouros, Dimitrios; Anderson, Peter; Glackin, Carlotta; Gronthos, Stan
Stem Cell Research & Therapy (2020), 11(1), 529 | Language: English, Database: CAplus and MEDLINE

Substances (0) Reactions (0) Cited By (6)

214

Histone demethylase Jmjd3 modulates osteoblast apoptosis induced by tumor necrosis factor-alpha through directly targeting RASSF5.

By: Sun, Hai-Yan; Yang, Di; Mi, Jing; Yu, Ya-Qiong; Qiu, Li-Hong
Connective tissue research (2020), 61(6), 517-525 | Language: English, Database: MEDLINE

Substance (1) Reactions (0) Cited By (4)

215

Histone demethylase JMJD3 protects against renal fibrosis by suppressing TGFβ and Notch signaling and preserving PTEN expression

By: Yu, Chao; Xiong, Chongxiang; Tang, Jinhua; Hou, Xiying; Liu, Na; Bayliss, George; Zhuang, Shougang
Theranostics (2021), 11(6), 2706-2721 | Language: English, Database: CAplus and MEDLINE

Substances (3) Reactions (0) Cited By (19)

216

Therapeutic potential of inhibiting histone 3 lysine 27 demethylases: a review of the literature

By: Abu-Hanna, Jeries; Patel, Jigisha A.; Anastasakis, Evangelos; Cohen, Richard; Clapp, Lucie H.; Loizidou, Marilena; Eddama, Mohammad M. R.
Clinical Epigenetics (2022), 14(1), 98 | Language: English, Database: CAplus and MEDLINE

Substance (1) Reactions (0) Cited By (0)

217

Effects of chronic stress on depressive-like behaviors and JMJD3 expression in the prefrontal cortex and hippocampus of C57BL/6 and ob/ob mice.

By: Wu, Huiran; Wang, Rui; Qin, Xiaqing; Liu, Dexiang; Wang, Wei; Xu, Jingjing; Jiang, Hong; Pan, Fang
Journal of psychiatric research (2021), 133, 142-155 | Language: English, Database: MEDLINE

Substances (0) Reactions (0) Cited By (4)

218

Design and discovery of new pyrimidine coupled nitrogen aromatic rings as chelating groups of JMJD3 inhibitors

By: Hu, Jianping; Wang, Xin; Chen, Lin; Huang, Min; Tang, Wei; Zuo, Jianping; Liu, Yu-Chih; Shi, Zhe; Liu, Rongfeng; Shen, Jingkang; et al
Bioorganic & Medicinal Chemistry Letters (2016), 26(3), 721-725 | Language: English, Database: CAplus and MEDLINE

Substances (130) Reactions (436) Cited By (14)

219

Targeting of Histone Demethylases KDM5A and KDM6B Inhibits the Proliferation of Temozolomide-Resistant Glioblastoma Cells.

By: Romani, Massimo; Daga, Antonio ; Forlani, Alessandra; Pistillo, Maria Pia; Banelli, Barbara
Cancers (2019), 11(6), | Language: English, Database: MEDLINE

Substances (0) Reactions (0) Cited By (16)

220

Histone demethylase Jmjd3 regulates the osteogenic differentiation and cytokine expressions of periodontal ligament cells

By: Yu, Bo; Wang, Rui; Luo, Huikun; Yang, Di; Wang, Simo; Yu, Yaqiong; Okamura, Hirohiko; Qiu, Lihong
Acta Medica Okayama (2022), 76(3), 281-290 | Language: English, Database: CAplus and MEDLINE

Substances (0) Reactions (0) Cited By (0)

221

JMJD3 downregulates IL4i1 aggravating lipopolysaccharide-induced acute lung injury via H3K27 and H3K4 demethylation

By: Gao, Yizhuo; Wang, Na; Jia, Dong
Environmental Toxicology, | Language: English, Database: CAplus

Substances (0) Reactions (0) Cited By (0)

222

Regulatory network analysis reveals the oncogenesis roles of feed-forward loops and therapeutic target in T-cell acute lymphoblastic leukemia.

By: Xia, Mengxuan; Zhang, Qiong; Luo, Mei; Li, Pan; Wang, Yingxue; Lei, Qian; Guo, An-Yuan
BMC medical genomics (2019), 12(1), 8 | Language: English, Database: MEDLINE

Substances (0) Reactions (0) Cited By (5)

223

Jmjd3 regulates inflammasome activation and aggravates DSS-induced colitis in mice

By: Huang, Mengwei; Wang, Qing; Long, Fen; Di, Yang; Wang, Jinghuan; Yi, Zhun Zhu; Liu, Xinhua
FASEB Journal (2020), 34(3), 4107-4119 | Language: English, Database: CAplus and MEDLINE

Substances (3) Reactions (0) Cited By (21)

224

The lysine demethylase kdm5b regulates islet function and glucose homeostasis

By: Backe, Marie Balslev ; Jin, Chunyu; Andreone, Luz ; Sankar, Aditya; Agger, Karl; Helin, Kristian; Madsen, Andreas Nygaard; Poulsen, Steen Seier; Bysani, Madhusudhan; Bacos, Karl; et al
Journal of Diabetes Research (2019), 5451038 | Language: English, Database: CAplus and MEDLINE

Substances (2) Reactions (0) Cited By (8)

225

hsa-miR-199b-3p prevents the epithelial-mesenchymal transition and dysfunction of the renal tubule by regulating E-cadherin through targeting KDM6A in diabetic nephropathy

By: Bai, Shoujun ; Xiong, Xiaoyan; Tang, Bo; Ji, Tingting; Li, Xiaoying; Qu, Xiaolei; Li, Weiliang
Oxidative Medicine and Cellular Longevity (2021), 8814163 | Language: English, Database: CAplus and MEDLINE

Substances (0) Reactions (0) Cited By (5)

226

Epigenetic profiling identifies LIF as a super-enhancer-controlled regulator of stem cell-like properties in osteosarcoma

By: Lu, Bing; He, Yangyang; He, Jincan; Wang, Li; Liu, Zhenguo; Yang, Jiayan; Gao, Zhuoxing; Lu, Guohao; Zou, Changye; Zhao, Wei
Molecular Cancer Research (2020), 18(1), 57-67 | Language: English, Database: CAplus and MEDLINE

Substances (0) Reactions (0) Cited By (20)

227

Histone demethylase UTX counteracts glucocorticoid deregulation of osteogenesis by modulating histone-dependent and -independent pathways

By: Wang, Feng-Sheng; Lian, Wei-Shiung; Lee, Mel S.; Weng, Wen-Tsan; Huang, Ying-Hsien; Chen, Yu-Shan; Sun, Yi-Chih; Wu, Shing-Long; Chuang, Pei-Chin; Ko, Jih-Yang
Journal of Molecular Medicine (Heidelberg, Germany) (2017), 95(5), 499-512 | Language: English, Database: CAplus and MEDLINE

Substances (7) Reactions (0) Cited By (10)

228

JMJD3 promotes esophageal squamous cell carcinoma pathogenesis through epigenetic regulation of MYC

By: Li, Shu-Man; He, Li-Ru; Chen, Jie-Wei; Zhou, Jie; Nie, Run-Cong; Jin, Xiao-Han; Wang, Xin; Fu, Jian-Hua; Wang, Feng-Wei; Xie, Dan
Signal Transduction and Targeted Therapy (2020), 5(1), 165 | Language: English, Database: CAplus and MEDLINE

Substances (4) Reactions (0) Cited By (3)

229

Effects of histone demethylase Jmjd3 on odontogenic differentiation of dental pulp stem cells

By: Wang Hui-jun; Yu Ya-qiong; Yang Di; Zhang Bei-di; Qiu Li-hong
Zhongguo Shiyong Kouqiangke Zazhi (2020), 13(5), 51-54 | Language: Chinese, Database: CAplus

Substances (0) Reactions (0) Cited By (0)

230

Histone demethylase JMJD3 downregulation protects against aberrant force-induced osteoarthritis through epigenetic control of NR4A1

By: Jin, Yu; Liu, Zhen; Li, Zhenxia; Li, Hairui; Zhu, Cheng; Li, Ruomei; Zhou, Ting; Fang, Bing
International Journal of Oral Science (2022), 14(1), 34 | Language: English, Database: CAplus and MEDLINE

Substances (0) Reactions (0) Cited By (0)

231

JMJD3 suppresses tumor progression in oral tongue squamous cell carcinoma patients receiving surgical resection.

By: Chen, Yen-Hao; Chen, Chang-Han; Chien, Chih-Yen; Su, Yan-Ye; Luo, Sheng-Dean; Li, Shau-Hsuan
PeerJ (2022), 10, e13759 | Language: English, Database: MEDLINE

Substances (0) Reactions (0) Cited By (0)

232

Adipocyte-mediated epigenomic instability in human T-ALL cells is cytotoxic and phenocopied by epigenetic-modifying drugs.

By: Lee, Miyoung; Geitgey, Delaney K; Hamilton, Jamie A G; Boss, Jeremy M; Scharer, Christopher D; Spangle, Jennifer M; Haynes, Karmella A; Henry, Curtis J
Frontiers in cell and developmental biology (2022), 10, 909557 | Language: English, Database: MEDLINE

Substances (0) Reactions (0) Cited By (0)

233

Study on the effect of JMJD3 on liver ischemia-reperfusion injury in mice

By: Zhou Qiang; Deng Minghua; Wu Yakun; Gong Jianping
Chongqing Yixue (2020), 49(18), 33-38, 43 | Language: Chinese, Database: CAplus

Substances (0) Reactions (0) Cited By (0)

234

Stretch-induced tenomodulin expression promotes tenocyte migration via F-actin and chromatin remodeling

By: Xu, Pu ; Deng, Bin; Zhang, Bingyu; Luo, Qing; Song, Guanbin
International Journal of Molecular Sciences (2021), 22(9), 4928 | Language: English, Database: CAplus and MEDLINE

Substances (4) Reactions (0) Cited By (4)

235

Screening method of small molecule inhibitor capable of inhibiting proliferation of Toxoplasma gondii and its application method

By: Liu, Shuxian; Tan, Feng; Zhang, Fangfei; Hua, Qianqian; Cao, Jiaxin; Wang, Yan; Hu, Xin
China, CN107884587 A 2018-04-06 | Language: Chinese, Database: CAplus

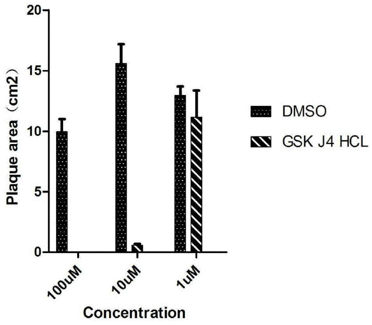

PatentPak available

Substance (1) | Reactions (0) | Cited By (0)

236

Epigenetic remodeling through downregulation of polycomb repressive complex 2 mediates chemotherapy resistance in testicular germ cell tumors

By: Singh, Ratnakar; Fazal, Zeeshan; Corbet, Andrea K.; Bikorimana, Emmanuel; Rodriguez, Jennifer C.; Khan, Ema M.; Shahid, Khadeeja; Freemantle, Sarah J.; Spinella, Michael J.
Cancers (2019), 11(6), 796 | Language: English, Database: CAplus and MEDLINE

Substances (0) | Reactions (0) | Cited By (15)

237

Downregulation of interleukin-1 beta via Jmjd3 inhibition improves post-myocardial infarction depression.

By: Tang, Xian'e; Liu, Ruhui; Zhang, Youyang; Zhu, Lingfeng; Shi, Weiqi; Shan, Yongmei; Wu, Shihao; Li, Yuhang; Liu, Guanghui; Ma, Wenlin
Cardiovascular diagnosis and therapy (2022), 12(3), 340-351 | Language: English, Database: MEDLINE

Substances (0) | Reactions (0) | Cited By (0)

238

The Epigenetic Regulation of OLIG2 by Histone Demethylase KDM6B in Glioma Cells

By: Sui, Aixia; Han, Biaogang; Ren, Wenjun; He, Weiliang; Gao, Chao; Han, Xiaohui; Liu, Shifeng; Zhang, Yan; Qi, Xueling; Guo, Xiaoqiang
Journal of Molecular Neuroscience (2022), 72(5), 939-946 | Language: English, Database: CAplus and MEDLINE

Substance (1) | Reactions (0) | Cited By (0)

239

Epigenetic reprogramming of H3K27me3 and DNA methylation during leaf-to-callus transition in peach.

By: Zheng, Beibei; Liu, Jingjing; Gao, Anqi; Chen, Xiaomei; Gao, Lingling; Liao, Liao; Luo, Binwen; Ogutu, Collins Otieno; Han, Yuepeng
Horticulture research (2022), 9, uhac132 | Language: English, Database: MEDLINE

Substances (0) Reactions (0) Cited By (1)

240

Heterochromatin protein 1γ deficiency decreases histone H3K27 methylation in mouse neurosphere neuronal genes

By: Naruse, Chie; Abe, Kanae; Yoshihara, Toru; Kato, Tomoaki; Nishiuchi, Takumi; Asano, Masahide
FASEB Journal (2020), 34(3), 3956-3968 | Language: English, Database: CAplus and MEDLINE

Substance (1) Reactions (0) Cited By (1)

241

Structural genomics in drug discovery: an overview

By: Sowjanya, Karri; Girish, Chandrashekaran
Journal of Pharmacology and Pharmacotherapeutics (2019), 10(1), 1-6 | Language: English, Database: CAplus

Substances (0) Reactions (0) Cited By (1)

242

Role of UTX Histone Demethylase in Regulation of MGMT, TRA2A, U2AF1, and RPS6KA2 Genes in Prostate Cancer Cell Lines

By: Houfaf Khoufaf, Fatma Zohra; Sanchez, Anna; Idrissou, Mouhamed; Boisnier, Tiphanie; Penault-Llorca, Frederique; Bignon, Yves-Jean; Guy, Laurent; Bernard-Gallon, Dominique
OMICS (2021), 25(2), 129-131 | Language: English, Database: CAplus and MEDLINE

Substances (6) Reactions (0) Cited By (1)

243

IL-4 alleviates ischaemia-reperfusion injury by inducing Kupffer cells M2 polarization via STAT6-JMJD3 pathway after rat liver transplantation

By: Deng, Minghua ; Wang, Jingyuan ; Wu, Hao ; Wang, Menghao; Cao, Ding; Li, Jinzheng; Wu, Yakun ; Gong, Jianping
BioMed Research International (2020), 2953068 | Language: English, Database: CAplus and MEDLINE

Substances (2) Reactions (0) Cited By (6)

244

Overexpression of UTX promotes tumor progression in Oral tongue squamous cell carcinoma patients receiving surgical resection: a case control study.

By: Chen, Yen-Hao; Chen, Chang-Han; Chien, Chih-Yen; Su, Yan-Ye; Luo, Sheng-Dean; Li, Shau-Hsuan
BMC cancer (2021), 21(1), 979 | Language: English, Database: MEDLINE

Substances (0) Reactions (0) Cited By (2)

245

Fingolimod Alleviates Cognitive Deficit in Type 2 Diabetes by Promoting Microglial M2 Polarization via the pSTAT3-jmjd3 Axis

By: Sood, Anika; Fernandes, Valencia; Preeti, Kumari; Khot, Mayuri; Khatri, Dharmendra Kumar; Singh, Shashi Bala
Molecular Neurobiology, | Language: English, Database: CAplus and MEDLINE

Substances (0) Reactions (0) Cited By (0)

246

Abnormally elevated EZH2-mediated H3K27me3 enhances osteogenesis in aortic valve interstitial cells by inhibiting SOCS3 expression

By: Xie, Kaiji; Zeng, Jingxin; Wen, Liming; Peng, Xin; Lin, Zhibin; Xian, Gaopeng; Guo, Yuyang; Yang, Xi; Li, Peixin; Xu, Dingli; et al
Atherosclerosis (Amsterdam, Netherlands) (2023), 364, 1-9 | Language: English, Database: CAplus and MEDLINE

Substances (0) Reactions (0) Cited By (4)

247

Bioinformatics analysis of the clinical expression of APOC1 in papillary thyroid carcinoma

By: Li Chaoyou; Luo Yaxing; Sun Xinmin; Xue Gang; Wu Jingfang
Zhongguo Er-Bi-Yanhou Ludi Waike Zazhi (2021), 27(4), 87-93 | Language: Chinese, Database: CAplus

Substances (0) Reactions (0) Cited By (0)

248

Mechanical stretch aggravates vascular smooth muscle cell apoptosis and vascular remodeling by downregulating EZH2

By: Zhong, Hong-yu; Yuan, Chong; Liu, Xiao-lin; Wang, Qian-qian; Li, Xiao; Zhao, Ya-chao; Li, Xuan; Liu, Dong-dong; Zheng, Teng-fei; Zhang, Mei
International Journal of Biochemistry & Cell Biology (2022), 151, 106278 | Language: English, Database: CAplus and MEDLINE

Substances (4) Reactions (0) Cited By (0)

249

Expression pattern of histone lysine-specific demethylase 6B in gastric cancer

By: Wang, Shujun; Wang, Yiping; Zhu, Hui; Chen, Miaohui; Zhang, Liang
Oncology Letters (2021), 21(6), 491 | Language: English, Database: CAplus and MEDLINE

Substances (0) Reactions (0) Cited By (0)

250

A comprehensive study of epigenetic alterations in hepatocellular carcinoma identifies potential therapeutic targets

By: Bayo, Juan ; Fiore, Esteban J.; Dominguez, Luciana M.; Real, Alejandrina; Malvicini, Mariana; Rizzo, Manglio; Atorrasagasti, Catalina; Garcia, Mariana G.; Argemi, Josepmaria; Martinez, Elisabeth D.; et al
Journal of Hepatology (2019), 71(1), 78-90 | Language: English, Database: CAplus and MEDLINE

Substances (0) Reactions (0) Cited By (53)

251

Dual G9A/EZH2 inhibition stimulates antitumor immune response in ovarian high-grade serous carcinoma

By: Spear, Sarah; Mirza, Hasan; Garner, Ian; McGarry, Lynn; Grundland-Freile, Fabio; Cheng, Zhao; Ennis, Darren P.; Iyer, Nayana; McNamara, Sophie; Natoli, Marina; et al
Molecular Cancer Therapeutics (2022), 21(4), 522-534 | Language: English, Database: CAplus and MEDLINE

Substances (0) Reactions (0) Cited By (6)

252

Mechanism of Alendronate Sodium Combined with LPS Induced Inflammatory Responses in Macrophages

By: Yang Xiao-jie; Wang Qing; Wang Guang-fei; Niu Xiao-yong; Ai Xue-min; Pan Jin-song
Xiandai Shengwuyixue Jinzhan (2020), 20(9), 23-28 | Language: Chinese, Database: CAplus

Substances (0) Reactions (0) Cited By (0)

253

The KDM Inhibitor GSKJ4 Triggers CREB Downregulation via a Protein Kinase A and Proteasome-Dependent Mechanism in Human Acute Myeloid Leukemia Cells.

By: Illiano, Michela; Conte, Mariarosaria; Salzillo, Alessia; Ragone, Angela; Spina, Annamaria; Nebbioso, Angela; Altucci, Lucia; Sapio, Luigi; Naviglio, Silvio
Frontiers in oncology (2020), 10, 799 | Language: English, Database: MEDLINE

Substances (0) Reactions (0) Cited By (8)

Copyright © 2023 American Chemical Society (ACS). All Rights Reserved.
Copyright © 2023 U.S. National Library of Medicine.
Internal use only. Redistribution is subject to the terms of your SciFinder^{n} License Agreement and CAS information Use Policies.

SciFinder^n

Task History

Initiating Search

January 11, 2023, 11:01PM

All:

Structure of GSK-J4 (right)

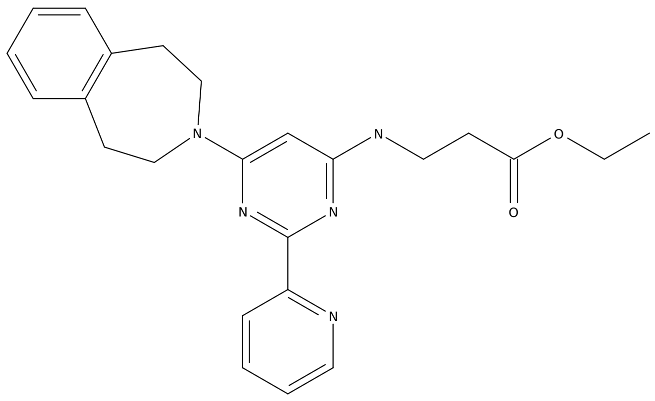

Search Tasks

Task	Search Type	View
Returned All Results	All	View Results
Exported: Retrieved Related Reference Results (164)	References	View Results

Copyright © 2023 American Chemical Society (ACS). All Rights Reserved.
Internal use only. Redistribution is subject to the terms of your SciFinder^n License Agreement and CAS information Use Policies.

CAS SciFinder^n

References (164)

View in SciFinder^n

1

Inhibition of demethylases by GSK-J1/J4

By: Heinemann, Bo; Nielsen, Jesper Morten; Hudlebusch, Heidi Rye; Lees, Michael J.; Larsen, Dorthe Vang; Boesen, Thomas; Labelle, Marc; Gerlach, Lars-Ole; Birk, Peter; Helin, Kristian
Nature (London, United Kingdom) (2014), 514(7520), E1-E2 | Language: English, Database: CAplus and MEDLINE

Substances (4) | Reactions (0) | Cited By (135)

2

GSKJ4, a selective Jumonji H3K27 demethylase inhibitor, effectively targets ovarian cancer stem cells

By: Sakaki, Hirotsugu; Okada, Masashi; Kuramoto, Kenta; Takeda, Hiroyuki; Watarai, Hikaru; Suzuki, Shuhei; Seino, Shizuka; Seino, Manabu; Ohta, Tsuyoshi; Nagase, Satoru; et al
Anticancer Research (2015), 35(12), 6607-6614 | Language: English, Database: CAplus and MEDLINE

Substance (1) | Reactions (0) | Cited By (45)

3

Inhibition of demethylase KDM6B sensitizes diffuse large B-cell lymphoma to chemotherapeutic drugs

By: Mathur, Rohit; Sehgal, Lalit; Havranek, Ondrej; Koehrer, Stefan; Khashab, Tamer; Jain, Neeraj; Burger, Jan A.; Neelapu, Sattva S.; Davis, R. Eric; Samaniego, Felipe
Haematologica (2017), 102(2), 373-380 | Language: English, Database: CAplus and MEDLINE

Substances (2) | Reactions (0) | Cited By (40)

4

Therapeutic potential of gsk-j4, a histone demethylase kdm6b/jmjd3 inhibitor, for acute myeloid leukemia

By: Li, Yunan; Zhang, Mingying; Sheng, Mengyao; Zhang, Peng; Chen, Zizhen; Xing, Wen; Bai, Jie; Cheng, Tao; Yang, Feng-Chun; Zhou, Yuan
Journal of Cancer Research and Clinical Oncology (2018), 144(6), 1065-1077 | Language: English, Database: CAplus and MEDLINE

Substances (3) | Reactions (0) | Cited By (48)

5

The histone demethylase inhibitor GSK-J4 limits inflammation through the induction of a tolerogenic phenotype on DCs

By: Donas, Cristian; Carrasco, Macarena; Fritz, Macarena; Prado, Carolina; Tejon, Gabriela; Osorio-Barrios, Francisco ; Manriquez, Valeria; Reyes, Paz; Pacheco, Rodrigo; Bono, Maria Rosa; et al
Journal of Autoimmunity (2016), 75, 105-117 | Language: English, Database: CAplus and MEDLINE

Substances (3) Reactions (0) Cited By (48)

6

A selective jumonji H3K27 demethylase inhibitor modulates the proinflammatory macrophage response

By: Kruidenier, Laurens; Chung, Chun-wa; Cheng, Zhongjun; Liddle, John; Che, Ka Hing; Joberty, Gerard; Bantscheff, Marcus; Bountra, Chas; Bridges, Angela; Diallo, Hawa; et al
Nature (London, United Kingdom) (2012), 488(7411), 404-408 | Language: English, Database: CAplus and MEDLINE

Substances (28) Reactions (53) Cited By (640)

7

The role and prospect of JMJD3 in stem cells and cancer

By: Yin, Xiaojiao; Yang, Siyu; Zhang, Mingyue; Yue, Ying
Biomedicine & Pharmacotherapy (2019), 118, 109384 | Language: English, Database: CAplus and MEDLINE

Substances (2) Reactions (0) Cited By (32)

8

The histone H3 lysine-27 demethylase Jmjd3 plays a critical role in specific regulation of Th17 cell differentiation

By: Liu, Zhi; Cao, Wei; Xu, Longxia; Chen, Xi; Zhan, Yu; Yang, Qian; Liu, Sanhong; Chen, Pengfei; Jiang, Yuhang; Sun, Xiaohua; et al
Journal of Molecular Cell Biology (Oxford, United Kingdom) (2015), 7(6), 505-516 | Language: English, Database: CAplus and MEDLINE

Substances (3) Reactions (0) Cited By (68)

9

Impact of H3K27 demethylase inhibitor GSKJ4 on NSCLC cells alone and in combination with metformin

By: Watarai, Hikaru; Okada, Masashi; Kuramoto, Kenta; Takeda, Hiroyuki; Sakaki, Hirotsugu; Suzuki, Shuhei; Seino, Shizuka; Oizumi, Hiroyuki; Sadahiro, Mitsuaki; Kitanaka, Chifumi
Anticancer Research (2016), 36(11), 6083-6092 | Language: English, Database: CAplus and MEDLINE

Substances (7) Reactions (0) Cited By (25)

10

GSKJ4, an H3K27me3 demethylase inhibitor, effectively suppresses the breast cancer stem cells

By: Yan, Ningning; Xu, Liang; Wu, Xiaobo; Zhang, Le; Fei, Xiaochun; Cao, Yali; Zhang, Fengchun
Experimental Cell Research (2017), 359(2), 405-414 | Language: English, Database: CAplus and MEDLINE

Substances (7) Reactions (0) Cited By (56)

11

Upregulation of H3K27 demethylase KDM6 during respiratory syncytial virus infection enhances proinflammatory responses and immunopathology

By: Malinczak, Carrie-Anne; Rasky, Andrew J.; Fonseca, Wendy; Schaller, Matthew A.; Allen, Ronald M.; Ptaschinski, Catherine; Morris, Susan; Lukacs, Nicholas W.
Journal of Immunology (2020), 204(1), 159-168 | Language: English, Database: CAplus and MEDLINE

Substances (4) Reactions (0) Cited By (17)

12

GSKJ4 protects mice against early sepsis via reducing proinflammatory factors and up-regulating MiR-146a

By: Pan, Yuchen; Wang, Jiali; Xue, Yaxian; Zhao, Jiaojiao; Li, Dan; Zhang, Shaolong; Li, Kuanyu; Hou, Yayi; Fan, Hongye
Frontiers in Immunology (2018), 9, 2272/1-2272/11 | Language: English, Database: CAplus and MEDLINE

Substances (2) Reactions (0) Cited By (25)

13

An epigenetic therapy for diffuse intrinsic pontine gliomas

By: Ramaswamy, Vijay; Remke, Marc; Taylor, Michael D.
Nature Medicine (New York, NY, United States) (2014), 20(12), 1378-1379 | Language: English, Database: CAplus and MEDLINE

Substances (2) Reactions (0) Cited By (24)

14

Taxane-Platin-Resistant Lung Cancers Co-develop Hypersensitivity to JumonjiC Demethylase Inhibitors

By: Dalvi, Maithili P.; Wang, Lei; Zhong, Rui; Kollipara, Rahul K.; Park, Hyunsil; Bayo, Juan; Yenerall, Paul; Zhou, Yunyun; Timmons, Brenda C.; Rodriguez-Canales, Jaime; et al
Cell Reports (2017), 19(8), 1669-1684 | Language: English, Database: CAplus and MEDLINE

Substances (8) Reactions (0) Cited By (56)

15

Cystathionine-γ-lyase ameliorates the histone demethylase JMJD3-mediated autoimmune response in rheumatoid arthritis

By: Wu, Weijun; Qin, Ming; Jia, Wanwan; Huang, Zheng; Li, Zhongzheng; Yang, Di; Huang, Mengwei; Xiao, Chenxi; Long, Fen; Mao, Jianchun; et al
Cellular & Molecular Immunology (2019), 16(8), 694-705 | Language: English, Database: CAplus and MEDLINE

Substances (4) Reactions (0) Cited By (27)

16

Histone demethylase UTX is a therapeutic target for diabetic kidney disease

By: Chen, Hong; Huang, Yixue; Zhu, Xiuqin; Liu, Chong; Yuan, Yangmian; Su, Hua; Zhang, Chun; Liu, Chengyu; Xiong, Mingrui; Qu, Yannan; et al
Journal of Physiology (Oxford, United Kingdom) (2019), 597(6), 1643-1660 | Language: English, Database: CAplus and MEDLINE

Substances (2) Reactions (0) Cited By (27)

17

Functionally defined therapeutic targets in diffuse intrinsic pontine glioma

By: Grasso, Catherine S.; Tang, Yujie; Truffaux, Nathalene; Berlow, Noah E.; Liu, Lining; Debily, Marie-Anne; Quist, Michael J.; Davis, Lara E.; Huang, Elaine C.; Woo, Pamelyn J.; et al
Nature Medicine (New York, NY, United States) (2015), 21(6), 555-559 | Language: English, Database: CAplus and MEDLINE

Substances (9) Reactions (0) Cited By (290)

18

Histone H3K27me3 demethylases regulate human Th17 cell development and effector functions by impacting on metabolism

By: Cribbs, Adam P. ; Terlecki-Zaniewicz, Stefan; Philpott, Martin; Baardman, Jeroen; Ahern, David; Lindow, Morten; Obad, Susanna; Oerum, Henrik; Sampey, Brante; Mander, Palwinder K.; et al
Proceedings of the National Academy of Sciences of the United States of America (2020), 117(11), 6056-6066 | Language: English, Database: CAplus and MEDLINE

Substances (3) Reactions (0) Cited By (39)

19

JMJD3 is involved in neutrophil membrane proteinase 3 overexpression during the hyperinflammatory response in early sepsis

By: Chen, Yang ; Liu, Zhaojun; Pan, Tingting; Chen, Erzhen; Mao, Enqiang; Chen, Ying; Tan, Ruoming; Wang, Xiaoli; Tian, Rui; Liu, Jialin ; et al
International Immunopharmacology (2018), 59, 40-46 | Language: English, Database: CAplus and MEDLINE

Substances (3) Reactions (0) Cited By (10)

20

PRC2 Epigenetically Silences Th1-Type Chemokines to Suppress Effector T-Cell Trafficking in Colon Cancer

By: Nagarsheth, Nisha; Peng, Dongjun; Kryczek, Ilona; Wu, Ke; Li, Wei; Zhao, Ende; Zhao, Lili; Wei, Shuang; Frankel, Timothy; Vatan, Linda; et al
Cancer Research (2016), 76(2), 275-282 | Language: English, Database: CAplus and MEDLINE

Substances (3) Reactions (0) Cited By (157)

21

Inhibition of demethylases by GSK-J1/J4

By: Kruidenier, Laurens; Chung, Chun-wa; Cheng, Zhongjun; Liddle, John; Che, KaHing; Joberty, Gerard; Bantscheff, Marcus; Bountra, Chas; Bridges, Angela; Diallo, Hawa; et al
Nature (London, United Kingdom) (2014), 514(7520), E2 | Language: English, Database: CAplus and MEDLINE

Substances (5) Reactions (0) Cited By (16)

22

GSK-J4 induces cell cycle arrest and apoptosis via ER stress and the synergism between GSK-J4 and decitabine in acute myeloid leukemia KG-1a cells

By: Chu, Xuan; Zhong, Liang; Yu, Lihua; Xiong, Ling; Li, Jian; Dan, Wenran; Ye, Jiao; Liu, Chen; Luo, Xu; Liu, Beizhong
Cancer Cell International (2020), 20(1), 209 | Language: English, Database: CAplus and MEDLINE

Substances (6) Reactions (0) Cited By (9)

23

Radiosensitization by histone H3 Demethylase inhibition in diffuse intrinsic pontine glioma

By: Katagi, Hiroaki; Louis, Nundia; Unruh, Dusten; Sasaki, Takahiro; He, Xingyao; Zhang, Ali; Ma, Quanhong; Piunti, Andrea; Shimazu, Yosuke; Lamano, Jonathan B.; et al
Clinical Cancer Research (2019), 25(18), 5572-5583 | Language: English, Database: CAplus and MEDLINE

Substances (2) Reactions (0) Cited By (28)

24

RNA sequencing reveals resistance of TLR4 ligand-activated microglial cells to inflammation mediated by the selective jumonji H3K27 demethylase inhibitor

By: Das, Amitabh; Arifuzzaman, Sarder; Yoon, Taeho; Kim, Sun Hwa; Chai, Jin Choul; Lee, Young Seek; Jung, Kyoung Hwa; Chai, Young Gyu
Scientific Reports (2017), 7(1), 1-19 | Language: English, Database: CAplus and MEDLINE

Substances (9) Reactions (0) Cited By (23)

25

KDM6B is an androgen regulated gene and plays oncogenic roles by demethylating H3K27me3 at cyclin D1 promoter in prostate cancer

By: Cao, Zhi ; Shi, Xiaolei; Tian, Feng; Fang, Yu; Wu, Jason Boyang ; Mrdenovic, Stefan; Nian, Xinwen; Ji, Jin; Xu, Huan; Kong, Chen; et al
Cell Death & Disease (2021), 12(1), 2 | Language: English, Database: CAplus and MEDLINE

Substances (5) Reactions (0) Cited By (16)

26

Role of JMJD3 Demethylase and Its Inhibitor GSK-J4 in Regulation of MGMT, TRA2A, RPS6KA2, and U2AF1 Genes in Prostate Cancer Cell Lines

By: Sanchez, Anna; El Ouardi, Driss; Houfaf Khoufaf, Fatma Zohra; Idrissou, Mouhamed; Boisnier, Tiphanie; Penault-Llorca, Frederique; Bignon, Yves-Jean; Guy, Laurent; Bernard-Gallon, Dominique
OMICS (2020), 24(8), 505-507 | Language: English, Database: CAplus and MEDLINE

Substances (3) Reactions (0) Cited By (4)

27

Jmjd3 is involved in the susceptibility to depression induced by maternal separation via enhancing the neuroinflammation in the prefrontal cortex and hippocampus of male rats

By: Wang, Rui; Wang, Wei; Xu, Jingjing; Liu, Dexiang; Wu, Huiran; Qin, Xiaqing; Jiang, Hong; Pan, Fang
Experimental Neurology (2020), 328, 113254 | Language: English, Database: CAplus and MEDLINE

Substances (3) Reactions (0) Cited By (22)

28

HDAC8 prevents anthrax lethal toxin-induced cell cycle arrest through silencing PTEN in human monocytic THP-1 cells

By: Ha, Soon-Duck; Cho, Woohyun; Kim, Sung Ouk
Toxins (2017), 9(5), 162/1-162/15 | Language: English, Database: CAplus and MEDLINE

Substances (3) Reactions (0) Cited By (12)

29

Inhibition of H3K27me3-specific histone demethylases JMJD3 and UTX blocks reactivation of herpes simplex virus 1 in trigeminal ganglion neurons

By: Messer, Harald G. P.; Jacobs, Derek; Dhummakupt, Adit; Bloom, David C.
Journal of Virology (2015), 89(6), 3417-3420 | Language: English, Database: CAplus and MEDLINE

Substances (3) Reactions (0) Cited By (34)

30

The histone demethylase KDM6B fine-tunes the host response to Streptococcus pneumoniae

By: Connor, Michael G.; Camarasa, Tiphaine M. N.; Patey, Emma; Rasid, Orhan; Barrio, Laura; Weight, Caroline M.; Miller, Daniel P.; Heyderman, Robert S.; Lamont, Richard J.; Enninga, Jost; et al
Nature Microbiology (2021), 6(2), 257-269 | Language: English, Database: CAplus and MEDLINE

Substances (4) Reactions (0) Cited By (7)

31

Histone H3K27 demethylase negatively controls the memory formation of antigen-stimulated CD8 $^{+}$ T cells

By: Yamada, Takeshi; Nabe, Shogo; Toriyama, Koji; Suzuki, Junpei; Inoue, Kazuki; Imai, Yuuki; Shiraishi, Atsushi; Takenaka, Katsuto; Yasukawa, Masaki; Yamashita, Masakatsu
Journal of Immunology (2019), 202(4), 1088-1098, S1-S4 | Language: English, Database: CAplus and MEDLINE

Substances (4) Reactions (0) Cited By (10)

32

UTX-1 regulates Tat-induced HIV-1 transactivation via changing the methylated status of histone H3

By: Zhang, Hong-Sheng; Du, Guang-Yuan; Liu, Yang; Zhang, Zhong-Guo; Zhou, Zhen; Li, Hu; Dai, Ke-Qing; Yu, Xiao-Ying; Gou, Xiao-Meng
International Journal of Biochemistry & Cell Biology (2016), 80, 51-56 | Language: English, Database: CAplus and MEDLINE

Substances (4) Reactions (0) Cited By (8)

33

Massively parallel combinatorial genetics for CRISPR

By: Lu, Timothy Kuan-Ta; Wong, Alan Siu Lun; Choi, Ching Gee
World Intellectual Property Organization, WO2016070037 A2 2016-05-06 | Language: English, Database: CAplus

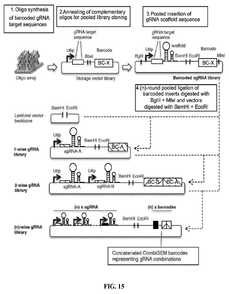

PatentPak available

Substances (4) | Reactions (0) | Cited By (5)

34

Inhibition of H3K27me3 demethylases promotes plasmablast formation

By: Kania, Anna K. ; Guo, Muyao ; Scharer, Christopher D. ; Boss, Jeremy M.
ImmunoHorizons (2021), 5(12), 918-930 | Language: English, Database: CAplus and MEDLINE

Substance (1) | Reactions (0) | Cited By (0)

35

H3K27me3 loss plays a vital role in CEMIP mediated carcinogenesis and progression of breast cancer with poor prognosis

By: Hsieh, I-yun; He, Jincan; Wang, Li ; Lin, Bo; Liang, Zhen; Lu, Bing; Chen, Weixin; Lu, Guohao; Li, Fuxi; Lv, Weiming; et al
Biomedicine & Pharmacotherapy (2020), 123, 109728 | Language: English, Database: CAplus and MEDLINE

Substance (1) | Reactions (0) | Cited By (40)

36

Jumonji domain containing-3 (JMJD3) inhibition attenuates IL-1β-induced chondrocytes damage in vitro and protects osteoarthritis cartilage in vivo

By: Zhou, Jun; Jin, Xinmeng; Yue, Liu; Zhi, Wang; Yan, Zhang; Yang, Tieyi; Tang, Jiangan
Inflammation Research (2020), 69(7), 657-666 | Language: English, Database: CAplus and MEDLINE

Substances (5) | Reactions (0) | Cited By (6)

37

The antischistosomal potential of GSK-J4, an H3K27 demethylase inhibitor: insights from molecular modeling, transcriptomics and in vitro assays

By: Lobo-Silva, Jessica; Cabral, Fernanda J.; Amaral, Murilo S.; Miyasato, Patricia A.; de Freitas, Rafaela Paula; Pereira, Adriana S. A.; Khouri, Mariana I.; Barbosa, Mayra M. F.; Ramos, Pablo I. P.; Leite, Luciana C. C.; et al
Parasites & Vectors (2020), 13(1), 140 | Language: English, Database: CAplus and MEDLINE

Substances (5) | Reactions (0) | Cited By (10)

38

Inhibition of H3K27me3 demethylases attenuates asthma by reversing the shift in airway smooth muscle phenotype

By: Yu, Qijun; Yu, Xiaowei; Zhao, Wenxue; Zhu, Manni; Wang, Zhengxia; Zhang, Jiaxiang; Huang, Mao ; Zeng, Xiaoning
Clinical & Experimental Allergy (2018), 48(11), 1439-1452 | Language: English, Database: CAplus and MEDLINE

Substances (5) | Reactions (0) | Cited By (11)

39

KDM2B promotes cell viability by enhancing DNA damage response in canine hemangiosarcoma

By: Gulay, Kevin Christian Montecillo ; Aoshima, Keisuke ; Shibata, Yuki ; Yasui, Hironobu ; Yan, Qin ; Kobayashi, Atsushi; Kimura, Takashi
Journal of Genetics and Genomics (2021), 48(7), 618-630 | Language: English, Database: CAplus and MEDLINE

Substances (2) | Reactions (0) | Cited By (6)

40

Application of H3K27me3 capable of regulating RUNX1 transcription in synthesis of steroid hormone in porcine ovarian granulosa cells

By: Zhong, Yuyi; Li, Jiaqi; Yuan, Xiaolong; Zhang, Zhe; Yun, Bing
China, CN111996194 A 2020-11-27 | Language: Chinese, Database: CAplus

PatentPak available

Substances (2) | Reactions (0) | Cited By (0)

41

Antiproliferative effect of the histone demethylase inhibitor GSK-J4 in chondrosarcomas

By: Lhuissier, Eva; Aury-Landas, Juliette; Allas, Lyess; Boittin, Martine; Boumediene, Karim; Bauge, Catherine
IUBMB Life (2019), 71(11), 1711-1719 | Language: English, Database: CAplus and MEDLINE

Substances (2) | Reactions (0) | Cited By (5)

42

Inhibition of the Histone H3K27 Demethylase UTX Enhances Tumor Cell Radiosensitivity

By: Rath, Barbara H.; Waung, Isabella; Camphausen, Kevin; Tofilon, Philip J.
Molecular Cancer Therapeutics (2018), 17(5), 1070-1078 | Language: English, Database: CAplus and MEDLINE

Substances (2) Reactions (0) Cited By (22)

43

Oncogenic KRAS sensitizes lung adenocarcinoma to GSK-J4-Induced metabolic and oxidative stress

By: Hong, Beom-Jin; Park, Woo-Yong; Kim, Hwa-Ryeon; Moon, Jin Woo; Lee, Ho Yeon; Park, Jun Hyung; Kim, Seon-Kyu; Oh, Youngbin; Roe, Jae-Seok; Kim, Mi-Young
Cancer Research (2019), 79(22), 5849-5859 | Language: English, Database: CAplus and MEDLINE

Substances (11) Reactions (0) Cited By (16)

44

The demethylase inhibitor GSK-J4 limits inflammatory colitis by promoting de novo synthesis of retinoic acid in dendritic cells

By: Donas, Cristian; Neira, Jocelyn; Osorio-Barrios, Francisco; Carrasco, Macarena; Fernandez, Dominique; Prado, Carolina; Loyola, Alejandra; Pacheco, Rodrigo; Rosemblatt, Mario
Scientific Reports (2021), 11(1), 1342 | Language: English, Database: CAplus and MEDLINE

Substances (7) Reactions (0) Cited By (5)

45

Compositions and methods for treating drug-tolerant glioblastoma

By: Liau, Brian B.; Patel, Anoop P.; Sievers, Cem; Bernstein, Bradley E.
World Intellectual Property Organization, WO2017099829 A1 2017-06-15 | Language: English, Database: CAplus

PatentPak available

Substances (7) Reactions (0) Cited By (2)

46

Lysyl oxidase expression is regulated by the H3K27 demethylase Jmjd3 in tumor-associated M2-like macrophages

By: Takemoto, Ryuhei; Kamiya, Tetsuro; Hara, Hirokazu; Adachi, Tetsuo
Journal of Clinical Biochemistry and Nutrition (2020), 66(2), 110-115 | Language: English, Database: CAplus and MEDLINE

Substances (8) Reactions (0) Cited By (4)

47

New BRAF knockin mice provide a pathogenetic mechanism of developmental defects and a therapeutic approach in cardio-facio-cutaneous syndrome

By: Inoue, Shin-ichi; Moriya, Mitsuji; Watanabe, Yusuke; Miyagawa-Tomita, Sachiko; Niihori, Tetsuya; Oba, Daiju; Ono, Masao; Kure, Shigeo; Ogura, Toshihiko; Matsubara, Yoichi; et al
Human Molecular Genetics (2014), 23(24), 6553-6566 | Language: English, Database: CAplus and MEDLINE

Substances (8) | Reactions (0) | Cited By (40)

48

Inhibition of histone H3 lysine-27 demethylase activity relieves rheumatoid arthritis symptoms via repression of IL6 transcription in macrophages

By: Zhao, Zhan; Zhang, Yazhuo; Gao, Danling; Zhang, Yidan; Han, Wenwei; Xu, Ximing; Song, Qiaoling; Zhao, Chenyang; Yang, Jinbo
Frontiers in Immunology (2022), 13, 818070 | Language: English, Database: CAplus and MEDLINE

Substances (3) | Reactions (0) | Cited By (1)

49

Lysine demethylase 6B regulates prostate cancer cell proliferation by controlling c-MYC expression

By: Yildirim-Buharalioglu, Gokce
Molecular Pharmacology (2022), 101(2), 106-119 | Language: English, Database: CAplus and MEDLINE

Substances (3) | Reactions (0) | Cited By (3)

50

Combined action of FOXO1 and superoxide dismutase 3 promotes MDA-MB-231 cell migration

By: Kamiya, Tetsuro ; Yamaguchi, Yuji; Oka, Manami; Hara, Hirokazu
Free Radical Research (2022), 56(1), 106-114 | Language: English, Database: CAplus and MEDLINE

Substances (3) | Reactions (0) | Cited By (1)

51

Nuclear pore protein NUP210 depletion suppresses metastasis through heterochromatin-mediated disruption of tumor cell mechanical response

By: Amin, Ruhul ; Shukla, Anjali; Zhu, Jacqueline Jufen ; Kim, Sohyoung ; Wang, Ping; Tian, Simon Zhongyuan; Tran, Andy D. ; Paul, Debasish; Cappell, Steven D. ; Burkett, Sandra ; et al
Nature Communications (2021), 12(1), 7216 | Language: English, Database: CAplus and MEDLINE

Substances (3) | Reactions (0) | Cited By (6)

52

Mucosal-associated invariant T cell effector function is an intrinsic cell property that can be augmented by the metabolic cofactor a-ketoglutarate

By: Howson, Lauren J. ; Li, Jasmine; von Borstel, Anouk ; Barugahare, Adele ; Mak, Jeffrey Y. W. ; Fairlie, David P. ; McCluskey, James ; Turner, Stephen J. ; Davey, Martin S. ; Rossjohn, Jamie
Journal of Immunology (2021), 206(7), 1425-1440 | Language: English, Database: CAplus and MEDLINE

Substances (3) Reactions (0) Cited By (3)

53

Upregulation of KDM6B contributes to lipopolysaccharide-induced anxiety-like behavior via modulation of VGLL4 in mice

By: Shentu, Yangping; Tian, Qiuyun; Yang, Jinge; Liu, Xiaoyuan; Han, Yujiao; Yang, Dichen; Zhang, Nan; Fan, Xiaofang; Wang, Ping; Ma, Jianshe; et al
Behavioural Brain Research (2021), 408, 113305 | Language: English, Database: CAplus and MEDLINE

Substances (3) Reactions (0) Cited By (3)

54

P53 affects epigenetic signature on SOCS1 promoter in response to TLR4 inhibition

By: Sheikh, Touseef; Sen, Ellora
Cytokine+ (2021), 140, 155418 | Language: English, Database: CAplus and MEDLINE

Substances (3) Reactions (0) Cited By (5)

55

Application of H3K27me3 capable of regulating Ssc-miR-143-3p transcription in synthesis of steroid hormone in porcine ovarian granulosa cells

By: Zhong, Yuyi; Yuan, Xiaolong; Li, Jiaqi; Zhang, Zhe; Li, Nian
China, CN111979242 A 2020-11-24 | Language: Chinese, Database: CAplus

PatentPak available

Substances (3) Reactions (0) Cited By (0)

56

Nanoparticle enhanced combination therapy for stem-like progenitors defined by single-cell transcriptomics in chemotherapy-resistant osteosarcoma

By: Wang, Li; Huang, Xiaojia; You, Xinru; Yi, Tianqi; Lu, Bing; Liu, Jiali; Lu, Guohao; Ma, Minglin; Zou, Changye; Wu, Jun; et al
Signal Transduction and Targeted Therapy (2020), 5(1), 196 | Language: English, Database: CAplus and MEDLINE

Substances (3) Reactions (0) Cited By (17)

57

H19-dependent transcriptional regulation of β3 and β4 integrins upon estrogen and hypoxia favors metastatic potential in prostate cancer

By: Bacci, Lorenza; Aiello, Aurora; Ripoli, Cristian; Loria, Rossella; Pugliese, Dario; Pierconti, Francesco; Rotili, Dante; Strigari, Lidia; Pinto, Francesco; Bassi, Pier Francesco; et al
International Journal of Molecular Sciences (2019), 20(16), 4012 | Language: English, Database: CAplus and MEDLINE

Substances (3) Reactions (0) Cited By (14)

58

Histone demethylase KDM6B regulates human podocyte differentiation in vitro

By: Guo, Yanyan; Xiong, Zuying; Guo, Xiaoqiang
Biochemical Journal (2019), 476(12), 1741-1751 | Language: English, Database: CAplus and MEDLINE

Substances (3) Reactions (0) Cited By (3)

59

Application of micromolecular inhibitor for chemosensitivity of colorectal cancer

By: Jiang, Yuhang
China, CN109498629 A 2019-03-22 | Language: Chinese, Database: CAplus

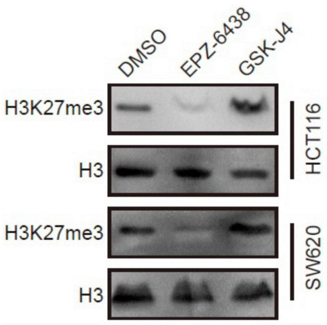

PatentPak available

Substances (3) Reactions (0) Cited By (0)

60

Pharmaceutical composition containing apatinib with synergistic anti-osteosarcoma effect and its application

By: Zhao, Wei; Zou, Changye
China, CN108186643 A 2018-06-22 | Language: Chinese, Database: CAplus

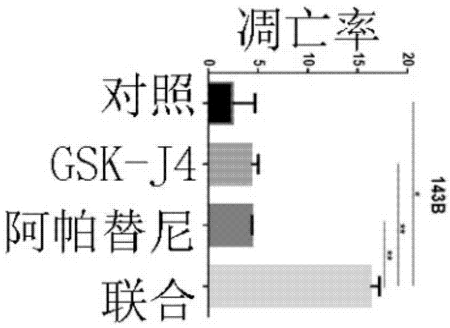

PatentPak available

Substances (3) | Reactions (0) | Cited By (0)

61

KDM6B promotes activation of the oncogenic CDK4/6-pRB-E2F pathway by maintaining enhancer activity in MYCN-amplified neuroblastoma

By: D'Oto, Alexandra; Fang, Jie; Jin, Hongjian; Xu, Beisi ; Singh, Shivendra; Mullasseril, Anoushka; Jones, Victoria; Abu-Zaid, Ahmed; von Buttlar, Xinyu; Cooke, Bailey; et al
Nature Communications (2021), 12(1), 7204 | Language: English, Database: CAplus and MEDLINE

Substances (9) | Reactions (0) | Cited By (6)

62

Histone lysine demethylase inhibition reprograms prostate cancer metabolism and mechanics

By: Chianese, Ugo ; Papulino, Chiara; Passaro, Eugenia; Evers, Tom MJ.; Babaei, Mehrad; Toraldo, Antonella; De Marchi, Tommaso; Nimeus, Emma; Carafa, Vincenzo; Nicoletti, Maria Maddalena; et al
Molecular Metabolism (2022), 64, 101561 | Language: English, Database: CAplus and MEDLINE

Substances (4) | Reactions (0) | Cited By (0)

63

KDM6B epigenetically regulated-interleukin-6 expression in the dorsal root ganglia and spinal dorsal horn contributes to the development and maintenance of neuropathic pain following peripheral nerve injury in male rats

By: Li, Liren; Bai, Liying; Yang, Kangli; Zhang, Jian; Gao, Yan; Jiang, Mingjun; Yang, Yin; Zhang, Xuan; Wang, Li; Wang, Xueli; et al
Brain, Behavior, and Immunity (2021), 98, 265-282 | Language: English, Database: CAplus and MEDLINE

Substances (4) | Reactions (0) | Cited By (3)

64

X-linked histone H3K27 demethylase Kdm6a regulates sexually dimorphic differentiation of hypothalamic neurons

By: Cabrera Zapata, Lucas E. ; Cisternas, Carla D. ; Sosa, Camila ; Garcia-Segura, Luis Miguel ; Arevalo, Maria Angeles ; Cambiasso, Maria Julia
Cellular and Molecular Life Sciences (2021), 78(21-22), 7043-7060 | Language: English, Database: CAplus and MEDLINE

Substances (4) Reactions (0) Cited By (4)

65

Targeted inhibition of KDM6 histone demethylases eradicates tumor-initiating cells via enhancer reprogramming in colorectal cancer

By: Zhang, Junbao; Ying, Ying; Li, Meiqi; Wang, Maolin; Huang, Xiaoyan; Jia, Min; Zeng, Junhui; Ma, Canjie; Zhang, Yixiang; Li, Chen; et al
Theranostics (2020), 10(22), 10016-10030 | Language: English, Database: CAplus and MEDLINE

Substances (4) Reactions (0) Cited By (10)

66

Dual regulation of histone methylation by mTOR complexes controls glioblastoma tumor cell growth via EZH2 and SAM

By: Harachi, Mio; Masui, Kenta; Honda, Hiroaki; Muragaki, Yoshihiro; Kawamata, Takakazu; Cavenee, Webster K.; Mischel, Paul S.; Shibata, Noriyuki
Molecular Cancer Research (2020), 18(8), 1142-1152 | Language: English, Database: CAplus and MEDLINE

Substances (4) Reactions (0) Cited By (12)

67

Palmitate-TLR4 signaling regulates the histone demethylase, JMJD3, in macrophages and impairs diabetic wound healing

By: Davis, Frank M. ; denDekker, Aaron; Joshi, Amrita D.; Wolf, Sonya J.; Audu, Christopher; Melvin, William J.; Mangum, Kevin; Riordan, Mary O.; Kunkel, Steven L.; Gallagher, Katherine A.
European Journal of Immunology (2020), 50(12), 1929-1940 | Language: English, Database: CAplus and MEDLINE

Substances (4) Reactions (0) Cited By (18)

68

Application of JMJD3 inhibitor in preparing medicine for preventing and treating pancreatitis

By: Wei, Xiawei; Zhang, Xiangxian; Wei, Yuquan
China, CN110339198 A 2019-10-18 | Language: Chinese, Database: CAplus

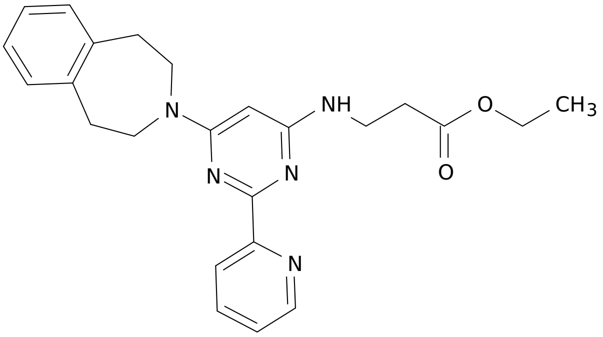

I

PatentPak available

Substances (4) Reactions (0) Cited By (0)

69

Targeting P16INK4A in uterine serous carcinoma through inhibition of histone demethylation

By: Xiao, Zhen; He, Yingying; Liu, Chongya; Xiang, Lin; Yi, Jingyan; Wang, Min; Shen, Tingting; Shen, Lanlin; Xue, Yijue; Shi, Hong; et al
Oncology Reports (2019), 41(5), 2667-2678 | Language: English, Database: CAplus and MEDLINE

Substances (4) Reactions (0) Cited By (3)

70

Critical role of histone demethylase Jumonji domain-containing protein 3 in the regulation of neointima formation following vascular injury

By: Luo, XiaoLing; Yang, Di; Wu, WeiJun; Long, Fen; Xiao, ChenXi; Qin, Ming; Law, Betty YuenKwan; Suguro, Rinkiko; Xu, Xin; Qu, LeFeng; et al
Cardiovascular Research (2018), 114(14), 1894-1906 | Language: English, Database: CAplus and MEDLINE

Substances (4) Reactions (0) Cited By (24)

71

KDM6B counteeacts EZH2-mediated suppression of IDFBP5 to confer resistance to PI3K/AKT inhibitor treatment in breast cancer

By: Wang, Wenyu; Lim, Keng Gat; Feng, Min; Bao, Yi; Lee, Puay Leng; Cai, Yu; Chen, Yufeng; Zhang, Hao; Marzese, Diego; Hoon, Dave S. B.; et al
Molecular Cancer Therapeutics (2018), 17(9), 1973-1983 | Language: English, Database: CAplus and MEDLINE

Substances (10) Reactions (0) Cited By (29)

72

Forskolin sensitizes human acute myeloid leukemia cells to H3K27me2/3 demethylases GSKJ4 inhibitor via protein kinase A

By: Illiano, Michela; Conte, Mariarosaria; Sapio, Luigi; Nebbioso, Angela; Spina, Annamaria; Altucci, Lucia; Naviglio, Silvio
Frontiers in Pharmacology (2018), 9, 792/1-792/9 | Language: English, Database: CAplus and MEDLINE

Substances (11) Reactions (0) Cited By (11)

73

The Histone Demethylase Inhibitor GSK-J4 Is a Therapeutic Target for the Kidney Fibrosis of Diabetic Kidney Disease via DKK1 Modulation

By: Hung, Peir-Haur; Hsu, Yung-Chien; Chen, Tsung-Hsien; Ho, Cheng; Lin, Chun-Liang
International Journal of Molecular Sciences (2022), 23(16), 9407 | Language: English, Database: CAplus and MEDLINE

Substances (5) Reactions (0) Cited By (0)

74

A KDM6 inhibitor potently induces ATF4 and its target gene expression through HRI activation and by UTX inhibition

By: Kitajima, Shojiro; Sun, Wendi; Lee, Kian Leong; Ho, Jolene Caifeng; Oyadomari, Seiichi; Okamoto, Takashi; Masai, Hisao; Poellinger, Lorenz; Kato, Hiroyuki
Scientific Reports (2021), 11(1), 4538 | Language: English, Database: CAplus and MEDLINE

Substances (5) Reactions (0) Cited By (0)

75

Small molecule for specifically regulating iron metabolism in nerve cell and application thereof

By: Ke, Ya; Mo, Mingdao; Qian, Zhongming
China, CN110063959 A 2019-07-30 | Language: Chinese, Database: CAplus

PatentPak available

Substances (5) Reactions (0) Cited By (0)

76

H3K27 demethylase inhibitors in pediatric and juvenile osteoporosis

By: Wan, Mei; Cao, Xu
World Intellectual Property Organization, WO2019046378 A1 2019-03-07 | Language: English, Database: CAplus

PatentPak available

Substances (5) Reactions (0) Cited By (0)

77

Micelle drug delivery system for targeting epigenetic modification site and preparation method thereof

By: Mo, Jingxin; Wei, Lili; Chen, Wei
China, CN108553418 A 2018-09-21 | Language: Chinese, Database: CAplus

PatentPak available

Substances (5) Reactions (0) Cited By (0)

78

Epigenetic drug histone demethylase inhibitor GSK-J4 for treating osteosarcoma

By: Zhao, Wei; Zou, Changye; Lu, Bing
China, CN108464984 A 2018-08-31 | Language: Chinese, Database: CAplus

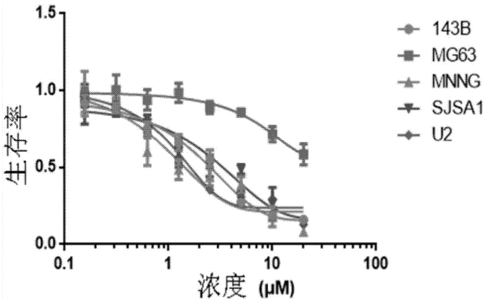

PatentPak available

Substances (5) Reactions (0) Cited By (0)

79

The Memory of Environmental Chemical Exposure in C. elegans Is Dependent on the Jumonji Demethylases jmjd-2 and jmjd-3/utx-1

By: Camacho, Jessica; Truong, Lisa; Kurt, Zeyneb; Chen, Yen-Wei; Morselli, Marco; Gutierrez, Gerardo; Pellegrini, Matteo; Yang, Xia; Allard, Patrick
Cell Reports (2018), 23(8), 2392-2404 | Language: English, Database: CAplus and MEDLINE

Substances (5) Reactions (0) Cited By (31)

80

Pharmaceutical compositions and methods for identification, assessment, prevention, and treatment of cancer using histone H3K27me2 biomarkers and modulators

By: Lane, Andrew; Weinstock, David

World Intellectual Property Organization, WO2014190035 A2 2014-11-27 | Language: English, Database: CAplus

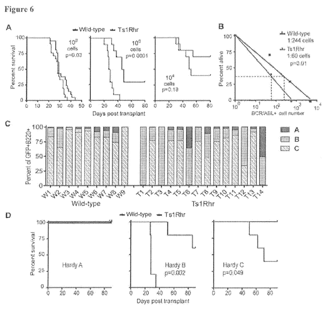

PatentPak available

Substances (5) | Reactions (0) | Cited By (0)

81

Cooperation between KDM6B overexpression and TET2 deficiency in the pathogenesis of chronic myelomonocytic leukemia

By: Wei, Yue; Kanagal-Shamanna, Rashmi ; Zheng, Hong; Bao, Naran; Lockyer, Pamela Pennington; Class, Caleb A. ; Darbaniyan, Faezeh; Lu, Yue; Lin, Kevin; Yang, Hui; et al

Leukemia (2022), 36(8), 2097-2107 | Language: English, Database: CAplus and MEDLINE

Substances (6) | Reactions (0) | Cited By (0)

82

Inhibition of the H3K27 demethylase UTX enhances the epigenetic silencing of HIV proviruses and induces HIV-1 DNA hypermethylation but fails to permanently block HIV reactivation

By: Nguyen, Kien ; Dobrowolski, Curtis ; Shukla, Meenakshi; Cho, Won-Kyung; Luttge, Benjamin ; Karn, Jonathan

PLoS Pathogens (2021), 17(10), e1010014 | Language: English, Database: CAplus and MEDLINE

Substances (6) | Reactions (0) | Cited By (8)

83

A unique bipartite Polycomb signature regulates stimulus-response transcription during development

By: Kitazawa, Taro; Machlab, Dania ; Joshi, Onkar; Maiorano, Nicola; Kohler, Hubertus; Ducret, Sebastien; Kessler, Sandra; Gezelius, Henrik; Soneson, Charlotte; Papasaikas, Panagiotis ; et al

Nature Genetics (2021), 53(3), 379-391 | Language: English, Database: CAplus and MEDLINE

Substances (6) | Reactions (0) | Cited By (8)

84

LncRNA H19 regulates macrophage polarization and promotes Freund's complete adjuvant-induced arthritis by upregulating KDM6A

By: Zhu, Xiaodong; Zhu, Ye; Ding, Chen; Zhang, Weiting; Guan, Huilin; Li, Chunmei; Lin, Xiao; Zhang, Yang; Huang, Chunyan; Zhang, Luyao; et al
International Immunopharmacology (2021), 93, 107402 | Language: English, Database: CAplus and MEDLINE

Substances (6) Reactions (0) Cited By (10)

85

Unlocking the Recovery Potential: JMJD3 Inhibition-Mediated SAPK/JNK Signaling Inactivation Supports Endogenous Oligodendrocyte-Lineage Commitment Post Mammalian Spinal Cord Injury

By: Zhang, Bo-Yin; Zhu, Qingsan; Ma, Yihang; Fan, Yang; Zhu, Yuhang; Chang, Pengyu
Neurochemical Research (2021), 46(4), 792-803 | Language: English, Database: CAplus and MEDLINE

Substances (6) Reactions (0) Cited By (0)

86

JMJD3 inhibition protects against isoproterenol-induced cardiac hypertrophy by suppressing β-MHC expression

By: Guo, Zhen; Lu, Jing ; Li, Jingyan; Wang, Panxia; Li, Zhenzhen; Zhong, Yao; Guo, Kaiteng; Wang, Junjian; Ye, Jiantao; Liu, Peiqing
Molecular and Cellular Endocrinology (2018), 477, 1-14 | Language: English, Database: CAplus and MEDLINE

Substances (6) Reactions (0) Cited By (17)

87

Drug-induced epigenetic remodeling to prevent fibrosis

By: Walker, Janice L.; Mazo, Alexander; Petruk, Svetlana; Menko, Sue A.
World Intellectual Property Organization, WO2018058034 A1 2018-03-29 | Language: English, Database: CAplus

PatentPak available

Substances (6) Reactions (0) Cited By (0)

88

Effects of GSK-J4 on JMJD3 histone demethylase in mouse prostate cancer xenografts

By: Sanchez, Anna; Penault-Llorca, Frederique; Bignon, Yves-Jean; Guy, Laurent; Bernard-Gallon, Dominique
Cancer Genomics & Proteomics (2022), 19(3), 339-349 | Language: English, Database: CAplus and MEDLINE

Substances (7) Reactions (0) Cited By (0)

89

HMGN1 plays a significant role in CRLF2 driven Down Syndrome leukemia and provides a potential therapeutic target in this high-risk cohort

By: Page, Elyse C.; Heatley, Susan L.; Eadie, Laura N.; McClure, Barbara J.; de Bock, Charles E.; Omari, Sofia; Yeung, David T.; Hughes, Timothy P.; Thomas, Paul Q.; White, Deborah L.
Oncogene (2022), 41(6), 797-808 | Language: English, Database: CAplus and MEDLINE

Substances (7) Reactions (0) Cited By (2)

90

Methods for treating glioblastoma with combination of KDM6B inhibitor and immunotherapy

By: Goswami, Sangeeta; Sharma, Padmanee; Allison, James
World Intellectual Property Organization, WO2021231405 A1 2021-11-18 | Language: English, Database: CAplus

PatentPak available

Substances (7) Reactions (0) Cited By (0)

91

An IFNγ/STAT1/JMJD3 axis induces ZEB1 expression and promotes aggressiveness in lung adenocarcinoma

By: Yang, Jianjian; Wang, Xue; Huang, Bing; Liu, Rong; Xiong, Hui; Ye, Fan; Zeng, Chenxi; Fu, Xiangning; Li, Lequn
Molecular Cancer Research (2021), 19(7), 1234-1246 | Language: English, Database: CAplus and MEDLINE

Substances (7) Reactions (0) Cited By (2)

92

SMARCA4 deficient tumours are vulnerable to KDM6A/UTX and KDM6B/JMJD3 blockade

By: Romero, Octavio A.; Vilarrubi, Andrea; Alburquerque-Bejar, Juan J.; Gomez, Antonio; Andrades, Alvaro; Trastulli, Deborah; Pros, Eva; Setien, Fernando; Verdura, Sara; Farre, Lourdes; et al
Nature Communications (2021), 12(1), 4319 | Language: English, Database: CAplus and MEDLINE

Substances (7) Reactions (0) Cited By (9)

93

KDM subfamily 6 protein inhibitor for use in the treatment of cancer

By: Sanchez Cespedes, Montse; Romero Ferraro, Octavio
World Intellectual Property Organization, WO2021105224 A1 2021-06-03 | Language: English, Database: CAplus

PatentPak available

Substances (7) Reactions (0) Cited By (0)

94

Jmjd3 Mediates Neuropathic Pain by Inducing Macrophage Infiltration and Activation in Lumbar Spinal Stenosis Animal Model

By: Lee, Jeeyoun; Choi, Haeyoung; Park, Chansol; Jeon, Sangryong; Yune, Taeyoung
International Journal of Molecular Sciences (2021), 22(24), 13426 | Language: English, Database: CAplus and MEDLINE

Substances (8) Reactions (0) Cited By (2)

95

Pharmaceutical interference of the EWS-FLI1-driven transcriptome by cotargeting H3K27ac and RNA polymerase activity in ewing sarcoma

By: Heisey, Daniel A. R.; Jacob, Sheeba; Lochmann, Timothy L.; Kurupi, Richard; Ghotra, Maninderjit S.; Calbert, Marissa L.; Shende, Mayuri; Maves, Yuki Kato; Koblinski, Jennifer E.; Dozmorov, Mikhail G.; et al
Molecular Cancer Therapeutics (2021), 20(10), 1868-1879 | Language: English, Database: CAplus and MEDLINE

Substances (8) Reactions (0) Cited By (4)

96

The inhibitors of KDM4 and KDM6 histone lysine demethylases enhance the anti-growth effects of erlotinib and HS-173 in head and neck cancer cells

By: Kleszcz, Robert; Skalski, Marcin; Krajka-Kuzniak, Violetta; Paluszczak, Jaroslaw
European Journal of Pharmaceutical Sciences (2021), 166, 105961 | Language: English, Database: CAplus and MEDLINE

Substances (8) Reactions (0) Cited By (4)

97

Identification of disulfiram as a potential antifungal drug by screening small molecular libraries

By: Hao, Weifeng; Qiao, Dan; Han, Ying; Du, Ning; Li, Xuefen; Fan, Yufeng; Ge, Xuejun; Zhang, Heyu
Journal of Infection and Chemotherapy (2021), 27(5), 696-701 | Language: English, Database: CAplus and MEDLINE
Analytical Methods available

Substances (8) Reactions (0) Cited By (10)

98

SAPCD2 promotes neuroblastoma progression by altering the subcellular distribution of E2F7

By: Zhang, Zi-Mu ; Cao, Hai-Bo; Li, Zhi-Heng; Zhuo, Ran; Tao, Yan-Fang; Li, Xiao-Lu; Li, Gen; Liao, Xin-Mei; Fang, Fang; Xie, Yi; et al
Cell Death & Disease (2022), 13(2), 174 | Language: English, Database: CAplus and MEDLINE

Substances (9) Reactions (0) Cited By (1)

99

KDM6B-dependent chromatin remodeling underpins effective virus-specific CD8+ T cell differentiation

By: Li, Jasmine; Hardy, Kristine; Olshansky, Moshe ; Barugahare, Adele; Gearing, Linden J.; Prier, Julia E.; Sng, Xavier Y. X. ; Nguyen, Michelle Ly Thai; Piovesan, Dana; Russ, Brendan E.; et al
Cell Reports (2021), 34(11), 108839 | Language: English, Database: CAplus and MEDLINE

Substances (9) Reactions (0) Cited By (8)

100

Preparation method of drug carrier and application thereof in treating osteosarcoma

By: Zhao, Wei; Wu, Jun; Wang, Li
China, CN110922587 A 2020-03-27 | Language: Chinese, Database: CAplus

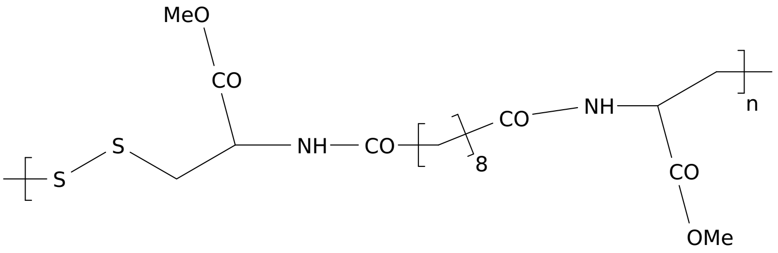

I

PatentPak available

Substances (9) Reaction (1) Cited By (0)

101

EZH2, JMJD3, and UTX epigenetically regulate hepatic plasticity inducing retro-differentiation and proliferation of liver cells

By: Pediconi, Natalia; Salerno, Debora; Lupacchini, Leonardo; Angrisani, Annapaola; Peruzzi, Giovanna ; De Smaele, Enrico ; Levrero, Massimo; Belloni, Laura
Cell Death & Disease (2019), 10(7), 1-13 | Language: English, Database: CAplus and MEDLINE

Substances (9) Reactions (0) Cited By (16)

102

Preparation of model mouse for CFC (cardiofaciocutaneous) syndrome and establishment of method for treating CFC syndrome

By: Inoue, Shinichi; Aoki, Yoko; Matsubara, Yoichi
Japan, JP2015181456 A 2015-10-22 | Language: Japanese, Database: CAplus

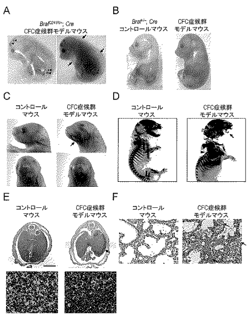

PatentPak available

Substances (9) | Reactions (0) | Cited By (0)

103

Method of predicting the tumor response to DNA methylation inhibitors and alternative therapeutic regimens for overcoming resistance

By: Agrawal, Khushboo; Dzubak, Petr; Hajduch, Marian; Frydrych, Ivo
European Patent Organization, EP2924125 A1 2015-09-30 | Language: English, Database: CAplus

PatentPak available

Substances (9) | Reactions (0) | Cited By (0)

104

Functionally defined therapeutic targets in diffuse intrinsic pontine glioma [Erratum to document cited in CA163:294035]

By: Grasso, Catherine S.; Tang, Yujie; Truffaux, Nathalene; Berlow, Noah E.; Liu, Lining; Debily, Marie-Anne; Quist, Michael J.; Davis, Lara E.; Huang, Elaine C.; Woo, Pamelyn J.; et al
Nature Medicine (New York, NY, United States) (2015), 21(7), 827 | Language: English, Database: CAplus and MEDLINE

Substances (9) | Reactions (0) | Cited By (73)

105

Inhibitors of Jumonji C domain-containing histone lysine demethylases overcome cisplatin and paclitaxel resistance in non-small cell lung cancer through APC/Cdh1-dependent degradation of CtIP and PAF15

By: Duan, Lei; Perez, Ricardo E.; Calhoun, Sarah; Maki, Carl G.
Cancer Biology & Therapy (2022), 23(1), 65-75 | Language: English, Database: CAplus and MEDLINE

Substances (10) | Reactions (0) | Cited By (1)

106

Disruption of the Plasmodium falciparum Life Cycle through Transcriptional Reprogramming by Inhibitors of Jumonji Demethylases

By: Matthews, Krista A.; Senagbe, Kossi M.; Notzel, Christopher; Gonzales, Christopher A.; Tong, Xinran; Rijo-Ferreira, Filipa; Bhanu, Natarajan V.; Miguel-Blanco, Celia; Lafuente-Monasterio, Maria Jose; Garcia, Benjamin A.; et al
ACS Infectious Diseases (2020), 6(5), 1058-1075 | Language: English, Database: CAplus and MEDLINE

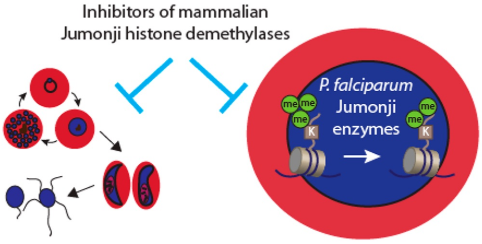

Substances (10) | Reactions (0) | Cited By (12)

107

Method and device for drug indication of acute myeloid leukemia

By: Guo, Anyuan; Lin, Shengyan; Chen, Zhichao; Miao, Yaru
China, CN107974506 A 2018-05-01 | Language: Chinese, Database: CAplus

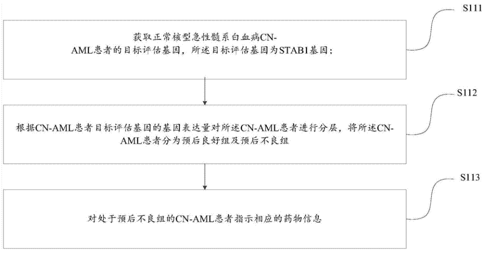

PatentPak available

Substances (10) | Reactions (0) | Cited By (0)

108

A transcriptomic dataset used to derive biomarkers of chemically induced histone deacetylase inhibition (HDACi) in human TK6 cells

By: Cho, Eunnara; Williams, Andrew; Yauk, Carole L.
Data in Brief (2021), 36, 107097 | Language: English, Database: CAplus and MEDLINE

Substances (11) | Reactions (0) | Cited By (0)

109

Histone Methylation: Achilles Heel and Powerful Mediator of Periodontal Homeostasis

By: Francis, M.; Gopinathan, G.; Foyle, D.; Fallah, P.; Gonzalez, M.; Luan, X.; Diekwisch, T. G. H.
Journal of Dental Research (2020), 99(12), 1332-1340 | Language: English, Database: CAplus and MEDLINE

Substances (11) | Reactions (0) | Cited By (9)

110

Structure of Nascent Chromatin Is Essential for Hematopoietic Lineage Specification

By: Petruk, Svetlana; Mariani, Samanta A.; De Dominici, Marco; Porazzi, Patrizia; Minieri, Valentina; Cai, Jingli; Iacovitti, Lorraine; Flomenberg, Neal; Calabretta, Bruno; Mazo, Alexander
Cell Reports (2017), 19(2), 295-306 | Language: English, Database: CAplus and MEDLINE

Substances (11) | Reactions (0) | Cited By (23)

111

Histone modification of pain-related gene expression in spinal cord neurons under a persistent postsurgical pain-like state by electrocautery

By: Katsuda, Yosuke; Tanaka, Kenichi; Mori, Tomohisa; Narita, Michiko; Takeshima, Hideyuki; Kondo, Takashige; Yamabe, Yoshiyuki; Matsufuji, Misa; Sato, Daisuke; Hamada, Yusuke; et al
Molecular Brain (2021), 14(1), 146 | Language: English, Database: CAplus and MEDLINE

Substances (12) | Reactions (0) | Cited By (0)

112

Activated Braf induces esophageal dilation and gastric epithelial hyperplasia in mice

By: Inoue, Shin-Ichi; Takahara, Shingo; Yoshikawa, Takeo; Niihori, Tetsuya; Yanai, Kazuhiko; Matsubara, Yoichi; Aoki, Yoko
Human Molecular Genetics (2017), 26(23), 4715-4727 | Language: English, Database: CAplus and MEDLINE

Substances (12) | Reactions (0) | Cited By (11)

113

Inhibition of H3K4 demethylation induces autophagy in cancer cell lines

By: Wang, Zhen; Long, Qiao-Yun; Chen, Lin; Fan, Jia-Dong; Wang, Zhao-Ning; Li, Lian-Yun; Wu, Min ; Chen, Xuefeng
Biochimica et Biophysica Acta, Molecular Cell Research (2017), 1864(12), 2428-2437 | Language: English, Database: CAplus and MEDLINE

Substances (12) | Reactions (0) | Cited By (20)

114

Methods of combinatorial drug screening and use of therapeutic targets thereof in cancer treatment

By: Bernstein, Bradley E.; Doench, John G.; Najm, Fadi J.

World Intellectual Property Organization, WO2020033585 A1 2020-02-13 | Language: English, Database: CAplus

PatentPak available

Substances (109) | Reactions (0) | Cited By (3)

115

Synergistic drug combinations predicted from genomic features and single-agent response profiles

By: Rees, Matthew; Johannessen, Cory

World Intellectual Property Organization, WO2019200224 A1 2019-10-17 | Language: English, Database: CAplus

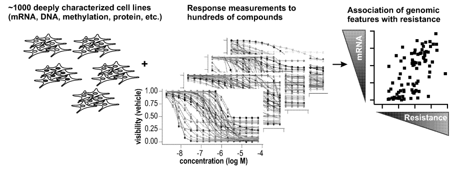

FIG. 1A

PatentPak available

Substances (134) | Reactions (0) | Cited By (3)

116

Treatment of cancers having alterations within the SWI/SNF chromatin remodeling complex

By: Fisher, David E.; Weng, Qing Yu; Kato, Shinichiro

World Intellectual Property Organization, WO2018049000 A1 2018-03-15 | Language: English, Database: CAplus

FIG. 2

Dose response of shControl vs shARID2 lines to G9a inhibitor

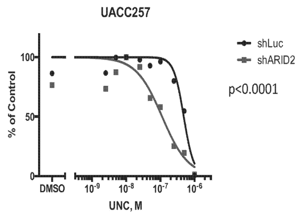

PatentPak available

Substances (46) | Reactions (0) | Cited By (1)

117

Use of Jumonji C demethylase inhibitors for the treatment and prevention of chemotherapy resistance and radioresistance in cancer

By: Dalvi, Maithili P.; Martinez, Elisabeth D.; Minna, John D.; Bayo-Fina, Juan; Das, Amit
World Intellectual Property Organization, WO2017190009 A1 2017-11-02 | Language: English, Database: CAplus

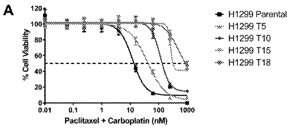

FIGS. 1A

PatentPak available

Substances (35) Reactions (0) Cited By (5)

118

The BRD9/7 Inhibitor TP-472 Blocks Melanoma Tumor Growth by Suppressing ECM-Mediated Oncogenic Signaling and Inducing Apoptosis

By: Mason, Lawrence David; Chava, Suresh; Reddi, Kiran Kumar; Gupta, Romi
Cancers (2021), 13(21), 5516 | Language: English, Database: CAplus and MEDLINE

Substances (13) Reactions (0) Cited By (5)

119

Forskolin potentiates the effects of GSKJ4 in human acute myeloid leukemia cells through protein kinase A pathway

By: Illiano, Michela; Conte, Mariarosaria; Sapio, Luigi; Nebbioso, Angela; Spina, Annamaria; Altucci, Lucia; Naviglio, Silvio
Pharmakeutike (2019), 31(2), 44-55 | Language: English, Database: CAplus

Substances (13) Reactions (0) Cited By (0)

120

Targeting histone acetylation dynamics and oncogenic transcription by catalytic P300/CBP inhibition

By: Hogg, Simon J.; Motorna, Olga; Cluse, Leonie A.; Johanson, Timothy M.; Coughlan, Hannah D.; Raviram, Ramya; Myers, Robert M.; Costacurta, Matteo; Todorovski, Izabela; Pijpers, Lizzy; et al
Molecular Cell (2021), 81(10), 2183-2200.e13 | Language: English, Database: CAplus and MEDLINE

Substances (13) Reactions (0) Cited By (22)

121

Systematic identification of biomarker-driven drug combinations to overcome resistance

By: Rees, Matthew G.; Brenan, Lisa; do Carmo, Mariana; Duggan, Patrick; Bajrami, Besnik; Arciprete, Michael; Boghossian, Andrew; Vaimberg, Emma; Ferrara, Steven J.; Lewis, Timothy A.; et al
Nature Chemical Biology (2022), 18(6), 615-624 | Language: English, Database: CAplus and MEDLINE

Substances (18) Reactions (0) Cited By (1)

122

Two old drugs, NVP-AEW541 and GSK-J4, repurposed against the Toxoplasma gondii RH strain

By: Liu, Shuxian; Wu, Mimi; Hua, Qianqian; Lu, Daiqiang; Tian, Yuan; Yu, Helin; Cheng, Linyan; Chen, Yinqi; Cao, Jiaxin; Hu, Xin; et al
Parasites & Vectors (2020), 13(1), 242 | Language: English, Database: CAplus and MEDLINE

Substances (18) Reactions (0) Cited By (6)

123

Methods of diagnosing and treating cancer with microRNAs

By: Chung, Leland W.K.; Wang, Fubo; Sun, Yinghao
World Intellectual Property Organization, WO2017193008 A1 2017-11-09 | Language: English, Database: CAplus

FIG. 1B

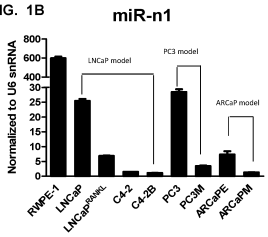

PatentPak available

Substances (18) Reactions (0) Cited By (0)

124

Increased H3K27 trimethylation contributes to cone survival in a mouse model of cone dystrophy

By: Miller, Annie L.; Fuller-Carter, Paula I.; Masarini, Klaudija; Samardzija, Marijana; Carter, Kim W.; Rashwan, Rabab; Lim, Xin Ru; Brunet, Alicia A.; Chopra, Abha; Ram, Ramesh; et al
Cellular and Molecular Life Sciences (2022), 79(8), 409 | Language: English, Database: CAplus and MEDLINE

Substances (19) Reactions (0) Cited By (0)

125

Novel anticancer compound and pharmaceutical composition comprising same as active ingredient

By: Kim, Yong Bae; Yun, Kyung Seop; Jang, Min Hee
World Intellectual Property Organization, WO2021020902 A1 2021-02-04 | Language: Korean, Database: CAplus

PatentPak available

Substances (19) Reactions (0) Cited By (0)

126

Acquired resistance during adoptive cell therapy by transcriptional silencing of immunogenic antigens

By: Wylie, Ben; Chee, Jonathan; Forbes, Catherine A.; Booth, Mitchell; Stone, Shane R.; Buzzai, Anthony; Abad, Ana; Foley, Bree; Cruickshank, Mark N.; Waithman, Jason
OncoImmunology (2019), 8(8), 1609874/1-1609874/11 | Language: English, Database: CAplus and MEDLINE

Substances (20) Reactions (0) Cited By (10)

127

Method of predicting the tumor response to DNA methylation inhibitors and alternative therapeutic regimen for overcoming resistance

By: Agrawal, Khushboo; Dzubak, Petr; Frydrych, Ivo; Hajduch, Marian
World Intellectual Property Organization, WO2015144102 A1 2015-10-01 | Language: English, Database: CAplus

PatentPak available

Substances (20) Reactions (0) Cited By (1)

128

The fungal metabolite chaetocin is a sensitizer for pro-apoptotic therapies in glioblastoma

By: Ozyerli-Goknar, Ezgi; Sur-Erdem, Ilknur; Seker, Fidan; Cingoz, Ahmet; Kayabolen, Alisan; Kahya-Yesil, Zeynep; Uyulur, Firat; Gezen, Melike; Tolay, Nazife; Erman, Batu; et al
Cell Death & Disease (2019), 10(12), 894 | Language: English, Database: CAplus and MEDLINE

Substances (21) Reactions (0) Cited By (11)

129

Morphological screening of mesenchymal mammary tumor organoids to identify drugs that reverse epithelial-mesenchymal transition

By: Zhao, Na ; Powell, Reid T.; Yuan, Xueying; Bae, Goeun; Roarty, Kevin P. ; Stossi, Fabio ; Strempfl, Martina ; Toneff, Michael J.; Johnson, Hannah L.; Mani, Sendurai A.; et al
Nature Communications (2021), 12(1), 4262 | Language: English, Database: CAplus and MEDLINE

Substances (22) Reactions (0) Cited By (10)

130

Analysis of characteristic genes and ceRNA regulation mechanism of endometriosis based on full transcriptional sequencing

By: Xie, Chengmao; Yin, Ziran; Liu, Yong
Frontiers in Genetics (2022), 13, 902329 | Language: English, Database: CAplus and MEDLINE

Substances (23) Reactions (0) Cited By (0)

131

Hypoxia induces HIF1α-dependent epigenetic vulnerability in triple negative breast cancer to confer immune effector dysfunction and resistance to anti-PD-1 immunotherapy

By: Ma, Shijun ; Zhao, Yue; Lee, Wee Chyan; Ong, Li-Teng; Lee, Puay Leng; Jiang, Zemin; Oguz, Gokce; Niu, Zhitong; Liu, Min; Goh, Jian Yuan; et al
Nature Communications (2022), 13(1), 4118 | Language: English, Database: CAplus and MEDLINE

Substances (23) Reactions (0) Cited By (3)

132

Methods for treating T-cell acute lymphoblastic leukemia (T-ALL)

By: Aifantis, Iannis; Ntziachristos, Panagiotis
United States, US20170042904 A1 2017-02-16 | Language: English, Database: CAplus

PatentPak available

Substances (25) Reactions (0) Cited By (0)

133

The N6-methylandenosine-related gene BIRC5 as a prognostic biomarker correlated with cell migration and immune cell infiltrates in low grade glioma

By: Jiang, Xiulin; Shi, Yulin; Chen, Xi; Xu, Haitao; Huang, Xiaobin; Li, Lihua; Pu, Jun
Frontiers in Molecular Biosciences (2022), 9, 773662 | Language: English, Database: CAplus and MEDLINE

Substances (31) Reactions (0) Cited By (1)

134

Ex vivo gastrointestinal biopsy platform to evaluate multi-omic signatures for screening candidate therapeutics

By: Iliopoulos, Dimitrios; Karagiannidis, Iordanis
World Intellectual Property Organization, WO2022031954 A1 2022-02-10 | Language: English, Database: CAplus

PatentPak available

Substances (33) Reactions (0) Cited By (0)

135

Compounds and compositions for treating, ameliorating, and/or preventing SARS-Cov-2 infection and/or complications thereof

By: Wilen, Craig
World Intellectual Property Organization, WO2021247367 A1 2021-12-09 | Language: English, Database: CAplus

PatentPak available

Substances (309) Reactions (0) Cited By (0)

136

Methods for induction of enteric neurons expressing NOS1 for use in cell therapy of enteric nervous system disorders and drug screening

By: Fattahi, Faranak
World Intellectual Property Organization, WO2021195439 A1 2021-09-30 | Language: English, Database: CAplus

PatentPak available

Substances (28) Reactions (0) Cited By (0)

137

Sequential targeting in crosslinking nano-theranostics for treating brain tumors

By: Li, Yuanpei; Wu, Hao; Lin, Tzu-Yin
World Intellectual Property Organization, WO2021126970 A1 2021-06-24 | Language: English, Database: CAplus

PatentPak available

Substances (58) Reactions (3) Cited By (0)

138

In silico identification of small molecules as new Cdc25 inhibitors through the correlation between chemosensitivity and protein expression pattern

By: Lauria, Antonino ; Martorana, Annamaria ; La Monica, Gabriele ; Mannino, Salvatore; Mannino, Giuseppe ; Peri, Daniele ; Gentile, Carla
International Journal of Molecular Sciences (2021), 22(7), 3714 | Language: English, Database: CAplus and MEDLINE

Substances (34) Reactions (0) Cited By (6)

139

Activity Comparison of Epigenetic Modulators against the Hemoprotozoan Parasites Babesia divergens and Plasmodium falciparum

By: Vanheer, Leen N.; Kafsack, Bjorn F. C.
ACS Infectious Diseases (2021), 7(8), 2277-2284 | Language: English, Database: CAplus and MEDLINE

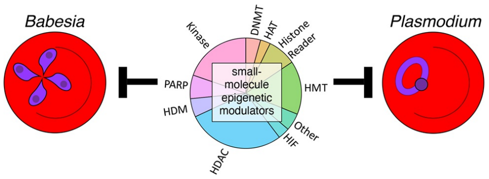

Substances (326) | Reactions (0) | Cited By (6)

140

Screening methods for identifying therapeutic agents for treating chronic inflammatory injury, metaplasia, dysplasia and cancers of epithelial tissues

By: McKeon, Frank; Duleba, Marcin; Zhang, Yanting; Xie, Jingzhong; Xian, Wa; Vincent, Matthew
World Intellectual Property Organization, WO2020219963 A1 2020-10-29 | Language: English, Database: CAplus

PatentPak available

Substances (113) | Reactions (0) | Cited By (2)

141

Methods of sensitizing cancer cells to tumor necrosis factor-α (TNFα)-induced cell death with BET inhibitors

By: Bacac, Marina; Fauti, Tanja Andrea; Hogg, Simon John; Johnstone, Ricky Wayne; Ruefli-Brasse, Astrid Alexandra; Rohle, Daniel Alan; Wellinger, Lisa Christina
World Intellectual Property Organization, WO2020169698 A1 2020-08-27 | Language: English, Database: CAplus

PatentPak available

Substances (65) | Reactions (0) | Cited By (2)

142

Novel glia-like cells differentiated from somatic cells, preparation method therefor, cocktail composition, cell therapy for preventing or treating neurological disorders

By: Kim, Kyeong-Kyu; De, Debojyoti
World Intellectual Property Organization, WO2020141648 A1 2020-07-09 | Language: Korean, Database: CAplus

PatentPak available

Substances (61) | Reactions (0) | Cited By (0)

143

Methods using protein arginine methyltransferase (PRMT) inhibitors for the treatment and prevention of hematopoietic disorders and malignancies

By: Shlush, Liran; Abdel-Wahab, Omar
World Intellectual Property Organization, WO2020089892 A1 2020-05-07 | Language: English, Database: CAplus

PatentPak available

Substances (73) | Reactions (0) | Cited By (0)

144

HDAC inhibition enhances the in vivo efficacy of MEK inhibitor therapy in Uveal melanoma

By: Faiao-Flores, Fernanda; Emmons, Michael F.; Durante, Michael A.; Kinose, Fumi; Saha, Biswarup; Fang, Bin; Koomen, John M.; Chellappan, Srikumar P.; Maria-Engler, Silvya Stuchi; Rix, Uwe; et al
Clinical Cancer Research (2019), 25(18), 5686-5701 | Language: English, Database: CAplus and MEDLINE

Substances (30) | Reactions (0) | Cited By (48)

145

Pluripotent stem cell microRNA and small mol. compound induction

By: Yu, Xiyong; Wang, Yigang; Liang, Jialiang
World Intellectual Property Organization, WO2020007036 A1 2020-01-09 | Language: Chinese, Database: CAplus

PatentPak available

Substances (95) | Reactions (0) | Cited By (0)

146

Compositions and methods for treating and preventing muscular disorders and dystrophies, steroid side effects, and inflammation

By: Heier, Christopher; Fiorillo, Alyson
World Intellectual Property Organization, WO2019232548 A2 2019-12-05 | Language: English, Database: CAplus

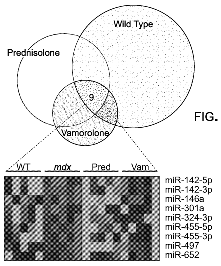

FIG. 1A
FIG. 1B

PatentPak available

Substances (95) | Reactions (0) | Cited By (1)

147

Composition and method to produce improved T cells with increased Ezh2 activity for treating cancer and chronic infection

By: Zhang, Yi; He, Shan

World Intellectual Property Organization, WO2019094404 A1 2019-05-16 | Language: English, Database: CAplus

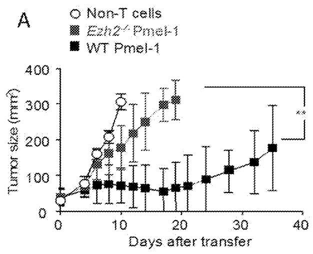

PatentPak available

Substances (92) Reactions (0) Cited By (0)

148

Methods for treating diseases and nerve injury by administering a composition comprising a myelination enhancing inhibitor

By: He, Xuelian; Lu, Qing; Zhang, Liguo

World Intellectual Property Organization, WO2019032652 A1 2019-02-14 | Language: English, Database: CAplus

FIG. 8

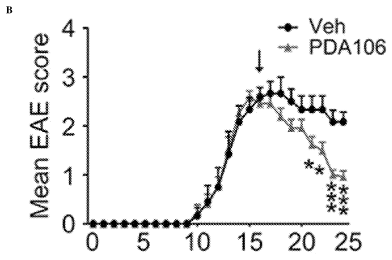

PatentPak available

Substances (33) Reactions (0) Cited By (0)

149

Engineering Lineage Potency and Plasticity of Stem Cells using Epigenetic Molecules

By: Dhaliwal, Anandika; Pelka, Sandra; Gray, David S.; Moghe, Prabhas V.

Scientific Reports (2018), 8(1), 1-13 | Language: English, Database: CAplus and MEDLINE

Substances (71) Reactions (0) Cited By (5)

150

Screening of a neuronal cell model of tau pathology for therapeutic compounds

By: Pickhardt, Marcus; Tassoni, Michele; Denner, Philip; Kurkowsky, Birgit; Kitanovic, Ana; Moehl, Christoph; Fava, Eugenio; Mandelkow, Eckhard

Neurobiology of Aging (2019), 76, 24-34 | Language: English, Database: CAplus and MEDLINE

Substances (36) Reactions (0) Cited By (5)

151

HIV signaling through CD4 and CCR5 activates Rho family GTPases that are required for optimal infection of primary CD4+ T cells

By: Lucera, Mark B.; Fleissner, Zach; Tabler, Caroline O.; Schlatzer, Daniela M.; Troyer, Zach; Tilton, John C.

Retrovirology (2017), 14, 4/1-4/13 | Language: English, Database: CAplus and MEDLINE

Substances (74) Reactions (0) Cited By (9)

152

Modulators of homeobox transcription factors, DUX4 (double homeobox 4) for regulation of muscle function

By: Rickard, Amanda; De La Garza, Anabel; Schmidt, Uli; Kiselyov, Alexander

World Intellectual Property Organization, WO2017201585 A1 2017-11-30 | Language: English, Database: CAplus

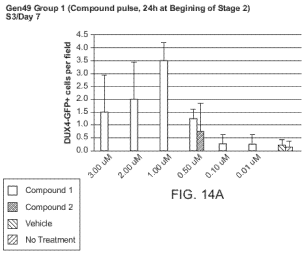

FIG. 14A

PatentPak available

Substances (241) Reactions (0) Cited By (3)

153

Retinoic acid receptor-α (RAR-R) agonists for the treatment of acute myelocytic leukemia (AML) and myelodysplastic syndrome (MDS)

By: McKeown, Michael Robert; Fiore, Christopher; Eaton, Matthew Lucas; Lee, Emily Payton; Fritz, Christian
World Intellectual Property Organization, WO2017177167 A1 2017-10-12 | Language: English, Database: CAplus

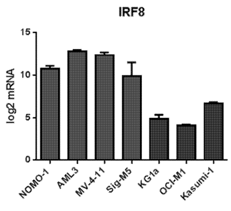

Figure 1

PatentPak available

Substances (31) | Reactions (0) | Cited By (0)

154

Discovery of novel drug sensitivities in T-PLL by high-throughput ex vivo drug testing and mutation profiling

By: Andersson, E. I.; Putzer, S.; Yadav, B.; Dufva, O.; Khan, S.; He, L.; Sellner, L. ; Schrader, A.; Crispatzu, G.; Oles, M.; et al
Leukemia (2018), 32(3), 774-787 | Language: English, Database: CAplus and MEDLINE

Substances (65) | Reactions (0) | Cited By (60)

155

Compositions and methods for modulating FMR1 expression

By: Green, Michael R.; Fang, Minggang
World Intellectual Property Organization, WO2017049192 A1 2017-03-23 | Language: English, Database: CAplus

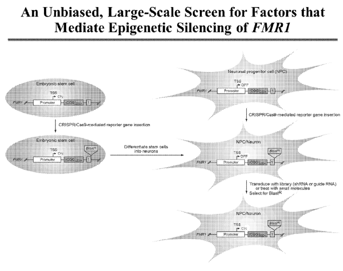

FIG. 5

PatentPak available

Substances (150) | Reactions (0) | Cited By (3)

156

Epigenetic silencing of N-myristoyltransferase 2 (NMT2) in cancer due to hypermethylation and treatment with inhibitors of NMT

By: Berthiaume, Luc G.; Beauchamp, Erwan

World Intellectual Property Organization, WO2017011907 A1 2017-01-26 | Language: English, Database: CAplus

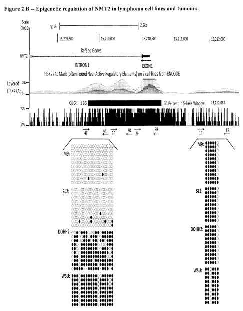

PatentPak available

Substances (76) | Reactions (0) | Cited By (3)

157

Systematic chemical and molecular profiling of MLL-rearranged infant acute lymphoblastic leukemia reveals efficacy of romidepsin

By: Cruickshank, M. N.; Ford, J.; Cheung, L. C.; Heng, J.; Singh, S.; Wells, J.; Failes, T. W.; Arndt, G. M.; Smithers, N.; Prinjha, R. K.; et al

Leukemia (2017), 31(1), 40-50 | Language: English, Database: CAplus and MEDLINE

Substances (32) | Reactions (0) | Cited By (18)

158

EZH2 inhibitors for treating lymphoma

By: Keilhack, Heike; Knutson, Sarah K.; Johnston, L. Danielle

World Intellectual Property Organization, WO2016201328 A1 2016-12-15 | Language: English, Database: CAplus

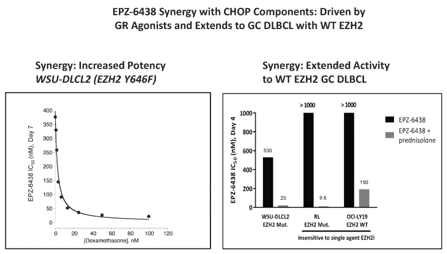

Figure 1A

Figure 1B

PatentPak available

Substances (43) | Reactions (0) | Cited By (3)

159

Methods of treating hepatitis B virus

By: Aguayo, Esmeralda; Appleby, Todd; Birkus, Gabriel; Cheng, Guofeng; Dornan, David; Kobayashi, Tetsuya; Mello, Christopher Charles; Schmitz, Uli; Willkom, Madeleine; Yu, Mei
World Intellectual Property Organization, WO2016168349 A1 2016-10-20 | Language: English, Database: CAplus

PatentPak available

Substances (51) Reactions (0) Cited By (1)

160

Methods for inhibiting alpha-v beta-3 expression on cancer stem cells and inhibiting progression to a cancer stem cell phenotype

By: Cheresh, David A.; Gozo, Maricel; Yebra, Mayra; Seguin, Laetitia
World Intellectual Property Organization, WO2016100858 A1 2016-06-23 | Language: English, Database: CAplus

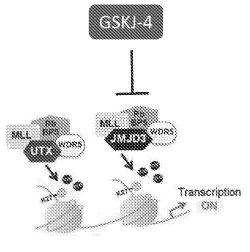

Fig. 11A

PatentPak available

Substances (33) Reactions (0) Cited By (2)

161

Epigenetic regulators of frataxin for use in treatment of Friedrich' s ataxia

By: Ozsolak, Fatih
World Intellectual Property Organization, WO2015023938 A1 2015-02-19 | Language: English, Database: CAplus

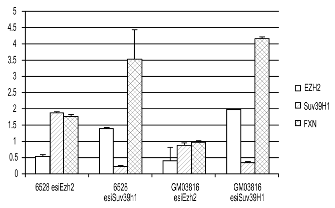

FIG. 3B

PatentPak available

Substances (268) Reactions (0) Cited By (1)

162

Transcriptional repression of ER through hMAPK dependent histone deacetylation by class I HDACs

By: Plotkin, Amy; Volmar, Claude-Henry; Wahlestedt, Claes; Ayad, Nagi; El-Ashry, Dorraya
Breast Cancer Research and Treatment (2014), 147(2), 249-263 | Language: English, Database: CAplus and MEDLINE

Substances (59) Reactions (0) Cited By (12)

163

Pyrmidinyl pyrimidines as affinity ligands for histone demethylases and their use identifying ligands and effectors of the demethylase

By: Atkinson, Stephen John; Barker, Michael David; Campbell, Matthew; Humphreys, Philip; Liddle, John; Sheppard, Robert John; Wilson, David; Joberty, Gerard
European Patent Organization, EP2592154 A1 2013-05-15 | Language: English, Database: CAplus

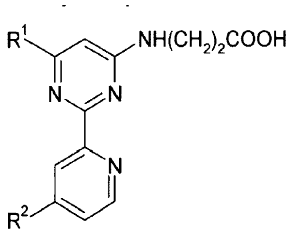

(I)

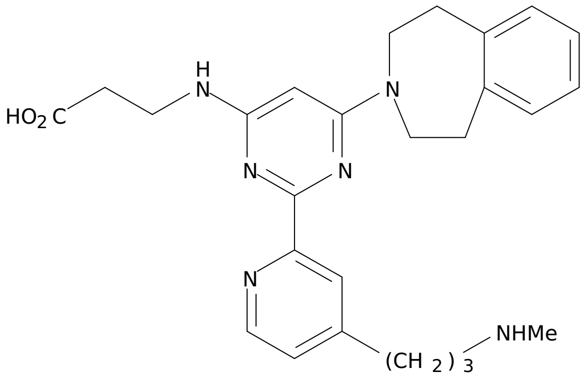

PatentPak available

Substances (56) Reactions (137) Cited By (1)

164

Preparation of N-(pyridinyl)pyrimidinyl-β-alanine derivatives for use as histone demethylase JMJD3 inhibitors

By: Atkinson, Stephen John; Barker, Michael David; Campbell, Matthew; Humphreys, Philip; Liddle, John; Sheppard, Robert John; Wilson, David
World Intellectual Property Organization, WO2012052390 A1 2012-04-26 | Language: English, Database: CAplus

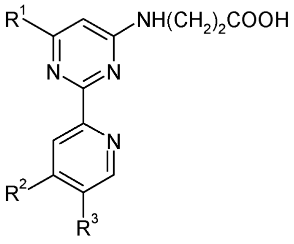

(I)

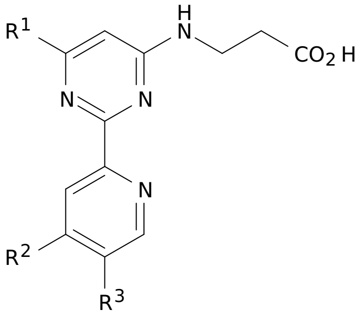

I

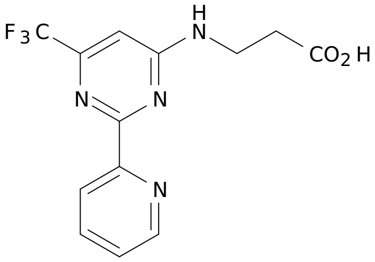

II

PatentPak available

Substances (387) Reactions (688) Cited By (3)

Copyright © 2023 American Chemical Society (ACS). All Rights Reserved.
Copyright © 2023 U.S. National Library of Medicine.
Internal use only. Redistribution is subject to the terms of your SciFindern License Agreement and CAS information Use Policies.

Results fo PubMed search: keyword A-485

1: Baldick CJ Jr, Moss B. Resistance of vaccinia virus to rifampicin conferred by a single nucleotide substitution near the predicted NH2 terminus of a gene encoding an Mr 62,000 polypeptide. Virology. 1987 Jan;156(1):138-45. doi: 10.1016/0042-6822(87)90444-2. PMID: 3811229.

2: Delort AM, Jeminet G, Sancelme M, Dauphin G. Microbial conversion of nigericin in three successive steps, by Sebekia benihana. J Antibiot (Tokyo). 1988 Jul;41(7):916-24. doi: 10.7164/antibiotics.41.916. PMID: 3417565.

3: Haggerty HG, Boise LH, Jordan SD, Holsapple MP. Differential effects of coadministration of aminoacetonitrile on immunosuppression and hepatotoxicity produced by dimethylnitrosamine. J Pharmacol Exp Ther. 1988 Nov;247(2):774-80. PMID: 3183971.

4: Delort AM, Dauphin G, Guyot J, Jeminet G. Study by NMR of the mode of action of monensin on Streptococcus faecalis de-energized and energized cells. Biochim Biophys Acta. 1989 Sep 4;1013(1):11-20. doi: 10.1016/0167-4889(89)90121-3. PMID: 2551382.

5: Brzović P, Holbrook EL, Greene RC, Dunn MF. Reaction mechanism of Escherichia coli cystathionine gamma-synthase: direct evidence for a pyridoxamine derivative of vinylglyoxylate as a key intermediate in pyridoxal phosphate dependent gamma-elimination and gamma-replacement reactions. Biochemistry. 1990 Jan 16;29(2):442-51. doi: 10.1021/bi00454a020. PMID: 2405904.

6: Vaufrey F, Delort AM, Jeminet G, Dauphin G. Bioconversion of monensin by a soil bacterium, Sebekia benihana. J Antibiot (Tokyo). 1990 Sep;43(9):1189-91. doi: 10.7164/antibiotics.43.1189. PMID: 2211382.

7: Nicolson L, Onions DE. The nucleotide sequence of the equine herpesvirus 4 gC gene homologue. Virology. 1990 Nov;179(1):378-87. doi: 10.1016/0042-6822(90)90305-b. PMID: 2171212.

8: Gitter MJ, Goldsmith SR, Dunbar DN, Sharkey SW. Cocaine and chest pain: clinical features and outcome of patients hospitalized to rule out myocardial infarction. Ann Intern Med. 1991 Aug 15;115(4):277-82. doi: 10.7326/0003-4819-115-4-277. PMID: 1854111.

9: Crozier YC, Koulianos S, Crozier RH. An improved test for Africanized honeybee mitochondrial DNA. Experientia. 1991 Sep 15;47(9):968-9. doi: 10.1007/BF01929894. PMID: 1915781.

10: David L, Loutheiller H, Bauchart D, Auboiron S, Asselineau J. Effects of exogenous methyl oleate on the biosynthesis of nigericin, a polyether carboxylic antibiotic, by Streptomyces hygroscopicus NRRL B-1865. Biosci Biotechnol Biochem. 1992 Feb;56(2):330. doi: 10.1271/bbb.56.330. PMID: 1368307.

11: Rabaste F, Jeminet G, Dauphin G, Delort AM. Conditions modulating the ionic selectivity of transport by monensin examined on Enterococcus hirae (Streptococcus faecalis) by 23Na-NMR and K+ atomic absorption. Biochim Biophys Acta. 1992 Jul 27;1108(2):177-82. doi: 10.1016/0005-2736(92)90023-f. PMID: 1637842.

12: Brady PS, Park EA, Liu JS, Hanson RW, Brady LJ. Isolation and characterization of the promoter for the gene coding for the 68 kDa carnitine palmitoyltransferase from the rat. Biochem J. 1992 Sep 15;286 (Pt 3)(Pt

3):779-83. doi: 10.1042/bj2860779. PMID: 1417736; PMCID: PMC1132971.

13: Svoboda M, Ciccarelli E, Tastenoy M, Cauvin A, Stiévenart M, Christophe J. Small introns in a hepatic cDNA encoding a new glucagon-like peptide 1-type receptor. Biochem Biophys Res Commun. 1993 Mar 15;191(2):479-86. doi: 10.1006/bbrc.1993.1243. PMID: 8384842.

14: Rowley KB, Clements DE, Mandel M, Humphreys T, Patil SS. Multiple copies of a DNA sequence from Pseudomonas syringae pathovar phaseolicola abolish thermoregulation of phaseolotoxin production. Mol Microbiol. 1993 May;8(3):625-35. doi: 10.1111/j.1365-2958.1993.tb01606.x. PMID: 8326870.

15: Rabaste F, Jeminet G, Dauphin G, Delort AM. Na+ and K+ transport by 4-chlorophenylurethane-monensin in Enterococcus hirae de-energized and energized cells studied by 23Na-NMR and K+ atomic absorption. Biochim Biophys Acta. 1993 Nov 7;1179(2):166-9. doi: 10.1016/0167-4889(93)90138-f. PMID: 8218359.

16: Semla TP, Palla K, Poddig B, Brauner DJ. Effect of the Omnibus Reconciliation Act 1987 on antipsychotic prescribing in nursing home residents. J Am Geriatr Soc. 1994 Jun;42(6):648-52. doi: 10.1111/j.1532-5415.1994.tb06865.x. PMID: 7911134.

17: Yu J, Nagarajan S, Liu J, Young N, Medof ME. Cloning and characterization of the mouse PIG-A gene. Biochim Biophys Acta. 1995 Apr 6;1255(3):344-50. doi: 10.1016/0005-2760(95)00015-5. PMID: 7734452.

18: Cook RJ, Wagner C. Enzymatic activities of rat liver cytosol 10-formyltetrahydrofolate dehydrogenase. Arch Biochem Biophys. 1995 Aug 20;321(2):336-44. doi: 10.1006/abbi.1995.1403. PMID: 7646059.

19: Burcelin R, Li J, Charron MJ. Cloning and sequence analysis of the murine glucagon receptor-encoding gene. Gene. 1995 Oct 27;164(2):305-10. doi: 10.1016/0378-1119(95)00472-i. PMID: 7590348.

20: Rochdi M, Delort AM, Guyot J, Sancelme M, Gibot S, Gourcy JG, Dauphin G, Gumila C, Vial H, Jeminet G. Ionophore properties of monensin derivatives studied on human erythrocytes by 23Na NMR and K+ and H+ potentiometry: relationship with antimicrobial and antimalarial activities. J Med Chem. 1996 Jan 19;39(2):588-95. doi: 10.1021/jm9505829. PMID: 8558531.

21: Howe DK, Vodkin MH, Novak RJ, Visvesvara G, McLaughlin GL. Identification of two genetic markers that distinguish pathogenic and nonpathogenic strains of Acanthamoeba spp. Parasitol Res. 1997;83(4):345-8. doi: 10.1007/s004360050259. PMID: 9134555.

22: Bowles DL. Relationship between the volume of total hip replacements performed by providers and the rates of postoperative complications in the state of Washington (79-A: 485-494, April 1997), by Kreder et al. J Bone Joint Surg Am. 1998 Apr;80(4):606. PMID: 9563393.

23: Bernstein J. Relationship between the volume of total hip replacements performed by providers and the rates of postoperative complications in the state of Washington (79-A:485-494, April 1997), by Kreder et al. J Bone Joint Surg Am. 1998 Apr;80(4):606-7. PMID: 9563394.

24: Rostaing L, Chatelut E, Payen JL, Izopet J, Thalamas C, Ton-That H, Pascal JP, Durand D, Canal P. Pharmacokinetics of alphaIFN-2b in chronic hepatitis C

virus patients undergoing chronic hemodialysis or with normal renal function: clinical implications. J Am Soc Nephrol. 1998 Dec;9(12):2344-8. doi: 10.1681/ASN.V9122344. PMID: 9848789.

25: Seki N, Hattori A, Sugano S, Suzuki Y, Nakagawara A, Ohhira M, Muramatsu M, Hori T, Saito T. Isolation, tissue expression, and chromosomal assignment of a novel human gene which encodes a protein with RING finger motif. J Hum Genet. 1998;43(4):272-4. doi: 10.1007/s100380050088. PMID: 9852682.

26: 'Grade D minimum' for all enrolled nurses. Emerg Nurse. 1999 Mar 1;6(10):4. doi: 10.7748/en.6.10.4.s6. PMID: 27238255.

27: Rowley KB, Xu R, Patil SS. Molecular analysis of thermoregulation of phaseolotoxin-resistant ornithine carbamoyltransferase (argK) from Pseudomonas syringae pv. phaseolicola. Mol Plant Microbe Interact. 2000 Oct;13(10):1071-80. doi: 10.1094/MPMI.2000.13.10.1071. PMID: 11043468.

28: Jiang H, Kendrick KE. Cloning and characterization of the gene encoding penicillin-binding protein A of Streptomyces griseus. FEMS Microbiol Lett. 2000 Dec 1;193(1):63-8. doi: 10.1111/j.1574-6968.2000.tb09403.x. PMID: 11094280.

29: Antes TJ, Goodart SA, Chen W, Levy-Wilson B. Human apolipoprotein B gene intestinal control region. Biochemistry. 2001 Jun 12;40(23):6720-30. doi: 10.1021/bi010073a. PMID: 11389586.

30: Liu Y, Chen YR. Analysis of visible reflectance spectra of stored, cooked and diseased chicken meats. Meat Sci. 2001 Aug;58(4):395-401. doi: 10.1016/s0309-1740(01)00041-9. PMID: 22062430.

31: Skrapari I, Perrea D, Ioannidis I, Karabina SA, Elisaf M, Tselepis AD, Karagiannacos P, Katsilambros N. Glibenclamide improves postprandial hypertriglyceridaemia in type 2 diabetic patients by reducing chylomicrons but not the very low-density lipoprotein subfraction levels. Diabet Med. 2001 Oct;18(10):781-5. doi: 10.1046/j.1464-5491.2001.00538.x. PMID: 11678967.

32: Singh K, Batuman OA, Akman HO, Kedees MH, Vakil V, Hussain MM. Differential, tissue-specific, transcriptional regulation of apolipoprotein B secretion by transforming growth factor beta. J Biol Chem. 2002 Oct 18;277(42):39515-24. doi: 10.1074/jbc.M205513200. Epub 2002 Aug 12. PMID: 12177061.

33: Liu Y, Fan X, Chen YR, Thayer DW. Changes in structure and color characteristics of irradiated chicken breasts as a function of dosage and storage time. Meat Sci. 2003 Mar;63(3):301-7. doi: 10.1016/s0309-1740(02)00086-4. PMID: 22062381.

34: Tazuke Y, Drongowski RA, Teitelbaum DH, Coran AG. The effect of hypoxia on permeability and bacterial translocation in Caco-2 adult and I-407 fetal enterocyte cell culture models. Pediatr Surg Int. 2003 Jul;19(5):316-20. doi: 10.1007/s00383-003-1002-9. Epub 2003 May 6. PMID: 12732937.

35: Miyatake R, Furukawa A, Matsushita S, Higuchi S, Suwaki H. Functional polymorphisms in the sigma1 receptor gene associated with alcoholism. Biol Psychiatry. 2004 Jan 1;55(1):85-90. doi: 10.1016/j.biopsych.2003.07.008. PMID: 14706429.

36: Schächinger H, Port J, Brody S, Linder L, Wilhelm FH, Huber PR, Cox D, Keller U. Increased high-frequency heart rate variability during insulin-induced

hypoglycaemia in healthy humans. Clin Sci (Lond). 2004 Jun;106(6):583-8. doi: 10.1042/CS20030337. PMID: 14717655.

37: Callen BL, Mahoney JE, Grieves CB, Wells TJ, Enloe M. Frequency of hallway ambulation by hospitalized older adults on medical units of an academic hospital. Geriatr Nurs. 2004 Jul-Aug;25(4):212-7. doi: 10.1016/j.gerinurse.2004.06.016. PMID: 15311196.

38: Puga I, Lainez B, Fernández-Real JM, Buxadé M, Broch M, Vendrell J, Espel E. A polymorphism in the 3' untranslated region of the gene for tumor necrosis factor receptor 2 modulates reporter gene expression. Endocrinology. 2005 May;146(5):2210-20. doi: 10.1210/en.2004-1366. Epub 2005 Jan 27. PMID: 15677760.

39: Tzimas C, Michailidou G, Arsenakis M, Kieff E, Mosialos G, Hatzivassiliou EG. Human ubiquitin specific protease 31 is a deubiquitinating enzyme implicated in activation of nuclear factor-kappaB. Cell Signal. 2006 Jan;18(1):83-92. doi: 10.1016/j.cellsig.2005.03.017. Epub 2005 Apr 26. PMID: 16214042.

40: D'Andrea TM, Zhang X, Jochnowitz EB, Lindeman TG, Simpson CJ, David DE, Curtiss TJ, Morris JR, Ellison GB. Oxidation of organic films by beams of hydroxyl radicals. J Phys Chem B. 2008 Jan 17;112(2):535-44. doi: 10.1021/jp7096108. Epub 2007 Dec 19. PMID: 18088112.

41: Ferreira KM, Lino e Silva O, Arias MC, Del Lama MA. Cytochrome-b variation in Apis mellifera samples and its association with COI-COII patterns. Genetica. 2009 Mar;135(2):149-55. doi: 10.1007/s10709-008-9264-8. Epub 2008 Mar 29. PMID: 18373204.

42: Eni AO, Kumar PL, Asiedu R, Alabi OJ, Naidu RA, Hughes J, Rey MEC. First Report of Cucumber mosaic virus in Yams (Dioscorea spp.) in Ghana, Togo, and Republic of Benin in West Africa. Plant Dis. 2008 May;92(5):833. doi: 10.1094/PDIS-92-5-0833B. PMID: 30769616.

43: Al-Otaibi F, Tucker AT, Johnston A, Perrett D. Rapid analysis of tetracaine for a tape stripping pharmacokinetic study using short-end capillary electrophoresis. Biomed Chromatogr. 2009 May;23(5):488-91. doi: 10.1002/bmc.1142. PMID: 19101917.

44: Intra J, Perotti ME, Pasini ME. Cloning, sequence identification and expression profile analysis of α-L-fucosidase gene from the Mediterranean fruit fly Ceratitis capitata. J Insect Physiol. 2011 Apr;57(4):452-61. doi: 10.1016/j.jinsphys.2011.01.007. Epub 2011 Jan 25. PMID: 21272587.

45: Li G, Park YJ. SCAR markers for discriminating species of two genera of medicinal plants, Liriope and Ophiopogon. Genet Mol Res. 2012 Aug 29;11(3):2987-96. doi: 10.4238/2012.May.18.14. PMID: 22653648.

46: Waugh JM, Aarssen LW. Size distributions and dispersions along a 485-year chronosequence for sand dune vegetation. Ecol Evol. 2012 Apr;2(4):719-26. doi: 10.1002/ece3.62. PMID: 22837820; PMCID: PMC3399194.

47: Inoue H, Motani-Saitoh H, Sakurada K, Ikegaya H, Yajima D, Nagasawa S, Iwase H. Genotypic polymorphisms of hepatitis B virus provide useful information for estimating geographical origin or place of long-term residence of unidentified cadavers. J Forensic Sci. 2014 Jan;59(1):236-41. doi: 10.1111/1556-4029.12257. Epub 2013 Sep 10. PMID: 24020429.

48: Trippel S, Mullins T, Müller NL, Kienitz JS, González-Férez R, Küpper J. Two-state wave packet for strong field-free molecular orientation. Phys Rev Lett. 2015 Mar 13;114(10):103003. doi: 10.1103/PhysRevLett.114.103003. Epub 2015 Mar 10. PMID: 25815928.

49: Soler R, Orenstein D, Honeycutt A, Bradley C, Trogdon J, Kent CK, Wile K, Haddix A, O'Neil D, Bunnell R; Communities Putting Prevention to Work Leadership Team. Community-Based Interventions to Decrease Obesity and Tobacco Exposure and Reduce Health Care Costs: Outcome Estimates From Communities Putting Prevention to Work for 2010-2020. Prev Chronic Dis. 2016 Apr 7;13:E47. doi: 10.5888/pcd13.150272. PMID: 27055264; PMCID: PMC4830258.

50: Chen JY, Pandey R, Nguyen TM. HAPPI-2: a Comprehensive and High-quality Map of Human Annotated and Predicted Protein Interactions. BMC Genomics. 2017 Feb 17;18(1):182. doi: 10.1186/s12864-017-3512-1. PMID: 28212602; PMCID: PMC5314692.

51: Ma GX, Zhou RQ, Hu L, Luo YL, Luo YF, Zhu HH. Molecular characterization and transcriptional analysis of the female-enriched chondroitin proteoglycan 2 of Toxocara canis. J Helminthol. 2018 Mar;92(2):154-160. doi: 10.1017/S0022149X17000359. Epub 2017 Apr 24. PMID: 28434412.

52: Lasko LM, Jakob CG, Edalji RP, Qiu W, Montgomery D, Digiammarino EL, Hansen TM, Risi RM, Frey R, Manaves V, Shaw B, Algire M, Hessler P, Lam LT, Uziel T, Faivre E, Ferguson D, Buchanan FG, Martin RL, Torrent M, Chiang GG, Karukurichi K, Langston JW, Weinert BT, Choudhary C, de Vries P, Van Drie JH, McElligott D, Kesicki E, Marmorstein R, Sun C, Cole PA, Rosenberg SH, Michaelides MR, Lai A, Bromberg KD. Discovery of a selective catalytic p300/CBP inhibitor that targets lineage-specific tumours. Nature. 2017 Oct 5;550(7674):128-132. doi: 10.1038/nature24028. Epub 2017 Sep 27. Erratum in: Nature. 2018 May 16;: PMID: 28953875; PMCID: PMC6050590.

53: Kodadek T. Another One (of the "Undruggable" Targets) Bites the Dust: Discovery of a Potent and Selective Inhibitor of the Histone Acetyl Transferase p300/CBP. Biochemistry. 2018 Feb 13;57(6):899-900. doi: 10.1021/acs.biochem.7b01179. Epub 2017 Dec 15. PMID: 29244478.

54: Zhang Y, Tang H, Li C, Lu G, Cai Y, Zhang J, Tan F. Design and Testing of a Flexible Inclinometer Probe for Model Tests of Landslide Deep Displacement Measurement. Sensors (Basel). 2018 Jan 14;18(1):224. doi: 10.3390/s18010224. PMID: 29342902; PMCID: PMC5796333.

55: Michaelides MR, Kluge A, Patane M, Van Drie JH, Wang C, Hansen TM, Risi RM, Mantei R, Hertel C, Karukurichi K, Nesterov A, McElligott D, de Vries P, Langston JW, Cole PA, Marmorstein R, Liu H, Lasko L, Bromberg KD, Lai A, Kesicki EA. Discovery of Spiro Oxazolidinediones as Selective, Orally Bioavailable Inhibitors of p300/CBP Histone Acetyltransferases. ACS Med Chem Lett. 2017 Dec 13;9(1):28-33. doi: 10.1021/acsmedchemlett.7b00395. PMID: 29348807; PMCID: PMC5767893.

56: Song K, Wen Z, Shang Y, Yang H, Lyu L, Liu G, Fang C, Du J, Zhao Y. Quantification of dissolved organic carbon (DOC) storage in lakes and reservoirs of mainland China. J Environ Manage. 2018 Jul 1;217:391-402. doi: 10.1016/j.jenvman.2018.03.121. Epub 2018 Apr 5. PMID: 29626842.

57: Weinert BT, Narita T, Satpathy S, Srinivasan B, Hansen BK, Schölz C, Hamilton WB, Zucconi BE, Wang WW, Liu WR, Brickman JM, Kesicki EA, Lai A, Bromberg KD, Cole PA, Choudhary C. Time-Resolved Analysis Reveals Rapid Dynamics

and Broad Scope of the CBP/p300 Acetylome. Cell. 2018 Jun 28;174(1):231-244.e12. doi: 10.1016/j.cell.2018.04.033. Epub 2018 May 24. PMID: 29804834; PMCID: PMC6078418.

58: Arshad M, Conzelmann C, Riaz MA, Noll T, Gündüz D. Inhibition of Cx43 attenuates ERK1/2 activation, enhances the expression of Cav-1 and suppresses cell proliferation. Int J Mol Med. 2018 Nov;42(5):2811-2818. doi: 10.3892/ijmm.2018.3828. Epub 2018 Aug 16. PMID: 30132504.

59: Wang R, He Y, Robinson V, Yang Z, Hessler P, Lasko LM, Lu X, Bhathena A, Lai A, Uziel T, Lam LT. Targeting Lineage-specific MITF Pathway in Human Melanoma Cell Lines by A-485, the Selective Small-molecule Inhibitor of p300/CBP. Mol Cancer Ther. 2018 Dec;17(12):2543-2550. doi: 10.1158/1535-7163.MCT-18-0511. Epub 2018 Sep 28. PMID: 30266801.

60: Bouabbache S, Pouradier F, Panhard S, Chaffiotte C, Loussouarn G. Exploring some characteristics (density, anagen ratio, growth rate) of human body hairs. Variations with skin sites, gender and ethnics. Int J Cosmet Sci. 2019 Feb;41(1):46-54. doi: 10.1111/ics.12510. Epub 2019 Jan 17. PMID: 30580453.

61: Zucconi BE, Makofske JL, Meyers DJ, Hwang Y, Wu M, Kuroda MI, Cole PA. Combination Targeting of the Bromodomain and Acetyltransferase Active Site of p300/CBP. Biochemistry. 2019 Apr 23;58(16):2133-2143. doi: 10.1021/acs.biochem.9b00160. Epub 2019 Apr 11. PMID: 30924641; PMCID: PMC6948846.

62: Tottone L, Zhdanovskaya N, Carmona Pestaña Á, Zampieri M, Simeoni F, Lazzari S, Ruocco V, Pelullo M, Caiafa P, Felli MP, Checquolo S, Bellavia D, Talora C, Screpanti I, Palermo R. Histone Modifications Drive Aberrant Notch3 Expression/Activity and Growth in T-ALL. Front Oncol. 2019 Apr 3;9:198. doi: 10.3389/fonc.2019.00198. PMID: 31001470; PMCID: PMC6456714.

63: Wang Z, Zhang Y, Huang S, Peng C, Hao Z, Li D. Nitrogen limitation significantly reduces the competitive advantage of toxic Microcystis at high light conditions. Chemosphere. 2019 Dec;237:124508. doi: 10.1016/j.chemosphere.2019.124508. Epub 2019 Aug 6. PMID: 31408798.

64: Peng J, Li J, Huang J, Xu P, Huang H, Liu Y, Yu L, Yang Y, Zhou B, Jiang H, Chen K, Dang Y, Zhang Y, Luo C, Li G. p300/CBP inhibitor A-485 alleviates acute liver injury by regulating macrophage activation and polarization. Theranostics. 2019 Oct 22;9(26):8344-8361. doi: 10.7150/thno.30707. PMID: 31754401; PMCID: PMC6857059.

65: Zhang X, Zegar T, Lucas A, Morrison-Smith C, Knox T, French CA, Knapp S, Müller S, Siveke JT. Therapeutic targeting of p300/CBP HAT domain for the treatment of NUT midline carcinoma. Oncogene. 2020 Jun;39(24):4770-4779. doi: 10.1038/s41388-020-1301-9. Epub 2020 May 4. PMID: 32366905; PMCID: PMC7286816.

66: Waddell AR, Liao D. Assays for Validating Histone Acetyltransferase Inhibitors. J Vis Exp. 2020 Aug 6;(162). doi: 10.3791/61289. PMID: 32831305.

67: Zhou F, Liu Q, Zhang L, Zhu Q, Wang S, Zhu K, Deng R, Liu Y, Yuan G, Wang X, Zhou L. Selective inhibition of CBP/p300 HAT by A-485 results in suppression of lipogenesis and hepatic gluconeogenesis. Cell Death Dis. 2020 Sep 11;11(9):745. doi: 10.1038/s41419-020-02960-6. PMID: 32917859; PMCID: PMC7486386.

68: Sharma AK, Razi MM, Prakash N, Sharma A, Sarraf S, Sinha S, Pandey U, Thakur

R, Verma CM, Krishna V. A comparative assessment of Dorsal radial artery access versus classical radial artery access for percutaneous coronary angiography-a randomized control trial (DORA trial). Indian Heart J. 2020 Sep-Oct;72(5):435-441. doi: 10.1016/j.ihj.2020.06.002. Epub 2020 Jun 18. PMID: 33189208; PMCID: PMC7670258.

69: Huo S, Liu X, Zhang S, Lyu Z, Zhang J, Wang Y, Nie B, Yue B. p300/CBP inhibitor A-485 inhibits the differentiation of osteoclasts and protects against osteoporotic bone loss. Int Immunopharmacol. 2021 May;94:107458. doi: 10.1016/j.intimp.2021.107458. Epub 2021 Feb 21. PMID: 33626422.

70: Ji Z, Clark RF, Bhat V, Matthew Hansen T, Lasko LM, Bromberg KD, Manaves V, Algire M, Martin R, Qiu W, Torrent M, Jakob CG, Liu H, Cole PA, Marmorstein R, Kesicki EA, Lai A, Michaelides MR. Discovery of spirohydantoins as selective, orally bioavailable inhibitors of p300/CBP histone acetyltransferases. Bioorg Med Chem Lett. 2021 May 1;39:127854. doi: 10.1016/j.bmcl.2021.127854. Epub 2021 Feb 23. PMID: 33631370; PMCID: PMC8392731.

71: Hsu E, Zemke NR, Berk AJ. Promoter-specific changes in initiation, elongation, and homeostasis of histone H3 acetylation during CBP/p300 inhibition. Elife. 2021 Mar 11;10:e63512. doi: 10.7554/eLife.63512. PMID: 33704060; PMCID: PMC8009678.

72: Zhou XR, Li X, Liao LP, Han J, Huang J, Li JC, Tao HR, Fan SJ, Chen ZF, Li Q, Chen SJ, Ding H, Yang YX, Zhou B, Jiang HL, Chen KX, Zhang YY, Huang CX, Luo C. P300/CBP inhibition sensitizes mantle cell lymphoma to PI3Kδ inhibitor idelalisib. Acta Pharmacol Sin. 2022 Feb;43(2):457-469. doi: 10.1038/s41401-021-00643-2. Epub 2021 Apr 13. PMID: 33850273; PMCID: PMC8791947.

73: Zhang L, Sheng C, Zhou F, Zhu K, Wang S, Liu Q, Yuan M, Xu Z, Liu Y, Lu J, Liu J, Zhou L, Wang X. CBP/p300 HAT maintains the gene network critical for β cell identity and functional maturity. Cell Death Dis. 2021 May 12;12(5):476. doi: 10.1038/s41419-021-03761-1. PMID: 33980820; PMCID: PMC8116341.

74: Mastracchio A, Lai C, Digiammarino E, Ready DB, Lasko LM, Bromberg KD, McClellan WJ, Montgomery D, Manaves V, Shaw B, Algire M, Patterson MJ, Sun CC, Rosenberg S, Lai A, Michaelides MR. Discovery of a Potent and Selective Covalent p300/CBP Inhibitor. ACS Med Chem Lett. 2021 Apr 5;12(5):726-731. doi: 10.1021/acsmedchemlett.0c00654. PMID: 34055218; PMCID: PMC8155265.

75: Feng L, Yu S, Wang H, Yang S, Li X, Dai H, Zhao L, Jiang C, Wang Y. Synthesis and Biological Evaluation of Spirocyclic Chromane Derivatives as a Potential Treatment of Prostate Cancer. Molecules. 2021 May 25;26(11):3162. doi: 10.3390/molecules26113162. PMID: 34070610; PMCID: PMC8198214.

76: Wang Y, Xie Q, Tan H, Liao M, Zhu S, Zheng LL, Huang H, Liu B. Targeting cancer epigenetic pathways with small-molecule compounds: Therapeutic efficacy and combination therapies. Pharmacol Res. 2021 Nov;173:105702. doi: 10.1016/j.phrs.2021.105702. Epub 2021 Jun 5. PMID: 34102228.

77: Hlushchuk I, Ruskoaho H, Domanskyi A, Airavaara M, Välimäki MJ. Domain-Independent Inhibition of CBP/p300 Attenuates α-Synuclein Aggregation. ACS Chem Neurosci. 2021 Jul 7;12(13):2273-2279. doi: 10.1021/acschemneuro.1c00215. Epub 2021 Jun 10. PMID: 34110772; PMCID: PMC8291498.

78: Waddell A, Mahmud I, Ding H, Huo Z, Liao D. Pharmacological Inhibition of CBP/p300 Blocks Estrogen Receptor Alpha (ERα) Function through Suppressing

Enhancer H3K27 Acetylation in Luminal Breast Cancer. Cancers (Basel). 2021 Jun 4;13(11):2799. doi: 10.3390/cancers13112799. PMID: 34199844; PMCID: PMC8200112.

79: Zheng Y, Cheng J, Zhang A, Wang Y, Dai C, Li J. Acetylation of histone 3 promotes miR-29a expression and downregulates STAT3 in sepsis. Injury. 2022 Feb;53(2):416-421. doi: 10.1016/j.injury.2021.09.018. Epub 2021 Sep 17. Erratum in: Injury. 2022 Nov;53(11):3864. PMID: 34615595.

80: Bosnakovski D, Ener ET, Cooper MS, Gearhart MD, Knights KA, Xu NC, Palumbo CA, Toso EA, Marsh GP, Maple HJ, Kyba M. Inactivation of the CIC-DUX4 oncogene through P300/CBP inhibition, a therapeutic approach for CIC-DUX4 sarcoma. Oncogenesis. 2021 Oct 12;10(10):68. doi: 10.1038/s41389-021-00357-4. PMID: 34642317; PMCID: PMC8511258.

81: Ding H, Pei Y, Li Y, Xu W, Mei L, Hou Z, Guang Y, Cao L, Li P, Cao H, Bian J, Chen K, Luo C, Zhou B, Zhang T, Li Z, Yang Y. Design, synthesis and biological evaluation of a novel spiro oxazolidinedione as potent p300/CBP HAT inhibitor for the treatment of ovarian cancer. Bioorg Med Chem. 2021 Dec 15;52:116512. doi: 10.1016/j.bmc.2021.116512. Epub 2021 Nov 10. PMID: 34801827.

82: Ji C, Xu W, Ding H, Chen Z, Shi C, Han J, Yu L, Qiao N, Zhang Y, Cao X, Zhou X, Cheng H, Feng H, Luo C, Li Z, Zhou B, Ye Z, Zhao Y. The p300 Inhibitor A-485 Exerts Antitumor Activity in Growth Hormone Pituitary Adenoma. J Clin Endocrinol Metab. 2022 May 17;107(6):e2291-e2300. doi: 10.1210/clinem/dgac128. PMID: 35247260; PMCID: PMC9113810.

83: Zhang R, Liu G, Xu H, Lou H, Zhai S, Chen A, Hao S, Xing J, Liu J, You M, Zhang Y, Xie C, Ma J, Liang R, Sun Q, Zhai H, Ni Z, Li B. Heat Stress Tolerance 2 confers basal heat stress tolerance in allohexaploid wheat (Triticum aestivum L.). J Exp Bot. 2022 Nov 2;73(19):6600-6614. doi: 10.1093/jxb/erac297. PMID: 35781562.

84: Brownsey DK, Rowley BC, Gorobets E, Mihara K, Maity R, Papatzimas JW, Gelfand BS, Hollenberg MD, Bahlis NJ, Derksen DJ. Identification of ligand linkage vectors for the development of p300/CBP degraders. RSC Med Chem. 2022 Apr 14;13(6):726-730. doi: 10.1039/d1md00070e. PMID: 35814928; PMCID: PMC9215131.

85: Ansari MSZ, Stagni V, Iuzzolino A, Rotili D, Mai A, Del Bufalo D, Lavia P, Degrassi F, Trisciuoglio D. Pharmacological targeting of CBP/p300 drives a redox/autophagy axis leading to senescence-induced growth arrest in non-small cell lung cancer cells. Cancer Gene Ther. 2023 Jan;30(1):124-136. doi: 10.1038/s41417-022-00524-8. Epub 2022 Sep 18. PMID: 36117234; PMCID: PMC9842509.

86: Vollan HS, Caugant DA, Eldholm V, Alfsnes K, Debech N, Brynildsrud O. Naturally occurring <i>Neisseria gonorrhoeae</i> can have large deletions in housekeeping gene <i>abcZ</i>, making them untypable with multilocus sequence typing. Microb Genom. 2022 Sep;8(9):mgen000889. doi: 10.1099/mgen.0.000889. PMID: 36136059; PMCID: PMC9676028.

87: Dance I. Understanding the tethered unhooking and rehooking of S2B in the reaction domain of FeMo-co, the active site of nitrogenase. Dalton Trans. 2022 Oct 18;51(40):15538-15554. doi: 10.1039/d2dt02571j. PMID: 36168836.

88: Hu Y, Mu H, Deng Z. H3K27 acetylation activated-CCS regulates autophagy and apoptosis of lung cancer by alleviating oxidative stress. Tissue Cell. 2023 Feb;80:101964. doi: 10.1016/j.tice.2022.101964. Epub 2022 Oct 28. PMID:

36402120.

89: Wei W, Song Z, Chiba M, Wu W, Jeong S, Zhang JP, Kadin ME, Nakagawa M, Yang Y. Analysis and therapeutic targeting of the EP300 and CREBBP acetyltransferases in anaplastic large cell lymphoma and Hodgkin lymphoma. Leukemia. 2023 Feb;37(2):396-407. doi: 10.1038/s41375-022-01774-z. Epub 2022 Dec 1. PMID: 36456744; PMCID: PMC9949602.

90: Rapchak K, Yagobian SD, Moore J, Khattri M, Shuda M. Merkel cell polyomavirus small T antigen is a viral transcription activator that is essential for viral genome maintenance. PLoS Pathog. 2022 Dec 27;18(12):e1011039. doi: 10.1371/journal.ppat.1011039. PMID: 36574443; PMCID: PMC9829177.

CAS SciFinder^n

Task History

Initiating Search

January 11, 2023, 10:18PM

All: **A-485**

Search Tasks

Task	Search Type	View
Returned All Results	All	View Results
Exported: Returned Reference Results + Filters (46)<br>Filtered By:<br>Document Type: Journal<br>Kept Selected Results<br>Removed Selected Results	References	View Results

Copyright © 2023 American Chemical Society (ACS). All Rights Reserved.
Internal use only. Redistribution is subject to the terms of your SciFinder^n License Agreement and CAS information Use Policies.

CAS SciFinder^n

References (46)

View in SciFinder^n

1

Discovery of a selective catalytic p300/CBP inhibitor that targets lineage-specific tumours Out

By: Lasko, Loren M.; Jakob, Clarissa G.; Edalji, Rohinton P.; Qiu, Wei; Montgomery, Debra; Digiammarino, Enrico L.; Hansen, T. Matt; Risi, Roberto M.; Frey, Robin; Manaves, Vlasios; et al
Nature (London, United Kingdom) (2017), 550(7674), 128-132 | Language: English, Database: CAplus and MEDLINE

Substances (38) | Reactions (96) | Cited By (345)

2

Selective inhibition of CBP/p300 HAT by A-485 results in suppression of lipogenesis and hepatic gluconeogenesis ✓

By: Zhou, Feiye; Liu, Qianqian; Zhang, Linlin; Zhu, Qin; Wang, Shushu; Zhu, Kecheng; Deng, Ruyuan; Liu, Yun; Yuan, Guoyue; Wang, Xiao; et al
Cell Death & Disease (2020), 11(9), 745 | Language: English, Database: CAplus and MEDLINE

Substances (9) | Reactions (0) | Cited By (10)

3

P300/CBP inhibitor A-485 alleviates acute liver injury by regulating macrophage activation and polarization ✓

By: Peng, Jinjin; Li, Jiacheng; Huang, Jing; Xu, Pan; Huang, Heming; Liu, Yanjun; Yu, Liang; Yang, Yaxi; Zhou, Bing; Jiang, Hualiang; et al
Theranostics (2019), 9(26), 8344-8361 | Language: English, Database: CAplus and MEDLINE

Substances (3) | Reactions (0) | Cited By (27)

4

Time-Resolved Analysis Reveals Rapid Dynamics and Broad Scope of the CBP/p300 Acetylome ✓

By: Weinert, Brian T.; Narita, Takeo; Satpathy, Shankha; Srinivasan, Balaji; Hansen, Bogi K.; Scholz, Christian; Hamilton, William B.; Zucconi, Beth E.; Wang, Wesley W.; Liu, Wenshe R.; et al
Cell (Cambridge, MA, United States) (2018), 174(1), 231-244.e12 | Language: English, Database: CAplus and MEDLINE

Substances (0) | Reactions (0) | Cited By (176)

5

Targeting lineage-specific MITF pathway in human melanoma cell lines by A-485, the selective small-molecule inhibitor of p300/CBP

By: Wang, Rui; He, Yupeng; Robinson, Valerie; Yang, Ziping; Hessler, Paul; Lasko, Loren M.; Lu, Xin; Bhathena, Anahita; Lai, Albert; Uziel, Tamar; et al
Molecular Cancer Therapeutics (2018), 17(12), 2543-2550 | Language: English, Database: CAplus and MEDLINE

Substances (3) | Reactions (0) | Cited By (37)

6

p300/CBP inhibitor A-485 inhibits the differentiation of osteoclasts and protects against osteoporotic bone loss

By: Huo, Shicheng; Liu, Xuesong; Zhang, Shutao; Lyu, Zhuocheng ; Zhang, Jue; Wang, You; Nie, Bin'en; Yue, Bing
International Immunopharmacology (2021), 94, 107458 | Language: English, Database: CAplus and MEDLINE

Substance (1) | Reactions (0) | Cited By (3)

7

Discovery of Spiro Oxazolidinediones as Selective, Orally Bioavailable Inhibitors of p300/CBP Histone Acetyltransferases

By: Michaelides, Michael R. ; Kluge, Arthur; Patane, Michael; Van Drie, John H.; Wang, Ce; Hansen, T. Matthew; Risi, Roberto M.; Mantei, Robert; Hertel, Carmen; Karukurichi, Kannan; et al
ACS Medicinal Chemistry Letters (2018), 9(1), 28-33 | Language: English, Database: CAplus and MEDLINE

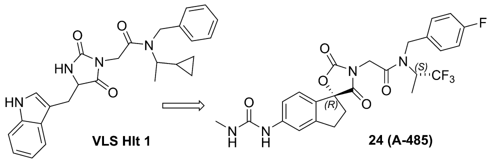

Out

Substances (98) | Reactions (312) | Cited By (46)

8

Therapeutic targeting of p300/CBP HAT domain for the treatment of NUT midline carcinoma

By: Zhang, Xin; Zegar, Tim; Lucas, Anais; Morrison-Smith, Chevaun; Knox, Tatiana; French, Christopher A.; Knapp, Stefan; Mueller, Susanne; Siveke, Jens T.
Oncogene (2020), 39(24), 4770-4779 | Language: English, Database: CAplus and MEDLINE

Substances (2) | Reactions (0) | Cited By (19)

9

TRPM3-induced gene transcription is under epigenetic control

By: Thiel, Gerald ; Roessler, Oliver G.
Pharmaceuticals (2022), 15(7), 846 | Language: English, Database: CAplus and MEDLINE

Substances (3) | Reactions (0) | Cited By (0)

10

Combination targeting of the bromodomain and acetyltransferase active site of p300/CBP

By: Zucconi, Beth E.; Makofske, Jessica L.; Meyers, David J.; Hwang, Yousang; Wu, Mingxuan; Kuroda, Mitzi I.; Cole, Philip A.
Biochemistry (2019), 58(16), 2133-2143 | Language: English, Database: CAplus and MEDLINE

Out

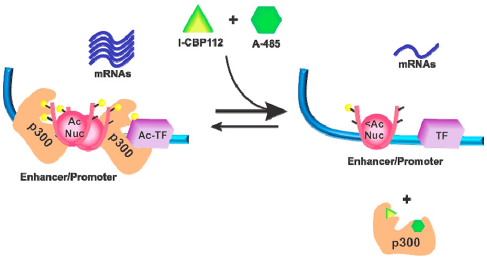

Substances (3) | Reactions (0) | Cited By (23)

11

A novel histone acetyltransferase inhibitor A485 improves sensitivity of non-small-cell lung carcinoma cells to TRAIL

By: Zhang, Baojie; Chen, Deng; Liu, Bin; Dekker, Frank J.; Quax, Wim J.
Biochemical Pharmacology (Amsterdam, Netherlands) (2020), 175, 113914 | Language: English, Database: CAplus and MEDLINE

Substances (2) | Reactions (0) | Cited By (12)

12

Histone acetyltransferases CBP/p300 in tumorigenesis and CBP/p300 inhibitors as promising novel anticancer agents

By: Chen, Qingjuan; Yang, Binhui; Liu, Xiaochen; Zhang, Xu D.; Zhang, Lirong; Liu, Tao
Theranostics (2022), 12(11), 4935-4948 | Language: English, Database: CAplus and MEDLINE

Substances (3) | Reactions (0) | Cited By (2)

13

Histone acetylation dynamics modulates chromatin conformation and allele-specific interactions at oncogenic loci

By: Sungalee, Stephanie ; Liu, Yuanlong; Lambuta, Ruxandra A.; Katanayeva, Natalya ; Donaldson Collier, Maria; Tavernari, Daniele ; Roulland, Sandrine; Ciriello, Giovanni ; Oricchio, Elisa
Nature Genetics (2021), 53(5), 650-662 | Language: English, Database: CAplus and MEDLINE

Substances (3) Reactions (0) Cited By (16)

14

P300/CBP inhibition sensitizes mantle cell lymphoma to PI3δ inhibitor idelalisib

By: Zhou, Xiao-ru; Li, Xiao; Liao, Li-ping; Han, Jie; Huang, Jing; Li, Jia-cheng; Tao, Hong-ru; Fan, Shi-jie; Chen, Zhi-feng; Li, Qi; et al
Acta Pharmacologica Sinica (2022), 43(2), 457-469 | Language: English, Database: CAplus and MEDLINE

Substances (13) Reactions (0) Cited By (4)

15

CBP/p300 HAT maintains the gene network critical for β cell identity and functional maturity

By: Zhang, Linlin; Sheng, Chunxiang; Zhou, Feiye; Zhu, Kecheng; Wang, Shushu; Liu, Qianqian; Yuan, Miaomiao; Xu, Zhaoqian; Liu, Yun; Lu, Jieli; et al
Cell Death & Disease (2021), 12(5), 476 | Language: English, Database: CAplus and MEDLINE

Substances (8) Reactions (0) Cited By (2)

16

Domain-Independent Inhibition of CBP/p300 Attenuates α-Synuclein Aggregation

By: Hlushchuk, Irena; Ruskoaho, Heikki ; Domanskyi, Andrii ; Airavaara, Mikko; Valimaki, Mika J.
ACS Chemical Neuroscience (2021), 12(13), 2273-2279 | Language: English, Database: CAplus and MEDLINE

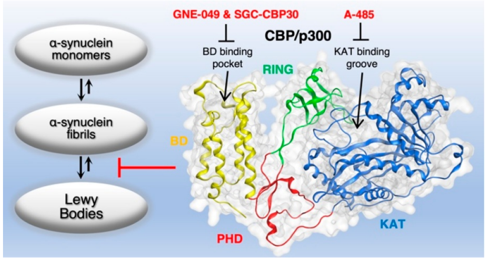

Substances (8) Reactions (0) Cited By (2)

17

Targeting histone acetylation dynamics and oncogenic transcription by catalytic P300/CBP inhibition

By: Hogg, Simon J.; Motorna, Olga; Cluse, Leonie A.; Johanson, Timothy M.; Coughlan, Hannah D.; Raviram, Ramya; Myers, Robert M.; Costacurta, Matteo; Todorovski, Izabela; Pijpers, Lizzy; et al
Molecular Cell (2021), 81(10), 2183-2200.e13 | Language: English, Database: CAplus and MEDLINE

Substances (13) | Reactions (0) | Cited By (22)

18

Discovery of spirohydantoins as selective, orally bioavailable inhibitors of p300/CBP histone acetyltransferases

Out

By: Ji, Zhiqin; Clark, Richard F.; Bhat, Vikram; Matthew Hansen, T.; Lasko, Loren M.; Bromberg, Kenneth D.; Manaves, Vlasios; Algire, Mikkel; Martin, Ruth; Qiu, Wei; et al
Bioorganic & Medicinal Chemistry Letters (2021), 39, 127854 | Language: English, Database: CAplus and MEDLINE

Substances (10) | Reactions (18) | Cited By (4)

19

Another One (of the "Undruggable" Targets) Bites the Dust: Discovery of a Potent and Selective Inhibitor of the Histone Acetyl Transferase p300/CBP

By: Kodadek, Thomas
Biochemistry (2018), 57(6), 899-900 | Language: English, Database: CAplus and MEDLINE

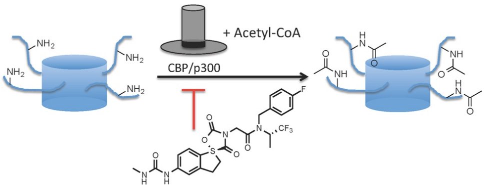

Substances (3) | Reactions (0) | Cited By (2)

20

Integrative genomic profiling uncovers therapeutic targets of acral melanoma in Asian populations

By: Shi, Qiong; Liu, Lin; Chen, Jianru; Zhang, Weigang; Guo, Weinan; Wang, Xiao; Wang, Huina; Guo, Sen; Yue, Qiao; Ma, Jingjing; et al
Clinical Cancer Research (2022), 28(12), 2690-2703 | Language: English, Database: CAplus and MEDLINE

Substances (3) | Reactions (0) | Cited By (2)

21

Report of the first international symposium on NUT carcinoma

By: French, Christopher A.; Cheng, Michael L.; Hanna, Glenn J.; DuBois, Steven G.; Chau, Nicole G.; Hann, Christine L.; Storck, Simone; Salgia, Ravi; Trucco, Matteo; Tseng, Jennifer; et al
Clinical Cancer Research (2022), 28(12), 2493-2505 | Language: English, Database: CAplus and MEDLINE

Substances (12) | Reactions (0) | Cited By (3)

22

Targeted degradation of the enhancer lysine acetyltransferases CBP and p300

By: Vannam, Raghu ; Sayilgan, Jan; Ojeda, Samuel ; Karakyriakou, Barbara ; Hu, Eileen; Kreuzer, Johannes ; Morris, Robert; Herrera Lopez, Xcanda Ixchel; Rai, Sumit; Haas, Wilhelm; et al
Cell Chemical Biology (2021), 28(4), 503-514.e12 | Language: English, Database: CAplus and MEDLINE

Substances (34) | Reactions (0) | Cited By (36)

23

Bromodomain inhibition of the coactivators CBP/EP300 facilitate cellular reprogramming

By: Ebrahimi, Ayyub ; Sevinc, Kenan; Gurhan Sevinc, Gulben; Cribbs, Adam P.; Philpott, Martin; Uyulur, Firat; Morova, Tunc; Dunford, James E. ; Goklemez, Sencer; Ari, Sule; et al
Nature Chemical Biology (2019), 15(5), 519-528 | Language: English, Database: CAplus and MEDLINE

Substances (7) | Reactions (0) | Cited By (44)

24

Assays for validating histone acetyltransferase inhibitors

By: Waddell, Aaron R.; Liao, Daiqing
Journal of Visualized Experiments (2020), (162), e61289 | Language: English, Database: CAplus and MEDLINE

Substances (7) | Reactions (0) | Cited By (1)

25

Large-scale RNAi screening uncovers therapeutic targets in the parasite Schistosoma mansoni

By: Wang, Jipeng ; Paz, Carlos ; Padalino, Gilda ; Coghlan, Avril ; Lu, Zhigang ; Gradinaru, Irina; Collins, Julie N. R.; Berriman, Matthew ; Hoffmann, Karl F.; Collins, James J. III
Science (Washington, DC, United States) (2020), 369(6511), 1649-1653 | Language: English, Database: CAplus and MEDLINE

Substances (16) | Reactions (0) | Cited By (26)

26

Discovery of a Potent and Selective Covalent p300/CBP Inhibitor

By: Mastracchio, Anthony ; Lai, Chunqiu; Digiammarino, Enrico; Ready, Damien B. ; Lasko, Loren M.; Bromberg, Kenneth D.; McClellan, William J.; Montgomery, Debra; Manaves, Vlasios; Shaw, Bailin; et al
ACS Medicinal Chemistry Letters (2021), 12(5), 726-731 | Language: English, Database: CAplus and MEDLINE

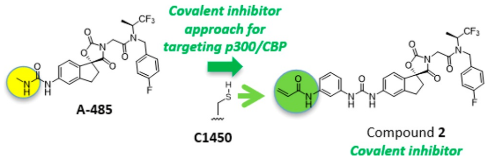

Out

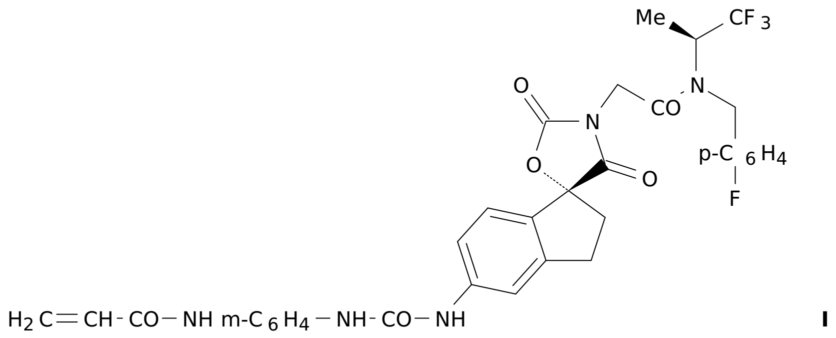

Substances (28) | Reactions (16) | Cited By (1)

27

Dual-functional polyetheretherketone surface modification for regulating immunity and bone metabolism

By: Huo, Shicheng; Wang, Fan; Lyu, Zhuocheng; Hong, Qimin; Nie, Bin'en; Wei, Jie; Wang, You; Zhang, Jue; Yue, Bing
Chemical Engineering Journal (Amsterdam, Netherlands) (2021), 426, 130806 | Language: English, Database: CAplus

Substances (13) | Reactions (0) | Cited By (11)

28

The p300 Inhibitor A-485 Exerts Antitumor Activity in Growth Hormone Pituitary Adenoma.

By: Ji, Chenxing ; Xu, Wen; Ding, Hong; Chen, Zhengyuan; Shi, Chengzhang; Han, Jie; Yu, Liang; Qiao, Nidan; Zhang, Yichao; Cao, Xiaoyun; et al
The Journal of clinical endocrinology and metabolism (2022), 107(6), e2291-e2300 | Language: English, Database: MEDLINE

Substances (2) | Reactions (0) | Cited By (2)

29

Targeted Protein Acetylation in Cells Using Heterobifunctional Molecules

By: Wang, Wesley W. ; Chen, Li-Yun; Wozniak, Jacob M.; Jadhav, Appaso M.; Anderson, Hayden; Malone, Taylor E.; Parker, Christopher G.
Journal of the American Chemical Society (2021), 143(40), 16700-16708 | Language: English, Database: CAplus and MEDLINE

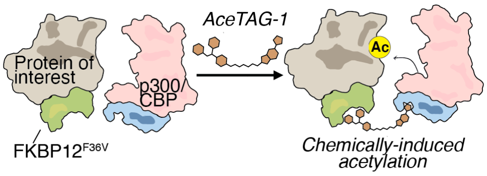

Substances (22) Reactions (0) Cited By (9)

30

Identification of potent inhibitors of SARS-CoV-2 infection by combined pharmacological evaluation and cellular network prioritization

By: Patten, J. J.; Keiser, Patrick T.; Morselli-Gysi, Deisy; Menichetti, Giulia; Mori, Hiroyuki; Donahue, Callie J.; Gan, Xiao; Valle, Italo do; Geoghegan-Barek, Kathleen; Anantpadma, Manu; et al
iScience (2022), 25(9), 104925 | Language: English, Database: CAplus and MEDLINE

Substances (44) Reactions (0) Cited By (2)

31

Interplay between protein acetylation and ubiquitination controls MCL1 protein stability

By: Shimizu, Kouhei; Gi, Min; Suzuki, Shugo; North, Brian J.; Watahiki, Asami; Fukumoto, Satoshi; Asara, John M.; Tokunaga, Fuminori ; Wei, Wenyi ; Inuzuka, Hiroyuki
Cell Reports (2021), 37(6), 109988 | Language: English, Database: CAplus and MEDLINE

Substances (12) Reactions (0) Cited By (4)

32

Therapeutic targeting of ATR yields durable regressions in small cell lung cancers with high replication stress

By: Thomas, Anish; Takahashi, Nobuyuki ; Rajapakse, Vinodh N.; Zhang, Xiaohu; Sun, Yilun; Ceribelli, Michele; Wilson, Kelli M.; Zhang, Yang; Beck, Erin; Sciuto, Linda; et al
Cancer Cell (2021), 39(4), 566-579.e7 | Language: English, Database: CAplus and MEDLINE

Substances (42) Reactions (0) Cited By (35)

33

Author Correction: Discovery of a selective catalytic p300/CBP inhibitor that targets lineage-specific tumours [Erratum to document cited in CA167:546652]

By: Lasko, Loren M.; Jakob, Clarissa G.; Edalji, Rohinton P.; Qiu, Wei; Montgomery, Debra; Digiammarino, Enrico L.; Hansen, T. Matt; Risi, Roberto M.; Frey, Robin; Manaves, Vlasios; et al
Nature (London, United Kingdom) (2018), 558(7710), E1 | Language: English, Database: CAplus and MEDLINE

Substances (27) Reactions (19) Cited By (3)

34

Pharmacological Inhibition of CBP/p300 Blocks Estrogen Receptor Alpha (ERα) Function through Suppressing Enhancer H3K27 Acetylation in Luminal Breast Cancer

By: Waddell, Aaron ; Mahmud, Iqbal ; Ding, Haocheng; Huo, Zhiguang ; Liao, Daiqing
Cancers (2021), 13(11), 2799 | Language: English, Database: CAplus and MEDLINE

Substances (0) Reactions (0) Cited By (11)

35

Histone Modifications Drive Aberrant Notch3 Expression/Activity and Growth in T-ALL.

By: Tottone, Luca; Zhdanovskaya, Nadezda; Carmona Pestaña, Álvaro; Zampieri, Michele; Simeoni, Fabrizio; Lazzari, Sara; Ruocco, Valeria; Pelullo, Maria; Caiafa, Paola; Felli, Maria Pia; et al
Frontiers in oncology (2019), 9, 198 | Language: English, Database: MEDLINE

Substances (0) Reactions (0) Cited By (25)

36

Pharmacological targeting of CBP/p300 drives a redox/autophagy axis leading to senescence-induced growth arrest in non-small cell lung cancer cells

By: Ansari, Mohammad Salik Zeya ; Stagni, Venturina; Iuzzolino, Angela ; Rotili, Dante; Mai, Antonello; Del Bufalo, Donatella; Lavia, Patrizia ; Degrassi, Francesca ; Trisciuoglio, Daniela
Cancer Gene Therapy, | Language: English, Database: CAplus and MEDLINE

Substances (0) Reactions (0) Cited By (0)

37

Synthesis and biological evaluation of spirocyclic chromane derivatives as a potential treatment of prostate cancer

By: Feng, Li; Yu, Shujia; Wang, Hai; Yang, Shengwei; Li, Xue; Dai, Hongjuan; Zhao, Liwen; Jiang, Cheng; Wang, Yazhou
Molecules (2021), 26(11), 3162 | Language: English, Database: CAplus and MEDLINE

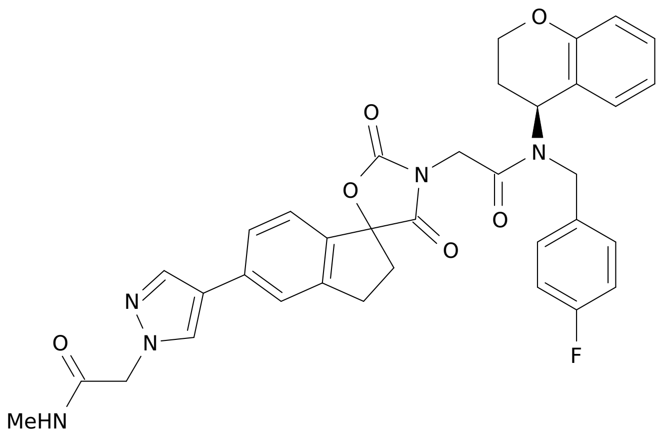

Out

Substances (160) | Reactions (418) | Cited By (3)

38

Inactivation of the CIC-DUX4 oncogene through P300/CBP inhibition, a therapeutic approach for CIC-DUX4 sarcoma

Out

By: Bosnakovski, Darko ; Ener, Elizabeth T. ; Cooper, Mark S.; Gearhart, Micah D. ; Knights, Kevin A.; Xu, Natalie C. ; Palumbo, Christian A. ; Toso, Erik A. ; Marsh, Graham P. ; Maple, Hannah J. ; et al
Oncogenesis (2021), 10(10), 68 | Language: English, Database: CAplus and MEDLINE

Substances (0) | Reactions (0) | Cited By (2)

39

Identification of ligand linkage vectors for the development of p300/CBP degraders

By: Brownsey, Duncan K.; Rowley, Ben C.; Gorobets, Evgueni; Mihara, Koichiro; Maity, Ranjan; Papatzimas, James W.; Gelfand, Benjamin S.; Hollenberg, Morley D.; Bahlis, Nizar J.; Derksen, Darren J.
RSC Medicinal Chemistry (2022), 13(6), 726-730 | Language: English, Database: CAplus and MEDLINE

Substances (28) | Reactions (0) | Cited By (0)

40

H3K27 acetylation activated-CCS regulates autophagy and apoptosis of lung cancer by alleviating oxidative stress

By: Hu, Yan; Mu, Hanshuo; Deng, Zhiping
Tissue & Cell, | Language: English, Database: CAplus and MEDLINE

Substances (0) Reactions (0) Cited By (0)

41

Design, synthesis and biological evaluation of a novel spiro oxazolidinedione as potent p300/CBP HAT inhibitor for the treatment of ovarian cancer

Out

By: Ding, Hong; Pei, Yuan; Li, Yuanqing; Xu, Wen; Mei, Lianghe; Hou, Zeng; Guang, Yiman; Cao, Liyuan; Li, Peizhuo; Cao, Haijing; et al
Bioorganic & Medicinal Chemistry (2021), 52, 116512 | Language: English, Database: CAplus and MEDLINE

Substances (152) Reactions (608) Cited By (0)

42

Promoter-specific changes in initiation, elongation, and homeostasis of histone H3 acetylation during CBP/p300 inhibition

By: Hsu, Emily; Zemke, Nathan R.; Berk, Arnold J.
eLife (2021), 10, e63512 | Language: English, Database: CAplus and MEDLINE

Substances (0) Reactions (0) Cited By (9)

43

Acetylation of histone 3 promotes miR-29a expression and downregulates STAT3 in sepsis.

By: Zheng, Yun; Cheng, Jun; Zhang, AFang; Wang, YuYang; Dai, ChengCai; Li, JiaBin
Injury (2022), 53(2), 416-421 | Language: English, Database: MEDLINE

Substances (0) Reactions (0) Cited By (0)

44

Analysis and therapeutic targeting of the EP300 and CREBBP acetyltransferases in anaplastic large cell lymphoma and Hodgkin lymphoma

By: Wei, Wei; Song, Zhihui; Chiba, Masahiro; Wu, Wenjun; Jeong, Subin; Zhang, Jing-Ping; Kadin, Marshall E.; Nakagawa, Masao; Yang, Yibin
Leukemia, | Language: English, Database: CAplus and MEDLINE

Substances (0) Reactions (0) Cited By (0)

45

Targeting cancer epigenetic pathways with small-molecule compounds: Therapeutic efficacy and combination therapies

Out

By: Wang, Yi ; Xie, Qiang; Tan, Huidan; Liao, Minru; Zhu, Shiou; Zheng, Ling-Li; Huang, Haixia; Liu, Bo
Pharmacological Research (2021), 173, 105702 | Language: English, Database: CAplus and MEDLINE

Substances (4) | Reactions (0) | Cited By (5)

46

The p300/CBP Inhibitor A485 Normalizes Psoriatic Fibroblast Gene Expression In Vitro and Reduces Psoriasis-Like Skin Inflammation In Vivo

By: Kim, Jihye ; He, Yuliang ; Tormen, Sabrina ; Kleindienst, Pascal ; Ducoli, Luca ; Restivo, Gaetana ; Drach, Mathias ; Levesque, Mitchell P. ; Navarini, Alexander A. ; Tacconi, Carlotta ; et al
Journal of Investigative Dermatology, | Language: English, Database: CAplus and MEDLINE

Substances (0) | Reactions (0) | Cited By (0)

Copyright © 2023 American Chemical Society (ACS). All Rights Reserved.
Copyright © 2023 U.S. National Library of Medicine.
Internal use only. Redistribution is subject to the terms of your SciFinder^n License Agreement and CAS information Use Policies.

CAS SciFinderⁿ

Task History

Initiating Search

January 11, 2023, 10:28PM

All:

Structure of A-485
(right)

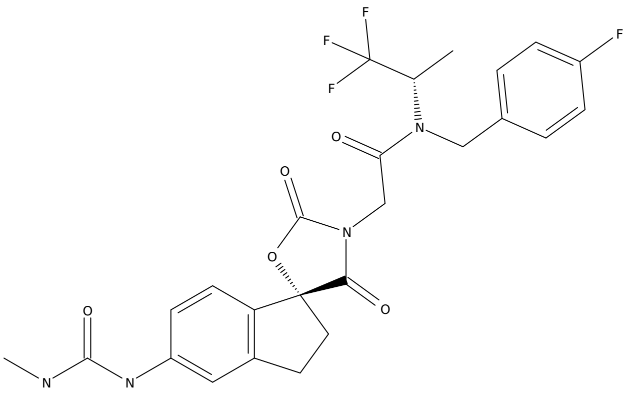

Search Tasks

Task	Search Type	View
Returned All Results	All	View Results
Exported: Returned Reference Results + Filters (27)<br>Filtered By:<br>Structure Match: As Drawn<br>Document Type: Journal	References	View Results

Copyright © 2023 American Chemical Society (ACS). All Rights Reserved.
Internal use only. Redistribution is subject to the terms of your SciFinderⁿ License Agreement and CAS information Use Policies.

CAS SciFinder^n

References (27)

View in SciFinder^n

1

Therapeutic targeting of p300/CBP HAT domain for the treatment of NUT midline carcinoma

By: Zhang, Xin; Zegar, Tim; Lucas, Anais; Morrison-Smith, Chevaun; Knox, Tatiana; French, Christopher A.; Knapp, Stefan; Mueller, Susanne; Siveke, Jens T.

Oncogene (2020), 39(24), 4770-4779 | Language: English, Database: CAplus and MEDLINE

Substances (2) Reactions (0) Cited By (19)

2

Discovery of a selective catalytic p300/CBP inhibitor that targets lineage-specific tumours

By: Lasko, Loren M.; Jakob, Clarissa G.; Edalji, Rohinton P.; Qiu, Wei; Montgomery, Debra; Digiammarino, Enrico L.; Hansen, T. Matt; Risi, Roberto M.; Frey, Robin; Manaves, Vlasios; et al

Nature (London, United Kingdom) (2017), 550(7674), 128-132 | Language: English, Database: CAplus and MEDLINE

Substances (38) Reactions (96) Cited By (345)

3

p300/CBP inhibitor A-485 inhibits the differentiation of osteoclasts and protects against osteoporotic bone loss

By: Huo, Shicheng; Liu, Xuesong; Zhang, Shutao; Lyu, Zhuocheng ; Zhang, Jue; Wang, You; Nie, Bin'en; Yue, Bing

International Immunopharmacology (2021), 94, 107458 | Language: English, Database: CAplus and MEDLINE

Substance (1) Reactions (0) Cited By (3)

4

Targeting lineage-specific MITF pathway in human melanoma cell lines by A-485, the selective small-molecule inhibitor of p300/CBP

By: Wang, Rui; He, Yupeng; Robinson, Valerie; Yang, Ziping; Hessler, Paul; Lasko, Loren M.; Lu, Xin; Bhathena, Anahita; Lai, Albert; Uziel, Tamar; et al

Molecular Cancer Therapeutics (2018), 17(12), 2543-2550 | Language: English, Database: CAplus and MEDLINE

Substances (3) Reactions (0) Cited By (37)

5

A novel histone acetyltransferase inhibitor A485 improves sensitivity of non-small-cell lung carcinoma cells to TRAIL

By: Zhang, Baojie; Chen, Deng; Liu, Bin; Dekker, Frank J.; Quax, Wim J.
Biochemical Pharmacology (Amsterdam, Netherlands) (2020), 175, 113914 | Language: English, Database: CAplus and MEDLINE

Substances (2) | Reactions (0) | Cited By (12)

6

Bromodomain inhibition of the coactivators CBP/EP300 facilitate cellular reprogramming

By: Ebrahimi, Ayyub ; Sevinc, Kenan; Gurhan Sevinc, Gulben; Cribbs, Adam P.; Philpott, Martin; Uyulur, Firat; Morova, Tunc; Dunford, James E. ; Goklemez, Sencer; Ari, Sule; et al
Nature Chemical Biology (2019), 15(5), 519-528 | Language: English, Database: CAplus and MEDLINE

Substances (7) | Reactions (0) | Cited By (44)

7

TRPM3-induced gene transcription is under epigenetic control

By: Thiel, Gerald ; Roessler, Oliver G.
Pharmaceuticals (2022), 15(7), 846 | Language: English, Database: CAplus and MEDLINE

Substances (3) | Reactions (0) | Cited By (0)

8

Histone acetyltransferases CBP/p300 in tumorigenesis and CBP/p300 inhibitors as promising novel anticancer agents

By: Chen, Qingjuan; Yang, Binhui; Liu, Xiaochen; Zhang, Xu D.; Zhang, Lirong; Liu, Tao
Theranostics (2022), 12(11), 4935-4948 | Language: English, Database: CAplus and MEDLINE

Substances (3) | Reactions (0) | Cited By (2)

9

Integrative genomic profiling uncovers therapeutic targets of acral melanoma in Asian populations

By: Shi, Qiong; Liu, Lin; Chen, Jianru; Zhang, Weigang; Guo, Weinan; Wang, Xiao; Wang, Huina; Guo, Sen; Yue, Qiao; Ma, Jingjing; et al
Clinical Cancer Research (2022), 28(12), 2690-2703 | Language: English, Database: CAplus and MEDLINE

Substances (3) | Reactions (0) | Cited By (2)

10

Histone acetylation dynamics modulates chromatin conformation and allele-specific interactions at oncogenic loci

By: Sungalee, Stephanie ; Liu, Yuanlong; Lambuta, Ruxandra A.; Katanayeva, Natalya ; Donaldson Collier, Maria; Tavernari, Daniele ; Roulland, Sandrine; Ciriello, Giovanni ; Oricchio, Elisa
Nature Genetics (2021), 53(5), 650-662 | Language: English, Database: CAplus and MEDLINE

Substances (3) | Reactions (0) | Cited By (16)

11

Selective inhibition of CBP/p300 HAT by A-485 results in suppression of lipogenesis and hepatic gluconeogenesis

By: Zhou, Feiye; Liu, Qianqian; Zhang, Linlin; Zhu, Qin; Wang, Shushu; Zhu, Kecheng; Deng, Ruyuan; Liu, Yun; Yuan, Guoyue; Wang, Xiao; et al
Cell Death & Disease (2020), 11(9), 745 | Language: English, Database: CAplus and MEDLINE

Substances (9) | Reactions (0) | Cited By (10)

12

Discovery of spirohydantoins as selective, orally bioavailable inhibitors of p300/CBP histone acetyltransferases

By: Ji, Zhiqin; Clark, Richard F.; Bhat, Vikram; Matthew Hansen, T.; Lasko, Loren M.; Bromberg, Kenneth D.; Manaves, Vlasios; Algire, Mikkel; Martin, Ruth; Qiu, Wei; et al
Bioorganic & Medicinal Chemistry Letters (2021), 39, 127854 | Language: English, Database: CAplus and MEDLINE

Substances (10) | Reactions (18) | Cited By (4)

13

Targeting histone acetylation dynamics and oncogenic transcription by catalytic P300/CBP inhibition

By: Hogg, Simon J. ; Motorna, Olga; Cluse, Leonie A. ; Johanson, Timothy M.; Coughlan, Hannah D. ; Raviram, Ramya; Myers, Robert M.; Costacurta, Matteo ; Todorovski, Izabela ; Pijpers, Lizzy; et al
Molecular Cell (2021), 81(10), 2183-2200.e13 | Language: English, Database: CAplus and MEDLINE

Substances (13) | Reactions (0) | Cited By (22)

14

Domain-Independent Inhibition of CBP/p300 Attenuates α-Synuclein Aggregation

By: Hlushchuk, Irena; Ruskoaho, Heikki ; Domanskyi, Andrii ; Airavaara, Mikko; Valimaki, Mika J.
ACS Chemical Neuroscience (2021), 12(13), 2273-2279 | Language: English, Database: CAplus and MEDLINE

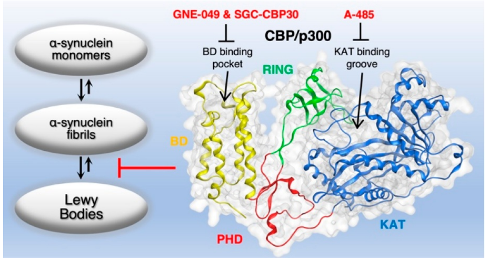

Substances (8) Reactions (0) Cited By (2)

15

CBP/p300 HAT maintains the gene network critical for β cell identity and functional maturity

By: Zhang, Linlin; Sheng, Chunxiang; Zhou, Feiye; Zhu, Kecheng; Wang, Shushu; Liu, Qianqian; Yuan, Miaomiao; Xu, Zhaoqian; Liu, Yun; Lu, Jieli; et al
Cell Death & Disease (2021), 12(5), 476 | Language: English, Database: CAplus and MEDLINE

Substances (8) Reactions (0) Cited By (2)

16

Discovery of Spiro Oxazolidinediones as Selective, Orally Bioavailable Inhibitors of p300/CBP Histone Acetyltransferases

By: Michaelides, Michael R. ; Kluge, Arthur; Patane, Michael; Van Drie, John H.; Wang, Ce; Hansen, T. Matthew; Risi, Roberto M.; Mantei, Robert; Hertel, Carmen; Karukurichi, Kannan; et al
ACS Medicinal Chemistry Letters (2018), 9(1), 28-33 | Language: English, Database: CAplus and MEDLINE

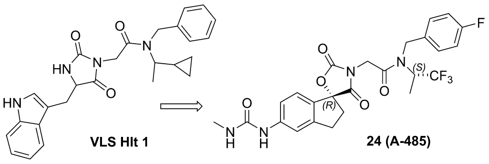

Substances (98) Reactions (312) Cited By (46)

17

Report of the first international symposium on NUT carcinoma

By: French, Christopher A.; Cheng, Michael L.; Hanna, Glenn J.; DuBois, Steven G.; Chau, Nicole G.; Hann, Christine L.; Storck, Simone; Salgia, Ravi; Trucco, Matteo; Tseng, Jennifer; et al
Clinical Cancer Research (2022), 28(12), 2493-2505 | Language: English, Database: CAplus and MEDLINE

Substances (12) Reactions (0) Cited By (3)

18

Interplay between protein acetylation and ubiquitination controls MCL1 protein stability

By: Shimizu, Kouhei; Gi, Min; Suzuki, Shugo; North, Brian J.; Watahiki, Asami; Fukumoto, Satoshi; Asara, John M.; Tokunaga, Fuminori ; Wei, Wenyi ; Inuzuka, Hiroyuki
Cell Reports (2021), 37(6), 109988 | Language: English, Database: CAplus and MEDLINE

Substances (12) | Reactions (0) | Cited By (4)

19

Dual-functional polyetheretherketone surface modification for regulating immunity and bone metabolism

By: Huo, Shicheng; Wang, Fan; Lyu, Zhuocheng; Hong, Qimin; Nie, Bin'en; Wei, Jie; Wang, You; Zhang, Jue; Yue, Bing
Chemical Engineering Journal (Amsterdam, Netherlands) (2021), 426, 130806 | Language: English, Database: CAplus

Substances (13) | Reactions (0) | Cited By (11)

20

P300/CBP inhibition sensitizes mantle cell lymphoma to PI3δ inhibitor idelalisib

By: Zhou, Xiao-ru; Li, Xiao; Liao, Li-ping; Han, Jie; Huang, Jing; Li, Jia-cheng; Tao, Hong-ru; Fan, Shi-jie; Chen, Zhi-feng; Li, Qi; et al
Acta Pharmacologica Sinica (2022), 43(2), 457-469 | Language: English, Database: CAplus and MEDLINE

Substances (13) | Reactions (0) | Cited By (4)

21

Large-scale RNAi screening uncovers therapeutic targets in the parasite Schistosoma mansoni

By: Wang, Jipeng ; Paz, Carlos ; Padalino, Gilda ; Coghlan, Avril ; Lu, Zhigang ; Gradinaru, Irina; Collins, Julie N. R.; Berriman, Matthew ; Hoffmann, Karl F.; Collins, James J. III
Science (Washington, DC, United States) (2020), 369(6511), 1649-1653 | Language: English, Database: CAplus and MEDLINE

Substances (16) | Reactions (0) | Cited By (26)

22

Targeted Protein Acetylation in Cells Using Heterobifunctional Molecules

By: Wang, Wesley W.; Chen, Li-Yun; Wozniak, Jacob M.; Jadhav, Appaso M.; Anderson, Hayden; Malone, Taylor E.; Parker, Christopher G.
Journal of the American Chemical Society (2021), 143(40), 16700-16708 | Language: English, Database: CAplus and MEDLINE

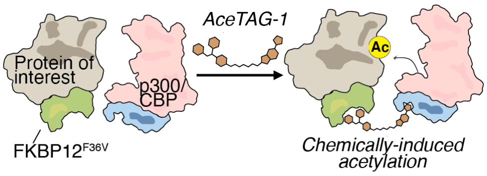

Substances (22) | Reactions (0) | Cited By (9)

23

Identification of potent inhibitors of SARS-CoV-2 infection by combined pharmacological evaluation and cellular network prioritization

By: Patten, J. J.; Keiser, Patrick T.; Morselli-Gysi, Deisy; Menichetti, Giulia; Mori, Hiroyuki; Donahue, Callie J.; Gan, Xiao; Valle, Italo do; Geoghegan-Barek, Kathleen; Anantpadma, Manu; et al
iScience (2022), 25(9), 104925 | Language: English, Database: CAplus and MEDLINE

Substances (44) | Reactions (0) | Cited By (2)

24

Therapeutic targeting of ATR yields durable regressions in small cell lung cancers with high replication stress

By: Thomas, Anish; Takahashi, Nobuyuki; Rajapakse, Vinodh N.; Zhang, Xiaohu; Sun, Yilun; Ceribelli, Michele; Wilson, Kelli M.; Zhang, Yang; Beck, Erin; Sciuto, Linda; et al
Cancer Cell (2021), 39(4), 566-579.e7 | Language: English, Database: CAplus and MEDLINE

Substances (42) | Reactions (0) | Cited By (35)

25

Discovery of a Potent and Selective Covalent p300/CBP Inhibitor

By: Mastracchio, Anthony ; Lai, Chunqiu; Digiammarino, Enrico; Ready, Damien B. ; Lasko, Loren M.; Bromberg, Kenneth D.; McClellan, William J.; Montgomery, Debra; Manaves, Vlasios; Shaw, Bailin; et al
ACS Medicinal Chemistry Letters (2021), 12(5), 726-731 | Language: English, Database: CAplus and MEDLINE

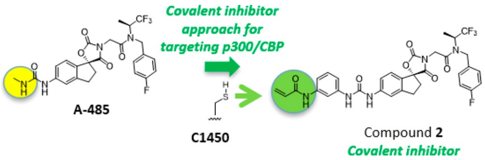

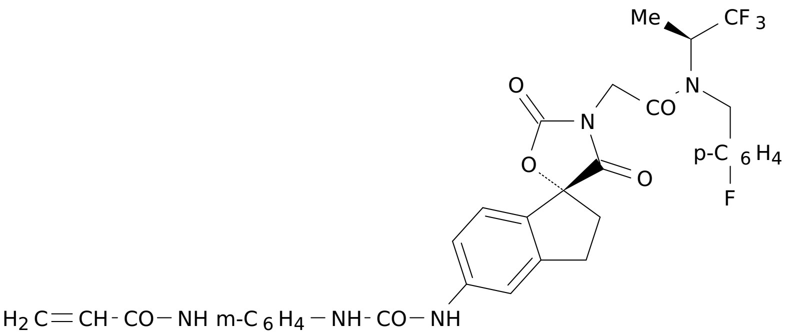

I

Substances (28) Reactions (16) Cited By (1)

26

Targeted degradation of the enhancer lysine acetyltransferases CBP and p300

By: Vannam, Raghu ; Sayilgan, Jan; Ojeda, Samuel ; Karakyriakou, Barbara ; Hu, Eileen; Kreuzer, Johannes ; Morris, Robert; Herrera Lopez, Xcanda Ixchel; Rai, Sumit; Haas, Wilhelm; et al
Cell Chemical Biology (2021), 28(4), 503-514.e12 | Language: English, Database: CAplus and MEDLINE

Substances (34) Reactions (0) Cited By (36)

27

Author Correction: Discovery of a selective catalytic p300/CBP inhibitor that targets lineage-specific tumours [Erratum to document cited in CA167:546652]

By: Lasko, Loren M.; Jakob, Clarissa G.; Edalji, Rohinton P.; Qiu, Wei; Montgomery, Debra; Digiammarino, Enrico L.; Hansen, T. Matt; Risi, Roberto M.; Frey, Robin; Manaves, Vlasios; et al
Nature (London, United Kingdom) (2018), 558(7710), E1 | Language: English, Database: CAplus and MEDLINE

Substances (27) Reactions (19) Cited By (3)

Copyright © 2023 American Chemical Society (ACS). All Rights Reserved.
Copyright © 2023 U.S. National Library of Medicine.
Internal use only. Redistribution is subject to the terms of your SciFinderⁿ License Agreement and CAS information Use Policies.

Results of PubMed search: keyword AMG900

1: Payton M, Bush TL, Chung G, Ziegler B, Eden P, McElroy P, Ross S, Cee VJ, Deak HL, Hodous BL, Nguyen HN, Olivieri PR, Romero K, Schenkel LB, Bak A, Stanton M, Dussault I, Patel VF, Geuns-Meyer S, Radinsky R, Kendall RL. Preclinical evaluation of AMG 900, a novel potent and highly selective pan-aurora kinase inhibitor with activity in taxane-resistant tumor cell lines. Cancer Res. 2010 Dec 1;70(23):9846-54. doi: 10.1158/0008-5472.CAN-10-3001. Epub 2010 Oct 8. PMID: 20935223.

2: Huang L, Be X, Berry L, Moore E, Janosky B, Wells M, Pan WJ, Zhao Z, Lin MH. In vitro and in vivo pharmacokinetic characterizations of AMG 900, an orally bioavailable small molecule inhibitor of aurora kinases. Xenobiotica. 2011 May;41(5):400-8. doi: 10.3109/00498254.2010.548534. Epub 2011 Feb 4. PMID: 21294625.

3: Waldon D, Berry L, Lin MH, Schenkel L, Hollis LS, Zhao Z. Gender effects on rat metabolism of AMG 900, an orally available small molecule aurora kinase inhibitor. Drug Metab Lett. 2011 Dec;5(4):290-7. doi: 10.2174/187231211798472557. PMID: 22022868.

4: Be X, Moore ES, Zhao Z, Wells MC. LC-MS/MS bioanalytical method development for AMG 900: resolution of an isobaric interference in rodent in vivo studies. J Pharm Biomed Anal. 2013 Feb 23;74:171-7. doi: 10.1016/j.jpba.2012.10.026. Epub 2012 Nov 2. PMID: 23245248.

5: Bush TL, Payton M, Heller S, Chung G, Hanestad K, Rottman JB, Loberg R, Friberg G, Kendall RL, Saffran D, Radinsky R. AMG 900, a small-molecule inhibitor of aurora kinases, potentiates the activity of microtubule-targeting agents in human metastatic breast cancer models. Mol Cancer Ther. 2013 Nov;12(11):2356-66. doi: 10.1158/1535-7163.MCT-12-1178. Epub 2013 Aug 29. PMID: 23990115.

6: Kalous O, Conklin D, Desai AJ, Dering J, Goldstein J, Ginther C, Anderson L, Lu M, Kolarova T, Eckardt MA, Langerød A, Børresen-Dale AL, Slamon DJ, Finn RS. AMG 900, pan-Aurora kinase inhibitor, preferentially inhibits the proliferation of breast cancer cell lines with dysfunctional p53. Breast Cancer Res Treat. 2013 Oct;141(3):397-408. doi: 10.1007/s10549-013-2702-z. Epub 2013 Oct 5. PMID: 24091768.

7: Paller CJ, Wissing MD, Mendonca J, Sharma A, Kim E, Kim HS, Kortenhorst MS, Gerber S, Rosen M, Shaikh F, Zahurak ML, Rudek MA, Hammers H, Rudin CM, Carducci MA, Kachhap SK. Combining the pan-aurora kinase inhibitor AMG 900 with histone deacetylase inhibitors enhances antitumor activity in prostate cancer. Cancer Med. 2014 Oct;3(5):1322-35. doi: 10.1002/cam4.289. Epub 2014 Jul 3. PMID: 24989836; PMCID: PMC4302682.

8: Juan G, Bush TL, Ma C, Manoukian R, Chung G, Hawkins JM, Zoog S, Kendall R, Radinsky R, Loberg R, Friberg G, Payton M. AMG 900, a potent inhibitor of aurora kinases causes pharmacodynamic changes in p-Histone H3 immunoreactivity in human tumor xenografts and proliferating mouse tissues. J Transl Med. 2014 Nov 4;12:307. doi: 10.1186/s12967-014-0307-x. PMID: 25367255; PMCID: PMC4221688.

9: Geuns-Meyer S, Cee VJ, Deak HL, Du B, Hodous BL, Nguyen HN, Olivieri PR, Schenkel LB, Vaida KR, Andrews P, Bak A, Be X, Beltran PJ, Bush TL, Chaves MK, Chung G, Dai Y, Eden P, Hanestad K, Huang L, Lin MH, Tang J, Ziegler B, Radinsky R, Kendall R, Patel VF, Payton M. Discovery of N-(4-(3-(2-aminopyrimidin-4-yl)py ridin-2-yloxy)phenyl)-4-(4-methylthiophen-2-yl)phthalazin-1-amine (AMG 900), a

highly selective, orally bioavailable inhibitor of aurora kinases with activity against multidrug-resistant cancer cell lines. J Med Chem. 2015 Jul 9;58(13):5189-207. doi: 10.1021/acs.jmedchem.5b00183. Epub 2015 May 31. PMID: 25970324.

10: Geron L, Borges KS, Andrade AF, Suazo VK, Scrideli CA, Tone LG. Antitumour activity of AMG 900 alone or in combination with histone deacetylase inhibitor SaHa on medulloblastoma cell lines. Neurol Res. 2015 Aug;37(8):703-11. doi: 10.1179/1743132815Y.0000000048. Epub 2015 May 22. PMID: 26000978.

11: Linardopoulos S, Blagg J. Aurora kinase inhibition: a new light in the sky? J Med Chem. 2015 Jul 9;58(13):5186-8. doi: 10.1021/acs.jmedchem.5b00918. Epub 2015 Jun 25. PMID: 26111271.

12: Kantarjian HM, Schuster MW, Jain N, Advani A, Jabbour E, Gamelin E, Rasmussen E, Juan G, Anderson A, Chow VF, Friberg G, Vogl FD, Sekeres MA. A phase 1 study of AMG 900, an orally administered pan-aurora kinase inhibitor, in adult patients with acute myeloid leukemia. Am J Hematol. 2017 Jul;92(7):660-667. doi: 10.1002/ajh.24736. Epub 2017 Jun 5. PMID: 28370201; PMCID: PMC5925751.

13: Borges KS, Andrade AF, Silveira VS, Marco Antonio DS, Vasconcelos EJR, Antonini SRR, Tone LG, Scrideli CA. The aurora kinase inhibitor AMG 900 increases apoptosis and induces chemosensitivity to anticancer drugs in the NCI-H295 adrenocortical carcinoma cell line. Anticancer Drugs. 2017 Jul;28(6):634-644. doi: 10.1097/CAD.0000000000000504. PMID: 28410270.

14: Noronha S, Alt LAC, Scimeca TE, Zarou O, Obrzut J, Zanotti B, Hayward EA, Pillai A, Mathur S, Rojas J, Salamah R, Chandar N, Fay MJ. Preclinical evaluation of the Aurora kinase inhibitors AMG 900, AZD1152-HQPA, and MK-5108 on SW-872 and 93T449 human liposarcoma cells. In Vitro Cell Dev Biol Anim. 2018 Jan;54(1):71-84. doi: 10.1007/s11626-017-0208-4. Epub 2017 Dec 1. PMID: 29197031.

15: Carducci M, Shaheen M, Markman B, Hurvitz S, Mahadevan D, Kotasek D, Goodman OB Jr, Rasmussen E, Chow V, Juan G, Friberg GR, Gamelin E, Vogl FD, Desai J. A phase 1, first-in-human study of AMG 900, an orally administered pan-Aurora kinase inhibitor, in adult patients with advanced solid tumors. Invest New Drugs. 2018 Dec;36(6):1060-1071. doi: 10.1007/s10637-018-0625-6. Epub 2018 Jul 7. PMID: 29980894; PMCID: PMC6639057.

16: Ryu J, Pyo J, Lee CW, Kim JE. An Aurora kinase inhibitor, AMG900, inhibits glioblastoma cell proliferation by disrupting mitotic progression. Cancer Med. 2018 Nov;7(11):5589-5603. doi: 10.1002/cam4.1771. Epub 2018 Sep 17. PMID: 30221846; PMCID: PMC6246935.

17: Payton M, Cheung HK, Ninniri MSS, Marinaccio C, Wayne WC, Hanestad K, Crispino JD, Juan G, Coxon A. Dual Targeting of Aurora Kinases with AMG 900 Exhibits Potent Preclinical Activity Against Acute Myeloid Leukemia with Distinct Post-Mitotic Outcomes. Mol Cancer Ther. 2018 Dec;17(12):2575-2585. doi: 10.1158/1535-7163.MCT-18-0186. Epub 2018 Sep 28. PMID: 30266802; PMCID: PMC6279493.

18: Takeda T, Tsubaki M, Genno S, Nemoto C, Onishi Y, Yamamoto Y, Imano M, Satou T, Nishida S. AT9283 exhibits antiproliferative effect on tyrosine kinase inhibitor-sensitive and -resistant chronic myeloid leukemia cells by inhibition of Aurora A and Aurora B. Oncol Rep. 2020 Nov;44(5):2211-2218. doi:

10.3892/or.2020.7739. Epub 2020 Aug 18. PMID: 33000229.

19: Fischer N, Seo EJ, Klinger A, Fleischer E, Efferth T. AMG900 as novel inhibitor of the translationally controlled tumor protein. Chem Biol Interact. 2021 Jan 25;334:109349. doi: 10.1016/j.cbi.2020.109349. Epub 2020 Nov 28. PMID: 33259807.

20: Maria AG, Silva Borges K, Lira RCP, Hassib Thomé C, Berthon A, Drougat L, Kiseljak-Vassiliades K, Wierman ME, Faucz FR, Faça VM, Tone LG, Stratakis CA. Inhibition of Aurora kinase A activity enhances the antitumor response of beta-catenin blockade in human adrenocortical cancer cells. Mol Cell Endocrinol. 2021 May 15;528:111243. doi: 10.1016/j.mce.2021.111243. Epub 2021 Mar 11. PMID: 33716050; PMCID: PMC8297658.

21: Lv G, Shi Q, Zhang T, Li J, Kalashova J, Long Y, Sun Y, Li C, Choudhry N, Li H, Yang C, Zhou X, Reddy MC, Anantoju KK, Jupelli R, Zhang S, Zhang J, Allen T, Liu H, Nimishetti N, Yang D. 2-Phenoxy-3, 4'-bipyridine derivatives inhibit AURKB-dependent mitotic processes by disrupting its localization. Eur J Med Chem. 2023 Jan 5;245(Pt 1):114904. doi: 10.1016/j.ejmech.2022.114904. Epub 2022 Nov 8. PMID: 36413818.

CAS SciFinder^{n}

Task History

Initiating Search

January 12, 2023, 4:28PM

All: **AMG900**

Search Tasks

Task	Search Type	View
Returned All Results	All	View Results
Exported: Returned Reference Results (17)	References	View Results

Copyright © 2023 American Chemical Society (ACS). All Rights Reserved.
Internal use only. Redistribution is subject to the terms of your SciFinder^{n} License Agreement and CAS information Use Policies.

CAS SciFinderⁿ

References (17)

View in SciFinderⁿ

1

An Aurora kinase inhibitor, AMG900, inhibits glioblastoma cell proliferation by disrupting mitotic progression

By: Ryu, Jaewook; Pyo, Jaehyuk; Lee, Chang-Woo; Kim, Ja-Eun
Cancer Medicine (2018), 7(11), 5589-5603 | Language: English, Database: CAplus and MEDLINE

Substances (5) | Reactions (0) | Cited By (8)

2

AMG900 as novel inhibitor of the translationally controlled tumor protein

By: Fischer, Nicolas; Seo, Ean-Jeong; Klinger, Anette; Fleischer, Edmond; Efferth, Thomas
Chemico-Biological Interactions (2021), 334, 109349 | Language: English, Database: CAplus and MEDLINE

Substances (4) | Reactions (0) | Cited By (3)

3

Preclinical Evaluation of AMG 900, a Novel Potent and Highly Selective Pan-Aurora Kinase Inhibitor with Activity in Taxane-Resistant Tumor Cell Lines

By: Payton, Marc; Bush, Tammy L.; Chung, Grace; Ziegler, Beth; Eden, Patrick; McElroy, Patricia; Ross, Sandra; Cee, Victor J.; Deak, Holly L.; Hodous, Brian L.; et al
Cancer Research (2010), 70(23), 9846-9854 | Language: English, Database: CAplus and MEDLINE

Substances (12) | Reactions (0) | Cited By (103)

4

AMG 900, a Small-Molecule Inhibitor of Aurora Kinases, Potentiates the Activity of Microtubule-Targeting Agents in Human Metastatic Breast Cancer Models

By: Bush, Tammy L.; Payton, Marc; Heller, Scott; Chung, Grace; Hanestad, Kelly; Rottman, James B.; Loberg, Robert; Friberg, Gregory; Kendall, Richard L.; Saffran, Douglas; et al
Molecular Cancer Therapeutics (2013), 12(11), 2356-2366 | Language: English, Database: CAplus and MEDLINE

Substances (6) | Reactions (0) | Cited By (32)

5

A phase 1 study of AMG 900, an orally administered pan-aurora kinase inhibitor, in adult patients with acute myeloid leukemia

By: Kantarjian, Hagop M.; Schuster, Michael W.; Jain, Nitin; Advani, Anjali; Jabbour, Elias; Gamelin, Erick; Rasmussen, Erik; Juan, Gloria; Anderson, Abraham; Chow, Vincent F.; et al
American Journal of Hematology (2017), 92(7), 660-667 | Language: English, Database: CAplus and MEDLINE

Substances (6) Reactions (0) Cited By (23)

6

AMG 900, pan-Aurora kinase inhibitor, preferentially inhibits the proliferation of breast cancer cell lines with dysfunctional p53

By: Kalous, Ondrej; Conklin, Dylan; Desai, Amrita J.; Dering, Judy; Goldstein, Jennifer; Ginther, Charles; Anderson, Lee; Lu, Ming; Kolarova, Teodora; Eckardt, Mark A.; et al
Breast Cancer Research and Treatment (2013), 141(3), 397-408 | Language: English, Database: CAplus and MEDLINE

Substances (5) Reactions (0) Cited By (19)

7

Aurora Kinase Inhibition: A New Light in the Sky?

By: Linardopoulos, Spiros; Blagg, Julian
Journal of Medicinal Chemistry (2015), 58(13), 5186-5188 | Language: English, Database: CAplus and MEDLINE

Substances (3) Reactions (0) Cited By (9)

8

Antitumour activity of AMG 900 alone or in combination with histone deacetylase inhibitor SaHa on medulloblastoma cell lines

By: Geron, Lenisa; Borges, Kleiton Silva; Andrade, Augusto Faria; Suazo, Veridiana Kill; Scrideli, Carlos Alberto; Tone, Luiz Gonzaga
Neurological Research (2015), 37(8), 703-711 | Language: English, Database: CAplus and MEDLINE

Substances (4) Reactions (0) Cited By (5)

9

2-Phenoxy-3, 4'-bipyridine derivatives inhibit AURKB-dependent mitotic processes by disrupting its localization

By: Lv, Gang; Shi, Qiong; Zhang, Ting; Li, Jinhua; Kalashova, Julia; Long, Yan; Sun, Yin; Li, Chao; Choudhry, Namrta; Li, Hongmei; et al
European Journal of Medicinal Chemistry (2023), 245(Part_1), 114904 | Language: English, Database: CAplus and MEDLINE

Substances (62) Reactions (0) Cited By (2)

10

AMG 900, a potent inhibitor of aurora kinases causes pharmacodynamic changes in p-Histone H3 immunoreactivity in human tumor xenografts and proliferating mouse tissues

By: Juan, Gloria; Bush, Tammy L.; Ma, Connie; Manoukian, Raffi; Chung, Grace; Hawkins, Jennifer M.; Zoog, Stephen; Kendall, Richard; Radinsky, Robert; Loberg, Robert; et al
Journal of Translational Medicine (2014), 12, 307/1-307/12 | Language: English, Database: CAplus and MEDLINE

Substances (3) | Reactions (0) | Cited By (7)

11

Dual targeting of aurora kinases with AMG 900 exhibits potent preclinical activity against acute myeloid leukemia with distinct post-mitotic outcomes

By: Payton, Marc; Cheung, Hung-Kam; Ninniri, Maria Stefania S.; Marinaccio, Christian; Wayne, William C.; Hanestad, Kelly; Crispino, John D.; Juan, Gloria; Coxon, Angela
Molecular Cancer Therapeutics (2018), 17(12), 2575-2585 | Language: English, Database: CAplus and MEDLINE

Substances (9) | Reactions (0) | Cited By (12)

12

Preclinical evaluation of the Aurora kinase inhibitors AMG 900, AZD1152-HQPA, and MK-5108 on SW-872 and 93T449 human liposarcoma cells

By: Noronha, Sandhya; Alt, Lauren A. C.; Scimeca, Taylor E.; Zarou, Omran; Obrzut, Justyna; Zanotti, Brian; Hayward, Elizabeth A.; Pillai, Akhil; Mathur, Shubha; Rojas, Joseph; et al
In Vitro Cellular & Developmental Biology: Animal (2018), 54(1), 71-84 | Language: English, Database: CAplus and MEDLINE

Substances (5) | Reactions (0) | Cited By (5)

13

Screening methods for identifying therapeutic agents for promoting motor neuron survival and treating and diagnosing neurodegenerative disorders

By: Rubin, Lee L.; Gupta, Shailesh K.
World Intellectual Property Organization, WO2015070071 A2 2015-05-14 | Language: English, Database: CAplus

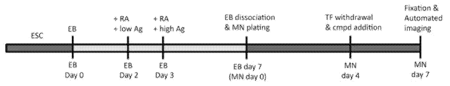

FIG. 1A

PatentPak available

Substances (30) | Reactions (0) | Cited By (0)

14

In silico binding mode analysis (molecular docking studies) and absorption, distribution, metabolism and excretion prediction of some novel inhibitors of Aurora kinase A in clinical trials

By: Deb, Pran Kishore; El-Rabie, Dina; Junaid, Ahmad; Nalaiya, Jeyashanthini A. P.; Siong, Lim Chee; Kulasingam, Kulasekar A. L.; Pichika, Mallikarjuna Rao
Asian Journal of Chemistry (2014), 26(18), 6221-6226 | Language: English, Database: CAplus

Substances (7) Reactions (0) Cited By (6)

15

Gender effects on rat metabolism of AMG 900, an orally available small molecule Aurora kinase inhibitor

By: Waldon, Daniel; Berry, Loren; Lin, Min-Hwa Jasmine; Schenkel, Laurie; Hollis, L. Steven; Zhao, Zhiyang
Drug Metabolism Letters (2011), 5(4), 290-297 | Language: English, Database: CAplus and MEDLINE

Substances (8) Reactions (0) Cited By (2)

16

AT9283 exhibits antiproliferative effect on tyrosine kinase inhibitor-sensitive and -resistant chronic myeloid leukemia cells by inhibition of Aurora A and Aurora B

By: Takeda, Tomoya; Tsubaki, Masanobu; Genno, Shuji; Nemoto, Chisato; Onishi, Yasuka; Yamamoto, Yuuta; Imano, Motohiro; Satou, Takao; Nishida, Shozo
Oncology Reports (2020), 44(5), 2211-2218 | Language: English, Database: CAplus and MEDLINE

Substances (9) Reactions (0) Cited By (2)

17

Preparation of crystalline forms of N-(4-((3-(2-amino-4-pyrimidinyl) - 2-pyridinyl)oxy)phenyl)-4-(4-methyl-2-thienyl)-1 -phthalazinamine pharmaceutically acceptable salts for the treatment of cancer

By: Stanton, Mary; Bio, Matthew; Peterson, Matthew

World Intellectual Property Organization, WO2015084649 A1 2015-06-11 | Language: English, Database: CAplus

5 FIGURE 4

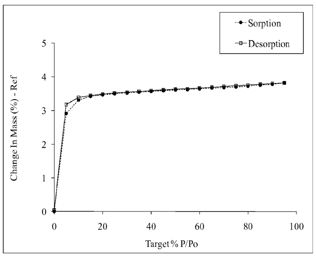

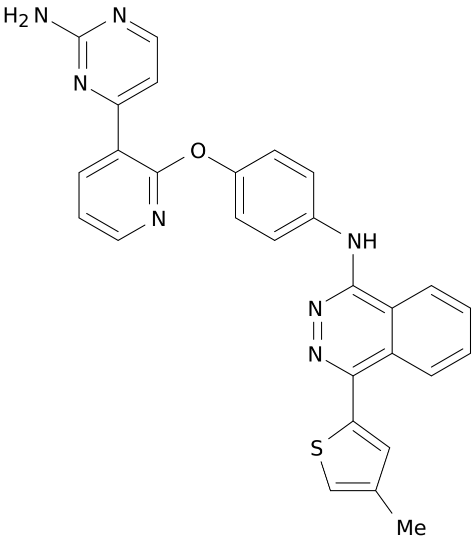

I

PatentPak available

Substances (80) Reactions (79) Cited By (1)

Copyright © 2023 American Chemical Society (ACS). All Rights Reserved.
Copyright © 2023 U.S. National Library of Medicine.
Internal use only. Redistribution is subject to the terms of your SciFinderⁿ License Agreement and CAS information Use Policies.

CAS SciFinder^n

Task History

Initiating Search

January 12, 2023, 4:36PM

All:

Structure of AMG900
(right)

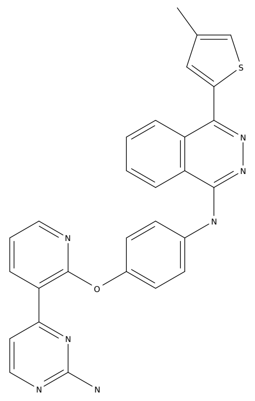

Search Tasks

Task	Search Type	View
Returned All Results	All	View Results
Exported: Retrieved Related Reference Results (96)	References	View Results

Copyright © 2023 American Chemical Society (ACS). All Rights Reserved.
Internal use only. Redistribution is subject to the terms of your SciFinder^n License Agreement and CAS information Use Policies.

CAS SciFinderⁿ

References (96)

View in SciFinderⁿ

1

Preclinical Evaluation of AMG 900, a Novel Potent and Highly Selective Pan-Aurora Kinase Inhibitor with Activity in Taxane-Resistant Tumor Cell Lines

By: Payton, Marc; Bush, Tammy L.; Chung, Grace; Ziegler, Beth; Eden, Patrick; McElroy, Patricia; Ross, Sandra; Cee, Victor J.; Deak, Holly L.; Hodous, Brian L.; et al
Cancer Research (2010), 70(23), 9846-9854 | Language: English, Database: CAplus and MEDLINE

Substances (12) Reactions (0) Cited By (103)

2

In vitro and in vivo pharmacokinetic characterizations of AMG 900, an orally bioavailable small molecule inhibitor of aurora kinases

By: Huang, Liyue; Be, Xuhai; Berry, Loren; Moore, Earl; Janosky, Brett; Wells, Mary; Pan, Wei-Jian; Zhao, Zhiyang; Lin, Min-Hwa Jasmine
Xenobiotica (2011), 41(5), 400-408 | Language: English, Database: CAplus and MEDLINE

Substances (2) Reactions (0) Cited By (9)

3

AMG 900, pan-Aurora kinase inhibitor, preferentially inhibits the proliferation of breast cancer cell lines with dysfunctional p53

By: Kalous, Ondrej; Conklin, Dylan; Desai, Amrita J.; Dering, Judy; Goldstein, Jennifer; Ginther, Charles; Anderson, Lee; Lu, Ming; Kolarova, Teodora; Eckardt, Mark A.; et al
Breast Cancer Research and Treatment (2013), 141(3), 397-408 | Language: English, Database: CAplus and MEDLINE

Substances (5) Reactions (0) Cited By (19)

4

AMG 900, a potent inhibitor of aurora kinases causes pharmacodynamic changes in p-Histone H3 immunoreactivity in human tumor xenografts and proliferating mouse tissues

By: Juan, Gloria; Bush, Tammy L.; Ma, Connie; Manoukian, Raffi; Chung, Grace; Hawkins, Jennifer M.; Zoog, Stephen; Kendall, Richard; Radinsky, Robert; Loberg, Robert; et al
Journal of Translational Medicine (2014), 12, 307/1-307/12 | Language: English, Database: CAplus and MEDLINE

Substances (3) Reactions (0) Cited By (7)

5

AMG 900, a Small-Molecule Inhibitor of Aurora Kinases, Potentiates the Activity of Microtubule-Targeting Agents in Human Metastatic Breast Cancer Models

By: Bush, Tammy L.; Payton, Marc; Heller, Scott; Chung, Grace; Hanestad, Kelly; Rottman, James B.; Loberg, Robert; Friberg, Gregory; Kendall, Richard L.; Saffran, Douglas; et al
Molecular Cancer Therapeutics (2013), 12(11), 2356-2366 | Language: English, Database: CAplus and MEDLINE

Substances (6) Reactions (0) Cited By (32)

6

A phase 1 study of AMG 900, an orally administered pan-aurora kinase inhibitor, in adult patients with acute myeloid leukemia

By: Kantarjian, Hagop M.; Schuster, Michael W.; Jain, Nitin; Advani, Anjali; Jabbour, Elias; Gamelin, Erick; Rasmussen, Erik; Juan, Gloria; Anderson, Abraham; Chow, Vincent F.; et al
American Journal of Hematology (2017), 92(7), 660-667 | Language: English, Database: CAplus and MEDLINE

Substances (6) Reactions (0) Cited By (23)

7

A phase 1, first-in-human study of AMG 900, an orally administered pan-Aurora kinase inhibitor, in adult patients with advanced solid tumors

By: Carducci, Michael; Shaheen, Montaser; Markman, Ben; Hurvitz, Sara; Mahadevan, Daruka; Kotasek, Dusan; Goodman, Jr Oscar B.; Rasmussen, Erik; Chow, Vincent; Juan, Gloria; et al
Investigational New Drugs (2018), 36(6), 1060-1071 | Language: English, Database: CAplus and MEDLINE

Substances (3) Reactions (0) Cited By (22)

8

Antitumour activity of AMG 900 alone or in combination with histone deacetylase inhibitor SaHa on medulloblastoma cell lines

By: Geron, Lenisa; Borges, Kleiton Silva; Andrade, Augusto Faria; Suazo, Veridiana Kill; Scrideli, Carlos Alberto; Tone, Luiz Gonzaga
Neurological Research (2015), 37(8), 703-711 | Language: English, Database: CAplus and MEDLINE

Substances (4) Reactions (0) Cited By (5)

9

Combining the pan-aurora kinase inhibitor AMG 900 with histone deacetylase inhibitors enhances antitumor activity in prostate cancer

By: Paller, Channing J.; Wissing, Michel D.; Mendonca, Janet; Sharma, Anup; Kim, Eugene; Kim, Hea-Soo; Kortenhorst, Madeleine S. Q.; Gerber, Stephanie; Rosen, Marc; Shaikh, Faraz; et al
Cancer Medicine (2014), 3(5), 1322-1335 | Language: English, Database: CAplus and MEDLINE

Substances (10) Reactions (0) Cited By (22)

10

The aurora kinase inhibitor AMG 900 increases apoptosis and induces chemosensitivity to anticancer drugs in the NCI-H295 adrenocortical carcinoma cell line

By: Borges, Kleiton S.; Andrade, Augusto F.; Silveira, Vanessa S.; Marco Antonio, David S.; Vasconcelos, Elton J. R.; Antonini, Sonir R. R.; Tone, Luiz G.; Scrideli, Carlos A.
Anti-Cancer Drugs (2017), 28(6), 634-644 | Language: English, Database: CAplus and MEDLINE

Substances (7) Reactions (0) Cited By (15)

11

Aurora Kinase Inhibition: A New Light in the Sky?

By: Linardopoulos, Spiros; Blagg, Julian
Journal of Medicinal Chemistry (2015), 58(13), 5186-5188 | Language: English, Database: CAplus and MEDLINE

Substances (3) Reactions (0) Cited By (9)

12

An Aurora kinase inhibitor, AMG900, inhibits glioblastoma cell proliferation by disrupting mitotic progression

By: Ryu, Jaewook; Pyo, Jaehyuk; Lee, Chang-Woo; Kim, Ja-Eun
Cancer Medicine (2018), 7(11), 5589-5603 | Language: English, Database: CAplus and MEDLINE

Substances (5) Reactions (0) Cited By (8)

13

Preclinical evaluation of the Aurora kinase inhibitors AMG 900, AZD1152-HQPA, and MK-5108 on SW-872 and 93T449 human liposarcoma cells

By: Noronha, Sandhya; Alt, Lauren A. C.; Scimeca, Taylor E.; Zarou, Omran; Obrzut, Justyna; Zanotti, Brian; Hayward, Elizabeth A.; Pillai, Akhil; Mathur, Shubha; Rojas, Joseph; et al
In Vitro Cellular & Developmental Biology: Animal (2018), 54(1), 71-84 | Language: English, Database: CAplus and MEDLINE

Substances (5) Reactions (0) Cited By (5)

14

Interlaboratory evaluation of a multiplexed high information content in vitro genotoxicity assay

By: Bryce, Steven M.; Bernacki, Derek T.; Bemis, Jeffrey C.; Spellman, Richard A.; Engel, Maria E.; Schuler, Maik; Lorge, Elisabeth; Heikkinen, Pekka T.; Hemmann, Ulrike; Thybaud, Veronique; et al
Environmental and Molecular Mutagenesis (2017), 58(3), 146-161 | Language: English, Database: CAplus and MEDLINE

Substances (84) Reactions (0) Cited By (43)

15

Experiments in the EpiDerm 3D skin in vitro model and minipigs in vivo indicate comparatively lower in vivo skin sensitivity of topically applied aneugenic compounds

By: Schuler, Maik; Tomlinson, Lindsay; Homiski, Michael; Cheung, Jennifer; Zhan, Yutian; Coffing, Stephanie; Engel, Maria; Rubitski, Elizabeth; Seitis, Gary; Hales, Katherine; et al
Toxicological Sciences (2021), 180(1), 103-121 | Language: English, Database: CAplus and MEDLINE

Substances (6) Reactions (0) Cited By (3)

16

LC-MS/MS bioanalytical method development for AMG 900: Resolution of an isobaric interference in rodent in vivo studies

By: Be, Xuhai; Moore, Earl S.; Zhao, Zhiyang; Wells, Mary C.
Journal of Pharmaceutical and Biomedical Analysis (2013), 74, 171-177 | Language: English, Database: CAplus and MEDLINE
Analytical Methods available

Substances (2) Reactions (0) Cited By (3)

17

Discovery of N-(4-(3-(2-Aminopyrimidin-4-yl)pyridin-2-yloxy)phenyl)-4-(4-methylthiophen-2-yl)phthalazin-1-amine (AMG 900), A Highly Selective, Orally Bioavailable Inhibitor of Aurora Kinases with Activity against Multidrug-Resistant Cancer Cell Lines

By: Geuns-Meyer, Stephanie; Cee, Victor J.; Deak, Holly L.; Du, Bingfan; Hodous, Brian L.; Nguyen, Hanh Nho; Olivieri, Philip R.; Schenkel, Laurie B.; Vaida, Karina R.; Andrews, Paul; et al
Journal of Medicinal Chemistry (2015), 58(13), 5189-5207 | Language: English, Database: CAplus and MEDLINE

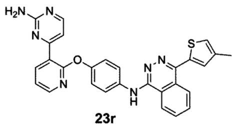

23r

Cell Line	EC_{50} (nM)
CAL51 (Non-MDR)	2
MES-SA Dx5 (MDR)	1
HCT15 (MDR)	2
SNU449 (MDR)	2
769P (MDR)	2

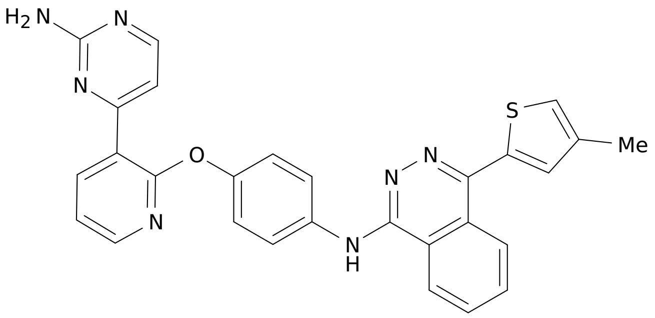

I

Substances (94) Reactions (138) Cited By (33)

18

Gender effects on rat metabolism of AMG 900, an orally available small molecule Aurora kinase inhibitor

By: Waldon, Daniel; Berry, Loren; Lin, Min-Hwa Jasmine; Schenkel, Laurie; Hollis, L. Steven; Zhao, Zhiyang
Drug Metabolism Letters (2011), 5(4), 290-297 | Language: English, Database: CAplus and MEDLINE

Substances (8) Reactions (0) Cited By (2)

19

Dual targeting of aurora kinases with AMG 900 exhibits potent preclinical activity against acute myeloid leukemia with distinct post-mitotic outcomes

By: Payton, Marc; Cheung, Hung-Kam; Ninniri, Maria Stefania S.; Marinaccio, Christian; Wayne, William C.; Hanestad, Kelly; Crispino, John D.; Juan, Gloria; Coxon, Angela
Molecular Cancer Therapeutics (2018), 17(12), 2575-2585 | Language: English, Database: CAplus and MEDLINE

Substances (9) Reactions (0) Cited By (12)

20

AMG900 as novel inhibitor of the translationally controlled tumor protein

By: Fischer, Nicolas; Seo, Ean-Jeong; Klinger, Anette; Fleischer, Edmond; Efferth, Thomas
Chemico-Biological Interactions (2021), 334, 109349 | Language: English, Database: CAplus and MEDLINE

Substances (4) Reactions (0) Cited By (3)

21

Compound for treating cerebral palsy and its application

By: Han, Bing; Peng, Limin
China, CN103720692 A 2014-04-16 | Language: Chinese, Database: CAplus

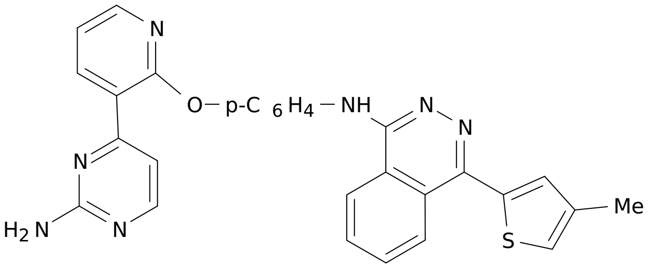

I

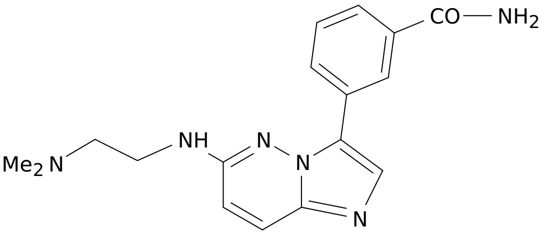

II

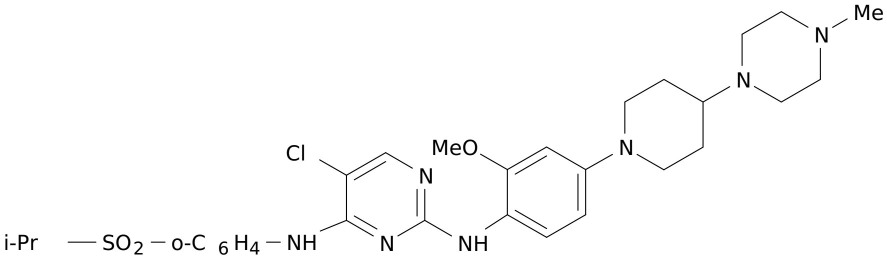

III

PatentPak available

Substances (4) Reactions (0) Cited By (0)

22

Report and Application of a Tool Compound Data Set

By: Butler, Kyle V.; MacDonald, Ian A.; Hathaway, Nathaniel A.; Jin, Jian
Journal of Chemical Information and Modeling (2017), 57(11), 2699-2706 | Language: English, Database: CAplus and MEDLINE

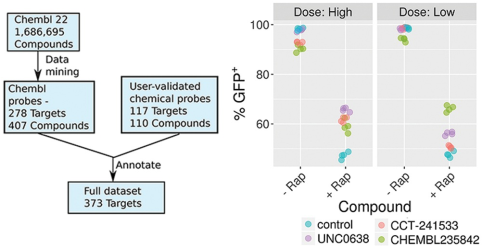

Substances (5) | Reactions (0) | Cited By (5)

23

Compounds for treating narrow angle between cornea and iris and application thereof

By: Yang, Zijiao; Hou, Kai
China, CN103664737 A 2014-03-26 | Language: Chinese, Database: CAplus

PatentPak available

Substances (5) | Reactions (0) | Cited By (0)

24

Aneugen molecular mechanism assay: proof-of-concept with 27 reference chemicals

By: Bernacki, Derek T.; Bryce, Steven M.; Bemis, Jeffrey C.; Dertinger, Stephen D.
Toxicological Sciences (2019), 170(2), 382-393 | Language: English, Database: CAplus and MEDLINE

Substances (28) | Reactions (0) | Cited By (9)

25

Inhibition of Aurora kinase A activity enhances the antitumor response of beta-catenin blockade in human adrenocortical cancer cells

By: Maria, Andrea Gutierrez; Silva Borges, Kleiton; Lira, R. C. P.; Hassib Thome, Carolina; Berthon, Annabel ; Drougat, Ludivine ; Kiseljak-Vassiliades, Katja; Wierman, Margaret E.; Faucz, Fabio R.; Faca, Vitor Marcel; et al
Molecular and Cellular Endocrinology (2021), 528, 111243 | Language: English, Database: CAplus and MEDLINE

Substances (7) | Reactions (0) | Cited By (4)

26

In silico binding mode analysis (molecular docking studies) and absorption, distribution, metabolism and excretion prediction of some novel inhibitors of Aurora kinase A in clinical trials

By: Deb, Pran Kishore; El-Rabie, Dina; Junaid, Ahmad; Nalaiya, Jeyashanthini A. P.; Siong, Lim Chee; Kulasingam, Kulasekar A. L.; Pichika, Mallikarjuna Rao
Asian Journal of Chemistry (2014), 26(18), 6221-6226 | Language: English, Database: CAplus

Substances (7) Reactions (0) Cited By (6)

27

Classification of in vitro genotoxicants using a novel multiplexed biomarker assay compared to the flow cytometric micronucleus test

By: Wilde, Sabrina; Dambowsky, Miriam; Hempt, Claudia; Sutter, Andreas; Queisser, Nina
Environmental and Molecular Mutagenesis (2017), 58(9), 662-677 | Language: English, Database: CAplus and MEDLINE

Substances (37) Reactions (0) Cited By (13)

28

Preparation of substituted phthalazinamines as Aurora kinase modulators

By: Cee, Victor J.; Deak, Holly L.; Du, Bingfan; Geuns-Meyer, Stephanie D.; Hodous, Brian L.; Nguyen, Hanh Nho; Olivieri, Philip R.; Patel, Vinod F.; Romero, Karina; Schenkel, Laurie
World Intellectual Property Organization, WO2007087276 A1 2007-08-02 | Language: English, Database: CAplus

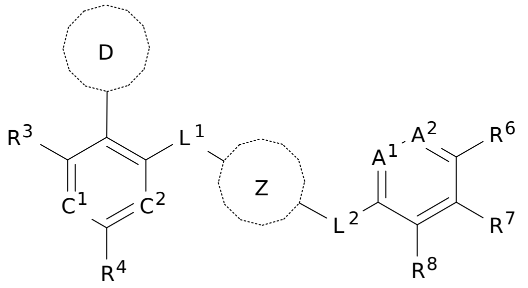

I

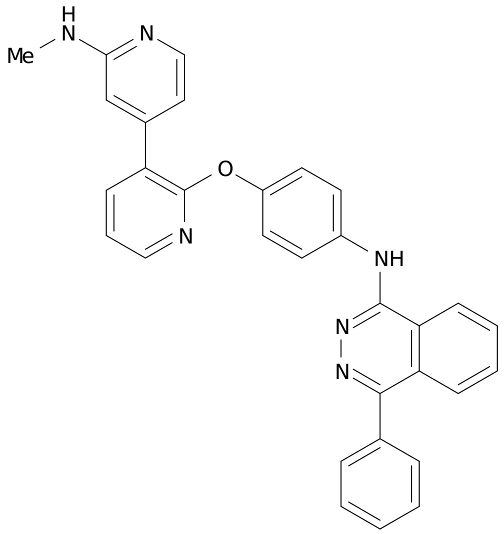

II

PatentPak available

Substances (722) Reactions (1,098) Cited By (11)

29

Aneugen versus clastogen evaluation and oxidative stress-related mode-of-action assessment of genotoxic compounds using the toxtracker reporter assay

By: Brandsma, Inger; Moelijker, Nynke; Derr, Remco; Hendriks, Giel
Toxicological Sciences (2020), 177(1), 202-213 | Language: English, Database: CAplus and MEDLINE

Substances (17) Reactions (0) Cited By (10)

30

Biologically Relevant Chemical Space Navigator: From Patent and Structure-Activity Relationship Analysis to Library Acquisition and Design

By: Rabal, Obdulia; Oyarzabal, Julen
Journal of Chemical Information and Modeling (2012), 52(12), 3123-3137 | Language: English, Database: CAplus and MEDLINE

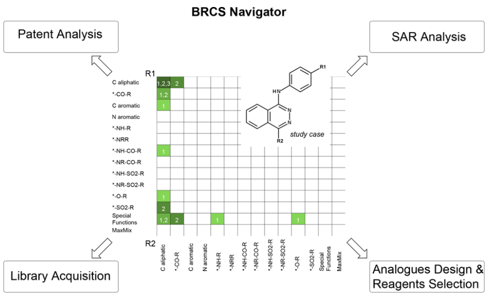

Substances (8) Reactions (0) Cited By (9)

31

Methods and compositions for treating liver cancer

By: Harrison, Stephen; Brew, Christine Taylor; Winther, Michael David; Shekaran, Asha
World Intellectual Property Organization, WO2021206633 A1 2021-10-14 | Language: English, Database: CAplus

PatentPak available

Substances (9) Reactions (0) Cited By (0)

32

Treating tumors with an alternating electrical field and an Aurora kinase inhibitor

By: Krex, Dietmar; Temme, Achim; Shnaiderman, Rosa S.; Giladi, Moshe
United States, US20190307781 A1 2019-10-10 | Language: English, Database: CAplus

PatentPak available

Substances (9) Reactions (0) Cited By (2)

33

N-(4-((3-(2-amino-4-pyrimidinyl)-2-pyridinyl)oxy)phenyl)-4-(4-methyl-2-thienyl)-1-phthalazinamine for use in the treatment of antimitotic agent resistant cancer

By: Payton, Marc; Kendall, Richard
World Intellectual Property Organization, WO2011031842 A1 2011-03-17 | Language: English, Database: CAplus

PatentPak available

Substances (41) Reactions (9) Cited By (3)

34

Compounds for treating traumatic brain injury disease and their application

By: Yang, Li; Yang, Yuxin; Li, Mai
China, CN103655575 A 2014-03-26 | Language: Chinese, Database: CAplus

PatentPak available

Substances (10) Reactions (0) Cited By (0)

35

Synthetic lethality of RB1 and aurora A is driven by stathmin-mediated disruption of microtubule dynamics

By: Lyu, Junfang; Yang, Eun Ju; Zhang, Baoyuan; Wu, Changjie; Pardeshi, Lakhansing; Shi, Changxiang; Mou, Pui Kei; Liu, Yifan; Tan, Kaeling; Shim, Joong Sup
Nature Communications (2020), 11(1), 5105 | Language: English, Database: CAplus and MEDLINE

Substances (22) Reactions (0) Cited By (17)

36

Aurora kinase A inhibition induces synthetic lethality in SMAD4-deficient colorectal cancer cells via spindle assembly checkpoint activation

By: Shi, Changxiang; Tao, Shishi; Ren, Guowen; Yang, Eun Ju; Shu, Xiaodong; Mou, Pui Kei; Liu, Yifan; Dang, Yongjun; Xu, Xiaoling; Shim, Joong Sup
Oncogene (2022), 41(19), 2734-2748 | Language: English, Database: CAplus and MEDLINE

Substances (11) Reactions (0) Cited By (1)

37

High-throughput screening of regulators of axonal transport

By: Shlevkov, Evgeny; Schwarz, Thomas L.; Bray, Mark-Anthony; Carpenter Van Dyk, Anne E.
World Intellectual Property Organization, WO2020149925 A1 2020-07-23 | Language: English, Database: CAplus

PatentPak available

Substances (11) Reactions (0) Cited By (0)

38

A new imaging platform (iScreen) allows for the concurrent assessment of micronucleus induction and genotoxic mode of action in human A375 cells

By: Sun, Xiaowen; Rubitski, Elizabeth; Spellman, Richard A.; Engel, Maria; Schuler, Maik
Environmental and Molecular Mutagenesis (2022), 63(5), 230-245 | Language: English, Database: CAplus and MEDLINE

Substances (12) Reactions (0) Cited By (0)

39

Real-time luminescence enables continuous drug-response analysis in adherent and suspension cell lines

By: Wandishin, Clayton M.; Robbins, Charles John; Tyson, Darren R.; Harris, Leonard A.; Quaranta, Vito
Cancer Biology & Therapy (2022), 23(1), 358-368 | Language: English, Database: CAplus and MEDLINE

Substances (12) Reactions (0) Cited By (0)

40

Quantitative conformational profiling of kinase inhibitors reveals origins of selectivity for Aurora kinase activation states

By: Lake, Eric W.; Muretta, Joseph M.; Thompson, Andrew R.; Rasmussen, Damien M.; Majumdar, Abir; Faber, Erik B.; Ruff, Emily F.; Thomas, David D.; Levinson, Nicholas M.
Proceedings of the National Academy of Sciences of the United States of America (2018), 115(51), E11894-E11903 | Language: English, Database: CAplus and MEDLINE

Substances (25) Reactions (0) Cited By (35)

41

Cell Panel Profiling Reveals Conserved Therapeutic Clusters and Differentiates the Mechanism of Action of Different PI3K/mTOR, Aurora Kinase and EZH2 Inhibitors

By: Uitdehaag, Joost C. M.; de Roos, Jeroen A. D. M.; Prinsen, Martine B. W.; Willemsen-Seegers, Nicole; de Vetter, Judith R. F.; Dylus, Jelle; van Doornmalen, Antoon M.; Kooijman, Jeffrey; Sawa, Masaaki; van Gerwen, Suzanne J. C.; et al
Molecular Cancer Therapeutics (2016), 15(12), 3097-3109 | Language: English, Database: CAplus and MEDLINE

Substances (130) Reactions (0) Cited By (13)

42

Therapeutic combinations for treating cancer comprising an irreversible mutant EGFR inhibitor and an antineoplastic agent such as an Aurora kinase inhibitor

By: Simmmons, Andrew D.; Harding, Thomas Christian
World Intellectual Property Organization, WO2015112705 A2 2015-07-30 | Language: English, Database: CAplus

PatentPak available

Substances (46) Reactions (0) Cited By (3)

43

RIPK1-dependent cell death: a novel target of the Aurora kinase inhibitor Tozasertib (VX-680)

By: Martens, Sofie; Goossens, Vera; Devisscher, Lars; Hofmans, Sam; Claeys, Polien; Vuylsteke, Marnik; Takahashi, Nozomi; Augustyns, Koen ; Vandenabeele, Peter
Cell Death & Disease (2018), 9(2), 1-13 | Language: English, Database: CAplus and MEDLINE

Substances (13) | Reactions (0) | Cited By (22)

44

Differential ABC transporter expression during hematopoiesis contributes to neutrophil-biased toxicity of Aurora kinase inhibitors

By: Chou, David B. ; Furlong, Brooke A. ; Posey, Ryan R.; Kyprianou, Christos ; O'Sullivan, Lucy R.; David, Rhiannon; Randle, Suzanne J.; Polanska, Urszula M.; Travers, Jon; Urosevic, Jelena; et al
Nature Communications (2022), 13(1), 6021 | Language: English, Database: CAplus and MEDLINE

Substances (14) | Reactions (0) | Cited By (0)

45

Image analysis of mechanistic protein biomarkers for the characterization of genotoxicants: Aneugens, clastogens, and reactive oxygen species inducers

By: Wilde, Sabrina; Queisser, Nina; Sutter, Andreas
Environmental and Molecular Mutagenesis (2020), 61(5), 534-550 | Language: English, Database: CAplus and MEDLINE

Substances (14) | Reactions (0) | Cited By (1)

46

Structural Biology Insight for the Design of Sub-type Selective Aurora Kinase Inhibitors

By: Sarvagalla, Sailu; Coumar, Mohane Selvaraj
Current Cancer Drug Targets (2015), 15(5), 375-393 | Language: English, Database: CAplus and MEDLINE

Substances (22) | Reactions (0) | Cited By (19)

47

Phthalazinone Scaffold: Emerging Tool in the Development of Target Based Novel Anticancer Agents

By: Singh, Jyoti ; Suryan, Amruta ; Kumar, Sandeep ; Sharma, Shweta
Anti-Cancer Agents in Medicinal Chemistry (2020), 20(18), 2228-2245 | Language: English, Database: CAplus and MEDLINE

Substances (15) | Reactions (0) | Cited By (2)

48

Use of AMG 900 for the treatment of cancer

By: Friberg, Gregory; Payton, Marc
World Intellectual Property Organization, WO2013181462 A1 2013-12-05 | Language: English, Database: CAplus

Figure 1-b
% Change

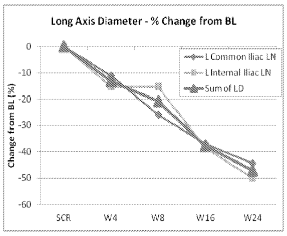

PatentPak available

Substances (85) | Reactions (8) | Cited By (1)

49

Definition of a novel cuproptosis-relevant lncRNA signature for uncovering distinct survival, genomic alterations, and treatment implications in lung adenocarcinoma

By: Wang, Zhuning; Yao, Junqiao; Dong, Tengyu; Niu, Xing
Journal of Immunology Research (2022), 2756611 | Language: English, Database: CAplus and MEDLINE

Substances (20) | Reactions (0) | Cited By (0)

50

Biomarkers for PSMA targeted therapy for prostate cancer

By: Olson, William C.; DiPippo, Vincent
World Intellectual Property Organization, WO2015073896 A2 2015-05-21 | Language: English, Database: CAplus

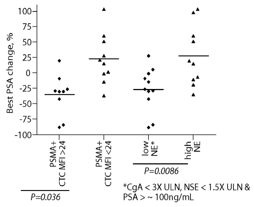

Fig. 2

PatentPak available

Substances (20) | Reactions (0) | Cited By (1)

51

TubulinTracker, a novel in vitro reporter assay to study intracellular microtubule dynamics, cell cycle progression, and aneugenicity

By: Geijer, Marit E. ; Moelijker, Nynke; Zhang, Gaonan; Derr, Remco; Osterlund, Torben; Hendriks, Giel ; Brandsma, Inger
Toxicological Sciences (2022), 186(2), 288-297 | Language: English, Database: CAplus and MEDLINE

Substances (25) Reactions (0) Cited By (2)

52

2-Phenoxy-3, 4'-bipyridine derivatives inhibit AURKB-dependent mitotic processes by disrupting its localization

By: Lv, Gang; Shi, Qiong; Zhang, Ting; Li, Jinhua; Kalashova, Julia; Long, Yan; Sun, Yin; Li, Chao; Choudhry, Namrta; Li, Hongmei; et al
European Journal of Medicinal Chemistry (2023), 245(Part_1), 114904 | Language: English, Database: CAplus and MEDLINE

Substances (62) Reactions (0) Cited By (2)

53

Methods for inhibiting KRAS oncoprotein through enhanced GTPase activity

By: Lito, Piro
World Intellectual Property Organization, WO2022217073 A1 2022-10-13 | Language: English, Database: CAplus

PatentPak available Substances (171) Reactions (0) Cited By (0)

54

Combination therapies including Myt1 inhibitors

By: Fourtounis, Jimmy; Young, Jordan; Bernier, Cynthia; Durocher, Daniel; Hustedt, Nicole
World Intellectual Property Organization, WO2022213204 A1 2022-10-13 | Language: English, Database: CAplus

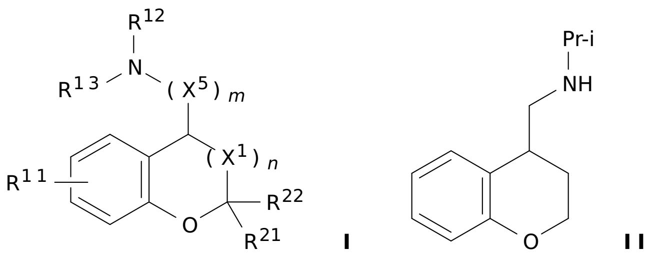

PatentPak available Substances (639) Reactions (455) Cited By (0)

55

Aurora kinase B inhibitors for use for treating cancer

By: Travers, Jon; Urosevic Despotovic, Jelena; Molina-Villa, Miguel-Angel; Bertran-Alamillo, Jordi
World Intellectual Property Organization, WO2022192139 A1 2022-09-15 | Language: English, Database: CAplus

PatentPak available

Substances (33) Reactions (0) Cited By (0)

56

N-myristoyl transferase (NMT) inhibitors for the treatment of senescence-associated diseases or disorders

By: Tate, Edward; Kallemeijn, Wouter; Gil, Jesus; McHugh, Domhnall
United States, US20220280484 A1 2022-09-08 | Language: English, Database: CAplus

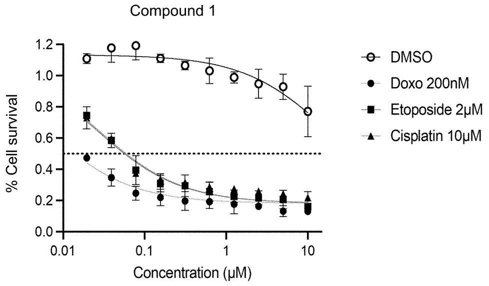

PatentPak available

Substances (58) Reactions (0) Cited By (0)

57

Biomarkers of DNA damage response improve in vitro micronucleus assays by revealing genotoxic mode of action and reducing the occurrence of irrelevant positive results

By: Avlasevich, Svetlana; Pellegrin, Tina; Godse, Manali; Bryce, Steven; Bemis, Jeffrey; Bajorski, Peter; Dertinger, Stephen
Mutagenesis (2021), 36(6), 407-418 | Language: English, Database: CAplus and MEDLINE

Substances (32) Reactions (0) Cited By (0)

58

MK2 activating compounds for use in treating vascular leak and endothelial barrier disorders

By: Tsaioun, Katya; Kayyali, Usamah; Villoutreix, Bruno
World Intellectual Property Organization, WO2022104097 A2 2022-05-19 | Language: English, Database: CAplus

PatentPak available

Substances (117) Reactions (0) Cited By (0)

59

N-myristoyl transferase (NMT) inhibitors for treating senescence-associated diseases or disorders

By: Tate, Edward; Kallemeijn, Wouter; Gil, Jesus; McHugh, Domhnall
World Intellectual Property Organization, WO2022090746 A1 2022-05-05 | Language: English, Database: CAplus

PatentPak available

Substances (65) Reactions (0) Cited By (0)

60

Methods for treating cancer with combination therapies

By: Ahsan, Aarif; Bisht, Kamlesh; Bjorklund, Chad; Flynt, Jennifer Erin; Hsu, Chih-Chao; Jeyaraju, Danny Vijey; Ortiz-Estevez, Maria; Pierceall, William Edward; Thakurta, Anjan; Towfic, Fadi George
World Intellectual Property Organization, WO2021262962 A1 2021-12-30 | Language: English, Database: CAplus

PatentPak available

Substances (95) Reactions (0) Cited By (0)

61

Use of TMPRSS2 inhibitors for the treatment of lung viral infections

By: Khatib, Abdel-Majid; Villoutreix, Bruno; Singh, Natesh
World Intellectual Property Organization, WO2021228762 A1 2021-11-18 | Language: English, Database: CAplus

PatentPak available

Substances (249) Reactions (0) Cited By (0)

62

Small molecule therapeutics for the treatment of viral infections

By: Arumugaswami, Vaithlingaraja; Christofk, Heather R.; Damoiseaux, Robert; Garcia, Gustavo; Mullen, Peter
World Intellectual Property Organization, WO2021207632 A1 2021-10-14 | Language: English, Database: CAplus

PatentPak available

Substances (146) Reactions (0) Cited By (2)

63

Compounds for the treatment of covid-19

By: Beccari, Andrea; Manelfi, Candida; Talarico, Carmine
World Intellectual Property Organization, WO2021176088 A1 2021-09-10 | Language: English, Database: CAplus

PatentPak available

Substances (122) Reactions (0) Cited By (0)

64

Compounds for the treatment of covid-19

By: Beccari, Andrea Rosario; Manelfi, Candida; Talarico, Carmine
European Patent Organization, EP3875078 A1 2021-09-08 | Language: English, Database: CAplus

PatentPak available

Substances (159) | Reactions (0) | Cited By (0)

65

Multi-functional chimeric molecules for treatment of cancer and their preparation

By: Choudhary, Amit; Lai, Sophia; Shoba, Veronika; Kokkonda, Praveen; Munkanatta Godage, Dhanushka; Siriwardena, Sachini
World Intellectual Property Organization, WO2021142351 A1 2021-07-15 | Language: English, Database: CAplus

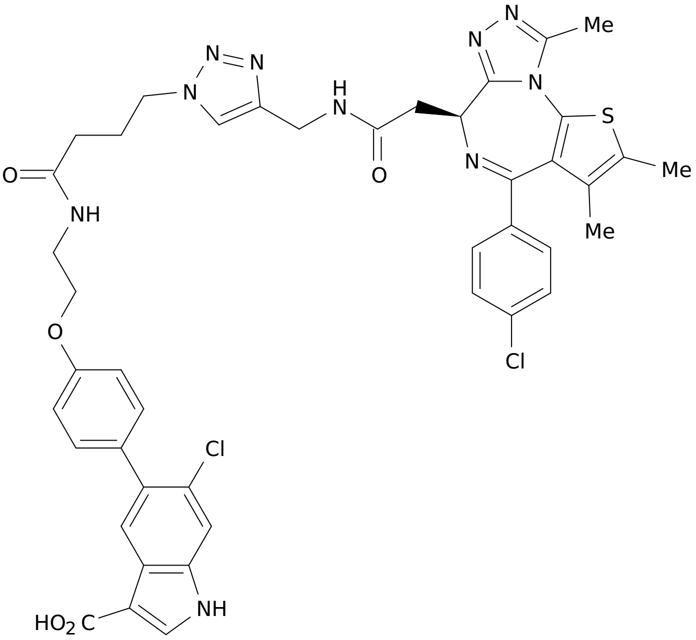

PatentPak available

Substances (178) | Reactions (383) | Cited By (0)

66

Substituted 4-aminoisoindoline-1,3-dione compounds and second active agents for combined use for treating hematological malignancies

By: Anton, Maria Soraya Carrancio; Buchholz, Tonia J.; Chang, Henry; Filvaroff, Ellen; Kasibhatla, Shailaja; Lopez-Girona, Antonia; Mohan, Adithi; Narla, Rama Krishna; Pourdehnad, Michael
United States, US20210113576 A1 2021-04-22 | Language: English, Database: CAplus

Sensitivity to Compound 1 single agent in DLBCL cell lines

sensitive to resistant

TMD-8	SU-DHL-2	SU-DHL-10	Pfeiffer	WSU-DLCL2	OCI-Ly10	Riva	U2932	SU-DHL-4	DB
58.67	108.4	123.7	288.2	362	403.5	496.3	577	585.3	670.8

PatentPak available

Substances (131) | Reactions (3) | Cited By (0)

67

Modeling and targeting of erythroleukemia by hematopoietic genome editing

By: Iacobucci, Ilaria; Qu, Chunxu; Varotto, Elena ; Janke, Laura J.; Yang, Xu; Seth, Aman; Shelat, Anang ; Friske, Jake D. ; Fukano, Reiji ; Yu, Jiyang ; et al
Blood (2021), 137(12), 1628-1640 | Language: English, Database: CAplus and MEDLINE

Substances (44) | Reactions (0) | Cited By (14)

68

Activity Comparison of Epigenetic Modulators against the Hemoprotozoan Parasites Babesia divergens and Plasmodium falciparum

By: Vanheer, Leen N.; Kafsack, Bjorn F. C.
ACS Infectious Diseases (2021), 7(8), 2277-2284 | Language: English, Database: CAplus and MEDLINE

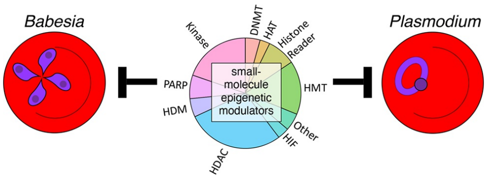

Substances (326) | Reactions (0) | Cited By (6)

69

The new paradigm of network medicine to analyze breast cancer phenotypes

By: Grimaldi, Anna Maria ; Conte, Federica ; Pane, Katia ; Fiscon, Giulia ; Mirabelli, Peppino; Baselice, Simona; Giannatiempo, Rosa; Messina, Francesco; Franzese, Monica ; Salvatore, Marco; et al
International Journal of Molecular Sciences (2020), 21(18), 6690 | Language: English, Database: CAplus and MEDLINE

Substances (30) | Reactions (0) | Cited By (16)

70

In silico identification of strong binders of the SARS-CoV-2 receptor-binding domain

By: Behloul, Nouredine; Baha, Sarra; Guo, Yuqian; Yang, Zhifang; Shi, Ruihua; Meng, Jihong
European Journal of Pharmacology (2021), 890, 173701 | Language: English, Database: CAplus and MEDLINE

Substances (51) | Reactions (0) | Cited By (10)

71

Aurora kinase as target for treating, preventing or curing HIV infection or aids

By: Este Araque, Jose Andres; Garcia Vidal, Edurne; Ballana Guix, Ester; Badia Corcoles, Roger; Riveira Munoz, Eva
World Intellectual Property Organization, WO2020049208 A1 2020-03-12 | Language: Spanish, Database: CAplus

PatentPak available

Substances (179) Reactions (0) Cited By (0)

72

High throughput drug screening of cancer stem cells for personalized anti-cancer treatment

By: Becker, Pamela; Lee, Su-In; Blau, Carl; Oehler, Vivian; Martins, Timothy; Monnat, Raymond J., Jr.
World Intellectual Property Organization, WO2020010262 A1 2020-01-09 | Language: English, Database: CAplus

PatentPak available

Substances (89) Reactions (0) Cited By (0)

73

Methods of treating or inhibiting onset of Huntington's disease with gene modulatory compounds

By: Goldman, Steven A.; Osipovitch, Mikhail
World Intellectual Property Organization, WO2019246262 A2 2019-12-26 | Language: English, Database: CAplus

PatentPak available

Substances (388) Reactions (0) Cited By (2)

74

High-throughput compound screen reveals mTOR inhibitors as potential therapeutics to reduce (auto)antibody production by human plasma cells

By: Tuijnenburg, Paul ; aan de Kerk, Daan J.; Jansen, Machiel H.; Morris, Ben; Lieftink, Cor; Beijersbergen, Roderick L.; van Leeuwen, Ester M. M.; Kuijpers, Taco W.
European Journal of Immunology (2020), 50(1), 73-85 | Language: English, Database: CAplus and MEDLINE

Substances (63) Reactions (0) Cited By (9)

75

Methods for treatment of cancers with EGFR activating mutations by administering a cyclin dependent kinase (CDK) inhibitor and/or spindle assembly checkpoint (SAC) component inhibitor

By: Heymach, John V.; Nilsson, Monique; Robichaux, Jacqulyne
World Intellectual Property Organization, WO2019178239 A1 2019-09-19 | Language: English, Database: CAplus

PatentPak available

Substances (57) Reactions (0) Cited By (1)

76

Inductive regeneration of the airway by transcriptional factor modulation of glandular myoepithelial stem cells using modulators of Lef-1/TCF/Wnt signaling

By: Engelhardt, John F.; Lynch, Thomas J.; Anderson, Preston J.; Shahin, Weam

World Intellectual Property Organization, WO2019178267 A2 2019-09-19 | Language: English, Database: CAplus

PatentPak available

Substances (1,589) | Reactions (0) | Cited By (0)

77

Methods of treating fibrosis using aurora kinase B (AURKB) inhibitors

By: Madala, Satish Kumar; Jegga, Anil Goud; Kasam, Rajesh K.

World Intellectual Property Organization, WO2019161000 A1 2019-08-22 | Language: English, Database: CAplus

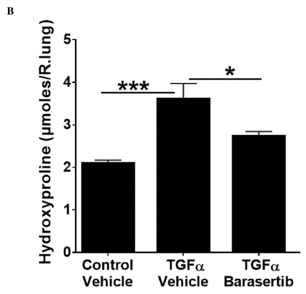

PatentPak available

Substances (46) | Reactions (0) | Cited By (0)

78

Optimization and biological evaluation of nicotinamide derivatives as Aurora kinase inhibitors

By: Qi, Baohui; Xu, Xingwei; Yang, Ying; He, Huan; Yue, Xupeng

Bioorganic & Medicinal Chemistry (2019), 27(17), 3825-3835 | Language: English, Database: CAplus and MEDLINE

Substances (75) | Reactions (130) | Cited By (3)

79

Induction of synthetic lethality with epigenetic therapy and screening for compounds that induce killing of cancer cells

By: Yegnasubramanian, Srinivasan; Nelson, William G.; Vaghasia, Ajay; Nuhn, Philipp
World Intellectual Property Organization, WO2018226802 A1 2018-12-13 | Language: English, Database: CAplus

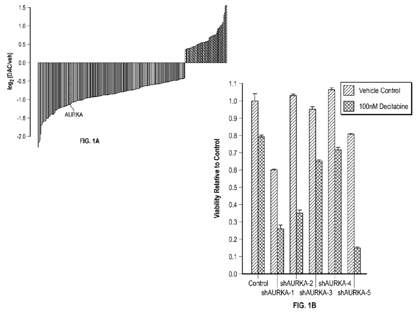

PatentPak available

Substances (35) | Reactions (0) | Cited By (0)

80

Epigenetic inhibitors for sensitizing hematologic or other malignancies to glucocorticoid therapy and Aurora Kinase B inhibitors

By: Stallcup, Michael R.; Poulard, Coralie; Pufall, Miles A.
World Intellectual Property Organization, WO2018213720 A2 2018-11-22 | Language: English, Database: CAplus

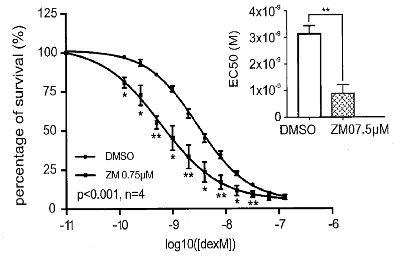

FIG. 40A

PatentPak available

Substances (31) | Reactions (0) | Cited By (0)

81

Refinement of in vitro methods for identification of aldehyde oxidase substrates reveals metabolites of kinase inhibitors

By: Dick, Ryan A.
Drug Metabolism & Disposition (2018), 46(6), 846-859 | Language: English, Database: CAplus and MEDLINE

Substances (58) | Reactions (0) | Cited By (18)

82

Classification and treatment of gastric cancer

By: Lauwers, Gregory Y.; Duda, Gabriel Dan
World Intellectual Property Organization, WO2017139470 A1 2017-08-17 | Language: English, Database: CAplus

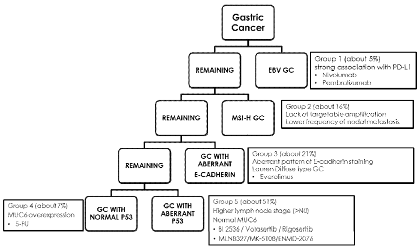

FIG. 8

PatentPak available

Substances (88) | Reactions (0) | Cited By (0)

83

Compound Selectivity and Target Residence Time of Kinase Inhibitors Studied with Surface Plasmon Resonance

By: Willemsen-Seegers, Nicole; Uitdehaag, Joost C. M.; Prinsen, Martine B. W.; de Vetter, Judith R. F.; de Man, Jos; Sawa, Masaaki; Kawase, Yusuke; Buijsman, Rogier C.; Zaman, Guido J. R.
Journal of Molecular Biology (2017), 429(4), 574-586 | Language: English, Database: CAplus and MEDLINE

Substances (62) | Reactions (0) | Cited By (23)

84

Molecular subtyping, prognosis and treatment of prostate cancer

By: Rubin, Mark A.; Beltran, Himisha
United States, US20140037647 A1 2014-02-06 | Language: English, Database: CAplus

PatentPak available

Substances (28) | Reactions (0) | Cited By (0)

85

Preparation of crystalline forms of N-(4-((3-(2-amino-4-pyrimidinyl) - 2-pyridinyl)oxy)phenyl)-4-(4-methyl-2-thienyl)-1 -phthalazinamine pharmaceutically acceptable salts for the treatment of cancer

By: Stanton, Mary; Bio, Matthew; Peterson, Matthew

World Intellectual Property Organization, WO2015084649 A1 2015-06-11 | Language: English, Database: CAplus

FIGURE 4

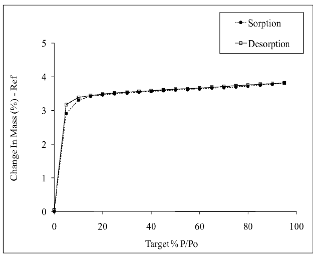

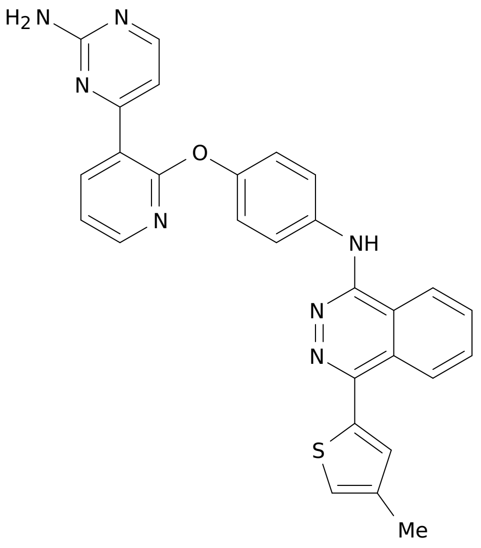

I

PatentPak available

Substances (80) Reactions (79) Cited By (1)

86

SnapShot: Kinase Inhibitors II

By: Wang, Jinhua; Gray, Nathanael S.

Molecular Cell (2015), 58(4), 710-710.e1 | Language: English, Database: CAplus and MEDLINE

Substances (87) Reactions (0) Cited By (10)

87

Screening methods for identifying therapeutic agents for promoting motor neuron survival and treating and diagnosing neurodegenerative disorders

By: Rubin, Lee L.; Gupta, Shailesh K.
World Intellectual Property Organization, WO2015070071 A2 2015-05-14 | Language: English, Database: CAplus

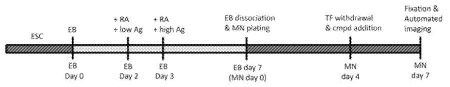

FIG. 1A

PatentPak available

Substances (30) | Reactions (0) | Cited By (0)

88

Use of N-(4-((3-(2-amino-4-pyrimidinyl)-2-pyridinyl)oxy)phenyl)-4-(4-methyl-2-thienyl)-1-phthalazinamine in combination with histone deacetylase inhibitors for treatment of cancer

By: Carducci, Michael; Kachhap, Sushant; Paller, Channing
United States, US20150072988 A1 2015-03-12 | Language: English, Database: CAplus

PatentPak available

Substances (31) | Reactions (8) | Cited By (0)

89

Preparing stealth nanoparticles and to their use in medicine

By: Schmid, Ruth; Stenstad, Per; Moerch, Yrr; Johnsen, Heidi
World Intellectual Property Organization, WO2014191502 A1 2014-12-04 | Language: English, Database: CAplus

PatentPak available

Substances (29) | Reactions (4) | Cited By (1)

90

Methods for modulating chemotherapeutic cytotoxicity

By: Roberts, David D.; Soto Pantoja, David R.
World Intellectual Property Organization, WO2014160183 A1 2014-10-02 | Language: English, Database: CAplus

PatentPak available

Substances (323) | Reactions (0) | Cited By (5)

91

New Affinity Probe Targeting VEGF Receptors for Kinase Inhibitor Selectivity Profiling by Chemical Proteomics

By: Ku, Xin; Heinzlmeir, Stephanie; Helm, Dominic; Medard, Guillaume; Kuster, Bernhard
Journal of Proteome Research (2014), 13(5), 2445-2452 | Language: English, Database: CAplus and MEDLINE

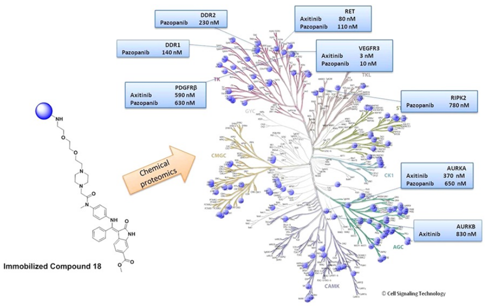

Substances (48) Reactions (39) Cited By (15)

92

Use of N-(4-((3-(2-amino-4-pyrimidinyl) -2-pyridinyl)oxy)phenyl)-4-(4-methyl-2-thienyl)-1-phthalazinamine in combination with histone deacetylase inhibitors for treatment of cancer

By: Carducci, Michael; Kachhap, Sushant; Paller, Channing
World Intellectual Property Organization, WO2013149026 A2 2013-10-03 | Language: English, Database: CAplus

PatentPak available

Substances (31) Reactions (8) Cited By (1)

93

A combined approach based on 3D pharmacophore and docking for identification of new aurora A kinase inhibitors

By: Joshi, Akshada J.; Gadhwal, Manoj K.; Joshi, Urmila J.
Medicinal Chemistry Research (2014), 23(3), 1414-1436 | Language: English, Database: CAplus

Substances (113) Reactions (0) Cited By (14)

94

SAR and evaluation of novel 5H-benzo[c][1,8]naphthyridin-6-one analogs as Aurora kinase inhibitors

By: Karra, Srinivasa; Xiao, Yufang; Chen, Xiaoling; Liu-Bujalski, Lesley; Huck, Bayard; Sutton, Amanda; Goutopoulos, Andreas; Askew, Ben; Josephson, Kristopher; Jiang, Xuliang; et al
Bioorganic & Medicinal Chemistry Letters (2013), 23(10), 3081-3087 | Language: English, Database: CAplus and MEDLINE

Substances (96) Reactions (122) Cited By (14)

95

Analysis of human genes AURKA, MYCN, and ERG for molecular subtyping, prognosis and treatment of metastatic prostate cancer

By: Rubin, Mark A.; Beltran, Himisha
World Intellectual Property Organization, WO2012145129 A2 2012-10-26 | Language: English, Database: CAplus

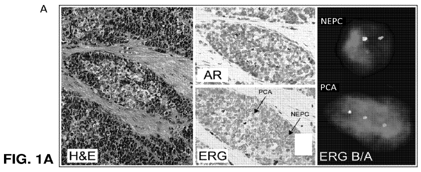

FIG. 1A

PatentPak available

Substances (28) | Reactions (0) | Cited By (0)

96

Preparation of substituted phthalazinamines as Aurora kinase modulators

By: Cee, Victor J.; Nguyen, Hanh Nho; Romero, Karina
United States, US7560551 B2 2009-07-14 | Language: English, Database: CAplus

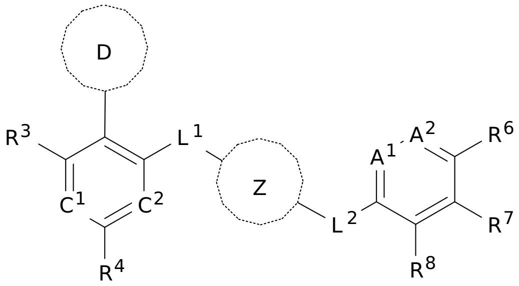
I

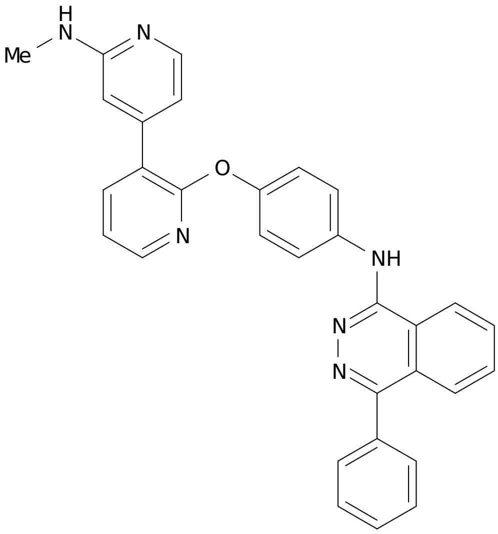
II

PatentPak available

Substances (615) | Reactions (537) | Cited By (1)

Copyright © 2023 American Chemical Society (ACS). All Rights Reserved.
Copyright © 2023 U.S. National Library of Medicine.
Internal use only. Redistribution is subject to the terms of your SciFinder^n License Agreement and CAS information Use Policies.

Results of PubMed search: keyword AZD1152

1: Mortlock AA, Foote KM, Heron NM, Jung FH, Pasquet G, Lohmann JJ, Warin N, Renaud F, De Savi C, Roberts NJ, Johnson T, Dousson CB, Hill GB, Perkins D, Hatter G, Wilkinson RW, Wedge SR, Heaton SP, Odedra R, Keen NJ, Crafter C, Brown E, Thompson K, Brightwell S, Khatri L, Brady MC, Kearney S, McKillop D, Rhead S, Parry T, Green S. Discovery, synthesis, and in vivo activity of a new class of pyrazoloquinazolines as selective inhibitors of aurora B kinase. J Med Chem. 2007 May 3;50(9):2213-24. doi: 10.1021/jm061335f. Epub 2007 Mar 21. PMID: 17373783.

2: Yang J, Ikezoe T, Nishioka C, Tasaka T, Taniguchi A, Kuwayama Y, Komatsu N, Bandobashi K, Togitani K, Koeffler HP, Taguchi H, Yokoyama A. AZD1152, a novel and selective aurora B kinase inhibitor, induces growth arrest, apoptosis, and sensitization for tubulin depolymerizing agent or topoisomerase II inhibitor in human acute leukemia cells in vitro and in vivo. Blood. 2007 Sep 15;110(6):2034-40. doi: 10.1182/blood-2007-02-073700. Epub 2007 May 10. PMID: 17495131.

3: Wilkinson RW, Odedra R, Heaton SP, Wedge SR, Keen NJ, Crafter C, Foster JR, Brady MC, Bigley A, Brown E, Byth KF, Barrass NC, Mundt KE, Foote KM, Heron NM, Jung FH, Mortlock AA, Boyle FT, Green S. AZD1152, a selective inhibitor of Aurora B kinase, inhibits human tumor xenograft growth by inducing apoptosis. Clin Cancer Res. 2007 Jun 15;13(12):3682-8. doi: 10.1158/1078-0432.CCR-06-2979. PMID: 17575233.

4: Evans RP, Naber C, Steffler T, Checkland T, Maxwell CA, Keats JJ, Belch AR, Pilarski LM, Lai R, Reiman T. The selective Aurora B kinase inhibitor AZD1152 is a potential new treatment for multiple myeloma. Br J Haematol. 2008 Feb;140(3):295-302. doi: 10.1111/j.1365-2141.2007.06913.x. Epub 2007 Dec 10. PMID: 18076711.

5: Tao Y, Zhang P, Girdler F, Frascogna V, Castedo M, Bourhis J, Kroemer G, Deutsch E. Enhancement of radiation response in p53-deficient cancer cells by the Aurora-B kinase inhibitor AZD1152. Oncogene. 2008 May 22;27(23):3244-55. doi: 10.1038/sj.onc.1210990. Epub 2007 Dec 17. PMID: 18084327.

6: Cummings J, Hodgkinson C, Odedra R, Sini P, Heaton SP, Mundt KE, Ward TH, Wilkinson RW, Growcott J, Hughes A, Dive C. Preclinical evaluation of M30 and M65 ELISAs as biomarkers of drug induced tumor cell death and antitumor activity. Mol Cancer Ther. 2008 Mar;7(3):455-63. doi: 10.1158/1535-7163.MCT-07-2136. PMID: 18347133.

7: Walsby E, Walsh V, Pepper C, Burnett A, Mills K. Effects of the aurora kinase inhibitors AZD1152-HQPA and ZM447439 on growth arrest and polyploidy in acute myeloid leukemia cell lines and primary blasts. Haematologica. 2008 May;93(5):662-9. doi: 10.3324/haematol.12148. Epub 2008 Mar 26. PMID: 18367484.

8: Li J, Anderson MG, Tucker LA, Shen Y, Glaser KB, Shah OJ. Inhibition of Aurora B kinase sensitizes a subset of human glioma cells to TRAIL concomitant with induction of TRAIL-R2. Cell Death Differ. 2009 Mar;16(3):498-511. doi: 10.1038/cdd.2008.174. Epub 2008 Dec 12. PMID: 19079141.

9: Guo J, Anderson MG, Tapang P, Palma JP, Rodriguez LE, Niquette A, Li J, Bouska JJ, Wang G, Semizarov D, Albert DH, Donawho CK, Glaser KB, Shah OJ. Identification of genes that confer tumor cell resistance to the aurora B kinase inhibitor, AZD1152. Pharmacogenomics J. 2009 Apr;9(2):90-102. doi: 10.1038/tpj.2008.20. Epub 2009 Feb 3. PMID: 19188929.

10: Nair JS, Ho AL, Tse AN, Coward J, Cheema H, Ambrosini G, Keen N, Schwartz GK. Aurora B kinase regulates the postmitotic endoreduplication checkpoint via phosphorylation of the retinoblastoma protein at serine 780. Mol Biol Cell. 2009 Apr;20(8):2218-28. doi: 10.1091/mbc.e08-08-0885. Epub 2009 Feb 18. PMID: 19225156; PMCID: PMC2669029.

11: Nair JS, de Stanchina E, Schwartz GK. The topoisomerase I poison CPT-11 enhances the effect of the aurora B kinase inhibitor AZD1152 both in vitro and in vivo. Clin Cancer Res. 2009 Mar 15;15(6):2022-30. doi: 10.1158/1078-0432.CCR-08-1826. Epub 2009 Mar 10. PMID: 19276280.

12: Anderson K, Lai Z, McDonald OB, Stuart JD, Nartey EN, Hardwicke MA, Newlander K, Dhanak D, Adams J, Patrick D, Copeland RA, Tummino PJ, Yang J. Biochemical characterization of GSK1070916, a potent and selective inhibitor of Aurora B and Aurora C kinases with an extremely long residence time1. Biochem J. 2009 May 13;420(2):259-65. doi: 10.1042/BJ20090121. PMID: 19284385.

13: Oke A, Pearce D, Wilkinson RW, Crafter C, Odedra R, Cavenagh J, Fitzgibbon J, Lister AT, Joel S, Bonnet D. AZD1152 rapidly and negatively affects the growth and survival of human acute myeloid leukemia cells in vitro and in vivo. Cancer Res. 2009 May 15;69(10):4150-8. doi: 10.1158/0008-5472.CAN-08-3203. Epub 2009 Apr 14. PMID: 19366807; PMCID: PMC2684546.

14: Lorenzo C, Liao Q, Hardwicke MA, Ducommun B. Pharmacological inhibition of aurora-A but not aurora-B impairs interphase microtubule dynamics. Cell Cycle. 2009 Jun 1;8(11):1733-7. doi: 10.4161/cc.8.11.8617. Epub 2009 Jun 2. PMID: 19395863.

15: Dancey JE. Kinase Inhibitor 4 Minisymposium summary. Expert Rev Anticancer Ther. 2009 Jul;9(7):891-4. doi: 10.1586/era.09.66. PMID: 19589028.

16: Pluim D, Beijnen JH, Schellens JH, van Tellingen O. Simultaneous determination of AZD1152 (prodrug) and AZD1152-hydroxyquinazoline pyrazol anilide by reversed phase liquid chromatography. J Chromatogr B Analyt Technol Biomed Life Sci. 2009 Nov 1;877(29):3549-55. doi: 10.1016/j.jchromb.2009.08.035. Epub 2009 Aug 28. PMID: 19744901.

17: Tao Y, Leteur C, Calderaro J, Girdler F, Zhang P, Frascogna V, Varna M, Opolon P, Castedo M, Bourhis J, Kroemer G, Deutsch E. The aurora B kinase inhibitor AZD1152 sensitizes cancer cells to fractionated irradiation and induces mitotic catastrophe. Cell Cycle. 2009 Oct 1;8(19):3172-81. doi: 10.4161/cc.8.19.9729. Epub 2009 Oct 4. PMID: 19755861.

18: Ikezoe T, Takeuchi T, Yang J, Adachi Y, Nishioka C, Furihata M, Koeffler HP, Yokoyama A. Analysis of Aurora B kinase in non-Hodgkin lymphoma. Lab Invest. 2009 Dec;89(12):1364-73. doi: 10.1038/labinvest.2009.106. Epub 2009 Oct 12. PMID: 19823168.

19: Aihara A, Tanaka S, Yasen M, Matsumura S, Mitsunori Y, Murakata A, Noguchi N, Kudo A, Nakamura N, Ito K, Arii S. The selective Aurora B kinase inhibitor AZD1152 as a novel treatment for hepatocellular carcinoma. J Hepatol. 2010 Jan;52(1):63-71. doi: 10.1016/j.jhep.2009.10.013. Epub 2009 Oct 29. PMID: 19913935.

20: Ikezoe T, Yang J, Nishioka C, Yokoyama A. p53 is critical for the Aurora B kinase inhibitor-mediated apoptosis in acute myelogenous leukemia cells. Int J Hematol. 2010 Jan;91(1):69-77. doi: 10.1007/s12185-009-0462-7. Epub 2009 Dec 16.

PMID: 20013323.

21: Lok W, Klein RQ, Saif MW. Aurora kinase inhibitors as anti-cancer therapy. Anticancer Drugs. 2010 Apr;21(4):339-50. doi: 10.1097/CAD.0b013e3283350dd1. PMID: 20016367.

22: Tomita M, Tanaka Y, Mori N. Aurora kinase inhibitor AZD1152 negatively affects the growth and survival of HTLV-1-infected T lymphocytes in vitro. Int J Cancer. 2010 Oct 1;127(7):1584-94. doi: 10.1002/ijc.25178. Retraction in: Int J Cancer. 2011 Dec 1;129(11):2762-3. PMID: 20091867.

23: Grundy M, Seedhouse C, Shang S, Richardson J, Russell N, Pallis M. The FLT3 internal tandem duplication mutation is a secondary target of the aurora B kinase inhibitor AZD1152-HQPA in acute myelogenous leukemia cells. Mol Cancer Ther. 2010 Mar;9(3):661-72. doi: 10.1158/1535-7163.MCT-09-1144. Epub 2010 Feb 16. PMID: 20159992.

24: Gully CP, Zhang F, Chen J, Yeung JA, Velazquez-Torres G, Wang E, Yeung SC, Lee MH. Antineoplastic effects of an Aurora B kinase inhibitor in breast cancer. Mol Cancer. 2010 Feb 22;9:42. doi: 10.1186/1476-4598-9-42. PMID: 20175926; PMCID: PMC2839967.

25: Zhu X, Ma Y, Liu D. Novel agents and regimens for acute myeloid leukemia: 2009 ASH annual meeting highlights. J Hematol Oncol. 2010 Apr 23;3:17. doi: 10.1186/1756-8722-3-17. PMID: 20416083; PMCID: PMC2880983.

26: Lordier L, Chang Y, Jalil A, Aurade F, Garçon L, Lécluse Y, Larbret F, Kawashima T, Kitamura T, Larghero J, Debili N, Vainchenker W. Aurora B is dispensable for megakaryocyte polyploidization, but contributes to the endomitotic process. Blood. 2010 Sep 30;116(13):2345-55. doi: 10.1182/blood-2010-01-265785. Epub 2010 Jun 14. PMID: 20548097.

27: Sheth PR, Ramanathan L, Ranchod A, Basso AD, Barrett D, Zhao J, Gray K, Liu YH, Zhang R, Le HV. Expression, purification, stability optimization and characterization of human Aurora B kinase domain from E. coli. Arch Biochem Biophys. 2010 Nov 15;503(2):191-201. doi: 10.1016/j.abb.2010.08.004. Epub 2010 Aug 10. PMID: 20699085.

28: Lin ZZ, Jeng YM, Hu FC, Pan HW, Tsao HW, Lai PL, Lee PH, Cheng AL, Hsu HC. Significance of Aurora B overexpression in hepatocellular carcinoma. Aurora B Overexpression in HCC. BMC Cancer. 2010 Aug 28;10:461. doi: 10.1186/1471-2407-10-461. PMID: 20799978; PMCID: PMC2940801.

29: Boss DS, Witteveen PO, van der Sar J, Lolkema MP, Voest EE, Stockman PK, Ataman O, Wilson D, Das S, Schellens JH. Clinical evaluation of AZD1152, an i.v. inhibitor of Aurora B kinase, in patients with solid malignant tumors. Ann Oncol. 2011 Feb;22(2):431-7. doi: 10.1093/annonc/mdq344. Epub 2010 Oct 5. PMID: 20924078.

30: Payton M, Bush TL, Chung G, Ziegler B, Eden P, McElroy P, Ross S, Cee VJ, Deak HL, Hodous BL, Nguyen HN, Olivieri PR, Romero K, Schenkel LB, Bak A, Stanton M, Dussault I, Patel VF, Geuns-Meyer S, Radinsky R, Kendall RL. Preclinical evaluation of AMG 900, a novel potent and highly selective pan-aurora kinase inhibitor with activity in taxane-resistant tumor cell lines. Cancer Res. 2010 Dec 1;70(23):9846-54. doi: 10.1158/0008-5472.CAN-10-3001. Epub 2010 Oct 8. PMID: 20935223.

31: Libertini S, Abagnale A, Passaro C, Botta G, Barbato S, Chieffi P, Portella G. AZD1152 negatively affects the growth of anaplastic thyroid carcinoma cells and enhances the effects of oncolytic virus dl922-947. Endocr Relat Cancer. 2011 Jan 13;18(1):129-41. doi: 10.1677/ERC-10-0234. PMID: 21071467.

32: Niermann KJ, Moretti L, Giacalone NJ, Sun Y, Schleicher SM, Kopsombut P, Mitchell LR, Kim KW, Lu B. Enhanced radiosensitivity of androgen-resistant prostate cancer: AZD1152-mediated Aurora kinase B inhibition. Radiat Res. 2011 Apr;175(4):444-51. doi: 10.1667/RR2317.1. Epub 2011 Jan 11. PMID: 21222513; PMCID: PMC3133747.

33: Moroz MA, Kochetkov T, Cai S, Wu J, Shamis M, Nair J, de Stanchina E, Serganova I, Schwartz GK, Banerjee D, Bertino JR, Blasberg RG. Imaging colon cancer response following treatment with AZD1152: a preclinical analysis of [18F]fluoro-2-deoxyglucose and 3'-deoxy-3'-[18F]fluorothymidine imaging. Clin Cancer Res. 2011 Mar 1;17(5):1099-110. doi: 10.1158/1078-0432.CCR-10-1430. Epub 2011 Jan 18. PMID: 21245090; PMCID: PMC3079195.

34: Azzariti A, Bocci G, Porcelli L, Fioravanti A, Sini P, Simone GM, Quatrale AE, Chiarappa P, Mangia A, Sebastian S, Del Bufalo D, Del Tacca M, Paradiso A. Aurora B kinase inhibitor AZD1152: determinants of action and ability to enhance chemotherapeutics effectiveness in pancreatic and colon cancer. Br J Cancer. 2011 Mar 1;104(5):769-80. doi: 10.1038/bjc.2011.21. Epub 2011 Feb 8. PMID: 21304529; PMCID: PMC3048212.

35: Mori N, Ishikawa C, Senba M, Kimura M, Okano Y. Effects of AZD1152, a selective Aurora B kinase inhibitor, on Burkitt's and Hodgkin's lymphomas. Biochem Pharmacol. 2011 May 1;81(9):1106-15. doi: 10.1016/j.bcp.2011.02.010. Epub 2011 Mar 1. Erratum in: Biochem Pharmacol. 2011 Nov 1;82(9):1252. PMID: 21371446.

36: Tsuboi K, Yokozawa T, Sakura T, Watanabe T, Fujisawa S, Yamauchi T, Uike N, Ando K, Kihara R, Tobinai K, Asou H, Hotta T, Miyawaki S. A Phase I study to assess the safety, pharmacokinetics and efficacy of barasertib (AZD1152), an Aurora B kinase inhibitor, in Japanese patients with advanced acute myeloid leukemia. Leuk Res. 2011 Oct;35(10):1384-9. doi: 10.1016/j.leukres.2011.04.008. Epub 2011 May 11. PMID: 21565405.

37: Keizer RJ, Zandvliet AS, Beijnen JH, Schellens JH, Huitema AD. Two-stage model-based design of cancer phase I dose escalation trials: evaluation using the phase I program of barasertib (AZD1152). Invest New Drugs. 2012 Aug;30(4):1519-30. doi: 10.1007/s10637-011-9694-5. Epub 2011 May 28. PMID: 21626115; PMCID: PMC3388254.

38: Grundy M, Seedhouse C, Russell NH, Pallis M. P-glycoprotein and breast cancer resistance protein in acute myeloid leukaemia cells treated with the aurora-B kinase inhibitor barasertib-hQPA. BMC Cancer. 2011 Jun 16;11:254. doi: 10.1186/1471-2407-11-254. PMID: 21679421; PMCID: PMC3146447.

39: Emery A, Sorrell DA, Lawrence S, Easthope E, Stockdale M, Jones DO, Zheleva D, Scaerou F, Glover DM. A novel cell-based, high-content assay for phosphorylation of Lats2 by Aurora A. J Biomol Screen. 2011 Sep;16(8):925-31. doi: 10.1177/1087057111413923. Epub 2011 Jul 25. PMID: 21788394.

40: Löwenberg B, Muus P, Ossenkoppele G, Rousselot P, Cahn JY, Ifrah N, Martinelli G, Amadori S, Berman E, Sonneveld P, Jongen-Lavrencic M, Rigaudeau S, Stockman P, Goudie A, Faderl S, Jabbour E, Kantarjian H. Phase 1/2 study to

assess the safety, efficacy, and pharmacokinetics of barasertib (AZD1152) in patients with advanced acute myeloid leukemia. Blood. 2011 Dec 1;118(23):6030-6. doi: 10.1182/blood-2011-07-366930. Epub 2011 Oct 5. PMID: 21976672; PMCID: PMC4186639.

41: Kwiatkowski N, Deng X, Wang J, Tan L, Villa F, Santaguida S, Huang HC, Mitchison T, Musacchio A, Gray N. Selective aurora kinase inhibitors identified using a taxol-induced checkpoint sensitivity screen. ACS Chem Biol. 2012 Jan 20;7(1):185-96. doi: 10.1021/cb200305u. Epub 2011 Oct 21. PMID: 21992004; PMCID: PMC3262907.

42: Holt SV, Logié A, Odedra R, Heier A, Heaton SP, Alferez D, Davies BR, Wilkinson RW, Smith PD. The MEK1/2 inhibitor, selumetinib (AZD6244; ARRY-142886), enhances anti-tumour efficacy when combined with conventional chemotherapeutic agents in human tumour xenograft models. Br J Cancer. 2012 Feb 28;106(5):858-66. doi: 10.1038/bjc.2012.8. Epub 2012 Feb 16. PMID: 22343622; PMCID: PMC3305954.

43: Sak A, Stuschke M, Groneberg M, Kübler D, Pöttgen C, Eberhardt WE. Inhibiting the aurora B kinase potently suppresses repopulation during fractionated irradiation of human lung cancer cell lines. Int J Radiat Oncol Biol Phys. 2012 Oct 1;84(2):492-9. doi: 10.1016/j.ijrobp.2011.12.021. Epub 2012 Feb 28. PMID: 22381900.

44: Diaz RJ, Golbourn B, Shekarforoush M, Smith CA, Rutka JT. Aurora kinase B/C inhibition impairs malignant glioma growth in vivo. J Neurooncol. 2012 Jul;108(3):349-60. doi: 10.1007/s11060-012-0835-2. Epub 2012 Mar 1. PMID: 22382783.

45: Schwartz GK, Carvajal RD, Midgley R, Rodig SJ, Stockman PK, Ataman O, Wilson D, Das S, Shapiro GI. Phase I study of barasertib (AZD1152), a selective inhibitor of Aurora B kinase, in patients with advanced solid tumors. Invest New Drugs. 2013 Apr;31(2):370-80. doi: 10.1007/s10637-012-9825-7. Epub 2012 Jun 2. PMID: 22661287.

46: Dennis M, Davies M, Oliver S, D'Souza R, Pike L, Stockman P. Phase I study of the Aurora B kinase inhibitor barasertib (AZD1152) to assess the pharmacokinetics, metabolism and excretion in patients with acute myeloid leukemia. Cancer Chemother Pharmacol. 2012 Sep;70(3):461-9. doi: 10.1007/s00280-012-1939-2. Epub 2012 Aug 4. PMID: 22864876; PMCID: PMC3428523.

47: Xu J, Li K, Smith RA, Waterton JC, Zhao P, Chen H, Does MD, Manning HC, Gore JC. Characterizing tumor response to chemotherapy at various length scales using temporal diffusion spectroscopy. PLoS One. 2012;7(7):e41714. doi: 10.1371/journal.pone.0041714. Epub 2012 Jul 24. PMID: 22911846; PMCID: PMC3404000.

48: Hartsink-Segers SA, Zwaan CM, Exalto C, Luijendijk MW, Calvert VS, Petricoin EF, Evans WE, Reinhardt D, de Haas V, Hedtjärn M, Hansen BR, Koch T, Caron HN, Pieters R, Den Boer ML. Aurora kinases in childhood acute leukemia: the promise of aurora B as therapeutic target. Leukemia. 2013 Mar;27(3):560-8. doi: 10.1038/leu.2012.256. Epub 2012 Sep 3. PMID: 22940834; PMCID: PMC3593181.

49: Marchetti S, Pluim D, van Eijndhoven M, van Tellingen O, Mazzanti R, Beijnen JH, Schellens JH. Effect of the drug transporters ABCG2, Abcg2, ABCB1 and ABCC2 on the disposition, brain accumulation and myelotoxicity of the aurora kinase B inhibitor barasertib and its more active form barasertib-hydroxy-QPA. Invest New

Drugs. 2013 Oct;31(5):1125-35. doi: 10.1007/s10637-013-9923-1. Epub 2013 Jan 13. Erratum in: Invest New Drugs. 2013 Jun;31(3):800. PMID: 23315030.

50: Ma Y, Weimer J, Fredrik R, Adam-Klages S, Sebens S, Caliebe A, Hilpert F, Eckmann-Scholz C, Arnold N, Schem C. Aurora kinase inhibitor AZD1152 has an additional effect of platinum on a sequential application at the human ovarian cancer cell line SKOV3. Arch Gynecol Obstet. 2013 Jul;288(1):173-82. doi: 10.1007/s00404-013-2719-x. Epub 2013 Feb 7. PMID: 23389245.

51: Cannon CM, Pozniak J, Scott MC, Ito D, Gorden BH, Graef AJ, Modiano JF. Canine osteosarcoma cells exhibit resistance to aurora kinase inhibitors. Vet Comp Oncol. 2015 Mar;13(1):48-59. doi: 10.1111/vco.12018. Epub 2013 Feb 15. PMID: 23410058; PMCID: PMC4672990.

52: Ma YX, Li XZ. [Effect of aurora kinase B inhibitor AZD1152 in the treatment of cisplatin-resistant ovarian carcinoma]. Zhonghua Fu Chan Ke Za Zhi. 2013 Jan;48(1):46-50. Chinese. PMID: 23531251.

53: Shah A, Seedhouse C. Frequency of TP53 Mutations and its Impact on Drug Sensitivity in Acute Myeloid Leukemia? Indian J Clin Biochem. 2012 Apr;27(2):121-6. doi: 10.1007/s12291-012-0203-1. Epub 2012 Mar 24. Retraction in: Indian J Clin Biochem. 2014 Apr;29(2):265. PMID: 23543587; PMCID: PMC3358364.

54: Yamauchi T, Uzui K, Shigemi H, Negoro E, Yoshida A, Ueda T. Aurora B inhibitor barasertib and cytarabine exert a greater-than-additive cytotoxicity in acute myeloid leukemia cells. Cancer Sci. 2013 Jul;104(7):926-33. doi: 10.1111/cas.12164. Epub 2013 May 12. PMID: 23557198; PMCID: PMC7657165.

55: Kantarjian HM, Martinelli G, Jabbour EJ, Quintás-Cardama A, Ando K, Bay JO, Wei A, Gröpper S, Papayannidis C, Owen K, Pike L, Schmitt N, Stockman PK, Giagounidis A; SPARK-AML1 Investigators. Stage I of a phase 2 study assessing the efficacy, safety, and tolerability of barasertib (AZD1152) versus low-dose cytosine arabinoside in elderly patients with acute myeloid leukemia. Cancer. 2013 Jul 15;119(14):2611-9. doi: 10.1002/cncr.28113. Epub 2013 Apr 19. PMID: 23605952; PMCID: PMC4132839.

56: Kantarjian HM, Sekeres MA, Ribrag V, Rousselot P, Garcia-Manero G, Jabbour EJ, Owen K, Stockman PK, Oliver SD. Phase I study assessing the safety and tolerability of barasertib (AZD1152) with low-dose cytosine arabinoside in elderly patients with AML. Clin Lymphoma Myeloma Leuk. 2013 Oct;13(5):559-67. doi: 10.1016/j.clml.2013.03.019. Epub 2013 Jun 10. PMID: 23763917; PMCID: PMC3775947.

57: Marampon F, Gravina GL, Popov VM, Scarsella L, Festuccia C, La Verghetta ME, Parente S, Cerasani M, Bruera G, Ficorella C, Ricevuto E, Tombolini V, Di Cesare E, Zani BM. Close correlation between MEK/ERK and Aurora-B signaling pathways in sustaining tumorigenic potential and radioresistance of gynecological cancer cell lines. Int J Oncol. 2014 Jan;44(1):285-94. doi: 10.3892/ijo.2013.2167. Epub 2013 Nov 5. PMID: 24189697.

58: Zhu XP, Liu ZL, Peng AF, Zhou YF, Long XH, Luo QF, Huang SH, Shu Y. Inhibition of Aurora-B suppresses osteosarcoma cell migration and invasion. Exp Ther Med. 2014 Mar;7(3):560-564. doi: 10.3892/etm.2014.1491. Epub 2014 Jan 20. PMID: 24520245; PMCID: PMC3919923.

59: Akiyama M, Izumi H, Wang KY, Yamaguchi T, Kuma A, Kitamura N, Harada Y, Oya

R, Yamaguchi K, Iwai Y, Kohno K. Hypersensitivity to aurora kinase inhibitors in cells resistant against platinum- containing anticancer agents. Anticancer Agents Med Chem. 2014;14(7):1042-50. doi: 10.2174/1871520614666140207154351. PMID: 24521151.

60: Hight MR, Cheung YY, Nickels ML, Dawson ES, Zhao P, Saleh S, Buck JR, Tang D, Washington MK, Coffey RJ, Manning HC. A peptide-based positron emission tomography probe for in vivo detection of caspase activity in apoptotic cells. Clin Cancer Res. 2014 Apr 15;20(8):2126-35. doi: 10.1158/1078-0432.CCR-13-2444. Epub 2014 Feb 26. PMID: 24573549; PMCID: PMC3989451.

61: Sessa F, Villa F. Structure of Aurora B-INCENP in complex with barasertib reveals a potential transinhibitory mechanism. Acta Crystallogr F Struct Biol Commun. 2014 Mar;70(Pt 3):294-8. doi: 10.1107/S2053230X14002118. Epub 2014 Feb 19. PMID: 24598913; PMCID: PMC3944688.

62: Chiou SK, Hoa N, Hodges A, Ge L, Jadus MR. Indomethacin promotes apoptosis in gastric cancer cells through concomitant degradation of Survivin and Aurora B kinase proteins. Apoptosis. 2014 Sep;19(9):1378-88. doi: 10.1007/s10495-014-1002-3. PMID: 24874838.

63: Cheung CH, Sarvagalla S, Lee JY, Huang YC, Coumar MS. Aurora kinase inhibitor patents and agents in clinical testing: an update (2011 - 2013). Expert Opin Ther Pat. 2014 Sep;24(9):1021-38. doi: 10.1517/13543776.2014.931374. Epub 2014 Jun 26. PMID: 24965505.

64: Baldini E, Tuccilli C, Prinzi N, Sorrenti S, Antonelli A, Gnessi L, Morrone S, Moretti C, Bononi M, Arlot-Bonnemains Y, D'Armiento M, Ulisse S. Effects of selective inhibitors of Aurora kinases on anaplastic thyroid carcinoma cell lines. Endocr Relat Cancer. 2014 Oct;21(5):797-811. doi: 10.1530/ERC-14-0299. Epub 2014 Jul 29. PMID: 25074669.

65: Boeckx C, Op de Beeck K, Wouters A, Deschoolmeester V, Limame R, Zwaenepoel K, Specenier P, Pauwels P, Vermorken JB, Peeters M, Van Camp G, Baay M, Lardon F. Overcoming cetuximab resistance in HNSCC: the role of AURKB and DUSP proteins. Cancer Lett. 2014 Nov 28;354(2):365-77. doi: 10.1016/j.canlet.2014.08.039. Epub 2014 Sep 2. PMID: 25192874.

66: Zekri A, Ghaffari SH, Ghanizadeh-Vesali S, Yaghmaie M, Salmaninejad A, Alimoghaddam K, Modarressi MH, Ghavamzadeh A. AZD1152-HQPA induces growth arrest and apoptosis in androgen-dependent prostate cancer cell line (LNCaP) via producing aneugenic micronuclei and polyploidy. Tumour Biol. 2015 Feb;36(2):623-32. doi: 10.1007/s13277-014-2664-8. Epub 2014 Oct 3. PMID: 25277659.

67: Ma Y, Cao H, Lou S, Shao X, Lv W, Qi X, Liu Y, Ying M, He Q, Yang X. Sequential treatment with aurora B inhibitors enhances cisplatin-mediated apoptosis via c-Myc. J Mol Med (Berl). 2015 Apr;93(4):427-38. doi: 10.1007/s00109-014-1228-0. Epub 2014 Nov 21. PMID: 25411027.

68: Matsunaga H, Tanaka S, Aihara A, Ogawa K, Matsumura S, Ban D, Ochiai T, Irie T, Kudo A, Nakamura N, Arii S, Tanabe M. A novel therapeutic combination sequentially targeting aurora B and Bcl-xL in hepatocellular carcinoma. Ann Surg Oncol. 2015 Sep;22(9):3079-86. doi: 10.1245/s10434-014-4292-3. Epub 2014 Dec 19. PMID: 25524010.

69: Pacaud R, Cheray M, Nadaradjane A, Vallette FM, Cartron PF. Histone H3

phosphorylation in GBM: a new rational to guide the use of kinase inhibitors in anti-GBM therapy. Theranostics. 2015 Jan 1;5(1):12-22. doi: 10.7150/thno.8799. PMID: 25553095; PMCID: PMC4265745.

70: Erba HP. Finding the optimal combination therapy for the treatment of newly diagnosed AML in older patients unfit for intensive therapy. Leuk Res. 2015 Feb;39(2):183-91. doi: 10.1016/j.leukres.2014.11.027. Epub 2014 Dec 9. PMID: 25577399.

71: Porcelli L, Guida G, Quatrale AE, Cocco T, Sidella L, Maida I, Iacobazzi RM, Ferretta A, Stolfa DA, Strippoli S, Guida S, Tommasi S, Guida M, Azzariti A. Aurora kinase B inhibition reduces the proliferation of metastatic melanoma cells and enhances the response to chemotherapy. J Transl Med. 2015 Jan 27;13:26. doi: 10.1186/s12967-015-0385-4. PMID: 25623468; PMCID: PMC4314759.

72: Hole S, Pedersen AM, Lykkesfeldt AE, Yde CW. Aurora kinase A and B as new treatment targets in aromatase inhibitor-resistant breast cancer cells. Breast Cancer Res Treat. 2015 Feb;149(3):715-26. doi: 10.1007/s10549-015-3284-8. Epub 2015 Feb 10. PMID: 25667100.

73: Collins GP, Eyre TA, Linton KM, Radford J, Vallance GD, Soilleux E, Hatton C. A phase II trial of AZD1152 in relapsed/refractory diffuse large B-cell lymphoma. Br J Haematol. 2015 Sep;170(6):886-90. doi: 10.1111/bjh.13333. Epub 2015 Feb 26. PMID: 25721307.

74: Diaz RJ, Golbourn B, Faria C, Picard D, Shih D, Raynaud D, Leadly M, MacKenzie D, Bryant M, Bebenek M, Smith CA, Taylor MD, Huang A, Rutka JT. Mechanism of action and therapeutic efficacy of Aurora kinase B inhibition in MYC overexpressing medulloblastoma. Oncotarget. 2015 Feb 20;6(5):3359-74. doi: 10.18632/oncotarget.3245. PMID: 25739120; PMCID: PMC4413659.

75: Zekri A, Ghaffari SH, Yaghmaie M, Estiar MA, Alimoghaddam K, Modarressi MH, Ghavamzadeh A. Inhibitor of Aurora Kinase B Induces Differentially Cell Death and Polyploidy via DNA Damage Response Pathways in Neurological Malignancy: Shedding New Light on the Challenge of Resistance to AZD1152-HQPA. Mol Neurobiol. 2016 Apr;53(3):1808-1823. doi: 10.1007/s12035-015-9139-9. Epub 2015 Mar 11. PMID: 25752998.

76: Li J, Hong MJ, Chow JP, Man WY, Mak JP, Ma HT, Poon RY. Co-inhibition of polo-like kinase 1 and Aurora kinases promotes mitotic catastrophe. Oncotarget. 2015 Apr 20;6(11):9327-40. doi: 10.18632/oncotarget.3313. PMID: 25871386; PMCID: PMC4496220.

77: Larsen SL, Yde CW, Laenkholm AV, Rasmussen BB, Duun-Henriksen AK, Bak M, Lykkesfeldt AE, Kirkegaard T. Aurora kinase B is important for antiestrogen resistant cell growth and a potential biomarker for tamoxifen resistant breast cancer. BMC Cancer. 2015 Apr 8;15:239. doi: 10.1186/s12885-015-1210-4. PMID: 25885472; PMCID: PMC4392616.

78: Sarvagalla S, Coumar MS. Structural Biology Insight for the Design of Sub-type Selective Aurora Kinase Inhibitors. Curr Cancer Drug Targets. 2015;15(5):375-93. doi: 10.2174/1568009615666150421110401. PMID: 25895501.

79: Tuccilli C, Baldini E, Prinzi N, Morrone S, Sorrenti S, Filippini A, Catania A, Alessandrini S, Rendina R, Coccaro C, D'Armiento M, Ulisse S. Preclinical testing of selective Aurora kinase inhibitors on a medullary thyroid carcinoma-derived cell line. Endocrine. 2016 May;52(2):287-95. doi:

10.1007/s12020-015-0700-0. Epub 2015 Jul 28. PMID: 26215279.

80: Polat AN, Karayel Ö, Giese SH, Harmanda B, Sanal E, Hu CK, Renard BY, Özlü N. Phosphoproteomic Analysis of Aurora Kinase Inhibition in Monopolar Cytokinesis. J Proteome Res. 2015 Sep 4;14(9):4087-98. doi: 10.1021/acs.jproteome.5b00645. Epub 2015 Aug 25. Erratum in: J Proteome Res. 2015 Dec 4;14(12):5408. PMID: 26270265.

81: Falchook GS, Bastida CC, Kurzrock R. Aurora Kinase Inhibitors in Oncology Clinical Trials: Current State of the Progress. Semin Oncol. 2015 Dec;42(6):832-48. doi: 10.1053/j.seminoncol.2015.09.022. Epub 2015 Sep 24. PMID: 26615129.

82: Zhelev Z, Ivanova D, Aoki I, Saga T, Bakalova R. 2-Deoxy-D-glucose Sensitizes Cancer Cells to Barasertib and Everolimus by ROS-independent Mechanism(s). Anticancer Res. 2015 Dec;35(12):6623-32. PMID: 26637878.

83: de Groot CO, Hsia JE, Anzola JV, Motamedi A, Yoon M, Wong YL, Jenkins D, Lee HJ, Martinez MB, Davis RL, Gahman TC, Desai A, Shiau AK. A Cell Biologist's Field Guide to Aurora Kinase Inhibitors. Front Oncol. 2015 Dec 21;5:285. doi: 10.3389/fonc.2015.00285. PMID: 26732741; PMCID: PMC4685510.

84: Baldini E, Tuccilli C, Prinzi N, Sorrenti S, Antonelli A, Fallahi P, Mian C, Barollo S, Catania A, Morrone S, Tartaglia F, Mascagni D, Coccaro C, Pepe M, Filippini A, D'Armiento M, Ulisse S. Selective inhibitors of aurora kinases inhibit proliferation, reduce cell viability and impair cell cycle progression in papillary thyroid carcinoma cells. J Biol Regul Homeost Agents. 2015 Oct-Dec;29(4):793-803. PMID: 26753639.

85: Ashton S, Song YH, Nolan J, Cadogan E, Murray J, Odedra R, Foster J, Hall PA, Low S, Taylor P, Ellston R, Polanska UM, Wilson J, Howes C, Smith A, Goodwin RJ, Swales JG, Strittmatter N, Takáts Z, Nilsson A, Andren P, Trueman D, Walker M, Reimer CL, Troiano G, Parsons D, De Witt D, Ashford M, Hrkach J, Zale S, Jewsbury PJ, Barry ST. Aurora kinase inhibitor nanoparticles target tumors with favorable therapeutic index in vivo. Sci Transl Med. 2016 Feb 10;8(325):325ra17. doi: 10.1126/scitranslmed.aad2355. PMID: 26865565.

86: Ju S, Peng X, Yang X, Sozar S, Muneri CW, Xu Y, Chen C, Cui P, Xu W, Rui R. Aurora B inhibitor barasertib prevents meiotic maturation and subsequent embryo development in pig oocytes. Theriogenology. 2016 Jul 15;86(2):503-15. doi: 10.1016/j.theriogenology.2016.01.030. Epub 2016 Feb 9. PMID: 26993175.

87: Song YH, Shin E, Wang H, Nolan J, Low S, Parsons D, Zale S, Ashton S, Ashford M, Ali M, Thrasher D, Boylan N, Troiano G. A novel in situ hydrophobic ion paring (HIP) formulation strategy for clinical product selection of a nanoparticle drug delivery system. J Control Release. 2016 May 10;229:106-119. doi: 10.1016/j.jconrel.2016.03.026. Epub 2016 Mar 18. PMID: 27001894.

88: Zhelev Z, Ivanova D, Lazarova D, Aoki I, Bakalova R, Saga T. Docosahexaenoic Acid Sensitizes Leukemia Lymphocytes to Barasertib and Everolimus by ROS-dependent Mechanism Without Affecting the Level of ROS and Viability of Normal Lymphocytes. Anticancer Res. 2016 Apr;36(4):1673-82. PMID: 27069145.

89: Ghanizadeh-Vesali S, Zekri A, Zaker F, Zaghal A, Yousefi M, Alimoghaddam K, Ghavamzadeh A, Ghaffari SH. Significance of AZD1152 as a potential treatment against Aurora B overexpression in acute promyelocytic leukemia. Ann Hematol. 2016 Jun;95(7):1031-42. doi: 10.1007/s00277-016-2670-6. Epub 2016 Apr 19. PMID:

27091351.

90: Weeks A, Agnihotri S, Lymer J, Chalil A, Diaz R, Isik S, Smith C, Rutka JT. Epithelial Cell Transforming 2 and Aurora Kinase B Modulate Formation of Stress Granule-Containing Transcripts from Diverse Cellular Pathways in Astrocytoma Cells. Am J Pathol. 2016 Jun;186(6):1674-87. doi: 10.1016/j.ajpath.2016.02.013. Epub 2016 Apr 20. PMID: 27106762.

91: Sarvagalla S, Hsieh HP, Coumar MS. Therapeutic polymeric nanoparticles and the methods of making and using thereof: a patent evaluation of WO2015036792. Expert Opin Ther Pat. 2016 Jul;26(7):751-5. doi: 10.1080/13543776.2016.1188919. Epub 2016 May 24. PMID: 27167102.

92: Bushby N, Bergin J, Harding J. [(14) C]-AZD1152 drug substance manufacture: challenges of an IV-infusion dosed human mass balance study in patients. J Labelled Comp Radiopharm. 2016 May 30;59(6):250-4. doi: 10.1002/jlcr.3376. PMID: 27169761.

93: Chhajer R, Bhattacharyya A, Didwania N, Shadab M, Das N, Palit P, Vaidya T, Ali N. Leishmania donovani Aurora kinase: A promising therapeutic target against visceral leishmaniasis. Biochim Biophys Acta. 2016 Sep;1860(9):1973-88. doi: 10.1016/j.bbagen.2016.06.005. Epub 2016 Jun 8. PMID: 27288586.

94: Helfrich BA, Kim J, Gao D, Chan DC, Zhang Z, Tan AC, Bunn PA Jr. Barasertib (AZD1152), a Small Molecule Aurora B Inhibitor, Inhibits the Growth of SCLC Cell Lines In Vitro and In Vivo. Mol Cancer Ther. 2016 Oct;15(10):2314-2322. doi: 10.1158/1535-7163.MCT-16-0298. Epub 2016 Aug 5. PMID: 27496133; PMCID: PMC5050114.

95: Wiedemuth R, Klink B, Fujiwara M, Schröck E, Tatsuka M, Schackert G, Temme A. Janus face-like effects of Aurora B inhibition: antitumoral mode of action versus induction of aneuploid progeny. Carcinogenesis. 2016 Oct;37(10):993-1003. doi: 10.1093/carcin/bgw083. Epub 2016 Aug 11. PMID: 27515963.

96: Sakai S, Izumi H, Yoshiura Y, Nakayama Y, Yamaguchi T, Harada Y, Koi C, Kurata H, Morimoto Y. <i>In vitro</i> evaluation of a combination treatment involving anticancer agents and an aurora kinase B inhibitor. Oncol Lett. 2016 Nov;12(5):4263-4269. doi: 10.3892/ol.2016.5156. Epub 2016 Sep 20. PMID: 27895801; PMCID: PMC5104265.

97: Xu J, Li K, Smith RA, Waterton JC, Zhao P, Ding Z, Does MD, Manning HC, Gore JC. A comparative assessment of preclinical chemotherapeutic response of tumors using quantitative non-Gaussian diffusion MRI. Magn Reson Imaging. 2017 Apr;37:195-202. doi: 10.1016/j.mri.2016.12.002. Epub 2016 Dec 3. PMID: 27919785; PMCID: PMC5316364.

98: Al-Khafaji AS, Davies MP, Risk JM, Marcus MW, Koffa M, Gosney JR, Shaw RJ, Field JK, Liloglou T. Aurora B expression modulates paclitaxel response in non-small cell lung cancer. Br J Cancer. 2017 Feb 28;116(5):592-599. doi: 10.1038/bjc.2016.453. Epub 2017 Jan 17. PMID: 28095398; PMCID: PMC5344288.

99: Chen C, Zhang Z, Cui P, Liao Y, Zhang Y, Yao L, Rui R, Ju S. Phosphorylation of histone H3 on Ser-10 by Aurora B is essential for chromosome condensation in porcine embryos during the first mitotic division. Histochem Cell Biol. 2017 Jul;148(1):73-83. doi: 10.1007/s00418-017-1546-8. Epub 2017 Feb 20. PMID: 28220245.

100: Floc'h N, Ashton S, Taylor P, Trueman D, Harris E, Odedra R, Maratea K, Derbyshire N, Caddy J, Jacobs VN, Hattersley M, Wen S, Curtis NJ, Pilling JE, Pease EJ, Barry ST. Optimizing Therapeutic Effect of Aurora B Inhibition in Acute Myeloid Leukemia with AZD2811 Nanoparticles. Mol Cancer Ther. 2017 Jun;16(6):1031-1040. doi: 10.1158/1535-7163.MCT-16-0580. Epub 2017 Mar 14. PMID: 28292940.

101: Zekri A, Mesbahi Y, Ghanizadeh-Vesali S, Alimoghaddam K, Ghavamzadeh A, Ghaffari SH. Reactive oxygen species generation and increase in mitochondrial copy number: new insight into the potential mechanism of cytotoxicity induced by aurora kinase inhibitor, AZD1152-HQPA. Anticancer Drugs. 2017 Sep;28(8):841-851. doi: 10.1097/CAD.0000000000000523. PMID: 28639950.

102: Noronha S, Alt LAC, Scimeca TE, Zarou O, Obrzut J, Zanotti B, Hayward EA, Pillai A, Mathur S, Rojas J, Salamah R, Chandar N, Fay MJ. Preclinical evaluation of the Aurora kinase inhibitors AMG 900, AZD1152-HQPA, and MK-5108 on SW-872 and 93T449 human liposarcoma cells. In Vitro Cell Dev Biol Anim. 2018 Jan;54(1):71-84. doi: 10.1007/s11626-017-0208-4. Epub 2017 Dec 1. PMID: 29197031.

103: Martens S, Goossens V, Devisscher L, Hofmans S, Claeys P, Vuylsteke M, Takahashi N, Augustyns K, Vandenabeele P. RIPK1-dependent cell death: a novel target of the Aurora kinase inhibitor Tozasertib (VX-680). Cell Death Dis. 2018 Feb 12;9(2):211. doi: 10.1038/s41419-017-0245-7. PMID: 29434255; PMCID: PMC5833749.

104: Gwee SSL, Radford RAW, Chow S, Syal MD, Morsch M, Formella I, Lee A, Don EK, Badrock AP, Cole NJ, West AK, Cheung SNS, Chung RS. Aurora kinase B regulates axonal outgrowth and regeneration in the spinal motor neurons of developing zebrafish. Cell Mol Life Sci. 2018 Dec;75(23):4269-4285. doi: 10.1007/s00018-018-2780-5. Epub 2018 Feb 21. PMID: 29468257.

105: Ivanova D, Zhelev Z, Lazarova D, Getsov P, Bakalova R, Aoki I. Vitamins C and K3: A Powerful Redox System for Sensitizing Leukemia Lymphocytes to Everolimus and Barasertib. Anticancer Res. 2018 Mar;38(3):1407-1414. doi: 10.21873/anticanres.12364. PMID: 29491065.

106: Jarocha D, Vo KK, Lyde RB, Hayes V, Camire RM, Poncz M. Enhancing functional platelet release in vivo from in vitro-grown megakaryocytes using small molecule inhibitors. Blood Adv. 2018 Mar 27;2(6):597-606. doi: 10.1182/bloodadvances.2017010975. PMID: 29545255; PMCID: PMC5873227.

107: Zekri A, Mesbahi Y, Boustanipour E, Sadr Z, Ghaffari SH. The Potential Contribution of microRNAs in Anti-cancer Effects of Aurora Kinase Inhibitor (AZD1152-HQPA). J Mol Neurosci. 2018 Aug;65(4):444-455. doi: 10.1007/s12031-018-1118-y. Epub 2018 Jul 26. PMID: 30051358.

108: Payton M, Cheung HK, Ninniri MSS, Marinaccio C, Wayne WC, Hanestad K, Crispino JD, Juan G, Coxon A. Dual Targeting of Aurora Kinases with AMG 900 Exhibits Potent Preclinical Activity Against Acute Myeloid Leukemia with Distinct Post-Mitotic Outcomes. Mol Cancer Ther. 2018 Dec;17(12):2575-2585. doi: 10.1158/1535-7163.MCT-18-0186. Epub 2018 Sep 28. PMID: 30266802; PMCID: PMC6279493.

109: He J, Qi Z, Zhang X, Yang Y, Liu F, Zhao G, Wang Z. Aurora kinase B inhibitor barasertib (AZD1152) inhibits glucose metabolism in gastric cancer cells. Anticancer Drugs. 2019 Jan;30(1):19-26. doi:

10.1097/CAD.0000000000000684. PMID: 30540594.

110: Floc'h N, Ashton S, Ferguson D, Taylor P, Carnevalli LS, Hughes AM, Harris E, Hattersley M, Wen S, Curtis NJ, Pilling JE, Young LA, Maratea K, Pease EJ, Barry ST. Modeling Dose and Schedule Effects of AZD2811 Nanoparticles Targeting Aurora B Kinase for Treatment of Diffuse Large B-cell Lymphoma. Mol Cancer Ther. 2019 May;18(5):909-919. doi: 10.1158/1535-7163.MCT-18-0577. Epub 2019 Mar 14. PMID: 30872381.

111: Wang S, Hwang EE, Guha R, O'Neill AF, Melong N, Veinotte CJ, Conway Saur A, Wuerthele K, Shen M, McKnight C, Alexe G, Lemieux ME, Wang A, Hughes E, Xu X, Boxer MB, Hall MD, Kung A, Berman JN, Davis MI, Stegmaier K, Crompton BD. High-throughput Chemical Screening Identifies Focal Adhesion Kinase and Aurora Kinase B Inhibition as a Synergistic Treatment Combination in Ewing Sarcoma. Clin Cancer Res. 2019 Jul 15;25(14):4552-4566. doi: 10.1158/1078-0432.CCR-17-0375. Epub 2019 Apr 12. PMID: 30979745; PMCID: PMC6634997.

112: Bertran-Alamillo J, Cattan V, Schoumacher M, Codony-Servat J, Giménez-Capitán A, Cantero F, Burbridge M, Rodríguez S, Teixidó C, Roman R, Castellví J, García-Román S, Codony-Servat C, Viteri S, Cardona AF, Karachaliou N, Rosell R, Molina-Vila MA. AURKB as a target in non-small cell lung cancer with acquired resistance to anti-EGFR therapy. Nat Commun. 2019 Apr 18;10(1):1812. doi: 10.1038/s41467-019-09734-5. PMID: 31000705; PMCID: PMC6472415.

113: Qi J, Gao X, Zhong X, Zhang N, Wang R, Zhang H, Pan T, Liu X, Yao Y, Wu Q, Niu M, Xu K. Selective inhibition of Aurora A and B kinases effectively induces cell cycle arrest in t(8;21) acute myeloid leukemia. Biomed Pharmacother. 2019 Sep;117:109113. doi: 10.1016/j.biopha.2019.109113. Epub 2019 Jun 14. PMID: 31207577.

114: Sumi NJ, Ctortecka C, Hu Q, Bryant AT, Fang B, Remsing Rix LL, Ayaz M, Kinose F, Welsh EA, Eschrich SA, Lawrence HR, Koomen JM, Haura EB, Rix U. Divergent Polypharmacology-Driven Cellular Activity of Structurally Similar Multi-Kinase Inhibitors through Cumulative Effects on Individual Targets. Cell Chem Biol. 2019 Sep 19;26(9):1240-1252.e11. doi: 10.1016/j.chembiol.2019.06.003. Epub 2019 Jun 27. PMID: 31257184; PMCID: PMC6754293.

115: Li J, Ha S, Li Z, Huang Y, Lin E, Xiao W. Aurora B prevents aneuploidy via MAD2 during the first mitotic cleavage in oxidatively damaged embryos. Cell Prolif. 2019 Sep;52(5):e12657. doi: 10.1111/cpr.12657. Epub 2019 Jul 1. PMID: 31264311; PMCID: PMC6797512.

116: Shaalan A, Proctor G. Salivary glands require Aurora Kinase B for regeneration after transient innate immune-mediated injury. Sci Rep. 2019 Aug 5;9(1):11339. doi: 10.1038/s41598-019-47762-9. PMID: 31383943; PMCID: PMC6683207.

117: Shen Y, Ding Z, Ma S, Zou Y, Yang X, Ding Z, Zhang Y, Zhu X, Schäfer MKE, Guo Q, Huang C. Targeting aurora kinase B alleviates spinal microgliosis and neuropathic pain in a rat model of peripheral nerve injury. J Neurochem. 2020 Jan;152(1):72-91. doi: 10.1111/jnc.14883. Epub 2019 Oct 20. PMID: 31563141.

118: Nie M, Wang Y, Yu Z, Li X, Deng Y, Wang Y, Yang D, Li Q, Zeng X, Ju J, Liu M, Zhao Q. AURKB promotes gastric cancer progression via activation of <i>CCND1</i> expression. Aging (Albany NY). 2020 Jan 25;12(2):1304-1321. doi: 10.18632/aging.102684. Epub 2020 Jan 25. PMID: 31982864; PMCID: PMC7053608.

119: Zhang J, Lin X, Wu L, Huang JJ, Jiang WQ, Kipps TJ, Zhang S. Aurora B induces epithelial-mesenchymal transition by stabilizing Snail1 to promote basal-like breast cancer metastasis. Oncogene. 2020 Mar;39(12):2550-2567. doi: 10.1038/s41388-020-1165-z. Epub 2020 Jan 29. PMID: 31996785.

120: Togar T, Desai S, Mishra R, Terwadkar P, Ramteke M, Ranjan M, Kawle D, Sahoo B, Pal A, Upadhyay P, Dutt A. Identifying cancer driver genes from functional genomics screens. Swiss Med Wkly. 2020 Feb 21;150:w20195. doi: 10.4414/smw.2020.20195. PMID: 32083704.

121: Krushkal J, Silvers T, Reinhold WC, Sonkin D, Vural S, Connelly J, Varma S, Meltzer PS, Kunkel M, Rapisarda A, Evans D, Pommier Y, Teicher BA. Epigenome-wide DNA methylation analysis of small cell lung cancer cell lines suggests potential chemotherapy targets. Clin Epigenetics. 2020 Jun 25;12(1):93. doi: 10.1186/s13148-020-00876-8. PMID: 32586373; PMCID: PMC7318526.

122: Sánchez-Molina S, Figuerola-Bou E, Blanco E, Sánchez-Jiménez M, Táboas P, Gómez S, Ballaré C, García-Domínguez DJ, Prada E, Hontecillas-Prieto L, M Carcaboso Á, Tirado ÓM, Hernández-Muñoz I, de Álava E, Lavarino C, Di Croce L, Mora J. RING1B recruits EWSR1-FLI1 and cooperates in the remodeling of chromatin necessary for Ewing sarcoma tumorigenesis. Sci Adv. 2020 Oct 23;6(43):eaba3058. doi: 10.1126/sciadv.aba3058. PMID: 33097530; PMCID: PMC7608835.

123: Borah NA, Sradhanjali S, Barik MR, Jha A, Tripathy D, Kaliki S, Rath S, Raghav SK, Patnaik S, Mittal R, Reddy MM. Aurora Kinase B Expression, Its Regulation and Therapeutic Targeting in Human Retinoblastoma. Invest Ophthalmol Vis Sci. 2021 Mar 1;62(3):16. doi: 10.1167/iovs.62.3.16. PMID: 33704359; PMCID: PMC7960835.

124: Sankhe K, Prabhu A, Khan T. Design strategies, SAR, and mechanistic insight of Aurora kinase inhibitors in cancer. Chem Biol Drug Des. 2021 Jul;98(1):73-93. doi: 10.1111/cbdd.13850. Epub 2021 May 15. PMID: 33934503.

125: Chen Y, Liu R, Wang W, Wang C, Zhang N, Shao X, He Q, Ying M. Advances in targeted therapy for osteosarcoma based on molecular classification. Pharmacol Res. 2021 Jul;169:105684. doi: 10.1016/j.phrs.2021.105684. Epub 2021 May 19. PMID: 34022396.

126: Nan J, Zhang S, Zhan P, Jiang L. Discovery of Novel GMPS Inhibitors of <i>Candidatus</i> Liberibacter Asiaticus by Structure Based Design and Enzyme Kinetic. Biology (Basel). 2021 Jun 28;10(7):594. doi: 10.3390/biology10070594. PMID: 34203217; PMCID: PMC8301025.

Results of PubMed search: keyword Barasertib

1: Mortlock AA, Foote KM, Heron NM, Jung FH, Pasquet G, Lohmann JJ, Warin N, Renaud F, De Savi C, Roberts NJ, Johnson T, Dousson CB, Hill GB, Perkins D, Hatter G, Wilkinson RW, Wedge SR, Heaton SP, Odedra R, Keen NJ, Crafter C, Brown E, Thompson K, Brightwell S, Khatri L, Brady MC, Kearney S, McKillop D, Rhead S, Parry T, Green S. Discovery, synthesis, and in vivo activity of a new class of pyrazoloquinazolines as selective inhibitors of aurora B kinase. J Med Chem. 2007 May 3;50(9):2213-24. doi: 10.1021/jm061335f. Epub 2007 Mar 21. PMID: 17373783.

2: Yang J, Ikezoe T, Nishioka C, Tasaka T, Taniguchi A, Kuwayama Y, Komatsu N, Bandobashi K, Togitani K, Koeffler HP, Taguchi H, Yokoyama A. AZD1152, a novel and selective aurora B kinase inhibitor, induces growth arrest, apoptosis, and sensitization for tubulin depolymerizing agent or topoisomerase II inhibitor in human acute leukemia cells in vitro and in vivo. Blood. 2007 Sep 15;110(6):2034-40. doi: 10.1182/blood-2007-02-073700. Epub 2007 May 10. PMID: 17495131.

3: Wilkinson RW, Odedra R, Heaton SP, Wedge SR, Keen NJ, Crafter C, Foster JR, Brady MC, Bigley A, Brown E, Byth KF, Barrass NC, Mundt KE, Foote KM, Heron NM, Jung FH, Mortlock AA, Boyle FT, Green S. AZD1152, a selective inhibitor of Aurora B kinase, inhibits human tumor xenograft growth by inducing apoptosis. Clin Cancer Res. 2007 Jun 15;13(12):3682-8. doi: 10.1158/1078-0432.CCR-06-2979. PMID: 17575233.

4: Evans RP, Naber C, Steffler T, Checkland T, Maxwell CA, Keats JJ, Belch AR, Pilarski LM, Lai R, Reiman T. The selective Aurora B kinase inhibitor AZD1152 is a potential new treatment for multiple myeloma. Br J Haematol. 2008 Feb;140(3):295-302. doi: 10.1111/j.1365-2141.2007.06913.x. Epub 2007 Dec 10. PMID: 18076711.

5: Tao Y, Zhang P, Girdler F, Frascogna V, Castedo M, Bourhis J, Kroemer G, Deutsch E. Enhancement of radiation response in p53-deficient cancer cells by the Aurora-B kinase inhibitor AZD1152. Oncogene. 2008 May 22;27(23):3244-55. doi: 10.1038/sj.onc.1210990. Epub 2007 Dec 17. PMID: 18084327.

6: Cummings J, Hodgkinson C, Odedra R, Sini P, Heaton SP, Mundt KE, Ward TH, Wilkinson RW, Growcott J, Hughes A, Dive C. Preclinical evaluation of M30 and M65 ELISAs as biomarkers of drug induced tumor cell death and antitumor activity. Mol Cancer Ther. 2008 Mar;7(3):455-63. doi: 10.1158/1535-7163.MCT-07-2136. PMID: 18347133.

7: Walsby E, Walsh V, Pepper C, Burnett A, Mills K. Effects of the aurora kinase inhibitors AZD1152-HQPA and ZM447439 on growth arrest and polyploidy in acute myeloid leukemia cell lines and primary blasts. Haematologica. 2008 May;93(5):662-9. doi: 10.3324/haematol.12148. Epub 2008 Mar 26. PMID: 18367484.

8: Guo J, Anderson MG, Tapang P, Palma JP, Rodriguez LE, Niquette A, Li J, Bouska JJ, Wang G, Semizarov D, Albert DH, Donawho CK, Glaser KB, Shah OJ. Identification of genes that confer tumor cell resistance to the aurora B kinase inhibitor, AZD1152. Pharmacogenomics J. 2009 Apr;9(2):90-102. doi: 10.1038/tpj.2008.20. Epub 2009 Feb 3. PMID: 19188929.

9: Nair JS, Ho AL, Tse AN, Coward J, Cheema H, Ambrosini G, Keen N, Schwartz GK.

Aurora B kinase regulates the postmitotic endoreduplication checkpoint via phosphorylation of the retinoblastoma protein at serine 780. Mol Biol Cell. 2009 Apr;20(8):2218-28. doi: 10.1091/mbc.e08-08-0885. Epub 2009 Feb 18. PMID: 19225156; PMCID: PMC2669029.

10: Nair JS, de Stanchina E, Schwartz GK. The topoisomerase I poison CPT-11 enhances the effect of the aurora B kinase inhibitor AZD1152 both in vitro and in vivo. Clin Cancer Res. 2009 Mar 15;15(6):2022-30. doi: 10.1158/1078-0432.CCR-08-1826. Epub 2009 Mar 10. PMID: 19276280.

11: Anderson K, Lai Z, McDonald OB, Stuart JD, Nartey EN, Hardwicke MA, Newlander K, Dhanak D, Adams J, Patrick D, Copeland RA, Tummino PJ, Yang J. Biochemical characterization of GSK1070916, a potent and selective inhibitor of Aurora B and Aurora C kinases with an extremely long residence time1. Biochem J. 2009 May 13;420(2):259-65. doi: 10.1042/BJ20090121. PMID: 19284385.

12: Oke A, Pearce D, Wilkinson RW, Crafter C, Odedra R, Cavenagh J, Fitzgibbon J, Lister AT, Joel S, Bonnet D. AZD1152 rapidly and negatively affects the growth and survival of human acute myeloid leukemia cells in vitro and in vivo. Cancer Res. 2009 May 15;69(10):4150-8. doi: 10.1158/0008-5472.CAN-08-3203. Epub 2009 Apr 14. PMID: 19366807; PMCID: PMC2684546.

13: Lorenzo C, Liao Q, Hardwicke MA, Ducommun B. Pharmacological inhibition of aurora-A but not aurora-B impairs interphase microtubule dynamics. Cell Cycle. 2009 Jun 1;8(11):1733-7. doi: 10.4161/cc.8.11.8617. Epub 2009 Jun 2. PMID: 19395863.

14: Pluim D, Beijnen JH, Schellens JH, van Tellingen O. Simultaneous determination of AZD1152 (prodrug) and AZD1152-hydroxyquinazoline pyrazol anilide by reversed phase liquid chromatography. J Chromatogr B Analyt Technol Biomed Life Sci. 2009 Nov 1;877(29):3549-55. doi: 10.1016/j.jchromb.2009.08.035. Epub 2009 Aug 28. PMID: 19744901.

15: Tao Y, Leteur C, Calderaro J, Girdler F, Zhang P, Frascogna V, Varna M, Opolon P, Castedo M, Bourhis J, Kroemer G, Deutsch E. The aurora B kinase inhibitor AZD1152 sensitizes cancer cells to fractionated irradiation and induces mitotic catastrophe. Cell Cycle. 2009 Oct 1;8(19):3172-81. doi: 10.4161/cc.8.19.9729. Epub 2009 Oct 4. PMID: 19755861.

16: Ikezoe T, Takeuchi T, Yang J, Adachi Y, Nishioka C, Furihata M, Koeffler HP, Yokoyama A. Analysis of Aurora B kinase in non-Hodgkin lymphoma. Lab Invest. 2009 Dec;89(12):1364-73. doi: 10.1038/labinvest.2009.106. Epub 2009 Oct 12. PMID: 19823168.

17: Aihara A, Tanaka S, Yasen M, Matsumura S, Mitsunori Y, Murakata A, Noguchi N, Kudo A, Nakamura N, Ito K, Arii S. The selective Aurora B kinase inhibitor AZD1152 as a novel treatment for hepatocellular carcinoma. J Hepatol. 2010 Jan;52(1):63-71. doi: 10.1016/j.jhep.2009.10.013. Epub 2009 Oct 29. PMID: 19913935.

18: Lok W, Klein RQ, Saif MW. Aurora kinase inhibitors as anti-cancer therapy. Anticancer Drugs. 2010 Apr;21(4):339-50. doi: 10.1097/CAD.0b013e3283350dd1. PMID: 20016367.

19: Tomita M, Tanaka Y, Mori N. Aurora kinase inhibitor AZD1152 negatively affects the growth and survival of HTLV-1-infected T lymphocytes in vitro. Int J Cancer. 2010 Oct 1;127(7):1584-94. doi: 10.1002/ijc.25178. Retraction in: Int J Cancer. 2011 Dec 1;129(11):2762-3. PMID: 20091867.

20: Gully CP, Zhang F, Chen J, Yeung JA, Velazquez-Torres G, Wang E, Yeung SC, Lee MH. Antineoplastic effects of an Aurora B kinase inhibitor in breast cancer. Mol Cancer. 2010 Feb 22;9:42. doi: 10.1186/1476-4598-9-42. PMID: 20175926; PMCID: PMC2839967.

21: Boss DS, Witteveen PO, van der Sar J, Lolkema MP, Voest EE, Stockman PK, Ataman O, Wilson D, Das S, Schellens JH. Clinical evaluation of AZD1152, an i.v. inhibitor of Aurora B kinase, in patients with solid malignant tumors. Ann Oncol. 2011 Feb;22(2):431-7. doi: 10.1093/annonc/mdq344. Epub 2010 Oct 5. PMID: 20924078.

22: Payton M, Bush TL, Chung G, Ziegler B, Eden P, McElroy P, Ross S, Cee VJ, Deak HL, Hodous BL, Nguyen HN, Olivieri PR, Romero K, Schenkel LB, Bak A, Stanton M, Dussault I, Patel VF, Geuns-Meyer S, Radinsky R, Kendall RL. Preclinical evaluation of AMG 900, a novel potent and highly selective pan-aurora kinase inhibitor with activity in taxane-resistant tumor cell lines. Cancer Res. 2010 Dec 1;70(23):9846-54. doi: 10.1158/0008-5472.CAN-10-3001. Epub 2010 Oct 8. PMID: 20935223.

23: Libertini S, Abagnale A, Passaro C, Botta G, Barbato S, Chieffi P, Portella G. AZD1152 negatively affects the growth of anaplastic thyroid carcinoma cells and enhances the effects of oncolytic virus dl922-947. Endocr Relat Cancer. 2011 Jan 13;18(1):129-41. doi: 10.1677/ERC-10-0234. PMID: 21071467.

24: Niermann KJ, Moretti L, Giacalone NJ, Sun Y, Schleicher SM, Kopsombut P, Mitchell LR, Kim KW, Lu B. Enhanced radiosensitivity of androgen-resistant prostate cancer: AZD1152-mediated Aurora kinase B inhibition. Radiat Res. 2011 Apr;175(4):444-51. doi: 10.1667/RR2317.1. Epub 2011 Jan 11. PMID: 21222513; PMCID: PMC3133747.

25: Moroz MA, Kochetkov T, Cai S, Wu J, Shamis M, Nair J, de Stanchina E, Serganova I, Schwartz GK, Banerjee D, Bertino JR, Blasberg RG. Imaging colon cancer response following treatment with AZD1152: a preclinical analysis of [18F]fluoro-2-deoxyglucose and 3'-deoxy-3'-[18F]fluorothymidine imaging. Clin Cancer Res. 2011 Mar 1;17(5):1099-110. doi: 10.1158/1078-0432.CCR-10-1430. Epub 2011 Jan 18. PMID: 21245090; PMCID: PMC3079195.

26: Azzariti A, Bocci G, Porcelli L, Fioravanti A, Sini P, Simone GM, Quatrale AE, Chiarappa P, Mangia A, Sebastian S, Del Bufalo D, Del Tacca M, Paradiso A. Aurora B kinase inhibitor AZD1152: determinants of action and ability to enhance chemotherapeutics effectiveness in pancreatic and colon cancer. Br J Cancer. 2011 Mar 1;104(5):769-80. doi: 10.1038/bjc.2011.21. Epub 2011 Feb 8. PMID: 21304529; PMCID: PMC3048212.

27: Mori N, Ishikawa C, Senba M, Kimura M, Okano Y. Effects of AZD1152, a selective Aurora B kinase inhibitor, on Burkitt's and Hodgkin's lymphomas. Biochem Pharmacol. 2011 May 1;81(9):1106-15. doi: 10.1016/j.bcp.2011.02.010. Epub 2011 Mar 1. Erratum in: Biochem Pharmacol. 2011 Nov 1;82(9):1252. PMID: 21371446.

28: Tsuboi K, Yokozawa T, Sakura T, Watanabe T, Fujisawa S, Yamauchi T, Uike N, Ando K, Kihara R, Tobinai K, Asou H, Hotta T, Miyawaki S. A Phase I study to assess the safety, pharmacokinetics and efficacy of barasertib (AZD1152), an Aurora B kinase inhibitor, in Japanese patients with advanced acute myeloid leukemia. Leuk Res. 2011 Oct;35(10):1384-9. doi: 10.1016/j.leukres.2011.04.008. Epub 2011 May 11. PMID: 21565405.

29: Keizer RJ, Zandvliet AS, Beijnen JH, Schellens JH, Huitema AD. Two-stage model-based design of cancer phase I dose escalation trials: evaluation using the phase I program of barasertib (AZD1152). Invest New Drugs. 2012 Aug;30(4):1519-30. doi: 10.1007/s10637-011-9694-5. Epub 2011 May 28. PMID: 21626115; PMCID: PMC3388254.

30: Grundy M, Seedhouse C, Russell NH, Pallis M. P-glycoprotein and breast cancer resistance protein in acute myeloid leukaemia cells treated with the aurora-B kinase inhibitor barasertib-hQPA. BMC Cancer. 2011 Jun 16;11:254. doi: 10.1186/1471-2407-11-254. PMID: 21679421; PMCID: PMC3146447.

31: Emery A, Sorrell DA, Lawrence S, Easthope E, Stockdale M, Jones DO, Zheleva D, Scaerou F, Glover DM. A novel cell-based, high-content assay for phosphorylation of Lats2 by Aurora A. J Biomol Screen. 2011 Sep;16(8):925-31. doi: 10.1177/1087057111413923. Epub 2011 Jul 25. PMID: 21788394.

32: LÃ¶wenberg B, Muus P, Ossenkoppele G, Rousselot P, Cahn JY, Ifrah N, Martinelli G, Amadori S, Berman E, Sonneveld P, Jongen-Lavrencic M, Rigaudeau S, Stockman P, Goudie A, Faderl S, Jabbour E, Kantarjian H. Phase 1/2 study to assess the safety, efficacy, and pharmacokinetics of barasertib (AZD1152) in patients with advanced acute myeloid leukemia. Blood. 2011 Dec 1;118(23):6030-6. doi: 10.1182/blood-2011-07-366930. Epub 2011 Oct 5. PMID: 21976672; PMCID: PMC4186639.

33: Kwiatkowski N, Deng X, Wang J, Tan L, Villa F, Santaguida S, Huang HC, Mitchison T, Musacchio A, Gray N. Selective aurora kinase inhibitors identified using a taxol-induced checkpoint sensitivity screen. ACS Chem Biol. 2012 Jan 20;7(1):185-96. doi: 10.1021/cb200305u. Epub 2011 Oct 21. PMID: 21992004; PMCID: PMC3262907.

34: Holt SV, LogiÃ© A, Odedra R, Heier A, Heaton SP, Alferez D, Davies BR, Wilkinson RW, Smith PD. The MEK1/2 inhibitor, selumetinib (AZD6244; ARRY-142886), enhances anti-tumour efficacy when combined with conventional chemotherapeutic agents in human tumour xenograft models. Br J Cancer. 2012 Feb 28;106(5):858-66. doi: 10.1038/bjc.2012.8. Epub 2012 Feb 16. PMID: 22343622; PMCID: PMC3305954.

35: Schwartz GK, Carvajal RD, Midgley R, Rodig SJ, Stockman PK, Ataman O, Wilson D, Das S, Shapiro GI. Phase I study of barasertib (AZD1152), a selective inhibitor of Aurora B kinase, in patients with advanced solid tumors. Invest New Drugs. 2013 Apr;31(2):370-80. doi: 10.1007/s10637-012-9825-7. Epub 2012 Jun 2. PMID: 22661287.

36: Alferez DG, Goodlad RA, Odedra R, Sini P, Crafter C, Ryan AJ, Wedge SR, Wright NA, Anderson E, Wilkinson RW. Inhibition of Aurora-B kinase activity confers antitumor efficacy in preclinical mouse models of early and advanced

gastrointestinal neoplasia. Int J Oncol. 2012 Oct;41(4):1475-85. doi: 10.3892/ijo.2012.1580. Epub 2012 Jul 31. PMID: 22858681.

37: Dennis M, Davies M, Oliver S, D'Souza R, Pike L, Stockman P. Phase I study of the Aurora B kinase inhibitor barasertib (AZD1152) to assess the pharmacokinetics, metabolism and excretion in patients with acute myeloid leukemia. Cancer Chemother Pharmacol. 2012 Sep;70(3):461-9. doi: 10.1007/s00280-012-1939-2. Epub 2012 Aug 4. PMID: 22864876; PMCID: PMC3428523.

38: Xu J, Li K, Smith RA, Waterton JC, Zhao P, Chen H, Does MD, Manning HC, Gore JC. Characterizing tumor response to chemotherapy at various length scales using temporal diffusion spectroscopy. PLoS One. 2012;7(7):e41714. doi: 10.1371/journal.pone.0041714. Epub 2012 Jul 24. PMID: 22911846; PMCID: PMC3404000.

39: Hartsink-Segers SA, Zwaan CM, Exalto C, Luijendijk MW, Calvert VS, Petricoin EF, Evans WE, Reinhardt D, de Haas V, HedtjÃ¤rn M, Hansen BR, Koch T, Caron HN, Pieters R, Den Boer ML. Aurora kinases in childhood acute leukemia: the promise of aurora B as therapeutic target. Leukemia. 2013 Mar;27(3):560-8. doi: 10.1038/leu.2012.256. Epub 2012 Sep 3. PMID: 22940834; PMCID: PMC3593181.

40: Marchetti S, Pluim D, van Eijndhoven M, van Tellingen O, Mazzanti R, Beijnen JH, Schellens JH. Effect of the drug transporters ABCG2, Abcg2, ABCB1 and ABCC2 on the disposition, brain accumulation and myelotoxicity of the aurora kinase B inhibitor barasertib and its more active form barasertib-hydroxy-QPA. Invest New Drugs. 2013 Oct;31(5):1125-35. doi: 10.1007/s10637-013-9923-1. Epub 2013 Jan 13. Erratum in: Invest New Drugs. 2013 Jun;31(3):800. PMID: 23315030.

41: Ma Y, Weimer J, Fredrik R, Adam-Klages S, Sebens S, Caliebe A, Hilpert F, Eckmann-Scholz C, Arnold N, Schem C. Aurora kinase inhibitor AZD1152 has an additional effect of platinum on a sequential application at the human ovarian cancer cell line SKOV3. Arch Gynecol Obstet. 2013 Jul;288(1):173-82. doi: 10.1007/s00404-013-2719-x. Epub 2013 Feb 7. PMID: 23389245.

42: Ma YX, Li XZ. [Effect of aurora kinase B inhibitor AZD1152 in the treatment of cisplatin-resistant ovarian carcinoma]. Zhonghua Fu Chan Ke Za Zhi. 2013 Jan;48(1):46-50. Chinese. PMID: 23531251.

43: Yamauchi T, Uzui K, Shigemi H, Negoro E, Yoshida A, Ueda T. Aurora B inhibitor barasertib and cytarabine exert a greater-than-additive cytotoxicity in acute myeloid leukemia cells. Cancer Sci. 2013 Jul;104(7):926-33. doi: 10.1111/cas.12164. Epub 2013 May 12. PMID: 23557198; PMCID: PMC7657165.

44: Kantarjian HM, Martinelli G, Jabbour EJ, QuintÃ¡s-Cardama A, Ando K, Bay JO, Wei A, GrÃ¶pper S, Papayannidis C, Owen K, Pike L, Schmitt N, Stockman PK, Giagounidis A; SPARK-AML1 Investigators. Stage I of a phase 2 study assessing the efficacy, safety, and tolerability of barasertib (AZD1152) versus low-dose cytosine arabinoside in elderly patients with acute myeloid leukemia. Cancer. 2013 Jul 15;119(14):2611-9. doi: 10.1002/cncr.28113. Epub 2013 Apr 19. PMID: 23605952; PMCID: PMC4132839.

45: Kantarjian HM, Sekeres MA, Ribrag V, Rousselot P, Garcia-Manero G, Jabbour EJ, Owen K, Stockman PK, Oliver SD. Phase I study assessing the safety and tolerability of barasertib (AZD1152) with low-dose cytosine arabinoside in

elderly patients with AML. Clin Lymphoma Myeloma Leuk. 2013 Oct;13(5):559-67. doi: 10.1016/j.clml.2013.03.019. Epub 2013 Jun 10. PMID: 23763917; PMCID: PMC3775947.

46: Marxer M, Ma HT, Man WY, Poon RY. p53 deficiency enhances mitotic arrest and slippage induced by pharmacological inhibition of Aurora kinases. Oncogene. 2014 Jul 3;33(27):3550-60. doi: 10.1038/onc.2013.325. Epub 2013 Aug 19. PMID: 23955083.

47: Akiyama M, Izumi H, Wang KY, Yamaguchi T, Kuma A, Kitamura N, Harada Y, Oya R, Yamaguchi K, Iwai Y, Kohno K. Hypersensitivity to aurora kinase inhibitors in cells resistant against platinum- containing anticancer agents. Anticancer Agents Med Chem. 2014;14(7):1042-50. doi: 10.2174/1871520614666140207154351. PMID: 24521151.

48: Hight MR, Cheung YY, Nickels ML, Dawson ES, Zhao P, Saleh S, Buck JR, Tang D, Washington MK, Coffey RJ, Manning HC. A peptide-based positron emission tomography probe for in vivo detection of caspase activity in apoptotic cells. Clin Cancer Res. 2014 Apr 15;20(8):2126-35. doi: 10.1158/1078-0432.CCR-13-2444. Epub 2014 Feb 26. PMID: 24573549; PMCID: PMC3989451.

49: Sessa F, Villa F. Structure of Aurora B-INCENP in complex with barasertib reveals a potential transinhibitory mechanism. Acta Crystallogr F Struct Biol Commun. 2014 Mar;70(Pt 3):294-8. doi: 10.1107/S2053230X14002118. Epub 2014 Feb 19. PMID: 24598913; PMCID: PMC3944688.

50: Umene K, Banno K, Kisu I, Yanokura M, Nogami Y, Tsuji K, Masuda K, Ueki A, Kobayashi Y, Yamagami W, Nomura H, Tominaga E, Susumu N, Aoki D. Aurora kinase inhibitors: Potential molecular-targeted drugs for gynecologic malignant tumors. Biomed Rep. 2013 May;1(3):335-340. doi: 10.3892/br.2013.91. Epub 2013 Mar 27. Erratum in: Biomed Rep. 2019 Dec;11(6):274. PMID: 24648944; PMCID: PMC3917679.

51: Chiou SK, Hoa N, Hodges A, Ge L, Jadus MR. Indomethacin promotes apoptosis in gastric cancer cells through concomitant degradation of Survivin and Aurora B kinase proteins. Apoptosis. 2014 Sep;19(9):1378-88. doi: 10.1007/s10495-014-1002-3. PMID: 24874838.

52: Baldini E, Tuccilli C, Prinzi N, Sorrenti S, Antonelli A, Gnessi L, Morrone S, Moretti C, Bononi M, Arlot-Bonnemains Y, D'Armiento M, Ulisse S. Effects of selective inhibitors of Aurora kinases on anaplastic thyroid carcinoma cell lines. Endocr Relat Cancer. 2014 Oct;21(5):797-811. doi: 10.1530/ERC-14-0299. Epub 2014 Jul 29. PMID: 25074669.

53: Montalban-Bravo G, Garcia-Manero G. Novel drugs for older patients with acute myeloid leukemia. Leukemia. 2015 Apr;29(4):760-9. doi: 10.1038/leu.2014.244. Epub 2014 Aug 21. PMID: 25142817.

54: Boeckx C, Op de Beeck K, Wouters A, Deschoolmeester V, Limame R, Zwaenepoel K, Specenier P, Pauwels P, Vermorken JB, Peeters M, Van Camp G, Baay M, Lardon F. Overcoming cetuximab resistance in HNSCC: the role of AURKB and DUSP proteins. Cancer Lett. 2014 Nov 28;354(2):365-77. doi: 10.1016/j.canlet.2014.08.039. Epub 2014 Sep 2. PMID: 25192874.

55: Zekri A, Ghaffari SH, Ghanizadeh-Vesali S, Yaghmaie M, Salmaninejad A,

Alimoghaddam K, Modarressi MH, Ghavamzadeh A. AZD1152-HQPA induces growth arrest and apoptosis in androgen-dependent prostate cancer cell line (LNCaP) via producing aneugenic micronuclei and polyploidy. Tumour Biol. 2015 Feb;36(2):623-32. doi: 10.1007/s13277-014-2664-8. Epub 2014 Oct 3. PMID: 25277659.

56: Ma Y, Cao H, Lou S, Shao X, Lv W, Qi X, Liu Y, Ying M, He Q, Yang X. Sequential treatment with aurora B inhibitors enhances cisplatin-mediated apoptosis via c-Myc. J Mol Med (Berl). 2015 Apr;93(4):427-38. doi: 10.1007/s00109-014-1228-0. Epub 2014 Nov 21. PMID: 25411027.

57: Pacaud R, Cheray M, Nadaradjane A, Vallette FM, Cartron PF. Histone H3 phosphorylation in GBM: a new rational to guide the use of kinase inhibitors in anti-GBM therapy. Theranostics. 2015 Jan 1;5(1):12-22. doi: 10.7150/thno.8799. PMID: 25553095; PMCID: PMC4265745.

58: Erba HP. Finding the optimal combination therapy for the treatment of newly diagnosed AML in older patients unfit for intensive therapy. Leuk Res. 2015 Feb;39(2):183-91. doi: 10.1016/j.leukres.2014.11.027. Epub 2014 Dec 9. PMID: 25577399.

59: Porcelli L, Guida G, Quatrale AE, Cocco T, Sidella L, Maida I, Iacobazzi RM, Ferretta A, Stolfa DA, Strippoli S, Guida S, Tommasi S, Guida M, Azzariti A. Aurora kinase B inhibition reduces the proliferation of metastatic melanoma cells and enhances the response to chemotherapy. J Transl Med. 2015 Jan 27;13:26. doi: 10.1186/s12967-015-0385-4. PMID: 25623468; PMCID: PMC4314759.

60: Hole S, Pedersen AM, Lykkesfeldt AE, Yde CW. Aurora kinase A and B as new treatment targets in aromatase inhibitor-resistant breast cancer cells. Breast Cancer Res Treat. 2015 Feb;149(3):715-26. doi: 10.1007/s10549-015-3284-8. Epub 2015 Feb 10. PMID: 25667100.

61: Collins GP, Eyre TA, Linton KM, Radford J, Vallance GD, Soilleux E, Hatton C. A phase II trial of AZD1152 in relapsed/refractory diffuse large B-cell lymphoma. Br J Haematol. 2015 Sep;170(6):886-90. doi: 10.1111/bjh.13333. Epub 2015 Feb 26. PMID: 25721307.

62: Zekri A, Ghaffari SH, Yaghmaie M, Estiar MA, Alimoghaddam K, Modarressi MH, Ghavamzadeh A. Inhibitor of Aurora Kinase B Induces Differentially Cell Death and Polyploidy via DNA Damage Response Pathways in Neurological Malignancy: Shedding New Light on the Challenge of Resistance to AZD1152-HQPA. Mol Neurobiol. 2016 Apr;53(3):1808-1823. doi: 10.1007/s12035-015-9139-9. Epub 2015 Mar 11. PMID: 25752998.

63: Li J, Hong MJ, Chow JP, Man WY, Mak JP, Ma HT, Poon RY. Co-inhibition of polo-like kinase 1 and Aurora kinases promotes mitotic catastrophe. Oncotarget. 2015 Apr 20;6(11):9327-40. doi: 10.18632/oncotarget.3313. PMID: 25871386; PMCID: PMC4496220.

64: Larsen SL, Yde CW, Laenkholm AV, Rasmussen BB, Duun-Henriksen AK, Bak M, Lykkesfeldt AE, Kirkegaard T. Aurora kinase B is important for antiestrogen resistant cell growth and a potential biomarker for tamoxifen resistant breast cancer. BMC Cancer. 2015 Apr 8;15:239. doi: 10.1186/s12885-015-1210-4. PMID: 25885472; PMCID: PMC4392616.

65: Tuccilli C, Baldini E, Prinzi N, Morrone S, Sorrenti S, Filippini A, Catania A, Alessandrini S, Rendina R, Coccaro C, D'Armiento M, Ulisse S. Preclinical testing of selective Aurora kinase inhibitors on a medullary thyroid carcinoma-derived cell line. Endocrine. 2016 May;52(2):287-95. doi: 10.1007/s12020-015-0700-0. Epub 2015 Jul 28. PMID: 26215279.

66: Bogen D, Wei JS, Azorsa DO, Ormanoglu P, Buehler E, Guha R, Keller JM, Mathews Griner LA, Ferrer M, Song YK, Liao H, Mendoza A, Gryder BE, Sindri S, He J, Wen X, Zhang S, Shern JF, Yohe ME, Taschner-Mandl S, Shohet JM, Thomas CJ, Martin SE, Ambros PF, Khan J. Aurora B kinase is a potent and selective target in MYCN-driven neuroblastoma. Oncotarget. 2015 Nov 3;6(34):35247-62. doi: 10.18632/oncotarget.6208. PMID: 26497213; PMCID: PMC4742102.

67: Falchook GS, Bastida CC, Kurzrock R. Aurora Kinase Inhibitors in Oncology Clinical Trials: Current State of the Progress. Semin Oncol. 2015 Dec;42(6):832-48. doi: 10.1053/j.seminoncol.2015.09.022. Epub 2015 Sep 24. PMID: 26615129.

68: Zhelev Z, Ivanova D, Aoki I, Saga T, Bakalova R. 2-Deoxy-D-glucose Sensitizes Cancer Cells to Barasertib and Everolimus by ROS-independent Mechanism(s). Anticancer Res. 2015 Dec;35(12):6623-32. PMID: 26637878.

69: Baldini E, Tuccilli C, Prinzi N, Sorrenti S, Antonelli A, Fallahi P, Mian C, Barollo S, Catania A, Morrone S, Tartaglia F, Mascagni D, Coccaro C, Pepe M, Filippini A, D'Armiento M, Ulisse S. Selective inhibitors of aurora kinases inhibit proliferation, reduce cell viability and impair cell cycle progression in papillary thyroid carcinoma cells. J Biol Regul Homeost Agents. 2015 Oct-Dec;29(4):793-803. PMID: 26753639.

70: Ashton S, Song YH, Nolan J, Cadogan E, Murray J, Odedra R, Foster J, Hall PA, Low S, Taylor P, Ellston R, Polanska UM, Wilson J, Howes C, Smith A, Goodwin RJ, Swales JG, Strittmatter N, TakÃ¡ts Z, Nilsson A, Andren P, Trueman D, Walker M, Reimer CL, Troiano G, Parsons D, De Witt D, Ashford M, Hrkach J, Zale S, Jewsbury PJ, Barry ST. Aurora kinase inhibitor nanoparticles target tumors with favorable therapeutic index in vivo. Sci Transl Med. 2016 Feb 10;8(325):325ra17. doi: 10.1126/scitranslmed.aad2355. PMID: 26865565.

71: Ju S, Peng X, Yang X, Sozar S, Muneri CW, Xu Y, Chen C, Cui P, Xu W, Rui R. Aurora B inhibitor barasertib prevents meiotic maturation and subsequent embryo development in pig oocytes. Theriogenology. 2016 Jul 15;86(2):503-15. doi: 10.1016/j.theriogenology.2016.01.030. Epub 2016 Feb 9. PMID: 26993175.

72: Song YH, Shin E, Wang H, Nolan J, Low S, Parsons D, Zale S, Ashton S, Ashford M, Ali M, Thrasher D, Boylan N, Troiano G. A novel in situ hydrophobic ion paring (HIP) formulation strategy for clinical product selection of a nanoparticle drug delivery system. J Control Release. 2016 May 10;229:106-119. doi: 10.1016/j.jconrel.2016.03.026. Epub 2016 Mar 18. PMID: 27001894.

73: Zhelev Z, Ivanova D, Lazarova D, Aoki I, Bakalova R, Saga T. Docosahexaenoic Acid Sensitizes Leukemia Lymphocytes to Barasertib and Everolimus by ROS-dependent Mechanism Without Affecting the Level of ROS and Viability of Normal Lymphocytes. Anticancer Res. 2016 Apr;36(4):1673-82. PMID: 27069145.

74: Ghanizadeh-Vesali S, Zekri A, Zaker F, Zaghal A, Yousefi M, Alimoghaddam K, Ghavamzadeh A, Ghaffari SH. Significance of AZD1152 as a potential treatment against Aurora B overexpression in acute promyelocytic leukemia. Ann Hematol. 2016 Jun;95(7):1031-42. doi: 10.1007/s00277-016-2670-6. Epub 2016 Apr 19. PMID: 27091351.

75: Sarvagalla S, Hsieh HP, Coumar MS. Therapeutic polymeric nanoparticles and the methods of making and using thereof: a patent evaluation of WO2015036792. Expert Opin Ther Pat. 2016 Jul;26(7):751-5. doi: 10.1080/13543776.2016.1188919. Epub 2016 May 24. PMID: 27167102.

76: Bushby N, Bergin J, Harding J. [(14) C]-AZD1152 drug substance manufacture: challenges of an IV-infusion dosed human mass balance study in patients. J Labelled Comp Radiopharm. 2016 May 30;59(6):250-4. doi: 10.1002/jlcr.3376. PMID: 27169761.

77: Chhajer R, Bhattacharyya A, Didwania N, Shadab M, Das N, Palit P, Vaidya T, Ali N. Leishmania donovani Aurora kinase: A promising therapeutic target against visceral leishmaniasis. Biochim Biophys Acta. 2016 Sep;1860(9):1973-88. doi: 10.1016/j.bbagen.2016.06.005. Epub 2016 Jun 8. PMID: 27288586.

78: Helfrich BA, Kim J, Gao D, Chan DC, Zhang Z, Tan AC, Bunn PA Jr. Barasertib (AZD1152), a Small Molecule Aurora B Inhibitor, Inhibits the Growth of SCLC Cell Lines In Vitro and In Vivo. Mol Cancer Ther. 2016 Oct;15(10):2314-2322. doi: 10.1158/1535-7163.MCT-16-0298. Epub 2016 Aug 5. PMID: 27496133; PMCID: PMC5050114.

79: Wiedemuth R, Klink B, Fujiwara M, SchrÃ¶ck E, Tatsuka M, Schackert G, Temme A. Janus face-like effects of Aurora B inhibition: antitumoral mode of action versus induction of aneuploid progeny. Carcinogenesis. 2016 Oct;37(10):993-1003. doi: 10.1093/carcin/bgw083. Epub 2016 Aug 11. PMID: 27515963.

80: Zhelev Z, Ivanova D, Bakalova R, Aoki I, Higashi T. Inhibition of the Pentose-phosphate Pathway Selectively Sensitizes Leukemia Lymphocytes to Chemotherapeutics by ROS-independent Mechanism. Anticancer Res. 2016 Nov;36(11):6011-6020. doi: 10.21873/anticanres.11190. PMID: 27793928.

81: Xu J, Li K, Smith RA, Waterton JC, Zhao P, Ding Z, Does MD, Manning HC, Gore JC. A comparative assessment of preclinical chemotherapeutic response of tumors using quantitative non-Gaussian diffusion MRI. Magn Reson Imaging. 2017 Apr;37:195-202. doi: 10.1016/j.mri.2016.12.002. Epub 2016 Dec 3. PMID: 27919785; PMCID: PMC5316364.

82: Zhelev Z, Ivanova D, Bakalova R, Aoki I, Higashi T. Synergistic Cytotoxicity of Melatonin and New-generation Anticancer Drugs Against Leukemia Lymphocytes But Not Normal Lymphocytes. Anticancer Res. 2017 Jan;37(1):149-159. doi: 10.21873/anticanres.11300. PMID: 28011485.

83: Al-Khafaji AS, Davies MP, Risk JM, Marcus MW, Koffa M, Gosney JR, Shaw RJ, Field JK, Liloglou T. Aurora B expression modulates paclitaxel response in non-small cell lung cancer. Br J Cancer. 2017 Feb 28;116(5):592-599. doi: 10.1038/bjc.2016.453. Epub 2017 Jan 17. PMID: 28095398; PMCID: PMC5344288.

84: Chen C, Zhang Z, Cui P, Liao Y, Zhang Y, Yao L, Rui R, Ju S. Phosphorylation

of histone H3 on Ser-10 by Aurora B is essential for chromosome condensation in porcine embryos during the first mitotic division. Histochem Cell Biol. 2017 Jul;148(1):73-83. doi: 10.1007/s00418-017-1546-8. Epub 2017 Feb 20. PMID: 28220245.

85: Floc'h N, Ashton S, Taylor P, Trueman D, Harris E, Odedra R, Maratea K, Derbyshire N, Caddy J, Jacobs VN, Hattersley M, Wen S, Curtis NJ, Pilling JE, Pease EJ, Barry ST. Optimizing Therapeutic Effect of Aurora B Inhibition in Acute Myeloid Leukemia with AZD2811 Nanoparticles. Mol Cancer Ther. 2017 Jun;16(6):1031-1040. doi: 10.1158/1535-7163.MCT-16-0580. Epub 2017 Mar 14. PMID: 28292940.

86: Zekri A, Mesbahi Y, Ghanizadeh-Vesali S, Alimoghaddam K, Ghavamzadeh A, Ghaffari SH. Reactive oxygen species generation and increase in mitochondrial copy number: new insight into the potential mechanism of cytotoxicity induced by aurora kinase inhibitor, AZD1152-HQPA. Anticancer Drugs. 2017 Sep;28(8):841-851. doi: 10.1097/CAD.0000000000000523. PMID: 28639950.

87: Gwee SSL, Radford RAW, Chow S, Syal MD, Morsch M, Formella I, Lee A, Don EK, Badrock AP, Cole NJ, West AK, Cheung SNS, Chung RS. Aurora kinase B regulates axonal outgrowth and regeneration in the spinal motor neurons of developing zebrafish. Cell Mol Life Sci. 2018 Dec;75(23):4269-4285. doi: 10.1007/s00018-018-2780-5. Epub 2018 Feb 21. PMID: 29468257.

88: Ivanova D, Zhelev Z, Lazarova D, Getsov P, Bakalova R, Aoki I. Vitamins C and K3: A Powerful Redox System for Sensitizing Leukemia Lymphocytes to Everolimus and Barasertib. Anticancer Res. 2018 Mar;38(3):1407-1414. doi: 10.21873/anticanres.12364. PMID: 29491065.

89: Baumann A, Buchberger AMS, Piontek G, SchÃ¼ttler D, Rudelius M, Reiter R, Gebel L, Piendl G, Brockhoff G, Pickhard A. The Aurora-Kinase A Phe31-Ile polymorphism as possible predictor of response to treatment in head and neck squamous cell carcinoma. Oncotarget. 2018 Jan 30;9(16):12769-12780. doi: 10.18632/oncotarget.24355. PMID: 29560108; PMCID: PMC5849172.

90: Zekri A, Mesbahi Y, Boustanipour E, Sadr Z, Ghaffari SH. The Potential Contribution of microRNAs in Anti-cancer Effects of Aurora Kinase Inhibitor (AZD1152-HQPA). J Mol Neurosci. 2018 Aug;65(4):444-455. doi: 10.1007/s12031-018-1118-y. Epub 2018 Jul 26. PMID: 30051358.

91: Payton M, Cheung HK, Ninniri MSS, Marinaccio C, Wayne WC, Hanestad K, Crispino JD, Juan G, Coxon A. Dual Targeting of Aurora Kinases with AMG 900 Exhibits Potent Preclinical Activity Against Acute Myeloid Leukemia with Distinct Post-Mitotic Outcomes. Mol Cancer Ther. 2018 Dec;17(12):2575-2585. doi: 10.1158/1535-7163.MCT-18-0186. Epub 2018 Sep 28. PMID: 30266802; PMCID: PMC6279493.

92: He J, Qi Z, Zhang X, Yang Y, Liu F, Zhao G, Wang Z. Aurora kinase B inhibitor barasertib (AZD1152) inhibits glucose metabolism in gastric cancer cells. Anticancer Drugs. 2019 Jan;30(1):19-26. doi: 10.1097/CAD.0000000000000684. PMID: 30540594.

93: Floc'h N, Ashton S, Ferguson D, Taylor P, Carnevalli LS, Hughes AM, Harris E, Hattersley M, Wen S, Curtis NJ, Pilling JE, Young LA, Maratea K, Pease EJ,

Barry ST. Modeling Dose and Schedule Effects of AZD2811 Nanoparticles Targeting Aurora B Kinase for Treatment of Diffuse Large B-cell Lymphoma. Mol Cancer Ther. 2019 May;18(5):909-919. doi: 10.1158/1535-7163.MCT-18-0577. Epub 2019 Mar 14. PMID: 30872381.

94: Wang S, Hwang EE, Guha R, O'Neill AF, Melong N, Veinotte CJ, Conway Saur A, Wuerthele K, Shen M, McKnight C, Alexe G, Lemieux ME, Wang A, Hughes E, Xu X, Boxer MB, Hall MD, Kung A, Berman JN, Davis MI, Stegmaier K, Crompton BD. High-throughput Chemical Screening Identifies Focal Adhesion Kinase and Aurora Kinase B Inhibition as a Synergistic Treatment Combination in Ewing Sarcoma. Clin Cancer Res. 2019 Jul 15;25(14):4552-4566. doi: 10.1158/1078-0432.CCR-17-0375. Epub 2019 Apr 12. PMID: 30979745; PMCID: PMC6634997.

95: Bertran-Alamillo J, Cattan V, Schoumacher M, Codony-Servat J, GimÃ©nez-CapitÃ¡n A, Cantero F, Burbridge M, RodrÃguez S, TeixidÃ³ C, Roman R, CastellvÃ J, GarcÃa-RomÃ¡n S, Codony-Servat C, Viteri S, Cardona AF, Karachaliou N, Rosell R, Molina-Vila MA. AURKB as a target in non-small cell lung cancer with acquired resistance to anti-EGFR therapy. Nat Commun. 2019 Apr 18;10(1):1812. doi: 10.1038/s41467-019-09734-5. PMID: 31000705; PMCID: PMC6472415.

96: Qi J, Gao X, Zhong X, Zhang N, Wang R, Zhang H, Pan T, Liu X, Yao Y, Wu Q, Niu M, Xu K. Selective inhibition of Aurora A and B kinases effectively induces cell cycle arrest in t(8;21) acute myeloid leukemia. Biomed Pharmacother. 2019 Sep;117:109113. doi: 10.1016/j.biopha.2019.109113. Epub 2019 Jun 14. PMID: 31207577.

97: Sumi NJ, Ctortecka C, Hu Q, Bryant AT, Fang B, Remsing Rix LL, Ayaz M, Kinose F, Welsh EA, Eschrich SA, Lawrence HR, Koomen JM, Haura EB, Rix U. Divergent Polypharmacology-Driven Cellular Activity of Structurally Similar Multi-Kinase Inhibitors through Cumulative Effects on Individual Targets. Cell Chem Biol. 2019 Sep 19;26(9):1240-1252.e11. doi: 10.1016/j.chembiol.2019.06.003. Epub 2019 Jun 27. PMID: 31257184; PMCID: PMC6754293.

98: Ivanova D, Zhelev Z, Semkova S, Aoki I, Bakalova R. Resveratrol Modulates the Redox-status and Cytotoxicity of Anticancer Drugs by Sensitizing Leukemic Lymphocytes and Protecting Normal Lymphocytes. Anticancer Res. 2019 Jul;39(7):3745-3755. doi: 10.21873/anticanres.13523. PMID: 31262901.

99: Li J, Ha S, Li Z, Huang Y, Lin E, Xiao W. Aurora B prevents aneuploidy via MAD2 during the first mitotic cleavage in oxidatively damaged embryos. Cell Prolif. 2019 Sep;52(5):e12657. doi: 10.1111/cpr.12657. Epub 2019 Jul 1. PMID: 31264311; PMCID: PMC6797512.

100: Shaalan A, Proctor G. Salivary glands require Aurora Kinase BÂ for regeneration after transient innate immune-mediated injury. Sci Rep. 2019 Aug 5;9(1):11339. doi: 10.1038/s41598-019-47762-9. PMID: 31383943; PMCID: PMC6683207.

101: Zhang J, Lin X, Wu L, Huang JJ, Jiang WQ, Kipps TJ, Zhang S. Aurora B induces epithelial-mesenchymal transition by stabilizing Snail1 to promote basal-like breast cancer metastasis. Oncogene. 2020 Mar;39(12):2550-2567. doi: 10.1038/s41388-020-1165-z. Epub 2020 Jan 29. PMID: 31996785.

102: Goto H, Yoshino Y, Ito M, Nagai J, Kumamoto T, Inukai T, Sakurai Y,

Miyagawa N, Keino D, Yokosuka T, Iwasaki F, Hamanoue S, Shiomi M, Goto S. Aurora B kinase as a therapeutic target in acute lymphoblastic leukemia. Cancer Chemother Pharmacol. 2020 Apr;85(4):773-783. doi: 10.1007/s00280-020-04045-9. Epub 2020 Mar 6. Erratum in: Cancer Chemother Pharmacol. 2021 Dec;88(6):1055-1056. PMID: 32144432.

103: Lakkaniga NR, Zhang L, Belachew B, Gunaganti N, Frett B, Li HY. Discovery of SP-96, the first non-ATP-competitive Aurora Kinase B inhibitor, for reduced myelosuppression. Eur J Med Chem. 2020 Oct 1;203:112589. doi: 10.1016/j.ejmech.2020.112589. Epub 2020 Jul 12. PMID: 32717530.

104: Kasam RK, Ghandikota S, Soundararajan D, Reddy GB, Huang SK, Jegga AG, Madala SK. Inhibition of Aurora Kinase B attenuates fibroblast activation and pulmonary fibrosis. EMBO Mol Med. 2020 Sep 7;12(9):e12131. doi: 10.15252/emmm.202012131. Epub 2020 Aug 6. PMID: 32761869; PMCID: PMC7507328.

105: Wang Y, Yella JK, Ghandikota S, Cherukuri TC, Ediga HH, Madala SK, Jegga AG. Pan-transcriptome-based candidate therapeutic discovery for idiopathic pulmonary fibrosis. Ther Adv Respir Dis. 2020 Jan-Dec;14:1753466620971143. doi: 10.1177/1753466620971143. PMID: 33167785; PMCID: PMC7659024.

106: Bakalova R, Semkova S, Ivanova D, Zhelev Z, Miller T, Takeshima T, Shibata S, Lazarova D, Aoki I, Higashi T. Selective Targeting of Cancerous Mitochondria and Suppression of Tumor Growth Using Redox-Active Treatment Adjuvant. Oxid Med Cell Longev. 2020 Nov 2;2020:6212935. doi: 10.1155/2020/6212935. PMID: 33204397; PMCID: PMC7652615.

107: Shaalan AK, Teshima THN, Tucker AS, Proctor GB. Inhibition of Aurora Kinase B activity disrupts development and differentiation of salivary glands. Cell Death Discov. 2021 Jan 18;7(1):16. doi: 10.1038/s41420-020-00393-w. PMID: 33462217; PMCID: PMC7814035.

108: Borah NA, Sradhanjali S, Barik MR, Jha A, Tripathy D, Kaliki S, Rath S, Raghav SK, Patnaik S, Mittal R, Reddy MM. Aurora Kinase B Expression, Its Regulation and Therapeutic Targeting in Human Retinoblastoma. Invest Ophthalmol Vis Sci. 2021 Mar 1;62(3):16. doi: 10.1167/iovs.62.3.16. PMID: 33704359; PMCID: PMC7960835.

109: Sankhe K, Prabhu A, Khan T. Design strategies, SAR, and mechanistic insight of Aurora kinase inhibitors in cancer. Chem Biol Drug Des. 2021 Jul;98(1):73-93. doi: 10.1111/cbdd.13850. Epub 2021 May 15. PMID: 33934503.

110: McNeal AS, Belote RL, Zeng H, Urquijo M, Barker K, Torres R, Curtin M, Shain AH, Andtbacka RH, Holmen S, Lum DH, McCalmont TH, VanBrocklin MW, Grossman D, Wei ML, Lang UE, Judson-Torres RL. BRAF^{V600E} induces reversible mitotic arrest in human melanocytes via microrna-mediated suppression of AURKB. Elife. 2021 Nov 23;10:e70385. doi: 10.7554/eLife.70385. PMID: 34812139; PMCID: PMC8610417.

111: Ivanova D, Zhelev Z, Zlateva G, Lazarova D, Yaneva Z, Panovska R, Aoki I, Bakalova R. Effect of Alpha-tocopheryl Succinate on the Cytotoxicity of Anticancer Drugs Towards Leukemia Lymphocytes. Anticancer Res. 2022 Jan;42(1):547-554. doi: 10.21873/anticanres.15512. PMID: 34969764.

112: Shimabukuro K, Fukazawa T, Kanai A, Kawai H, Mekata K, Hirohashi N, Kakimoto N, Tanimoto K. Low-Dose-Rate Irradiation Suppresses the Expression of Cell Cycle-Related Genes, Resulting in Modification of Sensitivity to Anti-Cancer Drugs. Cells. 2022 Jan 31;11(3):501. doi: 10.3390/cells11030501. PMID: 35159310; PMCID: PMC8833988.

113: Bhutia WD, Gupta S, Rani R, Batra K, Sethi K, Kumar S, Kumar R. In vitro and in vivo evaluation of kinase and protease inhibitors against Trypanosoma evansi. Vet Res Commun. 2022 Jun 25. doi: 10.1007/s11259-022-09964-x. Epub ahead of print. PMID: 35751782.

114: Garlapati C, Joshi S, Bhattarai S, Krishnamurthy J, Turaga RC, Nguyen T, Li X, Aneja R. PLK1 and AURKB phosphorylate survivin differentially to affect proliferation in racially distinct triple-negative breast cancer. Cell Death Dis. 2023 Jan 10;14(1):12. doi: 10.1038/s41419-022-05539-5. PMID: 36627281; PMCID: PMC9832024.

CAS SciFinder^{n}

Task History

Initiating Search

May 11, 2023, 9:41AM

References: azd1152

Filtered By:

Publication Year: 2004 to 2022

Search Tasks

Task	Search Type	View
Exported: Returned Reference Results + Filters (391)	References	View Results

Copyright © 2023 American Chemical Society (ACS). All Rights Reserved.
Internal use only. Redistribution is subject to the terms of your SciFinder^{n} License Agreement and CAS information Use Policies.

CAS SciFinderⁿ

References (391)

View in SciFinderⁿ

1

Dicer promotes genome stability via the bromodomain transcriptional co-activator BRD4

By: Gutbrod, M. J. ; Roche, B.; Steinberg, J. I.; Lakhani, A. A.; Chang, K.; Schorn, A. J.; Martienssen, R. A.
Nature Communications (2022), 13(1), 1001 | Language: English, Database: CAplus and MEDLINE

RNA interference is required for post-transcriptional silencing, but also has addnl. roles in transcri ptional silencing of centromeres and genome stability. However, these roles have been controversial in mammals. Striki ngly, we found that Dicer-deficient embryonic stem cells have strong proliferation and chromosome segregation defects as well as increased transcription of centro meric satellite repeats, which triggers the interferon response. We conducted a C RISPR-Cas9 genetic screen to restore viability and identified transcriptional activators, histone H3K9 methyltransferases, and chromosome segregation factors as suppressors, resembling Dicer suppressors identified in independent screens in fission yeast. The strongest suppre ssors were mutations in the transcriptional co-activator Brd4, which reversed the strand- specific transcription of major satellite repeats suppre ssing the interferon response, and in the histone acetyltransferase Elp3. We show that identical mutations in the second bromod omain of Brd4 rescue Dicer-dependent silencing and chromosome segregation defects in both mammalian cells and fission yeast. This remarkable conservation demonstrates that RNA interference has an ancient role in transcri ptional silencing and in particular of satellite repeats, which is essential for cell cycle progression and proper chromosome segregation. Our results have pharmacol. implications for cancer and autoimmune diseases charact erized by unregulated transcription of satellite repeats.

Keywords: Dicer genome stability bromodomain transcriptional coactivator BRD

Substances (17) | Reactions (0) | Citing (2)

2

Screening of compounds to identify novel epigenetic regulatory factors that affect innate immune memory in macrophages

By: Benjaskulluecha, Salisa; Boonmee, Atsadang; Pattarakankul, Thitiporn; Wongprom, Benjawan; Klomsing, Jeerameth; Palaga, Tanapat

Scientific Reports (2022), 12(1), 1912 | Language: English, Database: CAplus and MEDLINE

Trained immunity and tolerance are part of the innate immune memory that allow innate immune cells to differentially respond to a second encounter with stimuli by enhancing or suppressing responses. In trained immunity, treatment of macrop hages with β-glucan (BG) facilitates the production of proinflammatory cytokines upon lipopolysaccharide (LPS) stimulation. For the tolerance response, LPS stimulation leads to suppressed inflammatory responses during subsequent L PS exposure. Epigenetic reprogr amming plays crucial roles in both phenomena, which are tightly associated with metabolic flux. In this study, we performed a screening of an epigenetics compound library that affects trained immunity or L PS tolerance in macrophages using TNFα as a readout. Among the 181 compounds tested, one compound showed suppressive effects, while 2 compounds showed promoting effects on BG-trained TNFα production In contrast, various inhibitors targeting Aurora kinase, histone methyltra nsferase, histone demethylase, histone deacetylase and DNA methyltransferase showed inhibitory activity against LPS tolerance. Several proteins previously unknown to be involved in innate immune memory, such as MGMT, Aurora kinase, LSD1 and PRMT5, were revealed. Protein network anal. revealed that the trained immunity targets are linked via Trp53, while LPS tolerance targets form three clusters of histone-modifying enzymes, cell division and base- excision repair. In trained immunity, the histone lysine methyltra nsferase SETD7 was identified, and its expression was increased during B G treatment. Level of the histone lysine demethylase, LS D1, increased during L PS priming and si RNA-mediated reduction resulted in increased expression of Il1b in L PS tolerance. Taken together, this screening approach confirmed the importance of epigenetic modifications in innate immune memory and provided potential novel targets for intervention.

Keywords: innate immune memory macrophage epigenetic regulatory factor

Substances (37) Reactions (0) Citing (4)

3

Methods and systems for personalized therapies by identifying a drug target in relation to a disease gene expression signature

By: Ghiassian, Susan; Akmaev, Viatcheslav R.; Voitalov, Ivan

World Intellectual Property Organization, WO2022271717 A1 2022-12-29 | Language: English, Database: CAplus

Described are methods and systems for identifying a target for therapy and treating a subject that exhibits a disease gene expression signature, comprising identifying and adminis tering a therapy determined to revert a disease gene expression signature in a subject suffering from a disease, disorder, or condition toward a non-diseased expression signature (e.g., disease gene expression signature of a non-diseased subject).

Keywords: personalized therapy drug target disease gene expression profile

PatentPak available

Substances (564) Reactions (0) Citing (0)

4

Probes comprising human granzyme B-cleavable hexapeptide and nanoparticles embedded with fluorescent dye, linker, and quencher and anti-GzmB antibody for detection of human granzyme B and small molecule drug as well as diagnosis of cancer, acute transplant rejection or IBD

By: Vendrell, Marc; Scott, Jamie I.; Gordon, Doireann
World Intellectual Property Organization, WO2022258997 A1 2022-12-15 | Language: English, Database: CAplus

The present disclosure relates to the development of novel cleavable peptide probes for use in detecting granzyme B, as well as methods for using the probes in medical and biol. settings. The probes may comprise a fluorescent moiety conjugated or bound to the peptide, and quencher moiety conjugated or bound to the peptide and upon cleavage of the peptide, the fluorescent moiety becomes dequenched.

Keywords: sequence granzyme cleavable peptide fluorescent dye quencher linker conjugate; Gzm B antibody small mol drug I BD cancer transplant rejection

PatentPak available

Substances (63) | Reactions (0) | Citing (0)

5

Methods and compositions for interfering with FAN1

By: Pearson, Christopher E.; Sartori, Alessandro A.
World Intellectual Property Organization, WO2022234494 A2 2022-11-10 | Language: English, Database: CAplus

This disclosure describes methods of altering a FAN1 polypeptide using, without limitation, a polypeptide, an antibody, a nucleic acid, a small mol., a kinase inhibitor, an agonist of a kinase, an agonist of an exonuclease, or an antagonist of an exonuclease.

Keywords: FAN1 antagonists

PatentPak available

Substances (33) | Reactions (0) | Citing (0)

6

A field guide to Aurora kinase inhibitors: an oocyte perspective

By: Blengini, Cecilia S.; Jung, Gyu Ik; Aboelenain, Mansour; Schindler, Karen
Reproduction (Bristol, United Kingdom) (2022), 164(4), V5-V7 | Language: English, Database: CAplus and MEDLINE

A review. In brief: The Aurora protein kinases have critical functions in controlling oocyte meiotic maturation. In this study, we describe an assay for examining their activation state in oocytes and establish the best working doses of three commonly used inhibitors. Abstract: Several small mol. inhibitors exist for targeting Aurora kinase proteins in somatic cells. From this point of view, we evaluate the specificity of these inhibitors in mouse oocytes, and we demons trate that MLN 8237 and AZD 1152 are specific for Aurora kinase A and Aurora kinase C, resp., only when used at low concentrations

Keywords: review oocyte aurora kinase inhibitor

Substances (4) | Reactions (0) | Citing (0)

7

Combined TRIP13 and aurora kinase inhibition induces apoptosis in human papillomavirus-driven cancers

By: Ghosh, Soma; Mazumdar, Tuhina; Xu, Wei; Powell, Reid T.; Stephan, Clifford; Shen, Li; Shah, Pooja A.; Pickering, Curtis R.; Myers, Jeffery N.; Wang, Jing; et al

Clinical Cancer Research (2022), 28(20), 4479-4493 | Language: English, Database: CAplus and MEDLINE

Human papillomavirus (HPV) causes >5% of cancers, but no therapies uniquely target H PV-driven cancers. We tested the cytotoxic effect of 864 drugs in 16 HPV-pos. and 17 HPV-neg. human squamous cancer cell lines. We confirmed apoptosis in vitro and in vivo using patientderived xenografts. Mitotic pathway components were manipu lated with drugs, knockdown, and overexpr ession. Aurora kinase inhibitors were more effective in vitro and in vivo in HPV-pos. than in HPV-neg. models. We hypoth esized that the mechanism of sensitivity involves retinob lastoma (Rb) expression because the viral oncopr otein E7 leads to Rb protein degrad ation, and basal Rb protein expression correlates with Aurora inhibition-induced apoptosis. Manipulating Rb directly, or by inducing E7 expression, altered cells' sensitivity to Aurora kinase inhibitors. Rb affects expression of the mitotic checkpoint genes M AD2L1 and BUB1B, which we found to be highly expressed in H PV-pos. patient tumors. Knockdown of MAD2L1 or BUB1B reduced Aurora kinase inhibition-induced apoptosis, whereas depletion of the M AD2L1 regulator TRIP13 enhanced it. TRIP13 is a potentially druggable AAA-ATPase. Combining Aurora kinase inhibition with T RIP13 depletion led to extensive apoptosis in H PV-pos. cancer cells but not in HPV-neg. cancer cells. Our data support a model in which H PV-pos. cancer cells maintain a balance of M AD2L1 and T RIP13 to allow mitotic exit and survival in the absence of Rb. Because it does not affect cells with intact Rb function, this novel combination may have a wide therap eutic window, enabling the effective treatment of Rb- deficient cancers.

Keywords: human papillomavirus cancer TRIP aurora kinase inhibition induce apoptosis

Substances (13) | Reactions (0) | Citing (1)

8

Methods for inhibiting KRAS oncoprotein through enhanced GTPase activity

By: Lito, Piro

World Intellectual Property Organization, WO2022217073 A1 2022-10-13 | Language: English, Database: CAplus

The present disclosure relates to methods for determining whether a cancer patient harboring a constit utively active KRAS mutation will be responsive to treatment with a $KRAS^{G12C}$ inhibitor that selectively targets the inactive state of K RAS. These methods are based on assaying regulators of G-protein signaling (R GS) expression in the cancer patient.

Keywords: sequence KRAS oncoprotein GTPase

PatentPak available

Substances (171) | Reactions (0) | Citing (0)

9

Combination therapies including Myt1 inhibitors

By: Fourtounis, Jimmy; Young, Jordan; Bernier, Cynthia; Durocher, Daniel; Hustedt, Nicole
World Intellectual Property Organization, WO2022213204 A1 2022-10-13 | Language: English, Database: CAplus

The use of inhibitors of tyrosine and threonine- specific cdc2-inhibitory kinase (Myt1) in the treatment of cancer is disclosed. In preferred embodiments, the Mytl inhibitor is a carboxamide pyrrolopyrazine or carboxamide pyrrolopyridine of Formula I. The Myt1 inhibitors may be used in combination with a variety of other anti- cancer agents. Such agents include a WEE1 inhibitor, TOPI or TOP2A inhibitor, RRM1 or RRM2 inhibitor, AURKA or AURKB inhibitor, ATR inhibitor, TTK inhibitor, SOD1 or SOD2 inhibitor, BUB1 inhibitor, CDC7 inhibitor, SAE1 inhibitor, PLK1 inhibitor, UBA2 inhibitor, DUT inhibitor, HDAC3 inhibitor, CHEK1 inhibitor, MEN1 inhibitor, DOT1L inhibitor, CREBBP inhibitor, EZH2 inhibitor, PLK4 inhibitor, HASPIN inhibitor, METTL3 inhibitor, nucleoside analogs, and platinum-based DNA alkylating agents.

Keywords: Myt1 inhibitor preparation treatment cancer

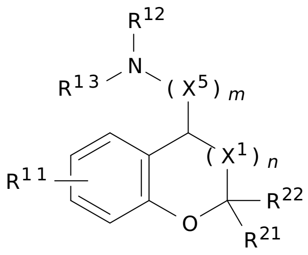

I

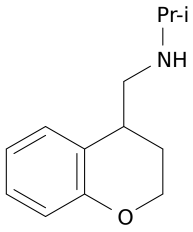

II

PatentPak available

Substances (639) | Reactions (455) | Citing (0)

10

MPS1 inhibition primes immunogenicity of KRAS-LKB1 mutant lung cancer

By: Kitajima, Shunsuke; Tani, Tetsuo; Springer, Benjamin F.; Campisi, Marco; Osaki, Tatsuya ; Haratani, Koji; Chen, Minyue; Knelson, Erik H. ; Mahadevan, Navin R. ; Ritter, Jessica; et al
Cancer Cell (2022), 40(10), 1128-1144.e8 | Language: English, Database: CAplus and MEDLINE

KRAS-LKB1 (KL) mutant lung cancers silence STING owing to intrinsic mitochondrial dysfunction, resulting in T cell exclusion and resistance to programmed cell death (ligand) 1 (PD-[L]1) blockade. Here we discover that KL cells also minimize intracellular accumu lation of 2'3'-cyclic GMP-AMP (2'3'-cGAMP) to further avoid downstream STING and STAT1 activation. An unbiased screen to co-opt this vulnerability reveals that transient MPS1 inhibition (MPS1i) potently re-engages this pathway in KL cells via micronuclei genera tion. This effect is markedly amplified by epigenetic de- repression of STING and only requires pulse MPS1i treatment, creating a therapeutic window compared with non- dividing cells. A single course of decitabine treatment followed by pulse M PS1i therapy restores T cell infiltration in vivo, enhances anti-PD-1 efficacy, and results in a durable response without evidence of signif icant toxicity.

Keywords: mutant lung cancer immunogenicity T cell infiltration mitochondrial dysfunction; 2’3’-cGAMP; DNMT1; EZH2; KRAS-LKB1 mutant lung adenocarcinoma; STING; cGAS; monopolar spindle kinase 1

Substances (31) | Reactions (0) | Citing (6)

11

Aurora kinase B inhibitors for use for treating cancer

By: Travers, Jon; Urosevic Despotovic, Jelena; Molina-Villa, Miguel-Angel; Bertran-Alamillo, Jordi
World Intellectual Property Organization, WO2022192139 A1 2022-09-15 | Language: English, Database: CAplus

Provided are methods for treating cancer with an Aurora kinase B inhibitor comprising determining if a cancer cell has a chromo somal amplification at location 22q11 on chromosome 22. Also provided are methods for treating cancer with an Aurora kinase B inhibitor comprising determining if a cancer cell has a gene with an increased copy number at location 22q11 on chromosome 22. Further provided are methods of determining if a cancer cell or a subject having cancer are amenable to treatment with an Aurora kinase B inhibitor.

Keywords: aurora kinase B inhibitor drug resistant cancer combination therapy

PatentPak available

Substances (33) Reactions (0) Citing (0)

12

N-myristoyl transferase (NMT) inhibitors for the treatment of senescence-associated diseases or disorders

By: Tate, Edward; Kallemeijn, Wouter; Gil, Jesus; McHugh, Domhnall
United States, US20220280484 A1 2022-09-08 | Language: English, Database: CAplus

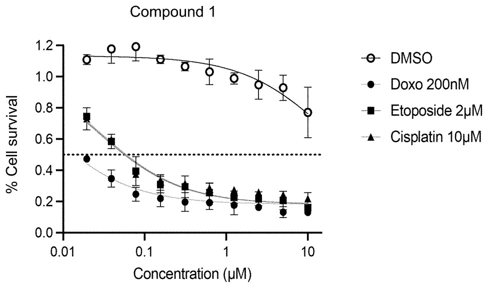

The present invention provides a method of prophylaxis or treatment of a senesc ence-associated disease or disorder by adminis tering to a subject in need thereof, a therapeu tically effective amount of a N-myristoyl transferase (NMT) inhibitor. A therapeu tically effective amount in this context means an amount of the NMT inhibitor sufficient to be effective in therapy e.g. in the treatment of cancer or other senescence-associated disease or disorder.

Keywords: senescence associated disease treatment N myristoyl transferase inhibitor

PatentPak available

Substances (58) Reactions (0) Citing (0)

13

Compounds, compositions and methods for treating age-related diseases and conditions

By: Tarkhov, Andrei Evgenevich; Avkhachev, Konstantin Aleksandrovich; Burmistrova, Olga Andreevna; Kholin, Maksim Nikolaevich; Fedichev, Petr Olegovich
World Intellectual Property Organization, WO2022173333 A2 2022-08-18 | Language: English, Database: CAplus

Antiaging compounds are selected according to the age-related disorder and the mol. targets associated with them by the disclosed compounds

Keywords: antiaging compounds drug targeting disease

PatentPak available

Substances (506) Reactions (0) Citing (0)

14

Methods for treating and ameliorating cancer

By: Jamieson, Catriona; Crews, Leslie; Burkart, Michael; Balaian, Larisa; Mondala, Phoebe; Mason, Cayla; Diep, Raymond H.; Laclair, James; Whisenant, Thomas; Chan, Warren C.; et al
World Intellectual Property Organization, WO2022165398 A1 2022-08-04 | Language: English, Database: CAplus

In alternative embodiments, provided are methods for treating and amelio rating a cancer, or recurrence of a cancer such as acute myeloid leukemia (AML) comprising administration to an individual in need thereof a pharmac eutical composition comprising 17S-F D-895 (or rebecsinib) and second drug such as an A TP-competitive protein tyrosine kinase inhibitor such as dasatinib. In altern ative embodiments, provided are methods for the in vivo inhibition of myeloprol iferative neoplasm (MPN) or AML stem cell propagation comprising administration to an individual in need thereof a pharmac eutical composition comprising 17S-FD-895 and second drug. In alternative embodiments, provided are methods for the in vivo inhibition pre- leukemia stem cell (pre-LSC) transformation into leukemia stem cells (LSCs) comprising administration to an individual in need thereof a pharmac eutical composition comprising 17 S-FD-895 and second drug.

Keywords: rebecsinib cancer

PatentPak available

Substances (166) Reactions (0) Citing (0)

15

Compounds for the treatment of an infection with an infectious microbe, and methods for identifying said compounds

By: Schurdak, Mark E.; Vogt, Andreas; Stern, Andrew Michael; Taylor, Douglass Lansing; Chen, Fangyuan; Pei, Fen; Cheng, Hongying; Bahar, Ivet; Shi, Qingya; Arditi, Moshe
World Intellectual Property Organization, WO2022159787 A1 2022-07-28 | Language: English, Database: CAplus

Disclosed herein are methods of identifying a compound for treating or preventing an infection with an infectious microbe, such as a coronavirus, in a subject in need thereof. Also disclosed herein are compounds and compos itions identified by said methods, and methods of use thereof. The methods comprise (a) obtaining transcriptomic data from cells infected with the infectious microbe, (b) identifying differentially expressed genes (DEGs), (c) characterizing host-targeted antimicrobial or anticytokine signature, (d) identi fying compounds that stimulate the antimic robial or -cytokine signature, (e) evaluating known and predicted targets of compounds identified in step (d), (f) constructing an infection host response protein-protein interaction (PPI) network and modules, (g) priori tizing compounds based on network proximity anal., (h) clustering of priori tized compounds associated with selected disease modules, (i) selecting representative compounds from each cluster for in vitro assays, and (j) analyzing the results of steps a- i to thereby identify the compound for treating or preventing the infection.

Keywords: microbial infection treatment drug screening method

PatentPak available

Substances (605) | Reactions (0) | Citing (0)

16

Methods for treating and ameliorating cancer

By: Jamieson, Catriona; Ma, Wenxue
World Intellectual Property Organization, WO2022159760 A1 2022-07-28 | Language: English, Database: CAplus

In alternative embodiments, provided are methods for treating and amelio rating a cancer such as a leukemia such as acute myeloid leukemia (AML) comprising administration to an individual in need thereof a pharmac eutical composition comprising imetelstat, or imetelstat and second drug such as dasatinib, or ruxolitinib, fedratinib, 8-aza-adenosine, raltegravir and/or dolutegravir or any combination thereof. In alternative embodiments, provided are methods for the in vivo inhibition of myeloprol iferative neoplasm (MPN) or AML stem cell propagation comprising administration to an individual in need thereof a pharmac eutical composition comprising imetelstat, or imetelstat and second drug.

Keywords: ameliorating cancer therapy; pharmaceutical composition myeloproliferative neoplasm stem cell propagation imetelstat drug

PatentPak available

Substances (172) | Reactions (0) | Citing (0)

17

Inducers of senescence, in combination with a selective death receptor 5 (DRr5) agonist, for use in a method of treating cancer

By: Bernards, Rene; Wang, Liqin

World Intellectual Property Organization, WO2022154664 A1 2022-07-21 | Language: English, Database: CAplus

The invention relates to an inducer of senescence, in combination with a short-lived, selective Death Receptor 5 (D R5) agonist, for use in a method of treating a patient suffering from a tumor. The invention further relates to a pharmaceutical composition, comprising a short-lived, selective Death Receptor 5 (D R5) agonist and, option ally, an inducer of senescence. The invention further relates to methods of treating a patient having a tumor, and to a selective, short-lived DR5 agonist, for use in a method of select ively killing senescent cells, including senescent cancer cells.

Keywords: DR5 agonist combin ation BRD2 inhibitor cancer

PatentPak available	Substances (12)	Reactions (0)	Citing (0)

18

Methods of potentiating antibiotic efficacy for treating bacterial infections

No inventor data available

World Intellectual Property Organization, WO2022153045 A1 2022-07-21 | Language: English, Database: CAplus

A method of potentiating or inducing antibiotic efficacy in one or more compos itions or compounds, said method including the step of inducing a substantially cell wall deficient (C WD) state in a bacteria by exposure to at least one antibiotic before and/or in combination with said one or more compos itions or compounds

Keywords: antibiotic combination chemotherapy bacterial infection treatment bacteria cell wall

PatentPak available	Substances (1,455)	Reactions (0)	Citing (0)

19

Sulfonamide derivatives, compositions comprising same and uses thereof in the treatment of cancers

By: Masutomi, Kenkichi; Yamashita, Taro; Kaneko, Shuichi; Yasukawa, Mami; Honma, Teruki; Koyama, Hiroo; Fukami, Takehiro

World Intellectual Property Organization, WO2022144780 A1 2022-07-07 | Language: English, Database: CAplus

Described herein are sulfonamide derivatives of Formula (I), where R1 and R2 are each indepen dently a halogen atom, and m and n are each independently an integer between 0 - 3, capable of inhibiting RNA dependent RNA polymerase (RdRP) activity of cancer cells. Also described are pharmaceutical compositions comprising these compounds and the uses thereof in the prophy laxis and/or treatment of cancers, including intractable solid cancers and stem cells cancer. The pharmac eutical composition may further comprise at least one of an hormonal therapeutic agent, a chemothe rapeutic agent, an immunothe rapeutic agent, a pharmac eutical agent inhibiting the action of cell growth factor and a pharmac eutical agent inhibiting the action of cell growth factor receptor.

Keywords: Sulfonamide derivative compos ition cancer

PatentPak available	Substances (158)	Reactions (0)	Citing (0)

20

A new imaging platform (iScreen) allows for the concurrent assessment of micronucleus induction and genotoxic mode of action in human A375 cells

By: Sun, Xiaowen; Rubitski, Elizabeth; Spellman, Richard A.; Engel, Maria; Schuler, Maik
Environmental and Molecular Mutagenesis (2022), 63(5), 230-245 | Language: English, Database: CAplus and MEDLINE

Genotoxicity testing guidelines require the assessment of the clasto genic and aneugenic potential of compounds While in vitro micronucleus assays detect both types of endpoints, it requires labor- intensive microscopic scoring and does not discri minate between the two modes of actions. Here, we present a novel high-content imaging platform in A375 human cells that addresses the need for rapid scoring while providing addnl. mechanistic information. We evaluated the new platform with 12 compounds, three compounds from each mechanistic class (clastogen, aneugen tubulin binder, aneugen aurora inhibitor, and nongenot oxicant) following 4- and 24-h compound treatments. The approach we developed is first discrim inating between genoto xicant and nongenotoxicant using an image anal. algorithm to quantify micron ucleus induction below a 60% cytoto xicity cutoff. Then it uses centromere protein A (CENPA) staining for the genotoxic compounds to discri minate between aneugens and clasto gens. Lastly, we use phosphorylated histone H2AX Ser139 (γH2AX) staining to confirm clastog enicity and changes in phospho rylated histone 3 Ser10 (pH 3) and increases in polyploidy in mitotic cells to discri minate between aneugens that bind tubulin from those that affect aurora kinases. All compounds were correctly classified, and we showed by using benchmark dose- response anal. that the imaging platform in A375 cells is at least as sensitive as the MicroFlow assay in TK6 cells for genoto xicant but appears to be more specific for the nongenotoxicants. A detailed comparison of the cell lines and a more compreh ensive validation with a much larger compound set, predictive and dose-response modeling will be presented in the future.

Keywords: clastogen aneugen tubulin binder aurora inhibitor micronucleus genotoxicity; aneuploidy; aurora kinase inhibitor; clastogen; image analysis; tubulin binder

Substances (12) | Reactions (0) | Citing (0)

21

Computational screening methods for identifying glutathione S-transferase P (GSTP1) inhibitors for treating pulmonary hypertension

By: Chan, Stephen Yu-Wah; Kim, Seungchan
World Intellectual Property Organization, WO2022103706 A1 2022-05-19 | Language: English, Database: CAplus

Disclosed are pathogenic mechanisms in pulmonary hypertension and mol. inhibitors of the same. Particu larly, GSTP1 (glutathione S-transferase P1) have been demons trated as having a role in regulating the endoth elial iron-sulfur cluster assembly enzyme ISCU function in pulmonary hypertension. Accordingly, methods for treating pulmonary hypert ension in a subject in need thereof comprising administering a therapeutically effective amount of a pharmac eutical compositions that inhibit glutathione S-transferase P (GSTP1) and/or increasing ISCU expression are disclosed. The GSTP1 inhibitor can comprise a piperlongumine analog, such as BR D-K34222889, or a derivative thereof.

Keywords: antihypertensive GSTP1 inhibitor piperlongumine analog BRDK34222889 pulmonary hypert ension treatment

PatentPak available

Substances (264) | Reactions (0) | Citing (0)

22

N-myristoyl transferase (NMT) inhibitors for treating senescence-associated diseases or disorders

By: Tate, Edward; Kallemeijn, Wouter; Gil, Jesus; McHugh, Domhnall

World Intellectual Property Organization, WO2022090746 A1 2022-05-05 | Language: English, Database: CAplus

The present invention provides a N-myristoyl transferase (NMT) inhibitors for use in the prophylaxis or treatment of a senescence-associated disease or disorder. The NMT inhibitor is selected from the group consisiting of 1-(5-(3,4-difluoro-2-(2-(1,3,5-trimethyl-1H-pyrazol-4-yl)ethoxy)phenyl)-1-methyl-1H-indazol-3-yl)-N,N-dimethylmethanamine, 4-(2-{2-[3-(2-aminoethyl)imidazo[1,2-a]pyridin-6-yl]-5-chlorophenoxy}ethyl)-N,N,1,5-tetramethyl-1H-pyrazole-3-carboxamide. In one aspect the senescence-associated disease or disorder is cancer, wherein said cancer comprises chemotherapy or radiotherapy induced senescent cells. In another aspect senescence-associated disease or disorder is one or more side effects of chemotherapy or radiotherapy, wherein said chemotherapy or radiotherapy induces senescence. One or more side effects of chemotherapy or radiotherapy are selected from weight loss, endocrine changes, hormone imbalance, changes in hormone signaling, cardiotoxicity, changes in body composition, a reduced ability to be phys. active, gastrointestinal toxicity, nausea, vomiting, constipation, anorexia, diarrhea, peripheral neuropathy, fatigue, malaise, low phys. activity, hematol. toxicity, anemia, hepatotoxicity, alopecia, pain, infection, mucositis, fluid retention, dermatol. toxicity, rashes, dermatitis, hyperpigmentation, urticaria, photosensitivity, nail changes, and mouth, gum and throat problems. In one aspect the senescence-associated disease or disorder is selected from a metabolic disease, an inflammatory or autoimmune disease or disorder, a cardiovascular disease or disorder, a pulmonary disease or disorder, a neurol. disease or disorder, an eye disease or disorder, and a dermatol. disease or disorder.

Keywords: senolytic agent NMT inhibitor aging senescence associated disease treatment

PatentPak available

Substances (65) | Reactions (0) | Citing (0)

23

Combination therapies for use in treating cancer

By: Li, Fu; Marks, Kevin; Marjon, Katya; Kalev, Petar; Hyer, Marc Lee

World Intellectual Property Organization, WO2022036067 A1 2022-02-17 | Language: English, Database: CAplus

The compound of Formula (I), or pharmaceutically acceptable salts thereof, is useful in, among other things, the treatment of cancer and provides a therapeutic advantage when used in combination with other agents as herein described compared to treatment with each agent when administered alone.

Keywords: antitumor cancer combination therapy

PatentPak available

Substances (170) | Reactions (0) | Citing (0)

24

Effect of alpha-tocopheryl succinate on the cytotoxicity of anticancer drugs towards leukemia lymphocytes

By: Ivanova, Donika; Zhelev, Zhivko; Zlateva, Genoveva; Lazarova, Dessislava; Yaneva, Zvezdelina; Panovska, Radmila; Aoki, Ichio; Bakalova, Rumiana

Anticancer Research (2022), 42(1), 547-554 | Language: English, Database: CAplus and MEDLINE

This study analyzed the effect of α-tocopheryl succinate (α-TS) on the redox-state of leukemia and normal lymphocytes, as well as their sensitization to fifteen anticancer drugs. Cell viability was analyzed by trypan blue staining and automated counting of live and dead cells. Apoptosis was analyzed by FITC-Annexin V test. Oxidative stress was evaluated by the intrace llular levels of reactive oxygen species (ROS) and protein-carbonyl products. Most combin ations (α-TS plus anticancer drug) exerted additive or antago nistic effects on the proliferation and viability of leukemia lymphocytes. α-TS combined with barasertib, bortezomib or lonafarnib showed a strong synergistic cytotoxic effect, which was best expressed in the case of barase stib. It was accompanied by impressive induction of apoptosis and increased production of ROS, but insignificant changes in protein-carbonyl levels. α-TS plus barasertib did not alter the viability and did not induce oxidative stress and apoptosis in normal lymphocytes. Alpha-TS could be a promising adjuvant in second-line anticancer therapy, particularly in acute lymphob lastic leukemia, to reduce the therapeutic doses of barase rtib, bortezomib, and lonafa rnib, increasing their effecti veness and minimizing their side effects.

Keywords: leukemia lymphocyte alpha tocopheryl succinate anticancer; Chemotherapy; alpha-tocopheryl succinate; apoptosis; oxidative stress

Substances (19) Reactions (0) Citing (1)

25

Pharmacophore Synergism in Diverse Scaffold Clinches in Aurora Kinase B

By: Masand, Vijay H.; Al-Hussain, Sami A.; Rathore, Mithilesh M.; Thakur, Sumer D.; Akasapu, Siddhartha; Samad, Abdul ; Al-Mutairi, Aamal A. ; Zaki, Magdi E. A.

International Journal of Molecular Sciences (2022), 23(23), 14527 | Language: English, Database: CAplus and MEDLINE

Aurora kinase B (AKB) is a crucial signaling kinase with an important role in cell division. Therefore, inhibition of A KB is an attractive approach to the treatment of cancer. In the present work, extensive quant. structure-activity relationships (QSAR) anal. has been performed using a set of 561 structurally diverse aurora kinase B inhibitors. The Organization for Economic Cooperation and Development (OECD) guidelines were used to develop a Q SAR model that has high statistical performance (R^2tr = 0.815, Q^2LMO = 0.808, R^2_{ex} = 0.814, CCC_{ex} = 0.899). The seven-variable-based newly developed QSAR model has an excellent balance of external predictive ability (Predictive QSAR) and mechanistic interpretation (Mechanistic QSAR). The QSAR anal. successfully identifies not only the visible pharmacophoric features but also the hidden features. The anal. indicates that the lipophilic and polar groups-especially the H-bond capable groups-must be present at a specific distance from each other. Moreover, the ring nitrogen and ring carbon atoms play important roles in determining the inhibitory activity for AKB. The anal. effectively captures reported as well as unreported pharmacophoric features. The results of the present anal. are also supported by the reported crystal structures of inhibitors bound to AKB.

Keywords: pharmacophore synergism diverse scaffold clinches aurora kinase B; Q SAR; aurora kinase B; pharmacophore modeling

Substances (5) Reactions (0) Citing (0)

26

Real-time luminescence enables continuous drug-response analysis in adherent and suspension cell lines

By: Wandishin, Clayton M.; Robbins, Charles John; Tyson, Darren R.; Harris, Leonard A.; Quaranta, Vito
Cancer Biology & Therapy (2022), 23(1), 358-368 | Language: English, Database: CAplus and MEDLINE

The drug-induced proliferation (DIP) rate is a metric of in vitro drug response that avoids inherent biases in commonly used metrics such as 72 h viability. However, DIP rate measurements rely on direct cell counting over time, a laborious task that is subject to numerous challenges, including the need to fluores cently label cells and automatically segment nuclei. Moreover, it is incredibly difficult to directly count cells and accurately measure DIP rates for cell popula tions in suspension. As an alternative, we use real-time luminescence measurements derived from the cellular activity of N AD(P)H oxidoreductase to efficiently estimate drug response in both adherent and suspension cell populations to a panel of known anticancer agents. For the adherent cell lines, we collect both luminescence reads and direct cell counts over time simulta neously to assess their congru ency. Our results demons trate that the proposed approach significantly speeds up data collec tion, avoids the need for cellular labels and image segment ation, and opens the door to signif icant advances in high-throughput screening of anticancer drugs.

Keywords: luminescence drug response analysis adherent suspension cell line; D IP rate; cell viability; continuous assay; drug response; drug screening; quantitative analysis; real-time luminescence; suspension cells

Substances (12) | Reactions (0) | Citing (0)

27

Using a gene network of pyroptosis to quantify the responses to immunotherapy and prognosis for neuroblastoma patients

By: Luo, Bingying; Wang, Limin; Gao, Weijing; Su, Yudong; Lu, Yao; Zheng, Jian; Yin, Jie; Zhao, Qiang; Li, Jie; Da, Yurong; et al
Frontiers in Immunology (2022), 13, 845757 | Language: English, Database: CAplus and MEDLINE

Pyroptosis, as an inflammatory form of cell death, is involved in many physiol. and pathol. processes. Neurobl astoma is the most common extracranial solid tumor in children. In this study, the relati onship between pyroptosis and tumor microenv ironment in neuroblastoma was systematically studied. We integrated four datasets of neuroblastomas. Through robust clustering of the m RNA expression profiles of 24 pyroptosis-related genes, a total of three pyroptosis patterns were identi fied. We then constructed a novel scoring method named as pyroscore to quantify the level of pyroptosis in neuroblastoma. Multi-omics data and single-cell RNA sequencing were used to accurately and comprehensively evaluate the effectiveness of pyroscore. Clin. data sets were used to evaluate the use of pyroscore to predict the responsiveness of immune checkpoint treatment. High pyroscore was associated with good prognosis, immune activation, and increased response to checkpoint blockade immunot herapy. Multivariate Cox anal. revealed that the pyroscore was an independent prognostic biomarker and could increase the accuracy of clin. prediction models. Etoposide, a drug picked up by our anal., could increase the sensitivity of neuroblastoma cells to pyroptosis. External verification using four cohorts of patients who had received immunotherapy showed that high pyroscore was signifi cantly associated with immunotherapy treatment benefit. Taken together, this study revealed that pyropt osis-related gene network could quantify the response of neuroblastoma to immune checkpoint blockade therapy and prognosis, and it may be helpful for clin. practit ioners to choose treatment strategies for neuroblastoma patients.

Keywords: human neuroblastoma prognosis immunotherapy gene network pyropt osis; etoposide; immunotherapy; neurobl astoma; prognosis; pyroptosis

Substances (22) | Reactions (0) | Citing (2)

28

Phenotypic screening of 1,953 FDA-approved drugs reveals 26 hits with potential for repurposing for Peyronie's disease

By: Ilg, Marcus M.; Lapthorn, Alice R.; Ralph, David J.; Cellek, Selim
PLoS One (2022), 17(12), e0277646 | Language: English, Database: CAplus and MEDLINE

Drug repurposing has been shown to bring safe medica tions to new patient populations, as recently evidenced by the C OVID-19 pandemic. We investigated whether we could use phenotypic screening to repurpose drugs for the treatment of Peyronie's disease (PD). PD is a fibrotic disease charact erized by continued myofibr oblast presence and activity leading to formation of a plaque in the penile tunica albuginea (TA) that can cause pain during erection, erectile dysfun ction, and penile deformity. P D affects 3-9% of men with treatment options limited to surgery or injection of collagenase which can only be utilized at late stages after the plaque is formed. Currently there are no approved medications that can be offered to patients presenting with early disease before the formation of the plaque. Drug repurposing may therefore be the ideal strategy to identify medical treatments to address this unmet medical need in early PD. We used primary human fibrob lasts from PD patients in a phenotypic screening assay that measures T GF-β1-induced myofibroblast transformation which is the main cellular phenotype that drives the pathol. in early P D. A library of FDA-approved 1953 drugs was screened in duplicate wells at a single concent ration (10μM) in presence of T GF-β1. The myofibroblast marker α-SMA was quantified after 72h incuba tion. A pos. control of S B-505124 (TGF-β1 receptor antagonist) was included on each plate. Hits were defined as showing >80% inhibition, while retaining >80% cell viability. 26 hits (1.3%) were identified which were divided into the following main groups: anti-cancer drugs, anti-inflammation, neurol., endocr inol., and imaging agents. Five of the top-ten drugs that increase myofibr oblast-transformation appear to act on VEGFR. This is the first phenotypic screening of F DA-approved drugs for P D and our results suggest that it is a viable method to predict drugs with potential for repurp osing to treat early PD.

Keywords: drug FDA phenotypic screening Peyronies disease

Substances (1,943) | Reactions (0) | Citing (0)

29

Explainable artificial intelligence for precision medicine in acute myeloid leukemia

By: Gimeno, Marian; San Jose-Eneriz, Edurne; Villar, Sara; Agirre, Xabier; Prosper, Felipe; Rubio, Angel; Carazo, Fernando
Frontiers in Immunology (2022), 13, 977358 | Language: English, Database: CAplus and MEDLINE

Artificial intelligence (AI) can unveil novel person alized treatments based on drug screening and whole- exome sequencing experi ments (WES). However, the concept of "black box" in A I limits the potential of this approach to be translated into the clin. practice. In contrast, explainable AI (XAI) focuses on making A I results underst andable to humans. Here, we present a novel X AI method -called multi-dimensional module optimization (MOM)- that associates drug screening with genetic events, while guaran teeing that predictions are interpretable and robust. We applied M OM to an acute myeloid leukemia (A ML) cohort of 319 ex-vivo tumor samples with 122 screened drugs and WES. MOM returned a therap eutic strategy based on the F LT3, CBFβ-MYH11, and N RAS status, which predicted AML patient response to Quizartinib, Trametinib, Selumetinib, and Crizotinib. We successfully validated the results in three different large-scale screening experiments We believe that XAI will help healthcare providers and drug regulators better understand AI medical decisions.

Keywords: acute myeloid leukemia precision medicine crizotinib selumetinib; assignation problem; biomarkers; drug repositioning; drug sensitivity; ex-vivo experiment; explainable artificial intelligence; large-scale screening; treatment selection

Substances (111) | Reactions (0) | Citing (3)

30

Integrative oncogene-dependency mapping identifies RIT1 vulnerabilities and synergies in lung cancer

By: Vichas, Athea; Riley, Amanda K. ; Nkinsi, Naomi T.; Kamlapurkar, Shriya; Parrish, Phoebe C. R. ; Lo, April; Duke, Fujiko; Chen, Jennifer; Fung, Iris; Watson, Jacqueline; et al
Nature Communications (2021), 12(1), 4789 | Language: English, Database: CAplus and MEDLINE

CRISPR-based cancer dependency maps are accelerating advances in cancer precision medicine, but adequate functional maps are limited to the most common oncogenes. To identify opportunities for therapeutic intervention in other rarer subsets of cancer, we investigate the oncogene-specific dependencies conferred by the lung cancer oncogene, R IT1. Here, genome-wide CRISPR screening in KRAS, EGFR, and RIT1-mutant isogenic lung cancer cells identifies shared and unique vulnerab ilities of each oncogene. Combining this genetic data with small-mol. sensitivity profiling, we identify a unique vulnera bility of RIT1-mutant cells to loss of spindle assembly checkpoint regulators. Oncogenic RIT1M90I weakens the spindle assembly checkpoint and perturbs mitotic timing, resulting in sensitivity to Aurora A inhibition. In addition, we observe synergy between mutant R IT1 and activation of Y AP1 in multiple models and frequent nuclear overexpression of YAP1 in human primary RIT1-mutant lung tumors. These results provide a genome-wide atlas of oncogenic R IT1 functional interactions and identify components of the R AS pathway, spindle assembly checkp oint, and Hippo/YAP1 network as candidate therapeutic targets in R IT1-mutant lung cancer.

Keywords: lung cancer integrative oncogene RIT vulnerability synergy

Substances (19) Reactions (0) Citing (9)

31

Methods for treating cancer with combination therapies

By: Ahsan, Aarif; Bisht, Kamlesh; Bjorklund, Chad; Flynt, Jennifer Erin; Hsu, Chih-Chao; Jeyaraju, Danny Vijey; Ortiz-Estevez, Maria; Pierceall, William Edward; Thakurta, Anjan; Towfic, Fadi George
World Intellectual Property Organization, WO2021262962 A1 2021-12-30 | Language: English, Database: CAplus

Provided herein are methods of using a compound provided herein (e.g., Compound 1, Compound 2, Compound 3, Compound 4, Compound 5, Compound 6, or Compound 7, or a stereoisomer or mixture of stereoi somers, pharmaceutically acceptable salt, tautomer, prodrug, solvate, hydrate, co-crystal, clathrate, or polymorph thereof), in combination with a second active agent for treating cancer. The second active agent is one or more of a PLK1 inhibitor, a B RD4 inhibitor, a B ET inhibitor, an N EK2 inhibitor, an A URKB inhibitor, an M EK inhibitor, a P HF19 inhibitor, a BTK inhibitor, an m TOR inhibitor, a P IM inhibitor, an I GF-1 R inhibitor, an X POI inhibitor, a DOT1 L inhibitor, an EZH2 inhibitor, a J AK2 inhibitor, a B IRC5 inhibitor, or a D NA methyltransferase inhibitor.

Keywords: antitumor agent combination therapy hematol cancer

PatentPak available

Substances (95) Reactions (0) Citing (0)

32

Combination of antibody-drug conjugate and aurora b inhibitor

By: Mettetal, Jerome Thomas, II; Durant, Stephen Thomas; Astaneh, Azadeh Cheraghchi Bashi
World Intellectual Property Organization, WO2021260582 A1 2021-12-30 | Language: English, Database: CAplus

A pharmaceutical product for administration of an anti-HER2 antibody-drug conjugate in combination with an Aurora B inhibitor is provided. The anti-HER2 antibody drug conjugate is an antibody- drug conjugate in which a drug- linker represented by the following formula (wherein A represents the connecting position to an antibody) is conjugated to an anti-HER2 antibody via a thioether bond. Also provided is a therapeutic use and method wherein the antibody- drug conjugate and the Aurora B inhibitor are administered in combination to a subject.

Keywords: HER2 cancer antitumor antibody drug conjugate aurora b sequence

PatentPak available

Substances (18) | Reactions (0) | Citing (0)

33

Compositions and methods for treating acute myeloid leukemia

By: Ishikawa, Fumihiko; Saito, Yoriko; Hashimoto, Mari; De Hoon, Michiel; Momozawa, Yukihide
World Intellectual Property Organization, WO2021255518 A1 2021-12-23 | Language: English, Database: CAplus

The present invention provides methods for identifying correlations between genetic abnormalities and effective combination treatments and treating a disease or disorder associated with inhibition of apoptosis, such as acute myeloid leukemia (AML) with compounds that block inhibition of apoptosis. The method of treating a subject with acute myeloid leukemia (AML) comprises (a) determining whether an AML cell of the subject is characterized by the presence or absence of one or more genetic mutations in F L T3, IDH1, CBL and/or NRAS, and (b) if one or more of the genetic mutations are present, individually or conjointly administering an effective amount of a XIAP inhibitor, BCL2 inhibitor, MCL1 inhibitor or aurora kinase B inhibitor to the subject. For example, the X IAP inhibitor includes AZD 5582.

Keywords: acute myeloid leukemia treatment gene mutation evaluation

PatentPak available

Substances (9) | Reactions (0) | Citing (0)

34

CNOT3 interacts with the Aurora B and MAPK/ERK kinases to promote survival of differentiating mesendodermal progenitor cells

By: Sarkar, Moumita; Martufi, Matteo; Roman-Trufero, Monica; Wang, Yi-Fang; Whilding, Chad; Dormann, Dirk; Sabbattini, Pierangela; Dillon, Niall

Molecular Biology of the Cell (2021), 32(22), ar40 | Language: English, Database: CAplus and MEDLINE

Mesendoderm cells are key intermediate progenitors that form at the early primitive streak (PrS) and give rise to mesoderm and endoderm in the gastrulating embryo. We have identified an interaction between CNOT3 and the cell cycle kinase Aurora B that requires sequences in the NOT box domain of CNOT3 and regulates MAPK/ERK signaling during mesendoderm differentiation. Aurora B phosphorylates CNOT3 at two sites located close to a nuclear localization signal and promotes localization of CNOT3 to the nuclei of mouse embryonic stem cells (ESCs) and metastatic lung cancer cells. ESCs that have both sites mutated give rise to embryoid bodies that are largely devoid of mesoderm and endoderm and are composed mainly of cells with ectodermal characteristics. The mutant ESCs are also compromised in their ability to differentiate into mesendoderm in response to FGF2, BMP4, and Wnt3 due to reduced survival and proliferation of differentiating mesendoderm cells. We also show that the double mutation alters the balance of interaction of CNOT3 with Aurora B and with ERK and reduces phosphorylation of ERK in response to FGF2. Our results identify a potential adaptor function for CNOT3 that regulates the Ras/MEK/ERK pathway during embryogenesis.

Keywords: CNOT Aurora B MAPK ERK kinase mesendodermal progenitor cell

Substances (9) | Reactions (0) | Citing (0)

35

Paradoxical implication of BAX/BAK in the persistence of tetraploid cells

By: Deng, Jiayin ; Gutierrez, Lucia G.; Stoll, Gautier ; Motino, Omar; Martins, Isabelle; Nunez, Lucia; Bravo-San Pedro, Jose Manuel; Humeau, Juliette; Bordenave, Chloe; Pan, Juncheng; et al

Cell Death & Disease (2021), 12(11), 1039 | Language: English, Database: CAplus and MEDLINE

Pro-apoptotic multi-domain proteins of the BCL2 family such as BAX and BAK are well known for their important role in the induction of mitochondrial outer membrane permeabilization (MOMP), which is the rate-limiting step of the intrinsic pathway of apoptosis. Human or mouse cells lacking both BAX and BAK (due to a double knockout, DKO) are notoriously resistant to MOMP and cell death induction. Here we report the surprising finding that BAX/BAK DKO cells proliferate less than control cells expressing both BAX and BAK (or either BAX or BAK) when they are driven into tetraploidy by transient exposure to the microtubule inhibitor nocodazole. Mechanistically, in contrast to their BAX/BAK-sufficient controls, tetraploid DKO cells activate a senescent program, as indicated by the overexpression of several cyclin-dependent kinase inhibitors and the activation of β-galactosidase. Moreover, DKO cells manifest alterations in ionomycin-mobilizable endoplasmic reticulum (ER) Ca^{2+} stores and store-operated Ca^{2+} entry that are affected by tetraploidization. DKO cells manifested reduced expression of endogenous sarcoplasmic/endoplasmic reticulum Ca^{2+} ATPase 2a (Serca2a) and transfection-enforced reintroduction of Serca2a, or reintroduction of an ER-targeted variant of BAK into DKO cells reestablished the same pattern of Ca^{2+} fluxes as observed in BAX/BAK-sufficient control cells. Serca2a reexpression and ER-targeted BAK also abolished the tetraploidy-induced senescence of DKO cells, placing ER Ca^{2+} fluxes downstream of the regulation of senescence by BAX/BAK. In conclusion, it appears that BAX/BAK prevent the induction of a tetraploidization-associated senescence program. Speculatively, this may contribute to the low incidence of cancers in BAX/BAK DKO mice and explain why human cancers rarely lose the expression of both BAX and BAK.

Keywords: BAX BAK expression tetraploid cell persistence

Substances (14) | Reactions (0) | Citing (3)

36

Use of TMPRSS2 inhibitors for the treatment of lung viral infections

By: Khatib, Abdel-Majid; Villoutreix, Bruno; Singh, Natesh
World Intellectual Property Organization, WO2021228762 A1 2021-11-18 | Language: English, Database: CAplus

In Dec. 2019, a new coronavirus was identified in the Hubei province of central china and named S ARS-CoV-2. This new virus induces COVID-19, a severe respiratory disease with high death rate. A putative target to interfere with the virus is the host transme mbrane serine protease family member II (TMPRSS2). This enzyme seems critical for the entry of coronav iruses into human cells by cleaving and activating the spike protein (S) of SARS-CoV-2. Repositioning approved, investigational and exptl. drugs on the serine protease domain of TMPRSS2 could thus be valuable. There is no exptl. structure for T MPRSS2 but it is possible to develop structural models for the serine protease domain using comparative modeling strategies. Beside the TMPRSS2 catalytic site, the inventors predicted on their structural models a main exosite that could be important for the binding of protein partners and/or substrates. To block the catalytic site or the exosite of TMPRSS2 they used structure-based virtual screening computations and two different collections of approved, investigational and exptl. drugs. The inventors propose a list of 156 mols. that could bind to the catalytic site and 100 compounds that may interact with the exosite. These small mols. could be used after exptl. validations for treatment purposes, to gain novel insights over the roles of TMPRSS2 or as starting point for the develo pment of second generation analogs.

Keywords: TMPRSS2 inhibitor treatment lung virus infection antiviral C OVID19

PatentPak available

Substances (249) Reactions (0) Citing (1)

37

Small molecule therapeutics for the treatment of viral infections

By: Arumugaswami, Vaithlingaraja; Christofk, Heather R.; Damoiseaux, Robert; Garcia, Gustavo; Mullen, Peter
World Intellectual Property Organization, WO2021207632 A1 2021-10-14 | Language: English, Database: CAplus

This disclosure relates to methods of treating viral infections, such as influenza or corona virus, with certain inhibitors, such as kinase inhibitors.

Keywords: protein kinase inhibitor viral infection COVID19 therapy

PatentPak available

Substances (146) Reactions (0) Citing (2)

38

Methods and compositions for treating liver cancer

By: Harrison, Stephen; Brew, Christine Taylor; Winther, Michael David; Shekaran, Asha
World Intellectual Property Organization, WO2021206633 A1 2021-10-14 | Language: English, Database: CAplus

Methods and compositions for treating cancer, such as liver cancer, are disclosed herein. The methods may comprise use of therapeutically effective amounts of one or more therap eutic agents to decrease expression or activity of Aurora kinase A (A URKA) in liver cancer cells that are deficient in AT-rich domain interactive domain 1A (ARID1A). In a preferred embodi ment, the one or more therapeutic agents comprises a D NA construct encoding Cas9 and a guide R NA targeting AURKA gene.

Keywords: Aurora kinase inhibitor liver cancer

PatentPak available

Substances (9) Reactions (0) Citing (0)

39

ALKBH5-mediated m6A-demethylation of USP1 regulated T-cell acute lymphoblastic leukemia cell glucocorticoid resistance by Aurora B

By: Gong, Hongtao; Liu, Liu ; Cui, Lina; Ma, Hongyan; Shen, Liyun
Molecular Carcinogenesis (2021), 60(9), 644-657 | Language: English, Database: CAplus and MEDLINE

Recent studies evidence that ubiquitin-specific proteases (USPs) are associated with the occurrence and chemores istance of T-cell acute lymphoblastic leukemia (T-ALL). N6-methyladenosine (m6A) demethylase AlkB homolog 5 (ALKBH5) exerts a carcinogenic effect in human cancers and improves the mRNA stability of USPs. Whether ubiquitin-specific protease 1 (USP1) controls chemores istance of T-ALL is unknown. Our study demonstrated that USP1 expression was upregulated in glucocorticoid (GC)-resistant T-ALL patients and cells (CEM-C1). High expression of USP1 was correlated to the poor prognosis in T-ALL patients. Silencing USP1 increased CEM-C1 cell sensitivity to dexamethasone (Dex), reduced cell invasion, promoted cell apoptosis, and amelio rated glucoco rticoid receptor (GR) expression. USP1 mediated T-ALL chemoresistance by interacting with and deubiquitination of Aurora B. Overexpression of USP1 reversed the amelioration effect of Aurora B inhibitor on CEM-C1 cell resistance to Dex. Mechanistically, AL KBH5 enhanced USP1 expression by reducing m6A level and mRNA stability in USP1 mRNA transcript. Downregulation of ALKBH5 reduced the levels of USP1 and Aurora B, facilitated CEM-C1 cell sensitivity to Dex, apoptosis, and GR expression, suppressed cell invasion. However, overexpression of USP1 reversed all the effects of ALKBH5 on CEM-C1 cells. In vivo results showed that tail vein injection of sh-USP1 resulted in a significant prolongation of mouse survival, suppressed tumor growth, maintained the normal weight of mice, reduced USP1 expression and facilitated GR expression. In conclusion, inhibition of ALKBH5-mediated m6A modifi cation decreased USP1 expression and downregulation of USP1 ameliorated GC resistance of T-ALL through suppressing Aurora B expression and elevating GR level.

Keywords: mediated demethylation acute lymphoblastic leukemia cell glucocorticoid; Aurora B; N6-methyladenosine demethylase; T-cell acute lymphoblastic leukemia; chemoresistance; ubiquitin-specific protease 1

Substances (7) Reactions (0) Citing (13)

40

CCT245718, a dual FLT3/Aurora A inhibitor overcomes D835Y-mediated resistance to FLT3 inhibitors in acute myeloid leukaemia cells

By: Tariq, Muhammad Usama; Furqan, Muhammad; Parveen, Hira; Ullah, Rahim; Muddassar, Muhammad; Saleem, Rahman Shah Zaib; Bavetsias, Vassilios; Linardopoulos, Spiros; Faisal, Amir
British Journal of Cancer (2021), 125(7), 966-974 | Language: English, Database: CAplus and MEDLINE

Activating mutations in the Fms-like tyrosine kinase 3 (FLT3) are among the most prevalent oncogenic mutations in acute myeloid leukemia. Inhibitors selectively targeting FLT3 kinase have shown promising clin. activity; their success in the clinic, however, has been limited due to the emergence of acquired resistance. CCT245718 was identified and characterized as a dual Aurora A/FLT3 inhibitor through cell-based and biochem. assays. The ability of CCT245718 to overcome TKD-mediated resistance was evaluated in a cell line-based model of drug resistance to FLT3 inhibitors. CCT245718 exhibits potent antiproliferative activity toward FLT3-ITD + AML cell lines and strongly binds to FLT3-ITD and TKD (D835Y) mutants in vitro. Activities of both FLT3-ITD and Aurora A are also inhibited in cells. Inhibition of FLT3 results in reduced phosphorylation of STAT5, downregulation of survivin and induction of apoptotic cell death. Moreover, CCT245718 overcomes TKD-mediated resistance in a MOLM-13-derived cell line containing FLT3 with both ITD and D835Y mutations. It also inhibits FLT3 signalling in both parental and resistant cell lines compared to F LT3-specific inhibitor MLN518, which is only active in the parental cell line. Our results demons trate that CCT245718 is a potent dual FL T3/Aurora A inhibitor that can overcome TKD-mediated acquired resistance.

Keywords: acute myeloid leukemia CCT245718 FLT3 Aurora A inhibitor D835Y

Substances (7) Reactions (0) Citing (7)

41

Suv420 enrichment at the centromere limits Aurora B localization and function

By: Herlihy, Conor P.; Hahn, Sabine; Hermance, Nicole M.; Crowley, Elizabeth A.; Manning, Amity L.
Journal of Cell Science (2021), 134(15), jcs249763 | Language: English, Database: CAplus and MEDLINE

Centromere structure and function are defined by the epigenetic modification of histones at centromeric and pericentromeric chromatin. The constitutive heterochromatin found at pericentromeric regions is highly enriched for H3K9me3 and H4K20me3. Although mis-expression of the methyltransferase enzymes that regulate these marks, Suv39 and Suv420, is common in disease, the consequences of such changes are not well understood. Our data show that increased centromere localization of Suv39 and Suv420 suppresses centromere transcription and compromises localization of the mitotic kinase Aurora B, decreasing microtubule dynamics and compromising chromosome alignment and segregation. We find that inhibition of Suv420 methyltransferase activity partially restores Aurora B localization to centromeres and that restoration of the Aurora B-containing chromosomal passenger complex to the centromere is sufficient to suppress mitotic errors that result when Suv420 and H4K20me3 is enriched at centromeres. Consistent with a role for Suv39 and Suv420 in neg. regulating Aurora B, high expression of these enzymes corresponds with increased sensitivity to Aurora kinase inhibition in human cancer cells, suggesting that increased H3 K9 and H4K20 methylation may be an underappreciated source of chromosome mis-segregation in cancer.

Keywords: human cancer centromere Aurora B localization chromosome instability; Centromere; Chromosome instability; Mitosis

Substances (15) Reactions (0) Citing (3)

42

The Phytochemical Scoulerine Inhibits Aurora Kinase Activity to Induce Mitotic and Cytokinetic Defects

By: Li, Jinhua; Yan, Ziqi; Li, Hongmei; Shi, Qiong; Ahire, Vidhula; Zhang, Shenqiu; Nimishetti, Naganna; Yang, Dun; Allen, Thaddeus D. ; Zhang, Jing

Journal of Natural Products (2021), 84(8), 2312-2320 | Language: English, Database: CAplus and MEDLINE

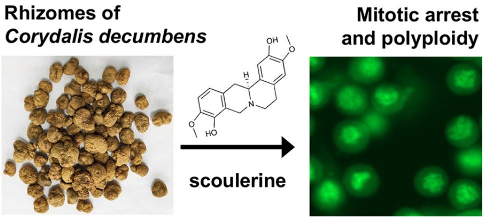

To identify novel bioactive compounds, an image-based, cell culture screening of natural product extracts was conducted. Specifi cally, our screen was designed to identify phytoc hems. that might phenocopy inhibition of the chromo somal passenger protein complex in eliciting mitotic and cytokinetic defects. A known alkaloid, scoule rine, was identified from the rhizomes of the plant Corydalis decumbens as being able to elicit a transient mitotic arrest followed by either apoptosis induction or polyploidy. In examining the mitotic abnormality further, we observed that scoulerine could elicit supernu merary centrosomes during mitosis, but not earlier in the cell cycle. The localization of NUMA1 at spindle poles was also inhibited, suggesting diminished potential for microtubule recruitment and spindle-pole focusing. Polyploid cells emerged subsequent to cytoki netic failure. The concent ration required for scoulerine to elicit all its cell division phenotypes was similar, and an examination of related compounds highlighted the requirement for proper positi oning of a hydroxyl and a methoxy group about an aromatic ring for activity. Mechanis tically, scoulerine inhibited AURKB activity at concent rations that elicited supernu merary centrosomes and polypl oidy. AURKA was only inhibited at higher concentrations, so AURKB inhibition is the likely mechanism by which scoulerine elicited division defects. A URKB inhibition was never complete, so scoulerine may be a suboptimal AURK inhibitor or work upstream of the chromo somal passenger protein complex to reduce AURKB activity. Scoulerine inhibited the viability of a variety of human cancer cell lines. Collect ively, these findings uncover a previously unknown activity of scoulerine that could facilitate targeting human cancers. Scoulerine, or a next-generation analog, may be useful as a nontoxic component of combin ation therapies where inhibiting the chromo somal passenger protein complex is desired.

Keywords: cancer AURKB inhibitor scoulerine screen Corydalis cytokinetic

Substances (8) Reactions (0) Citing (4)

43

Application of aurora kinase B inhibitor in preparation of drugs for treating or preventing kidney damage caused by autoimmune disease

By: Zhao, Yue; Liu, Zhihong

China, CN113304153 A 2021-08-27 | Language: Chinese, Database: CAplus

The present invention relates to an application of aurora kinase B inhibitor charact erized in that the dosage of the aurora kinase B inhibitor (AZD1152) is 5-150mg/kg/day. The administ ration of AZD115225 mg/kg starting from the age of 10 wk in M RL/lpr lupus mice for 8 wk of continuous treatment can significantly improve lymph node and enlarged spleen, serum inflam matory factors increase and other immune activation phenotypes, reduce the ratio of urine protein to creati nine, suggesting that AZD1152 treatment can inhibit autoimmune diseases.

Keywords: aurora kinase B inhibitor drug kidney damage autoimmune disease

PatentPak available

Substances (3) Reactions (0) Citing (0)

44

Activity Comparison of Epigenetic Modulators against the Hemoprotozoan Parasites Babesia divergens and Plasmodium falciparum

By: Vanheer, Leen N.; Kafsack, Bjorn F. C.

ACS Infectious Diseases (2021), 7(8), 2277-2284 | Language: English, Database: CAplus and MEDLINE

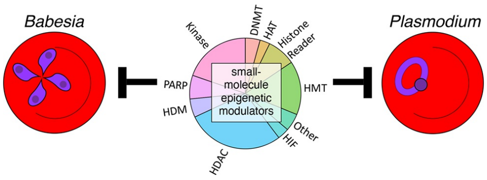

Babesiosis is a tick-borne parasitic disease of humans and livestock that has dramat ically increased in frequency and geog. range over the past few decades. Infection of cattle often causes large economic losses, and human infection can be fatal in immunocom promised patients. Unlike for malaria, another disease caused by hemopro tozoan parasites, limited treatment options exist for Babesia infections. As epigenetic regulation is a promising target for new antipar asitic drugs, we screened 324 epigenetic inhibitors against Babesia divergens blood stages and identified 75 (23%) and 17 (5%) compounds that displayed ≥90% inhibition at 10 and 1μ M, resp., including over a dozen compounds with activity in the low nanomolar range. We observed differ ential activity of some inhibitor classes against Babesia divergens and Plasmodium falciparum parasites and identified pairs of compounds with a high difference in activity despite a high similarity in chem. structure, highlighting new insights into the develo pment of epigenetic inhibitors as antiparasitic drugs.

Keywords: epigenetic modulator Babesia Plasmodium antiparasitic; Babesia; Plasmo dium; epigenetic; parasites; small molecule screen

Substances (326) Reactions (0) Citing (7)

45

Hyperbranched polyester polyol derivative as drug solubilizer

By: Haag, Rainer; Ferraro, Magda; Mohammadifar, Ehsan; Schirner, Michael
European Patent Organization, EP3858330 A1 2021-08-04 | Language: English, Database: CAplus

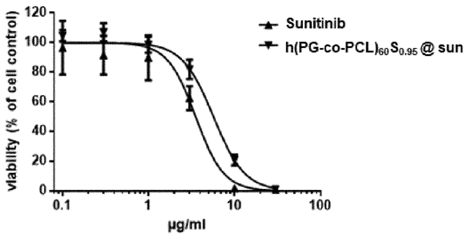

FIG 1

The present invention relates to a composition comprising a hyperbranched polyester polyol derivative and a small mol. comprising at least one cyclic amine. The hyperbranched polyester polyol derivative is obtainable by a method comprising the following steps: (a) reacting only glycidol and ε-caprolactone at a temperature lying in a range of between 40°C and 140°C to obtain a hyperbr anched polyester polyol derivative in which caprol actone residues and glycerol residues are randomly arranged; (b) reacting the hyperbranched polyester polyol derivative of step (a) with a sulfation reagent to obtain a sulfated hyperbr anched polyester polyol as hyperbranched polyester polyol derivative

Keywords: hyperbranched polyester polyol drug solubi lizer

PatentPak available

Substances (61) Reaction (1) Citing (0)

46

Hyperbranched polyester polyol derivative as drug solubilizer

By: Haag, Rainer; Ferraro, Magda; Mohammadifar, Ehsan; Schirner, Michael
World Intellectual Property Organization, WO2021152172 A1 2021-08-05 | Language: English, Database: CAplus

The present invention relates to a composition comprising a hyperbranched polyester polyol derivative and a small mol. comprising at least one cyclic amine. The hyperbranched polyester polyol derivative is obtainable by a method comprising the following steps: (a) reacting only glycidol and ε-caprolactone at a temperature lying in a range of between 40°C and 140°C to obtain a hyperbr anched polyester polyol derivative in which caprol actone residues and glycerol residues are randomly arranged; (b) reacting the hyperbranched polyester polyol derivative of step (a) with a sulfation reagent to obtain a sulfated hyperbr anched polyester polyol as hyperbranched polyester polyol derivative

Keywords: hyperbranched polyester polyol drug solubi lizer

PatentPak available

Substances (63) Reactions (0) Citing (0)

47

Chromosome oscillation promotes Aurora A-dependent Hec1 phosphorylation and mitotic fidelity

By: Iemura, Kenji ; Natsume, Toyoaki ; Maehara, Kayoko ; Kanemaki, Masato T. ; Tanaka, Kozo
Journal of Cell Biology (2021), 220(7), e202006116 | Language: English, Database: CAplus and MEDLINE

Most cancer cells show chromosomal instability, a condition where chromosome missegr egation occurs frequently. We found that chromosome oscillation, an iterative chromosome motion during metaphase, is attenuated in cancer cell lines. We also found that metaphase phosphorylation of Hec1 at serine 55, which is mainly dependent on Aurora A on the spindle, is reduced in cancer cell lines. The Aurora A-dependent Hec1-S55 phosphorylation level was regulated by the chromosome oscill ation amplitude and vice versa: Hec1-S55 and -S69 phosphorylation by Aurora A is required for efficient chromosome oscill ation. Furthermore, enhancement of chromosome oscillation reduced the number of erroneous kineto chore-microtubule attachments and chromosome missegre gation, whereas inhibition of Aurora A during metaphase increased such errors. We propose that Aurora A- mediated metaphase Hec1-S55 phosphorylation through chromosome oscillation, together with Hec1-S69 phosphorylation, ensures mitotic fidelity by eliminating erroneous kinetochore-microtubule attachments. Attenuated chromosome oscillation and the resulting reduced Hec1-S55 phosphorylation may be a cause of C IN in cancer cell lines.

Keywords: Aurora A Hec phosphorylation mitotic fidelity chromosome oscill ation

Substances (7) | Reactions (0) | Citing (11)

48

Advances in targeted therapy for osteosarcoma based on molecular classification

By: Chen, Yingqian; Liu, Runzhi ; Wang, Wei; Wang, Chen; Zhang, Ning; Shao, Xuejing; He, Qiaojun; Ying, Meidan
Pharmacological Research (2021), 169, 105684 | Language: English, Database: CAplus and MEDLINE

A review. Osteosarcoma, a highly malignant tumor, is charact erized by widespread and recurrent chromo somal and genetic abnormalities. In recent years, a number of elaborated sequencing analyses have made it possible to cluster the osteos arcoma based on the identification of candidate driver genes and develop targeted therapy. Here, we ed recent next- generation genome sequencing studies and advances in targeted therapies for osteosarcoma based on mol. classification. First, we stratified osteosa rcomas into ten mol. subtypes based on genetic changes. And we analyzed potential targeted therapies for osteos arcoma based on the identified mol. subtypes. Finally, the development of targeted therapies for osteosarcoma investigated in clin. trials were further summarized and discussed. Therefore, we indicated the importance of mol. classification on the targeted therapy for osteosarcoma. And the stratification of patients based on the genetic characteristics of osteosarcoma will help to obtain a better therap eutic response to targeted therapies, bringing us closer to the era of personalized medicine.

Keywords: review osteosarcoma targeted therapy; AT7519 (PubChem CID: 11338033); AZD1152 (PubChem CID: 11497983); Alisertib (PubChem CID: 24771867); Anlotinib (PubChem CID: 25017411); Dinaciclib (PubChem CID: 46926350); Everolimus (Pub Chem CID: 6442177); HOI-07 (PubChem CID: 72193858); JQ1 (PubChem CID: 46907787); Molecular classification; Nutlin-3a (Pub Chem CID: 11433190); Osteosarcoma; Palbociclib (PubChem CID: 5330286); Pazopanib (PubChem CID: 10113978); Personalized medicine; RG7112 (PubChem CID: 57406853); Rapamycin (PubChem CID: 5284616); Ridaforolimus (PubChem CID: 11520894); SA R405838 (PubChem CID: 53476877); Sorafenib (PubChem CID: 216239); Sunitinib (PubChem CID: 5329102); THZ1 (PubChem CID: 73602827); Targeted therapy; VX-680 (PubChem CID: 5494449); ZM447439 (PubChem CID: 9914412)

Substances (0) | Reactions (0) | Citing (12)

49

Inhibition of Aurora Kinase B activity disrupts development and differentiation of salivary glands

By: Shaalan, Abeer K.; Teshima, Tathyane H. N.; Tucker, Abigail S.; Proctor, Gordon B.
Cell Death Discovery (2021), 7(1), 16 | Language: English, Database: CAplus and MEDLINE

Abstract: Little is known about the key mols. that regulate cell division during organogenesis. Here we determine the role of the cell cycle promoter aurora kinase B (AURKB) during development, using embryonic salivary glands (E-SGs) as a model. AURKB is a serine/threonine kinase that regulates key events in mitosis, which makes it an attractive target for tailored anticancer therapy. Many reports have elaborated on the role of AURKB in neoplasia and cancer; however, no previous study has shown its role during organ development. Our previous experiments have highlighted the essential requirement for AURKB during adult exocrine regeneration. To investigate if AURKB is similarly required for progression during embryonic development, we pharmacol. inhibited AURKB in developing submandibular glands (SMGs) at embryonic day (E)13.5 and E16.5, using the highly potent and selective drug Barasertib. Inhibition of AURKB interfered with the expansion of the embryonic buds. Interestingly, this effect on SMG development was also seen when the mature explants (E16.5) were incubated for 24 h with another cell cycle inhibitor Aphidicolin. Barasertib prompted apoptosis, DNA damage and senescence, the markers of which (cleaved caspase 3, γH2AX, SA-βgal and p21, resp.), were predominantly seen in the developing buds. In addition to a reduction in cell cycling and prolife ration of the epithelial cells in response to AURKB inhibition, Barasertib treatment led to an excessive generation of reactive oxygen species (ROS) that resulted in downregulation of the acinar differentiation marker Mist1. Importantly, inhibition of ROS was able to rescue this loss of identity, with Mist1 expression maintained despite loss of AURKB. Together, these data identify AURKB as a key mol. in supporting embryonic development and differentiation, while inhibiting senescence-inducing signals during organogenesis.

Keywords: aurora Kinase B salivary gland

Substances (4) Reactions (0) Citing (3)

50

Substituted 4-aminoisoindoline-1,3-dione compounds and second active agents for combined use for treating hematological malignancies

By: Anton, Maria Soraya Carrancio; Buchholz, Tonia J.; Chang, Henry; Filvaroff, Ellen; Kasibhatla, Shailaja; Lopez-Girona, Antonia; Mohan, Adithi; Narla, Rama Krishna; Pourdehnad, Michael
United States, US20210113576 A1 2021-04-22 | Language: English, Database: CAplus

Sensitivity to Compound 1 single agent in DLBCL cell lines

sensitive to resistant

TMD-8	SU-DHL-2	SU-DHL-10	Pfeiffer	WSU-DLCL2	OCI-Ly10	Riva	U2932	SU-DHL-4	DB
58.67	108.4	123.7	288.2	362	403.5	496.3	577	585.3	670.8

Provided herein are methods of using (*S*)-2-(2,6-dioxopiperidin-3-yl)-4-((2-fluoro-4-((3-morpholinoazetidin-1-yl)methyl)benzyl) amino)isoindoline-1,3-dione, or an enantiomer, mixture of enantiomers, tautomer, isotopolog, or pharmaceutically acceptable salt thereof, in combination with a second active agent for treating, preventing or managing hematol. maligna ncies. The second active agent is one or more of an HDAC inhibitor, a BCL2 inhibitor, a BTK inhibitor, an mTOR inhibitor, a PI3K inhibitor, a PKCβ inhibitor, a SYK inhibitor, a JAK2 inhibitor, an Aurora kinase inhibitor, an EZH2 inhibitor, a BET inhibitor, a hypomethylating agent, a DOT1L inhibitor, a HAT inhibitor, a WDR5 inhibitor, a DNMT1 inhibitor, an LSD-1 inhibitor, a G9A inhibitor, a PRMT5 inhibitor, a BRD inhibitor, a SUV420H1/H2 inhibitor, a CARM1 inhibitor, a PLK1 inhibitor, an NEK2 inhibitor, an MEK inhibitor, a PHF19 inhibitor, a PIM inhibitor, an IGF-1R inhibitor, an XPO1 inhibitor, a BIRC5 inhibitor, or a chemotherapy.

Keywords: hematol neoplasm treatment aminoisoindolinedione compound active agent combination

PatentPak available

Substances (131) Reactions (3) Citing (0)

51

Predicting cell health phenotypes using image-based morphology profiling

By: Way, Gregory P.; Kost-Alimova, Maria; Shibue, Tsukasa; Harrington, William F.; Gill, Stanley; Piccioni, Federica; Becker, Tim; Shafqat-Abbasi, Hamdah; Hahn, William C.; Carpenter, Anne E.; et al
Molecular Biology of the Cell (2021), 32(9), 995-1005 | Language: English, Database: CAplus and MEDLINE

Genetic and chem. perturbations impact diverse cellular phenotypes, including multiple indicators of cell health. These readouts reveal toxicity and antitumorigenic effects relevant to drug discovery and personalized medicine. We developed two customized microscopy assays, one using four targeted reagents and the other three targeted reagents, to collectively measure 70 specific cell health phenotypes including proliferation, apoptosis, reactive oxygen species, DNA damage, and cell cycle stage. We then tested an approach to predict multiple cell health phenotypes using Cell Painting, an inexpensive and scalable image-based morphol. assay. In matched CRISPR perturbations of three cancer cell lines, we collected both Cell Painting and cell health data. We found that simple machine learning algorithms can predict many cell health readouts directly from Cell Painting images, at less than half the cost. We hypothesized that these models can be applied to accurately predict cell health assay outcomes for any future or existing Cell Painting dataset. For Cell Painting images from a set of 1500+ compound perturbations across multiple doses, we validated predic tions by orthogonal assay readouts. We provide a web app to browse predic tions: http://broad.io/cell-health-apparatus Our approach can be used to add cell health annotations to Cell Painting datasets.

Keywords: cell health phenotype morphol profiling deep machine learning

Substances (6) Reactions (0) Citing (33)

52

Targeting pan-essential genes in cancer: challenges and opportunities

By: Chang, Liang; Ruiz, Paloma ; Ito, Takahiro; Sellers, William R.
Cancer Cell (2021), 39(4), 466-479 | Language: English, Database: CAplus and MEDLINE

Despite remarkable successes in the clinic, cancer targeted therapy development remains challenging and the failure rate is disappointingly high. This problem is partly due to the misappl ication of the targeted therapy paradigm to therapeutics targeting pan-essential genes, which can result in therapeutics whereby efficacy is attenuated by dose-limiting toxicity. Here we summarize the key features of successful chemotherapy and targeted therapy agents, and use case studies to outline recurrent challenges to drug development efforts targeting pan-essential genes. Finally, we suggest strategies to avoid previous pitfalls for ongoing and future development of pan-essential therapeutics.

Keywords: human neoplasm therapeutic target chemotherapy antibody drug conjugate; drug develo pment; pan-essential genes; target identification; target validation; targeted therapies

Substances (22) Reactions (0) Citing (45)

53

Combined inhibition of XIAP and BCL2 drives maximal therapeutic efficacy in genetically diverse aggressive acute myeloid leukemia

By: Hashimoto, Mari; Saito, Yoriko; Nakagawa, Ryo; Ogahara, Ikuko; Takagi, Shinsuke ; Takata, Sadaaki; Amitani, Hanae; Endo, Mikiko; Yuki, Hitomi ; Ramilowski, Jordan A.; et al

Nature Cancer (2021), 2(3), 340-356 | Language: English, Database: CAplus and MEDLINE

Aggressive therapy-resistant and refractory acute myeloid leukemia (A ML) has an extremely poor outcome. By analyzing a large number of genetically complex and diverse, primary high- risk poor-outcome human AML samples, we identified specific pathways of therapeutic vulnerability. Through drug screens followed by extensive in vivo validation and genomic analyses, we found inhibition of cytosolic and mitochondrial anti-apoptotic proteins XIAP, BCL2 and MCL1, and a key regulator of mitosis, A URKB, as a vulnerability hub based on patient-specific genetic aberrations and transcriptional signatures. Combinatorial therapeutic inhibition of XIAP with an addnl. patient-specific vulnerability eliminated established AML in vivo in patient-derived xenografts (PDXs) bearing diverse genetic aberrations, with no signs of recurrence during off- treatment follow-up. By integrating genomic profiling and drug-sensitivity testing, this work provides a platform for a precision- medicine approach for treating aggressive AML with high unmet need.

Keywords: venetoclax S63845 anticancer agent XIAP BCL2 acute myeloid leukemia

Substances (10) Reactions (0) Citing (7)

54

Preparation of biaminoquinolines and nanoformulations for cancer treatment

By: Li, Yuanpei; Ma, Zhao
World Intellectual Property Organization, WO2021055705 A1 2021-03-25 | Language: English, Database: CAplus

The invention provides bisaminoquinoline compounds of formula **I**, wherein R^1 is H, C_{1-40} alkyl, C_{2-40} alkenyl, etc.; R^{2a}, R^{2b}, R^{3a}, R^{3b} are independently H, C_{1-40} alkyl, C_{2-40} alkenyl, etc.; m and n are indepen dently 1 to 10; p = 1 - 20; each X is absent or O; and pharmaceutically acceptable salt thereof, are claimed. Example compound **II** was prepared by a general procedure (procedure given). The present invention also provides nanoca rriers comprising compounds of the present invention, and methods of using the nanocarriers for treating diseases and as imaging agent.

Keywords: biaminoquinoline preparation nanoformulation cancer treatment imaging

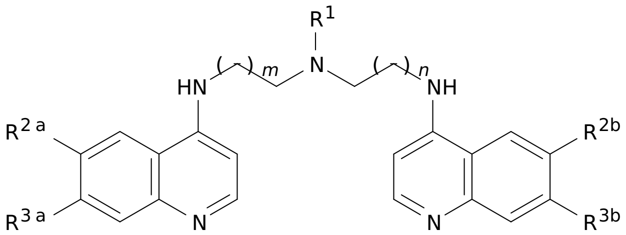

I

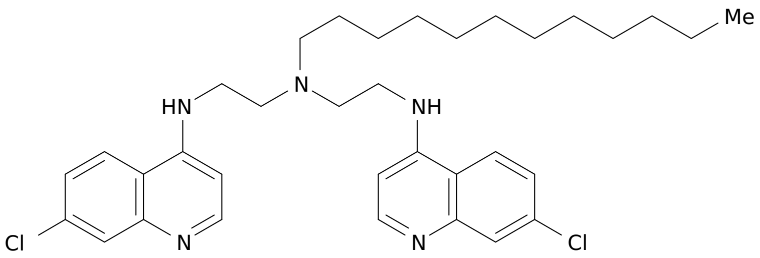

II

PatentPak available

Substances (342) | Reactions (90) | Citing (0)

55

Nanoencapsulated combination drug formulations

By: Baldwin, Paige; Sridhar, Srinivas; Singh, Bijay
United States, US20210085619 A1 2021-03-25 | Language: English, Database: CAplus

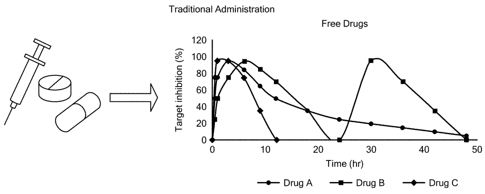

Pharmaceutical formulations (nanococktails) include two or more nanoparticulate delivery vehicles each including an active agent. The nanococktails can be formulated at any ratio of active agents by changing the ratio of nanopart iculate delivery vehicles in the nanococktail. The nanoparticulate delivery vehicles can have matched surface potential to prevent aggreg ation. The nanopart iculate delivery vehicles of the combined formul ation are designed to provide each active agent with desired, preferably overla pping therapeutic windows and a specif ically selected pharmaco kinetic and pharmaco dynamic profile when the nanoco cktail is admini stered to a subject. Each of the nanopart iculate delivery vehicles can be designed to enhance solubi lity, duration of action, targeting, stability, and to program release while preventing degradation of an active agent and preventing side effects, toxicity, and tolera bility issues in a subject.

Keywords: nanoencapsulation combination drug formul ation pharmaco dynamic

PatentPak available

Substances (51) Reactions (0) Citing (0)

56

QSAR Modeling of SARS-CoV M^{pro} Inhibitors Identifies Sufugolix, Cenicriviroc, Proglumetacin, and Other Drugs as Candidates for Repurposing against SARS-CoV-2

By: Alves, Vinicius M.; Bobrowski, Tesia; Melo-Filho, Cleber C.; Korn, Daniel; Auerbach, Scott; Schmitt, Charles; Muratov, Eugene N. ; Tropsha, Alexander
Molecular Informatics (2021), 40(1), 2000113 | Language: English, Database: CAplus and MEDLINE

The main protease (M^{pro}) of the SARS-CoV-2 has been proposed as one of the major drug targets for C OVID-19. We have identified the exptl. data on the inhibitory activity of compounds tested against the closely related (96% sequence identity, 100% active site conservation) M^{pro} of SARS-CoV. We developed QSAR models of these inhibitors and employed these models for virtual screening of all drugs in the DrugBank database. Similarity searching and mol. docking were explored in parallel, but docking failed to correctly discriminate between exptl. active and inactive compounds, so it was not relied upon for prospe ctive virtual screening. Forty-two compounds were identified by our models as consensus computa tional hits. Subsequent to our computa tional studies, N CATS reported the results of exptl. screening of their drug collection in S ARS-CoV-2 cytopathic effect assay (https: //opendata.ncats. nih.gov/covid19/). Coincidentally, NCATS tested 11 of our 42 hits, and three of them, cenicr iviroc (AC_{50} of 8.9μM), proglumetacin (tested twice independently, with AC_{50} of 8.9μM and 12.5μM), and sufugolix (AC_{50} 12.6μM), were shown to be active. These observ ations support the value of our modeling approaches and models for guiding the exptl. investi gations of putative anti-COVID-19 drug candidates. All data and models used in this study are publicly available via Supplem entary Materials, GitHub (https://github. com/alvesvm/sars-cov-mpro), and Chembench web portal (https: //chembench.mml.unc.edu/).

Keywords: SARS CoV2 coronavirus protease inhibitors drug repurp osing screening chemoinfo rmatics; SARS-CoV-2; SARS-CoV-2 Mpro.; cheminformatics; drug repurp osing; virtual screening

Substances (12) Reactions (0) Citing (37)

57

Loss of Aurora kinase signaling allows lung cancer cells to adopt endoreplication and form polyploid giant cancer cells that resist antimitotic drugs

By: Tagal, Vural; Roth, Michael G.

Cancer Research (2021), 81(2), 400-413 | Language: English, Database: CAplus and MEDLINE

Polyploid giant cancer cells (PGCC) are common in tumors and have been associated with resistance to cancer therapy, tumor relapse, malignancy, immunosuppression, metastasis, cancer stem cell production, and modulation of the tumor microenvi ronment. However, the mol. mechanisms that cause these cells to form are not yet known. In this study, we discover that Aurora kinases are synergistic determinants of a switch from the proliferative cell cycle to polyploid growth and multinuc leation in lung cancer cell lines. When Aurora kinases were inhibited together, lung cancer cells uniformly grew into multinucleated PGCCs. These cells adopted an endoreplication in which the genome replicates, mitosis is omitted, and cells grow in size. Consequ ently, such cells continued to safely grow in the presence of antimitotic agents. These PGCC re-entered the proliferative cell cycle and grew in cell number when treatment was terminated. Thus, PGCC formation might represent a fundam ental cellular response to Aurora kinase inhibitors and contributes to therapy resistance or tumor relapse.

Keywords: polyploid giant cancer Aurora kinase endoreplication antimitotic drug resistance

Substances (29) Reactions (0) Citing (15)

58

Experiments in the EpiDerm 3D skin in vitro model and minipigs in vivo indicate comparatively lower in vivo skin sensitivity of topically applied aneugenic compounds

By: Schuler, Maik; Tomlinson, Lindsay; Homiski, Michael; Cheung, Jennifer; Zhan, Yutian; Coffing, Stephanie; Engel, Maria; Rubitski, Elizabeth; Seitis, Gary; Hales, Katherine; et al

Toxicological Sciences (2021), 180(1), 103-121 | Language: English, Database: CAplus and MEDLINE

Risk management of in vitro aneugens for topically applied compounds is not clearly defined because there is no validated methodol. to accurately measure compound concentration in proliferating stratum basale keratin ocytes of the skin. Here, we exptl. tested several known aneugens in the EpiDerm reconstructed human skin in vitro micron ucleus assay and compared the results to flow cytometric mechanistic biomarkers (phospho-H3; MPM2, DNA content). We then evaluated similar biomarkers (Ki- 67, nuclear area) using immunohistochem. in skin sections of minipigs following topical exposure the potent aneugens, colchi cine, and hesper adin. Data from the Epi Derm model showed pos. micron ucleus responses for all aneugens tested following topical or direct media dosing with similar sensitivity when adjusted for applied dose. Quant. benchmark dose- response anal. exhibited increases in the mitotic index biomarkers phospho-H3 and MPM2 for tubulin binders and polyploidy for aurora kinase inhibitors are at least as sensitive as the micronucleus endpoint. By compar ison, the aneugens tested did not induce histop athol. changes, increases in Ki-67 immunolabeling or nuclear area in skin sections from the in vivo minipig study at doses in signif icant excess of those eliciting a response in vitro. Results indicate the EpiDerm in vitro micron ucleus assay is suitable for the hazard identif ication of aneugens. The lack of response in the minipig studies indicates that the barrier function of the minipig skin, which is comparable to human skin, protects from the effects of aneugens in vivo. These results provide a basis for conducting addnl. studies in the future to further refine this understanding.

Keywords: topical aneugen genotoxicant aneuploidy keratin ocyte; adverse outcome pathway; aneupl oidy; aurora kinase inhibitor; skin; tubulin binder

Substances (6) Reactions (0) Citing (3)

59

A cell cycle progression-derived gene signature to predict prognosis and therapeutic response in hepatocellular carcinoma

By: Hui, Yongfeng; Leng, Junzhi; Jin, Dong; Liu, Di; Wang, Genwang; Wang, Qi ; Wang, Yanyang
Disease Markers (2021), 1986159 | Language: English, Database: CAplus and MEDLINE

Dysregulation of cell cycle progression (CCP) is one of the hallmarks of cancer. Here, our study is aimed at developing a CCP-derived gene signature for predicting high-risk population of hepatocellular carcinoma (HCC). Our study retrospectively analyzed the transcriptome profiling and clin. information of HCC patients from The Cancer Genome Atlas (TCGA) and International Cancer Genome Consortium (ICGC) projects. Uni- and multivariate cox regression models were conducted for identifying which hallmarks of cancer were risk factors of HCC. CCP-derived gene signature was developed with LASSO method. The predictive efficacy was verified by ROC curves and subgroup analyses. A nomogram was then generated and validated by ROC, calibration, and decisive curves. Immune cell infiltration was estimated with ssGSEA method. Potential small mol. compounds were predicted via CTRP and CMap analyses. The response to chemotherapeutic agents was evaluated based on the GDSC project. Among hallmarks of cancer, CCP was identified as a dominant risk factor for HCC prognosis. CCP-derived gene signature displayed the favorable predictive efficacy in HCC prognosis independent of other clinicopathol. parameters. A nomogram was generated for optimizing risk stratification and quantifying risk evaluation. CCP-derived signature was in relation to immune cell infiltration, HLA, and immune checkpoint expression. Combining CTRP and CMap analyses, fluvastatin was identified as a promising therapeutic agent against HCC. Furthermore, CCP-derived signature might be applied for predicting the response to doxorubicin and gemcitabine. Collectively, CCP-derived gene signature was a promising marker in prediction of survival outcomes and therapeutic responses for HCC patients.

Keywords: ccp

Substances (6) Reactions (0) Citing (3)

60

BCL-XL blockage in TNBC models confers vulnerability to inhibition of specific cell cycle regulators

By: Castellanet, Olivier; Ahmad, Fahmida; Vinik, Yaron; Mills, Gordon B.; Habermann, Bianca; Borg, Jean-Paul; Lev, Sima; Lamballe, Fabienne; Maina, Flavio

Theranostics (2021), 11(19), 9180-9197 | Language: English, Database: CAplus and MEDLINE

Cell cycle regulators are frequently altered in Triple-Neg. Breast Cancer (TNBC). Emerging agents targeting these signals offer the possibility to design new combina torial therapies. However, preclin. models that recapitulate TNBC primary resistance and heterog eneity are essential to evaluate the potency of these combined treatm ents. Bioinformatic processing of human breast cancer datasets was used to analyze correlations between expression levels of cell cycle regulators and patient survival outcome. The M MT V-R26Met mouse model of TNBC resistance and heterog eneity was employed to analyze expression and targeting vulnera bility of cell cycle regulators in the presence of BCL-XL blockage. Robustness of outcomes and select ivity was further explored using a panel of human breast cancer cells. Orthotopic studies in nude mice were applied for preclin. evaluation of efficacy and toxicity. Altera tions of protein expression, phosphorylation, and/or cellular localisation were analyzed by western blots, reverse phase protein array, and immunocytochem. Bioinformatics was performed to highlight drug's mechanisms of action. We report that high expression levels of the BCL2L1 gene encoding BCL-XL and of specific cell cycle regulators correlate with poor survival outcomes of TNBC patients. Blockage of B CL-XL confers vulnera bility to drugs targeting C DK1/2/4, but not FOXM1, CDK4/6, Aurora A and Aurora B, to all MMTV-R26Met and human TNBC cell lines tested. Combined blockage of B CL-XL and CDK1/2/4 interfered with tumor growth in vivo. Mechanistically, we show that, co- targeting of BCL-XL and CDK1/2/4 synergistically inhibited cell viability by combina torial depletion of survival and R TK/AKT signals, and concomi tantly restoring FOXO3a tumor suppre ssion actions. This was accomp anied by an accumulation of DNA damage and conseq uently apoptosis. Our studies illustrate the possib ility to exploit the vulnera bility of TNBC cells to CDK1/2/4 inhibition by targeting B CL-XL. Moreover, they underline that specif icity matters in targeting cell cycle regulators for combinatorial anticancer therapies.

Keywords: triple neg breast cancer BCLXL blockage cell cycle; B CL-XL; M ET; cancer mouse model; cell cycle regula tors; triple-negative breast cancer

Substances (15)   Reactions (0)   Citing (2)

61

Discovery of Novel GMPS Inhibitors of Candidatus Liberibacter Asiaticus by Structure Based Design and Enzyme Kinetic

By: Nan, Jing ; Zhang, Shaoran; Zhan, Ping; Jiang, Ling

Biology (Basel, Switzerland) (2021), 10(7), 594 | Language: English, Database: CAplus and MEDLINE

Simple Summary: The spread of citrus Huanglongbing caused significant damage to the world's citrus industry. Thermotherapy and chem. agents were used to control this disease; however, the effectiveness of these treatments is frequently inconsistent. In addition, CLas cannot be cultured in vitro. Therefore, structure-based virtual screening is a novel method to find compounds that work against CLas. This study used CLas GMPS as a target for high-throughput screening and selected some compounds which have a higher binding affinity to test their inhibition of CLas GMPS. Finally, two mols. were identified as the lead compound to control citrus HLB. Abstract: Citrus production is facing an unprecedented problem because of huanglongbing (HLB) disease. Presently, no effective HLB-easing method is available when citrus becomes infected. GMP synthetase (GMPS) is a key protein in the de novo synthesis of guanine nucleotides. GMPS is used as an attractive target for developing agents that are effective against the patogen infection. In this research, homol. modeling, structure-based virtual screening, and mol. docking were used to discover the new inhibitors against CLas GMPS. Enzyme assay showed that folic acid and AZD1152 showed high inhibition at micromole concentrations, with AZD1152 being the most potent mol. The inhibition constant (Ki) value of folic acid and AZD1152 was 51.98 μM and 4.05 μM, resp. These results suggested that folic acid and AZD1152 could be considered as promising candidates for the development of CLas agents.

Keywords: Candidatus Liberibacter asiaticus; enzyme activity; guanosine 5'- monophosphate synthetase; molecular docking; virtual screening

Substances (0) Reactions (0) Citing (3)

62

Aurora B and C kinases regulate chromosome desynapsis and segregation during mouse and human spermatogenesis

By: Wellard, Stephen R.; Schindler, Karen; Jordan, Philip W.

Journal of Cell Science (2020), 133(23), jcs248831 | Language: English, Database: CAplus and MEDLINE

Precise control of chromosome dynamics during meiosis is critical for fertility. A gametocyte undergoing meiosis coordinates formation of the synaptonemal complex (SC) to promote efficient homologous chromosome recombination. Subsequent disassembly of the SC occurs prior to segregation of homologous chromosomes during meiosis I. We examined the requirements of the mammalian Aurora kinases (AURKA, AURKB and AURKC) during SC disassembly and chromosome segregation using a combination of chem. inhibition and gene deletion approaches. We find that both mouse and human spermatocytes fail to disassemble SC lateral elements when the kinase activity of AURKB and AURKC are chem. inhibited. Interestingly, both Aurkb conditional knockout and Aurkc knockout mouse spermatocytes successfully progress through meiosis, and the mice are fertile. In contrast, Aurkb, Aurkc double knockout spermatocytes fail to coordinate disassembly of SC lateral elements with chromosome condensation and segregation, resulting in delayed meiotic progression. In addition, deletion of Aurkb and Aurkc leads to an accumulation of metaphase spermatocytes, chromosome missegregation and aberrant cytokinesis. Collectively, our data demonstrate that AURKB and AURKC functionally compensate for one another ensuring successful mammalian spermatogenesis.

Keywords: auroraB auroraC kinase chromosome desynapsis segregation spermatogenesis; Aurora kinase; Chromosome segregation; Gametogenesis; Infertility; Meiosis; Synaptonemal complex

Substances (10) Reactions (0) Citing (15)

63

Computationally predicting clinical drug combination efficacy with cancer cell line screens and independent drug action

By: Ling, Alexander ; Huang, R. Stephanie

Nature Communications (2020), 11(1), 5848 | Language: English, Database: CAplus and MEDLINE

Evidence has recently emerged that many clin. cancer drug combinations may derive their efficacy from indepe ndent drug action (I DA), where patients only receive benefit from the single most effective drug in a drug combin ation. Here we present I DACombo, an I DA based method to predict the efficacy of drug combin ations using monotherapy data from high-throughput cancer cell line screens. We show that IDACombo predictions closely agree with measured drug combin ation efficacies both in vitro (Pearson's correlation = 0.93 when comparing predicted efficacies to measured efficacies for > 5000 combin ations) and in a systema tically selected set of clin. trials (accuracy > 84% for predicting statistically significant improvements in patient outcomes for 26 first line therapy trials). Finally, we demons trate how IDACombo can be used to systema tically prioritize combinations for development in specific cancer settings, providing a framework for quickly translating existing monotherapy cell line data into clin. meaningful predictions of drug combin ation efficacy.

Keywords: independent drug action clin combin ation cancer cell line

Substances (122) Reactions (0) Citing (19)

64

Epigenome-wide DNA methylation analysis of small cell lung cancer cell lines suggests potential chemotherapy targets

By: Krushkal, Julia ; Silvers, Thomas; Reinhold, William C.; Sonkin, Dmitriy; Vural, Suleyman; Connelly, John; Varma, Sudhir; Meltzer, Paul S.; Kunkel, Mark; Rapisarda, Annamaria; et al

Clinical Epigenetics (2020), 12(1), 93 | Language: English, Database: CAplus and MEDLINE

A potentially important association was observed for T REX1, which encodes the 3' exonuc lease I that serves as a S TING antagonist in the regulation of a cytosolic DNA-sensing pathway. Increased methyl ation and low expression of T REX1 were associated with the sensitivity to Aurora kinase inhibitors AZD-1152, SCH-1473759, SNS-314, and TAK-901; the CDK inhibitor R-547; the Vertex ATR inhibitor Cpd 45; and the mitotic spindle disruptor vinorelbine. Compared with cell lines of other cancer types, T REX1 had low m RN A expression and increased upstream region methyl ation in SCLC, suggesting a possible relati onship with SCLC sensitivity to Aurora kinase inhibitors. Methylation of the 3'UTR of C EP350 and MLPH, involved in centrosome machinery and microt ubule tracking, resp., was associated with response to Aurora kinase inhibitors and other agents. EPAS1 methylation was associated with response to Aurora kinase inhibitors, a PLK-1 inhibitor and a Bcl-2 inhibitor. Increased promoter methyl ation of SLFN11 was correlated with resistance to DNA damaging agents, as a result of low or no S LFN11 expression. The 5' UTR of the epigenetic modifier EZH2 was associated with response to Aurora kinase inhibitors and a FGFR inhibitor. Methyl ation and expression of YAP1 were correlated with response to an mTOR inhibitor. Among non- neuroendocrine markers, EPHA2 was associated with response to Aurora kinase inhibitors and a PLK-1 inhibitor and CD151 with Bcl-2 inhibitors.

Keywords: small cell lung cancer DNA methylation chemotherapy epigen omics; Chemotherapy; DNA methylation; Epigenetic regula tion; Gene expression; Small cell lung cancer

Substances (0) Reactions (0) Citing (25)

65

Methods for reducing tumor progression and fibrosis and increasing adaptive immunity in malignancies by administering dimethyl-β-hydroxycholenamide derivatives

By: Glazer, Robert I.; Levi, Moshe; Yuan, Hongyan; Ranjit, Suman
World Intellectual Property Organization, WO2020252198 A1 2020-12-17 | Language: English, Database: CAplus

Aspects of the technol. described herein relate to a method of treating a malignancy in a subject. This method involves selecting a subject having a malignancy and administering di-Meβ-hydroxy-cholenamide (DMHCA) or derivative thereof to the subject in an amount effective to treat the malignancy. Methods of reducing malign ancy- associated fibrosis in a subject and pharmac eutical combinations comprising (i) DMHCA or derivative thereof and (ii) one or more immune checkpoint inhibitors are also disclosed.

Keywords: tumor fibrosis treatment adaptive immunity increase cholenamide derivative

PatentPak available

Substances (62) | Reactions (0) | Citing (0)

66

Methods for treating small cell neuroendocrine (SCN) tumors and small-round-blue cell tumors (SRBCTs)

By: Graeber, Thomas G.; Balanis, Nikolas G.; Sheu, Katherine M.; Witte, Owen N.; Esedebe, Favour; Park, Jung Wook
World Intellectual Property Organization, WO2020243329 A1 2020-12-03 | Language: English, Database: CAplus

The current disclosure provides methods of identifying and treating small cell neuroen docrine (SCN) tumors and small-round-blue cell tumors (SRBCTs). Aspects of the disclosure relates to a method comprising measuring the level of expression of one or more biomarkers in a biol. sample from a cancer patient. Further aspects relate to a method for treating small cell neuroendocrine (SCN) cancer or small-round-blue cell tumor (S RBCT) in a patient comprising adminis tering a cancer treatment to the patient, wherein the cancer treatment is a treatment selected from ABT-263, PHA-793887, vorinostat, tubastatin A, CX-5461 and combin ations thereof.

Keywords: antitumor ABT263 vorinostat small cell neuroen docrine cancer treatment

PatentPak available

Substances (259) | Reactions (0) | Citing (0)

67

Nf1-mutant tumors undergo transcriptome and kinome remodeling after inhibition of either mTOR or MEK

By: Pucciarelli, Daniela; Angus, Steven P.; Huang, Benjamin; Zhang, Chi; Nakaoka, Hiroki J.; Krishnamurthi, Ganesh; Bandyopadhyay, Sourav; Clapp, D. Wade; Shannon, Kevin; Johnson, Gary L.; et al
Molecular Cancer Therapeutics (2020), 19(11), 2382-2395 | Language: English, Database: CAplus and MEDLINE

Loss of the tumor suppressor NF1 leads to activation of RAS effector pathways, which are therapeu tically targeted by inhibition of m TOR (mTORi) or MRK (MEKi). However, therapeutic inhibition of RAS effectors leads to the develo pment of drug resistance and ultimately disease progression. To investigate mol. signatures in the context of N F1 loss and subsequent acquired drug resist ance, we analyzed the exomes, transcriptomes, and kinomcs of Nf1-mutant mouse tumor cell lines and deriva tives of these lines that acquired resistance to either MEKi or mTORi. Biochem. comparisons of this unique panel of tumor cells, all of which arose in Nf1+/- mice, indicate that loss of heterozygosity of Nf1 as an initial genetic event does not confer a common biochem. signature or response to kinase inhibition. Although acquired drug resistance by Nf1- mutant tumor cells was accompanied by altered kinomes and irreversibly altered transcriptomes, functionally in multiple Nf1- mutant tumor cell lines, M EKi resistance was a stable phenotype, in contrast to mTORi resistance, which was reversible. Collectively, these findings demonstrate that Nf1-mutant tumors represent a heterogeneous group biochem. and undergo broader remodeling of kinome activity and gene expression in response to targeted kinase inhibition.

Keywords: anticancer agent NF1 mTOR MEK transcriptome kinome cancer

Substances (13) Reactions (0) Citing (1)

68

Inhibition of Aurora Kinase B attenuates fibroblast activation and pulmonary fibrosis

By: Kasam, Rajesh K.; Ghandikota, Sudhir ; Soundararajan, Divyalakshmi; Reddy, Geereddy B.; Huang, Steven K.; Jegga, Anil G. ; Madala, Satish K.
EMBO Molecular Medicine (2020), 12(9), e12131 | Language: English, Database: CAplus and MEDLINE

Fibroblast activation including proliferation, survival, and ECM production is central to initiation and mainte nance of fibrotic lesions in idiopathic pulmonary fibrosis (IPF). However, druggable mols. that target fibroblast activation remain limited. In this study, we show that multiple pro-fibrotic growth factors, including TGFα, CTGF, and IGF1, increase aurora kinase B (A URKB) expression and activity in fibroblasts. Mechanistically, we demonstrate that Wilms tumor 1 (WT1) is a key transcription factor that mediates TGFα-driven AURKB upregulation in fibroblasts. Importantly, we found that inhibition of AURKB expression or activity is sufficient to attenuate fibroblast activation. We show that fibrosis induced by T GFα is highly dependent on AURKB expression and treating TGFα mice with barasertib, an AURKB inhibitor, reverses fibroblast activa tion, and pulmonary fibrosis. Barasertib similarly attenuated fibrosis in the bleomycin model of pulmonary fibrosis. Together, our preclin. studies provide important proof-of-concept that demonstrate barasertib as a possible interv ention therapy for IPF.

Keywords: Aurora Kinase B fibroblast pulmonary fibrosis; Aurora Kinase B; Barasertib; Wilms’ tumor 1; fibroproli feration; pulmonary fibrosis

Substances (5) Reactions (0) Citing (18)

69

Dihydro-spiro[indoline-3:1'-isoquinolin]-2-ones and their analogs and derivatives and methods of treating cancer and other diseases and their preparation

By: Mabanyi, Lobe Maloba Mesembe; Efange, Simon Mbua Ngale
World Intellectual Property Organization, WO2020183307 A1 2020-09-17 | Language: English, Database: CAplus

The invention is directed to various 3',4'-dihydro-2'H-spiro[indoline-3:1'-isoquinolin]-2-one compounds of formula I and methods for treating disease states and/or conditions which are mediated through sphingosine-1-phosphate receptor(s). The invention is also directed to the use of these compounds as anticancer agents and as modulators of sphingosine-1-phosphate receptor function in the treatment of disease states and/or conditions which are mediated through these receptors. In addition, the invention relates to pharmaceutical compositions comprising one or more of these compounds alone or in combin ation with other therapeutic agents. The invention is also directed to methods of treatment of cancer and/or conditions that may respond to the modulation of sphingosine-1-phosphate receptor function and which employ compounds of the present invention or pharmac eutical compos itions comprising one or more of the compounds of this invention. Compounds of formula I wherein R^1 is H, OH, halo, C_{1-6} alkoxy, etc.; R^2 and R^3 are independently H, OH, C_{1-6} hydroxyalkyl, etc.; R^4 is H, OH, halo, C_{1-6} alkoxy, etc.; R^5 and R^6 are independently H, alkyl, C_{1-6} alkoxy, etc.; R^7 is H, alkyl, arylalkyl, etc.; R^8 is H, OH, halo, NO_2, etc.; R^9, R^{10} and R^{11} are independently H, OH, halo, C_{1-6} hydroxyalkyl, etc.; R^{12} is H, OH, hydroxyalkyl, arylalkyl, etc. ; R^{13} is O and S; and pharmace utically acceptable salts, stereoisomers, solvates and polymorphs thereof, are claimed. Example compound II as prepared by cycliz ation of isatin with 3-hydroxyphenethy lamine. The invention compounds were evaluated for their anticancer activity (some data given) .

Keywords: spiroindoline isoquinolinone preparation treatment cancer

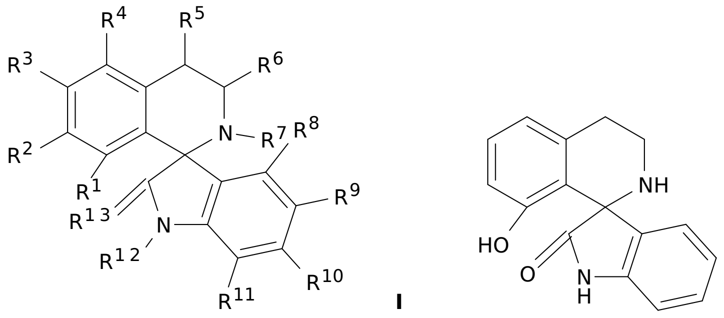

I II

PatentPak available

Substances (450) Reactions (239) Citing (3)

70

Methods of treating venetoclax-resistant acute myeloid leukemia

By: Tyner, Jeffrey W.; Nechiporuk, Tamilla; Kurtz, Stephen; McWeeny, Shannon
World Intellectual Property Organization, WO2020176686 A1 2020-09-03 | Language: English, Database: CAplus

The present invention concerns novel methods of treatment for venetoclax-resistant acute myeloid leukemia, partic ularly in subjects with a low expression of TP53 protein or expression of TP53 protein associated with a mutation of the T P53 gene, the methods comprising administering to the subject in need thereof a therapeu tically effective amount of a NTRK/ALK kinase/ROS1 kinase inhibitor, or a pharmaceutically acceptable salt thereof.

Keywords: venetoclax antitumor resistance acute myeloid leukemia treatment

PatentPak available

Substances (73) Reactions (0) Citing (0)

71

High-throughput screening of regulators of axonal transport

By: Shlevkov, Evgeny; Schwarz, Thomas L.; Bray, Mark-Anthony; Carpenter Van Dyk, Anne E.
World Intellectual Property Organization, WO2020149925 A1 2020-07-23 | Language: English, Database: CAplus

Provided are techniques for processing a sequence of images of neurons to identify stationary and motile organelles of the neurons, generating tracking data associated with movement of the one or more motile organelles across the sequence of images, and determining an overall percent motile based on the identified stationary organelles and motile organe lles. Also provided are compounds identified using the systems described, wherein the compounds, and pharmaceutically acceptable salts, and compos itions thereof are administered to a subject in need thereof in a sufficient amount to increase or promote organelle (e.g., mitocho ndrial) motility, or administered in a sufficient amount to treat a disease or disorder associated with decreased organelle motility. The compounds may be used in methods for increasing or promoting organelle (e.g., mitochondrial) motility in a cell, and methods for treating a disease. Also provided in the present disclosure are pharmaceutical compositions, kits, methods, and uses including or using a compound described herein.

Keywords: high throughput screening regulator axonal transport

PatentPak available

Substances (11) | Reactions (0) | Citing (0)

72

Antiproliferative compounds and second active agents for combined use for treating multiple myeloma

By: Wong, Lilly L.
United States, US20200215060 A1 2020-07-09 | Language: English, Database: CAplus

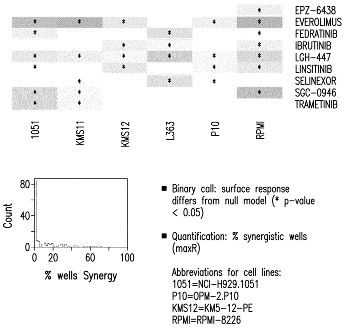

Provided herein are methods of using 4-(4-(4-(((2-(2,6-dioxopiperidin-3-yl)-1-oxoisoindolin-4-yl)oxy)methyl)benzyl)piperazin-1-yl)-3-fluorobenzonitrile, or an enantiomer, a mixture of enantiomers, a tautomer, or a pharmace utically acceptable salt thereof, in combination with a second active agent for treating, preventing or managing multiple myeloma. The second active agent is one or more of a BTK inhibitor, an mTOR inhibitor, a PIM inhibitor, an IGF-1R inhibitor, an MEK inhibitor, an XPO1 inhibitor, a DOT1L inhibitor, an EZH2 inhibitor, a JAK2 inhibitor, a BRD4 inhibitor, a PLK 1 inhibitor, an NEK2 inhibitor, an AURKB inhibitor, a BIRC5 inhibitor, a BET inhibitor, or a DNA methyltransferase inhibitor.

Keywords: antiproliferative compound combination multiple myeloma treatment

PatentPak available

Substances (76) | Reactions (42) | Citing (0)

73

Correction: Bromodomain and extraterminal protein inhibition blocks growth of triple-negative breast cancers through the suppression of aurora kinases. [Erratum to document cited in CA165:569636]

By: Sahni, Jennifer M.; Gayle, Sylvia S.; Bonk, Kristen L. Weber; Vite, Leslie Cuellar; Yori, Jennifer L.; Webb, Bryan; Ramos, Erika K.; Seachrist, Darcie D.; Landis, Melissa D.; Chang, Jenny C.; et al
Journal of Biological Chemistry (2020), 295(27), 9266 | Language: English, Database: CAplus and MEDLINE

There is no abstract available for this document.

Keywords: Aurora kinase BET protein inhibitor anticancer breast cancer erratum

Substances (4) Reactions (0) Citing (1)

74

Genome-wide CRISPR screen uncovers a synergistic effect of combining Haspin and Aurora kinase B inhibition

By: Huang, Min; Feng, Xu; Su, Dan ; Wang, Gang; Wang, Chao; Tang, Mengfan; Paulucci-Holthauzen, Adriana; Hart, Traver; Chen, Junjie
Oncogene (2020), 39(21), 4312-4322 | Language: English, Database: CAplus and MEDLINE

Abstract: Aurora kinases are a family of serine/threonine kinases vital for cell division. Because of the overexp ression of Aurora kinases in a broad range of cancers and their important roles in mitosis, inhibitors targeting Aurora kinases have attracted attention in cancer therapy. VX-680 is an effective pan-Aurora kinase inhibitor; however, its clin. efficacy was not satisf ying. In this study, we performed CRISPR/Cas9 screens to identify genes whose depletion shows synthetic lethality with V X-680. The top hit from these screens was GSG2 (also known as Haspin) , a serine/threonine kinase that phospho rylates histone H3 at Thr- 3 during mitosis. Moreover, both Haspin knockout and Haspin inhibitor-treated HCT116 cells were hypersensitive to VX-680. Furthermore, we showed that the synthetic lethal interaction between Haspin depletion and V X-680 was mediated by the inhibition of Haspin with Aurora kinase B (AURKB), but not with Aurora kinase A (A URKA). Strikingly, combined inhibition of Haspin and A URKB had a better efficacy than single-agent treatment in both head and neck squamous cell carcinoma and non- small cell lung cancer. Taken together, our findings have uncovered a synthetic lethal interaction between A URKB and Haspin, which provides a strong rationale for this combination therapy for cancer patients.

Keywords: Haspin AURKB NSCLC VX680 CHR6494 barasertib head neck carcinoma

Substances (7) Reactions (0) Citing (11)

75

Aurora B kinase as a therapeutic target in acute lymphoblastic leukemia

By: Goto, Hiroaki ; Yoshino, Yuki; Ito, Mieko; Nagai, Junichi; Kumamoto, Tadashi; Inukai, Takesi; Sakurai, Yukari; Miyagawa, Naoyuki; Keino, Dai; Yokosuka, Tomoko; et al
Cancer Chemotherapy and Pharmacology (2020), 85(4), 773-783 | Language: English, Database: CAplus and MEDLINE

Abstract: Purpose: Acute lymphoblastic leukemia (ALL) is curable with standardized chemotherapy. However, the development of novel therapies is still required, especially for patients with relapsed or refractory disease. By utilizing an in vitro drug screening system, active mol. targeting agents against ALL were explored in this study. Methods: By the in vitro drug sensit ivity test, 81 agents with various actions were screened for their cytotoxicity in a panel of 22 ALL cell lines and ALL clin. samples. The drug effect score (D ES) was calculated from the dose-response of each drug for comparison among drugs or samples. Normal peripheral blood mononuclear cells were also applied onto the drug screening to provide the reference control values. The drug combin ation effect was screened based on the Bliss independent model, and validated by the improved isobol ogram method. Results: On sensitivity screening in a cell line panel, barasertib-HQPA which is an active metabolite of barasertib, an aurora B kinase inhibitor, alisertib, an aurora A kinase inhibitor, and YM155, a survivin inhibitor, were effective against the broadest range of A LL cells. The DES of barase rtib-HQPA was significantly higher in ALL clin. samples compared to the reference value. There were signif icant correlations in DES between barasertib-HQPA and vincristine or docetaxel. In the drug combin ation assay, barasertib-HQPA and eribulin showed additive to synergistic effects. Conclusion: Aurora B kinase was identified to be an active therap eutic target in a broad range of A LL cells. Combination therapy of barasertib and a microtubule-targeting drug is of clin. interest.

Keywords: barasertib alisertib antitumor aurora B kinase inhibitor lymphoblastic leukemia; Acute lymphoblastic leukemia; Aurora B kinase; Barasertib; In vitro drug sensitivity test; Survivin

Substances (10) | Reactions (0) | Citing (4)

76

Methods of treatment of cancer comprising CDC7 inhibitors

By: Hassig, Christian Andrew; Hansen, Ryan James; Milutinovic, Snezana; Strouse, Bryan William
World Intellectual Property Organization, WO2020068347 A1 2020-04-02 | Language: English, Database: CAplus

Herein disclosed are methods of treatment administering SRA141 as a monotherapy or in a combination therapy useful for inhibiting the growth of tumors such as those in patients with cancer.

Keywords: treatment cancer CDC7 inhibitor SRA141

PatentPak available

Substances (14) | Reactions (0) | Citing (0)

77

Pan-transcriptome-based candidate therapeutic discovery for idiopathic pulmonary fibrosis

By: Wang, Yunguan; Yella, Jaswanth K.; Ghandikota, Sudhir; Cherukuri, Tejaswini C.; Ediga, Harshavardhana H.; Madala, Satish K.; Jegga, Anil G.
Therapeutic Advances in Respiratory Disease (2020), 14, 1753466620971143 | Language: English, Database: CAplus and MEDLINE

Two methods were used to calculate IPF-compound connectivity: gene expres sion-based connectivity and feature-based connect ivity. Identified compounds were further priori tized if their shared mechanism(s) of action were I PF-related. We found 77 compounds as potential candidate therapeutics for IPF. Of these, 39 compounds are either F DA-approved for other diseases or are currently in phase II/III clin. trials suggesting their repurp osing potential for I PF. Among these compounds are multiple receptor kinase inhibitors, aurora kinase inhibitor (barasertib), epidermal growth factor receptor inhibitors, calcium channel blocker (verapamil), phosphodiesterase inhibitors (roflumilast, sildenafil), PPAR agonists (pioglitazone), histone deacetylase inhibitors (entinostat), and opioid receptor antago nists (nalbuphine). As a proof of concept, we performed in vitro valida tions with verapamil using lung fibroblasts from I PF and show its potential benefits in pulmonary fibrosis. As about half of the candidates discovered in this study are either FDA-approved or are currently in clin. trials for other diseases, rapid transl ation of these compounds as potential IPF therapeutics is possible. Further, the integr ative connectivity anal. framework in this study can be adapted in early phase drug discovery for other common and rare diseases with transcriptomic profiles. The reviews of this paper are available via the supplemental material section.

Keywords: idiopathic pulmonary fibrosis transcriptome therapy; IPF; computational drug discovery; drug discovery; drug reposit ioning; idiopathic pulmonary fibrosis; pulmonary fibrosis; verapamil

Substances (12) Reactions (0) Citing (3)

78

Aurora kinase as target for treating, preventing or curing HIV infection or aids

By: Este Araque, Jose Andres; Garcia Vidal, Edurne; Ballana Guix, Ester; Badia Corcoles, Roger; Riveira Munoz, Eva
World Intellectual Property Organization, WO2020049208 A1 2020-03-12 | Language: Spanish, Database: CAplus

The present invention relates to the use of Aurora kinase (AURK) inhibitors for treating or preventing an H IV infection or AIDS in a subject. The present invention further relates to compositions containing AURK inhibitors and combin ations of AURK inhibitors with other active agents useful for treating or preventing an HIV infection or AIDS in a subject.

Keywords: aurora kinase inhibitors antiviral HIV AIDS

PatentPak available

Substances (179) Reactions (0) Citing (0)

79

Identifying cancer driver genes from functional genomics screens

By: Trupti, Togar; Sanket, Desai; Rohit, Mishra; Prachi, Terwadkar; Manoj, Ramteke; Malika, Ranjan; Dhananjay, Kawle; Bikram, Sahoo; Ankita, Pal; Pawan, Upadhyay; et al
Swiss Medical Weekly (2020), 150(Feb.), w20195 | Language: English, Database: CAplus and MEDLINE

With the emerging advances made in genomics and functional genomics approaches, there is a critical and growing unmet need to integrate plural datasets in order to identify driver genes in cancer. An integrative approach, with the convergence of multiple types of genetic evidence, can limit false positives through a posterior filtering strategy and reduce the need for multiple hypothesis testing to identify true cancer vulnerabilities. We performed a pooled shRNA screen against 906 human genes in the oral cancer cell line AW13516 in triplicate. The genes that were depleted in the screen were integrated with copy number alteration and gene expression data and ranked based on ROAST anal., using an integrative scoring system, DepRanker, to compute a Rank Impact Score (RIS) for each gene. The RIS-based ranking of candidate driver genes was used to identify the putative oncogenes AURKB and TK1 as essential for oral cancer cell proliferation. We validated the findings, showing that shRNA mediated genetic knockdown of TK1 or pharmacol. inhibition of AURKB by AZD-1152 HQPA in AW13516 cells could significantly impede their proliferation. Next we analyzed alterations in AURKB and TK1 genes in head and neck cancer and their association with prognosis using data on 528 patients obtained from TCGA. Patients harbouring alterations in AURKB and TK1 genes were associated with poor survival. To summarise, we present DepRanker as a simple yet robust package with no third- party dependencies for the identification of potential driver genes from a pooled shRNA functional genomic screen by integrating results from RNAi screens with gene expression and copy number data. Using DepRanker, we identify AURKB and TK1 as potential therapeutic targets in oral cancer.

Substances (0) | Reactions (0) | Citing (24)

80

Treatments for acute myeloid leukemia in the presence of gene mutations such as MPM1

By: Tyner, Jeffrey W.; Tognon, Cristina; Druker, Brian J.; Bottomly, Daniel; Wilmot, Beth; Kurtz, Stephen E.; Savage, Samantha; Long, Nicola; Reister-Schultz, Anna; Traer, Elie; et al
World Intellectual Property Organization, WO2020023921 A1 2020-01-30 | Language: English, Database: CAplus

The present invention relates to useful methods of treating Acute Myeloid Leukemia (AML) in a human. in the presence of gene mutations such as MPM1.

Keywords: acute myeloid leukemia treatment MPM1 gene mutation

PatentPak available

Substances (65) | Reactions (0) | Citing (1)

81

Structure-based discovery and bioactivity evaluation of novel Aurora-A kinase inhibitors as anticancer agents via docking-based comparative intermolecular contacts analysis (dbCICA)

By: Hijjawi, Majd S. ; Abutayeh, Reem Fawaz ; Taha, Mutasem O.
Molecules (2020), 25(24), 6003 | Language: English, Database: CAplus and MEDLINE

Aurora-A kinase plays a central role in mitosis, where aberrant activation contri butes to cancer by promoting cell cycle progre ssion, genomic instability, epithelial-mesenchymal transition, and cancer stemness. Aurora- A kinase inhibitors have shown encour aging results in clin. trials but have not gained Food and Drug Administration (FDA) approval. An innovative computational workflow named Docking-based Comparative Intermol. Contacts Anal. (db CICA) was applied-aiming to identify novel Aurora-A kinase inhibi tors-using seventy-nine reported Aurora- A kinase inhibitors to specify the best possible docking settings needed to fit into the active-site binding pocket of Aurora- A kinase crystal structure, in a process that only potent ligands contact critical binding- site spots, distinct from those occupied by less-active ligands. Optimal db CICA models were transformed into two corresp onding pharmacophores. The optimal one, in capturing active hits and discarding inactive ones, validated by receiver operating charact eristic anal., was used as a virtual in- silico search query for screening new mols. from the National Cancer Institute database. A fluorescence resonance energy transfer (FRET)-based assay was used to assess the activity of captured mols. and five promising Aurora-A kinase inhibitors were identi fied. The activity was next validated using a cell culture anti- proliferative assay (MTT) and revealed a most potent lead 85(NCI 14040) mol. after 72 h of incuba tion, scoring IC50 values of 3.5-11.0μM against PANC1 (pancreas), PC-3 (prostate), T-47D and MDA-MB-231 (breast)cancer cells, and showing favorable safety profiles (27.5μ M IC50 on fibroblasts). Our results provide new clues for further develo pment of Aurora-A kinase inhibitors as anticancer mols.

Keywords: danusertib CYC116 barasertib GSK1070916 MLN8237 auroraAkinase inhibitor anticancer cancer; AURKA; Aurora-A kinase; MDA-MB-231; MTT assay; PANC1; PC-3; T-47D; aurora inhibitors; docking-based comparative intermolecular contacts analysis; in-silico screening; molecular docking; structure- based drug design

Substances (7) | Reactions (0) | Citing (6)

82

Inhibition of BRD4 triggers cellular senescence through suppressing aurora kinases in oesophageal cancer cells

By: Xu, Jian-Ling ; Yuan, Ya-Jiao; Lv, Jiao; Qi, Di; Wu, Meng-Di; Lan, Jing; Liu, Sheng-Nan; Yang, Yong; Zhai, Jing; Jiang, Han-Ming
Journal of Cellular and Molecular Medicine (2020), 24(22), 13036-13045 | Language: English, Database: CAplus and MEDLINE

Oesophageal cancer is one of the most frequent solid malign ancies and the leading cause of cancer- related death around the world. It is urgent to develop novel therapy strategies to improve patient outcomes. Acetylation modification of histones has been extensively studied in epigen etics. BRD4, a reader of acetylated histone and non- histone proteins, has involved in tumorig enesis. It has emerged as a promising target for cancer therapy. BRD4 inhibitors, such as JQ1, have exerted efficacious anti-proliferation activities in diverse cancers. However, the effects of JQ1 on oesoph ageal cancer are still not fully described. Here, we demons trate that JQ1 suppresses cell growth and triggers cellular senescence in K YSE450 cells. Mechanistically, JQ1 up-regulates p21 level and decreases cyclin D1 resulting in G1 cycle arrest. The inhibitory effects of JQ1 on KYSE450 cells are independent on apoptosis. It activates cellular senescence by increasing SA-β-gal activity. BRD4 knockdown by sh RNA recapitulates cellular senescence. We also display that administration of JQ1 decreases recrui tment of BRD4 on the promoter of aurora kinases A and B. Inhibitors targeting at AURKA/B phenocopy JQ1 treatment in KYSE450 cells. These results identify a novel action manner of B RD4 in oesophageal cancer, which strengthens JQ1 as a candidate drug in oesoph ageal cancer chemotherapy.

Keywords: Inhibition BRD4 triggers cellular senescence aurora kinases oesoph ageal cancer; BRD4; aurora kinase; cellular senesc ence; oesoph ageal cancer

Substances (8) | Reactions (0) | Citing (3)

83

High-throughput compound screen reveals mTOR inhibitors as potential therapeutics to reduce (auto)antibody production by human plasma cells

By: Tuijnenburg, Paul ; aan de Kerk, Daan J.; Jansen, Machiel H.; Morris, Ben; Lieftink, Cor; Beijersbergen, Roderick L.; van Leeuwen, Ester M. M.; Kuijpers, Taco W.
European Journal of Immunology (2020), 50(1), 73-85 | Language: English, Database: CAplus and MEDLINE

Antibody production by the B cell compartment is a crucial part of the adaptive immune response. Dysreg ulated antibody production in the form of autoantibodies can cause autoimmune disease. To date, B- cell depletion with anti-CD20 antibodies is commonly applied in autoimmunity, but pre-existing plasma cells are not eliminated in this way. Altern ative ways of more selective inhibition of antibody production would add to the treatment of these autoimmune diseases. To explore novel therapeutic targets in signaling pathways essential for plasmablast formation and/or Ig produc tion, we performed a compound screen of almost 200 protein kinase inhibitors in a robust B-cell differen tiation culture system. This study yielded 35 small cell- permeable compounds with a reproducible inhibitory effect on B- cell activation and plasma blast formation, among which was the clin. applied mammalian target of rapamycin (mTOR) inhibitor rapamycin. Two addnl. compounds targeting the phosphoi nositide 3-kinase-AKT-mTOR pathway (BKM120 and WYE-354) did not affect prolife ration and plasmablast formation, but specif ically reduced the Ig production With this compound screen we successfully applied a method to investigate therapeutic targets for B- cell differen tiation and identified compounds in the phosphoinositide 3-kinase-AKT-mTOR pathway that could specif ically inhibit Ig production only. These drugs may well be explored to be of value in current B-cell-depleting treatment regimens in autoimmune disorders.

Keywords: IgM IgA activation differen tiation plasmablast rapamycin; B cells; B- cell activation and differen tiation; PI3K-AKT-mTOR; autoimmune disease; plasma cells

Substances (63) Reactions (0) Citing (9)

84

Synthesis of Aurora-B Kinase Inhibitor AZD1152-HQPA

By: Wang Li-li; Wang Xing-lan; Ma Cai-yu; Cheng Fei; Mao Yuan-hu; Tang Lei; Zhang Ji-quan
Guangzhou Huagong (2020), 48(8), 47-49 | Language: Chinese, Database: CAplus

AZD1152-HQPA is the prodrug of Aurora-B kinase selective inhibitor AZD1152(Barasertib) bearing a quinaz oline scaffold and this study presents the optimization of the synthetic route. Starting from 2- amino-4-fluorobenzoic acid, AZD1152-HQPA was synthe sized via cyclization, substitution, chloridi zation, ammoniation, amidation and final substit ution. The title compound was obtained with a total yield of 27.6% and purity of more than 99%. The optimization of the amidating condensation reaction made this synthetic route more materials-accessible, operations-handy and cost-effective, and it was suitable for the scale-up preparation of AZD1152-HQPA.

Substances (0) Reactions (0) Citing (0)

85

AURKB promotes gastric cancer progression via activation of CCND1 expression

By: Nie, Min; Wang, Yadong; Yu, Zenong; Li, Xinyu; Deng, Yexuan; Wang, Ying; Yang, Dongjun; Li, Qixiang; Zeng, Xiangwei; Ju, Junyi; et al
Aging (2020), 12(2), 1304-1321 | Language: English, Database: CAplus and MEDLINE

Aurora kinase B (AURKB) triggers the phosphorylation of serine 10 on histone H3 (H3S10ph), which is important for chromosome condensation and cytokinesis during mitosis in mammals. However, how exactly AURKB controls cell cycle and contributes to tumorigenesis as an oncoprotein under pathol. conditions remains largely unknown. Here, we report that AURKB promotes gastric cancer cell proliferation in vitro and in vivo. Silencing AURKB expression inhibits gastric cell proliferation and arrests the cell cycle in G_2/M phase. We demonstrate that cyclin D1 (CCND1) is a direct downstream target of AURKB that plays a key role in gastric cancer cell proliferation. AURKB is able to activate the expression of CCND1 through mediating H3S10ph in the promoter of the CCND1 gene. Furthermore, we show that AZD1152, a specific inhibitor of AURKB, can suppress the expression of CCND1 in the gastric cancer cells and inhibit cell proliferation in vitro and in vivo. Importantly, we found that high AURKB and CCND1 expression levels are correlated with shorter overall survival of gastric cancer patients. This study demonstrates that AURKB promotes gastric tumorigenesis potentially through epigenetically activating CCND1 expression, suggesting AURKB as a promising therapeutic target in gastric cancer.

Keywords: AURKB; CCND1; H3S10ph; gastric cancer; proliferation

Substance (1) Reactions (0) Citing (32)

86

The Correlation between Expression Level of Aurora B and Occurrence of Cervical Cancer

By: Peng Ruijuan; Song Dan; Mayire Nu'er'aihe'maiti; Amutika'ri Ma'muti; Mayi'nu'er Niyazi; Ma Zhenghai
Jiyinzuxue Yu Yingyong Shengwuxue (2020), 39(5), 417-423 | Language: Chinese, Database: CAplus

To investigate the expression level of Aurora B in cervical cancer tissues and the inhibitory effect of Aurora B on the proliferation, invasion and migration of cervical cancer cells. cervical cancer tissues and uterine fibroid tissues(control) without radiotherapy and chemotherapy were collected(control), tissues total RNA was extracted, the expression level of Aurora B in cervical cancer tissues was detected by RFQ-PCR, and the correlation between Aurora B and occurrence of cervical cancer was analyzed. Si Ha, HeLa and 293 FT cells(control) were treated with different doses of Aurora B inhibitor AZD1152-HQPA. The inhibition rate of cell proliferation were analyzed by CCK-8 cell proliferation assay. Transwell invasion assay and Transwell migration assay were used to detect cell invasion and migration ability. the expression of Aurora B mRNA in cervical cancer(0.003 88±0.000 560) was obviously higher ($p<0.01$) than that in uterine fibroids(0.001 03±0.000 161) and the expression was increased by 3.76 fold. When AZD1152-HQPA inhibited Aurora B kinase activity, the number of invasive cells and migratory cells in cervical cancer cell lines were decreased obviously than those in control group($p<0.01$); and the concentration of AZD1152-HQPA was increased from 50 nmoL to 100 nmo L, The number of invasive cells and the number of migrated cells significantly decreased($p<0.01$). The expression level of Aurora B in cervical cancer tissue is significantly increased, which may promote the occurrence of tumor by promoting the proliferation, invasion and migration of cancer cells, and is a potential target for the diagnosis and treatment of cervical cancer.

Substances (0) Reactions (0) Citing (0)

87

RING1B recruits EWSR1-FLI1 and cooperates in the remodeling of chromatin necessary for Ewing sarcoma tumorigenesis

By: Sanchez-Molina, Sara; Figuerola-Bou, Elisabet ; Blanco, Enrique ; Sanchez-Jimenez, Maria; Taboas, Pablo; Gomez, Soledad; Ballare, Cecilia; Garcia-Dominguez, Daniel J.; Prada, Estela; Hontecillas-Prieto, Lourdes; et al
Science Advances (2020), 6(43), eaba3058 | Language: English, Database: CAplus and MEDLINE

Ewing sarcoma (EwS) is an aggressive tumor that affects adoles cents and young adults. Ew S is defined by a chromo somal translo cation, EWSR1-FLI1 being the most common, that causes genome reprogr amming through remodeling of enhancers. Here, we describe an unexpected function of RING1B, which is highly expressed in Ew S. While retaining its repressive activity at Polycomb developmental regulated genes, RING1B colocalizes with EWSR1-FLI1 at active enhancers. We demons trate that RING1B is necessary for the expression of key EWSR1-FLI1 targets by facili tating oncogene recruitment to their enhancers. Knockdown of R IN G1B impairs growth of tumor xenografts and expression of genes regulated by E WSR1-FLI1 bound enhancers. Pharmacol. inhibition of AURKB with AZD1152 increases H2Aub levels causing down-regulation of RING1B/EWSR1-FLI1 common targets. Our findings demonstrate that RING1B is a critical modulator of E WSR1- FLI1-induced chromatin remodeling, and its inhibition is a potential therapeutic strategy for the treatment of these tumors.

Substances (0) Reactions (0) Citing (15)

88

Targeting aurora kinase B alleviates spinal microgliosis and neuropathic pain in a rat model of peripheral nerve injury

By: Shen, Yu; Ding, Zhuofeng; Ma, Shengyun; Zou, Yu; Yang, Xin; Ding, Zijin; Zhang, Yu; Zhu, Xiaoyan; Schaefer, Michael K. E.; Guo, Qulian; et al
Journal of Neurochemistry (2020), 152(1), 72-91 | Language: English, Database: CAplus and MEDLINE

Peripheral nerve injury elicits spinal microgliosis, contributing to neuropathic pain. The aurora kinases A (AURKA), B (AURKB), and C (AURKC) are potential therapeutic targets in prolife rating cells. However, their role has not been clarified in microglia. The aim of this study was to examine the regulation of aurora kinases and their roles and druggability in spinal microgliosis and neuropathic pain. Sprague-Dawley rats received chronic constriction injury (CCI). Gene expression of aurora kinases A-C was evaluated by quant. RT-PCR and western blot, resp., in spinal cords at 1, 3, 7, and 14 days after C CI. AURKB gene and protein expression was up-regulated concomitantly with the development of spinal microg liosis and neuropathic pain. Using lentiviral over- expression and adeno-associated viral knockdown approa ches, the function of AURKB was further investigated by western blot, immunohis tochem., RNA sequencing, and pain behavior tests. We found that A URKB over-expression in naive rats caused spinal microg liosis and pain hypersensitivity, whereas AURKB knockdown reduced microg liosis and alleviated CCI-induced neuropathic pain. Accord ingly, RNA sequencing data revealed down- regulation of genes critically involved in signaling pathways associated with spinal microgliosis and neuropathic pain after AURKB knockdown in CCI rats. To examine its therap eutic potential for treatment of neurop athic pain, animals were treated intrath ecally with the pharmacol. AURKB inhibitor AZD1152 -HQPA resulting in the alleviation of CC I-induced pain. Taken together, our findings indicated that A URKB plays a critical role in spinal microg liosis and neuropathic pain. Targeting AURKB may be an efficient method for treatment of neurop athic pain subsequent to peripheral nerve injury. Open science badges : This article has received a badge for *Open Materials* because it provided all relevant information to reproduce the study in the manuscript. The complete Open Science Disclosure form for this article can be found at the end of the article. More information about the Open Practices badges can be found at .

Keywords: target aurora kinase spinal microgliosis neuropathic pain nerve injury; aurora kinases; microgl iosis; neuropathic pain; peripheral nerve injury; spinal cord

Substances (2) Reactions (0) Citing (9)

89

AURKB as a target in non-small cell lung cancer with acquired resistance to anti-EGFR therapy

By: Bertran-Alamillo, Jordi ; Cattan, Valerie; Schoumacher, Marie ; Codony-Servat, Jordi; Gimenez-Capitan, Ana; Cantero, Frederique; Burbridge, Mike; Rodriguez, Sonia; Teixido, Cristina; Roman, Ruth; et al
Nature Communications (2019), 10(1), 1-14 | Language: English, Database: CAplus and MEDLINE

Non-small cell lung cancer (NSCLC) tumors harboring mutations in EGFR ultimately relapse to therapy with EGFR tyrosine kinase inhibitors (EGFR TKIs). Here, we show that resistant cells without the p. T790 M or other acquired mutations are sensitive to the Aurora B (AURKB) inhibitors barasertib and S49076. Phospho-histone H3 (pH3), a major product of AURKB, is increased in most resistant cells and treatment with AURKB inhibitors reduces the levels of pH3, triggering G1/S arrest and polyploidy. Senescence is subsequently induced in cells with acquired mutations while, in their absence, polyploidy is followed by cell death. Finally, in NSCLC patients, pH3 levels are increased after progression on EGFR TKIs and high pH3 baseline correlates with shorter survival. Our results reveal that AURKB activation is associated with acquired resistance to EGFR TKIs, and that AURKB constitutes a potential target in NSCLC progressing to anti-EGFR therapy and not carrying resistance mutations.

Keywords: lung cancer barasertib gefitinib anticancer AURKB point mutation

Substances (9) Reactions (0) Citing (73)

90

Aurora A-selective inhibitor LY3295668 Leads to dominant mitotic arrest, apoptosis in cancer cells, and shows potent preclinical antitumor efficacy

By: Du, Jian; Yan, Lei; Torres, Raquel; Gong, Xueqian; Bian, Huimin; Marugan, Carlos; Boehnke, Karsten; Baquero, Carmen; Hui, Yu-Hua; Chapman, Sonya C.; et al
Molecular Cancer Therapeutics (2019), 18(12), 2207-2219 | Language: English, Database: CAplus and MEDLINE

Although Aurora A, B, and C kinases share high sequence similarity, especially within the kinase domain, they function distinctly in cell-cycle progression. Aurora A depletion primarily leads to mitotic spindle formation defects and consequently prometaphase arrest, whereas Aurora B/C inactivation primarily induces polyploidy from cytokinesis failure. Aurora B/C inactivation phenotypes are also epistatic to those of Aurora A, such that the concomitant inactivation of Aurora A and B, or all Aurora isoforms by noniso form-selective Aurora inhibitors, demonstrates the Aurora B/C-dominant cytokinesis failure and polyploidy phenotypes. Several Aurora inhibitors are in clin. trials for T/B-cell lymphoma, multiple myeloma, leukemia, lung, and breast cancers. Here, we describe an Aurora A-selective inhibitor, LY3295668, which potently inhibits Aurora autophosphorylation and its kinase activity in vitro and in vivo, persistently arrests cancer cells in mitosis, and induces more profound apoptosis than Aurora B or Aurora A/B dual inhibitors without Aurora B inhibition-associated cytokinesis failure and aneuploidy. LY3295668 inhibits the growth of a broad panel of cancer cell lines, including small-cell lung and breast cancer cells. It demonstrates significant efficacy in small-cell lung cancer xenograft and patient-derived tumor preclin. models as a single agent and in combination with standard-of-care agents. LY3295668, as a highly Aurora A-selective inhibitor, may represent a preferred approach to the current pan- Aurora inhibitors as a cancer therapeutic agent.

Keywords: cancer cell aurora mitotic arrest antitumor efficacy

Substances (7) Reactions (0) Citing (21)

91

Salivary glands require Aurora Kinase B for regeneration after transient innate immune-mediated injury

By: Shaalan, Abeer ; Proctor, Gordon

Scientific Reports (2019), 9(1), 1-13 | Language: English, Database: CAplus and MEDLINE

The present study examined the initial mitogenic events marking the regenerative response of the murine submandibular gland (SMG), following innate immune-mediated injury. Local intraductal administration of the synthetic double stranded (ds) RNA polyinosinic-polycytidylic acid (poly (I: C)) widely, but transiently, depleted the acinar and progenitor cells, 24 h post poly (I: C) introduction. While the progenitor and duct cells started to proliferate and expand at 72 h, the Mist1- positve acinar cells did not re-appear until 96 h post poly (I:C) injury. The cellular replenishment during regeneration involved significant upregulation of the cell cycle promoter Aurora kinase B (AURKB). AURKB, which is expressed in healthy proliferating and cancerous cells, is a serine/threonine protein kinase, well known to orchestrate key events in cell division and cytokinesis. However, the expression and role of AURKB in regeneration of post mitotic salivary gland cells has not been previously explored. In vivo inhibition of AURKB using the selective inhibitor Barasertib (AZD1152-HQPA) interfered with SMG recovery from the transient, but severe poly (I: C)-mediated injury and cellular depletion. AURKB deficiency during regeneration of the injured tissues: disrupted cell cycle progression, repressed renewal of Mist1-pos. acinar cells and prevented recovery of salivary secretion.

Keywords: salivary gland progenitor acinar duct cell aurora kinase B

Substances (4) Reactions (0) Citing (4)

92

Screening methods for identifying pharmaceutical compounds and uses thereof for treating cancer

By: Klug, Christopher

World Intellectual Property Organization, WO2019241636 A1 2019-12-19 | Language: English, Database: CAplus

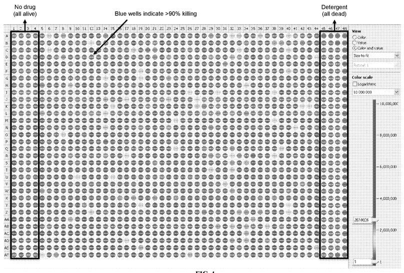

FIG. 1

Provided herein is a method for identifying compounds that inhibit cell growth or have cytotoxic activity against cancer cells, such as hematol. cancer cells or ovarian cancer cells, in a subject. Further provided is a method for identifying one or more compounds that sensitize refractory cancer cells from a subject. Also provided are compositions and methods for treating cancer, including refractory cancer, such as refractory acute myeloid leukemia (AML) or ovarian cancer.

Keywords: antitumor drug screening acute myeloid leukemia ovarian cancer treatment

PatentPak available

Substances (414) Reactions (0) Citing (0)

93

Analytical method considerations regarding carryover for monophosphate prodrugs for in vivo samples by liquid chromatography-tandem mass spectrometry

By: Heinle, Lance; Patel, Hardikkumar; Jenkins, Gary; Ruterbories, Kenneth
Journal of Chromatography A (2019), 1606, 460379 | Language: English, Database: CAplus and MEDLINE

Three different components that impact carryover in a liquid chromatog.-tandem mass spectrometry (LC-MS/MS) method were evaluated to establish baseline conditions for analyzing in vivo samples for twelve monophosphate prodrug compounds and their corresponding parent compounds The three components were: wash solvent modifier, column shell material (metal vs. metal free) , and tubing composition These components were tested for their impact on system carryover by using rat plasma extracted samples. It was determined that a wash solution containing hexylamine yielded the lowest average carryover of the solutions tested. In addition, metal free columns and PEEK (poly ether ether ketone) tubing yielded the lowest carryover when compared to metal columns, stainless steel tubing and nickel tubing. These conditions were also tested against the parent mols. for each prodrug in the test set, to ensure that changing the conditions for the prodrugs did not impact the ability to analyze the parent, since there is typically a desire to measure both compounds in study samples. Under all conditions, the carryover of the corresp onding parent mol. was not adversely impacted in these studies.

Keywords: monophosphate prodrugs carryover LC tandem MS PEEK tubing; Carryover; Liquid chromato graphy-tandem mass spectrometry; Monophosphate prodrugs; PEEK tubing

Substances (5)   Reactions (0)   Citing (1)

94

Bifunctional small molecules to target the selective degradation of circulating proteins

By: Spiegel, David; Caianiello, David; Zhang, Mengwen
World Intellectual Property Organization, WO2019199634 A1 2019-10-17 | Language: English, Database: CAplus

The present invention is directed to bifunctional small mols. which contain a circul ating protein binding moiety (CPBM) linked through a linker group to a cellular receptor binding moiety (CRBM) which is a membrane receptor of degrading cell such as a hepatocyte or other degrading cell. In embodiments, the (CRBM) is a moiety which binds to asialogly coprotein receptor (an asialogly coprotein receptor binding moiety, or ASGPRBM) of a hepato cyte. In addnl. embodiments, the (CRBM) is a moiety which binds to a receptor of other cells which can degrade proteins, such as a LRP1, LDLR, FcγRI, FcRN, Transferrin or Macrophage Scavenger receptor. Pharmaceutical compositions based upon these bifunc tional small mols. represent an addnl. aspect of the present invention. These compounds and/or compositions may be used to treat disease states and conditions by removing circul ating proteins through degradation in the hepatocytes or macrophages of a patient or subject in need of therapy. Methods of treating disease states and/or conditions in which circulating proteins are associated with the disease state and/or condition are also described herein.

Keywords: circulating protein targeting bifunc tional agent cancer autoimmune disease inflam mation

PatentPak available   Substances (881)   Reactions (2,119)   Citing (3)

95

Preparation of cereblon E3 ligase ligands and bifunctional PROTAC compounds for targeting and degradation of androgen receptor, estrogen receptor alpha, bromodomain-containing protein 4, and BRaf

By: Crew, Andrew P.; Crews, Craig M.; Dong, Hanqing; Hornberger, Keith R.; Wang, Jing; Qian, Yimin; Zimmermann, Kurt; Berlin, Michael; Snyder, Lawrence B.

World Intellectual Property Organization, WO2019199816 A1 2019-10-17 | Language: English, Database: CAplus

FIG. 1B

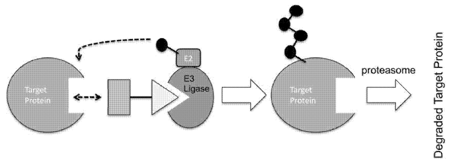

The invention relates to cereblon E3 ligase binding compounds, including bifunctional compounds comprising them, which find utility as modulators of targeted ubiquitination, especially inhibitors of a variety of polype ptides and other proteins which are degraded and/or otherwise inhibited by bifunctional compounds according to the present disclo sure. In particular, the invention provides compounds, which contain on one end a ligand which binds to the cereblon E3 ubiquitin ligase and on the other end a moiety which binds a target protein such that the target protein is placed in proximity to the ubiquitin ligase to effect degradation (and inhibition) of that protein. Specifically, the invention relates to bifunctional compounds CLM-L-PTM [PTM = small mol. containing a protein targeting moiety that binds bromodomain-containing protein 4, estrogen receptor (E R), or androgen receptor or BRaf; L = a bond, a chem. linking moiety covalently coupling the C LM and the PTM; CLM = a small mol. cereblon E3 ubiquitin ligase binding moiety that binds or targets a cereblon E3 ubiquitin ligase], their pharmaceutically acceptable salts, enantiomers, stereoi somers, solvates, polymorphs and prodrugs. Exempl ified compound **I** was prepared from a 12 step procedure (prepar ation given). The invention exhibits a broad range of pharmacol. activities associated with compounds according to the present invention, consistent with the degradation/inhibition of targeted polypeptides of nearly any type (data given). Diseases or disorders that result from aggregation or accumulation of the target protein are treated or prevented with compounds and compos itions of the invention.

Keywords: cereblon ligand chimeric mol preparation targeted protein degradation ubiquitination; PROTAC modulator preparation targeted ubiquitination estrogen receptor ERRa degradation; androgen receptor PROTAC degradation inhibition preparation antiproliferative; BRD4 BRaf PROTAC degradation inhibition cereblon E3 ligase antitumor

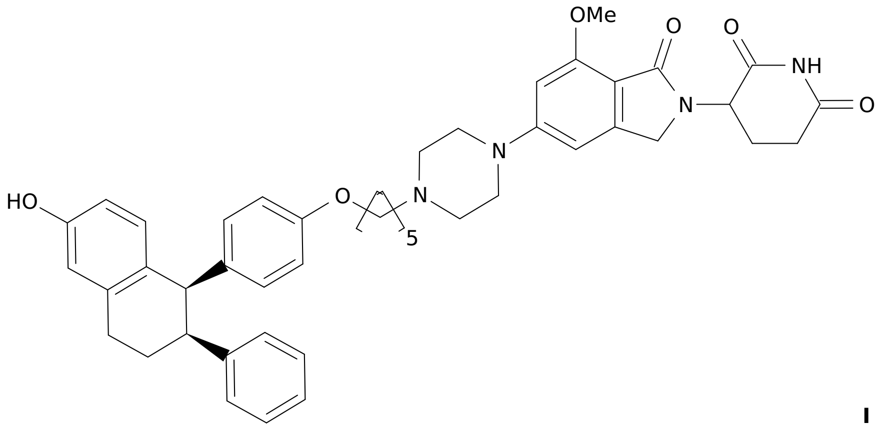

I

PatentPak available

Substances (727)

Reactions (1,470)

Citing (5)

96

Bi-functional molecules to degrade circulating proteins

By: Spiegel, David; Caianiello, David
World Intellectual Property Organization, WO2019199621 A1 2019-10-17 | Language: English, Database: CAplus

The present invention is directed to bi-functional compounds which find use as pharmaceutical agents in the treatment of disease states and/or conditions which are through macrophage migration inhibitory factor (MIF) or immunoglubin G (IgG). The present invention is also directed to pharmaceutical compositions which comprise these bi- functional compounds as well as methods for treating disease states and/or conditions which are mediated through MIF/IgG or where MIF/IgG is a contributing factor to the development and perpetuation of diseases and/or conditions, especially including autoimmune diseases and cancer, among others. The purpose of the present invention is to provide a mol. strategy to lower plasma MIF/IgG level in patients with autoimmune diseases or certain types of cancers. The b.i -functional mol. construct is comprised of a M IF/IgQ-targeting motif, that is derived from small mol. MIF/IgG ligands, and an ASGPr- targeting motif that binds to hepatocyte asialogly coprotein receptor (ASGPr). The compounds selectively bind MIF or IgG in plasma and subsequently engage the endo-lysosomal pathway of hepatocytes through AS GPr. As a conseq uence, MIF/igG is internalized and degraded by hepato cytes, thus resulting in potential attenu ation of corresp onding disease symptoms which are modulated through MIF/igG.

Keywords: bifunctional circulating protein migration inhibitory factor immunoglubin G cancer; autoimmune diseases circulating protein migration inhibitory factor immunoglubin G

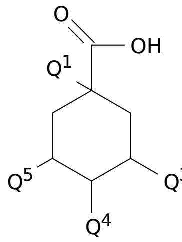

I

PatentPak available

Substances (97) Reactions (368) Citing (5)

97

Treating tumors with an alternating electrical field and an Aurora kinase inhibitor

By: Krex, Dietmar; Temme, Achim; Shnaiderman, Rosa S.; Giladi, Moshe
United States, US20190307781 A1 2019-10-10 | Language: English, Database: CAplus

The viability of cancer cells can be reduced by administering an Aurora kinase inhibitor (e.g., M LN8237 or another Aurora A kinase inhibitor, AZD1152 or another Aurora B kinase inhibitor) to the cancer cells, and applying an altern ating elec. field with a frequency between 100 and 300 kHz (e.g., 200 kHz) to the cancer cells. Furthermore, cancer (e.g., glioblastoma) in a subject may be treated by administering an Aurora kinase inhibitor to the subject, and applying an alternating elec. field with frequency between 100 and 300 kHz (e.g., 200 kHz) to a target region of the subject (e.g., the brain) .

Keywords: cancer treatment alternating elec field Aurora kinase inhibitor combination

PatentPak available

Substances (9) Reactions (0) Citing (2)

98

Selective inhibition of Aurora A and B kinases effectively induces cell cycle arrest in t(8;21) acute myeloid leukemia

By: Qi, Jialei; Gao, Xiang; Zhong, Xiaomin; Zhang, Ninghan; Wang, Rong; Zhang, Huihui; Pan, Ting; Liu, Xuejiao; Yao, Yao; Wu, Qingyun; et al

Biomedicine & Pharmacotherapy (2019), 117, 109113 | Language: English, Database: CAplus and MEDLINE

The fusion gene AML1-ETO initially dysregulates various cell cycle mols. in t(8;21) acute myeloid leukemia. Aurora kinases have shown great promise in treating tumors. However, the efficacy of Aurora kinase (AURK) A and B inhibition in t(8;21) AML remains unclear. We found that AURK-A inhibitor Alisertib and AURK-B inhibitor Barasertib strongly inhibited the growth and proliferation of t(8;21) AML cells. The quantity and size of cell colonies were markedly decreased after a 14- d drug exposure. The cell cycle distribution was blocked at the G2/M phase in both dose- and time- dependent manner. The expression of p53 family and cdc2- p34 significantly changed as well. Notably, we found that t (8;21) AML cells are more sensitive to Aurora B inhibition. In each set of experiments, Barasertib took less time or a lower concentration to achieve similar efficacy. Taken together, our data highlighted the potential role of Aurora kinases as promising cell cycle targets for the treatment of t(8;21) AML and hereby provided a theor. basis to guide relevant clin. trials.

Keywords: alisertib barasertib anticancer AURKA AURKB inhibitor acute myeloid leukemia; AML-ETO; Acute myeloid leukemia; Alisertib; Aurora kinase; Barasertib

Substances (3) Reactions (0) Citing (7)

99

Inductive regeneration of the airway by transcriptional factor modulation of glandular myoepithelial stem cells using modulators of Lef-1/TCF/Wnt signaling

By: Engelhardt, John F.; Lynch, Thomas J.; Anderson, Preston J.; Shahin, Weam

World Intellectual Property Organization, WO2019178267 A2 2019-09-19 | Language: English, Database: CAplus

Compositions and methods to modulate Lef-1/TCF/Wnt signaling ex vivo or in vivo, and assays to detect those modulators, are described. The disclosure provides a composition comprising an isolated transcription factor (Lymphoid enhancer factor 1 or Lef-1) that when introduced to or induced in a specific airway stem cell leads to self-limiting regenerative expansion of the airway and submucosal glands. Using genetically engineered mice, Lef-1 expression in glandular myoepithelial cells (MECs) was shown to either enhance airway repair following injury (with monoallelic Lef-1 expression) or spontaneously induce MEC-mediated airway regeneration (with biallelic Lef-1 expression). These findings open the door to enhancing the regenerative capacity of MECs ex vivo using cell therapy approaches and in vivo using small mols. that influence Lef-1 function and expression.

Keywords: airway regeneration transcription factor modulator glandular myoepithelium stem cell

PatentPak available

Substances (1,589) Reactions (0) Citing (0)

100

Divergent Polypharmacology-Driven Cellular Activity of Structurally Similar Multi-Kinase Inhibitors through Cumulative Effects on Individual Targets

By: Sumi, Natalia J.; Ctortecka, Claudia; Hu, Qianqian; Bryant, Annamarie T.; Fang, Bin; Remsing Rix, Lily L.; Ayaz, Muhammad; Kinose, Fumi; Welsh, Eric A.; Eschrich, Steven A.; et al
Cell Chemical Biology (2019), 26(9), 1240-1252.e11 | Language: English, Database: CAplus and MEDLINE

Despite recent successes of precision and immunotherapies there is a persisting need for novel targeted or multi- targeted approaches in complex diseases. Through a systems pharmacol. approach, including phenotypic screening, chem. and phosphopr oteomics, and RNA-seq, we elucidated the targets and mechanisms underlying the differ ential anticancer activity of two structurally related multi-kinase inhibitors, foretinib, and cabozantinib, in lung cancer cells. Biochem. and cellular target validation using probe mols. and RNAi revealed a polypharmacol. mechanism involving MEK1/2, FER, and AURKB, which were each more potently inhibited by foretinib than cabozantinib. Based on this, we developed a synerg istic combination of foretinib with barasertib, a more potent A URKB inhibitor, for MYC-amplified small-cell lung cancer. This systems pharmacol. approach showed that small structural changes of drugs can cumulatively, through multiple targets, result in pronounced anticancer activity differ ences and that detailed mechan istic understanding of polypharmacol. can enable repurposing opportunities for cancers with unmet medical need. The accession number for the chem. proteomics, tyrosine phosphoproteomics, global phosphoproteomics and ABPP data reported in this paper is PRIDE: PXD012961, PXD012962, PXD012963 and PXD012965.

Keywords: proteome foretinib cabozantinib barasertib anticancer agent synerg istic lung cancer; cabozantinib; chemoproteomics; drug combination; foretinib; kinase inhibitor; lung cancer; phosphopr oteomics; polypharmacology; systems pharmacology; target selectivity

Substances (48) Reactions (0) Citing (10)

101

Optimization and biological evaluation of nicotinamide derivatives as Aurora kinase inhibitors

By: Qi, Baohui; Xu, Xingwei; Yang, Ying; He, Huan; Yue, Xupeng
Bioorganic & Medicinal Chemistry (2019), 27(17), 3825-3835 | Language: English, Database: CAplus and MEDLINE

Aurora kinases are known to be overexpressed in various solid tumors and implicated in oncoge nesis and tumor progression. A series of nicotinamide derivatives were synthesized and their biol. activities were evaluated, including kinase inhibitory activity against Aur A and Aur B and in vitro antitumor activity against SW620, HT-29, NCI-H1975 and Hela cancer cell lines. In addition, the study of antiproliferation, cytotoxicity and apoptosis was performed meanwhile. As the most potent inhibitor of Aur A, 4- ((3-bromo-4-fluorophenyl)amino)-6-chloro-N-(4-((6,7-dimethoxyquinolin-4-yl)oxy)-3-fluorophenyl)nicotinamide (10l) showed excellent antitumor activity against SW620 and NCI-H1975 with IC_{50} values were 0.61 and 1.06 μ M, while the IC_{50} values of reference compound were 3.37 and 6.67 μM, resp. Furthermore, binding mode studies indicated that compound 10l forms better intera ction with Aur A.

Keywords: nicotinamide synthesis antitumor SAR Aurora kinase cancer; Antitumor; Aurora kinases; Kinase inhibi tors; Nicotinamide; SAR

Substances (75) Reactions (130) Citing (3)

102

Aneugen molecular mechanism assay: proof-of-concept with 27 reference chemicals

By: Bernacki, Derek T.; Bryce, Steven M.; Bemis, Jeffrey C.; Dertinger, Stephen D.
Toxicological Sciences (2019), 170(2), 382-393 | Language: English, Database: CAplus and MEDLINE

A tiered bioassay and data anal. scheme is described for elucidating the most common mol. targets respon sible for chem.-induced in vitro aneugenicity: tubulin destabilization, tubulin stabilization, and inhibition of mitotic kinase(s). To evaluate this strategy, T K6 cells were first exposed to each of 27 presumed aneugens over a range of concentrations After 4 and 24 h of treatment, γ H2AX, p53, phospho-histone H3 (p-H3), and polyploidization biomarkers were evaluated using the Multi Flow DNA Damage Assay Kit. The assay identified 27 of 27 chems. as genotoxic, with 25 exhibiting aneugenic signatures, 1 aneugenic and clastogenic, and 1 clasto genic. Subsequently, a newly described follow-up assay was employed to investigate the aneugenic agents' mol. targets. For these experiments, TK6 cells were exposed to each of 26 chems. in the presence of 488 Taxol. After 4 h, cells were lysed and the liberated nuclei and mitotic chromosomes were stained with a nucleic acid dye and labeled with fluore scent antibodies against p-H3 and Ki-67. Flow cytometric analyses revealed that altera tions to 488 Taxol-associated fluorescence were only observed with tubulin binders-increases in the case of tubulin stabilizers, decreases with destabi lizers. Mitotic kinase inhibitors with known Aurora kinase B inhibiting activity were the only aneugens that dramatically decreased the ratio of p-H3-pos. to Ki-67-pos. nuclei. Unsupervised hierarchical clustering based on 488 Taxol fluore scence and p-H3: Ki-67 ratios clearly distinguished compounds with these disparate mol. mechanisms. Furthermore, a classification algorithm based on an artificial neural network was found to effect ively predict mol. target, as leave-one-out cross-validation resulted in 25/26 agreement with a priori expecta tions. These results are encouraging, as they suggest that an adequate number of training set chems., in conjun ction with a machine learning algorithm based on 488 Taxol, p-H3, and Ki-67 responses, can reliably elucidate the most commonly encoun tered aneugenic mol. targets.

Keywords: aneugen mol mechanism assay artificial neural network classification algorithm; Mitotic kinase inhibitors; TK6 cells; aneugen; flow cytometry; spindle poisons

Substances (28) Reactions (0) Citing (9)

103

Methods of treatment of cancer comprising cell division control protein 7 (CDC7) inhibitors

By: Hassig, Christian Andrew; Hansen, Ryan James
World Intellectual Property Organization, WO2019165473 A1 2019-08-29 | Language: English, Database: CAplus

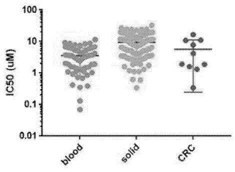

Fig. 3B

Herein disclosed are methods of treatment administering a cell division control protein 7 (C DC7) inhibitor as a monotherapy or in a combination therapy useful for inhibiting the growth of tumors such as those in patients with cancer.

Keywords: antitumor protein CDC7 kinase inhibitor furanone derivative cancer treatment

PatentPak available

Substances (18) Reactions (0) Citing (3)

104

Methods of treating fibrosis using aurora kinase B (AURKB) inhibitors

By: Madala, Satish Kumar; Jegga, Anil Goud; Kasam, Rajesh K.

World Intellectual Property Organization, WO2019161000 A1 2019-08-22 | Language: English, Database: CAplus

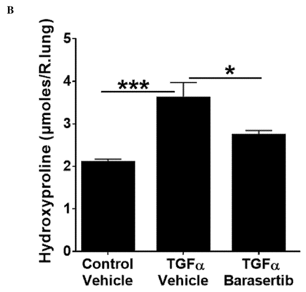

Some embodiments of the invention include methods for treating an animal for fibrosis comprising one or more administ rations of one or more compositions comprising one or more A URKB (Aurora kinase B) inhibitors. Other embodiments of the methods for treating further include other fibrosis treatments. Still other embodi ments of the invention include methods for treating a human for lung fibrosis or idiopathic pulmonary fibrosis, comprising administering one or more compos itions comprising AZD1152 or barasertib. Addnl. embodiments of the invention are also discussed herein.

Keywords: antifibrotic aurora kinase inhibitor barasertib AZD1152 pulmonary fibrosis treatment

PatentPak available

Substances (46) Reactions (0) Citing (0)

105

Cabazitaxel-resistant prostate cancer preventive or therapeutic agent

By: Kosaka, Takeo; Hongo, Hiroshi; Oya, Mototsugu

Japan, JP2019127437 A 2019-08-01 | Language: Japanese, Database: CAplus

The present invention relates to a preventive or therapeutic agent comprising one or more agent selected from a group consisting of pimozide or its salt, an Aurora kinase inhibitor, and a KIF20A inhibitor. The preventive or therap eutic agent comprises a low mol. weight compound that exerts an anticancer effect against cabazitaxel-resistant prostate cancer that can be manufa ctured relatively easily and inexpensively.

Keywords: Aurora kinase inhibitor cabazitaxel resistant prostate cancer antitumor; pimozide cabazi taxel resistant prostate cancer antitumor; KIF20A inhibitor cabazitaxel resistant prostate cancer antitumor

PatentPak available

Substances (5) Reactions (0) Citing (0)

106

Resveratrol modulates the redox-status and cytotoxicity of anticancer drugs by sensitizing leukemic lymphocytes and protecting normal lymphocytes

By: Ivanova, Donika; Zhelev, Zhivko; Semkova, Severina; Aokl, Iciiio; Bakalova, Rumiana
Anticancer Research (2019), 39(7), 3745-3755 | Language: English, Database: CAplus and MEDLINE

Background/Aim: The study is directed to the effect of resver atrol on the redox-status and viability of leukemic and normal lympho cytes, as well as its ability to sensitize leukemic lympho cytes to anticancer drugs. Materials and Methods: Cytoto xicity was analyzed by trypan blue staining, apoptosis - by Annexin V test, and oxidative stress - by the intracellular levels of reactive oxygen species (R O S) and protein-carbonyl products. Results: Incubation of resver atrol in combination with the majority of anticancer drugs resulted in higher toxicity than resveratrol or drug alone. In the case of leukemic lympho cytes treated with barasertib and everolimus in the presence of resveratrol, synergistic cytotoxicity was accompanied by strong induction of apoptosis, increased levels of hydrope roxides and insignificant changes in proteincarbonyl products. None of these parameters changed in normal lympho cytes. Conclu sion: Resveratrol is a promising supplem entary compound for anticancer therapy that may allow reduction of the therap eutic doses of barasertib and everolimus, minimizing their side- effects.

Keywords: leukemic lymphocyte cytotoxicity anticancer resveratrol; Cancer; apoptosis; chemotherapy; oxidative stress; resver atrol

Substances (8) Reactions (0) Citing (14)

107

High-throughput chemical screening identifies focal adhesion kinase and Aurora kinase B inhibition as a synergistic treatment combination in Ewing sarcoma

By: Wang, Sarah; Hwang, Elizabeth E.; Guha, Rajarshi; O'Neill, Allison F.; Melong, Nicole; Veinotte, Chansey J.; Saur, Amy Conway; Wuerthele, Kellsey; Shen, Min; McKnight, Crystal; et al
Clinical Cancer Research (2019), 25(14), 4552-4566 | Language: English, Database: CAplus and MEDLINE

Ewing sarcoma is an aggressive solid tumor malignancy of childhood. Although current treatment regimens cure approx. 70% of patients with localized disease, they are ineffective for most patients with metastases or relapse. New treatment combin ations are necessary for these patients. Ewing sarcoma cells are dependent on focal adhesion kinase (FAK) for growth. To identify candidate treatment combinations for Ewing sarcoma, we performed a small- mol. library screen to identify compounds syneg istic with FAK inhibitors in impairing Ewing cell growth. The activity of a top-scoring class of compounds was then validated across multiple Ewing cell lines in vitro and in multiple xenograft models of Ewing sarcoma. Numerous Aurora kinase inhibitors scored as synergistic with FAK inhibition in this screen. F AK and Aurora kinase B inhibitors synergis tically impair Ewing sarcoma cell viability and signifi cantly inhibit tumor progression. This study provides preclin. support for the conside ration of a clin. trial testing the safety and efficacy of this combination for patients with Ewing sarcoma.

Keywords: Ewing sarcoma FAK Aurora kinase B synergism

Substances (8) Reactions (0) Citing (22)

108

Aurora kinase inhibitor and application of alphavirus in preparing antitumor medicament

By: Yan, Guangmei; Lin, Yuan; Zhu, Wenbo; Zhang, Haipeng; Liang, Jiankai; Cai, Jing; Gong, Shoufang
China, CN109985239 A 2019-07-09 | Language: Chinese, Database: CAplus

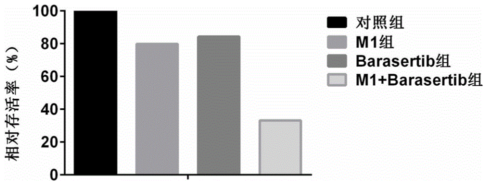

The invention belongs to the field of biomedicine, relates to aurora kinase inhibitor and applic ation of alphavirus in preparing antitumor medicament.The aurora kinase inhibitor disclosed by the invention can be used for preparing an alphavirus antitumor synergist for the first time.The invention also relates to a pharmaceutical composition comprising an aurora kinase inhibitor and an alphavirus, a pharmaceutical kit comprising the aurora kinase inhibitor and the alphav irus, and application of the aurora kinase inhibitor and the alphavirus in treating tumors, in particular tumors insensitive to the alphavirus.

Keywords: aurora kinase inhibitor alphavirus antitumor

PatentPak available

Substances (4) Reactions (0) Citing (0)

109

Use of aurora kinase inhibitor and alphavirus in preparing anti-tumor drug

By: Yan, Guangmei; Lin, Yuan; Zhu, Wenbo; Zhang, Haipeng; Liang, Jiankai; Cai, Jing; Gong, Shoufang
World Intellectual Property Organization, WO2019129234 A1 2019-07-04 | Language: Chinese, Database: CAplus

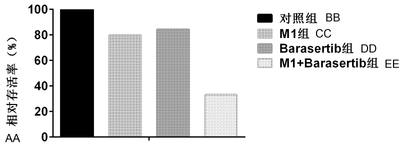

图 1

AA Relative survival rate (%)
BB Control group
CC M1 group
DD Barasertib group
EE M1+Barasertib group

Disclosed is the use of an aurora kinase inhibitor and an alphavirus in preparing an anti-tumor drug. The aurora kinase inhibitor can be used in preparing an alphavirus anti-tumor synergist. Disclosed are a pharmaceutical composition containing the aurora kinase inhibitor and the alphavirus, a drug kit containing the aurora kinase inhibitor and the alphav irus, and the use of the aurora kinase inhibitor and the alphavirus in treating tumors, especially those that are insensitive to the alphavirus.

Keywords: aurora kinase inhibitor alphavirus antitumor

PatentPak available

Substances (4) Reactions (0) Citing (2)

110

Modeling dose and schedule effects of AZD2811 nanoparticles targeting Aurora B kinase for treatment of diffuse large B-cell lymphoma

By: Floc'h, Nicolas; Ashton, Susan; Ferguson, Douglas; Taylor, Paula; Carnevalli, Larissa S.; Hughes, Adina M.; Harris, Emily; Hattersley, Maureen; Wen, Shenghua; Curtis, Nicola J.; et al
Molecular Cancer Therapeutics (2019), 18(5), 909-919 | Language: English, Database: CAplus and MEDLINE

Barasertib (AZD1152), a pro-drug of the highly potent and selective Aurora B kinase inhibitor A ZD2811, showed promising clin. activity in relapsed/refractory diffuse large B- cell lymphoma (DLBCL) patients administered as a 4- day infusion. To improve potential therapeutic benefit of Aurora B kinase inhibi tion, a nanopa rticle formulation of AZD2811 has been developed to address limita tions of repeated i.v. infusion. One of the challenges with the use of nanoparticles for chronic treatment of tumors is optimizing dose and schedule required to enable repeat administration to sustain tumor growth inhibi tion. AZD2811 gives potent cell growth inhibition across a range of DLBCL cells lines in vitro. In vivo, repeat adminis tration of the AZD2811 nanoparticle gave antitumor activity at half the dose intensity of AZD1152. Compared with AZD1152, a single dose of A ZD2811 nanoparticle gave less reduction in p HH3, but increased apoptosis and reduction of cells in G1 and G2-M, albeit at later time points, suggesting that duration and depth of target inhibition influence the nature of the tumor cell response to drug. Further exploration of the influence of dose and schedule on efficacy revealed that AZD2811 nanoparticle can be used flexibly with repeat adminis tration of 25 mg/kg admini stered up to 7 days apart being sufficient to maintain equivalent tumor control. Timing of repeat administration could be varied with 50 mg/kg every 2 wk controlling tumor control as effect ively as 25 mg/kg every week. A ZD2811 nanoparticle can be administered with very different doses and schedules to inhibit DLBCL tumor growth, although maximal tumor growth inhibition was achieved with the highest dose intensities.

Keywords: DLBCL cell growth aurora B kinase nanopa rticle therapeutics

Substances (2) Reactions (0) Citing (8)

111

Preparation of compounds and compositions for detecting caspase activity and/or apoptosis in vivo

By: Manning, H. Charles; Hight, Matthew R.
United States, US10294271 B1 2019-05-21 | Language: English, Database: CAplus

Mol. probe suitable for quantification of caspase activity in vivo using positron emission tomog. (P ET). Embodiments of the present invention can detect apoptosis in tumors and as a novel, potentially translatable biomarker for predicting response to person alized medicine.

Keywords: isotope labeled peptide substrate caspase activity apoptosis diagnosis preparation

PatentPak available

Substances (24) Reactions (16) Citing (0)

112

Identification and use of cytotoxic T lymphocyte (CTL) antigen-specific target cell killing enhancer agents

By: Lizotte, Patrick H.; Kirschmeier, Paul T.; Bittinger, Mark; Gray, Nathanael
World Intellectual Property Organization, WO2019014111 A1 2019-01-17 | Language: English, Database: CAplus

FIG. 2C

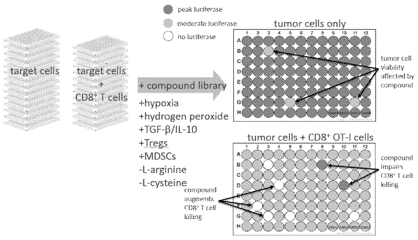

The present invention relates to screening methods for identification of agents (e.g., small mols.) that modulate cytotoxic T lymphocyte antigen-specific target (e.g., tumor) cell killing, as well as to uses of compounds identified thereby as immunomod ulatory, including use of EGFR inhibitors as immunomo dulatory agents.

Keywords: CD8 T lymphocyte antigen specific target cell killing enhancer

PatentPak available

Substances (220) Reactions (0) Citing (0)

113

Aurora kinase B inhibitor barasertib (AZD1152) inhibits glucose metabolism in gastric cancer cells

By: He, Jian; Qi, Zihao; Zhang, Xiaofei; Yang, Yufei; Liu, Fei; Zhao, Guangfa; Wang, Ziliang
Anti-Cancer Drugs (2019), 30(1), 19-26 | Language: English, Database: CAplus and MEDLINE

Barasertib is a highly selective Aurora kinase B (AURKB) inhibitor and has been widely applied in a variety of cancer cells to invest igate the regulatory function of AURKB. However, the effect of barasertib on glucose metabolism in gastric cancer (GC) remains illustrated. Here, barasertib was identified to effectively reduce glucose uptake and lactate production in GC cells in a dose-dependent and time-dependent manner. The expression levels of GLUT1, LDHA and HK2 were decreased by barasertib treatment of GC cells. Furthermore, we found that barasertib induced the expression of ribosomal protein S7 (RPS7), as a tumor suppressor, to regulate glucose metabolism Silencing of RPS7 rescued the effects of barasertib on glucose metabolism in GC cells. Overexpression of RPS7 suppressed the promoter activity of C-Myc, which has been identified as an important regulator of glucose metabolism in cancer cells. The clin. data showed that the expression level of AURKB in GC patients' sera and tissues were pos. correlated with those of C-Myc, GLUT1 and LDHA, but neg. with that of RPS7. Therefore, these findings provide new evidence that barasertib regulates GC cell glucose metabolism by inducing the RPS7/C-Myc signal pathway, and have important implications for the develo pment of therapeutic approaches using AURKB as a target protein to prevent tumor recurrence.

Keywords: barasertib AURKB inhibitor anticancer agent glucose metabolism gastric cancer

Substances (6) Reactions (0) Citing (8)

114

Aurora kinase: a target for cancer therapy

By: Pushpaveni, C.; Arun, Kumar C.; Visagaperumal, D.; Chandy, Vineeth
Research & Reviews: A Journal of Pharmacology (2019), 9(3), 10-17 | Language: English, Database: CAplus

Aurora kinases are the enzymes that belong to the family of serine/threonine kinases. It consists of Aurora- A (AURK-A), Aurora-B (AURK-B) and Aurora-C (AURK-C), which are the essential kinases for cell division through mitosis regulation especially for the process of chromosomal segregation. Aurora kinases are involved in meiosis regulation along with the regulation of mitosis. Inhibition of Aurora kinase leads to neglected cell division. Various cancer studies generally deal with gene amplification or overexpression. From the literature review, it is clear that inhibiting Aurora kinase will increase the effect of chemotherapies. Hence from the past decades, Aurora kinase inhibitors were developed, and they are now in clin. trials; however, these inhibitors effectively fail to give the complete therapy for cancer. This review mainly focuses on the Aurora kinase inhibitors and Aurora kinase as a target for anticancer therapy.

Keywords: baresertib anticancer agent aurora kinase cancer

Substances (3) Reactions (0) Citing (0)

115

Disassembly of actin and keratin networks by Aurora B kinase at the midplane of cleaving Xenopus laevis eggs

By: Field, Christine M.; Pelletier, James F.; Mitchison, Timothy J.
bioRxiv (2019), 1-24 | Language: English, Database: CAplus

We investigated how bulk cytoplasm prepares for cytoki nesis in Xenopus laevis eggs, which are large, rapidly dividing cells. The egg midplane is demarcated by Chromosomal Passenger Complex (CPC) localized on microtubule bundles between asters. Using an extract system and intact eggs we found that local kinase activity of the AURKB subunit of the CPC caused disassembly of F-actin and keratin between asters, and local softening of the cytoplasm as assayed by flow patterns. Beads coated with active CPC mimicked aster boundaries and caused AURKB-dependent disassembly of F-actin and keratin that propagated ~40μm without microtubules, and much farther with microt ubules present, due to C PC auto-activation. We propose that active C PC at aster boundaries locally reduces cytoplasmic stiffness by disasse mbling actin and keratin networks. This may help sister centro somes move apart after mitosis, prepare a soft path for furrow ingression and/or release G-actin to build the furrow cortex.

Keywords: IgG Factin keratin A URKB CCP disassembly zygote fertilization Xenopus

Substances (7) Reactions (0) Citing (0)

116

Aurora kinase B regulates axonal outgrowth and regeneration in the spinal motor neurons of developing zebrafish

By: Gwee, Serene S. L.; Radford, Rowan A. W.; Chow, Sharron; Syal, Monisha D.; Morsch, Marco; Formella, Isabel; Lee, Albert; Don, Emily K.; Badrock, Andrew P.; Cole, Nicholas J.; et al
Cellular and Molecular Life Sciences (2018), 75(23), 4269-4285 | Language: English, Database: CAplus and MEDLINE

Aurora kinase B (AurkB) is a serine/threonine protein kinase with a well- characterised role in orchestrating cell division and cytokinesis, and is prominently expressed in healthy proliferating and cancerous cells. However, the role of AurkB in differentiated and non-dividing cells has not been extensively explored. Previously, we have described a significant upregulation of AurkB expression in cultured cortical neurons following an exptl. axonal transection. This is somewhat surprising, as AurkB expression is generally associated only with dividing cells Frangini et al. (Mol Cell 51:647-661, 2013); Hegarat et al. (J Cell Biol 195: 1103-1113, 2011); Lu et al. (J Biol Chem 283:31785-31790, 2008); Trakala et al. (Cell Cycle 12: 1030-1041, 2014). Herein, we present the first description of a role for AurkB in terminally differentiated neurons. AurkB was prominently expressed within post-mitotic neurons of the zebrafish brain and spinal cord. The expression of AurkB varied during the development of the zebrafish spinal motor neurons. Utilizing pharmacol. and genetic manipulation to impair AurkB activity resulted in truncation and aberrant motor axon morphol., while overexpression of AurkB resulted in extended axonal outgrowth. Further pharmacol. inhibition of Aurk B activity in regenerating axons delayed their recovery following UV laser-mediated injury. Collectively, these results suggest a hitherto unreported role of AurkB in regulating neuronal development and axonal outgrowth.

Keywords: aurora kinase B neuronal development spinal motor neuron; Aurora kinase B; Axonal outgrowth; Axonal regeneration; Spinal motor neurons; Zebrafish

Substances (2) Reactions (0) Citing (12)

117

Recurrent 8q24 rearrangement in blastic plasmacytoid dendritic cell neoplasm: association with immunoblastoid cytomorphology, MYC expression, and drug response

By: Sakamoto, Kana; Katayama, Ryohei; Asaka, Reimi; Sakata, Seiji; Baba, Satoko; Nakasone, Hideki; Koike, Sumie; Tsuyama, Naoko; Dobashi, Akito; Sasaki, Makoto; et al
Leukemia (2018), 32(12), 2590-2603 | Language: English, Database: CAplus and MEDLINE

Blastic plasmacytoid dendritic cell neoplasm (BPDCN) is a rare skin- tropic hematol. malignancy of uncertain pathogenesis and poor prognosis. We examined 118 BPDCN cases for cytomorphol., MYC locus rearrangement, and MYC expression. Sixty-two (53%) and 41 (35%) cases showed the classic and immunoblastoid cytomorphol., resp. Forty-one (38%) MYC^{+}BPDCN (pos. for rearrangement and expression) and 59 (54%) MYC^{-}BPDCN (both neg.) cases were identified. Immunoblastoid cytomorphol. was significantly associated with MYC^{+}BPDCN. All examined MYC^{+}BPDCNs were neg. for MYB/MYBL1 rearrangement (0/36). Clin., MYC^{+}BPDCN showed older onset, poorer outcome, and localized skin tumors more commonly than MYC^{-}BPDCN. MYC was demonstrated by expression profiling as one of the clearest discriminators between CAL-1 (MYC^{+}BPDCN) and PMDC05 (MYC^{-}BPDCN) cell lines, and its shRNA knockdown suppressed CAL-1 viability. Inhibitors for bromodomain and extra-terminal protein (BETis), and aurora kinases (AKis) inhibited CAL-1 growth more effectively than PMDC05. We further showed that a BCL2 inhibitor was effective in both CAL-1 and PMDC05, indicating that this inhibitor can be used to treat MYC^{-}BPDCN, to which BETis and AKis are probably less effective. Our data will provide a rationale for the development of new treatment strategies for patients with BPDCN, in accordance with precision medicine.

Keywords: blastic plasmacytoid dendritic cell neoplasm MYC immunoblastoid cytomorphol

Substances (11) Reactions (0) Citing (31)

118

Dual targeting of aurora kinases with AMG 900 exhibits potent preclinical activity against acute myeloid leukemia with distinct post-mitotic outcomes

By: Payton, Marc; Cheung, Hung-Kam; Ninniri, Maria Stefania S.; Marinaccio, Christian; Wayne, William C.; Hanestad, Kelly; Crispino, John D.; Juan, Gloria; Coxon, Angela
Molecular Cancer Therapeutics (2018), 17(12), 2575-2585 | Language: English, Database: CAplus and MEDLINE

In this study, pan-aurora kinase inhibitor (AKI) AMG 900 distinguishes itself as an anti-leukemic agent that is more uniformly potent against a panel of acute myeloid leukemia (AML) cell lines than are isoform-selective AKIs and classic AML drugs. AMG 900 inhibited AML cell growth by inducing polyploi dization and/or apoptosis. AMG 900 and aurora-B-selective inhibitor AZD1152-hQPA showed comparable cellular effects on AML lines that do not harbor a FLT3-1TD mutation. AMG 900 was active against P-glycoprotein-expressing AML cells resistant to AZD1152-hQPA and was effective at inducing expression of megakar yocyte-lineage markers (CD41, CD42) on human CHRF-288-11 cells and mouse Jak2 $V^{617}F$ cells. In MOLM-13 cells, inhibition of p-histone H3 by AMG 900 was associated with polyploidy, extra centrosomes, accumulation of p53 protein, apoptosis, and cleavage of Bcl- 2 protein. AMG 900 inhibited the proliferation of primary human bone marrow cells in culture, with a better prolife ration recovery profile relative to classic antimitotic drug docetaxel. In vivo, AMG 900 significantly reduced tumor burden in a systemic MOLM-13 xenograft model where we demonstrate the utility of 3'-deoxy-3'-^{18}F-fluorothymidine [^{18}F]FLT positron emission tomog. (PET)-CT imaging to measure the antiproliferative effects of AMG 900 in skeletal tissues in mice.

Keywords: acute myeloid leukemia mitosis aurora kinase AMG900 antiproliferative

Substances (9) Reactions (0) Citing (12)

119

Methods of cancer treatment with ferroptosis inducer in combination with other antitumor agents

By: Chen, Ruihong; Jiang, Chun
World Intellectual Property Organization, WO2018218087 A1 2018-11-29 | Language: English, Database: CAplus

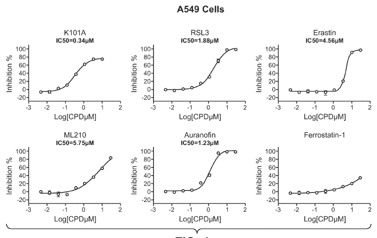

FIG. 1

The present disclosure relates to a method of treating a subject with cancer with a ferroptosis inducer, including use of the ferrop tosis inducer (e.g. erastin, auranofin, sulfasalazine) in combination with a second therap eutic agent (e.g. afatinib, afures ertib, alectinib, alisertib).

Keywords: ferroptosis inducer erastin antitumor afuresertib combination chemotherapy cancer treatment

PatentPak available Substances (150) Reactions (0) Citing (3)

120

Combination therapies of cancer with histone H3 lysine-9 N-methyltransferase EHMT2 inhibitors

By: Campbell, John Emmerson; Duncan, Kenneth William; Raimondi, Maria Alejandra; Klaus, Christine; Penebre, Elayne

World Intellectual Property Organization, WO2018195450 A1 2018-10-25 | Language: English, Database: CAplus

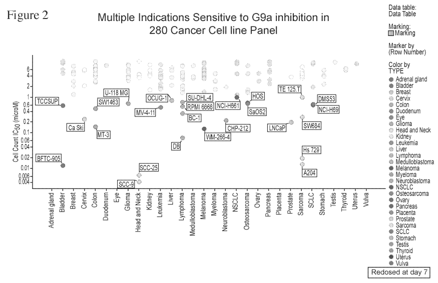

The present disclosure relates to a method of preventing or treating a cancer via administering an histone H3 lysine-9 N-methyltra nsferase EHMT2 inhibitor or a combin ation comprising an EHMT2 inhibitor compound and one or more addnl. therap eutic agent disclosed herein or a pharmaceutical composition thereof to subjects in need thereof. The present disclosure also relates to the use of such compounds or combinations for research or other non- therapeutic purposes.

Keywords: combination therapy antitumor EHMT2 inhibitor cancer treatment

PatentPak available

Substances (1,586) Reactions (0) Citing (4)

121

Method and compositions for treating cancerous tumors

By: Alliger, Howard

United States, US10105389 B1 2018-10-23 | Language: English, Database: CAplus

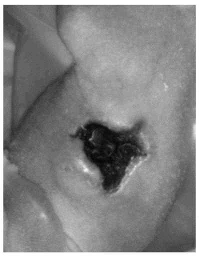

The present invention relates to the use of chlorine dioxide compositions for treating cancerous tumors. The present invention relates to compositions and methods for treating cancerous tumors, including naive, metastatic and recurrent cancers. The compos itions comprise chlorine dioxide in an effective amount, which is injected into the cancerous tumor at least once, and often at least several times over the course of treatment. The chlorine dioxide compositions are injected directly into the cancerous tumor and the resulting tumor is effectively eliminated from the patient or subject over a period of one to several days to a few weeks, often after a single injection, or multiple injections at one session into the tumor. Often, an initial injection or multiple injections at one session are sufficient to dissolve the cancerous tumor. Often the cancer is eliminated (as evidenced by no remission) in a period of no more than several days to about two-three months and does not recur.

Keywords: chlorine dioxide cancer

PatentPak available

Substances (294) Reactions (0) Citing (0)

122

Porous nanoparticle-supported lipid bilayer delivery of transcriptional gene modulators

By: Brinker, Charles Jeffrey; Agola, Jacob Ongudi; Muniz, Ayse; Butler, Kimberly

World Intellectual Property Organization, WO2018187287 A1 2018-10-11 | Language: English, Database: CAplus

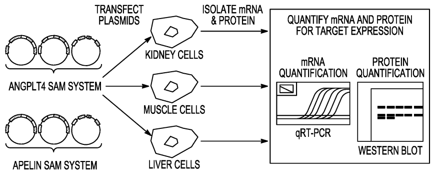

FIG. 15B

A porous protocell is provided comprising one or more non-integrating vectors comprising nucleic acid encoding a non-nucleolytic fusion protein comprising a protein sequence that specifically binds a sequence in a double strand nucleic acid mol. linked to a protein sequence that is a transcriptional mediator, and optionally guide RNA or a vector for expression of guide R NA, and methods of using the protocells.

Keywords: porous nanoparticle lipid bilayer transcription gene fusion protein sequence

PatentPak available

Substances (110) Reactions (0) Citing (0)

123

The Potential Contribution of microRNAs in Anti-cancer Effects of Aurora Kinase Inhibitor (AZD1152-HQPA)

By: Zekri, Ali; Mesbahi, Yashar ; Boustanipour, Elham; Sadr, Zahra; Ghaffari, Seyed H.

Journal of Molecular Neuroscience (2018), 65(4), 444-455 | Language: English, Database: CAplus and MEDLINE

In the present study, we first investigated the restrictive potentials of AZD1152-HQPA on cell viability, colony formation, nucleus morphol., polyploidy, and cell-cycle distribution. We then studied the expres sions level of 88 cancer-related miRNAs in untreated and AZD1152-HQPA-treated NB cell line (SK-N-MC) by real-time PCR using mi RNA cancer-array system. After normalizing, the fold change of miRNAs was calculated in the AZD1152-HQPA-treated cell as compared to untreated. Our results demons trate that the inhibition of AURKB by AZD1152-HQPA induced potent antitumor activity, suppressed cell survival, and triggered apoptosis and polyploidy in NB cells. AZD1152-HQPA, at a relevant concent ration, modulated a substantial number of cancer-related miRNAs in N B cell. Interestingly, by screening the litera ture, among the 7 top AZD1152-HQPA-induced upregulated mi RNAs (> 3-fold change; P < 0.01), all were potential tumor suppre ssors associated with cell apoptosis and cycle arrest, as well as inhibition of angioge nesis, invasion, and metastasis, while two downregulated miRNAs were known to have oncogenic function. Taken together, our study showed for the first time the potential contribution of mi RNAs in the anti-cancer effects of AZD1152-HQPA.

Keywords: neuroblastoma aurora kinase inhibitor anticancer micro RNA; AZD1152-HQPA; Aurora kinase B; Micro RNAs; Neurobl astoma; Polyploidy

Substance (1) Reactions (0) Citing (3)

124

Preparation of cereblon E3 ligase ligands and bifunctional PROTAC compounds for targeting and degradation of androgen receptor, estrogen receptor alpha, bromodomain-containing protein 4, and BRaf

By: Crew, Andrew P.; Crews, Craig M.; Dong, Hanqing; Hornberger, Keith R.; Wang, Jing; Qian, Yimin; Zimmermann, Kurt; Berlin, Michael; Snyder, Lawrence B.

United States, US20180228907 A1 2018-08-16 | Language: English, Database: CAplus

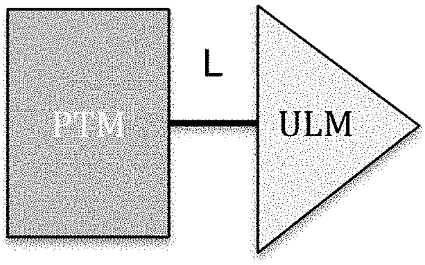

The invention relates to cereblon E3 ligase binding compounds, including bifunctional compounds comprising them, which find utility as modulators of targeted ubiquitination, especially inhibitors of a variety of polype ptides and other proteins which are degraded and/or otherwise inhibited by bifunctional compounds according to the present disclo sure. In particular, the invention provides compounds, which contain on one end a ligand which binds to the cereblon E3 ubiquitin ligase and on the other end a moiety which binds a target protein such that the target protein is placed in proximity to the ubiquitin ligase to effect degradation (and inhibition) of that protein. Specifically, the invention relates to bifunctional compounds CLM-L-PTM [PTM = small mol. containing a protein targeting moiety that binds bromodomain-containing protein 4, estrogen receptor (E R), or androgen receptor or Braf; L = a bond, a chem. linking moiety covalently coupling the CLM and the PTM; CLM = a small mol. cereblon E3 ubiquitin ligase binding moiety that binds or targets a cereblon E3 ubiquitin ligase], their pharmaceutically acceptable salts, enantiomers, stereoi somers, solvates, polymorphs and prodrugs. Exempl ified compound **I** was prepared from a 12 step procedure (prepar ation given). The invention exhibits a broad range of pharmacol. activities associated with compounds according to the present invention, consistent with the degradation/inhibition of targeted polypeptides of nearly any type (data given). Diseases or disorders that result from aggregation or accumulation of the target protein are treated or prevented with compounds and compos itions of the invention.

Keywords: cereblon ligand chimeric mol preparation targeted protein degradation ubiquitination; PROTAC modulator preparation targeted ubiquitination estrogen receptor E RRa degradation; androgen receptor PROTAC degradation inhibition preparation antiproliferative; BRD4 BRaf PROTAC degradation inhibition cereblon E3 ligase antitumor

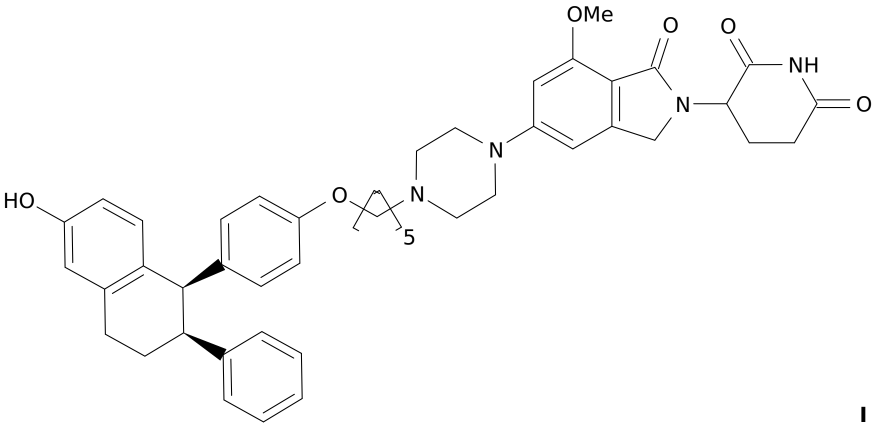

I

PatentPak available

Substances (731) Reactions (1,474) Citing (3)

125

Preparation of cereblon E3 ligase ligands and bifunctional PROTAC compounds comprising them for the degradation of targeted proteins including TANK-binding kinase 1, estrogen receptor alpha, bromodomain-containing protein 4 and tau protein

By: Crew, Andrew P.; Berlin, Michael; Dong, Hanqing; Homberger, Keith R.; Qian, Yimin; Snyder, Lawrence B.; Wang, Jing; Zimmermann, Kurt

United States, US20180215731 A1 2018-08-02 | Language: English, Database: CAplus

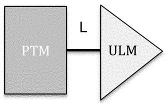

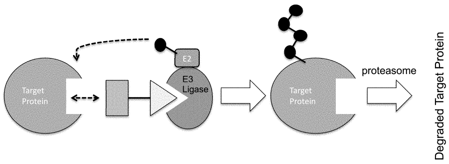

The invention relates to cereblon E3 ligase binding compounds, including bifunctional compounds comprising them, which find utility as modulators of targeted ubiquitination, especially inhibitors of a variety of polype ptides and other proteins which are degraded and/or otherwise inhibited by bifunctional compounds according to the present disclo sure. In particular, the invention provides compounds, which contain on one end a ligand which binds to the cereblon E3 ubiquitin ligase and on the other end a moiety which binds a target protein such that the target protein is placed in proximity to the ubiquitin ligase to effect degradation (and inhibition) of that protein. Specifically, the invention relates to bifunctional compounds CLM-L-PTM [PTM = small mol. containing a protein targeting moiety that binds bromodomain-containing protein 4, Tau protein, estrogen receptor (E R), or androgen receptor; L = a bond, a chem. linking moiety covalently coupling the CLM and the PTM; CLM = a small mol. cereblon E3 ubiquitin ligase binding moiety that binds or targets a cereblon E3 ubiquitin ligase], their pharmaceutically acceptable salts, enanti omers, stereoisomers, solvates, polymorphs and prodrugs. The invention exhibits a broad range of pharmacol. activities associated with compounds according to the present invention, consistent with the degradation/inhibition of targeted polypeptides of nearly any type (data given). Diseases or disorders that result from aggreg ation or accumulation of the target protein are treated or prevented with compounds and compositions of the invention.

Keywords: cereblon ligand chimeric mol preparation targeted protein degradation ubiquitination; PROTAC modulator preparation targeted ubiquitination estrogen receptor ERRa degradation; tau protein PROTAC degradation inhibition preparation antiproli ferative; BRD4 PROTAC degradation inhibition cereblon E3 ligase antitumor; preparation cereblon ligand Tank binding kinase 1 PRO TAC degradation; TBK1 degradation inhibition PROTAC modulator preparation targeted ubiquitination

PatentPak available

Substances (1,219) Reactions (1,874) Citing (3)

126

Hyperstabilized liposomes increase targeting of mitotic cells

By: Ng, Chang Zhi Adrian; Cheong, Shen-Yi Ian
World Intellectual Property Organization, WO2018136002 A1 2018-07-26 | Language: English, Database: CAplus

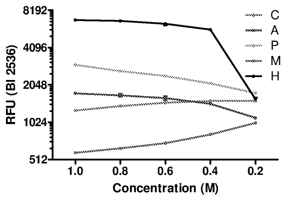

Fig. 2

Hyperstable liposome comprising an anti-mitotic agent, one or more anions and one or more cations entrapped in the inner milieu, wherein the entrapped anti-mitotic drug is released at a slow rate that is less than 0.6% in 12 h or less than 5% in 8 h when the liposomes are suspended in 600mM sucrose. These liposomes are useful in the treatment of cancer. In partic ular, HEPC:Chol:DSPE-PEG2000 (50:45:5) liposomes comprising BI2536 and citrate:phosphate in a ratio of 1:3.

Keywords: hyperstabilized phosphatidylcholine liposome target mitosis B I2536 antitumor neoplasm

PatentPak available

Substances (43) Reactions (0) Citing (0)

127

Preparation of functionalized pyrazoles and other nitrogen-containing heterocycles as inhibitors of MCT4 for the treatment of MCT4-mediated diseases

By: Parnell, Kenneth Mark; McCall, John; Romero, Donna

United States, US20180162822 A1 2018-06-14 | Language: English, Database: CAplus

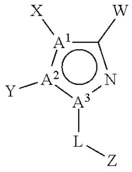

(I)

The invention relates to preparation of functionalized pyrazoles and other nitrogen heterocycles of formula **I** and compositions containing them as monocarboxylate transporter MCT4 inhibitors. Compounds **I** wherein A^1, A^2 and A^3 are independently chosen from N and C, wherein at least one of A^1, A^2 and A^3 is N; L is a bond, methylene, or ethylene; X is H or null; Y is alkenyl, alkenyl amino, alkyl, etc.; Z is alkyl, cycloa lkyl, aryl, etc., are claimed. The example compound **II** was prepared by multistep synthesis (procedure given). Compounds **I** were evaluated for their biol. activity (example - M CT4 inhibiting activity) (data given). Compounds **I** are useful in the treatment of MCT4-mediated diseases, such as proliferative and inflammatory diseases, such as cancer.

Keywords: pyrazole preparation MCT4 inhibitor antiproliferative antiinflammatory antitumor; nitrogen heterocycle preparation MC T4 disease treatment

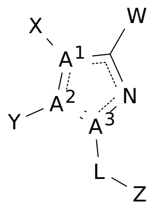

I

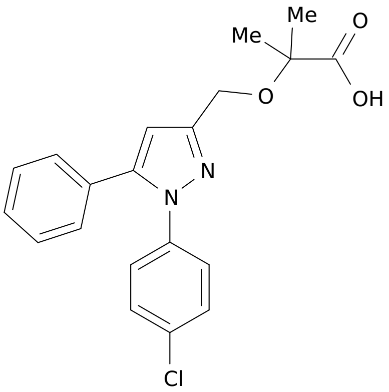

II

PatentPak available

Substances (1,145)   Reactions (4,041)   Citing (0)

128

Preparation of small molecule based antibody-recruiting compounds for cancer treatment

By: Spiegel, David; Rullo, Anthony
United States, US20180155332 A1 2018-06-07 | Language: English, Database: CAplus

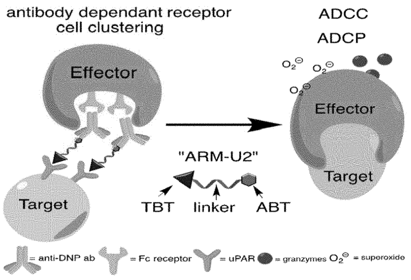

The present invention relates to chimeric (including bifunctional) compounds **I** [(UPAR$_B$M)$_m$-CT-(L^1)$_k$-CT-(L^2)$_j$-CT-(A$_B$M)$_n$; wherein UPA R$_B$M = moiety which binds to an active site of urokinase- type plasminogen activator receptor (u PAR) on the surface of cancer cells; A$_B$M = antibody binding moiety comprising a hapten which is capable of binding to an antibody; L ^{1} and L^2 = linkers; CT = (indepen dently) an optional connector mol.; j = 0- 5; k = 0-5; with the proviso that at least one C T is present when k and j are both 0 (preferably at least one of k and j is 1); each m and n = (indepen dently) 1-15 (preferably m = 1 and n = 1- 3)], compositions comprising those compounds and methods of treating cancer in a patient or subject, especially including metastatic cancer where cancer cells exhibit overexpression (heightened expression) of cell surface urokinase-type plasminogen activator receptor (urokinase receptor) compared to normal (non-cancerous) cells. The compounds **I** bind to the urokinase-type plasminogen activator receptor (uPAR) on the surface of a cancer cell, including a metastatic cancer cell, and conseq uently recruit native antibodies of the patient or subject where the antibodies can selectively degrade and/or deactivate targeted cancer cells through antibody-dependent cellular phagocytosis and antibody-dependent cellular cytotoxicity (ADCC) and/or complement dependent cytotoxicity (C DC) against a large number and variety of cancers, thus providing cancer cell death and an inhibition of growth, elabor ation and/or metastasis of the cancer, including remission and cure of the patient's cancer. E.g., a multi-step synthesis of compound **II**, starting from 5-hydroxy-1,4-naphthoquinone and 1-(trimethylsilyloxy)-1,3-butadiene, was described. Exemplified title compounds **I** were tested for their biol. activity (data given for representative compounds).

Keywords: chimeric bifunctional compound preparation antitumor uPAR modulator antibody recruiting; urokinase type plasmi nogen activator receptor chimeric bifunc tional compound preparation; antibody dependent cellular phagocytosis cancer chimeric bifunctional compound preparation; ADCC antibody dependent cellular cytotoxicity chimeric bifunctional compound preparation; C DC complement dependent cytotoxicity metastasis chimeric bifunc tional compound preparation

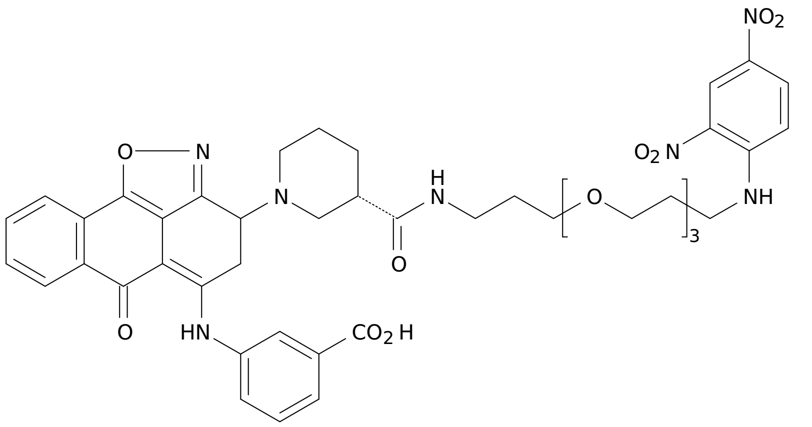

II

PatentPak available

Substances (389) Reactions (333) Citing (0)

129

Chemical Genetic Screens Identify Kinase Inhibitor Combinations that Target Anti-Apoptotic Proteins for Cancer Therapy

By: Contreras, Jacob I.; Robb, Caroline M.; King, Hannah M.; Baxter, Jared; Crawford, Ayrianne J.; Kour, Smit; Kizhake, Smitha; Sonawane, Yogesh A. ; Rana, Sandeep; Hollingsworth, Michael A.; et al
ACS Chemical Biology (2018), 13(5), 1148-1152 | Language: English, Database: CAplus and MEDLINE

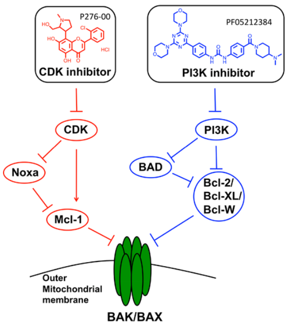

The study presented here provides a framework for the discovery of unique inhibitor combinations that target the apoptosis network for cancer therapy. A pair of doxycycline (Dox)-inducible cell lines that specifically report on the ability of an inhibitor to induce apoptosis by targeting either the Mcl-1 arm or the Bcl-2/Bcl-xL/Bcl-w arm were used. Cell-based assays were optimized for high throughput screening (HTS) with caspase 3/7 as a read out. H TS with a 355- member kinase inhibitor library and the panel of Dox-inducible cell lines revealed that cyclin dependent kinase (C DK) inhibitors induced apoptosis by targeting the Mcl- 1 arm, whereas PI3K inhibitors induced apoptosis by targeting the Bcl- 2/Bcl-xL/Bcl-w arm. Validation studies identified unique combin ations that synergistically inhibited growth and induced apoptosis in a panel of cancer cell lines. Since these inhibitors have been or are currently in clin. trials as single agents, the combinations can be rapidly translated to the clinics.

Keywords: antitumor combination synergy apoptosis cancer

Substances (15) | Reactions (0) | Citing (10)

130

Methods and compositions for identifying and treating patients with small cell lung cancer with aurora kinase inhibitors using transgenic animal models and cell lines

By: Oliver, Trudy; Sos, Martin; Wechsler-Reya, Rob
World Intellectual Property Organization, WO2018081575 A1 2018-05-03 | Language: English, Database: CAplus

Described herein are methods and compositions useful in detecting, diagnosing and treating small cell lung cancer. Transgenic animal models and cell lines are disclosed for the study of a small cell lung cancer subtype. Methods of screening and identifying active agents for the treatment of a small cell lung cancer subtype as well as methods of identifying patients susceptible to treatment with aurora kinase inhibitors are also provided.

Keywords: lung cancer aurora kinase inhibitor screening transgenic mouse

PatentPak available

Substances (14) | Reactions (0) | Citing (0)

131

Pharmaceutical combinations comprising a histone deacetylase inhibitor and an aurora kinase inhibitor and methods of use thereof

By: North, Brian; Quayle, Steven
World Intellectual Property Organization, WO2018081585 A1 2018-05-03 | Language: English, Database: CAplus

FIG. 1

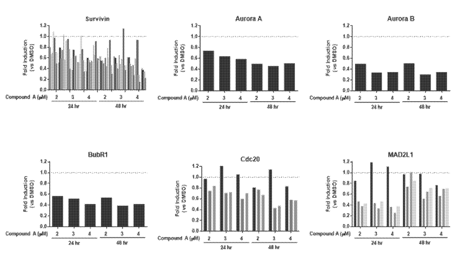

The present disclosure relates to a pharmaceutical combination comprising (a) a histone deacetylase 6 inhibitor and (b) an aurora kinase inhibitor, including combined preparations and pharmaceutical compositions thereof; uses of such combination in the treatment or prevention of cancer; and methods of treating or preventing cancer in a subject comprising administering a therapeutically effective amount of such combination.

Keywords: pharmaceutical combination histone deacetylase aurora kinase inhibitor

PatentPak available

Substances (26) | Reactions (10) | Citing (1)

132

Vitamins c and k3: a powerful redox system for sensitizing leukemia lymphocytes to everolimus and barasertib

By: Ivanova, Donika; Zhelev, Zhivko; Lazarova, Dessislava; Getsov, Plamen; Bakalova, Rumiana; Aoki, Ichio
Anticancer Research (2018), 38(3), 1407-1414 | Language: English, Database: CAplus and MEDLINE

Background/Aim: Recent studies provided convincing evidence for the anticancer activity of combined application of vitamin C and pro-vitamin K3 (menadione). The mol. pathways underlying this process are still not well established. The present study aimed to investigate the effect of the combination of vitamin C plus pro-vitamin K3 on the redox status of leukemia and normal lymphocytes, as well as their sensitizing effect for a variety of anticancer drugs. Materials and methods: Cytotoxicity of the substances was analyzed by trypan blue staining and automated counting of live and dead cells. Apoptosis was analyzed by fluorescein isothiocyanate-annexin V test. Oxidative stress was evaluated by the intracellular levels of reactive oxygen and nitrogen species and protein-carbonyl products. Results: Combined administration of 300µM vitamin C plus 3µM pro-vitamin K3 reduced the viability of leukemia lymphocytes by ~20%, but did not influence the viability of normal lymphocytes. All combinations of anticancer drug plus vitamins C and K3 were characterized by synergistic cytotoxicity towards Jurkat cells, compared to cells treated with drug alone for 24 h. In the case of barasertib and everolimus, this synergistic cytotoxicity increased within 72 h. It was accompanied by strong induction of apoptosis, but a reduction of level of hydroperoxides and moderately increased protein-carbonyl products in leukemia cells. Conclusion: Leukemia lymphocytes were more sensitive to combined administration of anticancer drug (everolimus or barasertib) plus vitamins C and K3, compared to normal lymphocytes. The combination of vitamin C plus K3 seems to be a powerful redox system that could specifically influence redox homeostasis of leukemia cells and sensitize them to conventional chemotherapy.

Keywords: vitamin redox system everolimus barasertib sensitizing leukemia lymphocyte; Jurkat; Vitamin C; apoptosis; leukemia lymphocytes; oxidative stress; pro-vitamin K3

Substances (7) | Reactions (0) | Citing (11)

133

RIPK1-dependent cell death: a novel target of the Aurora kinase inhibitor Tozasertib (VX-680)

By: Martens, Sofie; Goossens, Vera; Devisscher, Lars; Hofmans, Sam; Claeys, Polien; Vuylsteke, Marnik; Takahashi, Nozomi; Augustyns, Koen ; Vandenabeele, Peter
Cell Death & Disease (2018), 9(2), 1-13 | Language: English, Database: CAplus and MEDLINE

The Aurora kinase family (Aurora A, B and C) are crucial regulators of several mitotic events, including cytokinesis. Increased expression of these kinases is associated with tumorigenesis and several compounds targeting Aurora kinase are under evaluation in clin. trials (a.o. AT9283, AZD1152, Danusertib, MLN8054). Here, we demonstrate that the pan-Aurora kinase inhibitor Tozasertib (VX-680 and MK-0457) not only causes cytokinesis defects through Aurora kinase inhibition, but is also a potent inhibitor of necroptosis, a cell death process regulated and executed by the RIPK1, RIPK3 and MLKL signalling axis. Tozasertib's potency to inhibit RIPK1-dependent necroptosis and to block cytokinesis in cells is in the same concentration range, with an IC50 of 1.06μM and 0.554μM, resp. A structure activity relationship (SAR) anal. of 67 Tozasertib analogs, modified at 4 different positions, allowed the identification of analogs that showed increased specificity for either cytokinesis inhibition or for necroptosis inhibition, reflecting more specific inhibition of Aurora kinase or RIPK1, resp. These results also suggested that RIPK1 and Aurora kinases are functionally non-interacting targets of Tozasertib and its analogs. Indeed, more specific Aurora kinase inhibitors did not show any effect in necroptosis and Necrostatin-1s treatment did not result in cytokinesis defects, demonstrating that both cellular processes are not interrelated. Finally, Tozasertib inhibited recombinant human RIPK1, human Aurora A and human Aurora B kinase activity, but not RIPK3. The potency ranking of the newly derived Tozasertib analogs and their specificity profile, as observed in cellular assays, coincide with ADP-Glo recombinant kinase activity assays. Overall, we show that Tozasertib not only targets Aurora kinases but also RIPK1 independently, and that we could generate analogs with increased selectivity to RIPK1 or Aurora kinases, resp.

Keywords: rozasertib antiapoptotic agent RIPK1 RIPK3 fibroblast necroptosis

Substances (13) Reactions (0) Citing (27)

134

Preclinical evaluation of the Aurora kinase inhibitors AMG 900, AZD1152-HQPA, and MK-5108 on SW-872 and 93T449 human liposarcoma cells

By: Noronha, Sandhya; Alt, Lauren A. C.; Scimeca, Taylor E.; Zarou, Omran; Obrzut, Justyna; Zanotti, Brian; Hayward, Elizabeth A.; Pillai, Akhil; Mathur, Shubha; Rojas, Joseph; et al
In Vitro Cellular & Developmental Biology: Animal (2018), 54(1), 71-84 | Language: English, Database: CAplus and MEDLINE

Liposarcoma is a malignant soft tissue tumor that originates from adipose tissue and is one of the most frequently diagnosed soft tissue sarcomas in humans. There is great interest in identifying novel chemotherapeutic options for treating liposarcoma based upon mol. alterations in the cancer cells. The Aurora kinases have been identified as promising chemotherapeutic targets based on their altered expression in many human cancers and cellular roles in mitosis and cytokinesis. In this study, we investigated the effects of an Aurora kinase A inhibitor (MK-5108), an Aurora kinase B inhibitor (AZD1152-HQPA), and a pan-Aurora kinase inhibitor (AMG 900) on undifferentiated SW-872 and well-differentiated 93T449 human liposarcoma cells. Treatment of the SW-872 and 93T449 cells with MK-5108 (0-1000 nM), AZD1152-HQPA (0-1000 nM), and AMG 900 (0-1000 nM) for 72 h resulted in a dose- dependent decrease in the total viable cell number Based upon the EC_{50} values, the potency of the three Aurora kinase inhibitors in the SW-872 cells was as follows: AMG 900 (EC_{50} = 3.7 nM) > AZD1152-HQPA (EC_{50} = 43.4 nM) > MK-5108 (EC_{50} = 309.0 nM), while the potency in the 93T449 cells was as follows: AMG 900 (EC_{50} = 6.5 nM) > AZD1152-HQPA (EC_{50} = 74.5 nM) > MK-5108 (EC_{50} = 283.6 nM). The percentage of polyploidy after 72 h of drug treatment (0- 1000 nM) was determined by propidium iodide staining and flow cytometric anal. AMG 900 caused a significant increase in polyploidy starting at 25 nM in the SW-872 and 93T449 cells, and AZD1152-HQPA caused a significant increase starting at 100 nM in the SW-872 cells and 250 nM in the 93T449 cells. The Aurora kinase A inhibitor MK-5108 did not significantly increase the percentage of polyploid cells at any of the doses tested in either cell line. The expression of Aurora kinase A and B was evaluated in the SW-872 cells vs. differentiated adipocytes and human mesenchymal stem cells by real-time RT-PCR and Western blot anal. Aurora kinase A and B mRNA expression was significantly increased in the SW-872 cells vs. the differentiated adipocytes and human mesenchymal stem cells. Western blot anal. revealed a ~ 48 kDa immunoreactive band for Aurora kinase A that was not present in the differentiated adipocytes or the human mesenchymal stem cells. A ~ 39 kDa immunoreactive band for Aurora kinase B was detected in the SW-872 cells, differentiated adipocytes, and human mesenchymal stem cells. A smaller immunoreactive band for Aurora kinase B was detected in the SW-872 cells but not in the differentiated adipocytes and human mesenchymal stem cells, and this may reflect the expression of a truncated splice variant of Aurora kinase B that has been associated with poor patient prognosis. The 93T449 cells demonstrated decreased expression of Aurora kinase A and B mRNA and protein compared to the SW-872 cells, and also expressed the truncated form of Aurora kinase B. The results of these in vitro studies indicate that Aurora kinase inhibitors should be further investigated as possible chemotherapeutic agents for human liposarcoma.

Keywords: AMG900 AZD1152 HQPA MK5108 aurora kinase inhibitor liposarcoma; 93T449 cell line; AMG 900; AZD1152-HQPA; Aurora kinase inhibitors; Liposarcoma; MK-5108; SW-872 cell line

Substances (5) Reactions (0) Citing (6)

135

A mixed modality approach towards Xi reactivation for Rett syndrome and other X-linked disorders

By: Carrette, Lieselot L. G.; Wang, Chen-Yu; Wei, Chunyao; Press, William; Ma, Weiyuan; Kelleher, Raymond J. III; Lee, Jeannie T.
Proceedings of the National Academy of Sciences of the United States of America (2018), 115(4), E668-E675 | Language: English, Database: CAplus and MEDLINE

The X-chromosome harbors hundreds of disease genes whose associated diseases predomi nantly affect males. However, a subset, including neurodevelopmental disorders, Rett syndrome (RTT), fragile X syndrome, and CDKL5 syndrome, also affects females. These disorders lack disease-specific treatment. Because female cells carry two X chromo somes, an emerging treatment strategy has been to reawaken the healthy allele on the inactive X (Xi). Here, we focus on methyl-CpG binding protein 2 (MECP2) restoration for RTT and combinat orially target factors in the intera ctome of Xist, the noncoding RNA responsible for X inactivation. We identify a mixed modality approach combining an Xist antisense oligonucleotide and a small-mol. inhibitor of DNA methylation, which, together, achieve 30,000-fold MECP2 up-regulation from the Xi in cultured cells. Combining a brain- specific genetic Xist ablation with short-term 5-aza-2'-deoxycytidine (Aza) treatment models the synergy in vivo without evident toxicity. The Xi is select ively reacti vated. These experi ments provide proof of concept for a mixed modality approach for treating X- linked disorders in females.

Keywords: Xi reactivation Rett syndrome X linked disorder; LNA; Rett syndrome; X reactivation; Xist; antisense oligonucleotides

Substances (22) | Reactions (0) | Citing (49)

136

Enhancing functional platelet release in vivo from in vitro-grown megakaryocytes using small molecule inhibitors

By: Jarocha, Danuta; Vo, Karen K.; Lyde, Randolph B.; Hayes, Vincent; Camire, Rodney M.; Poncz, Mortimer
Blood Advances (2018), 2(6), 597-606 | Language: English, Database: CAplus and MEDLINE

In vitro-grown megakaryocytes for generating platelets may have value in meeting the increasing demand for platelet transfu sions. Remaining challenges have included the poor yield and quality of in vitro-generated platelets. We have shown that infusing megakaryocytes leads to intrapulmonary release of functional platelets. A Src kinase inhibitor (SU6656), a Rho-associated kinase inhibitor (Y27632), and an aurora B kinase inhibitor (AZD1152) have been shown to increase megakar yocyte ploidy and in vitro proplatelet release. We now tested whether megakar yocytes generated from CD34$^+$ hematopoietic cells in the presence of these inhibitors could enhance functional platelet yield following megakaryocyte infusion. As expected, all inhibitors increased megakar yocyte ploidy, size, and granul arity, but these inhibitors differed in whether they injured terminal megakary ocytes: SU6656 was protective, whereas Y27632 and AZD1152 increased injury. Upon infusion, inhibitor- treated megakaryocytes released threefold to ninefold more platelets per initial noninjured megakaryocyte relative to control, but only SU6656-treated megakaryocytes had a significant increase in platelet yield when calculated based on the number of initial CD34$^+$ cells; this was fourfold over nontreated megakaryocytes. The released platelets from drug- treated, but healthy, megakar yocytes contained similar percen tages of young, uninjured platelets that robustly responded to agonists and were well incorporated into a growing thrombus in vivo as controls. These studies suggest that drug screens that select megakaryocytes with enhanced ploidy, cell size, and granul arity may include a subset of drugs that can enhance the yield and function of platelets, and may have clin. application for ex vivo-generated megakar yocytes and platelet transfusion.

Keywords: platelet release megakaryocyte small mol inhibitor

Substances (3) | Reactions (0) | Citing (17)

137

Data-driven exploration of selectivity and off-target activities of designated chemical probes

By: Miljkovic, Filip; Bajorath, Juergen
Molecules (2018), 23(10), 1-12 | Language: English, Database: CAplus and MEDLINE

Chem. probes are of central relevance for chem. biol. To unambiguously explore the role of target proteins in triggering or mediating biol. functions, small mols. used as probes should ideally be target-specific; at least, they should have sufficiently high selectivity for a primary target. We present a thorough anal. of currently available activity data for designated chem. probes to address several key questions: How well defined are chem. probes What is their level of selectivity Is there evidence for addnl. activities Are some probes "better" thanothers. Therefore, highly curated chem. probes were collected and their select ivity was analyzed on the basis of publicly available compound activity data. Different selectivity patterns were observed, which distinguished designated high-quality probes.

Keywords: data driven exploration selectivity activity chem probe; bioactive compounds; chemical probes; hemical biology; molecular scaffold; off-target activity; promiscuity; target selectivity

Substances (13) | Reactions (0) | Citing (6)

138

Discovery of a novel hydrogen sulfide donor and the study of its structure-activity relationship

By: Gong, Sixu; Zhou, Yueyang; Wu, Fang; Yu, Jing
Shengwu Jishu (2018), 28(2), 185-191, 150 | Language: Chinese, Database: CAplus

Objective To discover a novel H_2S donor and to validate its cellular availa bility and mechanism. Methods It employed an in vitro H_2S assay method to identify novel H_2S donors from about 20000 compounds AZD1152 was found to dose-dependently promote the released amounts of H_2S, and enhanced the production of H_2S in cells as accessed by a lead acetate paper assay and a fluore scent probe-based H_2S imaging method. Results A new H_2S donor, AZD1152, was found to enhance the production of H_2S by reacting with Cys and Hcys. 100μmol/L AZD1152 was able to increase the production of H_2S by 2 and 1.5-fold in HEK293T and HepG2 cells, resp. Conclusion AZD1152 was a novel and bioactive donor of H_2S, which can promote intracellular H_2S production by more than 1.5-fold, whose phosphate groups were important for promoting the release of H_2S. It can provide not only mol. tools for the physiol. and pathol. research of endogenous H_2S, but also a drug lead for the treatments of related diseases.

Substances (0) | Reactions (0) | Citing (0)

139

Regioselective synthesis of N-substituted pyrazoles

By: Norman, Natalie; Mello, Tatsiana; Kneitschel, Nika; Cheon, Diane; Moeller, Olivia; Huang, Adrian
Abstracts of Papers, 255th ACS National Meeting & Exposition, New Orleans, LA, United States, March 18-22, 2018 (2018), MEDI-368 | Language: English, Database: CAplus

Pyrazole derivatives have received increasing attention in chem., pharmace utical, agrochem., and material science. In the pharmac eutical sphere, N-substituted pyrazoles have shown to be valuable as they possess inhibitory activities against various biol. targets such as PDE4, CCR2, ALK5, COX2, CRF1 receptors and c-Met protein kinase to name a few. Notably, the aminopy razole derivative AZ D1152 has been clin. evaluated as an anticancer agent. N- Substitution of pyrazoles is a common method to prepare pyrazole deriva tives However most of the literature reported N-substitution reactions gave a mixture of two regiois omers, which needs to be separated through tedious chromatog. Therefore, efficient and regioselective synthetic methods are important in drug discovery. Recently, our team has conducted a systematic study of the N-substitution reactions of pyrazoles under basic conditions. Using the K2CO3-DMSO system, N-substitution reactions occur at room temper ature to yield N1 regioi somers as the major products. Twenty-five X-ray crystallog. structures have been obtained to confirm the regiochem. of the major products. D FT calculations at the B3LY P/6-31G**(d) level suggest that the electronic effect of the C3 substi tuent plays an important role towards the regiochem. This study has thus led to a simple, robust and regioselective reaction protocol that functionalizes the pyrazole rings at the N1 position in good to excellent yields. With this method, the resulting functionalized pyrazoles will allow chemists to access the pyrazoles which were difficult to prepare via traditional cycloaddition methods.

Substances (0) Reactions (0) Citing (0)

140

Mitotic slippage and the subsequent cell fates after inhibition of Aurora B during tubulin-binding agent-induced mitotic arrest

By: Tsuda, Yasuo; Iimori, Makoto; Nakashima, Yuichiro; Nakanishi, Ryota; Ando, Koji; Ohgaki, Kippei; Kitao, Hiroyuki; Saeki, Hiroshi; Oki, Eiji; Maehara, Yoshihiko
Scientific Reports (2017), 7(1), 1-12 | Language: English, Database: CAplus and MEDLINE

Tubulin-binding agents (TBAs) are designed to target microtubule (MT) dynamics, resulting in compro mised mitotic spindles and an unsatisfied spindle assembly checkpoint. The activity of Aurora B kinase is indispe nsable for TBA-induced mitotic arrest, and its inhibition causes mitotic slippage and postmitotic endoredup lication. However, the precise phenomenon underlying mitotic slippage, which is caused by treatment with both Aurora B inhibitors and TBAs, and the cell fate after postmi totic slippage are not completely understood. Here, we found that He La and breast cancer cells treated with the different types of T BAs, such as paclitaxel and eribulin (MT-stabilizing and MT-destabilizing agents, resp.), exhibited distinct behaviors of mitotic slippage on inhibition of Aurora B. In such conditions, the cell fates after postmi totic slippage vastly differed with respect to cell morphol., cell proliferation, and cytotoxicity in short-term culture; i.e., the effects of inhibition of Aurora B were beneficial for cytoto xicity enhanc ement in eribulin treatment but not in paclit axel. However, in long-term culture, the cells that survived after mitotic slippage underwent endoreduplication and became giant cells in both cases, resulting in cellular senesc ence. We propose that MT-destabi lizing agents may be more approp riate than MT-stabilizing agents for treating cancer cells with a weakened Aurora B kinase activity.

Keywords: cervical cancer cell fate mitotic arrest slippage Aurora B

Substances (10) Reactions (0) Citing (25)

141

The target landscape of clinical kinase drugs

By: Klaeger, Susan ; Heinzlmeir, Stephanie ; Wilhelm, Mathias ; Polzer, Harald; Vick, Binje ; Koenig, Paul-Albert ; Reinecke, Maria; Ruprecht, Benjamin; Petzoldt, Svenja; Meng, Chen; et al
Science (Washington, DC, United States) (2017), 358(6367), 1148 | Language: English, Database: CAplus and MEDLINE

Kinase inhibitors are important cancer therapeutic agents. Polypharmacol. is commonly observed, requiring thorough target deconvolution to understand drug mechanism of action. Using chem. proteo mics, the authors analyzed the target spectrum of 243 clin. evaluated kinase drugs. The data revealed previously unknown targets for established drugs, offered a perspe ctive on the "druggable" kinome, highlighted (non)kinase off-targets, and suggested potential therapeutic applications. Integration of phosphop roteomic data refined drug-affected pathways, identified response markers, and streng thened rationale for combination treatm ents. The authors exemplify transla tional value by discov ering SIK2 (salt-inducible kinase 2) inhibitors that modulate cytokine production in primary cells, by identifying drugs against the lung cancer survival marker M ELK (maternal embryonic leucine zipper kinase), and by repurposing cabozantinib to treat FLT3-ITD-pos. acute myeloid leukemia. This resource, available via the Proteomics DB database, should facilitate basic, clin., and drug discovery research and aid clin. decision- making.

Keywords: protein kinase inhibitor drug target discovery Proteomics database antitumor

Substances (128) Reactions (0) Citing (461)

142

Modulators of homeobox transcription factors, DUX4 (double homeobox 4) for regulation of muscle function

By: Rickard, Amanda; De La Garza, Anabel; Schmidt, Uli; Kiselyov, Alexander
World Intellectual Property Organization, WO2017201585 A1 2017-11-30 | Language: English, Database: CAplus

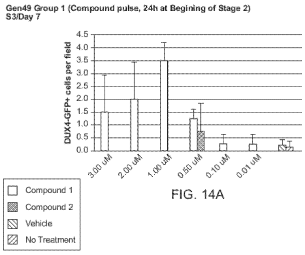

FIG. 14A

Disclosed herein are methods and compositions for the treatment of facioscapu lohumeral muscular dystrophy and other muscle diseases or disorders. In some cases, the methods and compositions involve the use of methyltra nsferase inhibitors (e.g. tazemet ostat, UNC-1999, EPZ005687, GSK343) to inhibit or repress D UX4 expression in muscle cells. Further disclosed herein are methods and cell based assays for screening compounds for the treatment of facioscapulohumeral muscular dystrophy and other muscle diseases.

Keywords: drug screening DUX4 modulator methyltransferase inhibitor muscle disease treatment

PatentPak available Substances (241) Reactions (0) Citing (3)

143

Targeting vcp enhances anticancer activity of oncolytic virus m1 in hepatocellular carcinoma

By: Zhang, Haipeng ; Li, Kai; Lin, Yuan ; Xing, Fan; Xiao, Xiao; Cai, Jing; Zhu, Wenbo; Liang, Jiankai; Tan, Yaqian; Fu, Liwu; et al
Science Translational Medicine (2017), 9(404), eaam7996/1-eaam7996/12 | Language: English, Database: CAplus and MEDLINE

Oncolytic virotherapy is rapidly progressing through clin. evaluation. However, the therapeutic efficacy of oncolytic viruses in humans has been less than expected from preclin. studies. We describe an anticancer drug screen for compounds that enhance M1 oncolytic virus activity in hepatocellular carcinoma (HCC). An inhibitor of the valosin-containing protein (VCP) was identified as the top sensitizer, selectively increasing potency of the oncolytic virus up to 3600- fold. Further investigation revealed that VCP inhibitors cooperated with M1 virus-suppressed inositol-requiring enzyme 1 α (I RE1 α)-X-box binding protein 1 (XBP1) pathway and triggered irresolvable endoplasmic reticulum (ER) stress, subsequently promoting robust apoptosis in H CC. We show that V CP inhibitor improved the oncolytic efficacy of M1 virus in several mouse models of HCC and primary HCC tissues. Finally, this combina torial therapeutic strategy was well tolerated in nonhuman primates. Our study identifies combined V CP inhibition and oncolytic virus as a potential treatment for HCC and demonstrates promising therapeutic potential.

Keywords: hepatocellular carcinoma oncolytic virus vcp anticancer activity

Substances (23) Reactions (0) Citing (287)

144

Diagnostic markers, test method, and therapeutic agents for blastic plasmacytoid dendritic cell neoplasm as rare hematopoietic tumor, and therapeutic agent screening method

By: Takeuchi, Kengo; Sakamoto, Kana; Katayama, Ryohei; Sakata, Seiji
World Intellectual Property Organization, WO2017138500 A1 2017-08-17 | Language: Japanese, Database: CAplus

Diagnosis markers that can provide diagnosis criteria for blastic plasmacytoid dendritic cell neoplasm (BPDCN) as rare hematop oietic tumor are searched, and the presence of an immunobl astoid-type cell form, the reconstruction of 8q24, and the expression of MYC are established as novel markers for sub-typing BPDCN. In addition, it is found that a BET bromo domain-selective inhibitor, an inhibitor capable of directly or indirectly inhibiting the expression or function of MYC or the signaling pathway of M YC such as aurora kinase inhibitor, a HDAC inhibitor or a BCL2 family protein inhibitor is effective as a therap eutic agent in a M YC-expressing group.

Keywords: blastic plasmacytoid dendritic cell neoplasm immunob lastoid diagnostic biomarker

PatentPak available

Substances (12) Reactions (0) Citing (0)

145

Compositions and methods for inhibiting kinase activity using an agent that binds to a PIF pocket of Aurora A kinase for cancer treatment

By: Kern, Dorothee; Zorba, Adelajda

World Intellectual Property Organization, WO2017139321 A1 2017-08-17 | Language: English, Database: CAplus

FIG. 1A

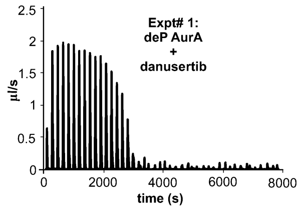

The present invention features therapeutic compositions comprising an agent that specifically binds to a PIF pocket of Aurora A kinase and an agent that specifically binds to an ATP-binding site of Aurora A kinase, and the use of the therap eutic compositions to modulate Aurora A kinase for the treatment of cancer.

Keywords: Aurora A kinase inhibitor PIF pocket binding cancer treatment

PatentPak available

Substances (23) Reactions (0) Citing (0)

146

DNA alkylation and cross-linking agents as compounds and payloads for targeted therapies

By: Herzon, Seth; Healy, Alan; Crawford, Jason; Vizcaino, Maria; Nikolayevskiy, Herman

World Intellectual Property Organization, WO2017132459 A1 2017-08-03 | Language: English, Database: CAplus

The present invention is directed to compounds related to precolibactin, pharmaceutical compositions based upon these compounds and methods of synthesis which are employed to provide intermediates and final compounds, which are alkylating agents and anticancer compounds The chem. synthetic approach disclosed facilitates the synthesis of numerous precolibactin analogs which can be used in the treatment of cancer. Example compound **I** was prepared by a multistep procedure. The invention compounds were evaluated for their anticancer activities (data given).

Keywords: thiazoline thiazole preparation DNA alkylation anticancer targeted therapy

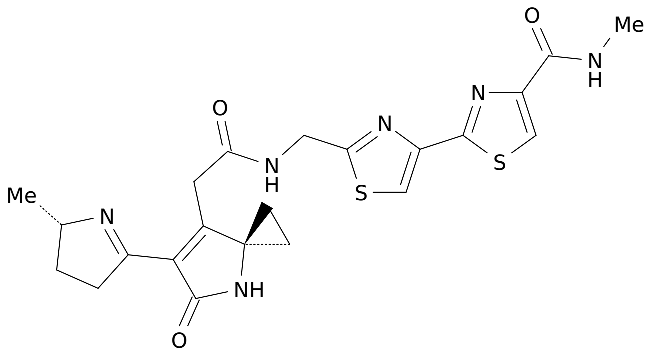

I

PatentPak available

Substances (425) Reactions (693) Citing (0)

147

In Vitro Sensitivity Profiling of Neuroblastoma Cells Against A Comprehensive Small Molecule Kinase Inhibitor Library to Identify Agents for Future Therapeutic Studies

By: Singh, Anjali; Meier-Stephenson, Vanessa; Jayanthan, Aarthi; Narendran, Aru
Current Cancer Drug Targets (2017), 17(6), 569-584 | Language: English, Database: CAplus and MEDLINE

Background: Neuroblastoma (NB) constitutes about 8% of all childhood tumors, yet accounts for more than 15% of deaths, with an unacceptable overall survival rate. These rates are despite the current multimodal therap eutic approaches involving surgery, radiation, chemotherapy and myeloablation with hematopoietic stem cell rescue. Hence, efforts have intens ified to identify new targets and novel therapeutic approaches to improve cure rates in these children. Numerous new agents for adult malign ancies are developed and evaluated for cancer each year, providing an invaluable resource, with the added advantage of available pharmacol. and toxicity data for consideration. Methods: To identify potential therap eutic targets, we screened a small mol. library of 151 small kinase inhibitors against NB cell lines. Based on our initial screening data, we further examined the potential of Bcr- Abl targeting small mol. inhibitors to affect the growth and survival of NB cells. Results: There is diverse activity among the currently available Bcr-Abl inhibitors, possibly reflecting the mol. heterog eneity and off-target activity in each combin ation. In depth analyses of ponatinib, an oral multi-target kinase inhibitor and effective agent in the treatment of refractory Philad elphia chromosome (Ph) pos. leukemia, show growth inhibition at sub-micromolar concentrations In addition, we also identified the potential of this agent to interfere with insulin-like growth factor-1 receptor (IGF-1R) signaling pathways and Src activity, inhibit cell migration and induce apoptosis. Conclusion: Our findings provide initial data on ponati nib's potential to target key growth regulatory pathways and provide the rationale for further studies and evaluation in future early phase clin. trials for the treatment of refractory NB.

Keywords: neuroblastoma cell small mol kinase inhibition therape utics; Bcr-Abl; IGF-1 Receptor; Neuroblastoma; Ponatinib; Src; small molecule kinase inhibitors

Substances (183) | Reactions (0) | Citing (2)

148

Osteotropic nanoparticles for delivering bioactive agents including anticancer agents and/or diagnostic agents for prevention or treatment of bone cancer metastases

By: Durfee, Paul N.; Brinker, Charles Jeffrey; Lin, Yu-Shen; Leong, Hon
World Intellectual Property Organization, WO2017120504 A1 2017-07-13 | Language: English, Database: CAplus

The present disclosure is directed to protocells or nanoparticles, which are optionally coated with a lipid bilayer, which can be used for targeting bone tissue for the delivery of bioactive agents useful in the treatment and/or diagnosis of bone cancer, often metastatic bone cancer which often occurs secondary to a primary cancer such as prostate cancer, breast cancer, lung cancer and ovarian cancer, among numerous others. These protocells or nanoparticles target bone cancer especially metastatic bone cancer with bioactive agents including anticancer agents and/or diagnostic agents for purposes of treating, diagnosing and/or monitoring the therapy of the bone cancer. Osteotropic protocells or nanoparticles, pharmaceutical compositions comprising a population of osteotropic protocells or nanoparticles and methods of diagno sing, treating and/or monitoring therapy of bone cancer are represe ntative aspects.

Keywords: osteotropic nanoparticle bone cancer metastasis targeting

PatentPak available

Substances (230) | Reactions (0) | Citing (4)

149

Optimizing Therapeutic Effect of Aurora B Inhibition in Acute Myeloid Leukemia with AZD2811 Nanoparticles

By: Floc'h, Nicolas; Ashton, Susan; Taylor, Paula; Trueman, Dawn; Harris, Emily; Odedra, Rajesh; Maratea, Kim; Derbyshire, Nicola; Caddy, Jacqueline; Jacobs, Vivien N.; et al
Molecular Cancer Therapeutics (2017), 16(6), 1031-1040 | Language: English, Database: CAplus and MEDLINE

Barasertib (AZD1152), a highly potent and selective aurora kinase B inhibitor, gave promising clin. activity in elderly acute myeloid leukemia (AML) patients. However, clin. utility was limited by the requirement for a 7-day infusion. Here we assessed the potential of a nanoparticle formulation of the selective Aurora kinase B inhibitor AZD2811 (formerly known as AZD1152-hQPA) in preclin. models of AML. When administered to HL-60 tumor xenografts at a single dose between 25 and 98.7 mg/kg, AZD2811 nanoparticle treatment delivered profound inhibition of tumor growth, exceeding the activity of AZD1152. The improved antitumor activity was associated with increased phospho-histone H3 inhibition, polyploidy, and tumor cell apoptosis. Moreover, AZD2811 nanoparticles increased antitumor activity when combined with cytosine arabinoside. By modifying dose of AZD2811 nanoparticle, therapeutic benefit in a range of preclin. models was further optimized. At high-dose, antitumor activity was seen in a range of models including the MOLM-13 disseminated model. At these higher doses, a transient reduction in bone marrow cellularity was observed demonstrating the potential for the formulation to target residual disease in the bone marrow, a key consideration when treating AML. Collectively, these data establish that AZD2811 nanoparticles have activity in preclin. models of AML. Targeting Aurora B kinase with AZD2811 nanoparticles is a novel approach to deliver a cell-cycle inhibitor in AML, and have potential to improve on the clin. activity seen with cell-cycle agents in this disease.

Keywords: AZD2811 nanoparticle anticancer aurora B inhibitor acute myeloid leukemia

Substances (4)   Reactions (0)   Citing (22)

150

Preparation of anti-human LAG3 antibodies, vaccines and/or therapeutic agents for treatment of cancer or infection

By: Malefyt, Rene de Waal; Fayadat-Dilman, Laurence; Liang, Linda
World Intellectual Property Organization, WO2017106129 A1 2017-06-22 | Language: English, Database: CAplus

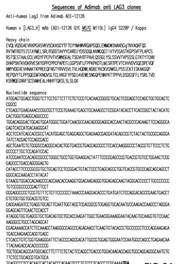
FIG.2A

The present invention includes antibodies and antigen-binding fragments thereof that specifically bind to human or cynomolgus monkey LAG3 as well as Ig chains thereof and polynucleotides encoding the same along with injection devices comprising such antibodies or fragments. Vaccines including such antibodies and fragments as well as compositions comprising the antibodies and fragments (e.g., including anti-PD1 antibodies) are included in the invention. Methods for treating or preventing cancer or infection using such compositions are also provided. In addition, methods for recombinant expression of the antibodies and fragments are part of the present invention.

Keywords: human LAG3 antibody vaccine therapeutic agent composition cancer infection

PatentPak available

Substances (367)   Reactions (0)   Citing (0)

151

Ex vivo drug response profiling detects recurrent sensitivity patterns in drug-resistant acute lymphoblastic leukemia

By: Frismantas, Viktoras; Dobay, Maria Pamela; Rinaldi, Anna; Tchinda, Joelle; Dunn, Samuel H.; Kunz, Joachim; Richter-Pechanska, Paulina; Marovca, Blerim; Pail, Orrin; Jenni, Silvia; et al
Blood (2017), 129(11), e26-e37 | Language: English, Database: CAplus and MEDLINE

Drug sensitivity and resistance testing on diagnostic leukemia samples should provide important functional inform ation to guide actionable target and biomarker discovery. We provide proof of concept data by profiling 60 drugs on 68 acute lymphoblastic leukemia (ALL) samples mostly from resistant disease in cocultures of bone marrow stromal cells. Patient- derived xenografts retained the original pattern of mutations found in the matched patient material. Stromal coculture did not prevent leukemia cell cycle activity, but a specific sensitivity profile to cell cycle-related drugs identified samples with higher cell prolife ration both in vitro and in vivo as leukemia xenografts. In patients with refractory relapses, individual patterns of marked drug resistance and except ional responses to new agents of immediate clin. relevance were detected. The B CL2-inhibitor venetoclax was highly active below 10 nM in B-cell precursor ALL (BCP-ALL) subsets, including MLL-AF4 and TCF3-HLF ALL, and in some T-cell ALLs (T-ALLs), predicting in vivo activity as a single agent and in combination with dexamethasone and vincristine. Unexpected sensitivity to dasatinib with half maximal inhibitory concentration values below 20 nM was detected in 2 indepe ndent T-ALL cohorts, which correlated with similar cytotoxic activity of the SRC inhibitor KX2-391 and inhibition of S RC phosphorylation. A patient with refractory T- ALL was treated with dasatinib on the basis of drug profiling information and achieved a 5- mo remission. Thus, drug profiling captures disease-relevant features and unexpected sensit ivity to relevant drugs, which warrants further explor ation of this functional assay in the context of clin. trials to develop drug repurposing strategies for patients with urgent medical needs.

Keywords: acute lymphoblastic leukemia drug resistance sensitivity

Substances (62) Reactions (0) Citing (123)

152

NPM-ALK expression levels identify two distinct subtypes of paediatric anaplastic large cell lymphoma

By: Pomari, E.; Basso, G.; Bresolin, S.; Pillon, M.; Carraro, E.; d'Amore, E. S.; Viola, G.; Frasson, C.; Basso, K.; Bonvini, P.; et al
Leukemia (2017), 31(2), 498-501 | Language: English, Database: CAplus and MEDLINE

Anaplastic large cell lymphoma (ALCL) is a distinct form of non-Hodgkin lymphoma that accounts for 10-15% of all childhood lymphomas. Differently from ALCL in adults, where about 30% of cases are A LK+, ALCL in children is nearly universally ALK+, and in almost all of the cases, it is characterized by the t(2;5)(p23;q35) translocation, which results in the expression of the hybrid oncogenic tyrosine kinase NPM-ALK. NPM-ALK fusion protein is respon sible for ALCL development, and throughout its constitutive tyrosine kinase activity, it regulates signalling pathways critical for proliferation, cell-cycle control and survival of A LCL cells. So far, few studies have investigated ALCL gene expression profiling, and most of them have used heterog eneous populations of paediatric and adult cases. Main aim of such studies was to identify whether ALK+ and ALK- ALCL had a different transcri ptional program, and although differences were found, similarities were also detected. Based on the fact that paediatric A LCL are almost exclusively AL K+, our study focussed on investi gating the potential heterogeneity inside ALK+ ALCL rather than differ ences between ALK+ and AL K- tumors. In order to investigate the transcriptional characteristics of ALK+ ALCL, we performed a global microa rray-based transcri ptional anal. on 23 paediatric tumor samples, 12 reactive lymph nodes and 4 A LCL cell lines (3 ALK+ and 1 ALK-) (Supplementary Materials). As previously reported, we found that A LK+ tumors displayed downreg ulation of both B-cell and T-cell receptor signalling mols. (such as ARNT2, CD3G, CD24, CD27, CD40, CD79A, NFATC2, PIK3CD, TNFRSF10A) in addition to T helper cell surface mols. (such as CD19, CD28, CTLA4), (Supplementary Table S1), consistent with the notion that this malignancy originates from undifferentiated normal CD4+ T lymphocytes.[5] To explore the mol. differ ences between clin. homoge neous ALK+ ALCL, we performed unsupervised clustering anal. of the tumor samples, identi fying two clearly distinct subgroups of patients. Indeed, supervised anal. identified two different gene expression signatures, both related to the diverse amount of N PM-ALK in ALCL cells (A LK-low and -high). Of note, A LCL cell lines clustered together and had a gene expression signatures more similar to A LK-high than to ALK-low samples (Figure 1a; Supplem entary Table S2). The ALK-high/low signatures were not affected by sample or patient selection bias, as our novel ALK group classif ication was independent of the lymph node infilt ration rate, as the percentage of ALK+ cells was about 40% in the large majority of cases and it could be applied to indipendent ALCL studies (Supplementary Figure S1). NPM-ALK levels were assessed by real-time quant. reverse transcriptase PCR (RQ-PCR) in the same set of patients showing a signif icant correl ation with gene expression profiling data (Pearson's correl ation coefficient 0.79, P<0.0001) (Figures 1b and c, resp.). Addnl. 19 paediatric ALCL cases were tested by R Q-PCR, and overall the full data set, including 42 samples, displayed a bimodal distri bution for NPM-ALK expression consistent with the presence of an A LK-low and an ALK-high group. The median expression values were

2.500 NPM-ALK copies/10.000 ABL in ALK-low cases and 125.000 N PM-ALK copies/10.000 ABL in ALK-high patients (Welch's t-test, P<0.0001 Figure 1d). In addition, when NPM-ALK protein expression was measured in a subset of our cases, selected based on tissue availability (Figure 1e), we found that expression and phosphorylation of NPM-ALK kinase correlated with mRNA levels of the two groups (Figure 1f), supporting the concept that ALK+ ALCL can be distinguished based on NPM-ALK endogenous expression level. We did not observe significant differences between the two subgroups based on clin. characteristics, although patients with central nervous system involvement (n=4) belonged to the ALK-high subgroup (Supplementary Table S3). Interestingly, 9 out of the 12 patients who experienced relapse were ALK-high cases and had a median time of relapse of 5 mo compared with the median time of relapse of 30 mo of the 3 ALK-low relapsed patients. Gene set enrichment anal. using the M Sig DB database (Mol. Signature Database, Oncogene signature C6, http://www.broad.mit.edu/gsea/) showed a pos. enrichment for genes involved in the interl eukin (IL) signalling pathway (specifically IL-2, IL-15 and IL-21) in ALK-low compared with ALK-high ALCL samples, while genes set associated with cell growth and division (CyclinD1, MYC and E2F1) were significantly upregulated in ALK-high cases (Figure 1g). The IL-2 signalling pathway is critical for CD4+ T-cell maturation and function,[6, 7] and its downregulation occurs in ALCL cell lines in response to NPM-ALK expression and activity. Consistently, we showed that IL-2Rγ was expressed in ALK- (FE-PD) ALCL cell line but not in ALK+ (KARPAS-299, SUD-HL-1 and SUP-M2) cell lines (Figure 2a). In order to evaluate the IL-2Rγ expression in ALCL primary tumor cases, we analyzed, by flow cytometry, nine patients (four ALK-low and five ALK-high), for whom tissue samples were available. The results showed that tumor cells in ALK-low patients ubiquitously expressed IL-2Rγ (ALK+/IL-2Rγ+ cells >95%), whereas ALK-high patients displayed a more heterogeneous pattern of expression (Figures 2b and c). Taking into account that ALK-high patients relapsed more frequently and rapidly than ALK-low patients, it seems that in ALK-low cases NPM-ALK is unable to make transformed lymphocytes completely 'deaf' to ILs, and IL signalling retention, in turn, contributes to a less aggressive tumor phenotype. Functional annotation anal. performed by gene set enrichment anal. in ALK-high tumor samples revealed increased expression of genes involved in cell growth and survival, such as cyclins and Aurora kinases A (AURKA) and B (AURKB). These genes are known not only to affect cell proliferation but also regulate chromosomes' segregation and mitosis. Hence, to validate our bioinformatics anal., we took advantage of com. available Aurora kinase inhibitors, which were used to treat a panel of ALK+ ALCL cell lines (KARPAS-299, SU-DHL-1 and SUP-M2) representative of the ALK-high subgroup. As expected, marked growth inhibition of ALCL cells was observed upon administration of AURKA and AURKB inhibitors Alisertib (MLN8237) and Barasertib (AZD1152), resp., and this was associated with cell-cycle arrest and induction of apoptosis (Figure 2d). In particular, the exposure of ALCL cells to increasing concentrations of MLN8237 and/or AZD1152 led to time-dependent inhibition of cell growth, which, consistent with Aurora kinases' involvement in mitotic entry and cytokinesis, was characterized by a dose-dependent 'growth plateau' typical of a cytostatic cell response (Figure 2d). In contrast, Aurora kinases inhibitors did not cause changes in healthy peripheral blood mononuclear cells' proliferation (stimulated with or without phytohaemagglutinin), when used in the same exptl. conditions (Figure 2e). Previous data have shown that Aurora inhibitors exert their activity leading to accumulation of polyploid cells with an endoreplication phenotype before mitotic catastrophe or apoptosis occur. Indeed, when drug- inhibitory effects were investigated in more detail, we found that both compounds, administered for 48 h as single agents or in combination, led to a dramatic increase of the polyploid cell content (8N) over the diploid (2N) and tetraploid (4N) cell population (Supplementary Figure S2A), whereas at earlier time points (24 h) they induced a provisional arrest in G2/M phase and accumulation of tetraploid cells only (data not shown). Prolonged drug exposure, instead, affected cell survival, as the fraction of the cells undergoing apoptosis (Annexin V-pos., AV^+) or necrosis (Propidium Iodide pos., PI^+) increased over time, after 72 h of treatment (Supplementary Figure S2B). AURKA and AURKB have been recently detected in non-Hodgkin lymphoma; however, their involvement in ALCL tumorigenesis, as well as their importance as therapeutic targets, has not been investigated. Herein we provide evidence that AURKA and AURKB may be considered novel drug targets in ALCL and that Aurora kinase inhibitors are effective agents in preclin. ALCL models. Their efficacy depends on their general mode of action and results in inhibiting proliferation, survival and cell-cycle progression of ALCL cells. Overall, our data suggest that the heterogeneous levels of aggressiveness observed in ALCL tumors may be explained by a NPM-ALK distinct gene expression and transcriptional program. High-level ALK+ cells seems to acquire a selective growth advantage compared with ALK-low cells. This concept merits attention to exploit novel therapeutic strategies in this malignancy that may increase curability and survival of children with ALCL.

Keywords: anaplastic large cell lymphoma NPM ALK fusion protein

Substances (4) Reactions (0) Citing (6)

153

Preparation of small molecule based antibody-recruiting compounds for cancer treatment

By: Spiegel, David; Rullo, Anthony

World Intellectual Property Organization, WO2017023994 A1 2017-02-09 | Language: English, Database: CAplus

The present invention relates to chimeric (including bifunctional) compounds **I** [$(UPAR_BM)_m$-CT-$(L^1)_k$-CT-$(L^2)_j$-CT-$(A_BM)_n$; wherein $UPAR_BM$ = moiety which binds to an active site of urokinase- type plasminogen activator receptor (u PAR) on the surface of cancer cells; A_BM = antibody binding moiety comprising a hapten which is capable of binding to an antibody; L^1 and L^2 = linkers; CT = (indepen dently) an optional connector mol.; j = 0- 5; k = 0-5; with the proviso that at least one C T is present when k and j are both 0 (preferably at least one of k and j is 1); each m and n = (indepen dently) 1-15 (preferably m = 1 and n = 1- 3)], compositions comprising those compounds and methods of treating cancer in a patient or subject, especially including metastatic cancer where cancer cells exhibit overexpression (heightened expression) of cell surface urokinase-type plasminogen activator receptor (urokinase receptor) compared to normal (non-cancerous) cells. The compounds **I** bind to the urokinase-type plasminogen activator receptor (uPAR) on the surface of a cancer cell, including a metastatic cancer cell, and conseq uently recruit native antibodies of the patient or subject where the antibodies can selectively degrade and/or deactivate targeted cancer cells through antibody-dependent cellular phagocytosis and antibody-dependent cellular cytotoxicity (ADCC) and/or complement dependent cytotoxicity (C DC) against a large number and variety of cancers, thus providing cancer cell death and an inhibition of growth, elabor ation and/or metastasis of the cancer, including remission and cure of the patient's cancer. E.g., a multi-step synthesis of compound **II**, starting from 5-hydroxy-1,4-naphthoquinone and 1-(trimethylsilyloxy)-1,3-butadiene, was described. Exemplified title compounds **I** were tested for their biol. activity (data given for representative compounds).

Keywords: chimeric bifunctional compound preparation antitumor u PAR modulator antibody recruiting; urokinase type plasmi nogen activator receptor chimeric bifunc tional compound preparation; antibody dependent cellular phagocytosis cancer chimeric bifunctional compound preparation; ADCC antibody dependent cellular cytotoxicity chimeric bifunctional compound preparation; C DC complement dependent cytotoxicity metastasis chimeric bifunc tional compound preparation

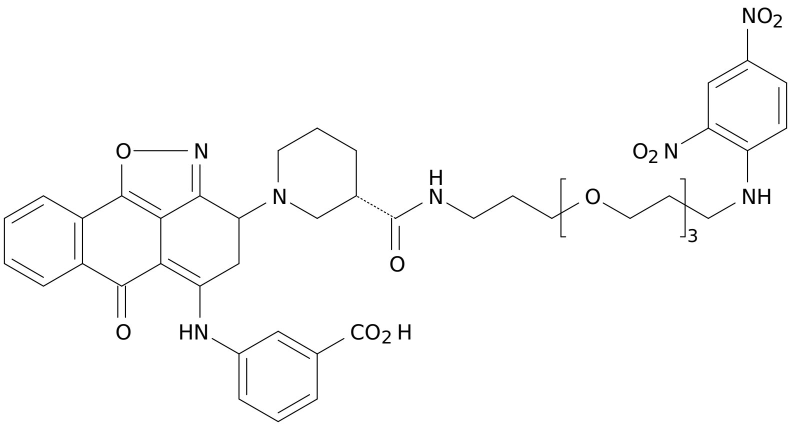

I

PatentPak available

Substances (374) | Reactions (318) | Citing (0)

154

Synergistic cytotoxicity of melatonin and new-generation anticancer drugs against leukemia lymphocytes but not normal lymphocytes

By: Zhelev, Zhivko; Ivanova, Donika; Bakalova, Rumiana; Aoki, Ichio; Higashi, Tatsuya
Anticancer Research (2017), 37(1), 149-160 | Language: English, Database: CAplus and MEDLINE

The present study demonstrates specific sensitization of leukemia lymphocytes towards anticancer drugs using melatonin and clarifies the role of reactive oxygen species (ROS) for induction of apoptosis. The study covers four conven tional and 11 new-generation anticancer drugs. Four parameters were analyzed simulta neously in leukemia and normal lympho cytes treated with drug, melatonin, or their combination: cell viability, induction of apoptosis, level of reactive oxygen species (R OS), and level of protein-carbonyl products. Almost all invest igated combinations of melatonin with new- generation anticancer drugs were charact erized by synerg istic cytotoxicity towards leukemia lymphocytes, while the combin ations with conventional drugs exhibited additive or antagonistic effects on cell viability. In leukemia lympho cytes, the additive cytoto xicity of doxorubicin plus melatonin was accomp anied by low levels of R OS and protein-carbonyl products, as well as by suppre ssion of apoptosis. In normal lymphocytes, none of the studied parameters changed significantly compared to cells treated with doxoru bicin only. The combin ations of everolimus plus melatonin and barasertib plus melatonin exhibited impressive synergistic cytotoxic effects on leukemia lymphocytes but did not affect the viability of normal lymphocytes. In leukemia cells, the synerg istic cytotoxicity was accompanied by strong induction of apoptosis but a decrease of ROS to a level below that of the control. In normal lympho cytes, these combin ations did not affect the level of ROS nor of protein- carbonyl products, and did not induce apoptosis. The data suggest that melatonin is a promising supplementary component in chemotherapy which allows the therap eutic doses of anticancer drugs to be reduced, minimizing their side-effects.

Keywords: anticancer agent melatonin cytotoxicity leukemia lymphocyte; Melatonin; anticancer drugs; apoptosis; leukemia lympho cytes; normal lympho cytes; reactive oxygen species

Substances (14) Reactions (0) Citing (29)

155

Methods and compositions for treatment of metastatic and refractory cancers and tumors

By: Conejo-Garcia, Jose R.; Allegrezza, Michael
United States, US20170020964 A1 2017-01-26 | Language: English, Database: CAplus

A method of treating a mammalian subject with cancer comprises administering to said subject having a cancer, e.g., a metastatic or refractory cancer or tumor, a small mol. inhibitor of a target signaling mol. of the MEK/MAPK pathway that impairs T cell activa tion, and administering to said subject a mol. that induces T cell prolife ration in the presence of said inhibitor. The combin ation of a small mol. inhibitor of a target of the MEK/MAPK pathway and the T cell prolife ration inducer reduces the proliferation of the cancer and tumor cells in vivo. Compositions of the invention include e.g. tramet inib, AZD6244, GDC0973, GDC0623, refametinib, bini metinib, or U0126.

Keywords: antitumor trametinib metastatic refractory cancer tumor treatment

PatentPak available

Substances (67) Reactions (0) Citing (0)

156

Preparation of alanine-based modulators of proteolysis and associated methods of use

By: Crew, Andrew P.; Berlin, Michael; Dong, Hanqing; Qian, Yimin
World Intellectual Property Organization, WO2017011590 A1 2017-01-19 | Language: English, Database: CAplus

FIGURE 1

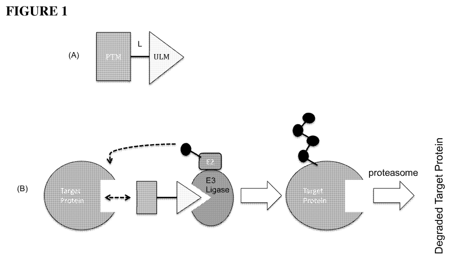

The invention is related to compounds PTM-L-ILM comprising an inhibitor of apoptosis protein (I AP) E3 ubiquitin ligase binding moiety (ILM); a linker (L) coupling I LM and a protein target moiety (P TM) that binds to a target protein or a target polype ptide, e.g., **I**, or a pharmaceutically acceptable salt, enantiomer, stereoisomer, solvate or polymorph thereof. Specifi cally, the invention is related to the preparation of bifunctional compounds, which find utility as modulators of targeted ubiquiti nation, especially inhibitors of a variety of polypeptides and other proteins which are degraded and/or otherwise inhibited by bifunc tional compounds according to the present invention. In particular, the invention is directed to compounds, which contain on one end a ligand which binds to the I AP E3 ubiquitin ligase and on the other end a moiety which binds a target protein such that the target protein is placed in proximity to the ubiquitin ligase to effect degradation (and inhibition) of that protein. Compounds that exhibited a broad range of pharmacol. activities consistent with the degradation/inhibition of targeted polypeptides were prepared Thus, **I** was prepared and at 1 μ M concentration displayed proteolytic modulatory activity (androgen receptor A R degradation between 30% and 60% in V CaP cells).

Keywords: chimeric peptidyl alanine modulator proteolysis targeted polypeptide protein; Iap E3 ligase directed proteo lysis targeting chimeric mol preparation; PROTAC modulator preparation targeted ubiquitination AR receptor degradation inhibition; alanine modulator preparation targeted EZH2 gene zeste protein degrad ation; targeted JNK1 JNK2 BRD4 degradation alanine PROTAC modulator preparation

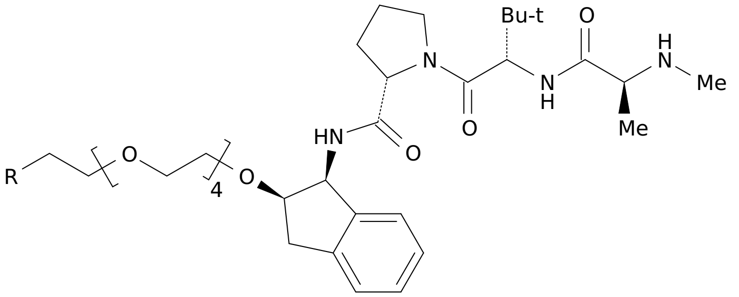

I

R =

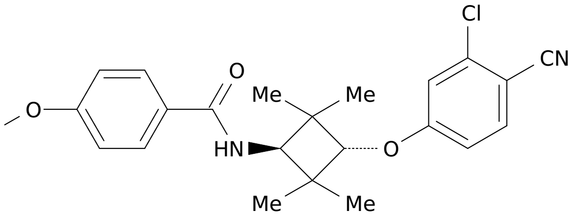

PatentPak available

Substances (539) Reactions (662) Citing (6)

157

Preparation of substituted diazepine derivatives and analogs for use as MDM2-based modulators

By: Crew, Andrew P.; Crews, Craig M.; Dong, Hanqing; Qian, Yimin; Wang, Jing

United States, US20170008904 A1 2017-01-12 | Language: English, Database: CAplus

Title compounds **I** [X = a protein targeting moiety; Y = a linker coupling; Z = a M DM2 E3 ubiquitin ligase binding moiety], and their pharmaceutically acceptable salts, are prepared and disclosed as M DM2-based modulators of proteolysis. Thus, e.g., **II** was prepared by a multistep procedure (preparation given). **I** were evaluated in AR ELISA assays, e.g., **II** demonstrated a degradation activity of 0 to 25% at 1 μM.

Keywords: diazepine preparation MDM2 modulator

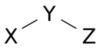

I

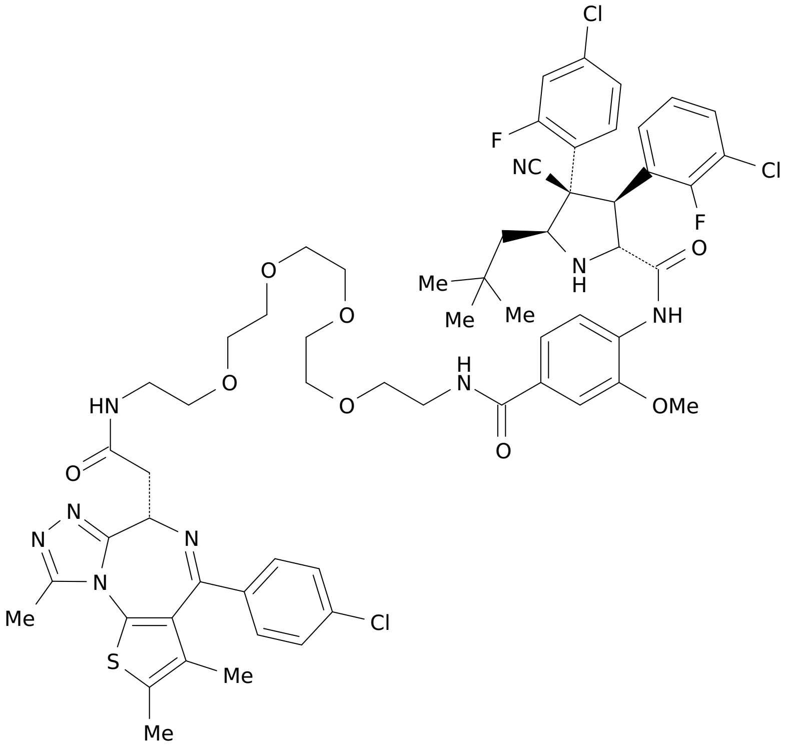

II

PatentPak available

Substances (430)

Reactions (565)

Citing (1)

158

Epidermal growth factor receptor (EGFR) structure-based bioactive pharmacophore models for identifying next-generation inhibitors against clinically relevant EGFR mutations

By: Panicker, Pooja S.; Melge, Anu R.; Biswas, Lalitha; Keechilat, Pavithran; Mohan, Chethampadi G.
Chemical Biology & Drug Design (2017), 90(4), 629-636 | Language: English, Database: CAplus and MEDLINE

Present work elucidates identification of next generation inhibitors for clin. relevant mutations of epidermal growth factor receptor (EGFR) using structure-based bioactive pharmacophore modeling followed by virtual screening (VS) techniques. Three-dimensional (3D) pharmacophore models of EGFR and its different mutants were generated. This includes seven 3D pharmacophoric points with three different chem. features (descriptors), i.e., one hydrogen bond donor, three hydrogen bond acceptors and three aromatic rings. Pharmacophore models were validated using decoy dataset, Receiver operating characteristic plot, and external dataset compounds The robust, bioactive 3D e-pharmacophore models were then used for VS of four different small compound databases: FDA approved, investigational, anticancer, and bioactive compounds collections of Selleck Chems. CUDC101 a multitargeted kinase inhibitor showed highest binding free energy and 3D pharmacophore fit value than the well known EGFR inhibitors, Gefitinib and Erlotinib. Further, we obtained ML167 as the second best hit on VS from bioactive database showing high binding energy and pharmacophore fit value with respect to EGFR receptor and its mutants. Optimistically, presented drug discovery based on the computational study serves as a foundation in identifying and designing of more potent EGFR next-generation kinase inhibitors and warrants further exptl. studies to fight against lung cancer.

Keywords: EGFR structure pharmacophore mutation; computer-aided drug design; epidermal growth factor receptor; erlotinib; next-generation EGFR inhibitors; non-small cell lung cancer; pharmacophore; virtual screening

Substances (9) Reactions (0) Citing (17)

159

Reactive oxygen species generation and increase in mitochondrial copy number: new insight into the potential mechanism of cytotoxicity induced by aurora kinase inhibitor, AZD1152-HQPA

By: Zekri, Ali; Mesbahi, Yashar; Ghanizadeh-Vesali, Samad; Alimoghaddam, Kamran; Ghavamzadeh, Ardeshir; Ghaffari, Seyed H.
Anti-Cancer Drugs (2017), 28(8), 841-851 | Language: English, Database: CAplus and MEDLINE

Aurora-B kinase overexpression plays important roles in the malignant progression of prostate cancer (PCa). AZD1152-HQPA, as an inhibitor of Aurora-B, has recently emerged as a promising agent for cancer treatment. In this study, we aimed to investigate the effects of AZD1152-HQPA on reactive oxygen species (ROS) generation and mitochondrial function in PCa. We used AZD1152-HQPA (Barasertib), a highly potent and selective inhibitor of Aurora-B kinase. The effects of AZD1152-HQPA on cell viability, DNA content, cell morphol., and ROS production were studied in the androgen-independent PC-3 PCa cell line. Moreover, the mitochondrial copy number and the expression of genes involved in cell survival and cancer stem cell maintenance were investigated. We found that AZD1152-HQPA treatment induced defective cell survival, polyploidy, micronuclei formation, cell enlargement, and cell death by significant overexpression of p73, p21 and downregulation of cell cycle-regulatory genes in a drug concentration-dependent manner. Moreover, AZD1152 treatment led to an excessive ROS generation and an increase in the mitochondrial copy number not only in PC-3 but also in several other malignant cells. AZD1152 treatment also led to downregulation of genes involved in the maintenance of cancer stem cells. Our results showed a functional relationship between the aurora kinase inhibition, an increase in mitochondrial copy number, and ROS generation in therapeutic modalities of cancer. This study suggests that the excessive ROS generation may be a novel mechanism of cytotoxicity induced by the aurora kinase inhibitor, AZD1152-HQPA.

Keywords: aurora kinase inhibitor ROS mitochondrial copy number cytotoxicity

Substances (11) Reactions (0) Citing (14)

160

Compositions and methods comprising protein phosphatase 2A (PP2A) activators and protein kinase inhibitors for treating cancer

By: Narla, Goutham
World Intellectual Property Organization, WO2016210134 A1 2016-12-29 | Language: English, Database: CAplus

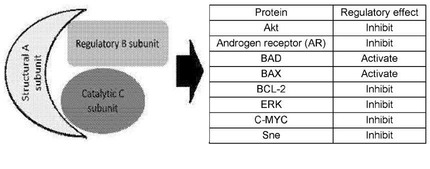

Fig. 1

A method of treating cancer in a subject in need thereof includes administering to the subject therapeutically effective amounts of a PP2A activator and a protein kinase inhibitor.

Keywords: antitumor protein phosphatase kinase inhibitor cancer treatment

PatentPak available

Substances (123) Reactions (0) Citing (0)

161

Inhibition of granulocyte colony stimulating factor in the treatment of cancer

By: Morris, Katherine Theresa; Beswick, Ellen Janine
United States, US20160368980 A1 2016-12-22 | Language: English, Database: CAplus

The present invention relates to the discovery that granulocyte colony stimulating factor (G-CSF) as well as granulocyte colony stimul ating factor receptor (G-CSFR) are highly expressed in cancer tissue and the inhibition of G- CSF using a G-CSF inhibitor, which term includes inhibitors of the G-CSF receptor, represents a viable approach to the treatment of cancer, including drug resistant cancers, metastatic cancers and recurrent cancers. G-CSF inhibitors as described herein may be used alone or in combin ation with an at least one addnl. anti-cancer agent for the treatment of cancer.

Keywords: granulocyte colony stimulating factor inhibitor cancer treatment anticancer combin ation

PatentPak available

Substances (307) Reactions (0) Citing (0)

162

MCT4 inhibitors for treating disease

By: Parnell, Kenneth Mark; McCall, John M.
World Intellectual Property Organization, WO2016201426 A1 2016-12-15 | Language: English, Database: CAplus

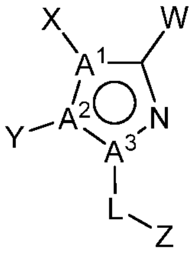

(I)

Disclosed are pyrazole compounds and compositions useful in the treatment of M CT4 mediated diseases, such as proliferative and inflammatory diseases, having the structure **I**, and methods of inhibiting M CT4 activity in a human or animal subject. Also disclosed is the preparation of compounds of the invention.

Keywords: pyrazole derivative preparation MCT4 inhibitor proliferative disease inflamation

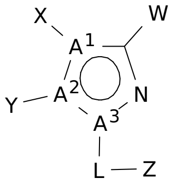

I

PatentPak available

Substances (300) | Reactions (245) | Citing (3)

163

Human GLUT5 specific inhibitors and methods of treatment

By: Oprea, Tudor I.; Bologa, Cristian George; Ursu, Oleg; Choe, Jun-Yong; Iancu, Cristina
World Intellectual Property Organization, WO2016201214 A1 2016-12-15 | Language: English, Database: CAplus

The present invention relates to compounds which have been discovered to be potent ligands (inhibitors) of human GLUT5 (glucose transporter type 5), a facilitative glucose transporter that transports fructose, and their use as ligands assays which can uncover addnl. ligands of GLUT5, having the potential for being used as drugs. In addition, the present invention is directed to compounds, chem. compositions and methods for treating disease states and conditions which are mediated through G LUT5, including such disease states and conditions as GLUT5 deficiency syndrome, diabetes (type I and I I), cancer, metabolic diseases including metabolic syndrome and fatty liver disease, among others.

Keywords: glucose transporter GLUT5 inhibitor diabetes cancer fatty liver disease

PatentPak available

Substances (347) | Reactions (0) | Citing (0)

164

Preparation of PROTAC compounds as TANK-binding kinase 1 inhibitors for modulation of proteolysis and associated methods of use

By: Crew, Andrew P.; Wang, Jing; Dong, Hanqing; Qian, Yimin; Siu, Kam; Ferraro, Caterina; Crews, Craig M.

World Intellectual Property Organization, WO2016197114 A1 2016-12-08 | Language: English, Database: CAplus

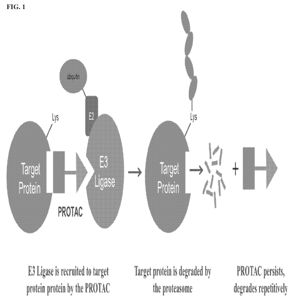

The invention is related to compounds TBM-L-PTM comprising an E3 ubiquitin ligase binding moiety (U LM), a TBK1 binding moiety (T BM) connected directly or through a chem. linker (L) , e.g., **I**, which find utility modulators of targeted ubiquitination and degradation of TANK-binding kinase (TBK1). In particular, the invention is directed to compounds, which contain on one end an E3 ubiquitin ligase binding moiety which binds to the ubiquitin ligase and on the other end a moiety which binds TBK1 such that TBK1 is placed in proximity to the ubiquitin ligase to effect degradation (and inhibition) of TBK1. Compounds that exhibited a broad range of pharmacol. activities consistent with the degradation/inhibition of TBK1 were prepared Thus, **I** was prepared and displayed proteo lytic modulatory activity (TBK1 degradation D_{max} = 96%).

Keywords: TANK binding kinase 1 P ROTAC chimeric mol prepar ation; PROTAC modulator preparation targeted TBK1 kinase degrad ation ubiquitination; Von Hippel Lindau ligand prepar ation TBK1 PROTAC degradation inhibition

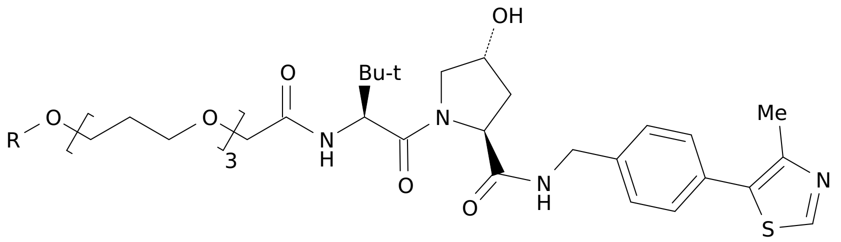

I

R =

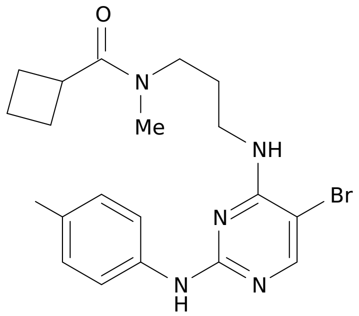

PatentPak available

Substances (439) Reactions (182) Citing (3)

165

Imide-based modulators of proteolysis and associated methods of use

By: Crews, Craig M.; Crew, Andrew P.; Dong, Hanqing; Wang, Jing; Qian, Yimin; Siu, Kam; Jin, Meizhong
World Intellectual Property Organization, WO2016197032 A1 2016-12-08 | Language: English, Database: CAplus

Figure 1B

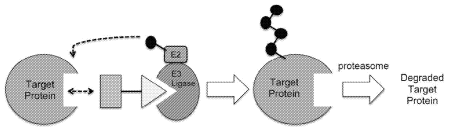

The description relates to imide- based compounds, including bifunctional. compounds comprising the same, which find utility as modulators of targeted ubiquitination, especially inhibitors of a variety of polypeptides and other proteins which are degraded and/or otherwise inhibited by bifunctional compounds according to the present invention. In particular, the description provides compounds, which contain on one end a ligand which binds to the cereblon E3 ubiquitin ligase and on the other end a moiety which binds a target protein such that the target protein is placed in proximity to the ubiquitin ligase to effect degradation (and inhibition) of that protein. Compounds can be synthesized that exhibit a broad range of pharmacol. activities consistent with the degradation/inhibition of targeted polypeptides of nearly any type.

Keywords: antitumor proteolysis modulator imide preparation cancer

PatentPak available

Substances (425) | Reactions (330) | Citing (5)

166

Inhibition of the pentose-phosphate pathway selectively sensitizes leukemia lymphocytes to chemotherapeutics by ROS-independent mechanism

By: Zhelev, Zhivko; Ivanova, Donika; Bakalova, Rumiana; Aoki, Ichio; Higashi, Tatsuya
Anticancer Research (2016), 36(11), 6011-6020 | Language: English, Database: CAplus and MEDLINE

The aim of the present study was to investigate: (i) the possibility of sensitizing leukemia lymphocytes to anticancer drugs by inhibiting pentose-phosphate pathway using 6-aminonicotinamide (6-ANA); (ii) to find combinations with synergistic cytotoxic effect on leukemia lymphocytes and to investigate their cytotoxicity towards normal lymphocytes; (iii) and to clarify the role of reactive oxygen species (ROS) in the induction of apoptosis by those combina tions. The study covers 15 anticancer drugs - conven tional and new-generation. The experiments were performed on Jurkat leukemia cell line and normal lympho cytes, isolated from clin. healthy blood donors. Four parameters were analyzed simultaneously in both cell suspen sions treated by drug or 6- ANA (sep., and in combination): cell viability, induction of apoptosis, level of R OS, and level of protein-carbonyl products. Most combin ations of drug plus 6-ANA were characterized by synergistic cytotoxic effects on Jurkat cells. The synergism increased with increasing incubation time. Upon combination of 6-ANA with conventional chemotherapeutic (e.g. doxorubicin), synergistic cytotoxic effects were also detected in normal lymphocytes. In both cell types, the cytoto xicity of the combination of doxorubicin plus 6-ANA was accompanied by increased induction of apoptosis, but by a slight reduction of ROS and protein-carbonyl products compared to cells treated with doxorubicin only. Upon combin ation of 6-ANA with new-generation anticancer drugs (e.g. everolimus or barasertib), the synergistic cytotoxic effect on leukemia lymphocytes was also accompanied by very strong induction of apoptosis through R OS-independent mechanism(s). Neither of these combin ations exhibited any cytotoxicity towards normal lymphocytes. The data suggest that 6- ANA could be used as a supplementary component in anticancer chemoth erapy, and would allows therapeutic doses of anticancer drugs to be reduced, thereby minimizing their side-effects.

Keywords: pentose phosphate pathway leukemia lymphocyte ROS mechanism chemother apeutics; 6-Aminonicotinamide; ROS; chemotherapeutics; leukemia lymphocytes

Substances (16) | Reactions (0) | Citing (11)

167

In vitro evaluation of a combination treatment involving anticancer agents and an aurora kinase B inhibitor

By: Sakai, Senna; Izumi, Hiroto; Yoshiura, Yukiko; Nakayama, Yoshifumi; Yamaguchi, Takahiro; Harada, Yoshikazu; Koi, Chiho; Kurata, Hiroyuki; Morimoto, Yasuo
Oncology Letters (2016), 12(5), 4263-4269 | Language: English, Database: CAplus and MEDLINE

Aurora kinase B (AURKB) inhibitors are regarded as potential mol.-targeting drugs for cancer therapy. The present study evaluated the cytotoxic effect of a combination of AZD1152-hQPA, an AURKB inhibitor, and various anticancer agents on the HeLa human cervical cancer cell line, as well as its cisplatin-resistant equivalent HCP4 cell line. It was demonstrated that AZD1152-hQPA had an antagonistic effect on the cytotoxicity of cisplatin, etoposide and doxorubicin, but had a synergistic effect on that of all-trans-retinoic acid (ATRA), Am80 and TAC-101, when tested on HeLa cells. Cisplatin, etoposide and doxorubicin were shown to increase the cellular expression of AURKB, while ATRA, Am80 and TAC-101 downregulated its expression. These results suggested that AURKB expression is regulated by these anticancer agents at the transcriptional level, and that the level of expression of AURKB may influence the cytotoxic effect of AZD1152-hQPA. Therefore, when using anticancer agents, decreasing the expression of AURKB using a mol.-targeting drug may be an optimal therapeutic strategy.

Keywords: anticancer agent; aurora kinase B; aurora kinase B inhibitor; combination index; combination treatment

Substances (0) Reactions (0) Citing (4)

168

Bromodomain and Extraterminal Protein Inhibition Blocks Growth of Triple-negative Breast Cancers through the Suppression of Aurora Kinases

By: Sahni, Jennifer M.; Gayle, Sylvia S.; Weber Bonk, Kristen L.; Vite, Leslie Cuellar; Yori, Jennifer L.; Webb, Bryan; Ramos, Erika K.; Seachrist, Darcie D.; Landis, Melissa D.; Chang, Jenny C.; et al
Journal of Biological Chemistry (2016), 291(45), 23756-23768 | Language: English, Database: CAplus and MEDLINE

Bromodomain and extraterminal (BET) proteins are epigenetic "readers" that recognize acetylated histones and mark areas of the genome for transcription. BRD4, a BET family member protein, has been implicated in a number of types of cancer, and BET protein inhibitors (BETi) are efficacious in many preclin. cancer models. However, the drivers of response to BETi vary depending on tumor type, and little is known regarding the target genes conveying BETi activity in triple-neg. breast cancer (TNBC). Here, we show that BETi repress growth of multiple in vitro and in vivo models of TNBC by inducing two terminal responses: apoptosis and senescence. Unlike in other cancers, response to BETi in TNBC is not dependent upon suppression of MYC. Instead, both end points are preceded by the appearance of polyploid cells caused by the suppression of Aurora kinases A and B (AURKA/B), which are critical mediators of mitosis. In addition, AURKA/B inhibitors phenocopy the effects of BETi. These results indicate that Aurora kinases play an important role in the growth suppressive activity of BETi in TNBC. Elucidating the mechanism of response to BETi in TNBC should (1) facilitate the prediction of how distinct TNBC tumors will respond to BETi and (2) inform the rational design of drug combination therapies.

Keywords: Aurora kinase BET protein inhibitor anticancer breast cancer; Aurora kinase; anticancer drug; apoptosis; breast cancer; bromodomain-containing protein 4 (BRD4); cellular senescence; inhibition mechanism

Substances (4) Reactions (0) Citing (44)

169

Barasertib (AZD1152), a Small Molecule Aurora B Inhibitor, Inhibits the Growth of SCLC Cell Lines In Vitro and In Vivo

By: Helfrich, Barbara A.; Kim, Jihye; Gao, Dexiang; Chan, Daniel C.; Zhang, Zhiyong; Tan, Aik-Choon; Bunn, Paul A. Jr.
Molecular Cancer Therapeutics (2016), 15(10), 2314-2322 | Language: English, Database: CAplus and MEDLINE

Small-cell lung cancer (SCLC) cells have rapid proliferation, universal Rb inactivation, and high rates of MYC family amplification, making aurora kinase inhibition a natural target. Preclin. studies have demonstrated activity for Aurora A and pan-Aurora inhibitors with some relationship to MYC family expression. A clin. trial showed activity for an Aurora kinase A inhibitor, but no biomarkers were evaluated. We screened a panel of 23 SCLC lines with and without MYC family gene amplification or high MYC family gene expression for growth inhibition by the highly potent, selective aurora kinase B inhibitor barasertib. Nine of the SCLC lines were very sensitive to growth inhibition by barasertib, with IC_{50} values of <50 nmol/L and >75% growth inhibition at 100 nmol/L. Growth inhibition correlated with cMYC amplification (P = 0.018) and cMYC gene expression (P = 0.026). Sensitive cell lines were also enriched in a published MYC gene signature (P = 0.042). In vivo, barasertib inhibited the growth of xenografts established from an SCLC line that had high cMYC gene expression, no cMYC amplification, and was pos. for the core MYC gene signature. Our studies suggest that SCLC tumors with cMYC amplification/high gene expression will frequently respond to Aurora B inhibitors and that clin. studies coupled with predictive biomarkers are indicated. Mol Cancer Ther; 15(10); 2314-22.

Keywords: barasertib aurora B inhibitor small cell lung cancer antitumor

Substances (2) | Reactions (0) | Citing (59)

170

Janus face-like effects of Aurora B inhibition: antitumoral mode of action versus induction of aneuploid progeny

By: Wiedemuth, Ralf; Klink, Barbara; Fujiwara, Mamoru; Schroeck, Evelin; Tatsuka, Masaaki; Schackert, Gabriele; Temme, Achim
Carcinogenesis (2016), 37(10), 993-1003 | Language: English, Database: CAplus and MEDLINE

The mitotic Aurora B kinase is overexpressed in tumors and various inhibitors for Aurora B are currently under clin. assessments. However, when considering Aurora B kinase inhibitors as anticancer drugs, their mode of action and the role of p53 status as a possible predictive factor for response still needs to be investigated. In this study, we analyzed the effects of selective Aurora B inhibition using AZD1152-HQPA/Barasertib (AZD1152) on HCT116 cells, U87-MG, corresponding isogenic p53-deficient cells and a primary glioblastoma cell line. AZD1152 treatment caused polyploidy and non-apoptotic cell death in all cell lines irresp. of p53 status and was accompanied by poly-merotelic kinetochore-microtubule attachments and DNA damage. In p53 wild-type cells a DNA damage response induced an inefficient pseudo-G1 cell cycle arrest, which was not able to halt ongoing endoreplication of cells. Of note, release of tumor cells from AZD1152 resulted in recovery of aneuploid progenies bearing numerical and structural chromosomal aberrations. Yet, AZD1152 treatment enhanced death receptor TRAIL-R2 levels in all tumor cell lines investigated. A concomitant increase of the activating natural killer (NK) cell ligand MIC A/B in p53-deficient cells and an induction of FAS/CD95 in cells containing p53 rendered AZD1152-treated cells more susceptible for NK-cell-mediated lysis. Our study mechanistically explains a p53-independent mode of action of a chem. Aurora B inhibitor and suggests a potential triggering of antitumoral immune responses, following polyploidization of tumor cells, which might constrain recovery of aneuploid tumor cells.

Keywords: aurora B kinase inhibitor antitumor aneuploidy

Substances (4) | Reactions (0) | Citing (7)

171

Preparation of dimeric quinacrine derivatives as autophagy inhibitors for cancer therapy

By: Amaravadi, Ravi K.; Winkler, Jeffrey
World Intellectual Property Organization, WO2016168721 A1 2016-10-20 | Language: English, Database: CAplus

The invention provides dimeric quinacrine derivatives and related compounds of formula I and compositions, methods of treatment and syntheses. The compounds exhibit unexpected anticancer activity and are useful in the treatment of a variety of autophagy-related disorders. Compounds of formula I wherein R^1, R^2, R^3, R^4, R^5, R^6, R^7, R^8, $R^{1'}$, $R^{2'}$, $R^{3'}$, $R^{4'}$, $R^{5'}$, $R^{6'}$, $R^{7'}$ and $R^{8'}$ are independently H, halo, C N, NO_2, OH, CO_2H, etc.; R and R' are indepen dently H, (un)substituted C_{1-6} alkyl, (un)substituted C_{1-6} alkoxy, (un)substituted C_{1-7} acyl, etc.; L is a linker which covalently links to two exocyclic amine groups; and pharmace utically acceptable salts, enantiomers, diastereoisomers, solvates and polymorphs thereof, are claimed. Example compound II was prepared by a general procedure (procedure given). The invention compounds were evaluated for their autophagy inhibitory activity (some data given).

Keywords: dimeric quinacrine preparation autophagy inhibitor treatment cancer

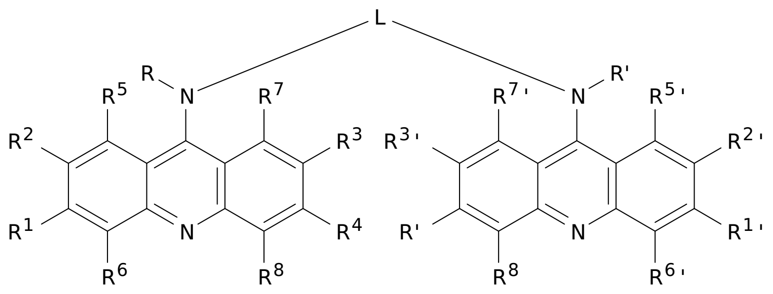

I

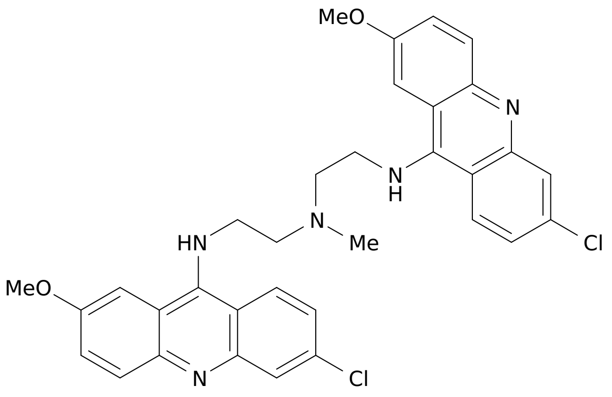

II

PatentPak available

Substances (335) Reactions (43) Citing (3)

172

Leishmania donovani Aurora kinase: A promising therapeutic target against visceral leishmaniasis

By: Chhajer, Rudra; Bhattacharyya, Anirban; Didwania, Nicky; Shadab, Md; Das, Nirupam; Palit, Partha; Vaidya, Tushar; Ali, Nahid
Biochimica et Biophysica Acta, General Subjects (2016), 1860(9), 1973-1988 | Language: English, Database: CAplus and MEDLINE

Aurora kinases are key mitotic kinases executing multiple aspects of eukaryotic cell-division. The apicomplexan homologs being essential for survival, suggest that the Leishmania homolog, annotated LdAIRK, may be equally important. Bioinformatics, stage-specific immunofluorescence microscopy, immunoblotting, RT-PCR, mol. docking, in-vitro kinase assay, anti-leishmanial activity assays, flow cytometry, fluorescence microscopy. Ldairk expression is seen to vary as the cell-cycle progresses from G1 through S and finally G2M and cytokinesis. Kinetic studies demonstrate their enzymic activity exhibiting a Km and Vmax of 6.12 μ M and 82.9 pmoles·min^{-1}mg^{-1} resp. against ATP using recombinant Leishmania donovani H3, its physiol. substrate. Due to the failure of Ld AIR K -/+ knock-out parasites to survive, we adopted a chem. knock- down approach. Based on the conservation of key active site residues, three mammalian Aurora kinase inhibitors were investigated to evaluate their potential as inhibitors of LdAIRK activity. Interestingly, the cell-cycle progressed unhindered, despite treatment with GSK-1070916 or Barasertib, inhibitors with greater potencies for the ATP-binding pocket compared to Hesperadin, which at nanomolar concentrations, severely compromised viability at IC_{50}s 105.9 and 36.4 n M for promastigotes and amastigotes, resp. Cell-cycle and morphol. studies implicated their role in both mitosis and cytokinesis. We identified an Aurora kinase homolog in L. donovani implicated in cell- cycle progression, whose inhibition led to aberrant changes in cell-cycle progression and reduced viability. Human homologs being actively pursued drug targets and the observations with LdAIRK in both promastigotes and amastigotes suggest their potential as therapeutic-targets. Importantly, our results encourage the exploration of other proteins identified herein as potential novel drug targets.

Keywords: Leishmania Aurora kinase visceral leishmaniasis development Hesperadin; Aurora kinase; Cell-cycle; Cytokinesis; Hesper adin; Leishmania donovani; Therapeutic target

Substances (5) | Reactions (0) | Citing (12)

173

Pericyte long non-coding RNA inhibitors for use in treatment of diseases mediated by PDGFR expression

By: Zehendner, Christoph Michael; Dimmeler, Stefanie; Zeiher, Andreas
World Intellectual Property Organization, WO2016150870 A1 2016-09-29 | Language: English, Database: CAplus

The present invention provides novel non-coding RNAs (lncRNA) that were identified to be expressed in pericytes upon hypoxia. The lncRNA of the invention pos. affect Platelet-derived Growth Factor Receptor (PDGFR) beta expression, pericytes proliferation and pericyte recruitment to endothelial cells. The invention provides inhibitors of the lncRNA for use in the treatment of diseases mediated by PDGFR expression. For example the invention described antisense approaches to target the lnc RNA of the invention. Furthermore, the invention provides lncRNA inhibitors as amplifier of therapeutic PDGFR inhibitors such as imatinib or other tyrosine kinase inhibitors. lncRNA inhibitors and methods for screening modulators of lnc RNA expression and/or function are provided.

Keywords: pericyte long non coding RNA human PDGFR disease

PatentPak available

Substances (28) | Reactions (0) | Citing (1)

174

Preparation of bifunctional PROTAC compounds and methods for the enhanced degradation of targeted proteins

By: Berlin, Michael; Zimmerman, Kurt; Snyder, Lawrence; Crew, Andrew P.; Crews, Craig M.; Chen, Xin; Dong, Hanqing; Ferraro, Caterina; Jin, Meizhong; Qian, Yimin; et al

World Intellectual Property Organization, WO2016149668 A1 2016-09-22 | Language: English, Database: CAplus

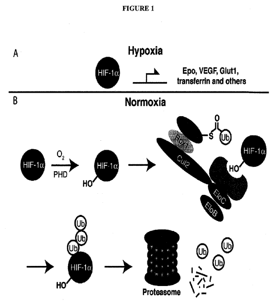

The invention is related to compounds ULM-L-PTM comprising a Von Hippel-Lindau (VHL) E3 ubiquitin ligase binding moiety (ULM), a protein targeting moiety (PTM) connected directly or through a chem. linker (L) , e.g., **I**, or a pharmaceutically acceptable salt, enanti omer, stereoisomer, solvate or polymorph thereof which find utility as modulators of targeted ubiquiti nation, especially inhibitors of a variety of polypeptides and other proteins which are degraded and/or otherwise inhibited by the bifunc tional compounds of the invention. In particular, the invention is directed to compounds, which contain on one end a V HL ligand which binds to the ubiquitin ligase and on the other end a moiety which binds a target protein such that the target protein is placed in proximity to the ubiquitin ligase to effect degradation (and inhibition) of that protein. Compounds that exhibited a broad range of pharmacol. activities consistent with the degradation/inhibition of targeted polypeptides were prepared Thus, **I** was prepared and displayed proteolytic modulatory activity (androgen receptor degradat ion51%≤ Dmax ≤70%; BRD4 degradation cMyc Imax ≥71%).

Keywords: chimeric mol preparation targeted protein degradation ubiquitination; PROTAC modulator preparation targeted ubiquit ination AR receptor degradation inhibition; Von Hippel Lindau ligand preparation BRD4 PROTAC degradation inhibition

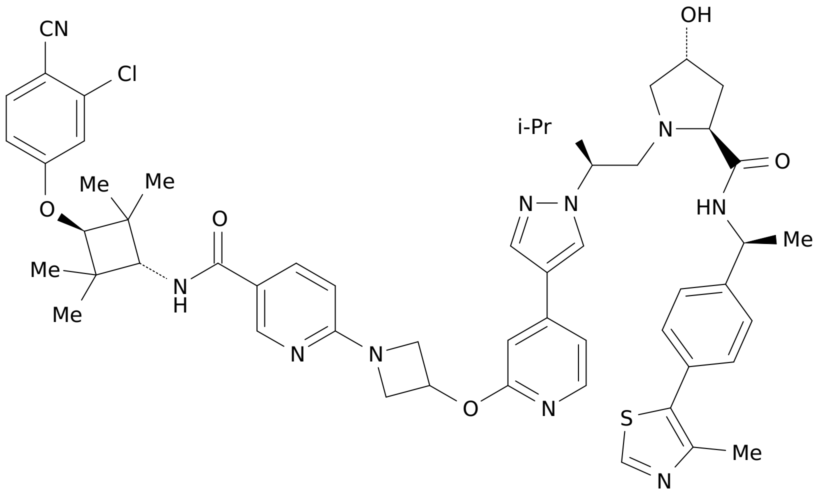

I

PatentPak available

Substances (590) Reactions (228) Citing (6)

175

Cholesteryl ester transfer protein (CEPT) inhibition in the treatment of cancer

By: Kaur, Mandeep; Esau, Luke E.; Sagar, Sunii
World Intellectual Property Organization, WO2016135633 A1 2016-09-01 | Language: English, Database: CAplus

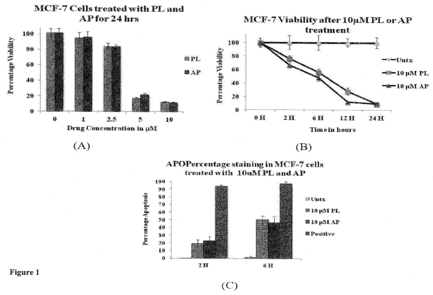

In one embodiment, the invention provides methods of treatment which use therapeu tically effective amounts of Choleste ryl Ester Transfer Protein (CETP) inhibitors to treat a variety of cancers. In certain embodi ments, the inhibitor is a CETP-inhibiting small mol., CETP-inhibiting antisense oligonucleotide, CETP-inhibiting siRNA or a C ETP- inhibiting antibody. Related pharmac eutical composi tions, kits, diagno stics and screens are also provided.

Keywords: cholesteryl ester transfer protein C EPT inhibition cancer

PatentPak available

Substances (362) Reactions (0) Citing (0)

176

Dye-stabilized nanoparticles and methods of their manufacture and therapeutic use

By: Heller, Daniel A.; Shamay, Yosef

World Intellectual Property Organization, WO2016130546 A1 2016-08-18 | Language: English, Database: CAplus

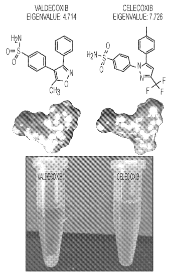

FIG. 18

Described herein are nanoparticles which are largely made of (e.g., 90 wt%) hydrop hobic drugs and are stabilized by water soluble dyes. The nanoparticles can range in size from 30 nm to 150 nm and have highly neg. surface charge (e.g., - 55 mV). These nanopar ticles are highly soluble in water, stable for days in P BS buffer and can be easily lyophi lized and reconstituted in water. Using quant. self-assembly prediction calculations, topochem. mol. descri ptors were identified and validated as highly predictive indicators of nano-assembly, nanoparticle size, and drug loading. The resulting nanopar ticles selectively targeted kinase inhibitors to caveolin-1-expressing human colon cancer and autocht honous liver cancer models to yield striking therap eutic effects while avoiding p ERK inhibition in healthy skin. The nanoparticles exhibited remarkable anti-tumor efficacy in vitro and in vivo in models of hepatoc ellular carcinoma. Thus, dye- encapsulated sorafenib nanoparticles (IR783-SFB) were synthesized using the nanopptn. method; 0.1 mL of sorafenib dissolved in D MSO (10 mg/m L) was added dropwise (20 µL per 15 s) to a 0.4 aqueous I R783 solution (1 mg/m L) containing 0.05 mM sodium bicarb onate; the solution was centrifuged twice and resuspension in 1 m L of sterile PBS; the suspension of nanoparticles was sonicated for 10 s with a probe sonicator at 40 % intensity; the resulted I R783-SFB nanoparticles had zeta potential of -52 mV and a size of 95 nm with a P DI of 0.1; by suspending the nanoparticles in lower volumes they were able to solubilize sorafenib up to 16 mg/mL in saline solution which is 2000 times better than free drug; the nanopar ticles were easily lyophilized with a saline/sucrose 5 % solution and reconst ituted in water at this concent ration

Keywords: dye stabilization nanoparticle hydrophobic drug self assembly cancer

PatentPak available

Substances (292) Reactions (0) Citing (0)

177

Aurora B inhibitor barasertib prevents meiotic maturation and subsequent embryo development in pig oocytes

By: Ju, Shiqiang; Peng, Xu; Yang, Xiaoliu; Sozar, Sparksi; Muneri, Caroline W.; Xu, Yaping; Chen, Changchao; Cui, Panpan; Xu, Weichao; Rui, Rong
Theriogenology (2016), 86(2), 503-515 | Language: English, Database: CAplus and MEDLINE

Barasertib, a highly selective Aurora B inhibitor, has been widely used in a variety of cells to invest igate the role of Aurora B kinase, which has been implicated in various functions in the mitotic process. However, effects of barasertib on the meiotic maturation process are not fully understood, particularly in porcine oocyte meiotic maturation. In the present study, the effects of barasertib on the meiotic maturation and developmental competence of pig oocytes were investi gated, and the possible roles of Aurora B were also evaluated in porcine oocytes undergoing meiosis. Initially, we examined the expression and subcellular localization of Aurora B using Western blot anal. and immunofluorescent staining. Aurora B was found to express and exhibit specific dynamic intracellular localization during porcine oocyte meiotic matura tion. Aurora B was observed around the chromo somes after germinal vesicle breakdown. Then it was transferred to the spindle region after metaphase I stage, and was partic ularly concentrated at the central spindles at telophase I stage. barasertib treatment resulted in the failure of polar body extrusion in pig oocytes, with a larger percentage of barasertib-treated oocytes remaining at the pro- metaphase I stage. Addnl. results reported that barasertib treatment had no effect on chromosome condensation but resulted in a signifi cantly higher percentage of the treated oocytes with aberrant spindles and misaligned chromosomes during the first meiotic division. In addition, inhibition of Aurora B with lower concent rations of barasertib during pig oocyte meiotic maturation decreased the subsequent embryo developmental competence. Thus, these results illustrate that barasertib has significant effects on porcine oocyte meiotic maturation and subsequent develo pment through Aurora B inhibition, and this regulation is related to its effects on spindle formation and chromosome alignment during the first meiotic division in porcine oocytes.

Keywords: barasertib oocyte aurora B inhibitor meiotic maturation embryo development; Aurora B; Barase rtib; Chromosome alignment; Oocytes maturation; Spindle formation

Substances (2) Reactions (0) Citing (10)

178

Significance of AZD1152 as a potential treatment against Aurora B overexpression in acute promyelocytic leukemia

By: Ghanizadeh-Vesali, Samad; Zekri, Ali; Zaker, Farhad; Zaghal, Azam; Yousefi, Meysam; Alimoghaddam, Kamran; Ghavamzadeh, Ardeshir; Ghaffari, Seyed H.
Annals of Hematology (2016), 95(7), 1031-1042 | Language: English, Database: CAplus and MEDLINE

Aurora B kinase as a chromosomal passenger protein plays multiple roles in regulating mitosis and cytoki nesis. The function of Aurora B in leukemic cells has made it an important treatment target. In this study, we explored the expressions of Aurora (A, B, and C) kinases in newly diagnosed acute promyelocytic leukemia (APL) patients. In addition, we investigated the effects of AZD1152 as a specific inhibitor of Aurora B on cell survival, DNA synthesis, nuclear morphol., apoptosis induction, cell cycle distrib ution, and gene expression in an APL-derived NB4 cell line. Our results showed that Aurora B was overexp ressed in 88 % of APL patients. AZ D1152 treatment of NB4 cells led to viability reduction and G2/M arrest followed by an increase in cell size and polyploidy induction. These giant cells showed morphol. evidence of mitotic catastrophe. AZD1152 treatment induced activation of G2/M checkpoint which in turn led to transient G2/M arrest in a p21-independent manner. Lack of functional p53 in N B4 cells might provide an opportunity to escape from G2/M block and to endure repeated rounds of replic ation and polyploidy. Treated cells were probably eliminated via p73-mediated overexpression of BAX, PUMA, and APAF1 and downregulation of survivin and MCL-1. In summary, AZD1152 treatment led to endomitosis and polyploidy in TP53-mutated NB4 cells. These giant polyploid cells might undergo mitotic catastrophe and p73-mediated apoptosis. It seems that induction of polyploidy via AZD1152 could be a novel form of anti-cancer therapy for APL that may be clin. accessible in the near future.

Keywords: AZD1152 anticancer aurora B apoptosis promyelocytic leukemia; AZD1152; Acute promyelocytic leukemia; Apoptosis; Aurora B kinase; Cell cycle arrest; p73

Substances (8) Reactions (0) Citing (10)

179

Epithelial Cell Transforming 2 and Aurora Kinase B Modulate Formation of Stress Granule-Containing Transcripts from Diverse Cellular Pathways in Astrocytoma Cells

By: Weeks, Adrienne; Agnihotri, Sameer; Lymer, Jennifer; Chalil, Alan; Diaz, Roberto; Isik, Semra; Smith, Christian; Rutka, James T.
American Journal of Pathology (2016), 186(6), 1674-1687 | Language: English, Database: CAplus and MEDLINE

Stress granules are small RNA-protein granules that modify the translational landscape during cellular stress to promote survival. The RhoGTPase RhoA is implicated in the formation of RNA stress granules. Our data demonstrate that the cytokinetic proteins epithelial cell transforming 2 and Aurora kinase B (Aurk B) are localized to stress granules in human astrocytoma cells. AurkB and its downstream target histone-3 are phosphorylated during arsenite-induced stress. Chem. (AZD1152-HQPA) and siRNA inhibition of AurkB results in fewer and smaller stress granules when analyzed using high- throughput fluorescent-based cellomics assays. RNA immunoprecipitation with the known stress granule aggregates T IAR and G3BP1 was performed on astrocytoma cells, and subsequent anal. revealed that astrocytoma stress granules harbor unique mRNAs for various cellular pathways, including cellular migration, metabolism, translation, and transcriptional regulation. Human astrocytoma cell stress granules contain mRNAs that are known to be involved in glioma signaling and the mammalian target of rapamycin pathway. These data provide evidence that RNA stress granules are a novel form of epigenetic regulation in astrocytoma cells, which may be targetable by chem. inhibitors and enhance astrocytoma susceptibility to conventional therapy, such as radiation and chemotherapy.

Keywords: astrocytoma cell ECT2 AurkB stress granule

Substances (13) Reactions (0) Citing (8)

180

Preparation of substituted bicyclic compounds as bromodomain inhibitors

By: Brown, Samuel David; Coburn, Craig Alan
World Intellectual Property Organization, WO2016097870 A1 2016-06-23 | Language: English, Database: CAplus

The title compounds I [W^1 = N and NH; W^2 and E^3 = (independently) C and N; R^1 = (un)substituted cycloalkyl, heterocyclyl; R^2 = H, alkyl (optionally substituted with halo and OH); R^3 = alkyl optionally substituted with halo and OH (with the proviso); R^4 = H, (un) substituted alkyl, cycloalkyl, etc.; X = CH_2 optionally substituted with 1-2 groups independently selected from R^5; R^5 = D, alkyl, alkoxy, etc.; Y = N and CH; with the provisos], useful for inhibition of BET protein function by binding to bromodo mains, were prepared E.g., a multi-step synthesis of **II**, starting from 5-bromopyridine-2,3-diamine and (3,5-dimethylisoxazol-4-yl)boronic acid, was described. Exemplified compounds **I** were tested in various biol. assays (data given) . Pharmaceutical compositions, comprising compound **I**, alone or in combination with other therapeutic agent, was disclosed.

Keywords: bicyclic compound imidazopyridine preparation BET bromodomain protein inhibitor

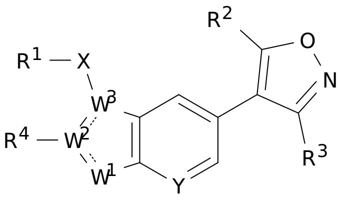

I

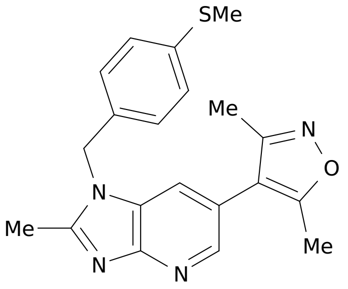

II

PatentPak available

Substances (116) Reactions (153) Citing (2)

181

Preparation of 4-heterocyclylisoxazole derivatives as inhibitors of bromodomains

By: Kharenko, Olesya; Young, Peter Ronald; Brown, Samuel David; Duffy, Bryan Cordell; Liu, Shuang; Guzzo, Peter
World Intellectual Property Organization, WO2016097863 A1 2016-06-23 | Language: English, Database: CAplus

The present disclosure relates to compounds **I** [R^1 = each (un)substituted C_{5-10} carbocyclyl or C_{2-10} heterocyclyl; R^2, R^3 = indepen dently C_{1-6} alkyl optionally substituted with halogen and hydroxy, with the proviso that if R^2 and R^3 are methyls, then R^1 is different from Q, Q^1, Q^2, 2-(difluoromethoxy)phenyl, 2,5-dichlorophenyl, or 2,5-dimethylphenyl; A = H, halogen, methoxy, cyano, NO_2, CO_2Me, or $CONMe_2$; R^4 = H, C_{1-10} alkyl, C_{3-10} carbocyclyl, or C_{2-10} heterocyclyl; X = (un)substituted CH_2; Y = N, CH, or $C(NH_2)$] or **II** (R^1, R^2, R^3, X = same as above; Z = H or NH_2) or stereoisomers, tautomers, pharmaceutically acceptable salts, or hydrates thereof. These compounds are capable of acting as covalent inhibitors of bromodomains and are useful for treating (A) an autoimmune or inflam matory disorder associated with bromodomain and extra terminal domain (BET) proteins, (B) an acute or chronic non-autoimmune inflammatory disorder characterized by disregulation of IL-6 and/or IL-17, (C) a cancer associated with overexpression, translo cation, amplification, or rearrangement of a myc family oncoprotein that is sensitive to BET inhibition, (D) cancer that relies on pTE Fb (Cdk9/cyclin T) and BET proteins to regulate oncogenes, (F) a cancer associated with upregu lation of BET responsive genes CDK6, Bcl2, TYRO3, MYB, and hTERT, (G) a cancer associated with a gene regulated by a super enhancer, (H) a cancer that is sensitive to effects of BET inhibition, (I) a cancer that is resistant to treatment with immunot herapy, hormone-deprivation therapy, and/or chemotherapy, (J) a benign proliferative or fibrotic disorder, or (K) a disease or disorder that benefits from upregu lation or ApoA-I transcription and protein expression. The autoimmune or inflammatory disorder is selected from acute disseminated encephalo myelitis, agammaglobulinemia, allergic disease, ankylosing spondylitis, anti-GBM/anti-TBM nephritis, anti-phospholipid syndrome, autoimmune aplastic anemia, autoimmune hepatitis, autoimmune inner ear disease, autoimmune myocarditis, autoimmune pancreatitis, autoimmune retinopathy, autoimmune thrombocytopenic purpura, Behcet's disease, Bullous pemphigoid, Castleman's disease, Celiac disease, Churg-Strauss syndrome, Crohn's disease, Cogan's syndrome, dry eye syndrome, essential mixed cryoglobu linemia, dermatomyositis, Devic's disease, encephalitis, eosinophlic esophagitis, eosinophilic fasciitis, erythema nodosum, giant cell arteritis, glomerulonephritis, Goodpasture's syndrome, granulomatosis with polyangiitis (Wegener's), Graves' disease, Guillain-Barre syndrome, Hashimoto's thyroiditis, hemolytic anemia, Henoch-Schonlein purpura, idiopathic pulmonary fibrosis, IgA nephropathy, inclusion body myositis, type I diabetes, interstitial cystitis, Kawasaki's disease, etc. The acute or chronic non-autoimmune inflam matory disorder is selected from sinusitis, pneumonitis, osteomyelitis, gastritis, enteritis, gingivitis, appendicitis, irritable bowel syndrome, tissue graft rejection, chronic obstructive pulmonary disease (COPD), septic shock, osteoarthritis, acute gout, acute lung

injury, acute renal failure, burns, Herxheimer reaction, and SIRS associated with viral infections. The benign proliferative or fibrotic disorder is selected from the group consisting of benign soft tissue tumors, bone tumors, brain tumors, spinal cord tumors, eyelid and orbital tumors, granuloma, lipoma, meningioma, multiple endocrine neoplasia, nasal polyps, pituitary tumors, prolactinoma, pseudotumor cerebri, seborrheic keratoses, stomach polyps, thyroid nodules, cystic neoplasms of the pancreas, hemangiomas, vocal cord nodules, polyps, and cysts, chronic pilonidal disease, dermatofibroma, pilar cyst, pyogenic granuloma, juvenile polyposis syndrome, renal fibrosis, postoperative stricture, keloid formation, and cardiac fibrosis. The other diseases include operative stricture, cardiovascular disease, dyslipidemia, atherosclerosis, hypercholesterolemia, metabolic syndrome, Alzheimer's disease, Parkinson's disease, Huntington disease, bipolar disorder, schizophrenia, Rubinstein-Taybi syndrome, and epilepsy. Thus, 4-bromo-3-fluorobenzyl alc. was stirred with methanesulfonyl chloride and pyridine in CH_2Cl_2 at room temperature for 16 h to give 95% 4-bromo-3-fluorobenzyl chloride. 5-(3,5-Dimethylisoxazol-4-yl)pyridin-2(1H)-one (**III**; R = H) was stirred with 4-bromo-3-fluorobenzyl chloride in the presence of K_2CO_3 in MeCN at 70° for 16 h to give 32% **III** (R = 4-bromo-3-fluorobenzyl). **III** (R = 4-bromo-3-fluoro benzyl) was coupled with tributyl(vinyl)stannane in the presence of $Pd(PPh_3)_2Cl_2$ in DMF under nitrogen at 85° for 16 h to give 53% **II I** (R = Q^3). A solution of 0.26 mmol **III** (R = Q^3) in water and acetone was treated with 0.29 mmol N-bromosuccinimide, stirred at for 6 h, treated with 0.52 mmol NaOH at 0°, and stirred at room temperature for 16 h to give 60% **III** (R = Q^4). **III** (R = Q^4) and 2-chloro-1-[4-[[6-(3,5-dimethylisoxazol-4-yl)-2-methyl-1H-imidazo[4,5-b]pyridin-1-yl]methyl]phenyl]ethanone (**IV**) inhibited the binding of tetra-acetyiated histone H4 to individual BET Bromodomains with IC_{50} of ≤0.3μM.

Keywords: oxazolylpyridinone oxazolylbenzimidazole preparation covalent inhibitor bromodomain; heterocyclylisoxazole preparation covalent inhibitor bromodomain; isoxazolylimidazopyridine preparation covalent inhibitor bromodomain; autoimmune disorder inflammatory disorder cancer treatment heterocyclylisoxazole preparation; benign tumor benign proliferative disorder treatment heterocyclylisoxazole preparation; fibrotic disorder cardiovascular disease treatment heterocyclylisoxazole preparation; bromodomain extra terminal domain BET protein

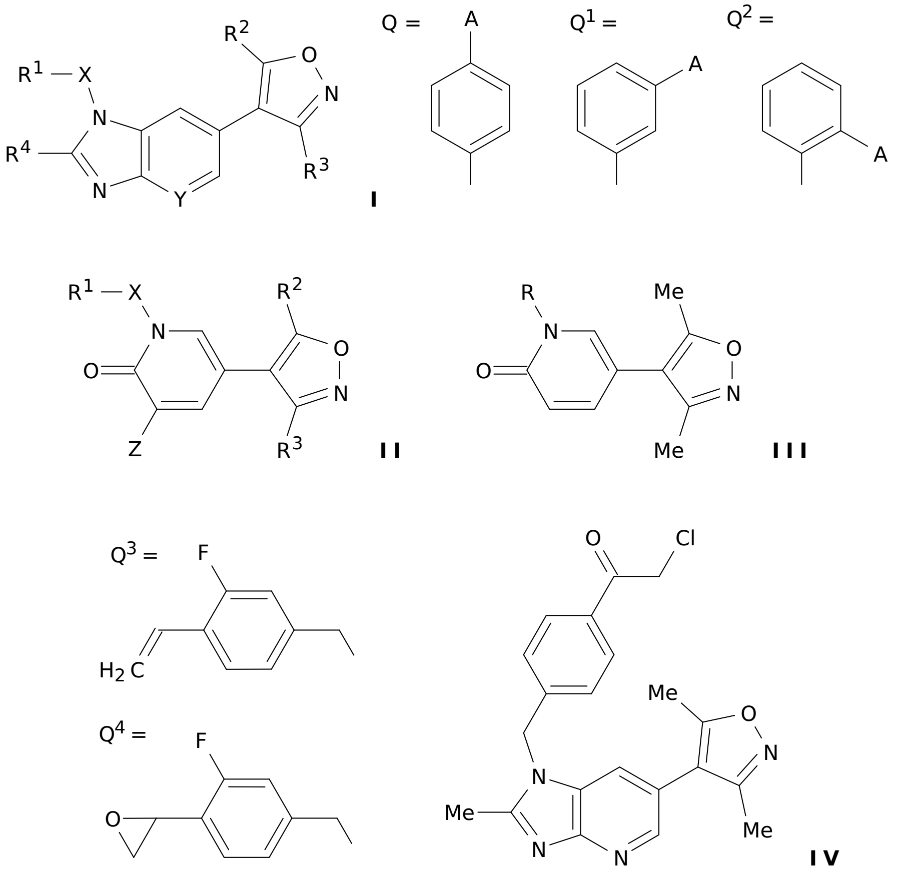

PatentPak available

Substances (84)   Reactions (62)   Citing (3)

182

Preparation of substituted imidazolyl triazoles as BET protein inhibitors via binding to bromodomains useful in therapy

By: Brown, Samuel David; Coburn, Craig Alan; Kharenko, Olesya
World Intellectual Property Organization, WO2016092375 A1 2016-06-16 | Language: English, Database: CAplus

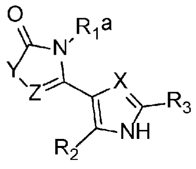

Formula I.

The invention relates to preparation of substituted imidazolyl triazoles of formula **I** wherein all the variables are as defined in the disclosure and pharmaceutical compositions thereof useful for the inhibition of B ET protein function by binding to bromodo mains. The example compound **II** was prepared by multistep synthesis (procedure shown) , was tested for inhibition of tetra- acetylated histone H4 binding individual BET bromodomains. Compounds **I** can be used in mono- and combin ation therapy of various diseases, like chemotherapy, immunotherapy and hormone- deprivation therapy resistant cancers and such.

Keywords: imidazolyl triazole preparation BET inhibitor anticancer antiproli ferative combination therapy

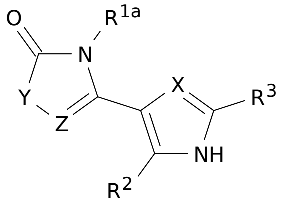

I

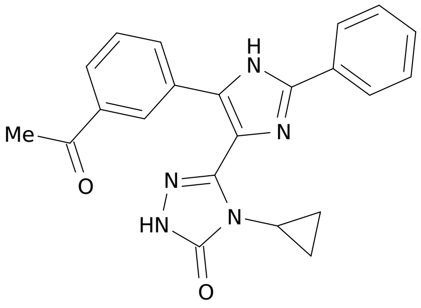

II

PatentPak available

Substances (159)   Reactions (304)   Citing (1)

183

Preparation of 5-(3,5-dialkylisoxazol-4-yl)pyridinones as bromodomain inhibitors

By: Jiang, May Xiaowu; Molino, Bruce Francis; Liu, Shuang; Wang, Ruifang; Duffy, Bryan Cordell; Quinn, John Frederick; Wagner, Gregory Steven

World Intellectual Property Organization, WO2016087936 A1 2016-06-09 | Language: English, Database: CAplus

The invention is related to the preparation of substituted pyridinones **I** [A = (un)substituted aryl, heteroaryl; L = CH_2, CH_2CH_2, CH (Me) optionally substituted with halogen; or L may be absent in which case A is connected to X via a covalent bond; X = O, N H; R^3, R^4 = independently C_1-C_6 alkyl optionally substituted with halogen and OH], their stereoisomers, tautomers, pharmaceutically acceptable salts, and hydrates, which are useful for inhibition of BET protein function by binding to bromodo mains, pharmaceutical compositions containing them., and ther use in therapy. Thus, Pd- coupling of 3-amino-5-bromo-2-methoxypyridine with 3,5-dimethyl-4-(4,4,5,5-tetramethyl-1,3,2-dioxaborolan-2-yl)isoxazole, amination of benzyl bromide with the resulting amine and treatment of 3-(benzylamino)-5-(3,5-dimethylisoxazol-4-yl)-2-methoxypyridine with HBr in EtOH gave **II**. Pyridinone **II** inhibited the tetracetylated histone H4 binding to Brd4 bromod omain 1, the cell proliferation in human AML MV-4-11 cells and the hIL-6 and hIL-17 mRNA transcription.

Keywords: isoxazolylpyridinone preparation bromodomain inhibitor

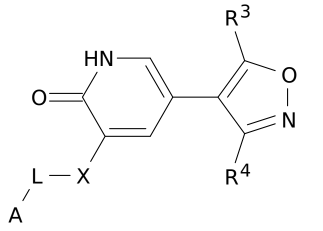

I

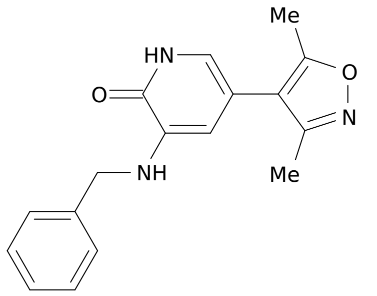

II

PatentPak available

Substances (136) Reactions (96) Citing (2)

184

Preparation of 5-(3,5-dialkylisoxazol-4-yl)pyridines as bromodomain inhibitors

By: Jiang, May Xiaowu; Molino, Bruce Francis; Liu, Shuang; Wang, Ruifang; Duffy, Bryan Cordell; Quinn, John Frederick; Wagner, Gregory Steven

World Intellectual Property Organization, WO2016087942 A1 2016-06-09 | Language: English, Database: CAplus

The invention is related to the preparation of substituted pyridinones **I** [A = (un)substituted aryl, heteroaryl; B = C_1-C_6 alkyl, benzyl, Ph optionally substituted with halogen; L = CH_2, CH(Me) optionally substituted with halogen; or L may be absent in which case A is connected to X via a covalent bond; X = O, NH; Y = O, NHMe, meaning if Y = NHMe then B is absent; R^3, R^4 = independently C_1-C_6 alkyl optionally substituted with halogen and OH], their stereoisomers, tautomers, pharmaceutically acceptable salts, and hydrates, which are useful for inhibition of BET protein function by binding to bromodomains, pharmaceutical compositions containing them., and ther use in therapy. Thus, Pd-coupling of 3-amino-5-bromo-2-methoxypyridine with 3,5-dimethyl-4-(4,4,5,5-tetramethyl-1,3,2-dioxaborolan-2-yl)isoxazole and amination of benzyl bromide with the 5-(3,5-dimethylisoxazol-4-yl)-2-methoxypyridin-3-amine gave **II**. Pyridine **II** inhibited the tetracetylated histone H4 binding to Brd4 bromodomain 1, the cell proliferation in human AML MV-4-11 cells and the hIL-6 and hIL-17 mRNA transcription.

Keywords: isoxazolylpyridine preparation bromodomain inhibitor

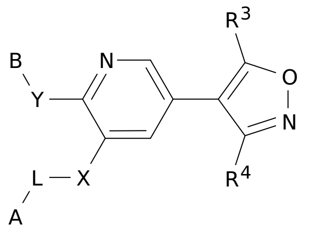

I

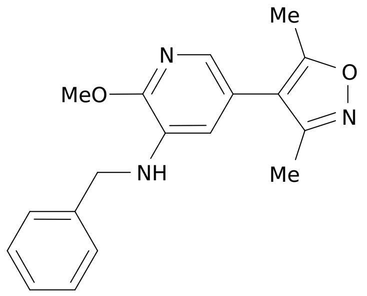

II

PatentPak available

Substances (190) Reactions (169) Citing (3)

185

Preclinical testing of selective Aurora kinase inhibitors on a medullary thyroid carcinoma-derived cell line

By: Tuccilli, Chiara; Baldini, Enke; Prinzi, Natalie; Morrone, Stefania; Sorrenti, Salvatore; Filippini, Angelo; Catania, Antonio; Alessandrini, Stefania; Rendina, Roberta; Coccaro, Carmela; et al

Endocrine (2016), 52(2), 287-295 | Language: English, Database: CAplus and MEDLINE

Deregulated expression of the Aurora kinases (Aurora-A, B, and C) is thought to be involved in cell malignant transfo rmation and genomic instability in several cancer types. Over the last decade, a number of small- mol. inhibitors of Aurora kinases have been developed, which have proved to efficiently restrain malignant cell growth and tumorige nicity. Regarding medullary thyroid carcinoma (MTC), we previously showed the efficacy of a pan-Aurora kinase inhibitor (MK-0457) in impairing growth and survival of the MTC-derived cell line TT. In the present study, we sought to establish if one of the Aurora kinases might represent a prefer ential target for MTC therapy. The effects of selective inhibitors of Aurora-A (MLN8237) and Aurora-B (AZD1152) were analyzed on TT cell proliferation, apoptosis, cell cycle, and ploidy. The two inhibitors reduced T T cell proliferation in a time- and dose-dependent manner, with IC_{50} of 19.0 ± 2.4 n M for M LN8237 and 401.6 ± 44.1 n M for AZD1152. Immunofluorescence experiments confirmed that AZD1152 inhibited phosphorylation of histone H3 (Ser10) by Aurora- B, while it did not affect Aurora-A autophosphorylation. M LN8237 inhibited Aurora-A autophosphorylation as expected, but at concent rations required to achieve the maximum antiproli ferative effects it also abolished H3 (Ser10) phosphor ylation. Cytofluo rimetry experiments showed that both inhibitors induced accumulation of cells in G2/M phase and increased the sub G0/G1 fraction and polypl oidy. Finally, both inhibitors triggered apoptosis. We demonstrated that inhibition of either Aurora-A or Aurora-B has antiproliferative effects on TT cells, and thus it would be worthwhile to further investigate the therapeutical potential of Aurora kinase inhibitors in M TC treatment.

Keywords: MLN8237 AZD1152 aurora kinase inhibitor anticancer medullary thyroid carcinoma; AZD1152; Aurora kinase inhibitors; Cell cycle; MLN8237; Medullary thyroid cancer; TT cell line

Substances (4) | Reactions (0) | Citing (6)

186

Irisin-related cancer treatments

By: Trujillo, Kristina; Gannon, Nicholas Patrick; Vaughan, Roger Alan

World Intellectual Property Organization, WO2016081603 A1 2016-05-26 | Language: English, Database: CAplus

In one embodiment, the invention provides methods of treating or preventing cancer, in particular aspects breast and/or prostate cancer, by administering to a subject in need thereof a therapeu tically-effective amount of irisin and, option ally, one or more adjuvant therapies (e.g. synergistic co-administration of an addnl. anti-cancer agent or chemotherapy). Related pharmaceutical compositions, assays and kits are also provided.

Keywords: irisin cancer FNDC5 gene theapy

PatentPak available

Substances (316) | Reactions (0) | Citing (1)

187

A novel in situ hydrophobic ion paring (HIP) formulation strategy for clinical product selection of a nanoparticle drug delivery system

By: Song, Young Ho; Shin, Eyoung; Wang, Hong; Nolan, Jim; Low, Susan; Parsons, Donald; Zale, Stephen; Ashton, Susan; Ashford, Marianne; Ali, Mir; et al

Journal of Controlled Release (2016), 229, 106-119 | Language: English, Database: CAplus and MEDLINE

The present studies were aimed at formulating AZD2811-loaded polylactic acid-polyethylene glycol (PLA-PEG) nanoparticles with adjustable release rates without altering the chem. structures of the polymer or active pharmaceutical ingredient (API). This was accomplished through the use of a hydrop hobic ion pairing approach. A series of A ZD2811-containing nanoparticles with a variety of hydrophobic counterions including oleic acid, 1- hydroxy-2-naphthoic acid, cholic acid, deoxyc holic acid, dioctylsul fosuccinic acid, and pamoic acid is described. The hydrophobicity of AZD2811 was increased through formation of ion pairs with these hydrop hobic counterions, producing nanoparticles with excepti onally high drug loading- up to five fold higher encapsu lation efficiency and drug loading compared to nanoparticles made without hydrop hobic ion pairs. Furthe rmore, the rate at which the drug was released from the nanoparticles could be controlled by employing counte rions with various hydropho bicities and structures, resulting in release half-lives ranging from about 2 to 120 h using the same polymer, nanopa rticle size, and nanoemulsion process. Process recipe variables affecting drug load and release rate were identified, including pH and molarity of quench buffer. Ion pair formation between AZD2811 and pamoic acid as a model counterion was invest igated using solubility enhancement as well as NMR spectr oscopy to demonstrate solution-state interactions. Further evidence for an ion pairing mechanism of controlled release was provided through the measurement of API and counterion release profiles using high- performance liquid chromatog., which had stoichiometric relationships. Finally, Raman spectra of an A ZD2811-pamoate salt compared well with those of the formulated nanoparticles, while single components (A ZD2811, pamoic acid) alone did not. A library of A ZD2811 batches was created for anal. and preclin. characterization. Dramatically improved preclin. efficacy and tolera bility data were generated for the pamoic acid lead formulation, which has been selected for evaluation in a Phase 1 clin. trial (Clinical Trials.gov Identifier N CT 02579226). This work clearly demonstrates the importance of assessing a wide range of drug release rates during formul ation screening as a critical step for new drug product development, and how utilizing hydrop hobic ion pairing enabled this promising nanopa rticle formulation to proceed into clin. development.

Keywords: hydrophobic ion pairing polylactic acid polyet hylene glycol nanoparticle; Adjustable release rate; Counte rions; Drug release kinetics; In situ hydrophobic ion paring; Nanopar ticles; PLA–PEG

Substances (18) | Reactions (0) | Citing (49)

188

IL15 Agonists Overcome the Immunosuppressive Effects of MEK Inhibitors

By: Allegrezza, Michael J.; Rutkowski, Melanie R.; Stephen, Tom L.; Svoronos, Nikolaos; Tesone, Amelia J.; Perales-Puchalt, Alfredo; Nguyen, Jenny M.; Sarmin, Fahmida; Sheen, Mee R.; Jeng, Emily K.; et al

Cancer Research (2016), 76(9), 2561-2572 | Language: English, Database: CAplus and MEDLINE

Many signal transduction inhibitors are being developed for cancer therapy target pathways that are also important for the proper function of antitumor lymphocytes, possibly weakening their therap eutic effects. Here we show that most inhibitors targeting multiple signaling pathways have especially strong neg. effects on T-cell activation at their active doses on cancer cells. In partic ular, we found that recently approved MEK inhibitors displayed potent suppre ssive effects on T cells in vitro. However, these effects could be attenuated by certain cytokines that can be administered to cancer patients. Among them, clin. available I L15 superag onists, which can activate PI3K selectively in T lympho cytes, synergized with M EK inhibitors in vivo to elicit potent and durable antitumor responses, including by a vaccine-like effect that generated resistance to tumor rechal lenge. Our work identifies a clin. actionable approach to overcome the T-cell-suppressive effects of MEK inhibitors and illust rates how to reconcile the defici encies of signal transduction inhibitors, which impede desired immunol. effects in vivo.

Keywords: T cell MEK inhibitor IL15 agonist immunosuppression

Substances (58) | Reactions (0) | Citing (22)

189

Docosahexaenoic acid sensitizes leukemia lymphocytes to barasertib and everolimus by ROS-dependent mechanism without affecting the level of ROS and viability of normal lymphocytes

By: Zhelev, Zhivko; Ivanova, Donika; Lazarova, Desislava; Aoki, Ichio; Bakalova, Rumiana; Saga, Tsuneo
Anticancer Research (2016), 36(4), 1673-1682 | Language: English, Database: CAplus and MEDLINE

The aim of the present study was: (i) to investigate the possibility of sensitizing leukemia lymphocytes to anticancer drugs using docosahexaenoic acid (DHA); (ii) to find combinations with synergistic cytotoxic effect on leukemia lymphocytes, without or with only very low cytotoxicity towards normal lymphocytes; (iii) and to clarify the role of reactive oxygen species (R OS) in the induction of apoptosis and cytotoxicity by such combinations. The study covered 15 anticancer drugs, conven tional and new-generation. Well-expressed synergistic cytotoxic effects were observed after treatment of leukemia lympho cytes (Jurkat) with DHA in combination with: barasertib, lonafarnib, everolimus, and palbociclib. We selected two synerg istic combinations, DHA with everolimus or barase rtib, and investigated their effects on viability of normal lymphocytes, as well as on the production of R OS and induction of apoptosis in both cell lines (leukemia and normal). At the selected concentr ations, DHA, everolimus and barasertib (applied sep.) were cytotoxic towards leukemia lymphocytes, but not normal lympho cytes. In leukemia cells, the cytotoxicity of combinations was accompanied by strong induction of apoptosis and production of R OS. In normal lymphocytes, drugs alone and in combin ation with DHA did not affect the level of R OS and did not induce apoptosis. To our knowledge, the present study is the first to report syneryg istic ROS-dependent cytotoxicity between DHA and new-generation anticancer drugs, such as everolimus and barase rtib, that is cancer cell-specific (particularly for acute lymphob lastic leukemia cells Jurkat). These combin ations are harmless to normal lympho cytes and do not induce abnormal production of R OS in these cells. The data suggest that D HA could be used as a supplem entary component in anticancer chemotherapy, allowing therapeutic doses of everolimus and barasertib to be reduced, minimizing their side-effects.

Keywords: leukemia lymphocyte docosahexaenoic acid barasertib everolimus R OS cell viability; D HA; Docosahe xaenoic acid; barase rtib; everolimus; leukemia lymphocytes; reactive oxygen species

Substances (12) Reactions (0) Citing (18)

190

Inhibitor of Aurora Kinase B Induces Differentially Cell Death and Polyploidy via DNA Damage Response Pathways in Neurological Malignancy: Shedding New Light on the Challenge of Resistance to AZD1152-HQPA

By: Zekri, Ali; Ghaffari, Seyed H.; Yaghmaie, Marjan; Estiar, Mehrdad Asghari; Alimoghaddam, Kamran; Modarressi, Mohammad Hossein; Ghavamzadeh, Ardeshir

Molecular Neurobiology (2016), 53(3), 1808-1823 | Language: English, Database: CAplus and MEDLINE

Aurora kinase B (AURKB), a crucial regulator of malignant mitosis, is involved in chromosome segreg ation and cytokinesis. AZ D1152-HQPA is a selective inhibitor for A URKB activity and currently bears clin. assessment for several maligna ncies. However, the effect of this drug still needs to be elucidated in neurol. malignancies. In this study, we invest igated the restrictive potentials of AZ D1152-HQPA in U87MG and SK-N-MC. AZD1152-HQPA treatment resulted in growth arrest, modifi cation of cell cycle, and inhibition of colony formation in both cell lines. Furthermore, lower concentrations of AZD1152-HQPA profoundly induced apoptosis in U87G M (p53/p73 wild type) cells in parallel with an upregu lation of p53 and its target genes B AX, BAD, APAF1, and PUMA. But remark ably, SK-N-MC (p53/p73 double null) responded to AZD1152-HQPA at much higher concent rations with an upregulation of genes involved in cell cycle progression, induction of excessive endoreduplication, and polyploidy rather than apoptosis. Although S K-N-M C was resistant to AZD1152-HQPA, we did not find a mutation in the coding sequence of Aurora B gene or overexpr essions of ABC G2 and ABCB1 as reported previously to be resistance mechanisms. However, our results suggest that p53/p73 status could be an important mechanism for the type of response and resistance of the tumor cells to AZD1152-HQPA. Collectively, inhibition of Aurora kinase B differentially induced cell death and polyploidy via D NA damage response pathways, depending on the status of p53/p73. We suggest p53/p73 could be a key regulator of sensitivity to AZD1152-HQPA and their status should be explored in clin. response to this ongoing drug in clin. trials.

Keywords: AURKB cell death polyploidy D NA damage neurol malignancy resist ance; AZD1152-HQPA; DNA damage response pathway; Mitotic catastrophe; Neurological malignancies; P53/p73 status

Substances (8) Reactions (0) Citing (17)

191

Screening methods for identifying compounds for cancer cell reprogramming and therapeutic uses thereof in the treatment of cancer

By: Chigaev, Alexandre; Sklar, Larry A.; Perez, Dominique

United States, US9314460 B1 2016-04-19 | Language: English, Database: CAplus

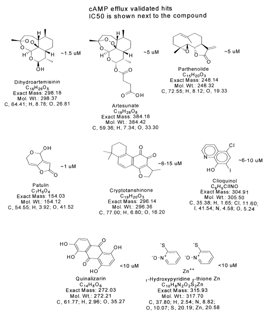

In one embodiment, the invention provides a method of inhibiting c AMP efflux and increasing intrace llular cAMP in a subject who suffers from, or who is at risk of developing, a cancer by adminis tering to the subject a therapeu tically-effective amount of a c AMP efflux inhibitor. Novel compounds, pharmaceutical compositions, diagno stics and screening methods are also provided.

Keywords: anticancer drug screening cancer cell apoptosis neoplasm treatment

PatentPak available

Substances (313) Reactions (0) Citing (2)

192

Method of testing systemic release of AZD 1152 hqpa or bioequivalents

By: Walker, Michael; Howes, Colin

World Intellectual Property Organization, WO2016059149 A1 2016-04-21 | Language: English, Database: CAplus

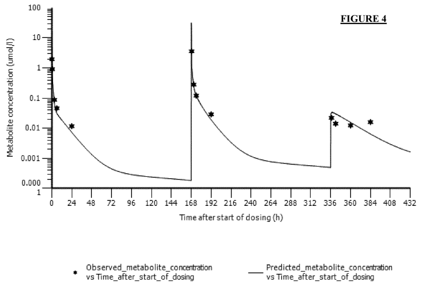

Methods of measuring systemic release, or in-vivo release profile of an active drug, e.g., AZD 1152 (barasertib), from a nanoparticle, and their use, in assessing bioequivalence are disclosed. The method involves detection of a metabolite of AZD 1152 hqpa (AZD 1152 hqpa desfluoroaniline (also referred to as AZ 12102238); AZD 1152 hqpa desfluoroaniline N-acetic acid; AZD 1152 hqpa N-acetic acid; AZD-1152 hqpa N-oxide; and AZD 1152 hqpa N-desethyl) by using mass spectrometry (LC-MS) and applying a pharmacokinetic model.

Keywords: AZD 1152 hqpa metabolism bioequivalence nanoparticle

PatentPak available

Substances (16) | Reaction (1) | Citing (0)

193

Map kinase activated protein kinase 2 (MK2) inhibitors in the treatment of cancer

By: Beswick, Ellen Janine; Morris, Katherine Theresa

World Intellectual Property Organization, WO2016032882 A1 2016-03-03 | Language: English, Database: CAplus

The present invention relates to the discovery that Map kinase activated protein kinase 2 (MK2) is highly expressed in cancer tissue and the inhibition of MK2 using an MK2 inhibitor (BIO-475863, PF-364402), represents a viable approach to the treatment of cancer, including drug resistant cancers, metastatic cancers and recurrent cancers. MK2 inhibitors as described herein may be used alone or in combination with an at least one addnl. anti- cancer agent for the treatment of cancer.

Keywords: antitumor Map kinase inhibitor combination chemotherapy cancer treatment

PatentPak available

Substances (311) | Reactions (0) | Citing (0)

194

Imide-based modulators of proteolysis and associated methods of use

By: Crew, Andrew P.; Crews, Craig; Dong, Hanqing; Wang, Jing; Qian, Yimin; Siu, Kam; Jin, Meizhong
United States, US20160058872 A1 2016-03-03 | Language: English, Database: CAplus

The description relates to imide-based compounds, including bifunctional compounds comprising the same, which find utility as modulators of targeted ubiquitination, especially inhibitors of a variety of polype ptides and other proteins which are degraded and/or otherwise inhibited by bifunctional compounds according to the present invention. In partic ular, the description provides compounds, which contain on one end a ligand which binds to the cereblon E3 ubiquitin ligase and on the other end a moiety which binds a target protein such that the target protein is placed in proximity to the ubiquitin ligase to effect degradation (and inhibition) of that protein. Compounds can be synthesized that exhibit a broad range of pharmacol. activities consistent with the degradation/inhibition of targeted polypeptides of nearly any type.

Keywords: antitumor proteolysis modulator imide preparation cancer

PatentPak available

Substances (425) | Reactions (330) | Citing (3)

195

Bromodomain inhibitors for treating disease

By: Palmer, Wylie; Jones, Philip; Liu, Gang; Petrocchi, Alessia; Reyna, Naphtali; Subrumanian, Govindan; Theroff, Jay; Yau, Anne
World Intellectual Property Organization, WO2016033416 A1 2016-03-03 | Language: English, Database: CAplus

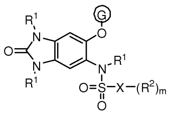

(I)

Disclosed herein are compounds and compositions useful in the treatment of bromod omain-containing protein-mediated diseases, such as cancer, and Methods of inhibiting activity of a bromodomain-containing protein in a human or animal subject are also provided.

Keywords: bromodomain inhibitor preparation cancer inflammation autoimmune CNS disease

PatentPak available

Substances (577) | Reactions (2,121) | Citing (1)

196

Anti-LAG3 antibodies and antigen-binding fragments

By: Liang, Linda; Fayadat-Dilman, Laurence; Malefyt, Rene de Waal; Raghunathan, Gopalan

World Intellectual Property Organization, WO2016028672 A1 2016-02-25 | Language: English, Database: CAplus

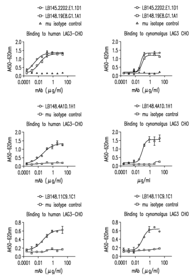

FIG.1

The present invention includes antibodies and antigen-binding fragments thereof that specifically bind to human or Cynomolgous monkey LAG3 as well as Ig chains thereof and polynucl eotides encoding the same along with injection devices comprising such antibodies or fragments. Vaccines including such antibodies and fragments as well as compositions comprising the antibodies and fragments (e.g., including anti-PD1 antibodies) are included in the invention. Methods for treating or preventing cancer or infection using such compositions are also provided. In addition, methods for recomb inant expression of the antibodies and fragments are part of the present invention.

Keywords: human LAG3 monoclonal antibody sequence humanized variant immunot herapy cancer

PatentPak available

Substances (595) Reactions (0) Citing (7)

197

Preparation of asymmetric bisaminoquinolines and bisaminoquinolines with varied linkers as autophagy inhibitors for cancer and other therapy

By: Amaravadi, Ravi K.; Winkler, Jeffrey

World Intellectual Property Organization, WO2016022956 A1 2016-02-11 | Language: English, Database: CAplus

The invention provides novel asym. and sym. bisaminoquinolmes and related compounds **I** [L = $(CH_2)_pN(R'')(CH_2)_p$, $(CH_2)_pN(R'')(CH_2)_{p1}N(R'')(CH_2)_p$, $(CH_2)_pO(CH_2)_p$, L^{1D}, or each (un)substituted $(CH_2Y)_n$-X-$(Y^1CH_2)_n$, or $A(CH_2CH_2$-$Z)_n$-A^1; R^1, R^1 = independently H, F Cl, Br, iodo, cyano, NO_2, or each (un)substituted C_{1-6} alkyl, O-C_{1-6} alkyl, C_{2-7} acyl, NH-acyl, or C_2-C_7 carboxy ester; R, R' = indepen dently H or each (un)substituted C_{1-6} alkyl, C_{1-7} acyl, or C_{2-7} carboxy ester (which forms a urethane group with the nitrogen atom to which R or R' is bonded); R'' = F, each (un) substituted cycloalkyl, aryl or heteroaryl, or $(C:O)_z$-G; G = each (un)substituted CO-C_{1-2} alkyl, alkene, or alkynyl; z = an integer of 0-5; R^M = independently H, F, Cl, Br, iodo, (un) substituted C_{1-12} alkyl, alkene, alkynyl or alkoxy group; p, p1 = an integer of 1-10; L^{1D} = (un)substituted C_{1-20} alkylene; X = absent, $(CH_2)_j$, O, S, or (un)substituted NH; Y, Y^1 = independently absent, CH_2, O, O, CHO, or (un)substituted NH; j = an integer of 1- 6; n = indepen dently an integer of 0- 10; Z = O or (un)substituted NH; A, A^1 = independently C(O) or (un)substituted $(CH_2)_j$] or pharmaceutically acceptable salts, enantiomers,s diastereomers, solvates, or polymorphs thereof. The novel compounds are autophagy inhibitors and are useful in the treatment of a variety of autophagy-related disorders, in particular (a) cancer including metastatic cancer, recurrent cancer, and drug- resistant cancer and (b) rheumatoid arthritis, malaria, antiphospholipid antibody syndrome, lupus, chronic urticaria or Sjogren's disease. The cancer is (1) carcinoma, (2) cancer of the esophagus, head, kidney, liver, lung, nasopharyngeal, neck, ovary, pancreas, prostate, and stomach, (3) a leukemia, a malignant lymphoma, a malignant melanoma, (4) myeloproliferative diseases, or (5) a sarcoma, a tumor of the central nervous system, a germ-line tumor, lung cancer, ovarian cancer, testicular cancer, thyroid cancer, astroc ytoma, esophageal cancer, pancreatic cancer, stomach cancer, liver cancer, colon cancer, melanoma, or a mixed type of neoplasia. Thus, to a microwave vial was added spermine 0.57, 4-bromo-7-chloroquinoline 1.32, BINAP 0.11, $Pd(OAc)_2$ 0.02, and K_3PO_4 1.80 g. The vial was sealed and placed under argon, followed by adding a solvent mixture of dioxane and water (10:1, 9 mL), and the resulting mixture was heated to 120° for 16 h to give, after workup, 73% N1,N1'-(butane-1,4-diyl)bis[N3-(7-chloroquinolin-4-yl)propane-1,3-diamine] (**II**). The compounds **I** were tested for anticancer activities. The increased cytotoxicity of longer linker and assyemtric compounds was confirmed in a 2 wk colony formation assay using 5 different cancer cell lines including A375, WM983B, CAPAN1, OANC1, and SKME L5 cells. Longer linker compounds localized to the lysosome. Longer linker compounds were associated with more profound autophagy modulation, reactive oxygen species (ROS) and apoptosis. Dimeric chloroquinolines with longer linkers impaired growth and invasion of melanoma in a 3 dimensional tissue like culture system.

Keywords: asym bisaminoquinoline preparation autophagy inhibitor treatment cancer; drug resistant cancer rheumatoid arthritis treatment asym bisaminoquinoline preparation; malaria treatment asym bisaminoq uinoline preparation; antiphospholipid antibody syndrome treatment asym bisaminoquinoline preparation; lupus chronic urticaria Sjogren disease treatment asym bisaminoquinoline preparation

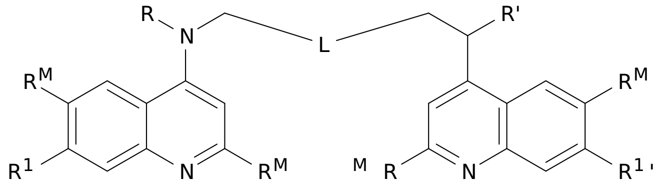

I

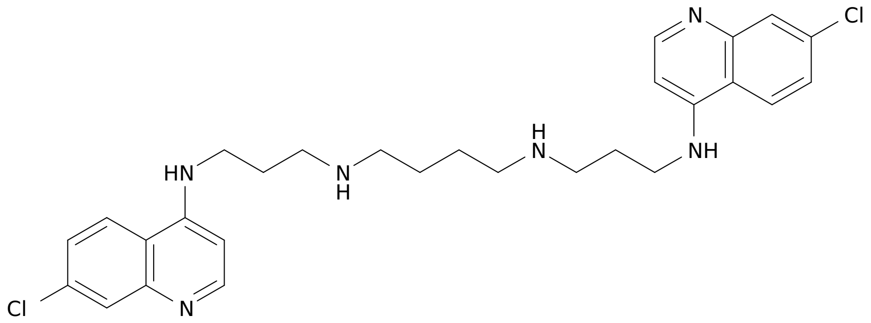

II

PatentPak available

Substances (418) Reactions (245) Citing (1)

198

Are Accurins the cure for Aurora kinase inhibitors?

By: Bearss, David J.
Science Translational Medicine (2016), 8(325), 325fs4/1-325fs4/3 | Language: English, Database: CAplus and MEDLINE

A review. A nanoparticle formulation of an Aurora B inhibitor increases antitumor efficacy and reduces toxicity, which may be a precedent for the use of this technol. with other small mols. (Ashton et al., this issue).

Keywords: review Accurins Aurora kinase inhibitor antitumor agent cancer

Substances (2) Reactions (0) Citing (3)

199

Aurora kinase inhibitor nanoparticles target tumors with favorable therapeutic index in vivo

By: Ashton, Susan; Song, Young Ho; Nolan, Jim; Cadogan, Elaine; Murray, Jim; Odedra, Rajesh; Foster, John; Hall, Peter A.; Low, Susan; Taylor, Paula; et al
Science Translational Medicine (2016), 8(325), 325ra17/1-325ra17/12 | Language: English, Database: CAplus and MEDLINE

Efforts to apply nanotechnol. in cancer have focused almost exclusively on the delivery of cytotoxic drugs to improve therapeutic index. There has been little consideration of molecularly targeted agents, in particular kinase inhibitors, which can also present considerable therapeutic index limitations. We describe the development of Accurin polymeric nanoparticles that encapsulate the clin. candidate AZD2811, an Aurora B kinase inhibitor, using an ion pairing approach. Accurins increase biodistribution to tumor sites and provide extended release of encapsulated drug payloads. AZD2811 nanoparticles containing pharmaceutically acceptable organic acids as ion pairing agents displayed continuous drug release for more than 1 wk in vitro and a corresponding extended pharmacodynamic reduction of tumor phosphorylated histone H3 levels in vivo for up to 96 h after a single administration. A specific AZD2811 nanoparticle formulation profile showed accumulation and retention in tumors with minimal impact on bone marrow pathol., and resulted in lower toxicity and increased efficacy in multiple tumor models at half the dose intensity of AZD1152, a water-soluble prodrug of AZD2811. These studies demonstrate that AZD2811 can be formulated in nanoparticles using ion pairing agents to give improved efficacy and tolerability in preclin. models with less frequent dosing. Accurins specifically, and nanotechnol. in general, can increase the therapeutic index of molecularly targeted agents, including kinase inhibitors targeting cell cycle and oncogenic signal transduction pathways, which have to date proved toxic in humans.

Keywords: aurora B kinase inhibitor accurin polymeric nanoparticle therapeutic index

Substances (8) Reactions (0) Citing (136)

200

Compounds useful for promoting protein degradation and methods using same

By: Crews, Craig M.; Gustafson, Jeffrey; Roth, Anke Gundula; Tae, Hyun Seop; Buckley, Dennis; Neklesa, Taavi
United States, US20160022642 A1 2016-01-28 | Language: English, Database: CAplus

The present description includes compounds that act as degraders of a target protein, wherein degrad ation is independent of the class of the target protein or its localization. In certain embodiments, the description includes a compound comprising a protein degradation moiety covalently bound to a linker, wherein the Clog P of the compound is equal to or higher than 1.5. The target protein contemplated within the description comprises an androgen receptor. Compounds of the present descri ption may be used to treat disease states wherein protein degradation is a viable therapeutic approach, such as cancer or any sort of oxidative stress disease state.

Keywords: protein degradation promoter preparation androgen receptor cancer oxidative stress

PatentPak available

Substances (439) | Reactions (226) | Citing (1)

201

[14C]-AZD1152 drug substance manufacture: challenges of an IV-infusion dosed human mass balance study in patients

By: Bushby, Nick ; Bergin, Julie; Harding, John
Journal of Labelled Compounds and Radiopharmaceuticals (2016), 59(6), 250-254 | Language: English, Database: CAplus and MEDLINE

[^{14}C]-AZD1152 (barasertib) drug substance was prepared in order to support a h ADME study that was to be dosed as an i.v. infusion to patients with acute myeloid leukemia. A long patient recruitment time (1-2 years) was expected, and because of its limited stability several batches of [^{14}C]-AZD1152 drug substance needed to be prepared in order to maintain a supply of [^{14}C]-AZD1152 in the clinic over this period. A method was developed to purify the [^{14}C]-AZD1152 to a GMP-like standard at high specific activity to provide material for each of these batches. This approach to the delivery of the drug substance was successful in supplying the study with radiolabeled drug for over 1 yr until all patients had been recruited.

Keywords: radiolabeled C14 AZD1152 synthesis; AZD1152; cGMP; hADME

Substances (16) | Reactions (30) | Citing (0)

202

Therapeutic polymeric nanoparticles and the methods of making and using thereof: a patent evaluation of WO2015036792

By: Sarvagalla, Sailu; Hsieh, Hsing Pang; Coumar, Mohane Selvaraj
Expert Opinion on Therapeutic Patents (2016), 26(7), 751-755 | Language: English, Database: CAplus and MEDLINE

Evaluation of the patent application WO2015036792 claiming therapeutic polymeric nanoparticles loaded with AZD1152-hqpa (aurora kinase inhibitor), and methods of making and using same for the treatment of cancer, is described. The claimed polymeric nano-formulations containing hydrophobic acid significantly improved the pharmacokinetic profiles (slow/sustained drug release profile) of the drug AZD1152-hqpa, as compared to the control agent (AZD1152). Drug efficacy and tolerability were also improved, and toxicity decreased in in vivo animal experiments, resulting in a better therapeutic index for the nano-formulation. Hence, the nano-formulated AZD1152-hqpa could be tested in the clinic at a dose level similar to, or higher than, that used for AZD1152, with lower incidence of toxicity.

Keywords: antitumor AZD1152 HQPA pharmacokinetic; AZD1152; AZD1152-hqpa; Aurora kinase inhibitor; cancer; drug delivery; nano-formulation; nano-medicine; nanoparticle; nanotechnology; therapeutic index

Substance (1) Reactions (0) Citing (2)

203

2-deoxy-D-glucose sensitizes cancer cells to barasertib and everolimus by ROS-independent mechanism(s)

By: Zhelev, Zhivko; Ivanova, Donika; Aoki, Ichjo; Saga, Tsuneo; Bakalova, Tana
Anticancer Research (2015), 35(12), 6623-6632 | Language: English, Database: CAplus and MEDLINE

The aim of the present study was to investigate: (i) the possibility of sensitizing cancer cells to anticancer drugs using the redox modulator 2-deoxy-D-glucose (2- DDG); (ii) to find such combinations with synergistic cytotoxic effect,- (iii) and to clarify the role of reactive oxygen species (ROS) for induction of apoptosis and cytotoxicity through these combinations. The study covers 15 anticancer drugs - both conventional and new-generation. Four parameters were analyzed simultaneously in Jurkat leukemia treated by drugs or 2-DUG (sep. or in combination): cell viability, induction of apoptosis, levels of R OS, and level of protein-carbonyl products.

Keywords: leukemia cell 2 deoxy glucose barasertib everolimus sensitivity ROS; 2-deoxy-D-glucose; Anticancer drugs; apoptosis; reactive oxygen species; synergism

Substances (22) Reactions (0) Citing (10)

204

Selective inhibitors of aurora kinases inhibit proliferation, reduce cell viability and impair cell cycle progression in papillary thyroid carcinoma cells

By: Baldini, E.; Tuccilli, C.; Prinzi, N.; Sorrenti, S.; Antonelli, A.; Fallahi, P.; Mian, C.; Barollo, S.; Catania, A.; Morrone, S.; et al

Journal of Biological Regulators and Homeostatic Agents (2015), 29(4), 793-803 | Language: English, Database: CAplus and MEDLINE

The three members of the Aurora kinase family, Aurora-A, -B and -C, regulate several aspects of the mitotic process, and their aberrant expression and/or function causes mitotic abnormalities leading either to cell death or aneupl oidy. They are found overexpressed in several human malignancies, including the papillary thyroid carcinoma (P TC). In the present study, we sought to establish whether Aurora kinase inhibition could be of any therapeutic value in the treatment of aggressive forms of P TC, enduring to radioactive iodide (RAI) ablation. To this end, the effects of selective inhibitors of Aurora- A (MLN8237) and Aurora- B (AZD1152) were analyzed on 3 human PTC cell lines expressing either wild-type (K1 and TPC1) or mutant p53 (B CPAP). The two inhibitors were capable of reducing cell proliferation in a time- and dose- dependent manner, with IC comprised between 65.4 and 114.9 n M for M L N8237, and between 26.6 and 484.6 n M for AZD1152. Immunofluorescence experiments confirmed that AZD1152 inhibited Aurora-B phosphorylation of histone H3 on Ser10, however, it did not affect Aurora- A autophosphorylation. MLN8237 inhibited Aurora-A autophosphorylation as expected, but at concent rations required to achieve the maximum antiproli ferative effects it also abolished H3 (Ser10) phosphorylation. Time-lapse videomicroscopy evidenced that both inhibitors prevented the completion of cytokinesis, and cytofluorimetric anal. showed accumulation of cells in G2/M phase and/or polypl oidy. Apoptosis was induced in all the cells by both inhibitors independently from the p53 status. In conclu sion, in the present preclin. study M LN8237 and AZD1152 have emerged as promising drug candidates for RAI-insensitive PTC.

Keywords: PTC cell prolife ration viability cycle progression aurora kinase inhibitor

Substances (7) Reactions (0) Citing (7)

205

A Cell Biologist's Field Guide to Aurora Kinase Inhibitors.

By: de Groot, Christian O; Hsia, Judy E; Anzola, John V; Motamedi, Amir; Yoon, Michelle; Wong, Yao Liang; Jenkins, David; Lee, Hyun J; Martinez, Mallory B; Davis, Robert L; et al
Frontiers in oncology (2015), 5, 285 | Language: English, Database: MEDLINE

Aurora kinases are essential for cell division and are frequently misregulated in human cancers. Based on their potential as cancer therapeutics, a plethora of small molecule Aurora kinase inhibitors have been developed, with a subset having been adopted as tools in cell biology. Here, we fill a gap in the characterization of Aurora kinase inhibitors by using bioche mical and cell-based assays to systematically profile a panel of 10 commer cially available compounds with reported selectivity for Aurora A (MLN8054, ML N8237, MK-5108, MK-8745, Genentech Aurora Inhibitor 1), Aurora B (Hesperadin, ZM447439, AZD1152-HQPA, GSK1070916), or Aurora A/B (VX-680). We quantify the in vitro effect of each inhibitor on the activity of Aurora A alone, as well as Aurora A and Aurora B bound to fragments of their activators, TPX2 and INCENP, respectively. We also report kinome profiling results for a subset of these compounds to highlight potential off-target effects. In a cellular context, we demons trate that immunofluorescence-based detection of LATS2 and histone H3 phospho-epitopes provides a facile and reliable means to assess potency and specif icity of Aurora A versus Aurora B inhibition, and that G2 duration measured in a live imaging assay is a specific readout of Aurora A activity. Our analysis also highlights variation between HeLa, U2OS, and hTERT-RPE1 cells that impacts selective Aurora A inhibi tion. For Aurora B, all four tested compounds exhibit excellent selectivity and do not signifi cantly inhibit Aurora A at effective doses. For Aurora A, MK-5108 and MK-8745 are significantly more selective than the commonly used inhibitors M LN8054 and MLN8237. A crystal structure of an Aurora A/MK-5108 complex that we determined suggests the chemical basis for this higher specif icity. Taken together, our quantitative biochemical and cell-based analyses indicate that AZD1152-HQPA and MK-8745 are the best current tools for selectively inhibiting Aurora B and Aurora A, respect ively. However, MK-8745 is not nearly as ideal as AZD1152-HQPA in that it requires high concentrations to achieve full inhibition in a cellular context, indicating a need for more potent Aurora A-selective inhibitors. We conclude with a set of "good practice" guidelines for the use of Aurora inhibitors in cell biology experi ments.

Keywords: AZD1152; Aurora kinase inhibi tors; Hesperadin; MK-5108; MK-8745; MLN8054; MLN8237; ZM447439

Substances (0) Reactions (0) Citing (58)

206

Phosphoproteomic analysis of Aurora kinase inhibition in monopolar cytokinesis [Erratum to document cited in CA163:327364]

By: Polat, Ayse Nur; Karayel, Ozge; Giese, Sven H.; Harmanda, Busra; Sanal, Erdem; Hu, Chi-Kuo; Renard, Bernhard Y.; Ozlu, Nurhan
Journal of Proteome Research (2015), 14(12), 5408 | Language: English, Database: CAplus and MEDLINE

Supplementary Figures 1-3 were omitted from the Supporting Information file; the corrected file is available online.

Keywords: erratum human Aurora kinase cytokinesis phosphoproteome target; phosphopeptide intermediate filament erratum

Substances (3) Reactions (0) Citing (0)

207

Management and novel drugs for older patients with acute myeloid leukemia

By: Yoshimoto, Goichi; Miyamoto, Toshihiro
Ketsueki Naika (2015), 71(5), 666-674 | Language: Japanese, Database: CAplus

A review. This article discusses on management and novel drugs for older patients with acute myeloid leukemia.

Keywords: human acute myeloid leukemia chemotherapy review

Substances (14) Reactions (0) Citing (0)

208

Sarcoma Cell Line Screen of Oncology Drugs and Investigational Agents Identifies Patterns Associated with Gene and microRNA Expression

By: Teicher, Beverly A.; Polley, Eric; Kunkel, Mark; Evans, David; Silvers, Thomas; Delosh, Rene; Laudeman, Julie; Ogle, Chad; Reinhart, Russell; Selby, Michael; et al
Molecular Cancer Therapeutics (2015), 14(11), 2452-2462 | Language: English, Database: CAplus and MEDLINE

The diversity in sarcoma phenotype and genotype make treatment of this family of diseases exceptionally challenging. Sixty-three human adult and pediatric sarcoma lines were screened with 100 FDA-approved oncol. agents and 345 investig ational agents. The investigational agents' library enabled comparison of several compounds targeting the same mol. entity allowing comparison of target specificity and heterogeneity of cell line response. Gene expression was derived from exon array data and micro RNA expression was derived from direct digital detection assays. The compounds were screened against each cell line at nine concent rations in triplicate with an exposure time of 96 h using Alamar blue as the endpoint. Results are presented for inhibitors of the following targets: aurora kinase, IGF-1R, MEK, BET bromodomain, and PARP1. Chem. structures, IC_{50} heat maps, concentration response curves, gene expression, and mi R expression heat maps are presented for selected examples. In addition, two cases of exceptional responders are presented. The drug and compound response, gene expres sion, and microRNA expression data are publicly available at http://sarcoma.cancer.gov. These data provide a unique resource to the cancer research community. Mol Cancer Ther; 14(11); 2452-62. ©2015 AACR.

Keywords: sarcoma cell IGF1R MEK1 PARP1 microRNA oncol drug

Substances (26) Reactions (0) Citing (49)

209

Gonadotropin-releasing hormone receptor-targeting peptides and their use to treat and diagnose cancer

By: Miao, Yubin
United States, US9180214 B1 2015-11-10 | Language: English, Database: CAplus

The present invention is directed to novel non-invasive diagnostic tools/compounds to image and treat cancers, especially cancers which overexpress GnRH such as breast cancer, prostate cancer and melanoma, among others, including metastatic breast cancer, prostate cancer and/or melanoma among numerous others in vivo. The novel imaging probes are capable of detecting cancerous cells, as well as their metastatic spread in tissues. The novel probes of the present invention will also be useful to initiate therapy for breast and/or prostate cancer, among other cancers, as well as monitor patients' response to chemotherapy treatments and other interventions or therapies used in the treatment of cancer, including metastatic cancer as otherwise described herein. Compounds according to the present invention may be used as diagnostic tools for diagnosing cancer as well as therapeutic agents for treating cancer and related secondary disease states and conditions.

Keywords: GnRh receptor targeting peptide cancer therapy diagnosis

PatentPak available

Substances (283) Reactions (0) Citing (0)

210

Combination therapies targeting mitochondria for cancer therapy

By: Altieri, Dario C.; Ghosh, Jagadish C.
World Intellectual Property Organization, WO2015168599 A1 2015-11-05 | Language: English, Database: CAplus

Fig 1A

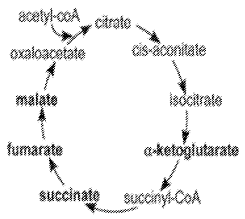

Pharmaceutical compositions for the treatment of cancer are provided. In one embodiment the compos ition comprises gamitrinib and a phosphatidylinositol 3-kinase (PI3K) inhibitor selected from PX-866, AZD6482, LY294002, BEZ235, GSK458, GDC0941, ZSTK474, BKM120, and GSK2636771B. Methods of treating cancer are also provided. The present invention is based in part on the inventor's discovery that combination therapy that targets mitochondria eliminates tumor adaptation induced by P13 K inhibition, and improves clin. outcome in cancer.

Keywords: antitumor gamitrinib BEZ235 combination therapy targeting mitochondria cancer treatment

PatentPak available

Substances (91) Reactions (0) Citing (0)

211

Methods for treating cancer using TOR kinase inhibitor combination therapy

By: Fultz, Kimberly Elizabeth; Tran, Tam Minh; Xu, Shuichan; Xu, Weiming; Trowe, Torsten; Filvaroff, Ellen
World Intellectual Property Organization, WO2015160868 A1 2015-10-22 | Language: English, Database: CAplus

Provided are methods for treating or preventing cancer, comprising administering an effective amount of a 3,4-dihydropyrazino[2,3-b]pyrazin-2(1H)-one TOR kinase inhibitor and an effective amount of a second active agent to a patient. Cancers treated by the method include solid tumors of the head, neck, breast, lung, ovaries, colon, rectum, stomach, prostate, urinary bladder, uterine, cervix, and other reproductive organs, wherein the tumors include relapsed, refractory or advanced solid tumors.

Keywords: pyrazinopyrazine TOR kinase inhibitor combination therapy cancer

PatentPak available

Substances (217) | Reactions (0) | Citing (0)

212

Quinazoline-benzimidazole hybrid as dual optical sensor for cyanide and Pb^{2+} ions and Aurora kinase inhibitor

By: Luxami, Vijay ; Rani, Richa; Sharma, Alka; Paul, Kamaldeep
Journal of Photochemistry and Photobiology, A: Chemistry (2015), 311, 68-75 | Language: English, Database: CAplus

Compounds 1 and 2 exhibited selective fluorescence response toward Pb^{2+} over the other metal ions like Zn^{2+}, Co^{2+}, Ca^{2+}, K^{+}, Ni^{2+}, Mn^{2+}, Fe^{2+}, Hg^{2+}, Na^{+}, etc. Compound 1 behaved as cyanide selective chromofluorescent sensor amongst other anions like F^{-}, Cl^{-}, Br^{-}, I^{-}, AcO^{-}, $H_2PO^{-}_4$, HSO^{-}_4, etc. The binding properties of compounds 1 and 2 with cyanide and Pb^{2+} were investigated via UV-vis, fluorescence spectroscopy, ^{1}H NMR titration experiment and DFT calculations Compound 1 has been used to estimate CN^{-} ion (2-500μM) and compound 2 used to estimate Pb^{2+} ion (0.05-1500μM) ratiometrically. Compounds 1 and 2 have also been used as Aurora kinase inhibitors with IC_{50} values of 7.50μM and 0.53μM, resp.

Keywords: cyanide lead quinazoline benzimidazole hybrid Aurora kinase optical sensor

Substances (19) | Reactions (0) | Citing (22)

213

Target inhibition map system for combination antitumor therapy design and methods of using same

By: Pal, Ranadip; Keller, Charles, III; Berlow, Noah E.; Seguin, Bernard
United States, US20150269307 A1 2015-09-24 | Language: English, Database: CAplus

Disclosed is a system and method for targeted therapy designs which model a cancer pathway for predicting the effectiveness of targeted anti-cancer drugs. The disclosed system and method includes utilization of a computer processor allowing for the selection of a set of drugs from available drugs using approximation algorithms utilizing cell viability data associated with a testable culture of a patient's tumor for generating a probabilistic target inhibition map (PTIM) from viability data for considering the selection of a set of drugs from available drugs, and further supports a wide variety of scenarios for personalized cancer therapy, related products and services.

Keywords: antitumor combination therapy design target map system algorithm modeling

PatentPak available

Substances (79) | Reactions (0) | Citing (0)

214

Phosphoproteomic analysis of Aurora kinase inhibition in monopolar cytokinesis

By: Polat, Ayse Nur; Karayel, Ozge; Giese, Sven H.; Harmanda, Busra; Sanal, Erdem; Hu, Chi-Kuo; Renard, Bernhard Y.; Ozlu, Nurhan
Journal of Proteome Research (2015), 14(9), 4087-4098 | Language: English, Database: CAplus and MEDLINE

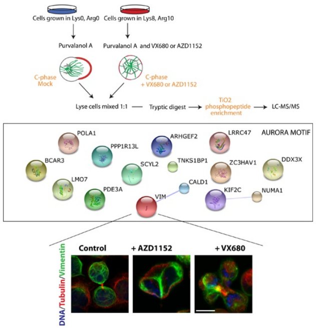

Cytokinesis is the last step of the cell cycle that requires coordi nated activities of the microtubule cytoskeleton, actin cytoskeleton, and membrane compartments. Aurora B kinase is one of the master regulatory kinases that orches trate multiple events during cytokinesis. To reveal targets of the Aurora B kinase, we combined quant. mass spectr ometry with chem. genetics. Using the quant. proteomic approach, SILAC (stable isotope labeling with amino acids in cell culture) , we analyzed the phosphoproteome of monopolar cytokinesis upon VX680- or AZD1152 -mediated aurora kinase inhibition. In total, our anal. quantified over 20 000 phosphopeptides in response to the Aurora-B kinase inhibition; 246 unique phosphopeptides were significantly down-regulated and 74 were up-regulated. Our data provide a broad anal. of downstream effectors of Aurora kinase and offer insights into how Aurora kinase regulates cytokinesis.

Keywords: human Aurora kinase cytokinesis phosphoproteome target phosphopeptide intermediate filament

Substances (3) | Reactions (0) | Citing (9)

215

Exosomes as a therapeutic for cancer

By: Trujillo, Kristina
World Intellectual Property Organization, WO2015120150 A1 2015-08-13 | Language: English, Database: CAplus

In one embodiment, the invention provides substantially purified exosomes which induce apoptosis in breast cancer cells and which are derived from a cultured medium of histol. normal breast tissue cells that are obtained from tumor-adjacent normal breast tissue. Related methods of treating breast cancer and pharmaceutical formulations are also provided.

Keywords: breast cancer antitumor autologous purified exosomes iv injection

PatentPak available

Substances (284) | Reactions (0) | Citing (4)

216

Therapeutic combinations for treating cancer comprising an irreversible mutant EGFR inhibitor and an antineoplastic agent such as an Aurora kinase inhibitor

By: Simmmons, Andrew D.; Harding, Thomas Christian

World Intellectual Property Organization, WO2015112705 A2 2015-07-30 | Language: English, Database: CAplus

The present invention relates to combination treatment methods for treating cancer, partic ularly non-small cell lung cancer (N SCLC) . More specifi cally, the invention relates to methods of treating or managing cancer, partic ularly NSCLC, in a subject, comprising administering an irreversible mutant E GFR inhibitor compound in combin ation with one or more antineo plastic agents, particularly an Aurora kinase inhibitor.

Keywords: cancer treatment EGFR inhibitor Aurora kinase inhibitor combin ation

PatentPak available

Substances (46) Reactions (0) Citing (3)

217

Discovery of N-(4-(3-(2-Aminopyrimidin-4-yl)pyridin-2-yloxy)phenyl)-4-(4-methylthiophen-2-yl)phthalazin-1-amine (AMG 900), A Highly Selective, Orally Bioavailable Inhibitor of Aurora Kinases with Activity against Multidrug-Resistant Cancer Cell Lines

By: Geuns-Meyer, Stephanie; Cee, Victor J.; Deak, Holly L.; Du, Bingfan; Hodous, Brian L.; Nguyen, Hanh Nho; Olivieri, Philip R.; Schenkel, Laurie B.; Vaida, Karina R.; Andrews, Paul; et al
Journal of Medicinal Chemistry (2015), 58(13), 5189-5207 | Language: English, Database: CAplus and MEDLINE

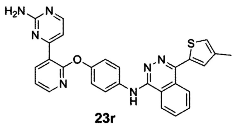

23r

Cell Line	EC_{50} (nM)
CAL51 (Non-MDR)	2
MES-SA Dx5 (MDR)	1
HCT15 (MDR)	2
SNU449 (MDR)	2
769P (MDR)	2

Efforts to improve upon the phys. properties and metabolic stability of pyridinyl-pyrimidine phthalazinamine Aurora kinase inhibitor revealed that potency against multidrug-resistant cell lines was compro mised by increased polarity. Despite its high in vitro metabolic intrinsic clearance, AMG 900 (**I**) showed acceptable pharmaco kinetic properties and robust pharmaco dynamic activity. Projecting from in vitro data to in vivo target coverage was not practical due to disjunctions between enzyme and cell data, complex and apparently contradictory indicators of binding kinetics, and unmeas urable free fraction in plasma. In contrast, it was straight forward to relate pharmaco kinetics to pharmacodynamics and efficacy by following the time above a threshold concent ration On the basis of its oral route of administration, a selectivity profile that favors Aurora-driven pharmacol. and its activity against multidrug-resistant cell lines, **I** was identified as a potential best- in-class Aurora kinase inhibitor. In phase 1 dose expansion studies with G-CSF support, **I** has shown promising single agent activity.

Keywords: pyridinylpyrimidine phthalazinamine AMG 900 analog prepar ation SAR Aurora kinase

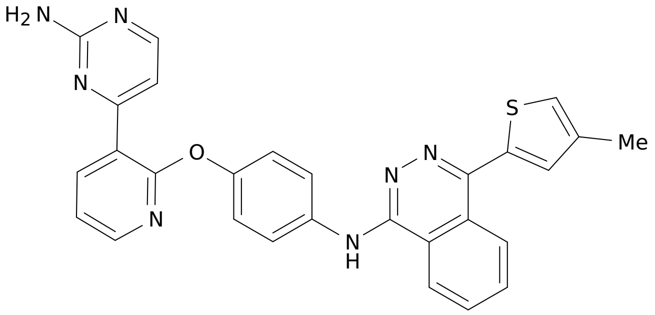

I

Substances (94) | Reactions (138) | Citing (36)

218

Structural Biology Insight for the Design of Sub-type Selective Aurora Kinase Inhibitors

By: Sarvagalla, Sailu; Coumar, Mohane Selvaraj
Current Cancer Drug Targets (2015), 15(5), 375-393 | Language: English, Database: CAplus and MEDLINE

A review. Aurora kinase A, B and C, are key regulators of mitosis and are over expressed in many of the human cancers, making them an ideal drug target for cancer chemotherapy. Currently, over a dozen of Aurora kinase inhibitors are in various phases of clin. development. The majority of the inhibitors (VX-680/MK-0457, PHA-739358, CYC116, SNS-314, AMG 900, AT-9283, SCH-1473759, ABT-348, PF-03814735, R-763/AS-703569, KW-2449 and TAK-901) are pan-selective (isoform non-selective) and few are Aurora A (MLN8054, MLN8237, VX-689/MK5108 and ENMD 2076) and Aurora B (AZD1152 and GSK1070916) sub-type selective. Despite the intensive research efforts in the past decade, no Aurora kinase inhibitor has reached the market. Recent evidence suggests that the sub-type selective Aurora kinase A inhibitor could possess advantages over pan- selective Aurora inhibitors, by avoiding Aurora B mediated neutropenia. However, sub-type selective Aurora kinase A inhibitor design is very challe nging due to the similarity in the active site among the isoforms. Structural biol. and computational aspects pertaining to the design of Aurora kinase inhibitors were analyzed and found that a possible means to develop sub-type selective inhibitor is by targeting Aurora A specific residues (Leu215, Thr217 and Arg220) or Aurora B specific residues (Arg159, Glu161 and Lys164), near the solvent exposed region of the protein. Particularly, a useful strategy for the design of sub- type selective Aurora A inhibitor could be by targeting Thr217 residue as in the case of MLN8054. Further preclin. and clin. studies with the sub- type selective Aurora inhibitors could help bring them to the market for the treatment of cancer.

Keywords: review Aurora kinase inhibitor design anticancer therapeutic target cancer

Substances (22) Reactions (0) Citing (20)

219

SnapShot: Kinase Inhibitors II

By: Wang, Jinhua; Gray, Nathanael S.
Molecular Cell (2015), 58(4), 710-710.e1 | Language: English, Database: CAplus and MEDLINE

Selective small-mol. inhibitors of protein kinases can serve as powerful tools to elucidate biol. function. Efforts to develop potential drug candidates have yielded a wealth of kinase inhibitors. However, selecting the optimal kinase inhibitor for a particular applic ation can be challenging. While the optimal inhibitor will be applic ation specific, we have attempted to summarize some of the best reported inhibitors for various kinases.

Keywords: protein kinase inhibition small mol

Substances (87) Reactions (0) Citing (10)

220

Biomarkers for PSMA targeted therapy for prostate cancer

By: Olson, William C.; DiPippo, Vincent
World Intellectual Property Organization, WO2015073896 A2 2015-05-21 | Language: English, Database: CAplus

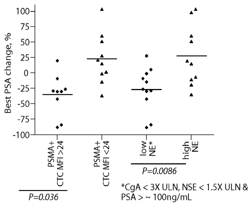

Fig. 2

Provided herein are a number of methods, assays and diagnostic tests based on one or more biomarkers as well as related kits and compositions that can be used to identify subjects or patients that would likely benefit from a treatment (or continued treatment) , such as a PSMA targeted therapy. Also provided are methods for treating the identified subjects or patients.

Keywords: PSMA targeted therapy prostate cancer biomarker

PatentPak available

Substances (20) Reactions (0) Citing (1)

221

Sequential treatment with aurora B inhibitors enhances cisplatin-mediated apoptosis via c-Myc

By: Ma, Yaxi; Cao, Handi; Lou, Siyue; Shao, Xuejing; Lv, Wen; Qi, Xiaotian; Liu, Yujia; Ying, Meidan; He, Qiaojun; Yang, Xiaochun
Journal of Molecular Medicine (Heidelberg, Germany) (2015), 93(4), 427-438 | Language: English, Database: CAplus and MEDLINE

Abstract: Platinum compound such as cisplatin is the first-line chemotherapy of choice in most patients with ovarian carcinoma. However, patients with inherent or acquired cisplatin resistance often experience relapse. Therefore, novel therapies are urgently required to treat drug-resistant ovarian carcinoma. Here, we showed that compared to the non- functional traditional simultaneous treatment, sequential combination of Aurora B inhibitors followed by cisplatin synergis tically enhanced apoptotic response in cisplatin-resistant OVCAR-8 cells. This effect was accomp anied by the induction of polyploidy in a c-Myc-dependent manner, as c-Myc knockdown reduced the efficacy of the combin ation by suppressing the expression of Aurora B and impairing cellular response to Aurora B inhibitor, as indicated by the decreased polyploidy and hyperphosphorylation of histone H1. In c-Myc-deficient SKOV3 cells, c-Myc overexpression restored Aurora B expression, induced polyploidy after inhibition of Aurora B, and sensitized cells to this combination therapy. Thus, our report reveals for the first time that sequential treatment of Aurora B inhibitors and cisplatin is essential to inhibit ovarian carcinoma by inducing polyploidy and downregulating c-Myc and that c-Myc is identified as a predictive biomarker to select cells responsive to chemotherapeutical combinations targeting Aurora B. Collect ively, these studies provide novel approaches to overcoming cisplatin chemotherapy resistance in ovarian cancer. Key Message: Pretre atment of Aurora B inhibitors augment apoptotic effects of cisplatin. The synergy of Aurora B inhibitor with cisplatin is dependent on c-Myc expression. c-Myc-dependent induction of polyploidy sensitizes cells to cisplatin.

Keywords: aurora B inhibitor cisplatin anticancer agent cMyc apoptosis cancer

Substances (7) Reactions (0) Citing (6)

222

Novel drugs for older patients with acute myeloid leukemia

By: Montalban-Bravo, G.; Garcia-Manero, G.
Leukemia (2015), 29(4), 760-769 | Language: English, Database: CAplus and MEDLINE

A review. Acute myeloid leukemia (AML) is the second most common form of leukemia and the most frequent cause of leukemia-related deaths in the United States. The incidence of A ML increases with advancing age and the prognosis for patients with A ML worsens substantially with increasing age. Many older patients are ineligible for intensive treatment and require other therap eutic approaches to optimize clin. outcome. To address this treatment gap, novel agents with varying mechanisms of action targeting different cellular processes are currently in development. Hypomethylating agents (azacitidine, decitabine, SGI-110), histone deacet ylase inhibitors (vorinostat, pracinostat, panobinostat), FMS-like tyrosine kinase receptor-3 inhibitors (quizartinib, sorafenib, midost aurin, crenolanib), cytotoxic agents (clofarabine, sapacitabine, vosaroxin), cell cycle inhibitors (barase rtib, volasertib, rigosertib) and monoclonal antibodies (gentuzumab ozogamicin, lintuzumab-Ac225) represent some of these promising new treatm ents. This review provides an overview of novel agents that have either completed or are currently in ongoing phase III trials in patients with previously untreated AML for whom intensive treatment is not an option. Other potential drugs in earlier stages of develo pment will also be addressed in this review.

Keywords: review anticancer agent acute myeloid leukemia

Substances (17) Reactions (0) Citing (54)

223

Anti-human VSTM5 antibodies, fragments and conjugates for therapy and diagnosis of cancer, infection and immune disease

By: Levine, Zurit; Rotman, Galit; Dassa, Liat; Levy, Ofer; Cojocaru, Gad S.; Toporik, Amir; Kliger, Yossef; Pow, Andrew; Liang, Spencer
World Intellectual Property Organization, WO2015037005 A1 2015-03-19 | Language: English, Database: CAplus

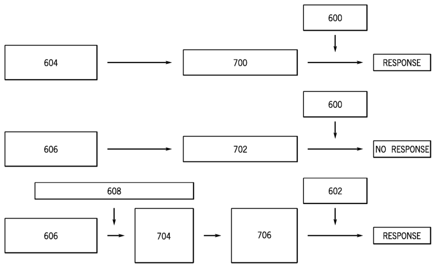

FIG. 1

The present invention relates to VSTM5-specific antibodies, antibody fragments, and VSTM5 polypeptides, conjugates and compos itions comprising same, for modulating (antago nizing or agonizing) one or more of the effects of VSTM5 expression on immunity. More specifically, the present invention relates to VSTM5-specific antibodies, antibody fragments, and VSTM5 polypeptides, conjugates and compositions comprising same for treating and aiding in the diagnosis of cancer, infectious diseases and immune related diseases, e.g., those associated with aberrant (higher or lower than normal) VSTM5 expression by diseased and/or immune cells and/or aberrant (increased or reduced) VSTM5-mediated effects on immunity.

Keywords: human VSTM5 antibody immunodiagnosis immunotherapy cancer infection immune disease

PatentPak available

Substances (424) Reactions (0) Citing (0)

224

Preparation of a therapeutic diblock polyester-based nanoparticles

By: Ashford, Marianne Bernice; Nolan, James Martin, III; Shin, Eyoung; Song, Young-Ho; Troiano, Greg; Wang, Hong
World Intellectual Property Organization, WO2015036792 A1 2015-03-19 | Language: English, Database: CAplus

Described herein are polymeric nanoparticles that include a therapeutic agent which is 2-(3-((7-(3-(ethyl(2-hydroxyethyl)amino)propoxy)quinazolin-4-yl)amino)-1H-pyrazolyl)-N-(3-fluorophenyl)acetamide (also known as AZD-1152) or a pharmaceutically acceptable salt thereof, and methods of making and using such therapeutic nanoparticles.

Keywords: therapeutic diblock polyester nanoparticle

PatentPak available

Substances (11)	Reactions (0)	Citing (1)

225

Aurora kinase A and B as new treatment targets in aromatase inhibitor-resistant breast cancer cells

By: Hole, Stine; Pedersen, Astrid M.; Lykkesfeldt, Anne E.; Yde, Christina W.
Breast Cancer Research and Treatment (2015), 149(3), 715-726 | Language: English, Database: CAplus and MEDLINE

Aromatase inhibitors (AIs) are used for treatment of estrogen receptor α (ER)-pos. breast cancer; however, resistance is a major obstacle for optimal outcome. This preclin. study aimed at identifying potential new treatment targets in AI-resistant breast cancer cells. Parental MCF-7 breast cancer cells and four newly established cell lines, resistant to the AIs exemestane or letrozole, were used for a functional kinase inhibitor screen. A library comprising 195 different compounds was tested for preferential growth inhibition of AI-resistant cell lines. Selected targets were validated by anal. of cell growth, cell cycle phase distribution, protein expression, and subcellular localization. We identified 24 compounds, including several inhibitors of Aurora kinases e.g., JNJ-7706621 and barasertib. Protein expression of Aurora kinase A and B was found upregulated in AI-resistant cells compared with MCF-7, and knockdown studies showed that Aurora kinase A was essential for AI-resistant cell growth. In AI-resistant cell lines, the clin. relevant Aurora kinase inhibitors alisertib and danusertib blocked cell cycle progression at the G2/M phase, interfered with chromosome alignment and spindle pole formation, and resulted in preferential growth inhibition compared with parental MCF-7 cells. Even further growth inhibition was obtained when combining the Aurora kinase inhibitors with the antiestrogen fulvestrant. Our study is the first to demonstrate that Aurora kinase A and B may be treatment targets in AI-resistant cells, and our data suggest that therapy targeting both ER and Aurora kinases may be a potent treatment strategy for overcoming AI resistance in breast cancer.

Keywords: Aurora kinase aromatase inhibitor resistance breast cancer treatment target

Substances (9)	Reactions (0)	Citing (31)

226

Finding the optimal combination therapy for the treatment of newly diagnosed AML in older patients unfit for intensive therapy

By: Erba, Harry P.

Leukemia Research (2015), 39(2), 183-191 | Language: English, Database: CAplus and MEDLINE

A review. There is no standard of care for older patients with newly diagnosed acute myeloid leukemia (AML) unfit for intensive therapy, and prognosis with currently recommended low-intensity therapies (decitabine, azacitidine, and low-dose cytarabine [LDA C]) remains poor. One promising strategy is to combine low- intensity treatments with novel agents. Gemtuzumab ozogam icin, tipifa rnib, and barasertib have been invest igated in phase 2/3 or 3 trials combined with LDAC, and phase 3 trials are currently investi gating sapacitabine plus decitabine, and volasertib plus LDAC in AML. This review discusses current treatment recommen dations and the development of combination therapies for older patients unfit for intensive therapy.

Keywords: combination intensive therapy diagnosis human AML review; Acute myeloid leukemia (AML); Combination; Low-intensity; Newly diagnosed; Novel agents; Older/elderly

Substances (8) Reactions (0) Citing (30)

227

AZD1152-HQPA induces growth arrest and apoptosis in androgen-dependent prostate cancer cell line (LNCaP) via producing aneugenic micronuclei and polyploidy

By: Zekri, Ali; Ghaffari, Seyed H.; Ghanizadeh-Vesali, Samad; Yaghmaie, Marjan; Salmaninejad, Arash; Alimoghaddam, Kamran; Modarressi, Mohammad H.; Ghavamzadeh, Ardeshir

Tumor Biology (2015), 36(2), 623-632 | Language: English, Database: CAplus and MEDLINE

Prostate cancer is the frequent non-cutaneous tumor with high mortality in men. Prostate tumors contain cells with different status of androgen receptor. Androgen receptor plays important roles in progression and treatment of prostate cancer. Aurora B kinase, with oncogenic potential, is involved in chromosome segregation and cytokinesis, and its inhibition is a promising anti- cancer therapy. In the present study, we aimed to investigate the effects of Aurora B inhibitor, AZD1152-HQPA, on survival and prolife ration of androgen receptor (AR)-pos. prostate cancer cells. LNCaP was used as androgen-dependent prostate cancer cell line. We explored the effects of AZD1152-HQPA on cell viability, DNA content, micronuclei formation, and expression of genes involved in apoptosis and cell cycle. Moreover, the expression of Aurora B and AR were investigated in 23 benign prostatic hyperplasia and 38 prostate cancer specimens. AZD1152-HQPA treatment induced defective cell survival, polypl oidy, and cell death in LNCaP cell line. Centromeric labeling with fluorescence in situ hybridization (FISH) showed that the loss of whole chromosomes is the origin of micronuclei, indicating on aneugenic action of AZD1152-HQPA. Treatment of AZD1152-HQPA decreased expression of AR. Moreover, we found weak pos. correlations between the expression of Aurora B and AR in both benign prostatic hyperp lasia and prostate cancer specimens (r = 0.25, r = 0.41). This is the first time to show that AZD1152-HQPA can be a useful therapeutic strategy for the treatment of androgen-dependent prostate cancer cell line. AZD1152-HQPA induces aneugenic mechanism of micron uclei production Taken together, this study provides new insight into the direction to overcome the therapeutic impediments against prostate cancer.

Keywords: prostate cancer AZD1152 HQPA cell arrest apoptosis androgen receptor

Substances (8) Reactions (0) Citing (12)

228

A phase II trial of AZD1152 in relapsed/refractory diffuse large B-cell lymphoma.

By: Collins, Graham P; Eyre, Toby A; Linton, Kim M; Radford, John; Vallance, Grant D; Soilleux, Elizabeth; Hatton, Chris
British journal of haematology (2015), 170(6), 886-90 | Language: English, Database: MEDLINE

There is no abstract available for this document.

Keywords: AZD1152; Aurora B kinase; barasertib; diffuse large B-cell lymphoma; lymphoid malignancies

Substances (0) Reactions (0) Citing (22)

229

Mechanism of action and therapeutic efficacy of Aurora kinase B inhibition in MYC overexpressing medulloblastoma.

By: Diaz, Roberto Jose; Golbourn, Brian; Faria, Claudia; Picard, Daniel; Shih, David; Raynaud, Denis; Leadly, Michael; MacKenzie, Danielle; Bryant, Melissa; Bebenek, Matthew; et al
Oncotarget (2015), 6(5), 3359-74 | Language: English, Database: MEDLINE

Medulloblastoma comprises four molecular subgroups of which Group 3 medulloblastoma is characterized by MYC amplification and MYC overexpression. Lymphoma cells expressing high levels of MYC are susceptible to apoptosis following treatment with inhibitors of mitosis. One of the key regulatory kinases involved in multiple stages of mitosis is Aurora kinase B. We hypothesized that medulloblastoma cells that overexpress MYC would be uniquely sensitized to the apoptotic effects of Aurora B inhibition. The specific inhibition of Aurora kinase B was achieved in MYC- overexpressing medulloblastoma cells with AZD1152-HQPA. MYC overexpression sensitized medulloblastoma cells to cell death upon Aurora B inhibition. This process was found to be independent of endoreplication. Using both flank and intracranial cerebellar xenografts we demonstrate that tumors formed from MYC-overexpressing medulloblastoma cells show a response to Aurora B inhibition including growth impairment and apoptosis induction. Lastly, we show the distribution of AZD1152-HQPA within the mouse brain and the ability to inhibit intracranial tumor growth and prolong survival in mice bearing tumors formed from MYC-overexpressing medulloblastoma cells. Our results suggest the potential for therapeutic application of Aurora kinase B inhibitors in the treatment of Group 3 medulloblastoma.

Substance (1) Reactions (0) Citing (18)

230

Novel substituted bicyclic compounds as bromodomain inhibitors

By: Liu, Shuang; Quinn, John Frederick; Duffy, Bryan Cordell; Wang, Ruifang; Jiang, May Xiaowu; Martin, Gregory Scott; Zhao, He; Ellis, Michael; Wagner, Gregory Steven; Young, Peter Ronald
World Intellectual Property Organization, WO2015004534 A2 2015-01-15 | Language: English, Database: CAplus

The invention relates to substituted bicyclic compounds, which are useful for inhibition of BET protein function by binding to bromodomains, pharmaceutical compositions comprising these compounds, and use of the compounds and compositions in therapy. Also disclosed is the preparation of compounds of the invention.

Keywords: bicyclic derivative preparation bromodomain

PatentPak available

Substances (145) Reactions (191) Citing (2)

231

Preparation of azabicyclic compounds as BET inhibitors and therapeutic uses thereof

By: Liu, Shuang; Quinn, John Frederick; Duffy, Bryan Cordell; Wang, Ruifang; Jiang, May Xiaowu; Martin, Gregory Scott; Zhao, He; Ellis, Michael; Wagner, Gregory Steven; Young, Peter Ronald

World Intellectual Property Organization, WO2015004533 A2 2015-01-15 | Language: English, Database: CAplus

The invention relates to substituted azabicyclic compounds of formula I which are useful for the inhibition of bromodomain and extra terminal domain (BET) proteins, pharmaceutical compositions comprising these compounds, and use of the compounds and compositions for treatment of BET-associated diseases such as cancer, inflammation, autoimmune disease, cardiovascular disease, benign proliferative and fibrotic disorders, and other diseases. Compounds of formulas I [wherein A is selected from 5- or 6-membered monocyclic carbocycle or heterocycles fused to ring B; B is a 6-membered carbocycle or heterocycle; Y is N or C; X is N H, alkyl, C(O)O, etc.; W^1 and W^2 are independently selected from N or C R; R at each occurrence is independently selected from H, NH_2, OH, etc.; W^3 and W^4 are independently selected from N, C H, and C with the proviso that both W^3 and W^4 cannot be N; R^3 is 4-7 membered carbocycle, 4-7 membered heterocycle, bicyclic carbocycle, etc.; D^1 is 5-membered monocyclic carbocycle or heterocycle connected to the B ring via a carbon-carbon bond with proviso] or a stereoisomer, tautomer, pharmaceutically acceptable salt, or hydrate thereof are claimed and exemplified. Reaction of 3,5-dimethyl-4-(4,4,5,5-tetramethyl-1,3,2-dioxaborolan-2-yl)isoxazole with 4,6-dibromo-2-methylbenzimidazole afforded II in 60% yield. I were evaluated for inhibition of tetra-acetylated histone H4 binding to BET bromodomains using TR-FRET anal. from which II was determined to inhibit the first bromodomain of Brd4 with an IC_{50} value of ≤ 0.3 μM.

Keywords: azabicyclic compound preparation BET inhibitor

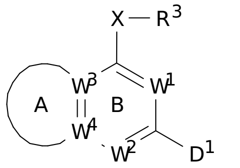

I

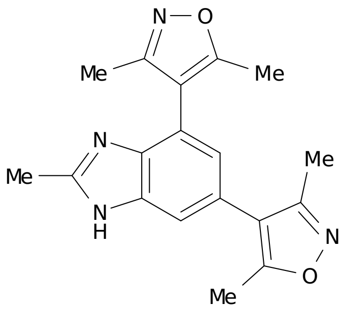

II

PatentPak available

Substances (542) Reactions (1,251) Citing (9)

232

Preparation of azabicyclic compounds as BET inhibitors and therapeutic uses thereof

By: Duffy, Bryan Cordell; Liu, Shuang; Quinn, John Frederick; Wang, Ruifang; Jiang, May Xiaowu; Martin, Gregory Scott; Wagner, Gregory Steven; Young, Peter Ronald

World Intellectual Property Organization, WO2015002754 A2 2015-01-08 | Language: English, Database: CAplus

The invention relates to substituted azabicyclic compounds of formula I which are useful for the inhibition of bromodomain and extra terminal domain (BET) proteins, pharmaceutical compositions comprising these compounds, and use of the compounds and compositions for treatment of BET-associated diseases such as cancer, inflammation, autoimmune disease, cardiovascular disease, benign proliferative and fibrotic disorders, and other diseases. Compounds of formulas I and II [wherein A is selected from 5- or 6-membered monocyclic heterocycles fused to ring B; B is a 6-membered aromatic carbocycle or heterocycle; Y is N or C; X is NH, alkyl, C(O)O, etc.; W^1, W^2, W^3 are independently selected from N or CR; R at each occurrence is independently selected from H, NH_2, halo, CN, etc.; R^4 is 3-7 membered carbocycle or heterocycle; D^1 is 5-membered monocyclic heterocycle with proviso] or a stereoisomer, tautomer, pharmaceutically acceptable salt, or hydrate thereof are claimed and exemplified. Benzylation of 2-chloroadenine provided 9-benzyl-2-chloroadenine which was reacted with 3,5-dimethyl-4-(4,4,5,5-tetramethyl-1,3,2-dioxaborolan-2-yl)isoxazole affording **III**. **I** were evaluated for inhibition of tetra-acetylated histone H4 binding to BET bromodomains using TR-FRET anal. from which **III** was determined to inhibit the first bromodomain of Brd4 with an IC_{50} value between 0.3 μM and 30 μM.

Keywords: azabicyclic compound preparation BET inhibitor

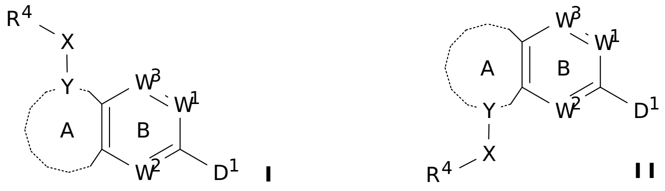

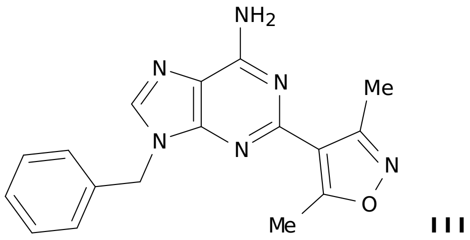

PatentPak available

Substances (556)

Reactions (982)

Citing (12)

233

Aurora kinase B inhibition reduces the proliferation of metastatic melanoma cells and enhances the response to chemotherapy

By: Porcelli, Letizia; Guida, Gabriella; Quatrale, Anna E.; Cocco, Tiziana; Sidella, Letizia; Maida, Immacolata; Iacobazzi, Rosa M.; Ferretta, Anna; Stolfa, Diana A.; Strippoli, Sabino; et al
Journal of Translational Medicine (2015), 13, 26/1-26/13 | Language: English, Database: CAplus and MEDLINE

Background: The poor response to chemotherapy and the brief response to vemurafenib in metastatic melanoma patients, make the identification of new therapeutic approaches an urgent need. Interestingly the increased expression and activity of the Aurora kinase B during melanoma progression suggests it as a promising therapeutic target. Methods: The efficacy of the Aurora B kinase inhibitor barasertib-HQPA was evaluated in BRAF mutated cells, sensitive and made resistant to vemura fenib after chronic exposure to the drug, and in BRAF wild type cells. The drug effectiveness has been evaluated as cell growth inhibition, cell cycle progression and cell migration. In addition, cellular effectors of drug resistance and response were investi gated. Results: The characterization of the effectors responsible for the resistance to vemurafenib evidenced the increased expression of MITF or the activation of Erk1/2 and p-38 kinases in the newly established cell lines with a phenotype resistant to vemura fenib. The sensitivity of cells to barasertib-HQPA was irresp. of BRAF mutational status. Barasertib-HQPA induced the mitotic catastrophe, ultimately causing apoptosis and necrosis of cells, inhibited cell migration and strongly affected the glycolytic metabolism of cells inducing the release of lactate. In association (i) with vemurafenib the gain in effectiveness was found only in BRAF(V600K) cells while (ii) with nab-paclit axel, the combination was more effective than each drug alone in all cells. Conclu sions: These findings suggest barasertib as a new therapeutic agent and as enhancer of chemotherapy in metastatic melanoma treatment.

Keywords: Aurora kinase B proliferation metastatic melanoma cell chemotherapy

Substances (10) Reactions (0) Citing (35)

234

Canine osteosarcoma cells exhibit resistance to aurora kinase inhibitors

By: Cannon, C. M.; Pozniak, J.; Scott, M. C.; Ito, D.; Gorden, B. H.; Graef, A. J.; Modiano, J. F.
Veterinary and Comparative Oncology (2015), 13(1), 48-59 | Language: English, Database: CAplus and MEDLINE

We evaluated the effect of Aurora kinase inhibitors AZD1152 and VX680 on canine osteosarcoma cells. Cytotoxicity was seen in all four cell lines; however, half-maximal inhibitory concentrations were significantly higher than in human leukemia and canine lymphoma cells. AZD1152 reduced Aurora kinase B phosphorylation, indicating resistance was not because of failure of target recognition. Efflux mediated by ABCB1 and ABCG2 transporters is one known mechanism of resistance against these drugs and verapamil enhanced AZD1152-induced apoptosis; however, these transporters were only expressed by a small percentage of cells in each line and the effects of verapamil were modest, suggesting other mechanisms contribute to resistance. Our results indicate that canine osteosarcoma cells are resistant to Aurora kinase inhibitors and suggest that these compounds are unlikely to be useful as single agents for this disease. Further investigation of these resistance mechanisms and the potential utility of Aurora kinase inhibitors in multi-agent protocols is warranted.

Keywords: canine osteosarcoma drug resistance aurora kinase inhibitor AZD1152 VX680; chemotherapy; comparative oncology; in vitro models; oncology; osteosarcoma

Substances (4) Reactions (0) Citing (4)

235

Aurora kinase B is important for antiestrogen resistant cell growth and a potential biomarker for tamoxifen resistant breast cancer

By: Larsen, Sarah L.; Yde, Christina W.; Laenkholm, Anne-Vibeke; Rasmussen, Birgitte B.; Duun-Henriksen, Anne Katrine; Bak, Martin; Lykkesfeldt, Anne E.; Kirkegaard, Tove
BMC Cancer (2015), 15, 1-15 | Language: English, Database: CAplus and MEDLINE

Background: Resistance to antiestrogen therapy is a major clin. challenge in the treatment of estrogen receptor α (E R)-pos. breast cancer. The aim of the study was to explore the growth promoting pathways of antiestrogen resistant breast cancer cells to identify biomarkers and novel treatment targets. Methods: Antiestrogen sensitive and resistant T47 D breast cancer cell lines were used as model systems. Parental and fulvestrant resistant cell lines were subjected to a kinase inhibitor library. Kinase inhibitors prefere ntially targeting growth of fulvestrant resistant cells were identified and the growth inhibitory effect verified by dose- response cell growth experiments Protein expression and phosphorylation were investigated by western blot anal. Cell cycle phase distri bution and cell death were analyzed by flow cytometry. To evaluate Aurora kinase B as a biomarker for endocrine resistance, immunohi stochem. was performed on archival primary tumor tissue from breast cancer patients who have received adjuvant endocrine treatment with tamoxifen. Results: The selective Aurora kinase B inhibitor barasertib was identified to preferentially inhibit growth of fulvestrant resistant T47 D breast cancer cell lines. Compared with parental cells, phosphor ylation of Aurora kinase B was higher in the fulvestrant resistant T47 D cells. Barasertib induced degradation of Aurora kinase B, caused mitotic errors, and induced apoptotic cell death as measured by accumulation of Sub G1 cells and PARP cleavage in the fulvestrant resistant cells. Barasertib also exerted preferential growth inhibition of tamoxifen resistant T47 D cell lines. Finally, high percentage of Aurora kinase B pos. tumor cells was significantly associated with reduced disease- free and overall survival in 261 E R-pos. breast cancer patients, who have received tamoxifen as first-line adjuvant endocrine treatment. Conclusions: Our results indicate that Aurora kinase B is a driving factor for growth of antiestrogen resistant T47 D breast cancer cell lines, and a biomarker for reduced benefit of tamoxifen treatment. Thus, inhibition of Aurora kinase B, e.g. with the highly selective kinase inhibitor barasertib, could be a candidate new treatment for breast cancer patients with acquired resistance to antiestrogens.

Keywords: barasertib anticancer aurora kinase B inhibitor therapeutic breast cancer

Substances (7)   Reactions (0)   Citing (20)

236

Histone H3 phosphorylation in GBM: a new rational to guide the use of kinase inhibitors in anti-GBM therapy

By: Pacaud, Romain; Cheray, Mathilde; Nadaradjane, Arulraj; Vallette, Francois M.; Cartron, Pierre-Francois
Theranostics (2015), 5(1), 12-22, 11 pp. | Language: English, Database: CAplus and MEDLINE

Histones post-translational modifications (PTMs) are crucial components of diverse processes that modulate chromatin. Among the histones PTMs, the histones phosphorylation appears such crucial since it plays a significant role into DNA repair structure, transcr iption and chromatin compaction during cell division and apoptosis. However, little is known about the prognostic value of the histone phosphorylation in human cancer. This point could be consid erate such as an important gap in anti-cancer therapy since the use of adequate kinase inhibitors could remedy to the aberrant histone phosphorylation associated with a poor prognosis factor. To remedy at this situation, we analyzed the phosphorylation level of histone H3 at the residues T3, T6, S10, S28, Y41 and T45 in a collection of 42 glioblastoma multiformes (GBM). Our data indicated that the high level of p H3T6, pH3S10 and pH3Y41 are signatures associated with a poor prognosis of overall survival (OS) of GBM treated with the "temozolomide and irradiation standard" treatment of GBM (named TMZ + Irad treatment). Our data also showed that these signatures are correlated with the high activity of kinases already described as writers of the pH3T6, pH3S10 and pH3Y41 i.e. the PKC, Aurora-B and JAK2, resp. Finally, our anal. revealed that the use of Enzastaurin, AZD1152, and AZD1480 abrogated the high level of pH3T6, pH3S10 and pH3Y41 while increasing the sensitivity to the "temozolomide and irradiation"-induced cell death. To conclude, it appears that this work provides biomarkers for patient stratification for a therapy including kinase inhibitors.

Keywords: glioblastoma histone H3 phosphorylation kinase inhibitor anti cancer therapy; apoptosis; epigenetic; glioma; histone; phosphorylation

Substances (9) Reactions (0) Citing (23)

237

Modeling the mitotic regulatory network identifies highly efficient anti-cancer drug combinations

By: Wu, Yiran; Zhuo, Xiaolong; Dai, Ziwei; Guo, Xiao; Wang, Yao; Zhang, Chuanmao; Lai, Luhua
Molecular BioSystems (2015), 11(2), 497-505 | Language: English, Database: CAplus and MEDLINE

Targeting mitotic regulation is recognized as an important strategy for cancer therapy. Aurora A/B kinase and polo-like kinase 1 (PLK1) are the key mitotic regulators, and many inhibitors have been developed. Combinations of these inhibitors are anticipated to be more effective therapeutics compared with single-inhibitor treatments; however, a systematic anal. of the combined effects is lacking. Here, we constructed the first mammalian cell mitotic regulation network model, which spans from mitotic entry to anaphase initiation, and contains all key mitotic kinase targets. The combined effects of different kinase inhibitors and microtubule inhibitors were systematically explored. Simultaneous inhibition of Aurora B and PLK1 strongly induces polyploidy. Microtubule inhibitor dosage can be significantly reduced when combined with a PLK1 inhibitor. The efficacy of these inhibitor combinations was validated by our exptl. results. The mitotic regulatory network model provides a platform to study the complex interactions during mitosis, enables identification of mitotic regulators, and determines targets for drug discovery research. The suggested use of combining microtubule inhibitors with PLK1 inhibitors is anticipated to enhance microtubule-inhibitor tolerance in a wide range of patients.

Keywords: anticancer discovery protein phosphorylation epithelial cell simulation computational model

Substances (8) Reactions (0) Citing (2)

238

A chemical screening approach to identify novel key mediators of erythroid enucleation

By: Wolwer, Christina B.; Pase, Luke B.; Pearson, Helen B.; Godde, Nathan J.; Lackovic, Kurt; Huang, David C. S.; Russell, Sarah M.; Humbert, Patrick O.
PLoS One (2015), 10(11), e0142655/1-e0142655/12 | Language: English, Database: CAplus and MEDLINE

Erythroid enucleation is critical for terminal differentiation of red blood cells, and involves extrusion of the nucleus by orthoch romatic erythroblasts to produce reticulocytes. Due to the difficulty of synchronizing erythroblasts, the mol. mechanisms underlying the enucleation process remain poorly understood. To elucidate the cellular program governing enucleation, we utilized a novel chem. screening approach whereby orthochromatic cells primed for enucleation were enriched ex vivo and subjected to a functional drug screen using a 324 compound library consisting of structurally diverse, medicinally active and cell permeable drugs. Using this approach, we have confirmed the role of HDACs, proteasomal regulators and MAPK in erythroid enucleation and introduce a new role for Cyclin-dependent kinases, in particular CDK9, in this process. Importantly, we demonstrate that when coupled with imaging anal., this approach provides a powerful means to identify and characterize rate limiting steps involved in the erythroid enucleation process.

Keywords: MAPK CDK9 HDAC PI3K orthochromatic erythroblast erythroid enucleation

Substances (294)   Reactions (0)   Citing (8)

239

A novel therapeutic combination sequentially targeting aurora B and Bcl-xL in hepatocellular carcinoma.

By: Matsunaga, Hiroko; Tanaka, Shinji; Aihara, Arihiro; Ogawa, Kousuke; Matsumura, Satoshi; Ban, Daisuke; Ochiai, Takanori; Irie, Takumi; Kudo, Atsushi; Nakamura, Noriaki; et al
Annals of surgical oncology (2015), 22(9), 3079-86 | Language: English, Database: MEDLINE

BACKGROUND: Effective therapeutic combinations targeting the oncogenic pathway still are unknown in human hepatoc ellular carcinoma (HCC). The authors previously identified aberrant expression of aurora B kinase as the indepe ndent predictor for the lethal recurrence of HCC, showing that AZD1152 induced in vitro and in vivo apoptosis with polyploidy in human H CC cells. In this preclinical study, the combined effects of molecular-targeted therapies were evaluated based on the cellular response of aurora B inhibition. METHODS: This study analyzed the expression of Bcl-2 family proteins in polyploidization induced by AZD1152 and the in vitro synergistic effects of AZD1152 with control of the Bcl-2 family pathway in human HCC cells. The in vivo effects of the combin ation therapy targeting the specific molecules were evaluated using subcutaneous tumor xenograft models. RESULTS: The findings showed that Bcl-xL was specifically overexpressed in AZD1152-induced polyploid HCC cells. The combination of AZD1152 followed by Bcl-xL/2 inhibitor ABT263 induced synergistically cellular apoptosis ($p < 0.001$) and growth inhibition ($p < 0.0001$) . Interestingly, the reverse sequential administration of AZD1152 combined with pretreatment of ABT263 was less effective than the original one. In vivo studies using tumor xenografts of human HCC cells showed that combination therapy of ABT263 after AZD1152 pretre atment induced significant intratumoral apoptosis ($p < 0.05$) and remarkable anti- tumor effects ($p < 0.05$) without a severe adverse effect compared with the monotherapy. CONCLUSION: Based on Bcl-xL overexpression in polyploidy induced by aurora B inhibi tion, the rationale for therapeutic combinations targeting aurora B and Bcl- xL was demonstrated in the authors' preclinical studies, leading to a promising novel approach for the mechanism-based treatment of human HCC.

Substance (1)   Reactions (0)   Citing (8)

240

Overcoming cetuximab resistance in HNSCC: The role of AURKB and DUSP proteins

By: Boeckx, Carolien; Op de Beeck, Ken; Wouters, An; Deschoolmeester, Vanessa; Limame, Ridha; Zwaenepoel, Karen; Specenier, Pol; Pauwels, Patrick; Vermorken, Jan B.; Peeters, Marc; et al
Cancer Letters (New York, NY, United States) (2014), 354(2), 365-377 | Language: English, Database: CAplus and MEDLINE

Unraveling the underlying mechanisms of cetuximab resistance in head and neck squamous cell carcinoma (HNSCC) is of major importance as many tumors remain non-responsive or become resistant. Our microarray results suggest that "resistant" cells still exhibit RAS-MAPK pathway signaling contributing to drug resistance, as witnessed by low expression of DUSP5 and DUSP6, neg. regulators of ERK1/2, and increased expression of AURKB, a key regulator of mitosis. Therefore, interrupting the RAS-MAPK pathway by an ERK1/2 inhibitor (apigenin) or an AURKB inhibitor (barasertib) might be a new strategy for overcoming cetuximab resistance in HNSCC.

Keywords: cetuximab anticancer AURKB DUSP head neck squamous cell carcinoma; Anti-EGFR therapy; Aurora kinase B; Cetuximab resistance; Dual-specificity phosphatase 5 and 6; Head and neck squamous cell carcinoma; Nano Pro 1000

Substances (10) Reactions (0) Citing (46)

241

Method of determination of cancer cell drug sensitivity towards aurora kinase inhibitors

By: Kollaredy, Madhusudhan Reddy; Hajduch, Marian; Dzubak, Petr; Srovnal, Josef; Hrabakova, Rita; Kovarova, Hana
United States, US20140336073 A1 2014-11-13 | Language: English, Database: CAplus

A method for determining the sensitivity and/or resistance of a patient suffering from a cancer disease to Aurora kinase inhibitor therapy, which comprises determining in vitro in the cancer cells or body fluids taken from the patient the expression of at least one gene selected from a particular group and/or determining in vitro in the cancer cells or body fluids taken from the patient the level of at least one protein selected from a particular group.

Keywords: cancer drug sensitivity Aurora kinase inhibitor gene protein expression

PatentPak available

Substances (55) Reactions (0) Citing (0)

242

Effects of selective inhibitors of Aurora kinases on anaplastic thyroid carcinoma cell lines

By: Baldini, Enke; Tuccilli, Chiara; Prinzi, Natalie; Sorrenti, Salvatore; Antonelli, Alessandro; Gnessi, Lucio; Morrone, Stefania; Moretti, Costanzo; Bononi, Marco; Arlot-Bonnemains, Yannick; et al
Endocrine-Related Cancer (2014), 21(5), 797-811 | Language: English, Database: CAplus and MEDLINE

Aurora kinases are serine/threonine kinases that play an essential role in cell division. Their aberrant expression and/or function induce severe mitotic abnormalities, resulting in either cell death or aneuploidy. Overexpression of Aurora kinases is often found in several malignancies, among which is anaplastic thyroid carcinoma (ATC). We have previously demonstrated the in vitro efficacy of Aurora kinase inhibitors in restraining cell growth and survival of different ATC cell lines. In this study, we sought to establish which Aurora might represent the preferential drug target for ATC. To this end, the effects of two selective inhibitors of Aurora-A (MLN8237) and Aurora-B (AZD1152) on four human ATC cell lines (CAL-62, BHT-101, 8305C, and 8505C) were analyzed. Both inhibitors reduced cell proliferation in a time- and dose-dependent manner, with IC_{50} ranges of 44.3-134.2 nM for MLN8237 and of 9.2-461.3 nM for AZD1152. Immunofluorescence experiments and time-lapse videomicroscopy yielded evidence that each inhibitor induced distinct mitotic phenotypes, but both of them prevented the completion of cytokinesis. As a result, poliploidy increased in all AZD1152-treated cells, and in two out of four cell lines treated with MLN8237. Apoptosis was induced in all the cells by MLN8237, and in BHT-101, 8305C, and 8505C by AZD1152, while CAL-62 exposed to AZD1152 died through necrosis after multiple rounds of endoreplication. Both inhibitors were capable of blocking anchorage-independent cell growth. In conclusion, we demonstrated that either Aurora-A or Aurora-B might represent therapeutic targets for the ATC treatment, but inhibition of Aurora-A appears more effective for suppressing ATC cell proliferation and for inducing the apoptotic pathway.

Keywords: Aurora kinase anaplastic thyroid carcinoma; AZD1152; Aurora inhibitors; Aurora kinases; MLN8237; anaplastic thyroid cancer; cell cycle; therapy

Substances (4) | Reactions (0) | Citing (26)

243

Methods of use of cleavable drug conjugates and compositions

By: Nguyen, Mark Quang
United States, US20140294851 A1 2014-10-02 | Language: English, Database: CAplus

Base-labile crosslinkers, base-labile conjugates comprising such crosslinkers, methods of their synthesis and use are disclosed for treatment of cancers, metabolic diseases and infections.

Keywords: cleavable drug conjugate preparation cancer metabolic disease infection

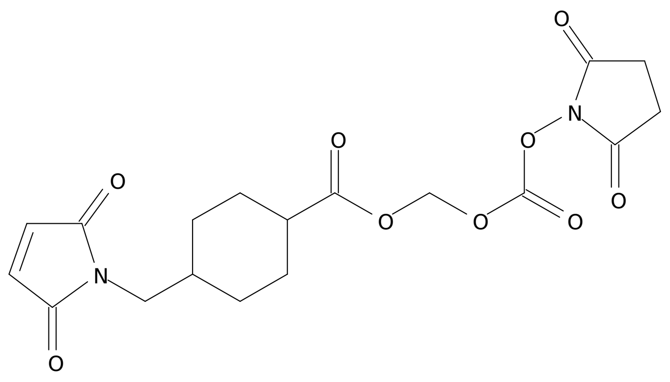

PatentPak available

Substances (286) | Reactions (29) | Citing (0)

244

Indomethacin promotes apoptosis in gastric cancer cells through concomitant degradation of Survivin and Aurora B kinase proteins

By: Chiou, Shiun-Kwei; Hoa, Neil; Hodges, Amy; Ge, Lishen; Jadus, Martin R.

Apoptosis (2014), 19(9), 1378-1388 | Language: English, Database: CAplus and MEDLINE

Regular usage of nonsteroidal anti-inflammatory drugs (NSAIDs) is associated with reduced incidence of a variety of cancers. The mol. mechanisms underlying these chemopreventive effects remain poorly understood. This current investigation showed that in gastric cancer cells: (1) Indomethacin treatment enhanced the degradation of chromosomal passenger proteins, Survivin and Aurora B kinase; (2) Indomethacin treatment down-regulated Aurora B kinase activity in a cell cycle- independent fashion; (3) siRNA knockdown of Survivin level promoted Aurora B kinase protein degradation, and vice versa; (4) ectopic overexp ression of Survivin blocked reduction of Aurora B kinase level and activity by indomethacin treatment, and vice versa; (5) siRNA knockdown of Aurora B kinase level and AZD1152 inhibition of its activity induced apoptosis, and overexp ression of Aurora B kinase inhibited indomet hacin-induced apoptosis; (6) indomethacin treatment reduced Aurora B kinase level, coinciding with reduction of Survivin level and induction of apoptosis, in KATO III and HT-29 cells, and in mouse gastric mucosa. A role for Aurora B kinase function in N SAID-induced apoptosis was not previously explored. Thus this report provides better underst anding of the mol. mechanisms underlying the anti-cancer effect of NSAIDs by elucidating a significant role for Aurora B kinase in indomet hacin-induced apoptosis.

Keywords: antitumor indomethacin apoptosis gastric cancer survivin aurora B kinase

Substances (6) Reactions (0) Citing (13)

245

Preparation of arylquinolines as anticancer agents

By: Courcambeck, Jerome; Bassissi, Firas; Brun, Sonia; Nicolas, Gregory; Beret, Antoine; Petit, Serge; Camus, Claire; Nallet, Jean Pierre; Halfon, Philippe

World Intellectual Property Organization, WO2014147611 A1 2014-09-25 | Language: English, Database: CAplus

The invention provides quinoline derivatives of general formula **I** (wherein R^1 is (un)substituted aryl, (un)substituted heteroaryl, or (un)substituted heteroaromatic 5 to 9-membered ring; R^2 is halo, (un)substituted C_{1-6} alkyl, OH, etc.; W is substituted piperidinyl, etc.; L^1 is a bond, carbonyl, O, etc.; and n = 0- 4) their manufacture, pharmaceutical compositions containing them, and their use as medicaments. The active compounds of the present invention are useful for the treatment of proliferative neoplastic and non-neoplastic diseases. Example compound **II**-2HCl was prepared in a multistep synthesis that culminates in reaction of intermediate **III** with 4-diethylaminopiperidine and subsequent conversion to the salt. The example compounds were tested extensively in anticancer screening assays both in vitro and in vivo. In cell viability assays on A-375, HCT-116, and MOLM-14AML cell lines, there was <20 % cell viability in the presence of **II** at a dose of 10 μ M.

Keywords: arylquinoline preparation anticancer agent

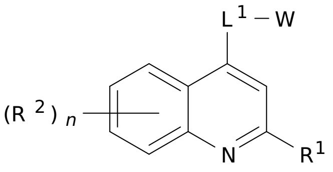

I

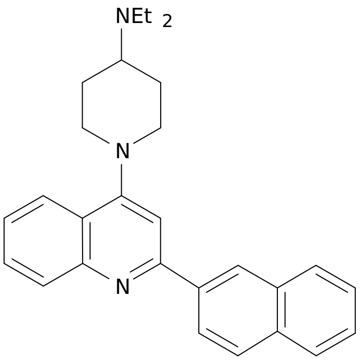

II

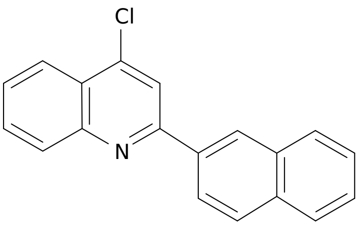

III

PatentPak available

Substances (510)

Reactions (472)

Citing (4)

246

Ligand efficiency based approach for efficient virtual screening of compound libraries

By: Ke, Yi-Yu; Coumar, Mohane Selvaraj ; Shiao, Hui-Yi; Wang, Wen-Chieh; Chen, Chieh-Wen; Song, Jen-Shin; Chen, Chun-Hwa; Lin, Wen-Hsing; Wu, Szu-Huei; Hsu, John T. A.; et al
European Journal of Medicinal Chemistry (2014), 83, 226-235 | Language: English, Database: CAplus and MEDLINE

Here we report for the first time the use of fit quality (FQ), a ligand efficiency (LE) based measure for virtual screening (VS) of compound libraries. The LE based VS protocol was used to screen an inhouse database of 125, 000 compounds to identify aurora kinase A inhibitors. First, 20 known aurora kinase inhibitors were docked to aurora kinase A crystal structure (P DB ID: 2W1C); and the conformations of docked ligand were used to create a pharmac ophore (PH) model. The PH model was used to screen the database compounds, and rank (PH rank) them based on the predicted IC_{50} values. Next, LE_Scale, a weight-dependant LE function, was derived from 294 known aurora kinase inhibitors. Using the fit quality (FQ = LE/LE_Scale) score derived from the LE_Scale function, the database compounds were reranked (PH_FQ rank) and the top 151 (0.12% of database) compounds were assessed for aurora kinase A inhibition biochem. This VS protocol led to the identification of 7 novel hits, with compound 5 showing aurora kinase A IC_{50} = 1.29 μM. Furthermore, testing of 5 against a panel of 31 kinase reveals that it is selective toward aurora kinase A & B, with <50% inhibition for other kinases at 10 μM concentrations and is a suitable candidate for further development. Incorporation of FQ score in the VS protocol not only helped identify a novel aurora kinase inhibitor, 5, but also increased the hit rate of the VS protocol by improving the enrichment factor (EF) for FQ based screening (EF = 828), compared to PH based screening (EF = 237) alone. The LE based VS protocol disclosed here could be applied to other targets for hit identification in an efficient manner.

Keywords: ligand efficiency virtual screening compound library; Aurora kinase inhibitor; Fit quality; Ligand efficiency; Pharmac ophore model; Virtual screening

Substances (93) Reactions (28) Citing (14)

247

p53 deficiency enhances mitotic arrest and slippage induced by pharmacological inhibition of Aurora kinases

By: Marxer, M.; Ma, H. T.; Man, W. Y.; Poon, R. Y. C.
Oncogene (2014), 33(27), 3550-3560 | Language: English, Database: CAplus and MEDLINE

A number of small-mol. inhibitors of Aurora kinases have been developed and are undergoing clin. trials for anti- cancer therapies. Different Aurora kinases, however, behave as very different targets: while inhibition of Aurora A (AURKA) induces a delay in mitotic exit, inhibition of Aurora B (AURKB) triggers mitotic slippage. Furthermore, while it is evident that p53 is regulated by Aurora kinase-dependent phosphorylation, how p53 may in turn regulate Aurora kinases remains mysterious. To address these issues, isogenic p53-containing and -neg. cells were exposed to classic inhibitors that target both AURKA and AURKB (Alisertib and ZM447439), as well as to new generation of inhibitors that target AURKA (MK-5108), AURKB (Barasertib) individually. The fate of individual cells was then tracked with time-lapse microscopy. Remarkably, loss of p53, either by gene disruption or small interfering RNA-mediated depletion, sensitized cells to inhibition of both AURKA and AURKB, promoting mitotic arrest and slippage resp. As the p53-dependent post-mitotic checkpoint is also important for preventing genome reduplication after mitotic slippage, these studies indicate that the loss of p53 in cancer cells represents a major opportunity for anti-cancer drugs targeting the Aurora kinases.

Keywords: p53 tumor suppressor mitosis slippage Aurora kinase inhibitor; Alisertib ZM447439 MK5108 Barasertib Aurora kinase inhibitor antitumor p53

Substances (7) Reactions (0) Citing (52)

248

Preparation of heterocyclic compounds as bromodomain inhibitors

By: Liu, Shuang; Duffy, Bryan Cordell; Quinn, John Frederick; Jiang, May Xiaowu; Wang, Ruifang; Martin, Gregory Scott; Zhao, He; Molino, Bruce Francis; Young, Peter Ronald

World Intellectual Property Organization, WO2014096965 A2 2014-06-26 | Language: English, Database: CAplus

The invention related to heterocyclic compounds of formula **I**, method for their preparation and their use as bromodomain inhibitors and use in therapy. Compounds of formula **I**, wherein W_1 is N and CR^5; W_2 is N and CR^4; W_3 is N and CR^3; R^1 is carbocycles and heterocycles; R^2 is 5- to 6-membered monocyclic carbocycle and heterocycle; R^3, R^4 and R^5 are independently H, alkyl, OH, carbamate, etc.; X is O and S; A is CO, SO_2, CH_2 and CH_2O, etc.; B is O, NH, S, SO, etc.; and their stereoisomers, tautomers, pharmaceutically acceptable salts, and hydrate, are claimed. Example compound **II** was prepared by benzylation of 6-chloro-2H-pyridazin-3-one to afford 2-benzyl-6-chloro-2H-pyridazin-3-one, which underwent cross coupling reaction with 3,5-dimethyl-4-(4,4,5,5-tetramethyl-1,3,2-dioxafooroian-2-yl)isoxazole to afford compound **II**. All the invention compounds were evaluated for their BET inhibitory activity and anticancer activity. From the assay, it was determined that example compound **II** exhibited IC_{50} value of < 30 μM.

Keywords: heterocycle preparation bromodomain inhibitor

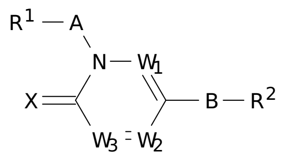

I

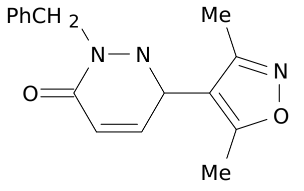

II

PatentPak available

Substances (596) Reactions (686) Citing (8)

249

Localization and function of Aurora-B in pig (Sus scrofa) oocytes

By: Peng, Xu; Ju, Shi-qiang; Xu, Ya-ping; Yang, Xiao-liu; Rui, Rong
Nongye Shengwu Jishu Xuebao (2014), 22(6), 681-689 | Language: Chinese, Database: CAplus

Aurora-B is a serine/threonine protein kinase which plays important regulatory role during mitotic cell cycle progre ssion, but its expression, localization and functionality during mammalian oocyte meiosis are seldom reported, and no related reports in pigs (Sus scrofa). In the present experiment, Aurora-B expression, subcellular localization, and its possible function during porcine oocyte meiotic maturation were investigated via confocal microscopic anal. and highly selective Aurora- B inhibitor treatment. Confocal microscopic anal. revealed that Aurora-B distributed uniformly in the cytoplasm at germinal vesicle (G V) stage. After germinal vesicle breakdown, Aurora-B expression was mainly associated with chromo somal. During M I period, with spindle assembly and sister chromatids were arranged in the equatorial plate, Aurora-B overlaped with chromatin. In AITI, chromosomes began to migrate to the poles under the spindle traction, then Aurora-B located in the center of chromo somes, but not overlaped with sister chromatids. Oocytes discharged the first polar body (pb I) after 44 h when Aurora- B enriched on the oocyte nucleus and pbI. After using inhibitors AZD-1152 treatment, the results showed that more and more oocytes arrested in G V stage with the increasing concentrations of AZD-1152, GVBD incidence suppressed; compared with control group, the oocytes arrested in Pro M I-AITI were significantly increased, arrested in M I I were significantly reduced. At the same time, M I I oocyte chromosome misali gned, some failed to align; abnormal spindle morphol., even disapp eared; actin filaments had a certain degree of fracture and dispersion. In addition, porcine parthenogenetic embryos development severely affected after suppressing the expression of Aurora-B by inhibitor on oocytes. Cleavage rate, blastocyst rate and the total number of blastocyst cells are signifi cantly lower with the increasing concentrations of AZD-1152 in each treatment group, and no blastocyst when the AZD-1152 concentration reached 50 nmol/L. Our study showed that Aurora-B might be a multifunctional kinase that plays pivotal regulatory roles in microt ubule assembly, chromosome segregation during porcine oocyte meiotic maturation, and also plays a role in the subsequent develo pment of oocytes.

Keywords: pig oocyte AuroraB localization function

Substance (1) Reactions (0) Citing (0)

250

Preparation of cyclic amines as BET inhibitors for treatment of cancer, autoimmune disorders, and other diseases

By: Fairfax, David John; Duffy, Bryan Cordell; Martin, Gregory Scott; Quinn, John Frederick; Liu, Shuang; Wagner, Gregory Steven; Young, Peter Ronald

United States, US20140142102 A1 2014-05-22 | Language: English, Database: CAplus

The present disclosure relates to compounds and pharmaceutical compositions thereof, which are useful as inhibitors of bromod omain and extra terminal domain (BET) protein function for treatment or prevention of cancer, autoimmune disorders, inflamm ation, and cardiovascular diseases. **I** [wherein W^1 is N or CR^1; W^2 is N or CR^2, W^3 is N or CR^3; W^4 is N or CR^4; each W may be the same or different from the others; A is N or CH; R^1, R^2, R^3, and R^4 are independently H, alkyl, aryloxy, etc.; two adjacent substi tuents selected from R^1, R^2, R^3, or R^4 may be connected in a 5- to 6- membered ring to form a bicyclic carbocycle or bicyclic hetero cycle; AR^1 is substituted Ph, substituted pyridinyl, substituted furanyl, etc.; B is aziridinyl, (un)substituted azetidinyl, (un)substituted piperidinyl, etc.] or a stereoisomer, tautomer, pharmaceutically acceptable salt, or hydrate thereof are claimed and exemplified. For example, **II** was prepared in a multistep synthesis (preparation given). **I** were assayed for the ability to inhibit binding of histone H4 and B ET bromodomains, inhibition of c-Myc expression and cell proliferation in cancer cell lines, and antitumor efficacy in a mouse model of acute myeloid leukemia (data given).

Keywords: cyclic amine preparation BET inhibitor cancer autoimmune disorder

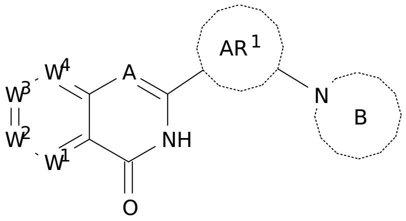

I

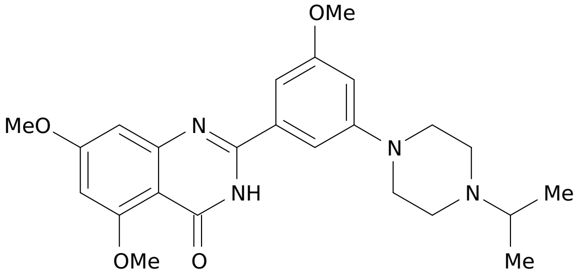

II

PatentPak available

Substances (180) Reactions (225) Citing (0)

251

Preparation of biaryl compounds as BET inhibitors for treatment of cancer, autoimmune disorders, and other diseases

By: Fairfax, David John; Martin, Gregory Scott; Quinn, John Frederick; Duffy, Bryan Cordell; Wagner, Gregory Steven; Young, Peter Ronald

United States, US20140140956 A1 2014-05-22 | Language: English, Database: CAplus

The present disclosure relates to compounds and pharmaceutical compositions thereof, which are useful as inhibitors of bromod omain and extra terminal domain (BET) protein function for treatment or prevention of cancer, autoimmune disorders, inflam matory disorders, and cardiovascular diseases. **I** [wherein W^1 is N or CR^1; W^2 is N or CR^2, W^3 is N or CR^3; W^4 is N or CR^4; each W may be the same or different from the others; A is N or CH; R^1, R^2, R^3, and R^4 are independently H, alkyl, aryloxy, etc.; two adjacent substi tuents selected from R^1, R^2, R^3, or R^4 may be connected in a 5- to 6- membered ring to form a bicyclic carbocycle or bicyclic hetero cycle; AR^1 is a substituted Ph, a substituted pyridinyl, a substituted furanyl, etc.; AR^2 is, for example, Ph, methylsulfonylphenyl, methylsulfinylphenyl, etc.] or a stereoisomer, tautomer, pharmaceutically acceptable salt, or hydrate thereof are claimed and exemplified. Thus, **II** was prepared in 67% yield by the cyclocond ensation of **III** with 2-amino-4,6-dimethoxybenzamide followed by deprotection. **I** were assayed for the ability to inhibit binding of histone H4 and BET bromodomains, inhibition of c-Myc expression and cell proliferation in cancer cell lines, and in vivo efficacy in a rat model of collagen- induced arthritis (data given).

Keywords: biaryl derivative BET inhibitor cancer autoimmune disorder

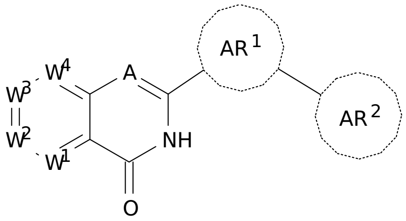

I

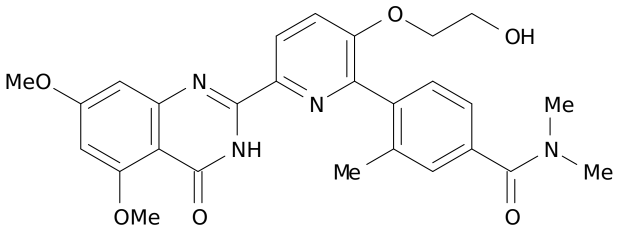

II

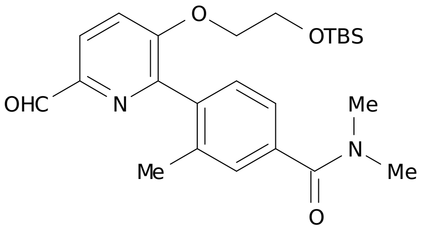

III

PatentPak available

Substances (276) Reactions (1,671) Citing (0)

252

Salt-inducible kinase 3 is a novel mitotic regulator and a target for enhancing antimitotic therapeutic-mediated cell death

By: Chen, H.; Huang, S.; Han, X.; Zhang, J.; Shan, C.; Tsang, Y. H.; Ma, H. T.; Poon, R. Y. C.
Cell Death & Disease (2014), 5(4), e1177 | Language: English, Database: CAplus and MEDLINE

Many mitotic kinases are both critical for maintaining genome stability and are important targets for anticancer therapies. We provide evidence that SIK3 (salt-inducible kinase 3), an AMP-activated protein kinase-related kinase, is important for mitosis to occur properly in mammalian cells. Downregulation of SIK3 resulted in an extension of mitosis in both mouse and human cells but did not affect the DNA damage checkp oint. Time-lapse microscopy and other approaches indicated that mitotic exit but not mitotic entry was delayed. Although repression of SIK3 alone simply delayed mitotic exit, it was able to sensitize cells to various antimi totic chems. Both mitotic arrest and cell death caused by spindle poisons were enhanced after SIK3 depletion. Likewise, the antimitotic effects due to pharmacol. inhibition of mitotic kinases including Aurora A, Aurora B, and polo-like kinase 1 were enhanced in the absence of SIK3. Finally, in addition to promoting the sensit ivity of a small-mol. inhibitor of the mitotic kinesin Eg5, S IK3 depletion was able to overcome cells that developed drug resistance. These results establish the importance of S IK3 as a mitotic regulator and underscore the potential of SIK3 as a druggable antimi totic target.

Keywords: SIK3 mitotic regulator antimitotic drug target mitosis cancer cell

Substances (24) Reactions (0) Citing (15)

253

Structure of Aurora B-INCENP in complex with barasertib reveals a potential transinhibitory mechanism

By: Sessa, Fabio; Villa, Fabrizio
Acta Crystallographica, Section F: Structural Biology Communications (2014), 70(3), 294-298 | Language: English, Database: CAplus and MEDLINE

The Aurora family is a well conserved and well characterized group of serine-threonine kinases involved in the normal progre ssion of mitosis. The deregulation of Aurora kinases impairs spindle assembly, checkpoint function and cell division. To date, many small mols. that compete with ATP for binding to Aurora kinases have been developed and charact erized. Here, the first structure of the Xenopus laevis Aurora B-INCENP complex bound to the clin. relevant small mol. barasertib was determined The binding properties of this inhibitor to the Aurora B active site are analyzed and reported. An unexpected crystal-packing contact in the Aurora B- INCEN P structure coordinated by an A TP analog is also reported, in which the I NCENP C-terminus occupies the substrate-binding region, resembling the protein kinase A inhibitory mechanism.

Keywords: Xenopus Aurora B kinase INCENP barasertib complex crystal structure; Aurora B INCENP AMP PNP complex crystal structure; Aurora kinases; PKA; barasertib; cancer; small-molecule inhibitors

Substances (3) Reactions (0) Citing (26)

254

Inhibition of Aurora-B suppresses osteosarcoma cell migration and invasion

By: Zhu, Xiao Ping; Liu, Zhi Li; Peng, Ai Fen; Zhou, Yun Fei; Long, Xin Hua; Luo, Qingfeng; Huang, Shan Hu; Shu, Yong
Experimental and Therapeutic Medicine (2014), 7(3), 560-564 | Language: English, Database: CAplus and MEDLINE

Previous studies have suggested that Aurora-B may be involved in cancer metastasis. However, its role has been poorly evaluated in osteosarcoma (OS). The aim of this study was to investigate the correlation between Aurora-B expression and metastasis in human OS. The human OS cell line, U2-OS, and OS biopsy specimens were used in the study. The expression of Aurora- B protein was examined using immunohistochem. and western blotting in OS tissues and U2-OS cells, resp. AZD1152-hydroxyquinazoline-pyrazol-anilide, an inhibitor of Aurora-B, was used to inhibit Aurora-B expression in U2-OS cells. The effect of Aurora- B inhibition on U2-OS cell proliferation, invasion and migration was assessed using MTT, colony formation, wound healing and Transwell assays. The results showed that pos. expression of the Aurora-B protein was observed in the nucleus, and that Aurora- B expression levels in the cases with pulmonary metastases were significantly higher than in those without metastasis. In vitro, the proliferation, invasion and migration of U2-OS cells were suppressed by the inhibition of Aurora- B. These results suggest that Aurora- B may be involved in OS metastasis, and may be a promising target in the treatment of O S metastasis.

Keywords: aurora kinase B inhibitor osteosarcoma antitumor cell migration invasion; AZD1152; Aurora-B; metastasis; osteos arcoma

Substances (2) Reactions (0) Citing (21)

255

Close correlation between MEK/ERK and Aurora-B signaling pathways in sustaining tumorigenic potential and radioresistance of gynecological cancer cell lines

By: Marampon, Francesco; Gravina, Giovanni Luca; Popov, Valdimir M.; Scarsella, Luca; Festuccia, Claudio; La Verghetta, Maria Emilia; Parente, Silvia; Cerasani, Manuela; Bruera, Gemma; Ficorella, Corrado; et al
International Journal of Oncology (2014), 44(1), 285-294 | Language: English, Database: CAplus and MEDLINE

Both Aurora-A and -B kinases have been implicated in tumorigenesis; and as such, they represent an attractive therapeutic target. Recent studies found that Aurora-A is a downstream target of mitogen- activated protein kinase 1/ERK2, while Aurora-B has been found to be a prognostic/predictive therapeutic target for epithelial cancer. In a wide range of human cancers, the Ras/Raf/M EK/ER K/MAP kinase pathway is enhanced and the cellular response to growth signals is known to increase. The purpose of this study was to investigate whether the MEK/ERK cascade regulates tumorigenic signaling and radioresistance via the Aurora-B-mediated pathway in a panel of gynecol. cancer cell lines. Exponentially growing human endometrial (Ishikawa), cervical (HeLa), cervical (CASK I) and vulva (SiHa) cancer cells were used in culture treated with either control or M EK/ERK inhibitor or AZD1152 before and after irradiation Western blotting, ERK1/2 siRNA transfection, growth assay in modified monolayer, Annexin V and migration /invasion assays were performed. The specific MEK/ERK inhibitor U0126 decreased the tumorigenic potential and improved the radiation response in all cellular models. The modulation of radioresponse upon U0126 treatment pos. correlated with the inhibition of phospho-ERKs and the reduction of Aurora- B kinase expression. In addition, upon U0126 treatment DNA-PKcs protein expression was found to be downregulated, indicating that the improved radiation response may be caused by decreased D NA double-strand damage repair mechanisms. The knockdown of ERK by siRNA confirmed the MEK/ERK-dependent Aurora-B kinase expression. The use of AZD1152, a selective Aurora- B inhibitor, counteracted tumorigenic potential and radioresistance phenotype by highly increasing apoptotic mechanisms in all gynecol. cancer cell lines used. Evidence from our experiments show that tumorigenic potential and radiation response in gynecol. cancer cells may ensue from a MEK/ERK or Aurora- B inhibition. Together with the close correlation of MEK/ERK and Aurora- B protein expression, this study underlines the potential role of a M EK/ERK/Aurora-B axis whose interruption recovers the antitumor effects of radiotherapy.

Keywords: radiotherapy MEK ERK AuroraB gynecol cancer

Substances (7) Reactions (0) Citing (35)

256

Aurora kinase inhibitor patents and agents in clinical testing: an update (2011 - 2013)

By: Cheung, Chun Hei Antonio; Sarvagalla, Sailu; Lee, Jane Ying-Chieh; Huang, Yi-Chun; Coumar, Mohane Selvaraj
Expert Opinion on Therapeutic Patents (2014), 24(9), 1021-1038 | Language: English, Database: CAplus and MEDLINE

A review. Introduction: Aurora kinase A, B and C, members of serine/t hreonine kinase family, are key regulators of mitosis. As Aurora kinases are overexpressed in many of the human cancers, small-mol. inhibitors of Aurora kinase have emerged as a possible treatment option for cancer. Areas covered: In 2009 and 2011, the literature pertaining to Aurora kinase inhibitors and their patents was reviewed. Here, the aim is to update the information for Aurora kinase inhibitors in clin. trials and the patents filed between the years 2011 and 2013. Pubmed, Scopus, Scifinder, USPTO, EPO and www.clinicaltrials.gov databases were used for searching the literature and patents for Aurora kinase inhibitors. Expert opinion: Even though both Aurora sub-type selective as well as pan-selective inhibitors show preclin. and clin. efficacy, so far no Aurora kinase inhibitor has been approved for clin. use. Particu larly, dose-limiting toxicity (neutropenia) is a key issue that needs to be addressed. Prelim inary evidence suggests that the use of selective Aurora A inhibitors could avoid Aurora B-mediated neutropenia in clin. settings. Also, use of adjunctive agents such as granulocyte stimulating factor to overcome neutropenia associated with Aurora B inhibition could be an answer to overcome the toxicity and bring Aurora inhibitors to market in the future.

Keywords: Aurora kinase inhibitor patent agent review; AMG 900 and KW-2449; AT-9283; AZD1152; Aurora kinase inhibitor; CY C116; ENMD-2076; GSK1070916; MLN8054; MLN8237; PF-3814735; PHA739358; R763; SNS-314; VX-680/MK0457; VX-689/MK-5108

Substances (14) Reactions (0) Citing (44)

257

Aurora kinases as druggable targets in pediatric leukemia: heterogeneity in target modulation activities and cytotoxicity by diverse novel therapeutic agents

By: Jayanthan, Aarthi; Ruan, Yibing; Truong, Tony H.; Narendran, Aru
PLoS One (2014), 9(7), e102741/1-e102741/12, 12 pp. | Language: English, Database: CAplus and MEDLINE

Leukemia is the most common pediatric malignancy, constituting more than 30% of all childhood cancers. Although cure rates have improved greatly, approx. one in five children relapse and poor survival rates post relapse remain a challenge. Given this, more effective and innovative therapeutic strategies are needed in order to improve prognosis. Aurora kinases, a family of serine/t hreonine kinases essential for the regulation of several mitotic processes, have been identified as potential targets for cancer therapeutics. Elevated expression of Aurora kinases has been demonstrated in several malignancies and is associated with aberrant mitotic activity, aneuploidy and alterations in chromosomal structure and genome instability. Based on this rationale, a number of small mol. inhibitors have been formulated and advanced to human studies in the recent past. A comparative anal. of these agents in cytotoxicity and target modulation analyses against a panel of leukemia cells provides novel insights into the unique mechanisms and codependent activity pathways involved in targeting Aurora kinases, consti tuting a distinctive preclin. exptl. framework to identify appropriate agents and combinations in future clin. studies.

Keywords: modulation cytotoxicity therapeutic agent aurora kinase pediatric leukemia heterog eneity

Substances (14) Reactions (0) Citing (13)

258

Identification of Aurora-A Inhibitors by Ligand and Structure-Based Virtual Screening

By: Morshed, Mohammad Neaz
Molecular Informatics (2014), 33(5), 369-381 | Language: English, Database: CAplus and MEDLINE

Aurora kinase A has been identified as one of the most attractive targets for cancer therapy because of its critical role in the regulation of the cell cycle. In order to identify active compounds with structural diversity we performed virtual screening. 3D-QSAR pharmacophore models were developed and the best model was used as a query for screening the databases. Ligand and structure-based virtual screening protocol was conducted sequentially by applying the common feature pharmacophore and mol. docking to discover potent Aurora-A inhibitors. A total of eighty-eight compounds were selected for the in vitro activities against various human cancer cell lines (DU145 and HT29). Considering the activity data, we have identified seven compounds to be considered for the next step, among which four compounds had high inhibition rate (above 50 %) at 10 μM with GI_{50} lower than 10 μM. Based on the cell line and enzyme assay (Aurora- A & B) result, these four compounds were used as templat e/query mol. for similarity search. The best result was obtained for similarity hit SH3. It had IC_{50} of 0.578 and 11.77 μ M for Aurora-A and B resp., which implies 20-fold selectivity over Aurora-B. The hits obtained from this screening scheme could be potential drug candidates after further optimization.

Keywords: Aurora A inhibitor anticancer drug discovery mol docking cancer; Anticancer; Aurora-A; Docking; Inhibitor; Pharmac ophore; Tumor cell; Virtual screening

Substances (45) Reactions (0) Citing (0)

259

Discovery of 7-Aryl-Substituted (1,5-Naphthyridin-4-yl)ureas as Aurora Kinase Inhibitors

By: Defaux, Julien; Antoine, Maud; Le Borgne, Marc; Schuster, Tilmann; Seipelt, Irene; Aicher, Babette; Teifel, Michael; Guenther, Eckhard; Gerlach, Matthias; Marchand, Pascal
ChemMedChem (2014), 9(1), 217-232 | Language: English, Database: CAplus and MEDLINE

As part of our research projects to identify new chem. entities of biol. interest, we developed a synthetic approach and the biol. evaluation of (7-aryl-1,5-naphthyridin-4-yl)ureas as a novel class of Aurora kinase inhibitors for the treatment of malignant diseases based on pathol. cell proliferation. 1,5-Naphthyridine derivatives showed excellent inhibitory activities toward Aurora kinases A and B, and the most active compound, 1-cyclopropyl-3-[7-(1-methyl-1H-pyrazol-4-yl)-1,5-naphthyridin-4-yl]urea (49), displayed IC_{50} values of 13 and 107 nm against Aurora kinases A and B, resp. In addition, the selectivity toward a panel of seven cancer-related protein kinases was highlighted. In vitro ADME properties were also determined in order to rationalize the difficulties in correlating antiproliferative activity with Aurora kinase inhibition. Finally, the good safety profile of these compounds imparts promising potential for their further development as anticancer agents.

Keywords: naphthyridinyl urea derivative preparation Aurora kinase inhibitor cancer; 1,5-naphthyridines; aurora kinases; heteroc ycles; inhibitors; ureas

Substances (95) Reactions (529) Citing (10)

260

Hypersensitivity to aurora kinase inhibitors in cells resistant against platinum- containing anticancer agents.

By: Akiyama, Masaki; Izumi, Hiroto; Wang, Ke-Yong; Yamaguchi, Takahiro; Kuma, Akihiro; Kitamura, Noriaki; Harada, Yoshikazu; Oya, Ryoichi; Yamaguchi, Koji; Iwai, Yoshiko; et al
Anti-cancer agents in medicinal chemistry (2014), 14(7), 1042-50 | Language: English, Database: MEDLINE

The aurora kinases are serine/threonine kinases that are essential for mitosis and contribute to tumorig enesis. Therefore, aurora kinases hold promise for molecularly targeted therapy. In the present study, we demons trated that aurora B kinase (A URKB) is overexpressed in both cisplatin- and oxalip latin-resistant cells. Downregulation of AURKB sensitized cells to both cisplatin and oxaliplatin, but not to paclit axel, 5-FU or hydrogen peroxide. Interestingly, we found that both cisplatin- and oxalip latin-resistant cells were hypersensitive to the AURKB specific inhibitors, AZD1152 HQPA and ZM447439, suggesting that both cisplatin- and oxaliplatinresistant cells develop an addiction to A URKB. These data provide evidence that aurora kinase inhibitors can overcome both cisplatin and oxaliplatin resistance. Therefore, AURKB inhibitors could offer potential benefits if used after first- line platinum-based chemotherapy.

Substances (2) | Reactions (0) | Citing (9)

261

Novel pharmaceutical combinations and methods for treating cancer

By: Cheok, Chit Fang; Lane, David
United States, US20130344140 A1 2013-12-26 | Language: English, Database: CAplus

Methods of selectively targeting a p53-deficient cancer cell, comprising administering to a patient suffering from cancer (i) a reversible cell cycle arrest-inducing agent for inducing cell cycle arrest in a p53- pos. cell; and (ii) an aurora kinase inhibitor, wherein said reversible cell cycle arrest-inducing agent is administered prior to administration of said aurora kinase inhibitor, and pharmac eutical combinations, kits and oral dosage forms for the same.

Keywords: p53 deficient cancer treatment pharmaceutical combination; aurora kinase inhibitor cell cycle arrest inducer cancer treatment

PatentPak available

Substances (27) | Reactions (0) | Citing (0)

262

Modulators of GTPase and use in relevant treatment

By: Wandinger-Ness, Angela; Sklar, Larry; Surviladze, Zurab; Oprea, Tudor; Hudson, Laurie; Aube, Jeffrey; Golden, Jennifer E.; Schroeder, Chad E.; Simpson, Denise S.; Noth, Julica J.
United States, US20130345277 A1 2013-12-26 | Language: English, Database: CAplus

The invention relates to pyrazole derivatives which function as selective modulators of the Ras- homologous (Rho) family of small G T Pases, in particular, Cdc42 GTPase and their use to treat diseases, for example cancers, including metastatic cancer, genetic and acquired diseases where activation of Cdc42 GTPase plays a pivotal role, such as neurodege nerative diseases, rheumatoid arthritis, atherosclerosis, diabetes type I, autosomal polycystic kidney disease, cystic kidney disease, precystic kidney disease, microbial infect ions, including Chlamydia infections, E. coli infections, H. pylori infections and its secondary effects including gastric ulcers, Coxiella Brunetti (Q-fever) infections and Strepto coccus pneumonia infections, fungal infections including Paracocc idiodes brasiliensis and Candida albicans and their secondary effects including lung edema. Addnl., compounds according to the invention may be used to inhibit rejection in transplant patients (pursuant to transplantation), to promote immunosuppression, anti-inflammatory response and to mobilize stem cell (migration) in patients in need. Also disclosed is the preparation of compounds of the invention.

Keywords: pyrazole derivative preparation GTPase cancer neurodegenerative disease microbial infection; transplant rejection inflammation immunosuppression stem cell migration pyrazole derivative

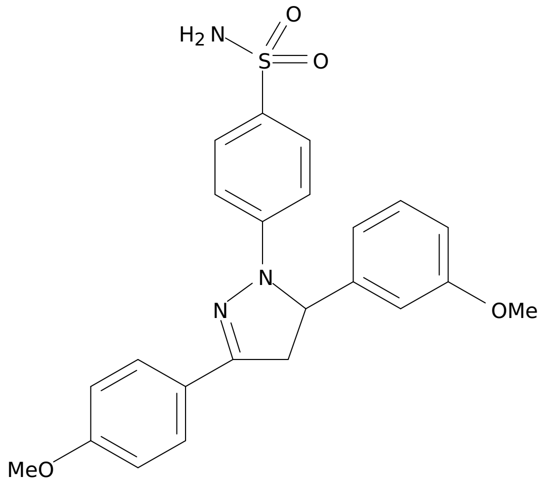

PatentPak available

Substances (325) | Reactions (25) | Citing (1)

263

Method for preparing barasertib

By: Xu, Xuenong
China, CN103435598 A 2013-12-11 | Language: Chinese, Database: CAplus

The invention relates to method the method for preparing barasertib. Barasertib was prepared via conden sation of 7-{3-[ethyl(2-hydroxyethyl)amino]propoxy}-3,4-dihydroquinazolin-4-one with 2-(5-amino-1H-pyrazol-3-yl)-N-(3-fluorophenyl)acetamide in the presences of organic base and condensing agent. The method has the advantages of simple process, mild conditions, good enviro nment, and high product quality, and is adapted to industrial production

Keywords: hydroxyethyl aminopropoxy quinazolinone amino pyrazolyl fluorophenyl acetamide condensation; barasertib prepar ation green chem

PatentPak available

Substances (3) | Reaction (1) | Citing (0)

264

Preparation of bifunctional compounds useful for promoting protein degradation for treatment of cancer and oxidative stress-associated diseases

By: Crews, Craig; Gustafson, Jeff; Roth, Anke Gundula; Tae, Hyun Seop; Buckley, Dennis; Neklesa, Taavi

World Intellectual Property Organization, WO2013170147 A1 2013-11-14 | Language: English, Database: CAplus

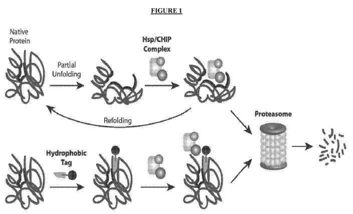

The invention relates to compounds that act as degraders of a target protein, independent of the protein class or its localization for use in treatment of diseases including cancer and oxidative stress-associated disorders. Compounds of formula L-DM [wherein DM is a protein degradation moiety covalently bound to a linker (L) which is a functional group forming a covalent bond to a protein binding moiety] and their pharmaceutically acceptable salts, solvates, or polymorphs thereof having a Clog P equal to or higher than 1.5 are claimed and prepared For example, the compound of formula **I** was prepared in 30% yield via the reaction of **II** with 2-(2-aminoethoxy)ethyl 2-(adamantan-1-yl)acetate. Compounds of the invention exhibit a broad range of pharmacol. activities consistent with the degradation/inhibition of targeted proteins. This was illustrated in an assay in which combined administration of selective androgen receptor degrader **I** (250 nM) and HSP90 inhibitor geldanamycin (100 nM) to LnCAP cells resulted in additive inhibition of androgen receptors compared to either compound alone.

Keywords: bifunctional compound preparation protein degradation cancer oxidative stress disease

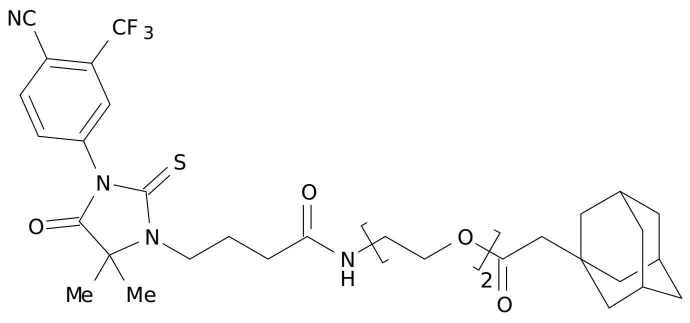

I

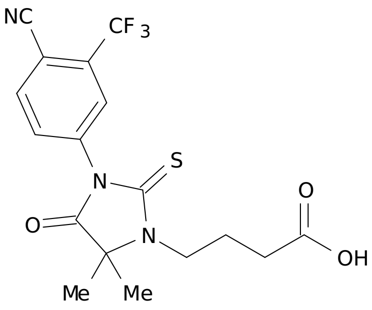

II

PatentPak available

Substances (569) Reactions (406) Citing (8)

265

Phase I Study Assessing the Safety and Tolerability of Barasertib (AZD1152) With Low-Dose Cytosine Arabinoside in Elderly Patients With AML

By: Kantarjian, Hagop M.; Sekeres, Mikkael A.; Ribrag, Vincent; Rousselot, Philippe; Garcia-Manero, Guillermo; Jabbour, Elias J.; Owen, Kate; Stockman, Paul K.; Oliver, Stuart D.
Clinical Lymphoma, Myeloma & Leukemia (2013), 13(5), 559-567 | Language: English, Database: CAplus and MEDLINE

Barasertib is the pro-drug of barasertib-hydroxy-quinazoline pyrazole anilide, a selective Aurora B kinase inhibitor that has demons trated preliminary anti-AML activity in the clin. setting. This Phase I dose- escalation study evaluated the safety and tolera bility of barasertib, combined with LDAC, in patients aged 60 years or older with de novo or secondary A ML. Barasertib (7-day continuous i.v. infusion) plus LDAC 20 mg (s.c. injection twice daily for 10 days) was admini stered in 28-day cycles. The MTD was defined as the highest dose at which ≤ 1 patient within a cohort of 6 experienced a dose-limiting toxicity (DLT) (clin. significant adverse event [AE] or laboratory abnormality considered related to barasertib). The MTD cohort was expanded to 12 patients. Twenty- two patients (median age, 71 years) received ≥ 1 treatment cycle (n = 6, 800 mg; n = 13, 1000 mg; n = 3, 1200 mg).. DLTs were reported in 2 patients (both, National Cancer Institute Common Terminol. Criteria for Adverse Events grade 3 stomatitis/mucositis; 1200 mg cohort). The most common A Es were infection (73%), febrile neutropenia (59%), nausea (50%), and diarrhea (46%). Barasertib plus L DAC resulted in an overall response rate (Interna tional Working Group criteria) of 45% (n = 10/22; according to invest igator opinion). The MTD of 1000 mg barasertib in combin ation with LDAC in older patients with A ML was associated with acceptable tolera bility and preliminary anti-AML activity.

Keywords: barasertib cytosine arabinoside antitumor pharmaco kinetics acute myeloid leukemia elderly; Acute myeloid leukemia; Barasertib-hQPA; Dose-escalation study

Substances (3)   Reactions (0)   Citing (40)

266

IGF1 biomarker for IGF1R inhibitor therapy of tumors

By: Sathyanarayanan, Sriram; Ayers, Mark
World Intellectual Property Organization, WO2013148568 A1 2013-10-03 | Language: English, Database: CAplus

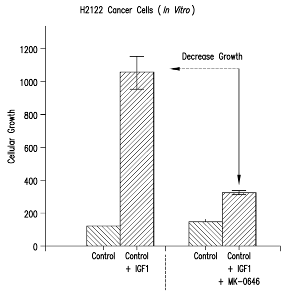

The present invention provides, inter alia, methods for treating tumors that are sensitive to an IGF1R inhibitor. The tumor are determined to be sensitive if the level of IGF1 mRNA expression in the tumor cells, relative to one or more reference genes, reaches a certain threshold level. Methods for evaluating patients as candidates for receipt of the IGF1R inhibitor therapy are also provided as well along with in vitro assay methods and kits for performing any of the methods.

Keywords: IGF1 biomarker IGF1R inhibitor therapy tumor

PatentPak available

Substances (349)   Reactions (0)   Citing (1)

267

Target inhibition networks: predicting selective combinations of druggable targets to block cancer survival pathways

By: Tang, Jing; Karhinen, Leena; Xu, Tao; Szwajda, Agnieszka; Yadav, Bhagwan; Wennerberg, Krister; Aittokallio, Tero
PLoS Computational Biology (2013), 9(9), e1003226 | Language: English, Database: CAplus and MEDLINE

A recent trend in drug development is to identify drug combin ations or multi-target agents that effectively modify multiple nodes of disease-associated networks. Such polypharmacol. effects may reduce the risk of emerging drug resistance by means of attacking the disease networks through synergistic and synthetic lethal interactions. However, due to the exponentially increasing number of potential drug and target combinations, systematic approaches are needed for priori tizing the most potent multi-target alternatives on a global network level. We took a functional systems pharmacol. approach toward the identification of selective target combin ations for specific cancer cells by combining large- scale screening data on drug treatment efficacies and drug- target binding affini ties. Our model-based prediction approach, named TIMMA, takes advantage of the polypha rmacol. effects of drugs and infers combinatorial drug efficacies through system- level target inhibition networks. Case studies in M CF-7 and MDA-MB-231 breast cancer and BxPC-3 pancreatic cancer cells demonstrated how the target inhibition modeling allows systematic explor ation of functional interactions between drugs and their targets to maximally inhibit multiple survival pathways in a given cancer type. The T I MMA prediction results were exptl. validated by means of systematic si RNA-mediated silencing of the selected targets and their pairwise combinations, showing increased ability to identify not only such druggable kinase targets that are essential for cancer survival either individually or in combination, but also synergistic interactions indicative of non-additive drug efficacies. These system-level analyses were enabled by a novel model constr uction method utilizing maximization and minimization rules, as well as a model selection algorithm based on sequential forward floating search. Compared with an existing computational solution, TIMM A showed both enhanced prediction accuracies in cross validation as well as signif icant reduction in computation times. Such cost-effective computational-exptl. design strategies have the potential to greatly speed- up the drug testing efforts by priori tizing those interventions and interactions warranting further study in individual cancer cases.

Keywords: algorithm anticancer drug target inhibition network prognosis breast carcinoma

Substances (56) Reactions (0) Citing (62)

268

Selectivity Data: Assessment, Predictions, Concordance, and Implications

By: Gao, Cen; Cahya, Suntara; Nicolaou, Christos A.; Wang, Jibo; Watson, Ian A.; Cummins, David J.; Iversen, Philip W.; Vieth, Michal
Journal of Medicinal Chemistry (2013), 56(17), 6991-7002 | Language: English, Database: CAplus and MEDLINE

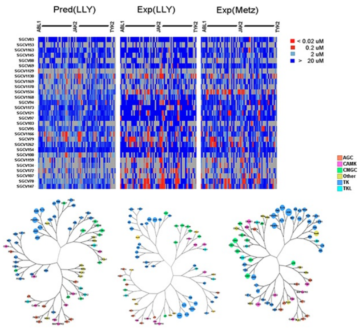

Could high-quality in silico predictions in drug discovery eventually replace part or most of exptl. testing. To evaluate the agreement of selectivity data from different exptl. or predictive sources, we introduce the new metric concor dance min. significant ratio (cMSR). Empowered by cMSR, we find the overall level of agreement between predicted and exptl. data to be comparable to that found between exptl. results from different sources. However, for mols. that are either highly selective or potent, the concordance between different exptl. sources is significantly higher than the concor dance between exptl. and predicted values. We also show that computational models built from one data set are less predictive for other data sources and highlight the importance of bias correction for assessing selectivity data. Finally, we show that small- mol. target space relatio nships derived from different data sources and predictive models share overall similarity but can significantly differ in details.

Keywords: selectivity data exptl predictive source drug discovery modeling

Substances (27)　Reactions (0)　Citing (10)

269

Aurora B inhibitor barasertib and cytarabine exert a greater-than-additive cytotoxicity in acute myeloid leukemia cells

By: Yamauchi, Takahiro; Uzui, Kanako; Shigemi, Hiroko; Negoro, Eiju; Yoshida, Akira; Ueda, Takanori
Cancer Science (2013), 104(7), 926-933 | Language: English, Database: CAplus and MEDLINE

Barasertib, an aurora B inhibitor, terminates cell division, introduces polypl oidy, and consequently causes apoptosis. In the present study, we evaluated the effect of the combination of barasertib and cytarabine (ara-C), a key agent for leukemia chemoth erapy, on leukemic cells in vitro. Human leukemia HL-60 cells and HL-60/ara-C20 cells, a 20- fold ara-C-resistant variant, were used. The 50% growth inhibitory concentrations of an active metabolite of barase rtib, barasertib-hydroxyquinazoline-pyrazol-aniline (Barasertib-H QPA), and ara-C were 51 n M and 300 n M for HL-60 cells and 70 n M and 5300 n M for HL-60/ara-C20 cells, resp. Barase rtib-HQPA induced polyploidy with a subsequent induction of sub-G1 phase apoptosis, indicating the M-phase specific cytotoxicity. Cells treated with the S-phase specific ara-C accumulated in S phase and subseq uently died through apoptosis. When H L-60 cells were treated with barasertib-HQPA and ara-C in combination, a greater-than-additive apoptosis was induced. This enhanc ement was obtained when the cells were treated with barasertib-HQPA prior to ara-C (37.9% sub-G1) or with both concur rently (31.2% sub-G1), but not with ara-C prior to barasertib-HQPA (17.8% sub-G1). The combin ation effects were similarly obtained in HL-60/ara-C20 cells with 19.7% sub-G1 for barasertib-HQPA→ara-C, 18.4% sub-G1 for both concurr ently, and 13.8% sub-G1 for ara-C→barasertib-HQPA, and another leukemic U937 cells with 25.4% sub-G1 for barasertib-HQPA→ara-C, 28.2% sub-G1 for both concurr ently, and 16.0% sub-G1 for ara-C→barasertib-HQPA. Barase rtib-HQPA inhibited aurora B autophosph orylation and histone H3 phosphor ylation in all the cell lines. Barasertib-HQPA did not inhibit D NA synthesis, allowing ara- C incorporation into D NA for its cytotox icity. Thus, barase rtib-HQPA and ara-C provided a greater- than-additive cytotoxicity in leukemic cells in vitro.

Keywords: barasertib hydroxyquinazoline cytarabine e additive acute myeloid leukemia antitumor

Substances (6) Reactions (0) Citing (22)

270

Compounds and methods for the enhanced degradation of targeted proteins and other polypeptides by an E3 ubiquitin ligase, particularly targeting the von Hippel-Lindau E3 ubiquitin ligase using hydroxyproline analogs to disrupt the VHL/HIF-1α interaction

By: Crews, Craig M.; Buckley, Dennis; Ciulli, Alessio; Jorgensen, William; Gareiss, Peter C.; Van Molle, Inge; Gustafson, Jeffrey; Tae, Hyun-Seop; Michel, Julien; Hoyer, Dentin Wade; et al

World Intellectual Property Organization, WO2013106643 A2 2013-07-18 | Language: English, Database: CAplus

The invention is related to bifunctional compounds, e.g., **I**, which find utility as modulators of targeted ubiquitination, especially inhibitors of a variety of polypeptides and other proteins which are degraded and/or otherwise inhibited by bifunctional compounds according to the present invention. In particular, the invention is related to compounds, which contain on one end a von Hippel-Lindau (VHL) ligand which binds to the ubiquitin ligase and on the other end a moiety which binds a target protein such that the target protein is placed in proximity to the ubiquitin ligase to effect degradation (and inhibition) of that protein. The invention exhibits a broad range of pharmacol. activities associated with the compounds of the invention, consistent with the degradation/inhibition of targeted polypeptides. Thus, **II** bound to VHL with a 4.1 μM IC_{50} value. **II** was cocrystd. with VHL and shown to bind at the HIF-1α binding site.

Keywords: hydroxyproline analog preparation ubiquitin ligase inhibitor; chimeric mol preparation targeted protein degradation ubiquitination

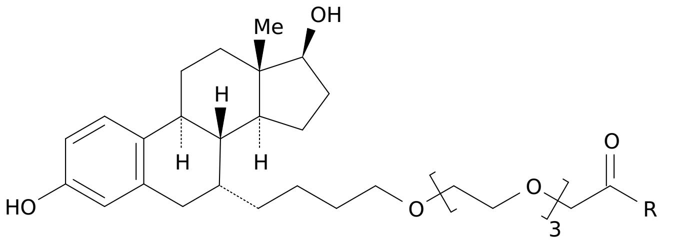

I

R =

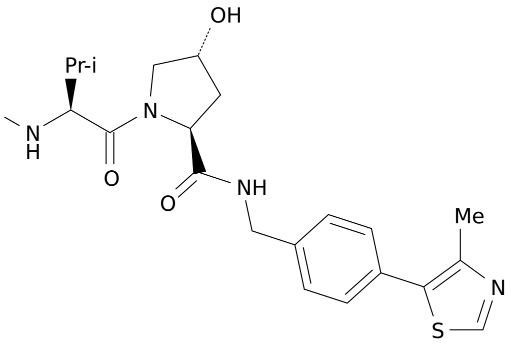

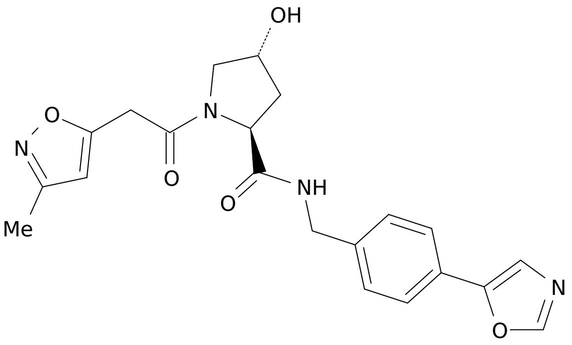

II

PatentPak available
Substances (1,211)
Reactions (2,697)
Citing (19)

271

Stage I of a phase 2 study assessing the efficacy, safety, and tolerability of barasertib (AZD1152) versus low-dose cytosine arabinoside in elderly patients with acute myeloid leukemia

By: Kantarjian, Hagop M.; Martinelli, Giovanni; Jabbour, Elias J.; Quintas-Cardama, Alfonso; Ando, Kiyoshi; Bay, Jacques-Olivier; Wei, Andrew; Groepper, Stefanie; Papayannidis, Cristina; Owen, Kate; et al
Cancer (Hoboken, NJ, United States) (2013), 119(14), 2611-2619 | Language: English, Database: CAplus and MEDLINE

BACKGROUND : In this phase 2 study, the authors evaluated the efficacy, safety, and tolera bility of the Aurora B kinase inhibitor barasertib compared with low-dose cytosine arabin oside (LDAC) in patients aged ≥60 years with acute myeloid leukemia (A ML). MET HODS : Patients were randomized 2: 1 to receive either open-label barasertib 1200 mg (as a 7- day i.v. infusion) or LDAC 20 mg (s.c. twice daily for 10 days) in 28-day cycles. The primary endpoint was the objective complete response rate (O CRR) (complete responses [CR] plus confirmed CRs with incomplete recovery of neutro phils or platelets [CRi] according to Cheson criteria [also requiring reconfirmation of CRi ≥21 days after the first appearance and associated with partial recovery of platelets and neutro phils]). Secondary endpoints included overall survival (O S) and safety. R ESULTS : In total, 74 patients (barase rtib, n=48; LDAC, n=26) completed ≥1 cycle of treatment. A significant improvement in the OCRR was observed with barasertib (35.4% vs 11.5%; differ ence, 23.9%; 95% confidence interval, 2.7%-39.9%; P<.05). Although the study was not formally sized to compare O S data, the median O S with barasertib was 8.2 mo vs. 4.5 mo with LDAC (hazard ratio, 0.88; 95% confidence interval, 0.49- 1.58; P=.663). Stomatitis and febrile neutropenia were the most common adverse events with barasertib vs. L DAC (71% vs 15% and 67% vs 19%, resp.) . CONCLUS IONS : Barasertib produced a signif icant improvement in the OCRR vs. LDAC and had a more toxic but manageable safety profile, consistent with previous studies. Cancer 2013;119:2611-2619. © 2013 American Cancer Society.

Keywords: antileukemic barasertib cytosine arabin oside dose efficacy safety tolera bility elderly; acute myeloid leukemia; AZD1152; barasertib; low-dose cytosine arabin oside

Substances (2) Reactions (0) Citing (76)

272

CRLF-2 binding peptides, protocells and viral-like particles useful in the treatment of cancer, including acute lymphoblastic leukemia

By: Brinker, C. Jeffrey; Peabody, David S.; Wharton, Walker; Chackerian, Bryce; Ashley, Carlee Erin; Willman, Cheryl L.; Carnes, Eric C.; Epler, Katherine; Castillo, Robert Eric
World Intellectual Property Organization, WO2013103614 A1 2013-07-11 | Language: English, Database: CAplus

The present invention relates to the use of which are attached or anchored phospholipid biolayers further modified by C RLF-2 and CD 19 binding peptides which may be used for delivering pharmac eutical cargos, to cells expressing CRLF-2 and CD 19, thereby treating cancer, in particular, acute lymphob lastic leukemia, including B-precursor acute lymphob lastic leukemia. Novel CRLF-2 binding peptides and CLRF-2 and CD19-binding viral-like particles (VLPs) useful in the treatment of cancer, including A LL are also provided.

Keywords: CRLF2 binding peptide protocell virus like particle cancer drug; acute lymphob lastic leukemia drug C RLF2 binding peptide

PatentPak available

Substances (335) Reactions (0) Citing (5)

273

Profiling pathway-specific novel therapeutics in preclinical assessment for central nervous system atypical teratoid rhabdoid tumors (CNS ATRT): Favorable activity of targeting EGFR- ErbB2 signaling with lapatinib

By: Singh, Anjali; Lun, Xueqing; Jayanthan, Aarthi; Obaid, Halah; Ruan, Yibing; Strother, Douglas; Chi, Susan N.; Smith, Amy; Forsyth, Peter; Narendran, Aru
Molecular Oncology (2013), 7(3), 497-512 | Language: English, Database: CAplus and MEDLINE

Despite intensifying multimodal treatments, children with central nervous system atypical teratoid/ rhabdoid tumor (CNS ATRT) continue to endure unacceptably high mortality rates. At present, concerted efforts are focusing on underst anding the charact eristic INI1 mutation and its implic ations for the growth and survival of these tumors. Addnl., pharmac eutical pipeline libraries constitute a significant source of potential agents that can be taken to clin. trials in a timely manner. However, this process requires efficient target validation and relevant preclin. studies. As an initial screening approach, a panel of 129 small mol. inhibitors from multiple pharmaceutical pipeline libraries was tested against three A TRT cell lines by in vitro cytoto xicity assays. Based on these data, agents that have strong activity and corresponding susceptible cellular pathways were identified. Target modulation, antibody array anal., drug combination and in vivo xenograft studies were performed on one of the pathway inhibitors found in this screening. Approx. 20% of agents in the library showed activity with IC_{50} values of 1 μM or less and many showed IC_{50} values less than 0.05 μM. Intra cell line variability was also noted among some of the drugs. However, it was determined that agents capable of affecting pathways constituting ErbB2, mTOR, proteasomes, Hsp90, Polo like kinases and Aurora kinases were univer sally effective against the three ATRT cell lines. The first target selected for further anal., the inhibition of Erb B2-EGFR pathway by the small mol. inhibitor lapatinib, indicated inhibition of cell migration properties and the initiation of apoptosis. Synergy between lapatinib and IG F-IR inhibition was also demonstrated by combin ation index (CI) values. Xenograft studies showed effective antitumor activity of lapatinib in vivo. We present an exptl. approach to identifying agents and drug combin ations for future clin. trials and provide evidence for the potential of lapatinib as an effective agent in the context of the biol. and heterogeneity of its targets in A TRT.

Keywords: therapeutic target central nervous system atypical teratoid rhabdoid tumor; anticancer lapatinib E GFR ErbB2 signaling

Substances (34) Reactions (0) Citing (29)

274

Method of determination of cancer cell drug sensitivity towards Aurora kinase inhibitors and overcoming their resistance comprising determination of gene or protein expression in patient sample

By: Kollaredy, Madhusudhan Reddy; Hajduch, Marian; Dzubak, Petr; Srovnal, Josef; Hrabakova, Rita; Kovarova, Hana
World Intellectual Property Organization, WO2013083098 A2 2013-06-13 | Language: English, Database: CAplus

The present invention relates to a method for determining the sensitivity and/or resistance of a patient suffering from a cancer disease to Aurora kinase inhibitor therapy, which comprises determining in vitro in the cancer cells or body fluids taken from the patient the expression of at least one gene selected from a particular group and/or determining in vitro in the cancer cells or body fluids taken from the patient the level of at least one protein selected from a particular group.

Keywords: cancer drug sensitivity Aurora kinase inhibitor gene protein expression

PatentPak available

Substances (54) Reactions (0) Citing (2)

275

Method of determination of cancer cell drug sensitivity towards Aurora kinase inhibitors and overcoming their resistance comprising determination of gene or protein expression in patient sample

By: Kollareddy, Madhusudhan Reddy; Hajduch, Marian; Dzubak, Petr; Srovnal, Josef; Hrabakova, Rita; Kovarova, Hana
European Patent Organization, EP2602330 A1 2013-06-12 | Language: English, Database: CAplus

The present invention relates to a method for determining the sensitivity and/or resistance of a patient suffering from a cancer disease to Aurora kinase inhibitor therapy, which comprises determining in vitro in the cancer cells or body fluids taken from the patient the expression of at least one gene selected from a particular group and/or determining in vitro in the cancer cells or body fluids taken from the patient the level of at least one protein selected from a particular group. Also, the invention relates to a group of compounds which allow overcoming the resistance.

Keywords: cancer drug sensitivity Aurora kinase inhibitor gene protein expression

PatentPak available

Substances (54) Reactions (0) Citing (0)

276

SAR and evaluation of novel 5H-benzo[c][1,8]naphthyridin-6-one analogs as Aurora kinase inhibitors

By: Karra, Srinivasa; Xiao, Yufang; Chen, Xiaoling; Liu-Bujalski, Lesley; Huck, Bayard; Sutton, Amanda; Goutopoulos, Andreas; Askew, Ben; Josephson, Kristopher; Jiang, Xuliang; et al
Bioorganic & Medicinal Chemistry Letters (2013), 23(10), 3081-3087 | Language: English, Database: CAplus and MEDLINE

Several potent Aurora kinase inhibitors derived from 5H-benzo[c][1,8]naphthyridin-6-one scaffold were identified. A crystal structure of Aurora kinase A in complex with an initial hit revealed a binding mode of the inhibitor within the ATP binding site and provided insight for structure-guided compound optimization. Subsequent SAR campaign provided a potent and selective pan Aurora inhibitor, which demonstrated potent target modulation and antiproliferative effects in the pancreatic cell line, MIAPaCa-2. Furthermore, this compound inhibited phosphorylation of histone H3 (pHH3) in mouse bone morrow upon oral administration, which is consistent with inhibition of Aurora kinase B activity.

Keywords: preparation structure benzo naphthyridinone analog Aurora kinase inhibitor antitumor

Substances (96) Reactions (122) Citing (15)

277

Reprogramming urokinase into an antibody-recruiting anticancer agent using peptides of arginine chloromethylketone

By: Spiegel, David A.; Jakobsche, Charles E.

World Intellectual Property Organization, WO2013070688 A1 2013-05-16 | Language: English, Database: CAplus

The invention relates to chimeric (preferably, bifunctional) compounds, e.g., **I**•2TFA, compositions comprising those compounds and methods of treating cancer in a patient or subject, especially including metastatic cancer where cancer cells exhibit overexpression (heightened expression) of cell surface urokinase-type plasminogen activator receptor (urokinase receptor) compared to normal (non-cancerous) cells. The compounds preferably covalently bind to the urokinase receptor and recruit native antibodies of the patient or subject where the antibodies can selectively degrade and/or deactivate targeted cancer cells through antibody-dependent cellular phagocytosis and/or antibody-dependent cellular cytotoxicity against a large number and variety of cancers, thus providing cancer cell death and/or an inhibition of growth, elaboration and/or metastasis of the cancer, including remission and cure of the patient's cancer. Thus, chloromethyl ketone **I**•2TFA was prepared by a multistep procedure and evaluated as an antibody-recruiting mol. that targets the urokinase receptor.

Keywords: peptide arginine chloromethylketone preparation reprogramming urokinase antibody recruiting anticancer

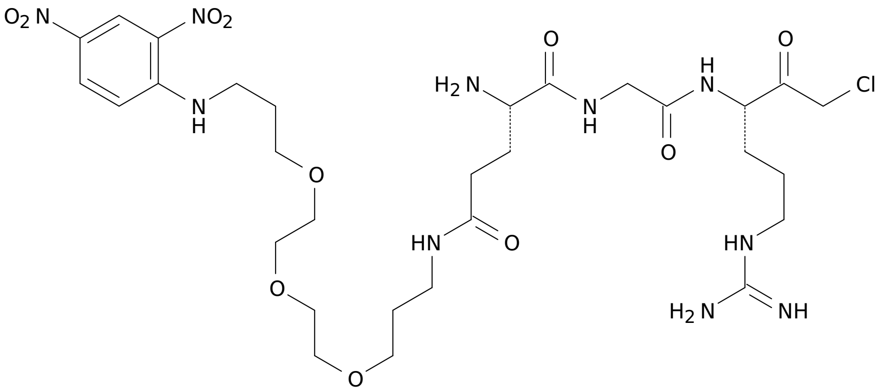

I

PatentPak available

Substances (346) Reactions (198) Citing (1)

278

Porous nanoparticle-supported lipid bilayers (protocells) for targeted delivery including transdermal delivery of cargo and methods thereof

By: Ashley, Carlee Erin; Brinker, C. Jeffrey; Carnes, Eric C.; Fekrazad, Mohammad Houman; Felton, Linda A.; Negrete, Oscar; Padilla, David Patrick; Wilkinson, Brian S.; Wilkinson, Dan C.; Willman, Cheryl L.
World Intellectual Property Organization, WO2013056132 A2 2013-04-18 | Language: English, Database: CAplus

The present invention is directed to protocells for specific targeting of hepatocellular and other cancer cells which comprise a nanoporous silica core with a supported lipid bilayer; at least one agent which facilitates cancer cell death, such as a traditional small mol., a macromol. cargo, e.g., siRNA, or a protein toxin, such as ricin toxin A-chain or diphtheria toxin A-chain, and/or a histone-packaged plasmid DNA disposed within the nanoporous silica core (preferably supercoiled in order to more efficiently package the DNA into protocells) which is optionally modified with a nuclear localization sequence to assist in localizing protocells within the nucleus of the cancer cell and the ability to express peptides involved in therapy (apoptosis/cell death) of the cancer cell or as a reporter, a targeting peptide which targets cancer cells in tissue to be treated such that binding of the protocell to the targeted cells is specific and enhanced and a fusogenic peptide that promotes endosomal escape of protocells and encapsulated DNA. Protocells according to the present invention may be used to treat cancer, especially including hepatocellular (liver) cancer using novel binding peptides (c-MET peptides) which selectively bind to hepatocellular tissue or to function in diagnosis of cancer, including cancer treatment and drug discovery. For example, the porous protocells were prepared via fusion of AEPTMS-modified silica cores and a liposomes composed of DOPC/DOPE/cholesterol/PEG2000 conjugated with a targeting peptide SP94 (H_2N-SFSIILTPILPLEEEGGC-COOH, SEQ ID NO: 8) for cellular delivery of siRNA. The ability of siRNA-loaded, SP94-targeted DOPC-containing protocells to selectively induce cytotoxicity of human hepatocellular carcinoma cells (Hep3B) was demonstrated.

Keywords: lipid bilayer nanoparticle protocell cancer targeting

PatentPak available

Substances (434) | Reactions (0) | Citing (15)

279

A molecular case report: Functional assay of tyrosine kinase inhibitors in cells from a patient's primary renal cell carcinoma

By: Kulesz-Martin, Molly; Lagowski, James; Olson, Susan; Wortham, Aaron; West, Toni; Thomas, George; Ryan, Christopher W.; Tyner, Jeffrey W.
Cancer Biology & Therapy (2013), 14(2), 95-99 | Language: English, Database: CAplus and MEDLINE

Current therapies for renal cell carcinoma favor vascular endothelial growth factor receptor (VEGF-R) tyrosine kinase (TK) inhibitors (TKIs). In theory, these are most applicable in tumors that have lost VHL-with subsequent stabilization of HIF and upregulation of VEGF. A subset of patients harbor primary-refractory disease, as in this case, where there was no evidence for loss of VHL or chromosome 3p. We evaluated mol. targeted agents in viable tumor cells cultured from a patient's clear cell renal cell carcinoma (RCC). Of 66 agents, only dasatinib, an inhibitor of Src tyrosine kinase, strongly reduced viability of the patient's cultured kidney tumor cells. Immunostaining of the original primary tumor revealed strong positivity for VHL and Src protein expression. Functional evaluation of a patient's tumor cells appears feasible in the setting of RCC.

Keywords: tyrosine kinase inhibitor renal cell carcinoma anticancer; molecular targeted therapy; renal cell carcinoma; tyrosine kinase

Substances (55) | Reactions (0) | Citing (2)

280

Effect of aurora kinase B inhibitor AZD1152 on cisplatin-resistant epithelial ovarian carcinoma Hey cells

By: Ma, Ya-xi; Li, Xiu-zhen
Zhonghua Fuchanke Zazhi (2013), 48(1), 46-50 | Language: Chinese, Database: CAplus and MEDLINE

This paper is to investigate the effect of aurora kinase B inhibitor AZD1152 on cisplatin-resistant epithelial ovarian carcinoma Hey cells. Hey (cisplatin-resistant ovarian cancer cell line) cells were analyzed. According to the treatment plan, Hey cells were divided into four groups (AZD group, cisplatin group, AZD+cisplatin group and control group). Me thiazolyl tetrazolium (MTT) assay was used to test the cell proliferation; caspase-3/7 activity anal. was used to analyze cells apoptosis; fluorescence in-situ hybridization (FISH) assay was used to determine the copy number of chromosome 7 and check the copy number of h TERC gene and C-myc gene. MTT test showed that proliferation in AZD group was lower than that in control group (P<0.01). The proliferation rates of cells treated with 10 and 20 nmol/L AZD for 24 h were (81.4±3.6) % and (81.4±3.6)% resp., and the proliferation rates of cells for 48 h were (43.1±2.0)% and (38.5±1.6)% resp., which was significantly lower than those in control group (100%, P<0.01); after cells were treated with same concentration of AZD, inhibition of proliferation was significantly enhanced as the time prolongation (P<0.01). Proliferation rate in AZD+cisplatin group was lower than that in cisplatin group (P<0.01), which suggest that there were additive effects after combining AZD with cisplatin. Compared with control group, caspase-3/7 activity in AZD group increased significantly (P=0.000), and the same result was seen between AZD±cisplatin group and cisplatin group or AZD group (all P<0.01). Compared with cisplatin group or control group, the copy numbers of hTERC, C-myc and the number of chromosome were significantly increased in AZD group and AZD+cisplatin group (all P<0.05). AZD could inhibit ovarian cancer cell proliferation and induce cell apoptosis significantly. AZD alone or in combination with cisplatin may result in the increased cell polyploidy.

Keywords: aurora kinase B inhibitor AZD1152 cisplatin resistant; epithelial ovarian carcinoma Hey cell

Substances (3) Reactions (0) Citing (7)

281

Phase I study of barasertib (AZD1152), a selective inhibitor of Aurora B kinase, in patients with advanced solid tumors

By: Schwartz, Gary K.; Carvajal, Richard D.; Midgley, Rachel; Rodig, Scott J.; Stockman, Paul K.; Ataman, Ozlem; Wilson, David; Das, Shampa; Shapiro, Geoffrey I.
Investigational New Drugs (2013), 31(2), 370-380 | Language: English, Database: CAplus and MEDLINE

The purpose of this study was to determine the maximum-tolerated dose (MTD), pharmacokinetics and safety profile for two different dosing regimens of barasertib, a selective inhibitor of Aurora B Kinase. In this Phase I trial, patients with advanced solid malignancies were treated with escalating doses of barasertib, administered as either a 48-h continuous infusion or as two 2-h infusions on consecutive days, both every 14 days of a 28- day cycle. Thirty-five patients were treated. The MTDs were 150 mg as a 48-h continuous infusion and 220 mg administered as two 2-h infusions (110 mg/day, days 1, 2, 15 and 16), with neutropenia the dose-limiting toxicity (DLT) of each schedule. Common Terminol. Criteria of Adverse Events (CTCAE) grade ≥ 3 neutropenia (with or without fever) occurred in 34 % of patients overall. Other adverse events, many of hematol. or gastrointestinal etiol., were of mild or moderate intensity. No objective tumor responses were observed, although stable disease was observed in 23 % of patients. Systemic exposure to barasertib-hQPA, the more active moiety to which barasertib is converted, was observed by 1 and 6 h into the 2-h and continuous infusion, resp., and exhibited linear pharmacokinetics. In summary, barasertib was generally well tolerated, with neutropenia the most frequent and dose-limiting toxicity, irresp. of schedule. Future development of barasertib will depend on better definition of its therapeutic index.

Keywords: barasertib AuroraB kinase inhibitor colon lung skin cancer; pharmacokinetics pharmacodynamics antitumor

Substances (3) Reactions (0) Citing (52)

282

Aurora kinase inhibitor AZD1152 has an additional effect of platinum on a sequential application at the human ovarian cancer cell line SKOV3

By: Ma, Yaxi; Weimer, Joerg; Fredrik, Regina; Adam-Klages, Sabine; Sebens, Susanne; Caliebe, Amke; Hilpert, Felix; Eckmann-Scholz, Christel; Arnold, Norbert; Schem, Christian
Archives of Gynecology and Obstetrics (2013), 288(1), 173-182 | Language: English, Database: CAplus and MEDLINE

Purpose: The treatment of ovarian tumors is carried out with platinum medicine which can lead to incompatibilities or resistances. Thus, it is of great interest to check new medicine suitability for its applic ation. AZD1152 is an aurora kinase inhibitor predomi nantly works against aurora kinase B involved in the chromosome segreg ation. Cells become polyploidy and reduce the prolife ration by this impair ment. To investigate whether AZD1152, may play a role in the treatment of ovarian carcinoma we serving it to the cisplatinum-resistant cell line S KOV3 alone and in combin ation with platinum. Methods: We look at the prolife ration, the ploidy, the phases of cell cycle and the apoptosis activity of the cells. Results and conclusion: We could show that the combin ation of both medicines in the preclin. experiment produces a working advantage.

Keywords: anticancer Aurora kinase inhibitor AZD1152 platinum ovarian cancer

Substances (6) Reactions (0) Citing (8)

283

Effect of the drug transporters ABCG2, Abcg2, ABCB1 and ABCC2 on the disposition, brain accumulation and myelotoxicity of the aurora kinase B inhibitor barasertib and its more active form barasertib-hydroxy-QPA

By: Marchetti, Serena; Pluim, Dick; Eijndhoven, Monique; Tellingen, Olaf; Mazzanti, Roberto; Beijnen, Jos H.; Schellens, Jan H. M.
Investigational New Drugs (2013), 31(5), 1125-1135 | Language: English, Database: CAplus and MEDLINE

We explored whether barasertib (AZD1152), a selective Aurora B kinase inhibitor, is a substrate for P- glycoprotein (Pgp, MDR1), breast cancer resistance protein (BCRP), and multidrug resistance protein 2 (M RP2) in vitro. Cell survival, drug transport, and competition experiments with barasertib pro-drug and the more active form of the drug (barase rtib-hQPA) were performed using MDCKII (wild type, M DR1, BCRP, and M RP2) and LLCPK (wild type and M DR1) cells and monolayers, and Sf9-BCRP membrane vesicles. Moreover we tested whether P-gp and BCRP affect the oral pharmacokinetics, tissue distribution, and myelotoxicity of barasertib in vivo using Bcrp1$^{-/-}$/Mdr1a/1b $^{-/-}$ (triple knockout) and wild type mice. In cell survival experi ments expression of BCRP and MDR1 resulted in signif icant resistance to barasertib. In transwell experiments, barasertib-hQPA was transported by B CRP and MDR1 efficiently. In Sf9-BCRP membrane vesicles, both barasertib and barase rtib-hQPA significantly inhibited the B CRP-mediated transport of methotrexate. In contrast, no active transport of barasertib by M RP2 was observed, and overexpression of MRP2 did not affect cytotoxicity of barasertib. In vivo, systemic exposure as well as bioavailability, brain penetration, kidney and liver distri bution and myelotoxicity of barasertib-hQPA were statistically significantly increased in Bcrp1$^{-/-}$/Mdr1a/1b$^{-/-}$ compared with wild type mice (p<0.001). Barasertib is transported efficiently by P-gp and BCRP/Bcrp1 in vitro. In vivo, genetic deletion of P-gp and BCRP in mice significantly affected pharmacokinetics, tissue distribution and myelotoxicity of barasertib-hQPA. Possible clin. conseq uences for the observed affinity of barasertib for P- gp and BCRP need to be explored.

Keywords: barasertib aurora kinase B inhibitor ABCG2 ABCB1 ABCC2 pharmacokinetics

Substances (4) Reactions (0) Citing (19)

284

Kinase Pathway Dependence in Primary Human Leukemias Determined by Rapid Inhibitor Screening

By: Tyner, Jeffrey W.; Yang, Wayne F.; Bankhead, Armand III; Fan, Guang; Fletcher, Luke B.; Bryant, Jade; Glover, Jason M.; Chang, Bill H.; Spurgeon, Stephen E.; Fleming, William H.; et al
Cancer Research (2013), 73(1), 285-296 | Language: English, Database: CAplus and MEDLINE

Kinases are dysregulated in most cancers, but the frequency of specific kinase mutations is low, indicating a complex etiol. in kinase dysregulation. Here, we report a strategy to rapidly identify functi onally important kinase targets, irresp. of the etiol. of kinase pathway dysregulation, ultimately enabling a correl ation of patient genetic profiles to clin. effective kinase inhibi tors. Our methodol. assessed the sensitivity of primary leukemia patient samples to a panel of 66 small- mol. kinase inhibitors over 3 days. Screening of 151 leukemia patient samples revealed a wide diversity of drug sensitivities, with 70% of the clin. specimens exhibiting hypersen sitivity to one or more drugs. From this data set, we developed an algorithm to predict kinase pathway dependence based on anal. of inhibitor sensitivity patterns. Applying this algorithm correctly identified pathway dependence in proof- of-principle specimens with known oncogenes, including a rare FLT3 mutation outside regions covered by standard mol. diagnostic tests. Interro gation of all 151 patient specimens with this algorithm identified a diversity of kinase targets and signaling pathways that could aid priorit ization of deep sequencing data sets, permitting a cumulative anal. to understand kinase pathway dependence within leukemia subsets. In a proof-of-principle case, we showed that in vitro drug sensit ivity could predict both a clin. response and the develo pment of drug resistance. Taken together, our results suggested that drug target scores derived from a compreh ensive kinase inhibitor panel could predict pathway dependence in cancer cells while simultaneously identifying potential therapeutic options.

Keywords: kinase inhibitor signaling leukemia drug screening target anticancer

Substances (231) Reactions (0) Citing (116)

285

Multiparametric cell-based assay for the evaluation of transcription inhibition by high-content imaging

By: Torres-Guzman, Raquel; Chu, Shaoyou; Velasco, Juan A.; Lallena, Maria Jose
Journal of Biomolecular Screening (2013), 18(5), 556-566 | Language: English, Database: CAplus and MEDLINE

Loss of normal cell cycle regulation is a hallmark of human cancer. Cyclin-dependent kinases (CDKs) are key regulators of the cell cycle and have been actively pursued as promising therapeutic targets. Likewise, members of the C DK family are functi onally related to transcriptional modulation, a mol. pathway suitable for therap eutic intervention. We used a set of 2500 compounds in the U2OS cell line to evaluate its effect in the cell division process. Interes tingly, out of this anal., we identified a subpopu lation of compounds that are able to inhibit RNA polymerase activity, thus interfering with gene transcription processes. After this finding, we developed, validated, and fully automated a multiparameter highcontent imaging (HCI) assay to measure phosphorylation of the RNA polymerase II carboxyl terminal domain (p CTD). Simultaneously, we measured both the D NA content and cell prolife ration index in the treated cells. The linear regression anal. comparing the IC50 for p CTD and the 4N EC50 for DNA content or IC50 for cell proliferation showed an excellent agreement (r^2 = 0.84 and r^2 = 0.94, resp.) . Our results confirm that this method allows discrim inating between cell cycle and transcr iption inhibition and confirms H CI as a powerful technol. for the identif ication of compounds with an effective and selective pathway phenotype.

Keywords: cancer multiparametric cell based assay transcription high content imaging; RNA pol II; assay development; cell cycle; gene transcription; high-content imaging

Substances (20) Reactions (0) Citing (6)

286

A new approach for prediction of tumor sensitivity to targeted drugs based on functional data

By: Berlow, Noah; Davis, Lara E.; Cantor, Emma L.; Seguin, Bernard; Keller, Charles; Pal, Ranadip
BMC Bioinformatics (2013), 14, 239 | Language: English, Database: CAplus and MEDLINE

Background: The success of targeted anti-cancer drugs are frequently hindered by the lack of knowledge of the individual pathway of the patient and the extreme data requirements on the estimation of the personalized genetic network of the patient's tumor. The prediction of tumor sensitivity to targeted drugs remains a major challenge in the design of optimal therapeutic strategies. The current sensitivity prediction approaches are primarily based on genetic characterizations of the tumor sample. We propose a novel sensitivity prediction approach based on functional perturbation data that incorporates the drug protein interaction information and sensitivities to a training set of drugs with known targets. Results: We illustrate the high prediction accuracy of our framework on synthetic data generated from the Kyoto Encyclopedia of Genes and Genomes (KEGG) and an exptl. dataset of four canine osteosarcoma tumor cultures following application of 60 targeted small-mol. drugs. We achieve a low leave one out cross validation error of <10% for the canine osteosarcoma tumor cultures using a drug screen consisting of 60 targeted drugs. Conclusions: The proposed framework provides a unique input-output based methodol. to model a cancer pathway and predict the effectiveness of targeted anti-cancer drugs. This framework can be developed as a viable approach for personalized cancer therapy.

Keywords: protein anticancer agent interaction osteosarcoma sensitivity math model

Substances (44) Reactions (0) Citing (22)

287

Aurora kinases in childhood acute leukemia: the promise of aurora B as therapeutic target

By: Hartsink-Segers, S. A.; Zwaan, C. M.; Exalto, C.; Luijendijk, M. W. J.; Calvert, V. S.; Petricoin, E. F.; Evans, W. E.; Reinhardt, D.; de Haas, V.; Hedtjaern, M.; et al
Leukemia (2013), 27(3), 560-568 | Language: English, Database: CAplus and MEDLINE

We investigated the effects of targeting the mitotic regulators aurora kinase A and B in pediatric acute lymphoblastic leukemia (ALL) and acute myeloid leukemia (AML). Aurora protein expression levels in pediatric ALL and AML patient samples were determined by western blot and reverse phase protein array. Both kinases were overexpressed in ALL and AML patients (P<0.0002), especially in E2A-PBX1-translocated ALL cases (P<0.002), compared with normal bone-marrow mononuclear cells. Aurora kinase expression was silenced in leukemic cell lines using short hairpin RNAs and locked nucleic acid-based mRNA antagonists. Aurora B knockdown resulted in proliferation arrest and apoptosis, whereas aurora A knockdown caused no or only minor growth delay. Most tested cell lines were highly sensitive to the AURKB-selective inhibitor barasertib-hydroxyquinazoline-pyrazol-anilide (AZD1152-HQPA) in the nanomolar range, as tested with an MTS (3-(4,5-dimethylthiazol-2-yl)-5-(3-carboxymethoxyphenyl)-2-(4-sulfophenyl)-2H-tetrazolium) assay. But most importantly, primary ALL cells with a high aurora B protein expression, especially E2A-PBX1-pos. cases, were sensitive as well. In adult AML early clin. trials, clear responses are observed with barasertib. Here we show that inhibition of aurora B, more than aurora A, has an antiproliferative and pro-apoptotic effect on acute leukemia cells, indicating that particularly targeting aurora B may offer a new strategy to treat pediatric ALL and AML. Leukemia (2013) 27, 560-568; doi:10.1038/leu.2012.256; published online 19 Oct. 2012.

Keywords: antiproliferative aurora kinase therapeutic target acute leukemia childhood

Substances (5) Reactions (0) Citing (53)

288

Combination therapy with antibodies targeting insulin-like growth factor 1 receptor (IGF1R)

By: Goetsch, Liliane; Hsu, Karl
World Intellectual Property Organization, WO2012158657 A1 2012-11-22 | Language: English, Database: CAplus

The authors disclose the use of an anti-IGF1R antibody (or antigen-binding fragment) in combination with one or more chemothe rapeutic agents for treatment of cancer. In one example, pancreatic cancer patients receiving dalotu zumab and gemcit abine exhibited greater progression free survival.

Keywords: combination therapy cancer insulinlike growth factor receptor antibody

PatentPak available

Substances (297) Reactions (0) Citing (1)

289

Preparation of bisaminoquinoline compounds as autophagy inhibitors useful in mono- and combination therapy of cancer and other diseases

By: Amaravadi, Ravi K.; Winkler, Jeffrey
World Intellectual Property Organization, WO2012149186 A2 2012-11-01 | Language: English, Database: CAplus

Title compounds **I** [R^1 and $R^{1'}$ independently = H, halo, C N, NO_2, (un)substituted alkyl, O-alkyl, acyl, oxycarbonyl ester or carboxy ester; R and R' indepen dently = H, (un)substituted alkyl, acyl, carboxy ester; L = $(CH_2Y)_n$-X-$(Y'CH_2)_n$ or A-$(CH_2$-CH_2-$Z)_n$-A'; X = absent, O, S, etc.; Y = absent, CH_2, O, CH_2O, etc.; Y' forms a stable bond when present; R" = H or (un) substituted alkyl; n = 0- 4; A = bond (absent), CH_2-CH_2, CH_2, etc.; Z = O or N- Rz; Rz = H or (un)substituted alkyl], and their pharmaceutically acceptable salts, enanti omers, diastereomers, solvates or polymorphs, are prepared for inhibiting autophagy in biol. systems. Methods of treating cancer in patients in need using compounds and/or compositions according to the present invention alone or in combin ation with at least one addnl. anticancer agent represent addnl. aspects of the invention. Methods of treating disease states and/or conditions in which inhibition of autophagy plays a favorable treatment role including rheumatoid arthritis, malaria, antiphospholipid antibody syndrome, lupus, chronic urticaria and Sjogren's disease, with compounds according to the present invention represent addnl. aspects of the invention.

Keywords: bisaminoquinoline autophagy inhibitor cancer

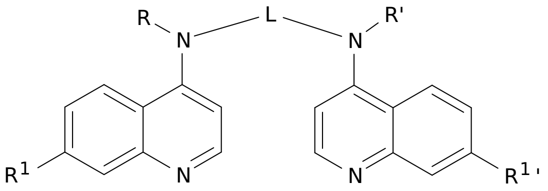

I

PatentPak available

Substances (376) Reactions (59) Citing (5)

290

Inhibition of Aurora-B kinase activity confers antitumor efficacy in preclinical mouse models of early and advanced gastrointestinal neoplasia

By: Alferez, Denis G.; Goodland, Roberta A.; Odedra, Rajesh; Sini, Patrizia; Crafter, Claire; Ryan, Anderson J.; Wedge, Stephen R.; Wright, Nicholas A.; Anderson, Elizabeth; Wilkinson, Robert W.
International Journal of Oncology (2012), 41(4), 1475-1485 | Language: English, Database: CAplus and MEDLINE

The Aurora family of kinases, play a fundamental role in cell division and are overexp ressed in several cancers including colon. The activity of barasertib-hQPA, a selective inhibitor of Aurora- B kinase (ABK) was investigated in a range of preclin. models of gastroin testinal cancer. Treatment with barasertib-hQPA produced anti-proliferative and cytotoxic effects across a panel of human colorectal cancer (CRC) cell lines in vitro. Prodrug, barasertib [48- h s.c. (s.c.) infusion; 150 mg/kg/day] inhibited the growth of S W620, Colo205, HCT116 human colorectal tumor xenografts in nude mice signifi cantly (Student's t-test, $P<0.05$, n=10-12 per group). Flow cytometric anal. of single cells from disaggregated barasertib-treated SW620 tumors revealed a decrease in phospho rylated histone H3 (phH3) and an increase in tumor cells with ≥4 N DNA content ($P<0.05$). The activity of barasertib was then examined in $Apc^{Min/+}$ mice, a spontaneous model of early intestinal neoplasia. Macroscopic evaluation of the small intestine revealed that barasertib treatment [25 mg/kg intra-peritoneal (i.p.) Q1Dx4 each week for 3 wk] of 8- wk old $Apc^{Min/+}$ mice produced a 39% reduction in macroadenoma number (P=0.02) and a 43% reduction in overall adenoma burden (P=0.02) compared with vehicle- treated controls. Quantification of microscopic adenomas revealed a >64% reduction in the number of adenomas spanning more than one villus. Histol. anal. of these adenomas revealed a number of distinct changes in barasertib-treated $Apc^{Min/+}$ mice, including a 94% reduction in the proportion of phosphohistone H3-pos. cells ($P<0.001$) and a 53% reduction in the number of cells per adenoma (P=0.001). These results provide a scientific rationale for investi gating ABK inhibitors as a treatment for intestinal cancer.

Keywords: antitumor auroraB kinase inhibitor barasertib APC signaling gastrointestinal neoplasia

Substances (4) Reactions (0) Citing (10)

291

Inhibiting the Aurora B Kinase Potently Suppresses Repopulation During Fractionated Irradiation of Human Lung Cancer Cell Lines

By: Sak, Ali; Stuschke, Martin; Groneberg, Michael; Kuebler, Dennis; Poettgen, Christoph; Eberhardt, Wilfried E. E.
International Journal of Radiation Oncology, Biology, Physics (2012), 84(2), 492-499 | Language: English, Database: CAplus and MEDLINE

The use of mol.-targeted agents during radiotherapy of non-small-cell lung cancer (NSCLC) is a promising strategy to inhibit repopul ation, thereby improving therap eutic outcome. We assessed the combined effecti veness of inhibiting Aurora B kinase and irradi ation on human NSCLC cell lines in vitro.NSCLC cell lines were exposed to concent rations of AZD1152-hydroxyquinazoline pyrazol anilide (AZD1152-HQPA) inhibiting colony formation by 50% (I $C50_{clone}$) in combination with single dose irradi ation or different fractionation schedules using multiple 2- Gy fractions per day up to total doses of 4- 40 Gy. The total irradi ation dose required to control growth of 50% of the plaque monolayers (TCD50) was determined Apoptosis, G2/M progression, and polyploi dization were also analyzed.TCD50 values after single dose irradi ation were similar for the H460 and H661 cell lines with 11.4 ± 0.2 Gy and 10.7 ± 0.3 Gy, resp. Fractionated irradiation using 3 × 2 Gy/day, 2 × 2 Gy/day, and 1 × 2 Gy/day schedules signifi cantly increased TCD50 values for both cell lines grown as plaque monolayers with increasing radiation treatment time. This could be explained by a repopu lation effect per day that counte racts 75 ± 8% and 27 ± 6% of the effect of a 2- Gy fraction in H460 and H661 cells, resp. AZD1152-H QPA treatment concom itant to radiotherapy significantly decreased the daily repopu lation effect (H460: 28 ± 5%, H661: 10 ± 4% of a 2-Gy fraction per day). Treatment with I$C50_{clone}$ AZD1152-HPQA did not induce apoptosis, prolong radiation- induced G2 arrest, or delay cell cycle progression before the spindle check point. However, polyploi dization was detected, especially in cell lines without functional p53.Inhibition of Aurora B kinase with low AZD1152-HQPA concentrations during irradi ation of NSCLC cell lines affects repopulation during radioth erapy. Thus, concom itant Aurora B kinase inhibition and irradi ation may be a promising strategy for fast repopulating tumors, which are difficult to cure by dose escalation based on conven tional fractionation.

Keywords: AZD1152 HQPA anticancer ABK inhibitor radiation lung cancer cell

Substances (2) Reactions (0) Citing (21)

292

Development of o-Chlorophenyl Substituted Pyrimidines as Exceptionally Potent Aurora Kinase Inhibitors

By: Lawrence, Harshani R.; Martin, Mathew P.; Luo, Yunting; Pireddu, Roberta; Yang, Hua; Gevariya, Harsukh; Ozcan, Sevil; Zhu, Jin-Yi; Kendig, Robert; Rodriguez, Mercedes; et al
Journal of Medicinal Chemistry (2012), 55(17), 7392-7416 | Language: English, Database: CAplus and MEDLINE

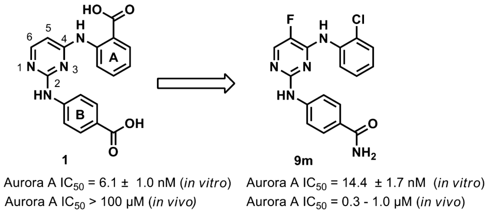

The o-carboxylic acid substituted bisanilinopyrimidine **I** was identified as a potent hit (Aurora A I C_{50} = 6.1 ± 1.0 n M) from inhouse screening. Detailed structure-activity relationship (SAR) studies indicated that polar substi tuents at the para position of the B- ring are critical for potent activity. X-ray crystallog. studies revealed that compound **I** is a type I inhibitor that binds the Aurora kinase active site in a DFG-in conformation. Structure-activity guided replacement of the A-ring carboxylic acid with halogens and incorpo ration of fluorine at the pyrimidine 5- position led to highly potent inhibitors of Aurora A that bind in a D FG-out conformation. B-Ring modifications were undertaken to improve the solubility and cell permeab ility. Compounds such as **II** with water-solubilizing moieties at the para position of the B-ring inhibited the autophosphorylation of Aurora A in M DA-MB-468 breast cancer cells.

Keywords: dianilinopyrimidine preparation SAR crystal structure Aurora kinase inhibitor

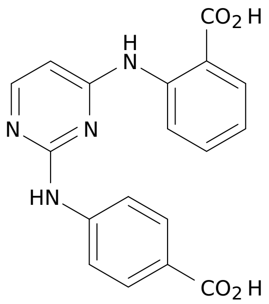

I

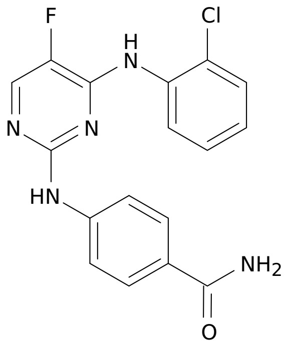

II

Substances (171) Reactions (197) Citing (53)

293

Novel pharmaceutical combinations and methods for treating cancer

By: Cheok, Chit Fang; Lane, David
World Intellectual Property Organization, WO2012121662 A1 2012-09-13 | Language: English, Database: CAplus

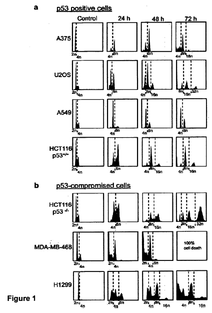

Methods of selectively targeting a p53-deficient cancer cell, comprising administering to a patient suffering from cancer (i) a reversible cell cycle arrest-inducing agent for inducing cell cycle arrest in a p53- pos. cell; and (ii) an aurora kinase inhibitor, wherein said reversible cell cycle arrest-inducing agent is administered prior to administration of said aurora kinase inhibitor, and pharmac eutical combinations, kits and oral dosage forms for the same.

Keywords: p53 deficient cancer treatment pharmaceutical combination; aurora kinase inhibitor cell cycle arrest inducer cancer treatment

PatentPak available

Substances (27) Reactions (0) Citing (0)

294

Two-stage model-based design of cancer phase I dose escalation trials: evaluation using the phase I program of barasertib (AZD1152)

By: Keizer, Ron J.; Zandvliet, Anthe S.; Beijnen, Jos H.; Schellens, Jan H. M.; Huitema, Alwin D. R.
Investigational New Drugs (2012), 30(4), 1519-1530 | Language: English, Database: CAplus and MEDLINE

Introduction. Modeling and simulation of pharmaco kinetics and pharmacodynamics has previously been shown to be potentially useful in designing Phase I programs of novel anti-cancer agents that show hematol. toxicity. In this anal., a two- stage model-based trial design was evaluated retrospectively using data from the Phase I program with the aurora kinase inhibitor barase rtib. Methods. Data from two Phase I trials and four regimens were used (n = 79). Using barasertib-hydroxy QPA plasma concentrations and neutrophil count data from only study 1A, a PKPD model was developed and subsequently used to predict the MTD and a safe starting dose for the other trials. Results. The PKPD model based on data from the first study adequately described the time course of neutrophil count fluctuation. The two-stage model-based design provided safe starting doses for subsequent phase I trials for barasertib. Predicted safe starting dose levels were higher than those used in two subsequent trials, but lower than used in the other trial. Discussion. The two-stage approach could have been applied safely to define starting doses for altern ative dosing strategies with barasertib. The limited improvement in efficiency for the phase I program of barasertib may have been due to the fact that starting doses for the studied phase I trials were already nearly optimal. Conclusion. Application of the two-stage model-based trial design in Phase I programs with novel anti-cancer drugs that cause haematol. toxicity is feasible, safe, and may lead to a reduction in the number of patient treated at sub-therapeutic dose-levels.

Keywords: barasertib pharmacokinetics pharmacodynamics cancer anticancer

Substances (2) Reactions (0) Citing (9)

295

Characterizing tumor response to chemotherapy at various length scales using temporal diffusion spectroscopy

By: Xu, Junzhong; Li, Ke; Smith, R. Adam; Waterton, John C.; Zhao, Ping; Chen, Heidi; Does, Mark D.; Manning, H. Charles; Gore, John C.
PLoS One (2012), 7(7), e41714 | Language: English, Database: CAplus and MEDLINE

Measurements of apparent diffusion coefficient (ADC) using magnetic resonance imaging (MRI) have been suggested as potential imaging biomarkers for monitoring tumor response to treatment. However, conventional pulsed-gradient spin echo (PGSE) methods incorporate relatively long diffusion times, and are usually sensitive to changes in cell d. and necrosis. Diffusion temporal spectroscopy using the oscillating gradient spin echo (OGSE) sequence is capable of probing short length scales, and may detect significant intracellular microstructural changes independent of gross cell d. changes following anti- cancer treatment. To test this hypothesis, SW620 xenografts were treated by barasertib (AZD1152), a selective inhibitor of Aurora B kinase which causes SW620 cancer cells to develop polyploidy and increase in size following treatment, ultimately leading to cell death through apoptosis. Following treatment, the ADC values obtained by both the PGSE and low frequency OGSE methods increased. However, the ADC values at high gradient frequency (i.e. short diffusion times) were significantly lower in treated tumors, consistent with increased intracellular restrictions/hindrances. This suggests that ADC values at long diffusion times are dominated by tumor microstructure at long length scales, and may not convey unambiguous information of subcellular space. While the diffusion temporal spectr oscopy provides more comprehensive means to probe tumor microstructure at various length scales. This work is the first study to probe intracellular microstructural variations due to polyploidy following treatment using diffusion MRI in vivo. It is also the first observation of post-treatment ADC changes occurring in opposite directions at short and long diffusion times. The current study suggests that temporal diffusion spectroscopy potentially provides pharmacodynamic biomarkers of tumor early response which distinguish microstructural variations following treatment at both the subcellular and supracellular length scales.

Keywords: barasertib antitumor colon cancer apoptosis spectroscopy

Substances (2) | Reactions (0) | Citing (29)

296

Exploring protein kinase inhibitors. Unveiling gemcitabine resistance in pancreatic cancer. Comments

By: Kim, Yeon Jeong; Hong, Young Bin; Cho, Chi Heum; Seong, Yeon-Sun; Bae, Insoo
Pancreas (Hagerstown, MD, United States) (2012), 41(5), 804-805 | Language: English, Database: CAplus and MEDLINE

Gemcitabine is one of first-line therapies for locally advanced pancreatic cancer, but severe resistance is respon sible for less than 6 mo of median survival and less than 20% of response rate. Although efforts to overcome gemcitabine resistance have been underway, the only combination treatment that has shown a small but statist ically significant outcome is gemcitabine with erlotinib, an epidermal growth factor receptor inhibitor. Improvements in pancreatic cancer therapy can be made by underst anding gemcit abine resistance. To enhance the treatment benefits of conven tional therapeutic drugs, targeting protein kinases in combin ation with DNA damaging drugs has been aggres sively attempted for several types of cancers. As part of this effort in pancreatic cancer, the present study explored 76 protein kinase inhibitors (PKIs) for probing new targetable protein kinases and found that several P K Is possess promising efficacy in combin ation with gemcitabine.

Keywords: protein kinase inhibitor gemcitabine resistance cytotoxic pancreatic cancer

Substances (21) | Reactions (0) | Citing (6)

297

Aurora kinase B/C inhibition impairs malignant glioma growth in vivo

By: Diaz, Roberto Jose; Golbourn, Brian; Shekarforoush, Maryam; Smith, Christian A.; Rutka, James T.
Journal of Neuro-Oncology (2012), 108(3), 349-360 | Language: English, Database: CAplus and MEDLINE

Inhibition of Aurora kinase B has been evaluated as a therapy to block solid tumor growth in breast cancer, hepatocellular carcinoma, lung adenocarcinoma, and colorectal cancer models. Aurora kinase inhibitors are in early clin. trials for the treatment of leukemia. We hypothesized that Aurora B inhibition would reduce malignant glioma cell viability and result in impaired tumor growth in vivo. Aurora B expression is greater in cultured malignant glioma U251 cells compared to proliferating normal human astrocytes, and expression is maintained in U251 flank xenogr afts. Aurora B inhibition with AZD1152-HQPA blocked cell division in four different p53-mutant glioma cell lines (U251, T98 G, U373, and U118). AZD1152-HQPA also inhibited Aurora C activation loop threonine autophosphorylation at the effective antiproli ferative concentrations in vitro. Reduction in cell viability of U251 (p53 ^{R273H}) cells was secondary to cytokinesis blockade and apoptosis induction following endorepl ication. AZD1152-HQPA inhibited the growth of U251 tumor xenografts and resulted in an increase in tumor cell apoptosis both in vitro and in vivo. S.c. administration of AZD1152-HQPA (25 mg/kg/day × 4 days; 2 cycles spaced 7 days apart) resulted in a prolon gation in median survival after intrac ranial inoculation of U251 cells in mice (P = 0.025). This is the first demonst ration that an Aurora kinase inhibitor can inhibit malignant glioma growth in vivo at drug doses that are clin. relevant.

Keywords: AZD1152HQPA aurora kinase inhibitor anticancer agent glioma cell growth

Substances (3) | Reactions (0) | Citing (33)

298

Frequency of TP53 Mutations and its Impact on Drug Sensitivity in Acute Myeloid Leukemia?

By: Shah, Ankur; Seedhouse, Claire
Indian Journal of Clinical Biochemistry (2012), 27(2), 121-126 | Language: English, Database: CAplus and MEDLINE

The purpose of this study is to find out the frequency of TP53 mutations in acute myeloid leukemia (AML) patients and correlate sensitivity of drug response with T P53 mutations. In AML more than 90 % of cases comprise of wild type T P53. 94.2 % of T P53 mutations are found within exon 5-8 of which 73 % are point mutations. T P53 mutations were analyzed with high resolution melting curve anal. We analyzed 106 AML samples of which we found nine mutations which represents 8.5 % mutation rate and found one rare SNP. The effect of TP53 mutations were studied on the chemosen sitivity of two new drugs AZD115 and RHPS4, an Aurora Kinase B inhibitor and Telomerase inhibitor resp. Four mutations were found out of 17 for RHPS4 stating signif icant (p = 0.002) increase in sensitivity and no mutation found in AZD1152 database, but need more study to get definite conclu sion.

Keywords: AZD1152 RHPS4 antitumor T P53 mutation chemosensitivity acute myeloid leukemia; Acute myeloid leukemia; Drug sensitivity; High resolution melting curve analysis; T P53

Substances (4) | Reactions (0) | Citing (3)

299

Possibility of aurora kinase inhibitors for AML

By: Tsuboi, Kosuke

Ketsueki Naika (2012), 64(3), 335-338 | Language: Japanese, Database: CAplus

A review. In this paper, (AZD1152)barasertib peptidase, possibility of Aurora kinase inhibitor for treatment of acute myeloid leukemia.

Keywords: review Aurora kinase antitumor acute myeloid leukemia barasertib

Substances (2) Reactions (0) Citing (0)

300

Probing the structural requirements of A-type Aurora kinase inhibitors using 3D-QSAR and molecular docking analysis

By: Zhang, Hui-xiao; Li, Yan; Wang, Xia; Wang, Yong-hua

Journal of Molecular Modeling (2012), 18(3), 1107-1122 | Language: English, Database: CAplus and MEDLINE

Aurora-A, the most widely studied isoform of Aurora kinase overexp ressed aberrantly in a wide variety of tumors, has been implicated in early mitotic entry, degradation of natural tumor suppressor p53 and centrosome maturation and separa tion; hence, potent inhibitors of Aurora-A may be therapeutically useful drugs in the treatment of various forms of cancer. Here, we report an in silico study on a group of 220 reported Aurora-A inhibitors with six different substru ctures. Three-dimensional quant. structure-activity relationship (3D-QSAR) studies were carried out using compar ative mol. field anal. (CoMFA) and comparative mol. similarity indexes anal. (CoMSIA) techniques on this series of mols. The resultant optimum 3 D-QSAR models exhibited an r value of 0.404-0.582 and their predictive ability was validated using an indepe ndent test set, ending in r 0.512- 0.985. In addition, docking studies were employed to explore these protein-inhibitor interactions at the mol. level. The results of 3 D-QSAR and docking analyses validated each other, and the key structural requirements affecting Aurora-A inhibitory activities, and the influential amino acids involved were identified. To the best of our knowledge, this is the first report on 3 D-QSAR modeling of Aurora-A inhibitors, and the results can be used to accurately predict the binding affinity of related analogs and also facilitate the rational design of novel inhibitors with more potent biol. activities.

Keywords: Aurora kinase inhibitor antitumor 3D QSAR mol docking

Substances (221) Reactions (0) Citing (12)

301

Evolution of resistance to Aurora kinase B inhibitors in leukaemia cells

By: Failes, Timothy W.; Mitic, Gorjana; Abdel-Halim, Heba; Po'uha, Sela T.; Liu, Marjorie; Hibbs, David E.; Kavallaris, Maria
PLoS One (2012), 7(2), e30734 | Language: English, Database: CAplus and MEDLINE

Aurora kinase inhibitors are new mitosis-targeting drugs currently in clin. trials for the treatment of haematol. and solid maligna ncies. However, knowledge of the mol. factors that influence sensit ivity and resistance remains limited. Herein, we developed and characterised an in vitro leukemia model of resistance to the Aurora B inhibitor Z M447439. Human T-cell acute lymphob lastic leukemia cells, CCRF-CEM, were selected for resistance in 4 μ M ZM447439. CEM/AKB4 cells showed no cross-resistance to tubulin-targeted and DNA-damaging agents, but were hypersensitive to an Aurora kinase A inhibitor. Sequencing revealed a mutation in the Aurora B kinase domain corresponding to a G160E amino acid substit ution. Mol. modeling of drug binding in Aurora B containing this mutation suggested that resistance is mediated by the glutamate substitution preventing formation of an active drug-binding motif. Progression of resistance in the more highly selected C EM/AKB8 and C EM/AKB16 cells, derived sequentially from CEM/AKB4 in 8 and 16 μM ZM447439 resp., was mediated by addnl. defects. These defects were indepe ndent of Aurora B and multi-drug resistance pathways and are associated with reduced apoptosis mostly likely due to reduced inhibition of the catalytic activity of Aurora kinase B in the presence of drug. Our findings are important in the context of the use of these new targeted agents in treatment regimes against leukemia and suggest resistance to therapy may arise through multiple independent mechanisms.

Keywords: ZM447439 anticancer aurora kinase B inhibitor leukemia cell

Substances (9) Reactions (0) Citing (12)

302

Selective Aurora Kinase Inhibitors Identified Using a Taxol-Induced Checkpoint Sensitivity Screen

By: Kwiatkowski, Nicholas; Deng, Xianming; Wang, Jinhua; Tan, Li; Villa, Fabrizio; Santaguida, Stefano; Huang, Hsiao-Chun; Mitchison, Tim; Musacchio, Andrea; Gray, Nathanael
ACS Chemical Biology (2012), 7(1), 185-196 | Language: English, Database: CAplus and MEDLINE

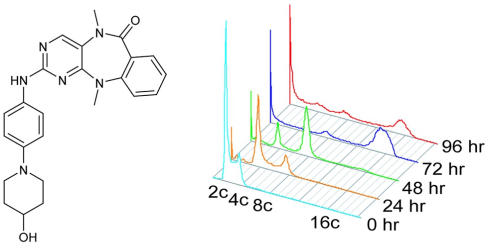

Compound **1**

The members of the Aurora kinase family play critical roles in the regulation of the cell cycle and mitotic spindle assembly and have been intensively investigated as potential targets for a new class of anticancer drugs. We describe a new highly potent and selective class of Aurora kinase inhibitors discovered using a phenotypic cellular screen. Optimized inhibitors display many of the hallmarks of Aurora inhibition including endoreduplication, polyploidy, and loss of cell viability in cancer cells. Structure- activity relationships with respect to kinome-wide selectivity and guided by an Aurora B co- crystal structure resulted in the identif ication of key selectivity determinants and discovery of a subseries with select ivity toward Aurora A. A direct comparison of biochem. and cellular profiles with respect to published Aurora inhibitors including VX-680, AZD1152, MLN8054, and a pyrimi dine-based compound from Genentech demonstrates that compounds 1 and 3 will become valuable addnl. pharmacol. probes of Aurora- dependent functions.

Keywords: aurora kinase inhibitor taxol checkpoint sensitivity screening

Substances (37) Reactions (0) Citing (22)

303

Aurora B Is Regulated by the Mitogen-activated Protein Kinase/Extracellular Signal-regulated Kinase (MAPK/ERK) Signaling Pathway and Is a Valuable Potential Target in Melanoma Cells

By: Bonet, Caroline; Giuliano, Sandy; Ohanna, Mickael; Bille, Karine; Allegra, Maryline; Lacour, Jean-Philippe; Bahadoran, Philippe; Rocchi, Stephane; Ballotti, Robert; Bertolotto, Corine
Journal of Biological Chemistry (2012), 287(35), 29887-29898 | Language: English, Database: CAplus and MEDLINE

Metastatic melanoma is a deadly skin cancer and is resistant to almost all existing treatment. Vemurafenib, which targets the BRAF V600E mutation, is one of the drugs that improves patient outcome, but the patients next develop secondary resistance and a return to cancer. Thus, new therapeutic strategies are needed to treat melanomas and to increase the duration of v- Raf murine sarcoma viral oncogene homolog B1 (BRAF) inhibitor response. The ERK pathway controls cell prolife ration, and Aurora B plays a pivotal role in cell division. Here, we confirm that Aurora B is highly expressed in metastatic melanoma cells and that Aurora B inhibition triggers both senescence-like phenotypes and cell death in melanoma cells. Furthe rmore, we show that the BRAF/ERK axis controls Aurora B expression at the transcriptional level, likely through the transcription factor FOXM1. Our results provide insight into the mechanism of Aurora B regulation and the first mol. basis of Aurora B regulation in melanoma cells. The inhibition of Aurora B expression that we observed in vemurafenib-sensitive melanoma cells was rescued in cells resistant to this drug. Consistently, these latter cells remain sensitive to the effect of the Aurora B inhibitor. Notewo rthy, wild-type BRAF melanoma cells are also sensitive to Aurora B inhibition. Collectively, our findings, showing that Aurora B is a potential target in melanoma cells, particularly in those vemurafenib-resistant, may open new avenues to improve the treatment of metastatic melanoma.

Keywords: Aurora B ERK melanoma metastasis FOXM1 vemurafenib resistance AZD1152

Substances (7) Reactions (0) Citing (60)

304

The MEK1/2 inhibitor, selumetinib (AZD6244; ARRY-142886), enhances anti-tumour efficacy when combined with conventional chemotherapeutic agents in human tumour xenograft models

By: Holt, S. V.; Logie, A.; Odedra, R.; Heier, A.; Heaton, S. P.; Alferez, D.; Davies, B. R.; Wilkinson, R. W.; Smith, P. D.
British Journal of Cancer (2012), 106(5), 858-866 | Language: English, Database: CAplus and MEDLINE

Background: The Ras/RAF/MEK/ERK pathway is frequently deregu lated in cancer and a number of inhibitors that target this pathway are currently in clin. development. It is likely that clin. testing of these agents will be in combin ation with standard therapies to harness the apoptotic potential of both the agents. To support this strategy, it has been widely observed that a number of chemotherapeutics stimulate the activation of several intrace llular signalling cascades including Ras/RAF/MEK/ERK. The MEK1/2 inhibitor selumetinib has been shown to have anti-tumor activity and induce apoptotic cell death as a monoth erapy. Methods: The aim of this study was to identify agents, which would be likely to offer clin. benefit when combined with selumetinib. Here, we used human tumor xenograft models and assessed the effects combining standard chemotherapeutic agents with selumetinib on tumor growth. In addition, we analyzed tumor tissue to determine the mechanistic effects of these combinations. Results: Combining selumetinib with the DNA-alkylating agent, temozolomide (TMZ), resulted in enhanced tumor growth inhibition compared with monotherapies. Biomarker studies highlighted an increase in γH2A.X suggesting that selumetinib is able to enhance the DNA damage induced by TMZ alone. In several models we observed that continuous exposure to selume tinib in combination with docetaxel results in tumor regression. Scheduling of docetaxel before selumetinib was more beneficial than when selume tinib was dosed before docetaxel and demonstrated a pro-apoptotic phenotype. Similar results were seen when selume tinib was combined with the Aurora B inhibitor barasertib. Conclusion: The data presented suggests that MEK inhibition in combin ation with several standard chemotherapeutics or an Aurora B kinase inhibitor is a promising clin. strategy. British Journal of Cancer (2012) 106, 858-866; doi:10.1038/bjc.2012.8 www.bjcancer.com Published online 16 Feb. 2012.

Keywords: selumetinib temozolomide docetaxel antitumor DNA damage apoptosis colorectal cancer

Substances (14) Reactions (0) Citing (85)

305

Phase I study of the Aurora B kinase inhibitor barasertib (AZD1152) to assess the pharmacokinetics, metabolism and excretion in patients with acute myeloid leukemia

By: Dennis, Mike; Davies, Michelle; Oliver, Stuart; D'Souza, Roy; Pike, Laura; Stockman, Paul
Cancer Chemotherapy and Pharmacology (2012), 70(3), 461-469 | Language: English, Database: CAplus and MEDLINE

Purpose. Barasertib (AZD1152) is a pro-drug that rapidly undergoes phosph atase-mediated cleavage in serum to release barase rtib-hQPA, a selective Aurora B kinase inhibitor that has shown prelim inary activity in clin. studies of patients with acute myeloid leukemia (AML). The pharmaco kinetic (PK), metabolic and excretion profiles of barasertib and barase rtib-hQPA were characterized in this open-label Phase I study. Methods. Five patients with poor prognosis A ML (newly diagnosed, relapsed or refractory) received barasertib 1,200 mg as a 7- day continuous infusion every 28 days. On Day 2 of Cycle 1 only, patients also received a 2- h infusion of [^{14}C]-barasertib. Blood, urine and feces samples were collected at various time points during Cycle 1. Safety and prelim inary efficacy were also assessed. Results. Barasertib-hQPA was extensively distributed to tissues, with a slow rate of total clearance (C L = 31.4 L/h). Overall, 72- 82 % of radioactivity was recovered, with approx. double the amount recovered in feces (mean = 51 %) compared with urine (mean = 27 %). The main metabolism pathways for barasertib were (1) cleavage of the phosphate group to form barase rtib-hQPA, followed by oxidation and (2) loss of the fluoroa niline moiety to form barase rtib-hQPA desfluoro aniline, followed by oxidation One of the four patients evaluable for response entered complete remission. No new or unexpected safety findings were observed; the most common adverse events were nausea and stomatitis. Conclusions. The PK profile of barasertib is similar to previous studies using the same dosing regimen in patients with AML. The majority of barasertib-hQPA clearance occurred via hepatic metabolic routes.

Keywords: antitumor aurora B kinase inhibitor barasertib pharmacokinetics metabolism; acute myeloid leukemia prodrug

Substances (2) Reactions (0) Citing (49)

306

Targeting Aurora kinases in cancer treatment

By: Kelly, Kevin R.; Ecsedy, Jeffrey; Mahalingam, Devalingam; Nawrocki, Steffan T.; Padmanabhan, Swaminathan; Giles, Francis J.; Carew, Jennifer S.
Current Drug Targets (2011), 12(14), 2067-2078 | Language: English, Database: CAplus and MEDLINE

A review. The Aurora family of serine/threonine kinases is essential for chromosome alignment, segreg ation, centrosomal matura tion, mitotic spindle formation, and cytoki nesis during mitosis. Their fundam ental role in cell cycle regulation and aberrant expression in a broad range of malignancies prompted the development of small mols. that selectively inhibit their activity. Recent studies have revealed new insights into the cellular effects of Aurora kinase inhibition. Moreover, early phase clin. studies have shown that these agents have therapeutic efficacy. In this review, we will outline the functions of Aurora kinases in normal cell division and in malignancy. We will focus on recent preclin. and clin. studies that have explored the mechanism of action and clin. effect of Aurora inhibitors in cancer treatment.

Keywords: review aurora kinase inhibitor cancer anticancer

Substances (12) Reactions (0) Citing (37)

307

Phase 1/2 study to assess the safety, efficacy, and pharmacokinetics of barasertib (AZD1152) in patients with advanced acute myeloid leukemia

By: Loewenberg, Bob; Muus, Petra; Ossenkoppele, Gert; Rousselot, Philippe; Cahn, Jean-Yves; Ifrah, Norbert; Martinelli, Giovanni; Amadori, Sergio; Berman, Ellin; Sonneveld, Pieter; et al
Blood (2011), 118(23), 6030-6036 | Language: English, Database: CAplus and MEDLINE

The primary objective of this 2-part phase 1/2 study was to determine the maximum-tolerated dose (M TD) of the potent and selective Aurora B kinase inhibitor barasertib (AZD1152) in patients with newly diagnosed or relapsed acute myeloid leukemia (A M L). Part A determined the M TD of barasertib administered as a continuous 7- day infusion every 21 days. In part B, the efficacy of barasertib was evaluated at the MTD. In part A, 32 patients were treated with barasertib 50 mg (n = 3) , 100 mg (n = 3), 200 mg (n = 3) , 400 mg (n = 4), 800 mg (n = 7), 1200 mg (n = 6), and 1600 mg (n = 6). Dose-limiting toxicities (stomatitis/mucosal inflammation events) were reported in the 800 mg (n = 1), 1200 mg (n = 1), and 1600 mg (n = 2) groups. The M TD was defined as 1200 mg. In part B, 32 patients received barasertib 1200 mg. In each part of the study, 8 of 32 patients had a hematol. response according to Cheson AML criteria. The most commonly reported grade ≥ 3 events were febrile neutro penia (n = 24) and stomatiti s/mucosal inflammation (n = 16). We concluded that the M TD of barasertib is 1200 mg in patients with relapsed or newly diagnosed A ML. Toxicity was manageable and barasertib treatment resulted in an overall hematol. response rate of 25%. This.

Keywords: barasertib anticancer agent toxicity pharmacokinetics acute myeloid leukemia

Substances (5) Reactions (0) Citing (55)

308

Aurora kinase inhibitor AZD1152 negatively affects the growth and survival of HTLV-1-infected T lymphocytes in vitro [Retraction of document cited in CA154:351346]

By: Tanaka, Tomita M.; Mori, N.
International Journal of Cancer (2011), 129(11), 2763 | Language: English, Database: CAplus

This article has been retracted by the authors, Editor-in-Chief, and publisher due to inappro priate duplication.

Keywords: retraction AZD1152 human T cell leukemia virus lymphocyte signaling

Substances (3) Reactions (0) Citing (0)

309

A Phase I study to assess the safety, pharmacokinetics and efficacy of barasertib (AZD1152), an Aurora B kinase inhibitor, in Japanese patients with advanced acute myeloid leukemia

By: Tsuboi, Kosuke; Yokozawa, Toshiya; Sakura, Toru; Watanabe, Takashi; Fujisawa, Shin; Yamauchi, Takahiro; Uike, Naokuni; Ando, Kiyoshi; Kihara, Rika; Tobinai, Kensei; et al
Leukemia Research (2011), 35(10), 1384-1389 | Language: English, Database: CAplus and MEDLINE

Barasertib (AZD1152) is a highly potent and selective Aurora B kinase inhibitor. The safety, efficacy and pharmaco kinetic (PK) profile of barasertib were investigated in Japanese patients with advanced acute myeloid leukemia. Barasertib (50- 1200 mg) was administered as a continuous 7-day i.v. infusion every 21 days. No dose-limiting toxicities were reported and barasertib 1200 mg was chosen for further evaluation in Japanese patients. Neutropenia and febrile neutropenia were the most commonly reported adverse events. The PK profile was similar to Western patients. A promising overall hematol. response rate of 19% was achieved, which warrants further investigation in these patients.

Keywords: barasertib acute myeloid leukemia pharmacokinetic antitumor Japanese

Substances (3) Reactions (0) Citing (41)

310

A novel cell-based, high-content assay for phosphorylation of Lats2 by Aurora A

By: Emery, Amy; Sorrell, David A.; Lawrence, Stacy; Easthope, Emma; Stockdale, Mark; Jones, David O.; Zheleva, Daniella; Scaerou, Frederic; Glover, David M.
Journal of Biomolecular Screening (2011), 16(8), 925-931 | Language: English, Database: CAplus and MEDLINE

Aurora A kinase is a key regulator of mitosis, which is upregulated in several human cancers, making it a potential target for anticancer therapeutics. Consequently, robust medium- to high-throughput cell-based assays to measure Aurora A kinase activity are critical for the development of small-mol. inhibitors. Here the authors compare measurement of the phosphorylation of two Aurora A substrates previously used in high-content screening Aurora A assays, Aurora A itself and T ACC3, with a novel substrate Lats2. Using antibodies directed against phosphorylated forms of Aurora A (pThr288), P-TACC3 (pSer558), and P-Lats2 (pSer83), the authors investigate their suitability in parallel for development of a cell-based assay using several reference Aurora inhibitors: ML N8054, VX680, and AZD1152-HQPA. They validate a combined assay of target-specific phosphorylation of Lats2 at the centrosome and an increase in mitotic index as a measure of Aurora A activity. The assay is both sensitive and robust and has acceptable assay performance for high-throughput screening or potency estimation from concentration-response assays. It has the advantage that it can be carried out using a com. available monoclonal antibody against phospho-Lats2 and the widely available Cellomics ArrayScan HCS reader and thus represents a significant addition to the tools available for the identification of Aurora A specific inhibitors.

Keywords: cell assay phosphorylation Lats2 Aurora A kinase T ACC3

Substances (3) Reactions (0) Citing (8)

311

Display of antibody fragments on virus-like particles of RNA bacteriophages

By: Peabody, David S.; Chackerian, Bryce
World Intellectual Property Organization, WO2011116226 A2 2011-09-22 | Language: English, Database: CAplus

The invention enables the display of antibody single-chain variable fragments (scFv's) on virus-like particles (VLPs) of bacteriophages such as MS2. The VLPs encapsidate mRNA encoding the coat protein from which it assembles, enabling the recovery by reverse transcription and PCR of affinity-selected sequences from scFv libraries. The antibody single-chain variable fragment is inserted at a site corresponding to amino acids 11-17 or more particularly 13-17, or at the C-terminus or C-terminus, of the coat protein of MS-2. The efficacy and rate of capsid assembly are influenced and maximized in the presence of the MS2 translational operator, a 19-nucleotide RNA stem-loop (pac site) that, via its interaction with coat protein, mediates exclusive encapsidation of the MS2 genome during bacteriophage replication. The VLP expresses or displays a scFv peptide that has an affinity for a cancer cell, including solid tumors and leukemias, or a cell infected with a virus. The invention may be used for cell-type specific delivery of drugs by employing VLPs which will deliver compounds to specific cells, or delivery of image contrast enhancing agents. Related virus- like particles, method for constructing a library of scFv-VLPs, drug delivery vehicles comprising one or more pharmaceu tically-active ingredients, biomedical imaging agents, assays, and kits are also provided.

Keywords: antibody display virus like particle RNA bacteriophage; drug delivery virus like particle R NA bacteriophage; single chain antibody display virus like particle RNA bacteriophage

PatentPak available

Substances (334) | Reactions (0) | Citing (7)

312

Combination therapy for treating cancer and diagnostic assays for use therein

By: Shah, Omar Jameel; Shen, Yu; Lin, Xiaoya; Anderson, Mark; Huang, Xiaoli; Li, Junling; Li, Leiming
World Intellectual Property Organization, WO2011068863 A1 2011-06-09 | Language: English, Database: CAplus

The present disclosure relates to a combination of therapeutic agents for use in treating a patient suffering from cancer, wherein said combination comprises at least one polyploidy inducing agent and at least one Bcl- 2 family protein inhibitor. In addition, the present disclosure also relates to diagnostic assays useful in classification of patients for treatment with one or more therap eutic agents.

Keywords: antitumor combination therapy cancer therapy diagnosis assay

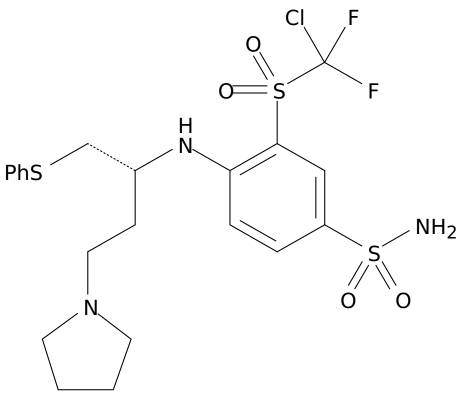

PatentPak available

Substances (181) | Reactions (803) | Citing (2)

313

IGF1R inhibitor based treatment of prostate cancer

By: Long, Brian; Groothuis, Patrick Gerhard; Hicklin, Daniel
World Intellectual Property Organization, WO2011057064 A1 2011-05-12 | Language: English, Database: CAplus

The present invention encompasses, inter alia, methods for treating prostate cancer comprising a chromo somal translocation with an IGF1R inhibitor such as an isolated antibody that binds specifically to IGF1R or a small organic compound The present invention provides a method for treating or preventing a TMPRSS2-ERG-pos. prostate cancer tumor or a prostate tumor comprising cells that overexpress the androgen receptor (AR), and/or for inducing apoptosis of cells of said tumor, in a subject, comprising adminis tering, to the subject, a therapeutically effective amount of IGF1R inhibitor; optionally, further comprising determining whether the tumor cells express IGF1R and whether the tumor cells comprise the TMPRSS2-ERG fusion and/or overexpress AR wherein (for example, by obtaining a sample of said tumor cells from the subject and analyzing them for the presence of said characteristics), if the tumor cells express IGF1R and if the tumor cells do comprise the TMPRSS2-ERG fusion and/or overexpress AR, then treatment with the IG F1R inhibitor is commenced or continued; and wherein if the tumor cells do not express I GF1R and/or if the tumor cells do not comprise the TMPRSS2-ERG fusion and/or overexpressed AR, then the treatment with the IGF1R inhibitor is discontinued.

Keywords: IGF1R inhibitor treatment prostate cancer TMPRSS2 ERG fusion; antibody IGF1R prostate cancer treatment

PatentPak available

Substances (323) | Reactions (0) | Citing (2)

314

Effects of AZD1152, a selective Aurora B kinase inhibitor, on Burkitt's and Hodgkin's lymphomas

By: Mori, Naoki; Ishikawa, Chie; Senba, Masachika; Kimura, Masashi; Okano, Yukio
Biochemical Pharmacology (2011), 81(9), 1106-1115 | Language: English, Database: CAplus and MEDLINE

We studied the effects of AZD1152, an Aurora B kinase inhibitor, on Burkitt's lymphoma (BL) and Hodgkin's lymphoma (HL) in human tissues and cell cultures and in a murine xenograft model of lymphoma. Aurora kinase A and B levels were assessed by RT-PCR and immunohistochem. They were aberrantly expressed in BL and HL cell lines, and in lymph nodes from patients with BL and HL. Next, activation of the Aurora B promoter was detected by reporter gene assays. The promoter activity of Aurora B kinase was high in BL cell lines and the Aurora B promoter contained a pos. regulatory region between - 74 and -104 from the transcription initiation site. AZD1152-hQPA had antiproliferative effects in the BL and HL cell lines studied; inhibited the phosphorylation of histone H3 and retinoblastoma proteins, and resulted in cells with >4 N DNA content. AZD1152-hQPA induced caspase-dependent apoptosis of some cell lines, demonstrated by loss of mitochondrial membrane potential, activation of caspase-9, followed by activation of caspase-3. This effect was accompanied by the inhibition of survivin expression. In vivo efficacy was determined in NOD/SCID/γc^{null} mice implanted with the Ramos human BL cell line. AZD1152 had anti-tumor effects in this murine xenograft model. There preclin. data suggest that the inhibition of Aurora B kinase is a potentially useful therapeutic strategy in BL and HL.

Keywords: Aurora B kinase inhibitor AZD1152 Burkitt's Hodgkin's lymphoma

Substances (7) | Reactions (0) | Citing (17)

315

Enhanced radiosensitivity of androgen-resistant prostate cancer: AZD1152-mediated Aurora kinase B inhibition

By: Niermann, Kenneth J.; Moretti, Luigi; Giacalone, Nicholas J.; Sun, Yunguang; Schleicher, Stephen M.; Kopsombut, Prapaporn; Mitchell, Lauren R.; Kim, Kwang Woon; Lu, Bo
Radiation Research (2011), 175(4), 444-451 | Language: English, Database: CAplus and MEDLINE

Aurora kinase B (AURKB) is critical to the process of mitosis, aiding in chromosome conden sation by phosphor ylating histone H3. We investigated the effects of AZD1152, an AURKB inhibitor, on radiosen sitivity of androgen-insensitive prostate cancer cells. The goal of this study was to test whether AZD1152 increases the susceptibility of hormone-refractory prostate cancer cells to radiation-induced DNA damage and to determine the conditions of AZD1152 treatment that maximize radiosensi tization. PC3 and DU145 cells were treated with various AZD1152 doses for various durations to elucidate the conditions that yielded maximal increases in G_2/M-phase and polyploid cells. To assess DNA damage, γ-H2AX phosphorylation was quantified for cells grown under radiosensitizing conditions and subjected to either no radiation or 5 Gy radiation. Radiosen sitivity was determined by clonogenic assays. Cell cycle effects in both cell lines were maximized by treatment with 60 nM AZD1152 for 48 h. AZD1152-treated cells exhibited significantly increased DNA damage 30 min postirra diation (PC3: 100% compared to 68%, P = 0.035; D U145: 100% compared to 69%, P = 0.034), with addnl. DNA damage 6 h postirra diation (PC3: 85% compared to 15%, P = 0.002; D U145: 67% compared to 21%, P = 0.012). Radiosensitivity was increased in both cell lines, with dose enhanc ement ratios of 1.53 for PC3 cells (P = 0.017) and 1.71 for DU145 cells (P = 0.02). This study identifies the optimal AZD1152 treatment conditions to maximize the radiosensitization of PC3 and DU145 cells. These results suggest a major role for D NA damage and impairment of D NA repair mechanisms in AZD1152-induced radiosensitization of prostate cancer cells.

Keywords: radiotherapy AZD1152 radiosensitizer androgen prostate cancer; Aurora kinase B signaling inhibitor

Substances (2) Reactions (0) Citing (25)

316

N-(4-((3-(2-amino-4-pyrimidinyl)-2-pyridinyl)oxy)phenyl)-4-(4-methyl-2-thienyl)-1-phthalazinamine for use in the treatment of antimitotic agent resistant cancer

By: Payton, Marc; Kendall, Richard
World Intellectual Property Organization, WO2011031842 A1 2011-03-17 | Language: English, Database: CAplus

The present invention relates to methods of using the compound, N-(4-((3-(2-amino-4-pyrimidinyl)-2-pyridinyl)oxy)phenyl)-4-(4-methyl-2-thienyl)-1-phthalazinamine or a pharmace utically acceptable salt thereof, to treat cancers, including solid tumors, which have become resistant to treatment with chemotherapeutic agents, including anti-mitotic agents such as taxanes, and/or other anti-cancer agents, including aurora kinase inhibiting agents. The invention also includes methods of treating cancers refractory to such treatments by administering a pharmaceutical composition, comprising the compound to a cancer subject.

Keywords: phthalazinamine derivative cancer treatment aurora kinase inhibitor

PatentPak available

Substances (41) Reactions (9) Citing (3)

317

Aurora B kinase inhibitor AZD1152: determinants of action and ability to enhance chemotherapeutics effectiveness in pancreatic and colon cancer

By: Azzariti, A.; Bocci, G.; Porcelli, L.; Fioravanti, A.; Sini, P.; Simone, G. M.; Quatrale, A. E.; Chiarappa, P.; Mangia, A.; Sebastian, S.; et al
British Journal of Cancer (2011), 104(5), 769-780 | Language: English, Database: CAplus and MEDLINE

Background: AZD1152, the prodrug for AZD1152-hydroxyquinazoline pyrazol anilide (HQPA), is a selective inhibitor of Aurora B kinase activity. Preclin. evaluation of AZD1152 has been reported in several human cancer models. The potent iality of this compound in combination therapy warrants further investigation in solid tumors. Exptl. design: This study explored the effects of AZD1152-HQPA in colon and pancreatic tumor cells. The antitumor properties of AZD1152, either as single agent or in combin ation with chemotherapeutics, were evaluated in each study model. The efficacy and the toxicity of AZD1152 alone and in combin ation with gemcitabine were validated in pancreatic tumor xenograft model. Results: AZD1152-HQPA treatment resulted in a dramatic increase of chromosome number, modification of cell cycle and induction of apoptosis. The most effective combin ation was that with chemotherapeutics given soon after AZD1152 in both tumor cell types. The effecti veness of the sequential schedule of AZD1152 with gemcit abine was confirmed in nude mice bearing Mia PaCa-2 tumors, showing inhibition of tumor volumes and delaying of tumor growth after the interruption of the treatments. Conclusion: Here we show that AZD1152-HQPA enhances oxalip latin and gemcitabine effectiveness in colon and pancreatic cancer, resp. First, we provide advances into adminis tration schedules and dosing regimens for the combination treatment in in vivo pancreatic tumor. British Journal of Cancer (2011) 104, 769- 780; doi: 10.1038/bjc.2011.21 www.bjcancer.com Published online 8 Feb. 2011.

Keywords: aurora B kinase inhibitor AZD1152 antitumor pancreas colon cancer; combin ation chemotherapy AZD1152 gemcitabine oxaliplatin antitumor pancreas colon cancer

Substances (6) Reactions (0) Citing (49)

318

Imaging Colon Cancer Response Following Treatment with AZD1152: A Preclinical Analysis of [^{18}F]Fluoro-2-deoxyglucose and 3'-deoxy-3'-[^{18}F]Fluorothymidine Imaging

By: Moroz, Maxim A.; Kochetkov, Tatiana; Cai, Shang-De; Wu, Ji-Yuan; Shamis, Mikhail; Nair, Jayasree; de Stanchina, Elisa; Serganova, Inna; Schwartz, Gary K.; Banerjee, Debabrata; et al
Clinical Cancer Research (2011), 17(5), 1099-1110 | Language: English, Database: CAplus and MEDLINE

Purpose: To determine whether treatment response to the Aurora B kinase inhibitor, AZD1152, could be monitored early in the course of therapy by noninvasive [^{18}F]-labeled fluoro-2-deoxyglucose, [^{18}F]FDG, and/or 3'-deoxy-3'-[^{18}F]fluorothymidine, [^{18}F]FLT, P ET imaging. Exptl. design: AZD1152-treated and control HCT116 and SW620 xenograft-bearing animals were monitored for tumor size and by [^{18}F]FDG, and [^{18}F]FLT PET imaging. Addnl. studies assessed the endogenous and exogenous contrib utions of thymidine synthesis in the two cell lines. Results: Both xenografts showed a significant volume-reduction to AZD1152. In contrast, [^{18}F]FDG uptake did not demonstrate a treatment response. [^{18}F]FLT uptake decreased to less than 20% of control values in AZD1152-treated HCT116 xenografts, whereas [^{18}F]FLT uptake was near background levels in both treated and untreated S W620 xenografts. The EC_{50} for AZD1152-HQPA was approx. 10 nmol/L in both S W620 and HCT116 cells; in contrast, SW620 cells were much more sensitive to methotrexate (MTX) and 5-Fluorouracil (5FU) than HCT116 cells. Immunoblot anal. demonstrated marginally lower expression of thymidine kinase in SW620 compared with HCT116 cells. The aforementioned results suggest that SW620 xenografts have a higher dependency on the de novo pathway of thymidine utilization than HCT116 xenografts. Conclusions: AZD1152 treatment showed antitumor efficacy in both colon cancer xenografts. Although [^{18}F]FDG PET was inadequate in monitoring treatment response, [^{18}F]FLT PET was very effective in monitoring response in HCT116 xenografts, but not in SW620 xenografts. These observations suggest that de novo thymidine synthesis could be a limitation and confou nding factor for [^{18}F]FLT PET imaging and quantification of tumor proliferation, and this may apply to some clin. studies as well.

Keywords: fluoride 18 FDG FLT PET imaging agent; AZD1152 antitumor colorectal cancer

Substances (8) Reactions (0) Citing (22)

319

AZD1152 negatively affects the growth of anaplastic thyroid carcinoma cells and enhances the effects of oncolytic virus dl922-947

By: Libertini, Silvana; Abagnale, Antonella; Passaro, Carmela; Botta, Ginevra; Barbato, Sara; Chieffi, Paolo; Portella, Giuseppe
Endocrine-Related Cancer (2011), 18(1), 129-141 | Language: English, Database: CAplus and MEDLINE

Novel therapeutic approaches are required for the treatment of anaplastic thyroid carcinoma (A TC), an incurable disease resistant to current available therapies. Aurora B is an important mitotic kinase involved in chromosome segregation and cytokinesis. It is overexpressed in many cancers including ATC and represents a potential target for chemoth erapy. The effects of AZD1152, a specific Aurora B kinase inhibitor, have been evaluated against ATC, showing G_2/M accumulation, polyploidy and subsequent cell death by mitotic catastrophe upon drug treatment. Only three administ rations of AZD1152 significantly reduced the growth of A TC tumor xenografts. Oncolytic viruses in associ ation with other forms of treatment have proven highly promising in preclin. and clin. reports. The oncolytic adenovirus dl922-947 is active against A TC cells, and we have evaluated the effects of the associ ation between AZD1152 and dl922-947. In cells treated with virus and drug, we report additive/s ynergistic killing effects. Interestingly, the phosphorylation of histone H3 (Ser10), the main Aurora B substrate, is inhibited by dl922- 947 in a dose-dependent manner, and completely abolished in association with AZD1152. The combined treatment signifi cantly inhibited the growth of A TC tumor xenografts with respect to single treatments. Our data demonstrate that the Aurora B inhibitor AZD1152, alone or in combination with oncolytic virus dl922-947, could represent a novel therap eutic option for the treatment of A TC.

Keywords: Aurora B AZD1152 oncolytic virus anaplastic thyroid carcinoma growth

Substances (4)	Reactions (0)	Citing (31)

320

A Robust High-Content Imaging Approach for Probing the Mechanism of Action and Phenotypic Outcomes of Cell-Cycle Modulators

By: Sutherland, Jeffrey J.; Low, Jonathan; Blosser, Wayne; Dowless, Michele; Engler, Thomas A.; Stancato, Louis F.
Molecular Cancer Therapeutics (2011), 10(2), 242-254 | Language: English, Database: CAplus and MEDLINE

High-content screening is increasingly used to elucidate changes in cellular biol. arising from treatment with small mols. and biol. probes. We describe a cell classifier for automated anal. of multiparametric data from immunofluorescence microscopy and charac terize the phenotypes of 41 cell-cycle modulators, including several protein kinase inhibitors in preclin. and clin. develo pment. This method produces a consistent assessment of treatment-induced phenotypes across experiments done by different biologists and highlights the prevalence of nonuniform and concentration-dependent cellular response to treatment. Contrasting cell phenotypes from high-content screening to kinase selectivity profiles from cell-free assays highlights the limited utility of enzyme potency ratios in understanding the mechanism of action for cell- cycle kinase inhibitors. Our cell-level approach for assessing phenotypic outcomes is reliable, reproducible and capable of supporting medium throughput analyses of a wide range of cellular perturb ations.

Keywords: cell cycle modulator phenotype high content imaging classification

Substances (40)	Reactions (0)	Citing (24)

321

P-glycoprotein and breast cancer resistance protein in acute myeloid leukaemia cells treated with the aurora-B kinase inhibitor barasertib-hQPA

By: Grundy, Martin; Seedhouse, Claire; Russell, Nigel H.; Pallis, Monica
BMC Cancer (2011), 11, 254 | Language: English, Database: CAplus and MEDLINE

Background: Aurora kinases play an essential role in orchestrating chromosome alignment, segregation and cytokinesis during mitotic progression, with both aurora-A and B frequently over-expressed in a variety of human malignancies. Over-expression of the ABC drug transporter proteins P-glycoprotein (Pgp) and Breast cancer resistance protein (BCRP) is a major obstacle for chemotherapy in many tumor types with Pgp conferring particularly poor prognosis in acute myeloid leukemia (AML). Barasertib-hQPA is a highly selective inhibitor of aurora-B kinase that has shown tumoricidal activity against a range tumor cell lines including those of leukemic AML origin. Methods: Effect of barasertib-hQPA on the pHH3 biomarker and cell viability was measured in a panel of leukemic cell lines and 37 primary AML samples by flow cytometry. Pgp status was determined by flow cytometry and BCRP status by flow cytometry and real-time PCR. Results: In this study we report the creation of the cell line OCI-AML3DNR, which over-expresses Pgp but not BCRP or multidrug resistance-associated protein (MRP), through prolonged treatment of OCI-AML3 cells with daunorubicin. We demonstrate that Pgp (OCI-AML3DNR and KG-1a) and BCRP (OCI-AML6.2) expressing AML cell lines are less sensitive to barasertib-hQPA induced pHH3 inhibition and subsequent loss of viability compared to transporter neg. cell lines. We also show that barasertib-hQPA resistance in these cell lines can be reversed using known Pgp and BCRP inhibitors. We report that barasertib-hQPA is not an inhibitor of Pgp or BCRP, but by using ^{14}[C]-barasertib-hQPA that it is effluxed by these transporters. Using phosphoHistone H3 (pHH3) as a biomarker of barasertib-hQPA responsiveness in primary AML blasts we determined that Pgp and BCRP pos. primary samples were less sensitive to barasertib-hQPA induced pHH3 inhibition ($p \leq 0.001$) than samples without these transporters. However, we demonstrate that IC_{50} inhibition of pHH3 by barasertib-hQPA was achieved in 94.6% of these samples after 1 h drug treatment, in contrast to the resistance of the cell lines. Conclusion: We conclude that Pgp and BCRP status and pHH3 down-regulation in patients treated with barasertib should be monitored in order to establish whether transporter-mediated efflux is sufficient to adversely impact on the efficacy of the agent.

Keywords: anticancer Aurora B kinase inhibitor barasertib hQPA Pgp BCRP; acute myeloid leukemia

Substances (3) Reactions (0) Citing (14)

322

Effects of AZD1152, a selective Aurora B kinase inhibitor, on Burkitt's and Hodgkin's lymphomas [Erratum to document cited in CA154:556930]

By: Mori, Naoki; Ishikawa, Chie; Senba, Masachika; Kimura, Masashi; Okano, Yukio
Biochemical Pharmacology (2011), 82(9), 1252 | Language: English, Database: CAplus

On page 1107, Section 2.4 contained an error; the correction is given.

Keywords: erratum Aurora B kinase inhibitor AZD1152 Burkitts Hodgkins lymphoma

Substances (6) Reactions (0) Citing (0)

323

A theoretical entropy score as a single value to express inhibitor selectivity

By: Uitdehaag, Joost C. M.; Zaman, Guido J. R.
BMC Bioinformatics (2011), 12, 94 | Language: English, Database: CAplus and MEDLINE

Designing maximally selective ligands that act on individual targets is the dominant paradigm in drug discovery. Poor selectivity can underlie toxicity and side effects in the clinic, and for this reason compound selectivity is increasingly monitored from very early on in the drug discovery process. To make sense of large amounts of profiling data, and to determine when a compound is sufficiently selective, there is a need for a proper quant. measure of selectivity. Here we propose a new theor. entropy score that can be calculated from a set of IC_{50} data. In contrast to previous measures such as the 'selec tivity score', Gini score, or partition index, the entropy score is non-arbitary, fully exploits IC_{50} data, and is not dependent on a reference kinase. In addition, the entropy score gives the most robust values with data from different sources, because it is less sensitive to errors. We apply the new score to kinase and nuclear receptor profiling data, and to high-throughput screening data. In addition, through analyzing profiles of clin. compounds, we show that a more selective inhibitor is not necessarily more drug-like. For quantifying selectivity from panel profiling, a theor. entropy score is the best method. It is valuable for studying the mol. mechanisms of selectivity, and to steer compound progression in drug discovery programs.

Keywords: theor entropy score allosteric inhibitor selectivity

Substances (42) Reactions (0) Citing (30)

324

Preclinical Evaluation of AMG 900, a Novel Potent and Highly Selective Pan-Aurora Kinase Inhibitor with Activity in Taxane-Resistant Tumor Cell Lines

By: Payton, Marc; Bush, Tammy L.; Chung, Grace; Ziegler, Beth; Eden, Patrick; McElroy, Patricia; Ross, Sandra; Cee, Victor J.; Deak, Holly L.; Hodous, Brian L.; et al
Cancer Research (2010), 70(23), 9846-9854 | Language: English, Database: CAplus and MEDLINE

In mammalian cells, the aurora kinases (aurora-A, -B, and -C) play essential roles in regulating cell division. The expression of aurora-A and -B is elevated in a variety of human cancers and is associated with high prolife ration rates and poor prognosis, making them attractive targets for anticancer therapy. AMG 900 is an orally bioavai lable, potent, and highly selective pan- aurora kinase inhibitor that is active in taxane-resistant tumor cell lines. In tumor cells, A MG 900 inhibited autophosph orylation of aurora-A and -B as well as phosphorylation of histone H3 on Ser^{10}, a proximal substrate of aurora- B. The predominant cellular response of tumor cells to AMG 900 treatment was aborted cell division without a prolonged mitotic arrest, which ultimately resulted in cell death. AMG 900 inhibited the prolife ration of 26 tumor cell lines, including cell lines resistant to the antimi totic drug paclitaxel and to other aurora kinase inhibitors (AZD1152, MK-0457, and PHA-739358), at low nanomolar concent rations Furthermore, AMG 900 was active in an AZD1152-resistant HCT116 variant cell line that harbors an aurora- B mutation (W221 L). Oral adminis tration of AM G 900 blocked the phosphor ylation of histone H3 in a dose- dependent manner and signifi cantly inhibited the growth of H CT116 tumor xenografts. Import antly, AMG 900 was broadly active in multiple xenograft models, including 3 multidrug- resistant xenograft models, representing 5 tumor types. A MG 900 has entered clin. evaluation in adult patients with advanced cancers and has the potential to treat tumors refractory to anticancer drugs such as the taxanes.

Keywords: antitumor AMG900 taxane resistance breast colon cancer

Substances (12) Reactions (0) Citing (104)

325

Methods for providing personalized medicine tests ex vivo for hematological neoplasms

By: Ballesteros, Juan; Bennett, Teresa; Primo, Daniel; Orfao, Alberto; Jackson, Coyt; Lago, Santiago; Matoses, Maria; Suarez, Lilia; Sapia, Sandra; Bosanquet, Andrew; et al
World Intellectual Property Organization, WO2010135468 A1 2010-11-25 | Language: English, Database: CAplus

Described herein are methods, devices, and compositions for providing person alized medicine tests for hematol. neoplasms. In some embodiments, the methods comprise measuring the efficacy of inducing apoptosis selectively in malignant cells using any number of potential alternative combination drug treatments. In some embodiments, the ex vivo testing is measured using a recently extracted patient hematol. samples. In other embodiments, the efficacy is measured ex vivo using an automated flow cytometry platform. For example, by using an automated flow cytometry platform, the evaluation of hundreds, or even thousands of drugs and compositions, can be made ex vivo. Thus, altern ative polytherapy treatments can be explored. Non-cytotoxic drugs surprisingly induce apoptosis selectively in malignant cells ex vivo. In some embodi ments, the methods described herein comprise evaluating non-cytotoxic drugs.

Keywords: bioassay hematol neoplasm drug personalized medicine; analytical device drug assay hematol neoplasm; automated flow cytometry drug combination assay blood; drug apoptosis blood neoplasm person alized test

PatentPak available

Substances (157) Reactions (0) Citing (3)

326

Expression, purification, stability optimization and characterization of human Aurora B kinase domain from E. coli

By: Sheth, Payal R.; Ramanathan, Lata; Ranchod, Ashwin; Basso, Andrea D.; Barrett, Dianah; Zhao, Jia; Gray, Kimberly; Liu, Yan-Hui; Zhang, Rumin; Le, Hung V.
Archives of Biochemistry and Biophysics (2010), 503(2), 191-201 | Language: English, Database: CAplus and MEDLINE

Aurora B kinase plays a critical role in regulating mitotic progression, and its dysregulation has been linked to tumorig enesis. The structure of the kinase domain of human Aurora B and the complementary information of binding thermodn. of known Aurora inhibitors is lacking. Towards that effort, we sought to identify a human Aurora B construct that would be amenable for large-scale protein production for biophys. and structural studies. Although the designed $AurB_{69-333}$ construct expressed at high levels in Escherichia coli, the purified protein was largely unstable and prone to aggreg ation. We employed thermal-shift assay for high-throughput screening of 192 conditions to identify optimal p H and salt conditions that increased the stability and minimized aggreg ation of $AurB_{69-333}$. Direct ligand binding analyses using temper ature-dependent CD (TdCD) and TR-FRET-based Lanthascreen binding assay showed that the purified protein was folded and functional. The affinity rank-order obtained using TdCD and Lantha screen binding assay correlated with enzymic IC_{50} values measured using full-length Aurora B protein for all the inhibitors tested except for AZD1152. The direct binding results support the hypothesis that the purified human Aur B_{69-333} fragment is a good surrogate for its full-length counterpart for biophys. and structural analyses.

Keywords: Aurora B kinase domain cloning Escherichia coli

Substances (6) Reactions (0) Citing (6)

327

Inhibition effect of AZD1152-HQPA, specific inhibitor of Aurora-B Kinase, on human Osteosarcoma cell line U2-OS cells

By: Liu, Zhi-li; Wang, Gao; Shu, Yong; Zou, Ping-an; Zhou, Yang; Huang, Shan-hu

Nanchang Daxue Xuebao, Yixueban (2010), 50(10), 30-32, 36 | Language: Chinese, Database: CAplus

The objective of this study was to explore the Aurora-B kinase inhibition effect AZD1152-HQPA specific inhibitor on human osteos arcoma cell line U2-OS cells. The human osteos arcoma cell line U2-OS cells were treated with AZD1152-HQPA at various concent ration (0, 10, 50, 100, 500 nmol·L^{-1}) in vitro for various time (24, 48,72 h). MTT assay was used to measure the inhibition effect of AZ D1152-HQPA on human osteos arcoma cell line U2-OS cells. The expression of Aurora- B kinase before and after of AZD1152-HQPA on human osteosarcoma cell line U2-OS cells were detected by Western Blotting. The results showed that the cells growth of human osteosarcoma cell line U2-OS were inhibited effetely by AZD1152-HQPA, showing the inhibition in time-and concentration-dependent manner. The expression of Aurora-B kinase in human osteos arcoma cell line U2-OS cells, treated with AZD1152-HQPA 50 nmol·L^{-1} for 24,48,72 h, was significantly reduced and the level of expression was lower as the time increased. It was concluded that the cells proliferation of U2-OS were inhibited by the selective Aurora- B kinase inhibitor AZD1152-HQPA in a time-and concent ration-dependent manner.

Keywords: osteosarcoma Aurora B kinase inhibitor AZD1152 HQPA

Substances (2) Reactions (0) Citing (0)

328

Fully human anti-VEGF antibodies and methods of using in cancer therapy

By: Ramachandra, Sumant; Huang, Chin-Yi; Masat, Linda; Huang, Chao Bai; Bishop, Walter Robert; Takeuchi, Toshihiko; Kantak, Seema; Horowitz, Jo Ann; Ladha, Mohamed H.

World Intellectual Property Organization, WO2010124009 A2 2010-10-28 | Language: English, Database: CAplus

Disclosed herein are fully human antibodies and antigen-binding fragments thereof that specifically bind human VEGF and inhibit V EGF binding to VEGF-R1 and VEGF-R2, and therefore inhibit VEGF signaling. The antibodies and antigen-binding fragments disclosed herein may be used, for example, to treat angiogenesis and conditions associated with angiog enesis both in vivo and in vitro.

Keywords: human VEGF antibody cancer therapy VEGFR antitumor conjugate

PatentPak available

Substances (357) Reactions (0) Citing (3)

329

Clinical evaluation of AZD1152, an i.v. inhibitor of Aurora B kinase, in patients with solid malignant tumors.

By: Boss, D S; Witteveen, P O; van der Sar, J; Lolkema, M P; Voest, E E; Stockman, P K; Ataman, O; Wilson, D; Das, S; Schellens, J H
Annals of oncology : official journal of the European Society for Medical Oncology (2011), 22(2), 431-7 | Language: English, Database: MEDLINE

BACKGROUND: To determine, for each of two dosing schedules, the dose-limiting toxicity (DLT) and maximum-tolerated dose (MTD) of AZD1152, an Aurora B kinase inhibitor, and to evaluate its safety, biologic activity and pharmaco kinetics (PK). PATIENTS AND ME THODS: Patients with advanced solid malign ancies were treated with escalating doses (100- 650 mg) of AZD1152, administered as a 2-h infusion every 7 days (A) or 14 days (B) . Adverse events (A Es), PK variables and tumor response were assessed. R ESULTS: Fifty-nine patients were treated; 19 in schedule A and 40 in schedule B. The M TDs were 200 and 450 mg, respect ively. Neutropenia (with/without fever) was the most frequent A E and DLT in each schedule. Common Termin ology Criteria of Adverse Events version 3.0 grade ≥3 neutropenia and leukopenia occurred in 58% and 11% of patients, respect ively, in schedule A and 43% and 20%, respectively, in schedule B. No objective tumor responses were observed at any dose or schedule, although stable disease, as defined by RECIST, was achieved in 15 patients (25%) overall. Systemic exposure to AZD1152-hQPA (active drug) was observed by 1 h into the infusion and exhibited linear PK. CONCLUSIONS: AZD1152 was generally well tolerated with neutro penia being the most frequently reported AE and DLT. Exposure to AZD1152-hQPA, the active drug of AZD1152, was linear.

Substances (0) Reactions (0) Citing (72)

330

Aurora kinase inhibitor AZD1152 negatively affects the growth and survival of HTLV-1-infected T lymphocytes in vitro

By: Tomita, Mariko; Tanaka, Yuetsu; Mori, Naoki
International Journal of Cancer (2010), 127(7), 1584-1594 | Language: English, Database: CAplus and MEDLINE

Aurora kinases play an essential role in regulating mitosis and cell division. Inhibition of Aurora kinases results in suppression of cell division, phosphorylation of histone H3 and induction of apoptosis in many cell types. These characte ristics have prompted the testing of Aurora kinase inhibitors as chemotherapeutic agents. In our study, we report the in vitro activities of AZD1152, a selective inhibitor of Aurora B kinase in human T-cell leukemia virus type 1 (HTLV-1), the causative agent of adult T-cell leukemia (ATL), -infected T-cell lines. Overexpression of Aurora B was noted in HTLV-1-infected T-cell lines compared to HTLV-1-uninfected T-cell lines. AZD1152 reduced the viability of HTLV-1-infected T-cell lines within 24 h but did not affect that of - uninfected T-cell lines. Although AZD1152 inhibited phosphorylation of histone H3 on Ser10 in both H TLV-1-infected and -uninfected T-cell lines, it induced polyploidy only in HTLV-1-uninfected T-cell lines. AZD1152 induced early apoptosis of HTLV-1-infected T-cells without induction of polyploidy. We have reported previously that a pan-Aurora kinase inhibitor induced apoptosis through inhibition of N F-κB signaling activity in HTLV-1-infected T-cell lines. In contrast, AZD1152 did not affect N F-κB activity in these cells. It induced p53 and p21 expression in HTLV-1-infected but not in HTLV-1-uninfected T-cell lines, suggesting that activation of p53- dependent postmitotic checkpoint might prevent polyploidy in HTLV-1-infected T-cells. Our results suggest that specific inhibition of Aurora B kinase is a potentially useful therapeutic strategy in the treatment of A TL and that further in vivo exploration is warranted.

Keywords: AZD1152 human T cell leukemia virus lymphocyte signaling

Substances (3) Reactions (0) Citing (6)

331

Aurora B is dispensable for megakaryocyte polyploidization, but contributes to the endomitotic process

By: Lordier, Larissa; Chang, Yunhua; Jalil, Abdelali; Aurade, Frederic; Garcon, Loic; Lecluse, Yann; Larbret, Frederic; Kawashima, Toshiyuki; Kitamura, Toshio; Larghero, Jerome; et al
Blood (2010), 116(13), 2345-2355 | Language: English, Database: CAplus and MEDLINE

Polyploidization of megakaryocytes (MKs), the platelet precursors, occurs by endomi tosis, a mitotic process that fails at late stages of cytokinesis. Expression and function of Aurora B kinase during endomi tosis remain controversial. Here, we report that Aurora B is normally expressed during the human MK endomitotic process. Aurora B localized normally in the midzone or midbody during anaphase and telophase in low ploidy megakaryocytes and in up to 16N rare endomitotic MKs was observed Aurora B was also functional during cytokinesis as attested by phosphor ylation of both its activation site and Mgc RacGAP, its main substrate. However, despite its activation, Aurora B did not prevent furrow regres sion. Inhibition of Aurora B by AZD1152-HQPA decreased cell cycle entry both in 2N to 4N and polyploid MKs and induced apoptosis mainly in 2N to 4N cells. In both MK classes, AZD1152-HQPA induced p53 activation and retinob lastoma hypophosphorylation. Resistance of polyploid MKs to apoptosis correlated to a high BclxL level. Aurora B inhibition did not impair MK polyploidization but profoundly modified the endomitotic process by inducing a mis-segregation of chromosomes and a mitotic failure in anaphase. This indicates that Aurora B is dispen sable for MK polyploidization but is necessary to achieve a normal endomi totic process.

Keywords: aurora endomitosis megakaryocyte polyploidization

Substances (4) Reactions (0) Citing (36)

332

System-Level Analysis of Neuroblastoma Tumor-Initiating Cells Implicates AURKB as a Novel Drug Target for Neuroblastoma

By: Morozova, Olena; Vojvodic, Milijana; Grinshtein, Natalie; Hansford, Loen M.; Blakely, Kim M.; Maslova, Alexandra; Hirst, Martin; Cezard, Timothee; Morin, Ryan D.; Moore, Richard; et al
Clinical Cancer Research (2010), 16(18), 4572-4582 | Language: English, Database: CAplus and MEDLINE

PURPOSE: Neuroblastoma (NB) is an aggressive tumor of the developing peripheral nervous system that remains difficult to cure in the advanced stages. The poor prognosis for high-risk NB patients is associated with common disease recurr ences that fail to respond to available therapies. NB tumor-initiating cells (TICs), isolated from metastases and primary tumors, may escape treatment and contribute to tumor relapse. New therapies that target the TICs may therefore prevent or treat tumor recurr ences. Exptl. Design: We undertook a system-level characterization of NB TICs to identify potential drug targets against recurrent N B. We used next-generation RNA sequencing and/or human exon arrays to profile the transcr iptomes of 11 NB TIC lines from six NB patients, revealing genes that are highly expressed in the TICs compared with normal neural crest- like cells and unrelated cancer tissues. We used gel-free two-dimensional liquid chromatog. coupled to shotgun tandem mass spectr ometry to confirm the presence of proteins corresponding to the most abundant TIC-enriched transcripts, thereby providing validation to the gene expression result. RESULTS: Our study revealed that genes in the BRCA1 signaling pathway are frequently misexp ressed in NB TICs and implicated Aurora B kinase as a potential drug target for NB therapy. Treatment with a selective AURKB inhibitor was cytotoxic to NB TICs but not to the normal neural crest- like cells. CONCLUSION: This work provides the first high- resolution system-level anal. of the transcriptomes of 11 primary human N B TICs and identifies a set of candidate N B TIC-enriched transcripts for further develo pment as therapeutic targets. Clin Cancer Res; 16(18); 4572-82.

Keywords: neuroblastoma tumor initiating cell A URKB therapeutic target

Substances (18) Reactions (0) Citing (37)

333

Combination therapy for treating cancer and diagnostic assays for use therein

By: Shah, Omar Jameel; Shen, Yu; Lin, Xiaoyu; Anderson, Mark; Huang, Xiaoli; Li, Junling; Li, Leiming
United States, US20100227838 A1 2010-09-09 | Language: English, Database: CAplus

The present disclosure relates to a combination of therapeutic agents for use in treating a patient a subject suffering from cancer. In addition, the present disclosure also relates to diagnostic assays useful in classification of patients for treatment with one or more therapeutic agents.

Keywords: antitumor combination therapy cancer therapy diagnosis assay

PatentPak available

Substances (174) | Reactions (802) | Citing (4)

334

Markers to predict and monitor response to Aurora kinase B inhibitor therapy of cancer

By: Semizarov, Dimitri; Shah, Jameel; Guo, Jun; Anderson, Mark
World Intellectual Property Organization, WO2010088470 A1 2010-08-05 | Language: English, Database: CAplus

The present invention relates to diagnostic assays useful in classification of patients for selection of cancer therapy with one or more Aurora kinase B inhibitors. In particular, the present invention relates to identifying the presence or absence of one or more copy number gains in the ABCB1 gene, the ABCB4 gene or combinations thereof, identifying patients eligible to receive Aurora kinase inhibitor therapy, either as monotherapy or as part of combination therapy, and monitoring patient response to such therapy.

Keywords: cancer therapy Aurora kinase B inhibitor biol marker

PatentPak available

Substances (327) | Reactions (0) | Citing (1)

335

Imidazo[4,5-b]pyridine Derivatives As Inhibitors of Aurora Kinases: Lead Optimization Studies toward the Identification of an Orally Bioavailable Preclinical Development Candidate

By: Bavetsias, Vassilios; Large, Jonathan M.; Sun, Chongbo; Bouloc, Nathalie; Kosmopoulou, Magda; Matteucci, Mizio; Wilsher, Nicola E.; Martins, Vanessa; Reynisson, Johannes; Atrash, Butrus; et al
Journal of Medicinal Chemistry (2010), 53(14), 5213-5228 | Language: English, Database: CAplus and MEDLINE

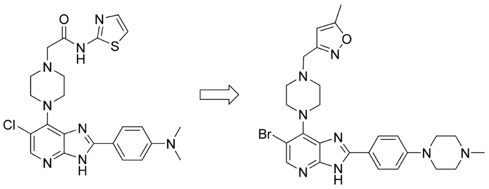

Lead optimization studies using an imidazo[4,5-b]pyridinylpiperazine as the starting point led to a new class of imidazo [4,5-b] pyridine-based inhibitors of Aurora kinases that possessed the 1-benzylpiperazinyl motif at the 7-position, and displayed favorable in vitro properties. Cocrystn. of Aurora-A with a morpholinylmethylphenylimidazopyridinyl chlorobenzyl piperazine (CCT137444) provided a clear understanding into the interactions of this novel class of inhibitors with the Aurora kinases. Subsequent physic ochem. property refinement by the incorpo ration of solubilizing groups led to the identification of 3-((4-(6-bromo-2-(4-(4-methylpi perazin-1-yl)phenyl)-3H-imidazo[4,5-b]pyridin-7-yl)piperazin-1-yl)methyl)-5-methylisoxazole (CCT137690)(**I**) which is a potent inhibitor of Aurora kinases (Aurora-A IC_{50} = 0.015±0.003 μM, Aurora-B IC_{50} = 0.025 μM, Aurora-C IC_{50} = 0.019 μM). **I** is highly orally bioavailable, and in in vivo efficacy studies it inhibited the growth of S W620 colon carcinoma xenografts following oral adminis tration with no observed toxicities as defined by body weight loss.

Keywords: imidazopyridine derivative arsenic inhibitor Aurora kinase orally bioava ilable development

Substances (234) Reactions (402) Citing (70)

336

Bcl-XL represents a druggable molecular vulnerability during aurora B inhibitor-mediated polyploidization

By: Shah, Jameel; Lin, Xiaoyu; Li, Leiming; Huang, Xiaoli; Li, Junling; Anderson, Mark G.; Tang, Hua; Rodriguez, Luis E.; Warder, Scott E.; Mcloughlin, Shaun; et al
Proceedings of the National Academy of Sciences of the United States of America (2010), 107(28), 12634-12639, S12634/1-S12634/10 | Language: English, Database: CAplus and MEDLINE

Aurora kinase B inhibitors induce apoptosis secondary to polyploidization and have entered clin. trials as an emerging class of neocytotoxic chemotherapeutics. We demonstrate here that polyploidization neutralizes Mcl-1 function, rendering cancer cells exquisitely dependent on Bcl-XL-2. This "addiction" can be exploited therapeutically by combining aurora kinase inhibitors and the orally bioavailable BH3 mimetic, ABT-263, which inhibits Bcl-XL, Bcl-2, and Bcl-w. The combination of ABT-263 with aurora B inhibitors produces a synergistic loss of viability in a range of cell lines of divergent tumor origin and exhibits more sustained tumor growth inhibition in vivo compared with aurora B inhibitor monotherapy. These data demonstrate that Bcl-XL/-2 is necessary to support viability during polyploidization in a variety of tumor models and represents a druggable mol. vulnera bility with potential therapeutic utility.

Keywords: Bcl XL aurora kinase B inhibitor polyploi dization cancer

Substances (4) Reactions (0) Citing (34)

337

Identification and classification of E2F-responsive gene overexpressing tumors for selection of therapies

By: Shackney, Stanley E.
World Intellectual Property Organization, WO2010056931 A1 2010-05-20 | Language: English, Database: CAplus

Methods for identifying a tumor as an E2 F-responsive gene over-expressing (ERGO) tumor are described. These methods can be used in diagnosis and to predict survival of the patient and in the selection and monitoring of therapies. These methods are applicable to ERGO tumors present in different human organs and tissues such as breast, lung, thyroid, ovary, and prostate. Transcr iption factor E2F regulates approx. 325 genes and abnormal levels of expression of a signif icant number of these in a tissue sample is diagnostic of an ERGO tumor.

Keywords: E2F transcription regulation neoplasm diagnosis therapy

Substances (376) Reactions (0) Citing (1)

338

The FLT3 Internal Tandem Duplication Mutation Is a Secondary Target of the Aurora B Kinase Inhibitor AZD1152-HQPA in Acute Myelogenous Leukemia Cells

By: Grundy, Martin; Seedhouse, Claire; Shang, Shilli; Richardson, Jaineeta; Russell, Nigel; Pallis, Monica
Molecular Cancer Therapeutics (2010), 9(3), 661-672 | Language: English, Database: CAplus and MEDLINE

Aurora kinases play an essential role in orchestrating chromosome alignment, segregation, and cytokinesis during mitotic progre ssion and both aurora- A and B are frequently overexp ressed in a variety of human maligna ncies. In this study, we report the effects of AZD1152-HQPA, a highly selective inhibitor of aurora- B kinase, in acute myeloid leukemia (AML) cell lines and primary samples. We show that AZD1152-HQPA inhibits the phosphorylation of Histone H3 (pHH3) on serine 10 resulting in polyploid cells, apoptosis, and loss of viability in a panel of AML cell lines. We also show that AZD1152-HQPA sensitivity in our cell lines is irresp. of p53 status and the FLT3-ITD-expressing MOLM-13 and MV4-11 cell lines are particularly sensitive to AZD1152-HQPA. Internal tandem duplications (ITD) within the FLT3 tyrosine kinase receptor are found in ~25% of A ML patients and are associated with a poor prognosis. Here, we report that AZD1152-HQPA directly targets phospho rylated FLT3 along with inhibiting its downstream target phospho-signal transducer and activator of transcr iption 5 (STAT5) in the FLT3-ITD cell lines. We show p HH3 expression in primary AML blasts and its inhibition by AZD1152-HQPA at low doses in all of our primary samples tested. AZD1152-HQPA inhibits the clonogenic potential of primary AML samples, with FLT3-ITD samples being the most sensitive (P = 0.029) . FLT3-ITD primary samples are also more sensitive to pHH3 inhibition (P = 0.022) and are partic ularly sensitive to pSTAT5 downregulation after treatment with AZD1152-HQPA compared with FLT3 wild-type samples (P = 0.007). We conclude that mutant FLT3 is a secondary target of AZD1152-HQPA and that FLT3-ITD primary samples are particularly sensitive to the drug. Mol Cancer Ther; 9 (3); 661-72.

Keywords: FLT3 tandem duplication mutation aurora kinase AZD1152 HQPA leukemia; myelogenous leukemia antitumor AZD1152 HQPA FLT3 tandem duplication mutation

Substances (2) Reactions (0) Citing (32)

339

The selective Aurora B kinase inhibitor AZD1152 as a novel treatment for hepatocellular carcinoma

By: Aihara, Arihiro; Tanaka, Shinji; Yasen, Mahmut; Matsumura, Satoshi; Mitsunori, Yusuke; Murakata, Ayano; Noguchi, Norio; Kudo, Atsushi; Nakamura, Noriaki; Ito, Koji; et al

Journal of Hepatology (2010), 52(1), 63-71 | Language: English, Database: CAplus and MEDLINE

Background & Aims: We previously identified that high Aurora B expression was associated with hepatocellular carcinoma (HCC) recurrence due to tumor dissemination. In this preclin. study, a novel inhibitor of Aurora B kinase was evaluated as a treatment for human HCC. Methods: AZD1152 is a selective inhibitor of Aurora B kinase. Twelve human H CC cell lines were analyzed for Aurora B kinase expression and the in vitro effects of AZD1152. The in vivo effects of AZD1152 were analyzed in a s.c. xenograft model and a novel orthotopic liver xenograft model. Results: Aurora B kinase expression varied among the human HCC cell lines and was found to correlate with inhibition of cell proliferation, accumulation of 4N DNA, and the proportion of polyploid cells following administration of AZD1152-hydroxyquinazoline-pyrazol-anilide (AZD1152-HQPA). AZD1152-HQPA suppressed histone H3 phosphorylation and induced cell death in a dose- dependent manner. Growth of s.c. human H CC xenografts was inhibited by AZD1152 administration. In an orthotopic hepatoma model, treatment with AZD1152 significantly decelerated tumor growth and increased survival. Pharmacobiol. anal. revealed that AZD1152 induced the rapid suppression of phosphohistone H3, followed by cellular apoptosis in the liver tumors but not in the normal tissues of the orthotopic models. Conclusions: Our preclin. studies indicate that AZD1152 is a promising novel therapeutic approach for the treatment of H CC.

Keywords: anticancer AZD1152 apoptosis phosphohistone H3 hepatocellular carcinoma growth

Substances (4) Reactions (0) Citing (53)

340

p53 is critical for the Aurora B kinase inhibitor-mediated apoptosis in acute myelogenous leukemia cells

By: Ikezoe, Takayuki; Yang, Jing; Nishioka, Chie; Yokoyama, Akihito

International Journal of Hematology (2010), 91(1), 69-77 | Language: English, Database: CAplus and MEDLINE

We previously showed that AZD1152-HQPA, the inhibitor of Aurora B kinase potently induced growth arrest and apoptosis of various types of human leukemia cells including MV4-11 acute myelogenous leukemia (AML) cells, although the mol. mechanisms by which this class of kinase inhibitors induces apoptosis remain to be fully elucidated. We have recently established the MV4-11 subline, designated as MV4-11 TP53 R248W, which possesses transcriptionally inactive R248W mutation in the TP53 gene. MV4-11 TP53 R248W cells were relatively resistant to AZD1152-HQPA-mediated growth arrest, as measured by MTT and clonogenic assays. AZD1152-HQPA (10-100 nM, 48 h) strikingly induced apoptosis of M V4-11 cells, as assessed by Annexin V binding, loss of mitochondrial outer membrane potential, and activation of caspase cascade, in parallel with up- regulation of p53 and its target mols. Bax and Noxa. Notably, AZD1152-HQPA (10-100 nM, 48 h) induced polyploidy rather than apoptosis in M V4-11 TP53 R248W cells. The polyploid cells were eventually eliminated via apoptosis at later time period (72-120 h) in association with up-regulation of p73. Taken together, p53 plays an important role in AZD1152-HQPA-induced growth arrest and early onset of apoptosis in A ML cells. P73 may mediate the late onset of apoptosis to eliminate the polyploid cells caused by the inhibitor of Aurora B kinase.

Keywords: p53 Aurora B kinase apoptosis acute myelogenous leukemia

Substances (4) Reactions (0) Citing (25)

341

Novel agents and regimens for acute myeloid leukemia: 2009 ASH annual meeting highlights

By: Zhu, Xiongpeng; Ma, Yuehua; Liu, Delong
Journal of Hematology & Oncology (2010), 3, No pp. given | Language: English, Database: CAplus and MEDLINE

A review. Prognostic markers, such as NPM1, Flt3-ITD, and cytoge netic abnormalities have made it possible to formulate aggressive treatment plans for unfavorable acute myeloid leukemia (AML). However, the long-term survival of AML with unfavorable factors remains unsatisfactory. The latest data indicate that the standard dose of daunor ubicin (DNR) at 45 mg/m^2 is inferior to high dose 90 mg/m^2 for induction therapy. The rates of complete remission and overall survival are signifi cantly better in the high dose induction regimen. New regimens exploring the new liposomal encapsulation of Ara-C and DNR as well as addition of gemtuzumab ozogamicin monoclonal antibody have been studied. New agents, including the nucleoside analogs (clofarabine, sapacitabine, elacytarabine), FLT3 inhibitor (sorafenib), farnesyl- transferase inhibitor (tipifarnib), histone deacetylase inhibitor (vorinostat), lenalid omide, as well as DNA methyltransferase inhibitors (decitabine, azacitidine), were recently reported for AML treatment in the 2009 A SH annual meeting. This review also summarizes the updates of the clin. trials on novel agents including voreloxin, A S1413, beheno ylara-C, ARRY520, ribavirin, AZD1152, AZD6244, and terameprocol (EM-1421) from the 2009 ASH annual meeting.

Keywords: review anticancer acute myeloid leukemia

Substances (22) Reactions (0) Citing (49)

342

Antineoplastic effects of an Aurora B kinase inhibitor in breast cancer

By: Gully, Christopher P.; Zhang, Fanmao; Chen, Jian; Yeung, James A.; Velazquez-Torres, Guermarie; Wang, Edward; Yeung, Sai-Ching Jim; Lee, Mong-Hong
Molecular Cancer (2010), 9, No pp. given | Language: English, Database: CAplus and MEDLINE

Background: Aurora B kinase is an important mitotic kinase involved in chromosome segreg ation and cytokinesis. It is overexp ressed in many cancers and thus may be an important mol. target for chemoth erapy. AZD1152 is the prodrug for AZD1152-HQPA, which is a selective inhibitor of Aurora B kinase activity. Preclin. antineoplastic activity of AZD1152 against acute myelog enous leukemia, multiple myeloma and colorectal cancer has been reported. However, this compound has not been evaluated in breast cancer, the second leading cause of cancer deaths among women. Results: The antineoplastic activity of AZD1152-HQPA in six human breast cancer cell lines, three of which overexpress HER2, is demonstrated. AZD1152-HQPA specifically inhibited Aurora B kinase activity in breast cancer cells, thereby causing mitotic catastrophe, polyploidy and apoptosis, which in turn led to apoptotic death. AZD1152 administration efficiently suppressed the tumor growth in a breast cancer cell xenograft model. In addition, AZ D1152 also inhibited pulmonary metastatic nodule formation in a metastatic breast cancer model. Notably, it was also found that the protein level of Aurora B kinase declined after inhibition of Aurora B kinase activity by AZD1152-HQPA in a time- and dose-dependent manner. Investigation of the underlying mechanism suggested that AZD1152-HQPA accelerated protein turnover of Aurora B via enhancing its ubiquitination. Conclusions: It was shown that AZD1152 is an effective antineo plastic agent for breast cancer, and our results define a novel mechanism for posttranscriptional regulation of Aurora B after AZD1152 treatment and provide insight into dosing regimen design for this kinase inhibitor in metastatic breast cancer treatment.

Keywords: antitumor AZD1152 hydroxyquinazoline pyrazol anilide Aurora kinase breast cancer

Substances (6) Reactions (0) Citing (72)

343

Significance of Aurora B overexpression in hepatocellular carcinoma

By: Lin, Zhong-Zhe; Jeng, Yung-Ming; Hu, Fu-Chang; Pan, Hung-Wei; Tsao, Hsin-Wei; Lai, Po-Lin; Lee, Po-Huang; Cheng, Ann-Lii; Hsu, Hey-Chi
BMC Cancer (2010), 10, No pp. given | Language: English, Database: CAplus and MEDLINE

Background: To investigate the significance of Aurora B expression in hepatocellular carcinoma (HCC). Methods: The Aurora B and Aurora A mRNA level was measured in 160 HCCs and the paired nontumorous liver tissues by reverse transcription-polymerase chain reaction. Mutations of the p53 and β-catenin genes were analyzed in 134 and 150 tumors, resp., by direct sequencing of exon 2 to exon 11 of p53 and exon 3 of β-catenin. Anticancer effects of AZD1152-HQPA, an Aurora B kinase selective inhibitor, were examined in Huh-7 and Hep3B cell lines. Results: Aurora B was overexpressed in 98 (61%) of 160 HCCs and in all 7 HCC cell lines examined The overexpression of Aurora B was associated with Aurora A overexpression (P = 0.0003) and p53 mutation (P = 0.002) and was inversely associated with β-catenin mutation (P = 0.002). Aurora B overexpression correlated with worse clinicopathol. characteristics. Multivariate anal. confirmed that Aurora B overexpression was an independent poor prognostic factor, despite its interaction with Aurora A overexpression and mutations of p53 and β-catenin. In Huh-7 and Hep3B cells, AZD1152-HQPA induced proliferation blockade, histone H3 (Ser10) dephosphorylation, cell cycle disturbance, and apoptosis. Conclusion: Aurora B overexpression is an independent mol. marker predicting tumor invasiveness and poor prognosis of HCC. Aurora B kinase selective inhibitors are potential therapeutic agents for HCC treatment.

Keywords: hepatocellular carcinoma aurora B gene mutation

Substances (3) Reactions (0) Citing (99)

344

Aurora kinase inhibitors: a new class of drugs targeting the regulatory mitotic system

By: Perez Fidalgo, Jose Alejandro; Roda, Desamparados; Rosello, Susana; Rodriguez-Braun, Edith; Cervantes, Andres
Clinical & Translational Oncology (2009), 11(12), 787-798 | Language: English, Database: CAplus and MEDLINE

The present review gives a perspective on the Aurora kinase family members, their function in normal cells, their role in cancer progression as well as their potential as target for anticancer treatment. Mitosis has been an important target for anticancer therapy development, leading to some specific drugs mainly addressing Tubulines, as a key structure of the mitotic spindle. Vinca alkaloids, taxanes or epotilones are good examples of conventionally developed antimitotic agents. However, novel classes of antineoplastic drugs are being studied, targeting the regulatory system that controls functional aspects of mitosis, such as Aurora or Polo-like kinases or Kinespondin inhibitors. The specific role of the different Aurora kinase proteins as regulator enzymes of the mitotic process in normal cells is discussed. Some of the mechanisms that link Aurora overexpression with cancer are also considered. Thereafter, the clin. and preclin. development of the different Aurora kinase inhibitors is presented. This is nowadays a very active area of therapeutic research and at least, sixteen new compounds are being studied as potential antineoplastic drugs. Most of them are in a very early phase of clin. development. However, we summarized the most recently published findings related with these drugs: main characteristics, way of administration, dose limiting toxicities and recommended doses for further studies. Another important aspect in Aurora kinase inhibition is the study and validation of potential biomarkers to optimize the clin. development. Several studies included pharmacodynamic assessments in normal blood cells, skin or/and tumor biopsies. Several proposals included a higher mitotic index, a decreased number of mitosis with bipolar spindles or normal alignment of chromosomes and inhibition of histone H3 phosphorylation. Future strategies and challenges for trials with Aurora kinase inhibitors are also discussed.

Keywords: review Aurora kinase inhibitor drug target mitosis cancer antineoplastic

Substances (5) Reactions (0) Citing (26)

345

Analysis of Aurora B kinase in non-Hodgkin lymphoma

By: Ikezoe, Takayuki; Takeuchi, Tamotsu; Yang, Jing; Adachi, Yoshihiro; Nishioka, Chie; Furihata, Mutsuo; Koeffler, H. Phillip; Yokoyama, Akihito
Laboratory Investigation (2009), 89(12), 1364-1373 | Language: English, Database: CAplus and MEDLINE

This study explored the levels of Aurora B, a key regulator of mitosis, in 71 lymph nodes and tumor specimens excised operatively from individuals with various types of non-Hodgkin lymphoma (NHLs). Immunohistochem. examination found that diffuse large B-cell lymphoma (10/21, 48%) and Burkitt lymphoma (B L) (6/7, 86%) cells highly (percentage of pos. cells, >20%) expressed Aurora B in their nuclei. On the other hand, none of the low-grade B-cell lymphoma (n=20), except for one case of follicular lymphoma, highly expressed this protein kinase, suggesting that levels of Aurora B correlated with histol. grade in B-cell NHLs (P<0.01). Exposure of B L/leukemia cells to AZD1152-HQPA in vitro, a selective inhibitor of Aurora B kinase, potently induced growth arrest and apoptosis in a caspase-dependent, as well as -independent manner. Moreover, AZD1152 synergistically enhanced the effects of vincristine (V CR) to induce growth arrest of these cells. Further experi ments found that VCR increased levels of the p-Aurora B through the activation of c-Jun N-terminal kinase, which was blocked in the presence of AZD1152-HQPA.

Keywords: aurora B kinase non Hodgkin lymphoma

Substances (7) Reactions (0) Citing (33)

346

Anti-IGF-1 receptor antibody combined with chemotherapeutic agent, antitumor agent, radiotherapy or surgery for treating or preventing colorectal cancer

By: Wang, Yaolin; Wang, Yan; Liu, Ming; Bishop, Walter Robert; Seidel-Dugan, Cynthia
World Intellectual Property Organization, WO2009142810 A2 2009-11-26 | Language: English, Database: CAplus

The present invention provides, for example, methods for treating or preventing colorectal cancer with an anti-IGF1R antibody in association with sunitinib or a combin ation of leucovorin and 5-fluorouracil.

Keywords: IGF1 receptor antibody chemotherapeutic antitumor radiotherapy surgery colorectal cancer

PatentPak available

Substances (343) Reactions (0) Citing (0)

347

Sequential administration of chemotherapeutic agents and anti-(insulin-like growth factor 1 receptor) for treatment of cancer

By: Wang, Yaolin; Wang, Yan; Lu, Brian Der-Hua; Liu, Ming; Seidel-Dugan, Cynthia; Yao, Siu-Long
World Intellectual Property Organization, WO2009137378 A2 2009-11-12 | Language: English, Database: CAplus

The present invention relates to the sequential administration of a cytotoxic agent followed by an insulin- like growth factor 1 receptor (IGF1R) antagonist (e.g., an antibody) for the treatment of hyperprol iferative disorders including cancer. Human monoclonal antibody clone 15H12/19D12 light and heavy chains targeting human I GF1R are provided. Thus, for example, sequential administration of irinotecan and then anti- IGF1R antibody for the treatment of human colorectal cancer in xenograft mice resulted in 77% tumor growth inhibition vs. 39% and 61% growth inhibition by irinotecan or anti-IGF1R antibody alone.

Keywords: cancer therapy chemotherapeutic agent I GF1 receptor antibody

PatentPak available

Substances (338) | Reactions (0) | Citing (2)

348

Simultaneous determination of AZD1152 (prodrug) and AZD1152-hydroxyquinazoline pyrazol anilide by reversed phase liquid chromatography

By: Pluim, Dick; Beijnen, Jos H.; Schellens, Jan H. M.; van Tellingen, Olaf
Journal of Chromatography B: Analytical Technologies in the Biomedical and Life Sciences (2009), 877(29), 3549-3555 | Language: English, Database: CAplus and MEDLINE
Analytical Methods available

A simple, selective and sensitive reversed phase liquid chromatog. method utilizing UV detection has been developed and validated for the simultaneous determination of the prodrug AZD1152 and its active product AZD1152-hydroxyquinazoline pyrazol anilide (h QPA) in human and mouse plasma and mouse tissues. Isocratic separation was achieved using an 5 μm Upti Sphere HDO C_{18} column (150 mm × 4.6 mm) with guard column in combination with a mobile phase comprised of phosphate buffered water (50 m M; pH 3.4) and acetonitrile (81.5: 18.5; volume/volume). UV detection at 318 nm was used. Sample prepar ation involved a single-step protein precipitation with ethanol. Ex vivo conversion of AZD1152 by endogenous phosphatases was prevented by immediate cooling of the samples in ice-water and addition of sodium fluoride and E DTA. The validation parameters included specif icity, recovery, accuracy, precision, sensitivity and stability. The lower limit of quantif ication in human plasma for AZD1152 and hQPA was 25 ng/mL. The applicability of the method was demonstrated by successful determi nation of AZD1152 and hQPA in human plasma and in plasma, brain, liver, kidney and ileum samples from mice dosed with AZD1152.

Keywords: AZD1152 pharmacokinetics tissue blood analysis H PLC

Substances (3) | Reactions (0) | Citing (7)

349

The Aurora B kinase inhibitor AZD1152 sensitizes cancer cells to fractionated irradiation and induces mitotic catastrophe

By: Tao, Yungan; Leteur, Celine; Calderaro, Julien; Girdler, Fiona; Zhang, Ping; Frascogna, Valerie; Varna, Mariana; Opolon, Paule; Castedo, Maria; Bourhis, Jean; et al
Cell Cycle (2009), 8(19), 3172-3181 | Language: English, Database: CAplus and MEDLINE

AZD1152, an Aurora kinase inhibitor with selectivity for Aurora B kinase, can enhance the effect of ionizing radiation (I R). The aim of this study was to evaluate and to mechanistically explore scheduling effects of AZD1152 on tumor responses to I R, in three different settings; neoadjuvant (AZD1152 before IR), adjuvant (IR before AZD1152), or concomitant treatments (AZD1152 plus one single IR dose). A more pronounced tumor growth delay was observed in the neoadj uvant and adjuvant schedules as compared to the concomitant schedule. However, AZD1152 enhanced the efficacy of IR when concomitant IR was fractionated over several days. Histopathol. examination revealed that AZD1152 + IR induced polyploidy, multinucleation and micronuclei in vivo. Time-lapse videomicroscopy confirmed that cell death induced by AZD1152 + IR was preceded by multinucleation and the formation of micron uclei, which both are hallmarks of mitotic catast rophe. Caspase inhibition or removal of the pro-apoptotic protein Bax did not ameliorate the long-term cell survival of AZD1152-treated cancer cells. In contrast, a chem. inhibitor of C HK1, Chir124, sensitized cancer cells to the lethal effect of AZD1152. Altogether, these data support the contention that AZD1152 mediates radiosens itization in vivo by enhancing mitotic catast rophe, which can be used as a biomarker of treatment efficacy.

Keywords: aurora B kinase inhibitor AZD1152 colon cancer radiotherapy anticancer

Substances (3)　Reactions (0)　Citing (44)

350

Gene expression profiling in the pulmonary artery in the selection of therapies for vascular-related diseases

By: Mann, David Marshall
World Intellectual Property Organization, WO2009121031 A1 2009-10-01 | Language: English, Database: CAplus

Methods of selecting treatment regimens for vascular-related diseases and disorders using gene expression profiles in affected blood vessels are described. Addnl., methods are disclosed involving diagnostic techniques to focus treatment regimens. Finally, methods of treating vascular-related disorder involving targeting micro RNAs are also disclosed. Therapies suitable for treatment at different stages of the disease are identified.

Keywords: pulmonary artery gene expression profiling vascular disease therapy; arterial hypertension lung diagnosis therapy

PatentPak available

Substances (522)　Reactions (0)　Citing (6)

351

Kinase Inhibitor 4 Minisymposium summary

By: Dancey, Janet E.
Expert Review of Anticancer Therapy (2009), 9(7), 891-894 | Language: English, Database: CAplus and MEDLINE

A review. In recent years no cancer therapeutic class has undergone more prolific or successful drug develo pment efforts than the kinase inhibitors. The robust research interest is evident in the number of kinase inhibitor present ations at the American Associ ation for Cancer Research Annual Meeting held in Denver (C O, USA) 18-22 Apr. 2009. Presen tation highlights from the Kinase Inhibitor 4 Minisymposium include the identification of FGF receptor genetic altera tions that correlate with sensitivity to the kinase inhibitor TKI258; evaluation of activity of mitotic kinase inhibitors G SK461364A and AZD1152 in breast and small- cell lung cancer and the identification of new inhibitors to PI3K/AKT/mTOR pathway.

Keywords: review kinase inhibitor 4 breast lung cancer anticancer signaling

Substances (5) Reactions (0) Citing (1)

352

Protein kinase genes showing altered levels of expression in breast cancer tissue and their diagnostic use

By: Bertucci, Francois; Birnbaum, Daniel; Finetti, Pascal
World Intellectual Property Organization, WO2009083780 A1 2009-07-09 | Language: English, Database: CAplus

The present invention relates to a method for analyzing cancer.e.g., breast cancer comprising detection of differ ential expression of at least one of the 16 genes encoding serine/threonine kinases listed in Table 1, or of said 16 genes, and to a polynuc leotide library comprising at least one said 16 genes. A method of diagnosing breast cancer by anal. of the levels of expression of members of a group of 16 protein kinase genes is described. Levels of expression of the genes can also be used in prognosis and in monitoring the effectiveness of therapies. The levels of expression of these genes were analyzed in 227 samples of breast cancer tissue as part of a larger anal. of gene expression in breast cancer. Validation of the use of these genes in diagnosis and in prognosis is demonst rated.

Keywords: protein kinase gene expression breast cancer diagnosis prognosis

PatentPak available

Substances (295) Reactions (0) Citing (2)

353

Gene expression biomarkers for detecting IGF1R inhibitor-resistant or -sensitive cancers and uses in anti-IGF1R antibody combination chemotherapy

By: Wang, Yan; Wang, Yaolin; Levitan, Diane; Seidel-Dugan, Cynthia; Liu, Ming; Ding, Wei

World Intellectual Property Organization, WO2009079587 A2 2009-06-25 | Language: English, Database: CAplus

The present invention provides, for example, methods for conveniently determining if a cancerous condition in a subject will be responsive to an IGF1R inhibitor. The invention includes patient selection methods and methods of treatment. The method comprises determining the expression levels of biomarker-genes associated with sensitivity or resistance to IGF1R inhibitor therapy. Thus, genes upregulated in sensitive cells relative to resistant cells are: T RE2, SMC4, TRIB2, TLE4, BMP7, PCDHGC3, AUTS2, C14orf132, CERK, HDGFRP3, TCF4, MEIS2, EML4, C7orf41, KIAA1450, ZNF136, D15F37, CDK6, TIMP, Clu, Prl1. Genes down-regulated in sensitive cells relative to resistant cells are: ACAT1, ALDOC, C6orf192, COL4A5, C1QBP, CRIP1, DEADC1, GSTK1, GSTO2, PPAPDC1B, TMEM107, JOSD3, TMEM77, MST1, MT1E, PARVB, PRDX4, RASGEF1A, RPL14, IFI30, ATF1, ACADVL, FBXO6, NQO2, TMEM64, ZFAND1, T MED5, PDIA5, MYO1C, GNPTAB, LACTB2, RPL22, TSPAN4, RPL15, PCCB, CRYZ, DNAJC10, C19orf54, HSPE1, hqp0376. Provided are sequences for anti-IGF1R antibodies to be used in combination chemotherapy.

Keywords: gene biomarker IGF1R inhibitor therapy resistance sensitivity cancer diagnosis; anticancer IGF1R antibody sequence combination chemotherapy

PatentPak available

Substances (339) | Reactions (0) | Citing (3)

354

Antitumor combination comprising a MEK inhibitor and an Aurora kinase inhibitor 188

By: Mundt, Kirsten Elisabeth; Smith, Paul David

World Intellectual Property Organization, WO2009074827 A2 2009-06-18 | Language: English, Database: CAplus

The present invention relates to a therapeutic combination comprising a MEK inhibitor and an Aurora kinase inhibitor, and to methods for the production of an anti-cancer effect in a patient, which is accordingly useful in the treatment of cancer in a patient. More specifically the present invention relates to: a therapeutic combination comprising a MEK inhibitor and an Aurora kinase inhibitor; a combination product comprising a MEK inhibitor and an Aurora kinase inhibitor, a kit of parts comprising a M EK inhibitor and an Aurora kinase inhibitor; use of a therapeutic combination, combination product or kit of parts in the treatment of cancer; a method of treating cancer comprising administering the therapeutic combination, combination product or kit of parts to a patient. The therapeutic combination and methods of the invention are also useful in the treatment of conditions in which the inhibition of MEK and/or Aurora kinase is beneficial.

Keywords: antitumor combination MEK inhibitor Aurora kinase

PatentPak available

Substances (6) | Reactions (0) | Citing (1)

355

Biochemical characterization of GSK1070916, a potent and selective inhibitor of Aurora B and Aurora C kinases with an extremely long residence time1

By: Anderson, Kelly; Lai, Zhihong; McDonald, Octerloney B.; Stuart, J. Darren; Nartey, Eldridge N.; Hardwicke, Mary Ann; Newlander, Ken; Dhanak, Dashyant; Adams, Jerry; Patrick, Denis; et al
Biochemical Journal (2009), 420(2), 259-265 | Language: English, Database: CAplus and MEDLINE

The Aurora kinases AurA, B and C are serine/threonine protein kinases that play essential roles in mitosis and cytoki nesis. Among them, AurB is required for maintaining proper chromosome alignment, separation and segreg ation during mitosis, and regulating a number of critical processes involved in cytokinesis. AurB overexpression has been observed in a variety of cancer cell lines, and inhibition of AurB has been shown to induce tumor regression in mouse xenograft models. In the present study we report the enzymic characterization of a potent and selective Aur B/AurC inhibitor. GSK1070916 is a reversible and A TP-competitive inhibitor of the AurB-INCENP (inner centromere protein) enzyme. It select ively inhibits Aur B-INCENP (Ki*=0.38±0.29 n M) and Aur C-INCENP (Ki*=1.5±0.4 n M) over Aur A-TPX2 (target protein for Xenopus kinesin- like protein 2) (Ki=490±60 n M). Inhibition of Aur B-INCENP and AurC-INCENP is time-dependent, with an enzyme-inhibitor dissociation half-life of >480 min and 270±28 min resp. The extremely slow rate of dissociation from the Aur B and Aur C enzymes distinguishes GSK1070916 from two other Aurora inhibitors in the clinic, AZD1152 and VX-680 (also known as M K-0457).

Keywords: GSK1070916 inhibitor Aurora B C kinase

Substances (6) Reactions (0) Citing (50)

356

Pharmacological inhibition of Aurora-A but not Aurora-B impairs interphase microtubule dynamics

By: Lorenzo, Corinne; Liao, Qiaoyin; Hardwicke, Mary Ann; Ducommun, Bernard
Cell Cycle (2009), 8(11), 1733-1737 | Language: English, Database: CAplus and MEDLINE

Aurora kinases are key cell cycle regulators and represent attractive new targets in cancer therapy. In this work we investigated the effect of specific inhibition of Aurora-A and Aurora-B on interphase microtubule dynamics using the GSK6000063A and AZD1152 H QPA compounds resp. We show that Aurora- A inhibition results in microt ubule network disorganization and bundling. Using video microscopy and laser-based photo ablation we demonstrate that Aurora-A inhibition decreases microt ubule shrinkage, growth rate, frequency rescue and nucleation. These results open new perspectives on the role of Aurora- A in interphase and might be worth considering in a pharmacol. perspective.

Keywords: GSK6000063A AZD1152 HQPA Aurora A kinase interphase microt ubule antitumor

Substances (4) Reactions (0) Citing (16)

357

Scutellaria barbata extract and combinations for the treatment of cancer

By: Cohen, Isaac

United States, US20090130118 A1 2009-05-21 | Language: English, Database: CAplus

An extract of Scutellaria barbata D. Don is effective in the arrest of cancer cell growth in the G1 phase, the induction of apoptosis in cancer cells and the shrinking of solid cancers. The extract may be prepared as a pharmaceutical composition for administration to mammals for the treatment of solid cancers, such as epithelial cancers. Such epithelial cancers include breast cancer and ovarian cancers. The extract is obtained from Scutellaria barbata D. Don by contacting aerial portions of a plant from the species Scutel laria barbata D. Don with an aqueous or alc. solvent.

Keywords: Scutellaria extract cancer combination treatment

PatentPak available

Substances (29) | Reactions (0) | Citing (1)

358

AZD1152 Rapidly and Negatively Affects the Growth and Survival of Human Acute Myeloid Leukemia Cells In vitro and In vivo

By: Oke, Adedayo; Pearce, Daniel; Wilkinson, Robert W.; Crafter, Claire; Odedra, Rajesh; Cavenagh, Jamie; Fitzgibbon, Jude; Lister, Andrew T.; Joel, Simon; Bonnet, Dominique

Cancer Research (2009), 69(10), 4150-4158 | Language: English, Database: CAplus and MEDLINE

Aurora kinases play a critical role in regulating mitosis and cell division, and their overexpression has been implicated in the survival and proliferation of human cancer. In this study, we report the in vitro and in vivo activities of AZD1152, a compound that has selectivity for aurora B kinase, in acute myeloid leukemia (A ML) cell lines, primary AML samples, and cord blood cells. AZD1152 exerted antiproliferative or cytotoxic effects in all cell lines studied, inhibited the phosphor ylation of histone H3 (p His H3) on Ser10 in a dose-dependent manner, and resulted in cells with >4 N DNA content. THP-1 cells treated with AZD1152 accumulated in a state of polyploidy and showed a senescent response to the drug, in contrast to the apoptotic response seen in other cell lines. Accord ingly, AZD1152 profoundly affected the growth of AML cell lines and primary AML in an in vivo xenotransplantation model. However, concentration-dependent effects on cell growth, apoptosis, and cell cycle progre ssion were also observed when human cord blood and primary lineage-neg. stem and progenitor cells were analyzed in vitro and in vivo. These data suggest that the inhibition of aurora B kinase may be a useful therapeutic strategy in the treatment of A ML and that further exploration of dosing and treatment schedules is warranted in clin. trials. [Cancer Res 2009;69(10):4150-8].

Keywords: AZD1152 antitumor prodrug aurora B kinase acute myeloid leukemia

Substances (3) | Reactions (0) | Citing (62)

359

Identification of genes that confer tumor cell resistance to the Aurora B kinase inhibitor, AZD1152

By: Guo, J.; Anderson, M. G.; Tapang, P.; Palma, J. P.; Rodriguez, L. E.; Niquette, A.; Li, J.; Bouska, J. J.; Wang, G.; Semizarov, D.; et al
Pharmacogenomics Journal (2009), 9(2), 90-102 | Language: English, Database: CAplus and MEDLINE

AZD1152 is a highly selective Aurora B kinase inhibitor currently undergoing Phase I and I I clin. evaluation in patients with acute myelogenous leukemia and advanced solid maligna ncies. We have established two AZD1152-resistant cell lines from S W620 colon and MiaPaCa pancreatic carcinoma lines, which are >100- fold resistant to the active metabolite of AZD1152, AZD1152 HQPA and interestingly, cross-resistant to the pan-Aurora kinase inhibitor, VX-680/MK0457. Using whole-genome microarray anal. and compar ative genomic hybridi zation, we were able to identify M DR1 and BCRP as the causative genes that underlie AZD1152 HQPA-resistance in these models. Furthe rmore, the upregulation of either of these genes is sufficient to render in vivo tumor growth insensitive to AZD1152. Finally, the upregu lation of M DR1 or BCRP is predictive of tumor cell sensit ivity to this agent, both in vitro and in vivo. The data provide a genetic basis for resistance to Aurora kinase inhibitors, which could be utilized to predict clin. response to therapy.

Keywords: AZD1152 antitumor resistance Aurora B kinase M DR1 BCRP

Substances (4) Reactions (0) Citing (32)

360

Human anti-VEGF antibodies and conjugates for treatment of angiogenesis conditions

By: Ramachandra, Sumant; Bishop, Robert Walter; Masat, Linda; Huang, Chao Bai; Takeuchi, Toshihiko; Kantak, Seema
World Intellectual Property Organization, WO2009055343 A2 2009-04-30 | Language: English, Database: CAplus

Disclosed herein are fully human antibodies and antigen-binding fragments thereof that specifically bind human VEGF and inhibit V EGF binding to VEGF-R1 and VEGF-R2, and therefore inhibit VEGF signaling. The antibodies and antigen-binding fragments disclosed herein may be used, for example, to treat angiogenesis and conditions associated with angiog enesis both in vivo and in vitro.

Keywords: human antibody fragment conjugate VEGF cancer diagnosis therapy

PatentPak available

Substances (346) Reactions (0) Citing (1)

361

Aurora B kinase regulates the postmitotic endoreduplication checkpoint via phosphorylation of the retinoblastoma protein at serine 780

By: Nair, Jayasree S.; Ho, Alan L.; Tse, Archie N.; Coward, Jesse; Cheema, Haider; Ambrosini, Grazia; Keen, Nicholas; Schwartz, Gary K.
Molecular Biology of the Cell (2009), 20(8), 2218-2228 | Language: English, Database: CAplus and MEDLINE

The phenotypic change characteristic of Aurora B inhibition is the induction of polypl oidy. Utilizing specific siRNA duplexes and a selective small mol. inhibitor (AZD1152) to inhibit Aurora B activity in tumor cells, we sought to elucidate the mechanism by which Aurora B inhibition results in polyploidy. Cells treated with AZD1152 progressed through mitosis with misaligned chromo somes and exited without cytokinesis and subsequently underwent endoredup lication of DNA despite activation of a p53-dependent pseudo G1 checkpoint. Concomitant with polyploid cell formation, we observed the appearance of Rb hypophosph orylation, an event that occurred independently of cyclin-dependent kinase inhibition. We went on to discover that Aurora B directly phospho rylates Rb at serine 780 both in vitro and in vivo. This novel intera ction plays a critical role in regulating the postmi totic checkpoint to prevent endoreduplication after an aberrant mitosis. Thus, we propose for the first time that Aurora B determines cellular fate after an aberrant mitosis by directly regulating the Rb tumor suppressor protein.

Keywords: Aurora B kinase Rb protein serine phosphorylation endoreduplication checkpoint

Substances (4) Reactions (0) Citing (75)

362

Inhibition of Aurora B kinase sensitizes a subset of human glioma cells to TRAIL concomitant with induction of TRAIL-R2

By: Li, J.; Anderson, M. G.; Tucker, L. A.; Shen, Y.; Glaser, K. B.; Shah, O. J.
Cell Death and Differentiation (2009), 16(3), 498-511 | Language: English, Database: CAplus and MEDLINE

Small-mol. inhibitors of the Aurora A and B kinases interfere with mitotic centrosome function and disrupt the mitotic spindle assembly checkpoint resulting in polyploidization and apoptosis of prolife rating cells. As such, several Aurora kinase inhibitors are at various stages of clin. development as anticancer agents. To identify candidate apoptosis-sensitizing genes that could be exploited in combination with Aurora kinase inhibitors in malignant glioma, we have carried out global gene expression anal. in a D54MG glioma cell derivative treated with three Aurora kinase inhibitors chosen for their distin ctive selectivities: MLN8054 (Aurora A-selective), AZD1152 (Aurora B-selective), and VX-680 (Aurora A/B). The modulation of apoptotic gene expression by p53 under these conditions was ascertained, as p53 expression can be toggled on and off in this D54 MG derivative by virtue of a stable, inducible, p53-targeting short hairpin RNA ($D54MG^{shp53}$). This anal. identified the tumor necrosis factor- related apoptosis-inducing ligand (TRAIL) death receptor, TRAIL receptor 2 (TRAIL-R2), as an apoptosis-sensitizing gene induced selectively following inhibition of Aurora B. In glioma cell lines where TRAIL-R2 was induced following polyploidization, the sensitivity, kinetics, and magnitude of T R AIL-mediated apoptosis were enhanced. Our data shed light on the apoptotic program induced during polyploi dization and suggest that TRAIL-R2 activation is a putative point of therap eutic intervention in combination with inhibitors of Aurora B.

Keywords: aurora B kinase inhibitor glioma TRAIL protein apoptosis cytotoxic

Substances (10) Reactions (0) Citing (27)

363

Inhibitors of aurora kinases

By: Pinel, S.; Barbault-Foucher, S.; Lott-Desroches, M.-C.; Astier, A.

Annales Pharmaceutiques Francaises (2009), 67(2), 69-77 | Language: French, Database: CAplus and MEDLINE

A review. Aurora kinases (A, B and C) are proteins expressed only in cells which divide actively and their increase is a factor of bad prognosis in cancer. They regulate the maturation of centrosomes, the separation and the conden sation of chromosomes, mitotic checkpoint and cytokinesis. The inhibition of aurora kinases, by powerful and selective inhibi tors, is due to the formation of abnormal cells which are eliminated by apoptosis. The purpose of this article is to present the role, the antitumor activity and the tolerability of these inhibitors. They can be administered orally or i.v., on weekly or monthly schedules. In our knowledge, twelve mols. are evaluated at the present time and will be discussed only the most advanced namely: VX-680, ZM 447439, MLN 8054, AZD 1152, PHA 739358, SU 6668 and AT 9283. The main indica tions are breast, colon, lung, pancreas and bladder cancers as well as hematol. tumors such as leukemia (ALL, AML, CML) and lymphoma. These inhibitors can be associated with other chemothe rapies. They seem well tolerated; the reported side effects are digestive disorders (diarrhea), fever, asthenia, alopecia, slumber, neutro penia, myelosuppression and disturbance of the biol. markers.

Keywords: review aurora kinase inhibitor antitumor toxicity cancer

Substances (8) Reactions (0) Citing (7)

364

The Topoisomerase I Poison CPT-11 Enhances the Effect of the Aurora B Kinase Inhibitor AZD1152 both In vitro and In vivo

By: Nair, Jayasree S.; de Stanchina, Elisa; Schwartz, Gary K.

Clinical Cancer Research (2009), 15(6), 2022-2030 | Language: English, Database: CAplus and MEDLINE

AZD1152 is an Aurora B kinase inhibitor currently in clin. trials. As the topoiso merase I poison CPT-11 induces a G_2 arrest, a mechanistic understanding of the cell cycle intera ctions between these agents may prove critical for combin ation therapy. AZD1152 was tested in vitro and in vivo with SN-38 and CPT-11 against HCT-116 cells. Inhibition of clonogenicity, induction of apoptosis, effects on polyploidy, and tumor growth were examined AZD1152 alone induced polyploidy of HCT-116 cells at low nanomolar concentrations The induction of apoptosis required prolonged exposure (48 h) and higher concent rations of drug. When SN-38 was given before or concomitantly with AZD1152, SN-38 blocked the AZD1152 effect by arresting cells in G_2 and inhibiting cells from undergoing polyploidy. With the reverse combin ation (AZD1152 followed by SN-38), there was a significant induction of polyploidy and apoptosis, even with shorter exposure (24 h) of AZD1152. In vivo, AZD1152 alone suppressed HCT-116 xenograft tumor growth in a dose-dependent manner with target inhibition of phospho H3, induction of multinu cleated giant cells, but without induction of apoptosis. In combination, both sequences in vivo (CPT->AZD, AZD->CPT, P = 0.008, AUC/d) proved superior to either single agent therapy. However, AZD->CPT still showed a greater increase in apoptosis and greater suppre ssion of tumor regrowth than CPT->AZD (P = 0.02, AUC/d). The results from these studies indicate a promising therap eutic strategy for combining AZD1152 with CPT-11, and suggest that the sequence of drug adminis tration is pivotal when an Aurora B kinase inhibitor is administered with a topoiso merase I poison.

Keywords: AZD1152 CPT11 combination chemotherapy antitumor

Substances (7) Reactions (0) Citing (31)

365

A panel of isogenic human cancer cells suggests a therapeutic approach for cancers with inactivated p53

By: Sur, Surojit; Pagliarini, Raymond; Bunz, Fred; Rago, Carlo; Diaz, Luis A. Jr.; Kinzler, Kenneth W.; Vogelstein, Bert; Papadopoulos, Nickolas
Proceedings of the National Academy of Sciences of the United States of America (2009), 106(10), 3964-3969 | Language: English, Database: CAplus and MEDLINE

Through targeted homologous recombination, we developed a panel of matched colorectal cancer cell lines that differ only with respect to their endogenous TP53 status. We then used these lines to define the genes whose expression was altered after D NA damage induced by ionizing radiation. Transcriptome analyses revealed a consistent upregu lation of polo-like kinase 1 (P LK1) as well as other genes controlling the G_2/M transition in the cells whose T P53 genes were inactivated compared with those with W T T P53 genes. This led to the hypothesis that the viability of stressed cells without W T TP53 depended on PLK1. This hypothesis was validated by demonstrating that stressed cancer cells without WT TP53 alleles were highly sensitive to P LK1 inhibitors, both in vivo and in vitro.

Keywords: colorectal cancer cell p53 PLK1 kinase

Substances (12) Reactions (0) Citing (248)

366

Structure-Based Drug Design of Novel Aurora Kinase A Inhibitors: Structural Basis for Potency and Specificity

By: Coumar, Mohane Selvaraj; Leou, Jiun-Shyang; Shukla, Paritosh; Wu, Jian-Sung; Dixit, Ajay Kumar; Lin, Wen-Hsing; Chang, Chun-Yu; Lien, Tzu-Wen; Tan, Uan-Kang; Chen, Chun-Hwa; et al
Journal of Medicinal Chemistry (2009), 52(4), 1050-1062 | Language: English, Database: CAplus and MEDLINE

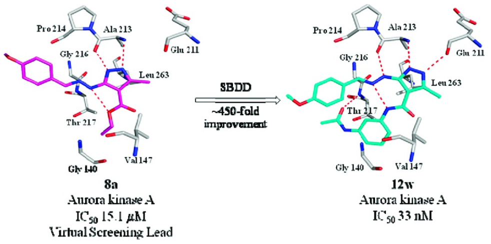

Aurora kinases have emerged as attractive targets for the design of anticancer drugs. Through structure-based virtual screening, novel pyrazole hit 8a was identified as Aurora kinase A inhibitor (IC_{50} = 15.1 μ M). X-ray cocrystal structure of 8a in complex with Aurora A protein revealed the C-4 position Et carbox ylate side chain as a possible modifi cation site for improving the potency. On the basis of this insight, bioisosteric replacement of the ester with amide linkage and changing the Et substi tuent to hydrophobic 3-acetamidophenyl ring led to the identification of 12w with a ~450-fold improved Aurora kinase A inhibition potency (I C_{50} = 33 n M), compared to 8a. Compound 12w showed selective inhibition of Aurora A kinase over Aurora B/C, which might be due to the presence of a unique H-bond interaction between the 3-acetamido group and the Aurora A noncon served Thr217 residue, which in Aurora B/C is Glu and found to sterically clash with the 3-acetamido group in modeling studies.

Keywords: structure aurora kinase A inhibitor acetamidophenyl derivative preparation

Substances (95) Reactions (90) Citing (61)

367

A specific pharmacophore model of aurora B kinase inhibitors and virtual screening studies based on it

By: Wang, Hui-Yuan; Li, Lin-Li; Cao, Zhi-Xing; Luo, Shi-Dong; Wei, Yu-Quan; Yang, Sheng-Yong
Chemical Biology & Drug Design (2009), 73(1), 115-126 | Language: English, Database: CAplus and MEDLINE

In this study, 3D-pharmacophore models of Aurora B kinase inhibitors have been developed by using Hip Hop and HypoGen modules in Catalyst software package. The best pharmacophore model, Hypo1, which has the highest correl ation coefficient (0.9911), consists of one hydrogen-bond acceptor, one hydrogen-bond donor, one hydrophobic aliphatic moiety and one ring aromatic feature. Hypo1 was validated by test set and cross-validation methods. Specificity of Hypo1 to Aurora B inhibitors was examined with the use of selective inhibitors against Aurora B and its paralogue Aurora A. The results clearly indicate that Hypo1 can differentiate selective inhibitors of Aurora B from those of Aurora A, and the ring aromatic feature likely plays some important roles for the specificity of Hypo1. Then Hypo1 was used as a 3 D query to screen several databases including Specs, N CI, Maybridge and Chinese Nature Product Database (CNPD) for identifying new inhibitors of Aurora B. The hit compounds were subseq uently subjected to filtering by Lipinski's rule of five and docking studies to refine the retrieved hits, and some compounds selected from the top ranked hits have been suggested for further exptl. assay studies.

Keywords: aurora B kinase inhibitor virtual screening pharmacophore model

Substances (70)   Reactions (0)   Citing (13)

368

Anti-insulin-like growth factor 1 receptor therapy

By: Wang, Yan; Pachter, Jonathan A.; Hailey, Judith Anne; Brams, Peter; Williams, Denise; Srinivasan, Mohan; Feingersh, Mary Diane
World Intellectual Property Organization, WO2009005673 A1 2009-01-08 | Language: English, Database: CAplus

The authors disclose the preparation and functional characterization of human antibodies to the type 1 insulin-like growth factor receptor. In one example, the growth of a human neuroblastoma was shown to be inhibited by an anti-IGF1R antibody in a xenograft model.

Keywords: insulinlike growth factor receptor antibody antitumor

PatentPak available

Substances (330)   Reactions (0)   Citing (0)

369

Discovery or AZD1152: a selective inhibitor of Aurora-B kinase with potent antitumor activity

By: Foote, Kevin M.; Mortlock, Andrew A.
Edited by Li, Rongshi; Stafford, Jeffrey A.
Kinase Inhibitor Drugs (2009), 309-332 | Language: English, Database: CAplus

A review. The Aurora kinases continue to generate significant interest as attractive drug targets in oncol. Through the work carried out at AstraZeneca, selective inhibitors of Aurora-B kinase have been profiled and have allowed a novel mechanism of action (failure of cell division) to be determined which is distinct from the classical antimitotic agents. The quinaz oline core has shown itself to be an excellent template in which to develop potent Aurora kinase inhibitors while an understanding of the binding mode has allowed rapid optimization of the series with respect to potency and select ivity for the Aurora kinases.

Keywords: review aurora B kinase inhibitor AZD1152 antitumor

Substances (4) Reactions (0) Citing (9)

370

Phenotypic fingerprinting of small molecule cell cycle kinase inhibitors for drug discovery

By: Low, Jonathan; Chakravartty, Arunava; Blosser, Wayne; Dowless, Michele; Chalfant, Christopher; Bragger, Patty; Stancato, Louis
Current Chemical Genomics (2009), 3, 13-21 | Language: English, Database: CAplus and MEDLINE

Phenotypic drug discovery, primarily abandoned in the 1980's in favor of targeted approaches to drug development, is once again demonstrating its value when used in conjun ction with new technol ogies. Phenotypic discovery has been brought back to the fore mainly due to recent advances in the field of high content imaging (HCI). HCI elucidates cellular responses using a combin ation of immunofluorescent assays and computer anal. which increase both the sensit ivity and throughput of phenotypic assays. Although HCI data characterize cellular responses in individual cells, these data are usually analyzed as an aggregate of the treated population and are unable to discern differentially responsive sub-populations. A collection of 44 kinase inhibitors affecting cell cycle and apoptosis were characterized with a number of univar iate, bivariate, and multiv ariate subpopulation analyses demonst rating that each level of complexity adds addnl. inform ation about the treated popula tions and often disting uishes between compounds with seemingly similar mechanisms of action. Finally, these subpopulation data were used to charac terize compounds as they relate in chem. space.

Keywords: cell cycle kinase inhibitor cervical colon cancer phenotype fingerprinting

Substances (25) Reactions (0) Citing (16)

371

Discovery and development of novel furano-pyrimidine analogs as Aurora kinase inhibitors

By: Hsieh, Hsing-Pang; Coumar, Mohane S.; Lin, Chen-Wei; Reddy, Gadarla Randheer; Tsai, Ming-Tsung; Lin, Wen-Hsing; Hsu, Tsu-An; Wu, Su-Ying
Abstracts of Papers, 237th ACS National Meeting, Salt Lake City, UT, United States, March 22-26, 2009 (2009), MEDI-221 | Language: English, Database: CAplus

Inhibition of Aurora kinase, a member of serine/threonine kinase involved in the regulation of cell division is emerging as a new mol. targeted cancer treatment option. Three isoforms of Aurora kinase, A, B and C are known. Both Aurora A and B are over expressed in many human cancers and are linked to chromosome instability, oncogenic transformation, tumor progression and development of chemoresistance. Inhibitors of Aurora kinase such as MK-0457/VX-680 (**1**), PHA-739358 (**2**), MLN8054 (**3**) and AZ D1152 (**4**), in various animal models had shown tumor regression and are now in different stages of clin. development for various cancers. Based on the current success of Aurora kinase inhibitors in the development of kinase-based cancer therapy, we have initiated an in house compound library screening program for the identification of novel Aurora kinase inhibitors. This led to the identification of furano-pyrimidine compound (**5**) BPR1K224 as an Aurora kinase A inhibitor with an IC_{50} of ~400 nM. Over 300 analogs of the lead compounds were rapidly synthesized and screened for Aurora kinase activity to identify 2nd generation lead compounds X-ray co-crystal structure of the lead compounds in complex with Aurora A protein revealed that the back pocket of Aurora protein is unoccupied. Based on this insight, rationale modification of one the 2nd generation lead had led to the identification of potent Aurora kinase A inhibitor BPR1K432 (IC_{50} ~50 nM), which possessed anti-proliferative activity in HCT-116 cell line (IC_{50} ~400 nM). Addnl. structural refinement led to the synthesis of several potent analogs.

Substances (0) Reactions (0) Citing (0)

372

Preparation of peptide prodrugs modified with a 1,2,3,4-cyclobutanetetracarboxylic acid derived moiety useful in treatment and diagnosis of tumors and inflammatory diseases

By: Matthieu, Michel; Dubois, Vincent; Tranchant, Isabelle; Kearsey, Jonathan
World Intellectual Property Organization, WO2008120098 A2 2008-10-09 | Language: English, Database: CAplus

The invention is related to the preparation of peptide prodrugs **I** [L^1, L^2 = independently a covalent bond or a linking moiety, the linking moiety should include at least two organic functional groups, one organic functional group that can bind to the 1,2,3,4-cyclobutanetetracarboxylic acid and a second organic functional group that allows binding to the oligopeptide moiety (L^1) or one functional group that can bind to the oligopeptide moiety and a second organic functional group that allows binding to D (L2) ; X = $(CH_2)_n$; X' = $(CH_2)_m$; X'' = $(CH_2)_p$; X''' = $(CH_2)_q$; n, m, p, q = independently 0-10; Y = cleavable oligopeptide moiety by at least one specific and/or selective peptidase that is present in the extracellular environment of target cells; D = therapeutic agent or marker] which improve the therapeutic index and the solubility of the therapeutic agent and are intended for the treatment and/or diagnosis of tumors and/or inflammatory reactions. Thus, tetraAcid-ALAL-doxorubicin **II** was prepared and showed stability in human plasma after 2 h incubation. **II** was evaluated for its in vivo efficacy in the LS 174T tumor model and in vitro reactivation into metabolites (leucyl-doxorubicin and doxorubicin) by tumor homogenates. A coumarin analog **III** was prepared for use as a marker for diagnosis purposes (no data).

Keywords: doxorubicin peptide prodrug cyclobutanetetracarboxylic acid modification preparation antitumor antiinflammatory; mitomycin C peptide prodrug cyclobutanetetracarboxylic acid modification preparation antitumor; paclitaxel peptide prodrug cyclobutanetetracarboxylic acid modification preparation cancer inflammation; coumarin peptide cyclobutanetetracarboxylic acid modification preparation marker tumor

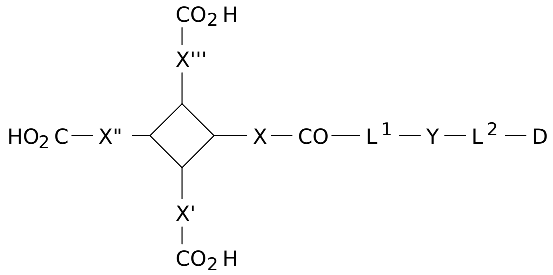

I

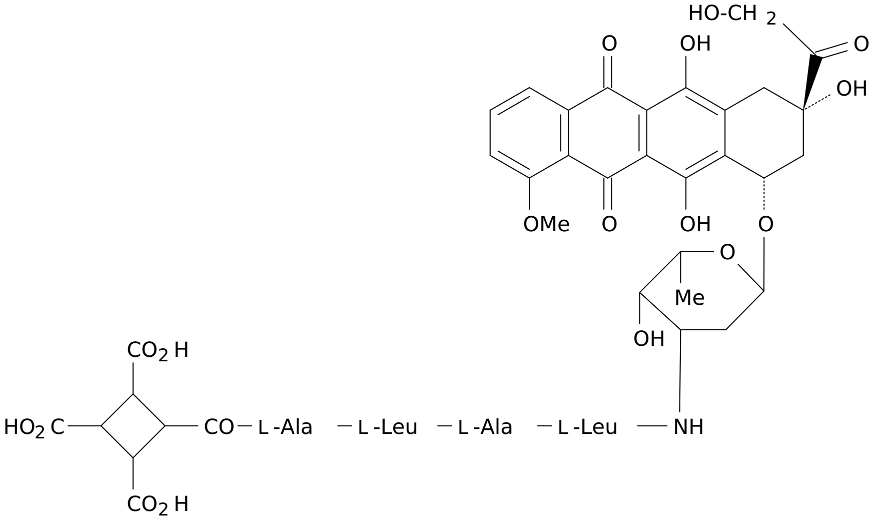

II

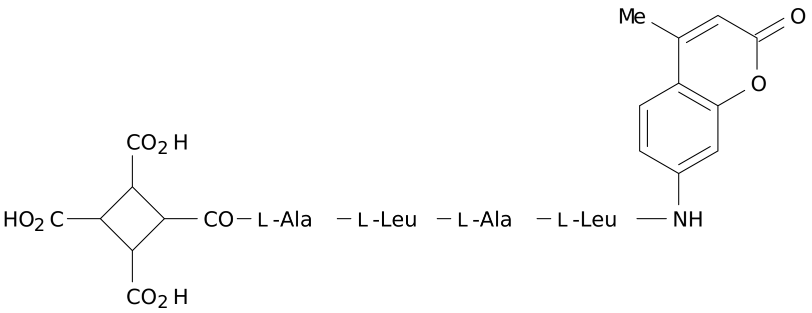

III

PatentPak available

Substances (316) Reactions (255) Citing (0)

373

Preparation of peptide prodrugs modified with a 1,2,3,4-cyclobutanetetracarboxylic acid derived moiety useful in treatment and diagnosis of tumors and inflammatory diseases

By: Matthieu, Michel; Dubois, Vincent; Tranchant, Isabelle; Kearsey, Jonathan

European Patent Organization, EP1977765 A1 2008-10-08 | Language: English, Database: CAplus

The invention is related to the preparation of peptide prodrugs **I** [L^1, L^2 = independently a covalent bond or a linking moiety; X = $(CH_2)_n$; X' = $(CH_2)_m$; X'' = $(CH_2)_p$; X''' = $(CH_2)_q$; n, m, p, q = independently 0-10; Y = cleavable oligopeptide moiety; D = therapeutic agent or marker] which improve the therapeutic index and the solubility of the therapeutic agent and are intended for the treatment and/or diagnosis of tumors and/or inflammatory reactions. Thus, tetraAcid-ALAL-doxorubicin **II** was prepared and showed stability in human plasma after 2 h incubation. **II** was evaluated for its in vivo efficacy in the LS 174T tumor model.

Keywords: peptide prodrug cyclobutanetetracarboxylic acid modification preparation cancer inflammation; doxorubicin peptide prodrug cyclobutanetetracarboxylic acid modification preparation antitumor antiinflammatory; mitomycin C peptide prodrug cyclobutanetetracarboxylic acid modification preparation antitumor; paclitaxel peptide prodrug cyclobutanetetracarboxylic acid modification preparation cancer inflammation

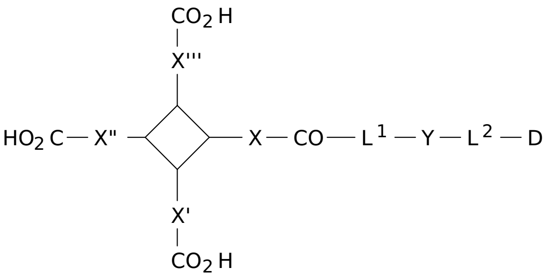

I

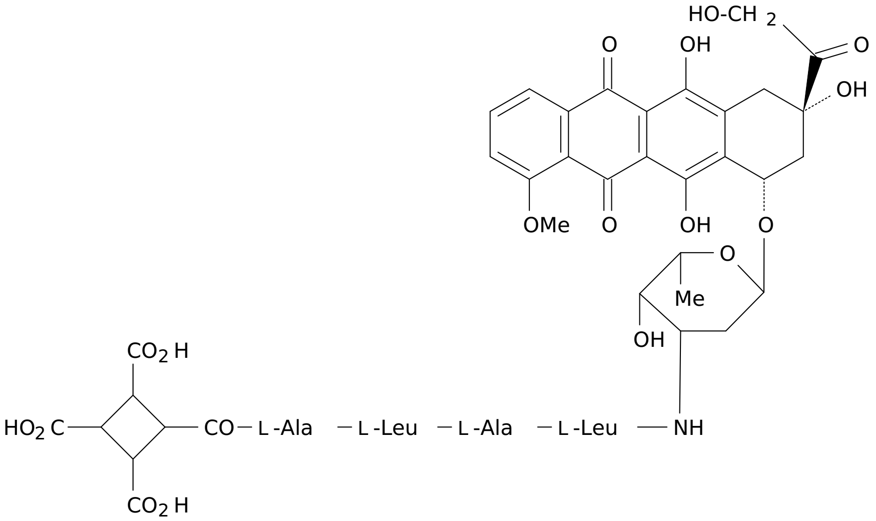

II

PatentPak available

Substances (150) Reactions (13) Citing (0)

374

Methods of treating cancer using IGF1R inhibitors

By: Wang, Yan; Zong, Chen; Seidel-Dugan, Cynthia; Wang, Yaolin; Yao, Siu-Long; Lu, Brian Der-Hua; Ladha, Mohamed H.
World Intellectual Property Organization, WO2008076278 A2 2008-06-26 | Language: English, Database: CAplus

The present invention provides IGF1R inhibitors and combin ations thereof that are effective at treating or preventing cancer. More specifically the IGF1R inhibitors are pyrrolo[2,3-d]pyrimidine derivatives or antibodies. The IGF1R inhibitors can be used in combin ation with other anticancer therapies, antiemetic agents, antianemic agents, or antimuc ositis agents.

Keywords: treatment cancer IGF1R inhibitor; antibody treatment cancer I GF1R inhibitor

PatentPak available

Substances (411) | Reactions (0) | Citing (3)

375

Effects of the aurora kinase inhibitors AZD1152-HQPA and ZM447439 on growth arrest and polyploidy in acute myeloid leukemia cell lines and primary blasts

By: Walsby, Elisabeth; Walsh, Val; Pepper, Chris; Burnett, Alan; Mills, Ken
Haematologica (2008), 93(5), 662-669 | Language: English, Database: CAplus and MEDLINE

Background: Aurora kinases play an essential role in the orchest ration of chromosome separation and cytoki nesis during mitosis. Small-mol. inhibition of the aurora kinases has been shown to result in inhibition of cell division, phosphor ylation of histone H3 and the induction of apoptosis in a number of cell systems. These characteristics have led aurora kinase inhibitors to be considered as potential therapeutic agents. Design and Methods: Aurora kinase gene expression profiles were assessed in 101 samples from patients with acute myeloid leukemia. Subsequently, aurora kinase inhibitors were invest igated for their in vitro effects on cell viability, histone H3 phosphorylation, cell cycle and morphol. in acute myeloid leukemia cell lines and primary acute myeloid leukemia samples. Results: The aurora kinase inhibitors AZD1152-HQPA and ZM447439 induced growth arrest and the accumu lation of hyperploid cells in acute myeloid leukemia cell lines and primary acute myeloid leukemia cultures. Furthe rmore, both agents inhibited histone H3 phosphorylation and this preceded perturb ations in cell cycle and the induction of apoptosis. Single cell cloning assays were performed on diploid and polyploid cells to investigate their colony-forming capacities. Although the polyploid cells showed a reduced capacity for colony formation when compared with their diploid counterparts, they were consistently able to form colonies. Conclusions: AZD1152-HQPA- and ZM447439 are effective apoptosis-inducing agents in acute myeloid leukemia cell lines and primary acute myeloid leukemia cultures. However, their propensity to induce polyploidy does not inevitably result in apoptosis.

Keywords: acute myeloid leukemia aurora kinase inhibitor phosphorylation apoptosis cyctoto xicity

Substances (3) | Reactions (0) | Citing (79)

376

Enhancement of radiation response in p53-deficient cancer cells by the Aurora-B kinase inhibitor AZD1152

By: Tao, Y.; Zhang, P.; Girdler, F.; Frascogna, V.; Castedo, M.; Bourhis, J.; Kroemer, G.; Deutsch, E.
Oncogene (2008), 27(23), 3244-3255 | Language: English, Database: CAplus and MEDLINE

Overexpression of the Aurora-B kinase correlates with oncogenic transfo rmation and poor prognosis. We evaluated the effects of the bona fide Aurora-B kinase inhibitor AZD1152 on tumor responses to ionizing radiation (I R). When $p53^{wt}$ HCT116 and A549 cells were pretreated with AZD1152-HQPA prior to IR, additive effects were observed Interes tingly, more pronounced tumoricidal effects were observed in p53-deficient HCT116 and HT29 cells, as well as A549 cells treated with the p53 inhibitor cyclic pifithrin- α. In vivo studies on xenografted mice confirmed enhanced tumor growth delay after the combin ation of IR plus AZD1152-IR as compared to IR alone. Again, this effect was more pronounced with $p53^{-/-}$ HCT116 and p53-mutant xenografts. The AZD1152-mediated radiosensitization was mimicked by knockdown of Aurora- B with a short interference RNA or by inhibition of Aurora- B by transf ection with an inducible kinase-dead Aurora-B. The radiosensitizing effect of AZD1152 was lost in $CHK2^{-/-}$ and $14\text{-}3\text{-}3^{-/-}$ HCT116 cells. Altogether, these data indicate that AZD1152 can radiose nsitize tumor cell lines in vitro and in vivo, the fact that these effects are exacerbated in p53-deficient cancer cells is of potential interest for further clin. develo pment.

Keywords: radiotherapy p53 cancer Aurora B kinase inhibitor AZD1152 radiosensitizer

Substances (3) Reactions (0) Citing (76)

377

Preclinical evaluation of M30 and M65 ELISAs as biomarkers of drug induced tumor cell death and antitumor activity

By: Cummings, Jeffrey; Hodgkinson, Cassandra; Odedra, Rajesh; Sini, Patrizia; Heaton, Simon P.; Mundt, Kirsten E.; Ward, Tim H.; Wilkinson, Robert W.; Growcott, Jim; Hughes, Andrew; et al
Molecular Cancer Therapeutics (2008), 7(3), 455-463 | Language: English, Database: CAplus and MEDLINE

M30 and M65 are ELISAs that detect different circul ating forms of cytokeratin 18. Using the aurora kinase inhibitor AZD1152 and the SW620 human colon cancer xenograft, experi ments were conducted to qualify preclinically both assays as serol. biomarkers of cell death. Using two different apoptotic markers, the kinetics of cell death induced by AZD1152 was first character ized in vitro in three different cell lines and shown to peak 5 to 7 days after drug addition Treatment of non-tumor-bearing rats with AZD1152 (25 mg/kg) produced no alterations in circulating baseline values of M30 and M65 antigens. In treated, tumor- bearing animals, M30 detected a 2- to 3-fold ($P < 0.05$) increase in plasma antigen levels by day 5 compared with controls. This correlated to a 3- fold increase in the number of apoptotic cells detected on day 5 in SW620 xenografts using immunohi stochem. By contrast, M65 did not detect a drug-induced increase in circul ating antigen levels at day 5. However, M65 plasma levels correlated to changes in tumor growth in control animals ($r^2 = 0.93$; $P < 0.01$) and also followed the magnitude of the temporal effect of AZD1152 on tumor growth. An intermediate but active dose of AZD1152 (12.5 mg/kg) produced a less signif icant increase in M30 plasma levels at day 5. It was also confirmed that the plasma profiles of M30 and M65 mirrored closely those measured in whole tumor lyzates. We conclude that M30 is a pharmacodynamic biomarker of AZD1152-induced apoptosis in the SW620 xenograft model, whereas M65 is a biomarker of therapeutic response.

Keywords: antitumor AZD1152 ELISA biomarker

Substances (2) Reactions (0) Citing (53)

378

The selective Aurora B kinase inhibitor AZD1152 is a potential new treatment for multiple myeloma

By: Evans, Robert P.; Naber, Claudia; Steffler, Tara; Checkland, Tamara; Maxwell, Christopher A.; Keats, Jonathan J.; Belch, Andrew R.; Pilarski, Linda M.; Lai, Raymond; Reiman, Tony
British Journal of Haematology (2008), 140(3), 295-302 | Language: English, Database: CAplus and MEDLINE

Aurora kinases are potential targets for cancer therapy. Previous studies have validated Aurora kinase A as a therapeutic target in multiple myeloma (MM), and have demonstrated in vitro anti-myeloma effects of small mol. Aurora kinase inhibitors that inhibit both Aurora A and B. This study demonstrated that Aurora B kinase was strongly expressed in myeloma cell lines and primary plasma cells. The selective Aurora B inhibitor AZD1152-induced apoptotic death in myeloma cell lines at nanomolar concentr ations, with a cell cycle phenotype consistent with that reported previously for Aurora B inhibition. In some cases, AZD1152 in combination with dexamethasone showed increased anti-myeloma activity compared with the use of either agent alone. AZD1152 was active against sorted CD138+ BM plasma cells from myeloma patients but also, as expected, was toxic to C D138- marrow cells from the same patients. In a murine myeloma xenograft model, AZD1152-inhibited tumor growth at well-tolerated doses and induced cell death in established tumors, with associated mild, transient leucop enia. AZD1152 shows promise in these preclin. studies as a novel treatment for MM.

Keywords: antimyeloma Aurora kinase B AZD1152 apoptosis dexamethasone multiple myeloma

Substances (3) Reactions (0) Citing (50)

379

3-Benzoylamino-1H-pyrazole-4-carboxamides as CDK kinase inhibitors, and their preparation, pharmaceutical combinations and use in the treatment of proliferative diseases

By: Lyons, John Francis; Squires, Matthew Simon; Thompson, Neil Thomas; Gallagher, Neil James; Curry, Jayne Elizabeth
World Intellectual Property Organization, WO2008007113 A2 2008-01-17 | Language: English, Database: CAplus

The invention provides a combination comprising an ancillary compound and a compound having the formula **I**: or salts or tautomers or N-oxides or solvates thereof/. Compounds of formula **I** wherein X is 5- to 6- membered (hetero/carbo)cyclic ring, amino, acylamino,sulfonylamino, etc.; Y is a bond and C_{1-3} alkylene; R^2 is H, halo, C_{1-4} alkoxy, (un)substituted C_{1-4} hydrocarbyl; R^3 is H, 3- to 12-membered (hetero/carbo)cyclic group; and their salts, tautomers, N- oxides and solvates thereof, are claimed. Example compound **II**•MsOH was prepared by esterification of 4-nitropyrazole-3-carboxylic acid; the resulting 4-nitropyrazole-3-carboxylic acid Me ester underwent hydrogenation to give 4-aminopyrazole-3-carboxylic acid Me ester, which underwent amidation with 2, 6-dichlorobenzoyl chloride to give 4-(2,6-dichlorobenzoylamino)pyrazole-3-carboxylic acid Me ester, which underwent hydrolysis to give 4-(2,6-dichlorobenzoylamino)pyrazole-3-carboxylic acid, which underwent chlorination to give the corresponding acid chloride, which underwent amidation with 4-amino-1-Boc-piperidine to give 1-Boc-piperidin-4-yl 4-(2,6-dichlorobenzoylamino)pyrazole-3-carboxamide, which underwent hydrolysis to give compound **II**•MsOH. All the invention compounds were evaluated for their C DK kinase inhibitory activity (some data given).

Keywords: benzoylamino pyrazolecarboxamide preparation CDK kinase inhibitor treatment prolife rative disease

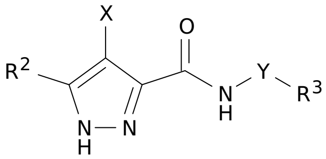

I

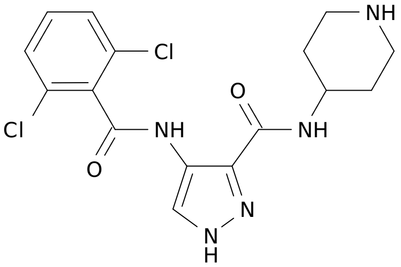

II

PatentPak available

Substances (298) Reactions (279) Citing (1)

380

Blood levels of insulin-like growth factor-binding protein 2 as a marker for monitoring the effectiveness of inhibitors of insulin-like growth factor I receptors in cancer therapy

By: Wang, Yan
World Intellectual Property Organization, WO2008005469 A2 2008-01-10 | Language: English, Database: CAplus

The present invention provides method for quickly and conveniently determining if a given treatment regimen of insulin- like growth factor I receptor (IGF1R) inhibitor is sufficient, e.g., to saturate IGF1 R receptors in the body of a subject. Blood levels of insulin- like growth factor-binding protein 2 (IGFBP2) are shown to be strongly correlated with the effectiveness of IGF1R receptor therapy. Several clin. relevant determinations may be made based on this point, including, for example, whether the dosage of the regimen is sufficient or should be increased. The relationship is demonstrated using animal xenograft models of neuroblastoma. Treatment with monoclonal antibodies to IGFR1 lowered the blood levels of IGFBP2. The level of IGFBP2 correlated with the tumor size.

Keywords: IGFBP2 marker cancer therapy IGF1 receptor inhibitor

PatentPak available

Substances (408) | Reactions (0) | Citing (2)

381

Readily activated and highly soluble pyrazoloquinazoline phosphate derivatives with potent and durable antitumor activity

By: Foote, Kevin M.
Abstracts of Papers, 235th ACS National Meeting, New Orleans, LA, United States, April 6-10, 2008 (2008), MEDI-198 | Language: English, Database: CAplus

The Aurora kinases have been shown to play critical roles in coordinating cell progression through mitosis by controlling chromosome segregation and cytokinesis. The irregular expression of Aurora A and B kinases have been implicated in tumor progression; therefore inhibitors of these proteins may have utility in the treatment of cancer. Several Aurora kinase inhibitors have shown potent anti-tumor activity in pre-clin. models and entered clin. evaluation. AZD1152 is a novel pyrazoloquinazoline-dihydrogen phosphate derivative with striking solubility (up to 25 mg/m L) in simple pH-adjusted aqueous vehicles making it suitable for parenteral administration. AZD1152 is readily converted to the active species (AZD1152HQPA), which is a highly selective inhibitor of Aurora B kinase. In human cancer xenograft models AZD1152 causes pharmacodynamic changes that result in durable anti-tumor growth inhibition at well-tolerated doses. AZD1152 has the potential for activity in a wide range of human tumors and is currently in Phase I/II clin. trials.

Substances (0) | Reactions (0) | Citing (0)

382

Emerging role of Aurora kinase inhibitors in chronic myeloid leukemia

By: Alvarado, Yesid; Cortes, Jorge E.
Clinical Leukemia (2007), 1(6), 325-330 | Language: English, Database: CAplus

A review. Resistance to imatinib and second-generation tyrosine kinase inhibitors is an ongoing problem most frequently mediated through mutations of the Bcr-Abl kinase domain. One mutation that affects respons iveness to all current available agents is T315I. Aurora proteins belong to a small family of serine/threonine kinases that are essential for proliferating cells and have been identified as key regulators of different steps in mitosis and meiosis, ranging from the formation of the mitotic spindle up to cytoki nesis. Unexpectedly, Aurora kinase inhibitors have been found to have activity against the T315 I bcr-abl mutation, and some of them might rise as important therapeutic options. The common mechanism of action for protein kinase inhibition is compet ition with ATP for the active site-binding pocket, which is very similar among the protein kinases, and this could explain the cross- reacti vity. Herein, we discuss the basics of imatinib resistance develo pment and Aurora kinase biol., and describe a selected group of Aurora kinase inhibitors with potential activity in this patient population.

Keywords: review chronic myeloid leukemia anticancer Aurora kinase inhibitor imatinib

Substances (7) Reactions (0) Citing (3)

383

Process for the preparation of pyrazolylaminoquinazoline derivatives comprising a phosphate group

By: Pittam, John David; Sependa, George Joseph
World Intellectual Property Organization, WO2007132210 A1 2007-11-22 | Language: English, Database: CAplus

The invention relates to a new process useful in the preparation of pharmaceutical compounds such as 2-{ethyl[3-({4-[(5-{2-[(3-fluorophenyl)amino]-2-oxoethyl}-1H-pyrazol-3-yl)amino]quinazolin-7-yl}oxy)propyl]amino}ethyl dihydrogen phosphate (AZD1152) and intermediates used therein.

Keywords: pyrazolyl amino quinazoline phosphate preparation; AZD1152 intermediate preparation; fluorophenyl amino oxoethyl pyrazolylamino quinazolinyloxypropyl dihydrogen phosphate AZD1152 intermediate

PatentPak available

Substances (16) Reactions (45) Citing (0)

384

Maleate co-crystal of AZD 1152 for dosage forms for treatment of hyperproliferative diseases

By: Sependa, George Joseph; Storey, Richard

World Intellectual Property Organization, WO2007132227 A1 2007-11-22 | Language: English, Database: CAplus

The present invention relates to a novel co-crystal form of 2-{ethyl[3-({4-[(5-{2-[(3-fluorophenyl)amino]-2-oxo-ethyl}-1H-pyrazol-3-yl) amino]quinazolin-7-yl}oxy)propyl]amino} Et dihydrogen phosphate (AZD 1152), an aurora kinase inhibitor useful in the treatment of hyperproliferative diseases, such as cancer. More specifi cally, the invention relates to a maleate co- crystal of AZD 1152, to a process for its preparation, its use in the manufac turing of a medicament for the treatment of hyperprol iferative diseases, and to methods of treating hyperproliferative diseases by adminis tering a therapeutically effective amount of a maleate co- crystal of AZD 1152. A particular crysta lline form of a maleate co- crystal of AZD 1152 is also described. Thus, crude AZD 1152 (preparation given, estimated at 7.44 g @ 100%, 11.61 mM) was added to DMSO (36 mL) and left at ambient temper ature to produce a pale brown solution To this solution was added a solution of maleic acid (1.76 g, 15.16 mM, 1.31 mol equivalent) in MeOH (36 mL) and the mixture left to stand overnight at ambient temperature Next day an aliquot of the clear solution was transf erred to a vial, scratched and left sealed for several hours. A deposit of white solid formed and this was transferred to the flask and left to stir. Gradually the solution turned turbid and solid deposited. The slurry was left to settle for several days and finally filtered. The cake was washed with a 1:1 mixture of DMSO/MeOH, slurried in situ with MeOH and then dried in vacuo. NMR confirmed the solid to be the maleate co-crystal of AZD1152 (yield of about 78.7%).

Keywords: AZD1152 maleate crystal form prepar ation dosage form; aurora kinase inhibitor AZD1152 maleate hyperprol iferative disease

PatentPak available

Substances (22) Reactions (30) Citing (1)

385

Quinazolin-4-ylaminopyrazolecarboxamides as aurora kinase inhibitors useful in bombination therapy for the treatment of cancer and their preparation

By: Keen, Nicholas John

World Intellectual Property Organization, WO2007132215 A1 2007-11-22 | Language: English, Database: CAplus

A combination comprising an aurora kinase inhibitor and an efflux transp orter inhibitor wherein the aurora kinase inhibitor is a compound of formula **I** or pharmaceutically acceptable salt thereof for use in the treatment of hyperprol iferative diseases such as cancer. Compounds of formula **I** wherein n is 0, 1, 2 and 3; R^1 is C_{1-4} hydroxyalkyl and C_{1-4} phosphonooxyalkyl; R^2 is H, C_{1-4} (hydroxy)alkyl, C_{1-4} alkoxy-C_{1-4} alkyl, and heterocyclyl; R^1R^2 together with nitrogen form a (un)substituted 4- to 6-membered hetero cyclic ring; R^3 is H and C_{1-4} alkoxy; R^4, R^6 and R^6 are independently H and C_{1-4} alkyl; R^5 is (un)substituted aryl; and their pharmace utically acceptable salts thereof. Example compound **II** was prepared by a multistep procedure (procedure given) . All the invention compounds were evaluated for their Aurora kinase inhibitory activity (some data given).

Keywords: quinazolinyl pyrazolylacetamide preparation aurora kinase inhibitor treatment cancer

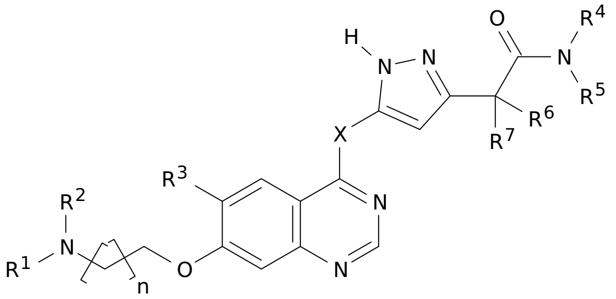

I

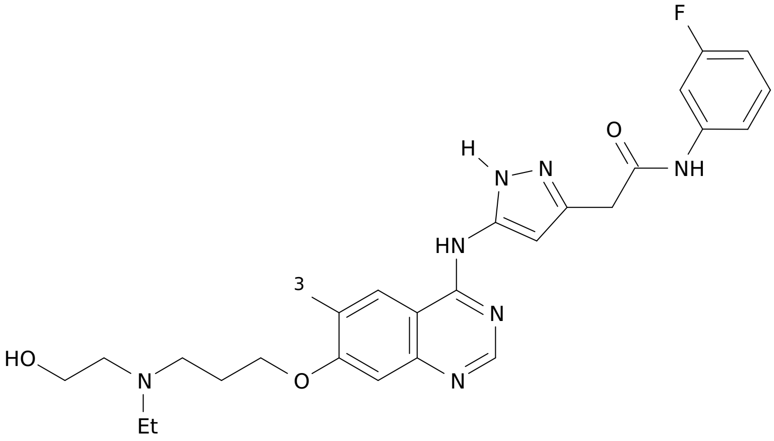

II

PatentPak available

Substances (65) Reactions (58) Citing (2)

386

Pharmaceutical combinations of diazole derivatives for cancer treatment and their preparation

By: Squires, Matthew Simon
World Intellectual Property Organization, WO2007129062 A1 2007-11-15 | Language: English, Database: CAplus

The invention provides a combination comprising (or consisting essentially of) an ancillary compound and a compound of the formula **I**, or salts, tautomers, solvates and N-oxides thereof. The combinations have activity as inhibitors of CDK kinases and inhibit the proliferation of cancer cells. Compounds of formula **I** wherein, R^1 is 2,6-dichlorophenyl; R^{2a} and R^{2b} are both H; R^3 is C_{1-4} alkyl-SO_2-piperidinyl; and their salts, tautomers, solvates, and N-oxides thereof, are claimed. Example compound **II** was prepared by methylation of 4-nitropyrazole-3-carboxylic acid; the resulting 4-nitropyrazole-3-carboxylic acid Me ester underwent hydrogenation to give 4-aminopyrazole-3-carboxylic acid Me ester, which underwent acylation with 2,6-dichlorobenzoyl chloride followed by hydrolysis to give 4-(2,6-dichlorobenzoylamino)-1H-pyrazole-3-carboxylic acid, which underwent amidation with 4-amino-1-Boc-piperidine, to give 4-[[4-(2,6-dichlorobenzoylamino)-1H-pyrazole-3-carbonyl]amino]piperidine-1-carboxylic acid tert-Bu ester, which underwent hydrolysis to give 4-(2,6-dichlorobenzoylamino)-1H-pyrazole-3-carboxylic acid piperidin-4-yl amide hydrochloride, which underwent sulfonylation with methanesulfonyl chloride to give compound **II**. The crystal structure of compound **II** was also determined The invention compounds were evaluated for their CDK kinase inhibitory activity (some data given).

Keywords: pyrazole derivative preparation CDK kinase inhibitor treatment cancer

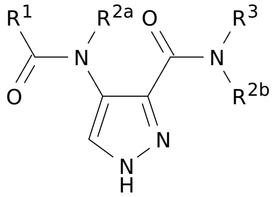

I

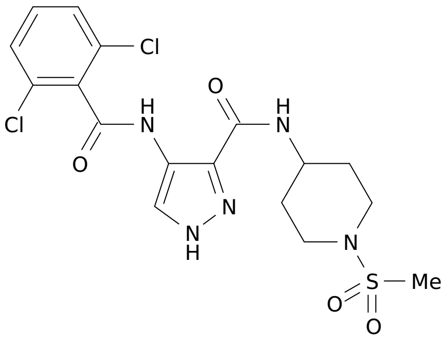

II

PatentPak available

Substances (143) Reactions (171) Citing (3)

387

AZD1152, a novel and selective aurora B kinase inhibitor, induces growth arrest, apoptosis, and sensitization for tubulin depolymerizing agent or topoisomerase II inhibitor in human acute leukemia cells in vitro and in vivo

By: Yang, Jing; Ikezoe, Takayuki; Nishioka, Chie; Tasaka, Taizo; Taniguchi, Ayuko; Kuwayama, Yoshio; Komatsu, Naoki; Bandobashi, Kentaro; Togitani, Kazuto; Koeffler, H. Phillip; et al
Blood (2007), 110(6), 2034-2040 | Language: English, Database: CAplus and MEDLINE

Aurora kinases play an important role in chromosome alignment, segregation, and cytokinesis during mitosis. We have recently shown that hematopoietic malignant cells including those from acute myeloid leukemia (AML) and acute lymphoblastic leukemia (A LL) aberrantly expressed Aurora A and B kinases, and Z M447439, a potent inhibitor of Aurora kinases, effectively induced growth arrest and apoptosis of a variety of leukemia cells. The present study explored the effect of AZD1152, a highly selective inhibitor of Aurora B kinase, on various types of human leukemia cells. AZD1152 inhibited the proliferation of AML lines (HL-60, NB4, MOLM13), ALL line (PALL-2), biphenotypic leukemia (MV4-11), acute eosinophilic leukemia (EOL-1), and the blast crisis of chronic myeloid leukemia K562 cells with an IC50 ranging from 3 nM to 40 nM, as measured by thymidine uptake on day 2 of culture. These cells had 4N/8N DNA content followed by apoptosis, as measured by cell- cycle anal. and annexin V staining, resp. Of note, AZD1152 synergistically enhanced the antiproliferative activity of vincristine, a tubulin depolymerizing agent, and daunorubicin, a topoiso merase II inhibitor, against the MOLM13 and PALL-2 cells in vitro. Furthermore, AZD1152 potentiated the action of vincristine and daunorubicin in a MOLM13 murine xenograft model. Taken together, AZD1152 is a promising new agent for treatment of indivi duals with leukemia. The combined administration of AZD1152 and conventional chemotherapeutic agent to patients with leukemia warrants further investigation.

Keywords: AZD1152 aurora B kinase inhibitor vincristine daunorubicin synergism leukemia

Substances (7) | Reactions (0) | Citing (221)

388

AZD1152, a Selective Inhibitor of Aurora B Kinase, Inhibits Human Tumor Xenograft Growth by Inducing Apoptosis

By: Wilkinson, Robert W.; Odedra, Rajesh; Heaton, Simon P.; Wedge, Stephen R.; Keen, Nicholas J.; Crafter, Claire; Foster, John R.; Brady, Madeleine C.; Bigley, Alison; Brown, Elaine; et al
Clinical Cancer Research (2007), 13(12), 3682-3688 | Language: English, Database: CAplus and MEDLINE

PURPOSE: In the current study, we examined the in vivo effects of AZD1152, a novel and specific inhibitor of Aurora kinase activity (with selectivity for Aurora B). Exptl. DESIGN: The pharmacodynamic effects and efficacy of AZD1152 were determined in a panel of human tumor xenograft models. AZD1152 was dosed via several parenteral (s.c. osmotic mini-pump, i.p., and i.v.) routes. RESULTS: AZD1152 potently inhibited the growth of human colon, lung, and hematol. tumor xenografts (mean tumor growth inhibition range, 55% to ≥100%; $P < 0.05$) in immunodeficient mice. Detailed pharmacodynamic anal. in colorectal SW620 tumor-bearing athymic rats treated i.v. with AZD1152 revealed a temporal sequence of phenotypic events in tumors: transient suppre ssion of histone H3 phosphorylation followed by accumulation of 4N DNA in cells (2.4-fold higher compared with controls) and then an increased proportion of polyploid cells (>4N DNA, 2.3-fold higher compared with controls). Histol. anal. showed aberrant cell division that was concurrent with an increase in apoptosis in AZD1152-treated tumors. Bone marrow analyses revealed transient myelosup pression with the drug that was fully reversible following cessation of AZD1152 treatment. CONCLUSIONS: These data suggest that selective targeting of Aurora B kinase may be a promising therapeutic approach for the treatment of a range of maligna ncies. In addition to the suppression of histone H3 phosphorylation, determination of tumor cell polyploidy and apoptosis may be useful biomarkers for this class of therapeutic agent. AZD1152 is currently in phase I trials.

Keywords: AZD1152 Aurora B kinase inhibitor antitumor tumor growth apoptosis

Substances (3) | Reactions (0) | Citing (303)

389

Anti-angiogenic activity of 2-methoxyestradiol in combination with anti-cancer agents

By: Plum, Stacy M.; Strawn, Steven J.; Lavallee, Theresa M.; Sidor, Carolyn F.; Fogler, William E.; Treston, Anthony M.
World Intellectual Property Organization, WO2007059111 A2 2007-05-24 | Language: English, Database: CAplus

The present invention relates generally to methods and compositions of treating disease characterized by abnormal cell prolife ration and/or abnormal or undesirable angiogenesis by administering antiangiogenic agents in combination with chemotherapeutic agents. More specifically, the present invention relates to a methods and compositions of treating diseases characterized by abnormal cell proliferation and/or abnormal or undesirable angiogenesis by administering 2-methoxyestradiol, in combination with chemotherapeutic agents.

Keywords: antiangiogenic agent methoxyestradiol combination therapy anticancer agent

PatentPak available

Substances (195) Reactions (0) Citing (2)

390

Discovery, Synthesis, and in Vivo Activity of a New Class of Pyrazolylamino Quinazolines as Selective Inhibitors of Aurora B Kinase

By: Mortlock, Andrew A.; Foote, Kevin M.; Heron, Nicola M.; Jung, Frederic H.; Pasquet, Georges; Lohmann, Jean-Jacques M.; Warin, Nicolas; Renaud, Fabrice; De Savi, Chris; Roberts, Nicola J.; et al
Journal of Medicinal Chemistry (2007), 50(9), 2213-2224 | Language: English, Database: CAplus and MEDLINE

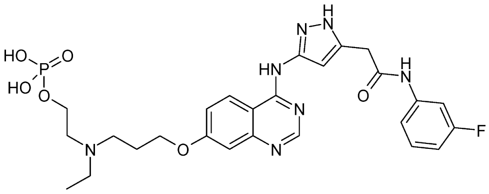

A series of pyrazolylamino-substituted quinazolines was synthesized and biol. evaluated as inhibitors of Aurora kinases, which have been the subject of considerable interest as targets for the development of new anticancer agents. Some of the products demons trated greater than 1000-fold selectivity for Aurora B over Aurora A kinase activity in recomb inant enzyme assays. These compounds have been designed for parenteral administration and achieve high levels of solubility by virtue of their ability to be delivered as readily activated phosphate derivatives The prodrugs are comprehensively converted to the des-phosphate form in vivo, and the active species have advantageous pharmacokinetic properties and safety pharmacol. profiles. The compounds display striking in vivo activity, and **I** (AZD1152) has been selected for clin. evaluation and is currently in phase 1 clin. trials.

Keywords: quinazoline pyrazolylamino preparation selective inhibitor Aurora kinase antitumor

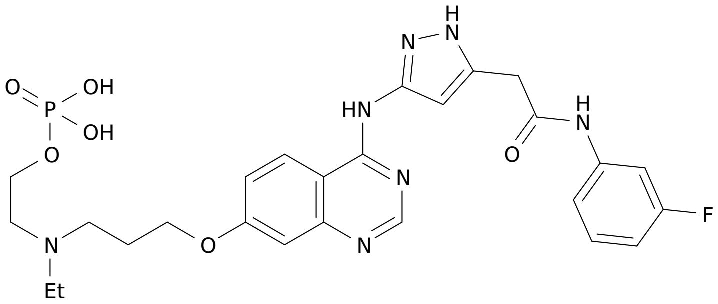

I

Substances (85) Reactions (245) Citing (238)

391

Preparation of phosphonooxy quinazoline derivatives and their pharmaceutical use

By: Heron, Nicola Murdoch; Jung, Frederic Henri; Pasquet, Georges Rene; Mortlock, Andrew Austen
World Intellectual Property Organization, WO2004058781 A1 2004-07-15 | Language: English, Database: CAplus

Preparation of phosphonooxy quinazoline derivatives, **I** (A = 5-membered heteroaryl containing a nitrogen atom and one or two further nitrogen atoms; X = O, S, S(O), $S(O)_2$, organoamino; m = 0-3; Z = organo amino, phosphonooxy, (un)substituted C_{3-6} cycloa lkyl, etc.; R^3 = H, halo, cyano, nitro, C_{1-6} alkoxy, C_{1-6} alkyl, alkoxycarbonyl, organoamido, sulfonylamido, etc.; R^4 = H, C_{1-4} alkyl, hetero aryl, heteroaryl C_{1-4} alkyl, aryl, etc.; R^5 = H, C_{1-4} alkyl, C_{2-4} alkenyl, C_{2-4} alkynyl, C_{3-6} cycloalkyl, etc.; R^6, R^7 = H, halo, C_{1-4} alkyl, C_{3-6} cycloalkyl, hydroxy, C_{1-4} alkoxy, etc.), and compositions containing them, processes for their prepar ation and their use in therapy is described. Thus, reaction of N-(3-fluorophenyl)-2-{3-[(7-{3-[4-(hydroxymethyl)piperidin-1-yl]propoxy}-6-methoxyquinazolin-4-yl) amino]-1H-pyrazol-5-yl}acetamide (preparation given) with di-tert-butyl-diethylphosphoramidite gave 70% di-tert-Bu {1-[3-({4-[(5-{2-[(3-fluorophenyl)amino]-2-oxoethyl}-1Hpyrazol-3-yl)amino]-6-methoxyquinazolin-7-yl}oxy)propyl]piperidin-4-yl}methyl phosphate which on acidic hydrolysis gave 94% title compound, di-tert-Bu {1-[3-({4-[(5-{2-[(3-fluorophenyl)amino]-2-oxoethyl}-1Hpyrazol-3-yl) amino]-6-methoxyquinazolin-7-yl}oxy)propyl]piperidin-4-yl}methyl dihydrogen phosphate. In vitro Aurora- A and Aurora-B kinase inhibition activity and cell proliferation and cycle anal. of the prepared compounds were determined

Keywords: phosphonooxy quinazoline derivative preparation Aurora A B kinase inhibition

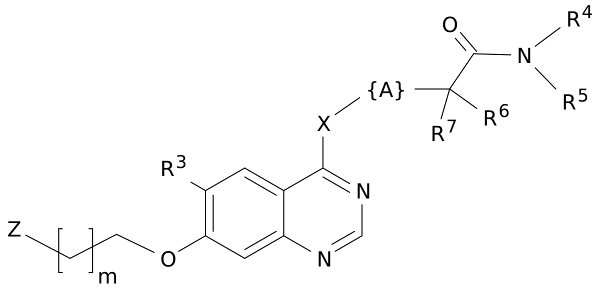

I

PatentPak available

Substances (268) | Reactions (1,525) | Citing (20)

Copyright © 2023 American Chemical Society (ACS). All Rights Reserved.
Copyright © 2023 U.S. National Library of Medicine.
Internal use only. Redistribution is subject to the terms of your SciFinderⁿ License Agreement and CAS information Use Policies.

CAS SciFinder^{n} Task History

Initiating Search

May 11, 2023, 9:41AM

References: **barasertib**

Filtered By:

Publication Year: 2004 to 2022

Search Tasks

Task	Search Type	View
Exported: Returned Reference Results + Filters (363)	**References**	View Results

Copyright © 2023 American Chemical Society (ACS). All Rights Reserved.
Internal use only. Redistribution is subject to the terms of your SciFinder^{n} License Agreement and CAS information Use Policies.

CAS SciFinderⁿ

References (363)

View in SciFinderⁿ

1

Dicer promotes genome stability via the bromodomain transcriptional co-activator BRD4

By: Gutbrod, M. J.; Roche, B.; Steinberg, J. I.; Lakhani, A. A.; Chang, K.; Schorn, A. J.; Martienssen, R. A.

Nature Communications (2022), 13(1), 1001 | Language: English, Database: CAplus and MEDLINE

RNA interference is required for post-transcriptional silencing, but also has addnl. roles in transcri ptional silencing of centromeres and genome stability. However, these roles have been controversial in mammals. Striki ngly, we found that Dicer-deficient embryonic stem cells have strong proliferation and chromosome segregation defects as well as increased transcription of centro meric satellite repeats, which triggers the interferon response. We conducted a C RISPR-Cas9 genetic screen to restore viability and identified transcriptional activators, histone H3K9 methyltransferases, and chromosome segregation factors as suppressors, resembling Dicer suppressors identified in independent screens in fission yeast. The strongest suppre ssors were mutations in the transcriptional co-activator Brd4, which reversed the strand- specific transcription of major satellite repeats suppre ssing the interferon response, and in the histone acetyltransferase Elp3. We show that identical mutations in the second bromod omain of Brd4 rescue Dicer-dependent silencing and chromosome segregation defects in both mammalian cells and fission yeast. This remarkable conservation demonstrates that RNA interference has an ancient role in transcri ptional silencing and in particular of satellite repeats, which is essential for cell cycle progression and proper chromosome segregation. Our results have pharmacol. implications for cancer and autoimmune diseases charact erized by unregulated transcription of satellite repeats.

Keywords: Dicer genome stability bromodomain transcriptional coactivator BRD

Substances (17) Reactions (0) Citing (2)

2

Screening of compounds to identify novel epigenetic regulatory factors that affect innate immune memory in macrophages

By: Benjaskulluecha, Salisa; Boonmee, Atsadang; Pattarakankul, Thitiporn; Wongprom, Benjawan; Klomsing, Jeerameth; Palaga, Tanapat
Scientific Reports (2022), 12(1), 1912 | Language: English, Database: CAplus and MEDLINE

Trained immunity and tolerance are part of the innate immune memory that allow innate immune cells to differentially respond to a second encounter with stimuli by enhancing or suppressing responses. In trained immunity, treatment of macrop hages with β-glucan (BG) facilitates the production of proinflammatory cytokines upon lipopolysaccharide (LPS) stimulation. For the tolerance response, LPS stimulation leads to suppressed inflammatory responses during subsequent L PS exposure. Epigenetic reprogr amming plays crucial roles in both phenomena, which are tightly associated with metabolic flux. In this study, we performed a screening of an epigenetics compound library that affects trained immunity or L PS tolerance in macrophages using TNFα as a readout. Among the 181 compounds tested, one compound showed suppressive effects, while 2 compounds showed promoting effects on BG-trained TNFα production In contrast, various inhibitors targeting Aurora kinase, histone methyltra nsferase, histone demethylase, histone deacetylase and DNA methyltransferase showed inhibitory activity against LPS tolerance. Several proteins previously unknown to be involved in innate immune memory, such as MGMT, Aurora kinase, LSD1 and PRMT5, were revealed. Protein network anal. revealed that the trained immunity targets are linked via Trp53, while LPS tolerance targets form three clusters of histone-modifying enzymes, cell division and base- excision repair. In trained immunity, the histone lysine methyltra nsferase SETD7 was identified, and its expression was increased during B G treatment. Level of the histone lysine demethylase, LS D1, increased during L PS priming and si RNA-mediated reduction resulted in increased expression of Il1b in L PS tolerance. Taken together, this screening approach confirmed the importance of epigenetic modifications in innate immune memory and provided potential novel targets for intervention.

Keywords: innate immune memory macrophage epigenetic regulatory factor

Substances (37) Reactions (0) Citing (4)

3

Methods and systems for personalized therapies by identifying a drug target in relation to a disease gene expression signature

By: Ghiassian, Susan; Akmaev, Viatcheslav R.; Voitalov, Ivan
World Intellectual Property Organization, WO2022271717 A1 2022-12-29 | Language: English, Database: CAplus

Described are methods and systems for identifying a target for therapy and treating a subject that exhibits a disease gene expression signature, comprising identifying and adminis tering a therapy determined to revert a disease gene expression signature in a subject suffering from a disease, disorder, or condition toward a non-diseased expression signature (e.g., disease gene expression signature of a non-diseased subject).

Keywords: personalized therapy drug target disease gene expression profile

PatentPak available

Substances (564) Reactions (0) Citing (0)

4

Probes comprising human granzyme B-cleavable hexapeptide and nanoparticles embedded with fluorescent dye, linker, and quencher and anti-GzmB antibody for detection of human granzyme B and small molecule drug as well as diagnosis of cancer, acute transplant rejection or IBD

By: Vendrell, Marc; Scott, Jamie I.; Gordon, Doireann
World Intellectual Property Organization, WO2022258997 A1 2022-12-15 | Language: English, Database: CAplus

The present disclosure relates to the development of novel cleavable peptide probes for use in detecting granzyme B, as well as methods for using the probes in medical and biol. settings. The probes may comprise a fluorescent moiety conjugated or bound to the peptide, and quencher moiety conjugated or bound to the peptide and upon cleavage of the peptide, the fluorescent moiety becomes dequenched.

Keywords: sequence granzyme cleavable peptide fluorescent dye quencher linker conjugate; Gzm B antibody small mol drug I BD cancer transplant rejection

PatentPak available

Substances (63) | Reactions (0) | Citing (0)

5

Methods and compositions for interfering with FAN1

By: Pearson, Christopher E.; Sartori, Alessandro A.
World Intellectual Property Organization, WO2022234494 A2 2022-11-10 | Language: English, Database: CAplus

This disclosure describes methods of altering a FAN1 polypeptide using, without limitation, a polypeptide, an antibody, a nucleic acid, a small mol., a kinase inhibitor, an agonist of a kinase, an agonist of an exonuclease, or an antagonist of an exonuclease.

Keywords: FAN1 antagonists

PatentPak available

Substances (33) | Reactions (0) | Citing (0)

6

A field guide to Aurora kinase inhibitors: an oocyte perspective

By: Blengini, Cecilia S.; Jung, Gyu Ik; Aboelenain, Mansour; Schindler, Karen
Reproduction (Bristol, United Kingdom) (2022), 164(4), V5-V7 | Language: English, Database: CAplus and MEDLINE

A review. In brief: The Aurora protein kinases have critical functions in controlling oocyte meiotic maturation. In this study, we describe an assay for examining their activation state in oocytes and establish the best working doses of three commonly used inhibitors. Abstract: Several small mol. inhibitors exist for targeting Aurora kinase proteins in somatic cells. From this point of view, we evaluate the specificity of these inhibitors in mouse oocytes, and we demons trate that MLN 8237 and AZD 1152 are specific for Aurora kinase A and Aurora kinase C, resp., only when used at low concentrations

Keywords: review oocyte aurora kinase inhibitor

Substances (4) | Reactions (0) | Citing (0)

7

Combined TRIP13 and aurora kinase inhibition induces apoptosis in human papillomavirus-driven cancers

By: Ghosh, Soma; Mazumdar, Tuhina; Xu, Wei; Powell, Reid T.; Stephan, Clifford; Shen, Li; Shah, Pooja A.; Pickering, Curtis R.; Myers, Jeffery N.; Wang, Jing; et al

Clinical Cancer Research (2022), 28(20), 4479-4493 | Language: English, Database: CAplus and MEDLINE

Human papillomavirus (HPV) causes >5% of cancers, but no therapies uniquely target H PV-driven cancers. We tested the cytotoxic effect of 864 drugs in 16 HPV-pos. and 17 HPV-neg. human squamous cancer cell lines. We confirmed apoptosis in vitro and in vivo using patientderived xenografts. Mitotic pathway components were manipu lated with drugs, knockdown, and overexpr ession. Aurora kinase inhibitors were more effective in vitro and in vivo in HPV-pos. than in HPV-neg. models. We hypoth esized that the mechanism of sensitivity involves retinob lastoma (Rb) expression because the viral oncopr otein E7 leads to Rb protein degrad ation, and basal Rb protein expression correlates with Aurora inhibition-induced apoptosis. Manipulating Rb directly, or by inducing E7 expression, altered cells' sensitivity to Aurora kinase inhibitors. Rb affects expression of the mitotic checkpoint genes M AD2L1 and BUB1B, which we found to be highly expressed in H PV-pos. patient tumors. Knockdown of MAD2L1 or BUB1B reduced Aurora kinase inhibition-induced apoptosis, whereas depletion of the M AD2L1 regulator TRIP13 enhanced it. TRIP13 is a potentially druggable AAA-ATPase. Combining Aurora kinase inhibition with T RIP13 depletion led to extensive apoptosis in H PV-pos. cancer cells but not in HPV-neg. cancer cells. Our data support a model in which H PV-pos. cancer cells maintain a balance of M AD2L1 and T RIP13 to allow mitotic exit and survival in the absence of Rb. Because it does not affect cells with intact Rb function, this novel combination may have a wide therap eutic window, enabling the effective treatment of Rb- deficient cancers.

Keywords: human papillomavirus cancer TRIP aurora kinase inhibition induce apoptosis

Substances (13) | Reactions (0) | Citing (1)

8

Methods for inhibiting KRAS oncoprotein through enhanced GTPase activity

By: Lito, Piro

World Intellectual Property Organization, WO2022217073 A1 2022-10-13 | Language: English, Database: CAplus

The present disclosure relates to methods for determining whether a cancer patient harboring a constit utively active KRAS mutation will be responsive to treatment with a $KRAS^{G12C}$ inhibitor that selectively targets the inactive state of K RAS. These methods are based on assaying regulators of G-protein signaling (R GS) expression in the cancer patient.

Keywords: sequence KRAS oncoprotein GTPase

PatentPak available

Substances (171) | Reactions (0) | Citing (0)

9

Combination therapies including Myt1 inhibitors

By: Fourtounis, Jimmy; Young, Jordan; Bernier, Cynthia; Durocher, Daniel; Hustedt, Nicole
World Intellectual Property Organization, WO2022213204 A1 2022-10-13 | Language: English, Database: CAplus

The use of inhibitors of tyrosine and threonine- specific cdc2-inhibitory kinase (Myt1) in the treatment of cancer is disclosed. In preferred embodiments, the Mytl inhibitor is a carboxamide pyrrolopyrazine or carboxamide pyrrolopyridine of Formula I. The Myt1 inhibitors may be used in combination with a variety of other anti- cancer agents. Such agents include a WEE1 inhibitor, TOPI or TOP2A inhibitor, RRM1 or RRM2 inhibitor, AURKA or AURKB inhibitor, ATR inhibitor, TTK inhibitor, SOD1 or SOD2 inhibitor, BUB1 inhibitor, CDC7 inhibitor, SAE1 inhibitor, PLK1 inhibitor, UBA2 inhibitor, DUT inhibitor, HDAC3 inhibitor, CHEK1 inhibitor, MEN1 inhibitor, DOT1L inhibitor, CREBBP inhibitor, EZH2 inhibitor, PLK4 inhibitor, HASPIN inhibitor, METTL3 inhibitor, nucleoside analogs, and platinum-based DNA alkylating agents.

Keywords: Myt1 inhibitor preparation treatment cancer

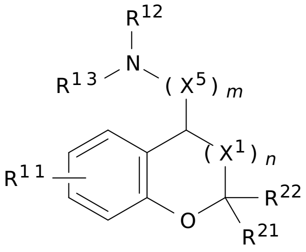

I

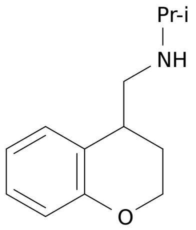

II

PatentPak available

Substances (639) | Reactions (455) | Citing (0)

10

MPS1 inhibition primes immunogenicity of KRAS-LKB1 mutant lung cancer

By: Kitajima, Shunsuke; Tani, Tetsuo; Springer, Benjamin F.; Campisi, Marco; Osaki, Tatsuya ; Haratani, Koji; Chen, Minyue; Knelson, Erik H. ; Mahadevan, Navin R. ; Ritter, Jessica; et al
Cancer Cell (2022), 40(10), 1128-1144.e8 | Language: English, Database: CAplus and MEDLINE

KRAS-LKB1 (KL) mutant lung cancers silence STING owing to intrinsic mitochondrial dysfunction, resulting in T cell exclusion and resistance to programmed cell death (ligand) 1 (PD-[L]1) blockade. Here we discover that KL cells also minimize intracellular accumu lation of 2'3'-cyclic GMP-AMP (2'3'-cGAMP) to further avoid downstream STING and STAT1 activation. An unbiased screen to co-opt this vulnerability reveals that transient MPS1 inhibition (MPS1i) potently re-engages this pathway in KL cells via micronuclei genera tion. This effect is markedly amplified by epigenetic de- repression of STING and only requires pulse MPS1i treatment, creating a therapeutic window compared with non- dividing cells. A single course of decitabine treatment followed by pulse M PS1i therapy restores T cell infiltration in vivo, enhances anti-PD-1 efficacy, and results in a durable response without evidence of signif icant toxicity.

Keywords: mutant lung cancer immunogenicity T cell infiltration mitochondrial dysfunction; 2’3’-cGAMP; DNMT1; EZH2; KRAS-LKB1 mutant lung adenocarcinoma; STING; cGAS; monopolar spindle kinase 1

Substances (31) | Reactions (0) | Citing (6)

11

Aurora kinase B inhibitors for use for treating cancer

By: Travers, Jon; Urosevic Despotovic, Jelena; Molina-Villa, Miguel-Angel; Bertran-Alamillo, Jordi
World Intellectual Property Organization, WO2022192139 A1 2022-09-15 | Language: English, Database: CAplus

Provided are methods for treating cancer with an Aurora kinase B inhibitor comprising determining if a cancer cell has a chromo somal amplification at location 22q11 on chromosome 22. Also provided are methods for treating cancer with an Aurora kinase B inhibitor comprising determining if a cancer cell has a gene with an increased copy number at location 22q11 on chromosome 22. Further provided are methods of determining if a cancer cell or a subject having cancer are amenable to treatment with an Aurora kinase B inhibitor.

Keywords: aurora kinase B inhibitor drug resistant cancer combination therapy

PatentPak available

Substances (33) Reactions (0) Citing (0)

12

N-myristoyl transferase (NMT) inhibitors for the treatment of senescence-associated diseases or disorders

By: Tate, Edward; Kallemeijn, Wouter; Gil, Jesus; McHugh, Domhnall
United States, US20220280484 A1 2022-09-08 | Language: English, Database: CAplus

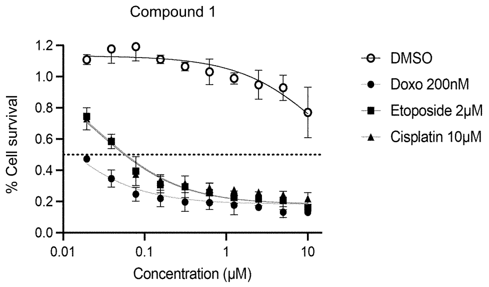

The present invention provides a method of prophylaxis or treatment of a senesc ence-associated disease or disorder by adminis tering to a subject in need thereof, a therapeu tically effective amount of a N-myristoyl transferase (NMT) inhibitor. A therapeu tically effective amount in this context means an amount of the NMT inhibitor sufficient to be effective in therapy e.g. in the treatment of cancer or other senescence-associated disease or disorder.

Keywords: senescence associated disease treatment N myristoyl transferase inhibitor

PatentPak available

Substances (58) Reactions (0) Citing (0)

13

Compounds, compositions and methods for treating age-related diseases and conditions

By: Tarkhov, Andrei Evgenevich; Avkhachev, Konstantin Aleksandrovich; Burmistrova, Olga Andreevna; Kholin, Maksim Nikolaevich; Fedichev, Petr Olegovich
World Intellectual Property Organization, WO2022173333 A2 2022-08-18 | Language: English, Database: CAplus

Antiaging compounds are selected according to the age-related disorder and the mol. targets associated with them by the disclosed compounds

Keywords: antiaging compounds drug targeting disease

PatentPak available

Substances (506) | Reactions (0) | Citing (0)

14

Methods for treating and ameliorating cancer

By: Jamieson, Catriona; Crews, Leslie; Burkart, Michael; Balaian, Larisa; Mondala, Phoebe; Mason, Cayla; Diep, Raymond H.; Laclair, James; Whisenant, Thomas; Chan, Warren C.; et al
World Intellectual Property Organization, WO2022165398 A1 2022-08-04 | Language: English, Database: CAplus

In alternative embodiments, provided are methods for treating and amelio rating a cancer, or recurrence of a cancer such as acute myeloid leukemia (AML) comprising administration to an individual in need thereof a pharmac eutical composition comprising 17S-F D-895 (or rebecsinib) and second drug such as an A TP-competitive protein tyrosine kinase inhibitor such as dasatinib. In altern ative embodiments, provided are methods for the in vivo inhibition of myeloprol iferative neoplasm (MPN) or AML stem cell propagation comprising administration to an individual in need thereof a pharmac eutical composition comprising 17S-FD-895 and second drug. In alternative embodiments, provided are methods for the in vivo inhibition pre- leukemia stem cell (pre-LSC) transformation into leukemia stem cells (LSCs) comprising administration to an individual in need thereof a pharmac eutical composition comprising 17 S-FD-895 and second drug.

Keywords: rebecsinib cancer

PatentPak available

Substances (166) | Reactions (0) | Citing (0)

15

Compounds for the treatment of an infection with an infectious microbe, and methods for identifying said compounds

By: Schurdak, Mark E.; Vogt, Andreas; Stern, Andrew Michael; Taylor, Douglass Lansing; Chen, Fangyuan; Pei, Fen; Cheng, Hongying; Bahar, Ivet; Shi, Qingya; Arditi, Moshe
World Intellectual Property Organization, WO2022159787 A1 2022-07-28 | Language: English, Database: CAplus

Disclosed herein are methods of identifying a compound for treating or preventing an infection with an infectious microbe, such as a coronavirus, in a subject in need thereof. Also disclosed herein are compounds and compos itions identified by said methods, and methods of use thereof. The methods comprise (a) obtaining transcriptomic data from cells infected with the infectious microbe, (b) identifying differentially expressed genes (DEGs), (c) characterizing host-targeted antimicrobial or anticytokine signature, (d) identi fying compounds that stimulate the antimic robial or -cytokine signature, (e) evaluating known and predicted targets of compounds identified in step (d), (f) constructing an infection host response protein-protein interaction (PPI) network and modules, (g) priori tizing compounds based on network proximity anal., (h) clustering of priori tized compounds associated with selected disease modules, (i) selecting representative compounds from each cluster for in vitro assays, and (j) analyzing the results of steps a- i to thereby identify the compound for treating or preventing the infection.

Keywords: microbial infection treatment drug screening method

PatentPak available

Substances (605) | Reactions (0) | Citing (0)

16

Methods for treating and ameliorating cancer

By: Jamieson, Catriona; Ma, Wenxue
World Intellectual Property Organization, WO2022159760 A1 2022-07-28 | Language: English, Database: CAplus

In alternative embodiments, provided are methods for treating and amelio rating a cancer such as a leukemia such as acute myeloid leukemia (AML) comprising administration to an individual in need thereof a pharmac eutical composition comprising imetelstat, or imetelstat and second drug such as dasatinib, or ruxolitinib, fedratinib, 8-aza-adenosine, raltegravir and/or dolutegravir or any combination thereof. In alternative embodiments, provided are methods for the in vivo inhibition of myeloprol iferative neoplasm (MPN) or AML stem cell propagation comprising administration to an individual in need thereof a pharmac eutical composition comprising imetelstat, or imetelstat and second drug.

Keywords: ameliorating cancer therapy; pharmaceutical composition myeloproliferative neoplasm stem cell propagation imetelstat drug

PatentPak available

Substances (172) | Reactions (0) | Citing (0)

17

Inducers of senescence, in combination with a selective death receptor 5 (DRr5) agonist, for use in a method of treating cancer

By: Bernards, Rene; Wang, Liqin
World Intellectual Property Organization, WO2022154664 A1 2022-07-21 | Language: English, Database: CAplus

The invention relates to an inducer of senescence, in combination with a short-lived, selective Death Receptor 5 (D R5) agonist, for use in a method of treating a patient suffering from a tumor. The invention further relates to a pharmaceutical composition, comprising a short-lived, selective Death Receptor 5 (D R5) agonist and, option ally, an inducer of senescence. The invention further relates to methods of treating a patient having a tumor, and to a selective, short-lived DR5 agonist, for use in a method of select ively killing senescent cells, including senescent cancer cells.

Keywords: DR5 agonist combin ation BRD2 inhibitor cancer

PatentPak available

Substances (12) | Reactions (0) | Citing (0)

18

Methods of potentiating antibiotic efficacy for treating bacterial infections

No inventor data available
World Intellectual Property Organization, WO2022153045 A1 2022-07-21 | Language: English, Database: CAplus

A method of potentiating or inducing antibiotic efficacy in one or more compos itions or compounds, said method including the step of inducing a substantially cell wall deficient (C WD) state in a bacteria by exposure to at least one antibiotic before and/or in combination with said one or more compos itions or compounds

Keywords: antibiotic combination chemotherapy bacterial infection treatment bacteria cell wall

PatentPak available

Substances (1,455) | Reactions (0) | Citing (0)

19

Sulfonamide derivatives, compositions comprising same and uses thereof in the treatment of cancers

By: Masutomi, Kenkichi; Yamashita, Taro; Kaneko, Shuichi; Yasukawa, Mami; Honma, Teruki; Koyama, Hiroo; Fukami, Takehiro
World Intellectual Property Organization, WO2022144780 A1 2022-07-07 | Language: English, Database: CAplus

Described herein are sulfonamide derivatives of Formula (I), where R1 and R2 are each indepen dently a halogen atom, and m and n are each independently an integer between 0 - 3, capable of inhibiting RNA dependent RNA polymerase (RdRP) activity of cancer cells. Also described are pharmaceutical compositions comprising these compounds and the uses thereof in the prophy laxis and/or treatment of cancers, including intractable solid cancers and stem cells cancer. The pharmac eutical composition may further comprise at least one of an hormonal therapeutic agent, a chemothe rapeutic agent, an immunothe rapeutic agent, a pharmac eutical agent inhibiting the action of cell growth factor and a pharmac eutical agent inhibiting the action of cell growth factor receptor.

Keywords: Sulfonamide derivative compos ition cancer

PatentPak available

Substances (158) | Reactions (0) | Citing (0)

20

A new imaging platform (iScreen) allows for the concurrent assessment of micronucleus induction and genotoxic mode of action in human A375 cells

By: Sun, Xiaowen; Rubitski, Elizabeth; Spellman, Richard A.; Engel, Maria; Schuler, Maik
Environmental and Molecular Mutagenesis (2022), 63(5), 230-245 | Language: English, Database: CAplus and MEDLINE

Genotoxicity testing guidelines require the assessment of the clasto genic and aneugenic potential of compounds While in vitro micronucleus assays detect both types of endpoints, it requires labor- intensive microscopic scoring and does not discri minate between the two modes of actions. Here, we present a novel high-content imaging platform in A375 human cells that addresses the need for rapid scoring while providing addnl. mechanistic information. We evaluated the new platform with 12 compounds, three compounds from each mechanistic class (clastogen, aneugen tubulin binder, aneugen aurora inhibitor, and nongenot oxicant) following 4- and 24-h compound treatments. The approach we developed is first discrim inating between genoto xicant and nongenotoxicant using an image anal. algorithm to quantify micron ucleus induction below a 60% cytoto xicity cutoff. Then it uses centromere protein A (CENPA) staining for the genotoxic compounds to discri minate between aneugens and clasto gens. Lastly, we use phosphorylated histone H2AX Ser139 (γH2AX) staining to confirm clastogenicity and changes in phospho rylated histone 3 Ser10 (pH 3) and increases in polyploidy in mitotic cells to discri minate between aneugens that bind tubulin from those that affect aurora kinases. All compounds were correctly classified, and we showed by using benchmark dose- response anal. that the imaging platform in A375 cells is at least as sensitive as the MicroFlow assay in TK6 cells for genoto xicant but appears to be more specific for the nongenotoxicants. A detailed comparison of the cell lines and a more compreh ensive validation with a much larger compound set, predictive and dose-response modeling will be presented in the future.

Keywords: clastogen aneugen tubulin binder aurora inhibitor micronucleus genotoxicity; aneuploidy; aurora kinase inhibitor; clastogen; image analysis; tubulin binder

Substances (12) Reactions (0) Citing (0)

21

In vitro and in vivo evaluation of kinase and protease inhibitors against Trypanosoma evansi.

By: Bhutia, Wangchuk Dorjee; Gupta, Snehil; Rani, Ruma; Batra, Kanisht; Sethi, Khushboo; Kumar, Sanjay; Kumar, Rajender
Veterinary research communications (2022), | Language: English, Database: MEDLINE

Trypanosoma evansi is a causative agent of chronic wasting and fatal disease of livestock and wild animals known as surra. In this study, repurposing approach based on drug target was used to invest igate the efficacy of kinase inhibitors (Barasertib -HQPA, BAR and Palbociclib isethionate, PAL) and protease inhibitors (Z- pro-prolinal, Z-PRO and Leupeptin hemisul phate, LEU) against T. evansi in HMI-9 medium. BAR, PAL and Z-PRO exhibited IC_{50} values of 13.52 μM, 0.6375 μM and 63.20 μM against T. evansi in terms of growth inhibition, in the contrary, LEU failed to exhibit a significant growth inhibition at any time interval. Furthe rmore, oligope ptidase B and aurora kinase genes of T. evansi were targeted to determine the effect of these drugs on quanti tative mRNA expres sion, which showed significant ($p < 0.01$) up-regulation of both genes in the B AR and PAL-exposed population at 12 h of exposure, whereas, Z-PRO showed only significant ($p < 0.05$) up-regulation of aurora kinase gene at 12 h interval. In cytoto xicity assay, BAR exhibited 52% and 41% cytotoxicity at 50 μM concentration (about five folds the IC_{50} value) on equine PBMC's and Vero cell line, respectively. Similarly, the cytotoxicity of 25% and 24% were recorded at 10 μ M concentration (about ten folds to the IC_{50} value) of P AL in equine PBMC's and Vero cell line, respect ively. Of these, BAR and PAL, which were found effective under in vitro trials, raised the longevity of mice at higher doses during in vivo trials. Data generated showed that kinase inhibitors have higher potential to explore therapeutic molecules against surra organism.

Keywords: India; Kinase inhibitors; Protease inhibitors; Surra; Trypanosoma evansi

Substances (0) Reactions (0) Citing (0)

22

Computational screening methods for identifying glutathione S-transferase P (GSTP1) inhibitors for treating pulmonary hypertension

By: Chan, Stephen Yu-Wah; Kim, Seungchan

World Intellectual Property Organization, WO2022103706 A1 2022-05-19 | Language: English, Database: CAplus

Disclosed are pathogenic mechanisms in pulmonary hypertension and mol. inhibitors of the same. Particu larly, GSTP1 (glutathione S-transferase P1) have been demons trated as having a role in regulating the endoth elial iron-sulfur cluster assembly enzyme I SCU function in pulmonary hypertension. Accordingly, methods for treating pulmonary hypert ension in a subject in need thereof comprising administering a therapeutically effective amount of a pharmac eutical compositions that inhibit glutathione S-transferase P (GSTP1) and/or increasing I SCU expression are disclosed. The GSTP1 inhibitor can comprise a piperlongumine analog, such as BR D-K34222889, or a derivative thereof.

Keywords: antihypertensive GSTP1 inhibitor piperlongumine analog BRDK34222889 pulmonary hypert ension treatment

PatentPak available

Substances (264) | Reactions (0) | Citing (0)

23

N-myristoyl transferase (NMT) inhibitors for treating senescence-associated diseases or disorders

By: Tate, Edward; Kallemeijn, Wouter; Gil, Jesus; McHugh, Domhnall

World Intellectual Property Organization, WO2022090746 A1 2022-05-05 | Language: English, Database: CAplus

The present invention provides a N-myristoyl transferase (NMT) inhibitors for use in the prophy laxis or treatment of a senesc ence-associated disease or disorder. The N MT inhibitor is selected from the group consis iting of 1-(5-(3,4-difluoro-2-(2-(1,3,5-trimethyl-1 H-pyrazol-4-yl)ethoxy)phenyl)-1-methyl-1H-indazol-3-yl)-N,N-dimethylmethanamine, 4-(2-{2-[3-(2-aminoethyl)imidazo[1,2-a]pyridin-6-yl]-5-chlorophenoxy}ethyl)-N,N,1,5-tetramethyl-1H-pyrazole-3-carboxamide. In one aspect the senescence-associated disease or disorder is cancer, wherein said cancer comprises chemotherapy or radiotherapy induced senescent cells. In another aspect senesc ence-associated disease or disorder is one or more side effects of chemot herapy or radioth erapy, wherein said chemotherapy or radiotherapy induces senescence. One or more side effects of chemot herapy or radiotherapy are selected from weight loss, endocrine changes, hormone imbalance, changes in hormone signaling, cardiotoxicity, changes in body compos ition, a reduced ability to be phys. active, gastrointestinal toxicity, nausea, vomiting, constip ation, anorexia, diarrhea, peripheral neurop athy, fatigue, malaise, low phys. activity, hematol. toxicity, anemia, hepatotoxicity, alopecia, pain, infection, mucositis, fluid retention, dermatol. toxicity, rashes, dermatitis, hyperpigmentation, urticaria, photosensitivity, nail changes, and mouth, gum and throat problems. In one aspect the senescence-associated disease or disorder is selected from a metabolic disease, an inflam matory or autoimmune disease or disorder, a cardiovascular disease or disorder, a pulmonary disease or disorder, a neurol. disease or disorder, an eye disease or disorder, and a dermatol. disease or disorder.

Keywords: senolytic agent NMT inhibitor aging senescence associated disease treatment

PatentPak available

Substances (65) | Reactions (0) | Citing (0)

24

Combination therapies for use in treating cancer

By: Li, Fu; Marks, Kevin; Marjon, Katya; Kalev, Petar; Hyer, Marc Lee
World Intellectual Property Organization, WO2022036067 A1 2022-02-17 | Language: English, Database: CAplus

The compound of Formula (I), or pharmaceutically acceptable salts thereof, is useful in, among other things, the treatment of cancer and provides a therapeutic advantage when used in combin ation with other agents as herein described compared to treatment with each agent when administered alone.

Keywords: antitumor cancer combination therapy

PatentPak available

Substances (170) | Reactions (0) | Citing (0)

25

Effect of alpha-tocopheryl succinate on the cytotoxicity of anticancer drugs towards leukemia lymphocytes

By: Ivanova, Donika; Zhelev, Zhivko; Zlateva, Genoveva; Lazarova, Dessislava; Yaneva, Zvezdelina; Panovska, Radmila; Aoki, Ichio; Bakalova, Rumiana
Anticancer Research (2022), 42(1), 547-554 | Language: English, Database: CAplus and MEDLINE

This study analyzed the effect of α-tocopheryl succinate (α-TS) on the redox-state of leukemia and normal lymphocytes, as well as their sensitization to fifteen anticancer drugs. Cell viability was analyzed by trypan blue staining and automated counting of live and dead cells. Apoptosis was analyzed by FITC-Annexin V test. Oxidative stress was evaluated by the intrace llular levels of reactive oxygen species (ROS) and protein-carbonyl products. Most combin ations (α-TS plus anticancer drug) exerted additive or antago nistic effects on the proliferation and viability of leukemia lymphocytes. α-TS combined with barasertib, bortezomib or lonafarnib showed a strong synergistic cytotoxic effect, which was best expressed in the case of barase stib. It was accompanied by impressive induction of apoptosis and increased production of ROS, but insignificant changes in protein-carbonyl levels. α-TS plus barasertib did not alter the viability and did not induce oxidative stress and apoptosis in normal lymphocytes. Alpha-TS could be a promising adjuvant in second-line anticancer therapy, particularly in acute lymphob lastic leukemia, to reduce the therapeutic doses of barasertib, bortezomib, and lonafa rnib, increasing their effecti veness and minimizing their side effects.

Keywords: leukemia lymphocyte alpha tocopheryl succinate anticancer; Chemotherapy; alpha-tocopheryl succinate; apoptosis; oxidative stress

Substances (19) | Reactions (0) | Citing (1)

26

Pharmacophore Synergism in Diverse Scaffold Clinches in Aurora Kinase B

By: Masand, Vijay H.; Al-Hussain, Sami A.; Rathore, Mithilesh M.; Thakur, Sumer D.; Akasapu, Siddhartha; Samad, Abdul ; Al-Mutairi, Aamal A. ; Zaki, Magdi E. A.
International Journal of Molecular Sciences (2022), 23(23), 14527 | Language: English, Database: CAplus and MEDLINE

Aurora kinase B (AKB) is a crucial signaling kinase with an important role in cell division. Therefore, inhibition of A KB is an attractive approach to the treatment of cancer. In the present work, extensive quant. structure-activity relationships (QSAR) anal. has been performed using a set of 561 structurally diverse aurora kinase B inhibitors. The Organization for Economic Cooperation and Development (OECD) guidelines were used to develop a QSAR model that has high statistical performance (R^2tr = 0.815, Q^2LMO = 0.808, R^2_{ex} = 0.814, CCC_{ex} = 0.899). The seven-variable-based newly developed QSAR model has an excellent balance of external predictive ability (Predictive QSAR) and mechanistic interpretation (Mechanistic QSAR). The QSAR anal. successfully identifies not only the visible pharmacophoric features but also the hidden features. The anal. indicates that the lipophilic and polar groups-especially the H-bond capable groups-must be present at a specific distance from each other. Moreover, the ring nitrogen and ring carbon atoms play important roles in determining the inhibitory activity for AKB. The anal. effectively captures reported as well as unreported pharmacophoric features. The results of the present anal. are also supported by the reported crystal structures of inhibitors bound to AKB.

Keywords: pharmacophore synergism diverse scaffold clinches aurora kinase B; Q SAR; aurora kinase B; pharmacophore modeling

Substances (5) Reactions (0) Citing (0)

27

Real-time luminescence enables continuous drug-response analysis in adherent and suspension cell lines

By: Wandishin, Clayton M. ; Robbins, Charles John; Tyson, Darren R.; Harris, Leonard A.; Quaranta, Vito
Cancer Biology & Therapy (2022), 23(1), 358-368 | Language: English, Database: CAplus and MEDLINE

The drug-induced proliferation (DIP) rate is a metric of in vitro drug response that avoids inherent biases in commonly used metrics such as 72 h viability. However, DIP rate measurements rely on direct cell counting over time, a laborious task that is subject to numerous challenges, including the need to fluorescently label cells and automatically segment nuclei. Moreover, it is incredibly difficult to directly count cells and accurately measure DIP rates for cell populations in suspension. As an alternative, we use real-time luminescence measurements derived from the cellular activity of NAD(P)H oxidoreductase to efficiently estimate drug response in both adherent and suspension cell populations to a panel of known anticancer agents. For the adherent cell lines, we collect both luminescence reads and direct cell counts over time simulta neously to assess their congru ency. Our results demons trate that the proposed approach significantly speeds up data collec tion, avoids the need for cellular labels and image segment ation, and opens the door to significant advances in high-throughput screening of anticancer drugs.

Keywords: luminescence drug response analysis adherent suspension cell line; D IP rate; cell viability; continuous assay; drug response; drug screening; quantitative analysis; real-time luminescence; suspension cells

Substances (12) Reactions (0) Citing (0)

28

Using a gene network of pyroptosis to quantify the responses to immunotherapy and prognosis for neuroblastoma patients

By: Luo, Bingying; Wang, Limin; Gao, Weijing; Su, Yudong; Lu, Yao; Zheng, Jian; Yin, Jie; Zhao, Qiang; Li, Jie; Da, Yurong; et al
Frontiers in Immunology (2022), 13, 845757 | Language: English, Database: CAplus and MEDLINE

Pyroptosis, as an inflammatory form of cell death, is involved in many physiol. and pathol. processes. Neurobl astoma is the most common extracranial solid tumor in children. In this study, the relati onship between pyroptosis and tumor microenv ironment in neuroblastoma was systematically studied. We integrated four datasets of neuroblastomas. Through robust clustering of the mRNA expression profiles of 24 pyroptosis-related genes, a total of three pyroptosis patterns were identi fied. We then constructed a novel scoring method named as pyroscore to quantify the level of pyroptosis in neuroblastoma. Multi-omics data and single-cell RNA sequencing were used to accurately and comprehensively evaluate the effectiveness of pyroscore. Clin. data sets were used to evaluate the use of pyroscore to predict the responsiveness of immune checkpoint treatment. High pyroscore was associated with good prognosis, immune activation, and increased response to checkpoint blockade immunotherapy. Multivariate Cox anal. revealed that the pyroscore was an independent prognostic biomarker and could increase the accuracy of clin. prediction models. Etoposide, a drug picked up by our anal., could increase the sensitivity of neuroblastoma cells to pyroptosis. External verification using four cohorts of patients who had received immunotherapy showed that high pyroscore was signifi cantly associated with immunotherapy treatment benefit. Taken together, this study revealed that pyropt osis-related gene network could quantify the response of neuroblastoma to immune checkpoint blockade therapy and prognosis, and it may be helpful for clin. practit ioners to choose treatment strategies for neuroblastoma patients.

Keywords: human neuroblastoma prognosis immunotherapy gene network pyropt osis; etoposide; immunotherapy; neurobl astoma; prognosis; pyroptosis

Substances (22) Reactions (0) Citing (2)

29

Phenotypic screening of 1,953 FDA-approved drugs reveals 26 hits with potential for repurposing for Peyronie's disease

By: Ilg, Marcus M.; Lapthorn, Alice R.; Ralph, David J.; Cellek, Selim
PLoS One (2022), 17(12), e0277646 | Language: English, Database: CAplus and MEDLINE

Drug repurposing has been shown to bring safe medications to new patient populations, as recently evidenced by the COVID-19 pandemic. We investigated whether we could use phenotypic screening to repurpose drugs for the treatment of Peyronie's disease (PD). PD is a fibrotic disease characterized by continued myofibroblast presence and activity leading to formation of a plaque in the penile tunica albuginea (TA) that can cause pain during erection, erectile dysfun ction, and penile deformity. PD affects 3-9% of men with treatment options limited to surgery or injection of collagenase which can only be utilized at late stages after the plaque is formed. Currently there are no approved medications that can be offered to patients presenting with early disease before the formation of the plaque. Drug repurposing may therefore be the ideal strategy to identify medical treatments to address this unmet medical need in early PD. We used primary human fibrob lasts from PD patients in a phenotypic screening assay that measures TGF-β1-induced myofibroblast transformation which is the main cellular phenotype that drives the pathol. in early PD. A library of FDA-approved 1953 drugs was screened in duplicate wells at a single concent ration (10μM) in presence of TGF-β1. The myofibroblast marker α-SMA was quantified after 72h incuba tion. A pos. control of SB-505124 (TGF-β1 receptor antagonist) was included on each plate. Hits were defined as showing >80% inhibition, while retaining >80% cell viability. 26 hits (1.3%) were identified which were divided into the following main groups: anti-cancer drugs, anti-inflammation, neurol., endocrinol., and imaging agents. Five of the top-ten drugs that increase myofibroblast-transformation appear to act on VEGFR. This is the first phenotypic screening of FDA-approved drugs for PD and our results suggest that it is a viable method to predict drugs with potential for repurp osing to treat early PD.

Keywords: drug FDA phenotypic screening Peyronies disease

Substances (1,943) Reactions (0) Citing (0)

30

Explainable artificial intelligence for precision medicine in acute myeloid leukemia

By: Gimeno, Marian; San Jose-Eneriz, Edurne; Villar, Sara; Agirre, Xabier; Prosper, Felipe; Rubio, Angel; Carazo, Fernando
Frontiers in Immunology (2022), 13, 977358 | Language: English, Database: CAplus and MEDLINE

Artificial intelligence (AI) can unveil novel personalized treatments based on drug screening and whole- exome sequencing experi ments (WES). However, the concept of "black box" in A I limits the potential of this approach to be translated into the clin. practice. In contrast, explainable AI (XAI) focuses on making A I results understandable to humans. Here, we present a novel X AI method - called multi-dimensional module optimization (MOM)- that associates drug screening with genetic events, while guaran teeing that predictions are interpretable and robust. We applied M OM to an acute myeloid leukemia (A ML) cohort of 319 ex-vivo tumor samples with 122 screened drugs and WES. MOM returned a therap eutic strategy based on the F LT3, CBFβ-MYH11, and N RAS status, which predicted AML patient response to Quizartinib, Trametinib, Selumetinib, and Crizotinib. We successfully validated the results in three different large-scale screening experiments We believe that XAI will help healthcare providers and drug regulators better understand AI medical decisions.

Keywords: acute myeloid leukemia precision medicine crizotinib selumetinib; assignation problem; biomarkers; drug repositioning; drug sensitivity; ex-vivo experiment; explainable artificial intelligence; large-scale screening; treatment selection

Substances (111) Reactions (0) Citing (3)

31

Low-Dose-Rate Irradiation Suppresses the Expression of Cell Cycle-Related Genes, Resulting in Modification of Sensitivity to Anti-Cancer Drugs

By: Shimabukuro, Kiichi; Fukazawa, Takahiro; Kanai, Akinori; Kawai, Hidehiko ; Mekata, Kengo; Hirohashi, Nobuyuki ; Kakimoto, Naoya ; Tanimoto, Keiji
Cells (2022), 11(3), 501 | Language: English, Database: CAplus and MEDLINE

The biol. effects of low-dose-rate (LDR) radiation exposure in nuclear power plant accidents and medical uses of ionizing radiation (I R), although being a social concern, remain unclear. In this study, we evaluated the effects of L DR-IR on global gene expression in human cells and aimed to clarify the mechanisms. RNA-seq analyses demonstrated that relatively low dose rates of I R modify gene expression levels in TIG-3 cells under normoxic conditions, but those effects were attenuated under hypoxia-mimicking conditions. Gene set enrichment anal. demonstrated that LDR-IR significantly decreased gene expression related to cell division, cell cycle, mitosis, and the Aurora kinase B and FOXM1 pathways. Quant. RT-PCR confirmed the down-regulation of AURKB and FOXM1 genes in TIG-3 cells with LDR-IR or hypoxia-mimicking treatments without any dose-rate effect. Knock-down experiments suggested that H IF-1α and HIF-2α, as well as DEC1, participated in down-regulation of AURKB and FOXM1 under DFOM treatments, but to a lesser extent under LDR-IR treatment. FACS and microscopic analyses demonstrated that LDR-IR induced G0/G1 arrest and increased micronucleus or chromosome condensation. Finally, MTT assays demonstrated that LDR-IR decreased sensitivity to paclitaxel or barasertib in TIG-3 cells but not in A549 cells. In conclu sion, LDR-IR modifies global gene expression and cell cycle control, resulting in a reduction of sensitivity to anti-cancer chemotherapy in non-cancer cells and thus a reduction in untoward effects (G A).

Keywords: AURKB; FOXM1; hypoxia; low-dose rate irradiation; paclitaxel

Substance (1) Reactions (0) Citing (1)

32

Integrative oncogene-dependency mapping identifies RIT1 vulnerabilities and synergies in lung cancer

By: Vichas, Athea; Riley, Amanda K.; Nkinsi, Naomi T.; Kamlapurkar, Shriya; Parrish, Phoebe C. R.; Lo, April; Duke, Fujiko; Chen, Jennifer; Fung, Iris; Watson, Jacqueline; et al
Nature Communications (2021), 12(1), 4789 | Language: English, Database: CAplus and MEDLINE

CRISPR-based cancer dependency maps are accelerating advances in cancer precision medicine, but adequate functional maps are limited to the most common oncogenes. To identify opportunities for therapeutic intervention in other rarer subsets of cancer, we investigate the oncogene-specific dependencies conferred by the lung cancer oncogene, RIT1. Here, genome-wide CRISPR screening in KRAS, EGFR, and RIT1-mutant isogenic lung cancer cells identifies shared and unique vulnerab ilities of each oncogene. Combining this genetic data with small-mol. sensitivity profiling, we identify a unique vulnera bility of RIT1-mutant cells to loss of spindle assembly checkpoint regulators. Oncogenic RIT1M90I weakens the spindle assembly checkpoint and perturbs mitotic timing, resulting in sensitivity to Aurora A inhibition. In addition, we observe synergy between mutant R IT1 and activation of YAP1 in multiple models and frequent nuclear overexpression of YAP1 in human primary RIT1-mutant lung tumors. These results provide a genome-wide atlas of oncogenic RIT1 functional interactions and identify components of the RAS pathway, spindle assembly checkp oint, and Hippo/YAP1 network as candidate therapeutic targets in RIT1-mutant lung cancer.

Keywords: lung cancer integrative oncogene RIT vulnerability synergy

Substances (19) | Reactions (0) | Citing (9)

33

Methods for treating cancer with combination therapies

By: Ahsan, Aarif; Bisht, Kamlesh; Bjorklund, Chad; Flynt, Jennifer Erin; Hsu, Chih-Chao; Jeyaraju, Danny Vijey; Ortiz-Estevez, Maria; Pierceall, William Edward; Thakurta, Anjan; Towfic, Fadi George
World Intellectual Property Organization, WO2021262962 A1 2021-12-30 | Language: English, Database: CAplus

Provided herein are methods of using a compound provided herein (e.g., Compound 1, Compound 2, Compound 3, Compound 4, Compound 5, Compound 6, or Compound 7, or a stereoisomer or mixture of stereoisomers, pharmaceutically acceptable salt, tautomer, prodrug, solvate, hydrate, co-crystal, clathrate, or polymorph thereof), in combination with a second active agent for treating cancer. The second active agent is one or more of a PLK1 inhibitor, a BRD4 inhibitor, a BET inhibitor, an NEK2 inhibitor, an AURKB inhibitor, an MEK inhibitor, a PHF19 inhibitor, a BTK inhibitor, an mTOR inhibitor, a PIM inhibitor, an IGF-1R inhibitor, an XPOI inhibitor, a DOT1L inhibitor, an EZH2 inhibitor, a JAK2 inhibitor, a BIRC5 inhibitor, or a DNA methyltransferase inhibitor.

Keywords: antitumor agent combination therapy hematol cancer

PatentPak available

Substances (95) | Reactions (0) | Citing (0)

34

Combination of antibody-drug conjugate and aurora b inhibitor

By: Mettetal, Jerome Thomas, II; Durant, Stephen Thomas; Astaneh, Azadeh Cheraghchi Bashi
World Intellectual Property Organization, WO2021260582 A1 2021-12-30 | Language: English, Database: CAplus

A pharmaceutical product for administration of an anti-HER2 antibody-drug conjugate in combination with an Aurora B inhibitor is provided. The anti-HER2 antibody drug conjugate is an antibody- drug conjugate in which a drug- linker represented by the following formula (wherein A represents the connecting position to an antibody) is conjugated to an anti-HER2 antibody via a thioether bond. Also provided is a therapeutic use and method wherein the antibody- drug conjugate and the Aurora B inhibitor are administered in combination to a subject.

Keywords: HER2 cancer antitumor antibody drug conjugate aurora b sequence

PatentPak available

Substances (18) | Reactions (0) | Citing (0)

35

Compositions and methods for treating acute myeloid leukemia

By: Ishikawa, Fumihiko; Saito, Yoriko; Hashimoto, Mari; De Hoon, Michiel; Momozawa, Yukihide
World Intellectual Property Organization, WO2021255518 A1 2021-12-23 | Language: English, Database: CAplus

The present invention provides methods for identifying correlations between genetic abnormalities and effective combination treatments and treating a disease or disorder associated with inhibition of apoptosis, such as acute myeloid leukemia (AML) with compounds that block inhibition of apoptosis. The method of treating a subject with acute myeloid leukemia (AML) comprises (a) determining whether an AML cell of the subject is characterized by the presence or absence of one or more genetic mutations in F L T3, IDH1, CBL and/or NRAS, and (b) if one or more of the genetic mutations are present, individually or conjointly administering an effective amount of a XIAP inhibitor, BCL2 inhibitor, MCL1 inhibitor or aurora kinase B inhibitor to the subject. For example, the X IAP inhibitor includes AZD 5582.

Keywords: acute myeloid leukemia treatment gene mutation evaluation

PatentPak available

Substances (9) | Reactions (0) | Citing (0)

36

CNOT3 interacts with the Aurora B and MAPK/ERK kinases to promote survival of differentiating mesendodermal progenitor cells

By: Sarkar, Moumita; Martufi, Matteo; Roman-Trufero, Monica; Wang, Yi-Fang; Whilding, Chad; Dormann, Dirk; Sabbattini, Pierangela; Dillon, Niall

Molecular Biology of the Cell (2021), 32(22), ar40 | Language: English, Database: CAplus and MEDLINE

Mesendoderm cells are key intermediate progenitors that form at the early primitive streak (PrS) and give rise to mesoderm and endoderm in the gastrulating embryo. We have identified an interaction between CNOT3 and the cell cycle kinase Aurora B that requires sequences in the NOT box domain of CNOT3 and regulates MAPK/ERK signaling during mesendoderm differentiation. Aurora B phosphorylates CNOT3 at two sites located close to a nuclear localization signal and promotes localization of CNOT3 to the nuclei of mouse embryonic stem cells (ESCs) and metastatic lung cancer cells. ESCs that have both sites mutated give rise to embryoid bodies that are largely devoid of mesoderm and endoderm and are composed mainly of cells with ectodermal characteristics. The mutant ESCs are also compromised in their ability to differentiate into mesendoderm in response to FGF2, BMP4, and Wnt3 due to reduced survival and proliferation of differentiating mesendoderm cells. We also show that the double mutation alters the balance of interaction of CNOT3 with Aurora B and with ERK and reduces phosphorylation of ERK in response to FGF2. Our results identify a potential adaptor function for CNOT3 that regulates the Ras/MEK/ERK pathway during embryogenesis.

Keywords: CNOT Aurora B MAPK ERK kinase mesendodermal progenitor cell

Substances (9) Reactions (0) Citing (0)

37

Paradoxical implication of BAX/BAK in the persistence of tetraploid cells

By: Deng, Jiayin ; Gutierrez, Lucia G.; Stoll, Gautier ; Motino, Omar; Martins, Isabelle; Nunez, Lucia; Bravo-San Pedro, Jose Manuel; Humeau, Juliette; Bordenave, Chloe; Pan, Juncheng; et al

Cell Death & Disease (2021), 12(11), 1039 | Language: English, Database: CAplus and MEDLINE

Pro-apoptotic multi-domain proteins of the BCL2 family such as BAX and BAK are well known for their important role in the induction of mitochondrial outer membrane permeabilization (MOMP), which is the rate-limiting step of the intrinsic pathway of apoptosis. Human or mouse cells lacking both BAX and BAK (due to a double knockout, DKO) are notoriously resistant to MOMP and cell death induction. Here we report the surprising finding that BAX/BAK DKO cells proliferate less than control cells expressing both BAX and BAK (or either BAX or BAK) when they are driven into tetraploidy by transient exposure to the microtubule inhibitor nocodazole. Mechanistically, in contrast to their BAX/BAK-sufficient controls, tetraploid DKO cells activate a senescent program, as indicated by the overexpression of several cyclin-dependent kinase inhibitors and the activation of β-galactosidase. Moreover, DKO cells manifest alterations in ionomycin-mobilizable endoplasmic reticulum (ER) Ca^{2+} stores and store-operated Ca^{2+} entry that are affected by tetraploidization. DKO cells manifested reduced expression of endogenous sarcoplasmic/endoplasmic reticulum Ca^{2+} ATPase 2a (Serca2a) and transfection-enforced reintroduction of Serca2a, or reintroduction of an ER-targeted variant of BAK into DKO cells reestablished the same pattern of Ca^{2+} fluxes as observed in BAX/BAK-sufficient control cells. Serca2a reexpression and ER-targeted BAK also abolished the tetraploidy-induced senescence of DKO cells, placing ER Ca^{2+} fluxes downstream of the regulation of senescence by BAX/BAK. In conclusion, it appears that BAX/BAK prevent the induction of a tetraploidization-associated senescence program. Speculatively, this may contribute to the low incidence of cancers in BAX/BAK DKO mice and explain why human cancers rarely lose the expression of both BAX and BAK.

Keywords: BAX BAK expression tetraploid cell persistence

Substances (14) Reactions (0) Citing (3)

38

Use of TMPRSS2 inhibitors for the treatment of lung viral infections

By: Khatib, Abdel-Majid; Villoutreix, Bruno; Singh, Natesh
World Intellectual Property Organization, WO2021228762 A1 2021-11-18 | Language: English, Database: CAplus

In Dec. 2019, a new coronavirus was identified in the Hubei province of central china and named S ARS-CoV-2. This new virus induces COVID-19, a severe respiratory disease with high death rate. A putative target to interfere with the virus is the host transme mbrane serine protease family member II (TMPRSS2). This enzyme seems critical for the entry of coronav iruses into human cells by cleaving and activating the spike protein (S) of SARS-CoV-2. Repositioning approved, investigational and exptl. drugs on the serine protease domain of TMPRSS2 could thus be valuable. There is no exptl. structure for T MPRSS2 but it is possible to develop structural models for the serine protease domain using comparative modeling strategies. Beside the TMPRSS2 catalytic site, the inventors predicted on their structural models a main exosite that could be important for the binding of protein partners and/or substrates. To block the catalytic site or the exosite of TMPRSS2 they used structure-based virtual screening computations and two different collections of approved, investigational and exptl. drugs. The inventors propose a list of 156 mols. that could bind to the catalytic site and 100 compounds that may interact with the exosite. These small mols. could be used after exptl. validations for treatment purposes, to gain novel insights over the roles of TMPRSS2 or as starting point for the develo pment of second generation analogs.

Keywords: TMPRSS2 inhibitor treatment lung virus infection antiviral C OVID19

PatentPak available

Substances (249) | Reactions (0) | Citing (1)

39

Small molecule therapeutics for the treatment of viral infections

By: Arumugaswami, Vaithlingaraja; Christofk, Heather R.; Damoiseaux, Robert; Garcia, Gustavo; Mullen, Peter
World Intellectual Property Organization, WO2021207632 A1 2021-10-14 | Language: English, Database: CAplus

This disclosure relates to methods of treating viral infections, such as influenza or corona virus, with certain inhibitors, such as kinase inhibitors.

Keywords: protein kinase inhibitor viral infection COVID19 therapy

PatentPak available

Substances (146) | Reactions (0) | Citing (2)

40

Methods and compositions for treating liver cancer

By: Harrison, Stephen; Brew, Christine Taylor; Winther, Michael David; Shekaran, Asha
World Intellectual Property Organization, WO2021206633 A1 2021-10-14 | Language: English, Database: CAplus

Methods and compositions for treating cancer, such as liver cancer, are disclosed herein. The methods may comprise use of therapeutically effective amounts of one or more therap eutic agents to decrease expression or activity of Aurora kinase A (A URKA) in liver cancer cells that are deficient in AT-rich domain interactive domain 1A (ARID1A). In a preferred embodi ment, the one or more therapeutic agents comprises a D NA construct encoding Cas9 and a guide R NA targeting AURKA gene.

Keywords: Aurora kinase inhibitor liver cancer

PatentPak available

Substances (9) | Reactions (0) | Citing (0)

41

ALKBH5-mediated m6A-demethylation of USP1 regulated T-cell acute lymphoblastic leukemia cell glucocorticoid resistance by Aurora B

By: Gong, Hongtao; Liu, Liu ; Cui, Lina; Ma, Hongyan; Shen, Liyun
Molecular Carcinogenesis (2021), 60(9), 644-657 | Language: English, Database: CAplus and MEDLINE

Recent studies evidence that ubiquitin-specific proteases (USPs) are associated with the occurrence and chemores istance of T-cell acute lymphoblastic leukemia (T-ALL). N6-methyladenosine (m6A) demethylase AlkB homolog 5 (ALKBH5) exerts a carcinogenic effect in human cancers and improves the mRNA stability of USPs. Whether ubiquitin-specific protease 1 (USP1) controls chemores istance of T-ALL is unknown. Our study demonstrated that USP1 expression was upregulated in glucocorticoid (GC)-resistant T-ALL patients and cells (CEM-C1). High expression of USP1 was correlated to the poor prognosis in T-ALL patients. Silencing USP1 increased CEM-C1 cell sensitivity to dexamethasone (Dex), reduced cell invasion, promoted cell apoptosis, and amelio rated glucoco rticoid receptor (GR) expression. USP1 mediated T-ALL chemoresistance by interacting with and deubiquitination of Aurora B. Overexpression of USP1 reversed the amelioration effect of Aurora B inhibitor on CEM-C1 cell resistance to Dex. Mechanistically, AL KBH5 enhanced USP1 expression by reducing m6A level and mRNA stability in USP1 mRNA transcript. Downregulation of ALKBH5 reduced the levels of USP1 and Aurora B, facilitated CEM-C1 cell sensitivity to Dex, apoptosis, and GR expression, suppressed cell invasion. However, overexpression of USP1 reversed all the effects of ALKBH5 on CEM-C1 cells. In vivo results showed that tail vein injection of sh-USP1 resulted in a significant prolongation of mouse survival, suppressed tumor growth, maintained the normal weight of mice, reduced USP1 expression and facilitated GR expression. In conclusion, inhibition of ALKBH5-mediated m6A modifi cation decreased USP1 expression and downregulation of USP1 ameliorated GC resistance of T-ALL through suppressing Aurora B expression and elevating GR level.

Keywords: mediated demethylation acute lymphoblastic leukemia cell glucocorticoid; Aurora B; N6-methyladenosine demethylase; T-cell acute lymphoblastic leukemia; chemoresistance; ubiquitin-specific protease 1

Substances (7) Reactions (0) Citing (13)

42

CCT245718, a dual FLT3/Aurora A inhibitor overcomes D835Y-mediated resistance to FLT3 inhibitors in acute myeloid leukaemia cells

By: Tariq, Muhammad Usama; Furqan, Muhammad; Parveen, Hira; Ullah, Rahim; Muddassar, Muhammad; Saleem, Rahman Shah Zaib; Bavetsias, Vassilios; Linardopoulos, Spiros; Faisal, Amir
British Journal of Cancer (2021), 125(7), 966-974 | Language: English, Database: CAplus and MEDLINE

Activating mutations in the Fms-like tyrosine kinase 3 (FLT3) are among the most prevalent oncogenic mutations in acute myeloid leukemia. Inhibitors selectively targeting FLT3 kinase have shown promising clin. activity; their success in the clinic, however, has been limited due to the emergence of acquired resistance. CCT245718 was identified and characterized as a dual Aurora A/FLT3 inhibitor through cell-based and biochem. assays. The ability of CCT245718 to overcome TKD-mediated resistance was evaluated in a cell line-based model of drug resistance to FLT3 inhibitors. CCT245718 exhibits potent antiproliferative activity toward FLT3-ITD + AML cell lines and strongly binds to FLT3-ITD and TKD (D835Y) mutants in vitro. Activities of both FLT3-ITD and Aurora A are also inhibited in cells. Inhibition of FLT3 results in reduced phosphorylation of STAT5, downregulation of survivin and induction of apoptotic cell death. Moreover, CCT245718 overcomes TKD-mediated resistance in a MOLM-13-derived cell line containing FLT3 with both ITD and D835Y mutations. It also inhibits FLT3 signalling in both parental and resistant cell lines compared to F LT3-specific inhibitor MLN518, which is only active in the parental cell line. Our results demons trate that CCT245718 is a potent dual FL T3/Aurora A inhibitor that can overcome TKD-mediated acquired resistance.

Keywords: acute myeloid leukemia CCT245718 FLT3 Aurora A inhibitor D835Y

Substances (7) Reactions (0) Citing (7)

43

Suv420 enrichment at the centromere limits Aurora B localization and function

By: Herlihy, Conor P.; Hahn, Sabine; Hermance, Nicole M.; Crowley, Elizabeth A.; Manning, Amity L.
Journal of Cell Science (2021), 134(15), jcs249763 | Language: English, Database: CAplus and MEDLINE

Centromere structure and function are defined by the epigenetic modification of histones at centromeric and pericentromeric chromatin. The constitutive heterochromatin found at pericentromeric regions is highly enriched for H3K9me3 and H4K20me3. Although mis-expression of the methyltransferase enzymes that regulate these marks, Suv39 and Suv420, is common in disease, the consequences of such changes are not well understood. Our data show that increased centromere localization of Suv39 and Suv420 suppresses centromere transcription and compromises localization of the mitotic kinase Aurora B, decreasing microtubule dynamics and compromising chromosome alignment and segregation. We find that inhibition of Suv420 methyltransferase activity partially restores Aurora B localization to centromeres and that restoration of the Aurora B-containing chromosomal passenger complex to the centromere is sufficient to suppress mitotic errors that result when Suv420 and H4K20me3 is enriched at centromeres. Consistent with a role for Suv39 and Suv420 in neg. regulating Aurora B, high expression of these enzymes corresponds with increased sensitivity to Aurora kinase inhibition in human cancer cells, suggesting that increased H3 K9 and H4K20 methylation may be an underappreciated source of chromosome mis-segregation in cancer.

Keywords: human cancer centromere Aurora B localization chromosome instability; Centromere; Chromosome instability; Mitosis

Substances (15) Reactions (0) Citing (3)

44

The Phytochemical Scoulerine Inhibits Aurora Kinase Activity to Induce Mitotic and Cytokinetic Defects

By: Li, Jinhua; Yan, Ziqi; Li, Hongmei; Shi, Qiong; Ahire, Vidhula; Zhang, Shenqiu; Nimishetti, Naganna; Yang, Dun; Allen, Thaddeus D. ; Zhang, Jing

Journal of Natural Products (2021), 84(8), 2312-2320 | Language: English, Database: CAplus and MEDLINE

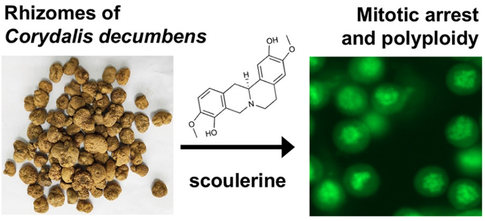

To identify novel bioactive compounds, an image-based, cell culture screening of natural product extracts was conducted. Specifi cally, our screen was designed to identify phytochems. that might phenocopy inhibition of the chromo somal passenger protein complex in eliciting mitotic and cytokinetic defects. A known alkaloid, scoule rine, was identified from the rhizomes of the plant Corydalis decumbens as being able to elicit a transient mitotic arrest followed by either apoptosis induction or polyploidy. In examining the mitotic abnormality further, we observed that scoulerine could elicit supernu merary centrosomes during mitosis, but not earlier in the cell cycle. The localization of NUMA1 at spindle poles was also inhibited, suggesting diminished potential for microtubule recruitment and spindle-pole focusing. Polyploid cells emerged subsequent to cytoki netic failure. The concentration required for scoulerine to elicit all its cell division phenotypes was similar, and an examination of related compounds highlighted the requirement for proper positi oning of a hydroxyl and a methoxy group about an aromatic ring for activity. Mechanis tically, scoulerine inhibited AURKB activity at concentrations that elicited supernu merary centrosomes and polypl oidy. AURKA was only inhibited at higher concentrations, so AURKB inhibition is the likely mechanism by which scoulerine elicited division defects. A URKB inhibition was never complete, so scoulerine may be a suboptimal AURK inhibitor or work upstream of the chromo somal passenger protein complex to reduce AURKB activity. Scoulerine inhibited the viability of a variety of human cancer cell lines. Collect ively, these findings uncover a previously unknown activity of scoulerine that could facilitate targeting human cancers. Scoulerine, or a next-generation analog, may be useful as a nontoxic component of combin ation therapies where inhibiting the chromo somal passenger protein complex is desired.

Keywords: cancer AURKB inhibitor scoulerine screen Corydalis cytokinetic

Substances (8) Reactions (0) Citing (4)

45

Application of aurora kinase B inhibitor in preparation of drugs for treating or preventing kidney damage caused by autoimmune disease

By: Zhao, Yue; Liu, Zhihong

China, CN113304153 A 2021-08-27 | Language: Chinese, Database: CAplus

The present invention relates to an application of aurora kinase B inhibitor charact erized in that the dosage of the aurora kinase B inhibitor (AZD1152) is 5-150mg/kg/day. The administration of AZD115225 mg/kg starting from the age of 10 wk in M RL/lpr lupus mice for 8 wk of continuous treatment can significantly improve lymph node and enlarged spleen, serum inflam matory factors increase and other immune activation phenotypes, reduce the ratio of urine protein to creati nine, suggesting that AZD1152 treatment can inhibit autoimmune diseases.

Keywords: aurora kinase B inhibitor drug kidney damage autoimmune disease

PatentPak available

Substances (3) Reactions (0) Citing (0)

46

Activity Comparison of Epigenetic Modulators against the Hemoprotozoan Parasites Babesia divergens and Plasmodium falciparum

By: Vanheer, Leen N.; Kafsack, Bjorn F. C.
ACS Infectious Diseases (2021), 7(8), 2277-2284 | Language: English, Database: CAplus and MEDLINE

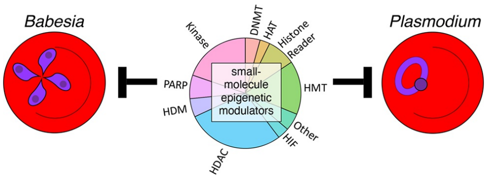

Babesiosis is a tick-borne parasitic disease of humans and livestock that has dramat ically increased in frequency and geog. range over the past few decades. Infection of cattle often causes large economic losses, and human infection can be fatal in immunocom promised patients. Unlike for malaria, another disease caused by hemopro tozoan parasites, limited treatment options exist for Babesia infections. As epigenetic regulation is a promising target for new antipar asitic drugs, we screened 324 epigenetic inhibitors against Babesia divergens blood stages and identified 75 (23%) and 17 (5%) compounds that displayed ≥90% inhibition at 10 and 1μ M, resp., including over a dozen compounds with activity in the low nanomolar range. We observed differ ential activity of some inhibitor classes against Babesia divergens and Plasmodium falciparum parasites and identified pairs of compounds with a high difference in activity despite a high similarity in chem. structure, highlighting new insights into the develo pment of epigenetic inhibitors as antiparasitic drugs.

Keywords: epigenetic modulator Babesia Plasmodium antiparasitic; Babesia; Plasmo dium; epigenetic; parasites; small molecule screen

Substances (326) Reactions (0) Citing (7)

47

Hyperbranched polyester polyol derivative as drug solubilizer

By: Haag, Rainer; Ferraro, Magda; Mohammadifar, Ehsan; Schirner, Michael
European Patent Organization, EP3858330 A1 2021-08-04 | Language: English, Database: CAplus

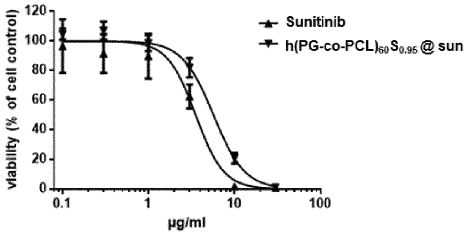

FIG 1

The present invention relates to a composition comprising a hyperbranched polyester polyol derivative and a small mol. comprising at least one cyclic amine. The hyperbranched polyester polyol derivative is obtainable by a method comprising the following steps: (a) reacting only glycidol and ε-caprolactone at a temper ature lying in a range of between 40°C and 140°C to obtain a hyperbr anched polyester polyol derivative in which caprol actone residues and glycerol residues are randomly arranged; (b) reacting the hyperbranched polyester polyol derivative of step (a) with a sulfation reagent to obtain a sulfated hyperbr anched polyester polyol as hyperbranched polyester polyol derivative

Keywords: hyperbranched polyester polyol drug solubi lizer

PatentPak available

Substances (61) Reaction (1) Citing (0)

48

Hyperbranched polyester polyol derivative as drug solubilizer

By: Haag, Rainer; Ferraro, Magda; Mohammadifar, Ehsan; Schirner, Michael
World Intellectual Property Organization, WO2021152172 A1 2021-08-05 | Language: English, Database: CAplus

The present invention relates to a composition comprising a hyperbranched polyester polyol derivative and a small mol. comprising at least one cyclic amine. The hyperbranched polyester polyol derivative is obtainable by a method comprising the following steps: (a) reacting only glycidol and ε-caprolactone at a temper ature lying in a range of between 40°C and 140°C to obtain a hyperbr anched polyester polyol derivative in which caprol actone residues and glycerol residues are randomly arranged; (b) reacting the hyperbranched polyester polyol derivative of step (a) with a sulfation reagent to obtain a sulfated hyperbr anched polyester polyol as hyperbranched polyester polyol derivative

Keywords: hyperbranched polyester polyol drug solubi lizer

PatentPak available

Substances (63) Reactions (0) Citing (0)

49

Chromosome oscillation promotes Aurora A-dependent Hec1 phosphorylation and mitotic fidelity

By: Iemura, Kenji ; Natsume, Toyoaki ; Maehara, Kayoko ; Kanemaki, Masato T. ; Tanaka, Kozo
Journal of Cell Biology (2021), 220(7), e202006116 | Language: English, Database: CAplus and MEDLINE

Most cancer cells show chromosomal instability, a condition where chromosome missegr egation occurs frequently. We found that chromosome oscillation, an iterative chromosome motion during metaphase, is attenuated in cancer cell lines. We also found that metaphase phosphorylation of Hec1 at serine 55, which is mainly dependent on Aurora A on the spindle, is reduced in cancer cell lines. The Aurora A-dependent Hec1-S55 phosphorylation level was regulated by the chromosome oscill ation amplitude and vice versa: Hec1-S55 and -S69 phosphorylation by Aurora A is required for efficient chromosome oscill ation. Furthermore, enhancement of chromosome oscillation reduced the number of erroneous kineto chore-microtubule attachments and chromosome missegre gation, whereas inhibition of Aurora A during metaphase increased such errors. We propose that Aurora A- mediated metaphase Hec1-S55 phosphorylation through chromosome oscillation, together with Hec1-S69 phosphorylation, ensures mitotic fidelity by eliminating erroneous kinetochore-microtubule attachments. Attenuated chromosome oscillation and the resulting reduced Hec1-S55 phosphorylation may be a cause of C IN in cancer cell lines.

Keywords: Aurora A Hec phosphorylation mitotic fidelity chromosome oscill ation

Substances (7) Reactions (0) Citing (11)

50

Inhibition of Aurora Kinase B activity disrupts development and differentiation of salivary glands

By: Shaalan, Abeer K. ; Teshima, Tathyane H. N.; Tucker, Abigail S.; Proctor, Gordon B.
Cell Death Discovery (2021), 7(1), 16 | Language: English, Database: CAplus and MEDLINE

Abstract: Little is known about the key mols. that regulate cell division during organogenesis. Here we determine the role of the cell cycle promoter aurora kinase B (AURKB) during development, using embryonic salivary glands (E-SGs) as a model. AURKB is a serine/threonine kinase that regulates key events in mitosis, which makes it an attractive target for tailored anticancer therapy. Many reports have elaborated on the role of AURKB in neoplasia and cancer; however, no previous study has shown its role during organ development. Our previous experiments have highlighted the essential requirement for AURKB during adult exocrine regeneration. To investigate if AURKB is similarly required for progression during embryonic development, we pharmacol. inhibited AURKB in developing submand ibular glands (SMGs) at embryonic day (E)13.5 and E16.5, using the highly potent and selective drug Barasertib. Inhibition of AURKB interfered with the expansion of the embryonic buds. Interestingly, this effect on S MG develo pment was also seen when the mature explants (E16.5) were incubated for 24 h with another cell cycle inhibitor Aphidi colin. Barasertib prompted apoptosis, DNA damage and senescence, the markers of which (cleaved caspase 3, γH2AX, SA-βgal and p21, resp.), were predominantly seen in the developing buds. In addition to a reduction in cell cycling and prolife ration of the epithelial cells in response to AURKB inhibition, Barasertib treatment led to an excessive generation of reactive oxygen species (R OS) that resulted in downregulation of the acinar differentiation marker Mist1. Importantly, inhibition of ROS was able to rescue this loss of identity, with Mist1 expression maintained despite loss of AURKB. Together, these data identify A URKB as a key mol. in supporting embryonic development and differentiation, while inhibiting senescence-inducing signals during organogenesis.

Keywords: aurora Kinase B salivary gland

Substances (4) Reactions (0) Citing (3)

51

Substituted 4-aminoisoindoline-1,3-dione compounds and second active agents for combined use for treating hematological malignancies

By: Anton, Maria Soraya Carrancio; Buchholz, Tonia J.; Chang, Henry; Filvaroff, Ellen; Kasibhatla, Shailaja; Lopez-Girona, Antonia; Mohan, Adithi; Narla, Rama Krishna; Pourdehnad, Michael

United States, US20210113576 A1 2021-04-22 | Language: English, Database: CAplus

Sensitivity to Compound 1 single agent in DLBCL cell lines

sensitive to resistant

TMD-8	SU-DHL-2	SU-DHL-10	Pfeiffer	WSU-DLCL2	OCI-Ly10	Riva	U2932	SU-DHL-4	DB
58.67	108.4	123.7	288.2	362	403.5	496.3	577	585.3	670.8

Provided herein are methods of using (*S*)-2-(2,6-dioxopiperidin-3-yl)-4-((2-fluoro-4-((3-morpholinoazetidin-1-yl)methyl)benzyl) amino)isoindoline-1,3-dione, or an enantiomer, mixture of enantiomers, tautomer, isotopolog, or pharmaceutically acceptable salt thereof, in combination with a second active agent for treating, preventing or managing hematol. malignancies. The second active agent is one or more of an HDAC inhibitor, a BCL2 inhibitor, a BTK inhibitor, an mTOR inhibitor, a PI3K inhibitor, a PKCβ inhibitor, a SYK inhibitor, a JAK2 inhibitor, an Aurora kinase inhibitor, an EZH2 inhibitor, a BET inhibitor, a hypomethylating agent, a DOT1L inhibitor, a HAT inhibitor, a WDR5 inhibitor, a DNMT1 inhibitor, an LSD-1 inhibitor, a G9A inhibitor, a PRMT5 inhibitor, a BRD inhibitor, a SUV420H1/H2 inhibitor, a CARM1 inhibitor, a PLK1 inhibitor, an NEK2 inhibitor, an MEK inhibitor, a PHF19 inhibitor, a PIM inhibitor, an IGF-1R inhibitor, an XPO1 inhibitor, a BIRC5 inhibitor, or a chemotherapy.

Keywords: hematol neoplasm treatment aminoisoindolinedione compound active agent combination

PatentPak available

Substances (131) | Reactions (3) | Citing (0)

52

Predicting cell health phenotypes using image-based morphology profiling

By: Way, Gregory P.; Kost-Alimova, Maria; Shibue, Tsukasa; Harrington, William F.; Gill, Stanley; Piccioni, Federica; Becker, Tim; Shafqat-Abbasi, Hamdah; Hahn, William C.; Carpenter, Anne E.; et al

Molecular Biology of the Cell (2021), 32(9), 995-1005 | Language: English, Database: CAplus and MEDLINE

Genetic and chem. perturbations impact diverse cellular phenotypes, including multiple indicators of cell health. These readouts reveal toxicity and antitumorigenic effects relevant to drug discovery and personalized medicine. We developed two customized microscopy assays, one using four targeted reagents and the other three targeted reagents, to collectively measure 70 specific cell health phenotypes including proliferation, apoptosis, reactive oxygen species, DNA damage, and cell cycle stage. We then tested an approach to predict multiple cell health phenotypes using Cell Painting, an inexpensive and scalable image-based morphol. assay. In matched CRISPR perturbations of three cancer cell lines, we collected both Cell Painting and cell health data. We found that simple machine learning algorithms can predict many cell health readouts directly from Cell Painting images, at less than half the cost. We hypothesized that these models can be applied to accurately predict cell health assay outcomes for any future or existing Cell Painting dataset. For Cell Painting images from a set of 1500+ compound perturbations across multiple doses, we validated predictions by orthogonal assay readouts. We provide a web app to browse predictions: http://broad.io/cell-health-apparatus Our approach can be used to add cell health annotations to Cell Painting datasets.

Keywords: cell health phenotype morphol profiling deep machine learning

Substances (6) | Reactions (0) | Citing (33)

53

Targeting pan-essential genes in cancer: challenges and opportunities

By: Chang, Liang; Ruiz, Paloma ; Ito, Takahiro; Sellers, William R.
Cancer Cell (2021), 39(4), 466-479 | Language: English, Database: CAplus and MEDLINE

Despite remarkable successes in the clinic, cancer targeted therapy development remains challenging and the failure rate is disappointingly high. This problem is partly due to the misappl ication of the targeted therapy paradigm to therapeutics targeting pan-essential genes, which can result in therapeutics whereby efficacy is attenuated by dose-limiting toxicity. Here we summarize the key features of successful chemotherapy and targeted therapy agents, and use case studies to outline recurrent challenges to drug development efforts targeting pan-essential genes. Finally, we suggest strategies to avoid previous pitfalls for ongoing and future development of pan-essential therapeutics.

Keywords: human neoplasm therapeutic target chemotherapy antibody drug conjugate; drug develo pment; pan-essential genes; target identification; target validation; targeted therapies

Substances (22) Reactions (0) Citing (45)

54

Combined inhibition of XIAP and BCL2 drives maximal therapeutic efficacy in genetically diverse aggressive acute myeloid leukemia

By: Hashimoto, Mari; Saito, Yoriko; Nakagawa, Ryo; Ogahara, Ikuko; Takagi, Shinsuke ; Takata, Sadaaki; Amitani, Hanae; Endo, Mikiko; Yuki, Hitomi ; Ramilowski, Jordan A.; et al
Nature Cancer (2021), 2(3), 340-356 | Language: English, Database: CAplus and MEDLINE

Aggressive therapy-resistant and refractory acute myeloid leukemia (A ML) has an extremely poor outcome. By analyzing a large number of genetically complex and diverse, primary high-risk poor-outcome human AML samples, we identified specific pathways of therapeutic vulnerability. Through drug screens followed by extensive in vivo validation and genomic analyses, we found inhibition of cytosolic and mitochondrial anti-apoptotic proteins XIAP, BCL2 and MCL1, and a key regulator of mitosis, A URKB, as a vulnerability hub based on patient-specific genetic aberrations and transcriptional signatures. Combinatorial therapeutic inhibition of XIAP with an addnl. patient-specific vulnerability eliminated established AML in vivo in patient-derived xenografts (P DXs) bearing diverse genetic aberrations, with no signs of recurrence during off-treatment follow-up. By integrating genomic profiling and drug-sensitivity testing, this work provides a platform for a precision- medicine approach for treating aggressive A ML with high unmet need.

Keywords: venetoclax S63845 anticancer agent XIAP BCL2 acute myeloid leukemia

Substances (10) Reactions (0) Citing (7)

55

Preparation of biaminoquinolines and nanoformulations for cancer treatment

By: Li, Yuanpei; Ma, Zhao

World Intellectual Property Organization, WO2021055705 A1 2021-03-25 | Language: English, Database: CAplus

The invention provides bisaminoquinoline compounds of formula I, wherein R^1 is H, C_{1-40} alkyl, C_{2-40} alkenyl, etc.; R^{2a}, R^{2b}, R^{3a}, R^{3b} are independently H, C_{1-40} alkyl, C_{2-40} alkenyl, etc.; m and n are indepen dently 1 to 10; p = 1 - 20; each X is absent or O; and pharmaceutically acceptable salt thereof, are claimed. Example compound II was prepared by a general procedure (procedure given). The present invention also provides nanoca rriers comprising compounds of the present invention, and methods of using the nanocarriers for treating diseases and as imaging agent.

Keywords: biaminoquinoline preparation nanoformulation cancer treatment imaging

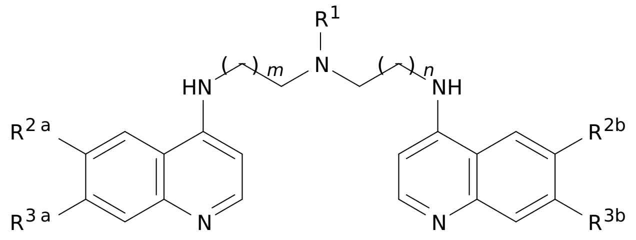

I

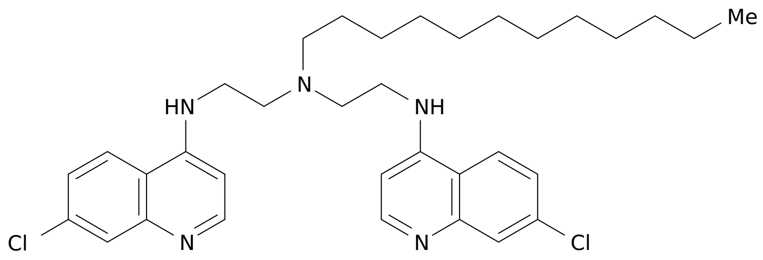

II

PatentPak available

Substances (342) Reactions (90) Citing (0)

56

Nanoencapsulated combination drug formulations

By: Baldwin, Paige; Sridhar, Srinivas; Singh, Bijay
United States, US20210085619 A1 2021-03-25 | Language: English, Database: CAplus

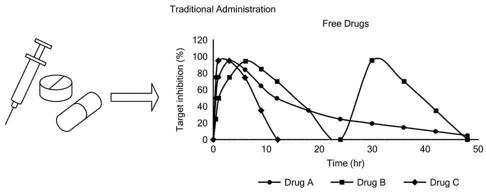

Pharmaceutical formulations (nanococktails) include two or more nanoparticulate delivery vehicles each including an active agent. The nanococktails can be formulated at any ratio of active agents by changing the ratio of nanopart iculate delivery vehicles in the nanococktail. The nanoparticulate delivery vehicles can have matched surface potential to prevent aggreg ation. The nanopart iculate delivery vehicles of the combined formul ation are designed to provide each active agent with desired, preferably overla pping therapeutic windows and a specif ically selected pharmaco kinetic and pharmaco dynamic profile when the nanoco cktail is admini stered to a subject. Each of the nanopart iculate delivery vehicles can be designed to enhance solubi lity, duration of action, targeting, stability, and to program release while preventing degradation of an active agent and preventing side effects, toxicity, and tolera bility issues in a subject.

Keywords: nanoencapsulation combination drug formul ation pharmaco dynamic

PatentPak available

Substances (51) Reactions (0) Citing (0)

57

QSAR Modeling of SARS-CoV M^{pro} Inhibitors Identifies Sufugolix, Cenicriviroc, Proglumetacin, and Other Drugs as Candidates for Repurposing against SARS-CoV-2

By: Alves, Vinicius M.; Bobrowski, Tesia; Melo-Filho, Cleber C.; Korn, Daniel; Auerbach, Scott; Schmitt, Charles; Muratov, Eugene N. ; Tropsha, Alexander
Molecular Informatics (2021), 40(1), 2000113 | Language: English, Database: CAplus and MEDLINE

The main protease (M^{pro}) of the SARS-CoV-2 has been proposed as one of the major drug targets for C OVID-19. We have identified the exptl. data on the inhibitory activity of compounds tested against the closely related (96% sequence identity, 100% active site conservation) M^{pro} of SARS-CoV. We developed QSAR models of these inhibitors and employed these models for virtual screening of all drugs in the DrugBank database. Similarity searching and mol. docking were explored in parallel, but docking failed to correctly discriminate between exptl. active and inactive compounds, so it was not relied upon for prospe ctive virtual screening. Forty-two compounds were identified by our models as consensus computa tional hits. Subsequent to our computa tional studies, N CATS reported the results of exptl. screening of their drug collection in S ARS-CoV-2 cytopathic effect assay (https: //opendata.ncats. nih.gov/covid19/). Coincidentally, NCATS tested 11 of our 42 hits, and three of them, cenicr iviroc (AC_{50} of 8.9μM), proglumetacin (tested twice independently, with AC_{50} of 8.9μM and 12.5μM), and sufugolix (AC_{50} 12.6μM), were shown to be active. These observ ations support the value of our modeling approaches and models for guiding the exptl. investi gations of putative anti-COVID-19 drug candidates. All data and models used in this study are publicly available via Supplem entary Materials, GitHub (https://github. com/alvesvm/sars-cov-mpro), and Chembench web portal (https: //chembench.mml.unc.edu/).

Keywords: SARS CoV2 coronavirus protease inhibitors drug repurp osing screening chemoinfo rmatics; SARS-CoV-2; SARS-CoV-2 Mpro.; cheminformatics; drug repurp osing; virtual screening

Substances (12) Reactions (0) Citing (37)

58

Loss of Aurora kinase signaling allows lung cancer cells to adopt endoreplication and form polyploid giant cancer cells that resist antimitotic drugs

By: Tagal, Vural; Roth, Michael G.
Cancer Research (2021), 81(2), 400-413 | Language: English, Database: CAplus and MEDLINE

Polyploid giant cancer cells (PGCC) are common in tumors and have been associated with resistance to cancer therapy, tumor relapse, malignancy, immunosuppression, metastasis, cancer stem cell production, and modulation of the tumor microenvi ronment. However, the mol. mechanisms that cause these cells to form are not yet known. In this study, we discover that Aurora kinases are synergistic determinants of a switch from the proliferative cell cycle to polyploid growth and multinucleation in lung cancer cell lines. When Aurora kinases were inhibited together, lung cancer cells uniformly grew into multinucleated PGCCs. These cells adopted an endoreplication in which the genome replicates, mitosis is omitted, and cells grow in size. Consequently, such cells continued to safely grow in the presence of antimitotic agents. These PGCC re-entered the proliferative cell cycle and grew in cell number when treatment was terminated. Thus, PGCC formation might represent a fundamental cellular response to Aurora kinase inhibitors and contributes to therapy resistance or tumor relapse.

Keywords: polyploid giant cancer Aurora kinase endoreplication antimitotic drug resistance

Substances (29) Reactions (0) Citing (15)

59

Experiments in the EpiDerm 3D skin in vitro model and minipigs in vivo indicate comparatively lower in vivo skin sensitivity of topically applied aneugenic compounds

By: Schuler, Maik; Tomlinson, Lindsay; Homiski, Michael; Cheung, Jennifer; Zhan, Yutian; Coffing, Stephanie; Engel, Maria; Rubitski, Elizabeth; Seitis, Gary; Hales, Katherine; et al
Toxicological Sciences (2021), 180(1), 103-121 | Language: English, Database: CAplus and MEDLINE

Risk management of in vitro aneugens for topically applied compounds is not clearly defined because there is no validated methodol. to accurately measure compound concentration in proliferating stratum basale keratinocytes of the skin. Here, we exptl. tested several known aneugens in the EpiDerm reconstructed human skin in vitro micronucleus assay and compared the results to flow cytometric mechanistic biomarkers (phospho-H3; MPM2, DNA content). We then evaluated similar biomarkers (Ki- 67, nuclear area) using immunohistochem. in skin sections of minipigs following topical exposure the potent aneugens, colchicine, and hesper adin. Data from the EpiDerm model showed pos. micronucleus responses for all aneugens tested following topical or direct media dosing with similar sensitivity when adjusted for applied dose. Quant. benchmark dose-response anal. exhibited increases in the mitotic index biomarkers phospho-H3 and MPM2 for tubulin binders and polyploidy for aurora kinase inhibitors are at least as sensitive as the micronucleus endpoint. By comparison, the aneugens tested did not induce histop athol. changes, increases in Ki-67 immunolabeling or nuclear area in skin sections from the in vivo minipig study at doses in significant excess of those eliciting a response in vitro. Results indicate the EpiDerm in vitro micronucleus assay is suitable for the hazard identification of aneugens. The lack of response in the minipig studies indicates that the barrier function of the minipig skin, which is comparable to human skin, protects from the effects of aneugens in vivo. These results provide a basis for conducting addnl. studies in the future to further refine this understanding.

Keywords: topical aneugen genotoxicant aneuploidy keratinocyte; adverse outcome pathway; aneuploidy; aurora kinase inhibitor; skin; tubulin binder

Substances (6) Reactions (0) Citing (3)

60

A cell cycle progression-derived gene signature to predict prognosis and therapeutic response in hepatocellular carcinoma

By: Hui, Yongfeng; Leng, Junzhi; Jin, Dong; Liu, Di; Wang, Genwang; Wang, Qi ; Wang, Yanyang
Disease Markers (2021), 1986159 | Language: English, Database: CAplus and MEDLINE

Dysregulation of cell cycle progre ssion (CCP) is one of the hallmarks of cancer. Here, our study is aimed at developing a C CP-derived gene signature for predicting high-risk population of hepatocellular carcinoma (HCC). Our study retrospe ctively analyzed the transcr iptome profiling and clin. information of HCC patients from The Cancer Genome Atlas (T CGA) and International Cancer Genome Consortium (ICGC) projects. Uni- and multiv ariate cox regression models were conducted for identi fying which hallmarks of cancer were risk factors of HCC. CCP-derived gene signature was developed with LASSO method. The predictive efficacy was verified by R O C curves and subgroup analyses. A nomogram was then generated and validated by R OC, calibration, and decisive curves. Immune cell infiltration was estimated with ssGSEA method. Potential small mol. compounds were predicted via C TRP and CMap analyses. The response to chemotherapeutic agents was evaluated based on the GDSC project. Among hallmarks of cancer, C CP was identified as a dominant risk factor for HCC prognosis. CCP-derived gene signature displayed the favorable predictive efficacy in HC C prognosis independent of other clinico pathol. parameters. A nomogram was generated for optimizing risk stratif ication and quantifying risk evalua tion. CCP-derived signature was in relation to immune cell infiltr ation, HLA, and immune checkpoint expres sion. Combining CTRP and CMap analyses, fluvastatin was identified as a promising therap eutic agent against HCC. Furthermore, CC P-derived signature might be applied for predicting the response to doxoru bicin and gemcit abine. Collectively, CCP-derived gene signature was a promising marker in prediction of survival outcomes and therapeutic responses for HCC patients.

Keywords: ccp

Substances (6) Reactions (0) Citing (3)

61

BCL-XL blockage in TNBC models confers vulnerability to inhibition of specific cell cycle regulators

By: Castellanet, Olivier; Ahmad, Fahmida; Vinik, Yaron; Mills, Gordon B.; Habermann, Bianca; Borg, Jean-Paul; Lev, Sima; Lamballe, Fabienne; Maina, Flavio
Theranostics (2021), 11(19), 9180-9197 | Language: English, Database: CAplus and MEDLINE

Cell cycle regulators are frequently altered in Triple-Neg. Breast Cancer (TNBC). Emerging agents targeting these signals offer the possibility to design new combina torial therapies. However, preclin. models that recapitulate TNBC primary resistance and heterog eneity are essential to evaluate the potency of these combined treatm ents. Bioinformatic processing of human breast cancer datasets was used to analyze correlations between expression levels of cell cycle regulators and patient survival outcome. The M MT V-R26Met mouse model of TNBC resistance and heterogeneity was employed to analyze expression and targeting vulnera bility of cell cycle regulators in the presence of BCL-XL blockage. Robustness of outcomes and select ivity was further explored using a panel of human breast cancer cells. Orthotopic studies in nude mice were applied for preclin. evaluation of efficacy and toxicity. Altera tions of protein expression, phosphorylation, and/or cellular localisation were analyzed by western blots, reverse phase protein array, and immunocytochem. Bioinformatics was performed to highlight drug's mechanisms of action. We report that high expression levels of the BCL2L1 gene encoding BCL-XL and of specific cell cycle regulators correlate with poor survival outcomes of TNBC patients. Blockage of BCL-XL confers vulnerability to drugs targeting CDK1/2/4, but not FOXM1, CDK4/6, Aurora A and Aurora B, to all MMTV-R26Met and human TNBC cell lines tested. Combined blockage of B CL-XL and CDK1/2/4 interfered with tumor growth in vivo. Mechanistically, we show that, co- targeting of BCL-XL and CDK1/2/4 synergistically inhibited cell viability by combina torial depletion of survival and RTK/AKT signals, and concomi tantly restoring FOXO3a tumor suppression actions. This was accomp anied by an accumulation of DNA damage and conseq uently apoptosis. Our studies illustrate the possib ility to exploit the vulnera bility of TNBC cells to CDK1/2/4 inhibition by targeting B CL-XL. Moreover, they underline that specificity matters in targeting cell cycle regulators for combinatorial anticancer therapies.

Keywords: triple neg breast cancer BCLXL blockage cell cycle; BCL-XL; MET; cancer mouse model; cell cycle regula tors; triple-negative breast cancer

Substances (15) | Reactions (0) | Citing (2)

62

Aurora B and C kinases regulate chromosome desynapsis and segregation during mouse and human spermatogenesis

By: Wellard, Stephen R.; Schindler, Karen; Jordan, Philip W.
Journal of Cell Science (2020), 133(23), jcs248831 | Language: English, Database: CAplus and MEDLINE

Precise control of chromosome dynamics during meiosis is critical for fertility. A gametocyte undergoing meiosis coordinates formation of the synaptonemal complex (SC) to promote efficient homologous chromosome recombi nation. Subsequent disass embly of the SC occurs prior to segregation of homologous chromosomes during meiosis I. We examined the requir ements of the mammalian Aurora kinases (AURKA, AURKB and AURKC) during SC disassembly and chromosome segregation using a combination of chem. inhibition and gene deletion approaches. We find that both mouse and human spermat ocytes fail to disass emble SC lateral elements when the kinase activity of AURKB and AURKC are chem. inhibited. Interestingly, both Aurkb condit ional knockout and Aurkc knockout mouse spermatocytes successfully progress through meiosis, and the mice are fertile. In contrast, Aurkb, Aurkc double knockout spermatocytes fail to coordinate disass embly of SC lateral elements with chromosome conden sation and segreg ation, resulting in delayed meiotic progre ssion. In addition, deletion of Aurkb and Aurkc leads to an accumu lation of metaphase spermatocytes, chromosome missegregation and aberrant cytoki nesis. Collectively, our data demonstrate that AURKB and AURKC functionally compensate for one another ensuring successful mammalian spermato genesis.

Keywords: auroraB auroraC kinase chromosome desynapsis segreg ation spermatogenesis; Aurora kinase; Chromosome segreg ation; Gametogenesis; Infertility; Meiosis; Synaptonemal complex

Substances (10) | Reactions (0) | Citing (15)

63

Computationally predicting clinical drug combination efficacy with cancer cell line screens and independent drug action

By: Ling, Alexander ; Huang, R. Stephanie

Nature Communications (2020), 11(1), 5848 | Language: English, Database: CAplus and MEDLINE

Evidence has recently emerged that many clin. cancer drug combinations may derive their efficacy from indepe ndent drug action (I DA), where patients only receive benefit from the single most effective drug in a drug combin ation. Here we present I DACombo, an I DA based method to predict the efficacy of drug combin ations using monotherapy data from high-throughput cancer cell line screens. We show that IDACombo predictions closely agree with measured drug combin ation efficacies both in vitro (Pearson's correlation = 0.93 when comparing predicted efficacies to measured efficacies for > 5000 combin ations) and in a systematically selected set of clin. trials (accuracy > 84% for predicting statistically significant improvements in patient outcomes for 26 first line therapy trials). Finally, we demonstrate how IDACombo can be used to systematically prioritize combinations for development in specific cancer settings, providing a framework for quickly translating existing monotherapy cell line data into clin. meaningful predictions of drug combination efficacy.

Keywords: independent drug action clin combin ation cancer cell line

Substances (122) | Reactions (0) | Citing (19)

64

Methods for reducing tumor progression and fibrosis and increasing adaptive immunity in malignancies by administering dimethyl-β-hydroxycholenamide derivatives

By: Glazer, Robert I.; Levi, Moshe; Yuan, Hongyan; Ranjit, Suman

World Intellectual Property Organization, WO2020252198 A1 2020-12-17 | Language: English, Database: CAplus

Aspects of the technol. described herein relate to a method of treating a malignancy in a subject. This method involves selecting a subject having a malignancy and administering di-Meβ-hydroxy-cholenamide (DMHCA) or derivative thereof to the subject in an amount effective to treat the malignancy. Methods of reducing malignancy- associated fibrosis in a subject and pharmac eutical combinations comprising (i) DMHCA or derivative thereof and (ii) one or more immune checkpoint inhibitors are also disclosed.

Keywords: tumor fibrosis treatment adaptive immunity increase cholenamide derivative

PatentPak available

Substances (62) | Reactions (0) | Citing (0)

65

Methods for treating small cell neuroendocrine (SCN) tumors and small-round-blue cell tumors (SRBCTs)

By: Graeber, Thomas G.; Balanis, Nikolas G.; Sheu, Katherine M.; Witte, Owen N.; Esedebe, Favour; Park, Jung Wook
World Intellectual Property Organization, WO2020243329 A1 2020-12-03 | Language: English, Database: CAplus

The current disclosure provides methods of identifying and treating small cell neuroen docrine (SCN) tumors and small-round-blue cell tumors (SRBCTs). Aspects of the disclosure relates to a method comprising measuring the level of expression of one or more biomarkers in a biol. sample from a cancer patient. Further aspects relate to a method for treating small cell neuroendocrine (SCN) cancer or small-round-blue cell tumor (S RBCT) in a patient comprising adminis tering a cancer treatment to the patient, wherein the cancer treatment is a treatment selected from ABT-263, PHA-793887, vorinostat, tubastatin A, CX-5461 and combinations thereof.

Keywords: antitumor ABT263 vorinostat small cell neuroen docrine cancer treatment

PatentPak available

Substances (259) | Reactions (0) | Citing (0)

66

Nf1-mutant tumors undergo transcriptome and kinome remodeling after inhibition of either mTOR or MEK

By: Pucciarelli, Daniela; Angus, Steven P.; Huang, Benjamin; Zhang, Chi; Nakaoka, Hiroki J.; Krishnamurthi, Ganesh; Bandyopadhyay, Sourav; Clapp, D. Wade; Shannon, Kevin; Johnson, Gary L.; et al
Molecular Cancer Therapeutics (2020), 19(11), 2382-2395 | Language: English, Database: CAplus and MEDLINE

Loss of the tumor suppressor NF1 leads to activation of RAS effector pathways, which are therapeu tically targeted by inhibition of m TOR (mTORi) or MRK (MEKi). However, therapeutic inhibition of RAS effectors leads to the develo pment of drug resistance and ultimately disease progression. To investigate mol. signatures in the context of N F1 loss and subsequent acquired drug resist ance, we analyzed the exomes, transcriptomes, and kinomcs of Nf1-mutant mouse tumor cell lines and deriva tives of these lines that acquired resistance to either MEKi or mTORi. Biochem. comparisons of this unique panel of tumor cells, all of which arose in Nf1+/- mice, indicate that loss of heterozygosity of Nf1 as an initial genetic event does not confer a common biochem. signature or response to kinase inhibition. Although acquired drug resistance by Nf1- mutant tumor cells was accompanied by altered kinomes and irreversibly altered transcriptomes, functionally in multiple Nf1- mutant tumor cell lines, M EKi resistance was a stable phenotype, in contrast to mTORi resistance, which was reversible. Collectively, these findings demonstrate that Nf1-mutant tumors represent a heterogeneous group biochem. and undergo broader remodeling of kinome activity and gene expression in response to targeted kinase inhibition.

Keywords: anticancer agent NF1 mTOR MEK transcriptome kinome cancer

Substances (13) | Reactions (0) | Citing (1)

67

Discovery of SP-96, the first non-ATP-competitive Aurora Kinase B inhibitor, for reduced myelosuppression

By: Lakkaniga, Naga Rajiv ; Zhang, Lingtian ; Belachew, Binyam; Gunaganti, Naresh ; Frett, Brendan ; Li, Hong-yu
European Journal of Medicinal Chemistry (2020), 203, 112589 | Language: English, Database: CAplus and MEDLINE

Aurora Kinase B is a serine-threonine kinase known to be overexpressed in several cancers, with no inhibitors approved for clin. use. Herein, we present the discovery and optimization of a series of novel quinazoline-based Aurora Kinase B inhibitors. The lead inhibitor SP-96 shows sub-nanomolar potency in Aurora B enzymic assays (IC_{50} = 0.316 ± 0.031 nM). We identified the important pharmacophore features resulting in selectivity against receptor tyrosine kinases. Particularly, SP-96 shows >2000 fold selectivity against FLT3 and KIT which is important for normal hematopoiesis. This could diminish the adverse effect of neutropenia reported in the clin. trials of the Aurora B inhibitor Barasertib, which inhibits FLT3 and KIT in addition to Aurora B. Enzyme kinetics of SP-96 shows non-ATP-competitive inhibition which makes it a first-in-class inhibitor. Further, SP-96 shows selective growth inhibition in NCI60 screening, including inhibition of MDA-MD-468, a Triple Neg. Breast Cancer cell line.

Keywords: quinazoline derivative aurora kinase inhibitor preparation breast cancer; Anti-Cancer drugs; Aurora kinase B; Kinase inhibitors; Non-ATP competitive Inhibition; Structure activity relationship (SAR); Synthetic lethal toxicity

Substances (2) Reactions (0) Citing (13)

68

Inhibition of Aurora Kinase B attenuates fibroblast activation and pulmonary fibrosis

By: Kasam, Rajesh K.; Ghandikota, Sudhir ; Soundararajan, Divyalakshmi; Reddy, Geereddy B.; Huang, Steven K.; Jegga, Anil G. ; Madala, Satish K.
EMBO Molecular Medicine (2020), 12(9), e12131 | Language: English, Database: CAplus and MEDLINE

Fibroblast activation including proliferation, survival, and ECM production is central to initiation and maintenance of fibrotic lesions in idiopathic pulmonary fibrosis (IPF). However, druggable mols. that target fibroblast activation remain limited. In this study, we show that multiple pro-fibrotic growth factors, including TGFα, CTGF, and IGF1, increase aurora kinase B (AURKB) expression and activity in fibroblasts. Mechanistically, we demonstrate that Wilms tumor 1 (WT1) is a key transcription factor that mediates TGFα-driven AURKB upregulation in fibroblasts. Importantly, we found that inhibition of AURKB expression or activity is sufficient to attenuate fibroblast activation. We show that fibrosis induced by TGFα is highly dependent on AURKB expression and treating TGFα mice with barasertib, an AURKB inhibitor, reverses fibroblast activation, and pulmonary fibrosis. Barasertib similarly attenuated fibrosis in the bleomycin model of pulmonary fibrosis. Together, our preclin. studies provide important proof-of-concept that demonstrate barasertib as a possible intervention therapy for IPF.

Keywords: Aurora Kinase B fibroblast pulmonary fibrosis; Aurora Kinase B; Barasertib; Wilms' tumor 1; fibroproliferation; pulmonary fibrosis

Substances (5) Reactions (0) Citing (18)

69

Dihydro-spiro[indoline-3:1'-isoquinolin]-2-ones and their analogs and derivatives and methods of treating cancer and other diseases and their preparation

By: Mabanyi, Lobe Maloba Mesembe; Efange, Simon Mbua Ngale
World Intellectual Property Organization, WO2020183307 A1 2020-09-17 | Language: English, Database: CAplus

The invention is directed to various 3',4'-dihydro-2'H-spiro[indoline-3:1'-isoquinolin]-2-one compounds of formula I and methods for treating disease states and/or conditions which are mediated through sphingosine-1-phosphate receptor(s). The invention is also directed to the use of these compounds as anticancer agents and as modulators of sphingosine-1-phosphate receptor function in the treatment of disease states and/or conditions which are mediated through these receptors. In addition, the invention relates to pharmaceutical compositions comprising one or more of these compounds alone or in combin ation with other therapeutic agents. The invention is also directed to methods of treatment of cancer and/or conditions that may respond to the modulation of sphingosine-1-phosphate receptor function and which employ compounds of the present invention or pharmac eutical compos itions comprising one or more of the compounds of this invention. Compounds of formula I wherein R^1 is H, OH, halo, C_{1-6} alkoxy, etc.; R^2 and R^3 are independently H, OH, C_{1-6} hydroxyalkyl, etc.; R^4 is H, OH, halo, C_{1-6} alkoxy, etc.; R^5 and R^6 are independently H, alkyl, C_{1-6} alkoxy, etc.; R^7 is H, alkyl, arylalkyl, etc.; R^8 is H, OH, halo, NO_2, etc.; R^9, R^{10} and R^{11} are independently H, OH, halo, C_{1-6} hydroxyalkyl, etc.; R^{12} is H, OH, hydroxyalkyl, arylalkyl, etc. ; R^{13} is O and S; and pharmace utically acceptable salts, stereoisomers, solvates and polymorphs thereof, are claimed. Example compound II as prepared by cycliz ation of isatin with 3-hydroxyphenethy lamine. The invention compounds were evaluated for their anticancer activity (some data given) .

Keywords: spiroindoline isoquinolinone preparation treatment cancer

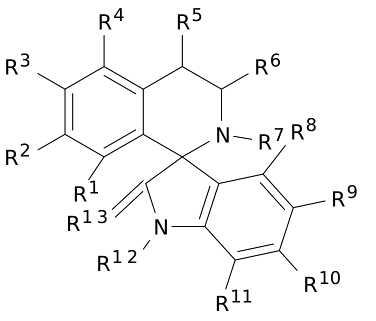

I

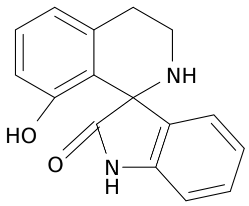

II

PatentPak available

Substances (450) | Reactions (239) | Citing (3)

70

Methods of treating venetoclax-resistant acute myeloid leukemia

By: Tyner, Jeffrey W.; Nechiporuk, Tamilla; Kurtz, Stephen; McWeeny, Shannon
World Intellectual Property Organization, WO2020176686 A1 2020-09-03 | Language: English, Database: CAplus

The present invention concerns novel methods of treatment for venetoclax-resistant acute myeloid leukemia, partic ularly in subjects with a low expression of TP53 protein or expression of TP53 protein associated with a mutation of the T P53 gene, the methods comprising administering to the subject in need thereof a therapeu tically effective amount of a NTRK/ALK kinase/ROS1 kinase inhibitor, or a pharmaceutically acceptable salt thereof.

Keywords: venetoclax antitumor resistance acute myeloid leukemia treatment

PatentPak available

Substances (73) | Reactions (0) | Citing (0)

71

High-throughput screening of regulators of axonal transport

By: Shlevkov, Evgeny; Schwarz, Thomas L.; Bray, Mark-Anthony; Carpenter Van Dyk, Anne E.
World Intellectual Property Organization, WO2020149925 A1 2020-07-23 | Language: English, Database: CAplus

Provided are techniques for processing a sequence of images of neurons to identify stationary and motile organelles of the neurons, generating tracking data associated with movement of the one or more motile organelles across the sequence of images, and determining an overall percent motile based on the identified stationary organelles and motile organe lles. Also provided are compounds identified using the systems described, wherein the compounds, and pharmaceutically acceptable salts, and compos itions thereof are administered to a subject in need thereof in a sufficient amount to increase or promote organelle (e.g., mitocho ndrial) motility, or administered in a sufficient amount to treat a disease or disorder associated with decreased organelle motility. The compounds may be used in methods for increasing or promoting organelle (e.g., mitochondrial) motility in a cell, and methods for treating a disease. Also provided in the present disclosure are pharmaceutical compositions, kits, methods, and uses including or using a compound described herein.

Keywords: high throughput screening regulator axonal transport

PatentPak available

Substances (11) Reactions (0) Citing (0)

72

Antiproliferative compounds and second active agents for combined use for treating multiple myeloma

By: Wong, Lilly L.
United States, US20200215060 A1 2020-07-09 | Language: English, Database: CAplus

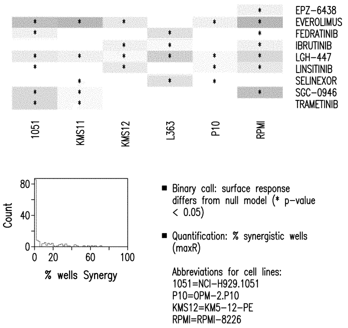

Provided herein are methods of using 4-(4-(4-(((2-(2,6-dioxopiperidin-3-yl)-1-oxoisoindolin-4-yl)oxy)methyl)benzyl)piperazin-1-yl)-3-fluorobenzonitrile, or an enantiomer, a mixture of enantiomers, a tautomer, or a pharmace utically acceptable salt thereof, in combination with a second active agent for treating, preventing or managing multiple myeloma. The second active agent is one or more of a BTK inhibitor, an mTOR inhibitor, a PIM inhibitor, an IGF-1R inhibitor, an MEK inhibitor, an XPO1 inhibitor, a DOT1L inhibitor, an EZH2 inhibitor, a JAK2 inhibitor, a BRD4 inhibitor, a PLK 1 inhibitor, an NEK2 inhibitor, an AURKB inhibitor, a BIRC5 inhibitor, a BET inhibitor, or a DNA methyltransferase inhibitor.

Keywords: antiproliferative compound combination multiple myeloma treatment

PatentPak available

Substances (76) Reactions (42) Citing (0)

73

Correction: Bromodomain and extraterminal protein inhibition blocks growth of triple-negative breast cancers through the suppression of aurora kinases. [Erratum to document cited in CA165:569636]

By: Sahni, Jennifer M.; Gayle, Sylvia S.; Bonk, Kristen L. Weber; Vite, Leslie Cuellar; Yori, Jennifer L.; Webb, Bryan; Ramos, Erika K.; Seachrist, Darcie D.; Landis, Melissa D.; Chang, Jenny C.; et al
Journal of Biological Chemistry (2020), 295(27), 9266 | Language: English, Database: CAplus and MEDLINE

There is no abstract available for this document.

Keywords: Aurora kinase BET protein inhibitor anticancer breast cancer erratum

Substances (4) Reactions (0) Citing (1)

74

Genome-wide CRISPR screen uncovers a synergistic effect of combining Haspin and Aurora kinase B inhibition

By: Huang, Min; Feng, Xu; Su, Dan ; Wang, Gang; Wang, Chao; Tang, Mengfan; Paulucci-Holthauzen, Adriana; Hart, Traver; Chen, Junjie
Oncogene (2020), 39(21), 4312-4322 | Language: English, Database: CAplus and MEDLINE

Abstract: Aurora kinases are a family of serine/threonine kinases vital for cell division. Because of the overexp ression of Aurora kinases in a broad range of cancers and their important roles in mitosis, inhibitors targeting Aurora kinases have attracted attention in cancer therapy. VX-680 is an effective pan-Aurora kinase inhibitor; however, its clin. efficacy was not satisf ying. In this study, we performed CRISPR/Cas9 screens to identify genes whose depletion shows synthetic lethality with V X-680. The top hit from these screens was GSG2 (also known as Haspin), a serine/threonine kinase that phospho rylates histone H3 at Thr- 3 during mitosis. Moreover, both Haspin knockout and Haspin inhibitor-treated HCT116 cells were hypersensitive to VX-680. Furthermore, we showed that the synthetic lethal interaction between Haspin depletion and VX-680 was mediated by the inhibition of Haspin with Aurora kinase B (AURKB), but not with Aurora kinase A (A URKA). Strikingly, combined inhibition of Haspin and AURKB had a better efficacy than single-agent treatment in both head and neck squamous cell carcinoma and non- small cell lung cancer. Taken together, our findings have uncovered a synthetic lethal interaction between AURKB and Haspin, which provides a strong rationale for this combination therapy for cancer patients.

Keywords: Haspin AURKB NSCLC VX680 CHR6494 barasertib head neck carcinoma

Substances (7) Reactions (0) Citing (11)

75

Aurora B kinase as a therapeutic target in acute lymphoblastic leukemia

By: Goto, Hiroaki ; Yoshino, Yuki; Ito, Mieko; Nagai, Junichi; Kumamoto, Tadashi; Inukai, Takesi; Sakurai, Yukari; Miyagawa, Naoyuki; Keino, Dai; Yokosuka, Tomoko; et al
Cancer Chemotherapy and Pharmacology (2020), 85(4), 773-783 | Language: English, Database: CAplus and MEDLINE

Abstract: Purpose: Acute lymphoblastic leukemia (ALL) is curable with standardized chemotherapy. However, the development of novel therapies is still required, especially for patients with relapsed or refractory disease. By utilizing an in vitro drug screening system, active mol. targeting agents against ALL were explored in this study. Methods: By the in vitro drug sensitivity test, 81 agents with various actions were screened for their cytotoxicity in a panel of 22 ALL cell lines and ALL clin. samples. The drug effect score (DES) was calculated from the dose-response of each drug for comparison among drugs or samples. Normal peripheral blood mononuclear cells were also applied onto the drug screening to provide the reference control values. The drug combination effect was screened based on the Bliss independent model, and validated by the improved isobologram method. Results: On sensitivity screening in a cell line panel, barasertib-HQPA which is an active metabolite of barasertib, an aurora B kinase inhibitor, alisertib, an aurora A kinase inhibitor, and YM155, a survivin inhibitor, were effective against the broadest range of ALL cells. The DES of barasertib-HQPA was significantly higher in ALL clin. samples compared to the reference value. There were significant correlations in DES between barasertib-HQPA and vincristine or docetaxel. In the drug combination assay, barasertib-HQPA and eribulin showed additive to synergistic effects. Conclusion: Aurora B kinase was identified to be an active therapeutic target in a broad range of ALL cells. Combination therapy of barasertib and a microtubule-targeting drug is of clin. interest.

Keywords: barasertib alisertib antitumor aurora B kinase inhibitor lymphoblastic leukemia; Acute lymphoblastic leukemia; Aurora B kinase; Barasertib; In vitro drug sensitivity test; Survivin

Substances (10) | Reactions (0) | Citing (4)

76

Methods of treatment of cancer comprising CDC7 inhibitors

By: Hassig, Christian Andrew; Hansen, Ryan James; Milutinovic, Snezana; Strouse, Bryan William
World Intellectual Property Organization, WO2020068347 A1 2020-04-02 | Language: English, Database: CAplus

Herein disclosed are methods of treatment administering SRA141 as a monotherapy or in a combination therapy useful for inhibiting the growth of tumors such as those in patients with cancer.

Keywords: treatment cancer CDC7 inhibitor SRA141

PatentPak available

Substances (14) | Reactions (0) | Citing (0)

77

Pan-transcriptome-based candidate therapeutic discovery for idiopathic pulmonary fibrosis

By: Wang, Yunguan; Yella, Jaswanth K.; Ghandikota, Sudhir; Cherukuri, Tejaswini C.; Ediga, Harshavardhana H.; Madala, Satish K.; Jegga, Anil G.
Therapeutic Advances in Respiratory Disease (2020), 14, 1753466620971143 | Language: English, Database: CAplus and MEDLINE

Two methods were used to calculate IPF-compound connectivity: gene expression-based connectivity and feature-based connectivity. Identified compounds were further prioritized if their shared mechanism(s) of action were IPF-related. We found 77 compounds as potential candidate therapeutics for IPF. Of these, 39 compounds are either FDA-approved for other diseases or are currently in phase II/III clin. trials suggesting their repurposing potential for IPF. Among these compounds are multiple receptor kinase inhibitors, aurora kinase inhibitor (barasertib), epidermal growth factor receptor inhibitors, calcium channel blocker (verapamil), phosphodiesterase inhibitors (roflumilast, sildenafil), PPAR agonists (pioglitazone), histone deacetylase inhibitors (entinostat), and opioid receptor antagonists (nalbuphine). As a proof of concept, we performed in vitro validations with verapamil using lung fibroblasts from IPF and show its potential benefits in pulmonary fibrosis. As about half of the candidates discovered in this study are either FDA-approved or are currently in clin. trials for other diseases, rapid translation of these compounds as potential IPF therapeutics is possible. Further, the integrative connectivity anal. framework in this study can be adapted in early phase drug discovery for other common and rare diseases with transcriptomic profiles. The reviews of this paper are available via the supplemental material section.

Keywords: idiopathic pulmonary fibrosis transcriptome therapy; IPF; computational drug discovery; drug discovery; drug repositioning; idiopathic pulmonary fibrosis; pulmonary fibrosis; verapamil

Substances (12) | Reactions (0) | Citing (3)

78

Aurora kinase as target for treating, preventing or curing HIV infection or aids

By: Este Araque, Jose Andres; Garcia Vidal, Edurne; Ballana Guix, Ester; Badia Corcoles, Roger; Riveira Munoz, Eva
World Intellectual Property Organization, WO2020049208 A1 2020-03-12 | Language: Spanish, Database: CAplus

The present invention relates to the use of Aurora kinase (AURK) inhibitors for treating or preventing an HIV infection or AIDS in a subject. The present invention further relates to compositions containing AURK inhibitors and combinations of AURK inhibitors with other active agents useful for treating or preventing an HIV infection or AIDS in a subject.

Keywords: aurora kinase inhibitors antiviral HIV AIDS

PatentPak available

Substances (179) | Reactions (0) | Citing (0)

79

Treatments for acute myeloid leukemia in the presence of gene mutations such as MPM1

By: Tyner, Jeffrey W.; Tognon, Cristina; Druker, Brian J.; Bottomly, Daniel; Wilmot, Beth; Kurtz, Stephen E.; Savage, Samantha; Long, Nicola; Reister-Schultz, Anna; Traer, Elie; et al

World Intellectual Property Organization, WO2020023921 A1 2020-01-30 | Language: English, Database: CAplus

The present invention relates to useful methods of treating Acute Myeloid Leukemia (AML) in a human. in the presence of gene mutations such as MPM1.

Keywords: acute myeloid leukemia treatment MPM1 gene mutation

PatentPak available

Substances (65) | Reactions (0) | Citing (1)

80

Structure-based discovery and bioactivity evaluation of novel Aurora-A kinase inhibitors as anticancer agents via docking-based comparative intermolecular contacts analysis (dbCICA)

By: Hijjawi, Majd S.; Abutayeh, Reem Fawaz; Taha, Mutasem O.

Molecules (2020), 25(24), 6003 | Language: English, Database: CAplus and MEDLINE

Aurora-A kinase plays a central role in mitosis, where aberrant activation contri butes to cancer by promoting cell cycle progre ssion, genomic instability, epithelial-mesenchymal transition, and cancer stemness. Aurora-A kinase inhibitors have shown encour aging results in clin. trials but have not gained Food and Drug Administration (FDA) approval. An innovative computational workflow named Docking-based Comparative Intermol. Contacts Anal. (dbCICA) was applied-aiming to identify novel Aurora-A kinase inhibi tors-using seventy-nine reported Aurora-A kinase inhibitors to specify the best possible docking settings needed to fit into the active-site binding pocket of Aurora-A kinase crystal structure, in a process that only potent ligands contact critical binding- site spots, distinct from those occupied by less-active ligands. Optimal dbCICA models were transformed into two corresp onding pharmacophores. The optimal one, in capturing active hits and discarding inactive ones, validated by receiver operating charact eristic anal., was used as a virtual in- silico search query for screening new mols. from the National Cancer Institute database. A fluorescence resonance energy transfer (FRET)-based assay was used to assess the activity of captured mols. and five promising Aurora-A kinase inhibitors were identi fied. The activity was next validated using a cell culture anti- proliferative assay (MTT) and revealed a most potent lead 85(NCI 14040) mol. after 72 h of incuba tion, scoring IC50 values of 3.5-11.0μM against PANC1 (pancreas), PC-3 (prostate), T-47D and MDA-MB-231 (breast)cancer cells, and showing favorable safety profiles (27.5μ M IC50 on fibroblasts). Our results provide new clues for further develo pment of Aurora-A kinase inhibitors as anticancer mols.

Keywords: danusertib CYC116 barasertib GSK1070916 MLN8237 auroraAkinase inhibitor anticancer cancer; AURKA; Aurora-A kinase; MDA-MB-231; MTT assay; PANC1; PC-3; T-47D; aurora inhibitors; docking-based comparative intermolecular contacts analysis; in-silico screening; molecular docking; structure- based drug design

Substances (7) | Reactions (0) | Citing (6)

81

Inhibition of BRD4 triggers cellular senescence through suppressing aurora kinases in oesophageal cancer cells

By: Xu, Jian-Ling ; Yuan, Ya-Jiao; Lv, Jiao; Qi, Di; Wu, Meng-Di; Lan, Jing; Liu, Sheng-Nan; Yang, Yong; Zhai, Jing; Jiang, Han-Ming
Journal of Cellular and Molecular Medicine (2020), 24(22), 13036-13045 | Language: English, Database: CAplus and MEDLINE

Oesophageal cancer is one of the most frequent solid malign ancies and the leading cause of cancer- related death around the world. It is urgent to develop novel therapy strategies to improve patient outcomes. Acetylation modification of histones has been extensively studied in epigen etics. BRD4, a reader of acetylated histone and non- histone proteins, has involved in tumorig enesis. It has emerged as a promising target for cancer therapy. BRD4 inhibitors, such as JQ1, have exerted efficacious anti-proliferation activities in diverse cancers. However, the effects of JQ1 on oesoph ageal cancer are still not fully described. Here, we demons trate that JQ1 suppresses cell growth and triggers cellular senescence in K YSE450 cells. Mechanistically, JQ1 up-regulates p21 level and decreases cyclin D1 resulting in G1 cycle arrest. The inhibitory effects of JQ1 on KYSE450 cells are independent on apoptosis. It activates cellular senescence by increasing SA-β-gal activity. BRD4 knockdown by sh RNA recapitulates cellular senescence. We also display that administration of JQ1 decreases recrui tment of BRD4 on the promoter of aurora kinases A and B. Inhibitors targeting at AURKA/B phenocopy JQ1 treatment in KYSE450 cells. These results identify a novel action manner of B RD4 in oesoph ageal cancer, which strengthens JQ1 as a candidate drug in oesoph ageal cancer chemotherapy.

Keywords: Inhibition BRD4 triggers cellular senescence aurora kinases oesoph ageal cancer; BRD4; aurora kinase; cellular senesc ence; oesoph ageal cancer

Substances (8) | Reactions (0) | Citing (3)

82

High-throughput compound screen reveals mTOR inhibitors as potential therapeutics to reduce (auto)antibody production by human plasma cells

By: Tuijnenburg, Paul ; aan de Kerk, Daan J.; Jansen, Machiel H.; Morris, Ben; Lieftink, Cor; Beijersbergen, Roderick L.; van Leeuwen, Ester M. M.; Kuijpers, Taco W.
European Journal of Immunology (2020), 50(1), 73-85 | Language: English, Database: CAplus and MEDLINE

Antibody production by the B cell compartment is a crucial part of the adaptive immune response. Dysreg ulated antibody production in the form of autoantibodies can cause autoimmune disease. To date, B- cell depletion with anti- CD20 antibodies is commonly applied in autoimmunity, but pre-existing plasma cells are not eliminated in this way. Altern ative ways of more selective inhibition of antibody production would add to the treatment of these autoimmune diseases. To explore novel therapeutic targets in signaling pathways essential for plasmablast formation and/or Ig produc tion, we performed a compound screen of almost 200 protein kinase inhibitors in a robust B-cell differen tiation culture system. This study yielded 35 small cell- permeable compounds with a reproducible inhibitory effect on B- cell activation and plasma blast formation, among which was the clin. applied mammalian target of rapamycin (mTOR) inhibitor rapamycin. Two addnl. compounds targeting the phosphoi nositide 3-kinase-AKT-mTOR pathway (BKM120 and WYE-354) did not affect prolife ration and plasmablast formation, but specif ically reduced the Ig production With this compound screen we successfully applied a method to invest igate therapeutic targets for B- cell differen tiation and identified compounds in the phosphoinositide 3-kinase-AKT-mTOR pathway that could specif ically inhibit Ig production only. These drugs may well be explored to be of value in current B-cell-depleting treatment regimens in autoimmune disorders.

Keywords: IgM IgA activation differen tiation plasmablast rapamycin; B cells; B- cell activation and differen tiation; PI3K-AKT-mTOR; autoimmune disease; plasma cells

Substances (63) | Reactions (0) | Citing (9)

83

Synthesis of Aurora-B Kinase Inhibitor AZD1152-HQPA

By: Wang Li-li; Wang Xing-lan; Ma Cai-yu; Cheng Fei; Mao Yuan-hu; Tang Lei; Zhang Ji-quan
Guangzhou Huagong (2020), 48(8), 47-49 | Language: Chinese, Database: CAplus

AZD1152-HQPA is the prodrug of Aurora-B kinase selective inhibitor AZD1152(Barasertib) bearing a quinazoline scaffold and this study presents the optimization of the synthetic route. Starting from 2- amino-4-fluorobenzoic acid, AZD1152-HQPA was synthesized via cyclization, substitution, chloridization, ammoniation, amidation and final substitution. The title compound was obtained with a total yield of 27.6% and purity of more than 99%. The optimization of the amidating condensation reaction made this synthetic route more materials-accessible, operations-handy and cost-effective, and it was suitable for the scale-up preparation of AZD1152-HQPA.

Substances (0) Reactions (0) Citing (0)

84

Selective targeting of cancerous mitochondria and suppression of tumor growth using redox-active treatment adjuvant

By: Bakalova, Rumiana; Semkova, Severina; Ivanova, Donika; Zhelev, Zhivko; Miller, Thomas; Takeshima, Tsuguhide; Shibata, Sayaka; Lazarova, Dessislava; Aoki, Ichio; Higashi, Tatsuya
Oxidative Medicine and Cellular Longevity (2020), 6212935 | Language: English, Database: CAplus and MEDLINE

Redox-active substances and their combinations, such as of quinone/ascorbate and in particular menadione/ascorbate (M/A; also named Apatone), attract attention with their unusual ability to kill cancer cells without affecting the viability of normal cells as well as with the synergistic anticancer effect of both mols. So far, the primary mechanism of M/A- mediated anticancer effects has not been linked to the mitochondria. The aim of our study was to clarify whether this "combi nation drug" affects mitochondrial functio nality specifically in cancer cells. Studies were conducted on cancer cells (Jurkat, Colon26, and M CF7) and normal cells (normal lymphocytes, FHC, and MCF10A), treated with different concentrations of menadione, ascorbate, and/or their combin ation (2/200, 3/300, 5/500, 10/1000, and 20/2000 μM/μM of M/A). M/A exhibited highly specific and synerg istic suppression on cancer cell growth but without adversely affecting the viability of normal cells at pharmacol. attainable concentrations In M/A-treated cancer cells, the cytostatic/cytotoxic effect is accompanied by (i) extremely high production of mitocho ndrial superoxide (up to 15-fold over the control level), (ii) a significant decrease of mitochondrial membrane potential, (iii) a decrease of the steady- state levels of ATP, succinate, NADH, and NAD+, and (iv) a decreased expression of programed cell death ligand 1 (P D-L1)-one of the major immune checkpoints. These effects were dose dependent. The inhibition of N QO1 by dicoumarol increased mitocho ndrial superoxide and sensitized cancer cells to M/A. In normal cells, M/A induced relatively low and dose-independent increase of mitocho ndrial superoxide and mild oxidative stress, which seems to be well tolerated. These data suggest that all anticancer effects of M/A result from a specific mechanism, tightly connected to the mitochondria of cancer cells. At low/tolerable doses of M/A (1/100-3/300 μM/μ M) attainable in cancer by oral and parenteral administ ration, M/A sensitized cancer cells to conventional anticancer drugs, exhibiting synergistic or additive cytotoxicity accompanied by impressive induction of apoptosis. Combinations of M/A with 13 anticancer drugs were investigated (ABT-737, barasertib, bleomycin, BEZ-235, bortezomib, cisplatin, everolimus, lomustine, lonafa rnib, MG-132, MLN-2238, palbociclib, and PI-103). Low/tolerable doses of M/A did not induce irreve rsible cytotoxicity in cancer cells but did cause irreversible metabolic changes, including: (i) a decrease of succinate and N ADH, (ii) depolarization of the mitocho ndrial membrane, and (iii) overpro duction of superoxide in the mitochondria of cancer cells only. In addition, M/A suppressed tumor growth in vivo after oral administration in mice with melanoma and the drug downreg ulated PD-L1 in melanoma cells. Exptl. data suggest a great potential for beneficial anticancer effects of M/A through increasing the sensitivity of cancer cells to conven tional anticancer therapy, as well as to the immune system, while sparing normal cells. We hypoth esize that M/A-mediated anticancer effects are triggered by redox cycling of both substances, specifically within dysfunctional mitochondria. M/A may also have a beneficial effect on the immune system, making cancer cells "visible" and more vulnerable to the native immune response.

Substances (2) Reactions (0) Citing (9)

85

AURKB as a target in non-small cell lung cancer with acquired resistance to anti-EGFR therapy

By: Bertran-Alamillo, Jordi ; Cattan, Valerie; Schoumacher, Marie ; Codony-Servat, Jordi; Gimenez-Capitan, Ana; Cantero, Frederique; Burbridge, Mike; Rodriguez, Sonia; Teixido, Cristina; Roman, Ruth; et al
Nature Communications (2019), 10(1), 1-14 | Language: English, Database: CAplus and MEDLINE

Non-small cell lung cancer (N SCLC) tumors harboring mutations in E GFR ultimately relapse to therapy with E GFR tyrosine kinase inhibitors (EGFR TKIs). Here, we show that resistant cells without the p. T790 M or other acquired mutations are sensitive to the Aurora B (AURKB) inhibitors barasertib and S49076. Phospho-histone H3 (pH3), a major product of AURKB, is increased in most resistant cells and treatment with AURKB inhibitors reduces the levels of pH3, triggering G1/S arrest and polypl oidy. Senescence is subsequently induced in cells with acquired mutations while, in their absence, polyploidy is followed by cell death. Finally, in N SCLC patients, pH3 levels are increased after progression on EGFR TKIs and high pH3 baseline correlates with shorter survival. Our results reveal that AURKB activation is associated with acquired resistance to E GFR TKIs, and that AURKB constitutes a potential target in NSCLC progressing to anti-EGFR therapy and not carrying resistance mutations.

Keywords: lung cancer barasertib gefitinib anticancer AURKB point mutation

Substances (9) Reactions (0) Citing (73)

86

Aurora A-selective inhibitor LY3295668 Leads to dominant mitotic arrest, apoptosis in cancer cells, and shows potent preclinical antitumor efficacy

By: Du, Jian; Yan, Lei; Torres, Raquel; Gong, Xueqian; Bian, Huimin; Marugan, Carlos; Boehnke, Karsten; Baquero, Carmen; Hui, Yu-Hua; Chapman, Sonya C.; et al
Molecular Cancer Therapeutics (2019), 18(12), 2207-2219 | Language: English, Database: CAplus and MEDLINE

Although Aurora A, B, and C kinases share high sequence similarity, especially within the kinase domain, they function distinctly in cell-cycle progression. Aurora A depletion primarily leads to mitotic spindle formation defects and conseq uently promet aphase arrest, whereas Aurora B/C inactivation primarily induces polyploidy from cytokinesis failure. Aurora B/C inactivation phenotypes are also epistatic to those of Aurora A, such that the concomitant inactivation of Aurora A and B, or all Aurora isoforms by noniso form-selective Aurora inhibitors, demonstrates the Aurora B/C-dominant cytokinesis failure and polyploidy phenotypes. Several Aurora inhibitors are in clin. trials for T/B-cell lymphoma, multiple myeloma, leukemia, lung, and breast cancers. Here, we describe an Aurora A-selective inhibitor, LY3295668, which potently inhibits Aurora autophosph orylation and its kinase activity in vitro and in vivo, persistently arrests cancer cells in mitosis, and induces more profound apoptosis than Aurora B or Aurora A/B dual inhibitors without Aurora B inhibition-associated cytokinesis failure and aneupl oidy. LY3295668 inhibits the growth of a broad panel of cancer cell lines, including small-cell lung and breast cancer cells. It demons trates significant efficacy in small-cell lung cancer xenograft and patient-derived tumor preclin. models as a single agent and in combin ation with standard-of-care agents. LY3295668, as a highly Aurora A-selective inhibitor, may represent a preferred approach to the current pan- Aurora inhibitors as a cancer therap eutic agent.

Keywords: cancer cell aurora mitotic arrest antitumor efficacy

Substances (7) Reactions (0) Citing (21)

87

Salivary glands require Aurora Kinase B for regeneration after transient innate immune-mediated injury

By: Shaalan, Abeer ; Proctor, Gordon
Scientific Reports (2019), 9(1), 1-13 | Language: English, Database: CAplus and MEDLINE

The present study examined the initial mitogenic events marking the regenerative response of the murine submandibular gland (S MG), following innate immune-mediated injury. Local intraductal administration of the synthetic double stranded (ds) R NA polyino sinic-polycytidylic acid (poly (I: C)) widely, but transiently, depleted the acinar and progenitor cells, 24 h post poly (I: C) introduction. While the progenitor and duct cells started to proliferate and expand at 72 h, the Mist1- positve acinar cells did not re- appear until 96 h post poly (I:C) injury. The cellular replenishment during regeneration involved significant upregulation of the cell cycle promoter Aurora kinase B (AURKB). AURKB, which is expressed in healthy proliferating and cancerous cells, is a serine/threonine protein kinase, well known to orchestrate key events in cell division and cytokinesis. However, the expression and role of AURKB in regene ration of post mitotic salivary gland cells has not been previously explored. In vivo inhibition of A URKB using the selective inhibitor Barasertib (AZD1152-HQPA) interfered with SMG recovery from the transient, but severe poly (I: C)-mediated injury and cellular depletion. AURKB deficiency during regeneration of the injured tissues: disrupted cell cycle progre ssion, repressed renewal of Mist1-pos. acinar cells and prevented recovery of salivary secretion.

Keywords: salivary gland progenitor acinar duct cell aurora kinase B

Substances (4) Reactions (0) Citing (4)

88

Screening methods for identifying pharmaceutical compounds and uses thereof for treating cancer

By: Klug, Christopher
World Intellectual Property Organization, WO2019241636 A1 2019-12-19 | Language: English, Database: CAplus

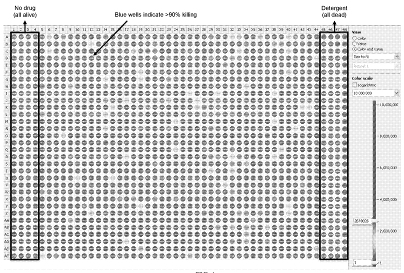

FIG. 1

Provided herein is a method for identifying compounds that inhibit cell growth or have cytotoxic activity against cancer cells, such as hematol. cancer cells or ovarian cancer cells, in a subject. Further provided is a method for identifying one or more compounds that sensitize refractory cancer cells from a subject. Also provided are compositions and methods for treating cancer, including refractory cancer, such as refractory acute myeloid leukemia (AML) or ovarian cancer.

Keywords: antitumor drug screening acute myeloid leukemia ovarian cancer treatment

PatentPak available

Substances (414) Reactions (0) Citing (0)

89

Analytical method considerations regarding carryover for monophosphate prodrugs for in vivo samples by liquid chromatography-tandem mass spectrometry

By: Heinle, Lance; Patel, Hardikkumar; Jenkins, Gary; Ruterbories, Kenneth
Journal of Chromatography A (2019), 1606, 460379 | Language: English, Database: CAplus and MEDLINE

Three different components that impact carryover in a liquid chromatog.-tandem mass spectrometry (LC-MS/MS) method were evaluated to establish baseline conditions for analyzing in vivo samples for twelve monophosphate prodrug compounds and their corresponding parent compounds The three components were: wash solvent modifier, column shell material (metal vs. metal free) , and tubing composition These components were tested for their impact on system carryover by using rat plasma extracted samples. It was determined that a wash solution containing hexylamine yielded the lowest average carryover of the solutions tested. In addition, metal free columns and PEEK (poly ether ether ketone) tubing yielded the lowest carryover when compared to metal columns, stainless steel tubing and nickel tubing. These conditions were also tested against the parent mols. for each prodrug in the test set, to ensure that changing the conditions for the prodrugs did not impact the ability to analyze the parent, since there is typically a desire to measure both compounds in study samples. Under all conditions, the carryover of the corresp onding parent mol. was not adversely impacted in these studies.

Keywords: monophosphate prodrugs carryover LC tandem MS PEEK tubing; Carryover; Liquid chromato graphy-tandem mass spectrometry; Monophosphate prodrugs; PEEK tubing

Substances (5) Reactions (0) Citing (1)

90

Bifunctional small molecules to target the selective degradation of circulating proteins

By: Spiegel, David; Caianiello, David; Zhang, Mengwen
World Intellectual Property Organization, WO2019199634 A1 2019-10-17 | Language: English, Database: CAplus

The present invention is directed to bifunctional small mols. which contain a circul ating protein binding moiety (CPBM) linked through a linker group to a cellular receptor binding moiety (CRBM) which is a membrane receptor of degrading cell such as a hepatocyte or other degrading cell. In embodiments, the (CRBM) is a moiety which binds to asialogly coprotein receptor (an asialogly coprotein receptor binding moiety, or ASGPRBM) of a hepato cyte. In addnl. embodiments, the (CRBM) is a moiety which binds to a receptor of other cells which can degrade proteins, such as a LRP1, LDLR, FcγRI, FcRN, Transferrin or Macrophage Scavenger receptor. Pharmaceutical compositions based upon these bifunc tional small mols. represent an addnl. aspect of the present invention. These compounds and/or compositions may be used to treat disease states and conditions by removing circul ating proteins through degradation in the hepatocytes or macrophages of a patient or subject in need of therapy. Methods of treating disease states and/or conditions in which circulating proteins are associated with the disease state and/or condition are also described herein.

Keywords: circulating protein targeting bifunc tional agent cancer autoimmune disease inflam mation

PatentPak available

Substances (881) Reactions (2,119) Citing (3)

91

Preparation of cereblon E3 ligase ligands and bifunctional PROTAC compounds for targeting and degradation of androgen receptor, estrogen receptor alpha, bromodomain-containing protein 4, and BRaf

By: Crew, Andrew P.; Crews, Craig M.; Dong, Hanqing; Hornberger, Keith R.; Wang, Jing; Qian, Yimin; Zimmermann, Kurt; Berlin, Michael; Snyder, Lawrence B.
World Intellectual Property Organization, WO2019199816 A1 2019-10-17 | Language: English, Database: CAplus

FIG. 1B

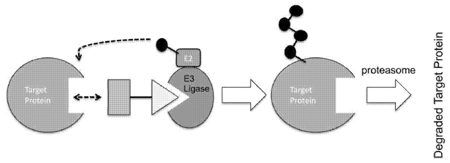

The invention relates to cereblon E3 ligase binding compounds, including bifunctional compounds comprising them, which find utility as modulators of targeted ubiquitination, especially inhibitors of a variety of polype ptides and other proteins which are degraded and/or otherwise inhibited by bifunctional compounds according to the present disclo sure. In particular, the invention provides compounds, which contain on one end a ligand which binds to the cereblon E3 ubiquitin ligase and on the other end a moiety which binds a target protein such that the target protein is placed in proximity to the ubiquitin ligase to effect degradation (and inhibition) of that protein. Specifically, the invention relates to bifunctional compounds CLM-L-PTM [PTM = small mol. containing a protein targeting moiety that binds bromodomain-containing protein 4, estrogen receptor (E R), or androgen receptor or BRaf; L = a bond, a chem. linking moiety covalently coupling the C LM and the PTM; CLM = a small mol. cereblon E3 ubiquitin ligase binding moiety that binds or targets a cereblon E3 ubiquitin ligase], their pharmaceutically acceptable salts, enantiomers, stereoi somers, solvates, polymorphs and prodrugs. Exempl ified compound **I** was prepared from a 12 step procedure (prepar ation given). The invention exhibits a broad range of pharmacol. activities associated with compounds according to the present invention, consistent with the degradation/inhibition of targeted polypeptides of nearly any type (data given). Diseases or disorders that result from aggregation or accumulation of the target protein are treated or prevented with compounds and compos itions of the invention.

Keywords: cereblon ligand chimeric mol preparation targeted protein degradation ubiquitination; PROTAC modulator preparation targeted ubiquitination estrogen receptor E RRa degradation; androgen receptor PROTAC degradation inhibition preparation antiproliferative; BRD4 BRaf PROTAC degradation inhibition cereblon E3 ligase antitumor

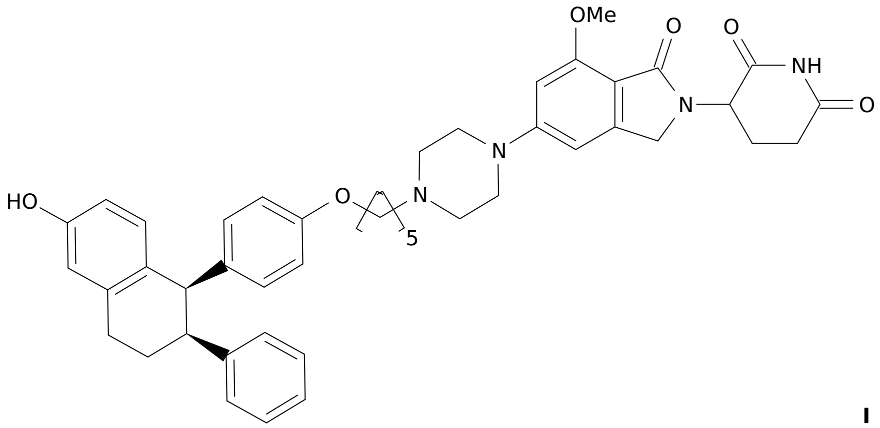

I

PatentPak available
Substances (727)
Reactions (1,470)
Citing (5)

92

Bi-functional molecules to degrade circulating proteins

By: Spiegel, David; Caianiello, David
World Intellectual Property Organization, WO2019199621 A1 2019-10-17 | Language: English, Database: CAplus

The present invention is directed to bi-functional compounds which find use as pharmaceutical agents in the treatment of disease states and/or conditions which are through macrophage migration inhibitory factor (MIF) or immunoglubin G (IgG). The present invention is also directed to pharmaceutical compositions which comprise these bi- functional compounds as well as methods for treating disease states and/or conditions which are mediated through MIF/lgG or where MIF/lgG is a contributing factor to the development and perpetuation of diseases and/or conditions, especially including autoimmune diseases and cancer, among others. The purpose of the present invention is to provide a mol. strategy to lower plasma MIF/lgG level in patients with autoimmune diseases or certain types of cancers. The b.i -functional mol. construct is comprised of a M IF/IgQ-targeting motif, that is derived from small mol. MIF/lgG ligands, and an ASGPr- targeting motif that binds to hepatocyte asialogly coprotein receptor (ASGPr). The compounds selectively bind MIF or IgG in plasma and subsequently engage the endo-lysosomal pathway of hepatocytes through AS GPr. As a conseq uence, MIF/igG is internalized and degraded by hepato cytes, thus resulting in potential attenu ation of corresp onding disease symptoms which are modulated through MIF/igG.

Keywords: bifunctional circulating protein migration inhibitory factor immunoglubin G cancer; autoimmune diseases circulating protein migration inhibitory factor immunoglubin G

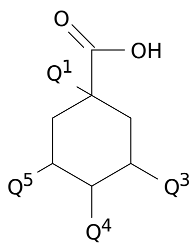

I

PatentPak available

Substances (97) Reactions (368) Citing (5)

93

Treating tumors with an alternating electrical field and an Aurora kinase inhibitor

By: Krex, Dietmar; Temme, Achim; Shnaiderman, Rosa S.; Giladi, Moshe
United States, US20190307781 A1 2019-10-10 | Language: English, Database: CAplus

The viability of cancer cells can be reduced by administering an Aurora kinase inhibitor (e.g., M LN8237 or another Aurora A kinase inhibitor, AZD1152 or another Aurora B kinase inhibitor) to the cancer cells, and applying an altern ating elec. field with a frequency between 100 and 300 kHz (e.g., 200 kHz) to the cancer cells. Furthermore, cancer (e.g., glioblastoma) in a subject may be treated by administering an Aurora kinase inhibitor to the subject, and applying an alternating elec. field with frequency between 100 and 300 kHz (e.g., 200 kHz) to a target region of the subject (e.g., the brain) .

Keywords: cancer treatment alternating elec field Aurora kinase inhibitor combination

PatentPak available

Substances (9) Reactions (0) Citing (2)

94

Selective inhibition of Aurora A and B kinases effectively induces cell cycle arrest in t(8;21) acute myeloid leukemia

By: Qi, Jialei; Gao, Xiang; Zhong, Xiaomin; Zhang, Ninghan; Wang, Rong; Zhang, Huihui; Pan, Ting; Liu, Xuejiao; Yao, Yao; Wu, Qingyun; et al

Biomedicine & Pharmacotherapy (2019), 117, 109113 | Language: English, Database: CAplus and MEDLINE

The fusion gene AML1-ETO initially dysregulates various cell cycle mols. in t(8;21) acute myeloid leukemia. Aurora kinases have shown great promise in treating tumors. However, the efficacy of Aurora kinase (AURK) A and B inhibition in t(8;21) AML remains unclear. We found that AURK-A inhibitor Alisertib and AURK-B inhibitor Barasertib strongly inhibited the growth and proliferation of t(8;21) AML cells. The quantity and size of cell colonies were markedly decreased after a 14- d drug exposure. The cell cycle distribution was blocked at the G2/M phase in both dose- and time- dependent manner. The expression of p53 family and cdc2- p34 significantly changed as well. Notably, we found that t (8;21) AML cells are more sensitive to Aurora B inhibition. In each set of experiments, Barasertib took less time or a lower concentration to achieve similar efficacy. Taken together, our data highlighted the potential role of Aurora kinases as promising cell cycle targets for the treatment of t(8;21) AML and hereby provided a theor. basis to guide relevant clin. trials.

Keywords: alisertib barasertib anticancer AURKA AURKB inhibitor acute myeloid leukemia; AML-ETO; Acute myeloid leukemia; Alisertib; Aurora kinase; Barasertib

Substances (3) Reactions (0) Citing (7)

95

Divergent Polypharmacology-Driven Cellular Activity of Structurally Similar Multi-Kinase Inhibitors through Cumulative Effects on Individual Targets

By: Sumi, Natalia J.; Ctortecka, Claudia; Hu, Qianqian; Bryant, Annamarie T.; Fang, Bin; Remsing Rix, Lily L.; Ayaz, Muhammad; Kinose, Fumi; Welsh, Eric A.; Eschrich, Steven A.; et al

Cell Chemical Biology (2019), 26(9), 1240-1252.e11 | Language: English, Database: CAplus and MEDLINE

Despite recent successes of precision and immunotherapies there is a persisting need for novel targeted or multi- targeted approaches in complex diseases. Through a systems pharmacol. approach, including phenotypic screening, chem. and phosphoproteomics, and RNA-seq, we elucidated the targets and mechanisms underlying the differential anticancer activity of two structurally related multi-kinase inhibitors, foretinib, and cabozantinib, in lung cancer cells. Biochem. and cellular target validation using probe mols. and RNAi revealed a polypharmacol. mechanism involving MEK1/2, FER, and AURKB, which were each more potently inhibited by foretinib than cabozantinib. Based on this, we developed a synergistic combination of foretinib with barasertib, a more potent AURKB inhibitor, for MYC-amplified small-cell lung cancer. This systems pharmacol. approach showed that small structural changes of drugs can cumulatively, through multiple targets, result in pronounced anticancer activity differences and that detailed mechanistic understanding of polypharmacol. can enable repurposing opportunities for cancers with unmet medical need. The accession number for the chem. proteomics, tyrosine phosphoproteomics, global phosphoproteomics and ABPP data reported in this paper is PRIDE: PXD012961, PXD012962, PXD012963 and PXD012965.

Keywords: proteome foretinib cabozantinib barasertib anticancer agent synergistic lung cancer; cabozantinib; chemoproteomics; drug combination; foretinib; kinase inhibitor; lung cancer; phosphoproteomics; polypharmacology; systems pharmacology; target selectivity

Substances (48) Reactions (0) Citing (10)

96

Inductive regeneration of the airway by transcriptional factor modulation of glandular myoepithelial stem cells using modulators of Lef-1/TCF/Wnt signaling

By: Engelhardt, John F.; Lynch, Thomas J.; Anderson, Preston J.; Shahin, Weam

World Intellectual Property Organization, WO2019178267 A2 2019-09-19 | Language: English, Database: CAplus

Compositions and methods to modulate Lef-1/TCF/Wnt signaling ex vivo or in vivo, and assays to detect those modula tors, are described. The disclosure provides a composition comprising an isolated transcription factor (Lymphoid enhancer factor 1 or Lef- 1) that when introduced to or induced in a specific airway stem cell leads to self-limiting regenerative expansion of the airway and submucosal glands. Using genetically engineered mice, Lef-1 expression in glandular myoepithelial cells (MECs) was shown to either enhance airway repair following injury (with monoallelic Lef-1 expression) or spontaneously induce MEC-mediated airway regene ration (with biallelic Lef-1 expression). These findings open the door to enhancing the regenerative capacity of MECs ex vivo using cell therapy approaches and in vivo using small mols. that influence Lef-1 function and expression.

Keywords: airway regeneration transcription factor modulator glandular myoepithelium stem cell

PatentPak available

Substances (1,589) | Reactions (0) | Citing (0)

97

Optimization and biological evaluation of nicotinamide derivatives as Aurora kinase inhibitors

By: Qi, Baohui; Xu, Xingwei; Yang, Ying; He, Huan; Yue, Xupeng

Bioorganic & Medicinal Chemistry (2019), 27(17), 3825-3835 | Language: English, Database: CAplus and MEDLINE

Aurora kinases are known to be overexpressed in various solid tumors and implicated in oncogenesis and tumor progression. A series of nicotinamide derivatives were synthesized and their biol. activities were evaluated, including kinase inhibitory activity against Aur A and Aur B and in vitro antitumor activity against SW620, HT-29, NCI-H1975 and Hela cancer cell lines. In addition, the study of antiproliferation, cytotoxicity and apoptosis was performed meanwhile. As the most potent inhibitor of Aur A, 4- ((3-bromo-4-fluorophenyl)amino)-6-chloro-N-(4-((6,7-dimethoxyquinolin-4-yl)oxy)-3-fluorophenyl)nicotinamide (10l) showed excellent antitumor activity against SW620 and NCI-H1975 with IC_{50} values were 0.61 and 1.06 μM, while the IC_{50} values of reference compound were 3.37 and 6.67 μM, resp. Furthermore, binding mode studies indicated that compound 10l forms better intera ction with Aur A.

Keywords: nicotinamide synthesis antitumor SAR Aurora kinase cancer; Antitumor; Aurora kinases; Kinase inhibi tors; Nicotinamide; SAR

Substances (75) | Reactions (130) | Citing (3)

98

Aneugen molecular mechanism assay: proof-of-concept with 27 reference chemicals

By: Bernacki, Derek T.; Bryce, Steven M.; Bemis, Jeffrey C.; Dertinger, Stephen D.
Toxicological Sciences (2019), 170(2), 382-393 | Language: English, Database: CAplus and MEDLINE

A tiered bioassay and data anal. scheme is described for elucidating the most common mol. targets respon sible for chem.-induced in vitro aneugenicity: tubulin destabilization, tubulin stabilization, and inhibition of mitotic kinase (s). To evaluate this strategy, T K6 cells were first exposed to each of 27 presumed aneugens over a range of concentrations After 4 and 24 h of treatment, γ H2AX, p53, phospho-histone H3 (p-H3), and polyploidization biomarkers were evaluated using the Multi Flow DNA Damage Assay Kit. The assay identified 27 of 27 chems. as genotoxic, with 25 exhibiting aneugenic signatures, 1 aneugenic and clasto genic, and 1 clasto genic. Subsequently, a newly described follow- up assay was employed to invest igate the aneugenic agents' mol. targets. For these experiments, TK6 cells were exposed to each of 26 chems. in the presence of 488 Taxol. After 4 h, cells were lysed and the liberated nuclei and mitotic chromosomes were stained with a nucleic acid dye and labeled with fluore scent antibodies against p-H3 and Ki-67. Flow cytometric analyses revealed that altera tions to 488 Taxol-associated fluorescence were only observed with tubulin binders-increases in the case of tubulin stabilizers, decreases with destabi lizers. Mitotic kinase inhibitors with known Aurora kinase B inhibiting activity were the only aneugens that dramatically decreased the ratio of p-H3-pos. to Ki-67-pos. nuclei. Unsupervised hierarchical clustering based on 488 Taxol fluore scence and p-H3: Ki-67 ratios clearly distinguished compounds with these disparate mol. mechanisms. Furthermore, a classification algorithm based on an artificial neural network was found to effect ively predict mol. target, as leave-one-out cross-validation resulted in 25/26 agreement with a priori expecta tions. These results are encouraging, as they suggest that an adequate number of training set chems., in conjun ction with a machine learning algorithm based on 488 Taxol, p-H3, and Ki- 67 responses, can reliably elucidate the most commonly encoun tered aneugenic mol. targets.

Keywords: aneugen mol mechanism assay artificial neural network classification algorithm; Mitotic kinase inhibi tors; TK6 cells; aneugen; flow cytometry; spindle poisons

Substances (28) Reactions (0) Citing (9)

99

Methods of treatment of cancer comprising cell division control protein 7 (CDC7) inhibitors

By: Hassig, Christian Andrew; Hansen, Ryan James
World Intellectual Property Organization, WO2019165473 A1 2019-08-29 | Language: English, Database: CAplus

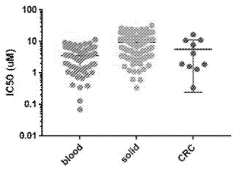

Fig. 3B

Herein disclosed are methods of treatment administering a cell division control protein 7 (C DC7) inhibitor as a monotherapy or in a combination therapy useful for inhibiting the growth of tumors such as those in patients with cancer.

Keywords: antitumor protein CDC7 kinase inhibitor furanone derivative cancer treatment

PatentPak available

Substances (18) Reactions (0) Citing (3)

100

Methods of treating fibrosis using aurora kinase B (AURKB) inhibitors

By: Madala, Satish Kumar; Jegga, Anil Goud; Kasam, Rajesh K.
World Intellectual Property Organization, WO2019161000 A1 2019-08-22 | Language: English, Database: CAplus

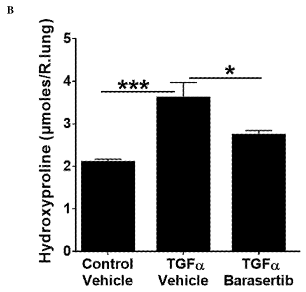

Some embodiments of the invention include methods for treating an animal for fibrosis comprising one or more administ rations of one or more compositions comprising one or more AURKB (Aurora kinase B) inhibitors. Other embodiments of the methods for treating further include other fibrosis treatments. Still other embodi ments of the invention include methods for treating a human for lung fibrosis or idiopathic pulmonary fibrosis, comprising administering one or more compositions comprising AZD1152 or barasertib. Addnl. embodiments of the invention are also discussed herein.

Keywords: antifibrotic aurora kinase inhibitor barasertib AZD1152 pulmonary fibrosis treatment

PatentPak available

Substances (46) Reactions (0) Citing (0)

101

Cabazitaxel-resistant prostate cancer preventive or therapeutic agent

By: Kosaka, Takeo; Hongo, Hiroshi; Oya, Mototsugu
Japan, JP2019127437 A 2019-08-01 | Language: Japanese, Database: CAplus

The present invention relates to a preventive or therapeutic agent comprising one or more agent selected from a group consisting of pimozide or its salt, an Aurora kinase inhibitor, and a KIF20A inhibitor. The preventive or therapeutic agent comprises a low mol. weight compound that exerts an anticancer effect against cabazitaxel-resistant prostate cancer that can be manufactured relatively easily and inexpensively.

Keywords: Aurora kinase inhibitor cabazitaxel resistant prostate cancer antitumor; pimozide cabazi taxel resistant prostate cancer antitumor; KIF20A inhibitor cabazitaxel resistant prostate cancer antitumor

PatentPak available

Substances (5) Reactions (0) Citing (0)

102

Resveratrol modulates the redox-status and cytotoxicity of anticancer drugs by sensitizing leukemic lymphocytes and protecting normal lymphocytes

By: Ivanova, Donika; Zhelev, Zhivko; Semkova, Severina; Aokl, Iciiio; Bakalova, Rumiana
Anticancer Research (2019), 39(7), 3745-3755 | Language: English, Database: CAplus and MEDLINE

Background/Aim: The study is directed to the effect of resver atrol on the redox-status and viability of leukemic and normal lympho cytes, as well as its ability to sensitize leukemic lympho cytes to anticancer drugs. Materials and Methods: Cytoto xicity was analyzed by trypan blue staining, apoptosis - by Annexin V test, and oxidative stress - by the intracellular levels of reactive oxygen species (R O S) and protein-carbonyl products. Results: Incubation of resver atrol in combination with the majority of anticancer drugs resulted in higher toxicity than resveratrol or drug alone. In the case of leukemic lympho cytes treated with barasertib and everolimus in the presence of resveratrol, synergistic cytotoxicity was accompanied by strong induction of apoptosis, increased levels of hydrope roxides and insignificant changes in proteincarbonyl products. None of these parameters changed in normal lympho cytes. Conclu sion: Resveratrol is a promising supplem entary compound for anticancer therapy that may allow reduction of the therap eutic doses of barasertib and everolimus, minimizing their side- effects.

Keywords: leukemic lymphocyte cytotoxicity anticancer resveratrol; Cancer; apoptosis; chemotherapy; oxidative stress; resver atrol

Substances (8) Reactions (0) Citing (14)

103

High-throughput chemical screening identifies focal adhesion kinase and Aurora kinase B inhibition as a synergistic treatment combination in Ewing sarcoma

By: Wang, Sarah; Hwang, Elizabeth E.; Guha, Rajarshi; O'Neill, Allison F.; Melong, Nicole; Veinotte, Chansey J.; Saur, Amy Conway; Wuerthele, Kellsey; Shen, Min; McKnight, Crystal; et al
Clinical Cancer Research (2019), 25(14), 4552-4566 | Language: English, Database: CAplus and MEDLINE

Ewing sarcoma is an aggressive solid tumor malignancy of childhood. Although current treatment regimens cure approx. 70% of patients with localized disease, they are ineffective for most patients with metastases or relapse. New treatment combin ations are necessary for these patients. Ewing sarcoma cells are dependent on focal adhesion kinase (FAK) for growth. To identify candidate treatment combinations for Ewing sarcoma, we performed a small- mol. library screen to identify compounds synerg istic with FAK inhibitors in impairing Ewing cell growth. The activity of a top-scoring class of compounds was then validated across multiple Ewing cell lines in vitro and in multiple xenograft models of Ewing sarcoma. Numerous Aurora kinase inhibitors scored as synergistic with FAK inhibition in this screen. F AK and Aurora kinase B inhibitors synergis tically impair Ewing sarcoma cell viability and signifi cantly inhibit tumor progression. This study provides preclin. support for the conside ration of a clin. trial testing the safety and efficacy of this combination for patients with Ewing sarcoma.

Keywords: Ewing sarcoma FAK Aurora kinase B synergism

Substances (8) Reactions (0) Citing (22)

104

Aurora kinase inhibitor and application of alphavirus in preparing antitumor medicament

By: Yan, Guangmei; Lin, Yuan; Zhu, Wenbo; Zhang, Haipeng; Liang, Jiankai; Cai, Jing; Gong, Shoufang

China, CN109985239 A 2019-07-09 | Language: Chinese, Database: CAplus

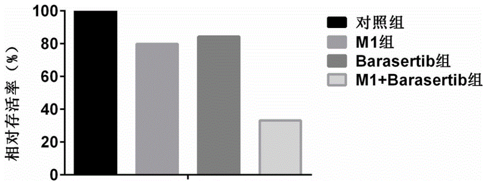

The invention belongs to the field of biomedicine, relates to aurora kinase inhibitor and applic ation of alphavirus in preparing antitumor medicament.The aurora kinase inhibitor disclosed by the invention can be used for preparing an alphavirus antitumor synergist for the first time.The invention also relates to a pharmaceutical composition comprising an aurora kinase inhibitor and an alphavirus, a pharmaceutical kit comprising the aurora kinase inhibitor and the alphav irus, and application of the aurora kinase inhibitor and the alphavirus in treating tumors, in particular tumors insensitive to the alphavirus.

Keywords: aurora kinase inhibitor alphavirus antitumor

PatentPak available

Substances (4) Reactions (0) Citing (0)

105

Use of aurora kinase inhibitor and alphavirus in preparing anti-tumor drug

By: Yan, Guangmei; Lin, Yuan; Zhu, Wenbo; Zhang, Haipeng; Liang, Jiankai; Cai, Jing; Gong, Shoufang

World Intellectual Property Organization, WO2019129234 A1 2019-07-04 | Language: Chinese, Database: CAplus

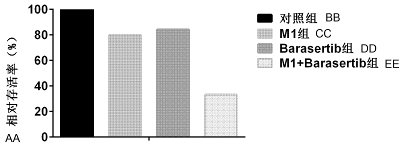

图 1

AA Relative survival rate (%)
BB Control group
CC M1 group
DD Barasertib group
EE M1+Barasertib group

Disclosed is the use of an aurora kinase inhibitor and an alphavirus in preparing an anti-tumor drug. The aurora kinase inhibitor can be used in preparing an alphavirus anti-tumor synergist. Disclosed are a pharmaceutical composition containing the aurora kinase inhibitor and the alphavirus, a drug kit containing the aurora kinase inhibitor and the alphav irus, and the use of the aurora kinase inhibitor and the alphavirus in treating tumors, especially those that are insensitive to the alphavirus.

Keywords: aurora kinase inhibitor alphavirus antitumor

PatentPak available

Substances (4) Reactions (0) Citing (2)

106

Modeling dose and schedule effects of AZD2811 nanoparticles targeting Aurora B kinase for treatment of diffuse large B-cell lymphoma

By: Floc'h, Nicolas; Ashton, Susan; Ferguson, Douglas; Taylor, Paula; Carnevalli, Larissa S.; Hughes, Adina M.; Harris, Emily; Hattersley, Maureen; Wen, Shenghua; Curtis, Nicola J.; et al
Molecular Cancer Therapeutics (2019), 18(5), 909-919 | Language: English, Database: CAplus and MEDLINE

Barasertib (AZD1152), a pro-drug of the highly potent and selective Aurora B kinase inhibitor A ZD2811, showed promising clin. activity in relapsed/refractory diffuse large B- cell lymphoma (DLBCL) patients administered as a 4- day infusion. To improve potential therapeutic benefit of Aurora B kinase inhibi tion, a nanopa rticle formulation of AZD2811 has been developed to address limita tions of repeated i.v. infusion. One of the challenges with the use of nanoparticles for chronic treatment of tumors is optimizing dose and schedule required to enable repeat administration to sustain tumor growth inhibi tion. AZD2811 gives potent cell growth inhibition across a range of DLBCL cells lines in vitro. In vivo, repeat adminis tration of the AZD2811 nanoparticle gave antitumor activity at half the dose intensity of AZD1152. Compared with A ZD1152, a single dose of A ZD2811 nanoparticle gave less reduction in p HH3, but increased apoptosis and reduction of cells in G1 and G2-M, albeit at later time points, suggesting that duration and depth of target inhibition influence the nature of the tumor cell response to drug. Further exploration of the influence of dose and schedule on efficacy revealed that AZD2811 nanoparticle can be used flexibly with repeat adminis tration of 25 mg/kg admini stered up to 7 days apart being sufficient to maintain equivalent tumor control. Timing of repeat administration could be varied with 50 mg/kg every 2 wk controlling tumor control as effect ively as 25 mg/kg every week. A ZD2811 nanoparticle can be administered with very different doses and schedules to inhibit DLBCL tumor growth, although maximal tumor growth inhibition was achieved with the highest dose intensities.

Keywords: DLBCL cell growth aurora B kinase nanopa rticle therapeutics

Substances (2) | Reactions (0) | Citing (8)

107

Preparation of compounds and compositions for detecting caspase activity and/or apoptosis in vivo

By: Manning, H. Charles; Hight, Matthew R.
United States, US10294271 B1 2019-05-21 | Language: English, Database: CAplus

Mol. probe suitable for quantification of caspase activity in vivo using positron emission tomog. (P ET). Embodiments of the present invention can detect apoptosis in tumors and as a novel, potentially translatable biomarker for predicting response to person alized medicine.

Keywords: isotope labeled peptide substrate caspase activity apoptosis diagnosis preparation

PatentPak available

Substances (24) | Reactions (16) | Citing (0)

108

Identification and use of cytotoxic T lymphocyte (CTL) antigen-specific target cell killing enhancer agents

By: Lizotte, Patrick H.; Kirschmeier, Paul T.; Bittinger, Mark; Gray, Nathanael
World Intellectual Property Organization, WO2019014111 A1 2019-01-17 | Language: English, Database: CAplus

FIG. 2C

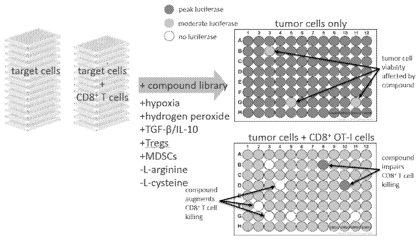

The present invention relates to screening methods for identification of agents (e.g., small mols.) that modulate cytotoxic T lymphocyte antigen-specific target (e.g., tumor) cell killing, as well as to uses of compounds identified thereby as immunomod ulatory, including use of EGFR inhibitors as immunomo dulatory agents.

Keywords: CD8 T lymphocyte antigen specific target cell killing enhancer

PatentPak available

Substances (220) Reactions (0) Citing (0)

109

Aurora kinase B inhibitor barasertib (AZD1152) inhibits glucose metabolism in gastric cancer cells

By: He, Jian; Qi, Zihao; Zhang, Xiaofei; Yang, Yufei; Liu, Fei; Zhao, Guangfa; Wang, Ziliang
Anti-Cancer Drugs (2019), 30(1), 19-26 | Language: English, Database: CAplus and MEDLINE

Barasertib is a highly selective Aurora kinase B (A URKB) inhibitor and has been widely applied in a variety of cancer cells to invest igate the regulatory function of AURKB. However, the effect of barasertib on glucose metabolism in gastric cancer (GC) remains illustrated. Here, barasertib was identified to effectively reduce glucose uptake and lactate production in GC cells in a dose-dependent and time-dependent manner. The expression levels of GLUT1, LDHA and HK2 were decreased by barasertib treatment of GC cells. Furthermore, we found that barasertib induced the expression of ribosomal protein S7 (R PS7), as a tumor suppressor, to regulate glucose metabolism Silencing of RPS7 rescued the effects of barasertib on glucose metabolism in GC cells. Overexp ression of RPS7 suppressed the promoter activity of C-Myc, which has been identified as an important regulator of glucose metabolism in cancer cells. The clin. data showed that the expression level of AURKB in GC patients' sera and tissues were pos. correlated with those of C-Myc, GLUT1 and LDHA, but neg. with that of R PS7. Therefore, these findings provide new evidence that barasertib regulates GC cell glucose metabolism by inducing the R PS7/C-Myc signal pathway, and have important implic ations for the development of therapeutic approaches using A URKB as a target protein to prevent tumor recurr ence.

Keywords: barasertib AURKB inhibitor anticancer agent glucose metabolism gastric cancer

Substances (6) Reactions (0) Citing (8)

110

Aurora kinase: a target for cancer therapy

By: Pushpaveni, C.; Arun, Kumar C.; Visagaperumal, D.; Chandy, Vineeth
Research & Reviews: A Journal of Pharmacology (2019), 9(3), 10-17 | Language: English, Database: CAplus

Aurora kinases are the enzymes that belong to the family of serine/threonine kinases. It consists of Aurora-A (AURK-A), Aurora-B (AURK-B) and Aurora-C (AURK-C), which are the essential kinases for cell division through mitosis regulation especially for the process of chromosomal segregation. Aurora kinases are involved in meiosis regulation along with the regulation of mitosis. Inhibition of Aurora kinase leads to neglected cell division. Various cancer studies generally deal with gene amplification or overexpression. From the literature review, it is clear that inhibiting Aurora kinase will increase the effect of chemotherapies. Hence from the past decades, Aurora kinase inhibitors were developed, and they are now in clin. trials; however, these inhibitors effectively fail to give the complete therapy for cancer. This review mainly focuses on the Aurora kinase inhibitors and Aurora kinase as a target for anticancer therapy.

Keywords: baresertib anticancer agent aurora kinase cancer

Substances (3) Reactions (0) Citing (0)

111

Disassembly of actin and keratin networks by Aurora B kinase at the midplane of cleaving Xenopus laevis eggs

By: Field, Christine M.; Pelletier, James F.; Mitchison, Timothy J.
bioRxiv (2019), 1-24 | Language: English, Database: CAplus

We investigated how bulk cytoplasm prepares for cytokinesis in Xenopus laevis eggs, which are large, rapidly dividing cells. The egg midplane is demarcated by Chromosomal Passenger Complex (CPC) localized on microtubule bundles between asters. Using an extract system and intact eggs we found that local kinase activity of the AURKB subunit of the CPC caused disassembly of F-actin and keratin between asters, and local softening of the cytoplasm as assayed by flow patterns. Beads coated with active CPC mimicked aster boundaries and caused AURKB-dependent disassembly of F-actin and keratin that propagated ~40μm without microtubules, and much farther with microtubules present, due to CPC auto-activation. We propose that active CPC at aster boundaries locally reduces cytoplasmic stiffness by disassembling actin and keratin networks. This may help sister centrosomes move apart after mitosis, prepare a soft path for furrow ingression and/or release G-actin to build the furrow cortex.

Keywords: IgG Factin keratin AURKB CCP disassembly zygote fertilization Xenopus

Substances (7) Reactions (0) Citing (0)

112

Aurora kinase B regulates axonal outgrowth and regeneration in the spinal motor neurons of developing zebrafish

By: Gwee, Serene S. L. ; Radford, Rowan A. W.; Chow, Sharron; Syal, Monisha D.; Morsch, Marco; Formella, Isabel; Lee, Albert; Don, Emily K.; Badrock, Andrew P.; Cole, Nicholas J.; et al
Cellular and Molecular Life Sciences (2018), 75(23), 4269-4285 | Language: English, Database: CAplus and MEDLINE

Aurora kinase B (AurkB) is a serine/threonine protein kinase with a well- characterised role in orchestrating cell division and cytokinesis, and is prominently expressed in healthy proliferating and cancerous cells. However, the role of AurkB in differentiated and non-dividing cells has not been extensively explored. Previously, we have described a significant upregulation of AurkB expression in cultured cortical neurons following an exptl. axonal transection. This is somewhat surprising, as AurkB expression is generally associated only with dividing cells Frangini et al. (Mol Cell 51:647-661, 2013); Hegarat et al. (J Cell Biol 195: 1103-1113, 2011); Lu et al. (J Biol Chem 283:31785-31790, 2008); Trakala et al. (Cell Cycle 12: 1030-1041, 2014). Herein, we present the first description of a role for AurkB in terminally differentiated neurons. AurkB was prominently expressed within post-mitotic neurons of the zebrafish brain and spinal cord. The expression of AurkB varied during the development of the zebrafish spinal motor neurons. Utilizing pharmacol. and genetic manipulation to impair AurkB activity resulted in truncation and aberrant motor axon morphol., while overexpression of AurkB resulted in extended axonal outgrowth. Further pharmacol. inhibition of Aurk B activity in regenerating axons delayed their recovery following UV laser-mediated injury. Collectively, these results suggest a hitherto unreported role of AurkB in regulating neuronal development and axonal outgrowth.

Keywords: aurora kinase B neuronal development spinal motor neuron; Aurora kinase B; Axonal outgrowth; Axonal regeneration; Spinal motor neurons; Zebrafish

Substances (2) Reactions (0) Citing (12)

113

Recurrent 8q24 rearrangement in blastic plasmacytoid dendritic cell neoplasm: association with immunoblastoid cytomorphology, MYC expression, and drug response

By: Sakamoto, Kana; Katayama, Ryohei ; Asaka, Reimi; Sakata, Seiji; Baba, Satoko; Nakasone, Hideki; Koike, Sumie; Tsuyama, Naoko; Dobashi, Akito; Sasaki, Makoto; et al
Leukemia (2018), 32(12), 2590-2603 | Language: English, Database: CAplus and MEDLINE

Blastic plasmacytoid dendritic cell neoplasm (BPDCN) is a rare skin- tropic hematol. malignancy of uncertain pathogenesis and poor prognosis. We examined 118 BPDCN cases for cytomorphol., MYC locus rearrangement, and MYC expression. Sixty-two (53%) and 41 (35%) cases showed the classic and immunoblastoid cytomorphol., resp. Forty-one (38%) MYC^{+}BPDCN (pos. for rearrangement and expression) and 59 (54%) MYC^{-}BPDCN (both neg.) cases were identified. Immunoblastoid cytomorphol. was significantly associated with MYC^{+}BPDCN. All examined MYC^{+}BPDCNs were neg. for MYB/MYBL1 rearrangement (0/36). Clin., MYC^{+}BPDCN showed older onset, poorer outcome, and localized skin tumors more commonly than MYC^{-}BPDCN. MYC was demonstrated by expression profiling as one of the clearest discriminators between CAL-1 (MYC^{+}BPDCN) and PMDC05 (MYC^{-}BPDCN) cell lines, and its shRNA knockdown suppressed CAL-1 viability. Inhibitors for bromodomain and extra-terminal protein (BETis), and aurora kinases (AKis) inhibited CAL-1 growth more effectively than PMDC05. We further showed that a BCL2 inhibitor was effective in both CAL-1 and PMDC05, indicating that this inhibitor can be used to treat MYC^{-}BPDCN, to which BETis and AKis are probably less effective. Our data will provide a rationale for the development of new treatment strategies for patients with BPDCN, in accordance with precision medicine.

Keywords: blastic plasmacytoid dendritic cell neoplasm MYC immunoblastoid cytomorphol

Substances (11) Reactions (0) Citing (31)

114

Methods of cancer treatment with ferroptosis inducer in combination with other antitumor agents

By: Chen, Ruihong; Jiang, Chun
World Intellectual Property Organization, WO2018218087 A1 2018-11-29 | Language: English, Database: CAplus

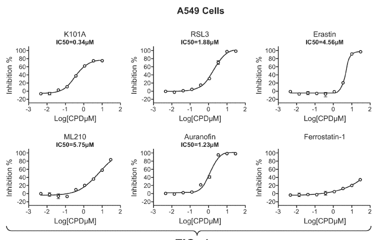

FIG. 1

The present disclosure relates to a method of treating a subject with cancer with a ferroptosis inducer, including use of the ferrop tosis inducer (e.g. erastin, auranofin, sulfasalazine) in combination with a second therap eutic agent (e.g. afatinib, afures ertib, alectinib, alisertib).

Keywords: ferroptosis inducer erastin antitumor afuresertib combination chemotherapy cancer treatment

PatentPak available

Substances (150) Reactions (0) Citing (3)

115

Epigenetic inhibitors for sensitizing hematologic or other malignancies to glucocorticoid therapy and Aurora Kinase B inhibitors

By: Stallcup, Michael R.; Poulard, Coralie; Pufall, Miles A.
World Intellectual Property Organization, WO2018213720 A2 2018-11-22 | Language: English, Database: CAplus

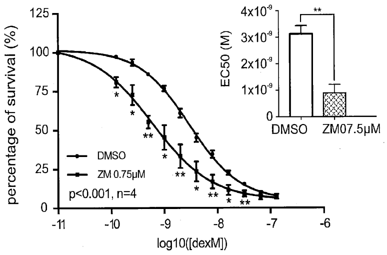

FIG. 40A

The present disclosure as disclosed in various embodiments is related to glucocorticoid compositions and glucocorticoid therapies for treating hematol. or other malignancies, methods and compos itions for enhancing the chemothe rapeutic effect of glucocor ticoids, methods for determ ining early relapse of a hematol. or other malignancy in a subject, and methods for treating relapse of a hematol. or other malignancy in a subject.

Keywords: epigenetics inhibitor chemosensitizer hematol malignancy glucocorticoid antitumor; chemosensitizer barasertib dexamethasone antitumor resistance neoplasm treatment

PatentPak available

Substances (31) Reactions (0) Citing (0)

116

Combination therapies of cancer with histone H3 lysine-9 N-methyltransferase EHMT2 inhibitors

By: Campbell, John Emmerson; Duncan, Kenneth William; Raimondi, Maria Alejandra; Klaus, Christine; Penebre, Elayne
World Intellectual Property Organization, WO2018195450 A1 2018-10-25 | Language: English, Database: CAplus

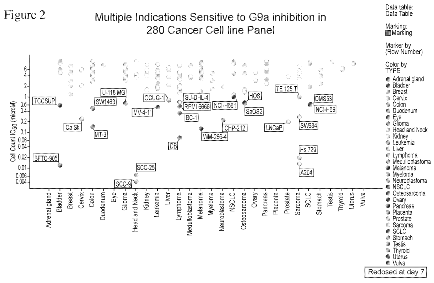

The present disclosure relates to a method of preventing or treating a cancer via administering an histone H3 lysine-9 N-methyltransferase EHMT2 inhibitor or a combination comprising an EHMT2 inhibitor compound and one or more addnl. therap eutic agent disclosed herein or a pharmaceutical composition thereof to subjects in need thereof. The present disclosure also relates to the use of such compounds or combinations for research or other non- therapeutic purposes.

Keywords: combination therapy antitumor EHMT2 inhibitor cancer treatment

PatentPak available

Substances (1,586)   Reactions (0)   Citing (4)

117

Method and compositions for treating cancerous tumors

By: Alliger, Howard
United States, US10105389 B1 2018-10-23 | Language: English, Database: CAplus

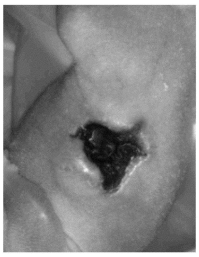

The present invention relates to the use of chlorine dioxide compositions for treating cancerous tumors. The present invention relates to compositions and methods for treating cancerous tumors, including naive, metastatic and recurrent cancers. The compositions comprise chlorine dioxide in an effective amount, which is injected into the cancerous tumor at least once, and often at least several times over the course of treatment. The chlorine dioxide compositions are injected directly into the cancerous tumor and the resulting tumor is effectively eliminated from the patient or subject over a period of one to several days to a few weeks, often after a single injection, or multiple injections at one session into the tumor. Often, an initial injection or multiple injections at one session are sufficient to dissolve the cancerous tumor. Often the cancer is eliminated (as evidenced by no remission) in a period of no more than several days to about two-three months and does not recur.

Keywords: chlorine dioxide cancer

PatentPak available

Substances (294)   Reactions (0)   Citing (0)

118

Porous nanoparticle-supported lipid bilayer delivery of transcriptional gene modulators

By: Brinker, Charles Jeffrey; Agola, Jacob Ongudi; Muniz, Ayse; Butler, Kimberly

World Intellectual Property Organization, WO2018187287 A1 2018-10-11 | Language: English, Database: CAplus

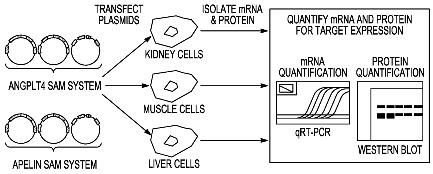

FIG. 15B

A porous protocell is provided comprising one or more non-integrating vectors comprising nucleic acid encoding a non- nucleolytic fusion protein comprising a protein sequence that specifically binds a sequence in a double strand nucleic acid mol. linked to a protein sequence that is a transcriptional mediator, and optionally guide RNA or a vector for expression of guide R NA, and methods of using the protocells.

Keywords: porous nanoparticle lipid bilayer transcription gene fusion protein sequence

PatentPak available

Substances (110) Reactions (0) Citing (0)

119

Preparation of cereblon E3 ligase ligands and bifunctional PROTAC compounds for targeting and degradation of androgen receptor, estrogen receptor alpha, bromodomain-containing protein 4, and BRaf

By: Crew, Andrew P.; Crews, Craig M.; Dong, Hanqing; Hornberger, Keith R.; Wang, Jing; Qian, Yimin; Zimmermann, Kurt; Berlin, Michael; Snyder, Lawrence B.
United States, US20180228907 A1 2018-08-16 | Language: English, Database: CAplus

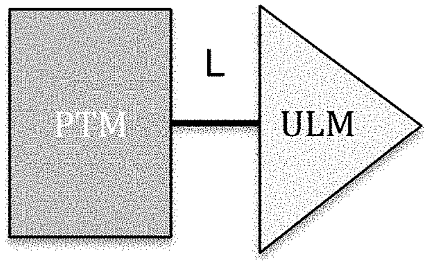

The invention relates to cereblon E3 ligase binding compounds, including bifunctional compounds comprising them, which find utility as modulators of targeted ubiquitination, especially inhibitors of a variety of polype ptides and other proteins which are degraded and/or otherwise inhibited by bifunctional compounds according to the present disclo sure. In particular, the invention provides compounds, which contain on one end a ligand which binds to the cereblon E3 ubiquitin ligase and on the other end a moiety which binds a target protein such that the target protein is placed in proximity to the ubiquitin ligase to effect degradation (and inhibition) of that protein. Specifically, the invention relates to bifunctional compounds CLM-L-PTM [PTM = small mol. containing a protein targeting moiety that binds bromodomain-containing protein 4, estrogen receptor (E R), or androgen receptor or Braf; L = a bond, a chem. linking moiety covalently coupling the CLM and the PTM; CLM = a small mol. cereblon E3 ubiquitin ligase binding moiety that binds or targets a cereblon E3 ubiquitin ligase], their pharmaceutically acceptable salts, enantiomers, stereoi somers, solvates, polymorphs and prodrugs. Exempl ified compound **I** was prepared from a 12 step procedure (prepar ation given). The invention exhibits a broad range of pharmacol. activities associated with compounds according to the present invention, consistent with the degradation/inhibition of targeted polypeptides of nearly any type (data given). Diseases or disorders that result from aggregation or accumulation of the target protein are treated or prevented with compounds and compos itions of the invention.

Keywords: cereblon ligand chimeric mol preparation targeted protein degradation ubiquitination; PROTAC modulator preparation targeted ubiquitination estrogen receptor E RRa degradation; androgen receptor PROTAC degradation inhibition preparation antiproliferative; BRD4 BRaf PROTAC degradation inhibition cereblon E3 ligase antitumor

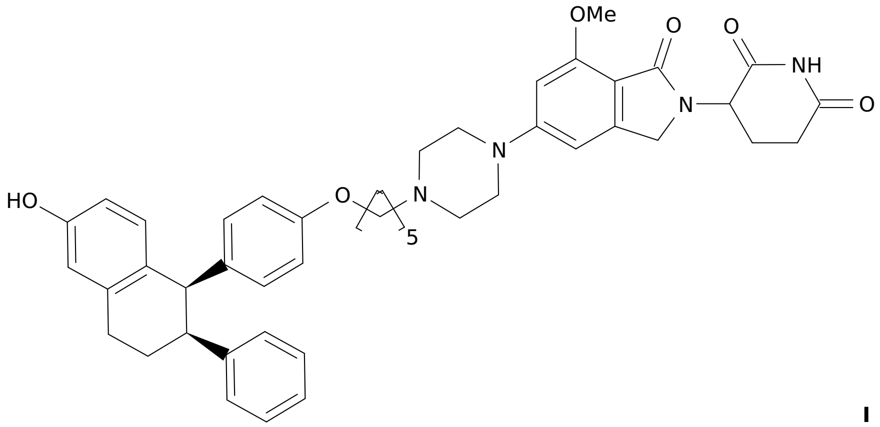

I

PatentPak available

Substances (731) Reactions (1,474) Citing (3)

120

Preparation of cereblon E3 ligase ligands and bifunctional PROTAC compounds comprising them for the degradation of targeted proteins including TANK-binding kinase 1, estrogen receptor alpha, bromodomain-containing protein 4 and tau protein

By: Crew, Andrew P.; Berlin, Michael; Dong, Hanqing; Homberger, Keith R.; Qian, Yimin; Snyder, Lawrence B.; Wang, Jing; Zimmermann, Kurt

United States, US20180215731 A1 2018-08-02 | Language: English, Database: CAplus

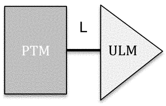

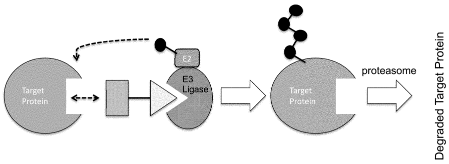

The invention relates to cereblon E3 ligase binding compounds, including bifunctional compounds comprising them, which find utility as modulators of targeted ubiquitination, especially inhibitors of a variety of polypeptides and other proteins which are degraded and/or otherwise inhibited by bifunctional compounds according to the present disclosure. In particular, the invention provides compounds, which contain on one end a ligand which binds to the cereblon E3 ubiquitin ligase and on the other end a moiety which binds a target protein such that the target protein is placed in proximity to the ubiquitin ligase to effect degradation (and inhibition) of that protein. Specifically, the invention relates to bifunctional compounds CLM-L-PTM [PTM = small mol. containing a protein targeting moiety that binds bromodomain-containing protein 4, Tau protein, estrogen receptor (E R), or androgen receptor; L = a bond, a chem. linking moiety covalently coupling the CLM and the PTM; CLM = a small mol. cereblon E3 ubiquitin ligase binding moiety that binds or targets a cereblon E3 ubiquitin ligase], their pharmaceutically acceptable salts, enanti omers, stereoisomers, solvates, polymorphs and prodrugs. The invention exhibits a broad range of pharmacol. activities associated with compounds according to the present invention, consistent with the degradation/inhibition of targeted polypeptides of nearly any type (data given). Diseases or disorders that result from aggreg ation or accumulation of the target protein are treated or prevented with compounds and compositions of the invention.

Keywords: cereblon ligand chimeric mol preparation targeted protein degradation ubiquitination; PROTAC modulator preparation targeted ubiquitination estrogen receptor ERRa degradation; tau protein PROTAC degradation inhibition preparation antiproli ferative; BRD4 PROTAC degradation inhibition cereblon E3 ligase antitumor; prepar ation cereblon ligand Tank binding kinase 1 PRO TAC degradation; TBK1 degradation inhibition PROTAC modulator preparation targeted ubiquitination

PatentPak available

Substances (1,219) | Reactions (1,874) | Citing (3)

121

Hyperstabilized liposomes increase targeting of mitotic cells

By: Ng, Chang Zhi Adrian; Cheong, Shen-Yi Ian
World Intellectual Property Organization, WO2018136002 A1 2018-07-26 | Language: English, Database: CAplus

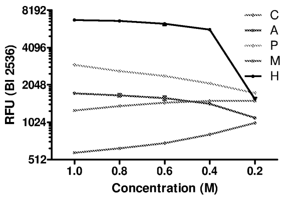

Fig. 2

Hyperstable liposome comprising an anti-mitotic agent, one or more anions and one or more cations entrapped in the inner milieu, wherein the entrapped anti-mitotic drug is released at a slow rate that is less than 0.6% in 12 h or less than 5% in 8 h when the liposomes are suspended in 600mM sucrose. These liposomes are useful in the treatment of cancer. In partic ular, HEPC:Chol:DSPE-PEG2000 (50:45:5) liposomes comprising BI2536 and citrate:phosphate in a ratio of 1:3.

Keywords: hyperstabilized phosphatidylcholine liposome target mitosis B I2536 antitumor neoplasm

PatentPak available

Substances (43)　Reactions (0)　Citing (0)

122

Preparation of functionalized pyrazoles and other nitrogen-containing heterocycles as inhibitors of MCT4 for the treatment of MCT4-mediated diseases

By: Parnell, Kenneth Mark; McCall, John; Romero, Donna
United States, US20180162822 A1 2018-06-14 | Language: English, Database: CAplus

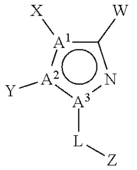

(I)

The invention relates to preparation of functionalized pyrazoles and other nitrogen heterocycles of formula **I** and compositions containing them as monocarboxylate transporter MCT4 inhibitors. Compounds **I** wherein A^1, A^2 and A^3 are independently chosen from N and C, wherein at least one of A^1, A^2 and A^3 is N; L is a bond, methylene, or ethylene; X is H or null; Y is alkenyl, alkenyl amino, alkyl, etc.; Z is alkyl, cycloa lkyl, aryl, etc., are claimed. The example compound **II** was prepared by multistep synthesis (procedure given). Compounds **I** were evaluated for their biol. activity (example - M CT4 inhibiting activity) (data given). Compounds **I** are useful in the treatment of MCT4-mediated diseases, such as proliferative and inflammatory diseases, such as cancer.

Keywords: pyrazole preparation MCT4 inhibitor antiproliferative antiinflammatory antitumor; nitrogen heterocycle preparation MC T4 disease treatment

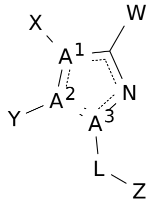

I

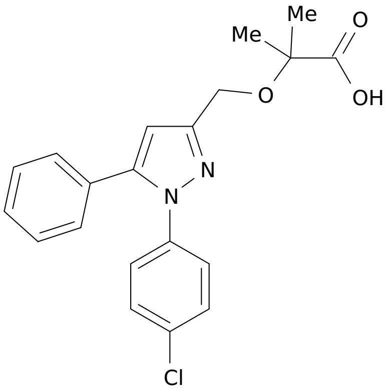

II

PatentPak available

Substances (1,145)   Reactions (4,041)   Citing (0)

123

Preparation of small molecule based antibody-recruiting compounds for cancer treatment

By: Spiegel, David; Rullo, Anthony
United States, US20180155332 A1 2018-06-07 | Language: English, Database: CAplus

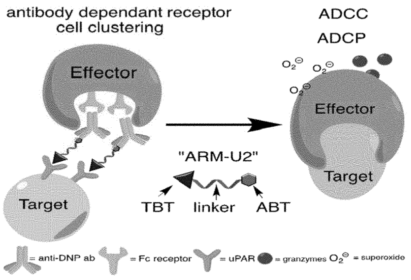

The present invention relates to chimeric (including bifunctional) compounds I [(UPAR$_B$M)$_m$-CT-(L^1)$_k$-CT-(L^2)$_j$-CT-(A$_B$M)$_n$; wherein UPA R$_B$M = moiety which binds to an active site of urokinase- type plasminogen activator receptor (u PAR) on the surface of cancer cells; A$_B$M = antibody binding moiety comprising a hapten which is capable of binding to an antibody; L ^{1} and L^2 = linkers; CT = (indepen dently) an optional connector mol.; j = 0- 5; k = 0-5; with the proviso that at least one C T is present when k and j are both 0 (preferably at least one of k and j is 1); each m and n = (indepen dently) 1-15 (preferably m = 1 and n = 1- 3)], compositions comprising those compounds and methods of treating cancer in a patient or subject, especially including metastatic cancer where cancer cells exhibit overexpression (heightened expression) of cell surface urokinase-type plasminogen activator receptor (urokinase receptor) compared to normal (non-cancerous) cells. The compounds I bind to the urokinase-type plasminogen activator receptor (uPAR) on the surface of a cancer cell, including a metastatic cancer cell, and conseq uently recruit native antibodies of the patient or subject where the antibodies can selectively degrade and/or deactivate targeted cancer cells through antibody-dependent cellular phagocytosis and antibody-dependent cellular cytotoxicity (ADCC) and/or complement dependent cytotoxicity (C DC) against a large number and variety of cancers, thus providing cancer cell death and an inhibition of growth, elabor ation and/or metastasis of the cancer, including remission and cure of the patient's cancer. E.g., a multi-step synthesis of compound II, starting from 5-hydroxy-1,4-naphthoquinone and 1-(trimethylsilyloxy)-1,3-butadiene, was described. Exemplified title compounds I were tested for their biol. activity (data given for representative compounds).

Keywords: chimeric bifunctional compound preparation antitumor u PAR modulator antibody recruiting; urokinase type plasmi nogen activator receptor chimeric bifunc tional compound preparation; antibody dependent cellular phagocytosis cancer chimeric bifunctional compound preparation; ADCC antibody dependent cellular cytotoxicity chimeric bifunctional compound preparation; C DC complement dependent cytotoxicity metastasis chimeric bifunc tional compound preparation

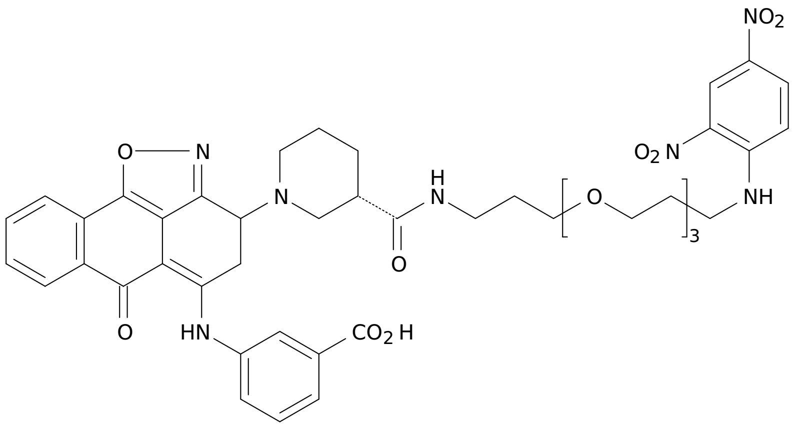

II

PatentPak available

Substances (389) Reactions (333) Citing (0)

124

Chemical Genetic Screens Identify Kinase Inhibitor Combinations that Target Anti-Apoptotic Proteins for Cancer Therapy

By: Contreras, Jacob I.; Robb, Caroline M.; King, Hannah M.; Baxter, Jared; Crawford, Ayrianne J.; Kour, Smit; Kizhake, Smitha; Sonawane, Yogesh A.; Rana, Sandeep; Hollingsworth, Michael A.; et al
ACS Chemical Biology (2018), 13(5), 1148-1152 | Language: English, Database: CAplus and MEDLINE

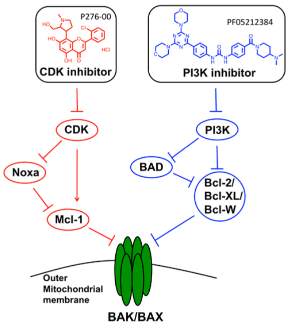

The study presented here provides a framework for the discovery of unique inhibitor combinations that target the apoptosis network for cancer therapy. A pair of doxycycline (Dox)-inducible cell lines that specifically report on the ability of an inhibitor to induce apoptosis by targeting either the Mcl-1 arm or the Bcl-2/Bcl-xL/Bcl-w arm were used. Cell-based assays were optimized for high throughput screening (HTS) with caspase 3/7 as a read out. H TS with a 355- member kinase inhibitor library and the panel of Dox-inducible cell lines revealed that cyclin dependent kinase (C DK) inhibitors induced apoptosis by targeting the Mcl- 1 arm, whereas PI3K inhibitors induced apoptosis by targeting the Bcl- 2/Bcl-xL/Bcl-w arm. Validation studies identified unique combin ations that synergistically inhibited growth and induced apoptosis in a panel of cancer cell lines. Since these inhibitors have been or are currently in clin. trials as single agents, the combinations can be rapidly translated to the clinics.

Keywords: antitumor combination synergy apoptosis cancer

Substances (15) Reactions (0) Citing (10)

125

Methods and compositions for identifying and treating patients with small cell lung cancer with aurora kinase inhibitors using transgenic animal models and cell lines

By: Oliver, Trudy; Sos, Martin; Wechsler-Reya, Rob
World Intellectual Property Organization, WO2018081575 A1 2018-05-03 | Language: English, Database: CAplus

Described herein are methods and compositions useful in detecting, diagnosing and treating small cell lung cancer. Transgenic animal models and cell lines are disclosed for the study of a small cell lung cancer subtype. Methods of screening and identifying active agents for the treatment of a small cell lung cancer subtype as well as methods of identifying patients susceptible to treatment with aurora kinase inhibitors are also provided.

Keywords: lung cancer aurora kinase inhibitor screening transgenic mouse

PatentPak available

Substances (14) Reactions (0) Citing (0)

126

Pharmaceutical combinations comprising a histone deacetylase inhibitor and an aurora kinase inhibitor and methods of use thereof

By: North, Brian; Quayle, Steven
World Intellectual Property Organization, WO2018081585 A1 2018-05-03 | Language: English, Database: CAplus

FIG. 1

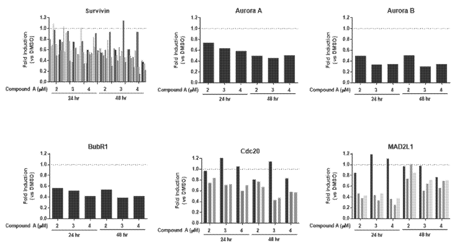

The present disclosure relates to a pharmaceutical combination comprising (a) a histone deacetylase 6 inhibitor and (b) an aurora kinase inhibitor, including combined preparations and pharmaceutical compositions thereof; uses of such combination in the treatment or prevention of cancer; and methods of treating or preventing cancer in a subject comprising administering a therapeu tically effective amount of such combination.

Keywords: pharmaceutical combination histone deacetylase aurora kinase inhibitor

PatentPak available

Substances (26) Reactions (10) Citing (1)

127

Vitamins c and k3: a powerful redox system for sensitizing leukemia lymphocytes to everolimus and barasertib

By: Ivanova, Donika; Zhelev, Zhivko; Lazarova, Dessislava; Getsov, Plamen; Bakalova, Rumiana; Aoki, Ichio
Anticancer Research (2018), 38(3), 1407-1414 | Language: English, Database: CAplus and MEDLINE

Background/Aim: Recent studies provided convincing evidence for the anticancer activity of combined applic ation of vitamin C and pro-vitamin K3 (menadione). The mol. pathways underlying this process are still not well establ ished. The present study aimed to investigate the effect of the combin ation of vitamin C plus pro-vitamin K3 on the redox status of leukemia and normal lympho cytes, as well as their sensitizing effect for a variety of anticancer drugs. Materials and methods: Cytoto xicity of the substances was analyzed by trypan blue staining and automated counting of live and dead cells. Apoptosis was analyzed by fluorescein isothioc yanate-annexin V test. Oxidative stress was evaluated by the intrace llular levels of reactive oxygen and nitrogen species and protein-carbonyl products. Results: Combined adminis tration of 300μM vitamin C plus 3μM pro-vitamin K3 reduced the viability of leukemia lymphocytes by ~20%, but did not influence the viability of normal lympho cytes. All combin ations of anticancer drug plus vitamins C and K3 were characterized by synerg istic cytotoxicity towards Jurkat cells, compared to cells treated with drug alone for 24 h. In the case of barasertib and everolimus, this synerg istic cytotoxicity increased within 72 h. It was accomp anied by strong induction of apoptosis, but a reduction of level of hydroperoxides and moderately increased protein-carbonyl products in leukemia cells. Conclusion: Leukemia lymphocytes were more sensitive to combined adminis tration of anticancer drug (everolimus or barasertib) plus vitamins C and K3, compared to normal lympho cytes. The combin ation of vitamin C plus K3 seems to be a powerful redox system that could specifically influence redox homeos tasis of leukemia cells and sensitize them to conven tional chemotherapy.

Keywords: vitamin redox system everolimus barasertib sensitizing leukemia lymphocyte; Jurkat; Vitamin C; apoptosis; leukemia lymphocytes; oxidative stress; pro-vitamin K3

Substances (7) Reactions (0) Citing (11)

128

RIPK1-dependent cell death: a novel target of the Aurora kinase inhibitor Tozasertib (VX-680)

By: Martens, Sofie; Goossens, Vera; Devisscher, Lars; Hofmans, Sam; Claeys, Polien; Vuylsteke, Marnik; Takahashi, Nozomi; Augustyns, Koen ; Vandenabeele, Peter
Cell Death & Disease (2018), 9(2), 1-13 | Language: English, Database: CAplus and MEDLINE

The Aurora kinase family (Aurora A, B and C) are crucial regulators of several mitotic events, including cytokinesis. Increased expression of these kinases is associated with tumorigenesis and several compounds targeting Aurora kinase are under evaluation in clin. trials (a.o. AT9283, AZD1152, Danusertib, MLN8054). Here, we demonstrate that the pan-Aurora kinase inhibitor Tozasertib (VX-680 and MK-0457) not only causes cytokinesis defects through Aurora kinase inhibition, but is also a potent inhibitor of necroptosis, a cell death process regulated and executed by the RIPK1, RIPK3 and MLKL signalling axis. Tozasertib's potency to inhibit RIPK1-dependent necroptosis and to block cytokinesis in cells is in the same concentration range, with an IC50 of 1.06μM and 0.554μM, resp. A structure activity relationship (SAR) anal. of 67 Tozasertib analogs, modified at 4 different positions, allowed the identification of analogs that showed increased specificity for either cytokinesis inhibition or for necroptosis inhibition, reflecting more specific inhibition of Aurora kinase or RIPK1, resp. These results also suggested that RIPK1 and Aurora kinases are functionally non-interacting targets of Tozasertib and its analogs. Indeed, more specific Aurora kinase inhibitors did not show any effect in necroptosis and Necrostatin-1s treatment did not result in cytokinesis defects, demonstrating that both cellular processes are not interrelated. Finally, Tozasertib inhibited recombinant human RIPK1, human Aurora A and human Aurora B kinase activity, but not RIPK3. The potency ranking of the newly derived Tozasertib analogs and their specificity profile, as observed in cellular assays, coincide with ADP-Glo recombinant kinase activity assays. Overall, we show that Tozasertib not only targets Aurora kinases but also RIPK1 independently, and that we could generate analogs with increased selectivity to RIPK1 or Aurora kinases, resp.

Keywords: rozasertib antiapoptotic agent RIPK1 RIPK3 fibroblast necroptosis

Substances (13) Reactions (0) Citing (27)

129

The Aurora-Kinase A Phe31-Ile polymorphism as possible predictor of response to treatment in head and neck squamous cell carcinoma.

By: Baumann, Alexander; Buchberger, Anna Maria S; Piontek, Guido; Schüttler, Dominik; Rudelius, Martina; Reiter, Rudolf; Gebel, Lena; Piendl, Gerhard; Brockhoff, Gero; Pickhard, Anja
Oncotarget (2018), 9(16), 12769-12780 | Language: English, Database: MEDLINE

Recently the Aurora-Kinases (Aurk) moved into the focus as novel disease related biomarkers and therapeutic targets. Elevated Aurora-Kinase expression has been found in a number of malignancies, amongst them HNSCC. For esophageal cancer, the AurkA Phe31-Ile polymorphism has previously been associated with tumor progression. Here we evaluated the treatment efficiency of HNSCC cell radiation as a function of Aurora- Kinases in HNSCC cell lines. Moreover, we investigated a potential sensitization to radiation by a cell treatment with the inhibitors Alisertib, Barasertib, Docetaxel and VX-680. In parallel the radiation dependent expression and regulation of AurkA/B, p-Akt Ser 473 and Survivin and the AurkA polymorphism were investigated in primary tumor samples. We identified a high-risk collective with elevated AurkA and Survivin or AurkA and p-Akt Ser 473 expression. High AurkA, AurkB, and p-Akt Ser 473 expression was exclusively found in the heterozygous cell line. We found a polymorphism dependent sensitivity to treatments with different Aurk inhibitors: The homozygous cell line UD-SCC-5 could be sensitized to radiation with Docetaxel in combination with any of the Aurora-Kinase inhibitors. In contrast, treatment with Docetaxel or radiation did not enhance the inhibitory effect of Barasertib or VX-680 in the heterozygous SAS cell line. These findings indicate that the Aurora-Kinase A Phe31-Ile-polymorphism is a possibly predictive factor for response to radiation in combination with Docetaxel and Aurora-Kinase inhibitor treatments.

Keywords: Aurora-Kinase; Aurora-Kinase a polymorphism; HNSCC; inhibition; radiation

Substances (0) Reactions (0) Citing (6)

130

Preparation method of barasertib as aurora kinase selective inhibitor

By: Hu, Zhangyan
China, CN107641115 A 2018-01-30 | Language: Chinese, Database: CAplus

The title preparation method of barasertib includes (1) substituting N-ethyl-n-propylamine (compound 1) with carbon tetrach loride and chlorine gas under light irradiation to obtain compound 2, and reacting with sodium hydroxide under catalysis of styrene iodide-divinylbenzene to obtain compound 3; (2) reacting 4- chloro-7-hydroxyquinazoline (compound 4) and ammonia gas under catalysis of copper oxide to obtain compound 5; (3) etherifying with compound 3 in presence of concen trated sulfuric acid to obtain compound 6, and condensing with 2-chloroethanol in presence of sodium hydroxide under catalysis of quaternary ammonium salt tetrabutylammonium hydrogen sulfate to obtain compound 7; (4) condensing with 5- chloro-3-bromo-1H-pyrazole in presence of sodium hydroxide under catalysis of quaternary ammonium salt tetrabutylammonium hydrogen sulfate to obtain compound 8; (5) reacting with acetyl chloride to obtain compound 9; (6) reducing 3-fluoronitrobenzene (compound 10) with hydrogen gas and hydrogen chloride under catalysis of iron powder to obtain compound 11; and (7) condensing with compound 9 in presence of sodium hydroxide under catalysis of quaternary ammonium salt tetrabutylammonium hydrogen sulfate to obtain 2-(5-((7-(3-(ethyl (2-hydroxyethyl)amino)propoxy)quinazoline-4-yl)amino)-1H-pyrazol-3-yl)-N-(3-fluorophenyl)acetamide (compound 12). The invention has easily available raw materials, high economy, environment friendliness, and reduced cost, and is suitable for large-scale production

Keywords: barasertib preparation aurora kinase selective inhibitor

PatentPak available

Substances (15) | Reactions (36) | Citing (0)

131

A mixed modality approach towards Xi reactivation for Rett syndrome and other X-linked disorders

By: Carrette, Lieselot L. G.; Wang, Chen-Yu; Wei, Chunyao; Press, William; Ma, Weiyuan; Kelleher, Raymond J. III; Lee, Jeannie T.
Proceedings of the National Academy of Sciences of the United States of America (2018), 115(4), E668-E675 | Language: English, Database: CAplus and MEDLINE

The X-chromosome harbors hundreds of disease genes whose associated diseases predomi nantly affect males. However, a subset, including neurodevelopmental disorders, Rett syndrome (RTT), fragile X syndrome, and CDKL5 syndrome, also affects females. These disorders lack disease-specific treatment. Because female cells carry two X chromo somes, an emerging treatment strategy has been to reawaken the healthy allele on the inactive X (Xi). Here, we focus on methyl-CpG binding protein 2 (MECP2) restoration for RTT and combinat orially target factors in the intera ctome of Xist, the noncoding RNA responsible for X inactivation. We identify a mixed modality approach combining an Xist antisense oligonucleotide and a small-mol. inhibitor of DNA methylation, which, together, achieve 30,000-fold MECP2 up-regulation from the Xi in cultured cells. Combining a brain- specific genetic Xist ablation with short-term 5-aza-2'-deoxycytidine (Aza) treatment models the synergy in vivo without evident toxicity. The Xi is select ively reacti vated. These experi ments provide proof of concept for a mixed modality approach for treating X- linked disorders in females.

Keywords: Xi reactivation Rett syndrome X linked disorder; LNA; Rett syndrome; X reactivation; Xist; antisense oligonucleotides

Substances (22) | Reactions (0) | Citing (49)

132

Enhancing functional platelet release in vivo from in vitro-grown megakaryocytes using small molecule inhibitors

By: Jarocha, Danuta; Vo, Karen K.; Lyde, Randolph B.; Hayes, Vincent; Camire, Rodney M.; Poncz, Mortimer

Blood Advances (2018), 2(6), 597-606 | Language: English, Database: CAplus and MEDLINE

In vitro-grown megakaryocytes for generating platelets may have value in meeting the increasing demand for platelet transfu sions. Remaining challenges have included the poor yield and quality of in vitro-generated platelets. We have shown that infusing megakaryocytes leads to intrapulmonary release of functional platelets. A Src kinase inhibitor (S U6656), a Rho-associated kinase inhibitor (Y27632), and an aurora B kinase inhibitor (AZD1152) have been shown to increase megakar yocyte ploidy and in vitro proplatelet release. We now tested whether megakar yocytes generated from C D34+ hematopoietic cells in the presence of these inhibitors could enhance functional platelet yield following megakaryocyte infusion. As expected, all inhibitors increased megakar yocyte ploidy, size, and granul arity, but these inhibitors differed in whether they injured terminal megakary ocytes: SU6656 was protective, whereas Y27632 and AZD1152 increased injury. Upon infusion, inhibitor- treated megakaryocytes released threefold to ninefold more platelets per initial noninjured megakaryocyte relative to control, but only S U6656-treated megakaryocytes had a significant increase in platelet yield when calculated based on the number of initial C D34+ cells; this was fourfold over nontreated megakaryocytes. The released platelets from drug- treated, but healthy, megakar yocytes contained similar percen tages of young, uninjured platelets that robustly responded to agonists and were well incorporated into a growing thrombus in vivo as controls. These studies suggest that drug screens that select megakaryocytes with enhanced ploidy, cell size, and granul arity may include a subset of drugs that can enhance the yield and function of platelets, and may have clin. application for ex vivo-generated megakar yocytes and platelet transfusion.

Keywords: platelet release megakaryocyte small mol inhibitor

Substances (3) Reactions (0) Citing (17)

133

Data-driven exploration of selectivity and off-target activities of designated chemical probes

By: Miljkovic, Filip; Bajorath, Juergen

Molecules (2018), 23(10), 1-12 | Language: English, Database: CAplus and MEDLINE

Chem. probes are of central relevance for chem. biol. To unambiguously explore the role of target proteins in triggering or mediating biol. functions, small mols. used as probes should ideally be target-specific; at least, they should have sufficiently high selectivity for a primary target. We present a thorough anal. of currently available activity data for designated chem. probes to address several key questions: How well defined are chem. probes What is their level of selectivity Is there evidence for addnl. activities Are some probes "better" thanothers. Therefore, highly curated chem. probes were collected and their select ivity was analyzed on the basis of publicly available compound activity data. Different selectivity patterns were observed, which distinguished designated high-quality probes.

Keywords: data driven exploration selectivity activity chem probe; bioactive compounds; chemical probes; hemical biology; molecular scaffold; off-target activity; promiscuity; target selectivity

Substances (13) Reactions (0) Citing (6)

134

Mitotic slippage and the subsequent cell fates after inhibition of Aurora B during tubulin-binding agent-induced mitotic arrest

By: Tsuda, Yasuo; Iimori, Makoto; Nakashima, Yuichiro; Nakanishi, Ryota; Ando, Koji; Ohgaki, Kippei; Kitao, Hiroyuki; Saeki, Hiroshi; Oki, Eiji; Maehara, Yoshihiko
Scientific Reports (2017), 7(1), 1-12 | Language: English, Database: CAplus and MEDLINE

Tubulin-binding agents (TBAs) are designed to target microtubule (MT) dynamics, resulting in compromised mitotic spindles and an unsatisfied spindle assembly checkpoint. The activity of Aurora B kinase is indispensable for TBA-induced mitotic arrest, and its inhibition causes mitotic slippage and postmitotic endoreduplication. However, the precise phenomenon underlying mitotic slippage, which is caused by treatment with both Aurora B inhibitors and TBAs, and the cell fate after postmitotic slippage are not completely understood. Here, we found that HeLa and breast cancer cells treated with the different types of TBAs, such as paclitaxel and eribulin (MT-stabilizing and MT-destabilizing agents, resp.), exhibited distinct behaviors of mitotic slippage on inhibition of Aurora B. In such conditions, the cell fates after postmitotic slippage vastly differed with respect to cell morphol., cell proliferation, and cytotoxicity in short-term culture; i.e., the effects of inhibition of Aurora B were beneficial for cytotoxicity enhancement in eribulin treatment but not in paclitaxel. However, in long-term culture, the cells that survived after mitotic slippage underwent endoreduplication and became giant cells in both cases, resulting in cellular senescence. We propose that MT-destabilizing agents may be more appropriate than MT-stabilizing agents for treating cancer cells with a weakened Aurora B kinase activity.

Keywords: cervical cancer cell fate mitotic arrest slippage Aurora B

Substances (10) Reactions (0) Citing (25)

135

The target landscape of clinical kinase drugs

By: Klaeger, Susan; Heinzlmeir, Stephanie; Wilhelm, Mathias; Polzer, Harald; Vick, Binje; Koenig, Paul-Albert; Reinecke, Maria; Ruprecht, Benjamin; Petzoldt, Svenja; Meng, Chen; et al
Science (Washington, DC, United States) (2017), 358(6367), 1148 | Language: English, Database: CAplus and MEDLINE

Kinase inhibitors are important cancer therapeutic agents. Polypharmacol. is commonly observed, requiring thorough target deconvolution to understand drug mechanism of action. Using chem. proteomics, the authors analyzed the target spectrum of 243 clin. evaluated kinase drugs. The data revealed previously unknown targets for established drugs, offered a perspective on the "druggable" kinome, highlighted (non)kinase off-targets, and suggested potential therapeutic applications. Integration of phosphoproteomic data refined drug-affected pathways, identified response markers, and strengthened rationale for combination treatments. The authors exemplify translational value by discovering SIK2 (salt-inducible kinase 2) inhibitors that modulate cytokine production in primary cells, by identifying drugs against the lung cancer survival marker MELK (maternal embryonic leucine zipper kinase), and by repurposing cabozantinib to treat FLT3-ITD-pos. acute myeloid leukemia. This resource, available via the ProteomicsDB database, should facilitate basic, clin., and drug discovery research and aid clin. decision- making.

Keywords: protein kinase inhibitor drug target discovery Proteomics database antitumor

Substances (128) Reactions (0) Citing (461)

136

Modulators of homeobox transcription factors, DUX4 (double homeobox 4) for regulation of muscle function

By: Rickard, Amanda; De La Garza, Anabel; Schmidt, Uli; Kiselyov, Alexander
World Intellectual Property Organization, WO2017201585 A1 2017-11-30 | Language: English, Database: CAplus

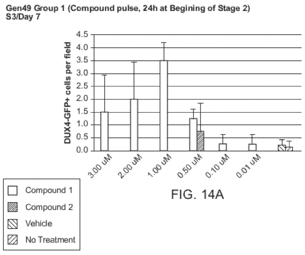

FIG. 14A

Disclosed herein are methods and compositions for the treatment of facioscapu lohumeral muscular dystrophy and other muscle diseases or disorders. In some cases, the methods and compositions involve the use of methyltransferase inhibitors (e.g. tazemet ostat, UNC-1999, EPZ005687, GSK343) to inhibit or repress DUX4 expression in muscle cells. Further disclosed herein are methods and cell based assays for screening compounds for the treatment of facioscapulohumeral muscular dystrophy and other muscle diseases.

Keywords: drug screening DUX4 modulator methyltransferase inhibitor muscle disease treatment

PatentPak available

Substances (241) Reactions (0) Citing (3)

137

Targeting vcp enhances anticancer activity of oncolytic virus m1 in hepatocellular carcinoma

By: Zhang, Haipeng ; Li, Kai; Lin, Yuan ; Xing, Fan; Xiao, Xiao; Cai, Jing; Zhu, Wenbo; Liang, Jiankai; Tan, Yaqian; Fu, Liwu; et al
Science Translational Medicine (2017), 9(404), eaam7996/1-eaam7996/12 | Language: English, Database: CAplus and MEDLINE

Oncolytic virotherapy is rapidly progressing through clin. evaluation. However, the therapeutic efficacy of oncolytic viruses in humans has been less than expected from preclin. studies. We describe an anticancer drug screen for compounds that enhance M1 oncolytic virus activity in hepatocellular carcinoma (HCC). An inhibitor of the valosin-containing protein (VCP) was identified as the top sensitizer, selectively increasing potency of the oncolytic virus up to 3600- fold. Further investigation revealed that VCP inhibitors cooperated with M1 virus-suppressed inositol-requiring enzyme 1 α (IRE1 α)-X-box binding protein 1 (XBP1) pathway and triggered irresolvable endoplasmic reticulum (ER) stress, subsequently promoting robust apoptosis in HCC. We show that VCP inhibitor improved the oncolytic efficacy of M1 virus in several mouse models of HCC and primary HCC tissues. Finally, this combinatorial therapeutic strategy was well tolerated in nonhuman primates. Our study identifies combined VCP inhibition and oncolytic virus as a potential treatment for HCC and demonstrates promising therapeutic potential.

Keywords: hepatocellular carcinoma oncolytic virus vcp anticancer activity

Substances (23) Reactions (0) Citing (287)

138

Diagnostic markers, test method, and therapeutic agents for blastic plasmacytoid dendritic cell neoplasm as rare hematopoietic tumor, and therapeutic agent screening method

By: Takeuchi, Kengo; Sakamoto, Kana; Katayama, Ryohei; Sakata, Seiji

World Intellectual Property Organization, WO2017138500 A1 2017-08-17 | Language: Japanese, Database: CAplus

Diagnosis markers that can provide diagnosis criteria for blastic plasmacytoid dendritic cell neoplasm (BPDCN) as rare hematop oietic tumor are searched, and the presence of an immunobl astoid-type cell form, the reconstruction of 8q24, and the expression of MYC are established as novel markers for sub-typing BPDCN. In addition, it is found that a BET bromo domain-selective inhibitor, an inhibitor capable of directly or indirectly inhibiting the expression or function of MYC or the signaling pathway of M YC such as aurora kinase inhibitor, a HDAC inhibitor or a BCL2 family protein inhibitor is effective as a therap eutic agent in a M YC-expressing group.

Keywords: blastic plasmacytoid dendritic cell neoplasm immunob lastoid diagnostic biomarker

PatentPak available

Substances (12) Reactions (0) Citing (0)

139

Compositions and methods for inhibiting kinase activity using an agent that binds to a PIF pocket of Aurora A kinase for cancer treatment

By: Kern, Dorothee; Zorba, Adelajda

World Intellectual Property Organization, WO2017139321 A1 2017-08-17 | Language: English, Database: CAplus

FIG. 1A

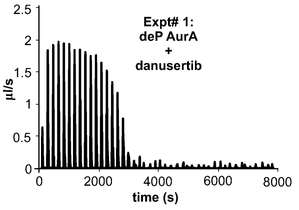

The present invention features therapeutic compositions comprising an agent that specifically binds to a PIF pocket of Aurora A kinase and an agent that specifically binds to an ATP-binding site of Aurora A kinase, and the use of the therap eutic compositions to modulate Aurora A kinase for the treatment of cancer.

Keywords: Aurora A kinase inhibitor PIF pocket binding cancer treatment

PatentPak available

Substances (23) Reactions (0) Citing (0)

140

DNA alkylation and cross-linking agents as compounds and payloads for targeted therapies

By: Herzon, Seth; Healy, Alan; Crawford, Jason; Vizcaino, Maria; Nikolayevskiy, Herman
World Intellectual Property Organization, WO2017132459 A1 2017-08-03 | Language: English, Database: CAplus

The present invention is directed to compounds related to precolibactin, pharmaceutical compositions based upon these compounds and methods of synthesis which are employed to provide intermediates and final compounds, which are alkylating agents and anticancer compounds The chem. synthetic approach disclosed facilitates the synthesis of numerous precolibactin analogs which can be used in the treatment of cancer. Example compound **I** was prepared by a multistep procedure. The invention compounds were evaluated for their anticancer activities (data given).

Keywords: thiazoline thiazole preparation DNA alkylation anticancer targeted therapy

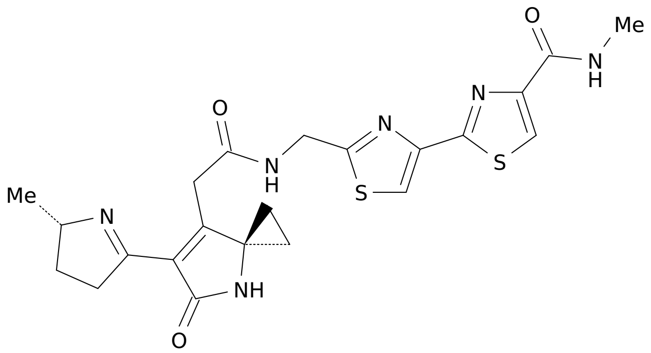

I

PatentPak available

Substances (425) Reactions (693) Citing (0)

141

In Vitro Sensitivity Profiling of Neuroblastoma Cells Against A Comprehensive Small Molecule Kinase Inhibitor Library to Identify Agents for Future Therapeutic Studies

By: Singh, Anjali; Meier-Stephenson, Vanessa; Jayanthan, Aarthi; Narendran, Aru
Current Cancer Drug Targets (2017), 17(6), 569-584 | Language: English, Database: CAplus and MEDLINE

Background: Neuroblastoma (NB) constitutes about 8% of all childhood tumors, yet accounts for more than 15% of deaths, with an unacceptable overall survival rate. These rates are despite the current multimodal therap eutic approaches involving surgery, radiation, chemotherapy and myeloablation with hematopoietic stem cell rescue. Hence, efforts have intens ified to identify new targets and novel therapeutic approaches to improve cure rates in these children. Numerous new agents for adult malign ancies are developed and evaluated for cancer each year, providing an invaluable resource, with the added advantage of available pharmacol. and toxicity data for consideration. Methods: To identify potential therapeutic targets, we screened a small mol. library of 151 small kinase inhibitors against NB cell lines. Based on our initial screening data, we further examined the potential of Bcr- Abl targeting small mol. inhibitors to affect the growth and survival of NB cells. Results: There is diverse activity among the currently available Bcr-Abl inhibitors, possibly reflecting the mol. heterogeneity and off-target activity in each combination. In depth analyses of ponatinib, an oral multi-target kinase inhibitor and effective agent in the treatment of refractory Philad elphia chromosome (Ph) pos. leukemia, show growth inhibition at sub-micromolar concentrations In addition, we also identified the potential of this agent to interfere with insulin-like growth factor-1 receptor (IGF-1R) signaling pathways and Src activity, inhibit cell migration and induce apoptosis. Conclusion: Our findings provide initial data on ponati nib's potential to target key growth regulatory pathways and provide the rationale for further studies and evaluation in future early phase clin. trials for the treatment of refractory NB.

Keywords: neuroblastoma cell small mol kinase inhibition therape utics; Bcr-Abl; IGF-1 Receptor; Neuroblastoma; Ponatinib; Src; small molecule kinase inhibitors

Substances (183) Reactions (0) Citing (2)

142

Phosphorylation of histone H3 on Ser-10 by Aurora B is essential for chromosome condensation in porcine embryos during the first mitotic division

By: Chen, Changchao; Zhang, Zixiao; Cui, Panpan; Liao, Yaya; Zhang, Yue; Yao, Lingyun; Rui, Rong; Ju, Shiqiang
Histochemistry and Cell Biology (2017), 148(1), 73-83 | Language: English, Database: CAplus and MEDLINE

Phosphorylation of histone H3 on Ser- 10 (H3S10ph) is involved in regulating mitotic chromosome conden sation and deconden sation, which plays an important regulatory role during mitotic cell cycle progre ssion in mammalian cells. However, whether H3 S10ph plays a similar role in early porcine embryos during the first mitotic division remains uncertain. In this study, the subcel lular localization and possible roles of H3 S10ph were evaluated in the first mitotic cell cycle progre ssion of porcine embryos using western blot, indirect immunofluorescence and barasertib (H3S10ph upstream regulator Aurora- B inhibitor) treatments. H3S10ph exhibited a dynamic localization pattern and was localized to chromosomes from promet aphase to anaphase stages. Treatment of porcine embryos with barasertib inhibited mitotic division at the prophase stage and was associated with a defect in chromosome condensation accompanied by the reduction of H3S10ph. These results indicated that H3 S10ph is involved in the first mitotic division in porcine embryos through its regulatory function in chromosome condensation, which further affects porcine embryo cell cycle progression during mitotic division.

Keywords: phosphorylation histone H3 Ser aurora essential chromosome conden sation porcine; Chromosome condensation; Embryos; H3S10ph; Mitotic division; Pig

Substances (2) | Reactions (0) | Citing (5)

143

Osteotropic nanoparticles for delivering bioactive agents including anticancer agents and/or diagnostic agents for prevention or treatment of bone cancer metastases

By: Durfee, Paul N.; Brinker, Charles Jeffrey; Lin, Yu-Shen; Leong, Hon
World Intellectual Property Organization, WO2017120504 A1 2017-07-13 | Language: English, Database: CAplus

The present disclosure is directed to protocells or nanoparticles, which are optionally coated with a lipid bilayer, which can be used for targeting bone tissue for the delivery of bioactive agents useful in the treatment and/or diagnosis of bone cancer, often metastatic bone cancer which often occurs secondary to a primary cancer such as prostate cancer, breast cancer, lung cancer and ovarian cancer, among numerous others. These protocells or nanoparticles target bone cancer especially metastatic bone cancer with bioactive agents including anticancer agents and/or diagnostic agents for purposes of treating, diagnosing and/or monitoring the therapy of the bone cancer. Osteotropic protocells or nanoparticles, pharmaceutical compositions comprising a population of osteotropic protocells or nanoparticles and methods of diagno sing, treating and/or monitoring therapy of bone cancer are represe ntative aspects.

Keywords: osteotropic nanoparticle bone cancer metastasis targeting

PatentPak available

Substances (230) | Reactions (0) | Citing (4)

144

Optimizing Therapeutic Effect of Aurora B Inhibition in Acute Myeloid Leukemia with AZD2811 Nanoparticles

By: Floc'h, Nicolas; Ashton, Susan; Taylor, Paula; Trueman, Dawn; Harris, Emily; Odedra, Rajesh; Maratea, Kim; Derbyshire, Nicola; Caddy, Jacqueline; Jacobs, Vivien N.; et al

Molecular Cancer Therapeutics (2017), 16(6), 1031-1040 | Language: English, Database: CAplus and MEDLINE

Barasertib (AZD1152), a highly potent and selective aurora kinase B inhibitor, gave promising clin. activity in elderly acute myeloid leukemia (AML) patients. However, clin. utility was limited by the requirement for a 7-day infusion. Here we assessed the potential of a nanoparticle formulation of the selective Aurora kinase B inhibitor AZD2811 (formerly known as AZD1152-hQPA) in preclin. models of AML. When administered to HL-60 tumor xenografts at a single dose between 25 and 98.7 mg/kg, AZD2811 nanoparticle treatment delivered profound inhibition of tumor growth, exceeding the activity of AZD1152. The improved antitumor activity was associated with increased phospho-histone H3 inhibition, polyploidy, and tumor cell apoptosis. Moreover, AZD2811 nanoparticles increased antitumor activity when combined with cytosine arabinoside. By modifying dose of AZD2811 nanoparticle, therapeutic benefit in a range of preclin. models was further optimized. At high-dose, antitumor activity was seen in a range of models including the MOLM-13 disseminated model. At these higher doses, a transient reduction in bone marrow cellularity was observed demonstrating the potential for the formulation to target residual disease in the bone marrow, a key consideration when treating AML. Collectively, these data establish that AZD2811 nanoparticles have activity in preclin. models of AML. Targeting Aurora B kinase with AZD2811 nanoparticles is a novel approach to deliver a cell-cycle inhibitor in AML, and have potential to improve on the clin. activity seen with cell-cycle agents in this disease.

Keywords: AZD2811 nanoparticle anticancer aurora B inhibitor acute myeloid leukemia

Substances (4) | Reactions (0) | Citing (22)

145

Preparation of anti-human LAG3 antibodies, vaccines and/or therapeutic agents for treatment of cancer or infection

By: Malefyt, Rene de Waal; Fayadat-Dilman, Laurence; Liang, Linda

World Intellectual Property Organization, WO2017106129 A1 2017-06-22 | Language: English, Database: CAplus

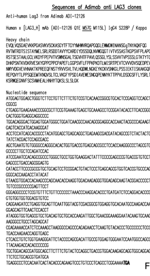

FIG.2A

The present invention includes antibodies and antigen-binding fragments thereof that specifically bind to human or cynomolgus monkey LAG3 as well as Ig chains thereof and polynucleotides encoding the same along with injection devices comprising such antibodies or fragments. Vaccines including such antibodies and fragments as well as compositions comprising the antibodies and fragments (e.g., including anti-PD1 antibodies) are included in the invention. Methods for treating or preventing cancer or infection using such compositions are also provided. In addition, methods for recombinant expression of the antibodies and fragments are part of the present invention.

Keywords: human LAG3 antibody vaccine therapeutic agent composition cancer infection

PatentPak available

Substances (367) | Reactions (0) | Citing (0)

146

Ex vivo drug response profiling detects recurrent sensitivity patterns in drug-resistant acute lymphoblastic leukemia

By: Frismantas, Viktoras; Dobay, Maria Pamela; Rinaldi, Anna; Tchinda, Joelle; Dunn, Samuel H.; Kunz, Joachim; Richter-Pechanska, Paulina; Marovca, Blerim; Pail, Orrin; Jenni, Silvia; et al
Blood (2017), 129(11), e26-e37 | Language: English, Database: CAplus and MEDLINE

Drug sensitivity and resistance testing on diagnostic leukemia samples should provide important functional inform ation to guide actionable target and biomarker discovery. We provide proof of concept data by profiling 60 drugs on 68 acute lymphoblastic leukemia (ALL) samples mostly from resistant disease in cocultures of bone marrow stromal cells. Patient- derived xenografts retained the original pattern of mutations found in the matched patient material. Stromal coculture did not prevent leukemia cell cycle activity, but a specific sensitivity profile to cell cycle-related drugs identified samples with higher cell prolife ration both in vitro and in vivo as leukemia xenografts. In patients with refractory relapses, individual patterns of marked drug resistance and except ional responses to new agents of immediate clin. relevance were detected. The B CL2-inhibitor venetoclax was highly active below 10 nM in B-cell precursor ALL (BCP-ALL) subsets, including MLL-AF4 and TCF3-HLF ALL, and in some T-cell ALLs (T-ALLs), predicting in vivo activity as a single agent and in combination with dexamethasone and vincristine. Unexpected sensitivity to dasatinib with half maximal inhibitory concentration values below 20 nM was detected in 2 indepe ndent T-ALL cohorts, which correlated with similar cytotoxic activity of the SRC inhibitor KX2-391 and inhibition of S RC phosphorylation. A patient with refractory T- ALL was treated with dasatinib on the basis of drug profiling information and achieved a 5- mo remission. Thus, drug profiling captures disease-relevant features and unexpected sensitivity to relevant drugs, which warrants further explor ation of this functional assay in the context of clin. trials to develop drug repurposing strategies for patients with urgent medical needs.

Keywords: acute lymphoblastic leukemia drug resistance sensitivity

Substances (62) Reactions (0) Citing (123)

147

NPM-ALK expression levels identify two distinct subtypes of paediatric anaplastic large cell lymphoma

By: Pomari, E.; Basso, G.; Bresolin, S.; Pillon, M.; Carraro, E.; d'Amore, E. S.; Viola, G.; Frasson, C.; Basso, K.; Bonvini, P.; et al
Leukemia (2017), 31(2), 498-501 | Language: English, Database: CAplus and MEDLINE

Anaplastic large cell lymphoma (ALCL) is a distinct form of non-Hodgkin lymphoma that accounts for 10-15% of all childhood lymphomas. Differently from ALCL in adults, where about 30% of cases are A LK+, ALCL in children is nearly universally ALK+, and in almost all of the cases, it is characterized by the t(2;5)(p23;q35) translocation, which results in the expression of the hybrid oncogenic tyrosine kinase NPM-ALK. NPM-ALK fusion protein is respon sible for ALCL development, and throughout its constitutive tyrosine kinase activity, it regulates signalling pathways critical for proliferation, cell-cycle control and survival of A LCL cells. So far, few studies have investigated ALCL gene expression profiling, and most of them have used heterog eneous populations of paediatric and adult cases. Main aim of such studies was to identify whether ALK+ and ALK- ALCL had a different transcri ptional program, and although differences were found, similarities were also detected. Based on the fact that paediatric A LCL are almost exclusively AL K+, our study focussed on investi gating the potential heterogeneity inside ALK+ ALCL rather than differ ences between ALK+ and AL K- tumors. In order to investigate the transcriptional characteristics of ALK+ ALCL, we performed a global microa rray-based transcri ptional anal. on 23 paediatric tumor samples, 12 reactive lymph nodes and 4 A LCL cell lines (3 ALK+ and 1 ALK-) (Supplementary Materials). As previously reported, we found that A LK+ tumors displayed downreg ulation of both B-cell and T-cell receptor signalling mols. (such as ARNT2, CD3G, CD24, CD27, CD40, CD79A, NFATC2, PIK3CD, TNFRSF10A) in addition to T helper cell surface mols. (such as CD19, CD28, CTLA4), (Supplementary Table S1), consistent with the notion that this malignancy originates from undifferentiated normal CD4+ T lymphocytes.[5] To explore the mol. differ ences between clin. homoge neous ALK+ ALCL, we performed unsupervised clustering anal. of the tumor samples, identi fying two clearly distinct subgroups of patients. Indeed, supervised anal. identified two different gene expression signatures, both related to the diverse amount of N PM-ALK in ALCL cells (A LK-low and -high). Of note, A LCL cell lines clustered together and had a gene expression signatures more similar to A LK-high than to ALK-low samples (Figure 1a; Supplem entary Table S2). The ALK-high/low signatures were not affected by sample or patient selection bias, as our novel ALK group classif ication was independent of the lymph node infilt ration rate, as the percentage of ALK+ cells was about 40% in the large majority of cases and it could be applied to indipendent ALCL studies (Supplementary Figure S1). N PM-ALK levels were assessed by real-time quant. reverse transcriptase PCR (RQ-PCR) in the same set of patients showing a signif icant correl ation with gene expression profiling data (Pearson's correl ation coefficient 0.79, P<0.0001) (Figures 1b and c, resp.). Addnl. 19 paediatric ALCL cases were tested by R Q-PCR, and overall the full data set, including 42 samples, displayed a bimodal distri bution for NPM-ALK expression consistent with the presence of an A LK-low and an ALK-high group. The median expression values were

2.500 NPM-ALK copies/10.000 ABL in ALK-low cases and 125.000 N PM-ALK copies/10.000 ABL in ALK-high patients (Welch's t-test, P<0.0001 Figure 1d). In addition, when NPM-ALK protein expression was measured in a subset of our cases, selected based on tissue availability (Figure 1e), we found that expression and phosphor ylation of NPM-ALK kinase correlated with m RNA levels of the two groups (Figure 1f), supporting the concept that ALK+ ALCL can be distinguished based on NPM-ALK endogenous expression level. We did not observe significant differences between the two subgroups based on clin. characte ristics, although patients with central nervous system involvement (n=4) belonged to the ALK-high subgroup (Supplementary Table S3). Interestingly, 9 out of the 12 patients who experienced relapse were ALK-high cases and had a median time of relapse of 5 mo compared with the median time of relapse of 30 mo of the 3 ALK-low relapsed patients. Gene set enrichment anal. using the M Sig DB database (Mol. Signature Database, Oncogene signature C6, http://www.broad.mit.edu/gsea/) showed a pos. enrichment for genes involved in the interl eukin (IL) signalling pathway (specifically IL-2, IL-15 and IL-21) in ALK-low compared with ALK-high ALCL samples, while genes set associated with cell growth and division (CyclinD1, MYC and E2F1) were significantly upregulated in ALK-high cases (Figure 1g). The I L-2 signalling pathway is critical for C D4+ T-cell maturation and function,[6, 7] and its downreg ulation occurs in ALCL cell lines in response to NPM-ALK expression and activity. Consist ently, we showed that IL-2Rγ was expressed in ALK- (FE-PD) ALCL cell line but not in ALK+ (KARPAS-299, SUD-HL-1 and SUP-M2) cell lines (Figure 2a). In order to evaluate the IL-2Rγ expression in ALCL primary tumor cases, we analyzed, by flow cytometry, nine patients (four ALK-low and five ALK-high), for whom tissue samples were available. The results showed that tumor cells in ALK-low patients ubiquitously expressed IL-2Rγ (ALK+/IL-2Rγ+ cells >95%), whereas ALK-high patients displayed a more heterog eneous pattern of expression (Figures 2b and c). Taking into account that A LK-high patients relapsed more frequently and rapidly than ALK-low patients, it seems that in ALK-low cases NPM-ALK is unable to make transformed lymphocytes completely 'deaf' to ILs, and IL signalling retention, in turn, contri butes to a less aggressive tumor phenotype. Functional annotation anal. performed by gene set enrichment anal. in ALK-high tumor samples revealed increased expression of genes involved in cell growth and survival, such as cyclins and Aurora kinases A (AURKA) and B (AURKB). These genes are known not only to affect cell proliferation but also regulate chromo somes' segregation and mitosis. Hence, to validate our bioinformatics anal., we took advantage of com. available Aurora kinase inhibi tors, which were used to treat a panel of ALK+ ALCL cell lines (KARPAS-299, SU-DHL-1 and SUP-M2) representative of the ALK-high subgroup. As expected, marked growth inhibition of A LCL cells was observed upon adminis tration of AURKA and AURKB inhibitors Alisertib (MLN8237) and Barasertib (AZD1152), resp., and this was associated with cell-cycle arrest and induction of apoptosis (Figure 2d). In particular, the exposure of ALCL cells to increasing concentrations of MLN8237 and/or AZD1152 led to time-dependent inhibition of cell growth, which, consistent with Aurora kinases' involvement in mitotic entry and cytoki nesis, was characterized by a dose-dependent 'growth plateau' typical of a cytostatic cell response (Figure 2d). In contrast, Aurora kinases inhibitors did not cause changes in healthy peripheral blood mononu clear cells' proliferation (stimulated with or without phytohaema gglutinin), when used in the same exptl. conditions (Figure 2e). Previous data have shown that Aurora inhibitors exert their activity leading to accumulation of polyploid cells with an endorepl ication phenotype before mitotic catastrophe or apoptosis occur. Indeed, when drug- inhibitory effects were investigated in more detail, we found that both compounds, administered for 48 h as single agents or in combin ation, led to a dramatic increase of the polyploid cell content (8N) over the diploid (2N) and tetraploid (4N) cell population (Supplem entary Figure S2A), whereas at earlier time points (24 h) they induced a provisional arrest in G2/M phase and accumu lation of tetraploid cells only (data not shown). Prolonged drug exposure, instead, affected cell survival, as the fraction of the cells undergoing apoptosis (Annexin V-pos., AV^+) or necrosis (Propidium Iodide pos., PI^+) increased over time, after 72 h of treatment (Supplem entary Figure S2B). AURKA and AURKB have been recently detected in non-Hodgkin lymphoma; however, their involv ement in ALCL tumorigenesis, as well as their importance as therapeutic targets, has not been investi gated. Herein we provide evidence that A URKA and AURKB may be considered novel drug targets in ALCL and that Aurora kinase inhibitors are effective agents in preclin. A LCL models. Their efficacy depends on their general mode of action and results in inhibiting proliferation, survival and cell-cycle progression of ALCL cells. Overall, our data suggest that the heterogeneous levels of aggressiveness observed in ALCL tumors may be explained by a N PM-ALK distinct gene expression and transcriptional program. High-level ALK+ cells seems to acquire a selective growth advantage compared with ALK-low cells. This concept merits attention to exploit novel therap eutic strategies in this malignancy that may increase curability and survival of children with ALCL.

Keywords: anaplastic large cell lymphoma NPM ALK fusion protein

Substances (4) Reactions (0) Citing (6)

148

Aurora B expression modulates paclitaxel response in non-small cell lung cancer

By: Al-Khafaji, Ahmed S. K.; Davies, Michael P. A.; Risk, Janet M.; Marcus, Michael W.; Koffa, Maria; Gosney, John R.; Shaw, Richard J.; Field, John K.; Liloglou, Triantafillos

British Journal of Cancer (2017), 116(5), 592-599 | Language: English, Database: CAplus and MEDLINE

Background: Taxanes are mitotic poisons widely used in the treatment of non- small cell lung cancer (NSCLC), however, little is known about potential mol. modulators of response to these compounds Aurora B (AURKB) is a critical regulator of the mitotic spindle assembly, previously shown overexpressed in NSCLC. Here we investigated the hypothesis that AURKB expression modulates the efficacy of taxanes in NSCLC cells. Methods: AURKB mRNA expression was determined by qPCR in 132 frozen NSCLC tissues and nine NSCLC cell lines. Aurora B expression was knocked down in cell lines using multiple sh RNA constructs. Barasertib was used to specifically inhibit AURKB activity, determined by the level of H3S10 phosphorylation. Results: Frequent AURKB mRNA upregulation was observed in NSCLC tissues (P<0.0001), being more prominent in squamous carcinomas (P<0.0001). Aurora B expression in cell lines strongly correlated with sensitivity to both docetaxel (P=0.004) and paclitaxel (P=0.007). Aurora B knockdown derivatives consistently showed a dose-dependent association between low-AURKB expression and resistance to paclitaxel. Specific chem. inhibition of Aurora B activity also demonstrated a strong dose-dependent efficiency in triggering paclitaxel resistance. Conclusions: Aurora B activity is an important modulator of taxane response in N SCLC cells. This may lead to further insights into taxane sensitivity of NSCLC tumors.

Keywords: non small cell lung cancer paclitaxel efficacy aurora B

Substances (3) Reactions (0) Citing (28)

149

Preparation of small molecule based antibody-recruiting compounds for cancer treatment

By: Spiegel, David; Rullo, Anthony

World Intellectual Property Organization, WO2017023994 A1 2017-02-09 | Language: English, Database: CAplus

The present invention relates to chimeric (including bifunctional) compounds **I** [$(UPAR_BM)_m$-CT-$(L^1)_k$-CT-$(L^2)_j$-CT-$(A_BM)_n$; wherein UPA R_BM = moiety which binds to an active site of urokinase- type plasminogen activator receptor (u PAR) on the surface of cancer cells; A_BM = antibody binding moiety comprising a hapten which is capable of binding to an antibody; L [1] and L^2 = linkers; CT = (indepen dently) an optional connector mol.; j = 0- 5; k = 0-5; with the proviso that at least one C T is present when k and j are both 0 (preferably at least one of k and j is 1); each m and n = (indepen dently) 1-15 (preferably m = 1 and n = 1- 3)], compositions comprising those compounds and methods of treating cancer in a patient or subject, especially including metastatic cancer where cancer cells exhibit overexpression (heightened expression) of cell surface urokinase-type plasminogen activator receptor (urokinase receptor) compared to normal (non-cancerous) cells. The compounds **I** bind to the urokinase-type plasminogen activator receptor (uPAR) on the surface of a cancer cell, including a metastatic cancer cell, and conseq uently recruit native antibodies of the patient or subject where the antibodies can selectively degrade and/or deactivate targeted cancer cells through antibody-dependent cellular phagocytosis and antibody-dependent cellular cytotoxicity (ADCC) and/or complement dependent cytotoxicity (C DC) against a large number and variety of cancers, thus providing cancer cell death and an inhibition of growth, elabor ation and/or metastasis of the cancer, including remission and cure of the patient's cancer. E.g., a multi-step synthesis of compound **II**, starting from 5-hydroxy-1,4-naphthoquinone and 1-(trimethylsilyloxy)-1,3-butadiene, was described. Exemplified title compounds **I** were tested for their biol. activity (data given for representative compounds).

Keywords: chimeric bifunctional compound preparation antitumor u PAR modulator antibody recruiting; urokinase type plasmi nogen activator receptor chimeric bifunc tional compound preparation; antibody dependent cellular phagocytosis cancer chimeric bifunctional compound preparation; ADCC antibody dependent cellular cytotoxicity chimeric bifunctional compound preparation; C DC complement dependent cytotoxicity metastasis chimeric bifunc tional compound preparation

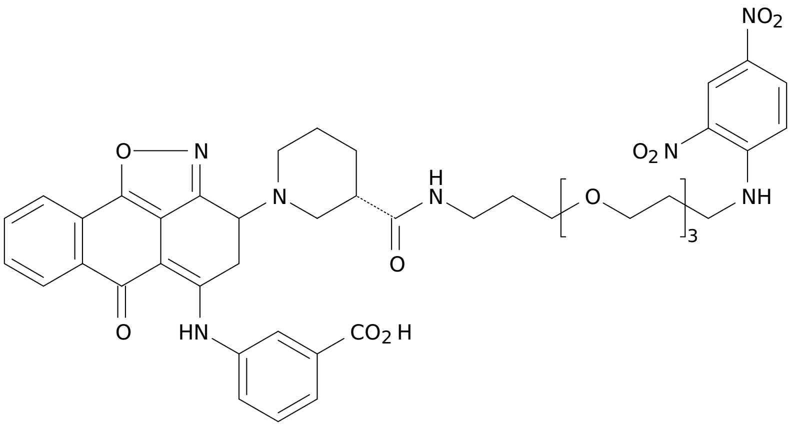

I

PatentPak available

Substances (374) | Reactions (318) | Citing (0)

150

Synergistic cytotoxicity of melatonin and new-generation anticancer drugs against leukemia lymphocytes but not normal lymphocytes

By: Zhelev, Zhivko; Ivanova, Donika; Bakalova, Rumiana; Aoki, Ichio; Higashi, Tatsuya
Anticancer Research (2017), 37(1), 149-160 | Language: English, Database: CAplus and MEDLINE

The present study demonstrates specific sensitization of leukemia lymphocytes towards anticancer drugs using melatonin and clarifies the role of reactive oxygen species (ROS) for induction of apoptosis. The study covers four conven tional and 11 new-generation anticancer drugs. Four parameters were analyzed simulta neously in leukemia and normal lympho cytes treated with drug, melatonin, or their combination: cell viability, induction of apoptosis, level of reactive oxygen species (R OS), and level of protein-carbonyl products. Almost all investigated combinations of melatonin with new-generation anticancer drugs were charact erized by synerg istic cytotoxicity towards leukemia lymphocytes, while the combin ations with conventional drugs exhibited additive or antagonistic effects on cell viability. In leukemia lympho cytes, the additive cytoto xicity of doxorubicin plus melatonin was accomp anied by low levels of R OS and protein-carbonyl products, as well as by suppre ssion of apoptosis. In normal lymphocytes, none of the studied parameters changed significantly compared to cells treated with doxoru bicin only. The combin ations of everolimus plus melatonin and barasertib plus melatonin exhibited impressive synerg istic cytotoxic effects on leukemia lymphocytes but did not affect the viability of normal lymphocytes. In leukemia cells, the synerg istic cytotoxicity was accompanied by strong induction of apoptosis but a decrease of ROS to a level below that of the control. In normal lympho cytes, these combin ations did not affect the level of ROS nor of protein- carbonyl products, and did not induce apoptosis. The data suggest that melatonin is a promising supplementary component in chemotherapy which allows the therap eutic doses of anticancer drugs to be reduced, minimizing their side-effects.

Keywords: anticancer agent melatonin cytotoxicity leukemia lymphocyte; Melatonin; anticancer drugs; apoptosis; leukemia lympho cytes; normal lympho cytes; reactive oxygen species

Substances (14) Reactions (0) Citing (29)

151

Methods and compositions for treatment of metastatic and refractory cancers and tumors

By: Conejo-Garcia, Jose R.; Allegrezza, Michael
United States, US20170020964 A1 2017-01-26 | Language: English, Database: CAplus

A method of treating a mammalian subject with cancer comprises administering to said subject having a cancer, e.g., a metastatic or refractory cancer or tumor, a small mol. inhibitor of a target signaling mol. of the MEK/MAPK pathway that impairs T cell activa tion, and administering to said subject a mol. that induces T cell prolife ration in the presence of said inhibitor. The combin ation of a small mol. inhibitor of a target of the MEK/MAPK pathway and the T cell prolife ration inducer reduces the proliferation of the cancer and tumor cells in vivo. Compositions of the invention include e.g. tramet inib, AZD6244, GDC0973, GDC0623, refametinib, binimetinib, or U0126.

Keywords: antitumor trametinib metastatic refractory cancer tumor treatment

PatentPak available

Substances (67) Reactions (0) Citing (0)

152

Preparation of alanine-based modulators of proteolysis and associated methods of use

By: Crew, Andrew P.; Berlin, Michael; Dong, Hanqing; Qian, Yimin
World Intellectual Property Organization, WO2017011590 A1 2017-01-19 | Language: English, Database: CAplus

FIGURE 1

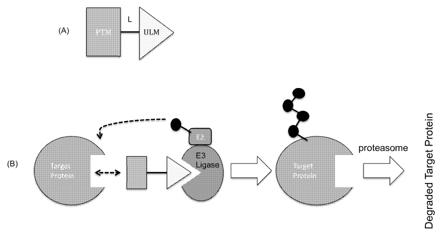

The invention is related to compounds PTM-L-ILM comprising an inhibitor of apoptosis protein (I AP) E3 ubiquitin ligase binding moiety (ILM); a linker (L) coupling I LM and a protein target moiety (P TM) that binds to a target protein or a target polype ptide, e.g., **I**, or a pharmaceutically acceptable salt, enantiomer, stereoisomer, solvate or polymorph thereof. Specifi cally, the invention is related to the preparation of bifunctional compounds, which find utility as modulators of targeted ubiquiti nation, especially inhibitors of a variety of polypeptides and other proteins which are degraded and/or otherwise inhibited by bifunc tional compounds according to the present invention. In particular, the invention is directed to compounds, which contain on one end a ligand which binds to the I AP E3 ubiquitin ligase and on the other end a moiety which binds a target protein such that the target protein is placed in proximity to the ubiquitin ligase to effect degradation (and inhibition) of that protein. Compounds that exhibited a broad range of pharmacol. activities consistent with the degradation/inhibition of targeted polypeptides were prepared Thus, **I** was prepared and at 1 μ M concentration displayed proteolytic modulatory activity (androgen receptor A R degradation between 30% and 60% in V CaP cells).

Keywords: chimeric peptidyl alanine modulator proteolysis targeted polypeptide protein; Iap E3 ligase directed proteo lysis targeting chimeric mol preparation; PROTAC modulator preparation targeted ubiquitination AR receptor degradation inhibition; alanine modulator preparation targeted EZH2 gene zeste protein degrad ation; targeted JNK1 JNK2 BRD4 degradation alanine PROTAC modulator preparation

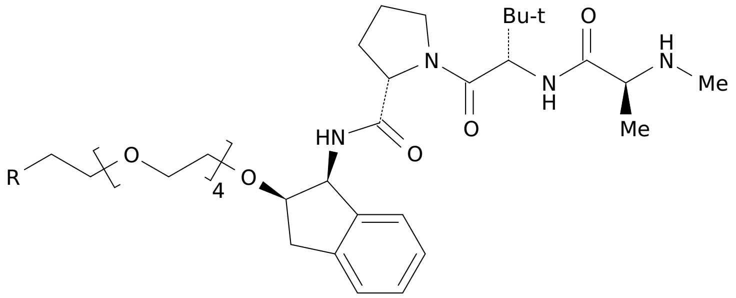

I

R =

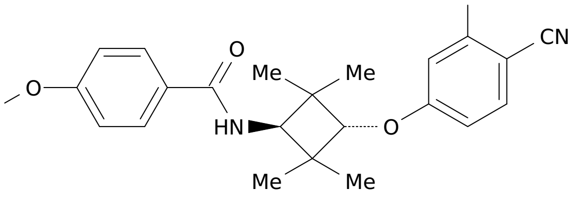

PatentPak available

Substances (539)   Reactions (662)   Citing (6)

153

Preparation of substituted diazepine derivatives and analogs for use as MDM2-based modulators

By: Crew, Andrew P.; Crews, Craig M.; Dong, Hanqing; Qian, Yimin; Wang, Jing
United States, US20170008904 A1 2017-01-12 | Language: English, Database: CAplus

Title compounds **I** [X = a protein targeting moiety; Y = a linker coupling; Z = a M DM2 E3 ubiquitin ligase binding moiety], and their pharmaceutically acceptable salts, are prepared and disclosed as M DM2-based modulators of proteolysis. Thus, e.g., **II** was prepared by a multistep procedure (preparation given). **I** were evaluated in AR ELISA assays, e.g., **II** demonstrated a degradation activity of 0 to 25% at 1 μM.

Keywords: diazepine preparation MDM2 modulator

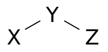

I

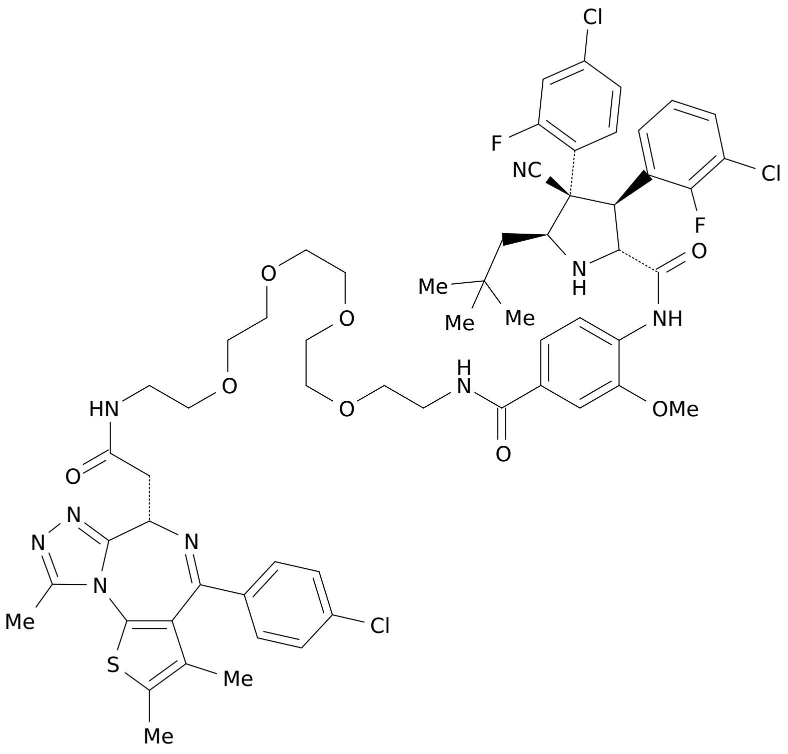

II

PatentPak available

Substances (430) Reactions (565) Citing (1)

154

Epidermal growth factor receptor (EGFR) structure-based bioactive pharmacophore models for identifying next-generation inhibitors against clinically relevant EGFR mutations

By: Panicker, Pooja S.; Melge, Anu R.; Biswas, Lalitha; Keechilat, Pavithran; Mohan, Chethampadi G.
Chemical Biology & Drug Design (2017), 90(4), 629-636 | Language: English, Database: CAplus and MEDLINE

Present work elucidates identification of next generation inhibitors for clin. relevant mutations of epidermal growth factor receptor (EGFR) using structure-based bioactive pharmacophore modeling followed by virtual screening (V S) techniques. Three-dimensional (3D) pharmacophore models of E GFR and its different mutants were generated. This includes seven 3 D pharmacophoric points with three different chem. features (descriptors), i.e., one hydrogen bond donor, three hydrogen bond acceptors and three aromatic rings. Pharmacophore models were validated using decoy dataset, Receiver operating charact eristic plot, and external dataset compounds The robust, bioactive 3D e-pharmacophore models were then used for V S of four different small compound databases: FDA approved, investig ational, anticancer, and bioactive compounds collec tions of Selleck Chems. C UDC101 a multita rgeted kinase inhibitor showed highest binding free energy and 3D pharmacophore fit value than the well known E GFR inhibitors, Gefitinib and Erlotinib. Further, we obtained ML167 as the second best hit on V S from bioactive database showing high binding energy and pharmacophore fit value with respect to E GFR receptor and its mutants. Optimist ically, presented drug discovery based on the computational study serves as a foundation in identi fying and designing of more potent E GFR next-generation kinase inhibitors and warrants further exptl. studies to fight against lung cancer.

Keywords: EGFR structure pharmac ophore mutation; computer- aided drug design; epidermal growth factor receptor; erlotinib; next-generation EGFR inhibitors; non-small cell lung cancer; pharmac ophore; virtual screening

Substances (9) Reactions (0) Citing (17)

155

Reactive oxygen species generation and increase in mitochondrial copy number: new insight into the potential mechanism of cytotoxicity induced by aurora kinase inhibitor, AZD1152-HQPA

By: Zekri, Ali; Mesbahi, Yashar; Ghanizadeh-Vesali, Samad; Alimoghaddam, Kamran; Ghavamzadeh, Ardeshir; Ghaffari, Seyed H.
Anti-Cancer Drugs (2017), 28(8), 841-851 | Language: English, Database: CAplus and MEDLINE

Aurora-B kinase overexp ression plays important roles in the malignant progre ssion of prostate cancer (P Ca). AZD1152-HQPA, as an inhibitor of Aurora-B, has recently emerged as a promising agent for cancer treatment. In this study, we aimed to invest igate the effects of AZD1152-HQPA on reactive oxygen species (R OS) generation and mitocho ndrial function in PCa. We used A ZD1152-HQPA (Barasertib), a highly potent and selective inhibitor of Aurora- B kinase. The effects of AZD1152-HQPA on cell viability, D NA content, cell morphol., and ROS production were studied in the androgen- independent PC-3 PCa cell line. Moreover, the mitocho ndrial copy number and the expression of genes involved in cell survival and cancer stem cell maintenance were investigated. We found that A Z D1152-HQPA treatment induced defective cell survival, polypl oidy, micronuclei formation, cell enlarg ement, and cell death by signif icant overexpression of p73, p21 and downreg ulation of cell cycle-regulatory genes in a drug concent ration-dependent manner. Moreover, AZD1152 treatment led to an excessive R OS generation and an increase in the mitocho ndrial copy number not only in P C-3 but also in several other malignant cells. A ZD1152 treatment also led to downreg ulation of genes involved in the mainte nance of cancer stem cells. Our results showed a functional relationship between the aurora kinase inhibi tion, an increase in mitocho ndrial copy number, and ROS generation in therap eutic modalities of cancer. This study suggests that the excessive R OS generation may be a novel mechanism of cytotoxicity induced by the aurora kinase inhibitor, A ZD1152-HQPA.

Keywords: aurora kinase inhibitor ROS mitocho ndrial copy number cytoto xicity

Substances (11) Reactions (0) Citing (14)

156

Compositions and methods comprising protein phosphatase 2A (PP2A) activators and protein kinase inhibitors for treating cancer

By: Narla, Goutham

World Intellectual Property Organization, WO2016210134 A1 2016-12-29 | Language: English, Database: CAplus

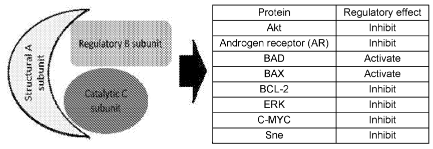

Fig. 1

A method of treating cancer in a subject in need thereof includes administering to the subject therapeutically effective amounts of a PP2A activator and a protein kinase inhibitor.

Keywords: antitumor protein phosphatase kinase inhibitor cancer treatment

PatentPak available

Substances (123) | Reactions (0) | Citing (0)

157

Inhibition of granulocyte colony stimulating factor in the treatment of cancer

By: Morris, Katherine Theresa; Beswick, Ellen Janine

United States, US20160368980 A1 2016-12-22 | Language: English, Database: CAplus

The present invention relates to the discovery that granulocyte colony stimulating factor (G-CSF) as well as granulocyte colony stimul ating factor receptor (G-CSFR) are highly expressed in cancer tissue and the inhibition of G- CSF using a G-CSF inhibitor, which term includes inhibitors of the G-CSF receptor, represents a viable approach to the treatment of cancer, including drug resistant cancers, metastatic cancers and recurrent cancers. G-CSF inhibitors as described herein may be used alone or in combin ation with an at least one addnl. anti-cancer agent for the treatment of cancer.

Keywords: granulocyte colony stimulating factor inhibitor cancer treatment anticancer combination

PatentPak available

Substances (307) | Reactions (0) | Citing (0)

158

MCT4 inhibitors for treating disease

By: Parnell, Kenneth Mark; McCall, John M.
World Intellectual Property Organization, WO2016201426 A1 2016-12-15 | Language: English, Database: CAplus

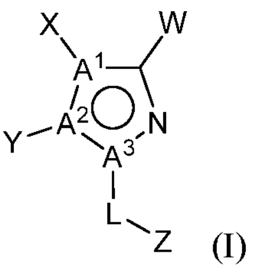

Disclosed are pyrazole compounds and compositions useful in the treatment of M CT4 mediated diseases, such as proliferative and inflammatory diseases, having the structure **I**, and methods of inhibiting M CT4 activity in a human or animal subject. Also disclosed is the preparation of compounds of the invention.

Keywords: pyrazole derivative preparation MCT4 inhibitor proliferative disease inflamation

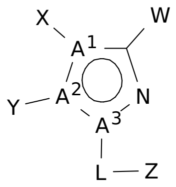

I

PatentPak available

Substances (300) | Reactions (245) | Citing (3)

159

Human GLUT5 specific inhibitors and methods of treatment

By: Oprea, Tudor I.; Bologa, Cristian George; Ursu, Oleg; Choe, Jun-Yong; Iancu, Cristina
World Intellectual Property Organization, WO2016201214 A1 2016-12-15 | Language: English, Database: CAplus

The present invention relates to compounds which have been discovered to be potent ligands (inhibitors) of human GLUT5 (glucose transporter type 5), a facilitative glucose transporter that transports fructose, and their use as ligands assays which can uncover addnl. ligands of GLUT5, having the potential for being used as drugs. In addition, the present invention is directed to compounds, chem. compositions and methods for treating disease states and conditions which are mediated through G LUT5, including such disease states and conditions as GLUT5 deficiency syndrome, diabetes (type I and I I), cancer, metabolic diseases including metabolic syndrome and fatty liver disease, among others.

Keywords: glucose transporter GLUT5 inhibitor diabetes cancer fatty liver disease

PatentPak available

Substances (347) | Reactions (0) | Citing (0)

160

Preparation of PROTAC compounds as TANK-binding kinase 1 inhibitors for modulation of proteolysis and associated methods of use

By: Crew, Andrew P.; Wang, Jing; Dong, Hanqing; Qian, Yimin; Siu, Kam; Ferraro, Caterina; Crews, Craig M.

World Intellectual Property Organization, WO2016197114 A1 2016-12-08 | Language: English, Database: CAplus

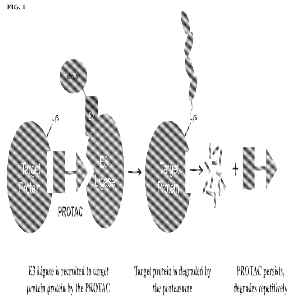

The invention is related to compounds TBM-L-PTM comprising an E3 ubiquitin ligase binding moiety (U LM), a TBK1 binding moiety (T BM) connected directly or through a chem. linker (L) , e.g., **I**, which find utility modulators of targeted ubiquitination and degradation of TANK-binding kinase (TBK1). In particular, the invention is directed to compounds, which contain on one end an E3 ubiquitin ligase binding moiety which binds to the ubiquitin ligase and on the other end a moiety which binds TBK1 such that TBK1 is placed in proximity to the ubiquitin ligase to effect degradation (and inhibition) of TBK1. Compounds that exhibited a broad range of pharmacol. activities consistent with the degradation/inhibition of TBK1 were prepared Thus, **I** was prepared and displayed proteo lytic modulatory activity (TBK1 degradation D_{max} = 96%).

Keywords: TANK binding kinase 1 P ROTAC chimeric mol prepar ation; PROTAC modulator preparation targeted TBK1 kinase degrad ation ubiquitination; Von Hippel Lindau ligand prepar ation TBK1 PROTAC degradation inhibition

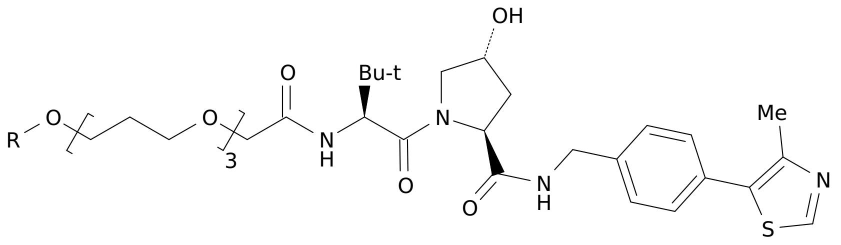

I

R =

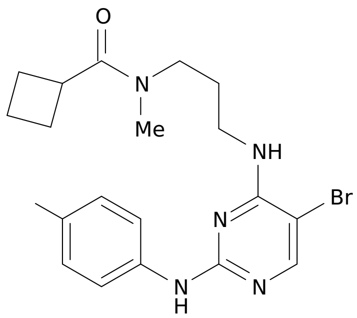

PatentPak available

Substances (439)   Reactions (182)   Citing (3)

161

Imide-based modulators of proteolysis and associated methods of use

By: Crews, Craig M.; Crew, Andrew P.; Dong, Hanqing; Wang, Jing; Qian, Yimin; Siu, Kam; Jin, Meizhong
World Intellectual Property Organization, WO2016197032 A1 2016-12-08 | Language: English, Database: CAplus

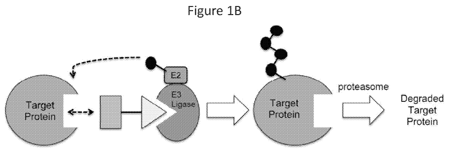

The description relates to imide- based compounds, including bifunctional. compounds comprising the same, which find utility as modulators of targeted ubiquitination, especially inhibitors of a variety of polype ptides and other proteins which are degraded and/or otherwise inhibited by bifunctional compounds according to the present invention. In partic ular, the description provides compounds, which contain on one end a ligand which binds to the cereblon E3 ubiquitin ligase and on the other end a moiety which binds a target protein such that the target protein is placed in proximity to the ubiquitin ligase to effect degradation (and inhibition) of that protein. Compounds can be synthesized that exhibit a broad range of pharmacol. activities consistent with the degradation/inhibition of targeted polypeptides of nearly any type.

Keywords: antitumor proteolysis modulator imide preparation cancer

PatentPak available

Substances (425) Reactions (330) Citing (5)

162

A comparative assessment of preclinical chemotherapeutic response of tumors using quantitative non-Gaussian diffusion MRI.

By: Xu, Junzhong ; Li, Ke; Smith, R Adam; Waterton, John C; Zhao, Ping; Ding, Zhaohua; Does, Mark D; Manning, H Charles; Gore, John C
Magnetic resonance imaging (2017), 37, 195-202 | Language: English, Database: MEDLINE

BACKGROUND: Diffusion-weighted MRI (DWI) signal attenuation is often not mono-exponential (i.e. non-Gaussian diffusion) with stronger diffusion weighting. Several non-Gaussian diffusion models have been developed and may provide new inform ation or higher sensitivity compared with the conventional apparent diffusion coefficient (ADC) method. However the relative merits of these models to detect tumor therapeutic response is not fully clear. METHODS: Conventional ADC, and three widely-used non-Gaussian models, (bi-exponential, stretched exponential, and statistical model), were implemented and compared for assessing S W620 human colon cancer xenografts responding to barasertib, an agent known to induce apoptosis via polypl oidy. Bayesian Information Criterion (BIC) was used for model selection among all three non- Gaussian models. RESULTS: All of tumor volume, histology, conventional ADC, and three non-Gaussian DWI models could show significant differences between control and treatment groups after four days of treatment. However, only the non-Gaussian models detected significant changes after two days of treatment. For any treatment or control group, over 65.7% of tumor voxels indicate the bi-exponential model is strongly or very strongly preferred. CONCLUSION: Non-Gaussian DWI model-derived biomarkers are capable of detecting tumor earlier chemothe rapeutic response of tumors compared with conventional ADC and tumor volume. The bi- exponential model provides better fitting compared with statistical and stretched exponential models for the tumor and treatment models used in the current work.

Keywords: BIC; Barasertib; Chemotherapeutic response; Diffusion; High b; MRI; Polyploidy; SW620

Substance (1) Reactions (0) Citing (4)

163

Inhibition of the pentose-phosphate pathway selectively sensitizes leukemia lymphocytes to chemotherapeutics by ROS-independent mechanism

By: Zhelev, Zhivko; Ivanova, Donika; Bakalova, Rumiana; Aoki, Ichio; Higashi, Tatsuya
Anticancer Research (2016), 36(11), 6011-6020 | Language: English, Database: CAplus and MEDLINE

The aim of the present study was to investigate: (i) the possibility of sensitizing leukemia lymphocytes to anticancer drugs by inhibiting pentose-phosphate pathway using 6-aminonicotinamide (6-ANA); (ii) to find combinations with synergistic cytotoxic effect on leukemia lymphocytes and to investigate their cytotoxicity towards normal lymphocytes; (iii) and to clarify the role of reactive oxygen species (ROS) in the induction of apoptosis by those combina tions. The study covers 15 anticancer drugs - conven tional and new-generation. The experiments were performed on Jurkat leukemia cell line and normal lympho cytes, isolated from clin. healthy blood donors. Four parameters were analyzed simultaneously in both cell suspen sions treated by drug or 6- ANA (sep., and in combination): cell viability, induction of apoptosis, level of R OS, and level of protein-carbonyl products. Most combin ations of drug plus 6-ANA were characterized by synergistic cytotoxic effects on Jurkat cells. The synergism increased with increasing incubation time. Upon combination of 6-ANA with conventional chemotherapeutic (e.g. doxorubicin), synergistic cytotoxic effects were also detected in normal lymphocytes. In both cell types, the cytoto xicity of the combination of doxorubicin plus 6-ANA was accompanied by increased induction of apoptosis, but by a slight reduction of ROS and protein-carbonyl products compared to cells treated with doxorubicin only. Upon combin ation of 6-ANA with new-generation anticancer drugs (e.g. everolimus or barasertib), the synergistic cytotoxic effect on leukemia lymphocytes was also accompanied by very strong induction of apoptosis through R OS-independent mechanism(s). Neither of these combin ations exhibited any cytotoxicity towards normal lymphocytes. The data suggest that 6-ANA could be used as a supplementary component in anticancer chemotherapy, and would allows therapeutic doses of anticancer drugs to be reduced, thereby minimizing their side-effects.

Keywords: pentose phosphate pathway leukemia lymphocyte ROS mechanism chemother apeutics; 6-Aminonicotinamide; ROS; chemotherapeutics; leukemia lymphocytes

Substances (16) Reactions (0) Citing (11)

164

Bromodomain and Extraterminal Protein Inhibition Blocks Growth of Triple-negative Breast Cancers through the Suppression of Aurora Kinases

By: Sahni, Jennifer M.; Gayle, Sylvia S.; Weber Bonk, Kristen L.; Vite, Leslie Cuellar; Yori, Jennifer L.; Webb, Bryan; Ramos, Erika K.; Seachrist, Darcie D.; Landis, Melissa D.; Chang, Jenny C.; et al
Journal of Biological Chemistry (2016), 291(45), 23756-23768 | Language: English, Database: CAplus and MEDLINE

Bromodomain and extraterminal (BET) proteins are epigenetic "readers" that recognize acetylated histones and mark areas of the genome for transcription. BRD4, a BET family member protein, has been implicated in a number of types of cancer, and B ET protein inhibitors (BETi) are efficacious in many preclin. cancer models. However, the drivers of response to BETi vary depending on tumor type, and little is known regarding the target genes conveying BETi activity in triple-neg. breast cancer (TNBC). Here, we show that BETi repress growth of multiple in vitro and in vivo models of T NBC by inducing two terminal responses: apoptosis and senesc ence. Unlike in other cancers, response to BETi in TNBC is not dependent upon suppression of MYC. Instead, both end points are preceded by the appearance of polyploid cells caused by the suppression of Aurora kinases A and B (AURKA/B), which are critical mediators of mitosis. In addition, AURKA/B inhibitors phenocopy the effects of BETi. These results indicate that Aurora kinases play an important role in the growth suppressive activity of BETi in TNBC. Elucidating the mechanism of response to BETi in TNBC should (1) facilitate the prediction of how distinct TNBC tumors will respond to BETi and (2) inform the rational design of drug combin ation therapies.

Keywords: Aurora kinase BET protein inhibitor anticancer breast cancer; Aurora kinase; anticancer drug; apoptosis; breast cancer; bromodomain-containing protein 4 (BRD4); cellular senescence; inhibition mechanism

Substances (4) Reactions (0) Citing (44)

165

Barasertib (AZD1152), a Small Molecule Aurora B Inhibitor, Inhibits the Growth of SCLC Cell Lines In Vitro and In Vivo

By: Helfrich, Barbara A.; Kim, Jihye; Gao, Dexiang; Chan, Daniel C.; Zhang, Zhiyong; Tan, Aik-Choon; Bunn, Paul A. Jr.
Molecular Cancer Therapeutics (2016), 15(10), 2314-2322 | Language: English, Database: CAplus and MEDLINE

Small-cell lung cancer (SCLC) cells have rapid proliferation, universal Rb inactivation, and high rates of MYC family amplification, making aurora kinase inhibition a natural target. Preclin. studies have demonstrated activity for Aurora A and pan-Aurora inhibitors with some relationship to MYC family expression. A clin. trial showed activity for an Aurora kinase A inhibitor, but no biomarkers were evaluated. We screened a panel of 23 SCLC lines with and without MYC family gene amplification or high MYC family gene expression for growth inhibition by the highly potent, selective aurora kinase B inhibitor barasertib. Nine of the SCLC lines were very sensitive to growth inhibition by barasertib, with IC_{50} values of <50 nmol/L and >75% growth inhibition at 100 nmol/L. Growth inhibition correlated with cMYC amplification (P = 0.018) and cMYC gene expression (P = 0.026). Sensitive cell lines were also enriched in a published MYC gene signature (P = 0.042). In vivo, barasertib inhibited the growth of xenografts established from an SCLC line that had high cMYC gene expression, no cMYC amplification, and was pos. for the core MYC gene signature. Our studies suggest that SCLC tumors with cMYC amplification/high gene expression will frequently respond to Aurora B inhibitors and that clin. studies coupled with predictive biomarkers are indicated. Mol Cancer Ther; 15(10); 2314-22.

Keywords: barasertib aurora B inhibitor small cell lung cancer antitumor

Substances (2) Reactions (0) Citing (59)

166

Janus face-like effects of Aurora B inhibition: antitumoral mode of action versus induction of aneuploid progeny

By: Wiedemuth, Ralf; Klink, Barbara; Fujiwara, Mamoru; Schroeck, Evelin; Tatsuka, Masaaki; Schackert, Gabriele; Temme, Achim
Carcinogenesis (2016), 37(10), 993-1003 | Language: English, Database: CAplus and MEDLINE

The mitotic Aurora B kinase is overexpressed in tumors and various inhibitors for Aurora B are currently under clin. assessments. However, when considering Aurora B kinase inhibitors as anticancer drugs, their mode of action and the role of p53 status as a possible predictive factor for response still needs to be investigated. In this study, we analyzed the effects of selective Aurora B inhibition using AZD1152-HQPA/Barasertib (AZD1152) on HCT116 cells, U87-MG, corresponding isogenic p53-deficient cells and a primary glioblastoma cell line. AZD1152 treatment caused polyploidy and non-apoptotic cell death in all cell lines irresp. of p53 status and was accompanied by poly-merotelic kinetochore-microtubule attachments and DNA damage. In p53 wild-type cells a DNA damage response induced an inefficient pseudo-G1 cell cycle arrest, which was not able to halt ongoing endoreplication of cells. Of note, release of tumor cells from AZD1152 resulted in recovery of aneuploid progenies bearing numerical and structural chromosomal aberrations. Yet, AZD1152 treatment enhanced death receptor TRAIL-R2 levels in all tumor cell lines investigated. A concomitant increase of the activating natural killer (NK) cell ligand MIC A/B in p53-deficient cells and an induction of FAS/CD95 in cells containing p53 rendered AZD1152-treated cells more susceptible for NK-cell-mediated lysis. Our study mechanistically explains a p53-independent mode of action of a chem. Aurora B inhibitor and suggests a potential triggering of antitumoral immune responses, following polyploidization of tumor cells, which might constrain recovery of aneuploid tumor cells.

Keywords: aurora B kinase inhibitor antitumor aneuploidy

Substances (4) Reactions (0) Citing (7)

167

Preparation of dimeric quinacrine derivatives as autophagy inhibitors for cancer therapy

By: Amaravadi, Ravi K.; Winkler, Jeffrey

World Intellectual Property Organization, WO2016168721 A1 2016-10-20 | Language: English, Database: CAplus

The invention provides dimeric quinacrine derivatives and related compounds of formula I and compositions, methods of treatment and syntheses. The compounds exhibit unexpected anticancer activity and are useful in the treatment of a variety of autophagy-related disorders. Compounds of formula I wherein R^1, R^2, R^3, R^4, R^5, R^6, R^7, R^8, $R^{1'}$, $R^{2'}$, $R^{3'}$, $R^{4'}$, $R^{5'}$, $R^{6'}$, $R^{7'}$ and $R^{8'}$ are independently H, halo, C N, NO_2, OH, CO_2H, etc.; R and R' are indepen dently H, (un)substituted C_{1-6} alkyl, (un)substituted C_{1-6} alkoxy, (un)substituted C_{1-7} acyl, etc.; L is a linker which covalently links to two exocyclic amine groups; and pharmace utically acceptable salts, enantiomers, diastereoisomers, solvates and polymorphs thereof, are claimed. Example compound II was prepared by a general procedure (procedure given). The invention compounds were evaluated for their autophagy inhibitory activity (some data given).

Keywords: dimeric quinacrine preparation autophagy inhibitor treatment cancer

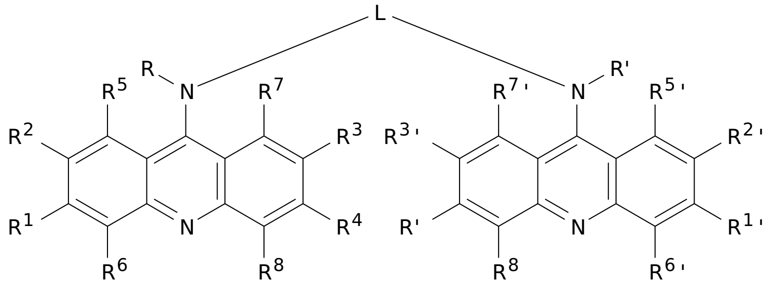

I

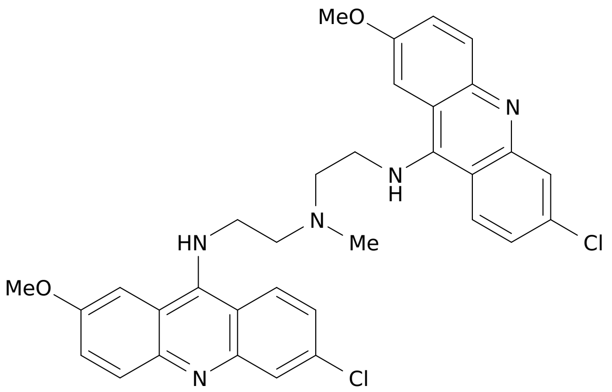

II

PatentPak available

Substances (335) Reactions (43) Citing (3)

168

Leishmania donovani Aurora kinase: A promising therapeutic target against visceral leishmaniasis

By: Chhajer, Rudra; Bhattacharyya, Anirban; Didwania, Nicky; Shadab, Md; Das, Nirupam; Palit, Partha; Vaidya, Tushar; Ali, Nahid
Biochimica et Biophysica Acta, General Subjects (2016), 1860(9), 1973-1988 | Language: English, Database: CAplus and MEDLINE

Aurora kinases are key mitotic kinases executing multiple aspects of eukaryotic cell-division. The apicomplexan homologs being essential for survival, suggest that the Leishmania homolog, annotated LdAIRK, may be equally important. Bioinformatics, stage-specific immunofluorescence microscopy, immunoblotting, RT-PCR, mol. docking, in-vitro kinase assay, anti-leishmanial activity assays, flow cytometry, fluorescence microscopy. Ldairk expression is seen to vary as the cell-cycle progresses from G1 through S and finally G2M and cytokinesis. Kinetic studies demonstrate their enzymic activity exhibiting a Km and Vmax of 6.12 μ M and 82.9 pmoles·min^{-1}mg^{-1} resp. against ATP using recombinant Leishmania donovani H3, its physiol. substrate. Due to the failure of Ld AIR K -/+ knock-out parasites to survive, we adopted a chem. knock- down approach. Based on the conservation of key active site residues, three mammalian Aurora kinase inhibitors were investigated to evaluate their potential as inhibitors of LdAIRK activity. Interestingly, the cell-cycle progressed unhindered, despite treatment with GSK-1070916 or Barasertib, inhibitors with greater potencies for the ATP-binding pocket compared to Hesperadin, which at nanomolar concentrations, severely compromised viability at IC_{50}s 105.9 and 36.4 n M for promastigotes and amastigotes, resp. Cell-cycle and morphol. studies implicated their role in both mitosis and cytokinesis. We identified an Aurora kinase homolog in L. donovani implicated in cell- cycle progression, whose inhibition led to aberrant changes in cell-cycle progression and reduced viability. Human homologs being actively pursued drug targets and the observations with LdAIRK in both promastigotes and amastigotes suggest their potential as therapeutic-targets. Importantly, our results encourage the exploration of other proteins identified herein as potential novel drug targets.

Keywords: Leishmania Aurora kinase visceral leishmaniasis development Hesperadin; Aurora kinase; Cell-cycle; Cytokinesis; Hesper adin; Leishmania donovani; Therapeutic target

Substances (5) | Reactions (0) | Citing (12)

169

Pericyte long non-coding RNA inhibitors for use in treatment of diseases mediated by PDGFR expression

By: Zehendner, Christoph Michael; Dimmeler, Stefanie; Zeiher, Andreas
World Intellectual Property Organization, WO2016150870 A1 2016-09-29 | Language: English, Database: CAplus

The present invention provides novel non-coding RNAs (lncRNA) that were identified to be expressed in pericytes upon hypoxia. The lncRNA of the invention pos. affect Platelet-derived Growth Factor Receptor (PDGFR) beta expression, pericytes proliferation and pericyte recruitment to endothelial cells. The invention provides inhibitors of the lncRNA for use in the treatment of diseases mediated by PDGFR expression. For example the invention described antisense approaches to target the lnc RNA of the invention. Furthermore, the invention provides lncRNA inhibitors as amplifier of therapeutic PDGFR inhibitors such as imatinib or other tyrosine kinase inhibitors. lncRNA inhibitors and methods for screening modulators of lnc RNA expression and/or function are provided.

Keywords: pericyte long non coding RNA human PDGFR disease

PatentPak available

Substances (28) | Reactions (0) | Citing (1)

170

Preparation of bifunctional PROTAC compounds and methods for the enhanced degradation of targeted proteins

By: Berlin, Michael; Zimmerman, Kurt; Snyder, Lawrence; Crew, Andrew P.; Crews, Craig M.; Chen, Xin; Dong, Hanqing; Ferraro, Caterina; Jin, Meizhong; Qian, Yimin; et al

World Intellectual Property Organization, WO2016149668 A1 2016-09-22 | Language: English, Database: CAplus

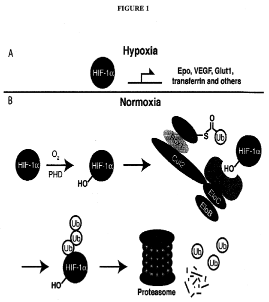

The invention is related to compounds ULM-L-PTM comprising a Von Hippel-Lindau (VHL) E3 ubiquitin ligase binding moiety (U LM), a protein targeting moiety (PTM) connected directly or through a chem. linker (L) , e.g., **I**, or a pharmaceutically acceptable salt, enanti omer, stereoisomer, solvate or polymorph thereof which find utility as modulators of targeted ubiquiti nation, especially inhibitors of a variety of polypeptides and other proteins which are degraded and/or otherwise inhibited by the bifunc tional compounds of the invention. In particular, the invention is directed to compounds, which contain on one end a V HL ligand which binds to the ubiquitin ligase and on the other end a moiety which binds a target protein such that the target protein is placed in proximity to the ubiquitin ligase to effect degradation (and inhibition) of that protein. Compounds that exhibited a broad range of pharmacol. activities consistent with the degradation/inhibition of targeted polypeptides were prepared Thus, **I** was prepared and displayed proteolytic modulatory activity (androgen receptor degradat ion51%≤ Dmax ≤70%; BRD4 degradation cMyc Imax ≥71%).

Keywords: chimeric mol preparation targeted protein degradation ubiquitination; PROTAC modulator preparation targeted ubiquit ination AR receptor degradation inhibition; Von Hippel Lindau ligand preparation BRD4 PROTAC degradation inhibition

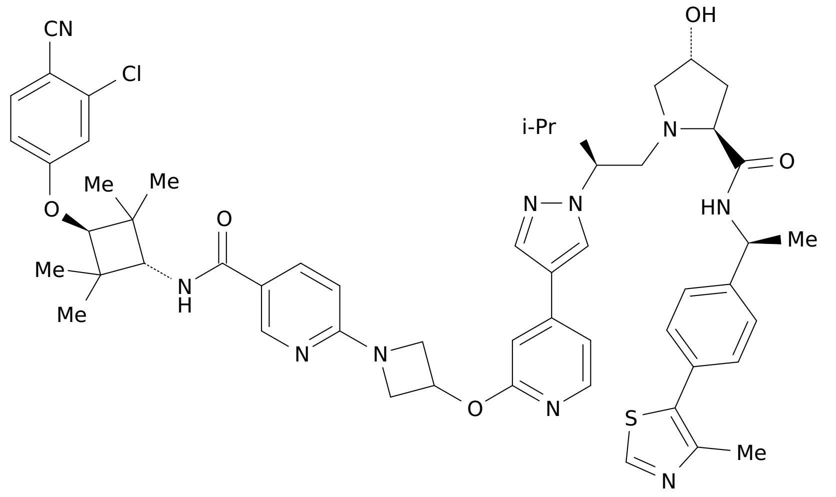

I

PatentPak available

Substances (590) Reactions (228) Citing (6)

171

Cholesteryl ester transfer protein (CEPT) inhibition in the treatment of cancer

By: Kaur, Mandeep; Esau, Luke E.; Sagar, Sunii
World Intellectual Property Organization, WO2016135633 A1 2016-09-01 | Language: English, Database: CAplus

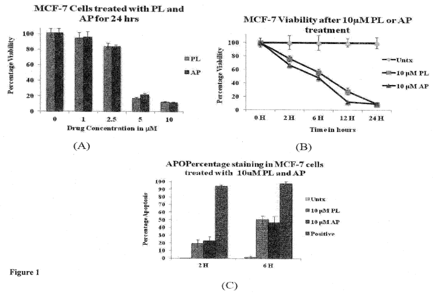

Figure 1

In one embodiment, the invention provides methods of treatment which use therapeu tically effective amounts of Choleste ryl Ester Transfer Protein (CETP) inhibitors to treat a variety of cancers. In certain embodi ments, the inhibitor is a CETP-inhibiting small mol., CETP-inhibiting antisense oligonucleotide, CETP-inhibiting siRNA or a CETP- inhibiting antibody. Related pharmaceutical composi tions, kits, diagno stics and screens are also provided.

Keywords: cholesteryl ester transfer protein CEPT inhibition cancer

PatentPak available

Substances (362) | Reactions (0) | Citing (0)

172

Dye-stabilized nanoparticles and methods of their manufacture and therapeutic use

By: Heller, Daniel A.; Shamay, Yosef

World Intellectual Property Organization, WO2016130546 A1 2016-08-18 | Language: English, Database: CAplus

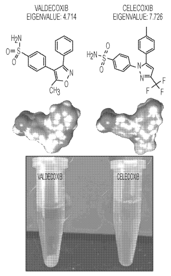

FIG. 18

Described herein are nanoparticles which are largely made of (e.g., 90 wt%) hydrophobic drugs and are stabilized by water soluble dyes. The nanoparticles can range in size from 30 nm to 150 nm and have highly neg. surface charge (e.g., - 55 mV). These nanoparticles are highly soluble in water, stable for days in PBS buffer and can be easily lyophilized and reconstituted in water. Using quant. self-assembly prediction calculations, topochem. mol. descriptors were identified and validated as highly predictive indicators of nano-assembly, nanoparticle size, and drug loading. The resulting nanoparticles selectively targeted kinase inhibitors to caveolin-1-expressing human colon cancer and autochthonous liver cancer models to yield striking therapeutic effects while avoiding pERK inhibition in healthy skin. The nanoparticles exhibited remarkable anti-tumor efficacy in vitro and in vivo in models of hepatocellular carcinoma. Thus, dye-encapsulated sorafenib nanoparticles (IR783-SFB) were synthesized using the nanopptn. method; 0.1 mL of sorafenib dissolved in DMSO (10 mg/mL) was added dropwise (20 µL per 15 s) to a 0.4 aqueous IR783 solution (1 mg/mL) containing 0.05 mM sodium bicarbonate; the solution was centrifuged twice and resuspension in 1 mL of sterile PBS; the suspension of nanoparticles was sonicated for 10 s with a probe sonicator at 40 % intensity; the resulted IR783-SFB nanoparticles had zeta potential of -52 mV and a size of 95 nm with a PDI of 0.1; by suspending the nanoparticles in lower volumes they were able to solubilize sorafenib up to 16 mg/mL in saline solution which is 2000 times better than free drug; the nanoparticles were easily lyophilized with a saline/sucrose 5 % solution and reconstituted in water at this concentration

Keywords: dye stabilization nanoparticle hydrophobic drug self assembly cancer

PatentPak available

Substances (292)   Reactions (0)   Citing (0)

173

Aurora B inhibitor barasertib prevents meiotic maturation and subsequent embryo development in pig oocytes

By: Ju, Shiqiang; Peng, Xu; Yang, Xiaoliu; Sozar, Sparksi; Muneri, Caroline W.; Xu, Yaping; Chen, Changchao; Cui, Panpan; Xu, Weichao; Rui, Rong
Theriogenology (2016), 86(2), 503-515 | Language: English, Database: CAplus and MEDLINE

Barasertib, a highly selective Aurora B inhibitor, has been widely used in a variety of cells to invest igate the role of Aurora B kinase, which has been implicated in various functions in the mitotic process. However, effects of barasertib on the meiotic maturation process are not fully understood, particularly in porcine oocyte meiotic maturation. In the present study, the effects of barasertib on the meiotic maturation and developmental competence of pig oocytes were investi gated, and the possible roles of Aurora B were also evaluated in porcine oocytes undergoing meiosis. Initially, we examined the expression and subcellular localization of Aurora B using Western blot anal. and immunofluorescent staining. Aurora B was found to express and exhibit specific dynamic intracellular localization during porcine oocyte meiotic matura tion. Aurora B was observed around the chromo somes after germinal vesicle breakdown. Then it was transferred to the spindle region after metaphase I stage, and was partic ularly concentrated at the central spindles at telophase I stage. barasertib treatment resulted in the failure of polar body extrusion in pig oocytes, with a larger percentage of barasertib-treated oocytes remaining at the pro-metaphase I stage. Addnl. results reported that barasertib treatment had no effect on chromosome condensation but resulted in a signifi cantly higher percentage of the treated oocytes with aberrant spindles and misaligned chromosomes during the first meiotic division. In addition, inhibition of Aurora B with lower concentrations of barasertib during pig oocyte meiotic maturation decreased the subsequent embryo develop mental competence. Thus, these results illustrate that barasertib has significant effects on porcine oocyte meiotic maturation and subsequent develo pment through Aurora B inhibition, and this regulation is related to its effects on spindle formation and chromosome alignment during the first meiotic division in porcine oocytes.

Keywords: barasertib oocyte aurora B inhibitor meiotic maturation embryo develo pment; Aurora B; Barasertib; Chromosome alignment; Oocytes maturation; Spindle formation

Substances (2) Reactions (0) Citing (10)

174

Significance of AZD1152 as a potential treatment against Aurora B overexpression in acute promyelocytic leukemia

By: Ghanizadeh-Vesali, Samad; Zekri, Ali; Zaker, Farhad; Zaghal, Azam; Yousefi, Meysam; Alimoghaddam, Kamran; Ghavamzadeh, Ardeshir; Ghaffari, Seyed H.

Annals of Hematology (2016), 95(7), 1031-1042 | Language: English, Database: CAplus and MEDLINE

Aurora B kinase as a chromosomal passenger protein plays multiple roles in regulating mitosis and cytoki nesis. The function of Aurora B in leukemic cells has made it an important treatment target. In this study, we explored the expressions of Aurora (A, B, and C) kinases in newly diagnosed acute promyelocytic leukemia (APL) patients. In addition, we investigated the effects of AZD1152 as a specific inhibitor of Aurora B on cell survival, DNA synthesis, nuclear morphol., apoptosis induction, cell cycle distrib ution, and gene expression in an APL-derived NB4 cell line. Our results showed that Aurora B was overexp ressed in 88 % of APL patients. AZ D1152 treatment of NB4 cells led to viability reduction and G2/M arrest followed by an increase in cell size and polyploidy induction. These giant cells showed morphol. evidence of mitotic catastrophe. AZD1152 treatment induced activation of G2/M checkpoint which in turn led to transient G2/M arrest in a p21-independent manner. Lack of functional p53 in N B4 cells might provide an opportunity to escape from G2/M block and to endure repeated rounds of replic ation and polyploidy. Treated cells were probably eliminated via p73-mediated overexpression of BAX, PUMA, and APAF1 and downregulation of survivin and MCL-1. In summary, AZ D1152 treatment led to endomitosis and polyploidy in TP53-mutated NB4 cells. These giant polyploid cells might undergo mitotic catastrophe and p73-mediated apoptosis. It seems that induction of polyploidy via AZD1152 could be a novel form of anti-cancer therapy for APL that may be clin. accessible in the near future.

Keywords: AZD1152 anticancer aurora B apoptosis promyelocytic leukemia; AZD1152; Acute promyelocytic leukemia; Apoptosis; Aurora B kinase; Cell cycle arrest; p73

Substances (8)   Reactions (0)   Citing (10)

175

Preparation of substituted bicyclic compounds as bromodomain inhibitors

By: Brown, Samuel David; Coburn, Craig Alan

World Intellectual Property Organization, WO2016097870 A1 2016-06-23 | Language: English, Database: CAplus

The title compounds **I** [W^1 = N and NH; W^2 and E^3 = (independently) C and N; R^1 = (un)substituted cycloalkyl, heterocyclyl; R^2 = H, alkyl (optionally substituted with halo and OH); R^3 = alkyl optionally substituted with halo and OH (with the proviso); R^4 = H, (un) substituted alkyl, cycloalkyl, etc.; X = CH_2 optionally substituted with 1-2 groups independently selected from R^5; R^5 = D, alkyl, alkoxy, etc.; Y = N and CH; with the provisos], useful for inhibition of BET protein function by binding to bromodo mains, were prepared E.g., a multi-step synthesis of **II**, starting from 5-bromopyridine-2,3-diamine and (3,5-dimethylisoxazol-4-yl)boronic acid, was described. Exemplified compounds **I** were tested in various biol. assays (data given) . Pharmaceutical compositions, comprising compound **I**, alone or in combination with other therapeutic agent, was disclosed.

Keywords: bicyclic compound imidazopyridine preparation BET bromodomain protein inhibitor

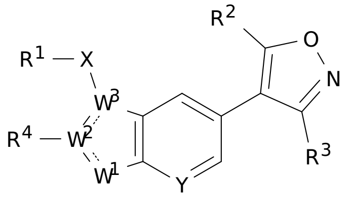

I

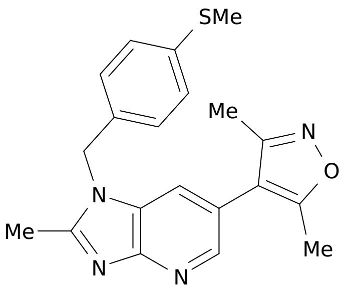

II

PatentPak available

Substances (116)   Reactions (153)   Citing (2)

176

Preparation of 4-heterocyclylisoxazole derivatives as inhibitors of bromodomains

By: Kharenko, Olesya; Young, Peter Ronald; Brown, Samuel David; Duffy, Bryan Cordell; Liu, Shuang; Guzzo, Peter
World Intellectual Property Organization, WO2016097863 A1 2016-06-23 | Language: English, Database: CAplus

The present disclosure relates to compounds **I** [R^1 = each (un)substituted C_{5-10} carbocyclyl or C_{2-10} heterocyclyl; R^2, R^3 = indepen dently C_{1-6} alkyl optionally substituted with halogen and hydroxy, with the proviso that if R^2 and R^3 are methyls, then R^1 is different from Q, Q^1, Q^2, 2-(difluoromethoxy)phenyl, 2,5-dichlorophenyl, or 2,5-dimethylphenyl; A = H, halogen, methoxy, cyano, NO_2, CO_2Me, or $CONMe_2$; R^4 = H, C_{1-10} alkyl, C_{3-10} carbocyclyl, or C_{2-10} heterocyclyl; X = (un)substituted CH_2; Y = N, CH, or $C(NH_2)$] or **II** (R^1, R^2, R^3, X = same as above; Z = H or NH_2) or stereoisomers, tautomers, pharmaceutically acceptable salts, or hydrates thereof. These compounds are capable of acting as covalent inhibitors of bromodomains and are useful for treating (A) an autoimmune or inflam matory disorder associated with bromodomain and extra terminal domain (BET) proteins, (B) an acute or chronic non-autoimmune inflammatory disorder characterized by disregulation of IL-6 and/or IL-17, (C) a cancer associated with overexpression, translo cation, amplification, or rearrangement of a myc family oncoprotein that is sensitive to BET inhibition, (D) cancer that relies on pTE Fb (Cdk9/cyclin T) and BET proteins to regulate oncogenes, (F) a cancer associated with upregu lation of BET responsive genes CDK6, Bcl2, TYRO3, MYB, and hTERT, (G) a cancer associated with a gene regulated by a super enhancer, (H) a cancer that is sensitive to effects of BET inhibition, (I) a cancer that is resistant to treatment with immunot herapy, hormone-deprivation therapy, and/or chemotherapy, (J) a benign proliferative or fibrotic disorder, or (K) a disease or disorder that benefits from upregu lation or ApoA-I transcription and protein expression. The autoimmune or inflammatory disorder is selected from acute disseminated encephalo myelitis, agammaglobulinemia, allergic disease, ankylosing spondylitis, anti-GBM/anti-TBM nephritis, anti-phospholipid syndrome, autoimmune aplastic anemia, autoimmune hepatitis, autoimmune inner ear disease, autoimmune myocarditis, autoimmune pancreatitis, autoimmune retinopathy, autoimmune thrombocytopenic purpura, Behcet's disease, Bullous pemphigoid, Castleman's disease, Celiac disease, Churg-Strauss syndrome, Crohn's disease, Cogan's syndrome, dry eye syndrome, essential mixed cryoglobu linemia, dermatomyositis, Devic's disease, encephalitis, eosinophlic esophagitis, eosinophilic fasciitis, erythema nodosum, giant cell arteritis, glomerulonephritis, Goodpasture's syndrome, granulomatosis with polyangiitis (Wegener's), Graves' disease, Guillain-Barre syndrome, Hashimoto's thyroiditis, hemolytic anemia, Henoch-Schonlein purpura, idiopathic pulmonary fibrosis, IgA nephropathy, inclusion body myositis, type I diabetes, interstitial cystitis, Kawasaki's disease, etc. The acute or chronic non-autoimmune inflam matory disorder is selected from sinusitis, pneumonitis, osteomyelitis, gastritis, enteritis, gingivitis, appendicitis, irritable bowel syndrome, tissue graft rejection, chronic obstructive pulmonary disease (COPD), septic shock, osteoarthritis, acute gout, acute lung injury, acute renal failure, burns, Herxheimer reaction, and SIRS associated with viral infections. The benign proliferative or fibrotic disorder is selected from the group consisting of benign soft tissue tumors, bone tumors, brain tumors, spinal cord tumors, eyelid and orbital tumors, granuloma, lipoma, meningioma, multiple endocrine neoplasia, nasal polyps, pituitary tumors, prolactinoma, pseudotumor cerebri, seborrheic keratoses, stomach polyps, thyroid nodules, cystic neoplasms of the pancreas, hemangiomas, vocal cord nodules, polyps, and cysts, chronic pilonidal disease, dermatofibroma, pilar cyst, pyogenic granuloma, juvenile polyposis syndrome, renal fibrosis, postoperative stricture, keloid formation, and cardiac fibrosis. The other diseases include operative stricture, cardiovascular disease, dyslipidemia, atherosclerosis, hypercholesterolemia, metabolic syndrome, Alzheimer's disease, Parkinson's disease, Huntington disease, bipolar disorder, schizophrenia, Rubinstein-Taybi syndrome, and epilepsy. Thus, 4-bromo-3-fluorobenzyl alc. was stirred with methanesulfonyl chloride and pyridine in CH_2Cl_2 at room temperature for 16 h to give 95% 4-bromo-3-fluorobenzyl chloride. 5-(3,5-Dimethylisoxazol-4-yl)pyridin-2(1H)-one (**III**; R = H) was stirred with 4-bromo-3-fluorobenzyl chloride in the presence of K_2CO_3 in MeCN at 70° for 16 h to give 32% **III** (R = 4-bromo-3-fluorobenzyl). **III** (R = 4-bromo-3-fluoro benzyl) was coupled with tributyl(vinyl)stannane in the presence of $Pd(PPh_3)_2Cl_2$ in DMF under nitrogen at 85° for 16 h to give 53% **III** (R = Q^3). A solution of 0.26 mmol **III** (R = Q^3) in water and acetone was treated with 0.29 mmol N-bromosuccinimide, stirred at for 6 h, treated with 0.52 mmol NaOH at 0°, and stirred at room temperature for 16 h to give 60% **III** (R = Q^4). **III** (R = Q^4) and 2-chloro-1-[4-[[6-(3,5-dimethylisoxazol-4-yl)-2-methyl-1H-imidazo[4,5-b]pyridin-1-yl]methyl]phenyl]ethanone (**IV**) inhibited the binding of tetra-acetyiated histone H4 to individual BET Bromodomains with IC_{50} of ≤0.3μM.

Keywords: oxazolylpyridinone oxazolylbenzimidazole preparation covalent inhibitor bromodomain; heterocyclylisoxazole prepar ation covalent inhibitor bromodomain; isoxazolylimidazopyridine preparation covalent inhibitor bromodomain; autoimmune disorder inflammatory disorder cancer treatment heterocyclylisoxazole preparation; benign tumor benign proliferative disorder treatment heterocyclylisoxazole preparation; fibrotic disorder cardiovascular disease treatment heterocyclylisoxazole preparation; bromodomain extra terminal domain BET protein

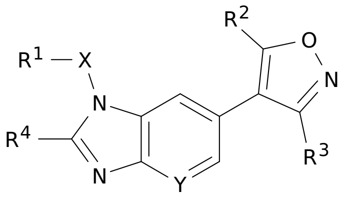

I

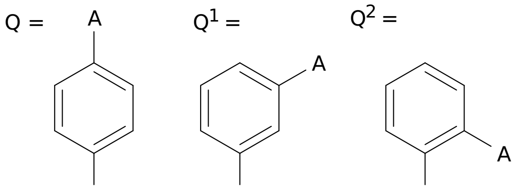

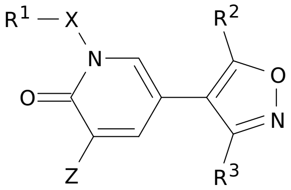

II

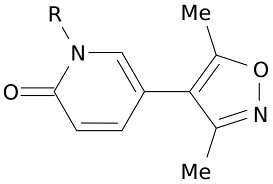

III

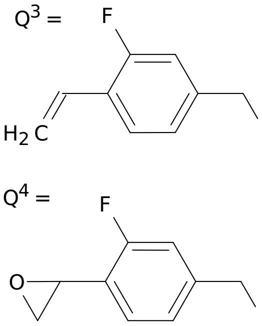

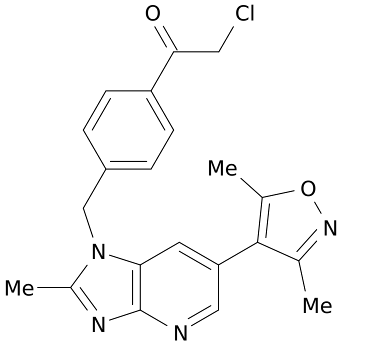

IV

PatentPak available

Substances (84) Reactions (62) Citing (3)

177

Preparation of substituted imidazolyl triazoles as BET protein inhibitors via binding to bromodomains useful in therapy

By: Brown, Samuel David; Coburn, Craig Alan; Kharenko, Olesya

World Intellectual Property Organization, WO2016092375 A1 2016-06-16 | Language: English, Database: CAplus

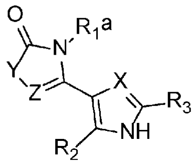

Formula I.

The invention relates to preparation of substituted imidazolyl triazoles of formula **I** wherein all the variables are as defined in the disclosure and pharmaceutical compositions thereof useful for the inhibition of B ET protein function by binding to bromodo mains. The example compound **II** was prepared by multistep synthesis (procedure shown) , was tested for inhibition of tetra- acetylated histone H4 binding individual BET bromodomains. Compounds **I** can be used in mono- and combin ation therapy of various diseases, like chemotherapy, immunotherapy and hormone- deprivation therapy resistant cancers and such.

Keywords: imidazolyl triazole preparation BET inhibitor anticancer antiproli ferative combination therapy

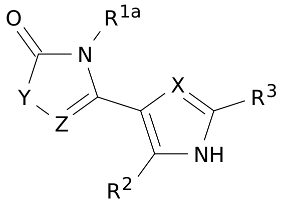

I

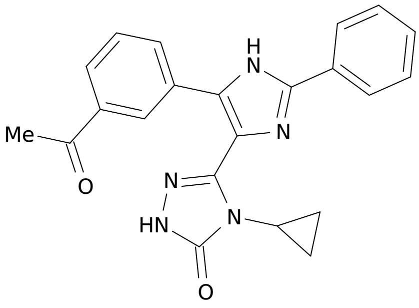

II

PatentPak available

Substances (159)

Reactions (304)

Citing (1)

178

Preparation of 5-(3,5-dialkylisoxazol-4-yl)pyridinones as bromodomain inhibitors

By: Jiang, May Xiaowu; Molino, Bruce Francis; Liu, Shuang; Wang, Ruifang; Duffy, Bryan Cordell; Quinn, John Frederick; Wagner, Gregory Steven

World Intellectual Property Organization, WO2016087936 A1 2016-06-09 | Language: English, Database: CAplus

The invention is related to the preparation of substituted pyridinones **I** [A = (un)substituted aryl, heteroaryl; L = CH_2, CH_2CH_2, CH (Me) optionally substituted with halogen; or L may be absent in which case A is connected to X via a covalent bond; X = O, N H; R^3, R^4 = independently C_1-C_6 alkyl optionally substituted with halogen and OH], their stereoisomers, tautomers, pharmaceutically acceptable salts, and hydrates, which are useful for inhibition of BET protein function by binding to bromodomains, pharmaceutical compositions containing them., and ther use in therapy. Thus, Pd- coupling of 3-amino-5-bromo-2-methoxypyridine with 3,5-dimethyl-4-(4,4,5,5-tetramethyl-1,3,2-dioxaborolan-2-yl)isoxazole, amination of benzyl bromide with the resulting amine and treatment of 3-(benzylamino)-5-(3,5-dimethylisoxazol-4-yl)-2-methoxypyridine with HBr in EtOH gave **II**. Pyridinone **II** inhibited the tetracetylated histone H4 binding to Brd4 bromodomain 1, the cell proliferation in human AML MV-4-11 cells and the hIL-6 and hIL-17 mRNA transcription.

Keywords: isoxazolylpyridinone preparation bromodomain inhibitor

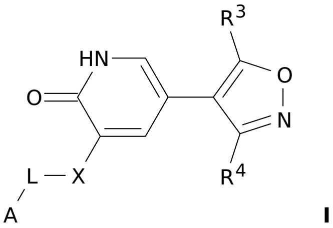

I

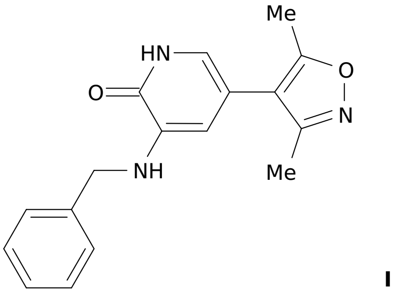

II

PatentPak available

Substances (136)

Reactions (96)

Citing (2)

179

Preparation of 5-(3,5-dialkylisoxazol-4-yl)pyridines as bromodomain inhibitors

By: Jiang, May Xiaowu; Molino, Bruce Francis; Liu, Shuang; Wang, Ruifang; Duffy, Bryan Cordell; Quinn, John Frederick; Wagner, Gregory Steven

World Intellectual Property Organization, WO2016087942 A1 2016-06-09 | Language: English, Database: CAplus

The invention is related to the preparation of substituted pyridinones **I** [A = (un)substituted aryl, heteroaryl; B = C_1-C_6 alkyl, benzyl, Ph optionally substituted with halogen; L = CH_2, CH(Me) optionally substituted with halogen; or L may be absent in which case A is connected to X via a covalent bond; X = O, NH; Y = O, NHMe, meaning if Y = NHMe then B is absent; R^3, R^4 = independently C_1-C_6 alkyl optionally substituted with halogen and OH], their stereoisomers, tautomers, pharmaceutically acceptable salts, and hydrates, which are useful for inhibition of BET protein function by binding to bromodomains, pharmaceutical compositions containing them., and ther use in therapy. Thus, Pd-coupling of 3-amino-5-bromo-2-methoxypyridine with 3,5-dimethyl-4-(4,4,5,5-tetramethyl-1,3,2-dioxaborolan-2-yl)isoxazole and amination of benzyl bromide with the 5-(3,5-dimethylisoxazol-4-yl)-2-methoxypyridin-3-amine gave **II**. Pyridine **II** inhibited the tetracetylated histone H4 binding to Brd4 bromodomain 1, the cell proliferation in human AML MV-4-11 cells and the hIL-6 and hIL-17 mRNA transcription.

Keywords: isoxazolylpyridine preparation bromodomain inhibitor

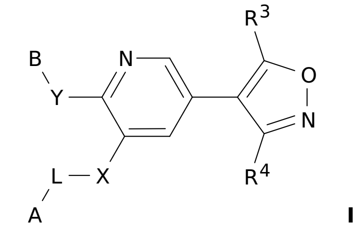

I

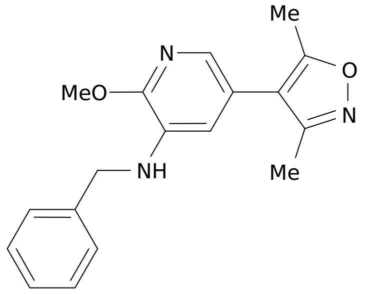

II

PatentPak available

Substances (190)

Reactions (169)

Citing (3)

180

Preclinical testing of selective Aurora kinase inhibitors on a medullary thyroid carcinoma-derived cell line

By: Tuccilli, Chiara; Baldini, Enke; Prinzi, Natalie; Morrone, Stefania; Sorrenti, Salvatore; Filippini, Angelo; Catania, Antonio; Alessandrini, Stefania; Rendina, Roberta; Coccaro, Carmela; et al
Endocrine (2016), 52(2), 287-295 | Language: English, Database: CAplus and MEDLINE

Deregulated expression of the Aurora kinases (Aurora-A, B, and C) is thought to be involved in cell malignant transfo rmation and genomic instability in several cancer types. Over the last decade, a number of small- mol. inhibitors of Aurora kinases have been developed, which have proved to efficiently restrain malignant cell growth and tumorige nicity. Regarding medullary thyroid carcinoma (MTC), we previously showed the efficacy of a pan-Aurora kinase inhibitor (MK-0457) in impairing growth and survival of the MTC-derived cell line TT. In the present study, we sought to establish if one of the Aurora kinases might represent a prefer ential target for MTC therapy. The effects of selective inhibitors of Aurora-A (MLN8237) and Aurora-B (AZD1152) were analyzed on TT cell proliferation, apoptosis, cell cycle, and ploidy. The two inhibitors reduced T T cell proliferation in a time- and dose-dependent manner, with IC_{50} of 19.0 ± 2.4 n M for MLN8237 and 401.6 ± 44.1 n M for AZD1152. Immunofluorescence experiments confirmed that AZD1152 inhibited phosphorylation of histone H3 (Ser10) by Aurora- B, while it did not affect Aurora-A autophosphorylation. ML N8237 inhibited Aurora-A autophosphorylation as expected, but at concentrations required to achieve the maximum antiproli ferative effects it also abolished H3 (Ser10) phosphorylation. Cytofluorimetry experiments showed that both inhibitors induced accumulation of cells in G2/M phase and increased the sub G0/G1 fraction and polyploidy. Finally, both inhibitors triggered apoptosis. We demonstrated that inhibition of either Aurora-A or Aurora-B has antiproliferative effects on TT cells, and thus it would be worthwhile to further investigate the therapeutical potential of Aurora kinase inhibitors in MTC treatment.

Keywords: MLN8237 AZD1152 aurora kinase inhibitor anticancer medullary thyroid carcinoma; A ZD1152; Aurora kinase inhibitors; Cell cycle; MLN8237; Medullary thyroid cancer; TT cell line

Substances (4) | Reactions (0) | Citing (6)

181

Irisin-related cancer treatments

By: Trujillo, Kristina; Gannon, Nicholas Patrick; Vaughan, Roger Alan
World Intellectual Property Organization, WO2016081603 A1 2016-05-26 | Language: English, Database: CAplus

In one embodiment, the invention provides methods of treating or preventing cancer, in particular aspects breast and/or prostate cancer, by administering to a subject in need thereof a therapeu tically-effective amount of irisin and, option ally, one or more adjuvant therapies (e.g. synergistic co-administration of an addnl. anti-cancer agent or chemotherapy). Related pharmaceutical compositions, assays and kits are also provided.

Keywords: irisin cancer FNDC5 gene theapy

PatentPak available

Substances (316) | Reactions (0) | Citing (1)

182

A novel in situ hydrophobic ion paring (HIP) formulation strategy for clinical product selection of a nanoparticle drug delivery system

By: Song, Young Ho; Shin, Eyoung; Wang, Hong; Nolan, Jim; Low, Susan; Parsons, Donald; Zale, Stephen; Ashton, Susan; Ashford, Marianne; Ali, Mir; et al
Journal of Controlled Release (2016), 229, 106-119 | Language: English, Database: CAplus and MEDLINE

The present studies were aimed at formulating AZD2811-loaded polylactic acid-polyethylene glycol (PLA-PEG) nanoparticles with adjustable release rates without altering the chem. structures of the polymer or active pharmaceutical ingredient (API). This was accomplished through the use of a hydrop hobic ion pairing approach. A series of A ZD2811-containing nanoparticles with a variety of hydrophobic counterions including oleic acid, 1- hydroxy-2-naphthoic acid, cholic acid, deoxyc holic acid, dioctylsul fosuccinic acid, and pamoic acid is described. The hydrophobicity of AZD2811 was increased through formation of ion pairs with these hydrop hobic counterions, producing nanoparticles with excepti onally high drug loading- up to five fold higher encapsu lation efficiency and drug loading compared to nanoparticles made without hydrop hobic ion pairs. Furthe rmore, the rate at which the drug was released from the nanoparticles could be controlled by employing counte rions with various hydropho bicities and structures, resulting in release half-lives ranging from about 2 to 120 h using the same polymer, nanopa rticle size, and nanoemulsion process. Process recipe variables affecting drug load and release rate were identified, including pH and molarity of quench buffer. Ion pair formation between AZD2811 and pamoic acid as a model counterion was invest igated using solubility enhancement as well as NMR spectr oscopy to demonstrate solution-state interactions. Further evidence for an ion pairing mechanism of controlled release was provided through the measurement of API and counterion release profiles using high- performance liquid chromatog., which had stoichiometric relationships. Finally, Raman spectra of an A ZD2811-pamoate salt compared well with those of the formulated nanoparticles, while single components (A ZD2811, pamoic acid) alone did not. A library of A ZD2811 batches was created for anal. and preclin. characterization. Dramatically improved preclin. efficacy and tolera bility data were generated for the pamoic acid lead formulation, which has been selected for evaluation in a Phase 1 clin. trial (Clinical Trials.gov Identifier N CT 02579226). This work clearly demonstrates the importance of assessing a wide range of drug release rates during formul ation screening as a critical step for new drug product development, and how utilizing hydrop hobic ion pairing enabled this promising nanopa rticle formulation to proceed into clin. development.

Keywords: hydrophobic ion pairing polylactic acid polyet hylene glycol nanoparticle; Adjustable release rate; Counte rions; Drug release kinetics; In situ hydrophobic ion paring; Nanopar ticles; PLA–PEG

Substances (18) | Reactions (0) | Citing (49)

183

IL15 Agonists Overcome the Immunosuppressive Effects of MEK Inhibitors

By: Allegrezza, Michael J.; Rutkowski, Melanie R.; Stephen, Tom L.; Svoronos, Nikolaos; Tesone, Amelia J.; Perales-Puchalt, Alfredo; Nguyen, Jenny M.; Sarmin, Fahmida; Sheen, Mee R.; Jeng, Emily K.; et al
Cancer Research (2016), 76(9), 2561-2572 | Language: English, Database: CAplus and MEDLINE

Many signal transduction inhibitors are being developed for cancer therapy target pathways that are also important for the proper function of antitumor lymphocytes, possibly weakening their therap eutic effects. Here we show that most inhibitors targeting multiple signaling pathways have especially strong neg. effects on T-cell activation at their active doses on cancer cells. In partic ular, we found that recently approved MEK inhibitors displayed potent suppre ssive effects on T cells in vitro. However, these effects could be attenuated by certain cytokines that can be administered to cancer patients. Among them, clin. available I L15 superag onists, which can activate PI3K selectively in T lympho cytes, synergized with M EK inhibitors in vivo to elicit potent and durable antitumor responses, including by a vaccine-like effect that generated resistance to tumor rechal lenge. Our work identifies a clin. actionable approach to overcome the T-cell-suppressive effects of M EK inhibitors and illust rates how to reconcile the defici encies of signal transduction inhibitors, which impede desired immunol. effects in vivo.

Keywords: T cell MEK inhibitor IL15 agonist immunosuppression

Substances (58) | Reactions (0) | Citing (22)

184

Docosahexaenoic acid sensitizes leukemia lymphocytes to barasertib and everolimus by ROS-dependent mechanism without affecting the level of ROS and viability of normal lymphocytes

By: Zhelev, Zhivko; Ivanova, Donika; Lazarova, Desislava; Aoki, Ichio; Bakalova, Rumiana; Saga, Tsuneo
Anticancer Research (2016), 36(4), 1673-1682 | Language: English, Database: CAplus and MEDLINE

The aim of the present study was: (i) to investigate the possibility of sensitizing leukemia lymphocytes to anticancer drugs using docosahexaenoic acid (DHA); (ii) to find combinations with synergistic cytotoxic effect on leukemia lymphocytes, without or with only very low cytotoxicity towards normal lymphocytes; (iii) and to clarify the role of reactive oxygen species (R OS) in the induction of apoptosis and cytotoxicity by such combinations. The study covered 15 anticancer drugs, conven tional and new-generation. Well-expressed synergistic cytotoxic effects were observed after treatment of leukemia lympho cytes (Jurkat) with DHA in combination with: barasertib, lonafarnib, everolimus, and palbociclib. We selected two synerg istic combinations, DHA with everolimus or barasertib, and investigated their effects on viability of normal lympho cytes, as well as on the production of R OS and induction of apoptosis in both cell lines (leukemia and normal). At the selected concentrations, DHA, everolimus and barasertib (applied sep.) were cytotoxic towards leukemia lymphocytes, but not normal lympho cytes. In leukemia cells, the cytotoxicity of combinations was accompanied by strong induction of apoptosis and production of R OS. In normal lymphocytes, drugs alone and in combin ation with DHA did not affect the level of R OS and did not induce apoptosis. To our knowledge, the present study is the first to report synerg istic ROS-dependent cytotoxicity between DHA and new-generation anticancer drugs, such as everolimus and barasertib, that is cancer cell-specific (particularly for acute lymphob lastic leukemia cells Jurkat). These combin ations are harmless to normal lympho cytes and do not induce abnormal production of R OS in these cells. The data suggest that D HA could be used as a supplem entary component in anticancer chemotherapy, allowing therapeutic doses of everolimus and barasertib to be reduced, minimizing their side-effects.

Keywords: leukemia lymphocyte docosahexaenoic acid barasertib everolimus ROS cell viability; DHA; Docosahexaenoic acid; barasertib; everolimus; leukemia lymphocytes; reactive oxygen species

Substances (12) Reactions (0) Citing (18)

185

Screening methods for identifying compounds for cancer cell reprogramming and therapeutic uses thereof in the treatment of cancer

By: Chigaev, Alexandre; Sklar, Larry A.; Perez, Dominique
United States, US9314460 B1 2016-04-19 | Language: English, Database: CAplus

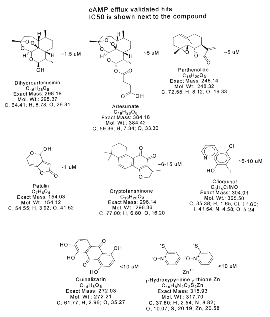

In one embodiment, the invention provides a method of inhibiting c AMP efflux and increasing intrace llular cAMP in a subject who suffers from, or who is at risk of developing, a cancer by adminis tering to the subject a therapeu tically-effective amount of a c AMP efflux inhibitor. Novel compounds, pharmaceutical compositions, diagno stics and screening methods are also provided.

Keywords: anticancer drug screening cancer cell apoptosis neoplasm treatment

PatentPak available

Substances (313) Reactions (0) Citing (2)

186

Method of testing systemic release of AZD 1152 hqpa or bioequivalents

By: Walker, Michael; Howes, Colin

World Intellectual Property Organization, WO2016059149 A1 2016-04-21 | Language: English, Database: CAplus

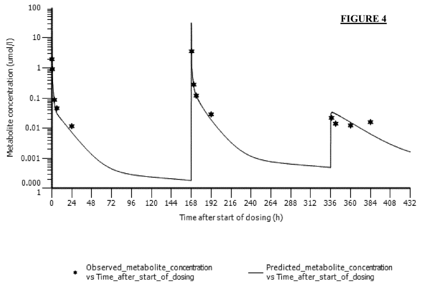

Methods of measuring systemic release, or in-vivo release profile of an active drug, e.g., AZD 1152 (barasertib), from a nanoparticle, and their use, in assessing bioequivalence are disclosed. The method involves detection of a metabolite of AZD 1152 hqpa (AZD 1152 hqpa desfluoroaniline (also referred to as AZ 12102238); AZD 1152 hqpa desfluoroaniline N-acetic acid; AZD 1152 hqpa N-acetic acid; AZD-1152 hqpa N-oxide; and AZD 1152 hqpa N-desethyl) by using mass spectrometry (LC-MS) and applying a pharmaco kinetic model.

Keywords: AZD 1152 hqpa metabolism bioequivalence nanoparticle

PatentPak available

Substances (16) | Reaction (1) | Citing (0)

187

Map kinase activated protein kinase 2 (MK2) inhibitors in the treatment of cancer

By: Beswick, Ellen Janine; Morris, Katherine Theresa

World Intellectual Property Organization, WO2016032882 A1 2016-03-03 | Language: English, Database: CAplus

The present invention relates to the discovery that Map kinase activated protein kinase 2 (MK2) is highly expressed in cancer tissue and the inhibition of MK2 using an MK2 inhibitor (BIO-475863, PF-364402), represents a viable approach to the treatment of cancer, including drug resistant cancers, metastatic cancers and recurrent cancers. MK2 inhibitors as described herein may be used alone or in combination with an at least one addnl. anti- cancer agent for the treatment of cancer.

Keywords: antitumor Map kinase inhibitor combination chemotherapy cancer treatment

PatentPak available

Substances (311) | Reactions (0) | Citing (0)

188

Imide-based modulators of proteolysis and associated methods of use

By: Crew, Andrew P.; Crews, Craig; Dong, Hanqing; Wang, Jing; Qian, Yimin; Siu, Kam; Jin, Meizhong
United States, US20160058872 A1 2016-03-03 | Language: English, Database: CAplus

The description relates to imide-based compounds, including bifunctional compounds comprising the same, which find utility as modulators of targeted ubiquitination, especially inhibitors of a variety of polype ptides and other proteins which are degraded and/or otherwise inhibited by bifunctional compounds according to the present invention. In partic ular, the description provides compounds, which contain on one end a ligand which binds to the cereblon E3 ubiquitin ligase and on the other end a moiety which binds a target protein such that the target protein is placed in proximity to the ubiquitin ligase to effect degradation (and inhibition) of that protein. Compounds can be synthesized that exhibit a broad range of pharmacol. activities consistent with the degradation/inhibition of targeted polypeptides of nearly any type.

Keywords: antitumor proteolysis modulator imide preparation cancer

PatentPak available

Substances (425) Reactions (330) Citing (3)

189

Bromodomain inhibitors for treating disease

By: Palmer, Wylie; Jones, Philip; Liu, Gang; Petrocchi, Alessia; Reyna, Naphtali; Subrumanian, Govindan; Theroff, Jay; Yau, Anne
World Intellectual Property Organization, WO2016033416 A1 2016-03-03 | Language: English, Database: CAplus

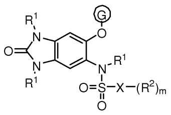

(I)

Disclosed herein are compounds and compositions useful in the treatment of bromod omain-containing protein-mediated diseases, such as cancer, and Methods of inhibiting activity of a bromodomain-containing protein in a human or animal subject are also provided.

Keywords: bromodomain inhibitor preparation cancer inflammation autoimmune CNS disease

PatentPak available

Substances (577) Reactions (2,121) Citing (1)

190

Anti-LAG3 antibodies and antigen-binding fragments

By: Liang, Linda; Fayadat-Dilman, Laurence; Malefyt, Rene de Waal; Raghunathan, Gopalan
World Intellectual Property Organization, WO2016028672 A1 2016-02-25 | Language: English, Database: CAplus

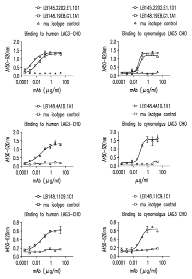
FIG.1

The present invention includes antibodies and antigen-binding fragments thereof that specifically bind to human or Cynomolgous monkey LAG3 as well as Ig chains thereof and polynucl eotides encoding the same along with injection devices comprising such antibodies or fragments. Vaccines including such antibodies and fragments as well as compositions comprising the antibodies and fragments (e.g., including anti-PD1 antibodies) are included in the invention. Methods for treating or preventing cancer or infection using such compositions are also provided. In addition, methods for recomb inant expression of the antibodies and fragments are part of the present invention.

Keywords: human LAG3 monoclonal antibody sequence humanized variant immunot herapy cancer

PatentPak available

Substances (595) Reactions (0) Citing (7)

191

Preparation of asymmetric bisaminoquinolines and bisaminoquinolines with varied linkers as autophagy inhibitors for cancer and other therapy

By: Amaravadi, Ravi K.; Winkler, Jeffrey

World Intellectual Property Organization, WO2016022956 A1 2016-02-11 | Language: English, Database: CAplus

The invention provides novel asym. and sym. bisaminoquinolmes and related compounds **I** [L = $(CH_2)_pN(R'')(CH_2)_p$, $(CH_2)_pN(R'')(CH_2)_{p1}N(R'')(CH_2)_p$, $(CH_2)_pO(CH_2)_p$, L^{1D}, or each (un)substituted $(CH_2Y)_n$-X-$(Y^1CH_2)_n$, or $A(CH_2CH_2$-$Z)_n$-A^1; R^1, R^1 = independently H, F Cl, Br, iodo, cyano, NO_2, or each (un)substituted C_{1-6} alkyl, O-C_{1-6} alkyl, C_{2-7} acyl, NH-acyl, or C_2-C_7 carboxy ester; R, R' = indepen dently H or each (un)substituted C_{1-6} alkyl, C_{1-7} acyl, or C_{2-7} carboxy ester (which forms a urethane group with the nitrogen atom to which R or R' is bonded); R'' = F, each (un) substituted cycloalkyl, aryl or heteroaryl, or $(C:O)_z$-G; G = each (un)substituted CO-C_{1-2} alkyl, alkene, or alkynyl; z = an integer of 0-5; R^M = indepen dently H, F, Cl, Br, iodo, (un) substituted C_{1-12} alkyl, alkene, alkynyl or alkoxy group; p, p1 = an integer of 1-10; L^{1D} = (un)substituted C_{1-20} alkylene; X = absent, $(CH_2)_j$, O, S, or (un)substituted NH; Y, Y^1 = indepen dently absent, CH_2, O, O, CHO, or (un)substituted NH; j = an integer of 1- 6; n = indepen dently an integer of 0- 10; Z = O or (un) substituted NH; A, A^1 = independently C(O) or (un)substituted $(CH_2)_j$] or pharmace utically acceptable salts, enanti omers,s diastereomers, solvates, or polymorphs thereof. The novel compounds are autophagy inhibitors and are useful in the treatment of a variety of autophagy-related disorders, in particular (a) cancer including metastatic cancer, recurrent cancer, and drug- resistant cancer and (b) rheumatoid arthritis, malaria, antiphospholipid antibody syndrome, lupus, chronic urticaria or Sjogren's disease. The cancer is (1) carcinoma, (2) cancer of the esophagus, head, kidney, liver, lung, nasopharyngeal, neck, ovary, pancreas, prostate, and stomach, (3) a leukemia, a malignant lymphoma, a malignant melanoma, (4) myeloproliferative diseases, or (5) a sarcoma, a tumor of the central nervous system, a germ-line tumor, lung cancer, ovarian cancer, testicular cancer, thyroid cancer, astroc ytoma, esophageal cancer, pancreatic cancer, stomach cancer, liver cancer, colon cancer, melanoma, or a mixed type of neoplasia. Thus, to a microwave vial was added spermine 0.57, 4-bromo-7-chloroquinoline 1.32, BINAP 0.11, $Pd(OAc)_2$ 0.02, and K_3PO_4 1.80 g. The vial was sealed and placed under argon, followed by adding a solvent mixture of dioxane and water (10:1, 9 mL), and the resulting mixture was heated to 120° for 16 h to give, after workup, 73% N1,N1'-(butane-1,4-diyl)bis[N3-(7-chloroquinolin-4-yl)propane-1,3-diamine] (**II**). The compounds **I** were tested for anticancer activities. The increased cytotoxicity of longer linker and assyemtric compounds was confirmed in a 2 wk colony formation assay using 5 different cancer cell lines including A375, WM983B, CAPAN1, OANC1, and SKME L5 cells. Longer linker compounds localized to the lysosome. Longer linker compounds were associated with more profound autophagy modulation, reactive oxygen species (ROS) and apoptosis. Dimeric chloroqu inolines with longer linkers impaired growth and invasion of melanoma in a 3 dimensional tissue like culture system.

Keywords: asym bisaminoquinoline preparation autophagy inhibitor treatment cancer; drug resistant cancer rheumatoid arthritis treatment asym bisaminoquinoline preparation; malaria treatment asym bisaminoq uinoline preparation; antiphospholipid antibody syndrome treatment asym bisaminoquinoline preparation; lupus chronic urticaria Sjogren disease treatment asym bisaminoquinoline preparation

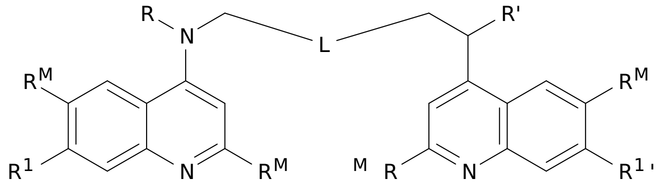

I

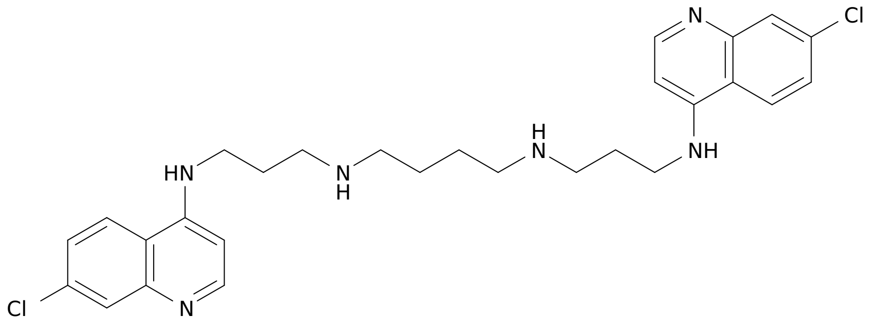

II

PatentPak available

Substances (418) Reactions (245) Citing (1)

192

Are Accurins the cure for Aurora kinase inhibitors?

By: Bearss, David J.
Science Translational Medicine (2016), 8(325), 325fs4/1-325fs4/3 | Language: English, Database: CAplus and MEDLINE

A review. A nanoparticle formulation of an Aurora B inhibitor increases antitumor efficacy and reduces toxicity, which may be a precedent for the use of this technol. with other small mols. (Ashton et al., this issue).

Keywords: review Accurins Aurora kinase inhibitor antitumor agent cancer

Substances (2) Reactions (0) Citing (3)

193

Compounds useful for promoting protein degradation and methods using same

By: Crews, Craig M.; Gustafson, Jeffrey; Roth, Anke Gundula; Tae, Hyun Seop; Buckley, Dennis; Neklesa, Taavi
United States, US20160022642 A1 2016-01-28 | Language: English, Database: CAplus

The present description includes compounds that act as degraders of a target protein, wherein degrad ation is independent of the class of the target protein or its localization. In certain embodiments, the description includes a compound comprising a protein degradation moiety covalently bound to a linker, wherein the Clog P of the compound is equal to or higher than 1.5. The target protein contemplated within the description comprises an androgen receptor. Compounds of the present descri ption may be used to treat disease states wherein protein degradation is a viable therapeutic approach, such as cancer or any sort of oxidative stress disease state.

Keywords: protein degradation promoter preparation androgen receptor cancer oxidative stress

PatentPak available

Substances (439) Reactions (226) Citing (1)

194

[^{14}C]-AZD1152 drug substance manufacture: challenges of an IV-infusion dosed human mass balance study in patients

By: Bushby, Nick ; Bergin, Julie; Harding, John
Journal of Labelled Compounds and Radiopharmaceuticals (2016), 59(6), 250-254 | Language: English, Database: CAplus and MEDLINE

[^{14}C]-AZD1152 (barasertib) drug substance was prepared in order to support a h ADME study that was to be dosed as an i.v. infusion to patients with acute myeloid leukemia. A long patient recruitment time (1-2 years) was expected, and because of its limited stability several batches of [^{14}C]-AZD1152 drug substance needed to be prepared in order to maintain a supply of [^{14}C]-AZ D1152 in the clinic over this period. A method was developed to purify the [^{14}C]-AZD1152 to a GMP-like standard at high specific activity to provide material for each of these batches. This approach to the delivery of the drug substance was successful in supplying the study with radiolabeled drug for over 1 yr until all patients had been recruited.

Keywords: radiolabeled C14 AZD1152 synthesis; AZD1152; cGMP; hADME

Substances (16) Reactions (30) Citing (0)

195

2-deoxy-D-glucose sensitizes cancer cells to barasertib and everolimus by ROS-independent mechanism(s)

By: Zhelev, Zhivko; Ivanova, Donika; Aoki, Ichjo; Saga, Tsuneo; Bakalova, Tana
Anticancer Research (2015), 35(12), 6623-6632 | Language: English, Database: CAplus and MEDLINE

The aim of the present study was to investigate: (i) the possibility of sensitizing cancer cells to anticancer drugs using the redox modulator 2-deoxy-D-glucose (2- DDG); (ii) to find such combinations with synergistic cytotoxic effect,- (iii) and to clarify the role of reactive oxygen species (ROS) for induction of apoptosis and cytoto xicity through these combinations. The study covers 15 anticancer drugs - both conventional and new-generation. Four parameters were analyzed simulta neously in Jurkat leukemia treated by drugs or 2-DUG (sep. or in combination): cell viability, induction of apoptosis, levels of R OS, and level of protein-carbonyl products.

Keywords: leukemia cell 2 deoxy glucose barasertib everolimus sensitivity ROS; 2-deoxy-D-glucose; Anticancer drugs; apoptosis; reactive oxygen species; synergism

Substances (22) | Reactions (0) | Citing (10)

196

Selective inhibitors of aurora kinases inhibit proliferation, reduce cell viability and impair cell cycle progression in papillary thyroid carcinoma cells

By: Baldini, E.; Tuccilli, C.; Prinzi, N.; Sorrenti, S.; Antonelli, A.; Fallahi, P.; Mian, C.; Barollo, S.; Catania, A.; Morrone, S.; et al
Journal of Biological Regulators and Homeostatic Agents (2015), 29(4), 793-803 | Language: English, Database: CAplus and MEDLINE

The three members of the Aurora kinase family, Aurora-A, -B and -C, regulate several aspects of the mitotic process, and their aberrant expression and/or function causes mitotic abnormalities leading either to cell death or aneupl oidy. They are found overexpressed in several human malignancies, including the papillary thyroid carcinoma (P TC). In the present study, we sought to establish whether Aurora kinase inhibition could be of any therapeutic value in the treatment of aggressive forms of P TC, enduring to radioactive iodide (RAI) ablation. To this end, the effects of selective inhibitors of Aurora- A (MLN8237) and Aurora- B (AZD1152) were analyzed on 3 human PTC cell lines expressing either wild-type (K1 and TPC1) or mutant p53 (B CPAP). The two inhibitors were capable of reducing cell proliferation in a time- and dose- dependent manner, with IC comprised between 65.4 and 114.9 n M for M L N8237, and between 26.6 and 484.6 n M for AZD1152. Immunofluorescence experiments confirmed that AZD1152 inhibited Aurora-B phosphorylation of histone H3 on Ser10, however, it did not affect Aurora- A autophosphorylation. MLN8237 inhibited Aurora- A autophosphorylation as expected, but at concent rations required to achieve the maximum antiproli ferative effects it also abolished H3 (Ser10) phosphorylation. Time-lapse videomicroscopy evidenced that both inhibitors prevented the completion of cytoki nesis, and cytofluorimetric anal. showed accumulation of cells in G2/M phase and/or polypl oidy. Apoptosis was induced in all the cells by both inhibitors independently from the p53 status. In conclu sion, in the present preclin. study M LN8237 and AZD1152 have emerged as promising drug candidates for RAI-insensitive PTC.

Keywords: PTC cell proliferation viability cycle progression aurora kinase inhibitor

Substances (7) | Reactions (0) | Citing (7)

197

Phosphoproteomic analysis of Aurora kinase inhibition in monopolar cytokinesis [Erratum to document cited in CA163:327364]

By: Polat, Ayse Nur; Karayel, Ozge; Giese, Sven H.; Harmanda, Busra; Sanal, Erdem; Hu, Chi-Kuo; Renard, Bernhard Y.; Ozlu, Nurhan
Journal of Proteome Research (2015), 14(12), 5408 | Language: English, Database: CAplus and MEDLINE

Supplementary Figures 1-3 were omitted from the Supporting Information file; the corrected file is available online.

Keywords: erratum human Aurora kinase cytokinesis phosphoproteome target; phosphopeptide intermediate filament erratum

Substances (3) | Reactions (0) | Citing (0)

198

Management and novel drugs for older patients with acute myeloid leukemia

By: Yoshimoto, Goichi; Miyamoto, Toshihiro
Ketsueki Naika (2015), 71(5), 666-674 | Language: Japanese, Database: CAplus

A review. This article discusses on management and novel drugs for older patients with acute myeloid leukemia.

Keywords: human acute myeloid leukemia chemotherapy review

Substances (14) | Reactions (0) | Citing (0)

199

Sarcoma Cell Line Screen of Oncology Drugs and Investigational Agents Identifies Patterns Associated with Gene and microRNA Expression

By: Teicher, Beverly A.; Polley, Eric; Kunkel, Mark; Evans, David; Silvers, Thomas; Delosh, Rene; Laudeman, Julie; Ogle, Chad; Reinhart, Russell; Selby, Michael; et al
Molecular Cancer Therapeutics (2015), 14(11), 2452-2462 | Language: English, Database: CAplus and MEDLINE

The diversity in sarcoma phenotype and genotype make treatment of this family of diseases exceptionally challenging. Sixty-three human adult and pediatric sarcoma lines were screened with 100 FDA-approved oncol. agents and 345 investigational agents. The investigational agents' library enabled comparison of several compounds targeting the same mol. entity allowing comparison of target specificity and heterogeneity of cell line response. Gene expression was derived from exon array data and micro RNA expression was derived from direct digital detection assays. The compounds were screened against each cell line at nine concent rations in triplicate with an exposure time of 96 h using Alamar blue as the endpoint. Results are presented for inhibitors of the following targets: aurora kinase, IGF-1R, MEK, BET bromodomain, and PARP1. Chem. structures, IC_{50} heat maps, concentration response curves, gene expression, and miR expression heat maps are presented for selected examples. In addition, two cases of exceptional responders are presented. The drug and compound response, gene expres sion, and microRNA expression data are publicly available at http://sarcoma.cancer.gov. These data provide a unique resource to the cancer research community. Mol Cancer Ther; 14(11); 2452-62. ©2015 AACR.

Keywords: sarcoma cell IGF1R MEK1 PARP1 microRNA oncol drug

Substances (26) | Reactions (0) | Citing (49)

200

Gonadotropin-releasing hormone receptor-targeting peptides and their use to treat and diagnose cancer

By: Miao, Yubin
United States, US9180214 B1 2015-11-10 | Language: English, Database: CAplus

The present invention is directed to novel non-invasive diagnostic tools/compounds to image and treat cancers, especially cancers which overexpress GnRH such as breast cancer, prostate cancer and melanoma, among others, including metastatic breast cancer, prostate cancer and/or melanoma among numerous others in vivo. The novel imaging probes are capable of detecting cancerous cells, as well as their metastatic spread in tissues. The novel probes of the present invention will also be useful to initiate therapy for breast and/or prostate cancer, among other cancers, as well as monitor patients' response to chemotherapy treatments and other interventions or therapies used in the treatment of cancer, including metastatic cancer as otherwise described herein. Compounds according to the present invention may be used as diagnostic tools for diagnosing cancer as well as therapeutic agents for treating cancer and related secondary disease states and conditions.

Keywords: GnRh receptor targeting peptide cancer therapy diagnosis

PatentPak available

Substances (283) | Reactions (0) | Citing (0)

201

Combination therapies targeting mitochondria for cancer therapy

By: Altieri, Dario C.; Ghosh, Jagadish C.
World Intellectual Property Organization, WO2015168599 A1 2015-11-05 | Language: English, Database: CAplus

Fig 1A

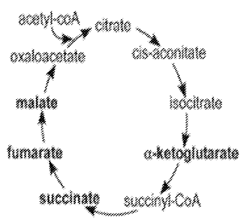

Pharmaceutical compositions for the treatment of cancer are provided. In one embodiment the compos ition comprises gamitrinib and a phosphatidylinositol 3-kinase (PI3K) inhibitor selected from PX-866, AZD6482, LY294002, BEZ235, GSK458, GDC0941, ZSTK474, BKM120, and GSK2636771B. Methods of treating cancer are also provided. The present invention is based in part on the inventor's discovery that combination therapy that targets mitochondria eliminates tumor adaptation induced by P13 K inhibition, and improves clin. outcome in cancer.

Keywords: antitumor gamitrinib BEZ235 combination therapy targeting mitochondria cancer treatment

PatentPak available

Substances (91) | Reactions (0) | Citing (0)

202

Aurora B kinase is a potent and selective target in MYCN-driven neuroblastoma.

By: Bogen, Dominik; Wei, Jun S; Azorsa, David O; Ormanoglu, Pinar; Buehler, Eugen; Guha, Rajarshi; Keller, Jonathan M; Mathews Griner, Lesley A; Ferrer, Marc; Song, Young K; et al
Oncotarget (2015), 6(34), 35247-62 | Language: English, Database: MEDLINE

Despite advances in multimodal treatment, neuroblastoma (NB) is often fatal for children with high- risk disease and many survivors need to cope with long-term side effects from high- dose chemotherapy and radiation. To identify new therap eutic targets, we performed an siRNA screen of the druggable genome combined with a small molecule screen of 465 compounds targeting 39 different mechanisms of actions in four NB cell lines. We identified 58 genes as targets, including A URKB, in at least one cell line. In the drug screen, aurora kinase inhibitors (nine molecules) and in particular the AURKB-selective compound, barasertib, were the most discriminatory with regard to sensitivity for MYCN-amplified cell lines. In an expanded panel of ten N B cell lines, those with M Y CN-amplification and wild-type TP53 were the most sensitive to low nanomolar concent rations of barasertib. Inhibition of the AURK B kinase activity resulted in decreased phosphor ylation of the known target, histone H3, and upregu lation of TP53 in MYCN-amplified, TP53 wild-type cells. However, both wild-type and TP53 mutant MYCN-amplified cell lines arrested in G2/M phase upon A URKB inhibition. Additionally, barasertib induced endoreduplication and apoptosis. Treatment of MYCN-amplified/TP53 wild-type neuroblastoma xenografts resulted in profound growth inhibition and tumor regres sion. Therefore, aurora B kinase inhibition is highly effective in aggressive neuroblastoma and warrants further investi gation in clinical trials.

Keywords: AURKB; MYCN; barasertib; high-throughput screening; neurobl astoma

Substances (0) | Reactions (0) | Citing (38)

203

Methods for treating cancer using TOR kinase inhibitor combination therapy

By: Fultz, Kimberly Elizabeth; Tran, Tam Minh; Xu, Shuichan; Xu, Weiming; Trowe, Torsten; Filvaroff, Ellen
World Intellectual Property Organization, WO2015160868 A1 2015-10-22 | Language: English, Database: CAplus

Provided are methods for treating or preventing cancer, comprising administering an effective amount of a 3,4-dihydropyrazino[2, 3-b]pyrazin-2(1H)-one TOR kinase inhibitor and an effective amount of a second active agent to a patient. Cancers treated by the method include solid tumors of the head, neck, breast, lung, ovaries, colon, rectum, stomach, prostate, urinary bladder, uterine, cervix, and other reproductive organs, wherein the tumors include relapsed, refractory or advanced solid tumors.

Keywords: pyrazinopyrazine TOR kinase inhibitor combin ation therapy cancer

PatentPak available

Substances (217) | Reactions (0) | Citing (0)

204

Quinazoline-benzimidazole hybrid as dual optical sensor for cyanide and Pb $^{2+}$ ions and Aurora kinase inhibitor

By: Luxami, Vijay ; Rani, Richa; Sharma, Alka; Paul, Kamaldeep
Journal of Photochemistry and Photobiology, A: Chemistry (2015), 311, 68-75 | Language: English, Database: CAplus

Compounds 1 and 2 exhibited selective fluorescence response toward Pb^{2+} over the other metal ions like Zn^{2+}, Co^{2+}, Ca^{2+}, K^+, Ni^{2+}, Mn^{2+}, Fe^{2+}, Hg^{2+}, Na^+, etc. Compound 1 behaved as cyanide selective chromofluorescent sensor amongst other anions like F^-, Cl^-, Br^-, I^-, AcO^-, $H_2PO^-_4$, HSO^-_4, etc. The binding properties of compounds 1 and 2 with cyanide and Pb^{2+} were investigated via UV-vis, fluorescence spectroscopy, ^{1}H NMR titration experiment and DFT calculations Compound 1 has been used to estimate CN^- ion (2-500μM) and compound 2 used to estimate Pb^{2+} ion (0.05-1500μM) ratiometrically. Compounds 1 and 2 have also been used as Aurora kinase inhibitors with IC_{50} values of 7.50μM and 0.53μM, resp.

Keywords: cyanide lead quinazoline benzimidazole hybrid Aurora kinase optical sensor

Substances (19) | Reactions (0) | Citing (22)

205

Target inhibition map system for combination antitumor therapy design and methods of using same

By: Pal, Ranadip; Keller, Charles, III; Berlow, Noah E.; Seguin, Bernard
United States, US20150269307 A1 2015-09-24 | Language: English, Database: CAplus

Disclosed is a system and method for targeted therapy designs which model a cancer pathway for predicting the effectiveness of targeted anti-cancer drugs. The disclosed system and method includes utilization of a computer processor allowing for the selection of a set of drugs from available drugs using approximation algorithms utilizing cell viability data associated with a testable culture of a patient's tumor for generating a probabilistic target inhibition map (PTIM) from viability data for considering the selection of a set of drugs from available drugs, and further supports a wide variety of scenarios for personalized cancer therapy, related products and services.

Keywords: antitumor combination therapy design target map system algorithm modeling

PatentPak available

Substances (79) | Reactions (0) | Citing (0)

206

Phosphoproteomic analysis of Aurora kinase inhibition in monopolar cytokinesis

By: Polat, Ayse Nur; Karayel, Ozge; Giese, Sven H.; Harmanda, Busra; Sanal, Erdem; Hu, Chi-Kuo; Renard, Bernhard Y.; Ozlu, Nurhan
Journal of Proteome Research (2015), 14(9), 4087-4098 | Language: English, Database: CAplus and MEDLINE

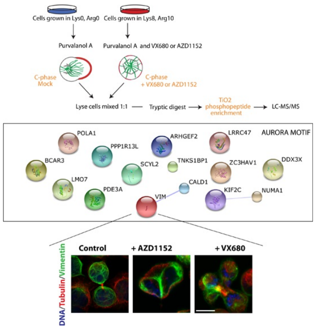

Cytokinesis is the last step of the cell cycle that requires coordi nated activities of the microtubule cytoskeleton, actin cytoskeleton, and membrane compartments. Aurora B kinase is one of the master regulatory kinases that orches trate multiple events during cytokinesis. To reveal targets of the Aurora B kinase, we combined quant. mass spectr ometry with chem. genetics. Using the quant. proteomic approach, SILAC (stable isotope labeling with amino acids in cell culture) , we analyzed the phosphoproteome of monopolar cytokinesis upon VX680- or AZD1152-mediated aurora kinase inhibition. In total, our anal. quantified over 20 000 phosphopeptides in response to the Aurora-B kinase inhibition; 246 unique phosphopeptides were significantly down-regulated and 74 were up-regulated. Our data provide a broad anal. of downstream effectors of Aurora kinase and offer insights into how Aurora kinase regulates cytokinesis.

Keywords: human Aurora kinase cytokinesis phosphoproteome target phosphopeptide intermediate filament

Substances (3) Reactions (0) Citing (9)

207

Exosomes as a therapeutic for cancer

By: Trujillo, Kristina
World Intellectual Property Organization, WO2015120150 A1 2015-08-13 | Language: English, Database: CAplus

In one embodiment, the invention provides substantially purified exosomes which induce apoptosis in breast cancer cells and which are derived from a cultured medium of histol. normal breast tissue cells that are obtained from tumor-adjacent normal breast tissue. Related methods of treating breast cancer and pharmaceutical formulations are also provided.

Keywords: breast cancer antitumor autologous purified exosomes iv injection

PatentPak available

Substances (284) Reactions (0) Citing (4)

208

Therapeutic combinations for treating cancer comprising an irreversible mutant EGFR inhibitor and an antineoplastic agent such as an Aurora kinase inhibitor

By: Simmmons, Andrew D.; Harding, Thomas Christian

World Intellectual Property Organization, WO2015112705 A2 2015-07-30 | Language: English, Database: CAplus

The present invention relates to combination treatment methods for treating cancer, partic ularly non-small cell lung cancer (N SCLC) . More specifi cally, the invention relates to methods of treating or managing cancer, partic ularly NSCLC, in a subject, comprising administering an irreversible mutant EGFR inhibitor compound in combin ation with one or more antineo plastic agents, particularly an Aurora kinase inhibitor.

Keywords: cancer treatment EGFR inhibitor Aurora kinase inhibitor combin ation

PatentPak available

Substances (46) Reactions (0) Citing (3)

209

Discovery of N-(4-(3-(2-Aminopyrimidin-4-yl)pyridin-2-yloxy)phenyl)-4-(4-methylthiophen-2-yl)phthalazin-1-amine (AMG 900), A Highly Selective, Orally Bioavailable Inhibitor of Aurora Kinases with Activity against Multidrug-Resistant Cancer Cell Lines

By: Geuns-Meyer, Stephanie; Cee, Victor J.; Deak, Holly L.; Du, Bingfan; Hodous, Brian L.; Nguyen, Hanh Nho; Olivieri, Philip R.; Schenkel, Laurie B.; Vaida, Karina R.; Andrews, Paul; et al
Journal of Medicinal Chemistry (2015), 58(13), 5189-5207 | Language: English, Database: CAplus and MEDLINE

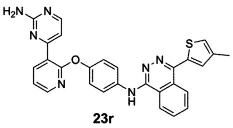

Cell Line	EC_{50} (nM)
CAL51 (Non-MDR)	2
MES-SA Dx5 (MDR)	1
HCT15 (MDR)	2
SNU449 (MDR)	2
769P (MDR)	2

Efforts to improve upon the phys. properties and metabolic stability of pyridinyl-pyrimidine phthalazinamine Aurora kinase inhibitor revealed that potency against multidrug-resistant cell lines was compro mised by increased polarity. Despite its high in vitro metabolic intrinsic clearance, AMG 900 (**I**) showed acceptable pharmaco kinetic properties and robust pharmaco dynamic activity. Projecting from in vitro data to in vivo target coverage was not practical due to disjunctions between enzyme and cell data, complex and apparently contradictory indicators of binding kinetics, and unmeas urable free fraction in plasma. In contrast, it was straight forward to relate pharmaco kinetics to pharmacodynamics and efficacy by following the time above a threshold concent ration On the basis of its oral route of administration, a selectivity profile that favors Aurora-driven pharmacol. and its activity against multidrug-resistant cell lines, **I** was identified as a potential best- in-class Aurora kinase inhibitor. In phase 1 dose expansion studies with G-CSF support, **I** has shown promising single agent activity.

Keywords: pyridinylpyrimidine phthalazinamine AMG 900 analog prepar ation SAR Aurora kinase

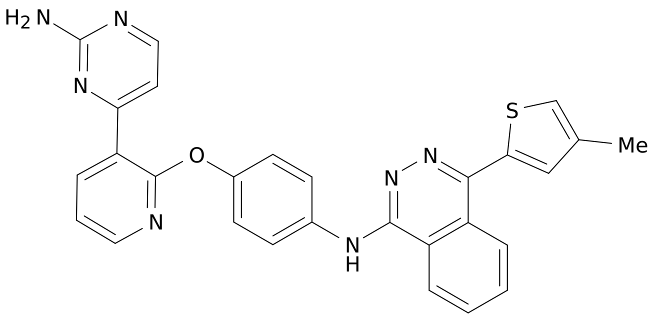

I

Substances (94)　Reactions (138)　Citing (36)

210

Structural Biology Insight for the Design of Sub-type Selective Aurora Kinase Inhibitors

By: Sarvagalla, Sailu; Coumar, Mohane Selvaraj
Current Cancer Drug Targets (2015), 15(5), 375-393 | Language: English, Database: CAplus and MEDLINE

A review. Aurora kinase A, B and C, are key regulators of mitosis and are over expressed in many of the human cancers, making them an ideal drug target for cancer chemotherapy. Currently, over a dozen of Aurora kinase inhibitors are in various phases of clin. development. The majority of the inhibitors (VX-680/MK-0457, PHA-739358, CYC116, SNS-314, AMG 900, AT-9283, SCH-1473759, ABT-348, PF-03814735, R-763/AS-703569, KW-2449 and TAK-901) are pan-selective (isoform non-selective) and few are Aurora A (MLN8054, MLN8237, VX-689/MK5108 and ENMD 2076) and Aurora B (AZD1152 and GSK1070916) sub-type selective. Despite the intensive research efforts in the past decade, no Aurora kinase inhibitor has reached the market. Recent evidence suggests that the sub-type selective Aurora kinase A inhibitor could possess advantages over pan- selective Aurora inhibitors, by avoiding Aurora B mediated neutropenia. However, sub-type selective Aurora kinase A inhibitor design is very challe nging due to the similarity in the active site among the isoforms. Structural biol. and computational aspects pertaining to the design of Aurora kinase inhibitors were analyzed and found that a possible means to develop sub-type selective inhibitor is by targeting Aurora A specific residues (Leu215, Thr217 and Arg220) or Aurora B specific residues (Arg159, Glu161 and Lys164), near the solvent exposed region of the protein. Particularly, a useful strategy for the design of sub- type selective Aurora A inhibitor could be by targeting Thr217 residue as in the case of MLN8054. Further preclin. and clin. studies with the sub- type selective Aurora inhibitors could help bring them to the market for the treatment of cancer.

Keywords: review Aurora kinase inhibitor design anticancer therapeutic target cancer

Substances (22) Reactions (0) Citing (20)

211

SnapShot: Kinase Inhibitors II

By: Wang, Jinhua; Gray, Nathanael S.
Molecular Cell (2015), 58(4), 710-710.e1 | Language: English, Database: CAplus and MEDLINE

Selective small-mol. inhibitors of protein kinases can serve as powerful tools to elucidate biol. function. Efforts to develop potential drug candidates have yielded a wealth of kinase inhibitors. However, selecting the optimal kinase inhibitor for a particular applic ation can be challenging. While the optimal inhibitor will be application specific, we have attempted to summarize some of the best reported inhibitors for various kinases.

Keywords: protein kinase inhibition small mol

Substances (87) Reactions (0) Citing (10)

212

Biomarkers for PSMA targeted therapy for prostate cancer

By: Olson, William C.; DiPippo, Vincent
World Intellectual Property Organization, WO2015073896 A2 2015-05-21 | Language: English, Database: CAplus

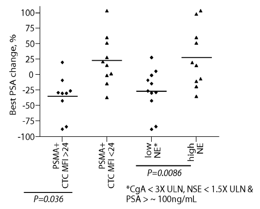
Fig. 2

Provided herein are a number of methods, assays and diagnostic tests based on one or more biomarkers as well as related kits and compositions that can be used to identify subjects or patients that would likely benefit from a treatment (or continued treatment) , such as a PSMA targeted therapy. Also provided are methods for treating the identified subjects or patients.

Keywords: PSMA targeted therapy prostate cancer biomarker

PatentPak available

Substances (20) | Reactions (0) | Citing (1)

213

Sequential treatment with aurora B inhibitors enhances cisplatin-mediated apoptosis via c-Myc

By: Ma, Yaxi; Cao, Handi; Lou, Siyue; Shao, Xuejing; Lv, Wen; Qi, Xiaotian; Liu, Yujia; Ying, Meidan; He, Qiaojun; Yang, Xiaochun
Journal of Molecular Medicine (Heidelberg, Germany) (2015), 93(4), 427-438 | Language: English, Database: CAplus and MEDLINE

Abstract: Platinum compound such as cisplatin is the first-line chemotherapy of choice in most patients with ovarian carcinoma. However, patients with inherent or acquired cisplatin resistance often experience relapse. Therefore, novel therapies are urgently required to treat drug-resistant ovarian carcinoma. Here, we showed that compared to the non- functional traditional simultaneous treatment, sequential combination of Aurora B inhibitors followed by cisplatin synergis tically enhanced apoptotic response in cisplatin-resistant OVCAR-8 cells. This effect was accomp anied by the induction of polyploidy in a c-Myc-dependent manner, as c-Myc knockdown reduced the efficacy of the combin ation by suppressing the expression of Aurora B and impairing cellular response to Aurora B inhibitor, as indicated by the decreased polyploidy and hyperphosphorylation of histone H1. In c-Myc-deficient SKOV3 cells, c-Myc overexpression restored Aurora B expression, induced polyploidy after inhibition of Aurora B, and sensitized cells to this combination therapy. Thus, our report reveals for the first time that sequential treatment of Aurora B inhibitors and cisplatin is essential to inhibit ovarian carcinoma by inducing polyploidy and downregulating c-Myc and that c-Myc is identified as a predictive biomarker to select cells responsive to chemotherapeutical combinations targeting Aurora B. Collect ively, these studies provide novel approaches to overcoming cisplatin chemotherapy resistance in ovarian cancer. Key Message: Pretre atment of Aurora B inhibitors augment apoptotic effects of cisplatin. The synergy of Aurora B inhibitor with cisplatin is dependent on c-Myc expression. c-Myc-dependent induction of polyploidy sensitizes cells to cisplatin.

Keywords: aurora B inhibitor cisplatin anticancer agent cMyc apoptosis cancer

Substances (7) | Reactions (0) | Citing (6)

214

Novel drugs for older patients with acute myeloid leukemia

By: Montalban-Bravo, G.; Garcia-Manero, G.
Leukemia (2015), 29(4), 760-769 | Language: English, Database: CAplus and MEDLINE

A review. Acute myeloid leukemia (AML) is the second most common form of leukemia and the most frequent cause of leukemia-related deaths in the United States. The incidence of A ML increases with advancing age and the prognosis for patients with A ML worsens substantially with increasing age. Many older patients are ineligible for intensive treatment and require other therap eutic approaches to optimize clin. outcome. To address this treatment gap, novel agents with varying mechanisms of action targeting different cellular processes are currently in development. Hypomethylating agents (azacitidine, decitabine, SGI-110), histone deacet ylase inhibitors (vorinostat, pracinostat, panobinostat), FMS-like tyrosine kinase receptor-3 inhibitors (quizartinib, sorafenib, midost aurin, crenolanib), cytotoxic agents (clofarabine, sapacitabine, vosaroxin), cell cycle inhibitors (barasertib, volasertib, rigosertib) and monoclonal antibodies (gentuzumab ozogamicin, lintuzumab-Ac225) represent some of these promising new treatm ents. This review provides an overview of novel agents that have either completed or are currently in ongoing phase III trials in patients with previously untreated AML for whom intensive treatment is not an option. Other potential drugs in earlier stages of develo pment will also be addressed in this review.

Keywords: review anticancer agent acute myeloid leukemia

Substances (17) Reactions (0) Citing (54)

215

Co-inhibition of polo-like kinase 1 and Aurora kinases promotes mitotic catastrophe.

By: Li, Jingjing; Hong, Myung Jin; Chow, Jeremy P H; Man, Wing Yu; Mak, Joyce P Y; Ma, Hoi Tang; Poon, Randy Y C
Oncotarget (2015), 6(11), 9327-40 | Language: English, Database: MEDLINE

Mitosis is choreographed by a number of protein kinases including polo- like kinases and Aurora kinases. As these kinases are frequently dysregulated in cancers, small-molecule inhibitors have been developed for targeted anticancer therapies. Given that P L K1 and Aurora kinases possess both unique functions as well as co- regulate multiple mitotic events, whether pharmaco logical inhibition of these kinases together can enhance mitotic catastrophe remains an outstanding issue to be determined. Using concentrations of inhibitors that did not induce severe mitotic defects on their own, we found that both the metaphase arrest and mitotic slippage induced by inhibitors targeting Aurora A and Aurora B (MK-5108 and Barasertib respectively) were enhanced by a PLK1 inhibitor (BI 2536). We found that P LK1 is overexpressed in cells from nasopharyngeal carcinoma, a highly invasive cancer with poor prognosis, in comparison to normal nasopharyngeal epithelial cells. Nasopharyngeal carcinoma cells were more sensitive to B I 2536 as a single agent and co-inhibition with Aurora kinases than normal cells. These observ ations underscore the mechanism and potential benefits of targeting PLK1 and Aurora kinases to induce mitotic catast rophe in cancer cells.

Keywords: anticancer drugs; antimitotic drugs; kinases; mitosis; mitotic slippage

Substances (0) Reactions (0) Citing (25)

216

Anti-human VSTM5 antibodies, fragments and conjugates for therapy and diagnosis of cancer, infection and immune disease

By: Levine, Zurit; Rotman, Galit; Dassa, Liat; Levy, Ofer; Cojocaru, Gad S.; Toporik, Amir; Kliger, Yossef; Pow, Andrew; Liang, Spencer
World Intellectual Property Organization, WO2015037005 A1 2015-03-19 | Language: English, Database: CAplus

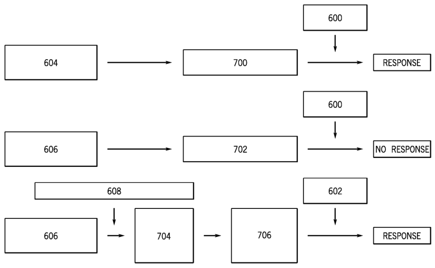

FIG. 1

The present invention relates to VSTM5-specific antibodies, antibody fragments, and VSTM5 polypeptides, conjugates and compos itions comprising same, for modulating (antago nizing or agonizing) one or more of the effects of VSTM5 expression on immunity. More specifically, the present invention relates to VSTM5-specific antibodies, antibody fragments, and VSTM5 polypeptides, conjugates and compositions comprising same for treating and aiding in the diagnosis of cancer, infectious diseases and immune related diseases, e.g., those associated with aberrant (higher or lower than normal) VSTM5 expression by diseased and/or immune cells and/or aberrant (increased or reduced) VSTM5-mediated effects on immunity.

Keywords: human VSTM5 antibody immunodiagnosis immunotherapy cancer infection immune disease

PatentPak available

Substances (424) | Reactions (0) | Citing (0)

217

Aurora kinase A and B as new treatment targets in aromatase inhibitor-resistant breast cancer cells

By: Hole, Stine; Pedersen, Astrid M.; Lykkesfeldt, Anne E.; Yde, Christina W.
Breast Cancer Research and Treatment (2015), 149(3), 715-726 | Language: English, Database: CAplus and MEDLINE

Aromatase inhibitors (AIs) are used for treatment of estrogen receptor α (E R)-pos. breast cancer; however, resistance is a major obstacle for optimal outcome. This preclin. study aimed at identifying potential new treatment targets in A I-resistant breast cancer cells. Parental MCF-7 breast cancer cells and four newly establ ished cell lines, resistant to the A Is exemestane or letrozole, were used for a functional kinase inhibitor screen. A library comprising 195 different compounds was tested for preferential growth inhibition of AI-resistant cell lines. Selected targets were validated by anal. of cell growth, cell cycle phase distrib ution, protein expression, and subcel lular localization. We identified 24 compounds, including several inhibitors of Aurora kinases e.g., J NJ-7706621 and barasertib. Protein expression of Aurora kinase A and B was found upregu lated in AI-resistant cells compared with M CF-7, and knockdown studies showed that Aurora kinase A was essential for A I-resistant cell growth. In A I-resistant cell lines, the clin. relevant Aurora kinase inhibitors alisertib and danusertib blocked cell cycle progression at the G2/M phase, interfered with chromosome alignment and spindle pole formation, and resulted in preferential growth inhibition compared with parental M CF-7 cells. Even further growth inhibition was obtained when combining the Aurora kinase inhibitors with the antiestrogen fulvestrant. Our study is the first to demonstrate that Aurora kinase A and B may be treatment targets in A I-resistant cells, and our data suggest that therapy targeting both ER and Aurora kinases may be a potent treatment strategy for overcoming A I resistance in breast cancer.

Keywords: Aurora kinase aromatase inhibitor resistance breast cancer treatment target

Substances (9) | Reactions (0) | Citing (31)

218

Finding the optimal combination therapy for the treatment of newly diagnosed AML in older patients unfit for intensive therapy

By: Erba, Harry P.
Leukemia Research (2015), 39(2), 183-191 | Language: English, Database: CAplus and MEDLINE

A review. There is no standard of care for older patients with newly diagnosed acute myeloid leukemia (AML) unfit for intensive therapy, and prognosis with currently recommended low-intensity therapies (decitabine, azacitidine, and low-dose cytarabine [LDA C]) remains poor. One promising strategy is to combine low- intensity treatments with novel agents. Gemtuzumab ozogam icin, tipifa rnib, and barasertib have been investigated in phase 2/3 or 3 trials combined with LDAC, and phase 3 trials are currently investi gating sapacitabine plus decitabine, and volasertib plus LDAC in AML. This review discusses current treatment recommen dations and the development of combination therapies for older patients unfit for intensive therapy.

Keywords: combination intensive therapy diagnosis human AML review; Acute myeloid leukemia (AML); Combination; Low-intensity; Newly diagnosed; Novel agents; Older/elderly

Substances (8) | Reactions (0) | Citing (30)

219

A phase II trial of AZD1152 in relapsed/refractory diffuse large B-cell lymphoma.

By: Collins, Graham P; Eyre, Toby A; Linton, Kim M; Radford, John; Vallance, Grant D; Soilleux, Elizabeth; Hatton, Chris
British journal of haematology (2015), 170(6), 886-90 | Language: English, Database: MEDLINE

There is no abstract available for this document.

Keywords: AZD1152; Aurora B kinase; barasertib; diffuse large B- cell lymphoma; lymphoid malign ancies

Substances (0) | Reactions (0) | Citing (22)

220

Novel substituted bicyclic compounds as bromodomain inhibitors

By: Liu, Shuang; Quinn, John Frederick; Duffy, Bryan Cordell; Wang, Ruifang; Jiang, May Xiaowu; Martin, Gregory Scott; Zhao, He; Ellis, Michael; Wagner, Gregory Steven; Young, Peter Ronald
World Intellectual Property Organization, WO2015004534 A2 2015-01-15 | Language: English, Database: CAplus

The invention relates to substituted bicyclic compounds, which are useful for inhibition of B ET protein function by binding to bromodomains, pharmaceutical compositions comprising these compounds, and use of the compounds and compos itions in therapy. Also disclosed is the preparation of compounds of the invention.

Keywords: bicyclic derivative preparation bromodomain

PatentPak available

Substances (145) | Reactions (191) | Citing (2)

221

Preparation of azabicyclic compounds as BET inhibitors and therapeutic uses thereof

By: Liu, Shuang; Quinn, John Frederick; Duffy, Bryan Cordell; Wang, Ruifang; Jiang, May Xiaowu; Martin, Gregory Scott; Zhao, He; Ellis, Michael; Wagner, Gregory Steven; Young, Peter Ronald

World Intellectual Property Organization, WO2015004533 A2 2015-01-15 | Language: English, Database: CAplus

The invention relates to substituted azabicyclic compounds of formula I which are useful for the inhibition of bromodomain and extra terminal domain (BET) proteins, pharmaceutical compositions comprising these compounds, and use of the compounds and compositions for treatment of BET-associated diseases such as cancer, inflammation, autoimmune disease, cardiovascular disease, benign proliferative and fibrotic disorders, and other diseases. Compounds of formulas I [wherein A is selected from 5- or 6-membered monocyclic carbocycle or heterocycles fused to ring B; B is a 6-membered carbocycle or heterocycle; Y is N or C; X is NH, alkyl, C(O)O, etc.; W^1 and W^2 are independently selected from N or CR; R at each occurrence is independently selected from H, NH_2, OH, etc.; W^3 and W^4 are independently selected from N, CH, and C with the proviso that both W^3 and W^4 cannot be N; R^3 is 4-7 membered carbocycle, 4-7 membered heterocycle, bicyclic carbocycle, etc.; D^1 is 5-membered monocyclic carbocycle or heterocycle connected to the B ring via a carbon-carbon bond with proviso] or a stereoisomer, tautomer, pharmaceutically acceptable salt, or hydrate thereof are claimed and exemplified. Reaction of 3,5-dimethyl-4-(4,4,5,5-tetramethyl-1,3,2-dioxaborolan-2-yl)isoxazole with 4,6-dibromo-2-methylbenzimidazole afforded II in 60% yield. I were evaluated for inhibition of tetra-acetylated histone H4 binding to BET bromodomains using TR-FRET anal. from which II was determined to inhibit the first bromodomain of Brd4 with an IC_{50} value of ≤ 0.3 μM.

Keywords: azabicyclic compound preparation BET inhibitor

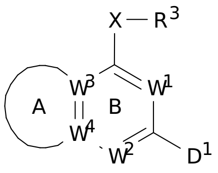

I

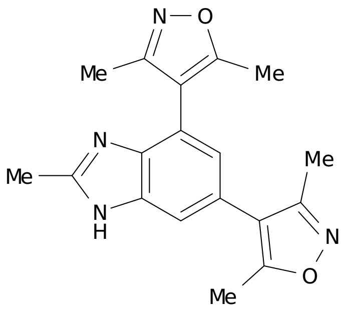

II

PatentPak available

Substances (542)   Reactions (1,251)   Citing (9)

222

Preparation of azabicyclic compounds as BET inhibitors and therapeutic uses thereof

By: Duffy, Bryan Cordell; Liu, Shuang; Quinn, John Frederick; Wang, Ruifang; Jiang, May Xiaowu; Martin, Gregory Scott; Wagner, Gregory Steven; Young, Peter Ronald

World Intellectual Property Organization, WO2015002754 A2 2015-01-08 | Language: English, Database: CAplus

The invention relates to substituted azabicyclic compounds of formula I which are useful for the inhibition of bromodomain and extra terminal domain (BET) proteins, pharmaceutical compositions comprising these compounds, and use of the compounds and compositions for treatment of BET-associated diseases such as cancer, inflammation, autoimmune disease, cardiovascular disease, benign proliferative and fibrotic disorders, and other diseases. Compounds of formulas I and II [wherein A is selected from 5- or 6-membered monocyclic heterocycles fused to ring B; B is a 6-membered aromatic carbocycle or heterocycle; Y is N or C; X is NH, alkyl, C(O)O, etc.; W^1, W^2, W^3 are independently selected from N or CR; R at each occurrence is independently selected from H, NH_2, halo, CN, etc.; R^4 is 3-7 membered carbocycle or heterocycle; D^1 is 5-membered monocyclic heterocycle with proviso] or a stereoisomer, tautomer, pharmaceutically acceptable salt, or hydrate thereof are claimed and exemplified. Benzylation of 2-chloroadenine provided 9-benzyl-2-chloroadenine which was reacted with 3,5-dimethyl-4-(4,4,5,5-tetramethyl-1,3,2-dioxaborolan-2-yl)isoxazole affording **III**. **I** were evaluated for inhibition of tetra-acetylated histone H4 binding to BET bromodomains using TR-FRET anal. from which **III** was determined to inhibit the first bromodomain of Brd4 with an IC_{50} value between 0.3 μM and 30 μM.

Keywords: azabicyclic compound preparation BET inhibitor

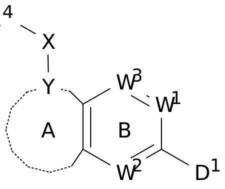

I

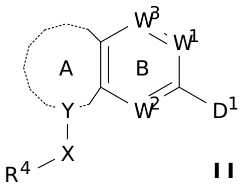

II

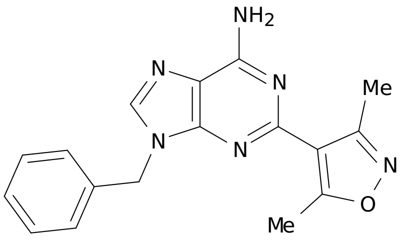

III

PatentPak available

Substances (556)

Reactions (982)

Citing (12)

223

Aurora kinase B inhibition reduces the proliferation of metastatic melanoma cells and enhances the response to chemotherapy

By: Porcelli, Letizia; Guida, Gabriella; Quatrale, Anna E.; Cocco, Tiziana; Sidella, Letizia; Maida, Immacolata; Iacobazzi, Rosa M.; Ferretta, Anna; Stolfa, Diana A.; Strippoli, Sabino; et al

Journal of Translational Medicine (2015), 13, 26/1-26/13 | Language: English, Database: CAplus and MEDLINE

Background: The poor response to chemotherapy and the brief response to vemurafenib in metastatic melanoma patients, make the identification of new therapeutic approaches an urgent need. Interestingly the increased expression and activity of the Aurora kinase B during melanoma progression suggests it as a promising therapeutic target. Methods: The efficacy of the Aurora B kinase inhibitor barasertib-HQPA was evaluated in BRAF mutated cells, sensitive and made resistant to vemura fenib after chronic exposure to the drug, and in BRAF wild type cells. The drug effectiveness has been evaluated as cell growth inhibition, cell cycle progression and cell migration. In addition, cellular effectors of drug resistance and response were investi gated. Results: The characterization of the effectors responsible for the resistance to vemurafenib evidenced the increased expression of MITF or the activation of Erk1/2 and p-38 kinases in the newly established cell lines with a phenotype resistant to vemura fenib. The sensitivity of cells to barasertib-HQPA was irresp. of BRAF mutational status. Barasertib-HQPA induced the mitotic catastrophe, ultimately causing apoptosis and necrosis of cells, inhibited cell migration and strongly affected the glycolytic metabolism of cells inducing the release of lactate. In association (i) with vemurafenib the gain in effectiveness was found only in BRAF(V600K) cells while (ii) with nab-paclitaxel, the combination was more effective than each drug alone in all cells. Conclu sions: These findings suggest barasertib as a new therapeutic agent and as enhancer of chemotherapy in metastatic melanoma treatment.

Keywords: Aurora kinase B proliferation metastatic melanoma cell chemotherapy

Substances (10) Reactions (0) Citing (35)

224

Aurora kinase B is important for antiestrogen resistant cell growth and a potential biomarker for tamoxifen resistant breast cancer

By: Larsen, Sarah L.; Yde, Christina W.; Laenkholm, Anne-Vibeke; Rasmussen, Birgitte B.; Duun-Henriksen, Anne Katrine; Bak, Martin; Lykkesfeldt, Anne E.; Kirkegaard, Tove
BMC Cancer (2015), 15, 1-15 | Language: English, Database: CAplus and MEDLINE

Background: Resistance to antiestrogen therapy is a major clin. challenge in the treatment of estrogen receptor α (E R)-pos. breast cancer. The aim of the study was to explore the growth promoting pathways of antiestrogen resistant breast cancer cells to identify biomarkers and novel treatment targets. Methods: Antiestrogen sensitive and resistant T47 D breast cancer cell lines were used as model systems. Parental and fulvestrant resistant cell lines were subjected to a kinase inhibitor library. Kinase inhibitors prefere ntially targeting growth of fulvestrant resistant cells were identified and the growth inhibitory effect verified by dose- response cell growth experiments Protein expression and phosphorylation were investigated by western blot anal. Cell cycle phase distri bution and cell death were analyzed by flow cytometry. To evaluate Aurora kinase B as a biomarker for endocrine resistance, immunohi stochem. was performed on archival primary tumor tissue from breast cancer patients who have received adjuvant endocrine treatment with tamoxifen. Results: The selective Aurora kinase B inhibitor barasertib was identified to preferentially inhibit growth of fulvestrant resistant T47D breast cancer cell lines. Compared with parental cells, phosphor ylation of Aurora kinase B was higher in the fulvestrant resistant T47D cells. Barasertib induced degradation of Aurora kinase B, caused mitotic errors, and induced apoptotic cell death as measured by accumulation of SubG1 cells and PARP cleavage in the fulvestrant resistant cells. Barasertib also exerted preferential growth inhibition of tamoxifen resistant T47 D cell lines. Finally, high percentage of Aurora kinase B pos. tumor cells was significantly associated with reduced disease-free and overall survival in 261 E R-pos. breast cancer patients, who have received tamoxifen as first-line adjuvant endocrine treatment. Conclusions: Our results indicate that Aurora kinase B is a driving factor for growth of antiestrogen resistant T47D breast cancer cell lines, and a biomarker for reduced benefit of tamoxifen treatment. Thus, inhibition of Aurora kinase B, e.g. with the highly selective kinase inhibitor barasertib, could be a candidate new treatment for breast cancer patients with acquired resistance to antiestrogens.

Keywords: barasertib anticancer aurora kinase B inhibitor therapeutic breast cancer

Substances (7) Reactions (0) Citing (20)

225

Canine osteosarcoma cells exhibit resistance to aurora kinase inhibitors

By: Cannon, C. M.; Pozniak, J.; Scott, M. C.; Ito, D.; Gorden, B. H.; Graef, A. J.; Modiano, J. F.
Veterinary and Comparative Oncology (2015), 13(1), 48-59 | Language: English, Database: CAplus and MEDLINE

We evaluated the effect of Aurora kinase inhibitors AZD1152 and VX680 on canine osteosarcoma cells. Cytotoxicity was seen in all four cell lines; however, half-maximal inhibitory concentrations were significantly higher than in human leukemia and canine lymphoma cells. AZD1152 reduced Aurora kinase B phosphor ylation, indicating resistance was not because of failure of target recognition. Efflux mediated by A BCB1 and ABCG2 transporters is one known mechanism of resistance against these drugs and verapamil enhanced AZD1152-induced apoptosis; however, these transporters were only expressed by a small percentage of cells in each line and the effects of verapamil were modest, suggesting other mechanisms contribute to resistance. Our results indicate that canine osteosarcoma cells are resistant to Aurora kinase inhibitors and suggest that these compounds are unlikely to be useful as single agents for this disease. Further investigation of these resistance mechanisms and the potential utility of Aurora kinase inhibitors in multi-agent protocols is warranted.

Keywords: canine osteosarcoma drug resistance aurora kinase inhibitor A ZD1152 VX680; chemotherapy; comparative oncology; in vitro models; oncology; osteosarcoma

Substances (4) Reactions (0) Citing (4)

226

Modeling the mitotic regulatory network identifies highly efficient anti-cancer drug combinations

By: Wu, Yiran; Zhuo, Xiaolong; Dai, Ziwei; Guo, Xiao; Wang, Yao; Zhang, Chuanmao; Lai, Luhua
Molecular BioSystems (2015), 11(2), 497-505 | Language: English, Database: CAplus and MEDLINE

Targeting mitotic regulation is recognized as an important strategy for cancer therapy. Aurora A/B kinase and polo-like kinase 1 (PLK1) are the key mitotic regulators, and many inhibitors have been developed. Combinations of these inhibitors are anticipated to be more effective therapeutics compared with single-inhibitor treatments; however, a systematic anal. of the combined effects is lacking. Here, we constructed the first mammalian cell mitotic regulation network model, which spans from mitotic entry to anaphase initiation, and contains all key mitotic kinase targets. The combined effects of different kinase inhibitors and microtubule inhibitors were systematically explored. Simultaneous inhibition of Aurora B and PLK1 strongly induces polyploidy. Microtubule inhibitor dosage can be significantly reduced when combined with a PLK1 inhibitor. The efficacy of these inhibitor combinations was validated by our exptl. results. The mitotic regulatory network model provides a platform to study the complex interactions during mitosis, enables identification of mitotic regulators, and determines targets for drug discovery research. The suggested use of combining microtubule inhibitors with PLK1 inhibitors is anticipated to enhance microtubule-inhibitor tolerance in a wide range of patients.

Keywords: anticancer discovery protein phosphorylation epithelial cell simulation computational model

Substances (8) Reactions (0) Citing (2)

227

Histone H3 phosphorylation in GBM: a new rational to guide the use of kinase inhibitors in anti-GBM therapy

By: Pacaud, Romain; Cheray, Mathilde; Nadaradjane, Arulraj; Vallette, Francois M.; Cartron, Pierre-Francois
Theranostics (2015), 5(1), 12-22, 11 pp. | Language: English, Database: CAplus and MEDLINE

Histones post-translational modifications (PTMs) are crucial components of diverse processes that modulate chromatin. Among the histones PTMs, the histones phosphorylation appears such crucial since it plays a significant role into DNA repair structure, transcription and chromatin compaction during cell division and apoptosis. However, little is known about the prognostic value of the histone phosphorylation in human cancer. This point could be considerate such as an important gap in anti-cancer therapy since the use of adequate kinase inhibitors could remedy to the aberrant histone phosphorylation associated with a poor prognosis factor. To remedy at this situation, we analyzed the phosphorylation level of histone H3 at the residues T3, T6, S10, S28, Y41 and T45 in a collection of 42 glioblastoma multiformes (GBM). Our data indicated that the high level of pH3T6, pH3S10 and pH3Y41 are signatures associated with a poor prognosis of overall survival (OS) of GBM treated with the "temozolomide and irradiation standard" treatment of GBM (named TMZ + Irad treatment). Our data also showed that these signatures are correlated with the high activity of kinases already described as writers of the pH3T6, pH3S10 and pH3Y41 i.e. the PKC, Aurora-B and JAK2, resp. Finally, our anal. revealed that the use of Enzastaurin, AZD1152, and AZD1480 abrogated the high level of pH3T6, pH3S10 and pH3Y41 while increasing the sensitivity to the "temozolomide and irradiation"-induced cell death. To conclude, it appears that this work provides biomarkers for patient stratification for a therapy including kinase inhibitors.

Keywords: glioblastoma histone H3 phosphorylation kinase inhibitor anti cancer therapy; apoptosis; epigenetic; glioma; histone; phosphorylation

Substances (9) Reactions (0) Citing (23)

228

A chemical screening approach to identify novel key mediators of erythroid enucleation

By: Wolwer, Christina B.; Pase, Luke B.; Pearson, Helen B.; Godde, Nathan J.; Lackovic, Kurt; Huang, David C. S.; Russell, Sarah M.; Humbert, Patrick O.
PLoS One (2015), 10(11), e0142655/1-e0142655/12 | Language: English, Database: CAplus and MEDLINE

Erythroid enucleation is critical for terminal differentiation of red blood cells, and involves extrusion of the nucleus by orthoch romatic erythroblasts to produce reticulocytes. Due to the difficulty of synchronizing erythroblasts, the mol. mechanisms underlying the enucleation process remain poorly understood. To elucidate the cellular program governing enucleation, we utilized a novel chem. screening approach whereby orthochromatic cells primed for enucleation were enriched ex vivo and subjected to a functional drug screen using a 324 compound library consisting of structurally diverse, medicinally active and cell permeable drugs. Using this approach, we have confirmed the role of HDACs, proteasomal regulators and MAPK in erythroid enucleation and introduce a new role for Cyclin-dependent kinases, in particular CDK9, in this process. Importantly, we demonstrate that when coupled with imaging anal., this approach provides a powerful means to identify and characterize rate limiting steps involved in the erythroid enucleation process.

Keywords: MAPK CDK9 HDAC PI3K orthochromatic erythroblast erythroid enucleation

Substances (294) Reactions (0) Citing (8)

229

Overcoming cetuximab resistance in HNSCC: The role of AURKB and DUSP proteins

By: Boeckx, Carolien; Op de Beeck, Ken; Wouters, An; Deschoolmeester, Vanessa; Limame, Ridha; Zwaenepoel, Karen; Specenier, Pol; Pauwels, Patrick; Vermorken, Jan B.; Peeters, Marc; et al
Cancer Letters (New York, NY, United States) (2014), 354(2), 365-377 | Language: English, Database: CAplus and MEDLINE

Unraveling the underlying mechanisms of cetuximab resistance in head and neck squamous cell carcinoma (HNSCC) is of major importance as many tumors remain non-responsive or become resistant. Our microarray results suggest that "resistant" cells still exhibit RAS-MAPK pathway signaling contributing to drug resistance, as witnessed by low expression of DUSP5 and DUSP6, neg. regulators of ERK1/2, and increased expression of AURKB, a key regulator of mitosis. Therefore, interrupting the RAS-MAPK pathway by an ERK1/2 inhibitor (apigenin) or an AURKB inhibitor (barasertib) might be a new strategy for overcoming cetuximab resistance in HNSCC.

Keywords: cetuximab anticancer AURKB DUSP head neck squamous cell carcinoma; Anti-EGFR therapy; Aurora kinase B; Cetuximab resistance; Dual-specificity phosphatase 5 and 6; Head and neck squamous cell carcinoma; Nano Pro 1000

Substances (10) Reactions (0) Citing (46)

230

Method of determination of cancer cell drug sensitivity towards aurora kinase inhibitors

By: Kollaredy, Madhusudhan Reddy; Hajduch, Marian; Dzubak, Petr; Srovnal, Josef; Hrabakova, Rita; Kovarova, Hana
United States, US20140336073 A1 2014-11-13 | Language: English, Database: CAplus

A method for determining the sensitivity and/or resistance of a patient suffering from a cancer disease to Aurora kinase inhibitor therapy, which comprises determining in vitro in the cancer cells or body fluids taken from the patient the expression of at least one gene selected from a particular group and/or determining in vitro in the cancer cells or body fluids taken from the patient the level of at least one protein selected from a particular group.

Keywords: cancer drug sensitivity Aurora kinase inhibitor gene protein expression

PatentPak available

Substances (55) | Reactions (0) | Citing (0)

231

Effects of selective inhibitors of Aurora kineases on anaplastic thyroid carcinoma cell lines

By: Baldini, Enke; Tuccilli, Chiara; Prinzi, Natalie; Sorrenti, Salvatore; Antonelli, Alessandro; Gnessi, Lucio; Morrone, Stefania; Moretti, Costanzo; Bononi, Marco; Arlot-Bonnemains, Yannick; et al
Endocrine-Related Cancer (2014), 21(5), 797-811 | Language: English, Database: CAplus and MEDLINE

Aurora kinases are serine/threonine kinases that play an essential role in cell division. Their aberrant expression and/or function induce severe mitotic abnormalities, resulting in either cell death or aneupl oidy. Overexpression of Aurora kinases is often found in several malignancies, among which is anaplastic thyroid carcinoma (A TC). We have previously demons trated the in vitro efficacy of Aurora kinase inhibitors in restraining cell growth and survival of different A TC cell lines. In this study, we sought to establish which Aurora might represent the preferential drug target for A TC. To this end, the effects of two selective inhibitors of Aurora- A (ML N8237) and Aurora- B (AZD1152) on four human ATC cell lines (CAL-62, BHT-101, 8305C, and 8505C) were analyzed. Both inhibitors reduced cell proliferation in a time- and dose- dependent manner, with IC_{50} ranges of 44.3-134.2 nM for M LN8237 and of 9.2-461.3 nM for AZD1152. Immunofluorescence experiments and time-lapse videomicroscopy yielded evidence that each inhibitor induced distinct mitotic phenotypes, but both of them prevented the completion of cytoki nesis. As a result, poliploidy increased in all A Z D1152-treated cells, and in two out of four cell lines treated with M LN8237. Apoptosis was induced in all the cells by M LN8237, and in BHT-101, 8305C, and 8505C by AZD1152, while CAL-62 exposed to AZD1152 died through necrosis after multiple rounds of endoreplication. Both inhibitors were capable of blocking anchorage- independent cell growth. In conclu sion, we demons trated that either Aurora-A or Aurora- B might represent therap eutic targets for the A TC treatment, but inhibition of Aurora- A appears more effective for suppressing ATC cell prolife ration and for inducing the apoptotic pathway.

Keywords: Aurora kinase anaplastic thyroid carcinoma; AZD1152; Aurora inhibitors; Aurora kinases; MLN8237; anaplastic thyroid cancer; cell cycle; therapy

Substances (4) | Reactions (0) | Citing (26)

232

Methods of use of cleavable drug conjugates and compositions

By: Nguyen, Mark Quang
United States, US20140294851 A1 2014-10-02 | Language: English, Database: CAplus

Base-labile crosslinkers, base-labile conjugates comprising such crosslinkers, methods of their synthesis and use are disclosed for treatment of cancers, metabolic diseases and infections.

Keywords: cleavable drug conjugate preparation cancer metabolic disease infection

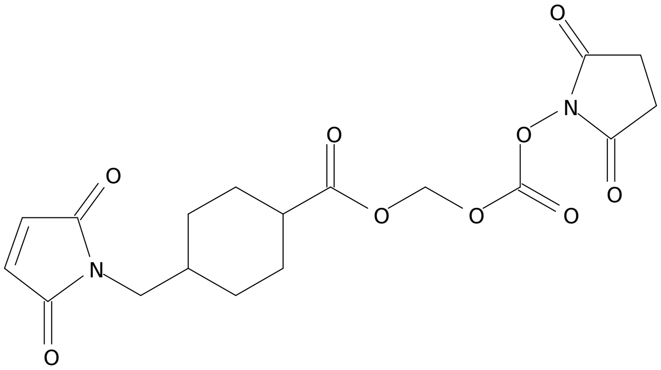

PatentPak available

Substances (286)   Reactions (29)   Citing (0)

233

Indomethacin promotes apoptosis in gastric cancer cells through concomitant degradation of Survivin and Aurora B kinase proteins

By: Chiou, Shiun-Kwei; Hoa, Neil; Hodges, Amy; Ge, Lishen; Jadus, Martin R.
Apoptosis (2014), 19(9), 1378-1388 | Language: English, Database: CAplus and MEDLINE

Regular usage of nonsteroidal anti-inflammatory drugs (NSAIDs) is associated with reduced incidence of a variety of cancers. The mol. mechanisms underlying these chemopreventive effects remain poorly understood. This current investigation showed that in gastric cancer cells: (1) Indomethacin treatment enhanced the degradation of chromosomal passenger proteins, Survivin and Aurora B kinase; (2) Indomethacin treatment down-regulated Aurora B kinase activity in a cell cycle- independent fashion; (3) siRNA knockdown of Survivin level promoted Aurora B kinase protein degradation, and vice versa; (4) ectopic overexp ression of Survivin blocked reduction of Aurora B kinase level and activity by indomethacin treatment, and vice versa; (5) si RNA knockdown of Aurora B kinase level and AZD1152 inhibition of its activity induced apoptosis, and overexp ression of Aurora B kinase inhibited indomet hacin-induced apoptosis; (6) indomethacin treatment reduced Aurora B kinase level, coinciding with reduction of Survivin level and induction of apoptosis, in KATO III and HT-29 cells, and in mouse gastric mucosa. A role for Aurora B kinase function in N SAID-induced apoptosis was not previously explored. Thus this report provides better underst anding of the mol. mechanisms underlying the anti-cancer effect of NSAIDs by elucid ating a significant role for Aurora B kinase in indomet hacin-induced apoptosis.

Keywords: antitumor indomethacin apoptosis gastric cancer survivin aurora B kinase

Substances (6)   Reactions (0)   Citing (13)

234

Preparation of arylquinolines as anticancer agents

By: Courcambeck, Jerome; Bassissi, Firas; Brun, Sonia; Nicolas, Gregory; Beret, Antoine; Petit, Serge; Camus, Claire; Nallet, Jean Pierre; Halfon, Philippe

World Intellectual Property Organization, WO2014147611 A1 2014-09-25 | Language: English, Database: CAplus

The invention provides quinoline derivatives of general formula **I** (wherein R^1 is (un)substituted aryl, (un)substituted heteroaryl, or (un)substituted heteroaromatic 5 to 9-membered ring; R^2 is halo, (un)substituted C_{1-6} alkyl, OH, etc.; W is substituted piperidinyl, etc.; L^1 is a bond, carbonyl, O, etc.; and n = 0- 4) their manufacture, pharmaceutical compositions containing them, and their use as medicaments. The active compounds of the present invention are useful for the treatment of proliferative neoplastic and non-neoplastic diseases. Example compound **II**-2HCl was prepared in a multistep synthesis that culminates in reaction of intermediate **III** with 4-diethylaminopiperidine and subsequent conversion to the salt. The example compounds were tested extensively in anticancer screening assays both in vitro and in vivo. In cell viability assays on A-375, HCT-116, and MOLM-14AML cell lines, there was <20 % cell viability in the presence of **II** at a dose of 10 μ M.

Keywords: arylquinoline preparation anticancer agent

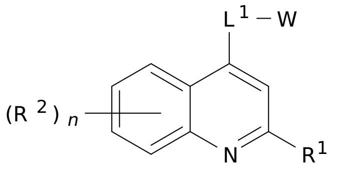

I

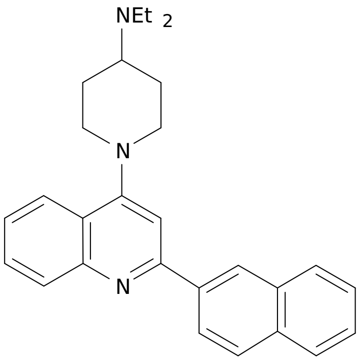

II

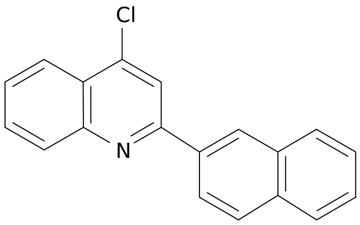

III

PatentPak available

Substances (510)

Reactions (472)

Citing (4)

235

Ligand efficiency based approach for efficient virtual screening of compound libraries

By: Ke, Yi-Yu; Coumar, Mohane Selvaraj ; Shiao, Hui-Yi; Wang, Wen-Chieh; Chen, Chieh-Wen; Song, Jen-Shin; Chen, Chun-Hwa; Lin, Wen-Hsing; Wu, Szu-Huei; Hsu, John T. A.; et al

European Journal of Medicinal Chemistry (2014), 83, 226-235 | Language: English, Database: CAplus and MEDLINE

Here we report for the first time the use of fit quality (FQ), a ligand efficiency (LE) based measure for virtual screening (VS) of compound libraries. The LE based VS protocol was used to screen an inhouse database of 125, 000 compounds to identify aurora kinase A inhibitors. First, 20 known aurora kinase inhibitors were docked to aurora kinase A crystal structure (P DB ID: 2W1C); and the conformations of docked ligand were used to create a pharmac ophore (PH) model. The PH model was used to screen the database compounds, and rank (PH rank) them based on the predicted IC_{50} values. Next, LE_Scale, a weight-dependant LE function, was derived from 294 known aurora kinase inhibitors. Using the fit quality (FQ = LE/LE_Scale) score derived from the LE_Scale function, the database compounds were reranked (PH_FQ rank) and the top 151 (0.12% of database) compounds were assessed for aurora kinase A inhibition biochem. This VS protocol led to the identif ication of 7 novel hits, with compound 5 showing aurora kinase A IC_{50} = 1.29 μM. Furthermore, testing of 5 against a panel of 31 kinase reveals that it is selective toward aurora kinase A & B, with <50% inhibition for other kinases at 10 μM concentrations and is a suitable candidate for further develo pment. Incorporation of FQ score in the VS protocol not only helped identify a novel aurora kinase inhibitor, 5, but also increased the hit rate of the V S protocol by improving the enrichment factor (EF) for FQ based screening (EF = 828), compared to PH based screening (EF = 237) alone. The LE based VS protocol disclosed here could be applied to other targets for hit identif ication in an efficient manner.

Keywords: ligand efficiency virtual screening compound library; Aurora kinase inhibitor; Fit quality; Ligand efficiency; Pharmac ophore model; Virtual screening

Substances (93) Reactions (28) Citing (14)

236

p53 deficiency enhances mitotic arrest and slippage induced by pharmacological inhibition of Aurora kinases

By: Marxer, M.; Ma, H. T.; Man, W. Y.; Poon, R. Y. C.

Oncogene (2014), 33(27), 3550-3560 | Language: English, Database: CAplus and MEDLINE

A number of small-mol. inhibitors of Aurora kinases have been developed and are undergoing clin. trials for anti- cancer therapies. Different Aurora kinases, however, behave as very different targets: while inhibition of Aurora A (AURKA) induces a delay in mitotic exit, inhibition of Aurora B (AURKB) triggers mitotic slippage. Furthe rmore, while it is evident that p53 is regulated by Aurora kinase-dependent phosphorylation, how p53 may in turn regulate Aurora kinases remains myster ious. To address these issues, isogenic p53-containing and -neg. cells were exposed to classic inhibitors that target both A URKA and AURKB (Alisertib and ZM447439), as well as to new generation of inhibitors that target AURKA (MK-5108), AURKB (Barasertib) individually. The fate of individual cells was then tracked with time-lapse microscopy. Remarkably, loss of p53, either by gene disruption or small interf ering RNA-mediated depletion, sensitized cells to inhibition of both AURKA and AURKB, promoting mitotic arrest and slippage resp. As the p53-dependent post-mitotic checkpoint is also important for preventing genome redupli cation after mitotic slippage, these studies indicate that the loss of p53 in cancer cells represents a major opportunity for anti-cancer drugs targeting the Aurora kinases.

Keywords: p53 tumor suppressor mitosis slippage Aurora kinase inhibitor; Alisertib ZM447439 MK5108 Barasertib Aurora kinase inhibitor antitumor p53

Substances (7) Reactions (0) Citing (52)

237

Preparation of heterocyclic compounds as bromodomain inhibitors

By: Liu, Shuang; Duffy, Bryan Cordell; Quinn, John Frederick; Jiang, May Xiaowu; Wang, Ruifang; Martin, Gregory Scott; Zhao, He; Molino, Bruce Francis; Young, Peter Ronald

World Intellectual Property Organization, WO2014096965 A2 2014-06-26 | Language: English, Database: CAplus

The invention related to heterocyclic compounds of formula I, method for their preparation and their use as bromodomain inhibitors and use in therapy. Compounds of formula I, wherein W_1 is N and CR^5; W_2 is N and CR^4; W_3 is N and CR^3; R^1 is carbocycles and heterocycles; R^2 is 5- to 6-membered monocyclic carbocycle and heterocycle; R^3, R^4 and R^5 are independently H, alkyl, OH, carbamate, etc.; X is O and S; A is CO, SO_2, CH_2 and CH_2O, etc.; B is O, NH, S, SO, etc.; and their stereoisomers, tautomers, pharmaceutically acceptable salts, and hydrate, are claimed. Example compound II was prepared by benzylation of 6-chloro-2H-pyridazin-3-one to afford 2-benzyl-6-chloro-2H-pyridazin-3-one, which underwent cross coupling reaction with 3,5-dimethyl-4-(4,4,5,5-tetramethyl-1,3,2-dioxafooroian-2-yl)isoxazole to afford compound II. All the invention compounds were evaluated for their BET inhibitory activity and anticancer activity. From the assay, it was determined that example compound II exhibited IC_{50} value of < 30 μM.

Keywords: heterocycle preparation bromodomain inhibitor

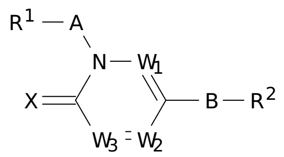

I

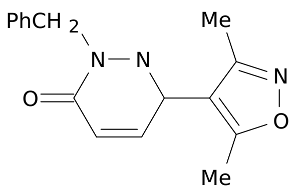

II

PatentPak available

Substances (596) Reactions (686) Citing (8)

238

Preparation of cyclic amines as BET inhibitors for treatment of cancer, autoimmune disorders, and other diseases

By: Fairfax, David John; Duffy, Bryan Cordell; Martin, Gregory Scott; Quinn, John Frederick; Liu, Shuang; Wagner, Gregory Steven; Young, Peter Ronald

United States, US20140142102 A1 2014-05-22 | Language: English, Database: CAplus

The present disclosure relates to compounds and pharmaceutical compositions thereof, which are useful as inhibitors of bromod omain and extra terminal domain (B ET) protein function for treatment or prevention of cancer, autoimmune disorders, inflamm ation, and cardiov ascular diseases. **I** [wherein W^1 is N or CR^1; W^2 is N or CR^2, W^3 is N or CR^3; W^4 is N or CR^4; each W may be the same or different from the others; A is N or CH; R^1, R^2, R^3, and R^4 are indepen dently H, alkyl, aryloxy, etc.; two adjacent substi tuents selected from R^1, R^2, R^3, or R^4 may be connected in a 5- to 6- membered ring to form a bicyclic carbocycle or bicyclic hetero cycle; AR^1 is substituted Ph, substituted pyridinyl, substi tuted furanyl, etc.; B is azirid inyl, (un)substituted azetidinyl, (un)substituted piperi dinyl, etc.] or a stereoisomer, tautomer, pharmace utically acceptable salt, or hydrate thereof are claimed and exempl ified. For example, **II** was prepared in a multistep synthesis (preparation given). **I** were assayed for the ability to inhibit binding of histone H4 and B ET bromodomains, inhibition of c-Myc expression and cell prolife ration in cancer cell lines, and antitumor efficacy in a mouse model of acute myeloid leukemia (data given).

Keywords: cyclic amine preparation BET inhibitor cancer autoimmune disorder

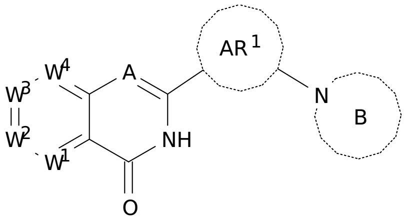

I

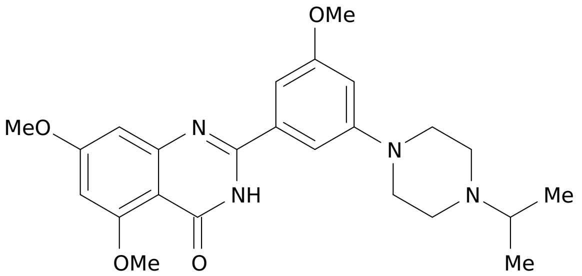

II

PatentPak available

Substances (180) Reactions (225) Citing (0)

239

Preparation of biaryl compounds as BET inhibitors for treatment of cancer, autoimmune disorders, and other diseases

By: Fairfax, David John; Martin, Gregory Scott; Quinn, John Frederick; Duffy, Bryan Cordell; Wagner, Gregory Steven; Young, Peter Ronald

United States, US20140140956 A1 2014-05-22 | Language: English, Database: CAplus

The present disclosure relates to compounds and pharmaceutical compositions thereof, which are useful as inhibitors of bromod omain and extra terminal domain (BET) protein function for treatment or prevention of cancer, autoimmune disorders, inflam matory disorders, and cardiovascular diseases. **I** [wherein W^1 is N or CR^1; W^2 is N or CR^2, W^3 is N or CR^3; W^4 is N or CR^4; each W may be the same or different from the others; A is N or CH; R^1, R^2, R^3, and R^4 are independently H, alkyl, aryloxy, etc.; two adjacent substi tuents selected from R^1, R^2, R^3, or R^4 may be connected in a 5- to 6- membered ring to form a bicyclic carbocycle or bicyclic hetero cycle; AR^1 is a substituted Ph, a substituted pyridinyl, a substituted furanyl, etc.; AR^2 is, for example, Ph, methylsulfonylphenyl, methylsulfinylphenyl, etc.] or a stereoisomer, tautomer, pharmaceutically acceptable salt, or hydrate thereof are claimed and exemplified. Thus, **II** was prepared in 67% yield by the cyclocondensation of **III** with 2-amino-4,6-dimethoxybenzamide followed by deprotection. **I** were assayed for the ability to inhibit binding of histone H4 and BET bromodomains, inhibition of c-Myc expression and cell proliferation in cancer cell lines, and in vivo efficacy in a rat model of collagen- induced arthritis (data given).

Keywords: biaryl derivative BET inhibitor cancer autoimmune disorder

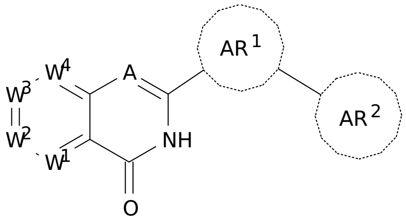

I

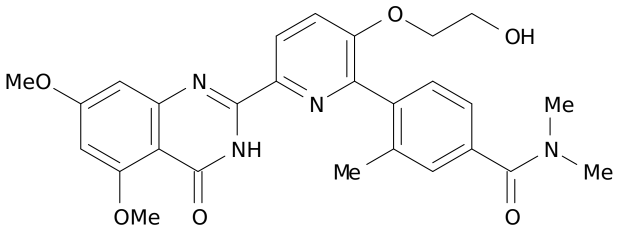

II

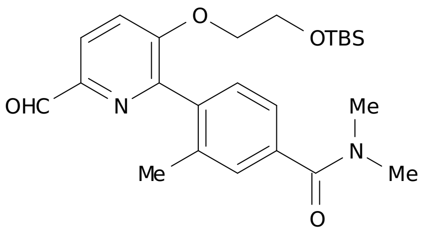

III

PatentPak available

Substances (276) Reactions (1,671) Citing (0)

240

Salt-inducible kinase 3 is a novel mitotic regulator and a target for enhancing antimitotic therapeutic-mediated cell death

By: Chen, H.; Huang, S.; Han, X.; Zhang, J.; Shan, C.; Tsang, Y. H.; Ma, H. T.; Poon, R. Y. C.
Cell Death & Disease (2014), 5(4), e1177 | Language: English, Database: CAplus and MEDLINE

Many mitotic kinases are both critical for maintaining genome stability and are important targets for anticancer therapies. We provide evidence that SIK3 (salt-inducible kinase 3), an AMP-activated protein kinase-related kinase, is important for mitosis to occur properly in mammalian cells. Downregulation of SIK3 resulted in an extension of mitosis in both mouse and human cells but did not affect the DNA damage checkp oint. Time-lapse microscopy and other approaches indicated that mitotic exit but not mitotic entry was delayed. Although repression of SIK3 alone simply delayed mitotic exit, it was able to sensitize cells to various antimi totic chems. Both mitotic arrest and cell death caused by spindle poisons were enhanced after SIK3 depletion. Likewise, the antimitotic effects due to pharmacol. inhibition of mitotic kinases including Aurora A, Aurora B, and polo-like kinase 1 were enhanced in the absence of SIK3. Finally, in addition to promoting the sensit ivity of a small-mol. inhibitor of the mitotic kinesin Eg5, S IK3 depletion was able to overcome cells that developed drug resistance. These results establish the importance of S IK3 as a mitotic regulator and underscore the potential of SIK3 as a druggable antimi totic target.

Keywords: SIK3 mitotic regulator antimitotic drug target mitosis cancer cell

Substances (24) Reactions (0) Citing (15)

241

Structure of Aurora B-INCENP in complex with barasertib reveals a potential transinhibitory mechanism

By: Sessa, Fabio; Villa, Fabrizio
Acta Crystallographica, Section F: Structural Biology Communications (2014), 70(3), 294-298 | Language: English, Database: CAplus and MEDLINE

The Aurora family is a well conserved and well characterized group of serine-threonine kinases involved in the normal progre ssion of mitosis. The deregulation of Aurora kinases impairs spindle assembly, checkpoint function and cell division. To date, many small mols. that compete with ATP for binding to Aurora kinases have been developed and charact erized. Here, the first structure of the Xenopus laevis Aurora B-INCENP complex bound to the clin. relevant small mol. barasertib was determined The binding properties of this inhibitor to the Aurora B active site are analyzed and reported. An unexpected crystal-packing contact in the Aurora B-INCEN P structure coordinated by an ATP analog is also reported, in which the INCENP C-terminus occupies the substrate-binding region, resembling the protein kinase A inhibitory mechanism.

Keywords: Xenopus Aurora B kinase INCENP barasertib complex crystal structure; Aurora B INCENP AMP PNP complex crystal structure; Aurora kinases; PKA; barasertib; cancer; small-molecule inhibitors

Substances (3) Reactions (0) Citing (26)

242

Close correlation between MEK/ERK and Aurora-B signaling pathways in sustaining tumorigenic potential and radioresistance of gynecological cancer cell lines

By: Marampon, Francesco; Gravina, Giovanni Luca; Popov, Valdimir M.; Scarsella, Luca; Festuccia, Claudio; La Verghetta, Maria Emilia; Parente, Silvia; Cerasani, Manuela; Bruera, Gemma; Ficorella, Corrado; et al
International Journal of Oncology (2014), 44(1), 285-294 | Language: English, Database: CAplus and MEDLINE

Both Aurora-A and -B kinases have been implicated in tumorigenesis; and as such, they represent an attractive therapeutic target. Recent studies found that Aurora-A is a downstream target of mitogen-activated protein kinase 1/ERK2, while Aurora-B has been found to be a prognostic/predictive therapeutic target for epithelial cancer. In a wide range of human cancers, the Ras/Raf/MEK/ERK/MAP kinase pathway is enhanced and the cellular response to growth signals is known to increase. The purpose of this study was to investigate whether the MEK/ERK cascade regulates tumorigenic signaling and radioresistance via the Aurora-B-mediated pathway in a panel of gynecol. cancer cell lines. Exponentially growing human endometrial (Ishikawa), cervical (HeLa), cervical (CASKI) and vulva (SiHa) cancer cells were used in culture treated with either control or MEK/ERK inhibitor or AZD1152 before and after irradiation Western blotting, ERK1/2 siRNA transfection, growth assay in modified monolayer, Annexin V and migration/invasion assays were performed. The specific MEK/ERK inhibitor U0126 decreased the tumorigenic potential and improved the radiation response in all cellular models. The modulation of radioresponse upon U0126 treatment pos. correlated with the inhibition of phospho-ERKs and the reduction of Aurora-B kinase expression. In addition, upon U0126 treatment DNA-PKcs protein expression was found to be downregulated, indicating that the improved radiation response may be caused by decreased DNA double-strand damage repair mechanisms. The knockdown of ERK by siRNA confirmed the MEK/ERK-dependent Aurora-B kinase expression. The use of AZD1152, a selective Aurora-B inhibitor, counteracted tumorigenic potential and radioresistance phenotype by highly increasing apoptotic mechanisms in all gynecol. cancer cell lines used. Evidence from our experiments show that tumorigenic potential and radiation response in gynecol. cancer cells may ensue from a MEK/ERK or Aurora-B inhibition. Together with the close correlation of MEK/ERK and Aurora-B protein expression, this study underlines the potential role of a MEK/ERK/Aurora-B axis whose interruption recovers the antitumor effects of radiotherapy.

Keywords: radiotherapy MEK ERK AuroraB gynecol cancer

Substances (7) Reactions (0) Citing (35)

243

Aurora kinase inhibitor patents and agents in clinical testing: an update (2011 - 2013)

By: Cheung, Chun Hei Antonio; Sarvagalla, Sailu; Lee, Jane Ying-Chieh; Huang, Yi-Chun; Coumar, Mohane Selvaraj
Expert Opinion on Therapeutic Patents (2014), 24(9), 1021-1038 | Language: English, Database: CAplus and MEDLINE

A review. Introduction: Aurora kinase A, B and C, members of serine/threonine kinase family, are key regulators of mitosis. As Aurora kinases are overexpressed in many of the human cancers, small-mol. inhibitors of Aurora kinase have emerged as a possible treatment option for cancer. Areas covered: In 2009 and 2011, the literature pertaining to Aurora kinase inhibitors and their patents was reviewed. Here, the aim is to update the information for Aurora kinase inhibitors in clin. trials and the patents filed between the years 2011 and 2013. Pubmed, Scopus, Scifinder, USPTO, EPO and www.clinicaltrials.gov databases were used for searching the literature and patents for Aurora kinase inhibitors. Expert opinion: Even though both Aurora sub-type selective as well as pan-selective inhibitors show preclin. and clin. efficacy, so far no Aurora kinase inhibitor has been approved for clin. use. Particularly, dose-limiting toxicity (neutropenia) is a key issue that needs to be addressed. Preliminary evidence suggests that the use of selective Aurora A inhibitors could avoid Aurora B-mediated neutropenia in clin. settings. Also, use of adjunctive agents such as granulocyte stimulating factor to overcome neutropenia associated with Aurora B inhibition could be an answer to overcome the toxicity and bring Aurora inhibitors to market in the future.

Keywords: Aurora kinase inhibitor patent agent review; AMG 900 and KW-2449; AT-9283; AZD1152; Aurora kinase inhibitor; CYC116; ENMD-2076; GSK1070916; MLN8054; MLN8237; PF-3814735; PHA739358; R763; SNS-314; VX-680/MK0457; VX-689/MK-5108

Substances (14) Reactions (0) Citing (44)

244

Aurora kinases as druggable targets in pediatric leukemia: heterogeneity in target modulation activities and cytotoxicity by diverse novel therapeutic agents

By: Jayanthan, Aarthi; Ruan, Yibing; Truong, Tony H.; Narendran, Aru

PLoS One (2014), 9(7), e102741/1-e102741/12, 12 pp. | Language: English, Database: CAplus and MEDLINE

Leukemia is the most common pediatric malignancy, constituting more than 30% of all childhood cancers. Although cure rates have improved greatly, approx. one in five children relapse and poor survival rates post relapse remain a challenge. Given this, more effective and innovative therapeutic strategies are needed in order to improve prognosis. Aurora kinases, a family of serine/t hreonine kinases essential for the regulation of several mitotic processes, have been identified as potential targets for cancer therapeutics. Elevated expression of Aurora kinases has been demonstrated in several malignancies and is associated with aberrant mitotic activity, aneuploidy and alterations in chromosomal structure and genome instability. Based on this rationale, a number of small mol. inhibitors have been formulated and advanced to human studies in the recent past. A comparative anal. of these agents in cytotoxicity and target modulation analyses against a panel of leukemia cells provides novel insights into the unique mechanisms and codependent activity pathways involved in targeting Aurora kinases, consti tuting a distinctive preclin. exptl. framework to identify appropriate agents and combinations in future clin. studies.

Keywords: modulation cytotoxicity therapeutic agent aurora kinase pediatric leukemia heterog eneity

Substances (14) Reactions (0) Citing (13)

245

Identification of Aurora-A Inhibitors by Ligand and Structure-Based Virtual Screening

By: Morshed, Mohammad Neaz

Molecular Informatics (2014), 33(5), 369-381 | Language: English, Database: CAplus and MEDLINE

Aurora kinase A has been identified as one of the most attractive targets for cancer therapy because of its critical role in the regulation of the cell cycle. In order to identify active compounds with structural diversity we performed virtual screening. 3D-QSAR pharmacophore models were developed and the best model was used as a query for screening the databases. Ligand and structure-based virtual screening protocol was conducted sequentially by applying the common feature pharmacophore and mol. docking to discover potent Aurora-A inhibitors. A total of eighty-eight compounds were selected for the in vitro activities against various human cancer cell lines (DU145 and HT29). Considering the activity data, we have identified seven compounds to be considered for the next step, among which four compounds had high inhibition rate (above 50 %) at 10 μM with GI_{50} lower than 10 μM. Based on the cell line and enzyme assay (Aurora- A & B) result, these four compounds were used as templat e/query mol. for similarity search. The best result was obtained for similarity hit SH3. It had IC_{50} of 0.578 and 11.77 μ M for Aurora-A and B resp., which implies 20-fold selectivity over Aurora-B. The hits obtained from this screening scheme could be potential drug candidates after further optimization.

Keywords: Aurora A inhibitor anticancer drug discovery mol docking cancer; Anticancer; Aurora-A; Docking; Inhibitor; Pharmac ophore; Tumor cell; Virtual screening

Substances (45) Reactions (0) Citing (0)

246

Discovery of 7-Aryl-Substituted (1,5-Naphthyridin-4-yl)ureas as Aurora Kinase Inhibitors

By: Defaux, Julien; Antoine, Maud; Le Borgne, Marc; Schuster, Tilmann; Seipelt, Irene; Aicher, Babette; Teifel, Michael; Guenther, Eckhard; Gerlach, Matthias; Marchand, Pascal
ChemMedChem (2014), 9(1), 217-232 | Language: English, Database: CAplus and MEDLINE

As part of our research projects to identify new chem. entities of biol. interest, we developed a synthetic approach and the biol. evaluation of (7-aryl-1,5-naphthyridin-4-yl)ureas as a novel class of Aurora kinase inhibitors for the treatment of malignant diseases based on pathol. cell proliferation. 1,5-Naphthyridine derivatives showed excellent inhibitory activities toward Aurora kinases A and B, and the most active compound, 1-cyclopropyl-3-[7-(1-methyl-1H-pyrazol-4-yl)-1,5-naphthyridin-4-yl]urea (49), displayed IC_{50} values of 13 and 107 nm against Aurora kinases A and B, resp. In addition, the selectivity toward a panel of seven cancer-related protein kinases was highlighted. In vitro ADME properties were also determined in order to rationalize the difficulties in correlating antiproliferative activity with Aurora kinase inhibition. Finally, the good safety profile of these compounds imparts promising potential for their further development as anticancer agents.

Keywords: naphthyridinyl urea derivative preparation Aurora kinase inhibitor cancer; 1,5-naphthyridines; aurora kinases; heterocycles; inhibitors; ureas

Substances (95) | Reactions (529) | Citing (10)

247

Novel pharmaceutical combinations and methods for treating cancer

By: Cheok, Chit Fang; Lane, David
United States, US20130344140 A1 2013-12-26 | Language: English, Database: CAplus

Methods of selectively targeting a p53-deficient cancer cell, comprising administering to a patient suffering from cancer (i) a reversible cell cycle arrest-inducing agent for inducing cell cycle arrest in a p53- pos. cell; and (ii) an aurora kinase inhibitor, wherein said reversible cell cycle arrest-inducing agent is administered prior to administration of said aurora kinase inhibitor, and pharmaceutical combinations, kits and oral dosage forms for the same.

Keywords: p53 deficient cancer treatment pharmaceutical combination; aurora kinase inhibitor cell cycle arrest inducer cancer treatment

PatentPak available

Substances (27) | Reactions (0) | Citing (0)

248

Modulators of GTPase and use in relevant treatment

By: Wandinger-Ness, Angela; Sklar, Larry; Surviladze, Zurab; Oprea, Tudor; Hudson, Laurie; Aube, Jeffrey; Golden, Jennifer E.; Schroeder, Chad E.; Simpson, Denise S.; Noth, Julica J.
United States, US20130345277 A1 2013-12-26 | Language: English, Database: CAplus

The invention relates to pyrazole derivatives which function as selective modulators of the Ras- homologous (Rho) family of small G T Pases, in particular, Cdc42 GTPase and their use to treat diseases, for example cancers, including metastatic cancer, genetic and acquired diseases where activation of Cdc42 GTPase plays a pivotal role, such as neurodege nerative diseases, rheumatoid arthritis, atherosclerosis, diabetes type I, autosomal polycystic kidney disease, cystic kidney disease, precystic kidney disease, microbial infect ions, including Chlamydia infections, E. coli infections, H. pylori infections and its secondary effects including gastric ulcers, Coxiella Brunetti (Q-fever) infections and Strepto coccus pneumonia infections, fungal infections including Paracocc idiodes brasiliensis and Candida albicans and their secondary effects including lung edema. Addnl., compounds according to the invention may be used to inhibit rejection in transplant patients (pursuant to transplantation), to promote immunosuppression, anti-inflammatory response and to mobilize stem cell (migration) in patients in need. Also disclosed is the preparation of compounds of the invention.

Keywords: pyrazole derivative preparation GTPase cancer neurodegenerative disease microbial infection; transplant rejection inflammation immunosuppression stem cell migration pyrazole derivative

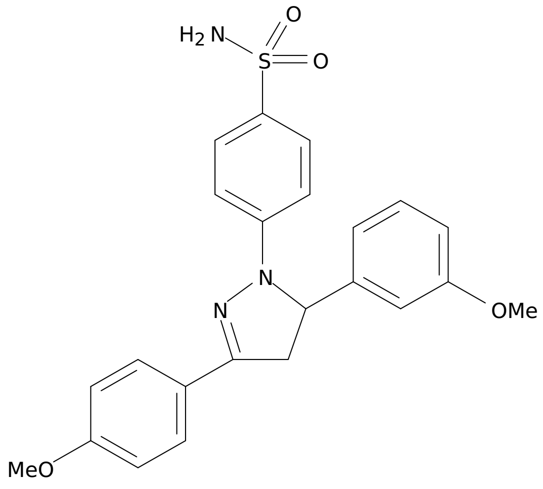

PatentPak available

Substances (325) Reactions (25) Citing (1)

249

Method for preparing barasertib

By: Xu, Xuenong
China, CN103435598 A 2013-12-11 | Language: Chinese, Database: CAplus

The invention relates to method the method for preparing barasertib. Barasertib was prepared via conden sation of 7-{3-[ethyl(2-hydroxyethyl)amino]propoxy}-3,4-dihydroquinazolin-4-one with 2-(5-amino-1H-pyrazol-3-yl)-N-(3-fluorophenyl)acetamide in the presences of organic base and condensing agent. The method has the advantages of simple process, mild conditions, good enviro nment, and high product quality, and is adapted to industrial production

Keywords: hydroxyethyl aminopropoxy quinazolinone amino pyrazolyl fluorophenyl acetamide condensation; barasertib prepar ation green chem

PatentPak available

Substances (3) Reaction (1) Citing (0)

250

Preparation of bifunctional compounds useful for promoting protein degradation for treatment of cancer and oxidative stress-associated diseases

By: Crews, Craig; Gustafson, Jeff; Roth, Anke Gundula; Tae, Hyun Seop; Buckley, Dennis; Neklesa, Taavi
World Intellectual Property Organization, WO2013170147 A1 2013-11-14 | Language: English, Database: CAplus

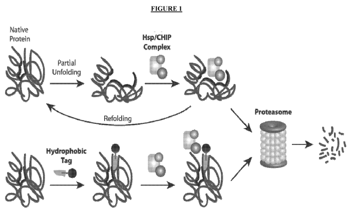

The invention relates to compounds that act as degraders of a target protein, independent of the protein class or its localization for use in treatment of diseases including cancer and oxidative stress-associated disorders. Compounds of formula L-DM [wherein DM is a protein degradation moiety covalently bound to a linker (L) which is a functional group forming a covalent bond to a protein binding moiety] and their pharmaceutically acceptable salts, solvates, or polymorphs thereof having a Clog P equal to or higher than 1.5 are claimed and prepared For example, the compound of formula **I** was prepared in 30% yield via the reaction of **II** with 2-(2-aminoethoxy)ethyl 2-(adamantan-1-yl)acetate. Compounds of the invention exhibit a broad range of pharmacol. activities consistent with the degradation/inhibition of targeted proteins. This was illustrated in an assay in which combined administration of selective androgen receptor degrader **I** (250 nM) and HSP90 inhibitor geldanamycin (100 nM) to LnCAP cells resulted in additive inhibition of androgen receptors compared to either compound alone.

Keywords: bifunctional compound preparation protein degradation cancer oxidative stress disease

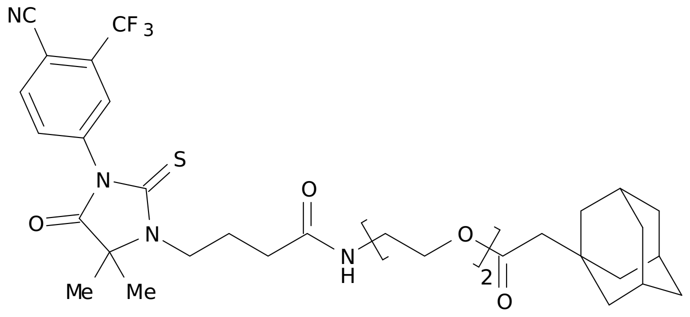

I

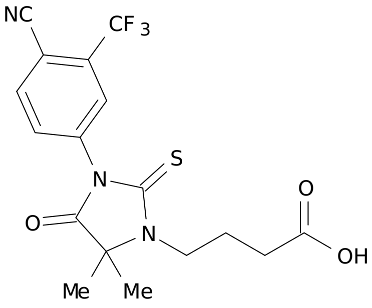

II

PatentPak available
Substances (569)
Reactions (406)
Citing (8)

251

Phase I Study Assessing the Safety and Tolerability of Barasertib (AZD1152) With Low-Dose Cytosine Arabinoside in Elderly Patients With AML

By: Kantarjian, Hagop M.; Sekeres, Mikkael A.; Ribrag, Vincent; Rousselot, Philippe; Garcia-Manero, Guillermo; Jabbour, Elias J.; Owen, Kate; Stockman, Paul K.; Oliver, Stuart D.

Clinical Lymphoma, Myeloma & Leukemia (2013), 13(5), 559-567 | Language: English, Database: CAplus and MEDLINE

Barasertib is the pro-drug of barasertib-hydroxy-quinazoline pyrazole anilide, a selective Aurora B kinase inhibitor that has demonstrated preliminary anti-AML activity in the clin. setting. This Phase I dose- escalation study evaluated the safety and tolera bility of barasertib, combined with LDAC, in patients aged 60 years or older with de novo or secondary A ML. Barasertib (7-day continuous i.v. infusion) plus LDAC 20 mg (s.c. injection twice daily for 10 days) was admini stered in 28-day cycles. The MTD was defined as the highest dose at which ≤ 1 patient within a cohort of 6 experienced a dose-limiting toxicity (DLT) (clin. significant adverse event [AE] or laboratory abnormality considered related to barasertib). The MTD cohort was expanded to 12 patients. Twenty-two patients (median age, 71 years) received ≥ 1 treatment cycle (n = 6, 800 mg; n = 13, 1000 mg; n = 3, 1200 mg) .. DLTs were reported in 2 patients (both, National Cancer Institute Common Terminol. Criteria for Adverse Events grade 3 stomatitis/muc ositis; 1200 mg cohort). The most common A Es were infection (73%), febrile neutropenia (59%), nausea (50%), and diarrhea (46%). Barasertib plus LDAC resulted in an overall response rate (Interna tional Working Group criteria) of 45% (n = 10/22; according to investigator opinion). The MTD of 1000 mg barasertib in combination with LDAC in older patients with A ML was associated with acceptable tolerability and preliminary anti-AML activity.

Keywords: barasertib cytosine arabinoside antitumor pharmaco kinetics acute myeloid leukemia elderly; Acute myeloid leukemia; Barasertib-hQPA; Dose-escalation study

Substances (3) Reactions (0) Citing (40)

252

IGF1 biomarker for IGF1R inhibitor therapy of tumors

By: Sathyanarayanan, Sriram; Ayers, Mark

World Intellectual Property Organization, WO2013148568 A1 2013-10-03 | Language: English, Database: CAplus

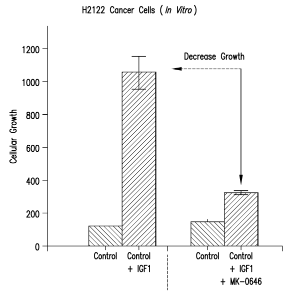

The present invention provides, inter alia, methods for treating tumors that are sensitive to an IGF1R inhibitor. The tumor are determined to be sensitive if the level of IGF1 mRNA expression in the tumor cells, relative to one or more reference genes, reaches a certain threshold level. Methods for evaluating patients as candidates for receipt of the IGF1R inhibitor therapy are also provided as well along with in vitro assay methods and kits for performing any of the methods.

Keywords: IGF1 biomarker IGF1R inhibitor therapy tumor

PatentPak available

Substances (349) Reactions (0) Citing (1)

253

Target inhibition networks: predicting selective combinations of druggable targets to block cancer survival pathways

By: Tang, Jing; Karhinen, Leena; Xu, Tao; Szwajda, Agnieszka; Yadav, Bhagwan; Wennerberg, Krister; Aittokallio, Tero
PLoS Computational Biology (2013), 9(9), e1003226 | Language: English, Database: CAplus and MEDLINE

A recent trend in drug development is to identify drug combin ations or multi-target agents that effectively modify multiple nodes of disease-associated networks. Such polypharmacol. effects may reduce the risk of emerging drug resistance by means of attacking the disease networks through synergistic and synthetic lethal interactions. However, due to the exponentially increasing number of potential drug and target combinations, systematic approaches are needed for priori tizing the most potent multi-target alternatives on a global network level. We took a functional systems pharmacol. approach toward the identification of selective target combin ations for specific cancer cells by combining large- scale screening data on drug treatment efficacies and drug- target binding affini ties. Our model-based prediction approach, named TIMMA, takes advantage of the polypha rmacol. effects of drugs and infers combinatorial drug efficacies through system- level target inhibition networks. Case studies in M CF-7 and MDA-MB-231 breast cancer and BxPC-3 pancreatic cancer cells demonstrated how the target inhibition modeling allows systematic explor ation of functional interactions between drugs and their targets to maximally inhibit multiple survival pathways in a given cancer type. The T I MMA prediction results were exptl. validated by means of systematic si RNA-mediated silencing of the selected targets and their pairwise combinations, showing increased ability to identify not only such druggable kinase targets that are essential for cancer survival either individually or in combination, but also synergistic interactions indicative of non-additive drug efficacies. These system-level analyses were enabled by a novel model constr uction method utilizing maximization and minimization rules, as well as a model selection algorithm based on sequential forward floating search. Compared with an existing computational solution, TIMM A showed both enhanced prediction accuracies in cross validation as well as signif icant reduction in computation times. Such cost-effective computational-exptl. design strategies have the potential to greatly speed- up the drug testing efforts by priori tizing those interventions and interactions warranting further study in individual cancer cases.

Keywords: algorithm anticancer drug target inhibition network prognosis breast carcinoma

Substances (56) Reactions (0) Citing (62)

254

Selectivity Data: Assessment, Predictions, Concordance, and Implications

By: Gao, Cen; Cahya, Suntara; Nicolaou, Christos A.; Wang, Jibo; Watson, Ian A.; Cummins, David J.; Iversen, Philip W.; Vieth, Michal
Journal of Medicinal Chemistry (2013), 56(17), 6991-7002 | Language: English, Database: CAplus and MEDLINE

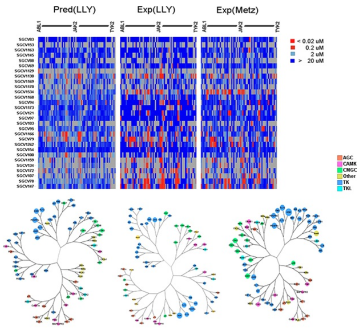

Could high-quality in silico predictions in drug discovery eventually replace part or most of exptl. testing. To evaluate the agreement of selectivity data from different exptl. or predictive sources, we introduce the new metric concor dance min. significant ratio (cMSR). Empowered by cMSR, we find the overall level of agreement between predicted and exptl. data to be comparable to that found between exptl. results from different sources. However, for mols. that are either highly selective or potent, the concordance between different exptl. sources is significantly higher than the concor dance between exptl. and predicted values. We also show that computational models built from one data set are less predictive for other data sources and highlight the importance of bias correction for assessing selectivity data. Finally, we show that small- mol. target space relatio nships derived from different data sources and predictive models share overall similarity but can significantly differ in details.

Keywords: selectivity data exptl predictive source drug discovery modeling

Substances (27) Reactions (0) Citing (10)

255

Aurora B inhibitor barasertib and cytarabine exert a greater-than-additive cytotoxicity in acute myeloid leukemia cells

By: Yamauchi, Takahiro; Uzui, Kanako; Shigemi, Hiroko; Negoro, Eiju; Yoshida, Akira; Ueda, Takanori

Cancer Science (2013), 104(7), 926-933 | Language: English, Database: CAplus and MEDLINE

Barasertib, an aurora B inhibitor, terminates cell division, introduces polypl oidy, and consequently causes apoptosis. In the present study, we evaluated the effect of the combination of barasertib and cytarabine (ara-C), a key agent for leukemia chemoth erapy, on leukemic cells in vitro. Human leukemia HL-60 cells and HL-60/ara-C20 cells, a 20- fold ara-C-resistant variant, were used. The 50% growth inhibitory concentrations of an active metabolite of barasertib, barasertib-hydroxyquinazoline-pyrazol-aniline (Barasertib-HQPA), and ara-C were 51 nM and 300 nM for HL-60 cells and 70 nM and 5300 nM for HL-60/ara-C20 cells, resp. Barasertib-HQPA induced polyploidy with a subsequent induction of sub-G1 phase apoptosis, indicating the M-phase specific cytotoxicity. Cells treated with the S-phase specific ara-C accumulated in S phase and subseq uently died through apoptosis. When HL-60 cells were treated with barasertib-HQPA and ara-C in combination, a greater-than-additive apoptosis was induced. This enhanc ement was obtained when the cells were treated with barasertib-HQPA prior to ara-C (37.9% sub-G1) or with both concur rently (31.2% sub-G1), but not with ara-C prior to barasertib-HQPA (17.8% sub-G1). The combination effects were similarly obtained in HL-60/ara-C20 cells with 19.7% sub-G1 for barasertib-HQPA→ara-C, 18.4% sub-G1 for both concurrently, and 13.8% sub-G1 for ara-C→barasertib-HQP A, and another leukemic U937 cells with 25.4% sub-G1 for barasertib-HQPA→ara-C, 28.2% sub-G1 for both concurrently, and 16.0% sub-G1 for ara-C→barasertib-HQPA. Barasertib-HQPA inhibited aurora B autophosph orylation and histone H3 phosphor ylation in all the cell lines. Barasertib-HQPA did not inhibit DNA synthesis, allowing ara-C incorporation into DNA for its cytotox icity. Thus, barasertib-HQPA and ara-C provided a greater-than-additive cytotoxicity in leukemic cells in vitro.

Keywords: barasertib hydroxyquinazoline cytarabine e additive acute myeloid leukemia antitumor

Substances (6) Reactions (0) Citing (22)

256

Compounds and methods for the enhanced degradation of targeted proteins and other polypeptides by an E3 ubiquitin ligase, particularly targeting the von Hippel-Lindau E3 ubiquitin ligase using hydroxyproline analogs to disrupt the VHL/HIF-1α interaction

By: Crews, Craig M.; Buckley, Dennis; Ciulli, Alessio; Jorgensen, William; Gareiss, Peter C.; Van Molle, Inge; Gustafson, Jeffrey; Tae, Hyun-Seop; Michel, Julien; Hoyer, Dentin Wade; et al
World Intellectual Property Organization, WO2013106643 A2 2013-07-18 | Language: English, Database: CAplus

The invention is related to bifunctional compounds, e.g., **I**, which find utility as modulators of targeted ubiquitination, especially inhibitors of a variety of polypeptides and other proteins which are degraded and/or otherwise inhibited by bifunctional compounds according to the present invention. In particular, the invention is related to compounds, which contain on one end a von Hippel-Lindau (VHL) ligand which binds to the ubiquitin ligase and on the other end a moiety which binds a target protein such that the target protein is placed in proximity to the ubiquitin ligase to effect degradation (and inhibition) of that protein. The invention exhibits a broad range of pharmacol. activities associated with the compounds of the invention, consistent with the degradation/inhibition of targeted polypeptides. Thus, **II** bound to VHL with a 4.1 μM IC_{50} value. **II** was cocrystd. with VHL and shown to bind at the HIF-1α binding site.

Keywords: hydroxyproline analog preparation ubiquitin ligase inhibitor; chimeric mol preparation targeted protein degradation ubiquitination

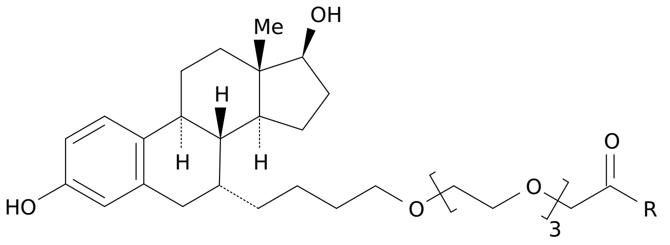

I

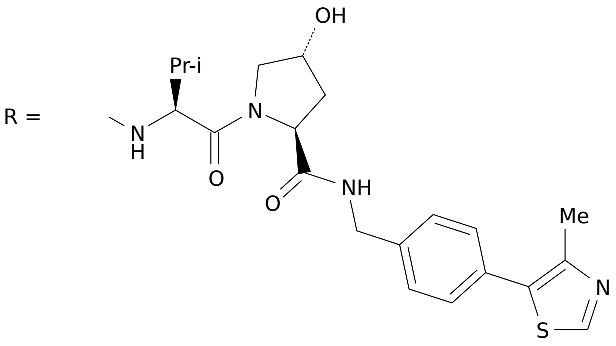

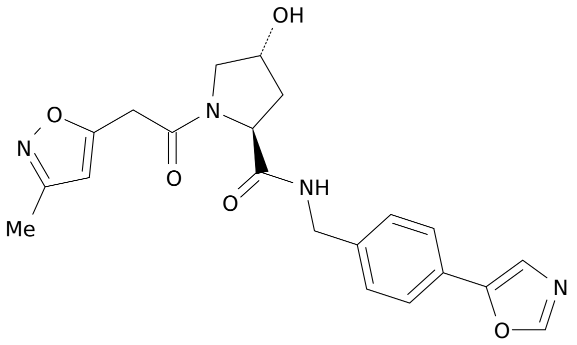

II

PatentPak available

Substances (1,211)   Reactions (2,697)   Citing (19)

257

Stage I of a phase 2 study assessing the efficacy, safety, and tolerability of barasertib (AZD1152) versus low-dose cytosine arabinoside in elderly patients with acute myeloid leukemia

By: Kantarjian, Hagop M.; Martinelli, Giovanni; Jabbour, Elias J.; Quintas-Cardama, Alfonso; Ando, Kiyoshi; Bay, Jacques-Olivier; Wei, Andrew; Groepper, Stefanie; Papayannidis, Cristina; Owen, Kate; et al

Cancer (Hoboken, NJ, United States) (2013), 119(14), 2611-2619 | Language: English, Database: CAplus and MEDLINE

BACKGROUND : In this phase 2 study, the authors evaluated the efficacy, safety, and tolera bility of the Aurora B kinase inhibitor barasertib compared with low-dose cytosine arabin oside (LDAC) in patients aged ≥60 years with acute myeloid leukemia (A ML). ME THODS : Patients were randomized 2: 1 to receive either open-label barasertib 1200 mg (as a 7- day i.v. infusion) or LDAC 20 mg (s.c. twice daily for 10 days) in 28-day cycles. The primary endpoint was the objective complete response rate (O CRR) (complete responses [CR] plus confirmed CRs with incomplete recovery of neutro phils or platelets [CRi] according to Cheson criteria [also requiring reconfirmation of CRi ≥21 days after the first appearance and associated with partial recovery of platelets and neutro phils]). Secondary endpoints included overall survival (O S) and safety. R ESULTS : In total, 74 patients (barasertib, n=48; LDAC, n=26) completed ≥1 cycle of treatment. A significant improvement in the OCRR was observed with barasertib (35.4% vs 11.5%; differ ence, 23.9%; 95% confidence interval, 2.7%-39.9%; P<.05). Although the study was not formally sized to compare O S data, the median O S with barasertib was 8.2 mo vs. 4.5 mo with L DAC (hazard ratio, 0.88; 95% confidence interval, 0.49- 1.58; P=.663). Stomatitis and febrile neutropenia were the most common adverse events with barasertib vs. LDAC (71% vs 15% and 67% vs 19%, resp.) . CONCLU SIONS : Barasertib produced a signif icant improvement in the OCRR vs. LDAC and had a more toxic but manageable safety profile, consistent with previous studies. Cancer 2013;119:2611-2619. © 2013 American Cancer Society.

Keywords: antileukemic barasertib cytosine arabin oside dose efficacy safety tolera bility elderly; acute myeloid leukemia; A ZD1152; barasertib; low-dose cytosine arabin oside

Substances (2) Reactions (0) Citing (76)

258

CRLF-2 binding peptides, protocells and viral-like particles useful in the treatment of cancer, including acute lymphoblastic leukemia

By: Brinker, C. Jeffrey; Peabody, David S.; Wharton, Walker; Chackerian, Bryce; Ashley, Carlee Erin; Willman, Cheryl L.; Carnes, Eric C.; Epler, Katherine; Castillo, Robert Eric

World Intellectual Property Organization, WO2013103614 A1 2013-07-11 | Language: English, Database: CAplus

The present invention relates to the use of which are attached or anchored phospholipid biolayers further modified by C RLF-2 and CD 19 binding peptides which may be used for delivering pharmac eutical cargos, to cells expressing CRLF-2 and CD 19, thereby treating cancer, in particular, acute lymphob lastic leukemia, including B- precursor acute lymphob lastic leukemia. Novel CRLF-2 binding peptides and CLRF-2 and CD19-binding viral-like particles (VLPs) useful in the treatment of cancer, including A LL are also provided.

Keywords: CRLF2 binding peptide protocell virus like particle cancer drug; acute lymphob lastic leukemia drug C RLF2 binding peptide

PatentPak available

Substances (335) Reactions (0) Citing (5)

259

Profiling pathway-specific novel therapeutics in preclinical assessment for central nervous system atypical teratoid rhabdoid tumors (CNS ATRT): Favorable activity of targeting EGFR- ErbB2 signaling with lapatinib

By: Singh, Anjali; Lun, Xueqing; Jayanthan, Aarthi; Obaid, Halah; Ruan, Yibing; Strother, Douglas; Chi, Susan N.; Smith, Amy; Forsyth, Peter; Narendran, Aru
Molecular Oncology (2013), 7(3), 497-512 | Language: English, Database: CAplus and MEDLINE

Despite intensifying multimodal treatments, children with central nervous system atypical teratoid/ rhabdoid tumor (CNS ATRT) continue to endure unacceptably high mortality rates. At present, concerted efforts are focusing on underst anding the charact eristic INI1 mutation and its implic ations for the growth and survival of these tumors. Addnl., pharmac eutical pipeline libraries constitute a significant source of potential agents that can be taken to clin. trials in a timely manner. However, this process requires efficient target validation and relevant preclin. studies. As an initial screening approach, a panel of 129 small mol. inhibitors from multiple pharmaceutical pipeline libraries was tested against three A TRT cell lines by in vitro cytoto xicity assays. Based on these data, agents that have strong activity and corresponding susceptible cellular pathways were identified. Target modulation, antibody array anal., drug combination and in vivo xenograft studies were performed on one of the pathway inhibitors found in this screening. Approx. 20% of agents in the library showed activity with IC_{50} values of 1 μM or less and many showed IC_{50} values less than 0.05 μM. Intra cell line variability was also noted among some of the drugs. However, it was determined that agents capable of affecting pathways constituting ErbB2, mTOR, proteasomes, Hsp90, Polo like kinases and Aurora kinases were univer sally effective against the three ATRT cell lines. The first target selected for further anal., the inhibition of Erb B2-EGFR pathway by the small mol. inhibitor lapatinib, indicated inhibition of cell migration properties and the initiation of apoptosis. Synergy between lapatinib and IG F-IR inhibition was also demonstrated by combin ation index (CI) values. Xenograft studies showed effective antitumor activity of lapatinib in vivo. We present an exptl. approach to identifying agents and drug combin ations for future clin. trials and provide evidence for the potential of lapatinib as an effective agent in the context of the biol. and heterogeneity of its targets in A TRT.

Keywords: therapeutic target central nervous system atypical teratoid rhabdoid tumor; anticancer lapatinib E GFR ErbB2 signaling

Substances (34)   Reactions (0)   Citing (29)

260

Aurora kinase inhibitors: potential molecular-targeted drugs for gynecologic malignant tumors (review)

By: Umene, Kiyoko; Banno, Kouji; Kisu, Iori; Yanokura, Megumi; Nagomi, Yuya; Tsuji, Kosuke; Masuda, Kenta; Ueki, Arisa; Kobayashi, Yusuke; Yamagami, Wataru; et al
Biomedical Reports (2013), 1(3), 335-340 | Language: English, Database: CAplus and MEDLINE

A review. Chemotherapy and surgery are important treatment strategies for gynecol. malignant tumors such as ovarian, cervical and endometrial cancer. However, many anticancer drugs currently available are cytotoxic and cause strong adverse reactions in patients. Aurora kinases have attracted increasing attention in recent years as serine/threonine kinases with various roles in cell division, including chromosomal agglutination and segregation, functions of centro meres, centrosomal maturation, spindle formation and cytokinesis. Aurora kinases are overexpressed in a number of cancers and recent studies have shown that they are involved in oncogenesis and cause an aberrant increase in centrosome number, emergence of polykar yocytes and failure of cancer inhibition mechanisms. Thus, drugs that inhibit Aurora kinases are likely to exert anticancer effects in various fields, including the gynecol. field. Aurora kinase inhibitors exert antitumor effects in monotherapy and synerg istic effects in combination therapy with taxane-based anticancer agents for gynecol. tumors and are likely to increase the efficacy of existing anticancer drugs. Current Aurora kinase inhibitors include ZM447439, Hesperadin, VX-680/MK-0457, AT9283 and Barasertib , and clin. trials are ongoing to verify the effects of these inhibitors.

Keywords: review aurora kinase inhibitor target gynecol malignant tumor; Aurora kinase A; Aurora kinase inhibitor; VX-680; Z M447439; gynecologic cancer; hesperadin

Substance (1)   Reactions (0)   Citing (25)

261

Method of determination of cancer cell drug sensitivity towards Aurora kinase inhibitors and overcoming their resistance comprising determination of gene or protein expression in patient sample

By: Kollaredy, Madhusudhan Reddy; Hajduch, Marian; Dzubak, Petr; Srovnal, Josef; Hrabakova, Rita; Kovarova, Hana
World Intellectual Property Organization, WO2013083098 A2 2013-06-13 | Language: English, Database: CAplus

The present invention relates to a method for determining the sensitivity and/or resistance of a patient suffering from a cancer disease to Aurora kinase inhibitor therapy, which comprises determining in vitro in the cancer cells or body fluids taken from the patient the expression of at least one gene selected from a particular group and/or determining in vitro in the cancer cells or body fluids taken from the patient the level of at least one protein selected from a particular group.

Keywords: cancer drug sensitivity Aurora kinase inhibitor gene protein expression

PatentPak available

Substances (54) | Reactions (0) | Citing (2)

262

Method of determination of cancer cell drug sensitivity towards Aurora kinase inhibitors and overcoming their resistance comprising determination of gene or protein expression in patient sample

By: Kollareddy, Madhusudhan Reddy; Hajduch, Marian; Dzubak, Petr; Srovnal, Josef; Hrabakova, Rita; Kovarova, Hana
European Patent Organization, EP2602330 A1 2013-06-12 | Language: English, Database: CAplus

The present invention relates to a method for determining the sensitivity and/or resistance of a patient suffering from a cancer disease to Aurora kinase inhibitor therapy, which comprises determining in vitro in the cancer cells or body fluids taken from the patient the expression of at least one gene selected from a particular group and/or determining in vitro in the cancer cells or body fluids taken from the patient the level of at least one protein selected from a particular group. Also, the invention relates to a group of compounds which allow overcoming the resistance.

Keywords: cancer drug sensitivity Aurora kinase inhibitor gene protein expression

PatentPak available

Substances (54) | Reactions (0) | Citing (0)

263

SAR and evaluation of novel 5H-benzo[c][1,8]naphthyridin-6-one analogs as Aurora kinase inhibitors

By: Karra, Srinivasa; Xiao, Yufang; Chen, Xiaoling; Liu-Bujalski, Lesley; Huck, Bayard; Sutton, Amanda; Goutopoulos, Andreas; Askew, Ben; Josephson, Kristopher; Jiang, Xuliang; et al
Bioorganic & Medicinal Chemistry Letters (2013), 23(10), 3081-3087 | Language: English, Database: CAplus and MEDLINE

Several potent Aurora kinase inhibitors derived from 5H-benzo[c][1,8]naphthyridin-6-one scaffold were identified. A crystal structure of Aurora kinase A in complex with an initial hit revealed a binding mode of the inhibitor within the ATP binding site and provided insight for structure-guided compound optimization. Subsequent SAR campaign provided a potent and selective pan Aurora inhibitor, which demonstrated potent target modulation and antiproliferative effects in the pancreatic cell line, MIAPaCa-2. Furthermore, this compound inhibited phosphorylation of histone H3 (pHH3) in mouse bone morrow upon oral administration, which is consistent with inhibition of Aurora kinase B activity.

Keywords: preparation structure benzo naphthyridinone analog Aurora kinase inhibitor antitumor

Substances (96) Reactions (122) Citing (15)

264

Reprogramming urokinase into an antibody-recruiting anticancer agent using peptides of arginine chloromethylketone

By: Spiegel, David A.; Jakobsche, Charles E.
World Intellectual Property Organization, WO2013070688 A1 2013-05-16 | Language: English, Database: CAplus

The invention relates to chimeric (preferably, bifunctional) compounds, e.g., **I**•2TFA, compositions comprising those compounds and methods of treating cancer in a patient or subject, especially including metastatic cancer where cancer cells exhibit overexpression (heightened expression) of cell surface urokinase-type plasminogen activator receptor (urokinase receptor) compared to normal (non-cancerous) cells. The compounds preferably covalently bind to the urokinase receptor and recruit native antibodies of the patient or subject where the antibodies can selectively degrade and/or deactivate targeted cancer cells through antibody-dependent cellular phagocytosis and/or antibody-dependent cellular cytotoxicity against a large number and variety of cancers, thus providing cancer cell death and/or an inhibition of growth, elaboration and/or metastasis of the cancer, including remission and cure of the patient's cancer. Thus, chloromethyl ketone **I**•2TFA was prepared by a multistep procedure and evaluated as an antibody-recruiting mol. that targets the urokinase receptor.

Keywords: peptide arginine chloromethylketone preparation reprogramming urokinase antibody recruiting anticancer

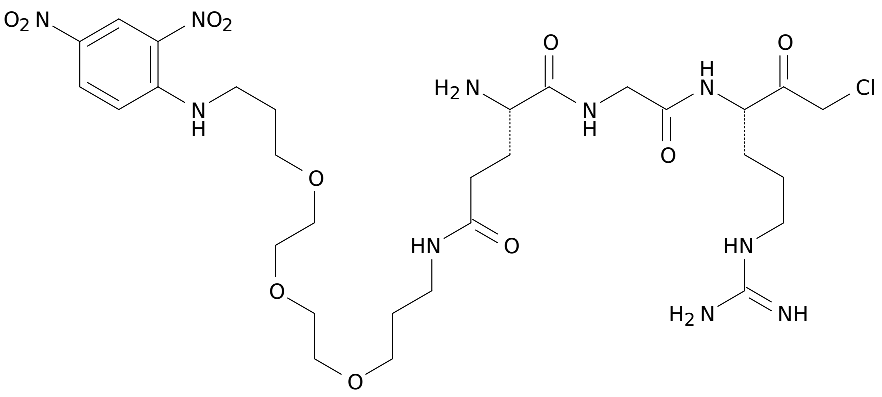

I

PatentPak available

Substances (346) Reactions (198) Citing (1)

265

Porous nanoparticle-supported lipid bilayers (protocells) for targeted delivery including transdermal delivery of cargo and methods thereof

By: Ashley, Carlee Erin; Brinker, C. Jeffrey; Carnes, Eric C.; Fekrazad, Mohammad Houman; Felton, Linda A.; Negrete, Oscar; Padilla, David Patrick; Wilkinson, Brian S.; Wilkinson, Dan C.; Willman, Cheryl L.
World Intellectual Property Organization, WO2013056132 A2 2013-04-18 | Language: English, Database: CAplus

The present invention is directed to protocells for specific targeting of hepatocellular and other cancer cells which comprise a nanoporous silica core with a supported lipid bilayer; at least one agent which facilitates cancer cell death, such as a traditional small mol., a macromol. cargo, e.g., siRNA, or a protein toxin, such as ricin toxin A-chain or diphtheria toxin A-chain, and/or a histone-packaged plasmid DNA disposed within the nanoporous silica core (preferably superc oiled in order to more efficiently package the DNA into protocells) which is optionally modified with a nuclear locali zation sequence to assist in localizing protocells within the nucleus of the cancer cell and the ability to express peptides involved in therapy (apoptosis/cell death) of the cancer cell or as a reporter, a targeting peptide which targets cancer cells in tissue to be treated such that binding of the protocell to the targeted cells is specific and enhanced and a fusogenic peptide that promotes endosomal escape of protocells and encapsulated D NA. Protocells according to the present invention may be used to treat cancer, especially including hepatoc ellular (liver) cancer using novel binding peptides (c-MET peptides) which selectively bind to hepatocellular tissue or to function in diagnosis of cancer, including cancer treatment and drug discovery. For example, the porous protocells were prepared via fusion of AEPTMS-modified silica cores and a liposomes composed of DOPC/DOPE/cholesterol/PEG2000 conjugated with a targeting peptide S P94 (H_2N-SFSIILTP ILPLEEEGGC-COOH, SEQ ID NO: 8) for cellular delivery of si RNA. The ability of si RNA-loaded, SP94-targeted DOPC-containing protocells to selectively induce cytotoxicity of human hepatoc ellular carcinoma cells (Hep3 B) was demonstrated.

Keywords: lipid bilayer nanoparticle protocell cancer targeting

PatentPak available

Substances (434) Reactions (0) Citing (15)

266

A molecular case report: Functional assay of tyrosine kinase inhibitors in cells from a patient's primary renal cell carcinoma

By: Kulesz-Martin, Molly; Lagowski, James; Olson, Susan; Wortham, Aaron; West, Toni; Thomas, George; Ryan, Christopher W.; Tyner, Jeffrey W.
Cancer Biology & Therapy (2013), 14(2), 95-99 | Language: English, Database: CAplus and MEDLINE

Current therapies for renal cell carcinoma favor vascular endothelial growth factor receptor (VEGF-R) tyrosine kinase (T K) inhibitors (TKIs). In theory, these are most applicable in tumors that have lost V HL-with subsequent stabili zation of HIF and upregulation of VE GF. A subset of patients harbor primary- refractory disease, as in this case, where there was no evidence for loss of V HL or chromosome 3p. We evaluated mol. targeted agents in viable tumor cells cultured from a patient's clear cell renal cell carcinoma (R CC). Of 66 agents, only dasatinib, an inhibitor of Src tyrosine kinase, strongly reduced viability of the patient's cultured kidney tumor cells. Immunostaining of the original primary tumor revealed strong positivity for V HL and Src protein expres sion. Functional evaluation of a patient's tumor cells appears feasible in the setting of RCC.

Keywords: tyrosine kinase inhibitor renal cell carcinoma anticancer; molecular targeted therapy; renal cell carcinoma; tyrosine kinase

Substances (55) Reactions (0) Citing (2)

267

Effect of aurora kinase B inhibitor AZD1152 on cisplatin-resistant epithelial ovarian carcinoma Hey cells

By: Ma, Ya-xi; Li, Xiu-zhen
Zhonghua Fuchanke Zazhi (2013), 48(1), 46-50 | Language: Chinese, Database: CAplus and MEDLINE

This paper is to investigate the effect of aurora kinase B inhibitor A ZD1152 on cisplatin-resistant epithelial ovarian carcinoma Hey cells. Hey (cisplatin-resistant ovarian cancer cell line) cells were analyzed. According to the treatment plan, Hey cells were divided into four groups (AZD group, cisplatin group, AZD+cisplatin group and control group). Me thiazolyl tetrazolium (MTT) assay was used to test the cell proliferation; caspase-3/7 activity anal. was used to analyze cells apoptosis; fluorescence in-situ hybridization (FI SH) assay was used to determine the copy number of chromosome 7 and check the copy number of h TERC gene and C-myc gene. MTT test showed that proliferation in AZD group was lower than that in control group(P<0.01). The proliferation rates of cells treated with 10 and 20 nmol/L AZD for 24 h were (81.4±3.6)% and (81.4±3.6)% resp., and the proliferation rates of cells for 48 h were (43.1±2.0)% and (38.5±1.6)% resp., which was significantly lower than those in control group (100%, P<0.01); after cells were treated with same concentration of AZD, inhibition of proliferation was significantly enhanced as the time prolongation (P<0.01). Proliferation rate in AZD+cisplatin group was lower than that in cisplatin group (P<0.01), which suggest that there were additive effects after combining AZD with cisplatin. Compared with control group, caspase-3/7 activity in AZD group increased significantly (P=0.000), and the same result was seen between A ZD±cisplatin group and cisplatin group or A ZD group (all P<0.01). Compared with cisplatin group or control group, the copy numbers of hTERC, C-myc and the number of chromosome were significantly increased in AZD group and AZD+cisplatin group (all P<0.05). AZD could inhibit ovarian cancer cell proliferation and induce cell apoptosis significantly. AZD alone or in combination with cisplatin may result in the increased cell polypl oidy.

Keywords: aurora kinase B inhibitor AZD1152 cisplatin resistant; epithelial ovarian carcinoma Hey cell

Substances (3) Reactions (0) Citing (7)

268

Phase I study of barasertib (AZD1152), a selective inhibitor of Aurora B kinase, in patients with advanced solid tumors

By: Schwartz, Gary K.; Carvajal, Richard D.; Midgley, Rachel; Rodig, Scott J.; Stockman, Paul K.; Ataman, Ozlem; Wilson, David; Das, Shampa; Shapiro, Geoffrey I.
Investigational New Drugs (2013), 31(2), 370-380 | Language: English, Database: CAplus and MEDLINE

The purpose of this study was to determine the maximum-tolerated dose (MTD), pharmacokinetics and safety profile for two different dosing regimens of barasertib, a selective inhibitor of Aurora B Kinase. In this Phase I trial, patients with advanced solid malignancies were treated with escalating doses of barasertib, administered as either a 48-h continuous infusion or as two 2-h infusions on consecutive days, both every 14 days of a 28- day cycle. Thirty-five patients were treated. The MTDs were 150 mg as a 48-h continuous infusion and 220 mg admini stered as two 2-h infusions (110 mg/day, days 1, 2, 15 and 16), with neutropenia the dose-limiting toxicity (DLT) of each schedule. Common Terminol. Criteria of Adverse Events (C TCAE) grade ≥ 3 neutropenia (with or without fever) occurred in 34 % of patients overall. Other adverse events, many of hematol. or gastrointestinal etiol., were of mild or moderate intensity. No objective tumor responses were observed, although stable disease was observed in 23 % of patients. Systemic exposure to barasertib-hQPA, the more active moiety to which barasertib is converted, was observed by 1 and 6 h into the 2-h and continuous infusion, resp., and exhibited linear pharmacok inetics. In summary, barasertib was generally well tolerated, with neutropenia the most frequent and dose-limiting toxicity, irresp. of schedule. Future development of barasertib will depend on better definition of its therapeutic index.

Keywords: barasertib AuroraB kinase inhibitor colon lung skin cancer; pharmaco kinetics pharmacodynamics antitumor

Substances (3) Reactions (0) Citing (52)

269

Effect of the drug transporters ABCG2, Abcg2, ABCB1 and ABCC2 on the disposition, brain accumulation and myelotoxicity of the aurora kinase B inhibitor barasertib and its more active form barasertib-hydroxy-QPA

By: Marchetti, Serena; Pluim, Dick; Eijndhoven, Monique; Tellingen, Olaf; Mazzanti, Roberto; Beijnen, Jos H.; Schellens, Jan H. M.
Investigational New Drugs (2013), 31(5), 1125-1135 | Language: English, Database: CAplus and MEDLINE

We explored whether barasertib (AZD1152), a selective Aurora B kinase inhibitor, is a substrate for P- glycoprotein (Pgp, MDR1), breast cancer resistance protein (BCRP), and multidrug resistance protein 2 (MRP2) in vitro. Cell survival, drug transport, and competition experiments with barasertib pro-drug and the more active form of the drug (barasertib-hQPA) were performed using MDCKII (wild type, MDR1, BCRP, and MRP2) and LLCPK (wild type and MDR1) cells and monolayers, and Sf9-BCRP membrane vesicles. Moreover we tested whether P-gp and BCRP affect the oral pharmacokinetics, tissue distribution, and myelotoxicity of barasertib in vivo using Bcrp1$^{-/-}$/Mdr1a/1b$^{-/-}$ (triple knockout) and wild type mice. In cell survival experi ments expression of BCRP and MDR1 resulted in significant resistance to barasertib. In transwell experiments, barasertib-hQPA was transported by BCRP and MDR1 efficiently. In Sf9-BCRP membrane vesicles, both barasertib and barasertib-hQPA significantly inhibited the BCRP-mediated transport of methotrexate. In contrast, no active transport of barasertib by MRP2 was observed, and overexpression of MRP2 did not affect cytotoxicity of barasertib. In vivo, systemic exposure as well as bioavailability, brain penetration, kidney and liver distri bution and myelotoxicity of barasertib-hQPA were statistically significantly increased in Bcrp1$^{-/-}$/Mdr1a/1b$^{-/-}$ compared with wild type mice (p<0.001). Barasertib is transported efficiently by P-gp and BCRP/Bcrp1 in vitro. In vivo, genetic deletion of P-gp and BCR P in mice significantly affected pharmacokinetics, tissue distribution and myelotoxicity of barasertib-hQPA. Possible clin. conseq uences for the observed affinity of barasertib for P-gp and BCRP need to be explored.

Keywords: barasertib aurora kinase B inhibitor ABCG2 ABCB1 ABCC2 pharmacokinetics

Substances (4) | Reactions (0) | Citing (19)

270

Aurora kinase inhibitor AZD1152 has an additional effect of platinum on a sequential application at the human ovarian cancer cell line SKOV3

By: Ma, Yaxi; Weimer, Joerg; Fredrik, Regina; Adam-Klages, Sabine; Sebens, Susanne; Caliebe, Amke; Hilpert, Felix; Eckmann-Scholz, Christel; Arnold, Norbert; Schem, Christian
Archives of Gynecology and Obstetrics (2013), 288(1), 173-182 | Language: English, Database: CAplus and MEDLINE

Purpose: The treatment of ovarian tumors is carried out with platinum medicine which can lead to incompatibilities or resistances. Thus, it is of great interest to check new medicine suitability for its applic ation. AZD1152 is an aurora kinase inhibitor predomi nantly works against aurora kinase B involved in the chromosome segregation. Cells become polyploidy and reduce the proliferation by this impairment. To investigate whether AZD1152, may play a role in the treatment of ovarian carcinoma we serving it to the cispla tinum-resistant cell line SKOV3 alone and in combination with platinum. Methods: We look at the prolife ration, the ploidy, the phases of cell cycle and the apoptosis activity of the cells. Results and conclusion: We could show that the combin ation of both medicines in the preclin. experiment produces a working advantage.

Keywords: anticancer Aurora kinase inhibitor AZD1152 platinum ovarian cancer

Substances (6) | Reactions (0) | Citing (8)

271

Kinase Pathway Dependence in Primary Human Leukemias Determined by Rapid Inhibitor Screening

By: Tyner, Jeffrey W.; Yang, Wayne F.; Bankhead, Armand III; Fan, Guang; Fletcher, Luke B.; Bryant, Jade; Glover, Jason M.; Chang, Bill H.; Spurgeon, Stephen E.; Fleming, William H.; et al
Cancer Research (2013), 73(1), 285-296 | Language: English, Database: CAplus and MEDLINE

Kinases are dysregulated in most cancers, but the frequency of specific kinase mutations is low, indicating a complex etiol. in kinase dysregulation. Here, we report a strategy to rapidly identify functi onally important kinase targets, irresp. of the etiol. of kinase pathway dysregulation, ultimately enabling a correl ation of patient genetic profiles to clin. effective kinase inhibi tors. Our methodol. assessed the sensitivity of primary leukemia patient samples to a panel of 66 small- mol. kinase inhibitors over 3 days. Screening of 151 leukemia patient samples revealed a wide diversity of drug sensitivities, with 70% of the clin. specimens exhibiting hypersen sitivity to one or more drugs. From this data set, we developed an algorithm to predict kinase pathway dependence based on anal. of inhibitor sensitivity patterns. Applying this algorithm correctly identified pathway dependence in proof- of-principle specimens with known oncogenes, including a rare FLT3 mutation outside regions covered by standard mol. diagnostic tests. Interro gation of all 151 patient specimens with this algorithm identified a diversity of kinase targets and signaling pathways that could aid priorit ization of deep sequencing data sets, permitting a cumulative anal. to understand kinase pathway dependence within leukemia subsets. In a proof-of-principle case, we showed that in vitro drug sensit ivity could predict both a clin. response and the develo pment of drug resistance. Taken together, our results suggested that drug target scores derived from a compreh ensive kinase inhibitor panel could predict pathway dependence in cancer cells while simultaneously identifying potential therapeutic options.

Keywords: kinase inhibitor signaling leukemia drug screening target anticancer

Substances (231) | Reactions (0) | Citing (116)

272

Multiparametric cell-based assay for the evaluation of transcription inhibition by high-content imaging

By: Torres-Guzman, Raquel; Chu, Shaoyou; Velasco, Juan A.; Lallena, Maria Jose
Journal of Biomolecular Screening (2013), 18(5), 556-566 | Language: English, Database: CAplus and MEDLINE

Loss of normal cell cycle regulation is a hallmark of human cancer. Cyclin-dependent kinases (CDKs) are key regulators of the cell cycle and have been actively pursued as promising therapeutic targets. Likewise, members of the C DK family are functi onally related to transcriptional modulation, a mol. pathway suitable for therap eutic intervention. We used a set of 2500 compounds in the U2OS cell line to evaluate its effect in the cell division process. Interes tingly, out of this anal., we identified a subpopu lation of compounds that are able to inhibit RNA polymerase activity, thus interfering with gene transcription processes. After this finding, we developed, validated, and fully automated a multiparameter highcontent imaging (HCI) assay to measure phosphorylation of the RNA polymerase II carboxyl terminal domain (p CTD). Simultaneously, we measured both the D NA content and cell prolife ration index in the treated cells. The linear regression anal. comparing the IC50 for p CTD and the 4N EC50 for DNA content or IC50 for cell proliferation showed an excellent agreement ($r2 = 0.84$ and $r2 = 0.94$, resp.) . Our results confirm that this method allows discrim inating between cell cycle and transcr iption inhibition and confirms HCI as a powerful technol. for the identif ication of compounds with an effective and selective pathway phenotype.

Keywords: cancer multiparametric cell based assay transcription high content imaging; RNA pol II; assay development; cell cycle; gene transcription; high-content imaging

Substances (20) | Reactions (0) | Citing (6)

273

A new approach for prediction of tumor sensitivity to targeted drugs based on functional data

By: Berlow, Noah; Davis, Lara E.; Cantor, Emma L.; Seguin, Bernard; Keller, Charles; Pal, Ranadip
BMC Bioinformatics (2013), 14, 239 | Language: English, Database: CAplus and MEDLINE

Background: The success of targeted anti-cancer drugs are frequently hindered by the lack of knowledge of the individual pathway of the patient and the extreme data requirements on the estimation of the personalized genetic network of the patient's tumor. The prediction of tumor sensitivity to targeted drugs remains a major challenge in the design of optimal therapeutic strategies. The current sensitivity prediction approaches are primarily based on genetic characterizations of the tumor sample. We propose a novel sensitivity prediction approach based on functional perturbation data that incorporates the drug protein interaction information and sensitivities to a training set of drugs with known targets. Results: We illustrate the high prediction accuracy of our framework on synthetic data generated from the Kyoto Encyclopedia of Genes and Genomes (KEGG) and an exptl. dataset of four canine osteosarcoma tumor cultures following application of 60 targeted small-mol. drugs. We achieve a low leave one out cross validation error of <10% for the canine osteosarcoma tumor cultures using a drug screen consisting of 60 targeted drugs. Conclusions: The proposed framework provides a unique input-output based methodol. to model a cancer pathway and predict the effectiveness of targeted anti-cancer drugs. This framework can be developed as a viable approach for personalized cancer therapy.

Keywords: protein anticancer agent interaction osteosarcoma sensitivity math model

Substances (44) Reactions (0) Citing (22)

274

Aurora kinases in childhood acute leukemia: the promise of aurora B as therapeutic target

By: Hartsink-Segers, S. A.; Zwaan, C. M.; Exalto, C.; Luijendijk, M. W. J.; Calvert, V. S.; Petricoin, E. F.; Evans, W. E.; Reinhardt, D.; de Haas, V.; Hedtjaern, M.; et al
Leukemia (2013), 27(3), 560-568 | Language: English, Database: CAplus and MEDLINE

We investigated the effects of targeting the mitotic regulators aurora kinase A and B in pediatric acute lymphoblastic leukemia (ALL) and acute myeloid leukemia (AML). Aurora protein expression levels in pediatric ALL and AML patient samples were determined by western blot and reverse phase protein array. Both kinases were overexpressed in ALL and AML patients (P<0.0002), especially in E2A-PBX1-translocated ALL cases (P<0.002), compared with normal bone-marrow mononuclear cells. Aurora kinase expression was silenced in leukemic cell lines using short hairpin RNAs and locked nucleic acid-based mRNA antagonists. Aurora B knockdown resulted in proliferation arrest and apoptosis, whereas aurora A knockdown caused no or only minor growth delay. Most tested cell lines were highly sensitive to the AURKB-selective inhibitor barasertib-hydroxyquinazoline-pyrazol-anilide (AZD1152-HQPA) in the nanomolar range, as tested with an MTS (3-(4,5-dimethylthiazol-2-yl)-5-(3-carboxymethoxyphenyl)-2-(4-sulfophenyl)-2H-tetrazolium) assay. But most importantly, primary ALL cells with a high aurora B protein expression, especially E2A-PBX1-pos. cases, were sensitive as well. In adult AML early clin. trials, clear responses are observed with barasertib. Here we show that inhibition of aurora B, more than aurora A, has an antiproliferative and pro-apoptotic effect on acute leukemia cells, indicating that particularly targeting aurora B may offer a new strategy to treat pediatric ALL and AML. Leukemia (2013) 27, 560-568; doi:10.1038/leu.2012.256; published online 19 Oct. 2012.

Keywords: antiproliferative aurora kinase therapeutic target acute leukemia childhood

Substances (5) Reactions (0) Citing (53)

275

Combination therapy with antibodies targeting insulin-like growth factor 1 receptor (IGF1R)

By: Goetsch, Liliane; Hsu, Karl
World Intellectual Property Organization, WO2012158657 A1 2012-11-22 | Language: English, Database: CAplus

The authors disclose the use of an anti-IGF1R antibody (or antigen-binding fragment) in combination with one or more chemothe rapeutic agents for treatment of cancer. In one example, pancreatic cancer patients receiving dalotu zumab and gemcit abine exhibited greater progression free survival.

Keywords: combination therapy cancer insulinlike growth factor receptor antibody

PatentPak available

Substances (297) | Reactions (0) | Citing (1)

276

Preparation of bisaminoquinoline compounds as autophagy inhibitors useful in mono- and combination therapy of cancer and other diseases

By: Amaravadi, Ravi K.; Winkler, Jeffrey
World Intellectual Property Organization, WO2012149186 A2 2012-11-01 | Language: English, Database: CAplus

Title compounds **I** [R^1 and $R^{1'}$ independently = H, halo, C N, NO_2, (un)substituted alkyl, O-alkyl, acyl, oxycarbonyl ester or carboxy ester; R and R' indepen dently = H, (un)substituted alkyl, acyl, carboxy ester; L = $(CH_2Y)_n$-X-$(Y'CH_2)_n$ or A-$(CH_2$-CH_2-$Z)_n$-A'; X = absent, O, S, etc.; Y = absent, CH_2, O, CH_2O, etc.; Y' forms a stable bond when present; R" = H or (un) substituted alkyl; n = 0- 4; A = bond (absent), CH_2-CH_2, CH_2, etc.; Z = O or N- Rz; Rz = H or (un)substituted alkyl], and their pharmaceutically acceptable salts, enanti omers, diastereomers, solvates or polymorphs, are prepared for inhibiting autophagy in biol. systems. Methods of treating cancer in patients in need using compounds and/or compositions according to the present invention alone or in combin ation with at least one addnl. anticancer agent represent addnl. aspects of the invention. Methods of treating disease states and/or conditions in which inhibition of autophagy plays a favorable treatment role including rheumatoid arthritis, malaria, antiphospholipid antibody syndrome, lupus, chronic urticaria and Sjogren's disease, with compounds according to the present invention represent addnl. aspects of the invention.

Keywords: bisaminoquinoline autophagy inhibitor cancer

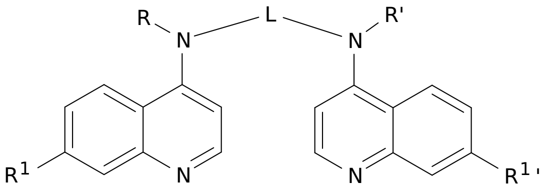

I

PatentPak available

Substances (376) | Reactions (59) | Citing (5)

277

Inhibition of Aurora-B kinase activity confers antitumor efficacy in preclinical mouse models of early and advanced gastrointestinal neoplasia

By: Alferez, Denis G.; Goodland, Roberta A.; Odedra, Rajesh; Sini, Patrizia; Crafter, Claire; Ryan, Anderson J.; Wedge, Stephen R.; Wright, Nicholas A.; Anderson, Elizabeth; Wilkinson, Robert W.
International Journal of Oncology (2012), 41(4), 1475-1485 | Language: English, Database: CAplus and MEDLINE

The Aurora family of kinases, play a fundamental role in cell division and are overexp ressed in several cancers including colon. The activity of barasertib-hQPA, a selective inhibitor of Aurora-B kinase (ABK) was investigated in a range of preclin. models of gastroin testinal cancer. Treatment with barasertib-hQPA produced anti-proliferative and cytotoxic effects across a panel of human colorectal cancer (CRC) cell lines in vitro. Prodrug, barasertib [48-h s.c. (s.c.) infusion; 150 mg/kg/day] inhibited the growth of S W620, Colo205, HCT116 human colorectal tumor xenografts in nude mice signifi cantly (Student's t-test, P<0.05, n=10-12 per group). Flow cytometric anal. of single cells from disaggregated barasertib-treated SW620 tumors revealed a decrease in phospho rylated histone H3 (phH3) and an increase in tumor cells with ≥4 N DNA content (P<0.05). The activity of barasertib was then examined in $Apc^{Min/+}$ mice, a spontaneous model of early intestinal neoplasia. Macroscopic evaluation of the small intestine revealed that barasertib treatment [25 mg/kg intra- peritoneal (i.p.) Q1 Dx4 each week for 3 wk] of 8- wk old $Apc^{Min/+}$ mice produced a 39% reduction in macroadenoma number (P=0.02) and a 43% reduction in overall adenoma burden (P=0.02) compared with vehicle-treated controls. Quantification of microscopic adenomas revealed a >64% reduction in the number of adenomas spanning more than one villus. Histol. anal. of these adenomas revealed a number of distinct changes in barasertib-treated $Apc^{Min/+}$ mice, including a 94% reduction in the proportion of phosphohistone H3-pos. cells (P<0.001) and a 53% reduction in the number of cells per adenoma (P=0.001). These results provide a scientific rationale for investi gating ABK inhibitors as a treatment for intestinal cancer.

Keywords: antitumor auroraB kinase inhibitor barasertib APC signaling gastrointestinal neoplasia

Substances (4) Reactions (0) Citing (10)

278

Development of o-Chlorophenyl Substituted Pyrimidines as Exceptionally Potent Aurora Kinase Inhibitors

By: Lawrence, Harshani R.; Martin, Mathew P.; Luo, Yunting; Pireddu, Roberta; Yang, Hua; Gevariya, Harsukh; Ozcan, Sevil; Zhu, Jin-Yi; Kendig, Robert; Rodriguez, Mercedes; et al

Journal of Medicinal Chemistry (2012), 55(17), 7392-7416 | Language: English, Database: CAplus and MEDLINE

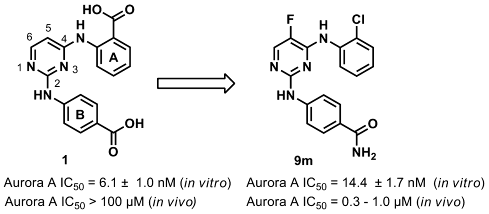

The o-carboxylic acid substituted bisanilinopyrimidine **I** was identified as a potent hit (Aurora A IC_{50} = 6.1 ± 1.0 nM) from inhouse screening. Detailed structure-activity relationship (SAR) studies indicated that polar substituents at the para position of the B- ring are critical for potent activity. X-ray crystallog. studies revealed that compound **I** is a type I inhibitor that binds the Aurora kinase active site in a DFG-in conformation. Structure-activity guided replacement of the A-ring carboxylic acid with halogens and incorporation of fluorine at the pyrimidine 5- position led to highly potent inhibitors of Aurora A that bind in a DFG-out conformation. B-Ring modifications were undertaken to improve the solubility and cell permeability. Compounds such as **II** with water-solubilizing moieties at the para position of the B-ring inhibited the autophosphorylation of Aurora A in MDA-MB-468 breast cancer cells.

Keywords: dianilinopyrimidine preparation SAR crystal structure Aurora kinase inhibitor

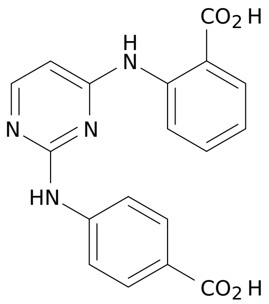
I

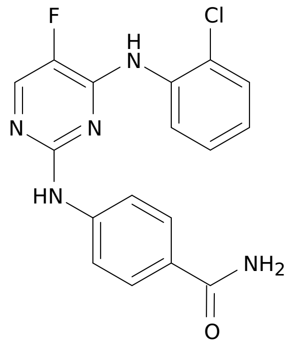
II

Substances (171) Reactions (197) Citing (53)

279

Novel pharmaceutical combinations and methods for treating cancer

By: Cheok, Chit Fang; Lane, David

World Intellectual Property Organization, WO2012121662 A1 2012-09-13 | Language: English, Database: CAplus

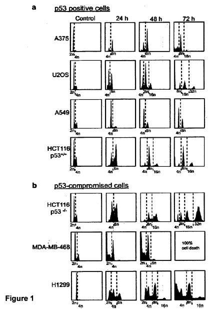

Methods of selectively targeting a p53-deficient cancer cell, comprising administering to a patient suffering from cancer (i) a reversible cell cycle arrest-inducing agent for inducing cell cycle arrest in a p53- pos. cell; and (ii) an aurora kinase inhibitor, wherein said reversible cell cycle arrest-inducing agent is administered prior to administration of said aurora kinase inhibitor, and pharmac eutical combinations, kits and oral dosage forms for the same.

Keywords: p53 deficient cancer treatment pharmaceutical combination; aurora kinase inhibitor cell cycle arrest inducer cancer treatment

PatentPak available

Substances (27) Reactions (0) Citing (0)

280

Two-stage model-based design of cancer phase I dose escalation trials: evaluation using the phase I program of barasertib (AZD1152)

By: Keizer, Ron J.; Zandvliet, Anthe S.; Beijnen, Jos H.; Schellens, Jan H. M.; Huitema, Alwin D. R.

Investigational New Drugs (2012), 30(4), 1519-1530 | Language: English, Database: CAplus and MEDLINE

Introduction. Modeling and simulation of pharmaco kinetics and pharmaco dynamics has previously been shown to be potentially useful in designing Phase I programs of novel anti-cancer agents that show hematol. toxicity. In this anal., a two- stage model-based trial design was evaluated retrospectively using data from the Phase I program with the aurora kinase inhibitor barasertib. Methods. Data from two Phase I trials and four regimens were used (n = 79). Using barasertib-hydroxy QPA plasma concentrations and neutrophil count data from only study 1A, a PKPD model was developed and subsequently used to predict the MTD and a safe starting dose for the other trials. Results. The PKPD model based on data from the first study adequately described the time course of neutrophil count fluctuation. The two-stage model-based design provided safe starting doses for subsequent phase I trials for barasertib. Predicted safe starting dose levels were higher than those used in two subsequent trials, but lower than used in the other trial. Discussion. The two-stage approach could have been applied safely to define starting doses for altern ative dosing strategies with barasertib. The limited improvement in efficiency for the phase I program of barasertib may have been due to the fact that starting doses for the studied phase I trials were already nearly optimal. Conclusion. Application of the two-stage model-based trial design in Phase I programs with novel anti-cancer drugs that cause haematol. toxicity is feasible, safe, and may lead to a reduction in the number of patient treated at sub-therapeutic dose-levels.

Keywords: barasertib pharmacokinetics pharmacodynamics cancer anticancer

Substances (2) Reactions (0) Citing (9)

281

Characterizing tumor response to chemotherapy at various length scales using temporal diffusion spectroscopy

By: Xu, Junzhong; Li, Ke; Smith, R. Adam; Waterton, John C.; Zhao, Ping; Chen, Heidi; Does, Mark D.; Manning, H. Charles; Gore, John C.
PLoS One (2012), 7(7), e41714 | Language: English, Database: CAplus and MEDLINE

Measurements of apparent diffusion coefficient (ADC) using magnetic resonance imaging (M RI) have been suggested as potential imaging biomarkers for monitoring tumor response to treatment. However, conventional pulsed-gradient spin echo (P GSE) methods incorporate relatively long diffusion times, and are usually sensitive to changes in cell d. and necrosis. Diffusion temporal spectroscopy using the oscillating gradient spin echo (O GSE) sequence is capable of probing short length scales, and may detect significant intracellular microstructural changes independent of gross cell d. changes following anti- cancer treatment. To test this hypothesis, SW620 xenografts were treated by barasertib (AZD1152), a selective inhibitor of Aurora B kinase which causes S W620 cancer cells to develop polyploidy and increase in size following treatment, ultimately leading to cell death through apoptosis. Following treatment, the ADC values obtained by both the P GSE and low frequency O GSE methods increased. However, the A DC values at high gradient frequency (i.e. short diffusion times) were significantly lower in treated tumors, consistent with increased intracellular restrictions/hindrances. This suggests that A DC values at long diffusion times are dominated by tumor microst ructure at long length scales, and may not convey unambiguous information of subcellular space. While the diffusion temporal spectr oscopy provides more comprehensive means to probe tumor microstructure at various length scales. This work is the first study to probe intracellular microstructural variations due to polyploidy following treatment using diffusion M RI in vivo. It is also the first observation of post-treatment ADC changes occurring in opposite directions at short and long diffusion times. The current study suggests that temporal diffusion spectroscopy potentially provides pharmacodynamic biomarkers of tumor early response which distinguish microstructural variations following treatment at both the subcel lular and supracellular length scales.

Keywords: barasertib antitumor colon cancer apoptosis spectr oscopy

Substances (2) Reactions (0) Citing (29)

282

Exploring protein kinase inhibitors. Unveiling gemcitabine resistance in pancreatic cancer. Comments

By: Kim, Yeon Jeong; Hong, Young Bin; Cho, Chi Heum; Seong, Yeon-Sun; Bae, Insoo
Pancreas (Hagerstown, MD, United States) (2012), 41(5), 804-805 | Language: English, Database: CAplus and MEDLINE

Gemcitabine is one of first-line therapies for locally advanced pancreatic cancer, but severe resistance is respon sible for less than 6 mo of median survival and less than 20% of response rate. Although efforts to overcome gemcitabine resistance have been underway, the only combination treatment that has shown a small but statist ically significant outcome is gemcitabine with erlotinib, an epidermal growth factor receptor inhibitor. Improvements in pancreatic cancer therapy can be made by underst anding gemcit abine resistance. To enhance the treatment benefits of conven tional therapeutic drugs, targeting protein kinases in combin ation with DNA damaging drugs has been aggres sively attempted for several types of cancers. As part of this effort in pancreatic cancer, the present study explored 76 protein kinase inhibitors (PKIs) for probing new targetable protein kinases and found that several P K Is possess promising efficacy in combin ation with gemcitabine.

Keywords: protein kinase inhibitor gemcitabine resistance cytotoxic pancreatic cancer

Substances (21) Reactions (0) Citing (6)

283

Aurora kinase inhibitor

By: Yoshida, Kozue; Nagai, Tadashi
Saishin Igaku (2012), 67(6), 1504-1513 | Language: Japanese, Database: CAplus

A review on role of aurora kinase in tumorigenesis discussing structure and function of aurora kinase- A, -B, and -C as serine-threonine kinases and overexpression in solid tumor or hemato genic organ tumors. Clin. efficacy and safety of aurora kinase inhibitors (alisertib, barasertib, danusertib) as low-mol. antitumor agents for tumor treatment in mono- or combin ation chemot herapy are also discussed.

Keywords: review aurora kinase inhibitor

Substance (1) Reactions (0) Citing (0)

284

Frequency of TP53 Mutations and its Impact on Drug Sensitivity in Acute Myeloid Leukemia?

By: Shah, Ankur; Seedhouse, Claire
Indian Journal of Clinical Biochemistry (2012), 27(2), 121-126 | Language: English, Database: CAplus and MEDLINE

The purpose of this study is to find out the frequency of TP53 mutations in acute myeloid leukemia (AML) patients and correlate sensitivity of drug response with T P53 mutations. In AML more than 90 % of cases comprise of wild type T P53. 94.2 % of T P53 mutations are found within exon 5-8 of which 73 % are point mutations. T P53 mutations were analyzed with high resolution melting curve anal. We analyzed 106 AML samples of which we found nine mutations which represents 8.5 % mutation rate and found one rare SNP. The effect of TP53 mutations were studied on the chemosen sitivity of two new drugs AZD115 and RHPS4, an Aurora Kinase B inhibitor and Telomerase inhibitor resp. Four mutations were found out of 17 for RHPS4 stating significant (p = 0.002) increase in sensitivity and no mutation found in A ZD1152 database, but need more study to get definite conclu sion.

Keywords: AZD1152 RHPS4 antitumor T P53 mutation chemosensitivity acute myeloid leukemia; Acute myeloid leukemia; Drug sensitivity; High resolution melting curve analysis; T P53

Substances (4) Reactions (0) Citing (3)

285

Possibility of aurora kinase inhibitors for AML

By: Tsuboi, Kosuke
Ketsueki Naika (2012), 64(3), 335-338 | Language: Japanese, Database: CAplus

A review. In this paper, (AZD1152)barasertib peptidase, possibility of Aurora kinase inhibitor for treatment of acute myeloid leukemia.

Keywords: review Aurora kinase antitumor acute myeloid leukemia barasertib

Substances (2) Reactions (0) Citing (0)

286

Probing the structural requirements of A-type Aurora kinase inhibitors using 3D-QSAR and molecular docking analysis

By: Zhang, Hui-xiao; Li, Yan; Wang, Xia; Wang, Yong-hua
Journal of Molecular Modeling (2012), 18(3), 1107-1122 | Language: English, Database: CAplus and MEDLINE

Aurora-A, the most widely studied isoform of Aurora kinase overexp ressed aberrantly in a wide variety of tumors, has been implicated in early mitotic entry, degradation of natural tumor suppressor p53 and centrosome maturation and separa tion; hence, potent inhibitors of Aurora-A may be therapeu tically useful drugs in the treatment of various forms of cancer. Here, we report an in silico study on a group of 220 reported Aurora-A inhibitors with six different substru ctures. Three-dimensional quant. structure-activity relationship (3D-QSAR) studies were carried out using compar ative mol. field anal. (CoMFA) and comparative mol. similarity indexes anal. (CoMSIA) techniques on this series of mols. The resultant optimum 3 D-QSAR models exhibited an r value of 0.404-0.582 and their predictive ability was validated using an indepe ndent test set, ending in r 0.512- 0.985. In addition, docking studies were employed to explore these protein-inhibitor interactions at the mol. level. The results of 3 D-QSAR and docking analyses validated each other, and the key structural requirements affecting Aurora-A inhibitory activities, and the influential amino acids involved were identified. To the best of our knowledge, this is the first report on 3 D-QSAR modeling of Aurora- A inhibitors, and the results can be used to accurately predict the binding affinity of related analogs and also facilitate the rational design of novel inhibitors with more potent biol. activities.

Keywords: Aurora kinase inhibitor antitumor 3D QSAR mol docking

Substances (221) Reactions (0) Citing (12)

287

Evolution of resistance to Aurora kinase B inhibitors in leukaemia cells

By: Failes, Timothy W.; Mitic, Gorjana; Abdel-Halim, Heba; Po'uha, Sela T.; Liu, Marjorie; Hibbs, David E.; Kavallaris, Maria
PLoS One (2012), 7(2), e30734 | Language: English, Database: CAplus and MEDLINE

Aurora kinase inhibitors are new mitosis-targeting drugs currently in clin. trials for the treatment of haematol. and solid maligna ncies. However, knowledge of the mol. factors that influence sensit ivity and resistance remains limited. Herein, we developed and characterised an in vitro leukemia model of resistance to the Aurora B inhibitor Z M447439. Human T-cell acute lymphob lastic leukemia cells, CCRF-CEM, were selected for resistance in 4 μ M ZM447439. CEM/AKB4 cells showed no cross- resistance to tubulin-targeted and DNA-damaging agents, but were hyperse nsitive to an Aurora kinase A inhibitor. Sequencing revealed a mutation in the Aurora B kinase domain corresponding to a G160E amino acid substit ution. Mol. modeling of drug binding in Aurora B containing this mutation suggested that resistance is mediated by the glutamate substitution preventing formation of an active drug- binding motif. Progression of resistance in the more highly selected C EM/AKB8 and CEM/AKB16 cells, derived sequen tially from CEM/AKB4 in 8 and 16 μM ZM447439 resp., was mediated by addnl. defects. These defects were indepe ndent of Aurora B and multi- drug resistance pathways and are associated with reduced apoptosis mostly likely due to reduced inhibition of the catalytic activity of Aurora kinase B in the presence of drug. Our findings are important in the context of the use of these new targeted agents in treatment regimes against leukemia and suggest resistance to therapy may arise through multiple independent mechanisms.

Keywords: ZM447439 anticancer aurora kinase B inhibitor leukemia cell

Substances (9) Reactions (0) Citing (12)

288

Selective Aurora Kinase Inhibitors Identified Using a Taxol-Induced Checkpoint Sensitivity Screen

By: Kwiatkowski, Nicholas; Deng, Xianming; Wang, Jinhua; Tan, Li; Villa, Fabrizio; Santaguida, Stefano; Huang, Hsiao-Chun; Mitchison, Tim; Musacchio, Andrea; Gray, Nathanael
ACS Chemical Biology (2012), 7(1), 185-196 | Language: English, Database: CAplus and MEDLINE

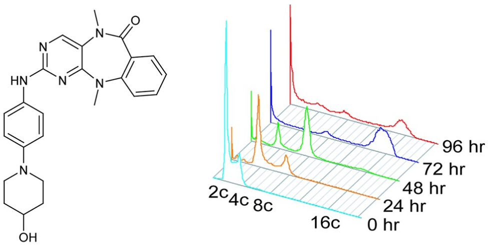

Compound **1**

The members of the Aurora kinase family play critical roles in the regulation of the cell cycle and mitotic spindle assembly and have been intensively investigated as potential targets for a new class of anticancer drugs. We describe a new highly potent and selective class of Aurora kinase inhibitors discovered using a phenotypic cellular screen. Optimized inhibitors display many of the hallmarks of Aurora inhibition including endoreduplication, polyploidy, and loss of cell viability in cancer cells. Structure- activity relationships with respect to kinome-wide selectivity and guided by an Aurora B co- crystal structure resulted in the identification of key selectivity determinants and discovery of a subseries with selectivity toward Aurora A. A direct comparison of biochem. and cellular profiles with respect to published Aurora inhibitors including VX-680, AZD1152, MLN8054, and a pyrimidine-based compound from Genentech demonstrates that compounds 1 and 3 will become valuable addnl. pharmacol. probes of Aurora- dependent functions.

Keywords: aurora kinase inhibitor taxol checkpoint sensitivity screening

Substances (37) Reactions (0) Citing (22)

289

Aurora B Is Regulated by the Mitogen-activated Protein Kinase/Extracellular Signal-regulated Kinase (MAPK/ERK) Signaling Pathway and Is a Valuable Potential Target in Melanoma Cells

By: Bonet, Caroline; Giuliano, Sandy; Ohanna, Mickael; Bille, Karine; Allegra, Maryline; Lacour, Jean-Philippe; Bahadoran, Philippe; Rocchi, Stephane; Ballotti, Robert; Bertolotto, Corine
Journal of Biological Chemistry (2012), 287(35), 29887-29898 | Language: English, Database: CAplus and MEDLINE

Metastatic melanoma is a deadly skin cancer and is resistant to almost all existing treatment. Vemurafenib, which targets the BRAF V600E mutation, is one of the drugs that improves patient outcome, but the patients next develop secondary resistance and a return to cancer. Thus, new therapeutic strategies are needed to treat melanomas and to increase the duration of v- Raf murine sarcoma viral oncogene homolog B1 (BRAF) inhibitor response. The ERK pathway controls cell proliferation, and Aurora B plays a pivotal role in cell division. Here, we confirm that Aurora B is highly expressed in metastatic melanoma cells and that Aurora B inhibition triggers both senescence-like phenotypes and cell death in melanoma cells. Furthermore, we show that the BRAF/ERK axis controls Aurora B expression at the transcriptional level, likely through the transcription factor FOXM1. Our results provide insight into the mechanism of Aurora B regulation and the first mol. basis of Aurora B regulation in melanoma cells. The inhibition of Aurora B expression that we observed in vemurafenib-sensitive melanoma cells was rescued in cells resistant to this drug. Consistently, these latter cells remain sensitive to the effect of the Aurora B inhibitor. Notewo rthy, wild-type BRAF melanoma cells are also sensitive to Aurora B inhibition. Collectively, our findings, showing that Aurora B is a potential target in melanoma cells, particularly in those vemurafenib-resistant, may open new avenues to improve the treatment of metastatic melanoma.

Keywords: Aurora B ERK melanoma metastasis FOXM1 vemurafenib resistance AZD1152

Substances (7) Reactions (0) Citing (60)

290

The MEK1/2 inhibitor, selumetinib (AZD6244; ARRY-142886), enhances anti-tumour efficacy when combined with conventional chemotherapeutic agents in human tumour xenograft models

By: Holt, S. V.; Logie, A.; Odedra, R.; Heier, A.; Heaton, S. P.; Alferez, D.; Davies, B. R.; Wilkinson, R. W.; Smith, P. D.
British Journal of Cancer (2012), 106(5), 858-866 | Language: English, Database: CAplus and MEDLINE

Background: The Ras/RAF/MEK/ERK pathway is frequently deregulated in cancer and a number of inhibitors that target this pathway are currently in clin. development. It is likely that clin. testing of these agents will be in combination with standard therapies to harness the apoptotic potential of both the agents. To support this strategy, it has been widely observed that a number of chemotherapeutics stimulate the activation of several intracellular signalling cascades including Ras/RAF/MEK/ERK. The MEK1/2 inhibitor selumetinib has been shown to have anti-tumor activity and induce apoptotic cell death as a monotherapy. Methods: The aim of this study was to identify agents, which would be likely to offer clin. benefit when combined with selumetinib. Here, we used human tumor xenograft models and assessed the effects combining standard chemotherapeutic agents with selumetinib on tumor growth. In addition, we analyzed tumor tissue to determine the mechanistic effects of these combinations. Results: Combining selumetinib with the DNA-alkylating agent, temozolomide (TMZ), resulted in enhanced tumor growth inhibition compared with monotherapies. Biomarker studies highlighted an increase in γH2A.X suggesting that selumetinib is able to enhance the DNA damage induced by TMZ alone. In several models we observed that continuous exposure to selumetinib in combination with docetaxel results in tumor regression. Scheduling of docetaxel before selumetinib was more beneficial than when selumetinib was dosed before docetaxel and demonstrated a pro-apoptotic phenotype. Similar results were seen when selumetinib was combined with the Aurora B inhibitor barasertib. Conclusion: The data presented suggests that MEK inhibition in combination with several standard chemotherapeutics or an Aurora B kinase inhibitor is a promising clin. strategy. British Journal of Cancer (2012) 106, 858-866; doi:10.1038/bjc.2012.8 www.bjcancer.com Published online 16 Feb. 2012.

Keywords: selumetinib temozolomide docetaxel antitumor DNA damage apoptosis colorectal cancer

Substances (14) Reactions (0) Citing (85)

291

Phase I study of the Aurora B kinase inhibitor barasertib (AZD1152) to assess the pharmacokinetics, metabolism and excretion in patients with acute myeloid leukemia

By: Dennis, Mike; Davies, Michelle; Oliver, Stuart; D'Souza, Roy; Pike, Laura; Stockman, Paul
Cancer Chemotherapy and Pharmacology (2012), 70(3), 461-469 | Language: English, Database: CAplus and MEDLINE

Purpose. Barasertib (AZD1152) is a pro-drug that rapidly undergoes phosphatase-mediated cleavage in serum to release barasertib-hQPA, a selective Aurora B kinase inhibitor that has shown preliminary activity in clin. studies of patients with acute myeloid leukemia (AML). The pharmacokinetic (PK), metabolic and excretion profiles of barasertib and barasertib-hQPA were characterized in this open-label Phase I study. Methods. Five patients with poor prognosis AML (newly diagnosed, relapsed or refractory) received barasertib 1,200 mg as a 7-day continuous infusion every 28 days. On Day 2 of Cycle 1 only, patients also received a 2-h infusion of [^{14}C]-barasertib. Blood, urine and feces samples were collected at various time points during Cycle 1. Safety and preliminary efficacy were also assessed. Results. Barasertib-hQPA was extensively distributed to tissues, with a slow rate of total clearance (CL = 31.4 L/h). Overall, 72-82 % of radioactivity was recovered, with approx. double the amount recovered in feces (mean = 51 %) compared with urine (mean = 27 %). The main metabolism pathways for barasertib were (1) cleavage of the phosphate group to form barasertib-hQPA, followed by oxidation and (2) loss of the fluoroaniline moiety to form barasertib-hQPA desfluoroaniline, followed by oxidation One of the four patients evaluable for response entered complete remission. No new or unexpected safety findings were observed; the most common adverse events were nausea and stomatitis. Conclusions. The PK profile of barasertib is similar to previous studies using the same dosing regimen in patients with AML. The majority of barasertib-hQPA clearance occurred via hepatic metabolic routes.

Keywords: antitumor aurora B kinase inhibitor barasertib pharmacokinetics metabolism; acute myeloid leukemia prodrug

Substances (2) Reactions (0) Citing (49)

292

Targeting Aurora kinases in cancer treatment

By: Kelly, Kevin R.; Ecsedy, Jeffrey; Mahalingam, Devalingam; Nawrocki, Steffan T.; Padmanabhan, Swaminathan; Giles, Francis J.; Carew, Jennifer S.

Current Drug Targets (2011), 12(14), 2067-2078 | Language: English, Database: CAplus and MEDLINE

A review. The Aurora family of serine/threonine kinases is essential for chromosome alignment, segregation, centrosomal matura tion, mitotic spindle formation, and cytoki nesis during mitosis. Their fundamental role in cell cycle regulation and aberrant expression in a broad range of malignancies prompted the development of small mols. that selectively inhibit their activity. Recent studies have revealed new insights into the cellular effects of Aurora kinase inhibition. Moreover, early phase clin. studies have shown that these agents have therapeutic efficacy. In this review, we will outline the functions of Aurora kinases in normal cell division and in malignancy. We will focus on recent preclin. and clin. studies that have explored the mechanism of action and clin. effect of Aurora inhibitors in cancer treatment.

Keywords: review aurora kinase inhibitor cancer anticancer

Substances (12) Reactions (0) Citing (37)

293

Phase 1/2 study to assess the safety, efficacy, and pharmacokinetics of barasertib (AZD1152) in patients with advanced acute myeloid leukemia

By: Loewenberg, Bob; Muus, Petra; Ossenkoppele, Gert; Rousselot, Philippe; Cahn, Jean-Yves; Ifrah, Norbert; Martinelli, Giovanni; Amadori, Sergio; Berman, Ellin; Sonneveld, Pieter; et al

Blood (2011), 118(23), 6030-6036 | Language: English, Database: CAplus and MEDLINE

The primary objective of this 2-part phase 1/2 study was to determine the maximum-tolerated dose (MTD) of the potent and selective Aurora B kinase inhibitor barasertib (AZD1152) in patients with newly diagnosed or relapsed acute myeloid leukemia (A M L). Part A determined the MTD of barasertib administered as a continuous 7-day infusion every 21 days. In part B, the efficacy of barasertib was evaluated at the MTD. In part A, 32 patients were treated with barasertib 50 mg (n = 3), 100 mg (n = 3), 200 mg (n = 3), 400 mg (n = 4), 800 mg (n = 7), 1200 mg (n = 6), and 1600 mg (n = 6). Dose-limiting toxicities (stomatitis/mucosal inflammation events) were reported in the 800 mg (n = 1), 1200 mg (n = 1), and 1600 mg (n = 2) groups. The M TD was defined as 1200 mg. In part B, 32 patients received barasertib 1200 mg. In each part of the study, 8 of 32 patients had a hematol. response according to Cheson AML criteria. The most commonly reported grade ≥ 3 events were febrile neutro penia (n = 24) and stomatiti s/mucosal inflammation (n = 16). We concluded that the MTD of barasertib is 1200 mg in patients with relapsed or newly diagnosed A ML. Toxicity was manageable and barasertib treatment resulted in an overall hematol. response rate of 25%. This.

Keywords: barasertib anticancer agent toxicity pharmaco kinetics acute myeloid leukemia

Substances (5) Reactions (0) Citing (55)

294

Aurora kinase inhibitor AZD1152 negatively affects the growth and survival of HTLV-1-infected T lymphocytes in vitro [Retraction of document cited in CA154:351346]

By: Tanaka, Tomita M.; Mori, N.

International Journal of Cancer (2011), 129(11), 2763 | Language: English, Database: CAplus

This article has been retracted by the authors, Editor-in-Chief, and publisher due to inappro priate duplication.

Keywords: retraction AZD1152 human T cell leukemia virus lymphocyte signaling

Substances (3) | Reactions (0) | Citing (0)

295

A Phase I study to assess the safety, pharmacokinetics and efficacy of barasertib (AZD1152), an Aurora B kinase inhibitor, in Japanese patients with advanced acute myeloid leukemia

By: Tsuboi, Kosuke; Yokozawa, Toshiya; Sakura, Toru; Watanabe, Takashi; Fujisawa, Shin; Yamauchi, Takahiro; Uike, Naokuni; Ando, Kiyoshi; Kihara, Rika; Tobinai, Kensei; et al

Leukemia Research (2011), 35(10), 1384-1389 | Language: English, Database: CAplus and MEDLINE

Barasertib (AZD1152) is a highly potent and selective Aurora B kinase inhibitor. The safety, efficacy and pharmaco kinetic (PK) profile of barasertib were investigated in Japanese patients with advanced acute myeloid leukemia. Barasertib (50-1200 mg) was administered as a continuous 7-day i.v. infusion every 21 days. No dose-limiting toxicities were reported and barasertib 1200 mg was chosen for further evaluation in Japanese patients. Neutropenia and febrile neutropenia were the most commonly reported adverse events. The PK profile was similar to Western patients. A promising overall hematol. response rate of 19% was achieved, which warrants further investigation in these patients.

Keywords: barasertib acute myeloid leukemia pharmaco kinetic antitumor Japanese

Substances (3) | Reactions (0) | Citing (41)

296

Display of antibody fragments on virus-like particles of RNA bacteriophages

By: Peabody, David S.; Chackerian, Bryce

World Intellectual Property Organization, WO2011116226 A2 2011-09-22 | Language: English, Database: CAplus

The invention enables the display of antibody single-chain variable fragments (scFv's) on virus-like particles (VLPs) of bacteriophages such as MS2. The VLPs encapsidate mRNA encoding the coat protein from which it assembles, enabling the recovery by reverse transcription and PCR of affinity-selected sequences from scFv libraries. The antibody single-chain variable fragment is inserted at a site corresponding to amino acids 11-17 or more particularly 13-17, or at the C-terminus or C-terminus, of the coat protein of MS-2. The efficacy and rate of capsid assembly are influenced and maximized in the presence of the MS2 translational operator, a 19-nucleotide RNA stem-loop (pac site) that, via its interaction with coat protein, mediates exclusive encapsidation of the MS2 genome during bacteriophage replication. The VLP expresses or displays a scFv peptide that has an affinity for a cancer cell, including solid tumors and leukemias, or a cell infected with a virus. The invention may be used for cell-type specific delivery of drugs by employing VLPs which will deliver compounds to specific cells, or delivery of image contrast enhancing agents. Related virus- like particles, method for constructing a library of scFv-VLPs, drug delivery vehicles comprising one or more pharmaceutically-active ingredients, biomedical imaging agents, assays, and kits are also provided.

Keywords: antibody display virus like particle RNA bacteriophage; drug delivery virus like particle RNA bacteriophage; single chain antibody display virus like particle RNA bacteriophage

PatentPak available

Substances (334) Reactions (0) Citing (7)

297

Combination therapy for treating cancer and diagnostic assays for use therein

By: Shah, Omar Jameel; Shen, Yu; Lin, Xiaoya; Anderson, Mark; Huang, Xiaoli; Li, Junling; Li, Leiming

World Intellectual Property Organization, WO2011068863 A1 2011-06-09 | Language: English, Database: CAplus

The present disclosure relates to a combination of therapeutic agents for use in treating a patient suffering from cancer, wherein said combination comprises at least one polyploidy inducing agent and at least one Bcl- 2 family protein inhibitor. In addition, the present disclosure also relates to diagnostic assays useful in classification of patients for treatment with one or more therapeutic agents.

Keywords: antitumor combination therapy cancer therapy diagnosis assay

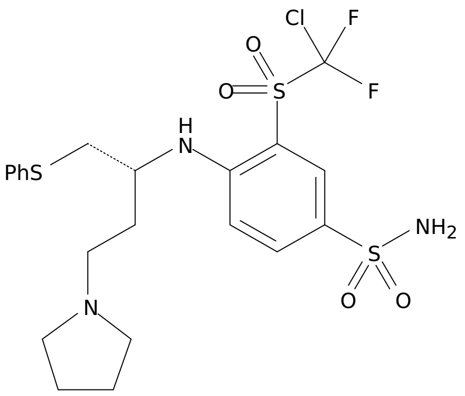

PatentPak available

Substances (181) Reactions (803) Citing (2)

298

IGF1R inhibitor based treatment of prostate cancer

By: Long, Brian; Groothuis, Patrick Gerhard; Hicklin, Daniel
World Intellectual Property Organization, WO2011057064 A1 2011-05-12 | Language: English, Database: CAplus

The present invention encompasses, inter alia, methods for treating prostate cancer comprising a chromo somal translocation with an IGF1R inhibitor such as an isolated antibody that binds specifically to IGF1R or a small organic compound The present invention provides a method for treating or preventing a TMPRSS2-ERG-pos. prostate cancer tumor or a prostate tumor comprising cells that overexpress the androgen receptor (AR), and/or for inducing apoptosis of cells of said tumor, in a subject, comprising adminis tering, to the subject, a therapeutically effective amount of IGF1R inhibitor; optionally, further comprising determining whether the tumor cells express IGF1R and whether the tumor cells comprise the TMPRSS2-ERG fusion and/or overexpress AR wherein (for example, by obtaining a sample of said tumor cells from the subject and analyzing them for the presence of said characteristics), if the tumor cells express IGF1R and if the tumor cells do comprise the TMPRSS2-ERG fusion and/or overexpress AR, then treatment with the IG F1R inhibitor is commenced or continued; and wherein if the tumor cells do not express I GF1R and/or if the tumor cells do not comprise the TMPRSS2-ERG fusion and/or overexpressed AR, then the treatment with the IGF1R inhibitor is discontinued.

Keywords: IGF1R inhibitor treatment prostate cancer TMPRSS2 ERG fusion; antibody IGF1R prostate cancer treatment

PatentPak available

Substances (323) Reactions (0) Citing (2)

299

Effects of AZD1152, a selective Aurora B kinase inhibitor, on Burkitt's and Hodgkin's lymphomas

By: Mori, Naoki; Ishikawa, Chie; Senba, Masachika; Kimura, Masashi; Okano, Yukio
Biochemical Pharmacology (2011), 81(9), 1106-1115 | Language: English, Database: CAplus and MEDLINE

We studied the effects of AZD1152, an Aurora B kinase inhibitor, on Burkitt's lymphoma (BL) and Hodgkin's lymphoma (HL) in human tissues and cell cultures and in a murine xenograft model of lymphoma. Aurora kinase A and B levels were assessed by RT-PCR and immunohistochem. They were aberrantly expressed in BL and HL cell lines, and in lymph nodes from patients with BL and HL. Next, activation of the Aurora B promoter was detected by reporter gene assays. The promoter activity of Aurora B kinase was high in BL cell lines and the Aurora B promoter contained a pos. regulatory region between -74 and -104 from the transcription initiation site. AZD1152-hQPA had antiproliferative effects in the BL and HL cell lines studied; inhibited the phosphorylation of histone H3 and retinoblastoma proteins, and resulted in cells with >4 N DNA content. AZD1152-hQPA induced caspase-dependent apoptosis of some cell lines, demonstrated by loss of mitochondrial membrane potential, activation of caspase-9, followed by activation of caspase-3. This effect was accompanied by the inhibition of survivin expression. In vivo efficacy was determined in NOD/SCID/γc^{null} mice implanted with the Ramos human BL cell line. AZD1152 had anti-tumor effects in this murine xenograft model. There preclin. data suggest that the inhibition of Aurora B kinase is a potentially useful therapeutic strategy in BL and HL.

Keywords: Aurora B kinase inhibitor AZD1152 Burkitt's Hodgkin's lymphoma

Substances (7) Reactions (0) Citing (17)

300

Enhanced radiosensitivity of androgen-resistant prostate cancer: AZD1152-mediated Aurora kinase B inhibition

By: Niermann, Kenneth J.; Moretti, Luigi; Giacalone, Nicholas J.; Sun, Yunguang; Schleicher, Stephen M.; Kopsombut, Prapaporn; Mitchell, Lauren R.; Kim, Kwang Woon; Lu, Bo
Radiation Research (2011), 175(4), 444-451 | Language: English, Database: CAplus and MEDLINE

Aurora kinase B (AURKB) is critical to the process of mitosis, aiding in chromosome conden sation by phosphor ylating histone H3. We investigated the effects of AZD1152, an AURKB inhibitor, on radiosen sitivity of androgen-insensitive prostate cancer cells. The goal of this study was to test whether AZD1152 increases the susceptibility of hormone-refractory prostate cancer cells to radiation-induced DNA damage and to determine the conditions of A ZD1152 treatment that maximize radiosensi tization. PC3 and DU145 cells were treated with various AZD1152 doses for various durations to elucidate the conditions that yielded maximal increases in G_2/M-phase and polyploid cells. To assess DNA damage, γ-H2AX phosphorylation was quantified for cells grown under radiosen sitizing conditions and subjected to either no radiation or 5 Gy radiation. Radiosen sitivity was determined by clonogenic assays. Cell cycle effects in both cell lines were maximized by treatment with 60 nM AZD1152 for 48 h. AZD1152-treated cells exhibited signifi cantly increased DNA damage 30 min postirra diation (PC3: 100% compared to 68%, P = 0.035; D U145: 100% compared to 69%, P = 0.034), with addnl. DNA damage 6 h postirra diation (PC3: 85% compared to 15%, P = 0.002; D U145: 67% compared to 21%, P = 0.012). Radiosensitivity was increased in both cell lines, with dose enhanc ement ratios of 1.53 for PC3 cells (P = 0.017) and 1.71 for DU145 cells (P = 0.02). This study identifies the optimal A ZD1152 treatment conditions to maximize the radiosens itization of PC3 and DU145 cells. These results suggest a major role for D NA damage and impairment of D NA repair mechanisms in AZD1152-induced radiosensitization of prostate cancer cells.

Keywords: radiotherapy AZD1152 radiosensitizer androgen prostate cancer; Aurora kinase B signaling inhibitor

Substances (2) Reactions (0) Citing (25)

301

N-(4-((3-(2-amino-4-pyrimidinyl)-2-pyridinyl)oxy)phenyl)-4-(4-methyl-2-thienyl)-1-phthalazinamine for use in the treatment of antimitotic agent resistant cancer

By: Payton, Marc; Kendall, Richard
World Intellectual Property Organization, WO2011031842 A1 2011-03-17 | Language: English, Database: CAplus

The present invention relates to methods of using the compound, N-(4-((3-(2-amino-4-pyrimidinyl)-2-pyridinyl)oxy)phenyl)-4-(4-methyl-2-thienyl)-1-phthalazinamine or a pharmace utically acceptable salt thereof, to treat cancers, including solid tumors, which have become resistant to treatment with chemotherapeutic agents, including anti-mitotic agents such as taxanes, and/or other anti-cancer agents, including aurora kinase inhibiting agents. The invention also includes methods of treating cancers refractory to such treatments by administering a pharmaceutical composition, comprising the compound to a cancer subject.

Keywords: phthalazinamine derivative cancer treatment aurora kinase inhibitor

PatentPak available

Substances (41) Reactions (9) Citing (3)

302

Aurora B kinase inhibitor AZD1152: determinants of action and ability to enhance chemotherapeutics effectiveness in pancreatic and colon cancer

By: Azzariti, A.; Bocci, G.; Porcelli, L.; Fioravanti, A.; Sini, P.; Simone, G. M.; Quatrale, A. E.; Chiarappa, P.; Mangia, A.; Sebastian, S.; et al
British Journal of Cancer (2011), 104(5), 769-780 | Language: English, Database: CAplus and MEDLINE

Background: AZD1152, the prodrug for AZD1152-hydroxyquinazoline pyrazol anilide (HQPA), is a selective inhibitor of Aurora B kinase activity. Preclin. evaluation of AZD1152 has been reported in several human cancer models. The potent iality of this compound in combination therapy warrants further investigation in solid tumors. Exptl. design: This study explored the effects of A ZD1152-HQPA in colon and pancreatic tumor cells. The antitumor properties of A ZD1152, either as single agent or in combin ation with chemotherapeutics, were evaluated in each study model. The efficacy and the toxicity of A ZD1152 alone and in combination with gemcitabine were validated in pancreatic tumor xenograft model. Results: A ZD1152-HQPA treatment resulted in a dramatic increase of chromosome number, modification of cell cycle and induction of apoptosis. The most effective combin ation was that with chemotherapeutics given soon after AZD1152 in both tumor cell types. The effecti veness of the sequential schedule of AZ D1152 with gemcitabine was confirmed in nude mice bearing Mia PaCa-2 tumors, showing inhibition of tumor volumes and delaying of tumor growth after the interruption of the treatments. Conclusion: Here we show that A ZD1152-HQPA enhances oxaliplatin and gemcitabine effectiveness in colon and pancreatic cancer, resp. First, we provide advances into adminis tration schedules and dosing regimens for the combination treatment in in vivo pancreatic tumor. British Journal of Cancer (2011) 104, 769- 780; doi: 10.1038/bjc.2011.21 www.bjcancer.com Published online 8 Feb. 2011.

Keywords: aurora B kinase inhibitor AZD1152 antitumor pancreas colon cancer; combin ation chemotherapy AZD1152 gemcitabine oxaliplatin antitumor pancreas colon cancer

Substances (6) Reactions (0) Citing (49)

303

Imaging Colon Cancer Response Following Treatment with AZD1152: A Preclinical Analysis of [^{18}F]Fluoro-2-deoxyglucose and 3'-deoxy-3'-[^{18}F]Fluorothymidine Imaging

By: Moroz, Maxim A.; Kochetkov, Tatiana; Cai, Shang-De; Wu, Ji-Yuan; Shamis, Mikhail; Nair, Jayasree; de Stanchina, Elisa; Serganova, Inna; Schwartz, Gary K.; Banerjee, Debabrata; et al
Clinical Cancer Research (2011), 17(5), 1099-1110 | Language: English, Database: CAplus and MEDLINE

Purpose: To determine whether treatment response to the Aurora B kinase inhibitor, AZD1152, could be monitored early in the course of therapy by noninvasive [^{18}F]-labeled fluoro-2-deoxyglucose, [^{18}F]FDG, and/or 3'-deoxy-3'-[^{18}F]fluorothymidine, [^{18}F]FLT, P ET imaging. Exptl. design: AZD1152-treated and control HCT116 and SW620 xenograft-bearing animals were monitored for tumor size and by [^{18}F]FDG, and [^{18}F]FLT PET imaging. Addnl. studies assessed the endogenous and exogenous contrib utions of thymidine synthesis in the two cell lines. Results: Both xenografts showed a significant volume-reduction to AZD1152. In contrast, [^{18}F]FDG uptake did not demonstrate a treatment response. [^{18}F]FLT uptake decreased to less than 20% of control values in A ZD1152-treated HCT116 xenografts, whereas [^{18}F]FLT uptake was near background levels in both treated and untreated S W620 xenografts. The EC_{50} for AZD1152-HQPA was approx. 10 nmol/L in both S W620 and HCT116 cells; in contrast, SW620 cells were much more sensitive to methotrexate (MTX) and 5-Fluorouracil (5FU) than HCT116 cells. Immunoblot anal. demonstrated marginally lower expression of thymidine kinase in SW620 compared with HCT116 cells. The aforementioned results suggest that SW620 xenografts have a higher dependency on the de novo pathway of thymidine utilization than HCT116 xenografts. Conclusions: AZD1152 treatment showed antitumor efficacy in both colon cancer xenografts. Although [^{18}F]FDG PET was inadequate in monitoring treatment response, [^{18}F]FLT PET was very effective in monitoring response in HCT116 xenografts, but not in SW620 xenografts. These observations suggest that de novo thymidine synthesis could be a limitation and confou nding factor for [^{18}F]FLT PET imaging and quantification of tumor proliferation, and this may apply to some clin. studies as well.

Keywords: fluoride 18 FDG FLT PET imaging agent; AZD1152 antitumor colorectal cancer

Substances (8) Reactions (0) Citing (22)

304

AZD1152 negatively affects the growth of anaplastic thyroid carcinoma cells and enhances the effects of oncolytic virus dl922-947

By: Libertini, Silvana; Abagnale, Antonella; Passaro, Carmela; Botta, Ginevra; Barbato, Sara; Chieffi, Paolo; Portella, Giuseppe
Endocrine-Related Cancer (2011), 18(1), 129-141 | Language: English, Database: CAplus and MEDLINE

Novel therapeutic approaches are required for the treatment of anaplastic thyroid carcinoma (A TC), an incurable disease resistant to current available therapies. Aurora B is an important mitotic kinase involved in chromosome segregation and cytokinesis. It is overexpressed in many cancers including ATC and represents a potential target for chemoth erapy. The effects of AZD1152, a specific Aurora B kinase inhibitor, have been evaluated against ATC, showing G_2/M accumulation, polyploidy and subsequent cell death by mitotic catastrophe upon drug treatment. Only three administ rations of AZD1152 significantly reduced the growth of A TC tumor xenografts. Oncolytic viruses in associ ation with other forms of treatment have proven highly promising in preclin. and clin. reports. The oncolytic adenovirus dl922-947 is active against A TC cells, and we have evaluated the effects of the associ ation between AZD1152 and dl922-947. In cells treated with virus and drug, we report additive/s ynergistic killing effects. Interestingly, the phosphorylation of histone H3 (Ser10) , the main Aurora B substrate, is inhibited by dl922- 947 in a dose-dependent manner, and completely abolished in association with AZD1152. The combined treatment signifi cantly inhibited the growth of A TC tumor xenografts with respect to single treatments. Our data demonstrate that the Aurora B inhibitor AZD1152, alone or in combin ation with oncolytic virus dl922-947, could represent a novel therap eutic option for the treatment of A TC.

Keywords: Aurora B AZD1152 oncolytic virus anaplastic thyroid carcinoma growth

Substances (4) Reactions (0) Citing (31)

305

A Robust High-Content Imaging Approach for Probing the Mechanism of Action and Phenotypic Outcomes of Cell-Cycle Modulators

By: Sutherland, Jeffrey J.; Low, Jonathan; Blosser, Wayne; Dowless, Michele; Engler, Thomas A.; Stancato, Louis F.
Molecular Cancer Therapeutics (2011), 10(2), 242-254 | Language: English, Database: CAplus and MEDLINE

High-content screening is increasingly used to elucidate changes in cellular biol. arising from treatment with small mols. and biol. probes. We describe a cell classifier for automated anal. of multiparametric data from immunofluorescence microscopy and charac terize the phenotypes of 41 cell-cycle modulators, including several protein kinase inhibitors in preclin. and clin. develo pment. This method produces a consistent assessment of treatment-induced phenotypes across experiments done by different biologists and highlights the prevalence of nonuniform and concentration-dependent cellular response to treatment. Contrasting cell phenotypes from high-content screening to kinase selectivity profiles from cell-free assays highlights the limited utility of enzyme potency ratios in understanding the mechanism of action for cell- cycle kinase inhibitors. Our cell-level approach for assessing phenotypic outcomes is reliable, reproducible and capable of supporting medium throughput analyses of a wide range of cellular perturb ations.

Keywords: cell cycle modulator phenotype high content imaging classification

Substances (40) Reactions (0) Citing (24)

306

P-glycoprotein and breast cancer resistance protein in acute myeloid leukaemia cells treated with the aurora-B kinase inhibitor barasertib-hQPA

By: Grundy, Martin; Seedhouse, Claire; Russell, Nigel H.; Pallis, Monica
BMC Cancer (2011), 11, 254 | Language: English, Database: CAplus and MEDLINE

Background: Aurora kinases play an essential role in orchestrating chromosome alignment, segregation and cytokinesis during mitotic progression, with both aurora-A and B frequently over-expressed in a variety of human malignancies. Over-expression of the ABC drug transporter proteins P-glycoprotein (Pgp) and Breast cancer resistance protein (BCRP) is a major obstacle for chemotherapy in many tumor types with Pgp conferring particularly poor prognosis in acute myeloid leukemia (AML). Barasertib-hQPA is a highly selective inhibitor of aurora-B kinase that has shown tumoricidal activity against a range tumor cell lines including those of leukemic AML origin. Methods: Effect of barasertib-hQPA on the pHH3 biomarker and cell viability was measured in a panel of leukemic cell lines and 37 primary AML samples by flow cytometry. Pgp status was determined by flow cytometry and BCRP status by flow cytometry and real-time PCR. Results: In this study we report the creation of the cell line OCI-AML3DNR, which over-expresses Pgp but not BCRP or multidrug resistance-associated protein (MRP), through prolonged treatment of OCI-AML3 cells with daunorubicin. We demonstrate that Pgp (OCI-AML3DNR and KG-1a) and BCRP (OCI-AML6.2) expressing AML cell lines are less sensitive to barasertib-hQPA induced pHH3 inhibition and subsequent loss of viability compared to transporter neg. cell lines. We also show that barasertib-hQPA resistance in these cell lines can be reversed using known Pgp and BCRP inhibitors. We report that barasertib-hQPA is not an inhibitor of Pgp or BCRP, but by using ^{14}[C]-barasertib-hQPA that it is effluxed by these transporters. Using phosphoHistone H3 (pHH3) as a biomarker of barasertib-hQPA responsiveness in primary AML blasts we determined that Pgp and BCRP pos. primary samples were less sensitive to barasertib-hQPA induced pHH3 inhibition ($p \leq 0.001$) than samples without these transporters. However, we demonstrate that IC_{50} inhibition of pHH3 by barasertib-hQPA was achieved in 94.6% of these samples after 1 h drug treatment, in contrast to the resistance of the cell lines. Conclusion: We conclude that Pgp and BCRP status and pHH3 down-regulation in patients treated with barasertib should be monitored in order to establish whether transporter-mediated efflux is sufficient to adversely impact on the efficacy of the agent.

Keywords: anticancer Aurora B kinase inhibitor barasertib hQPA Pgp BCRP; acute myeloid leukemia

Substances (3) Reactions (0) Citing (14)

307

Effects of AZD1152, a selective Aurora B kinase inhibitor, on Burkitt's and Hodgkin's lymphomas [Erratum to document cited in CA154:556930]

By: Mori, Naoki; Ishikawa, Chie; Senba, Masachika; Kimura, Masashi; Okano, Yukio
Biochemical Pharmacology (2011), 82(9), 1252 | Language: English, Database: CAplus

On page 1107, Section 2.4 contained an error; the correction is given.

Keywords: erratum Aurora B kinase inhibitor AZD1152 Burkitts Hodgkins lymphoma

Substances (6) Reactions (0) Citing (0)

308

A theoretical entropy score as a single value to express inhibitor selectivity

By: Uitdehaag, Joost C. M.; Zaman, Guido J. R.
BMC Bioinformatics (2011), 12, 94 | Language: English, Database: CAplus and MEDLINE

Designing maximally selective ligands that act on individual targets is the dominant paradigm in drug discovery. Poor selectivity can underlie toxicity and side effects in the clinic, and for this reason compound selectivity is increasingly monitored from very early on in the drug discovery process. To make sense of large amounts of profiling data, and to determine when a compound is sufficiently selective, there is a need for a proper quant. measure of selectivity. Here we propose a new theor. entropy score that can be calculated from a set of IC_{50} data. In contrast to previous measures such as the 'selec tivity score', Gini score, or partition index, the entropy score is non-arbitary, fully exploits IC_{50} data, and is not dependent on a reference kinase. In addition, the entropy score gives the most robust values with data from different sources, because it is less sensitive to errors. We apply the new score to kinase and nuclear receptor profiling data, and to high-throughput screening data. In addition, through analyzing profiles of clin. compounds, we show that a more selective inhibitor is not necessarily more drug-like. For quantifying selectivity from panel profiling, a theor. entropy score is the best method. It is valuable for studying the mol. mechanisms of selectivity, and to steer compound progression in drug discovery programs.

Keywords: theor entropy score allosteric inhibitor selectivity

Substances (42) Reactions (0) Citing (30)

309

Preclinical Evaluation of AMG 900, a Novel Potent and Highly Selective Pan-Aurora Kinase Inhibitor with Activity in Taxane-Resistant Tumor Cell Lines

By: Payton, Marc; Bush, Tammy L.; Chung, Grace; Ziegler, Beth; Eden, Patrick; McElroy, Patricia; Ross, Sandra; Cee, Victor J.; Deak, Holly L.; Hodous, Brian L.; et al
Cancer Research (2010), 70(23), 9846-9854 | Language: English, Database: CAplus and MEDLINE

In mammalian cells, the aurora kinases (aurora-A, -B, and -C) play essential roles in regulating cell division. The expression of aurora-A and -B is elevated in a variety of human cancers and is associated with high prolife ration rates and poor prognosis, making them attractive targets for anticancer therapy. AMG 900 is an orally bioavai lable, potent, and highly selective pan- aurora kinase inhibitor that is active in taxane-resistant tumor cell lines. In tumor cells, A MG 900 inhibited autophosph orylation of aurora-A and -B as well as phosphorylation of histone H3 on Ser^{10}, a proximal substrate of aurora- B. The predominant cellular response of tumor cells to AMG 900 treatment was aborted cell division without a prolonged mitotic arrest, which ultimately resulted in cell death. AMG 900 inhibited the proliferation of 26 tumor cell lines, including cell lines resistant to the antimi totic drug paclitaxel and to other aurora kinase inhibitors (AZD1152, MK-0457, and PHA-739358), at low nanomolar concent rations Furthermore, AMG 900 was active in an AZD1152-resistant HCT116 variant cell line that harbors an aurora- B mutation (W221L). Oral adminis tration of AMG 900 blocked the phosphorylation of histone H3 in a dose- dependent manner and signifi cantly inhibited the growth of HCT116 tumor xenografts. Importantly, AMG 900 was broadly active in multiple xenograft models, including 3 multidrug- resistant xenograft models, representing 5 tumor types. AMG 900 has entered clin. evaluation in adult patients with advanced cancers and has the potential to treat tumors refractory to anticancer drugs such as the taxanes.

Keywords: antitumor AMG900 taxane resistance breast colon cancer

Substances (12) Reactions (0) Citing (104)

310

Methods for providing personalized medicine tests ex vivo for hematological neoplasms

By: Ballesteros, Juan; Bennett, Teresa; Primo, Daniel; Orfao, Alberto; Jackson, Coyt; Lago, Santiago; Matoses, Maria; Suarez, Lilia; Sapia, Sandra; Bosanquet, Andrew; et al
World Intellectual Property Organization, WO2010135468 A1 2010-11-25 | Language: English, Database: CAplus

Described herein are methods, devices, and compositions for providing personalized medicine tests for hematol. neoplasms. In some embodiments, the methods comprise measuring the efficacy of inducing apoptosis selectively in malignant cells using any number of potential alternative combination drug treatments. In some embodiments, the ex vivo testing is measured using a recently extracted patient hematol. samples. In other embodiments, the efficacy is measured ex vivo using an automated flow cytometry platform. For example, by using an automated flow cytometry platform, the evaluation of hundreds, or even thousands of drugs and compositions, can be made ex vivo. Thus, alternative polytherapy treatments can be explored. Non-cytotoxic drugs surprisingly induce apoptosis selectively in malignant cells ex vivo. In some embodiments, the methods described herein comprise evaluating non-cytotoxic drugs.

Keywords: bioassay hematol neoplasm drug personalized medicine; analytical device drug assay hematol neoplasm; automated flow cytometry drug combination assay blood; drug apoptosis blood neoplasm personalized test

PatentPak available

Substances (157) Reactions (0) Citing (3)

311

Expression, purification, stability optimization and characterization of human Aurora B kinase domain from E. coli

By: Sheth, Payal R.; Ramanathan, Lata; Ranchod, Ashwin; Basso, Andrea D.; Barrett, Dianah; Zhao, Jia; Gray, Kimberly; Liu, Yan-Hui; Zhang, Rumin; Le, Hung V.
Archives of Biochemistry and Biophysics (2010), 503(2), 191-201 | Language: English, Database: CAplus and MEDLINE

Aurora B kinase plays a critical role in regulating mitotic progression, and its dysregulation has been linked to tumorigenesis. The structure of the kinase domain of human Aurora B and the complementary information of binding thermodn. of known Aurora inhibitors is lacking. Towards that effort, we sought to identify a human Aurora B construct that would be amenable for large-scale protein production for biophys. and structural studies. Although the designed $AurB_{69-333}$ construct expressed at high levels in Escherichia coli, the purified protein was largely unstable and prone to aggregation. We employed thermal-shift assay for high-throughput screening of 192 conditions to identify optimal pH and salt conditions that increased the stability and minimized aggregation of $AurB_{69-333}$. Direct ligand binding analyses using temperature-dependent CD (TdCD) and TR-FRET-based Lanthascreen binding assay showed that the purified protein was folded and functional. The affinity rank-order obtained using TdCD and Lantha screen binding assay correlated with enzymic IC_{50} values measured using full-length Aurora B protein for all the inhibitors tested except for AZD1152. The direct binding results support the hypothesis that the purified human $AurB_{69-333}$ fragment is a good surrogate for its full-length counterpart for biophys. and structural analyses.

Keywords: Aurora B kinase domain cloning Escherichia coli

Substances (6) Reactions (0) Citing (6)

312

Fully human anti-VEGF antibodies and methods of using in cancer therapy

By: Ramachandra, Sumant; Huang, Chin-Yi; Masat, Linda; Huang, Chao Bai; Bishop, Walter Robert; Takeuchi, Toshihiko; Kantak, Seema; Horowitz, Jo Ann; Ladha, Mohamed H.
World Intellectual Property Organization, WO2010124009 A2 2010-10-28 | Language: English, Database: CAplus

Disclosed herein are fully human antibodies and antigen-binding fragments thereof that specifically bind human VEGF and inhibit V EGF binding to VEGF-R1 and VEGF-R2, and therefore inhibit VEGF signaling. The antibodies and antigen-binding fragments disclosed herein may be used, for example, to treat angiogenesis and conditions associated with angiog enesis both in vivo and in vitro.

Keywords: human VEGF antibody cancer therapy VEGFR antitumor conjugate

PatentPak available

Substances (357) | Reactions (0) | Citing (3)

313

Aurora kinase inhibitor AZD1152 negatively affects the growth and survival of HTLV-1-infected T lymphocytes in vitro

By: Tomita, Mariko; Tanaka, Yuetsu; Mori, Naoki
International Journal of Cancer (2010), 127(7), 1584-1594 | Language: English, Database: CAplus and MEDLINE

Aurora kinases play an essential role in regulating mitosis and cell division. Inhibition of Aurora kinases results in suppression of cell division, phosphorylation of histone H3 and induction of apoptosis in many cell types. These characte ristics have prompted the testing of Aurora kinase inhibitors as chemotherapeutic agents. In our study, we report the in vitro activities of A ZD1152, a selective inhibitor of Aurora B kinase in human T-cell leukemia virus type 1 (HTLV-1), the causative agent of adult T-cell leukemia (ATL), -infected T-cell lines. Overexpression of Aurora B was noted in HTLV-1-infected T-cell lines compared to HTLV-1-uninfected T-cell lines. AZD1152 reduced the viability of HTLV-1-infected T-cell lines within 24 h but did not affect that of - uninfected T-cell lines. Although AZD1152 inhibited phosphorylation of histone H3 on Ser10 in both H TLV-1-infected and -uninfected T-cell lines, it induced polyploidy only in HTLV-1-uninfected T-cell lines. AZD1152 induced early apoptosis of HTLV-1-infected T-cells without induction of polyploidy. We have reported previously that a pan- Aurora kinase inhibitor induced apoptosis through inhibition of N F-κB signaling activity in HTLV-1-infected T-cell lines. In contrast, AZD1152 did not affect N F-κB activity in these cells. It induced p53 and p21 expression in HTLV-1-infected but not in HTLV-1-uninfected T-cell lines, suggesting that activation of p53- dependent postmitotic checkpoint might prevent polyploidy in HTLV-1-infected T-cells. Our results suggest that specific inhibition of Aurora B kinase is a potentially useful therapeutic strategy in the treatment of A TL and that further in vivo exploration is warranted.

Keywords: AZD1152 human T cell leukemia virus lymphocyte signaling

Substances (3) | Reactions (0) | Citing (6)

314

System-Level Analysis of Neuroblastoma Tumor-Initiating Cells Implicates AURKB as a Novel Drug Target for Neuroblastoma

By: Morozova, Olena; Vojvodic, Milijana; Grinshtein, Natalie; Hansford, Loen M.; Blakely, Kim M.; Maslova, Alexandra; Hirst, Martin; Cezard, Timothee; Morin, Ryan D.; Moore, Richard; et al
Clinical Cancer Research (2010), 16(18), 4572-4582 | Language: English, Database: CAplus and MEDLINE

PURPOSE: Neuroblastoma (NB) is an aggressive tumor of the developing peripheral nervous system that remains difficult to cure in the advanced stages. The poor prognosis for high-risk NB patients is associated with common disease recurr ences that fail to respond to available therapies. NB tumor-initiating cells (TICs), isolated from metastases and primary tumors, may escape treatment and contribute to tumor relapse. New therapies that target the TICs may therefore prevent or treat tumor recurr ences. Exptl. Design: We undertook a system-level characterization of NB TICs to identify potential drug targets against recurrent N B. We used next-generation RNA sequencing and/or human exon arrays to profile the transcr iptomes of 11 NB TIC lines from six N B patients, revealing genes that are highly expressed in the TICs compared with normal neural crest- like cells and unrelated cancer tissues. We used gel-free two-dimensional liquid chromatog. coupled to shotgun tandem mass spectr ometry to confirm the presence of proteins corresponding to the most abundant TIC-enriched transcripts, thereby providing validation to the gene expression result. RESULTS: Our study revealed that genes in the B RCA1 signaling pathway are frequently misexp ressed in NB TICs and implicated Aurora B kinase as a potential drug target for NB therapy. Treatment with a selective A URKB inhibitor was cytotoxic to NB TICs but not to the normal neural crest- like cells. CONCLUSION: This work provides the first high- resolution system-level anal. of the transcriptomes of 11 primary human N B TICs and identifies a set of candidate N B TIC-enriched transcripts for further develo pment as therapeutic targets. Clin Cancer Res; 16(18); 4572-82.

Keywords: neuroblastoma tumor initiating cell A URKB therapeutic target

Substances (18) | Reactions (0) | Citing (37)

315

Combination therapy for treating cancer and diagnostic assays for use therein

By: Shah, Omar Jameel; Shen, Yu; Lin, Xiaoyu; Anderson, Mark; Huang, Xiaoli; Li, Junling; Li, Leiming
United States, US20100227838 A1 2010-09-09 | Language: English, Database: CAplus

The present disclosure relates to a combination of therapeutic agents for use in treating a patient a subject suffering from cancer. In addition, the present disclosure also relates to diagnostic assays useful in classification of patients for treatment with one or more therapeutic agents.

Keywords: antitumor combination therapy cancer therapy diagnosis assay

PatentPak available

Substances (174) | Reactions (802) | Citing (4)

316

Markers to predict and monitor response to Aurora kinase B inhibitor therapy of cancer

By: Semizarov, Dimitri; Shah, Jameel; Guo, Jun; Anderson, Mark
World Intellectual Property Organization, WO2010088470 A1 2010-08-05 | Language: English, Database: CAplus

The present invention relates to diagnostic assays useful in classification of patients for selection of cancer therapy with one or more Aurora kinase B inhibitors. In particular, the present invention relates to identifying the presence or absence of one or more copy number gains in the ABCB1 gene, the ABCB4 gene or combinations thereof, identifying patients eligible to receive Aurora kinase inhibitor therapy, either as monotherapy or as part of combination therapy, and monitoring patient response to such therapy.

Keywords: cancer therapy Aurora kinase B inhibitor biol marker

PatentPak available

Substances (327) Reactions (0) Citing (1)

317

Imidazo[4,5-b]pyridine Derivatives As Inhibitors of Aurora Kinases: Lead Optimization Studies toward the Identification of an Orally Bioavailable Preclinical Development Candidate

By: Bavetsias, Vassilios; Large, Jonathan M.; Sun, Chongbo; Bouloc, Nathalie; Kosmopoulou, Magda; Matteucci, Mizio; Wilsher, Nicola E.; Martins, Vanessa; Reynisson, Johannes; Atrash, Butrus; et al
Journal of Medicinal Chemistry (2010), 53(14), 5213-5228 | Language: English, Database: CAplus and MEDLINE

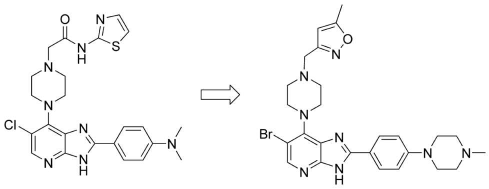

Lead optimization studies using an imidazo[4,5-b]pyridinylpiperazine as the starting point led to a new class of imidazo[4,5-b]pyridine-based inhibitors of Aurora kinases that possessed the 1-benzylpiperazinyl motif at the 7-position, and displayed favorable in vitro properties. Cocrystn. of Aurora-A with a morpholinylmethylphenylimidazopyridinyl chlorobenzyl piperazine (CCT137444) provided a clear understanding into the interactions of this novel class of inhibitors with the Aurora kinases. Subsequent physicochem. property refinement by the incorporation of solubilizing groups led to the identification of 3-((4-(6-bromo-2-(4-(4-methylpiperazin-1-yl)phenyl)-3H-imidazo[4,5-b]pyridin-7-yl)piperazin-1-yl)methyl)-5-methylisoxazole (CCT137690)(**I**) which is a potent inhibitor of Aurora kinases (Aurora-A IC_{50} = 0.015±0.003 μM, Aurora-B IC_{50} = 0.025 μM, Aurora-C IC_{50} = 0.019 μM). **I** is highly orally bioavailable, and in in vivo efficacy studies it inhibited the growth of SW620 colon carcinoma xenografts following oral administration with no observed toxicities as defined by body weight loss.

Keywords: imidazopyridine derivative arsenic inhibitor Aurora kinase orally bioavailable development

Substances (234) Reactions (402) Citing (70)

318

Bcl-XL represents a druggable molecular vulnerability during aurora B inhibitor-mediated polyploidization

By: Shah, Jameel; Lin, Xiaoyu; Li, Leiming; Huang, Xiaoli; Li, Junling; Anderson, Mark G.; Tang, Hua; Rodriguez, Luis E.; Warder, Scott E.; Mcloughlin, Shaun; et al
Proceedings of the National Academy of Sciences of the United States of America (2010), 107(28), 12634-12639, S12634/1-S12634/10 | Language: English, Database: CAplus and MEDLINE

Aurora kinase B inhibitors induce apoptosis secondary to polyploidization and have entered clin. trials as an emerging class of neocytotoxic chemotherapeutics. We demonstrate here that polyploidization neutralizes Mcl-1 function, rendering cancer cells exquisitely dependent on Bcl-XL-2. This "addiction" can be exploited therapeutically by combining aurora kinase inhibitors and the orally bioavailable BH3 mimetic, ABT-263, which inhibits Bcl-XL, Bcl-2, and Bcl-w. The combination of ABT-263 with aurora B inhibitors produces a synergistic loss of viability in a range of cell lines of divergent tumor origin and exhibits more sustained tumor growth inhibition in vivo compared with aurora B inhibitor monotherapy. These data demonstrate that Bcl-XL/-2 is necessary to support viability during polyploidization in a variety of tumor models and represents a druggable mol. vulnerability with potential therapeutic utility.

Keywords: Bcl XL aurora kinase B inhibitor polyploidization cancer

Substances (4) Reactions (0) Citing (34)

319

Identification and classification of E2F-responsive gene overexpressing tumors for selection of therapies

By: Shackney, Stanley E.
World Intellectual Property Organization, WO2010056931 A1 2010-05-20 | Language: English, Database: CAplus

Methods for identifying a tumor as an E2F-responsive gene over-expressing (ERGO) tumor are described. These methods can be used in diagnosis and to predict survival of the patient and in the selection and monitoring of therapies. These methods are applicable to ERGO tumors present in different human organs and tissues such as breast, lung, thyroid, ovary, and prostate. Transcription factor E2F regulates approx. 325 genes and abnormal levels of expression of a significant number of these in a tissue sample is diagnostic of an ERGO tumor.

Keywords: E2F transcription regulation neoplasm diagnosis therapy

Substances (376) Reactions (0) Citing (1)

320

The selective Aurora B kinase inhibitor AZD1152 as a novel treatment for hepatocellular carcinoma

By: Aihara, Arihiro; Tanaka, Shinji; Yasen, Mahmut; Matsumura, Satoshi; Mitsunori, Yusuke; Murakata, Ayano; Noguchi, Norio; Kudo, Atsushi; Nakamura, Noriaki; Ito, Koji; et al
Journal of Hepatology (2010), 52(1), 63-71 | Language: English, Database: CAplus and MEDLINE

Background & Aims: We previously identified that high Aurora B expression was associated with hepatocellular carcinoma (HCC) recurrence due to tumor dissemination. In this preclin. study, a novel inhibitor of Aurora B kinase was evaluated as a treatment for human HCC. Methods: AZD1152 is a selective inhibitor of Aurora B kinase. Twelve human H CC cell lines were analyzed for Aurora B kinase expression and the in vitro effects of AZD1152. The in vivo effects of AZD1152 were analyzed in a s.c. xenograft model and a novel orthotopic liver xenograft model. Results: Aurora B kinase expression varied among the human HCC cell lines and was found to correlate with inhibition of cell proliferation, accumulation of 4N DNA, and the proportion of polyploid cells following adminis tration of AZD1152-hydroxyquinazoline-pyrazol-anilide (AZD1152-HQPA). AZD1152-HQPA suppressed histone H3 phosphor ylation and induced cell death in a dose-dependent manner. Growth of s.c. human H CC xenografts was inhibited by A ZD1152 administ ration. In an orthotopic hepatoma model, treatment with A ZD1152 significantly decelerated tumor growth and increased survival. Pharmacobiol. anal. revealed that AZD1152 induced the rapid suppression of phosphohistone H3, followed by cellular apoptosis in the liver tumors but not in the normal tissues of the orthotopic models. Conclusions: Our preclin. studies indicate that AZD1152 is a promising novel therapeutic approach for the treatment of H CC.

Keywords: anticancer AZD1152 apoptosis phosphohistone H3 hepatocellular carcinoma growth

Substances (4) Reactions (0) Citing (53)

321

Novel agents and regimens for acute myeloid leukemia: 2009 ASH annual meeting highlights

By: Zhu, Xiongpeng; Ma, Yuehua; Liu, Delong
Journal of Hematology & Oncology (2010), 3, No pp. given | Language: English, Database: CAplus and MEDLINE

A review. Prognostic markers, such as NPM1, Flt3-ITD, and cytogenetic abnormalities have made it possible to formulate aggressive treatment plans for unfavorable acute myeloid leukemia (AML). However, the long-term survival of AML with unfavorable factors remains unsatisfactory. The latest data indicate that the standard dose of daunor ubicin (DNR) at 45 mg/m^2 is inferior to high dose 90 mg/m^2 for induction therapy. The rates of complete remission and overall survival are signifi cantly better in the high dose induction regimen. New regimens exploring the new liposomal encapsulation of Ara-C and DNR as well as addition of gemtuzumab ozogamicin monoclonal antibody have been studied. New agents, including the nucleoside analogs (clofarabine, sapacitabine, elacytarabine), FLT3 inhibitor (sorafenib), farnesyl- transferase inhibitor (tipifarnib), histone deacetylase inhibitor (vorinostat), lenalid omide, as well as DNA methyltransferase inhibitors (decitabine, azacitidine), were recently reported for AML treatment in the 2009 A SH annual meeting. This review also summarizes the updates of the clin. trials on novel agents including voreloxin, A S1413, beheno ylara-C, ARRY520, ribavirin, AZD1152, AZD6244, and terameprocol (EM-1421) from the 2009 ASH annual meeting.

Keywords: review anticancer acute myeloid leukemia

Substances (22) Reactions (0) Citing (49)

322

Aurora kinase inhibitors: a new class of drugs targeting the regulatory mitotic system

By: Perez Fidalgo, Jose Alejandro; Roda, Desamparados; Rosello, Susana; Rodriguez-Braun, Edith; Cervantes, Andres
Clinical & Translational Oncology (2009), 11(12), 787-798 | Language: English, Database: CAplus and MEDLINE

The present review gives a perspective on the Aurora kinase family members, their function in normal cells, their role in cancer progression as well as their potential as target for anticancer treatment. Mitosis has been an important target for anticancer therapy development, leading to some specific drugs mainly addressing Tubulines, as a key structure of the mitotic spindle. Vinca alkaloids, taxanes or epotilones are good examples of conventionally developed antimitotic agents. However, novel classes of antineoplastic drugs are being studied, targeting the regulatory system that controls functional aspects of mitosis, such as Aurora or Polo-like kinases or Kinespondin inhibitors. The specific role of the different Aurora kinase proteins as regulator enzymes of the mitotic process in normal cells is discussed. Some of the mechanisms that link Aurora overexpression with cancer are also consid ered. Thereafter, the clin. and preclin. develo pment of the different Aurora kinase inhibitors is presented. This is nowadays a very active area of therapeutic research and at least, sixteen new compounds are being studied as potential antineo plastic drugs. Most of them are in a very early phase of clin. development. However, we summarized the most recently published findings related with these drugs: main characteristics, way of administ ration, dose limiting toxicities and recomm ended doses for further studies. Another important aspect in Aurora kinase inhibition is the study and validation of potential biomarkers to optimize the clin. develo pment. Several studies included pharmaco dynamic assessments in normal blood cells, skin or/and tumor biopsies. Several proposals included a higher mitotic index, a decreased number of mitosis with bipolar spindles or normal alignment of chromo somes and inhibition of histone H3 phosphor ylation. Future strategies and challenges for trials with Aurora kinase inhibitors are also discussed.

Keywords: review Aurora kinase inhibitor drug target mitosis cancer antineoplastic

Substances (5) | Reactions (0) | Citing (26)

323

Anti-IGF-1 receptor antibody combined with chemotherapeutic agent, antitumor agent, radiotherapy or surgery for treating or preventing colorectal cancer

By: Wang, Yaolin; Wang, Yan; Liu, Ming; Bishop, Walter Robert; Seidel-Dugan, Cynthia
World Intellectual Property Organization, WO2009142810 A2 2009-11-26 | Language: English, Database: CAplus

The present invention provides, for example, methods for treating or preventing colorectal cancer with an anti-IGF1R antibody in association with sunitinib or a combin ation of leucovorin and 5-fluorouracil.

Keywords: IGF1 receptor antibody chemotherapeutic antitumor radiot herapy surgery colorectal cancer

PatentPak available

Substances (343) | Reactions (0) | Citing (0)

324

Sequential administration of chemotherapeutic agents and anti-(insulin-like growth factor 1 receptor) for treatment of cancer

By: Wang, Yaolin; Wang, Yan; Lu, Brian Der-Hua; Liu, Ming; Seidel-Dugan, Cynthia; Yao, Siu-Long
World Intellectual Property Organization, WO2009137378 A2 2009-11-12 | Language: English, Database: CAplus

The present invention relates to the sequential administration of a cytotoxic agent followed by an insulin- like growth factor 1 receptor (IGF1R) antagonist (e.g., an antibody) for the treatment of hyperprol iferative disorders including cancer. Human monoclonal antibody clone 15H12/19D12 light and heavy chains targeting human I GF1R are provided. Thus, for example, sequential administration of irinotecan and then anti- IGF1R antibody for the treatment of human colorectal cancer in xenograft mice resulted in 77% tumor growth inhibition vs. 39% and 61% growth inhibition by irinotecan or anti-IGF1R antibody alone.

Keywords: cancer therapy chemotherapeutic agent I GF1 receptor antibody

PatentPak available

Substances (338) Reactions (0) Citing (2)

325

Simultaneous determination of AZD1152 (prodrug) and AZD1152-hydroxyquinazoline pyrazol anilide by reversed phase liquid chromatography

By: Pluim, Dick; Beijnen, Jos H.; Schellens, Jan H. M.; van Tellingen, Olaf
Journal of Chromatography B: Analytical Technologies in the Biomedical and Life Sciences (2009), 877(29), 3549-3555 | Language: English, Database: CAplus and MEDLINE
Analytical Methods available

A simple, selective and sensitive reversed phase liquid chromatog. method utilizing UV detection has been developed and validated for the simultaneous determination of the prodrug AZD1152 and its active product AZD1152-hydroxyquinazoline pyrazol anilide (hQ PA) in human and mouse plasma and mouse tissues. Isocratic separation was achieved using an 5 µm Upti Sphere HDO C_{18} column (150 mm × 4.6 mm) with guard column in combination with a mobile phase comprised of phosphate buffered water (50 m M; pH 3.4) and acetonitrile (81.5: 18.5; volume/volume). UV detection at 318 nm was used. Sample prepar ation involved a single-step protein precipitation with ethanol. Ex vivo conversion of AZD1152 by endogenous phosphatases was prevented by immediate cooling of the samples in ice-water and addition of sodium fluoride and E DTA. The validation parameters included specif icity, recovery, accuracy, precision, sensitivity and stability. The lower limit of quantif ication in human plasma for AZD1152 and hQPA was 25 ng/mL. The applicability of the method was demonstrated by successful determi nation of AZD1152 and hQPA in human plasma and in plasma, brain, liver, kidney and ileum samples from mice dosed with AZD1152.

Keywords: AZD1152 pharmacokinetics tissue blood analysis H PLC

Substances (3) Reactions (0) Citing (7)

326

The Aurora B kinase inhibitor AZD1152 sensitizes cancer cells to fractionated irradiation and induces mitotic catastrophe

By: Tao, Yungan; Leteur, Celine; Calderaro, Julien; Girdler, Fiona; Zhang, Ping; Frascogna, Valerie; Varna, Mariana; Opolon, Paule; Castedo, Maria; Bourhis, Jean; et al

Cell Cycle (2009), 8(19), 3172-3181 | Language: English, Database: CAplus and MEDLINE

AZD1152, an Aurora kinase inhibitor with selectivity for Aurora B kinase, can enhance the effect of ionizing radiation (I R). The aim of this study was to evaluate and to mechanistically explore scheduling effects of AZD1152 on tumor responses to IR, in three different settings; neoadjuvant (AZD1152 before IR), adjuvant (IR before AZD1152), or concomitant treatments (AZD1152 plus one single IR dose). A more pronounced tumor growth delay was observed in the neoadj uvant and adjuvant schedules as compared to the concomitant schedule. However, AZD1152 enhanced the efficacy of IR when concomitant IR was fractionated over several days. Histopathol. examination revealed that AZD1152 + IR induced polyploidy, multinucleation and micronuclei in vivo. Time-lapse videomicroscopy confirmed that cell death induced by A ZD1152 + IR was preceded by multinucleation and the formation of micron uclei, which both are hallmarks of mitotic catast rophe. Caspase inhibition or removal of the pro-apoptotic protein Bax did not ameliorate the long-term cell survival of A ZD1152-treated cancer cells. In contrast, a chem. inhibitor of C HK1, Chir124, sensitized cancer cells to the lethal effect of AZD1152. Altogether, these data support the contention that A ZD1152 mediates radiosensitization in vivo by enhancing mitotic catastrophe, which can be used as a biomarker of treatment efficacy.

Keywords: aurora B kinase inhibitor AZD1152 colon cancer radiotherapy anticancer

Substances (3) | Reactions (0) | Citing (44)

327

Gene expression profiling in the pulmonary artery in the selection of therapies for vascular-related diseases

By: Mann, David Marshall

World Intellectual Property Organization, WO2009121031 A1 2009-10-01 | Language: English, Database: CAplus

Methods of selecting treatment regimens for vascular-related diseases and disorders using gene expression profiles in affected blood vessels are described. Addnl., methods are disclosed involving diagnostic techniques to focus treatment regimens. Finally, methods of treating vascular-related disorder involving targeting microRNAs are also disclosed. Therapies suitable for treatment at different stages of the disease are identified.

Keywords: pulmonary artery gene expression profiling vascular disease therapy; arterial hypertension lung diagnosis therapy

PatentPak available

Substances (522) | Reactions (0) | Citing (6)

328

Kinase Inhibitor 4 Minisymposium summary

By: Dancey, Janet E.
Expert Review of Anticancer Therapy (2009), 9(7), 891-894 | Language: English, Database: CAplus and MEDLINE

A review. In recent years no cancer therapeutic class has undergone more prolific or successful drug develo pment efforts than the kinase inhibitors. The robust research interest is evident in the number of kinase inhibitor present ations at the American Associ ation for Cancer Research Annual Meeting held in Denver (C O, USA) 18-22 Apr. 2009. Presen tation highlights from the Kinase Inhibitor 4 Minisymposium include the identification of FGF receptor genetic altera tions that correlate with sensitivity to the kinase inhibitor TKI258; evaluation of activity of mitotic kinase inhibitors G SK461364A and AZD1152 in breast and small- cell lung cancer and the identification of new inhibitors to PI3K/AKT/mTOR pathway.

Keywords: review kinase inhibitor 4 breast lung cancer anticancer signaling

Substances (5) Reactions (0) Citing (1)

329

Protein kinase genes showing altered levels of expression in breast cancer tissue and their diagnostic use

By: Bertucci, Francois; Birnbaum, Daniel; Finetti, Pascal
World Intellectual Property Organization, WO2009083780 A1 2009-07-09 | Language: English, Database: CAplus

The present invention relates to a method for analyzing cancer.e.g., breast cancer comprising detection of differ ential expression of at least one of the 16 genes encoding serine/threonine kinases listed in Table 1, or of said 16 genes, and to a polynuc leotide library comprising at least one said 16 genes. A method of diagnosing breast cancer by anal. of the levels of expression of members of a group of 16 protein kinase genes is described. Levels of expression of the genes can also be used in prognosis and in monitoring the effectiveness of therapies. The levels of expression of these genes were analyzed in 227 samples of breast cancer tissue as part of a larger anal. of gene expression in breast cancer. Validation of the use of these genes in diagnosis and in prognosis is demonst rated.

Keywords: protein kinase gene expression breast cancer diagnosis prognosis

PatentPak available

Substances (295) Reactions (0) Citing (2)

330

Gene expression biomarkers for detecting IGF1R inhibitor-resistant or -sensitive cancers and uses in anti-IGF1R antibody combination chemotherapy

By: Wang, Yan; Wang, Yaolin; Levitan, Diane; Seidel-Dugan, Cynthia; Liu, Ming; Ding, Wei
World Intellectual Property Organization, WO2009079587 A2 2009-06-25 | Language: English, Database: CAplus

The present invention provides, for example, methods for conveniently determining if a cancerous condition in a subject will be responsive to an IGF1R inhibitor. The invention includes patient selection methods and methods of treatment. The method comprises determining the expression levels of biomarker-genes associated with sensitivity or resistance to IGF1R inhibitor therapy. Thus, genes upregulated in sensitive cells relative to resistant cells are: T RE2, SMC4, TRIB2, TLE4, BMP7, PCDHGC3, AUTS2, C14orf132, CERK, HDGFRP3, TCF4, MEIS2, EML4, C7orf41, KIAA1450, ZNF136, D15F37, CDK6, TIMP, Clu, Prl1. Genes down-regulated in sensitive cells relative to resistant cells are: ACAT1, ALDOC, C6orf192, COL4A5, C1QBP, CRIP1, DEADC1, GSTK1, GSTO2, PPAPDC1B, TMEM107, JOSD3, TMEM77, MST1, MT1E, PARVB, PRDX4, RASGEF1A, RPL14, IFI30, ATF1, ACADVL, FBXO6, NQO2, TMEM64, ZFAND1, T MED5, PDIA5, MYO1C, GNPTAB, LACTB2, RPL22, TSPAN4, RPL15, PCCB, CRYZ, DNAJC10, C19orf54, HSPE1, hqp0376. Provided are sequences for anti-IGF1R antibodies to be used in combination chemotherapy.

Keywords: gene biomarker IGF1R inhibitor therapy resistance sensitivity cancer diagnosis; anticancer IGF1R antibody sequence combination chemotherapy

PatentPak available

Substances (339) Reactions (0) Citing (3)

331

Antitumor combination comprising a MEK inhibitor and an Aurora kinase inhibitor 188

By: Mundt, Kirsten Elisabeth; Smith, Paul David
World Intellectual Property Organization, WO2009074827 A2 2009-06-18 | Language: English, Database: CAplus

The present invention relates to a therapeutic combination comprising a MEK inhibitor and an Aurora kinase inhibitor, and to methods for the production of an anti-cancer effect in a patient, which is accordingly useful in the treatment of cancer in a patient. More specifically the present invention relates to: a therapeutic combination comprising a MEK inhibitor and an Aurora kinase inhibitor; a combination product comprising a MEK inhibitor and an Aurora kinase inhibitor, a kit of parts comprising a M EK inhibitor and an Aurora kinase inhibitor; use of a therapeutic combination, combination product or kit of parts in the treatment of cancer; a method of treating cancer comprising administering the therapeutic combination, combination product or kit of parts to a patient. The therapeutic combination and methods of the invention are also useful in the treatment of conditions in which the inhibition of MEK and/or Aurora kinase is beneficial.

Keywords: antitumor combination MEK inhibitor Aurora kinase

PatentPak available

Substances (6) Reactions (0) Citing (1)

332

Scutellaria barbata extract and combinations for the treatment of cancer

By: Cohen, Isaac
United States, US20090130118 A1 2009-05-21 | Language: English, Database: CAplus

An extract of Scutellaria barbata D. Don is effective in the arrest of cancer cell growth in the G1 phase, the induction of apoptosis in cancer cells and the shrinking of solid cancers. The extract may be prepared as a pharmaceutical composition for administration to mammals for the treatment of solid cancers, such as epithelial cancers. Such epithelial cancers include breast cancer and ovarian cancers. The extract is obtained from Scutellaria barbata D. Don by contacting aerial portions of a plant from the species Scutel laria barbata D. Don with an aqueous or alc. solvent.

Keywords: Scutellaria extract cancer combination treatment

PatentPak available

Substances (29) | Reactions (0) | Citing (1)

333

AZD1152 Rapidly and Negatively Affects the Growth and Survival of Human Acute Myeloid Leukemia Cells In vitro and In vivo

By: Oke, Adedayo; Pearce, Daniel; Wilkinson, Robert W.; Crafter, Claire; Odedra, Rajesh; Cavenagh, Jamie; Fitzgibbon, Jude; Lister, Andrew T.; Joel, Simon; Bonnet, Dominique
Cancer Research (2009), 69(10), 4150-4158 | Language: English, Database: CAplus and MEDLINE

Aurora kinases play a critical role in regulating mitosis and cell division, and their overexpression has been implicated in the survival and proliferation of human cancer. In this study, we report the in vitro and in vivo activities of A ZD1152, a compound that has select ivity for aurora B kinase, in acute myeloid leukemia (A ML) cell lines, primary AML samples, and cord blood cells. A ZD1152 exerted antiproliferative or cytotoxic effects in all cell lines studied, inhibited the phosphor ylation of histone H3 (p His H3) on Ser10 in a dose-dependent manner, and resulted in cells with >4 N DNA content. THP-1 cells treated with AZD1152 accumulated in a state of polyploidy and showed a senescent response to the drug, in contrast to the apoptotic response seen in other cell lines. Accordingly, AZD1152 profoundly affected the growth of AML cell lines and primary AML in an in vivo xenotransplantation model. However, concentration-dependent effects on cell growth, apoptosis, and cell cycle progre ssion were also observed when human cord blood and primary lineage-neg. stem and progenitor cells were analyzed in vitro and in vivo. These data suggest that the inhibition of aurora B kinase may be a useful therapeutic strategy in the treatment of A ML and that further exploration of dosing and treatment schedules is warranted in clin. trials. [Cancer Res 2009;69(10):4150-8].

Keywords: AZD1152 antitumor prodrug aurora B kinase acute myeloid leukemia

Substances (3) | Reactions (0) | Citing (62)

334

Identification of genes that confer tumor cell resistance to the Aurora B kinase inhibitor, AZD1152

By: Guo, J.; Anderson, M. G.; Tapang, P.; Palma, J. P.; Rodriguez, L. E.; Niquette, A.; Li, J.; Bouska, J. J.; Wang, G.; Semizarov, D.; et al
Pharmacogenomics Journal (2009), 9(2), 90-102 | Language: English, Database: CAplus and MEDLINE

AZD1152 is a highly selective Aurora B kinase inhibitor currently undergoing Phase I and I I clin. evaluation in patients with acute myelogenous leukemia and advanced solid maligna ncies. We have established two AZD1152-resistant cell lines from S W620 colon and MiaPaCa pancreatic carcinoma lines, which are >100- fold resistant to the active metabolite of A ZD1152, AZD1152 HQPA and interestingly, cross-resistant to the pan-Aurora kinase inhibitor, VX-680/MK0457. Using whole-genome microarray anal. and compar ative genomic hybridi zation, we were able to identify M DR1 and B CRP as the causative genes that underlie A ZD1152 HQPA-resistance in these models. Furthe rmore, the upregulation of either of these genes is sufficient to render in vivo tumor growth insensitive to AZD1152. Finally, the upregu lation of M DR1 or BCRP is predictive of tumor cell sensit ivity to this agent, both in vitro and in vivo. The data provide a genetic basis for resistance to Aurora kinase inhibitors, which could be utilized to predict clin. response to therapy.

Keywords: AZD1152 antitumor resistance Aurora B kinase M DR1 BCRP

Substances (4) | Reactions (0) | Citing (32)

335

Human anti-VEGF antibodies and conjugates for treatment of angiogenesis conditions

By: Ramachandra, Sumant; Bishop, Robert Walter; Masat, Linda; Huang, Chao Bai; Takeuchi, Toshihiko; Kantak, Seema
World Intellectual Property Organization, WO2009055343 A2 2009-04-30 | Language: English, Database: CAplus

Disclosed herein are fully human antibodies and antigen-binding fragments thereof that specifically bind human VEGF and inhibit V EGF binding to VEGF-R1 and VEGF-R2, and therefore inhibit V EGF signaling. The antibodies and antigen-binding fragments disclosed herein may be used, for example, to treat angiogenesis and conditions associated with angiog enesis both in vivo and in vitro.

Keywords: human antibody fragment conjugate VEGF cancer diagnosis therapy

PatentPak available

Substances (346) | Reactions (0) | Citing (1)

336

Aurora B kinase regulates the postmitotic endoreduplication checkpoint via phosphorylation of the retinoblastoma protein at serine 780

By: Nair, Jayasree S.; Ho, Alan L.; Tse, Archie N.; Coward, Jesse; Cheema, Haider; Ambrosini, Grazia; Keen, Nicholas; Schwartz, Gary K.
Molecular Biology of the Cell (2009), 20(8), 2218-2228 | Language: English, Database: CAplus and MEDLINE

The phenotypic change characteristic of Aurora B inhibition is the induction of polypl oidy. Utilizing specific siRNA duplexes and a selective small mol. inhibitor (AZD1152) to inhibit Aurora B activity in tumor cells, we sought to elucidate the mechanism by which Aurora B inhibition results in polyploidy. Cells treated with AZD1152 progressed through mitosis with misaligned chromo somes and exited without cytokinesis and subsequently underwent endoredup lication of DNA despite activation of a p53- dependent pseudo G1 checkpoint. Concomitant with polyploid cell formation, we observed the appearance of Rb hypophosph orylation, an event that occurred independently of cyclin- dependent kinase inhibition. We went on to discover that Aurora B directly phospho rylates Rb at serine 780 both in vitro and in vivo. This novel interaction plays a critical role in regulating the postmi totic checkpoint to prevent endoreduplication after an aberrant mitosis. Thus, we propose for the first time that Aurora B determines cellular fate after an aberrant mitosis by directly regulating the Rb tumor suppressor protein.

Keywords: Aurora B kinase Rb protein serine phosphorylation endoreduplication checkpoint

Substances (4) Reactions (0) Citing (75)

337

Inhibition of Aurora B kinase sensitizes a subset of human glioma cells to TRAIL concomitant with induction of TRAIL-R2

By: Li, J.; Anderson, M. G.; Tucker, L. A.; Shen, Y.; Glaser, K. B.; Shah, O. J.
Cell Death and Differentiation (2009), 16(3), 498-511 | Language: English, Database: CAplus and MEDLINE

Small-mol. inhibitors of the Aurora A and B kinases interfere with mitotic centrosome function and disrupt the mitotic spindle assembly checkpoint resulting in polyploidization and apoptosis of prolife rating cells. As such, several Aurora kinase inhibitors are at various stages of clin. development as anticancer agents. To identify candidate apoptosis- sensitizing genes that could be exploited in combination with Aurora kinase inhibitors in malignant glioma, we have carried out global gene expression anal. in a D54MG glioma cell derivative treated with three Aurora kinase inhibitors chosen for their distin ctive selectivities: MLN8054 (Aurora A-selective), AZD1152 (Aurora B-selective), and VX-680 (Aurora A/B). The modulation of apoptotic gene expression by p53 under these conditions was ascertained, as p53 expression can be toggled on and off in this D54 MG derivative by virtue of a stable, inducible, p53-targeting short hairpin RNA ($D54MG^{shp53}$). This anal. identified the tumor necrosis factor- related apoptosis-inducing ligand (TRAIL) death receptor, TRAIL receptor 2 (TRAIL-R2), as an apoptosis-sensitizing gene induced selectively following inhibition of Aurora B. In glioma cell lines where TRAIL-R2 was induced following polyploidization, the sensitivity, kinetics, and magnitude of T R AIL-mediated apoptosis were enhanced. Our data shed light on the apoptotic program induced during polyploi dization and suggest that TRAIL-R2 activation is a putative point of therap eutic intervention in combination with inhibitors of Aurora B.

Keywords: aurora B kinase inhibitor glioma TRAIL protein apoptosis cytotoxic

Substances (10) Reactions (0) Citing (27)

338

Inhibitors of aurora kinases

By: Pinel, S.; Barbault-Foucher, S.; Lott-Desroches, M.-C.; Astier, A.
Annales Pharmaceutiques Francaises (2009), 67(2), 69-77 | Language: French, Database: CAplus and MEDLINE

A review. Aurora kinases (A, B and C) are proteins expressed only in cells which divide actively and their increase is a factor of bad prognosis in cancer. They regulate the maturation of centrosomes, the separation and the conden sation of chromosomes, mitotic checkpoint and cytokinesis. The inhibition of aurora kinases, by powerful and selective inhibi tors, is due to the formation of abnormal cells which are eliminated by apoptosis. The purpose of this article is to present the role, the antitumor activity and the tolerability of these inhibitors. They can be administered orally or i.v., on weekly or monthly schedules. In our knowledge, twelve mols. are evaluated at the present time and will be discussed only the most advanced namely: VX-680, ZM 447439, MLN 8054, AZD 1152, PHA 739358, SU 6668 and AT 9283. The main indica tions are breast, colon, lung, pancreas and bladder cancers as well as hematol. tumors such as leukemia (ALL, AML, CML) and lymphoma. These inhibitors can be associated with other chemothe rapies. They seem well tolerated; the reported side effects are digestive disorders (diarrhea), fever, asthenia, alopecia, slumber, neutro penia, myelosuppression and disturbance of the biol. markers.

Keywords: review aurora kinase inhibitor antitumor toxicity cancer

Substances (8) Reactions (0) Citing (7)

339

The Topoisomerase I Poison CPT-11 Enhances the Effect of the Aurora B Kinase Inhibitor AZD1152 both In vitro and In vivo

By: Nair, Jayasree S.; de Stanchina, Elisa; Schwartz, Gary K.
Clinical Cancer Research (2009), 15(6), 2022-2030 | Language: English, Database: CAplus and MEDLINE

AZD1152 is an Aurora B kinase inhibitor currently in clin. trials. As the topoiso merase I poison CPT-11 induces a G_2 arrest, a mechan istic understanding of the cell cycle intera ctions between these agents may prove critical for combin ation therapy. AZD1152 was tested in vitro and in vivo with SN-38 and CPT-11 against HCT-116 cells. Inhibition of clonoge nicity, induction of apoptosis, effects on polyploidy, and tumor growth were examined A ZD1152 alone induced polyploidy of HCT-116 cells at low nanomolar concent rations The induction of apoptosis required prolonged exposure (48 h) and higher concent rations of drug. When SN-38 was given before or concomitantly with AZD1152, SN-38 blocked the AZD1152 effect by arresting cells in G_2 and inhibiting cells from undergoing polyploidy. With the reverse combin ation (AZD1152 followed by SN-38), there was a significant induction of polyploidy and apoptosis, even with shorter exposure (24 h) of AZD1152. In vivo, AZD1152 alone suppressed HCT-116 xenograft tumor growth in a dose-dependent manner with target inhibition of phospho H3, induction of multinu cleated giant cells, but without induction of apoptosis. In combination, both sequences in vivo (C PT->AZD, AZD->CPT, P = 0.008, AUC/d) proved superior to either single agent therapy. However, AZD->CPT still showed a greater increase in apoptosis and greater suppre ssion of tumor regrowth than C PT->AZ D (P = 0.02, AUC/d). The results from these studies indicate a promising therap eutic strategy for combining A ZD1152 with CPT-11, and suggest that the sequence of drug administration is pivotal when an Aurora B kinase inhibitor is admini stered with a topoiso merase I poison.

Keywords: AZD1152 CPT11 combination chemotherapy antitumor

Substances (7) Reactions (0) Citing (31)

340

A panel of isogenic human cancer cells suggests a therapeutic approach for cancers with inactivated p53

By: Sur, Surojit; Pagliarini, Raymond; Bunz, Fred; Rago, Carlo; Diaz, Luis A. Jr.; Kinzler, Kenneth W.; Vogelstein, Bert; Papadopoulos, Nickolas
Proceedings of the National Academy of Sciences of the United States of America (2009), 106(10), 3964-3969 | Language: English, Database: CAplus and MEDLINE

Through targeted homologous recombination, we developed a panel of matched colorectal cancer cell lines that differ only with respect to their endogenous TP53 status. We then used these lines to define the genes whose expression was altered after D NA damage induced by ionizing radiation. Transcriptome analyses revealed a consistent upregu lation of polo-like kinase 1 (P LK1) as well as other genes controlling the G_2/M transition in the cells whose T P53 genes were inactivated compared with those with W T T P53 genes. This led to the hypothesis that the viability of stressed cells without W T TP53 depended on PLK1. This hypothesis was validated by demonstrating that stressed cancer cells without WT TP53 alleles were highly sensitive to P LK1 inhibitors, both in vivo and in vitro.

Keywords: colorectal cancer cell p53 PLK1 kinase

Substances (12) Reactions (0) Citing (248)

341

Structure-Based Drug Design of Novel Aurora Kinase A Inhibitors: Structural Basis for Potency and Specificity

By: Coumar, Mohane Selvaraj; Leou, Jiun-Shyang; Shukla, Paritosh; Wu, Jian-Sung; Dixit, Ajay Kumar; Lin, Wen-Hsing; Chang, Chun-Yu; Lien, Tzu-Wen; Tan, Uan-Kang; Chen, Chun-Hwa; et al
Journal of Medicinal Chemistry (2009), 52(4), 1050-1062 | Language: English, Database: CAplus and MEDLINE

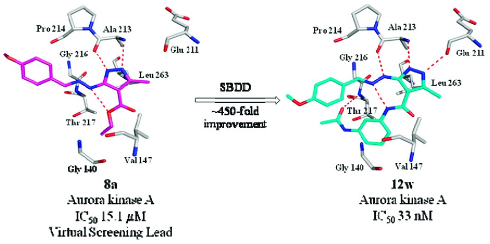

Aurora kinases have emerged as attractive targets for the design of anticancer drugs. Through structure-based virtual screening, novel pyrazole hit 8a was identified as Aurora kinase A inhibitor (IC_{50} = 15.1 μ M). X-ray cocrystal structure of 8a in complex with Aurora A protein revealed the C-4 position Et carbox ylate side chain as a possible modifi cation site for improving the potency. On the basis of this insight, bioisosteric replacement of the ester with amide linkage and changing the Et substi tuent to hydrophobic 3-acetamidophenyl ring led to the identification of 12w with a ~450-fold improved Aurora kinase A inhibition potency (IC_{50} = 33 n M), compared to 8a. Compound 12w showed selective inhibition of Aurora A kinase over Aurora B/C, which might be due to the presence of a unique H-bond interaction between the 3-acetamido group and the Aurora A noncon served Thr217 residue, which in Aurora B/C is Glu and found to sterically clash with the 3-acetamido group in modeling studies.

Keywords: structure aurora kinase A inhibitor acetamidophenyl derivative preparation

Substances (95) Reactions (90) Citing (61)

342

A specific pharmacophore model of aurora B kinase inhibitors and virtual screening studies based on it

By: Wang, Hui-Yuan; Li, Lin-Li; Cao, Zhi-Xing; Luo, Shi-Dong; Wei, Yu-Quan; Yang, Sheng-Yong
Chemical Biology & Drug Design (2009), 73(1), 115-126 | Language: English, Database: CAplus and MEDLINE

In this study, 3D-pharmacophore models of Aurora B kinase inhibitors have been developed by using Hip Hop and HypoGen modules in Catalyst software package. The best pharmacophore model, Hypo1, which has the highest correl ation coefficient (0.9911), consists of one hydrogen-bond acceptor, one hydrogen-bond donor, one hydrophobic aliphatic moiety and one ring aromatic feature. Hypo1 was validated by test set and cross-validation methods. Specificity of Hypo1 to Aurora B inhibitors was examined with the use of selective inhibitors against Aurora B and its paralogue Aurora A. The results clearly indicate that Hypo1 can differentiate selective inhibitors of Aurora B from those of Aurora A, and the ring aromatic feature likely plays some important roles for the specificity of Hypo1. Then Hypo1 was used as a 3 D query to screen several databases including Specs, N CI, Maybridge and Chinese Nature Product Database (CNPD) for identifying new inhibitors of Aurora B. The hit compounds were subseq uently subjected to filtering by Lipinski's rule of five and docking studies to refine the retrieved hits, and some compounds selected from the top ranked hits have been suggested for further exptl. assay studies.

Keywords: aurora B kinase inhibitor virtual screening pharmacophore model

Substances (70) | Reactions (0) | Citing (13)

343

Anti-insulin-like growth factor 1 receptor therapy

By: Wang, Yan; Pachter, Jonathan A.; Hailey, Judith Anne; Brams, Peter; Williams, Denise; Srinivasan, Mohan; Feingersh, Mary Diane
World Intellectual Property Organization, WO2009005673 A1 2009-01-08 | Language: English, Database: CAplus

The authors disclose the preparation and functional characterization of human antibodies to the type 1 insulin-like growth factor receptor. In one example, the growth of a human neuroblastoma was shown to be inhibited by an anti-IGF1R antibody in a xenograft model.

Keywords: insulinlike growth factor receptor antibody antitumor

PatentPak available

Substances (330) | Reactions (0) | Citing (0)

344

Discovery or AZD1152: a selective inhibitor of Aurora-B kinase with potent antitumor activity

By: Foote, Kevin M.; Mortlock, Andrew A.
Edited by Li, Rongshi; Stafford, Jeffrey A.
Kinase Inhibitor Drugs (2009), 309-332 | Language: English, Database: CAplus

A review. The Aurora kinases continue to generate significant interest as attractive drug targets in oncol. Through the work carried out at AstraZeneca, selective inhibitors of Aurora-B kinase have been profiled and have allowed a novel mechanism of action (failure of cell division) to be determined which is distinct from the classical antimitotic agents. The quinazoline core has shown itself to be an excellent template in which to develop potent Aurora kinase inhibitors while an understanding of the binding mode has allowed rapid optimization of the series with respect to potency and selectivity for the Aurora kinases.

Keywords: review aurora B kinase inhibitor AZD1152 antitumor

Substances (4) Reactions (0) Citing (9)

345

Phenotypic fingerprinting of small molecule cell cycle kinase inhibitors for drug discovery

By: Low, Jonathan; Chakravartty, Arunava; Blosser, Wayne; Dowless, Michele; Chalfant, Christopher; Bragger, Patty; Stancato, Louis
Current Chemical Genomics (2009), 3, 13-21 | Language: English, Database: CAplus and MEDLINE

Phenotypic drug discovery, primarily abandoned in the 1980's in favor of targeted approaches to drug development, is once again demonstrating its value when used in conjunction with new technologies. Phenotypic discovery has been brought back to the fore mainly due to recent advances in the field of high content imaging (HCI). HCI elucidates cellular responses using a combination of immunofluorescent assays and computer anal. which increase both the sensitivity and throughput of phenotypic assays. Although HCI data characterize cellular responses in individual cells, these data are usually analyzed as an aggregate of the treated population and are unable to discern differentially responsive sub-populations. A collection of 44 kinase inhibitors affecting cell cycle and apoptosis were characterized with a number of univariate, bivariate, and multivariate subpopulation analyses demonstrating that each level of complexity adds addnl. information about the treated populations and often distinguishes between compounds with seemingly similar mechanisms of action. Finally, these subpopulation data were used to characterize compounds as they relate in chem. space.

Keywords: cell cycle kinase inhibitor cervical colon cancer phenotype fingerprinting

Substances (25) Reactions (0) Citing (16)

346

Preparation of peptide prodrugs modified with a 1,2,3,4-cyclobutanetetracarboxylic acid derived moiety useful in treatment and diagnosis of tumors and inflammatory diseases

By: Matthieu, Michel; Dubois, Vincent; Tranchant, Isabelle; Kearsey, Jonathan
World Intellectual Property Organization, WO2008120098 A2 2008-10-09 | Language: English, Database: CAplus

The invention is related to the preparation of peptide prodrugs **I** [L^1, L^2 = independently a covalent bond or a linking moiety, the linking moiety should include at least two organic functional groups, one organic functional group that can bind to the 1,2,3,4-cyclobutanetetracarboxylic acid and a second organic functional group that allows binding to the oligopeptide moiety (L^1) or one functional group that can bind to the oligopeptide moiety and a second organic functional group that allows binding to D (L2) ; X = $(CH_2)_n$; X' = $(CH_2)_m$; X'' = $(CH_2)_p$; X''' = $(CH_2)_q$; n, m, p, q = independently 0-10; Y = cleavable oligopeptide moiety by at least one specific and/or selective peptidase that is present in the extracellular environment of target cells; D = therapeutic agent or marker] which improve the therapeutic index and the solubility of the therapeutic agent and are intended for the treatment and/or diagnosis of tumors and/or inflammatory reactions. Thus, tetraAcid-ALAL-doxorubicin **II** was prepared and showed stability in human plasma

after 2 h incubation. **II** was evaluated for its in vivo efficacy in the L S 174T tumor model and in vitro reactivation into metabolites (leucyl-doxorubicin and doxorubicin) by tumor homogenates. A coumarin analog **III** was prepared for use as a marker for diagnosis purposes (no data).

Keywords: doxorubicin peptide prodrug cyclobutanetetracarboxylic acid modification preparation antitumor antiinflammatory; mitomycin C peptide prodrug cyclobutanetetracarboxylic acid modification preparation antitumor; paclitaxel peptide prodrug cyclobutanetetracarboxylic acid modification preparation cancer inflammation; coumarin peptide cyclobutanetetracarboxylic acid modification preparation marker tumor

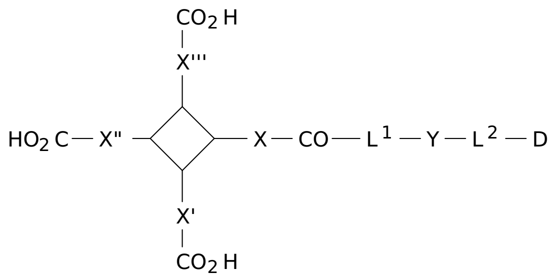

I

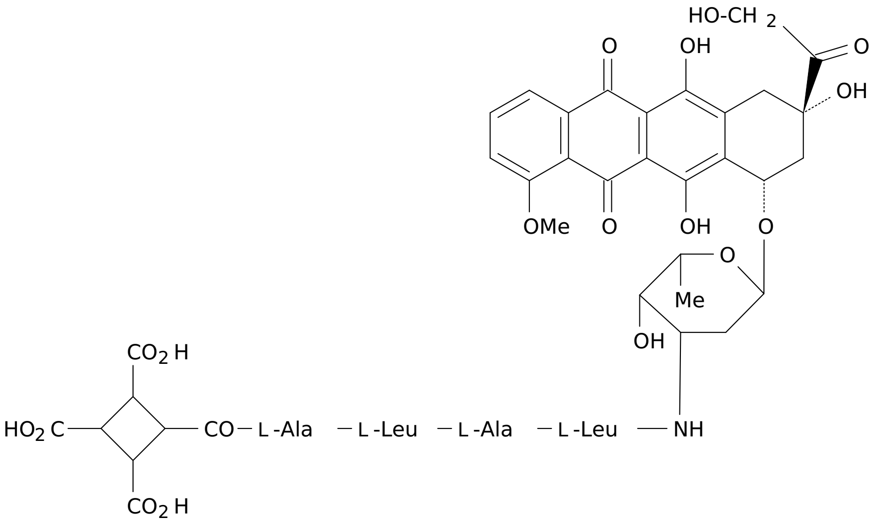

II

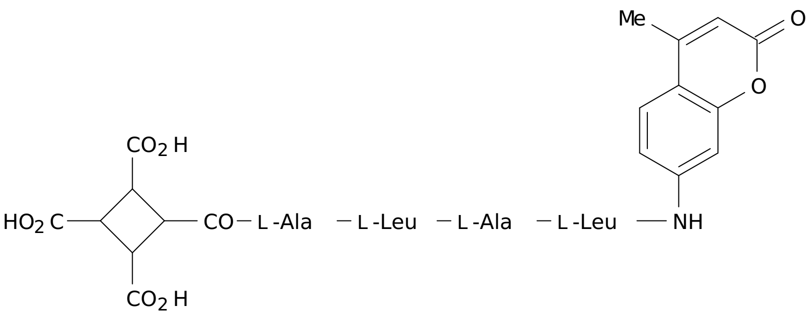

III

PatentPak available

Substances (316) Reactions (255) Citing (0)

347

Preparation of peptide prodrugs modified with a 1,2,3,4-cyclobutanetetracarboxylic acid derived moiety useful in treatment and diagnosis of tumors and inflammatory diseases

By: Matthieu, Michel; Dubois, Vincent; Tranchant, Isabelle; Kearsey, Jonathan

European Patent Organization, EP1977765 A1 2008-10-08 | Language: English, Database: CAplus

The invention is related to the preparation of peptide prodrugs **I** [L^1, L^2 = independently a covalent bond or a linking moiety; X = $(CH_2)_n$; X' = $(CH_2)_m$; X'' = $(CH_2)_p$; X''' = $(CH_2)_q$; n, m, p, q = independently 0-10; Y = cleavable oligopeptide moiety; D = therapeutic agent or marker] which improve the therapeutic index and the solubility of the therapeutic agent and are intended for the treatment and/or diagnosis of tumors and/or inflammatory reactions. Thus, tetraAcid-ALAL-doxorubicin **II** was prepared and showed stability in human plasma after 2 h incubation. **II** was evaluated for its in vivo efficacy in the LS 174T tumor model.

Keywords: peptide prodrug cyclobutanetetracarboxylic acid modification preparation cancer inflammation; doxorubicin peptide prodrug cyclobutanetetracarboxylic acid modification preparation antitumor antiinflammatory; mitomycin C peptide prodrug cyclobutanetetracarboxylic acid modification preparation antitumor; paclitaxel peptide prodrug cyclobutanetetracarboxylic acid modification preparation cancer inflammation

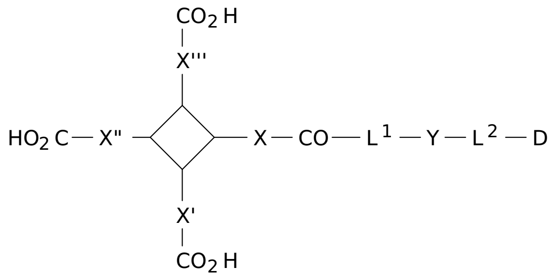

I

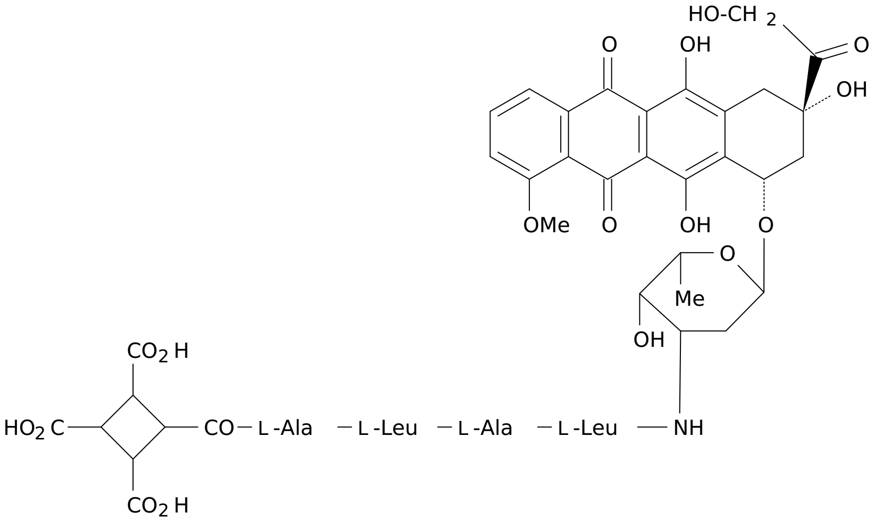

II

PatentPak available

Substances (150) Reactions (13) Citing (0)

348

Methods of treating cancer using IGF1R inhibitors

By: Wang, Yan; Zong, Chen; Seidel-Dugan, Cynthia; Wang, Yaolin; Yao, Siu-Long; Lu, Brian Der-Hua; Ladha, Mohamed H.
World Intellectual Property Organization, WO2008076278 A2 2008-06-26 | Language: English, Database: CAplus

The present invention provides IGF1R inhibitors and combinations thereof that are effective at treating or preventing cancer. More specifically the IGF1R inhibitors are pyrrolo[2,3-d]pyrimidine derivatives or antibodies. The IGF1R inhibitors can be used in combination with other anticancer therapies, antiemetic agents, antianemic agents, or antimucositis agents.

Keywords: treatment cancer IGF1R inhibitor; antibody treatment cancer IGF1R inhibitor

PatentPak available

Substances (411) | Reactions (0) | Citing (3)

349

Enhancement of radiation response in p53-deficient cancer cells by the Aurora-B kinase inhibitor AZD1152

By: Tao, Y.; Zhang, P.; Girdler, F.; Frascogna, V.; Castedo, M.; Bourhis, J.; Kroemer, G.; Deutsch, E.
Oncogene (2008), 27(23), 3244-3255 | Language: English, Database: CAplus and MEDLINE

Overexpression of the Aurora-B kinase correlates with oncogenic transformation and poor prognosis. We evaluated the effects of the bona fide Aurora-B kinase inhibitor AZD1152 on tumor responses to ionizing radiation (IR). When $p53^{wt}$ HCT116 and A549 cells were pretreated with AZD1152-HQPA prior to IR, additive effects were observed Interestingly, more pronounced tumoricidal effects were observed in p53-deficient HCT116 and HT29 cells, as well as A549 cells treated with the p53 inhibitor cyclic pifithrin- α. In vivo studies on xenografted mice confirmed enhanced tumor growth delay after the combination of IR plus AZD1152-IR as compared to IR alone. Again, this effect was more pronounced with $p53^{-/-}$ HCT116 and p53-mutant xenografts. The AZD1152-mediated radiosensitization was mimicked by knockdown of Aurora- B with a short interference RNA or by inhibition of Aurora- B by transfection with an inducible kinase-dead Aurora-B. The radiosensitizing effect of AZD1152 was lost in $CHK2^{-/-}$ and $14\text{-}3\text{-}3^{-/-}$ HCT116 cells. Altogether, these data indicate that AZD1152 can radiosensitize tumor cell lines in vitro and in vivo, the fact that these effects are exacerbated in p53-deficient cancer cells is of potential interest for further clin. development.

Keywords: radiotherapy p53 cancer Aurora B kinase inhibitor AZD1152 radiosensitizer

Substances (3) | Reactions (0) | Citing (76)

350

Preclinical evaluation of M30 and M65 ELISAs as biomarkers of drug induced tumor cell death and antitumor activity

By: Cummings, Jeffrey; Hodgkinson, Cassandra; Odedra, Rajesh; Sini, Patrizia; Heaton, Simon P.; Mundt, Kirsten E.; Ward, Tim H.; Wilkinson, Robert W.; Growcott, Jim; Hughes, Andrew; et al
Molecular Cancer Therapeutics (2008), 7(3), 455-463 | Language: English, Database: CAplus and MEDLINE

M30 and M65 are ELISAs that detect different circulating forms of cytokeratin 18. Using the aurora kinase inhibitor A ZD1152 and the SW620 human colon cancer xenograft, experi ments were conducted to qualify preclinically both assays as serol. biomarkers of cell death. Using two different apoptotic markers, the kinetics of cell death induced by AZD1152 was first characterized in vitro in three different cell lines and shown to peak 5 to 7 days after drug addition Treatment of non-tumor-bearing rats with AZD1152 (25 mg/kg) produced no alterations in circulating baseline values of M30 and M65 antigens. In treated, tumor-bearing animals, M30 detected a 2- to 3-fold ($P < 0.05$) increase in plasma antigen levels by day 5 compared with controls. This correlated to a 3- fold increase in the number of apoptotic cells detected on day 5 in SW620 xenografts using immunohistochem. By contrast, M65 did not detect a drug-induced increase in circulating antigen levels at day 5. However, M65 plasma levels correlated to changes in tumor growth in control animals ($r^2 = 0.93$; $P < 0.01$) and also followed the magnitude of the temporal effect of A ZD1152 on tumor growth. An intermediate but active dose of A ZD1152 (12.5 mg/kg) produced a less signif icant increase in M30 plasma levels at day 5. It was also confirmed that the plasma profiles of M30 and M65 mirrored closely those measured in whole tumor lyzates. We conclude that M30 is a pharmacodynamic biomarker of AZD1152-induced apoptosis in the SW620 xenograft model, whereas M65 is a biomarker of therapeutic response.

Keywords: antitumor AZD1152 ELISA biomarker

Substances (2) | Reactions (0) | Citing (53)

351

The selective Aurora B kinase inhibitor AZD1152 is a potential new treatment for multiple myeloma

By: Evans, Robert P.; Naber, Claudia; Steffler, Tara; Checkland, Tamara; Maxwell, Christopher A.; Keats, Jonathan J.; Belch, Andrew R.; Pilarski, Linda M.; Lai, Raymond; Reiman, Tony
British Journal of Haematology (2008), 140(3), 295-302 | Language: English, Database: CAplus and MEDLINE

Aurora kinases are potential targets for cancer therapy. Previous studies have validated Aurora kinase A as a therapeutic target in multiple myeloma (MM), and have demonstrated in vitro anti-myeloma effects of small mol. Aurora kinase inhibitors that inhibit both Aurora A and B. This study demonstrated that Aurora B kinase was strongly expressed in myeloma cell lines and primary plasma cells. The selective Aurora B inhibitor AZD1152-induced apoptotic death in myeloma cell lines at nanomolar concentr ations, with a cell cycle phenotype consistent with that reported previously for Aurora B inhibition. In some cases, AZD1152 in combination with dexamethasone showed increased anti-myeloma activity compared with the use of either agent alone. A ZD1152 was active against sorted CD138+ BM plasma cells from myeloma patients but also, as expected, was toxic to C D138$^-$ marrow cells from the same patients. In a murine myeloma xenograft model, AZD1152-inhibited tumor growth at well-tolerated doses and induced cell death in established tumors, with associated mild, transient leucop enia. AZD1152 shows promise in these preclin. studies as a novel treatment for MM.

Keywords: antimyeloma Aurora kinase B A ZD1152 apoptosis dexamethasone multiple myeloma

Substances (3) | Reactions (0) | Citing (50)

352

3-Benzoylamino-1H-pyrazole-4-carboxamides as CDK kinase inhibitors, and their preparation, pharmaceutical combinations and use in the treatment of proliferative diseases

By: Lyons, John Francis; Squires, Matthew Simon; Thompson, Neil Thomas; Gallagher, Neil James; Curry, Jayne Elizabeth
World Intellectual Property Organization, WO2008007113 A2 2008-01-17 | Language: English, Database: CAplus

The invention provides a combination comprising an ancillary compound and a compound having the formula **I**: or salts or tautomers or N-oxides or solvates thereof/. Compounds of formula **I** wherein X is 5- to 6- membered (hetero/carbo)cyclic ring, amino, acylamino,sulfonylamino, etc.; Y is a bond and C_{1-3} alkylene; R^2 is H, halo, C_{1-4} alkoxy, (un)substituted C_{1-4} hydrocarbyl; R^3 is H, 3- to 12-membered (hetero/carbo)cyclic group; and their salts, tautomers, N- oxides and solvates thereof, are claimed. Example compound **II**•MsOH was prepared by esterification of 4-nitropyrazole-3-carboxylic acid; the resulting 4-nitropyrazole-3-carboxylic acid Me ester underwent hydrogenation to give 4-aminopyrazole-3-carboxylic acid Me ester, which underwent amidation with 2,6-dichlorobenzoyl chloride to give 4-(2,6-dichlorobenzoylamino)pyrazole-3-carboxylic acid Me ester, which underwent hydrolysis to give 4-(2,6-dichlorobenzoylamino)pyrazole-3-carboxylic acid, which underwent chlorination to give the corresponding acid chloride, which underwent amidation with 4-amino-1-Boc-piperidine to give 1-Boc-piperidin-4-yl 4-(2,6-dichlorobenzoylamino)pyrazole-3-carboxamide, which underwent hydrolysis to give compound **II**•MsOH. All the invention compounds were evaluated for their C DK kinase inhibitory activity (some data given).

Keywords: benzoylamino pyrazolecarboxamide preparation CDK kinase inhibitor treatment proliferative disease

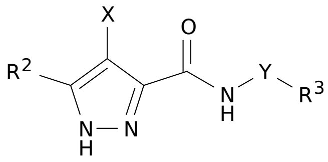

I

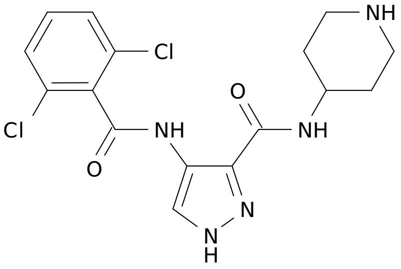

II

PatentPak available

Substances (298) | Reactions (279) | Citing (1)

353

Blood levels of insulin-like growth factor-binding protein 2 as a marker for monitoring the effectiveness of inhibitors of insulin-like growth factor I receptors in cancer therapy

By: Wang, Yan
World Intellectual Property Organization, WO2008005469 A2 2008-01-10 | Language: English, Database: CAplus

The present invention provides method for quickly and conveniently determining if a given treatment regimen of insulin- like growth factor I receptor (IGF1R) inhibitor is sufficient, e.g., to saturate IGF1 R receptors in the body of a subject. Blood levels of insulin- like growth factor-binding protein 2 (IGFBP2) are shown to be strongly correlated with the effectiveness of IGF1R receptor therapy. Several clin. relevant determinations may be made based on this point, including, for example, whether the dosage of the regimen is sufficient or should be increased. The relationship is demonstrated using animal xenograft models of neuroblastoma. Treatment with monoclonal antibodies to IGFR1 lowered the blood levels of IGFBP2. The level of IGFBP2 correlated with the tumor size.

Keywords: IGFBP2 marker cancer therapy IGF1 receptor inhibitor

PatentPak available

Substances (408) | Reactions (0) | Citing (2)

354

Emerging role of Aurora kinase inhibitors in chronic myeloid leukemia

By: Alvarado, Yesid; Cortes, Jorge E.
Clinical Leukemia (2007), 1(6), 325-330 | Language: English, Database: CAplus

A review. Resistance to imatinib and second-generation tyrosine kinase inhibitors is an ongoing problem most frequently mediated through mutations of the Bcr-Abl kinase domain. One mutation that affects respons iveness to all current available agents is T315I. Aurora proteins belong to a small family of serine/threonine kinases that are essential for proliferating cells and have been identified as key regulators of different steps in mitosis and meiosis, ranging from the formation of the mitotic spindle up to cytoki nesis. Unexpectedly, Aurora kinase inhibitors have been found to have activity against the T315 I bcr-abl mutation, and some of them might rise as important therapeutic options. The common mechanism of action for protein kinase inhibition is compet ition with ATP for the active site-binding pocket, which is very similar among the protein kinases, and this could explain the cross- reacti vity. Herein, we discuss the basics of imatinib resistance develo pment and Aurora kinase biol., and describe a selected group of Aurora kinase inhibitors with potential activity in this patient population.

Keywords: review chronic myeloid leukemia anticancer Aurora kinase inhibitor imatinib

Substances (7) Reactions (0) Citing (3)

355

Process for the preparation of pyrazolylaminoquinazoline derivatives comprising a phosphate group

By: Pittam, John David; Sependa, George Joseph
World Intellectual Property Organization, WO2007132210 A1 2007-11-22 | Language: English, Database: CAplus

The invention relates to a new process useful in the preparation of pharmaceutical compounds such as 2-{ethyl[3-({4-[(5-{2-[(3-fluorophenyl)amino]-2-oxoethyl}-1H-pyrazol-3-yl)amino]quinazolin-7-yl}oxy)propyl]amino}ethyl dihydrogen phosphate (AZD1152) and intermediates used therein.

Keywords: pyrazolyl amino quinazoline phosphate preparation; AZD1152 intermediate preparation; fluorophenyl amino oxoethyl pyrazolylamino quinazolinyloxypropyl dihydrogen phosphate AZD1152 intermediate

PatentPak available

Substances (16) Reactions (45) Citing (0)

356

Maleate co-crystal of AZD 1152 for dosage forms for treatment of hyperproliferative diseases

By: Sependa, George Joseph; Storey, Richard

World Intellectual Property Organization, WO2007132227 A1 2007-11-22 | Language: English, Database: CAplus

The present invention relates to a novel co-crystal form of 2-{ethyl[3-({4-[(5-{2-[(3-fluorophenyl)amino]-2-oxo-ethyl}-1H-pyrazol-3-yl) amino]quinazolin-7-yl}oxy)propyl]amino} Et dihydrogen phosphate (A ZD 1152), an aurora kinase inhibitor useful in the treatment of hyperproliferative diseases, such as cancer. More specifi cally, the invention relates to a maleate co- crystal of AZD 1152, to a process for its preparation, its use in the manufacturing of a medicament for the treatment of hyperprol iferative diseases, and to methods of treating hyperproliferative diseases by adminis tering a therapeutically effective amount of a maleate co- crystal of AZD 1152. A particular crystalline form of a maleate co- crystal of AZD 1152 is also described. Thus, crude A ZD 1152 (preparation given, estimated at 7.44 g @ 100%, 11.61 mM) was added to DMSO (36 mL) and left at ambient temper ature to produce a pale brown solution To this solution was added a solution of maleic acid (1.76 g, 15.16 mM, 1.31 mol equivalent) in Me OH (36 mL) and the mixture left to stand overnight at ambient temperature Next day an aliquot of the clear solution was transf erred to a vial, scratched and left sealed for several hours. A deposit of white solid formed and this was transferred to the flask and left to stir. Gradually the solution turned turbid and solid deposited. The slurry was left to settle for several days and finally filtered. The cake was washed with a 1:1 mixture of DMSO/MeOH, slurried in situ with Me OH and then dried in vacuo. N MR confirmed the solid to be the maleate co- crystal of AZ D1152 (yield of about 78.7%).

Keywords: AZD1152 maleate crystal form prepar ation dosage form; aurora kinase inhibitor A ZD1152 maleate hyperprol iferative disease

PatentPak available

Substances (22) Reactions (30) Citing (1)

357

Quinazolin-4-ylaminopyrazolecarboxamides as aurora kinase inhibitors useful in bombination therapy for the treatment of cancer and their preparation

By: Keen, Nicholas John

World Intellectual Property Organization, WO2007132215 A1 2007-11-22 | Language: English, Database: CAplus

A combination comprising an aurora kinase inhibitor and an efflux transp orter inhibitor wherein the aurora kinase inhibitor is a compound of formula **I** or pharmaceutically acceptable salt thereof for use in the treatment of hyperprol iferative diseases such as cancer. Compounds of formula **I** wherein n is 0, 1, 2 and 3; R ^{1} is C_{1-4} hydroxyalkyl and C_{1-4} phosphonooxyalkyl; R^2 is H, C_{1-4} (hydroxy)alkyl, C_{1-4} alkoxy-C_{1-4} alkyl, and heterocyclyl; R^1R^2 together with nitrogen form a (un)substituted 4- to 6-membered hetero cyclic ring; R^3 is H and C_{1-4} alkoxy; R^4, R^6 and R^6 are independently H and C_{1-4} alkyl; R^5 is (un)substituted aryl; and their pharmace utically acceptable salts thereof. Example compound **II** was prepared by a multistep procedure (procedure given) . All the invention compounds were evaluated for their Aurora kinase inhibitory activity (some data given).

Keywords: quinazolinyl pyrazolylacetamide preparation aurora kinase inhibitor treatment cancer

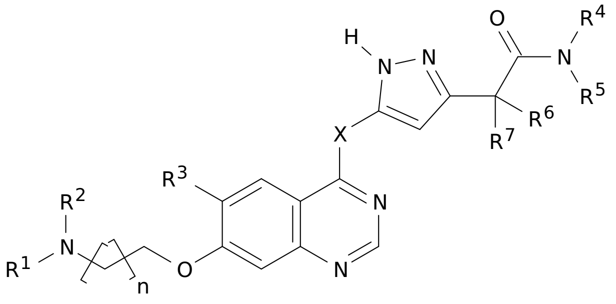

I

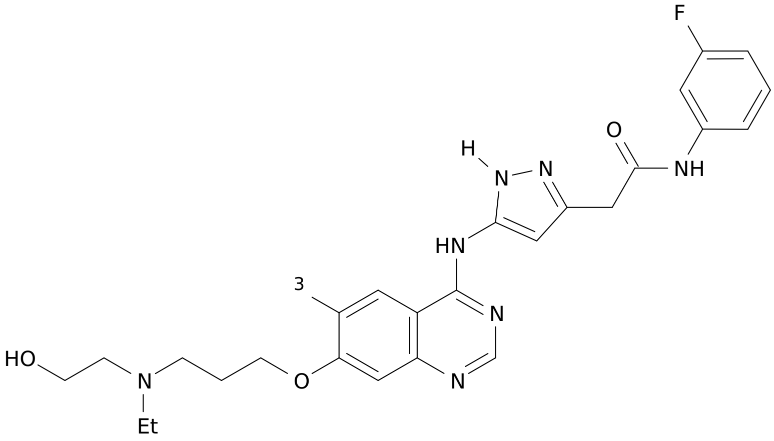

II

PatentPak available

Substances (65) Reactions (58) Citing (2)

358

Pharmaceutical combinations of diazole derivatives for cancer treatment and their preparation

By: Squires, Matthew Simon
World Intellectual Property Organization, WO2007129062 A1 2007-11-15 | Language: English, Database: CAplus

The invention provides a combination comprising (or consisting essentially of) an ancillary compound and a compound of the formula **I**, or salts, tautomers, solvates and N-oxides thereof. The combinations have activity as inhibitors of CDK kinases and inhibit the proliferation of cancer cells. Compounds of formula **I** wherein, R^1 is 2,6-dichlorophenyl; R^{2a} and R^{2b} are both H; R^3 is C_{1-4} alkyl-S O_2-piperidinyl; and their salts, tautomers, solvates, and N-oxides thereof, are claimed. Example compound **II** was prepared by methylation of 4-nitropyrazole-3-carboxylic acid; the resulting 4-nitropyrazole-3-carboxylic acid Me ester underwent hydrogenation to give 4-aminopyrazole-3-carboxylic acid Me ester, which underwent acylation with 2,6-dichlorobenzoyl chloride followed by hydrolysis to give 4-(2,6-dichlorobenzoylamino)-1H-pyrazole-3-carboxylic acid, which underwent amidation with 4-amino-1-Boc-piperidine, to give 4-[[4-(2,6-dichlorobenzoylamino)-1H-pyrazole-3-carbonyl]amino]piperidine-1-carboxylic acid tert-Bu ester, which underwent hydrolysis to give 4-(2,6-dichlorobenzoylamino)-1H-pyrazole-3-carboxylic acid piperidin-4-yl amide hydrochloride, which underwent sulfonylation with methanesulfonyl chloride to give compound **II**. The crystal structure of compound **II** was also determined The invention compounds were evaluated for their CDK kinase inhibitory activity (some data given).

Keywords: pyrazole derivative preparation CDK kinase inhibitor treatment cancer

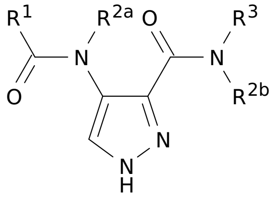

I

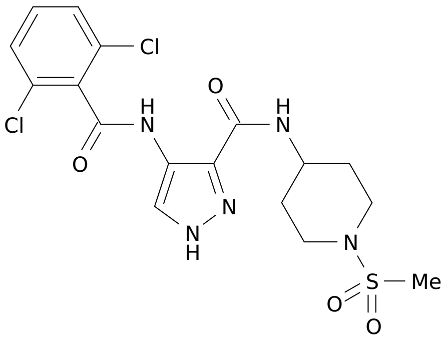

II

PatentPak available

Substances (143) Reactions (171) Citing (3)

359

AZD1152, a novel and selective aurora B kinase inhibitor, induces growth arrest, apoptosis, and sensitization for tubulin depolymerizing agent or topoisomerase II inhibitor in human acute leukemia cells in vitro and in vivo

By: Yang, Jing; Ikezoe, Takayuki; Nishioka, Chie; Tasaka, Taizo; Taniguchi, Ayuko; Kuwayama, Yoshio; Komatsu, Naoki; Bandobashi, Kentaro; Togitani, Kazuto; Koeffler, H. Phillip; et al
Blood (2007), 110(6), 2034-2040 | Language: English, Database: CAplus and MEDLINE

Aurora kinases play an important role in chromosome alignment, segregation, and cytokinesis during mitosis. We have recently shown that hematopoietic malignant cells including those from acute myeloid leukemia (AML) and acute lymphoblastic leukemia (A LL) aberrantly expressed Aurora A and B kinases, and ZM447439, a potent inhibitor of Aurora kinases, effectively induced growth arrest and apoptosis of a variety of leukemia cells. The present study explored the effect of AZD1152, a highly selective inhibitor of Aurora B kinase, on various types of human leukemia cells. AZD1152 inhibited the proliferation of AML lines (HL-60, NB4, MOLM13), ALL line (PALL-2), biphenotypic leukemia (MV4-11), acute eosinophilic leukemia (EOL-1), and the blast crisis of chronic myeloid leukemia K562 cells with an IC50 ranging from 3 nM to 40 nM, as measured by thymidine uptake on day 2 of culture. These cells had 4N/8N DNA content followed by apoptosis, as measured by cell- cycle anal. and annexin V staining, resp. Of note, AZD1152 synergistically enhanced the antiproliferative activity of vincristine, a tubulin depolymerizing agent, and daunorubicin, a topoiso merase II inhibitor, against the MOLM13 and PALL-2 cells in vitro. Furthermore, AZD1152 potentiated the action of vincristine and daunorubicin in a MOLM13 murine xenograft model. Taken together, AZD1152 is a promising new agent for treatment of indivi duals with leukemia. The combined administration of AZD1152 and conventional chemotherapeutic agent to patients with leukemia warrants further investigation.

Keywords: AZD1152 aurora B kinase inhibitor vincristine daunorubicin synergism leukemia

Substances (7) Reactions (0) Citing (221)

360

AZD1152, a Selective Inhibitor of Aurora B Kinase, Inhibits Human Tumor Xenograft Growth by Inducing Apoptosis

By: Wilkinson, Robert W.; Odedra, Rajesh; Heaton, Simon P.; Wedge, Stephen R.; Keen, Nicholas J.; Crafter, Claire; Foster, John R.; Brady, Madeleine C.; Bigley, Alison; Brown, Elaine; et al
Clinical Cancer Research (2007), 13(12), 3682-3688 | Language: English, Database: CAplus and MEDLINE

PURPOSE: In the current study, we examined the in vivo effects of AZD1152, a novel and specific inhibitor of Aurora kinase activity (with selectivity for Aurora B). Exptl. DESIGN: The pharmacodynamic effects and efficacy of AZD1152 were determined in a panel of human tumor xenograft models. AZD1152 was dosed via several parenteral (s.c. osmotic mini-pump, i.p., and i.v.) routes. RESULTS: AZD1152 potently inhibited the growth of human colon, lung, and hematol. tumor xenografts (mean tumor growth inhibition range, 55% to ≥100%; $P < 0.05$) in immunodeficient mice. Detailed pharmacodynamic anal. in colorectal SW620 tumor-bearing athymic rats treated i.v. with AZD1152 revealed a temporal sequence of phenotypic events in tumors: transient suppre ssion of histone H3 phosphorylation followed by accumulation of 4N DNA in cells (2.4-fold higher compared with controls) and then an increased proportion of polyploid cells (>4N DNA, 2.3-fold higher compared with controls). Histol. anal. showed aberrant cell division that was concurrent with an increase in apoptosis in AZD1152-treated tumors. Bone marrow analyses revealed transient myelosup pression with the drug that was fully reversible following cessation of AZD1152 treatment. CONCLUSIONS: These data suggest that selective targeting of Aurora B kinase may be a promising therapeutic approach for the treatment of a range of maligna ncies. In addition to the suppression of histone H3 phosphorylation, determination of tumor cell polyploidy and apoptosis may be useful biomarkers for this class of therapeutic agent. AZD1152 is currently in phase I trials.

Keywords: AZD1152 Aurora B kinase inhibitor antitumor tumor growth apoptosis

Substances (3) Reactions (0) Citing (303)

361

Anti-angiogenic activity of 2-methoxyestradiol in combination with anti-cancer agents

By: Plum, Stacy M.; Strawn, Steven J.; Lavallee, Theresa M.; Sidor, Carolyn F.; Fogler, William E.; Treston, Anthony M.
World Intellectual Property Organization, WO2007059111 A2 2007-05-24 | Language: English, Database: CAplus

The present invention relates generally to methods and compositions of treating disease characterized by abnormal cell prolife ration and/or abnormal or undesirable angiogenesis by administering antiangiogenic agents in combination with chemotherapeutic agents. More specifically, the present invention relates to a methods and compositions of treating diseases characterized by abnormal cell proliferation and/or abnormal or undesirable angiogenesis by administering 2-methoxyestradiol, in combination with chemotherapeutic agents.

Keywords: antiangiogenic agent methoxyestradiol combination therapy anticancer agent

PatentPak available

Substances (195) | Reactions (0) | Citing (2)

362

Discovery, Synthesis, and in Vivo Activity of a New Class of Pyrazolylamino Quinazolines as Selective Inhibitors of Aurora B Kinase

By: Mortlock, Andrew A.; Foote, Kevin M.; Heron, Nicola M.; Jung, Frederic H.; Pasquet, Georges; Lohmann, Jean-Jacques M.; Warin, Nicolas; Renaud, Fabrice; De Savi, Chris; Roberts, Nicola J.; et al
Journal of Medicinal Chemistry (2007), 50(9), 2213-2224 | Language: English, Database: CAplus and MEDLINE

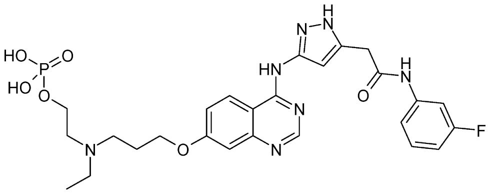

A series of pyrazolylamino-substituted quinazolines was synthesized and biol. evaluated as inhibitors of Aurora kinases, which have been the subject of considerable interest as targets for the development of new anticancer agents. Some of the products demons trated greater than 1000-fold selectivity for Aurora B over Aurora A kinase activity in recomb inant enzyme assays. These compounds have been designed for parenteral administration and achieve high levels of solubility by virtue of their ability to be delivered as readily activated phosphate derivatives The prodrugs are comprehensively converted to the des-phosphate form in vivo, and the active species have advantageous pharmacokinetic properties and safety pharmacol. profiles. The compounds display striking in vivo activity, and **I** (AZD1152) has been selected for clin. evaluation and is currently in phase 1 clin. trials.

Keywords: quinazoline pyrazolylamino preparation selective inhibitor Aurora kinase antitumor

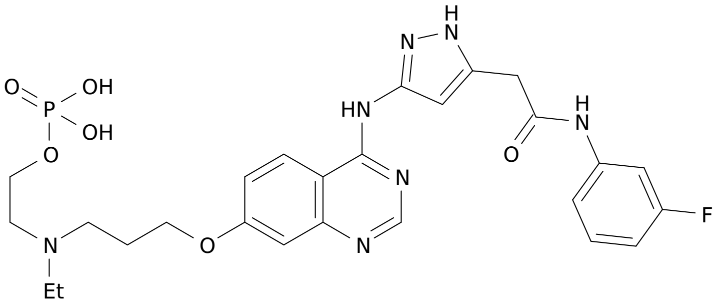

I

Substances (85) | Reactions (245) | Citing (238)

363

Preparation of phosphonooxy quinazoline derivatives and their pharmaceutical use

By: Heron, Nicola Murdoch; Jung, Frederic Henri; Pasquet, Georges Rene; Mortlock, Andrew Austen

World Intellectual Property Organization, WO2004058781 A1 2004-07-15 | Language: English, Database: CAplus

Preparation of phosphonooxy quinazoline derivatives, **I** (A = 5-membered heteroaryl containing a nitrogen atom and one or two further nitrogen atoms; X = O, S, S(O), $S(O)_2$, organoamino; m = 0-3; Z = organo amino, phosphonooxy, (un)substituted C_{3-6} cycloa lkyl, etc.; R^3 = H, halo, cyano, nitro, C_{1-6} alkoxy, C_{1-6} alkyl, alkoxycarbonyl, organoamido, sulfonylamido, etc.; R^4 = H, C_{1-4} alkyl, hetero aryl, heteroaryl C_{1-4} alkyl, aryl, etc.; R^5 = H, C_{1-4} alkyl, C_{2-4} alkenyl, C_{2-4} alkynyl, C_{3-6} cycloalkyl, etc.; R^6, R^7 = H, halo, C_{1-4} alkyl, C_{3-6} cycloalkyl, hydroxy, C_{1-4} alkoxy, etc.), and compositions containing them, processes for their prepar ation and their use in therapy is described. Thus, reaction of N-(3-fluorophenyl)-2-{3-[(7-{3-[4-(hydroxymethyl)piperidin-1-yl]propoxy}-6-methoxyquinazolin-4-yl) amino]-1H-pyrazol-5-yl}acetamide (preparation given) with di-tert-butyl-diethylphosphoramidite gave 70% di-tert-Bu {1-[3-({4-[(5-{2-[(3-fluorophenyl)amino]-2-oxoethyl}-1Hpyrazol-3-yl)amino]-6-methoxyquinazolin-7-yl}oxy)propyl]piperidin-4-yl}methyl phosphate which on acidic hydrolysis gave 94% title compound, di-tert-Bu {1-[3-({4-[(5-{2-[(3-fluorophenyl)amino]-2-oxoethyl}-1Hpyrazol-3-yl) amino]-6-methoxyquinazolin-7-yl}oxy)propyl]piperidin-4-yl}methyl dihydrogen phosphate. In vitro Aurora- A and Aurora-B kinase inhibition activity and cell proliferation and cycle anal. of the prepared compounds were determined

Keywords: phosphonooxy quinazoline derivative preparation Aurora A B kinase inhibition

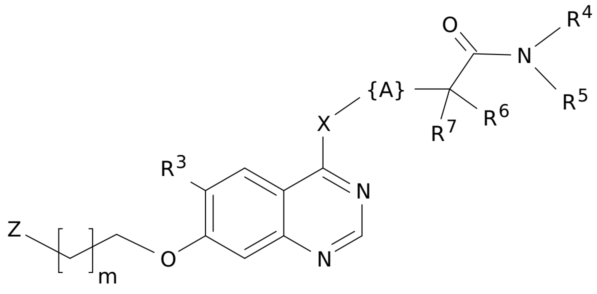

I

PatentPak available

Substances (268) Reactions (1,525) Citing (20)

Copyright © 2023 American Chemical Society (ACS). All Rights Reserved.
Copyright © 2023 U.S. National Library of Medicine.
Internal use only. Redistribution is subject to the terms of your SciFinderⁿ License Agreement and CAS information Use Policies.

CAS SciFinderⁿ

Task History

Initiating Search

May 11, 2023, 9:57AM

All:

Structure of AZD1152 (right)

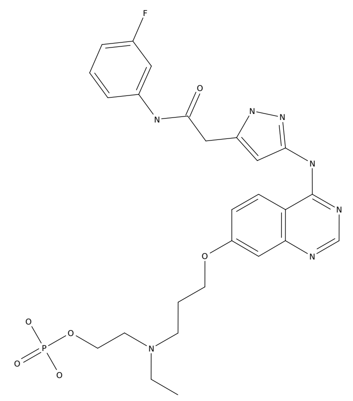

Search Tasks

Task	Search Type	View
Returned All Results	All	View Results
Viewed Substance Detail	Substances	View Detail
Exported: Retrieved Related Reference Results + Filters (338)<br>Filtered By:<br>Publication Year: 2004 to 2022	References	View Results

Copyright © 2023 American Chemical Society (ACS). All Rights Reserved.
Internal use only. Redistribution is subject to the terms of your SciFinderⁿ License Agreement and CAS information Use Policies.

CAS SciFinderⁿ

References (338)

View in SciFinderⁿ

1

Dicer promotes genome stability via the bromodomain transcriptional co-activator BRD4

By: Gutbrod, M. J. ; Roche, B.; Steinberg, J. I.; Lakhani, A. A.; Chang, K.; Schorn, A. J.; Martienssen, R. A.

Nature Communications (2022), 13(1), 1001 | Language: English, Database: CAplus and MEDLINE

RNA interference is required for post-transcriptional silencing, but also has addnl. roles in transcri ptional silencing of centromeres and genome stability. However, these roles have been controversial in mammals. Striki ngly, we found that Dicer-deficient embryonic stem cells have strong proliferation and chromosome segregation defects as well as increased transcription of centro meric satellite repeats, which triggers the interferon response. We conducted a C RISPR-Cas9 genetic screen to restore viability and identified transcriptional activators, histone H3K9 methyltransferases, and chromosome segregation factors as suppressors, resembling Dicer suppressors identified in independent screens in fission yeast. The strongest suppre ssors were mutations in the transcriptional co-activator Brd4, which reversed the strand- specific transcription of major satellite repeats suppre ssing the interferon response, and in the histone acetyltransferase Elp3. We show that identical mutations in the second bromod omain of Brd4 rescue Dicer-dependent silencing and chromosome segregation defects in both mammalian cells and fission yeast. This remarkable conservation demonstrates that RNA interference has an ancient role in transcri ptional silencing and in particular of satellite repeats, which is essential for cell cycle progression and proper chromosome segregation. Our results have pharmacol. implications for cancer and autoimmune diseases charact erized by unregulated transcription of satellite repeats.

Keywords: Dicer genome stability bromodomain transcriptional coactivator BRD

Substances (17) Reactions (0) Citing (2)

2

Screening of compounds to identify novel epigenetic regulatory factors that affect innate immune memory in macrophages

By: Benjaskulluecha, Salisa; Boonmee, Atsadang; Pattarakankul, Thitiporn; Wongprom, Benjawan; Klomsing, Jeerameth; Palaga, Tanapat

Scientific Reports (2022), 12(1), 1912 | Language: English, Database: CAplus and MEDLINE

Trained immunity and tolerance are part of the innate immune memory that allow innate immune cells to differentially respond to a second encounter with stimuli by enhancing or suppressing responses. In trained immunity, treatment of macrop hages with β-glucan (BG) facilitates the production of proinflammatory cytokines upon lipopolysaccharide (LPS) stimulation. For the tolerance response, LPS stimulation leads to suppressed inflammatory responses during subsequent L PS exposure. Epigenetic reprogr amming plays crucial roles in both phenomena, which are tightly associated with metabolic flux. In this study, we performed a screening of an epigenetics compound library that affects trained immunity or L PS tolerance in macrophages using TNFα as a readout. Among the 181 compounds tested, one compound showed suppressive effects, while 2 compounds showed promoting effects on BG-trained TNFα production In contrast, various inhibitors targeting Aurora kinase, histone methyltra nsferase, histone demethylase, histone deacetylase and DNA methyltransferase showed inhibitory activity against LPS tolerance. Several proteins previously unknown to be involved in innate immune memory, such as MGMT, Aurora kinase, LSD1 and PRMT5, were revealed. Protein network anal. revealed that the trained immunity targets are linked via Trp53, while LPS tolerance targets form three clusters of histone-modifying enzymes, cell division and base- excision repair. In trained immunity, the histone lysine methyltra nsferase SETD7 was identified, and its expression was increased during B G treatment. Level of the histone lysine demethylase, LS D1, increased during L PS priming and si RNA-mediated reduction resulted in increased expression of Il1b in L PS tolerance. Taken together, this screening approach confirmed the importance of epigenetic modifications in innate immune memory and provided potential novel targets for intervention.

Keywords: innate immune memory macrophage epigenetic regulatory factor

Substances (37) | Reactions (0) | Citing (4)

3

Methods and systems for personalized therapies by identifying a drug target in relation to a disease gene expression signature

By: Ghiassian, Susan; Akmaev, Viatcheslav R.; Voitalov, Ivan

World Intellectual Property Organization, WO2022271717 A1 2022-12-29 | Language: English, Database: CAplus

Described are methods and systems for identifying a target for therapy and treating a subject that exhibits a disease gene expression signature, comprising identifying and adminis tering a therapy determined to revert a disease gene expression signature in a subject suffering from a disease, disorder, or condition toward a non-diseased expression signature (e.g., disease gene expression signature of a non-diseased subject).

Keywords: personalized therapy drug target disease gene expression profile

PatentPak available

Substances (564) | Reactions (0) | Citing (0)

4

Probes comprising human granzyme B-cleavable hexapeptide and nanoparticles embedded with fluorescent dye, linker, and quencher and anti-GzmB antibody for detection of human granzyme B and small molecule drug as well as diagnosis of cancer, acute transplant rejection or IBD

By: Vendrell, Marc; Scott, Jamie I.; Gordon, Doireann
World Intellectual Property Organization, WO2022258997 A1 2022-12-15 | Language: English, Database: CAplus

The present disclosure relates to the development of novel cleavable peptide probes for use in detecting granzyme B, as well as methods for using the probes in medical and biol. settings. The probes may comprise a fluorescent moiety conjugated or bound to the peptide, and quencher moiety conjugated or bound to the peptide and upon cleavage of the peptide, the fluorescent moiety becomes dequenched.

Keywords: sequence granzyme cleavable peptide fluorescent dye quencher linker conjugate; Gzm B antibody small mol drug I BD cancer transplant rejection

PatentPak available

Substances (63) Reactions (0) Citing (0)

5

Methods and compositions for interfering with FAN1

By: Pearson, Christopher E.; Sartori, Alessandro A.
World Intellectual Property Organization, WO2022234494 A2 2022-11-10 | Language: English, Database: CAplus

This disclosure describes methods of altering a FAN1 polypeptide using, without limitation, a polypeptide, an antibody, a nucleic acid, a small mol., a kinase inhibitor, an agonist of a kinase, an agonist of an exonuclease, or an antagonist of an exonuclease.

Keywords: FAN1 antagonists

PatentPak available

Substances (33) Reactions (0) Citing (0)

6

A field guide to Aurora kinase inhibitors: an oocyte perspective

By: Blengini, Cecilia S.; Jung, Gyu Ik; Aboelenain, Mansour; Schindler, Karen
Reproduction (Bristol, United Kingdom) (2022), 164(4), V5-V7 | Language: English, Database: CAplus and MEDLINE

A review. In brief: The Aurora protein kinases have critical functions in controlling oocyte meiotic maturation. In this study, we describe an assay for examining their activation state in oocytes and establish the best working doses of three commonly used inhibitors. Abstract: Several small mol. inhibitors exist for targeting Aurora kinase proteins in somatic cells. From this point of view, we evaluate the specificity of these inhibitors in mouse oocytes, and we demons trate that MLN 8237 and AZD 1152 are specific for Aurora kinase A and Aurora kinase C, resp., only when used at low concentrations

Keywords: review oocyte aurora kinase inhibitor

Substances (4) Reactions (0) Citing (0)

7

Combined TRIP13 and aurora kinase inhibition induces apoptosis in human papillomavirus-driven cancers

By: Ghosh, Soma; Mazumdar, Tuhina; Xu, Wei; Powell, Reid T.; Stephan, Clifford; Shen, Li; Shah, Pooja A.; Pickering, Curtis R.; Myers, Jeffery N.; Wang, Jing; et al

Clinical Cancer Research (2022), 28(20), 4479-4493 | Language: English, Database: CAplus and MEDLINE

Human papillomavirus (HPV) causes >5% of cancers, but no therapies uniquely target H PV-driven cancers. We tested the cytotoxic effect of 864 drugs in 16 HPV-pos. and 17 HPV-neg. human squamous cancer cell lines. We confirmed apoptosis in vitro and in vivo using patientderived xenografts. Mitotic pathway components were manipu lated with drugs, knockdown, and overexpr ession. Aurora kinase inhibitors were more effective in vitro and in vivo in HPV-pos. than in HPV-neg. models. We hypoth esized that the mechanism of sensitivity involves retinob lastoma (Rb) expression because the viral oncopr otein E7 leads to Rb protein degrad ation, and basal Rb protein expression correlates with Aurora inhibition-induced apoptosis. Manipulating Rb directly, or by inducing E7 expression, altered cells' sensitivity to Aurora kinase inhibitors. Rb affects expression of the mitotic checkpoint genes M AD2L1 and BUB1B, which we found to be highly expressed in H PV-pos. patient tumors. Knockdown of MAD2L1 or BUB1B reduced Aurora kinase inhibition-induced apoptosis, whereas depletion of the M AD2L1 regulator TRIP13 enhanced it. TRIP13 is a potentially druggable AAA-ATPase. Combining Aurora kinase inhibition with T RIP13 depletion led to extensive apoptosis in H PV-pos. cancer cells but not in HPV-neg. cancer cells. Our data support a model in which H PV-pos. cancer cells maintain a balance of M AD2L1 and T RIP13 to allow mitotic exit and survival in the absence of Rb. Because it does not affect cells with intact Rb function, this novel combination may have a wide therap eutic window, enabling the effective treatment of Rb- deficient cancers.

Keywords: human papillomavirus cancer TRIP aurora kinase inhibition induce apoptosis

Substances (13) | Reactions (0) | Citing (1)

8

Methods for inhibiting KRAS oncoprotein through enhanced GTPase activity

By: Lito, Piro

World Intellectual Property Organization, WO2022217073 A1 2022-10-13 | Language: English, Database: CAplus

The present disclosure relates to methods for determining whether a cancer patient harboring a constit utively active KRAS mutation will be responsive to treatment with a $KRAS^{G12C}$ inhibitor that selectively targets the inactive state of K RAS. These methods are based on assaying regulators of G-protein signaling (R GS) expression in the cancer patient.

Keywords: sequence KRAS oncoprotein GTPase

PatentPak available

Substances (171) | Reactions (0) | Citing (0)

9

Combination therapies including Myt1 inhibitors

By: Fourtounis, Jimmy; Young, Jordan; Bernier, Cynthia; Durocher, Daniel; Hustedt, Nicole
World Intellectual Property Organization, WO2022213204 A1 2022-10-13 | Language: English, Database: CAplus

The use of inhibitors of tyrosine and threonine- specific cdc2-inhibitory kinase (Myt1) in the treatment of cancer is disclosed. In preferred embodiments, the Mytl inhibitor is a carboxamide pyrrolopyrazine or carboxamide pyrrolopyridine of Formula I. The Myt1 inhibitors may be used in combination with a variety of other anti- cancer agents. Such agents include a WEE1 inhibitor, TOPI or TOP2A inhibitor, RRM1 or RRM2 inhibitor, AURKA or AURKB inhibitor, ATR inhibitor, TTK inhibitor, SOD1 or SOD2 inhibitor, BUB1 inhibitor, CDC7 inhibitor, SAE1 inhibitor, PLK1 inhibitor, UBA2 inhibitor, DUT inhibitor, HDAC3 inhibitor, CHEK1 inhibitor, MEN1 inhibitor, DOT1L inhibitor, CREBBP inhibitor, EZH2 inhibitor, PLK4 inhibitor, HASPIN inhibitor, METTL3 inhibitor, nucleoside analogs, and platinum-based DNA alkylating agents.

Keywords: Myt1 inhibitor preparation treatment cancer

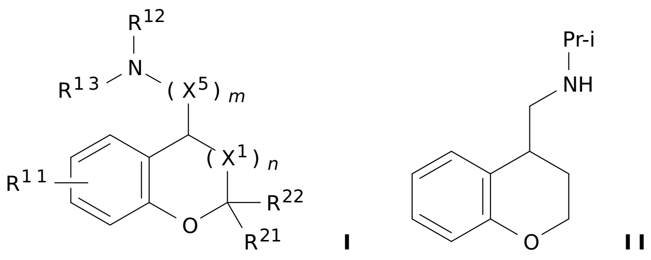

PatentPak available

Substances (639) Reactions (455) Citing (0)

10

MPS1 inhibition primes immunogenicity of KRAS-LKB1 mutant lung cancer

By: Kitajima, Shunsuke; Tani, Tetsuo; Springer, Benjamin F.; Campisi, Marco; Osaki, Tatsuya ; Haratani, Koji; Chen, Minyue; Knelson, Erik H. ; Mahadevan, Navin R. ; Ritter, Jessica; et al
Cancer Cell (2022), 40(10), 1128-1144.e8 | Language: English, Database: CAplus and MEDLINE

KRAS-LKB1 (KL) mutant lung cancers silence STING owing to intrinsic mitochondrial dysfunction, resulting in T cell exclusion and resistance to programmed cell death (ligand) 1 (PD-[L]1) blockade. Here we discover that KL cells also minimize intracellular accumu lation of 2'3'-cyclic GMP-AMP (2'3'-cGAMP) to further avoid downstream STING and STAT1 activation. An unbiased screen to co-opt this vulnerability reveals that transient MPS1 inhibition (MPS1i) potently re-engages this pathway in KL cells via micronuclei genera tion. This effect is markedly amplified by epigenetic de- repression of STING and only requires pulse MPS1i treatment, creating a therapeutic window compared with non- dividing cells. A single course of decitabine treatment followed by pulse M PS1i therapy restores T cell infiltration in vivo, enhances anti-PD-1 efficacy, and results in a durable response without evidence of signif icant toxicity.

Keywords: mutant lung cancer immunogenicity T cell infiltration mitochondrial dysfunction; 2’3’-cGAMP; DNMT1; EZH2; KRAS-LKB1 mutant lung adenocarcinoma; STING; cGAS; monopolar spindle kinase 1

Substances (31) Reactions (0) Citing (6)

11

Aurora kinase B inhibitors for use for treating cancer

By: Travers, Jon; Urosevic Despotovic, Jelena; Molina-Villa, Miguel-Angel; Bertran-Alamillo, Jordi
World Intellectual Property Organization, WO2022192139 A1 2022-09-15 | Language: English, Database: CAplus

Provided are methods for treating cancer with an Aurora kinase B inhibitor comprising determining if a cancer cell has a chromo somal amplification at location 22q11 on chromosome 22. Also provided are methods for treating cancer with an Aurora kinase B inhibitor comprising determining if a cancer cell has a gene with an increased copy number at location 22q11 on chromosome 22. Further provided are methods of determining if a cancer cell or a subject having cancer are amenable to treatment with an Aurora kinase B inhibitor.

Keywords: aurora kinase B inhibitor drug resistant cancer combination therapy

PatentPak available

Substances (33)   Reactions (0)   Citing (0)

12

N-myristoyl transferase (NMT) inhibitors for the treatment of senescence-associated diseases or disorders

By: Tate, Edward; Kallemeijn, Wouter; Gil, Jesus; McHugh, Domhnall
United States, US20220280484 A1 2022-09-08 | Language: English, Database: CAplus

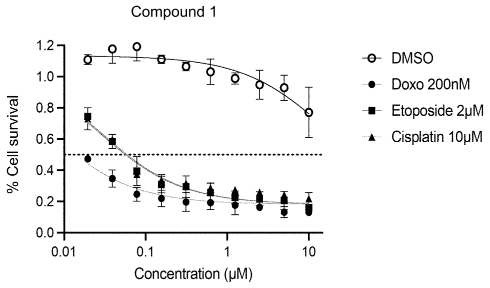

The present invention provides a method of prophylaxis or treatment of a senesc ence-associated disease or disorder by adminis tering to a subject in need thereof, a therapeu tically effective amount of a N-myristoyl transferase (NMT) inhibitor. A therapeu tically effective amount in this context means an amount of the NMT inhibitor sufficient to be effective in therapy e.g. in the treatment of cancer or other senescence-associated disease or disorder.

Keywords: senescence associated disease treatment N myristoyl transferase inhibitor

PatentPak available

Substances (58)   Reactions (0)   Citing (0)

13

Compounds, compositions and methods for treating age-related diseases and conditions

By: Tarkhov, Andrei Evgenevich; Avkhachev, Konstantin Aleksandrovich; Burmistrova, Olga Andreevna; Kholin, Maksim Nikolaevich; Fedichev, Petr Olegovich
World Intellectual Property Organization, WO2022173333 A2 2022-08-18 | Language: English, Database: CAplus

Antiaging compounds are selected according to the age-related disorder and the mol. targets associated with them by the disclosed compounds

Keywords: antiaging compounds drug targeting disease

PatentPak available

Substances (506) | Reactions (0) | Citing (0)

14

Methods for treating and ameliorating cancer

By: Jamieson, Catriona; Crews, Leslie; Burkart, Michael; Balaian, Larisa; Mondala, Phoebe; Mason, Cayla; Diep, Raymond H.; Laclair, James; Whisenant, Thomas; Chan, Warren C.; et al
World Intellectual Property Organization, WO2022165398 A1 2022-08-04 | Language: English, Database: CAplus

In alternative embodiments, provided are methods for treating and amelio rating a cancer, or recurrence of a cancer such as acute myeloid leukemia (AML) comprising administration to an individual in need thereof a pharmac eutical composition comprising 17S-F D-895 (or rebecsinib) and second drug such as an A TP-competitive protein tyrosine kinase inhibitor such as dasatinib. In altern ative embodiments, provided are methods for the in vivo inhibition of myeloprol iferative neoplasm (MPN) or AML stem cell propagation comprising administration to an individual in need thereof a pharmac eutical composition comprising 17S-FD-895 and second drug. In alternative embodiments, provided are methods for the in vivo inhibition pre- leukemia stem cell (pre-LSC) transformation into leukemia stem cells (LSCs) comprising administration to an individual in need thereof a pharmac eutical composition comprising 17 S-FD-895 and second drug.

Keywords: rebecsinib cancer

PatentPak available

Substances (166) | Reactions (0) | Citing (0)

15

Compounds for the treatment of an infection with an infectious microbe, and methods for identifying said compounds

By: Schurdak, Mark E.; Vogt, Andreas; Stern, Andrew Michael; Taylor, Douglass Lansing; Chen, Fangyuan; Pei, Fen; Cheng, Hongying; Bahar, Ivet; Shi, Qingya; Arditi, Moshe

World Intellectual Property Organization, WO2022159787 A1 2022-07-28 | Language: English, Database: CAplus

Disclosed herein are methods of identifying a compound for treating or preventing an infection with an infectious microbe, such as a coronavirus, in a subject in need thereof. Also disclosed herein are compounds and compos itions identified by said methods, and methods of use thereof. The methods comprise (a) obtaining transcriptomic data from cells infected with the infectious microbe, (b) identifying differentially expressed genes (DEGs), (c) characterizing host-targeted antimicrobial or anticytokine signature, (d) identi fying compounds that stimulate the antimic robial or -cytokine signature, (e) evaluating known and predicted targets of compounds identified in step (d), (f) constructing an infection host response protein-protein interaction (PPI) network and modules, (g) priori tizing compounds based on network proximity anal., (h) clustering of priori tized compounds associated with selected disease modules, (i) selecting representative compounds from each cluster for in vitro assays, and (j) analyzing the results of steps a- i to thereby identify the compound for treating or preventing the infection.

Keywords: microbial infection treatment drug screening method

PatentPak available

Substances (605) | Reactions (0) | Citing (0)

16

Methods for treating and ameliorating cancer

By: Jamieson, Catriona; Ma, Wenxue

World Intellectual Property Organization, WO2022159760 A1 2022-07-28 | Language: English, Database: CAplus

In alternative embodiments, provided are methods for treating and amelio rating a cancer such as a leukemia such as acute myeloid leukemia (AML) comprising administration to an individual in need thereof a pharmac eutical composition comprising imetelstat, or imetelstat and second drug such as dasatinib, or ruxolitinib, fedratinib, 8-aza-adenosine, raltegravir and/or dolutegravir or any combination thereof. In alternative embodiments, provided are methods for the in vivo inhibition of myeloprol iferative neoplasm (MPN) or AML stem cell propagation comprising administration to an individual in need thereof a pharmac eutical composition comprising imetelstat, or imetelstat and second drug.

Keywords: ameliorating cancer therapy; pharmaceutical composition myeloproliferative neoplasm stem cell propagation imetelstat drug

PatentPak available

Substances (172) | Reactions (0) | Citing (0)

17

Inducers of senescence, in combination with a selective death receptor 5 (DRr5) agonist, for use in a method of treating cancer

By: Bernards, Rene; Wang, Liqin
World Intellectual Property Organization, WO2022154664 A1 2022-07-21 | Language: English, Database: CAplus

The invention relates to an inducer of senescence, in combination with a short-lived, selective Death Receptor 5 (D R5) agonist, for use in a method of treating a patient suffering from a tumor. The invention further relates to a pharmaceutical composition, comprising a short-lived, selective Death Receptor 5 (D R5) agonist and, option ally, an inducer of senescence. The invention further relates to methods of treating a patient having a tumor, and to a selective, short-lived DR5 agonist, for use in a method of select ively killing senescent cells, including senescent cancer cells.

Keywords: DR5 agonist combin ation BRD2 inhibitor cancer

PatentPak available

Substances (12) | Reactions (0) | Citing (0)

18

Methods of potentiating antibiotic efficacy for treating bacterial infections

No inventor data available
World Intellectual Property Organization, WO2022153045 A1 2022-07-21 | Language: English, Database: CAplus

A method of potentiating or inducing antibiotic efficacy in one or more compos itions or compounds, said method including the step of inducing a substantially cell wall deficient (C WD) state in a bacteria by exposure to at least one antibiotic before and/or in combination with said one or more compos itions or compounds

Keywords: antibiotic combination chemotherapy bacterial infection treatment bacteria cell wall

PatentPak available

Substances (1,455) | Reactions (0) | Citing (0)

19

Sulfonamide derivatives, compositions comprising same and uses thereof in the treatment of cancers

By: Masutomi, Kenkichi; Yamashita, Taro; Kaneko, Shuichi; Yasukawa, Mami; Honma, Teruki; Koyama, Hiroo; Fukami, Takehiro
World Intellectual Property Organization, WO2022144780 A1 2022-07-07 | Language: English, Database: CAplus

Described herein are sulfonamide derivatives of Formula (I), where R1 and R2 are each indepen dently a halogen atom, and m and n are each independently an integer between 0 - 3, capable of inhibiting RNA dependent RNA polymerase (RdRP) activity of cancer cells. Also described are pharmaceutical compositions comprising these compounds and the uses thereof in the prophy laxis and/or treatment of cancers, including intractable solid cancers and stem cells cancer. The pharmac eutical composition may further comprise at least one of an hormonal therapeutic agent, a chemothe rapeutic agent, an immunothe rapeutic agent, a pharmac eutical agent inhibiting the action of cell growth factor and a pharmac eutical agent inhibiting the action of cell growth factor receptor.

Keywords: Sulfonamide derivative compos ition cancer

PatentPak available

Substances (158) | Reactions (0) | Citing (0)

20

A new imaging platform (iScreen) allows for the concurrent assessment of micronucleus induction and genotoxic mode of action in human A375 cells

By: Sun, Xiaowen; Rubitski, Elizabeth; Spellman, Richard A.; Engel, Maria; Schuler, Maik
Environmental and Molecular Mutagenesis (2022), 63(5), 230-245 | Language: English, Database: CAplus and MEDLINE

Genotoxicity testing guidelines require the assessment of the clasto genic and aneugenic potential of compounds While in vitro micronucleus assays detect both types of endpoints, it requires labor- intensive microscopic scoring and does not discri minate between the two modes of actions. Here, we present a novel high-content imaging platform in A375 human cells that addresses the need for rapid scoring while providing addnl. mechanistic information. We evaluated the new platform with 12 compounds, three compounds from each mechanistic class (clastogen, aneugen tubulin binder, aneugen aurora inhibitor, and nongenot oxicant) following 4- and 24-h compound treatments. The approach we developed is first discrim inating between genoto xicant and nongenotoxicant using an image anal. algorithm to quantify micron ucleus induction below a 60% cytoto xicity cutoff. Then it uses centromere protein A (CENPA) staining for the genotoxic compounds to discri minate between aneugens and clasto gens. Lastly, we use phosphorylated histone H2AX Ser139 (γH2AX) staining to confirm clastog enicity and changes in phospho rylated histone 3 Ser10 (pH 3) and increases in polyploidy in mitotic cells to discri minate between aneugens that bind tubulin from those that affect aurora kinases. All compounds were correctly classified, and we showed by using benchmark dose- response anal. that the imaging platform in A375 cells is at least as sensitive as the MicroFlow assay in TK6 cells for genoto xicant but appears to be more specific for the nongenotoxicants. A detailed comparison of the cell lines and a more compreh ensive validation with a much larger compound set, predictive and dose-response modeling will be presented in the future.

Keywords: clastogen aneugen tubulin binder aurora inhibitor micronucleus genotoxicity; aneuploidy; aurora kinase inhibitor; clastogen; image analysis; tubulin binder

Substances (12) | Reactions (0) | Citing (0)

21

Computational screening methods for identifying glutathione S-transferase P (GSTP1) inhibitors for treating pulmonary hypertension

By: Chan, Stephen Yu-Wah; Kim, Seungchan
World Intellectual Property Organization, WO2022103706 A1 2022-05-19 | Language: English, Database: CAplus

Disclosed are pathogenic mechanisms in pulmonary hypertension and mol. inhibitors of the same. Particu larly, GSTP1 (glutathione S-transferase P1) have been demons trated as having a role in regulating the endoth elial iron-sulfur cluster assembly enzyme ISCU function in pulmonary hypertension. Accordingly, methods for treating pulmonary hypert ension in a subject in need thereof comprising administering a therapeutically effective amount of a pharmac eutical compositions that inhibit glutathione S-transferase P (GSTP1) and/or increasing ISCU expression are disclosed. The GSTP1 inhibitor can comprise a piperlongumine analog, such as BR D-K34222889, or a derivative thereof.

Keywords: antihypertensive GSTP1 inhibitor piperlongumine analog BRDK34222889 pulmonary hypert ension treatment

PatentPak available

Substances (264) | Reactions (0) | Citing (0)

22

N-myristoyl transferase (NMT) inhibitors for treating senescence-associated diseases or disorders

By: Tate, Edward; Kallemeijn, Wouter; Gil, Jesus; McHugh, Domhnall
World Intellectual Property Organization, WO2022090746 A1 2022-05-05 | Language: English, Database: CAplus

The present invention provides a N-myristoyl transferase (NMT) inhibitors for use in the prophylaxis or treatment of a senescence-associated disease or disorder. The NMT inhibitor is selected from the group consisiting of 1-(5-(3,4-difluoro-2-(2-(1,3,5-trimethyl-1H-pyrazol-4-yl)ethoxy)phenyl)-1-methyl-1H-indazol-3-yl)-N,N-dimethylmethanamine, 4-(2-{2-[3-(2-aminoethyl)imidazo[1,2-a]pyridin-6-yl]-5-chlorophenoxy}ethyl)-N,N,1,5-tetramethyl-1H-pyrazole-3-carboxamide. In one aspect the senescence-associated disease or disorder is cancer, wherein said cancer comprises chemotherapy or radiotherapy induced senescent cells. In another aspect senescence-associated disease or disorder is one or more side effects of chemotherapy or radiotherapy, wherein said chemotherapy or radiotherapy induces senescence. One or more side effects of chemotherapy or radiotherapy are selected from weight loss, endocrine changes, hormone imbalance, changes in hormone signaling, cardiotoxicity, changes in body composition, a reduced ability to be phys. active, gastrointestinal toxicity, nausea, vomiting, constipation, anorexia, diarrhea, peripheral neuropathy, fatigue, malaise, low phys. activity, hematol. toxicity, anemia, hepatotoxicity, alopecia, pain, infection, mucositis, fluid retention, dermatol. toxicity, rashes, dermatitis, hyperpigmentation, urticaria, photosensitivity, nail changes, and mouth, gum and throat problems. In one aspect the senescence-associated disease or disorder is selected from a metabolic disease, an inflammatory or autoimmune disease or disorder, a cardiovascular disease or disorder, a pulmonary disease or disorder, a neurol. disease or disorder, an eye disease or disorder, and a dermatol. disease or disorder.

Keywords: senolytic agent NMT inhibitor aging senescence associated disease treatment

PatentPak available

Substances (65) | Reactions (0) | Citing (0)

23

Combination therapies for use in treating cancer

By: Li, Fu; Marks, Kevin; Marjon, Katya; Kalev, Petar; Hyer, Marc Lee
World Intellectual Property Organization, WO2022036067 A1 2022-02-17 | Language: English, Database: CAplus

The compound of Formula (I), or pharmaceutically acceptable salts thereof, is useful in, among other things, the treatment of cancer and provides a therapeutic advantage when used in combination with other agents as herein described compared to treatment with each agent when administered alone.

Keywords: antitumor cancer combination therapy

PatentPak available

Substances (170) | Reactions (0) | Citing (0)

24

Effect of alpha-tocopheryl succinate on the cytotoxicity of anticancer drugs towards leukemia lymphocytes

By: Ivanova, Donika; Zhelev, Zhivko; Zlateva, Genoveva; Lazarova, Dessislava; Yaneva, Zvezdelina; Panovska, Radmila; Aoki, Ichio; Bakalova, Rumiana

Anticancer Research (2022), 42(1), 547-554 | Language: English, Database: CAplus and MEDLINE

This study analyzed the effect of α-tocopheryl succinate (α-TS) on the redox-state of leukemia and normal lymphocytes, as well as their sensitization to fifteen anticancer drugs. Cell viability was analyzed by trypan blue staining and automated counting of live and dead cells. Apoptosis was analyzed by FITC-Annexin V test. Oxidative stress was evaluated by the intrace llular levels of reactive oxygen species (ROS) and protein-carbonyl products. Most combin ations (α-TS plus anticancer drug) exerted additive or antago nistic effects on the proliferation and viability of leukemia lymphocytes. α-TS combined with barasertib, bortezomib or lonafarnib showed a strong synergistic cytotoxic effect, which was best expressed in the case of barase stib. It was accompanied by impressive induction of apoptosis and increased production of ROS, but insignificant changes in protein-carbonyl levels. α-TS plus barasertib did not alter the viability and did not induce oxidative stress and apoptosis in normal lymphocytes. Alpha-TS could be a promising adjuvant in second-line anticancer therapy, particularly in acute lymphob lastic leukemia, to reduce the therapeutic doses of barase rtib, bortezomib, and lonafarnib, increasing their effecti veness and minimizing their side effects.

Keywords: leukemia lymphocyte alpha tocopheryl succinate anticancer; Chemotherapy; alpha-tocopheryl succinate; apoptosis; oxidative stress

Substances (19) Reactions (0) Citing (1)

25

Pharmacophore Synergism in Diverse Scaffold Clinches in Aurora Kinase B

By: Masand, Vijay H.; Al-Hussain, Sami A.; Rathore, Mithilesh M.; Thakur, Sumer D.; Akasapu, Siddhartha; Samad, Abdul ; Al-Mutairi, Aamal A. ; Zaki, Magdi E. A.

International Journal of Molecular Sciences (2022), 23(23), 14527 | Language: English, Database: CAplus and MEDLINE

Aurora kinase B (AKB) is a crucial signaling kinase with an important role in cell division. Therefore, inhibition of A KB is an attractive approach to the treatment of cancer. In the present work, extensive quant. structure-activity relationships (QSAR) anal. has been performed using a set of 561 structurally diverse aurora kinase B inhibitors. The Organization for Economic Cooperation and Development (OECD) guidelines were used to develop a QSAR model that has high statistical performance (R^2tr = 0.815, Q^2LMO = 0.808, R^2_{ex} = 0.814, CCC_{ex} = 0.899). The seven-variable-based newly developed QSAR model has an excellent balance of external predictive ability (Predictive QSAR) and mechanistic interpretation (Mechanistic QSAR). The QSAR anal. successfully identifies not only the visible pharmacophoric features but also the hidden features. The anal. indicates that the lipophilic and polar groups-especially the H-bond capable groups-must be present at a specific distance from each other. Moreover, the ring nitrogen and ring carbon atoms play important roles in determining the inhibitory activity for AKB. The anal. effectively captures reported as well as unreported pharmacophoric features. The results of the present anal. are also supported by the reported crystal structures of inhibitors bound to AKB.

Keywords: pharmacophore synergism diverse scaffold clinches aurora kinase B; Q SAR; aurora kinase B; pharmacophore modeling

Substances (5) Reactions (0) Citing (0)

26

Real-time luminescence enables continuous drug-response analysis in adherent and suspension cell lines

By: Wandishin, Clayton M. ; Robbins, Charles John; Tyson, Darren R.; Harris, Leonard A.; Quaranta, Vito
Cancer Biology & Therapy (2022), 23(1), 358-368 | Language: English, Database: CAplus and MEDLINE

The drug-induced proliferation (DIP) rate is a metric of in vitro drug response that avoids inherent biases in commonly used metrics such as 72 h viability. However, DIP rate measurements rely on direct cell counting over time, a laborious task that is subject to numerous challenges, including the need to fluores cently label cells and automatically segment nuclei. Moreover, it is incredibly difficult to directly count cells and accurately measure DIP rates for cell popula tions in suspension. As an alternative, we use real-time luminescence measurements derived from the cellular activity of N AD(P)H oxidoreductase to efficiently estimate drug response in both adherent and suspension cell populations to a panel of known anticancer agents. For the adherent cell lines, we collect both luminescence reads and direct cell counts over time simulta neously to assess their congru ency. Our results demons trate that the proposed approach significantly speeds up data collec tion, avoids the need for cellular labels and image segment ation, and opens the door to signif icant advances in high-throughput screening of anticancer drugs.

Keywords: luminescence drug response analysis adherent suspension cell line; D IP rate; cell viability; continuous assay; drug response; drug screening; quantitative analysis; real-time luminescence; suspension cells

Substances (12) Reactions (0) Citing (0)

27

Using a gene network of pyroptosis to quantify the responses to immunotherapy and prognosis for neuroblastoma patients

By: Luo, Bingying; Wang, Limin; Gao, Weijing; Su, Yudong; Lu, Yao; Zheng, Jian; Yin, Jie; Zhao, Qiang; Li, Jie; Da, Yurong; et al
Frontiers in Immunology (2022), 13, 845757 | Language: English, Database: CAplus and MEDLINE

Pyroptosis, as an inflammatory form of cell death, is involved in many physiol. and pathol. processes. Neurobl astoma is the most common extracranial solid tumor in children. In this study, the relati onship between pyroptosis and tumor microenv ironment in neuroblastoma was systematically studied. We integrated four datasets of neuroblastomas. Through robust clustering of the m RNA expression profiles of 24 pyroptosis-related genes, a total of three pyroptosis patterns were identi fied. We then constructed a novel scoring method named as pyroscore to quantify the level of pyroptosis in neuroblastoma. Multi-omics data and single-cell RNA sequencing were used to accurately and comprehensively evaluate the effectiveness of pyroscore. Clin. data sets were used to evaluate the use of pyroscore to predict the responsiveness of immune checkpoint treatment. High pyroscore was associated with good prognosis, immune activation, and increased response to checkpoint blockade immunot herapy. Multivariate Cox anal. revealed that the pyroscore was an independent prognostic biomarker and could increase the accuracy of clin. prediction models. Etoposide, a drug picked up by our anal., could increase the sensitivity of neuroblastoma cells to pyroptosis. External verification using four cohorts of patients who had received immunotherapy showed that high pyroscore was signifi cantly associated with immunotherapy treatment benefit. Taken together, this study revealed that pyropt osis-related gene network could quantify the response of neuroblastoma to immune checkpoint blockade therapy and prognosis, and it may be helpful for clin. practit ioners to choose treatment strategies for neuroblastoma patients.

Keywords: human neuroblastoma prognosis immunotherapy gene network pyropt osis; etoposide; immunotherapy; neurobl astoma; prognosis; pyroptosis

Substances (22) Reactions (0) Citing (2)

28

Phenotypic screening of 1,953 FDA-approved drugs reveals 26 hits with potential for repurposing for Peyronie's disease

By: Ilg, Marcus M.; Lapthorn, Alice R.; Ralph, David J.; Cellek, Selim
PLoS One (2022), 17(12), e0277646 | Language: English, Database: CAplus and MEDLINE

Drug repurposing has been shown to bring safe medications to new patient populations, as recently evidenced by the COVID-19 pandemic. We investigated whether we could use phenotypic screening to repurpose drugs for the treatment of Peyronie's disease (PD). PD is a fibrotic disease characterized by continued myofibroblast presence and activity leading to formation of a plaque in the penile tunica albuginea (TA) that can cause pain during erection, erectile dysfunction, and penile deformity. PD affects 3-9% of men with treatment options limited to surgery or injection of collagenase which can only be utilized at late stages after the plaque is formed. Currently there are no approved medications that can be offered to patients presenting with early disease before the formation of the plaque. Drug repurposing may therefore be the ideal strategy to identify medical treatments to address this unmet medical need in early PD. We used primary human fibroblasts from PD patients in a phenotypic screening assay that measures TGF-β1-induced myofibroblast transformation which is the main cellular phenotype that drives the pathol. in early PD. A library of FDA-approved 1953 drugs was screened in duplicate wells at a single concentration (10µM) in presence of TGF-β1. The myofibroblast marker α-SMA was quantified after 72h incubation. A pos. control of SB-505124 (TGF-β1 receptor antagonist) was included on each plate. Hits were defined as showing >80% inhibition, while retaining >80% cell viability. 26 hits (1.3%) were identified which were divided into the following main groups: anti-cancer drugs, anti-inflammation, neurol., endocrinol., and imaging agents. Five of the top-ten drugs that increase myofibroblast-transformation appear to act on VEGFR. This is the first phenotypic screening of FDA-approved drugs for PD and our results suggest that it is a viable method to predict drugs with potential for repurposing to treat early PD.

Keywords: drug FDA phenotypic screening Peyronies disease

Substances (1,943) | Reactions (0) | Citing (0)

29

Explainable artificial intelligence for precision medicine in acute myeloid leukemia

By: Gimeno, Marian; San Jose-Eneriz, Edurne; Villar, Sara; Agirre, Xabier; Prosper, Felipe; Rubio, Angel; Carazo, Fernando
Frontiers in Immunology (2022), 13, 977358 | Language: English, Database: CAplus and MEDLINE

Artificial intelligence (AI) can unveil novel personalized treatments based on drug screening and whole- exome sequencing experiments (WES). However, the concept of "black box" in AI limits the potential of this approach to be translated into the clin. practice. In contrast, explainable AI (XAI) focuses on making AI results understandable to humans. Here, we present a novel XAI method - called multi-dimensional module optimization (MOM)- that associates drug screening with genetic events, while guaranteeing that predictions are interpretable and robust. We applied MOM to an acute myeloid leukemia (AML) cohort of 319 ex-vivo tumor samples with 122 screened drugs and WES. MOM returned a therapeutic strategy based on the FLT3, CBFβ-MYH11, and NRAS status, which predicted AML patient response to Quizartinib, Trametinib, Selumetinib, and Crizotinib. We successfully validated the results in three different large-scale screening experiments We believe that XAI will help healthcare providers and drug regulators better understand AI medical decisions.

Keywords: acute myeloid leukemia precision medicine crizotinib selumetinib; assignation problem; biomarkers; drug repositioning; drug sensitivity; ex-vivo experiment; explainable artificial intelligence; large-scale screening; treatment selection

Substances (111) | Reactions (0) | Citing (3)

30

Integrative oncogene-dependency mapping identifies RIT1 vulnerabilities and synergies in lung cancer

By: Vichas, Athea; Riley, Amanda K. ; Nkinsi, Naomi T.; Kamlapurkar, Shriya; Parrish, Phoebe C. R. ; Lo, April; Duke, Fujiko; Chen, Jennifer; Fung, Iris; Watson, Jacqueline; et al
Nature Communications (2021), 12(1), 4789 | Language: English, Database: CAplus and MEDLINE

CRISPR-based cancer dependency maps are accelerating advances in cancer precision medicine, but adequate functional maps are limited to the most common oncogenes. To identify opportunities for therapeutic intervention in other rarer subsets of cancer, we investigate the oncogene-specific dependencies conferred by the lung cancer oncogene, R IT1. Here, genome-wide CRISPR screening in KRAS, EGFR, and RIT1-mutant isogenic lung cancer cells identifies shared and unique vulnerab ilities of each oncogene. Combining this genetic data with small-mol. sensitivity profiling, we identify a unique vulnera bility of RIT1-mutant cells to loss of spindle assembly checkpoint regulators. Oncogenic RIT1M90I weakens the spindle assembly checkpoint and perturbs mitotic timing, resulting in sensitivity to Aurora A inhibition. In addition, we observe synergy between mutant R IT1 and activation of Y AP1 in multiple models and frequent nuclear overexpression of YAP1 in human primary R IT1-mutant lung tumors. These results provide a genome-wide atlas of oncogenic R IT1 functional interactions and identify components of the R AS pathway, spindle assembly checkp oint, and Hippo/YAP1 network as candidate therapeutic targets in R IT1-mutant lung cancer.

Keywords: lung cancer integrative oncogene RIT vulnerability synergy

Substances (19) Reactions (0) Citing (9)

31

Combination of antibody-drug conjugate and aurora b inhibitor

By: Mettetal, Jerome Thomas, II; Durant, Stephen Thomas; Astaneh, Azadeh Cheraghchi Bashi
World Intellectual Property Organization, WO2021260582 A1 2021-12-30 | Language: English, Database: CAplus

A pharmaceutical product for administration of an anti-HER2 antibody-drug conjugate in combin ation with an Aurora B inhibitor is provided. The anti-HER2 antibody drug conjugate is an antibody- drug conjugate in which a drug- linker represented by the following formula (wherein A represents the connecting position to an antibody) is conjugated to an anti-HER2 antibody via a thioether bond. Also provided is a therapeutic use and method wherein the antibody- drug conjugate and the Aurora B inhibitor are admini stered in combination to a subject.

Keywords: HER2 cancer antitumor antibody drug conjugate aurora b sequence

PatentPak available

Substances (18) Reactions (0) Citing (0)

32

Methods for treating cancer with combination therapies

By: Ahsan, Aarif; Bisht, Kamlesh; Bjorklund, Chad; Flynt, Jennifer Erin; Hsu, Chih-Chao; Jeyaraju, Danny Vijey; Ortiz-Estevez, Maria; Pierceall, William Edward; Thakurta, Anjan; Towfic, Fadi George
World Intellectual Property Organization, WO2021262962 A1 2021-12-30 | Language: English, Database: CAplus

Provided herein are methods of using a compound provided herein (e.g., Compound 1, Compound 2, Compound 3, Compound 4, Compound 5, Compound 6, or Compound 7, or a stereoisomer or mixture of stereoisomers, pharmaceutically acceptable salt, tautomer, prodrug, solvate, hydrate, co-crystal, clathrate, or polymorph thereof), in combination with a second active agent for treating cancer. The second active agent is one or more of a PLK1 inhibitor, a BRD4 inhibitor, a BET inhibitor, an NEK2 inhibitor, an A URKB inhibitor, an MEK inhibitor, a PHF19 inhibitor, a BTK inhibitor, an mTOR inhibitor, a PIM inhibitor, an IGF-1R inhibitor, an XPOl inhibitor, a DOT1L inhibitor, an EZH2 inhibitor, a JAK2 inhibitor, a BIRC5 inhibitor, or a DNA methyltransferase inhibitor.

Keywords: antitumor agent combination therapy hematol cancer

PatentPak available

Substances (95) Reactions (0) Citing (0)

33

Compositions and methods for treating acute myeloid leukemia

By: Ishikawa, Fumihiko; Saito, Yoriko; Hashimoto, Mari; De Hoon, Michiel; Momozawa, Yukihide
World Intellectual Property Organization, WO2021255518 A1 2021-12-23 | Language: English, Database: CAplus

The present invention provides methods for identifying correlations between genetic abnormalities and effective combination treatments and treating a disease or disorder associated with inhibition of apoptosis, such as acute myeloid leukemia (AML) with compounds that block inhibition of apoptosis. The method of treating a subject with acute myeloid leukemia (AML) comprises (a) determining whether an AML cell of the subject is characterized by the presence or absence of one or more genetic mutations in F L T3, IDH1, CBL and/or NRAS, and (b) if one or more of the genetic mutations are present, indivi dually or conjointly administering an effective amount of a XIAP inhibitor, BCL2 inhibitor, MCL1 inhibitor or aurora kinase B inhibitor to the subject. For example, the XIAP inhibitor includes AZD 5582.

Keywords: acute myeloid leukemia treatment gene mutation evaluation

PatentPak available

Substances (9) Reactions (0) Citing (0)

34

CNOT3 interacts with the Aurora B and MAPK/ERK kinases to promote survival of differentiating mesendodermal progenitor cells

By: Sarkar, Moumita; Martufi, Matteo; Roman-Trufero, Monica; Wang, Yi-Fang; Whilding, Chad; Dormann, Dirk; Sabbattini, Pierangela; Dillon, Niall

Molecular Biology of the Cell (2021), 32(22), ar40 | Language: English, Database: CAplus and MEDLINE

Mesendoderm cells are key intermediate progenitors that form at the early primitive streak (PrS) and give rise to mesoderm and endoderm in the gastrulating embryo. We have identified an interaction between CNOT3 and the cell cycle kinase Aurora B that requires sequences in the NOT box domain of CNOT3 and regulates MAPK/ERK signaling during mesendoderm differentiation. Aurora B phosphorylates CNOT3 at two sites located close to a nuclear localization signal and promotes localization of CNOT3 to the nuclei of mouse embryonic stem cells (ESCs) and metastatic lung cancer cells. ESCs that have both sites mutated give rise to embryoid bodies that are largely devoid of mesoderm and endoderm and are composed mainly of cells with ectodermal characteristics. The mutant ESCs are also compromised in their ability to differentiate into mesendoderm in response to FGF2, BMP4, and Wnt3 due to reduced survival and proliferation of differentiating mesendoderm cells. We also show that the double mutation alters the balance of interaction of CNOT3 with Aurora B and with ERK and reduces phosphorylation of ERK in response to FGF2. Our results identify a potential adaptor function for CNOT3 that regulates the Ras/MEK/ERK pathway during embryogenesis.

Keywords: CNOT Aurora B MAPK ERK kinase mesendodermal progenitor cell

Substances (9) | Reactions (0) | Citing (0)

35

Paradoxical implication of BAX/BAK in the persistence of tetraploid cells

By: Deng, Jiayin ; Gutierrez, Lucia G.; Stoll, Gautier ; Motino, Omar; Martins, Isabelle; Nunez, Lucia; Bravo-San Pedro, Jose Manuel; Humeau, Juliette; Bordenave, Chloe; Pan, Juncheng; et al

Cell Death & Disease (2021), 12(11), 1039 | Language: English, Database: CAplus and MEDLINE

Pro-apoptotic multi-domain proteins of the BCL2 family such as BAX and BAK are well known for their important role in the induction of mitochondrial outer membrane permeabilization (MOMP), which is the rate-limiting step of the intrinsic pathway of apoptosis. Human or mouse cells lacking both BAX and BAK (due to a double knockout, DKO) are notoriously resistant to MOMP and cell death induction. Here we report the surprising finding that BAX/BAK DKO cells proliferate less than control cells expressing both BAX and BAK (or either BAX or BAK) when they are driven into tetraploidy by transient exposure to the microtubule inhibitor nocodazole. Mechanistically, in contrast to their BAX/BAK-sufficient controls, tetraploid DKO cells activate a senescent program, as indicated by the overexpression of several cyclin-dependent kinase inhibitors and the activation of β-galactosidase. Moreover, DKO cells manifest alterations in ionomycin-mobilizable endoplasmic reticulum (ER) Ca2+ stores and store-operated Ca2+ entry that are affected by tetraploidization. DKO cells manifested reduced expression of endogenous sarcoplasmic/endoplasmic reticulum Ca2+ ATPase 2a (Serca2a) and transfection-enforced reintroduction of Serca2a, or reintroduction of an ER-targeted variant of BAK into DKO cells reestablished the same pattern of Ca2+ fluxes as observed in BAX/BAK-sufficient control cells. Serca2a reexpression and ER-targeted BAK also abolished the tetraploidy-induced senescence of DKO cells, placing ER Ca2+ fluxes downstream of the regulation of senescence by BAX/BAK. In conclusion, it appears that BAX/BAK prevent the induction of a tetraploidization-associated senescence program. Speculatively, this may contribute to the low incidence of cancers in BAX/BAK DKO mice and explain why human cancers rarely lose the expression of both BAX and BAK.

Keywords: BAX BAK expression tetraploid cell persistence

Substances (14) | Reactions (0) | Citing (3)

36

Use of TMPRSS2 inhibitors for the treatment of lung viral infections

By: Khatib, Abdel-Majid; Villoutreix, Bruno; Singh, Natesh
World Intellectual Property Organization, WO2021228762 A1 2021-11-18 | Language: English, Database: CAplus

In Dec. 2019, a new coronavirus was identified in the Hubei province of central china and named S ARS-CoV-2. This new virus induces COVID-19, a severe respiratory disease with high death rate. A putative target to interfere with the virus is the host transme mbrane serine protease family member II (TMPRSS2). This enzyme seems critical for the entry of coronav iruses into human cells by cleaving and activating the spike protein (S) of SARS-CoV-2. Repositioning approved, investigational and exptl. drugs on the serine protease domain of TMPRSS2 could thus be valuable. There is no exptl. structure for T MPRSS2 but it is possible to develop structural models for the serine protease domain using comparative modeling strategies. Beside the TMPRSS2 catalytic site, the inventors predicted on their structural models a main exosite that could be important for the binding of protein partners and/or substrates. To block the catalytic site or the exosite of TMPRSS2 they used structure-based virtual screening computations and two different collections of approved, investigational and exptl. drugs. The inventors propose a list of 156 mols. that could bind to the catalytic site and 100 compounds that may interact with the exosite. These small mols. could be used after exptl. validations for treatment purposes, to gain novel insights over the roles of TMPRSS2 or as starting point for the develo pment of second generation analogs.

Keywords: TMPRSS2 inhibitor treatment lung virus infection antiviral C OVID19

PatentPak available

Substances (249) | Reactions (0) | Citing (1)

37

Methods and compositions for treating liver cancer

By: Harrison, Stephen; Brew, Christine Taylor; Winther, Michael David; Shekaran, Asha
World Intellectual Property Organization, WO2021206633 A1 2021-10-14 | Language: English, Database: CAplus

Methods and compositions for treating cancer, such as liver cancer, are disclosed herein. The methods may comprise use of therapeutically effective amounts of one or more therap eutic agents to decrease expression or activity of Aurora kinase A (A URKA) in liver cancer cells that are deficient in AT-rich domain interactive domain 1A (ARID1A). In a preferred embodi ment, the one or more therapeutic agents comprises a D NA construct encoding Cas9 and a guide R NA targeting AURKA gene.

Keywords: Aurora kinase inhibitor liver cancer

PatentPak available

Substances (9) | Reactions (0) | Citing (0)

38

Small molecule therapeutics for the treatment of viral infections

By: Arumugaswami, Vaithlingaraja; Christofk, Heather R.; Damoiseaux, Robert; Garcia, Gustavo; Mullen, Peter
World Intellectual Property Organization, WO2021207632 A1 2021-10-14 | Language: English, Database: CAplus

This disclosure relates to methods of treating viral infections, such as influenza or corona virus, with certain inhibitors, such as kinase inhibitors.

Keywords: protein kinase inhibitor viral infection COVID19 therapy

PatentPak available

Substances (146) | Reactions (0) | Citing (2)

39

ALKBH5-mediated m6A-demethylation of USP1 regulated T-cell acute lymphoblastic leukemia cell glucocorticoid resistance by Aurora B

By: Gong, Hongtao; Liu, Liu ; Cui, Lina; Ma, Hongyan; Shen, Liyun
Molecular Carcinogenesis (2021), 60(9), 644-657 | Language: English, Database: CAplus and MEDLINE

Recent studies evidence that ubiquitin-specific proteases (USPs) are associated with the occurrence and chemores istance of T-cell acute lymphoblastic leukemia (T-ALL). N6-methyladenosine (m6A) demethylase AlkB homolog 5 (ALKBH5) exerts a carcinogenic effect in human cancers and improves the mRNA stability of USPs. Whether ubiquitin-specific protease 1 (USP1) controls chemores istance of T-ALL is unknown. Our study demonstrated that USP1 expression was upregulated in glucocorticoid (GC)-resistant T-ALL patients and cells (CEM-C1). High expression of USP1 was correlated to the poor prognosis in T-ALL patients. Silencing USP1 increased CEM-C1 cell sensitivity to dexamethasone (Dex), reduced cell invasion, promoted cell apoptosis, and amelio rated glucoco rticoid receptor (GR) expression. USP1 mediated T-ALL chemoresistance by interacting with and deubiquitination of Aurora B. Overexpression of USP1 reversed the amelioration effect of Aurora B inhibitor on CEM-C1 cell resistance to Dex. Mechanistically, AL KBH5 enhanced USP1 expression by reducing m6A level and mRNA stability in USP1 mRNA transcript. Downregulation of ALKBH5 reduced the levels of USP1 and Aurora B, facilitated CEM-C1 cell sensitivity to Dex, apoptosis, and GR expression, suppressed cell invasion. However, overexpression of USP1 reversed all the effects of ALKBH5 on CEM-C1 cells. In vivo results showed that tail vein injection of sh-USP1 resulted in a significant prolongation of mouse survival, suppressed tumor growth, maintained the normal weight of mice, reduced USP1 expression and facilitated GR expression. In conclusion, inhibition of ALKBH5-mediated m6A modifi cation decreased USP1 expression and downregulation of USP1 ameliorated GC resistance of T-ALL through suppressing Aurora B expression and elevating GR level.

Keywords: mediated demethylation acute lymphoblastic leukemia cell glucocorticoid; Aurora B; N6-methyladenosine demethylase; T-cell acute lymphoblastic leukemia; chemoresistance; ubiquitin-specific protease 1

Substances (7) Reactions (0) Citing (13)

40

CCT245718, a dual FLT3/Aurora A inhibitor overcomes D835Y-mediated resistance to FLT3 inhibitors in acute myeloid leukaemia cells

By: Tariq, Muhammad Usama; Furqan, Muhammad; Parveen, Hira; Ullah, Rahim; Muddassar, Muhammad; Saleem, Rahman Shah Zaib; Bavetsias, Vassilios; Linardopoulos, Spiros; Faisal, Amir
British Journal of Cancer (2021), 125(7), 966-974 | Language: English, Database: CAplus and MEDLINE

Activating mutations in the Fms-like tyrosine kinase 3 (FLT3) are among the most prevalent oncogenic mutations in acute myeloid leukemia. Inhibitors selectively targeting FLT3 kinase have shown promising clin. activity; their success in the clinic, however, has been limited due to the emergence of acquired resistance. CCT245718 was identified and characterized as a dual Aurora A/FLT3 inhibitor through cell-based and biochem. assays. The ability of CCT245718 to overcome TKD-mediated resistance was evaluated in a cell line-based model of drug resistance to FLT3 inhibitors. CCT245718 exhibits potent antiproliferative activity toward FLT3-ITD + AML cell lines and strongly binds to FLT3-ITD and TKD (D835Y) mutants in vitro. Activities of both FLT3-ITD and Aurora A are also inhibited in cells. Inhibition of FLT3 results in reduced phosphorylation of STAT5, downregulation of survivin and induction of apoptotic cell death. Moreover, CCT245718 overcomes TKD-mediated resistance in a MOLM-13-derived cell line containing FLT3 with both ITD and D835Y mutations. It also inhibits FLT3 signalling in both parental and resistant cell lines compared to F LT3-specific inhibitor MLN518, which is only active in the parental cell line. Our results demons trate that CCT245718 is a potent dual FL T3/Aurora A inhibitor that can overcome TKD-mediated acquired resistance.

Keywords: acute myeloid leukemia CCT245718 FLT3 Aurora A inhibitor D835Y

Substances (7) Reactions (0) Citing (7)

41

Suv420 enrichment at the centromere limits Aurora B localization and function

By: Herlihy, Conor P.; Hahn, Sabine; Hermance, Nicole M.; Crowley, Elizabeth A.; Manning, Amity L.
Journal of Cell Science (2021), 134(15), jcs249763 | Language: English, Database: CAplus and MEDLINE

Centromere structure and function are defined by the epigenetic modification of histones at centromeric and pericentromeric chromatin. The constitutive heterochromatin found at pericentromeric regions is highly enriched for H3K9me3 and H4K20me3. Although mis-expression of the methyltransferase enzymes that regulate these marks, Suv39 and Suv420, is common in disease, the consequences of such changes are not well understood. Our data show that increased centromere localization of Suv39 and Suv420 suppresses centromere transcription and compromises localization of the mitotic kinase Aurora B, decreasing microtubule dynamics and compromising chromosome alignment and segregation. We find that inhibition of Suv420 methyltransferase activity partially restores Aurora B localization to centromeres and that restoration of the Aurora B-containing chromosomal passenger complex to the centromere is sufficient to suppress mitotic errors that result when Suv420 and H4K20me3 is enriched at centromeres. Consistent with a role for Suv39 and Suv420 in neg. regulating Aurora B, high expression of these enzymes corresponds with increased sensitivity to Aurora kinase inhibition in human cancer cells, suggesting that increased H3 K9 and H4K20 methylation may be an underappreciated source of chromosome mis-segregation in cancer.

Keywords: human cancer centromere Aurora B localization chromosome instability; Centromere; Chromosome instability; Mitosis

Substances (15) Reactions (0) Citing (3)

42

Application of aurora kinase B inhibitor in preparation of drugs for treating or preventing kidney damage caused by autoimmune disease

By: Zhao, Yue; Liu, Zhihong
China, CN113304153 A 2021-08-27 | Language: Chinese, Database: CAplus

The present invention relates to an application of aurora kinase B inhibitor characterized in that the dosage of the aurora kinase B inhibitor (AZD1152) is 5-150mg/kg/day. The administration of AZD115225 mg/kg starting from the age of 10 wk in M RL/lpr lupus mice for 8 wk of continuous treatment can significantly improve lymph node and enlarged spleen, serum inflam matory factors increase and other immune activation phenotypes, reduce the ratio of urine protein to creatinine, suggesting that AZD1152 treatment can inhibit autoimmune diseases.

Keywords: aurora kinase B inhibitor drug kidney damage autoimmune disease

PatentPak available

Substances (3) Reactions (0) Citing (0)

43

The Phytochemical Scoulerine Inhibits Aurora Kinase Activity to Induce Mitotic and Cytokinetic Defects

By: Li, Jinhua; Yan, Ziqi; Li, Hongmei; Shi, Qiong; Ahire, Vidhula; Zhang, Shenqiu; Nimishetti, Naganna; Yang, Dun; Allen, Thaddeus D. ; Zhang, Jing

Journal of Natural Products (2021), 84(8), 2312-2320 | Language: English, Database: CAplus and MEDLINE

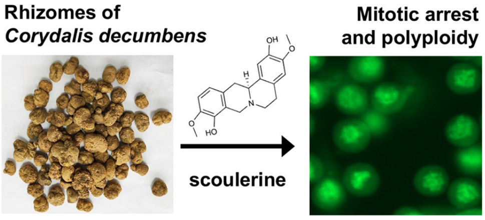

To identify novel bioactive compounds, an image-based, cell culture screening of natural product extracts was conducted. Specifi cally, our screen was designed to identify phytoc hems. that might phenocopy inhibition of the chromo somal passenger protein complex in eliciting mitotic and cytokinetic defects. A known alkaloid, scoule rine, was identified from the rhizomes of the plant Corydalis decumbens as being able to elicit a transient mitotic arrest followed by either apoptosis induction or polyploidy. In examining the mitotic abnormality further, we observed that scoulerine could elicit supernu merary centrosomes during mitosis, but not earlier in the cell cycle. The localization of N UMA1 at spindle poles was also inhibited, suggesting diminished potential for microtubule recruitment and spindle-pole focusing. Polyploid cells emerged subsequent to cytoki netic failure. The concent ration required for scoulerine to elicit all its cell division phenotypes was similar, and an examination of related compounds highlighted the requirement for proper positi oning of a hydroxyl and a methoxy group about an aromatic ring for activity. Mechanis tically, scoulerine inhibited AURKB activity at concent rations that elicited supernu merary centrosomes and polypl oidy. AURKA was only inhibited at higher concentrations, so AURKB inhibition is the likely mechanism by which scoulerine elicited division defects. A URKB inhibition was never complete, so scoulerine may be a suboptimal AURK inhibitor or work upstream of the chromo somal passenger protein complex to reduce AURKB activity. Scoulerine inhibited the viability of a variety of human cancer cell lines. Collect ively, these findings uncover a previously unknown activity of scoulerine that could facilitate targeting human cancers. Scoulerine, or a next-generation analog, may be useful as a nontoxic component of combin ation therapies where inhibiting the chromo somal passenger protein complex is desired.

Keywords: cancer AURKB inhibitor scoulerine screen Corydalis cytokinetic

Substances (8) Reactions (0) Citing (4)

44

Activity Comparison of Epigenetic Modulators against the Hemoprotozoan Parasites Babesia divergens and Plasmodium falciparum

By: Vanheer, Leen N.; Kafsack, Bjorn F. C.

ACS Infectious Diseases (2021), 7(8), 2277-2284 | Language: English, Database: CAplus and MEDLINE

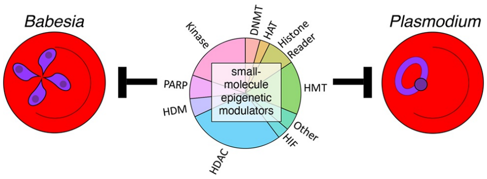

Babesiosis is a tick-borne parasitic disease of humans and livestock that has dramat ically increased in frequency and geog. range over the past few decades. Infection of cattle often causes large economic losses, and human infection can be fatal in immunocom promised patients. Unlike for malaria, another disease caused by hemopro tozoan parasites, limited treatment options exist for Babesia infections. As epigenetic regulation is a promising target for new antipar asitic drugs, we screened 324 epigenetic inhibitors against Babesia divergens blood stages and identified 75 (23%) and 17 (5%) compounds that displayed ≥90% inhibition at 10 and 1μ M, resp., including over a dozen compounds with activity in the low nanomolar range. We observed differ ential activity of some inhibitor classes against Babesia divergens and Plasmodium falciparum parasites and identified pairs of compounds with a high difference in activity despite a high similarity in chem. structure, highlighting new insights into the develo pment of epigenetic inhibitors as antiparasitic drugs.

Keywords: epigenetic modulator Babesia Plasmodium antiparasitic; Babesia; Plasmo dium; epigenetic; parasites; small molecule screen

Substances (326) Reactions (0) Citing (7)

45

Hyperbranched polyester polyol derivative as drug solubilizer

By: Haag, Rainer; Ferraro, Magda; Mohammadifar, Ehsan; Schirner, Michael
European Patent Organization, EP3858330 A1 2021-08-04 | Language: English, Database: CAplus

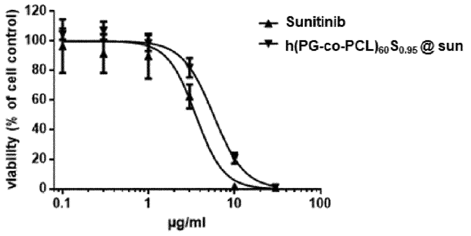

FIG 1

The present invention relates to a composition comprising a hyperbranched polyester polyol derivative and a small mol. comprising at least one cyclic amine. The hyperbranched polyester polyol derivative is obtainable by a method comprising the following steps: (a) reacting only glycidol and ε-caprolactone at a temper ature lying in a range of between 40°C and 140°C to obtain a hyperbr anched polyester polyol derivative in which caprol actone residues and glycerol residues are randomly arranged; (b) reacting the hyperbranched polyester polyol derivative of step (a) with a sulfation reagent to obtain a sulfated hyperbr anched polyester polyol as hyperbranched polyester polyol derivative

Keywords: hyperbranched polyester polyol drug solubi lizer

PatentPak available

Substances (61) | Reaction (1) | Citing (0)

46

Hyperbranched polyester polyol derivative as drug solubilizer

By: Haag, Rainer; Ferraro, Magda; Mohammadifar, Ehsan; Schirner, Michael
World Intellectual Property Organization, WO2021152172 A1 2021-08-05 | Language: English, Database: CAplus

The present invention relates to a composition comprising a hyperbranched polyester polyol derivative and a small mol. comprising at least one cyclic amine. The hyperbranched polyester polyol derivative is obtainable by a method comprising the following steps: (a) reacting only glycidol and ε-caprolactone at a temper ature lying in a range of between 40°C and 140°C to obtain a hyperbr anched polyester polyol derivative in which caprol actone residues and glycerol residues are randomly arranged; (b) reacting the hyperbranched polyester polyol derivative of step (a) with a sulfation reagent to obtain a sulfated hyperbr anched polyester polyol as hyperbranched polyester polyol derivative

Keywords: hyperbranched polyester polyol drug solubi lizer

PatentPak available

Substances (63) | Reactions (0) | Citing (0)

47

Chromosome oscillation promotes Aurora A-dependent Hec1 phosphorylation and mitotic fidelity

By: Iemura, Kenji ; Natsume, Toyoaki ; Maehara, Kayoko ; Kanemaki, Masato T. ; Tanaka, Kozo
Journal of Cell Biology (2021), 220(7), e202006116 | Language: English, Database: CAplus and MEDLINE

Most cancer cells show chromosomal instability, a condition where chromosome missegr egation occurs frequently. We found that chromosome oscillation, an iterative chromosome motion during metaphase, is attenuated in cancer cell lines. We also found that metaphase phosphorylation of Hec1 at serine 55, which is mainly dependent on Aurora A on the spindle, is reduced in cancer cell lines. The Aurora A-dependent Hec1-S55 phosphorylation level was regulated by the chromosome oscill ation amplitude and vice versa: Hec1-S55 and -S69 phosphorylation by Aurora A is required for efficient chromosome oscill ation. Furthermore, enhancement of chromosome oscillation reduced the number of erroneous kineto chore-microtubule attachments and chromosome missegre gation, whereas inhibition of Aurora A during metaphase increased such errors. We propose that Aurora A- mediated metaphase Hec1-S55 phosphorylation through chromosome oscillation, together with Hec1-S69 phosphorylation, ensures mitotic fidelity by eliminating erroneous kinetochore-microtubule attachments. Attenuated chromosome oscillation and the resulting reduced Hec1-S55 phosphorylation may be a cause of C IN in cancer cell lines.

Keywords: Aurora A Hec phosphorylation mitotic fidelity chromosome oscill ation

Substances (7) Reactions (0) Citing (11)

48

Inhibition of Aurora Kinase B activity disrupts development and differentiation of salivary glands

By: Shaalan, Abeer K. ; Teshima, Tathyane H. N.; Tucker, Abigail S.; Proctor, Gordon B.
Cell Death Discovery (2021), 7(1), 16 | Language: English, Database: CAplus and MEDLINE

Abstract: Little is known about the key mols. that regulate cell division during organogenesis. Here we determine the role of the cell cycle promoter aurora kinase B (AURKB) during development, using embryonic salivary glands (E-SGs) as a model. AURKB is a serine/threonine kinase that regulates key events in mitosis, which makes it an attractive target for tailored anticancer therapy. Many reports have elaborated on the role of AURKB in neoplasia and cancer; however, no previous study has shown its role during organ development. Our previous experiments have highlighted the essential requirement for AURKB during adult exocrine regeneration. To investigate if AURKB is similarly required for progression during embryonic development, we pharmacol. inhibited AURKB in developing submandibular glands (SMGs) at embryonic day (E)13.5 and E16.5, using the highly potent and selective drug Barasertib. Inhibition of AURKB interfered with the expansion of the embryonic buds. Interestingly, this effect on S MG development was also seen when the mature explants (E16.5) were incubated for 24 h with another cell cycle inhibitor Aphidicolin. Barasertib prompted apoptosis, DNA damage and senescence, the markers of which (cleaved caspase 3, γH2AX, SA-βgal and p21, resp.), were predominantly seen in the developing buds. In addition to a reduction in cell cycling and prolife ration of the epithelial cells in response to AURKB inhibition, Barasertib treatment led to an excessive generation of reactive oxygen species (R OS) that resulted in downregulation of the acinar differentiation marker Mist1. Importantly, inhibition of ROS was able to rescue this loss of identity, with Mist1 expression maintained despite loss of AURKB. Together, these data identify A URKB as a key mol. in supporting embryonic development and differentiation, while inhibiting senescence-inducing signals during organogenesis.

Keywords: aurora Kinase B salivary gland

Substances (4) Reactions (0) Citing (3)

49

Substituted 4-aminoisoindoline-1,3-dione compounds and second active agents for combined use for treating hematological malignancies

By: Anton, Maria Soraya Carrancio; Buchholz, Tonia J.; Chang, Henry; Filvaroff, Ellen; Kasibhatla, Shailaja; Lopez-Girona, Antonia; Mohan, Adithi; Narla, Rama Krishna; Pourdehnad, Michael

United States, US20210113576 A1 2021-04-22 | Language: English, Database: CAplus

Sensitivity to Compound 1 single agent in DLBCL cell lines

sensitive to resistant

TMD-8	SU-DHL-2	SU-DHL-10	Pfeiffer	WSU-DLCL2	OCI-Ly10	Riva	U2932	SU-DHL-4	DB
58.67	108.4	123.7	288.2	362	403.5	496.3	577	585.3	670.8

Provided herein are methods of using (*S*)-2-(2,6-dioxopiperidin-3-yl)-4-((2-fluoro-4-((3-morpholinoazetidin-1-yl)methyl)benzyl) amino)isoindoline-1,3-dione, or an enantiomer, mixture of enantiomers, tautomer, isotopolog, or pharmaceutically acceptable salt thereof, in combination with a second active agent for treating, preventing or managing hematol. malignancies. The second active agent is one or more of an HDAC inhibitor, a BCL2 inhibitor, a BTK inhibitor, an mTOR inhibitor, a PI3K inhibitor, a PKCβ inhibitor, a SYK inhibitor, a JAK2 inhibitor, an Aurora kinase inhibitor, an EZH2 inhibitor, a BET inhibitor, a hypomethylating agent, a DOT1L inhibitor, a HAT inhibitor, a WDR5 inhibitor, a DNMT1 inhibitor, an LSD-1 inhibitor, a G9A inhibitor, a PRMT5 inhibitor, a BRD inhibitor, a SUV420H1/H2 inhibitor, a CARM1 inhibitor, a PLK1 inhibitor, an NEK2 inhibitor, an MEK inhibitor, a PHF19 inhibitor, a PIM inhibitor, an IGF-1R inhibitor, an XPO1 inhibitor, a BIRC5 inhibitor, or a chemotherapy.

Keywords: hematol neoplasm treatment aminoisoindolinedione compound active agent combination

PatentPak available

Substances (131) Reactions (3) Citing (0)

50

Predicting cell health phenotypes using image-based morphology profiling

By: Way, Gregory P.; Kost-Alimova, Maria; Shibue, Tsukasa; Harrington, William F.; Gill, Stanley; Piccioni, Federica; Becker, Tim; Shafqat-Abbasi, Hamdah; Hahn, William C.; Carpenter, Anne E.; et al

Molecular Biology of the Cell (2021), 32(9), 995-1005 | Language: English, Database: CAplus and MEDLINE

Genetic and chem. perturbations impact diverse cellular phenotypes, including multiple indicators of cell health. These readouts reveal toxicity and antitumorigenic effects relevant to drug discovery and personalized medicine. We developed two customized microscopy assays, one using four targeted reagents and the other three targeted reagents, to collectively measure 70 specific cell health phenotypes including proliferation, apoptosis, reactive oxygen species, DNA damage, and cell cycle stage. We then tested an approach to predict multiple cell health phenotypes using Cell Painting, an inexpensive and scalable image-based morphol. assay. In matched CRISPR perturbations of three cancer cell lines, we collected both Cell Painting and cell health data. We found that simple machine learning algorithms can predict many cell health readouts directly from Cell Painting images, at less than half the cost. We hypothesized that these models can be applied to accurately predict cell health assay outcomes for any future or existing Cell Painting dataset. For Cell Painting images from a set of 1500+ compound perturbations across multiple doses, we validated predictions by orthogonal assay readouts. We provide a web app to browse predictions: http://broad.io/cell-health-apparatus Our approach can be used to add cell health annotations to Cell Painting datasets.

Keywords: cell health phenotype morphol profiling deep machine learning

Substances (6) Reactions (0) Citing (33)

51

Targeting pan-essential genes in cancer: challenges and opportunities

By: Chang, Liang; Ruiz, Paloma ; Ito, Takahiro; Sellers, William R.
Cancer Cell (2021), 39(4), 466-479 | Language: English, Database: CAplus and MEDLINE

Despite remarkable successes in the clinic, cancer targeted therapy development remains challenging and the failure rate is disappointingly high. This problem is partly due to the misapplication of the targeted therapy paradigm to therapeutics targeting pan-essential genes, which can result in therapeutics whereby efficacy is attenuated by dose-limiting toxicity. Here we summarize the key features of successful chemotherapy and targeted therapy agents, and use case studies to outline recurrent challenges to drug development efforts targeting pan-essential genes. Finally, we suggest strategies to avoid previous pitfalls for ongoing and future development of pan-essential therapeutics.

Keywords: human neoplasm therapeutic target chemotherapy antibody drug conjugate; drug development; pan-essential genes; target identification; target validation; targeted therapies

Substances (22) Reactions (0) Citing (45)

52

Combined inhibition of XIAP and BCL2 drives maximal therapeutic efficacy in genetically diverse aggressive acute myeloid leukemia

By: Hashimoto, Mari; Saito, Yoriko; Nakagawa, Ryo; Ogahara, Ikuko; Takagi, Shinsuke ; Takata, Sadaaki; Amitani, Hanae; Endo, Mikiko; Yuki, Hitomi ; Ramilowski, Jordan A.; et al
Nature Cancer (2021), 2(3), 340-356 | Language: English, Database: CAplus and MEDLINE

Aggressive therapy-resistant and refractory acute myeloid leukemia (AML) has an extremely poor outcome. By analyzing a large number of genetically complex and diverse, primary high-risk poor-outcome human AML samples, we identified specific pathways of therapeutic vulnerability. Through drug screens followed by extensive in vivo validation and genomic analyses, we found inhibition of cytosolic and mitochondrial anti-apoptotic proteins XIAP, BCL2 and MCL1, and a key regulator of mitosis, AURKB, as a vulnerability hub based on patient-specific genetic aberrations and transcriptional signatures. Combinatorial therapeutic inhibition of XIAP with an addnl. patient-specific vulnerability eliminated established AML in vivo in patient-derived xenografts (PDXs) bearing diverse genetic aberrations, with no signs of recurrence during off-treatment follow-up. By integrating genomic profiling and drug-sensitivity testing, this work provides a platform for a precision-medicine approach for treating aggressive AML with high unmet need.

Keywords: venetoclax S63845 anticancer agent XIAP BCL2 acute myeloid leukemia

Substances (10) Reactions (0) Citing (7)

53

Preparation of biaminoquinolines and nanoformulations for cancer treatment

By: Li, Yuanpei; Ma, Zhao
World Intellectual Property Organization, WO2021055705 A1 2021-03-25 | Language: English, Database: CAplus

The invention provides bisaminoquinoline compounds of formula **I**, wherein R^1 is H, C_{1-40} alkyl, C_{2-40} alkenyl, etc.; R^{2a}, R^{2b}, R^{3a}, R^{3b} are independently H, C_{1-40} alkyl, C_{2-40} alkenyl, etc.; m and n are indepen dently 1 to 10; p = 1 - 20; each X is absent or O; and pharmaceutically acceptable salt thereof, are claimed. Example compound **II** was prepared by a general procedure (procedure given). The present invention also provides nanoca rriers comprising compounds of the present invention, and methods of using the nanocarriers for treating diseases and as imaging agent.

Keywords: biaminoquinoline preparation nanoformulation cancer treatment imaging

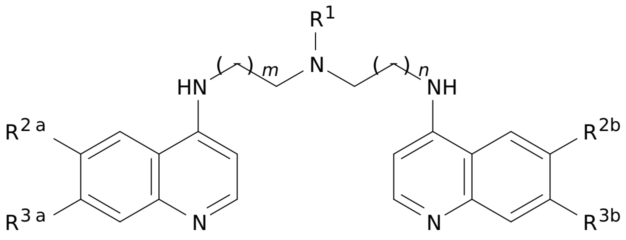

I

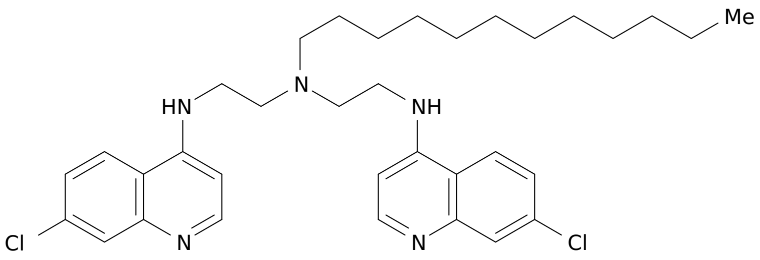

II

PatentPak available

Substances (342) Reactions (90) Citing (0)

54

Nanoencapsulated combination drug formulations

By: Baldwin, Paige; Sridhar, Srinivas; Singh, Bijay
United States, US20210085619 A1 2021-03-25 | Language: English, Database: CAplus

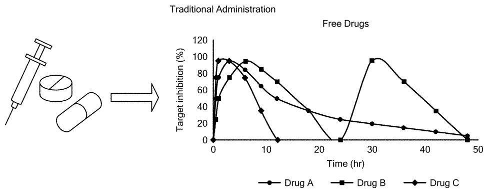

Pharmaceutical formulations (nanococktails) include two or more nanoparticulate delivery vehicles each including an active agent. The nanococktails can be formulated at any ratio of active agents by changing the ratio of nanopart iculate delivery vehicles in the nanococktail. The nanoparticulate delivery vehicles can have matched surface potential to prevent aggreg ation. The nanopart iculate delivery vehicles of the combined formul ation are designed to provide each active agent with desired, preferably overla pping therapeutic windows and a specif ically selected pharmaco kinetic and pharmaco dynamic profile when the nanoco cktail is admini stered to a subject. Each of the nanopart iculate delivery vehicles can be designed to enhance solubi lity, duration of action, targeting, stability, and to program release while preventing degradation of an active agent and preventing side effects, toxicity, and tolera bility issues in a subject.

Keywords: nanoencapsulation combination drug formul ation pharmaco dynamic

PatentPak available

Substances (51) Reactions (0) Citing (0)

55

QSAR Modeling of SARS-CoV M^pro Inhibitors Identifies Sufugolix, Cenicriviroc, Proglumetacin, and Other Drugs as Candidates for Repurposing against SARS-CoV-2

By: Alves, Vinicius M.; Bobrowski, Tesia; Melo-Filho, Cleber C.; Korn, Daniel; Auerbach, Scott; Schmitt, Charles; Muratov, Eugene N. ; Tropsha, Alexander
Molecular Informatics (2021), 40(1), 2000113 | Language: English, Database: CAplus and MEDLINE

The main protease (M^{pro}) of the SARS-CoV-2 has been proposed as one of the major drug targets for C OVID-19. We have identified the exptl. data on the inhibitory activity of compounds tested against the closely related (96% sequence identity, 100% active site conservation) M^{pro} of SARS-CoV. We developed QSAR models of these inhibitors and employed these models for virtual screening of all drugs in the DrugBank database. Similarity searching and mol. docking were explored in parallel, but docking failed to correctly discriminate between exptl. active and inactive compounds, so it was not relied upon for prospe ctive virtual screening. Forty-two compounds were identified by our models as consensus computa tional hits. Subsequent to our computa tional studies, N CATS reported the results of exptl. screening of their drug collection in S ARS-CoV-2 cytopathic effect assay (https: //opendata.ncats. nih.gov/covid19/). Coincidentally, NCATS tested 11 of our 42 hits, and three of them, cenicr iviroc (AC_{50} of 8.9μM), proglumetacin (tested twice independently, with AC_{50} of 8.9μM and 12.5μM), and sufugolix (AC_{50} 12.6μM), were shown to be active. These observ ations support the value of our modeling approaches and models for guiding the exptl. investi gations of putative anti-COVID-19 drug candidates. All data and models used in this study are publicly available via Supplem entary Materials, GitHub (https://github. com/alvesvm/sars-cov-mpro), and Chembench web portal (https: //chembench.mml.unc.edu/).

Keywords: SARS CoV2 coronavirus protease inhibitors drug repurp osing screening chemoinfo rmatics; SARS-CoV-2; SARS-CoV-2 Mpro.; cheminformatics; drug repurp osing; virtual screening

Substances (12) Reactions (0) Citing (37)

56

Loss of Aurora kinase signaling allows lung cancer cells to adopt endoreplication and form polyploid giant cancer cells that resist antimitotic drugs

By: Tagal, Vural; Roth, Michael G.
Cancer Research (2021), 81(2), 400-413 | Language: English, Database: CAplus and MEDLINE

Polyploid giant cancer cells (PGCC) are common in tumors and have been associated with resistance to cancer therapy, tumor relapse, malignancy, immunosuppression, metastasis, cancer stem cell produc tion, and modulation of the tumor microenvi ronment. However, the mol. mechanisms that cause these cells to form are not yet known. In this study, we discover that Aurora kinases are synergistic determinants of a switch from the prolife rative cell cycle to polyploid growth and multinuc leation in lung cancer cell lines. When Aurora kinases were inhibited together, lung cancer cells uniformly grew into multinucleated PGCCs. These cells adopted an endoreplication in which the genome replicates, mitosis is omitted, and cells grow in size. Consequ ently, such cells continued to safely grow in the presence of antimitotic agents. These PGCC re-entered the proliferative cell cycle and grew in cell number when treatment was terminated. Thus, PGCC formation might represent a fundam ental cellular response to Aurora kinase inhibitors and contributes to therapy resistance or tumor relapse.

Keywords: polyploid giant cancer Aurora kinase endoreplication antimitotic drug resistance

Substances (29) Reactions (0) Citing (15)

57

Experiments in the EpiDerm 3D skin in vitro model and minipigs in vivo indicate comparatively lower in vivo skin sensitivity of topically applied aneugenic compounds

By: Schuler, Maik; Tomlinson, Lindsay; Homiski, Michael; Cheung, Jennifer; Zhan, Yutian; Coffing, Stephanie; Engel, Maria; Rubitski, Elizabeth; Seitis, Gary; Hales, Katherine; et al
Toxicological Sciences (2021), 180(1), 103-121 | Language: English, Database: CAplus and MEDLINE

Risk management of in vitro aneugens for topically applied compounds is not clearly defined because there is no validated methodol. to accurately measure compound concentration in proliferating stratum basale keratin ocytes of the skin. Here, we exptl. tested several known aneugens in the EpiDerm reconstructed human skin in vitro micron ucleus assay and compared the results to flow cytometric mechanistic biomarkers (phospho-H3; MPM2, DNA content). We then evaluated similar biomarkers (Ki- 67, nuclear area) using immunohistochem. in skin sections of minipigs following topical exposure the potent aneugens, colchi cine, and hesper adin. Data from the Epi Derm model showed pos. micron ucleus responses for all aneugens tested following topical or direct media dosing with similar sensitivity when adjusted for applied dose. Quant. benchmark dose- response anal. exhibited increases in the mitotic index biomarkers phospho-H3 and MPM2 for tubulin binders and polyploidy for aurora kinase inhibitors are at least as sensitive as the micronucleus endpoint. By compar ison, the aneugens tested did not induce histop athol. changes, increases in Ki-67 immunolabeling or nuclear area in skin sections from the in vivo minipig study at doses in signif icant excess of those eliciting a response in vitro. Results indicate the EpiDerm in vitro micron ucleus assay is suitable for the hazard identif ication of aneugens. The lack of response in the minipig studies indicates that the barrier function of the minipig skin, which is comparable to human skin, protects from the effects of aneugens in vivo. These results provide a basis for conducting addnl. studies in the future to further refine this understanding.

Keywords: topical aneugen genotoxicant aneuploidy keratin ocyte; adverse outcome pathway; aneupl oidy; aurora kinase inhibitor; skin; tubulin binder

Substances (6) Reactions (0) Citing (3)

58

A cell cycle progression-derived gene signature to predict prognosis and therapeutic response in hepatocellular carcinoma

By: Hui, Yongfeng; Leng, Junzhi; Jin, Dong; Liu, Di; Wang, Genwang; Wang, Qi ; Wang, Yanyang
Disease Markers (2021), 1986159 | Language: English, Database: CAplus and MEDLINE

Dysregulation of cell cycle progre ssion (CCP) is one of the hallmarks of cancer. Here, our study is aimed at developing a C CP-derived gene signature for predicting high-risk population of hepatocellular carcinoma (HCC). Our study retrospe ctively analyzed the transcr iptome profiling and clin. information of HCC patients from The Cancer Genome Atlas (T CGA) and International Cancer Genome Consortium (ICGC) projects. Uni- and multiv ariate cox regression models were conducted for identi fying which hallmarks of cancer were risk factors of HCC. CCP-derived gene signature was developed with LASSO method. The predictive efficacy was verified by R O C curves and subgroup analyses. A nomogram was then generated and validated by R OC, calibration, and decisive curves. Immune cell infiltration was estimated with ssGSEA method. Potential small mol. compounds were predicted via C TRP and CMap analyses. The response to chemotherapeutic agents was evaluated based on the GDSC project. Among hallmarks of cancer, C CP was identified as a dominant risk factor for HCC prognosis. CCP-derived gene signature displayed the favorable predictive efficacy in HC C prognosis independent of other clinico pathol. parameters. A nomogram was generated for optimizing risk stratif ication and quantifying risk evaluation. CCP-derived signature was in relation to immune cell infiltr ation, HLA, and immune checkpoint expres sion. Combining CTRP and CMap analyses, fluvastatin was identified as a promising therap eutic agent against HCC. Furthermore, CC P-derived signature might be applied for predicting the response to doxoru bicin and gemcitabine. Collectively, CCP-derived gene signature was a promising marker in prediction of survival outcomes and therapeutic responses for HCC patients.

Keywords: ccp

Substances (6) Reactions (0) Citing (3)

59

BCL-XL blockage in TNBC models confers vulnerability to inhibition of specific cell cycle regulators

By: Castellanet, Olivier; Ahmad, Fahmida; Vinik, Yaron; Mills, Gordon B.; Habermann, Bianca; Borg, Jean-Paul; Lev, Sima; Lamballe, Fabienne; Maina, Flavio
Theranostics (2021), 11(19), 9180-9197 | Language: English, Database: CAplus and MEDLINE

Cell cycle regulators are frequently altered in Triple-Neg. Breast Cancer (TNBC). Emerging agents targeting these signals offer the possibility to design new combina torial therapies. However, preclin. models that recapitulate TNBC primary resistance and heterog eneity are essential to evaluate the potency of these combined treatm ents. Bioinformatic processing of human breast cancer datasets was used to analyze correlations between expression levels of cell cycle regulators and patient survival outcome. The M MTV-R26Met mouse model of TNBC resistance and heterogeneity was employed to analyze expression and targeting vulnera bility of cell cycle regulators in the presence of BCL-XL blockage. Robustness of outcomes and select ivity was further explored using a panel of human breast cancer cells. Orthotopic studies in nude mice were applied for preclin. evaluation of efficacy and toxicity. Altera tions of protein expression, phosphorylation, and/or cellular localisation were analyzed by western blots, reverse phase protein array, and immunocytochem. Bioinformatics was performed to highlight drug's mechanisms of action. We report that high expression levels of the BCL2L1 gene encoding BCL-XL and of specific cell cycle regulators correlate with poor survival outcomes of TNBC patients. Blockage of BCL-XL confers vulnerability to drugs targeting CDK1/2/4, but not FOXM1, CDK4/6, Aurora A and Aurora B, to all MMTV-R26Met and human TNBC cell lines tested. Combined blockage of B CL-XL and CDK1/2/4 interfered with tumor growth in vivo. Mechanistically, we show that, co- targeting of BCL-XL and CDK1/2/4 synergistically inhibited cell viability by combina torial depletion of survival and RTK/AKT signals, and concomitantly restoring FOXO3a tumor suppression actions. This was accomp anied by an accumulation of DNA damage and conseq uently apoptosis. Our studies illustrate the possibility to exploit the vulnera bility of TNBC cells to CDK1/2/4 inhibition by targeting B CL-XL. Moreover, they underline that specificity matters in targeting cell cycle regulators for combinatorial anticancer therapies.

Keywords: triple neg breast cancer BCLXL blockage cell cycle; BCL-XL; MET; cancer mouse model; cell cycle regula tors; triple-negative breast cancer

Substances (15) Reactions (0) Citing (2)

60

Aurora B and C kinases regulate chromosome desynapsis and segregation during mouse and human spermatogenesis

By: Wellard, Stephen R.; Schindler, Karen; Jordan, Philip W.
Journal of Cell Science (2020), 133(23), jcs248831 | Language: English, Database: CAplus and MEDLINE

Precise control of chromosome dynamics during meiosis is critical for fertility. A gametocyte undergoing meiosis coordinates formation of the synaptonemal complex (SC) to promote efficient homologous chromosome recombi nation. Subsequent disass embly of the SC occurs prior to segregation of homologous chromosomes during meiosis I. We examined the requir ements of the mammalian Aurora kinases (AURKA, AURKB and AURKC) during SC disassembly and chromosome segregation using a combination of chem. inhibition and gene deletion approaches. We find that both mouse and human spermat ocytes fail to disass emble SC lateral elements when the kinase activity of AURKB and AURKC are chem. inhibited. Interestingly, both Aurkb condit ional knockout and Aurkc knockout mouse spermatocytes successfully progress through meiosis, and the mice are fertile. In contrast, Aurkb, Aurkc double knockout spermatocytes fail to coordinate disass embly of SC lateral elements with chromosome conden sation and segreg ation, resulting in delayed meiotic progre ssion. In addition, deletion of Aurkb and Aurkc leads to an accumu lation of metaphase spermatocytes, chromosome missegregation and aberrant cytoki nesis. Collectively, our data demonstrate that AURKB and AURKC functionally compensate for one another ensuring successful mammalian spermato genesis.

Keywords: auroraB auroraC kinase chromosome desynapsis segreg ation spermatogenesis; Aurora kinase; Chromosome segreg ation; Gametogenesis; Infertility; Meiosis; Synaptonemal complex

Substances (10) Reactions (0) Citing (15)

61

Computationally predicting clinical drug combination efficacy with cancer cell line screens and independent drug action

By: Ling, Alexander ; Huang, R. Stephanie
Nature Communications (2020), 11(1), 5848 | Language: English, Database: CAplus and MEDLINE

Evidence has recently emerged that many clin. cancer drug combinations may derive their efficacy from indepe ndent drug action (I DA), where patients only receive benefit from the single most effective drug in a drug combin ation. Here we present I DACombo, an I DA based method to predict the efficacy of drug combin ations using monotherapy data from high-throughput cancer cell line screens. We show that IDACombo predictions closely agree with measured drug combin ation efficacies both in vitro (Pearson's correlation = 0.93 when comparing predicted efficacies to measured efficacies for > 5000 combin ations) and in a systema tically selected set of clin. trials (accuracy > 84% for predicting statistically significant improvements in patient outcomes for 26 first line therapy trials). Finally, we demons trate how IDACombo can be used to systema tically prioritize combinations for development in specific cancer settings, providing a framework for quickly translating existing monotherapy cell line data into clin. meaningful predictions of drug combin ation efficacy.

Keywords: independent drug action clin combin ation cancer cell line

Substances (122) Reactions (0) Citing (19)

62

Methods for reducing tumor progression and fibrosis and increasing adaptive immunity in malignancies by administering dimethyl-β-hydroxycholenamide derivatives

By: Glazer, Robert I.; Levi, Moshe; Yuan, Hongyan; Ranjit, Suman
World Intellectual Property Organization, WO2020252198 A1 2020-12-17 | Language: English, Database: CAplus

Aspects of the technol. described herein relate to a method of treating a malignancy in a subject. This method involves selecting a subject having a malignancy and administering di-Meβ-hydroxy-cholenamide (DMHCA) or derivative thereof to the subject in an amount effective to treat the malignancy. Methods of reducing malign ancy- associated fibrosis in a subject and pharmac eutical combinations comprising (i) DMHCA or derivative thereof and (ii) one or more immune checkpoint inhibitors are also disclosed.

Keywords: tumor fibrosis treatment adaptive immunity increase cholenamide derivative

PatentPak available

Substances (62) Reactions (0) Citing (0)

63

Methods for treating small cell neuroendocrine (SCN) tumors and small-round-blue cell tumors (SRBCTs)

By: Graeber, Thomas G.; Balanis, Nikolas G.; Sheu, Katherine M.; Witte, Owen N.; Esedebe, Favour; Park, Jung Wook
World Intellectual Property Organization, WO2020243329 A1 2020-12-03 | Language: English, Database: CAplus

The current disclosure provides methods of identifying and treating small cell neuroen docrine (SCN) tumors and small-round-blue cell tumors (SRBCTs). Aspects of the disclosure relates to a method comprising measuring the level of expression of one or more biomarkers in a biol. sample from a cancer patient. Further aspects relate to a method for treating small cell neuroendocrine (SCN) cancer or small-round-blue cell tumor (S RBCT) in a patient comprising adminis tering a cancer treatment to the patient, wherein the cancer treatment is a treatment selected from ABT-263, PHA-793887, vorinostat, tubastatin A, CX-5461 and combinations thereof.

Keywords: antitumor ABT263 vorinostat small cell neuroen docrine cancer treatment

PatentPak available

Substances (259) | Reactions (0) | Citing (0)

64

Nf1-mutant tumors undergo transcriptome and kinome remodeling after inhibition of either mTOR or MEK

By: Pucciarelli, Daniela; Angus, Steven P.; Huang, Benjamin; Zhang, Chi; Nakaoka, Hiroki J.; Krishnamurthi, Ganesh; Bandyopadhyay, Sourav; Clapp, D. Wade; Shannon, Kevin; Johnson, Gary L.; et al
Molecular Cancer Therapeutics (2020), 19(11), 2382-2395 | Language: English, Database: CAplus and MEDLINE

Loss of the tumor suppressor NF1 leads to activation of RAS effector pathways, which are therapeu tically targeted by inhibition of m TOR (mTORi) or MRK (MEKi). However, therapeutic inhibition of RAS effectors leads to the develo pment of drug resistance and ultimately disease progression. To investigate mol. signatures in the context of N F1 loss and subsequent acquired drug resist ance, we analyzed the exomes, transcriptomes, and kinomcs of Nf1-mutant mouse tumor cell lines and deriva tives of these lines that acquired resistance to either MEKi or mTORi. Biochem. comparisons of this unique panel of tumor cells, all of which arose in Nf1+/- mice, indicate that loss of heterozygosity of Nf1 as an initial genetic event does not confer a common biochem. signature or response to kinase inhibition. Although acquired drug resistance by Nf1- mutant tumor cells was accompanied by altered kinomes and irreversibly altered transcriptomes, functionally in multiple Nf1- mutant tumor cell lines, M EKi resistance was a stable phenotype, in contrast to mTORi resistance, which was reversible. Collectively, these findings demonstrate that Nf1-mutant tumors represent a heterogeneous group biochem. and undergo broader remodeling of kinome activity and gene expression in response to targeted kinase inhibition.

Keywords: anticancer agent NF1 mTOR MEK transcriptome kinome cancer

Substances (13) | Reactions (0) | Citing (1)

65

Inhibition of Aurora Kinase B attenuates fibroblast activation and pulmonary fibrosis

By: Kasam, Rajesh K.; Ghandikota, Sudhir ; Soundararajan, Divyalakshmi; Reddy, Geereddy B.; Huang, Steven K.; Jegga, Anil G. ; Madala, Satish K.
EMBO Molecular Medicine (2020), 12(9), e12131 | Language: English, Database: CAplus and MEDLINE

Fibroblast activation including proliferation, survival, and ECM production is central to initiation and maintenance of fibrotic lesions in idiopathic pulmonary fibrosis (IPF). However, druggable mols. that target fibroblast activation remain limited. In this study, we show that multiple pro-fibrotic growth factors, including TGFα, CTGF, and IGF1, increase aurora kinase B (AURKB) expression and activity in fibroblasts. Mechanistically, we demonstrate that Wilms tumor 1 (WT1) is a key transcription factor that mediates TGFα-driven AURKB upregulation in fibroblasts. Importantly, we found that inhibition of AURKB expression or activity is sufficient to attenuate fibroblast activation. We show that fibrosis induced by TGFα is highly dependent on AURKB expression and treating TGFα mice with barasertib, an AURKB inhibitor, reverses fibroblast activation, and pulmonary fibrosis. Barasertib similarly attenuated fibrosis in the bleomycin model of pulmonary fibrosis. Together, our preclin. studies provide important proof-of-concept that demonstrate barasertib as a possible intervention therapy for IPF.

Keywords: Aurora Kinase B fibroblast pulmonary fibrosis; Aurora Kinase B; Barasertib; Wilms’ tumor 1; fibroproliferation; pulmonary fibrosis

Substances (5) Reactions (0) Citing (18)

66

Dihydro-spiro[indoline-3:1'-isoquinolin]-2-ones and their analogs and derivatives and methods of treating cancer and other diseases and their preparation

By: Mabanyi, Lobe Maloba Mesembe; Efange, Simon Mbua Ngale
World Intellectual Property Organization, WO2020183307 A1 2020-09-17 | Language: English, Database: CAplus

The invention is directed to various 3',4'-dihydro-2'H-spiro[indoline-3:1'-isoquinolin]-2-one compounds of formula I and methods for treating disease states and/or conditions which are mediated through sphingosine-1-phosphate receptor(s). The invention is also directed to the use of these compounds as anticancer agents and as modulators of sphingosine-1-phosphate receptor function in the treatment of disease states and/or conditions which are mediated through these receptors. In addition, the invention relates to pharmaceutical compositions comprising one or more of these compounds alone or in combin ation with other therapeutic agents. The invention is also directed to methods of treatment of cancer and/or conditions that may respond to the modulation of sphingosine-1-phosphate receptor function and which employ compounds of the present invention or pharmac eutical compos itions comprising one or more of the compounds of this invention. Compounds of formula I wherein R^1 is H, OH, halo, C_{1-6} alkoxy, etc.; R^2 and R^3 are independently H, OH, C_{1-6} hydroxyalkyl, etc.; R^4 is H, OH, halo, C_{1-6} alkoxy, etc.; R^5 and R^6 are independently H, alkyl, C_{1-6} alkoxy, etc.; R^7 is H, alkyl, arylalkyl, etc.; R^8 is H, OH, halo, NO_2, etc.; R^9, R^{10} and R^{11} are independently H, OH, halo, C_{1-6} hydroxyalkyl, etc.; R^{12} is H, OH, hydroxyalkyl, arylalkyl, etc. ; R^{13} is O and S; and pharmace utically acceptable salts, stereoi somers, solvates and polymorphs thereof, are claimed. Example compound II as prepared by cycliz ation of isatin with 3-hydroxyphenethy lamine. The invention compounds were evaluated for their anticancer activity (some data given) .

Keywords: spiroindoline isoquinolinone preparation treatment cancer

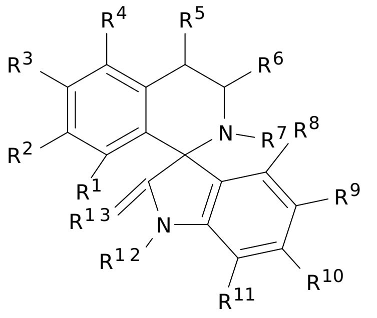

I

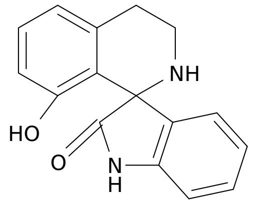

II

PatentPak available

Substances (450) | Reactions (239) | Citing (3)

67

Methods of treating venetoclax-resistant acute myeloid leukemia

By: Tyner, Jeffrey W.; Nechiporuk, Tamilla; Kurtz, Stephen; McWeeny, Shannon
World Intellectual Property Organization, WO2020176686 A1 2020-09-03 | Language: English, Database: CAplus

The present invention concerns novel methods of treatment for venetoclax-resistant acute myeloid leukemia, partic ularly in subjects with a low expression of TP53 protein or expression of TP53 protein associated with a mutation of the T P53 gene, the methods comprising administering to the subject in need thereof a therapeu tically effective amount of a NTRK/ALK kinase/ROS1 kinase inhibitor, or a pharmaceutically acceptable salt thereof.

Keywords: venetoclax antitumor resistance acute myeloid leukemia treatment

PatentPak available

Substances (73) | Reactions (0) | Citing (0)

68

High-throughput screening of regulators of axonal transport

By: Shlevkov, Evgeny; Schwarz, Thomas L.; Bray, Mark-Anthony; Carpenter Van Dyk, Anne E.

World Intellectual Property Organization, WO2020149925 A1 2020-07-23 | Language: English, Database: CAplus

Provided are techniques for processing a sequence of images of neurons to identify stationary and motile organelles of the neurons, generating tracking data associated with movement of the one or more motile organelles across the sequence of images, and determining an overall percent motile based on the identified stationary organelles and motile organe lles. Also provided are compounds identified using the systems described, wherein the compounds, and pharmaceutically acceptable salts, and compos itions thereof are administered to a subject in need thereof in a sufficient amount to increase or promote organelle (e.g., mitocho ndrial) motility, or administered in a sufficient amount to treat a disease or disorder associated with decreased organelle motility. The compounds may be used in methods for increasing or promoting organelle (e.g., mitochondrial) motility in a cell, and methods for treating a disease. Also provided in the present disclosure are pharmaceutical compositions, kits, methods, and uses including or using a compound described herein.

Keywords: high throughput screening regulator axonal transport

PatentPak available

Substances (11) | Reactions (0) | Citing (0)

69

Antiproliferative compounds and second active agents for combined use for treating multiple myeloma

By: Wong, Lilly L.

United States, US20200215060 A1 2020-07-09 | Language: English, Database: CAplus

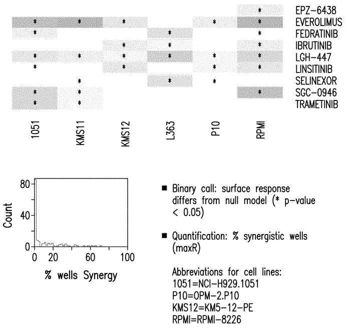

Provided herein are methods of using 4-(4-(4-(((2-(2,6-dioxopiperidin-3-yl)-1-oxoisoindolin-4-yl)oxy)methyl)benzyl)piperazin-1-yl)-3-fluorobenzonitrile, or an enantiomer, a mixture of enantiomers, a tautomer, or a pharmace utically acceptable salt thereof, in combination with a second active agent for treating, preventing or managing multiple myeloma. The second active agent is one or more of a BTK inhibitor, an mTOR inhibitor, a PIM inhibitor, an IGF-1R inhibitor, an MEK inhibitor, an XPO1 inhibitor, a DOT1L inhibitor, an EZH2 inhibitor, a JAK2 inhibitor, a BRD4 inhibitor, a PLK 1 inhibitor, an NEK2 inhibitor, an AURKB inhibitor, a BIRC5 inhibitor, a BET inhibitor, or a DNA methyltransferase inhibitor.

Keywords: antiproliferative compound combination multiple myeloma treatment

PatentPak available

Substances (76) | Reactions (42) | Citing (0)

70

Correction: Bromodomain and extraterminal protein inhibition blocks growth of triple-negative breast cancers through the suppression of aurora kinases. [Erratum to document cited in CA165:569636]

By: Sahni, Jennifer M.; Gayle, Sylvia S.; Bonk, Kristen L. Weber; Vite, Leslie Cuellar; Yori, Jennifer L.; Webb, Bryan; Ramos, Erika K.; Seachrist, Darcie D.; Landis, Melissa D.; Chang, Jenny C.; et al
Journal of Biological Chemistry (2020), 295(27), 9266 | Language: English, Database: CAplus and MEDLINE

There is no abstract available for this document.

Keywords: Aurora kinase BET protein inhibitor anticancer breast cancer erratum

Substances (4) Reactions (0) Citing (1)

71

Genome-wide CRISPR screen uncovers a synergistic effect of combining Haspin and Aurora kinase B inhibition

By: Huang, Min; Feng, Xu; Su, Dan ; Wang, Gang; Wang, Chao; Tang, Mengfan; Paulucci-Holthauzen, Adriana; Hart, Traver; Chen, Junjie
Oncogene (2020), 39(21), 4312-4322 | Language: English, Database: CAplus and MEDLINE

Abstract: Aurora kinases are a family of serine/threonine kinases vital for cell division. Because of the overexp ression of Aurora kinases in a broad range of cancers and their important roles in mitosis, inhibitors targeting Aurora kinases have attracted attention in cancer therapy. VX-680 is an effective pan-Aurora kinase inhibitor; however, its clin. efficacy was not satisf ying. In this study, we performed CRISPR/Cas9 screens to identify genes whose depletion shows synthetic lethality with V X-680. The top hit from these screens was GSG2 (also known as Haspin), a serine/threonine kinase that phospho rylates histone H3 at Thr- 3 during mitosis. Moreover, both Haspin knockout and Haspin inhibitor-treated HCT116 cells were hypersensitive to VX-680. Furthermore, we showed that the synthetic lethal interaction between Haspin depletion and VX-680 was mediated by the inhibition of Haspin with Aurora kinase B (AURKB), but not with Aurora kinase A (A URKA). Strikingly, combined inhibition of Haspin and AURKB had a better efficacy than single-agent treatment in both head and neck squamous cell carcinoma and non- small cell lung cancer. Taken together, our findings have uncovered a synthetic lethal interaction between AURKB and Haspin, which provides a strong rationale for this combination therapy for cancer patients.

Keywords: Haspin AURKB NSCLC VX680 CHR6494 barasertib head neck carcinoma

Substances (7) Reactions (0) Citing (11)

72

Aurora B kinase as a therapeutic target in acute lymphoblastic leukemia

By: Goto, Hiroaki ; Yoshino, Yuki; Ito, Mieko; Nagai, Junichi; Kumamoto, Tadashi; Inukai, Takesi; Sakurai, Yukari; Miyagawa, Naoyuki; Keino, Dai; Yokosuka, Tomoko; et al
Cancer Chemotherapy and Pharmacology (2020), 85(4), 773-783 | Language: English, Database: CAplus and MEDLINE

Abstract: Purpose: Acute lymphoblastic leukemia (ALL) is curable with standardized chemotherapy. However, the development of novel therapies is still required, especially for patients with relapsed or refractory disease. By utilizing an in vitro drug screening system, active mol. targeting agents against ALL were explored in this study. Methods: By the in vitro drug sensit ivity test, 81 agents with various actions were screened for their cytotoxicity in a panel of 22 A LL cell lines and A LL clin. samples. The drug effect score (D ES) was calculated from the dose-response of each drug for comparison among drugs or samples. Normal peripheral blood mononuclear cells were also applied onto the drug screening to provide the reference control values. The drug combin ation effect was screened based on the Bliss independent model, and validated by the improved isobol ogram method. Results: On sensit ivity screening in a cell line panel, barasertib-HQPA which is an active metabolite of barase rtib, an aurora B kinase inhibitor, alisertib, an aurora A kinase inhibitor, and YM155, a survivin inhibitor, were effective against the broadest range of A LL cells. The DES of barase rtib-HQPA was significantly higher in A LL clin. samples compared to the reference value. There were signif icant correlations in DES between barasertib-HQPA and vincristine or docetaxel. In the drug combin ation assay, barasertib-HQPA and eribulin showed additive to synergistic effects. Conclusion: Aurora B kinase was identified to be an active therap eutic target in a broad range of A LL cells. Combination therapy of barasertib and a microt ubule-targeting drug is of clin. interest.

Keywords: barasertib alisertib antitumor aurora B kinase inhibitor lymphoblastic leukemia; Acute lymphob lastic leukemia; Aurora B kinase; Barasertib; In vitro drug sensit ivity test; Survivin

Substances (10) Reactions (0) Citing (4)

73

Methods of treatment of cancer comprising CDC7 inhibitors

By: Hassig, Christian Andrew; Hansen, Ryan James; Milutinovic, Snezana; Strouse, Bryan William
World Intellectual Property Organization, WO2020068347 A1 2020-04-02 | Language: English, Database: CAplus

Herein disclosed are methods of treatment administering SRA141 as a monoth erapy or in a combin ation therapy useful for inhibiting the growth of tumors such as those in patients with cancer.

Keywords: treatment cancer CDC7 inhibitor SRA141

PatentPak available

Substances (14) Reactions (0) Citing (0)

74

Pan-transcriptome-based candidate therapeutic discovery for idiopathic pulmonary fibrosis

By: Wang, Yunguan; Yella, Jaswanth K.; Ghandikota, Sudhir; Cherukuri, Tejaswini C.; Ediga, Harshavardhana H.; Madala, Satish K.; Jegga, Anil G.
Therapeutic Advances in Respiratory Disease (2020), 14, 1753466620971143 | Language: English, Database: CAplus and MEDLINE

Two methods were used to calculate IPF-compound connectivity: gene expres sion-based connectivity and feature-based connect ivity. Identified compounds were further priori tized if their shared mechanism(s) of action were I PF-related. We found 77 compounds as potential candidate therapeutics for IPF. Of these, 39 compounds are either F DA-approved for other diseases or are currently in phase II/III clin. trials suggesting their repurp osing potential for I PF. Among these compounds are multiple receptor kinase inhibitors, aurora kinase inhibitor (barasertib), epidermal growth factor receptor inhibitors, calcium channel blocker (verapamil), phosphodiesterase inhibitors (roflumilast, sildenafil), PPAR agonists (pioglitazone), histone deacetylase inhibitors (entinostat), and opioid receptor antago nists (nalbuphine). As a proof of concept, we performed in vitro valida tions with verapamil using lung fibroblasts from I PF and show its potential benefits in pulmonary fibrosis. As about half of the candidates discovered in this study are either FDA-approved or are currently in clin. trials for other diseases, rapid transl ation of these compounds as potential IPF therapeutics is possible. Further, the integr ative connectivity anal. framework in this study can be adapted in early phase drug discovery for other common and rare diseases with transcriptomic profiles. The reviews of this paper are available via the supplemental material section.

Keywords: idiopathic pulmonary fibrosis transcriptome therapy; IPF; computational drug discovery; drug discovery; drug reposit ioning; idiopathic pulmonary fibrosis; pulmonary fibrosis; verapamil

Substances (12) Reactions (0) Citing (3)

75

Aurora kinase as target for treating, preventing or curing HIV infection or aids

By: Este Araque, Jose Andres; Garcia Vidal, Edurne; Ballana Guix, Ester; Badia Corcoles, Roger; Riveira Munoz, Eva
World Intellectual Property Organization, WO2020049208 A1 2020-03-12 | Language: Spanish, Database: CAplus

The present invention relates to the use of Aurora kinase (AURK) inhibitors for treating or preventing an H IV infection or AIDS in a subject. The present invention further relates to compositions containing AURK inhibitors and combin ations of AURK inhibitors with other active agents useful for treating or preventing an HIV infection or AIDS in a subject.

Keywords: aurora kinase inhibitors antiviral HIV AIDS

PatentPak available

Substances (179) Reactions (0) Citing (0)

76

Treatments for acute myeloid leukemia in the presence of gene mutations such as MPM1

By: Tyner, Jeffrey W.; Tognon, Cristina; Druker, Brian J.; Bottomly, Daniel; Wilmot, Beth; Kurtz, Stephen E.; Savage, Samantha; Long, Nicola; Reister-Schultz, Anna; Traer, Elie; et al
World Intellectual Property Organization, WO2020023921 A1 2020-01-30 | Language: English, Database: CAplus

The present invention relates to useful methods of treating Acute Myeloid Leukemia (AML) in a human. in the presence of gene mutations such as MPM1.

Keywords: acute myeloid leukemia treatment MPM1 gene mutation

PatentPak available

Substances (65) Reactions (0) Citing (1)

77

Structure-based discovery and bioactivity evaluation of novel Aurora-A kinase inhibitors as anticancer agents via docking-based comparative intermolecular contacts analysis (dbCICA)

By: Hijjawi, Majd S.; Abutayeh, Reem Fawaz; Taha, Mutasem O.
Molecules (2020), 25(24), 6003 | Language: English, Database: CAplus and MEDLINE

Aurora-A kinase plays a central role in mitosis, where aberrant activation contri butes to cancer by promoting cell cycle progre ssion, genomic instability, epithelial-mesenchymal transition, and cancer stemness. Aurora-A kinase inhibitors have shown encour aging results in clin. trials but have not gained Food and Drug Administration (FDA) approval. An innovative computational workflow named Docking-based Comparative Intermol. Contacts Anal. (db CICA) was applied-aiming to identify novel Aurora-A kinase inhibi tors-using seventy-nine reported Aurora-A kinase inhibitors to specify the best possible docking settings needed to fit into the active-site binding pocket of Aurora-A kinase crystal structure, in a process that only potent ligands contact critical binding- site spots, distinct from those occupied by less-active ligands. Optimal db CICA models were transformed into two corresp onding pharmacophores. The optimal one, in capturing active hits and discarding inactive ones, validated by receiver operating charact eristic anal., was used as a virtual in- silico search query for screening new mols. from the National Cancer Institute database. A fluorescence resonance energy transfer (FRET)-based assay was used to assess the activity of captured mols. and five promising Aurora-A kinase inhibitors were identi fied. The activity was next validated using a cell culture anti- proliferative assay (MTT) and revealed a most potent lead 85(NCI 14040) mol. after 72 h of incuba tion, scoring IC50 values of 3.5-11.0μM against PANC1 (pancreas), PC-3 (prostate), T-47D and MDA-MB-231 (breast)cancer cells, and showing favorable safety profiles (27.5μ M IC50 on fibroblasts). Our results provide new clues for further develo pment of Aurora-A kinase inhibitors as anticancer mols.

Keywords: danusertib CYC116 barasertib GSK1070916 MLN8237 auroraAkinase inhibitor anticancer cancer; AURKA; Aurora-A kinase; MDA-MB-231; MTT assay; PANC1; PC-3; T-47D; aurora inhibitors; docking-based comparative intermolecular contacts analysis; in-silico screening; molecular docking; structure- based drug design

Substances (7) Reactions (0) Citing (6)

78

Inhibition of BRD4 triggers cellular senescence through suppressing aurora kinases in oesophageal cancer cells

By: Xu, Jian-Ling ; Yuan, Ya-Jiao; Lv, Jiao; Qi, Di; Wu, Meng-Di; Lan, Jing; Liu, Sheng-Nan; Yang, Yong; Zhai, Jing; Jiang, Han-Ming
Journal of Cellular and Molecular Medicine (2020), 24(22), 13036-13045 | Language: English, Database: CAplus and MEDLINE

Oesophageal cancer is one of the most frequent solid malign ancies and the leading cause of cancer- related death around the world. It is urgent to develop novel therapy strategies to improve patient outcomes. Acetylation modification of histones has been extensively studied in epigen etics. BRD4, a reader of acetylated histone and non- histone proteins, has involved in tumorig enesis. It has emerged as a promising target for cancer therapy. BRD4 inhibitors, such as JQ1, have exerted efficacious anti-proliferation activities in diverse cancers. However, the effects of JQ1 on oesoph ageal cancer are still not fully described. Here, we demons trate that JQ1 suppresses cell growth and triggers cellular senescence in K YSE450 cells. Mechanistically, JQ1 up-regulates p21 level and decreases cyclin D1 resulting in G1 cycle arrest. The inhibitory effects of JQ1 on KYSE450 cells are indepe ndent on apoptosis. It activates cellular senescence by increasing SA-β-gal activity. BRD4 knockdown by sh RNA recapitulates cellular senescence. We also display that administration of JQ1 decreases recrui tment of BRD4 on the promoter of aurora kinases A and B. Inhibitors targeting at AURKA/B phenocopy JQ1 treatment in KYSE450 cells. These results identify a novel action manner of B RD4 in oesoph ageal cancer, which strengthens JQ1 as a candidate drug in oesoph ageal cancer chemotherapy.

Keywords: Inhibition BRD4 triggers cellular senescence aurora kinases oesoph ageal cancer; BRD4; aurora kinase; cellular senesc ence; oesoph ageal cancer

Substances (8) Reactions (0) Citing (3)

79

High-throughput compound screen reveals mTOR inhibitors as potential therapeutics to reduce (auto)antibody production by human plasma cells

By: Tuijnenburg, Paul ; aan de Kerk, Daan J.; Jansen, Machiel H.; Morris, Ben; Lieftink, Cor; Beijersbergen, Roderick L.; van Leeuwen, Ester M. M.; Kuijpers, Taco W.
European Journal of Immunology (2020), 50(1), 73-85 | Language: English, Database: CAplus and MEDLINE

Antibody production by the B cell compartment is a crucial part of the adaptive immune response. Dysreg ulated antibody production in the form of autoantibodies can cause autoimmune disease. To date, B- cell depletion with anti- CD20 antibodies is commonly applied in autoimmunity, but pre- existing plasma cells are not eliminated in this way. Altern ative ways of more selective inhibition of antibody production would add to the treatment of these autoimmune diseases. To explore novel therapeutic targets in signaling pathways essential for plasmablast formation and/or Ig produc tion, we performed a compound screen of almost 200 protein kinase inhibitors in a robust B-cell differen tiation culture system. This study yielded 35 small cell- permeable compounds with a reproducible inhibitory effect on B- cell activation and plasma blast formation, among which was the clin. applied mammalian target of rapamycin (mTOR) inhibitor rapamycin. Two addnl. compounds targeting the phosphoi nositide 3-kinase-AKT-mTOR pathway (BKM120 and WYE-354) did not affect prolife ration and plasmablast formation, but specif ically reduced the Ig production With this compound screen we successfully applied a method to invest igate therapeutic targets for B- cell differen tiation and identified compounds in the phosphoinositide 3-kinase-AKT-mTOR pathway that could specif ically inhibit Ig production only. These drugs may well be explored to be of value in current B-cell-depleting treatment regimens in autoimmune disorders.

Keywords: IgM IgA activation differen tiation plasmablast rapamycin; B cells; B- cell activation and differen tiation; PI3K-AKT-mTOR; autoimmune disease; plasma cells

Substances (63) Reactions (0) Citing (9)

80

AURKB as a target in non-small cell lung cancer with acquired resistance to anti-EGFR therapy

By: Bertran-Alamillo, Jordi ; Cattan, Valerie; Schoumacher, Marie ; Codony-Servat, Jordi; Gimenez-Capitan, Ana; Cantero, Frederique; Burbridge, Mike; Rodriguez, Sonia; Teixido, Cristina; Roman, Ruth; et al
Nature Communications (2019), 10(1), 1-14 | Language: English, Database: CAplus and MEDLINE

Non-small cell lung cancer (N SCLC) tumors harboring mutations in E GFR ultimately relapse to therapy with E GFR tyrosine kinase inhibitors (EGFR TKIs). Here, we show that resistant cells without the p. T790 M or other acquired mutations are sensitive to the Aurora B (AURKB) inhibitors barasertib and S49076. Phospho-histone H3 (p H3), a major product of AURKB, is increased in most resistant cells and treatment with AURKB inhibitors reduces the levels of p H3, triggering G1/S arrest and polypl oidy. Senescence is subsequently induced in cells with acquired mutations while, in their absence, polyploidy is followed by cell death. Finally, in N SCLC patients, pH3 levels are increased after progression on E GFR TKIs and high p H3 baseline correlates with shorter survival. Our results reveal that AURKB activation is associated with acquired resistance to E GFR TKIs, and that AURKB constitutes a potential target in NSCLC progressing to anti-EGFR therapy and not carrying resistance mutations.

Keywords: lung cancer barasertib gefitinib anticancer AURKB point mutation

Substances (9) Reactions (0) Citing (73)

81

Aurora A-selective inhibitor LY3295668 Leads to dominant mitotic arrest, apoptosis in cancer cells, and shows potent preclinical antitumor efficacy

By: Du, Jian; Yan, Lei; Torres, Raquel; Gong, Xueqian; Bian, Huimin; Marugan, Carlos; Boehnke, Karsten; Baquero, Carmen; Hui, Yu-Hua; Chapman, Sonya C.; et al
Molecular Cancer Therapeutics (2019), 18(12), 2207-2219 | Language: English, Database: CAplus and MEDLINE

Although Aurora A, B, and C kinases share high sequence similarity, especially within the kinase domain, they function distinctly in cell-cycle progression. Aurora A depletion primarily leads to mitotic spindle formation defects and conseq uently promet aphase arrest, whereas Aurora B/C inactivation primarily induces polyploidy from cytokinesis failure. Aurora B/C inactivation phenotypes are also epistatic to those of Aurora A, such that the concomitant inactivation of Aurora A and B, or all Aurora isoforms by noniso form-selective Aurora inhibitors, demonstrates the Aurora B/C- dominant cytokinesis failure and polyploidy phenotypes. Several Aurora inhibitors are in clin. trials for T/B-cell lymphoma, multiple myeloma, leukemia, lung, and breast cancers. Here, we describe an Aurora A-selective inhibitor, LY3295668, which potently inhibits Aurora autophosph orylation and its kinase activity in vitro and in vivo, persistently arrests cancer cells in mitosis, and induces more profound apoptosis than Aurora B or Aurora A/B dual inhibitors without Aurora B inhibition-associated cytokinesis failure and aneupl oidy. LY3295668 inhibits the growth of a broad panel of cancer cell lines, including small-cell lung and breast cancer cells. It demons trates significant efficacy in small-cell lung cancer xenograft and patient-derived tumor preclin. models as a single agent and in combin ation with standard-of-care agents. LY3295668, as a highly Aurora A-selective inhibitor, may represent a preferred approach to the current pan- Aurora inhibitors as a cancer therap eutic agent.

Keywords: cancer cell aurora mitotic arrest antitumor efficacy

Substances (7) Reactions (0) Citing (21)

82

Screening methods for identifying pharmaceutical compounds and uses thereof for treating cancer

By: Klug, Christopher
World Intellectual Property Organization, WO2019241636 A1 2019-12-19 | Language: English, Database: CAplus

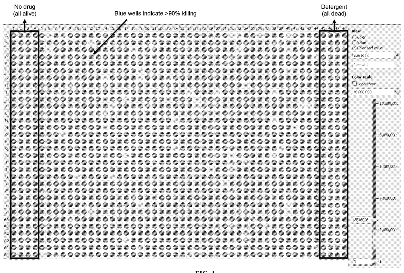

FIG. 1

Provided herein is a method for identifying compounds that inhibit cell growth or have cytotoxic activity against cancer cells, such as hematol. cancer cells or ovarian cancer cells, in a subject. Further provided is a method for identifying one or more compounds that sensitize refractory cancer cells from a subject. Also provided are compositions and methods for treating cancer, including refractory cancer, such as refractory acute myeloid leukemia (AML) or ovarian cancer.

Keywords: antitumor drug screening acute myeloid leukemia ovarian cancer treatment

PatentPak available

Substances (414) Reactions (0) Citing (0)

83

Analytical method considerations regarding carryover for monophosphate prodrugs for in vivo samples by liquid chromatography-tandem mass spectrometry

By: Heinle, Lance; Patel, Hardikkumar; Jenkins, Gary; Ruterbories, Kenneth
Journal of Chromatography A (2019), 1606, 460379 | Language: English, Database: CAplus and MEDLINE

Three different components that impact carryover in a liquid chromatog.-tandem mass spectrometry (LC-MS/MS) method were evaluated to establish baseline conditions for analyzing in vivo samples for twelve monophosphate prodrug compounds and their corresponding parent compounds The three components were: wash solvent modifier, column shell material (metal vs. metal free) , and tubing composition These components were tested for their impact on system carryover by using rat plasma extracted samples. It was determined that a wash solution containing hexylamine yielded the lowest average carryover of the solutions tested. In addition, metal free columns and PEEK (poly ether ether ketone) tubing yielded the lowest carryover when compared to metal columns, stainless steel tubing and nickel tubing. These conditions were also tested against the parent mols. for each prodrug in the test set, to ensure that changing the conditions for the prodrugs did not impact the ability to analyze the parent, since there is typically a desire to measure both compounds in study samples. Under all conditions, the carryover of the corresp onding parent mol. was not adversely impacted in these studies.

Keywords: monophosphate prodrugs carryover LC tandem MS PEEK tubing; Carryover; Liquid chromato graphy-tandem mass spectrometry; Monophosphate prodrugs; PEEK tubing

Substances (5) Reactions (0) Citing (1)

84

Bifunctional small molecules to target the selective degradation of circulating proteins

By: Spiegel, David; Caianiello, David; Zhang, Mengwen

World Intellectual Property Organization, WO2019199634 A1 2019-10-17 | Language: English, Database: CAplus

The present invention is directed to bifunctional small mols. which contain a circul ating protein binding moiety (CPBM) linked through a linker group to a cellular receptor binding moiety (CRBM) which is a membrane receptor of degrading cell such as a hepatocyte or other degrading cell. In embodiments, the (CRBM) is a moiety which binds to asialogly coprotein receptor (an asialogly coprotein receptor binding moiety, or ASGPRBM) of a hepatocyte. In addnl. embodiments, the (CRBM) is a moiety which binds to a receptor of other cells which can degrade proteins, such as a LRP1, LDLR, FcγRI, FcRN, Transferrin or Macrophage Scavenger receptor. Pharmaceutical compositions based upon these bifunctional small mols. represent an addnl. aspect of the present invention. These compounds and/or compositions may be used to treat disease states and conditions by removing circul ating proteins through degradation in the hepatocytes or macrophages of a patient or subject in need of therapy. Methods of treating disease states and/or conditions in which circulating proteins are associated with the disease state and/or condition are also described herein.

Keywords: circulating protein targeting bifunctional agent cancer autoimmune disease inflam mation

PatentPak available

Substances (881) | Reactions (2,119) | Citing (3)

85

Preparation of cereblon E3 ligase ligands and bifunctional PROTAC compounds for targeting and degradation of androgen receptor, estrogen receptor alpha, bromodomain-containing protein 4, and BRaf

By: Crew, Andrew P.; Crews, Craig M.; Dong, Hanqing; Hornberger, Keith R.; Wang, Jing; Qian, Yimin; Zimmermann, Kurt; Berlin, Michael; Snyder, Lawrence B.

World Intellectual Property Organization, WO2019199816 A1 2019-10-17 | Language: English, Database: CAplus

FIG. 1B

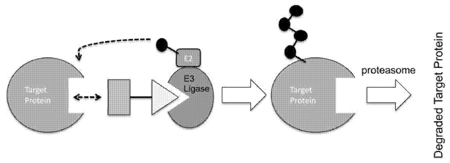

The invention relates to cereblon E3 ligase binding compounds, including bifunctional compounds comprising them, which find utility as modulators of targeted ubiquitination, especially inhibitors of a variety of polype ptides and other proteins which are degraded and/or otherwise inhibited by bifunctional compounds according to the present disclo sure. In particular, the invention provides compounds, which contain on one end a ligand which binds to the cereblon E3 ubiquitin ligase and on the other end a moiety which binds a target protein such that the target protein is placed in proximity to the ubiquitin ligase to effect degradation (and inhibition) of that protein. Specifically, the invention relates to bifunctional compounds C LM-L-PTM [PTM = small mol. containing a protein targeting moiety that binds bromodomain-containing protein 4, estrogen receptor (E R), or androgen receptor or BRaf; L = a bond, a chem. linking moiety covalently coupling the C LM and the PTM; CLM = a small mol. cereblon E3 ubiquitin ligase binding moiety that binds or targets a cereblon E3 ubiquitin ligase], their pharmaceutically acceptable salts, enanti omers, stereoi somers, solvates, polymorphs and prodrugs. Exempl ified compound **I** was prepared from a 12 step procedure (prepar ation given). The invention exhibits a broad range of pharmacol. activities associated with compounds according to the present invention, consistent with the degradation/inhibition of targeted polypeptides of nearly any type (data given). Diseases or disorders that result from aggregation or accumulation of the target protein are treated or prevented with compounds and compos itions of the invention.

Keywords: cereblon ligand chimeric mol preparation targeted protein degradation ubiquitination; PROTAC modulator preparation targeted ubiquitination estrogen receptor E RRa degradation; androgen receptor PROTAC degradation inhibition preparation antiproliferative; BRD4 BRaf PROTAC degradation inhibition cereblon E3 ligase antitumor

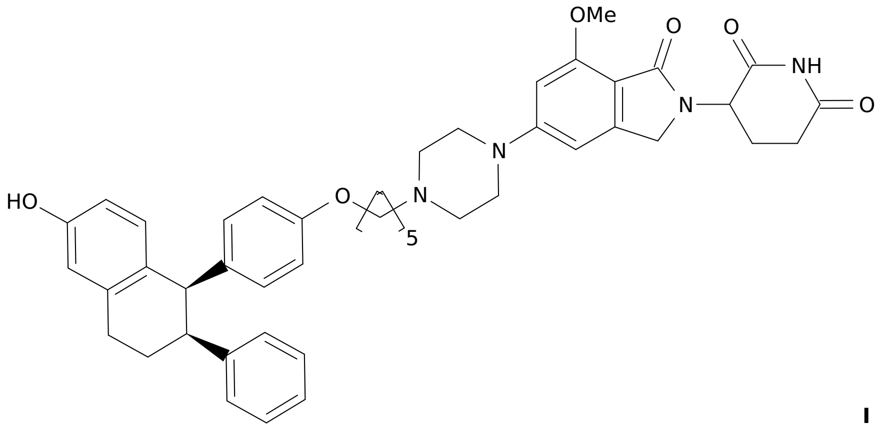

I

PatentPak available

Substances (727)

Reactions (1,470)

Citing (5)

86

Bi-functional molecules to degrade circulating proteins

By: Spiegel, David; Caianiello, David
World Intellectual Property Organization, WO2019199621 A1 2019-10-17 | Language: English, Database: CAplus

The present invention is directed to bi-functional compounds which find use as pharmaceutical agents in the treatment of disease states and/or conditions which are through macrophage migration inhibitory factor (MIF) or immunoglubin G (IgG). The present invention is also directed to pharmaceutical compositions which comprise these bi- functional compounds as well as methods for treating disease states and/or conditions which are mediated through MIF/lgG or where MIF/lgG is a contributing factor to the development and perpetuation of diseases and/or conditions, especially including autoimmune diseases and cancer, among others. The purpose of the present invention is to provide a mol. strategy to lower plasma MIF/lgG level in patients with autoimmune diseases or certain types of cancers. The b.i -functional mol. construct is comprised of a M IF/IgQ-targeting motif, that is derived from small mol. MIF/lgG ligands, and an ASGPr- targeting motif that binds to hepatocyte asialogly coprotein receptor (ASGPr). The compounds selectively bind MIF or IgG in plasma and subsequently engage the endo-lysosomal pathway of hepatocytes through AS GPr. As a conseq uence, MIF/igG is internalized and degraded by hepato cytes, thus resulting in potential attenu ation of corresp onding disease symptoms which are modulated through MIF/igG.

Keywords: bifunctional circulating protein migration inhibitory factor immunoglubin G cancer; autoimmune diseases circulating protein migration inhibitory factor immunoglubin G

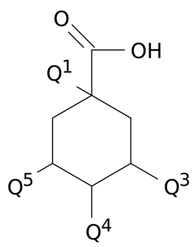

I

PatentPak available

Substances (97) Reactions (368) Citing (5)

87

Treating tumors with an alternating electrical field and an Aurora kinase inhibitor

By: Krex, Dietmar; Temme, Achim; Shnaiderman, Rosa S.; Giladi, Moshe
United States, US20190307781 A1 2019-10-10 | Language: English, Database: CAplus

The viability of cancer cells can be reduced by administering an Aurora kinase inhibitor (e.g., M LN8237 or another Aurora A kinase inhibitor, AZD1152 or another Aurora B kinase inhibitor) to the cancer cells, and applying an altern ating elec. field with a frequency between 100 and 300 kHz (e.g., 200 kHz) to the cancer cells. Furthermore, cancer (e.g., glioblastoma) in a subject may be treated by administering an Aurora kinase inhibitor to the subject, and applying an alternating elec. field with frequency between 100 and 300 kHz (e.g., 200 kHz) to a target region of the subject (e.g., the brain) .

Keywords: cancer treatment alternating elec field Aurora kinase inhibitor combination

PatentPak available

Substances (9) Reactions (0) Citing (2)

88

Selective inhibition of Aurora A and B kinases effectively induces cell cycle arrest in t(8;21) acute myeloid leukemia

By: Qi, Jialei; Gao, Xiang; Zhong, Xiaomin; Zhang, Ninghan; Wang, Rong; Zhang, Huihui; Pan, Ting; Liu, Xuejiao; Yao, Yao; Wu, Qingyun; et al

Biomedicine & Pharmacotherapy (2019), 117, 109113 | Language: English, Database: CAplus and MEDLINE

The fusion gene AML1-ETO initially dysregulates various cell cycle mols. in t(8;21) acute myeloid leukemia. Aurora kinases have shown great promise in treating tumors. However, the efficacy of Aurora kinase (AURK) A and B inhibition in t(8;21) AML remains unclear. We found that AURK-A inhibitor Alisertib and AURK-B inhibitor Barasertib strongly inhibited the growth and proliferation of t(8;21) AML cells. The quantity and size of cell colonies were markedly decreased after a 14- d drug exposure. The cell cycle distribution was blocked at the G2/M phase in both dose- and time- dependent manner. The expression of p53 family and cdc2- p34 significantly changed as well. Notably, we found that t(8;21) AML cells are more sensitive to Aurora B inhibition. In each set of experiments, Barasertib took less time or a lower concentration to achieve similar efficacy. Taken together, our data highlighted the potential role of Aurora kinases as promising cell cycle targets for the treatment of t(8;21) AML and hereby provided a theor. basis to guide relevant clin. trials.

Keywords: alisertib barasertib anticancer AURKA AURKB inhibitor acute myeloid leukemia; AML-ETO; Acute myeloid leukemia; Alisertib; Aurora kinase; Barasertib

Substances (3) | Reactions (0) | Citing (7)

89

Inductive regeneration of the airway by transcriptional factor modulation of glandular myoepithelial stem cells using modulators of Lef-1/TCF/Wnt signaling

By: Engelhardt, John F.; Lynch, Thomas J.; Anderson, Preston J.; Shahin, Weam

World Intellectual Property Organization, WO2019178267 A2 2019-09-19 | Language: English, Database: CAplus

Compositions and methods to modulate Lef-1/TCF/Wnt signaling ex vivo or in vivo, and assays to detect those modulators, are described. The disclosure provides a composition comprising an isolated transcription factor (Lymphoid enhancer factor 1 or Lef-1) that when introduced to or induced in a specific airway stem cell leads to self-limiting regenerative expansion of the airway and submucosal glands. Using genetically engineered mice, Lef-1 expression in glandular myoepithelial cells (MECs) was shown to either enhance airway repair following injury (with monoallelic Lef-1 expression) or spontaneously induce MEC-mediated airway regeneration (with biallelic Lef-1 expression). These findings open the door to enhancing the regenerative capacity of MECs ex vivo using cell therapy approaches and in vivo using small mols. that influence Lef-1 function and expression.

Keywords: airway regeneration transcription factor modulator glandular myoepithelium stem cell

PatentPak available

Substances (1,589) | Reactions (0) | Citing (0)

90

Divergent Polypharmacology-Driven Cellular Activity of Structurally Similar Multi-Kinase Inhibitors through Cumulative Effects on Individual Targets

By: Sumi, Natalia J.; Ctortecka, Claudia; Hu, Qianqian; Bryant, Annamarie T.; Fang, Bin; Remsing Rix, Lily L.; Ayaz, Muhammad; Kinose, Fumi; Welsh, Eric A.; Eschrich, Steven A.; et al
Cell Chemical Biology (2019), 26(9), 1240-1252.e11 | Language: English, Database: CAplus and MEDLINE

Despite recent successes of precision and immunotherapies there is a persisting need for novel targeted or multi- targeted approaches in complex diseases. Through a systems pharmacol. approach, including phenotypic screening, chem. and phosphopr oteomics, and RNA-seq, we elucidated the targets and mechanisms underlying the differ ential anticancer activity of two structurally related multi-kinase inhibitors, foretinib, and cabozantinib, in lung cancer cells. Biochem. and cellular target validation using probe mols. and RNAi revealed a polypharmacol. mechanism involving MEK1/2, FER, and AURKB, which were each more potently inhibited by foretinib than cabozantinib. Based on this, we developed a synerg istic combination of foretinib with barasertib, a more potent A URKB inhibitor, for MYC-amplified small-cell lung cancer. This systems pharmacol. approach showed that small structural changes of drugs can cumulatively, through multiple targets, result in pronounced anticancer activity differ ences and that detailed mechan istic understanding of polypharmacol. can enable repurposing opportunities for cancers with unmet medical need. The accession number for the chem. proteomics, tyrosine phosphoproteomics, global phosphoproteomics and ABPP data reported in this paper is PRIDE: PXD012961, PXD012962, PXD012963 and PXD012965.

Keywords: proteome foretinib cabozantinib barasertib anticancer agent synerg istic lung cancer; cabozantinib; chemoproteomics; drug combination; foretinib; kinase inhibitor; lung cancer; phosphopr oteomics; polypharmacology; systems pharmacology; target selectivity

Substances (48) Reactions (0) Citing (10)

91

Optimization and biological evaluation of nicotinamide derivatives as Aurora kinase inhibitors

By: Qi, Baohui; Xu, Xingwei; Yang, Ying; He, Huan; Yue, Xupeng
Bioorganic & Medicinal Chemistry (2019), 27(17), 3825-3835 | Language: English, Database: CAplus and MEDLINE

Aurora kinases are known to be overexpressed in various solid tumors and implicated in oncoge nesis and tumor progression. A series of nicotinamide derivatives were synthesized and their biol. activities were evaluated, including kinase inhibitory activity against Aur A and Aur B and in vitro antitumor activity against SW620, HT-29, NCI-H1975 and Hela cancer cell lines. In addition, the study of antiproliferation, cytotoxicity and apoptosis was performed meanwhile. As the most potent inhibitor of Aur A, 4- ((3-bromo-4-fluorophenyl)amino)-6-chloro-N-(4-((6,7-dimethoxyquinolin-4-yl)oxy)-3-fluorophenyl)nicotinamide (10l) showed excellent antitumor activity against SW620 and NCI-H1975 with IC_{50} values were 0.61 and 1.06 μM, while the IC_{50} values of reference compound were 3.37 and 6.67 μM, resp. Furthermore, binding mode studies indicated that compound 10l forms better intera ction with Aur A.

Keywords: nicotinamide synthesis antitumor SAR Aurora kinase cancer; Antitumor; Aurora kinases; Kinase inhibi tors; Nicotinamide; SAR

Substances (75) Reactions (130) Citing (3)

92

Aneugen molecular mechanism assay: proof-of-concept with 27 reference chemicals

By: Bernacki, Derek T.; Bryce, Steven M.; Bemis, Jeffrey C.; Dertinger, Stephen D.
Toxicological Sciences (2019), 170(2), 382-393 | Language: English, Database: CAplus and MEDLINE

A tiered bioassay and data anal. scheme is described for elucidating the most common mol. targets respon sible for chem.-induced in vitro aneugenicity: tubulin destabilization, tubulin stabilization, and inhibition of mitotic kinase(s). To evaluate this strategy, T K6 cells were first exposed to each of 27 presumed aneugens over a range of concentrations After 4 and 24 h of treatment, γ H2AX, p53, phospho-histone H3 (p-H3), and polyploidization biomarkers were evaluated using the Multi Flow DNA Damage Assay Kit. The assay identified 27 of 27 chems. as genotoxic, with 25 exhibiting aneugenic signatures, 1 aneugenic and clasto genic, and 1 clasto genic. Subsequently, a newly described follow-up assay was employed to invest igate the aneugenic agents' mol. targets. For these experiments, TK6 cells were exposed to each of 26 chems. in the presence of 488 Taxol. After 4 h, cells were lysed and the liberated nuclei and mitotic chromosomes were stained with a nucleic acid dye and labeled with fluore scent antibodies against p-H3 and Ki-67. Flow cytometric analyses revealed that altera tions to 488 Taxol-associated fluorescence were only observed with tubulin binders-increases in the case of tubulin stabilizers, decreases with destabi lizers. Mitotic kinase inhibitors with known Aurora kinase B inhibiting activity were the only aneugens that dramatically decreased the ratio of p-H3-pos. to Ki-67-pos. nuclei. Unsupervised hierarchical clustering based on 488 Taxol fluore scence and p-H3: Ki-67 ratios clearly distinguished compounds with these disparate mol. mechanisms. Furthermore, a classification algorithm based on an artificial neural network was found to effect ively predict mol. target, as leave-one-out cross-validation resulted in 25/26 agreement with a priori expecta tions. These results are encouraging, as they suggest that an adequate number of training set chems., in conjun ction with a machine learning algorithm based on 488 Taxol, p-H3, and Ki-67 responses, can reliably elucidate the most commonly encoun tered aneugenic mol. targets.

Keywords: aneugen mol mechanism assay artificial neural network classification algorithm; Mitotic kinase inhibi tors; TK6 cells; aneugen; flow cytometry; spindle poisons

Substances (28)	Reactions (0)	Citing (9)

93

Methods of treatment of cancer comprising cell division control protein 7 (CDC7) inhibitors

By: Hassig, Christian Andrew; Hansen, Ryan James
World Intellectual Property Organization, WO2019165473 A1 2019-08-29 | Language: English, Database: CAplus

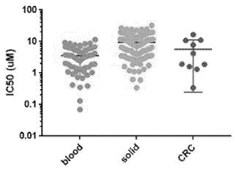

Fig. 3B

Herein disclosed are methods of treatment administering a cell division control protein 7 (C DC7) inhibitor as a monotherapy or in a combination therapy useful for inhibiting the growth of tumors such as those in patients with cancer.

Keywords: antitumor protein CDC7 kinase inhibitor furanone derivative cancer treatment

PatentPak available

Substances (18)	Reactions (0)	Citing (3)

94

Methods of treating fibrosis using aurora kinase B (AURKB) inhibitors

By: Madala, Satish Kumar; Jegga, Anil Goud; Kasam, Rajesh K.

World Intellectual Property Organization, WO2019161000 A1 2019-08-22 | Language: English, Database: CAplus

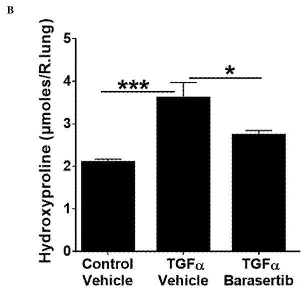

Some embodiments of the invention include methods for treating an animal for fibrosis comprising one or more administ rations of one or more compositions comprising one or more A URKB (Aurora kinase B) inhibitors. Other embodiments of the methods for treating further include other fibrosis treatments. Still other embodi ments of the invention include methods for treating a human for lung fibrosis or idiopathic pulmonary fibrosis, comprising administering one or more compos itions comprising AZD1152 or barasertib. Addnl. embodiments of the invention are also discussed herein.

Keywords: antifibrotic aurora kinase inhibitor barasertib A ZD1152 pulmonary fibrosis treatment

PatentPak available

Substances (46) | Reactions (0) | Citing (0)

95

Cabazitaxel-resistant prostate cancer preventive or therapeutic agent

By: Kosaka, Takeo; Hongo, Hiroshi; Oya, Mototsugu

Japan, JP2019127437 A 2019-08-01 | Language: Japanese, Database: CAplus

The present invention relates to a preventive or therapeutic agent comprising one or more agent selected from a group consisting of pimozide or its salt, an Aurora kinase inhibitor, and a KIF20A inhibitor. The preventive or therap eutic agent comprises a low mol. weight compound that exerts an anticancer effect against cabazitaxel-resistant prostate cancer that can be manufa ctured relatively easily and inexpensively.

Keywords: Aurora kinase inhibitor cabazitaxel resistant prostate cancer antitumor; pimozide cabazi taxel resistant prostate cancer antitumor; KIF20A inhibitor cabazitaxel resistant prostate cancer antitumor

PatentPak available

Substances (5) | Reactions (0) | Citing (0)

96

Resveratrol modulates the redox-status and cytotoxicity of anticancer drugs by sensitizing leukemic lymphocytes and protecting normal lymphocytes

By: Ivanova, Donika; Zhelev, Zhivko; Semkova, Severina; Aokl, Iciiio; Bakalova, Rumiana
Anticancer Research (2019), 39(7), 3745-3755 | Language: English, Database: CAplus and MEDLINE

Background/Aim: The study is directed to the effect of resver atrol on the redox-status and viability of leukemic and normal lympho cytes, as well as its ability to sensitize leukemic lympho cytes to anticancer drugs. Materials and Methods: Cytoto xicity was analyzed by trypan blue staining, apoptosis - by Annexin V test, and oxidative stress - by the intracellular levels of reactive oxygen species (R O S) and protein-carbonyl products. Results: Incubation of resver atrol in combination with the majority of anticancer drugs resulted in higher toxicity than resveratrol or drug alone. In the case of leukemic lympho cytes treated with barasertib and everolimus in the presence of resveratrol, synergistic cytotoxicity was accompanied by strong induction of apoptosis, increased levels of hydrope roxides and insignificant changes in proteincarbonyl products. None of these parameters changed in normal lympho cytes. Conclu sion: Resveratrol is a promising supplem entary compound for anticancer therapy that may allow reduction of the therap eutic doses of barasertib and everolimus, minimizing their side- effects.

Keywords: leukemic lymphocyte cytotoxicity anticancer resveratrol; Cancer; apoptosis; chemotherapy; oxidative stress; resveratrol

Substances (8) Reactions (0) Citing (14)

97

High-throughput chemical screening identifies focal adhesion kinase and Aurora kinase B inhibition as a synergistic treatment combination in Ewing sarcoma

By: Wang, Sarah; Hwang, Elizabeth E.; Guha, Rajarshi; O'Neill, Allison F.; Melong, Nicole; Veinotte, Chansey J.; Saur, Amy Conway; Wuerthele, Kellsey; Shen, Min; McKnight, Crystal; et al
Clinical Cancer Research (2019), 25(14), 4552-4566 | Language: English, Database: CAplus and MEDLINE

Ewing sarcoma is an aggressive solid tumor malignancy of childhood. Although current treatment regimens cure approx. 70% of patients with localized disease, they are ineffective for most patients with metastases or relapse. New treatment combin ations are necessary for these patients. Ewing sarcoma cells are dependent on focal adhesion kinase (FAK) for growth. To identify candidate treatment combinations for Ewing sarcoma, we performed a small- mol. library screen to identify compounds syner gistic with FAK inhibitors in impairing Ewing cell growth. The activity of a top-scoring class of compounds was then validated across multiple Ewing cell lines in vitro and in multiple xenograft models of Ewing sarcoma. Numerous Aurora kinase inhibitors scored as synergistic with FAK inhibition in this screen. F AK and Aurora kinase B inhibitors synergis tically impair Ewing sarcoma cell viability and signifi cantly inhibit tumor progression. This study provides preclin. support for the conside ration of a clin. trial testing the safety and efficacy of this combination for patients with Ewing sarcoma.

Keywords: Ewing sarcoma FAK Aurora kinase B synergism

Substances (8) Reactions (0) Citing (22)

98

Aurora kinase inhibitor and application of alphavirus in preparing antitumor medicament

By: Yan, Guangmei; Lin, Yuan; Zhu, Wenbo; Zhang, Haipeng; Liang, Jiankai; Cai, Jing; Gong, Shoufang
China, CN109985239 A 2019-07-09 | Language: Chinese, Database: CAplus

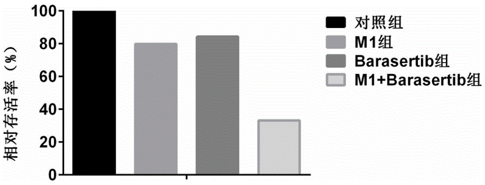

The invention belongs to the field of biomedicine, relates to aurora kinase inhibitor and applic ation of alphavirus in preparing antitumor medicament.The aurora kinase inhibitor disclosed by the invention can be used for preparing an alphavirus antitumor synergist for the first time.The invention also relates to a pharmaceutical composition comprising an aurora kinase inhibitor and an alphavirus, a pharmaceutical kit comprising the aurora kinase inhibitor and the alphav irus, and application of the aurora kinase inhibitor and the alphavirus in treating tumors, in particular tumors insensitive to the alphavirus.

Keywords: aurora kinase inhibitor alphavirus antitumor

PatentPak available

Substances (4) Reactions (0) Citing (0)

99

Use of aurora kinase inhibitor and alphavirus in preparing anti-tumor drug

By: Yan, Guangmei; Lin, Yuan; Zhu, Wenbo; Zhang, Haipeng; Liang, Jiankai; Cai, Jing; Gong, Shoufang
World Intellectual Property Organization, WO2019129234 A1 2019-07-04 | Language: Chinese, Database: CAplus

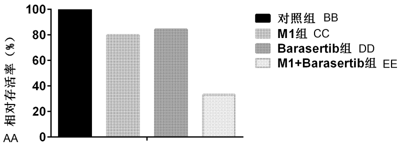

图 1

AA Relative survival rate (%)
BB Control group
CC M1 group
DD Barasertib group
EE M1+Barasertib group

Disclosed is the use of an aurora kinase inhibitor and an alphavirus in preparing an anti-tumor drug. The aurora kinase inhibitor can be used in preparing an alphavirus anti-tumor synergist. Disclosed are a pharmaceutical composition containing the aurora kinase inhibitor and the alphavirus, a drug kit containing the aurora kinase inhibitor and the alphav irus, and the use of the aurora kinase inhibitor and the alphavirus in treating tumors, especially those that are insensitive to the alphavirus.

Keywords: aurora kinase inhibitor alphavirus antitumor

PatentPak available

Substances (4) Reactions (0) Citing (2)

100

Preparation of compounds and compositions for detecting caspase activity and/or apoptosis in vivo

By: Manning, H. Charles; Hight, Matthew R.
United States, US10294271 B1 2019-05-21 | Language: English, Database: CAplus

Mol. probe suitable for quantification of caspase activity in vivo using positron emission tomog. (P ET). Embodiments of the present invention can detect apoptosis in tumors and as a novel, potentially translatable biomarker for predicting response to person alized medicine.

Keywords: isotope labeled peptide substrate caspase activity apoptosis diagnosis preparation

PatentPak available

Substances (24) | Reactions (16) | Citing (0)

101

Identification and use of cytotoxic T lymphocyte (CTL) antigen-specific target cell killing enhancer agents

By: Lizotte, Patrick H.; Kirschmeier, Paul T.; Bittinger, Mark; Gray, Nathanael
World Intellectual Property Organization, WO2019014111 A1 2019-01-17 | Language: English, Database: CAplus

FIG. 2C

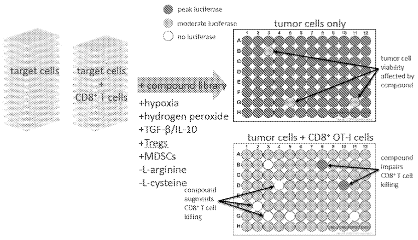

The present invention relates to screening methods for identification of agents (e.g., small mols.) that modulate cytotoxic T lymphocyte antigen-specific target (e.g., tumor) cell killing, as well as to uses of compounds identified thereby as immunomod ulatory, including use of EGFR inhibitors as immunomo dulatory agents.

Keywords: CD8 T lymphocyte antigen specific target cell killing enhancer

PatentPak available

Substances (220) | Reactions (0) | Citing (0)

102

Aurora kinase B inhibitor barasertib (AZD1152) inhibits glucose metabolism in gastric cancer cells

By: He, Jian; Qi, Zihao; Zhang, Xiaofei; Yang, Yufei; Liu, Fei; Zhao, Guangfa; Wang, Ziliang
Anti-Cancer Drugs (2019), 30(1), 19-26 | Language: English, Database: CAplus and MEDLINE

Barasertib is a highly selective Aurora kinase B (AURKB) inhibitor and has been widely applied in a variety of cancer cells to invest igate the regulatory function of A URKB. However, the effect of barasertib on glucose metabolism in gastric cancer (G C) remains illustrated. Here, barasertib was identified to effect ively reduce glucose uptake and lactate production in G C cells in a dose-dependent and time-dependent manner. The expression levels of GLUT1, LDHA and HK2 were decreased by barasertib treatment of GC cells. Furthe rmore, we found that barasertib induced the expression of ribosomal protein S7 (R PS7), as a tumor suppre ssor, to regulate glucose metabolism Silencing of RPS7 rescued the effects of barasertib on glucose metabolism in G C cells. Overexpression of RPS7 suppressed the promoter activity of C- Myc, which has been identified as an important regulator of glucose metabolism in cancer cells. The clin. data showed that the expression level of AURKB in GC patients' sera and tissues were pos. correlated with those of C-Myc, GLUT1 and LDHA, but neg. with that of R PS7. Therefore, these findings provide new evidence that barasertib regulates GC cell glucose metabolism by inducing the R PS7/C-Myc signal pathway, and have important implic ations for the develo pment of therapeutic approaches using A URKB as a target protein to prevent tumor recurr ence.

Keywords: barasertib AURKB inhibitor anticancer agent glucose metabolism gastric cancer

Substances (6) | Reactions (0) | Citing (8)

103

Aurora kinase: a target for cancer therapy

By: Pushpaveni, C.; Arun, Kumar C.; Visagaperumal, D.; Chandy, Vineeth
Research & Reviews: A Journal of Pharmacology (2019), 9(3), 10-17 | Language: English, Database: CAplus

Aurora kinases are the enzymes that belong to the family of serine/threonine kinases. It consists of Aurora- A (AURK-A), Aurora-B (AU RK-B) and Aurora- C (AURK-C), which are the essential kinases for cell division through mitosis regulation especially for the process of chromosomal segregation. Aurora kinases are involved in meiosis regulation along with the regulation of mitosis. Inhibition of Aurora kinase leads to neglected cell division. Various cancer studies generally deal with gene amplification or overexpression. From the literature review, it is clear that inhibiting Aurora kinase will increase the effect of chemotherapies. Hence from the past decades, Aurora kinase inhibitors were developed, and they are now in clin. trials; however, these inhibitors effectively fail to give the complete therapy for cancer. This review mainly focuses on the Aurora kinase inhibitors and Aurora kinase as a target for anticancer therapy.

Keywords: baresertib anticancer agent aurora kinase cancer

Substances (3) | Reactions (0) | Citing (0)

104

Disassembly of actin and keratin networks by Aurora B kinase at the midplane of cleaving Xenopus laevis eggs

By: Field, Christine M.; Pelletier, James F.; Mitchison, Timothy J.
bioRxiv (2019), 1-24 | Language: English, Database: CAplus

We investigated how bulk cytoplasm prepares for cytoki nesis in Xenopus laevis eggs, which are large, rapidly dividing cells. The egg midplane is demarcated by Chromosomal Passenger Complex (CPC) localized on microtubule bundles between asters. Using an extract system and intact eggs we found that local kinase activity of the AURKB subunit of the CPC caused disassembly of F-actin and keratin between asters, and local softening of the cytoplasm as assayed by flow patterns. Beads coated with active CPC mimicked aster boundaries and caused AURKB-dependent disassembly of F-actin and keratin that propagated ~40μm without microtubules, and much farther with microt ubules present, due to C PC auto-activation. We propose that active C PC at aster boundaries locally reduces cytoplasmic stiffness by disasse mbling actin and keratin networks. This may help sister centro somes move apart after mitosis, prepare a soft path for furrow ingression and/or release G-actin to build the furrow cortex.

Keywords: IgG Factin keratin A URKB CCP disassembly zygote fertilization Xenopus

Substances (7) Reactions (0) Citing (0)

105

Aurora kinase B regulates axonal outgrowth and regeneration in the spinal motor neurons of developing zebrafish

By: Gwee, Serene S. L. ; Radford, Rowan A. W.; Chow, Sharron; Syal, Monisha D.; Morsch, Marco; Formella, Isabel; Lee, Albert; Don, Emily K.; Badrock, Andrew P.; Cole, Nicholas J.; et al
Cellular and Molecular Life Sciences (2018), 75(23), 4269-4285 | Language: English, Database: CAplus and MEDLINE

Aurora kinase B (AurkB) is a serine/threonine protein kinase with a well- characterised role in orchest rating cell division and cytoki nesis, and is promin ently expressed in healthy proliferating and cancerous cells. However, the role of Aurk B in differentiated and non-dividing cells has not been extens ively explored. Previo usly, we have described a signif icant upregulation of AurkB expression in cultured cortical neurons following an exptl. axonal transection. This is somewhat surpri sing, as AurkB expression is generally associated only with dividing cells Frangini et al. (Mol Cell 51:647-661, 2013); Hegarat et al. (J Cell Biol 195: 1103-1113, 2011); Lu et al. (J Biol Chem 283:31785-31790, 2008); Trakala et al. (Cell Cycle 12: 1030-1041, 2014). Herein, we present the first descri ption of a role for AurkB in terminally differe ntiated neurons. AurkB was prominently expressed within post-mitotic neurons of the zebrafish brain and spinal cord. The expression of AurkB varied during the development of the zebrafish spinal motor neurons. Utilizing pharmacol. and genetic manipulation to impair AurkB activity resulted in truncation and aberrant motor axon morphol., while overexpression of AurkB resulted in extended axonal outgrowth. Further pharmacol. inhibition of Aurk B activity in regenerating axons delayed their recovery following UV laser-mediated injury. Collectively, these results suggest a hitherto unreported role of AurkB in regulating neuronal develo pment and axonal outgrowth.

Keywords: aurora kinase B neuronal development spinal motor neuron; Aurora kinase B; Axonal outgrowth; Axonal regener ation; Spinal motor neurons; Zebrafish

Substances (2) Reactions (0) Citing (12)

106

Recurrent 8q24 rearrangement in blastic plasmacytoid dendritic cell neoplasm: association with immunoblastoid cytomorphology, MYC expression, and drug response

By: Sakamoto, Kana; Katayama, Ryohei ; Asaka, Reimi; Sakata, Seiji; Baba, Satoko; Nakasone, Hideki; Koike, Sumie; Tsuyama, Naoko; Dobashi, Akito; Sasaki, Makoto; et al
Leukemia (2018), 32(12), 2590-2603 | Language: English, Database: CAplus and MEDLINE

Blastic plasmacytoid dendritic cell neoplasm (BPDCN) is a rare skin-tropic hematol. malignancy of uncertain pathogenesis and poor prognosis. We examined 118 BPDCN cases for cytomorphol., MYC locus rearrangement, and MYC expression. Sixty-two (53%) and 41 (35%) cases showed the classic and immunoblastoid cytomorphol., resp. Forty-one (38%) MYC^+BPDCN (pos. for rearrangement and expression) and 59 (54%) MYC^-BPDCN (both neg.) cases were identified. Immunoblastoid cytomorphol. was significantly associated with MYC^+BPDCN. All examined MYC^+BPDCNs were neg. for MYB/MYBL1 rearrangement (0/36). Clin., MYC^+BPDCN showed older onset, poorer outcome, and localized skin tumors more commonly than MYC^-BPDCN. MYC was demonstrated by expression profiling as one of the clearest discriminators between CAL-1 (MYC^+BPDCN) and PMDC05 (MYC^-BPDCN) cell lines, and its shRNA knockdown suppressed CAL-1 viability. Inhibitors for bromodomain and extra-terminal protein (BETis), and aurora kinases (AKis) inhibited CAL-1 growth more effectively than PMDC05. We further showed that a BCL2 inhibitor was effective in both CAL-1 and PMDC05, indicating that this inhibitor can be used to treat MYC^-BPDCN, to which BETis and AKis are probably less effective. Our data will provide a rationale for the development of new treatment strategies for patients with BPDCN, in accordance with precision medicine.

Keywords: blastic plasmacytoid dendritic cell neoplasm MYC immunoblastoid cytomorphol

Substances (11) Reactions (0) Citing (31)

107

Methods of cancer treatment with ferroptosis inducer in combination with other antitumor agents

By: Chen, Ruihong; Jiang, Chun
World Intellectual Property Organization, WO2018218087 A1 2018-11-29 | Language: English, Database: CAplus

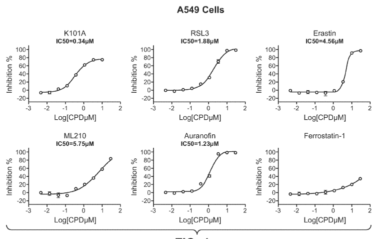

FIG. 1

The present disclosure relates to a method of treating a subject with cancer with a ferroptosis inducer, including use of the ferroptosis inducer (e.g. erastin, auranofin, sulfasalazine) in combination with a second therapeutic agent (e.g. afatinib, afuresertib, alectinib, alisertib).

Keywords: ferroptosis inducer erastin antitumor afuresertib combination chemotherapy cancer treatment

PatentPak available

Substances (150) Reactions (0) Citing (3)

108

Combination therapies of cancer with histone H3 lysine-9 N-methyltransferase EHMT2 inhibitors

By: Campbell, John Emmerson; Duncan, Kenneth William; Raimondi, Maria Alejandra; Klaus, Christine; Penebre, Elayne
World Intellectual Property Organization, WO2018195450 A1 2018-10-25 | Language: English, Database: CAplus

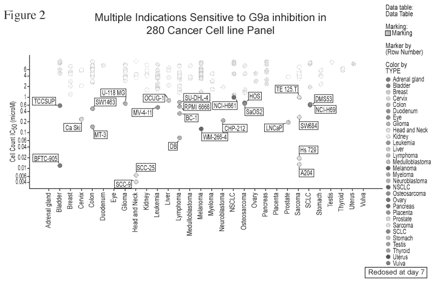

The present disclosure relates to a method of preventing or treating a cancer via administering an histone H3 lysine-9 N-methyltra nsferase EHMT2 inhibitor or a combin ation comprising an EHMT2 inhibitor compound and one or more addnl. therap eutic agent disclosed herein or a pharmaceutical composition thereof to subjects in need thereof. The present disclosure also relates to the use of such compounds or combinations for research or other non- therapeutic purposes.

Keywords: combination therapy antitumor EHMT2 inhibitor cancer treatment

PatentPak available Substances (1,586) Reactions (0) Citing (4)

109

Method and compositions for treating cancerous tumors

By: Alliger, Howard
United States, US10105389 B1 2018-10-23 | Language: English, Database: CAplus

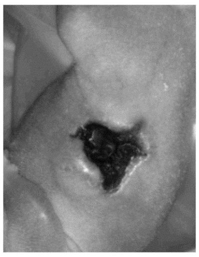

The present invention relates to the use of chlorine dioxide compositions for treating cancerous tumors. The present invention relates to compositions and methods for treating cancerous tumors, including naive, metastatic and recurrent cancers. The compos itions comprise chlorine dioxide in an effective amount, which is injected into the cancerous tumor at least once, and often at least several times over the course of treatment. The chlorine dioxide compositions are injected directly into the cancerous tumor and the resulting tumor is effectively eliminated from the patient or subject over a period of one to several days to a few weeks, often after a single injection, or multiple injections at one session into the tumor. Often, an initial injection or multiple injections at one session are sufficient to dissolve the cancerous tumor. Often the cancer is eliminated (as evidenced by no remission) in a period of no more than several days to about two-three months and does not recur.

Keywords: chlorine dioxide cancer

PatentPak available Substances (294) Reactions (0) Citing (0)

110

Porous nanoparticle-supported lipid bilayer delivery of transcriptional gene modulators

By: Brinker, Charles Jeffrey; Agola, Jacob Ongudi; Muniz, Ayse; Butler, Kimberly

World Intellectual Property Organization, WO2018187287 A1 2018-10-11 | Language: English, Database: CAplus

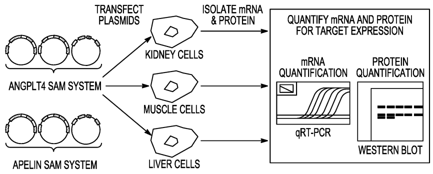

FIG. 15B

A porous protocell is provided comprising one or more non-integrating vectors comprising nucleic acid encoding a non- nucleolytic fusion protein comprising a protein sequence that specifically binds a sequence in a double strand nucleic acid mol. linked to a protein sequence that is a transcriptional mediator, and optionally guide RNA or a vector for expression of guide R NA, and methods of using the protocells.

Keywords: porous nanoparticle lipid bilayer transcription gene fusion protein sequence

PatentPak available

Substances (110) Reactions (0) Citing (0)

111

Preparation of cereblon E3 ligase ligands and bifunctional PROTAC compounds for targeting and degradation of androgen receptor, estrogen receptor alpha, bromodomain-containing protein 4, and BRaf

By: Crew, Andrew P.; Crews, Craig M.; Dong, Hanqing; Hornberger, Keith R.; Wang, Jing; Qian, Yimin; Zimmermann, Kurt; Berlin, Michael; Snyder, Lawrence B.
United States, US20180228907 A1 2018-08-16 | Language: English, Database: CAplus

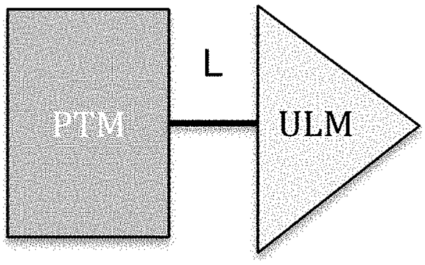

The invention relates to cereblon E3 ligase binding compounds, including bifunctional compounds comprising them, which find utility as modulators of targeted ubiquitination, especially inhibitors of a variety of polype ptides and other proteins which are degraded and/or otherwise inhibited by bifunctional compounds according to the present disclo sure. In particular, the invention provides compounds, which contain on one end a ligand which binds to the cereblon E3 ubiquitin ligase and on the other end a moiety which binds a target protein such that the target protein is placed in proximity to the ubiquitin ligase to effect degradation (and inhibition) of that protein. Specifically, the invention relates to bifunctional compounds CLM-L-PTM [PTM = small mol. containing a protein targeting moiety that binds bromodomain-containing protein 4, estrogen receptor (E R), or androgen receptor or Braf; L = a bond, a chem. linking moiety covalently coupling the CLM and the PTM; CLM = a small mol. cereblon E3 ubiquitin ligase binding moiety that binds or targets a cereblon E3 ubiquitin ligase], their pharmaceutically acceptable salts, enantiomers, stereoi somers, solvates, polymorphs and prodrugs. Exempl ified compound **I** was prepared from a 12 step procedure (prepar ation given). The invention exhibits a broad range of pharmacol. activities associated with compounds according to the present invention, consistent with the degradation/inhibition of targeted polypeptides of nearly any type (data given). Diseases or disorders that result from aggregation or accumulation of the target protein are treated or prevented with compounds and compos itions of the invention.

Keywords: cereblon ligand chimeric mol preparation targeted protein degradation ubiquitination; PROTAC modulator preparation targeted ubiquitination estrogen receptor ERRa degradation; androgen receptor PROTAC degradation inhibition preparation antiproliferative; BRD4 BRaf PROTAC degradation inhibition cereblon E3 ligase antitumor

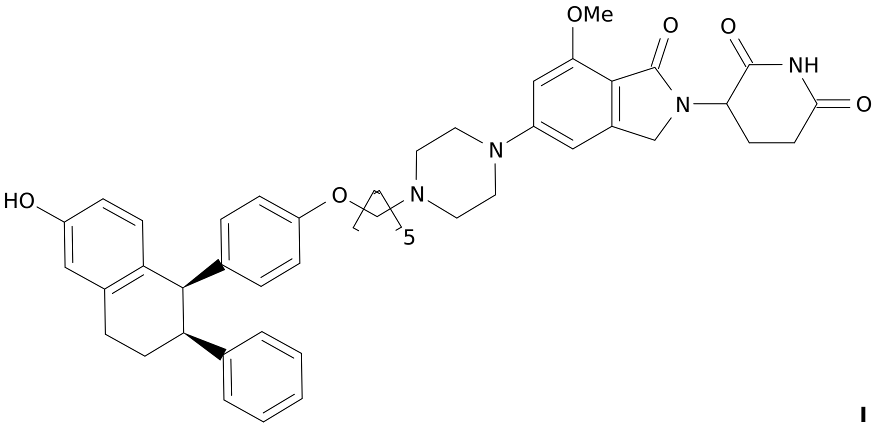

I

PatentPak available

Substances (731) | Reactions (1,474) | Citing (3)

112

Preparation of cereblon E3 ligase ligands and bifunctional PROTAC compounds comprising them for the degradation of targeted proteins including TANK-binding kinase 1, estrogen receptor alpha, bromodomain-containing protein 4 and tau protein

By: Crew, Andrew P.; Berlin, Michael; Dong, Hanqing; Homberger, Keith R.; Qian, Yimin; Snyder, Lawrence B.; Wang, Jing; Zimmermann, Kurt

United States, US20180215731 A1 2018-08-02 | Language: English, Database: CAplus

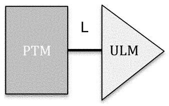

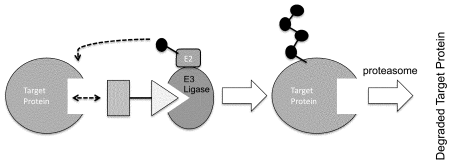

The invention relates to cereblon E3 ligase binding compounds, including bifunctional compounds comprising them, which find utility as modulators of targeted ubiquitination, especially inhibitors of a variety of polype ptides and other proteins which are degraded and/or otherwise inhibited by bifunctional compounds according to the present disclo sure. In particular, the invention provides compounds, which contain on one end a ligand which binds to the cereblon E3 ubiquitin ligase and on the other end a moiety which binds a target protein such that the target protein is placed in proximity to the ubiquitin ligase to effect degradation (and inhibition) of that protein. Specifically, the invention relates to bifunctional compounds CLM-L-PTM [PTM = small mol. containing a protein targeting moiety that binds bromodomain-containing protein 4, Tau protein, estrogen receptor (E R), or androgen receptor; L = a bond, a chem. linking moiety covalently coupling the CLM and the PTM; CLM = a small mol. cereblon E3 ubiquitin ligase binding moiety that binds or targets a cereblon E3 ubiquitin ligase], their pharmaceutically acceptable salts, enanti omers, stereoisomers, solvates, polymorphs and prodrugs. The invention exhibits a broad range of pharmacol. activities associated with compounds according to the present invention, consistent with the degradation/inhibition of targeted polypeptides of nearly any type (data given). Diseases or disorders that result from aggreg ation or accumulation of the target protein are treated or prevented with compounds and compositions of the invention.

Keywords: cereblon ligand chimeric mol preparation targeted protein degradation ubiquitination; PROTAC modulator preparation targeted ubiquitination estrogen receptor ERRa degradation; tau protein PROTAC degradation inhibition preparation antiproli ferative; BRD4 PROTAC degradation inhibition cereblon E3 ligase antitumor; preparation cereblon ligand Tank binding kinase 1 PRO TAC degradation; TBK1 degradation inhibition PROTAC modulator preparation targeted ubiquitination

PatentPak available

Substances (1,219)   Reactions (1,874)   Citing (3)

113

Hyperstabilized liposomes increase targeting of mitotic cells

By: Ng, Chang Zhi Adrian; Cheong, Shen-Yi Ian
World Intellectual Property Organization, WO2018136002 A1 2018-07-26 | Language: English, Database: CAplus

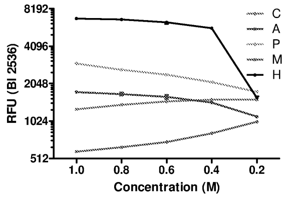

Fig. 2

Hyperstable liposome comprising an anti-mitotic agent, one or more anions and one or more cations entrapped in the inner milieu, wherein the entrapped anti-mitotic drug is released at a slow rate that is less than 0.6% in 12 h or less than 5% in 8 h when the liposomes are suspended in 600mM sucrose. These liposomes are useful in the treatment of cancer. In partic ular, HEPC:Chol:DSPE-PEG2000 (50:45:5) liposomes comprising BI2536 and citrate:phosphate in a ratio of 1:3.

Keywords: hyperstabilized phosphatidylcholine liposome target mitosis B I2536 antitumor neoplasm

PatentPak available

Substances (43) Reactions (0) Citing (0)

114

Preparation of functionalized pyrazoles and other nitrogen-containing heterocycles as inhibitors of MCT4 for the treatment of MCT4-mediated diseases

By: Parnell, Kenneth Mark; McCall, John; Romero, Donna
United States, US20180162822 A1 2018-06-14 | Language: English, Database: CAplus

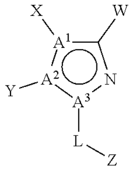

(I)

The invention relates to preparation of functionalized pyrazoles and other nitrogen heterocycles of formula **I** and compositions containing them as monocarboxylate transporter MCT4 inhibitors. Compounds **I** wherein A^1, A^2 and A^3 are independently chosen from N and C, wherein at least one of A^1, A^2 and A^3 is N; L is a bond, methylene, or ethylene; X is H or null; Y is alkenyl, alkenyl amino, alkyl, etc.; Z is alkyl, cycloa lkyl, aryl, etc., are claimed. The example compound **II** was prepared by multistep synthesis (procedure given). Compounds **I** were evaluated for their biol. activity (example - M CT4 inhibiting activity) (data given). Compounds **I** are useful in the treatment of MCT4-mediated diseases, such as proliferative and inflammatory diseases, such as cancer.

Keywords: pyrazole preparation MCT4 inhibitor antiproliferative antiinflammatory antitumor; nitrogen heterocycle preparation MC T4 disease treatment

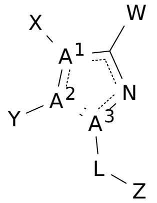

I

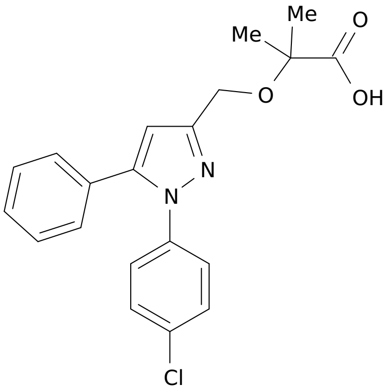

II

PatentPak available

Substances (1,145) Reactions (4,041) Citing (0)

115

Preparation of small molecule based antibody-recruiting compounds for cancer treatment

By: Spiegel, David; Rullo, Anthony
United States, US20180155332 A1 2018-06-07 | Language: English, Database: CAplus

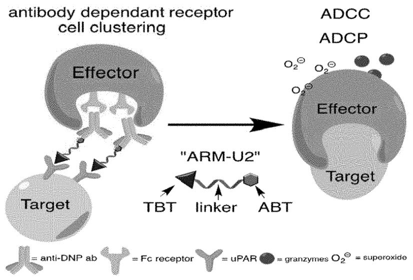

The present invention relates to chimeric (including bifunctional) compounds **I** [$(UPAR_BM)_m$-CT-$(L^1)_k$-CT-$(L^2)_j$-CT-$(A_BM)_n$; wherein UPA R_BM = moiety which binds to an active site of urokinase- type plasminogen activator receptor (u PAR) on the surface of cancer cells; A_BM = antibody binding moiety comprising a hapten which is capable of binding to an antibody; L^1 and L^2 = linkers; CT = (indepen dently) an optional connector mol.; j = 0- 5; k = 0-5; with the proviso that at least one C T is present when k and j are both 0 (preferably at least one of k and j is 1); each m and n = (indepen dently) 1-15 (preferably m = 1 and n = 1- 3)], compositions comprising those compounds and methods of treating cancer in a patient or subject, especially including metastatic cancer where cancer cells exhibit overexpression (heightened expression) of cell surface urokinase-type plasminogen activator receptor (urokinase receptor) compared to normal (non-cancerous) cells. The compounds **I** bind to the urokinase-type plasminogen activator receptor (uPAR) on the surface of a cancer cell, including a metastatic cancer cell, and conseq uently recruit native antibodies of the patient or subject where the antibodies can selectively degrade and/or deactivate targeted cancer cells through antibody-dependent cellular phagocytosis and antibody-dependent cellular cytotoxicity (ADCC) and/or complement dependent cytotoxicity (C DC) against a large number and variety of cancers, thus providing cancer cell death and an inhibition of growth, elabor ation and/or metastasis of the cancer, including remission and cure of the patient's cancer. E.g., a multi-step synthesis of compound **II**, starting from 5-hydroxy-1,4-naphthoquinone and 1-(trimethylsilyloxy)-1,3-butadiene, was described. Exemplified title compounds **I** were tested for their biol. activity (data given for representative compounds).

Keywords: chimeric bifunctional compound preparation antitumor u PAR modulator antibody recruiting; urokinase type plasmi nogen activator receptor chimeric bifunc tional compound preparation; antibody dependent cellular phagocytosis cancer chimeric bifunctional compound preparation; ADCC antibody dependent cellular cytotoxicity chimeric bifunctional compound preparation; C DC complement dependent cytotoxicity metastasis chimeric bifunc tional compound preparation

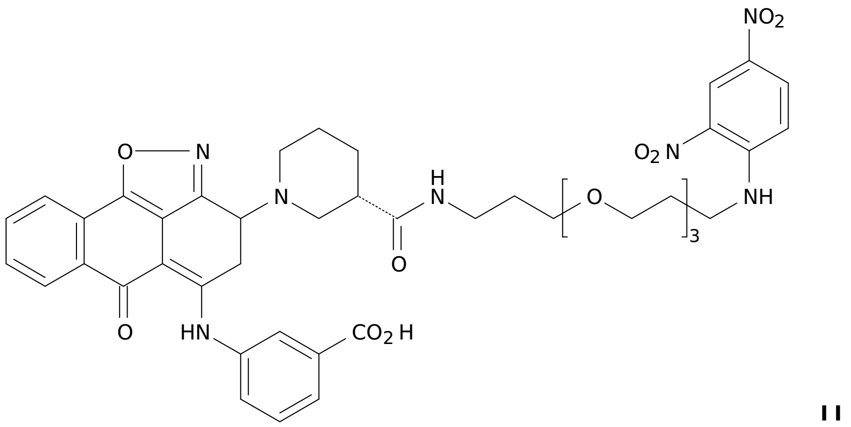

II

PatentPak available

Substances (389) Reactions (333) Citing (0)

116

Chemical Genetic Screens Identify Kinase Inhibitor Combinations that Target Anti-Apoptotic Proteins for Cancer Therapy

By: Contreras, Jacob I.; Robb, Caroline M.; King, Hannah M.; Baxter, Jared; Crawford, Ayrianne J.; Kour, Smit; Kizhake, Smitha; Sonawane, Yogesh A.; Rana, Sandeep; Hollingsworth, Michael A.; et al
ACS Chemical Biology (2018), 13(5), 1148-1152 | Language: English, Database: CAplus and MEDLINE

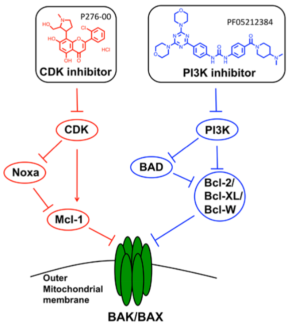

The study presented here provides a framework for the discovery of unique inhibitor combinations that target the apoptosis network for cancer therapy. A pair of doxycycline (Dox)-inducible cell lines that specifically report on the ability of an inhibitor to induce apoptosis by targeting either the Mcl-1 arm or the Bcl-2/Bcl-xL/Bcl-w arm were used. Cell-based assays were optimized for high throughput screening (HTS) with caspase 3/7 as a read out. H TS with a 355- member kinase inhibitor library and the panel of Dox-inducible cell lines revealed that cyclin dependent kinase (C DK) inhibitors induced apoptosis by targeting the Mcl- 1 arm, whereas PI3K inhibitors induced apoptosis by targeting the Bcl- 2/Bcl-xL/Bcl-w arm. Validation studies identified unique combin ations that synergistically inhibited growth and induced apoptosis in a panel of cancer cell lines. Since these inhibitors have been or are currently in clin. trials as single agents, the combinations can be rapidly translated to the clinics.

Keywords: antitumor combination synergy apoptosis cancer

Substances (15) Reactions (0) Citing (10)

117

Methods and compositions for identifying and treating patients with small cell lung cancer with aurora kinase inhibitors using transgenic animal models and cell lines

By: Oliver, Trudy; Sos, Martin; Wechsler-Reya, Rob
World Intellectual Property Organization, WO2018081575 A1 2018-05-03 | Language: English, Database: CAplus

Described herein are methods and compositions useful in detecting, diagnosing and treating small cell lung cancer. Transgenic animal models and cell lines are disclosed for the study of a small cell lung cancer subtype. Methods of screening and identifying active agents for the treatment of a small cell lung cancer subtype as well as methods of identifying patients susceptible to treatment with aurora kinase inhibitors are also provided.

Keywords: lung cancer aurora kinase inhibitor screening transgenic mouse

PatentPak available

Substances (14) Reactions (0) Citing (0)

118

Pharmaceutical combinations comprising a histone deacetylase inhibitor and an aurora kinase inhibitor and methods of use thereof

By: North, Brian; Quayle, Steven
World Intellectual Property Organization, WO2018081585 A1 2018-05-03 | Language: English, Database: CAplus

FIG. 1

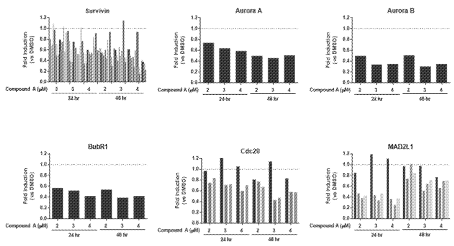

The present disclosure relates to a pharmaceutical combination comprising (a) a histone deacetylase 6 inhibitor and (b) an aurora kinase inhibitor, including combined preparations and pharmaceutical compositions thereof; uses of such combination in the treatment or prevention of cancer; and methods of treating or preventing cancer in a subject comprising administering a therapeutically effective amount of such combination.

Keywords: pharmaceutical combination histone deacetylase aurora kinase inhibitor

PatentPak available

Substances (26) Reactions (10) Citing (1)

119

Vitamins c and k3: a powerful redox system for sensitizing leukemia lymphocytes to everolimus and barasertib

By: Ivanova, Donika; Zhelev, Zhivko; Lazarova, Dessislava; Getsov, Plamen; Bakalova, Rumiana; Aoki, Ichio
Anticancer Research (2018), 38(3), 1407-1414 | Language: English, Database: CAplus and MEDLINE

Background/Aim: Recent studies provided convincing evidence for the anticancer activity of combined application of vitamin C and pro-vitamin K3 (menadione). The mol. pathways underlying this process are still not well established. The present study aimed to investigate the effect of the combination of vitamin C plus pro-vitamin K3 on the redox status of leukemia and normal lymphocytes, as well as their sensitizing effect for a variety of anticancer drugs. Materials and methods: Cytotoxicity of the substances was analyzed by trypan blue staining and automated counting of live and dead cells. Apoptosis was analyzed by fluorescein isothiocyanate-annexin V test. Oxidative stress was evaluated by the intracellular levels of reactive oxygen and nitrogen species and protein-carbonyl products. Results: Combined administration of 300μM vitamin C plus 3μM pro-vitamin K3 reduced the viability of leukemia lymphocytes by ~20%, but did not influence the viability of normal lymphocytes. All combinations of anticancer drug plus vitamins C and K3 were characterized by synergistic cytotoxicity towards Jurkat cells, compared to cells treated with drug alone for 24 h. In the case of barasertib and everolimus, this synergistic cytotoxicity increased within 72 h. It was accompanied by strong induction of apoptosis, but a reduction of level of hydroperoxides and moderately increased protein-carbonyl products in leukemia cells. Conclusion: Leukemia lymphocytes were more sensitive to combined administration of anticancer drug (everolimus or barasertib) plus vitamins C and K3, compared to normal lymphocytes. The combination of vitamin C plus K3 seems to be a powerful redox system that could specifically influence redox homeostasis of leukemia cells and sensitize them to conventional chemotherapy.

Keywords: vitamin redox system everolimus barasertib sensitizing leukemia lymphocyte; Jurkat; Vitamin C; apoptosis; leukemia lymphocytes; oxidative stress; pro-vitamin K3

Substances (7) Reactions (0) Citing (11)

120

RIPK1-dependent cell death: a novel target of the Aurora kinase inhibitor Tozasertib (VX-680)

By: Martens, Sofie; Goossens, Vera; Devisscher, Lars; Hofmans, Sam; Claeys, Polien; Vuylsteke, Marnik; Takahashi, Nozomi; Augustyns, Koen ; Vandenabeele, Peter
Cell Death & Disease (2018), 9(2), 1-13 | Language: English, Database: CAplus and MEDLINE

The Aurora kinase family (Aurora A, B and C) are crucial regulators of several mitotic events, including cytokinesis. Increased expression of these kinases is associated with tumorigenesis and several compounds targeting Aurora kinase are under evaluation in clin. trials (a.o. AT9283, AZD1152, Danusertib, MLN8054). Here, we demonstrate that the pan-Aurora kinase inhibitor Tozasertib (V X-680 and MK-0457) not only causes cytokinesis defects through Aurora kinase inhibition, but is also a potent inhibitor of necrop tosis, a cell death process regulated and executed by the R IPK1, RIPK3 and MLKL signalling axis. Tozasertib's potency to inhibit RIP K1-dependent necroptosis and to block cytokinesis in cells is in the same concentration range, with an IC50 of 1.06μM and 0.554μ M, resp. A structure activity relationship (SAR) anal. of 67 Tozasertib analogs, modified at 4 different positions, allowed the identif ication of analogs that showed increased specificity for either cytokinesis inhibition or for necroptosis inhibition, reflecting more specific inhibition of Aurora kinase or RIPK1, resp. These results also suggested that RIPK1 and Aurora kinases are functionally non-interacting targets of Tozasertib and its analogs. Indeed, more specific Aurora kinase inhibitors did not show any effect in necrop tosis and Necrostatin-1s treatment did not result in cytokinesis defects, demonstrating that both cellular processes are not interre lated. Finally, Tozasertib inhibited recombinant human RIPK1, human Aurora A and human Aurora B kinase activity, but not R IPK3. The potency ranking of the newly derived Tozasertib analogs and their specificity profile, as observed in cellular assays, coincide with ADP-Glo recombinant kinase activity assays. Overall, we show that Tozasertib not only targets Aurora kinases but also R IPK1 independently, and that we could generate analogs with increased selectivity to RIPK1 or Aurora kinases, resp.

Keywords: rozasertib antiapoptotic agent RIPK1 RIPK3 fibroblast necroptosis

Substances (13) Reactions (0) Citing (27)

121

A mixed modality approach towards Xi reactivation for Rett syndrome and other X-linked disorders

By: Carrette, Lieselot L. G.; Wang, Chen-Yu; Wei, Chunyao; Press, William; Ma, Weiyuan; Kelleher, Raymond J. III; Lee, Jeannie T.
Proceedings of the National Academy of Sciences of the United States of America (2018), 115(4), E668-E675 | Language: English, Database: CAplus and MEDLINE

The X-chromosome harbors hundreds of disease genes whose associated diseases predominantly affect males. However, a subset, including neurodevelopmental disorders, Rett syndrome (RTT), fragile X syndrome, and CDKL5 syndrome, also affects females. These disorders lack disease-specific treatment. Because female cells carry two X chromosomes, an emerging treatment strategy has been to reawaken the healthy allele on the inactive X (Xi). Here, we focus on methyl-CpG binding protein 2 (MECP2) restoration for RTT and combinatorially target factors in the interactome of Xist, the noncoding RNA responsible for X inactivation. We identify a mixed modality approach combining an Xist antisense oligonucleotide and a small-mol. inhibitor of DNA methylation, which, together, achieve 30,000-fold MECP2 up-regulation from the Xi in cultured cells. Combining a brain- specific genetic Xist ablation with short-term 5-aza-2'-deoxycytidine (Aza) treatment models the synergy in vivo without evident toxicity. The Xi is selectively reacti vated. These experiments provide proof of concept for a mixed modality approach for treating X- linked disorders in females.

Keywords: Xi reactivation Rett syndrome X linked disorder; LNA; Rett syndrome; X reactivation; Xist; antisense oligonucleotides

Substances (22) Reactions (0) Citing (49)

122

Enhancing functional platelet release in vivo from in vitro-grown megakaryocytes using small molecule inhibitors

By: Jarocha, Danuta; Vo, Karen K.; Lyde, Randolph B.; Hayes, Vincent; Camire, Rodney M.; Poncz, Mortimer
Blood Advances (2018), 2(6), 597-606 | Language: English, Database: CAplus and MEDLINE

In vitro-grown megakaryocytes for generating platelets may have value in meeting the increasing demand for platelet transfu sions. Remaining challenges have included the poor yield and quality of in vitro-generated platelets. We have shown that infusing megakaryocytes leads to intrapulmonary release of functional platelets. A Src kinase inhibitor (S U6656), a Rho-associated kinase inhibitor (Y27632), and an aurora B kinase inhibitor (AZD1152) have been shown to increase megakar yocyte ploidy and in vitro proplatelet release. We now tested whether megakar yocytes generated from C D34$^+$ hematopoietic cells in the presence of these inhibitors could enhance functional platelet yield following megakaryocyte infusion. As expected, all inhibitors increased megakar yocyte ploidy, size, and granul arity, but these inhibitors differed in whether they injured terminal megakary ocytes: SU6656 was protective, whereas Y27632 and AZD1152 increased injury. Upon infusion, inhibitor- treated megakaryocytes released threefold to ninefold more platelets per initial noninjured megakaryocyte relative to control, but only S U6656-treated megakaryocytes had a significant increase in platelet yield when calculated based on the number of initial C D34$^+$ cells; this was fourfold over nontreated megakaryocytes. The released platelets from drug- treated, but healthy, megakar yocytes contained similar percen tages of young, uninjured platelets that robustly responded to agonists and were well incorporated into a growing thrombus in vivo as controls. These studies suggest that drug screens that select megakaryocytes with enhanced ploidy, cell size, and granul arity may include a subset of drugs that can enhance the yield and function of platelets, and may have clin. application for ex vivo-generated megakar yocytes and platelet transf usion.

Keywords: platelet release megakaryocyte small mol inhibitor

Substances (3) Reactions (0) Citing (17)

123

Data-driven exploration of selectivity and off-target activities of designated chemical probes

By: Miljkovic, Filip; Bajorath, Juergen
Molecules (2018), 23(10), 1-12 | Language: English, Database: CAplus and MEDLINE

Chem. probes are of central relevance for chem. biol. To unambiguously explore the role of target proteins in triggering or mediating biol. functions, small mols. used as probes should ideally be target-specific; at least, they should have sufficiently high selectivity for a primary target. We present a thorough anal. of currently available activity data for designated chem. probes to address several key questions: How well defined are chem. probes What is their level of selectivity Is there evidence for addnl. activities Are some probes "better" thanothers. Therefore, highly curated chem. probes were collected and their select ivity was analyzed on the basis of publicly available compound activity data. Different selectivity patterns were observed, which distinguished designated high-quality probes.

Keywords: data driven exploration selectivity activity chem probe; bioactive compounds; chemical probes; hemical biology; molecular scaffold; off-target activity; promiscuity; target selectivity

Substances (13) Reactions (0) Citing (6)

124

Mitotic slippage and the subsequent cell fates after inhibition of Aurora B during tubulin-binding agent-induced mitotic arrest

By: Tsuda, Yasuo; Iimori, Makoto; Nakashima, Yuichiro; Nakanishi, Ryota; Ando, Koji; Ohgaki, Kippei; Kitao, Hiroyuki; Saeki, Hiroshi; Oki, Eiji; Maehara, Yoshihiko
Scientific Reports (2017), 7(1), 1-12 | Language: English, Database: CAplus and MEDLINE

Tubulin-binding agents (TBAs) are designed to target microtubule (MT) dynamics, resulting in compromised mitotic spindles and an unsatisfied spindle assembly checkpoint. The activity of Aurora B kinase is indispensable for TBA-induced mitotic arrest, and its inhibition causes mitotic slippage and postmitotic endoreduplication. However, the precise phenomenon underlying mitotic slippage, which is caused by treatment with both Aurora B inhibitors and TBAs, and the cell fate after postmitotic slippage are not completely understood. Here, we found that HeLa and breast cancer cells treated with the different types of T BAs, such as paclitaxel and eribulin (MT-stabilizing and MT-destabilizing agents, resp.), exhibited distinct behaviors of mitotic slippage on inhibition of Aurora B. In such conditions, the cell fates after postmitotic slippage vastly differed with respect to cell morphol., cell proliferation, and cytotoxicity in short-term culture; i.e., the effects of inhibition of Aurora B were beneficial for cytoto xicity enhanc ement in eribulin treatment but not in paclit axel. However, in long-term culture, the cells that survived after mitotic slippage underwent endoreduplication and became giant cells in both cases, resulting in cellular senesc ence. We propose that MT-destabi lizing agents may be more appropriate than MT-stabilizing agents for treating cancer cells with a weakened Aurora B kinase activity.

Keywords: cervical cancer cell fate mitotic arrest slippage Aurora B

Substances (10) Reactions (0) Citing (25)

125

The target landscape of clinical kinase drugs

By: Klaeger, Susan; Heinzlmeir, Stephanie; Wilhelm, Mathias; Polzer, Harald; Vick, Binje; Koenig, Paul-Albert; Reinecke, Maria; Ruprecht, Benjamin; Petzoldt, Svenja; Meng, Chen; et al
Science (Washington, DC, United States) (2017), 358(6367), 1148 | Language: English, Database: CAplus and MEDLINE

Kinase inhibitors are important cancer therapeutic agents. Polypharmacol. is commonly observed, requiring thorough target deconvolution to understand drug mechanism of action. Using chem. proteo mics, the authors analyzed the target spectrum of 243 clin. evaluated kinase drugs. The data revealed previously unknown targets for established drugs, offered a perspe ctive on the "druggable" kinome, highlighted (non)kinase off-targets, and suggested potential therapeutic applications. Integration of phosphop roteomic data refined drug-affected pathways, identified response markers, and strengthened rationale for combination treatm ents. The authors exemplify translational value by discovering SIK2 (salt-inducible kinase 2) inhibitors that modulate cytokine production in primary cells, by identifying drugs against the lung cancer survival marker M ELK (maternal embryonic leucine zipper kinase), and by repurposing cabozantinib to treat FLT3-ITD-pos. acute myeloid leukemia. This resource, available via the Proteomics DB database, should facilitate basic, clin., and drug discovery research and aid clin. decision- making.

Keywords: protein kinase inhibitor drug target discovery Proteomics database antitumor

Substances (128) Reactions (0) Citing (461)

126

Modulators of homeobox transcription factors, DUX4 (double homeobox 4) for regulation of muscle function

By: Rickard, Amanda; De La Garza, Anabel; Schmidt, Uli; Kiselyov, Alexander
World Intellectual Property Organization, WO2017201585 A1 2017-11-30 | Language: English, Database: CAplus

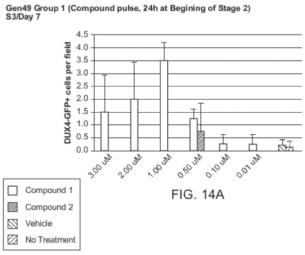

FIG. 14A

Disclosed herein are methods and compositions for the treatment of facioscapu lohumeral muscular dystrophy and other muscle diseases or disorders. In some cases, the methods and compositions involve the use of methyltransferase inhibitors (e.g. tazemet ostat, UNC-1999, EPZ005687, GSK343) to inhibit or repress DUX4 expression in muscle cells. Further disclosed herein are methods and cell based assays for screening compounds for the treatment of facioscapulohumeral muscular dystrophy and other muscle diseases.

Keywords: drug screening DUX4 modulator methyltransferase inhibitor muscle disease treatment

PatentPak available

Substances (241) | Reactions (0) | Citing (3)

127

Targeting vcp enhances anticancer activity of oncolytic virus m1 in hepatocellular carcinoma

By: Zhang, Haipeng ; Li, Kai; Lin, Yuan ; Xing, Fan; Xiao, Xiao; Cai, Jing; Zhu, Wenbo; Liang, Jiankai; Tan, Yaqian; Fu, Liwu; et al
Science Translational Medicine (2017), 9(404), eaam7996/1-eaam7996/12 | Language: English, Database: CAplus and MEDLINE

Oncolytic virotherapy is rapidly progressing through clin. evaluation. However, the therapeutic efficacy of oncolytic viruses in humans has been less than expected from preclin. studies. We describe an anticancer drug screen for compounds that enhance M1 oncolytic virus activity in hepatocellular carcinoma (HCC). An inhibitor of the valosin-containing protein (VCP) was identified as the top sensitizer, selectively increasing potency of the oncolytic virus up to 3600- fold. Further investigation revealed that VCP inhibitors cooperated with M1 virus-suppressed inositol-requiring enzyme 1 α (IRE1 α)-X-box binding protein 1 (XBP1) pathway and triggered irresolvable endoplasmic reticulum (ER) stress, subsequently promoting robust apoptosis in HCC. We show that VCP inhibitor improved the oncolytic efficacy of M1 virus in several mouse models of HCC and primary HCC tissues. Finally, this combina torial therapeutic strategy was well tolerated in nonhuman primates. Our study identifies combined V CP inhibition and oncolytic virus as a potential treatment for HCC and demonstrates promising therapeutic potential.

Keywords: hepatocellular carcinoma oncolytic virus vcp anticancer activity

Substances (23) | Reactions (0) | Citing (287)

128

Diagnostic markers, test method, and therapeutic agents for blastic plasmacytoid dendritic cell neoplasm as rare hematopoietic tumor, and therapeutic agent screening method

By: Takeuchi, Kengo; Sakamoto, Kana; Katayama, Ryohei; Sakata, Seiji
World Intellectual Property Organization, WO2017138500 A1 2017-08-17 | Language: Japanese, Database: CAplus

Diagnosis markers that can provide diagnosis criteria for blastic plasmacytoid dendritic cell neoplasm (BPDCN) as rare hematop oietic tumor are searched, and the presence of an immunobl astoid-type cell form, the reconstruction of 8q24, and the expression of MYC are established as novel markers for sub-typing BPDCN. In addition, it is found that a BET bromo domain-selective inhibitor, an inhibitor capable of directly or indirectly inhibiting the expression or function of MYC or the signaling pathway of M YC such as aurora kinase inhibitor, a HDAC inhibitor or a BCL2 family protein inhibitor is effective as a therap eutic agent in a M YC-expressing group.

Keywords: blastic plasmacytoid dendritic cell neoplasm immunob lastoid diagnostic biomarker

PatentPak available

Substances (12) | Reactions (0) | Citing (0)

129

Compositions and methods for inhibiting kinase activity using an agent that binds to a PIF pocket of Aurora A kinase for cancer treatment

By: Kern, Dorothee; Zorba, Adelajda
World Intellectual Property Organization, WO2017139321 A1 2017-08-17 | Language: English, Database: CAplus

FIG. 1A

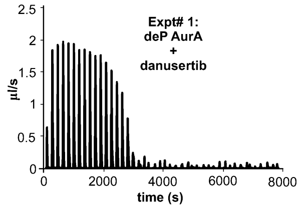

The present invention features therapeutic compositions comprising an agent that specifically binds to a PIF pocket of Aurora A kinase and an agent that specifically binds to an ATP-binding site of Aurora A kinase, and the use of the therap eutic compositions to modulate Aurora A kinase for the treatment of cancer.

Keywords: Aurora A kinase inhibitor PIF pocket binding cancer treatment

PatentPak available

Substances (23) | Reactions (0) | Citing (0)

130

DNA alkylation and cross-linking agents as compounds and payloads for targeted therapies

By: Herzon, Seth; Healy, Alan; Crawford, Jason; Vizcaino, Maria; Nikolayevskiy, Herman
World Intellectual Property Organization, WO2017132459 A1 2017-08-03 | Language: English, Database: CAplus

The present invention is directed to compounds related to precolibactin, pharmaceutical compositions based upon these compounds and methods of synthesis which are employed to provide intermediates and final compounds, which are alkylating agents and anticancer compounds The chem. synthetic approach disclosed facilitates the synthesis of numerous precolibactin analogs which can be used in the treatment of cancer. Example compound **I** was prepared by a multistep procedure. The invention compounds were evaluated for their anticancer activities (data given).

Keywords: thiazoline thiazole preparation DNA alkylation anticancer targeted therapy

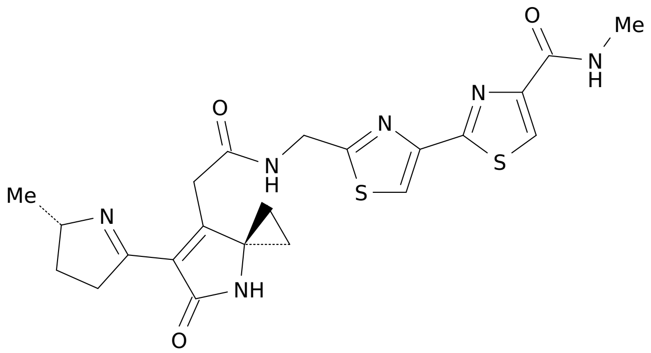

I

PatentPak available

Substances (425) Reactions (693) Citing (0)

131

In Vitro Sensitivity Profiling of Neuroblastoma Cells Against A Comprehensive Small Molecule Kinase Inhibitor Library to Identify Agents for Future Therapeutic Studies

By: Singh, Anjali; Meier-Stephenson, Vanessa; Jayanthan, Aarthi; Narendran, Aru
Current Cancer Drug Targets (2017), 17(6), 569-584 | Language: English, Database: CAplus and MEDLINE

Background: Neuroblastoma (NB) constitutes about 8% of all childhood tumors, yet accounts for more than 15% of deaths, with an unacceptable overall survival rate. These rates are despite the current multimodal therap eutic approaches involving surgery, radiation, chemotherapy and myeloablation with hematopoietic stem cell rescue. Hence, efforts have intens ified to identify new targets and novel therapeutic approaches to improve cure rates in these children. Numerous new agents for adult malign ancies are developed and evaluated for cancer each year, providing an invaluable resource, with the added advantage of available pharmacol. and toxicity data for consideration. Methods: To identify potential therapeutic targets, we screened a small mol. library of 151 small kinase inhibitors against NB cell lines. Based on our initial screening data, we further examined the potential of Bcr- Abl targeting small mol. inhibitors to affect the growth and survival of NB cells. Results: There is diverse activity among the currently available Bcr-Abl inhibitors, possibly reflecting the mol. heterogeneity and off-target activity in each combination. In depth analyses of ponatinib, an oral multi-target kinase inhibitor and effective agent in the treatment of refractory Philad elphia chromosome (Ph) pos. leukemia, show growth inhibition at sub-micromolar concentrations In addition, we also identified the potential of this agent to interfere with insulin-like growth factor-1 receptor (IGF-1R) signaling pathways and Src activity, inhibit cell migration and induce apoptosis. Conclusion: Our findings provide initial data on ponati nib's potential to target key growth regulatory pathways and provide the rationale for further studies and evaluation in future early phase clin. trials for the treatment of refractory NB.

Keywords: neuroblastoma cell small mol kinase inhibition therape utics; Bcr-Abl; IGF-1 Receptor; Neuroblastoma; Ponatinib; Src; small molecule kinase inhibitors

Substances (183) Reactions (0) Citing (2)

132

Osteotropic nanoparticles for delivering bioactive agents including anticancer agents and/or diagnostic agents for prevention or treatment of bone cancer metastases

By: Durfee, Paul N.; Brinker, Charles Jeffrey; Lin, Yu-Shen; Leong, Hon
World Intellectual Property Organization, WO2017120504 A1 2017-07-13 | Language: English, Database: CAplus

The present disclosure is directed to protocells or nanoparticles, which are optionally coated with a lipid bilayer, which can be used for targeting bone tissue for the delivery of bioactive agents useful in the treatment and/or diagnosis of bone cancer, often metastatic bone cancer which often occurs secondary to a primary cancer such as prostate cancer, breast cancer, lung cancer and ovarian cancer, among numerous others. These protocells or nanoparticles target bone cancer especially metastatic bone cancer with bioactive agents including anticancer agents and/or diagnostic agents for purposes of treating, diagnosing and/or monitoring the therapy of the bone cancer. Osteotropic protocells or nanoparticles, pharmaceutical compositions comprising a population of osteotropic protocells or nanoparticles and methods of diagnosing, treating and/or monitoring therapy of bone cancer are represe ntative aspects.

Keywords: osteotropic nanoparticle bone cancer metastasis targeting

PatentPak available

Substances (230) | Reactions (0) | Citing (4)

133

Preparation of anti-human LAG3 antibodies, vaccines and/or therapeutic agents for treatment of cancer or infection

By: Malefyt, Rene de Waal; Fayadat-Dilman, Laurence; Liang, Linda
World Intellectual Property Organization, WO2017106129 A1 2017-06-22 | Language: English, Database: CAplus

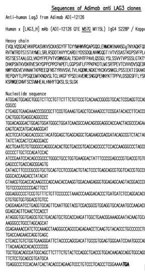

FIG.2A

The present invention includes antibodies and antigen-binding fragments thereof that specifically bind to human or cynomolgus monkey LAG3 as well as Ig chains thereof and polynucl eotides encoding the same along with injection devices comprising such antibodies or fragments. Vaccines including such antibodies and fragments as well as compositions comprising the antibodies and fragments (e.g., including anti-PD1 antibodies) are included in the invention. Methods for treating or preventing cancer or infection using such compositions are also provided. In addition, methods for recombinant expression of the antibodies and fragments are part of the present invention.

Keywords: human LAG3 antibody vaccine therapeutic agent composition cancer infection

PatentPak available

Substances (367) | Reactions (0) | Citing (0)

134

Ex vivo drug response profiling detects recurrent sensitivity patterns in drug-resistant acute lymphoblastic leukemia

By: Frismantas, Viktoras; Dobay, Maria Pamela; Rinaldi, Anna; Tchinda, Joelle; Dunn, Samuel H.; Kunz, Joachim; Richter-Pechanska, Paulina; Marovca, Blerim; Pail, Orrin; Jenni, Silvia; et al
Blood (2017), 129(11), e26-e37 | Language: English, Database: CAplus and MEDLINE

Drug sensitivity and resistance testing on diagnostic leukemia samples should provide important functional inform ation to guide actionable target and biomarker discovery. We provide proof of concept data by profiling 60 drugs on 68 acute lymphoblastic leukemia (ALL) samples mostly from resistant disease in cocultures of bone marrow stromal cells. Patient- derived xenografts retained the original pattern of mutations found in the matched patient material. Stromal coculture did not prevent leukemia cell cycle activity, but a specific sensitivity profile to cell cycle-related drugs identified samples with higher cell prolife ration both in vitro and in vivo as leukemia xenografts. In patients with refractory relapses, individual patterns of marked drug resistance and except ional responses to new agents of immediate clin. relevance were detected. The B CL2-inhibitor venetoclax was highly active below 10 nM in B-cell precursor ALL (BCP-ALL) subsets, including MLL-AF4 and TCF3-HLF ALL, and in some T-cell ALLs (T-ALLs), predicting in vivo activity as a single agent and in combination with dexamethasone and vincristine. Unexpected sensitivity to dasatinib with half maximal inhibitory concentration values below 20 nM was detected in 2 indepe ndent T-ALL cohorts, which correlated with similar cytotoxic activity of the SRC inhibitor KX2-391 and inhibition of S RC phosphorylation. A patient with refractory T- ALL was treated with dasatinib on the basis of drug profiling information and achieved a 5- mo remission. Thus, drug profiling captures disease-relevant features and unexpected sensitivity to relevant drugs, which warrants further explor ation of this functional assay in the context of clin. trials to develop drug repurposing strategies for patients with urgent medical needs.

Keywords: acute lymphoblastic leukemia drug resistance sensitivity

Substances (62) Reactions (0) Citing (123)

135

NPM-ALK expression levels identify two distinct subtypes of paediatric anaplastic large cell lymphoma

By: Pomari, E.; Basso, G.; Bresolin, S.; Pillon, M.; Carraro, E.; d'Amore, E. S.; Viola, G.; Frasson, C.; Basso, K.; Bonvini, P.; et al
Leukemia (2017), 31(2), 498-501 | Language: English, Database: CAplus and MEDLINE

Anaplastic large cell lymphoma (ALCL) is a distinct form of non-Hodgkin lymphoma that accounts for 10-15% of all childhood lymphomas. Differently from ALCL in adults, where about 30% of cases are A LK+, ALCL in children is nearly universally ALK+, and in almost all of the cases, it is characterized by the t(2;5)(p23;q35) translocation, which results in the expression of the hybrid oncogenic tyrosine kinase NPM-ALK. NPM-ALK fusion protein is respon sible for ALCL development, and throughout its constitutive tyrosine kinase activity, it regulates signalling pathways critical for proliferation, cell-cycle control and survival of A LCL cells. So far, few studies have investigated ALCL gene expression profiling, and most of them have used heterog eneous populations of paediatric and adult cases. Main aim of such studies was to identify whether ALK+ and ALK- ALCL had a different transcri ptional program, and although differences were found, similarities were also detected. Based on the fact that paediatric A LCL are almost exclusively AL K+, our study focussed on investi gating the potential heterogeneity inside ALK+ ALCL rather than differ ences between ALK+ and AL K- tumors. In order to investigate the transcriptional characteristics of ALK+ ALCL, we performed a global microa rray-based transcri ptional anal. on 23 paediatric tumor samples, 12 reactive lymph nodes and 4 A LCL cell lines (3 ALK+ and 1 ALK-) (Supplementary Materials). As previously reported, we found that A LK+ tumors displayed downreg ulation of both B-cell and T-cell receptor signalling mols. (such as ARNT2, CD3G, CD24, CD27, CD40, CD79A, NFATC2, PIK3CD, TNFRSF10A) in addition to T helper cell surface mols. (such as CD19, CD28, CTLA4), (Supplementary Table S1), consistent with the notion that this malignancy originates from undifferentiated normal CD4+ T lymphocytes.[5] To explore the mol. differ ences between clin. homoge neous ALK+ ALCL, we performed unsupervised clustering anal. of the tumor samples, identi fying two clearly distinct subgroups of patients. Indeed, supervised anal. identified two different gene expression signatures, both related to the diverse amount of N PM-ALK in ALCL cells (A LK-low and -high). Of note, A LCL cell lines clustered together and had a gene expression signatures more similar to A LK-high than to ALK-low samples (Figure 1a; Supplem entary Table S2). The ALK-high/low signatures were not affected by sample or patient selection bias, as our novel ALK group classif ication was independent of the lymph node infilt ration rate, as the percentage of ALK+ cells was about 40% in the large majority of cases and it could be applied to indipendent ALCL studies (Supplementary Figure S1). NPM-ALK levels were assessed by real-time quant. reverse transcriptase PCR (RQ-PCR) in the same set of patients showing a signif icant correl ation with gene expression profiling data (Pearson's correl ation coefficient 0.79, P<0.0001) (Figures 1b and c, resp.). Addnl. 19 paediatric ALCL cases were tested by R Q-PCR, and overall the full data set, including 42 samples, displayed a bimodal distri bution for NPM-ALK expression consistent with the presence of an A LK-low and an ALK-high group. The median expression values were

2.500 NPM-ALK copies/10.000 ABL in ALK-low cases and 125.000 N PM-ALK copies/10.000 ABL in ALK-high patients (Welch's t-test, P<0.0001 Figure 1d). In addition, when NPM-ALK protein expression was measured in a subset of our cases, selected based on tissue availability (Figure 1e), we found that expression and phosphor ylation of NPM-ALK kinase correlated with m RNA levels of the two groups (Figure 1f), supporting the concept that ALK+ ALCL can be distinguished based on NPM-ALK endogenous expression level. We did not observe significant differences between the two subgroups based on clin. characte ristics, although patients with central nervous system involvement (n=4) belonged to the ALK-high subgroup (Supplementary Table S3). Interestingly, 9 out of the 12 patients who experienced relapse were ALK-high cases and had a median time of relapse of 5 mo compared with the median time of relapse of 30 mo of the 3 ALK-low relapsed patients. Gene set enrichment anal. using the M Sig DB database (Mol. Signature Database, Oncogene signature C6, http://www.broad.mit.edu/gsea/) showed a pos. enrichment for genes involved in the interl eukin (IL) signalling pathway (specifically IL-2, IL-15 and IL-21) in ALK-low compared with ALK-high ALCL samples, while genes set associated with cell growth and division (CyclinD1, MYC and E2F1) were significantly upregulated in ALK-high cases (Figure 1g). The I L-2 signalling pathway is critical for C D4+ T-cell maturation and function,[6, 7] and its downregulation occurs in ALCL cell lines in response to NPM-ALK expression and activity. Consistently, we showed that IL-2Rγ was expressed in ALK- (FE-PD) ALCL cell line but not in ALK+ (KARPAS-299, SUD-HL-1 and SUP-M2) cell lines (Figure 2a). In order to evaluate the IL-2Rγ expression in ALCL primary tumor cases, we analyzed, by flow cytometry, nine patients (four ALK-low and five ALK-high), for whom tissue samples were available. The results showed that tumor cells in ALK-low patients ubiquitously expressed IL-2Rγ (ALK+/IL-2Rγ+ cells >95%), whereas ALK-high patients displayed a more heterogeneous pattern of expression (Figures 2b and c). Taking into account that A LK-high patients relapsed more frequently and rapidly than ALK-low patients, it seems that in ALK-low cases NPM-ALK is unable to make transformed lymphocytes completely 'deaf' to ILs, and IL signalling retention, in turn, contributes to a less aggressive tumor phenotype. Functional annotation anal. performed by gene set enrichment anal. in ALK-high tumor samples revealed increased expression of genes involved in cell growth and survival, such as cyclins and Aurora kinases A (AURKA) and B (AURKB). These genes are known not only to affect cell proliferation but also regulate chromosomes' segregation and mitosis. Hence, to validate our bioinformatics anal., we took advantage of com. available Aurora kinase inhibi tors, which were used to treat a panel of ALK+ ALCL cell lines (KARPAS-299, SU-DHL-1 and SUP-M2) representative of the ALK-high subgroup. As expected, marked growth inhibition of A LCL cells was observed upon administration of AURKA and AURKB inhibitors Alisertib (MLN8237) and Barasertib (AZD1152), resp., and this was associated with cell-cycle arrest and induction of apoptosis (Figure 2d). In particular, the exposure of ALCL cells to increasing concentrations of MLN8237 and/or AZD1152 led to time-dependent inhibition of cell growth, which, consistent with Aurora kinases' involvement in mitotic entry and cytokinesis, was characterized by a dose-dependent 'growth plateau' typical of a cytostatic cell response (Figure 2d). In contrast, Aurora kinases inhibitors did not cause changes in healthy peripheral blood mononu clear cells' proliferation (stimulated with or without phytohaema gglutinin), when used in the same exptl. conditions (Figure 2e). Previous data have shown that Aurora inhibitors exert their activity leading to accumulation of polyploid cells with an endorepl ication phenotype before mitotic catastrophe or apoptosis occur. Indeed, when drug- inhibitory effects were investigated in more detail, we found that both compounds, administered for 48 h as single agents or in combin ation, led to a dramatic increase of the polyploid cell content (8N) over the diploid (2N) and tetraploid (4N) cell population (Supplementary Figure S2A), whereas at earlier time points (24 h) they induced a provisional arrest in G2/M phase and accumulation of tetraploid cells only (data not shown). Prolonged drug exposure, instead, affected cell survival, as the fraction of the cells undergoing apoptosis (Annexin V-pos., AV^+) or necrosis (Propidium Iodide pos., PI^+) increased over time, after 72 h of treatment (Supplementary Figure S2B). AURKA and AURKB have been recently detected in non-Hodgkin lymphoma; however, their involvement in ALCL tumorigenesis, as well as their importance as therapeutic targets, has not been investigated. Herein we provide evidence that A URKA and AURKB may be considered novel drug targets in ALCL and that Aurora kinase inhibitors are effective agents in preclin. ALCL models. Their efficacy depends on their general mode of action and results in inhibiting proliferation, survival and cell-cycle progression of ALCL cells. Overall, our data suggest that the heterogeneous levels of aggressiveness observed in ALCL tumors may be explained by a N PM-ALK distinct gene expression and transcriptional program. High-level ALK+ cells seems to acquire a selective growth advantage compared with ALK-low cells. This concept merits attention to exploit novel therap eutic strategies in this malignancy that may increase curability and survival of children with ALCL.

Keywords: anaplastic large cell lymphoma NPM ALK fusion protein

Substances (4) Reactions (0) Citing (6)

136

Preparation of small molecule based antibody-recruiting compounds for cancer treatment

By: Spiegel, David; Rullo, Anthony

World Intellectual Property Organization, WO2017023994 A1 2017-02-09 | Language: English, Database: CAplus

The present invention relates to chimeric (including bifunctional) compounds **I** [$(UPAR_BM)_m$-CT-$(L^1)_k$-CT-$(L^2)_j$-CT-$(A_BM)_n$; wherein UPA R_BM = moiety which binds to an active site of urokinase- type plasminogen activator receptor (u PAR) on the surface of cancer cells; A_BM = antibody binding moiety comprising a hapten which is capable of binding to an antibody; L ^{1} and L^2 = linkers; CT = (indepen dently) an optional connector mol.; j = 0- 5; k = 0-5; with the proviso that at least one C T is present when k and j are both 0 (preferably at least one of k and j is 1); each m and n = (indepen dently) 1-15 (preferably m = 1 and n = 1- 3)], compositions comprising those compounds and methods of treating cancer in a patient or subject, especially including metastatic cancer where cancer cells exhibit overexpression (heightened expression) of cell surface urokinase- type plasminogen activator receptor (urokinase receptor) compared to normal (non-cancerous) cells. The compounds **I** bind to the urokinase-type plasminogen activator receptor (uPAR) on the surface of a cancer cell, including a metastatic cancer cell, and conseq uently recruit native antibodies of the patient or subject where the antibodies can selectively degrade and/or deactivate targeted cancer cells through antibody-dependent cellular phagocytosis and antibody-dependent cellular cytotoxicity (ADCC) and/or complement dependent cytotoxicity (C DC) against a large number and variety of cancers, thus providing cancer cell death and an inhibition of growth, elabor ation and/or metastasis of the cancer, including remission and cure of the patient's cancer. E.g., a multi-step synthesis of compound **II**, starting from 5-hydroxy-1,4-naphthoquinone and 1-(trimethylsilyloxy)-1,3-butadiene, was described. Exemplified title compounds **I** were tested for their biol. activity (data given for representative compounds).

Keywords: chimeric bifunctional compound preparation antitumor u PAR modulator antibody recruiting; urokinase type plasmi nogen activator receptor chimeric bifunc tional compound preparation; antibody dependent cellular phagocytosis cancer chimeric bifunctional compound preparation; ADCC antibody dependent cellular cytotoxicity chimeric bifunctional compound preparation; C DC complement dependent cytotoxicity metastasis chimeric bifunc tional compound preparation

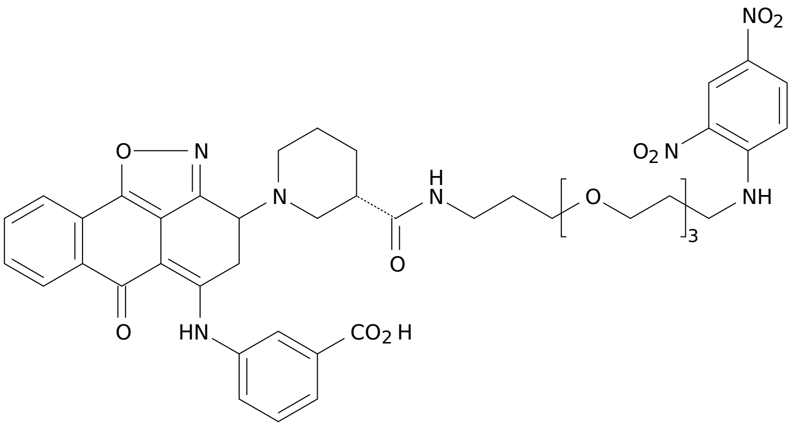

I

PatentPak available

Substances (374) Reactions (318) Citing (0)

137

Synergistic cytotoxicity of melatonin and new-generation anticancer drugs against leukemia lymphocytes but not normal lymphocytes

By: Zhelev, Zhivko; Ivanova, Donika; Bakalova, Rumiana; Aoki, Ichio; Higashi, Tatsuya
Anticancer Research (2017), 37(1), 149-160 | Language: English, Database: CAplus and MEDLINE

The present study demonstrates specific sensitization of leukemia lymphocytes towards anticancer drugs using melatonin and clarifies the role of reactive oxygen species (ROS) for induction of apoptosis. The study covers four conven tional and 11 new-generation anticancer drugs. Four parameters were analyzed simulta neously in leukemia and normal lympho cytes treated with drug, melatonin, or their combination: cell viability, induction of apoptosis, level of reactive oxygen species (R OS), and level of protein-carbonyl products. Almost all invest igated combinations of melatonin with new- generation anticancer drugs were charact erized by synerg istic cytotoxicity towards leukemia lymphocytes, while the combin ations with conventional drugs exhibited additive or antagonistic effects on cell viability. In leukemia lympho cytes, the additive cytoto xicity of doxorubicin plus melatonin was accomp anied by low levels of R OS and protein-carbonyl products, as well as by suppre ssion of apoptosis. In normal lymphocytes, none of the studied parameters changed significantly compared to cells treated with doxoru bicin only. The combin ations of everolimus plus melatonin and barasertib plus melatonin exhibited impressive synergistic cytotoxic effects on leukemia lymphocytes but did not affect the viability of normal lymphocytes. In leukemia cells, the synerg istic cytotoxicity was accompanied by strong induction of apoptosis but a decrease of ROS to a level below that of the control. In normal lympho cytes, these combin ations did not affect the level of ROS nor of protein- carbonyl products, and did not induce apoptosis. The data suggest that melatonin is a promising supplementary component in chemotherapy which allows the therap eutic doses of anticancer drugs to be reduced, minimizing their side-effects.

Keywords: anticancer agent melatonin cytotoxicity leukemia lymphocyte; Melatonin; anticancer drugs; apoptosis; leukemia lympho cytes; normal lympho cytes; reactive oxygen species

Substances (14) Reactions (0) Citing (29)

138

Methods and compositions for treatment of metastatic and refractory cancers and tumors

By: Conejo-Garcia, Jose R.; Allegrezza, Michael
United States, US20170020964 A1 2017-01-26 | Language: English, Database: CAplus

A method of treating a mammalian subject with cancer comprises administering to said subject having a cancer, e.g., a metastatic or refractory cancer or tumor, a small mol. inhibitor of a target signaling mol. of the MEK/MAPK pathway that impairs T cell activa tion, and administering to said subject a mol. that induces T cell prolife ration in the presence of said inhibitor. The combin ation of a small mol. inhibitor of a target of the MEK/MAPK pathway and the T cell prolife ration inducer reduces the proliferation of the cancer and tumor cells in vivo. Compositions of the invention include e.g. tramet inib, AZD6244, GDC0973, GDC0623, refametinib, binimetinib, or U0126.

Keywords: antitumor trametinib metastatic refractory cancer tumor treatment

PatentPak available

Substances (67) Reactions (0) Citing (0)

139

Preparation of alanine-based modulators of proteolysis and associated methods of use

By: Crew, Andrew P.; Berlin, Michael; Dong, Hanqing; Qian, Yimin

World Intellectual Property Organization, WO2017011590 A1 2017-01-19 | Language: English, Database: CAplus

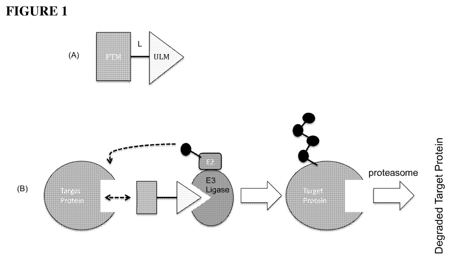

The invention is related to compounds PTM-L-ILM comprising an inhibitor of apoptosis protein (I AP) E3 ubiquitin ligase binding moiety (ILM); a linker (L) coupling I LM and a protein target moiety (P TM) that binds to a target protein or a target polype ptide, e.g., **I**, or a pharmaceutically acceptable salt, enantiomer, stereoisomer, solvate or polymorph thereof. Specifi cally, the invention is related to the preparation of bifunctional compounds, which find utility as modulators of targeted ubiquiti nation, especially inhibitors of a variety of polypeptides and other proteins which are degraded and/or otherwise inhibited by bifunc tional compounds according to the present invention. In particular, the invention is directed to compounds, which contain on one end a ligand which binds to the I AP E3 ubiquitin ligase and on the other end a moiety which binds a target protein such that the target protein is placed in proximity to the ubiquitin ligase to effect degradation (and inhibition) of that protein. Compounds that exhibited a broad range of pharmacol. activities consistent with the degradation/inhibition of targeted polypeptides were prepared Thus, **I** was prepared and at 1 μ M concentration displayed proteolytic modulatory activity (androgen receptor A R degradation between 30% and 60% in V CaP cells).

Keywords: chimeric peptidyl alanine modulator proteolysis targeted polypeptide protein; Iap E3 ligase directed proteo lysis targeting chimeric mol preparation; PROTAC modulator preparation targeted ubiquitination AR receptor degrad ation inhibition; alanine modulator preparation targeted EZH2 gene zeste protein degrad ation; targeted JNK1 JNK2 BRD4 degradation alanine PROTAC modulator preparation

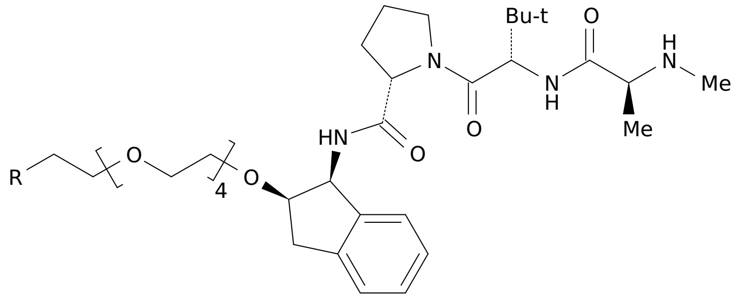

I

R =

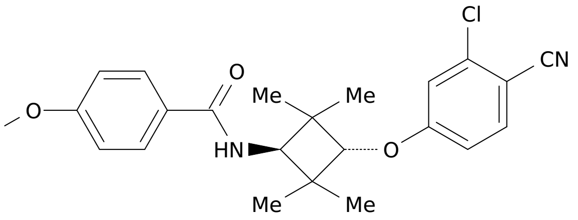

PatentPak available

Substances (539) Reactions (662) Citing (6)

140

Preparation of substituted diazepine derivatives and analogs for use as MDM2-based modulators

By: Crew, Andrew P.; Crews, Craig M.; Dong, Hanqing; Qian, Yimin; Wang, Jing

United States, US20170008904 A1 2017-01-12 | Language: English, Database: CAplus

Title compounds **I** [X = a protein targeting moiety; Y = a linker coupling; Z = a M DM2 E3 ubiquitin ligase binding moiety], and their pharmaceutically acceptable salts, are prepared and disclosed as M DM2-based modulators of proteolysis. Thus, e.g., **II** was prepared by a multistep procedure (preparation given). **I** were evaluated in AR ELISA assays, e.g., **II** demonstrated a degradation activity of 0 to 25% at 1 μM.

Keywords: diazepine preparation MDM2 modulator

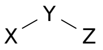

I

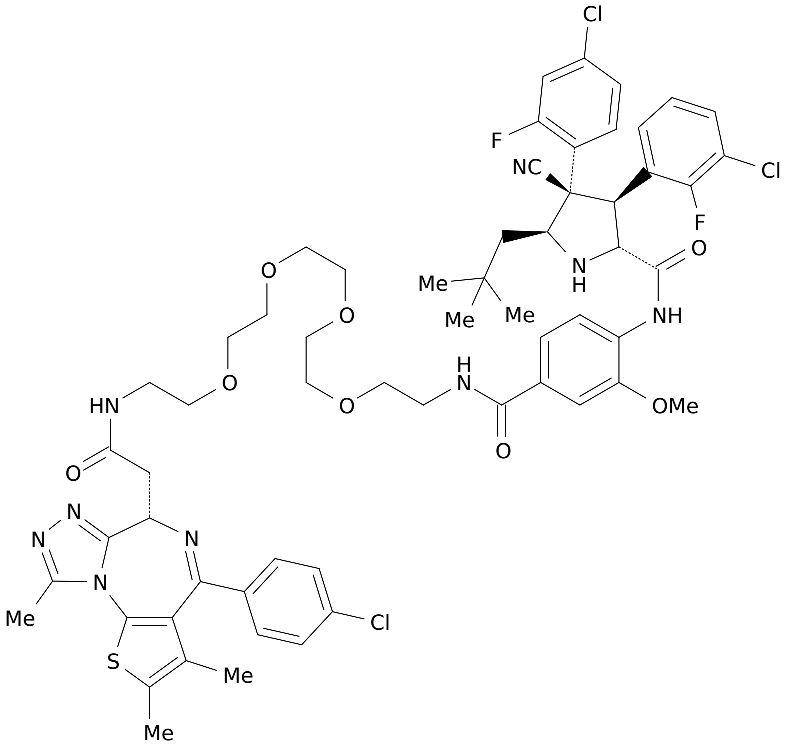

II

PatentPak available

Substances (430)

Reactions (565)

Citing (1)

141

Epidermal growth factor receptor (EGFR) structure-based bioactive pharmacophore models for identifying next-generation inhibitors against clinically relevant EGFR mutations

By: Panicker, Pooja S.; Melge, Anu R.; Biswas, Lalitha; Keechilat, Pavithran; Mohan, Chethampadi G.
Chemical Biology & Drug Design (2017), 90(4), 629-636 | Language: English, Database: CAplus and MEDLINE

Present work elucidates identification of next generation inhibitors for clin. relevant mutations of epidermal growth factor receptor (EGFR) using structure-based bioactive pharmacophore modeling followed by virtual screening (V S) techniques. Three-dimensional (3D) pharmacophore models of EGFR and its different mutants were generated. This includes seven 3 D pharmacophoric points with three different chem. features (descriptors), i.e., one hydrogen bond donor, three hydrogen bond acceptors and three aromatic rings. Pharmacophore models were validated using decoy dataset, Receiver operating charact eristic plot, and external dataset compounds The robust, bioactive 3D e-pharmacophore models were then used for V S of four different small compound databases: FDA approved, investigational, anticancer, and bioactive compounds collec tions of Selleck Chems. CUDC101 a multitargeted kinase inhibitor showed highest binding free energy and 3D pharmacophore fit value than the well known E GFR inhibitors, Gefitinib and Erlotinib. Further, we obtained ML167 as the second best hit on VS from bioactive database showing high binding energy and pharmacophore fit value with respect to E GFR receptor and its mutants. Optimist ically, presented drug discovery based on the computational study serves as a foundation in identi fying and designing of more potent E GFR next-generation kinase inhibitors and warrants further exptl. studies to fight against lung cancer.

Keywords: EGFR structure pharmac ophore mutation; computer-aided drug design; epidermal growth factor receptor; erlotinib; next-generation EGFR inhibitors; non-small cell lung cancer; pharmac ophore; virtual screening

Substances (9) Reactions (0) Citing (17)

142

Compositions and methods comprising protein phosphatase 2A (PP2A) activators and protein kinase inhibitors for treating cancer

By: Narla, Goutham
World Intellectual Property Organization, WO2016210134 A1 2016-12-29 | Language: English, Database: CAplus

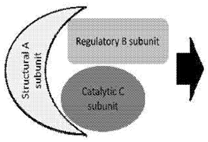

Protein	Regulatory effect
Akt	Inhibit
Androgen receptor (AR)	Inhibit
BAD	Activate
BAX	Activate
BCL-2	Inhibit
ERK	Inhibit
C-MYC	Inhibit
Sne	Inhibit

Fig. 1

A method of treating cancer in a subject in need thereof includes administering to the subject therapeutically effective amounts of a PP2A activator and a protein kinase inhibitor.

Keywords: antitumor protein phosphatase kinase inhibitor cancer treatment

PatentPak available

Substances (123) Reactions (0) Citing (0)

143

Inhibition of granulocyte colony stimulating factor in the treatment of cancer

By: Morris, Katherine Theresa; Beswick, Ellen Janine

United States, US20160368980 A1 2016-12-22 | Language: English, Database: CAplus

The present invention relates to the discovery that granulocyte colony stimulating factor (G-CSF) as well as granulocyte colony stimul ating factor receptor (G-CSFR) are highly expressed in cancer tissue and the inhibition of G- CSF using a G-CSF inhibitor, which term includes inhibitors of the G-CSF receptor, represents a viable approach to the treatment of cancer, including drug resistant cancers, metastatic cancers and recurrent cancers. G-CSF inhibitors as described herein may be used alone or in combin ation with an at least one addnl. anti-cancer agent for the treatment of cancer.

Keywords: granulocyte colony stimulating factor inhibitor cancer treatment anticancer combination

PatentPak available

Substances (307) | Reactions (0) | Citing (0)

144

MCT4 inhibitors for treating disease

By: Parnell, Kenneth Mark; McCall, John M.

World Intellectual Property Organization, WO2016201426 A1 2016-12-15 | Language: English, Database: CAplus

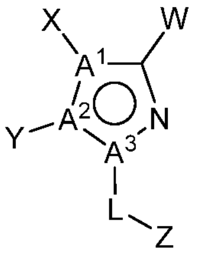

(I)

Disclosed are pyrazole compounds and compositions useful in the treatment of M CT4 mediated diseases, such as proliferative and inflammatory diseases, having the structure **I**, and methods of inhibiting MCT4 activity in a human or animal subject. Also disclosed is the preparation of compounds of the invention.

Keywords: pyrazole derivative preparation MCT4 inhibitor proliferative disease inflamation

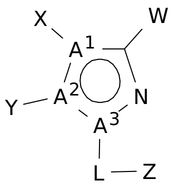

I

PatentPak available

Substances (300) | Reactions (245) | Citing (3)

145

Human GLUT5 specific inhibitors and methods of treatment

By: Oprea, Tudor I.; Bologa, Cristian George; Ursu, Oleg; Choe, Jun-Yong; Iancu, Cristina

World Intellectual Property Organization, WO2016201214 A1 2016-12-15 | Language: English, Database: CAplus

The present invention relates to compounds which have been discovered to be potent ligands (inhibitors) of human GLUT5 (glucose transporter type 5), a facilitative glucose transporter that transports fructose, and their use as ligands assays which can uncover addnl. ligands of GLUT5, having the potential for being used as drugs. In addition, the present invention is directed to compounds, chem. compositions and methods for treating disease states and conditions which are mediated through G LUT5, including such disease states and conditions as GLUT5 deficiency syndrome, diabetes (type I and I I), cancer, metabolic diseases including metabolic syndrome and fatty liver disease, among others.

Keywords: glucose transporter GLUT5 inhibitor diabetes cancer fatty liver disease

PatentPak available

Substances (347) Reactions (0) Citing (0)

146

Preparation of PROTAC compounds as TANK-binding kinase 1 inhibitors for modulation of proteolysis and associated methods of use

By: Crew, Andrew P.; Wang, Jing; Dong, Hanqing; Qian, Yimin; Siu, Kam; Ferraro, Caterina; Crews, Craig M.

World Intellectual Property Organization, WO2016197114 A1 2016-12-08 | Language: English, Database: CAplus

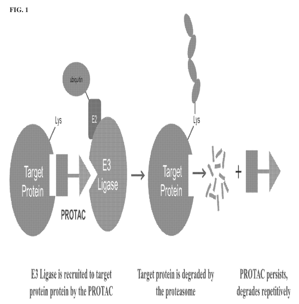

The invention is related to compounds TBM-L-PTM comprising an E3 ubiquitin ligase binding moiety (U LM), a TBK1 binding moiety (T BM) connected directly or through a chem. linker (L) , e.g., **I**, which find utility modulators of targeted ubiquitination and degradation of TANK-binding kinase (TBK1). In particular, the invention is directed to compounds, which contain on one end an E3 ubiquitin ligase binding moiety which binds to the ubiquitin ligase and on the other end a moiety which binds TBK1 such that TBK1 is placed in proximity to the ubiquitin ligase to effect degradation (and inhibition) of TBK1. Compounds that exhibited a broad range of pharmacol. activities consistent with the degradation/inhibition of TBK1 were prepared Thus, **I** was prepared and displayed proteo lytic modulatory activity (TBK1 degradation D_{max} = 96%).

Keywords: TANK binding kinase 1 P ROTAC chimeric mol prepar ation; PROTAC modulator preparation targeted TBK1 kinase degrad ation ubiquitination; Von Hippel Lindau ligand prepar ation TBK1 PROTAC degradation inhibition

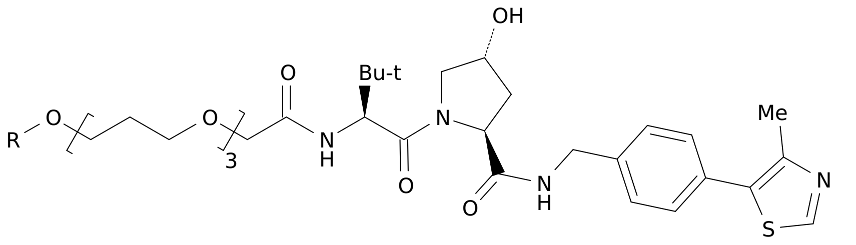

I

R =

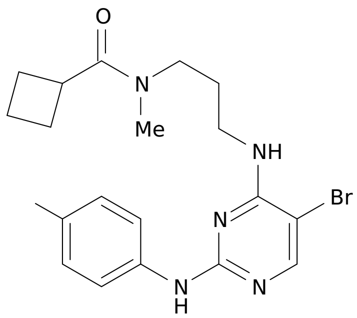

PatentPak available

Substances (439) Reactions (182) Citing (3)

147

Imide-based modulators of proteolysis and associated methods of use

By: Crews, Craig M.; Crew, Andrew P.; Dong, Hanqing; Wang, Jing; Qian, Yimin; Siu, Kam; Jin, Meizhong
World Intellectual Property Organization, WO2016197032 A1 2016-12-08 | Language: English, Database: CAplus

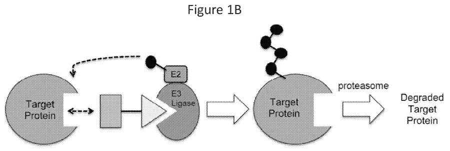

The description relates to imide- based compounds, including bifunctional. compounds comprising the same, which find utility as modulators of targeted ubiquitination, especially inhibitors of a variety of polypeptides and other proteins which are degraded and/or otherwise inhibited by bifunctional compounds according to the present invention. In particular, the description provides compounds, which contain on one end a ligand which binds to the cereblon E3 ubiquitin ligase and on the other end a moiety which binds a target protein such that the target protein is placed in proximity to the ubiquitin ligase to effect degradation (and inhibition) of that protein. Compounds can be synthesized that exhibit a broad range of pharmacol. activities consistent with the degradation/inhibition of targeted polypeptides of nearly any type.

Keywords: antitumor proteolysis modulator imide preparation cancer

PatentPak available

Substances (425) | Reactions (330) | Citing (5)

148

Inhibition of the pentose-phosphate pathway selectively sensitizes leukemia lymphocytes to chemotherapeutics by ROS-independent mechanism

By: Zhelev, Zhivko; Ivanova, Donika; Bakalova, Rumiana; Aoki, Ichio; Higashi, Tatsuya
Anticancer Research (2016), 36(11), 6011-6020 | Language: English, Database: CAplus and MEDLINE

The aim of the present study was to investigate: (i) the possibility of sensitizing leukemia lymphocytes to anticancer drugs by inhibiting pentose-phosphate pathway using 6-aminonicotinamide (6-ANA); (ii) to find combinations with synergistic cytotoxic effect on leukemia lymphocytes and to investigate their cytotoxicity towards normal lymphocytes; (iii) and to clarify the role of reactive oxygen species (ROS) in the induction of apoptosis by those combinations. The study covers 15 anticancer drugs - conventional and new-generation. The experiments were performed on Jurkat leukemia cell line and normal lymphocytes, isolated from clin. healthy blood donors. Four parameters were analyzed simultaneously in both cell suspensions treated by drug or 6- ANA (sep., and in combination): cell viability, induction of apoptosis, level of ROS, and level of protein-carbonyl products. Most combinations of drug plus 6-ANA were characterized by synergistic cytotoxic effects on Jurkat cells. The synergism increased with increasing incubation time. Upon combination of 6-ANA with conventional chemotherapeutic (e.g. doxorubicin), synergistic cytotoxic effects were also detected in normal lymphocytes. In both cell types, the cytotoxicity of the combination of doxorubicin plus 6-ANA was accompanied by increased induction of apoptosis, but by a slight reduction of ROS and protein-carbonyl products compared to cells treated with doxorubicin only. Upon combination of 6-ANA with new-generation anticancer drugs (e.g. everolimus or barasertib), the synergistic cytotoxic effect on leukemia lymphocytes was also accompanied by very strong induction of apoptosis through ROS-independent mechanism(s). Neither of these combinations exhibited any cytotoxicity towards normal lymphocytes. The data suggest that 6-ANA could be used as a supplementary component in anticancer chemotherapy, and would allows therapeutic doses of anticancer drugs to be reduced, thereby minimizing their side-effects.

Keywords: pentose phosphate pathway leukemia lymphocyte ROS mechanism chemotherapeutics; 6-Aminonicotinamide; ROS; chemotherapeutics; leukemia lymphocytes

Substances (16) | Reactions (0) | Citing (11)

149

Bromodomain and Extraterminal Protein Inhibition Blocks Growth of Triple-negative Breast Cancers through the Suppression of Aurora Kinases

By: Sahni, Jennifer M.; Gayle, Sylvia S.; Weber Bonk, Kristen L.; Vite, Leslie Cuellar; Yori, Jennifer L.; Webb, Bryan; Ramos, Erika K.; Seachrist, Darcie D.; Landis, Melissa D.; Chang, Jenny C.; et al
Journal of Biological Chemistry (2016), 291(45), 23756-23768 | Language: English, Database: CAplus and MEDLINE

Bromodomain and extraterminal (BET) proteins are epigenetic "readers" that recognize acetylated histones and mark areas of the genome for transcription. BRD4, a BET family member protein, has been implicated in a number of types of cancer, and BET protein inhibitors (BETi) are efficacious in many preclin. cancer models. However, the drivers of response to BETi vary depending on tumor type, and little is known regarding the target genes conveying BETi activity in triple-neg. breast cancer (TNBC). Here, we show that BETi repress growth of multiple in vitro and in vivo models of TNBC by inducing two terminal responses: apoptosis and senescence. Unlike in other cancers, response to BETi in TNBC is not dependent upon suppression of MYC. Instead, both end points are preceded by the appearance of polyploid cells caused by the suppression of Aurora kinases A and B (AURKA/B), which are critical mediators of mitosis. In addition, AURKA/B inhibitors phenocopy the effects of BETi. These results indicate that Aurora kinases play an important role in the growth suppressive activity of BETi in TNBC. Elucidating the mechanism of response to BETi in TNBC should (1) facilitate the prediction of how distinct TNBC tumors will respond to BETi and (2) inform the rational design of drug combination therapies.

Keywords: Aurora kinase BET protein inhibitor anticancer breast cancer; Aurora kinase; anticancer drug; apoptosis; breast cancer; bromodomain-containing protein 4 (BRD4); cellular senescence; inhibition mechanism

Substances (4) Reactions (0) Citing (44)

150

Barasertib (AZD1152), a Small Molecule Aurora B Inhibitor, Inhibits the Growth of SCLC Cell Lines In Vitro and In Vivo

By: Helfrich, Barbara A.; Kim, Jihye; Gao, Dexiang; Chan, Daniel C.; Zhang, Zhiyong; Tan, Aik-Choon; Bunn, Paul A. Jr.
Molecular Cancer Therapeutics (2016), 15(10), 2314-2322 | Language: English, Database: CAplus and MEDLINE

Small-cell lung cancer (SCLC) cells have rapid proliferation, universal Rb inactivation, and high rates of MYC family amplification, making aurora kinase inhibition a natural target. Preclin. studies have demonstrated activity for Aurora A and pan-Aurora inhibitors with some relationship to MYC family expression. A clin. trial showed activity for an Aurora kinase A inhibitor, but no biomarkers were evaluated. We screened a panel of 23 SCLC lines with and without MYC family gene amplification or high MYC family gene expression for growth inhibition by the highly potent, selective aurora kinase B inhibitor barasertib. Nine of the SCLC lines were very sensitive to growth inhibition by barasertib, with IC_{50} values of <50 nmol/L and >75% growth inhibition at 100 nmol/L. Growth inhibition correlated with cMYC amplification (P = 0.018) and cMYC gene expression (P = 0.026). Sensitive cell lines were also enriched in a published MYC gene signature (P = 0.042). In vivo, barasertib inhibited the growth of xenografts established from an SCLC line that had high cMYC gene expression, no cMYC amplification, and was pos. for the core MYC gene signature. Our studies suggest that SCLC tumors with cMYC amplification/high gene expression will frequently respond to Aurora B inhibitors and that clin. studies coupled with predictive biomarkers are indicated. Mol Cancer Ther; 15(10); 2314-22.

Keywords: barasertib aurora B inhibitor small cell lung cancer antitumor

Substances (2) Reactions (0) Citing (59)

151

Preparation of dimeric quinacrine derivatives as autophagy inhibitors for cancer therapy

By: Amaravadi, Ravi K.; Winkler, Jeffrey

World Intellectual Property Organization, WO2016168721 A1 2016-10-20 | Language: English, Database: CAplus

The invention provides dimeric quinacrine derivatives and related compounds of formula I and compositions, methods of treatment and syntheses. The compounds exhibit unexpected anticancer activity and are useful in the treatment of a variety of autophagy-related disorders. Compounds of formula I wherein R^1, R^2, R^3, R^4, R^5, R^6, R^7, R^8, $R^{1'}$, $R^{2'}$, $R^{3'}$, $R^{4'}$, $R^{5'}$, $R^{6'}$, $R^{7'}$ and $R^{8'}$ are independently H, halo, C N, NO_2, OH, CO_2H, etc.; R and R' are indepen dently H, (un)substituted C_{1-6} alkyl, (un)substituted C_{1-6} alkoxy, (un)substituted C_{1-7} acyl, etc.; L is a linker which covalently links to two exocyclic amine groups; and pharmace utically acceptable salts, enantiomers, diastereoisomers, solvates and polymorphs thereof, are claimed. Example compound II was prepared by a general procedure (procedure given). The invention compounds were evaluated for their autophagy inhibitory activity (some data given).

Keywords: dimeric quinacrine preparation autophagy inhibitor treatment cancer

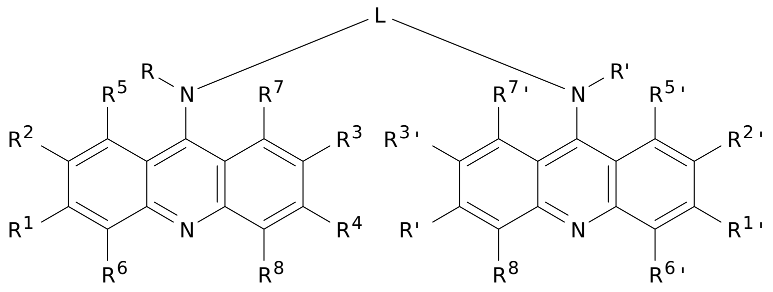

I

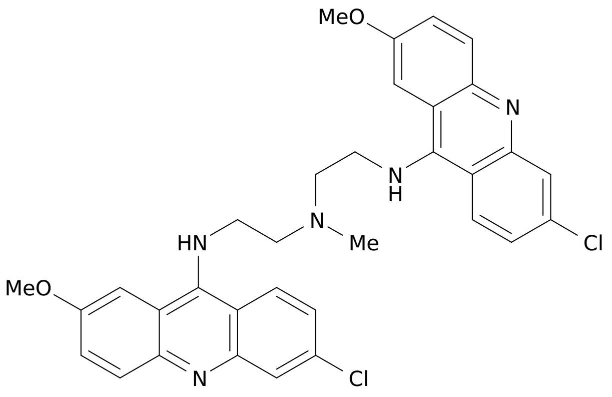

II

PatentPak available

Substances (335) Reactions (43) Citing (3)

152

Leishmania donovani Aurora kinase: A promising therapeutic target against visceral leishmaniasis

By: Chhajer, Rudra; Bhattacharyya, Anirban; Didwania, Nicky; Shadab, Md; Das, Nirupam; Palit, Partha; Vaidya, Tushar; Ali, Nahid
Biochimica et Biophysica Acta, General Subjects (2016), 1860(9), 1973-1988 | Language: English, Database: CAplus and MEDLINE

Aurora kinases are key mitotic kinases executing multiple aspects of eukaryotic cell-division. The apicomplexan homologs being essential for survival, suggest that the Leishmania homolog, annotated LdAIRK, may be equally important. Bioinformatics, stage-specific immunofluorescence microscopy, immunoblotting, RT-PCR, mol. docking, in-vitro kinase assay, anti-leishmanial activity assays, flow cytometry, fluorescence microscopy. Ldairk expression is seen to vary as the cell-cycle progresses from G1 through S and finally G2M and cytokinesis. Kinetic studies demonstrate their enzymic activity exhibiting a Km and Vmax of 6.12 μ M and 82.9 pmoles·min^{-1}mg^{-1} resp. against ATP using recombinant Leishmania donovani H3, its physiol. substrate. Due to the failure of Ld AIR K -/+ knock-out parasites to survive, we adopted a chem. knock- down approach. Based on the conservation of key active site residues, three mammalian Aurora kinase inhibitors were investigated to evaluate their potential as inhibitors of LdAIRK activity. Interestingly, the cell-cycle progressed unhindered, despite treatment with GSK-1070916 or Barasertib, inhibitors with greater potencies for the ATP-binding pocket compared to Hesperadin, which at nanomolar concentrations, severely compromised viability at IC_{50}s 105.9 and 36.4 n M for promastigotes and amastigotes, resp. Cell-cycle and morphol. studies implicated their role in both mitosis and cytokinesis. We identified an Aurora kinase homolog in L. donovani implicated in cell- cycle progression, whose inhibition led to aberrant changes in cell-cycle progression and reduced viability. Human homologs being actively pursued drug targets and the observations with LdAIRK in both promastigotes and amastigotes suggest their potential as therapeutic-targets. Importantly, our results encourage the exploration of other proteins identified herein as potential novel drug targets.

Keywords: Leishmania Aurora kinase visceral leishmaniasis development Hesperadin; Aurora kinase; Cell-cycle; Cytokinesis; Hesper adin; Leishmania donovani; Therapeutic target

Substances (5) | Reactions (0) | Citing (12)

153

Pericyte long non-coding RNA inhibitors for use in treatment of diseases mediated by PDGFR expression

By: Zehendner, Christoph Michael; Dimmeler, Stefanie; Zeiher, Andreas
World Intellectual Property Organization, WO2016150870 A1 2016-09-29 | Language: English, Database: CAplus

The present invention provides novel non-coding RNAs (lncRNA) that were identified to be expressed in pericytes upon hypoxia. The lncRNA of the invention pos. affect Platelet-derived Growth Factor Receptor (P DGFR) beta expression, pericytes proliferation and pericyte recruitment to endothelial cells. The invention provides inhibitors of the lnc RNA for use in the treatment of diseases mediated by PDGFR expression. For example the invention described antisense approaches to target the lnc RNA of the invention. Furthermore, the invention provides lncRNA inhibitors as amplifier of therapeutic PDGFR inhibitors such as imatinib or other tyrosine kinase inhibitors. lncRNA inhibitors and methods for screening modulators of lnc RNA expression and/or function are provided.

Keywords: pericyte long non coding RNA human PDGFR disease

PatentPak available

Substances (28) | Reactions (0) | Citing (1)

154

Preparation of bifunctional PROTAC compounds and methods for the enhanced degradation of targeted proteins

By: Berlin, Michael; Zimmerman, Kurt; Snyder, Lawrence; Crew, Andrew P.; Crews, Craig M.; Chen, Xin; Dong, Hanqing; Ferraro, Caterina; Jin, Meizhong; Qian, Yimin; et al

World Intellectual Property Organization, WO2016149668 A1 2016-09-22 | Language: English, Database: CAplus

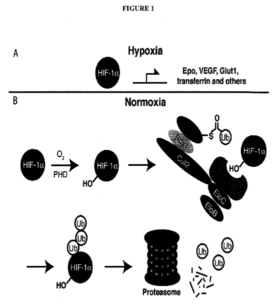

The invention is related to compounds ULM-L-PTM comprising a Von Hippel-Lindau (VHL) E3 ubiquitin ligase binding moiety (U LM), a protein targeting moiety (PTM) connected directly or through a chem. linker (L) , e.g., **I**, or a pharmaceutically acceptable salt, enanti omer, stereoisomer, solvate or polymorph thereof which find utility as modulators of targeted ubiquiti nation, especially inhibitors of a variety of polypeptides and other proteins which are degraded and/or otherwise inhibited by the bifunc tional compounds of the invention. In particular, the invention is directed to compounds, which contain on one end a V HL ligand which binds to the ubiquitin ligase and on the other end a moiety which binds a target protein such that the target protein is placed in proximity to the ubiquitin ligase to effect degradation (and inhibition) of that protein. Compounds that exhibited a broad range of pharmacol. activities consistent with the degradation/inhibition of targeted polypeptides were prepared Thus, **I** was prepared and displayed proteolytic modulatory activity (androgen receptor degradat ion51%≤ Dmax ≤70%; BRD4 degradation cMyc Imax ≥71%).

Keywords: chimeric mol preparation targeted protein degradation ubiquitination; PROTAC modulator preparation targeted ubiquit ination AR receptor degradation inhibition; Von Hippel Lindau ligand preparation BRD4 PROTAC degradation inhibition

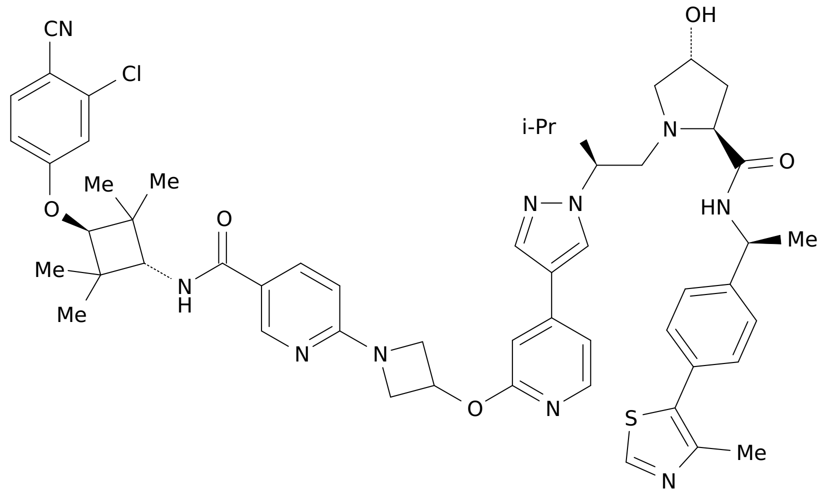

I

PatentPak available

Substances (590) Reactions (228) Citing (6)

155

Cholesteryl ester transfer protein (CEPT) inhibition in the treatment of cancer

By: Kaur, Mandeep; Esau, Luke E.; Sagar, Sunii
World Intellectual Property Organization, WO2016135633 A1 2016-09-01 | Language: English, Database: CAplus

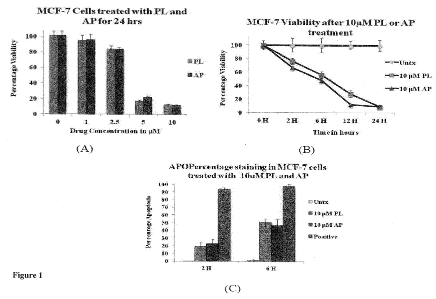

In one embodiment, the invention provides methods of treatment which use therapeu tically effective amounts of Choleste ryl Ester Transfer Protein (CETP) inhibitors to treat a variety of cancers. In certain embodi ments, the inhibitor is a CETP-inhibiting small mol., CETP-inhibiting antisense oligonucleotide, CETP-inhibiting siRNA or a C ETP- inhibiting antibody. Related pharmac eutical composi tions, kits, diagno stics and screens are also provided.

Keywords: cholesteryl ester transfer protein C EPT inhibition cancer

PatentPak available

Substances (362) Reactions (0) Citing (0)

156

Dye-stabilized nanoparticles and methods of their manufacture and therapeutic use

By: Heller, Daniel A.; Shamay, Yosef

World Intellectual Property Organization, WO2016130546 A1 2016-08-18 | Language: English, Database: CAplus

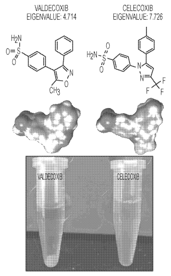

FIG. 18

Described herein are nanoparticles which are largely made of (e.g., 90 wt%) hydrop hobic drugs and are stabilized by water soluble dyes. The nanoparticles can range in size from 30 nm to 150 nm and have highly neg. surface charge (e.g., - 55 mV). These nanopar ticles are highly soluble in water, stable for days in P BS buffer and can be easily lyophi lized and reconstituted in water. Using quant. self-assembly prediction calculations, topochem. mol. descri ptors were identified and validated as highly predictive indicators of nano-assembly, nanoparticle size, and drug loading. The resulting nanopar ticles selectively targeted kinase inhibitors to caveolin-1-expressing human colon cancer and autocht honous liver cancer models to yield striking therap eutic effects while avoiding p ERK inhibition in healthy skin. The nanoparticles exhibited remarkable anti-tumor efficacy in vitro and in vivo in models of hepatoc ellular carcinoma. Thus, dye- encapsulated sorafenib nanoparticles (IR783-SFB) were synthesized using the nanopptn. method; 0.1 mL of sorafenib dissolved in D MSO (10 mg/m L) was added dropwise (20 µL per 15 s) to a 0.4 aqueous I R783 solution (1 mg/m L) containing 0.05 mM sodium bicarb onate; the solution was centrifuged twice and resuspension in 1 m L of sterile PBS; the suspension of nanoparticles was sonicated for 10 s with a probe sonicator at 40 % intensity; the resulted I R783-SFB nanoparticles had zeta potential of -52 mV and a size of 95 nm with a P DI of 0.1; by suspending the nanoparticles in lower volumes they were able to solubilize sorafenib up to 16 mg/mL in saline solution which is 2000 times better than free drug; the nanopar ticles were easily lyophilized with a saline/sucrose 5 % solution and reconst ituted in water at this concent ration

Keywords: dye stabilization nanoparticle hydrophobic drug self assembly cancer

PatentPak available

Substances (292) Reactions (0) Citing (0)

157

Aurora B inhibitor barasertib prevents meiotic maturation and subsequent embryo development in pig oocytes

By: Ju, Shiqiang; Peng, Xu; Yang, Xiaoliu; Sozar, Sparksi; Muneri, Caroline W.; Xu, Yaping; Chen, Changchao; Cui, Panpan; Xu, Weichao; Rui, Rong

Theriogenology (2016), 86(2), 503-515 | Language: English, Database: CAplus and MEDLINE

Barasertib, a highly selective Aurora B inhibitor, has been widely used in a variety of cells to invest igate the role of Aurora B kinase, which has been implicated in various functions in the mitotic process. However, effects of barasertib on the meiotic maturation process are not fully understood, particularly in porcine oocyte meiotic maturation. In the present study, the effects of barasertib on the meiotic maturation and developmental competence of pig oocytes were investi gated, and the possible roles of Aurora B were also evaluated in porcine oocytes undergoing meiosis. Initially, we examined the expression and subcellular localization of Aurora B using Western blot anal. and immunofluorescent staining. Aurora B was found to express and exhibit specific dynamic intracellular localization during porcine oocyte meiotic matura tion. Aurora B was observed around the chromo somes after germinal vesicle breakdown. Then it was transferred to the spindle region after metaphase I stage, and was partic ularly concentrated at the central spindles at telophase I stage. barasertib treatment resulted in the failure of polar body extrusion in pig oocytes, with a larger percentage of barasertib-treated oocytes remaining at the pro- metaphase I stage. Addnl. results reported that barasertib treatment had no effect on chromosome condensation but resulted in a signifi cantly higher percentage of the treated oocytes with aberrant spindles and misaligned chromosomes during the first meiotic division. In addition, inhibition of Aurora B with lower concent rations of barasertib during pig oocyte meiotic maturation decreased the subsequent embryo developmental competence. Thus, these results illustrate that barasertib has significant effects on porcine oocyte meiotic maturation and subsequent develo pment through Aurora B inhibition, and this regulation is related to its effects on spindle formation and chromosome alignment during the first meiotic division in porcine oocytes.

Keywords: barasertib oocyte aurora B inhibitor meiotic maturation embryo development; Aurora B; Barase rtib; Chromosome alignment; Oocytes maturation; Spindle formation

Substances (2) Reactions (0) Citing (10)

158

Significance of AZD1152 as a potential treatment against Aurora B overexpression in acute promyelocytic leukemia

By: Ghanizadeh-Vesali, Samad; Zekri, Ali; Zaker, Farhad; Zaghal, Azam; Yousefi, Meysam; Alimoghaddam, Kamran; Ghavamzadeh, Ardeshir; Ghaffari, Seyed H.
Annals of Hematology (2016), 95(7), 1031-1042 | Language: English, Database: CAplus and MEDLINE

Aurora B kinase as a chromosomal passenger protein plays multiple roles in regulating mitosis and cytokinesis. The function of Aurora B in leukemic cells has made it an important treatment target. In this study, we explored the expressions of Aurora (A, B, and C) kinases in newly diagnosed acute promyelocytic leukemia (APL) patients. In addition, we investigated the effects of AZD1152 as a specific inhibitor of Aurora B on cell survival, DNA synthesis, nuclear morphol., apoptosis induction, cell cycle distribution, and gene expression in an APL-derived NB4 cell line. Our results showed that Aurora B was overexpressed in 88 % of APL patients. AZD1152 treatment of NB4 cells led to viability reduction and G2/M arrest followed by an increase in cell size and polyploidy induction. These giant cells showed morphol. evidence of mitotic catastrophe. AZD1152 treatment induced activation of G2/M checkpoint which in turn led to transient G2/M arrest in a p21-independent manner. Lack of functional p53 in NB4 cells might provide an opportunity to escape from G2/M block and to endure repeated rounds of replication and polyploidy. Treated cells were probably eliminated via p73-mediated overexpression of BAX, PUMA, and APAF1 and downregulation of survivin and MCL-1. In summary, AZD1152 treatment led to endomitosis and polyploidy in TP53-mutated NB4 cells. These giant polyploid cells might undergo mitotic catastrophe and p73-mediated apoptosis. It seems that induction of polyploidy via AZD1152 could be a novel form of anti-cancer therapy for APL that may be clin. accessible in the near future.

Keywords: AZD1152 anticancer aurora B apoptosis promyelocytic leukemia; AZD1152; Acute promyelocytic leukemia; Apoptosis; Aurora B kinase; Cell cycle arrest; p73

Substances (8) Reactions (0) Citing (10)

159

Preparation of substituted bicyclic compounds as bromodomain inhibitors

By: Brown, Samuel David; Coburn, Craig Alan
World Intellectual Property Organization, WO2016097870 A1 2016-06-23 | Language: English, Database: CAplus

The title compounds **I** [W^1 = N and NH; W^2 and E^3 = (independently) C and N; R^1 = (un)substituted cycloalkyl, heterocyclyl; R^2 = H, alkyl (optionally substituted with halo and OH); R^3 = alkyl optionally substituted with halo and OH (with the proviso); R^4 = H, (un)substituted alkyl, cycloalkyl, etc.; X = CH_2 optionally substituted with 1-2 groups independently selected from R^5; R^5 = D, alkyl, alkoxy, etc.; Y = N and CH; with the provisos], useful for inhibition of BET protein function by binding to bromodomains, were prepared E.g., a multi-step synthesis of **II**, starting from 5-bromopyridine-2,3-diamine and (3,5-dimethylisoxazol-4-yl)boronic acid, was described. Exemplified compounds **I** were tested in various biol. assays (data given) . Pharmaceutical compositions, comprising compound **I**, alone or in combination with other therapeutic agent, was disclosed.

Keywords: bicyclic compound imidazopyridine preparation BET bromodomain protein inhibitor

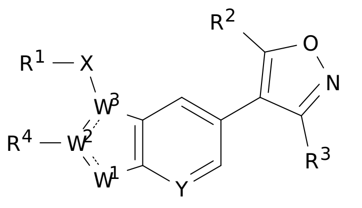

I

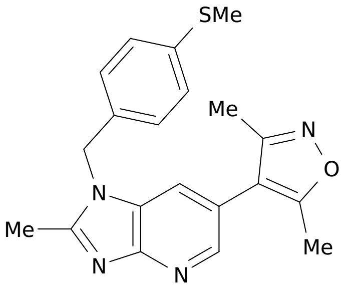

II

PatentPak available

Substances (116) Reactions (153) Citing (2)

160

Preparation of 4-heterocyclylisoxazole derivatives as inhibitors of bromodomains

By: Kharenko, Olesya; Young, Peter Ronald; Brown, Samuel David; Duffy, Bryan Cordell; Liu, Shuang; Guzzo, Peter
World Intellectual Property Organization, WO2016097863 A1 2016-06-23 | Language: English, Database: CAplus

The present disclosure relates to compounds **I** [R^1 = each (un)substituted C_{5-10} carbocyclyl or C_{2-10} heterocyclyl; R^2, R^3 = indepen dently C_{1-6} alkyl optionally substituted with halogen and hydroxy, with the proviso that if R^2 and R^3 are methyls, then R^1 is different from Q, Q^1, Q^2, 2-(difluoromethoxy)phenyl, 2,5-dichlorophenyl, or 2,5-dimethylphenyl; A = H, halogen, methoxy, cyano, NO_2, CO_2Me, or $CONMe_2$; R^4 = H, C_{1-10} alkyl, C_{3-10} carbocyclyl, or C_{2-10} heterocyclyl; X = (un)substituted CH_2; Y = N, CH, or $C(NH_2)$] or **II** (R^1, R^2, R^3, X = same as above; Z = H or NH_2) or stereoisomers, tautomers, pharmaceutically acceptable salts, or hydrates thereof. These compounds are capable of acting as covalent inhibitors of bromodomains and are useful for treating (A) an autoimmune or inflam matory disorder associated with bromodomain and extra terminal domain (BET) proteins, (B) an acute or chronic non-autoimmune inflammatory disorder characterized by disregulation of IL-6 and/or IL-17, (C) a cancer associated with overexpression, translo cation, amplification, or rearrangement of a myc family oncoprotein that is sensitive to BET inhibition, (D) cancer that relies on pTE Fb (Cdk9/cyclin T) and BET proteins to regulate oncogenes, (F) a cancer associated with upregu lation of BET responsive genes CDK6, Bcl2, TYRO3, MYB, and hTERT, (G) a cancer associated with a gene regulated by a super enhancer, (H) a cancer that is sensitive to effects of BET inhibition, (I) a cancer that is resistant to treatment with immunot herapy, hormone-deprivation therapy, and/or chemotherapy, (J) a benign proliferative or fibrotic disorder, or (K) a disease or disorder that benefits from upregu lation or ApoA-I transcription and protein expression. The autoimmune or inflammatory disorder is selected from acute dissem inated encephalo myelitis, agammaglobulinemia, allergic disease, ankylosing spondylitis, anti-GBM/anti-TBM nephritis, anti-phospholipid syndrome, autoimmune aplastic anemia, autoimmune hepatitis, autoimmune inner ear disease, autoimmune myocarditis, autoimmune pancreatitis, autoimmune retinopathy, autoimmune thrombocytopenic purpura, Behcet's disease, Bullous pemphigoid, Castleman's disease, Celiac disease, Churg-Strauss syndrome, Crohn's disease, Cogan's syndrome, dry eye syndrome, essential mixed cryoglobu linemia, dermatomyositis, Devic's disease, encephalitis, eosinophlic esophagitis, eosinophilic fasciitis, erythema nodosum, giant cell arteritis, glomerulonephritis, Goodpasture's syndrome, granulomatosis with polyangiitis (Wegener's), Graves' disease, Guillain-Barre syndrome, Hashimoto's thyroiditis, hemolytic anemia, Henoch-Schonlein purpura, idiopathic pulmonary fibrosis, IgA nephropathy, inclusion body myositis, type I diabetes, interstitial cystitis, Kawasaki's disease, etc. The acute or chronic non-autoimmune inflam matory disorder is selected from sinusitis, pneumonitis, osteomyelitis, gastritis, enteritis, gingivitis, appendicitis, irritable bowel syndrome, tissue graft rejection, chronic obstructive pulmonary disease (COPD), septic shock, osteoarthritis, acute gout, acute lung injury, acute renal failure, burns, Herxheimer reaction, and SIRS associated with viral infections. The benign proliferative or fibrotic disorder is selected from the group consisting of benign soft tissue tumors, bone tumors, brain tumors, spinal cord tumors, eyelid and orbital tumors, granuloma, lipoma, meningioma, multiple endocrine neoplasia, nasal polyps, pituitary tumors, prolactinoma, pseudotumor cerebri, seborrheic keratoses, stomach polyps, thyroid nodules, cystic neoplasms of the pancreas, hemangiomas, vocal cord nodules, polyps, and cysts, chronic pilonidal disease, dermatofibroma, pilar cyst, pyogenic granuloma, juvenile polyposis syndrome, renal fibrosis, postoperative stricture, keloid formation, and cardiac fibrosis. The other diseases include operative stricture, cardiovascular disease, dyslipidemia, atherosclerosis, hypercholesterolemia, metabolic syndrome, Alzheimer's disease, Parkinson's disease, Huntington disease, bipolar disorder, schizophrenia, Rubinstein-Taybi syndrome, and epilepsy. Thus, 4-bromo-3-fluorobenzyl alc. was stirred with methanesulfonyl chloride and pyridine in CH_2Cl_2 at room temperature for 16 h to give 95% 4-bromo-3-fluorobenzyl chloride. 5-(3,5-Dimethylisoxazol-4-yl)pyridin-2(1H)-one (**III**; R = H) was stirred with 4-bromo-3-fluorobenzyl chloride in the presence of K_2CO_3 in MeCN at 70° for 16 h to give 32% **III** (R = 4-bromo-3-fluorobenzyl). **III** (R = 4-bromo-3-fluoro benzyl) was coupled with tributyl(vinyl)stannane in the presence of $Pd(PPh_3)_2Cl_2$ in DMF under nitrogen at 85° for 16 h to give 53% **II I** (R = Q^3). A solution of 0.26 mmol **III** (R = Q^3) in water and acetone was treated with 0.29 mmol N-bromosuccinimide, stirred at for 6 h, treated with 0.52 mmol NaOH at 0°, and stirred at room temperature for 16 h to give 60% **III** (R = Q^4). **III** (R = Q^4) and 2-chloro-1-[4-[[6-(3,5-dimethylisoxazol-4-yl)-2-methyl-1H-imidazo[4,5-b]pyridin-1-yl]methyl]phenyl]ethanone (**IV**) inhibited the binding of tetra-acetyiated histone H4 to individual BET Bromodomains with IC_{50} of ≤0.3μM.

Keywords: oxazolylpyridinone oxazolylbenzimidazole preparation covalent inhibitor bromodomain; heterocyclylisoxazole prepar ation covalent inhibitor bromodomain; isoxazolylimidazopyridine preparation covalent inhibitor bromodomain; autoimmune disorder inflammatory disorder cancer treatment heterocyclylisoxazole preparation; benign tumor benign proliferative disorder treatment heterocyclylisoxazole preparation; fibrotic disorder cardiovascular disease treatment heterocyclylisoxazole preparation; bromodomain extra terminal domain BET protein

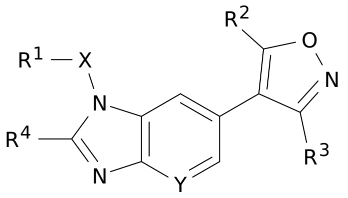

I

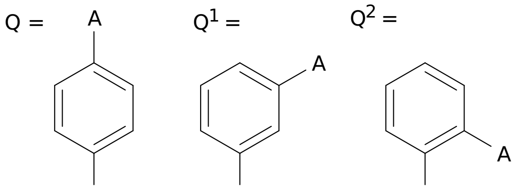

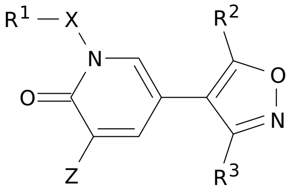

II

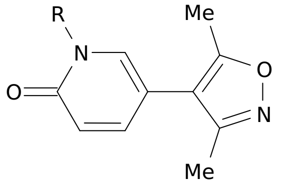

III

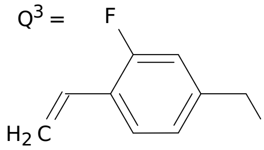

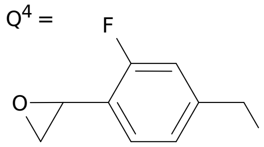

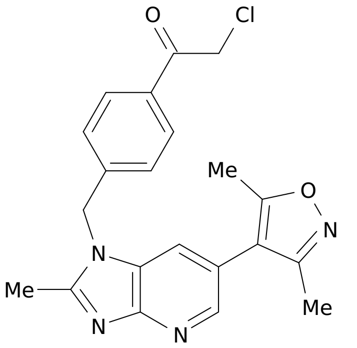

IV

PatentPak available

Substances (84) Reactions (62) Citing (3)

161

Preparation of substituted imidazolyl triazoles as BET protein inhibitors via binding to bromodomains useful in therapy

By: Brown, Samuel David; Coburn, Craig Alan; Kharenko, Olesya
World Intellectual Property Organization, WO2016092375 A1 2016-06-16 | Language: English, Database: CAplus

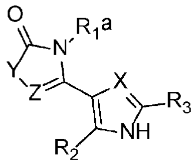

Formula I.

The invention relates to preparation of substituted imidazolyl triazoles of formula I wherein all the variables are as defined in the disclosure and pharmaceutical compositions thereof useful for the inhibition of B ET protein function by binding to bromodo mains. The example compound II was prepared by multistep synthesis (procedure shown) , was tested for inhibition of tetra- acetylated histone H4 binding individual BET bromodomains. Compounds I can be used in mono- and combin ation therapy of various diseases, like chemotherapy, immunotherapy and hormone- deprivation therapy resistant cancers and such.

Keywords: imidazolyl triazole preparation BET inhibitor anticancer antiproli ferative combination therapy

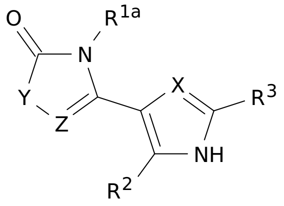

I

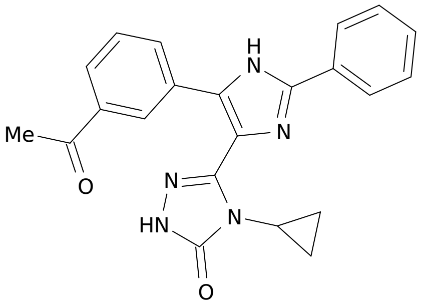

II

PatentPak available

Substances (159) Reactions (304) Citing (1)

162

Preparation of 5-(3,5-dialkylisoxazol-4-yl)pyridinones as bromodomain inhibitors

By: Jiang, May Xiaowu; Molino, Bruce Francis; Liu, Shuang; Wang, Ruifang; Duffy, Bryan Cordell; Quinn, John Frederick; Wagner, Gregory Steven

World Intellectual Property Organization, WO2016087936 A1 2016-06-09 | Language: English, Database: CAplus

The invention is related to the preparation of substituted pyridinones **I** [A = (un)substituted aryl, heteroaryl; L = CH_2, CH_2CH_2, CH (Me) optionally substituted with halogen; or L may be absent in which case A is connected to X via a covalent bond; X = O, N H; R^3, R^4 = independently C_1-C_6 alkyl optionally substituted with halogen and OH], their stereoisomers, tautomers, pharmaceutically acceptable salts, and hydrates, which are useful for inhibition of BET protein function by binding to bromodomains, pharmaceutical compositions containing them., and ther use in therapy. Thus, Pd- coupling of 3-amino-5-bromo-2-methoxypyridine with 3,5-dimethyl-4-(4,4,5,5-tetramethyl-1,3,2-dioxaborolan-2-yl)isoxazole, amination of benzyl bromide with the resulting amine and treatment of 3-(benzylamino)-5-(3,5-dimethylisoxazol-4-yl)-2-methoxypyridine with HBr in EtOH gave **II**. Pyridinone **II** inhibited the tetracetylated histone H4 binding to Brd4 bromodomain 1, the cell proliferation in human AML MV-4-11 cells and the hIL-6 and hIL-17 mRNA transcription.

Keywords: isoxazolylpyridinone preparation bromodomain inhibitor

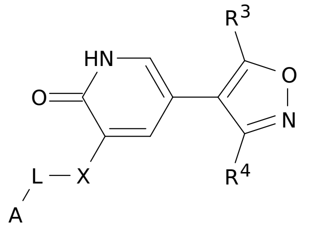

I

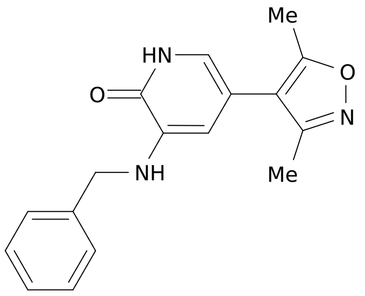

II

PatentPak available

Substances (136) Reactions (96) Citing (2)

163

Preparation of 5-(3,5-dialkylisoxazol-4-yl)pyridines as bromodomain inhibitors

By: Jiang, May Xiaowu; Molino, Bruce Francis; Liu, Shuang; Wang, Ruifang; Duffy, Bryan Cordell; Quinn, John Frederick; Wagner, Gregory Steven

World Intellectual Property Organization, WO2016087942 A1 2016-06-09 | Language: English, Database: CAplus

The invention is related to the preparation of substituted pyridinones **I** [A = (un)substituted aryl, heteroaryl; B = C_1-C_6 alkyl, benzyl, Ph optionally substituted with halogen; L = CH_2, CH(Me) optionally substituted with halogen; or L may be absent in which case A is connected to X via a covalent bond; X = O, NH; Y = O, NHMe, meaning if Y = NHMe then B is absent; R^3, R^4 = independently C_1-C_6 alkyl optionally substituted with halogen and OH], their stereoisomers, tautomers, pharmaceutically acceptable salts, and hydrates, which are useful for inhibition of BET protein function by binding to bromodomains, pharmaceutical compositions containing them., and ther use in therapy. Thus, Pd-coupling of 3-amino-5-bromo-2-methoxypyridine with 3,5-dimethyl-4-(4,4,5,5-tetramethyl-1,3,2-dioxaborolan-2-yl)isoxazole and amination of benzyl bromide with the 5-(3,5-dimethylisoxazol-4-yl)-2-methoxypyridin-3-amine gave **II**. Pyridine **II** inhibited the tetracetylated histone H4 binding to Brd4 bromodomain 1, the cell proliferation in human AML MV-4-11 cells and the hIL-6 and hIL-17 mRNA transcription.

Keywords: isoxazolylpyridine preparation bromodomain inhibitor

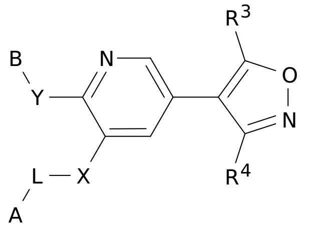

I

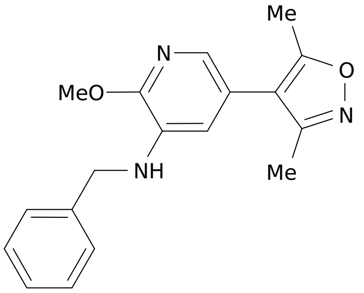

II

PatentPak available

Substances (190) Reactions (169) Citing (3)

164

Preclinical testing of selective Aurora kinase inhibitors on a medullary thyroid carcinoma-derived cell line

By: Tuccilli, Chiara; Baldini, Enke; Prinzi, Natalie; Morrone, Stefania; Sorrenti, Salvatore; Filippini, Angelo; Catania, Antonio; Alessandrini, Stefania; Rendina, Roberta; Coccaro, Carmela; et al
Endocrine (2016), 52(2), 287-295 | Language: English, Database: CAplus and MEDLINE

Deregulated expression of the Aurora kinases (Aurora-A, B, and C) is thought to be involved in cell malignant transfo rmation and genomic instability in several cancer types. Over the last decade, a number of small- mol. inhibitors of Aurora kinases have been developed, which have proved to efficiently restrain malignant cell growth and tumorige nicity. Regarding medullary thyroid carcinoma (MTC), we previously showed the efficacy of a pan-Aurora kinase inhibitor (MK-0457) in impairing growth and survival of the MTC-derived cell line TT. In the present study, we sought to establish if one of the Aurora kinases might represent a prefer ential target for MTC therapy. The effects of selective inhibitors of Aurora-A (MLN8237) and Aurora-B (AZD1152) were analyzed on TT cell proliferation, apoptosis, cell cycle, and ploidy. The two inhibitors reduced T T cell proliferation in a time- and dose-dependent manner, with IC_{50} of 19.0 ± 2.4 n M for M LN8237 and 401.6 ± 44.1 n M for AZD1152. Immunofluorescence experiments confirmed that AZD1152 inhibited phosphorylation of histone H3 (Ser10) by Aurora- B, while it did not affect Aurora-A autophosphorylation. ML N8237 inhibited Aurora-A autophosphorylation as expected, but at concent rations required to achieve the maximum antiproli ferative effects it also abolished H3 (Ser10) phosphor ylation. Cytofluo rimetry experiments showed that both inhibitors induced accumulation of cells in G2/M phase and increased the sub G0/G1 fraction and polypl oidy. Finally, both inhibitors triggered apoptosis. We demonstrated that inhibition of either Aurora-A or Aurora-B has antiproli ferative effects on TT cells, and thus it would be worthwhile to further investigate the therapeutical potential of Aurora kinase inhibitors in M TC treatment.

Keywords: MLN8237 AZD1152 aurora kinase inhibitor anticancer medullary thyroid carcinoma; A ZD1152; Aurora kinase inhibitors; Cell cycle; MLN8237; Medullary thyroid cancer; TT cell line

Substances (4)   Reactions (0)   Citing (6)

165

Irisin-related cancer treatments

By: Trujillo, Kristina; Gannon, Nicholas Patrick; Vaughan, Roger Alan
World Intellectual Property Organization, WO2016081603 A1 2016-05-26 | Language: English, Database: CAplus

In one embodiment, the invention provides methods of treating or preventing cancer, in particular aspects breast and/or prostate cancer, by administering to a subject in need thereof a therapeu tically-effective amount of irisin and, option ally, one or more adjuvant therapies (e.g. synergistic co-administration of an addnl. anti-cancer agent or chemotherapy). Related pharmaceutical compositions, assays and kits are also provided.

Keywords: irisin cancer FNDC5 gene theapy

PatentPak available

Substances (316)   Reactions (0)   Citing (1)

166

A novel in situ hydrophobic ion paring (HIP) formulation strategy for clinical product selection of a nanoparticle drug delivery system

By: Song, Young Ho; Shin, Eyoung; Wang, Hong; Nolan, Jim; Low, Susan; Parsons, Donald; Zale, Stephen; Ashton, Susan; Ashford, Marianne; Ali, Mir; et al
Journal of Controlled Release (2016), 229, 106-119 | Language: English, Database: CAplus and MEDLINE

The present studies were aimed at formulating AZD2811-loaded polylactic acid-polyethylene glycol (PLA-PEG) nanoparticles with adjustable release rates without altering the chem. structures of the polymer or active pharmaceutical ingredient (API). This was accomplished through the use of a hydrophobic ion pairing approach. A series of AZD2811-containing nanoparticles with a variety of hydrophobic counterions including oleic acid, 1-hydroxy-2-naphthoic acid, cholic acid, deoxycholic acid, dioctylsulfosuccinic acid, and pamoic acid is described. The hydrophobicity of AZD2811 was increased through formation of ion pairs with these hydrophobic counterions, producing nanoparticles with exceptionally high drug loading- up to five fold higher encapsulation efficiency and drug loading compared to nanoparticles made without hydrophobic ion pairs. Furthermore, the rate at which the drug was released from the nanoparticles could be controlled by employing counterions with various hydrophobicities and structures, resulting in release half-lives ranging from about 2 to 120 h using the same polymer, nanoparticle size, and nanoemulsion process. Process recipe variables affecting drug load and release rate were identified, including pH and molarity of quench buffer. Ion pair formation between AZD2811 and pamoic acid as a model counterion was investigated using solubility enhancement as well as NMR spectroscopy to demonstrate solution-state interactions. Further evidence for an ion pairing mechanism of controlled release was provided through the measurement of API and counterion release profiles using high-performance liquid chromatog., which had stoichiometric relationships. Finally, Raman spectra of an AZD2811-pamoate salt compared well with those of the formulated nanoparticles, while single components (AZD2811, pamoic acid) alone did not. A library of AZD2811 batches was created for anal. and preclin. characterization. Dramatically improved preclin. efficacy and tolerability data were generated for the pamoic acid lead formulation, which has been selected for evaluation in a Phase 1 clin. trial (Clinical Trials.gov Identifier NCT 02579226). This work clearly demonstrates the importance of assessing a wide range of drug release rates during formulation screening as a critical step for new drug product development, and how utilizing hydrophobic ion pairing enabled this promising nanoparticle formulation to proceed into clin. development.

Keywords: hydrophobic ion pairing polylactic acid polyethylene glycol nanoparticle; Adjustable release rate; Counterions; Drug release kinetics; In situ hydrophobic ion paring; Nanoparticles; PLA–PEG

Substances (18) Reactions (0) Citing (49)

167

IL15 Agonists Overcome the Immunosuppressive Effects of MEK Inhibitors

By: Allegrezza, Michael J.; Rutkowski, Melanie R.; Stephen, Tom L.; Svoronos, Nikolaos; Tesone, Amelia J.; Perales-Puchalt, Alfredo; Nguyen, Jenny M.; Sarmin, Fahmida; Sheen, Mee R.; Jeng, Emily K.; et al
Cancer Research (2016), 76(9), 2561-2572 | Language: English, Database: CAplus and MEDLINE

Many signal transduction inhibitors are being developed for cancer therapy target pathways that are also important for the proper function of antitumor lymphocytes, possibly weakening their therapeutic effects. Here we show that most inhibitors targeting multiple signaling pathways have especially strong neg. effects on T-cell activation at their active doses on cancer cells. In particular, we found that recently approved MEK inhibitors displayed potent suppressive effects on T cells in vitro. However, these effects could be attenuated by certain cytokines that can be administered to cancer patients. Among them, clin. available IL15 superagonists, which can activate PI3K selectively in T lymphocytes, synergized with MEK inhibitors in vivo to elicit potent and durable antitumor responses, including by a vaccine-like effect that generated resistance to tumor rechallenge. Our work identifies a clin. actionable approach to overcome the T-cell-suppressive effects of MEK inhibitors and illustrates how to reconcile the deficiencies of signal transduction inhibitors, which impede desired immunol. effects in vivo.

Keywords: T cell MEK inhibitor IL15 agonist immunosuppression

Substances (58) Reactions (0) Citing (22)

168

Docosahexaenoic acid sensitizes leukemia lymphocytes to barasertib and everolimus by ROS-dependent mechanism without affecting the level of ROS and viability of normal lymphocytes

By: Zhelev, Zhivko; Ivanova, Donika; Lazarova, Desislava; Aoki, Ichio; Bakalova, Rumiana; Saga, Tsuneo
Anticancer Research (2016), 36(4), 1673-1682 | Language: English, Database: CAplus and MEDLINE

The aim of the present study was: (i) to investigate the possibility of sensitizing leukemia lymphocytes to anticancer drugs using docosahexaenoic acid (DHA); (ii) to find combinations with synergistic cytotoxic effect on leukemia lymphocytes, without or with only very low cytotoxicity towards normal lymphocytes; (iii) and to clarify the role of reactive oxygen species (R OS) in the induction of apoptosis and cytotoxicity by such combinations. The study covered 15 anticancer drugs, conven tional and new-generation. Well-expressed synergistic cytotoxic effects were observed after treatment of leukemia lympho cytes (Jurkat) with DHA in combination with: barasertib, lonafarnib, everolimus, and palbociclib. We selected two synerg istic combinations, DHA with everolimus or barase rtib, and investigated their effects on viability of normal lymphocytes, as well as on the production of R OS and induction of apoptosis in both cell lines (leukemia and normal). At the selected concentrations, DHA, everolimus and barasertib (applied sep.) were cytotoxic towards leukemia lymphocytes, but not normal lympho cytes. In leukemia cells, the cytotoxicity of combinations was accompanied by strong induction of apoptosis and production of R OS. In normal lymphocytes, drugs alone and in combin ation with DHA did not affect the level of R OS and did not induce apoptosis. To our knowledge, the present study is the first to report synerg istic ROS-dependent cytotoxicity between DHA and new-generation anticancer drugs, such as everolimus and barase rtib, that is cancer cell-specific (particularly for acute lymphob lastic leukemia cells Jurkat). These combin ations are harmless to normal lympho cytes and do not induce abnormal production of R OS in these cells. The data suggest that D HA could be used as a supplem entary component in anticancer chemotherapy, allowing therapeutic doses of everolimus and barasertib to be reduced, minimizing their side-effects.

Keywords: leukemia lymphocyte docosahexaenoic acid barasertib everolimus R OS cell viability; D HA; Docosahexaenoic acid; barase rtib; everolimus; leukemia lymphocytes; reactive oxygen species

Substances (12) Reactions (0) Citing (18)

169

Screening methods for identifying compounds for cancer cell reprogramming and therapeutic uses thereof in the treatment of cancer

By: Chigaev, Alexandre; Sklar, Larry A.; Perez, Dominique
United States, US9314460 B1 2016-04-19 | Language: English, Database: CAplus

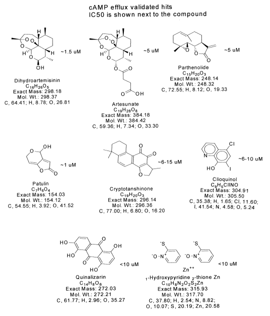

In one embodiment, the invention provides a method of inhibiting c AMP efflux and increasing intrace llular cAMP in a subject who suffers from, or who is at risk of developing, a cancer by adminis tering to the subject a therapeu tically-effective amount of a c AMP efflux inhibitor. Novel compounds, pharmaceutical compositions, diagnostics and screening methods are also provided.

Keywords: anticancer drug screening cancer cell apoptosis neoplasm treatment

PatentPak available

Substances (313) Reactions (0) Citing (2)

170

Map kinase activated protein kinase 2 (MK2) inhibitors in the treatment of cancer

By: Beswick, Ellen Janine; Morris, Katherine Theresa
World Intellectual Property Organization, WO2016032882 A1 2016-03-03 | Language: English, Database: CAplus

The present invention relates to the discovery that Map kinase activated protein kinase 2 (MK2) is highly expressed in cancer tissue and the inhibition of MK2 using an MK2 inhibitor (BIO-475863, PF-364402), represents a viable approach to the treatment of cancer, including drug resistant cancers, metastatic cancers and recurrent cancers. MK2 inhibitors as described herein may be used alone or in combination with an at least one addnl. anti- cancer agent for the treatment of cancer.

Keywords: antitumor Map kinase inhibitor combination chemotherapy cancer treatment

PatentPak available

Substances (311) | Reactions (0) | Citing (0)

171

Imide-based modulators of proteolysis and associated methods of use

By: Crew, Andrew P.; Crews, Craig; Dong, Hanqing; Wang, Jing; Qian, Yimin; Siu, Kam; Jin, Meizhong
United States, US20160058872 A1 2016-03-03 | Language: English, Database: CAplus

The description relates to imide-based compounds, including bifunctional compounds comprising the same, which find utility as modulators of targeted ubiquitination, especially inhibitors of a variety of polypeptides and other proteins which are degraded and/or otherwise inhibited by bifunctional compounds according to the present invention. In particular, the description provides compounds, which contain on one end a ligand which binds to the cereblon E3 ubiquitin ligase and on the other end a moiety which binds a target protein such that the target protein is placed in proximity to the ubiquitin ligase to effect degradation (and inhibition) of that protein. Compounds can be synthesized that exhibit a broad range of pharmacol. activities consistent with the degradation/inhibition of targeted polypeptides of nearly any type.

Keywords: antitumor proteolysis modulator imide preparation cancer

PatentPak available

Substances (425) | Reactions (330) | Citing (3)

172

Bromodomain inhibitors for treating disease

By: Palmer, Wylie; Jones, Philip; Liu, Gang; Petrocchi, Alessia; Reyna, Naphtali; Subrumanian, Govindan; Theroff, Jay; Yau, Anne
World Intellectual Property Organization, WO2016033416 A1 2016-03-03 | Language: English, Database: CAplus

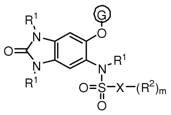

Disclosed herein are compounds and compositions useful in the treatment of bromod omain-containing protein-mediated diseases, such as cancer, and Methods of inhibiting activity of a bromodomain-containing protein in a human or animal subject are also provided.

Keywords: bromodomain inhibitor preparation cancer inflammation autoimmune CNS disease

PatentPak available   Substances (577)   Reactions (2,121)   Citing (1)

173

Anti-LAG3 antibodies and antigen-binding fragments

By: Liang, Linda; Fayadat-Dilman, Laurence; Malefyt, Rene de Waal; Raghunathan, Gopalan
World Intellectual Property Organization, WO2016028672 A1 2016-02-25 | Language: English, Database: CAplus

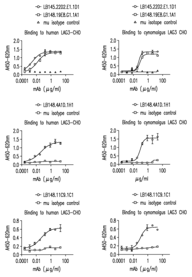
FIG.1

The present invention includes antibodies and antigen-binding fragments thereof that specifically bind to human or Cynomolgous monkey LAG3 as well as Ig chains thereof and polynucl eotides encoding the same along with injection devices comprising such antibodies or fragments. Vaccines including such antibodies and fragments as well as compositions comprising the antibodies and fragments (e.g., including anti-PD1 antibodies) are included in the invention. Methods for treating or preventing cancer or infection using such compositions are also provided. In addition, methods for recomb inant expression of the antibodies and fragments are part of the present invention.

Keywords: human LAG3 monoclonal antibody sequence humanized variant immunot herapy cancer

PatentPak available   Substances (595)   Reactions (0)   Citing (7)

174

Preparation of asymmetric bisaminoquinolines and bisaminoquinolines with varied linkers as autophagy inhibitors for cancer and other therapy

By: Amaravadi, Ravi K.; Winkler, Jeffrey

World Intellectual Property Organization, WO2016022956 A1 2016-02-11 | Language: English, Database: CAplus

The invention provides novel asym. and sym. bisaminoquinolmes and related compounds **I** [L = $(CH_2)_pN(R'')(CH_2)_p$, $(CH_2)_pN(R'')(CH_2)_{p1}N(R'')(CH_2)_p$, $(CH_2)_pO(CH_2)_p$, L^{1D}, or each (un)substituted $(CH_2Y)_n$-X-$(Y^1CH_2)_n$, or $A(CH_2CH_2\text{-}Z)_n\text{-}A^1$; R^1, R^1 = independently H, F Cl, Br, iodo, cyano, NO_2, or each (un)substituted C_{1-6} alkyl, O-C_{1-6} alkyl, C_{2-7} acyl, NH-acyl, or C_2-C_7 carboxy ester; R, R' = indepen dently H or each (un)substituted C_{1-6} alkyl, C_{1-7} acyl, or C_{2-7} carboxy ester (which forms a urethane group with the nitrogen atom to which R or R' is bonded); R'' = F, each (un) substituted cycloalkyl, aryl or heteroaryl, or $(C:O)_z$-G; G = each (un)substituted CO-C_{1-2} alkyl, alkene, or alkynyl; z = an integer of 0-5; R^M = indepen dently H, F, Cl, Br, iodo, (un) substituted C_{1-12} alkyl, alkene, alkynyl or alkoxy group; p, p1 = an integer of 1-10; L^{1D} = (un)substituted C_{1-20} alkylene; X = absent, $(CH_2)_j$, O, S, or (un)substituted NH; Y, Y^1 = indepen dently absent, CH_2, O, O, CHO, or (un)substituted NH; j = an integer of 1- 6; n = indepen dently an integer of 0- 10; Z = O or (un) substituted NH; A, A^1 = independently C(O) or (un)substituted $(CH_2)_j$] or pharmace utically acceptable salts, enanti omers,s diastereomers, solvates, or polymorphs thereof. The novel compounds are autophagy inhibitors and are useful in the treatment of a variety of autophagy-related disorders, in particular (a) cancer including metastatic cancer, recurrent cancer, and drug- resistant cancer and (b) rheumatoid arthritis, malaria, antiphospholipid antibody syndrome, lupus, chronic urticaria or Sjogren's disease. The cancer is (1) carcinoma, (2) cancer of the esophagus, head, kidney, liver, lung, nasopharyngeal, neck, ovary, pancreas, prostate, and stomach, (3) a leukemia, a malignant lymphoma, a malignant melanoma, (4) myeloproliferative diseases, or (5) a sarcoma, a tumor of the central nervous system, a germ-line tumor, lung cancer, ovarian cancer, testicular cancer, thyroid cancer, astroc ytoma, esophageal cancer, pancreatic cancer, stomach cancer, liver cancer, colon cancer, melanoma, or a mixed type of neoplasia. Thus, to a microwave vial was added spermine 0.57, 4-bromo-7-chloroquinoline 1.32, BINAP 0.11, $Pd(OAc)_2$ 0.02, and K_3PO_4 1.80 g. The vial was sealed and placed under argon, followed by adding a solvent mixture of dioxane and water (10:1, 9 mL), and the resulting mixture was heated to 120° for 16 h to give, after workup, 73% N1,N1'-(butane-1,4-diyl)bis[N3-(7-chloroquinolin-4-yl)propane-1,3-diamine] (**II**). The compounds **I** were tested for anticancer activi ties. The increased cytotoxicity of longer linker and assyemtric compounds was confirmed in a 2 wk colony formation assay using 5 different cancer cell lines including A375, WM983B, CAPAN1, OANC1, and SKME L5 cells. Longer linker compounds localized to the lysosome. Longer linker compounds were associated with more profound autophagy modulation, reactive oxygen species (R OS) and apoptosis. Dimeric chloroqu inolines with longer linkers impaired growth and invasion of melanoma in a 3 dimensional tissue like culture system.

Keywords: asym bisaminoquinoline preparation autophagy inhibitor treatment cancer; drug resistant cancer rheumatoid arthritis treatment asym bisaminoquinoline preparation; malaria treatment asym bisaminoq uinoline preparation; antiphospholipid antibody syndrome treatment asym bisaminoquinoline preparation; lupus chronic urticaria Sjogren disease treatment asym bisaminoquinoline preparation

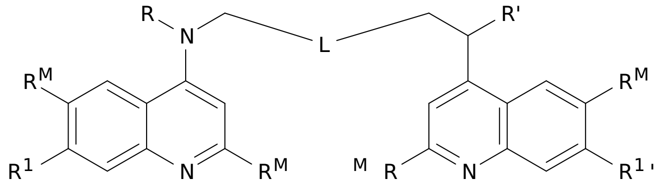

I

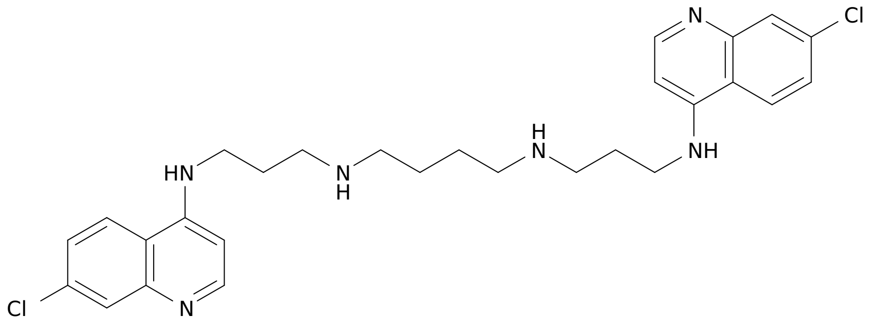

II

PatentPak available

Substances (418) Reactions (245) Citing (1)

175

Are Accurins the cure for Aurora kinase inhibitors?

By: Bearss, David J.
Science Translational Medicine (2016), 8(325), 325fs4/1-325fs4/3 | Language: English, Database: CAplus and MEDLINE

A review. A nanoparticle formulation of an Aurora B inhibitor increases antitumor efficacy and reduces toxicity, which may be a precedent for the use of this technol. with other small mols. (Ashton et al., this issue).

Keywords: review Accurins Aurora kinase inhibitor antitumor agent cancer

Substances (2) Reactions (0) Citing (3)

176

Compounds useful for promoting protein degradation and methods using same

By: Crews, Craig M.; Gustafson, Jeffrey; Roth, Anke Gundula; Tae, Hyun Seop; Buckley, Dennis; Neklesa, Taavi
United States, US20160022642 A1 2016-01-28 | Language: English, Database: CAplus

The present description includes compounds that act as degraders of a target protein, wherein degrad ation is independent of the class of the target protein or its localization. In certain embodiments, the description includes a compound comprising a protein degradation moiety covalently bound to a linker, wherein the Clog P of the compound is equal to or higher than 1.5. The target protein contemplated within the description comprises an androgen receptor. Compounds of the present descri ption may be used to treat disease states wherein protein degradation is a viable therapeutic approach, such as cancer or any sort of oxidative stress disease state.

Keywords: protein degradation promoter preparation androgen receptor cancer oxidative stress

PatentPak available

Substances (439) Reactions (226) Citing (1)

177

2-deoxy-D-glucose sensitizes cancer cells to barasertib and everolimus by ROS-independent mechanism(s)

By: Zhelev, Zhivko; Ivanova, Donika; Aoki, Ichjo; Saga, Tsuneo; Bakalova, Tana
Anticancer Research (2015), 35(12), 6623-6632 | Language: English, Database: CAplus and MEDLINE

The aim of the present study was to investigate: (i) the possibility of sensitizing cancer cells to anticancer drugs using the redox modulator 2-deoxy-D-glucose (2- DDG); (ii) to find such combinations with synergistic cytotoxic effect,- (iii) and to clarify the role of reactive oxygen species (ROS) for induction of apoptosis and cytoto xicity through these combinations. The study covers 15 anticancer drugs - both conventional and new-generation. Four parameters were analyzed simulta neously in Jurkat leukemia treated by drugs or 2-DUG (sep. or in combin ation): cell viability, induction of apoptosis, levels of R OS, and level of protein-carbonyl products.

Keywords: leukemia cell 2 deoxy glucose barasertib everolimus sensitivity ROS; 2-deoxy-D-glucose; Anticancer drugs; apoptosis; reactive oxygen species; synergism

Substances (22) Reactions (0) Citing (10)

178

Selective inhibitors of aurora kinases inhibit proliferation, reduce cell viability and impair cell cycle progression in papillary thyroid carcinoma cells

By: Baldini, E.; Tuccilli, C.; Prinzi, N.; Sorrenti, S.; Antonelli, A.; Fallahi, P.; Mian, C.; Barollo, S.; Catania, A.; Morrone, S.; et al
Journal of Biological Regulators and Homeostatic Agents (2015), 29(4), 793-803 | Language: English, Database: CAplus and MEDLINE

The three members of the Aurora kinase family, Aurora-A, -B and -C, regulate several aspects of the mitotic process, and their aberrant expression and/or function causes mitotic abnormalities leading either to cell death or aneupl oidy. They are found overexpressed in several human malignancies, including the papillary thyroid carcinoma (P TC). In the present study, we sought to establish whether Aurora kinase inhibition could be of any therapeutic value in the treatment of aggressive forms of P TC, enduring to radioactive iodide (RAI) ablation. To this end, the effects of selective inhibitors of Aurora- A (MLN8237) and Aurora- B (AZD1152) were analyzed on 3 human PTC cell lines expressing either wild-type (K1 and TPC1) or mutant p53 (B CPAP). The two inhibitors were capable of reducing cell proliferation in a time- and dose- dependent manner, with IC comprised between 65.4 and 114.9 n M for M L N8237, and between 26.6 and 484.6 n M for AZD1152. Immunofluorescence experiments confirmed that AZD1152 inhibited Aurora-B phosphorylation of histone H3 on Ser10, however, it did not affect Aurora- A autophosphorylation. MLN8237 inhibited Aurora- A autophosphorylation as expected, but at concent rations required to achieve the maximum antiproli ferative effects it also abolished H3 (Ser10) phosphorylation. Time-lapse videomicroscopy evidenced that both inhibitors prevented the completion of cytoki nesis, and cytofluorimetric anal. showed accumulation of cells in G2/M phase and/or polypl oidy. Apoptosis was induced in all the cells by both inhibitors independently from the p53 status. In conclu sion, in the present preclin. study M LN8237 and AZD1152 have emerged as promising drug candidates for RAI-insensitive PTC.

Keywords: PTC cell prolife ration viability cycle progression aurora kinase inhibitor

Substances (7) | Reactions (0) | Citing (7)

179

Phosphoproteomic analysis of Aurora kinase inhibition in monopolar cytokinesis [Erratum to document cited in CA163:327364]

By: Polat, Ayse Nur; Karayel, Ozge; Giese, Sven H.; Harmanda, Busra; Sanal, Erdem; Hu, Chi-Kuo; Renard, Bernhard Y.; Ozlu, Nurhan
Journal of Proteome Research (2015), 14(12), 5408 | Language: English, Database: CAplus and MEDLINE

Supplementary Figures 1-3 were omitted from the Supporting Information file; the corrected file is available online.

Keywords: erratum human Aurora kinase cytokinesis phosphoproteome target; phosphopeptide intermediate filament erratum

Substances (3) | Reactions (0) | Citing (0)

180

Management and novel drugs for older patients with acute myeloid leukemia

By: Yoshimoto, Goichi; Miyamoto, Toshihiro
Ketsueki Naika (2015), 71(5), 666-674 | Language: Japanese, Database: CAplus

A review. This article discusses on management and novel drugs for older patients with acute myeloid leukemia.

Keywords: human acute myeloid leukemia chemotherapy review

Substances (14) | Reactions (0) | Citing (0)

181

Sarcoma Cell Line Screen of Oncology Drugs and Investigational Agents Identifies Patterns Associated with Gene and microRNA Expression

By: Teicher, Beverly A.; Polley, Eric; Kunkel, Mark; Evans, David; Silvers, Thomas; Delosh, Rene; Laudeman, Julie; Ogle, Chad; Reinhart, Russell; Selby, Michael; et al
Molecular Cancer Therapeutics (2015), 14(11), 2452-2462 | Language: English, Database: CAplus and MEDLINE

The diversity in sarcoma phenotype and genotype make treatment of this family of diseases exceptionally challenging. Sixty-three human adult and pediatric sarcoma lines were screened with 100 FDA-approved oncol. agents and 345 investig ational agents. The investigational agents' library enabled comparison of several compounds targeting the same mol. entity allowing comparison of target specificity and heterogeneity of cell line response. Gene expression was derived from exon array data and micro RNA expression was derived from direct digital detection assays. The compounds were screened against each cell line at nine concent rations in triplicate with an exposure time of 96 h using Alamar blue as the endpoint. Results are presented for inhibitors of the following targets: aurora kinase, IGF-1R, MEK, BET bromodomain, and PARP1. Chem. structures, IC_{50} heat maps, concentration response curves, gene expression, and mi R expression heat maps are presented for selected examples. In addition, two cases of exceptional responders are presented. The drug and compound response, gene expres sion, and microRNA expression data are publicly available at http://sarcoma.cancer.gov. These data provide a unique resource to the cancer research community. Mol Cancer Ther; 14(11); 2452-62. ©2015 AACR.

Keywords: sarcoma cell IGF1R MEK1 PARP1 microRNA oncol drug

Substances (26) Reactions (0) Citing (49)

182

Gonadotropin-releasing hormone receptor-targeting peptides and their use to treat and diagnose cancer

By: Miao, Yubin
United States, US9180214 B1 2015-11-10 | Language: English, Database: CAplus

The present invention is directed to novel non-invasive diagnostic tools/compounds to image and treat cancers, especially cancers which overexpress GnRH such as breast cancer, prostate cancer and melanoma, among others, including metastatic breast cancer, prostate cancer and/or melanoma among numerous others in vivo. The novel imaging probes are capable of detecting cancerous cells, as well as their metastatic spread in tissues. The novel probes of the present invention will also be useful to initiate therapy for breast and/or prostate cancer, among other cancers, as well as monitor patients' response to chemotherapy treatments and other interventions or therapies used in the treatment of cancer, including metastatic cancer as otherwise described herein. Compounds according to the present invention may be used as diagnostic tools for diagnosing cancer as well as therapeutic agents for treating cancer and related secondary disease states and conditions.

Keywords: GnRh receptor targeting peptide cancer therapy diagnosis

PatentPak available

Substances (283) Reactions (0) Citing (0)

183

Combination therapies targeting mitochondria for cancer therapy

By: Altieri, Dario C.; Ghosh, Jagadish C.
World Intellectual Property Organization, WO2015168599 A1 2015-11-05 | Language: English, Database: CAplus

Fig 1A

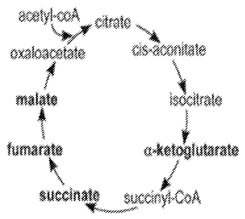

Pharmaceutical compositions for the treatment of cancer are provided. In one embodiment the compos ition comprises gamitrinib and a phosphatidylinositol 3-kinase (PI3K) inhibitor selected from PX-866, AZD6482, LY294002, BEZ235, GSK458, GDC0941, ZSTK474, BKM120, and GSK2636771B. Methods of treating cancer are also provided. The present invention is based in part on the inventor's discovery that combination therapy that targets mitochondria eliminates tumor adaptation induced by P13 K inhibition, and improves clin. outcome in cancer.

Keywords: antitumor gamitrinib BEZ235 combination therapy targeting mitochondria cancer treatment

PatentPak available

Substances (91) Reactions (0) Citing (0)

184

Methods for treating cancer using TOR kinase inhibitor combination therapy

By: Fultz, Kimberly Elizabeth; Tran, Tam Minh; Xu, Shuichan; Xu, Weiming; Trowe, Torsten; Filvaroff, Ellen
World Intellectual Property Organization, WO2015160868 A1 2015-10-22 | Language: English, Database: CAplus

Provided are methods for treating or preventing cancer, comprising administering an effective amount of a 3,4-dihydropyrazino[2, 3-b]pyrazin-2(1H)-one TOR kinase inhibitor and an effective amount of a second active agent to a patient. Cancers treated by the method include solid tumors of the head, neck, breast, lung, ovaries, colon, rectum, stomach, prostate, urinary bladder, uterine, cervix, and other reproductive organs, wherein the tumors include relapsed, refractory or advanced solid tumors.

Keywords: pyrazinopyrazine TOR kinase inhibitor combin ation therapy cancer

PatentPak available

Substances (217) Reactions (0) Citing (0)

185

Quinazoline-benzimidazole hybrid as dual optical sensor for cyanide and Pb $^{2+}$ ions and Aurora kinase inhibitor

By: Luxami, Vijay ; Rani, Richa; Sharma, Alka; Paul, Kamaldeep
Journal of Photochemistry and Photobiology, A: Chemistry (2015), 311, 68-75 | Language: English, Database: CAplus

Compounds 1 and 2 exhibited selective fluorescence response toward Pb^{2+} over the other metal ions like Zn^{2+}, Co^{2+}, Ca^{2+}, K^{+}, Ni^{2+}, Mn^{2+}, Fe^{2+}, Hg^{2+}, Na^{+}, etc. Compound 1 behaved as cyanide selective chromofluorescent sensor amongst other anions like F^{-}, Cl^{-}, Br^{-}, I^{-}, AcO^{-}, $H_2PO^{-}{}_4$, $HSO^{-}{}_4$, etc. The binding properties of compounds 1 and 2 with cyanide and Pb $^{2+}$ were investigated via UV-vis, fluorescence spectroscopy, ^{1}H NMR titration experiment and DFT calculations Compound 1 has been used to estimate CN^{-} ion (2-500μM) and compound 2 used to estimate Pb $^{2+}$ ion (0.05-1500μM) ratiometrically. Compounds 1 and 2 have also been used as Aurora kinase inhibitors with IC_{50} values of 7.50μM and 0.53μM, resp.

Keywords: cyanide lead quinazoline benzimidazole hybrid Aurora kinase optical sensor

Substances (19) Reactions (0) Citing (22)

186

Target inhibition map system for combination antitumor therapy design and methods of using same

By: Pal, Ranadip; Keller, Charles, III; Berlow, Noah E.; Seguin, Bernard
United States, US20150269307 A1 2015-09-24 | Language: English, Database: CAplus

Disclosed is a system and method for targeted therapy designs which model a cancer pathway for predicting the effectiveness of targeted anti-cancer drugs. The disclosed system and method includes utilization of a computer processor allowing for the selection of a set of drugs from available drugs using approximation algorithms utilizing cell viability data associated with a testable culture of a patient's tumor for generating a probabilistic target inhibition map (PTIM) from viability data for considering the selection of a set of drugs from available drugs, and further supports a wide variety of scenarios for personalized cancer therapy, related products and services.

Keywords: antitumor combination therapy design target map system algorithm modeling

PatentPak available

Substances (79) Reactions (0) Citing (0)

187

Phosphoproteomic analysis of Aurora kinase inhibition in monopolar cytokinesis

By: Polat, Ayse Nur; Karayel, Ozge; Giese, Sven H.; Harmanda, Busra; Sanal, Erdem; Hu, Chi-Kuo; Renard, Bernhard Y.; Ozlu, Nurhan
Journal of Proteome Research (2015), 14(9), 4087-4098 | Language: English, Database: CAplus and MEDLINE

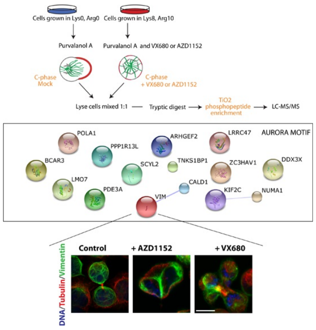

Cytokinesis is the last step of the cell cycle that requires coordi nated activities of the microtubule cytoskeleton, actin cytoskeleton, and membrane compartments. Aurora B kinase is one of the master regulatory kinases that orches trate multiple events during cytokinesis. To reveal targets of the Aurora B kinase, we combined quant. mass spectr ometry with chem. genetics. Using the quant. proteomic approach, SILAC (stable isotope labeling with amino acids in cell culture), we analyzed the phosphoproteome of monopolar cytokinesis upon VX680- or AZD1152-mediated aurora kinase inhibition. In total, our anal. quantified over 20 000 phosphopeptides in response to the Aurora-B kinase inhibition; 246 unique phosphopeptides were significantly down-regulated and 74 were up-regulated. Our data provide a broad anal. of downstream effectors of Aurora kinase and offer insights into how Aurora kinase regulates cytokinesis.

Keywords: human Aurora kinase cytokinesis phosphoproteome target phosphopeptide intermediate filament

Substances (3) Reactions (0) Citing (9)

188

Exosomes as a therapeutic for cancer

By: Trujillo, Kristina
World Intellectual Property Organization, WO2015120150 A1 2015-08-13 | Language: English, Database: CAplus

In one embodiment, the invention provides substantially purified exosomes which induce apoptosis in breast cancer cells and which are derived from a cultured medium of histol. normal breast tissue cells that are obtained from tumor-adjacent normal breast tissue. Related methods of treating breast cancer and pharmaceutical formulations are also provided.

Keywords: breast cancer antitumor autologous purified exosomes iv injection

PatentPak available

Substances (284) Reactions (0) Citing (4)

189

Therapeutic combinations for treating cancer comprising an irreversible mutant EGFR inhibitor and an antineoplastic agent such as an Aurora kinase inhibitor

By: Simmmons, Andrew D.; Harding, Thomas Christian

World Intellectual Property Organization, WO2015112705 A2 2015-07-30 | Language: English, Database: CAplus

The present invention relates to combination treatment methods for treating cancer, partic ularly non-small cell lung cancer (N SCLC) . More specifi cally, the invention relates to methods of treating or managing cancer, partic ularly NSCLC, in a subject, comprising administering an irreversible mutant E GFR inhibitor compound in combin ation with one or more antineo plastic agents, particularly an Aurora kinase inhibitor.

Keywords: cancer treatment EGFR inhibitor Aurora kinase inhibitor combin ation

PatentPak available

Substances (46) Reactions (0) Citing (3)

190

Discovery of N-(4-(3-(2-Aminopyrimidin-4-yl)pyridin-2-yloxy)phenyl)-4-(4-methylthiophen-2-yl)phthalazin-1-amine (AMG 900), A Highly Selective, Orally Bioavailable Inhibitor of Aurora Kinases with Activity against Multidrug-Resistant Cancer Cell Lines

By: Geuns-Meyer, Stephanie; Cee, Victor J.; Deak, Holly L.; Du, Bingfan; Hodous, Brian L.; Nguyen, Hanh Nho; Olivieri, Philip R.; Schenkel, Laurie B.; Vaida, Karina R.; Andrews, Paul; et al
Journal of Medicinal Chemistry (2015), 58(13), 5189-5207 | Language: English, Database: CAplus and MEDLINE

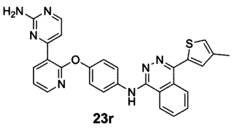

23r

Cell Line	EC_{50} (nM)
CAL51 (Non-MDR)	2
MES-SA Dx5 (MDR)	1
HCT15 (MDR)	2
SNU449 (MDR)	2
769P (MDR)	2

Efforts to improve upon the phys. properties and metabolic stability of pyridinyl-pyrimidine phthalazinamine Aurora kinase inhibitor revealed that potency against multidrug-resistant cell lines was compro mised by increased polarity. Despite its high in vitro metabolic intrinsic clearance, AMG 900 (**I**) showed acceptable pharmaco kinetic properties and robust pharmaco dynamic activity. Projecting from in vitro data to in vivo target coverage was not practical due to disjunctions between enzyme and cell data, complex and apparently contradictory indicators of binding kinetics, and unmeas urable free fraction in plasma. In contrast, it was straight forward to relate pharmaco kinetics to pharmacodynamics and efficacy by following the time above a threshold concent ration On the basis of its oral route of administration, a selectivity profile that favors Aurora-driven pharmacol. and its activity against multidrug-resistant cell lines, **I** was identified as a potential best- in-class Aurora kinase inhibitor. In phase 1 dose expansion studies with G-CSF support, **I** has shown promising single agent activity.

Keywords: pyridinylpyrimidine phthalazinamine AMG 900 analog prepar ation SAR Aurora kinase

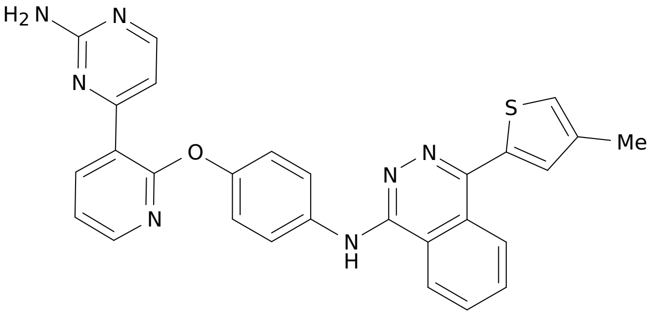

I

Substances (94) Reactions (138) Citing (36)

191

Structural Biology Insight for the Design of Sub-type Selective Aurora Kinase Inhibitors

By: Sarvagalla, Sailu; Coumar, Mohane Selvaraj
Current Cancer Drug Targets (2015), 15(5), 375-393 | Language: English, Database: CAplus and MEDLINE

A review. Aurora kinase A, B and C, are key regulators of mitosis and are over expressed in many of the human cancers, making them an ideal drug target for cancer chemotherapy. Currently, over a dozen of Aurora kinase inhibitors are in various phases of clin. development. The majority of the inhibitors (VX-680/MK-0457, PHA-739358, CYC116, SNS-314, AMG 900, AT-9283, SCH-1473759, ABT-348, PF-03814735, R-763/AS-703569, KW-2449 and TAK-901) are pan-selective (isoform non-selective) and few are Aurora A (MLN8054, MLN8237, VX-689/MK5108 and ENMD 2076) and Aurora B (AZD1152 and GSK1070916) sub-type selective. Despite the intensive research efforts in the past decade, no Aurora kinase inhibitor has reached the market. Recent evidence suggests that the sub-type selective Aurora kinase A inhibitor could possess advantages over pan- selective Aurora inhibitors, by avoiding Aurora B mediated neutropenia. However, sub-type selective Aurora kinase A inhibitor design is very challe nging due to the similarity in the active site among the isoforms. Structural biol. and computational aspects pertaining to the design of Aurora kinase inhibitors were analyzed and found that a possible means to develop sub-type selective inhibitor is by targeting Aurora A specific residues (Leu215, Thr217 and Arg220) or Aurora B specific residues (Arg159, Glu161 and Lys164), near the solvent exposed region of the protein. Particularly, a useful strategy for the design of sub- type selective Aurora A inhibitor could be by targeting Thr217 residue as in the case of MLN8054. Further preclin. and clin. studies with the sub- type selective Aurora inhibitors could help bring them to the market for the treatment of cancer.

Keywords: review Aurora kinase inhibitor design anticancer therapeutic target cancer

Substances (22) Reactions (0) Citing (20)

192

Biomarkers for PSMA targeted therapy for prostate cancer

By: Olson, William C.; DiPippo, Vincent
World Intellectual Property Organization, WO2015073896 A2 2015-05-21 | Language: English, Database: CAplus

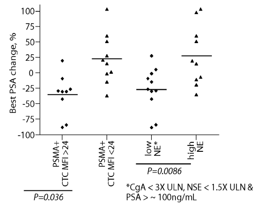

Fig. 2

Provided herein are a number of methods, assays and diagnostic tests based on one or more biomarkers as well as related kits and compositions that can be used to identify subjects or patients that would likely benefit from a treatment (or continued treatment) , such as a PSMA targeted therapy. Also provided are methods for treating the identified subjects or patients.

Keywords: PSMA targeted therapy prostate cancer biomarker

PatentPak available

Substances (20) Reactions (0) Citing (1)

193

SnapShot: Kinase Inhibitors II

By: Wang, Jinhua; Gray, Nathanael S.
Molecular Cell (2015), 58(4), 710-710.e1 | Language: English, Database: CAplus and MEDLINE

Selective small-mol. inhibitors of protein kinases can serve as powerful tools to elucidate biol. function. Efforts to develop potential drug candidates have yielded a wealth of kinase inhibitors. However, selecting the optimal kinase inhibitor for a particular applic ation can be challenging. While the optimal inhibitor will be applic ation specific, we have attempted to summarize some of the best reported inhibitors for various kinases.

Keywords: protein kinase inhibition small mol

Substances (87) Reactions (0) Citing (10)

194

Sequential treatment with aurora B inhibitors enhances cisplatin-mediated apoptosis via c-Myc

By: Ma, Yaxi; Cao, Handi; Lou, Siyue; Shao, Xuejing; Lv, Wen; Qi, Xiaotian; Liu, Yujia; Ying, Meidan; He, Qiaojun; Yang, Xiaochun
Journal of Molecular Medicine (Heidelberg, Germany) (2015), 93(4), 427-438 | Language: English, Database: CAplus and MEDLINE

Abstract: Platinum compound such as cisplatin is the first-line chemotherapy of choice in most patients with ovarian carcinoma. However, patients with inherent or acquired cisplatin resistance often experience relapse. Therefore, novel therapies are urgently required to treat drug-resistant ovarian carcinoma. Here, we showed that compared to the non- functional traditional simultaneous treatment, sequential combination of Aurora B inhibitors followed by cisplatin synergis tically enhanced apoptotic response in cisplatin-resistant OVCAR-8 cells. This effect was accomp anied by the induction of polyploidy in a c- Myc-dependent manner, as c-Myc knockdown reduced the efficacy of the combin ation by suppressing the expression of Aurora B and impairing cellular response to Aurora B inhibitor, as indicated by the decreased polyploidy and hyperphosphorylation of histone H1. In c- Myc-deficient SKOV3 cells, c-Myc overexpression restored Aurora B expression, induced polyploidy after inhibition of Aurora B, and sensitized cells to this combination therapy. Thus, our report reveals for the first time that sequential treatment of Aurora B inhibitors and cisplatin is essential to inhibit ovarian carcinoma by inducing polyploidy and downregulating c-Myc and that c-Myc is identified as a predictive biomarker to select cells responsive to chemotherapeutical combinations targeting Aurora B. Collect ively, these studies provide novel approaches to overcoming cisplatin chemotherapy resistance in ovarian cancer. Key Message: Pretre atment of Aurora B inhibitors augment apoptotic effects of cisplatin. The synergy of Aurora B inhibitor with cisplatin is dependent on c-Myc expression. c-Myc-dependent induction of polyploidy sensitizes cells to cisplatin.

Keywords: aurora B inhibitor cisplatin anticancer agent cMyc apoptosis cancer

Substances (7) Reactions (0) Citing (6)

195

Novel drugs for older patients with acute myeloid leukemia

By: Montalban-Bravo, G.; Garcia-Manero, G.
Leukemia (2015), 29(4), 760-769 | Language: English, Database: CAplus and MEDLINE

A review. Acute myeloid leukemia (AML) is the second most common form of leukemia and the most frequent cause of leukemia-related deaths in the United States. The incidence of A ML increases with advancing age and the prognosis for patients with A ML worsens substantially with increasing age. Many older patients are ineligible for intensive treatment and require other therap eutic approaches to optimize clin. outcome. To address this treatment gap, novel agents with varying mechanisms of action targeting different cellular processes are currently in development. Hypomethylating agents (azacitidine, decitabine, SGI-110), histone deacet ylase inhibitors (vorinostat, pracinostat, panobinostat), FMS-like tyrosine kinase receptor-3 inhibitors (quizartinib, sorafenib, midost aurin, crenolanib), cytotoxic agents (clofarabine, sapacitabine, vosaroxin), cell cycle inhibitors (barase rtib, volasertib, rigosertib) and monoclonal antibodies (gentuzumab ozogamicin, lintuzumab-Ac225) represent some of these promising new treatm ents. This review provides an overview of novel agents that have either completed or are currently in ongoing phase III trials in patients with previously untreated AML for whom intensive treatment is not an option. Other potential drugs in earlier stages of develo pment will also be addressed in this review.

Keywords: review anticancer agent acute myeloid leukemia

Substances (17) Reactions (0) Citing (54)

196

Anti-human VSTM5 antibodies, fragments and conjugates for therapy and diagnosis of cancer, infection and immune disease

By: Levine, Zurit; Rotman, Galit; Dassa, Liat; Levy, Ofer; Cojocaru, Gad S.; Toporik, Amir; Kliger, Yossef; Pow, Andrew; Liang, Spencer
World Intellectual Property Organization, WO2015037005 A1 2015-03-19 | Language: English, Database: CAplus

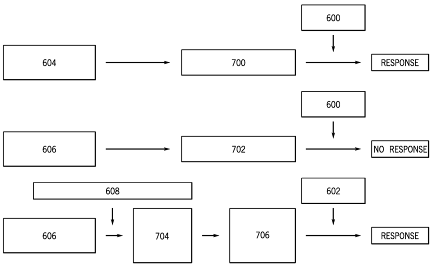

FIG. 1

The present invention relates to VSTM5-specific antibodies, antibody fragments, and VSTM5 polypeptides, conjugates and compos itions comprising same, for modulating (antago nizing or agonizing) one or more of the effects of VSTM5 expression on immunity. More specifically, the present invention relates to VSTM5-specific antibodies, antibody fragments, and VSTM5 polypeptides, conjugates and compositions comprising same for treating and aiding in the diagnosis of cancer, infectious diseases and immune related diseases, e.g., those associated with aberrant (higher or lower than normal) VSTM5 expression by diseased and/or immune cells and/or aberrant (increased or reduced) VSTM5-mediated effects on immunity.

Keywords: human VSTM5 antibody immunodiagnosis immunotherapy cancer infection immune disease

PatentPak available

Substances (424) Reactions (0) Citing (0)

197

Aurora kinase A and B as new treatment targets in aromatase inhibitor-resistant breast cancer cells

By: Hole, Stine; Pedersen, Astrid M.; Lykkesfeldt, Anne E.; Yde, Christina W.
Breast Cancer Research and Treatment (2015), 149(3), 715-726 | Language: English, Database: CAplus and MEDLINE

Aromatase inhibitors (AIs) are used for treatment of estrogen receptor α (E R)-pos. breast cancer; however, resistance is a major obstacle for optimal outcome. This preclin. study aimed at identifying potential new treatment targets in A I-resistant breast cancer cells. Parental MCF-7 breast cancer cells and four newly establ ished cell lines, resistant to the A Is exemestane or letrozole, were used for a functional kinase inhibitor screen. A library comprising 195 different compounds was tested for preferential growth inhibition of AI-resistant cell lines. Selected targets were validated by anal. of cell growth, cell cycle phase distrib ution, protein expression, and subcel lular localization. We identified 24 compounds, including several inhibitors of Aurora kinases e.g., J NJ-7706621 and barasertib. Protein expression of Aurora kinase A and B was found upregu lated in A I-resistant cells compared with M C F-7, and knockdown studies showed that Aurora kinase A was essential for A I-resistant cell growth. In A I-resistant cell lines, the clin. relevant Aurora kinase inhibitors alisertib and danusertib blocked cell cycle progression at the G2/M phase, interfered with chromosome alignment and spindle pole formation, and resulted in preferential growth inhibition compared with parental M CF-7 cells. Even further growth inhibition was obtained when combining the Aurora kinase inhibitors with the antiestrogen fulvestrant. Our study is the first to demonstrate that Aurora kinase A and B may be treatment targets in A I-resistant cells, and our data suggest that therapy targeting both ER and Aurora kinases may be a potent treatment strategy for overcoming A I resistance in breast cancer.

Keywords: Aurora kinase aromatase inhibitor resistance breast cancer treatment target

Substances (9) Reactions (0) Citing (31)

198

Finding the optimal combination therapy for the treatment of newly diagnosed AML in older patients unfit for intensive therapy

By: Erba, Harry P.
Leukemia Research (2015), 39(2), 183-191 | Language: English, Database: CAplus and MEDLINE

A review. There is no standard of care for older patients with newly diagnosed acute myeloid leukemia (AML) unfit for intensive therapy, and prognosis with currently recommended low-intensity therapies (decitabine, azacitidine, and low-dose cytarabine [LDA C]) remains poor. One promising strategy is to combine low- intensity treatments with novel agents. Gemtuzumab ozogam icin, tipifa rnib, and barasertib have been invest igated in phase 2/3 or 3 trials combined with L DAC, and phase 3 trials are currently investi gating sapacitabine plus decitabine, and volasertib plus LDAC in AML. This review discusses current treatment recommen dations and the development of combination therapies for older patients unfit for intensive therapy.

Keywords: combination intensive therapy diagnosis human A ML review; Acute myeloid leukemia (A ML); Combination; Low-intensity; Newly diagnosed; Novel agents; Older/elderly

Substances (8) Reactions (0) Citing (30)

199

Novel substituted bicyclic compounds as bromodomain inhibitors

By: Liu, Shuang; Quinn, John Frederick; Duffy, Bryan Cordell; Wang, Ruifang; Jiang, May Xiaowu; Martin, Gregory Scott; Zhao, He; Ellis, Michael; Wagner, Gregory Steven; Young, Peter Ronald
World Intellectual Property Organization, WO2015004534 A2 2015-01-15 | Language: English, Database: CAplus

The invention relates to substituted bicyclic compounds, which are useful for inhibition of B ET protein function by binding to bromodomains, pharmaceutical compositions comprising these compounds, and use of the compounds and compos itions in therapy. Also disclosed is the preparation of compounds of the invention.

Keywords: bicyclic derivative preparation bromodomain

PatentPak available

Substances (145) | Reactions (191) | Citing (2)

200

Preparation of azabicyclic compounds as BET inhibitors and therapeutic uses thereof

By: Liu, Shuang; Quinn, John Frederick; Duffy, Bryan Cordell; Wang, Ruifang; Jiang, May Xiaowu; Martin, Gregory Scott; Zhao, He; Ellis, Michael; Wagner, Gregory Steven; Young, Peter Ronald
World Intellectual Property Organization, WO2015004533 A2 2015-01-15 | Language: English, Database: CAplus

The invention relates to substituted azabicyclic compounds of formula I which are useful for the inhibition of bromod omain and extra terminal domain (BET) proteins, pharmaceutical compositions comprising these compounds, and use of the compounds and compositions for treatment of B ET-associated diseases such as cancer, inflamm ation, autoimmune disease, cardiovascular disease, benign proliferative and fibrotic disorders, and other diseases. Compounds of formulas I [wherein A is selected from 5- or 6-membered monocyclic carbocycle or heterocycles fused to ring B; B is a 6-membered carbocycle or heterocycle; Y is N or C; X is N H, alkyl, C(O)O, etc.; W^1 and W^2 are independently selected from N or C R; R at each occurrence is indepen dently selected from H, N H_2, OH, etc.; W^3 and W^4 are independently selected from N, C H, and C with the proviso that both W^3 and W^4 cannot be N; R^3 is 4-7 membered carbocycle, 4-7 membered heterocycle, bicyclic carbocycle, etc.; D^1 is 5-membered monocyclic carbocycle or heterocycle connected to the B ring via a carbon-carbon bond with proviso] or a stereoi somer, tautomer, pharmaceutically acceptable salt, or hydrate thereof are claimed and exemplified. Reaction of 3,5-dimethyl-4-(4,4,5,5-tetramethyl-1,3,2-dioxaborolan-2-yl)isoxazole with 4,6-dibromo-2-methylbenzimidazole afforded II in 60% yield. I were evaluated for inhibition of tetra- acetylated histone H4 binding to BET bromodomains using TR-FRET anal. from which II was determined to inhibit the first bromod omain of Brd4 with an IC_{50} value of ≤ 0.3 μM.

Keywords: azabicyclic compound preparation BET inhibitor

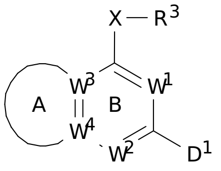

I

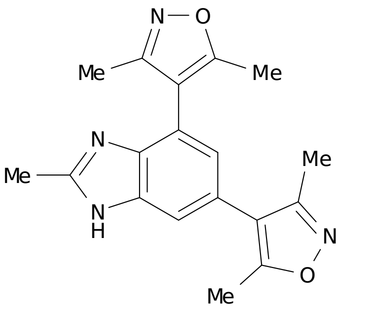

II

PatentPak available

Substances (542) | Reactions (1,251) | Citing (9)

201

Preparation of azabicyclic compounds as BET inhibitors and therapeutic uses thereof

By: Duffy, Bryan Cordell; Liu, Shuang; Quinn, John Frederick; Wang, Ruifang; Jiang, May Xiaowu; Martin, Gregory Scott; Wagner, Gregory Steven; Young, Peter Ronald

World Intellectual Property Organization, WO2015002754 A2 2015-01-08 | Language: English, Database: CAplus

The invention relates to substituted azabicyclic compounds of formula I which are useful for the inhibition of bromodomain and extra terminal domain (BET) proteins, pharmaceutical compositions comprising these compounds, and use of the compounds and compositions for treatment of BET-associated diseases such as cancer, inflammation, autoimmune disease, cardiovascular disease, benign proliferative and fibrotic disorders, and other diseases. Compounds of formulas I and II [wherein A is selected from 5- or 6-membered monocyclic heterocycles fused to ring B; B is a 6-membered aromatic carbocycle or heterocycle; Y is N or C; X is NH, alkyl, C(O)O, etc.; W^1, W^2, W^3 are independently selected from N or CR; R at each occurrence is independently selected from H, NH_2, halo, CN, etc.; R^4 is 3-7 membered carbocycle or heterocycle; D^1 is 5-membered monocyclic heterocycle with proviso] or a stereoisomer, tautomer, pharmaceutically acceptable salt, or hydrate thereof are claimed and exemplified. Benzylation of 2-chloroadenine provided 9-benzyl-2-chloroadenine which was reacted with 3,5-dimethyl-4-(4,4,5,5-tetramethyl-1,3,2-dioxaborolan-2-yl)isoxazole affording **III**. **I** were evaluated for inhibition of tetra-acetylated histone H4 binding to BET bromodomains using TR-FRET anal. from which **III** was determined to inhibit the first bromodomain of Brd4 with an IC_{50} value between 0.3 μM and 30 μM.

Keywords: azabicyclic compound preparation BET inhibitor

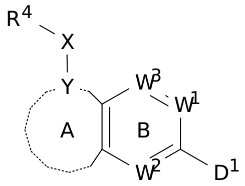

I

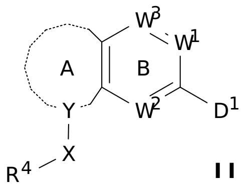

II

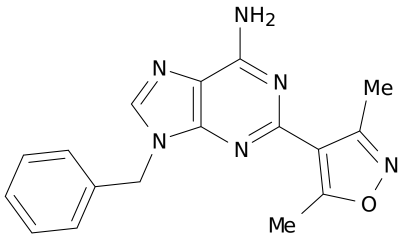

III

PatentPak available

Substances (556)　Reactions (982)　Citing (12)

202

Aurora kinase B inhibition reduces the proliferation of metastatic melanoma cells and enhances the response to chemotherapy

By: Porcelli, Letizia; Guida, Gabriella; Quatrale, Anna E.; Cocco, Tiziana; Sidella, Letizia; Maida, Immacolata; Iacobazzi, Rosa M.; Ferretta, Anna; Stolfa, Diana A.; Strippoli, Sabino; et al
Journal of Translational Medicine (2015), 13, 26/1-26/13 | Language: English, Database: CAplus and MEDLINE

Background: The poor response to chemotherapy and the brief response to vemura fenib in metastatic melanoma patients, make the identification of new therapeutic approaches an urgent need. Interes tingly the increased expression and activity of the Aurora kinase B during melanoma progression suggests it as a promising therap eutic target. Methods: The efficacy of the Aurora B kinase inhibitor barasertib-HQPA was evaluated in BRAF mutated cells, sensitive and made resistant to vemura fenib after chronic exposure to the drug, and in BRAF wild type cells. The drug effecti veness has been evaluated as cell growth inhibi tion, cell cycle progression and cell migration. In addition, cellular effectors of drug resistance and response were investi gated. Results: The characterization of the effectors respon sible for the resistance to vemura fenib evidenced the increased expression of MITF or the activation of Erk1/2 and p-38 kinases in the newly established cell lines with a phenotype resistant to vemura fenib. The sensitivity of cells to barasertib-HQPA was irresp. of BRAF mutational status. Barasertib-HQPA induced the mitotic catast rophe, ultimately causing apoptosis and necrosis of cells, inhibited cell migration and strongly affected the glycolytic metabolism of cells inducing the release of lactate. In association (i) with vemura fenib the gain in effectiveness was found only in BRAF(V600K) cells while (ii) with nab-paclit axel, the combination was more effective than each drug alone in all cells. Conclu sions: These findings suggest barasertib as a new therapeutic agent and as enhancer of chemot herapy in metastatic melanoma treatment.

Keywords: Aurora kinase B proliferation metastatic melanoma cell chemot herapy

Substances (10) Reactions (0) Citing (35)

203

Aurora kinase B is important for antiestrogen resistant cell growth and a potential biomarker for tamoxifen resistant breast cancer

By: Larsen, Sarah L.; Yde, Christina W.; Laenkholm, Anne-Vibeke; Rasmussen, Birgitte B.; Duun-Henriksen, Anne Katrine; Bak, Martin; Lykkesfeldt, Anne E.; Kirkegaard, Tove
BMC Cancer (2015), 15, 1-15 | Language: English, Database: CAplus and MEDLINE

Background: Resistance to antiestrogen therapy is a major clin. challenge in the treatment of estrogen receptor α (E R)-pos. breast cancer. The aim of the study was to explore the growth promoting pathways of antiestrogen resistant breast cancer cells to identify biomarkers and novel treatment targets. Methods: Antiestrogen sensitive and resistant T47 D breast cancer cell lines were used as model systems. Parental and fulvestrant resistant cell lines were subjected to a kinase inhibitor library. Kinase inhibitors prefere ntially targeting growth of fulvestrant resistant cells were identified and the growth inhibitory effect verified by dose- response cell growth experiments Protein expression and phosphorylation were investigated by western blot anal. Cell cycle phase distri bution and cell death were analyzed by flow cytometry. To evaluate Aurora kinase B as a biomarker for endocrine resistance, immunohi stochem. was performed on archival primary tumor tissue from breast cancer patients who have received adjuvant endocrine treatment with tamoxifen. Results: The selective Aurora kinase B inhibitor barasertib was identified to preferentially inhibit growth of fulvestrant resistant T47 D breast cancer cell lines. Compared with parental cells, phosphor ylation of Aurora kinase B was higher in the fulvestrant resistant T47 D cells. Barasertib induced degradation of Aurora kinase B, caused mitotic errors, and induced apoptotic cell death as measured by accumulation of Sub G1 cells and PARP cleavage in the fulvestrant resistant cells. Barasertib also exerted preferential growth inhibition of tamoxifen resistant T47 D cell lines. Finally, high percentage of Aurora kinase B pos. tumor cells was significantly associated with reduced disease- free and overall survival in 261 E R-pos. breast cancer patients, who have received tamoxifen as first-line adjuvant endocrine treatment. Conclu sions: Our results indicate that Aurora kinase B is a driving factor for growth of antiestrogen resistant T47 D breast cancer cell lines, and a biomarker for reduced benefit of tamoxifen treatment. Thus, inhibition of Aurora kinase B, e.g. with the highly selective kinase inhibitor barasertib, could be a candidate new treatment for breast cancer patients with acquired resistance to antiestrogens.

Keywords: barasertib anticancer aurora kinase B inhibitor therapeutic breast cancer

Substances (7) | Reactions (0) | Citing (20)

204

Canine osteosarcoma cells exhibit resistance to aurora kinase inhibitors

By: Cannon, C. M.; Pozniak, J.; Scott, M. C.; Ito, D.; Gorden, B. H.; Graef, A. J.; Modiano, J. F.
Veterinary and Comparative Oncology (2015), 13(1), 48-59 | Language: English, Database: CAplus and MEDLINE

We evaluated the effect of Aurora kinase inhibitors AZD1152 and VX680 on canine osteosarcoma cells. Cytotoxicity was seen in all four cell lines; however, half-maximal inhibitory concentrations were significantly higher than in human leukemia and canine lymphoma cells. AZD1152 reduced Aurora kinase B phosphor ylation, indicating resistance was not because of failure of target recognition. Efflux mediated by A BCB1 and ABCG2 transporters is one known mechanism of resistance against these drugs and verapamil enhanced AZD1152-induced apoptosis; however, these transporters were only expressed by a small percentage of cells in each line and the effects of verapamil were modest, suggesting other mechanisms contribute to resistance. Our results indicate that canine osteosarcoma cells are resistant to Aurora kinase inhibitors and suggest that these compounds are unlikely to be useful as single agents for this disease. Further investigation of these resistance mechanisms and the potential utility of Aurora kinase inhibitors in multi-agent protocols is warranted.

Keywords: canine osteosarcoma drug resistance aurora kinase inhibitor A ZD1152 VX680; chemotherapy; comparative oncology; in vitro models; oncology; osteosarcoma

Substances (4) | Reactions (0) | Citing (4)

205

Modeling the mitotic regulatory network identifies highly efficient anti-cancer drug combinations

By: Wu, Yiran; Zhuo, Xiaolong; Dai, Ziwei; Guo, Xiao; Wang, Yao; Zhang, Chuanmao; Lai, Luhua
Molecular BioSystems (2015), 11(2), 497-505 | Language: English, Database: CAplus and MEDLINE

Targeting mitotic regulation is recognized as an important strategy for cancer therapy. Aurora A/B kinase and polo-like kinase 1 (PLK1) are the key mitotic regulators, and many inhibitors have been developed. Combinations of these inhibitors are anticipated to be more effective therapeutics compared with single-inhibitor treatments; however, a systematic anal. of the combined effects is lacking. Here, we constructed the first mammalian cell mitotic regulation network model, which spans from mitotic entry to anaphase initiation, and contains all key mitotic kinase targets. The combined effects of different kinase inhibitors and microtubule inhibitors were systematically explored. Simultaneous inhibition of Aurora B and PLK1 strongly induces polyploidy. Microtubule inhibitor dosage can be significantly reduced when combined with a PLK1 inhibitor. The efficacy of these inhibitor combinations was validated by our exptl. results. The mitotic regulatory network model provides a platform to study the complex interactions during mitosis, enables identification of mitotic regulators, and determines targets for drug discovery research. The suggested use of combining microtubule inhibitors with PLK1 inhibitors is anticipated to enhance microtubule-inhibitor tolerance in a wide range of patients.

Keywords: anticancer discovery protein phosphorylation epithelial cell simulation computational model

Substances (8) Reactions (0) Citing (2)

206

Histone H3 phosphorylation in GBM: a new rational to guide the use of kinase inhibitors in anti-GBM therapy

By: Pacaud, Romain; Cheray, Mathilde; Nadaradjane, Arulraj; Vallette, Francois M.; Cartron, Pierre-Francois
Theranostics (2015), 5(1), 12-22, 11 pp. | Language: English, Database: CAplus and MEDLINE

Histones post-translational modifications (PTMs) are crucial components of diverse processes that modulate chromatin. Among the histones PTMs, the histones phosphorylation appears such crucial since it plays a significant role into DNA repair structure, transcription and chromatin compaction during cell division and apoptosis. However, little is known about the prognostic value of the histone phosphorylation in human cancer. This point could be considerate such as an important gap in anti-cancer therapy since the use of adequate kinase inhibitors could remedy to the aberrant histone phosphorylation associated with a poor prognosis factor. To remedy at this situation, we analyzed the phosphorylation level of histone H3 at the residues T3, T6, S10, S28, Y41 and T45 in a collection of 42 glioblastoma multiformes (GBM). Our data indicated that the high level of pH3T6, pH3S10 and pH3Y41 are signatures associated with a poor prognosis of overall survival (OS) of GBM treated with the "temozolomide and irradiation standard" treatment of GBM (named TMZ + Irad treatment). Our data also showed that these signatures are correlated with the high activity of kinases already described as writers of the pH3T6, pH3S10 and pH3Y41 i.e. the PKC, Aurora-B and JAK2, resp. Finally, our anal. revealed that the use of Enzastaurin, AZD1152, and AZD1480 abrogated the high level of pH3T6, pH3S10 and pH3Y41 while increasing the sensitivity to the "temozolomide and irradiation"-induced cell death. To conclude, it appears that this work provides biomarkers for patient stratification for a therapy including kinase inhibitors.

Keywords: glioblastoma histone H3 phosphorylation kinase inhibitor anti cancer therapy; apoptosis; epigenetic; glioma; histone; phosphorylation

Substances (9) Reactions (0) Citing (23)

207

A chemical screening approach to identify novel key mediators of erythroid enucleation

By: Wolwer, Christina B.; Pase, Luke B.; Pearson, Helen B.; Godde, Nathan J.; Lackovic, Kurt; Huang, David C. S.; Russell, Sarah M.; Humbert, Patrick O.
PLoS One (2015), 10(11), e0142655/1-e0142655/12 | Language: English, Database: CAplus and MEDLINE

Erythroid enucleation is critical for terminal differen tiation of red blood cells, and involves extrusion of the nucleus by orthoch romatic erythroblasts to produce reticulocytes. Due to the difficulty of synchro nizing erythroblasts, the mol. mechanisms underlying the enucleation process remain poorly unders tood. To elucidate the cellular program governing enucle ation, we utilized a novel chem. screening approach whereby orthochromatic cells primed for enucle ation were enriched ex vivo and subjected to a functional drug screen using a 324 compound library consisting of structurally diverse, medicinally active and cell permeable drugs. Using this approach, we have confirmed the role of HDACs, proteasomal regulators and MAPK in erythroid enucle ation and introduce a new role for Cyclin-dependent kinases, in particular CDK9, in this process. Import antly, we demonstrate that when coupled with imaging anal., this approach provides a powerful means to identify and characterize rate limiting steps involved in the erythroid enucleation process.

Keywords: MAPK CDK9 HDAC PI3K orthochromatic erythroblast erythroid enucleation

Substances (294) Reactions (0) Citing (8)

208

Overcoming cetuximab resistance in HNSCC: The role of AURKB and DUSP proteins

By: Boeckx, Carolien; Op de Beeck, Ken; Wouters, An; Deschoolmeester, Vanessa; Limame, Ridha; Zwaenepoel, Karen; Specenier, Pol; Pauwels, Patrick; Vermorken, Jan B.; Peeters, Marc; et al
Cancer Letters (New York, NY, United States) (2014), 354(2), 365-377 | Language: English, Database: CAplus and MEDLINE

Unraveling the underlying mechanisms of cetuximab resistance in head and neck squamous cell carcinoma (HNSCC) is of major importance as many tumors remain non-responsive or become resistant. Our microarray results suggest that "resis tant" cells still exhibit RAS-MAPK pathway signaling contri buting to drug resistance, as witnessed by low expression of DUSP5 and DUSP6, neg. regulators of ERK1/2, and increased expression of AURKB, a key regulator of mitosis. Therefore, interr upting the RAS-MAPK pathway by an ERK1/2 inhibitor (apigenin) or an AURKB inhibitor (barasertib) might be a new strategy for overcoming cetuximab resistance in HNSCC.

Keywords: cetuximab anticancer AURKB DUSP head neck squamous cell carcinoma; Anti-EGFR therapy; Aurora kinase B; Cetuximab resistance; Dual-specificity phosphatase 5 and 6; Head and neck squamous cell carcinoma; Nano Pro 1000

Substances (10) Reactions (0) Citing (46)

209

Method of determination of cancer cell drug sensitivity towards aurora kinase inhibitors

By: Kollaredy, Madhusudhan Reddy; Hajduch, Marian; Dzubak, Petr; Srovnal, Josef; Hrabakova, Rita; Kovarova, Hana
United States, US20140336073 A1 2014-11-13 | Language: English, Database: CAplus

A method for determining the sensitivity and/or resistance of a patient suffering from a cancer disease to Aurora kinase inhibitor therapy, which comprises determining in vitro in the cancer cells or body fluids taken from the patient the expression of at least one gene selected from a particular group and/or determining in vitro in the cancer cells or body fluids taken from the patient the level of at least one protein selected from a particular group.

Keywords: cancer drug sensitivity Aurora kinase inhibitor gene protein expression

PatentPak available

Substances (55) | Reactions (0) | Citing (0)

210

Effects of selective inhibitors of Aurora kineases on anaplastic thyroid carcinoma cell lines

By: Baldini, Enke; Tuccilli, Chiara; Prinzi, Natalie; Sorrenti, Salvatore; Antonelli, Alessandro; Gnessi, Lucio; Morrone, Stefania; Moretti, Costanzo; Bononi, Marco; Arlot-Bonnemains, Yannick; et al
Endocrine-Related Cancer (2014), 21(5), 797-811 | Language: English, Database: CAplus and MEDLINE

Aurora kinases are serine/threonine kinases that play an essential role in cell division. Their aberrant expression and/or function induce severe mitotic abnormalities, resulting in either cell death or aneupl oidy. Overexpression of Aurora kinases is often found in several malignancies, among which is anaplastic thyroid carcinoma (A TC). We have previously demons trated the in vitro efficacy of Aurora kinase inhibitors in restraining cell growth and survival of different A TC cell lines. In this study, we sought to establish which Aurora might represent the preferential drug target for A TC. To this end, the effects of two selective inhibitors of Aurora- A (ML N8237) and Aurora- B (AZD1152) on four human ATC cell lines (CAL-62, BHT-101, 8305C, and 8505C) were analyzed. Both inhibitors reduced cell proliferation in a time- and dose- dependent manner, with IC_{50} ranges of 44.3-134.2 nM for M LN8237 and of 9.2-461.3 nM for AZD1152. Immunofluorescence experiments and time-lapse videomicroscopy yielded evidence that each inhibitor induced distinct mitotic phenotypes, but both of them prevented the completion of cytoki nesis. As a result, poliploidy increased in all A Z D1152-treated cells, and in two out of four cell lines treated with M LN8237. Apoptosis was induced in all the cells by M LN8237, and in BHT-101, 8305C, and 8505C by AZD1152, while CAL-62 exposed to AZD1152 died through necrosis after multiple rounds of endoreplication. Both inhibitors were capable of blocking anchorage- independent cell growth. In conclu sion, we demons trated that either Aurora-A or Aurora- B might represent therap eutic targets for the A TC treatment, but inhibition of Aurora- A appears more effective for suppressing ATC cell prolife ration and for inducing the apoptotic pathway.

Keywords: Aurora kinase anaplastic thyroid carcinoma; AZD1152; Aurora inhibitors; Aurora kinases; MLN8237; anaplastic thyroid cancer; cell cycle; therapy

Substances (4) | Reactions (0) | Citing (26)

211

Methods of use of cleavable drug conjugates and compositions

By: Nguyen, Mark Quang
United States, US20140294851 A1 2014-10-02 | Language: English, Database: CAplus

Base-labile crosslinkers, base-labile conjugates comprising such crosslinkers, methods of their synthesis and use are disclosed for treatment of cancers, metabolic diseases and infections.

Keywords: cleavable drug conjugate preparation cancer metabolic disease infection

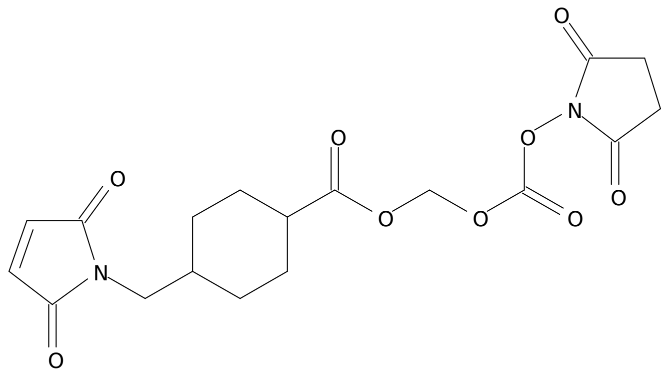

PatentPak available

Substances (286) | Reactions (29) | Citing (0)

212

Indomethacin promotes apoptosis in gastric cancer cells through concomitant degradation of Survivin and Aurora B kinase proteins

By: Chiou, Shiun-Kwei; Hoa, Neil; Hodges, Amy; Ge, Lishen; Jadus, Martin R.
Apoptosis (2014), 19(9), 1378-1388 | Language: English, Database: CAplus and MEDLINE

Regular usage of nonsteroidal anti-inflammatory drugs (NSAIDs) is associated with reduced incidence of a variety of cancers. The mol. mechanisms underlying these chemopreventive effects remain poorly understood. This current investigation showed that in gastric cancer cells: (1) Indomethacin treatment enhanced the degradation of chromosomal passenger proteins, Survivin and Aurora B kinase; (2) Indomethacin treatment down-regulated Aurora B kinase activity in a cell cycle- independent fashion; (3) siRNA knockdown of Survivin level promoted Aurora B kinase protein degradation, and vice versa; (4) ectopic overexp ression of Survivin blocked reduction of Aurora B kinase level and activity by indomethacin treatment, and vice versa; (5) si RNA knockdown of Aurora B kinase level and AZD1152 inhibition of its activity induced apoptosis, and overexp ression of Aurora B kinase inhibited indomet hacin-induced apoptosis; (6) indomethacin treatment reduced Aurora B kinase level, coinciding with reduction of Survivin level and induction of apoptosis, in KATO III and HT-29 cells, and in mouse gastric mucosa. A role for Aurora B kinase function in N SAID-induced apoptosis was not previously explored. Thus this report provides better underst anding of the mol. mechanisms underlying the anti-cancer effect of NSAIDs by elucid ating a significant role for Aurora B kinase in indomet hacin-induced apoptosis.

Keywords: antitumor indomethacin apoptosis gastric cancer survivin aurora B kinase

Substances (6) | Reactions (0) | Citing (13)

213

Preparation of arylquinolines as anticancer agents

By: Courcambeck, Jerome; Bassissi, Firas; Brun, Sonia; Nicolas, Gregory; Beret, Antoine; Petit, Serge; Camus, Claire; Nallet, Jean Pierre; Halfon, Philippe

World Intellectual Property Organization, WO2014147611 A1 2014-09-25 | Language: English, Database: CAplus

The invention provides quinoline derivatives of general formula **I** (wherein R^1 is (un)substituted aryl, (un)substituted heteroaryl, or (un)substituted heteroaromatic 5 to 9-membered ring; R^2 is halo, (un)substituted C_{1-6} alkyl, OH, etc.; W is substituted piperidinyl, etc.; L^1 is a bond, carbonyl, O, etc.; and n = 0- 4) their manufacture, pharmaceutical compositions containing them, and their use as medicaments. The active compounds of the present invention are useful for the treatment of proliferative neoplastic and non-neoplastic diseases. Example compound **II**-2HCl was prepared in a multistep synthesis that culminates in reaction of intermediate **III** with 4-diethylaminopiperidine and subsequent conversion to the salt. The example compounds were tested extensively in anticancer screening assays both in vitro and in vivo. In cell viability assays on A-375, HCT-116, and MOLM-14AML cell lines, there was <20 % cell viability in the presence of **II** at a dose of 10 μ M.

Keywords: arylquinoline preparation anticancer agent

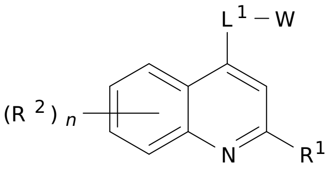

I

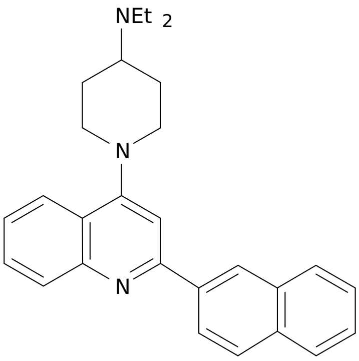

II

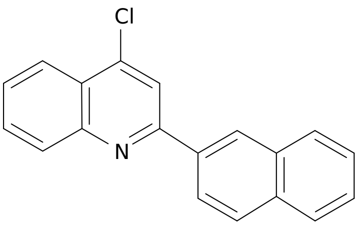

III

PatentPak available

Substances (510) Reactions (472) Citing (4)

214

Ligand efficiency based approach for efficient virtual screening of compound libraries

By: Ke, Yi-Yu; Coumar, Mohane Selvaraj ; Shiao, Hui-Yi; Wang, Wen-Chieh; Chen, Chieh-Wen; Song, Jen-Shin; Chen, Chun-Hwa; Lin, Wen-Hsing; Wu, Szu-Huei; Hsu, John T. A.; et al
European Journal of Medicinal Chemistry (2014), 83, 226-235 | Language: English, Database: CAplus and MEDLINE

Here we report for the first time the use of fit quality (FQ), a ligand efficiency (LE) based measure for virtual screening (VS) of compound libraries. The LE based VS protocol was used to screen an inhouse database of 125, 000 compounds to identify aurora kinase A inhibitors. First, 20 known aurora kinase inhibitors were docked to aurora kinase A crystal structure (P DB ID: 2W1C); and the conformations of docked ligand were used to create a pharmac ophore (PH) model. The PH model was used to screen the database compounds, and rank (PH rank) them based on the predicted IC_{50} values. Next, LE_Scale, a weight-dependant LE function, was derived from 294 known aurora kinase inhibitors. Using the fit quality (FQ = LE/LE_Scale) score derived from the LE_Scale function, the database compounds were reranked (PH_FQ rank) and the top 151 (0.12% of database) compounds were assessed for aurora kinase A inhibition biochem. This VS protocol led to the identif ication of 7 novel hits, with compound 5 showing aurora kinase A IC_{50} = 1.29 μM. Furthermore, testing of 5 against a panel of 31 kinase reveals that it is selective toward aurora kinase A & B, with <50% inhibition for other kinases at 10 μM concentrations and is a suitable candidate for further develo pment. Incorporation of FQ score in the VS protocol not only helped identify a novel aurora kinase inhibitor, 5, but also increased the hit rate of the V S protocol by improving the enrichment factor (EF) for FQ based screening (EF = 828), compared to PH based screening (EF = 237) alone. The LE based VS protocol disclosed here could be applied to other targets for hit identif ication in an efficient manner.

Keywords: ligand efficiency virtual screening compound library; Aurora kinase inhibitor; Fit quality; Ligand efficiency; Pharmac ophore model; Virtual screening

Substances (93) Reactions (28) Citing (14)

215

p53 deficiency enhances mitotic arrest and slippage induced by pharmacological inhibition of Aurora kinases

By: Marxer, M.; Ma, H. T.; Man, W. Y.; Poon, R. Y. C.
Oncogene (2014), 33(27), 3550-3560 | Language: English, Database: CAplus and MEDLINE

A number of small-mol. inhibitors of Aurora kinases have been developed and are undergoing clin. trials for anti- cancer therapies. Different Aurora kinases, however, behave as very different targets: while inhibition of Aurora A (AURKA) induces a delay in mitotic exit, inhibition of Aurora B (AURKB) triggers mitotic slippage. Furthe rmore, while it is evident that p53 is regulated by Aurora kinase-dependent phosphorylation, how p53 may in turn regulate Aurora kinases remains myster ious. To address these issues, isogenic p53-containing and -neg. cells were exposed to classic inhibitors that target both A URKA and AURKB (Alisertib and ZM447439), as well as to new generation of inhibitors that target AURKA (MK-5108), AURKB (Barasertib) individually. The fate of individual cells was then tracked with time-lapse microscopy. Remarkably, loss of p53, either by gene disruption or small interf ering RNA-mediated depletion, sensitized cells to inhibition of both AURKA and AURKB, promoting mitotic arrest and slippage resp. As the p53-dependent post-mitotic checkpoint is also important for preventing genome redupli cation after mitotic slippage, these studies indicate that the loss of p53 in cancer cells represents a major opportunity for anti-cancer drugs targeting the Aurora kinases.

Keywords: p53 tumor suppressor mitosis slippage Aurora kinase inhibitor; Alisertib ZM447439 MK5108 Barasertib Aurora kinase inhibitor antitumor p53

Substances (7) Reactions (0) Citing (52)

216

Preparation of heterocyclic compounds as bromodomain inhibitors

By: Liu, Shuang; Duffy, Bryan Cordell; Quinn, John Frederick; Jiang, May Xiaowu; Wang, Ruifang; Martin, Gregory Scott; Zhao, He; Molino, Bruce Francis; Young, Peter Ronald
World Intellectual Property Organization, WO2014096965 A2 2014-06-26 | Language: English, Database: CAplus

The invention related to heterocyclic compounds of formula **I**, method for their preparation and their use as bromodomain inhibitors and use in therapy. Compounds of formula **I**, wherein W_1 is N and CR^5; W_2 is N and CR^4; W_3 is N and CR^3; R^1 is carbocycles and heterocycles; R^2 is 5- to 6-membered monocyclic carbocycle and heterocycle; R^3, R^4 and R^5 are independently H, alkyl, OH, carbamate, etc.; X is O and S; A is CO, SO_2, CH_2 and CH_2O, etc.; B is O, NH, S, SO, etc.; and their stereoisomers, tautomers, pharmaceutically acceptable salts, and hydrate, are claimed. Example compound **II** was prepared by benzylation of 6-chloro-2H-pyridazin-3-one to afford 2-benzyl-6-chloro-2H-pyridazin-3-one, which underwent cross coupling reaction with 3,5-dimethyl-4-(4,4,5,5-tetramethyl-1,3,2-dioxafooroian-2-yl)isoxazole to afford compound **II**. All the invention compounds were evaluated for their BET inhibitory activity and anticancer activity. From the assay, it was determined that example compound **II** exhibited IC_{50} value of < 30 μM.

Keywords: heterocycle preparation bromodomain inhibitor

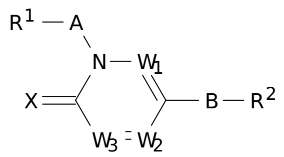

I

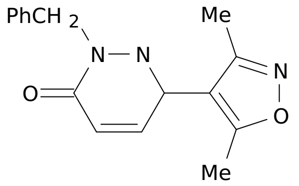

II

PatentPak available

Substances (596) Reactions (686) Citing (8)

217

Preparation of cyclic amines as BET inhibitors for treatment of cancer, autoimmune disorders, and other diseases

By: Fairfax, David John; Duffy, Bryan Cordell; Martin, Gregory Scott; Quinn, John Frederick; Liu, Shuang; Wagner, Gregory Steven; Young, Peter Ronald

United States, US20140142102 A1 2014-05-22 | Language: English, Database: CAplus

The present disclosure relates to compounds and pharmaceutical compositions thereof, which are useful as inhibitors of bromod omain and extra terminal domain (B ET) protein function for treatment or prevention of cancer, autoimmune disorders, inflamm ation, and cardiov ascular diseases. **I** [wherein W^1 is N or C R^1; W^2 is N or C R^2, W^3 is N or C R^3; W^4 is N or C R^4; each W may be the same or different from the others; A is N or CH; R^1, R^2, R^3, and R^4 are indepen dently H, alkyl, aryloxy, etc.; two adjacent substi tuents selected from R^1, R^2, R^3, or R^4 may be connected in a 5- to 6- membered ring to form a bicyclic carbocycle or bicyclic hetero cycle; AR^1 is substituted Ph, substituted pyridinyl, substi tuted furanyl, etc.; B is azirid inyl, (un)substituted azetidinyl, (un)substituted piperi dinyl, etc.] or a stereoisomer, tautomer, pharmace utically acceptable salt, or hydrate thereof are claimed and exempl ified. For example, **II** was prepared in a multistep synthesis (preparation given). **I** were assayed for the ability to inhibit binding of histone H4 and B ET bromodomains, inhibition of c-Myc expression and cell prolife ration in cancer cell lines, and antitumor efficacy in a mouse model of acute myeloid leukemia (data given).

Keywords: cyclic amine preparation BET inhibitor cancer autoimmune disorder

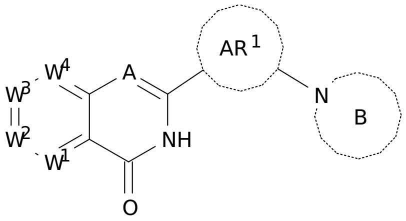

I

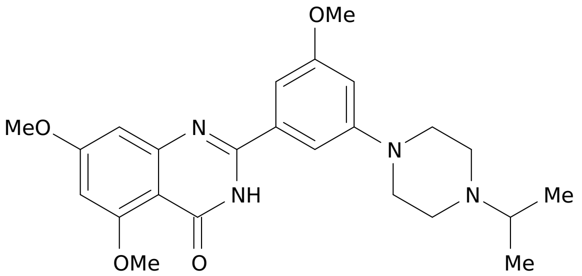

II

PatentPak available

Substances (180) Reactions (225) Citing (0)

218

Preparation of biaryl compounds as BET inhibitors for treatment of cancer, autoimmune disorders, and other diseases

By: Fairfax, David John; Martin, Gregory Scott; Quinn, John Frederick; Duffy, Bryan Cordell; Wagner, Gregory Steven; Young, Peter Ronald

United States, US20140140956 A1 2014-05-22 | Language: English, Database: CAplus

The present disclosure relates to compounds and pharmaceutical compositions thereof, which are useful as inhibitors of bromod omain and extra terminal domain (BET) protein function for treatment or prevention of cancer, autoimmune disorders, inflam matory disorders, and cardiovascular diseases. **I** [wherein W^1 is N or CR^1; W^2 is N or CR^2, W^3 is N or CR^3; W^4 is N or CR^4; each W may be the same or different from the others; A is N or CH; R^1, R^2, R^3, and R^4 are independently H, alkyl, aryloxy, etc.; two adjacent substi tuents selected from R^1, R^2, R^3, or R^4 may be connected in a 5- to 6- membered ring to form a bicyclic carbocycle or bicyclic hetero cycle; AR^1 is a substituted Ph, a substituted pyridinyl, a substituted furanyl, etc.; AR^2 is, for example, Ph, methylsulfonylphenyl, methylsulfinylphenyl, etc.] or a stereoisomer, tautomer, pharmaceutically acceptable salt, or hydrate thereof are claimed and exemplified. Thus, **II** was prepared in 67% yield by the cyclocond ensation of **III** with 2-amino-4,6-dimethoxybenzamide followed by deprotection. **I** were assayed for the ability to inhibit binding of histone H4 and BET bromodomains, inhibition of c-Myc expression and cell proliferation in cancer cell lines, and in vivo efficacy in a rat model of collagen- induced arthritis (data given).

Keywords: biaryl derivative BET inhibitor cancer autoimmune disorder

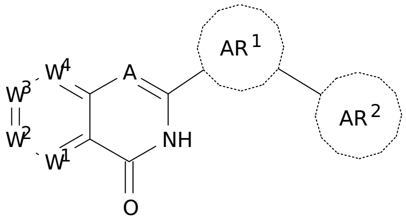

I

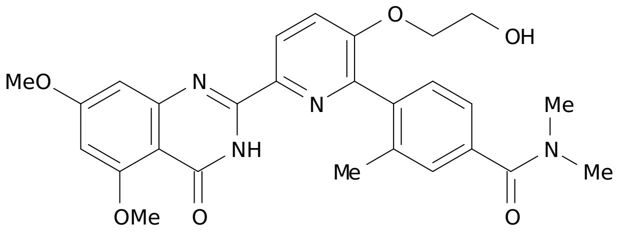

II

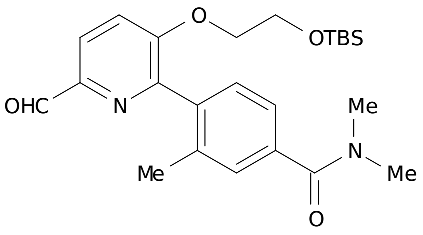

III

PatentPak available

Substances (276) Reactions (1,671) Citing (0)

219

Salt-inducible kinase 3 is a novel mitotic regulator and a target for enhancing antimitotic therapeutic-mediated cell death

By: Chen, H.; Huang, S.; Han, X.; Zhang, J.; Shan, C.; Tsang, Y. H.; Ma, H. T.; Poon, R. Y. C.
Cell Death & Disease (2014), 5(4), e1177 | Language: English, Database: CAplus and MEDLINE

Many mitotic kinases are both critical for maintaining genome stability and are important targets for anticancer therapies. We provide evidence that SIK3 (salt-inducible kinase 3), an AMP-activated protein kinase-related kinase, is important for mitosis to occur properly in mammalian cells. Downregulation of SIK3 resulted in an extension of mitosis in both mouse and human cells but did not affect the DNA damage checkp oint. Time-lapse microscopy and other approaches indicated that mitotic exit but not mitotic entry was delayed. Although repression of SIK3 alone simply delayed mitotic exit, it was able to sensitize cells to various antimi totic chems. Both mitotic arrest and cell death caused by spindle poisons were enhanced after SIK3 depletion. Likewise, the antimitotic effects due to pharmacol. inhibition of mitotic kinases including Aurora A, Aurora B, and polo-like kinase 1 were enhanced in the absence of SIK3. Finally, in addition to promoting the sensit ivity of a small-mol. inhibitor of the mitotic kinesin Eg5, S IK3 depletion was able to overcome cells that developed drug resistance. These results establish the importance of S IK3 as a mitotic regulator and underscore the potential of SIK3 as a druggable antimi totic target.

Keywords: SIK3 mitotic regulator antimitotic drug target mitosis cancer cell

Substances (24) Reactions (0) Citing (15)

220

Structure of Aurora B-INCENP in complex with barasertib reveals a potential transinhibitory mechanism

By: Sessa, Fabio; Villa, Fabrizio
Acta Crystallographica, Section F: Structural Biology Communications (2014), 70(3), 294-298 | Language: English, Database: CAplus and MEDLINE

The Aurora family is a well conserved and well characterized group of serine-threonine kinases involved in the normal progre ssion of mitosis. The deregulation of Aurora kinases impairs spindle assembly, checkpoint function and cell division. To date, many small mols. that compete with ATP for binding to Aurora kinases have been developed and charact erized. Here, the first structure of the Xenopus laevis Aurora B-INCENP complex bound to the clin. relevant small mol. barasertib was determined The binding properties of this inhibitor to the Aurora B active site are analyzed and reported. An unexpected crystal-packing contact in the Aurora B- INCEN P structure coordinated by an A TP analog is also reported, in which the I NCENP C-terminus occupies the substrate-binding region, resembling the protein kinase A inhibitory mechanism.

Keywords: Xenopus Aurora B kinase INCENP barasertib complex crystal structure; Aurora B INCENP AMP PNP complex crystal structure; Aurora kinases; PKA; barasertib; cancer; small-molecule inhibitors

Substances (3) Reactions (0) Citing (26)

221

Close correlation between MEK/ERK and Aurora-B signaling pathways in sustaining tumorigenic potential and radioresistance of gynecological cancer cell lines

By: Marampon, Francesco; Gravina, Giovanni Luca; Popov, Valdimir M.; Scarsella, Luca; Festuccia, Claudio; La Verghetta, Maria Emilia; Parente, Silvia; Cerasani, Manuela; Bruera, Gemma; Ficorella, Corrado; et al
International Journal of Oncology (2014), 44(1), 285-294 | Language: English, Database: CAplus and MEDLINE

Both Aurora-A and -B kinases have been implicated in tumorigenesis; and as such, they represent an attractive therapeutic target. Recent studies found that Aurora-A is a downstream target of mitogen-activated protein kinase 1/ERK2, while Aurora-B has been found to be a prognostic/predictive therapeutic target for epithelial cancer. In a wide range of human cancers, the Ras/Raf/MEK/ERK/MAP kinase pathway is enhanced and the cellular response to growth signals is known to increase. The purpose of this study was to investigate whether the MEK/ERK cascade regulates tumorigenic signaling and radioresistance via the Aurora-B-mediated pathway in a panel of gynecol. cancer cell lines. Exponentially growing human endometrial (Ishikawa), cervical (HeLa), cervical (CASKI) and vulva (SiHa) cancer cells were used in culture treated with either control or MEK/ERK inhibitor or AZD1152 before and after irradiation Western blotting, ERK1/2 siRNA transfection, growth assay in modified monolayer, Annexin V and migration/invasion assays were performed. The specific MEK/ERK inhibitor U0126 decreased the tumorigenic potential and improved the radiation response in all cellular models. The modulation of radioresponse upon U0126 treatment pos. correlated with the inhibition of phospho-ERKs and the reduction of Aurora-B kinase expression. In addition, upon U0126 treatment DNA-PKcs protein expression was found to be downregulated, indicating that the improved radiation response may be caused by decreased DNA double-strand damage repair mechanisms. The knockdown of ERK by siRNA confirmed the MEK/ERK-dependent Aurora-B kinase expression. The use of AZD1152, a selective Aurora-B inhibitor, counteracted tumorigenic potential and radioresistance phenotype by highly increasing apoptotic mechanisms in all gynecol. cancer cell lines used. Evidence from our experiments show that tumorigenic potential and radiation response in gynecol. cancer cells may ensue from a MEK/ERK or Aurora-B inhibition. Together with the close correlation of MEK/ERK and Aurora-B protein expression, this study underlines the potential role of a MEK/ERK/Aurora-B axis whose interruption recovers the antitumor effects of radiotherapy.

Keywords: radiotherapy MEK ERK AuroraB gynecol cancer

Substances (7) Reactions (0) Citing (35)

222

Aurora kinase inhibitor patents and agents in clinical testing: an update (2011 - 2013)

By: Cheung, Chun Hei Antonio; Sarvagalla, Sailu; Lee, Jane Ying-Chieh; Huang, Yi-Chun; Coumar, Mohane Selvaraj
Expert Opinion on Therapeutic Patents (2014), 24(9), 1021-1038 | Language: English, Database: CAplus and MEDLINE

A review. Introduction: Aurora kinase A, B and C, members of serine/threonine kinase family, are key regulators of mitosis. As Aurora kinases are overexpressed in many of the human cancers, small-mol. inhibitors of Aurora kinase have emerged as a possible treatment option for cancer. Areas covered: In 2009 and 2011, the literature pertaining to Aurora kinase inhibitors and their patents was reviewed. Here, the aim is to update the information for Aurora kinase inhibitors in clin. trials and the patents filed between the years 2011 and 2013. Pubmed, Scopus, Scifinder, USPTO, EPO and www.clinicaltrials.gov databases were used for searching the literature and patents for Aurora kinase inhibitors. Expert opinion: Even though both Aurora sub-type selective as well as pan-selective inhibitors show preclin. and clin. efficacy, so far no Aurora kinase inhibitor has been approved for clin. use. Particu larly, dose-limiting toxicity (neutropenia) is a key issue that needs to be addressed. Preliminary evidence suggests that the use of selective Aurora A inhibitors could avoid Aurora B-mediated neutropenia in clin. settings. Also, use of adjunctive agents such as granulocyte stimulating factor to overcome neutropenia associated with Aurora B inhibition could be an answer to overcome the toxicity and bring Aurora inhibitors to market in the future.

Keywords: Aurora kinase inhibitor patent agent review; AMG 900 and KW-2449; AT-9283; AZD1152; Aurora kinase inhibitor; CYC116; ENMD-2076; GSK1070916; MLN8054; MLN8237; PF-3814735; PHA739358; R763; SNS-314; VX-680/MK0457; VX-689/MK-5108

Substances (14) Reactions (0) Citing (44)

223

Aurora kinases as druggable targets in pediatric leukemia: heterogeneity in target modulation activities and cytotoxicity by diverse novel therapeutic agents

By: Jayanthan, Aarthi; Ruan, Yibing; Truong, Tony H.; Narendran, Aru

PLoS One (2014), 9(7), e102741/1-e102741/12, 12 pp. | Language: English, Database: CAplus and MEDLINE

Leukemia is the most common pediatric malignancy, constituting more than 30% of all childhood cancers. Although cure rates have improved greatly, approx. one in five children relapse and poor survival rates post relapse remain a challenge. Given this, more effective and innovative therapeutic strategies are needed in order to improve prognosis. Aurora kinases, a family of serine/t hreonine kinases essential for the regulation of several mitotic processes, have been identified as potential targets for cancer therapeutics. Elevated expression of Aurora kinases has been demonstrated in several malignancies and is associated with aberrant mitotic activity, aneuploidy and alterations in chromosomal structure and genome instability. Based on this rationale, a number of small mol. inhibitors have been formulated and advanced to human studies in the recent past. A comparative anal. of these agents in cytotoxicity and target modulation analyses against a panel of leukemia cells provides novel insights into the unique mechanisms and codependent activity pathways involved in targeting Aurora kinases, consti tuting a distinctive preclin. exptl. framework to identify appropriate agents and combinations in future clin. studies.

Keywords: modulation cytotoxicity therapeutic agent aurora kinase pediatric leukemia heterog eneity

Substances (14) | Reactions (0) | Citing (13)

224

Identification of Aurora-A Inhibitors by Ligand and Structure-Based Virtual Screening

By: Morshed, Mohammad Neaz

Molecular Informatics (2014), 33(5), 369-381 | Language: English, Database: CAplus and MEDLINE

Aurora kinase A has been identified as one of the most attractive targets for cancer therapy because of its critical role in the regulation of the cell cycle. In order to identify active compounds with structural diversity we performed virtual screening. 3D-QSAR pharmacophore models were developed and the best model was used as a query for screening the databases. Ligand and structure-based virtual screening protocol was conducted sequentially by applying the common feature pharmacophore and mol. docking to discover potent Aurora-A inhibitors. A total of eighty-eight compounds were selected for the in vitro activities against various human cancer cell lines (DU145 and HT29). Considering the activity data, we have identified seven compounds to be considered for the next step, among which four compounds had high inhibition rate (above 50 %) at 10 μM with GI_{50} lower than 10 μM. Based on the cell line and enzyme assay (Aurora- A & B) result, these four compounds were used as templat e/query mol. for similarity search. The best result was obtained for similarity hit SH3. It had IC_{50} of 0.578 and 11.77 μM for Aurora-A and B resp., which implies 20-fold selectivity over Aurora-B. The hits obtained from this screening scheme could be potential drug candidates after further optimization.

Keywords: Aurora A inhibitor anticancer drug discovery mol docking cancer; Anticancer; Aurora-A; Docking; Inhibitor; Pharmac ophore; Tumor cell; Virtual screening

Substances (45) | Reactions (0) | Citing (0)

225

Discovery of 7-Aryl-Substituted (1,5-Naphthyridin-4-yl)ureas as Aurora Kinase Inhibitors

By: Defaux, Julien; Antoine, Maud; Le Borgne, Marc; Schuster, Tilmann; Seipelt, Irene; Aicher, Babette; Teifel, Michael; Guenther, Eckhard; Gerlach, Matthias; Marchand, Pascal
ChemMedChem (2014), 9(1), 217-232 | Language: English, Database: CAplus and MEDLINE

As part of our research projects to identify new chem. entities of biol. interest, we developed a synthetic approach and the biol. evaluation of (7-aryl-1,5-naphthyridin-4-yl)ureas as a novel class of Aurora kinase inhibitors for the treatment of malignant diseases based on pathol. cell proliferation. 1,5-Naphthyridine derivatives showed excellent inhibitory activities toward Aurora kinases A and B, and the most active compound, 1-cyclopropyl-3-[7-(1-methyl-1H-pyrazol-4-yl)-1,5-naphthyridin-4-yl]urea (49), displayed IC_{50} values of 13 and 107 nm against Aurora kinases A and B, resp. In addition, the selectivity toward a panel of seven cancer-related protein kinases was highlighted. In vitro ADME properties were also determined in order to rationalize the difficulties in correlating antiproliferative activity with Aurora kinase inhibition. Finally, the good safety profile of these compounds imparts promising potential for their further development as anticancer agents.

Keywords: naphthyridinyl urea derivative preparation Aurora kinase inhibitor cancer; 1,5-naphthyridines; aurora kinases; heterocycles; inhibitors; ureas

Substances (95) | Reactions (529) | Citing (10)

226

Novel pharmaceutical combinations and methods for treating cancer

By: Cheok, Chit Fang; Lane, David
United States, US20130344140 A1 2013-12-26 | Language: English, Database: CAplus

Methods of selectively targeting a p53-deficient cancer cell, comprising administering to a patient suffering from cancer (i) a reversible cell cycle arrest-inducing agent for inducing cell cycle arrest in a p53- pos. cell; and (ii) an aurora kinase inhibitor, wherein said reversible cell cycle arrest-inducing agent is administered prior to administration of said aurora kinase inhibitor, and pharmaceutical combinations, kits and oral dosage forms for the same.

Keywords: p53 deficient cancer treatment pharmaceutical combination; aurora kinase inhibitor cell cycle arrest inducer cancer treatment

PatentPak available

Substances (27) | Reactions (0) | Citing (0)

227

Modulators of GTPase and use in relevant treatment

By: Wandinger-Ness, Angela; Sklar, Larry; Surviladze, Zurab; Oprea, Tudor; Hudson, Laurie; Aube, Jeffrey; Golden, Jennifer E.; Schroeder, Chad E.; Simpson, Denise S.; Noth, Julica J.
United States, US20130345277 A1 2013-12-26 | Language: English, Database: CAplus

The invention relates to pyrazole derivatives which function as selective modulators of the Ras- homologous (Rho) family of small G T Pases, in particular, Cdc42 GTPase and their use to treat diseases, for example cancers, including metastatic cancer, genetic and acquired diseases where activation of Cdc42 GTPase plays a pivotal role, such as neurodege nerative diseases, rheumatoid arthritis, atherosclerosis, diabetes type I, autosomal polycystic kidney disease, cystic kidney disease, precystic kidney disease, microbial infect ions, including Chlamydia infections, E. coli infections, H. pylori infections and its secondary effects including gastric ulcers, Coxiella Brunetti (Q-fever) infections and Strepto coccus pneumonia infections, fungal infections including Paracocc idiodes brasiliensis and Candida albicans and their secondary effects including lung edema. Addnl., compounds according to the invention may be used to inhibit rejection in transplant patients (pursuant to transplantation), to promote immunosuppression, anti-inflammatory response and to mobilize stem cell (migration) in patients in need. Also disclosed is the preparation of compounds of the invention.

Keywords: pyrazole derivative preparation GTPase cancer neurodegenerative disease microbial infection; transplant rejection inflammation immunosuppression stem cell migration pyrazole derivative

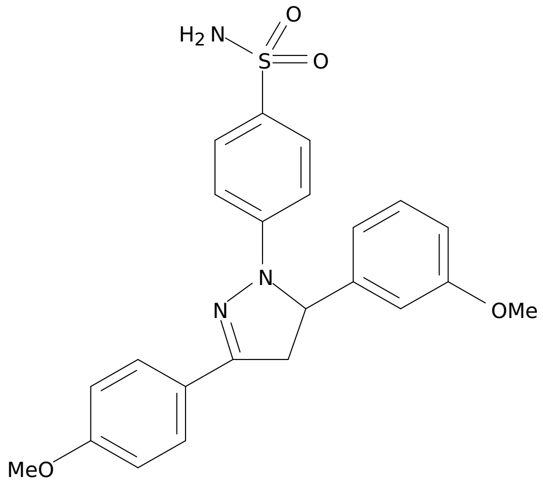

PatentPak available

Substances (325) Reactions (25) Citing (1)

228

Method for preparing barasertib

By: Xu, Xuenong
China, CN103435598 A 2013-12-11 | Language: Chinese, Database: CAplus

The invention relates to method the method for preparing barasertib. Barasertib was prepared via conden sation of 7-{3-[ethyl(2-hydroxyethyl)amino]propoxy}-3,4-dihydroquinazolin-4-one with 2-(5-amino-1H-pyrazol-3-yl)-N-(3-fluorophenyl)acetamide in the presences of organic base and condensing agent. The method has the advantages of simple process, mild conditions, good enviro nment, and high product quality, and is adapted to industrial production

Keywords: hydroxyethyl aminopropoxy quinazolinone amino pyrazolyl fluorophenyl acetamide condensation; barasertib prepar ation green chem

PatentPak available

Substances (3) Reaction (1) Citing (0)

229

Preparation of bifunctional compounds useful for promoting protein degradation for treatment of cancer and oxidative stress-associated diseases

By: Crews, Craig; Gustafson, Jeff; Roth, Anke Gundula; Tae, Hyun Seop; Buckley, Dennis; Neklesa, Taavi

World Intellectual Property Organization, WO2013170147 A1 2013-11-14 | Language: English, Database: CAplus

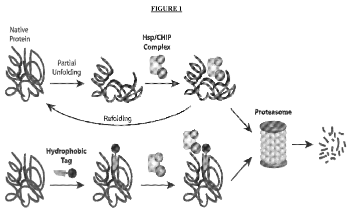

The invention relates to compounds that act as degraders of a target protein, independent of the protein class or its localization for use in treatment of diseases including cancer and oxidative stress-associated disorders. Compounds of formula L-DM [wherein DM is a protein degradation moiety covalently bound to a linker (L) which is a functional group forming a covalent bond to a protein binding moiety] and their pharmaceutically acceptable salts, solvates, or polymorphs thereof having a Clog P equal to or higher than 1.5 are claimed and prepared For example, the compound of formula **I** was prepared in 30% yield via the reaction of **II** with 2-(2-aminoethoxy)ethyl 2-(adamantan-1-yl)acetate. Compounds of the invention exhibit a broad range of pharmacol. activities consistent with the degradation/inhibition of targeted proteins. This was illustrated in an assay in which combined administration of selective androgen receptor degrader **I** (250 nM) and HSP90 inhibitor geldanamycin (100 nM) to LnCAP cells resulted in additive inhibition of androgen receptors compared to either compound alone.

Keywords: bifunctional compound preparation protein degradation cancer oxidative stress disease

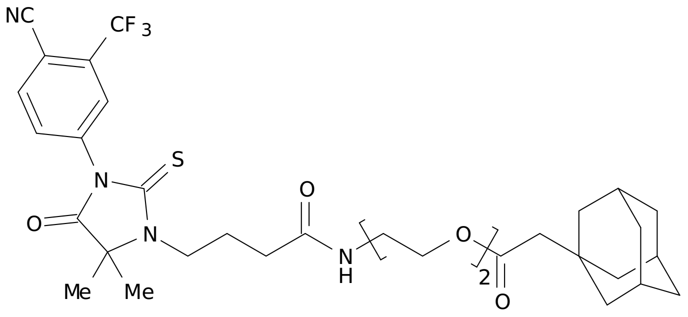

I

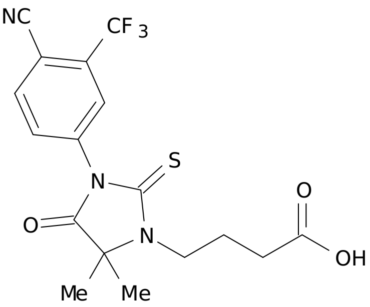

II

PatentPak available

Substances (569) Reactions (406) Citing (8)

230

Phase I Study Assessing the Safety and Tolerability of Barasertib (AZD1152) With Low-Dose Cytosine Arabinoside in Elderly Patients With AML

By: Kantarjian, Hagop M.; Sekeres, Mikkael A.; Ribrag, Vincent; Rousselot, Philippe; Garcia-Manero, Guillermo; Jabbour, Elias J.; Owen, Kate; Stockman, Paul K.; Oliver, Stuart D.
Clinical Lymphoma, Myeloma & Leukemia (2013), 13(5), 559-567 | Language: English, Database: CAplus and MEDLINE

Barasertib is the pro-drug of barasertib-hydroxy-quinazoline pyrazole anilide, a selective Aurora B kinase inhibitor that has demons trated preliminary anti-AML activity in the clin. setting. This Phase I dose- escalation study evaluated the safety and tolera bility of barasertib, combined with LDAC, in patients aged 60 years or older with de novo or secondary A ML. Barasertib (7-day continuous i.v. infusion) plus LDAC 20 mg (s.c. injection twice daily for 10 days) was admini stered in 28-day cycles. The MTD was defined as the highest dose at which ≤ 1 patient within a cohort of 6 experienced a dose-limiting toxicity (DLT) (clin. significant adverse event [AE] or laboratory abnormality considered related to barasertib). The MTD cohort was expanded to 12 patients. Twenty- two patients (median age, 71 years) received ≥ 1 treatment cycle (n = 6, 800 mg; n = 13, 1000 mg; n = 3, 1200 mg).. DLTs were reported in 2 patients (both, National Cancer Institute Common Terminol. Criteria for Adverse Events grade 3 stomatitis/mucositis; 1200 mg cohort). The most common A Es were infection (73%), febrile neutropenia (59%), nausea (50%), and diarrhea (46%). Barasertib plus L DAC resulted in an overall response rate (Interna tional Working Group criteria) of 45% (n = 10/22; according to invest igator opinion). The MTD of 1000 mg barasertib in combin ation with LDAC in older patients with A ML was associated with acceptable tolera bility and preliminary anti-AML activity.

Keywords: barasertib cytosine arabinoside antitumor pharmaco kinetics acute myeloid leukemia elderly; Acute myeloid leukemia; Barasertib-hQPA; Dose-escalation study

Substances (3) Reactions (0) Citing (40)

231

IGF1 biomarker for IGF1R inhibitor therapy of tumors

By: Sathyanarayanan, Sriram; Ayers, Mark
World Intellectual Property Organization, WO2013148568 A1 2013-10-03 | Language: English, Database: CAplus

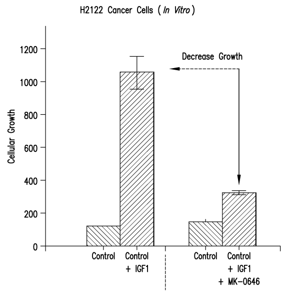

The present invention provides, inter alia, methods for treating tumors that are sensitive to an IGF1R inhibitor. The tumor are determined to be sensitive if the level of IGF1 mRNA expression in the tumor cells, relative to one or more reference genes, reaches a certain threshold level. Methods for evaluating patients as candidates for receipt of the IGF1R inhibitor therapy are also provided as well along with in vitro assay methods and kits for performing any of the methods.

Keywords: IGF1 biomarker IGF1R inhibitor therapy tumor

PatentPak available

Substances (349) Reactions (0) Citing (1)

232

Target inhibition networks: predicting selective combinations of druggable targets to block cancer survival pathways

By: Tang, Jing; Karhinen, Leena; Xu, Tao; Szwajda, Agnieszka; Yadav, Bhagwan; Wennerberg, Krister; Aittokallio, Tero
PLoS Computational Biology (2013), 9(9), e1003226 | Language: English, Database: CAplus and MEDLINE

A recent trend in drug development is to identify drug combin ations or multi-target agents that effectively modify multiple nodes of disease-associated networks. Such polypharmacol. effects may reduce the risk of emerging drug resistance by means of attacking the disease networks through synergistic and synthetic lethal interactions. However, due to the exponentially increasing number of potential drug and target combinations, systematic approaches are needed for priori tizing the most potent multi-target alternatives on a global network level. We took a functional systems pharmacol. approach toward the identification of selective target combin ations for specific cancer cells by combining large- scale screening data on drug treatment efficacies and drug- target binding affini ties. Our model-based prediction approach, named TIMMA, takes advantage of the polypha rmacol. effects of drugs and infers combinatorial drug efficacies through system- level target inhibition networks. Case studies in M CF-7 and MDA-MB-231 breast cancer and BxPC-3 pancreatic cancer cells demonstrated how the target inhibition modeling allows systematic explor ation of functional interactions between drugs and their targets to maximally inhibit multiple survival pathways in a given cancer type. The T I MMA prediction results were exptl. validated by means of systematic si RNA-mediated silencing of the selected targets and their pairwise combinations, showing increased ability to identify not only such druggable kinase targets that are essential for cancer survival either individually or in combination, but also synergistic interactions indicative of non-additive drug efficacies. These system-level analyses were enabled by a novel model constr uction method utilizing maximization and minimization rules, as well as a model selection algorithm based on sequential forward floating search. Compared with an existing computational solution, TIMM A showed both enhanced prediction accuracies in cross validation as well as signif icant reduction in computation times. Such cost-effective computational-exptl. design strategies have the potential to greatly speed- up the drug testing efforts by priori tizing those interventions and interactions warranting further study in individual cancer cases.

Keywords: algorithm anticancer drug target inhibition network prognosis breast carcinoma

Substances (56) Reactions (0) Citing (62)

233

Selectivity Data: Assessment, Predictions, Concordance, and Implications

By: Gao, Cen; Cahya, Suntara; Nicolaou, Christos A.; Wang, Jibo; Watson, Ian A.; Cummins, David J.; Iversen, Philip W.; Vieth, Michal
Journal of Medicinal Chemistry (2013), 56(17), 6991-7002 | Language: English, Database: CAplus and MEDLINE

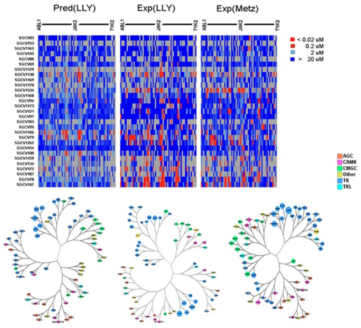

Could high-quality in silico predictions in drug discovery eventually replace part or most of exptl. testing. To evaluate the agreement of selectivity data from different exptl. or predictive sources, we introduce the new metric concor dance min. significant ratio (cMSR). Empowered by cMSR, we find the overall level of agreement between predicted and exptl. data to be comparable to that found between exptl. results from different sources. However, for mols. that are either highly selective or potent, the concordance between different exptl. sources is significantly higher than the concor dance between exptl. and predicted values. We also show that computational models built from one data set are less predictive for other data sources and highlight the importance of bias correction for assessing selectivity data. Finally, we show that small- mol. target space relatio nships derived from different data sources and predictive models share overall similarity but can significantly differ in details.

Keywords: selectivity data exptl predictive source drug discovery modeling

Substances (27)   Reactions (0)   Citing (10)

234

Aurora B inhibitor barasertib and cytarabine exert a greater-than-additive cytotoxicity in acute myeloid leukemia cells

By: Yamauchi, Takahiro; Uzui, Kanako; Shigemi, Hiroko; Negoro, Eiju; Yoshida, Akira; Ueda, Takanori
Cancer Science (2013), 104(7), 926-933 | Language: English, Database: CAplus and MEDLINE

Barasertib, an aurora B inhibitor, terminates cell division, introduces polypl oidy, and consequently causes apoptosis. In the present study, we evaluated the effect of the combination of barasertib and cytarabine (ara-C), a key agent for leukemia chemoth erapy, on leukemic cells in vitro. Human leukemia HL-60 cells and HL-60/ara-C20 cells, a 20- fold ara-C-resistant variant, were used. The 50% growth inhibitory concentrations of an active metabolite of barase rtib, barasertib-hydroxyquinazoline-pyrazol-aniline (Barasertib-H QPA), and ara-C were 51 n M and 300 n M for H L-60 cells and 70 n M and 5300 n M for H L-60/ara-C20 cells, resp. Barase rtib-HQPA induced polyploidy with a subsequent induction of sub-G1 phase apoptosis, indicating the M-phase specific cytotoxicity. Cells treated with the S-phase specific ara-C accumulated in S phase and subseq uently died through apoptosis. When H L-60 cells were treated with barasertib-HQPA and ara-C in combination, a greater-than-additive apoptosis was induced. This enhanc ement was obtained when the cells were treated with barasertib-HQPA prior to ara-C (37.9% sub-G1) or with both concur rently (31.2% sub-G1), but not with ara-C prior to barasertib-HQPA (17.8% sub-G1). The combin ation effects were similarly obtained in HL-60/ara-C20 cells with 19.7% sub-G1 for barasertib-HQPA→ara-C, 18.4% sub-G1 for both concurr ently, and 13.8% sub-G1 for ara-C→barasertib-HQPA, and another leukemic U937 cells with 25.4% sub-G1 for barasertib-HQPA→ara-C, 28.2% sub-G1 for both concurr ently, and 16.0% sub-G1 for ara-C→barasertib-HQPA. Barase rtib-HQPA inhibited aurora B autophosph orylation and histone H3 phosphor ylation in all the cell lines. Barasertib-HQPA did not inhibit D NA synthesis, allowing ara- C incorporation into D NA for its cytotox icity. Thus, barase rtib-HQPA and ara-C provided a greater-than-additive cytotoxicity in leukemic cells in vitro.

Keywords: barasertib hydroxyquinazoline cytarabine e additive acute myeloid leukemia antitumor

Substances (6) Reactions (0) Citing (22)

235

Compounds and methods for the enhanced degradation of targeted proteins and other polypeptides by an E3 ubiquitin ligase, particularly targeting the von Hippel-Lindau E3 ubiquitin ligase using hydroxyproline analogs to disrupt the VHL/HIF-1α interaction

By: Crews, Craig M.; Buckley, Dennis; Ciulli, Alessio; Jorgensen, William; Gareiss, Peter C.; Van Molle, Inge; Gustafson, Jeffrey; Tae, Hyun-Seop; Michel, Julien; Hoyer, Dentin Wade; et al
World Intellectual Property Organization, WO2013106643 A2 2013-07-18 | Language: English, Database: CAplus

The invention is related to bifunctional compounds, e.g., **I**, which find utility as modulators of targeted ubiquitination, especially inhibitors of a variety of polypeptides and other proteins which are degraded and/or otherwise inhibited by bifunctional compounds according to the present invention. In particular, the invention is related to compounds, which contain on one end a von Hippel-Lindau (VHL) ligand which binds to the ubiquitin ligase and on the other end a moiety which binds a target protein such that the target protein is placed in proximity to the ubiquitin ligase to effect degradation (and inhibition) of that protein. The invention exhibits a broad range of pharmacol. activities associated with the compounds of the invention, consistent with the degradation/inhibition of targeted polypeptides. Thus, **II** bound to VHL with a 4.1 μM IC_{50} value. **II** was cocrystd. with VHL and shown to bind at the HIF-1α binding site.

Keywords: hydroxyproline analog preparation ubiquitin ligase inhibitor; chimeric mol preparation targeted protein degradation ubiquitination

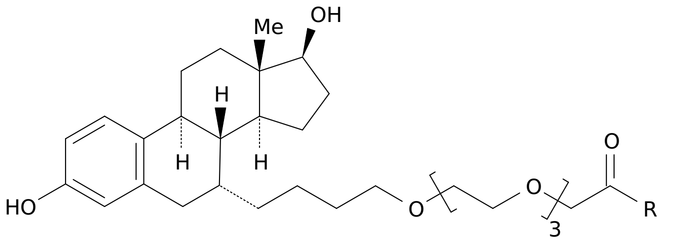

I

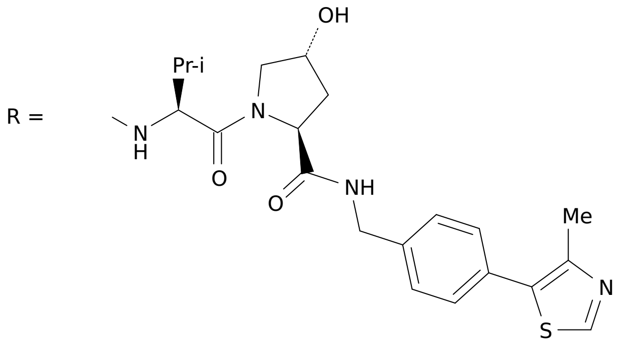

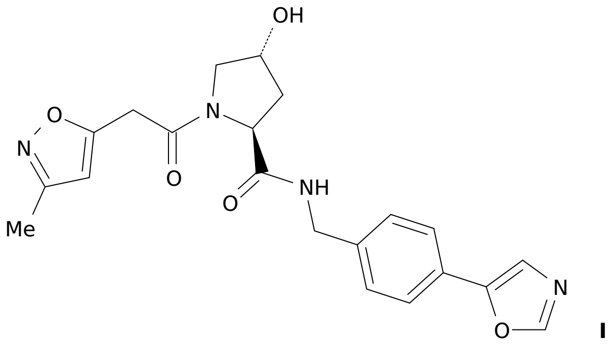

II

PatentPak available

Substances (1,211) Reactions (2,697) Citing (19)

236

Stage I of a phase 2 study assessing the efficacy, safety, and tolerability of barasertib (AZD1152) versus low-dose cytosine arabinoside in elderly patients with acute myeloid leukemia

By: Kantarjian, Hagop M.; Martinelli, Giovanni; Jabbour, Elias J.; Quintas-Cardama, Alfonso; Ando, Kiyoshi; Bay, Jacques-Olivier; Wei, Andrew; Groepper, Stefanie; Papayannidis, Cristina; Owen, Kate; et al
Cancer (Hoboken, NJ, United States) (2013), 119(14), 2611-2619 | Language: English, Database: CAplus and MEDLINE

BACKGROUND : In this phase 2 study, the authors evaluated the efficacy, safety, and tolera bility of the Aurora B kinase inhibitor barasertib compared with low-dose cytosine arabin oside (LDAC) in patients aged ≥60 years with acute myeloid leukemia (A ML). MET HODS : Patients were randomized 2: 1 to receive either open-label barasertib 1200 mg (as a 7- day i.v. infusion) or LDAC 20 mg (s.c. twice daily for 10 days) in 28-day cycles. The primary endpoint was the objective complete response rate (O CRR) (complete responses [CR] plus confirmed CRs with incomplete recovery of neutro phils or platelets [CRi] according to Cheson criteria [also requiring reconfirmation of CRi ≥21 days after the first appearance and associated with partial recovery of platelets and neutro phils]). Secondary endpoints included overall survival (O S) and safety. R ESULTS : In total, 74 patients (barase rtib, n=48; LDAC, n=26) completed ≥1 cycle of treatment. A significant improvement in the OCRR was observed with barasertib (35.4% vs 11.5%; differ ence, 23.9%; 95% confidence interval, 2.7%-39.9%; P<.05). Although the study was not formally sized to compare O S data, the median O S with barasertib was 8.2 mo vs. 4.5 mo with LDAC (hazard ratio, 0.88; 95% confidence interval, 0.49- 1.58; P=.663). Stomatitis and febrile neutropenia were the most common adverse events with barasertib vs. L DAC (71% vs 15% and 67% vs 19%, resp.) . CONCLUS IONS : Barasertib produced a signif icant improvement in the OCRR vs. LDAC and had a more toxic but manageable safety profile, consistent with previous studies. Cancer 2013;119:2611-2619. © 2013 American Cancer Society.

Keywords: antileukemic barasertib cytosine arabin oside dose efficacy safety tolera bility elderly; acute myeloid leukemia; A ZD1152; barasertib; low-dose cytosine arabin oside

Substances (2) Reactions (0) Citing (76)

237

CRLF-2 binding peptides, protocells and viral-like particles useful in the treatment of cancer, including acute lymphoblastic leukemia

By: Brinker, C. Jeffrey; Peabody, David S.; Wharton, Walker; Chackerian, Bryce; Ashley, Carlee Erin; Willman, Cheryl L.; Carnes, Eric C.; Epler, Katherine; Castillo, Robert Eric
World Intellectual Property Organization, WO2013103614 A1 2013-07-11 | Language: English, Database: CAplus

The present invention relates to the use of which are attached or anchored phospholipid biolayers further modified by C RLF-2 and CD 19 binding peptides which may be used for delivering pharmac eutical cargos, to cells expressing CRLF-2 and CD 19, thereby treating cancer, in particular, acute lymphob lastic leukemia, including B-precursor acute lymphob lastic leukemia. Novel CRLF-2 binding peptides and CLRF-2 and CD19-binding viral-like particles (VLPs) useful in the treatment of cancer, including A LL are also provided.

Keywords: CRLF2 binding peptide protocell virus like particle cancer drug; acute lymphob lastic leukemia drug C RLF2 binding peptide

PatentPak available

Substances (335) Reactions (0) Citing (5)

238

Profiling pathway-specific novel therapeutics in preclinical assessment for central nervous system atypical teratoid rhabdoid tumors (CNS ATRT): Favorable activity of targeting EGFR- ErbB2 signaling with lapatinib

By: Singh, Anjali; Lun, Xueqing; Jayanthan, Aarthi; Obaid, Halah; Ruan, Yibing; Strother, Douglas; Chi, Susan N.; Smith, Amy; Forsyth, Peter; Narendran, Aru
Molecular Oncology (2013), 7(3), 497-512 | Language: English, Database: CAplus and MEDLINE

Despite intensifying multimodal treatments, children with central nervous system atypical teratoid/ rhabdoid tumor (CNS ATRT) continue to endure unacceptably high mortality rates. At present, concerted efforts are focusing on underst anding the charact eristic INI1 mutation and its implic ations for the growth and survival of these tumors. Addnl., pharmac eutical pipeline libraries constitute a significant source of potential agents that can be taken to clin. trials in a timely manner. However, this process requires efficient target validation and relevant preclin. studies. As an initial screening approach, a panel of 129 small mol. inhibitors from multiple pharmaceutical pipeline libraries was tested against three A TRT cell lines by in vitro cytoto xicity assays. Based on these data, agents that have strong activity and corresponding susceptible cellular pathways were identified. Target modulation, antibody array anal., drug combination and in vivo xenograft studies were performed on one of the pathway inhibitors found in this screening. Approx. 20% of agents in the library showed activity with IC_{50} values of 1 μM or less and many showed IC_{50} values less than 0.05 μM. Intra cell line variability was also noted among some of the drugs. However, it was determined that agents capable of affecting pathways constituting ErbB2, mTOR, proteasomes, Hsp90, Polo like kinases and Aurora kinases were univer sally effective against the three ATRT cell lines. The first target selected for further anal., the inhibition of Erb B2-EGFR pathway by the small mol. inhibitor lapatinib, indicated inhibition of cell migration properties and the initiation of apoptosis. Synergy between lapatinib and IG F-IR inhibition was also demonstrated by combin ation index (CI) values. Xenograft studies showed effective antitumor activity of lapatinib in vivo. We present an exptl. approach to identifying agents and drug combin ations for future clin. trials and provide evidence for the potential of lapatinib as an effective agent in the context of the biol. and heterogeneity of its targets in A TRT.

Keywords: therapeutic target central nervous system atypical teratoid rhabdoid tumor; anticancer lapatinib E GFR ErbB2 signaling

Substances (34) | Reactions (0) | Citing (29)

239

Method of determination of cancer cell drug sensitivity towards Aurora kinase inhibitors and overcoming their resistance comprising determination of gene or protein expression in patient sample

By: Kollaredy, Madhusudhan Reddy; Hajduch, Marian; Dzubak, Petr; Srovnal, Josef; Hrabakova, Rita; Kovarova, Hana
World Intellectual Property Organization, WO2013083098 A2 2013-06-13 | Language: English, Database: CAplus

The present invention relates to a method for determining the sensitivity and/or resistance of a patient suffering from a cancer disease to Aurora kinase inhibitor therapy, which comprises determining in vitro in the cancer cells or body fluids taken from the patient the expression of at least one gene selected from a particular group and/or determining in vitro in the cancer cells or body fluids taken from the patient the level of at least one protein selected from a particular group.

Keywords: cancer drug sensitivity Aurora kinase inhibitor gene protein expression

PatentPak available

Substances (54) | Reactions (0) | Citing (2)

240

Method of determination of cancer cell drug sensitivity towards Aurora kinase inhibitors and overcoming their resistance comprising determination of gene or protein expression in patient sample

By: Kollareddy, Madhusudhan Reddy; Hajduch, Marian; Dzubak, Petr; Srovnal, Josef; Hrabakova, Rita; Kovarova, Hana
European Patent Organization, EP2602330 A1 2013-06-12 | Language: English, Database: CAplus

The present invention relates to a method for determining the sensitivity and/or resistance of a patient suffering from a cancer disease to Aurora kinase inhibitor therapy, which comprises determining in vitro in the cancer cells or body fluids taken from the patient the expression of at least one gene selected from a particular group and/or determining in vitro in the cancer cells or body fluids taken from the patient the level of at least one protein selected from a particular group. Also, the invention relates to a group of compounds which allow overcoming the resistance.

Keywords: cancer drug sensitivity Aurora kinase inhibitor gene protein expression

PatentPak available

Substances (54) | Reactions (0) | Citing (0)

241

SAR and evaluation of novel 5H-benzo[c][1,8]naphthyridin-6-one analogs as Aurora kinase inhibitors

By: Karra, Srinivasa; Xiao, Yufang; Chen, Xiaoling; Liu-Bujalski, Lesley; Huck, Bayard; Sutton, Amanda; Goutopoulos, Andreas; Askew, Ben; Josephson, Kristopher; Jiang, Xuliang; et al
Bioorganic & Medicinal Chemistry Letters (2013), 23(10), 3081-3087 | Language: English, Database: CAplus and MEDLINE

Several potent Aurora kinase inhibitors derived from 5H-benzo[c][1,8]naphthyridin-6-one scaffold were identified. A crystal structure of Aurora kinase A in complex with an initial hit revealed a binding mode of the inhibitor within the ATP binding site and provided insight for structure-guided compound optimization. Subsequent SAR campaign provided a potent and selective pan Aurora inhibitor, which demonstrated potent target modulation and antiproliferative effects in the pancreatic cell line, MIAPaCa-2. Furthermore, this compound inhibited phosphorylation of histone H3 (pHH3) in mouse bone morrow upon oral administration, which is consistent with inhibition of Aurora kinase B activity.

Keywords: preparation structure benzo naphthyridinone analog Aurora kinase inhibitor antitumor

Substances (96) | Reactions (122) | Citing (15)

242

Reprogramming urokinase into an antibody-recruiting anticancer agent using peptides of arginine chloromethylketone

By: Spiegel, David A.; Jakobsche, Charles E.

World Intellectual Property Organization, WO2013070688 A1 2013-05-16 | Language: English, Database: CAplus

The invention relates to chimeric (preferably, bifunctional) compounds, e.g., **I**•2TFA, compositions comprising those compounds and methods of treating cancer in a patient or subject, especially including metastatic cancer where cancer cells exhibit overexpression (heightened expression) of cell surface urokinase-type plasminogen activator receptor (urokinase receptor) compared to normal (non-cancerous) cells. The compounds preferably covalently bind to the urokinase receptor and recruit native antibodies of the patient or subject where the antibodies can selectively degrade and/or deactivate targeted cancer cells through antibody-dependent cellular phagocytosis and/or antibody-dependent cellular cytotoxicity against a large number and variety of cancers, thus providing cancer cell death and/or an inhibition of growth, elaboration and/or metastasis of the cancer, including remission and cure of the patient's cancer. Thus, chloromethyl ketone **I**•2TFA was prepared by a multistep procedure and evaluated as an antibody-recruiting mol. that targets the urokinase receptor.

Keywords: peptide arginine chloromethylketone preparation reprogramming urokinase antibody recruiting anticancer

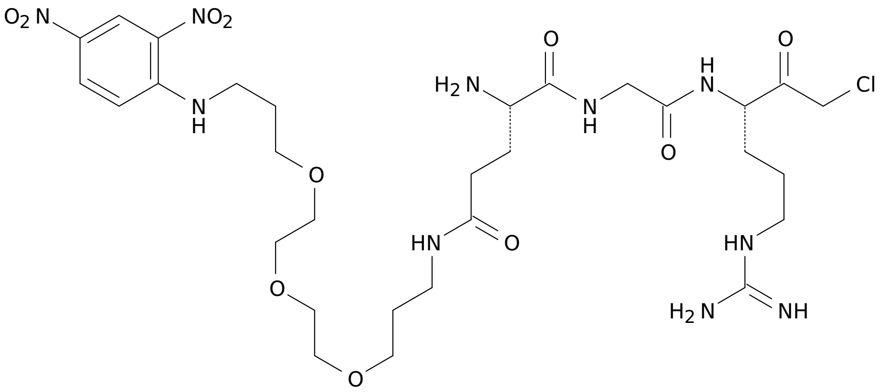

I

PatentPak available

Substances (346) | Reactions (198) | Citing (1)

243

Porous nanoparticle-supported lipid bilayers (protocells) for targeted delivery including transdermal delivery of cargo and methods thereof

By: Ashley, Carlee Erin; Brinker, C. Jeffrey; Carnes, Eric C.; Fekrazad, Mohammad Houman; Felton, Linda A.; Negrete, Oscar; Padilla, David Patrick; Wilkinson, Brian S.; Wilkinson, Dan C.; Willman, Cheryl L.
World Intellectual Property Organization, WO2013056132 A2 2013-04-18 | Language: English, Database: CAplus

The present invention is directed to protocells for specific targeting of hepatocellular and other cancer cells which comprise a nanoporous silica core with a supported lipid bilayer; at least one agent which facilitates cancer cell death, such as a traditional small mol., a macromol. cargo, e.g., siRNA, or a protein toxin, such as ricin toxin A-chain or diphtheria toxin A-chain, and/or a histone-packaged plasmid DNA disposed within the nanoporous silica core (preferably superc oiled in order to more efficiently package the DNA into protocells) which is optionally modified with a nuclear locali zation sequence to assist in localizing protocells within the nucleus of the cancer cell and the ability to express peptides involved in therapy (apoptosis/cell death) of the cancer cell or as a reporter, a targeting peptide which targets cancer cells in tissue to be treated such that binding of the protocell to the targeted cells is specific and enhanced and a fusogenic peptide that promotes endosomal escape of protocells and encapsulated D NA. Protocells according to the present invention may be used to treat cancer, especially including hepatoc ellular (liver) cancer using novel binding peptides (c-MET peptides) which selectively bind to hepatocellular tissue or to function in diagnosis of cancer, including cancer treatment and drug discovery. For example, the porous protocells were prepared via fusion of AEPTMS-modified silica cores and a liposomes composed of DOPC/DOPE/cholesterol/PEG2000 conjugated with a targeting peptide S P94 (H_2N-SFSIILTP ILPLEEEGGC-COOH, SEQ ID NO: 8) for cellular delivery of si RNA. The ability of si RNA-loaded, SP94-targeted DOPC-containing protocells to selectively induce cytotoxicity of human hepatoc ellular carcinoma cells (Hep3 B) was demonstrated.

Keywords: lipid bilayer nanoparticle protocell cancer targeting

PatentPak available

Substances (434) Reactions (0) Citing (15)

244

A molecular case report: Functional assay of tyrosine kinase inhibitors in cells from a patient's primary renal cell carcinoma

By: Kulesz-Martin, Molly; Lagowski, James; Olson, Susan; Wortham, Aaron; West, Toni; Thomas, George; Ryan, Christopher W.; Tyner, Jeffrey W.
Cancer Biology & Therapy (2013), 14(2), 95-99 | Language: English, Database: CAplus and MEDLINE

Current therapies for renal cell carcinoma favor vascular endothelial growth factor receptor (VEGF-R) tyrosine kinase (T K) inhibitors (TKIs). In theory, these are most applicable in tumors that have lost V HL-with subsequent stabili zation of HIF and upregulation of VE GF. A subset of patients harbor primary- refractory disease, as in this case, where there was no evidence for loss of V HL or chromosome 3p. We evaluated mol. targeted agents in viable tumor cells cultured from a patient's clear cell renal cell carcinoma (R CC). Of 66 agents, only dasatinib, an inhibitor of Src tyrosine kinase, strongly reduced viability of the patient's cultured kidney tumor cells. Immunostaining of the original primary tumor revealed strong positivity for V HL and Src protein expres sion. Functional evaluation of a patient's tumor cells appears feasible in the setting of RCC.

Keywords: tyrosine kinase inhibitor renal cell carcinoma anticancer; molecular targeted therapy; renal cell carcinoma; tyrosine kinase

Substances (55) Reactions (0) Citing (2)

245

Effect of aurora kinase B inhibitor AZD1152 on cisplatin-resistant epithelial ovarian carcinoma Hey cells

By: Ma, Ya-xi; Li, Xiu-zhen
Zhonghua Fuchanke Zazhi (2013), 48(1), 46-50 | Language: Chinese, Database: CAplus and MEDLINE

This paper is to investigate the effect of aurora kinase B inhibitor A ZD1152 on cisplatin-resistant epithelial ovarian carcinoma Hey cells. Hey (cisplatin-resistant ovarian cancer cell line) cells were analyzed. According to the treatment plan, Hey cells were divided into four groups (AZD group, cisplatin group, AZD+cisplatin group and control group). Me thiazolyl tetrazolium (MTT) assay was used to test the cell proliferation; caspase-3/7 activity anal. was used to analyze cells apoptosis; fluorescence in-situ hybridization (FI SH) assay was used to determine the copy number of chromosome 7 and check the copy number of h TERC gene and C-myc gene. MTT test showed that proliferation in AZD group was lower than that in control group (P<0.01). The proliferation rates of cells treated with 10 and 20 nmol/L AZD for 24 h were (81.4±3.6)% and (81.4±3.6)% resp., and the proliferation rates of cells for 48 h were (43.1±2.0)% and (38.5±1.6)% resp., which was significantly lower than those in control group (100%, P<0.01); after cells were treated with same concentration of AZD, inhibition of proliferation was significantly enhanced as the time prolongation (P<0.01). Proliferation rate in AZD+cisplatin group was lower than that in cisplatin group (P<0.01), which suggest that there were additive effects after combining AZD with cisplatin. Compared with control group, caspase-3/7 activity in AZD group increased significantly (P=0.000), and the same result was seen between A ZD±cisplatin group and cisplatin group or A ZD group (all P<0.01). Compared with cisplatin group or control group, the copy numbers of hTERC, C-myc and the number of chromosome were significantly increased in AZD group and AZD+cisplatin group (all P<0.05). AZD could inhibit ovarian cancer cell proliferation and induce cell apoptosis significantly. AZD alone or in combination with cisplatin may result in the increased cell polypl oidy.

Keywords: aurora kinase B inhibitor AZD1152 cisplatin resistant; epithelial ovarian carcinoma Hey cell

Substances (3) Reactions (0) Citing (7)

246

Phase I study of barasertib (AZD1152), a selective inhibitor of Aurora B kinase, in patients with advanced solid tumors

By: Schwartz, Gary K.; Carvajal, Richard D.; Midgley, Rachel; Rodig, Scott J.; Stockman, Paul K.; Ataman, Ozlem; Wilson, David; Das, Shampa; Shapiro, Geoffrey I.
Investigational New Drugs (2013), 31(2), 370-380 | Language: English, Database: CAplus and MEDLINE

The purpose of this study was to determine the maximum-tolerated dose (MTD), pharmacokinetics and safety profile for two different dosing regimens of barasertib, a selective inhibitor of Aurora B Kinase. In this Phase I trial, patients with advanced solid malignancies were treated with escalating doses of barasertib, administered as either a 48-h continuous infusion or as two 2-h infusions on consecutive days, both every 14 days of a 28- day cycle. Thirty-five patients were treated. The MTDs were 150 mg as a 48-h continuous infusion and 220 mg admini stered as two 2-h infusions (110 mg/day, days 1, 2, 15 and 16), with neutropenia the dose-limiting toxicity (DLT) of each schedule. Common Terminol. Criteria of Adverse Events (C TCAE) grade ≥ 3 neutropenia (with or without fever) occurred in 34 % of patients overall. Other adverse events, many of hematol. or gastrointestinal etiol., were of mild or moderate intensity. No objective tumor responses were observed, although stable disease was observed in 23 % of patients. Systemic exposure to barasertib-hQPA, the more active moiety to which barasertib is converted, was observed by 1 and 6 h into the 2-h and continuous infusion, resp., and exhibited linear pharmacok inetics. In summary, barasertib was generally well tolerated, with neutropenia the most frequent and dose-limiting toxicity, irresp. of schedule. Future development of barasertib will depend on better definition of its therapeutic index.

Keywords: barasertib AuroraB kinase inhibitor colon lung skin cancer; pharmaco kinetics pharmacodynamics antitumor

Substances (3) Reactions (0) Citing (52)

247

Aurora kinase inhibitor AZD1152 has an additional effect of platinum on a sequential application at the human ovarian cancer cell line SKOV3

By: Ma, Yaxi; Weimer, Joerg; Fredrik, Regina; Adam-Klages, Sabine; Sebens, Susanne; Caliebe, Amke; Hilpert, Felix; Eckmann-Scholz, Christel; Arnold, Norbert; Schem, Christian
Archives of Gynecology and Obstetrics (2013), 288(1), 173-182 | Language: English, Database: CAplus and MEDLINE

Purpose: The treatment of ovarian tumors is carried out with platinum medicine which can lead to incompatibilities or resistances. Thus, it is of great interest to check new medicine suitability for its applic ation. AZD1152 is an aurora kinase inhibitor predomi nantly works against aurora kinase B involved in the chromosome segregation. Cells become polyploidy and reduce the proliferation by this impairment. To investigate whether AZD1152, may play a role in the treatment of ovarian carcinoma we serving it to the cispla tinum-resistant cell line S KOV3 alone and in combination with platinum. Methods: We look at the prolife ration, the ploidy, the phases of cell cycle and the apoptosis activity of the cells. Results and conclusion: We could show that the combin ation of both medicines in the preclin. experiment produces a working advantage.

Keywords: anticancer Aurora kinase inhibitor AZD1152 platinum ovarian cancer

Substances (6) Reactions (0) Citing (8)

248

Effect of the drug transporters ABCG2, Abcg2, ABCB1 and ABCC2 on the disposition, brain accumulation and myelotoxicity of the aurora kinase B inhibitor barasertib and its more active form barasertib-hydroxy-QPA

By: Marchetti, Serena; Pluim, Dick; Eijndhoven, Monique; Tellingen, Olaf; Mazzanti, Roberto; Beijnen, Jos H.; Schellens, Jan H. M.
Investigational New Drugs (2013), 31(5), 1125-1135 | Language: English, Database: CAplus and MEDLINE

We explored whether barasertib (AZD1152), a selective Aurora B kinase inhibitor, is a substrate for P- glycoprotein (Pgp, MDR1), breast cancer resistance protein (BCRP), and multidrug resistance protein 2 (M RP2) in vitro. Cell survival, drug transport, and competition experiments with barasertib pro-drug and the more active form of the drug (barase rtib-hQPA) were performed using MDCKII (wild type, M DR1, BCRP, and M RP2) and LLCPK (wild type and M DR1) cells and monolayers, and Sf9-BCRP membrane vesicles. Moreover we tested whether P-gp and BCRP affect the oral pharmacokinetics, tissue distribution, and myelotoxicity of barasertib in vivo using Bcrp1$^{-/-}$/Mdr1a/1b $^{-/-}$ (triple knockout) and wild type mice. In cell survival experi ments expression of BCRP and MDR1 resulted in signif icant resistance to barasertib. In transwell experiments, barasertib-hQPA was transported by B CRP and MDR1 efficiently. In Sf9-BCRP membrane vesicles, both barasertib and barase rtib-hQPA significantly inhibited the B CRP-mediated transport of methotrexate. In contrast, no active transport of barasertib by M RP2 was observed, and overexpression of MRP2 did not affect cytotoxicity of barasertib. In vivo, systemic exposure as well as bioavailability, brain penetration, kidney and liver distri bution and myelotoxicity of barasertib-hQPA were statistically significantly increased in Bcrp1$^{-/-}$/Mdr1a/1b$^{-/-}$ compared with wild type mice ($p<0.001$). Barasertib is transported efficiently by P-gp and BCRP/Bcrp1 in vitro. In vivo, genetic deletion of P-gp and BCRP in mice significantly affected pharmacokinetics, tissue distribution and myelotoxicity of barasertib-hQPA. Possible clin. conseq uences for the observed affinity of barasertib for P- gp and BCRP need to be explored.

Keywords: barasertib aurora kinase B inhibitor ABCG2 ABCB1 ABCC2 pharmacokinetics

Substances (4) Reactions (0) Citing (19)

249

Kinase Pathway Dependence in Primary Human Leukemias Determined by Rapid Inhibitor Screening

By: Tyner, Jeffrey W.; Yang, Wayne F.; Bankhead, Armand III; Fan, Guang; Fletcher, Luke B.; Bryant, Jade; Glover, Jason M.; Chang, Bill H.; Spurgeon, Stephen E.; Fleming, William H.; et al
Cancer Research (2013), 73(1), 285-296 | Language: English, Database: CAplus and MEDLINE

Kinases are dysregulated in most cancers, but the frequency of specific kinase mutations is low, indicating a complex etiol. in kinase dysregulation. Here, we report a strategy to rapidly identify functi onally important kinase targets, irresp. of the etiol. of kinase pathway dysregulation, ultimately enabling a correl ation of patient genetic profiles to clin. effective kinase inhibi tors. Our methodol. assessed the sensitivity of primary leukemia patient samples to a panel of 66 small- mol. kinase inhibitors over 3 days. Screening of 151 leukemia patient samples revealed a wide diversity of drug sensitivities, with 70% of the clin. specimens exhibiting hypersen sitivity to one or more drugs. From this data set, we developed an algorithm to predict kinase pathway dependence based on anal. of inhibitor sensitivity patterns. Applying this algorithm correctly identified pathway dependence in proof- of-principle specimens with known oncogenes, including a rare FLT3 mutation outside regions covered by standard mol. diagnostic tests. Interro gation of all 151 patient specimens with this algorithm identified a diversity of kinase targets and signaling pathways that could aid priorit ization of deep sequencing data sets, permitting a cumulative anal. to understand kinase pathway dependence within leukemia subsets. In a proof-of-principle case, we showed that in vitro drug sensit ivity could predict both a clin. response and the develo pment of drug resistance. Taken together, our results suggested that drug target scores derived from a compreh ensive kinase inhibitor panel could predict pathway dependence in cancer cells while simultaneously identifying potential therapeutic options.

Keywords: kinase inhibitor signaling leukemia drug screening target anticancer

Substances (231) Reactions (0) Citing (116)

250

Multiparametric cell-based assay for the evaluation of transcription inhibition by high-content imaging

By: Torres-Guzman, Raquel; Chu, Shaoyou; Velasco, Juan A.; Lallena, Maria Jose
Journal of Biomolecular Screening (2013), 18(5), 556-566 | Language: English, Database: CAplus and MEDLINE

Loss of normal cell cycle regulation is a hallmark of human cancer. Cyclin-dependent kinases (CDKs) are key regulators of the cell cycle and have been actively pursued as promising therapeutic targets. Likewise, members of the C DK family are functi onally related to transcriptional modulation, a mol. pathway suitable for therap eutic intervention. We used a set of 2500 compounds in the U2OS cell line to evaluate its effect in the cell division process. Interes tingly, out of this anal., we identified a subpopu lation of compounds that are able to inhibit RNA polymerase activity, thus interfering with gene transcription processes. After this finding, we developed, validated, and fully automated a multiparameter highcontent imaging (HCI) assay to measure phosphorylation of the RNA polymerase II carboxyl terminal domain (p CTD). Simultaneously, we measured both the D NA content and cell prolife ration index in the treated cells. The linear regression anal. comparing the IC50 for p CTD and the 4N EC50 for DNA content or IC50 for cell proliferation showed an excellent agreement ($r2 = 0.84$ and $r2 = 0.94$, resp.) . Our results confirm that this method allows discrim inating between cell cycle and transcr iption inhibition and confirms H CI as a powerful technol. for the identif ication of compounds with an effective and selective pathway phenotype.

Keywords: cancer multiparametric cell based assay transcription high content imaging; RNA pol II; assay development; cell cycle; gene transcription; high-content imaging

Substances (20) Reactions (0) Citing (6)

251

A new approach for prediction of tumor sensitivity to targeted drugs based on functional data

By: Berlow, Noah; Davis, Lara E.; Cantor, Emma L.; Seguin, Bernard; Keller, Charles; Pal, Ranadip
BMC Bioinformatics (2013), 14, 239 | Language: English, Database: CAplus and MEDLINE

Background: The success of targeted anti-cancer drugs are frequently hindered by the lack of knowledge of the individual pathway of the patient and the extreme data requirements on the estimation of the personalized genetic network of the patient's tumor. The prediction of tumor sensitivity to targeted drugs remains a major challenge in the design of optimal therapeutic strategies. The current sensitivity prediction approaches are primarily based on genetic characterizations of the tumor sample. We propose a novel sensitivity prediction approach based on functional perturbation data that incorporates the drug protein interaction information and sensitivities to a training set of drugs with known targets. Results: We illustrate the high prediction accuracy of our framework on synthetic data generated from the Kyoto Encyclopedia of Genes and Genomes (KEGG) and an exptl. dataset of four canine osteosarcoma tumor cultures following application of 60 targeted small-mol. drugs. We achieve a low leave one out cross validation error of <10% for the canine osteosarcoma tumor cultures using a drug screen consisting of 60 targeted drugs. Conclusions: The proposed framework provides a unique input-output based methodol. to model a cancer pathway and predict the effectiveness of targeted anti-cancer drugs. This framework can be developed as a viable approach for personalized cancer therapy.

Keywords: protein anticancer agent interaction osteosarcoma sensitivity math model

Substances (44) Reactions (0) Citing (22)

252

Combination therapy with antibodies targeting insulin-like growth factor 1 receptor (IGF1R)

By: Goetsch, Liliane; Hsu, Karl
World Intellectual Property Organization, WO2012158657 A1 2012-11-22 | Language: English, Database: CAplus

The authors disclose the use of an anti-IGF1R antibody (or antigen-binding fragment) in combination with one or more chemotherapeutic agents for treatment of cancer. In one example, pancreatic cancer patients receiving dalotuzumab and gemcitabine exhibited greater progression free survival.

Keywords: combination therapy cancer insulinlike growth factor receptor antibody

PatentPak available

Substances (297) Reactions (0) Citing (1)

253

Preparation of bisaminoquinoline compounds as autophagy inhibitors useful in mono- and combination therapy of cancer and other diseases

By: Amaravadi, Ravi K.; Winkler, Jeffrey
World Intellectual Property Organization, WO2012149186 A2 2012-11-01 | Language: English, Database: CAplus

Title compounds **I** [R^1 and $R^{1'}$ independently = H, halo, C N, NO_2, (un)substituted alkyl, O-alkyl, acyl, oxycarbonyl ester or carboxy ester; R and R' indepen dently = H, (un)substituted alkyl, acyl, carboxy ester; L = $(CH_2Y)_n$-X-$(Y'CH_2)_n$ or A-$(CH_2$-CH_2-$Z)_n$-A'; X = absent, O, S, etc.; Y = absent, CH_2, O, CH_2O, etc.; Y' forms a stable bond when present; R" = H or (un) substituted alkyl; n = 0- 4; A = bond (absent), CH_2-CH_2, CH_2, etc.; Z = O or N- Rz; Rz = H or (un)substituted alkyl], and their pharmaceutically acceptable salts, enanti omers, diastereomers, solvates or polymorphs, are prepared for inhibiting autophagy in biol. systems. Methods of treating cancer in patients in need using compounds and/or compositions according to the present invention alone or in combin ation with at least one addnl. anticancer agent represent addnl. aspects of the invention. Methods of treating disease states and/or conditions in which inhibition of autophagy plays a favorable treatment role including rheumatoid arthritis, malaria, antiphospholipid antibody syndrome, lupus, chronic urticaria and Sjogren's disease, with compounds according to the present invention represent addnl. aspects of the invention.

Keywords: bisaminoquinoline autophagy inhibitor cancer

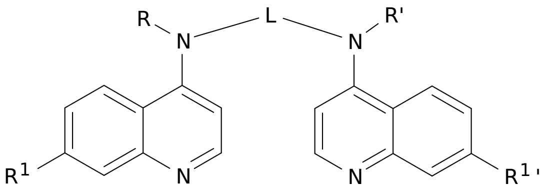

I

PatentPak available

Substances (376)   Reactions (59)   Citing (5)

254

Inhibition of Aurora-B kinase activity confers antitumor efficacy in preclinical mouse models of early and advanced gastrointestinal neoplasia

By: Alferez, Denis G.; Goodland, Roberta A.; Odedra, Rajesh; Sini, Patrizia; Crafter, Claire; Ryan, Anderson J.; Wedge, Stephen R.; Wright, Nicholas A.; Anderson, Elizabeth; Wilkinson, Robert W.
International Journal of Oncology (2012), 41(4), 1475-1485 | Language: English, Database: CAplus and MEDLINE

The Aurora family of kinases, play a fundamental role in cell division and are overexp ressed in several cancers including colon. The activity of barasertib-hQPA, a selective inhibitor of Aurora- B kinase (ABK) was investigated in a range of preclin. models of gastroin testinal cancer. Treatment with barasertib-hQPA produced anti-proliferative and cytotoxic effects across a panel of human colorectal cancer (CRC) cell lines in vitro. Prodrug, barasertib [48- h s.c. (s.c.) infusion; 150 mg/kg/day] inhibited the growth of S W620, Colo205, HCT116 human colorectal tumor xenografts in nude mice signifi cantly (Student's t-test, P<0.05, n=10-12 per group). Flow cytometric anal. of single cells from disaggregated barasertib-treated SW620 tumors revealed a decrease in phospho rylated histone H3 (phH3) and an increase in tumor cells with ≥4 N DNA content (P<0.05). The activity of barasertib was then examined in $Apc^{Min/+}$ mice, a spontaneous model of early intestinal neoplasia. Macroscopic evaluation of the small intestine revealed that barasertib treatment [25 mg/kg intra-peritoneal (i.p.) Q1Dx4 each week for 3 wk] of 8- wk old $Apc^{Min/+}$ mice produced a 39% reduction in macroadenoma number (P=0.02) and a 43% reduction in overall adenoma burden (P=0.02) compared with vehicle- treated controls. Quantification of microscopic adenomas revealed a >64% reduction in the number of adenomas spanning more than one villus. Histol. anal. of these adenomas revealed a number of distinct changes in barasertib-treated $Apc^{Min/+}$ mice, including a 94% reduction in the proportion of phosphohistone H3-pos. cells (P<0.001) and a 53% reduction in the number of cells per adenoma (P=0.001). These results provide a scientific rationale for investi gating ABK inhibitors as a treatment for intestinal cancer.

Keywords: antitumor auroraB kinase inhibitor barasertib APC signaling gastrointestinal neoplasia

Substances (4) Reactions (0) Citing (10)

255

Development of o-Chlorophenyl Substituted Pyrimidines as Exceptionally Potent Aurora Kinase Inhibitors

By: Lawrence, Harshani R.; Martin, Mathew P.; Luo, Yunting; Pireddu, Roberta; Yang, Hua; Gevariya, Harsukh; Ozcan, Sevil; Zhu, Jin-Yi; Kendig, Robert; Rodriguez, Mercedes; et al

Journal of Medicinal Chemistry (2012), 55(17), 7392-7416 | Language: English, Database: CAplus and MEDLINE

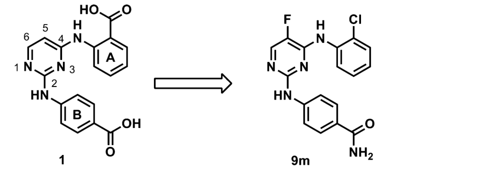

Aurora A IC_{50} = 6.1 ± 1.0 nM (*in vitro*) Aurora A IC_{50} = 14.4 ± 1.7 nM (*in vitro*)
Aurora A IC_{50} > 100 μM (*in vivo*) Aurora A IC_{50} = 0.3 - 1.0 μM (*in vivo*)

The o-carboxylic acid substituted bisanilinopyrimidine **I** was identified as a potent hit (Aurora A I C_{50} = 6.1 ± 1.0 n M) from inhouse screening. Detailed structure-activity relationship (SAR) studies indicated that polar substi tuents at the para position of the B- ring are critical for potent activity. X-ray crystallog. studies revealed that compound **I** is a type I inhibitor that binds the Aurora kinase active site in a DFG-in conformation. Structure-activity guided replacement of the A-ring carboxylic acid with halogens and incorpo ration of fluorine at the pyrimidine 5- position led to highly potent inhibitors of Aurora A that bind in a D FG-out conformation. B-Ring modifications were undertaken to improve the solubility and cell permeab ility. Compounds such as **II** with water-solubilizing moieties at the para position of the B-ring inhibited the autophosphorylation of Aurora A in M DA-MB-468 breast cancer cells.

Keywords: dianilinopyrimidine preparation SAR crystal structure Aurora kinase inhibitor

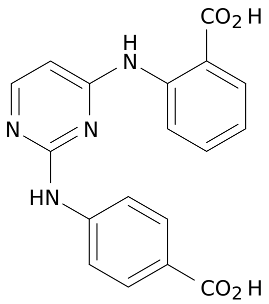

I

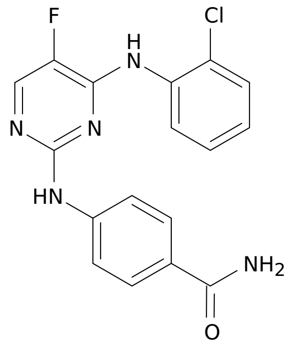

I I

Substances (171) Reactions (197) Citing (53)

256

Novel pharmaceutical combinations and methods for treating cancer

By: Cheok, Chit Fang; Lane, David
World Intellectual Property Organization, WO2012121662 A1 2012-09-13 | Language: English, Database: CAplus

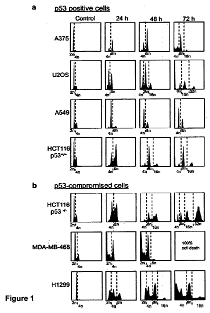

Methods of selectively targeting a p53-deficient cancer cell, comprising administering to a patient suffering from cancer (i) a reversible cell cycle arrest-inducing agent for inducing cell cycle arrest in a p53- pos. cell; and (ii) an aurora kinase inhibitor, wherein said reversible cell cycle arrest-inducing agent is administered prior to administration of said aurora kinase inhibitor, and pharmac eutical combinations, kits and oral dosage forms for the same.

Keywords: p53 deficient cancer treatment pharmaceutical combination; aurora kinase inhibitor cell cycle arrest inducer cancer treatment

PatentPak available

Substances (27) | Reactions (0) | Citing (0)

257

Two-stage model-based design of cancer phase I dose escalation trials: evaluation using the phase I program of barasertib (AZD1152)

By: Keizer, Ron J.; Zandvliet, Anthe S.; Beijnen, Jos H.; Schellens, Jan H. M.; Huitema, Alwin D. R.
Investigational New Drugs (2012), 30(4), 1519-1530 | Language: English, Database: CAplus and MEDLINE

Introduction. Modeling and simulation of pharmaco kinetics and pharmaco dynamics has previously been shown to be potentially useful in designing Phase I programs of novel anti-cancer agents that show hematol. toxicity. In this anal., a two- stage model-based trial design was evaluated retrospectively using data from the Phase I program with the aurora kinase inhibitor barase rtib. Methods. Data from two Phase I trials and four regimens were used (n = 79). Using barasertib-hydroxy QPA plasma concentrations and neutrophil count data from only study 1A, a PKPD model was developed and subsequently used to predict the MTD and a safe starting dose for the other trials. Results. The PKPD model based on data from the first study adequately described the time course of neutrophil count fluctuation. The two-stage model-based design provided safe starting doses for subsequent phase I trials for barasertib. Predicted safe starting dose levels were higher than those used in two subsequent trials, but lower than used in the other trial. Discussion. The two-stage approach could have been applied safely to define starting doses for altern ative dosing strategies with barasertib. The limited improvement in efficiency for the phase I program of barasertib may have been due to the fact that starting doses for the studied phase I trials were already nearly optimal. Conclusion. Application of the two-stage model-based trial design in Phase I programs with novel anti-cancer drugs that cause haematol. toxicity is feasible, safe, and may lead to a reduction in the number of patient treated at sub-therapeutic dose-levels.

Keywords: barasertib pharmacokinetics pharmacodynamics cancer anticancer

Substances (2) | Reactions (0) | Citing (9)

258

Characterizing tumor response to chemotherapy at various length scales using temporal diffusion spectroscopy

By: Xu, Junzhong; Li, Ke; Smith, R. Adam; Waterton, John C.; Zhao, Ping; Chen, Heidi; Does, Mark D.; Manning, H. Charles; Gore, John C.
PLoS One (2012), 7(7), e41714 | Language: English, Database: CAplus and MEDLINE

Measurements of apparent diffusion coeffi cient (ADC) using magnetic resonance imaging (M RI) have been suggested as potential imaging biomarkers for monitoring tumor response to treatment. However, conventional pulsed-gradient spin echo (P GSE) methods incorporate relatively long diffusion times, and are usually sensitive to changes in cell d. and necrosis. Diffusion temporal spectroscopy using the oscillating gradient spin echo (O GSE) sequence is capable of probing short length scales, and may detect significant intracellular microstru ctural changes indepe ndent of gross cell d. changes following anti- cancer treatment. To test this hypothesis, SW620 xenografts were treated by barasertib (A ZD1152), a selective inhibitor of Aurora B kinase which causes S W620 cancer cells to develop polyploidy and increase in size following treatment, ultimately leading to cell death through apoptosis. Following treatment, the ADC values obtained by both the P GSE and low frequency O GSE methods increased. However, the A DC values at high gradient frequency (i.e. short diffusion times) were significantly lower in treated tumors, consistent with increased intracellular restrictions/hindrances. This suggests that A DC values at long diffusion times are dominated by tumor microst ructure at long length scales, and may not convey unambiguous information of subcellular space. While the diffusion temporal spectr oscopy provides more comprehe nsive means to probe tumor microstr ucture at various length scales. This work is the first study to probe intracellular microstru ctural variations due to polyploidy following treatment using diffusion M RI in vivo. It is also the first observation of post-treatment ADC changes occurring in opposite directions at short and long diffusion times. The current study suggests that temporal diffusion spectroscopy potentially provides pharmacodynamic biomarkers of tumor early response which distinguish microstru ctural variations following treatment at both the subcel lular and suprace llular length scales.

Keywords: barasertib antitumor colon cancer apoptosis spectroscopy

Substances (2) | Reactions (0) | Citing (29)

259

Exploring protein kinase inhibitors. Unveiling gemcitabine resistance in pancreatic cancer. Comments

By: Kim, Yeon Jeong; Hong, Young Bin; Cho, Chi Heum; Seong, Yeon-Sun; Bae, Insoo
Pancreas (Hagerstown, MD, United States) (2012), 41(5), 804-805 | Language: English, Database: CAplus and MEDLINE

Gemcitabine is one of first-line therapies for locally advanced pancreatic cancer, but severe resistance is respon sible for less than 6 mo of median survival and less than 20% of response rate. Although efforts to overcome gemcitabine resistance have been underway, the only combination treatment that has shown a small but statist ically significant outcome is gemcit abine with erlotinib, an epidermal growth factor receptor inhibitor. Improvements in pancreatic cancer therapy can be made by underst anding gemcit abine resistance. To enhance the treatment benefits of conven tional therapeutic drugs, targeting protein kinases in combin ation with DNA damaging drugs has been aggres sively attempted for several types of cancers. As part of this effort in pancreatic cancer, the present study explored 76 protein kinase inhibitors (PKIs) for probing new targetable protein kinases and found that several P K Is possess promising efficacy in combin ation with gemcit abine.

Keywords: protein kinase inhibitor gemcitabine resistance cytotoxic pancreatic cancer

Substances (21) | Reactions (0) | Citing (6)

260

Frequency of TP53 Mutations and its Impact on Drug Sensitivity in Acute Myeloid Leukemia?

By: Shah, Ankur; Seedhouse, Claire

Indian Journal of Clinical Biochemistry (2012), 27(2), 121-126 | Language: English, Database: CAplus and MEDLINE

The purpose of this study is to find out the frequency of TP53 mutations in acute myeloid leukemia (AML) patients and correlate sensitivity of drug response with T P53 mutations. In AML more than 90 % of cases comprise of wild type T P53. 94.2 % of T P53 mutations are found within exon 5-8 of which 73 % are point mutations. T P53 mutations were analyzed with high resolution melting curve anal. We analyzed 106 AML samples of which we found nine mutations which represents 8.5 % mutation rate and found one rare SNP. The effect of T P53 mutations were studied on the chemosen sitivity of two new drugs AZD115 and RHPS4, an Aurora Kinase B inhibitor and Telomerase inhibitor resp. Four mutations were found out of 17 for RHPS4 stating signif icant (p = 0.002) increase in sensitivity and no mutation found in A ZD1152 database, but need more study to get definite conclu sion.

Keywords: AZD1152 RHPS4 antitumor T P53 mutation chemosensitivity acute myeloid leukemia; Acute myeloid leukemia; Drug sensitivity; High resolution melting curve analysis; T P53

Substances (4) Reactions (0) Citing (3)

261

Possibility of aurora kinase inhibitors for AML

By: Tsuboi, Kosuke

Ketsueki Naika (2012), 64(3), 335-338 | Language: Japanese, Database: CAplus

A review. In this paper, (AZD1152)barasertib peptidase, possibility of Aurora kinase inhibitor for treatment of acute myeloid leukemia.

Keywords: review Aurora kinase antitumor acute myeloid leukemia barasertib

Substances (2) Reactions (0) Citing (0)

262

Probing the structural requirements of A-type Aurora kinase inhibitors using 3D-QSAR and molecular docking analysis

By: Zhang, Hui-xiao; Li, Yan; Wang, Xia; Wang, Yong-hua
Journal of Molecular Modeling (2012), 18(3), 1107-1122 | Language: English, Database: CAplus and MEDLINE

Aurora-A, the most widely studied isoform of Aurora kinase overexp ressed aberrantly in a wide variety of tumors, has been implicated in early mitotic entry, degradation of natural tumor suppressor p53 and centrosome maturation and separa tion; hence, potent inhibitors of Aurora-A may be therapeutically useful drugs in the treatment of various forms of cancer. Here, we report an in silico study on a group of 220 reported Aurora-A inhibitors with six different substru ctures. Three-dimensional quant. structure-activity relationship (3D-QSAR) studies were carried out using compar ative mol. field anal. (CoMFA) and comparative mol. similarity indexes anal. (CoMSIA) techniques on this series of mols. The resultant optimum 3 D-QSAR models exhibited an r value of 0.404-0.582 and their predictive ability was validated using an indepe ndent test set, ending in r 0.512- 0.985. In addition, docking studies were employed to explore these protein-inhibitor interactions at the mol. level. The results of 3 D-QSAR and docking analyses validated each other, and the key structural requirements affecting Aurora-A inhibitory activities, and the influential amino acids involved were identified. To the best of our knowledge, this is the first report on 3 D-QSAR modeling of Aurora- A inhibitors, and the results can be used to accurately predict the binding affinity of related analogs and also facilitate the rational design of novel inhibitors with more potent biol. activities.

Keywords: Aurora kinase inhibitor antitumor 3D QSAR mol docking

Substances (221) | Reactions (0) | Citing (12)

263

Evolution of resistance to Aurora kinase B inhibitors in leukaemia cells

By: Failes, Timothy W.; Mitic, Gorjana; Abdel-Halim, Heba; Po'uha, Sela T.; Liu, Marjorie; Hibbs, David E.; Kavallaris, Maria
PLoS One (2012), 7(2), e30734 | Language: English, Database: CAplus and MEDLINE

Aurora kinase inhibitors are new mitosis-targeting drugs currently in clin. trials for the treatment of haematol. and solid maligna ncies. However, knowledge of the mol. factors that influence sensit ivity and resistance remains limited. Herein, we developed and characterised an in vitro leukemia model of resistance to the Aurora B inhibitor Z M447439. Human T-cell acute lymphob lastic leukemia cells, CCRF-CEM, were selected for resistance in 4 μ M ZM447439. CEM/AKB4 cells showed no cross- resistance to tubulin-targeted and DNA-damaging agents, but were hypersensitive to an Aurora kinase A inhibitor. Sequencing revealed a mutation in the Aurora B kinase domain corresponding to a G160E amino acid substit ution. Mol. modeling of drug binding in Aurora B containing this mutation suggested that resistance is mediated by the glutamate substitution preventing formation of an active drug- binding motif. Progression of resistance in the more highly selected C EM/AKB8 and CEM/AKB16 cells, derived sequentially from CEM/AKB4 in 8 and 16 μM ZM447439 resp., was mediated by addnl. defects. These defects were indepe ndent of Aurora B and multi- drug resistance pathways and are associated with reduced apoptosis mostly likely due to reduced inhibition of the catalytic activity of Aurora kinase B in the presence of drug. Our findings are important in the context of the use of these new targeted agents in treatment regimes against leukemia and suggest resistance to therapy may arise through multiple independent mechanisms.

Keywords: ZM447439 anticancer aurora kinase B inhibitor leukemia cell

Substances (9) | Reactions (0) | Citing (12)

264

Selective Aurora Kinase Inhibitors Identified Using a Taxol-Induced Checkpoint Sensitivity Screen

By: Kwiatkowski, Nicholas; Deng, Xianming; Wang, Jinhua; Tan, Li; Villa, Fabrizio; Santaguida, Stefano; Huang, Hsiao-Chun; Mitchison, Tim; Musacchio, Andrea; Gray, Nathanael
ACS Chemical Biology (2012), 7(1), 185-196 | Language: English, Database: CAplus and MEDLINE

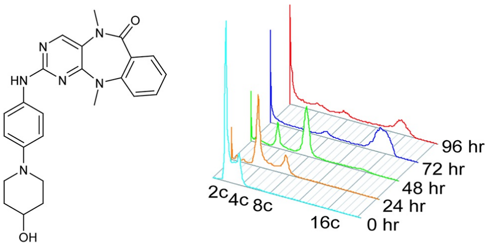

Compound **1**

The members of the Aurora kinase family play critical roles in the regulation of the cell cycle and mitotic spindle assembly and have been intensively investigated as potential targets for a new class of anticancer drugs. We describe a new highly potent and selective class of Aurora kinase inhibitors discovered using a phenotypic cellular screen. Optimized inhibitors display many of the hallmarks of Aurora inhibition including endoreduplication, polyploidy, and loss of cell viability in cancer cells. Structure- activity relationships with respect to kinome-wide selectivity and guided by an Aurora B co- crystal structure resulted in the identification of key selectivity determinants and discovery of a subseries with selectivity toward Aurora A. A direct comparison of biochem. and cellular profiles with respect to published Aurora inhibitors including VX-680, AZD1152, MLN8054, and a pyrimidine-based compound from Genentech demonstrates that compounds 1 and 3 will become valuable addnl. pharmacol. probes of Aurora- dependent functions.

Keywords: aurora kinase inhibitor taxol checkpoint sensitivity screening

Substances (37) Reactions (0) Citing (22)

265

Aurora B Is Regulated by the Mitogen-activated Protein Kinase/Extracellular Signal-regulated Kinase (MAPK/ERK) Signaling Pathway and Is a Valuable Potential Target in Melanoma Cells

By: Bonet, Caroline; Giuliano, Sandy; Ohanna, Mickael; Bille, Karine; Allegra, Maryline; Lacour, Jean-Philippe; Bahadoran, Philippe; Rocchi, Stephane; Ballotti, Robert; Bertolotto, Corine
Journal of Biological Chemistry (2012), 287(35), 29887-29898 | Language: English, Database: CAplus and MEDLINE

Metastatic melanoma is a deadly skin cancer and is resistant to almost all existing treatment. Vemurafenib, which targets the BRAF V600E mutation, is one of the drugs that improves patient outcome, but the patients next develop secondary resistance and a return to cancer. Thus, new therapeutic strategies are needed to treat melanomas and to increase the duration of v- Raf murine sarcoma viral oncogene homolog B1 (BRAF) inhibitor response. The ERK pathway controls cell proliferation, and Aurora B plays a pivotal role in cell division. Here, we confirm that Aurora B is highly expressed in metastatic melanoma cells and that Aurora B inhibition triggers both senescence-like phenotypes and cell death in melanoma cells. Furthermore, we show that the BRAF/ERK axis controls Aurora B expression at the transcriptional level, likely through the transcription factor FOXM1. Our results provide insight into the mechanism of Aurora B regulation and the first mol. basis of Aurora B regulation in melanoma cells. The inhibition of Aurora B expression that we observed in vemurafenib-sensitive melanoma cells was rescued in cells resistant to this drug. Consistently, these latter cells remain sensitive to the effect of the Aurora B inhibitor. Noteworthy, wild-type BRAF melanoma cells are also sensitive to Aurora B inhibition. Collectively, our findings, showing that Aurora B is a potential target in melanoma cells, particularly in those vemurafenib-resistant, may open new avenues to improve the treatment of metastatic melanoma.

Keywords: Aurora B ERK melanoma metastasis FOXM1 vemurafenib resistance AZD1152

Substances (7) Reactions (0) Citing (60)

266

The MEK1/2 inhibitor, selumetinib (AZD6244; ARRY-142886), enhances anti-tumour efficacy when combined with conventional chemotherapeutic agents in human tumour xenograft models

By: Holt, S. V.; Logie, A.; Odedra, R.; Heier, A.; Heaton, S. P.; Alferez, D.; Davies, B. R.; Wilkinson, R. W.; Smith, P. D.
British Journal of Cancer (2012), 106(5), 858-866 | Language: English, Database: CAplus and MEDLINE

Background: The Ras/RAF/MEK/ERK pathway is frequently deregu lated in cancer and a number of inhibitors that target this pathway are currently in clin. development. It is likely that clin. testing of these agents will be in combin ation with standard therapies to harness the apoptotic potential of both the agents. To support this strategy, it has been widely observed that a number of chemotherapeutics stimulate the activation of several intrace llular signalling cascades including Ras/R AF/MEK/ERK. The MEK1/2 inhibitor selumetinib has been shown to have anti-tumor activity and induce apoptotic cell death as a monoth erapy. Methods: The aim of this study was to identify agents, which would be likely to offer clin. benefit when combined with selumetinib. Here, we used human tumor xenograft models and assessed the effects combining standard chemotherapeutic agents with selumetinib on tumor growth. In addition, we analyzed tumor tissue to determine the mechanistic effects of these combinations. Results: Combining selumetinib with the DNA-alkylating agent, temozolomide (TMZ), resulted in enhanced tumor growth inhibition compared with monotherapies. Biomarker studies highlighted an increase in γ H2A.X suggesting that selumetinib is able to enhance the D NA damage induced by TMZ alone. In several models we observed that continuous exposure to selume tinib in combination with docetaxel results in tumor regression. Scheduling of docetaxel before selumetinib was more beneficial than when selume tinib was dosed before docetaxel and demonstrated a pro-apoptotic phenotype. Similar results were seen when selume tinib was combined with the Aurora B inhibitor barasertib. Conclusion: The data presented suggests that M EK inhibition in combin ation with several standard chemotherapeutics or an Aurora B kinase inhibitor is a promising clin. strategy. British Journal of Cancer (2012) 106, 858-866; doi:10.1038/bjc.2012.8 www.bjcancer.com Published online 16 Feb. 2012.

Keywords: selumetinib temozolomide docetaxel antitumor D NA damage apoptosis colorectal cancer

Substances (14) Reactions (0) Citing (85)

267

Targeting Aurora kinases in cancer treatment

By: Kelly, Kevin R.; Ecsedy, Jeffrey; Mahalingam, Devalingam; Nawrocki, Steffan T.; Padmanabhan, Swaminathan; Giles, Francis J.; Carew, Jennifer S.
Current Drug Targets (2011), 12(14), 2067-2078 | Language: English, Database: CAplus and MEDLINE

A review. The Aurora family of serine/threonine kinases is essential for chromosome alignment, segreg ation, centrosomal matura tion, mitotic spindle formation, and cytoki nesis during mitosis. Their fundam ental role in cell cycle regulation and aberrant expression in a broad range of malignancies prompted the development of small mols. that selectively inhibit their activity. Recent studies have revealed new insights into the cellular effects of Aurora kinase inhibition. Moreover, early phase clin. studies have shown that these agents have therapeutic efficacy. In this review, we will outline the functions of Aurora kinases in normal cell division and in malignancy. We will focus on recent preclin. and clin. studies that have explored the mechanism of action and clin. effect of Aurora inhibitors in cancer treatment.

Keywords: review aurora kinase inhibitor cancer anticancer

Substances (12) Reactions (0) Citing (37)

268

Phase 1/2 study to assess the safety, efficacy, and pharmacokinetics of barasertib (AZD1152) in patients with advanced acute myeloid leukemia

By: Loewenberg, Bob; Muus, Petra; Ossenkoppele, Gert; Rousselot, Philippe; Cahn, Jean-Yves; Ifrah, Norbert; Martinelli, Giovanni; Amadori, Sergio; Berman, Ellin; Sonneveld, Pieter; et al
Blood (2011), 118(23), 6030-6036 | Language: English, Database: CAplus and MEDLINE

The primary objective of this 2-part phase 1/2 study was to determine the maximum-tolerated dose (MTD) of the potent and selective Aurora B kinase inhibitor barasertib (AZD1152) in patients with newly diagnosed or relapsed acute myeloid leukemia (AML). Part A determined the MTD of barasertib administered as a continuous 7-day infusion every 21 days. In part B, the efficacy of barasertib was evaluated at the MTD. In part A, 32 patients were treated with barasertib 50 mg (n = 3), 100 mg (n = 3), 200 mg (n = 3), 400 mg (n = 4), 800 mg (n = 7), 1200 mg (n = 6), and 1600 mg (n = 6). Dose-limiting toxicities (stomatitis/mucosal inflammation events) were reported in the 800 mg (n = 1), 1200 mg (n = 1), and 1600 mg (n = 2) groups. The MTD was defined as 1200 mg. In part B, 32 patients received barasertib 1200 mg. In each part of the study, 8 of 32 patients had a hematol. response according to Cheson AML criteria. The most commonly reported grade ≥ 3 events were febrile neutropenia (n = 24) and stomatitis/mucosal inflammation (n = 16). We concluded that the MTD of barasertib is 1200 mg in patients with relapsed or newly diagnosed AML. Toxicity was manageable and barasertib treatment resulted in an overall hematol. response rate of 25%. This.

Keywords: barasertib anticancer agent toxicity pharmacokinetics acute myeloid leukemia

Substances (5) Reactions (0) Citing (55)

269

Aurora kinase inhibitor AZD1152 negatively affects the growth and survival of HTLV-1-infected T lymphocytes in vitro [Retraction of document cited in CA154:351346]

By: Tanaka, Tomita M.; Mori, N.
International Journal of Cancer (2011), 129(11), 2763 | Language: English, Database: CAplus

This article has been retracted by the authors, Editor-in-Chief, and publisher due to inappropriate duplication.

Keywords: retraction AZD1152 human T cell leukemia virus lymphocyte signaling

Substances (3) Reactions (0) Citing (0)

270

A Phase I study to assess the safety, pharmacokinetics and efficacy of barasertib (AZD1152), an Aurora B kinase inhibitor, in Japanese patients with advanced acute myeloid leukemia

By: Tsuboi, Kosuke; Yokozawa, Toshiya; Sakura, Toru; Watanabe, Takashi; Fujisawa, Shin; Yamauchi, Takahiro; Uike, Naokuni; Ando, Kiyoshi; Kihara, Rika; Tobinai, Kensei; et al

Leukemia Research (2011), 35(10), 1384-1389 | Language: English, Database: CAplus and MEDLINE

Barasertib (AZD1152) is a highly potent and selective Aurora B kinase inhibitor. The safety, efficacy and pharmaco kinetic (PK) profile of barasertib were investigated in Japanese patients with advanced acute myeloid leukemia. Barasertib (50- 1200 mg) was admini stered as a continuous 7- day i.v. infusion every 21 days. No dose- limiting toxicities were reported and barasertib 1200 mg was chosen for further evaluation in Japanese patients. Neutropenia and febrile neutropenia were the most commonly reported adverse events. The PK profile was similar to Western patients. A promising overall hematol. response rate of 19% was achieved, which warrants further investigation in these patients.

Keywords: barasertib acute myeloid leukemia pharmacokinetic antitumor Japanese

Substances (3) Reactions (0) Citing (41)

271

Display of antibody fragments on virus-like particles of RNA bacteriophages

By: Peabody, David S.; Chackerian, Bryce

World Intellectual Property Organization, WO2011116226 A2 2011-09-22 | Language: English, Database: CAplus

The invention enables the display of antibody single-chain variable fragments (scFv's) on virus-like particles (VLPs) of bacteriophages such as MS2. The VLPs encapsidate mRNA encoding the coat protein from which it assembles, enabling the recovery by reverse transcription and PCR of affinity-selected sequences from scFv libraries. The antibody single-chain variable fragment is inserted at a site corresponding to amino acids 11-17 or more particularly 13-17, or at the C-terminus or C-terminus, of the coat protein of MS-2. The efficacy and rate of capsid assembly are influenced and maximized in the presence of the MS2 translational operator, a 19-nucleotide RNA stem-loop (pac site) that, via its interaction with coat protein, mediates exclusive encapsidation of the MS2 genome during bacteriophage replication. The VLP expresses or displays a scFv peptide that has an affinity for a cancer cell, including solid tumors and leukemias, or a cell infected with a virus. The invention may be used for cell-type specific delivery of drugs by employing VLPs which will deliver compounds to specific cells, or delivery of image contrast enhancing agents. Related virus- like particles, method for constructing a library of scFv-VLPs, drug delivery vehicles comprising one or more pharmaceu tically-active ingredients, biomedical imaging agents, assays, and kits are also provided.

Keywords: antibody display virus like particle RNA bacteriophage; drug delivery virus like particle R NA bacteriophage; single chain antibody display virus like particle RNA bacteriophage

PatentPak available

Substances (334) Reactions (0) Citing (7)

272

Combination therapy for treating cancer and diagnostic assays for use therein

By: Shah, Omar Jameel; Shen, Yu; Lin, Xiaoya; Anderson, Mark; Huang, Xiaoli; Li, Junling; Li, Leiming
World Intellectual Property Organization, WO2011068863 A1 2011-06-09 | Language: English, Database: CAplus

The present disclosure relates to a combination of therapeutic agents for use in treating a patient suffering from cancer, wherein said combination comprises at least one polyploidy inducing agent and at least one Bcl- 2 family protein inhibitor. In addition, the present disclosure also relates to diagnostic assays useful in classification of patients for treatment with one or more therap eutic agents.

Keywords: antitumor combination therapy cancer therapy diagnosis assay

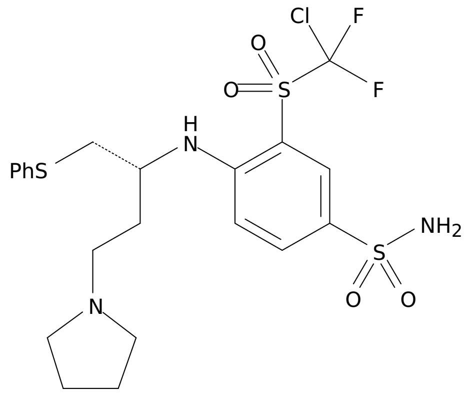

PatentPak available

Substances (181) Reactions (803) Citing (2)

273

IGF1R inhibitor based treatment of prostate cancer

By: Long, Brian; Groothuis, Patrick Gerhard; Hicklin, Daniel
World Intellectual Property Organization, WO2011057064 A1 2011-05-12 | Language: English, Database: CAplus

The present invention encompasses, inter alia, methods for treating prostate cancer comprising a chromo somal translocation with an IGF1R inhibitor such as an isolated antibody that binds specifically to IGF1R or a small organic compound The present invention provides a method for treating or preventing a TMPRSS2-ERG-pos. prostate cancer tumor or a prostate tumor comprising cells that overexpress the androgen receptor (AR), and/or for inducing apoptosis of cells of said tumor, in a subject, comprising adminis tering, to the subject, a therapeutically effective amount of IGF1R inhibitor; option ally, further comprising determining whether the tumor cells express IGF1R and whether the tumor cells comprise the T MPRSS2-ERG fusion and/or overex press AR wherein (for example, by obtaining a sample of said tumor cells from the subject and analyzing them for the presence of said characteristics), if the tumor cells express IGF1R and if the tumor cells do comprise the T MPRSS2-ERG fusion and/or overex press AR, then treatment with the IG F1R inhibitor is commenced or continued; and wherein if the tumor cells do not express I GF1R and/or if the tumor cells do not comprise the TMPRSS2-ERG fusion and/or overexp ressed AR, then the treatment with the IGF1R inhibitor is discontinued.

Keywords: IGF1R inhibitor treatment prostate cancer T MPRSS2 ERG fusion; antibody IGF1R prostate cancer treatment

PatentPak available

Substances (323) Reactions (0) Citing (2)

274

Effects of AZD1152, a selective Aurora B kinase inhibitor, on Burkitt's and Hodgkin's lymphomas

By: Mori, Naoki; Ishikawa, Chie; Senba, Masachika; Kimura, Masashi; Okano, Yukio
Biochemical Pharmacology (2011), 81(9), 1106-1115 | Language: English, Database: CAplus and MEDLINE

We studied the effects of AZD1152, an Aurora B kinase inhibitor, on Burkitt's lymphoma (B L) and Hodgkin's lymphoma (HL) in human tissues and cell cultures and in a murine xenograft model of lymphoma. Aurora kinase A and B levels were assessed by RT-P CR and immunohistochem. They were aberrantly expressed in BL and HL cell lines, and in lymph nodes from patients with B L and HL. Next, activation of the Aurora B promoter was detected by reporter gene assays. The promoter activity of Aurora B kinase was high in BL cell lines and the Aurora B promoter contained a pos. regulatory region between - 74 and -104 from the transcription initiation site. AZD1152-hQPA had antiproliferative effects in the BL and HL cell lines studied; inhibited the phosphorylation of histone H3 and retinoblastoma proteins, and resulted in cells with >4 N D NA content. AZD1152-hQPA induced caspase-dependent apoptosis of some cell lines, demonstrated by loss of mitochondrial membrane potential, activation of caspase-9, followed by activation of caspase-3. This effect was accompanied by the inhibition of survivin expression. In vivo efficacy was determined in NO D/SCID/γc^{null} mice implanted with the Ramos human B L cell line. AZD1152 had anti-tumor effects in this murine xenograft model. There preclin. data suggest that the inhibition of Aurora B kinase is a potentially useful therapeutic strategy in BL and HL.

Keywords: Aurora B kinase inhibitor AZD1152 Burkitt's Hodgkin's lymphoma

Substances (7)   Reactions (0)   Citing (17)

275

Enhanced radiosensitivity of androgen-resistant prostate cancer: AZD1152-mediated Aurora kinase B inhibition

By: Niermann, Kenneth J.; Moretti, Luigi; Giacalone, Nicholas J.; Sun, Yunguang; Schleicher, Stephen M.; Kopsombut, Prapaporn; Mitchell, Lauren R.; Kim, Kwang Woon; Lu, Bo
Radiation Research (2011), 175(4), 444-451 | Language: English, Database: CAplus and MEDLINE

Aurora kinase B (AURKB) is critical to the process of mitosis, aiding in chromosome condensation by phosphorylating histone H3. We investigated the effects of AZD1152, an AURKB inhibitor, on radiosensitivity of androgen-insensitive prostate cancer cells. The goal of this study was to test whether AZD1152 increases the susceptibility of hormone-refractory prostate cancer cells to radiation-induced DNA damage and to determine the conditions of A ZD1152 treatment that maximize radiosensitization. PC3 and DU145 cells were treated with various AZD1152 doses for various durations to elucidate the conditions that yielded maximal increases in G_2/M-phase and polyploid cells. To assess DNA damage, γ-H2AX phosphorylation was quantified for cells grown under radiosen sitizing conditions and subjected to either no radiation or 5 Gy radiation. Radiosen sitivity was determined by clonogenic assays. Cell cycle effects in both cell lines were maximized by treatment with 60 nM AZD1152 for 48 h. AZD1152-treated cells exhibited signifi cantly increased DNA damage 30 min postirradiation (PC3: 100% compared to 68%, P = 0.035; D U145: 100% compared to 69%, P = 0.034), with addnl. DNA damage 6 h postirradiation (PC3: 85% compared to 15%, P = 0.002; D U145: 67% compared to 21%, P = 0.012). Radiosensitivity was increased in both cell lines, with dose enhancement ratios of 1.53 for PC3 cells (P = 0.017) and 1.71 for DU145 cells (P = 0.02). This study identifies the optimal A ZD1152 treatment conditions to maximize the radiosensitization of PC3 and DU145 cells. These results suggest a major role for D NA damage and impairment of D NA repair mechanisms in AZD1152-induced radiosensitization of prostate cancer cells.

Keywords: radiotherapy AZD1152 radiosensitizer androgen prostate cancer; Aurora kinase B signaling inhibitor

Substances (2)   Reactions (0)   Citing (25)

276

N-(4-((3-(2-amino-4-pyrimidinyl)-2-pyridinyl)oxy)phenyl)-4-(4-methyl-2-thienyl)-1-phthalazinamine for use in the treatment of antimitotic agent resistant cancer

By: Payton, Marc; Kendall, Richard
World Intellectual Property Organization, WO2011031842 A1 2011-03-17 | Language: English, Database: CAplus

The present invention relates to methods of using the compound, N-(4-((3-(2-amino-4-pyrimidinyl)-2-pyridinyl)oxy)phenyl)-4-(4-methyl-2-thienyl)-1-phthalazinamine or a pharmace utically acceptable salt thereof, to treat cancers, including solid tumors, which have become resistant to treatment with chemotherapeutic agents, including anti-mitotic agents such as taxanes, and/or other anti-cancer agents, including aurora kinase inhibiting agents. The invention also includes methods of treating cancers refractory to such treatments by administering a pharmaceutical composition, comprising the compound to a cancer subject.

Keywords: phthalazinamine derivative cancer treatment aurora kinase inhibitor

PatentPak available

Substances (41) | Reactions (9) | Citing (3)

277

Aurora B kinase inhibitor AZD1152: determinants of action and ability to enhance chemotherapeutics effectiveness in pancreatic and colon cancer

By: Azzariti, A.; Bocci, G.; Porcelli, L.; Fioravanti, A.; Sini, P.; Simone, G. M.; Quatrale, A. E.; Chiarappa, P.; Mangia, A.; Sebastian, S.; et al
British Journal of Cancer (2011), 104(5), 769-780 | Language: English, Database: CAplus and MEDLINE

Background: AZD1152, the prodrug for AZD1152-hydroxyquinazoline pyrazol anilide (HQPA), is a selective inhibitor of Aurora B kinase activity. Preclin. evaluation of AZD1152 has been reported in several human cancer models. The potent iality of this compound in combination therapy warrants further investigation in solid tumors. Exptl. design: This study explored the effects of A ZD1152-HQPA in colon and pancreatic tumor cells. The antitumor properties of A ZD1152, either as single agent or in combin ation with chemotherapeutics, were evaluated in each study model. The efficacy and the toxicity of A ZD1152 alone and in combination with gemcitabine were validated in pancreatic tumor xenograft model. Results: A ZD1152-HQPA treatment resulted in a dramatic increase of chromosome number, modification of cell cycle and induction of apoptosis. The most effective combin ation was that with chemotherapeutics given soon after A ZD1152 in both tumor cell types. The effecti veness of the sequential schedule of AZ D1152 with gemcitabine was confirmed in nude mice bearing Mia PaCa-2 tumors, showing inhibition of tumor volumes and delaying of tumor growth after the interruption of the treatments. Conclusion: Here we show that A ZD1152-HQPA enhances oxaliplatin and gemcitabine effectiveness in colon and pancreatic cancer, resp. First, we provide advances into adminis tration schedules and dosing regimens for the combination treatment in in vivo pancreatic tumor. British Journal of Cancer (2011) 104, 769- 780; doi: 10.1038/bjc.2011.21 www.bjcancer.com Published online 8 Feb. 2011.

Keywords: aurora B kinase inhibitor AZD1152 antitumor pancreas colon cancer; combin ation chemotherapy AZD1152 gemcitabine oxaliplatin antitumor pancreas colon cancer

Substances (6) | Reactions (0) | Citing (49)

278

Imaging Colon Cancer Response Following Treatment with AZD1152: A Preclinical Analysis of [18F]Fluoro-2-deoxyglucose and 3'-deoxy-3'-[18F]Fluorothymidine Imaging

By: Moroz, Maxim A.; Kochetkov, Tatiana; Cai, Shang-De; Wu, Ji-Yuan; Shamis, Mikhail; Nair, Jayasree; de Stanchina, Elisa; Serganova, Inna; Schwartz, Gary K.; Banerjee, Debabrata; et al
Clinical Cancer Research (2011), 17(5), 1099-1110 | Language: English, Database: CAplus and MEDLINE

Purpose: To determine whether treatment response to the Aurora B kinase inhibitor, AZD1152, could be monitored early in the course of therapy by noninvasive [18F]-labeled fluoro-2-deoxyglucose, [18F]FDG, and/or 3'-deoxy-3'-[18F]fluorothymidine, [18F]FLT, P ET imaging. Exptl. design: AZD1152-treated and control HCT116 and SW620 xenograft-bearing animals were monitored for tumor size and by [18F]FDG, and [18F]FLT PET imaging. Addnl. studies assessed the endogenous and exogenous contrib utions of thymidine synthesis in the two cell lines. Results: Both xenografts showed a significant volume-reduction to AZD1152. In contrast, [18F]FDG uptake did not demonstrate a treatment response. [18F]FLT uptake decreased to less than 20% of control values in A ZD1152-treated HCT116 xenografts, whereas [18F]FLT uptake was near background levels in both treated and untreated S W620 xenografts. The EC_{50} for AZD1152-HQPA was approx. 10 nmol/L in both S W620 and HCT116 cells; in contrast, SW620 cells were much more sensitive to methotrexate (MTX) and 5-Fluorouracil (5FU) than HCT116 cells. Immunoblot anal. demonstrated marginally lower expression of thymidine kinase in SW620 compared with HCT116 cells. The aforementioned results suggest that SW620 xenografts have a higher dependency on the de novo pathway of thymidine utilization than HCT116 xenografts. Conclusions: AZD1152 treatment showed antitumor efficacy in both colon cancer xenografts. Although [18F]FDG PET was inadequate in monitoring treatment response, [18F]FLT PET was very effective in monitoring response in HCT116 xenografts, but not in SW620 xenografts. These observations suggest that de novo thymidine synthesis could be a limitation and confou nding factor for [18F]FLT PET imaging and quantification of tumor proliferation, and this may apply to some clin. studies as well.

Keywords: fluoride 18 FDG FLT PET imaging agent; AZD1152 antitumor colorectal cancer

Substances (8) Reactions (0) Citing (22)

279

AZD1152 negatively affects the growth of anaplastic thyroid carcinoma cells and enhances the effects of oncolytic virus dl922-947

By: Libertini, Silvana; Abagnale, Antonella; Passaro, Carmela; Botta, Ginevra; Barbato, Sara; Chieffi, Paolo; Portella, Giuseppe
Endocrine-Related Cancer (2011), 18(1), 129-141 | Language: English, Database: CAplus and MEDLINE

Novel therapeutic approaches are required for the treatment of anaplastic thyroid carcinoma (A TC), an incurable disease resistant to current available therapies. Aurora B is an important mitotic kinase involved in chromosome segregation and cytokinesis. It is overexpressed in many cancers including ATC and represents a potential target for chemoth erapy. The effects of AZD1152, a specific Aurora B kinase inhibitor, have been evaluated against ATC, showing G_2/M accumulation, polyploidy and subsequent cell death by mitotic catastrophe upon drug treatment. Only three administ rations of AZD1152 significantly reduced the growth of ATC tumor xenografts. Oncolytic viruses in associ ation with other forms of treatment have proven highly promising in preclin. and clin. reports. The oncolytic adenovirus dl922-947 is active against ATC cells, and we have evaluated the effects of the associ ation between AZD1152 and dl922-947. In cells treated with virus and drug, we report additive/s ynergistic killing effects. Interestingly, the phosphorylation of histone H3 (Ser10), the main Aurora B substrate, is inhibited by dl922- 947 in a dose-dependent manner, and completely abolished in association with AZD1152. The combined treatment signifi cantly inhibited the growth of ATC tumor xenografts with respect to single treatments. Our data demonstrate that the Aurora B inhibitor AZD1152, alone or in combin ation with oncolytic virus dl922-947, could represent a novel therap eutic option for the treatment of A TC.

Keywords: Aurora B AZD1152 oncolytic virus anaplastic thyroid carcinoma growth

Substances (4) Reactions (0) Citing (31)

280

A Robust High-Content Imaging Approach for Probing the Mechanism of Action and Phenotypic Outcomes of Cell-Cycle Modulators

By: Sutherland, Jeffrey J.; Low, Jonathan; Blosser, Wayne; Dowless, Michele; Engler, Thomas A.; Stancato, Louis F.
Molecular Cancer Therapeutics (2011), 10(2), 242-254 | Language: English, Database: CAplus and MEDLINE

High-content screening is increasingly used to elucidate changes in cellular biol. arising from treatment with small mols. and biol. probes. We describe a cell classifier for automated anal. of multiparametric data from immunofluorescence microscopy and charac terize the phenotypes of 41 cell-cycle modulators, including several protein kinase inhibitors in preclin. and clin. develo pment. This method produces a consistent assessment of treatment-induced phenotypes across experiments done by different biologists and highlights the prevalence of nonuniform and concentration-dependent cellular response to treatment. Contrasting cell phenotypes from high-content screening to kinase selectivity profiles from cell-free assays highlights the limited utility of enzyme potency ratios in understanding the mechanism of action for cell- cycle kinase inhibitors. Our cell-level approach for assessing phenotypic outcomes is reliable, reproducible and capable of supporting medium throughput analyses of a wide range of cellular perturb ations.

Keywords: cell cycle modulator phenotype high content imaging classification

Substances (40) | Reactions (0) | Citing (24)

281

P-glycoprotein and breast cancer resistance protein in acute myeloid leukaemia cells treated with the aurora-B kinase inhibitor barasertib-hQPA

By: Grundy, Martin; Seedhouse, Claire; Russell, Nigel H.; Pallis, Monica
BMC Cancer (2011), 11, 254 | Language: English, Database: CAplus and MEDLINE

Background: Aurora kinases play an essential role in orchest rating chromosome alignment, segregation and cytokinesis during mitotic progression, with both aurora-A and B frequently over-expressed in a variety of human maligna ncies. Over-expression of the ABC drug transporter proteins P-glycoprotein (Pgp) and Breast cancer resistance protein (BCRP) is a major obstacle for chemot herapy in many tumor types with Pgp conferring partic ularly poor prognosis in acute myeloid leukemia (AML). Barasertib-hQPA is a highly selective inhibitor of aurora-B kinase that has shown tumoricidal activity against a range tumor cell lines including those of leukemic AML origin. Methods: Effect of barasertib-hQPA on the pHH3 biomarker and cell viability was measured in a panel of leukemic cell lines and 37 primary AML samples by flow cytometry. Pgp status was determined by flow cytometry and B CRP status by flow cytometry and real-time PCR. Results: In this study we report the creation of the cell line O CI-AML3DNR, which over-expresses Pgp but not BCRP or multidrug resistance-associated protein (MRP), through prolonged treatment of OCI-AML3 cells with daunorubicin. We demonstrate that Pgp (OCI-AML3DNR and KG-1a) and BCRP (OCI-AML6.2) expressing AML cell lines are less sensitive to barasertib-hQPA induced pHH3 inhibition and subsequent loss of viability compared to transp orter neg. cell lines. We also show that barasertib-hQPA resistance in these cell lines can be reversed using known Pgp and B CRP inhibitors. We report that barasertib-hQPA is not an inhibitor of Pgp or BCRP, but by using ^{14}[C]-barasertib-hQPA that it is effluxed by these transpo rters. Using phosphoHistone H3 (pHH3) as a biomarker of barasertib-hQPA responsiveness in primary AML blasts we determined that Pgp and BCRP pos. primary samples were less sensitive to barasertib-hQPA induced pHH3 inhibition ($p \leq 0.001$) than samples without these transporters. However, we demonstrate that IC_{50} inhibition of pHH3 by barasertib-hQPA was achieved in 94.6% of these samples after 1 h drug treatment, in contrast to the resistance of the cell lines. Conclusion: We conclude that Pgp and B CRP status and pHH3 down-regulation in patients treated with barasertib should be monitored in order to establish whether transp orter-mediated efflux is sufficient to adversely impact on the efficacy of the agent.

Keywords: anticancer Aurora B kinase inhibitor barasertib hQPA Pgp BCRP; acute myeloid leukemia

Substances (3) | Reactions (0) | Citing (14)

282

Effects of AZD1152, a selective Aurora B kinase inhibitor, on Burkitt's and Hodgkin's lymphomas [Erratum to document cited in CA154:556930]

By: Mori, Naoki; Ishikawa, Chie; Senba, Masachika; Kimura, Masashi; Okano, Yukio
Biochemical Pharmacology (2011), 82(9), 1252 | Language: English, Database: CAplus

On page 1107, Section 2.4 contained an error; the correction is given.

Keywords: erratum Aurora B kinase inhibitor AZD1152 Burkitts Hodgkins lymphoma

Substances (6) Reactions (0) Citing (0)

283

A theoretical entropy score as a single value to express inhibitor selectivity

By: Uitdehaag, Joost C. M.; Zaman, Guido J. R.
BMC Bioinformatics (2011), 12, 94 | Language: English, Database: CAplus and MEDLINE

Designing maximally selective ligands that act on individual targets is the dominant paradigm in drug discovery. Poor selectivity can underlie toxicity and side effects in the clinic, and for this reason compound selectivity is increasingly monitored from very early on in the drug discovery process. To make sense of large amounts of profiling data, and to determine when a compound is sufficiently selective, there is a need for a proper quant. measure of selectivity. Here we propose a new theor. entropy score that can be calculated from a set of IC_{50} data. In contrast to previous measures such as the 'selec tivity score', Gini score, or partition index, the entropy score is non-arbitary, fully exploits IC_{50} data, and is not dependent on a reference kinase. In addition, the entropy score gives the most robust values with data from different sources, because it is less sensitive to errors. We apply the new score to kinase and nuclear receptor profiling data, and to high-throughput screening data. In addition, through analyzing profiles of clin. compounds, we show that a more selective inhibitor is not necessarily more drug-like. For quantifying selectivity from panel profiling, a theor. entropy score is the best method. It is valuable for studying the mol. mechanisms of selectivity, and to steer compound progression in drug discovery programs.

Keywords: theor entropy score allosteric inhibitor selectivity

Substances (42) Reactions (0) Citing (30)

284

Preclinical Evaluation of AMG 900, a Novel Potent and Highly Selective Pan-Aurora Kinase Inhibitor with Activity in Taxane-Resistant Tumor Cell Lines

By: Payton, Marc; Bush, Tammy L.; Chung, Grace; Ziegler, Beth; Eden, Patrick; McElroy, Patricia; Ross, Sandra; Cee, Victor J.; Deak, Holly L.; Hodous, Brian L.; et al

Cancer Research (2010), 70(23), 9846-9854 | Language: English, Database: CAplus and MEDLINE

In mammalian cells, the aurora kinases (aurora-A, -B, and -C) play essential roles in regulating cell division. The expression of aurora-A and -B is elevated in a variety of human cancers and is associated with high prolife ration rates and poor prognosis, making them attractive targets for anticancer therapy. AMG 900 is an orally bioavailable, potent, and highly selective pan-aurora kinase inhibitor that is active in taxane-resistant tumor cell lines. In tumor cells, AMG 900 inhibited autophosphorylation of aurora-A and -B as well as phosphorylation of histone H3 on Ser^{10}, a proximal substrate of aurora-B. The predominant cellular response of tumor cells to AMG 900 treatment was aborted cell division without a prolonged mitotic arrest, which ultimately resulted in cell death. AMG 900 inhibited the proliferation of 26 tumor cell lines, including cell lines resistant to the antimitotic drug paclitaxel and to other aurora kinase inhibitors (AZD1152, MK-0457, and PHA-739358), at low nanomolar concentrations Furthermore, AMG 900 was active in an AZD1152-resistant HCT116 variant cell line that harbors an aurora-B mutation (W221L). Oral administration of AMG 900 blocked the phosphorylation of histone H3 in a dose-dependent manner and significantly inhibited the growth of HCT116 tumor xenografts. Importantly, AMG 900 was broadly active in multiple xenograft models, including 3 multidrug- resistant xenograft models, representing 5 tumor types. AMG 900 has entered clin. evaluation in adult patients with advanced cancers and has the potential to treat tumors refractory to anticancer drugs such as the taxanes.

Keywords: antitumor AMG900 taxane resistance breast colon cancer

Substances (12) | Reactions (0) | Citing (104)

285

Methods for providing personalized medicine tests ex vivo for hematological neoplasms

By: Ballesteros, Juan; Bennett, Teresa; Primo, Daniel; Orfao, Alberto; Jackson, Coyt; Lago, Santiago; Matoses, Maria; Suarez, Lilia; Sapia, Sandra; Bosanquet, Andrew; et al

World Intellectual Property Organization, WO2010135468 A1 2010-11-25 | Language: English, Database: CAplus

Described herein are methods, devices, and compositions for providing personalized medicine tests for hematol. neoplasms. In some embodiments, the methods comprise measuring the efficacy of inducing apoptosis selectively in malignant cells using any number of potential alternative combination drug treatments. In some embodiments, the ex vivo testing is measured using a recently extracted patient hematol. samples. In other embodiments, the efficacy is measured ex vivo using an automated flow cytometry platform. For example, by using an automated flow cytometry platform, the evaluation of hundreds, or even thousands of drugs and compositions, can be made ex vivo. Thus, alternative polytherapy treatments can be explored. Non-cytotoxic drugs surprisingly induce apoptosis selectively in malignant cells ex vivo. In some embodiments, the methods described herein comprise evaluating non-cytotoxic drugs.

Keywords: bioassay hematol neoplasm drug personalized medicine; analytical device drug assay hematol neoplasm; automated flow cytometry drug combination assay blood; drug apoptosis blood neoplasm personalized test

PatentPak available

Substances (157) | Reactions (0) | Citing (3)

286

Expression, purification, stability optimization and characterization of human Aurora B kinase domain from E. coli

By: Sheth, Payal R.; Ramanathan, Lata; Ranchod, Ashwin; Basso, Andrea D.; Barrett, Dianah; Zhao, Jia; Gray, Kimberly; Liu, Yan-Hui; Zhang, Rumin; Le, Hung V.
Archives of Biochemistry and Biophysics (2010), 503(2), 191-201 | Language: English, Database: CAplus and MEDLINE

Aurora B kinase plays a critical role in regulating mitotic progression, and its dysregulation has been linked to tumorigenesis. The structure of the kinase domain of human Aurora B and the complementary information of binding thermodn. of known Aurora inhibitors is lacking. Towards that effort, we sought to identify a human Aurora B construct that would be amenable for large-scale protein production for biophys. and structural studies. Although the designed $AurB_{69-333}$ construct expressed at high levels in Escherichia coli, the purified protein was largely unstable and prone to aggreg ation. We employed thermal-shift assay for high-throughput screening of 192 conditions to identify optimal p H and salt conditions that increased the stability and minimized aggreg ation of $AurB_{69-333}$. Direct ligand binding analyses using temper ature-dependent CD (TdCD) and TR-FRET-based Lanthascreen binding assay showed that the purified protein was folded and functional. The affinity rank-order obtained using TdCD and Lantha screen binding assay correlated with enzymic IC_{50} values measured using full-length Aurora B protein for all the inhibitors tested except for AZD1152. The direct binding results support the hypothesis that the purified human Aur B_{69-333} fragment is a good surrogate for its full-length counterpart for biophys. and structural analyses.

Keywords: Aurora B kinase domain cloning Escherichia coli

Substances (6) | Reactions (0) | Citing (6)

287

Fully human anti-VEGF antibodies and methods of using in cancer therapy

By: Ramachandra, Sumant; Huang, Chin-Yi; Masat, Linda; Huang, Chao Bai; Bishop, Walter Robert; Takeuchi, Toshihiko; Kantak, Seema; Horowitz, Jo Ann; Ladha, Mohamed H.
World Intellectual Property Organization, WO2010124009 A2 2010-10-28 | Language: English, Database: CAplus

Disclosed herein are fully human antibodies and antigen-binding fragments thereof that specifically bind human VEGF and inhibit V EGF binding to VEGF-R1 and VEGF-R2, and therefore inhibit VEGF signaling. The antibodies and antigen-binding fragments disclosed herein may be used, for example, to treat angiogenesis and conditions associated with angiog enesis both in vivo and in vitro.

Keywords: human VEGF antibody cancer therapy VEGFR antitumor conjugate

PatentPak available

Substances (357) | Reactions (0) | Citing (3)

288

Aurora kinase inhibitor AZD1152 negatively affects the growth and survival of HTLV-1-infected T lymphocytes in vitro

By: Tomita, Mariko; Tanaka, Yuetsu; Mori, Naoki
International Journal of Cancer (2010), 127(7), 1584-1594 | Language: English, Database: CAplus and MEDLINE

Aurora kinases play an essential role in regulating mitosis and cell division. Inhibition of Aurora kinases results in suppression of cell division, phosphorylation of histone H3 and induction of apoptosis in many cell types. These characte ristics have prompted the testing of Aurora kinase inhibitors as chemotherapeutic agents. In our study, we report the in vitro activities of A ZD1152, a selective inhibitor of Aurora B kinase in human T-cell leukemia virus type 1 (HTLV-1), the causative agent of adult T-cell leukemia (ATL), -infected T-cell lines. Overexpression of Aurora B was noted in HTLV-1-infected T-cell lines compared to HTLV-1-uninfected T-cell lines. AZD1152 reduced the viability of HTLV-1-infected T-cell lines within 24 h but did not affect that of - uninfected T-cell lines. Although AZD1152 inhibited phosphorylation of histone H3 on Ser10 in both H TLV-1-infected and -uninfected T-cell lines, it induced polyploidy only in HTLV-1-uninfected T-cell lines. AZD1152 induced early apoptosis of HTLV-1-infected T-cells without induction of polyploidy. We have reported previously that a pan- Aurora kinase inhibitor induced apoptosis through inhibition of N F-κB signaling activity in HTLV-1-infected T-cell lines. In contrast, AZD1152 did not affect N F-κB activity in these cells. It induced p53 and p21 expression in HTLV-1-infected but not in HTLV-1-uninfected T-cell lines, suggesting that activation of p53- dependent postmitotic checkpoint might prevent polyploidy in HTLV-1-infected T-cells. Our results suggest that specific inhibition of Aurora B kinase is a potentially useful therapeutic strategy in the treatment of A TL and that further in vivo exploration is warranted.

Keywords: AZD1152 human T cell leukemia virus lymphocyte signaling

Substances (3) Reactions (0) Citing (6)

289

System-Level Analysis of Neuroblastoma Tumor-Initiating Cells Implicates AURKB as a Novel Drug Target for Neuroblastoma

By: Morozova, Olena; Vojvodic, Milijana; Grinshtein, Natalie; Hansford, Loen M.; Blakely, Kim M.; Maslova, Alexandra; Hirst, Martin; Cezard, Timothee; Morin, Ryan D.; Moore, Richard; et al
Clinical Cancer Research (2010), 16(18), 4572-4582 | Language: English, Database: CAplus and MEDLINE

PURPOSE: Neuroblastoma (NB) is an aggressive tumor of the developing peripheral nervous system that remains difficult to cure in the advanced stages. The poor prognosis for high-risk NB patients is associated with common disease recurr ences that fail to respond to available therapies. NB tumor-initiating cells (TICs), isolated from metastases and primary tumors, may escape treatment and contribute to tumor relapse. New therapies that target the TICs may therefore prevent or treat tumor recurr ences. Exptl. Design: We undertook a system-level characterization of NB TICs to identify potential drug targets against recurrent N B. We used next-generation RNA sequencing and/or human exon arrays to profile the transcr iptomes of 11 NB TIC lines from six N B patients, revealing genes that are highly expressed in the TICs compared with normal neural crest- like cells and unrelated cancer tissues. We used gel-free two-dimensional liquid chromatog. coupled to shotgun tandem mass spectr ometry to confirm the presence of proteins corresponding to the most abundant TIC-enriched transcripts, thereby providing validation to the gene expression result. RESULTS: Our study revealed that genes in the B RCA1 signaling pathway are frequently misexp ressed in NB TICs and implicated Aurora B kinase as a potential drug target for NB therapy. Treatment with a selective A URKB inhibitor was cytotoxic to NB TICs but not to the normal neural crest- like cells. CONCLUSION: This work provides the first high- resolution system-level anal. of the transcriptomes of 11 primary human N B TICs and identifies a set of candidate N B TIC-enriched transcripts for further develo pment as therapeutic targets. Clin Cancer Res; 16(18); 4572-82.

Keywords: neuroblastoma tumor initiating cell A URKB therapeutic target

Substances (18) Reactions (0) Citing (37)

290

Combination therapy for treating cancer and diagnostic assays for use therein

By: Shah, Omar Jameel; Shen, Yu; Lin, Xiaoyu; Anderson, Mark; Huang, Xiaoli; Li, Junling; Li, Leiming
United States, US20100227838 A1 2010-09-09 | Language: English, Database: CAplus

The present disclosure relates to a combination of therapeutic agents for use in treating a patient a subject suffering from cancer. In addition, the present disclosure also relates to diagnostic assays useful in classification of patients for treatment with one or more therapeutic agents.

Keywords: antitumor combination therapy cancer therapy diagnosis assay

PatentPak available

Substances (174) | Reactions (802) | Citing (4)

291

Markers to predict and monitor response to Aurora kinase B inhibitor therapy of cancer

By: Semizarov, Dimitri; Shah, Jameel; Guo, Jun; Anderson, Mark
World Intellectual Property Organization, WO2010088470 A1 2010-08-05 | Language: English, Database: CAplus

The present invention relates to diagnostic assays useful in classification of patients for selection of cancer therapy with one or more Aurora kinase B inhibitors. In particular, the present invention relates to identifying the presence or absence of one or more copy number gains in the ABCB1 gene, the ABCB4 gene or combinations thereof, identifying patients eligible to receive Aurora kinase inhibitor therapy, either as monotherapy or as part of combination therapy, and monitoring patient response to such therapy.

Keywords: cancer therapy Aurora kinase B inhibitor biol marker

PatentPak available

Substances (327) | Reactions (0) | Citing (1)

292

Imidazo[4,5-b]pyridine Derivatives As Inhibitors of Aurora Kinases: Lead Optimization Studies toward the Identification of an Orally Bioavailable Preclinical Development Candidate

By: Bavetsias, Vassilios; Large, Jonathan M.; Sun, Chongbo; Bouloc, Nathalie; Kosmopoulou, Magda; Matteucci, Mizio; Wilsher, Nicola E.; Martins, Vanessa; Reynisson, Johannes; Atrash, Butrus; et al
Journal of Medicinal Chemistry (2010), 53(14), 5213-5228 | Language: English, Database: CAplus and MEDLINE

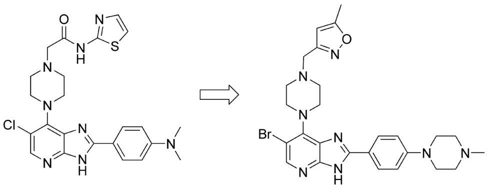

Lead optimization studies using an imidazo[4,5-b]pyridinylpiperazine as the starting point led to a new class of imidazo[4,5-b]pyridine-based inhibitors of Aurora kinases that possessed the 1-benzylpiperazinyl motif at the 7-position, and displayed favorable in vitro properties. Cocrystn. of Aurora-A with a morpholinylmethylphenylimidazopyridinyl chlorobenzyl piperazine (CCT137444) provided a clear understanding into the interactions of this novel class of inhibitors with the Aurora kinases. Subsequent physicochem. property refinement by the incorporation of solubilizing groups led to the identification of 3-((4-(6-bromo-2-(4-(4-methylpiperazin-1-yl)phenyl)-3H-imidazo[4,5-b]pyridin-7-yl)piperazin-1-yl)methyl)-5-methylisoxazole (CCT137690)(**I**) which is a potent inhibitor of Aurora kinases (Aurora-A IC_{50} = 0.015±0.003 μM, Aurora-B IC_{50} = 0.025 μM, Aurora-C IC_{50} = 0.019 μM). **I** is highly orally bioavailable, and in in vivo efficacy studies it inhibited the growth of SW620 colon carcinoma xenografts following oral administration with no observed toxicities as defined by body weight loss.

Keywords: imidazopyridine derivative arsenic inhibitor Aurora kinase orally bioavailable development

Substances (234) Reactions (402) Citing (70)

293

Bcl-XL represents a druggable molecular vulnerability during aurora B inhibitor-mediated polyploidization

By: Shah, Jameel; Lin, Xiaoyu; Li, Leiming; Huang, Xiaoli; Li, Junling; Anderson, Mark G.; Tang, Hua; Rodriguez, Luis E.; Warder, Scott E.; Mcloughlin, Shaun; et al
Proceedings of the National Academy of Sciences of the United States of America (2010), 107(28), 12634-12639, S12634/1-S12634/10 | Language: English, Database: CAplus and MEDLINE

Aurora kinase B inhibitors induce apoptosis secondary to polyploidization and have entered clin. trials as an emerging class of neocytotoxic chemotherapeutics. We demonstrate here that polyploidization neutralizes Mcl-1 function, rendering cancer cells exquisitely dependent on Bcl-XL-2. This "addiction" can be exploited therapeutically by combining aurora kinase inhibitors and the orally bioavailable BH3 mimetic, ABT-263, which inhibits Bcl-XL, Bcl-2, and Bcl-w. The combination of ABT-263 with aurora B inhibitors produces a synergistic loss of viability in a range of cell lines of divergent tumor origin and exhibits more sustained tumor growth inhibition in vivo compared with aurora B inhibitor monotherapy. These data demonstrate that Bcl-XL/-2 is necessary to support viability during polyploidization in a variety of tumor models and represents a druggable mol. vulnerability with potential therapeutic utility.

Keywords: Bcl XL aurora kinase B inhibitor polyploidization cancer

Substances (4) Reactions (0) Citing (34)

294

Identification and classification of E2F-responsive gene overexpressing tumors for selection of therapies

By: Shackney, Stanley E.
World Intellectual Property Organization, WO2010056931 A1 2010-05-20 | Language: English, Database: CAplus

Methods for identifying a tumor as an E2 F-responsive gene over-expressing (ERGO) tumor are described. These methods can be used in diagnosis and to predict survival of the patient and in the selection and monitoring of therapies. These methods are applicable to ERGO tumors present in different human organs and tissues such as breast, lung, thyroid, ovary, and prostate. Transcr iption factor E2F regulates approx. 325 genes and abnormal levels of expression of a signif icant number of these in a tissue sample is diagnostic of an ERGO tumor.

Keywords: E2F transcription regulation neoplasm diagnosis therapy

Substances (376) | Reactions (0) | Citing (1)

295

The selective Aurora B kinase inhibitor AZD1152 as a novel treatment for hepatocellular carcinoma

By: Aihara, Arihiro; Tanaka, Shinji; Yasen, Mahmut; Matsumura, Satoshi; Mitsunori, Yusuke; Murakata, Ayano; Noguchi, Norio; Kudo, Atsushi; Nakamura, Noriaki; Ito, Koji; et al
Journal of Hepatology (2010), 52(1), 63-71 | Language: English, Database: CAplus and MEDLINE

Background & Aims: We previously identified that high Aurora B expression was associated with hepatocellular carcinoma (HCC) recurrence due to tumor dissemination. In this preclin. study, a novel inhibitor of Aurora B kinase was evaluated as a treatment for human HCC. Methods: AZD1152 is a selective inhibitor of Aurora B kinase. Twelve human H CC cell lines were analyzed for Aurora B kinase expression and the in vitro effects of AZD1152. The in vivo effects of AZD1152 were analyzed in a s.c. xenograft model and a novel orthotopic liver xenograft model. Results: Aurora B kinase expression varied among the human HCC cell lines and was found to correlate with inhibition of cell proliferation, accumulation of 4N DNA, and the proportion of polyploid cells following adminis tration of AZD1152-hydroxyquinazoline-pyrazol-anilide (AZD1152-HQPA). AZD1152-HQPA suppressed histone H3 phosphor ylation and induced cell death in a dose-dependent manner. Growth of s.c. human H CC xenografts was inhibited by A ZD1152 administ ration. In an orthotopic hepatoma model, treatment with A ZD1152 significantly decelerated tumor growth and increased survival. Pharmacobiol. anal. revealed that AZD1152 induced the rapid suppression of phosphohistone H3, followed by cellular apoptosis in the liver tumors but not in the normal tissues of the orthotopic models. Conclusions: Our preclin. studies indicate that A ZD1152 is a promising novel therapeutic approach for the treatment of H CC.

Keywords: anticancer AZD1152 apoptosis phosphohistone H3 hepatocellular carcinoma growth

Substances (4) | Reactions (0) | Citing (53)

296

Novel agents and regimens for acute myeloid leukemia: 2009 ASH annual meeting highlights

By: Zhu, Xiongpeng; Ma, Yuehua; Liu, Delong
Journal of Hematology & Oncology (2010), 3, No pp. given | Language: English, Database: CAplus and MEDLINE

A review. Prognostic markers, such as NPM1, Flt3-ITD, and cytoge netic abnormalities have made it possible to formulate aggressive treatment plans for unfavorable acute myeloid leukemia (AML). However, the long-term survival of AML with unfavorable factors remains unsatisfactory. The latest data indicate that the standard dose of daunor ubicin (DNR) at 45 mg/m^2 is inferior to high dose 90 mg/m^2 for induction therapy. The rates of complete remission and overall survival are signifi cantly better in the high dose induction regimen. New regimens exploring the new liposomal encapsulation of Ara-C and DNR as well as addition of gemtuzumab ozogamicin monoclonal antibody have been studied. New agents, including the nucleoside analogs (clofarabine, sapacitabine, elacytarabine), FLT3 inhibitor (sorafenib), farnesyl- transferase inhibitor (tipifarnib), histone deacetylase inhibitor (vorinostat), lenalid omide, as well as DNA methyltransferase inhibitors (decitabine, azacitidine), were recently reported for AML treatment in the 2009 A SH annual meeting. This review also summarizes the updates of the clin. trials on novel agents including voreloxin, A S1413, beheno ylara-C, ARRY520, ribavirin, AZD1152, AZD6244, and terameprocol (EM-1421) from the 2009 ASH annual meeting.

Keywords: review anticancer acute myeloid leukemia

Substances (22) Reactions (0) Citing (49)

297

Aurora kinase inhibitors: a new class of drugs targeting the regulatory mitotic system

By: Perez Fidalgo, Jose Alejandro; Roda, Desamparados; Rosello, Susana; Rodriguez-Braun, Edith; Cervantes, Andres
Clinical & Translational Oncology (2009), 11(12), 787-798 | Language: English, Database: CAplus and MEDLINE

The present review gives a perspective on the Aurora kinase family members, their function in normal cells, their role in cancer progression as well as their potential as target for anticancer treatment. Mitosis has been an important target for anticancer therapy development, leading to some specific drugs mainly addressing Tubulines, as a key structure of the mitotic spindle. Vinca alkaloids, taxanes or epotilones are good examples of conventionally developed antimitotic agents. However, novel classes of antineoplastic drugs are being studied, targeting the regulatory system that controls functional aspects of mitosis, such as Aurora or Polo-like kinases or Kinespondin inhibitors. The specific role of the different Aurora kinase proteins as regulator enzymes of the mitotic process in normal cells is discussed. Some of the mechanisms that link Aurora overexpression with cancer are also consid ered. Thereafter, the clin. and preclin. develo pment of the different Aurora kinase inhibitors is presented. This is nowadays a very active area of therapeutic research and at least, sixteen new compounds are being studied as potential antineo plastic drugs. Most of them are in a very early phase of clin. development. However, we summarized the most recently published findings related with these drugs: main characteristics, way of administ ration, dose limiting toxicities and recomm ended doses for further studies. Another important aspect in Aurora kinase inhibition is the study and validation of potential biomarkers to optimize the clin. develo pment. Several studies included pharmaco dynamic assessments in normal blood cells, skin or/and tumor biopsies. Several proposals included a higher mitotic index, a decreased number of mitosis with bipolar spindles or normal alignment of chromo somes and inhibition of histone H3 phosphor ylation. Future strategies and challenges for trials with Aurora kinase inhibitors are also discussed.

Keywords: review Aurora kinase inhibitor drug target mitosis cancer antineoplastic

Substances (5) Reactions (0) Citing (26)

298

Anti-IGF-1 receptor antibody combined with chemotherapeutic agent, antitumor agent, radiotherapy or surgery for treating or preventing colorectal cancer

By: Wang, Yaolin; Wang, Yan; Liu, Ming; Bishop, Walter Robert; Seidel-Dugan, Cynthia
World Intellectual Property Organization, WO2009142810 A2 2009-11-26 | Language: English, Database: CAplus

The present invention provides, for example, methods for treating or preventing colorectal cancer with an anti-IGF1R antibody in association with sunitinib or a combin ation of leucovorin and 5-fluorouracil.

Keywords: IGF1 receptor antibody chemotherapeutic antitumor radiotherapy surgery colorectal cancer

PatentPak available

Substances (343) Reactions (0) Citing (0)

299

Sequential administration of chemotherapeutic agents and anti-(insulin-like growth factor 1 receptor) for treatment of cancer

By: Wang, Yaolin; Wang, Yan; Lu, Brian Der-Hua; Liu, Ming; Seidel-Dugan, Cynthia; Yao, Siu-Long
World Intellectual Property Organization, WO2009137378 A2 2009-11-12 | Language: English, Database: CAplus

The present invention relates to the sequential administration of a cytotoxic agent followed by an insulin- like growth factor 1 receptor (IGF1R) antagonist (e.g., an antibody) for the treatment of hyperprol iferative disorders including cancer. Human monoclonal antibody clone 15H12/19D12 light and heavy chains targeting human I GF1R are provided. Thus, for example, sequential administration of irinotecan and then anti- IGF1R antibody for the treatment of human colorectal cancer in xenograft mice resulted in 77% tumor growth inhibition vs. 39% and 61% growth inhibition by irinotecan or anti-IGF1R antibody alone.

Keywords: cancer therapy chemotherapeutic agent I GF1 receptor antibody

PatentPak available

Substances (338) Reactions (0) Citing (2)

300

Simultaneous determination of AZD1152 (prodrug) and AZD1152-hydroxyquinazoline pyrazol anilide by reversed phase liquid chromatography

By: Pluim, Dick; Beijnen, Jos H.; Schellens, Jan H. M.; van Tellingen, Olaf
Journal of Chromatography B: Analytical Technologies in the Biomedical and Life Sciences (2009), 877(29), 3549-3555 | Language: English, Database: CAplus and MEDLINE
Analytical Methods available

A simple, selective and sensitive reversed phase liquid chromatog. method utilizing UV detection has been developed and validated for the simultaneous determination of the prodrug AZD1152 and its active product AZD1152-hydroxyquinazoline pyrazol anilide (hQPA) in human and mouse plasma and mouse tissues. Isocratic separation was achieved using an 5 μm Upti Sphere HDO C_{18} column (150 mm × 4.6 mm) with guard column in combination with a mobile phase comprised of phosphate buffered water (50 m M; pH 3.4) and acetonitrile (81.5: 18.5; volume/volume). UV detection at 318 nm was used. Sample preparation involved a single-step protein precipitation with ethanol. Ex vivo conversion of AZD1152 by endogenous phosphatases was prevented by immediate cooling of the samples in ice-water and addition of sodium fluoride and EDTA. The validation parameters included specificity, recovery, accuracy, precision, sensitivity and stability. The lower limit of quantification in human plasma for AZD1152 and hQPA was 25 ng/mL. The applicability of the method was demonstrated by successful determination of AZD1152 and hQPA in human plasma and in plasma, brain, liver, kidney and ileum samples from mice dosed with AZD1152.

Keywords: AZD1152 pharmacokinetics tissue blood analysis HPLC

Substances (3) Reactions (0) Citing (7)

301

The Aurora B kinase inhibitor AZD1152 sensitizes cancer cells to fractionated irradiation and induces mitotic catastrophe

By: Tao, Yungan; Leteur, Celine; Calderaro, Julien; Girdler, Fiona; Zhang, Ping; Frascogna, Valerie; Varna, Mariana; Opolon, Paule; Castedo, Maria; Bourhis, Jean; et al
Cell Cycle (2009), 8(19), 3172-3181 | Language: English, Database: CAplus and MEDLINE

AZD1152, an Aurora kinase inhibitor with selectivity for Aurora B kinase, can enhance the effect of ionizing radiation (IR). The aim of this study was to evaluate and to mechanistically explore scheduling effects of AZD1152 on tumor responses to IR, in three different settings; neoadjuvant (AZD1152 before IR), adjuvant (IR before AZD1152), or concomitant treatments (AZD1152 plus one single IR dose). A more pronounced tumor growth delay was observed in the neoadj uvant and adjuvant schedules as compared to the concomitant schedule. However, AZD1152 enhanced the efficacy of IR when concomitant IR was fractionated over several days. Histopathol. examination revealed that AZD1152 + IR induced polyploidy, multinucleation and micronuclei in vivo. Time-lapse videomicroscopy confirmed that cell death induced by AZD1152 + IR was preceded by multinucleation and the formation of micron uclei, which both are hallmarks of mitotic catastrophe. Caspase inhibition or removal of the pro-apoptotic protein Bax did not ameliorate the long-term cell survival of AZD1152-treated cancer cells. In contrast, a chem. inhibitor of CHK1, Chir124, sensitized cancer cells to the lethal effect of AZD1152. Altogether, these data support the contention that AZD1152 mediates radiosensitization in vivo by enhancing mitotic catastrophe, which can be used as a biomarker of treatment efficacy.

Keywords: aurora B kinase inhibitor AZD1152 colon cancer radiotherapy anticancer

Substances (3) Reactions (0) Citing (44)

302

Gene expression profiling in the pulmonary artery in the selection of therapies for vascular-related diseases

By: Mann, David Marshall

World Intellectual Property Organization, WO2009121031 A1 2009-10-01 | Language: English, Database: CAplus

Methods of selecting treatment regimens for vascular-related diseases and disorders using gene expression profiles in affected blood vessels are described. Addnl., methods are disclosed involving diagnostic techniques to focus treatment regimens. Finally, methods of treating vascular-related disorder involving targeting micro RNAs are also disclosed. Therapies suitable for treatment at different stages of the disease are identified.

Keywords: pulmonary artery gene expression profiling vascular disease therapy; arterial hypertension lung diagnosis therapy

PatentPak available

Substances (522) Reactions (0) Citing (6)

303

Kinase Inhibitor 4 Minisymposium summary

By: Dancey, Janet E.

Expert Review of Anticancer Therapy (2009), 9(7), 891-894 | Language: English, Database: CAplus and MEDLINE

A review. In recent years no cancer therapeutic class has undergone more prolific or successful drug develo pment efforts than the kinase inhibitors. The robust research interest is evident in the number of kinase inhibitor present ations at the American Associ ation for Cancer Research Annual Meeting held in Denver (C O, USA) 18-22 Apr. 2009. Presen tation highlights from the Kinase Inhibitor 4 Minisymposium include the identification of FGF receptor genetic altera tions that correlate with sensitivity to the kinase inhibitor TKI258; evaluation of activity of mitotic kinase inhibitors G SK461364A and AZD1152 in breast and small- cell lung cancer and the identification of new inhibitors to PI3K/AKT/mTOR pathway.

Keywords: review kinase inhibitor 4 breast lung cancer anticancer signaling

Substances (5) Reactions (0) Citing (1)

304

Protein kinase genes showing altered levels of expression in breast cancer tissue and their diagnostic use

By: Bertucci, Francois; Birnbaum, Daniel; Finetti, Pascal
World Intellectual Property Organization, WO2009083780 A1 2009-07-09 | Language: English, Database: CAplus

The present invention relates to a method for analyzing cancer.e.g., breast cancer comprising detection of differ ential expression of at least one of the 16 genes encoding serine/threonine kinases listed in Table 1, or of said 16 genes, and to a polynuc leotide library comprising at least one said 16 genes. A method of diagnosing breast cancer by anal. of the levels of expression of members of a group of 16 protein kinase genes is described. Levels of expression of the genes can also be used in prognosis and in monitoring the effectiveness of therapies. The levels of expression of these genes were analyzed in 227 samples of breast cancer tissue as part of a larger anal. of gene expression in breast cancer. Validation of the use of these genes in diagnosis and in prognosis is demonst rated.

Keywords: protein kinase gene expression breast cancer diagnosis prognosis

PatentPak available

Substances (295) | Reactions (0) | Citing (2)

305

Gene expression biomarkers for detecting IGF1R inhibitor-resistant or -sensitive cancers and uses in anti-IGF1R antibody combination chemotherapy

By: Wang, Yan; Wang, Yaolin; Levitan, Diane; Seidel-Dugan, Cynthia; Liu, Ming; Ding, Wei
World Intellectual Property Organization, WO2009079587 A2 2009-06-25 | Language: English, Database: CAplus

The present invention provides, for example, methods for conveniently determining if a cancerous condition in a subject will be responsive to an IGF1R inhibitor. The invention includes patient selection methods and methods of treatment. The method comprises determining the expression levels of biomarker-genes associated with sensit ivity or resistance to IGF1R inhibitor therapy. Thus, genes upregulated in sensitive cells relative to resistant cells are: T RE2, SMC4, TRIB2, TLE4, BMP7, PCDHGC3, AUTS2, C14orf132, CERK, HDGFRP3, TCF4, MEIS2, EML4, C7orf41, KIAA1450, ZNF136, D15F37, CDK6, TIMP, Clu, Prl1. Genes down-regulated in sensitive cells relative to resistant cells are: ACAT1, ALDOC, C6orf192, COL4A5, C1QBP, CRIP1, DEADC1, GSTK1, GSTO2, PPAPDC1B, TMEM107, JOSD3, TMEM77, MST1, MT1E, PARVB, PRDX4, RASGEF1A, RPL14, IFI30, ATF1, ACADVL, FBXO6, NQO2, TMEM64, ZFAND1, T MED5, PDIA5, MYO1C, GNPTAB, LACTB2, RPL22, TSPAN4, RPL15, PCCB, CRYZ, DNAJC10, C19orf54, HSPE1, hqp0376. Provided are sequences for anti-IGF1R antibodies to be used in combin ation chemotherapy.

Keywords: gene biomarker IGF1R inhibitor therapy resistance sensitivity cancer diagnosis; anticancer IGF1R antibody sequence combination chemotherapy

PatentPak available

Substances (339) | Reactions (0) | Citing (3)

306

Antitumor combination comprising a MEK inhibitor and an Aurora kinase inhibitor 188

By: Mundt, Kirsten Elisabeth; Smith, Paul David
World Intellectual Property Organization, WO2009074827 A2 2009-06-18 | Language: English, Database: CAplus

The present invention relates to a therapeutic combination comprising a MEK inhibitor and an Aurora kinase inhibitor, and to methods for the production of an anti-cancer effect in a patient, which is accord ingly useful in the treatment of cancer in a patient. More specifically the present invention relates to: a therapeutic combination comprising a MEK inhibitor and an Aurora kinase inhibitor; a combination product comprising a MEK inhibitor and an Aurora kinase inhibitor, a kit of parts comprising a M EK inhibitor and an Aurora kinase inhibitor; use of a therapeutic combination, combination product or kit of parts in the treatment of cancer; a method of treating cancer comprising administering the therapeutic combination, combination product or kit of parts to a patient. The therapeutic combination and methods of the invention are also useful in the treatment of conditions in which the inhibition of MEK and/or Aurora kinase is benefi cial.

Keywords: antitumor combination MEK inhibitor Aurora kinase

PatentPak available

Substances (6) | Reactions (0) | Citing (1)

307

Scutellaria barbata extract and combinations for the treatment of cancer

By: Cohen, Isaac
United States, US20090130118 A1 2009-05-21 | Language: English, Database: CAplus

An extract of Scutellaria barbata D. Don is effective in the arrest of cancer cell growth in the G1 phase, the induction of apoptosis in cancer cells and the shrinking of solid cancers. The extract may be prepared as a pharmaceutical composition for administration to mammals for the treatment of solid cancers, such as epithelial cancers. Such epithelial cancers include breast cancer and ovarian cancers. The extract is obtained from Scutellaria barbata D. Don by contacting aerial portions of a plant from the species Scutel laria barbata D. Don with an aqueous or alc. solvent.

Keywords: Scutellaria extract cancer combination treatment

PatentPak available

Substances (29) | Reactions (0) | Citing (1)

308

AZD1152 Rapidly and Negatively Affects the Growth and Survival of Human Acute Myeloid Leukemia Cells In vitro and In vivo

By: Oke, Adedayo; Pearce, Daniel; Wilkinson, Robert W.; Crafter, Claire; Odedra, Rajesh; Cavenagh, Jamie; Fitzgibbon, Jude; Lister, Andrew T.; Joel, Simon; Bonnet, Dominique
Cancer Research (2009), 69(10), 4150-4158 | Language: English, Database: CAplus and MEDLINE

Aurora kinases play a critical role in regulating mitosis and cell division, and their overexpression has been implicated in the survival and proliferation of human cancer. In this study, we report the in vitro and in vivo activities of A ZD1152, a compound that has select ivity for aurora B kinase, in acute myeloid leukemia (A ML) cell lines, primary A ML samples, and cord blood cells. A ZD1152 exerted antiproliferative or cytotoxic effects in all cell lines studied, inhibited the phosphor ylation of histone H3 (p His H3) on Ser10 in a dose-dependent manner, and resulted in cells with >4 N DNA content. THP-1 cells treated with A ZD1152 accumulated in a state of polyploidy and showed a senescent response to the drug, in contrast to the apoptotic response seen in other cell lines. Accordingly, AZD1152 profoundly affected the growth of AML cell lines and primary A ML in an in vivo xenotransplantation model. However, concentration-dependent effects on cell growth, apoptosis, and cell cycle progre ssion were also observed when human cord blood and primary lineage-neg. stem and progenitor cells were analyzed in vitro and in vivo. These data suggest that the inhibition of aurora B kinase may be a useful therapeutic strategy in the treatment of A ML and that further exploration of dosing and treatment schedules is warranted in clin. trials. [Cancer Res 2009;69(10):4150-8].

Keywords: AZD1152 antitumor prodrug aurora B kinase acute myeloid leukemia

Substances (3) Reactions (0) Citing (62)

309

Identification of genes that confer tumor cell resistance to the Aurora B kinase inhibitor, AZD1152

By: Guo, J.; Anderson, M. G.; Tapang, P.; Palma, J. P.; Rodriguez, L. E.; Niquette, A.; Li, J.; Bouska, J. J.; Wang, G.; Semizarov, D.; et al
Pharmacogenomics Journal (2009), 9(2), 90-102 | Language: English, Database: CAplus and MEDLINE

AZD1152 is a highly selective Aurora B kinase inhibitor currently undergoing Phase I and I I clin. evaluation in patients with acute myelogenous leukemia and advanced solid maligna ncies. We have established two AZD1152-resistant cell lines from S W620 colon and MiaPaCa pancreatic carcinoma lines, which are >100- fold resistant to the active metabolite of A ZD1152, AZD1152 HQPA and interestingly, cross-resistant to the pan-Aurora kinase inhibitor, VX-680/MK0457. Using whole-genome microarray anal. and compar ative genomic hybridi zation, we were able to identify M DR1 and BCRP as the causative genes that underlie A ZD1152 HQPA-resistance in these models. Furthe rmore, the upregulation of either of these genes is sufficient to render in vivo tumor growth insensitive to AZD1152. Finally, the upregu lation of MDR1 or BCRP is predictive of tumor cell sensit ivity to this agent, both in vitro and in vivo. The data provide a genetic basis for resistance to Aurora kinase inhibitors, which could be utilized to predict clin. response to therapy.

Keywords: AZD1152 antitumor resistance Aurora B kinase M DR1 BCRP

Substances (4) Reactions (0) Citing (32)

310

Human anti-VEGF antibodies and conjugates for treatment of angiogenesis conditions

By: Ramachandra, Sumant; Bishop, Robert Walter; Masat, Linda; Huang, Chao Bai; Takeuchi, Toshihiko; Kantak, Seema
World Intellectual Property Organization, WO2009055343 A2 2009-04-30 | Language: English, Database: CAplus

Disclosed herein are fully human antibodies and antigen-binding fragments thereof that specifically bind human VEGF and inhibit V EGF binding to VEGF-R1 and VEGF-R2, and therefore inhibit VEGF signaling. The antibodies and antigen-binding fragments disclosed herein may be used, for example, to treat angiogenesis and conditions associated with angiog enesis both in vivo and in vitro.

Keywords: human antibody fragment conjugate VEGF cancer diagnosis therapy

PatentPak available

Substances (346) Reactions (0) Citing (1)

311

Aurora B kinase regulates the postmitotic endoreduplication checkpoint via phosphorylation of the retinoblastoma protein at serine 780

By: Nair, Jayasree S.; Ho, Alan L.; Tse, Archie N.; Coward, Jesse; Cheema, Haider; Ambrosini, Grazia; Keen, Nicholas; Schwartz, Gary K.
Molecular Biology of the Cell (2009), 20(8), 2218-2228 | Language: English, Database: CAplus and MEDLINE

The phenotypic change characteristic of Aurora B inhibition is the induction of polypl oidy. Utilizing specific siRNA duplexes and a selective small mol. inhibitor (AZD1152) to inhibit Aurora B activity in tumor cells, we sought to elucidate the mechanism by which Aurora B inhibition results in polyploidy. Cells treated with AZD1152 progressed through mitosis with misaligned chromo somes and exited without cytokinesis and subsequently underwent endoredup lication of DNA despite activation of a p53-dependent pseudo G1 checkpoint. Concomitant with polyploid cell formation, we observed the appearance of Rb hypophosph orylation, an event that occurred independently of cyclin-dependent kinase inhibition. We went on to discover that Aurora B directly phospho rylates Rb at serine 780 both in vitro and in vivo. This novel interaction plays a critical role in regulating the postmi totic checkpoint to prevent endoreduplication after an aberrant mitosis. Thus, we propose for the first time that Aurora B determines cellular fate after an aberrant mitosis by directly regulating the Rb tumor suppressor protein.

Keywords: Aurora B kinase Rb protein serine phosphorylation endoreduplication checkpoint

Substances (4) Reactions (0) Citing (75)

312

Inhibition of Aurora B kinase sensitizes a subset of human glioma cells to TRAIL concomitant with induction of TRAIL-R2

By: Li, J.; Anderson, M. G.; Tucker, L. A.; Shen, Y.; Glaser, K. B.; Shah, O. J.
Cell Death and Differentiation (2009), 16(3), 498-511 | Language: English, Database: CAplus and MEDLINE

Small-mol. inhibitors of the Aurora A and B kinases interfere with mitotic centrosome function and disrupt the mitotic spindle assembly checkpoint resulting in polyploidization and apoptosis of proliferating cells. As such, several Aurora kinase inhibitors are at various stages of clin. development as anticancer agents. To identify candidate apoptosis-sensitizing genes that could be exploited in combination with Aurora kinase inhibitors in malignant glioma, we have carried out global gene expression anal. in a D54MG glioma cell derivative treated with three Aurora kinase inhibitors chosen for their distin ctive selectivities: MLN8054 (Aurora A-selective), AZD1152 (Aurora B-selective), and VX-680 (Aurora A/B). The modulation of apoptotic gene expression by p53 under these conditions was ascertained, as p53 expression can be toggled on and off in this D54 MG derivative by virtue of a stable, inducible, p53-targeting short hairpin RNA ($D54MG^{shp53}$). This anal. identified the tumor necrosis factor- related apoptosis-inducing ligand (TRAIL) death receptor, TRAIL receptor 2 (TRAIL-R2), as an apoptosis-sensitizing gene induced selectively following inhibition of Aurora B. In glioma cell lines where TRAIL-R2 was induced following polyploidization, the sensitivity, kinetics, and magnitude of T R AIL-mediated apoptosis were enhanced. Our data shed light on the apoptotic program induced during polyploi dization and suggest that TRAIL-R2 activation is a putative point of therap eutic intervention in combination with inhibitors of Aurora B.

Keywords: aurora B kinase inhibitor glioma TRAIL protein apoptosis cytotoxic

Substances (10) Reactions (0) Citing (27)

313

Inhibitors of aurora kinases

By: Pinel, S.; Barbault-Foucher, S.; Lott-Desroches, M.-C.; Astier, A.
Annales Pharmaceutiques Francaises (2009), 67(2), 69-77 | Language: French, Database: CAplus and MEDLINE

A review. Aurora kinases (A, B and C) are proteins expressed only in cells which divide actively and their increase is a factor of bad prognosis in cancer. They regulate the maturation of centrosomes, the separation and the conden sation of chromosomes, mitotic checkpoint and cytokinesis. The inhibition of aurora kinases, by powerful and selective inhibi tors, is due to the formation of abnormal cells which are eliminated by apoptosis. The purpose of this article is to present the role, the antitumor activity and the tolerability of these inhibitors. They can be administered orally or i.v., on weekly or monthly schedules. In our knowledge, twelve mols. are evaluated at the present time and will be discussed only the most advanced namely: VX-680, ZM 447439, MLN 8054, AZD 1152, PHA 739358, SU 6668 and AT 9283. The main indica tions are breast, colon, lung, pancreas and bladder cancers as well as hematol. tumors such as leukemia (ALL, AML, CML) and lymphoma. These inhibitors can be associated with other chemothe rapies. They seem well tolerated; the reported side effects are digestive disorders (diarrhea), fever, asthenia, alopecia, slumber, neutro penia, myelosuppression and disturbance of the biol. markers.

Keywords: review aurora kinase inhibitor antitumor toxicity cancer

Substances (8) Reactions (0) Citing (7)

314

The Topoisomerase I Poison CPT-11 Enhances the Effect of the Aurora B Kinase Inhibitor AZD1152 both In vitro and In vivo

By: Nair, Jayasree S.; de Stanchina, Elisa; Schwartz, Gary K.
Clinical Cancer Research (2009), 15(6), 2022-2030 | Language: English, Database: CAplus and MEDLINE

AZD1152 is an Aurora B kinase inhibitor currently in clin. trials. As the topoiso merase I poison CPT-11 induces a G_2 arrest, a mechan istic understanding of the cell cycle intera ctions between these agents may prove critical for combin ation therapy. AZD1152 was tested in vitro and in vivo with SN-38 and CPT-11 against HCT-116 cells. Inhibition of clonogenicity, induction of apoptosis, effects on polyploidy, and tumor growth were examined A ZD1152 alone induced polyploidy of HCT-116 cells at low nanomolar concent rations The induction of apoptosis required prolonged exposure (48 h) and higher concent rations of drug. When SN-38 was given before or concomitantly with AZD1152, SN-38 blocked the AZD1152 effect by arresting cells in G_2 and inhibiting cells from undergoing polyploidy. With the reverse combin ation (AZD1152 followed by SN-38), there was a significant induction of polyploidy and apoptosis, even with shorter exposure (24 h) of AZD1152. In vivo, AZD1152 alone suppressed HCT-116 xenograft tumor growth in a dose-dependent manner with target inhibition of phospho H3, induction of multinucleated giant cells, but without induction of apoptosis. In combination, both sequences in vivo (C PT->AZD, AZD->CPT, P = 0.008, AUC/d) proved superior to either single agent therapy. However, AZD->CPT still showed a greater increase in apoptosis and greater suppre ssion of tumor regrowth than C PT->AZ D (P = 0.02, AUC/d). The results from these studies indicate a promising therap eutic strategy for combining A ZD1152 with CPT-11, and suggest that the sequence of drug administration is pivotal when an Aurora B kinase inhibitor is admini stered with a topoiso merase I poison.

Keywords: AZD1152 CPT11 combination chemotherapy antitumor

Substances (7) Reactions (0) Citing (31)

315

A panel of isogenic human cancer cells suggests a therapeutic approach for cancers with inactivated p53

By: Sur, Surojit; Pagliarini, Raymond; Bunz, Fred; Rago, Carlo; Diaz, Luis A. Jr.; Kinzler, Kenneth W.; Vogelstein, Bert; Papadopoulos, Nickolas
Proceedings of the National Academy of Sciences of the United States of America (2009), 106(10), 3964-3969 | Language: English, Database: CAplus and MEDLINE

Through targeted homologous recombination, we developed a panel of matched colorectal cancer cell lines that differ only with respect to their endogenous TP53 status. We then used these lines to define the genes whose expression was altered after D NA damage induced by ionizing radiation. Transcriptome analyses revealed a consistent upregu lation of polo-like kinase 1 (PLK1) as well as other genes controlling the G_2/M transition in the cells whose TP53 genes were inactivated compared with those with WT T P53 genes. This led to the hypothesis that the viability of stressed cells without W T TP53 depended on PLK1. This hypothesis was validated by demonstrating that stressed cancer cells without WT TP53 alleles were highly sensitive to PLK1 inhibitors, both in vivo and in vitro.

Keywords: colorectal cancer cell p53 PLK1 kinase

Substances (12) Reactions (0) Citing (248)

316

Structure-Based Drug Design of Novel Aurora Kinase A Inhibitors: Structural Basis for Potency and Specificity

By: Coumar, Mohane Selvaraj; Leou, Jiun-Shyang; Shukla, Paritosh; Wu, Jian-Sung; Dixit, Ajay Kumar; Lin, Wen-Hsing; Chang, Chun-Yu; Lien, Tzu-Wen; Tan, Uan-Kang; Chen, Chun-Hwa; et al

Journal of Medicinal Chemistry (2009), 52(4), 1050-1062 | Language: English, Database: CAplus and MEDLINE

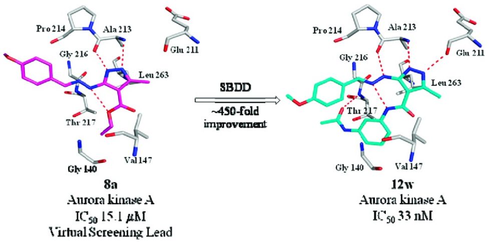

Aurora kinases have emerged as attractive targets for the design of anticancer drugs. Through structure-based virtual screening, novel pyrazole hit 8a was identified as Aurora kinase A inhibitor (IC_{50} = 15.1 μM). X-ray cocrystal structure of 8a in complex with Aurora A protein revealed the C-4 position Et carboxylate side chain as a possible modification site for improving the potency. On the basis of this insight, bioisosteric replacement of the ester with amide linkage and changing the Et substituent to hydrophobic 3-acetamidophenyl ring led to the identification of 12w with a ~450-fold improved Aurora kinase A inhibition potency (IC_{50} = 33 nM), compared to 8a. Compound 12w showed selective inhibition of Aurora A kinase over Aurora B/C, which might be due to the presence of a unique H-bond interaction between the 3-acetamido group and the Aurora A nonconserved Thr217 residue, which in Aurora B/C is Glu and found to sterically clash with the 3-acetamido group in modeling studies.

Keywords: structure aurora kinase A inhibitor acetamidophenyl derivative preparation

Substances (95) | Reactions (90) | Citing (61)

317

A specific pharmacophore model of aurora B kinase inhibitors and virtual screening studies based on it

By: Wang, Hui-Yuan; Li, Lin-Li; Cao, Zhi-Xing; Luo, Shi-Dong; Wei, Yu-Quan; Yang, Sheng-Yong

Chemical Biology & Drug Design (2009), 73(1), 115-126 | Language: English, Database: CAplus and MEDLINE

In this study, 3D-pharmacophore models of Aurora B kinase inhibitors have been developed by using HipHop and HypoGen modules in Catalyst software package. The best pharmacophore model, Hypo1, which has the highest correlation coefficient (0.9911), consists of one hydrogen-bond acceptor, one hydrogen-bond donor, one hydrophobic aliphatic moiety and one ring aromatic feature. Hypo1 was validated by test set and cross-validation methods. Specificity of Hypo1 to Aurora B inhibitors was examined with the use of selective inhibitors against Aurora B and its paralogue Aurora A. The results clearly indicate that Hypo1 can differentiate selective inhibitors of Aurora B from those of Aurora A, and the ring aromatic feature likely plays some important roles for the specificity of Hypo1. Then Hypo1 was used as a 3D query to screen several databases including Specs, NCI, Maybridge and Chinese Nature Product Database (CNPD) for identifying new inhibitors of Aurora B. The hit compounds were subsequently subjected to filtering by Lipinski's rule of five and docking studies to refine the retrieved hits, and some compounds selected from the top ranked hits have been suggested for further exptl. assay studies.

Keywords: aurora B kinase inhibitor virtual screening pharmacophore model

Substances (70) | Reactions (0) | Citing (13)

318

Anti-insulin-like growth factor 1 receptor therapy

By: Wang, Yan; Pachter, Jonathan A.; Hailey, Judith Anne; Brams, Peter; Williams, Denise; Srinivasan, Mohan; Feingersh, Mary Diane
World Intellectual Property Organization, WO2009005673 A1 2009-01-08 | Language: English, Database: CAplus

The authors disclose the preparation and functional characterization of human antibodies to the type 1 insulin- like growth factor receptor. In one example, the growth of a human neuroblastoma was shown to be inhibited by an anti- IGF1R antibody in a xenograft model.

Keywords: insulinlike growth factor receptor antibody antitumor

PatentPak available

Substances (330) Reactions (0) Citing (0)

319

Discovery or AZD1152: a selective inhibitor of Aurora-B kinase with potent antitumor activity

By: Foote, Kevin M.; Mortlock, Andrew A.
Edited by Li, Rongshi; Stafford, Jeffrey A.
Kinase Inhibitor Drugs (2009), 309-332 | Language: English, Database: CAplus

A review. The Aurora kinases continue to generate significant interest as attractive drug targets in oncol. Through the work carried out at AstraZeneca, selective inhibitors of Aurora- B kinase have been profiled and have allowed a novel mechanism of action (failure of cell division) to be determined which is distinct from the classical antimitotic agents. The quinaz oline core has shown itself to be an excellent template in which to develop potent Aurora kinase inhibitors while an understanding of the binding mode has allowed rapid optimization of the series with respect to potency and select ivity for the Aurora kinases.

Keywords: review aurora B kinase inhibitor AZD1152 antitumor

Substances (4) Reactions (0) Citing (9)

320

Phenotypic fingerprinting of small molecule cell cycle kinase inhibitors for drug discovery

By: Low, Jonathan; Chakravartty, Arunava; Blosser, Wayne; Dowless, Michele; Chalfant, Christopher; Bragger, Patty; Stancato, Louis
Current Chemical Genomics (2009), 3, 13-21 | Language: English, Database: CAplus and MEDLINE

Phenotypic drug discovery, primarily abandoned in the 1980's in favor of targeted approaches to drug development, is once again demonstrating its value when used in conjun ction with new technol ogies. Phenotypic discovery has been brought back to the fore mainly due to recent advances in the field of high content imaging (HCI). HCI elucidates cellular responses using a combin ation of immunofluorescent assays and computer anal. which increase both the sensit ivity and throughput of phenotypic assays. Although HCI data characterize cellular responses in individual cells, these data are usually analyzed as an aggregate of the treated population and are unable to discern differentially responsive sub-populations. A collection of 44 kinase inhibitors affecting cell cycle and apoptosis were characterized with a number of univar iate, bivariate, and multiv ariate subpopulation analyses demonst rating that each level of complexity adds addnl. inform ation about the treated popula tions and often distinguishes between compounds with seemingly similar mechanisms of action. Finally, these subpopulation data were used to charac terize compounds as they relate in chem. space.

Keywords: cell cycle kinase inhibitor cervical colon cancer phenotype fingerprinting

Substances (25) Reactions (0) Citing (16)

321

Preparation of peptide prodrugs modified with a 1,2,3,4-cyclobutanetetracarboxylic acid derived moiety useful in treatment and diagnosis of tumors and inflammatory diseases

By: Matthieu, Michel; Dubois, Vincent; Tranchant, Isabelle; Kearsey, Jonathan
World Intellectual Property Organization, WO2008120098 A2 2008-10-09 | Language: English, Database: CAplus

The invention is related to the preparation of peptide prodrugs **I** [L^1, L^2 = indepen dently a covalent bond or a linking moiety, the linking moiety should include at least two organic functional groups, one organic functional group that can bind to the 1,2,3,4-cyclobutanetetracarboxylic acid and a second organic functional group that allows binding to the oligop eptide moiety (L^1) or one functional group that can bind to the oligopeptide moiety and a second organic functional group that allows binding to D (L2) ; X = $(CH_2)_n$; X' = $(CH_2)_m$; X'' = $(CH_2)_p$; X''' = $(CH_2)_q$; n, m, p, q = indepen dently 0-10; Y = cleavable oligop eptide moiety by at least one specific and/or selective peptidase that is present in the extracellular environment of target cells; D = therap eutic agent or marker] which improve the therapeutic index and the solubility of the therap eutic agent and are intended for the treatment and/or diagnosis of tumors and/or inflammatory reactions. Thus, tetra Acid-ALAL-doxorubicin **II** was prepared and showed stability in human plasma after 2 h incubation. **II** was evaluated for its in vivo efficacy in the L S 174T tumor model and in vitro reacti vation into metabo lites (leucyl-doxorubicin and doxorubicin) by tumor homoge nates. A coumarin analog **III** was prepared for use as a marker for diagnosis purposes (no data).

Keywords: doxorubicin peptide prodrug cyclobuta netetracarboxylic acid modifi cation preparation antitumor antiinfla mmatory; mitomycin C peptide prodrug cyclobutanetetracarboxylic acid modifi cation preparation antitumor; paclitaxel peptide prodrug cyclobutanetetracarboxylic acid modifi cation preparation cancer inflammation; coumarin peptide cyclobuta netetracarboxylic acid modification preparation marker tumor

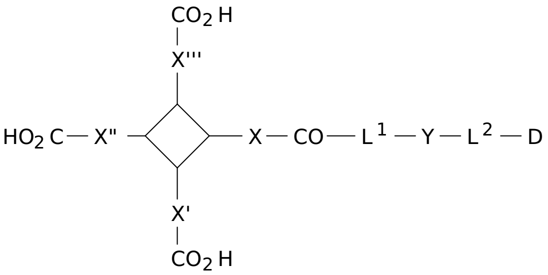

I

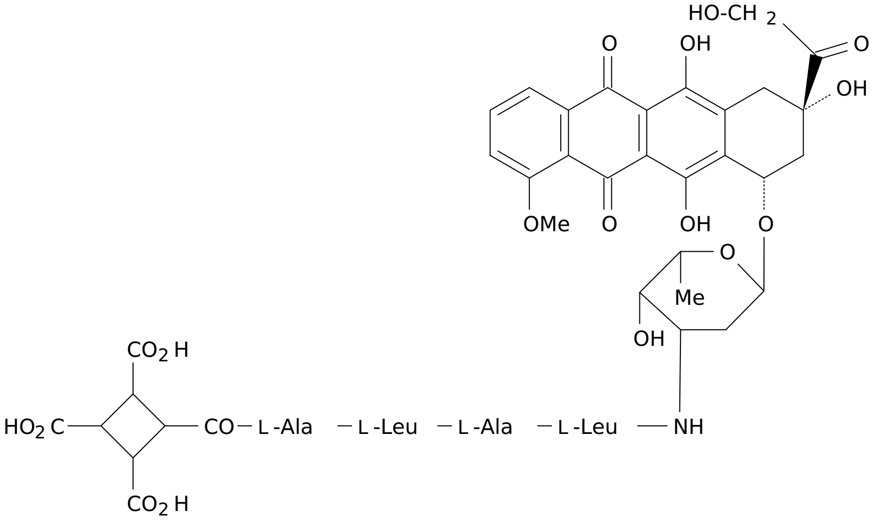

II

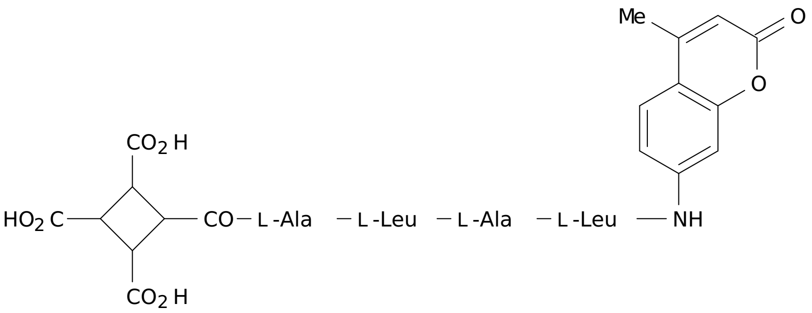

III

PatentPak available

Substances (316) | Reactions (255) | Citing (0)

322

Preparation of peptide prodrugs modified with a 1,2,3,4-cyclobutanetetracarboxylic acid derived moiety useful in treatment and diagnosis of tumors and inflammatory diseases

By: Matthieu, Michel; Dubois, Vincent; Tranchant, Isabelle; Kearsey, Jonathan

European Patent Organization, EP1977765 A1 2008-10-08 | Language: English, Database: CAplus

The invention is related to the preparation of peptide prodrugs **I** [L^1, L^2 = independently a covalent bond or a linking moiety; X = $(CH_2)_n$; X' = $(CH_2)_m$; X'' = $(CH_2)_p$; X''' = $(CH_2)_q$; n, m, p, q = independently 0-10; Y = cleavable oligopeptide moiety; D = therapeutic agent or marker] which improve the therapeutic index and the solubility of the therapeutic agent and are intended for the treatment and/or diagnosis of tumors and/or inflammatory reactions. Thus, tetraAcid-ALAL-doxorubicin **II** was prepared and showed stability in human plasma after 2 h incubation. **II** was evaluated for its in vivo efficacy in the LS 174T tumor model.

Keywords: peptide prodrug cyclobutanetetracarboxylic acid modification preparation cancer inflammation; doxorubicin peptide prodrug cyclobutanetetracarboxylic acid modification preparation antitumor antiinflammatory; mitomycin C peptide prodrug cyclobutanetetracarboxylic acid modification preparation antitumor; paclitaxel peptide prodrug cyclobutanetetracarboxylic acid modification preparation cancer inflammation

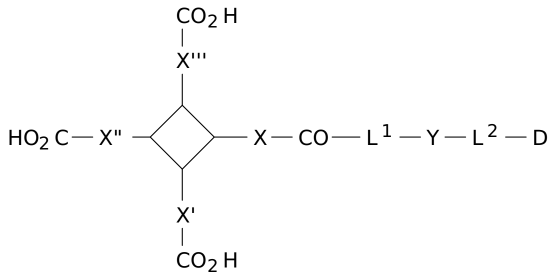

I

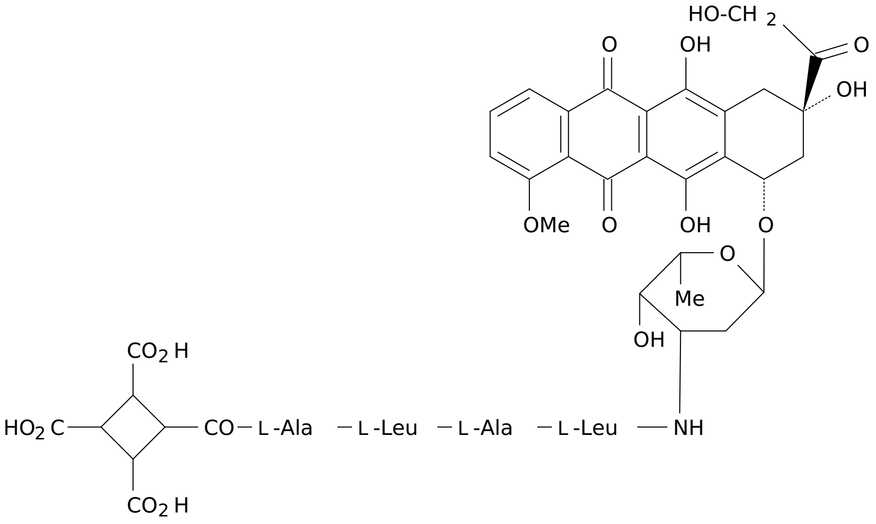

II

PatentPak available

Substances (150)   Reactions (13)   Citing (0)

323

Methods of treating cancer using IGF1R inhibitors

By: Wang, Yan; Zong, Chen; Seidel-Dugan, Cynthia; Wang, Yaolin; Yao, Siu-Long; Lu, Brian Der-Hua; Ladha, Mohamed H.
World Intellectual Property Organization, WO2008076278 A2 2008-06-26 | Language: English, Database: CAplus

The present invention provides IGF1R inhibitors and combin ations thereof that are effective at treating or preventing cancer. More specifically the IGF1R inhibitors are pyrrolo[2,3-d]pyrimidine derivatives or antibodies. The IGF1R inhibitors can be used in combin ation with other anticancer therapies, antiemetic agents, antianemic agents, or antimuc ositis agents.

Keywords: treatment cancer IGF1R inhibitor; antibody treatment cancer I GF1R inhibitor

PatentPak available

Substances (411) | Reactions (0) | Citing (3)

324

Enhancement of radiation response in p53-deficient cancer cells by the Aurora-B kinase inhibitor AZD1152

By: Tao, Y.; Zhang, P.; Girdler, F.; Frascogna, V.; Castedo, M.; Bourhis, J.; Kroemer, G.; Deutsch, E.
Oncogene (2008), 27(23), 3244-3255 | Language: English, Database: CAplus and MEDLINE

Overexpression of the Aurora-B kinase correlates with oncogenic transfo rmation and poor prognosis. We evaluated the effects of the bona fide Aurora-B kinase inhibitor AZD1152 on tumor responses to ionizing radiation (I R). When $p53^{wt}$ HCT116 and A549 cells were pretreated with AZD1152-HQPA prior to IR, additive effects were observed Interestingly, more pronounced tumoricidal effects were observed in p53-deficient HCT116 and HT29 cells, as well as A549 cells treated with the p53 inhibitor cyclic pifithrin- α. In vivo studies on xenografted mice confirmed enhanced tumor growth delay after the combin ation of IR plus AZD1152-IR as compared to IR alone. Again, this effect was more pronounced with $p53^{-/-}$ HCT116 and p53-mutant xenografts. The AZD1152-mediated radiosens itization was mimicked by knockdown of Aurora- B with a short interference RNA or by inhibition of Aurora- B by transfection with an inducible kinase-dead Aurora-B. The radiosensitizing effect of AZD1152 was lost in $CHK2^{-/-}$ and $14\text{-}3\text{-}3^{-/-}$ HCT116 cells. Altogether, these data indicate that AZD1152 can radiosensitize tumor cell lines in vitro and in vivo, the fact that these effects are exacer bated in p53-deficient cancer cells is of potential interest for further clin. develo pment.

Keywords: radiotherapy p53 cancer Aurora B kinase inhibitor A ZD1152 radiosensitizer

Substances (3) | Reactions (0) | Citing (76)

325

Preclinical evaluation of M30 and M65 ELISAs as biomarkers of drug induced tumor cell death and antitumor activity

By: Cummings, Jeffrey; Hodgkinson, Cassandra; Odedra, Rajesh; Sini, Patrizia; Heaton, Simon P.; Mundt, Kirsten E.; Ward, Tim H.; Wilkinson, Robert W.; Growcott, Jim; Hughes, Andrew; et al
Molecular Cancer Therapeutics (2008), 7(3), 455-463 | Language: English, Database: CAplus and MEDLINE

M30 and M65 are ELISAs that detect different circulating forms of cytokeratin 18. Using the aurora kinase inhibitor A ZD1152 and the SW620 human colon cancer xenograft, experiments were conducted to qualify preclinically both assays as serol. biomarkers of cell death. Using two different apoptotic markers, the kinetics of cell death induced by AZD1152 was first characterized in vitro in three different cell lines and shown to peak 5 to 7 days after drug addition Treatment of non-tumor-bearing rats with AZD1152 (25 mg/kg) produced no alterations in circulating baseline values of M30 and M65 antigens. In treated, tumor-bearing animals, M30 detected a 2- to 3-fold ($P < 0.05$) increase in plasma antigen levels by day 5 compared with controls. This correlated to a 3- fold increase in the number of apoptotic cells detected on day 5 in SW620 xenografts using immunohistochem. By contrast, M65 did not detect a drug-induced increase in circulating antigen levels at day 5. However, M65 plasma levels correlated to changes in tumor growth in control animals ($r^2 = 0.93$; $P < 0.01$) and also followed the magnitude of the temporal effect of A ZD1152 on tumor growth. An intermediate but active dose of A ZD1152 (12.5 mg/kg) produced a less significant increase in M30 plasma levels at day 5. It was also confirmed that the plasma profiles of M30 and M65 mirrored closely those measured in whole tumor lyzates. We conclude that M30 is a pharmacodynamic biomarker of AZD1152-induced apoptosis in the SW620 xenograft model, whereas M65 is a biomarker of therapeutic response.

Keywords: antitumor AZD1152 ELISA biomarker

Substances (2) Reactions (0) Citing (53)

326

The selective Aurora B kinase inhibitor AZD1152 is a potential new treatment for multiple myeloma

By: Evans, Robert P.; Naber, Claudia; Steffler, Tara; Checkland, Tamara; Maxwell, Christopher A.; Keats, Jonathan J.; Belch, Andrew R.; Pilarski, Linda M.; Lai, Raymond; Reiman, Tony
British Journal of Haematology (2008), 140(3), 295-302 | Language: English, Database: CAplus and MEDLINE

Aurora kinases are potential targets for cancer therapy. Previous studies have validated Aurora kinase A as a therapeutic target in multiple myeloma (MM), and have demonstrated in vitro anti-myeloma effects of small mol. Aurora kinase inhibitors that inhibit both Aurora A and B. This study demonstrated that Aurora B kinase was strongly expressed in myeloma cell lines and primary plasma cells. The selective Aurora B inhibitor AZD1152-induced apoptotic death in myeloma cell lines at nanomolar concentr ations, with a cell cycle phenotype consistent with that reported previously for Aurora B inhibition. In some cases, AZD1152 in combination with dexamethasone showed increased anti-myeloma activity compared with the use of either agent alone. A ZD1152 was active against sorted CD138+ BM plasma cells from myeloma patients but also, as expected, was toxic to C $D138^-$ marrow cells from the same patients. In a murine myeloma xenograft model, AZD1152-inhibited tumor growth at well-tolerated doses and induced cell death in established tumors, with associated mild, transient leucop enia. AZD1152 shows promise in these preclin. studies as a novel treatment for MM.

Keywords: antimyeloma Aurora kinase B A ZD1152 apoptosis dexamethasone multiple myeloma

Substances (3) Reactions (0) Citing (50)

327

3-Benzoylamino-1H-pyrazole-4-carboxamides as CDK kinase inhibitors, and their preparation, pharmaceutical combinations and use in the treatment of proliferative diseases

By: Lyons, John Francis; Squires, Matthew Simon; Thompson, Neil Thomas; Gallagher, Neil James; Curry, Jayne Elizabeth
World Intellectual Property Organization, WO2008007113 A2 2008-01-17 | Language: English, Database: CAplus

The invention provides a combination comprising an ancillary compound and a compound having the formula **I**: or salts or tautomers or N-oxides or solvates thereof/. Compounds of formula **I** wherein X is 5- to 6- membered (hetero/carbo)cyclic ring, amino, acylamino,sulfonylamino, etc.; Y is a bond and C_{1-3} alkylene; R^2 is H, halo, C_{1-4} alkoxy, (un)substituted C_{1-4} hydrocarbyl; R^3 is H, 3- to 12-membered (hetero/carbo)cyclic group; and their salts, tautomers, N- oxides and solvates thereof, are claimed. Example compound **II**•MsOH was prepared by esterification of 4-nitropyrazole-3-carboxylic acid; the resulting 4-nitropyrazole-3-carboxylic acid Me ester underwent hydrogenation to give 4-aminopyrazole-3-carboxylic acid Me ester, which underwent amidation with 2,6-dichlorobenzoyl chloride to give 4-(2,6-dichlorobenzoylamino)pyrazole-3-carboxylic acid Me ester, which underwent hydrolysis to give 4-(2,6-dichlorobenzoylamino)pyrazole-3-carboxylic acid, which underwent chlorination to give the corresponding acid chloride, which underwent amidation with 4-amino-1-Boc-piperidine to give 1-Boc-piperidin-4-yl 4-(2,6-dichlorobenzoylamino)pyrazole-3-carboxamide, which underwent hydrolysis to give compound **II**•MsOH. All the invention compounds were evaluated for their C DK kinase inhibitory activity (some data given).

Keywords: benzoylamino pyrazolecarboxamide preparation CDK kinase inhibitor treatment prolife rative disease

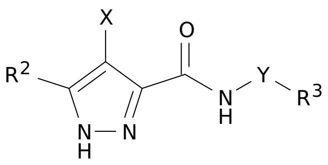

I

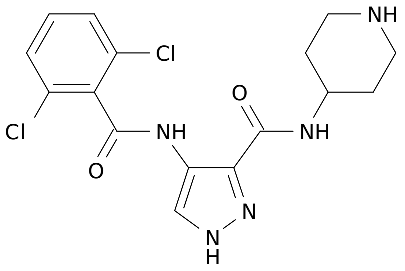

II

PatentPak available

Substances (298) | Reactions (279) | Citing (1)

328

Blood levels of insulin-like growth factor-binding protein 2 as a marker for monitoring the effectiveness of inhibitors of insulin-like growth factor I receptors in cancer therapy

By: Wang, Yan
World Intellectual Property Organization, WO2008005469 A2 2008-01-10 | Language: English, Database: CAplus

The present invention provides method for quickly and conveniently determining if a given treatment regimen of insulin- like growth factor I receptor (IGF1R) inhibitor is sufficient, e.g., to saturate IGF1 R receptors in the body of a subject. Blood levels of insulin- like growth factor-binding protein 2 (IGFBP2) are shown to be strongly correlated with the effecti veness of IGF1R receptor therapy. Several clin. relevant determinations may be made based on this point, including, for example, whether the dosage of the regimen is sufficient or should be increased. The relationship is demonstrated using animal xenograft models of neurobl astoma. Treatment with monoclonal antibodies to IGFR1 lowered the blood levels of IGFBP2. The level of IGFBP2 correlated with the tumor size.

Keywords: IGFBP2 marker cancer therapy IGF1 receptor inhibitor

PatentPak available

Substances (408) | Reactions (0) | Citing (2)

329

Emerging role of Aurora kinase inhibitors in chronic myeloid leukemia

By: Alvarado, Yesid; Cortes, Jorge E.
Clinical Leukemia (2007), 1(6), 325-330 | Language: English, Database: CAplus

A review. Resistance to imatinib and second-generation tyrosine kinase inhibitors is an ongoing problem most frequently mediated through mutations of the Bcr-Abl kinase domain. One mutation that affects respons iveness to all current available agents is T315I. Aurora proteins belong to a small family of serine/threonine kinases that are essential for proliferating cells and have been identified as key regulators of different steps in mitosis and meiosis, ranging from the formation of the mitotic spindle up to cytoki nesis. Unexpectedly, Aurora kinase inhibitors have been found to have activity against the T315 I bcr-abl mutation, and some of them might rise as important therapeutic options. The common mechanism of action for protein kinase inhibition is compet ition with ATP for the active site-binding pocket, which is very similar among the protein kinases, and this could explain the cross- reacti vity. Herein, we discuss the basics of imatinib resistance develo pment and Aurora kinase biol., and describe a selected group of Aurora kinase inhibitors with potential activity in this patient population.

Keywords: review chronic myeloid leukemia anticancer Aurora kinase inhibitor imatinib

Substances (7) Reactions (0) Citing (3)

330

Process for the preparation of pyrazolylaminoquinazoline derivatives comprising a phosphate group

By: Pittam, John David; Sependa, George Joseph
World Intellectual Property Organization, WO2007132210 A1 2007-11-22 | Language: English, Database: CAplus

The invention relates to a new process useful in the preparation of pharmaceutical compounds such as 2-{ethyl[3-({4-[(5-{2-[(3-fluorophenyl)amino]-2-oxoethyl}-1H-pyrazol-3-yl)amino]quinazolin-7-yl}oxy)propyl]amino}ethyl dihydrogen phosphate (AZD1152) and intermediates used therein.

Keywords: pyrazolyl amino quinazoline phosphate preparation; AZD1152 intermediate preparation; fluorophenyl amino oxoethyl pyrazolylamino quinazolinyloxypropyl dihydrogen phosphate AZD1152 intermediate

PatentPak available

Substances (16) Reactions (45) Citing (0)

331

Maleate co-crystal of AZD 1152 for dosage forms for treatment of hyperproliferative diseases

By: Sependa, George Joseph; Storey, Richard
World Intellectual Property Organization, WO2007132227 A1 2007-11-22 | Language: English, Database: CAplus

The present invention relates to a novel co-crystal form of 2-{ethyl[3-({4-[(5-{2-[(3-fluorophenyl)amino]-2-oxo-ethyl}-1H-pyrazol-3-yl)amino]quinazolin-7-yl}oxy)propyl]amino} Et dihydrogen phosphate (A ZD 1152), an aurora kinase inhibitor useful in the treatment of hyperproliferative diseases, such as cancer. More specifi cally, the invention relates to a maleate co- crystal of AZD 1152, to a process for its preparation, its use in the manufacturing of a medicament for the treatment of hyperprol iferative diseases, and to methods of treating hyperproliferative diseases by adminis tering a therapeutically effective amount of a maleate co- crystal of AZD 1152. A particular crystalline form of a maleate co- crystal of AZD 1152 is also described. Thus, crude A ZD 1152 (preparation given, estimated at 7.44 g @ 100%, 11.61 mM) was added to DMSO (36 mL) and left at ambient temper ature to produce a pale brown solution To this solution was added a solution of maleic acid (1.76 g, 15.16 mM, 1.31 mol equivalent) in Me OH (36 mL) and the mixture left to stand overnight at ambient temperature Next day an aliquot of the clear solution was transf erred to a vial, scratched and left sealed for several hours. A deposit of white solid formed and this was transferred to the flask and left to stir. Gradually the solution turned turbid and solid deposited. The slurry was left to settle for several days and finally filtered. The cake was washed with a 1:1 mixture of DMSO/MeOH, slurried in situ with Me OH and then dried in vacuo. N MR confirmed the solid to be the maleate co- crystal of AZ D1152 (yield of about 78.7%).

Keywords: AZD1152 maleate crystal form prepar ation dosage form; aurora kinase inhibitor A ZD1152 maleate hyperprol iferative disease

PatentPak available

Substances (22) Reactions (30) Citing (1)

332

Quinazolin-4-ylaminopyrazolecarboxamides as aurora kinase inhibitors useful in bombination therapy for the treatment of cancer and their preparation

By: Keen, Nicholas John

World Intellectual Property Organization, WO2007132215 A1 2007-11-22 | Language: English, Database: CAplus

A combination comprising an aurora kinase inhibitor and an efflux transp orter inhibitor wherein the aurora kinase inhibitor is a compound of formula **I** or pharmaceutically acceptable salt thereof for use in the treatment of hyperprol iferative diseases such as cancer. Compounds of formula **I** wherein n is 0, 1, 2 and 3; R ^{1} is C_{1-4} hydroxyalkyl and C_{1-4} phosphonooxyalkyl; R^2 is H, C_{1-4} (hydroxy)alkyl, C_{1-4} alkoxy-C_{1-4} alkyl, and heterocyclyl; R^1R^2 together with nitrogen form a (un)substituted 4- to 6-membered hetero cyclic ring; R^3 is H and C_{1-4} alkoxy; R^4, R^6 and R^6 are independently H and C_{1-4} alkyl; R^5 is (un)substituted aryl; and their pharmace utically acceptable salts thereof. Example compound **II** was prepared by a multistep procedure (procedure given) . All the invention compounds were evaluated for their Aurora kinase inhibitory activity (some data given).

Keywords: quinazolinyl pyrazolylacetamide preparation aurora kinase inhibitor treatment cancer

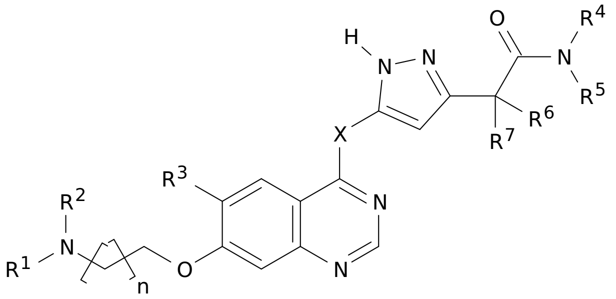

I

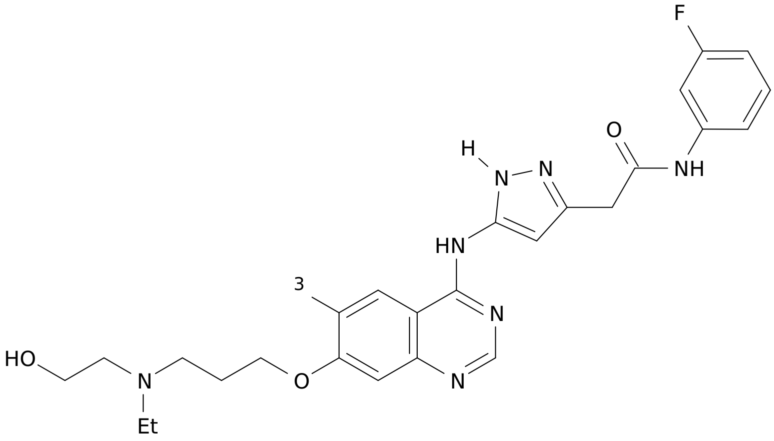

II

PatentPak available

Substances (65) Reactions (58) Citing (2)

333

Pharmaceutical combinations of diazole derivatives for cancer treatment and their preparation

By: Squires, Matthew Simon
World Intellectual Property Organization, WO2007129062 A1 2007-11-15 | Language: English, Database: CAplus

The invention provides a combination comprising (or consisting essentially of) an ancillary compound and a compound of the formula **I**, or salts, tautomers, solvates and N-oxides thereof. The combinations have activity as inhibitors of CDK kinases and inhibit the proliferation of cancer cells. Compounds of formula **I** wherein, R^1 is 2,6-dichlorophenyl; R^{2a} and R^{2b} are both H; R^3 is C_{1-4} alkyl-S O_2-piperidinyl; and their salts, tautomers, solvates, and N-oxides thereof, are claimed. Example compound **II** was prepared by methylation of 4-nitropyrazole-3-carboxylic acid; the resulting 4-nitropyrazole-3-carboxylic acid Me ester underwent hydrogenation to give 4-aminopyrazole-3-carboxylic acid Me ester, which underwent acylation with 2,6-dichlorobenzoyl chloride followed by hydrolysis to give 4-(2,6-dichlorobenzoylamino)-1H-pyrazole-3-carboxylic acid, which underwent amidation with 4-amino-1-Boc-piperidine, to give 4-[[4-(2,6-dichlorobenzoylamino)-1H-pyrazole-3-carbonyl]amino]piperidine-1-carboxylic acid tert-Bu ester, which underwent hydrolysis to give 4-(2,6-dichlorobenzoylamino)-1H-pyrazole-3-carboxylic acid piperidin-4-yl amide hydrochloride, which underwent sulfonylation with methanesulfonyl chloride to give compound **II**. The crystal structure of compound **II** was also determined The invention compounds were evaluated for their CDK kinase inhibitory activity (some data given).

Keywords: pyrazole derivative preparation CDK kinase inhibitor treatment cancer

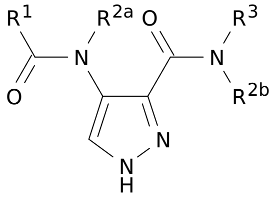

I

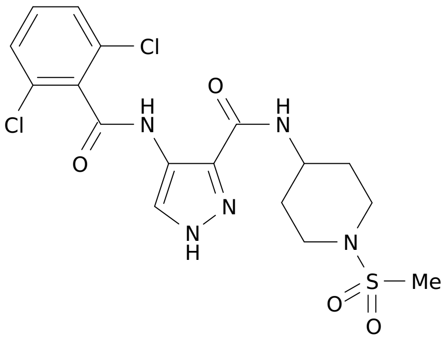

II

PatentPak available

Substances (143) Reactions (171) Citing (3)

334

AZD1152, a novel and selective aurora B kinase inhibitor, induces growth arrest, apoptosis, and sensitization for tubulin depolymerizing agent or topoisomerase II inhibitor in human acute leukemia cells in vitro and in vivo

By: Yang, Jing; Ikezoe, Takayuki; Nishioka, Chie; Tasaka, Taizo; Taniguchi, Ayuko; Kuwayama, Yoshio; Komatsu, Naoki; Bandobashi, Kentaro; Togitani, Kazuto; Koeffler, H. Phillip; et al
Blood (2007), 110(6), 2034-2040 | Language: English, Database: CAplus and MEDLINE

Aurora kinases play an important role in chromosome alignment, segregation, and cytokinesis during mitosis. We have recently shown that hematopoietic malignant cells including those from acute myeloid leukemia (AML) and acute lymphoblastic leukemia (A LL) aberrantly expressed Aurora A and B kinases, and Z M447439, a potent inhibitor of Aurora kinases, effectively induced growth arrest and apoptosis of a variety of leukemia cells. The present study explored the effect of AZD1152, a highly selective inhibitor of Aurora B kinase, on various types of human leukemia cells. AZD1152 inhibited the proliferation of AML lines (HL-60, NB4, MOLM13), ALL line (PALL-2), biphenotypic leukemia (MV4-11), acute eosinophilic leukemia (EOL-1), and the blast crisis of chronic myeloid leukemia K562 cells with an IC50 ranging from 3 nM to 40 nM, as measured by thymidine uptake on day 2 of culture. These cells had 4N/8N DNA content followed by apoptosis, as measured by cell- cycle anal. and annexin V staining, resp. Of note, A ZD1152 synergistically enhanced the antiproliferative activity of vincristine, a tubulin depolymerizing agent, and daunorubicin, a topoiso merase II inhibitor, against the MOLM13 and PALL-2 cells in vitro. Furthermore, AZD1152 potentiated the action of vincristine and daunorubicin in a MOLM13 murine xenograft model. Taken together, AZD1152 is a promising new agent for treatment of indivi duals with leukemia. The combined administration of AZD1152 and conventional chemotherapeutic agent to patients with leukemia warrants further investigation.

Keywords: AZD1152 aurora B kinase inhibitor vincristine daunorubicin synergism leukemia

Substances (7) Reactions (0) Citing (221)

335

AZD1152, a Selective Inhibitor of Aurora B Kinase, Inhibits Human Tumor Xenograft Growth by Inducing Apoptosis

By: Wilkinson, Robert W.; Odedra, Rajesh; Heaton, Simon P.; Wedge, Stephen R.; Keen, Nicholas J.; Crafter, Claire; Foster, John R.; Brady, Madeleine C.; Bigley, Alison; Brown, Elaine; et al
Clinical Cancer Research (2007), 13(12), 3682-3688 | Language: English, Database: CAplus and MEDLINE

PURPOSE: In the current study, we examined the in vivo effects of A ZD1152, a novel and specific inhibitor of Aurora kinase activity (with selectivity for Aurora B). Exptl. DESIGN: The pharmacodynamic effects and efficacy of AZD1152 were determined in a panel of human tumor xenograft models. AZD1152 was dosed via several parenteral (s.c. osmotic mini-pump, i.p., and i.v.) routes. RESULTS: AZD1152 potently inhibited the growth of human colon, lung, and hematol. tumor xenografts (mean tumor growth inhibition range, 55% to ≥100%; $P < 0.05$) in immunodeficient mice. Detailed pharmacodynamic anal. in colorectal SW620 tumor-bearing athymic rats treated i.v. with AZD1152 revealed a temporal sequence of phenotypic events in tumors: transient suppre ssion of histone H3 phosphorylation followed by accumulation of 4N DNA in cells (2.4-fold higher compared with controls) and then an increased proportion of polyploid cells (>4N DNA, 2.3-fold higher compared with controls). Histol. anal. showed aberrant cell division that was concurrent with an increase in apoptosis in AZD1152-treated tumors. Bone marrow analyses revealed transient myelosup pression with the drug that was fully reversible following cessation of AZD1152 treatment. CONCLUSIONS: These data suggest that selective targeting of Aurora B kinase may be a promising therapeutic approach for the treatment of a range of maligna ncies. In addition to the suppression of histone H3 phosphorylation, determination of tumor cell polyploidy and apoptosis may be useful biomarkers for this class of therapeutic agent. AZD1152 is currently in phase I trials.

Keywords: AZD1152 Aurora B kinase inhibitor antitumor tumor growth apoptosis

Substances (3) Reactions (0) Citing (303)

336

Anti-angiogenic activity of 2-methoxyestradiol in combination with anti-cancer agents

By: Plum, Stacy M.; Strawn, Steven J.; Lavallee, Theresa M.; Sidor, Carolyn F.; Fogler, William E.; Treston, Anthony M.
World Intellectual Property Organization, WO2007059111 A2 2007-05-24 | Language: English, Database: CAplus

The present invention relates generally to methods and compositions of treating disease characterized by abnormal cell prolife ration and/or abnormal or undesirable angiogenesis by administering antiangiogenic agents in combination with chemotherapeutic agents. More specifically, the present invention relates to a methods and compositions of treating diseases characterized by abnormal cell proliferation and/or abnormal or undesirable angiogenesis by administering 2-methoxyestradiol, in combination with chemotherapeutic agents.

Keywords: antiangiogenic agent methoxyestradiol combination therapy anticancer agent

PatentPak available

Substances (195) Reactions (0) Citing (2)

337

Discovery, Synthesis, and in Vivo Activity of a New Class of Pyrazolylamino Quinazolines as Selective Inhibitors of Aurora B Kinase

By: Mortlock, Andrew A.; Foote, Kevin M.; Heron, Nicola M.; Jung, Frederic H.; Pasquet, Georges; Lohmann, Jean-Jacques M.; Warin, Nicolas; Renaud, Fabrice; De Savi, Chris; Roberts, Nicola J.; et al
Journal of Medicinal Chemistry (2007), 50(9), 2213-2224 | Language: English, Database: CAplus and MEDLINE

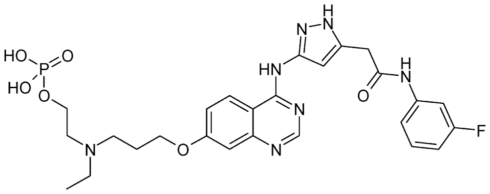

A series of pyrazolylamino-substituted quinazolines was synthesized and biol. evaluated as inhibitors of Aurora kinases, which have been the subject of considerable interest as targets for the development of new anticancer agents. Some of the products demons trated greater than 1000-fold selectivity for Aurora B over Aurora A kinase activity in recomb inant enzyme assays. These compounds have been designed for parenteral administration and achieve high levels of solubility by virtue of their ability to be delivered as readily activated phosphate derivatives The prodrugs are comprehensively converted to the des-phosphate form in vivo, and the active species have advantageous pharmacokinetic properties and safety pharmacol. profiles. The compounds display striking in vivo activity, and **I** (AZD1152) has been selected for clin. evaluation and is currently in phase 1 clin. trials.

Keywords: quinazoline pyrazolylamino preparation selective inhibitor Aurora kinase antitumor

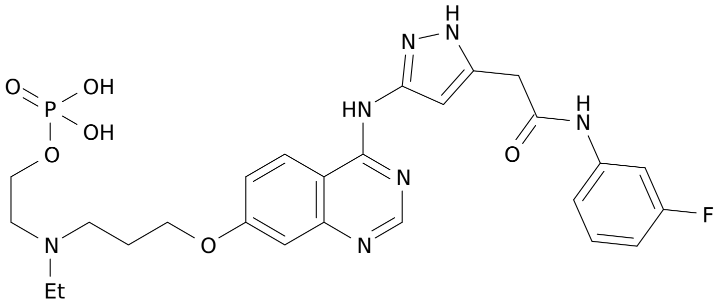

I

Substances (85) Reactions (245) Citing (238)

338

Preparation of phosphonooxy quinazoline derivatives and their pharmaceutical use

By: Heron, Nicola Murdoch; Jung, Frederic Henri; Pasquet, Georges Rene; Mortlock, Andrew Austen
World Intellectual Property Organization, WO2004058781 A1 2004-07-15 | Language: English, Database: CAplus

Preparation of phosphonooxy quinazoline derivatives, **I** (A = 5-membered heteroaryl containing a nitrogen atom and one or two further nitrogen atoms; X = O, S, S(O), $S(O)_2$, organoamino; m = 0-3; Z = organoamino, phosphonooxy, (un)substituted C_{3-6} cycloalkyl, etc.; R^3 = H, halo, cyano, nitro, C_{1-6} alkoxy, C_{1-6} alkyl, alkoxycarbonyl, organoamido, sulfonylamido, etc.; R^4 = H, C_{1-4} alkyl, heteroaryl, heteroaryl C_{1-4} alkyl, aryl, etc.; R^5 = H, C_{1-4} alkyl, C_{2-4} alkenyl, C_{2-4} alkynyl, C_{3-6} cycloalkyl, etc.; R^6, R^7 = H, halo, C_{1-4} alkyl, C_{3-6} cycloalkyl, hydroxy, C_{1-4} alkoxy, etc.), and compositions containing them, processes for their preparation and their use in therapy is described. Thus, reaction of N-(3-fluorophenyl)-2-{3-[(7-{3-[4-(hydroxymethyl)piperidin-1-yl]propoxy}-6-methoxyquinazolin-4-yl)amino]-1H-pyrazol-5-yl}acetamide (preparation given) with di-tert-butyl-diethylphosphoramidite gave 70% di-tert-Bu {1-[3-({4-[(5-{2-[(3-fluorophenyl)amino]-2-oxoethyl}-1Hpyrazol-3-yl)amino]-6-methoxyquinazolin-7-yl}oxy)propyl]piperidin-4-yl}methyl phosphate which on acidic hydrolysis gave 94% title compound, di-tert-Bu {1-[3-({4-[(5-{2-[(3-fluorophenyl)amino]-2-oxoethyl}-1Hpyrazol-3-yl)amino]-6-methoxyquinazolin-7-yl}oxy)propyl]piperidin-4-yl}methyl dihydrogen phosphate. In vitro Aurora- A and Aurora-B kinase inhibition activity and cell proliferation and cycle anal. of the prepared compounds were determined

Keywords: phosphonooxy quinazoline derivative preparation Aurora A B kinase inhibition

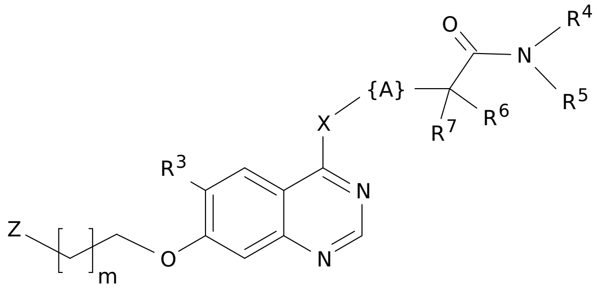

I

PatentPak available

Substances (268) Reactions (1,525) Citing (20)

Copyright © 2023 American Chemical Society (ACS). All Rights Reserved.
Copyright © 2023 U.S. National Library of Medicine.
Internal use only. Redistribution is subject to the terms of your SciFinderⁿ License Agreement and CAS information Use Policies.

SciFinderⁿ

Task History

Initiating Search

May 11, 2023, 10:08AM

References:

Filtered By:

Publication Year: 2004 to 2022

Structure of AZD1152-HQPA (right)

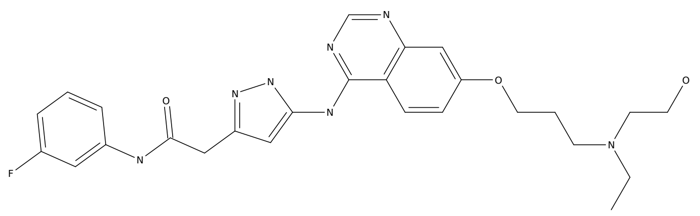

Structure Match: **As Drawn**

Search Tasks

Task	Search Type	View
Exported: Returned Reference Results + Filters (133)	References	View Results

Copyright © 2023 American Chemical Society (ACS). All Rights Reserved.
Internal use only. Redistribution is subject to the terms of your SciFinderⁿ License Agreement and CAS information Use Policies.

CAS SciFinderⁿ

References (133)

View in SciFinderⁿ

1

Differential ABC transporter expression during hematopoiesis contributes to neutrophil-biased toxicity of Aurora kinase inhibitors

By: Chou, David B. ; Furlong, Brooke A. ; Posey, Ryan R.; Kyprianou, Christos ; O'Sullivan, Lucy R.; David, Rhiannon; Randle, Suzanne J.; Polanska, Urszula M.; Travers, Jon; Urosevic, Jelena; et al
Nature Communications (2022), 13(1), 6021 | Language: English, Database: CAplus and MEDLINE

Drug-induced cytopenias are a prevalent and significant issue that worsens clin. outcomes and hinders the effective treatment of cancer. While reductions in blood cell numbers are classically associated with traditional cytotoxic chemotherapies, they also occur with newer targeted small mols. and the factors that detect the hematotoxicity profiles of oncol. drugs are not fully understood. Here, we explore why some Aurora kinase inhibitors cause preferential neutropenia. By studying drug responses of healthy human hematopoietic cells in vitro and analyzing existing gene expression datasets, we provide evidence that the enhanced vulnera bility of neutrophil-lineage cells to Aurora kinase inhibition is caused by early develop mental changes in ATP-binding cassette (ABC) transp orter expression. These data show that hematop oietic cell-intrinsic expression of ABC transporters may be an important factor that determines how some Aurora kinase inhibitors affect the bone marrow.

Keywords: aurora kinase inhibitor toxicity ABC transporter hematopoiesis cancer

Substances (14) | Reactions (0) | Citing (0)

2

Methods for inhibiting KRAS oncoprotein through enhanced GTPase activity

By: Lito, Piro
World Intellectual Property Organization, WO2022217073 A1 2022-10-13 | Language: English, Database: CAplus

The present disclosure relates to methods for determining whether a cancer patient harboring a constit utively active KRAS mutation will be responsive to treatment with a $KRAS^{G12C}$ inhibitor that selectively targets the inactive state of K RAS. These methods are based on assaying regulators of G-protein signaling (R GS) expression in the cancer patient.

Keywords: sequence KRAS oncoprotein GTPase

PatentPak available

Substances (171) | Reactions (0) | Citing (0)

3

Aurora kinase B inhibitors for use for treating cancer

By: Travers, Jon; Urosevic Despotovic, Jelena; Molina-Villa, Miguel-Angel; Bertran-Alamillo, Jordi
World Intellectual Property Organization, WO2022192139 A1 2022-09-15 | Language: English, Database: CAplus

Provided are methods for treating cancer with an Aurora kinase B inhibitor comprising determining if a cancer cell has a chromo somal amplification at location 22q11 on chromosome 22. Also provided are methods for treating cancer with an Aurora kinase B inhibitor comprising determining if a cancer cell has a gene with an increased copy number at location 22q11 on chromosome 22. Further provided are methods of determining if a cancer cell or a subject having cancer are amenable to treatment with an Aurora kinase B inhibitor.

Keywords: aurora kinase B inhibitor drug resistant cancer combination therapy

PatentPak available

Substances (33) | Reactions (0) | Citing (0)

4

Hormad1 therapeutics

By: Tutt, Andrew; Walker, Callum; Weekes, Daniel; Pines, Jon; Lord, Christopher
World Intellectual Property Organization, WO2022122938 A1 2022-06-16 | Language: English, Database: CAplus

The present invention relates to the treatment of cancer and, in particular to the treatment of patients whose cancer expresses HO RMAD1 with an agent that modulates mitotic processes, and diagnostic methods thereof.

Keywords: Hormad1 expression cancer diagnosis mitotsis kinase inhibition antitumor chemotherapy

PatentPak available

Substances (10) | Reactions (0) | Citing (0)

5

Amphiphilic peptides, peptide amphiphile lipid micelles and chemotherapeutic agent or imaging agent formulations for cancer treatment or diagnosis

By: Homan, Reynold
World Intellectual Property Organization, WO2022035818 A2 2022-02-17 | Language: English, Database: CAplus

Novel amphiphilic peptide, peptide amphiphile lipid micelles, processes for making peptide amphiphile lipid micelles comprising an amphiphilic peptide and phospholipid and optionally comprising a cargo mol., and methods of use.

Keywords: amphiphilic peptide amphiphile lipid micelle chemothe rapeutic formulation cancer therapy

PatentPak available

Substances (67) | Reactions (26) | Citing (0)

6

Multi-modal molecular imaging maps the correlation between tumor microenvironments and nanomedicine distribution

By: Strittmatter, Nicole; Moss, Jennifer I.; Race, Alan M.; Sutton, Daniel; Canales, Jaime Rodriguez; Ling, Stephanie; Wong, Edmond; Wilson, Joanne; Smith, Aaron; Howes, Colin; et al
Theranostics (2022), 12(5), 2162-2174 | Language: English, Database: CAplus and MEDLINE

Gaining insight into the heterogeneity of nanoparticle drug distribution within tumors would improve both design and clin. transl ation of nanomedicines. There is little data showing the spatio-temporal behavior of nanomedicines in tissues as current methods are not able to provide a comprehensive view of the nanomedicine distribution, released drug or its effects in the context of a complex tissue microenvironment. A new exptl. approach which integrates the mol. imaging and bioanal. techno logies MSI and IMC was developed to determine the biodistribution of total drug and drug metabolite delivered via P LA-PEG nanoparticles and to overlay this with imaging of the nanomedicine in the context of detailed tumor microenv ironment markers. This was used to assess the nanomedicine AZD2811 in animals bearing three different pre- clin. PDX tumors. This new approach delivered new insights into the nanoparticle/drug biodistribution. Mass spectrometry imaging was able to differe ntiate the tumor distri bution of co-dosed deuterated non-nanoparticle-formulated free drug alongside the nanopar ticle-formulated drug by directly visual izing both delivery approaches within the same animal or tissue. While the IV delivered free drug was uniformly distri buted, the nanomedicine delivered drug was heterogeneous. By staining for multiple biomarkers of the tumor microenv ironment on the same tumor sections using imaging mass cytometry, co-registering and integrating data from both imaging modalities it was possible to determine the features in regions with highest nanomedicine distribution. Nanomedicine delivered drug was associated with regions higher in macrophages, as well as more stromal regions of the tumor. Such a comparison of complem entary mol. data allows delineation of drug abundance in individual cell types and in stroma. This multi- modal imaging solution offers resear chers a better understanding of drug and nanoca rrier distribution in complex tissues and enables data- driven drug carrier design.

Keywords: mol imaging tumor microenvironment nanomedicine; mass spectrometry imaging; nanomedicine; tumor distri bution

Substance (1) Reactions (0) Citing (4)

7

Phenotypic screening of 1,953 FDA-approved drugs reveals 26 hits with potential for repurposing for Peyronie's disease

By: Ilg, Marcus M.; Lapthorn, Alice R.; Ralph, David J.; Cellek, Selim
PLoS One (2022), 17(12), e0277646 | Language: English, Database: CAplus and MEDLINE

Drug repurposing has been shown to bring safe medications to new patient populations, as recently evidenced by the COVID-19 pandemic. We investigated whether we could use phenotypic screening to repurpose drugs for the treatment of Peyronie's disease (PD). PD is a fibrotic disease characterized by continued myofibroblast presence and activity leading to formation of a plaque in the penile tunica albuginea (TA) that can cause pain during erection, erectile dysfunction, and penile deformity. PD affects 3-9% of men with treatment options limited to surgery or injection of collagenase which can only be utilized at late stages after the plaque is formed. Currently there are no approved medications that can be offered to patients presenting with early disease before the formation of the plaque. Drug repurposing may therefore be the ideal strategy to identify medical treatments to address this unmet medical need in early PD. We used primary human fibroblasts from PD patients in a phenotypic screening assay that measures TGF-β1-induced myofibroblast transformation which is the main cellular phenotype that drives the pathol. in early PD. A library of FDA-approved 1953 drugs was screened in duplicate wells at a single concentration (10μM) in presence of TGF-β1. The myofibroblast marker α-SMA was quantified after 72h incubation. A pos. control of SB-505124 (TGF-β1 receptor antagonist) was included on each plate. Hits were defined as showing >80% inhibition, while retaining >80% cell viability. 26 hits (1.3%) were identified which were divided into the following main groups: anti-cancer drugs, anti-inflammation, neurol., endocrinol., and imaging agents. Five of the top-ten drugs that increase myofibroblast-transformation appear to act on VEGFR. This is the first phenotypic screening of FDA-approved drugs for PD and our results suggest that it is a viable method to predict drugs with potential for repurposing to treat early PD.

Keywords: drug FDA phenotypic screening Peyronies disease

Substances (1,943) Reactions (0) Citing (0)

8

Viability fingerprint of glioblastoma cell lines: roles of mitotic, proliferative, and epigenetic targets

By: Lavogina, Darja; Laasfeld, Tonis; Vardja, Markus; Lust, Helen; Jaal, Jana
Scientific Reports (2021), 11(1), 20338 | Language: English, Database: CAplus and MEDLINE

Despite the use of multimodal treatment combinations, the prognosis of glioblastoma (GB) is still poor. To prevent rapid tumor recurrence, targeted strategies for the treatment of GB are widely sought. Here, we compared the efficacy of focused modulation of a set of signaling pathways in two GB cell lines, U-251 MG and T98-G, using a panel of thirteen compounds targeting cell cycle progression, proliferation, epigenetic modifications, and DNA repair mechanism. In parallel, we tested combinations of these compounds with temozolomide and lomustine, the standard chemotherapy agents used in GB treatment. Two major trends were found: within individual compounds, the lowest IC_{50} values were exhibited by the Aurora kinase inhibitors, whereas in the case of mixtures, the addition of DNA methyltransferase 1 inhibitor azacytidine to lomustine proved the most beneficial. The efficacy of cell cycle-targeting compounds was further augmented by combination with radiation therapy using two different treatment regimes. The potency of azacytidine and lomustine mixtures was validated using a unique assay pipeline that utilizes automated imaging and machine learning-based data anal. algorithm for assessment of cell number and DNA damage extent. Based on our results, the combination of azacytidine and lomustine should be tested in GB clin. trials.

Keywords: glioblastoma cell viability mitotic proliferative epigenetic target

Substances (13) Reactions (0) Citing (2)

9

Butyrophilin-like 9 expression is associated with outcome in lung adenocarcinoma

By: Ma, Weishuang; Liang, Jiaming; Mo, Junjian; Zhang, Siyuan; Hu, Ningdong; Tian, Dongbo; Chen, Zisheng
BMC Cancer (2021), 21(1), 1096 | Language: English, Database: CAplus and MEDLINE

Lung adenocarcinoma (LUAD) is the most prevalent non-small cell lung cancer (N SCLC). Patients with L UAD have a poor 5-yr survival rate. The use of immune checkpoint inhibitors (ICIs) for the treatment of L UAD has been on the rise in the past decade. This study explored the prognostic role of butyrophilin-like 9 (BTNL9) in LUAD. Gene expression profile of buytrophilins (BTNs) was determined using the GEPIA database. The effect of B TNL9 on the survival of LUAD patients was assessed using Kaplan-Meier plotter and OncoLnc. Correlation between BTNL9 expression and tumor-infiltrating immune cells (TILs) was explored using TIMER and GEPIA databases. Further, the relationship between BTNL9 expression and drug response was evaluated using C ARE. Besides, construction and evaluation of nomogram based on B TNL9 expression and TNM stage. BTNL9 expression was downregulated in LU AD and was associated with a poor probab ility of 1, 3, 5-years overall survival (O S). In addition, BTNL9 expression was regulated at epigenetic and post-transcriptional modification levels. Moreover, BTNL9 expression was significantly pos. correlated with Immune Score and ESTIMATEScore. Furthermore, BTNL9 expression was pos. associated with infiltration levels of B cells, C D4+ T cells, and macrophages. Kaplan-Meier anal. showed that BTNL9 expression in B cells and dendritic cells (D Cs) was significantly associated with OS. BTNL9 expression was significantly pos. correlated with C ARE scores. These findings show that B TNL9 is a potential prognostic biomarker for LUAD. Low BTNL9 expression levels associated with low infilt ration levels of naive B cells, and D Cs in the tumor microenvironment are unfavorable for OS in LUAD patients.

Keywords: lung adenocarcinoma butyrophilin immune cell gene expres sion; B cells; Butyrophilin-like 9; Dendritic cells; Lung adenocarcinoma; Prognosis

Substances (115) Reactions (0) Citing (1)

10

High-throughput compound screening identifies navitoclax combined with irradiation as a candidate therapy for HPV-negative head and neck squamous cell carcinoma

By: Tuomainen, Katja; Hyytiainen, Aini; Al-Samadi, Ahmed; Ianevski, Philipp; Ianevski, Aleksandr; Potdar, Swapnil; Turunen, Laura; Saarela, Jani; Kuznetsov, Sergey; Wahbi, Wafa; et al
Scientific Reports (2021), 11(1), 14755 | Language: English, Database: CAplus and MEDLINE

Conventional chemotherapeutic agents are nonselective, often resulting in severe side effects and the develo pment of resistance. Therefore, new mol.-targeted therapies are urgently needed to be integrated into existing treatment regimens. Here, we performed a high-throughput compound screen to identify a synerg istic interaction between ionizing radiation and 396 anticancer compounds The assay was run using five human papillomavirus (HPV)-neg. head and neck squamous cell carcinoma (H NSCC) cell lines cultured on the human tumor-derived matrix Myogel. Our screen identified several compounds with strong synerg istic and antagonistic effects, which we further investigated using multiple irradiation doses. Navitoclax, which emerged as the most promising radiosen sitizer, exhibited synergy with irradi ation regardless of the p53 mutation status in all 13 H NSCC cell lines. We performed a live cell apoptosis assay for two representative HNSCC cell lines to examine the effects of navitoclax and irradi ation As a single agent, navitoclax reduced proliferation and induced apoptosis in a dose- dependent manner, whereas the navitoclax-irradiation combin ation arrested cell cycle progre ssion and resulted in substan tially elevated apoptosis. Overall, we demonstrated that combining navitoclax with irradiation resulted in synergistic in vitro antitumor effects in H NSCC cell lines, possibly indicating the therap eutic potential for HNSCC patients.

Keywords: navitoclax ionizing radiation HPV head neck squamous cell carcinoma

Substances (37) Reactions (0) Citing (5)

11

Combination of antibody-drug conjugate and aurora b inhibitor

By: Mettetal, Jerome Thomas, II; Durant, Stephen Thomas; Astaneh, Azadeh Cheraghchi Bashi
World Intellectual Property Organization, WO2021260582 A1 2021-12-30 | Language: English, Database: CAplus

A pharmaceutical product for administration of an anti-HER2 antibody-drug conjugate in combination with an Aurora B inhibitor is provided. The anti-HER2 antibody drug conjugate is an antibody- drug conjugate in which a drug- linker represented by the following formula (wherein A represents the connecting position to an antibody) is conjugated to an anti-HER2 antibody via a thioether bond. Also provided is a therapeutic use and method wherein the antibody- drug conjugate and the Aurora B inhibitor are administered in combination to a subject.

Keywords: HER2 cancer antitumor antibody drug conjugate aurora b sequence

PatentPak available

Substances (18) | Reactions (0) | Citing (0)

12

Methods for treating cancer with combination therapies

By: Ahsan, Aarif; Bisht, Kamlesh; Bjorklund, Chad; Flynt, Jennifer Erin; Hsu, Chih-Chao; Jeyaraju, Danny Vijey; Ortiz-Estevez, Maria; Pierceall, William Edward; Thakurta, Anjan; Towfic, Fadi George
World Intellectual Property Organization, WO2021262962 A1 2021-12-30 | Language: English, Database: CAplus

Provided herein are methods of using a compound provided herein (e.g., Compound 1, Compound 2, Compound 3, Compound 4, Compound 5, Compound 6, or Compound 7, or a stereoisomer or mixture of stereoisomers, pharmaceutically acceptable salt, tautomer, prodrug, solvate, hydrate, co-crystal, clathrate, or polymorph thereof), in combination with a second active agent for treating cancer. The second active agent is one or more of a PLK1 inhibitor, a BRD4 inhibitor, a BET inhibitor, an NEK2 inhibitor, an AURKB inhibitor, an MEK inhibitor, a PHF19 inhibitor, a BTK inhibitor, an mTOR inhibitor, a PIM inhibitor, an IGF-1R inhibitor, an XPOI inhibitor, a DOT1L inhibitor, an EZH2 inhibitor, a JAK2 inhibitor, a BIRC5 inhibitor, or a DNA methyltransferase inhibitor.

Keywords: antitumor agent combination therapy hematol cancer

PatentPak available

Substances (95) | Reactions (0) | Citing (0)

13

Measuring the Rate of In-vitro Drug Release From Polymeric Nanoparticles by ^{19}F Solution State NMR Spectroscopy

By: Wren, Stephen; Collins, Laura; Hughes, Leslie P.; Jones, Ian
Journal of Pharmaceutical Sciences (Philadelphia, PA, United States) (2021), 110(11), 3546-3549 | Language: English, Database: CAplus and MEDLINE

We report what we believe is the first use of 19F NMR spectroscopy to directly measure in-vitro release (IVR) from polymeric nanoparticles (PNPs). Using 19F NMR we selectively measured IVR of AZD2811 from PNPs. Due to rapid nuclear relaxation in solid-like environments only AZD2811 in solution is detected, and phys. separation from the P NPs isnt required. The NMR approach and ultra-centrifugation/UHPLC were shown to be equivalent The selectivity of 19F NMR means it is readily applied to complex IVR media such as recombinant human serum albumin (r HSA).

Keywords: fluorine polymeric nanoparticle delivery system NMR spectrum; Analytical chemistry; N MR; Nanomedicines; Nanopar ticles; PLA

Substances (2) | Reactions (0) | Citing (0)

14

Combination therapy for treating cancer

By: Travers, Jon
World Intellectual Property Organization, WO2021224381 A1 2021-11-11 | Language: English, Database: CAplus

Disclosed are methods of treating cancer comprising administering to a subject in need thereof comprising adminis tering to the subject an effective amount of a pharmaceutical composition comprising a plurality of AZD2811 nanoparticles and an effective amount of olaparib.

Keywords: antitumor AZD2811 nanoparticles olaparib

PatentPak available

Substances (2) | Reactions (0) | Citing (0)

15

Antitumor Combination therapy

By: Samatar, Ahmed Abdi; Li, Jiali; Ma, Jianhui; Huang, Peter Qinhua; Hegde, Sayee Gajanan; Bunker, Kevin Duane; Donate, Fernando
World Intellectual Property Organization, WO2021127043 A1 2021-06-24 | Language: English, Database: CAplus

Disclosed herein are combinations of compounds for treating a disease or condition, such as cancer. A combin ation of compounds for treating a disease or condition can include a SERD inhibitor and another inhibitor (such as a C DK4/6 inhibitor, a selective aromatase inhibitor, a non-selective aromatase inhibitor, a mTOR inhibitor, a PI3K inhibitor, an ERK or a MEK inhibitor, AKT inhibitor, a BET inhibitor and an Aurora inhibitor), along with pharmaceutically acceptable salts of any of the foregoing.

Keywords: aromatase mTOR PI3K MEK inhibitor SERD combination therapy cancer

PatentPak available

Substances (180) | Reactions (0) | Citing (1)

16

Epidermal growth factor receptor tyrosine kinase inhibitors for the treatment of cancer

By: Floch, Nicolas; Smith, Paul David; Martin, Matthew Joseph
World Intellectual Property Organization, WO2021094379 A1 2021-05-20 | Language: English, Database: CAplus

The specification relates to epidermal growth factor receptor (EGFR) tyrosine kinase inhibitors (TKIs) (e.g. osimertinib) for use in the treatment of cancer, wherein the EGFR TKI is administered in combination with AZD2811.

Keywords: epidermal growth factor receptor tyrosine kinase inhibitor cancer treatment; antitumor EGFR inhibitor osimertinib AZ D2811 combination chemotherapy cancer treatment

PatentPak available

Substances (18) | Reactions (0) | Citing (0)

17

RUNX1 mutations in blast-phase chronic myeloid leukemia associate with distinct phenotypes, transcriptional profiles, and drug responses

By: Adnan Awad, Shady ; Dufva, Olli ; Ianevski, Aleksandr; Ghimire, Bishwa ; Koski, Jan ; Maliniemi, Pilvi; Thomson, Daniel; Schreiber, Andreas; Heckman, Caroline A.; Koskenvesa, Perttu; et al
Leukemia (2021), 35(4), 1087-1099 | Language: English, Database: CAplus and MEDLINE

Blast-phase chronic myeloid leukemia (BP-CML) is associated with addnl. chromosomal aberrations, RUNX1 mutations being one of the most common. Tyrosine kinase inhibitor therapy has only limited efficacy in BP-CML, and characterization of more defined mol. subtypes is warranted in order to design better treatment modalities for this poor prognosis patient group. Using whole-exome and RNA sequencing we demonstrate that PHF6 and BCORL1 mutations, IKZF1 deletions, and AID/RAG-mediated rearrangements are enriched in RUNX1mut BP-CML leading to typical mutational signature. On transcriptional level interferon and TNF signaling were deregulated in primary RUNX1mut CML cells and stem cell and B-lymphoid factors upregulated giving a rise to distinct phenotype. This was accompanied with the sensitivity of RUNX1mut blasts to CD19-CAR T cells in ex vivo assays. High-throughput drug sensitivity and resistance testing revealed leukemia cells from RUNX1mut patients to be highly responsive for mTOR-, BCL2-, and VEGFR inhibitors and glucocorticoids. These findings were further investigated and confirmed in CRISPR/Cas9-edited homozygous $^{-/-}$ and heterozygous RUNX1$^{-/mut}$ BCR-ABL pos. cell lines. Overall, our study provides insights into the pathogenic role of RUNX1 mutations and highlights personalized targeted therapy and CAR T-cell immunotherapy as potentially promising strategies for treating RUNX1mut BP-CML patients.

Keywords: RUNX mutation BP CML phenotype transcriptional profile drug response

Substances (22) | Reactions (0) | Citing (18)

18

Substituted 4-aminoisoindoline-1,3-dione compounds and second active agents for combined use for treating hematological malignancies

By: Anton, Maria Soraya Carrancio; Buchholz, Tonia J.; Chang, Henry; Filvaroff, Ellen; Kasibhatla, Shailaja; Lopez-Girona, Antonia; Mohan, Adithi; Narla, Rama Krishna; Pourdehnad, Michael

United States, US20210113576 A1 2021-04-22 | Language: English, Database: CAplus

Sensitivity to Compound 1 single agent in DLBCL cell lines

sensitive to resistant

TMD-8	SU-DHL-2	SU-DHL-10	Pfeiffer	WSU-DLCL2	OCI-Ly10	Riva	U2932	SU-DHL-4	DB
58.67	108.4	123.7	288.2	362	403.5	496.3	577	585.3	670.8

Provided herein are methods of using (*S*)-2-(2,6-dioxopiperidin-3-yl)-4-((2-fluoro-4-((3-morpholinoazetidin-1-yl)methyl)benzyl)amino)isoindoline-1,3-dione, or an enantiomer, mixture of enantiomers, tautomer, isotopolog, or pharmaceutically acceptable salt thereof, in combination with a second active agent for treating, preventing or managing hematol. malignancies. The second active agent is one or more of an HDAC inhibitor, a BCL2 inhibitor, a BTK inhibitor, an mTOR inhibitor, a PI3K inhibitor, a PKCβ inhibitor, a SYK inhibitor, a JAK2 inhibitor, an Aurora kinase inhibitor, an EZH2 inhibitor, a BET inhibitor, a hypomethylating agent, a DOT1L inhibitor, a HAT inhibitor, a WDR5 inhibitor, a DNMT1 inhibitor, an LSD-1 inhibitor, a G9A inhibitor, a PRMT5 inhibitor, a BRD inhibitor, a SUV420H1/H2 inhibitor, a CARM1 inhibitor, a PLK1 inhibitor, an NEK2 inhibitor, an MEK inhibitor, a PHF19 inhibitor, a PIM inhibitor, an IGF-1R inhibitor, an XPO1 inhibitor, a BIRC5 inhibitor, or a chemotherapy.

Keywords: hematol neoplasm treatment aminoisoindolinedione compound active agent combination

PatentPak available

Substances (131) | Reactions (3) | Citing (0)

19

Combination of AZD2811 nanoparticles, 5-azacitidine and venetoclax for use in the treatment of cancer

By: Brugger, Wolfram

World Intellectual Property Organization, WO2021037933 A1 2021-03-04 | Language: English, Database: CAplus

Disclosed are methods of treating cancer comprising administering to a subject in need thereof an effective amount of a pharmaceutical composition comprising a plurality of AZD2811 nanoparticles; an effective amount of 5-azacitidine; and an effective amount of venetoclax.

Keywords: AZD2811 nanoparticle azacitidine venetoclax antitumor

PatentPak available

Substances (3) | Reactions (0) | Citing (0)

20

Visualization of the distribution of nanoparticle-formulated AZD2811 in mouse tumor model using matrix-assisted laser desorption ionization mass spectrometry imaging

By: Ryu, Shoraku; Ohuchi, Mayu; Yagishita, Shigehiro; Shimoi, Tatsunori; Yonemori, Kan; Tamura, Kenji; Fujiwara, Yasuhiro; Hamada, Akinobu
Scientific Reports (2020), 10(1), 15535 | Language: English, Database: CAplus and MEDLINE

Penetration of nanoparticles into viable tumor regions is essential for an effective response. Mass spectr ometry imaging (MSI) is a novel method for evaluating the intratumoral pharmacokinetics (PK) of a drug in terms of spatial distrib ution. The application of MSI for anal. of nanomedicine PK remains in its infancy. In this study, we evaluated the applica bility of MALDI-MSI for nanoparticle-formulated drug visuali zation in tumors and biopsies, with an aim toward future applic ation in clin. nanomedicine research. We established an analytic method for the free drug (AZD2811) and then applied it to visualize nanoparticle-formulated AZD2811. MSI anal. demonstrated heterogeneous intratumoral drug distri bution in three xenograft tumors. The intensity of M SI signals correlated well with total drug concentration in tumors, indicating that drug distri bution can be monitored quant. Anal. of tumor biopsies indicated that MSI is applicable for analyzing the distri bution of nanoparticle-formulated drugs in tumor biopsies, suggesting clin. applicability.

Keywords: nanoparticle matrix assisted laser desorption mass spectr ometry imaging

Substance (1) | Reactions (0) | Citing (5)

21

Aurora-B knockdown inhibits osteosarcoma metastasis by inducing autophagy via the mTOR/ULK1 pathway

By: Wu, Xin; Liu, Jia-ming; Song, Hong-hai; Yang, Qi-kun; Ying, Hui; Tong, Wei-lai; Zhou, Yang; Liu, Zhi-li
Cancer Cell International (2020), 20(1), 575 | Language: English, Database: CAplus and MEDLINE

Autophagy plays an essential role in metastasis of malignancies. Although our studies showed that Aurora- B facilitate pulmonary metastasis in OS, the mechanism of Aurora- B kinase on autophagy and metastasis in O S has not been explored. Clin.- pathol. parameters and follow-up information was collected in OS patients. Immunohi stochem. staining was performed to detect Aurora- B and LC3 protein in OS tissues. Short hairpin RNA transfection was used to silence Aurora- B in OS cells. Real-time quant. PCR (RT-qP CR) was performed to detect Aurora- B mRNA expression in OS cells. Aurora- B and autophagy related protein were measured by Western blot. Transmission electron microscopy and laser scanning confocal microscopy were performed to observe the formation of autophagosomes and autolys osomes. Migratory and invasive ability of O S cells were measured by Wound healing and transwell assays. Orthotopic xenograft model was used to evaluate the effect of autophagy mediated by Aurora-B inhibition on pulmonary metastasis of OS. The elevated expression of Aurora-B protein in OS tissues neg. associated with the overall survival of O S patients. Further investigation has found that Aurora- B expression was neg. correl ative with autophagy related protein LC3 in OS patient tissues. Knockdown Aurora-B stimulates autophagy and inhibits migratory and invasive ability of O S cells. Mechanistically, Aurora-B knockdown suppressed the mTOR/ULK1 signaling pathway and reacti vation of the mTOR/ULK1 pathway decreased autophagy level. Furthermore, the inhibition effect of silencing Aurora- B on migration and invasion of OS was reversed by chloro quine and mTOR activator in vitro and vivo. Our results suggest that silencing of Aurora-B stimulate autophagy via decreasing mTOR/ULK1 and result in inhibiting OS metastasis. Targeted Aurora-B/mTOR/ULK1 pathway may be a promising treatment strategy for O S patients.

Keywords: AuroraB mTOR ULK1 pathway autophagy osteos arcoma metastasis; Aurora-B; Autophagy; Metast asis; Osteosarcoma; The mTOR/ULK1 pathway

Substances (8) | Reactions (0) | Citing (13)

22

Synthetic Lethal Interaction between the ESCRT Paralog Enzymes VPS4A and VPS4B in Cancers Harboring Loss of Chromosome 18q or 16q

By: Neggers, Jasper E. ; Paolella, Brenton R.; Asfaw, Adhana; Rothberg, Michael V.; Skipper, Thomas A.; Yang, Annan; Kalekar, Radha L.; Krill-Burger, John M.; Dharia, Neekesh V.; Kugener, Guillaume; et al
Cell Reports (2020), 33(11), 108493 | Language: English, Database: CAplus and MEDLINE

Few therapies target the loss of tumor suppressor genes in cancer. We examine CRISPR-SpCas9 and RNA-interference loss-of-function screens to identify new therapeutic targets associated with genomic loss of tumor suppressor genes. The endosomal sorting complexes required for transport (ESCRT) ATPases VPS4A and VPS4B score as strong synthetic lethal dependencies. VPS4A is essential in cancers harboring loss of VPS4B adjacent to SMAD4 on chromosome 18q and VPS4B is required in tumors with co-deletion of VPS4A and CDH1 (E-cadherin) on chromosome 16q. We demonstrate that more than 30% of cancers selectively require V PS4A or VPS4B. VPS4A suppression in VPS4B-deficient cells selectively leads to ESCRT-III filament accumulation, cytokinesis defects, nuclear deformation, G2/M arrest, apoptosis, and potent tumor regression. CRISPR-SpCas9 screening and integrative genomic anal. reveal other ESCRT members, regulators of abscission, and interferon signaling as modifiers of VPS4A dependency. We describe a compendium of synthetic lethal vulnerabilities and nominate VPS4A and VPS4B as high-priority therapeutic targets for cancers with 18q or 16q loss.

Keywords: VPS4A VPS4B ESCRTIII CHMP4B paclitaxel AZD2811 IFNAR2 cancer; 16q loss; 18q loss; ESCRT; VPS4A; VPS4B; cancer; drug target; functional genomics; synthetic lethality; target identification

Substances (5) Reactions (0) Citing (15)

23

Combination of 3-(cyclohex-1-en-1-yl)-6-(4-methoxyphenyl)-2-phenyl-5-(pyridine-3-ylamino)pyrzolo[1,5-a]pyrimidin-7(4H)-one and other chemotherapeutic agents for treatment of cancer

By: Hyer, Marc Lee; Kalev, Petar; Marjon, Katya; Marks, Kevin
World Intellectual Property Organization, WO2020168032 A1 2020-08-20 | Language: English, Database: CAplus

The invention relates to compounds (e.g. 3-(cyclohex-1-en-1-yl)-6-(4-methoxyphenyl)-2-phenyl-5-(pyridine-3-ylamino)pyrazolo[1,5-a]pyrimidin-7(4H)-one), or pharmaceutically acceptable salts thereof, for use in the treatment of MTAP-deficient lung cancer, such as non-small-cell lung carcinoma (NSCLC), or MTAP-deficient pancreatic cancer, such as pancreatic ductal adenocarcinoma (PDAC), or MTAP-deficient esophageal cancer and provides a therapeutic advantage when used in combination with other agents (e.g. docetaxel) as herein described compared to treatment with each agent when administered alone.

Keywords: antitumor MAT2A inhibitor combination chemotherapy synergy docetaxel cancer treatment

PatentPak available

Substances (19) Reactions (0) Citing (0)

24

Evaluation of Particle Size Techniques to Support the Development of Manufacturing Scale Nanoparticles for Application in Pharmaceuticals

By: Wren, Stephen; Minelli, Caterina; Pei, Yiwen; Akhtar, Nadim
Journal of Pharmaceutical Sciences (Philadelphia, PA, United States) (2020), 109(7), 2284-2293 | Language: English, Database: CAplus and MEDLINE

The measurement of nanoparticle size, and size distrib ution, is important to the develo pment of pharmaceutical nanoparticle products and their manufacturing processes. In this work we report on the use of four widely- used liquid-phase techniques, DLS, D CS, PTA, and TRPS to measure four different batches of AZD2811NPs. The techniques rely on different phys. principles to measure nanoparticle size. The batches cover a range of different manufac turing scales and different sites of manufa cture, and were made to support toxicity, clin., and engineering studies. The results from the different techniques and different batches are compared in terms of the average size, and size distribution, measured. In addition, we discuss the suitab ility of techniques for different applic ations e.g. QC and process underst anding.

Keywords: antitumor drug AZD2811 nanoparticle nanoemulsion formulation quality control; Batch processing; Cancer; Drug delivery system; Formulation; Injectable; Nanomedicine; Nanoparticles; Particle size; Physical characterisation; Polymer

Substances (2) Reactions (0) Citing (5)

25

Antiproliferative compounds and second active agents for combined use for treating multiple myeloma

By: Wong, Lilly L.
United States, US20200215060 A1 2020-07-09 | Language: English, Database: CAplus

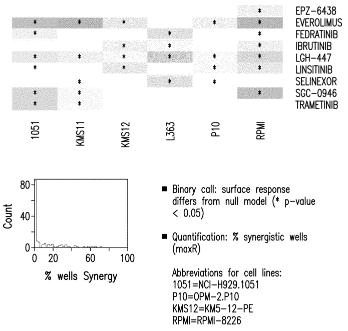

Provided herein are methods of using 4-(4-(4-(((2-(2,6-dioxopiperidin-3-yl)-1-oxoisoindolin-4-yl)oxy)methyl)benzyl)piperazin-1-yl)-3-fluorobenzonitrile, or an enantiomer, a mixture of enantiomers, a tautomer, or a pharmace utically acceptable salt thereof, in combination with a second active agent for treating, preventing or managing multiple myeloma. The second active agent is one or more of a BTK inhibitor, an mTOR inhibitor, a PIM inhibitor, an IGF-1R inhibitor, an MEK inhibitor, an XPO1 inhibitor, a DOT1L inhibitor, an EZH2 inhibitor, a JAK2 inhibitor, a BRD4 inhibitor, a PLK 1 inhibitor, an NEK2 inhibitor, an AURKB inhibitor, a BIRC5 inhibitor, a BET inhibitor, or a DNA methyltransferase inhibitor.

Keywords: antiproliferative compound combination multiple myeloma treatment

PatentPak available

Substances (76) Reactions (42) Citing (0)

26

Aurora B kinase as a therapeutic target in acute lymphoblastic leukemia

By: Goto, Hiroaki ; Yoshino, Yuki; Ito, Mieko; Nagai, Junichi; Kumamoto, Tadashi; Inukai, Takesi; Sakurai, Yukari; Miyagawa, Naoyuki; Keino, Dai; Yokosuka, Tomoko; et al
Cancer Chemotherapy and Pharmacology (2020), 85(4), 773-783 | Language: English, Database: CAplus and MEDLINE

Abstract: Purpose: Acute lymphoblastic leukemia (ALL) is curable with standardized chemotherapy. However, the development of novel therapies is still required, especially for patients with relapsed or refractory disease. By utilizing an in vitro drug screening system, active mol. targeting agents against ALL were explored in this study. Methods: By the in vitro drug sensit ivity test, 81 agents with various actions were screened for their cytotoxicity in a panel of 22 ALL cell lines and ALL clin. samples. The drug effect score (D ES) was calculated from the dose-response of each drug for comparison among drugs or samples. Normal peripheral blood mononuclear cells were also applied onto the drug screening to provide the reference control values. The drug combin ation effect was screened based on the Bliss independent model, and validated by the improved isobol ogram method. Results: On sensitivity screening in a cell line panel, barasertib-HQPA which is an active metabolite of barasertib, an aurora B kinase inhibitor, alisertib, an aurora A kinase inhibitor, and YM155, a survivin inhibitor, were effective against the broadest range of A LL cells. The DES of barase rtib-HQPA was significantly higher in ALL clin. samples compared to the reference value. There were signif icant correlations in DES between barasertib-HQPA and vincristine or docetaxel. In the drug combin ation assay, barasertib-HQPA and eribulin showed additive to synergistic effects. Conclusion: Aurora B kinase was identified to be an active therap eutic target in a broad range of A LL cells. Combination therapy of barasertib and a microtubule-targeting drug is of clin. interest.

Keywords: barasertib alisertib antitumor aurora B kinase inhibitor lymphoblastic leukemia; Acute lymphoblastic leukemia; Aurora B kinase; Barasertib; In vitro drug sensitivity test; Survivin

Substances (10) | Reactions (0) | Citing (4)

27

Combination therapy using AZD2811 and 5-azacytidine for treating hematological cancer

By: Brugger, Wolfram
World Intellectual Property Organization, WO2020026102 A1 2020-02-06 | Language: English, Database: CAplus

Disclosed are methods of treating cancer comprising administering to a subject in need thereof comprising adminis tering to the subject an effective amount of a pharmaceutical composition comprising a plurality of AZD2811 nanoparticles and an effective amount of 5-azacytidine.

Keywords: antitumor AZD2811 nanoparticle azacytidine combination therapy hematol cancer

PatentPak available

Substances (3) | Reactions (0) | Citing (1)

28

Combination of AZD2811 and venetoclax for treating cancer

By: Brugger, Wolfram

World Intellectual Property Organization, WO2020026100 A1 2020-02-06 | Language: English, Database: CAplus

Disclosed are methods of treating cancer comprising administering to a subject in need thereof comprising adminis tering to the subject an effective amount of a pharmaceutical composition comprising a plurality of AZD2811 nanoparticles and venetoclax.

Keywords: antitumor combination chemotherapy AZD2811 nanoparticle venetoclax ABT199 cancer treatment

PatentPak available

Substances (3) Reactions (0) Citing (1)

29

Biomarker-driven phase 2 umbrella trial study for patients with recurrent small cell lung cancer failing platinum-based chemotherapy

By: Park, Sehhoon; Shim, Joonho ; Mortimer, Peter G. S.; Smith, Simon A.; Godin, Robert E.; Hollingsworth, Simon J.; Kim, Hee-Jung; Jung, Hyun Ae; Sun, Jong-Mu; Park, Woong-Yang; et al

Cancer (Hoboken, NJ, United States) (2020), 126(17), 4002-4012 | Language: English, Database: CAplus and MEDLINE

A high percentage of small cell lung cancer (SCLC) cases harbor cell cycle- related gene mutations and RICTOR amplification. Based on underlying somatic mutations, the authors have conducted a phase 2 biomarker-driven, multiarm umbrella study. The S CLC Umbrella Korea StudiES (SUKSES) is an adaptive platform trial that undergoes continual modifi cation according to the observed outcomes. This study included 286 patients with SCLC who failed platinum therapy and who had known genomic profiles based on a predesigned screening trial. Patients with MYC amplification or CDKN2A and TP53 co-alterations were allocated to adavosertib (SU KSES protocol C [SUKSES-C]; 7 patients) and those with RICTOR amplification were allocated to vistusertib (SUKSES-D; 4 patients). Alternatively, patients who were without any predefined biomarkers were assigned to a non- biomarker-selected arm: adavosertib (S UKSES-N1; 21 patients) or AZD2811NP (SUKSES-N3; 15 patients). Patients in the SUKSES-C and SUKSES-N1 arms demonstrated no objective response. Three patients presented with stable disease (SD) in SUKSES-C and 6 patients in SUKSES-N1. The median progre ssion-free survival (PFS) was 1.3 mo (95% confidence interval, 0.9 mo to not available) for S UKSES-C and 1.2 mo (95% CI, 1.1-1.4 mo) for SUKSES-N1. Patients in the SUKSES-D arm demonstrated no objective response and no SD, with a PFS of 1.2 mo (95% CI, 1.0 mo to not available). The SUKSES-N3 arm had 5 patients with S D and a PFS of 1.6 mo (95% CI, 0.9-1.7 mo), without an objective response. Grade≥3 adverse events (graded according to National Cancer Institute Common Terminol. Criteria for Adverse Events [version 4.03]) were observed as follows: 3.2% in the SUKSES-C and SUKSES-N1 arms and 50.0% in the SUKSES-D arm. Target-related neutropenia (grade≥3) was observed in approx. 60.0% of patients in the AZD2811NP arm using the current dosing schedule. To the best of the authors' knowledge, the current study is the first biomarker-driven umbrella study conducted in patients with recurrent SCLC. Although the current study demonstrated the limited clin. efficacy of monotherapy, novel biomarker approaches using other cell cycle inhibitor(s) or combinations warrant further investigation.

Keywords: biomarker recurrent lung cancer failing platinum chemotherapy; AZD2811NP; adavosertib; small cell lung carcinoma; vistusertib

Substances (4) Reactions (0) Citing (9)

30

Targeting aurora kinase B alleviates spinal microgliosis and neuropathic pain in a rat model of peripheral nerve injury

By: Shen, Yu; Ding, Zhuofeng; Ma, Shengyun; Zou, Yu; Yang, Xin; Ding, Zijin; Zhang, Yu; Zhu, Xiaoyan; Schaefer, Michael K. E.; Guo, Qulian; et al

Journal of Neurochemistry (2020), 152(1), 72-91 | Language: English, Database: CAplus and MEDLINE

Peripheral nerve injury elicits spinal microgliosis, contributing to neuropathic pain. The aurora kinases A (AURKA), B (AURKB), and C (AURKC) are potential therapeutic targets in proliferating cells. However, their role has not been clarified in microglia. The aim of this study was to examine the regulation of aurora kinases and their roles and druggability in spinal microgliosis and neuropathic pain. Sprague-Dawley rats received chronic constriction injury (CCI). Gene expression of aurora kinases A-C was evaluated by quant. RT-PCR and western blot, resp., in spinal cords at 1, 3, 7, and 14 days after C CI. AURKB gene and protein expression was up-regulated concomitantly with the development of spinal microgliosis and neuropathic pain. Using lentiviral over-expression and adeno-associated viral knockdown approaches, the function of AURKB was further investigated by western blot, immunohis tochem., RNA sequencing, and pain behavior tests. We found that AURKB over-expression in naive rats caused spinal microgliosis and pain hypersensitivity, whereas AURKB knockdown reduced microgliosis and alleviated CCI-induced neuropathic pain. Accord ingly, RNA sequencing data revealed down-regulation of genes critically involved in signaling pathways associated with spinal microgliosis and neuropathic pain after AURKB knockdown in CCI rats. To examine its therapeutic potential for treatment of neurop athic pain, animals were treated intrathecally with the pharmacol. AURKB inhibitor AZD1152-HQPA resulting in the alleviation of CCI-induced pain. Taken together, our findings indicated that AURKB plays a critical role in spinal microg liosis and neuropathic pain. Targeting AURKB may be an efficient method for treatment of neurop athic pain subsequent to peripheral nerve injury. Open science badges : This article has received a badge for *Open Materials* because it provided all relevant information to reproduce the study in the manuscript. The complete Open Science Disclosure form for this article can be found at the end of the article. More information about the Open Practices badges can be found at .

Keywords: target aurora kinase spinal microgliosis neuropathic pain nerve injury; aurora kinases; microgliosis; neuropathic pain; peripheral nerve injury; spinal cord

Substances (2) | Reactions (0) | Citing (9)

31

Salivary glands require Aurora Kinase B for regeneration after transient innate immune-mediated injury

By: Shaalan, Abeer ; Proctor, Gordon

Scientific Reports (2019), 9(1), 1-13 | Language: English, Database: CAplus and MEDLINE

The present study examined the initial mitogenic events marking the regenerative response of the murine submandibular gland (S MG), following innate immune-mediated injury. Local intraductal administration of the synthetic double stranded (ds) R NA polyino sinic-polycytidylic acid (poly (I: C)) widely, but transiently, depleted the acinar and progenitor cells, 24 h post poly (I: C) introduction. While the progenitor and duct cells started to proliferate and expand at 72 h, the Mist1- positve acinar cells did not re-appear until 96 h post poly (I:C) injury. The cellular replenishment during regeneration involved significant upregulation of the cell cycle promoter Aurora kinase B (AURKB). AURKB, which is expressed in healthy proliferating and cancerous cells, is a serine/threonine protein kinase, well known to orchestrate key events in cell division and cytokinesis. However, the expression and role of AURKB in regene ration of post mitotic salivary gland cells has not been previously explored. In vivo inhibition of A URKB using the selective inhibitor Barasertib (AZD1152-HQPA) interfered with SMG recovery from the transient, but severe poly (I: C)-mediated injury and cellular depletion. AURKB deficiency during regeneration of the injured tissues: disrupted cell cycle progre ssion, repressed renewal of Mist1-pos. acinar cells and prevented recovery of salivary secretion.

Keywords: salivary gland progenitor acinar duct cell aurora kinase B

Substances (4) | Reactions (0) | Citing (4)

32

Modeling dose and schedule effects of AZD2811 nanoparticles targeting Aurora B kinase for treatment of diffuse large B-cell lymphoma

By: Floc'h, Nicolas; Ashton, Susan; Ferguson, Douglas; Taylor, Paula; Carnevalli, Larissa S.; Hughes, Adina M.; Harris, Emily; Hattersley, Maureen; Wen, Shenghua; Curtis, Nicola J.; et al
Molecular Cancer Therapeutics (2019), 18(5), 909-919 | Language: English, Database: CAplus and MEDLINE

Barasertib (AZD1152), a pro-drug of the highly potent and selective Aurora B kinase inhibitor A ZD2811, showed promising clin. activity in relapsed/refractory diffuse large B- cell lymphoma (DLBCL) patients administered as a 4- day infusion. To improve potential therapeutic benefit of Aurora B kinase inhibi tion, a nanopa rticle formulation of AZD2811 has been developed to address limita tions of repeated i.v. infusion. One of the challenges with the use of nanoparticles for chronic treatment of tumors is optimizing dose and schedule required to enable repeat administration to sustain tumor growth inhibi tion. AZD2811 gives potent cell growth inhibition across a range of DLBCL cells lines in vitro. In vivo, repeat adminis tration of the AZD2811 nanoparticle gave antitumor activity at half the dose intensity of AZD1152. Compared with A ZD1152, a single dose of A ZD2811 nanoparticle gave less reduction in p HH3, but increased apoptosis and reduction of cells in G1 and G2-M, albeit at later time points, suggesting that duration and depth of target inhibition influence the nature of the tumor cell response to drug. Further exploration of the influence of dose and schedule on efficacy revealed that AZD2811 nanoparticle can be used flexibly with repeat adminis tration of 25 mg/kg admini stered up to 7 days apart being sufficient to maintain equivalent tumor control. Timing of repeat administration could be varied with 50 mg/kg every 2 wk controlling tumor control as effect ively as 25 mg/kg every week. A ZD2811 nanoparticle can be administered with very different doses and schedules to inhibit DLBCL tumor growth, although maximal tumor growth inhibition was achieved with the highest dose intensities.

Keywords: DLBCL cell growth aurora B kinase nanopa rticle therapeutics

Substances (2)	Reactions (0)	Citing (8)

33

Use of aurora kinase inhibitors for prophylaxis and treatment of malaria

By: Chakrabarti, Debopam; Chakrabarti, Ratna
United States, US20190077768 A1 2019-03-14 | Language: English, Database: CAplus

Compound 001 (LAS 31077790)

- Dd2 IC50: 82.5nM
- HepG2 IC50: 233.9nM
- 3T3 IC50: 1754nM
- Selectivity: 2.8/21.3

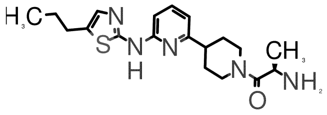

The invention provides Aurora kinase inhibitor compositions and methods for prevention and/or treatment of malaria.

Keywords: Aurora kinase inhibitor antimalarial

PatentPak available	Substances (17)	Reactions (0)	Citing (0)

34

Machine learning models for the prediction of chemotherapy-induced peripheral neuropathy

By: Bloomingdale, Peter; Mager, Donald E.

Pharmaceutical Research (2019), 36(2), 1-12 | Language: English, Database: CAplus and MEDLINE

Chemotherapy-induced peripheral neuropathy (CIPN) is a common adverse side effect of cancer chemot herapy that can be life debilitating and cause extreme pain. The multifa ctorial and poorly understood mechanisms of toxicity have impeded the identif ication of novel treatment strategies. Computational models of drug neuroto xicity could be implem ented in early drug discovery to screen for high-risk compounds and select safer drug candidates for further develo pment. Quant.-structure toxicity relationship (Q STR) models were developed to predict the incidence of PN. A manually curated library of 95 approved drugs were used to develop the model. Mol. descriptors sensitive to the incidence of PN were identified to provide insights into structural modific ations to reduce neurotoxicity. The incidence of PN was predicted for 60 antineo plastic drug candidates currently under clin. investi gation. The number of aromatic nitrogens was identified as the most important mol. descriptor. The chem. transformation of aromatic nitrogens to carbons reduced the predicted PN incidence of bortezomib from 32.3% to 21.1%. Antineo plastic drug candidates were categorized into three groups (high, medium, low) based on their predicted P N incidence. QSTR models were developed to link physicochem. descriptors of compounds with PN incidence, which can be utilized during drug candidate selection to reduce neurotoxicity.

Keywords: quant structure toxicity relationship antitumor induced peripheral neurop athy; ADME ADMET predictor machine learning; ADMET predictor; QSAR; chemotherapy-induced peripheral neuropathy; machine learning; toxicity

Substances (60) | Reactions (0) | Citing (10)

35

Relapse-associated AURKB blunts the glucocorticoid sensitivity of B cell acute lymphoblastic leukemia

By: Poulard, Coralie; Kim, Hye Na; Fang, Mimi; Kruth, Karina; Gagnieux, Celine; Gerke, Daniel S.; Bhojwani, Deepa; Kim, Yong-Mi; Kampmann, Martin ; Stallcup, Michael R. ; et al

Proceedings of the National Academy of Sciences of the United States of America (2019), 116(8), 3052-3061 | Language: English, Database: CAplus and MEDLINE

Glucocorticoids (GCs) are used in combin ation chemotherapies as front-line treatment for B cell acute lymphob lastic leukemia (B-AL L). Although effective, many patients relapse and become resistant to chemot herapy and GCs in particular. Why these patients relapse is not clear. We took a comprehensive, functional genomics approach to identify sources of G C resistance. A genome-wide shRNA screen identified the transcri ptional coactivators EHMT2, EHMT1, and CBX3 as important contri butors to GC-induced cell death. This complex selectively supports GC-induced expression of genes contri buting to cell death. A metaanal. of gene expression data from B-ALL patient specimens revealed that Aurora kinase B (AURKB), which restrains GC signaling by phosphor ylating EHMT1-2, is overexpressed in relapsed B-ALL, suggesting it as a potential contri butor to relapse. Inhibition of AURKB enhanced GC-induced expression of cell death genes, resulting in potentiation of GC cytotoxicity in cell lines and relapsed B- ALL patient samples. This function for AURKB is distinct from its canonical role in the cell cycle. These results show the utility of functional genomics in understanding mechanisms of resistance and rapidly identi fying combin ation chemotherapeutics.

Keywords: glucocorticoid Aurora kinase cell death drug resistance leukemia; A URKB; cell death; coregu lator; glucocorticoid; leukemia

Substances (5) | Reactions (0) | Citing (23)

36

CDK1 phosphorylates ULK1-ATG13 complex to regulate mitotic autophagy and taxol chemosensitivity

By: Li, Zhiyuan; Tian, Xiaofei ; Ji, Xinmiao ; Wang, Dongmei; Zhan, Xin
bioRxiv (2019), 1-38 | Language: English, Database: CAplus

ULK1-ATG13 is the most upstream autophagy initiation complex that is phosphorylated by mTORC1 and AMPK to induce autophagy in asynchronous conditions. However, the phospho-regulation and function of ULK1-ATG13 in mitosis and cell cycle remains unknown. Here we show that ULK1-ATG13 complex is differentially regulated throughout the cell cycle. Notably, in mitosis, both UL K1 and ATG13 are highly phosphorylated by CDK1/cyclin B, the key cell cycle machinery. Combining mass spectrometry and site-directed mutagenesis, we found that CDK1-induced ULK1-ATG13 phosphorylation pos. regulates mitotic autophagy and Taxol chemosensitivity, and some phosphorylation sites occur in cancer patients. Moreover, double knockout of ULK1 and ATG13 could block cell cycle progression and significantly decrease cancer cell proliferation in cell line and mouse models. Our results not only bridge the mutual regulation between the core machineries of autophagy and mitosis, illustrate the mitotic autophagy regulation mechanism, but also provide ULK1-ATG13 as potential targets for cancer therapy.

Keywords: CDK phosphorylation ULK ATG complex mitotic autophagy taxol chemosensitivity

Substances (17) Reactions (0) Citing (0)

37

Dual targeting of aurora kinases with AMG 900 exhibits potent preclinical activity against acute myeloid leukemia with distinct post-mitotic outcomes

By: Payton, Marc; Cheung, Hung-Kam; Ninniri, Maria Stefania S.; Marinaccio, Christian; Wayne, William C.; Hanestad, Kelly; Crispino, John D.; Juan, Gloria; Coxon, Angela
Molecular Cancer Therapeutics (2018), 17(12), 2575-2585 | Language: English, Database: CAplus and MEDLINE

In this study, pan-aurora kinase inhibitor (AKI) AMG 900 distinguishes itself as an anti-leukemic agent that is more uniformly potent against a panel of acute myeloid leukemia (AML) cell lines than are isoform-selective AKIs and classic AML drugs. AMG 900 inhibited AML cell growth by inducing polyploidization and/or apoptosis. AMG 900 and aurora-B-selective inhibitor AZD1152-hQPA showed comparable cellular effects on AML lines that do not harbor a FLT3-1TD mutation. AMG 900 was active against P-glycoprotein-expressing AML cells resistant to AZD1152-hQPA and was effective at inducing expression of megakaryocyte-lineage markers (CD41, CD42) on human CHRF-288-11 cells and mouse Jak2 $V^{617}F$ cells. In MOLM-13 cells, inhibition of p-histone H3 by AMG 900 was associated with polyploidy, extra centrosomes, accumulation of p53 protein, apoptosis, and cleavage of Bcl-2 protein. AMG 900 inhibited the proliferation of primary human bone marrow cells in culture, with a better proliferation recovery profile relative to classic antimitotic drug docetaxel. In vivo, AMG 900 significantly reduced tumor burden in a systemic MOLM-13 xenograft model where we demonstrate the utility of 3'-deoxy-3'-^{18}F-fluorothymidine [^{18}F]FLT positron emission tomog. (PET)-CT imaging to measure the antiproliferative effects of AMG 900 in skeletal tissues in mice.

Keywords: acute myeloid leukemia mitosis aurora kinase AMG900 antiproliferative

Substances (9) Reactions (0) Citing (12)

38

Compositions and methods for treating a clinical condition through the use of hematopoietic stem cells

By: Broxmeyer, Hal Edward; Capitano, Maegan Lynn; Cooper, Scott Harrison; Cai, Qingchun
World Intellectual Property Organization, WO2018232272 A1 2018-12-20 | Language: English, Database: CAplus

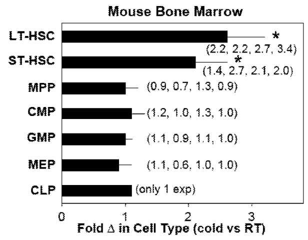

Figure 1A

Various aspects and embodiments disclosed herein relate generally to the modeling, treatment, reducing resistance to the treatment, prevention, and diagnosis of a condition/disease associated with insufficient quantity and/or quality of hematopoietic stem cells (HSCs) and differentiated blood cells thereof. Embodiments include compositions and methods for treating the condition /disease, comprising the step of providing to a subject at least one therapeutically ED of a composition disclosed herein. Other embodiments include methods for generating and/or collecting hematopoietic stem cells from a subject.

Keywords: hematopoietic stem cell therapy cancer autoimmune diseases antitumor

PatentPak available

Substances (18) Reactions (0) Citing (0)

39

Epigenetic inhibitors for sensitizing hematologic or other malignancies to glucocorticoid therapy and Aurora Kinase B inhibitors

By: Stallcup, Michael R.; Poulard, Coralie; Pufall, Miles A.
World Intellectual Property Organization, WO2018213720 A2 2018-11-22 | Language: English, Database: CAplus

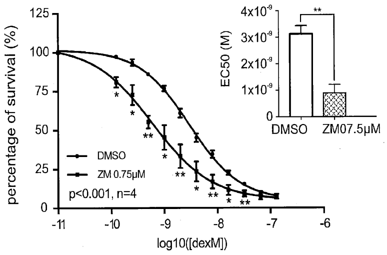

FIG. 40A

The present disclosure as disclosed in various embodiments is related to glucocorticoid compositions and glucocorticoid therapies for treating hematol. or other malignancies, methods and compositions for enhancing the chemotherapeutic effect of glucocor ticoids, methods for determining early relapse of a hematol. or other malignancy in a subject, and methods for treating relapse of a hematol. or other malignancy in a subject.

Keywords: epigenetics inhibitor chemosensitizer hematol malignancy glucocorticoid antitumor; chemosensitizer barasertib dexamethasone antitumor resistance neoplasm treatment

PatentPak available

Substances (31) Reactions (0) Citing (0)

40

The Potential Contribution of microRNAs in Anti-cancer Effects of Aurora Kinase Inhibitor (AZD1152-HQPA)

By: Zekri, Ali; Mesbahi, Yashar ; Boustanipour, Elham; Sadr, Zahra; Ghaffari, Seyed H.
Journal of Molecular Neuroscience (2018), 65(4), 444-455 | Language: English, Database: CAplus and MEDLINE

In the present study, we first investigated the restrictive potentials of AZD1152-HQPA on cell viability, colony formation, nucleus morphol., polyploidy, and cell-cycle distribution. We then studied the expressions level of 88 cancer-related miRNAs in untreated and AZD1152-HQPA-treated NB cell line (SK-N-MC) by real-time PCR using miRNA cancer-array system. After normalizing, the fold change of miRNAs was calculated in the AZD1152-HQPA-treated cell as compared to untreated. Our results demonstrate that the inhibition of AURKB by AZD1152-HQPA induced potent antitumor activity, suppressed cell survival, and triggered apoptosis and polyploidy in NB cells. AZD1152-HQPA, at a relevant concentration, modulated a substantial number of cancer-related miRNAs in NB cell. Interestingly, by screening the literature, among the 7 top AZD1152-HQPA-induced upregulated miRNAs (> 3-fold change; P < 0.01), all were potential tumor suppressors associated with cell apoptosis and cycle arrest, as well as inhibition of angiogenesis, invasion, and metastasis, while two downregulated miRNAs were known to have oncogenic function. Taken together, our study showed for the first time the potential contribution of miRNAs in the anti-cancer effects of AZD1152-HQPA.

Keywords: neuroblastoma aurora kinase inhibitor anticancer microRNA; AZD1152-HQPA; Aurora kinase B; MicroRNAs; Neuroblastoma; Polyploidy

Substance (1) Reactions (0) Citing (3)

41

Drug composition for the treatment of colon cancer

By: Zhang, Shoufen
China, CN108272801 A 2018-07-13 | Language: Chinese, Database: CAplus

The invention discloses a drug composition for the treatment of colon cancer, which has the advantages of good cancer inhibition effect, safety, and less dosage. The drug composition for the treatment of colon cancer comprises the following raw materials: 2- {3-[(7-{3-[ethyl(2-hydroxy ethyl)amino]propoxy}quinazoline-4-yl)amino]-1H-pyrazol-5-yl}-N-(3-fluorophenyl)acetamide and 1,8-dihydroxy-3-carboxyl anthraquinone.

Keywords: colon cancer drug

PatentPak available

Substances (11) Reactions (0) Citing (0)

42

Preparation of azoloquinoline and benzonaphthyridine compounds and method for blocking transmission of malarial parasite

By: Huang, Wenwei; Li, Hao; Sun, Wei; Huang, Xiuli; Patel, Paresma R.; Sun, Hangmao; Zheng, Wei; Lu, Xiao; Sanderson, Philip E.; Kim, Myunghoon; et al

World Intellectual Property Organization, WO2018071836 A1 2018-04-19 | Language: English, Database: CAplus

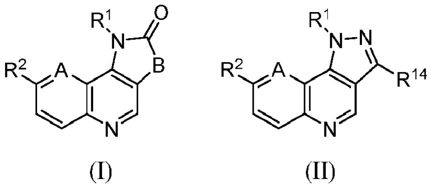

Disclosed are compounds of formula **I**,. Also disclosed is a method of blocking transm ission of a Plasmodium parasite and a method of treating or preventing malaria comprising administering to an animal an effective amount of a first compound of formula **I** either alone or in combination with a second antima larial compound Compounds of formula **I** wherein A is CR^5 and N; B is CR^3:CR^4 and N H and derivatives; R^1 is (un)substituted C_{6-10} aryl and (un)substituted heteroaryl; R^2 is (un)substituted pyridinyl, (un)substituted pyrimidinyl, quinolinyl, etc.; R^3 and R^4 are independently H, O H and derivatives, halo, etc.; R^5 is H, C_{1-12} alkyl, C_{6-10} aryl, halo and OH and derivatives; and pharmaceutically acceptable salts thereof, are claimed. Example compound **II** was prepared by a general procedure (procedure given). The invention compounds were evaluated for their antima larial activity (data given).

Keywords: azoloquinoline benzonaphthyridine preparation blocking transmission malarial parasite antima larial

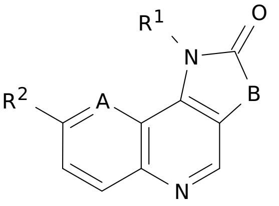

I

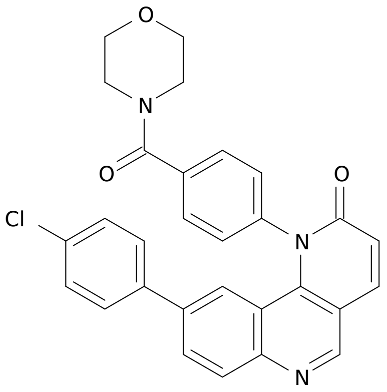

II

PatentPak available

Substances (751) Reactions (189) Citing (0)

43

Preclinical evaluation of the Aurora kinase inhibitors AMG 900, AZD1152-HQPA, and MK-5108 on SW-872 and 93T449 human liposarcoma cells

By: Noronha, Sandhya; Alt, Lauren A. C.; Scimeca, Taylor E.; Zarou, Omran; Obrzut, Justyna; Zanotti, Brian; Hayward, Elizabeth A.; Pillai, Akhil; Mathur, Shubha; Rojas, Joseph; et al

In Vitro Cellular & Developmental Biology: Animal (2018), 54(1), 71-84 | Language: English, Database: CAplus and MEDLINE

Liposarcoma is a malignant soft tissue tumor that originates from adipose tissue and is one of the most frequently diagnosed soft tissue sarcomas in humans. There is great interest in identifying novel chemotherapeutic options for treating liposa rcoma based upon mol. alterations in the cancer cells. The Aurora kinases have been identified as promising chemothe rapeutic targets based on their altered expression in many human cancers and cellular roles in mitosis and cytokinesis. In this study, we invest igated the effects of an Aurora kinase A inhibitor (MK-5108), an Aurora kinase B inhibitor (AZD1152-HQPA), and a pan-Aurora kinase inhibitor (AMG 900) on undifferentiated SW-872 and well-differentiated 93T449 human liposa rcoma cells. Treatment of the SW-872 and 93 T449 cells with MK-5108 (0-1000 nM), AZD1152-HQPA (0-1000 nM), and AMG 900 (0-1000 nM) for 72 h resulted in a dose- dependent decrease in the total viable cell number Based upon the EC_{50} values, the potency of the three Aurora kinase inhibitors in the S W-872 cells was as follows: AMG 900 (EC_{50} = 3.7 n M) > AZD1152-HQPA (EC_{50} = 43.4 n M) > MK-5108 (EC_{50} = 309.0 n M), while the potency in the 93T449 cells was as follows: AMG 900 (EC_{50} = 6.5 n M) > AZD1152-HQPA (EC_{50} = 74.5 n M) > MK-5108 (EC_{50} = 283.6 n M). The percentage of polyploidy after 72 h of drug treatment (0- 1000 nM) was determined by propidium iodide staining and flow cytometric anal. AMG 900 caused a signif icant increase in polyploidy starting at 25 n M in the SW-872 and 93T449 cells, and AZ D1152-HQPA caused a signif icant increase starting at 100 n M in the SW-872 cells and 250 n M in the 93T449 cells. The Aurora kinase A inhibitor MK-5108 did not signifi cantly increase the percentage of polyploid cells at any of the doses tested in either cell line. The expression of Aurora kinase A and B was evaluated in the SW-872 cells vs. differentiated adipocytes and human mesenc hymal stem cells by real-time RT-PCR and Western blot anal. Aurora kinase A and B m RNA expression was significantly increased in the SW-872 cells vs. the differentiated adipocytes and human mesenc hymal stem cells. Western blot anal. revealed a ~ 48 k Da immunoreactive band for Aurora kinase A that was not present in the differentiated adipocytes or the human mesenc hymal stem cells. A ~ 39 kDa immunoreactive band for Aurora kinase B was detected in the S W-872 cells, differe ntiated adipocytes, and human mesenc hymal stem cells. A smaller immunoreactive band for Aurora kinase B was detected in the S W-872 cells but not in the differe ntiated adipocytes and human mesenchymal stem cells, and this may reflect the expression of a truncated splice variant of Aurora kinase B that has been associated with poor patient prognosis. The 93T449 cells demonstrated decreased expression of Aurora kinase A and B mRNA and protein compared to the S W-872 cells, and also expressed the truncated form of Aurora kinase B. The results of these in vitro studies indicate that Aurora kinase inhibitors should be further investigated as possible chemotherapeutic agents for human liposarcoma.

Keywords: AMG900 AZD1152 HQPA MK5108 aurora kinase inhibitor liposa rcoma; 93T449 cell line; AMG 900; AZD1152-HQPA; Aurora kinase inhibitors; Liposarcoma; MK-5108; SW-872 cell line

Substances (5) Reactions (0) Citing (6)

44

Preparation method of barasertib as aurora kinase selective inhibitor

By: Hu, Zhangyan
China, CN107641115 A 2018-01-30 | Language: Chinese, Database: CAplus

The title preparation method of barasertib includes (1) substituting N-ethyl-n-propylamine (compound 1) with carbon tetrach loride and chlorine gas under light irradiation to obtain compound 2, and reacting with sodium hydroxide under catalysis of styrene iodide-divinylbenzene to obtain compound 3; (2) reacting 4- chloro-7-hydroxyquinazoline (compound 4) and ammonia gas under catalysis of copper oxide to obtain compound 5; (3) etherifying with compound 3 in presence of concen trated sulfuric acid to obtain compound 6, and condensing with 2-chloroethanol in presence of sodium hydroxide under catalysis of quaternary ammonium salt tetrabutylammonium hydrogen sulfate to obtain compound 7; (4) condensing with 5- chloro-3-bromo-1H-pyrazole in presence of sodium hydroxide under catalysis of quaternary ammonium salt tetrabutylammonium hydrogen sulfate to obtain compound 8; (5) reacting with acetyl chloride to obtain compound 9; (6) reducing 3-fluoronitrobenzene (compound 10) with hydrogen gas and hydrogen chloride under catalysis of iron powder to obtain compound 11; and (7) condensing with compound 9 in presence of sodium hydroxide under catalysis of quaternary ammonium salt tetrabutylammonium hydrogen sulfate to obtain 2-(5-((7-(3-(ethyl (2-hydroxyethyl)amino)propoxy)quinazoline-4-yl)amino)-1H-pyrazol-3-yl)-N-(3-fluorophenyl)acetamide (compound 12). The invention has easily available raw materials, high economy, environment friendliness, and reduced cost, and is suitable for large-scale production

Keywords: barasertib preparation aurora kinase selective inhibitor

PatentPak available

Substances (15) | Reactions (36) | Citing (0)

45

The target landscape of clinical kinase drugs

By: Klaeger, Susan ; Heinzlmeir, Stephanie ; Wilhelm, Mathias ; Polzer, Harald; Vick, Binje ; Koenig, Paul-Albert ; Reinecke, Maria; Ruprecht, Benjamin; Petzoldt, Svenja; Meng, Chen; et al
Science (Washington, DC, United States) (2017), 358(6367), 1148 | Language: English, Database: CAplus and MEDLINE

Kinase inhibitors are important cancer therapeutic agents. Polypharmacol. is commonly observed, requiring thorough target deconvolution to understand drug mechanism of action. Using chem. proteo mics, the authors analyzed the target spectrum of 243 clin. evaluated kinase drugs. The data revealed previously unknown targets for established drugs, offered a perspe ctive on the "druggable" kinome, highlighted (non)kinase off-targets, and suggested potential therapeutic applications. Integration of phosphop roteomic data refined drug-affected pathways, identified response markers, and streng thened rationale for combination treatm ents. The authors exemplify transla tional value by discov ering SIK2 (salt-inducible kinase 2) inhibitors that modulate cytokine production in primary cells, by identifying drugs against the lung cancer survival marker M ELK (maternal embryonic leucine zipper kinase), and by repurp osing cabozantinib to treat FLT3-ITD-pos. acute myeloid leukemia. This resource, available via the Proteomics DB database, should facilitate basic, clin., and drug discovery research and aid clin. decision- making.

Keywords: protein kinase inhibitor drug target discovery Proteomics database antitumor

Substances (128) | Reactions (0) | Citing (461)

46

Classification and treatment of gastric cancer

By: Lauwers, Gregory Y.; Duda, Gabriel Dan
World Intellectual Property Organization, WO2017139470 A1 2017-08-17 | Language: English, Database: CAplus

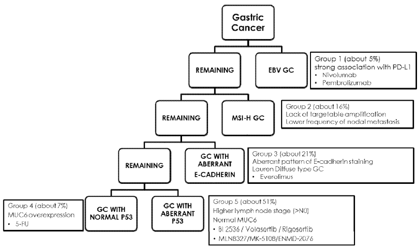

FIG. 8

Protein and mRNA expression based methods for classification of gastric cancer, and methods of treatment based thereon.

Keywords: gastric cancer diagnosis therapeutic biomarker

PatentPak available

Substances (88) Reactions (0) Citing (0)

47

JAK1/2 and BCL2 inhibitors synergize to counteract bone marrow stromal cell-induced protection of AML

By: Karjalainen, Riikka; Pemovska, Tea; Popa, Mihaela; Liu, Minxia; Javarappa, Komal K.; Majumder, Muntasir M.; Yadav, Bhagwan; Tamborero, David; Tang, Jing; Bychkov, Dmitrii; et al
Blood (2017), 130(6), 789-802 | Language: English, Database: CAplus and MEDLINE

The bone marrow (BM) provides a protective microenvironment to support the survival of leukemic cells and influence their response to therapeutic agents. In acute myeloid leukemia (AML), the high rate of relapse may in part be a result of the inability of current treatment to effectively overcome the protective influence of the BM niche. To better understand the effect of the BM microenvironment on drug responses in AML, we conducted a comprehensive evaluation of 304 inhibitors, including approved and investigational agents, comparing ex vivo responses of primary AML cells in BM stroma-derived and standard culture conditions. In the stroma-based conditions, the AML patient cells exhibited significantly reduced sensitivity to 12% of the tested compounds, including topoisomerase II, B-cell chronic lymphocytic leukemia/lymphoma 2 (BCL2), and many tyrosine kinase inhibitors (TKIs). The loss of TKI sensitivity was most pronounced in patient samples harboring FLT3 or PDGFRB alterations. In contrast, the stroma-derived conditions enhanced sensitivity to Janus kinase (JAK) inhibitors. Increased cell viability and resistance to specific drug classes in the BM stroma-derived conditions was a result of activation of alternative signaling pathways mediated by factors secreted by BM stromal cells and involved a switch from BCL2 to BCLXL-dependent cell survival. Moreover, the JAK1/2 inhibitor ruxolitinib restored sensitivity to the BCL2 inhibitor venetoclax in AML patient cells ex vivo in different model systems and in vivo in an AML xenograft mouse model. These findings highlight the potential of JAK inhibitors to counteract stroma-induced resistance to BCL2 inhibitors in AML. (Blood. 2017;130(6):789-802).

Keywords: anticancer agent JAK BCL2 inhibitor cytokine acute myeloid leukemia

Substances (270) Reactions (0) Citing (67)

48

Inhibition of p38MAPK for the treatment of cancer

By: Mani, Sendurai A.; Werden, Steven J.; Paranjape, Anurag N.; Soundararajan, Rama; Sphyris, Natalia; Sun, Xiaoping; Mallampati, Saradhi

World Intellectual Property Organization, WO2017117182 A1 2017-07-06 | Language: English, Database: CAplus

Methods are provided for treating cancer patients who have elevated expression of FOXC2. In certain aspects, patients having elevated FOXC2 are treated with an anti-cancer therapy (e.g. docetaxel) in conjunction with a p38 MAPK inhibitor (e.g. SB203580). Methods of identifying cancer patients to be treated with p38 MAPK inhibitors are also provided. Further provided herein are methods for treating and/or preventing breast cancer metastasis by the administration of a p38 MAPK inhibitor. In certain aspects, patients are treated with an anti-cancer therapy in conjunction with a p38 MAPK inhibitor. In addition, methods are provided for the treatment of BCR-ABL pos. cancers, such as acute lymphoblastic leukemia, by the administration of a p38 MAPK inhibitor and/or a glucocorticoid inhibitor in combination with a tyrosine kinase inhibitor.

Keywords: antitumor p38MAPK inhibitor combination chemotherapy cancer treatment

PatentPak available

Substances (187) | Reactions (0) | Citing (5)

49

Optimizing Therapeutic Effect of Aurora B Inhibition in Acute Myeloid Leukemia with AZD2811 Nanoparticles

By: Floc'h, Nicolas; Ashton, Susan; Taylor, Paula; Trueman, Dawn; Harris, Emily; Odedra, Rajesh; Maratea, Kim; Derbyshire, Nicola; Caddy, Jacqueline; Jacobs, Vivien N.; et al

Molecular Cancer Therapeutics (2017), 16(6), 1031-1040 | Language: English, Database: CAplus and MEDLINE

Barasertib (AZD1152), a highly potent and selective aurora kinase B inhibitor, gave promising clin. activity in elderly acute myeloid leukemia (AML) patients. However, clin. utility was limited by the requirement for a 7-day infusion. Here we assessed the potential of a nanoparticle formulation of the selective Aurora kinase B inhibitor AZD2811 (formerly known as AZD1152-hQPA) in preclin. models of AML. When administered to HL-60 tumor xenografts at a single dose between 25 and 98.7 mg/kg, AZD2811 nanoparticle treatment delivered profound inhibition of tumor growth, exceeding the activity of AZD1152. The improved antitumor activity was associated with increased phospho-histone H3 inhibition, polyploidy, and tumor cell apoptosis. Moreover, AZD2811 nanoparticles increased antitumor activity when combined with cytosine arabinoside. By modifying dose of AZD2811 nanoparticle, therapeutic benefit in a range of preclin. models was further optimized. At high-dose, antitumor activity was seen in a range of models including the MOLM-13 disseminated model. At these higher doses, a transient reduction in bone marrow cellularity was observed demonstrating the potential for the formulation to target residual disease in the bone marrow, a key consideration when treating AML. Collectively, these data establish that AZD2811 nanoparticles have activity in preclin. models of AML. Targeting Aurora B kinase with AZD2811 nanoparticles is a novel approach to deliver a cell-cycle inhibitor in AML, and have potential to improve on the clin. activity seen with cell-cycle agents in this disease.

Keywords: AZD2811 nanoparticle anticancer aurora B inhibitor acute myeloid leukemia

Substances (4) | Reactions (0) | Citing (22)

50

Methods of determining features of polymeric nanoparticles that include a therapeutic agent

By: Troiano, Gregory Charles; Parsons, Donald Maxwell
World Intellectual Property Organization, WO2017081606 A1 2017-05-18 | Language: English, Database: CAplus

The present disclosure generally relates to methods of determining the structural features corresp onding to the release rate of a therapeutic agent in a therap eutic nanoparticle. Such nanoparticles may include the therapeutic agent. In partic ular, the application discloses a method of determining the structural features corresp onding to the release rate of a therapeutic agent in a therap eutic nanoparticle that includes the therapeutic agent and a hydrop hobic counterion, comprising: obtaining a Raman spectra of the nanoparticle; comparing the Raman spectra of the nanopa rticle to a Raman spectra of at least one of the therap eutic agent alone, the hydrophobic counterion alone, a nanopa rticle that does not include the therap eutic agent, and a salt formed from the therap eutic agent and the hydrop hobic counterion to determine whether the therapeutic agent and the hydrop hobic counterion in therap eutic nanoparticle occur as a salt.

Keywords: antitumor drug AZD2811 pamoate targeted controlled release nanoparticle

PatentPak available

Substances (4) | Reactions (0) | Citing (0)

51

Treating neuromuscular or neurologic disease through reducing GABAergic and/or glycinergic inhibitory neurotransmitter overstimulation

By: Tuk, Lambertus
World Intellectual Property Organization, WO2017065602 A1 2017-04-20 | Language: English, Database: CAplus

The disclosure provides novel methods of treating neuromuscular or neurol. disease, for example, amyotr ophic lateral sclerosis (AL S), through reducing GABAergic and Glycin ergic inhibitory neurotransmitter activity overstimulation. Pharmaceutical compositions comprising one or more compounds capable of reducing glycinergic activity and/or one or more compounds capable of reducing G ABAergic activity are also provided. Pharmaceutical compositions comprising Penicillin G and, option ally, glucocorticoid are especially useful in the present invention. Methods for treating one or more symptoms caused by inhibitory neurotransmitter overstimulation (e.g., muscle wasting, loss of muscle function, loss of muscle coordin ation, respiratory depression, dysphagia, dysarthria, eye movement difficu lties, oculomotor gaze palsy, supran uclear gaze palsy, bladder dysfun ction and gastrointestinal dysfunction) in an individual afflicted with ALS or an ALS-like disorder are also provided.

Keywords: neuromuscular neurol disease treatment G ABAergic glycinergic inhibitory neurotransmitter overstimulation

PatentPak available

Substances (708) | Reactions (0) | Citing (0)

52

Reactive oxygen species generation and increase in mitochondrial copy number: new insight into the potential mechanism of cytotoxicity induced by aurora kinase inhibitor, AZD1152-HQPA

By: Zekri, Ali; Mesbahi, Yashar; Ghanizadeh-Vesali, Samad; Alimoghaddam, Kamran; Ghavamzadeh, Ardeshir; Ghaffari, Seyed H.
Anti-Cancer Drugs (2017), 28(8), 841-851 | Language: English, Database: CAplus and MEDLINE

Aurora-B kinase overexp ression plays important roles in the malignant progre ssion of prostate cancer (P Ca). AZD1152-HQPA, as an inhibitor of Aurora-B, has recently emerged as a promising agent for cancer treatment. In this study, we aimed to invest igate the effects of AZD1152-HQPA on reactive oxygen species (R OS) generation and mitocho ndrial function in PCa. We used A ZD1152-HQPA (Barasertib), a highly potent and selective inhibitor of Aurora- B kinase. The effects of AZD1152-HQPA on cell viability, D NA content, cell morphol., and ROS production were studied in the androgen- independent PC-3 PCa cell line. Moreover, the mitocho ndrial copy number and the expression of genes involved in cell survival and cancer stem cell maintenance were investigated. We found that A Z D1152-HQPA treatment induced defective cell survival, polypl oidy, micronuclei formation, cell enlarg ement, and cell death by signif icant overexpression of p73, p21 and downreg ulation of cell cycle-regulatory genes in a drug concent ration-dependent manner. Moreover, AZD1152 treatment led to an excessive R OS generation and an increase in the mitocho ndrial copy number not only in P C-3 but also in several other malignant cells. A ZD1152 treatment also led to downreg ulation of genes involved in the mainte nance of cancer stem cells. Our results showed a functional relationship between the aurora kinase inhibi tion, an increase in mitocho ndrial copy number, and ROS generation in therap eutic modalities of cancer. This study suggests that the excessive R OS generation may be a novel mechanism of cytotoxicity induced by the aurora kinase inhibitor, A ZD1152-HQPA.

Keywords: aurora kinase inhibitor ROS mitocho ndrial copy number cytoto xicity

Substances (11) Reactions (0) Citing (14)

53

Janus face-like effects of Aurora B inhibition: antitumoral mode of action versus induction of aneuploid progeny

By: Wiedemuth, Ralf; Klink, Barbara; Fujiwara, Mamoru; Schroeck, Evelin; Tatsuka, Masaaki; Schackert, Gabriele; Temme, Achim
Carcinogenesis (2016), 37(10), 993-1003 | Language: English, Database: CAplus and MEDLINE

The mitotic Aurora B kinase is overexpressed in tumors and various inhibitors for Aurora B are currently under clin. assess ments. However, when considering Aurora B kinase inhibitors as anticancer drugs, their mode of action and the role of p53 status as a possible predictive factor for response still needs to be investigated. In this study, we analyzed the effects of selective Aurora B inhibition using AZD1152-HQPA/Barasertib (AZD1152) on HCT116 cells, U87- MG, corresp onding isogenic p53-deficient cells and a primary glioblastoma cell line. A ZD1152 treatment caused polyploidy and non- apoptotic cell death in all cell lines irresp. of p53 status and was accompanied by poly-merotelic kineto chore-microtubule attachments and DNA damage. In p53 wild-type cells a DN A damage response induced an ineffi cient pseudo-G1 cell cycle arrest, which was not able to halt ongoing endorepl ication of cells. Of note, release of tumor cells from AZD1152 resulted in recovery of aneuploid progenies bearing numerical and structural chromo somal aberrations. Yet, AZD1152 treatment enhanced death receptor T RAIL-R2 levels in all tumor cell lines investi gated. A concom itant increase of the activating natural killer (N K) cell ligand M IC A/B in p53-deficient cells and an induction of FAS/CD95 in cells containing p53 rendered AZD1152-treated cells more suscep tible for NK-cell-mediated lysis. Our study mechanis tically explains a p53-independent mode of action of a chem. Aurora B inhibitor and suggests a potential triggering of antitu moral immune responses, following polyploidization of tumor cells, which might constrain recovery of aneuploid tumor cells.

Keywords: aurora B kinase inhibitor antitumor aneuploidy

Substances (4) Reactions (0) Citing (7)

54

Treating neuromuscular or neurologic disease through reducing GABAergic and/or glycinergic inhibitory neurotransmitter overstimulation

By: Tuk, Lambertus

World Intellectual Property Organization, WO2016114655 A1 2016-07-21 | Language: English, Database: CAplus

The disclosure provides novel methods of treating neuromuscular or neurol. disease, for example, amyotrophic lateral sclerosis (AL S), through reducing GABAergic and glycinergic inhibitory neurotransmitter activity overstimulation. Pharmaceutical compositions (e.g., riluzole) comprising one or more compounds capable of reducing glycinergic activity and/or one or more compounds capable of reducing GABAergic activity are also provided. Methods for treating one or more symptoms caused by inhibitory neurotra nsmitter overstimulation (e.g., muscle wasting, loss of muscle function, loss of muscle coordin ation, respiratory depression, dysphagia, dysarthria, eye movement difficulties, oculomotor gaze palsy, supran uclear gaze palsy, bladder dysfun ction and gastroin testinal dysfunction) in an individual afflicted with ALS or an ALS-like disorder are also provided.

Keywords: neuromuscular neurol disease treatment G ABAergic glycinergic inhibitory neurotransmitter overstimulation

PatentPak available

Substances (652) Reactions (0) Citing (5)

55

Epithelial Cell Transforming 2 and Aurora Kinase B Modulate Formation of Stress Granule-Containing Transcripts from Diverse Cellular Pathways in Astrocytoma Cells

By: Weeks, Adrienne; Agnihotri, Sameer; Lymer, Jennifer; Chalil, Alan; Diaz, Roberto; Isik, Semra; Smith, Christian; Rutka, James T.

American Journal of Pathology (2016), 186(6), 1674-1687 | Language: English, Database: CAplus and MEDLINE

Stress granules are small RNA-protein granules that modify the translational landscape during cellular stress to promote survival. The RhoGTPase RhoA is implicated in the formation of R NA stress granules. Our data demons trate that the cytokinetic proteins epithelial cell transforming 2 and Aurora kinase B (Aurk B) are localized to stress granules in human astroc ytoma cells. AurkB and its downstream target histone-3 are phosphorylated during arsenite-induced stress. Chem. (AZD1152-HQPA) and siRNA inhibition of AurkB results in fewer and smaller stress granules when analyzed using high- throughput fluore scent-based cellomics assays. RNA immunoprecipitation with the known stress granule aggregates T IAR and G3BP1 was performed on astrocytoma cells, and subsequent anal. revealed that astrocytoma stress granules harbor unique m RNAs for various cellular pathways, including cellular migration, metabolism, translation, and transcri ptional regulation. Human astrocytoma cell stress granules contain m RNAs that are known to be involved in glioma signaling and the mammalian target of rapamycin pathway. These data provide evidence that RNA stress granules are a novel form of epigenetic regulation in astrocytoma cells, which may be targetable by chem. inhibitors and enhance astrocytoma susceptibility to conventional therapy, such as radiation and chemoth erapy.

Keywords: astrocytoma cell E CT2 AurkB stress granule

Substances (13) Reactions (0) Citing (8)

56

A novel in situ hydrophobic ion paring (HIP) formulation strategy for clinical product selection of a nanoparticle drug delivery system

By: Song, Young Ho; Shin, Eyoung; Wang, Hong; Nolan, Jim; Low, Susan; Parsons, Donald; Zale, Stephen; Ashton, Susan; Ashford, Marianne; Ali, Mir; et al

Journal of Controlled Release (2016), 229, 106-119 | Language: English, Database: CAplus and MEDLINE

The present studies were aimed at formulating AZD2811-loaded polylactic acid-polyethylene glycol (PLA-PEG) nanoparticles with adjustable release rates without altering the chem. structures of the polymer or active pharmaceutical ingredient (API). This was accomplished through the use of a hydrophobic ion pairing approach. A series of AZD2811-containing nanoparticles with a variety of hydrophobic counterions including oleic acid, 1-hydroxy-2-naphthoic acid, cholic acid, deoxycholic acid, dioctylsulfosuccinic acid, and pamoic acid is described. The hydrophobicity of AZD2811 was increased through formation of ion pairs with these hydrophobic counterions, producing nanoparticles with exceptionally high drug loading-up to five fold higher encapsulation efficiency and drug loading compared to nanoparticles made without hydrophobic ion pairs. Furthermore, the rate at which the drug was released from the nanoparticles could be controlled by employing counterions with various hydrophobicities and structures, resulting in release half-lives ranging from about 2 to 120 h using the same polymer, nanoparticle size, and nanoemulsion process. Process recipe variables affecting drug load and release rate were identified, including pH and molarity of quench buffer. Ion pair formation between AZD2811 and pamoic acid as a model counterion was investigated using solubility enhancement as well as NMR spectroscopy to demonstrate solution-state interactions. Further evidence for an ion pairing mechanism of controlled release was provided through the measurement of API and counterion release profiles using high-performance liquid chromatog., which had stoichiometric relationships. Finally, Raman spectra of an AZD2811-pamoate salt compared well with those of the formulated nanoparticles, while single components (AZD2811, pamoic acid) alone did not. A library of AZD2811 batches was created for anal. and preclin. characterization. Dramatically improved preclin. efficacy and tolerability data were generated for the pamoic acid lead formulation, which has been selected for evaluation in a Phase 1 clin. trial (Clinical Trials.gov Identifier NCT 02579226). This work clearly demonstrates the importance of assessing a wide range of drug release rates during formulation screening as a critical step for new drug product development, and how utilizing hydrophobic ion pairing enabled this promising nanoparticle formulation to proceed into clin. development.

Keywords: hydrophobic ion pairing polylactic acid polyethylene glycol nanoparticle; Adjustable release rate; Counterions; Drug release kinetics; In situ hydrophobic ion paring; Nanoparticles; PLA–PEG

Substances (18) Reactions (0) Citing (49)

57

Inhibitor of Aurora Kinase B Induces Differentially Cell Death and Polyploidy via DNA Damage Response Pathways in Neurological Malignancy: Shedding New Light on the Challenge of Resistance to AZD1152-HQPA

By: Zekri, Ali; Ghaffari, Seyed H.; Yaghmaie, Marjan; Estiar, Mehrdad Asghari; Alimoghaddam, Kamran; Modarressi, Mohammad Hossein; Ghavamzadeh, Ardeshir
Molecular Neurobiology (2016), 53(3), 1808-1823 | Language: English, Database: CAplus and MEDLINE

Aurora kinase B (AURKB), a crucial regulator of malignant mitosis, is involved in chromosome segreg ation and cytokinesis. AZD1152-HQPA is a selective inhibitor for A URKB activity and currently bears clin. assessment for several maligna ncies. However, the effect of this drug still needs to be elucidated in neurol. malignancies. In this study, we invest igated the restrictive potentials of AZD1152-HQP A in U87MG and SK-N-MC. AZD1152-HQPA treatment resulted in growth arrest, modifi cation of cell cycle, and inhibition of colony formation in both cell lines. Furthermore, lower concentrations of AZD1152-HQPA profoundly induced apoptosis in U87 GM (p53/p73 wild type) cells in parallel with an upregulation of p53 and its target genes B AX, BAD, APAF1, and PUMA. But remarkably, S K-N-MC (p53/p73 double null) responded to A ZD1152-HQPA at much higher concent rations with an upregulation of genes involved in cell cycle progression, induction of excessive endoreduplication, and polyploidy rather than apoptosis. Although S K-N-MC was resistant to AZD1152-HQPA, we did not find a mutation in the coding sequence of Aurora B gene or overexpr essions of ABCG2 and ABCB1 as reported previously to be resistance mechanisms. However, our results suggest that p53/p73 status could be an important mechanism for the type of response and resistance of the tumor cells to AZD1152-HQPA. Collectively, inhibition of Aurora kinase B differentially induced cell death and polyploidy via D NA damage response pathways, depending on the status of p53/p73. We suggest p53/p73 could be a key regulator of sensitivity to AZD1152-HQPA and their status should be explored in clin. response to this ongoing drug in clin. trials.

Keywords: AURKB cell death polyploidy D NA damage neurol malignancy resist ance; AZD1152-HQPA; DNA damage response pathway; Mitotic catastrophe; Neurol ogical malignancies; P53/p73 status

Substances (8) | Reactions (0) | Citing (17)

58

Method of testing systemic release of AZD 1152 hqpa or bioequivalents

By: Walker, Michael; Howes, Colin
World Intellectual Property Organization, WO2016059149 A1 2016-04-21 | Language: English, Database: CAplus

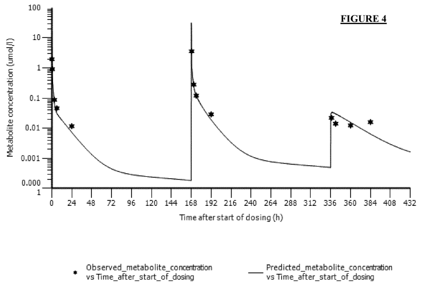

Methods of measuring systemic release, or in-vivo release profile of an active drug, e.g., AZD 1152 (barasertib), from a nanoparticle, and their use, in assessing bioequivalence are disclosed. The method involves detection of a metabolite of AZD 1152 hqpa (AZD 1152 hqpa desfluoroaniline (also referred to as AZ 12102238); AZD 1152 hqpa desfluoroaniline N-acetic acid; AZD 1152 hqpa N-acetic acid; AZD-1152 hqpa N-oxide; and AZD 1152 hqpa N-desethyl) by using mass spectrometry (LC-MS) and applying a pharmaco kinetic model.

Keywords: AZD 1152 hqpa metabolism bioequivalence nanoparticle

PatentPak available

Substances (16) | Reaction (1) | Citing (0)

59

Aurora kinase inhibitor nanoparticles target tumors with favorable therapeutic index in vivo

By: Ashton, Susan; Song, Young Ho; Nolan, Jim; Cadogan, Elaine; Murray, Jim; Odedra, Rajesh; Foster, John; Hall, Peter A.; Low, Susan; Taylor, Paula; et al
Science Translational Medicine (2016), 8(325), 325ra17/1-325ra17/12 | Language: English, Database: CAplus and MEDLINE

Efforts to apply nanotechnol. in cancer have focused almost exclusively on the delivery of cytotoxic drugs to improve therapeutic index. There has been little consideration of molecularly targeted agents, in particular kinase inhibitors, which can also present considerable therapeutic index limitations. We describe the development of Accurin polymeric nanoparticles that encapsulate the clin. candidate AZD2811, an Aurora B kinase inhibitor, using an ion pairing approach. Accurins increase biodistribution to tumor sites and provide extended release of encapsulated drug payloads. AZD2811 nanoparticles containing pharmaceutically acceptable organic acids as ion pairing agents displayed continuous drug release for more than 1 wk in vitro and a corresponding extended pharmacodynamic reduction of tumor phosphorylated histone H3 levels in vivo for up to 96 h after a single administration. A specific AZD2811 nanoparticle formulation profile showed accumulation and retention in tumors with minimal impact on bone marrow pathol., and resulted in lower toxicity and increased efficacy in multiple tumor models at half the dose intensity of AZD1152, a water-soluble prodrug of AZD2811. These studies demonstrate that AZD2811 can be formulated in nanoparticles using ion pairing agents to give improved efficacy and tolerability in preclin. models with less frequent dosing. Accurins specifically, and nanotechnol. in general, can increase the therapeutic index of molecularly targeted agents, including kinase inhibitors targeting cell cycle and oncogenic signal transduction pathways, which have to date proved toxic in humans.

Keywords: aurora B kinase inhibitor accurin polymeric nanoparticle therapeutic index

Substances (8) | Reactions (0) | Citing (136)

60

Human genome-wide RNAi screen reveals host factors required for enterovirus 71 replication

By: Wu, Kan Xing; Phuektes, Patchara; Kumar, Pankaj; Goh, Germaine Yen Lin; Moreau, Dimitri ; Chow, Vincent Tak Kwong; Bard, Frederic; Chu, Justin Jang Hann
Nature Communications (2016), 7, 13150 | Language: English, Database: CAplus and MEDLINE

Enterovirus 71 (EV71) is a neurotropic enterovirus without antivirals or vaccine, and its host-pathogen interactions remain poorly understood. Here we use a human genome- wide RNAi screen to identify 256 host factors involved in E V71 replication in human rhabdomyosarcoma cells. Enrichment analyses reveal overrepre sentation in processes like mitotic cell cycle and transcri ptional regulation. We have carried out orthogonal experi ments to characterize the roles of selected factors involved in cell cycle regulation and endoplasmatic reticulum-associated degradation We demonstrate nuclear egress of CDK6 in EV71 infected cells, and identify C DK6 and AURKB as resistance factors. NGLY1, which co-localizes with EV71 replication complexes at the endoplasmatic reticulum, supports EV71 replication. We confirm importance of these factors for E V71 replication in a human neuronal cell line and for coxsackievirus A16 infection. A small mol. inhibitor of N GLY1 reduces EV71 replication. This study provides a compreh ensive map of EV71 host factors and reveals potential antiviral targets.

Keywords: human genome RNAi enterovirus replication

Substances (4) Reactions (0) Citing (34)

61

Therapeutic polymeric nanoparticles and the methods of making and using thereof: a patent evaluation of WO2015036792

By: Sarvagalla, Sailu; Hsieh, Hsing Pang; Coumar, Mohane Selvaraj
Expert Opinion on Therapeutic Patents (2016), 26(7), 751-755 | Language: English, Database: CAplus and MEDLINE

Evaluation of the patent application WO2015036792 claiming therapeutic polymeric nanoparticles loaded with AZD1152-hqpa (aurora kinase inhibitor), and methods of making and using same for the treatment of cancer, is described. The claimed polymeric nano-formulations containing hydrophobic acid signifi cantly improved the pharmaco kinetic profiles (slow/sustained drug release profile) of the drug AZD1152-hqpa, as compared to the control agent (AZD1152). Drug efficacy and tolera bility were also improved, and toxicity decreased in in vivo animal experiments, resulting in a better therap eutic index for the nano- formulation. Hence, the nano-formulated AZD1152-hqpa could be tested in the clinic at a dose level similar to, or higher than, that used for A ZD1152, with lower incidence of toxicity.

Keywords: antitumor AZD1152 HQPA pharmaco kinetic; AZD1152; AZD1152-hqpa; Aurora kinase inhibitor; cancer; drug delivery; nano-formulation; nano-medicine; nanoparticle; nanotechnology; therapeutic index

Substance (1) Reactions (0) Citing (2)

62

Identification of selective cytotoxic and synthetic lethal drug responses in triple negative breast cancer cells

By: Gautam, Prson; Karhinen, Leena; Szwajda, Agnieszka; Kumar, Jha Sawan; Yadav, Bhagwan; Aittokallio, Tero; Wennerberg, Krister
Molecular Cancer (2016), 15, 34/1-34/16 | Language: English, Database: CAplus and MEDLINE

Background: Triple neg. breast cancer (T NBC) is a highly heterog eneous and aggressive type of cancer that lacks effective targeted therapy. Despite detailed mol. profiling, no targeted therapy has been established. Hence, with the aim of gaining deeper underst anding of the functional differences of TNBC subtypes and how that may relate to potential novel therap eutic strategies, we studied comprehensive anticancer-agent responses among a panel of TNBC cell lines. Method: The responses of 301 approved and investig ational oncol. compounds were measured in 16 T NBC cell lines applying a functional profiling approach. To go beyond the standard drug viability effect profiling, which has been used in most chemosensitivity studies, we utilized a multip lexed readout for both cell viability and cytotoxicity, allowing us to differentiate between cytostatic and cytotoxic responses. Results: Our approach revealed that most single-agent anti-cancer compounds that showed activity for the viability readout had no or little cytotoxic effects. Major compound classes that exhibited this type of response included anti-mitotics, mTOR, CDK, and metabolic inhibitors, as well as many agents selectively inhibiting oncogene-activated pathways. However, within the broad viability-acting classes of compounds, there were often subsets of cell lines that responded by cell death, suggesting that these cells are particularly vulnerable to the tested substance. In those cases we could identify differential levels of protein markers associated with cytotoxic responses. For example, PAI-1, MAPK phosphatase and Notch-3 levels associated with cytotoxic responses to mitotic and proteasome inhibi tors, suggesting that these might serve as markers of response also in clin. settings. Furthermore, the cytotoxicity readout highlighted selective synergistic and synthetic lethal drug combin ations that were missed by the cell viability readouts. For instance, the M EK inhibitor trametinib synergized with PARP inhibitors. Similarly, combination of two non-cytotoxic compounds, the rapamycin analog everolimus and an ATP-competitive mTOR inhibitor dactolisib, showed synthetic lethality in several m TOR-addicted cell lines. Conclu sions: Taken together, by studying the combin ation of cytotoxic and cytostatic drug responses, we identified a deeper spectrum of cellular responses both to single agents and combinations that may be highly relevant for identi fying precision medicine approaches in TNBC as well as in other types of cancers.

Keywords: triple neg breast cancer mTOR drug response cytotox icity; Antagonism; Chemosensitivity; Cytotoxicity; DSRT; DSS; Heterogeneity; Synergy; TNBC; Viability

Substances (257) Reactions (0) Citing (47)

63

[^{14}C]-AZD1152 drug substance manufacture: challenges of an IV-infusion dosed human mass balance study in patients

By: Bushby, Nick ; Bergin, Julie; Harding, John
Journal of Labelled Compounds and Radiopharmaceuticals (2016), 59(6), 250-254 | Language: English, Database: CAplus and MEDLINE

[^{14}C]-AZD1152 (barasertib) drug substance was prepared in order to support a h ADME study that was to be dosed as an i.v. infusion to patients with acute myeloid leukemia. A long patient recruitment time (1-2 years) was expected, and because of its limited stability several batches of [^{14}C]-AZD1152 drug substance needed to be prepared in order to maintain a supply of [^{14}C]-AZD1152 in the clinic over this period. A method was developed to purify the [^{14}C]-AZD1152 to a GMP-like standard at high specific activity to provide material for each of these batches. This approach to the delivery of the drug substance was successful in supplying the study with radiolabeled drug for over 1 yr until all patients had been recruited.

Keywords: radiolabeled C14 AZD1152 synthesis; AZD1152; cGMP; hADME

Substances (16) Reactions (30) Citing (0)

64

Methods for treating cancer using TOR kinase inhibitor combination therapy

By: Fultz, Kimberly Elizabeth; Tran, Tam Minh; Xu, Shuichan; Xu, Weiming; Trowe, Torsten; Filvaroff, Ellen
World Intellectual Property Organization, WO2015160868 A1 2015-10-22 | Language: English, Database: CAplus

Provided are methods for treating or preventing cancer, comprising administering an effective amount of a 3,4-dihydropyrazino[2, 3-b]pyrazin-2(1H)-one TOR kinase inhibitor and an effective amount of a second active agent to a patient. Cancers treated by the method include solid tumors of the head, neck, breast, lung, ovaries, colon, rectum, stomach, prostate, urinary bladder, uterine, cervix, and other reproductive organs, wherein the tumors include relapsed, refractory or advanced solid tumors.

Keywords: pyrazinopyrazine TOR kinase inhibitor combination therapy cancer

PatentPak available Substances (217) Reactions (0) Citing (0)

65

Target inhibition map system for combination antitumor therapy design and methods of using same

By: Pal, Ranadip; Keller, Charles, III; Berlow, Noah E.; Seguin, Bernard
United States, US20150269307 A1 2015-09-24 | Language: English, Database: CAplus

Disclosed is a system and method for targeted therapy designs which model a cancer pathway for predicting the effectiveness of targeted anti-cancer drugs. The disclosed system and method includes utiliz ation of a computer processor allowing for the selection of a set of drugs from available drugs using approximation algorithms utilizing cell viability data associated with a testable culture of a patient's tumor for generating a probabilistic target inhibition map (P TIM) from viability data for considering the selection of a set of drugs from available drugs, and further supports a wide variety of scenarios for personalized cancer therapy, related products and services.

Keywords: antitumor combination therapy design target map system algorithm modeling

PatentPak available Substances (79) Reactions (0) Citing (0)

66

Therapeutic combinations for treating cancer comprising an irreversible mutant EGFR inhibitor and an antineoplastic agent such as an Aurora kinase inhibitor

By: Simmmons, Andrew D.; Harding, Thomas Christian
World Intellectual Property Organization, WO2015112705 A2 2015-07-30 | Language: English, Database: CAplus

The present invention relates to combination treatment methods for treating cancer, partic ularly non-small cell lung cancer (N SCLC) . More specifi cally, the invention relates to methods of treating or managing cancer, partic ularly NSCLC, in a subject, comprising administering an irreversible mutant EGFR inhibitor compound in combination with one or more antineo plastic agents, particularly an Aurora kinase inhibitor.

Keywords: cancer treatment EGFR inhibitor Aurora kinase inhibitor combination

PatentPak available Substances (46) Reactions (0) Citing (3)

67

Discovery of N-(4-(3-(2-Aminopyrimidin-4-yl)pyridin-2-yloxy)phenyl)-4-(4-methylthiophen-2-yl)phthalazin-1-amine (AMG 900), A Highly Selective, Orally Bioavailable Inhibitor of Aurora Kinases with Activity against Multidrug-Resistant Cancer Cell Lines

By: Geuns-Meyer, Stephanie; Cee, Victor J.; Deak, Holly L.; Du, Bingfan; Hodous, Brian L.; Nguyen, Hanh Nho; Olivieri, Philip R.; Schenkel, Laurie B.; Vaida, Karina R.; Andrews, Paul; et al

Journal of Medicinal Chemistry (2015), 58(13), 5189-5207 | Language: English, Database: CAplus and MEDLINE

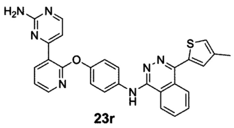

23r

Cell Line	EC_{50} (nM)
CAL51 (Non-MDR)	2
MES-SA Dx5 (MDR)	1
HCT15 (MDR)	2
SNU449 (MDR)	2
769P (MDR)	2

Efforts to improve upon the phys. properties and metabolic stability of pyridinyl-pyrimidine phthalazinamine Aurora kinase inhibitor revealed that potency against multidrug-resistant cell lines was compro mised by increased polarity. Despite its high in vitro metabolic intrinsic clearance, AMG 900 (**I**) showed acceptable pharmaco kinetic properties and robust pharmaco dynamic activity. Projecting from in vitro data to in vivo target coverage was not practical due to disjunctions between enzyme and cell data, complex and apparently contradictory indicators of binding kinetics, and unmeas urable free fraction in plasma. In contrast, it was straight forward to relate pharmaco kinetics to pharmacodynamics and efficacy by following the time above a threshold concent ration On the basis of its oral route of administration, a selectivity profile that favors Aurora-driven pharmacol. and its activity against multidrug-resistant cell lines, **I** was identified as a potential best- in-class Aurora kinase inhibitor. In phase 1 dose expansion studies with G-CSF support, **I** has shown promising single agent activity.

Keywords: pyridinylpyrimidine phthalazinamine AMG 900 analog prepar ation SAR Aurora kinase

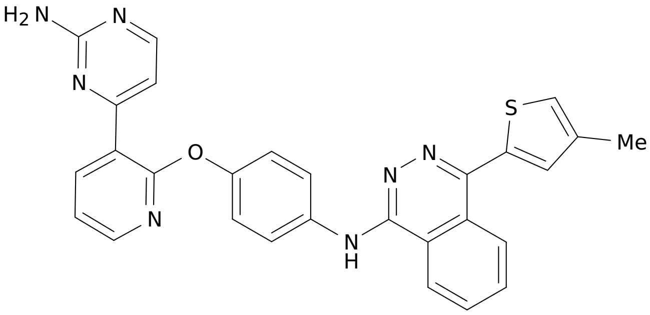

I

Substances (94) Reactions (138) Citing (36)

68

Method of blocking transmission of malarial parasites

By: McKew, John C.; Zheng, Wei; Williamson, Kim C.; Huang, Wenwei; Sun, Wei; Tanaka, Takeshi; Dehdashti, Seameen Jean; Southall, Noel Terrence; Magle, Crystal Tobin; Huang, Xiuli; et al

World Intellectual Property Organization, WO2015073804 A2 2015-05-21 | Language: English, Database: CAplus

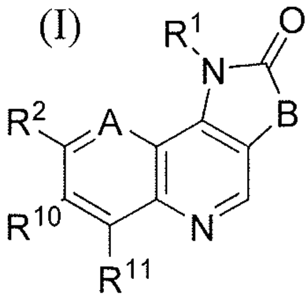

The invention provides a method of blocking transmission of a Plasmodium parasite and a method of treating or preventing malaria comprising administering to an animal an effective amount of a first compound I, wherein A, B, R^1, R^2, R^{10}, and R^{11} are described herein, either alone or in combination with a second compound selected from elescl omol, NSC 174938, NVP-AUY922, Maduramicin, Narasin, Alvespimycin, Omacetaxine, Thiram, Zinc pyrithione, Phanquinone, Bortezomib, Salinomycin sodium, Monensin sodium, Dipyrithione, Dicyclopentamethylene-thiuram disulfide, YM155, Withaferin A, Adriamycin, Romidepsin, AZD-1 152-HQPA, CAY10581, Plicamycin, CUDC-101, Auranofin, Trametinib, GSK-458, Afatinib, and Panobinostat.

Keywords: Plasmodium parasite antimalarial

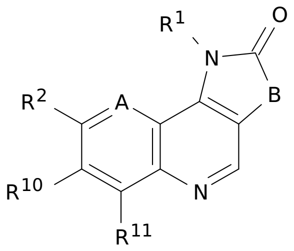

I

PatentPak available

Substances (279) | Reactions (397) | Citing (0)

69

Preparation of a therapeutic diblock polyester-based nanoparticles

By: Ashford, Marianne Bernice; Nolan, James Martin, III; Shin, Eyoung; Song, Young-Ho; Troiano, Greg; Wang, Hong

World Intellectual Property Organization, WO2015036792 A1 2015-03-19 | Language: English, Database: CAplus

Described herein are polymeric nanoparticles that include a therapeutic agent which is 2-(3-((7-(3-(ethyl(2-hydroxyethyl)amino) propoxy)quinazolin-4-yl)amino)-1H-pyrazolyl)-N-(3-fluorophenyl)acetamide (also known as AZD-1152) or a pharmaceutically acceptable salt thereof, and methods of making and using such therapeutic nanoparticles.

Keywords: therapeutic diblock polyester nanopa rticle

PatentPak available

Substances (11) | Reactions (0) | Citing (1)

70

AZD1152-HQPA induces growth arrest and apoptosis in androgen-dependent prostate cancer cell line (LNCaP) via producing aneugenic micronuclei and polyploidy

By: Zekri, Ali; Ghaffari, Seyed H.; Ghanizadeh-Vesali, Samad; Yaghmaie, Marjan; Salmaninejad, Arash; Alimoghaddam, Kamran; Modarressi, Mohammad H.; Ghavamzadeh, Ardeshir
Tumor Biology (2015), 36(2), 623-632 | Language: English, Database: CAplus and MEDLINE

Prostate cancer is the frequent non-cutaneous tumor with high mortality in men. Prostate tumors contain cells with different status of androgen receptor. Androgen receptor plays important roles in progression and treatment of prostate cancer. Aurora B kinase, with oncogenic potential, is involved in chromosome segregation and cytokinesis, and its inhibition is a promising anti- cancer therapy. In the present study, we aimed to investigate the effects of Aurora B inhibitor, AZD1152-HQPA, on survival and proliferation of androgen receptor (AR)-pos. prostate cancer cells. LNCaP was used as androgen-dependent prostate cancer cell line. We explored the effects of AZD1152-HQPA on cell viability, DNA content, micronuclei formation, and expression of genes involved in apoptosis and cell cycle. Moreover, the expression of Aurora B and AR were investigated in 23 benign prostatic hyperplasia and 38 prostate cancer specimens. AZD1152-HQPA treatment induced defective cell survival, polyploidy, and cell death in LNCaP cell line. Centromeric labeling with fluorescence in situ hybridization (FISH) showed that the loss of whole chromosomes is the origin of micronuclei, indicating on aneugenic action of AZD1152-HQPA. Treatment of AZD1152-HQPA decreased expression of AR. Moreover, we found weak pos. correlations between the expression of Aurora B and AR in both benign prostatic hyperplasia and prostate cancer specimens (r = 0.25, r = 0.41). This is the first time to show that AZD1152-HQPA can be a useful therapeutic strategy for the treatment of androgen-dependent prostate cancer cell line. AZD1152-HQPA induces aneugenic mechanism of micronuclei production Taken together, this study provides new insight into the direction to overcome the therapeutic impediments against prostate cancer.

Keywords: prostate cancer AZD1152 HQPA cell arrest apoptosis androgen receptor

Substances (8) Reactions (0) Citing (12)

71

Mechanism of action and therapeutic efficacy of Aurora kinase B inhibition in MYC overexpressing medulloblastoma.

By: Diaz, Roberto Jose; Golbourn, Brian; Faria, Claudia; Picard, Daniel; Shih, David; Raynaud, Denis; Leadly, Michael; MacKenzie, Danielle; Bryant, Melissa; Bebenek, Matthew; et al
Oncotarget (2015), 6(5), 3359-74 | Language: English, Database: MEDLINE

Medulloblastoma comprises four molecular subgroups of which Group 3 medulloblastoma is characterized by MYC amplification and MYC overexpression. Lymphoma cells expressing high levels of MYC are susceptible to apoptosis following treatment with inhibitors of mitosis. One of the key regulatory kinases involved in multiple stages of mitosis is Aurora kinase B. We hypothesized that medulloblastoma cells that overexpress MYC would be uniquely sensitized to the apoptotic effects of Aurora B inhibition. The specific inhibition of Aurora kinase B was achieved in MYC- overexpressing medulloblastoma cells with AZD1152-HQPA. MYC overexpression sensitized medulloblastoma cells to cell death upon Aurora B inhibition. This process was found to be independent of endoreplication. Using both flank and intracranial cerebellar xenografts we demonstrate that tumors formed from MYC-overexpressing medulloblastoma cells show a response to Aurora B inhibition including growth impairment and apoptosis induction. Lastly, we show the distribution of AZD1152-HQPA within the mouse brain and the ability to inhibit intracranial tumor growth and prolong survival in mice bearing tumors formed from MYC-overexpressing medulloblastoma cells. Our results suggest the potential for therapeutic application of Aurora kinase B inhibitors in the treatment of Group 3 medulloblastoma.

Substance (1) Reactions (0) Citing (18)

72

Aurora kinase B inhibition reduces the proliferation of metastatic melanoma cells and enhances the response to chemotherapy

By: Porcelli, Letizia; Guida, Gabriella; Quatrale, Anna E.; Cocco, Tiziana; Sidella, Letizia; Maida, Immacolata; Iacobazzi, Rosa M.; Ferretta, Anna; Stolfa, Diana A.; Strippoli, Sabino; et al
Journal of Translational Medicine (2015), 13, 26/1-26/13 | Language: English, Database: CAplus and MEDLINE

Background: The poor response to chemotherapy and the brief response to vemura fenib in metastatic melanoma patients, make the identification of new therapeutic approaches an urgent need. Interes tingly the increased expression and activity of the Aurora kinase B during melanoma progression suggests it as a promising therap eutic target. Methods: The efficacy of the Aurora B kinase inhibitor barasertib-HQPA was evaluated in BRAF mutated cells, sensitive and made resistant to vemura fenib after chronic exposure to the drug, and in BRAF wild type cells. The drug effecti veness has been evaluated as cell growth inhibi tion, cell cycle progression and cell migration. In addition, cellular effectors of drug resistance and response were investi gated. Results: The characterization of the effectors respon sible for the resistance to vemura fenib evidenced the increased expression of MITF or the activation of Erk1/2 and p-38 kinases in the newly established cell lines with a phenotype resistant to vemura fenib. The sensitivity of cells to barasertib-HQPA was irresp. of BRAF mutational status. Barasertib-HQPA induced the mitotic catastrophe, ultimately causing apoptosis and necrosis of cells, inhibited cell migration and strongly affected the glycolytic metabolism of cells inducing the release of lactate. In association (i) with vemura fenib the gain in effectiveness was found only in BRAF(V600K) cells while (ii) with nab-paclit axel, the combination was more effective than each drug alone in all cells. Conclu sions: These findings suggest barasertib as a new therapeutic agent and as enhancer of chemot herapy in metastatic melanoma treatment.

Keywords: Aurora kinase B proliferation metastatic melanoma cell chemotherapy

Substances (10) Reactions (0) Citing (35)

73

Patient-derived models of acquired resistance can identify effective drug combinations for cancer

By: Crystal, Adam S.; Shaw, Alice T.; Sequist, Lecia V.; Friboulet, Luc; Niederst, Matthew J.; Lockerman, Elizabeth L.; Frias, Rosa L.; Gainor, Justin F.; Amzallag, Arnaud; Greninger, Patricia; et al
Science (Washington, DC, United States) (2014), 346(6216), 1480-1486 | Language: English, Database: CAplus and MEDLINE

Targeted cancer therapies have produced substantial clin. responses, but most tumors develop resistance to these drugs. Here, we describe a pharmacogenomic platform that facili tates rapid discovery of drug combin ations that can overcome resistance. We established cell culture models derived from biopsy samples of lung cancer patients whose disease had progressed while on treatment with epidermal growth factor receptor (EGFR) or anaplastic lymphoma kinase (ALK) tyrosine kinase inhibitors and then subjected these cells to genetic analyses and a pharmacol. screen. Multiple effective drug combinations were identified. For example, the combination of ALK and MAPK kinase (MEK) inhibitors was active in an ALK-pos. resistant tumor that had developed a MAP2K1 activating mutation, and the combin ation of EGFR and fibroblast growth factor receptor (FGFR) inhibitors was active in an E GFR mutant resistant cancer with a mutation in FGFR3. Combined ALK and SRC (pp60c-src) inhibition was effective in several ALK-driven patient-derived models, a result not predicted by genetic anal. alone. With further refine ments, this strategy could help direct therapeutic choices for individual patients.

Keywords: patient model acquired resistance effective drug cancer

Substances (15) Reactions (0) Citing (510)

74

Efflux inhibitor compositions containing effect transporter protein inhibitors

By: Bunt, Antonius Martinus Gustave; Van Tellingen, Olaf
United States, US20140235631 A1 2014-08-21 | Language: English, Database: CAplus

The present invention relates to efflux inhibitor compositions and methods of using these agents for treating conditions where the activity of efflux transporter proteins (e.g., breast cancer resistance protein (B CRP) and P-glycoprotein (P-GP)) inhibit effective delivery of a therapeutic agent to a target tissue (e.g., brain, spinal cord, nerves, cerebro spinal fluid, testis, eyeballs, retina, inner ear, placenta, mammary gland, liver, biliary tract, kidney, intestines, lung, adrenal cortex, endometrium, hematopoietic cells, and/or stem cells). For example, elacridar nanoparticles were prepared

Keywords: efflux inhibitor pharmaceutical containing efflux transporter protein inhibitors

PatentPak available

Substances (249) Reactions (0) Citing (2)

75

Inhibition of Aurora-B suppresses osteosarcoma cell migration and invasion

By: Zhu, Xiao Ping; Liu, Zhi Li; Peng, Ai Fen; Zhou, Yun Fei; Long, Xin Hua; Luo, Qingfeng; Huang, Shan Hu; Shu, Yong
Experimental and Therapeutic Medicine (2014), 7(3), 560-564 | Language: English, Database: CAplus and MEDLINE

Previous studies have suggested that Aurora-B may be involved in cancer metastasis. However, its role has been poorly evaluated in osteosarcoma (OS). The aim of this study was to investigate the correlation between Aurora-B expression and metastasis in human OS. The human OS cell line, U2-OS, and OS biopsy specimens were used in the study. The expression of Aurora- B protein was examined using immunohistochem. and western blotting in OS tissues and U2-OS cells, resp. AZD1152-hydroxyquinazoline-pyrazol-anilide, an inhibitor of Aurora-B, was used to inhibit Aurora-B expression in U2-OS cells. The effect of Aurora- B inhibition on U2-OS cell proliferation, invasion and migration was assessed using MTT, colony formation, wound healing and Transwell assays. The results showed that pos. expression of the Aurora-B protein was observed in the nucleus, and that Aurora- B expression levels in the cases with pulmonary metastases were significantly higher than in those without metastasis. In vitro, the proliferation, invasion and migration of U2-OS cells were suppressed by the inhibition of Aurora- B. These results suggest that Aurora- B may be involved in OS metastasis, and may be a promising target in the treatment of OS metastasis.

Keywords: aurora kinase B inhibitor osteosarcoma antitumor cell migration invasion; AZD1152; Aurora-B; metastasis; osteosarcoma

Substances (2) Reactions (0) Citing (21)

76

Compounds for treating eye narrow angle and application thereof

By: Yang, Zijiao; Hou, Kai
China, CN103664797 A 2014-03-26 | Language: Chinese, Database: CAplus

The present invention discloses a class of compounds and pharmaceutical compositions and application thereof in preparing drug for treating narrow angle. The above compounds have significant therapeutic effect on narrow angle.

Keywords: eye narrow angle drug formulation

PatentPak available

Substances (3) Reactions (0) Citing (0)

77

Compounds for treating traumatic brain injury disease and their application

By: Yang, Li; Yang, Yuxin; Li, Mai
China, CN103655575 A 2014-03-26 | Language: Chinese, Database: CAplus

The present invention discloses a class of compounds and their pharmaceutical compositions and application. The compounds are given in the claim 1 (A-D), and applied in preparation of drugs for treating traumatic brain injury disease. These compounds have significant effects on treating traumatic brain injury disease.

Keywords: compound traumatic brain injury

PatentPak available

Substances (10) Reactions (0) Citing (0)

78

Efflux inhibitor compositions and methods of treatment using the same

By: Bunt, Antonius Martinus Gustave; Van Tellingen, Olaf
World Intellectual Property Organization, WO2014018932 A2 2014-01-30 | Language: English, Database: CAplus

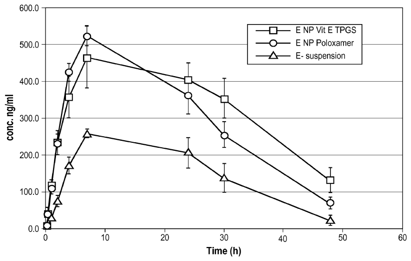

Fig. 3

The present invention relates to efflux inhibitor compositions and methods of using these agents for treating conditions where the activity of efflux transporter proteins (e.g., Breast Cancer Resistance Protein (BCRP) and P-Glycoprotein (P-GP)) inhibit effective delivery of a therapeutic agent to a target tissue (e.g., brain, spinal cord, nerves, cerebro spinal fluid, testis, eyeballs, retina, inner ear, placenta, mammary gland, liver, biliary tract, kidney, intestines, lung, adrenal cortex, endometrium, hematopoietic cells, and/or stem cells).

Keywords: BCRP P Glycoprotein efflux inhibitor elacridar TPGS nanoparticle

PatentPak available

Substances (278) Reactions (0) Citing (5)

79

A High Content Clonogenic Survival Drug Screen Identifies MEK Inhibitors as Potent Radiation Sensitizers for KRAS Mutant Non-Small-Cell Lung Cancer

By: Lin, Steven H.; Zhang, Jing; Giri, Uma; Stephan, Clifford; Sobieski, Mary; Zhong, Ling; Mason, Kathy A.; Molkentine, Jessica; Thames, Howard D.; Yoo, Stephen S.; et al
Journal of Thoracic Oncology (2014), 9(7), 965-973 | Language: English, Database: CAplus and MEDLINE

Introduction: Traditional clonogenic survival and high throughput colori metric assays are inadequate as drug screens to identify novel radiation sensitizers. We developed a method that we call the high content clonogenic survival assay (H CSA) that will allow screening of drug libraries to identify candidate radiation sensitizers. Methods: Drug screen using HCSA was done in 96 well plates. After drug treatment, irradiation, and incubation, colonies were stained with crystal violet and imaged on the I NCell 6000 (GE Health). Colonies achieving 50 or more cells were enumerated using the I NCell Developer image anal. software. A proof- of-principle screen was done on the KRAS mutant lung cancer cell line H460 and a Custom Clin. Collection (146 compounds) . Results: Multiple drugs of the same class were found to be radiation sensitizers and levels of potency seemed to reflect the clin. relevance of these drugs. For instance, several PARP inhibitors were identified as good radiation sensitizers in the HCSA screen. However, there were also a few PARP inhibitors not found to be sensitizing that have either not made it into clin. develo pment, or in the case of BSI-201, was proven to not even be a PARP inhibitor. We discovered that inhibitors of pathways downstream of activated mutant K RAS (PI3K, AKT, mTOR, and MEK1/2) sensitized H460 cells to radiation. Furthe rmore, the potent MEK1/2 inhibitor tramenitib selectively enhanced radiation effects in KRAS mutant but not wild- type lung cancer cells. Conclusions: Drug screening for novel radiation sensitizers is feasible using the HCSA approach. This is an enabling technol. that will help accelerate the discovery of novel radiosen sitizers for clin. testing.

Keywords: anticancer MEK inhibitor radiosensitizer clonogenic survival drug screening N SCLC

Substances (56) Reactions (0) Citing (24)

80

Chemical signatures and new drug targets for gametocytocidal drug development

By: Sun, Wei; Tanaka, Takeshi Q.; Magle, Crystal T.; Huang, Wenwei; Southall, Noel; Huang, Ruili; Dehdashti, Seameen J.; McKew, John C.; Williamson, Kim C.; Zheng, Wei
Scientific Reports (2014), 4, 3743/1-3743/11 | Language: English, Database: CAplus and MEDLINE

Control of parasite transmission is critical for the eradication of malaria. However, most antimalarial drugs are not active against P. falciparum gametocytes, responsible for the spread of malaria. Consequ ently, patients can remain infectious for weeks after the clearance of asexual parasites and clin. symptoms. Here we report the identification of 27 potent gametocytocidal compounds (IC50 < 1 μM) from screening 5,215 known drugs and compounds All these compounds were active against three strains of gameto cytes with different drug sensitivities and geog. origins, 3D7, HB3 and Dd2. Cheminformatic anal. revealed chem. signatures for P. falciparum sexual and asexual stages indicative of druggability and suggesting potential targets. Torin 2, a top lead compound (I C50 = 8 nM against gameto cytes in vitro), completely blocked oocyst formation in a mouse model of transmi ssion. These results provide critical new leads and potential targets to expand the repertoire of malaria transmission-blocking reagents.

Keywords: antimalarial agent therapeutic target gametocyte Plasmodium malaria

Substances (42) Reactions (0) Citing (78)

81

Facile Identification of Dual FLT3-Aurora A Inhibitors: A Computer-Guided Drug Design Approach

By: Chang Hsu, Yung; Ke, Yi-Yu; Shiao, Hui-Yi; Lee, Chieh-Chien; Lin, Wen-Hsing; Chen, Chun-Hwa; Yen, Kuei-Jung; Hsu, John T.-A.; Chang, Chungming; Hsieh, Hsing-Pang
ChemMedChem (2014), 9(5), 953-961 | Language: English, Database: CAplus and MEDLINE

Computer-guided drug design is a powerful tool for drug discovery. Herein we disclose the use of this approach for the discovery of dual FMS-like receptor tyrosine kinase-3 (FLT3)-Aurora A inhibitors against cancer. An Aurora hit compound was selected as a starting point, from which 288 virtual mols. were screened. Subsequently, some of these were synthesized and evaluated for their capacity to inhibit FLT3 and Aurora kinase A. To further enhance FLT3 inhibition, structure-activity relationship studies of the lead compound were conducted through a simplification strategy and bioisosteric replacement, followed by the use of computer-guided drug design to prioritize mols. bearing a variety of different terminal groups in terms of favorable binding energy. Selected compounds were then synthesized, and their bioactivity was evaluated. Of these, one novel inhibitor was found to exhibit excellent inhibition of FLT3 and Aurora kinase A and exert a dramatic antiproliferative effect on MOLM-13 and MV4-11 cells, with an IC_{50} value of 7 nM. Accordingly, it is considered a highly promising candidate for further development.

Keywords: computer drug design preparation dual FLT3 aurora kinase inhibitor; cancer antitumor dual FLT3 aurora A kinase inhibitor; Aurora kinase A; FLT3; drug design; inhibitors; leukemia

Substances (106) Reactions (68) Citing (7)

82

Phase I study of barasertib (AZD1152), a selective inhibitor of Aurora B kinase, in patients with advanced solid tumors

By: Schwartz, Gary K.; Carvajal, Richard D.; Midgley, Rachel; Rodig, Scott J.; Stockman, Paul K.; Ataman, Ozlem; Wilson, David; Das, Shampa; Shapiro, Geoffrey I.
Investigational New Drugs (2013), 31(2), 370-380 | Language: English, Database: CAplus and MEDLINE

The purpose of this study was to determine the maximum-tolerated dose (MTD), pharmacokinetics and safety profile for two different dosing regimens of barasertib, a selective inhibitor of Aurora B Kinase. In this Phase I trial, patients with advanced solid malignancies were treated with escalating doses of barasertib, administered as either a 48-h continuous infusion or as two 2-h infusions on consecutive days, both every 14 days of a 28- day cycle. Thirty-five patients were treated. The MTDs were 150 mg as a 48-h continuous infusion and 220 mg administered as two 2-h infusions (110 mg/day, days 1, 2, 15 and 16), with neutropenia the dose-limiting toxicity (DLT) of each schedule. Common Terminol. Criteria of Adverse Events (CTCAE) grade ≥ 3 neutropenia (with or without fever) occurred in 34 % of patients overall. Other adverse events, many of hematol. or gastrointestinal etiol., were of mild or moderate intensity. No objective tumor responses were observed, although stable disease was observed in 23 % of patients. Systemic exposure to barasertib-hQPA, the more active moiety to which barasertib is converted, was observed by 1 and 6 h into the 2-h and continuous infusion, resp., and exhibited linear pharmacokinetics. In summary, barasertib was generally well tolerated, with neutropenia the most frequent and dose-limiting toxicity, irresp. of schedule. Future development of barasertib will depend on better definition of its therapeutic index.

Keywords: barasertib AuroraB kinase inhibitor colon lung skin cancer; pharmacokinetics pharmacodynamics antitumor

Substances (3) Reactions (0) Citing (52)

83

Aurora kinases in childhood acute leukemia: the promise of aurora B as therapeutic target

By: Hartsink-Segers, S. A.; Zwaan, C. M.; Exalto, C.; Luijendijk, M. W. J.; Calvert, V. S.; Petricoin, E. F.; Evans, W. E.; Reinhardt, D.; de Haas, V.; Hedtjaern, M.; et al
Leukemia (2013), 27(3), 560-568 | Language: English, Database: CAplus and MEDLINE

We investigated the effects of targeting the mitotic regulators aurora kinase A and B in pediatric acute lymphob lastic leukemia (ALL) and acute myeloid leukemia (AML). Aurora protein expression levels in pediatric A LL and AML patient samples were determined by western blot and reverse phase protein array. Both kinases were overexpressed in ALL and AML patients (P<0.0002), especially in E2 A-PBX1-translocated ALL cases (P<0.002), compared with normal bone-marrow mononu clear cells. Aurora kinase expression was silenced in leukemic cell lines using short hairpin RNAs and locked nucleic acid-based mRNA antagonists. Aurora B knockdown resulted in proliferation arrest and apoptosis, whereas aurora A knockdown caused no or only minor growth delay. Most tested cell lines were highly sensitive to the AURKB-selective inhibitor barasertib-hydroxyquinazoline-pyrazol-anilide (AZD1152-HQPA) in the nanomolar range, as tested with an MTS (3-(4,5-dimethylthiazol-2-yl)-5-(3-carboxymethoxyphenyl)-2-(4-sulfophenyl)-2H-tetraz olium) assay. But most import antly, primary ALL cells with a high aurora B protein expres sion, especially E2A-PBX1-pos. cases, were sensitive as well. In adult AML early clin. trials, clear responses are observed with barase rtib. Here we show that inhibition of aurora B, more than aurora A, has an antiproliferative and pro-apoptotic effect on acute leukemia cells, indicating that partic ularly targeting aurora B may offer a new strategy to treat pediatric ALL and AML. Leukemia (2013) 27, 560-568; doi:10.1038/leu.2012.256; published online 19 Oct. 2012.

Keywords: antiproliferative aurora kinase therapeutic target acute leukemia childhood

Substances (5) Reactions (0) Citing (53)

84

Effect of the drug transporters ABCG2, Abcg2, ABCB1 and ABCC2 on the disposition, brain accumulation and myelotoxicity of the aurora kinase B inhibitor barasertib and its more active form barasertib-hydroxy-QPA

By: Marchetti, Serena; Pluim, Dick; Eijndhoven, Monique; Tellingen, Olaf; Mazzanti, Roberto; Beijnen, Jos H.; Schellens, Jan H. M.
Investigational New Drugs (2013), 31(5), 1125-1135 | Language: English, Database: CAplus and MEDLINE

We explored whether barasertib (AZD1152), a selective Aurora B kinase inhibitor, is a substrate for P- glycoprotein (Pgp, MDR1), breast cancer resistance protein (BCRP), and multidrug resistance protein 2 (M RP2) in vitro. Cell survival, drug transport, and competition experiments with barasertib pro-drug and the more active form of the drug (barase rtib-hQPA) were performed using MDCKII (wild type, M DR1, BCRP, and M RP2) and LLCPK (wild type and M DR1) cells and monolayers, and Sf9-BCRP membrane vesicles. Moreover we tested whether P-gp and BCRP affect the oral pharmacokinetics, tissue distribution, and myelotoxicity of barasertib in vivo using Bcrp1$^{-/-}$/Mdr1a/1b $^{-/-}$ (triple knockout) and wild type mice. In cell survival experi ments expression of BCRP and MDR1 resulted in signif icant resistance to barasertib. In transwell experiments, barasertib-hQPA was transported by BCRP and MDR1 efficiently. In Sf9-BCRP membrane vesicles, both barasertib and barase rtib-hQPA significantly inhibited the BCRP-mediated transport of methotrexate. In contrast, no active transport of barasertib by M RP2 was observed, and overexpression of MRP2 did not affect cytotoxicity of barasertib. In vivo, systemic exposure as well as bioavailability, brain penetration, kidney and liver distri bution and myelotoxicity of barasertib-hQPA were statistically significantly increased in Bcrp1$^{-/-}$/Mdr1a/1b$^{-/-}$ compared with wild type mice (p<0.001). Barasertib is transported efficiently by P-gp and BCRP/Bcrp1 in vitro. In vivo, genetic deletion of P-gp and BCRP in mice significantly affected pharmacokinetics, tissue distribution and myelotoxicity of barasertib-hQPA. Possible clin. conseq uences for the observed affinity of barasertib for P- gp and BCRP need to be explored.

Keywords: barasertib aurora kinase B inhibitor ABCG2 ABCB1 ABCC2 pharmacokinetics

Substances (4) Reactions (0) Citing (19)

85

Cytofluorometric Assessment of Cell Cycle Progression

By: Vitale, Ilio; Jemaa, Mohamed; Galluzzi, Lorenzo; Metivier, Didier; Castedo, Maria; Kroemer, Guido
Methods in Molecular Biology (New York, NY, United States) (2013), 965, 93-120 | Language: English, Database: CAplus and MEDLINE

One of the most prominent features of cellular senescence, a stress response that prevents the propagation of cells that have accumulated potentially oncogenic alterations, is a permanent loss of proliferative potential. Thus, at odds with quiescent cells, which resume proliferation when stimulated to do so, senescent cells cannot proceed through the cell cycle even in the presence of mitogenic factors. Here, we describe a set of cytofluorometric techniques for studying how chem. and/or phys. stimuli alter the cell cycle in vitro, in both qual. and quant. terms. Taken together, these methods allow for the identification of bona fide cytostatic effects as well as for a refined characterization of cell cycle distributions, providing information on proliferation, DNA content as well as on the presence of cell cycle phase-specific markers. At the end of the chapter, a set of guidelines is offered to assist researchers that approach the study of the cell cycle with the interpretation of results.

Keywords: cell cycle progression cytofluorometric technique

Substances (5) | Reactions (0) | Citing (5)

86

Combining machine learning systems and multiple docking simulation packages to improve docking prediction reliability for network pharmacology

By: Hsin, Kun-Yi; Ghosh, Samik; Kitano, Hiroaki
PLoS One (2013), 8(12), e83922/1-e83922/9, 9 pp. | Language: English, Database: CAplus and MEDLINE

Increased availability of bioinformatics resources is creating opportunities for the application of network pharmacol. to predict drug effects and toxicity resulting from multi-target interactions. Here we present a high- precision computational prediction approach that combines two elaborately built machine learning systems and multiple mol. docking tools to assess binding potentials of a test compound against proteins involved in a complex mol. network. One of the two machine learning systems is a re-scoring function to evaluate binding modes generated by docking tools. The second is a binding mode selection function to identify the most predictive binding mode. Results from a series of benchmark validations and a case study show that this approach surpasses the prediction reliability of other techniques and that it also identifies either primary or off- targets of kinase inhibitors. Integrating this approach with mol. network maps makes it possible to address drug safety issues by comprehensively investigating network-dependent effects of a drug or drug candidate.

Keywords: mol docking binding affinity signaling protein ligand

Substances (38) | Reactions (0) | Citing (163)

87

Inhibition of Aurora-B kinase activity confers antitumor efficacy in preclinical mouse models of early and advanced gastrointestinal neoplasia

By: Alferez, Denis G.; Goodland, Roberta A.; Odedra, Rajesh; Sini, Patrizia; Crafter, Claire; Ryan, Anderson J.; Wedge, Stephen R.; Wright, Nicholas A.; Anderson, Elizabeth; Wilkinson, Robert W.

International Journal of Oncology (2012), 41(4), 1475-1485 | Language: English, Database: CAplus and MEDLINE

The Aurora family of kinases, play a fundamental role in cell division and are overexp ressed in several cancers including colon. The activity of barasertib-hQPA, a selective inhibitor of Aurora- B kinase (ABK) was investigated in a range of preclin. models of gastroin testinal cancer. Treatment with barasertib-hQPA produced anti-proliferative and cytotoxic effects across a panel of human colorectal cancer (CRC) cell lines in vitro. Prodrug, barasertib [48- h s.c. (s.c.) infusion; 150 mg/kg/day] inhibited the growth of S W620, Colo205, HCT116 human colorectal tumor xenografts in nude mice signifi cantly (Student's t-test, $P<0.05$, $n=10-12$ per group). Flow cytometric anal. of single cells from disaggregated barasertib-treated SW620 tumors revealed a decrease in phospho rylated histone H3 (phH3) and an increase in tumor cells with ≥4 N DNA content ($P<0.05$). The activity of barasertib was then examined in $Apc^{Min/+}$ mice, a spontaneous model of early intestinal neoplasia. Macroscopic evaluation of the small intestine revealed that barasertib treatment [25 mg/kg intra-peritoneal (i.p.) Q1Dx4 each week for 3 wk] of 8- wk old $Apc^{Min/+}$ mice produced a 39% reduction in macroadenoma number ($P=0.02$) and a 43% reduction in overall adenoma burden ($P=0.02$) compared with vehicle- treated controls. Quantification of microscopic adenomas revealed a >64% reduction in the number of adenomas spanning more than one villus. Histol. anal. of these adenomas revealed a number of distinct changes in barasertib-treated $Apc^{Min/+}$ mice, including a 94% reduction in the proportion of phosphohistone H3-pos. cells ($P<0.001$) and a 53% reduction in the number of cells per adenoma ($P=0.001$). These results provide a scientific rationale for investi gating ABK inhibitors as a treatment for intestinal cancer.

Keywords: antitumor auroraB kinase inhibitor barasertib APC signaling gastrointestinal neoplasia

Substances (4) Reactions (0) Citing (10)

88

Inhibiting the Aurora B Kinase Potently Suppresses Repopulation During Fractionated Irradiation of Human Lung Cancer Cell Lines

By: Sak, Ali; Stuschke, Martin; Groneberg, Michael; Kuebler, Dennis; Poettgen, Christoph; Eberhardt, Wilfried E. E.
International Journal of Radiation Oncology, Biology, Physics (2012), 84(2), 492-499 | Language: English, Database: CAplus and MEDLINE

The use of mol.-targeted agents during radiotherapy of non-small-cell lung cancer (NSCLC) is a promising strategy to inhibit repopul ation, thereby improving therap eutic outcome. We assessed the combined effecti veness of inhibiting Aurora B kinase and irradi ation on human NSCLC cell lines in vitro.NSCLC cell lines were exposed to concent rations of AZD1152-hydroxyquinazoline pyrazol anilide (AZD1152-HQPA) inhibiting colony formation by 50% ($IC50_{clone}$) in combination with single dose irradi ation or different fractionation schedules using multiple 2- Gy fractions per day up to total doses of 4- 40 Gy. The total irradi ation dose required to control growth of 50% of the plaque monolayers (TCD50) was determined Apoptosis, G2/M progression, and polyploidization were also analyzed.TCD50 values after single dose irradi ation were similar for the H460 and H661 cell lines with 11.4 ± 0.2 Gy and 10.7 ± 0.3 Gy, resp. Fractionated irradiation using 3 × 2 Gy/day, 2 × 2 Gy/day, and 1 × 2 Gy/day schedules signifi cantly increased TCD50 values for both cell lines grown as plaque monolayers with increasing radiation treatment time. This could be explained by a repopu lation effect per day that counte racts 75 ± 8% and 27 ± 6% of the effect of a 2- Gy fraction in H460 and H661 cells, resp. AZD1152-HQ PA treatment concom itant to radiotherapy significantly decreased the daily repopu lation effect (H460: 28 ± 5%, H661: 10 ± 4% of a 2-Gy fraction per day). Treatment with $IC50_{clone}$ AZD1152-HPQA did not induce apoptosis, prolong radiation- induced G2 arrest, or delay cell cycle progression before the spindle check point. However, polyploi dization was detected, especially in cell lines without functional p53.Inhibition of Aurora B kinase with low AZD1152-HQPA concentrations during irradi ation of NSCLC cell lines affects repopulation during radioth erapy. Thus, concom itant Aurora B kinase inhibition and irradi ation may be a promising strategy for fast repopulating tumors, which are difficult to cure by dose escalation based on conven tional fractionation.

Keywords: AZD1152 HQPA anticancer ABK inhibitor radiation lung cancer cell

Substances (2) Reactions (0) Citing (21)

89

Aurora kinase B/C inhibition impairs malignant glioma growth in vivo

By: Diaz, Roberto Jose; Golbourn, Brian; Shekarforoush, Maryam; Smith, Christian A.; Rutka, James T.
Journal of Neuro-Oncology (2012), 108(3), 349-360 | Language: English, Database: CAplus and MEDLINE

Inhibition of Aurora kinase B has been evaluated as a therapy to block solid tumor growth in breast cancer, hepatocellular carcinoma, lung adenocarcinoma, and colorectal cancer models. Aurora kinase inhibitors are in early clin. trials for the treatment of leukemia. We hypothesized that Aurora B inhibition would reduce malignant glioma cell viability and result in impaired tumor growth in vivo. Aurora B expression is greater in cultured malignant glioma U251 cells compared to proliferating normal human astrocytes, and expression is maintained in U251 flank xenogr afts. Aurora B inhibition with AZD1152-HQPA blocked cell division in four different p53-mutant glioma cell lines (U251, T98 G, U373, and U118). AZD1152-HQPA also inhibited Aurora C activation loop threonine autophosphorylation at the effective antiproli ferative concentrations in vitro. Reduction in cell viability of U251 ($p53^{R273H}$) cells was secondary to cytokinesis blockade and apoptosis induction following endorepl ication. AZD1152-HQPA inhibited the growth of U251 tumor xenografts and resulted in an increase in tumor cell apoptosis both in vitro and in vivo. S.c. administration of AZ D1152-HQPA (25 mg/kg/day × 4 days; 2 cycles spaced 7 days apart) resulted in a prolon gation in median survival after intracranial inoculation of U251 cells in mice (P = 0.025). This is the first demonst ration that an Aurora kinase inhibitor can inhibit malignant glioma growth in vivo at drug doses that are clin. relevant.

Keywords: AZD1152HQPA aurora kinase inhibitor anticancer agent glioma cell growth

Substances (3) Reactions (0) Citing (33)

90

Aurora B kinase phosphorylates and instigates degradation of p53

By: Gully, Chris P.; Velazquez-Torres, Guermarie; Shin, Ji-Hyun; Fuentes-Mattei, Enrique; Wang, Edward; Carlock, Colin; Chen, Jian; Rothenberg, Daniel; Adams, Henry P.; Choi, Hyun Ho; et al
Proceedings of the National Academy of Sciences of the United States of America (2012), 109(24), E1513-E1522, SE1513/1-SE1513/6 | Language: English, Database: CAplus and MEDLINE

Aurora B is a mitotic checkpoint kinase that plays a pivotal role in the cell cycle, ensuring correct chromosome segregation and normal progression through mitosis. Aurora B is overexp ressed in many types of human cancers, which has made it an attractive target for cancer therapies. Tumor suppressor p53 is a genome guardian and important neg. regulator of the cell cycle. Whether Aurora B and p53 are coordinately regulated during the cell cycle is not known. We report that Aurora B directly interacts with p53 at different subcellular localizations and during different phases of the cell cycle (for instance, at the nucleus in interphase and the centromeres in prometaphase of mitosis). We show that Aurora B phospho rylates p53 at S183, T211, and S215 to accelerate the degradation of p53 through the polyubiquitination-proteasome pathway, thus functionally suppressing the expression of p53 target genes involved in cell cycle inhibition and apoptosis (e.g., p21 and PUMA). Pharmacol. inhibition of Aurora B in cancer cells with W T p53 increased p53 protein level and expression of p53 target genes to inhibit tumor growth. Together, these results define a mechanism of p53 inactivation during the cell cycle and imply that oncogenic hyperact ivation or overexpression of Aurora B may compromise the tumor suppressor function of p53. We have elucidated the antineoplastic mechanism for Aurora B kinase inhibitors in cancer cells with WT p53.

Keywords: Aurora B kinase p53 protein phosphorylation cancer antineoplastic signaling

Substances (7) Reactions (0) Citing (121)

91

CEP-32496: A Novel Orally Active BRAF^{V600E} Inhibitor with Selective Cellular and In Vivo Antitumor Activity

By: James, Joyce; Ruggeri, Bruce; Armstrong, Robert C.; Rowbottom, Martin W.; Jones-Bolin, Susan; Gunawardane, Ruwanthi N.; Dobrzanski, Pawel; Gardner, Michael F.; Zhao, Hugh; Cramer, Merryl D.; et al
Molecular Cancer Therapeutics (2012), 11(4), 930-941 | Language: English, Database: CAplus and MEDLINE

Mutations in the BRAF gene have been identified in approx. 7% of cancers, including 60% to 70% of melanomas, 29% to 83% of papillary thyroid carcinomas, 4% to 16% colorectal cancers, and a lesser extent in serous ovarian and non- small cell lung cancers. The V600E mutation is found in the vast majority of cases and is an activating mutation, conferring transf orming and immortal ization potential to cells. CEP-32496 is a potent BRAF inhibitor in an in vitro binding assay for mutated B RAF^{V600E} (K_d BRAF^{V600E} = 14 nmol/L) and in a mitogen-activated protein (MAP)/extracellular signal-regulated (ER) kinase (MEK) phosphorylation (pMEK) inhibition assay in human melanoma (A375) and colorectal cancer (Colo-205) cell lines (IC_{50} = 78 and 60 nmol/L). In vitro, CEP-32496 has multikinase binding activity at other cancer targets of interest; however, it exhibits selective cellular cytoto xicity for BRAF^{V600E} vs. wild-type cells. CEP-32496 is orally bioavailable in multiple preclin. species (>95% in rats, dogs, and monkeys) and has single oral dose pharmacodynamic inhibition (10-55 mg/kg) of both p MEK and p ERK in BRAF^{V600E} colon carcinoma xenografts in nude mice. Sustained tumor stasis and regressions are observed with oral adminis tration (30-100 mg/kg twice daily) against B RAF^{V600E} melanoma and colon carcinoma xenografts, with no adverse effects. Little or no epithelial hyperp lasia was observed in rodents and primates with prolonged oral administration and sustained exposure. CEP-32496 benchmarks favorably with respect to other kinase inhibitors, including RAF-265 (phase I), sorafenib, (approved), and vemurafenib (PLX4032/RG7204, approved). CEP-32496 represents a novel and pharmacol. active BRAF inhibitor with a favorable side effect profile currently in clin. develo pment. Mol Cancer Ther; 11(4); 930-41.

Keywords: antitumor CEP32496 BRAF inhibitor gene mutation melanoma colon carcinoma

Substances (49) Reactions (0) Citing (40)

92

Probing the structural requirements of A-type Aurora kinase inhibitors using 3D-QSAR and molecular docking analysis

By: Zhang, Hui-xiao; Li, Yan; Wang, Xia; Wang, Yong-hua
Journal of Molecular Modeling (2012), 18(3), 1107-1122 | Language: English, Database: CAplus and MEDLINE

Aurora-A, the most widely studied isoform of Aurora kinase overexp ressed aberrantly in a wide variety of tumors, has been implicated in early mitotic entry, degradation of natural tumor suppressor p53 and centrosome maturation and separa tion; hence, potent inhibitors of Aurora-A may be therapeu tically useful drugs in the treatment of various forms of cancer. Here, we report an in silico study on a group of 220 reported Aurora-A inhibitors with six different substru ctures. Three-dimensional quant. structure-activity relationship (3D-QSAR) studies were carried out using compar ative mol. field anal. (CoMFA) and comparative mol. similarity indexes anal. (CoMSIA) techniques on this series of mols. The resultant optimum 3 D-QSAR models exhibited an r value of 0.404-0.582 and their predictive ability was validated using an indepe ndent test set, ending in r 0.512- 0.985. In addition, docking studies were employed to explore these protein-inhibitor interactions at the mol. level. The results of 3 D-QSAR and docking analyses validated each other, and the key structural requirements affecting Aurora-A inhibitory activities, and the influential amino acids involved were identified. To the best of our knowledge, this is the first report on 3 D-QSAR modeling of Aurora- A inhibitors, and the results can be used to accurately predict the binding affinity of related analogs and also facilitate the rational design of novel inhibitors with more potent biol. activities.

Keywords: Aurora kinase inhibitor antitumor 3D QSAR mol docking

Substances (221) Reactions (0) Citing (12)

93

Identification of Binding Specificity-Determining Features in Protein Families

By: Anderson, Peter C.; De Sapio, Vincent; Turner, Kevin B.; Elmer, Sidney P.; Roe, Diana C.; Schoeniger, Joseph S.
Journal of Medicinal Chemistry (2012), 55(5), 1926-1939 | Language: English, Database: CAplus and MEDLINE

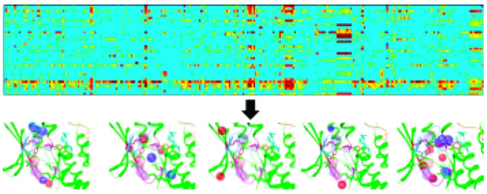

The authors present a new approach for identifying features of ligand-protein binding interfaces that predict binding select ivity and demonstrate its effectiveness for predicting kinase inhibitor specif icity. The authors analyzed a large set of human kinases and kinase inhibitors using clustering of exptl. determined inhibition constants (to define specificity classes of kinases and inhibitors) and virtual ligand docking (to extract structural and chem. features of the ligand-protein binding interfaces). The authors then used statistical methods to identify features charact eristic of each class. Machine learning was employed to determine which combin ations of characteristic features were predictive of class membership and to predict binding specifi cities and affinities of new compounds Experiments showed predictions were 70% accurate. The authors' method can automat ically pinpoint on the three-dimensional binding interfaces pharmacophore-like features that act as selectivity filters. The method is not restricted to kinases, requires no prior hypotheses about specific interactions, and can be applied to any protein families for which sets of structures and ligand binding data are available.

Keywords: ligand binding specificity determination protein kinase

Substances (47) Reactions (0) Citing (4)

94

Phase I study of the Aurora B kinase inhibitor barasertib (AZD1152) to assess the pharmacokinetics, metabolism and excretion in patients with acute myeloid leukemia

By: Dennis, Mike; Davies, Michelle; Oliver, Stuart; D'Souza, Roy; Pike, Laura; Stockman, Paul
Cancer Chemotherapy and Pharmacology (2012), 70(3), 461-469 | Language: English, Database: CAplus and MEDLINE

Purpose. Barasertib (AZD1152) is a pro-drug that rapidly undergoes phosph atase-mediated cleavage in serum to release barasertib-hQPA, a selective Aurora B kinase inhibitor that has shown prelim inary activity in clin. studies of patients with acute myeloid leukemia (AML). The pharmaco kinetic (PK), metabolic and excretion profiles of barasertib and barase rtib-hQPA were characterized in this open-label Phase I study. Methods. Five patients with poor prognosis A ML (newly diagnosed, relapsed or refractory) received barasertib 1,200 mg as a 7- day continuous infusion every 28 days. On Day 2 of Cycle 1 only, patients also received a 2- h infusion of [^{14}C]-barasertib. Blood, urine and feces samples were collected at various time points during Cycle 1. Safety and prelim inary efficacy were also assessed. Results. Barasertib-hQPA was extensively distributed to tissues, with a slow rate of total clearance (C L = 31.4 L/h). Overall, 72- 82 % of radioactivity was recovered, with approx. double the amount recovered in feces (mean = 51 %) compared with urine (mean = 27 %). The main metabolism pathways for barasertib were (1) cleavage of the phosphate group to form barase rtib-hQPA, followed by oxidation and (2) loss of the fluoroa niline moiety to form barase rtib-hQPA desfluoro aniline, followed by oxidation One of the four patients evaluable for response entered complete remission. No new or unexpected safety findings were observed; the most common adverse events were nausea and stomatitis. Conclusions. The PK profile of barasertib is similar to previous studies using the same dosing regimen in patients with AML. The majority of barasertib-hQPA clearance occurred via hepatic metabolic routes.

Keywords: antitumor aurora B kinase inhibitor barasertib pharmacokinetics metabolism; acute myeloid leukemia prodrug

Substances (2) | Reactions (0) | Citing (49)

95

Comprehensive analysis of kinase inhibitor selectivity

By: Davis, Mindy I.; Hunt, Jeremy P.; Herrgard, Sanna; Ciceri, Pietro; Wodicka, Lisa M.; Pallares, Gabriel; Hocker, Michael; Treiber, Daniel K.; Zarrinkar, Patrick P.
Nature Biotechnology (2011), 29(11), 1046-1051 | Language: English, Database: CAplus and MEDLINE

We tested the interaction of 72 kinase inhibitors with 442 kinases covering >80% of the human catalytic protein kinome. Our data show that, as a class, type II inhibitors are more selective than type I inhibi tors, but that there are important exceptions to this trend. The data further illustrate that selective inhibitors have been developed against the majority of kinases targeted by the compounds tested. Anal. of the interaction patterns reveals a class of 'group- selective' inhibitors broadly active against a single subfamily of kinases, but selective outside that subfamily. The data set suggests compounds to use as tools to study kinases for which no dedicated inhibitors exist. It also provides a foundation for further exploring kinase inhibitor biol. and toxicity, as well as for studying the structural basis of the observed interaction patterns. Our findings will help to realize the direct enabling potential of genomics for drug development and basic research about cellular signaling.

Keywords: protein kinase inhibitor drug development signaling

Substances (144) | Reactions (0) | Citing (1,284)

96

A Phase I study to assess the safety, pharmacokinetics and efficacy of barasertib (AZD1152), an Aurora B kinase inhibitor, in Japanese patients with advanced acute myeloid leukemia

By: Tsuboi, Kosuke; Yokozawa, Toshiya; Sakura, Toru; Watanabe, Takashi; Fujisawa, Shin; Yamauchi, Takahiro; Uike, Naokuni; Ando, Kiyoshi; Kihara, Rika; Tobinai, Kensei; et al
Leukemia Research (2011), 35(10), 1384-1389 | Language: English, Database: CAplus and MEDLINE

Barasertib (AZD1152) is a highly potent and selective Aurora B kinase inhibitor. The safety, efficacy and pharmaco kinetic (PK) profile of barasertib were investigated in Japanese patients with advanced acute myeloid leukemia. Barasertib (50- 1200 mg) was admini stered as a continuous 7- day i.v. infusion every 21 days. No dose- limiting toxicities were reported and barasertib 1200 mg was chosen for further evaluation in Japanese patients. Neutropenia and febrile neutropenia were the most commonly reported adverse events. The PK profile was similar to Western patients. A promising overall hematol. response rate of 19% was achieved, which warrants further investigation in these patients.

Keywords: barasertib acute myeloid leukemia pharmacokinetic antitumor Japanese

Substances (3) | Reactions (0) | Citing (41)

97

Docking study and three-dimensional quantitative structure-activity relationship (3D-QSAR) analyses and novel molecular design of a series of 4-aminoquinazolines as inhibitors of Aurora B kinase

By: Sun, Haopeng; Zhu, Jia; Chen, Yadong; Sun, Yuan; Zhi, Huijing; Li, Hao; You, Qidong
Chinese Journal of Chemistry (2011), 29(9), 1785-1799 | Language: English, Database: CAplus

The Aurora proteins are critical regulators of major mitotic events and attractive targets for anticancer therapy. 3D-QSAR studies based on mol. docking were performed on a dataset of 40 4-aminoquinazolines compounds The CoMSIA model produced significantly better results than CoMFA model, with $q^2 = 0.652$ and $r^2 = 0.991$. The contours anal. provides useful information about the structural requirements for 4- aminoquinazolines for inhibiting Aurora B. Scaffold hopping method was used to generate new structures based on the maximum common substructure of the training and test set compounds The ADMET property, binding affinity and inhibitory activity of the new designed compounds were predicted, resp. Finally 16 compounds were identified as the novel inhibitors for Aurora B kinase.

Keywords: aurora B kinase inhibitor 3D QSAR aminoquinazoline mol docking

Substances (42) | Reactions (0) | Citing (11)

98

Effects of AZD1152, a selective Aurora B kinase inhibitor, on Burkitt's and Hodgkin's lymphomas

By: Mori, Naoki; Ishikawa, Chie; Senba, Masachika; Kimura, Masashi; Okano, Yukio
Biochemical Pharmacology (2011), 81(9), 1106-1115 | Language: English, Database: CAplus and MEDLINE

We studied the effects of AZD1152, an Aurora B kinase inhibitor, on Burkitt's lymphoma (B L) and Hodgkin's lymphoma (HL) in human tissues and cell cultures and in a murine xenograft model of lymphoma. Aurora kinase A and B levels were assessed by RT-P CR and immunohistochem. They were aberrantly expressed in BL and HL cell lines, and in lymph nodes from patients with B L and HL. Next, activation of the Aurora B promoter was detected by reporter gene assays. The promoter activity of Aurora B kinase was high in BL cell lines and the Aurora B promoter contained a pos. regulatory region between - 74 and -104 from the transcription initiation site. AZD1152-hQPA had antiproliferative effects in the BL and HL cell lines studied; inhibited the phosphorylation of histone H3 and retinoblastoma proteins, and resulted in cells with >4 N D NA content. AZD1152-hQPA induced caspase-dependent apoptosis of some cell lines, demonstrated by loss of mitochondrial membrane potential, activation of caspase-9, followed by activation of caspase-3. This effect was accompanied by the inhibition of survivin expression. In vivo efficacy was determined in NO D/SCID/γc^{null} mice implanted with the Ramos human B L cell line. AZD1152 had anti-tumor effects in this murine xenograft model. There preclin. data suggest that the inhibition of Aurora B kinase is a potentially useful therapeutic strategy in BL and HL.

Keywords: Aurora B kinase inhibitor AZD1152 Burkitt's Hodgkin's lymphoma

Substances (7) Reactions (0) Citing (17)

99

Prediction of biological activity of Aurora-A kinase inhibitors by multilinear regression analysis and support vector machine

By: Yan, Aixia; Chong, Yang; Wang, Liyu; Hu, Xiaoying; Wang, Kai
Bioorganic & Medicinal Chemistry Letters (2011), 21(8), 2238-2243 | Language: English, Database: CAplus and MEDLINE

Several QSAR (quant. structure-activity relationships) models for predicting the inhibitory activity of 117 Aurora- A kinase inhibitors were developed. The whole dataset was split into a training set and a test set based on two different methods, (1) by a random selection; and (2) on the basis of a Kohonen's self-organizing map (SOM). Then the inhibitory activity of 117 Aurora- A kinase inhibitors was predicted using multilinear regression (MLR) anal. and support vector machine (S VM) methods, resp. For the two M LR models and the two SVM models, for the test sets, the correlation coefficients of over 0.92 were achieved.

Keywords: aurora protein kinase inhibitor multilinear regression analysis vector machine; drug design Q SAR aurora kinase inhibitor simulation modeling

Substances (116) Reactions (0) Citing (16)

100

Aurora B kinase inhibitor AZD1152: determinants of action and ability to enhance chemotherapeutics effectiveness in pancreatic and colon cancer

By: Azzariti, A.; Bocci, G.; Porcelli, L.; Fioravanti, A.; Sini, P.; Simone, G. M.; Quatrale, A. E.; Chiarappa, P.; Mangia, A.; Sebastian, S.; et al
British Journal of Cancer (2011), 104(5), 769-780 | Language: English, Database: CAplus and MEDLINE

Background: AZD1152, the prodrug for AZD1152-hydroxyquinazoline pyrazol anilide (HQPA), is a selective inhibitor of Aurora B kinase activity. Preclin. evaluation of AZD1152 has been reported in several human cancer models. The potent iality of this compound in combination therapy warrants further investigation in solid tumors. Exptl. design: This study explored the effects of A ZD1152-HQPA in colon and pancreatic tumor cells. The antitumor properties of A ZD1152, either as single agent or in combin ation with chemotherapeutics, were evaluated in each study model. The efficacy and the toxicity of A ZD1152 alone and in combination with gemcitabine were validated in pancreatic tumor xenograft model. Results: A ZD1152-HQPA treatment resulted in a dramatic increase of chromosome number, modification of cell cycle and induction of apoptosis. The most effective combin ation was that with chemotherapeutics given soon after AZD1152 in both tumor cell types. The effecti veness of the sequential schedule of AZ D1152 with gemcitabine was confirmed in nude mice bearing Mia PaCa-2 tumors, showing inhibition of tumor volumes and delaying of tumor growth after the interruption of the treatments. Conclusion: Here we show that A ZD1152-HQPA enhances oxaliplatin and gemcitabine effectiveness in colon and pancreatic cancer, resp. First, we provide advances into adminis tration schedules and dosing regimens for the combination treatment in in vivo pancreatic tumor. British Journal of Cancer (2011) 104, 769- 780; doi: 10.1038/bjc.2011.21 www.bjcancer.com Published online 8 Feb. 2011.

Keywords: aurora B kinase inhibitor AZD1152 antitumor pancreas colon cancer; combin ation chemotherapy AZD1152 gemcitabine oxaliplatin antitumor pancreas colon cancer

Substances (6) Reactions (0) Citing (49)

101

P-glycoprotein and breast cancer resistance protein in acute myeloid leukaemia cells treated with the aurora-B kinase inhibitor barasertib-hQPA

By: Grundy, Martin; Seedhouse, Claire; Russell, Nigel H.; Pallis, Monica
BMC Cancer (2011), 11, 254 | Language: English, Database: CAplus and MEDLINE

Background: Aurora kinases play an essential role in orchestrating chromosome alignment, segregation and cytokinesis during mitotic progression, with both aurora-A and B frequently over-expressed in a variety of human malignancies. Over-expression of the ABC drug transporter proteins P-glycoprotein (Pgp) and Breast cancer resistance protein (BCRP) is a major obstacle for chemotherapy in many tumor types with Pgp conferring particularly poor prognosis in acute myeloid leukemia (AML). Barasertib-hQPA is a highly selective inhibitor of aurora-B kinase that has shown tumoricidal activity against a range tumor cell lines including those of leukemic AML origin. Methods: Effect of barasertib-hQPA on the pHH3 biomarker and cell viability was measured in a panel of leukemic cell lines and 37 primary AML samples by flow cytometry. Pgp status was determined by flow cytometry and BCRP status by flow cytometry and real-time PCR. Results: In this study we report the creation of the cell line OCI-AML3DNR, which over-expresses Pgp but not BCRP or multidrug resistance-associated protein (MRP), through prolonged treatment of OCI-AML3 cells with daunorubicin. We demonstrate that Pgp (OCI-AML3DNR and KG-1a) and BCRP (OCI-AML6.2) expressing AML cell lines are less sensitive to barasertib-hQPA induced pHH3 inhibition and subsequent loss of viability compared to transporter neg. cell lines. We also show that barasertib-hQPA resistance in these cell lines can be reversed using known Pgp and BCRP inhibitors. We report that barasertib-hQPA is not an inhibitor of Pgp or BCRP, but by using ^{14}[C]-barasertib-hQPA that it is effluxed by these transporters. Using phosphoHistone H3 (pHH3) as a biomarker of barasertib-hQPA responsiveness in primary AML blasts we determined that Pgp and BCRP pos. primary samples were less sensitive to barasertib-hQPA induced pHH3 inhibition ($p \le 0.001$) than samples without these transporters. However, we demonstrate that IC_{50} inhibition of pHH3 by barasertib-hQPA was achieved in 94.6% of these samples after 1 h drug treatment, in contrast to the resistance of the cell lines. Conclusion: We conclude that Pgp and BCRP status and pHH3 down-regulation in patients treated with barasertib should be monitored in order to establish whether transporter-mediated efflux is sufficient to adversely impact on the efficacy of the agent.

Keywords: anticancer Aurora B kinase inhibitor barasertib hQPA Pgp BCRP; acute myeloid leukemia

Substances (3) | Reactions (0) | Citing (14)

102

A theoretical entropy score as a single value to express inhibitor selectivity

By: Uitdehaag, Joost C. M.; Zaman, Guido J. R.
BMC Bioinformatics (2011), 12, 94 | Language: English, Database: CAplus and MEDLINE

Designing maximally selective ligands that act on individual targets is the dominant paradigm in drug discovery. Poor selectivity can underlie toxicity and side effects in the clinic, and for this reason compound selectivity is increasingly monitored from very early on in the drug discovery process. To make sense of large amounts of profiling data, and to determine when a compound is sufficiently selective, there is a need for a proper quant. measure of selectivity. Here we propose a new theor. entropy score that can be calculated from a set of IC_{50} data. In contrast to previous measures such as the 'selectivity score', Gini score, or partition index, the entropy score is non-arbitary, fully exploits IC_{50} data, and is not dependent on a reference kinase. In addition, the entropy score gives the most robust values with data from different sources, because it is less sensitive to errors. We apply the new score to kinase and nuclear receptor profiling data, and to high-throughput screening data. In addition, through analyzing profiles of clin. compounds, we show that a more selective inhibitor is not necessarily more drug-like. For quantifying selectivity from panel profiling, a theor. entropy score is the best method. It is valuable for studying the mol. mechanisms of selectivity, and to steer compound progression in drug discovery programs.

Keywords: theor entropy score allosteric inhibitor selectivity

Substances (42) | Reactions (0) | Citing (30)

103

Inhibition effect of AZD1152-HQPA, specific inhibitor of Aurora-B Kinase, on human Osteosarcoma cell line U2-OS cells

By: Liu, Zhi-li; Wang, Gao; Shu, Yong; Zou, Ping-an; Zhou, Yang; Huang, Shan-hu
Nanchang Daxue Xuebao, Yixueban (2010), 50(10), 30-32, 36 | Language: Chinese, Database: CAplus

The objective of this study was to explore the Aurora-B kinase inhibition effect A ZD1152-HQPA specific inhibitor on human osteos arcoma cell line U2-OS cells. The human osteos arcoma cell line U2-OS cells were treated with A ZD1152-HQPA at various concent ration (0, 10, 50, 100, 500 nmol·L^{-1}) in vitro for various time (24, 48,72 h). MTT assay was used to measure the inhibition effect of A Z D1152-HQPA on human osteos arcoma cell line U2-OS cells. The expression of Aurora- B kinase before and after of A ZD1152-HQPA on human osteosarcoma cell line U2-OS cells were detected by Western Blotting. The results showed that the cells growth of human osteosarcoma cell line U2-OS were inhibited effetely by A ZD1152-HQPA, showing the inhibition in time-and concentration-dependent manner. The expression of Aurora- B kinase in human osteos arcoma cell line U2-OS cells, treated with A ZD1152-HQPA 50 nmol·L^{-1} for 24,48,72 h, was significantly reduced and the level of expression was lower as the time increased. It was concluded that the cells proliferation of U2-OS were inhibited by the selective Aurora- B kinase inhibitor AZD1152-HQPA in a time- and concent ration-dependent manner.

Keywords: osteosarcoma Aurora B kinase inhibitor AZD1152 HQPA

Substances (2) | Reactions (0) | Citing (0)

104

Aurora B is dispensable for megakaryocyte polyploidization, but contributes to the endomitotic process

By: Lordier, Larissa; Chang, Yunhua; Jalil, Abdelali; Aurade, Frederic; Garcon, Loic; Lecluse, Yann; Larbret, Frederic; Kawashima, Toshiyuki; Kitamura, Toshio; Larghero, Jerome; et al
Blood (2010), 116(13), 2345-2355 | Language: English, Database: CAplus and MEDLINE

Polyploidization of megakaryocytes (MKs), the platelet precursors, occurs by endomi tosis, a mitotic process that fails at late stages of cytokinesis. Expression and function of Aurora B kinase during endomi tosis remain controversial. Here, we report that Aurora B is normally expressed during the human MK endomitotic process. Aurora B localized normally in the midzone or midbody during anaphase and telophase in low ploidy megakaryocytes and in up to 16N rare endomitotic MKs was observed Aurora B was also functional during cytokinesis as attested by phosphor ylation of both its activation site and Mgc RacGAP, its main substrate. However, despite its activation, Aurora B did not prevent furrow regres sion. Inhibition of Aurora B by A ZD1152-HQPA decreased cell cycle entry both in 2N to 4N and polyploid MKs and induced apoptosis mainly in 2N to 4N cells. In both M K classes, AZD1152-HQPA induced p53 activation and retinoblastoma hypophosphorylation. Resistance of polyploid MKs to apoptosis correlated to a high Bclx L level. Aurora B inhibition did not impair M K polyploi dization but profoundly modified the endomitotic process by inducing a mis-segregation of chromosomes and a mitotic failure in anaphase. This indicates that Aurora B is dispen sable for MK polyploi dization but is necessary to achieve a normal endomitotic process.

Keywords: aurora endomitosis megakaryocyte polyploidization

Substances (4) | Reactions (0) | Citing (36)

105

Preparation of novel prodrug compounds containing a kinase inhibitor and a reductively-activated fragmenting aromatic nitroheterocycle or aromatic nitrocarbocycle trigger and their use as anti-proliferative agents

By: Smaill, Jeffrey Bruce; Patterson, Adam Vorn; Denny, William Alexander; Wilson, William Robert; Lu, Guo-Liang; Anderson, Robert Forbes; Lee, Ho Huat; Ashoorzaden, Amir

World Intellectual Property Organization, WO2010104406 A1 2010-09-16 | Language: English, Database: CAplus

The invention provides novel prodrug compounds comprising a kinase inhibitor and a reductively-activated fragmenting aromatic nitroheterocycle or aromatic nitrocarbocycle trigger, where the compound carries a pos. charge. Title compounds **I** [where X = neg. charged counterion; R^1 = group of the formula -$(CH_2)_n$Tr, wherein Tr = an aromatic nitroheterocycle or aromatic nitrocarbocycle and -$(CH_2)_n$Tr acts as a reductively-activated fragmenting trigger, and n = 0-6; R^2, R^3, and R^4 independently = aliphatic or aromatic groups of a tertiary amine kinase inhibitor, $(R^2)(R^3)(R^4)$N; or two of R^2, R^3, and R^4 may form an aliphatic or aromatic heterocyclic amine ring of a kinase inhibitor; or one of R^2, R^3, and R^4 may be absent and two of R^2, R^3, and R^4 form an aromatic heterocyclic amine ring of a kinase inhibitor] are claimed. For example, compound **II** was prepared via alkylation of (2E)-N-[4-(3-bromoanilino)-6-quinazolinyl]-4-(dimethylamino)-2-butenamide with 2-nitrobenzyl bromide. Select **I** were assayed for their ability to inhibit erbB1 tyrosine kinase and compound **II** was found to possess an IC_{50} value of 0.20 nM. The compounds of the invention are useful in treating proliferative diseases such as cancer.

Keywords: alkylammonium bromide derivative preparation kinase inhibitor antiproliferative agent; alkyl ammonium trifluoroacetate derivative preparation kinase inhibitor treatment cancer

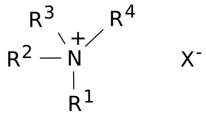

I

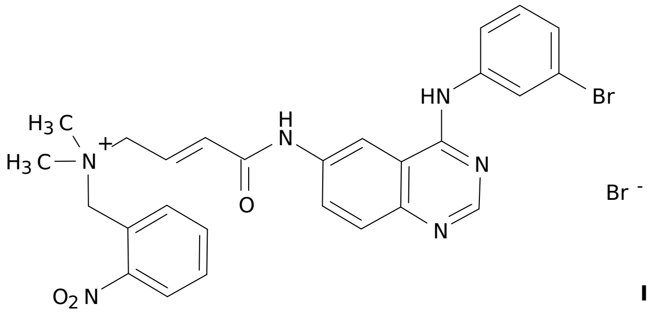

II

PatentPak available

Substances (187) Reactions (534) Citing (7)

106

Markers to predict and monitor response to Aurora kinase B inhibitor therapy of cancer

By: Semizarov, Dimitri; Shah, Jameel; Guo, Jun; Anderson, Mark
World Intellectual Property Organization, WO2010088470 A1 2010-08-05 | Language: English, Database: CAplus

The present invention relates to diagnostic assays useful in classification of patients for selection of cancer therapy with one or more Aurora kinase B inhibitors. In particular, the present invention relates to identifying the presence or absence of one or more copy number gains in the ABCB1 gene, the ABCB4 gene or combinations thereof, identifying patients eligible to receive Aurora kinase inhibitor therapy, either as monotherapy or as part of combination therapy, and monitoring patient response to such therapy.

Keywords: cancer therapy Aurora kinase B inhibitor biol marker

PatentPak available

Substances (327) | Reactions (0) | Citing (1)

107

Analysis of Kinase Inhibitor Selectivity using a Thermodynamics-Based Partition Index

By: Cheng, Alan C.; Eksterowicz, John; Geuns-Meyer, Stephanie; Sun, Yaxiong
Journal of Medicinal Chemistry (2010), 53(11), 4502-4510 | Language: English, Database: CAplus and MEDLINE

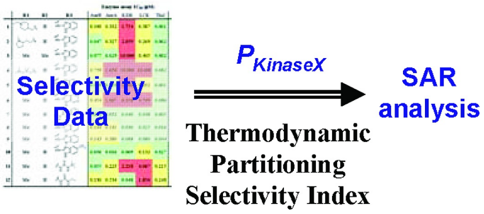

In the quest for safe, efficacious kinase inhibitors as drugs, selectivity is often assessed early using kinase profiling panels. Here we present a selectivity index based on thermodn. principles that can help in anal. of the resulting data. The "parti tion" selectivity index is easy to calculate and is applicable in certain situations where other widely used indexes are not. It is uniquely useful in anal. of small, focused selectivity panel data frequently encountered in medicinal chem. hit-to-lead and lead optimization. For larger "kinome" panels, the partition index allows assessment of selectivity relative to a kinase or multiple kinases of interest.

Keywords: thermodn partition index kinase inhibitor selectivity

Substances (55) | Reactions (0) | Citing (43)

108

The FLT3 Internal Tandem Duplication Mutation Is a Secondary Target of the Aurora B Kinase Inhibitor AZD1152-HQPA in Acute Myelogenous Leukemia Cells

By: Grundy, Martin; Seedhouse, Claire; Shang, Shilli; Richardson, Jaineeta; Russell, Nigel; Pallis, Monica
Molecular Cancer Therapeutics (2010), 9(3), 661-672 | Language: English, Database: CAplus and MEDLINE

Aurora kinases play an essential role in orchestrating chromosome alignment, segregation, and cytokinesis during mitotic progre ssion and both aurora- A and B are frequently overexp ressed in a variety of human maligna ncies. In this study, we report the effects of AZD1152-HQPA, a highly selective inhibitor of aurora- B kinase, in acute myeloid leukemia (A ML) cell lines and primary samples. We show that AZD1152-HQPA inhibits the phosphorylation of Histone H3 (pHH3) on serine 10 resulting in polyploid cells, apoptosis, and loss of viability in a panel of AML cell lines. We also show that A ZD1152-HQPA sensitivity in our cell lines is irresp. of p53 status and the FLT3-ITD-expressing MOLM-13 and MV4-11 cell lines are particularly sensitive to AZD1152-HQPA. Internal tandem duplic ations (ITD) within the FLT3 tyrosine kinase receptor are found in ~25% of A ML patients and are associated with a poor prognosis. Here, we report that AZD1152-HQPA directly targets phospho rylated FLT3 along with inhibiting its downstream target phospho-signal transducer and activator of transcr iption 5 (STAT5) in the FLT3-ITD cell lines. We show p HH3 expression in primary AML blasts and its inhibition by AZD1152-HQPA at low doses in all of our primary samples tested. A ZD1152-HQPA inhibits the clonogenic potential of primary AML samples, with FLT3-ITD samples being the most sensitive (P = 0.029) . FLT3-ITD primary samples are also more sensitive to pHH3 inhibition (P = 0.022) and are partic ularly sensitive to pSTAT5 downregulation after treatment with AZD1152-HQPA compared with FLT3 wild-type samples (P = 0.007). We conclude that mutant FLT3 is a secondary target of AZD1152-HQPA and that FLT3-ITD primary samples are particularly sensitive to the drug. Mol Cancer Ther; 9 (3); 661-72.

Keywords: FLT3 tandem duplication mutation aurora kinase AZD1152 HQPA leukemia; myelogenous leukemia antitumor AZD1152 H QPA FLT3 tandem duplication mutation

Substances (2) Reactions (0) Citing (32)

109

Structural studies of B-type Aurora kinase inhibitors using computational methods

By: Neaz, M. M.; Muddassar, M.; Pasha, F. A.; Cho, Seung Joo
Acta Pharmacologica Sinica (2010), 31(2), 244-258 | Language: English, Database: CAplus and MEDLINE

The aim of the study was to characterize the structural features of quinazoline-based Aurora B inhibitors that influence its inhibitor activity. Two geometrical methods, Method 1 and Method 2, were used to develop the 3 D-QSAR models. The most active ligand was used as the template for the alignment of all the ligands in Method 1, and a conformer of the cocrystal ligand was used as the template for the alignment of all the ligands in Method 2. The models suggest that highly active ligands can be designed by varying the R1 substituent at position 7 of the quinaz oline ring with pos. charged, bulky, hydrop hobic groups, while bulky and hydrop hobic groups around the thiazole ring are desirable for higher activity. This study emphasizes that the bioactive conformer is rather different from the min. The steric, electrostatic, and hydrophobic field effects contribute to its inhibitory activity.

Keywords: Aurora B kinase inhibitor QSAR drug design

Substances (46) Reactions (0) Citing (11)

110

The selective Aurora B kinase inhibitor AZD1152 as a novel treatment for hepatocellular carcinoma

By: Aihara, Arihiro; Tanaka, Shinji; Yasen, Mahmut; Matsumura, Satoshi; Mitsunori, Yusuke; Murakata, Ayano; Noguchi, Norio; Kudo, Atsushi; Nakamura, Noriaki; Ito, Koji; et al
Journal of Hepatology (2010), 52(1), 63-71 | Language: English, Database: CAplus and MEDLINE

Background & Aims: We previously identified that high Aurora B expression was associated with hepatocellular carcinoma (HCC) recurrence due to tumor dissemination. In this preclin. study, a novel inhibitor of Aurora B kinase was evaluated as a treatment for human HCC. Methods: AZD1152 is a selective inhibitor of Aurora B kinase. Twelve human H CC cell lines were analyzed for Aurora B kinase expression and the in vitro effects of AZD1152. The in vivo effects of AZD1152 were analyzed in a s.c. xenograft model and a novel orthotopic liver xenograft model. Results: Aurora B kinase expression varied among the human HCC cell lines and was found to correlate with inhibition of cell proliferation, accumulation of 4N DNA, and the proportion of polyploid cells following adminis tration of AZD1152-hydroxyquinazoline-pyrazol-anilide (AZD1152-HQPA). AZD1152-HQPA suppressed histone H3 phosphor ylation and induced cell death in a dose-dependent manner. Growth of s.c. human H CC xenografts was inhibited by A ZD1152 administ ration. In an orthotopic hepatoma model, treatment with A ZD1152 significantly decelerated tumor growth and increased survival. Pharmacobiol. anal. revealed that AZD1152 induced the rapid suppression of phosphohistone H3, followed by cellular apoptosis in the liver tumors but not in the normal tissues of the orthotopic models. Conclusions: Our preclin. studies indicate that AZD1152 is a promising novel therapeutic approach for the treatment of H CC.

Keywords: anticancer AZD1152 apoptosis phosphohistone H3 hepatocellular carcinoma growth

Substances (4) | Reactions (0) | Citing (53)

111

p53 is critical for the Aurora B kinase inhibitor-mediated apoptosis in acute myelogenous leukemia cells

By: Ikezoe, Takayuki; Yang, Jing; Nishioka, Chie; Yokoyama, Akihito
International Journal of Hematology (2010), 91(1), 69-77 | Language: English, Database: CAplus and MEDLINE

We previously showed that AZD1152-HQPA, the inhibitor of Aurora B kinase potently induced growth arrest and apoptosis of various types of human leukemia cells including MV4-11 acute myelogenous leukemia (AML) cells, although the mol. mechanisms by which this class of kinase inhibitors induces apoptosis remain to be fully elucidated. We have recently established the MV4-11 subline, designated as MV4-11 TP53 R248W, which possesses transcriptionally inactive R248W mutation in the TP53 gene. MV4-11 T P53 R248W cells were relatively resistant to A ZD1152-HQPA-mediated growth arrest, as measured by MTT and clonogenic assays. A ZD1152-HQPA (10-100 nM, 48 h) strikingly induced apoptosis of M V4-11 cells, as assessed by Annexin V binding, loss of mitocho ndrial outer membrane potential, and activation of caspase cascade, in parallel with up- regulation of p53 and its target mols. Bax and Noxa. Notably, AZD1152-HQPA (10-100 nM, 48 h) induced polyploidy rather than apoptosis in M V4-11 TP53 R248W cells. The polyploid cells were eventually eliminated via apoptosis at later time period (72-120 h) in association with up-regulation of p73. Taken together, p53 plays an important role in AZD1152-HQPA-induced growth arrest and early onset of apoptosis in A ML cells. P73 may mediate the late onset of apoptosis to eliminate the polyploid cells caused by the inhibitor of Aurora B kinase.

Keywords: p53 Aurora B kinase apoptosis acute myelogenous leukemia

Substances (4) | Reactions (0) | Citing (25)

112

Antineoplastic effects of an Aurora B kinase inhibitor in breast cancer

By: Gully, Christopher P.; Zhang, Fanmao; Chen, Jian; Yeung, James A.; Velazquez-Torres, Guermarie; Wang, Edward; Yeung, Sai-Ching Jim; Lee, Mong-Hong
Molecular Cancer (2010), 9, No pp. given | Language: English, Database: CAplus and MEDLINE

Background: Aurora B kinase is an important mitotic kinase involved in chromosome segreg ation and cytokinesis. It is overexp ressed in many cancers and thus may be an important mol. target for chemoth erapy. AZD1152 is the prodrug for AZD1152-HQPA, which is a selective inhibitor of Aurora B kinase activity. Preclin. antineoplastic activity of AZD1152 against acute myelog enous leukemia, multiple myeloma and colorectal cancer has been reported. However, this compound has not been evaluated in breast cancer, the second leading cause of cancer deaths among women. Results: The antineoplastic activity of AZD1152-HQPA in six human breast cancer cell lines, three of which overexpress HER2, is demonstrated. AZD1152-HQPA specifically inhibited Aurora B kinase activity in breast cancer cells, thereby causing mitotic catastrophe, polyploidy and apoptosis, which in turn led to apoptotic death. AZD1152 administration efficiently suppressed the tumor growth in a breast cancer cell xenograft model. In addition, A Z D1152 also inhibited pulmonary metastatic nodule formation in a metastatic breast cancer model. Notably, it was also found that the protein level of Aurora B kinase declined after inhibition of Aurora B kinase activity by AZD1152-HQPA in a time- and dose-dependent manner. Investigation of the underlying mechanism suggested that A ZD1152-HQPA accelerated protein turnover of Aurora B via enhancing its ubiquitination. Conclusions: It was shown that A ZD1152 is an effective antineoplastic agent for breast cancer, and our results define a novel mechanism for posttranscriptional regulation of Aurora B after A ZD1152 treatment and provide insight into dosing regimen design for this kinase inhibitor in metastatic breast cancer treatment.

Keywords: antitumor AZD1152 hydroxyquinazoline pyrazol anilide Aurora kinase breast cancer

Substances (6) Reactions (0) Citing (72)

113

Significance of Aurora B overexpression in hepatocellular carcinoma

By: Lin, Zhong-Zhe; Jeng, Yung-Ming; Hu, Fu-Chang; Pan, Hung-Wei; Tsao, Hsin-Wei; Lai, Po-Lin; Lee, Po-Huang; Cheng, Ann-Lii; Hsu, Hey-Chi
BMC Cancer (2010), 10, No pp. given | Language: English, Database: CAplus and MEDLINE

Background: To investigate the significance of Aurora B expression in hepatoc ellular carcinoma (HCC). Methods: The Aurora B and Aurora A mRNA level was measured in 160 H CCs and the paired nontum orous liver tissues by reverse transcr iption-polymerase chain reaction. Mutations of the p53 and β-catenin genes were analyzed in 134 and 150 tumors, resp., by direct sequencing of exon 2 to exon 11 of p53 and exon 3 of β-catenin. Anticancer effects of AZD1152-HQPA, an Aurora B kinase selective inhibitor, were examined in Huh-7 and Hep3B cell lines. Results: Aurora B was overexp ressed in 98 (61%) of 160 H CCs and in all 7 H CC cell lines examined The overexpression of Aurora B was associated with Aurora A overexp ression (P = 0.0003) and p53 mutation (P = 0.002) and was inversely associated with β-catenin mutation (P = 0.002) . Aurora B overexp ression correlated with worse clinico pathol. characteristics. Multivariate anal. confirmed that Aurora B overexp ression was an independent poor prognostic factor, despite its interaction with Aurora A overexp ression and mutations of p53 and β- catenin. In Huh-7 and Hep3B cells, AZD1152-HQPA induced proliferation blockade, histone H3 (Ser10) dephospho rylation, cell cycle distur bance, and apoptosis. Conclusion: Aurora B overexp ression is an independent mol. marker predicting tumor invasi veness and poor prognosis of H CC. Aurora B kinase selective inhibitors are potential therapeutic agents for HCC treatment.

Keywords: hepatocellular carcinoma aurora B gene mutation

Substances (3) Reactions (0) Citing (99)

114

Analysis of Aurora B kinase in non-Hodgkin lymphoma

By: Ikezoe, Takayuki; Takeuchi, Tamotsu; Yang, Jing; Adachi, Yoshihiro; Nishioka, Chie; Furihata, Mutsuo; Koeffler, H. Phillip; Yokoyama, Akihito
Laboratory Investigation (2009), 89(12), 1364-1373 | Language: English, Database: CAplus and MEDLINE

This study explored the levels of Aurora B, a key regulator of mitosis, in 71 lymph nodes and tumor specimens excised operatively from individuals with various types of non-Hodgkin lymphoma (NHLs). Immunohistochem. examination found that diffuse large B-cell lymphoma (10/21, 48%) and Burkitt lymphoma (B L) (6/7, 86%) cells highly (percentage of pos. cells, >20%) expressed Aurora B in their nuclei. On the other hand, none of the low-grade B-cell lymphoma (n=20), except for one case of follicular lymphoma, highly expressed this protein kinase, suggesting that levels of Aurora B correlated with histol. grade in B-cell NHLs (P<0.01). Exposure of B L/leukemia cells to AZD1152-HQPA in vitro, a selective inhibitor of Aurora B kinase, potently induced growth arrest and apoptosis in a caspase-dependent, as well as -independent manner. Moreover, AZD1152 synergistically enhanced the effects of vincristine (VCR) to induce growth arrest of these cells. Further experiments found that VCR increased levels of the p-Aurora B through the activation of c-Jun N-terminal kinase, which was blocked in the presence of AZD1152-HQPA.

Keywords: aurora B kinase non Hodgkin lymphoma

Substances (7) | Reactions (0) | Citing (33)

115

Simultaneous determination of AZD1152 (prodrug) and AZD1152-hydroxyquinazoline pyrazol anilide by reversed phase liquid chromatography

By: Pluim, Dick; Beijnen, Jos H.; Schellens, Jan H. M.; van Tellingen, Olaf
Journal of Chromatography B: Analytical Technologies in the Biomedical and Life Sciences (2009), 877(29), 3549-3555 | Language: English, Database: CAplus and MEDLINE
Analytical Methods available

A simple, selective and sensitive reversed phase liquid chromatog. method utilizing UV detection has been developed and validated for the simultaneous determination of the prodrug AZD1152 and its active product AZD1152-hydroxyquinazoline pyrazol anilide (hQ PA) in human and mouse plasma and mouse tissues. Isocratic separation was achieved using an 5 μm Upti Sphere HDO C_{18} column (150 mm × 4.6 mm) with guard column in combination with a mobile phase comprised of phosphate buffered water (50 m M; pH 3.4) and acetonitrile (81.5: 18.5; volume/volume). UV detection at 318 nm was used. Sample preparation involved a single-step protein precipitation with ethanol. Ex vivo conversion of AZD1152 by endogenous phosphatases was prevented by immediate cooling of the samples in ice-water and addition of sodium fluoride and EDTA. The validation parameters included specificity, recovery, accuracy, precision, sensitivity and stability. The lower limit of quantification in human plasma for AZD1152 and hQPA was 25 ng/mL. The applicability of the method was demonstrated by successful determination of AZD1152 and hQPA in human plasma and in plasma, brain, liver, kidney and ileum samples from mice dosed with AZD1152.

Keywords: AZD1152 pharmacokinetics tissue blood analysis HPLC

Substances (3) | Reactions (0) | Citing (7)

116

Method using a combination of an aurora kinase inhibitor and a TRAIL inhibitor for the treatment of glioblastoma

By: Glaser, Keith B.; Shah, Omar Jameel
United States, US20090203671 A1 2009-08-13 | Language: English, Database: CAplus

Methods of treating cancer in patients comprising administering thereto aurora kinase inhibitors and T RAIL (tumor necrosis factor (TNF)-related apoptosis inducing ligand) inhibitors is disclosed. The invention discloses methods for treating cancer in patients, comprising administering thereto aurora kinase inhibitors and T RAIL (tumor necrosis factor (T NF)-related apoptosis inducing ligand) inhibitors. To identify candidate apoptosis-sensitizing genes that could be exploited in combin ation with Aurora kinase inhibitors in malignant glioma, global gene expression anal. in a D54MG glioma cell derivative treated with three Aurora kinases inhibitors chosen for their distinctive selectivities was carried out namely with M LN8054 [[4-[9-chloro-7-(2,6-dif-luorophenyl)-5H-pyrimidol [5, 4-d][2]benzazepin-2-yl]amino]benzoic acid, Aurora-A-selective], AZD 1152-HQPA (Aurora-B-selective), and VXX-680 (Aurora-A/B). This anal. identified the TRAIL death receptor, DR5, as an apoptosis sensitizing gene induced selectively following inhibition of Aurora- B kinase cancer cells. In glioma cell lines where DR5 was induced following polyploidization, the sensitivity, kinetics, and magnitude of TRAIL-mediated apoptosis were enhanced. These data shed light on the apoptotic program induced during polyploi dization and suggest that the combination of TRAIL and inhibitors of Aurora B kinase can select ively enhance TRAIL-induced apoptosis and antiproliferative effects through the upregulation of DR5, the TRAIL death receptor.

Keywords: aurora kinase TRAIL inhibitor combination chemotherapy glioblastoma cancer

PatentPak available

Substances (6) | Reactions (0) | Citing (1)

117

Method using a combination of an aurora kinase inhibitor and a TRAIL inhibitor for the treatment of cancer

By: Glaser, Keith B.; Shah, Omar Jameel
World Intellectual Property Organization, WO2009070652 A1 2009-06-04 | Language: English, Database: CAplus

The invention discloses methods for treating cancer in patients, comprising administering thereto aurora kinase inhibitors and T RAI L (tumor necrosis factor (T NF)-related apoptosis inducing ligand) inhibi tors.

Keywords: aurora kinase TRAIL inhibitor combination caner treatment

PatentPak available

Substances (6) | Reactions (0) | Citing (1)

118

Pharmacological inhibition of Aurora-A but not Aurora-B impairs interphase microtubule dynamics

By: Lorenzo, Corinne; Liao, Qiaoyin; Hardwicke, Mary Ann; Ducommun, Bernard
Cell Cycle (2009), 8(11), 1733-1737 | Language: English, Database: CAplus and MEDLINE

Aurora kinases are key cell cycle regulators and represent attractive new targets in cancer therapy. In this work we investigated the effect of specific inhibition of Aurora-A and Aurora-B on interphase microtubule dynamics using the GSK6000063A and AZD1152 H QPA compounds resp. We show that Aurora- A inhibition results in microt ubule network disorganization and bundling. Using video microscopy and laser-based photo ablation we demonstrate that Aurora-A inhibition decreases microt ubule shrinkage, growth rate, frequency rescue and nucleation. These results open new perspectives on the role of Aurora- A in interphase and might be worth considering in a pharmacol. perspe ctive.

Keywords: GSK6000063A AZD1152 HQPA Aurora A kinase interphase microt ubule antitumor

Substances (4) Reactions (0) Citing (16)

119

AZD1152 Rapidly and Negatively Affects the Growth and Survival of Human Acute Myeloid Leukemia Cells In vitro and In vivo

By: Oke, Adedayo; Pearce, Daniel; Wilkinson, Robert W.; Crafter, Claire; Odedra, Rajesh; Cavenagh, Jamie; Fitzgibbon, Jude; Lister, Andrew T.; Joel, Simon; Bonnet, Dominique
Cancer Research (2009), 69(10), 4150-4158 | Language: English, Database: CAplus and MEDLINE

Aurora kinases play a critical role in regulating mitosis and cell division, and their overexpression has been implicated in the survival and proliferation of human cancer. In this study, we report the in vitro and in vivo activities of A ZD1152, a compound that has select ivity for aurora B kinase, in acute myeloid leukemia (A ML) cell lines, primary AML samples, and cord blood cells. A ZD1152 exerted antiproliferative or cytotoxic effects in all cell lines studied, inhibited the phosphor ylation of histone H3 (p His H3) on Ser10 in a dose-dependent manner, and resulted in cells with >4 N DNA content. THP-1 cells treated with AZD1152 accumulated in a state of polyploidy and showed a senescent response to the drug, in contrast to the apoptotic response seen in other cell lines. Accordingly, AZD1152 profoundly affected the growth of AML cell lines and primary AML in an in vivo xenotransplantation model. However, concentration-dependent effects on cell growth, apoptosis, and cell cycle progre ssion were also observed when human cord blood and primary lineage-neg. stem and progenitor cells were analyzed in vitro and in vivo. These data suggest that the inhibition of aurora B kinase may be a useful therapeutic strategy in the treatment of A ML and that further exploration of dosing and treatment schedules is warranted in clin. trials. [Cancer Res 2009;69(10):4150-8].

Keywords: AZD1152 antitumor prodrug aurora B kinase acute myeloid leukemia

Substances (3) Reactions (0) Citing (62)

120

Identification of genes that confer tumor cell resistance to the Aurora B kinase inhibitor, AZD1152

By: Guo, J.; Anderson, M. G.; Tapang, P.; Palma, J. P.; Rodriguez, L. E.; Niquette, A.; Li, J.; Bouska, J. J.; Wang, G.; Semizarov, D.; et al
Pharmacogenomics Journal (2009), 9(2), 90-102 | Language: English, Database: CAplus and MEDLINE

AZD1152 is a highly selective Aurora B kinase inhibitor currently undergoing Phase I and I I clin. evaluation in patients with acute myelogenous leukemia and advanced solid maligna ncies. We have established two AZD1152-resistant cell lines from S W620 colon and MiaPaCa pancreatic carcinoma lines, which are >100- fold resistant to the active metabolite of A ZD1152, AZD1152 HQPA and interestingly, cross-resistant to the pan-Aurora kinase inhibitor, VX-680/MK0457. Using whole-genome microarray anal. and compar ative genomic hybridi zation, we were able to identify M DR1 and B CRP as the causative genes that underlie A ZD1152 HQPA-resistance in these models. Furthe rmore, the upregulation of either of these genes is sufficient to render in vivo tumor growth insensitive to AZD1152. Finally, the upregu lation of M DR1 or B CRP is predictive of tumor cell sensit ivity to this agent, both in vitro and in vivo. The data provide a genetic basis for resistance to Aurora kinase inhibitors, which could be utilized to predict clin. response to therapy.

Keywords: AZD1152 antitumor resistance Aurora B kinase M DR1 BCRP

Substances (4) Reactions (0) Citing (32)

121

The Topoisomerase I Poison CPT-11 Enhances the Effect of the Aurora B Kinase Inhibitor AZD1152 both In vitro and In vivo

By: Nair, Jayasree S.; de Stanchina, Elisa; Schwartz, Gary K.
Clinical Cancer Research (2009), 15(6), 2022-2030 | Language: English, Database: CAplus and MEDLINE

AZD1152 is an Aurora B kinase inhibitor currently in clin. trials. As the topoiso merase I poison C PT-11 induces a G_2 arrest, a mechan istic understanding of the cell cycle intera ctions between these agents may prove critical for combin ation therapy. AZD1152 was tested in vitro and in vivo with SN-38 and CPT-11 against HCT-116 cells. Inhibition of clonoge nicity, induction of apoptosis, effects on polyploidy, and tumor growth were examined A ZD1152 alone induced polyploidy of HCT-116 cells at low nanomolar concent rations The induction of apoptosis required prolonged exposure (48 h) and higher concent rations of drug. When S N-38 was given before or concomitantly with AZD1152, SN-38 blocked the AZD1152 effect by arresting cells in G_2 and inhibiting cells from undergoing polyploidy. With the reverse combin ation (AZD1152 followed by S N-38), there was a signif icant induction of polyploidy and apoptosis, even with shorter exposure (24 h) of AZD1152. In vivo, AZD1152 alone suppressed HCT-116 xenograft tumor growth in a dose-dependent manner with target inhibition of phospho H3, induction of multinu cleated giant cells, but without induction of apoptosis. In combination, both sequences in vivo (C PT->AZD, AZD->CPT, P = 0.008, A UC/d) proved superior to either single agent therapy. However, AZD->CPT still showed a greater increase in apoptosis and greater suppre ssion of tumor regrowth than C PT->AZ D (P = 0.02, A UC/d). The results from these studies indicate a promising therap eutic strategy for combining A ZD1152 with CPT-11, and suggest that the sequence of drug administration is pivotal when an Aurora B kinase inhibitor is admini stered with a topoiso merase I poison.

Keywords: AZD1152 CPT11 combin ation chemotherapy antitumor

Substances (7) Reactions (0) Citing (31)

122

A specific pharmacophore model of aurora B kinase inhibitors and virtual screening studies based on it

By: Wang, Hui-Yuan; Li, Lin-Li; Cao, Zhi-Xing; Luo, Shi-Dong; Wei, Yu-Quan; Yang, Sheng-Yong
Chemical Biology & Drug Design (2009), 73(1), 115-126 | Language: English, Database: CAplus and MEDLINE

In this study, 3D-pharmacophore models of Aurora B kinase inhibitors have been developed by using Hip Hop and HypoGen modules in Catalyst software package. The best pharmacophore model, Hypo1, which has the highest correl ation coefficient (0.9911), consists of one hydrogen-bond acceptor, one hydrogen-bond donor, one hydrophobic aliphatic moiety and one ring aromatic feature. Hypo1 was validated by test set and cross-validation methods. Specificity of Hypo1 to Aurora B inhibitors was examined with the use of selective inhibitors against Aurora B and its paralogue Aurora A. The results clearly indicate that Hypo1 can differentiate selective inhibitors of Aurora B from those of Aurora A, and the ring aromatic feature likely plays some important roles for the specificity of Hypo1. Then Hypo1 was used as a 3 D query to screen several databases including Specs, N CI, Maybridge and Chinese Nature Product Database (CNPD) for identifying new inhibitors of Aurora B. The hit compounds were subseq uently subjected to filtering by Lipinski's rule of five and docking studies to refine the retrieved hits, and some compounds selected from the top ranked hits have been suggested for further exptl. assay studies.

Keywords: aurora B kinase inhibitor virtual screening pharmacophore model

Substances (70) Reactions (0) Citing (13)

123

Discovery or AZD1152: a selective inhibitor of Aurora-B kinase with potent antitumor activity

By: Foote, Kevin M.; Mortlock, Andrew A.
Edited by Li, Rongshi; Stafford, Jeffrey A.
Kinase Inhibitor Drugs (2009), 309-332 | Language: English, Database: CAplus

A review. The Aurora kinases continue to generate significant interest as attractive drug targets in oncol. Through the work carried out at AstraZeneca, selective inhibitors of Aurora-B kinase have been profiled and have allowed a novel mechanism of action (failure of cell division) to be determined which is distinct from the classical antimitotic agents. The quinazoline core has shown itself to be an excellent template in which to develop potent Aurora kinase inhibitors while an understanding of the binding mode has allowed rapid optimization of the series with respect to potency and selectivity for the Aurora kinases.

Keywords: review aurora B kinase inhibitor AZD1152 antitumor

Substances (4) Reactions (0) Citing (9)

124

Effects of the aurora kinase inhibitors AZD1152-HQPA and ZM447439 on growth arrest and polyploidy in acute myeloid leukemia cell lines and primary blasts

By: Walsby, Elisabeth; Walsh, Val; Pepper, Chris; Burnett, Alan; Mills, Ken
Haematologica (2008), 93(5), 662-669 | Language: English, Database: CAplus and MEDLINE

Background: Aurora kinases play an essential role in the orchest ration of chromosome separation and cytoki nesis during mitosis. Small-mol. inhibition of the aurora kinases has been shown to result in inhibition of cell division, phosphor ylation of histone H3 and the induction of apoptosis in a number of cell systems. These characteristics have led aurora kinase inhibitors to be considered as potential therapeutic agents. Design and Methods: Aurora kinase gene expression profiles were assessed in 101 samples from patients with acute myeloid leukemia. Subsequently, aurora kinase inhibitors were invest igated for their in vitro effects on cell viability, histone H3 phosphorylation, cell cycle and morphol. in acute myeloid leukemia cell lines and primary acute myeloid leukemia samples. Results: The aurora kinase inhibitors AZD1152-HQPA and ZM447439 induced growth arrest and the accumu lation of hyperploid cells in acute myeloid leukemia cell lines and primary acute myeloid leukemia cultures. Furthe rmore, both agents inhibited histone H3 phosphorylation and this preceded perturb ations in cell cycle and the induction of apoptosis. Single cell cloning assays were performed on diploid and polyploid cells to investigate their colony-forming capacities. Although the polyploid cells showed a reduced capacity for colony formation when compared with their diploid counterparts, they were consistently able to form colonies. Conclusions: AZD1152-HQPA- and ZM447439 are effective apoptosis-inducing agents in acute myeloid leukemia cell lines and primary acute myeloid leukemia cultures. However, their propensity to induce polyploidy does not inevitably result in apoptosis.

Keywords: acute myeloid leukemia aurora kinase inhibitor phosphorylation apoptosis cyctoto xicity

Substances (3) Reactions (0) Citing (79)

125

Enhancement of radiation response in p53-deficient cancer cells by the Aurora-B kinase inhibitor AZD1152

By: Tao, Y.; Zhang, P.; Girdler, F.; Frascogna, V.; Castedo, M.; Bourhis, J.; Kroemer, G.; Deutsch, E.
Oncogene (2008), 27(23), 3244-3255 | Language: English, Database: CAplus and MEDLINE

Overexpression of the Aurora-B kinase correlates with oncogenic transfo rmation and poor prognosis. We evaluated the effects of the bona fide Aurora-B kinase inhibitor AZD1152 on tumor responses to ionizing radiation (I R). When $p53^{wt}$ HCT116 and A549 cells were pretreated with AZD1152-HQPA prior to IR, additive effects were observed Interestingly, more pronounced tumori cidal effects were observed in p53-deficient HCT116 and HT29 cells, as well as A549 cells treated with the p53 inhibitor cyclic pifithrin- α. In vivo studies on xenografted mice confirmed enhanced tumor growth delay after the combin ation of IR plus AZD1152-IR as compared to IR alone. Again, this effect was more pronounced with $p53^{-/-}$ HCT116 and p53-mutant xenografts. The AZD1152-mediated radiosens itization was mimicked by knockdown of Aurora- B with a short interference RNA or by inhibition of Aurora- B by transfection with an inducible kinase-dead Aurora-B. The radiosensitizing effect of AZD1152 was lost in $CHK2^{-/-}$ and $14\text{-}3\text{-}3^{-/-}$ HCT116 cells. Altogether, these data indicate that AZD1152 can radiosensitize tumor cell lines in vitro and in vivo, the fact that these effects are exacer bated in p53-deficient cancer cells is of potential interest for further clin. develo pment.

Keywords: radiotherapy p53 cancer Aurora B kinase inhibitor A ZD1152 radiosensitizer

Substances (3) Reactions (0) Citing (76)

126

Discovery of Selective Aminothiazole Aurora Kinase Inhibitors

By: Andersen, Carsten B.; Wan, Yongqin; Chang, Jae W.; Riggs, Blake; Lee, Christian; Liu, Yi; Sessa, Fabio; Villa, Fabrizio; Kwiatkowski, Nicholas; Suzuki, Melissa; et al

ACS Chemical Biology (2008), 3(3), 180-192 | Language: English, Database: CAplus and MEDLINE

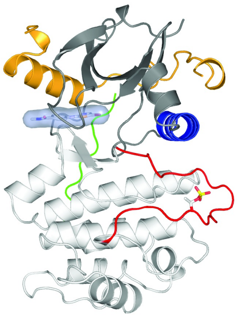

Aurora family kinases regulate important events during mitosis including centrosome maturation and separation, mitotic spindle assembly, and chromosome segregation. Misregulation of Aurora kinases due to genetic amplifi cation and protein overexp ression results in aneuploidy and may contribute to tumorigenesis. Here we report the discovery of new small mol. aminoth iazole inhibitors of Aurora kinases with exceptional kinase selectivity and report a 1.7 Å cocrystal structure with the Aurora B: INCENP complex from Xenopus laevis. The compounds recapitulate the hallmarks of Aurora kinase inhibi tion, including decreased histone H3 serine 10 phosphorylation, failure to complete cytoki nesis, and endoredup lication.

Keywords: aminothiazole inhibitor Aurora kinase active site crystal structure mitosis; mitotic spindle histone H3 phosphor ylation

Substances (211) | Reactions (251) | Citing (34)

127

A quantitative analysis of kinase inhibitor selectivity

By: Karaman, Mazen W.; Herrgard, Sanna; Treiber, Daniel K.; Gallant, Paul; Atteridge, Corey E.; Campbell, Brian T.; Chan, Katrina W.; Ciceri, Pietro; Davis, Mindy I.; Edeen, Philip T.; et al

Nature Biotechnology (2008), 26(1), 127-132 | Language: English, Database: CAplus and MEDLINE

Kinase inhibitors are a new class of therapeutics with a propensity to inhibit multiple targets. The biol. conseq uences of multikinase activity are poorly defined, and an important step toward understanding the relationship between selectivity, efficacy and safety is the exploration of how inhibitors interact with the human kinome. The authors present intera ction maps for 38 kinase inhibitors across a panel of 317 kinases representing >50% of the predicted human protein kinome. The data constitute the most compreh ensive study of kinase inhibitor selectivity to date and reveal a wide diversity of intera ction patterns. To enable a global anal. of the results, the authors introduce the concept of a selectivity score as a general tool to quantify and differe ntiate the observed intera ction patterns. The authors further invest igate the impact of panel size and find that small assay panels do not provide a robust measure of selectivity.

Keywords: protein kinase inhibitor selectivity

Substances (256) | Reactions (0) | Citing (1,907)

128

Process for the preparation of pyrazolylaminoquinazoline derivatives comprising a phosphate group

By: Pittam, John David; Sependa, George Joseph
World Intellectual Property Organization, WO2007132210 A1 2007-11-22 | Language: English, Database: CAplus

The invention relates to a new process useful in the preparation of pharmaceutical compounds such as 2-{ethyl[3-({4-[(5-{2-[(3-fluorophenyl)amino]-2-oxoethyl}-1H-pyrazol-3-yl)amino]quinazolin-7-yl}oxy)propyl]amino}ethyl dihydrogen phosphate (AZD1152) and intermediates used therein.

Keywords: pyrazolyl amino quinazoline phosphate preparation; AZD1152 intermediate preparation; fluorophenyl amino oxoethyl pyrazolylamino quinazolinyloxypropyl dihydrogen phosphate AZD1152 intermediate

PatentPak available

Substances (16) | Reactions (45) | Citing (0)

129

Maleate co-crystal of AZD 1152 for dosage forms for treatment of hyperproliferative diseases

By: Sependa, George Joseph; Storey, Richard
World Intellectual Property Organization, WO2007132227 A1 2007-11-22 | Language: English, Database: CAplus

The present invention relates to a novel co-crystal form of 2-{ethyl[3-({4-[(5-{2-[(3-fluorophenyl)amino]-2-oxo-ethyl}-1H-pyrazol-3-yl) amino]quinazolin-7-yl}oxy)propyl]amino} Et dihydrogen phosphate (AZD 1152), an aurora kinase inhibitor useful in the treatment of hyperproliferative diseases, such as cancer. More specifi cally, the invention relates to a maleate co- crystal of AZD 1152, to a process for its preparation, its use in the manufacturing of a medicament for the treatment of hyperprol iferative diseases, and to methods of treating hyperproliferative diseases by adminis tering a therapeutically effective amount of a maleate co- crystal of AZD 1152. A particular crystalline form of a maleate co- crystal of AZD 1152 is also described. Thus, crude AZD 1152 (preparation given, estimated at 7.44 g @ 100%, 11.61 mM) was added to DMSO (36 mL) and left at ambient temper ature to produce a pale brown solution To this solution was added a solution of maleic acid (1.76 g, 15.16 mM, 1.31 mol equivalent) in MeOH (36 mL) and the mixture left to stand overnight at ambient temperature Next day an aliquot of the clear solution was transf erred to a vial, scratched and left sealed for several hours. A deposit of white solid formed and this was transferred to the flask and left to stir. Gradually the solution turned turbid and solid deposited. The slurry was left to settle for several days and finally filtered. The cake was washed with a 1:1 mixture of DMSO/MeOH, slurried in situ with MeOH and then dried in vacuo. NMR confirmed the solid to be the maleate co- crystal of AZ D1152 (yield of about 78.7%).

Keywords: AZD1152 maleate crystal form preparation dosage form; aurora kinase inhibitor AZD1152 maleate hyperproliferative disease

PatentPak available

Substances (22) | Reactions (30) | Citing (1)

130

Quinazolin-4-ylaminopyrazolecarboxamides as aurora kinase inhibitors useful in bombination therapy for the treatment of cancer and their preparation

By: Keen, Nicholas John

World Intellectual Property Organization, WO2007132215 A1 2007-11-22 | Language: English, Database: CAplus

A combination comprising an aurora kinase inhibitor and an efflux transp orter inhibitor wherein the aurora kinase inhibitor is a compound of formula **I** or pharmaceutically acceptable salt thereof for use in the treatment of hyperprol iferative diseases such as cancer. Compounds of formula **I** wherein n is 0, 1, 2 and 3; R^1 is C_{1-4} hydroxyalkyl and C_{1-4} phosphonooxyalkyl; R^2 is H, C_{1-4} (hydroxy)alkyl, C_{1-4} alkoxy-C_{1-4} alkyl, and heterocyclyl; R^1R^2 together with nitrogen form a (un)substituted 4- to 6-membered hetero cyclic ring; R^3 is H and C_{1-4} alkoxy; R^4, R^6 and R^6 are independently H and C_{1-4} alkyl; R^5 is (un)substituted aryl; and their pharmace utically acceptable salts thereof. Example compound **II** was prepared by a multistep procedure (procedure given) . All the invention compounds were evaluated for their Aurora kinase inhibitory activity (some data given).

Keywords: quinazolinyl pyrazolylacetamide preparation aurora kinase inhibitor treatment cancer

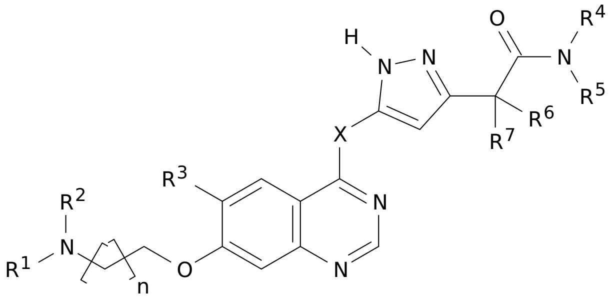

I

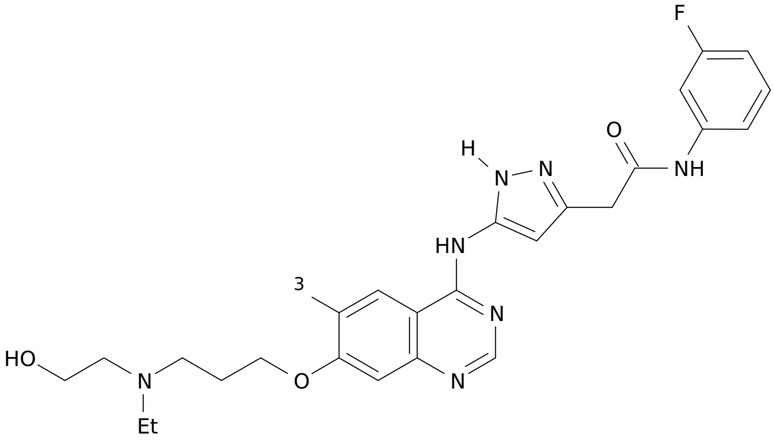

II

PatentPak available

Substances (65) Reactions (58) Citing (2)

131

AZD1152, a novel and selective aurora B kinase inhibitor, induces growth arrest, apoptosis, and sensitization for tubulin depolymerizing agent or topoisomerase II inhibitor in human acute leukemia cells in vitro and in vivo

By: Yang, Jing; Ikezoe, Takayuki; Nishioka, Chie; Tasaka, Taizo; Taniguchi, Ayuko; Kuwayama, Yoshio; Komatsu, Naoki; Bandobashi, Kentaro; Togitani, Kazuto; Koeffler, H. Phillip; et al
Blood (2007), 110(6), 2034-2040 | Language: English, Database: CAplus and MEDLINE

Aurora kinases play an important role in chromosome alignment, segregation, and cytokinesis during mitosis. We have recently shown that hematopoietic malignant cells including those from acute myeloid leukemia (AML) and acute lymphoblastic leukemia (A LL) aberrantly expressed Aurora A and B kinases, and ZM447439, a potent inhibitor of Aurora kinases, effectively induced growth arrest and apoptosis of a variety of leukemia cells. The present study explored the effect of AZD1152, a highly selective inhibitor of Aurora B kinase, on various types of human leukemia cells. AZD1152 inhibited the proliferation of AML lines (HL-60, NB4, MOLM13), ALL line (PALL-2), biphenotypic leukemia (MV4-11), acute eosinophilic leukemia (EOL-1), and the blast crisis of chronic myeloid leukemia K562 cells with an IC50 ranging from 3 nM to 40 nM, as measured by thymidine uptake on day 2 of culture. These cells had 4N/8N DNA content followed by apoptosis, as measured by cell- cycle anal. and annexin V staining, resp. Of note, AZD1152 synergistically enhanced the antiproliferative activity of vincristine, a tubulin depolymerizing agent, and daunorubicin, a topoiso merase II inhibitor, against the MOLM13 and PALL-2 cells in vitro. Furthermore, AZD1152 potentiated the action of vincristine and daunorubicin in a MOLM13 murine xenograft model. Taken together, AZD1152 is a promising new agent for treatment of indivi duals with leukemia. The combined administration of AZD1152 and conventional chemotherapeutic agent to patients with leukemia warrants further investigation.

Keywords: AZD1152 aurora B kinase inhibitor vincristine daunorubicin synergism leukemia

Substances (7) Reactions (0) Citing (221)

132

Discovery, Synthesis, and in Vivo Activity of a New Class of Pyrazolylamino Quinazolines as Selective Inhibitors of Aurora B Kinase

By: Mortlock, Andrew A.; Foote, Kevin M.; Heron, Nicola M.; Jung, Frederic H.; Pasquet, Georges; Lohmann, Jean-Jacques M.; Warin, Nicolas; Renaud, Fabrice; De Savi, Chris; Roberts, Nicola J.; et al
Journal of Medicinal Chemistry (2007), 50(9), 2213-2224 | Language: English, Database: CAplus and MEDLINE

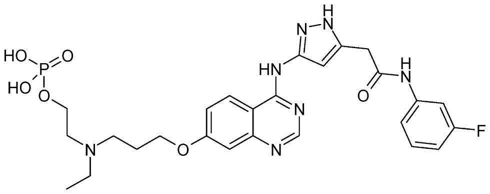

A series of pyrazolylamino-substituted quinazolines was synthesized and biol. evaluated as inhibitors of Aurora kinases, which have been the subject of considerable interest as targets for the development of new anticancer agents. Some of the products demons trated greater than 1000-fold selectivity for Aurora B over Aurora A kinase activity in recomb inant enzyme assays. These compounds have been designed for parenteral administration and achieve high levels of solubility by virtue of their ability to be delivered as readily activated phosphate derivatives The prodrugs are comprehensively converted to the des-phosphate form in vivo, and the active species have advantageous pharmacokinetic properties and safety pharmacol. profiles. The compounds display striking in vivo activity, and **I** (AZD1152) has been selected for clin. evaluation and is currently in phase 1 clin. trials.

Keywords: quinazoline pyrazolylamino preparation selective inhibitor Aurora kinase antitumor

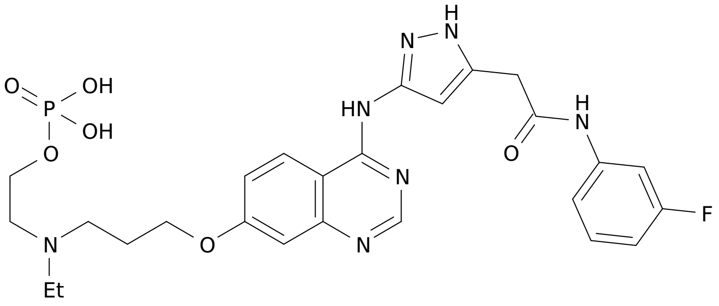

I

Substances (85) Reactions (245) Citing (238)

133

Preparation of phosphonooxy quinazoline derivatives and their pharmaceutical use

By: Heron, Nicola Murdoch; Jung, Frederic Henri; Pasquet, Georges Rene; Mortlock, Andrew Austen
World Intellectual Property Organization, WO2004058781 A1 2004-07-15 | Language: English, Database: CAplus

Preparation of phosphonooxy quinazoline derivatives, **I** (A = 5-membered heteroaryl containing a nitrogen atom and one or two further nitrogen atoms; X = O, S, S(O), $S(O)_2$, organoamino; m = 0-3; Z = organo amino, phosphonooxy, (un)substituted C_{3-6} cycloa lkyl, etc.; R^3 = H, halo, cyano, nitro, C_{1-6} alkoxy, C_{1-6} alkyl, alkoxyca rbonyl, organoamido, sulfony lamido, etc.; R^4 = H, C_{1-4} alkyl, hetero aryl, heteroaryl C_{1-4} alkyl, aryl, etc.; R^5 = H, C_{1-4} alkyl, C_{2-4} alkenyl, C_{2-4} alkynyl, C_{3-6} cycloalkyl, etc.; R^6, R^7 = H, halo, C_{1-4} alkyl, C_{3-6} cycloalkyl, hydroxy, C_{1-4} alkoxy, etc.), and compositions containing them, processes for their prepar ation and their use in therapy is described. Thus, reaction of N-(3-fluorophenyl)-2-{3-[(7-{3-[4-(hydroxymethyl)piperidin-1-yl]propoxy}-6-methoxyquinazolin-4-yl) amino]-1H-pyrazol-5-yl}acetamide (preparation given) with di-tert-butyl-diethylphosphoramidite gave 70% di-tert-Bu {1-[3-({4-[(5-{2-[(3-fluorophenyl)amino]-2-oxoethyl}-1Hpyrazol-3-yl)amino]-6-methoxyquinazolin-7-yl}oxy)propyl]piperidin-4-yl}methyl phosphate which on acidic hydrolysis gave 94% title compound, di-tert-Bu {1-[3-({4-[(5-{2-[(3-fluorophenyl)amino]-2-oxoethyl}-1Hpyrazol-3-yl) amino]-6-methoxyquinazolin-7-yl}oxy)propyl]piperidin-4-yl}methyl dihydrogen phosphate. In vitro Aurora- A and Aurora- B kinase inhibition activity and cell proliferation and cycle anal. of the prepared compounds were determined

Keywords: phosphonooxy quinazoline derivative preparation Aurora A B kinase inhibition

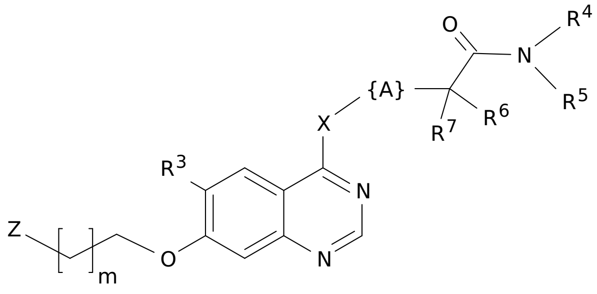

I

PatentPak available

Substances (268) Reactions (1,525) Citing (20)

Copyright © 2023 American Chemical Society (ACS). All Rights Reserved.
Copyright © 2023 U.S. National Library of Medicine.
Internal use only. Redistribution is subject to the terms of your SciFinderⁿ License Agreement and CAS information Use Policies.

Results of PubMed search: keyword AZD2014

1: Pike KG, Malagu K, Hummersone MG, Menear KA, Duggan HM, Gomez S, Martin NM, Ruston L, Pass SL, Pass M. Optimization of potent and selective dual mTORC1 and mTORC2 inhibitors: the discovery of AZD8055 and AZD2014. Bioorg Med Chem Lett. 2013 Mar 1;23(5):1212-6. doi: 10.1016/j.bmcl.2013.01.019. Epub 2013 Jan 18. PMID: 23375793.

2: Rastogi R, Jiang Z, Ahmad N, Rosati R, Liu Y, Beuret L, Monks R, Charron J, Birnbaum MJ, Samavati L. Rapamycin induces mitogen-activated protein (MAP) kinase phosphatase-1 (MKP-1) expression through activation of protein kinase B and mitogen-activated protein kinase kinase pathways. J Biol Chem. 2013 Nov 22;288(47):33966-33977. doi: 10.1074/jbc.M113.492702. Epub 2013 Oct 14. PMID: 24126911; PMCID: PMC3837136.

3: Huo HZ, Zhou ZY, Wang B, Qin J, Liu WY, Gu Y. Dramatic suppression of colorectal cancer cell growth by the dual mTORC1 and mTORC2 inhibitor AZD-2014. Biochem Biophys Res Commun. 2014 Jan 10;443(2):406-12. doi: 10.1016/j.bbrc.2013.11.099. Epub 2013 Dec 2. PMID: 24309100.

4: Kahn J, Hayman TJ, Jamal M, Rath BH, Kramp T, Camphausen K, Tofilon PJ. The mTORC1/mTORC2 inhibitor AZD2014 enhances the radiosensitivity of glioblastoma stem-like cells. Neuro Oncol. 2014 Jan;16(1):29-37. doi: 10.1093/neuonc/not139. Epub 2013 Dec 4. PMID: 24311635; PMCID: PMC3870843.

5: Eyre TA, Collins GP, Goldstone AH, Cwynarski K. Time now to TORC the TORC? New developments in mTOR pathway inhibition in lymphoid malignancies. Br J Haematol. 2014 Aug;166(3):336-51. doi: 10.1111/bjh.12945. Epub 2014 May 19. PMID: 24842496.

6: Li C, Wang Q, Wang JF. Transforming growth factor-β (TGF-β) induces the expression of chondrogenesis-related genes through TGF-β receptor II (TGFRII)-AKT-mTOR signaling in primary cultured mouse precartilaginous stem cells. Biochem Biophys Res Commun. 2014 Jul 18;450(1):646-51. doi: 10.1016/j.bbrc.2014.06.030. Epub 2014 Jun 16. PMID: 24946212.

7: Ezell SA, Mayo M, Bihani T, Tepsuporn S, Wang S, Passino M, Grosskurth SE, Collins M, Parmentier J, Reimer C, Byth KF. Synergistic induction of apoptosis by combination of BTK and dual mTORC1/2 inhibitors in diffuse large B cell lymphoma. Oncotarget. 2014 Jul 15;5(13):4990-5001. doi: 10.18632/oncotarget.2071. PMID: 24970801; PMCID: PMC4148116.

8: Martin D, Li Y, Yang J, Wang G, Margariti A, Jiang Z, Yu H, Zampetaki A, Hu Y, Xu Q, Zeng L. Unspliced X-box-binding protein 1 (XBP1) protects endothelial cells from oxidative stress through interaction with histone deacetylase 3. J Biol Chem. 2014 Oct 31;289(44):30625-30634. doi: 10.1074/jbc.M114.571984. Epub 2014 Sep 4. PMID: 25190803; PMCID: PMC4215241.

9: Zhong H, Fazenbaker C, Breen S, Chen C, Huang J, Morehouse C, Yao Y, Hollingsworth RE. MEDI-573, alone or in combination with mammalian target of rapamycin inhibitors, targets the insulin-like growth factor pathway in sarcomas. Mol Cancer Ther. 2014 Nov;13(11):2662-73. doi: 10.1158/1535-7163.MCT-14-0144. Epub 2014 Sep 5. Erratum in: Mol Cancer Ther. 2015 Mar;14(3):844. PMID: 25193511.

10: Zheng B, Mao JH, Qian L, Zhu H, Gu DH, Pan XD, Yi F, Ji DM. Pre-clinical evaluation of AZD-2014, a novel mTORC1/2 dual inhibitor, against renal cell carcinoma. Cancer Lett. 2015 Feb 28;357(2):468-75. doi:

10.1016/j.canlet.2014.11.012. Epub 2014 Nov 12. PMID: 25444920.

11: Liao H, Huang Y, Guo B, Liang B, Liu X, Ou H, Jiang C, Li X, Yang D. Dramatic antitumor effects of the dual mTORC1 and mTORC2 inhibitor AZD2014 in hepatocellular carcinoma. Am J Cancer Res. 2014 Dec 15;5(1):125-39. PMID: 25628925; PMCID: PMC4300717.

12: Mohindra NA, Platanias LC. Catalytic mammalian target of rapamycin inhibitors as antineoplastic agents. Leuk Lymphoma. 2015;56(9):2518-23. doi: 10.3109/10428194.2015.1026816. Epub 2015 May 12. PMID: 25747970.

13: Basu B, Dean E, Puglisi M, Greystoke A, Ong M, Burke W, Cavallin M, Bigley G, Womack C, Harrington EA, Green S, Oelmann E, de Bono JS, Ranson M, Banerji U. First-in-Human Pharmacokinetic and Pharmacodynamic Study of the Dual m-TORC 1/2 Inhibitor AZD2014. Clin Cancer Res. 2015 Aug 1;21(15):3412-9. doi: 10.1158/1078-0432.CCR-14-2422. Epub 2015 Mar 24. PMID: 25805799; PMCID: PMC4512239.

14: Leung EY, Askarian-Amiri M, Finlay GJ, Rewcastle GW, Baguley BC. Potentiation of Growth Inhibitory Responses of the mTOR Inhibitor Everolimus by Dual mTORC1/2 Inhibitors in Cultured Breast Cancer Cell Lines. PLoS One. 2015 Jul 6;10(7):e0131400. doi: 10.1371/journal.pone.0131400. PMID: 26148118; PMCID: PMC4492962.

15: Jiang S, Zou Z, Nie P, Wen R, Xiao Y, Tang J. Synergistic Effects between mTOR Complex 1/2 and Glycolysis Inhibitors in Non-Small-Cell Lung Carcinoma Cells. PLoS One. 2015 Jul 15;10(7):e0132880. doi: 10.1371/journal.pone.0132880. PMID: 26176608; PMCID: PMC4503566.

16: Beauchamp RL, James MF, DeSouza PA, Wagh V, Zhao WN, Jordan JT, Stemmer-Rachamimov A, Plotkin SR, Gusella JF, Haggarty SJ, Ramesh V. A high-throughput kinome screen reveals serum/glucocorticoid-regulated kinase 1 as a therapeutic target for NF2-deficient meningiomas. Oncotarget. 2015 Jul 10;6(19):16981-97. doi: 10.18632/oncotarget.4858. PMID: 26219339; PMCID: PMC4627286.

17: Guichard SM, Curwen J, Bihani T, D'Cruz CM, Yates JW, Grondine M, Howard Z, Davies BR, Bigley G, Klinowska T, Pike KG, Pass M, Chresta CM, Polanska UM, McEwen R, Delpuech O, Green S, Cosulich SC. AZD2014, an Inhibitor of mTORC1 and mTORC2, Is Highly Effective in ER+ Breast Cancer When Administered Using Intermittent or Continuous Schedules. Mol Cancer Ther. 2015 Nov;14(11):2508-18. doi: 10.1158/1535-7163.MCT-15-0365. Epub 2015 Sep 10. PMID: 26358751.

18: Singleton KR, Hinz TK, Kleczko EK, Marek LA, Kwak J, Harp T, Kim J, Tan AC, Heasley LE. Kinome RNAi Screens Reveal Synergistic Targeting of MTOR and FGFR1 Pathways for Treatment of Lung Cancer and HNSCC. Cancer Res. 2015 Oct 15;75(20):4398-406. doi: 10.1158/0008-5472.CAN-15-0509. Epub 2015 Sep 10. PMID: 26359452; PMCID: PMC4609283.

19: Powles T, Wheater M, Din O, Geldart T, Boleti E, Stockdale A, Sundar S, Robinson A, Ahmed I, Wimalasingham A, Burke W, Sarker SJ, Hussain S, Ralph C. A Randomised Phase 2 Study of AZD2014 Versus Everolimus in Patients with VEGF-Refractory Metastatic Clear Cell Renal Cancer. Eur Urol. 2016 Mar;69(3):450-6. doi: 10.1016/j.eururo.2015.08.035. Epub 2015 Sep 11. PMID: 26364551.

20: Harada M, Benito J, Yamamoto S, Kaur S, Arslan D, Ramirez S, Jacamo R, Platanias L, Matsushita H, Fujimura T, Kazuno S, Kojima K, Tabe Y, Konopleva M. The novel combination of dual mTOR inhibitor AZD2014 and pan-PIM inhibitor

AZD1208 inhibits growth in acute myeloid leukemia via HSF pathway suppression. Oncotarget. 2015 Nov 10;6(35):37930-47. doi: 10.18632/oncotarget.6122. PMID: 26473447; PMCID: PMC4741975.

21: Vandamme T, Beyens M, de Beeck KO, Dogan F, van Koetsveld PM, Pauwels P, Mortier G, Vangestel C, de Herder W, Van Camp G, Peeters M, Hofland LJ. Long-term acquired everolimus resistance in pancreatic neuroendocrine tumours can be overcome with novel PI3K-AKT-mTOR inhibitors. Br J Cancer. 2016 Mar 15;114(6):650-8. doi: 10.1038/bjc.2016.25. PMID: 26978006; PMCID: PMC4800296.

22: Lynch JT, McEwen R, Crafter C, McDermott U, Garnett MJ, Barry ST, Davies BR. Identification of differential PI3K pathway target dependencies in T-cell acute lymphoblastic leukemia through a large cancer cell panel screen. Oncotarget. 2016 Apr 19;7(16):22128-39. doi: 10.18632/oncotarget.8031. PMID: 26989080; PMCID: PMC5008349.

23: Yu CC, Huang HB, Hung SK, Liao HF, Lee CC, Lin HY, Li SC, Ho HC, Hung CL, Su YC. AZD2014 Radiosensitizes Oral Squamous Cell Carcinoma by Inhibiting AKT/mTOR Axis and Inducing G1/G2/M Cell Cycle Arrest. PLoS One. 2016 Mar 31;11(3):e0151942. doi: 10.1371/journal.pone.0151942. PMID: 27031247; PMCID: PMC4816280.

24: Kim HK, Kim SY, Lee SJ, Kang M, Kim ST, Jang J, Rath O, Schueler J, Lee DW, Park WY, Kim SJ, Park SH, Lee J. BEZ235 (PIK3/mTOR inhibitor) Overcomes Pazopanib Resistance in Patient-Derived Refractory Soft Tissue Sarcoma Cells. Transl Oncol. 2016 Jun;9(3):197-202. doi: 10.1016/j.tranon.2016.03.008. Epub 2016 May 12. PMID: 27267837; PMCID: PMC4907899.

25: Peng X, Zhang D, Li Z, Fu M, Liu H. mTOR inhibition sensitizes human hepatocellular carcinoma cells to resminostat. Biochem Biophys Res Commun. 2016 Sep 2;477(4):556-562. doi: 10.1016/j.bbrc.2016.06.060. Epub 2016 Jun 14. PMID: 27311860.

26: Li Y, Cui JT. Inhibition of Bcl-2 potentiates AZD-2014-induced anti-head and neck squamous cell carcinoma cell activity. Biochem Biophys Res Commun. 2016 Sep 2;477(4):607-613. doi: 10.1016/j.bbrc.2016.06.100. Epub 2016 Jun 22. PMID: 27343560.

27: Zhong H, Fazenbaker C, Chen C, Breen S, Huang J, Yao X, Ren P, Yao Y, Herbst R, Hollingsworth RE. Overproduction of IGF-2 drives a subset of colorectal cancer cells, which specifically respond to an anti-IGF therapeutic antibody and combination therapies. Oncogene. 2017 Feb 9;36(6):797-806. doi: 10.1038/onc.2016.248. Epub 2016 Jul 11. PMID: 27399333.

28: Broutin S, Stewart A, Thavasu P, Paci A, Bidart JM, Banerji U. Insights into significance of combined inhibition of MEK and m-TOR signalling output in KRAS mutant non-small-cell lung cancer. Br J Cancer. 2016 Aug 23;115(5):549-52. doi: 10.1038/bjc.2016.220. Epub 2016 Jul 21. PMID: 27441499; PMCID: PMC4997545.

29: Roskoski R Jr. Ibrutinib inhibition of Bruton protein-tyrosine kinase (BTK) in the treatment of B cell neoplasms. Pharmacol Res. 2016 Nov;113(Pt A):395-408. doi: 10.1016/j.phrs.2016.09.011. Epub 2016 Sep 15. PMID: 27641927.

30: Suryo Rahmanto A, Savov V, Brunner A, Bolin S, Weishaupt H, Malyukova A, Rosén G, Čančer M, Hutter S, Sundström A, Kawauchi D, Jones DT, Spruck C, Taylor MD, Cho YJ, Pfister SM, Kool M, Korshunov A, Swartling FJ, Sangfelt O. FBW7 suppression leads to SOX9 stabilization and increased malignancy in

medulloblastoma. EMBO J. 2016 Oct 17;35(20):2192-2212. doi: 10.15252/embj.201693889. Epub 2016 Sep 13. PMID: 27625374; PMCID: PMC5069553.

31: Sakre N, Wildey G, Behtaj M, Kresak A, Yang M, Fu P, Dowlati A. RICTOR amplification identifies a subgroup in small cell lung cancer and predicts response to drugs targeting mTOR. Oncotarget. 2017 Jan 24;8(4):5992-6002. doi: 10.18632/oncotarget.13362. PMID: 27863413; PMCID: PMC5351607.

32: Kim ST, Kim SY, Klempner SJ, Yoon J, Kim N, Ahn S, Bang H, Kim KM, Park W, Park SH, Park JO, Park YS, Lim HY, Lee SH, Park K, Kang WK, Lee J. Rapamycin-insensitive companion of mTOR (RICTOR) amplification defines a subset of advanced gastric cancer and is sensitive to AZD2014-mediated mTORC1/2 inhibition. Ann Oncol. 2017 Mar 1;28(3):547-554. doi: 10.1093/annonc/mdw669. PMID: 28028034.

33: Yang B, Xu QY, Guo CY, Huang JW, Wang SM, Li YM, Tu Y, He L, Bi ZG, Ji C, Cheng B. MHY1485 ameliorates UV-induced skin cell damages via activating mTOR-Nrf2 signaling. Oncotarget. 2017 Feb 21;8(8):12775-12783. doi: 10.18632/oncotarget.14299. PMID: 28061443; PMCID: PMC5355053.

34: Sekihara K, Saitoh K, Han L, Ciurea S, Yamamoto S, Kikkawa M, Kazuno S, Taka H, Kaga N, Arai H, Miida T, Andreeff M, Konopleva M, Tabe Y. Targeting mantle cell lymphoma metabolism and survival through simultaneous blockade of mTOR and nuclear transporter exportin-1. Oncotarget. 2017 May 23;8(21):34552-34564. doi: 10.18632/oncotarget.16602. Erratum in: Oncotarget. 2019 Nov 26;10(63):6842. PMID: 28388555; PMCID: PMC5470990.

35: Divac Rankov A, Ljujić M, Petrić M, Radojković D, Pešić M, Dinić J. Targeting autophagy to modulate cell survival: a comparative analysis in cancer, normal and embryonic cells. Histochem Cell Biol. 2017 Nov;148(5):529-544. doi: 10.1007/s00418-017-1590-4. Epub 2017 Jun 29. PMID: 28664293.

36: Mastronikolis NS, Tsiambas E, Papadas TA, Fotiades PP, Papadas AT, Mastronikolis SN, Kastanioudakis I, Ragos V. mTOR deregulation in oral cavity squamous cell carcinoma. J BUON. 2017 May-Jun;22(3):610-613. PMID: 28730764.

37: Fantus D, Dai H, Ono Y, Watson A, Yokota S, Mohib K, Yoshida O, Ross MA, Watkins SC, Ramaswami B, Valusjkikh A, Rothstein DM, Thomson AW. Influence of the Novel ATP-Competitive Dual mTORC1/2 Inhibitor AZD2014 on Immune Cell Populations and Heart Allograft Rejection. Transplantation. 2017 Dec;101(12):2830-2840. doi: 10.1097/TP.0000000000001933. PMID: 28885497; PMCID: PMC5709200.

38: Flannery PC, DeSisto JA, Amani V, Venkataraman S, Lemma RT, Prince EW, Donson A, Moroze EE, Hoffman L, Levy JMM, Foreman N, Vibhakar R, Green AL. Preclinical analysis of MTOR complex 1/2 inhibition in diffuse intrinsic pontine glioma. Oncol Rep. 2018 Feb;39(2):455-464. doi: 10.3892/or.2017.6122. Epub 2017 Nov 29. PMID: 29207163; PMCID: PMC5783612.

39: Wong Te Fong AC, Thavasu P, Gagrica S, Swales KE, Leach MO, Cosulich SC, Chung YL, Banerji U. Evaluation of the combination of the dual m-TORC1/2 inhibitor vistusertib (AZD2014) and paclitaxel in ovarian cancer models. Oncotarget. 2017 Dec 6;8(69):113874-113884. doi: 10.18632/oncotarget.23022. PMID: 29371953; PMCID: PMC5768370.

40: Earwaker P, Anderson C, Willenbrock F, Harris AL, Protheroe AS, Macaulay VM. RAPTOR up-regulation contributes to resistance of renal cancer cells to

PI3K-mTOR inhibition. PLoS One. 2018 Feb 1;13(2):e0191890. doi: 10.1371/journal.pone.0191890. PMID: 29389967; PMCID: PMC5794101.

41: Wang JY, Jin X, Zhang X, Li XF. CC-223 inhibits human head and neck squamous cell carcinoma cell growth. Biochem Biophys Res Commun. 2018 Feb 19;496(4):1191-1196. doi: 10.1016/j.bbrc.2018.01.168. Epub 2018 Jan 31. PMID: 29402408.

42: Michaloglou C, Crafter C, Siersbaek R, Delpuech O, Curwen JO, Carnevalli LS, Staniszewska AD, Polanska UM, Cheraghchi-Bashi A, Lawson M, Chernukhin I, McEwen R, Carroll JS, Cosulich SC. Combined Inhibition of mTOR and CDK4/6 Is Required for Optimal Blockade of E2F Function and Long-term Growth Inhibition in Estrogen Receptor-positive Breast Cancer. Mol Cancer Ther. 2018 May;17(5):908-920. doi: 10.1158/1535-7163.MCT-17-0537. Epub 2018 Feb 26. PMID: 29483206; PMCID: PMC6485624.

43: Zhang Q, Shi C, Han L, Jain N, Roberts KG, Ma H, Cai T, Cavazos A, Tabe Y, Jacamo RO, Mu H, Zhao Y, Wang J, Wu SC, Cao F, Zeng Z, Zhou J, Mi Y, Jabbour EJ, Levine R, Tasian SK, Mullighan CG, Weinstock DM, Fruman DA, Konopleva M. Inhibition of mTORC1/C2 signaling improves anti-leukemia efficacy of JAK/STAT blockade in <i>CRLF2</i> rearranged and/or <i>JAK</i> driven Philadelphia chromosome-like acute B-cell lymphoblastic leukemia. Oncotarget. 2018 Jan 17;9(8):8027-8041. doi: 10.18632/oncotarget.24261. PMID: 29487712; PMCID: PMC5814279.

44: Teh JLF, Cheng PF, Purwin TJ, Nikbakht N, Patel P, Chervoneva I, Ertel A, Fortina PM, Kleiber I, HooKim K, Davies MA, Kwong LN, Levesque MP, Dummer R, Aplin AE. <i>In Vivo</i> E2F Reporting Reveals Efficacious Schedules of MEK1/2-CDK4/6 Targeting and mTOR-S6 Resistance Mechanisms. Cancer Discov. 2018 May;8(5):568-581. doi: 10.1158/2159-8290.CD-17-0699. Epub 2018 Mar 1. Erratum in: Cancer Discov. 2018 Dec;8(12):1654. PMID: 29496664; PMCID: PMC6858088.

45: Yin H, Ma J, Chen L, Piao S, Zhang Y, Zhang S, Ma H, Li Y, Qu Y, Wang X, Xu Q. MiR-99a Enhances the Radiation Sensitivity of Non-Small Cell Lung Cancer by Targeting mTOR. Cell Physiol Biochem. 2018;46(2):471-481. doi: 10.1159/000488615. Epub 2018 Mar 26. Erratum in: Cell Physiol Biochem. 2022 Aug 31;56(4):451-452. PMID: 29614485.

46: Shi X, Liu HY, Li SP, Xu HB. Keratinocyte growth factor protects endometrial cells from oxygen glucose deprivation/re-oxygenation via activating Nrf2 signaling. Biochem Biophys Res Commun. 2018 Jun 18;501(1):178-185. doi: 10.1016/j.bbrc.2018.04.208. Epub 2018 May 5. PMID: 29709474.

47: Decaudin D, El Botty R, Diallo B, Massonnet G, Fleury J, Naguez A, Raymondie C, Davies E, Smith A, Wilson J, Howes C, Smith PD, Cassoux N, Piperno-Neumann S, Roman-Roman S, Némati F. Selumetinib-based therapy in uveal melanoma patient-derived xenografts. Oncotarget. 2018 Apr 24;9(31):21674-21686. doi: 10.18632/oncotarget.24670. PMID: 29774094; PMCID: PMC5955168.

48: Milošević Z, Banković J, Dinić J, Tsimplouli C, Sereti E, Dragoj M, Paunović V, Milovanović Z, Stepanović M, Tanić N, Dimas K, Pešić M. Potential of the dual mTOR kinase inhibitor AZD2014 to overcome paclitaxel resistance in anaplastic thyroid carcinoma. Cell Oncol (Dordr). 2018 Aug;41(4):409-426. doi: 10.1007/s13402-018-0380-x. Epub 2018 May 22. PMID: 29790111.

49: Conway JRW, Warren SC, Herrmann D, Murphy KJ, Cazet AS, Vennin C, Shearer RF, Killen MJ, Magenau A, Mélénec P, Pinese M, Nobis M, Zaratzian A,

Boulghourjian A, Da Silva AM, Del Monte-Nieto G, Adam ASA, Harvey RP, Haigh JJ, Wang Y, Croucher DR, Sansom OJ, Pajic M, Caldon CE, Morton JP, Timpson P. Intravital Imaging to Monitor Therapeutic Response in Moving Hypoxic Regions Resistant to PI3K Pathway Targeting in Pancreatic Cancer. Cell Rep. 2018 Jun 12;23(11):3312-3326. doi: 10.1016/j.celrep.2018.05.038. PMID: 29898401; PMCID: PMC6019737.

50: Zięba S, Kowalik A, Zalewski K, Rusetska N, Goryca K, Piaścik A, Misiek M, Bakuła-Zalewska E, Kopczyński J, Kowalski K, Radziszewski J, Bidziński M, Góźdź S, Kowalewska M. Somatic mutation profiling of vulvar cancer: Exploring therapeutic targets. Gynecol Oncol. 2018 Sep;150(3):552-561. doi: 10.1016/j.ygyno.2018.06.026. Epub 2018 Jul 3. PMID: 29980281.

51: Basu B, Krebs MG, Sundar R, Wilson RH, Spicer J, Jones R, Brada M, Talbot DC, Steele N, Ingles Garces AH, Brugger W, Harrington EA, Evans J, Hall E, Tovey H, de Oliveira FM, Carreira S, Swales K, Ruddle R, Raynaud FI, Purchase B, Dawes JC, Parmar M, Turner AJ, Tunariu N, Banerjee S, de Bono JS, Banerji U. Vistusertib (dual m-TORC1/2 inhibitor) in combination with paclitaxel in patients with high-grade serous ovarian and squamous non-small-cell lung cancer. Ann Oncol. 2018 Sep 1;29(9):1918-1925. doi: 10.1093/annonc/mdy245. PMID: 30016392; PMCID: PMC6158767.

52: Lynch JT, Polanska UM, Hancox U, Delpuech O, Maynard J, Trigwell C, Eberlein C, Lenaghan C, Polanski R, Avivar-Valderas A, Cumberbatch M, Klinowska T, Critchlow SE, Cruzalegui F, Barry ST. Combined Inhibition of PI3Kβ and mTOR Inhibits Growth of PTEN-null Tumors. Mol Cancer Ther. 2018 Nov;17(11):2309-2319. doi: 10.1158/1535-7163.MCT-18-0183. Epub 2018 Aug 10. PMID: 30097489.

53: Tang X, Xie C, Jiang Z, Li A, Cai S, Hou C, Wang J, Liang Y, Ma D. Rituximab (anti-CD20)-modified AZD-2014-encapsulated nanoparticles killing of B lymphoma cells. Artif Cells Nanomed Biotechnol. 2018;46(sup2):1063-1073. doi: 10.1080/21691401.2018.1478844. Epub 2018 Sep 8. PMID: 30198340.

54: Mattoo AR, Joun A, Jessup JM. Repurposing of mTOR Complex Inhibitors Attenuates MCL-1 and Sensitizes to PARP Inhibition. Mol Cancer Res. 2019 Jan;17(1):42-53. doi: 10.1158/1541-7786.MCR-18-0650. Epub 2018 Sep 10. PMID: 30201826.

55: Langdon S, Hughes A, Taylor MA, Kuczynski EA, Mele DA, Delpuech O, Jarvis L, Staniszewska A, Cosulich S, Carnevalli LS, Sinclair C. Combination of dual mTORC1/2 inhibition and immune-checkpoint blockade potentiates anti-tumour immunity. Oncoimmunology. 2018 May 7;7(8):e1458810. doi: 10.1080/2162402X.2018.1458810. PMID: 30221055; PMCID: PMC6136876.

56: Cosimo E, Tarafdar A, Moles MW, Holroyd AK, Malik N, Catherwood MA, Hay J, Dunn KM, Macdonald AM, Guichard SM, O'Rourke D, Leach MT, Sansom OJ, Cosulich SC, McCaig AM, Michie AM. AKT/mTORC2 Inhibition Activates FOXO1 Function in CLL Cells Reducing B-Cell Receptor-Mediated Survival. Clin Cancer Res. 2019 Mar 1;25(5):1574-1587. doi: 10.1158/1078-0432.CCR-18-2036. Epub 2018 Dec 17. PMID: 30559170; PMCID: PMC6398589.

57: Babic T, Dinic J, Buric SS, Hadzic S, Pesic M, Radojkovic D, Rankov AD. Comparative toxicity evaluation of targeted anticancer therapeutics in embryonic zebrafish and sea urchin models. Acta Biol Hung. 2018 Dec;69(4):395-410. doi: 10.1556/018.69.2018.4.3. PMID: 30587022.

58: MacDonald A, Scarfe G, Magirr D, Sarvotham T, Charlton J, Brugger W, Dean E.

Phase I study of orally administered ¹⁴Carbon-isotope labelled-vistusertib (AZD2014), a dual TORC1/2 kinase inhibitor, to assess the absorption, metabolism, excretion, and pharmacokinetics in patients with advanced solid malignancies. Cancer Chemother Pharmacol. 2019 Apr;83(4):787-795. doi: 10.1007/s00280-019-03781-x. Epub 2019 Feb 13. PMID: 30758651.

59: Hadley G, Beard DJ, Alexopoulou Z, Sutherland BA, Buchan AM. Investigation of the novel mTOR inhibitor AZD2014 in neuronal ischemia. Neurosci Lett. 2019 Jul 27;706:223-230. doi: 10.1016/j.neulet.2019.05.023. Epub 2019 May 14. PMID: 31100427.

60: Jones AT, Yang J, Narov K, Henske EP, Sampson JR, Shen MH. Allosteric and ATP-Competitive Inhibitors of mTOR Effectively Suppress Tumor Progression-Associated Epithelial-Mesenchymal Transition in the Kidneys of Tsc2^{+/-} Mice. Neoplasia. 2019 Aug;21(8):731-739. doi: 10.1016/j.neo.2019.05.003. Epub 2019 Jun 14. PMID: 31207499; PMCID: PMC6580094.

61: de Oliveira AA, Neves BJ, Silva LDC, Soares CMA, Andrade CH, Pereira M. Drug Repurposing for Paracoccidioidomycosis Through a Computational Chemogenomics Framework. Front Microbiol. 2019 Jun 12;10:1301. doi: 10.3389/fmicb.2019.01301. PMID: 31244810; PMCID: PMC6581699.

62: Mizutani Y, Inase A, Maimaitili Y, Miyata Y, Kitao A, Matsumoto H, Kawaguchi K, Higashime A, Goto H, Kurata K, Yakushijin K, Minami H, Matsuoka H. An mTORC1/2 dual inhibitor, AZD2014, acts as a lysosomal function activator and enhances gemtuzumab ozogamicin-induced apoptosis in primary human leukemia cells. Int J Hematol. 2019 Oct;110(4):490-499. doi: 10.1007/s12185-019-02701-2. Epub 2019 Jul 8. PMID: 31286402.

63: Li N, Lu XY, Shi WY, Mao FJ, Yang XY, Luo YB, Li W. Combined mTOR/MEK inhibition prevents proliferation and induces apoptosis in NF2-mutant tumors. Eur Rev Med Pharmacol Sci. 2019 Jul;23(13):5874-5883. doi: 10.26355/eurrev_201907_18331. PMID: 31298338.

64: Lee J, Kim ST, Kim K, Lee H, Kozarewa I, Mortimer PGS, Odegaard JI, Harrington EA, Lee J, Lee T, Oh SY, Kang JH, Kim JH, Kim Y, Ji JH, Kim YS, Lee KE, Kim J, Sohn TS, An JY, Choi MG, Lee JH, Bae JM, Kim S, Kim JJ, Min YW, Min BH, Kim NKD, Luke S, Kim YH, Hong JY, Park SH, Park JO, Park YS, Lim HY, Talasaz A, Hollingsworth SJ, Kim KM, Kang WK. Tumor Genomic Profiling Guides Patients with Metastatic Gastric Cancer to Targeted Treatment: The VIKTORY Umbrella Trial. Cancer Discov. 2019 Oct;9(10):1388-1405. doi: 10.1158/2159-8290.CD-19-0442. Epub 2019 Jul 17. PMID: 31315834.

65: Boussios S, Karihtala P, Moschetta M, Karathanasi A, Sadauskaite A, Rassy E, Pavlidis N. Combined Strategies with Poly (ADP-Ribose) Polymerase (PARP) Inhibitors for the Treatment of Ovarian Cancer: A Literature Review. Diagnostics (Basel). 2019 Aug 1;9(3):87. doi: 10.3390/diagnostics9030087. PMID: 31374917; PMCID: PMC6787707.

66: Eyre TA, Hildyard C, Hamblin A, Ali AS, Houlton A, Hopkins L, Royston D, Linton KM, Pettitt A, Rule S, Cwynarski K, Barrington SF, Warbey V, Wrench D, Barrans S, Hirst CS, Panchal A, Roudier MP, Harrington EA, Davies A, Collins GP. A phase II study to assess the safety and efficacy of the dual mTORC1/2 inhibitor vistusertib in relapsed, refractory DLBCL. Hematol Oncol. 2019 Oct;37(4):352-359. doi: 10.1002/hon.2662. Epub 2019 Sep 9. PMID: 31385336.

67: Ki DH, Oppel F, Durbin AD, Look AT. Mechanisms underlying synergy between

DNA topoisomerase I-targeted drugs and mTOR kinase inhibitors in NF1-associated malignant peripheral nerve sheath tumors. Oncogene. 2019 Sep;38(39):6585-6598. doi: 10.1038/s41388-019-0965-5. Epub 2019 Aug 23. PMID: 31444410.

68: Schmid P, Zaiss M, Harper-Wynne C, Ferreira M, Dubey S, Chan S, Makris A, Nemsadze G, Brunt AM, Kuemmel S, Ruiz I, Perelló A, Kendall A, Brown J, Kristeleit H, Conibear J, Saura C, Grenier J, Máhr K, Schenker M, Sohn J, Lee KS, Shepherd CJ, Oelmann E, Sarker SJ, Prendergast A, Marosics P, Moosa A, Lawrence C, Coetzee C, Mousa K, Cortés J. Fulvestrant Plus Vistusertib vs Fulvestrant Plus Everolimus vs Fulvestrant Alone for Women With Hormone Receptor-Positive Metastatic Breast Cancer: The MANTA Phase 2 Randomized Clinical Trial. JAMA Oncol. 2019 Nov 1;5(11):1556-1564. doi: 10.1001/jamaoncol.2019.2526. Erratum in: JAMA Oncol. 2021 Feb 1;7(2):312. PMID: 31465093; PMCID: PMC6865233.

69: Liao HF, Lin YZ, Yu CC, Tai TS, Hung SK, Yang CC, Su YC. mTORC1/2 Inhibitor Served as a More Ideal Agent Against the Growth of Mouse Lymphocytic Leukemia Both <i>In Vitro</i> and <i>In Vivo</i>. Anticancer Res. 2019 Sep;39(9):4829-4835. doi: 10.21873/anticanres.13668. PMID: 31519585.

70: Reita D, Bour C, Benbrika R, Groh A, Pencreach E, Guérin E, Guenot D. Synergistic Anti-Tumor Effect of mTOR Inhibitors with Irinotecan on Colon Cancer Cells. Cancers (Basel). 2019 Oct 17;11(10):1581. doi: 10.3390/cancers11101581. PMID: 31627299; PMCID: PMC6826690.

71: Lapointe S, Mason W, MacNeil M, Harlos C, Tsang R, Sederias J, Luchman HA, Weiss S, Rossiter JP, Tu D, Seymour L, Smoragiewicz M. A phase I study of vistusertib (dual mTORC1/2 inhibitor) in patients with previously treated glioblastoma multiforme: a CCTG study. Invest New Drugs. 2020 Aug;38(4):1137-1144. doi: 10.1007/s10637-019-00875-4. Epub 2019 Nov 9. PMID: 31707687.

72: Kraus D, Palasuberniam P, Chen B. Therapeutic Enhancement of Verteporfin-mediated Photodynamic Therapy by mTOR Inhibitors. Photochem Photobiol. 2020 Mar;96(2):358-364. doi: 10.1111/php.13187. Epub 2019 Dec 17. PMID: 31769520; PMCID: PMC7138740.

73: Pancholi S, Leal MF, Ribas R, Simigdala N, Schuster E, Chateau-Joubert S, Zabaglo L, Hills M, Dodson A, Gao Q, Johnston SR, Dowsett M, Cosulich SC, Maragoni E, Martin LA. Combination of mTORC1/2 inhibitor vistusertib plus fulvestrant in vitro and in vivo targets oestrogen receptor-positive endocrine-resistant breast cancer. Breast Cancer Res. 2019 Dec 4;21(1):135. doi: 10.1186/s13058-019-1222-0. Erratum in: Breast Cancer Res. 2020 Jan 31;22(1):14. PMID: 31801615; PMCID: PMC6894349.

74: Pancholi S, Leal MF, Ribas R, Simigdala N, Schuster E, Chateau-Joubert S, Zabaglo L, Hills M, Dodson A, Gao Q, Johnston SR, Dowsett M, Cosulich SC, Marangoni E, Martin LA. Correction to: Combination of mTORC1/2 inhibitor vistusertib plus fulvestrant in vitro and in vivo targets oestrogen receptor-positive endocrine-resistant breast cancer. Breast Cancer Res. 2020 Jan 31;22(1):14. doi: 10.1186/s13058-020-1254-5. Erratum for: Breast Cancer Res. 2019 Dec 4;21(1):135. PMID: 32005287; PMCID: PMC6993431.

75: Li F, Li Z, Han Q, Cheng Y, Ji W, Yang Y, Lu S, Xia W. Enhanced autocrine FGF19/FGFR4 signaling drives the progression of lung squamous cell carcinoma, which responds to mTOR inhibitor AZD2104. Oncogene. 2020 Apr;39(17):3507-3521. doi: 10.1038/s41388-020-1227-2. Epub 2020 Feb 28. PMID: 32111983; PMCID:

PMC7176586.

76: Shaik A, Kirubakaran S. Evolution of PIKK family kinase inhibitors: A new age cancer therapeutics. Front Biosci (Landmark Ed). 2020 Mar 1;25(8):1510-1537. doi: 10.2741/4866. PMID: 32114443.

77: Sagers JE, Beauchamp RL, Zhang Y, Vasilijic S, Wu L, DeSouza P, Seist R, Zhou W, Xu L, Ramesh V, Stankovic KM. Combination therapy with mTOR kinase inhibitor and dasatinib as a novel therapeutic strategy for vestibular schwannoma. Sci Rep. 2020 Mar 6;10(1):4211. doi: 10.1038/s41598-020-60156-6. PMID: 32144278; PMCID: PMC7060236.

78: Acar A, Nichol D, Fernandez-Mateos J, Cresswell GD, Barozzi I, Hong SP, Trahearn N, Spiteri I, Stubbs M, Burke R, Stewart A, Caravagna G, Werner B, Vlachogiannis G, Maley CC, Magnani L, Valeri N, Banerji U, Sottoriva A. Exploiting evolutionary steering to induce collateral drug sensitivity in cancer. Nat Commun. 2020 Apr 21;11(1):1923. doi: 10.1038/s41467-020-15596-z. PMID: 32317663; PMCID: PMC7174377.

79: Kim H, Lee SJ, Lee IK, Min SC, Sung HH, Jeong BC, Lee J, Park SH. Synergistic Effects of Combination Therapy with AKT and mTOR Inhibitors on Bladder Cancer Cells. Int J Mol Sci. 2020 Apr 18;21(8):2825. doi: 10.3390/ijms21082825. PMID: 32325639; PMCID: PMC7215775.

80: Park S, Shim J, Mortimer PGS, Smith SA, Godin RE, Hollingsworth SJ, Kim HJ, Jung HA, Sun JM, Park WY, Ahn JS, Ahn MJ, Lee SH, Park K. Biomarker-driven phase 2 umbrella trial study for patients with recurrent small cell lung cancer failing platinum-based chemotherapy. Cancer. 2020 Sep 1;126(17):4002-4012. doi: 10.1002/cncr.33048. Epub 2020 Jun 25. PMID: 32584426.

81: Silva MC, Nandi GA, Tentarelli S, Gurrell IK, Jamier T, Lucente D, Dickerson BC, Brown DG, Brandon NJ, Haggarty SJ. Prolonged tau clearance and stress vulnerability rescue by pharmacological activation of autophagy in tauopathy neurons. Nat Commun. 2020 Jun 26;11(1):3258. doi: 10.1038/s41467-020-16984-1. PMID: 32591533; PMCID: PMC7320012.

82: Zormpas-Petridis K, Poon E, Clarke M, Jerome NP, Boult JKR, Blackledge MD, Carceller F, Koers A, Barone G, Pearson ADJ, Moreno L, Anderson J, Sebire N, McHugh K, Koh DM, Chesler L, Yuan Y, Robinson SP, Jamin Y. Noninvasive MRI Native T₁ Mapping Detects Response to <i>MYCN</i>-targeted Therapies in the Th-<i>MYCN</i> Model of Neuroblastoma. Cancer Res. 2020 Aug 15;80(16):3424-3435. doi: 10.1158/0008-5472.CAN-20-0133. Epub 2020 Jun 28. PMID: 32595135.

83: MacCallum PE, Blundell J. The mTORC1 inhibitor rapamycin and the mTORC1/2 inhibitor AZD2014 impair the consolidation and persistence of contextual fear memory. Psychopharmacology (Berl). 2020 Sep;237(9):2795-2808. doi: 10.1007/s00213-020-05573-1. Epub 2020 Jun 29. PMID: 32601986.

84: Oh S, Yeom J, Cho HJ, Kim JH, Yoon SJ, Kim H, Sa JK, Ju S, Lee H, Oh MJ, Lee W, Kwon Y, Li H, Choi S, Han JH, Chang JH, Choi E, Kim J, Her NG, Kim SH, Kang SG, Paek E, Nam DH, Lee C, Kim HS. Integrated pharmaco-proteogenomics defines two subgroups in isocitrate dehydrogenase wild-type glioblastoma with prognostic and therapeutic opportunities. Nat Commun. 2020 Jul 3;11(1):3288. doi: 10.1038/s41467-020-17139-y. PMID: 32620753; PMCID: PMC7335111.

85: Anderson E, Havener TM, Zorn KM, Foil DH, Lane TR, Capuzzi SJ, Morris D,

Hickey AJ, Drewry DH, Ekins S. Synergistic drug combinations and machine learning for drug repurposing in chordoma. Sci Rep. 2020 Jul 31;10(1):12982. doi: 10.1038/s41598-020-70026-w. PMID: 32737414; PMCID: PMC7395084.

86: Ahmed AR, Candeo A, D'Abrantes S, Needham SR, Yadav RB, Botchway SW, Parker AW. Directly imaging the localisation and photosensitization properties of the pan-mTOR inhibitor, AZD2014, in living cancer cells. J Photochem Photobiol B. 2020 Dec;213:112055. doi: 10.1016/j.jphotobiol.2020.112055. Epub 2020 Oct 15. PMID: 33142217; PMCID: PMC7762844.

87: Turnock S, Turton DR, Martins CD, Chesler L, Wilson TC, Gouverneur V, Smith G, Kramer-Marek G. ¹⁸F-meta-fluorobenzylguanidine (¹⁸F-mFBG) to monitor changes in norepinephrine transporter expression in response to therapeutic intervention in neuroblastoma models. Sci Rep. 2020 Dec 1;10(1):20918. doi: 10.1038/s41598-020-77788-3. PMID: 33262374; PMCID: PMC7708446.

88: Beauchamp RL, Erdin S, Witt L, Jordan JT, Plotkin SR, Gusella JF, Ramesh V. mTOR kinase inhibition disrupts neuregulin 1-ERBB3 autocrine signaling and sensitizes NF2-deficient meningioma cellular models to IGF1R inhibition. J Biol Chem. 2021 Jan-Jun;296:100157. doi: 10.1074/jbc.RA120.014960. Epub 2020 Dec 9. PMID: 33273014; PMCID: PMC7949095.

89: Chaube UJ, Rawal R, Jha AB, Variya B, Bhatt HG. Design and development of Tetrahydro-Quinoline derivatives as dual mTOR-C1/C2 inhibitors for the treatment of lung cancer. Bioorg Chem. 2021 Jan;106:104501. doi: 10.1016/j.bioorg.2020.104501. Epub 2020 Nov 24. PMID: 33280832.

90: Chuang FC, Wang CC, Chen JH, Hwang TZ, Yeh SA, Su YC. PI3k inhibitors (BKM120 and BYL719) as radiosensitizers for head and neck squamous cell carcinoma during radiotherapy. PLoS One. 2021 Jan 20;16(1):e0245715. doi: 10.1371/journal.pone.0245715. PMID: 33471836; PMCID: PMC7817006.

91: Li S, Sheng J, Liu Z, Fan Y, Zhang C, Lv T, Hu S, Jin J, Yu W, Song Y. Potent antitumour of the mTORC1/2 dual inhibitor AZD2014 in docetaxel-sensitive and docetaxel-resistant castration-resistant prostate cancer cells. J Cell Mol Med. 2021 Mar;25(5):2436-2449. doi: 10.1111/jcmm.16155. Epub 2021 Jan 28. PMID: 33507584; PMCID: PMC7933970.

92: Mullen PJ, Garcia G Jr, Purkayastha A, Matulionis N, Schmid EW, Momcilovic M, Sen C, Langerman J, Ramaiah A, Shackelford DB, Damoiseaux R, French SW, Plath K, Gomperts BN, Arumugaswami V, Christofk HR. SARS-CoV-2 infection rewires host cell metabolism and is potentially susceptible to mTORC1 inhibition. Nat Commun. 2021 Mar 25;12(1):1876. doi: 10.1038/s41467-021-22166-4. PMID: 33767183; PMCID: PMC7994801.

93: Georgopoulou D, Callari M, Rueda OM, Shea A, Martin A, Giovannetti A, Qosaj F, Dariush A, Chin SF, Carnevalli LS, Provenzano E, Greenwood W, Lerda G, Esmaeilishirazifard E, O'Reilly M, Serra V, Bressan D; IMAXT Consortium; Mills GB, Ali HR, Cosulich SS, Hannon GJ, Bruna A, Caldas C. Landscapes of cellular phenotypic diversity in breast cancer xenografts and their impact on drug response. Nat Commun. 2021 Mar 31;12(1):1998. doi: 10.1038/s41467-021-22303-z. PMID: 33790302; PMCID: PMC8012607.

94: Bensalem J, Fourrier C, Hein LK, Hassiotis S, Proud CG, Sargeant TJ. Inhibiting mTOR activity using AZD2014 increases autophagy in the mouse cerebral cortex. Neuropharmacology. 2021 Jun 1;190:108541. doi:

10.1016/j.neuropharm.2021.108541. Epub 2021 Mar 30. PMID: 33794244.

95: Ooft SN, Weeber F, Schipper L, Dijkstra KK, McLean CM, Kaing S, van de Haar J, Prevoo W, van Werkhoven E, Snaebjornsson P, Hoes LR, Chalabi M, van der Velden D, van Leerdam M, Boot H, Grootscholten C, Huitema ADR, Bloemendal HJ, Cuppen E, Voest EE. Prospective experimental treatment of colorectal cancer patients based on organoid drug responses. ESMO Open. 2021 Jun;6(3):100103. doi: 10.1016/j.esmoop.2021.100103. Epub 2021 Apr 19. PMID: 33887686; PMCID: PMC8086019.

96: Powles T, Carroll D, Chowdhury S, Gravis G, Joly F, Carles J, Fléchon A, Maroto P, Petrylak D, Rolland F, Cook N, Balar AV, Sridhar SS, Galsky MD, Grivas P, Ravaud A, Jones R, Cosaert J, Hodgson D, Kozarewa I, Mather R, McEwen R, Mercier F, Landers D. An adaptive, biomarker-directed platform study of durvalumab in combination with targeted therapies in advanced urothelial cancer. Nat Med. 2021 May;27(5):793-801. doi: 10.1038/s41591-021-01317-6. Epub 2021 May 3. PMID: 33941921.

97: Collins GP, Clevenger TN, Burke KA, Yang B, MacDonald A, Cunningham D, Fox CP, Goy A, Gribben J, Nowakowski GS, Roschewski M, Vose JM, Vallurupalli A, Cheung J, Raymond A, Nuttall B, Stetson D, Dougherty BA, Schalkwijk S, Carnevalli LS, Willis B, Tao L, Harrington EA, Hamdy A, Izumi R, Pease JE, Frigault MM, Flinn I. A phase 1/2 study of the combination of acalabrutinib and vistusertib in patients with relapsed/refractory B-cell malignancies. Leuk Lymphoma. 2021 Nov;62(11):2625-2636. doi: 10.1080/10428194.2021.1938027. Epub 2021 Jul 16. PMID: 34269152.

98: Liao H, Wang Y, Xu X, Zhou C, Zhang J, Zhong K, Yang D. [Antitumor effects of AZD2014, a dual mTORC1/2 inhibitor, against human hepatocellular carcinoma xenograft in nude mice]. Nan Fang Yi Ke Da Xue Xue Bao. 2021 Jul 20;41(7):1056-1061. Chinese. doi: 10.12122/j.issn.1673-4254.2021.07.13. PMID: 34308856; PMCID: PMC8329683.

99: Wang F, Song D, Zou F, Zhao H, Zhao X. Quantitation of vistusertib by UHPLC-MS/MS in rat plasma and its application to a pharmacokinetic study. Bioanalysis. 2021 Sep;13(17):1333-1341. doi: 10.4155/bio-2021-0152. Epub 2021 Sep 2. PMID: 34470474.

100: Morscher RJ, Brard C, Berlanga P, Marshall LV, André N, Rubino J, Aerts I, De Carli E, Corradini N, Nebchi S, Paoletti X, Mortimer P, Lacroix L, Pierron G, Schleiermacher G, Vassal G, Geoerger B. First-in-child phase I/II study of the dual mTORC1/2 inhibitor vistusertib (AZD2014) as monotherapy and in combination with topotecan-temozolomide in children with advanced malignancies: arms E and F of the AcSé-ESMART trial. Eur J Cancer. 2021 Nov;157:268-277. doi: 10.1016/j.ejca.2021.08.010. Epub 2021 Sep 17. PMID: 34543871.

101: Pi R, Yang Y, Hu X, Li H, Shi H, Liu Y, Wang X, Tong A, Lu T, Wei Y, Zhao X, Wei X. Dual mTORC1/2 inhibitor AZD2014 diminishes myeloid-derived suppressor cells accumulation in ovarian cancer and delays tumor growth. Cancer Lett. 2021 Dec 28;523:72-81. doi: 10.1016/j.canlet.2021.09.017. Epub 2021 Sep 21. PMID: 34560229.

102: Cha BH, Jung M, Kim AS, Lepak VC, Colson BA, Bull DA, Won Y. AZD2014, a dual mTOR inhibitor, attenuates cardiac hypertrophy in vitro and in vivo. J Biol Eng. 2021 Oct 21;15(1):24. doi: 10.1186/s13036-021-00276-3. PMID: 34674743; PMCID: PMC8529824.

103: Scott SC, Anders NM, Scardina M, Hann CL, Rudek MA. Validation of a rapid liquid chromatography tandem mass spectrometric method for the quantitative analysis of vistusertib. J Pharm Biomed Anal. 2022 Jan 20;208:114436. doi: 10.1016/j.jpba.2021.114436. Epub 2021 Oct 22. PMID: 34735991; PMCID: PMC8693366.

104: Pinker B, Barciszewska AM. mTOR Signaling and Potential Therapeutic Targeting in Meningioma. Int J Mol Sci. 2022 Feb 10;23(4):1978. doi: 10.3390/ijms23041978. PMID: 35216092; PMCID: PMC8876623.

105: Choudhury AD, Higano CS, de Bono JS, Cook N, Rathkopf DE, Wisinski KB, Martin-Liberal J, Linch M, Heath EI, Baird RD, García-Carbacho J, Quintela-Fandino M, Barry ST, de Bruin EC, Colebrook S, Hawkins G, Klinowska T, Maroj B, Moorthy G, Mortimer PG, Moschetta M, Nikolaou M, Sainsbury L, Shapiro GI, Siu LL, Hansen AR. A Phase I Study Investigating AZD8186, a Potent and Selective Inhibitor of PI3Kβ/δ, in Patients with Advanced Solid Tumors. Clin Cancer Res. 2022 Jun 1;28(11):2257-2269. doi: 10.1158/1078-0432.CCR-21-3087. PMID: 35247924; PMCID: PMC9662946.

106: Liao H, Wang Y, Xu X, Zhou C, Zhang J, Zhong K, Yang D. [The dual mTORC1/2 inhibitor AZD2014 inhibits acute graft rejection in a rat liver transplantation model]. Nan Fang Yi Ke Da Xue Xue Bao. 2022 Apr 20;42(4):598-603. Chinese. doi: 10.12122/j.issn.1673-4254.2022.04.17. PMID: 35527497; PMCID: PMC9085585.

107: Heudel P, Frenel JS, Dalban C, Bazan F, Joly F, Arnaud A, Abdeddaim C, Chevalier-Place A, Augereau P, Pautier P, Chakiba C, You B, Lancry-Lecomte L, Garin G, Marcel V, Diaz JJ, Treilleux I, Pérol D, Fabbro M, Ray-Coquard I. Safety and Efficacy of the mTOR Inhibitor, Vistusertib, Combined With Anastrozole in Patients With Hormone Receptor-Positive Recurrent or Metastatic Endometrial Cancer: The VICTORIA Multicenter, Open-label, Phase 1/2 Randomized Clinical Trial. JAMA Oncol. 2022 Jul 1;8(7):1001-1009. doi: 10.1001/jamaoncol.2022.1047. PMID: 35551299; PMCID: PMC9100474.

108: Combination Vistusertib and Anastrozole Shows Benefit in Endometrial Cancer. Cancer Discov. 2022 Jul 6;12(7):OF20. doi: 10.1158/2159-8290.CD-RW2022-093. PMID: 35593584.

109: Tang W, Zhou J, Yang W, Feng Y, Wu H, Mok MTS, Zhang L, Liang Z, Liu X, Xiong Z, Zeng X, Wang J, Lu J, Li J, Sun H, Tian X, Yeung PC, Hou Y, Lee HM, Lam CCH, Leung HHW, Chan AWH, To KF, Wong J, Lai PBS, Ng KKC, Wong SKH, Wong VWS, Kong APS, Sung JJY, Cheng ASL. Aberrant cholesterol metabolic signaling impairs antitumor immunosurveillance through natural killer T cell dysfunction in obese liver. Cell Mol Immunol. 2022 Jul;19(7):834-847. doi: 10.1038/s41423-022-00872-3. Epub 2022 May 20. PMID: 35595819; PMCID: PMC9243114.

110: Barlesi F, Tomasini P, Karimi M, Michiels S, Raimbourg J, Daniel C, Janicot H, Madroszyk A, Audigier-Valette C, Quoix E, Mazieres J, Moro-Sibilot D, Dansin E, Molinier O, Morel H, Pichon E, Cortot A, Otto J, Chomy F, Souquet PJ, Cloarec N, Giroux-Leprieur E, Bieche I, Lacroix L, Boyault S, Attignon V, Soubeyran I, Morel A, Tran-Dien A, Jacquet A, Dall'Olio FG, Jimenez M, Soria JC, Besse B. Comprehensive Genome Profiling in Patients With Metastatic Non-Small Cell Lung Cancer: The Precision Medicine Phase II Randomized SAFIR02-Lung/IFCT 1301 Trial. Clin Cancer Res. 2022 Sep 15;28(18):4018-4026. doi: 10.1158/1078-0432.CCR-22-0371. PMID: 35802649.

111: Xu W, Berning P, Erdmann T, Grau M, Bettazová N, Zapukhlyak M, Frontzek F, Kosnopfel C, Lenz P, Grondine M, Willis B, Lynch JT, Klener P, Hailfinger S, Barry ST, Lenz G. mTOR inhibition amplifies the anti-lymphoma effect of PI3Kβ/δ

blockage in diffuse large B-cell lymphoma. Leukemia. 2023 Jan;37(1):178-189. doi: 10.1038/s41375-022-01749-0. Epub 2022 Nov 9. PMID: 36352190; PMCID: PMC9883168.

112: Mourksi NE, Dalban C, Colombe-Vermorel A, Odeyer L, Simioni V, Frenel JS, Fabbro M, Bazan F, Abadie-Lacourtoisie S, Coquan E, Martinez S, Garin G, Tabone-Eglinger S, Treilleux I, Chabaud S, Pérol D, Ray-Coquard I, Heudel PE, Diaz JJ, Marcel V. Ribosome biogenesis-based predictive biomarkers in endocrine therapy (Anastrozole) combined with mTOR inhibitor (Vistusertib) in endometrial cancer: translational study from the VICTORIA trial in collaboration with the GINECO group. Mol Oncol. 2023 Jan;17(1):27-36. doi: 10.1002/1878-0261.13340. Epub 2022 Dec 7. PMID: 36370117; PMCID: PMC9812831.

113: Zhuo BB, Zhu LQ, Yao C, Wang XH, Li SX, Wang R, Li Y, Ling ZY. ADCK1 is a potential therapeutic target of osteosarcoma. Cell Death Dis. 2022 Nov 12;13(11):954. doi: 10.1038/s41419-022-05401-8. PMID: 36371387; PMCID: PMC9653483.

CAS SciFinder^{n}

Task History

Initiating Search

January 12, 2023, 10:12PM

All: **AZD2014**

Search Tasks

Task	Search Type	View
Returned All Results	All	View Results
Exported: Returned Reference Results + Filters (169)<br>Filtered By:<br>Document Type: Journal	References	View Results

Copyright © 2023 American Chemical Society (ACS). All Rights Reserved.
Internal use only. Redistribution is subject to the terms of your SciFinder^{n} License Agreement and CAS information Use Policies.

CAS SciFinderⁿ

References (169)

View in SciFinderⁿ

1

First-in-Human Pharmacokinetic and Pharmacodynamic Study of the Dual m-TORC 1/2 Inhibitor AZD2014

Out

By: Basu, Bristi; Dean, Emma; Puglisi, Martina; Greystoke, Alastair; Ong, Michael; Burke, Wendy; Cavallin, Maria; Bigley, Graham; Womack, Christopher; Harrington, Elizabeth A.; et al
Clinical Cancer Research (2015), 21(15), 3412-3419 | Language: English, Database: CAplus and MEDLINE

Substances (2) | Reactions (0) | Cited By (91)

2

Optimization of potent and selective dual mTORC1 and mTORC2 inhibitors: The discovery of AZD8055 and AZD2014

Out

By: Pike, Kurt G.; Malagu, Karine; Hummersone, Marc G.; Menear, Keith A.; Duggan, Heather M. E.; Gomez, Sylvie; Martin, Niall M. B.; Ruston, Linette; Pass, Sarah L.; Pass, Martin
Bioorganic & Medicinal Chemistry Letters (2013), 23(5), 1212-1216 | Language: English, Database: CAplus and MEDLINE

Substances (53) | Reactions (125) | Cited By (161)

3

AZD2014, an Inhibitor of mTORC1 and mTORC2, Is Highly Effective in ER^+ Breast Cancer When Administered Using Intermittent or Continuous Schedules

✓

By: Guichard, Sylvie M.; Curwen, Jon; Bihani, Teeru; D'Cruz, Celina M.; Yates, James W. T.; Grondine, Michael; Howard, Zoe; Davies, Barry R.; Bigley, Graham; Klinowska, Teresa; et al
Molecular Cancer Therapeutics (2015), 14(11), 2508-2518 | Language: English, Database: CAplus and MEDLINE

Substances (10) | Reactions (0) | Cited By (90)

4

The mTORC1/mTORC2 inhibitor AZD2014 enhances the radiosensitivity of glioblastoma stem-like cells

By: Kahn, Jenna; Hayman, Thomas J.; Jamal, Muhammad; Rath, Barbara H.; Kramp, Tamalee; Camphausen, Kevin; Tofilon, Philip J.
Neuro-Oncology (Cary, NC, United States) (2014), 16(1), 29-37 | Language: English, Database: CAplus and MEDLINE

✓

Substances (2) | Reactions (0) | Cited By (76)

5

A Randomised Phase 2 Study of AZD2014 Versus Everolimus in Patients with VEGF-Refractory Metastatic Clear Cell Renal Cancer

Out

By: Powles, Thomas; Wheater, Matthew; Din, Omar; Geldart, Thomas; Boleti, Ekaterini; Stockdale, Andrew; Sundar, Santhanam; Robinson, Angus; Ahmed, Imtiaz; Wimalasingham, Akhila; et al
European Urology (2016), 69(3), 450-456 | Language: English, Database: CAplus and MEDLINE

Substances (4) | Reactions (0) | Cited By (71)

6

Pre-clinical evaluation of AZD-2014, a novel mTORC1/2 dual inhibitor, against renal cell carcinoma

✓

By: Zheng, Bing; Mao, Jia-Hui; Qian, Lin; Zhu, Hua; Gu, Dong-hua; Pan, Xiao-dong; Yi, Fang; Ji, Dong-mei
Cancer Letters (New York, NY, United States) (2015), 357(2), 468-475 | Language: English, Database: CAplus and MEDLINE

Substances (8) | Reactions (0) | Cited By (90)

7

Dramatic suppression of colorectal cancer cell growth by the dual mTORC1 and mTORC2 inhibitor AZD-2014

✓

By: Huo, Hai-zhong; Zhou, Zhi-yuan; Wang, Bing; Qin, Jian; Liu, Wen-yong; Gu, Yan
Biochemical and Biophysical Research Communications (2014), 443(2), 406-412 | Language: English, Database: CAplus and MEDLINE

Substances (5) | Reactions (0) | Cited By (42)

8

The novel combination of dual mTOR inhibitor AZD2014 and pan-PIM inhibitor AZD1208 inhibits growth in acute myeloid leukemia via HSF pathway suppression.

✓

By: Harada, Masako; Benito, Juliana; Yamamoto, Shinichi; Kaur, Surinder; Arslan, Dirim; Ramirez, Santiago; Jacamo, Rodrigo; Platanias, Leonidas; Matsushita, Hiromichi; Fujimura, Tsutomu; et al
Oncotarget (2015), 6(35), 37930-47 | Language: English, Database: MEDLINE

Substance (1) | Reactions (0) | Cited By (28)

9

Rapamycin-insensitive companion of mTOR (RICTOR) amplification defines a subset of advanced gastric cancer and is sensitive to AZD2014-mediated mTORC1/2 inhibition.

Out

By: Kim, S T; Kim, S Y; Klempner, S J; Yoon, J; Kim, N; Ahn, S; Bang, H; Kim, K-M; Park, W; Park, S H; et al
Annals of oncology : official journal of the European Society for Medical Oncology (2017), 28(3), 547-554 | Language: English, Database: MEDLINE

Substances (3) | Reactions (0) | Cited By (40)

10

A high-throughput kinome screen reveals serum/glucocorticoid-regulated kinase 1 as a therapeutic target for NF2-deficient meningiomas.

By: Beauchamp, Roberta L; James, Marianne F; DeSouza, Patrick A; Wagh, Vilas; Zhao, Wen-Ning; Jordan, Justin T; Stemmer-Rachamimov, Anat; Plotkin, Scott R; Gusella, James F; Haggarty, Stephen J; et al
Oncotarget (2015), 6(19), 16981-97 | Language: English, Database: MEDLINE

√

Substance (1) | Reactions (0) | Cited By (37)

11

Potential of the dual mTOR kinase inhibitor AZD2014 to overcome paclitaxel resistance in anaplastic thyroid carcinoma.

√

By: Milošević, Zorica; Banković, Jasna; Dinić, Jelena; Tsimplouli, Chrisiida; Sereti, Evangelia; Dragoj, Miodrag; Paunović, Verica; Milovanović, Zorka; Stepanović, Marija; Tanić, Nikola; et al
Cellular oncology (Dordrecht) (2018), 41(4), 409-426 | Language: English, Database: MEDLINE

Substances (2) | Reactions (0) | Cited By (13)

12

Phase I study of orally administered ^{14}Carbon-isotope labelled-vistusertib (AZD2014), a dual TORC1/2 kinase inhibitor, to assess the absorption, metabolism, excretion, and pharmacokinetics in patients with advanced solid malignancies

Out

By: MacDonald, Alexander; Scarfe, Graeme; Magirr, Dominic; Sarvotham, Tinnu; Charlton, Julie; Brugger, Wolfram; Dean, Emma
Cancer Chemotherapy and Pharmacology (2019), 83(4), 787-795 | Language: English, Database: CAplus and MEDLINE

Substances (9) | Reactions (0) | Cited By (4)

13

AZD2014 radiosensitizes oral squamous cell carcinoma by inhibiting AKT/mTOR axis and inducing G1/G2/M cell cycle arrest

√

By: Yu, Chih-Chia; Huang, Hsien-bin; Hung, Shih-Kai; Liao, Hui-Fen; Lee, Ching-Chih; Lin, Hon-Yi; Li, Szu-Chin; Ho, Hsu-Chueh; Hung, Chung-Lin; Su, Yu-Chieh
PLoS One (2016), 11(3), e0151942/1-e0151942/15 | Language: English, Database: CAplus and MEDLINE

Substances (10) | Reactions (0) | Cited By (15)

14

Potent antitumour of the mTORC1/2 dual inhibitor AZD2014 in docetaxel-sensitive and docetaxel-resistant castration-resistant prostate cancer cells

By: Li, Senmao; Sheng, Jindong; Liu, Zhenhua; Fan, Yu; Zhang, Cuijian; Lv, Tianjing; Hu, Shuai; Jin, Jie; Yu, Wei; Song, Yi
Journal of Cellular and Molecular Medicine (2021), 25(5), 2436-2449 | Language: English, Database: CAplus and MEDLINE

Substances (4) Reactions (0) Cited By (7)

15

Synergistic induction of apoptosis by combination of BTK and dual mTORC1/2 inhibitors in diffuse large B cell lymphoma.

By: Ezell, Scott A; Mayo, Michele; Bihani, Teeru; Tepsuporn, Suprawee; Wang, Suping; Passino, Melissa; Grosskurth, Shaun E; Collins, Mike; Parmentier, Julie; Reimer, Corinne; et al
Oncotarget (2014), 5(13), 4990-5001 | Language: English, Database: MEDLINE

Substances (4) Reactions (0) Cited By (39)

16

Directly imaging the localisation and photosensitization properties of the pan-mTOR inhibitor, AZD2014, in living cancer cells

By: Ahmed, Abdullah R.; Candeo, Alessia; D'Abrantes, Sofia; Needham, Sarah R.; Yadav, Rahul B.; Botchway, Stanley W.; Parker, Anthony W.
Journal of Photochemistry and Photobiology, B: Biology (2020), 213, 112055 | Language: English, Database: CAplus and MEDLINE

Substances (5) Reactions (0) Cited By (2)

17

A phase II study to assess the safety and efficacy of the dual mTORC1/2 inhibitor vistusertib in relapsed, refractory DLBCL

Out

By: Eyre, Toby A.; Hildyard, Catherine; Hamblin, Angela; Ali, Ayesha S.; Houlton, Aimee; Hopkins, Louise; Royston, Daniel; Linton, Kim M.; Pettitt, Andrew; Rule, Simon; et al
Hematological Oncology (2019), 37(4), 352-359 | Language: English, Database: CAplus and MEDLINE

Substances (3) Reactions (0) Cited By (13)

18

The mTORC1 inhibitor rapamycin and the mTORC1/2 inhibitor AZD2014 impair the consolidation and persistence of contextual fear memory

Out

By: MacCallum, Phillip E.; Blundell, Jacqueline
Psychopharmacology (Heidelberg, Germany) (2020), 237(9), 2795-2808 | Language: English, Database: CAplus and MEDLINE

Substances (4) Reactions (0) Cited By (4)

19

Practical synthesis of Vistusertib (AZD2014), an ATP competitive mTOR inhibitor

By: Shen, Guobing; Liu, Miaoqing; Lu, Jianjun; Meng, Tao
Tetrahedron Letters (2019), 60(52), 151333 | Language: English, Database: CAplus

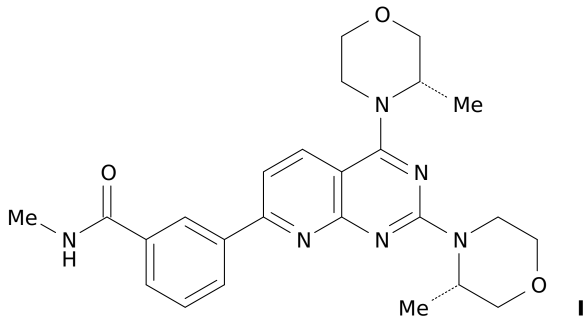

Substances (13) Reactions (21) Cited By (4)

20

An mTORC1/2 dual inhibitor, AZD2014, acts as a lysosomal function activator and enhances gemtuzumab ozogamicin-induced apoptosis in primary human leukemia cells

✓

By: Mizutani, Yu; Inase, Aki; Maimaitili, Yimamu; Miyata, Yoshiharu; Kitao, Akihito; Matsumoto, Hisayuki; Kawaguchi, Koji; Higashime, Ako; Goto, Hideaki; Kurata, Keiji; et al
International Journal of Hematology (2019), 110(4), 490-499 | Language: English, Database: CAplus and MEDLINE

Substances (4) Reactions (0) Cited By (12)

21

Allosteric and ATP-Competitive Inhibitors of mTOR Effectively Suppress Tumor Progression-Associated Epithelial-Mesenchymal Transition in the Kidneys of Tsc2$^{+/-}$ Mice

Out

By: Jones, Ashley T.; Yang, Jian; Narov, Kalin; Henske, Elizabeth P.; Sampson, Julian R.; Shen, Ming Hong
Neoplasia (New York, NY, United States) (2019), 21(8), 731-739 | Language: English, Database: CAplus and MEDLINE

Substances (5) Reactions (0) Cited By (8)

22

Targeting mantle cell lymphoma metabolism and survival through simultaneous blockade of mTOR and nuclear transporter exportin-1.

By: Sekihara, Kazumasa; Saitoh, Kaori; Han, Lina; Ciurea, Stefan; Yamamoto, Shinichi; Kikkawa, Mika; Kazuno, Saiko; Taka, Hikari; Kaga, Naoko; Arai, Hajime; et al
Oncotarget (2017), 8(21), 34552-34564 | Language: English, Database: MEDLINE

Substance (1) | Reactions (0) | Cited By (6)

23

Dual mTORC1/2 inhibitor AZD2014 diminishes myeloid-derived suppressor cells accumulation in ovarian cancer and delays tumor growth

By: Pi, Ruyu; Yang, Yang; Hu, Xiaoyi; Li, Hongyi; Shi, Houhui; Liu, Yu; Wang, Xi; Tong, An; Lu, Tianqi; Wei, Yuquan; et al
Cancer Letters (New York, NY, United States) (2021), 523, 72-81 | Language: English, Database: CAplus and MEDLINE

Substances (2) | Reactions (0) | Cited By (2)

24

Preclinical analysis of MTOR complex 1/2 inhibition in diffuse intrinsic pontine glioma

By: Flannery, Patrick C.; DeSisto, John A.; Amani, Vladimir; Venkataraman, Sujatha; Lemma, Rakeb T.; Prince, Eric W.; Donson, Andrew; Moroze, Erin E.; Hoffman, Lindsey; Mulcahy Levy, Jean M.; et al
Oncology Reports (2018), 39(2), 455-464 | Language: English, Database: CAplus and MEDLINE

Substances (4) | Reactions (0) | Cited By (16)

25

Vistusertib (dual m-TORC1/2 inhibitor) in combination with paclitaxel in patients with high-grade serous ovarian and squamous non-small-cell lung cancer.

Out

By: Basu, B; Krebs, M G; Sundar, R; Wilson, R H; Spicer, J; Jones, R; Brada, M; Talbot, D C; Steele, N; Ingles Garces, A H; et al
Annals of oncology : official journal of the European Society for Medical Oncology (2018), 29(9), 1918-1925 | Language: English, Database: MEDLINE

Substances (2) | Reactions (0) | Cited By (16)

26

Rapamycin induces mitogen-activated protein (MAP) kinase phosphatase-1 (MKP-1) expression through activation of protein kinase B and mitogen-activated protein kinase kinase pathways

By: Rastogi, Ruchi; Jiang, Zhongliang; Ahmad, Nisar; Rosati, Rita; Liu, Yusen; Beuret, Laurent; Monks, Robert; Charron, Jean; Birnbaum, Morris J.; Samavati, Lobelia
Journal of Biological Chemistry (2013), 288(47), 33966-33977 | Language: English, Database: CAplus and MEDLINE

Substances (8) | Reactions (0) | Cited By (43)

27

mTORC1/2 inhibitor served as a more ideal agent against the growth of mouse lymphocytic leukemia both in vitro and in vivo

By: Liao, Hui-Fen; Lin, You-Zhu; Yu, Chih-Chia; Tai, Tzong-Shyuan; Hung, Shih-Kai; Yang, Ching-Chieh; Su, Yu-Chieh
Anticancer Research (2019), 39(9), 4829-4835 | Language: English, Database: CAplus and MEDLINE

Substances (7) | Reactions (0) | Cited By (3)

28

Investigation of the novel mTOR inhibitor AZD2014 in neuronal ischemia

By: Hadley, Gina; Beard, Daniel J.; Alexopoulou, Zoi; Sutherland, Brad A.; Buchan, Alastair M.
Neuroscience Letters (2019), 706, 223-230 | Language: English, Database: CAplus and MEDLINE

Substances (2) | Reactions (0) | Cited By (5)

29

MEDI-573, Alone or in Combination with Mammalian Target of Rapamycin Inhibitors, Targets the Insulin-like Growth Factor Pathway in Sarcomas

By: Zhong, Haihong; Fazenbaker, Christine; Breen, Shannon; Chen, Cui; Huang, Jiaqi; Morehouse, Christopher; Yao, Yihong; Hollingsworth, Robert E.
Molecular Cancer Therapeutics (2014), 13(11), 2662-2673 | Language: English, Database: CAplus and MEDLINE

Substances (7) | Reactions (0) | Cited By (18)

30

Noninvasive MRI native T_1 mapping detects response to MYCN-targeted therapies in the Th-MYCN model of neuroblastoma

Out

By: Zormpas-Petridis, Konstantinos; Poon, Evon; Clarke, Matthew; Jerome, Neil P.; Boult, Jessica K. R.; Blackledge, Matthew D.; Carceller, Fernando; Koers, Alexander; Barone, Giuseppe; Pearson, Andrew D. J.; et al
Cancer Research (2020), 80(16), 3424-3435 | Language: English, Database: CAplus and MEDLINE

Substances (5) | Reactions (0) | Cited By (10)

31

Antitumor effects of AZD2014, a dual mTORC1/2 inhibitor, against human hepatocellular carcinoma xenograft in nude mice

Out

By: Liao, Hui; Wang, Yi; Xu, Xiaoping; Zhou, Chenjie; Zhang, Jianmin; Zhong, Kebo; Yang, Dinghua
Nanfang Yike Daxue Xuebao (2021), 41(7), 1056-1061 | Language: Chinese, Database: CAplus and MEDLINE

Substances (3) | Reactions (0) | Cited By (0)

32

Inhibiting mTOR activity using AZD2014 increases autophagy in the mouse cerebral cortex

✓

By: Bensalem, Julien; Fourrier, Celia; Hein, Leanne K.; Hassiotis, Sofia; Proud, Christopher G.; Sargeant, Timothy J.
Neuropharmacology (2021), 190, 108541 | Language: English, Database: CAplus and MEDLINE

Substances (8) | Reactions (0) | Cited By (5)

33

A phase I study of vistusertib (dual mTORC1/2 inhibitor) in patients with previously treated glioblastoma multiforme: a CCTG study

Out

By: Lapointe, Sarah; Mason, Warren; MacNeil, Mary; Harlos, Craig; Tsang, Roger; Sederias, Joana; Luchman, H. Artee; Weiss, Samuel; Rossiter, John P.; Tu, Dongsheng; et al
Investigational New Drugs (2020), 38(4), 1137-1144 | Language: English, Database: CAplus and MEDLINE

Substances (6) | Reactions (0) | Cited By (14)

34

RICTOR amplification identifies a subgroup in small cell lung cancer and predicts response to drugs targeting mTOR.

✓

By: Sakre, Nneha; Wildey, Gary; Behtaj, Mohadese; Kresak, Adam; Yang, Michael; Fu, Pingfu; Dowlati, Afshin
Oncotarget (2017), 8(4), 5992-6002 | Language: English, Database: MEDLINE

Substances (3) | Reactions (0) | Cited By (34)

35

Influence of the novel ATP-competitive dual mTORC1/2 inhibitor AZD2014 on immune cell populations and heart allograft rejection

✓

By: Fantus, Daniel; Dai, Helong; Ono, Yoshihiro; Watson, Alicia; Yokota, Shinichiro; Mohib, Kanishka; Yoshida, Osamu; Ross, Mark A.; Watkins, Simon C.; Ramaswami, Bala; et al
Transplantation (2017), 101(12), 2830-2840 | Language: English, Database: CAplus and MEDLINE

Substances (3) | Reactions (0) | Cited By (10)

36

Combination therapy with mTOR kinase inhibitor and dasatinib as a novel therapeutic strategy for vestibular schwannoma

By: Sagers, Jessica E.; Beauchamp, Roberta L.; Zhang, Yanling; Vasilijic, Sasa; Wu, Limeng; De Souza, Patrick; Seist, Richard; Zhou, Wenjianlong; Xu, Lei ; Ramesh, Vijaya; et al
Scientific Reports (2020), 10(1), 4211 | Language: English, Database: CAplus and MEDLINE

Substances (5) | Reactions (0) | Cited By (8)

37

Targeting autophagy to modulate cell survival: a comparative analysis in cancer, normal and embryonic cells

By: Divac Rankov, Aleksandra; Ljujic, Mila; Petric, Marija; Radojkovic, Dragica; Pesic, Milica; Dinic, Jelena
Histochemistry and Cell Biology (2017), 148(5), 529-544 | Language: English, Database: CAplus and MEDLINE

Substances (5) | Reactions (0) | Cited By (10)

38

Inhibition of Bcl-2 potentiates AZD-2014-induced anti-head and neck squamous cell carcinoma cell activity

By: Li, Yi; Cui, Jiang-Tao
Biochemical and Biophysical Research Communications (2016), 477(4), 607-613 | Language: English, Database: CAplus and MEDLINE

Substances (6) | Reactions (0) | Cited By (10)

39

F-metafuorobenzylguanidine (18F-mFBG) to monitor changes in norepinephrine transporter expression in response to therapeutic intervention in neuroblastoma models

By: Turnock, Stephen ; Turton, David R.; Martins, Carlos Daniel; Chesler, Louis; Wilson, Thomas C.; Gouverneur, Veronique; Smith, Graham; Kramer-Marek, Gabriela
Scientific Reports (2020), 10(1), 20918 | Language: English, Database: CAplus and MEDLINE

Substances (2) | Reactions (0) | Cited By (3)

40

AZD2014, a dual mTOR inhibitor, attenuates cardiac hypertrophy in vitro and in vivo

By: Cha, Byung-Hyun; Jung, Minjin; Kim, Angela S.; Lepak, Victoria C.; Colson, Brett A.; Bull, David A.; Won, Youngwook
Journal of Biological Engineering (2021), 15(1), 24 | Language: English, Database: CAplus and MEDLINE

Substances (4) | Reactions (0) | Cited By (0)

41

Validation of a rapid liquid chromatography tandem mass spectrometric method for the quantitative analysis of vistusertib

Out

By: Scott, Susan C.; Anders, Nicole M.; Scardina, Morgan; Hann, Christine L.; Rudek, Michelle A.
Journal of Pharmaceutical and Biomedical Analysis (2022), 208, 114436 | Language: English, Database: CAplus and MEDLINE

Substance (1) | Reactions (0) | Cited By (0)

42

mTOR deregulation in oral cavity squamous cell carcinoma.

Out

By: Mastronikolis, Nicholas S; Tsiambas, Evangelos; Papadas, Theodoros A; Fotiades, Panagiotis P; Papadas, Athanasios T; Mastronikolis, Stylianos N; Kastanioudakis, Ioannis; Ragos, Vasileios
Journal of B.U.ON. : official journal of the Balkan Union of Oncology (2017), 22(3), 610-613 | Language: English, Database: MEDLINE

Substance (1) | Reactions (0) | Cited By (1)

43

The dual mTORC1/2 inhibitor AZD2014 inhibits acute graft rejection in a rat liver transplantation model

Out

By: Liao, Hui; Wang, Yi; Xu, Xiaoping; Zhou, Chenjie; Zhang, Jianmin; Zhong, Kebo; Yang, Dinghua
Nanfang Yike Daxue Xuebao (2022), 42(4), 598-603 | Language: Chinese, Database: CAplus and MEDLINE

Substances (4) | Reactions (0) | Cited By (0)

44

Combination of dual mTORC1/2 inhibition and immune-checkpoint blockade potentiates anti-tumor immunity

Out

By: Langdon, Sophie; Hughes, Adina; Taylor, Molly A.; Kuczynski, Elizabeth A.; Mele, Deanna A.; Delpuech, Oona; Jarvis, Laura; Staniszewska, Anna; Cosulich, Sabina; Carnevalli, Larissa S.; et al
OncoImmunology (2018), 7(8), e1458810/1-e1458810/12 | Language: English, Database: CAplus and MEDLINE

Substances (2) | Reactions (0) | Cited By (21)

45

MiR-99a Enhances the Radiation Sensitivity of Non-Small Cell Lung Cancer by Targeting mTOR ✓

By: Yin, Hang; Ma, Jianqun; Chen, Lin; Piao, Shiqi; Zhang, Yu; Zhang, Siliang; Ma, Hongyu; Li, Yang; Qu, Yuanyuan; Wang, Xiaoyuan; et al
Cellular Physiology and Biochemistry (2018), 46(2), 471-481 | Language: English, Database: CAplus and MEDLINE

Substances (2) | Reactions (0) | Cited By (43)

46

An adaptive, biomarker-directed platform study of durvalumab in combination with targeted therapies in advanced urothelial cancer

Out

By: Powles, Thomas ; Carroll, Danielle; Chowdhury, Simon; Gravis, Gwenaelle; Joly, Florence; Carles, Joan; Flechon, Aude; Maroto, Pablo; Petrylak, Daniel; Rolland, Frederic ; et al
Nature Medicine (New York, NY, United States) (2021), 27(5), 793-801 | Language: English, Database: CAplus and MEDLINE

Substances (4) | Reactions (0) | Cited By (24)

47

Combined Inhibition of mTOR and CDK4/6 Is Required for Optimal Blockade of E2F Function and Long-term Growth Inhibition in Estrogen Receptor-positive Breast Cancer

By: Michaloglou, Chrysiis; Crafter, Claire; Siersbaek, Rasmus; Delpuech, Oona; Curwen, Jon O.; Carnevalli, Larissa S.; Staniszewska, Anna D.; Polanska, Urszula M.; Cheraghchi-Bashi, Azadeh; Lawson, Mandy; et al
Molecular Cancer Therapeutics (2018), 17(5), 908-920 | Language: English, Database: CAplus and MEDLINE

Substances (9) | Reactions (0) | Cited By (90)

48

Rituximab (anti-CD20)-modified AZD-2014-encapsulated nanoparticles killing of B lymphoma cells

Out

By: Tang, Xiaolong; Xie, Chunmei; Jiang, Zhenyou; Li, Amin; Cai, Shiyu; Hou, Changhao; Wang, Jian; Liang, Yong; Ma, Dong
Artificial Cells, Nanomedicine, and Biotechnology (2018), 46(sup2), 1063-1073 | Language: English, Database: CAplus and MEDLINE

Substances (4) | Reactions (0) | Cited By (6)

49

Aberrant cholesterol metabolic signaling impairs antitumor immunosurveillance through natural killer T cell dysfunction in obese liver

Out

By: Tang, Wenshu; Zhou, Jingying ; Yang, Weiqin; Feng, Yu; Wu, Haoran; Mok, Myth T. S.; Zhang, Lingyun; Liang, Zhixian; Liu, Xiaoyu; Xiong, Zhewen; et al
Cellular & Molecular Immunology (2022), 19(7), 834-847 | Language: English, Database: CAplus and MEDLINE

Substances (2) | Reactions (0) | Cited By (2)

50

Combination Vistusertib and Anastrozole Shows Benefit in Endometrial Cancer.

Out

No author and editor data available.
Cancer discovery (2022), 12(7), OF20 | Language: English, Database: MEDLINE

Substances (2) | Reactions (0) | Cited By (0)

51

Safety and Efficacy of the mTOR Inhibitor, Vistusertib, Combined With Anastrozole in Patients With Hormone Receptor-Positive Recurrent or Metastatic Endometrial Cancer: The VICTORIA Multicenter, Open-label, Phase 1/2 Randomized Clinical Trial.

Out

By: Heudel, Pierre; Frenel, Jean-Sébastien; Dalban, Cécile; Bazan, Fernando; Joly, Florence; Arnaud, Antoine; Abdeddaim, Cyril; Chevalier-Place, Annick; Augereau, Paule; Pautier, Patricia; et al
JAMA oncology (2022), 8(7), 1001-1009 | Language: English, Database: MEDLINE

Substances (2) | Reactions (0) | Cited By (5)

52

Combined mTOR/MEK inhibition prevents proliferation and induces apoptosis in NF2-mutant tumors.

Out

By: Li, N; Lu, X-Y; Shi, W-Y; Mao, F-J; Yang, X-Y; Luo, Y-B; Li, W
European review for medical and pharmacological sciences (2019), 23(13), 5874-5883 | Language: English, Database: MEDLINE

Substances (2) | Reactions (0) | Cited By (5)

53

Evolution of PIKK family kinase inhibitors: A new age cancer therapeutics.

Out

By: Shaik, Althaf; Kirubakaran, Sivapriya
Frontiers in bioscience (Landmark edition) (2020), 25(8), 1510-1537 | Language: English, Database: MEDLINE

Substances (3) | Reactions (0) | Cited By (6)

54

Intravital Imaging to Monitor Therapeutic Response in Moving Hypoxic Regions Resistant to PI3K Pathway Targeting in Pancreatic Cancer

✓

By: Conway, James R. W.; Warren, Sean C.; Herrmann, David; Murphy, Kendelle J.; Cazet, Aurelie S.; Vennin, Claire; Shearer, Robert F.; Killen, Monica J.; Magenau, Astrid; Melenec, Pauline; et al
Cell Reports (2018), 23(11), 3312-3326 | Language: English, Database: CAplus and MEDLINE

Substances (9) | Reactions (0) | Cited By (50)

55

Combination of mTORC1/2 inhibitor vistusertib plus fulvestrant in vitro and in vivo targets oestrogen receptor-positive endocrine-resistant breast cancer

✓

By: Pancholi, Sunil; Leal, Mariana Ferreira; Ribas, Ricardo; Simigdala, Nikiana; Schuster, Eugene; Chateau-Joubert, Sophie; Zabaglo, Lila; Hills, Margaret; Dodson, Andrew; Gao, Qiong; et al
Breast Cancer Research (2019), 21(1), 135 | Language: English, Database: CAplus and MEDLINE

Substances (5) | Reactions (0) | Cited By (5)

56

Kinome RNAi Screens Reveal Synergistic Targeting of MTOR and FGFR1 Pathways for Treatment of Lung Cancer and HNSCC

By: Singleton, Katherine R.; Hinz, Trista K.; Kleczko, Emily K.; Marek, Lindsay A.; Kwak, Jeff; Harp, Taylor; Kim, Jihye; Tan, Aik Choon; Heasley, Lynn E.
Cancer Research (2015), 75(20), 4398-4406 | Language: English, Database: CAplus and MEDLINE

Substances (14) | Reactions (0) | Cited By (46)

57

Synergistic effects of combination therapy with AKT and mTOR inhibitors on bladder cancer cells

By: Kim, Hyera; Lee, Su Jin; Lee, In Kyoung; Min, Suejean C.; Sung, Hyun Hwan; Jeong, Byong Chang; Lee, Jeeyun; Park, Se Hoon
International Journal of Molecular Sciences (2020), 21(8), 2825 | Language: English, Database: CAplus and MEDLINE

Substances (5) | Reactions (0) | Cited By (6)

58

Inducing and exploiting vulnerabilities for the treatment of liver cancer

By: Wang, Cun; Vegna, Serena; Jin, Haojie; Benedict, Bente; Lieftink, Cor; Ramirez, Christel; Leite de Oliveira, Rodrigo; Ben, Morris; Gadiot, Jules; Wang, Wei; et al
Nature (London, United Kingdom) (2019), 574(7777), 268-272 | Language: English, Database: CAplus and MEDLINE

Substances (6) | Reactions (0) | Cited By (148)

59

mTOR inhibition sensitizes human hepatocellular carcinoma cells to resminostat

By: Peng, Xingang; Zhang, Donghui; Li, Zhengling; Fu, Meili; Liu, Haiyan
Biochemical and Biophysical Research Communications (2016), 477(4), 556-562 | Language: English, Database: CAplus and MEDLINE

Substances (12) | Reactions (0) | Cited By (9)

60

Quantitation of vistusertib by UHPLC-MS/MS in rat plasma and its application to a pharmacokinetic study

By: Wang, Fuqi; Song, Danni; Zou, Fengmao; Zhao, Honghui; Zhao, Xu
Bioanalysis (2021), 13(17), 1333-1341 | Language: English, Database: CAplus and MEDLINE
Analytical Methods available

Substances (3) | Reactions (0) | Cited By (0)

61

mTORC2 Signaling Drives the Development and Progression of Pancreatic Cancer

By: Driscoll, David R.; Karim, Saadia A.; Sano, Makoto; Gay, David M.; Jacob, Wright; Yu, Jun; Mizukami, Yusuke; Gopinathan, Aarthi; Jodrell, Duncan I.; Evans, T. R. Jeffry; et al
Cancer Research (2016), 76(23), 6911-6923 | Language: English, Database: CAplus and MEDLINE

Substances (10) Reactions (0) Cited By (51)

62

Synergistic drug combinations and machine learning for drug repurposing in chordoma

By: Anderson, Edward; Havener, Tammy M.; Zorn, Kimberley M.; Foil, Daniel H.; Lane, Thomas R.; Capuzzi, Stephen J.; Morris, Dave; Hickey, Anthony J.; Drewry, David H.; Ekins, Sean
Scientific Reports (2020), 10(1), 12982 | Language: English, Database: CAplus and MEDLINE

Out

Substances (6) Reactions (0) Cited By (16)

63

EPH receptor signaling as a novel therapeutic target in NF2-deficient meningioma

By: Angus, Steven P.; Oblinger, Janet L.; Stuhlmiller, Timothy J.; DeSouza, Patrick A.; Beauchamp, Roberta L.; Witt, Luke; Chen, Xin; Jordan, Justin T.; Gilbert, Thomas S. K.; Stemmer-Rachamimov, Anat; et al
Neuro-Oncology (Cary, NC, United States) (2018), 20(9), 1185-1196 | Language: English, Database: CAplus and MEDLINE

Substances (12) Reactions (0) Cited By (14)

64

Integrated pharmaco-proteogenomics defines two subgroups in isocitrate dehydrogenase wild-type glioblastoma with prognostic and therapeutic opportunities

By: Oh, Sejin; Yeom, Jeonghun; Cho, Hee Jin; Kim, Ju-Hwa; Yoon, Seon-Jin; Kim, Hakhyun; Sa, Jason K.; Ju, Shinyeong; Lee, Hwanho; Oh, Myung Joon; et al
Nature Communications (2020), 11(1), 3288 | Language: English, Database: CAplus and MEDLINE

Substances (5) Reactions (0) Cited By (23)

65

Insights into significance of combined inhibition of MEK and m-TOR signalling output in KRAS mutant non-small-cell lung cancer

By: Broutin, Sophie; Stewart, Adam; Thavasu, Parames; Paci, Angelo; Bidart, Jean-Michel; Banerji, Udai
British Journal of Cancer (2016), 115(5), 549-552 | Language: English, Database: CAplus and MEDLINE

Substances (5) Reactions (0) Cited By (9)

66

Biomarker-driven phase 2 umbrella trial study for patients with recurrent small cell lung cancer failing platinum-based chemotherapy

Out

By: Park, Sehhoon; Shim, Joonho ; Mortimer, Peter G. S.; Smith, Simon A.; Godin, Robert E.; Hollingsworth, Simon J.; Kim, Hee-Jung; Jung, Hyun Ae; Sun, Jong-Mu; Park, Woong-Yang; et al
Cancer (Hoboken, NJ, United States) (2020), 126(17), 4002-4012 | Language: English, Database: CAplus and MEDLINE

Substances (4) Reactions (0) Cited By (6)

67

Catalytic mammalian target of rapamycin inhibitors as antineoplastic agents

By: Mohindra, Nisha A.; Platanias, Leonidas C.
Leukemia & Lymphoma, | Language: English, Database: CAplus and MEDLINE

Out

Substances (4) Reactions (0) Cited By (0)

68

Mechanisms underlying synergy between DNA topoisomerase I-targeted drugs and mTOR kinase inhibitors in NF1-associated malignant peripheral nerve sheath tumors

✓

By: Ki, Dong Hyuk ; Oppel, Felix ; Durbin, Adam D. ; Look, A. Thomas
Oncogene (2019), 38(39), 6585-6598 | Language: English, Database: CAplus and MEDLINE

Substances (6) Reactions (0) Cited By (12)

69

CC-223 inhibits human head and neck squamous cell carcinoma cell growth

By: Wang, Jun-ying; Jin, Xin; Zhang, Xin; Li, Xiao-feng

Out

Biochemical and Biophysical Research Communications (2018), 496(4), 1191-1196 | Language: English, Database: CAplus and MEDLINE

Substances (13) Reactions (0) Cited By (7)

70

PI3k inhibitors (BKM120 and BYL719) as radiosensitizers for head and neck squamous cell carcinoma during radiotherapy

By: Chuang, Fu-Cheng; Wang, Chih-Chun; Chen, Jian-Han; Hwang, Tzer-Zen; Yeh, Shyh-An; Su, Yu-Chieh

✓

PLoS One (2021), 16(1), e0245715 | Language: English, Database: CAplus and MEDLINE

Substances (6) Reactions (0) Cited By (6)

71

Phosphorylated ribosomal S6 (p-rpS6) as a post-treatment indicator of HER2 signalling targeted drug resistance

By: Yang-Kolodji, Gloria; Mumenthaler, Shannon M.; Mehta, Arjun; Ji, Lingyun; Tripathy, Debu
Biomarkers (2015), 20(5), 313-322 | Language: English, Database: CAplus and MEDLINE

Substances (9) Reactions (0) Cited By (7)

72

MEDI-573, Alone or in Combination with Mammalian Target of Rapamycin Inhibitors, Targets the Insulin-like Growth Factor Pathway in Sarcomas [Erratum to document cited in CA162:419021]

By: Zhong, Haihong; Fazenbaker, Christine; Breen, Shannon; Chen, Cui; Huang, Jiaqi; Morehouse, Christopher; Yao, Yihong; Hollingsworth, Robert E.
Molecular Cancer Therapeutics (2015), 14(3), 844 | Language: English, Database: CAplus and MEDLINE

Substances (7) Reactions (0) Cited By (0)

73

Reconciling Selectivity Trends from a Comprehensive Kinase Inhibitor Profiling Campaign with Known Activity Data

By: Miljkovic, Filip; Bajorath, Juergen
ACS Omega (2018), 3(3), 3113-3119 | Language: English, Database: CAplus and MEDLINE

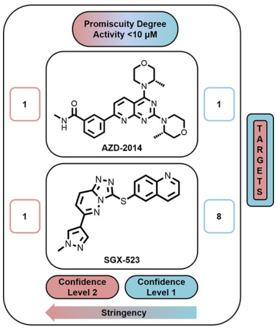

Substances (5) Reactions (0) Cited By (12)

74

Chemo-protective and regenerative effects of diarylheptanoids from the bark of black alder (Alnus glutinosa) in human normal keratinocytes

By: Dinic, Jelena; Randjelovic, Teodora; Stankovic, Tijana; Dragoj, Miodrag; Isakovic, Aleksandra; Novakovic, Miroslav; Pesic, Milica
Fitoterapia (2015), 105, 169-176 | Language: English, Database: CAplus and MEDLINE

Substances (5) Reactions (0) Cited By (13)

75

Context-dependent intravital imaging of therapeutic response using intramolecular FRET biosensors

By: Conway, James R. W.; Warren, Sean C.; Timpson, Paul
Methods (Amsterdam, Netherlands) (2017), 128, 78-94 | Language: English, Database: CAplus and MEDLINE

Substances (10) | Reactions (0) | Cited By (27)

76

Intermittent High-Dose Scheduling of AZD8835, a Novel Selective Inhibitor of PI3Kα and PI3Kδ, Demonstrates Treatment Strategies for PIK3CA-Dependent Breast Cancers

By: Hudson, Kevin; Hancox, Urs J.; Trigwell, Cath; McEwen, Robert; Polanska, Urszula M.; Nikolaou, Myria; Morentin Gutierrez, Pablo; Avivar-Valderas, Alvaro; Delpuech, Oona; Dudley, Phillippa; et al
Molecular Cancer Therapeutics (2016), 15(5), 877-889 | Language: English, Database: CAplus and MEDLINE

Substances (14) | Reactions (0) | Cited By (34)

77

Enhanced autocrine FGF19/FGFR4 signaling drives the progression of lung squamous cell carcinoma, which responds to mTOR inhibitor AZD2104

By: Li, Fan; Li, Ziming; Han, Qing; Cheng, Yirui; Ji, Wenxiang; Yang, Ying; Lu, Shun; Xia, Weiliang
Oncogene (2020), 39(17), 3507-3521 | Language: English, Database: CAplus and MEDLINE

Substances (7) | Reactions (0) | Cited By (14)

78

Repurposing of mtor complex inhibitors attenuates MCL-1 and sensitizes to PARP inhibition

By: Mattoo, Abid R.; Joun, Alex; Jessup, J. Milburn
Molecular Cancer Research (2019), 17(1), 42 | Language: English, Database: CAplus and MEDLINE

Substances (7) | Reactions (0) | Cited By (11)

79

Comparative toxicity evaluation of targeted anticancer therapeutics in embryonic zebrafish and sea urchin models

Out

By: Babic, Tamara; Dinic, Jelena; Buric, Sonja Stojkovic; Hadzic, Stefan; Pesic, Milica; Radojkovic, Dragica; Rankov, Aleksandra Divac
Acta Biologica Hungarica (2018), 69(4), 395-410 | Language: English, Database: CAplus and MEDLINE

Substances (8) | Reactions (0) | Cited By (1)

80

An mTOR and DNA-PK dual inhibitor CC-115 hinders non-small cell lung cancer cell growth

By: Chen, Fagui; Zhao, Huasi; Li, Chenhui; Li, Ping ; Zhang, Qichuan
Cell Death Discovery (2022), 8(1), 293 | Language: English, Database: CAplus and MEDLINE

Substances (6) | Reactions (0) | Cited By (1)

81

Impact of baseline culture conditions of cancer organoids when determining therapeutic response and tumor heterogeneity

By: DeStefanis, Rebecca A.; Kratz, Jeremy D.; Olson, Autumn M.; Sunil, Aishwarya; DeZeeuw, Alyssa K.; Gillette, Amani A.; Sha, Gioia C.; Johnson, Katherine A.; Pasch, Cheri A.; Clipson, Linda; et al
Scientific Reports (2022), 12(1), 5205 | Language: English, Database: CAplus and MEDLINE

Substances (6) | Reactions (0) | Cited By (3)

82

A phase 1/2 study of the combination of acalabrutinib and vistusertib in patients with relapsed/refractory B-cell malignancies

Out

By: Collins, Graham P.; Clevenger, Tracy N.; Burke, Kathleen A.; Yang, Buyue; MacDonald, Alex; Cunningham, David; Fox, Christopher P.; Goy, Andre; Gribben, John; Nowakowski, Grzegorz S.; et al
Leukemia & Lymphoma (2021), 62(11), 2625-2636 | Language: English, Database: CAplus and MEDLINE

Substances (6) | Reactions (0) | Cited By (3)

83

Tumor genomic profiling guides patients with metastatic gastric cancer to targeted treatment: the VIKTORY umbrella trial

Out

By: Lee, Jeeyun; Kim, Seung Tae; Kim, Kyung; Lee, Hyuk; Kozarewa, Iwanka; Mortimer, Peter G. S.; Odegaard, Justin I.; Harrington, Elizabeth A.; Lee, Juyoung; Lee, Taehyang; et al
Cancer Discovery (2019), 9(10), 1388-1405 | Language: English, Database: CAplus and MEDLINE

Substances (6) | Reactions (0) | Cited By (90)

84

Therapeutic Enhancement of Verteporfin-mediated Photodynamic Therapy by mTOR Inhibitors.

By: Kraus, Daniel; Palasuberniam, Pratheeba; Chen, Bin
Photochemistry and Photobiology (2020), 96(2), 358-364 | Language: English, Database: CAplus and MEDLINE

Substances (8) | Reactions (0) | Cited By (3)

85

Overproduction of IGF-2 drives a subset of colorectal cancer cells, which specifically respond to an anti-IGF therapeutic antibody and combination therapies

By: Zhong, H.; Fazenbaker, C.; Chen, C.; Breen, S.; Huang, J.; Yao, X.; Ren, P.; Yao, Y.; Herbst, R.; Hollingsworth, R. E.
Oncogene (2017), 36(6), 797-806 | Language: English, Database: CAplus and MEDLINE

Substances (11) | Reactions (0) | Cited By (17)

86

First-in-child phase I/II study of the dual mTORC1/2 inhibitor vistusertib (AZD2014) as monotherapy and in combination with topotecan-temozolomide in children with advanced malignancies: arms E and F of the AcS-ESMART trial

Out

By: Morscher, Raphael J.; Brard, Caroline ; Berlanga, Pablo ; Marshall, Lynley V. ; Andre, Nicolas; Rubino, Jonathan ; Aerts, Isabelle; De Carli, Emilie ; Corradini, Nadege; Nebchi, Souad; et al
European Journal of Cancer (2021), 157, 268-277 | Language: English, Database: CAplus and MEDLINE

Substances (17) | Reactions (0) | Cited By (3)

87

Prolonged tau clearance and stress vulnerability rescue by pharmacological activation of autophagy in tauopathy neurons

By: Silva, M. Catarina ; Nandi, Ghata A. ; Tentarelli, Sharon; Gurrell, Ian K.; Jamier, Tanguy; Lucente, Diane; Dickerson, Bradford C.; Brown, Dean G.; Brandon, Nicholas J.; Haggarty, Stephen J.
Nature Communications (2020), 11(1), 3258 | Language: English, Database: CAplus and MEDLINE

Substances (16) | Reactions (0) | Cited By (53)

88

mTOR inhibition amplifies the anti-lymphoma effect of PI3Kβ/δ blockage in diffuse large B-cell lymphoma

By: Xu, Wendan; Berning, Philipp; Erdmann, Tabea; Grau, Michael; Bettazova, Nardjas; Zapukhlyak, Myroslav; Frontzek, Fabian ; Kosnopfel, Corinna ; Lenz, Peter; Grondine, Michael; et al
Leukemia, | Language: English, Database: CAplus and MEDLINE

Substances (9) | Reactions (0) | Cited By (0)

89

SARS-CoV-2 infection rewires host cell metabolism and is potentially susceptible to mTORC1 inhibition

By: Mullen, Peter J.; Garcia, Gustavo Jr; Purkayastha, Arunima; Matulionis, Nedas; Schmid, Ernst W.; Momcilovic, Milica; Sen, Chandani; Langerman, Justin; Ramaiah, Arunachalam; Shackelford, David B.; et al
Nature Communications (2021), 12(1), 1876 | Language: English, Database: CAplus and MEDLINE

Substances (12) | Reactions (0) | Cited By (56)

90

GDC-0349 inhibits non-small cell lung cancer cell growth

By: Yang, Han; Zhao, Jun; Zhao, Mengjing; Zhao, Lihao; Zhou, Li-na; Duan, Yuxia; Li, Gang
Cell Death & Disease (2020), 11(11), 951 | Language: English, Database: CAplus and MEDLINE

Substances (15) | Reactions (0) | Cited By (12)

91

Sensitivity to pi3k and akt inhibitors is mediated by divergent molecular mechanisms in subtypes of dlbcl

By: Erdmann, Tabea; Klener, Pavel; Lynch, James T.; Grau, Michael; Vockova, Petra; Molinsky, Jan; Tuskova, Diana; Hudson, Kevin; Polanska, Urszula M.; Grondine, Michael; et al
Blood (2017), 130(3), 310-322 | Language: English, Database: CAplus and MEDLINE

Substances (23) | Reactions (0) | Cited By (59)

92

Learning from BISCAY: The future of biomarker-based trial design in bladder cancer

By: Meza, Luis; Salgia, Nicholas J.; Patel, Kalen C.; Pal, Sumanta K.
Cancer Cell (2021), 39(7), 910-912 | Language: English, Database: CAplus and MEDLINE

Substances (8) | Reactions (0) | Cited By (1)

93

Sustained mTORC1 activity during palbociclib-induced growth arrest triggers senescence in ER+ breast cancer cells

By: Maskey, Reeja S.; Wang, Fang; Lehman, Elyssa; Wang, Yiqun; Emmanuel, Natasha; Zhong, Wenyan; Jin, Guixian; Abraham, Robert T.; Arndt, Kim T.; Myers, Jeremy S.; et al
Cell Cycle (2021), 20(1), 65-80 | Language: English, Database: CAplus and MEDLINE

Substances (8) | Reactions (0) | Cited By (10)

94

A phase I study investigating AZD8186, a potent and selective inhibitor of PI3κβ/δ, in patients with advanced solid tumors

Out

By: Choudhury, Atish D.; Higano, Celestia S.; de Bono, Johann S.; Cook, Natalie; Rathkopf, Dana E.; Wisinski, Kari B.; Martin-Liberal, Juan; Linch, Mark; Heath, Elisabeth I.; Baird, Richard D.; et al
Clinical Cancer Research (2022), 28(11), 2257-2269 | Language: English, Database: CAplus and MEDLINE

Substances (16) Reactions (0) Cited By (1)

95

Drug Efficacy Comparison of 3D Forming and Preforming Sphere Models with a Micropillar and Microwell Chip Platform

By: Doh, Il; Kwon, Yong-Jun; Ku, Bosung; Lee, Dong Woo
SLAS Discovery (2019), 24(4), 476-483 | Language: English, Database: CAplus and MEDLINE

Substances (11) Reactions (0) Cited By (6)

96

PIK3CA mutations enable targeting of a breast tumor dependency through mTOR-mediated MCL-1 translation

By: Anderson, Grace R.; Wardell, Suzanne E.; Cakir, Merve; Crawford, Lorin; Leeds, Jim C.; Nussbaum, Daniel P.; Shankar, Pallavi S.; Soderquist, Ryan S.; Stein, Elizabeth M.; Tingley, Jennifer P.; et al
Science Translational Medicine (2016), 8(369), 369ra175/1-369ra175/15 | Language: English, Database: CAplus and MEDLINE

Substances (16) Reactions (0) Cited By (36)

97

Dramatic antitumor effects of the dual mTORC1 and mTORC2 inhibitor AZD2014 in hepatocellular carcinoma.

By: Liao, Hui; Huang, Yu; Guo, Botang; Liang, Bo; Liu, Xincheng; Ou, Huohui; Jiang, Chenglong; Li, Xianghong; Yang, Dinghua
American journal of cancer research (2015), 5(1), 125-39 | Language: English, Database: MEDLINE

Substances (0) Reactions (0) Cited By (44)

98

High-throughput dynamic BH3 profiling may quickly and accurately predict effective therapies in solid tumors

By: Bhola, Patrick D.; Ahmed, Eman; Guerriero, Jennifer L.; Sicinska, Ewa; Su, Emily; Lavrova, Elizaveta; Ni, Jing; Chipashvili, Otari; Hagan, Timothy; Pioso, Marissa S.; et al
Science Signaling (2020), 13(636), eaay1451 | Language: English, Database: CAplus and MEDLINE

Substances (31) | Reactions (0) | Cited By (23)

99

mTOR kinase inhibition disrupts neuregulin 1-ERBB3 autocrine signaling and sensitizes NF2-deficient meningioma cellular models to IGF1R inhibition

By: Beauchamp, Roberta L.; Erdin, Serkan; Witt, Luke; Jordan, Justin T.; Plotkin, Scott R.; Gusella, James F.; Ramesh, Vijaya
Journal of Biological Chemistry (2021), 296, 100157 | Language: English, Database: CAplus and MEDLINE

Substances (9) | Reactions (0) | Cited By (1)

100

Synergistic effects between mTOR complex 1/2 and glycolysis inhibitors in non-small-cell lung carcinoma cells

By: Jiang, Suhua; Zou, Zhengzhi; Nie, Peipei; Wen, Ruiling; Xiao, Yingying; Tang, Jun
PLoS One (2015), 10(7), e0132880/1-e0132880/19 | Language: English, Database: CAplus and MEDLINE

Substances (9) | Reactions (0) | Cited By (15)

101

Drug-Dependent Morphological Transitions in Spherical and Worm-Like Polymeric Micelles Define Stability and Pharmacological Performance of Micellar Drugs

By: Lim, Chaemin; Ramsey, Jacob D.; Hwang, Duhyeong; Teixeira, Susana C. M.; Poon, Chi-Duen; Strauss, Joshua D.; Rosen, Elias P.; Sokolsky-Papkov, Marina; Kabanov, Alexander V.
Small (2022), 18(4), 2103552 | Language: English, Database: CAplus and MEDLINE

Substances (17) | Reactions (0) | Cited By (9)

102

Reciprocal effects of mTOR inhibitors on pro-survival proteins dictate therapeutic responses in tuberous sclerosis complex

By: McNamara, Molly C.; Hosios, Aaron M.; Torrence, Margaret E.; Zhao, Ting; Fraser, Cameron; Wilkinson, Meghan; Kwiatkowski, David J.; Henske, Elizabeth P.; Wu, Chin-Lee; Sarosiek, Kristopher A.; et al
iScience (2022), 25(11), 105458 | Language: English, Database: CAplus and MEDLINE

Substances (10) Reactions (0) Cited By (0)

103

Identification and analysis of DNA methylation-driven signatures for prognostic and immune microenvironments evaluation in hepatocellular carcinoma

By: Shen, Bingbing; Wen, Zhen; Lv, Gang; Wang, Jianguo; Han, Ruijie; Jiang, Jianxin
Frontiers in Genetics (2022), 13, 1022078 | Language: English, Database: CAplus and MEDLINE

Substances (10) Reactions (0) Cited By (0)

104

Pre-clinical activity of combined LSD1 and mTORC1 inhibition in MLL-translocated acute myeloid leukaemia

By: Deb, Gauri; Wingelhofer, Bettina; Amaral, Fabio M. R.; Maiques-Diaz, Alba; Chadwick, John A.; Spencer, Gary J.; Williams, Emma L.; Leong, Hui-Sun; Maes, Tamara; Somervaille, Tim C. P.
Leukemia (2020), 34(5), 1266-1277 | Language: English, Database: CAplus and MEDLINE

Substances (10) Reactions (0) Cited By (17)

105

A Phase 1 study of ADI-PEG20 (pegargiminase) combined with cisplatin and pemetrexed in ASS1-negative metastatic uveal melanoma

By: Chan, Pui Ying ; Phillips, Melissa M.; Ellis, Stephen; Johnston, Amanda; Feng, Xiaoxing; Arora, Amit; Hay, Gordon; Cohen, Victoria M. L.; Sagoo, Mandeep S.; Bomalaski, John S.; et al
Pigment Cell & Melanoma Research (2022), 35(4), 461-470 | Language: English, Database: CAplus and MEDLINE

Substances (13) Reactions (0) Cited By (1)

106

One size doesn't fit all in Ph-like ALL

By: Post, Sean M.
Blood (2017), 129(2), 140-141 | Language: English, Database: CAplus and MEDLINE

Substances (13) Reactions (0) Cited By (0)

107

Atractylenolide I inhibits colorectal cancer cell proliferation by affecting metabolism and stemness via AKT/mTOR signaling

By: Wang, Kuilong; Huang, Wei; Sang, Xianan; Wu, Xin ; Shan, Qiyuan; Tang, Dongxin; Xu, Xiaofen; Cao, Gang
Phytomedicine (2020), 68, 153191 | Language: English, Database: CAplus and MEDLINE

Substances (11) Reactions (0) Cited By (20)

108

Dual blockade of the PI3K/Akt/mTOR pathway inhibits posttransplant Epstein-Barr virus B cell lymphomas and promotes allograft survival

By: Sang, Adam X. ; McPherson, Marla C.; Ivison, Geoffrey T.; Qu, Xiumei; Rigdon, Joseph; Esquivel, Carlos O.; Krams, Sheri M.; Martinez, Olivia M.
American Journal of Transplantation (2019), 19(5), 1305-1314 | Language: English, Database: CAplus and MEDLINE

Substances (11) Reactions (0) Cited By (12)

109

Extra-virgin olive oil contains a metabolo-epigenetic inhibitor of cancer stem cells

By: Corominas-Faja, Bruna; Cuyas, Elisabet; Lozano-Sanchez, Jesus; Cufi, Silvia; Verdura, Sara; Fernandez-Arroyo, Salvador; Borras-Linares, Isabel; Martin-Castillo, Begona; Martin, Angel G.; Lupu, Ruth; et al
Carcinogenesis (2018), 39(4), 601-613 | Language: English, Database: CAplus and MEDLINE

Substances (11) Reactions (0) Cited By (38)

110

Somatic mutation profiling of vulvar cancer: Exploring therapeutic targets

By: Zieba, Sebastian; Kowalik, Artur ; Zalewski, Kamil; Rusetska, Natalia; Goryca, Krzysztof; Piascik, Agata; Misiek, Marcin; Bakula-Zalewska, Elwira; Kopczynski, Janusz; Kowalski, Kamil; et al
Gynecologic Oncology (2018), 150(3), 552-561 | Language: English, Database: CAplus and MEDLINE

Substances (11) Reactions (0) Cited By (29)

111

FBW7 suppression leads to SOX9 stabilization and increased malignancy in medulloblastoma

By: Suryo Rahmanto, Aldwin ; Savov, Vasil; Brunner, Andrae; Bolin, Sara; Weishaupt, Holger; Malyukova, Alena; Rosen, Gabriela; Cancer, Matko; Hutter, Sonja; Sundstroem, Anders; et al
EMBO Journal (2016), 35(20), 2192-2212 | Language: English, Database: CAplus and MEDLINE

Substances (11) Reactions (0) Cited By (39)

112

Keratinocyte growth factor protects endometrial cells from oxygen glucose deprivation/re-oxygenation via activating Nrf2 signaling

By: Shi, Xuting; Liu, Hai-yan; Li, Shu-ping; Xu, Hong-bin
Biochemical and Biophysical Research Communications (2018), 501(1), 178-185 | Language: English, Database: CAplus and MEDLINE

Substances (13) | Reactions (0) | Cited By (22)

113

mTOR inhibition via displacement of phosphatidic acid induces enhanced cytotoxicity specifically in cancer cells

By: Nguyen, Tra-Ly; Nokin, Marie-Julie; Egorov, Maxim; Tome, Mercedes; Bodineau, Clement; Primo, Carmelo Di; Minder, Laetitia; Wdzieczak-Bakala, Joanna; Garcia-Alvarez, Maria Concepcion; Bignon, Jerome; et al
Cancer Research (2018), 78(18), 5384-5397 | Language: English, Database: CAplus and MEDLINE

Substances (19) | Reactions (0) | Cited By (11)

114

High Throughput 3D Cell Migration Assay Using Micropillar/Microwell Chips

By: Lee, Sang-Yun ; Park, Lily M.; Oh, Yoo Jung; Choi, Dong Hyuk; Lee, Dong Woo
Molecules (2022), 27(16), 5306 | Language: English, Database: CAplus and MEDLINE

Substances (70) | Reactions (0) | Cited By (0)

115

Colon cancer organoids using monoclonal organoids established in four different lesions of one cancer patient reveal tumor heterogeneity and different real-time responsiveness to anti-cancer drugs

By: Song, Myoung-Hyun; Park, Ji Won; Kim, Min Jung; Shin, Young-Kyoung; Kim, Soon-Chan; Jeong, Seung-Yong; Ku, Ja-Lok
Biomedicine & Pharmacotherapy (2022), 152, 113260 | Language: English, Database: CAplus and MEDLINE

Substances (15) | Reactions (0) | Cited By (1)

116

Design, synthesis and antitumor evaluation of novel quinazoline analogs in hepatocellular carcinoma cell

By: Yu, Pan ; Cao, Weiya; Yang, Shilong; Wang, Yuan; Xia, Aixin; Tan, Xinlan; Wang, Luyi
Journal of Molecular Structure (2022), 1268, 133718 | Language: English, Database: CAplus

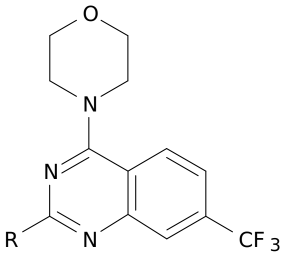

I

Substances (37) Reactions (22) Cited By (1)

117

High-risk neuroblastoma with NF1 loss of function is targetable using SHP2 inhibition

By: Cai, Jinyang; Jacob, Sheeba; Kurupi, Richard; Dalton, Krista M.; Coon, Colin; Greninger, Patricia; Egan, Regina K.; Stein, Giovanna T.; Murchie, Ellen; McClanaghan, Joseph; et al
Cell Reports (2022), 40(4), 111095 | Language: English, Database: CAplus and MEDLINE

Substances (15) Reactions (0) Cited By (1)

118

HSP90 inhibitors induce GPNMB cell-surface expression by modulating lysosomal positioning and sensitize breast cancer cells to glembatumumab vedotin

By: Biondini, Marco; Kiepas, Alex; El-Houjeiri, Leeanna; Annis, Matthew G.; Hsu, Brian E.; Fortier, Anne-Marie; Morin, Genevieve; Martina, Jose A.; Sirois, Isabelle; Aguilar-Mahecha, Adriana; et al
Oncogene (2022), 41(12), 1701-1717 | Language: English, Database: CAplus and MEDLINE

Substances (20) Reactions (0) Cited By (1)

119

Dynamic BH3 profiling identifies active BH3 mimetic combinations in non-small cell lung cancer

By: Potter, Danielle S.; Du, Ruochen; Bhola, Patrick; Bueno, Raphael; Letai, Anthony
Cell Death & Disease (2021), 12(8), 741 | Language: English, Database: CAplus and MEDLINE

Substances (26) Reactions (0) Cited By (4)

120

Screening reveals sterol derivatives with pro-differentiation, pro-survival, or potent cytotoxic effects on oligodendrocyte progenitor cells

By: Sax, Joel L.; Hubler, Zita; Allimuthu, Dharmaraja ; Adams, Drew J.
ACS Chemical Biology (2021), 16(7), 1288-1297 | Language: English, Database: CAplus and MEDLINE

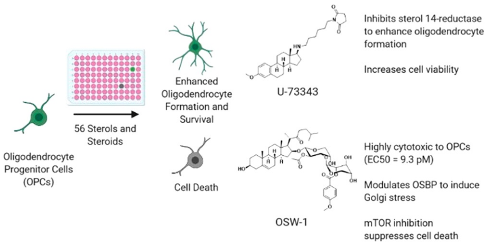

Substances (31) | Reactions (0) | Cited By (3)

121

Landscapes of cellular phenotypic diversity in breast cancer xenografts and their impact on drug response

By: Georgopoulou, Dimitra; Callari, Maurizio; Rueda, Oscar M.; Shea, Abigail; Martin, Alistair; Giovannetti, Agnese ; Qosaj, Fatime; Dariush, Ali; Chin, Suet-Feung ; Carnevalli, Larissa S. ; et al
Nature Communications (2021), 12(1), 1998 | Language: English, Database: CAplus and MEDLINE

Substances (23) | Reactions (0) | Cited By (16)

122

Mastering the Complex Targeted Therapy for Non-small Cell Lung Cancer

By: Devarakonda, Siddhartha; Govindan, Ramaswamy; Morgensztern, Daniel
Cancer Cell (2020), 38(3), 320-322 | Language: English, Database: CAplus and MEDLINE

Substances (12) | Reactions (0) | Cited By (5)

123

Ex vivo modelling of drug efficacy in a rare metastatic urachal carcinoma

By: Makela, Rami; Arjonen, Antti; Harma, Ville; Rintanen, Nina; Paasonen, Lauri; Paprotka, Tobias; Ronsch, Kerstin; Kuopio, Teijo; Kononen, Juha; Rantala, Juha K.
BMC Cancer (2020), 20(1), 590 | Language: English, Database: CAplus and MEDLINE

Substances (46) | Reactions (0) | Cited By (3)

124

Establishment and characterization of 18 human colorectal cancer cell lines

By: Kim, Soon-Chan; Kim, Hyun-Soo; Kim, Jae Hyeon; Jeong, Nahyun; Shin, Young-Kyoung; Kim, Min Jung; Park, Ji Won; Jeong, Seung-Yong; Ku, Ja-Lok
Scientific Reports (2020), 10(1), 6801 | Language: English, Database: CAplus and MEDLINE

Substances (23) | Reactions (0) | Cited By (2)

125

Discovery of Novel Autophagy Inhibitors and Their Sensitization Abilities for Vincristine-Resistant Esophageal Cancer Cell Line Eca109/VCR

By: Shan, Changyu; Hui, Wenqi; Li, Hongwei; Wang, Zheng; Guo, Chunling; Peng, Ruikun; Gu, Jing; Chen, Yingchun; Ouyang, Qin
ChemMedChem (2020), 15(11), 970-981 | Language: English, Database: CAplus and MEDLINE

Substances (51) | Reactions (0) | Cited By (5)

126

Drug repurposing screening identifies bortezomib and panobinostat as drugs targeting cancer associated fibroblasts (CAFs) by synergistic induction of apoptosis

By: Lee, Hak-Min; Lee, Eunmyong; Yeo, So-Young; Shin, Sang; Park, Hyun-Kyu; Nam, Do-Hyun; Kim, Seok-Hyung
Investigational New Drugs (2018), 36(4), 545-560 | Language: English, Database: CAplus and MEDLINE

Substances (67) | Reactions (0) | Cited By (10)

127

JAK1/2 and BCL2 inhibitors synergize to counteract bone marrow stromal cell-induced protection of AML

By: Karjalainen, Riikka; Pemovska, Tea; Popa, Mihaela; Liu, Minxia; Javarappa, Komal K.; Majumder, Muntasir M.; Yadav, Bhagwan; Tamborero, David; Tang, Jing; Bychkov, Dmitrii; et al
Blood (2017), 130(6), 789-802 | Language: English, Database: CAplus and MEDLINE

Substances (270) | Reactions (0) | Cited By (66)

128

Identification of selective cytotoxic and synthetic lethal drug responses in triple negative breast cancer cells

By: Gautam, Prson; Karhinen, Leena; Szwajda, Agnieszka; Kumar, Jha Sawan; Yadav, Bhagwan; Aittokallio, Tero; Wennerberg, Krister
Molecular Cancer (2016), 15, 34/1-34/16 | Language: English, Database: CAplus and MEDLINE

Substances (257) | Reactions (0) | Cited By (46)

129

Chemical Proteomics Reveals Ferrochelatase as a Common Off-target of Kinase Inhibitors

By: Klaeger, Susan; Gohlke, Bjoern; Perrin, Jessica; Gupta, Vipul; Heinzlmeir, Stephanie; Helm, Dominic; Qiao, Huichao; Bergamini, Giovanna; Handa, Hiroshi; Savitski, Mikhail M.; et al

ACS Chemical Biology (2016), 11(5), 1245-1254 | Language: English, Database: CAplus and MEDLINE

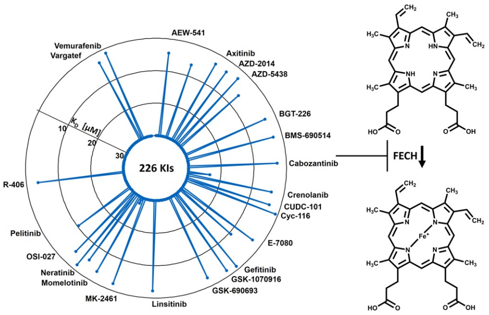

Substances (32) | Reactions (0) | Cited By (62)

130

Macropinocytosis Renders a Subset of Pancreatic Tumor Cells Resistant to mTOR Inhibition

By: Michalopoulou, Evdokia; Auciello, Francesca R.; Bulusu, Vinay; Strachan, David; Campbell, Andrew D.; Tait-Mulder, Jacqueline; Karim, Saadia A.; Morton, Jennifer P.; Sansom, Owen J.; Kamphorst, Jurre J.

Cell Reports (2020), 30(8), 2729-2742.e4 | Language: English, Database: CAplus and MEDLINE

Substances (12) | Reactions (0) | Cited By (16)

131

Overcoming resistance to mTOR inhibition for enhanced strategies in clinical trials

By: Naing, Aung

Expert Opinion on Investigational Drugs (2013), 22(6), 679-685 | Language: English, Database: CAplus and MEDLINE

Substances (15) | Reactions (0) | Cited By (6)

132

Antiviral drug screen identifies DNA-damage response inhibitor as potent blocker of SARS-CoV-2 replication

By: Garcia, Gustavo; Sharma, Arun; Ramaiah, Arunachalam; Sen, Chandani; Purkayastha, Arunima; Kohn, Donald B.; Parcells, Mark S.; Beck, Sebastian; Kim, Heeyoung; Bakowski, Malina A.; et al

Cell Reports (2021), 35(1), 108940 | Language: English, Database: CAplus and MEDLINE

Substances (13) | Reactions (0) | Cited By (43)

133

RAPTOR up-regulation contributes to resistance of renal cancer cells to PI3K-mTOR inhibition

By: Earwaker, Philip; Anderson, Caroline; Willenbrock, Frances; Harris, Adrian L.; Protheroe, Andrew S.; Macaulay, Valentine M.
PLoS One (2018), 13(2), e0191890/1-e0191890/22 | Language: English, Database: CAplus and MEDLINE

Substances (18) Reactions (0) Cited By (19)

134

A cell-based high-throughput screen identifies inhibitors that overcome P-glycoprotein (Pgp)-mediated multidrug resistance

By: Zahra, Rida; Furqan, Muhammad; Ullah, Rahim; Mithani, Aziz ; Saleem, Rahman Shah Zaib ; Faisal, Amir
PLoS One (2020), 15(6), e0233993 | Language: English, Database: CAplus and MEDLINE

Substances (16) Reactions (0) Cited By (12)

135

Implementation of a Multiplex and Quantitative Proteomics Platform for Assessing Protein Lysates Using DNA-Barcoded Antibodies

By: Lee, Jinho; Geiss, Gary K.; Demirkan, Gokhan; Vellano, Christopher P.; Filanoski, Brian; Lu, Yiling; Ju, Zhenlin; Yu, Shuangxing; Guo, Huifang; Bogatzki, Lisa Y.; et al
Molecular & Cellular Proteomics (2018), 17(6), 1245-1258 | Language: English, Database: CAplus and MEDLINE

Substances (17) Reactions (0) Cited By (13)

136

A Conformational Restriction Strategy for the Identification of a Highly Selective Pyrimido-pyrrolo-oxazine mTOR Inhibitor

By: Borsari, Chiara ; Rageot, Denise ; Dall'Asen, Alix; Bohnacker, Thomas; Melone, Anna; Sele, Alexander M. ; Jackson, Eileen; Langlois, Jean-Baptiste; Beaufils, Florent; Hebeisen, Paul; et al
Journal of Medicinal Chemistry (2019), 62(18), 8609-8630 | Language: English, Database: CAplus and MEDLINE

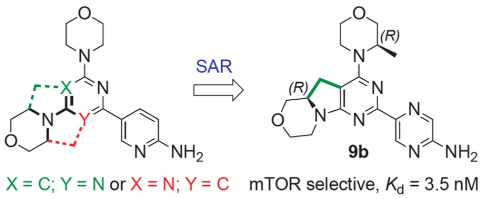

Substances (120) Reactions (421) Cited By (19)

137

3D Cell-Based High-Content Screening (HCS) Using a Micropillar and Microwell Chip Platform

By: Lee, Sang-Yun; Doh, Il; Nam, Do-Hyun; Lee, Dong Woo
Analytical Chemistry (Washington, DC, United States) (2018), 90(14), 8354-8361 | Language: English, Database: CAplus and MEDLINE

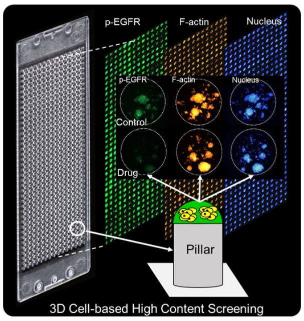

Substances (78) Reactions (0) Cited By (15)

138

Evaluation of the combination of the dual m-TORC1/2 inhibitor vistusertib (AZD2014) and paclitaxel in ovarian cancer models.

By: Wong Te Fong, Anne-Christine; Thavasu, Parames; Gagrica, Sladjana; Swales, Karen E; Leach, Martin O; Cosulich, Sabina C; Chung, Yuen-Li; Banerji, Udai
Oncotarget (2017), 8(69), 113874-113884 | Language: English, Database: MEDLINE

Substances (0) Reactions (0) Cited By (15)

139

Methuosis Contributes to Jaspine-B-Induced Cell Death

By: Bielsa, Nuria; Casasampere, Mireia; Abad, Jose Luis; Enrich, Carlos ; Delgado, Antonio; Fabrias, Gemma; Lizcano, Jose M. ; Casas, Josefina
International Journal of Molecular Sciences (2022), 23(13), 7257 | Language: English, Database: CAplus and MEDLINE

Substances (18) Reactions (0) Cited By (0)

140

Enhanced MAPK signaling drives ETS1-mediated induction of miR-29b leading to downregulation of TET1 and changes in epigenetic modifications in a subset of lung SCC

By: Taylor, M. A.; Wappett, M.; Delpuech, O.; Brown, H.; Chresta, C. M.
Oncogene (2016), 35(33), 4345-4357 | Language: English, Database: CAplus and MEDLINE

Substances (18) Reactions (0) Cited By (26)

141

A genome-scale CRISPR screen identifies the ERBB and mTOR signaling networks as key determinants of response to PI3K inhibition in pancreatic cancer

By: Milton, Charlotte K.; Self, Annette J.; Clarke, Paul A.; Banerji, Udai; Piccioni, Federica; Root, David E.; Whittaker, Steven R.
Molecular Cancer Therapeutics (2020), 19(7), 1423-1435 | Language: English, Database: CAplus and MEDLINE

Substances (19) Reactions (0) Cited By (8)

142

Diverse resistance mechanisms to the third-generation ALK inhibitor lorlatinib in alk-rearranged lung cancer

By: Recondo, Gonzalo; Mezquita, Laura; Facchinetti, Francesco; Planchard, David; Gazzah, Anas; Bigot, Ludovic; Rizvi, Ahsan Z.; Frias, Rosa L.; Thiery, Jean Paul; Scoazec, Jean-Yves; et al
Clinical Cancer Research (2020), 26(1), 242-255 | Language: English, Database: CAplus and MEDLINE

Substances (20) Reactions (0) Cited By (63)

143

Selective colony area method for heterogeneous patient-derived tumor cell lines in anti-cancer drug screening system

By: Cho, Jang Ho ; Kim, Ju-Sun; Kim, Seung Tae; Hong, Jung Yong ; Park, Joon Oh; Park, Young Suk; Nam, Do-Hyun; Lee, Dong Woo; Lee, Jeeyun
PLoS One (2019), 14(4), e0215080 | Language: English, Database: CAplus and MEDLINE

Substances (23) Reactions (0) Cited By (1)

144

Palbociclib-based high-throughput combination drug screening identifies synergistic therapeutic options in HPV-negative head and neck squamous cell carcinoma

By: Gu, Ziyue; Shi, Chaoji; Li, Jiayi; Han, Yong; Sun, Bao; Zhang, Wuchang; Wu, Jing; Zhou, Guoyu; Ye, Weimin; Li, Jiang; et al
BMC Medicine (2022), 20(1), 175 | Language: English, Database: CAplus and MEDLINE

Substances (24) Reactions (0) Cited By (0)

145

Molecular taxonomy of systemic lupus erythematosus through data-driven patient stratification: molecular endotypes and cluster-tailored drugs

By: Garantziotis, Panagiotis; Nikolakis, Dimitrios; Doumas, Stavros; Frangou, Eleni; Sentis, George; Filia, Anastasia; Fanouriakis, Antonis; Bertsias, George; Boumpas, Dimitrios T.
Frontiers in Immunology (2022), 13, 860726 | Language: English, Database: CAplus and MEDLINE

Substances (37) Reactions (0) Cited By (0)

146

Pyrimidine inhibitors synergize with nucleoside analogues to block SARS-CoV-2

By: Schultz, David C. ; Johnson, Robert M. ; Ayyanathan, Kasirajan; Miller, Jesse ; Whig, Kanupriya ; Kamalia, Brinda; Dittmar, Mark ; Weston, Stuart; Hammond, Holly L.; Dillen, Carly; et al
Nature (London, United Kingdom) (2022), 604(7904), 134-140 | Language: English, Database: CAplus and MEDLINE

Substances (112) Reactions (0) Cited By (45)

147

Ponatinib, lestaurtinib, and mTOR/PI3K inhibitors are promising repurposing candidates against Entamoeba histolytica

By: Kangussu-Marcolino, Monica M.; Singh, Upinder
Antimicrobial Agents and Chemotherapy (2022), 66(2), e01207-21 | Language: English, Database: CAplus

Substances (50) Reactions (0) Cited By (2)

148

SHMT2 inhibition disrupts the TCF3 transcriptional survival program in Burkitt lymphoma

By: Wilke, Anne C.; Doebele, Carmen; Zindel, Alena; Lee, Kwang Seok ; Rieke, Sara A. ; Ceribelli, Michele; Comoglio, Federico ; Phelan, James D.; Wang, James Q.; Pikman, Yana; et al
Blood (2022), 139(4), 538-553 | Language: English, Database: CAplus and MEDLINE

Substances (31) Reactions (0) Cited By (7)

149

4-(Difluoromethyl)-5-(4-((3R,5S)-3,5-dimethylmorpholino)-6-((R)-3-methylmorpholino)-1,3,5-triazin-2-yl)pyridin-2-amine (PQR626), a Potent, Orally Available, and Brain-Penetrant mTOR Inhibitor for the Treatment of Neurological Disorders

By: Borsari, Chiara ; Keles, Erhan; Rageot, Denise; Treyer, Andrea ; Bohnacker, Thomas; Bissegger, Lukas; De Pascale, Martina; Melone, Anna; Sriramaratnam, Rohitha; Beaufils, Florent; et al
Journal of Medicinal Chemistry (2020), 63(22), 13595-13617 | Language: English, Database: CAplus and MEDLINE

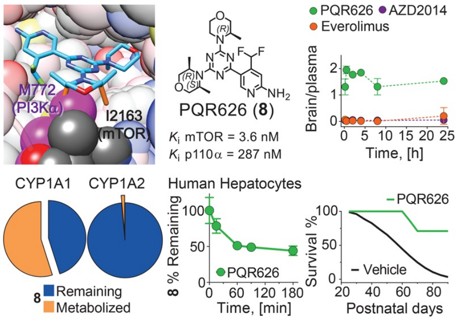

Substances (78) Reactions (107) Cited By (11)

150

Machine learning models for the prediction of chemotherapy-induced peripheral neuropathy

By: Bloomingdale, Peter; Mager, Donald E.
Pharmaceutical Research (2019), 36(2), 1-12 | Language: English, Database: CAplus and MEDLINE

Substances (60) Reactions (0) Cited By (8)

151

Effect of mTOR inhibitor AZD2014 on proliferation of hepatoma HCC cells

By: Lin, Kaihuang; Cai, Gaoyang; Liu, Xincheng
Aibian, Jibian, Tubian (2020), 32(6), 448-451 | Language: Chinese, Database: CAplus

Substances (0) Reactions (0) Cited By (0)

152

Dual mTORC1/2 inhibitor AZD2014 inhibits proliferation of HCCLM3 cells through induction of autophagy

By: Liao Hui; Xu Xiao-ping; Zhou Chen-jie; Zhang Jian-min; Zhong Ke-bo; Yang Ding-hua
Jiefangjun Yixue Zazhi (2020), 45(5), 61-64 | Language: Chinese, Database: CAplus

Substances (0) Reactions (0) Cited By (0)

153

Time now to TORC the TORC? New developments in mTOR pathway inhibition in lymphoid malignancies

By: Eyre, Toby A.; Collins, Graham P.; Goldstone, Anthony H.; Cwynarski, Kate
British Journal of Haematology (2014), 166(3), 336-351 | Language: English, Database: CAplus and MEDLINE

Substance (1) | Reactions (0) | Cited By (37)

154

Long-term acquired everolimus resistance in pancreatic neuroendocrine tumours can be overcome with novel PI3K-AKT-mTOR inhibitors

By: Vandamme, Timon; Beyens, Matthias; Op de Beeck, Ken; Dogan, Fadime; van Koetsveld, Peter M.; Pauwels, Patrick; Mortier, Geert; Vangestel, Christel; de Herder, Wouter; Van Camp, Guy; et al
British Journal of Cancer (2016), 114(6), 650-658 | Language: English, Database: CAplus and MEDLINE

Substances (2) | Reactions (0) | Cited By (57)

155

Synergistic anti-tumor effect of mTOR inhibitors with irinotecan on colon cancer cells

By: Reita, Damien ; Bour, Cyril; Benbrika, Radhia; Groh, Audrey ; Pencreach, Erwan; Guerin, Eric; Guenot, Dominique
Cancers (2019), 11(10), 1581 | Language: English, Database: CAplus and MEDLINE

Substances (0) | Reactions (0) | Cited By (15)

156

AKT/mTORC2 inhibition activates FOXO1 function in CLL cells reducing B-cell receptor-mediated survival

Out

By: Cosimo, Emilio; Tarafdar, Anuradha; Moles, Michael W.; Holroyd, Ailsa K.; Malik, Natasha; Catherwood, Mark A.; Hay, Jodie; Dunn, Karen M.; Macdonald, Alan M.; Guichard, Sylvie M.; et al
Clinical Cancer Research (2019), 25(5), 1574-1587 | Language: English, Database: CAplus and MEDLINE

Substances (4) | Reactions (0) | Cited By (15)

157

Potentiation of growth inhibitory responses of the mTOR inhibitor Everolimus by dual mTORC1/2 inhibitors in cultured breast cancer cell lines

By: Leung, Euphemia Y.; Askarian-Amiri, Marjan; Finlay, Graeme J.; Rewcastle, Gordon W.; Baguley, Bruce C.
PLoS One (2015), 10(7), e0131400/1-e0131400/18 | Language: English, Database: CAplus and MEDLINE

Substances (8) | Reactions (0) | Cited By (28)

158

BEZ235 (PIK3/mTOR inhibitor) Overcomes Pazopanib Resistance in Patient-Derived Refractory Soft Tissue Sarcoma Cells.

By: Kim, Hee Kyung; Kim, Sun Young; Lee, Su Jin; Kang, Mihyeon ; Kim, Seung Tae; Jang, Jiryeon; Rath, Oliver; Schueler, Julia; Lee, Dong Woo; Park, Woong Yang; et al
Translational oncology (2016), 9(3), 197-202 | Language: English, Database: MEDLINE

Substances (0) Reactions (0) Cited By (9)

159

In Vivo E2F Reporting Reveals Efficacious Schedules of MEK1/2-CDK4/6 Targeting and mTOR-S6 Resistance Mechanisms

Out

By: Teh, Jessica L. F.; Cheng, Phil F.; Purwin, Timothy J.; Nikbakht, Neda; Patel, Prem; Chervoneva, Inna; Ertel, Adam; Fortina, Paolo M.; Kleiber, Ines; HooKim, Kim; et al
Cancer Discovery (2018), 8(5), 568-581 | Language: English, Database: CAplus and MEDLINE

Substances (0) Reactions (0) Cited By (47)

160

Inhibition of mTORC1/C2 signaling improves anti-leukemia efficacy of JAK/STAT blockade in *CRLF2* rearranged and/or *JAK* driven Philadelphia chromosome-like acute B-cell lymphoblastic leukemia.

By: Zhang, Qi; Shi, Ce; Han, Lina; Jain, Nitin; Roberts, Kathryn G; Ma, Helen; Cai, Tianyu; Cavazos, Antonio; Tabe, Yoko; Jacamo, Rodrigo O; et al
Oncotarget (2018), 9(8), 8027-8041 | Language: English, Database: MEDLINE

Substances (0) Reactions (0) Cited By (27)

161

Optimization of Synthesis Process of 2-amino-6-chioronatenic Acidnic Acid

By: Dai Zhengyi; Lu Yuhang; Huang Wenjing; Zhang Mei; Jiang Xi; Le Yi; Zhou Zhixu
Guangdong Huagong (2020), 47(15), 16 | Language: Chinese, Database: CAplus

Substances (0) Reactions (0) Cited By (0)

162

Identification of differential PI3K pathway target dependencies in T-cell acute lymphoblastic leukemia through a large cancer cell panel screen.

By: Lynch, James T; McEwen, Robert; Crafter, Claire; McDermott, Ultan; Garnett, Mathew J; Barry, Simon T; Davies, Barry R
Oncotarget (2016), 7(16), 22128-39 | Language: English, Database: MEDLINE

Substances (0) Reactions (0) Cited By (16)

163

Selumetinib-based therapy in uveal melanoma patient-derived xenografts.

Out

By: Decaudin, Didier; El Botty, Rania; Diallo, Béré; Massonnet, Gerald; Fleury, Justine; Naguez, Adnan; Raymondie, Chloé; Davies, Emma; Smith, Aaron; Wilson, Joanne; et al
Oncotarget (2018), 9(31), 21674-21686 | Language: English, Database: MEDLINE

Substances (0) Reactions (0) Cited By (17)

164

Evolution of PIKK family kinase inhibitors: a new age cancer therapeutics

Repeat paper

By: Shaik, Althaf; Kirubakaran, Sivapriya
Frontiers in Bioscience, Landmark Edition (2020), 25(8), 1510-1537 | Language: English, Database: CAplus

Substances (0) Reactions (0) Cited By (6)

165

ADCK1 is a potential therapeutic target of osteosarcoma

By: Zhuo, Bao-biao; Zhu, Lun-qing; Yao, Chen; Wang, Xi-hua; Li, Shi-xian; Wang, Rong; Li, Yuan; Ling, Zhuo-yan
Cell Death & Disease (2022), 13(11), 954 | Language: English, Database: CAplus and MEDLINE

Substance (1) Reactions (0) Cited By (0)

166

Unspliced X-box-binding Protein 1 (XBP1) Protects Endothelial Cells from Oxidative Stress through Interaction with Histone Deacetylase 3

By: Martin, Daniel; Li, Yi; Yang, Junyao; Wang, Gang; Margariti, Andriana; Jiang, Zhixin; Yu, Hui; Zampetaki, Anna; Hu, Yanhua; Xu, Qingbo; et al
Journal of Biological Chemistry (2014), 289(44), 30625-30634 | Language: English, Database: CAplus and MEDLINE

Substances (4) Reactions (0) Cited By (60)

167

Transforming growth factor-β (TGF-β) induces the expression of chondrogenesis-related genes through TGF-β receptor II (TGFRII)-AKT-mTOR signaling in primary cultured mouse precartilaginous stem cells

By: Li, Cheng; Wang, Qiong; Wang, Jun-fang
Biochemical and Biophysical Research Communications (2014), 450(1), 646-651 | Language: English, Database: CAplus and MEDLINE

Substances (3) Reactions (0) Cited By (18)

168

MHY1485 ameliorates UV-induced skin cell damages via activating mTOR-Nrf2 signaling.

By: Yang, Bo; Xu, Qiu-Yun; Guo, Chun-Yan; Huang, Jin-Wen; Wang, Shu-Mei; Li, Yong-Mei; Tu, Ying; He, Li; Bi, Zhi-Gang; Ji, Chao; et al
Oncotarget (2017), 8(8), 12775-12783 | Language: English, Database: MEDLINE

Substances (0) | Reactions (0) | Cited By (8)

169

Design and development of Tetrahydro-Quinoline derivatives as dual mTOR-C1/C2 inhibitors for the treatment of lung cancer

By: Chaube, Udit J.; Rawal, Rakesh; Jha, Abhishek B.; Variya, Bhavesh; Bhatt, Hardik G.
Bioorganic Chemistry (2021), 106, 104501 | Language: English, Database: CAplus and MEDLINE

Substances (64) | Reactions (221) | Cited By (4)

Copyright © 2023 American Chemical Society (ACS). All Rights Reserved.
Copyright © 2023 U.S. National Library of Medicine.
Internal use only. Redistribution is subject to the terms of your SciFinderⁿ License Agreement and CAS information Use Policies.

CAS SciFinderⁿ

Task History

Initiating Search

January 12, 2023, 9:59PM

All:

Structure of AZD2014 (right)

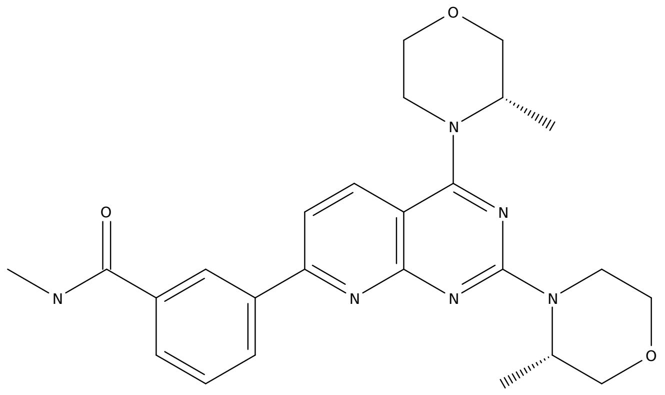

Search Tasks

Task	Search Type	View
Returned All Results	All	View Results
Exported: Retrieved Related Reference Results + Filters (148)<br>Filtered By:<br>Document Type: Journal	References	View Results

Copyright © 2023 American Chemical Society (ACS). All Rights Reserved.
Internal use only. Redistribution is subject to the terms of your SciFinderⁿ License Agreement and CAS information Use Policies.

CAS SciFinderⁿ

References (148)

View in SciFinderⁿ

1

First-in-Human Pharmacokinetic and Pharmacodynamic Study of the Dual m-TORC 1/2 Inhibitor AZD2014

By: Basu, Bristi; Dean, Emma; Puglisi, Martina; Greystoke, Alastair; Ong, Michael; Burke, Wendy; Cavallin, Maria; Bigley, Graham; Womack, Christopher; Harrington, Elizabeth A.; et al
Clinical Cancer Research (2015), 21(15), 3412-3419 | Language: English, Database: CAplus and MEDLINE

Substances (2) | Reactions (0) | Cited By (91)

2

The mTORC1/mTORC2 inhibitor AZD2014 enhances the radiosensitivity of glioblastoma stem-like cells

By: Kahn, Jenna; Hayman, Thomas J.; Jamal, Muhammad; Rath, Barbara H.; Kramp, Tamalee; Camphausen, Kevin; Tofilon, Philip J.
Neuro-Oncology (Cary, NC, United States) (2014), 16(1), 29-37 | Language: English, Database: CAplus and MEDLINE

Substances (2) | Reactions (0) | Cited By (76)

3

AZD2014, an Inhibitor of mTORC1 and mTORC2, Is Highly Effective in ER^+ Breast Cancer When Administered Using Intermittent or Continuous Schedules

By: Guichard, Sylvie M.; Curwen, Jon; Bihani, Teeru; D'Cruz, Celina M.; Yates, James W. T.; Grondine, Michael; Howard, Zoe; Davies, Barry R.; Bigley, Graham; Klinowska, Teresa; et al
Molecular Cancer Therapeutics (2015), 14(11), 2508-2518 | Language: English, Database: CAplus and MEDLINE

Substances (10) | Reactions (0) | Cited By (90)

4

A Randomised Phase 2 Study of AZD2014 Versus Everolimus in Patients with VEGF-Refractory Metastatic Clear Cell Renal Cancer

By: Powles, Thomas; Wheater, Matthew; Din, Omar; Geldart, Thomas; Boleti, Ekaterini; Stockdale, Andrew; Sundar, Santhanam; Robinson, Angus; Ahmed, Imtiaz; Wimalasingham, Akhila; et al
European Urology (2016), 69(3), 450-456 | Language: English, Database: CAplus and MEDLINE

Substances (4) | Reactions (0) | Cited By (71)

5

Pre-clinical evaluation of AZD-2014, a novel mTORC1/2 dual inhibitor, against renal cell carcinoma

By: Zheng, Bing; Mao, Jia-Hui; Qian, Lin; Zhu, Hua; Gu, Dong-hua; Pan, Xiao-dong; Yi, Fang; Ji, Dong-mei
Cancer Letters (New York, NY, United States) (2015), 357(2), 468-475 | Language: English, Database: CAplus and MEDLINE

Substances (8) Reactions (0) Cited By (90)

6

A high-throughput kinome screen reveals serum/glucocorticoid-regulated kinase 1 as a therapeutic target for NF2-deficient meningiomas.

By: Beauchamp, Roberta L; James, Marianne F; DeSouza, Patrick A; Wagh, Vilas; Zhao, Wen-Ning; Jordan, Justin T; Stemmer-Rachamimov, Anat; Plotkin, Scott R; Gusella, James F; Haggarty, Stephen J; et al
Oncotarget (2015), 6(19), 16981-97 | Language: English, Database: MEDLINE

Substance (1) Reactions (0) Cited By (37)

7

Optimization of potent and selective dual mTORC1 and mTORC2 inhibitors: The discovery of AZD8055 and AZD2014

By: Pike, Kurt G.; Malagu, Karine; Hummersone, Marc G.; Menear, Keith A.; Duggan, Heather M. E.; Gomez, Sylvie; Martin, Niall M. B.; Ruston, Linette; Pass, Sarah L.; Pass, Martin
Bioorganic & Medicinal Chemistry Letters (2013), 23(5), 1212-1216 | Language: English, Database: CAplus and MEDLINE

Substances (53) Reactions (125) Cited By (161)

8

Dramatic suppression of colorectal cancer cell growth by the dual mTORC1 and mTORC2 inhibitor AZD-2014

By: Huo, Hai-zhong; Zhou, Zhi-yuan; Wang, Bing; Qin, Jian; Liu, Wen-yong; Gu, Yan
Biochemical and Biophysical Research Communications (2014), 443(2), 406-412 | Language: English, Database: CAplus and MEDLINE

Substances (5) Reactions (0) Cited By (42)

9

Vistusertib (dual m-TORC1/2 inhibitor) in combination with paclitaxel in patients with high-grade serous ovarian and squamous non-small-cell lung cancer.

By: Basu, B; Krebs, M G; Sundar, R; Wilson, R H; Spicer, J; Jones, R; Brada, M; Talbot, D C; Steele, N; Ingles Garces, A H; et al
Annals of oncology : official journal of the European Society for Medical Oncology (2018), 29(9), 1918-1925 | Language: English, Database: MEDLINE

Substances (2) Reactions (0) Cited By (16)

10

The novel combination of dual mTOR inhibitor AZD2014 and pan-PIM inhibitor AZD1208 inhibits growth in acute myeloid leukemia via HSF pathway suppression.

By: Harada, Masako; Benito, Juliana; Yamamoto, Shinichi; Kaur, Surinder; Arslan, Dirim; Ramirez, Santiago; Jacamo, Rodrigo; Platanias, Leonidas; Matsushita, Hiromichi; Fujimura, Tsutomu; et al
Oncotarget (2015), 6(35), 37930-47 | Language: English, Database: MEDLINE

Substance (1) | Reactions (0) | Cited By (28)

11

A phase II study to assess the safety and efficacy of the dual mTORC1/2 inhibitor vistusertib in relapsed, refractory DLBCL

By: Eyre, Toby A.; Hildyard, Catherine; Hamblin, Angela; Ali, Ayesha S.; Houlton, Aimee; Hopkins, Louise; Royston, Daniel; Linton, Kim M.; Pettitt, Andrew; Rule, Simon; et al
Hematological Oncology (2019), 37(4), 352-359 | Language: English, Database: CAplus and MEDLINE

Substances (3) | Reactions (0) | Cited By (13)

12

Rapamycin-insensitive companion of mTOR (RICTOR) amplification defines a subset of advanced gastric cancer and is sensitive to AZD2014-mediated mTORC1/2 inhibition.

By: Kim, S T; Kim, S Y; Klempner, S J; Yoon, J; Kim, N; Ahn, S; Bang, H; Kim, K-M; Park, W; Park, S H; et al
Annals of oncology : official journal of the European Society for Medical Oncology (2017), 28(3), 547-554 | Language: English, Database: MEDLINE

Substances (3) | Reactions (0) | Cited By (40)

13

Synergistic induction of apoptosis by combination of BTK and dual mTORC1/2 inhibitors in diffuse large B cell lymphoma.

By: Ezell, Scott A; Mayo, Michele; Bihani, Teeru; Tepsuporn, Suprawee; Wang, Suping; Passino, Melissa; Grosskurth, Shaun E; Collins, Mike; Parmentier, Julie; Reimer, Corinne; et al
Oncotarget (2014), 5(13), 4990-5001 | Language: English, Database: MEDLINE

Substances (4) | Reactions (0) | Cited By (39)

14

Targeting mantle cell lymphoma metabolism and survival through simultaneous blockade of mTOR and nuclear transporter exportin-1.

By: Sekihara, Kazumasa; Saitoh, Kaori; Han, Lina; Ciurea, Stefan; Yamamoto, Shinichi; Kikkawa, Mika; Kazuno, Saiko; Taka, Hikari; Kaga, Naoko; Arai, Hajime; et al
Oncotarget (2017), 8(21), 34552-34564 | Language: English, Database: MEDLINE

Substance (1) | Reactions (0) | Cited By (6)

15

Allosteric and ATP-Competitive Inhibitors of mTOR Effectively Suppress Tumor Progression-Associated Epithelial-Mesenchymal Transition in the Kidneys of Tsc2$^{+/-}$ Mice

By: Jones, Ashley T.; Yang, Jian; Narov, Kalin; Henske, Elizabeth P.; Sampson, Julian R.; Shen, Ming Hong
Neoplasia (New York, NY, United States) (2019), 21(8), 731-739 | Language: English, Database: CAplus and MEDLINE

Substances (5) | Reactions (0) | Cited By (8)

16

Preclinical analysis of MTOR complex 1/2 inhibition in diffuse intrinsic pontine glioma

By: Flannery, Patrick C.; DeSisto, John A.; Amani, Vladimir; Venkataraman, Sujatha; Lemma, Rakeb T.; Prince, Eric W.; Donson, Andrew; Moroze, Erin E.; Hoffman, Lindsey; Mulcahy Levy, Jean M.; et al
Oncology Reports (2018), 39(2), 455-464 | Language: English, Database: CAplus and MEDLINE

Substances (4) | Reactions (0) | Cited By (16)

17

Potential of the dual mTOR kinase inhibitor AZD2014 to overcome paclitaxel resistance in anaplastic thyroid carcinoma.

By: Milošević, Zorica; Banković, Jasna; Dinić, Jelena; Tsimplouli, Chrisiida; Sereti, Evangelia; Dragoj, Miodrag; Paunović, Verica; Milovanović, Zorka; Stepanović, Marija; Tanić, Nikola; et al
Cellular oncology (Dordrecht) (2018), 41(4), 409-426 | Language: English, Database: MEDLINE

Substances (2) | Reactions (0) | Cited By (13)

18

Phase I study of orally administered ^{14}Carbon-isotope labelled-vistusertib (AZD2014), a dual TORC1/2 kinase inhibitor, to assess the absorption, metabolism, excretion, and pharmacokinetics in patients with advanced solid malignancies

By: MacDonald, Alexander; Scarfe, Graeme; Magirr, Dominic; Sarvotham, Tinnu; Charlton, Julie; Brugger, Wolfram; Dean, Emma
Cancer Chemotherapy and Pharmacology (2019), 83(4), 787-795 | Language: English, Database: CAplus and MEDLINE

Substances (9) Reactions (0) Cited By (4)

19

MEDI-573, Alone or in Combination with Mammalian Target of Rapamycin Inhibitors, Targets the Insulin-like Growth Factor Pathway in Sarcomas

By: Zhong, Haihong; Fazenbaker, Christine; Breen, Shannon; Chen, Cui; Huang, Jiaqi; Morehouse, Christopher; Yao, Yihong; Hollingsworth, Robert E.
Molecular Cancer Therapeutics (2014), 13(11), 2662-2673 | Language: English, Database: CAplus and MEDLINE

Substances (7) Reactions (0) Cited By (18)

20

Noninvasive MRI native T_1 mapping detects response to MYCN-targeted therapies in the Th-MYCN model of neuroblastoma

By: Zormpas-Petridis, Konstantinos; Poon, Evon; Clarke, Matthew; Jerome, Neil P.; Boult, Jessica K. R.; Blackledge, Matthew D.; Carceller, Fernando; Koers, Alexander; Barone, Giuseppe; Pearson, Andrew D. J.; et al
Cancer Research (2020), 80(16), 3424-3435 | Language: English, Database: CAplus and MEDLINE

Substances (5) Reactions (0) Cited By (10)

21

AZD2014 radiosensitizes oral squamous cell carcinoma by inhibiting AKT/mTOR axis and inducing G1/G2/M cell cycle arrest

By: Yu, Chih-Chia; Huang, Hsien-bin; Hung, Shih-Kai; Liao, Hui-Fen; Lee, Ching-Chih; Lin, Hon-Yi; Li, Szu-Chin; Ho, Hsu-Chueh; Hung, Chung-Lin; Su, Yu-Chieh
PLoS One (2016), 11(3), e0151942/1-e0151942/15 | Language: English, Database: CAplus and MEDLINE

Substances (10) Reactions (0) Cited By (15)

22

Rapamycin induces mitogen-activated protein (MAP) kinase phosphatase-1 (MKP-1) expression through activation of protein kinase B and mitogen-activated protein kinase kinase pathways

By: Rastogi, Ruchi; Jiang, Zhongliang; Ahmad, Nisar; Rosati, Rita; Liu, Yusen; Beuret, Laurent; Monks, Robert; Charron, Jean; Birnbaum, Morris J.; Samavati, Lobelia
Journal of Biological Chemistry (2013), 288(47), 33966-33977 | Language: English, Database: CAplus and MEDLINE

Substances (8) Reactions (0) Cited By (43)

23

RICTOR amplification identifies a subgroup in small cell lung cancer and predicts response to drugs targeting mTOR.

By: Sakre, Nneha; Wildey, Gary; Behtaj, Mohadese; Kresak, Adam; Yang, Michael; Fu, Pingfu; Dowlati, Afshin
Oncotarget (2017), 8(4), 5992-6002 | Language: English, Database: MEDLINE

Substances (3) Reactions (0) Cited By (34)

24

A phase I study of vistusertib (dual mTORC1/2 inhibitor) in patients with previously treated glioblastoma multiforme: a CCTG study

By: Lapointe, Sarah; Mason, Warren; MacNeil, Mary; Harlos, Craig; Tsang, Roger; Sederias, Joana; Luchman, H. Artee; Weiss, Samuel; Rossiter, John P.; Tu, Dongsheng; et al
Investigational New Drugs (2020), 38(4), 1137-1144 | Language: English, Database: CAplus and MEDLINE

Substances (6) Reactions (0) Cited By (14)

25

Combined Inhibition of mTOR and CDK4/6 Is Required for Optimal Blockade of E2F Function and Long-term Growth Inhibition in Estrogen Receptor-positive Breast Cancer

By: Michaloglou, Chrysiis; Crafter, Claire; Siersbaek, Rasmus; Delpuech, Oona; Curwen, Jon O.; Carnevalli, Larissa S.; Staniszewska, Anna D.; Polanska, Urszula M.; Cheraghchi-Bashi, Azadeh; Lawson, Mandy; et al
Molecular Cancer Therapeutics (2018), 17(5), 908-920 | Language: English, Database: CAplus and MEDLINE

Substances (9) Reactions (0) Cited By (90)

26

mTORC1/2 inhibitor served as a more ideal agent against the growth of mouse lymphocytic leukemia both in vitro and in vivo

By: Liao, Hui-Fen; Lin, You-Zhu; Yu, Chih-Chia; Tai, Tzong-Shyuan; Hung, Shih-Kai; Yang, Ching-Chieh; Su, Yu-Chieh
Anticancer Research (2019), 39(9), 4829-4835 | Language: English, Database: CAplus and MEDLINE

Substances (7) Reactions (0) Cited By (3)

27

An adaptive, biomarker-directed platform study of durvalumab in combination with targeted therapies in advanced urothelial cancer

By: Powles, Thomas ; Carroll, Danielle; Chowdhury, Simon; Gravis, Gwenaelle; Joly, Florence; Carles, Joan; Flechon, Aude; Maroto, Pablo; Petrylak, Daniel; Rolland, Frederic ; et al
Nature Medicine (New York, NY, United States) (2021), 27(5), 793-801 | Language: English, Database: CAplus and MEDLINE

Substances (4) Reactions (0) Cited By (24)

28

Potent antitumour of the mTORC1/2 dual inhibitor AZD2014 in docetaxel-sensitive and docetaxel-resistant castration-resistant prostate cancer cells

By: Li, Senmao ; Sheng, Jindong; Liu, Zhenhua; Fan, Yu; Zhang, Cuijian; Lv, Tianjing; Hu, Shuai; Jin, Jie; Yu, Wei; Song, Yi
Journal of Cellular and Molecular Medicine (2021), 25(5), 2436-2449 | Language: English, Database: CAplus and MEDLINE

Substances (4) Reactions (0) Cited By (7)

29

The mTORC1 inhibitor rapamycin and the mTORC1/2 inhibitor AZD2014 impair the consolidation and persistence of contextual fear memory

By: MacCallum, Phillip E.; Blundell, Jacqueline
Psychopharmacology (Heidelberg, Germany) (2020), 237(9), 2795-2808 | Language: English, Database: CAplus and MEDLINE

Substances (4) Reactions (0) Cited By (4)

30

An mTORC1/2 dual inhibitor, AZD2014, acts as a lysosomal function activator and enhances gemtuzumab ozogamicin-induced apoptosis in primary human leukemia cells

By: Mizutani, Yu; Inase, Aki; Maimaitili, Yimamu; Miyata, Yoshiharu; Kitao, Akihito; Matsumoto, Hisayuki; Kawaguchi, Koji; Higashime, Ako; Goto, Hideaki; Kurata, Keiji; et al
International Journal of Hematology (2019), 110(4), 490-499 | Language: English, Database: CAplus and MEDLINE

Substances (4) Reactions (0) Cited By (12)

31

Validation of a rapid liquid chromatography tandem mass spectrometric method for the quantitative analysis of vistusertib

By: Scott, Susan C.; Anders, Nicole M.; Scardina, Morgan; Hann, Christine L.; Rudek, Michelle A.
Journal of Pharmaceutical and Biomedical Analysis (2022), 208, 114436 | Language: English, Database: CAplus and MEDLINE

Substance (1) Reactions (0) Cited By (0)

32

mTOR deregulation in oral cavity squamous cell carcinoma.

By: Mastronikolis, Nicholas S; Tsiambas, Evangelos; Papadas, Theodoros A; Fotiades, Panagiotis P; Papadas, Athanasios T; Mastronikolis, Stylianos N; Kastanioudakis, Ioannis; Ragos, Vasileios
Journal of B.U.ON. : official journal of the Balkan Union of Oncology (2017), 22(3), 610-613 | Language: English, Database: MEDLINE

Substance (1) Reactions (0) Cited By (1)

33

Directly imaging the localisation and photosensitization properties of the pan-mTOR inhibitor, AZD2014, in living cancer cells

By: Ahmed, Abdullah R.; Candeo, Alessia; D'Abrantes, Sofia; Needham, Sarah R.; Yadav, Rahul B.; Botchway, Stanley W.; Parker, Anthony W.
Journal of Photochemistry and Photobiology, B: Biology (2020), 213, 112055 | Language: English, Database: CAplus and MEDLINE

Substances (5) Reactions (0) Cited By (2)

34

Combination therapy with mTOR kinase inhibitor and dasatinib as a novel therapeutic strategy for vestibular schwannoma

By: Sagers, Jessica E.; Beauchamp, Roberta L.; Zhang, Yanling; Vasilijic, Sasa; Wu, Limeng; De Souza, Patrick; Seist, Richard; Zhou, Wenjianlong; Xu, Lei ; Ramesh, Vijaya; et al
Scientific Reports (2020), 10(1), 4211 | Language: English, Database: CAplus and MEDLINE

Substances (5) | Reactions (0) | Cited By (8)

35

Combination of mTORC1/2 inhibitor vistusertib plus fulvestrant in vitro and in vivo targets oestrogen receptor-positive endocrine-resistant breast cancer

By: Pancholi, Sunil; Leal, Mariana Ferreira; Ribas, Ricardo; Simigdala, Nikiana; Schuster, Eugene; Chateau-Joubert, Sophie; Zabaglo, Lila; Hills, Margaret; Dodson, Andrew; Gao, Qiong; et al
Breast Cancer Research (2019), 21(1), 135 | Language: English, Database: CAplus and MEDLINE

Substances (5) | Reactions (0) | Cited By (5)

36

Targeting autophagy to modulate cell survival: a comparative analysis in cancer, normal and embryonic cells

By: Divac Rankov, Aleksandra; Ljujic, Mila; Petric, Marija; Radojkovic, Dragica; Pesic, Milica; Dinic, Jelena
Histochemistry and Cell Biology (2017), 148(5), 529-544 | Language: English, Database: CAplus and MEDLINE

Substances (5) | Reactions (0) | Cited By (10)

37

mTORC2 Signaling Drives the Development and Progression of Pancreatic Cancer

By: Driscoll, David R.; Karim, Saadia A.; Sano, Makoto; Gay, David M.; Jacob, Wright; Yu, Jun; Mizukami, Yusuke; Gopinathan, Aarthi; Jodrell, Duncan I.; Evans, T. R. Jeffry; et al
Cancer Research (2016), 76(23), 6911-6923 | Language: English, Database: CAplus and MEDLINE

Substances (10) | Reactions (0) | Cited By (51)

38

Inhibition of Bcl-2 potentiates AZD-2014-induced anti-head and neck squamous cell carcinoma cell activity

By: Li, Yi; Cui, Jiang-Tao
Biochemical and Biophysical Research Communications (2016), 477(4), 607-613 | Language: English, Database: CAplus and MEDLINE

Substances (6) | Reactions (0) | Cited By (10)

39

Aberrant cholesterol metabolic signaling impairs antitumor immunosurveillance through natural killer T cell dysfunction in obese liver

By: Tang, Wenshu; Zhou, Jingying ; Yang, Weiqin; Feng, Yu; Wu, Haoran; Mok, Myth T. S.; Zhang, Lingyun; Liang, Zhixian; Liu, Xiaoyu; Xiong, Zhewen; et al
Cellular & Molecular Immunology (2022), 19(7), 834-847 | Language: English, Database: CAplus and MEDLINE

Substances (2) | Reactions (0) | Cited By (2)

40

Dual mTORC1/2 inhibitor AZD2014 diminishes myeloid-derived suppressor cells accumulation in ovarian cancer and delays tumor growth

By: Pi, Ruyu; Yang, Yang; Hu, Xiaoyi; Li, Hongyi; Shi, Houhui; Liu, Yu; Wang, Xi; Tong, An; Lu, Tianqi; Wei, Yuquan; et al
Cancer Letters (New York, NY, United States) (2021), 523, 72-81 | Language: English, Database: CAplus and MEDLINE

Substances (2) | Reactions (0) | Cited By (2)

41

F-metafuorobenzylguanidine (18F-mFBG) to monitor changes in norepinephrine transporter expression in response to therapeutic intervention in neuroblastoma models

By: Turnock, Stephen ; Turton, David R.; Martins, Carlos Daniel; Chesler, Louis; Wilson, Thomas C.; Gouverneur, Veronique; Smith, Graham; Kramer-Marek, Gabriela
Scientific Reports (2020), 10(1), 20918 | Language: English, Database: CAplus and MEDLINE

Substances (2) | Reactions (0) | Cited By (3)

42

Investigation of the novel mTOR inhibitor AZD2014 in neuronal ischemia

By: Hadley, Gina; Beard, Daniel J.; Alexopoulou, Zoi; Sutherland, Brad A.; Buchan, Alastair M.
Neuroscience Letters (2019), 706, 223-230 | Language: English, Database: CAplus and MEDLINE

Substances (2) | Reactions (0) | Cited By (5)

43

Combination of dual mTORC1/2 inhibition and immune-checkpoint blockade potentiates anti-tumor immunity

By: Langdon, Sophie; Hughes, Adina; Taylor, Molly A.; Kuczynski, Elizabeth A.; Mele, Deanna A.; Delpuech, Oona; Jarvis, Laura; Staniszewska, Anna; Cosulich, Sabina; Carnevalli, Larissa S.; et al
OncoImmunology (2018), 7(8), e1458810/1-e1458810/12 | Language: English, Database: CAplus and MEDLINE

Substances (2) | Reactions (0) | Cited By (21)

44

MiR-99a Enhances the Radiation Sensitivity of Non-Small Cell Lung Cancer by Targeting mTOR

By: Yin, Hang; Ma, Jianqun; Chen, Lin; Piao, Shiqi; Zhang, Yu; Zhang, Siliang; Ma, Hongyu; Li, Yang; Qu, Yuanyuan; Wang, Xiaoyuan; et al
Cellular Physiology and Biochemistry (2018), 46(2), 471-481 | Language: English, Database: CAplus and MEDLINE

Substances (2) Reactions (0) Cited By (43)

45

Combination Vistusertib and Anastrozole Shows Benefit in Endometrial Cancer.

No author and editor data available.
Cancer discovery (2022), 12(7), OF20 | Language: English, Database: MEDLINE

Substances (2) Reactions (0) Cited By (0)

46

Safety and Efficacy of the mTOR Inhibitor, Vistusertib, Combined With Anastrozole in Patients With Hormone Receptor-Positive Recurrent or Metastatic Endometrial Cancer: The VICTORIA Multicenter, Open-label, Phase 1/2 Randomized Clinical Trial.

By: Heudel, Pierre; Frenel, Jean-Sébastien; Dalban, Cécile; Bazan, Fernando; Joly, Florence; Arnaud, Antoine; Abdeddaim, Cyril; Chevalier-Place, Annick; Augereau, Paule; Pautier, Patricia; et al
JAMA oncology (2022), 8(7), 1001-1009 | Language: English, Database: MEDLINE

Substances (2) Reactions (0) Cited By (5)

47

Combined mTOR/MEK inhibition prevents proliferation and induces apoptosis in NF2-mutant tumors.

By: Li, N; Lu, X-Y; Shi, W-Y; Mao, F-J; Yang, X-Y; Luo, Y-B; Li, W
European review for medical and pharmacological sciences (2019), 23(13), 5874-5883 | Language: English, Database: MEDLINE

Substances (2) Reactions (0) Cited By (5)

48

EPH receptor signaling as a novel therapeutic target in NF2-deficient meningioma

By: Angus, Steven P.; Oblinger, Janet L.; Stuhlmiller, Timothy J.; DeSouza, Patrick A.; Beauchamp, Roberta L.; Witt, Luke; Chen, Xin; Jordan, Justin T.; Gilbert, Thomas S. K.; Stemmer-Rachamimov, Anat; et al
Neuro-Oncology (Cary, NC, United States) (2018), 20(9), 1185-1196 | Language: English, Database: CAplus and MEDLINE

Substances (12) Reactions (0) Cited By (14)

49

Inhibiting mTOR activity using AZD2014 increases autophagy in the mouse cerebral cortex

By: Bensalem, Julien; Fourrier, Celia; Hein, Leanne K.; Hassiotis, Sofia; Proud, Christopher G.; Sargeant, Timothy J.
Neuropharmacology (2021), 190, 108541 | Language: English, Database: CAplus and MEDLINE

Substances (8) | Reactions (0) | Cited By (5)

50

Antitumor effects of AZD2014, a dual mTORC1/2 inhibitor, against human hepatocellular carcinoma xenograft in nude mice

By: Liao, Hui; Wang, Yi; Xu, Xiaoping; Zhou, Chenjie; Zhang, Jianmin; Zhong, Kebo; Yang, Dinghua
Nanfang Yike Daxue Xuebao (2021), 41(7), 1056-1061 | Language: Chinese, Database: CAplus and MEDLINE

Substances (3) | Reactions (0) | Cited By (0)

51

Quantitation of vistusertib by UHPLC-MS/MS in rat plasma and its application to a pharmacokinetic study

By: Wang, Fuqi; Song, Danni; Zou, Fengmao; Zhao, Honghui; Zhao, Xu
Bioanalysis (2021), 13(17), 1333-1341 | Language: English, Database: CAplus and MEDLINE
Analytical Methods available

Substances (3) | Reactions (0) | Cited By (0)

52

Influence of the novel ATP-competitive dual mTORC1/2 inhibitor AZD2014 on immune cell populations and heart allograft rejection

By: Fantus, Daniel; Dai, Helong; Ono, Yoshihiro; Watson, Alicia; Yokota, Shinichiro; Mohib, Kanishka; Yoshida, Osamu; Ross, Mark A.; Watkins, Simon C.; Ramaswami, Bala; et al
Transplantation (2017), 101(12), 2830-2840 | Language: English, Database: CAplus and MEDLINE

Substances (3) | Reactions (0) | Cited By (10)

53

Evolution of PIKK family kinase inhibitors: A new age cancer therapeutics.

By: Shaik, Althaf; Kirubakaran, Sivapriya
Frontiers in bioscience (Landmark edition) (2020), 25(8), 1510-1537 | Language: English, Database: MEDLINE

Substances (3) | Reactions (0) | Cited By (6)

54

Kinome RNAi Screens Reveal Synergistic Targeting of MTOR and FGFR1 Pathways for Treatment of Lung Cancer and HNSCC

By: Singleton, Katherine R.; Hinz, Trista K.; Kleczko, Emily K.; Marek, Lindsay A.; Kwak, Jeff; Harp, Taylor; Kim, Jihye; Tan, Aik Choon; Heasley, Lynn E.
Cancer Research (2015), 75(20), 4398-4406 | Language: English, Database: CAplus and MEDLINE

Substances (14) Reactions (0) Cited By (46)

55

Intravital Imaging to Monitor Therapeutic Response in Moving Hypoxic Regions Resistant to PI3K Pathway Targeting in Pancreatic Cancer

By: Conway, James R. W.; Warren, Sean C.; Herrmann, David; Murphy, Kendelle J.; Cazet, Aurelie S.; Vennin, Claire; Shearer, Robert F.; Killen, Monica J.; Magenau, Astrid; Melenec, Pauline; et al
Cell Reports (2018), 23(11), 3312-3326 | Language: English, Database: CAplus and MEDLINE

Substances (9) Reactions (0) Cited By (50)

56

The dual mTORC1/2 inhibitor AZD2014 inhibits acute graft rejection in a rat liver transplantation model

By: Liao, Hui; Wang, Yi; Xu, Xiaoping; Zhou, Chenjie; Zhang, Jianmin; Zhong, Kebo; Yang, Dinghua
Nanfang Yike Daxue Xuebao (2022), 42(4), 598-603 | Language: Chinese, Database: CAplus and MEDLINE

Substances (4) Reactions (0) Cited By (0)

57

AZD2014, a dual mTOR inhibitor, attenuates cardiac hypertrophy in vitro and in vivo

By: Cha, Byung-Hyun; Jung, Minjin; Kim, Angela S.; Lepak, Victoria C.; Colson, Brett A.; Bull, David A.; Won, Youngwook
Journal of Biological Engineering (2021), 15(1), 24 | Language: English, Database: CAplus and MEDLINE

Substances (4) Reactions (0) Cited By (0)

58

Biomarker-driven phase 2 umbrella trial study for patients with recurrent small cell lung cancer failing platinum-based chemotherapy

By: Park, Sehhoon; Shim, Joonho ; Mortimer, Peter G. S.; Smith, Simon A.; Godin, Robert E.; Hollingsworth, Simon J.; Kim, Hee-Jung; Jung, Hyun Ae; Sun, Jong-Mu; Park, Woong-Yang; et al
Cancer (Hoboken, NJ, United States) (2020), 126(17), 4002-4012 | Language: English, Database: CAplus and MEDLINE

Substances (4) Reactions (0) Cited By (6)

59

Rituximab (anti-CD20)-modified AZD-2014-encapsulated nanoparticles killing of B lymphoma cells

By: Tang, Xiaolong; Xie, Chunmei; Jiang, Zhenyou; Li, Amin; Cai, Shiyu; Hou, Changhao; Wang, Jian; Liang, Yong; Ma, Dong
Artificial Cells, Nanomedicine, and Biotechnology (2018), 46(sup2), 1063-1073 | Language: English, Database: CAplus and MEDLINE

Substances (4) | Reactions (0) | Cited By (6)

60

Catalytic mammalian target of rapamycin inhibitors as antineoplastic agents

By: Mohindra, Nisha A.; Platanias, Leonidas C.
Leukemia & Lymphoma, | Language: English, Database: CAplus and MEDLINE

Substances (4) | Reactions (0) | Cited By (0)

61

Context-dependent intravital imaging of therapeutic response using intramolecular FRET biosensors

By: Conway, James R. W.; Warren, Sean C.; Timpson, Paul
Methods (Amsterdam, Netherlands) (2017), 128, 78-94 | Language: English, Database: CAplus and MEDLINE

Substances (10) | Reactions (0) | Cited By (27)

62

Synergistic effects of combination therapy with AKT and mTOR inhibitors on bladder cancer cells

By: Kim, Hyera; Lee, Su Jin; Lee, In Kyoung; Min, Suejean C.; Sung, Hyun Hwan; Jeong, Byong Chang; Lee, Jeeyun; Park, Se Hoon
International Journal of Molecular Sciences (2020), 21(8), 2825 | Language: English, Database: CAplus and MEDLINE

Substances (5) | Reactions (0) | Cited By (6)

63

Integrated pharmaco-proteogenomics defines two subgroups in isocitrate dehydrogenase wild-type glioblastoma with prognostic and therapeutic opportunities

By: Oh, Sejin ; Yeom, Jeonghun ; Cho, Hee Jin; Kim, Ju-Hwa; Yoon, Seon-Jin ; Kim, Hakhyun ; Sa, Jason K. ; Ju, Shinyeong ; Lee, Hwanho ; Oh, Myung Joon; et al
Nature Communications (2020), 11(1), 3288 | Language: English, Database: CAplus and MEDLINE

Substances (5) | Reactions (0) | Cited By (23)

64

Reconciling Selectivity Trends from a Comprehensive Kinase Inhibitor Profiling Campaign with Known Activity Data

By: Miljkovic, Filip; Bajorath, Juergen
ACS Omega (2018), 3(3), 3113-3119 | Language: English, Database: CAplus and MEDLINE

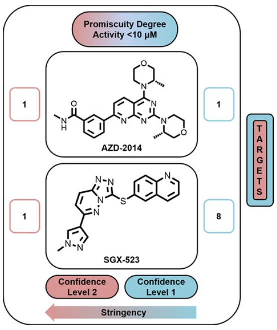

Substances (5) Reactions (0) Cited By (12)

65

Insights into significance of combined inhibition of MEK and m-TOR signalling output in KRAS mutant non-small-cell lung cancer

By: Broutin, Sophie; Stewart, Adam; Thavasu, Parames; Paci, Angelo; Bidart, Jean-Michel; Banerji, Udai
British Journal of Cancer (2016), 115(5), 549-552 | Language: English, Database: CAplus and MEDLINE

Substances (5) Reactions (0) Cited By (9)

66

Chemo-protective and regenerative effects of diarylheptanoids from the bark of black alder (Alnus glutinosa) in human normal keratinocytes

By: Dinic, Jelena; Randjelovic, Teodora; Stankovic, Tijana; Dragoj, Miodrag; Isakovic, Aleksandra; Novakovic, Miroslav; Pesic, Milica
Fitoterapia (2015), 105, 169-176 | Language: English, Database: CAplus and MEDLINE

Substances (5) Reactions (0) Cited By (13)

67

SARS-CoV-2 infection rewires host cell metabolism and is potentially susceptible to mTORC1 inhibition

By: Mullen, Peter J.; Garcia, Gustavo Jr; Purkayastha, Arunima; Matulionis, Nedas ; Schmid, Ernst W.; Momcilovic, Milica; Sen, Chandani; Langerman, Justin; Ramaiah, Arunachalam; Shackelford, David B. ; et al
Nature Communications (2021), 12(1), 1876 | Language: English, Database: CAplus and MEDLINE

Substances (12) Reactions (0) Cited By (56)

68

mTOR inhibition sensitizes human hepatocellular carcinoma cells to resminostat

By: Peng, Xingang; Zhang, Donghui; Li, Zhengling; Fu, Meili; Liu, Haiyan
Biochemical and Biophysical Research Communications (2016), 477(4), 556-562 | Language: English, Database: CAplus and MEDLINE

Substances (12) Reactions (0) Cited By (9)

69

An mTOR and DNA-PK dual inhibitor CC-115 hinders non-small cell lung cancer cell growth

By: Chen, Fagui; Zhao, Huasi; Li, Chenhui; Li, Ping ; Zhang, Qichuan
Cell Death Discovery (2022), 8(1), 293 | Language: English, Database: CAplus and MEDLINE

Substances (6) Reactions (0) Cited By (1)

70

Impact of baseline culture conditions of cancer organoids when determining therapeutic response and tumor heterogeneity

By: DeStefanis, Rebecca A.; Kratz, Jeremy D.; Olson, Autumn M.; Sunil, Aishwarya; DeZeeuw, Alyssa K.; Gillette, Amani A.; Sha, Gioia C.; Johnson, Katherine A.; Pasch, Cheri A.; Clipson, Linda; et al
Scientific Reports (2022), 12(1), 5205 | Language: English, Database: CAplus and MEDLINE

Substances (6) Reactions (0) Cited By (3)

71

A phase 1/2 study of the combination of acalabrutinib and vistusertib in patients with relapsed/refractory B-cell malignancies

By: Collins, Graham P.; Clevenger, Tracy N.; Burke, Kathleen A.; Yang, Buyue; MacDonald, Alex; Cunningham, David; Fox, Christopher P.; Goy, Andre; Gribben, John; Nowakowski, Grzegorz S.; et al
Leukemia & Lymphoma (2021), 62(11), 2625-2636 | Language: English, Database: CAplus and MEDLINE

Substances (6) Reactions (0) Cited By (3)

72

PI3k inhibitors (BKM120 and BYL719) as radiosensitizers for head and neck squamous cell carcinoma during radiotherapy

By: Chuang, Fu-Cheng; Wang, Chih-Chun; Chen, Jian-Han; Hwang, Tzer-Zen; Yeh, Shyh-An; Su, Yu-Chieh
PLoS One (2021), 16(1), e0245715 | Language: English, Database: CAplus and MEDLINE

Substances (6) Reactions (0) Cited By (6)

73

Tumor genomic profiling guides patients with metastatic gastric cancer to targeted treatment: the VIKTORY umbrella trial

By: Lee, Jeeyun; Kim, Seung Tae; Kim, Kyung; Lee, Hyuk; Kozarewa, Iwanka; Mortimer, Peter G. S.; Odegaard, Justin I.; Harrington, Elizabeth A.; Lee, Juyoung; Lee, Taehyang; et al
Cancer Discovery (2019), 9(10), 1388-1405 | Language: English, Database: CAplus and MEDLINE

Substances (6) Reactions (0) Cited By (90)

74

Synergistic drug combinations and machine learning for drug repurposing in chordoma

By: Anderson, Edward; Havener, Tammy M.; Zorn, Kimberley M.; Foil, Daniel H.; Lane, Thomas R.; Capuzzi, Stephen J.; Morris, Dave; Hickey, Anthony J.; Drewry, David H.; Ekins, Sean
Scientific Reports (2020), 10(1), 12982 | Language: English, Database: CAplus and MEDLINE

Substances (6) Reactions (0) Cited By (16)

75

Inducing and exploiting vulnerabilities for the treatment of liver cancer

By: Wang, Cun; Vegna, Serena; Jin, Haojie; Benedict, Bente; Lieftink, Cor; Ramirez, Christel; Leite de Oliveira, Rodrigo; Ben, Morris; Gadiot, Jules; Wang, Wei; et al
Nature (London, United Kingdom) (2019), 574(7777), 268-272 | Language: English, Database: CAplus and MEDLINE

Substances (6) Reactions (0) Cited By (148)

76

Mechanisms underlying synergy between DNA topoisomerase I-targeted drugs and mTOR kinase inhibitors in NF1-associated malignant peripheral nerve sheath tumors

By: Ki, Dong Hyuk; Oppel, Felix; Durbin, Adam D.; Look, A. Thomas
Oncogene (2019), 38(39), 6585-6598 | Language: English, Database: CAplus and MEDLINE

Substances (6) Reactions (0) Cited By (12)

77

Intermittent High-Dose Scheduling of AZD8835, a Novel Selective Inhibitor of PI3Kα and PI3Kδ, Demonstrates Treatment Strategies for PIK3CA-Dependent Breast Cancers

By: Hudson, Kevin; Hancox, Urs J.; Trigwell, Cath; McEwen, Robert; Polanska, Urszula M.; Nikolaou, Myria; Morentin Gutierrez, Pablo; Avivar-Valderas, Alvaro; Delpuech, Oona; Dudley, Phillippa; et al
Molecular Cancer Therapeutics (2016), 15(5), 877-889 | Language: English, Database: CAplus and MEDLINE

Substances (14) Reactions (0) Cited By (34)

78

GDC-0349 inhibits non-small cell lung cancer cell growth

By: Yang, Han; Zhao, Jun; Zhao, Mengjing; Zhao, Lihao; Zhou, Li-na; Duan, Yuxia ; Li, Gang
Cell Death & Disease (2020), 11(11), 951 | Language: English, Database: CAplus and MEDLINE

Substances (15) | Reactions (0) | Cited By (12)

79

Enhanced autocrine FGF19/FGFR4 signaling drives the progression of lung squamous cell carcinoma, which responds to mTOR inhibitor AZD2104

By: Li, Fan; Li, Ziming; Han, Qing; Cheng, Yirui; Ji, Wenxiang; Yang, Ying; Lu, Shun; Xia, Weiliang
Oncogene (2020), 39(17), 3507-3521 | Language: English, Database: CAplus and MEDLINE

Substances (7) | Reactions (0) | Cited By (14)

80

Repurposing of mtor complex inhibitors attenuates MCL-1 and sensitizes to PARP inhibition

By: Mattoo, Abid R.; Joun, Alex; Jessup, J. Milburn
Molecular Cancer Research (2019), 17(1), 42 | Language: English, Database: CAplus and MEDLINE

Substances (7) | Reactions (0) | Cited By (11)

81

MEDI-573, Alone or in Combination with Mammalian Target of Rapamycin Inhibitors, Targets the Insulin-like Growth Factor Pathway in Sarcomas [Erratum to document cited in CA162:419021]

By: Zhong, Haihong; Fazenbaker, Christine; Breen, Shannon; Chen, Cui; Huang, Jiaqi; Morehouse, Christopher; Yao, Yihong; Hollingsworth, Robert E.
Molecular Cancer Therapeutics (2015), 14(3), 844 | Language: English, Database: CAplus and MEDLINE

Substances (7) | Reactions (0) | Cited By (0)

82

Prolonged tau clearance and stress vulnerability rescue by pharmacological activation of autophagy in tauopathy neurons

By: Silva, M. Catarina ; Nandi, Ghata A. ; Tentarelli, Sharon; Gurrell, Ian K.; Jamier, Tanguy; Lucente, Diane; Dickerson, Bradford C.; Brown, Dean G.; Brandon, Nicholas J.; Haggarty, Stephen J.
Nature Communications (2020), 11(1), 3258 | Language: English, Database: CAplus and MEDLINE

Substances (16) | Reactions (0) | Cited By (53)

83

PIK3CA mutations enable targeting of a breast tumor dependency through mTOR-mediated MCL-1 translation

By: Anderson, Grace R.; Wardell, Suzanne E.; Cakir, Merve; Crawford, Lorin; Leeds, Jim C.; Nussbaum, Daniel P.; Shankar, Pallavi S.; Soderquist, Ryan S.; Stein, Elizabeth M.; Tingley, Jennifer P.; et al
Science Translational Medicine (2016), 8(369), 369ra175/1-369ra175/15 | Language: English, Database: CAplus and MEDLINE

Substances (16) Reactions (0) Cited By (36)

84

Learning from BISCAY: The future of biomarker-based trial design in bladder cancer

By: Meza, Luis; Salgia, Nicholas J.; Patel, Kalen C.; Pal, Sumanta K.
Cancer Cell (2021), 39(7), 910-912 | Language: English, Database: CAplus and MEDLINE

Substances (8) Reactions (0) Cited By (1)

85

Sustained mTORC1 activity during palbociclib-induced growth arrest triggers senescence in ER+ breast cancer cells

By: Maskey, Reeja S.; Wang, Fang; Lehman, Elyssa ; Wang, Yiqun; Emmanuel, Natasha ; Zhong, Wenyan; Jin, Guixian; Abraham, Robert T.; Arndt, Kim T.; Myers, Jeremy S.; et al
Cell Cycle (2021), 20(1), 65-80 | Language: English, Database: CAplus and MEDLINE

Substances (8) Reactions (0) Cited By (10)

86

Therapeutic Enhancement of Verteporfin-mediated Photodynamic Therapy by mTOR Inhibitors.

By: Kraus, Daniel; Palasuberniam, Pratheeba; Chen, Bin
Photochemistry and Photobiology (2020), 96(2), 358-364 | Language: English, Database: CAplus and MEDLINE

Substances (8) Reactions (0) Cited By (3)

87

Comparative toxicity evaluation of targeted anticancer therapeutics in embryonic zebrafish and sea urchin models

By: Babic, Tamara; Dinic, Jelena; Buric, Sonja Stojkovic; Hadzic, Stefan; Pesic, Milica; Radojkovic, Dragica; Rankov, Aleksandra Divac
Acta Biologica Hungarica (2018), 69(4), 395-410 | Language: English, Database: CAplus and MEDLINE

Substances (8) Reactions (0) Cited By (1)

88

mTOR inhibition amplifies the anti-lymphoma effect of PI3Kβ/δ blockage in diffuse large B-cell lymphoma

By: Xu, Wendan; Berning, Philipp; Erdmann, Tabea; Grau, Michael; Bettazova, Nardjas; Zapukhlyak, Myroslav; Frontzek, Fabian ; Kosnopfel, Corinna ; Lenz, Peter; Grondine, Michael; et al
Leukemia, | Language: English, Database: CAplus and MEDLINE

Substances (9) Reactions (0) Cited By (0)

89

mTOR kinase inhibition disrupts neuregulin 1-ERBB3 autocrine signaling and sensitizes NF2-deficient meningioma cellular models to IGF1R inhibition

By: Beauchamp, Roberta L.; Erdin, Serkan; Witt, Luke; Jordan, Justin T.; Plotkin, Scott R.; Gusella, James F.; Ramesh, Vijaya
Journal of Biological Chemistry (2021), 296, 100157 | Language: English, Database: CAplus and MEDLINE

Substances (9) Reactions (0) Cited By (1)

90

Phosphorylated ribosomal S6 (p-rpS6) as a post-treatment indicator of HER2 signalling targeted drug resistance

By: Yang-Kolodji, Gloria; Mumenthaler, Shannon M.; Mehta, Arjun; Ji, Lingyun; Tripathy, Debu
Biomarkers (2015), 20(5), 313-322 | Language: English, Database: CAplus and MEDLINE

Substances (9) Reactions (0) Cited By (7)

91

Synergistic effects between mTOR complex 1/2 and glycolysis inhibitors in non-small-cell lung carcinoma cells

By: Jiang, Suhua; Zou, Zhengzhi; Nie, Peipei; Wen, Ruiling; Xiao, Yingying; Tang, Jun
PLoS One (2015), 10(7), e0132880/1-e0132880/19 | Language: English, Database: CAplus and MEDLINE

Substances (9) Reactions (0) Cited By (15)

92

Reciprocal effects of mTOR inhibitors on pro-survival proteins dictate therapeutic responses in tuberous sclerosis complex

By: McNamara, Molly C.; Hosios, Aaron M.; Torrence, Margaret E.; Zhao, Ting; Fraser, Cameron; Wilkinson, Meghan; Kwiatkowski, David J.; Henske, Elizabeth P.; Wu, Chin-Lee; Sarosiek, Kristopher A.; et al
iScience (2022), 25(11), 105458 | Language: English, Database: CAplus and MEDLINE

Substances (10) Reactions (0) Cited By (0)

93

Identification and analysis of DNA methylation-driven signatures for prognostic and immune microenvironments evaluation in hepatocellular carcinoma

By: Shen, Bingbing; Wen, Zhen; Lv, Gang; Wang, Jianguo; Han, Ruijie; Jiang, Jianxin
Frontiers in Genetics (2022), 13, 1022078 | Language: English, Database: CAplus and MEDLINE

Substances (10) Reactions (0) Cited By (0)

94

Pre-clinical activity of combined LSD1 and mTORC1 inhibition in MLL-translocated acute myeloid leukaemia

By: Deb, Gauri; Wingelhofer, Bettina; Amaral, Fabio M. R.; Maiques-Diaz, Alba; Chadwick, John A.; Spencer, Gary J.; Williams, Emma L.; Leong, Hui-Sun; Maes, Tamara; Somervaille, Tim C. P.
Leukemia (2020), 34(5), 1266-1277 | Language: English, Database: CAplus and MEDLINE

Substances (10) Reactions (0) Cited By (17)

95

Sensitivity to pi3k and akt inhibitors is mediated by divergent molecular mechanisms in subtypes of dlbcl

By: Erdmann, Tabea; Klener, Pavel; Lynch, James T.; Grau, Michael; Vockova, Petra; Molinsky, Jan; Tuskova, Diana; Hudson, Kevin; Polanska, Urszula M.; Grondine, Michael; et al
Blood (2017), 130(3), 310-322 | Language: English, Database: CAplus and MEDLINE

Substances (23) Reactions (0) Cited By (59)

96

Atractylenolide I inhibits colorectal cancer cell proliferation by affecting metabolism and stemness via AKT/mTOR signaling

By: Wang, Kuilong; Huang, Wei; Sang, Xianan; Wu, Xin ; Shan, Qiyuan; Tang, Dongxin; Xu, Xiaofen; Cao, Gang
Phytomedicine (2020), 68, 153191 | Language: English, Database: CAplus and MEDLINE

Substances (11) Reactions (0) Cited By (20)

97

Dual blockade of the PI3K/Akt/mTOR pathway inhibits posttransplant Epstein-Barr virus B cell lymphomas and promotes allograft survival

By: Sang, Adam X. ; McPherson, Marla C.; Ivison, Geoffrey T.; Qu, Xiumei; Rigdon, Joseph; Esquivel, Carlos O.; Krams, Sheri M.; Martinez, Olivia M.
American Journal of Transplantation (2019), 19(5), 1305-1314 | Language: English, Database: CAplus and MEDLINE

Substances (11) Reactions (0) Cited By (12)

98

Drug Efficacy Comparison of 3D Forming and Preforming Sphere Models with a Micropillar and Microwell Chip Platform

By: Doh, Il; Kwon, Yong-Jun; Ku, Bosung; Lee, Dong Woo
SLAS Discovery (2019), 24(4), 476-483 | Language: English, Database: CAplus and MEDLINE

Substances (11) Reactions (0) Cited By (6)

99

Extra-virgin olive oil contains a metabolo-epigenetic inhibitor of cancer stem cells

By: Corominas-Faja, Bruna; Cuyas, Elisabet; Lozano-Sanchez, Jesus; Cufi, Silvia; Verdura, Sara; Fernandez-Arroyo, Salvador; Borras-Linares, Isabel; Martin-Castillo, Begona; Martin, Angel G.; Lupu, Ruth; et al
Carcinogenesis (2018), 39(4), 601-613 | Language: English, Database: CAplus and MEDLINE

Substances (11) Reactions (0) Cited By (38)

100

Somatic mutation profiling of vulvar cancer: Exploring therapeutic targets

By: Zieba, Sebastian; Kowalik, Artur ; Zalewski, Kamil; Rusetska, Natalia; Goryca, Krzysztof; Piascik, Agata; Misiek, Marcin; Bakula-Zalewska, Elwira; Kopczynski, Janusz; Kowalski, Kamil; et al
Gynecologic Oncology (2018), 150(3), 552-561 | Language: English, Database: CAplus and MEDLINE

Substances (11) Reactions (0) Cited By (29)

101

Overproduction of IGF-2 drives a subset of colorectal cancer cells, which specifically respond to an anti-IGF therapeutic antibody and combination therapies

By: Zhong, H.; Fazenbaker, C.; Chen, C.; Breen, S.; Huang, J.; Yao, X.; Ren, P.; Yao, Y.; Herbst, R.; Hollingsworth, R. E.
Oncogene (2017), 36(6), 797-806 | Language: English, Database: CAplus and MEDLINE

Substances (11) Reactions (0) Cited By (17)

102

FBW7 suppression leads to SOX9 stabilization and increased malignancy in medulloblastoma

By: Suryo Rahmanto, Aldwin ; Savov, Vasil; Brunner, Andrae; Bolin, Sara; Weishaupt, Holger; Malyukova, Alena; Rosen, Gabriela; Cancer, Matko; Hutter, Sonja; Sundstroem, Anders; et al
EMBO Journal (2016), 35(20), 2192-2212 | Language: English, Database: CAplus and MEDLINE

Substances (11) Reactions (0) Cited By (39)

103

Mastering the Complex Targeted Therapy for Non-small Cell Lung Cancer

By: Devarakonda, Siddhartha; Govindan, Ramaswamy; Morgensztern, Daniel
Cancer Cell (2020), 38(3), 320-322 | Language: English, Database: CAplus and MEDLINE

Substances (12) Reactions (0) Cited By (5)

104

Macropinocytosis Renders a Subset of Pancreatic Tumor Cells Resistant to mTOR Inhibition

By: Michalopoulou, Evdokia; Auciello, Francesca R.; Bulusu, Vinay; Strachan, David; Campbell, Andrew D.; Tait-Mulder, Jacqueline; Karim, Saadia A.; Morton, Jennifer P.; Sansom, Owen J.; Kamphorst, Jurre J.
Cell Reports (2020), 30(8), 2729-2742.e4 | Language: English, Database: CAplus and MEDLINE

Substances (12) Reactions (0) Cited By (16)

105

High-throughput dynamic BH3 profiling may quickly and accurately predict effective therapies in solid tumors

By: Bhola, Patrick D. ; Ahmed, Eman; Guerriero, Jennifer L. ; Sicinska, Ewa; Su, Emily ; Lavrova, Elizaveta; Ni, Jing; Chipashvili, Otari ; Hagan, Timothy ; Pioso, Marissa S.; et al
Science Signaling (2020), 13(636), eaay1451 | Language: English, Database: CAplus and MEDLINE

Substances (31) Reactions (0) Cited By (23)

106

3D Cell-Based High-Content Screening (HCS) Using a Micropillar and Microwell Chip Platform

By: Lee, Sang-Yun; Doh, Il; Nam, Do-Hyun; Lee, Dong Woo
Analytical Chemistry (Washington, DC, United States) (2018), 90(14), 8354-8361 | Language: English, Database: CAplus and MEDLINE

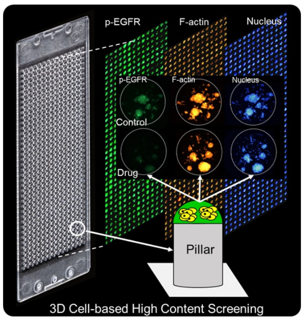

Substances (78) Reactions (0) Cited By (15)

107

A Phase 1 study of ADI-PEG20 (pegargiminase) combined with cisplatin and pemetrexed in ASS1-negative metastatic uveal melanoma

By: Chan, Pui Ying ; Phillips, Melissa M.; Ellis, Stephen; Johnston, Amanda; Feng, Xiaoxing; Arora, Amit; Hay, Gordon; Cohen, Victoria M. L.; Sagoo, Mandeep S.; Bomalaski, John S.; et al
Pigment Cell & Melanoma Research (2022), 35(4), 461-470 | Language: English, Database: CAplus and MEDLINE

Substances (13) Reactions (0) Cited By (1)

108

Antiviral drug screen identifies DNA-damage response inhibitor as potent blocker of SARS-CoV-2 replication

By: Garcia, Gustavo; Sharma, Arun; Ramaiah, Arunachalam; Sen, Chandani; Purkayastha, Arunima; Kohn, Donald B.; Parcells, Mark S.; Beck, Sebastian; Kim, Heeyoung; Bakowski, Malina A.; et al
Cell Reports (2021), 35(1), 108940 | Language: English, Database: CAplus and MEDLINE

Substances (13) Reactions (0) Cited By (43)

109

Practical synthesis of Vistusertib (AZD2014), an ATP competitive mTOR inhibitor

By: Shen, Guobing; Liu, Miaoqing; Lu, Jianjun; Meng, Tao
Tetrahedron Letters (2019), 60(52), 151333 | Language: English, Database: CAplus

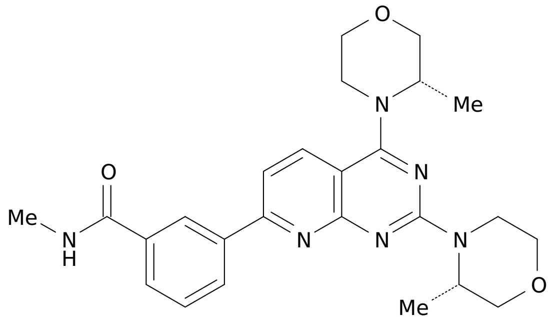

I

Substances (13) | Reactions (21) | Cited By (4)

110

Keratinocyte growth factor protects endometrial cells from oxygen glucose deprivation/re-oxygenation via activating Nrf2 signaling

By: Shi, Xuting; Liu, Hai-yan; Li, Shu-ping; Xu, Hong-bin
Biochemical and Biophysical Research Communications (2018), 501(1), 178-185 | Language: English, Database: CAplus and MEDLINE

Substances (13) | Reactions (0) | Cited By (22)

111

CC-223 inhibits human head and neck squamous cell carcinoma cell growth

By: Wang, Jun-ying; Jin, Xin; Zhang, Xin; Li, Xiao-feng
Biochemical and Biophysical Research Communications (2018), 496(4), 1191-1196 | Language: English, Database: CAplus and MEDLINE

Substances (13) | Reactions (0) | Cited By (7)

112

One size doesn't fit all in Ph-like ALL

By: Post, Sean M.
Blood (2017), 129(2), 140-141 | Language: English, Database: CAplus and MEDLINE

Substances (13) | Reactions (0) | Cited By (0)

113

Colon cancer organoids using monoclonal organoids established in four different lesions of one cancer patient reveal tumor heterogeneity and different real-time responsiveness to anti-cancer drugs

By: Song, Myoung-Hyun; Park, Ji Won; Kim, Min Jung; Shin, Young-Kyoung; Kim, Soon-Chan; Jeong, Seung-Yong; Ku, Ja-Lok
Biomedicine & Pharmacotherapy (2022), 152, 113260 | Language: English, Database: CAplus and MEDLINE

Substances (15) Reactions (0) Cited By (1)

114

High-risk neuroblastoma with NF1 loss of function is targetable using SHP2 inhibition

By: Cai, Jinyang; Jacob, Sheeba; Kurupi, Richard; Dalton, Krista M.; Coon, Colin; Greninger, Patricia; Egan, Regina K.; Stein, Giovanna T.; Murchie, Ellen; McClanaghan, Joseph; et al
Cell Reports (2022), 40(4), 111095 | Language: English, Database: CAplus and MEDLINE

Substances (15) Reactions (0) Cited By (1)

115

Overcoming resistance to mTOR inhibition for enhanced strategies in clinical trials

By: Naing, Aung
Expert Opinion on Investigational Drugs (2013), 22(6), 679-685 | Language: English, Database: CAplus and MEDLINE

Substances (15) Reactions (0) Cited By (6)

116

A phase I study investigating AZD8186, a potent and selective inhibitor of PI3κβ/δ, in patients with advanced solid tumors

By: Choudhury, Atish D.; Higano, Celestia S.; de Bono, Johann S.; Cook, Natalie; Rathkopf, Dana E.; Wisinski, Kari B.; Martin-Liberal, Juan; Linch, Mark; Heath, Elisabeth I.; Baird, Richard D.; et al
Clinical Cancer Research (2022), 28(11), 2257-2269 | Language: English, Database: CAplus and MEDLINE

Substances (16) Reactions (0) Cited By (1)

117

A cell-based high-throughput screen identifies inhibitors that overcome P-glycoprotein (Pgp)-mediated multidrug resistance

By: Zahra, Rida; Furqan, Muhammad; Ullah, Rahim; Mithani, Aziz; Saleem, Rahman Shah Zaib; Faisal, Amir
PLoS One (2020), 15(6), e0233993 | Language: English, Database: CAplus and MEDLINE

Substances (16) Reactions (0) Cited By (12)

118

A Conformational Restriction Strategy for the Identification of a Highly Selective Pyrimido-pyrrolo-oxazine mTOR Inhibitor

By: Borsari, Chiara ; Rageot, Denise ; Dall'Asen, Alix; Bohnacker, Thomas; Melone, Anna; Sele, Alexander M. ; Jackson, Eileen; Langlois, Jean-Baptiste; Beaufils, Florent; Hebeisen, Paul; et al
Journal of Medicinal Chemistry (2019), 62(18), 8609-8630 | Language: English, Database: CAplus and MEDLINE

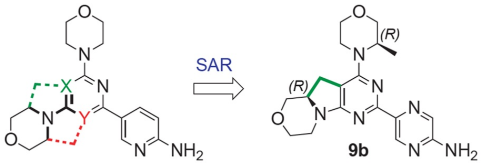

Substances (120) | Reactions (421) | Cited By (19)

119

First-in-child phase I/II study of the dual mTORC1/2 inhibitor vistusertib (AZD2014) as monotherapy and in combination with topotecan-temozolomide in children with advanced malignancies: arms E and F of the AcS-ESMART trial

By: Morscher, Raphael J.; Brard, Caroline ; Berlanga, Pablo ; Marshall, Lynley V. ; Andre, Nicolas; Rubino, Jonathan ; Aerts, Isabelle; De Carli, Emilie ; Corradini, Nadege; Nebchi, Souad; et al
European Journal of Cancer (2021), 157, 268-277 | Language: English, Database: CAplus and MEDLINE

Substances (17) | Reactions (0) | Cited By (3)

120

Drug-Dependent Morphological Transitions in Spherical and Worm-Like Polymeric Micelles Define Stability and Pharmacological Performance of Micellar Drugs

By: Lim, Chaemin; Ramsey, Jacob D.; Hwang, Duhyeong; Teixeira, Susana C. M.; Poon, Chi-Duen; Strauss, Joshua D.; Rosen, Elias P.; Sokolsky-Papkov, Marina; Kabanov, Alexander V.
Small (2022), 18(4), 2103552 | Language: English, Database: CAplus and MEDLINE

Substances (17) | Reactions (0) | Cited By (9)

121

Implementation of a Multiplex and Quantitative Proteomics Platform for Assessing Protein Lysates Using DNA-Barcoded Antibodies

By: Lee, Jinho; Geiss, Gary K.; Demirkan, Gokhan; Vellano, Christopher P.; Filanoski, Brian; Lu, Yiling; Ju, Zhenlin; Yu, Shuangxing; Guo, Huifang; Bogatzki, Lisa Y.; et al
Molecular & Cellular Proteomics (2018), 17(6), 1245-1258 | Language: English, Database: CAplus and MEDLINE

Substances (17) | Reactions (0) | Cited By (13)

122

Methuosis Contributes to Jaspine-B-Induced Cell Death

By: Bielsa, Nuria; Casasampere, Mireia; Abad, Jose Luis; Enrich, Carlos ; Delgado, Antonio; Fabrias, Gemma; Lizcano, Jose M. ; Casas, Josefina
International Journal of Molecular Sciences (2022), 23(13), 7257 | Language: English, Database: CAplus and MEDLINE

Substances (18) | Reactions (0) | Cited By (0)

123

RAPTOR up-regulation contributes to resistance of renal cancer cells to PI3K-mTOR inhibition

By: Earwaker, Philip; Anderson, Caroline; Willenbrock, Frances; Harris, Adrian L.; Protheroe, Andrew S.; Macaulay, Valentine M.
PLoS One (2018), 13(2), e0191890/1-e0191890/22 | Language: English, Database: CAplus and MEDLINE

Substances (18) | Reactions (0) | Cited By (19)

124

Enhanced MAPK signaling drives ETS1-mediated induction of miR-29b leading to downregulation of TET1 and changes in epigenetic modifications in a subset of lung SCC

By: Taylor, M. A.; Wappett, M.; Delpuech, O.; Brown, H.; Chresta, C. M.
Oncogene (2016), 35(33), 4345-4357 | Language: English, Database: CAplus and MEDLINE

Substances (18) | Reactions (0) | Cited By (26)

125

A genome-scale CRISPR screen identifies the ERBB and mTOR signaling networks as key determinants of response to PI3K inhibition in pancreatic cancer

By: Milton, Charlotte K.; Self, Annette J.; Clarke, Paul A.; Banerji, Udai; Piccioni, Federica; Root, David E.; Whittaker, Steven R.
Molecular Cancer Therapeutics (2020), 19(7), 1423-1435 | Language: English, Database: CAplus and MEDLINE

Substances (19) | Reactions (0) | Cited By (8)

126

mTOR inhibition via displacement of phosphatidic acid induces enhanced cytotoxicity specifically in cancer cells

By: Nguyen, Tra-Ly; Nokin, Marie-Julie; Egorov, Maxim; Tome, Mercedes; Bodineau, Clement; Primo, Carmelo Di; Minder, Laetitia; Wdzieczak-Bakala, Joanna; Garcia-Alvarez, Maria Concepcion; Bignon, Jerome; et al
Cancer Research (2018), 78(18), 5384-5397 | Language: English, Database: CAplus and MEDLINE

Substances (19) | Reactions (0) | Cited By (11)

127

HSP90 inhibitors induce GPNMB cell-surface expression by modulating lysosomal positioning and sensitize breast cancer cells to glembatumumab vedotin

By: Biondini, Marco; Kiepas, Alex; El-Houjeiri, Leeanna; Annis, Matthew G.; Hsu, Brian E.; Fortier, Anne-Marie; Morin, Genevieve; Martina, Jose A.; Sirois, Isabelle; Aguilar-Mahecha, Adriana; et al
Oncogene (2022), 41(12), 1701-1717 | Language: English, Database: CAplus and MEDLINE

Substances (20) Reactions (0) Cited By (1)

128

Diverse resistance mechanisms to the third-generation ALK inhibitor lorlatinib in alk-rearranged lung cancer

By: Recondo, Gonzalo; Mezquita, Laura; Facchinetti, Francesco; Planchard, David; Gazzah, Anas; Bigot, Ludovic; Rizvi, Ahsan Z.; Frias, Rosa L.; Thiery, Jean Paul; Scoazec, Jean-Yves; et al
Clinical Cancer Research (2020), 26(1), 242-255 | Language: English, Database: CAplus and MEDLINE

Substances (20) Reactions (0) Cited By (63)

129

Landscapes of cellular phenotypic diversity in breast cancer xenografts and their impact on drug response

By: Georgopoulou, Dimitra; Callari, Maurizio; Rueda, Oscar M.; Shea, Abigail; Martin, Alistair; Giovannetti, Agnese ; Qosaj, Fatime; Dariush, Ali; Chin, Suet-Feung ; Carnevalli, Larissa S. ; et al
Nature Communications (2021), 12(1), 1998 | Language: English, Database: CAplus and MEDLINE

Substances (23) Reactions (0) Cited By (16)

130

Establishment and characterization of 18 human colorectal cancer cell lines

By: Kim, Soon-Chan; Kim, Hyun-Soo; Kim, Jae Hyeon; Jeong, Nahyun; Shin, Young-Kyoung; Kim, Min Jung; Park, Ji Won; Jeong, Seung-Yong; Ku, Ja-Lok
Scientific Reports (2020), 10(1), 6801 | Language: English, Database: CAplus and MEDLINE

Substances (23) Reactions (0) Cited By (2)

131

Selective colony area method for heterogeneous patient-derived tumor cell lines in anti-cancer drug screening system

By: Cho, Jang Ho; Kim, Ju-Sun; Kim, Seung Tae; Hong, Jung Yong; Park, Joon Oh; Park, Young Suk; Nam, Do-Hyun; Lee, Dong Woo; Lee, Jeeyun
PLoS One (2019), 14(4), e0215080 | Language: English, Database: CAplus and MEDLINE

Substances (23) Reactions (0) Cited By (1)

132

Palbociclib-based high-throughput combination drug screening identifies synergistic therapeutic options in HPV-negative head and neck squamous cell carcinoma

By: Gu, Ziyue; Shi, Chaoji; Li, Jiayi; Han, Yong; Sun, Bao; Zhang, Wuchang; Wu, Jing; Zhou, Guoyu; Ye, Weimin; Li, Jiang; et al
BMC Medicine (2022), 20(1), 175 | Language: English, Database: CAplus and MEDLINE

Substances (24) Reactions (0) Cited By (0)

133

High Throughput 3D Cell Migration Assay Using Micropillar/Microwell Chips

By: Lee, Sang-Yun; Park, Lily M.; Oh, Yoo Jung; Choi, Dong Hyuk; Lee, Dong Woo
Molecules (2022), 27(16), 5306 | Language: English, Database: CAplus and MEDLINE

Substances (70) Reactions (0) Cited By (0)

134

Design, synthesis and antitumor evaluation of novel quinazoline analogs in hepatocellular carcinoma cell

By: Yu, Pan; Cao, Weiya; Yang, Shilong; Wang, Yuan; Xia, Aixin; Tan, Xinlan; Wang, Luyi
Journal of Molecular Structure (2022), 1268, 133718 | Language: English, Database: CAplus

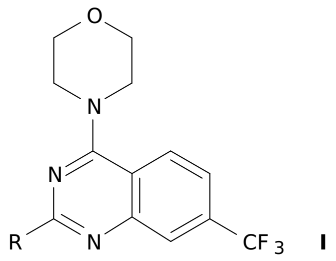

Substances (37) Reactions (22) Cited By (1)

135

Molecular taxonomy of systemic lupus erythematosus through data-driven patient stratification: molecular endotypes and cluster-tailored drugs

By: Garantziotis, Panagiotis; Nikolakis, Dimitrios; Doumas, Stavros; Frangou, Eleni; Sentis, George; Filia, Anastasia; Fanouriakis, Antonis; Bertsias, George; Boumpas, Dimitrios T.
Frontiers in Immunology (2022), 13, 860726 | Language: English, Database: CAplus and MEDLINE

Substances (37) Reactions (0) Cited By (0)

136

Pyrimidine inhibitors synergize with nucleoside analogues to block SARS-CoV-2

By: Schultz, David C. ; Johnson, Robert M. ; Ayyanathan, Kasirajan; Miller, Jesse ; Whig, Kanupriya ; Kamalia, Brinda; Dittmar, Mark ; Weston, Stuart; Hammond, Holly L.; Dillen, Carly; et al
Nature (London, United Kingdom) (2022), 604(7904), 134-140 | Language: English, Database: CAplus and MEDLINE

Substances (112) Reactions (0) Cited By (45)

137

Ponatinib, lestaurtinib, and mTOR/PI3K inhibitors are promising repurposing candidates against Entamoeba histolytica

By: Kangussu-Marcolino, Monica M.; Singh, Upinder
Antimicrobial Agents and Chemotherapy (2022), 66(2), e01207-21 | Language: English, Database: CAplus

Substances (50) Reactions (0) Cited By (2)

138

SHMT2 inhibition disrupts the TCF3 transcriptional survival program in Burkitt lymphoma

By: Wilke, Anne C.; Doebele, Carmen; Zindel, Alena; Lee, Kwang Seok ; Rieke, Sara A. ; Ceribelli, Michele; Comoglio, Federico ; Phelan, James D.; Wang, James Q.; Pikman, Yana; et al
Blood (2022), 139(4), 538-553 | Language: English, Database: CAplus and MEDLINE

Substances (31) Reactions (0) Cited By (7)

139

Dynamic BH3 profiling identifies active BH3 mimetic combinations in non-small cell lung cancer

By: Potter, Danielle S.; Du, Ruochen; Bhola, Patrick; Bueno, Raphael; Letai, Anthony
Cell Death & Disease (2021), 12(8), 741 | Language: English, Database: CAplus and MEDLINE

Substances (26) Reactions (0) Cited By (4)

140

Screening reveals sterol derivatives with pro-differentiation, pro-survival, or potent cytotoxic effects on oligodendrocyte progenitor cells

By: Sax, Joel L.; Hubler, Zita; Allimuthu, Dharmaraja; Adams, Drew J.
ACS Chemical Biology (2021), 16(7), 1288-1297 | Language: English, Database: CAplus and MEDLINE

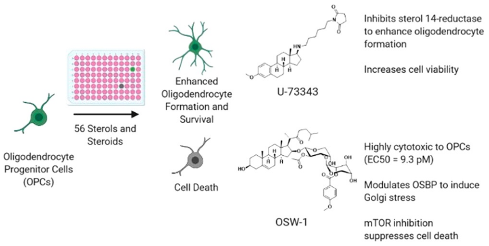

Substances (31) Reactions (0) Cited By (3)

141

4-(Difluoromethyl)-5-(4-((3R,5S)-3,5-dimethylmorpholino)-6-((R)-3-methylmorpholino)-1,3,5-triazin-2-yl)pyridin-2-amine (PQR626), a Potent, Orally Available, and Brain-Penetrant mTOR Inhibitor for the Treatment of Neurological Disorders

By: Borsari, Chiara; Keles, Erhan; Rageot, Denise; Treyer, Andrea; Bohnacker, Thomas; Bissegger, Lukas; De Pascale, Martina; Melone, Anna; Sriramaratnam, Rohitha; Beaufils, Florent; et al
Journal of Medicinal Chemistry (2020), 63(22), 13595-13617 | Language: English, Database: CAplus and MEDLINE

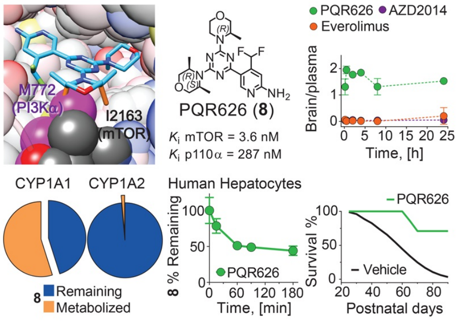

Substances (78) Reactions (107) Cited By (11)

142

Ex vivo modelling of drug efficacy in a rare metastatic urachal carcinoma

By: Makela, Rami; Arjonen, Antti; Harma, Ville; Rintanen, Nina; Paasonen, Lauri; Paprotka, Tobias; Ronsch, Kerstin; Kuopio, Teijo; Kononen, Juha; Rantala, Juha K.
BMC Cancer (2020), 20(1), 590 | Language: English, Database: CAplus and MEDLINE

Substances (46) Reactions (0) Cited By (3)

143

Discovery of Novel Autophagy Inhibitors and Their Sensitization Abilities for Vincristine-Resistant Esophageal Cancer Cell Line Eca109/VCR

By: Shan, Changyu; Hui, Wenqi; Li, Hongwei; Wang, Zheng; Guo, Chunling; Peng, Ruikun; Gu, Jing; Chen, Yingchun; Ouyang, Qin
ChemMedChem (2020), 15(11), 970-981 | Language: English, Database: CAplus and MEDLINE

Substances (51) | Reactions (0) | Cited By (5)

144

Machine learning models for the prediction of chemotherapy-induced peripheral neuropathy

By: Bloomingdale, Peter; Mager, Donald E.
Pharmaceutical Research (2019), 36(2), 1-12 | Language: English, Database: CAplus and MEDLINE

Substances (60) | Reactions (0) | Cited By (8)

145

Drug repurposing screening identifies bortezomib and panobinostat as drugs targeting cancer associated fibroblasts (CAFs) by synergistic induction of apoptosis

By: Lee, Hak-Min; Lee, Eunmyong; Yeo, So-Young; Shin, Sang; Park, Hyun-Kyu; Nam, Do-Hyun; Kim, Seok-Hyung
Investigational New Drugs (2018), 36(4), 545-560 | Language: English, Database: CAplus and MEDLINE

Substances (67) | Reactions (0) | Cited By (10)

146

JAK1/2 and BCL2 inhibitors synergize to counteract bone marrow stromal cell-induced protection of AML

By: Karjalainen, Riikka; Pemovska, Tea; Popa, Mihaela; Liu, Minxia; Javarappa, Komal K.; Majumder, Muntasir M.; Yadav, Bhagwan; Tamborero, David; Tang, Jing; Bychkov, Dmitrii; et al
Blood (2017), 130(6), 789-802 | Language: English, Database: CAplus and MEDLINE

Substances (270) | Reactions (0) | Cited By (66)

147

Identification of selective cytotoxic and synthetic lethal drug responses in triple negative breast cancer cells

By: Gautam, Prson; Karhinen, Leena; Szwajda, Agnieszka; Kumar, Jha Sawan; Yadav, Bhagwan; Aittokallio, Tero; Wennerberg, Krister
Molecular Cancer (2016), 15, 34/1-34/16 | Language: English, Database: CAplus and MEDLINE

Substances (257) | Reactions (0) | Cited By (46)

148

Chemical Proteomics Reveals Ferrochelatase as a Common Off-target of Kinase Inhibitors

By: Klaeger, Susan; Gohlke, Bjoern; Perrin, Jessica; Gupta, Vipul; Heinzlmeir, Stephanie; Helm, Dominic; Qiao, Huichao; Bergamini, Giovanna; Handa, Hiroshi; Savitski, Mikhail M.; et al

ACS Chemical Biology (2016), 11(5), 1245-1254 | Language: English, Database: CAplus and MEDLINE

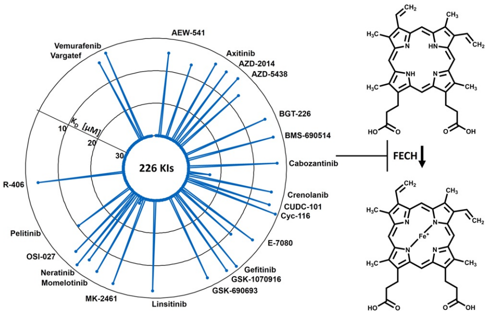

Substances (32) Reactions (0) Cited By (62)

Copyright © 2023 American Chemical Society (ACS). All Rights Reserved.
Copyright © 2023 U.S. National Library of Medicine.
Internal use only. Redistribution is subject to the terms of your SciFinderⁿ License Agreement and CAS information Use Policies.

Results of PubMed search: keyword THZ1

1: Sauge S, Swillo M, Albert-Seifried S, Xavier GB, WaldebÃ¤ck J, Tengner M, Ljunggren D, Karlsson A. Narrowband polarization-entangled photon pairs distributed over a WDM link for qubit networks. Opt Express. 2007 May 28;15(11):6926-33. doi: 10.1364/oe.15.006926. PMID: 19547007.

2: Sauge S, Swillo M, Tengner M, Karlsson A. A single-crystal source of path-polarization entangled photons at non-degenerate wavelengths. Opt Express. 2008 Jun 23;16(13):9701-7. doi: 10.1364/oe.16.009701. PMID: 18575538.

3: Li Z, Zhang ZH, Zhao XY, Su HX, Yan F. [Extracting THz absorption coefficient spectrum based on accurate determination of sample thickness]. Guang Pu Xue Yu Guang Pu Fen Xi. 2012 Apr;32(4):1043-6. Chinese. PMID: 22715781.

4: Kwiatkowski N, Zhang T, Rahl PB, Abraham BJ, Reddy J, Ficarro SB, Dastur A, Amzallag A, Ramaswamy S, Tesar B, Jenkins CE, Hannett NM, McMillin D, Sanda T, Sim T, Kim ND, Look T, Mitsiades CS, Weng AP, Brown JR, Benes CH, Marto JA, Young RA, Gray NS. Targeting transcription regulation in cancer with a covalent CDK7 inhibitor. Nature. 2014 Jul 31;511(7511):616-20. doi: 10.1038/nature13393. Epub 2014 Jun 22. PMID: 25043025; PMCID: PMC4244910.

5: Cao K, Shilatifard A. Inhibit globally, act locally: CDK7 inhibitors in cancer therapy. Cancer Cell. 2014 Aug 11;26(2):158-9. doi: 10.1016/j.ccr.2014.07.020. PMID: 25117707.

6: Chipumuro E, Marco E, Christensen CL, Kwiatkowski N, Zhang T, Hatheway CM, Abraham BJ, Sharma B, Yeung C, Altabef A, Perez-Atayde A, Wong KK, Yuan GC, Gray NS, Young RA, George RE. CDK7 inhibition suppresses super-enhancer-linked oncogenic transcription in MYCN-driven cancer. Cell. 2014 Nov 20;159(5):1126-1139. doi: 10.1016/j.cell.2014.10.024. Epub 2014 Nov 6. PMID: 25416950; PMCID: PMC4243043.

7: Augert A, MacPherson D. Treating transcriptional addiction in small cell lung cancer. Cancer Cell. 2014 Dec 8;26(6):783-784. doi: 10.1016/j.ccell.2014.11.012. PMID: 25490443.

8: Christensen CL, Kwiatkowski N, Abraham BJ, Carretero J, Al-Shahrour F, Zhang T, Chipumuro E, Herter-Sprie GS, Akbay EA, Altabef A, Zhang J, Shimamura T, Capelletti M, Reibel JB, Cavanaugh JD, Gao P, Liu Y, Michaelsen SR, Poulsen HS, Aref AR, Barbie DA, Bradner JE, George RE, Gray NS, Young RA, Wong KK. Targeting transcriptional addictions in small cell lung cancer with a covalent CDK7 inhibitor. Cancer Cell. 2014 Dec 8;26(6):909-922. doi: 10.1016/j.ccell.2014.10.019. Erratum in: Cancer Cell. 2015 Jan 12;27(1):149. PMID: 25490451; PMCID: PMC4261156.

9: Nilson KA, Guo J, Turek ME, Brogie JE, Delaney E, Luse DS, Price DH. THZ1 Reveals Roles for Cdk7 in Co-transcriptional Capping and Pausing. Mol Cell. 2015 Aug 20;59(4):576-87. doi: 10.1016/j.molcel.2015.06.032. Epub 2015 Aug 6. PMID: 26257281; PMCID: PMC4546572.

10: Coin F, Egly JM. Revisiting the Function of CDK7 in Transcription by Virtue of a Recently Described TFIIH Kinase Inhibitor. Mol Cell. 2015 Aug 20;59(4):513-4. doi: 10.1016/j.molcel.2015.08.006. PMID: 26295956.

11: Franco HL, Kraus WL. No driver behind the wheel? Targeting transcription in cancer. Cell. 2015 Sep 24;163(1):28-30. doi: 10.1016/j.cell.2015.09.013. PMID: 26406367.

12: Posternak V, Cole MD. Strategically targeting MYC in cancer. F1000Res. 2016 Mar 29;5:F1000 Faculty Rev-408. doi: 10.12688/f1000research.7879.1. PMID: 27081479; PMCID: PMC4813636.

13: Jiang YY, Lin DC, Mayakonda A, Hazawa M, Ding LW, Chien WW, Xu L, Chen Y, Xiao JF, Senapedis W, Baloglu E, Kanojia D, Shang L, Xu X, Yang H, Tyner JW, Wang MR, Koeffler HP. Targeting super-enhancer-associated oncogenes in oesophageal squamous cell carcinoma. Gut. 2017 Aug;66(8):1358-1368. doi: 10.1136/gutjnl-2016-311818. Epub 2016 May 10. PMID: 27196599; PMCID: PMC5912916.

14: Compe E, Egly JM. Nucleotide Excision Repair and Transcriptional Regulation: TFIIH and Beyond. Annu Rev Biochem. 2016 Jun 2;85:265-90. doi: 10.1146/annurev-biochem-060815-014857. PMID: 27294439.

15: Harrod A, Fulton J, Nguyen VTM, Periyasamy M, Ramos-Garcia L, Lai CF, Metodieva G, de Giorgio A, Williams RL, Santos DB, Gomez PJ, Lin ML, Metodiev MV, Stebbing J, Castellano L, Magnani L, Coombes RC, Buluwela L, Ali S. Genomic modelling of the ESR1 Y537S mutation for evaluating function and new therapeutic approaches for metastatic breast cancer. Oncogene. 2017 Apr 20;36(16):2286-2296. doi: 10.1038/onc.2016.382. Epub 2016 Oct 17. PMID: 27748765; PMCID: PMC5245767.

16: Wang H, Jo YJ, Sun TY, Namgoong S, Cui XS, Oh JS, Kim NH. Inhibition of CDK7 bypasses spindle assembly checkpoint via premature cyclin B degradation during oocyte meiosis. Biochim Biophys Acta. 2016 Dec;1863(12):2993-3000. doi: 10.1016/j.bbamcr.2016.09.020. PMID: 27693251.

17: Cayrol F, Praditsuktavorn P, Fernando TM, Kwiatkowski N, Marullo R, Calvo-Vidal MN, Phillip J, Pera B, Yang SN, Takpradit K, Roman L, Gaudiano M, Crescenzo R, Ruan J, Inghirami G, Zhang T, Cremaschi G, Gray NS, Cerchietti L. THZ1 targeting CDK7 suppresses STAT transcriptional activity and sensitizes T-cell lymphomas to BCL2 inhibitors. Nat Commun. 2017 Jan 30;8:14290. doi: 10.1038/ncomms14290. Erratum in: Nat Commun. 2017 Feb 20;8:14747. PMID: 28134252; PMCID: PMC5290269.

18: Cayrol F, Praditsuktavorn P, Fernando TM, Kwiatkowski N, Marullo R, Calvo-Vidal MN, Phillip J, Pera B, Yang SN, Takpradit K, Roman L, Gaudiano M, Crescenzo R, Ruan J, Inghirami G, Zhang T, Cremaschi G, Gray NS, Cerchietti L. Corrigendum: THZ1 targeting CDK7 suppresses STAT transcriptional activity and sensitizes T-cell lymphomas to BCL2 inhibitors. Nat Commun. 2017 Feb 20;8:14747. doi: 10.1038/ncomms14747. Erratum for: Nat Commun. 2017 Jan 30;8:14290. PMID: 28218232; PMCID: PMC5321718.

19: Francavilla C, Lupia M, Tsafou K, Villa A, Kowalczyk K, Rakownikow Jersie-Christensen R, Bertalot G, Confalonieri S, Brunak S, Jensen LJ, Cavallaro U, Olsen JV. Phosphoproteomics of Primary Cells Reveals Druggable Kinase Signatures in Ovarian Cancer. Cell Rep. 2017 Mar 28;18(13):3242-3256. doi: 10.1016/j.celrep.2017.03.015. PMID: 28355574; PMCID: PMC5382236.

20: Kavarthapu R, Dufau ML. Essential role of endogenous prolactin and CDK7 in estrogen-induced upregulation of the prolactin receptor in breast cancer cells.

Oncotarget. 2017 Apr 18;8(16):27353-27363. doi: 10.18632/oncotarget.16040. PMID: 28423697; PMCID: PMC5432340.

21: Nagaraja S, Vitanza NA, Woo PJ, Taylor KR, Liu F, Zhang L, Li M, Meng W, Ponnuswami A, Sun W, Ma J, Hulleman E, Swigut T, Wysocka J, Tang Y, Monje M. Transcriptional Dependencies in Diffuse Intrinsic Pontine Glioma. Cancer Cell. 2017 May 8;31(5):635-652.e6. doi: 10.1016/j.ccell.2017.03.011. Epub 2017 Apr 20. PMID: 28434841; PMCID: PMC5462626.

22: Li B, Ni Chonghaile T, Fan Y, Madden SF, Klinger R, O'Connor AE, Walsh L, O'Hurley G, Mallya Udupi G, Joseph J, Tarrant F, Conroy E, Gaber A, Chin SF, Bardwell HA, Provenzano E, Crown J, Dubois T, Linn S, Jirstrom K, Caldas C, O'Connor DP, Gallagher WM. Therapeutic Rationale to Target Highly Expressed CDK7 Conferring Poor Outcomes in Triple-Negative Breast Cancer. Cancer Res. 2017 Jul 15;77(14):3834-3845. doi: 10.1158/0008-5472.CAN-16-2546. Epub 2017 Apr 28. PMID: 28455421.

23: Greenall SA, Lim YC, Mitchell CB, Ensbey KS, Stringer BW, Wilding AL, O'Neill GM, McDonald KL, Gough DJ, Day BW, Johns TG. Cyclin-dependent kinase 7 is a therapeutic target in high-grade glioma. Oncogenesis. 2017 May 15;6(5):e336. doi: 10.1038/oncsis.2017.33. PMID: 28504693; PMCID: PMC5523066.

24: Kandanur SGS, Nanduri S, Golakoti NR. Synthesis and biological evaluation of new C-12(Î±/Î²)-(N-) sulfamoyl-phenylamino-14-deoxy-andrographolide derivatives as potent anti-cancer agents. Bioorg Med Chem Lett. 2017 Jul 1;27(13):2854-2862. doi: 10.1016/j.bmcl.2017.04.033. Epub 2017 Apr 13. PMID: 28527822.

25: Zhang Z, Peng H, Wang X, Yin X, Ma P, Jing Y, Cai MC, Liu J, Zhang M, Zhang S, Shi K, Gao WQ, Di W, Zhuang G. Preclinical Efficacy and Molecular Mechanism of Targeting CDK7-Dependent Transcriptional Addiction in Ovarian Cancer. Mol Cancer Ther. 2017 Sep;16(9):1739-1750. doi: 10.1158/1535-7163.MCT-17-0078. Epub 2017 Jun 1. PMID: 28572168.

26: Ebmeier CC, Erickson B, Allen BL, Allen MA, Kim H, Fong N, Jacobsen JR, Liang K, Shilatifard A, Dowell RD, Old WM, Bentley DL, Taatjes DJ. Human TFIIH Kinase CDK7 Regulates Transcription-Associated Chromatin Modifications. Cell Rep. 2017 Aug 1;20(5):1173-1186. doi: 10.1016/j.celrep.2017.07.021. PMID: 28768201; PMCID: PMC5564226.

27: Yuan J, Jiang YY, Mayakonda A, Huang M, Ding LW, Lin H, Yu F, Lu Y, Loh TKS, Chow M, Savage S, Tyner JW, Lin DC, Koeffler HP. Super-Enhancers Promote Transcriptional Dysregulation in Nasopharyngeal Carcinoma. Cancer Res. 2017 Dec 1;77(23):6614-6626. doi: 10.1158/0008-5472.CAN-17-1143. Epub 2017 Sep 26. PMID: 28951465; PMCID: PMC6637769.

28: Wong RWJ, Ngoc PCT, Leong WZ, Yam AWY, Zhang T, Asamitsu K, Iida S, Okamoto T, Ueda R, Gray NS, Ishida T, Sanda T. Enhancer profiling identifies critical cancer genes and characterizes cell identity in adult T-cell leukemia. Blood. 2017 Nov 23;130(21):2326-2338. doi: 10.1182/blood-2017-06-792184. Epub 2017 Oct 4. PMID: 28978570; PMCID: PMC5701524.

29: Kalan S, Amat R, Schachter MM, Kwiatkowski N, Abraham BJ, Liang Y, Zhang T, Olson CM, Larochelle S, Young RA, Gray NS, Fisher RP. Activation of the p53 Transcriptional Program Sensitizes Cancer Cells to Cdk7 Inhibitors. Cell Rep.

2017 Oct 10;21(2):467-481. doi: 10.1016/j.celrep.2017.09.056. PMID: 29020632; PMCID: PMC5687273.

30: Rusan M, Li K, Li Y, Christensen CL, Abraham BJ, Kwiatkowski N, Buczkowski KA, Bockorny B, Chen T, Li S, Rhee K, Zhang H, Chen W, Terai H, Tavares T, Leggett AL, Li T, Wang Y, Zhang T, Kim TJ, Hong SH, Poudel-Neupane N, Silkes M, Mudianto T, Tan L, Shimamura T, Meyerson M, Bass AJ, Watanabe H, Gray NS, Young RA, Wong KK, Hammerman PS. Suppression of Adaptive Responses to Targeted Cancer Therapy by Transcriptional Repression. Cancer Discov. 2018 Jan;8(1):59-73. doi: 10.1158/2159-8290.CD-17-0461. Epub 2017 Oct 20. PMID: 29054992; PMCID: PMC5819998.

31: Sahni JM, Keri RA. Targeting bromodomain and extraterminal proteins in breast cancer. Pharmacol Res. 2018 Mar;129:156-176. doi: 10.1016/j.phrs.2017.11.015. Epub 2017 Nov 16. PMID: 29154989; PMCID: PMC5828951.

32: He G, Yang X, Wang G, Qi J, Mao R, Wu Z, Zhou Z. Cdk7 Is Required for Activity-Dependent Neuronal Gene Expression, Long-Lasting Synaptic Plasticity and Long-Term Memory. Front Mol Neurosci. 2017 Nov 7;10:365. doi: 10.3389/fnmol.2017.00365. PMID: 29163040; PMCID: PMC5681959.

33: Terai H, Kitajima S, Potter DS, Matsui Y, Quiceno LG, Chen T, Kim TJ, Rusan M, Thai TC, Piccioni F, Donovan KA, Kwiatkowski N, Hinohara K, Wei G, Gray NS, Fischer ES, Wong KK, Shimamura T, Letai A, Hammerman PS, Barbie DA. ER Stress Signaling Promotes the Survival of Cancer "Persister Cells" Tolerant to EGFR Tyrosine Kinase Inhibitors. Cancer Res. 2018 Feb 15;78(4):1044-1057. doi: 10.1158/0008-5472.CAN-17-1904. Epub 2017 Dec 19. PMID: 29259014; PMCID: PMC5815936.

34: Gao Y, Zhang T, Terai H, Ficarro SB, Kwiatkowski N, Hao MF, Sharma B, Christensen CL, Chipumuro E, Wong KK, Marto JA, Hammerman PS, Gray NS, George RE. Overcoming Resistance to the THZ Series of Covalent Transcriptional CDK Inhibitors. Cell Chem Biol. 2018 Feb 15;25(2):135-142.e5. doi: 10.1016/j.chembiol.2017.11.007. Epub 2017 Dec 21. PMID: 29276047; PMCID: PMC6389506.

35: Carugo A, Draetta GF. Collapsing the Tumor Ecosystem: Preventing Adaptive Response to Treatment by Inhibiting Transcription. Cancer Discov. 2018 Jan;8(1):17-19. doi: 10.1158/2159-8290.CD-17-1245. PMID: 29311224.

36: Iniguez AB, Stolte B, Wang EJ, Conway AS, Alexe G, Dharia NV, Kwiatkowski N, Zhang T, Abraham BJ, Mora J, Kalev P, Leggett A, Chowdhury D, Benes CH, Young RA, Gray NS, Stegmaier K. EWS/FLI Confers Tumor Cell Synthetic Lethality to CDK12 Inhibition in Ewing Sarcoma. Cancer Cell. 2018 Feb 12;33(2):202-216.e6. doi: 10.1016/j.ccell.2017.12.009. Epub 2018 Jan 18. PMID: 29358035; PMCID: PMC5846483.

37: Eliades P, Abraham BJ, Ji Z, Miller DM, Christensen CL, Kwiatkowski N, Kumar R, Njauw CN, Taylor M, Miao B, Zhang T, Wong KK, Gray NS, Young RA, Tsao H. High MITF Expression Is Associated with Super-Enhancers and Suppressed by CDK7 Inhibition in Melanoma. J Invest Dermatol. 2018 Jul;138(7):1582-1590. doi: 10.1016/j.jid.2017.09.056. Epub 2018 Feb 8. PMID: 29408204; PMCID: PMC6019629.

38: Chen D, Zhao Z, Huang Z, Chen DC, Zhu XX, Wang YZ, Yan YW, Tang S, Madhavan

S, Ni W, Huang ZP, Li W, Ji W, Shen H, Lin S, Jiang YZ. Super enhancer inhibitors suppress MYC driven transcriptional amplification and tumor progression in osteosarcoma. Bone Res. 2018 Apr 4;6:11. doi: 10.1038/s41413-018-0009-8. PMID: 29644114; PMCID: PMC5884797.

39: Rimel JK, Taatjes DJ. The essential and multifunctional TFIIH complex. Protein Sci. 2018 Jun;27(6):1018-1037. doi: 10.1002/pro.3424. Epub 2018 Apr 27. PMID: 29664212; PMCID: PMC5980561.

40: Mbonye U, Wang B, Gokulrangan G, Shi W, Yang S, Karn J. Cyclin-dependent kinase 7 (CDK7)-mediated phosphorylation of the CDK9 activation loop promotes P-TEFb assembly with Tat and proviral HIV reactivation. J Biol Chem. 2018 Jun 29;293(26):10009-10025. doi: 10.1074/jbc.RA117.001347. Epub 2018 May 9. PMID: 29743242; PMCID: PMC6028954.

41: Henry KL, Kellner D, Bajrami B, Anderson JE, Beyna M, Bhisetti G, Cameron T, Capacci AG, Bertolotti-Ciarlet A, Feng J, Gao B, Hopkins B, Jenkins T, Li K, May-Dracka T, Murugan P, Wei R, Zeng W, Allaire N, Buckler A, Loh C, Juhasz P, Lucas B, Ennis KA, Vollman E, Cahir-McFarland E, Hett EC, Ols ML. CDK12-mediated transcriptional regulation of noncanonical NF-Î°B components is essential for signaling. Sci Signal. 2018 Jul 31;11(541):eaam8216. doi: 10.1126/scisignal.aam8216. PMID: 30065029.

42: Valenciaga A, Saji M, Yu L, Zhang X, Bumrah C, Yilmaz AS, Knippler CM, Miles W, Giordano TJ, Cote GJ, Ringel MD. Transcriptional targeting of oncogene addiction in medullary thyroid cancer. JCI Insight. 2018 Aug 23;3(16):e122225. doi: 10.1172/jci.insight.122225. PMID: 30135308; PMCID: PMC6141185.

43: Zhong L, Yang S, Jia Y, Lei K. Inhibition of cyclin-dependent kinase 7 suppresses human hepatocellular carcinoma by inducing apoptosis. J Cell Biochem. 2018 Dec;119(12):9742-9751. doi: 10.1002/jcb.27292. Epub 2018 Aug 26. PMID: 30145799.

44: Ma X, Kuang X, Xia Q, Huang Z, Fan Y, Ning J, Wen J, Zhang H, Yan J, Zhang Q, Shen H, Long C. Covalent CDK7 Inhibitor THZ1 Inhibits Myogenic Differentiation. J Cancer. 2018 Aug 6;9(17):3149-3155. doi: 10.7150/jca.25395. PMID: 30210638; PMCID: PMC6134829.

45: Wang D, Su X, Du Y, Tian Q, Liang Y, Niu L, Huang W, Gai W, Yan L, Tang C, Antipov S. Non-perturbing THz generation at the Tsinghua University Accelerator Laboratory 31 MeV electron beamline. Rev Sci Instrum. 2018 Sep;89(9):093301. doi: 10.1063/1.5042006. PMID: 30278713.

46: Zeng M, Kwiatkowski NP, Zhang T, Nabet B, Xu M, Liang Y, Quan C, Wang J, Hao M, Palakurthi S, Zhou S, Zeng Q, Kirschmeier PT, Meghani K, Leggett AL, Qi J, Shapiro GI, Liu JF, Matulonis UA, Lin CY, Konstantinopoulos PA, Gray NS. Targeting MYC dependency in ovarian cancer through inhibition of CDK7 and CDK12/13. Elife. 2018 Nov 13;7:e39030. doi: 10.7554/eLife.39030. PMID: 30422115; PMCID: PMC6251623.

47: Cheng ZJ, Miao DL, Su QY, Tang XL, Wang XL, Deng LB, Shi HD, Xin HB. THZ1 suppresses human non-small-cell lung cancer cells in vitro through interference with cancer metabolism. Acta Pharmacol Sin. 2019 Jun;40(6):814-822. doi: 10.1038/s41401-018-0187-3. Epub 2018 Nov 16. PMID: 30446732; PMCID: PMC6786356.

48: Decaesteker B, Denecker G, Van Neste C, Dolman EM, Van Loocke W, Gartlgruber M, Nunes C, De Vloed F, Depuydt P, Verboom K, Rombaut D, Loontiens S, De Wyn J, Kholosy WM, Koopmans B, Essing AHW, Herrmann C, Dreidax D, Durinck K, Deforce D, Van Nieuwerburgh F, Henssen A, Versteeg R, Boeva V, Schleiermacher G, van Nes J, Mestdagh P, Vanhauwaert S, Schulte JH, Westermann F, Molenaar JJ, De Preter K, Speleman F. TBX2 is a neuroblastoma core regulatory circuitry component enhancing MYCN/FOXM1 reactivation of DREAM targets. Nat Commun. 2018 Nov 19;9(1):4866. doi: 10.1038/s41467-018-06699-9. PMID: 30451831; PMCID: PMC6242972.

49: Zhou Y, Lu L, Jiang G, Chen Z, Li J, An P, Chen L, Du J, Wang H. Targeting CDK7 increases the stability of Snail to promote the dissemination of colorectal cancer. Cell Death Differ. 2019 Aug;26(8):1442-1452. doi: 10.1038/s41418-018-0222-4. Epub 2018 Nov 19. PMID: 30451989; PMCID: PMC6748077.

50: Browne CM, Jiang B, Ficarro SB, Doctor ZM, Johnson JL, Card JD, Sivakumaren SC, Alexander WM, Yaron TM, Murphy CJ, Kwiatkowski NP, Zhang T, Cantley LC, Gray NS, Marto JA. A Chemoproteomic Strategy for Direct and Proteome-Wide Covalent Inhibitor Target-Site Identification. J Am Chem Soc. 2019 Jan 9;141(1):191-203. doi: 10.1021/jacs.8b07911. Epub 2018 Dec 20. PMID: 30518210; PMCID: PMC6487859.

51: Meng W, Wang J, Wang B, Liu F, Li M, Zhao Y, Zhang C, Li Q, Chen J, Zhang L, Tang Y, Ma J. CDK7 inhibition is a novel therapeutic strategy against GBM both in vitro and in vivo. Cancer Manag Res. 2018 Nov 15;10:5747-5758. doi: 10.2147/CMAR.S183696. PMID: 30532595; PMCID: PMC6245350.

52: Sharifnia T, Wawer MJ, Chen T, Huang QY, Weir BA, Sizemore A, Lawlor MA, Goodale A, Cowley GS, Vazquez F, Ott CJ, Francis JM, Sassi S, Cogswell P, Sheppard HE, Zhang T, Gray NS, Clarke PA, Blagg J, Workman P, Sommer J, Hornicek F, Root DE, Hahn WC, Bradner JE, Wong KK, Clemons PA, Lin CY, Kotz JD, Schreiber SL. Small-molecule targeting of brachyury transcription factor addiction in chordoma. Nat Med. 2019 Feb;25(2):292-300. doi: 10.1038/s41591-018-0312-3. Epub 2019 Jan 21. PMID: 30664779; PMCID: PMC6633917.

53: Lu P, Geng J, Zhang L, Wang Y, Niu N, Fang Y, Liu F, Shi J, Zhang ZG, Sun YW, Wang LW, Tang Y, Xue J. THZ1 reveals CDK7-dependent transcriptional addictions in pancreatic cancer. Oncogene. 2019 May;38(20):3932-3945. doi: 10.1038/s41388-019-0701-1. Epub 2019 Jan 28. PMID: 30692639.

54: Wang J, Li Z, Mei H, Zhang D, Wu G, Zhang T, Lin Z. Antitumor effects of a covalent cyclin-dependent kinase 7 inhibitor in colorectal cancer. Anticancer Drugs. 2019 Jun;30(5):466-474. doi: 10.1097/CAD.0000000000000749. PMID: 30694816.

55: Tsang FH, Law CT, Tang TC, Cheng CL, Chin DW, Tam WV, Wei L, Wong CC, Ng IO, Wong CM. Aberrant Super-Enhancer Landscape in Human Hepatocellular Carcinoma. Hepatology. 2019 Jun;69(6):2502-2517. doi: 10.1002/hep.30544. Epub 2019 Apr 16. PMID: 30723918.

56: Sampathi S, Acharya P, Zhao Y, Wang J, Stengel KR, Liu Q, Savona MR, Hiebert SW. The CDK7 inhibitor THZ1 alters RNA polymerase dynamics at the 5' and 3' ends of genes. Nucleic Acids Res. 2019 May 7;47(8):3921-3936. doi: 10.1093/nar/gkz127. PMID: 30805632; PMCID: PMC6486546.

57: Hur JY, Kim HR, Lee JY, Park S, Hwang JA, Kim WS, Yoon S, Choi CM, Rho JK, Lee JC. CDK7 inhibition as a promising therapeutic strategy for lung squamous cell carcinomas with a SOX2 amplification. Cell Oncol (Dordr). 2019 Aug;42(4):449-458. doi: 10.1007/s13402-019-00434-2. Epub 2019 Mar 5. PMID: 30838525.

58: Stretton C, Lipina C, Hyde R, Cwiklinski E, Hoffmann TM, Taylor PM, Hundal HS. CDK7 is a component of the integrated stress response regulating SNAT2 (SLC38A2)/System A adaptation in response to cellular amino acid deprivation. Biochim Biophys Acta Mol Cell Res. 2019 Jun;1866(6):978-991. doi: 10.1016/j.bbamcr.2019.03.002. Epub 2019 Mar 8. PMID: 30857869; PMCID: PMC6456927.

59: Olson CM, Liang Y, Leggett A, Park WD, Li L, Mills CE, Elsarrag SZ, Ficarro SB, Zhang T, DÃ¼ster R, Geyer M, Sim T, Marto JA, Sorger PK, Westover KD, Lin CY, Kwiatkowski N, Gray NS. Development of a Selective CDK7 Covalent Inhibitor Reveals Predominant Cell-Cycle Phenotype. Cell Chem Biol. 2019 Jun 20;26(6):792-803.e10. doi: 10.1016/j.chembiol.2019.02.012. Epub 2019 Mar 21. PMID: 30905681; PMCID: PMC6588464.

60: Cao X, Dang L, Zheng X, Lu Y, Lu Y, Ji R, Zhang T, Ruan X, Zhi J, Hou X, Yi X, Li MJ, Gu T, Gao M, Zhang L, Chen Y. Targeting Super-Enhancer-Driven Oncogenic Transcription by CDK7 Inhibition in Anaplastic Thyroid Carcinoma. Thyroid. 2019 Jun;29(6):809-823. doi: 10.1089/thy.2018.0550. Epub 2019 May 3. PMID: 30924726.

61: Zhong S, Zhang Y, Yin X, Di W. CDK7 inhibitor suppresses tumor progression through blocking the cell cycle at the G2/M phase and inhibiting transcriptional activity in cervical cancer. Onco Targets Ther. 2019 Mar 22;12:2137-2147. doi: 10.2147/OTT.S195655. PMID: 30962695; PMCID: PMC6434917.

62: Compe E, Genes CM, Braun C, Coin F, Egly JM. TFIIE orchestrates the recruitment of the TFIIH kinase module at promoter before release during transcription. Nat Commun. 2019 May 7;10(1):2084. doi: 10.1038/s41467-019-10131-1. PMID: 31064989; PMCID: PMC6504876.

63: Zhao X, Ren Y, Lawlor M, Shah BD, Park PMC, Lwin T, Wang X, Liu K, Wang M, Gao J, Li T, Xu M, Silva AS, Lee K, Zhang T, Koomen JM, Jiang H, Sudalagunta PR, Meads MB, Cheng F, Bi C, Fu K, Fan H, Dalton WS, Moscinski LC, Shain KH, Sotomayor EM, Wang GG, Gray NS, Cleveland JL, Qi J, Tao J. BCL2 Amplicon Loss and Transcriptional Remodeling Drives ABT-199 Resistance in B Cell Lymphoma Models. Cancer Cell. 2019 May 13;35(5):752-766.e9. doi: 10.1016/j.ccell.2019.04.005. PMID: 31085176; PMCID: PMC6945775.

64: Liu F, Jiang W, Sui Y, Meng W, Hou L, Li T, Li M, Zhang L, Mo J, Wang J, Zhao Y, Zhang L, Ma J, Tang Y. CDK7 inhibition suppresses aberrant hedgehog pathway and overcomes resistance to smoothened antagonists. Proc Natl Acad Sci U S A. 2019 Jun 25;116(26):12986-12995. doi: 10.1073/pnas.1815780116. Epub 2019 Jun 10. PMID: 31182587; PMCID: PMC6600918.

65: Ning J, Ma X, Long C, Mao Y, Kuang X, Huang Z, Fan Y, Zhang H, Xia Q, Wang R, Liang Y, Lin S, Zhang Q, Shen H. Anti-tumor Drug THZ1 Suppresses TGFÎ²2-mediated EMT in Lens Epithelial Cells via Notch and TGFÎ²/Smad Signaling

Pathway. J Cancer. 2019 Jun 9;10(16):3778-3788. doi: 10.7150/jca.30359. PMID: 31333795; PMCID: PMC6636287.

66: Wong M, Sun Y, Xi Z, Milazzo G, Poulos RC, Bartenhagen C, Bell JL, Mayoh C, Ho N, Tee AE, Chen X, Li Y, Ciaccio R, Liu PY, Jiang CC, Lan Q, Jayatilleke N, Cheung BB, Haber M, Norris MD, Zhang XD, Marshall GM, Wang JY, HÃ¼ttelmaier S, Fischer M, Wong JWH, Xu H, Perini G, Dong Q, George RE, Liu T. JMJD6 is a tumorigenic factor and therapeutic target in neuroblastoma. Nat Commun. 2019 Jul 25;10(1):3319. doi: 10.1038/s41467-019-11132-w. PMID: 31346162; PMCID: PMC6658504.

67: Zhang Y, Zhou L, Bandyopadhyay D, Sharma K, Allen AJ, Kmieciak M, Grant S. The Covalent CDK7 Inhibitor THZ1 Potently Induces Apoptosis in Multiple Myeloma Cells <i>In Vitro</i> and <i>In Vivo</i>. Clin Cancer Res. 2019 Oct 15;25(20):6195-6205. doi: 10.1158/1078-0432.CCR-18-3788. Epub 2019 Jul 29. PMID: 31358538; PMCID: PMC6801078.

68: Chen CH, Yang N, Zhang Y, Ding J, Zhang W, Liu R, Liu W, Chen C. Inhibition of super enhancer downregulates the expression of KLF5 in basal-like breast cancers. Int J Biol Sci. 2019 Jun 10;15(8):1733-1742. doi: 10.7150/ijbs.35138. PMID: 31360115; PMCID: PMC6643226.

69: Huang T, Ding X, Xu G, Chen G, Cao Y, Peng C, Shen S, Lv Y, Wang L, Zou X. CDK7 inhibitor THZ1 inhibits MCL1 synthesis and drives cholangiocarcinoma apoptosis in combination with BCL2/BCL-XL inhibitor ABT-263. Cell Death Dis. 2019 Aug 9;10(8):602. doi: 10.1038/s41419-019-1831-7. PMID: 31399555; PMCID: PMC6688996.

70: Sun B, Mason S, Wilson RC, Hazard SE, Wang Y, Fang R, Wang Q, Yeh ES, Yang M, Roberts TM, Zhao JJ, Wang Q. Inhibition of the transcriptional kinase CDK7 overcomes therapeutic resistance in HER2-positive breast cancers. Oncogene. 2020 Jan;39(1):50-63. doi: 10.1038/s41388-019-0953-9. Epub 2019 Aug 28. PMID: 31462705; PMCID: PMC6937212.

71: Rasool RU, Natesan R, Deng Q, Aras S, Lal P, Sander Effron S, Mitchell-Velasquez E, Posimo JM, Carskadon S, Baca SC, Pomerantz MM, Siddiqui J, Schwartz LE, Lee DJ, Palanisamy N, Narla G, Den RB, Freedman ML, Brady DC, Asangani IA. CDK7 Inhibition Suppresses Castration-Resistant Prostate Cancer through MED1 Inactivation. Cancer Discov. 2019 Nov;9(11):1538-1555. doi: 10.1158/2159-8290.CD-19-0189. Epub 2019 Aug 29. PMID: 31466944; PMCID: PMC7202356.

72: Zarei M, Du H, Nassar AH, Yan RE, Giannikou K, Johnson SH, Lam HC, Henske EP, Wang Y, Zhang T, Asara J, Kwiatkowski DJ. Tumors with TSC mutations are sensitive to CDK7 inhibition through NRF2 and glutathione depletion. J Exp Med. 2019 Nov 4;216(11):2635-2652. doi: 10.1084/jem.20190251. Epub 2019 Sep 10. PMID: 31506280; PMCID: PMC6829598.

73: Sava GP, Fan H, Fisher RA, Lusvarghi S, Pancholi S, Ambudkar SV, Martin LA, Charles Coombes R, Buluwela L, Ali S. ABC-transporter upregulation mediates resistance to the CDK7 inhibitors THZ1 and ICEC0942. Oncogene. 2020 Jan;39(3):651-663. doi: 10.1038/s41388-019-1008-y. Epub 2019 Sep 17. PMID: 31530935; PMCID: PMC6962093.

74: Li J, Ji H, Porter DC, Broude EV, Roninson IB, Chen M. Characterizing CDK8/19 Inhibitors through a NFÎºB-Dependent Cell-Based Assay. Cells. 2019 Oct 6;8(10):1208. doi: 10.3390/cells8101208. PMID: 31590445; PMCID: PMC6830309.

75: Russo JW, Nouri M, Balk SP. Androgen Receptor Interaction with Mediator Complex Is Enhanced in Castration-Resistant Prostate Cancer by CDK7 Phosphorylation of MED1. Cancer Discov. 2019 Nov;9(11):1490-1492. doi: 10.1158/2159-8290.CD-19-1028. PMID: 31676563; PMCID: PMC6830511.

76: Shi CS, Kuo KL, Chen MS, Chow PM, Liu SH, Chang YW, Lin WC, Liao SM, Hsu CH, Hsu FS, Chang HC, Huang KH. Suppression of Angiogenesis by Targeting Cyclin-Dependent Kinase 7 in Human Umbilical Vein Endothelial Cells and Renal Cell Carcinoma: An In Vitro and In Vivo Study. Cells. 2019 Nov 19;8(11):1469. doi: 10.3390/cells8111469. PMID: 31752390; PMCID: PMC6912535.

77: Zhang W, Ge H, Jiang Y, Huang R, Wu Y, Wang D, Guo S, Li S, Wang Y, Jiang H, Cheng J. Combinational therapeutic targeting of BRD4 and CDK7 synergistically induces anticancer effects in head and neck squamous cell carcinoma. Cancer Lett. 2020 Jan 28;469:510-523. doi: 10.1016/j.canlet.2019.11.027. Epub 2019 Nov 22. PMID: 31765738.

78: Kim J, Cho YJ, Ryu JY, Hwang I, Han HD, Ahn HJ, Kim WY, Cho H, Chung JY, Hewitt SM, Kim JH, Kim BG, Bae DS, Choi CH, Lee JW. CDK7 is a reliable prognostic factor and novel therapeutic target in epithelial ovarian cancer. Gynecol Oncol. 2020 Jan;156(1):211-221. doi: 10.1016/j.ygyno.2019.11.004. Epub 2019 Nov 24. PMID: 31776040; PMCID: PMC8861881.

79: Ghezzi C, Wong A, Chen BY, Ribalet B, Damoiseaux R, Clark PM. A high-throughput screen identifies that CDK7 activates glucose consumption in lung cancer cells. Nat Commun. 2019 Nov 29;10(1):5444. doi: 10.1038/s41467-019-13334-8. PMID: 31784510; PMCID: PMC6884612.

80: Chow PM, Liu SH, Chang YW, Kuo KL, Lin WC, Huang KH. The covalent CDK7 inhibitor THZ1 enhances temsirolimus-induced cytotoxicity via autophagy suppression in human renal cell carcinoma. Cancer Lett. 2020 Feb 28;471:27-37. doi: 10.1016/j.canlet.2019.12.005. Epub 2019 Dec 6. PMID: 31812697.

81: Cho YS, Li S, Wang X, Zhu J, Zhuo S, Han Y, Yue T, Yang Y, Jiang J. CDK7 regulates organ size and tumor growth by safeguarding the Hippo pathway effector Yki/Yap/Taz in the nucleus. Genes Dev. 2020 Jan 1;34(1-2):53-71. doi: 10.1101/gad.333146.119. Epub 2019 Dec 19. PMID: 31857346; PMCID: PMC6938674.

82: Li BB, Wang B, Zhu CM, Tang D, Pang J, Zhao J, Sun CH, Qiu MJ, Qian ZR. Cyclin-dependent kinase 7 inhibitor THZ1 in cancer therapy. Chronic Dis Transl Med. 2019 Oct 18;5(3):155-169. doi: 10.1016/j.cdtm.2019.08.006. PMID: 31891127; PMCID: PMC6926117.

83: Guo L, Li J, Zeng H, Guzman AG, Li T, Lee M, Zhou Y, Goodell MA, Stephan C, Davies PJA, Dawson MA, Sun D, Huang Y. A combination strategy targeting enhancer plasticity exerts synergistic lethality against BETi-resistant leukemia cells. Nat Commun. 2020 Feb 6;11(1):740. doi: 10.1038/s41467-020-14604-6. PMID: 32029739; PMCID: PMC7005144.

84: Tee AE, Ciampa OC, Wong M, Fletcher JI, Kamili A, Chen J, Ho N, Sun Y,

Carter DR, Cheung BB, Marshall GM, Liu PY, Liu T. Combination therapy with the CDK7 inhibitor and the tyrosine kinase inhibitor exerts synergistic anticancer effects against MYCN-amplified neuroblastoma. Int J Cancer. 2020 Oct 1;147(7):1928-1938. doi: 10.1002/ijc.32936. Epub 2020 Mar 16. PMID: 32086952.

85: Zhang B, Zhong X, Sauane M, Zhao Y, Zheng ZL. Modulation of the Pol II CTD Phosphorylation Code by Rac1 and Cdc42 Small GTPases in Cultured Human Cancer Cells and Its Implication for Developing a Synthetic-Lethal Cancer Therapy. Cells. 2020 Mar 4;9(3):621. doi: 10.3390/cells9030621. PMID: 32143485; PMCID: PMC7140432.

86: McDermott MSJ, Sharko AC, Munie J, Kassler S, Melendez T, Lim CU, Broude EV. CDK7 Inhibition is Effective in all the Subtypes of Breast Cancer: Determinants of Response and Synergy with EGFR Inhibition. Cells. 2020 Mar 6;9(3):638. doi: 10.3390/cells9030638. PMID: 32155786; PMCID: PMC7140476.

87: Chen HD, Huang CS, Xu QC, Li F, Huang XT, Wang JQ, Li SJ, Zhao W, Yin XY. Therapeutic Targeting of CDK7 Suppresses Tumor Progression in Intrahepatic Cholangiocarcinoma. Int J Biol Sci. 2020 Feb 10;16(7):1207-1217. doi: 10.7150/ijbs.39779. PMID: 32174795; PMCID: PMC7053328.

88: Huang CS, You X, Dai C, Xu QC, Li F, Wang L, Huang XT, Wang JQ, Li SJ, Gao Z, Wu J, Yin XY, Zhao W. Targeting Super-Enhancers via Nanoparticle-Facilitated BRD4 and CDK7 Inhibitors Synergistically Suppresses Pancreatic Ductal Adenocarcinoma. Adv Sci (Weinh). 2020 Feb 16;7(7):1902926. doi: 10.1002/advs.201902926. PMID: 32274304; PMCID: PMC7140991.

89: Attia YM, Shouman SA, Salama SA, Ivan C, Elsayed AM, Amero P, Rodriguez-Aguayo C, Lopez-Berestein G. Blockade of CDK7 Reverses Endocrine Therapy Resistance in Breast Cancer. Int J Mol Sci. 2020 Apr 23;21(8):2974. doi: 10.3390/ijms21082974. PMID: 32340192; PMCID: PMC7215326.

90: Tang L, Jin J, Xu K, Wang X, Tang J, Guan X. SOX9 interacts with FOXC1 to activate MYC and regulate CDK7 inhibitor sensitivity in triple-negative breast cancer. Oncogenesis. 2020 May 12;9(5):47. doi: 10.1038/s41389-020-0232-1. PMID: 32398735; PMCID: PMC7217837.

91: Wang QY, Peng L, Chen Y, Liao LD, Chen JX, Li M, Li YY, Qian FC, Zhang YX, Wang F, Li CQ, Lin DC, Xu LY, Li EM. Characterization of super-enhancer-associated functional lncRNAs acting as ceRNAs in ESCC. Mol Oncol. 2020 Sep;14(9):2203-2230. doi: 10.1002/1878-0261.12726. Epub 2020 Jun 20. PMID: 32460441; PMCID: PMC7463357.

92: Uriostegui-Arcos M, Aguayo-Ortiz R, Valencia-Morales MDP, Melchy-PÃ©rez E, Rosenstein Y, Dominguez L, Zurita M. Disruption of TFIIH activities generates a stress gene expression response and reveals possible new targets against cancer. Open Biol. 2020 Jun;10(6):200050. doi: 10.1098/rsob.200050. Epub 2020 Jun 17. PMID: 32543350; PMCID: PMC7333893.

93: Paliouras AR, Buzzetti M, Shi L, Donaldson IJ, Magee P, Sahoo S, Leong HS, Fassan M, Carter M, Di Leva G, Krebs MG, Blackhall F, Lovly CM, Garofalo M. Vulnerability of drug-resistant EML4-ALK rearranged lung cancer to transcriptional inhibition. EMBO Mol Med. 2020 Jul 7;12(7):e11099. doi: 10.15252/emmm.201911099. Epub 2020 Jun 17. PMID: 32558295; PMCID: PMC7338803.

94: Shan W, Yuan J, Hu Z, Jiang J, Wang Y, Loo N, Fan L, Tang Z, Zhang T, Xu M, Pan Y, Lu J, Long M, Tanyi JL, Montone KT, Fan Y, Hu X, Zhang Y, Zhang L. Systematic Characterization of Recurrent Genomic Alterations in Cyclin-Dependent Kinases Reveals Potential Therapeutic Strategies for Cancer Treatment. Cell Rep. 2020 Jul 14;32(2):107884. doi: 10.1016/j.celrep.2020.107884. PMID: 32668240; PMCID: PMC7391471.

95: Wang J, Zhang R, Lin Z, Zhang S, Chen Y, Tang J, Hong J, Zhou X, Zong Y, Xu Y, Meng R, Xu S, Liu L, Zhang T, Yang K, Dong X, Wu G. CDK7 inhibitor THZ1 enhances antiPD-1 therapy efficacy via the p38Î±/MYC/PD-L1 signaling in non-small cell lung cancer. J Hematol Oncol. 2020 Jul 20;13(1):99. doi: 10.1186/s13045-020-00926-x. PMID: 32690037; PMCID: PMC7370470.

96: Mi Z, Song Y, Cao X, Lu Y, Liu Z, Zhu X, Geng M, Sun Y, Lan B, He C, Xiong H, Zhang L, Chen Y. Super-enhancer-driven metabolic reprogramming promotes cystogenesis in autosomal dominant polycystic kidney disease. Nat Metab. 2020 Aug;2(8):717-731. doi: 10.1038/s42255-020-0227-4. Epub 2020 Jul 13. PMID: 32694829.

97: Shang E, Nguyen TTT, Shu C, Westhoff MA, Karpel-Massler G, Siegelin MD. Epigenetic Targeting of Mcl-1 Is Synthetically Lethal with Bcl-xL/Bcl-2 Inhibition in Model Systems of Glioblastoma. Cancers (Basel). 2020 Aug 1;12(8):2137. doi: 10.3390/cancers12082137. PMID: 32752193; PMCID: PMC7464325.

98: Greber BJ, Perez-Bertoldi JM, Lim K, Iavarone AT, Toso DB, Nogales E. The cryoelectron microscopy structure of the human CDK-activating kinase. Proc Natl Acad Sci U S A. 2020 Sep 15;117(37):22849-22857. doi: 10.1073/pnas.2009627117. Epub 2020 Aug 27. PMID: 32855301; PMCID: PMC7502720.

99: Traylor JI, Sheppard HE, Ravikumar V, Breshears J, Raza SM, Lin CY, Patel SR, DeMonte F. Computational Drug Repositioning Identifies Potentially Active Therapies for Chordoma. Neurosurgery. 2021 Jan 13;88(2):428-436. doi: 10.1093/neuros/nyaa398. PMID: 33017025; PMCID: PMC7803434.

100: Yan D, Meng M, Li J, Li J, Li X. Vanadium dioxide-assisted broadband absorption and linear-to-circular polarization conversion based on a single metasurface design for the terahertz wave. Opt Express. 2020 Sep 28;28(20):29843-29854. doi: 10.1364/OE.404829. PMID: 33114874.

101: Ji W, Choi YJ, Kang MH, Sung KJ, Kim DH, Jung S, Choi CM, Lee JC, Rho JK. Efficacy of the CDK7 Inhibitor on EMT-Associated Resistance to 3rd Generation EGFR-TKIs in Non-Small Cell Lung Cancer Cell Lines. Cells. 2020 Dec 3;9(12):2596. doi: 10.3390/cells9122596. PMID: 33287368; PMCID: PMC7761809.

102: Harris ZB, Katletz S, Khani ME, Virk A, Arbab MH. Design and characterization of telecentric f-<i>Î¸</i> scanning lenses for broadband terahertz frequency systems. AIP Adv. 2020 Dec 11;10(12):125313. doi: 10.1063/5.0030110. PMID: 33344001; PMCID: PMC7733541.

103: Wei Y, Li C, Bian H, Qian W, Jin K, Xu T, Guo X, Lu X, Su F. Targeting CDK7 suppresses super enhancer-linked inflammatory genes and alleviates CAR T cell-induced cytokine release syndrome. Mol Cancer. 2021 Jan 4;20(1):5. doi: 10.1186/s12943-020-01301-7. PMID: 33397398; PMCID: PMC7780220.

104: Ge H, Yao Y, Jiang Y, Wu X, Wang Y. Pharmacological inhibition of CDK7 by THZ1 impairs tumor growth in p53-mutated HNSCC. Oral Dis. 2022 Apr;28(3):611-620. doi: 10.1111/odi.13783. Epub 2021 Feb 6. PMID: 33503275.

105: Kuo KL, Lin WC, Liu SH, Hsu FS, Kuo Y, Liao SM, Yang SP, Wang ZH, Hsu CH, Huang KH. THZ1, a covalent CDK7 inhibitor, enhances gemcitabine-induced cytotoxicity via suppression of Bcl-2 in urothelial carcinoma. Am J Cancer Res. 2021 Jan 1;11(1):171-180. PMID: 33520367; PMCID: PMC7840706.

106: Jia Y, Zhou J, Tan TK, Chung TH, Wong RWJ, Chooi JY, Lim JSL, Sanda T, Ooi M, De Mel S, Soekojo C, Chen Y, Zhang E, Cai Z, Shen P, Ruan J, Chng WJ. Myeloma-specific superenhancers affect genes of biological and clinical relevance in myeloma. Blood Cancer J. 2021 Feb 12;11(2):32. doi: 10.1038/s41408-021-00421-7. PMID: 33579893; PMCID: PMC7881003.

107: Chow PM, Chang YW, Kuo KL, Lin WC, Liu SH, Huang KH. CDK7 inhibition by THZ1 suppresses cancer stemness in both chemonaÃ¯ve and chemoresistant urothelial carcinoma via the hedgehog signaling pathway. Cancer Lett. 2021 Jun 1;507:70-79. doi: 10.1016/j.canlet.2021.03.012. Epub 2021 Mar 16. PMID: 33741425.

108: Xia L, Zheng Z, Liu JY, Chen YJ, Ding J, Hu GS, Hu YH, Liu S, Luo WX, Xia NS, Liu W. Targeting Triple-Negative Breast Cancer with Combination Therapy of EGFR CAR T Cells and CDK7 Inhibition. Cancer Immunol Res. 2021 Jun;9(6):707-722. doi: 10.1158/2326-6066.CIR-20-0405. Epub 2021 Apr 19. PMID: 33875483.

109: Abudureheman T, Xia J, Li MH, Zhou H, Zheng WW, Zhou N, Shi RY, Zhu JM, Yang LT, Chen L, Zheng L, Xue K, Qing K, Duan CW. CDK7 Inhibitor THZ1 Induces the Cell Apoptosis of B-Cell Acute Lymphocytic Leukemia by Perturbing Cellular Metabolism. Front Oncol. 2021 Apr 6;11:663360. doi: 10.3389/fonc.2021.663360. PMID: 33889549; PMCID: PMC8056175.

110: Yang Y, Jiang D, Zhou Z, Xiong H, Yang X, Peng G, Xia W, Wang S, Lei H, Zhao J, Qian Z, Wu S, Pang J. CDK7 blockade suppresses super-enhancer-associated oncogenes in bladder cancer. Cell Oncol (Dordr). 2021 Aug;44(4):871-887. doi: 10.1007/s13402-021-00608-x. Epub 2021 Apr 27. PMID: 33905040.

111: Chen Y, Liu R, Wang W, Wang C, Zhang N, Shao X, He Q, Ying M. Advances in targeted therapy for osteosarcoma based on molecular classification. Pharmacol Res. 2021 Jul;169:105684. doi: 10.1016/j.phrs.2021.105684. Epub 2021 May 19. PMID: 34022396.

112: Schoenwaelder N, Salewski I, Engel N, Krause M, Schneider B, MÃ¼ller M, Riess C, Lemcke H, Skorska A, Grosse-Thie C, Junghanss C, Maletzki C. The Individual Effects of Cyclin-Dependent Kinase Inhibitors on Head and Neck Cancer Cells-A Systematic Analysis. Cancers (Basel). 2021 May 15;13(10):2396. doi: 10.3390/cancers13102396. PMID: 34063457; PMCID: PMC8157193.

113: Juric V, DÃ¼ssmann H, Lamfers MLM, Prehn JHM, Rehm M, Murphy BM. Transcriptional CDK Inhibitors CYC065 and THZ1 Induce Apoptosis in Glioma Stem Cells Derived from Recurrent GBM. Cells. 2021 May 12;10(5):1182. doi: 10.3390/cells10051182. PMID: 34066147; PMCID: PMC8151379.

114: Peng J, Yang M, Bi R, Wang Y, Wang C, Wei X, Zhang Z, Xie X, Wei W.

Targeting Mutated p53 Dependency in Triple-Negative Breast Cancer Cells Through CDK7 Inhibition. Front Oncol. 2021 May 24;11:664848. doi: 10.3389/fonc.2021.664848. PMID: 34109118; PMCID: PMC8183379.

115: Choi YJ, Lee H, Kim DS, Kim DH, Kang MH, Cho YH, Choi CM, Yoo J, Lee KO, Choi EK, Lee JC, Rho JK. Discovery of a novel CDK7 inhibitor YPN-005 in small cell lung cancer. Eur J Pharmacol. 2021 Sep 15;907:174298. doi: 10.1016/j.ejphar.2021.174298. Epub 2021 Jul 3. PMID: 34224696.

116: Heisey DAR, Jacob S, Lochmann TL, Kurupi R, Ghotra MS, Calbert ML, Shende M, Maves YK, Koblinski JE, Dozmorov MG, Boikos SA, Benes CH, Faber AC. Pharmaceutical Interference of the EWS-FLI1-driven Transcriptome By Cotargeting H3K27ac and RNA Polymerase Activity in Ewing Sarcoma. Mol Cancer Ther. 2021 Oct;20(10):1868-1879. doi: 10.1158/1535-7163.MCT-20-0489. Epub 2021 Jul 26. PMID: 34315769.

117: Juric V, Hudson L, Fay J, Richards CE, Jahns H, Verreault M, Bielle F, Idbaih A, Lamfers MLM, Hopkins AM, Rehm M, Murphy BM. Transcriptional CDK inhibitors, CYC065 and THZ1 promote Bim-dependent apoptosis in primary and recurrent GBM through cell cycle arrest and Mcl-1 downregulation. Cell Death Dis. 2021 Aug 3;12(8):763. doi: 10.1038/s41419-021-04050-7. PMID: 34344865; PMCID: PMC8333061.

118: Huang CS, Xu QC, Dai C, Wang L, Tien YC, Li F, Su Q, Huang XT, Wu J, Zhao W, Yin XY. Nanomaterial-Facilitated Cyclin-Dependent Kinase 7 Inhibition Suppresses Gallbladder Cancer Progression via Targeting Transcriptional Addiction. ACS Nano. 2021 Sep 28;15(9):14744-14755. doi: 10.1021/acsnano.1c04570. Epub 2021 Aug 18. PMID: 34405985.

119: Wang J, Ma X, Ma J. Identification of Four Enhancer-Associated Genes as Risk Signature for Diffuse Glioma Patients. J Mol Neurosci. 2022 Feb;72(2):410-419. doi: 10.1007/s12031-021-01861-3. Epub 2021 Aug 31. PMID: 34462884.

120: Webb BM, Bryson BL, Williams-Medina E, Bobbitt JR, Seachrist DD, Anstine LJ, Keri RA. TGF-Î²/activin signaling promotes CDK7 inhibitor resistance in triple-negative breast cancer cells through upregulation of multidrug transporters. J Biol Chem. 2021 Oct;297(4):101162. doi: 10.1016/j.jbc.2021.101162. Epub 2021 Sep 2. PMID: 34481843; PMCID: PMC8498470.

121: Zhou J, Wang S, Nie D, Lai P, Li Y, Li Y, Jin Y, Pan J. Super-enhancer landscape reveals leukemia stem cell reliance on X-box binding protein 1 as a therapeutic vulnerability. Sci Transl Med. 2021 Sep 22;13(612):eabh3462. doi: 10.1126/scitranslmed.abh3462. Epub 2021 Sep 22. PMID: 34550724.

122: Kumar V, Parate S, Thakur G, Lee G, Ro HS, Kim Y, Kim HJ, Kim MO, Lee KW. Identification of CDK7 Inhibitors from Natural Sources Using Pharmacoinformatics and Molecular Dynamics Simulations. Biomedicines. 2021 Sep 10;9(9):1197. doi: 10.3390/biomedicines9091197. PMID: 34572383; PMCID: PMC8468199.

123: Attia YM, Salama SA, Shouman SA, Ivan C, Elsayed AM, Amero P, Rodriguez-Aguayo C, Lopez-Berestein G. Targeting CDK7 reverses tamoxifen resistance through regulating stemness in ER+ breast cancer. Pharmacol Rep. 2022 Apr;74(2):366-378. doi: 10.1007/s43440-021-00346-9. Epub 2022 Jan 9. PMID:

35000145.

124: Zhong M, Li X, Zhao F, Huang Y, Long Y, Chen K, Tian X, Liu M, Ma X. Natural compound library screening identifies Sanguinarine chloride for the treatment of SCLC by upregulating CDKN1A. Transl Oncol. 2022 Jan 20;17:101345. doi: 10.1016/j.tranon.2022.101345. Epub ahead of print. PMID: 35066462; PMCID: PMC8789530.

125: Gao L, Xia S, Zhang K, Lin C, He X, Zhang Y. Gene expression profile of THZ1-treated nasopharyngeal carcinoma cell lines indicates its involvement in the inhibition of the cell cycle. Transl Cancer Res. 2021 Jan;10(1):445-460. doi: 10.21037/tcr-19-2888. PMID: 35116274; PMCID: PMC8799269.

126: Huang X, Qiu M, Wang T, Li B, Zhang S, Zhang T, Liu P, Wang Q, Qian ZR, Zhu C, Wu M, Zhao J. Carrier-free multifunctional nanomedicine for intraperitoneal disseminated ovarian cancer therapy. J Nanobiotechnology. 2022 Feb 22;20(1):93. doi: 10.1186/s12951-022-01300-4. PMID: 35193583; PMCID: PMC8864853.

127: Gao Y, Volegova M, Nasholm N, Das S, Kwiatkowski N, Abraham BJ, Zhang T, Gray NS, Gustafson C, Krajewska M, George RE. Synergistic Anti-Tumor Effect of Combining Selective CDK7 and BRD4 Inhibition in Neuroblastoma. Front Oncol. 2022 Jan 27;11:773186. doi: 10.3389/fonc.2021.773186. PMID: 35198433; PMCID: PMC8859926.

128: Funke K, DÃ¼ster R, Wilson PD, ArÃ©valo L, Geyer M, Schorle H. Transcriptional CDK Inhibitors as Potential Treatment Option for Testicular Germ Cell Tumors. Cancers (Basel). 2022 Mar 26;14(7):1690. doi: 10.3390/cancers14071690. PMID: 35406461; PMCID: PMC8997165.

129: Xie G, Zhu A, Gu X. Converged DNA Damage Response Renders Human Hepatocellular Carcinoma Sensitive to CDK7 Inhibition. Cancers (Basel). 2022 Mar 28;14(7):1714. doi: 10.3390/cancers14071714. PMID: 35406486; PMCID: PMC8996977.

130: Schoenwaelder N, Krause M, Freitag T, Schneider B, Zonnur S, Zimpfer A, Becker AS, Salewski I, StrÃ¼der DF, Lemcke H, Grosse-Thie C, Junghanss C, Maletzki C. Preclinical Head and Neck Squamous Cell Carcinoma Models for Combined Targeted Therapy Approaches. Cancers (Basel). 2022 May 18;14(10):2484. doi: 10.3390/cancers14102484. PMID: 35626088; PMCID: PMC9139292.

131: Sriha J, Louis-Brennetot C, Pierre-EugÃ¨ne C, Baulande S, Raynal V, Kramdi A, Adameyko I, Ernsberger U, Deller T, Delattre O, Janoueix-Lerosey I, Rohrer H. BET and CDK Inhibition Reveal Differences in the Proliferation Control of Sympathetic Ganglion Neuroblasts and Adrenal Chromaffin Cells. Cancers (Basel). 2022 Jun 1;14(11):2755. doi: 10.3390/cancers14112755. PMID: 35681734; PMCID: PMC9179499.

132: Moser R, Annis J, Nikolova O, Whatcott C, Gurley K, Mendez E, Moran-Jones K, Dorrell C, Sears RC, Kuo C, Han H, Biankin A, Grandori C, Von Hoff DD, Kemp CJ. Pharmacologic Targeting of TFIIH Suppresses KRAS-Mutant Pancreatic Ductal Adenocarcinoma and Synergizes with TRAIL. Cancer Res. 2022 Sep 16;82(18):3375-3393. doi: 10.1158/0008-5472.CAN-21-4222. PMID: 35819261; PMCID: PMC9481717.

133: Doolittle WKL, Zhao L, Cheng SY. Blocking CDK7-Mediated NOTCH1-cMYC

Signaling Attenuates Cancer Stem Cell Activity in Anaplastic Thyroid Cancer. Thyroid. 2022 Aug;32(8):937-948. doi: 10.1089/thy.2022.0087. PMID: 35822558; PMCID: PMC9419935.

134: Sun Y, Zhang Y, Schultz CW, Pommier Y, Thomas A. CDK7 Inhibition Synergizes with Topoisomerase I Inhibition in Small Cell Lung Cancer Cells by Inducing Ubiquitin-Mediated Proteolysis of RNA Polymerase II. Mol Cancer Ther. 2022 Sep 6;21(9):1430-1438. doi: 10.1158/1535-7163.MCT-21-0891. PMID: 35830858.

135: Hsu A, Duan Q, Day DS, Luo X, McMahon S, Huang Y, Feldman ZB, Jiang Z, Zhang T, Liang Y, Alexanian M, Padmanabhan A, Brown JD, Lin CY, Gray NS, Young RA, Bruneau BG, Haldar SM. Targeting transcription in heart failure via CDK7/12/13 inhibition. Nat Commun. 2022 Jul 27;13(1):4345. doi: 10.1038/s41467-022-31541-8. PMID: 35896549; PMCID: PMC9329381.

136: Zeng S, Lan B, Ren X, Zhang S, Schreyer D, Eckstein M, Yang H, Britzen-Laurent N, Dahl A, Mukhopadhyay D, Chang D, Kutschick I, Pfeffer S, Bailey P, Biankin A, GrÃ¼tzmann R, Pilarsky C. CDK7 inhibition augments response to multidrug chemotherapy in pancreatic cancer. J Exp Clin Cancer Res. 2022 Aug 10;41(1):241. doi: 10.1186/s13046-022-02443-w. PMID: 35945614; PMCID: PMC9364549.

137: Piipponen M, RiihilÃ¤ P, Knuutila JS, Kallajoki M, KÃ¤hÃ¤ri VM, Nissinen L. Super Enhancer-Regulated <i>LINC00094</i> (<i>SERLOC</i>) Upregulates the Expression of MMP-1 and MMP-13 and Promotes Invasion of Cutaneous Squamous Cell Carcinoma. Cancers (Basel). 2022 Aug 17;14(16):3980. doi: 10.3390/cancers14163980. PMID: 36010973; PMCID: PMC9406669.

138: Zhang T, Li J, Yang M, Ma X, Wang Z, Ma X, Sun M, Sun W, Xu J, Hua Y, Cai Z. CDK7/GRP78 signaling axis contributes to tumor growth and metastasis in osteosarcoma. Oncogene. 2022 Sep;41(40):4524-4536. doi: 10.1038/s41388-022-02446-z. Epub 2022 Aug 30. PMID: 36042349.

139: Wang Y, Zhang Z, Mi X, Li M, Huang D, Song T, Qi X, Yang M. Elevation of effective p53 expression sensitizes wild-type p53 breast cancer cells to CDK7 inhibitor THZ1. Cell Commun Signal. 2022 Sep 5;20(1):96. doi: 10.1186/s12964-022-00837-z. PMID: 36058938; PMCID: PMC9442925.

140: Sun J, Zhang Q, Sun X, Xue A, Gao X, Shen K. THZ1 targeting CDK7 suppresses c-KIT transcriptional activity in gastrointestinal stromal tumours. Cell Commun Signal. 2022 Sep 8;20(1):138. doi: 10.1186/s12964-022-00928-x. PMID: 36076237; PMCID: PMC9454178.

141: GÃ³mez-Cabello D, Pappas G, Aguilar-Morante D, Dinant C, Bartek J. CtIP-dependent nascent RNA expression flanking DNA breaks guides the choice of DNA repair pathway. Nat Commun. 2022 Sep 9;13(1):5303. doi: 10.1038/s41467-022-33027-z. PMID: 36085345; PMCID: PMC9463442.

142: Nguyen AP, Yamagata K, Iwata S, Trimova G, Zhang T, Shan Y, Nguyen MP, Sonomoto K, Nakayamada S, Kato S, Tanaka Y. Enhancer RNA commits osteogenesis via microRNA-3129 expression in human bone marrow-derived mesenchymal stem cells. Inflamm Regen. 2022 Sep 16;42(1):43. doi: 10.1186/s41232-022-00228-4. PMID: 36114571; PMCID: PMC9479228.

143: Li Y, Zeng L, Zhang H, Zhang D, Xia K, Zhang L. Multifunctional and tunable metastructure based on VO₂ for polarization conversion and absorption. Opt Express. 2022 Sep 12;30(19):34586-34600. doi: 10.1364/OE.470910. PMID: 36242468.

144: Yang B, Zhang H, Li N, Gao L, Jiang H, Kan W, Yuan H, Li J, Zhao D, Xiong B, Zhou Y, Guo D, Liu T. Discovery of Novel <i>N</i>-(5-(Pyridin-3-yl)-1<i>H</i>-indazol-3-yl)benzamide Derivatives as Potent Cyclin-Dependent Kinase 7 Inhibitors for the Treatment of Autosomal Dominant Polycystic Kidney Disease. J Med Chem. 2022 Dec 8;65(23):15770-15788. doi: 10.1021/acs.jmedchem.2c01334. Epub 2022 Nov 17. PMID: 36384292.

145: Sung K, Kurowski A, Lansiquot C, Wan KK, Patnaik S, Walsh MJ, Lazarus MB. Selective Inhibitors of Autophagy Reveal New Link between the Cell Cycle and Autophagy and Lead to Discovery of Novel Synergistic Drug Combinations. ACS Chem Biol. 2022 Dec 16;17(12):3290-3297. doi: 10.1021/acschembio.2c00710. Epub 2022 Dec 5. PMID: 36469692; PMCID: PMC9879295.

146: Li X, Tang L, Chen Q, Cheng X, Liu Y, Wang C, Zhu C, Xu K, Gao F, Huang J, Wang R, Guan X. Inhibition of MYC suppresses programmed cell death ligand-1 expression and enhances immunotherapy in triple-negative breast cancer. Chin Med J (Engl). 2022 Oct 20;135(20):2436-2445. doi: 10.1097/CM9.0000000000002329. PMID: 36583862; PMCID: PMC9945371.

147: Zhang S, Feng R, Bai J, Ning S, Xu X, Sun J, Wu M, Liu H. CDK7 inhibition induces apoptosis in acute myeloid leukemia cells and exerts synergistic antileukemic effects with azacitidine <i>inÂ vitro</i> and <i>inÂ vivo</i>. Leuk Lymphoma. 2023 Mar;64(3):639-650. doi: 10.1080/10428194.2023.2169045. Epub 2023 Jan 19. PMID: 36657437.

CAS SciFinder^n

Task History

Initiating Search

January 12, 2023, 10:19PM

All: **THZ1**

Search Tasks

Task	Search Type	View
Returned All Results	All	View Results
Exported: Returned Reference Results + Filters (170)<br>Filtered By:<br>Document Type: **Journal**	**References**	View Results

Copyright © 2023 American Chemical Society (ACS). All Rights Reserved.
Internal use only. Redistribution is subject to the terms of your SciFinder^n License Agreement and CAS information Use Policies.

CAS SciFinderⁿ

References (170)

View in SciFinderⁿ

1

THZ1 Reveals Roles for Cdk7 in Co-transcriptional Capping and Pausing

By: Nilson, Kyle A.; Guo, Jiannan; Turek, Michael E.; Brogie, John E.; Delaney, Elizabeth; Luse, Donal S.; Price, David H.
Molecular Cell (2015), 59(4), 576-587 | Language: English, Database: CAplus and MEDLINE

Substances (2) Reactions (0) Cited By (112)

2

THZ1 targeting CDK7 suppresses STAT transcriptional activity and sensitizes T-cell lymphomas to BCL2 inhibitors

By: Cayrol, Florencia; Praditsuktavorn, Pannee; Fernando, Tharu M.; Kwiatkowski, Nicholas; Marullo, Rosella; Calvo-Vidal, M. Nieves; Phillip, Jude; Pera, Benet; Yang, Shao Ning; Takpradit, Kaipol; et al
Nature Communications (2017), 8, 14290 | Language: English, Database: CAplus and MEDLINE

Substances (23) Reactions (0) Cited By (63)

3

CDK7 Inhibition Suppresses Super-Enhancer-Linked Oncogenic Transcription in MYCN-Driven Cancer

By: Chipumuro, Edmond; Marco, Eugenio; Christensen, Camilla L.; Kwiatkowski, Nicholas; Zhang, Tinghu; Hatheway, Clark M.; Abraham, Brian J.; Sharma, Bandana; Yeung, Caleb; Altabef, Abigail; et al
Cell (Cambridge, MA, United States) (2014), 159(5), 1126-1139 | Language: English, Database: CAplus and MEDLINE

Substance (1) Reactions (0) Cited By (394)

4

THZ1 reveals CDK7-dependent transcriptional addictions in pancreatic cancer

By: Lu, Ping; Geng, Jing; Zhang, Lei; Wang, Yu; Niu, Ningning; Fang, Yuan; Liu, Fang; Shi, Juanjuan; Zhang, Zhi-Gang ; Sun, Yong-Wei; et al
Oncogene (2019), 38(20), 3932-3945 | Language: English, Database: CAplus and MEDLINE

Substances (2) Reactions (0) Cited By (30)

5

Targeting super-enhancer-associated oncogenes in oesophageal squamous cell carcinoma

By: Jiang, Yan-Yi; Lin, De-Chen; Mayakonda, Anand; Hazawa, Masaharu; Ding, Ling-Wen; Chien, Wen-Wen; Xu, Liang; Chen, Ye; Xiao, Jin-Fen; Senapedis, William; et al
Gut (2017), 66(8), 1358-1368 | Language: English, Database: CAplus and MEDLINE

Substances (4) | Reactions (0) | Cited By (134)

6

Preclinical Efficacy and Molecular Mechanism of Targeting CDK7-Dependent Transcriptional Addiction in Ovarian Cancer

By: Zhang, Zhenfeng; Peng, Huixin; Wang, Xiaojie; Yin, Xia; Ma, Pengfei; Jing, Ying; Cai, Mei-Chun; Liu, Jin; Zhang, Meiying; Zhang, Shengzhe; et al
Molecular Cancer Therapeutics (2017), 16(9), 1739-1750 | Language: English, Database: CAplus and MEDLINE

Substances (40) | Reactions (0) | Cited By (48)

7

Therapeutic Rationale to Target Highly Expressed CDK7 Conferring Poor Outcomes in Triple-Negative Breast Cancer

By: Li, Bo; Ni Chonghaile, Triona; Fan, Yue; Madden, Stephen F.; Klinger, Rut; O'Connor, Aisling E.; Walsh, Louise; O'Hurley, Gillian; Mallya Udupi, Girish; Joseph, Jesuchristopher; et al
Cancer Research (2017), 77(14), 3834-3845 | Language: English, Database: CAplus and MEDLINE

Substances (6) | Reactions (0) | Cited By (60)

8

CDK7 inhibitor THZ1 inhibits MCL1 synthesis and drives cholangiocarcinoma apoptosis in combination with BCL2/BCL-XL inhibitor ABT-263

By: Huang, Tianlu; Ding, Xiwei ; Xu, Guifang; Chen, Gang; Cao, Yu; Peng, Chunyan; Shen, Shanshan; Lv, Ying; Wang, Lei; Zou, Xiaoping
Cell Death & Disease (2019), 10(8), 1-14 | Language: English, Database: CAplus and MEDLINE

Substances (7) | Reactions (0) | Cited By (25)

9

THZ1 suppresses human non-small-cell lung cancer cells in vitro through interference with cancer metabolism

By: Cheng, Zhu-Jun; Miao, Du-Ling; Su, Qiu-Yun; Tang, Xiao-Li; Wang, Xiao-Lei; Deng, Li-Bin; Shi, Hui-Dong; Xin, Hong-Bo
Acta Pharmacologica Sinica (2019), 40(6), 814-822 | Language: English, Database: CAplus and MEDLINE

Substances (24) Reactions (0) Cited By (27)

10

Targeting MYC dependency in ovarian cancer through inhibition of CDK7 and CDK12/13.

By: Zeng, Mei ; Kwiatkowski, Nicholas P; Zhang, Tinghu; Nabet, Behnam ; Xu, Mousheng; Liang, Yanke; Quan, Chunshan; Wang, Jinhua; Hao, Mingfeng; Palakurthi, Sangeetha; et al
eLife (2018), 7, | Language: English, Database: MEDLINE

Substances (0) Reactions (0) Cited By (90)

11

The covalent CDK7 inhibitor THZI potently induces apoptosis in multiple myeloma cells in vitro and in vivo

By: Zhang, Yu; Zhou, Liang; Bandyopadhyay, Dipankar; Sharma, Kanika; Allen, Alexander Joseph; Kmieciak, Maciej; Grant, Steven
Clinical Cancer Research (2019), 25(20), 6195-6205 | Language: English, Database: CAplus and MEDLINE

Substances (11) Reactions (0) Cited By (31)

12

Super-Enhancers Promote Transcriptional Dysregulation in Nasopharyngeal Carcinoma

By: Yuan, Jiang; Jiang, Yan-Yi; Mayakonda, Anand; Huang, Moli; Ding, Ling-Wen; Lin, Han; Yu, Fenggang; Lu, Yanan; Loh, Thomas Kwok Seng; Chow, Marilynn; et al
Cancer Research (2017), 77(23), 6614-6626 | Language: English, Database: CAplus and MEDLINE

Substances (4) Reactions (0) Cited By (69)

13

Activation of the p53 Transcriptional Program Sensitizes Cancer Cells to Cdk7 Inhibitors

By: Kalan, Sampada; Amat, Ramon; Schachter, Miriam Merzel; Kwiatkowski, Nicholas; Abraham, Brian J.; Liang, Yanke; Zhang, Tinghu; Olson, Calla M.; Larochelle, Stephane; Young, Richard A.; et al
Cell Reports (2017), 21(2), 467-481 | Language: English, Database: CAplus and MEDLINE

Substances (10) Reactions (0) Cited By (51)

14

Nucleotide Excision Repair and Transcriptional Regulation: TFIIH and Beyond

Out

By: Compe, Emmanuel; Egly, Jean-Marc
Annual Review of Biochemistry (2016), 85, 265-290 | Language: English, Database: CAplus and MEDLINE

Substances (3) | Reactions (0) | Cited By (103)

15

Transcriptional Dependencies in Diffuse Intrinsic Pontine Glioma

✓

By: Nagaraja, Surya; Vitanza, Nicholas A.; Woo, Pamelyn J.; Taylor, Kathryn R.; Liu, Fang; Zhang, Lei; Li, Meng; Meng, Wei; Ponnuswami, Anitha; Sun, Wenchao; et al
Cancer Cell (2017), 31(5), 635-652.e6 | Language: English, Database: CAplus and MEDLINE

Substances (22) | Reactions (0) | Cited By (181)

16

Overcoming Resistance to the THZ Series of Covalent Transcriptional CDK Inhibitors

Out

By: Gao, Yang; Zhang, Tinghu; Terai, Hideki; Ficarro, Scott B.; Kwiatkowski, Nicholas; Hao, Ming-Feng; Sharma, Bandana; Christensen, Camilla L.; Chipumuro, Edmond; Wong, Kwok-kin; et al
Cell Chemical Biology (2018), 25(2), 135-142.e5 | Language: English, Database: CAplus and MEDLINE

Substances (19) | Reactions (5) | Cited By (47)

17

Enhancer profiling identifies critical cancer genes and characterizes cell identity in adult T-cell leukemia

✓

By: Wong, Regina Wan Ju; Ngoc, Phuong Cao Thi; Leong, Wei Zhong; Yam, Alice Wei Yee; Zhang, Tinghu; Asamitsu, Tinghu; Iida, Shinsuke; Okamoto, Takashi; Ueda, Ryuzo; Gray, Nathanael S.; et al
Blood (2017), 130(21), 2326-2338 | Language: English, Database: CAplus and MEDLINE

Substances (4) | Reactions (0) | Cited By (52)

18

The covalent CDK7 inhibitor THZ1 enhances temsirolimus-induced cytotoxicity via autophagy suppression in human renal cell carcinoma

✓

By: Chow, Po-Ming; Liu, Shing-Hwa; Chang, Yu-Wei; Kuo, Kuan-Lin; Lin, Wei-Chou; Huang, Kuo-How
Cancer Letters (New York, NY, United States) (2020), 471, 27-37 | Language: English, Database: CAplus and MEDLINE

Substances (4) | Reactions (0) | Cited By (16)

19

The CDK7 inhibitor THZ1 alters RNA polymerase dynamics at the 5' and 3' ends of genes

By: Sampathi, Shilpa; Acharya, Pankaj; Zhao, Yue; Wang, Jing; Stengel, Kristy R.; Liu, Qi; Savona, Michael R.; Hiebert, Scott W.
Nucleic Acids Research (2019), 47(8), 3921-3936 | Language: English, Database: CAplus and MEDLINE

Substances (6) | Reactions (0) | Cited By (19)

20

Inhibition of the transcriptional kinase CDK7 overcomes therapeutic resistance in HER2-positive breast cancers

By: Sun, Bowen; Mason, Seth; Wilson, Robert C.; Hazard, Starr E.; Wang, Yubao; Fang, Rong; Wang, Qiwei; Yeh, Elizabeth S.; Yang, Meixiang; Roberts, Thomas M.; et al
Oncogene (2020), 39(1), 50-63 | Language: English, Database: CAplus and MEDLINE

Substances (7) | Reactions (0) | Cited By (33)

21

EWS/FLI Confers Tumor Cell Synthetic Lethality to CDK12 Inhibition in Ewing Sarcoma

By: Iniguez, Amanda Balboni; Stolte, Bjorn; Wang, Emily Jue; Conway, Amy Saur; Alexe, Gabriela; Dharia, Neekesh V.; Kwiatkowski, Nicholas; Zhang, Tinghu; Abraham, Brian J.; Mora, Jaume; et al
Cancer Cell (2018), 33(2), 202-216.e6 | Language: English, Database: CAplus and MEDLINE

Substances (18) | Reactions (0) | Cited By (80)

22

Inhibition of cyclin-dependent kinase 7 suppresses human hepatocellular carcinoma by inducing apoptosis

By: Zhong, Liqiang; Yang, Sihao; Jia, Yuming; Lei, Kaijian
Journal of Cellular Biochemistry (2018), 119(12), 9742-9751 | Language: English, Database: CAplus and MEDLINE

Substances (4) | Reactions (0) | Cited By (16)

23

CDK7 inhibition is effective in all the subtypes of breast cancer: determinants of response and synergy with EGFR inhibition

By: McDermott, Martina S. J.; Sharko, Amanda C.; Munie, Jessica; Kassler, Susannah; Melendez, Theresa; Lim, Chang-uk; Broude, Eugenia V.
Cells (2020), 9(3), 638 | Language: English, Database: CAplus and MEDLINE

Substance (1) | Reactions (0) | Cited By (16)

24

CDK7 inhibition as a promising therapeutic strategy for lung squamous cell carcinomas with a SOX2 amplification

By: Hur, Jae Young; Kim, Hyeong Ryul; Lee, Jung Yeon; Park, Sojung; Hwang, Ji An; Kim, Woo Sung; Yoon, Shinkyo; Choi, Chang-Min; Rho, Jin Kyung; Lee, Jae Cheol
Cellular Oncology (2019), 42(4), 449-458 | Language: English, Database: CAplus and MEDLINE

Substances (3) Reactions (0) Cited By (11)

25

High MITF Expression Is Associated with Super-Enhancers and Suppressed by CDK7 Inhibition in Melanoma

By: Eliades, Philip; Abraham, Brian J.; Ji, Zhenyu; Miller, David M.; Christensen, Camilla L.; Kwiatkowski, Nicholas; Kumar, Raj; Njauw, Ching Ni; Taylor, Michael; Miao, Benchun; et al
Journal of Investigative Dermatology (2018), 138(7), 1582-1590 | Language: English, Database: CAplus and MEDLINE

Substances (3) Reactions (0) Cited By (32)

26

CDK7 inhibitor THZ1 enhances antiPD-1 therapy efficacy via the p38α/MYC/PD-L1 signaling in non-small cell lung cancer

By: Wang, Jian ; Zhang, Ruiguang; Lin, Zhenyu; Zhang, Sheng; Chen, Yaobing; Tang, Jing; Hong, Jiaxin; Zhou, Xiaoshu; Zong, Yan; Xu, Yingzhuo; et al
Journal of Hematology & Oncology, | Language: English, Database: CAplus and MEDLINE

Substances (8) Reactions (0) Cited By (29)

27

CDK7 inhibition suppresses castration-resistant prostate cancer through MED1 inactivation

By: ur Rasool, Reyaz; Natesan, Ramakrishnan; Deng, Qu; Aras, Shweta; Lal, Priti; Effron, Samuel Sander; Mitchell-Velasquez, Erick; Posimo, Jessica M.; Carskadon, Shannon; Baca, Sylvan C.; et al
Cancer Discovery (2019), 9(11), 1538-1555 | Language: English, Database: CAplus and MEDLINE

Substances (2) Reactions (0) Cited By (42)

28

ABC-transporter upregulation mediates resistance to the CDK7 inhibitors THZ1 and ICEC0942

By: Sava, Georgina P.; Fan, Hailing; Fisher, Rosemary A.; Lusvarghi, Sabrina; Pancholi, Sunil ; Ambudkar, Suresh V. ; Martin, Lesley-Ann; Charles Coombes, R.; Buluwela, Lakjaya; Ali, Simak
Oncogene (2020), 39(3), 651-663 | Language: English, Database: CAplus and MEDLINE

Substances (5) | Reactions (0) | Cited By (10)

29

CDK7 is a reliable prognostic factor and novel therapeutic target in epithelial ovarian cancer

By: Kim, Jihye; Cho, Young-Jae; Ryu, Ji-Yoon; Hwang, Ilseon; Han, Hee Dong; Ahn, Hyung Jun; Kim, Woo Young; Cho, Hanbyoul; Chung, Joon-Yong; Hewitt, Stephen M.; et al
Gynecologic Oncology (2020), 156(1), 211-221 | Language: English, Database: CAplus and MEDLINE

Substances (3) | Reactions (0) | Cited By (10)

30

Combination therapy with the CDK7 inhibitor and the tyrosine kinase inhibitor exerts synergistic anticancer effects against MYCN-amplified neuroblastoma

By: Tee, Andrew E.; Ciampa, Olivia C.; Wong, Matthew; Fletcher, Jamie I. ; Kamili, Alvin; Chen, Jingwei; Ho, Nicholas; Sun, Yuting; Carter, Daniel R.; Cheung, Belamy B.; et al
International Journal of Cancer (2020), 147(7), 1928-1938 | Language: English, Database: CAplus and MEDLINE

Substances (25) | Reactions (0) | Cited By (18)

31

CDK7 inhibition suppresses aberrant hedgehog pathway and overcomes resistance to smoothened antagonists

By: Liu, Fang; Jiang, Wenyan; Sui, Yi; Meng, Wei; Hou, Linjun; Li, Tiantian; Li, Meng; Zhang, Lei; Mo, Jialin; Wang, Jiajia; et al
Proceedings of the National Academy of Sciences of the United States of America (2019), 116(26), 12986-12995 | Language: English, Database: CAplus and MEDLINE

Substances (2) | Reactions (0) | Cited By (15)

32

Antitumor effects of a covalent cyclin-dependent kinase 7 inhibitor in colorectal cancer

By: Wang, Jian; Li, Zhenyu; Mei, Hong; Zhang, Dejun; Wu, Gang; Zhang, Tao; Lin, Zhenyu
Anti-Cancer Drugs (2019), 30(5), 466-474 | Language: English, Database: CAplus and MEDLINE

Substances (6) | Reactions (0) | Cited By (5)

33

CDK7 inhibition by THZ1 suppresses cancer stemness in both chemonaive and chemoresistant urothelial carcinoma via the hedgehog signaling pathway

By: Chow, Po-Ming; Chang, Yu-Wei; Kuo, Kuan-Lin; Lin, Wei-Chou ; Liu, Shing-Hwa; Huang, Kuo-How
Cancer Letters (New York, NY, United States) (2021), 507, 70-79 | Language: English, Database: CAplus and MEDLINE

Substances (3) | Reactions (0) | Cited By (4)

34

A high-throughput screen identifies that CDK7 activates glucose consumption in lung cancer cells

By: Ghezzi, Chiara; Wong, Alicia; Chen, Bao Ying; Ribalet, Bernard; Damoiseaux, Robert; Clark, Peter M.
Nature Communications (2019), 10(1), 1-15 | Language: English, Database: CAplus and MEDLINE

Substances (13) | Reactions (0) | Cited By (15)

35

Disruption of TFIIH activities generates a stress gene expression response and reveals possible new targets against cancer

By: Uriostegui-Arcos, Maritere; Aguayo-Ortiz, Rodrigo; del Pilar Valencia-Morales, Maria; Melchy-Perez, Erika; Rosenstein, Yvonne; Dominguez, Laura; Zurita, Mario
Open Biology (2020), 10(6), 200050 | Language: English, Database: CAplus and MEDLINE

Substances (6) | Reactions (0) | Cited By (2)

36

Inhibition of CDK7 bypasses spindle assembly checkpoint via premature cyclin B degradation during oocyte meiosis

By: Wang, HaiYang; Jo, Yu-Jin; Sun, Tian-Yi; Namgoong, Suk; Cui, Xiang-Shun; Oh, Jeong Su ; Kim, Nam-Hyung
Biochimica et Biophysica Acta, Molecular Cell Research (2016), 1863(12), 2993-3000 | Language: English, Database: CAplus and MEDLINE

Substance (1) | Reactions (0) | Cited By (7)

37

Tumors with TSC mutations are sensitive to CDK7 inhibition through NRF2 and glutathione depletion

By: Zarei, Mahsa; Du, Heng; Nassar, Amin H. ; Yan, Rachel E. ; Giannikou, Krinio; Johnson, Sneha H.; Lam, Hilaire C.; Henske, Elizabeth P.; Wang, Yubao; Zhang, Tinghu; et al
Journal of Experimental Medicine (2019), 216(11), 2635-2652 | Language: English, Database: CAplus and MEDLINE

Substances (4) | Reactions (0) | Cited By (12)

38

Systematic Characterization of Recurrent Genomic Alterations in Cyclin-Dependent Kinases Reveals Potential Therapeutic Strategies for Cancer Treatment

By: Shan, Weiwei ; Yuan, Jiao ; Hu, Zhongyi; Jiang, Junjie; Wang, Yueying; Loo, Nicki; Fan, Lingling; Tang, Zhaoqing; Zhang, Tianli ; Xu, Mu; et al
Cell Reports (2020), 32(2), 107884 | Language: English, Database: CAplus and MEDLINE

Substances (4) | Reactions (0) | Cited By (13)

39

TBX2 is a neuroblastoma core regulatory circuitry component enhancing MYCN/FOXM1 reactivation of DREAM targets

By: Decaesteker, Bieke ; Denecker, Geertrui ; Van Neste, Christophe ; Dolman, Emmy M.; Van Loocke, Wouter; Gartlgruber, Moritz; Nunes, Carolina ; De Vloed, Fanny ; Depuydt, Pauline ; Verboom, Karen ; et al
Nature Communications (2018), 9(1), 1-17 | Language: English, Database: CAplus and MEDLINE

Substances (6) | Reactions (0) | Cited By (48)

40

A combination strategy targeting enhancer plasticity exerts synergistic lethality against BETi-resistant leukemia cells

By: Guo, Lei; Li, Jia; Zeng, Hongxiang; Guzman, Anna G.; Li, Tingting; Lee, Minjung; Zhou, Yubin ; Goodell, Margaret A.; Stephan, Clifford; Davies, Peter J. A.; et al
Nature Communications (2020), 11(1), 740 | Language: English, Database: CAplus and MEDLINE

Substances (5) | Reactions (0) | Cited By (27)

41

THZ1 targeting CDK7 suppresses c-KIT transcriptional activity in gastrointestinal stromal tumours

By: Sun, Jianyi; Zhang, Qiang; Sun, Xiangfei; Xue, Anwei; Gao, Xiaodong; Shen, Kuntang
Cell Communication and Signaling (2022), 20(1), 138 | Language: English, Database: CAplus and MEDLINE

Substances (0) | Reactions (0) | Cited By (0)

42

Transcriptional CDK inhibitors, CYC065 and THZ1 promote Bim-dependent apoptosis in primary and recurrent GBM through cell cycle arrest and Mcl-1 downregulation

By: Juric, Viktorija ; Hudson, Lance; Fay, Joanna; Richards, Cathy E.; Jahns, Hanne; Verreault, Maite; Bielle, Franck; Idbaih, Ahmed; Lamfers, Martine L. M.; Hopkins, Ann M.; et al
Cell Death & Disease (2021), 12(8), 763 | Language: English, Database: CAplus and MEDLINE

Substances (10) Reactions (0) Cited By (3)

43

Super-enhancer-driven metabolic reprogramming promotes cystogenesis in autosomal dominant polycystic kidney disease

By: Mi, Zeyun ; Song, Yandong; Cao, Xinyi; Lu, Yi; Liu, Zhiheng; Zhu, Xu; Geng, Meijuan ; Sun, Yongzhan; Lan, Bingxue; He, Chaoran; et al
Nature Metabolism (2020), 2(8), 717-731 | Language: English, Database: CAplus and MEDLINE

Substances (8) Reactions (0) Cited By (11)

44

CDK7 regulates organ size and tumor growth by safeguarding the Hippo pathway effector Yki/Yap/Taz in the nucleus

By: Cho, Yong Suk; Li, Shuang; Wang, Xiaohui; Zhu, Jian; Zhuo, Shu; Han, Yuhong; Yue, Tao; Yang, Yingzi; Jiang, Jin
Genes & Development (2020), 34(1-2), 53-71 | Language: English, Database: CAplus and MEDLINE

Substances (3) Reactions (0) Cited By (32)

45

Nanomaterial-Facilitated Cyclin-Dependent Kinase 7 Inhibition Suppresses Gallbladder Cancer Progression via Targeting Transcriptional Addiction

By: Huang, Chen-Song; Xu, Qiong-Cong; Dai, Chunlei; Wang, Liying; Tien, Yi-Chih; Li, Fuxi; Su, Qiao; Huang, Xi-Tai; Wu, Jun ; Zhao, Wei; et al
ACS Nano (2021), 15(9), 14744-14755 | Language: English, Database: CAplus and MEDLINE

Out

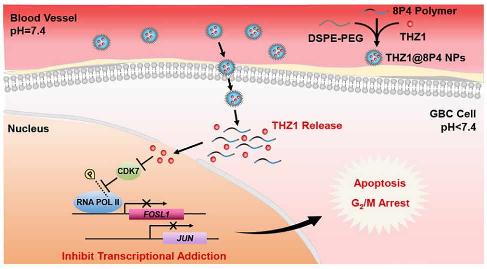

Substances (20) Reactions (8) Cited By (4)

46

Targeting CDK7 suppresses super enhancer-linked inflammatory genes and alleviates CAR T cell-induced cytokine release syndrome

By: Wei, Ye; Li, Chong; Bian, Huifang; Qian, Wei; Jin, Kairui; Xu, Tingting; Guo, Xiaomao; Lu, Xueguan; Su, Fengtao

Molecular Cancer (2021), 20(1), 5 | Language: English, Database: CAplus and MEDLINE

Substance (1) | Reactions (0) | Cited By (9)

47

CDK7 blockade suppresses super-enhancer-associated oncogenes in bladder cancer

By: Yang, Yafei; Jiang, Donggen; Zhou, Ziyu; Xiong, Haiyun; Yang, Xiangwei; Peng, Guoyu; Xia, Wuchao; Wang, Shang; Lei, Hanqi; Zhao, Jing; et al

Cellular Oncology (2021), 44(4), 871-887 | Language: English, Database: CAplus and MEDLINE

Substances (3) | Reactions (0) | Cited By (2)

48

Inhibition of super enhancer downregulates the expression of KLF5 in basal-like breast cancers

By: Chen, Chuan-Huizi; Yang, Nong; Zhang, Yongchang; Ding, Jiancheng; Zhang, Wenjuan; Liu, Rong; Liu, Wen; Chen, Ceshi

International Journal of Biological Sciences (2019), 15(8), 1733-1742 | Language: English, Database: CAplus and MEDLINE

Substances (3) | Reactions (0) | Cited By (18)

49

Cyclin-dependent kinase 7 inhibitor THZ1 in cancer therapy.

Out

By: Li, Bin-Bin; Wang, Bo; Zhu, Cheng-Ming; Tang, Di; Pang, Jun; Zhao, Jing; Sun, Chun-Hui; Qiu, Miao-Juan; Qian, Zhi-Rong

Chronic diseases and translational medicine (2019), 5(3), 155-169 | Language: English, Database: MEDLINE

Substances (0) | Reactions (0) | Cited By (12)

50

Combinational therapeutic targeting of BRD4 and CDK7 synergistically induces anticancer effects in head and neck squamous cell carcinoma

By: Zhang, Wei; Ge, Han; Jiang, Yue; Huang, Rong; Wu, Yaping; Wang, Dongmiao; Guo, Songsong; Li, Sheng; Wang, Yanling; Jiang, Hongbing; et al

Cancer Letters (New York, NY, United States) (2020), 469, 510-523 | Language: English, Database: CAplus and MEDLINE

Substances (0) | Reactions (0) | Cited By (27)

51

CDK7 is a component of the integrated stress response regulating SNAT2 (SLC38A2)/System A adaptation in response to cellular amino acid deprivation

By: Stretton, Clare; Lipina, Christopher; Hyde, Russell; Cwiklinski, Emma; Hoffmann, Thorsten M.; Taylor, Peter M.; Hundal, Harinder S.
Biochimica et Biophysica Acta, Molecular Cell Research (2019), 1866(6), 978-991 | Language: English, Database: CAplus and MEDLINE

Substances (13) | Reactions (0) | Cited By (5)

52

Pharmaceutical interference of the EWS-FLI1-driven transcriptome by cotargeting H3K27ac and RNA polymerase activity in ewing sarcoma

By: Heisey, Daniel A. R.; Jacob, Sheeba; Lochmann, Timothy L.; Kurupi, Richard; Ghotra, Maninderjit S.; Calbert, Marissa L.; Shende, Mayuri; Maves, Yuki Kato; Koblinski, Jennifer E.; Dozmorov, Mikhail G.; et al
Molecular Cancer Therapeutics (2021), 20(10), 1868-1879 | Language: English, Database: CAplus and MEDLINE

Substances (8) | Reactions (0) | Cited By (4)

53

Anti-tumor drug THZ1 suppresses TGFβ2-mediated EMT in lens epithelial cells via notch and TGFβ/Smad signaling pathway

By: Ning, Jie; Ma, Xinqi; Long, Chongde; Mao, Yuxiang; Kuang, Xielan; Huang, Zixin; Fan, Yuting; Zhang, Han; Xia, Qing; Wang, Renchun; et al
Journal of Cancer (Wyoming, Australia) (2019), 10(16), 3778-3788 | Language: English, Database: CAplus and MEDLINE

Substances (0) | Reactions (0) | Cited By (10)

54

Covalent CDK7 inhibitor THZ1 inhibits myogenic differentiation

By: Ma, Xinqi; Kuang, Xielan; Xia, Qing; Huang, Zixin; Fan, Yuting; Ning, Jie; Wen, Jiajie; Zhang, Han; Yan, Jianhua; Zhang, Qingjiong; et al
Journal of Cancer (Wyoming, Australia) (2018), 9(17), 1-7 | Language: English, Database: CAplus and MEDLINE

Substances (0) | Reactions (0) | Cited By (5)

55

THZ1, a covalent CDK7 inhibitor, enhances gemcitabine-induced cytotoxicity via suppression of Bcl- 2 in urothelial carcinoma.

✓

By: Kuo, Kuan-Lin; Lin, Wei-Chou; Liu, Shing-Hwa; Hsu, Fu-Shun; Kuo, Yu; Liao, Shih-Ming; Yang, Shao-Ping; Wang, Zuo-He; Hsu, Chen-Hsun; Huang, Kuo-How
American journal of cancer research (2021), 11(1), 171-180 | Language: English, Database: MEDLINE

Substances (0) | Reactions (0) | Cited By (2)

56

THZ1, a covalent CDK7 inhibitor, enhances gemcitabine-induced cytotoxicity via suppression of Bcl- 2 in urothelial carcinoma

✓

By: Kuo, Kuan-Lin; Lin, Wei-Chou; Liu, Shing-Hwa; Hsu, Fu-Shun; Kuo, Yu.; Liao, Shih-Ming; Yang, Shao-Ping; Wang, Zuo-He; Hsu, Chen-Hsun; Huang, Kuo-How
American Journal of Cancer Research (2021), 11(1), 171-180 | Language: English, Database: CAplus

Substances (0) | Reactions (0) | Cited By (2)

57

Targeting transcription regulation in cancer with a covalent CDK7 inhibitor

Out

By: Kwiatkowski, Nicholas; Zhang, Tinghu; Rahl, Peter B.; Abraham, Brian J.; Reddy, Jessica; Ficarro, Scott B.; Dastur, Anahita; Amzallag, Arnaud; Ramaswamy, Sridhar; Tesar, Bethany; et al
Nature (London, United Kingdom) (2014), 511(7511), 616-620 | Language: English, Database: CAplus and MEDLINE

Substances (17) | Reactions (23) | Cited By (536)

58

Corrigendum: THZ1 targeting CDK7 suppresses STAT transcriptional activity and sensitizes T-cell lymphomas to BCL2 inhibitors [Erratum to document cited in CA168:274984]

Out

By: Cayrol, Florencia; Praditsuktavorn, Pannee; Fernando, Tharu M.; Kwiatkowski, Nicholas; Marullo, Rossella; Calvo-Vidal, M. Nieves; Phillip, Jude; Pera, Benet; Yang, Shao Ning; Takpradit, Kaipol; et al
Nature Communications (2017), 8, 14747 | Language: English, Database: CAplus and MEDLINE

Substances (22) | Reactions (0) | Cited By (3)

59

CDK7 Inhibitor THZ1 Induces the Cell Apoptosis of B-Cell Acute Lymphocytic Leukemia by Perturbing Cellular Metabolism.

By: Abudureheman, Tuersunayi; Xia, Jing; Li, Ming-Hao; Zhou, Hang; Zheng, Wei-Wei; Zhou, Neng; Shi, Rong-Yi; Zhu, Jian-Min; Yang, Li-Ting; Chen, Li; et al
Frontiers in oncology (2021), 11, 663360 | Language: English, Database: MEDLINE

Substances (0) Reactions (0) Cited By (5)

60

Pharmacological inhibition of CDK7 by THZ1 impairs tumor growth in p53-mutated HNSCC.

By: Ge, Han; Yao, Yuan; Jiang, Yue; Wu, Xiang; Wang, Yanling
Oral diseases (2022), 28(3), 611-620 | Language: English, Database: MEDLINE

Substances (0) Reactions (0) Cited By (2)

61

CDK7 inhibitor THZ1 increases the radiosensitivity of human glioma cell line U251

By: Li Wenqing; Ye Lan; Jiang Yuhua
Shandong Daxue Xuebao, Yixueban (2021), 59(1), 14-19, 33 | Language: Chinese, Database: CAplus

Substances (0) Reactions (0) Cited By (0)

62

Elevation of effective p53 expression sensitizes wild-type p53 breast cancer cells to CDK7 inhibitor THZ1.

By: Wang, Yueyuan; Zhang, Zhihao; Mi, Xuguang; Li, Mingxi; Huang, Dan; Song, Tingting; Qi, Xiaoyan; Yang, Ming
Cell communication and signaling : CCS (2022), 20(1), 96 | Language: English, Database: MEDLINE

Substances (0) Reactions (0) Cited By (0)

63

Targeting Transcriptional Addictions in Small Cell Lung Cancer with a Covalent CDK7 Inhibitor

By: Christensen, Camilla L.; Kwiatkowski, Nicholas; Abraham, Brian J.; Carretero, Julian; Al-Shahrour, Fatima; Zhang, Tinghu; Chipumuro, Edmond; Herter-Sprie, Grit S.; Akbay, Esra A.; Altabef, Abigail; et al
Cancer Cell (2014), 26(6), 909-922 | Language: English, Database: CAplus and MEDLINE

Substances (3) Reactions (0) Cited By (295)

64

Gene expression profile of THZ1-treated nasopharyngeal carcinoma cell lines indicates its involvement in the inhibition of the cell cycle

By: Gao, Lijuan; Xia, Shuang; Zhang, Kunyi; Lin, Chengguang; He, Xuyu; Zhang, Ying
Translational Cancer Research (2021), 10(1), 445-460 | Language: English, Database: CAplus and MEDLINE

Substance (1) | Reactions (0) | Cited By (0)

65

Transcriptional CDK inhibitors CYC065 and THZ1 induce apoptosis in glioma stem cells derived from recurrent GBM

By: Juric, Viktorija ; Duessmann, Heiko; Lamfers, Martine L. M. ; Prehn, Jochen H. M.; Rehm, Markus; Murphy, Brona M.
Cells (2021), 10(5), 1182 | Language: English, Database: CAplus and MEDLINE

Substances (2) | Reactions (0) | Cited By (2)

66

Cyclin-dependent kinase 7 is a therapeutic target in high-grade glioma

By: Greenall, S. A.; Lim, Y. C.; Mitchell, C. B.; Ensbey, K. S.; Stringer, B. W.; Wilding, A. L.; O'Neill, G. M.; McDonald, K. L.; Gough, D. J.; Day, B. W.; et al
Oncogenesis (2017), 6(5), e336 | Language: English, Database: CAplus and MEDLINE

Substances (5) | Reactions (0) | Cited By (46)

67

No Driver behind the Wheel? Targeting Transcription in Cancer

Out

By: Franco, Hector L.; Kraus, W. Lee
Cell (Cambridge, MA, United States) (2015), 163(1), 28-30 | Language: English, Database: CAplus and MEDLINE

Substances (2) | Reactions (0) | Cited By (24)

68

Suppression of Adaptive Responses to Targeted Cancer Therapy by Transcriptional Repression

By: Rusan, Maria; Li, Kapsok; Li, Yvonne; Christensen, Camilla L.; Abraham, Brian J.; Kwiatkowski, Nicholas; Buczkowski, Kevin A.; Bockorny, Bruno; Chen, Ting; Li, Shuai; et al
Cancer Discovery (2018), 8(1), 59-73 | Language: English, Database: CAplus and MEDLINE

Substances (2) | Reactions (0) | Cited By (65)

69

CDK7 inhibition is a novel therapeutic strategy against GBM both in vitro and in vivo ✓

By: Meng, Wei; Wang, Jiajia; Wang, Baocheng; Liu, Fang; Li, Meng; Zhao, Yang; Zhang, Chenran; Li, Qifeng; Chen, Juxiang; Zhang, Liye; et al
Cancer Management and Research (2018), 10, 5747-5758 | Language: English, Database: CAplus and MEDLINE

Substances (0) | Reactions (0) | Cited By (19)

70

Therapeutic targeting of CDK7 suppresses tumor progression in intrahepatic cholangiocarcinoma ✓

By: Chen, Hua-Dong; Huang, Chen-Song; Xu, Qiong-Cong; Li, Fuxi; Huang, Xi-Tai; Wang, Jie-Qin; Li, Shi-Jin; Zhao, Wei; Yin, Xiao-Yu
International Journal of Biological Sciences (2020), 16(7), 1207-1217 | Language: English, Database: CAplus and MEDLINE

Substances (6) | Reactions (0) | Cited By (10)

71

Targeting Super-Enhancer-Driven Oncogenic Transcription by CDK7 Inhibition in Anaplastic Thyroid Carcinoma ✓

By: Cao, Xinyi; Dang, Lin; Zheng, Xiangqian; Lu, Yi; Lu, Yumei; Ji, Rongjie; Zhang, Tianye; Ruan, Xianhui; Zhi, Jingtai; Hou, Xiukun; et al
Thyroid (2019), 29(6), 809-823 | Language: English, Database: CAplus and MEDLINE

Substances (2) | Reactions (0) | Cited By (33)

72

Development of a Selective CDK7 Covalent Inhibitor Reveals Predominant Cell-Cycle Phenotype ✓

By: Olson, Calla M.; Liang, Yanke; Leggett, Alan; Park, Woojun D.; Li, Lianbo; Mills, Caitlin E.; Elsarrag, Selma Z.; Ficarro, Scott B.; Zhang, Tinghu; Duster, Robert; et al
Cell Chemical Biology (2019), 26(6), 792-803.e10 | Language: English, Database: CAplus and MEDLINE

Substances (3) | Reactions (0) | Cited By (69)

73

Targeting Super-Enhancers via Nanoparticle-Facilitated BRD4 and CDK7 Inhibitors Synergistically Suppresses Pancreatic Ductal Adenocarcinoma

Out

By: Huang, Chen-Song; You, Xinru; Dai, Chunlei; Xu, Qiong-Cong; Li, Fuxi; Wang, Li; Huang, Xi-Tai; Wang, Jie-Qin; Li, Shi-Jin; Gao, Zhuoxing; et al
Advanced Science (Weinheim, Germany) (2020), 7(7), 1902926 | Language: English, Database: CAplus and MEDLINE

Substances (4) | Reactions (0) | Cited By (18)

74

Inhibit Globally, Act Locally: CDK7 Inhibitors in Cancer Therapy

Out

By: Cao, Kaixiang; Shilatifard, Ali
Cancer Cell (2014), 26(2), 158-159 | Language: English, Database: CAplus and MEDLINE

Substance (1) | Reactions (0) | Cited By (24)

75

Efficacy of the CDK7 inhibitor on EMT-associated resistance to 3rd generation EGFR-TKIs in non-small cell lung cancer cell lines

✓

By: Ji, Wonjun; Choi, Yun Jung; Kang, Myoung-Hee; Sung, Ki Jung; Kim, Dong Ha; Jung, Sangyong; Choi, Chang-Min; Lee, Jae Cheol; Rho, Jin Kyung
Cells (2020), 9(12), 2596 | Language: English, Database: CAplus and MEDLINE

Substances (4) | Reactions (0) | Cited By (9)

76

Targeting CDK7 increases the stability of Snail to promote the dissemination of colorectal cancer ✓

By: Zhou, Yan; Lu, Linlin; Jiang, Guanmin; Chen, Zhuojia; Li, Jiexin; An, Panpan; Chen, Likun; Du, Jun; Wang, Hongsheng
Cell Death & Differentiation (2019), 26(8), 1442-1452 | Language: English, Database: CAplus and MEDLINE

Substances (3) | Reactions (0) | Cited By (29)

77

A CRISPR screen identifies CDK7 as a therapeutic target in hepatocellular carcinoma

By: Wang, Cun; Jin, Haojie; Gao, Dongmei; Wang, Liqin; Evers, Bastiaan; Xue, Zheng; Jin, Guangzhi; Lieftink, Cor; Beijersbergen, Roderick L.; Qin, Wenxin; et al
Cell Research (2018), 28(6), 690-692 | Language: English, Database: CAplus and MEDLINE

Substances (4) | Reactions (0) | Cited By (35)

78

Revisiting the function of CDK7 in transcription by virtue of a recently described TFIIH kinase inhibitor

Out

By: Coin, Frederic; Egly, Jean-Marc
Molecular Cell (2015), 59(4), 513-514 | Language: English, Database: CAplus and MEDLINE

Substances (2) | Reactions (0) | Cited By (17)

79

Targeting MYC dependency in ovarian cancer through inhibition of CDK7 and CDK12/13

By: Zeng, Mei; Kwiatkowski, Nicholas P.; Zhang, Tinghu; Nabet, Behnam; Xu, Mousheng; Liang, Yanke; Quan, Chunshan; Wang, Jinhua; Hao, Mingfeng; Palakurthi, Sangeetha; et al
eLife (2018), 7, e39030/1-e39030/20 | Language: English, Database: CAplus

Repeat article

Substances (0) | Reactions (0) | Cited By (34)

80

Cdk7 is required for activity-dependent neuronal gene expression, long-lasting synaptic plasticity and long-term memory

By: He, Guiqin; Yang, Xiangyu; Wang, Guo; Qi, Junxia; Mao, Rui; Wu, Zhengping; Zhou, Zikai
Frontiers in Molecular Neuroscience (2017), 10, 365/1-365/12 | Language: English, Database: CAplus and MEDLINE

Substances (0) | Reactions (0) | Cited By (10)

81

Epigenetic targeting of Mcl-1 is synthetically lethal with Bcl-xL/Bcl- 2 inhibition in model systems of glioblastoma

By: Shang, Enyuan; Nguyen, Trang T. T.; Shu, Chang; Westhoff, Mike-Andrew; Karpel-Massler, Georg; Siegelin, Markus D.
Cancers (2020), 12(8), 2137 | Language: English, Database: CAplus and MEDLINE

Substances (0) | Reactions (0) | Cited By (10)

82

Treating Transcriptional Addiction in Small Cell Lung Cancer

Out

By: Augert, Arnaud; MacPherson, David
Cancer Cell (2014), 26(6), 783-784 | Language: English, Database: CAplus and MEDLINE

Substances (0) | Reactions (0) | Cited By (14)

83

SOX9 interacts with FOXC1 to activate MYC and regulate CDK7 inhibitor sensitivity in triple-negative breast cancer

By: Tang, Lin; Jin, Juan; Xu, Kun; Wang, Xin; Tang, Jinhai; Guan, Xiaoxiang
Oncogenesis (2020), 9(5), 47 | Language: English, Database: CAplus and MEDLINE

Substance (1) | Reactions (0) | Cited By (16)

84

CDK7 inhibitor suppresses tumor progression through blocking the cell cycle at the G2/M phase and inhibiting transcriptional activity in cervical cancer

By: Zhong, Shanshan; Zhang, Yi; Yin, Xia; Wen, Di-
OncoTargets and Therapy (2019), 12, 2137-2147 | Language: English, Database: CAplus and MEDLINE

Substances (0) Reactions (0) Cited By (16)

85

Super enhancer inhibitors suppress MYC driven transcriptional amplification and tumor progression in osteosarcoma

By: Chen, Demeng; Zhao, Zhiqiang; Huang, Zixin; Chen, Du-Chu; Zhu, Xin-Xing; Wang, Yi-Ze; Yan, Ya-Wei; Tang, Shaojun; Madhavan, Subha; Ni, Weiyi; et al
Bone Research (2018), 6(1), 1-7 | Language: English, Database: CAplus and MEDLINE

Substances (3) Reactions (0) Cited By (66)

86

Aberrant Super-Enhancer Landscape in Human Hepatocellular Carcinoma

By: Tsang, Felice Ho-Ching; Law, Cheuk-Ting; Tang, Tsz-Ching Chloe; Cheng, Carol Lai-Hung; Chin, Don Wai-Ching; Tam, Wing-Sum Vincy; Wei, Lai; Wong, Carmen Chak-Lui; Ng, Irene Oi-Lin ; Wong, Chun-Ming
Hepatology (Hoboken, NJ, United States) (2019), 69(6), 2502-2517 | Language: English, Database: CAplus and MEDLINE

Substances (4) Reactions (0) Cited By (53)

87

Genomic modeling of the ESR1 Y537S mutation for evaluating function and new therapeutic approaches for metastatic breast cancer

By: Harrod, A.; Fulton, J.; Nguyen, V. T. M.; Periyasamy, M.; Ramos-Garcia, L.; Lai, C.-F.; Metodieva, G.; de Giorgio, A.; Williams, R. L.; Santos, D. B.; et al
Oncogene (2017), 36(16), 2286-2296 | Language: English, Database: CAplus and MEDLINE

Substances (7) Reactions (0) Cited By (101)

88

Blockade of CDK7 reverses endocrine therapy resistance in breast cancer

By: Attia, Yasmin M.; Shouman, Samia A. ; Salama, Salama A.; Ivan, Cristina; Elsayed, Abdelrahman M.; Amero, Paola ; Rodriguez-Aguayo, Cristian ; Lopez-Berestein, Gabriel
International Journal of Molecular Sciences (2020), 21(8), 2974 | Language: English, Database: CAplus and MEDLINE

Substances (3) Reactions (0) Cited By (8)

89

Human TFIIH Kinase CDK7 Regulates Transcription-Associated Chromatin Modifications

By: Ebmeier, Christopher C.; Erickson, Benjamin; Allen, Benjamin L.; Allen, Mary A.; Kim, Hyunmin; Fong, Nova; Jacobsen, Jeremy R.; Liang, Kaiwei; Shilatifard, Ali; Dowell, Robin D.; et al
Cell Reports (2017), 20(5), 1173-1186 | Language: English, Database: CAplus and MEDLINE

Substances (9) | Reactions (0) | Cited By (83)

90

The essential and multifunctional TFIIH complex

Out

By: Rimel, Jenna K.; Taatjes, Dylan J.
Protein Science (2018), 27(6), 1018-1037 | Language: English, Database: CAplus and MEDLINE

Substances (2) | Reactions (0) | Cited By (58)

91

Targeting Mutated p53 Dependency in Triple-Negative Breast Cancer Cells Through CDK7 Inhibition.

By: Peng, Jingyu; Yang, Ming; Bi, Ran; Wang, Yueyuan; Wang, Chunxi; Wei, Xue; Zhang, Zhihao; Xie, Xiao; Wei, Wei
Frontiers in oncology (2021), 11, 664848 | Language: English, Database: MEDLINE

Substances (0) | Reactions (0) | Cited By (4)

92

The cryoelectron microscopy structure of the human CDK-activating kinase

Out

By: Greber, Basil J.; Perez-Bertoldi, Juan M.; Lim, Kif; Iavarone, Anthony T.; Toso, Daniel B.; Nogales, Eva
Proceedings of the National Academy of Sciences of the United States of America (2020), 117(37), 22849-22857 | Language: English, Database: CAplus and MEDLINE

Substance (1) | Reactions (0) | Cited By (30)

93

Phosphoproteomics of Primary Cells Reveals Druggable Kinase Signatures in Ovarian Cancer

By: Francavilla, Chiara; Lupia, Michela; Tsafou, Kalliopi; Villa, Alessandra; Kowalczyk, Katarzyna; Rakownikow Jersie-Christensen, Rosa; Bertalot, Giovanni; Confalonieri, Stefano; Brunak, Soeren; Jensen, Lars J.; et al
Cell Reports (2017), 18(13), 3242-3256 | Language: English, Database: CAplus and MEDLINE

Substances (6) | Reactions (0) | Cited By (67)

94

Converged DNA Damage Response Renders Human Hepatocellular Carcinoma Sensitive to CDK7 Inhibition

By: Xie, Guiqin ; Zhu, Ailin; Gu, Xinbin
Cancers (2022), 14(7), 1714 | Language: English, Database: CAplus and MEDLINE

Substances (0) Reactions (0) Cited By (0)

95

Strategically targeting MYC in cancer[version 1; referees: 2 approved]

Out

By: Posternak, Valeriya; Cole, Michael D.
F1000Research (2016), 5, 408/1-408/7 | Language: English, Database: CAplus and MEDLINE

Substances (0) Reactions (0) Cited By (65)

96

Strategically targeting MYC in cancer [version 1; referees: 2 approved]

Repeat article

By: Posternak, Valeriya; Cole, Michael D.
F1000 Faculty Reviews (2016), 5, 408/1-408/7 | Language: English, Database: CAplus and MEDLINE

Substances (0) Reactions (0) Cited By (65)

97

Suppression of angiogenesis by targeting cyclin-dependent kinase 7 in human umbilical vein endothelial cells and renal cell carcinoma: an in vitro and in vivo study

By: Shi, Chung-Sheng ; Kuo, Kuan-Lin; Chen, Mei-Sin; Chow, Po-Ming; Liu, Shing-Hwa ; Chang, Yu-Wei; Lin, Wei-Chou; Liao, Shih-Ming; Hsu, Chen-Hsun; Hsu, Fu-Shun ; et al
Cells (2019), 8(11), 1469 | Language: English, Database: CAplus and MEDLINE

Substances (0) Reactions (0) Cited By (8)

98

The Individual Effects of Cyclin-Dependent Kinase Inhibitors on Head and Neck Cancer Cells-A Systematic Analysis

By: Schoenwaelder, Nina; Salewski, Inken; Engel, Nadja ; Krause, Mareike ; Schneider, Bjoern ; Mueller, Michael; Riess, Christin ; Lemcke, Heiko ; Skorska, Anna ; Grosse-Thie, Christina ; et al
Cancers (2021), 13(10), 2396 | Language: English, Database: CAplus and MEDLINE

Substances (0) Reactions (0) Cited By (1)

99

Collapsing the Tumor Ecosystem: Preventing Adaptive Response to Treatment by Inhibiting Transcription.

Out

By: Carugo, Alessandro; Draetta, Giulio F
Cancer discovery (2018), 8(1), 17-19 | Language: English, Database: MEDLINE

Substances (0) Reactions (0) Cited By (2)

100

JMJD6 is a tumorigenic factor and therapeutic target in neuroblastoma

By: Wong, Matthew; Sun, Yuting; Xi, Zhichao; Milazzo, Giorgio; Poulos, Rebecca C.; Bartenhagen, Christoph; Bell, Jessica L.; Mayoh, Chelsea; Ho, Nicholas; Tee, Andrew E.; et al
Nature Communications (2019), 10(1), 1-15 | Language: English, Database: CAplus and MEDLINE

Substances (2) Reactions (0) Cited By (48)

101

Identification of Four Enhancer-Associated Genes as Risk Signature for Diffuse Glioma Patients

By: Wang, Jiajia; Ma, Xin; Ma, Jie
Journal of Molecular Neuroscience (2022), 72(2), 410-419 | Language: English, Database: CAplus and MEDLINE

Substances (4) Reactions (0) Cited By (2)

102

Essential role of endogenous prolactin and CDK7 in estrogen-induced upregulation of the prolactin receptor in breast cancer cells.

By: Kavarthapu, Raghuveer; Dufau, Maria L
Oncotarget (2017), 8(16), 27353-27363 | Language: English, Database: MEDLINE

Substances (2) Reactions (0) Cited By (12)

103

Characterization of super-enhancer-associated functional lncRNAs acting as ceRNAs in ESCC

By: Wang, Qiu-Yu; Peng, Liu; Chen, Yang; Liao, Lian-Di; Chen, Jia-Xin; Li, Meng; Li, Yan-Yu; Qian, Feng-Cui; Zhang, Yue-Xin; Wang, Fan; et al
Molecular Oncology (2020), 14(9), 2203-2230 | Language: English, Database: CAplus and MEDLINE

Substance (1) Reactions (0) Cited By (19)

104

Modulation of the pol II CTD phosphorylation code by Rac1 and Cdc42 small GTPases in cultured human cancer cells and its implication for developing a synthetic-lethal cancer therapy

By: Zhang, Bo; Zhong, Xuelin; Sauane, Moira; Zhao, Yihong; Zheng, Zhi-Liang
Cells (2020), 9(3), 621 | Language: English, Database: CAplus and MEDLINE

Substances (0) | Reactions (0) | Cited By (2)

105

ER Stress Signaling Promotes the Survival of Cancer "Persister Cells" Tolerant to EGFR Tyrosine Kinase Inhibitors

By: Terai, Hideki; Kitajima, Shunsuke; Potter, Danielle S.; Matsui, Yusuke; Quiceno, Laura Gutierrez; Chen, Ting; Kim, Tae-jung; Rusan, Maria; Thai, Tran C.; Piccioni, Federica; et al
Cancer Research (2018), 78(4), 1044-1057 | Language: English, Database: CAplus and MEDLINE

Substances (2) | Reactions (0) | Cited By (62)

106

CDK7 inhibition augments response to multidrug chemotherapy in pancreatic cancer

By: Zeng, Siyuan; Lan, Bin; Ren, Xiaofan; Zhang, Shuman; Schreyer, Daniel; Eckstein, Markus; Yang, Hai; Britzen-Laurent, Nathalie; Dahl, Andreas; Mukhopadhyay, Debabrata; et al
Journal of Experimental & Clinical Cancer Research (2022), 41(1), 241 | Language: English, Database: CAplus and MEDLINE

Substances (16) | Reactions (0) | Cited By (0)

107

Carrier-free multifunctional nanomedicine for intraperitoneal disseminated ovarian cancer therapy

By: Huang, Xiuyu; Qiu, Miaojuan; Wang, Tianqi; Li, Binbin; Zhang, Shiqiang; Zhang, Tianzhi; Liu, Peng; Wang, Qiang; Qian, Zhi Rong; Zhu, Chengming; et al
Journal of Nanobiotechnology (2022), 20(1), 93 | Language: English, Database: CAplus and MEDLINE

Substances (5) | Reactions (0) | Cited By (5)

108

A chemoproteomic strategy for direct and proteome-wide covalent inhibitor target-site identification

By: Browne, Christopher M. ; Jiang, Baishan; Ficarro, Scott B.; Doctor, Zainab M.; Johnson, Jared L.; Card, Joseph D.; Sivakumaren, Sindhu Carmen; Alexander, William M.; Yaron, Tomer M.; Murphy, Charles J.; et al
Journal of the American Chemical Society (2019), 141(1), 191-203 | Language: English, Database: CAplus and MEDLINE

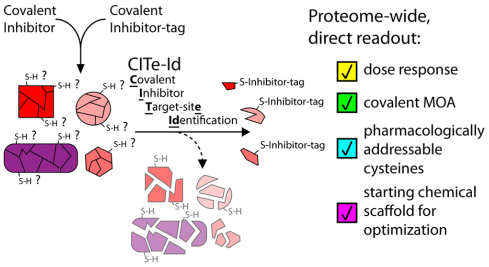

Substances (24) Reactions (19) Cited By (48)

109

Super-enhancer landscape reveals leukemia stem cell reliance on X-box binding protein 1 as a therapeutic vulnerability

By: Zhou, Jingfeng ; Wang, Shubo ; Nie, Danian; Lai, Peilong; Li, Yiqing; Li, Yangqiu; Jin, Yanli ; Pan, Jingxuan
Science Translational Medicine (2021), 13(612), eabh3462 | Language: English, Database: CAplus and MEDLINE

Substances (8) Reactions (0) Cited By (7)

110

Cyclin-dependent kinase 7 (CDK7)-mediated phosphorylation of the CDK9 activation loop promotes P-TEFb assembly with Tat and proviral HIV reactivation

By: Mbonye, Uri; Wang, Benlian; Gokulrangan, Giridharan; Shi, Wuxian; Yang, Sichun; Karn, Jonathan
Journal of Biological Chemistry (2018), 293(26), 10009-10025 | Language: English, Database: CAplus and MEDLINE

Substances (5) Reactions (0) Cited By (29)

111

Targeting transcription in heart failure via CDK7/12/13 inhibition

By: Hsu, Austin ; Duan, Qiming; Day, Daniel S.; Luo, Xin; McMahon, Sarah; Huang, Yu; Feldman, Zachary B.; Jiang, Zhen; Zhang, Tinghu; Liang, Yanke; et al
Nature Communications (2022), 13(1), 4345 | Language: English, Database: CAplus and MEDLINE

Substances (6) Reactions (0) Cited By (0)

112

Cyclin-dependent kinase 7 (CDK7) expression in human hepatocellular carcinoma: association with HCC progression, prognosis and cell proliferative capacity

By: Liu, Zheran; Liu, Dajiao; Zhong, Rongrong; Su, Qiuyun; Zhao, Tiancheng; Fu, Fen; Liu, Jinsheng; Xu, Derong; Zeng, Changqing
Translational Cancer Research (2018), 7(3), 472-479, S1-S2 | Language: English, Database: CAplus

Substances (2) | Reactions (0) | Cited By (1)

113

Blocking CDK7-Mediated NOTCH1-cMYC Signaling Attenuates Cancer Stem Cell Activity in Anaplastic Thyroid Cancer

By: Doolittle, Woo Kyung Lee ; Zhao, Li; Cheng, Sheue-Yann
Thyroid (2022), 32(8), 937-948 | Language: English, Database: CAplus and MEDLINE

Substances (0) | Reactions (0) | Cited By (0)

114

CDK7 inhibition synergizes with topoisomerase I inhibition in small cell lung cancer cells by inducing ubiquitin-mediated proteolysis of RNA polymerase II

By: Sun, Yilun; Zhang, Yang; Schultz, Christopher W.; Pommier, Yves; Thomas, Anish
Molecular Cancer Therapeutics (2022), 21(9), 1430-1438 | Language: English, Database: CAplus and MEDLINE

Substance (1) | Reactions (0) | Cited By (1)

115

YBCO-constriction hot spot modeling: DC and RF descriptions for HEB THz mixer noise temperature and conversion gain

Not related

By: Ladret, Romain G.; Kreisler, Alain J.; Degardin, Annick F.
IEEE Transactions on Applied Superconductivity (2015), 25(3), 1-5 | Language: English, Database: CAplus

Substances (2) | Reactions (0) | Cited By (0)

116

Cryostats for SQUID magnetometers

Not related

By: Testard, O. A.; Locatelli, M.
Cryogenics (1983), 23(6), 299-302 | Language: English, Database: CAplus

Substances (0) | Reactions (0) | Cited By (0)

117

High-Sensitivity Magnetometer for Studying Magnetic Flux Capture in High-Temperature Superconductors

Not related

By: Rostami, Kh. R.
Measurement Techniques (2017), 59(12), 1297-1303 | Language: English, Database: CAplus

Substances (0) Reactions (0) Cited By (1)

118

The Space-Time Characteristics of High-Temperature Superconductors and A Hall Microscope for Investigating Them

Not related

By: Rostami, Kh. R.
Measurement Techniques (2015), 57(12), 1443-1450 | Language: English, Database: CAplus

Substances (2) Reactions (0) Cited By (0)

119

BET and CDK Inhibition Reveal Differences in the Proliferation Control of Sympathetic Ganglion Neuroblasts and Adrenal Chromaffin Cells ✓

By: Sriha, Jessica; Louis-Brennetot, Caroline; Pierre-Eugene, Cecile; Baulande, Sylvain; Raynal, Virginie; Kramdi, Amira; Adameyko, Igor; Ernsberger, Uwe; Deller, Thomas; Delattre, Olivier; et al
Cancers (2022), 14(11), 2755 | Language: English, Database: CAplus and MEDLINE

Substances (0) Reactions (0) Cited By (0)

120

Preclinical Head and Neck Squamous Cell Carcinoma Models for Combined Targeted Therapy Approaches ✓

By: Schoenwaelder, Nina; Krause, Mareike; Freitag, Thomas; Schneider, Bjoern ; Zonnur, Sarah; Zimpfer, Annette; Becker, Anne Sophie ; Salewski, Inken; Strueder, Daniel Fabian; Lemcke, Heiko ; et al
Cancers (2022), 14(10), 2484 | Language: English, Database: CAplus and MEDLINE

Substances (0) Reactions (0) Cited By (0)

121

Targeting triple-negative breast cancer with combination therapy of EGFR CAR T cells and CDK7 inhibition

✓

By: Xia, Lin; Zheng, Zaozao; Liu, Jun-yi; Chen, Yu-jie; Ding, Jiancheng; Hu, Guo-sheng; Hu, Ya-hong; Liu, Suling; Luo, Wen-xin; Xia, Ning-shao; et al
Cancer Immunology Research (2021), 9(6), 707-722 | Language: English, Database: CAplus and MEDLINE

Substances (0) Reactions (0) Cited By (19)

122

Synergistic Anti-Tumor Effect of Combining Selective CDK7 and BRD4 Inhibition in Neuroblastoma.

By: Gao, Yang; Volegova, Marina; Nasholm, Nicole; Das, Sanjukta; Kwiatkowski, Nicholas; Abraham, Brian J; Zhang, Tinghu; Gray, Nathanael S; Gustafson, Clay; Krajewska, Malgorzata; et al
Frontiers in oncology (2021), 11, 773186 | Language: English, Database: MEDLINE

✓

Substances (0) Reactions (0) Cited By (2)

123

A thin film magnetic field sensor of sub-pT resolution and magnetocardiogram (MCG) measurement at room temperature

Not related

By: Yabukami, S.; Kato, K.; Ohtomo, Y.; Ozawa, T.; Arai, K. I.
Journal of Magnetism and Magnetic Materials (2009), 321(7), 675-678 | Language: English, Database: CAplus

Substance (1) Reactions (0) Cited By (16)

124

Androgen receptor interaction with mediator complex is enhanced in castration-resistant prostate cancer by CDK7 phosphorylation of MED1

Out

By: Russo, Joshua W.; Nouri, Mannan; Balk, Steven P.
Cancer Discovery (2019), 9(11), 1490-1492 | Language: English, Database: CAplus and MEDLINE

Substances (3) Reactions (0) Cited By (10)

125

CDK12-mediated transcriptional regulation of noncanonical NF-κB components is essential for signaling

✓

By: Henry, Kate L.; Kellner, Debra; Bajrami, Bekim; Anderson, John E.; Beyna, Mercedes; Bhisetti, Govinda; Cameron, Tom; Capacci, Andrew G.; Bertolotti-Ciarlet, Andrea; Feng, Jun; et al
Science Signaling (2018), 11(541), eaam8216/1-eaam8216/12 | Language: English, Database: CAplus and MEDLINE

Substances (3) Reactions (0) Cited By (18)

126

SQUID operating at liquid nitrogen temperatures

Not related

By: Bobrakov, V. F.; Polushkin, V. N.; Vasiliev, B. V.
Modern Physics Letters B (1989), 3(5), 415-19 | Language: English, Database: CAplus

Substance (1) | Reactions (0) | Cited By (0)

127

S/N study of Si-implanted InP Hall devices

Not related

By: Sugiyama, Yoshinobu
Sensors and Materials (1994), 5(6), 317-28 | Language: English, Database: CAplus

Substances (2) | Reactions (0) | Cited By (0)

128

UHF SQUID-magnetometer at 77 K

Not related

By: Timofeev, Valery P.; Khvostov, Sergej S.; Tsoi, Georgy M.; Shnyrkov, Vladimir I.
Cryogenics (1992), 32(ICEC Suppl.), 517-20 | Language: English, Database: CAplus

Substance (1) | Reactions (0) | Cited By (0)

129

A measurement of magnetocardiogram (MCG) by planar type sensor using CoNbZr film

By: Ohtomo, Y.; Yabukami, S.; Kato, K.; Ozawa, T.; Arai, K. I.
Journal of the Magnetics Society of Japan (2009), 33(3), 283-286 | Language: Japanese, Database: CAplus

Not related

Substances (2) | Reactions (0) | Cited By (0)

130

Identification of CDK7 Inhibitors from Natural Sources Using Pharmacoinformatics and Molecular Dynamics Simulations

Out

By: Kumar, Vikas ; Parate, Shraddha ; Thakur, Gunjan; Lee, Gihwan; Ro, Hyeon-Su ; Kim, Yongseong; Kim, Hong Ja; Kim, Myeong Ok; Lee, Keun Woo
Biomedicines (2021), 9(9), 1197 | Language: English, Database: CAplus and MEDLINE

Substances (0) | Reactions (0) | Cited By (5)

131

A single-crystal source of path-polarization entangled photons at non-degenerate wavelengths.

By: Sauge, S; Swillo, M; Tengner, M; Karlsson, A
Optics express (2008), 16(13), 9701-7 | Language: English, Database: MEDLINE

Not related
Repeat paper

Substances (0) | Reactions (0) | Cited By (9)

132

Study of terahertz intensity dependence on time resolved dynamic fringes in the interferometric autocorrelation setup

Not related

By: Venkatesh, M.; Chaudhary, A. K.
AIP Conference Proceedings (2014), 1620(1), 616-621 | Language: English, Database: CAplus

Substance (1) | Reactions (0) | Cited By (1)

133

A single-crystal source of path/polarization entanglement at non-degenerate wavelengths

Out

By: Sauge, S.; Swillo, M.
Optics and Spectroscopy (2010), 108(2), 165-169 | Language: English, Database: CAplus

Not related

Substances (3) | Reactions (0) | Cited By (0)

134

Nuclear-resonance magnetometer with flowing liquid for superstrong inhomogeneous fields measuring

Not related

By: Davydov, V. V.; Dudkin, V. I.; Vologdin, V. A.
International Journal of Modern Physics: Conference Series (2016), 41, 1660146/1-1660146/8 | Language: English, Database: CAplus

Substances (0) | Reactions (0) | Cited By (0)

135

Narrowband polarization-entangled photon pairs distributed over a WDM link for qubit networks.

By: Sauge, S; Swillo, M; Albert-Seifried, S; Xavier, G B; Waldebäck, J; Tengner, M; Ljunggren, D; Karlsson, A
Optics express (2007), 15(11), 6926-33 | Language: English, Database: MEDLINE

Not related
Out

Substances (0) | Reactions (0) | Cited By (22)

136

A measurement of magneto-cardiogram (MCG) using a high-frequency carrier-type thin-film field sensor

Not related

By: Yabukami, S.; Kato, K.; Kamo, Y.; Ozawa, T.; Arai, K. I.
Journal of the Magnetics Society of Japan (2008), 32(4), 483-486 | Language: Japanese, Database: CAplus

Substance (1) | Reactions (0) | Cited By (0)

137

Targeting bromodomain and extraterminal proteins in breast cancer

Out

By: Sahni, Jennifer M.; Keri, Ruth A.
Pharmacological Research (2018), 129, 156-176 | Language: English, Database: CAplus and MEDLINE

Substance (1) | Reactions (0) | Cited By (31)

138

TFIIE orchestrates the recruitment of the TFIIH kinase module at promoter before release during transcription

√

By: Compe, Emmanuel; Genes, Carlos M.; Braun, Cathy; Coin, Frederic; Egly, Jean-Marc
Nature Communications (2019), 10(1), 1-14 | Language: English, Database: CAplus and MEDLINE

Substances (4) | Reactions (0) | Cited By (21)

139

Synthesis and biological evaluation of new C-12(α/β)-(N-) sulfamoyl-phenylamino-14-deoxy-andrographolide derivatives as potent anti-cancer agents

By: Kandanur, Sai Giridhar Sarma; Nanduri, Srinivas; Golakoti, Nageswara Rao
Bioorganic & Medicinal Chemistry Letters (2017), 27(13), 2854-2862 | Language: English, Database: CAplus and MEDLINE

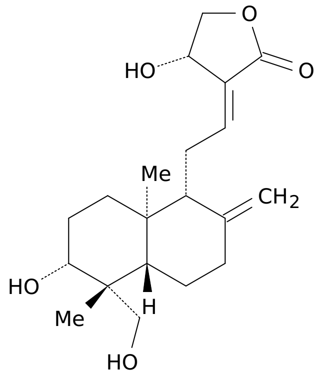

Out

I

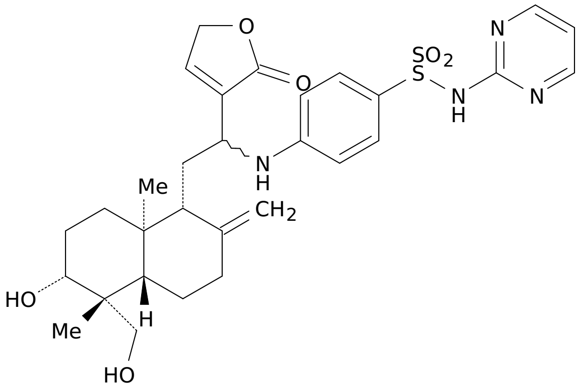

II

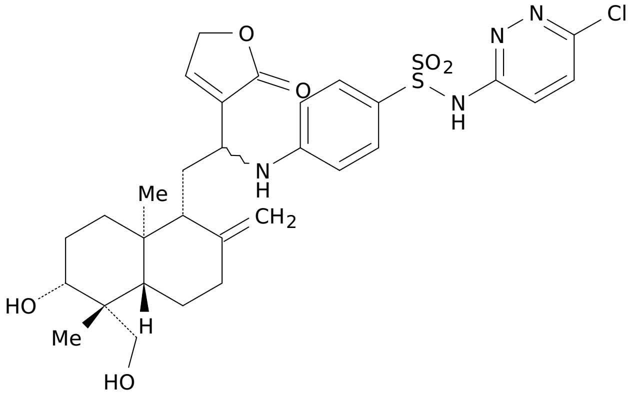

III

Substances (21) Reactions (6) Cited By (16)

140

High-frequency carrier-type magnetic field sensor with a sub-pT resolution using a magnetic film and a transmission line

Not related

By: Murayama, Y.; Ozawa, T.; Yabukami, S.; Ishiyama, K.; Arai, K. I.
Nippon Oyo Jiki Gakkaishi (2007), 31(1), 17-22 | Language: Japanese, Database: CAplus

Substance (1) Reactions (0) Cited By (4)

141

A TlBaCaCuO thin film planar dc-SQUID magnetometer

Not related

By: Chen, Lie; Zhang, Lihua; Weng, Yaojun; Yang, Tao; Liu, Dong; Shao, Kai; Yan, Shaolin
Chinese Physics Letters (1993), 10(3), 132-4 | Language: English, Database: CAplus

Substance (1) Reactions (0) Cited By (0)

142

Functional analysis of degrading astringence strains

Not related

By: Li, Qiu-tong; Su, Wei; Mu, Ying-chun
Shipin Yanjiu Yu Kaifa (2019), 40(15), 171-176 | Language: Chinese, Database: CAplus

Substances (0) Reactions (0) Cited By (0)

143

Competition of collimated ultraviolet lights generated by four-wave mixing process in Cs vapor

By: Hu, Shu; Gai, Baodong; Li, Juntao; Chen, Ying; Cai, Xianglong; Wang, Pengyuan; Liu, Jinbo; Guo, Jingwei

Not related

Optics Communications (2023), 527, 128960 | Language: English, Database: CAplus

Substance (1) Reactions (0) Cited By (0)

144

SQUID behavior at liquid nitrogen temperature in high-T_c superconductors of the type yttrium barium copper oxide

Not related

By: Tichy, R.; Odehnal, M.; Petricek, V.; Safrata, S.; Pollert, E.; Kamarad, J.; Hejtmanek, J.; Smrckova, O.; Sykorova, D.
Journal of Low Temperature Physics (1988), 70(1-2), 187-90 | Language: English, Database: CAplus

Substance (1) Reactions (0) Cited By (0)

145

CtIP-dependent nascent RNA expression flanking DNA breaks guides the choice of DNA repair pathway

By: Gomez-Cabello, Daniel; Pappas, George ; Aguilar-Morante, Diana ; Dinant, Christoffel; Bartek, Jiri
Nature Communications (2022), 13(1), 5303 | Language: English, Database: CAplus and MEDLINE

Substances (5) | Reactions (0) | Cited By (0)

146

High birefringence, low loss, and flattened dispersion photonic crystal fiber for terahertz application

By: Wang, Dou-Dou; Mu, Chang-Long; Kong, De-Peng; Guo, Chen-Yu.
Chinese Physics B (2019), 28(11), 118701 | Language: English, Database: CAplus

Not related

Out

Substances (0) | Reactions (0) | Cited By (1)

147

Selective Inhibitors of Autophagy Reveal New Link between the Cell Cycle and Autophagy and Lead to Discovery of Novel Synergistic Drug Combinations

By: Sung, Kisa; Kurowski, Agata ; Lansiquot, Carisse ; Wan, Kanny K.; Patnaik, Samarjit ; Walsh, Martin J.; Lazarus, Michael B.
ACS Chemical Biology (2022), 17(12), 3290-3297 | Language: English, Database: CAplus and MEDLINE

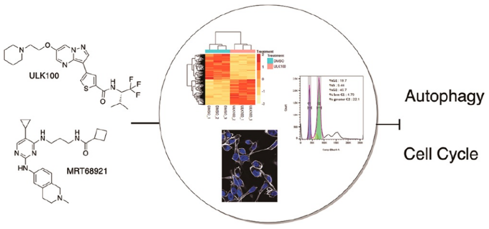

Substances (18) | Reactions (2) | Cited By (0)

148

Vulnerability of drug-resistant EML4-ALK rearranged lung cancer to transcriptional inhibition

By: Paliouras, Athanasios R.; Buzzetti, Marta; Shi, Lei; Donaldson, Ian J.; Magee, Peter; Sahoo, Sudhakar; Leong, Hui-Sun; Fassan, Matteo; Carter, Matthew; Di Leva, Gianpiero; et al
EMBO Molecular Medicine (2020), 12(7), e11099 | Language: English, Database: CAplus and MEDLINE

Substances (21) | Reactions (0) | Cited By (7)

149

Design and characterization of telecentric f- θ scanning lenses for broadband terahertz frequency systems.

Not related

Out

By: Harris, Zachery B; Katletz, Stefan; Khani, Mahmoud E; Virk, Arjun; Arbab, M Hassan
AIP advances (2020), 10(12), 125313 | Language: English, Database: MEDLINE

Substances (0) | Reactions (0) | Cited By (10)

150

Characterizing CDK8/19 inhibitors through a NFκB-dependent cell-based assay

By: Li, Jing; Ji, Hao; Porter, Donald C.; Broude, Eugenia V.; Roninson, Igor B.; Chen, Mengqian
Cells (2019), 8(10), 1208 | Language: English, Database: CAplus and MEDLINE

Substances (0) | Reactions (0) | Cited By (7)

151

Discovery of a novel CDK7 inhibitor YPN-005 in small cell lung cancer

By: Choi, Yun Jung; Lee, Hyeonjeong; Kim, Da-Som; Kim, Dong Ha; Kang, Myoung-Hee; Cho, Yong-Hee; Choi, Chang-Min; Yoo, Jakyung; Lee, Kwang-Ok; Choi, Eun Kyung; et al
European Journal of Pharmacology (2021), 907, 174298 | Language: English, Database: CAplus and MEDLINE

Substances (5) | Reactions (0) | Cited By (3)

152

Discovery of Novel N-(5-(Pyridin-3-yl)-1H-indazol-3-yl)benzamide Derivatives as Potent Cyclin-Dependent Kinase 7 Inhibitors for the Treatment of Autosomal Dominant Polycystic Kidney Disease

By: Yang, Bowen; Zhang, Haoran; Li, Na; Gao, Lixin; Jiang, Hong; Kan, Weijuan; Yuan, Haoxing; Li, Jia; Zhao, Dongmei; Xiong, Bing; et al
Journal of Medicinal Chemistry, | Language: English, Database: CAplus and MEDLINE

Substances (0) | Reactions (0) | Cited By (0)

153

CDK7/GRP78 signaling axis contributes to tumor growth and metastasis in osteosarcoma

By: Zhang, Tao; Li, Jingjie; Yang, Mengkai; Ma, Xinglong; Wang, Zhuoying; Ma, Xiaojun; Sun, Mengxiong; Sun, Wei; Xu, Jing; Hua, Yingqi; et al
Oncogene (2022), 41(40), 4524-4536 | Language: English, Database: CAplus and MEDLINE

Substances (3) | Reactions (0) | Cited By (0)

154

Transcriptional targeting of oncogene addiction in medullary thyroid cancer.

By: Valenciaga, Anisley ; Saji, Motoyasu; Yu, Lianbo; Zhang, Xiaoli; Bumrah, Ceimoani; Yilmaz, Ayse S; Knippler, Christina M; Miles, Wayne; Giordano, Thomas J; Cote, Gilbert J; et al
JCI insight (2018), 3(16), | Language: English, Database: MEDLINE

Substances (3) | Reactions (0) | Cited By (15)

155

Myeloma-specific superenhancers affect genes of biological and clinical relevance in myeloma.

By: Jia, Yunlu; Zhou, Jianbiao ; Tan, Tze King; Chung, Tae-Hoon; Wong, Regina Wan Ju; Chooi, Jing-Yuan; Lim, Julia Sze Lynn; Sanda, Takaomi ; Ooi, Melissa; De Mel, Sanjay; et al
Blood cancer journal (2021), 11(2), 32 | Language: English, Database: MEDLINE

Substances (0) | Reactions (0) | Cited By (5)

156

Theory and experiment of entanglement in a quasi-phase-matched two-crystal source

Not related

By: Ljunggren, Daniel; Tengner, Maria; Marsden, Philip; Pelton, Matthew
Physical Review A: Atomic, Molecular, and Optical Physics (2006), 73(3), 032326/1-032326/12 | Language: English, Database: CAplus

Substances (2) | Reactions (0) | Cited By (3)

157

Low-loss polarization-maintaining hollowcore anti-resonant terahertz fiber

Not related

By: Xiao, Han ; Li, Haisu ; Wu, Beilei; Dong, Yue; Xiao, Shiying; Jian, Shuisheng
Journal of Optics (Bristol, United Kingdom) (2019), 21(8), 085708 | Language: English, Database: CAplus

Substances (0) | Reactions (0) | Cited By (0)

158

Broadband Single-Mode Hybrid Photonic Crystal Waveguides for Terahertz Integration on a Chip

Not related

By: Li, Haisu ; Low, Mei Xian; Ako, Rajour Tanyi; Bhaskaran, Madhu; Sriram, Sharath; Withayachumnankul, Withawat; Kuhlmey, Boris T.; Atakaramians, Shaghik
Advanced Materials Technologies (Weinheim, Germany) (2020), 5(7), 2000117 | Language: English, Database: CAplus

Substance (1) | Reactions (0) | Cited By (8)

159

Super Enhancer-Regulated LINC00094 (SERLOC) Upregulates the Expression of MMP-1 and MMP-13 and Promotes Invasion of Cutaneous Squamous Cell Carcinoma

By: Piipponen, Minna; Riihila, Pilvi; Knuutila, Jaakko S.; Kallajoki, Markku; Kahari, Veli-Matti; Nissinen, Liisa
Cancers (2022), 14(16), 3980 | Language: English, Database: CAplus and MEDLINE

Substances (0) Reactions (0) Cited By (0)

160

Transcriptional CDK Inhibitors as Potential Treatment Option for Testicular Germ Cell Tumors

By: Funke, Kai; Duester, Robert; Wilson, Prince De-Graft; Arevalo, Lena; Geyer, Matthias; Schorle, Hubert
Cancers (2022), 14(7), 1690 | Language: English, Database: CAplus and MEDLINE

Substances (0) Reactions (0) Cited By (0)

161

TGF-β/activin signaling promotes CDK7 inhibitor resistance in triple-negative breast cancer cells through upregulation of multidrug transporters

By: Webb, Bryan M.; Bryson, Benjamin L.; Williams-Medina, Eduardo; Bobbitt, Jessica R.; Seachrist, Darcie D.; Anstine, Lindsey J.; Keri, Ruth A.
Journal of Biological Chemistry (2021), 297(4), 101162 | Language: English, Database: CAplus and MEDLINE

Substances (4) Reactions (0) Cited By (1)

162

Computational Drug Repositioning Identifies Potentially Active Therapies for Chordoma.

By: Traylor, Jeffrey I; Sheppard, Hadley E; Ravikumar, Visweswaran; Breshears, Jonathan; Raza, Shaan M; Lin, Charles Y; Patel, Shreyaskumar R; DeMonte, Franco
Neurosurgery (2021), 88(2), 428-436 | Language: English, Database: MEDLINE

Out

Substances (0) Reactions (0) Cited By (1)

163

Natural compound library screening identifies Sanguinarine chloride for the treatment of SCLC by upregulating CDKN1A

By: Zhong, Mingtian; Li, Xun; Zhao, Fengyun; Huang, Yanni; Long, Yihao; Chen, Kaizhao; Tian, Xuemei; Liu, Ming; Ma, Xiaodong
Translational Oncology (2022), 17, 101345 | Language: English, Database: CAplus and MEDLINE

Substances (5) Reactions (0) Cited By (0)

164

Targeting CDK7 reverses tamoxifen resistance through regulating stemness in ER+ breast cancer

By: Attia, Yasmin M.; Salama, Salama A. ; Shouman, Samia A.; Ivan, Cristina; Elsayed, Abdelrahman M.; Amero, Paola; Rodriguez-Aguayo, Cristian; Lopez-Berestein, Gabriel
Pharmacological Reports (2022), 74(2), 366-378 | Language: English, Database: CAplus and MEDLINE

Substances (3) | Reactions (0) | Cited By (1)

165

Enhancer RNA commits osteogenesis via microRNA-3129 expression in human bone marrow-derived mesenchymal stem cells

By: Nguyen, Anh Phuong; Yamagata, Kaoru; Iwata, Shigeru; Trimova, Gulzhan; Zhang, Tong; Shan, Yu; Nguyen, Mai-Phuong; Sonomoto, Koshiro; Nakayamada, Shingo; Kato, Shigeaki; et al
Inflammation and Regeneration (2022), 42(1), 43 | Language: English, Database: CAplus and MEDLINE

Substances (2) | Reactions (0) | Cited By (0)

166

Pharmacologic targeting of TFIIH suppresses KRAS-mutant pancreatic ductal adenocarcinoma and synergizes with TRAIL

By: Moser, Russell; Annis, James; Nikolova, Olga; Whatcott, Cliff; Gurley, Kay; Mendez, Eduardo; Moran-Jones, Kim; Dorrell, Craig; Sears, Rosalie C.; Kuo, Calvin; et al
Cancer Research (2022), 82(18), 3375-3393 | Language: English, Database: CAplus and MEDLINE

Substances (0) | Reactions (0) | Cited By (0)

167

Magnetoencephalography with a Cs-based high-sensitivity compact atomic magnetometer

Not related

By: Sheng, Jingwei; Wan, Shuangai; Sun, Yifan; Dou, Rongshe; Guo, Yuhao; Wei, Kequan; He, Kaiyan; Qin, Jie; Gao, Jia-Hong
Review of Scientific Instruments (2017), 88(9), 094304/1-094304/5 | Language: English, Database: CAplus and MEDLINE

Substance (1) | Reactions (0) | Cited By (14)

168

Advances in targeted therapy for osteosarcoma based on molecular classification

Out

By: Chen, Yingqian; Liu, Runzhi ; Wang, Wei; Wang, Chen; Zhang, Ning; Shao, Xuejing; He, Qiaojun; Ying, Meidan
Pharmacological Research (2021), 169, 105684 | Language: English, Database: CAplus and MEDLINE

Substances (0) | Reactions (0) | Cited By (9)

169

Inhibition of retinoic acid receptor α phosphorylation represses the progression of triple-negative breast cancer via transactivating miR-3074-5p to target DHRS3

By: Lou, Siyue; Gao, Hang; Hong, Huanwu; Zhu, Zhihui; Zhao, Huajun

Journal of Experimental & Clinical Cancer Research (2021), 40(1), 141 | Language: English, Database: CAplus and MEDLINE

Substances (10) Reactions (0) Cited By (2)

170

BCL2 Amplicon Loss and Transcriptional Remodeling Drives ABT-199 Resistance in B Cell Lymphoma Models

By: Zhao, Xiaohong; Ren, Yuan; Lawlor, Matthew; Shah, Bijal D.; Park, Paul M. C.; Lwin, Tint; Wang, Xuefeng; Liu, Kenian; Wang, Michelle; Gao, Jing; et al

Cancer Cell (2019), 35(5), 752-766.e9 | Language: English, Database: CAplus and MEDLINE

Substances (9) Reactions (0) Cited By (41)

Copyright © 2023 American Chemical Society (ACS). All Rights Reserved.

Copyright © 2023 U.S. National Library of Medicine.

Internal use only. Redistribution is subject to the terms of your SciFinderⁿ License Agreement and CAS information Use Policies.

CAS SciFinder^{n}

Task History

Initiating Search

January 12, 2023, 10:23PM

Substances:

Filtered By:

Structure of THZ1
(right)

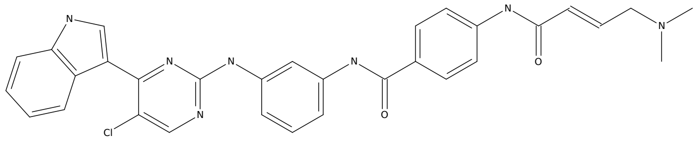

Structure Match: **As Drawn**

Search Tasks

Task	Search Type	View
Returned Substance Results + Filters (5)	Substances	View Results
Exported: Retrieved Related Reference Results + Filters (93)	References	View Results
Filtered By:		
Document Type: Journal		

Copyright © 2023 American Chemical Society (ACS). All Rights Reserved.
Internal use only. Redistribution is subject to the terms of your SciFinder^{n} License Agreement and CAS information Use Policies.

SciFinder^{n}

References (93)

View in SciFinder^{n}

1

CDK7-dependent transcriptional addiction in triple-negative breast cancer

By: Wang, Yubao; Zhang, Tinghu; Kwiatkowski, Nicholas; Abraham, Brian J.; Lee, Tong Ihn; Xie, Shaozhen; Yuzugullu, Haluk; Von, Thanh; Li, Heyuan; Lin, Ziao; et al
Cell (Cambridge, MA, United States) (2015), 163(1), 174-186 | Language: English, Database: CAplus and MEDLINE

Substances (8) | Reactions (0) | Cited By (279)

2

Targeting transcription regulation in cancer with a covalent CDK7 inhibitor

By: Kwiatkowski, Nicholas; Zhang, Tinghu; Rahl, Peter B.; Abraham, Brian J.; Reddy, Jessica; Ficarro, Scott B.; Dastur, Anahita; Amzallag, Arnaud; Ramaswamy, Sridhar; Tesar, Bethany; et al
Nature (London, United Kingdom) (2014), 511(7511), 616-620 | Language: English, Database: CAplus and MEDLINE

Substances (17) | Reactions (23) | Cited By (536)

3

Targeting super-enhancer-associated oncogenes in oesophageal squamous cell carcinoma

By: Jiang, Yan-Yi; Lin, De-Chen; Mayakonda, Anand; Hazawa, Masaharu; Ding, Ling-Wen; Chien, Wen-Wen; Xu, Liang; Chen, Ye; Xiao, Jin-Fen; Senapedis, William; et al
Gut (2017), 66(8), 1358-1368 | Language: English, Database: CAplus and MEDLINE

Substances (4) | Reactions (0) | Cited By (134)

4

Suppression of Adaptive Responses to Targeted Cancer Therapy by Transcriptional Repression

By: Rusan, Maria; Li, Kapsok; Li, Yvonne; Christensen, Camilla L.; Abraham, Brian J.; Kwiatkowski, Nicholas; Buczkowski, Kevin A.; Bockorny, Bruno; Chen, Ting; Li, Shuai; et al
Cancer Discovery (2018), 8(1), 59-73 | Language: English, Database: CAplus and MEDLINE

Substances (2) | Reactions (0) | Cited By (65)

5

Cyclin-dependent kinase 7 is a therapeutic target in high-grade glioma

By: Greenall, S. A.; Lim, Y. C.; Mitchell, C. B.; Ensbey, K. S.; Stringer, B. W.; Wilding, A. L.; O'Neill, G. M.; McDonald, K. L.; Gough, D. J.; Day, B. W.; et al
Oncogenesis (2017), 6(5), e336 | Language: English, Database: CAplus and MEDLINE

Substances (5) Reactions (0) Cited By (46)

6

Therapeutic Rationale to Target Highly Expressed CDK7 Conferring Poor Outcomes in Triple-Negative Breast Cancer

By: Li, Bo; Ni Chonghaile, Triona; Fan, Yue; Madden, Stephen F.; Klinger, Rut; O'Connor, Aisling E.; Walsh, Louise; O'Hurley, Gillian; Mallya Udupi, Girish; Joseph, Jesuchristopher; et al
Cancer Research (2017), 77(14), 3834-3845 | Language: English, Database: CAplus and MEDLINE

Substances (6) Reactions (0) Cited By (60)

7

THZ1 reveals CDK7-dependent transcriptional addictions in pancreatic cancer

By: Lu, Ping; Geng, Jing; Zhang, Lei; Wang, Yu; Niu, Ningning; Fang, Yuan; Liu, Fang; Shi, Juanjuan; Zhang, Zhi-Gang ; Sun, Yong-Wei; et al
Oncogene (2019), 38(20), 3932-3945 | Language: English, Database: CAplus and MEDLINE

Substances (2) Reactions (0) Cited By (30)

8

High MITF Expression Is Associated with Super-Enhancers and Suppressed by CDK7 Inhibition in Melanoma

By: Eliades, Philip; Abraham, Brian J.; Ji, Zhenyu; Miller, David M.; Christensen, Camilla L.; Kwiatkowski, Nicholas; Kumar, Raj; Njauw, Ching Ni; Taylor, Michael; Miao, Benchun; et al
Journal of Investigative Dermatology (2018), 138(7), 1582-1590 | Language: English, Database: CAplus and MEDLINE

Substances (3) Reactions (0) Cited By (32)

9

No Driver behind the Wheel? Targeting Transcription in Cancer

By: Franco, Hector L.; Kraus, W. Lee
Cell (Cambridge, MA, United States) (2015), 163(1), 28-30 | Language: English, Database: CAplus and MEDLINE

Substances (2) Reactions (0) Cited By (24)

10

CDK7 inhibition suppresses castration-resistant prostate cancer through MED1 inactivation

By: ur Rasool, Reyaz; Natesan, Ramakrishnan; Deng, Qu; Aras, Shweta; Lal, Priti; Effron, Samuel Sander; Mitchell-Velasquez, Erick; Posimo, Jessica M.; Carskadon, Shannon; Baca, Sylvan C.; et al
Cancer Discovery (2019), 9(11), 1538-1555 | Language: English, Database: CAplus and MEDLINE

Substances (2) Reactions (0) Cited By (42)

11

Enhancer profiling identifies critical cancer genes and characterizes cell identity in adult T-cell leukemia

By: Wong, Regina Wan Ju; Ngoc, Phuong Cao Thi; Leong, Wei Zhong; Yam, Alice Wei Yee; Zhang, Tinghu; Asamitsu, Tinghu; Iida, Shinsuke; Okamoto, Takashi; Ueda, Ryuzo; Gray, Nathanael S.; et al
Blood (2017), 130(21), 2326-2338 | Language: English, Database: CAplus and MEDLINE

Substances (4) Reactions (0) Cited By (52)

12

Inhibition of cyclin-dependent kinase 7 suppresses human hepatocellular carcinoma by inducing apoptosis

By: Zhong, Liqiang; Yang, Sihao; Jia, Yuming; Lei, Kaijian
Journal of Cellular Biochemistry (2018), 119(12), 9742-9751 | Language: English, Database: CAplus and MEDLINE

Substances (4) Reactions (0) Cited By (16)

13

CDK7 inhibition as a promising therapeutic strategy for lung squamous cell carcinomas with a SOX2 amplification

By: Hur, Jae Young; Kim, Hyeong Ryul; Lee, Jung Yeon; Park, Sojung; Hwang, Ji An; Kim, Woo Sung; Yoon, Shinkyo; Choi, Chang-Min; Rho, Jin Kyung; Lee, Jae Cheol
Cellular Oncology (2019), 42(4), 449-458 | Language: English, Database: CAplus and MEDLINE

Substances (3) Reactions (0) Cited By (11)

14

CDK7 inhibition suppresses aberrant hedgehog pathway and overcomes resistance to smoothened antagonists

By: Liu, Fang; Jiang, Wenyan; Sui, Yi; Meng, Wei; Hou, Linjun; Li, Tiantian; Li, Meng; Zhang, Lei; Mo, Jialin; Wang, Jiajia; et al
Proceedings of the National Academy of Sciences of the United States of America (2019), 116(26), 12986-12995 | Language: English, Database: CAplus and MEDLINE

Substances (2) Reactions (0) Cited By (15)

15

THZ1 targeting CDK7 suppresses STAT transcriptional activity and sensitizes T-cell lymphomas to BCL2 inhibitors

By: Cayrol, Florencia; Praditsuktavorn, Pannee; Fernando, Tharu M.; Kwiatkowski, Nicholas; Marullo, Rosella; Calvo-Vidal, M. Nieves; Phillip, Jude; Pera, Benet; Yang, Shao Ning; Takpradit, Kaipol; et al
Nature Communications (2017), 8, 14290 | Language: English, Database: CAplus and MEDLINE

Substances (23) Reactions (0) Cited By (63)

16

CDK7 inhibitor THZ1 inhibits MCL1 synthesis and drives cholangiocarcinoma apoptosis in combination with BCL2/BCL-XL inhibitor ABT-263

By: Huang, Tianlu; Ding, Xiwei ; Xu, Guifang; Chen, Gang; Cao, Yu; Peng, Chunyan; Shen, Shanshan; Lv, Ying; Wang, Lei; Zou, Xiaoping
Cell Death & Disease (2019), 10(8), 1-14 | Language: English, Database: CAplus and MEDLINE

Substances (7) Reactions (0) Cited By (25)

17

A CRISPR screen identifies CDK7 as a therapeutic target in hepatocellular carcinoma

By: Wang, Cun; Jin, Haojie; Gao, Dongmei; Wang, Liqin; Evers, Bastiaan; Xue, Zheng; Jin, Guangzhi; Lieftink, Cor; Beijersbergen, Roderick L.; Qin, Wenxin; et al
Cell Research (2018), 28(6), 690-692 | Language: English, Database: CAplus and MEDLINE

Substances (4) Reactions (0) Cited By (35)

18

Targeting Super-Enhancers via Nanoparticle-Facilitated BRD4 and CDK7 Inhibitors Synergistically Suppresses Pancreatic Ductal Adenocarcinoma

By: Huang, Chen-Song; You, Xinru; Dai, Chunlei; Xu, Qiong-Cong; Li, Fuxi; Wang, Li; Huang, Xi-Tai; Wang, Jie-Qin; Li, Shi-Jin; Gao, Zhuoxing; et al
Advanced Science (Weinheim, Germany) (2020), 7(7), 1902926 | Language: English, Database: CAplus and MEDLINE

Substances (4) Reactions (0) Cited By (18)

19

Aberrant Super-Enhancer Landscape in Human Hepatocellular Carcinoma

By: Tsang, Felice Ho-Ching; Law, Cheuk-Ting; Tang, Tsz-Ching Chloe; Cheng, Carol Lai-Hung; Chin, Don Wai-Ching; Tam, Wing-Sum Vincy; Wei, Lai; Wong, Carmen Chak-Lui; Ng, Irene Oi-Lin ; Wong, Chun-Ming
Hepatology (Hoboken, NJ, United States) (2019), 69(6), 2502-2517 | Language: English, Database: CAplus and MEDLINE

Substances (4) Reactions (0) Cited By (53)

20

Super enhancer inhibitors suppress MYC driven transcriptional amplification and tumor progression in osteosarcoma

By: Chen, Demeng; Zhao, Zhiqiang; Huang, Zixin; Chen, Du-Chu; Zhu, Xin-Xing; Wang, Yi-Ze; Yan, Ya-Wei; Tang, Shaojun; Madhavan, Subha; Ni, Weiyi; et al
Bone Research (2018), 6(1), 1-7 | Language: English, Database: CAplus and MEDLINE

Substances (3) Reactions (0) Cited By (66)

21

Targeting CDK7 increases the stability of Snail to promote the dissemination of colorectal cancer

By: Zhou, Yan; Lu, Linlin; Jiang, Guanmin; Chen, Zhuojia; Li, Jiexin; An, Panpan; Chen, Likun; Du, Jun; Wang, Hongsheng
Cell Death & Differentiation (2019), 26(8), 1442-1452 | Language: English, Database: CAplus and MEDLINE

Substances (3) Reactions (0) Cited By (29)

22

Antitumor effects of a covalent cyclin-dependent kinase 7 inhibitor in colorectal cancer

By: Wang, Jian; Li, Zhenyu; Mei, Hong; Zhang, Dejun; Wu, Gang; Zhang, Tao; Lin, Zhenyu
Anti-Cancer Drugs (2019), 30(5), 466-474 | Language: English, Database: CAplus and MEDLINE

Substances (6) Reactions (0) Cited By (5)

23

Tumors with TSC mutations are sensitive to CDK7 inhibition through NRF2 and glutathione depletion

By: Zarei, Mahsa; Du, Heng; Nassar, Amin H. ; Yan, Rachel E. ; Giannikou, Krinio; Johnson, Sneha H.; Lam, Hilaire C.; Henske, Elizabeth P.; Wang, Yubao; Zhang, Tinghu; et al
Journal of Experimental Medicine (2019), 216(11), 2635-2652 | Language: English, Database: CAplus and MEDLINE

Substances (4) Reactions (0) Cited By (12)

24

Transcriptional Dependencies in Diffuse Intrinsic Pontine Glioma

By: Nagaraja, Surya; Vitanza, Nicholas A.; Woo, Pamelyn J.; Taylor, Kathryn R.; Liu, Fang; Zhang, Lei; Li, Meng; Meng, Wei; Ponnuswami, Anitha; Sun, Wenchao; et al
Cancer Cell (2017), 31(5), 635-652.e6 | Language: English, Database: CAplus and MEDLINE

Substances (22) Reactions (0) Cited By (181)

25

Super-enhancer-associated MEIS1 promotes transcriptional dysregulation in Ewing sarcoma in co-operation with EWS-FLI1

By: Lin, Lehang; Huang, Moli; Shi, Xianping; Mayakonda, Anand; Hu, Kaishun; Jiang, Yan-Yi; Guo, Xiao; Chen, Li; Pang, Brendan; Doan, Ngan; et al
Nucleic Acids Research (2019), 47(3), 1255-1267 | Language: English, Database: CAplus and MEDLINE

Substances (2) Reactions (0) Cited By (35)

26

JMJD6 is a tumorigenic factor and therapeutic target in neuroblastoma

By: Wong, Matthew ; Sun, Yuting; Xi, Zhichao ; Milazzo, Giorgio ; Poulos, Rebecca C. ; Bartenhagen, Christoph; Bell, Jessica L. ; Mayoh, Chelsea ; Ho, Nicholas ; Tee, Andrew E.; et al
Nature Communications (2019), 10(1), 1-15 | Language: English, Database: CAplus and MEDLINE

Substances (2) Reactions (0) Cited By (48)

27

ER Stress Signaling Promotes the Survival of Cancer "Persister Cells" Tolerant to EGFR Tyrosine Kinase Inhibitors

By: Terai, Hideki; Kitajima, Shunsuke; Potter, Danielle S.; Matsui, Yusuke; Quiceno, Laura Gutierrez; Chen, Ting; Kim, Tae-jung; Rusan, Maria; Thai, Tran C.; Piccioni, Federica; et al
Cancer Research (2018), 78(4), 1044-1057 | Language: English, Database: CAplus and MEDLINE

Substances (2) Reactions (0) Cited By (62)

28

The covalent CDK7 inhibitor THZI potently induces apoptosis in multiple myeloma cells in vitro and in vivo

By: Zhang, Yu; Zhou, Liang; Bandyopadhyay, Dipankar; Sharma, Kanika; Allen, Alexander Joseph; Kmieciak, Maciej; Grant, Steven
Clinical Cancer Research (2019), 25(20), 6195-6205 | Language: English, Database: CAplus and MEDLINE

Substances (11) Reactions (0) Cited By (31)

29

Preclinical Efficacy and Molecular Mechanism of Targeting CDK7-Dependent Transcriptional Addiction in Ovarian Cancer

By: Zhang, Zhenfeng; Peng, Huixin; Wang, Xiaojie; Yin, Xia; Ma, Pengfei; Jing, Ying; Cai, Mei-Chun; Liu, Jin; Zhang, Meiying; Zhang, Shengzhe; et al
Molecular Cancer Therapeutics (2017), 16(9), 1739-1750 | Language: English, Database: CAplus and MEDLINE

Substances (40) Reactions (0) Cited By (48)

30

Activation of the p53 Transcriptional Program Sensitizes Cancer Cells to Cdk7 Inhibitors

By: Kalan, Sampada; Amat, Ramon; Schachter, Miriam Merzel; Kwiatkowski, Nicholas; Abraham, Brian J.; Liang, Yanke; Zhang, Tinghu; Olson, Calla M.; Larochelle, Stephane; Young, Richard A.; et al
Cell Reports (2017), 21(2), 467-481 | Language: English, Database: CAplus and MEDLINE

Substances (10) Reactions (0) Cited By (51)

31

ABC-transporter upregulation mediates resistance to the CDK7 inhibitors THZ1 and ICEC0942

By: Sava, Georgina P.; Fan, Hailing; Fisher, Rosemary A.; Lusvarghi, Sabrina; Pancholi, Sunil ; Ambudkar, Suresh V. ; Martin, Lesley-Ann; Charles Coombes, R.; Buluwela, Lakjaya; Ali, Simak
Oncogene (2020), 39(3), 651-663 | Language: English, Database: CAplus and MEDLINE

Substances (5) Reactions (0) Cited By (10)

32

CDK7 inhibition by THZ1 suppresses cancer stemness in both chemonaive and chemoresistant urothelial carcinoma via the hedgehog signaling pathway

By: Chow, Po-Ming; Chang, Yu-Wei; Kuo, Kuan-Lin; Lin, Wei-Chou ; Liu, Shing-Hwa; Huang, Kuo-How
Cancer Letters (New York, NY, United States) (2021), 507, 70-79 | Language: English, Database: CAplus and MEDLINE

Substances (3) Reactions (0) Cited By (4)

33

CDK7 is a reliable prognostic factor and novel therapeutic target in epithelial ovarian cancer

By: Kim, Jihye; Cho, Young-Jae; Ryu, Ji-Yoon; Hwang, Ilseon; Han, Hee Dong; Ahn, Hyung Jun; Kim, Woo Young; Cho, Hanbyoul; Chung, Joon-Yong; Hewitt, Stephen M.; et al
Gynecologic Oncology (2020), 156(1), 211-221 | Language: English, Database: CAplus and MEDLINE

Substances (3) Reactions (0) Cited By (10)

34

The CDK7 inhibitor THZ1 alters RNA polymerase dynamics at the 5' and 3' ends of genes

By: Sampathi, Shilpa; Acharya, Pankaj; Zhao, Yue; Wang, Jing; Stengel, Kristy R.; Liu, Qi; Savona, Michael R.; Hiebert, Scott W.
Nucleic Acids Research (2019), 47(8), 3921-3936 | Language: English, Database: CAplus and MEDLINE

Substances (6) Reactions (0) Cited By (19)

35

Discovery and characterization of SY-1365, a selective, covalent inhibitor of CDK7

By: Hu, Shanhu; Marineau, Jason J.; Rajagopal, Nisha; Hamman, Kristin B.; Choi, Yoon Jong; Schmidt, Darby R.; Ke, Nan; Johannessen, Liv; Bradley, Michael J.; Orlando, David A.; et al
Cancer Research (2019), 79(13), 3480-3492 | Language: English, Database: CAplus and MEDLINE

Substances (21) Reactions (0) Cited By (66)

36

Overcoming Resistance to the THZ Series of Covalent Transcriptional CDK Inhibitors

By: Gao, Yang; Zhang, Tinghu; Terai, Hideki; Ficarro, Scott B.; Kwiatkowski, Nicholas; Hao, Ming-Feng; Sharma, Bandana; Christensen, Camilla L.; Chipumuro, Edmond; Wong, Kwok-kin; et al
Cell Chemical Biology (2018), 25(2), 135-142.e5 | Language: English, Database: CAplus and MEDLINE

Substances (19) Reactions (5) Cited By (47)

37

Inhibition of the transcriptional kinase CDK7 overcomes therapeutic resistance in HER2-positive breast cancers

By: Sun, Bowen; Mason, Seth; Wilson, Robert C.; Hazard, Starr E. ; Wang, Yubao; Fang, Rong; Wang, Qiwei; Yeh, Elizabeth S.; Yang, Meixiang; Roberts, Thomas M.; et al
Oncogene (2020), 39(1), 50-63 | Language: English, Database: CAplus and MEDLINE

Substances (7) Reactions (0) Cited By (33)

38

The covalent CDK7 inhibitor THZ1 enhances temsirolimus-induced cytotoxicity via autophagy suppression in human renal cell carcinoma

By: Chow, Po-Ming; Liu, Shing-Hwa; Chang, Yu-Wei; Kuo, Kuan-Lin; Lin, Wei-Chou; Huang, Kuo-How
Cancer Letters (New York, NY, United States) (2020), 471, 27-37 | Language: English, Database: CAplus and MEDLINE

Substances (4) Reactions (0) Cited By (16)

39

Gene expression profile of THZ1-treated nasopharyngeal carcinoma cell lines indicates its involvement in the inhibition of the cell cycle

By: Gao, Lijuan; Xia, Shuang; Zhang, Kunyi; Lin, Chengguang; He, Xuyu; Zhang, Ying
Translational Cancer Research (2021), 10(1), 445-460 | Language: English, Database: CAplus and MEDLINE

Substance (1) Reactions (0) Cited By (0)

40

EWS/FLI Confers Tumor Cell Synthetic Lethality to CDK12 Inhibition in Ewing Sarcoma

By: Iniguez, Amanda Balboni; Stolte, Bjorn; Wang, Emily Jue; Conway, Amy Saur; Alexe, Gabriela; Dharia, Neekesh V.; Kwiatkowski, Nicholas; Zhang, Tinghu; Abraham, Brian J.; Mora, Jaume; et al
Cancer Cell (2018), 33(2), 202-216.e6 | Language: English, Database: CAplus and MEDLINE

Substances (18) Reactions (0) Cited By (80)

41

CDK7 inhibitor THZ1 enhances antiPD-1 therapy efficacy via the p38α/MYC/PD-L1 signaling in non-small cell lung cancer

By: Wang, Jian ; Zhang, Ruiguang; Lin, Zhenyu; Zhang, Sheng; Chen, Yaobing; Tang, Jing; Hong, Jiaxin; Zhou, Xiaoshu; Zong, Yan; Xu, Yingzhuo; et al
Journal of Hematology & Oncology, | Language: English, Database: CAplus and MEDLINE

Substances (8) Reactions (0) Cited By (29)

42

Enhancer RNA commits osteogenesis via microRNA-3129 expression in human bone marrow-derived mesenchymal stem cells

By: Nguyen, Anh Phuong; Yamagata, Kaoru; Iwata, Shigeru; Trimova, Gulzhan; Zhang, Tong; Shan, Yu; Nguyen, Mai-Phuong; Sonomoto, Koshiro; Nakayamada, Shingo; Kato, Shigeaki; et al
Inflammation and Regeneration (2022), 42(1), 43 | Language: English, Database: CAplus and MEDLINE

Substances (2) Reactions (0) Cited By (0)

43

Cyclin-dependent kinase 7 (CDK7) expression in human hepatocellular carcinoma: association with HCC progression, prognosis and cell proliferative capacity

By: Liu, Zheran; Liu, Dajiao; Zhong, Rongrong; Su, Qiuyun; Zhao, Tiancheng; Fu, Fen; Liu, Jinsheng; Xu, Derong; Zeng, Changqing
Translational Cancer Research (2018), 7(3), 472-479, S1-S2 | Language: English, Database: CAplus

Substances (2) | Reactions (0) | Cited By (1)

44

THZ1 suppresses human non-small-cell lung cancer cells in vitro through interference with cancer metabolism

By: Cheng, Zhu-Jun; Miao, Du-Ling; Su, Qiu-Yun; Tang, Xiao-Li; Wang, Xiao-Lei; Deng, Li-Bin; Shi, Hui-Dong; Xin, Hong-Bo
Acta Pharmacologica Sinica (2019), 40(6), 814-822 | Language: English, Database: CAplus and MEDLINE

Substances (24) | Reactions (0) | Cited By (27)

45

Transcriptional control of DNA repair networks by CDK7 regulates sensitivity to radiation in MYC-driven medulloblastoma

By: Veo, Bethany; Danis, Etienne ; Pierce, Angela; Wang, Dong; Fosmire, Susan; Sullivan, Kelly D.; Joshi, Molishree; Khanal, Santosh; Dahl, Nathan; Karam, Sana; et al
Cell Reports (2021), 35(4), 109013 | Language: English, Database: CAplus and MEDLINE

Substances (7) | Reactions (0) | Cited By (12)

46

CDK7/GRP78 signaling axis contributes to tumor growth and metastasis in osteosarcoma

By: Zhang, Tao ; Li, Jingjie; Yang, Mengkai; Ma, Xinglong; Wang, Zhuoying; Ma, Xiaojun; Sun, Mengxiong; Sun, Wei; Xu, Jing; Hua, Yingqi; et al
Oncogene (2022), 41(40), 4524-4536 | Language: English, Database: CAplus and MEDLINE

Substances (3) | Reactions (0) | Cited By (0)

47

CDK7 blockade suppresses super-enhancer-associated oncogenes in bladder cancer

By: Yang, Yafei; Jiang, Donggen; Zhou, Ziyu; Xiong, Haiyun; Yang, Xiangwei; Peng, Guoyu; Xia, Wuchao; Wang, Shang; Lei, Hanqi; Zhao, Jing; et al
Cellular Oncology (2021), 44(4), 871-887 | Language: English, Database: CAplus and MEDLINE

Substances (3) | Reactions (0) | Cited By (2)

48

Blockade of CDK7 reverses endocrine therapy resistance in breast cancer

By: Attia, Yasmin M.; Shouman, Samia A.; Salama, Salama A.; Ivan, Cristina; Elsayed, Abdelrahman M.; Amero, Paola; Rodriguez-Aguayo, Cristian; Lopez-Berestein, Gabriel
International Journal of Molecular Sciences (2020), 21(8), 2974 | Language: English, Database: CAplus and MEDLINE

Substances (3) | Reactions (0) | Cited By (8)

49

Androgen receptor interaction with mediator complex is enhanced in castration-resistant prostate cancer by CDK7 phosphorylation of MED1

By: Russo, Joshua W.; Nouri, Mannan; Balk, Steven P.
Cancer Discovery (2019), 9(11), 1490-1492 | Language: English, Database: CAplus and MEDLINE

Substances (3) | Reactions (0) | Cited By (10)

50

Inhibition of super enhancer downregulates the expression of KLF5 in basal-like breast cancers

By: Chen, Chuan-Huizi; Yang, Nong; Zhang, Yongchang; Ding, Jiancheng; Zhang, Wenjuan; Liu, Rong; Liu, Wen; Chen, Ceshi
International Journal of Biological Sciences (2019), 15(8), 1733-1742 | Language: English, Database: CAplus and MEDLINE

Substances (3) | Reactions (0) | Cited By (18)

51

Transcription without XPB Establishes a Unified Helicase-Independent Mechanism of Promoter Opening in Eukaryotic Gene Expression

By: Alekseev, Sergey; Nagy, Zita; Sandoz, Jeremy; Weiss, Amelie; Egly, Jean-Marc; Le May, Nicolas; Coin, Frederic
Molecular Cell (2017), 65(3), 504-514.e4 | Language: English, Database: CAplus and MEDLINE

Substances (9) | Reactions (0) | Cited By (47)

52

Identification of Four Enhancer-Associated Genes as Risk Signature for Diffuse Glioma Patients

By: Wang, Jiajia; Ma, Xin; Ma, Jie
Journal of Molecular Neuroscience (2022), 72(2), 410-419 | Language: English, Database: CAplus and MEDLINE

Substances (4) | Reactions (0) | Cited By (2)

53

Systematic Characterization of Recurrent Genomic Alterations in Cyclin-Dependent Kinases Reveals Potential Therapeutic Strategies for Cancer Treatment

By: Shan, Weiwei ; Yuan, Jiao ; Hu, Zhongyi; Jiang, Junjie; Wang, Yueying; Loo, Nicki; Fan, Lingling; Tang, Zhaoqing; Zhang, Tianli ; Xu, Mu; et al
Cell Reports (2020), 32(2), 107884 | Language: English, Database: CAplus and MEDLINE

Substances (4) Reactions (0) Cited By (13)

54

TFIIE orchestrates the recruitment of the TFIIH kinase module at promoter before release during transcription

By: Compe, Emmanuel; Genes, Carlos M.; Braun, Cathy; Coin, Frederic; Egly, Jean-Marc
Nature Communications (2019), 10(1), 1-14 | Language: English, Database: CAplus and MEDLINE

Substances (4) Reactions (0) Cited By (21)

55

Natural compound library screening identifies Sanguinarine chloride for the treatment of SCLC by upregulating CDKN1A

By: Zhong, Mingtian; Li, Xun; Zhao, Fengyun; Huang, Yanni; Long, Yihao; Chen, Kaizhao; Tian, Xuemei; Liu, Ming; Ma, Xiaodong
Translational Oncology (2022), 17, 101345 | Language: English, Database: CAplus and MEDLINE

Substances (5) Reactions (0) Cited By (0)

56

Carrier-free multifunctional nanomedicine for intraperitoneal disseminated ovarian cancer therapy

By: Huang, Xiuyu; Qiu, Miaojuan; Wang, Tianqi; Li, Binbin; Zhang, Shiqiang; Zhang, Tianzhi; Liu, Peng; Wang, Qiang; Qian, Zhi Rong; Zhu, Chengming; et al
Journal of Nanobiotechnology (2022), 20(1), 93 | Language: English, Database: CAplus and MEDLINE

Substances (5) Reactions (0) Cited By (5)

57

Discovery of a novel CDK7 inhibitor YPN-005 in small cell lung cancer

By: Choi, Yun Jung; Lee, Hyeonjeong ; Kim, Da-Som; Kim, Dong Ha; Kang, Myoung-Hee; Cho, Yong-Hee; Choi, Chang-Min ; Yoo, Jakyung; Lee, Kwang-Ok; Choi, Eun Kyung; et al
European Journal of Pharmacology (2021), 907, 174298 | Language: English, Database: CAplus and MEDLINE

Substances (5) Reactions (0) Cited By (3)

58

A combination strategy targeting enhancer plasticity exerts synergistic lethality against BETi-resistant leukemia cells

By: Guo, Lei; Li, Jia; Zeng, Hongxiang; Guzman, Anna G.; Li, Tingting; Lee, Minjung; Zhou, Yubin ; Goodell, Margaret A.; Stephan, Clifford; Davies, Peter J. A.; et al
Nature Communications (2020), 11(1), 740 | Language: English, Database: CAplus and MEDLINE

Substances (5) Reactions (0) Cited By (27)

59

Targeting transcription in heart failure via CDK7/12/13 inhibition

By: Hsu, Austin ; Duan, Qiming; Day, Daniel S.; Luo, Xin; McMahon, Sarah; Huang, Yu; Feldman, Zachary B.; Jiang, Zhen; Zhang, Tinghu; Liang, Yanke; et al
Nature Communications (2022), 13(1), 4345 | Language: English, Database: CAplus and MEDLINE

Substances (6) Reactions (0) Cited By (0)

60

Disruption of TFIIH activities generates a stress gene expression response and reveals possible new targets against cancer

By: Uriostegui-Arcos, Maritere; Aguayo-Ortiz, Rodrigo; del Pilar Valencia-Morales, Maria; Melchy-Perez, Erika; Rosenstein, Yvonne; Dominguez, Laura; Zurita, Mario
Open Biology (2020), 10(6), 200050 | Language: English, Database: CAplus and MEDLINE

Substances (6) Reactions (0) Cited By (2)

61

CDK9 activity is critical for maintaining MDM4 overexpression in tumor cells

By: Stetkova, Monika; Growkova, Katerina; Fojtik, Petr; Valcikova, Barbora; Palusova, Veronika; Verlande, Amandine; Jorda, Radek ; Krystof, Vladimir; Hejret, Vaclav; Alexiou, Panagiotis; et al
Cell Death & Disease (2020), 11(9), 754 | Language: English, Database: CAplus and MEDLINE

Substances (14) Reactions (0) Cited By (16)

62

Global and transcription-coupled repair of 8-oxoG is initiated by nucleotide excision repair proteins

By: Kumar, Namrata; Theil, Arjan F. ; Roginskaya, Vera; Ali, Yasmin; Calderon, Michael; Watkins, Simon C.; Barnes, Ryan P.; Opresko, Patricia L. ; Pines, Alex; Lans, Hannes ; et al
Nature Communications (2022), 13(1), 974 | Language: English, Database: CAplus and MEDLINE

Substances (7) Reactions (0) Cited By (3)

63

Pharmaceutical interference of the EWS-FLI1-driven transcriptome by cotargeting H3K27ac and RNA polymerase activity in ewing sarcoma

By: Heisey, Daniel A. R.; Jacob, Sheeba; Lochmann, Timothy L.; Kurupi, Richard; Ghotra, Maninderjit S.; Calbert, Marissa L.; Shende, Mayuri; Maves, Yuki Kato; Koblinski, Jennifer E.; Dozmorov, Mikhail G.; et al
Molecular Cancer Therapeutics (2021), 20(10), 1868-1879 | Language: English, Database: CAplus and MEDLINE

Substances (8) | Reactions (0) | Cited By (4)

64

Super-enhancer landscape reveals leukemia stem cell reliance on X-box binding protein 1 as a therapeutic vulnerability

By: Zhou, Jingfeng ; Wang, Shubo ; Nie, Danian; Lai, Peilong; Li, Yiqing; Li, Yangqiu; Jin, Yanli ; Pan, Jingxuan
Science Translational Medicine (2021), 13(612), eabh3462 | Language: English, Database: CAplus and MEDLINE

Substances (8) | Reactions (0) | Cited By (7)

65

Trib1 promotes acute myeloid leukemia progression by modulating the transcriptional programs of Hoxa9

By: Yoshino, Seiko; Yokoyama, Takashi; Sunami, Yoshitaka; Takahara, Tomoko; Nakamura, Aya; Yamazaki, Yukari; Tsutsumi, Shuichi; Aburatani, Hiroyuki; Nakamura, Takuro
Blood (2021), 137(1), 75-88 | Language: English, Database: CAplus and MEDLINE

Substances (8) | Reactions (0) | Cited By (17)

66

Measurement and models accounting for cell death capture hidden variation in compound response

By: Bae, Song Yi; Guan, Ning ; Yan, Rui ; Warner, Katrina ; Taylor, Scott D.; Meyer, Aaron S.
Cell Death & Disease (2020), 11(4), 255 | Language: English, Database: CAplus and MEDLINE

Substances (8) | Reactions (0) | Cited By (5)

67

Transcriptional CDK inhibitors, CYC065 and THZ1 promote Bim-dependent apoptosis in primary and recurrent GBM through cell cycle arrest and Mcl-1 downregulation

By: Juric, Viktorija ; Hudson, Lance; Fay, Joanna; Richards, Cathy E.; Jahns, Hanne; Verreault, Maite; Bielle, Franck; Idbaih, Ahmed; Lamfers, Martine L. M.; Hopkins, Ann M.; et al
Cell Death & Disease (2021), 12(8), 763 | Language: English, Database: CAplus and MEDLINE

Substances (10) | Reactions (0) | Cited By (3)

68

Inhibition of retinoic acid receptor α phosphorylation represses the progression of triple-negative breast cancer via transactivating miR-3074-5p to target DHRS3

By: Lou, Siyue; Gao, Hang; Hong, Huanwu; Zhu, Zhihui; Zhao, Huajun
Journal of Experimental & Clinical Cancer Research (2021), 40(1), 141 | Language: English, Database: CAplus and MEDLINE

Substances (10) | Reactions (0) | Cited By (2)

69

Leveraging Gas-Phase Fragmentation Pathways for Improved Identification and Selective Detection of Targets Modified by Covalent Probes

By: Ficarro, Scott B.; Browne, Christopher M.; Card, Joseph D.; Alexander, William M.; Zhang, Tinghu; Park, Eunyoung; McNally, Randall; Dhe-Paganon, Sirano; Seo, Hyuk-Soo; Lamberto, Ilaria; et al
Analytical Chemistry (Washington, DC, United States) (2016), 88(24), 12248-12254 | Language: English, Database: CAplus and MEDLINE

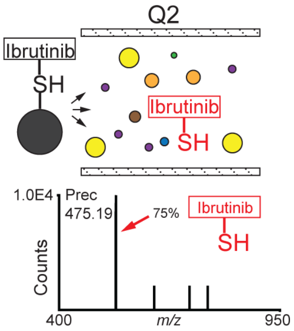

Substances (20) | Reactions (0) | Cited By (22)

70

Corrigendum: THZ1 targeting CDK7 suppresses STAT transcriptional activity and sensitizes T-cell lymphomas to BCL2 inhibitors [Erratum to document cited in CA168:274984]

By: Cayrol, Florencia; Praditsuktavorn, Pannee; Fernando, Tharu M.; Kwiatkowski, Nicholas; Marullo, Rossella; Calvo-Vidal, M. Nieves; Phillip, Jude; Pera, Benet; Yang, Shao Ning; Takpradit, Kaipol; et al
Nature Communications (2017), 8, 14747 | Language: English, Database: CAplus and MEDLINE

Substances (22) | Reactions (0) | Cited By (3)

71

A chemoproteomic strategy for direct and proteome-wide covalent inhibitor target-site identification

By: Browne, Christopher M. ; Jiang, Baishan; Ficarro, Scott B.; Doctor, Zainab M.; Johnson, Jared L.; Card, Joseph D.; Sivakumaren, Sindhu Carmen; Alexander, William M.; Yaron, Tomer M.; Murphy, Charles J.; et al
Journal of the American Chemical Society (2019), 141(1), 191-203 | Language: English, Database: CAplus and MEDLINE

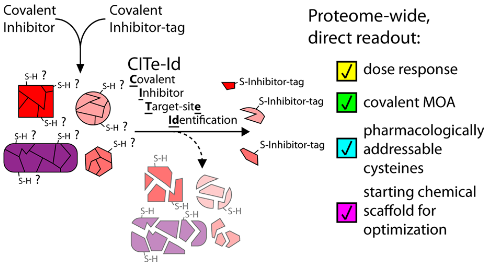

Substances (24) Reactions (19) Cited By (48)

72

Partitioning of cancer therapeutics in nuclear condensates

By: Klein, Isaac A. ; Boija, Ann ; Afeyan, Lena K.; Hawken, Susana Wilson; Fan, Mengyang ; Dall'Agnese, Alessandra ; Oksuz, Ozgur ; Henninger, Jonathan E. ; Shrinivas, Krishna ; Sabari, Benjamin R. ; et al
Science (Washington, DC, United States) (2020), 368(6497), 1386-1392 | Language: English, Database: CAplus and MEDLINE

Substances (12) Reactions (0) Cited By (153)

73

Combination therapy with the CDK7 inhibitor and the tyrosine kinase inhibitor exerts synergistic anticancer effects against MYCN-amplified neuroblastoma

By: Tee, Andrew E.; Ciampa, Olivia C.; Wong, Matthew; Fletcher, Jamie I. ; Kamili, Alvin; Chen, Jingwei; Ho, Nicholas; Sun, Yuting; Carter, Daniel R.; Cheung, Belamy B.; et al
International Journal of Cancer (2020), 147(7), 1928-1938 | Language: English, Database: CAplus and MEDLINE

Substances (25) Reactions (0) Cited By (18)

74

SQLE inhibition suppresses the development of pancreatic ductal adenocarcinoma and enhances its sensitivity to chemotherapeutic agents in vitro

By: Zhao, Fengyun ; Huang, Yanni; Zhang, Yingheng; Li, Xun; Chen, Kaizhao; Long, Yihao; Li, Fugui; Ma, Xiaodong
Molecular Biology Reports (2022), 49(7), 6613-6621 | Language: English, Database: CAplus and MEDLINE

Substances (13) Reactions (0) Cited By (0)

75

Systematic Analysis of Drug Vulnerabilities Conferred by Tumor Suppressor Loss

By: Ding, Hongyu; Zhao, Jie; Zhang, Yanli; Yu, Jiao; Liu, Mingxian; Li, Xiaoxi; Xu, Liang; Lin, Minghui; Liu, Chuan; He, Zhengjin; et al
Cell Reports (2019), 27(11), 3331-3344.e6 | Language: English, Database: CAplus and MEDLINE

Substances (13) Reactions (0) Cited By (14)

76

Phosphatase PP2A enhances MCL-1 protein half-life in multiple myeloma cells

By: Slomp, Anne; Moesbergen, Laura M.; Eldering, Eric; Kersten, Marie Jose; Minnema, Monique C.; Peperzak, Victor
Cell Death & Disease (2021), 12(3), 229 | Language: English, Database: CAplus and MEDLINE

Substances (14) Reactions (0) Cited By (5)

77

Synthetic Lethality of PARP Inhibitors in Combination with MYC Blockade Is Independent of BRCA Status in Triple-Negative Breast Cancer

By: Carey, Jason. P. W.; Karakas, Cansu; Bui, Tuyen; Chen, Xian; Vijayaraghavan, Smruthi; Zhao, Yang; Wang, Jing; Mikule, Keith; Litton, Jennifer K.; Hunt, Kelly K.; et al
Cancer Research (2018), 78(3), 742-757 | Language: English, Database: CAplus and MEDLINE

Substances (14) Reactions (0) Cited By (74)

78

Reimagining high-throughput profiling of reactive cysteines for cell-based screening of large electrophile libraries

By: Kuljanin, Miljan; Mitchell, Dylan C.; Schweppe, Devin K.; Gikandi, Ajami S.; Nusinow, David P.; Bulloch, Nathan J.; Vinogradova, Ekaterina V.; Wilson, David L.; Kool, Eric T.; Mancias, Joseph D.; et al
Nature Biotechnology (2021), 39(5), 630-641 | Language: English, Database: CAplus and MEDLINE

Substances (15) Reactions (0) Cited By (50)

79

CDK7 inhibition augments response to multidrug chemotherapy in pancreatic cancer

By: Zeng, Siyuan; Lan, Bin; Ren, Xiaofan; Zhang, Shuman; Schreyer, Daniel; Eckstein, Markus; Yang, Hai; Britzen-Laurent, Nathalie; Dahl, Andreas; Mukhopadhyay, Debabrata; et al
Journal of Experimental & Clinical Cancer Research (2022), 41(1), 241 | Language: English, Database: CAplus and MEDLINE

Substances (16) Reactions (0) Cited By (0)

80

Selective Inhibitors of Autophagy Reveal New Link between the Cell Cycle and Autophagy and Lead to Discovery of Novel Synergistic Drug Combinations

By: Sung, Kisa; Kurowski, Agata ; Lansiquot, Carisse ; Wan, Kanny K.; Patnaik, Samarjit ; Walsh, Martin J.; Lazarus, Michael B.

ACS Chemical Biology (2022), 17(12), 3290-3297 | Language: English, Database: CAplus and MEDLINE

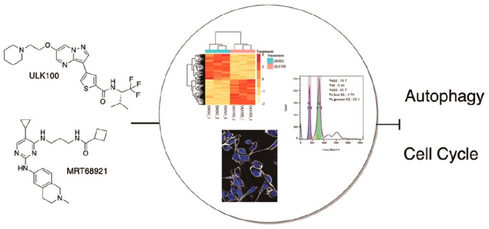

Substances (18) Reactions (2) Cited By (0)

81

Nanomaterial-Facilitated Cyclin-Dependent Kinase 7 Inhibition Suppresses Gallbladder Cancer Progression via Targeting Transcriptional Addiction

By: Huang, Chen-Song; Xu, Qiong-Cong; Dai, Chunlei; Wang, Liying; Tien, Yi-Chih; Li, Fuxi; Su, Qiao; Huang, Xi-Tai; Wu, Jun ; Zhao, Wei; et al

ACS Nano (2021), 15(9), 14744-14755 | Language: English, Database: CAplus and MEDLINE

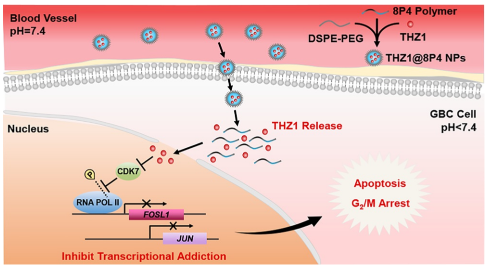

Substances (20) Reactions (8) Cited By (4)

82

Dynamic regulation of P-TEFb by 7SK snRNP is integral to the DNA damage response to regulate chemotherapy sensitivity

By: Fang, Yin; Wang, Yan; Spector, Benjamin M.; Xiao, Xue; Yang, Chao; Li, Ping; Yuan, Yuan; Ding, Ping; Xiao, Zhi-Xiong; Zhang, Peixuan; et al

iScience (2022), 25(9), 104844 | Language: English, Database: CAplus and MEDLINE

Substances (21) Reactions (0) Cited By (0)

83

Functionally active cyclin-dependent kinase 9 is essential for porcine reproductive and respiratory syndrome virus subgenomic RNA synthesis

By: Wang, Meng-Di; Yang, Le; Meng, Jie-Jie; Pan, Jia-Jia; Zhang, Chao; Wan, Bo; Sun, Yu; Wang, Jiang; Chu, Bei-Bei; Zhang, Gai-Ping
Molecular Immunology (2021), 135, 351-364 | Language: English, Database: CAplus and MEDLINE

Substances (21) | Reactions (0) | Cited By (0)

84

Vulnerability of drug-resistant EML4-ALK rearranged lung cancer to transcriptional inhibition

By: Paliouras, Athanasios R.; Buzzetti, Marta; Shi, Lei; Donaldson, Ian J.; Magee, Peter; Sahoo, Sudhakar; Leong, Hui-Sun; Fassan, Matteo; Carter, Matthew; Di Leva, Gianpiero; et al
EMBO Molecular Medicine (2020), 12(7), e11099 | Language: English, Database: CAplus and MEDLINE

Substances (21) | Reactions (0) | Cited By (7)

85

Multiplexed and reproducible high content screening of live and fixed cells using Dye Drop

By: Mills, Caitlin E.; Subramanian, Kartik; Hafner, Marc; Niepel, Mario; Gerosa, Luca; Chung, Mirra; Victor, Chiara; Gaudio, Benjamin; Yapp, Clarence; Nirmal, Ajit J.; et al
Nature Communications (2022), 13(1), 6918 | Language: English, Database: CAplus and MEDLINE

Substances (30) | Reactions (0) | Cited By (0)

86

Patient-derived models recapitulate heterogeneity of molecular signatures and drug response in pediatric high-grade glioma

By: He, Chen; Xu, Ke; Zhu, Xiaoyan; Dunphy, Paige S.; Gudenas, Brian; Lin, Wenwei; Twarog, Nathaniel; Hover, Laura D.; Kwon, Chang-Hyuk; Kasper, Lawryn H.; et al
Nature Communications (2021), 12(1), 4089 | Language: English, Database: CAplus and MEDLINE

Substances (31) | Reactions (0) | Cited By (11)

87

An integrative multi-omics approach uncovers the regulatory role of CDK7 and CDK4 in autophagy activation induced by silica nanoparticles

By: Ruan, Chen; Wang, Chenwei; Gong, Xuanqing; Zhang, Ying; Deng, Wankun; Zhou, Jiaqi; Huang, Dengtong; Wang, Zining; Zhang, Qiong; Guo, Anyuan; et al
Autophagy (2021), 17(6), 1426-1447 | Language: English, Database: CAplus and MEDLINE

Substances (26) | Reactions (0) | Cited By (12)

88

Therapeutic targeting of ATR yields durable regressions in small cell lung cancers with high replication stress

By: Thomas, Anish; Takahashi, Nobuyuki ; Rajapakse, Vinodh N.; Zhang, Xiaohu; Sun, Yilun; Ceribelli, Michele; Wilson, Kelli M.; Zhang, Yang; Beck, Erin; Sciuto, Linda; et al
Cancer Cell (2021), 39(4), 566-579.e7 | Language: English, Database: CAplus and MEDLINE

Substances (42) Reactions (0) Cited By (35)

89

Modeling and targeting of erythroleukemia by hematopoietic genome editing

By: Iacobucci, Ilaria; Qu, Chunxu; Varotto, Elena ; Janke, Laura J.; Yang, Xu; Seth, Aman; Shelat, Anang ; Friske, Jake D. ; Fukano, Reiji ; Yu, Jiyang ; et al
Blood (2021), 137(12), 1628-1640 | Language: English, Database: CAplus and MEDLINE

Substances (44) Reactions (0) Cited By (14)

90

Modulating Androgen Receptor-Driven Transcription in Prostate Cancer with Selective CDK9 Inhibitors

By: Richters, Andre; Doyle, Shelby K. ; Freeman, David B. ; Lee, Christina; Leifer, Becky S.; Jagannathan, Sajjeev; Kabinger, Florian ; Koren, Jost Vrabic; Struntz, Nicholas B.; Urgiles, Julie ; et al
Cell Chemical Biology (2021), 28(2), 134-147.e14 | Language: English, Database: CAplus and MEDLINE

Substances (29) Reactions (0) Cited By (27)

91

Quantifying CDK inhibitor selectivity in live cells

By: Wells, Carrow I. ; Vasta, James D.; Corona, Cesear R.; Wilkinson, Jennifer; Zimprich, Chad A.; Ingold, Morgan R.; Pickett, Julie E. ; Drewry, David H. ; Pugh, Kathryn M.; Schwinn, Marie K.; et al
Nature Communications (2020), 11(1), 2743 | Language: English, Database: CAplus and MEDLINE

Substances (44) Reactions (5) Cited By (21)

92

CDK7 Inhibitors in Cancer Therapy: The Sweet Smell of Success?

By: Diab, Sarah; Yu, Mingfeng; Wang, Shudong
Journal of Medicinal Chemistry (2020), 63(14), 7458-7474 | Language: English, Database: CAplus and MEDLINE

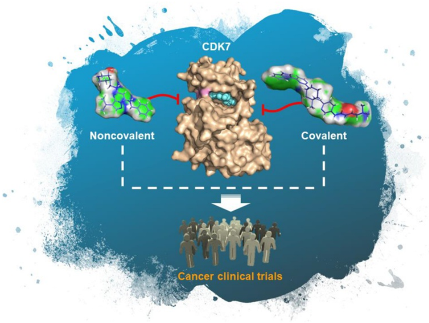

Substances (48) | Reactions (7) | Cited By (28)

93

Refinement of in vitro methods for identification of aldehyde oxidase substrates reveals metabolites of kinase inhibitors

By: Dick, Ryan A.
Drug Metabolism & Disposition (2018), 46(6), 846-859 | Language: English, Database: CAplus and MEDLINE

Substances (58) | Reactions (0) | Cited By (18)

Copyright © 2023 American Chemical Society (ACS). All Rights Reserved.
Copyright © 2023 U.S. National Library of Medicine.
Internal use only. Redistribution is subject to the terms of your SciFinder^n License Agreement and CAS information Use Policies.
